# Altered Genes and Biological Functions in Response to Severe Burns

**DOI:** 10.1155/2021/8836243

**Published:** 2021-05-24

**Authors:** Xinheng Liu, Yongxian Rong, Donglin Huang, Zhijie Liang, Xiaolin Yi, Fangxiao Wu, Dandan Zhu, Steven Mo, Wenhai Nong, Hongmian Li

**Affiliations:** ^1^Department of Burn and Plastic Surgery, Guiping People's Hospital, Ren-Min West Road, Guiping, Guangxi 537200, China; ^2^Department of Plastic and Aesthetic Surgery, The Fifth Affiliated Hospital of Guangxi Medical University & The First People's Hospital of Nanning, NO.89, Qi-Xing Road, Nanning, Guangxi 530022, China; ^3^CAS Center for Excellence in Brain Science and Intelligence Technology, Shanghai 200031, China; ^4^Department of Orthopedics, Binyang County People's Hospital, Binyang, China

## Abstract

Severe burns are acute wounds caused by local heat exposure, resulting in life-threatening systemic effects and poor survival. However, the specific molecular mechanisms remain unclear. First, we downloaded gene expression data related to severe burns from the GEO database (GSE19743, GSE37069, and GSE77791). Then, a gene expression analysis was performed to identify differentially expressed genes (DEGs) and construct protein-protein interaction (PPI) network. The molecular mechanism was identified by enrichment analysis and Gene Set Enrichment Analysis. In addition, STEM software was used to screen for genes persistently expressed during response to severe burns, and receiver operating characteristic (ROC) curve was used to identify key DEGs. A total of 2631 upregulated and 3451 downregulated DEGs were identified. PPI network analysis clustered these DEGs into 13 modules. Importantly, module genes mostly related with immune responses and metabolism. In addition, we identified genes persistently altered during the response to severe burns corresponding to survival and death status. Among the genes with high area under the ROC curve in the PPI network gene, CCL5 and LCK were identified as key DEGs, which may affect the prognosis of burn patients. Gene set variation analysis showed that the immune response was inhibited and several types of immune cells were decreased, while the metabolic response was enhanced. The results showed that persistent gene expression changes occur in response to severe burns, which may underlie chronic alterations in physiological pathways. Identifying the key altered genes may reveal potential therapeutic targets for mitigating the effects of severe burns.

## 1. Introduction

Severe burns are serious injuries with global influence. According to the latest report of the World Health Organization (WHO), it is estimated that 265,000 people die of burns every year. More than 500,000 people seek treatment each year in the United States, 40,000 are hospitalized, and 4000 die because of severe burns [1]. The annual cost of treating these burns is estimated to exceed one billion dollars worldwide, excluding the indirect costs of disability and rehabilitation [2]. Improvements in treatments have increased the survival rate of many severely burned people.

Burn rehabilitation is a complex and dynamic process [3]. In recent years, great progress has been made in the identification of clinical biomarkers for severely burned patients [4–6]. The response to severe burns affects almost every organ [7]. Inflammation, hypermetabolism, muscle wasting, and insulin resistance are all markers of pathophysiological response after severe burn [8]. Burn patients differ from patients with other forms of trauma in their resuscitation requirements, metabolic pressure, complications, and determinants of prognosis [9]. Severely burned patients are divided into several stages for management and treatment, and each stage also has different molecular and cellular mechanisms [10]. More studies and effective strategies are needed to stratify severe burn patients for treatment and for predicting prognosis.

Severe burn patients may enter the “burn shock” state, which is characterized by poor tissue perfusion, serious capillary leakage, occult coagulation disease, and a large release of inflammatory mediators [11]. The high levels or the inhibitory activity of some of immune system are related to adverse outcomes after burn [12]. After severe burn, T helper cells gradually enter a state of immunosuppression [13]. High Toll-like receptor reactivity after burn promotes the production of proinflammatory cytokines [14]. Tumor necrosis factor-*α* (TNF-*α*) is also highly expressed in burn patients, with low TNF-*α* levels being related to good prognosis [15]. Therefore, the immune inflammatory response plays an important role in the regulation of severe burns and therefore is closely related to the survival and rehabilitation of patients.

To increase our understanding of the complex responses to severe burn, the present study used an array of bioinformatic techniques to examine molecular mechanisms in response to severe burn. In addition to revealing basic insights into the burn process and the body's response, our results may help identify potential markers for patient stratification and prognosis prediction as well as potential therapeutic targets.

## 2. Materials and Methods

### 2.1. GEO Datasets

The gene expression microarray datasets GSE19743, GSE37069, and GSE77791 were downloaded from the GEO database (http://www.ncbi.nlm.nih.gov/geo/) [16]. The GSE19743 series (GPL570 platform) contained a total of 120 white blood cells, including 114 arrays for 57 patients (two time points per patient) and 63 arrays for 63 healthy controls. The GSE37069 series (GPL570 platform) contained a total of 279 white blood cell samples (244 severe burns patients and 35 healthy subjects). The GSE77791 series (GPL570 platform) contained a total of 117 whole blood samples (15 burn patients, 15 healthy controls, 15 burn patients receiving hydrocortisone (CB), and 15 patients receiving placebo (PB)).

### 2.2. DEG Analysis

First, probe information was converted into gene symbols. For burn patients and healthy subjects, the mRNA levels of DEGs were identified using the *limma* package [17]. *P* criterion of <0.05 is signature. A similar analysis was applied to find DEGs in different phases of the burn response.

### 2.3. PPI Network Construction and Module Analysis

Protein interaction data were obtained from the Search Tool for the Retrieval of Interacting Genes/Proteins (STRING) database [18]. Next, a PPI network was constructed including DEGs with the selected gene signatures using Cytoscape [19]. Subsequently, we identified the subnetwork with strongly interacting genes as a module using the MCODE clustering algorithm with Kcore = 7 [20]. The AUC of module genes was calculated using plotROC [21].

### 2.4. Functional and Pathway Analyses

To explore the biological characteristics of module genes, we performed GO and KEGG pathway enrichment analyses with the *clusterProfiler* package in R [22]. *P* < 0.05 was considered as significant.

### 2.5. Gene Set Enrichment Analysis (GSEA)

GSEA [23] was performed to identify KEGG pathways. We used the results of KEGG enrichment as the background set. To evaluate the enrichment of the same pathway in the different groups, we used gene set variation analysis (GSVA), a gene set enrichment method that estimates variation of pathway activity over a sample population in an unsupervised manner [24]. Single sample GSEA (ssGSEA) classifies gene sets with common biological functions, chromosomal localization, and physiological regulation [23]. We used ssGSEA to quantify the types of immune cells present in each burn stage based on analysis of 24 genes of immune cell marker genes [25].

### 2.6. Persistent DEGs

The STEM software was used to detect coexpressed genes in different stages of burn patients in order to identify the genes whose expression was persistently altered from early to late stages. The obtained genes were clustered by cm function to identify up- and downregulated genes.

### 2.7. Nomogram

The hub genes were included in a logistic regression analysis to determine whether their expression was associated with the prognosis of burn patients. The logistic regression analysis was used to build a nomograph [26]. Based on the logistic model, a risk prediction model was established by using all the risk factors related to burn. The score was used to assess association with prognosis.

### 2.8. Quantitative Real-Time Polymerase Chain Reaction (qRT-PCR)

The whole blood samples were collected from 10 burn patients and 10 healthy controls. Written, informed consent was obtained from each patient. This study was approved by the Ethics Committee of the General Hospital of Xinjiang Military Command. The total RNA was isolated with TRIzol (Thermo Fisher) and quantified by NanoDrop. The cDNA was created using the cDNA synthesis kit (Invitrogen). The cDNA was transcribed into DNA through SYBR Green PCR Master Mix (Thermo Fisher) using a real-time PCR machine (Applied Biosystems). The resulting Ct values were relative to a GAPDH reference gene, and the 2^-*ΔΔ*Ct^ was obtained. Primers were used as follows: CCL5 forward, 5′-AGATCTCTGCAGCTGCCCTCA-3′ and reverse, 5′-GGAGCACTTGCTGCTGGTGTAG-3′; LCK forward, 5′-CACGGATGACAGCTCTGAAA-3′ and reverse, 5′-ATGGAGAACGGGAGCCTA GT-3′; GAPDH forward, 5′-GACTAACCCTFCFCTCCTG-3′ and reverse, 5′-GCCCAATACGACCAAATCAG-3′.

## 3. Results

### 3.1. Differentially Expressed Genes (DEGs) Associated with Severe Burn

**Figure 1:**
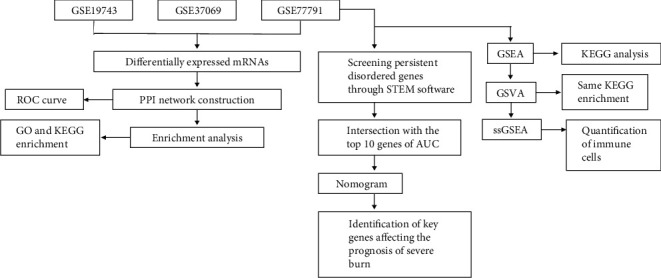
Flow chart of the study.

**Figure 2:**
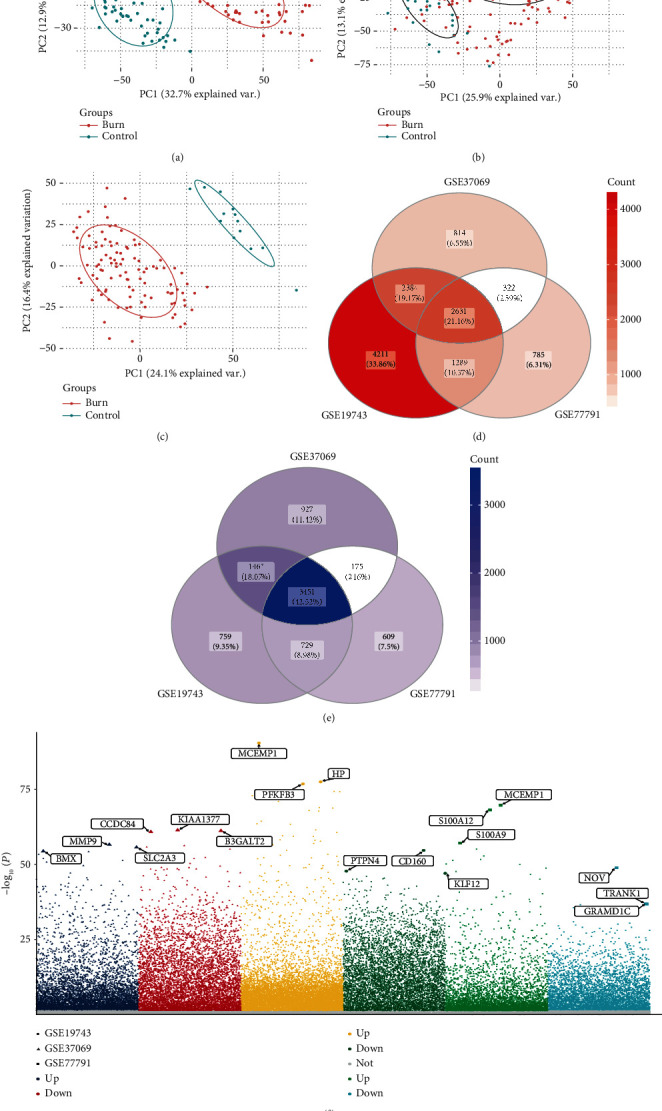
Differentially expressed genes (DEGs) between severe burn and controls in three datasets. Principal component analysis (PCA) results for (a) GSE19743, (b) GSE37069, and (c) GSE77791. (d) Upregulated genes common to the three datasets. Darker color indicates a higher number of upregulated genes. (e) Downregulated genes common to the three datasets. Darker color indicates a higher number of downregulated genes. (f) Manhattan map of common up- and downregulated DEGs.

In this study, we conducted a comprehensive bioinformatic analysis of gene expression data of severe burn patients to determine the key DEGs as potential biomarkers of severe burn (Figure 1). First of all, we conducted a principal component analysis (PCA) on the GSE19743, GSE37069, and GSE77791 datasets. PCA revealed that burn patients and healthy control samples in the three datasets showed a significantly different gene expression profile (Figures 2(a)–2(c)). We identified 3101 DEGs in GSE19743 (Table S1), 12171 DEGs in GSE37069 (Table S2), and 9991 DEGs in GSE77791 (Table S3). In order to further evaluate gene expression changes caused by severe burns, we obtained the intersection of the three groups of DEGs (Figures 2(d) and 2(e)) and found 2631 common upregulated genes (Table S4) and 3451 common downregulated genes (Table S5). The expression levels of common genes differed across the datasets, but across all datasets, upregulated common DEGs showed a significantly greater change in expression than common downregulated DEGs (Figure 2(f)).

### 3.2. PPI Network Construction and Functional Enrichment Analysis of Common DEGs

**Figure 3:**
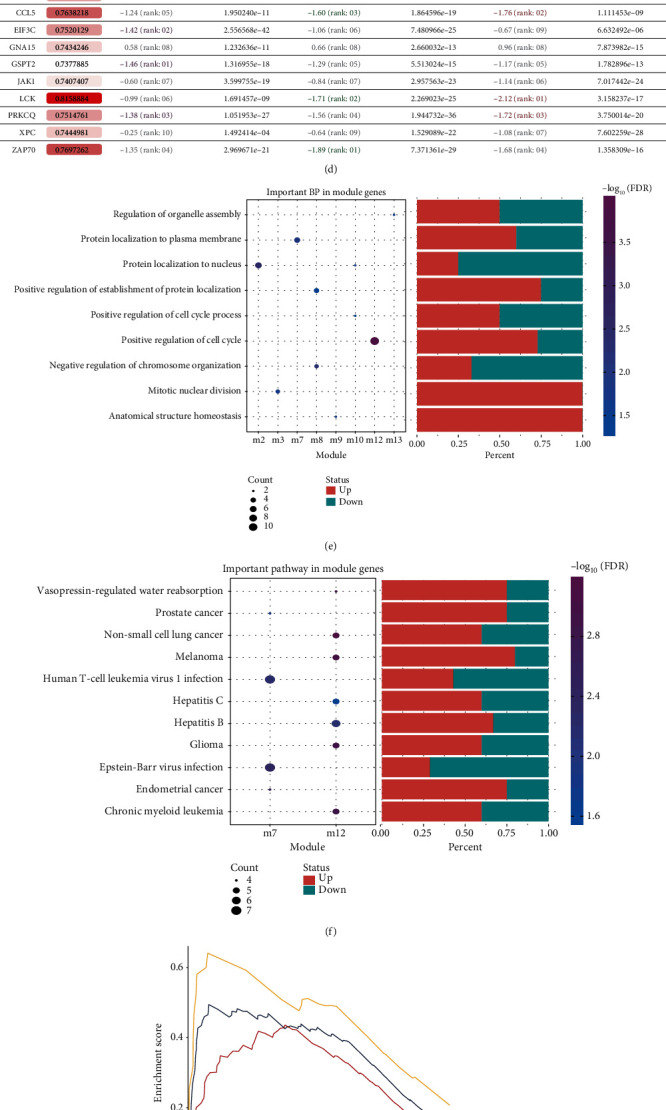
Protein-protein interaction (PPI) network and biological function of common differentially expressed genes (DEGs) in the three datasets. (a) Common DEGs formed a PPI network and clustered into 13 modules. (b) The coupling between transcription regulators and module genes. (c) Receiver operating characteristic curves for the top 10 module genes showing higher area under the curve (AUC). (d) AUCs of the top 10 genes and their expression in different datasets. (e) The biological processes (BPs) involving module genes. (f) The Kyoto Encyclopedia of Genes and Genomes (KEGG) pathways involving module genes. (g–i) Gene Set Enrichment Analysis to identify KEGG pathways involving DEGs in the datasets (g) GSE19743, (h) GSE37069, or (i) GSE77791.

In order to explore the interaction of DEGs caused by burn, we constructed a PPI network for common DEGs. The network contained 695 genes and was divided into 13 modules using MCODE (Table S6, Figure 3(a)). The expression heatmaps of module genes in each dataset are shown in Figure S1. We found that module 5 genes all were downregulated, while there was a large proportion of upregulated genes in module 3. With the transcription factors, we found the transcription factors REST and COPS5, which may have the ability to jointly regulate target genes (Figure 3(b)).

In order to identify the hub genes in each module, we screened the top 10 genes with highest area under the receiver operating characteristic curve (AUC) (Figures 3(c) and 3(d)). The over- or underexpression trend of these 10 genes was consistent across the three datasets (Figure S2).

In order to explore the biological function of the PPI network, Gene Ontology (GO) and Kyoto Encyclopedia of Genes and Genomes (KEGG) enrichment analysis were performed for DEGs in the modules (Table S7). We obtained 4490 biological processes (BP), 600 cell components (CC), and 713 mobile functions (MF) involving, for example, acute phase response, regulation of p38/MAPK cascade, and positive regulation of cell cycle (Figure 3(e)). We also found 203 KEGG pathways, including p53, T cell receptor, and Toll-like receptor signaling pathways (Figure 3(f)). Gene Set Enrichment Analysis (GSEA) showed that complement and coagulation cascades, PPAR signaling pathway, starch, and sucrose metabolism had significantly higher expression in burn patients than in the control group (Figures 3(g)–3(i)). Therefore, we believe that severe burn causes not only a strong immune inflammatory response, but also enhances metabolism.

### 3.3. Persistent DEGs during Severe Burn

**Figure 4:**
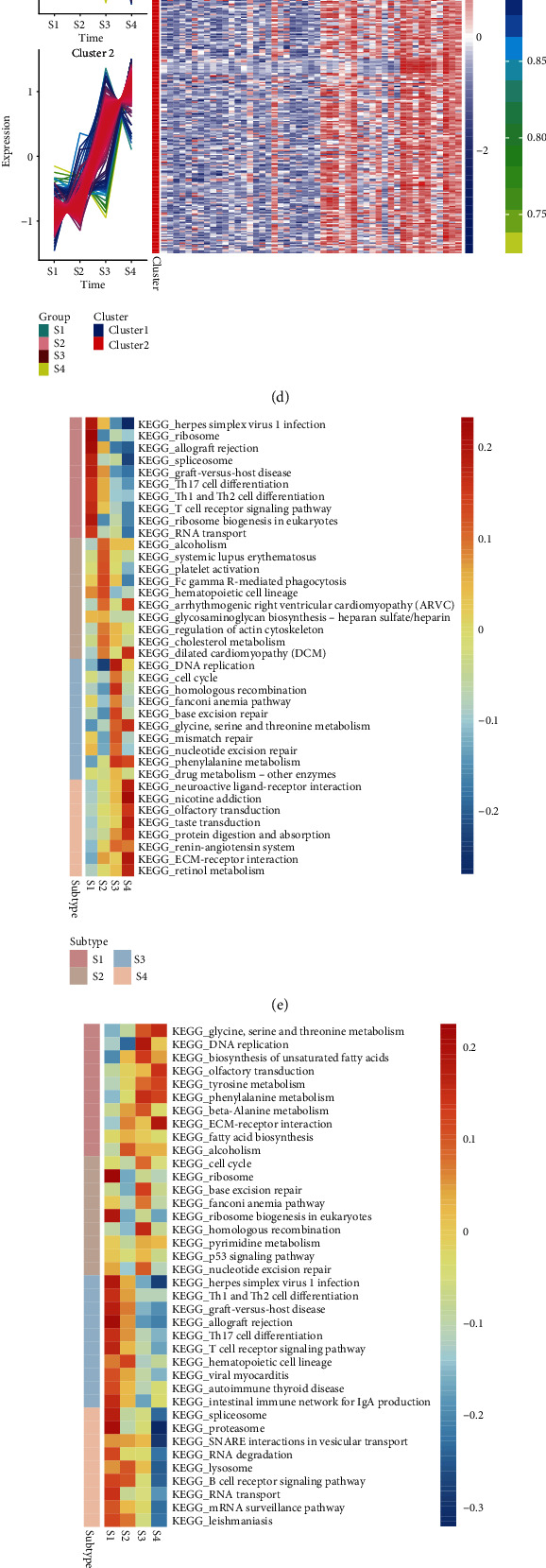
Genes and signaling pathways that are persistently altered during severe burn. (a, b) Persistently differentially expressed genes (DEGs) in patients who (a) died or (b) survived in the CB group were clustered into persistently up- or downregulated genes. (c, d) The same analysis was performed for persistent DEGs in placebo (PB) patients who (c) died or (d) survived. Red represents upregulated genes, and blue represents downregulated genes. In the CB group, the signaling pathways with the (e) highest or (f) lowest activity during four burn stages are shown. Analogously, in the PB group, the signaling pathways with the (g) highest or (h) lowest activity during four burn stages are shown. Signaling pathway activity is represented on a color gradient from blue (lower) to red (higher). Subtype refers to S1-S4. (i) Levels of different immune cell types during burn response in patients treated with hydrocortisone (CB) or placebo (PB). A change from blue to red indicates a gradual increase in the number of cells. S1-S4 refer to before treatment administration, one day after treatment administration, 120 h after treatment administration, and 168 h after treatment administration.

In order to further explore the effect of DEGs on severe burn, we compared the gene expression of GSE77791 samples in different times after the severe burn. We compared the expression profiles in a hydrocortisone (CB) treatment and a placebo (PB) group between 120 h and 24 h and between 168 h and 120 h, respectively. Then, we screened the DEGs that were persistently altered based on analysis with the STEM software. In the CB group, 644 persistent DEGs identified in patients who died were clustered into two modules by cm function, namely, a “persistent upregulation” module and a “persistent downregulation” module (Figure 4(a)). A total of 456 persistent DEGs were identified in patients who survived in the CB group (Figure 4(b)), while 597 persistent DEGs were observed in patients who died in the PB group (Figure 4(c)). On the other hand, 330 persistent DEGs were identified in the survivors in the PB group (Figure 4(d)). In the CB and PB groups, there were 166 common genes persistently upregulated and 83 genes persistently downregulated in death state; the corresponding numbers in survival state were 117 and 70.

Next, we performed gene set variation analysis (GSVA) to explore the potential biological effects of persistent DEGs. In the CB group, we found the most active signaling pathways (Figure 4(e)) and least active signaling pathways (Figure 4(f)) during each burn period. We did the same for the PB group (Figures 4(g) and 4(h)). Interestingly, we found that similar signaling pathways were activated or inhibited during a given burn period in both treatment groups.

By quantifying the types of immune infiltrating cells, we found that CD8 T cells, Tem, and cytotoxic cells gradually decreased, while mass cells, neutrophils, and T helper 2 (Th2) cells gradually increased (Figure 4(i)). In other words, over the course of the burn response, immune response gradually weakened, while metabolism and repair functions became gradually stronger.

### 3.4. Identification of Key Prognostic Genes

**Figure 5:**
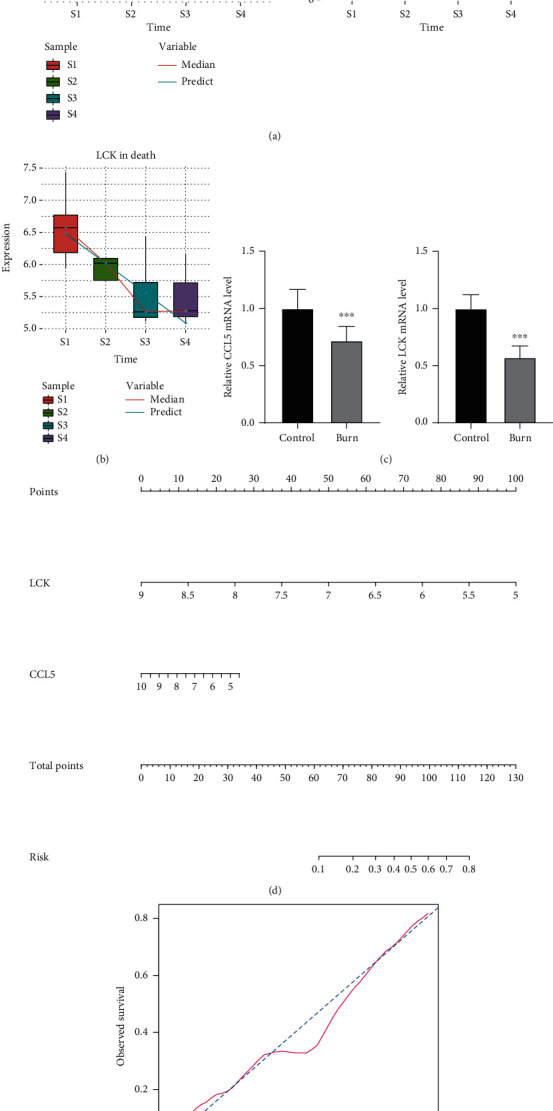
Potential prognostic genes for severe burns. (a) CCL5 expression over time in severe burn patients who died or survived. (b) LCK expression over time in patients with severe burn who died. S1-S4 refer to before treatment administration, one day after treatment administration, 120 h after treatment administration, and 168 h after treatment administration. (c) The expression of CCL5 and LCK in burn patients and healthy controls was determined using qRT-PCR. ^∗^*P* < 0.05. (d) Nomogram to evaluate the risk of death in severely burned patients. (e) Plots depicting the agreement between predicted and real outcomes for each model.

In order to identify the key genes that affect the prognosis of severe burns, we analyzed the interaction between persistent DEGs in whole blood of burn patients and the top 10 genes with the highest AUC value in the PPI network. Two hub genes were identified: chemokine ligand 5 (CCL5, AUC 0.76) and lymphocyte-specific protein tyrosine kinase (LCK, AUC 0.82). Patients who died showed stronger downregulation of CCL5 (Figure 5(a)) and LCK (Figure 5(b)) than survivors. Importantly, we validated that CCL5 and LCK were significantly downexpressed in burn patients using qRT-PCR, compared with healthy controls (Figure 5(c)).

We used the logistic regression coefficient to generate a nomogram, which indicated that the higher the expression of CCL5 and LCK, the lower the risk of burn-related death (Figure 5(d)). Calibration showed that the nomogram performed well compared with the ideal model (Figure 5(e)). Therefore, our results suggest that CCL5 and LCK can be used to estimate the risk of death in patients with severe burns.

## 4. Discussion

Burns, especially when severe, are associated with high mortality [27]. Based on published sequencing data from severe burn patients, this study explored the key genes that can affect the prognosis. The commonly expressed genes in white blood after severe burn from three patients' datasets can be regarded as potential DEGs whose over or underexpression may affect the survival or death of burn patients.

Through a PPI network, we identified these DEGs as a subnetwork of 13 interacting genes. Each subnetwork may subserve pathways and processes with clinical significance [28]. Enrichment analysis showed that most PPI network genes were involved in the immune inflammatory response.

In the enrichment results, a large number of immune inflammatory reactions were involved. Previous studies described a role for some of these genes in burn patients. For example, the expression and phosphorylation level of p38 were significantly increased in burn models, and this kinase was also an effective target to alleviate burn reaction [29, 30]. Mitogen-activated protein kinase (MAPK) can aggravate the oxidative stress and inflammatory response of burn patients, promote proliferation, reduce the differentiation of keratinocytes, and thus inhibit the healing of skin wounds [31, 32]. As a well-known apoptotic signaling pathway, p53 signaling pathway plays an important role contributing to the prognosis of burn [33–35]. Burn can activate the Toll-like receptor and stimulate the secretion of cytokines, which usually lead to extreme system dynamic balance and may lead to life-threatening multiple organ dysfunction syndrome [36]. The PPAR signaling pathway exerts anti-inflammatory, antifibrosis, and antiangiogenesis effects in response to burns [37].

Our study found that the metabolic rate of burn patients was significantly increased compared with controls. This increase was accompanied by an acute inflammatory response to injury, leading to a higher risk of death [38]. After severe burn, the adipose tissue changes from white to beige, the number of mitochondria increases, and the metabolic function of adipose tissue changes [39, 40]. Therefore, the DEG network identified in this study may play an important regulatory role in response to burns.

Importantly, through the STEM software, we identified genes that are persistently altered during the response to severe burns. We predict that these genes can have an important influence on prognosis. Burn patients receiving either placebo or medication showed persistent DEGs, and among PPI network genes, the hub genes CCL5 and LCK showed the highest AUCs for predicting survival. These changes of CCL5 and LCK may have prognostic value.

CCL5 is a chemokine that plays a role in the peripheral immune system, it helps regulate synaptic activity, and it protects against a variety of neurotoxins [41]. There is evidence that CCL5 is expressed in the skin after burns and may constitute a drug target [42, 43]. On the other hand, LCK is involved in the development, function, and differentiation of T cells [44]. Consistent with our analysis, LCK was significantly downregulated after burn, which constituted a risk factor for poor prognosis [45].

Interestingly, we found that several kinds of immunoregulatory effects gradually weakened after burn, based on the types of immune cells present and the signaling pathways activated. This has been confirmed in other studies observing that deep burn can lead to severe immunosuppression and may induce sepsis and multiple organ failure [46]. Moreover, the destruction of the skin barrier and the blood vessel supply, as well as systemic immunosuppression, are risk factors for infection in burn patients [47].

Our study presents several limitations. First, the results were based on bioinformatic analysis, and require more experimental confirmation. Second, the samples used in the analysis were all blood samples and not tissue, and the results may need more clinical samples to verify.

## 5. Conclusions

The survival or death of burn patients involves a series of complex processes, which need to be further investigated to improve the risk stratification of burn patients. In summary, we found that the persistent DEGs, especially CCL5 and LCK, may be key factors affecting the prognosis of severely burned patients and may have a clinical utility as prognostic biomarkers.

## Data Availability

The research data used to support the findings of this study are included within the supplementary information files.

## Supplementary Materials

Supplementary 1 Figure S1: (A) heatmaps of differentially expressed genes (DEGs) classified into modules in the datasets (A) GSE19743, (B) GSE37069, or (C) GSE77791.

Supplementary 2 Figure S2: expression of the top 10 genes with highest area under the receiver operating characteristic curve (AUC) for predicting survival.

Supplementary 3 Table S1: 3101 DEGs in GSE19743, DEG_sort_0.05.

Supplementary 4 Table S2: 12171 DEGs in GSE37069, DEG_sort_0.05.

Supplementary 5 Table S3: 9991 DEGs in GSE77791, DEG_sort_0.05.

Supplementary 6 Table S4: 2631 common upregulated genes.

Supplementary 7 Table S5: 3451 common downregulated genes.

Supplementary 8 Table S6: module_gene.

Supplementary 9 Table S7: Gene Ontology (GO) and Kyoto Encyclopedia of Genes and Genomes (KEGG) enrichment analysis, enrichment_all_0.05.
