# Corrigendum to “Altered Genes and Biological Functions in Response to Severe Burns”

**DOI:** 10.1155/2021/9858140

**Published:** 2021-10-12

**Authors:** Xinheng Liu, Yongxian Rong, Donglin Huang, Zhijie Liang, Xiaolin Yi, Fangxiao Wu, Dandan Zhu, Wenjian Xu, Steven Mo, Wenhai Nong, Hongmian Li

**Affiliations:** ^1^Department of Burn and Plastic Surgery, Guiping People's Hospital, Ren-Min West Road, Guiping, Guangxi 537200, China; ^2^Department of Plastic and Aesthetic Surgery, The Fifth Affiliated Hospital of Guangxi Medical University & the First People's Hospital of Nanning, No. 89, Qi-Xing Road, Nanning, Guangxi 530022, China; ^3^Department of Dermatology, General Hospital of Xinjiang Military Command, Urumqi, Xinjiang 830000, China; ^4^YuanDong International Academy of Life Sciences, Nanning, 530229 Guangxi, China; ^5^Department of Orthopedics, Binyang County People's Hospital, Binyang, China

In the article titled “Altered Genes and Biological Functions in Response to Severe Burns” [1], the authors wish to add Wenjian Xu to the author list, who provided the samples used in the qRT-PCR experiment. Additionally, the affiliation for Steven Mo was incorrect on the original publication. The corrected author list and affiliation is as above.
