# Supplementary figures and images for: Altered Genes and Biological Functions in Response to Severe Burns
Source: Biomed Res Int. 2021 May 24;2021:8836243. doi: 10.1155/2021/8836243 (PMC8168476; doi:10.1155/2021/8836243)

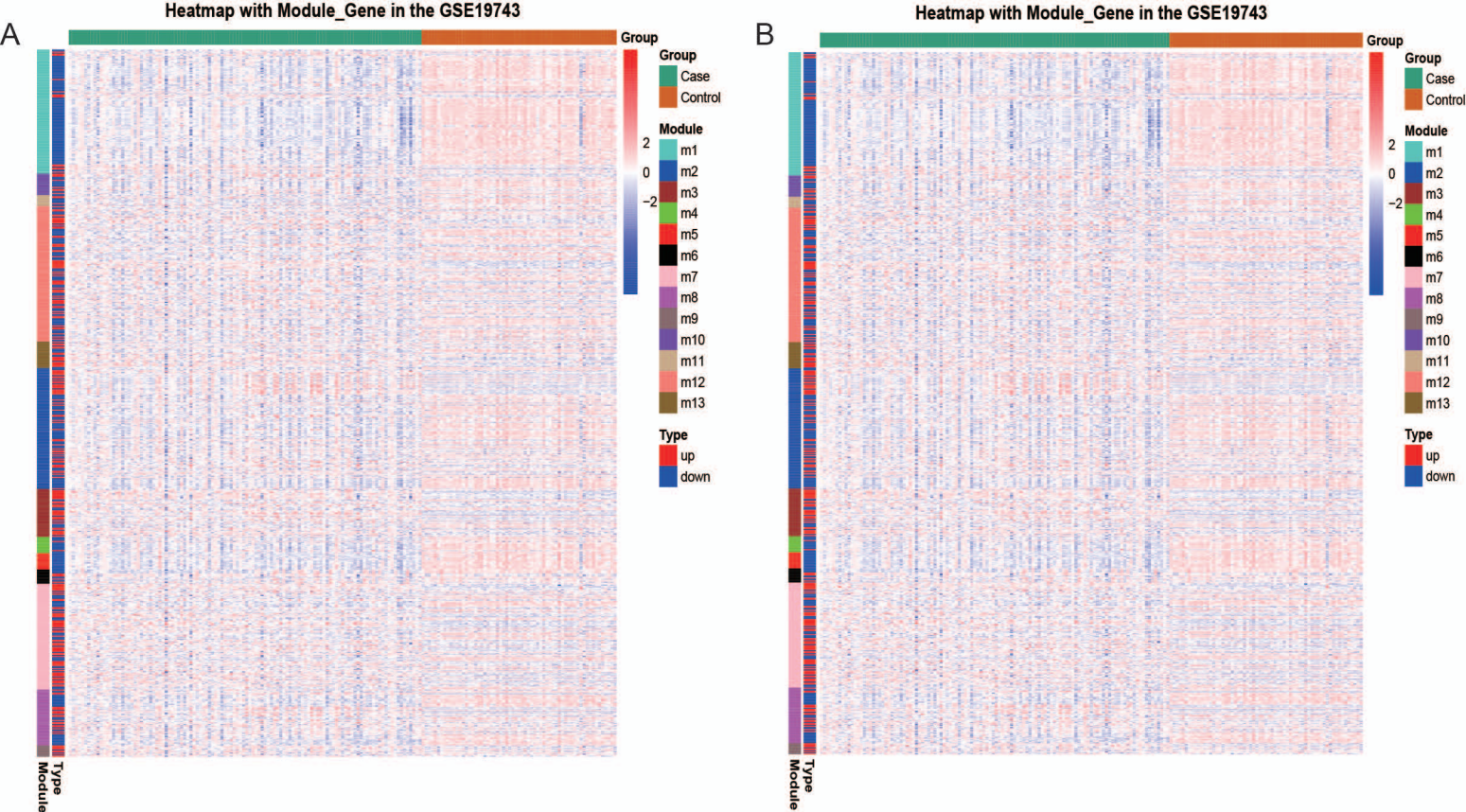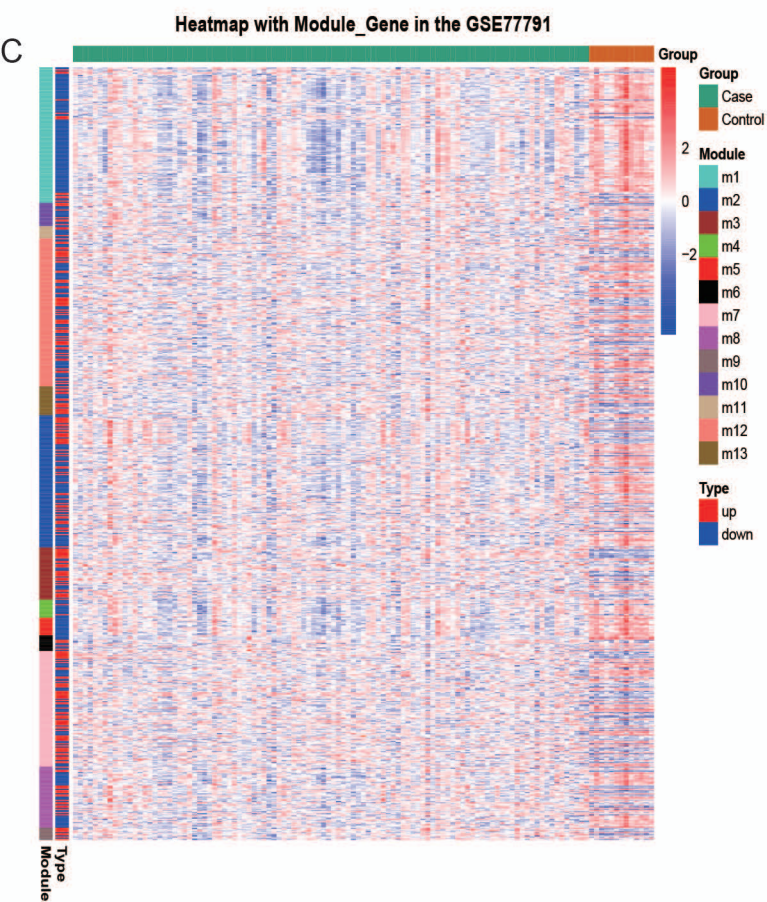

Supplement: Supplementary 1 — Figure S1: (A) heatmaps of differentially expressed genes (DEGs) classified into modules in the datasets (A) GSE19743, (B) GSE37069, or (C) GSE77791. [file 8836243.f1.pdf]
