# Supplementary material for: Altered Genes and Biological Functions in Response to Severe Burns
Source: Biomed Res Int. 2021 May 24;2021:8836243. doi: 10.1155/2021/8836243 (PMC8168476; doi:10.1155/2021/8836243)
Supplement: Supplementary 2 — Figure S2: expression of the top 10 genes with highest area under the receiver operating characteristic curve (AUC) for predicting survival. [file 8836243.f2.pdf]

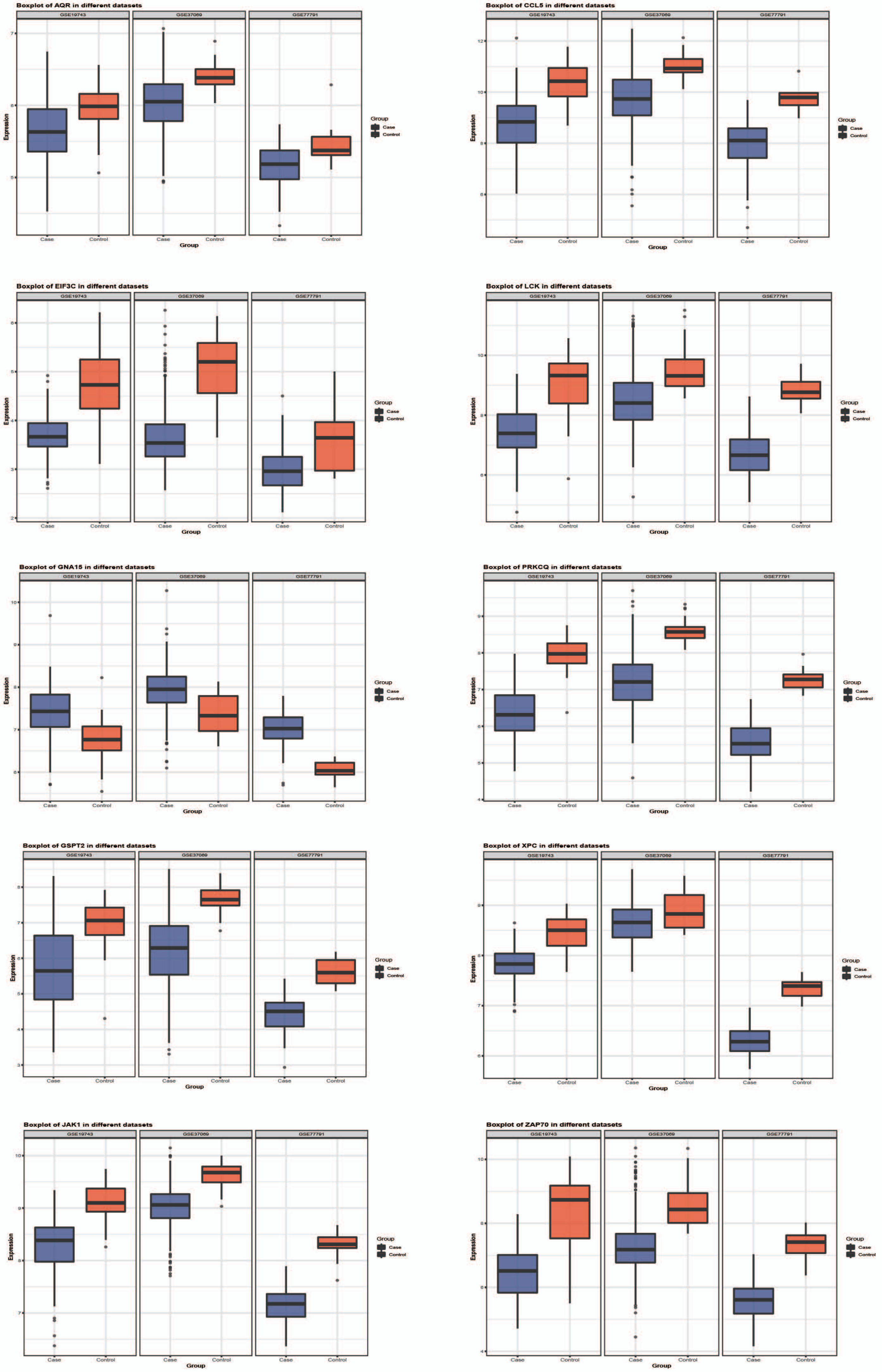
Boxplot of AQR in different datasets
Boxplot of CCL5 in different datasets
Boxplot of EIF3C in different datasets
Boxplot of LCK in different datasets
Boxplot of GNA15 in different datasets
Boxplot of PRKCQ in different datasets
Boxplot of GSPT2 in different datasets
Boxplot of XPC in different datasets
Boxplot of JAK1 in different datasets
Boxplot of ZAP70 in different datasets
GSE19743
GSE37069
GSE77791
Expression
Group
Case
Control
