# Supplementary material for: Altered Genes and Biological Functions in Response to Severe Burns
Source: Biomed Res Int. 2021 May 24;2021:8836243. doi: 10.1155/2021/8836243 (PMC8168476; doi:10.1155/2021/8836243)
Supplement: Supplementary 3 — Table S1: 3101 DEGs in GSE19743, DEG_sort_0.05. [file 8836243.f3.pdf]

Table S1 3101 DEGs in GSE19743, DEG_sort_0.05

| DEG | logFC | AveExpr | t | P.Value | adj.P.Val | B |
|---|---|---|---|---|---|---|
| DEFA4 | 2.917749 | 9.538176 | 9.017929 | 4.98E-15 | 3.4E-11 | 23.436 |
| MS4A3 | 2.802203 | 7.926795 | 10.16052 | 1.06E-17 | 2.17E-13 | 29.24118 |
| CEACAM6 | 2.755338 | 8.107644 | 9.597659 | 2.22E-16 | 2.27E-12 | 26.37592 |
| ELANE | 2.555761 | 7.889911 | 7.019605 | 1.65E-10 | 1.69E-07 | 13.58882 |
| CEACAM8 | 2.405668 | 9.494383 | 7.711364 | 4.86E-12 | 1.11E-08 | 16.92328 |
| MPO | 2.18245 | 7.439552 | 7.125769 | 9.67E-11 | 1.29E-07 | 14.09357 |
| HBD | 2.133692 | 8.90723 | 6.357205 | 4.28E-09 | 2.3E-06 | 10.50909 |
| CTSG | 2.042749 | 6.625717 | 7.875552 | 2.08E-12 | 8.5E-09 | 17.72874 |
| IFIT1 | 1.980085 | 7.047256 | 4.677521 | 7.97E-06 | 0.000642 | 3.429907 |
| RRM2 | 1.905885 | 7.450741 | 7.98581 | 1.17E-12 | 5.99E-09 | 18.27218 |
| AZU1 | 1.856897 | 6.357174 | 5.437844 | 3.07E-07 | 5.19E-05 | 6.481414 |
| PRTN3 | 1.82964 | 5.021449 | 4.751527 | 5.88E-06 | 0.000514 | 3.714012 |
| ABCA13 | 1.797087 | 4.088101 | 7.686593 | 5.53E-12 | 1.13E-08 | 16.80218 |
| OLR1 | 1.772985 | 4.441725 | 6.938833 | 2.47E-10 | 2.4E-07 | 13.2067 |
| IFI44L | 1.757242 | 5.566188 | 4.789292 | 5.03E-06 | 0.000459 | 3.860127 |
| RSAD2 | 1.675836 | 6.050606 | 4.545056 | 1.36E-05 | 0.000937 | 2.928879 |
| AHSP | 1.551543 | 6.732975 | 5.897426 | 3.77E-08 | 1.07E-05 | 8.454151 |
| PTX3 | 1.526261 | 7.147322 | 5.99468 | 2.4E-08 | 7.54E-06 | 8.882442 |
| HBM | 1.509663 | 7.87325 | 5.64443 | 1.21E-07 | 2.52E-05 | 7.35733 |
| CD24 | 1.457336 | 9.105952 | 6.32687 | 4.95E-09 | 2.53E-06 | 10.37125 |
| CMPK2 | 1.403303 | 7.39291 | 4.210391 | 5.08E-05 | 0.002589 | 1.707886 |
| KIF11 | 1.383507 | 4.71655 | 5.79155 | 6.16E-08 | 1.58E-05 | 7.992026 |
| ZWINT | 1.364731 | 6.723896 | 6.106731 | 1.41E-08 | 5.01E-06 | 9.380262 |
| E2F8 | 1.357363 | 4.635966 | 6.079559 | 1.61E-08 | 5.31E-06 | 9.259121 |
| BEX1 | 1.35055 | 5.988077 | 5.712019 | 8.88E-08 | 2.02E-05 | 7.647823 |
| CLC | 1.310888 | 10.27496 | 5.224225 | 7.88E-07 | 0.000112 | 5.59562 |
| RNASE3 | 1.300328 | 7.694687 | 5.679546 | 1.03E-07 | 2.24E-05 | 7.50802 |
| TMEM52B | 1.271457 | 3.782417 | 6.009936 | 2.23E-08 | 7.13E-06 | 8.949949 |
| PRG2 | 1.269306 | 5.248139 | 6.40501 | 3.4E-09 | 1.94E-06 | 10.72694 |
| SUCNR1 | 1.267404 | 5.454886 | 7.292238 | 4.17E-11 | 6.56E-08 | 14.89046 |
| IFI44 | 1.236635 | 5.957745 | 4.645372 | 9.09E-06 | 0.00071 | 3.307416 |
| LTF | 1.222838 | 11.56249 | 4.579326 | 1.19E-05 | 0.000833 | 3.057562 |
| DTL | 1.209845 | 4.947292 | 7.114771 | 1.02E-10 | 1.29E-07 | 14.04115 |
| EPB42 | 1.194802 | 5.993956 | 5.30684 | 5.48E-07 | 8.5E-05 | 5.935708 |
| CA1 | 1.192677 | 5.398481 | 7.54756 | 1.13E-11 | 2.1E-08 | 16.1246 |
| SELENBP1 | 1.191996 | 5.788523 | 5.111086 | 1.29E-06 | 0.000159 | 5.135127 |
| TCN1 | 1.184615 | 9.931183 | 5.320872 | 5.15E-07 | 8.05E-05 | 5.993785 |
| CHI3L1 | 1.158206 | 6.534276 | 5.831878 | 5.11E-08 | 1.38E-05 | 8.167532 |
| NUSAP1 | 1.131123 | 6.462043 | 6.30564 | 5.48E-09 | 2.67E-06 | 10.27495 |
| MX1 | 1.116084 | 8.51235 | 4.481404 | 1.76E-05 | 0.001135 | 2.69163 |
| UHRF1 | 1.10569 | 5.366824 | 5.423783 | 3.27E-07 | 5.48E-05 | 6.422474 |
| HERC5 | 1.104657 | 6.777953 | 3.480204 | 0.000709 | 0.018903 | -0.71207 |
| DLGAP5 | 1.070375 | 4.91755 | 4.874158 | 3.53E-06 | 0.000355 | 4.191228 |
| CEBPE | 1.062846 | 6.451088 | 5.766194 | 6.93E-08 | 1.69E-05 | 7.882012 |
| SHCBP1 | 1.043571 | 5.152206 | 4.948277 | 2.58E-06 | 0.000279 | 4.483463 |
| TTK | 1.026412 | 3.916289 | 5.54558 | 1.89E-07 | 3.46E-05 | 6.935917 |
| KIAA0101 | 1.024378 | 6.372184 | 7.711556 | 4.86E-12 | 1.11E-08 | 16.92422 |
| KIF15 | 1.022249 | 4.258342 | 5.78031 | 6.49E-08 | 1.64E-05 | 7.943227 |
| IFIT3 | 1.013647 | 7.899127 | 3.404029 | 0.000914 | 0.022436 | -0.94368 |
| ZNF367 | 0.992828 | 5.235792 | 5.800884 | 5.9E-08 | 1.53E-05 | 8.03259 |
| ANLN | 0.984523 | 3.794339 | 5.844431 | 4.83E-08 | 1.32E-05 | 8.222293 |
| IFI6 | 0.98072 | 7.664475 | 3.424266 | 0.000855 | 0.021463 | -0.88255 |
| IFIT2 | 0.975919 | 8.11622 | 3.443895 | 0.000801 | 0.020657 | -0.82299 |
| BUB1B | 0.96703 | 5.574532 | 4.774687 | 5.34E-06 | 0.000479 | 3.803529 |
| CD38 | 0.96581 | 6.302529 | 5.694893 | 9.6E-08 | 2.14E-05 | 7.574038 |
| ATP8B4 | 0.962555 | 6.801725 | 4.701754 | 7.21E-06 | 0.00059 | 3.522611 |

| | | | | | | |
|---|---|---|---|---|---|---|
| MELK | 0.962146 | 4.774276 | 5.163215 | 1.03E-06 | 0.000131 | 5.346536 |
| PRC1 | 0.961743 | 5.059938 | 4.964733 | 2.41E-06 | 0.000265 | 4.548726 |
| TYMS | 0.96083 | 5.672778 | 7.393682 | 2.49E-11 | 4.24E-08 | 15.37916 |
| PCNA | 0.959781 | 8.097368 | 5.920268 | 3.39E-08 | 9.92E-06 | 8.55442 |
| CCNA1 | 0.956464 | 4.805948 | 4.284075 | 3.82E-05 | 0.002092 | 1.97103 |
| ISG15 | 0.949194 | 7.960205 | 3.48812 | 0.00069 | 0.018494 | -0.68777 |
| SLC4A1 | 0.948206 | 5.942641 | 5.552705 | 1.83E-07 | 3.44E-05 | 6.966154 |
| ALAS2 | 0.946036 | 5.230463 | 6.234964 | 7.69E-09 | 3.35E-06 | 9.955488 |
| NCAPG | 0.943923 | 4.69304 | 6.468215 | 2.5E-09 | 1.68E-06 | 11.01609 |
| GJB6 | 0.943869 | 6.215892 | 3.386536 | 0.000969 | 0.023384 | -0.99628 |
| PRSS33 | 0.943634 | 5.948215 | 5.386583 | 3.85E-07 | 6.21E-05 | 6.266961 |
| RAD51AP1 | 0.933996 | 5.000204 | 4.604981 | 1.07E-05 | 0.000784 | 3.154329 |
| EPSTI1 | 0.932435 | 6.251681 | 3.635343 | 0.000416 | 0.012699 | -0.22775 |
| FRMD3 | 0.931765 | 5.966697 | 6.1241 | 1.3E-08 | 4.85E-06 | 9.457833 |
| NUF2 | 0.92366 | 4.461929 | 5.151963 | 1.08E-06 | 0.000136 | 5.300792 |
| CPA3 | 0.915669 | 4.764295 | 5.05772 | 1.62E-06 | 0.000195 | 4.920068 |
| RAB13 | 0.913594 | 8.125207 | 6.285378 | 6.04E-09 | 2.85E-06 | 10.18319 |
| XK | 0.904184 | 6.213774 | 3.187242 | 0.00185 | 0.037997 | -1.57982 |
| DDX60 | 0.90287 | 7.2884 | 3.871641 | 0.00018 | 0.006853 | 0.541576 |
| SLPI | 0.897011 | 8.349706 | 4.456283 | 1.95E-05 | 0.001245 | 2.598633 |
| SERPING1 | 0.889041 | 5.159764 | 3.413876 | 0.000885 | 0.02203 | -0.91397 |
| INHBA | 0.885937 | 4.12628 | 7.100202 | 1.1E-10 | 1.29E-07 | 13.97176 |
| TOP2A | 0.883208 | 4.606731 | 5.983355 | 2.53E-08 | 7.72E-06 | 8.832383 |
| CDK1 | 0.879144 | 4.641083 | 7.093862 | 1.14E-10 | 1.29E-07 | 13.94158 |
| LCN2 | 0.87706 | 11.03352 | 3.029139 | 0.003028 | 0.054023 | -2.02174 |
| GINS1 | 0.876834 | 4.689471 | 4.638336 | 9.35E-06 | 0.000719 | 3.280683 |
| PIWIL4 | 0.876353 | 5.63101 | 5.379302 | 3.98E-07 | 6.36E-05 | 6.236593 |
| CDKN3 | 0.871969 | 4.895562 | 5.190721 | 9.11E-07 | 0.000122 | 5.458614 |
| PCOLCE2 | 0.86834 | 5.899964 | 2.831195 | 0.005475 | 0.081769 | -2.548 |
| OLIG1 | 0.859918 | 7.138032 | 4.052922 | 9.22E-05 | 0.004228 | 1.156699 |
| NFE4 | 0.852437 | 8.139625 | 5.822062 | 5.35E-08 | 1.41E-05 | 8.124756 |
| FAM46C | 0.852264 | 7.409984 | 5.201703 | 8.69E-07 | 0.000118 | 5.503466 |
| OIP5 | 0.846727 | 4.575254 | 6.180149 | 9.98E-09 | 3.93E-06 | 9.708893 |
| CCNE2 | 0.846309 | 4.077393 | 6.1944 | 9.33E-09 | 3.74E-06 | 9.772904 |
| NDC80 | 0.84321 | 4.878113 | 5.562767 | 1.75E-07 | 3.35E-05 | 7.00889 |
| HMMR | 0.841779 | 5.234174 | 4.313527 | 3.41E-05 | 0.001924 | 2.077125 |
| RGCC | 0.832285 | 5.521807 | 7.804611 | 3E-12 | 8.78E-09 | 17.38014 |
| SLC27A2 | 0.828139 | 4.787411 | 6.836267 | 4.11E-10 | 3.82E-07 | 12.72394 |
| CTNNAL1 | 0.818145 | 5.781889 | 4.790539 | 5E-06 | 0.000459 | 3.864965 |
| KIF14 | 0.812375 | 4.595478 | 4.882085 | 3.41E-06 | 0.000346 | 4.222347 |
| CCNA2 | 0.810524 | 4.930748 | 5.30218 | 5.6E-07 | 8.61E-05 | 5.916443 |
| XAF1 | 0.808165 | 7.014022 | 3.727202 | 0.000302 | 0.010172 | 0.066857 |
| C12orf75 | 0.807583 | 6.708779 | 3.780316 | 0.00025 | 0.008864 | 0.239808 |
| BCL2L15 | 0.803572 | 4.734335 | 6.456021 | 2.65E-09 | 1.7E-06 | 10.9602 |
| GGH | 0.78579 | 6.604753 | 4.060108 | 8.98E-05 | 0.004147 | 1.181514 |
| TPX2 | 0.783154 | 4.914033 | 4.496085 | 1.66E-05 | 0.001075 | 2.746147 |
| GMNN | 0.780531 | 6.590789 | 4.319528 | 3.33E-05 | 0.001894 | 2.098804 |
| MKI67 | 0.777416 | 5.339197 | 5.726995 | 8.29E-08 | 1.94E-05 | 7.712442 |
| CYP4F3 | 0.77666 | 10.80677 | 5.950341 | 2.95E-08 | 8.75E-06 | 8.686736 |
| NCAPG2 | 0.77644 | 5.419618 | 5.22166 | 7.96E-07 | 0.000112 | 5.585114 |
| IFI27 | 0.775457 | 4.408339 | 2.775587 | 0.006433 | 0.090401 | -2.69032 |
| GMPR | 0.770926 | 5.762715 | 3.860777 | 0.000187 | 0.007055 | 0.505391 |
| GGTA1P | 0.769144 | 7.024923 | 4.301091 | 3.58E-05 | 0.001996 | 2.032262 |
| MCM2 | 0.766285 | 6.378073 | 5.446456 | 2.95E-07 | 5.07E-05 | 6.517558 |
| CENPU | 0.759901 | 4.576777 | 6.464582 | 2.55E-09 | 1.68E-06 | 10.99943 |
| PRKAR2B | 0.755701 | 9.008652 | 3.005274 | 0.003257 | 0.056674 | -2.08679 |
| VLDLR | 0.755607 | 3.92052 | 5.220613 | 8E-07 | 0.000112 | 5.580825 |
| IGJ | 0.750178 | 9.730902 | 2.741777 | 0.007087 | 0.096358 | -2.77565 |

| | | | | | | |
|---|---|---|---|---|---|---|
| NEIL3 | 0.745625 | 3.794969 | 5.653286 | 1.16E-07 | 2.45E-05 | 7.395282 |
| KLF5 | 0.745336 | 6.12654 | 5.482383 | 2.51E-07 | 4.4E-05 | 6.668693 |
| ATAD2 | 0.744896 | 4.854447 | 5.549191 | 1.86E-07 | 3.46E-05 | 6.951239 |
| GBP1 | 0.742456 | 7.227343 | 3.420666 | 0.000865 | 0.021616 | -0.89345 |
| SERPINB1( | 0.740802 | 5.587385 | 3.097776 | 0.00245 | 0.046376 | -1.83221 |
| RACGAP1 | 0.737978 | 6.27271 | 3.864366 | 0.000185 | 0.006985 | 0.517337 |
| CENPK | 0.734724 | 5.099798 | 3.113505 | 0.002333 | 0.044753 | -1.78827 |
| OASL | 0.732755 | 6.284605 | 3.358029 | 0.001065 | 0.025013 | -1.08152 |
| SPC25 | 0.729466 | 3.25118 | 5.254788 | 6.89E-07 | 0.000101 | 5.721062 |
| CAMP | 0.722484 | 8.958653 | 3.569052 | 0.000524 | 0.01507 | -0.43675 |
| SCARF1 | 0.722408 | 5.906289 | 6.619553 | 1.2E-09 | 9.79E-07 | 11.71349 |
| PLS1 | 0.712582 | 4.144718 | 4.757226 | 5.74E-06 | 0.000504 | 3.736013 |
| DHFR | 0.711827 | 6.785358 | 5.390449 | 3.79E-07 | 6.2E-05 | 6.283094 |
| FBXO5 | 0.710182 | 5.240307 | 4.819871 | 4.43E-06 | 0.000425 | 3.978992 |
| SGOL2 | 0.707706 | 5.266251 | 4.252501 | 4.32E-05 | 0.002302 | 1.85787 |
| ASPM | 0.707322 | 4.131321 | 5.595276 | 1.51E-07 | 2.97E-05 | 7.147262 |
| OAS3 | 0.704095 | 5.343213 | 4.030471 | 0.0001 | 0.004511 | 1.079375 |
| TNFRSF17 | 0.703608 | 5.90344 | 2.312508 | 0.022527 | 0.207443 | -3.77806 |
| TREML1 | 0.701284 | 6.679001 | 2.97727 | 0.003546 | 0.060168 | -2.16257 |
| PSAT1 | 0.699455 | 5.953615 | 5.685691 | 1E-07 | 2.2E-05 | 7.534441 |
| CYP4F2 | 0.698921 | 7.639776 | 5.747061 | 7.56E-08 | 1.8E-05 | 7.799167 |
| TRIM6 | 0.698725 | 4.508655 | 3.908881 | 0.000157 | 0.006243 | 0.666207 |
| MZB1 | 0.697617 | 6.275645 | 3.356266 | 0.001071 | 0.025095 | -1.08678 |
| KIF20A | 0.696188 | 4.055959 | 4.169201 | 5.95E-05 | 0.002932 | 1.562226 |
| MMRN1 | 0.691167 | 4.572043 | 2.711416 | 0.007726 | 0.101935 | -2.85149 |
| STAT1 | 0.690849 | 7.789464 | 5.215771 | 8.17E-07 | 0.000113 | 5.560999 |
| FANCI | 0.687616 | 5.00409 | 6.118615 | 1.34E-08 | 4.88E-06 | 9.433328 |
| CEP55 | 0.685865 | 4.795834 | 2.704674 | 0.007875 | 0.103157 | -2.86823 |
| FEN1 | 0.685849 | 5.964082 | 5.63439 | 1.27E-07 | 2.59E-05 | 7.314337 |
| MYB | 0.680554 | 4.632689 | 6.449528 | 2.74E-09 | 1.7E-06 | 10.93046 |
| CD274 | 0.677598 | 6.551972 | 4.351602 | 2.94E-05 | 0.00169 | 2.215047 |
| MLC1 | 0.674497 | 5.287763 | 4.989295 | 2.17E-06 | 0.000242 | 4.646394 |
| KIF18B | 0.674119 | 5.496701 | 4.770042 | 5.44E-06 | 0.000482 | 3.785554 |
| GALNT14 | 0.665806 | 7.700618 | 4.557928 | 1.3E-05 | 0.000898 | 2.977138 |
| GLRX5 | 0.664459 | 8.935576 | 5.178454 | 9.61E-07 | 0.000126 | 5.408584 |
| NUCB2 | 0.662757 | 7.491098 | 4.915009 | 2.97E-06 | 0.000309 | 4.351944 |
| GALM | 0.660506 | 6.34852 | 5.768346 | 6.86E-08 | 1.69E-05 | 7.891341 |
| DEPDC1B | 0.657892 | 3.443403 | 4.31861 | 3.34E-05 | 0.001896 | 2.095489 |
| TK1 | 0.654167 | 5.463315 | 6.15279 | 1.14E-08 | 4.39E-06 | 9.586206 |
| TSPAN2 | 0.64777 | 7.524507 | 4.040297 | 9.67E-05 | 0.004397 | 1.113178 |
| FUT4 | 0.647404 | 6.439442 | 5.265656 | 6.57E-07 | 9.81E-05 | 5.765774 |
| TCF19 | 0.646723 | 5.463644 | 6.406549 | 3.37E-09 | 1.94E-06 | 10.73396 |
| TGFB1I1 | 0.645151 | 5.165396 | 2.622011 | 0.009923 | 0.120975 | -3.07052 |
| AIM2 | 0.645128 | 9.098672 | 4.144273 | 6.54E-05 | 0.003201 | 1.474582 |
| TIPIN | 0.643264 | 4.613006 | 3.521883 | 0.000615 | 0.016966 | -0.58361 |
| STIL | 0.64167 | 4.564462 | 5.060701 | 1.6E-06 | 0.000195 | 4.932045 |
| LOC64307 | 0.639661 | 7.323337 | 5.167259 | 1.01E-06 | 0.00013 | 5.362992 |
| IFIT5 | 0.639367 | 7.272846 | 2.781081 | 0.006332 | 0.089411 | -2.67636 |
| MYL9 | 0.636446 | 5.79691 | 4.691688 | 7.52E-06 | 0.000613 | 3.484063 |
| SNCA | 0.633654 | 7.025837 | 3.972346 | 0.000124 | 0.005271 | 0.880679 |
| LAPTM4B | 0.633163 | 5.720159 | 4.301691 | 3.57E-05 | 0.001996 | 2.034425 |
| VRK1 | 0.631664 | 6.963095 | 4.281203 | 3.87E-05 | 0.002105 | 1.960711 |
| HJURP | 0.630926 | 4.579074 | 6.588725 | 1.39E-09 | 1.09E-06 | 11.57086 |
| C15orf65 | 0.629448 | 5.339864 | 4.004528 | 0.00011 | 0.004819 | 0.990424 |
| EGF | 0.626984 | 4.815473 | 3.568491 | 0.000525 | 0.015077 | -0.43851 |
| DDX58 | 0.626269 | 7.276951 | 4.428458 | 2.17E-05 | 0.001339 | 2.496049 |
| CDC20 | 0.625872 | 5.356453 | 4.26768 | 4.08E-05 | 0.002194 | 1.912196 |
| SAMD9L | 0.625032 | 8.075071 | 3.66007 | 0.000382 | 0.011935 | -0.14901 |

| | | | | | | |
|---|---|---|---|---|---|---|
| CPNE3 | 0.621044 | 9.430289 | 4.771061 | 5.42E-06 | 0.000482 | 3.789495 |
| SMC2 | 0.616368 | 4.682211 | 3.901342 | 0.000161 | 0.00639 | 0.640902 |
| RPH3A | 0.616332 | 6.03482 | 2.498173 | 0.013895 | 0.153608 | -3.36316 |
| GYPB | 0.613821 | 5.298627 | 5.706308 | 9.11E-08 | 2.05E-05 | 7.623202 |
| KAZN | 0.613206 | 5.785035 | 7.068981 | 1.29E-10 | 1.39E-07 | 13.82323 |
| WSB2 | 0.612147 | 9.818107 | 6.10594 | 1.42E-08 | 5.01E-06 | 9.376731 |
| BPGM | 0.609769 | 7.305715 | 3.533469 | 0.000591 | 0.016472 | -0.54768 |
| PROS1 | 0.605881 | 5.656036 | 2.86259 | 0.004993 | 0.076765 | -2.46657 |
| CENPE | 0.604149 | 4.528665 | 4.054163 | 9.18E-05 | 0.004222 | 1.160982 |
| RTP4 | 0.603356 | 6.098994 | 2.971418 | 0.00361 | 0.061141 | -2.17833 |
| COL17A1 | 0.602148 | 5.090327 | 3.615625 | 0.000446 | 0.01336 | -0.29023 |
| CKS2 | 0.601373 | 6.896135 | 3.56043 | 0.000539 | 0.015414 | -0.46371 |
| GP6 | 0.600854 | 7.36717 | 3.247765 | 0.001524 | 0.032832 | -1.40569 |
| DSC2 | 0.597733 | 6.809391 | 3.883873 | 0.000172 | 0.006675 | 0.582412 |
| CHAC2 | 0.593887 | 4.93669 | 2.847309 | 0.005223 | 0.079219 | -2.5063 |
| RRAGD | 0.593323 | 9.010735 | 5.906572 | 3.62E-08 | 1.04E-05 | 8.494273 |
| CMTM5 | 0.591536 | 6.961594 | 3.108395 | 0.00237 | 0.045159 | -1.80257 |
| PTTG1 | 0.590531 | 7.551962 | 3.980092 | 0.000121 | 0.005154 | 0.907032 |
| RHAG | 0.590465 | 4.078246 | 4.893811 | 3.25E-06 | 0.000334 | 4.268438 |
| DHCR24 | 0.587116 | 5.365818 | 4.081612 | 8.28E-05 | 0.003852 | 1.255973 |
| GYPA | 0.586862 | 3.463464 | 4.81738 | 4.47E-06 | 0.000428 | 3.969292 |
| ANKRD55 | 0.582458 | 8.90536 | 3.114082 | 0.002329 | 0.044753 | -1.78666 |
| MCM10 | 0.581776 | 3.715351 | 6.377039 | 3.89E-09 | 2.15E-06 | 10.59939 |
| IGLL5 | 0.58021 | 6.962729 | 2.739671 | 0.00713 | 0.096746 | -2.78093 |
| C9orf40 | 0.580071 | 5.898192 | 5.56866 | 1.7E-07 | 3.29E-05 | 7.033943 |
| FOXM1 | 0.575845 | 5.048631 | 4.716707 | 6.78E-06 | 0.000564 | 3.57997 |
| EZH2 | 0.575821 | 5.351307 | 3.190668 | 0.00183 | 0.037737 | -1.57003 |
| RHOBTB1 | 0.575308 | 4.934846 | 2.614189 | 0.01014 | 0.122699 | -3.08938 |
| LOC10272 | 0.574484 | 4.08318 | 4.210101 | 5.09E-05 | 0.002589 | 1.706857 |
| STRADB | 0.572745 | 7.44092 | 3.154312 | 0.002053 | 0.041095 | -1.67342 |
| SPTA1 | 0.569704 | 3.408416 | 4.502075 | 1.62E-05 | 0.001056 | 2.768422 |
| TMEM92 | 0.568217 | 5.257064 | 6.40384 | 3.42E-09 | 1.94E-06 | 10.7216 |
| SPIN4 | 0.567014 | 5.216638 | 2.907231 | 0.004375 | 0.070276 | -2.34945 |
| TTC7B | 0.564556 | 6.20982 | 3.091437 | 0.002499 | 0.046952 | -1.84986 |
| SLC28A3 | 0.564276 | 4.249124 | 4.27429 | 3.97E-05 | 0.00215 | 1.935896 |
| CCNB1 | 0.562755 | 4.157732 | 3.241628 | 0.001555 | 0.033345 | -1.42347 |
| NR2E1 | 0.560909 | 5.711215 | 3.881358 | 0.000174 | 0.006696 | 0.574008 |
| CDC6 | 0.558893 | 3.490751 | 4.958503 | 2.47E-06 | 0.000269 | 4.523999 |
| HPSE | 0.558487 | 8.169534 | 4.027472 | 0.000101 | 0.004551 | 1.069072 |
| KIF2C | 0.55824 | 4.746828 | 6.072409 | 1.66E-08 | 5.4E-06 | 9.22729 |
| RFC4 | 0.557671 | 5.850569 | 3.378168 | 0.000996 | 0.023843 | -1.02136 |
| APOL6 | 0.553595 | 5.809075 | 3.878882 | 0.000175 | 0.006733 | 0.565739 |
| KIF4A | 0.55291 | 4.714171 | 4.390048 | 2.53E-05 | 0.001513 | 2.35518 |
| NGFRAP1 | 0.549688 | 9.130506 | 2.828001 | 0.005526 | 0.082234 | -2.55624 |
| SKA2 | 0.54766 | 6.197351 | 3.632817 | 0.00042 | 0.012792 | -0.23576 |
| SLC2A5 | 0.547093 | 4.764503 | 6.512599 | 2.02E-09 | 1.47E-06 | 11.21989 |
| CDC45 | 0.546544 | 4.669334 | 6.146442 | 1.17E-08 | 4.44E-06 | 9.55778 |
| SELP | 0.546318 | 7.645673 | 3.066505 | 0.002699 | 0.049672 | -1.919 |
| DEPDC1 | 0.543956 | 3.475258 | 4.780313 | 5.22E-06 | 0.000472 | 3.825317 |
| HEMGN | 0.54229 | 4.749621 | 2.902719 | 0.004435 | 0.070976 | -2.36136 |
| DDIAS | 0.542078 | 7.722866 | 3.696656 | 0.000336 | 0.01095 | -0.03175 |
| SLC14A1 | 0.542076 | 3.682291 | 3.591492 | 0.000485 | 0.014168 | -0.36634 |
| UAP1 | 0.538669 | 6.330552 | 2.503178 | 0.013711 | 0.152636 | -3.35157 |
| SPTSSA | 0.536464 | 7.767151 | 3.98029 | 0.000121 | 0.005154 | 0.907706 |
| ARHGAP2: | 0.534992 | 5.494465 | 4.249838 | 4.37E-05 | 0.002302 | 1.848352 |
| CKS1B | 0.533607 | 6.051939 | 3.19272 | 0.001818 | 0.03753 | -1.56417 |
| POLE2 | 0.53213 | 4.791507 | 4.393832 | 2.49E-05 | 0.001503 | 2.369022 |
| CAV1 | 0.531698 | 3.717296 | 4.519897 | 1.51E-05 | 0.001003 | 2.834828 |

| | | | | | | |
|---|---|---|---|---|---|---|
| USP18 | 0.529382 | 4.721372 | 3.469381 | 0.000735 | 0.01937 | -0.74523 |
| CCDC125 | 0.52688 | 6.132131 | 4.08315 | 8.24E-05 | 0.003845 | 1.261307 |
| PRTFDC1 | 0.524558 | 4.013599 | 2.998295 | 0.003327 | 0.057548 | -2.10574 |
| CHIT1 | 0.523348 | 6.075152 | 2.614101 | 0.010142 | 0.122699 | -3.08959 |
| CENPW | 0.522367 | 6.362444 | 3.890132 | 0.000168 | 0.006567 | 0.603346 |
| CDCA7L | 0.521606 | 7.233669 | 2.475524 | 0.01476 | 0.159866 | -3.41531 |
| FAM83D | 0.521264 | 4.322602 | 5.174216 | 9.79E-07 | 0.000128 | 5.39132 |
| FHL1 | 0.521012 | 5.25282 | 4.549162 | 1.34E-05 | 0.000924 | 2.944263 |
| CDCA5 | 0.520947 | 5.460746 | 4.811745 | 4.58E-06 | 0.000434 | 3.94736 |
| ISOC1 | 0.520711 | 6.733998 | 2.840933 | 0.005321 | 0.08 | -2.52282 |
| UBE2C | 0.520324 | 6.575334 | 4.822743 | 4.37E-06 | 0.000424 | 3.990182 |
| ERG | 0.519691 | 3.958839 | 5.765174 | 6.96E-08 | 1.69E-05 | 7.877593 |
| IFIH1 | 0.517999 | 4.68645 | 4.291895 | 3.71E-05 | 0.002046 | 1.999151 |
| SH3BGRL2 | 0.517509 | 6.455868 | 2.635711 | 0.009553 | 0.118615 | -3.03738 |
| SPAG5 | 0.517191 | 5.351122 | 4.743141 | 6.08E-06 | 0.000527 | 3.68167 |
| IGLL3P | 0.516727 | 7.803779 | 3.389474 | 0.00096 | 0.023285 | -0.98746 |
| CDT1 | 0.516674 | 5.016992 | 4.670201 | 8.21E-06 | 0.000656 | 3.401965 |
| SPRY2 | 0.507978 | 4.387859 | 2.61973 | 0.009986 | 0.121549 | -3.07603 |
| PBK | 0.506694 | 3.404115 | 3.76636 | 0.000263 | 0.009154 | 0.19418 |
| UBE2T | 0.505133 | 4.757477 | 5.210968 | 8.34E-07 | 0.000115 | 5.541345 |
| GBP3 | 0.500605 | 7.475073 | 2.001427 | 0.047698 | 0.321476 | -4.40745 |
| BIRC5 | 0.500424 | 5.123128 | 4.8692 | 3.6E-06 | 0.000359 | 4.171779 |
| FAR2 | 0.500285 | 7.047871 | 3.940053 | 0.00014 | 0.005734 | 0.771224 |
| CDCA3 | 0.49977 | 5.73941 | 6.08136 | 1.59E-08 | 5.31E-06 | 9.267141 |
| HIST1H2A | 0.499082 | 5.050341 | 3.645157 | 0.000402 | 0.012421 | -0.19655 |
| OAS2 | 0.497266 | 5.96687 | 2.707613 | 0.00781 | 0.10257 | -2.86094 |
| OR52K3P | 0.497063 | 6.067864 | 2.730498 | 0.007319 | 0.098333 | -2.80391 |
| DUT | 0.493449 | 7.914878 | 5.168748 | 1E-06 | 0.00013 | 5.369052 |
| BATF2 | 0.493166 | 5.374422 | 3.082117 | 0.002572 | 0.04789 | -1.87576 |
| MAD2L1 | 0.491944 | 4.113142 | 4.455255 | 1.95E-05 | 0.001245 | 2.594836 |
| IGK | 0.491904 | 5.984008 | 3.031289 | 0.003008 | 0.053824 | -2.01586 |
| TCTEX1D1 | 0.490867 | 3.627244 | 3.113406 | 0.002334 | 0.044753 | -1.78855 |
| CEP97 | 0.489421 | 5.003963 | 4.367904 | 2.76E-05 | 0.00162 | 2.27436 |
| DPY19L1P | 0.489243 | 3.590326 | 4.514516 | 1.54E-05 | 0.001015 | 2.814759 |
| TACSTD2 | 0.489145 | 4.588404 | 5.579683 | 1.62E-07 | 3.16E-05 | 7.080836 |
| OAS1 | 0.489063 | 6.760842 | 2.048747 | 0.04276 | 0.301104 | -4.31712 |
| GFI1 | 0.487466 | 5.056492 | 4.133621 | 6.81E-05 | 0.00332 | 1.437247 |
| NEK2 | 0.483575 | 3.605074 | 4.578422 | 1.19E-05 | 0.000833 | 3.05416 |
| RMI2 | 0.482936 | 5.370703 | 4.251476 | 4.34E-05 | 0.002302 | 1.854206 |
| RRM1 | 0.481921 | 5.810509 | 4.532594 | 1.43E-05 | 0.000969 | 2.88225 |
| CAV2 | 0.481353 | 3.633067 | 4.114358 | 7.32E-05 | 0.003501 | 1.369911 |
| CDCA2 | 0.480987 | 4.508494 | 4.820852 | 4.41E-06 | 0.000425 | 3.982815 |
| NRN1 | 0.479984 | 4.61146 | 2.81361 | 0.005762 | 0.084445 | -2.59327 |
| FBXO6 | 0.479613 | 7.900619 | 2.276575 | 0.02466 | 0.219575 | -3.85501 |
| SLC35D3 | 0.475686 | 3.162786 | 3.675469 | 0.000362 | 0.011421 | -0.09977 |
| CKAP2L | 0.472004 | 5.2293 | 5.46722 | 2.69E-07 | 4.66E-05 | 6.604837 |
| HBQ1 | 0.471837 | 5.699421 | 2.999125 | 0.003319 | 0.057452 | -2.10349 |
| MXI1 | 0.471779 | 9.775722 | 3.679881 | 0.000356 | 0.011291 | -0.08563 |
| TMCC2 | 0.470928 | 5.747476 | 3.420885 | 0.000865 | 0.021616 | -0.89279 |
| UBE2L6 | 0.470803 | 8.157657 | 3.058661 | 0.002766 | 0.050302 | -1.94066 |
| ANKRD33I | 0.469776 | 6.840289 | 4.384541 | 2.58E-05 | 0.001537 | 2.335054 |
| CDC7 | 0.46873 | 5.260283 | 2.814641 | 0.005745 | 0.084332 | -2.59062 |
| FBXL13 | 0.466584 | 4.591116 | 5.444602 | 2.98E-07 | 5.07E-05 | 6.509774 |
| SLFN13 | 0.466579 | 4.593803 | 4.120566 | 7.15E-05 | 0.003427 | 1.391587 |
| TRIM58 | 0.465172 | 5.599605 | 2.643549 | 0.009347 | 0.116413 | -3.01835 |
| ZWILCH | 0.464263 | 5.179661 | 3.06471 | 0.002714 | 0.04977 | -1.92396 |
| CPNE2 | 0.463504 | 8.51559 | 2.801012 | 0.005977 | 0.086069 | -2.62555 |
| NCAPH | 0.463199 | 4.837325 | 4.428454 | 2.17E-05 | 0.001339 | 2.496034 |

| | | | | | | |
|---|---|---|---|---|---|---|
| TMEM158 | 0.462835 | 5.819094 | 2.340178 | 0.020997 | 0.19863 | -3.71806 |
| TNFAIP6 | 0.459729 | 10.23974 | 3.47917 | 0.000711 | 0.018919 | -0.71524 |
| BAMBI | 0.456935 | 4.901127 | 3.972608 | 0.000124 | 0.005271 | 0.881571 |
| C1orf198 | 0.455 | 6.11378 | 3.28749 | 0.001341 | 0.029755 | -1.28993 |
| DACH1 | 0.453254 | 7.440157 | 3.059409 | 0.002759 | 0.050275 | -1.93859 |
| NT5C3A | 0.451979 | 10.24836 | 3.927963 | 0.000146 | 0.005947 | 0.730418 |
| CHMP5 | 0.450373 | 9.604827 | 4.533805 | 1.43E-05 | 0.000967 | 2.886777 |
| HIST1H2A | 0.449494 | 4.820095 | 3.688973 | 0.000345 | 0.011074 | -0.05645 |
| PRIM1 | 0.44868 | 6.065568 | 2.902579 | 0.004436 | 0.070976 | -2.36173 |
| MCM6 | 0.447912 | 5.311905 | 4.718717 | 6.73E-06 | 0.000562 | 3.587692 |
| HIST1H1C | 0.444241 | 8.063754 | 3.161366 | 0.002007 | 0.040388 | -1.65343 |
| AGPS | 0.443886 | 6.296463 | 3.292851 | 0.001318 | 0.029498 | -1.27421 |
| COL6A3 | 0.442786 | 5.034991 | 2.619043 | 0.010005 | 0.121708 | -3.07768 |
| MCU | 0.441759 | 7.277459 | 3.779007 | 0.000251 | 0.008887 | 0.235525 |
| IFI35 | 0.44156 | 7.985828 | 2.186878 | 0.030774 | 0.250329 | -4.04228 |
| FECH | 0.441426 | 5.258498 | 3.502867 | 0.000656 | 0.017786 | -0.64237 |
| PPBP | 0.44097 | 11.89882 | 2.388675 | 0.018535 | 0.183574 | -3.61134 |
| VSTM1 | 0.440728 | 6.911992 | 2.685981 | 0.008301 | 0.10737 | -2.91446 |
| RFC5 | 0.439904 | 5.531142 | 5.046119 | 1.7E-06 | 0.000202 | 4.873505 |
| TOR1B | 0.438351 | 8.557374 | 4.082702 | 8.25E-05 | 0.003845 | 1.259752 |
| LOC10050 | 0.435975 | 8.758171 | 3.604699 | 0.000463 | 0.013758 | -0.32474 |
| HIST1H2B( | 0.435118 | 7.354683 | 2.480785 | 0.014555 | 0.158471 | -3.40323 |
| SLC22A16 | 0.434076 | 4.834638 | 4.208538 | 5.12E-05 | 0.002598 | 1.701313 |
| ZBP1 | 0.43347 | 5.755538 | 2.798883 | 0.006014 | 0.086358 | -2.63099 |
| PARP14 | 0.432853 | 5.830408 | 4.19927 | 5.3E-05 | 0.002674 | 1.668458 |
| SPAG1 | 0.432151 | 4.701399 | 2.290114 | 0.023836 | 0.215524 | -3.82614 |
| GUCY1B3 | 0.43187 | 5.51123 | 2.255483 | 0.025992 | 0.225459 | -3.89966 |
| MASTL | 0.431539 | 4.5735 | 2.979822 | 0.003519 | 0.059804 | -2.15569 |
| KLF1 | 0.430816 | 4.48547 | 3.495077 | 0.000674 | 0.018165 | -0.66637 |
| MND1 | 0.429796 | 3.23166 | 4.730835 | 6.4E-06 | 0.000541 | 3.634278 |
| TRIM16 | 0.428374 | 4.744594 | 4.431369 | 2.15E-05 | 0.001336 | 2.506761 |
| FAS | 0.427307 | 8.264364 | 3.166925 | 0.001972 | 0.03996 | -1.63766 |
| PRRG4 | 0.427157 | 7.425465 | 2.986757 | 0.003446 | 0.059002 | -2.13697 |
| HIST1H2BI | 0.426048 | 6.929115 | 3.382252 | 0.000983 | 0.023579 | -1.00913 |
| TPM1 | 0.42463 | 5.494645 | 4.621399 | 1E-05 | 0.000751 | 3.216447 |
| ZCCHC2 | 0.423597 | 6.27851 | 3.132975 | 0.002195 | 0.042804 | -1.73363 |
| CITED2 | 0.423174 | 6.930349 | 6.086028 | 1.56E-08 | 5.31E-06 | 9.28794 |
| LGALS12 | 0.422645 | 6.338636 | 3.257453 | 0.001477 | 0.032162 | -1.37757 |
| EIF2AK2 | 0.422121 | 7.489072 | 3.693243 | 0.00034 | 0.011046 | -0.04272 |
| RNASE2 | 0.422088 | 10.92004 | 2.532463 | 0.012673 | 0.143973 | -3.28339 |
| BRI3BP | 0.420545 | 8.145178 | 3.122798 | 0.002266 | 0.043784 | -1.76222 |
| KIFC1 | 0.419105 | 6.483357 | 4.636901 | 9.41E-06 | 0.00072 | 3.275234 |
| PROM1 | 0.417368 | 3.349078 | 3.560775 | 0.000539 | 0.015414 | -0.46263 |
| CYP27A1 | 0.41712 | 6.775499 | 3.717553 | 0.000312 | 0.010405 | 0.035642 |
| SAMD9 | 0.417103 | 8.228951 | 3.252734 | 0.0015 | 0.032551 | -1.39127 |
| SIGLEC1 | 0.417092 | 4.577403 | 3.221055 | 0.001661 | 0.034915 | -1.48287 |
| LOC10106 | 0.416462 | 6.99703 | 3.701482 | 0.00033 | 0.010845 | -0.01621 |
| TMEM55A | 0.416427 | 9.362318 | 3.810027 | 0.000225 | 0.008165 | 0.337376 |
| FANCL | 0.414957 | 4.769571 | 2.655445 | 0.009043 | 0.114288 | -2.98937 |
| IGLJ3 | 0.414568 | 6.177321 | 2.857971 | 0.005062 | 0.077466 | -2.4786 |
| RHD | 0.413274 | 4.844832 | 4.665143 | 8.38E-06 | 0.000665 | 3.382677 |
| OPTN | 0.413105 | 6.418479 | 3.226811 | 0.00163 | 0.034403 | -1.46628 |
| ITGA2B | 0.41271 | 5.951324 | 2.090758 | 0.03875 | 0.28266 | -4.23528 |
| IGLV1-44 | 0.412105 | 6.041863 | 2.785323 | 0.006255 | 0.088629 | -2.66557 |
| IGF2BP3 | 0.411467 | 4.719688 | 3.061859 | 0.002738 | 0.050075 | -1.93183 |
| MSRB3 | 0.410715 | 5.408483 | 4.857937 | 3.77E-06 | 0.000373 | 4.127649 |
| MT2A | 0.410429 | 8.60444 | 3.806 | 0.000228 | 0.008254 | 0.324117 |
| ANP32E | 0.409913 | 8.344048 | 2.709749 | 0.007763 | 0.102214 | -2.85563 |

| | | | | | | |
|---|---|---|---|---|---|---|
| ASF1B | 0.409894 | 6.231197 | 3.578724 | 0.000507 | 0.014701 | -0.40645 |
| FZD6 | 0.409479 | 3.615897 | 2.996515 | 0.003345 | 0.057763 | -2.11056 |
| CD46 | 0.409353 | 8.960873 | 3.417461 | 0.000874 | 0.021821 | -0.90314 |
| MCM4 | 0.408858 | 4.762179 | 5.72264 | 8.46E-08 | 1.94E-05 | 7.693643 |
| LOC10192 | 0.408624 | 5.399539 | 3.638499 | 0.000412 | 0.012586 | -0.21772 |
| ABCB10 | 0.408375 | 7.036825 | 2.35333 | 0.020302 | 0.19394 | -3.68932 |
| NETO2 | 0.408193 | 5.225711 | 3.289183 | 0.001333 | 0.029689 | -1.28497 |
| ADCY3 | 0.408017 | 5.801499 | 2.634781 | 0.009578 | 0.118818 | -3.03963 |
| GCLM | 0.407315 | 8.1649 | 2.908485 | 0.004359 | 0.070157 | -2.34614 |
| TMEM56 | 0.407024 | 3.231248 | 4.617585 | 1.02E-05 | 0.00076 | 3.202001 |
| AURKB | 0.406312 | 4.481321 | 5.672236 | 1.06E-07 | 2.28E-05 | 7.47661 |
| EPAS1 | 0.406178 | 5.383322 | 4.683511 | 7.77E-06 | 0.000629 | 3.452792 |
| PLK4 | 0.40443 | 3.524151 | 5.630012 | 1.29E-07 | 2.59E-05 | 7.295605 |
| DMTN | 0.404406 | 7.170931 | 2.228278 | 0.027804 | 0.235493 | -3.9567 |
| FHL2 | 0.403622 | 4.917081 | 3.33913 | 0.001133 | 0.026254 | -1.13772 |
| RBPMS2 | 0.403385 | 5.052242 | 3.294322 | 0.001311 | 0.029422 | -1.2699 |
| ANXA4 | 0.401856 | 8.42161 | 3.563595 | 0.000534 | 0.01529 | -0.45382 |
| SLAMF7 | 0.401303 | 6.161205 | 2.692066 | 0.00816 | 0.105949 | -2.89944 |
| HOXA10 | 0.400579 | 4.766105 | 5.545593 | 1.89E-07 | 3.46E-05 | 6.935975 |
| SPARC | 0.40003 | 6.480204 | 2.091335 | 0.038697 | 0.282505 | -4.23414 |
| BRIP1 | 0.399928 | 3.351914 | 4.226829 | 4.77E-05 | 0.002465 | 1.766305 |
| H2BFS | 0.399897 | 9.224814 | 2.127585 | 0.035505 | 0.269275 | -4.16228 |
| NET1 | 0.398734 | 5.039093 | 3.372 | 0.001017 | 0.024137 | -1.03982 |
| AURKA | 0.398479 | 4.604457 | 3.995762 | 0.000114 | 0.004957 | 0.960464 |
| SIAH2 | 0.396803 | 8.355608 | 2.908328 | 0.004361 | 0.070157 | -2.34655 |
| GBP4 | 0.396415 | 5.076995 | 2.788125 | 0.006204 | 0.088159 | -2.65844 |
| ERV3-2 | 0.395865 | 7.327332 | 2.185593 | 0.03087 | 0.250651 | -4.04491 |
| TRIM21 | 0.395863 | 8.409455 | 3.174876 | 0.001923 | 0.039123 | -1.61506 |
| ITGB3 | 0.395851 | 5.346346 | 3.015595 | 0.003156 | 0.055601 | -2.05871 |
| SLC1A4 | 0.395219 | 5.504356 | 4.591416 | 1.13E-05 | 0.000804 | 3.103119 |
| HDC | 0.394661 | 5.449228 | 4.436867 | 2.1E-05 | 0.001315 | 2.527003 |
| EPX | 0.390581 | 4.363921 | 4.585437 | 1.16E-05 | 0.000815 | 3.080581 |
| APP | 0.390445 | 7.704334 | 4.366666 | 2.77E-05 | 0.00162 | 2.26985 |
| TEX2 | 0.389493 | 7.094481 | 4.121429 | 7.13E-05 | 0.003424 | 1.394601 |
| GUCY1A3 | 0.388817 | 4.587015 | 2.615321 | 0.010108 | 0.122528 | -3.08665 |
| TRIP13 | 0.388399 | 5.019776 | 3.623147 | 0.000434 | 0.013054 | -0.26643 |
| NCAPD3 | 0.387038 | 6.612313 | 4.871858 | 3.56E-06 | 0.000357 | 4.182203 |
| ESPL1 | 0.386802 | 5.193253 | 5.216838 | 8.13E-07 | 0.000113 | 5.565367 |
| CHEK1 | 0.386759 | 3.987731 | 5.557599 | 1.79E-07 | 3.39E-05 | 6.986937 |
| CORO2A | 0.386221 | 6.742025 | 2.536347 | 0.012541 | 0.1428 | -3.27429 |
| KIF18A | 0.386114 | 3.154136 | 3.992524 | 0.000115 | 0.005006 | 0.949411 |
| PRG3 | 0.386023 | 4.123183 | 4.403946 | 2.39E-05 | 0.001453 | 2.406053 |
| GADD45G | 0.385176 | 5.467277 | 4.359186 | 2.85E-05 | 0.001645 | 2.24262 |
| B4GALT5 | 0.384651 | 10.80114 | 4.43696 | 2.1E-05 | 0.001315 | 2.527347 |
| EMB | 0.38459 | 9.055746 | 4.405585 | 2.38E-05 | 0.001448 | 2.412059 |
| CLEC5A | 0.384347 | 8.256134 | 2.001479 | 0.047692 | 0.321476 | -4.40735 |
| CDK2AP1 | 0.384217 | 9.301075 | 3.059551 | 0.002758 | 0.050275 | -1.9382 |
| POLQ | 0.383856 | 4.578875 | 5.177801 | 9.64E-07 | 0.000126 | 5.405926 |
| DCAF10 | 0.383368 | 6.359742 | 3.474816 | 0.000722 | 0.019125 | -0.72859 |
| GFI1B | 0.38312 | 5.403751 | 3.023371 | 0.003082 | 0.054794 | -2.0375 |
| SLC12A2 | 0.382261 | 4.445413 | 3.449182 | 0.000786 | 0.020371 | -0.80689 |
| TSPAN5 | 0.380418 | 5.718495 | 2.904922 | 0.004406 | 0.070649 | -2.35555 |
| HIST1H2BI | 0.377878 | 9.22244 | 3.103045 | 0.00241 | 0.045833 | -1.81751 |
| ABLIM3 | 0.377708 | 5.517326 | 3.182363 | 0.001878 | 0.038437 | -1.59374 |
| SMC4 | 0.377612 | 6.212432 | 3.487844 | 0.000691 | 0.018494 | -0.68862 |
| SMS | 0.376906 | 7.57021 | 3.273735 | 0.001402 | 0.030876 | -1.33014 |
| LINC0126ꞔ | 0.373043 | 3.399404 | 3.670295 | 0.000369 | 0.011569 | -0.11633 |
| YEATS4 | 0.370649 | 5.082778 | 2.845618 | 0.005249 | 0.079317 | -2.51068 |

| | | | | | | |
|---|---|---|---|---|---|---|
| E2F7 | 0.37055 | 4.072175 | 5.631215 | 1.28E-07 | 2.59E-05 | 7.300753 |
| FSTL3 | 0.369363 | 5.713968 | 3.513121 | 0.000634 | 0.017342 | -0.61072 |
| PDE2A | 0.368445 | 5.189801 | 3.570725 | 0.000521 | 0.015025 | -0.43151 |
| HELLS | 0.36833 | 3.76427 | 4.190387 | 5.49E-05 | 0.002751 | 1.637017 |
| RAD51 | 0.367453 | 4.55236 | 5.763277 | 7.02E-08 | 1.69E-05 | 7.869372 |
| HIST1H2BI | 0.367133 | 11.3121 | 4.127375 | 6.97E-05 | 0.003372 | 1.415387 |
| CDCA8 | 0.367037 | 6.020009 | 5.243731 | 7.23E-07 | 0.000105 | 5.675632 |
| CDCA7 | 0.366862 | 4.557688 | 3.288381 | 0.001337 | 0.029734 | -1.28732 |
| MYO10 | 0.366452 | 4.423214 | 4.269776 | 4.04E-05 | 0.002182 | 1.919709 |
| IRF2BP2 | 0.365991 | 7.832278 | 6.351224 | 4.4E-09 | 2.31E-06 | 10.48189 |
| HIST1H2BI | 0.364327 | 7.73631 | 3.05946 | 0.002759 | 0.050275 | -1.93845 |
| ANKRD9 | 0.363946 | 4.982627 | 3.949933 | 0.000135 | 0.005552 | 0.804639 |
| FAM117A | 0.363724 | 8.184337 | 4.612243 | 1.04E-05 | 0.000774 | 3.181785 |
| ASRGL1 | 0.363492 | 6.50035 | 3.649022 | 0.000397 | 0.012292 | -0.18424 |
| MCM7 | 0.362812 | 6.318268 | 2.626071 | 0.009812 | 0.120367 | -3.06072 |
| SUSD3 | 0.362648 | 5.806875 | 4.725867 | 6.53E-06 | 0.00055 | 3.615171 |
| HYAL3 | 0.36236 | 4.455002 | 4.227916 | 4.75E-05 | 0.002461 | 1.770175 |
| COL9A3 | 0.362121 | 6.416293 | 3.1409 | 0.002141 | 0.042098 | -1.7113 |
| ST6GALNA | 0.361063 | 7.469694 | 2.863208 | 0.004984 | 0.076683 | -2.46496 |
| CETP | 0.360267 | 4.620418 | 2.952657 | 0.00382 | 0.063856 | -2.22868 |
| ISCA1 | 0.359503 | 7.41495 | 3.328242 | 0.001174 | 0.027011 | -1.16997 |
| GCH1 | 0.358045 | 10.2193 | 3.258982 | 0.00147 | 0.032072 | -1.37312 |
| TNFAIP8 | 0.357481 | 9.120562 | 4.59375 | 1.12E-05 | 0.000799 | 3.111922 |
| FAM101B | 0.356037 | 11.58226 | 3.616841 | 0.000444 | 0.013323 | -0.28638 |
| YPEL4 | 0.35439 | 4.051776 | 3.166151 | 0.001977 | 0.039979 | -1.63986 |
| CENPM | 0.353966 | 5.291934 | 4.219705 | 4.9E-05 | 0.002514 | 1.740968 |
| GFOD1 | 0.353933 | 5.261458 | 4.881785 | 3.42E-06 | 0.000346 | 4.221166 |
| LOC10193 | 0.35256 | 3.663032 | 2.426689 | 0.016788 | 0.173495 | -3.52631 |
| HBBP1 | 0.352362 | 4.176368 | 4.181515 | 5.67E-05 | 0.002825 | 1.605664 |
| SDC1 | 0.351103 | 4.365815 | 4.455196 | 1.95E-05 | 0.001245 | 2.594617 |
| ARG2 | 0.350252 | 4.485695 | 3.324546 | 0.001188 | 0.02726 | -1.1809 |
| LOC10029 | 0.35003 | 4.382741 | 2.847304 | 0.005223 | 0.079219 | -2.50631 |
| JAG1 | 0.34993 | 5.181812 | 6.28259 | 6.12E-09 | 2.85E-06 | 10.17058 |
| NCOA7 | 0.349036 | 5.894261 | 6.088306 | 1.54E-08 | 5.31E-06 | 9.298088 |
| KCNK5 | 0.34768 | 4.292395 | 4.734778 | 6.3E-06 | 0.000536 | 3.649454 |
| DTWD2 | 0.347596 | 5.454995 | 5.60238 | 1.46E-07 | 2.91E-05 | 7.177559 |
| LAMP3 | 0.347432 | 3.918974 | 2.040216 | 0.043617 | 0.304292 | -4.33355 |
| FAXDC2 | 0.346313 | 7.06269 | 3.08884 | 0.002519 | 0.047226 | -1.85709 |
| CLEC11A | 0.3456 | 5.4238 | 3.575292 | 0.000513 | 0.01482 | -0.41721 |
| MTFMT | 0.345325 | 5.982767 | 3.25848 | 0.001473 | 0.03209 | -1.37458 |
| AUNIP | 0.345253 | 4.101808 | 4.774813 | 5.34E-06 | 0.000479 | 3.804019 |
| ORC6 | 0.344377 | 6.930795 | 3.140829 | 0.002141 | 0.042098 | -1.7115 |
| PNPT1 | 0.344266 | 6.521076 | 2.088812 | 0.038928 | 0.28347 | -4.2391 |
| GNG8 | 0.344148 | 4.621149 | 2.509868 | 0.013467 | 0.150747 | -3.33606 |
| C10orf128 | 0.343646 | 5.030597 | 3.877109 | 0.000176 | 0.006744 | 0.559822 |
| MYBL2 | 0.343253 | 5.996556 | 3.376643 | 0.001001 | 0.023908 | -1.02593 |
| RAB6B | 0.343203 | 5.285539 | 4.807332 | 4.66E-06 | 0.000439 | 3.930191 |
| GSR | 0.342246 | 8.133743 | 3.415313 | 0.000881 | 0.021951 | -0.90963 |
| PLAU | 0.341374 | 4.55509 | 5.236074 | 7.48E-07 | 0.000107 | 5.644202 |
| STARD4 | 0.341272 | 5.905553 | 2.884221 | 0.004685 | 0.073741 | -2.41001 |
| GBE1 | 0.340711 | 9.337216 | 2.922019 | 0.004187 | 0.067993 | -2.31031 |
| FAM46A | 0.340518 | 6.242644 | 4.597491 | 1.1E-05 | 0.00079 | 3.126039 |
| DONSON | 0.340059 | 5.570415 | 2.723138 | 0.007474 | 0.099605 | -2.82229 |
| VAT1 | 0.339717 | 7.633784 | 2.694381 | 0.008107 | 0.10546 | -2.89372 |
| IGLC1 | 0.339615 | 7.086861 | 2.723845 | 0.007459 | 0.09949 | -2.82053 |
| LTBP1 | 0.339161 | 6.509959 | 2.41334 | 0.017384 | 0.177414 | -3.55631 |
| PPAP2B | 0.337899 | 4.452739 | 4.1445 | 6.53E-05 | 0.003201 | 1.475379 |
| SCD | 0.337033 | 5.461477 | 4.69086 | 7.54E-06 | 0.000613 | 3.480894 |

| | | | | | | |
|---|---|---|---|---|---|---|
| NCAPD2 | 0.336559 | 6.643627 | 3.658916 | 0.000384 | 0.011965 | -0.15269 |
| RBM11 | 0.336512 | 3.169407 | 3.442618 | 0.000804 | 0.020704 | -0.82687 |
| HP | 0.336013 | 10.53482 | 2.159939 | 0.032851 | 0.257643 | -4.09717 |
| ND6 | 0.335833 | 8.2917 | 2.157022 | 0.033083 | 0.258473 | -4.10308 |
| TMEM123 | 0.335368 | 10.50415 | 3.203033 | 0.001759 | 0.036614 | -1.53465 |
| MTURN | 0.335318 | 6.08749 | 2.127627 | 0.035501 | 0.269275 | -4.16219 |
| CASC5 | 0.33526 | 3.566659 | 4.554504 | 1.31E-05 | 0.000908 | 2.964289 |
| PXMP2 | 0.334047 | 5.167916 | 3.936944 | 0.000142 | 0.005777 | 0.760721 |
| SPX | 0.33391 | 4.994308 | 2.449161 | 0.015826 | 0.166407 | -3.47548 |
| ID2 | 0.333908 | 9.237323 | 3.769727 | 0.000259 | 0.009076 | 0.205179 |
| SMIM24 | 0.333359 | 5.349613 | 3.844388 | 0.000199 | 0.007358 | 0.450947 |
| EXO1 | 0.332634 | 4.372363 | 4.095576 | 7.86E-05 | 0.003702 | 1.304476 |
| SMIM5 | 0.332593 | 6.134942 | 3.084241 | 0.002555 | 0.047619 | -1.86987 |
| SH3RF1 | 0.331668 | 4.04852 | 3.66522 | 0.000375 | 0.01174 | -0.13256 |
| KNTC1 | 0.33092 | 4.584857 | 3.917014 | 0.000152 | 0.006117 | 0.693546 |
| MCUR1 | 0.330691 | 5.984813 | 2.062484 | 0.041412 | 0.29453 | -4.29053 |
| ZKSCAN7 | 0.330519 | 5.109177 | 3.920258 | 0.000151 | 0.006069 | 0.704462 |
| BRCA2 | 0.330298 | 3.141751 | 4.589648 | 1.14E-05 | 0.000807 | 3.096451 |
| DSCC1 | 0.329692 | 4.812257 | 5.030995 | 1.81E-06 | 0.000211 | 4.812899 |
| C1orf106 | 0.328748 | 4.134375 | 4.097167 | 7.81E-05 | 0.003692 | 1.310009 |
| PDE6H | 0.328386 | 3.374413 | 3.456889 | 0.000766 | 0.020003 | -0.7834 |
| SLC26A8 | 0.328175 | 7.603092 | 2.370682 | 0.019417 | 0.189103 | -3.65117 |
| ETV7 | 0.327588 | 4.108271 | 3.499629 | 0.000664 | 0.017935 | -0.65235 |
| TMEM45B | 0.327304 | 6.300598 | 2.085419 | 0.039241 | 0.284975 | -4.24577 |
| MSANTD3 | 0.32522 | 5.706189 | 3.062931 | 0.002729 | 0.049977 | -1.92888 |
| ACOT7 | 0.32495 | 5.303161 | 4.644324 | 9.13E-06 | 0.00071 | 3.30343 |
| STON1 | 0.324363 | 3.994612 | 3.149403 | 0.002085 | 0.041572 | -1.6873 |
| XRCC6BP1 | 0.324142 | 5.114139 | 4.037963 | 9.75E-05 | 0.004425 | 1.105143 |
| PECR | 0.323957 | 7.9254 | 3.06655 | 0.002699 | 0.049672 | -1.91888 |
| TMEM38A | 0.3239 | 5.366348 | 4.113216 | 7.35E-05 | 0.003507 | 1.365924 |
| FANCG | 0.323379 | 6.120634 | 2.946986 | 0.003885 | 0.064424 | -2.24385 |
| PTGR1 | 0.322399 | 4.333912 | 4.299574 | 3.6E-05 | 0.002002 | 2.026798 |
| GLDN | 0.322184 | 4.986487 | 4.222756 | 4.85E-05 | 0.002497 | 1.751815 |
| RAD54L | 0.319625 | 5.086974 | 4.182819 | 5.65E-05 | 0.002818 | 1.61027 |
| DNA2 | 0.319335 | 5.164561 | 2.88245 | 0.004709 | 0.073896 | -2.41466 |
| CYBRD1 | 0.319156 | 6.433509 | 2.409944 | 0.017539 | 0.1782 | -3.56392 |
| MT1X | 0.319117 | 7.481537 | 2.942226 | 0.003941 | 0.06506 | -2.25655 |
| YWHAH | 0.318941 | 5.000869 | 4.922533 | 2.88E-06 | 0.000302 | 4.381637 |
| IGKC | 0.318857 | 5.682502 | 3.146566 | 0.002103 | 0.041771 | -1.69531 |
| KIAA1324 | 0.31737 | 5.695027 | 2.18174 | 0.031161 | 0.251786 | -4.0528 |
| XPO7 | 0.316866 | 5.962312 | 2.723918 | 0.007457 | 0.09949 | -2.82035 |
| SCP2 | 0.316558 | 9.221661 | 3.348322 | 0.001099 | 0.025648 | -1.11042 |
| GSTO1 | 0.316343 | 10.11126 | 3.30001 | 0.001287 | 0.029042 | -1.2532 |
| PEAR1 | 0.3162 | 5.120794 | 2.122825 | 0.035911 | 0.270768 | -4.17178 |
| SMARCD3 | 0.316013 | 5.487619 | 3.894421 | 0.000166 | 0.00649 | 0.617708 |
| HNRNPLL | 0.315582 | 6.050974 | 3.238021 | 0.001573 | 0.0336 | -1.43391 |
| GINS3 | 0.315565 | 4.101082 | 3.643276 | 0.000405 | 0.012465 | -0.20253 |
| HMGN2 | 0.314926 | 11.73096 | 3.387395 | 0.000966 | 0.023345 | -0.9937 |
| VEGFA | 0.314686 | 5.853231 | 4.671896 | 8.15E-06 | 0.000654 | 3.408435 |
| IDNK | 0.314302 | 6.951723 | 2.587633 | 0.010908 | 0.128819 | -3.15302 |
| CLU | 0.314154 | 6.58772 | 2.309919 | 0.022675 | 0.208619 | -3.78364 |
| PGRMC1 | 0.313412 | 7.723423 | 2.608374 | 0.010303 | 0.124073 | -3.10336 |
| WDR76 | 0.313384 | 3.706527 | 4.66278 | 8.46E-06 | 0.000669 | 3.373671 |
| LOXL3 | 0.312878 | 6.235389 | 4.173349 | 5.85E-05 | 0.002894 | 1.576848 |
| CCL23 | 0.312404 | 3.377153 | 3.542828 | 0.000573 | 0.016079 | -0.51859 |
| MST4 | 0.311954 | 7.513758 | 3.690644 | 0.000343 | 0.011074 | -0.05108 |
| CENPL | 0.310877 | 4.43816 | 3.252001 | 0.001504 | 0.032571 | -1.3934 |
| SLC2A1 | 0.309411 | 5.08986 | 4.019141 | 0.000105 | 0.004654 | 1.040475 |

| | | | | | | |
|---|---|---|---|---|---|---|
| SLA2 | 0.309177 | 5.888326 | 3.283075 | 0.00136 | 0.030054 | -1.30285 |
| FAM132B | 0.308167 | 4.535371 | 3.01713 | 0.003142 | 0.055463 | -2.05453 |
| ITM2C | 0.306527 | 6.247905 | 3.033397 | 0.002989 | 0.053556 | -2.01008 |
| RSPH9 | 0.306516 | 4.896961 | 3.362371 | 0.00105 | 0.024772 | -1.06858 |
| OXTR | 0.306291 | 3.882557 | 3.747738 | 0.000281 | 0.009682 | 0.133504 |
| ABCC4 | 0.306265 | 4.396552 | 2.556203 | 0.011884 | 0.137508 | -3.2276 |
| MICB | 0.305596 | 8.205265 | 2.782512 | 0.006305 | 0.089227 | -2.67273 |
| TSPO2 | 0.305348 | 4.874478 | 4.078877 | 8.37E-05 | 0.003883 | 1.246484 |
| S100A4 | 0.304803 | 12.6659 | 3.376878 | 0.001001 | 0.023908 | -1.02523 |
| STMN1 | 0.304783 | 4.87231 | 4.221304 | 4.87E-05 | 0.002505 | 1.746654 |
| GBAP1 | 0.304543 | 8.465675 | 2.24004 | 0.027008 | 0.231618 | -3.93212 |
| JAZF1 | 0.304444 | 8.158298 | 3.473566 | 0.000725 | 0.019158 | -0.73242 |
| EPDR1 | 0.303583 | 4.606763 | 2.515071 | 0.01328 | 0.148974 | -3.32397 |
| KREMEN1 | 0.303295 | 5.481461 | 2.320379 | 0.022082 | 0.205379 | -3.76106 |
| IL17RB | 0.303075 | 4.739831 | 4.390757 | 2.52E-05 | 0.001513 | 2.357775 |
| PLD1 | 0.302932 | 4.779995 | 3.367889 | 0.001031 | 0.024374 | -1.05211 |
| PTH2R | 0.302917 | 3.682676 | 2.372123 | 0.019345 | 0.188545 | -3.64799 |
| CXCL10 | 0.302636 | 4.144719 | 2.983831 | 0.003476 | 0.059279 | -2.14488 |
| FAM65C | 0.302521 | 4.644534 | 3.410208 | 0.000896 | 0.022122 | -0.92505 |
| FAM8A1 | 0.3022 | 10.4204 | 3.981439 | 0.00012 | 0.00515 | 0.91162 |
| CDC14B | 0.30121 | 4.258891 | 3.895145 | 0.000165 | 0.006485 | 0.620133 |
| HDGF | 0.300805 | 9.106062 | 2.986277 | 0.003451 | 0.059038 | -2.13827 |
| JAK2 | 0.299814 | 7.720281 | 2.537911 | 0.012488 | 0.142356 | -3.27063 |
| RPIA | 0.299146 | 7.778835 | 2.321193 | 0.022036 | 0.205329 | -3.7593 |
| KIF2A | 0.298209 | 7.015645 | 2.113012 | 0.03676 | 0.273935 | -4.19131 |
| SERPINI1 | 0.297127 | 5.452848 | 2.015895 | 0.046139 | 0.314491 | -4.38004 |
| CRNDE | 0.29685 | 3.07666 | 3.409011 | 0.000899 | 0.022173 | -0.92866 |
| HIST1H4J | 0.295686 | 6.127946 | 3.085677 | 0.002544 | 0.047514 | -1.86588 |
| KRT1 | 0.295661 | 4.574788 | 2.809347 | 0.005834 | 0.084668 | -2.60421 |
| ANK1 | 0.295604 | 5.034475 | 3.506276 | 0.000649 | 0.01761 | -0.63186 |
| DUSP13 | 0.29417 | 6.590748 | 2.526872 | 0.012866 | 0.145364 | -3.29646 |
| CENPF | 0.293725 | 3.958318 | 4.24443 | 4.46E-05 | 0.002345 | 1.829041 |
| KIF23 | 0.293661 | 3.100796 | 4.454353 | 1.96E-05 | 0.001246 | 2.591503 |
| SNRNP25 | 0.293647 | 7.281063 | 2.494287 | 0.01404 | 0.154639 | -3.37213 |
| PLK1 | 0.293141 | 4.479002 | 3.251887 | 0.001504 | 0.032571 | -1.39373 |
| MPP7 | 0.291293 | 6.263931 | 3.162051 | 0.002003 | 0.040341 | -1.65149 |
| KCNJ15 | 0.290748 | 8.667786 | 2.592737 | 0.010756 | 0.127631 | -3.14083 |
| SMOX | 0.28956 | 5.784992 | 3.394038 | 0.000945 | 0.023027 | -0.97375 |
| RNF138 | 0.289543 | 7.982113 | 2.772029 | 0.006499 | 0.090956 | -2.69934 |
| MREG | 0.288529 | 5.142465 | 2.959946 | 0.003737 | 0.063085 | -2.20915 |
| WEE1 | 0.287323 | 5.490238 | 2.537073 | 0.012516 | 0.142599 | -3.27259 |
| SEC14L5 | 0.287082 | 4.678645 | 2.263985 | 0.025448 | 0.222566 | -3.8817 |
| CDK14 | 0.286362 | 6.581884 | 3.965357 | 0.000128 | 0.005332 | 0.856932 |
| AIG1 | 0.286319 | 4.852124 | 3.165043 | 0.001984 | 0.04008 | -1.643 |
| TMEM60 | 0.284629 | 8.229254 | 3.029428 | 0.003026 | 0.054022 | -2.02095 |
| RNASEH2/ | 0.284036 | 5.434812 | 3.204633 | 0.00175 | 0.036465 | -1.53006 |
| FAM210B | 0.283981 | 7.726005 | 2.012436 | 0.046508 | 0.315847 | -4.38661 |
| HTRA3 | 0.283639 | 4.777238 | 3.999553 | 0.000113 | 0.004898 | 0.973414 |
| GINS2 | 0.283449 | 4.928535 | 5.889583 | 3.91E-08 | 1.1E-05 | 8.419768 |
| AACS | 0.283359 | 6.019316 | 2.875195 | 0.004811 | 0.074808 | -2.43366 |
| TRIM24 | 0.283311 | 5.142852 | 3.547277 | 0.000564 | 0.01599 | -0.50474 |
| TRPM6 | 0.282993 | 6.167919 | 3.388523 | 0.000963 | 0.023285 | -0.99032 |
| GNAZ | 0.28262 | 6.263398 | 2.213358 | 0.028844 | 0.240904 | -3.98771 |
| HMGB3 | 0.282549 | 5.315351 | 3.19784 | 0.001788 | 0.037073 | -1.54952 |
| RIOK3 | 0.282455 | 8.254445 | 4.723729 | 6.59E-06 | 0.000553 | 3.606951 |
| ZNF770 | 0.281013 | 6.525568 | 2.908478 | 0.004359 | 0.070157 | -2.34616 |
| CLIC4 | 0.280944 | 6.120008 | 2.813441 | 0.005765 | 0.084445 | -2.5937 |
| LOC10192 | 0.280897 | 3.421813 | 2.765134 | 0.006629 | 0.092021 | -2.71679 |

| | | | | | | |
|---|---|---|---|---|---|---|
| GPSM2 | 0.280591 | 5.325737 | 3.133643 | 0.00219 | 0.042766 | -1.73175 |
| CCND2 | 0.280456 | 5.610758 | 3.601954 | 0.000468 | 0.013824 | -0.3334 |
| HYI | 0.279005 | 5.794066 | 4.4476 | 2.01E-05 | 0.001274 | 2.566573 |
| ELL2 | 0.278229 | 5.503902 | 2.736955 | 0.007185 | 0.097176 | -2.78774 |
| SPNS3 | 0.278212 | 4.988226 | 2.230011 | 0.027686 | 0.235195 | -3.95308 |
| AMFR | 0.27749 | 6.403667 | 2.044857 | 0.043149 | 0.302679 | -4.32462 |
| WDR34 | 0.276894 | 5.18913 | 3.510873 | 0.000639 | 0.017425 | -0.61766 |
| MT1F | 0.276482 | 7.134916 | 3.517178 | 0.000625 | 0.017193 | -0.59817 |
| BNIP3L | 0.276304 | 10.09599 | 3.018616 | 0.003127 | 0.055308 | -2.05048 |
| RBBP8 | 0.276243 | 7.017331 | 1.986074 | 0.049401 | 0.328198 | -4.43634 |
| GCSAML | 0.275701 | 2.907396 | 2.983544 | 0.003479 | 0.059282 | -2.14565 |
| ATP8A1 | 0.275012 | 5.081177 | 4.374063 | 2.69E-05 | 0.001587 | 2.296811 |
| BUB1 | 0.275007 | 3.672301 | 4.524237 | 1.48E-05 | 0.000994 | 2.851027 |
| ZNFX1 | 0.274565 | 9.002096 | 2.262405 | 0.025548 | 0.223023 | -3.88505 |
| ARL6IP6 | 0.273998 | 7.407123 | 3.848578 | 0.000196 | 0.007261 | 0.46485 |
| DHX58 | 0.273619 | 5.610531 | 2.87596 | 0.0048 | 0.074753 | -2.43166 |
| ALOX15 | 0.273308 | 4.193196 | 2.531607 | 0.012702 | 0.144077 | -3.28539 |
| SLC22A15 | 0.273228 | 8.65904 | 2.724982 | 0.007434 | 0.099363 | -2.81769 |
| C9orf78 | 0.272814 | 7.459438 | 2.873653 | 0.004833 | 0.075036 | -2.43769 |
| TGM3 | 0.271938 | 4.92924 | 2.338238 | 0.021101 | 0.199153 | -3.72229 |
| LONRF3 | 0.271173 | 5.147992 | 2.932407 | 0.004059 | 0.066409 | -2.28272 |
| LOC10050 | 0.270615 | 6.80039 | 2.241037 | 0.026941 | 0.231239 | -3.93003 |
| SYNM | 0.270515 | 5.112923 | 2.402872 | 0.017865 | 0.179806 | -3.57973 |
| PIK3CB | 0.270093 | 7.467903 | 2.777514 | 0.006397 | 0.090148 | -2.68543 |
| TLK1 | 0.269222 | 4.990592 | 5.049029 | 1.68E-06 | 0.000201 | 4.88518 |
| TMOD1 | 0.269156 | 5.55825 | 2.647854 | 0.009236 | 0.115659 | -3.00788 |
| BZRAP1-A | 0.26902 | 6.707608 | 2.051628 | 0.042474 | 0.299901 | -4.31156 |
| GTSE1 | 0.26847 | 5.040677 | 4.866884 | 3.64E-06 | 0.000361 | 4.162701 |
| SMIM1 | 0.266861 | 5.113018 | 2.91669 | 0.004254 | 0.068755 | -2.32444 |
| GPD2 | 0.266166 | 5.37244 | 3.5457 | 0.000567 | 0.016054 | -0.50965 |
| OSBP2 | 0.265906 | 5.101932 | 3.138013 | 0.00216 | 0.042345 | -1.71944 |
| HMBS | 0.265781 | 6.42008 | 2.478687 | 0.014636 | 0.159047 | -3.40805 |
| ADSS | 0.265734 | 5.638757 | 4.012533 | 0.000107 | 0.004728 | 1.017825 |
| DENND2C | 0.265687 | 4.478244 | 2.808522 | 0.005848 | 0.084751 | -2.60632 |
| MICU1 | 0.264933 | 8.40636 | 2.320784 | 0.022059 | 0.205379 | -3.76018 |
| TDP2 | 0.264896 | 10.66175 | 2.811334 | 0.005801 | 0.0846 | -2.59911 |
| RARA-AS1 | 0.26472 | 8.301405 | 2.296753 | 0.023441 | 0.213082 | -3.81193 |
| CDC25A | 0.26465 | 4.175402 | 4.036363 | 9.81E-05 | 0.004442 | 1.099637 |
| CCNB2 | 0.263642 | 4.715492 | 4.651456 | 8.86E-06 | 0.000695 | 3.330551 |
| BEND7 | 0.263394 | 4.622957 | 4.539944 | 1.39E-05 | 0.00095 | 2.90974 |
| E2F1 | 0.263278 | 5.441264 | 3.920427 | 0.000151 | 0.006069 | 0.705032 |
| GCNT1 | 0.262788 | 6.809307 | 2.754534 | 0.006833 | 0.094095 | -2.74356 |
| ANKRD36 | 0.262466 | 3.676784 | 2.370184 | 0.019442 | 0.189136 | -3.65227 |
| POLA1 | 0.262238 | 4.861873 | 2.793047 | 0.006117 | 0.087339 | -2.64589 |
| IL5RA | 0.26202 | 4.240902 | 4.362642 | 2.82E-05 | 0.001627 | 2.255199 |
| NCF1 | 0.26193 | 11.11872 | 3.195342 | 0.001803 | 0.037293 | -1.55667 |
| HSH2D | 0.261504 | 5.922962 | 2.063529 | 0.04131 | 0.29422 | -4.2885 |
| HADH | 0.261193 | 5.634815 | 2.861006 | 0.005017 | 0.077008 | -2.4707 |
| TDRD7 | 0.261123 | 7.853137 | 2.747092 | 0.00698 | 0.09535 | -2.76229 |
| CBX1 | 0.259929 | 8.3953 | 2.593917 | 0.010721 | 0.127368 | -3.13801 |
| HMGN5 | 0.259424 | 3.981643 | 3.543764 | 0.000571 | 0.016061 | -0.51568 |
| SOCS2 | 0.259134 | 4.43777 | 2.776511 | 0.006415 | 0.090278 | -2.68797 |
| ANKRD18/ | 0.258847 | 3.257537 | 4.294249 | 3.68E-05 | 0.002033 | 2.007623 |
| PARVB | 0.25803 | 5.590194 | 2.302755 | 0.023089 | 0.211006 | -3.79905 |
| DCAF12 | 0.257292 | 8.914827 | 2.667119 | 0.008752 | 0.111397 | -2.96082 |
| HIST1H2B: | 0.256555 | 3.858923 | 2.493536 | 0.014069 | 0.154822 | -3.37387 |
| LOC28518 | 0.255745 | 3.697367 | 2.901034 | 0.004457 | 0.071136 | -2.3658 |
| TAP1 | 0.255097 | 8.992053 | 2.333707 | 0.021346 | 0.200911 | -3.73215 |

| | | | | | | |
|---|---|---|---|---|---|---|
| LRRK2 | 0.254954 | 10.49439 | 2.288435 | 0.023937 | 0.21634 | -3.82973 |
| PKP2 | 0.254152 | 3.866579 | 3.091988 | 0.002495 | 0.046931 | -1.84833 |
| VEPH1 | 0.254047 | 4.011482 | 3.776954 | 0.000253 | 0.008937 | 0.228805 |
| CLDN12 | 0.254016 | 3.836661 | 2.735301 | 0.007219 | 0.097442 | -2.79188 |
| GLDC | 0.25362 | 4.417974 | 2.083913 | 0.03938 | 0.285395 | -4.24872 |
| TP53INP2 | 0.253504 | 6.9987 | 2.124248 | 0.035789 | 0.270262 | -4.16894 |
| RILP | 0.253253 | 7.454923 | 2.979274 | 0.003525 | 0.059854 | -2.15717 |
| VPS13A | 0.252956 | 4.537479 | 2.408065 | 0.017625 | 0.178535 | -3.56812 |
| KNSTRN | 0.252849 | 5.999996 | 2.418541 | 0.01715 | 0.175988 | -3.54464 |
| POLE | 0.252109 | 5.995641 | 2.850734 | 0.00517 | 0.078894 | -2.49741 |
| KIAA1524 | 0.251577 | 2.962621 | 3.271418 | 0.001412 | 0.030995 | -1.3369 |
| MPC2 | 0.251409 | 6.872247 | 3.06957 | 0.002674 | 0.049376 | -1.91053 |
| RAP1GAP | 0.250999 | 5.754288 | 3.821245 | 0.000216 | 0.007912 | 0.374369 |
| ORC1 | 0.250359 | 3.51166 | 3.827889 | 0.000211 | 0.007739 | 0.396316 |
| CLTCL1 | 0.249586 | 6.028427 | 2.146099 | 0.033964 | 0.261959 | -4.12513 |
| EIF4E3 | 0.249521 | 8.551429 | 2.465202 | 0.015169 | 0.161747 | -3.43894 |
| PKMYT1 | 0.249245 | 5.722004 | 3.372958 | 0.001014 | 0.024088 | -1.03696 |
| LOC81691 | 0.249222 | 4.235199 | 4.110993 | 7.42E-05 | 0.003529 | 1.35817 |
| CD9 | 0.249176 | 5.350538 | 3.764561 | 0.000264 | 0.009182 | 0.188308 |
| LRRC4 | 0.249027 | 8.29025 | 2.580251 | 0.01113 | 0.130965 | -3.17061 |
| PPP2R5A | 0.248581 | 7.185729 | 3.739746 | 0.000289 | 0.009889 | 0.107532 |
| SMPD3 | 0.248394 | 4.983212 | 2.662627 | 0.008863 | 0.112504 | -2.97182 |
| CLEC4E | 0.247471 | 9.495375 | 2.181514 | 0.031178 | 0.251786 | -4.05326 |
| WIPI1 | 0.247316 | 8.730761 | 2.046187 | 0.043016 | 0.302159 | -4.32205 |
| STON2 | 0.247135 | 4.222156 | 2.328324 | 0.02164 | 0.202936 | -3.74384 |
| KPNA2 | 0.247111 | 8.403439 | 2.693792 | 0.008121 | 0.105568 | -2.89518 |
| MMP1 | 0.246302 | 3.015567 | 2.112221 | 0.036829 | 0.274237 | -4.19288 |
| LOXL1 | 0.24617 | 4.83864 | 2.552541 | 0.012003 | 0.138446 | -3.23623 |
| ELOVL6 | 0.246093 | 3.22675 | 4.277425 | 3.92E-05 | 0.00213 | 1.947146 |
| PMM2 | 0.246027 | 6.38005 | 2.257898 | 0.025837 | 0.224583 | -3.89457 |
| OLIG2 | 0.24588 | 3.752561 | 3.423385 | 0.000857 | 0.021499 | -0.88522 |
| C17orf99 | 0.245845 | 3.683882 | 2.939842 | 0.003969 | 0.065396 | -2.26291 |
| GTPBP2 | 0.24529 | 5.531808 | 2.634138 | 0.009595 | 0.118846 | -3.04119 |
| CTDSPL | 0.244601 | 4.917011 | 2.893119 | 0.004563 | 0.072431 | -2.38665 |
| CYP4F12 | 0.243833 | 6.243323 | 2.430491 | 0.016622 | 0.172472 | -3.51774 |
| PKP4 | 0.242642 | 4.840545 | 3.021129 | 0.003103 | 0.055075 | -2.04362 |
| CDCA4 | 0.24244 | 6.118616 | 3.449822 | 0.000785 | 0.020353 | -0.80494 |
| ZYG11B | 0.242408 | 7.906162 | 2.378698 | 0.01902 | 0.186387 | -3.63346 |
| RPA3 | 0.241445 | 7.069567 | 2.045935 | 0.043041 | 0.302232 | -4.32254 |
| BARD1 | 0.241258 | 5.202808 | 2.066204 | 0.041053 | 0.292894 | -4.2833 |
| ERCC6L | 0.241141 | 3.796503 | 3.970256 | 0.000125 | 0.00529 | 0.873574 |
| FAM111A | 0.240887 | 5.560862 | 4.791535 | 4.98E-06 | 0.000459 | 3.868831 |
| NDC1 | 0.240544 | 5.568364 | 2.182796 | 0.031081 | 0.251574 | -4.05064 |
| KLHDC8B | 0.240419 | 5.696694 | 2.471015 | 0.014937 | 0.160614 | -3.42564 |
| PHF19 | 0.240273 | 6.126274 | 3.655907 | 0.000388 | 0.012055 | -0.1623 |
| PARPBP | 0.240202 | 3.083016 | 4.564103 | 1.26E-05 | 0.000879 | 3.00032 |
| RNF213 | 0.240122 | 7.103516 | 2.146216 | 0.033955 | 0.261959 | -4.1249 |
| MT1HL1 | 0.240068 | 8.096694 | 3.23908 | 0.001567 | 0.033549 | -1.43084 |
| USP1 | 0.239608 | 8.595546 | 2.61689 | 0.010064 | 0.122289 | -3.08287 |
| TMEM38B | 0.239087 | 4.967364 | 2.224167 | 0.028088 | 0.237003 | -3.96526 |
| PRR11 | 0.23906 | 9.326553 | 2.99487 | 0.003362 | 0.058003 | -2.11502 |
| CENPN | 0.238819 | 4.372707 | 4.30343 | 3.55E-05 | 0.001989 | 2.040694 |
| NAV3 | 0.238748 | 3.120795 | 4.701765 | 7.21E-06 | 0.00059 | 3.522652 |
| OXCT1 | 0.238493 | 5.771697 | 2.047744 | 0.04286 | 0.30148 | -4.31905 |
| DTX3L | 0.237957 | 9.518187 | 2.592616 | 0.01076 | 0.127631 | -3.14112 |
| ABCC13 | 0.237856 | 3.526696 | 3.076255 | 0.002619 | 0.048591 | -1.89202 |
| PER2 | 0.237817 | 5.863888 | 3.009367 | 0.003217 | 0.056307 | -2.07567 |
| FUT8 | 0.237194 | 5.091832 | 2.302577 | 0.023099 | 0.211007 | -3.79943 |

| | | | | | | |
|---|---|---|---|---|---|---|
| LOC10050 | 0.236813 | 3.654877 | 3.534339 | 0.00059 | 0.016472 | -0.54498 |
| TMX1 | 0.236545 | 8.845321 | 2.871184 | 0.004869 | 0.075242 | -2.44414 |
| CMC2 | 0.236012 | 7.668003 | 2.216388 | 0.028631 | 0.239606 | -3.98143 |
| ARHGEF17 | 0.235876 | 6.2633 | 3.908752 | 0.000157 | 0.006243 | 0.665774 |
| MTFR2 | 0.23491 | 3.734255 | 3.144407 | 0.002118 | 0.041864 | -1.70141 |
| PCMT1 | 0.234753 | 10.10504 | 3.011943 | 0.003192 | 0.05601 | -2.06866 |
| DNAJC9 | 0.233883 | 5.343648 | 2.538311 | 0.012474 | 0.142281 | -3.26969 |
| TMEM106 | 0.233852 | 5.499991 | 3.424962 | 0.000853 | 0.021463 | -0.88045 |
| PCDHB12 | 0.232544 | 3.979573 | 2.066675 | 0.041007 | 0.292803 | -4.28238 |
| HSPA4L | 0.231978 | 2.74312 | 2.818711 | 0.005678 | 0.08364 | -2.58016 |
| EBP | 0.231603 | 5.544135 | 4.437205 | 2.1E-05 | 0.001315 | 2.528249 |
| DIAPH3 | 0.230748 | 3.51887 | 3.987603 | 0.000118 | 0.005066 | 0.932625 |
| BRCA1 | 0.230667 | 5.552415 | 4.255665 | 4.27E-05 | 0.002281 | 1.869183 |
| C10orf11 | 0.230216 | 5.795543 | 2.956018 | 0.003781 | 0.063565 | -2.21968 |
| SCN9A | 0.230138 | 3.37836 | 3.062787 | 0.002731 | 0.049977 | -1.92927 |
| CCNF | 0.230048 | 4.844545 | 4.599607 | 1.09E-05 | 0.000786 | 3.13403 |
| COL10A1 | 0.229319 | 3.759889 | 3.887263 | 0.00017 | 0.006623 | 0.59375 |
| IL3RA | 0.228418 | 5.998526 | 2.416779 | 0.017229 | 0.176572 | -3.5486 |
| HBE1 | 0.22827 | 4.657896 | 2.447071 | 0.015913 | 0.166897 | -3.48023 |
| FLJ36848 | 0.228187 | 4.29064 | 2.812936 | 0.005774 | 0.084448 | -2.595 |
| FIGNL1 | 0.228103 | 4.591777 | 1.987728 | 0.049215 | 0.327409 | -4.43324 |
| DAPP1 | 0.227858 | 8.319694 | 2.390556 | 0.018445 | 0.182948 | -3.60716 |
| ZC3HAV1L | 0.227772 | 3.625738 | 2.478019 | 0.014662 | 0.15916 | -3.40959 |
| TPRG1L | 0.227447 | 8.681483 | 2.485704 | 0.014365 | 0.157443 | -3.39192 |
| HERC6 | 0.227421 | 4.131284 | 2.650249 | 0.009175 | 0.115315 | -3.00204 |
| SLCO4C1 | 0.227265 | 6.680467 | 3.328859 | 0.001172 | 0.026999 | -1.16814 |
| TPST2 | 0.22711 | 10.30766 | 2.166597 | 0.032326 | 0.256262 | -4.08367 |
| NCK1-AS1 | 0.226953 | 5.173301 | 2.201909 | 0.029665 | 0.244603 | -4.01138 |
| UBN1 | 0.226821 | 9.429497 | 2.352325 | 0.020354 | 0.194349 | -3.69152 |
| NLK | 0.226512 | 6.589104 | 2.042502 | 0.043386 | 0.303459 | -4.32915 |
| TNS1 | 0.226495 | 4.982948 | 2.038746 | 0.043766 | 0.304719 | -4.33637 |
| GNB4 | 0.226454 | 7.360182 | 2.924434 | 0.004157 | 0.067612 | -2.3039 |
| SKA1 | 0.226421 | 2.849817 | 3.757654 | 0.000271 | 0.009362 | 0.165784 |
| LAMP2 | 0.226329 | 9.686698 | 2.447928 | 0.015877 | 0.166863 | -3.47828 |
| HIST1H2A | 0.226026 | 6.603628 | 2.509908 | 0.013466 | 0.150747 | -3.33597 |
| MUC1 | 0.225857 | 5.059912 | 3.690646 | 0.000343 | 0.011074 | -0.05107 |
| SLC40A1 | 0.225024 | 8.532639 | 3.328035 | 0.001175 | 0.027011 | -1.17058 |
| LOC10050 | 0.224758 | 5.996986 | 2.058843 | 0.041765 | 0.296182 | -4.29759 |
| ANO10 | 0.224693 | 7.508498 | 2.286416 | 0.024058 | 0.216769 | -3.83404 |
| RNF11 | 0.224558 | 10.03582 | 2.801082 | 0.005976 | 0.086069 | -2.62537 |
| SAMD14 | 0.22434 | 4.927875 | 3.296268 | 0.001303 | 0.029301 | -1.26419 |
| ADIPOR1 | 0.22388 | 10.03757 | 2.385035 | 0.018711 | 0.184686 | -3.61942 |
| DNAJC13 | 0.223818 | 5.728628 | 2.883832 | 0.00469 | 0.073741 | -2.41104 |
| AP1M2 | 0.223636 | 5.120043 | 3.860569 | 0.000187 | 0.007055 | 0.504697 |
| AZI2 | 0.222956 | 6.603588 | 2.312235 | 0.022542 | 0.207493 | -3.77865 |
| RNF208 | 0.222912 | 4.736072 | 2.369979 | 0.019452 | 0.189136 | -3.65272 |
| MCM3 | 0.222713 | 6.530306 | 2.104939 | 0.037471 | 0.27749 | -4.20731 |
| ITGA9 | 0.222132 | 3.944057 | 3.330299 | 0.001166 | 0.026903 | -1.16388 |
| IGSF10 | 0.22128 | 4.400266 | 3.410352 | 0.000895 | 0.022122 | -0.92461 |
| DZIP1L | 0.2205 | 4.569199 | 2.161341 | 0.03274 | 0.25697 | -4.09433 |
| MAOB | 0.220469 | 3.510714 | 4.929305 | 2.8E-06 | 0.000298 | 4.408391 |
| MYL6B | 0.22046 | 6.102804 | 2.402337 | 0.01789 | 0.179884 | -3.58092 |
| SLC30A1 | 0.219789 | 6.240582 | 2.751744 | 0.006888 | 0.094532 | -2.75058 |
| IFI27L1 | 0.21944 | 5.779764 | 2.825459 | 0.005567 | 0.082645 | -2.56279 |
| TMEM11 | 0.219394 | 7.663239 | 2.560817 | 0.011736 | 0.13629 | -3.2167 |
| JAM3 | 0.219329 | 4.791288 | 3.153284 | 0.002059 | 0.041188 | -1.67632 |
| PLA2G16 | 0.219223 | 5.520336 | 3.466537 | 0.000742 | 0.019514 | -0.75393 |
| CITED4 | 0.219127 | 5.804104 | 2.392022 | 0.018375 | 0.18252 | -3.60391 |

| | | | | | | |
|---|---|---|---|---|---|---|
| TNFSF10 | 0.21907 | 10.71635 | 2.595674 | 0.01067 | 0.126932 | -3.13381 |
| LETM2 | 0.218958 | 4.640546 | 2.279778 | 0.024462 | 0.218522 | -3.84819 |
| MCTP1 | 0.218656 | 7.624731 | 2.036328 | 0.044012 | 0.306005 | -4.34101 |
| HEPACAM | 0.218592 | 2.633899 | 2.704462 | 0.00788 | 0.103157 | -2.86876 |
| CHRM5 | 0.21841 | 3.818777 | 2.901376 | 0.004452 | 0.071119 | -2.3649 |
| SUV39H2 | 0.217655 | 3.702547 | 3.021574 | 0.003099 | 0.055048 | -2.04241 |
| PLCL2 | 0.217357 | 6.906249 | 3.399897 | 0.000927 | 0.022691 | -0.95612 |
| CDK6 | 0.216604 | 4.907205 | 4.607756 | 1.06E-05 | 0.000782 | 3.164817 |
| HMGB3P1 | 0.215456 | 5.511488 | 3.739385 | 0.000289 | 0.009889 | 0.106362 |
| TROAP | 0.215155 | 5.262243 | 2.932301 | 0.00406 | 0.066409 | -2.283 |
| KIAA1211 | 0.215149 | 3.054357 | 3.878593 | 0.000175 | 0.006733 | 0.564773 |
| CD200R1 | 0.214703 | 4.369318 | 3.734018 | 0.000295 | 0.010029 | 0.088945 |
| NXPE3 | 0.214401 | 6.351688 | 2.091157 | 0.038713 | 0.282509 | -4.2345 |
| PML | 0.214381 | 5.309173 | 3.290334 | 0.001328 | 0.029675 | -1.28159 |
| STT3A | 0.214232 | 7.346584 | 2.231267 | 0.0276 | 0.234825 | -3.95046 |
| LINC0052{ | 0.213919 | 6.91414 | 2.20026 | 0.029785 | 0.245276 | -4.01478 |
| PALLD | 0.21372 | 4.399044 | 2.020943 | 0.045606 | 0.312132 | -4.37043 |
| IGF2BP2 | 0.213224 | 5.45176 | 2.340959 | 0.020955 | 0.198507 | -3.71636 |
| CENPO | 0.212738 | 4.71388 | 4.746142 | 6.01E-06 | 0.000523 | 3.69324 |
| BTG3 | 0.212704 | 4.559628 | 2.930715 | 0.004079 | 0.066645 | -2.28722 |
| MT1E | 0.211202 | 7.215718 | 2.878006 | 0.004771 | 0.074473 | -2.4263 |
| WDHD1 | 0.211115 | 3.17273 | 3.038401 | 0.002943 | 0.052972 | -1.99637 |
| CLIP1 | 0.210838 | 6.026533 | 2.647379 | 0.009248 | 0.115661 | -3.00903 |
| SGPP2 | 0.21072 | 3.685999 | 3.551826 | 0.000555 | 0.01583 | -0.49056 |
| FAM185A | 0.210191 | 5.26328 | 3.32338 | 0.001193 | 0.027333 | -1.18434 |
| HIATL1 | 0.210114 | 6.745099 | 2.306036 | 0.022898 | 0.210001 | -3.792 |
| BTBD6 | 0.209938 | 7.090034 | 2.367493 | 0.019577 | 0.189667 | -3.6582 |
| TDRP | 0.209781 | 4.414808 | 2.531964 | 0.01269 | 0.144018 | -3.28456 |
| NATD1 | 0.209069 | 7.509328 | 2.664474 | 0.008817 | 0.112133 | -2.9673 |
| UCHL1 | 0.208935 | 4.336952 | 3.881132 | 0.000174 | 0.006696 | 0.573253 |
| MXRA7 | 0.208767 | 5.728421 | 2.082428 | 0.039518 | 0.286037 | -4.25163 |
| XRN1 | 0.208746 | 7.000858 | 2.13048 | 0.03526 | 0.268014 | -4.15649 |
| ANP32B | 0.208584 | 9.953061 | 2.457339 | 0.015488 | 0.163993 | -3.45688 |
| WHSC1 | 0.208145 | 4.729274 | 3.506198 | 0.000649 | 0.01761 | -0.6321 |
| CASP3 | 0.20806 | 7.896552 | 2.305766 | 0.022914 | 0.210001 | -3.79258 |
| PTGDR2 | 0.20738 | 4.793643 | 2.753038 | 0.006863 | 0.094363 | -2.74733 |
| STAC | 0.206926 | 5.674033 | 2.022696 | 0.045422 | 0.311155 | -4.36709 |
| DEGS1 | 0.206764 | 9.841046 | 2.722978 | 0.007477 | 0.099605 | -2.82269 |
| PPM1A | 0.206669 | 7.536151 | 2.409737 | 0.017548 | 0.1782 | -3.56438 |
| PP13439 | 0.205953 | 4.315119 | 2.403899 | 0.017817 | 0.179806 | -3.57744 |
| THBS4 | 0.205543 | 5.355507 | 2.362076 | 0.019851 | 0.191243 | -3.67012 |
| PRDX2 | 0.205005 | 6.394078 | 3.957758 | 0.000131 | 0.005434 | 0.83115 |
| C1orf226 | 0.204241 | 4.708343 | 2.406304 | 0.017706 | 0.179178 | -3.57206 |
| APOBEC3( | 0.203634 | 7.345188 | 2.745235 | 0.007018 | 0.09573 | -2.76696 |
| UPB1 | 0.203484 | 5.183893 | 2.89709 | 0.004509 | 0.071862 | -2.37619 |
| MYL4 | 0.203127 | 5.941146 | 2.424937 | 0.016865 | 0.174117 | -3.53026 |
| ARNTL2 | 0.20229 | 3.727372 | 5.388014 | 3.83E-07 | 6.21E-05 | 6.272929 |
| RANBP9 | 0.202168 | 6.930168 | 2.027343 | 0.044937 | 0.308764 | -4.35822 |
| CALD1 | 0.201888 | 3.798039 | 3.869655 | 0.000181 | 0.00689 | 0.534956 |
| CCPG1 | 0.201791 | 9.114828 | 2.43058 | 0.016618 | 0.172472 | -3.51754 |
| MALL | 0.201221 | 4.446679 | 2.353723 | 0.020282 | 0.19394 | -3.68845 |
| HIRA | 0.201116 | 5.348463 | 3.11591 | 0.002315 | 0.044611 | -1.78154 |
| CCRN4L | 0.200983 | 4.51195 | 2.964965 | 0.003681 | 0.06224 | -2.19568 |
| NCOA4 | 0.200413 | 11.90953 | 2.652886 | 0.009107 | 0.114777 | -2.99561 |
| PHGDH | 0.199736 | 5.788252 | 2.843938 | 0.005274 | 0.079474 | -2.51504 |
| PCYT1B | 0.199722 | 4.086102 | 3.380858 | 0.000987 | 0.02366 | -1.0133 |
| PLEKHG3 | 0.199592 | 5.85726 | 2.799116 | 0.00601 | 0.086358 | -2.6304 |
| EFCAB11 | 0.199407 | 3.57376 | 3.297568 | 0.001298 | 0.029241 | -1.26037 |

| | | | | | | |
|---|---|---|---|---|---|---|
| LOC33966 | 0.199273 | 5.03265 | 2.35047 | 0.020451 | 0.195002 | -3.69558 |
| TMEM154 | 0.19921 | 10.57737 | 1.988871 | 0.049087 | 0.327304 | -4.43109 |
| CENPJ | 0.198585 | 3.728549 | 4.099883 | 7.73E-05 | 0.003663 | 1.319463 |
| RAB11A | 0.198361 | 8.485582 | 2.062904 | 0.041371 | 0.29453 | -4.28971 |
| RHOQ | 0.198249 | 7.940203 | 2.373698 | 0.019266 | 0.188087 | -3.64451 |
| HIST1H1B | 0.197237 | 3.333201 | 2.763918 | 0.006652 | 0.092155 | -2.71987 |
| VCPIP1 | 0.196572 | 6.687999 | 2.044214 | 0.043214 | 0.302925 | -4.32586 |
| IGLV6-57 | 0.196291 | 4.314986 | 2.38233 | 0.018842 | 0.185616 | -3.62542 |
| PGM1 | 0.196135 | 9.320252 | 2.120571 | 0.036104 | 0.271306 | -4.17627 |
| FHDC1 | 0.195919 | 5.578399 | 2.27673 | 0.02465 | 0.219575 | -3.85467 |
| STARD13 | 0.195145 | 3.690804 | 2.762333 | 0.006682 | 0.09245 | -2.72387 |
| TFRC | 0.194921 | 5.828529 | 2.249874 | 0.026357 | 0.227275 | -3.91147 |
| NEK7 | 0.194701 | 10.41061 | 2.400358 | 0.017982 | 0.180219 | -3.58534 |
| VEGFC | 0.194294 | 3.555016 | 2.342248 | 0.020886 | 0.198002 | -3.71355 |
| TOPORS-/ | 0.19404 | 7.618674 | 2.415851 | 0.017271 | 0.176697 | -3.55068 |
| FAM160B1 | 0.193805 | 6.523715 | 3.802669 | 0.000231 | 0.008318 | 0.313158 |
| SLC38A5 | 0.193578 | 5.089829 | 2.180493 | 0.031255 | 0.252167 | -4.05535 |
| KANK1 | 0.19271 | 5.039734 | 3.53335 | 0.000592 | 0.016472 | -0.54805 |
| KANK2 | 0.192231 | 4.664 | 2.925117 | 0.004148 | 0.067527 | -2.30209 |
| SYNE1 | 0.19222 | 6.122107 | 1.981317 | 0.049939 | 0.32976 | -4.44525 |
| TEX30 | 0.192055 | 5.085508 | 2.168919 | 0.032145 | 0.255711 | -4.07895 |
| SKA3 | 0.191468 | 4.790323 | 2.62423 | 0.009862 | 0.120482 | -3.06516 |
| LIPH | 0.191337 | 3.834449 | 2.80938 | 0.005834 | 0.084668 | -2.60412 |
| CEP63 | 0.191307 | 6.357014 | 2.209071 | 0.029149 | 0.242461 | -3.99658 |
| ATP2C2 | 0.190853 | 5.029564 | 2.114394 | 0.036639 | 0.273718 | -4.18856 |
| TDRG1 | 0.190548 | 6.02027 | 2.504125 | 0.013676 | 0.1525 | -3.34938 |
| MT1H | 0.190477 | 7.85519 | 2.55745 | 0.011844 | 0.137356 | -3.22465 |
| MANSC1 | 0.19046 | 10.09507 | 2.400544 | 0.017973 | 0.180219 | -3.58493 |
| CDC42BP/ | 0.190446 | 4.338752 | 5.137463 | 1.15E-06 | 0.000144 | 5.241936 |
| PDLIM5 | 0.190157 | 5.055939 | 3.694232 | 0.000339 | 0.011026 | -0.03954 |
| KCNH2 | 0.18985 | 4.651835 | 3.181533 | 0.001883 | 0.038459 | -1.5961 |
| STBD1 | 0.189516 | 3.281069 | 3.57515 | 0.000513 | 0.01482 | -0.41765 |
| MIS18A | 0.18933 | 4.576481 | 2.156401 | 0.033132 | 0.258662 | -4.10434 |
| FBXO34 | 0.189068 | 8.908975 | 2.376728 | 0.019116 | 0.187017 | -3.63782 |
| ZRANB1 | 0.188984 | 7.235634 | 1.988792 | 0.049096 | 0.327304 | -4.43124 |
| ABCG2 | 0.188955 | 3.494758 | 2.780333 | 0.006345 | 0.08948 | -2.67827 |
| QPRT | 0.188772 | 4.2456 | 3.22757 | 0.001626 | 0.034403 | -1.46409 |
| SPATS2 | 0.188763 | 4.217142 | 2.916746 | 0.004253 | 0.068755 | -2.32429 |
| C14orf1 | 0.188248 | 5.017343 | 2.677922 | 0.008491 | 0.109299 | -2.9343 |
| CEACAM3 | 0.188137 | 7.086109 | 2.116835 | 0.036427 | 0.272452 | -4.18371 |
| LDLR | 0.187905 | 5.746814 | 2.150229 | 0.033629 | 0.260752 | -4.11681 |
| SLC45A4 | 0.187761 | 8.01507 | 1.987557 | 0.049235 | 0.327409 | -4.43356 |
| FAM107B | 0.187696 | 10.39184 | 2.234884 | 0.027355 | 0.233171 | -3.9429 |
| GRAMD1B | 0.187247 | 5.912189 | 2.752835 | 0.006867 | 0.094363 | -2.74784 |
| ACKR1 | 0.187082 | 4.293474 | 2.444362 | 0.016027 | 0.167662 | -3.48637 |
| INCENP | 0.186884 | 4.37989 | 2.974827 | 0.003573 | 0.060564 | -2.16916 |
| SYNJ2 | 0.186562 | 4.964574 | 4.238253 | 4.57E-05 | 0.002377 | 1.807003 |
| GSTM5 | 0.185487 | 5.303474 | 2.415649 | 0.01728 | 0.176701 | -3.55113 |
| GABARAPI | 0.18539 | 11.40366 | 2.588933 | 0.010869 | 0.12848 | -3.14992 |
| ELOVL3 | 0.185107 | 3.504538 | 2.350787 | 0.020435 | 0.194935 | -3.69488 |
| HHEX | 0.184746 | 9.562093 | 2.061824 | 0.041476 | 0.294832 | -4.29181 |
| DSTN | 0.184676 | 6.509319 | 2.661606 | 0.008888 | 0.112686 | -2.97432 |
| RMND5A | 0.183157 | 7.169973 | 2.828455 | 0.005519 | 0.082186 | -2.55507 |
| GPRC5D | 0.183022 | 5.092599 | 2.89368 | 0.004555 | 0.072367 | -2.38517 |
| RUNDC3A | 0.182797 | 5.349766 | 2.649532 | 0.009193 | 0.115403 | -3.00379 |
| SLC44A1 | 0.18245 | 6.279398 | 2.117694 | 0.036353 | 0.272393 | -4.182 |
| RAD54B | 0.182159 | 3.807015 | 2.575397 | 0.011279 | 0.132318 | -3.18215 |
| ADI1 | 0.181975 | 7.003379 | 2.729056 | 0.007349 | 0.098476 | -2.80751 |

| | | | | | | |
|---|---|---|---|---|---|---|
| C15orf52 | 0.180929 | 5.197944 | 2.140722 | 0.034406 | 0.264268 | -4.13595 |
| ARHGEF5 | 0.180461 | 4.920151 | 2.95592 | 0.003782 | 0.063565 | -2.21994 |
| CSF1 | 0.180263 | 5.230258 | 4.36373 | 2.8E-05 | 0.001625 | 2.25916 |
| SLC17A5 | 0.180135 | 6.964059 | 2.162524 | 0.032646 | 0.256841 | -4.09193 |
| PRPSAP1 | 0.179777 | 6.324728 | 2.983075 | 0.003484 | 0.059317 | -2.14692 |
| DDX11 | 0.179748 | 5.283397 | 2.71427 | 0.007664 | 0.101371 | -2.84439 |
| GSG2 | 0.17974 | 4.725107 | 2.607444 | 0.01033 | 0.124096 | -3.1056 |
| ZNF660 | 0.179411 | 3.574666 | 3.251152 | 0.001508 | 0.032613 | -1.39586 |
| CENPH | 0.178496 | 4.557799 | 2.249268 | 0.026397 | 0.227521 | -3.91275 |
| VWF | 0.178452 | 4.728181 | 2.556086 | 0.011888 | 0.137508 | -3.22787 |
| TSTD1 | 0.178133 | 8.524781 | 2.403171 | 0.017851 | 0.179806 | -3.57906 |
| KDELC1 | 0.178008 | 3.782933 | 3.05063 | 0.002835 | 0.051334 | -1.96278 |
| GPR157 | 0.177882 | 4.798165 | 3.347613 | 0.001102 | 0.025678 | -1.11253 |
| LRRC20 | 0.177672 | 5.045697 | 2.362739 | 0.019818 | 0.191187 | -3.66866 |
| H2AFX | 0.177507 | 5.25458 | 2.909755 | 0.004343 | 0.070067 | -2.34278 |
| CA3 | 0.177313 | 2.620223 | 2.136313 | 0.034771 | 0.26543 | -4.1448 |
| LOC10192 | 0.177257 | 3.315486 | 2.134018 | 0.034963 | 0.266448 | -4.14941 |
| RNASEL | 0.176862 | 8.147662 | 2.112179 | 0.036833 | 0.274237 | -4.19296 |
| APLN | 0.17659 | 5.295974 | 2.392448 | 0.018355 | 0.182424 | -3.60296 |
| EME1 | 0.175904 | 5.119465 | 2.040874 | 0.04355 | 0.30406 | -4.33228 |
| LOC10159 | 0.175729 | 4.588536 | 2.274926 | 0.024762 | 0.219809 | -3.85851 |
| STOX2 | 0.175496 | 3.571778 | 4.173515 | 5.85E-05 | 0.002894 | 1.577435 |
| FOXC1 | 0.175419 | 4.627616 | 1.982756 | 0.049776 | 0.32976 | -4.44255 |
| 4-Sep | 0.174922 | 4.183584 | 2.301803 | 0.023144 | 0.211146 | -3.8011 |
| C4orf32 | 0.174755 | 5.783572 | 2.389528 | 0.018494 | 0.183347 | -3.60945 |
| TRIM5 | 0.174594 | 5.255481 | 2.22686 | 0.027902 | 0.235984 | -3.95965 |
| TRIB3 | 0.174075 | 5.181578 | 2.891227 | 0.004588 | 0.072629 | -2.39162 |
| CENPA | 0.173805 | 4.925192 | 2.496852 | 0.013945 | 0.153985 | -3.36621 |
| DERL3 | 0.173197 | 5.269873 | 2.764324 | 0.006644 | 0.092111 | -2.71884 |
| AMPD1 | 0.173061 | 3.194205 | 3.500343 | 0.000662 | 0.017915 | -0.65015 |
| KIF1B | 0.172952 | 7.802192 | 2.321606 | 0.022013 | 0.205207 | -3.7584 |
| FANCA | 0.172883 | 5.041401 | 3.473535 | 0.000725 | 0.019158 | -0.73251 |
| ADAR | 0.172597 | 11.69393 | 2.362192 | 0.019846 | 0.191243 | -3.66986 |
| C11orf21 | 0.172125 | 6.920939 | 2.505973 | 0.013608 | 0.151913 | -3.3451 |
| PARP2 | 0.172038 | 5.13398 | 2.151191 | 0.033551 | 0.260538 | -4.11487 |
| GFOD2 | 0.171958 | 6.565863 | 2.116887 | 0.036423 | 0.272452 | -4.18361 |
| IRAK2 | 0.171251 | 4.325952 | 3.582869 | 0.000499 | 0.014534 | -0.39344 |
| HIST1H2B( | 0.169937 | 3.309835 | 2.422237 | 0.016985 | 0.175174 | -3.53633 |
| RAD51C | 0.169341 | 5.519622 | 2.012559 | 0.046495 | 0.315847 | -4.38638 |
| SLCO3A1 | 0.169338 | 8.299328 | 1.995528 | 0.048347 | 0.324244 | -4.41857 |
| ARHGAP1: | 0.169201 | 3.087851 | 3.085539 | 0.002545 | 0.047514 | -1.86626 |
| MYCT1 | 0.169108 | 4.180456 | 2.094372 | 0.038421 | 0.281275 | -4.22817 |
| PLEK2 | 0.168975 | 6.006947 | 2.1741 | 0.031744 | 0.254526 | -4.0684 |
| TTF2 | 0.168633 | 3.764511 | 2.21959 | 0.028406 | 0.238802 | -3.97478 |
| CKAP2 | 0.168425 | 4.447132 | 2.223625 | 0.028125 | 0.237026 | -3.96639 |
| IL15RA | 0.167868 | 6.362565 | 2.031202 | 0.044538 | 0.30795 | -4.35084 |
| RAB11FIP5 | 0.167425 | 5.556378 | 3.018643 | 0.003127 | 0.055308 | -2.0504 |
| DAPK2 | 0.167303 | 6.543804 | 2.248875 | 0.026423 | 0.227549 | -3.91357 |
| VSIG10 | 0.167116 | 3.850314 | 2.416397 | 0.017246 | 0.176618 | -3.54945 |
| LINC00899 | 0.166971 | 4.2591 | 2.145548 | 0.034009 | 0.262208 | -4.12625 |
| LRRCC1 | 0.166776 | 3.861699 | 2.096469 | 0.038231 | 0.280889 | -4.22404 |
| LYPD3 | 0.166623 | 5.566841 | 2.606977 | 0.010343 | 0.12413 | -3.10672 |
| RSPH1 | 0.166084 | 4.284031 | 2.494256 | 0.014042 | 0.154639 | -3.37221 |
| SPNS2 | 0.165554 | 5.966305 | 2.175429 | 0.031642 | 0.253906 | -4.06569 |
| TIMELESS | 0.165171 | 4.540495 | 2.872632 | 0.004848 | 0.075105 | -2.44036 |
| CASP10 | 0.164316 | 5.46042 | 3.451111 | 0.000781 | 0.020291 | -0.80102 |
| CHAF1A | 0.163162 | 4.988292 | 3.404195 | 0.000914 | 0.022436 | -0.94318 |
| AFF2 | 0.161782 | 4.129336 | 3.582362 | 0.0005 | 0.014539 | -0.39503 |

| | | | | | | |
|---|---|---|---|---|---|---|
| CLGN | 0.161772 | 2.785254 | 2.323893 | 0.021885 | 0.204298 | -3.75345 |
| SPATS2L | 0.161585 | 3.876808 | 2.3515 | 0.020397 | 0.194669 | -3.69332 |
| MYO1B | 0.161567 | 3.715075 | 3.548002 | 0.000563 | 0.01599 | -0.50248 |
| REXO2 | 0.161213 | 4.973149 | 2.184677 | 0.030939 | 0.25071 | -4.04679 |
| CNKSR1 | 0.161146 | 4.243026 | 2.349135 | 0.020521 | 0.195396 | -3.6985 |
| CDR2L | 0.161141 | 3.733386 | 2.447241 | 0.015906 | 0.166897 | -3.47984 |
| PDXK | 0.161134 | 7.027943 | 3.07343 | 0.002642 | 0.048884 | -1.89984 |
| CR1L | 0.160758 | 3.810018 | 2.027407 | 0.04493 | 0.308764 | -4.3581 |
| PHLDA1 | 0.16012 | 4.489091 | 3.093661 | 0.002482 | 0.0468 | -1.84367 |
| LOC64648 | 0.16012 | 3.36644 | 2.945326 | 0.003905 | 0.064671 | -2.24828 |
| EGLN3 | 0.160059 | 4.302224 | 2.139602 | 0.034498 | 0.264582 | -4.1382 |
| IL2RA | 0.159802 | 4.572802 | 2.456089 | 0.015539 | 0.164237 | -3.45972 |
| HIAT1 | 0.1598 | 9.527229 | 2.185925 | 0.030845 | 0.25061 | -4.04423 |
| MAP7D2 | 0.159758 | 3.313187 | 3.127091 | 0.002236 | 0.043363 | -1.75017 |
| TNPO1 | 0.159214 | 7.332664 | 2.948333 | 0.00387 | 0.064372 | -2.24025 |
| NXF3 | 0.159203 | 3.764094 | 2.097221 | 0.038163 | 0.28049 | -4.22255 |
| GSN | 0.158976 | 5.609871 | 2.153376 | 0.033375 | 0.259862 | -4.11046 |
| NAV1 | 0.158862 | 4.522103 | 3.612214 | 0.000451 | 0.013499 | -0.30101 |
| DIAPH2 | 0.158608 | 5.593786 | 2.093163 | 0.038531 | 0.281676 | -4.23055 |
| OTUD1 | 0.158437 | 6.617752 | 2.282245 | 0.024312 | 0.21809 | -3.84294 |
| GML | 0.158158 | 3.781272 | 2.16864 | 0.032167 | 0.255711 | -4.07951 |
| GTF3C5 | 0.158131 | 6.495951 | 2.155272 | 0.033223 | 0.258973 | -4.10662 |
| C7orf43 | 0.158113 | 7.981682 | 2.056275 | 0.042017 | 0.297418 | -4.30257 |
| EPB41 | 0.157817 | 5.897376 | 2.654022 | 0.009079 | 0.114671 | -2.99284 |
| ARL8B | 0.157613 | 8.982342 | 2.107846 | 0.037214 | 0.276396 | -4.20155 |
| MAST1 | 0.157576 | 4.202582 | 2.251312 | 0.026263 | 0.22656 | -3.90845 |
| YAF2 | 0.157443 | 4.389342 | 1.988687 | 0.049108 | 0.327304 | -4.43143 |
| PRPF18 | 0.157442 | 6.030043 | 2.437541 | 0.016317 | 0.169925 | -3.50182 |
| PROP1 | 0.157387 | 4.61916 | 2.328088 | 0.021653 | 0.202965 | -3.74436 |
| TRIM35 | 0.15698 | 5.482787 | 2.140536 | 0.034421 | 0.264287 | -4.13633 |
| LOC10012 | 0.15642 | 3.780111 | 2.648813 | 0.009211 | 0.115493 | -3.00554 |
| SERPINE1 | 0.15635 | 3.921267 | 3.513074 | 0.000634 | 0.017342 | -0.61086 |
| UPK1A | 0.155549 | 3.358131 | 2.894839 | 0.00454 | 0.072287 | -2.38212 |
| PDE6A | 0.1549 | 4.002605 | 2.399029 | 0.018044 | 0.180546 | -3.5883 |
| RCOR1 | 0.154763 | 7.65661 | 2.935427 | 0.004022 | 0.065963 | -2.27468 |
| SEC11C | 0.154115 | 6.034729 | 2.104698 | 0.037493 | 0.27749 | -4.20779 |
| ZC3H12C | 0.153952 | 3.140342 | 2.408818 | 0.01759 | 0.178361 | -3.56644 |
| SPC24 | 0.153609 | 4.353389 | 2.797456 | 0.006039 | 0.086533 | -2.63464 |
| PRPH2 | 0.153265 | 4.747112 | 2.697108 | 0.008045 | 0.104898 | -2.88698 |
| APOL5 | 0.153245 | 4.184166 | 2.058606 | 0.041789 | 0.296182 | -4.29805 |
| CYBB | 0.153192 | 6.70874 | 2.849545 | 0.005188 | 0.078934 | -2.5005 |
| RNF217 | 0.152557 | 3.801595 | 2.326013 | 0.021768 | 0.203945 | -3.74886 |
| LEP | 0.152445 | 3.975193 | 2.151515 | 0.033525 | 0.260538 | -4.11421 |
| GLRX2 | 0.151122 | 7.334235 | 2.184728 | 0.030935 | 0.25071 | -4.04668 |
| FANCE | 0.149841 | 6.039858 | 2.495009 | 0.014013 | 0.154578 | -3.37047 |
| PCBP4 | 0.149383 | 4.874086 | 2.410725 | 0.017503 | 0.178095 | -3.56217 |
| LOC10012 | 0.149103 | 4.595964 | 2.122388 | 0.035948 | 0.270768 | -4.17265 |
| GOT1 | 0.148973 | 5.791159 | 2.038795 | 0.043761 | 0.304719 | -4.33628 |
| 11-Sep | 0.148898 | 4.451987 | 2.040324 | 0.043606 | 0.304292 | -4.33334 |
| TMEM217 | 0.148848 | 3.721539 | 2.208609 | 0.029182 | 0.242638 | -3.99754 |
| GLRX | 0.148729 | 10.64977 | 2.363404 | 0.019784 | 0.190951 | -3.6672 |
| DGKG | 0.14845 | 4.047731 | 2.562086 | 0.011696 | 0.135975 | -3.2137 |
| CCNE1 | 0.148175 | 4.698833 | 2.838073 | 0.005366 | 0.080376 | -2.53023 |
| PRR15L | 0.14794 | 4.365415 | 2.820761 | 0.005644 | 0.083442 | -2.57489 |
| SLC18A2 | 0.147432 | 3.989478 | 3.126171 | 0.002242 | 0.043447 | -1.75276 |
| KLHDC8A | 0.147374 | 4.414741 | 2.861326 | 0.005012 | 0.076993 | -2.46986 |
| TRMT5 | 0.147364 | 7.338766 | 2.197195 | 0.030009 | 0.24621 | -4.02109 |
| DNAJC6 | 0.146786 | 3.087178 | 2.099145 | 0.037989 | 0.279835 | -4.21876 |

| | | | | | | |
|---|---|---|---|---|---|---|
| GATA2 | 0.146481 | 5.781036 | 3.144442 | 0.002117 | 0.041864 | -1.70131 |
| NMI | 0.14619 | 10.96207 | 2.166867 | 0.032305 | 0.256214 | -4.08312 |
| AIM1L | 0.145961 | 4.858404 | 3.307134 | 0.001258 | 0.028565 | -1.23226 |
| VKORC1L1 | 0.145709 | 4.645173 | 2.198299 | 0.029928 | 0.245842 | -4.01882 |
| CYAT1 | 0.14509 | 4.880716 | 2.109546 | 0.037064 | 0.275582 | -4.19818 |
| MT1G | 0.144592 | 5.144125 | 2.894189 | 0.004548 | 0.072367 | -2.38383 |
| LOC10050 | 0.144354 | 5.417308 | 2.305994 | 0.022901 | 0.210001 | -3.79209 |
| FAM201A | 0.144274 | 2.96926 | 2.990316 | 0.003409 | 0.058564 | -2.12735 |
| ABCG4 | 0.143806 | 5.646178 | 2.362512 | 0.019829 | 0.191209 | -3.66916 |
| RAD51D | 0.143767 | 4.304802 | 2.573184 | 0.011347 | 0.132901 | -3.1874 |
| PSG6 | 0.143241 | 3.639811 | 3.965121 | 0.000128 | 0.005332 | 0.856131 |
| TIMD4 | 0.143138 | 3.455361 | 2.238604 | 0.027104 | 0.232246 | -3.93512 |
| LIPE | 0.142877 | 4.946222 | 1.99667 | 0.04822 | 0.32361 | -4.41642 |
| SPTB | 0.142551 | 5.638723 | 2.04213 | 0.043423 | 0.303459 | -4.32987 |
| EXOC3L2 | 0.142193 | 7.272437 | 2.864138 | 0.004971 | 0.076589 | -2.46253 |
| GUSBP11 | 0.141958 | 6.781386 | 2.729202 | 0.007346 | 0.098476 | -2.80715 |
| SLC7A11 | 0.141614 | 3.919094 | 2.24871 | 0.026434 | 0.227549 | -3.91392 |
| IQGAP2 | 0.141454 | 7.046214 | 2.229219 | 0.02774 | 0.235233 | -3.95474 |
| CDC25C | 0.141164 | 3.442638 | 3.195736 | 0.0018 | 0.037284 | -1.55554 |
| SLC16A1 | 0.141079 | 4.141268 | 1.992932 | 0.048634 | 0.325746 | -4.42346 |
| HTATIP2 | 0.14039 | 7.103616 | 2.583522 | 0.011031 | 0.130022 | -3.16282 |
| DNM1 | 0.140361 | 4.324965 | 2.22082 | 0.02832 | 0.238276 | -3.97222 |
| ACTA2 | 0.140332 | 5.168076 | 2.32548 | 0.021797 | 0.204128 | -3.75001 |
| LOC10192 | 0.140002 | 3.835204 | 2.840322 | 0.005331 | 0.080025 | -2.5244 |
| LOC10192 | 0.139863 | 4.809134 | 2.421751 | 0.017007 | 0.175309 | -3.53743 |
| LOC14970 | 0.139679 | 4.067972 | 2.676384 | 0.008528 | 0.109484 | -2.93808 |
| IQGAP3 | 0.139542 | 3.835384 | 3.26352 | 0.001449 | 0.031708 | -1.35992 |
| CDKL1 | 0.138586 | 4.004767 | 2.676352 | 0.008529 | 0.109484 | -2.93816 |
| AMOTL1 | 0.138584 | 4.322451 | 3.090324 | 0.002507 | 0.047071 | -1.85296 |
| DARS2 | 0.138431 | 5.097821 | 2.18179 | 0.031157 | 0.251786 | -4.05269 |
| ZBTB7C | 0.138105 | 3.963036 | 3.148972 | 0.002087 | 0.041588 | -1.68852 |
| NEURL1B | 0.138038 | 4.62423 | 2.035818 | 0.044064 | 0.306263 | -4.34199 |
| CRYAB | 0.137975 | 4.236227 | 2.466487 | 0.015118 | 0.16168 | -3.436 |
| SMIM10 | 0.13783 | 3.389574 | 3.397803 | 0.000934 | 0.022795 | -0.96242 |
| DEFA6 | 0.137729 | 5.02618 | 2.461458 | 0.01532 | 0.162511 | -3.44749 |
| LOC10050 | 0.137688 | 4.692444 | 2.625349 | 0.009832 | 0.120408 | -3.06246 |
| C1orf112 | 0.137257 | 3.369864 | 2.257906 | 0.025836 | 0.224583 | -3.89455 |
| SYCE2 | 0.137066 | 4.228662 | 2.317434 | 0.022247 | 0.20589 | -3.76742 |
| SLC30A3 | 0.136681 | 6.587649 | 2.187412 | 0.030734 | 0.250329 | -4.04118 |
| STX1B | 0.136063 | 5.626717 | 2.463628 | 0.015233 | 0.162158 | -3.44253 |
| LRRTM2 | 0.135829 | 3.120045 | 2.516689 | 0.013223 | 0.1485 | -3.3202 |
| PPEF1 | 0.135585 | 3.864693 | 2.217631 | 0.028543 | 0.239227 | -3.97885 |
| SNTB1 | 0.135105 | 5.026762 | 2.346289 | 0.020671 | 0.196641 | -3.70472 |
| SVOPL | 0.134481 | 3.338331 | 2.629365 | 0.009723 | 0.119913 | -3.05275 |
| SLC35C2 | 0.13438 | 5.870055 | 2.447367 | 0.015901 | 0.166897 | -3.47955 |
| GALNT3 | 0.134309 | 6.581783 | 2.116401 | 0.036465 | 0.272613 | -4.18457 |
| ST3GAL6 | 0.133784 | 5.074491 | 2.101746 | 0.037756 | 0.278794 | -4.21362 |
| TMEM17 | 0.133703 | 3.278603 | 2.158967 | 0.032928 | 0.258149 | -4.09914 |
| ZNF385D | 0.13346 | 4.074779 | 2.006437 | 0.047153 | 0.318749 | -4.39798 |
| LOC10192 | 0.13342 | 3.471218 | 2.16485 | 0.032463 | 0.256336 | -4.08721 |
| C14orf119 | 0.133291 | 8.934475 | 2.165639 | 0.032401 | 0.256336 | -4.08561 |
| KRTAP4-9 | 0.133231 | 3.373879 | 3.141969 | 0.002134 | 0.042064 | -1.70829 |
| LOC72817 | 0.133182 | 3.593277 | 2.696715 | 0.008054 | 0.104902 | -2.88795 |
| GSX2 | 0.132976 | 4.543198 | 2.138885 | 0.034558 | 0.264739 | -4.13964 |
| RAB3IP | 0.132955 | 3.999201 | 2.367735 | 0.019565 | 0.189667 | -3.65766 |
| RUFY1 | 0.132801 | 6.178549 | 2.05795 | 0.041853 | 0.296533 | -4.29932 |
| ERI2 | 0.132701 | 3.553697 | 2.420953 | 0.017042 | 0.175321 | -3.53922 |
| CD8B | 0.13204 | 3.732729 | 1.985622 | 0.049452 | 0.328429 | -4.43718 |

| | | | | | | |
|---|---|---|---|---|---|---|
| WDR26 | 0.131913 | 8.098392 | 2.165449 | 0.032416 | 0.256336 | -4.086 |
| KEL | 0.131503 | 5.554725 | 2.012201 | 0.046533 | 0.315913 | -4.38706 |
| SPTBN1 | 0.13125 | 4.534839 | 3.367632 | 0.001032 | 0.024374 | -1.05287 |
| PAQR4 | 0.131043 | 6.382441 | 2.126358 | 0.035609 | 0.269758 | -4.16473 |
| LOC10192 | 0.130841 | 3.22417 | 2.952496 | 0.003821 | 0.063856 | -2.22911 |
| SLC6A7 | 0.130747 | 5.470374 | 2.124194 | 0.035794 | 0.270262 | -4.16905 |
| LOC10050 | 0.130655 | 3.29231 | 3.410602 | 0.000895 | 0.022122 | -0.92386 |
| RASL11A | 0.130056 | 4.525704 | 2.027211 | 0.044951 | 0.308764 | -4.35847 |
| N4BP1 | 0.129951 | 9.316597 | 2.292295 | 0.023705 | 0.214629 | -3.82148 |
| RAB10 | 0.129798 | 10.84636 | 2.013692 | 0.046374 | 0.31546 | -4.38423 |
| AKAP12 | 0.129741 | 3.447615 | 3.052109 | 0.002822 | 0.051146 | -1.95871 |
| SLC13A5 | 0.129332 | 4.122349 | 2.188862 | 0.030625 | 0.249963 | -4.03821 |
| PELI2 | 0.129299 | 6.602439 | 2.238395 | 0.027118 | 0.232269 | -3.93556 |
| AFAP1L2 | 0.129227 | 3.272637 | 2.270283 | 0.025051 | 0.221102 | -3.86837 |
| HOXD4 | 0.129097 | 3.649963 | 2.162157 | 0.032675 | 0.256841 | -4.09268 |
| ZNF521 | 0.128628 | 2.877364 | 2.478249 | 0.014653 | 0.159148 | -3.40906 |
| SCG5 | 0.128347 | 3.139037 | 2.278945 | 0.024514 | 0.218847 | -3.84996 |
| ST7-AS1 | 0.127891 | 3.559641 | 2.170035 | 0.032059 | 0.255711 | -4.07667 |
| KIAA2018 | 0.12736 | 5.722005 | 2.542623 | 0.01233 | 0.141182 | -3.25957 |
| HRASLS5 | 0.127252 | 4.602524 | 2.209882 | 0.029092 | 0.242177 | -3.99491 |
| MS4A2 | 0.126935 | 3.243823 | 2.957884 | 0.00376 | 0.063426 | -2.21468 |
| POM121L8 | 0.126905 | 3.257894 | 2.320442 | 0.022078 | 0.205379 | -3.76092 |
| LMAN1 | 0.12678 | 5.212008 | 2.040837 | 0.043554 | 0.30406 | -4.33235 |
| LINC01282 | 0.12655 | 3.407265 | 2.027198 | 0.044952 | 0.308764 | -4.3585 |
| CRHBP | 0.126164 | 3.681472 | 2.482106 | 0.014504 | 0.158433 | -3.4002 |
| DRD5 | 0.125889 | 4.668798 | 2.484251 | 0.014421 | 0.1578 | -3.39526 |
| SHISA2 | 0.125713 | 2.661062 | 3.1763 | 0.001915 | 0.038986 | -1.61101 |
| HARBI1 | 0.125309 | 5.011505 | 2.349409 | 0.020507 | 0.19535 | -3.6979 |
| FLJ12825 | 0.125261 | 3.415198 | 2.245814 | 0.026624 | 0.228901 | -3.92 |
| ECSCR | 0.124946 | 3.921361 | 2.017908 | 0.045926 | 0.313768 | -4.37621 |
| DCAF6 | 0.124861 | 5.818527 | 2.868062 | 0.004914 | 0.075766 | -2.4523 |
| PPIC | 0.12449 | 3.190464 | 2.737537 | 0.007173 | 0.097144 | -2.78628 |
| TRAM2 | 0.124339 | 5.641948 | 2.23512 | 0.027339 | 0.233171 | -3.94241 |
| LRIG1 | 0.124229 | 4.091083 | 3.530542 | 0.000597 | 0.016607 | -0.55677 |
| HESX1 | 0.124184 | 3.410278 | 2.670475 | 0.008671 | 0.110678 | -2.95259 |
| SSTR2 | 0.123586 | 3.759377 | 2.603078 | 0.010455 | 0.124963 | -3.11608 |
| MRAP2 | 0.123561 | 3.146923 | 2.680131 | 0.008439 | 0.108875 | -2.92887 |
| NAT6 | 0.123027 | 5.854924 | 2.205346 | 0.029417 | 0.243793 | -4.00428 |
| ANKRD18B | 0.123008 | 4.126024 | 2.412995 | 0.0174 | 0.177486 | -3.55708 |
| CCDC144C | 0.122909 | 2.69011 | 3.275112 | 0.001396 | 0.030772 | -1.32612 |
| SLC8A3 | 0.122888 | 4.595192 | 2.403491 | 0.017836 | 0.179806 | -3.57835 |
| LY6G6C | 0.122832 | 5.328939 | 2.002924 | 0.047535 | 0.320921 | -4.40462 |
| S100A16 | 0.122483 | 5.862665 | 2.374608 | 0.019221 | 0.187825 | -3.6425 |
| TTLL7 | 0.122172 | 3.218222 | 3.143612 | 0.002123 | 0.041928 | -1.70365 |
| CYP1A2 | 0.122109 | 4.652535 | 3.0658 | 0.002705 | 0.049692 | -1.92095 |
| LOC28612 | 0.121737 | 3.744737 | 2.042252 | 0.043411 | 0.303459 | -4.32963 |
| MED12L | 0.12154 | 3.882033 | 2.751985 | 0.006883 | 0.09453 | -2.74998 |
| LOC10192 | 0.121447 | 4.709092 | 3.012681 | 0.003184 | 0.055932 | -2.06665 |
| KIF26A | 0.121425 | 3.476295 | 2.702257 | 0.007929 | 0.103564 | -2.87423 |
| CHDH | 0.121078 | 4.551659 | 2.387458 | 0.018594 | 0.183817 | -3.61404 |
| ACAT1 | 0.119808 | 4.592342 | 2.792138 | 0.006133 | 0.087385 | -2.64821 |
| CLSPN | 0.119215 | 3.233582 | 3.808158 | 0.000226 | 0.008205 | 0.331221 |
| P2RY4 | 0.119136 | 3.946483 | 2.200109 | 0.029796 | 0.245276 | -4.01509 |
| ESCO2 | 0.119091 | 3.023196 | 4.284883 | 3.81E-05 | 0.002092 | 1.973933 |
| STMN4 | 0.118539 | 4.250784 | 2.595796 | 0.010666 | 0.126932 | -3.13352 |
| HIST1H3F | 0.118487 | 3.339244 | 2.947173 | 0.003883 | 0.064424 | -2.24335 |
| PSG9 | 0.118353 | 4.388784 | 2.617295 | 0.010053 | 0.122224 | -3.0819 |
| CENPI | 0.118221 | 3.032029 | 3.737266 | 0.000291 | 0.009931 | 0.099484 |

| | | | | | | |
|---|---|---|---|---|---|---|
| SLC43A1 | 0.118165 | 5.25516 | 2.07412 | 0.040298 | 0.289832 | -4.26787 |
| NDFIP2 | 0.117473 | 3.524 | 2.090254 | 0.038796 | 0.282718 | -4.23627 |
| CCL1 | 0.11746 | 4.33865 | 2.016471 | 0.046078 | 0.314345 | -4.37894 |
| PHACTR3 | 0.117435 | 3.064557 | 2.235931 | 0.027284 | 0.233125 | -3.94072 |
| FAM26E | 0.117377 | 5.181417 | 2.465234 | 0.015168 | 0.161747 | -3.43887 |
| PCDHGB5 | 0.116748 | 5.207788 | 2.826673 | 0.005547 | 0.082494 | -2.55966 |
| LOC14679 | 0.11661 | 3.646205 | 2.228685 | 0.027777 | 0.235445 | -3.95585 |
| ZBTB8A | 0.116572 | 2.901125 | 2.484852 | 0.014398 | 0.157716 | -3.39388 |
| C18orf12 | 0.115135 | 3.7192 | 2.234864 | 0.027356 | 0.233171 | -3.94295 |
| TIMP3 | 0.114907 | 4.29117 | 2.429315 | 0.016673 | 0.172761 | -3.5204 |
| CMBL | 0.114712 | 6.020202 | 1.989079 | 0.049064 | 0.327304 | -4.4307 |
| VDR | 0.11453 | 6.337017 | 2.737203 | 0.00718 | 0.097172 | -2.78712 |
| LOC10192 | 0.11426 | 4.387834 | 2.000104 | 0.047843 | 0.321922 | -4.40995 |
| AKAP4 | 0.114162 | 3.945621 | 1.981147 | 0.049959 | 0.32976 | -4.44556 |
| LOC72794 | 0.11408 | 3.69963 | 2.104385 | 0.037521 | 0.27749 | -4.20841 |
| EAF2 | 0.114066 | 4.190325 | 2.133279 | 0.035025 | 0.266721 | -4.15089 |
| TRIM10 | 0.113999 | 3.862432 | 2.744765 | 0.007027 | 0.095789 | -2.76814 |
| LOC10050 | 0.113762 | 5.430908 | 2.076183 | 0.040103 | 0.288933 | -4.26385 |
| MUC6 | 0.113676 | 4.107045 | 2.534121 | 0.012616 | 0.14358 | -3.27951 |
| NPR3 | 0.113585 | 3.692192 | 2.439909 | 0.016216 | 0.169118 | -3.49646 |
| RNF186 | 0.113569 | 4.342366 | 2.530807 | 0.01273 | 0.144229 | -3.28726 |
| HIST1H2BI | 0.113221 | 3.996384 | 2.399183 | 0.018037 | 0.180546 | -3.58796 |
| MAMDC2 | 0.11318 | 2.504542 | 3.002419 | 0.003286 | 0.057001 | -2.09455 |
| CD34 | 0.113075 | 5.06982 | 2.085653 | 0.039219 | 0.284975 | -4.24531 |
| HIST1H2A | 0.113039 | 3.013812 | 2.020196 | 0.045684 | 0.312431 | -4.37186 |
| PINLYP | 0.113031 | 4.240987 | 2.068568 | 0.040826 | 0.291989 | -4.2787 |
| HSF2BP | 0.112789 | 4.020879 | 2.263571 | 0.025474 | 0.222566 | -3.88258 |
| AGBL5 | 0.112486 | 4.468741 | 2.359653 | 0.019975 | 0.192075 | -3.67544 |
| KIF24 | 0.112363 | 2.80589 | 2.984113 | 0.003473 | 0.059279 | -2.14411 |
| LOC28493 | 0.112247 | 4.301498 | 2.730489 | 0.007319 | 0.098333 | -2.80393 |
| CARHSP1 | 0.112108 | 6.059022 | 2.167155 | 0.032283 | 0.256143 | -4.08253 |
| SPRY4 | 0.111702 | 4.987107 | 2.631027 | 0.009678 | 0.119514 | -3.04873 |
| KCNN3 | 0.111668 | 3.954901 | 2.218466 | 0.028485 | 0.239168 | -3.97711 |
| C21orf58 | 0.111658 | 4.340902 | 2.81114 | 0.005804 | 0.0846 | -2.59961 |
| DNAH2 | 0.111349 | 4.139814 | 2.700317 | 0.007973 | 0.104039 | -2.87903 |
| ICA1 | 0.11069 | 4.581599 | 2.672229 | 0.008628 | 0.11048 | -2.94829 |
| LOC10192 | 0.110574 | 5.118127 | 2.011464 | 0.046612 | 0.316239 | -4.38845 |
| AQP1 | 0.109961 | 3.744574 | 2.280621 | 0.024411 | 0.218462 | -3.8464 |
| SH2D4A | 0.109923 | 4.749821 | 2.06261 | 0.041399 | 0.29453 | -4.29028 |
| OR2S2 | 0.109907 | 4.157919 | 2.185839 | 0.030852 | 0.25061 | -4.04441 |
| LOC33980 | 0.109777 | 4.660109 | 2.401442 | 0.017931 | 0.180034 | -3.58292 |
| GLRA1 | 0.109407 | 3.25665 | 1.991663 | 0.048775 | 0.326255 | -4.42585 |
| ARSD | 0.109279 | 5.608081 | 1.983829 | 0.049655 | 0.329468 | -4.44054 |
| RCL1 | 0.109263 | 4.892985 | 2.68798 | 0.008255 | 0.106903 | -2.90953 |
| LINC01351 | 0.108336 | 3.206424 | 2.085361 | 0.039246 | 0.284975 | -4.24588 |
| GPRIN2 | 0.108044 | 5.028469 | 2.633296 | 0.009617 | 0.118979 | -3.04323 |
| OPN1SW | 0.107879 | 3.639214 | 2.061522 | 0.041505 | 0.294832 | -4.29239 |
| LINC00997 | 0.107675 | 3.5346 | 2.625082 | 0.009839 | 0.120408 | -3.06311 |
| NRIP3 | 0.107425 | 4.620491 | 2.474739 | 0.01479 | 0.159959 | -3.41711 |
| SLC6A4 | 0.107195 | 3.284621 | 2.097897 | 0.038102 | 0.280412 | -4.22122 |
| HMGB2 | 0.106988 | 8.032505 | 2.049964 | 0.042639 | 0.300547 | -4.31477 |
| LOC28581 | 0.106209 | 3.694097 | 2.392411 | 0.018357 | 0.182424 | -3.60304 |
| LOC10192 | 0.106058 | 3.270449 | 2.224873 | 0.028039 | 0.236688 | -3.96379 |
| OR10D3 | 0.106009 | 4.61348 | 2.136224 | 0.034779 | 0.26543 | -4.14498 |
| AAMDC | 0.105984 | 4.722327 | 2.231719 | 0.027569 | 0.234661 | -3.94952 |
| ART4 | 0.105856 | 3.103544 | 2.083815 | 0.03939 | 0.285395 | -4.24891 |
| HIST1H3C | 0.105639 | 4.358199 | 2.217532 | 0.02855 | 0.239227 | -3.97905 |
| BDH1 | 0.1055 | 4.803889 | 2.086477 | 0.039143 | 0.284646 | -4.24369 |

| | | | | | | |
|---|---|---|---|---|---|---|
| TRIM69 | 0.104831 | 5.574207 | 2.169399 | 0.032108 | 0.255711 | -4.07797 |
| HIST1H4A | 0.104493 | 3.650446 | 2.331564 | 0.021463 | 0.201639 | -3.73681 |
| THBS2 | 0.104015 | 3.779102 | 2.50298 | 0.013718 | 0.152636 | -3.35203 |
| FAM162B | 0.103848 | 3.359965 | 2.46996 | 0.014979 | 0.160979 | -3.42806 |
| AR | 0.10318 | 3.934161 | 2.880308 | 0.004739 | 0.074195 | -2.42027 |
| CAPN13 | 0.102975 | 4.10833 | 2.576963 | 0.011231 | 0.131844 | -3.17843 |
| OSMR | 0.102413 | 3.311221 | 3.295624 | 0.001306 | 0.02933 | -1.26608 |
| CASC17 | 0.102383 | 3.548938 | 2.528595 | 0.012806 | 0.144851 | -3.29243 |
| C15orf27 | 0.102215 | 3.3689 | 2.199669 | 0.029828 | 0.245276 | -4.01599 |
| EFNB3 | 0.10221 | 4.732089 | 2.434469 | 0.016449 | 0.170942 | -3.50876 |
| BOD1L2 | 0.10208 | 3.669395 | 2.213931 | 0.028804 | 0.240762 | -3.98652 |
| LOC10192 | 0.101877 | 2.71375 | 2.171709 | 0.031929 | 0.255523 | -4.07327 |
| LINC0118( | 0.101614 | 3.740229 | 2.124871 | 0.035736 | 0.270126 | -4.1677 |
| C3orf49 | 0.101504 | 4.036513 | 2.370407 | 0.019431 | 0.189136 | -3.65177 |
| STARD13- | 0.10068 | 3.675757 | 2.647638 | 0.009242 | 0.115659 | -3.0084 |
| CSN2 | 0.100162 | 3.279439 | 2.178065 | 0.03144 | 0.252982 | -4.06031 |
| LOC10192 | 0.100144 | 5.460757 | 2.186789 | 0.03078 | 0.250329 | -4.04246 |
| PRKG1 | 0.100082 | 3.393546 | 2.712871 | 0.007694 | 0.101642 | -2.84787 |
| VANGL2 | 0.100079 | 3.23367 | 2.383757 | 0.018773 | 0.185209 | -3.62226 |
| LOC10192 | 0.099713 | 3.111202 | 2.780662 | 0.006339 | 0.089457 | -2.67743 |
| DMC1 | 0.09909 | 3.038684 | 3.33418 | 0.001151 | 0.026593 | -1.15239 |
| HSPA1L | 0.09878 | 6.416896 | 2.054919 | 0.04215 | 0.297975 | -4.30519 |
| A2MP1 | 0.098722 | 4.419641 | 1.98686 | 0.049313 | 0.327823 | -4.43486 |
| EXOSC3 | 0.098586 | 5.269297 | 2.186244 | 0.030821 | 0.250562 | -4.04358 |
| IGF1 | 0.098216 | 3.943043 | 2.211702 | 0.028962 | 0.241507 | -3.99114 |
| C10orf10 | 0.097905 | 4.309475 | 2.176103 | 0.03159 | 0.25379 | -4.06431 |
| VCAM1 | 0.097903 | 3.046284 | 2.097556 | 0.038132 | 0.280421 | -4.22189 |
| ABCC6P1 | 0.097851 | 2.935856 | 2.300168 | 0.02324 | 0.211632 | -3.80461 |
| RAB23 | 0.097603 | 3.311698 | 2.167869 | 0.032227 | 0.255966 | -4.08108 |
| CDH1 | 0.097348 | 3.136188 | 2.069961 | 0.040693 | 0.291547 | -4.27598 |
| ICMT | 0.097125 | 5.055641 | 2.180792 | 0.031233 | 0.252104 | -4.05474 |
| TTTY13 | 0.096613 | 3.308215 | 2.339431 | 0.021037 | 0.198639 | -3.71969 |
| SGOL1 | 0.09651 | 3.429796 | 2.15341 | 0.033372 | 0.259862 | -4.11039 |
| STEAP2 | 0.096481 | 3.459314 | 2.117568 | 0.036364 | 0.272393 | -4.18225 |
| LINC0031i | 0.096071 | 4.352133 | 2.309201 | 0.022716 | 0.208904 | -3.78519 |
| NXPH2 | 0.096055 | 3.481294 | 2.871658 | 0.004862 | 0.075193 | -2.4429 |
| TBC1D22B | 0.095987 | 4.824104 | 2.39346 | 0.018307 | 0.182105 | -3.60071 |
| KY | 0.095839 | 4.293946 | 1.993114 | 0.048614 | 0.325717 | -4.42312 |
| LOC64676 | 0.095707 | 4.420273 | 2.284515 | 0.024174 | 0.217401 | -3.8381 |
| GNA14 | 0.095122 | 3.619662 | 2.308234 | 0.022771 | 0.209321 | -3.78727 |
| SLC22A23 | 0.094437 | 5.186203 | 2.442351 | 0.016112 | 0.168293 | -3.49093 |
| BCL2L13 | 0.094407 | 5.332176 | 2.626132 | 0.00981 | 0.120367 | -3.06057 |
| CEP128 | 0.094388 | 3.8453 | 2.089282 | 0.038885 | 0.283257 | -4.23818 |
| GXYLT2 | 0.094039 | 3.148096 | 2.465752 | 0.015147 | 0.16168 | -3.43768 |
| ONECUT1 | 0.093821 | 3.769379 | 2.072825 | 0.04042 | 0.290408 | -4.2704 |
| PCDH18 | 0.093403 | 4.476335 | 2.451295 | 0.015737 | 0.165814 | -3.47063 |
| PERP | 0.09332 | 3.56774 | 2.428963 | 0.016688 | 0.172814 | -3.52119 |
| LOC10192 | 0.092979 | 4.066491 | 2.134621 | 0.034913 | 0.266162 | -4.1482 |
| LINC0011: | 0.092337 | 2.954962 | 2.324308 | 0.021862 | 0.204192 | -3.75255 |
| LINC0094 | 0.091722 | 3.844401 | 2.769955 | 0.006538 | 0.091251 | -2.70459 |
| PRICKLE2 | 0.091511 | 3.487742 | 2.412547 | 0.01742 | 0.177605 | -3.55809 |
| MAP1B | 0.090716 | 3.329942 | 2.037472 | 0.043895 | 0.305298 | -4.33882 |
| PRELID2 | 0.090503 | 4.297831 | 2.111842 | 0.036862 | 0.274283 | -4.19363 |
| GINS4 | 0.089892 | 3.830669 | 2.705636 | 0.007854 | 0.103013 | -2.86585 |
| PTGER3 | 0.089761 | 3.369019 | 2.474939 | 0.014783 | 0.159959 | -3.41665 |
| MEP1B | 0.089468 | 3.509491 | 2.029573 | 0.044706 | 0.308523 | -4.35396 |
| ARHGEF12 | 0.089328 | 3.431646 | 2.772267 | 0.006494 | 0.090956 | -2.69874 |
| LINC0092: | 0.089101 | 3.208419 | 2.071365 | 0.040559 | 0.291098 | -4.27325 |

| | | | | | | |
|---|---|---|---|---|---|---|
| GREB1L | 0.088844 | 3.939447 | 2.667545 | 0.008742 | 0.111382 | -2.95978 |
| GIF | 0.088793 | 3.003419 | 2.504596 | 0.013659 | 0.152391 | -3.34829 |
| LINC0088[illegible] | 0.088641 | 4.368941 | 2.120637 | 0.036099 | 0.271306 | -4.17614 |
| SLC5A4 | 0.088627 | 3.762039 | 2.070502 | 0.040641 | 0.291279 | -4.27493 |
| LINC0133[illegible] | 0.088229 | 3.016456 | 2.131081 | 0.03521 | 0.267729 | -4.15529 |
| IGHG1 | 0.088117 | 3.907944 | 2.293327 | 0.023644 | 0.214357 | -3.81927 |
| MICALL2 | 0.088005 | 4.242202 | 2.184535 | 0.03095 | 0.25071 | -4.04708 |
| DIAPH2-A | 0.087861 | 3.151837 | 2.616319 | 0.01008 | 0.122409 | -3.08425 |
| ATRNL1 | 0.087448 | 3.118222 | 2.840508 | 0.005328 | 0.080025 | -2.52392 |
| FGFR1 | 0.086669 | 5.065685 | 2.704777 | 0.007873 | 0.103157 | -2.86798 |
| C3orf80 | 0.086525 | 2.407747 | 2.688003 | 0.008254 | 0.106903 | -2.90947 |
| DDAH1 | 0.086427 | 3.504397 | 3.040715 | 0.002923 | 0.052722 | -1.99002 |
| SHROOM[illegible] | 0.085825 | 4.348571 | 2.094709 | 0.03839 | 0.281151 | -4.2275 |
| FUT2 | 0.085618 | 4.770701 | 2.113916 | 0.036681 | 0.273829 | -4.18951 |
| SULT1C4 | 0.085359 | 3.169552 | 2.010293 | 0.046738 | 0.316566 | -4.39067 |
| FAT1 | 0.084474 | 2.863619 | 2.153835 | 0.033338 | 0.259772 | -4.10953 |
| C16orf95 | 0.083941 | 4.880465 | 2.524406 | 0.012951 | 0.145882 | -3.30222 |
| LOC65299 | 0.083654 | 3.037749 | 2.402989 | 0.017859 | 0.179806 | -3.57947 |
| CLDN10-[illegible] | 0.083322 | 2.755294 | 2.216718 | 0.028607 | 0.239606 | -3.98074 |
| IQCG | 0.083095 | 3.979695 | 2.082083 | 0.039551 | 0.286168 | -4.25231 |
| TTC22 | 0.082745 | 4.166933 | 2.121257 | 0.036045 | 0.271162 | -4.17491 |
| TUSC7 | 0.082161 | 3.036389 | 2.234878 | 0.027355 | 0.233171 | -3.94292 |
| BRD7P3 | 0.081676 | 3.487846 | 2.143296 | 0.034194 | 0.263332 | -4.13078 |
| LRTM1 | 0.081484 | 3.381452 | 2.025771 | 0.0451 | 0.309679 | -4.36122 |
| DTYMK | 0.081164 | 4.570474 | 2.044501 | 0.043185 | 0.302826 | -4.3253 |
| PPP1R3C | 0.08095 | 3.248036 | 2.142646 | 0.034247 | 0.263545 | -4.13208 |
| LHX2 | 0.080741 | 3.191292 | 2.192537 | 0.030352 | 0.24813 | -4.03067 |
| VSNL1 | 0.080606 | 4.020719 | 2.068774 | 0.040806 | 0.291989 | -4.2783 |
| CASZ1 | 0.080505 | 4.808972 | 2.472047 | 0.014897 | 0.160466 | -3.42328 |
| CNPY1 | 0.080448 | 2.63092 | 2.471956 | 0.0149 | 0.160466 | -3.42349 |
| LRTOMT | 0.080157 | 4.009618 | 2.214418 | 0.028769 | 0.240577 | -3.98551 |
| PTRF | 0.079981 | 4.82024 | 2.093366 | 0.038512 | 0.281641 | -4.23015 |
| PABPC4L | 0.079597 | 2.721169 | 2.012802 | 0.046469 | 0.315845 | -4.38592 |
| POM121L[illegible] | 0.07937 | 3.551789 | 2.008777 | 0.046901 | 0.317356 | -4.39355 |
| PANX3 | 0.079066 | 2.6411 | 2.183477 | 0.03103 | 0.251257 | -4.04924 |
| CRISPLD1 | 0.078606 | 2.801585 | 2.313175 | 0.022489 | 0.207279 | -3.77662 |
| L2HGDH | 0.078077 | 3.604944 | 2.429584 | 0.016661 | 0.172761 | -3.51979 |
| FKBP14 | 0.078044 | 3.765219 | 1.987754 | 0.049212 | 0.327409 | -4.43319 |
| PDCD1LG[illegible] | 0.077827 | 3.603855 | 2.264628 | 0.025407 | 0.222543 | -3.88035 |
| SBF2-AS1 | 0.077691 | 3.4347 | 2.13245 | 0.035094 | 0.267152 | -4.15255 |
| PELO | 0.077347 | 4.326402 | 1.981374 | 0.049933 | 0.32976 | -4.44514 |
| CLDN8 | 0.07701 | 2.718923 | 2.09358 | 0.038493 | 0.2816 | -4.22973 |
| ABHD17B | 0.076521 | 5.690328 | 2.000108 | 0.047842 | 0.321922 | -4.40994 |
| KERA | 0.075988 | 2.504352 | 2.129242 | 0.035365 | 0.268709 | -4.15897 |
| CCSER1 | 0.075633 | 2.976433 | 1.981288 | 0.049943 | 0.32976 | -4.4453 |
| COL6A6 | 0.075393 | 2.781309 | 2.696953 | 0.008049 | 0.104898 | -2.88736 |
| C8orf49 | 0.075177 | 2.637465 | 2.027254 | 0.044946 | 0.308764 | -4.35839 |
| LRRN2 | 0.075149 | 3.42349 | 2.09024 | 0.038797 | 0.282718 | -4.2363 |
| GYPE | 0.074523 | 2.657354 | 2.277584 | 0.024597 | 0.219307 | -3.85286 |
| PARD6B | 0.073838 | 3.09442 | 2.103015 | 0.037643 | 0.278167 | -4.21111 |
| ACSM5 | 0.073452 | 3.681643 | 2.095767 | 0.038294 | 0.280977 | -4.22542 |
| TMPRSS1[illegible] | 0.073404 | 2.68286 | 2.229709 | 0.027706 | 0.235195 | -3.95371 |
| RBM24 | 0.073122 | 2.54597 | 2.226429 | 0.027932 | 0.235984 | -3.96055 |
| OVOL2 | 0.072731 | 3.435073 | 2.233836 | 0.027426 | 0.233601 | -3.9451 |
| XG | 0.071484 | 3.347602 | 2.281063 | 0.024384 | 0.21845 | -3.84545 |
| ZNF876P | 0.071156 | 2.366399 | 2.287329 | 0.024003 | 0.216752 | -3.83209 |
| LINC0096[illegible] | 0.070917 | 3.109116 | 2.019491 | 0.045759 | 0.3128 | -4.3732 |
| PAK7 | 0.070901 | 3.682896 | 2.008838 | 0.046894 | 0.317356 | -4.39343 |

| | | | | | | |
|---|---|---|---|---|---|---|
| CATR1 | 0.070894 | 2.615939 | 2.095281 | 0.038338 | 0.280977 | -4.22638 |
| STXBP6 | 0.070894 | 3.34639 | 2.181475 | 0.031181 | 0.251786 | -4.05334 |
| PCSK6 | 0.068632 | 3.908778 | 2.061434 | 0.041513 | 0.294832 | -4.29256 |
| LOC34018 | 0.068177 | 2.570738 | 2.091314 | 0.038699 | 0.282505 | -4.23419 |
| LRRC2 | 0.067316 | 2.496657 | 2.38824 | 0.018556 | 0.183693 | -3.61231 |
| MIR31HG | 0.06709 | 4.272165 | 2.076823 | 0.040043 | 0.288607 | -4.2626 |
| RBBP9 | 0.06626 | 3.963022 | 2.171051 | 0.03198 | 0.255711 | -4.07461 |
| LRRC28 | 0.065996 | 3.967867 | 2.317805 | 0.022226 | 0.205789 | -3.76662 |
| LOC10192 | 0.065956 | 2.495145 | 1.991398 | 0.048805 | 0.326255 | -4.42634 |
| AKR1D1 | 0.065291 | 2.670043 | 2.001216 | 0.047721 | 0.321514 | -4.40785 |
| MET | 0.064783 | 3.725905 | 2.26043 | 0.025674 | 0.223647 | -3.88922 |
| USP49 | 0.061553 | 4.116889 | 2.201845 | 0.02967 | 0.244603 | -4.01151 |
| TAF7L | 0.061473 | 3.118622 | 2.139749 | 0.034486 | 0.264582 | -4.13791 |
| GPX5 | 0.060904 | 3.068882 | 1.980824 | 0.049995 | 0.329895 | -4.44617 |
| GRID1 | 0.060868 | 3.820939 | 2.028621 | 0.044804 | 0.308682 | -4.35578 |
| MTBP | 0.060778 | 2.890789 | 2.567646 | 0.01152 | 0.134468 | -3.20054 |
| SLC6A1 | 0.060574 | 2.796718 | 2.029181 | 0.044746 | 0.308682 | -4.35471 |
| ZFHX4 | 0.059416 | 3.325663 | 2.545867 | 0.012222 | 0.140419 | -3.25194 |
| ATP6V0D2 | 0.054548 | 4.401773 | 2.078103 | 0.039922 | 0.288146 | -4.26009 |
| LOC64242 | 0.048715 | 2.3151 | 2.097477 | 0.03814 | 0.280421 | -4.22205 |
| LUM | 0.045818 | 2.450111 | 2.071046 | 0.040589 | 0.291214 | -4.27387 |
| LOC40109 | -0.057 | 2.52386 | -1.98362 | 0.049678 | 0.329501 | -4.44093 |
| ZNF7 | -0.05919 | 4.35987 | -2.27064 | 0.025028 | 0.221102 | -3.8676 |
| LOC10050 | -0.06008 | 2.553001 | -2.31791 | 0.02222 | 0.205789 | -3.7664 |
| EIF4G2 | -0.06046 | 6.882062 | -2.25454 | 0.026054 | 0.225703 | -3.90166 |
| AXDND1 | -0.06241 | 2.713485 | -2.2568 | 0.025907 | 0.224912 | -3.89689 |
| TTC12 | -0.06427 | 4.299659 | -2.14273 | 0.03424 | 0.263545 | -4.13192 |
| EIF4A2 | -0.06503 | 2.744983 | -2.28052 | 0.024417 | 0.218462 | -3.84662 |
| ZNF568 | -0.06526 | 3.109035 | -2.62373 | 0.009876 | 0.120571 | -3.06637 |
| RGMB | -0.06594 | 3.721899 | -2.2123 | 0.028919 | 0.241332 | -3.98989 |
| GH1 | -0.06597 | 4.293977 | -1.98321 | 0.049724 | 0.329624 | -4.4417 |
| CTTNBP2 | -0.06827 | 2.493674 | -2.13608 | 0.034791 | 0.26543 | -4.14527 |
| CNTNAP2 | -0.06882 | 3.598706 | -2.14023 | 0.034446 | 0.26438 | -4.13694 |
| LOC37519 | -0.06899 | 2.86616 | -2.02893 | 0.044772 | 0.308682 | -4.35519 |
| FGD6 | -0.06965 | 3.281991 | -2.01659 | 0.046066 | 0.314345 | -4.37872 |
| SCARB1 | -0.06997 | 4.395065 | -2.20708 | 0.029292 | 0.243052 | -4.00069 |
| OTUD7A | -0.07041 | 3.601442 | -2.0858 | 0.039206 | 0.284975 | -4.24502 |
| AGBL3 | -0.07307 | 2.48668 | -2.28479 | 0.024157 | 0.217368 | -3.83751 |
| BTBD9 | -0.07435 | 4.923976 | -2.03386 | 0.044264 | 0.307028 | -4.34574 |
| IL16 | -0.07509 | 6.88625 | -2.03488 | 0.04416 | 0.306618 | -4.3438 |
| ZNF362 | -0.07512 | 5.185214 | -2.02803 | 0.044865 | 0.308764 | -4.35691 |
| TMEM231 | -0.07584 | 3.411924 | -2.40464 | 0.017783 | 0.1796 | -3.57578 |
| RTKN2 | -0.07599 | 2.585201 | -2.30583 | 0.02291 | 0.210001 | -3.79244 |
| PTGR2 | -0.07672 | 2.396741 | -2.12826 | 0.035448 | 0.269042 | -4.16094 |
| ZFP28 | -0.07672 | 3.768717 | -3.11517 | 0.002321 | 0.044672 | -1.78362 |
| CDHR3 | -0.07711 | 3.298421 | -2.26447 | 0.025417 | 0.222543 | -3.88068 |
| HPS4 | -0.07738 | 4.912945 | -2.40342 | 0.017839 | 0.179806 | -3.5785 |
| TSC22D2 | -0.07788 | 4.118911 | -2.19461 | 0.030199 | 0.247282 | -4.0264 |
| RPS8 | -0.07806 | 3.295353 | -2.11707 | 0.036407 | 0.272452 | -4.18325 |
| LOC10050 | -0.07949 | 3.475189 | -2.09912 | 0.037992 | 0.279835 | -4.21881 |
| CYTH3 | -0.08084 | 4.631153 | -2.02331 | 0.045357 | 0.310922 | -4.36592 |
| ZCCHC4 | -0.08089 | 3.395678 | -2.64118 | 0.009409 | 0.11704 | -3.0241 |
| PAXBP1-A | -0.08095 | 3.85728 | -2.0209 | 0.04561 | 0.312132 | -4.37051 |
| KCNN2 | -0.08102 | 2.94432 | -2.22932 | 0.027733 | 0.235233 | -3.95452 |
| TNRC6C | -0.08104 | 4.98461 | -2.27797 | 0.024574 | 0.219192 | -3.85204 |
| SAMM50 | -0.0818 | 5.638709 | -2.05623 | 0.042021 | 0.297418 | -4.30266 |
| STK35 | -0.08207 | 5.544689 | -2.51172 | 0.013401 | 0.150166 | -3.33177 |
| ZBTB40 | -0.08238 | 5.478693 | -2.26536 | 0.025361 | 0.22224 | -3.8788 |

| | | | | | | |
|---|---|---|---|---|---|---|
| WNT16 | -0.08309 | 3.498877 | -2.78917 | 0.006185 | 0.087954 | -2.65577 |
| RPAIN | -0.08327 | 4.033969 | -2.7455 | 0.007012 | 0.095721 | -2.76629 |
| MLLT4 | -0.08363 | 3.628336 | -2.16816 | 0.032205 | 0.255911 | -4.08049 |
| STARD9 | -0.08364 | 4.106553 | -2.2835 | 0.024235 | 0.217789 | -3.84027 |
| SLC38A7 | -0.08378 | 4.978377 | -2.31325 | 0.022484 | 0.207279 | -3.77646 |
| CNR1 | -0.0844 | 3.733508 | -2.16354 | 0.032566 | 0.256688 | -4.08986 |
| CCAR2 | -0.08447 | 4.317485 | -2.36373 | 0.019767 | 0.190882 | -3.66648 |
| RALGAPA1 | -0.08454 | 4.222794 | -2.05108 | 0.042529 | 0.300051 | -4.31262 |
| FAM45A | -0.08465 | 3.117454 | -2.12217 | 0.035967 | 0.270768 | -4.17308 |
| SLC25A53 | -0.08475 | 2.923442 | -2.05865 | 0.041784 | 0.296182 | -4.29797 |
| ZNF555 | -0.08523 | 3.860552 | -2.04872 | 0.042763 | 0.301104 | -4.31718 |
| KLHDC4 | -0.08569 | 4.766 | -1.99881 | 0.047984 | 0.322344 | -4.41238 |
| CROT | -0.08607 | 3.771781 | -2.16212 | 0.032678 | 0.256841 | -4.09276 |
| URB1 | -0.0862 | 3.860997 | -1.98921 | 0.049049 | 0.327304 | -4.43044 |
| LINC01098 | -0.08629 | 2.634317 | -2.01037 | 0.04673 | 0.316566 | -4.39053 |
| ZNF649 | -0.08652 | 3.274478 | -2.20242 | 0.029628 | 0.244456 | -4.01032 |
| AAK1 | -0.08719 | 5.493538 | -2.38138 | 0.018888 | 0.185895 | -3.62753 |
| DGKE | -0.0874 | 3.628506 | -2.00577 | 0.047226 | 0.31905 | -4.39925 |
| HMCN1 | -0.0874 | 2.385859 | -2.81542 | 0.005732 | 0.084206 | -2.58862 |
| FAM184A | -0.08745 | 3.677296 | -2.03926 | 0.043713 | 0.304695 | -4.33538 |
| SENP5 | -0.08755 | 5.395039 | -2.40214 | 0.017899 | 0.179884 | -3.58137 |
| STK24 | -0.08763 | 6.637936 | -2.03858 | 0.043782 | 0.304719 | -4.33668 |
| BTN2A2 | -0.08778 | 4.439047 | -2.09988 | 0.037924 | 0.279638 | -4.21732 |
| CIITA | -0.08789 | 5.06074 | -2.11093 | 0.036942 | 0.274775 | -4.19543 |
| FMN1 | -0.08794 | 3.983235 | -2.12351 | 0.035852 | 0.270506 | -4.17042 |
| CARS2 | -0.0881 | 4.936854 | -2.35605 | 0.020161 | 0.193316 | -3.68336 |
| TLDC1 | -0.08907 | 4.098658 | -2.61159 | 0.010213 | 0.123141 | -3.09564 |
| TCEB3 | -0.08908 | 5.56892 | -2.14183 | 0.034314 | 0.263861 | -4.13372 |
| DYNC1H1 | -0.08938 | 4.917696 | -2.40859 | 0.017601 | 0.178378 | -3.56694 |
| FLJ38379 | -0.08953 | 2.744891 | -2.16494 | 0.032456 | 0.256336 | -4.08702 |
| LCE2B | -0.08963 | 3.695022 | -2.31893 | 0.022163 | 0.205769 | -3.76418 |
| TRAF1 | -0.09029 | 4.592488 | -1.98317 | 0.049729 | 0.329624 | -4.44177 |
| ZNF346 | -0.09063 | 4.92096 | -2.22222 | 0.028222 | 0.237747 | -3.9693 |
| LINC00473 | -0.09087 | 3.577156 | -2.25824 | 0.025815 | 0.224583 | -3.89385 |
| LOC10272 | -0.0911 | 2.624049 | -2.56239 | 0.011686 | 0.135975 | -3.21299 |
| DDX31 | -0.09125 | 4.714491 | -2.73263 | 0.007274 | 0.098056 | -2.79856 |
| SDK2 | -0.09126 | 4.876807 | -2.5266 | 0.012875 | 0.145389 | -3.29709 |
| SEMA4F | -0.09129 | 4.967007 | -2.16912 | 0.03213 | 0.255711 | -4.07855 |
| PIGG | -0.09221 | 5.231752 | -2.84683 | 0.00523 | 0.079245 | -2.50753 |
| NCL | -0.09235 | 6.536192 | -2.01056 | 0.046709 | 0.316566 | -4.39018 |
| CXCL2 | -0.09303 | 3.788597 | -2.00574 | 0.047229 | 0.31905 | -4.3993 |
| FIRRE | -0.09307 | 3.057818 | -2.02845 | 0.044822 | 0.308684 | -4.35611 |
| ZNF142 | -0.09401 | 5.847373 | -2.05922 | 0.041729 | 0.296064 | -4.29686 |
| ZNF594 | -0.09471 | 3.979882 | -2.05937 | 0.041714 | 0.296062 | -4.29656 |
| GDPD1 | -0.09538 | 3.352969 | -2.0786 | 0.039876 | 0.287956 | -4.25913 |
| CTGF | -0.09539 | 2.854955 | -2.45655 | 0.01552 | 0.164123 | -3.45868 |
| PI4K2A | -0.09568 | 5.159294 | -2.7908 | 0.006156 | 0.087601 | -2.65162 |
| CARF | -0.0959 | 3.216072 | -2.16233 | 0.032662 | 0.256841 | -4.09234 |
| TSEN54 | -0.09599 | 6.243363 | -2.05295 | 0.042344 | 0.299185 | -4.309 |
| DLGAP4 | -0.09617 | 6.288138 | -2.40941 | 0.017563 | 0.178265 | -3.56512 |
| PACS2 | -0.09647 | 6.068066 | -2.07059 | 0.040633 | 0.291279 | -4.27475 |
| MAP3K9 | -0.09659 | 4.320166 | -2.27562 | 0.024718 | 0.219809 | -3.85703 |
| ZNF252P-1 | -0.09691 | 3.008589 | -2.22743 | 0.027863 | 0.235787 | -3.95847 |
| NPC1 | -0.09714 | 4.39696 | -2.37701 | 0.019102 | 0.187017 | -3.63718 |
| EXOSC2 | -0.09726 | 4.988975 | -2.1477 | 0.033834 | 0.261542 | -4.1219 |
| NLN | -0.09735 | 5.062161 | -2.06364 | 0.0413 | 0.29422 | -4.28829 |
| UST | -0.09736 | 3.541626 | -2.47536 | 0.014766 | 0.159866 | -3.41569 |
| RNF216 | -0.09753 | 5.462056 | -2.08816 | 0.038989 | 0.283808 | -4.24039 |

| | | | | | | |
|---|---|---|---|---|---|---|
| IRGQ | -0.09781 | 4.822347 | -3.2892 | 0.001333 | 0.029689 | -1.28491 |
| SF3B2 | -0.09817 | 7.17504 | -2.01201 | 0.046553 | 0.315945 | -4.38741 |
| TCL6 | -0.09822 | 4.032832 | -2.39744 | 0.018119 | 0.180938 | -3.59184 |
| LINC01355 | -0.09847 | 3.835436 | -2.07445 | 0.040267 | 0.289712 | -4.26724 |
| WARS2 | -0.09871 | 3.720237 | -2.0437 | 0.043266 | 0.302978 | -4.32685 |
| RMDN2 | -0.09912 | 3.975826 | -2.47255 | 0.014877 | 0.160417 | -3.42213 |
| ATP1B3 | -0.09928 | 5.424533 | -2.474 | 0.014819 | 0.160188 | -3.4188 |
| DOCK9 | -0.09936 | 3.999224 | -2.10631 | 0.03735 | 0.276903 | -4.2046 |
| HS3ST1 | -0.09952 | 3.75767 | -2.51587 | 0.013252 | 0.148745 | -3.32211 |
| RIMKLB | -0.0998 | 3.930896 | -2.02833 | 0.044835 | 0.308684 | -4.35634 |
| ZNF416 | -0.10001 | 3.732237 | -2.17168 | 0.031931 | 0.255523 | -4.07333 |
| RPL7 | -0.10019 | 9.431875 | -2.30571 | 0.022917 | 0.210001 | -3.7927 |
| MMAA | -0.10033 | 3.462938 | -2.59983 | 0.010548 | 0.125782 | -3.12385 |
| ZNF85 | -0.10036 | 4.891168 | -2.05119 | 0.042517 | 0.300051 | -4.31239 |
| UQCC1 | -0.10043 | 4.801605 | -2.8298 | 0.005497 | 0.081982 | -2.55159 |
| DHX57 | -0.1006 | 4.385784 | -2.31794 | 0.022219 | 0.205789 | -3.76633 |
| ZDHHC9 | -0.10083 | 4.926601 | -2.09063 | 0.038762 | 0.28266 | -4.23553 |
| NBEAL1 | -0.10139 | 3.465849 | -2.34358 | 0.020815 | 0.197548 | -3.71064 |
| USP40 | -0.10142 | 4.878024 | -2.27069 | 0.025025 | 0.221102 | -3.86751 |
| HGSNAT | -0.10145 | 5.193809 | -2.13631 | 0.034772 | 0.26543 | -4.14481 |
| OSBPL10 | -0.10145 | 4.726807 | -2.24524 | 0.026662 | 0.229038 | -3.92122 |
| PHC2 | -0.10158 | 7.663549 | -2.01059 | 0.046706 | 0.316566 | -4.39012 |
| TTC7A | -0.10186 | 5.052018 | -2.04849 | 0.042786 | 0.301104 | -4.31761 |
| ZNF345 | -0.10198 | 3.895931 | -2.76952 | 0.006546 | 0.091303 | -2.7057 |
| BMS1P5 | -0.10217 | 4.246994 | -2.24745 | 0.026516 | 0.228068 | -3.91656 |
| WDR52 | -0.10259 | 3.416962 | -2.46963 | 0.014992 | 0.161037 | -3.42882 |
| PTPDC1 | -0.10276 | 3.9567 | -2.6001 | 0.010541 | 0.125782 | -3.12321 |
| SERPINB6 | -0.10313 | 4.593128 | -2.54439 | 0.012271 | 0.140902 | -3.25541 |
| USP36 | -0.10315 | 5.010852 | -2.67772 | 0.008496 | 0.109299 | -2.93479 |
| TCL1B | -0.10351 | 5.436716 | -2.28314 | 0.024257 | 0.217869 | -3.84103 |
| PTPN9 | -0.10356 | 4.686031 | -2.27982 | 0.02446 | 0.218522 | -3.8481 |
| THRB | -0.10369 | 3.481471 | -2.14937 | 0.033698 | 0.260793 | -4.11855 |
| LOC64373 | -0.10398 | 3.92921 | -2.25139 | 0.026258 | 0.22656 | -3.90828 |
| COG8 | -0.10429 | 5.631494 | -2.61353 | 0.010158 | 0.122699 | -3.09096 |
| FMNL3 | -0.10494 | 4.273527 | -2.62737 | 0.009777 | 0.120151 | -3.05758 |
| KIAA0020 | -0.10519 | 4.838147 | -2.61372 | 0.010153 | 0.122699 | -3.0905 |
| ZNF333 | -0.10565 | 5.076378 | -2.0297 | 0.044692 | 0.308523 | -4.3537 |
| PHF20 | -0.10577 | 6.45514 | -2.46843 | 0.01504 | 0.161115 | -3.43156 |
| ACAA2 | -0.10583 | 5.845997 | -2.04382 | 0.043253 | 0.302978 | -4.32661 |
| CYP2U1 | -0.10632 | 3.688728 | -2.64212 | 0.009385 | 0.116807 | -3.02183 |
| MYEF2 | -0.10649 | 3.364642 | -2.60746 | 0.010329 | 0.124096 | -3.10555 |
| PCBP1-AS | -0.10665 | 4.6843 | -2.80854 | 0.005848 | 0.084751 | -2.60629 |
| MAP3K6 | -0.10668 | 4.273017 | -2.16161 | 0.032719 | 0.256902 | -4.09379 |
| CDC42BPE | -0.10669 | 3.774011 | -2.62618 | 0.009809 | 0.120367 | -3.06046 |
| EME2 | -0.10673 | 5.526216 | -2.27821 | 0.024559 | 0.219156 | -3.85153 |
| GNRH1 | -0.10694 | 3.770761 | -2.43667 | 0.016355 | 0.17013 | -3.50379 |
| SP140L | -0.107 | 5.248605 | -2.14114 | 0.034371 | 0.264099 | -4.1351 |
| NOP16 | -0.10705 | 4.916547 | -2.80528 | 0.005904 | 0.085371 | -2.61464 |
| NTMT1 | -0.10712 | 5.355442 | -2.0206 | 0.045642 | 0.312247 | -4.37109 |
| CAMSAP1 | -0.10734 | 4.106505 | -3.17908 | 0.001898 | 0.038682 | -1.60308 |
| PHKG2 | -0.10739 | 4.729748 | -1.98249 | 0.049807 | 0.32976 | -4.44306 |
| MAP3K13 | -0.10745 | 4.571357 | -2.41621 | 0.017254 | 0.176618 | -3.54987 |
| HEBP2 | -0.10769 | 7.682437 | -2.2348 | 0.02736 | 0.233171 | -3.94308 |
| BRPF1 | -0.10815 | 6.392336 | -2.2561 | 0.025952 | 0.225207 | -3.89835 |
| ZNF112 | -0.10832 | 2.776218 | -3.06098 | 0.002746 | 0.050167 | -1.93427 |
| VPRBP | -0.10845 | 5.032675 | -2.59199 | 0.010778 | 0.127674 | -3.14261 |
| ABCF2 | -0.10862 | 5.448498 | -2.60821 | 0.010308 | 0.124073 | -3.10375 |
| NDUFB4 | -0.10876 | 6.849141 | -2.11895 | 0.036244 | 0.271855 | -4.1795 |

| | | | | | | |
|---|---|---|---|---|---|---|
| IFT172 | -0.10881 | 4.473402 | -2.28697 | 0.024025 | 0.216752 | -3.83285 |
| SPATA13 | -0.10889 | 6.263522 | -2.25486 | 0.026033 | 0.225703 | -3.90098 |
| CNDP2 | -0.10889 | 5.981348 | -2.46172 | 0.015309 | 0.16248 | -3.44688 |
| HKR1 | -0.10893 | 3.4876 | -3.02739 | 0.003045 | 0.054266 | -2.02653 |
| ZNF621 | -0.10898 | 4.566537 | -2.04721 | 0.042913 | 0.301645 | -4.32007 |
| ZNF441 | -0.10918 | 2.813566 | -2.46126 | 0.015328 | 0.162512 | -3.44794 |
| TRNAU1AI | -0.10944 | 5.10931 | -3.40493 | 0.000912 | 0.022422 | -0.94095 |
| HSD17B1 | -0.10964 | 4.929619 | -2.28438 | 0.024182 | 0.217401 | -3.83838 |
| HNRNPA2 | -0.10988 | 8.576735 | -2.20365 | 0.029539 | 0.244103 | -4.00778 |
| USP7 | -0.10992 | 7.332333 | -2.28271 | 0.024283 | 0.217932 | -3.84195 |
| RBM15 | -0.10993 | 5.923774 | -2.27427 | 0.024802 | 0.219942 | -3.85989 |
| C12orf65 | -0.10999 | 5.482883 | -2.21862 | 0.028474 | 0.239168 | -3.97678 |
| CDK12 | -0.11028 | 5.835712 | -1.99761 | 0.048117 | 0.323021 | -4.41465 |
| LOC15506 | -0.1103 | 5.857987 | -2.2709 | 0.025012 | 0.221102 | -3.86705 |
| MAP9 | -0.1104 | 3.36545 | -2.20764 | 0.029252 | 0.242829 | -3.99955 |
| SUGP1 | -0.11061 | 6.410505 | -1.98381 | 0.049657 | 0.329468 | -4.44058 |
| UNKL | -0.11062 | 6.123018 | -2.70903 | 0.007779 | 0.102292 | -2.85743 |
| NSUN4 | -0.11076 | 4.446226 | -2.1978 | 0.029964 | 0.245942 | -4.01983 |
| SEPT7P2 | -0.11077 | 3.76684 | -2.17358 | 0.031784 | 0.254748 | -4.06945 |
| LOC40095 | -0.11117 | 2.929325 | -2.57792 | 0.011201 | 0.131727 | -3.17616 |
| PTTG1IP | -0.11137 | 11.12969 | -1.99257 | 0.048674 | 0.325907 | -4.42414 |
| GGA1 | -0.11146 | 6.735022 | -2.16177 | 0.032706 | 0.256901 | -4.09346 |
| SYNE4 | -0.11148 | 4.264384 | -1.9883 | 0.049151 | 0.327304 | -4.43215 |
| CDC42EP4 | -0.11175 | 5.441457 | -2.09605 | 0.038268 | 0.280965 | -4.22486 |
| SZT2 | -0.11214 | 4.596514 | -2.42927 | 0.016675 | 0.172761 | -3.52049 |
| NHP2L1 | -0.11217 | 5.821132 | -2.25722 | 0.02588 | 0.22477 | -3.89599 |
| MRPS25 | -0.11218 | 5.267219 | -2.34302 | 0.020845 | 0.19774 | -3.71186 |
| UNK | -0.11232 | 5.297822 | -2.1071 | 0.03728 | 0.276584 | -4.20303 |
| NANS | -0.1124 | 6.099337 | -2.16238 | 0.032658 | 0.256841 | -4.09223 |
| RABEP1 | -0.11276 | 5.578874 | -2.13624 | 0.034777 | 0.26543 | -4.14495 |
| GOPC | -0.11287 | 5.537561 | -2.10435 | 0.037524 | 0.27749 | -4.20848 |
| ZZEF1 | -0.11291 | 5.995384 | -2.15099 | 0.033567 | 0.260568 | -4.11528 |
| ZNF785 | -0.11302 | 3.793851 | -2.15243 | 0.033451 | 0.260356 | -4.11237 |
| ZNF605 | -0.11322 | 3.488144 | -1.98139 | 0.049931 | 0.32976 | -4.4451 |
| BRD8 | -0.11324 | 5.573403 | -2.82254 | 0.005615 | 0.083131 | -2.5703 |
| NRF1 | -0.11347 | 5.313107 | -2.89224 | 0.004575 | 0.072508 | -2.38896 |
| TBC1D32 | -0.11373 | 2.718682 | -2.05718 | 0.041928 | 0.296968 | -4.30082 |
| BRD4 | -0.11375 | 7.061829 | -2.27476 | 0.024772 | 0.219809 | -3.85886 |
| VAV2 | -0.11381 | 4.922552 | -2.32169 | 0.022008 | 0.205207 | -3.75822 |
| FBRSL1 | -0.11415 | 5.653526 | -2.88454 | 0.00468 | 0.073741 | -2.40918 |
| PFKP | -0.11421 | 5.484767 | -2.02418 | 0.045266 | 0.310502 | -4.36426 |
| COX19 | -0.11465 | 6.032105 | -2.12831 | 0.035444 | 0.269042 | -4.16084 |
| KRT73 | -0.1149 | 4.158491 | -2.95556 | 0.003786 | 0.063565 | -2.22092 |
| PCNXL4 | -0.11496 | 3.918619 | -2.44953 | 0.01581 | 0.16633 | -3.47464 |
| TPCN2 | -0.11497 | 5.853002 | -2.39876 | 0.018056 | 0.180553 | -3.5889 |
| PLD4 | -0.11515 | 4.268835 | -2.47247 | 0.01488 | 0.160417 | -3.42231 |
| LPHN1 | -0.11527 | 5.214331 | -2.94768 | 0.003877 | 0.064416 | -2.242 |
| MRPL41 | -0.11553 | 5.00429 | -2.28019 | 0.024437 | 0.218522 | -3.84731 |
| SGCE | -0.11613 | 3.451125 | -2.26789 | 0.025201 | 0.221631 | -3.87343 |
| TSPYL1 | -0.11657 | 4.730333 | -2.17197 | 0.031909 | 0.255523 | -4.07273 |
| IL18BP | -0.11683 | 5.487873 | -2.61321 | 0.010167 | 0.122735 | -3.09173 |
| FLJ35934 | -0.11687 | 3.519998 | -2.02873 | 0.044793 | 0.308682 | -4.35557 |
| SYT17 | -0.11715 | 4.115151 | -2.71527 | 0.007642 | 0.101214 | -2.8419 |
| TSPAN3 | -0.11718 | 4.823393 | -3.0036 | 0.003274 | 0.056916 | -2.09135 |
| IFNLR1 | -0.11752 | 5.509949 | -2.16425 | 0.03251 | 0.256547 | -4.08843 |
| PIEZO1 | -0.1177 | 5.689959 | -1.99911 | 0.047952 | 0.322231 | -4.41182 |
| KCNJ2-AS | -0.11809 | 2.93102 | -2.23596 | 0.027282 | 0.233125 | -3.94065 |
| RPS28 | -0.1183 | 7.704196 | -2.87412 | 0.004827 | 0.074989 | -2.43647 |

| | | | | | | |
|---|---|---|---|---|---|---|
| NSDHL | -0.11904 | 5.06277 | -2.14246 | 0.034262 | 0.263561 | -4.13245 |
| CPSF7 | -0.1191 | 5.467439 | -2.67064 | 0.008667 | 0.110678 | -2.95219 |
| KCTD15 | -0.11941 | 4.368391 | -2.38116 | 0.018899 | 0.185895 | -3.62801 |
| SPIDR | -0.11958 | 6.152568 | -2.31826 | 0.022201 | 0.205789 | -3.76564 |
| BMS1 | -0.11966 | 6.408914 | -2.19107 | 0.030461 | 0.248821 | -4.03368 |
| EFTUD2 | -0.1199 | 6.461192 | -2.05035 | 0.042601 | 0.300382 | -4.31403 |
| POU2F1 | -0.11994 | 5.3922 | -2.95075 | 0.003841 | 0.06401 | -2.23377 |
| DHX30 | -0.11996 | 4.964819 | -3.6046 | 0.000463 | 0.013758 | -0.32506 |
| SCYL1 | -0.12022 | 5.108585 | -2.68734 | 0.008269 | 0.107027 | -2.9111 |
| SLC11A2 | -0.12039 | 4.668437 | -2.1756 | 0.031629 | 0.253902 | -4.06534 |
| SYNRG | -0.12045 | 6.646261 | -2.47962 | 0.0146 | 0.158796 | -3.40591 |
| RECK | -0.12054 | 4.059201 | -2.48325 | 0.01446 | 0.158051 | -3.39756 |
| PDE4A | -0.12098 | 4.36392 | -2.04216 | 0.04342 | 0.303459 | -4.3298 |
| STK36 | -0.12127 | 5.387506 | -2.16585 | 0.032385 | 0.256336 | -4.08518 |
| MZF1 | -0.12158 | 6.443674 | -2.54058 | 0.012398 | 0.141649 | -3.26437 |
| ZNF790-A | -0.12185 | 4.09222 | -2.54271 | 0.012327 | 0.141182 | -3.25937 |
| PKNOX1 | -0.12187 | 5.050804 | -3.18181 | 0.001882 | 0.038459 | -1.5953 |
| FAM193B | -0.12215 | 6.209758 | -2.07604 | 0.040116 | 0.288933 | -4.26412 |
| BLVRA | -0.12217 | 5.955835 | -2.08681 | 0.039112 | 0.284607 | -4.24303 |
| C1orf115 | -0.12262 | 4.103349 | -2.38254 | 0.018831 | 0.185611 | -3.62494 |
| C14orf64 | -0.12264 | 4.664948 | -2.62894 | 0.009734 | 0.119913 | -3.05377 |
| C8orf33 | -0.12268 | 4.575716 | -2.27338 | 0.024858 | 0.220189 | -3.8618 |
| MEI1 | -0.12287 | 5.440148 | -2.13922 | 0.03453 | 0.264626 | -4.13897 |
| SBF1 | -0.12288 | 5.592103 | -1.98193 | 0.04987 | 0.32976 | -4.4441 |
| FBXL12 | -0.1231 | 5.695509 | -2.87731 | 0.004781 | 0.074569 | -2.42811 |
| ZFYVE28 | -0.12358 | 4.355113 | -2.95676 | 0.003773 | 0.063565 | -2.2177 |
| TFAM | -0.12361 | 5.512218 | -2.09547 | 0.038321 | 0.280977 | -4.226 |
| RPS27A | -0.1239 | 6.988008 | -2.18538 | 0.030886 | 0.250651 | -4.04534 |
| RANGRF | -0.12406 | 5.474572 | -2.01698 | 0.046024 | 0.314333 | -4.37797 |
| WDR19 | -0.12408 | 3.551143 | -3.3743 | 0.001009 | 0.024038 | -1.03295 |
| DPYD | -0.12411 | 6.232994 | -2.26186 | 0.025583 | 0.223032 | -3.88619 |
| NAPSA | -0.12427 | 4.80597 | -2.1582 | 0.032989 | 0.25833 | -4.10069 |
| UTP23 | -0.12431 | 5.097588 | -2.03833 | 0.043808 | 0.304793 | -4.33717 |
| LOC10192 | -0.12447 | 4.859717 | -2.40975 | 0.017548 | 0.1782 | -3.56435 |
| ZNF138 | -0.1245 | 3.797974 | -2.13745 | 0.034676 | 0.265198 | -4.14252 |
| NOMO3 | -0.12467 | 6.261258 | -2.02415 | 0.045269 | 0.310502 | -4.36431 |
| MSR1 | -0.12483 | 3.449159 | -2.60306 | 0.010455 | 0.124963 | -3.11612 |
| CEP95 | -0.12486 | 4.461386 | -2.1168 | 0.03643 | 0.272452 | -4.18377 |
| RPP30 | -0.12495 | 4.135465 | -2.50196 | 0.013755 | 0.152864 | -3.35439 |
| ZNF677 | -0.12517 | 3.286964 | -2.30394 | 0.02302 | 0.21062 | -3.79651 |
| CEP104 | -0.12519 | 4.838355 | -2.28726 | 0.024008 | 0.216752 | -3.83224 |
| INPP5F | -0.12538 | 4.24243 | -2.22658 | 0.027921 | 0.235984 | -3.96023 |
| FAM135A | -0.12548 | 3.265359 | -2.35016 | 0.020468 | 0.195066 | -3.69626 |
| EMC10 | -0.12562 | 5.9663 | -2.00228 | 0.047605 | 0.32127 | -4.40583 |
| SLC8A1 | -0.12569 | 4.574403 | -1.9883 | 0.049151 | 0.327304 | -4.43217 |
| BMF | -0.12578 | 5.904665 | -2.15671 | 0.033108 | 0.258568 | -4.10371 |
| EFNA4 | -0.12582 | 5.000797 | -2.30017 | 0.02324 | 0.211632 | -3.8046 |
| NOC2L | -0.12595 | 5.069194 | -2.07935 | 0.039806 | 0.28771 | -4.25766 |
| HNRNPU | -0.12618 | 6.852844 | -2.17038 | 0.032032 | 0.255711 | -4.07597 |
| BCAS4 | -0.12622 | 4.295607 | -2.64415 | 0.009332 | 0.11639 | -3.01689 |
| HIGD2A | -0.1263 | 7.31889 | -2.30791 | 0.02279 | 0.209398 | -3.78796 |
| SLC30A4 | -0.12632 | 3.749595 | -2.39793 | 0.018095 | 0.180795 | -3.59074 |
| CCDC142 | -0.12654 | 3.957021 | -2.04522 | 0.043112 | 0.302602 | -4.32391 |
| ERVK13-1 | -0.12662 | 4.458152 | -3.18744 | 0.001848 | 0.037997 | -1.57926 |
| TMEM198I | -0.12663 | 5.733224 | -2.63466 | 0.009581 | 0.118818 | -3.03993 |
| KIAA0141 | -0.12689 | 5.625627 | -2.16975 | 0.03208 | 0.255711 | -4.07725 |
| ZNF500 | -0.12713 | 5.197155 | -2.48577 | 0.014363 | 0.157443 | -3.39177 |
| MIR4435-: | -0.12718 | 4.860273 | -2.1938 | 0.030259 | 0.247561 | -4.02806 |

| | | | | | | |
|---|---|---|---|---|---|---|
| TP53BP1 | -0.1272 | 4.906451 | -3.15555 | 0.002045 | 0.041015 | -1.66992 |
| LOC10192 | -0.12726 | 3.583136 | -2.05237 | 0.042401 | 0.299484 | -4.31012 |
| EHD4 | -0.12736 | 4.43492 | -3.70793 | 0.000323 | 0.010694 | 0.004576 |
| GPN2 | -0.12753 | 5.512264 | -2.26371 | 0.025465 | 0.222566 | -3.88228 |
| PPP6R2 | -0.12763 | 5.871386 | -3.68941 | 0.000345 | 0.011074 | -0.05505 |
| ZNF607 | -0.12774 | 3.536166 | -2.57747 | 0.011215 | 0.131742 | -3.17722 |
| ZBED5 | -0.12782 | 6.533643 | -3.21763 | 0.001679 | 0.035126 | -1.49272 |
| LOC10192 | -0.1279 | 6.786017 | -1.98995 | 0.048966 | 0.327114 | -4.42906 |
| ADAM19 | -0.12809 | 6.885471 | -2.74273 | 0.007068 | 0.09616 | -2.77325 |
| EFHC1 | -0.12846 | 4.519728 | -2.16582 | 0.032387 | 0.256336 | -4.08525 |
| PEG10 | -0.12847 | 3.962518 | -2.48176 | 0.014517 | 0.158433 | -3.40099 |
| HEATR6 | -0.12857 | 5.330689 | -2.34578 | 0.020698 | 0.196803 | -3.70582 |
| MATN1-A | -0.12863 | 3.368066 | -2.85748 | 0.005069 | 0.077521 | -2.47989 |
| TRIO | -0.12901 | 4.31097 | -4.50055 | 1.63E-05 | 0.001059 | 2.762747 |
| HMCES | -0.12966 | 5.783744 | -2.40994 | 0.017539 | 0.1782 | -3.56393 |
| PRO1082 | -0.12967 | 3.486248 | -2.46193 | 0.015301 | 0.162479 | -3.44642 |
| IRAK1 | -0.12982 | 6.774534 | -2.03137 | 0.04452 | 0.30795 | -4.35051 |
| DOPEY1 | -0.13008 | 4.155802 | -2.55385 | 0.01196 | 0.138061 | -3.23314 |
| KLHL20 | -0.1301 | 4.842575 | -2.05097 | 0.04254 | 0.300051 | -4.31283 |
| ELAC1 | -0.13098 | 4.049931 | -2.84898 | 0.005197 | 0.079006 | -2.50196 |
| WDR81 | -0.13104 | 7.00308 | -2.53091 | 0.012726 | 0.144229 | -3.28701 |
| ANKRD37 | -0.13112 | 4.373429 | -2.62852 | 0.009746 | 0.119913 | -3.0548 |
| TBL3 | -0.13139 | 5.26451 | -3.50981 | 0.000641 | 0.017465 | -0.62095 |
| ACTR5 | -0.13142 | 4.848852 | -2.55732 | 0.011848 | 0.137356 | -3.22495 |
| TIMM50 | -0.1318 | 5.175896 | -2.18692 | 0.030771 | 0.250329 | -4.04219 |
| TCF25 | -0.13188 | 7.103932 | -2.24215 | 0.026867 | 0.230696 | -3.92768 |
| ZNF175 | -0.1321 | 4.339225 | -2.84636 | 0.005237 | 0.079262 | -2.50876 |
| HMBOX1 | -0.13222 | 6.329425 | -2.07715 | 0.040012 | 0.288487 | -4.26196 |
| LOC93444 | -0.13222 | 4.935147 | -2.17029 | 0.032038 | 0.255711 | -4.07615 |
| CD84 | -0.13225 | 5.325925 | -3.03628 | 0.002963 | 0.053271 | -2.00219 |
| C9orf114 | -0.13264 | 5.385803 | -2.31903 | 0.022157 | 0.205769 | -3.76398 |
| ZNF502 | -0.13292 | 4.637571 | -2.78129 | 0.006328 | 0.089411 | -2.67584 |
| MAFK | -0.13308 | 5.940818 | -2.12551 | 0.035681 | 0.270026 | -4.16642 |
| MDN1 | -0.13338 | 4.194226 | -2.1192 | 0.036222 | 0.271794 | -4.179 |
| LIMD1 | -0.13397 | 5.044327 | -4.20339 | 5.22E-05 | 0.002643 | 1.683047 |
| RPL27 | -0.13417 | 12.57627 | -2.23625 | 0.027262 | 0.233125 | -3.94005 |
| MID1 | -0.13425 | 3.848505 | -2.73871 | 0.00715 | 0.096885 | -2.78335 |
| CD40 | -0.13439 | 5.319969 | -2.35643 | 0.020141 | 0.193218 | -3.68252 |
| NAT9 | -0.13446 | 6.193072 | -2.00717 | 0.047075 | 0.318321 | -4.3966 |
| PDCD6 | -0.13456 | 7.007906 | -2.04728 | 0.042906 | 0.301645 | -4.31994 |
| FEM1A | -0.13495 | 6.111178 | -3.02484 | 0.003068 | 0.054643 | -2.03349 |
| PROSER3 | -0.13501 | 4.878486 | -2.6752 | 0.008556 | 0.10977 | -2.94099 |
| RAB3GAP2 | -0.13502 | 3.723128 | -2.49341 | 0.014073 | 0.154822 | -3.37416 |
| LAMB2 | -0.1351 | 4.226768 | -2.07302 | 0.040402 | 0.290375 | -4.27001 |
| LRIG2 | -0.13514 | 4.345186 | -3.09187 | 0.002495 | 0.046931 | -1.84865 |
| FBXO32 | -0.13517 | 3.813576 | -2.71299 | 0.007692 | 0.101642 | -2.84758 |
| PLXNA1 | -0.13555 | 4.903318 | -3.71076 | 0.00032 | 0.010623 | 0.013703 |
| TCEA2 | -0.13556 | 4.52326 | -2.29637 | 0.023464 | 0.213195 | -3.81276 |
| ZNF627 | -0.13559 | 5.093398 | -2.10744 | 0.03725 | 0.276562 | -4.20236 |
| SLC25A14 | -0.13571 | 6.072558 | -2.48085 | 0.014552 | 0.158471 | -3.40309 |
| SCAMP3 | -0.13586 | 7.670461 | -1.98229 | 0.049828 | 0.32976 | -4.44342 |
| SFI1 | -0.13594 | 5.802972 | -2.15783 | 0.033019 | 0.258339 | -4.10146 |
| LOC33998 | -0.13617 | 6.138772 | -2.42676 | 0.016785 | 0.173495 | -3.52616 |
| EIF4E2 | -0.13632 | 6.817784 | -2.37991 | 0.01896 | 0.186072 | -3.63077 |
| NDUFB8 | -0.13634 | 7.599092 | -2.19786 | 0.02996 | 0.245942 | -4.01972 |
| ZNF235 | -0.13644 | 4.023765 | -2.93058 | 0.004081 | 0.066645 | -2.28757 |
| AKAP8 | -0.13718 | 6.529507 | -2.61199 | 0.010201 | 0.123076 | -3.09466 |
| RGPD4-AS | -0.13718 | 2.671214 | -2.38102 | 0.018906 | 0.185895 | -3.62831 |

| | | | | | | |
|---|---|---|---|---|---|---|
| HLA-F-AS | -0.13724 | 4.034199 | -2.22642 | 0.027932 | 0.235984 | -3.96058 |
| CYP1B1-A | -0.13758 | 3.769565 | -2.98209 | 0.003495 | 0.059443 | -2.14956 |
| KAT2A | -0.13762 | 6.593644 | -1.99106 | 0.048843 | 0.326395 | -4.42698 |
| LOC38964 | -0.13775 | 4.485658 | -2.76699 | 0.006594 | 0.091787 | -2.71211 |
| PPM1N | -0.13808 | 5.196936 | -2.03026 | 0.044635 | 0.308344 | -4.35264 |
| FPGS | -0.13821 | 5.528929 | -2.26692 | 0.025262 | 0.221743 | -3.8755 |
| AP1AR | -0.13851 | 3.562496 | -2.11942 | 0.036204 | 0.271794 | -4.17857 |
| CCDC94 | -0.13877 | 5.56815 | -2.14668 | 0.033917 | 0.261892 | -4.12397 |
| SH3BP5-A | -0.1388 | 5.849316 | -2.11554 | 0.03654 | 0.273073 | -4.18629 |
| ELK4 | -0.13899 | 4.76055 | -2.26216 | 0.025564 | 0.223032 | -3.88556 |
| TMTC4 | -0.13911 | 4.268169 | -2.7768 | 0.00641 | 0.090272 | -2.68725 |
| GLB1L2 | -0.13932 | 5.565221 | -2.19443 | 0.030212 | 0.247282 | -4.02678 |
| ZNF485 | -0.13948 | 2.708728 | -2.42102 | 0.017039 | 0.175321 | -3.53907 |
| MON2 | -0.13961 | 5.254758 | -1.9823 | 0.049828 | 0.32976 | -4.4434 |
| CYP2S1 | -0.13969 | 4.348174 | -2.14948 | 0.03369 | 0.260793 | -4.11833 |
| ADPRH | -0.13985 | 4.813432 | -2.73123 | 0.007304 | 0.098256 | -2.80209 |
| ODF2L | -0.13997 | 3.615726 | -2.99052 | 0.003407 | 0.058564 | -2.12679 |
| PNPO | -0.14005 | 5.402781 | -2.27017 | 0.025058 | 0.221102 | -3.86861 |
| ZMYM6 | -0.14012 | 4.32201 | -2.39658 | 0.018159 | 0.181223 | -3.59377 |
| CST4 | -0.14024 | 5.357794 | -2.50133 | 0.013779 | 0.15298 | -3.35586 |
| IL11RA | -0.14055 | 5.146992 | -2.31609 | 0.022323 | 0.206315 | -3.77034 |
| UQCRQ | -0.14065 | 8.814303 | -2.06649 | 0.041026 | 0.292803 | -4.28275 |
| CWF19L2 | -0.14066 | 4.265488 | -2.43697 | 0.016342 | 0.170083 | -3.50311 |
| ALKBH2 | -0.14089 | 4.143446 | -2.47746 | 0.014684 | 0.159313 | -3.41087 |
| FAM3A | -0.14107 | 6.143725 | -2.22127 | 0.028289 | 0.238162 | -3.9713 |
| TARBP2 | -0.14109 | 6.145607 | -2.46826 | 0.015047 | 0.161115 | -3.43195 |
| ZNF569 | -0.14119 | 3.90159 | -2.25242 | 0.026191 | 0.226223 | -3.90611 |
| PRDM4 | -0.14177 | 6.779555 | -2.43448 | 0.016449 | 0.170942 | -3.50874 |
| GIGYF1 | -0.14182 | 5.877445 | -2.40195 | 0.017907 | 0.179884 | -3.58178 |
| SERPINB9F | -0.14193 | 5.212349 | -2.09145 | 0.038686 | 0.282505 | -4.23391 |
| SLC7A6 | -0.14217 | 5.77582 | -2.16868 | 0.032164 | 0.255711 | -4.07943 |
| ITPRIPL2 | -0.14228 | 5.386511 | -2.42374 | 0.016918 | 0.174574 | -3.53295 |
| QRICH1 | -0.14246 | 6.739661 | -2.60461 | 0.010411 | 0.124648 | -3.11239 |
| SIN3B | -0.14249 | 5.868868 | -2.31655 | 0.022297 | 0.206258 | -3.76933 |
| VASH1 | -0.14313 | 4.534173 | -2.7354 | 0.007217 | 0.097442 | -2.79165 |
| FAM168B | -0.14332 | 6.319279 | -2.61606 | 0.010087 | 0.122411 | -3.08488 |
| KDM2A | -0.14337 | 8.426082 | -2.02986 | 0.044676 | 0.308523 | -4.3534 |
| RNPS1 | -0.14338 | 8.25731 | -2.75718 | 0.006782 | 0.093574 | -2.73688 |
| LOC64551 | -0.14344 | 4.115289 | -2.16203 | 0.032686 | 0.256841 | -4.09294 |
| ZNF638 | -0.14367 | 7.55483 | -2.05608 | 0.042036 | 0.297418 | -4.30294 |
| LOC10099 | -0.14368 | 3.987414 | -2.04379 | 0.043256 | 0.302978 | -4.32667 |
| SOX4 | -0.14368 | 4.732358 | -2.70599 | 0.007846 | 0.102976 | -2.86497 |
| PAM16 | -0.1438 | 6.51981 | -2.2522 | 0.026205 | 0.22625 | -3.90657 |
| TNFRSF10/ | -0.1439 | 6.176138 | -2.01511 | 0.046223 | 0.314854 | -4.38154 |
| ASB1 | -0.14406 | 4.72607 | -2.5733 | 0.011344 | 0.132901 | -3.18713 |
| KCNH8 | -0.14407 | 3.311392 | -2.6489 | 0.009209 | 0.115493 | -3.00534 |
| FUOM | -0.14413 | 5.405376 | -2.46324 | 0.015248 | 0.162165 | -3.44341 |
| ARHGEF9 | -0.1442 | 4.37477 | -2.36116 | 0.019898 | 0.191514 | -3.67213 |
| GTF3C1 | -0.14449 | 6.902391 | -2.17958 | 0.031325 | 0.252351 | -4.05721 |
| ZXDB | -0.14459 | 3.430923 | -2.28657 | 0.024049 | 0.216769 | -3.83371 |
| IKBKE | -0.14459 | 6.178199 | -2.42542 | 0.016844 | 0.173985 | -3.52917 |
| LOC10027 | -0.14463 | 4.619519 | -2.36864 | 0.019519 | 0.189491 | -3.65567 |
| RAD51-AS | -0.14466 | 4.530135 | -2.38779 | 0.018578 | 0.183817 | -3.61331 |
| GALNT6 | -0.14479 | 5.034972 | -2.01475 | 0.046261 | 0.314904 | -4.38222 |
| RBM48 | -0.14513 | 4.805829 | -2.26691 | 0.025263 | 0.221743 | -3.87551 |
| RPUSD2 | -0.14527 | 4.359954 | -2.44678 | 0.015926 | 0.166941 | -3.4809 |
| OCEL1 | -0.14527 | 6.805971 | -2.20622 | 0.029354 | 0.243372 | -4.00248 |
| TMEM51 | -0.1455 | 4.56132 | -2.54328 | 0.012308 | 0.141143 | -3.25802 |

| | | | | | | |
|---|---|---|---|---|---|---|
| SURF1 | -0.14589 | 7.303202 | -2.89085 | 0.004594 | 0.072639 | -2.39262 |
| DYM | -0.14602 | 5.678351 | -2.31339 | 0.022476 | 0.207279 | -3.77616 |
| FAM127A | -0.14639 | 8.181863 | -2.03445 | 0.044204 | 0.306819 | -4.34462 |
| ZNF415 | -0.14642 | 2.864878 | -2.73184 | 0.007291 | 0.098208 | -2.80054 |
| MED30 | -0.14667 | 6.060576 | -2.28195 | 0.02433 | 0.218156 | -3.84357 |
| GGA2 | -0.14685 | 5.314097 | -3.36783 | 0.001031 | 0.024374 | -1.05228 |
| THAP4 | -0.14692 | 6.181877 | -2.87257 | 0.004849 | 0.075105 | -2.44052 |
| TBL2 | -0.147 | 5.599776 | -2.56299 | 0.011667 | 0.135873 | -3.21156 |
| FAM200A | -0.14718 | 2.959514 | -2.79611 | 0.006063 | 0.08675 | -2.63808 |
| CSK | -0.1473 | 10.17873 | -2.19941 | 0.029847 | 0.245276 | -4.01653 |
| ZNF540 | -0.14738 | 3.787816 | -3.69282 | 0.000341 | 0.011046 | -0.04408 |
| ZNF844 | -0.14741 | 4.77653 | -2.11751 | 0.036369 | 0.272393 | -4.18237 |
| ZNF783 | -0.14753 | 6.284949 | -2.81934 | 0.005667 | 0.08364 | -2.57854 |
| AS3MT | -0.14765 | 3.379038 | -2.55528 | 0.011914 | 0.13773 | -3.22977 |
| TICAM1 | -0.14793 | 5.728537 | -2.09779 | 0.038111 | 0.280412 | -4.22142 |
| AFG3L1P | -0.14807 | 5.305035 | -2.25386 | 0.026097 | 0.225889 | -3.90307 |
| ANAPC16 | -0.14812 | 9.151226 | -2.20368 | 0.029537 | 0.244103 | -4.00773 |
| GDPD5 | -0.1483 | 6.256075 | -2.28139 | 0.024364 | 0.218367 | -3.84476 |
| ZNF736 | -0.14832 | 3.482348 | -2.16647 | 0.032336 | 0.256262 | -4.08393 |
| NGRN | -0.14839 | 6.539401 | -1.98201 | 0.049861 | 0.32976 | -4.44395 |
| ATL2 | -0.14841 | 4.649759 | -2.4966 | 0.013954 | 0.154006 | -3.3668 |
| ZNF224 | -0.14856 | 5.115098 | -2.21353 | 0.028832 | 0.2409 | -3.98735 |
| LRRK1 | -0.14866 | 5.244958 | -2.12514 | 0.035713 | 0.270126 | -4.16716 |
| ERI3 | -0.14874 | 5.126232 | -2.13738 | 0.034682 | 0.265198 | -4.14266 |
| CEP164 | -0.14882 | 5.415129 | -3.66821 | 0.000371 | 0.011636 | -0.123 |
| BRWD1 | -0.14894 | 4.926631 | -2.13353 | 0.035004 | 0.266659 | -4.15038 |
| ACKR3 | -0.14918 | 3.74866 | -2.25771 | 0.025849 | 0.224592 | -3.89496 |
| ZNF526 | -0.1492 | 4.321752 | -2.68329 | 0.008364 | 0.108048 | -2.92108 |
| DDX56 | -0.14937 | 5.827621 | -3.00769 | 0.003233 | 0.056499 | -2.08023 |
| PCNXL2 | -0.14938 | 4.350041 | -3.43257 | 0.000831 | 0.021145 | -0.8574 |
| RPL35A | -0.14952 | 7.394431 | -2.69267 | 0.008146 | 0.105835 | -2.89795 |
| ZNF134 | -0.14956 | 5.398198 | -2.16967 | 0.032087 | 0.255711 | -4.07741 |
| ZNF678 | -0.15004 | 3.592687 | -2.45556 | 0.015561 | 0.164381 | -3.46093 |
| UBE2G2 | -0.15012 | 5.803949 | -3.09397 | 0.002479 | 0.046799 | -1.84282 |
| RGP1 | -0.15062 | 6.289878 | -2.65571 | 0.009036 | 0.114274 | -2.98873 |
| CDK5RAP1 | -0.15066 | 6.533169 | -2.46886 | 0.015023 | 0.161113 | -3.43059 |
| LACC1 | -0.15081 | 3.287833 | -2.37665 | 0.01912 | 0.187017 | -3.63799 |
| SHARPIN | -0.15089 | 6.312762 | -2.20791 | 0.029233 | 0.242829 | -3.99899 |
| URGCP | -0.15098 | 5.683189 | -2.25337 | 0.026129 | 0.225976 | -3.90412 |
| ZNF296 | -0.15112 | 4.980512 | -2.17179 | 0.031922 | 0.255523 | -4.0731 |
| PAF1 | -0.15116 | 6.658408 | -2.29286 | 0.023672 | 0.214515 | -3.82027 |
| SND1 | -0.15166 | 7.234122 | -2.17739 | 0.031492 | 0.253197 | -4.06168 |
| PLA2G15 | -0.1518 | 5.110832 | -2.31892 | 0.022164 | 0.205769 | -3.76422 |
| DICER1 | -0.15194 | 6.841026 | -2.93398 | 0.00404 | 0.066182 | -2.27852 |
| ZNF585B | -0.152 | 3.821181 | -2.20361 | 0.029542 | 0.244103 | -4.00787 |
| LYVE1 | -0.15246 | 3.739619 | -2.63294 | 0.009627 | 0.119025 | -3.04409 |
| ZNF570 | -0.15255 | 3.856287 | -2.93766 | 0.003995 | 0.065667 | -2.26874 |
| HDAC1 | -0.15291 | 8.799531 | -2.27481 | 0.024769 | 0.219809 | -3.85876 |
| INTS9 | -0.15325 | 5.734023 | -3.5203 | 0.000619 | 0.017035 | -0.58851 |
| MPRIP | -0.15338 | 5.880285 | -3.96477 | 0.000128 | 0.005332 | 0.854946 |
| ADAT2 | -0.15347 | 3.889767 | -2.74455 | 0.007031 | 0.095789 | -2.76867 |
| FER | -0.15349 | 4.514821 | -3.65207 | 0.000393 | 0.01218 | -0.17453 |
| MADD | -0.15352 | 6.980038 | -2.49942 | 0.013849 | 0.153264 | -3.36028 |
| SIN3A | -0.15373 | 5.195821 | -2.31854 | 0.022185 | 0.205789 | -3.76504 |
| ZNF76 | -0.15373 | 6.103728 | -2.50656 | 0.013587 | 0.151913 | -3.34373 |
| ZNF511 | -0.15376 | 6.593641 | -2.31974 | 0.022117 | 0.205618 | -3.76244 |
| LOC10013 | -0.15382 | 4.314779 | -2.12681 | 0.035571 | 0.269584 | -4.16382 |
| GLIDR | -0.15386 | 5.073217 | -2.58924 | 0.01086 | 0.128446 | -3.14919 |

| | | | | | | |
|---|---|---|---|---|---|---|
| ANKLE2 | -0.15408 | 5.045693 | -2.94757 | 0.003878 | 0.064416 | -2.2423 |
| SMAGP | -0.15413 | 4.947898 | -1.99437 | 0.048475 | 0.324997 | -4.42075 |
| MUM1 | -0.15431 | 5.476593 | -2.59186 | 0.010782 | 0.127674 | -3.14293 |
| ELP2 | -0.15434 | 5.109995 | -2.96619 | 0.003667 | 0.062062 | -2.19239 |
| SLC5A9 | -0.15437 | 4.224237 | -3.0244 | 0.003073 | 0.05467 | -2.0347 |
| ZFP14 | -0.15438 | 3.669577 | -2.36188 | 0.019861 | 0.191249 | -3.67055 |
| OTUD3 | -0.15449 | 4.793912 | -2.01668 | 0.046056 | 0.314345 | -4.37856 |
| RLN2 | -0.15454 | 3.324533 | -2.55469 | 0.011933 | 0.137874 | -3.23117 |
| LOC10099 | -0.15454 | 3.409521 | -2.64823 | 0.009227 | 0.115611 | -3.00697 |
| PNPLA4 | -0.15465 | 4.298393 | -2.80549 | 0.0059 | 0.085371 | -2.61408 |
| XCL1 | -0.1547 | 4.185366 | -2.18177 | 0.031159 | 0.251786 | -4.05274 |
| BCDIN3D | -0.15474 | 5.622564 | -2.29055 | 0.02381 | 0.215381 | -3.8252 |
| LOC10050 | -0.15495 | 3.303118 | -2.42679 | 0.016784 | 0.173495 | -3.52608 |
| COL9A2 | -0.1551 | 5.394192 | -2.08375 | 0.039395 | 0.285395 | -4.24903 |
| SMARCC2 | -0.15515 | 5.950908 | -2.283 | 0.024266 | 0.217869 | -3.84133 |
| ZNF682 | -0.15521 | 3.157128 | -3.16828 | 0.001964 | 0.039829 | -1.63382 |
| MRPS18B | -0.15549 | 6.757274 | -2.13785 | 0.034643 | 0.265162 | -4.14171 |
| ZBTB10 | -0.15584 | 3.423654 | -3.43383 | 0.000828 | 0.021113 | -0.85356 |
| SLC25A3 | -0.15587 | 7.997485 | -2.29265 | 0.023685 | 0.214534 | -3.82073 |
| ZNF256 | -0.1563 | 3.29494 | -2.71964 | 0.007548 | 0.100358 | -2.83103 |
| DNPEP | -0.15645 | 5.131138 | -2.35777 | 0.020072 | 0.192825 | -3.67958 |
| KIAA1407 | -0.15651 | 4.979076 | -2.02404 | 0.045281 | 0.310502 | -4.36452 |
| LOC10013 | -0.15663 | 3.255799 | -2.49969 | 0.013839 | 0.153236 | -3.35965 |
| ATG16L1 | -0.1568 | 4.966231 | -2.58121 | 0.011101 | 0.130697 | -3.16833 |
| ZNF317 | -0.15711 | 5.495198 | -2.26886 | 0.02514 | 0.221389 | -3.87139 |
| SND1-IT1 | -0.15713 | 5.036915 | -2.45314 | 0.015661 | 0.16518 | -3.46644 |
| FCF1 | -0.15731 | 4.517272 | -2.01385 | 0.046357 | 0.315447 | -4.38392 |
| INO80 | -0.15735 | 6.635864 | -2.76144 | 0.0067 | 0.092626 | -2.72614 |
| DTWD1 | -0.15736 | 3.559745 | -2.87996 | 0.004744 | 0.074214 | -2.42118 |
| SON | -0.1574 | 9.459229 | -2.62141 | 0.009939 | 0.121059 | -3.07198 |
| EVI5 | -0.15766 | 4.954787 | -2.66182 | 0.008883 | 0.112686 | -2.97379 |
| IGIP | -0.15779 | 5.498788 | -2.26836 | 0.025171 | 0.221528 | -3.87244 |
| MICALCL | -0.15785 | 3.827282 | -2.21788 | 0.028526 | 0.23922 | -3.97833 |
| SLC4A7 | -0.15789 | 4.021625 | -2.58418 | 0.011011 | 0.129865 | -3.16126 |
| MLLT6 | -0.1579 | 5.85869 | -2.27732 | 0.024613 | 0.219354 | -3.85341 |
| MGC1627! | -0.15796 | 4.234219 | -2.38928 | 0.018506 | 0.183378 | -3.61001 |
| MAPK7 | -0.15805 | 5.905438 | -2.21 | 0.029083 | 0.242177 | -3.99466 |
| RPLP1 | -0.15813 | 8.698882 | -2.95218 | 0.003825 | 0.063856 | -2.22996 |
| ZNF814 | -0.15827 | 3.995478 | -2.29481 | 0.023556 | 0.213936 | -3.81609 |
| METTL17 | -0.15853 | 6.426438 | -2.14681 | 0.033907 | 0.261892 | -4.12371 |
| PC | -0.15861 | 4.010426 | -2.21654 | 0.02862 | 0.239606 | -3.98111 |
| VPS36 | -0.15868 | 5.433109 | -2.22962 | 0.027713 | 0.235195 | -3.9539 |
| ATG101 | -0.15871 | 6.515725 | -2.01528 | 0.046204 | 0.314829 | -4.3812 |
| POLG2 | -0.15882 | 5.577747 | -2.22367 | 0.028122 | 0.237026 | -3.9663 |
| SUPT5H | -0.15892 | 7.30882 | -1.98897 | 0.049076 | 0.327304 | -4.4309 |
| TTBK2 | -0.15894 | 3.931354 | -3.51379 | 0.000632 | 0.017342 | -0.60865 |
| LOC10106 | -0.15897 | 3.053391 | -2.22782 | 0.027836 | 0.235653 | -3.95764 |
| POLR2B | -0.15905 | 7.362106 | -3.22211 | 0.001655 | 0.034877 | -1.47984 |
| ZMYM2 | -0.15906 | 5.450702 | -2.14952 | 0.033686 | 0.260793 | -4.11825 |
| RNASET2 | -0.15911 | 7.525425 | -2.18722 | 0.030748 | 0.250329 | -4.04158 |
| ASXL1 | -0.15927 | 6.018148 | -2.17979 | 0.031309 | 0.252351 | -4.05678 |
| FTSJ1 | -0.15929 | 6.229537 | -2.56977 | 0.011454 | 0.133921 | -3.19551 |
| WDR35 | -0.15946 | 4.205945 | -2.26879 | 0.025145 | 0.221389 | -3.87154 |
| SPRED1 | -0.15968 | 2.858543 | -3.20132 | 0.001769 | 0.03674 | -1.53956 |
| EIF3K | -0.1603 | 8.4398 | -2.67421 | 0.00858 | 0.110006 | -2.94343 |
| LOC10065 | -0.16033 | 4.304941 | -2.46346 | 0.015239 | 0.162158 | -3.44293 |
| HTT | -0.1604 | 6.62066 | -2.88368 | 0.004692 | 0.073741 | -2.41143 |
| KATNAL1 | -0.16044 | 3.963656 | -2.40041 | 0.017979 | 0.180219 | -3.58522 |

| | | | | | | |
|---|---|---|---|---|---|---|
| RAPH1 | -0.16045 | 3.311841 | -3.08508 | 0.002549 | 0.047538 | -1.86754 |
| MYO5A | -0.16055 | 5.804947 | -2.07853 | 0.039882 | 0.287956 | -4.25925 |
| ZNF382 | -0.16058 | 3.650318 | -2.54314 | 0.012313 | 0.141143 | -3.25835 |
| EML3 | -0.16063 | 6.035488 | -2.1587 | 0.032949 | 0.258149 | -4.09968 |
| VPS13D | -0.16088 | 5.48545 | -2.88517 | 0.004672 | 0.07374 | -2.40753 |
| ZNF443 | -0.1609 | 4.513048 | -2.62512 | 0.009838 | 0.120408 | -3.06302 |
| TMED8 | -0.161 | 7.713912 | -2.09834 | 0.038062 | 0.280253 | -4.22035 |
| PEX5 | -0.16111 | 5.430897 | -2.82426 | 0.005587 | 0.082775 | -2.56588 |
| KLF12 | -0.16117 | 4.486644 | -2.80964 | 0.005829 | 0.084668 | -2.60345 |
| PLBD2 | -0.16124 | 5.813326 | -2.70217 | 0.007931 | 0.103564 | -2.87445 |
| SOCS7 | -0.16129 | 5.40368 | -2.13804 | 0.034628 | 0.265162 | -4.14134 |
| LOC10050 | -0.16158 | 3.294308 | -2.37826 | 0.019041 | 0.186511 | -3.63444 |
| ANKRD40 | -0.16159 | 5.114287 | -3.7283 | 0.000301 | 0.01017 | 0.070406 |
| AMDHD1 | -0.1616 | 3.176068 | -2.64384 | 0.00934 | 0.11639 | -3.01765 |
| SNAPC5 | -0.16166 | 6.012418 | -2.62752 | 0.009773 | 0.120151 | -3.05721 |
| KIFC2 | -0.16194 | 5.725407 | -2.15218 | 0.033471 | 0.260413 | -4.11287 |
| RAD50 | -0.16207 | 4.837802 | -2.03498 | 0.04415 | 0.306618 | -4.3436 |
| TMEM70 | -0.16223 | 6.32555 | -2.29426 | 0.023588 | 0.214043 | -3.81727 |
| CHN2 | -0.16231 | 4.600305 | -2.726 | 0.007413 | 0.099142 | -2.81516 |
| CCDC88A | -0.16233 | 4.456673 | -2.15865 | 0.032953 | 0.258149 | -4.09979 |
| BRICD5 | -0.16245 | 5.37133 | -2.12472 | 0.035749 | 0.270126 | -4.168 |
| RPL39 | -0.16246 | 13.10582 | -2.60577 | 0.010378 | 0.124326 | -3.10963 |
| CASK | -0.16264 | 4.027557 | -3.76998 | 0.000259 | 0.009076 | 0.206015 |
| PPP2CA | -0.16288 | 6.814131 | -2.1268 | 0.035572 | 0.269584 | -4.16386 |
| ZNF548 | -0.16313 | 4.931416 | -2.1688 | 0.032155 | 0.255711 | -4.07919 |
| RPL37 | -0.16315 | 12.40443 | -2.59699 | 0.010631 | 0.126694 | -3.13065 |
| SFSWAP | -0.16329 | 5.888718 | -2.32468 | 0.021842 | 0.204188 | -3.75174 |
| NUBP2 | -0.16339 | 6.313818 | -2.31411 | 0.022435 | 0.207069 | -3.7746 |
| RASGRF2 | -0.16345 | 5.262916 | -2.0029 | 0.047537 | 0.320921 | -4.40467 |
| SYS1 | -0.16362 | 5.847053 | -2.8042 | 0.005922 | 0.085564 | -2.61739 |
| DCHS1 | -0.16428 | 4.577775 | -3.01023 | 0.003208 | 0.056256 | -2.07332 |
| XRCC1 | -0.16449 | 6.033827 | -2.04305 | 0.043331 | 0.303226 | -4.3281 |
| KBTBD3 | -0.16455 | 4.326932 | -2.06751 | 0.040927 | 0.292507 | -4.28075 |
| MRPL24 | -0.16464 | 6.377968 | -2.09359 | 0.038492 | 0.2816 | -4.22971 |
| CYB5A | -0.16465 | 5.042947 | -2.26571 | 0.025338 | 0.222139 | -3.87805 |
| ZFP36L1 | -0.16469 | 6.528667 | -2.1134 | 0.036726 | 0.273869 | -4.19055 |
| C11orf31 | -0.16471 | 6.673479 | -2.9527 | 0.003819 | 0.063856 | -2.22856 |
| NT5C3B | -0.16493 | 6.196744 | -2.0847 | 0.039307 | 0.285266 | -4.24717 |
| KLF8 | -0.165 | 3.559634 | -3.86555 | 0.000184 | 0.006968 | 0.521283 |
| LDB1 | -0.16507 | 7.29576 | -2.46356 | 0.015235 | 0.162158 | -3.4427 |
| ARIH2 | -0.16514 | 5.765204 | -2.42775 | 0.016742 | 0.173276 | -3.52392 |
| IMPDH1 | -0.16539 | 8.900016 | -2.81885 | 0.005675 | 0.08364 | -2.57981 |
| KLF9 | -0.16554 | 4.870327 | -2.73163 | 0.007295 | 0.098208 | -2.80108 |
| RPS15A | -0.16562 | 7.566847 | -3.78612 | 0.000245 | 0.008739 | 0.258813 |
| PDCD11 | -0.16572 | 5.712238 | -2.94209 | 0.003943 | 0.06506 | -2.2569 |
| SYNGR3 | -0.16576 | 3.892515 | -3.30198 | 0.001279 | 0.028891 | -1.24742 |
| NECAP2 | -0.16602 | 6.499939 | -2.92885 | 0.004102 | 0.066831 | -2.29217 |
| CNOT6L | -0.16617 | 5.299943 | -2.77119 | 0.006514 | 0.091051 | -2.70146 |
| TMEM129 | -0.16637 | 5.235541 | -2.62952 | 0.009719 | 0.119913 | -3.05237 |
| PIK3IP1 | -0.16638 | 6.311597 | -2.24872 | 0.026433 | 0.227549 | -3.91389 |
| LPP | -0.16669 | 5.840033 | -2.48464 | 0.014406 | 0.157721 | -3.39437 |
| TAF1C | -0.16675 | 5.834697 | -3.02001 | 0.003114 | 0.055193 | -2.04668 |
| NKTR | -0.16689 | 5.720012 | -2.00064 | 0.047784 | 0.321737 | -4.40893 |
| PIM1 | -0.16702 | 9.314136 | -2.11924 | 0.036219 | 0.271794 | -4.17893 |
| 9-Sep | -0.1671 | 6.946791 | -2.30219 | 0.023122 | 0.211117 | -3.80026 |
| RBMX2 | -0.16716 | 6.06895 | -2.29388 | 0.023611 | 0.214152 | -3.81808 |
| CD244 | -0.16735 | 4.856809 | -3.10092 | 0.002426 | 0.046095 | -1.82345 |
| SLC41A1 | -0.16739 | 5.335735 | -2.39627 | 0.018174 | 0.181223 | -3.59445 |

| | | | | | | |
|---|---|---|---|---|---|---|
| TPT1-AS1 | -0.16755 | 4.04585 | -3.13367 | 0.00219 | 0.042766 | -1.73167 |
| NEDD9 | -0.16757 | 6.285947 | -2.15534 | 0.033217 | 0.258973 | -4.10649 |
| FAM102A | -0.16764 | 5.157791 | -2.46764 | 0.015072 | 0.161295 | -3.43337 |
| NEK8 | -0.16777 | 6.354758 | -1.99801 | 0.048073 | 0.322834 | -4.41391 |
| SDHAF1 | -0.16779 | 5.963783 | -2.40304 | 0.017857 | 0.179806 | -3.57934 |
| PPP1CB | -0.16787 | 9.509779 | -2.00987 | 0.046783 | 0.316767 | -4.39147 |
| DGKD | -0.16789 | 8.725783 | -2.12136 | 0.036037 | 0.271162 | -4.1747 |
| ZFHX3 | -0.16796 | 4.459084 | -3.23398 | 0.001593 | 0.033925 | -1.44558 |
| ZNF202 | -0.16805 | 4.346016 | -2.28491 | 0.02415 | 0.217368 | -3.83726 |
| OLFML2B | -0.16811 | 4.166214 | -2.77262 | 0.006488 | 0.090925 | -2.69783 |
| SUN2 | -0.16812 | 8.474122 | -2.27024 | 0.025053 | 0.221102 | -3.86846 |
| MYO7A | -0.16813 | 4.403107 | -2.68236 | 0.008386 | 0.108265 | -2.92339 |
| PGLS | -0.16856 | 6.8502 | -2.79797 | 0.00603 | 0.086465 | -2.63332 |
| YBEY | -0.16861 | 5.682807 | -2.10547 | 0.037424 | 0.277355 | -4.20626 |
| MRPL52 | -0.16868 | 5.515697 | -2.32508 | 0.02182 | 0.204148 | -3.75087 |
| ARHGEF18 | -0.16908 | 9.227199 | -2.4167 | 0.017233 | 0.176572 | -3.54878 |
| GPR17 | -0.16909 | 3.509515 | -4.00521 | 0.00011 | 0.004819 | 0.992755 |
| IFFO1 | -0.16945 | 7.724705 | -2.09624 | 0.038251 | 0.280938 | -4.22448 |
| EIF2B4 | -0.16946 | 6.915968 | -2.54104 | 0.012383 | 0.141552 | -3.26329 |
| NTPCR | -0.16948 | 5.635997 | -2.40205 | 0.017903 | 0.179884 | -3.58157 |
| ZNF467 | -0.16966 | 7.265278 | -2.56226 | 0.01169 | 0.135975 | -3.2133 |
| EXOSC5 | -0.16974 | 6.279967 | -2.32949 | 0.021576 | 0.202519 | -3.74131 |
| UBE4A | -0.16999 | 9.604866 | -2.19513 | 0.030161 | 0.247157 | -4.02534 |
| CHMP7 | -0.17004 | 7.23665 | -2.24807 | 0.026476 | 0.227815 | -3.91526 |
| UXT | -0.17008 | 9.461397 | -2.27971 | 0.024467 | 0.218522 | -3.84833 |
| UBE2I | -0.17021 | 6.185664 | -3.82864 | 0.00021 | 0.007732 | 0.398796 |
| B3GALT6 | -0.17025 | 5.282117 | -3.63032 | 0.000424 | 0.012861 | -0.24368 |
| ARHGAP24 | -0.17068 | 5.736163 | -2.10843 | 0.037162 | 0.276112 | -4.20039 |
| C12orf45 | -0.17091 | 5.784201 | -2.32298 | 0.021936 | 0.204681 | -3.75543 |
| FOXO1 | -0.17112 | 6.120608 | -2.62561 | 0.009825 | 0.120408 | -3.06184 |
| DDX27 | -0.17136 | 7.561315 | -2.84311 | 0.005287 | 0.079607 | -2.51718 |
| CTC1 | -0.1715 | 5.373364 | -3.23967 | 0.001564 | 0.033521 | -1.42914 |
| L3HYPDH | -0.17156 | 4.355685 | -3.11133 | 0.002349 | 0.044914 | -1.79435 |
| LOC28616 | -0.17158 | 4.6012 | -2.40495 | 0.017768 | 0.179544 | -3.57509 |
| BCL7A | -0.17168 | 4.995843 | -2.60224 | 0.010479 | 0.125172 | -3.11808 |
| BDH2 | -0.17175 | 4.409666 | -2.59202 | 0.010777 | 0.127674 | -3.14255 |
| RPL41 | -0.17179 | 13.30891 | -3.07554 | 0.002625 | 0.048611 | -1.89401 |
| FAM129B | -0.17188 | 5.716473 | -2.50016 | 0.013822 | 0.153211 | -3.35856 |
| RPS29 | -0.17192 | 8.768836 | -3.14322 | 0.002125 | 0.041939 | -1.70475 |
| STX18 | -0.17206 | 6.67568 | -2.08343 | 0.039425 | 0.285461 | -4.24966 |
| FSCN1 | -0.17238 | 3.792015 | -2.8154 | 0.005733 | 0.084206 | -2.58867 |
| XPR1 | -0.17266 | 5.292954 | -2.03204 | 0.044451 | 0.307804 | -4.34923 |
| PLIN2 | -0.17276 | 5.725705 | -2.47948 | 0.014605 | 0.158796 | -3.40623 |
| RPL38 | -0.17306 | 8.691479 | -2.06858 | 0.040825 | 0.291989 | -4.27867 |
| ZNF606 | -0.17308 | 5.514103 | -3.0774 | 0.00261 | 0.048462 | -1.88884 |
| PTGDR | -0.17324 | 3.872734 | -2.09237 | 0.038603 | 0.282105 | -4.23211 |
| CHD6 | -0.17327 | 4.172962 | -3.41237 | 0.000889 | 0.022087 | -0.91852 |
| PPP2R2D | -0.17331 | 5.789657 | -2.33982 | 0.021016 | 0.19863 | -3.71884 |
| ZNF767P | -0.17342 | 5.224538 | -2.03347 | 0.044304 | 0.307204 | -4.3465 |
| PET112 | -0.17345 | 5.496336 | -2.80065 | 0.005983 | 0.086097 | -2.62647 |
| FKRP | -0.17348 | 4.678954 | -2.60003 | 0.010543 | 0.125782 | -3.12339 |
| ZNF880 | -0.17353 | 3.86344 | -2.44165 | 0.016142 | 0.168497 | -3.49251 |
| CCNJ | -0.17389 | 4.365745 | -2.44462 | 0.016016 | 0.167634 | -3.48579 |
| WDR45B | -0.17409 | 8.378474 | -3.27138 | 0.001413 | 0.030995 | -1.33701 |
| ST8SIA1 | -0.17448 | 2.955021 | -3.56795 | 0.000526 | 0.015084 | -0.44021 |
| CD55 | -0.17501 | 11.65648 | -2.26421 | 0.025433 | 0.222566 | -3.88123 |
| PAPD7 | -0.17502 | 6.949391 | -2.15773 | 0.033026 | 0.258339 | -4.10164 |
| NGDN | -0.17523 | 5.287032 | -3.09657 | 0.002459 | 0.046507 | -1.83557 |

| | | | | | | |
|---|---|---|---|---|---|---|
| MLXIP | -0.17545 | 5.700841 | -2.13775 | 0.034652 | 0.265162 | -4.14192 |
| TMEM9 | -0.1756 | 5.863872 | -2.73441 | 0.007238 | 0.097626 | -2.79412 |
| UNC5CL | -0.1756 | 4.642978 | -3.64169 | 0.000407 | 0.012515 | -0.20759 |
| IFNAR2 | -0.17566 | 8.241882 | -3.0334 | 0.002989 | 0.053556 | -2.01008 |
| EEF1A1 | -0.1759 | 10.48622 | -2.03494 | 0.044154 | 0.306618 | -4.34368 |
| FZD1 | -0.17592 | 3.919419 | -2.74032 | 0.007117 | 0.096631 | -2.7793 |
| ATP6V0E2 | -0.17642 | 6.072161 | -2.10373 | 0.037579 | 0.277797 | -4.2097 |
| RAB11FIP3 | -0.17653 | 5.215505 | -3.1846 | 0.001865 | 0.038278 | -1.58735 |
| IGHV5-78 | -0.17692 | 3.343951 | -2.84498 | 0.005258 | 0.079407 | -2.51234 |
| EIF3F | -0.1772 | 6.093304 | -2.73592 | 0.007207 | 0.097399 | -2.79033 |
| GLG1 | -0.17725 | 8.045052 | -2.58518 | 0.010981 | 0.129583 | -3.15887 |
| PTPN2 | -0.17732 | 5.277593 | -4.64091 | 9.25E-06 | 0.000714 | 3.290473 |
| STAG3 | -0.17734 | 5.662449 | -2.55674 | 0.011867 | 0.137495 | -3.22632 |
| CAPRIN2 | -0.17736 | 5.505389 | -2.42098 | 0.017041 | 0.175321 | -3.53917 |
| SPRYD3 | -0.17751 | 6.752558 | -2.15043 | 0.033613 | 0.260752 | -4.11641 |
| HIC2 | -0.17774 | 4.917116 | -3.87756 | 0.000176 | 0.006744 | 0.561331 |
| TMEM63C | -0.17784 | 4.565342 | -2.2194 | 0.028419 | 0.238816 | -3.97517 |
| ZNF124 | -0.17791 | 4.520294 | -2.11206 | 0.036843 | 0.274237 | -4.19319 |
| TPPP3 | -0.17803 | 4.873775 | -2.34802 | 0.02058 | 0.195865 | -3.70095 |
| PPARD | -0.17815 | 4.89044 | -4.02272 | 0.000103 | 0.004612 | 1.052753 |
| FUK | -0.17825 | 6.195188 | -2.28705 | 0.02402 | 0.216752 | -3.83268 |
| LOC64465 | -0.17842 | 4.904457 | -2.3151 | 0.022379 | 0.206735 | -3.77246 |
| ZNF566 | -0.17844 | 2.87661 | -2.78683 | 0.006227 | 0.088367 | -2.66174 |
| SH2D3A | -0.17857 | 4.306853 | -3.38878 | 0.000962 | 0.023285 | -0.98953 |
| ZNF75A | -0.17871 | 6.374304 | -2.40911 | 0.017577 | 0.178315 | -3.56579 |
| REXO4 | -0.17878 | 5.895388 | -3.29237 | 0.00132 | 0.029512 | -1.27563 |
| CREBZF | -0.17904 | 5.03781 | -2.86343 | 0.004981 | 0.076683 | -2.46439 |
| AGO3 | -0.1793 | 5.410688 | -2.17085 | 0.031996 | 0.255711 | -4.07502 |
| CCNT2 | -0.17932 | 5.718509 | -2.21817 | 0.028505 | 0.23922 | -3.97772 |
| ARF1 | -0.17938 | 6.815385 | -2.27539 | 0.024733 | 0.219809 | -3.85753 |
| LOC10028 | -0.17957 | 4.660336 | -2.26191 | 0.02558 | 0.223032 | -3.88609 |
| DAPK1 | -0.17966 | 5.58072 | -2.35412 | 0.020261 | 0.19394 | -3.68758 |
| RCN3 | -0.17983 | 5.855365 | -2.06975 | 0.040713 | 0.291589 | -4.2764 |
| SNX21 | -0.17987 | 5.194703 | -2.16024 | 0.032827 | 0.257556 | -4.09657 |
| RNF113A | -0.17993 | 6.868638 | -1.98228 | 0.04983 | 0.32976 | -4.44344 |
| RPS27 | -0.18006 | 9.458769 | -2.01273 | 0.046477 | 0.315845 | -4.38606 |
| LINC00662 | -0.18009 | 4.295747 | -3.49105 | 0.000683 | 0.018371 | -0.67875 |
| AGPAT6 | -0.18013 | 5.328012 | -3.72368 | 0.000306 | 0.010257 | 0.055447 |
| RELL2 | -0.18025 | 6.029288 | -2.72953 | 0.007339 | 0.098476 | -2.80634 |
| ABHD6 | -0.18043 | 4.224602 | -4.38113 | 2.62E-05 | 0.001553 | 2.32261 |
| MECP2 | -0.18049 | 6.402383 | -2.67751 | 0.008501 | 0.109299 | -2.93531 |
| MCOLN2 | -0.18049 | 4.603957 | -2.16714 | 0.032284 | 0.256143 | -4.08256 |
| FBLN5 | -0.18092 | 4.447963 | -2.74311 | 0.00706 | 0.096119 | -2.77229 |
| ERCC5 | -0.18123 | 7.485631 | -2.07462 | 0.04025 | 0.289695 | -4.2669 |
| PURA | -0.18123 | 5.298881 | -2.54409 | 0.012281 | 0.140938 | -3.25612 |
| PSMD2 | -0.18137 | 8.269392 | -1.99559 | 0.04834 | 0.324244 | -4.41846 |
| CPNE8 | -0.18162 | 4.425755 | -2.17768 | 0.03147 | 0.25312 | -4.0611 |
| LCN10 | -0.18191 | 5.159049 | -2.66923 | 0.008701 | 0.110995 | -2.95564 |
| PLB1 | -0.18197 | 5.307418 | -2.51878 | 0.013149 | 0.147747 | -3.31533 |
| WDR3 | -0.18198 | 4.409028 | -2.1562 | 0.033149 | 0.25869 | -4.10475 |
| SLC16A4 | -0.182 | 3.119136 | -2.45701 | 0.015502 | 0.164009 | -3.45763 |
| ZNF212 | -0.18223 | 6.521323 | -2.4376 | 0.016315 | 0.169925 | -3.50169 |
| SIGLEC17P | -0.18247 | 5.143374 | -2.03105 | 0.044553 | 0.30795 | -4.35113 |
| HIP1R | -0.18258 | 5.920241 | -3.81271 | 0.000222 | 0.00813 | 0.346225 |
| NFE2L1 | -0.18319 | 7.117789 | -2.71849 | 0.007573 | 0.10062 | -2.83388 |
| ZNF792 | -0.18355 | 4.964883 | -2.33311 | 0.021378 | 0.201124 | -3.73345 |
| ZNF592 | -0.1838 | 6.845741 | -3.2154 | 0.001691 | 0.035341 | -1.49914 |
| HEG1 | -0.18382 | 5.77009 | -2.25341 | 0.026127 | 0.225976 | -3.90404 |

| | | | | | | |
|---|---|---|---|---|---|---|
| PCED1B-A | -0.18389 | 5.498081 | -2.48154 | 0.014525 | 0.158433 | -3.40149 |
| AAR2 | -0.18391 | 6.505668 | -2.65673 | 0.00901 | 0.114019 | -2.98623 |
| METTL3 | -0.18404 | 6.489647 | -2.18036 | 0.031265 | 0.252167 | -4.05561 |
| SFPQ | -0.18405 | 7.080517 | -3.00678 | 0.003242 | 0.056584 | -2.08269 |
| ACVR2A | -0.18411 | 4.952054 | -2.15162 | 0.033516 | 0.260538 | -4.114 |
| LYRM4 | -0.1843 | 5.282468 | -2.74945 | 0.006934 | 0.094964 | -2.75637 |
| AAMP | -0.18437 | 7.559105 | -2.04913 | 0.042723 | 0.30103 | -4.31639 |
| PHLPP1 | -0.18447 | 3.742066 | -2.80401 | 0.005925 | 0.085564 | -2.61787 |
| WDR54 | -0.18449 | 6.09159 | -2.18748 | 0.030729 | 0.250329 | -4.04104 |
| LINC00963 | -0.18479 | 5.372364 | -2.26906 | 0.025128 | 0.221389 | -3.87097 |
| GYLTL1B | -0.18481 | 5.831062 | -3.42888 | 0.000842 | 0.021265 | -0.86858 |
| KIF13B | -0.18513 | 6.266503 | -3.68316 | 0.000352 | 0.011197 | -0.07512 |
| SMARCC1 | -0.18525 | 6.520059 | -2.16775 | 0.032237 | 0.255966 | -4.08133 |
| GTF3A | -0.18542 | 9.003238 | -2.11299 | 0.036762 | 0.273935 | -4.19136 |
| RPL32P3 | -0.18576 | 5.951695 | -2.71533 | 0.007641 | 0.101214 | -2.84176 |
| COG2 | -0.18581 | 5.066548 | -3.00586 | 0.003251 | 0.056621 | -2.08519 |
| 10-Sep | -0.18589 | 3.670217 | -2.09502 | 0.038361 | 0.281042 | -4.22688 |
| HNRNPA3 | -0.1863 | 8.279946 | -2.13202 | 0.035131 | 0.267227 | -4.15341 |
| SUGP2 | -0.18657 | 6.297495 | -3.45639 | 0.000768 | 0.020011 | -0.78494 |
| RPS15 | -0.1866 | 7.979272 | -2.59528 | 0.010681 | 0.126997 | -3.13476 |
| RRN3P1 | -0.18662 | 4.956572 | -2.89381 | 0.004553 | 0.072367 | -2.38482 |
| RAB29 | -0.18695 | 7.317719 | -2.20321 | 0.029571 | 0.244103 | -4.0087 |
| HOOK2 | -0.18703 | 5.567213 | -3.78022 | 0.00025 | 0.008864 | 0.239488 |
| SNORD89 | -0.18705 | 9.852156 | -2.00208 | 0.047627 | 0.321312 | -4.40622 |
| CLTB | -0.18707 | 6.185619 | -2.67153 | 0.008645 | 0.110627 | -2.95 |
| WDR37 | -0.18708 | 6.035468 | -2.39585 | 0.018193 | 0.181331 | -3.59538 |
| CORO1B | -0.18713 | 6.411758 | -2.08168 | 0.039588 | 0.28628 | -4.2531 |
| ZNF786 | -0.18733 | 5.27463 | -2.53841 | 0.012471 | 0.142281 | -3.26945 |
| TBCC | -0.1874 | 8.20653 | -2.39523 | 0.018223 | 0.181538 | -3.59678 |
| SELM | -0.18776 | 4.580113 | -2.24555 | 0.026642 | 0.228956 | -3.92056 |
| DUSP11 | -0.18788 | 7.746512 | -2.19982 | 0.029817 | 0.245276 | -4.01568 |
| BEND5 | -0.18795 | 3.987765 | -3.12906 | 0.002222 | 0.043137 | -1.74463 |
| WDR41 | -0.18799 | 5.641166 | -2.29846 | 0.02334 | 0.212358 | -3.80828 |
| CHD4 | -0.1881 | 7.711103 | -2.12218 | 0.035966 | 0.270768 | -4.17306 |
| PUF60 | -0.18811 | 8.105739 | -2.20808 | 0.02922 | 0.242829 | -3.99863 |
| CTNNBL1 | -0.18858 | 6.848938 | -2.30528 | 0.022942 | 0.210135 | -3.79363 |
| PHB | -0.18864 | 6.037288 | -2.6334 | 0.009615 | 0.118979 | -3.04299 |
| MOB3B | -0.18887 | 4.911427 | -2.33613 | 0.021215 | 0.199952 | -3.72689 |
| ERCC6L2 | -0.1889 | 5.18867 | -2.54648 | 0.012202 | 0.140264 | -3.2505 |
| DUSP22 | -0.18904 | 9.385642 | -2.9385 | 0.003985 | 0.065607 | -2.26649 |
| CBR4 | -0.18924 | 6.19728 | -2.15138 | 0.033536 | 0.260538 | -4.11449 |
| CLCN6 | -0.18967 | 5.207722 | -2.79661 | 0.006054 | 0.086685 | -2.63681 |
| ACPP | -0.18983 | 5.803457 | -2.09567 | 0.038303 | 0.280977 | -4.2256 |
| ACAP3 | -0.18984 | 5.477922 | -2.09527 | 0.038339 | 0.280977 | -4.22639 |
| C19orf43 | -0.18991 | 7.896292 | -2.35667 | 0.020129 | 0.193218 | -3.682 |
| FKBP15 | -0.18991 | 6.056631 | -2.50773 | 0.013544 | 0.151529 | -3.34101 |
| GNPTG | -0.18999 | 7.055424 | -2.50627 | 0.013598 | 0.151913 | -3.34441 |
| ECH1 | -0.19004 | 8.008319 | -2.72781 | 0.007375 | 0.098762 | -2.81064 |
| RNMT | -0.19005 | 4.845548 | -2.46225 | 0.015288 | 0.162422 | -3.44567 |
| FTSJ2 | -0.19026 | 6.268208 | -2.52082 | 0.013077 | 0.147102 | -3.31058 |
| ZNF587 | -0.19032 | 6.794235 | -2.18975 | 0.030559 | 0.249524 | -4.03639 |
| ZNF226 | -0.19034 | 4.115708 | -3.35599 | 0.001072 | 0.025095 | -1.08759 |
| EIF3C | -0.19048 | 3.704467 | -2.23507 | 0.027342 | 0.233171 | -3.94252 |
| CCDC117 | -0.19049 | 6.415588 | -2.36475 | 0.019716 | 0.190562 | -3.66424 |
| SLC39A13 | -0.19074 | 5.962032 | -2.24004 | 0.027008 | 0.231618 | -3.93212 |
| EXOC3 | -0.19108 | 6.873583 | -2.52687 | 0.012866 | 0.145364 | -3.29646 |
| DDX24 | -0.19132 | 7.154749 | -2.2719 | 0.02495 | 0.220814 | -3.86494 |
| PAN3 | -0.19155 | 9.960233 | -2.75073 | 0.006908 | 0.09473 | -2.75314 |

| | | | | | | |
|---|---|---|---|---|---|---|
| INTS10 | -0.19156 | 4.896939 | -3.89114 | 0.000168 | 0.006555 | 0.606735 |
| THAP5 | -0.1916 | 5.335988 | -2.41478 | 0.017319 | 0.177014 | -3.55308 |
| SECISBP2 | -0.1916 | 7.408691 | -2.10078 | 0.037843 | 0.279242 | -4.21553 |
| PPP1R2 | -0.19163 | 6.268165 | -2.63883 | 0.009471 | 0.117663 | -3.02981 |
| PRL | -0.19184 | 4.209192 | -2.03985 | 0.043654 | 0.304449 | -4.33426 |
| PMVK | -0.19189 | 5.553986 | -2.1209 | 0.036076 | 0.271294 | -4.17562 |
| LOC10028 | -0.19206 | 3.316121 | -2.15599 | 0.033166 | 0.258724 | -4.10518 |
| AKAP7 | -0.19219 | 5.48016 | -2.14426 | 0.034115 | 0.262825 | -4.12885 |
| SIAH1 | -0.19222 | 5.85357 | -2.15729 | 0.033061 | 0.258473 | -4.10253 |
| NACA | -0.1923 | 9.716842 | -2.81231 | 0.005784 | 0.084482 | -2.59661 |
| PSMG3 | -0.19238 | 6.067648 | -2.12268 | 0.035924 | 0.270768 | -4.17208 |
| PFKFB3 | -0.19245 | 11.17172 | -2.38074 | 0.01892 | 0.185943 | -3.62895 |
| HDDC2 | -0.19245 | 5.767934 | -2.16516 | 0.032439 | 0.256336 | -4.08658 |
| ZNF397 | -0.19305 | 4.429331 | -3.45977 | 0.000759 | 0.019884 | -0.77461 |
| RBM8A | -0.19311 | 6.91209 | -3.19889 | 0.001782 | 0.036988 | -1.54653 |
| CORO2B | -0.19316 | 3.562438 | -2.7763 | 0.006419 | 0.090278 | -2.68852 |
| FYCO1 | -0.19334 | 6.11077 | -3.85018 | 0.000194 | 0.007246 | 0.470156 |
| COX7C | -0.19359 | 9.354171 | -2.7679 | 0.006576 | 0.091667 | -2.7098 |
| RNASEH1- | -0.19396 | 3.831673 | -2.84979 | 0.005185 | 0.078934 | -2.49986 |
| ZSWIM7 | -0.19404 | 5.759295 | -2.36051 | 0.019932 | 0.191745 | -3.67357 |
| PRICKLE1 | -0.19404 | 4.119294 | -2.564 | 0.011635 | 0.135652 | -3.20916 |
| RPL24 | -0.1942 | 11.30291 | -2.32517 | 0.021815 | 0.204148 | -3.75069 |
| BCS1L | -0.19439 | 5.668808 | -2.50181 | 0.013761 | 0.152864 | -3.35474 |
| TNFRSF25 | -0.19448 | 5.350216 | -2.61588 | 0.010092 | 0.122411 | -3.0853 |
| DHRS7B | -0.19462 | 6.642725 | -1.99139 | 0.048806 | 0.326255 | -4.42636 |
| ARHGEF1( | -0.19466 | 4.793156 | -3.65788 | 0.000385 | 0.01199 | -0.156 |
| CIAPIN1 | -0.19481 | 5.589052 | -3.25276 | 0.0015 | 0.032551 | -1.39118 |
| CBX6 | -0.19498 | 8.021472 | -2.81313 | 0.005771 | 0.084448 | -2.5945 |
| SLC22A5 | -0.19514 | 4.232701 | -3.85581 | 0.00019 | 0.007112 | 0.488864 |
| DNAJC7 | -0.19517 | 6.736879 | -3.12959 | 0.002218 | 0.043121 | -1.74315 |
| ITPA | -0.19538 | 7.067283 | -2.98745 | 0.003439 | 0.058928 | -2.13511 |
| PDIA3 | -0.19549 | 6.685735 | -2.45507 | 0.015581 | 0.16451 | -3.46205 |
| RPL30 | -0.19557 | 12.60759 | -2.65789 | 0.008981 | 0.11372 | -2.98339 |
| FAM178A | -0.19574 | 5.454462 | -2.45098 | 0.01575 | 0.165865 | -3.47134 |
| FAM213B | -0.19589 | 5.532577 | -2.3686 | 0.019521 | 0.189491 | -3.65577 |
| CYP2R1 | -0.19608 | 4.368291 | -3.68972 | 0.000344 | 0.011074 | -0.05405 |
| PCM1 | -0.19622 | 6.513554 | -2.90214 | 0.004442 | 0.071014 | -2.36289 |
| TOB2 | -0.1967 | 5.33013 | -3.73067 | 0.000298 | 0.010115 | 0.078107 |
| PITPNC1 | -0.19696 | 5.591219 | -3.62998 | 0.000424 | 0.012861 | -0.24478 |
| UBE2H | -0.19704 | 6.848148 | -2.44314 | 0.016079 | 0.168114 | -3.48913 |
| TMEM99 | -0.19704 | 5.265126 | -2.33658 | 0.02119 | 0.199813 | -3.7259 |
| SUMF1 | -0.19715 | 8.566181 | -2.26171 | 0.025593 | 0.223032 | -3.88653 |
| DFFA | -0.19727 | 5.897833 | -3.33722 | 0.00114 | 0.026389 | -1.14337 |
| MRPL21 | -0.19752 | 6.712702 | -2.27518 | 0.024746 | 0.219809 | -3.85796 |
| C9orf64 | -0.19777 | 8.108445 | -2.14447 | 0.034098 | 0.262792 | -4.12842 |
| IQCE | -0.19793 | 5.068808 | -2.47631 | 0.014729 | 0.159651 | -3.41351 |
| CCNL2 | -0.19829 | 6.69303 | -2.67096 | 0.008659 | 0.110678 | -2.9514 |
| LTBP3 | -0.19833 | 5.284751 | -3.00636 | 0.003246 | 0.056584 | -2.08386 |
| ATXN1L | -0.19835 | 7.626617 | -2.23659 | 0.027239 | 0.233111 | -3.93933 |
| RPP14 | -0.19837 | 5.093194 | -2.7813 | 0.006328 | 0.089411 | -2.67582 |
| GAS7 | -0.19847 | 8.410842 | -2.04121 | 0.043517 | 0.304006 | -4.33164 |
| SPOCK2 | -0.19863 | 6.707266 | -2.32991 | 0.021553 | 0.202399 | -3.74041 |
| ATP2A2 | -0.19888 | 6.591346 | -3.63835 | 0.000412 | 0.012586 | -0.21818 |
| HECW2 | -0.19906 | 4.034392 | -2.18761 | 0.030719 | 0.250329 | -4.04077 |
| IKBKB | -0.19938 | 6.152251 | -2.19953 | 0.029839 | 0.245276 | -4.01629 |
| WDR91 | -0.19944 | 4.943741 | -4.42477 | 2.2E-05 | 0.00135 | 2.482486 |
| ATR | -0.19957 | 5.054054 | -2.09935 | 0.037971 | 0.279835 | -4.21836 |
| RSAD1 | -0.19964 | 6.561984 | -2.36747 | 0.019578 | 0.189667 | -3.65824 |

| | | | | | | |
|---|---|---|---|---|---|---|
| C5orf45 | -0.19976 | 5.7623 | -2.91204 | 0.004313 | 0.069659 | -2.33673 |
| VEGFB | -0.20002 | 4.675291 | -2.37425 | 0.019239 | 0.187907 | -3.64328 |
| ZNF160 | -0.20005 | 5.445989 | -3.13275 | 0.002196 | 0.042804 | -1.73426 |
| GAK | -0.20017 | 8.582786 | -2.74056 | 0.007112 | 0.096629 | -2.7787 |
| DIS3L2 | -0.20022 | 4.719843 | -2.67969 | 0.008449 | 0.108941 | -2.92995 |
| ZNF550 | -0.20025 | 4.658813 | -2.63202 | 0.009652 | 0.119258 | -3.04633 |
| ST8SIA4 | -0.20041 | 7.08713 | -2.07515 | 0.0402 | 0.289434 | -4.26585 |
| ABCB1 | -0.20046 | 3.891951 | -3.3448 | 0.001112 | 0.025887 | -1.12089 |
| C11orf24 | -0.20059 | 5.843074 | -2.99711 | 0.003339 | 0.057707 | -2.10894 |
| PHC1 | -0.2007 | 5.911839 | -1.98829 | 0.049153 | 0.327304 | -4.43219 |
| YPEL1 | -0.20085 | 4.740774 | -3.62501 | 0.000432 | 0.013008 | -0.26052 |
| SPEN | -0.20107 | 7.109731 | -2.30744 | 0.022817 | 0.209552 | -3.78897 |
| RPL19 | -0.20112 | 11.93643 | -2.2116 | 0.028969 | 0.241507 | -3.99134 |
| ANK3 | -0.20139 | 4.19688 | -3.12467 | 0.002253 | 0.043569 | -1.75696 |
| TP53TG1 | -0.20174 | 5.04057 | -3.43959 | 0.000812 | 0.020878 | -0.83608 |
| EIF4EBP1 | -0.20209 | 6.065263 | -2.20401 | 0.029513 | 0.244103 | -4.00703 |
| SGSM2 | -0.20212 | 6.532552 | -3.7937 | 0.000238 | 0.00852 | 0.283698 |
| ATRN | -0.20235 | 5.733752 | -3.23365 | 0.001595 | 0.033926 | -1.44655 |
| HEIH | -0.2026 | 8.048156 | -2.47294 | 0.014861 | 0.160417 | -3.42123 |
| ZNF763 | -0.20274 | 3.890311 | -2.34007 | 0.021002 | 0.19863 | -3.71829 |
| MCEMP1 | -0.20279 | 12.42478 | -2.17663 | 0.03155 | 0.253564 | -4.06323 |
| ZFYVE27 | -0.20282 | 5.982555 | -2.36695 | 0.019604 | 0.189753 | -3.65939 |
| LILRB2 | -0.20282 | 10.35046 | -2.64696 | 0.009259 | 0.115664 | -3.01004 |
| FAM3C | -0.20306 | 4.112102 | -2.02284 | 0.045407 | 0.311155 | -4.36681 |
| ARHGAP3 | -0.20311 | 3.907604 | -3.68613 | 0.000349 | 0.011158 | -0.06558 |
| SUN1 | -0.20323 | 4.251707 | -3.2216 | 0.001658 | 0.034897 | -1.48129 |
| SREBF1 | -0.20341 | 5.074226 | -3.55662 | 0.000546 | 0.015594 | -0.47561 |
| ULK2 | -0.20362 | 4.488776 | -3.28764 | 0.00134 | 0.029755 | -1.28949 |
| ZFC3H1 | -0.20362 | 5.501894 | -2.8071 | 0.005873 | 0.085042 | -2.60998 |
| OPHN1 | -0.20365 | 8.571818 | -2.13228 | 0.035109 | 0.267161 | -4.15289 |
| LOC10050 | -0.20366 | 5.612938 | -2.1356 | 0.034831 | 0.265638 | -4.14623 |
| ITPR1 | -0.20367 | 5.660007 | -2.45177 | 0.015717 | 0.165691 | -3.46954 |
| ZNF567 | -0.20437 | 4.355597 | -2.20322 | 0.029571 | 0.244103 | -4.00868 |
| PPM1F | -0.20459 | 6.797961 | -2.74844 | 0.006954 | 0.095109 | -2.75889 |
| MRFAP1L1 | -0.20468 | 8.422682 | -2.1513 | 0.033542 | 0.260538 | -4.11465 |
| KIAA0247 | -0.20494 | 9.772393 | -2.04317 | 0.043319 | 0.303226 | -4.32787 |
| RPS7 | -0.20503 | 11.74155 | -1.99936 | 0.047925 | 0.322155 | -4.41136 |
| RPS16 | -0.20523 | 10.25613 | -2.50264 | 0.013731 | 0.152693 | -3.35283 |
| LUC7L3 | -0.20529 | 7.608532 | -2.20377 | 0.029531 | 0.244103 | -4.00754 |
| CCAR1 | -0.20538 | 6.682132 | -2.00109 | 0.047735 | 0.321514 | -4.40809 |
| LOC10012 | -0.20543 | 6.312608 | -2.10846 | 0.037159 | 0.276112 | -4.20033 |
| C15orf48 | -0.20552 | 3.491297 | -2.77347 | 0.006472 | 0.090828 | -2.69568 |
| HOXA1 | -0.20555 | 4.056066 | -2.35351 | 0.020293 | 0.19394 | -3.68893 |
| SH3BGRL | -0.20558 | 10.57742 | -2.48679 | 0.014324 | 0.157156 | -3.38942 |
| MPV17 | -0.20578 | 6.492816 | -2.03233 | 0.044421 | 0.307736 | -4.34867 |
| CCNT1 | -0.20608 | 7.159751 | -2.19666 | 0.030049 | 0.246435 | -4.0222 |
| RPL9 | -0.20615 | 12.24795 | -2.69159 | 0.008171 | 0.106023 | -2.90061 |
| CAMLG | -0.20618 | 8.648568 | -2.37201 | 0.01935 | 0.188545 | -3.64823 |
| FAM193A | -0.20629 | 7.170421 | -2.03096 | 0.044563 | 0.30795 | -4.3513 |
| TGIF2 | -0.20644 | 5.448903 | -2.31488 | 0.022392 | 0.206759 | -3.77294 |
| ELL3 | -0.20648 | 4.675208 | -3.27122 | 0.001413 | 0.030995 | -1.33748 |
| LRP1 | -0.20658 | 4.896565 | -2.99342 | 0.003377 | 0.058211 | -2.11895 |
| ZNF831 | -0.20676 | 3.523853 | -3.04029 | 0.002926 | 0.052722 | -1.99119 |
| DUSP7 | -0.2071 | 5.453653 | -3.06932 | 0.002676 | 0.049376 | -1.91123 |
| WDR73 | -0.20716 | 6.201504 | -3.47952 | 0.00071 | 0.018919 | -0.71416 |
| PMPCA | -0.20733 | 6.319858 | -2.19565 | 0.030123 | 0.246944 | -4.02427 |
| PANK4 | -0.20733 | 5.892158 | -2.06695 | 0.040981 | 0.292789 | -4.28184 |
| EEF1D | -0.20736 | 7.283241 | -4.01729 | 0.000105 | 0.004676 | 1.034144 |

| | | | | | | |
|---|---|---|---|---|---|---|
| MRPS30 | -0.20743 | 5.323586 | -2.00143 | 0.047698 | 0.321476 | -4.40744 |
| UQCRC2 | -0.20744 | 6.988302 | -2.07225 | 0.040475 | 0.290598 | -4.27153 |
| ZNF252P | -0.2075 | 5.09111 | -3.83382 | 0.000206 | 0.007617 | 0.415925 |
| LOC10192 | -0.20774 | 3.975871 | -2.88031 | 0.004739 | 0.074195 | -2.42026 |
| SPHK1 | -0.20782 | 5.190817 | -1.99949 | 0.04791 | 0.322155 | -4.41111 |
| SEMA3C | -0.20829 | 3.903972 | -2.19346 | 0.030284 | 0.24767 | -4.02877 |
| ATF6 | -0.20837 | 7.51292 | -2.15716 | 0.033072 | 0.258473 | -4.1028 |
| ELMSAN1 | -0.20853 | 5.31298 | -2.82467 | 0.00558 | 0.082737 | -2.56483 |
| DFNA5 | -0.20856 | 4.072978 | -2.02421 | 0.045264 | 0.310502 | -4.36421 |
| PDCD4 | -0.20861 | 3.329963 | -2.30179 | 0.023145 | 0.211146 | -3.80113 |
| FAIM | -0.20868 | 4.080851 | -1.98762 | 0.049228 | 0.327409 | -4.43344 |
| PROK2 | -0.20911 | 12.26454 | -2.54186 | 0.012355 | 0.141357 | -3.26137 |
| AGO1 | -0.20926 | 6.068021 | -3.84945 | 0.000195 | 0.007251 | 0.467741 |
| IFT20 | -0.20943 | 8.666835 | -2.34214 | 0.020892 | 0.198002 | -3.71379 |
| NDUFA12 | -0.20955 | 9.245946 | -2.22827 | 0.027805 | 0.235493 | -3.95672 |
| GRHPR | -0.20994 | 6.676123 | -2.52551 | 0.012913 | 0.145735 | -3.29965 |
| UFM1 | -0.20994 | 6.260637 | -2.17958 | 0.031325 | 0.252351 | -4.05722 |
| C15orf39 | -0.21002 | 6.904545 | -2.17456 | 0.031709 | 0.254343 | -4.06746 |
| FBXO21 | -0.21007 | 5.741103 | -2.38216 | 0.01885 | 0.185616 | -3.62579 |
| SDCCAG3 | -0.21018 | 5.366325 | -3.77246 | 0.000257 | 0.009034 | 0.214103 |
| SCML4 | -0.2102 | 5.280498 | -2.26367 | 0.025468 | 0.222566 | -3.88237 |
| YTHDC1 | -0.21021 | 5.395298 | -3.13852 | 0.002157 | 0.042317 | -1.718 |
| CEBPB | -0.21028 | 12.73826 | -3.52827 | 0.000602 | 0.016691 | -0.56382 |
| R3HDM2 | -0.21039 | 8.002472 | -3.28487 | 0.001352 | 0.029912 | -1.29761 |
| AIF1 | -0.21046 | 9.40673 | -3.35463 | 0.001077 | 0.025178 | -1.09164 |
| LOC72908 | -0.21048 | 2.976026 | -3.92483 | 0.000148 | 0.005992 | 0.719876 |
| RPS17 | -0.21072 | 12.25496 | -2.64481 | 0.009315 | 0.116289 | -3.01529 |
| ST14 | -0.21097 | 5.065427 | -2.76697 | 0.006594 | 0.091787 | -2.71216 |
| LOC10012 | -0.21123 | 4.660538 | -3.23105 | 0.001608 | 0.034139 | -1.45406 |
| KIF3A | -0.21126 | 4.054527 | -2.65277 | 0.00911 | 0.114777 | -2.9959 |
| SF1 | -0.21133 | 6.549382 | -2.16475 | 0.032471 | 0.256336 | -4.08741 |
| SERTAD1 | -0.21156 | 6.64312 | -2.88311 | 0.0047 | 0.073809 | -2.41293 |
| DHPS | -0.21159 | 6.652389 | -2.45029 | 0.015779 | 0.166082 | -3.47291 |
| CD53 | -0.21183 | 8.167894 | -2.66392 | 0.008831 | 0.112175 | -2.96865 |
| LOC10272 | -0.21185 | 3.360864 | -2.95552 | 0.003787 | 0.063565 | -2.22102 |
| PARP15 | -0.2119 | 3.482163 | -3.38963 | 0.000959 | 0.023285 | -0.987 |
| TULP4 | -0.21226 | 4.135329 | -3.30319 | 0.001274 | 0.028838 | -1.24385 |
| CCDC130 | -0.21229 | 6.63551 | -2.98815 | 0.003431 | 0.058852 | -2.13321 |
| FUBP1 | -0.21237 | 6.087434 | -2.9915 | 0.003397 | 0.058452 | -2.12414 |
| DISP1 | -0.21274 | 3.507908 | -4.08998 | 8.03E-05 | 0.003758 | 1.285031 |
| FLJ38717 | -0.21278 | 5.63727 | -2.10035 | 0.037881 | 0.279423 | -4.21638 |
| ENGASE | -0.2128 | 7.002807 | -3.11304 | 0.002336 | 0.044753 | -1.78956 |
| RYR1 | -0.21307 | 3.603542 | -2.94517 | 0.003906 | 0.064671 | -2.24869 |
| FAM159A | -0.2135 | 4.449524 | -3.76168 | 0.000267 | 0.00926 | 0.178925 |
| MRFAP1 | -0.21351 | 11.34628 | -3.2852 | 0.001351 | 0.029912 | -1.29664 |
| CSNK1D | -0.21352 | 7.043139 | -2.52994 | 0.01276 | 0.144486 | -3.28929 |
| ABI3 | -0.21356 | 6.957957 | -2.80968 | 0.005829 | 0.084668 | -2.60336 |
| FBXO25 | -0.21358 | 6.191916 | -2.81061 | 0.005813 | 0.084659 | -2.60098 |
| NOB1 | -0.21377 | 6.317393 | -2.27376 | 0.024834 | 0.220071 | -3.86098 |
| LOC10050 | -0.21383 | 5.570818 | -2.57299 | 0.011353 | 0.132901 | -3.18787 |
| ZNF91 | -0.21413 | 4.356863 | -3.42936 | 0.00084 | 0.021257 | -0.86711 |
| RFTN1 | -0.21426 | 5.052593 | -3.05904 | 0.002762 | 0.050288 | -1.93962 |
| KLHL36 | -0.21481 | 7.068501 | -3.72727 | 0.000302 | 0.010172 | 0.067065 |
| FNBP4 | -0.21503 | 4.418788 | -2.87213 | 0.004855 | 0.075144 | -2.44166 |
| ATP2B4 | -0.21512 | 7.0301 | -2.42045 | 0.017065 | 0.175465 | -3.54036 |
| CEPT1 | -0.21534 | 6.398976 | -2.01035 | 0.046732 | 0.316566 | -4.39057 |
| WBP11 | -0.21553 | 6.074558 | -2.23036 | 0.027662 | 0.235195 | -3.95235 |
| VPS45 | -0.21555 | 5.468593 | -4.09523 | 7.87E-05 | 0.003702 | 1.303256 |

| | | | | | | |
|---|---|---|---|---|---|---|
| SAFB2 | -0.21555 | 7.073268 | -3.59484 | 0.000479 | 0.014047 | -0.35581 |
| RPL15 | -0.21586 | 8.35454 | -2.8341 | 0.005428 | 0.081136 | -2.54049 |
| SRSF7 | -0.21605 | 7.143565 | -2.06885 | 0.040799 | 0.291989 | -4.27816 |
| RNF111 | -0.21613 | 8.732174 | -2.06265 | 0.041396 | 0.29453 | -4.29021 |
| RPS4X | -0.21637 | 11.97921 | -2.79256 | 0.006125 | 0.087354 | -2.64715 |
| CXCR5 | -0.2169 | 4.981193 | -3.08058 | 0.002584 | 0.048074 | -1.88002 |
| PQLC3 | -0.21693 | 6.413083 | -2.36424 | 0.019741 | 0.190721 | -3.66536 |
| CRTC2 | -0.217 | 6.832203 | -2.31627 | 0.022313 | 0.206312 | -3.76994 |
| FRMD4B | -0.21707 | 4.654279 | -3.56979 | 0.000522 | 0.015053 | -0.43445 |
| SSBP4 | -0.21744 | 5.919914 | -3.12949 | 0.002219 | 0.043121 | -1.74344 |
| HRAS | -0.21758 | 5.45063 | -2.36986 | 0.019458 | 0.189136 | -3.65298 |
| ERO1LB | -0.21777 | 5.089511 | -2.23589 | 0.027286 | 0.233125 | -3.94079 |
| MAN2B2 | -0.21811 | 7.473779 | -2.17569 | 0.031622 | 0.253902 | -4.06516 |
| ZNF253 | -0.21818 | 4.750281 | -2.49029 | 0.014191 | 0.156033 | -3.38136 |
| PTPRK | -0.2182 | 3.054975 | -2.47105 | 0.014936 | 0.160614 | -3.42555 |
| SLC25A25 | -0.2188 | 5.372995 | -2.84409 | 0.005272 | 0.079474 | -2.51465 |
| DET1 | -0.21891 | 4.639429 | -2.71765 | 0.007591 | 0.100733 | -2.83597 |
| EIF3L | -0.21898 | 11.03205 | -2.64406 | 0.009334 | 0.11639 | -3.01712 |
| MACROD2 | -0.2191 | 3.497212 | -4.01146 | 0.000108 | 0.004737 | 1.014162 |
| SDR39U1 | -0.21937 | 7.265824 | -2.03156 | 0.044501 | 0.30794 | -4.35016 |
| ERV3-1 | -0.2195 | 3.848912 | -2.65112 | 0.009152 | 0.115177 | -2.99992 |
| WASF2 | -0.21956 | 8.43985 | -1.99396 | 0.04852 | 0.325196 | -4.42153 |
| SLC3A2 | -0.21956 | 6.824428 | -2.69 | 0.008208 | 0.106431 | -2.90454 |
| TMSB10 | -0.22023 | 11.97458 | -2.14836 | 0.033781 | 0.261231 | -4.12059 |
| ZNF587B | -0.22035 | 6.537014 | -2.04671 | 0.042964 | 0.301896 | -4.32105 |
| PCGF3 | -0.22058 | 6.158731 | -2.87859 | 0.004763 | 0.074459 | -2.42477 |
| LOC15357 | -0.22065 | 5.060324 | -2.10665 | 0.03732 | 0.276781 | -4.20393 |
| USP53 | -0.22079 | 3.65391 | -2.4812 | 0.014539 | 0.158471 | -3.40228 |
| ZNF764 | -0.22086 | 5.629618 | -2.20475 | 0.029459 | 0.244048 | -4.00551 |
| POLR1C | -0.22096 | 5.40695 | -3.90018 | 0.000162 | 0.006392 | 0.63702 |
| XPA | -0.22099 | 6.277959 | -2.16984 | 0.032074 | 0.255711 | -4.07708 |
| KCNK6 | -0.22114 | 6.130158 | -2.79813 | 0.006027 | 0.086465 | -2.6329 |
| PLEKHG4 | -0.22211 | 5.098656 | -3.70926 | 0.000321 | 0.010662 | 0.008879 |
| CDC37L1 | -0.22212 | 4.491019 | -2.55862 | 0.011806 | 0.137028 | -3.22188 |
| YPEL2 | -0.22238 | 6.195115 | -2.86857 | 0.004906 | 0.075708 | -2.45096 |
| SLC25A12 | -0.22248 | 5.868705 | -2.34359 | 0.020815 | 0.197548 | -3.71062 |
| IFT88 | -0.22266 | 6.223613 | -2.30125 | 0.023177 | 0.211243 | -3.80228 |
| JADE2 | -0.22296 | 5.364393 | -3.7282 | 0.000301 | 0.01017 | 0.070089 |
| EIF3H | -0.22354 | 7.363501 | -2.11358 | 0.03671 | 0.273849 | -4.19018 |
| ZFP90 | -0.22367 | 5.336391 | -2.29863 | 0.02333 | 0.212358 | -3.8079 |
| BRAT1 | -0.2238 | 6.12255 | -2.25258 | 0.026181 | 0.226223 | -3.90578 |
| HSBP1 | -0.22402 | 8.886302 | -4.1319 | 6.85E-05 | 0.00333 | 1.431214 |
| ZNF529 | -0.22412 | 4.513486 | -2.15771 | 0.033028 | 0.258339 | -4.10169 |
| PRR24 | -0.22424 | 6.334276 | -2.1653 | 0.032428 | 0.256336 | -4.0863 |
| HTRA1 | -0.22435 | 5.603238 | -2.11361 | 0.036708 | 0.273849 | -4.19012 |
| NOTCH2N | -0.22438 | 10.35916 | -2.38002 | 0.018955 | 0.186072 | -3.63054 |
| NT5E | -0.22448 | 3.746389 | -3.70142 | 0.00033 | 0.010845 | -0.01639 |
| TARDBP | -0.22453 | 7.539055 | -2.18176 | 0.031159 | 0.251786 | -4.05276 |
| ENDOG | -0.22466 | 5.224882 | -2.65259 | 0.009115 | 0.114777 | -2.99634 |
| HIVEP1 | -0.22471 | 5.847109 | -1.98657 | 0.049345 | 0.327933 | -4.43541 |
| GHDC | -0.22473 | 5.842455 | -2.016 | 0.046128 | 0.314491 | -4.37984 |
| RRS1 | -0.22474 | 6.727095 | -2.62476 | 0.009848 | 0.120445 | -3.0639 |
| ERMN | -0.22505 | 3.556229 | -2.42123 | 0.01703 | 0.175321 | -3.53861 |
| LOC10192 | -0.2251 | 6.815616 | -3.35968 | 0.001059 | 0.024963 | -1.07659 |
| PTPRO | -0.2251 | 6.136279 | -3.45267 | 0.000777 | 0.02021 | -0.79625 |
| AHSA2 | -0.22518 | 6.338672 | -2.70812 | 0.007799 | 0.102489 | -2.85968 |
| MRPS6 | -0.22518 | 7.152176 | -2.16572 | 0.032395 | 0.256336 | -4.08544 |
| LOC15368 | -0.22525 | 3.603749 | -2.48172 | 0.014519 | 0.158433 | -3.40108 |

| | | | | | | |
|---|---|---|---|---|---|---|
| GCN1L1 | -0.22559 | 6.24485 | -3.24835 | 0.001521 | 0.032805 | -1.40399 |
| TBRG1 | -0.22569 | 5.866038 | -3.13748 | 0.002164 | 0.042375 | -1.72095 |
| ANG | -0.22581 | 5.056899 | -2.36815 | 0.019544 | 0.189616 | -3.65674 |
| SCRIB | -0.22595 | 6.112648 | -2.27568 | 0.024715 | 0.219809 | -3.85691 |
| BTBD18 | -0.22598 | 4.155526 | -2.26975 | 0.025084 | 0.221235 | -3.86949 |
| MFSD8 | -0.22598 | 4.736234 | -2.22363 | 0.028125 | 0.237026 | -3.96638 |
| NME3 | -0.2261 | 5.993051 | -2.26677 | 0.025272 | 0.221743 | -3.87581 |
| TSPYL2 | -0.22614 | 7.042828 | -3.68359 | 0.000352 | 0.011197 | -0.07372 |
| PTEN | -0.22621 | 9.358115 | -2.755 | 0.006824 | 0.094034 | -2.74239 |
| GAB1 | -0.22624 | 4.805352 | -3.91836 | 0.000152 | 0.006099 | 0.698062 |
| LRRC57 | -0.22658 | 5.608313 | -2.21441 | 0.02877 | 0.240577 | -3.98553 |
| ZNF470 | -0.22665 | 3.312053 | -3.89873 | 0.000163 | 0.006413 | 0.632161 |
| THEM4 | -0.22686 | 5.091059 | -2.5894 | 0.010855 | 0.128446 | -3.14881 |
| AFF3 | -0.22702 | 3.754598 | -4.92756 | 2.82E-06 | 0.000299 | 4.401483 |
| GPR133 | -0.22728 | 3.514831 | -3.09833 | 0.002446 | 0.046374 | -1.83067 |
| GALNT10 | -0.22759 | 5.936193 | -2.48842 | 0.014262 | 0.156642 | -3.38566 |
| SRSF11 | -0.22759 | 6.489135 | -2.22118 | 0.028295 | 0.238162 | -3.97147 |
| ZNF26 | -0.22763 | 4.683654 | -2.4415 | 0.016148 | 0.168497 | -3.49285 |
| FAM13A | -0.22768 | 5.091198 | -3.15267 | 0.002063 | 0.041227 | -1.67806 |
| PPP1R17 | -0.22829 | 4.63254 | -3.73275 | 0.000296 | 0.010057 | 0.084822 |
| NUDT9 | -0.22849 | 5.903241 | -2.08463 | 0.039314 | 0.285266 | -4.24732 |
| RPL6 | -0.22859 | 11.70519 | -2.32464 | 0.021844 | 0.204188 | -3.75182 |
| TMEM167/ | -0.22869 | 9.744055 | -3.79839 | 0.000234 | 0.008423 | 0.299095 |
| PTRH2 | -0.22888 | 6.45831 | -2.18743 | 0.030732 | 0.250329 | -4.04114 |
| DDX18 | -0.22912 | 6.706295 | -2.32851 | 0.02163 | 0.202934 | -3.74344 |
| PDE8A | -0.22916 | 4.814078 | -5.48448 | 2.49E-07 | 4.39E-05 | 6.677555 |
| RPL31 | -0.22919 | 8.931426 | -2.55639 | 0.011878 | 0.137508 | -3.22715 |
| LOC10028 | -0.22945 | 5.528926 | -2.56318 | 0.011661 | 0.135873 | -3.21111 |
| TFEB | -0.22959 | 7.909096 | -2.41698 | 0.01722 | 0.176572 | -3.54814 |
| XPO6 | -0.22969 | 10.46012 | -2.1996 | 0.029833 | 0.245276 | -4.01613 |
| SLC25A45 | -0.22983 | 4.886733 | -2.55378 | 0.011963 | 0.138061 | -3.23332 |
| NCKAP5L | -0.23002 | 5.112939 | -4.00742 | 0.000109 | 0.004798 | 1.000329 |
| ZCCHC7 | -0.23023 | 6.093025 | -2.06136 | 0.041521 | 0.294832 | -4.29271 |
| CR2 | -0.23049 | 3.641285 | -2.93803 | 0.003991 | 0.065647 | -2.26775 |
| ERICH1 | -0.23059 | 5.853267 | -5.0295 | 1.83E-06 | 0.000211 | 4.8069 |
| ZNF581 | -0.23084 | 7.232993 | -2.6774 | 0.008504 | 0.109299 | -2.93558 |
| TMEM106/ | -0.23097 | 4.542309 | -4.76886 | 5.47E-06 | 0.000482 | 3.780991 |
| LINC0018! | -0.23101 | 3.196965 | -2.60796 | 0.010315 | 0.124085 | -3.10435 |
| ZNF34 | -0.23117 | 4.190059 | -3.70521 | 0.000326 | 0.010774 | -0.00421 |
| NR3C2 | -0.23124 | 4.065771 | -2.06555 | 0.041116 | 0.29324 | -4.28457 |
| IKZF3 | -0.23156 | 5.410728 | -2.44467 | 0.016014 | 0.167634 | -3.48567 |
| ERGIC1 | -0.23164 | 6.75284 | -2.28062 | 0.024411 | 0.218462 | -3.8464 |
| PRPF38B | -0.23167 | 7.877836 | -2.33452 | 0.021302 | 0.200589 | -3.73039 |
| PIK3R1 | -0.2318 | 7.645122 | -2.21049 | 0.029048 | 0.242015 | -3.99366 |
| ZCCHC18 | -0.23191 | 4.758923 | -3.54397 | 0.000571 | 0.016061 | -0.51505 |
| NOP14 | -0.23194 | 5.755168 | -2.23372 | 0.027433 | 0.233601 | -3.94534 |
| TRPV2 | -0.23261 | 6.028881 | -2.23963 | 0.027035 | 0.231752 | -3.93297 |
| ZNF324 | -0.23263 | 5.954903 | -2.47278 | 0.014867 | 0.160417 | -3.42159 |
| HPD | -0.233 | 3.805646 | -2.23724 | 0.027196 | 0.232837 | -3.93798 |
| SLC39A4 | -0.23314 | 5.645028 | -2.65362 | 0.009089 | 0.114728 | -2.99382 |
| TRAF3IP3 | -0.23324 | 7.724682 | -2.19189 | 0.0304 | 0.248419 | -4.03199 |
| LOC10013 | -0.23325 | 6.126329 | -2.33542 | 0.021253 | 0.200221 | -3.72843 |
| FPR3 | -0.23356 | 3.801252 | -4.32338 | 3.28E-05 | 0.001871 | 2.11275 |
| RPS25 | -0.23374 | 11.3525 | -2.91803 | 0.004237 | 0.068644 | -2.32089 |
| LUC7L | -0.23379 | 5.844394 | -4.03115 | 0.0001 | 0.004509 | 1.081722 |
| ABHD15 | -0.23388 | 5.343018 | -3.10917 | 0.002365 | 0.045144 | -1.80039 |
| NISCH | -0.23393 | 6.875989 | -4.02159 | 0.000104 | 0.004621 | 1.04889 |
| NUBP1 | -0.23457 | 6.736021 | -2.41138 | 0.017473 | 0.177878 | -3.56069 |

| | | | | | | |
|---|---|---|---|---|---|---|
| CD4 | -0.23477 | 5.377524 | -3.68947 | 0.000345 | 0.011074 | -0.05484 |
| WDR82 | -0.23478 | 10.01125 | -4.05258 | 9.24E-05 | 0.004228 | 1.155531 |
| C15orf54 | -0.23527 | 3.33883 | -3.59862 | 0.000473 | 0.01394 | -0.34389 |
| CEBPG | -0.23531 | 6.315719 | -2.12472 | 0.035749 | 0.270126 | -4.16801 |
| CMKLR1 | -0.23536 | 4.627668 | -4.29472 | 3.67E-05 | 0.002033 | 2.00932 |
| TNS3 | -0.23544 | 5.421992 | -2.71764 | 0.007591 | 0.100733 | -2.83601 |
| VPS37C | -0.2355 | 7.150341 | -5.04144 | 1.74E-06 | 0.000203 | 4.854738 |
| MEGF6 | -0.23576 | 5.936319 | -2.78614 | 0.00624 | 0.088482 | -2.6635 |
| PRDM1 | -0.2361 | 6.684684 | -2.05483 | 0.042158 | 0.297975 | -4.30536 |
| CST3 | -0.23624 | 6.51487 | -2.57066 | 0.011426 | 0.13367 | -3.19338 |
| NOP2 | -0.23637 | 6.921854 | -3.28936 | 0.001333 | 0.029689 | -1.28446 |
| CPT1A | -0.23691 | 5.296414 | -3.31788 | 0.001215 | 0.027736 | -1.2006 |
| DENND1C | -0.23705 | 7.630108 | -3.76832 | 0.000261 | 0.009106 | 0.200583 |
| ALG13 | -0.23753 | 6.148366 | -2.75054 | 0.006912 | 0.09473 | -2.75361 |
| MIOS | -0.23755 | 5.396981 | -2.85997 | 0.005032 | 0.077127 | -2.47339 |
| FAM134B | -0.23773 | 4.51819 | -3.43242 | 0.000832 | 0.021145 | -0.85783 |
| ZNF573 | -0.23791 | 4.008516 | -2.67352 | 0.008597 | 0.110148 | -2.94511 |
| LFNG | -0.23846 | 6.140967 | -2.7712 | 0.006514 | 0.091051 | -2.70145 |
| TRPT1 | -0.2385 | 6.236743 | -3.59602 | 0.000477 | 0.01403 | -0.35211 |
| RPUSD4 | -0.23867 | 6.692537 | -2.92132 | 0.004196 | 0.068081 | -2.31216 |
| U2AF1L4 | -0.23883 | 5.965041 | -2.20675 | 0.029316 | 0.243156 | -4.00139 |
| CELF6 | -0.23894 | 4.438541 | -3.18402 | 0.001869 | 0.038311 | -1.58902 |
| STK25 | -0.23932 | 5.514655 | -2.956 | 0.003781 | 0.063565 | -2.21973 |
| TGIF1 | -0.23952 | 4.508365 | -3.34063 | 0.001127 | 0.026154 | -1.13326 |
| ZNF84 | -0.2396 | 5.254577 | -2.53229 | 0.012679 | 0.143973 | -3.2838 |
| ZNF18 | -0.23999 | 6.276339 | -2.4969 | 0.013943 | 0.153985 | -3.36609 |
| TNFRSF1B | -0.24009 | 9.755092 | -2.39107 | 0.01842 | 0.182793 | -3.60602 |
| ZXDA | -0.24039 | 4.361582 | -2.64993 | 0.009183 | 0.115347 | -3.00283 |
| TMCC1 | -0.24066 | 5.972374 | -2.62855 | 0.009745 | 0.119913 | -3.05472 |
| NAGS | -0.24067 | 4.776501 | -3.79661 | 0.000236 | 0.008462 | 0.293253 |
| LOC15476 | -0.24077 | 6.113813 | -2.12846 | 0.035431 | 0.269042 | -4.16053 |
| ZNF385A | -0.24092 | 6.23292 | -2.6639 | 0.008832 | 0.112175 | -2.96871 |
| OSGEPL1 | -0.24112 | 5.070702 | -2.14985 | 0.033659 | 0.260793 | -4.11756 |
| VPS35 | -0.24118 | 8.181441 | -2.45373 | 0.015636 | 0.165009 | -3.4651 |
| TNFSF13 | -0.24128 | 8.356257 | -2.13717 | 0.0347 | 0.265234 | -4.14308 |
| RPL23A | -0.24145 | 12.7682 | -3.09198 | 0.002495 | 0.046931 | -1.84836 |
| FAM179A | -0.24148 | 4.537989 | -3.11805 | 0.0023 | 0.044356 | -1.77555 |
| ZHX2 | -0.24174 | 6.140936 | -3.1198 | 0.002287 | 0.044154 | -1.77063 |
| UTP3 | -0.24197 | 7.602939 | -2.08368 | 0.039402 | 0.285395 | -4.24917 |
| ZNF862 | -0.24222 | 6.447509 | -3.16066 | 0.002012 | 0.040438 | -1.65543 |
| MTCH1 | -0.24228 | 9.810631 | -3.29707 | 0.0013 | 0.029257 | -1.26184 |
| ZNF322 | -0.24241 | 5.705382 | -2.33963 | 0.021026 | 0.19863 | -3.71926 |
| ATP7A | -0.24258 | 6.676588 | -2.56109 | 0.011727 | 0.136268 | -3.21606 |
| GATAD2B | -0.24259 | 6.856871 | -2.72471 | 0.00744 | 0.099375 | -2.81838 |
| LILRB4 | -0.24261 | 4.278774 | -3.78493 | 0.000246 | 0.008761 | 0.25491 |
| DOK2 | -0.24283 | 7.179909 | -2.03868 | 0.043773 | 0.304719 | -4.3365 |
| GABPB1-A | -0.24296 | 4.992843 | -2.4461 | 0.015954 | 0.167152 | -3.48243 |
| TNIP2 | -0.24321 | 7.498105 | -3.48515 | 0.000697 | 0.018638 | -0.6969 |
| TTYH2 | -0.24336 | 5.425067 | -3.10796 | 0.002374 | 0.045179 | -1.8038 |
| FMNL2 | -0.24336 | 3.82286 | -3.0128 | 0.003183 | 0.055932 | -2.06632 |
| LINC0127( | -0.24343 | 4.297065 | -2.0318 | 0.044476 | 0.307875 | -4.3497 |
| NUFIP2 | -0.24344 | 8.240984 | -2.49995 | 0.01383 | 0.153211 | -3.35905 |
| BCL2A1 | -0.24348 | 12.054 | -3.23525 | 0.001587 | 0.033857 | -1.44191 |
| AGFG1 | -0.24353 | 7.974616 | -2.82574 | 0.005563 | 0.082645 | -2.56208 |
| UBXN1 | -0.24371 | 6.546513 | -3.37482 | 0.001007 | 0.024024 | -1.03138 |
| PRKAG2-A | -0.24427 | 4.494101 | -5.00682 | 2.01E-06 | 0.000226 | 4.716272 |
| CLNS1A | -0.24462 | 6.811217 | -2.39985 | 0.018005 | 0.180337 | -3.58647 |
| SLC22A18 | -0.24531 | 6.863337 | -2.44275 | 0.016095 | 0.168202 | -3.49002 |

| | | | | | | |
|---|---|---|---|---|---|---|
| SETD6 | -0.2454 | 4.785854 | -3.37357 | 0.001012 | 0.024068 | -1.03514 |
| RPL22 | -0.2459 | 10.40073 | -2.78696 | 0.006225 | 0.088367 | -2.66141 |
| JOSD1 | -0.24605 | 8.185884 | -1.99215 | 0.048721 | 0.326112 | -4.42492 |
| MAGEF1 | -0.24608 | 5.343316 | -3.42143 | 0.000863 | 0.021614 | -0.89115 |
| MTMR1 | -0.24615 | 6.767758 | -2.36672 | 0.019616 | 0.189777 | -3.6599 |
| C11orf80 | -0.24629 | 4.135354 | -2.68546 | 0.008313 | 0.107459 | -2.91574 |
| SNHG17 | -0.24664 | 5.588461 | -2.84668 | 0.005232 | 0.079245 | -2.50792 |
| LINC00667 | -0.24666 | 5.17195 | -4.01483 | 0.000106 | 0.004708 | 1.025699 |
| C2orf42 | -0.24668 | 6.051404 | -2.37919 | 0.018995 | 0.18625 | -3.63236 |
| PUS3 | -0.24673 | 7.127578 | -2.27299 | 0.024882 | 0.22031 | -3.86263 |
| INPP5E | -0.247 | 6.51561 | -3.0787 | 0.0026 | 0.048312 | -1.88524 |
| KCNMA1 | -0.24738 | 3.797608 | -3.31349 | 0.001232 | 0.028104 | -1.21352 |
| SPDYE2 | -0.24741 | 5.582242 | -2.30313 | 0.023067 | 0.210903 | -3.79825 |
| TLR2 | -0.24747 | 10.92029 | -2.50008 | 0.013825 | 0.153211 | -3.35874 |
| ZNF273 | -0.24758 | 4.125839 | -2.11826 | 0.036304 | 0.272206 | -4.18088 |
| TNFSF13B | -0.24764 | 11.58783 | -2.89759 | 0.004503 | 0.071811 | -2.37488 |
| RPL37A | -0.2481 | 8.014607 | -2.70908 | 0.007778 | 0.102292 | -2.8573 |
| SEPW1 | -0.2484 | 8.445856 | -2.2672 | 0.025244 | 0.221743 | -3.8749 |
| DNLZ | -0.24855 | 4.918705 | -2.01288 | 0.046461 | 0.315845 | -4.38577 |
| RLIM | -0.24859 | 7.76736 | -2.10161 | 0.037768 | 0.278794 | -4.21389 |
| TBC1D30 | -0.24867 | 4.491514 | -3.85701 | 0.00019 | 0.007094 | 0.492862 |
| FLJ12120 | -0.2491 | 3.469985 | -2.7217 | 0.007504 | 0.099902 | -2.82589 |
| C20orf194 | -0.24929 | 5.728644 | -2.66873 | 0.008713 | 0.111083 | -2.95688 |
| TTC9C | -0.2494 | 5.611537 | -2.80273 | 0.005947 | 0.085761 | -2.62114 |
| SNRNP70 | -0.24948 | 6.296559 | -2.54743 | 0.01217 | 0.139983 | -3.24827 |
| HADHB | -0.2496 | 9.660945 | -4.80105 | 4.79E-06 | 0.000447 | 3.905782 |
| RPL10A | -0.24966 | 11.43014 | -2.61363 | 0.010155 | 0.122699 | -3.09072 |
| ZNF671 | -0.24975 | 5.659783 | -3.28526 | 0.00135 | 0.029912 | -1.29645 |
| SGPL1 | -0.24981 | 5.810566 | -2.54175 | 0.012359 | 0.141357 | -3.26161 |
| CPM | -0.25006 | 4.526663 | -3.96776 | 0.000127 | 0.005328 | 0.865105 |
| KIAA1147 | -0.25048 | 6.002218 | -2.7486 | 0.00695 | 0.095109 | -2.7585 |
| PCSK7 | -0.25084 | 6.176965 | -3.23493 | 0.001588 | 0.033858 | -1.44286 |
| SPATA6 | -0.25128 | 3.36369 | -4.60776 | 1.06E-05 | 0.000782 | 3.164842 |
| CLSTN1 | -0.25136 | 6.410634 | -3.43775 | 0.000817 | 0.020954 | -0.84165 |
| ZNF689 | -0.25141 | 5.336243 | -2.90725 | 0.004375 | 0.070276 | -2.34939 |
| MRPS21 | -0.2517 | 7.418354 | -2.88524 | 0.00467 | 0.07374 | -2.40734 |
| COMMD9 | -0.25185 | 7.278653 | -2.19446 | 0.03021 | 0.247282 | -4.02672 |
| XYLT1 | -0.25188 | 5.187994 | -3.16631 | 0.001976 | 0.039979 | -1.63941 |
| GTPBP6 | -0.25217 | 5.897207 | -2.50352 | 0.013698 | 0.152636 | -3.35078 |
| CYB561A3 | -0.25252 | 6.919964 | -2.85405 | 0.00512 | 0.078246 | -2.48879 |
| LOC10272 | -0.25257 | 4.810546 | -3.01986 | 0.003115 | 0.055193 | -2.04709 |
| ZGPAT | -0.25265 | 6.696797 | -2.46907 | 0.015014 | 0.161113 | -3.43009 |
| SEC14L1P1 | -0.25269 | 4.515246 | -3.24943 | 0.001516 | 0.032725 | -1.40086 |
| ZNF766 | -0.25274 | 6.429215 | -2.25932 | 0.025745 | 0.224168 | -3.89156 |
| RPS23 | -0.25275 | 8.974436 | -3.31963 | 0.001208 | 0.027609 | -1.19543 |
| SFT2D2 | -0.25277 | 7.485435 | -2.91673 | 0.004253 | 0.068755 | -2.32432 |
| PELI3 | -0.25301 | 5.655964 | -3.19425 | 0.001809 | 0.037386 | -1.5598 |
| ZNF254 | -0.25325 | 4.343357 | -3.93408 | 0.000143 | 0.005827 | 0.751058 |
| LAPTM4A | -0.25331 | 10.61695 | -4.51891 | 1.52E-05 | 0.001003 | 2.831136 |
| RASSF4 | -0.25334 | 5.154739 | -2.92357 | 0.004168 | 0.067733 | -2.3062 |
| SIPA1L1 | -0.25373 | 5.593983 | -2.33975 | 0.021019 | 0.19863 | -3.71899 |
| HLA-DRB6 | -0.25388 | 5.802368 | -3.70076 | 0.000331 | 0.010845 | -0.01854 |
| LMBR1L | -0.25393 | 6.530404 | -2.84004 | 0.005335 | 0.080032 | -2.52513 |
| MAP3K14 | -0.25408 | 4.691275 | -3.60392 | 0.000464 | 0.013771 | -0.32719 |
| 9-Mar | -0.2541 | 4.753491 | -4.93547 | 2.72E-06 | 0.000292 | 4.432768 |
| CHTOP | -0.25466 | 7.523617 | -3.73808 | 0.00029 | 0.009918 | 0.102135 |
| MPZL1 | -0.2549 | 5.388967 | -3.1702 | 0.001952 | 0.039666 | -1.62835 |
| ZNF667-A | -0.25498 | 4.282695 | -2.46408 | 0.015214 | 0.162143 | -3.4415 |

| | | | | | | |
|---|---|---|---|---|---|---|
| ZNF337 | -0.255 | 5.979248 | -4.78677 | 5.08E-06 | 0.000462 | 3.850333 |
| RPL12 | -0.25501 | 11.25279 | -3.00842 | 0.003226 | 0.056421 | -2.07823 |
| EPM2AIP1 | -0.25525 | 6.414046 | -2.03401 | 0.044249 | 0.307026 | -4.34546 |
| PHF1 | -0.25544 | 7.089298 | -3.03078 | 0.003013 | 0.053846 | -2.01725 |
| ZNF703 | -0.25599 | 4.519578 | -2.92978 | 0.004091 | 0.066751 | -2.28969 |
| PLXND1 | -0.25607 | 6.189419 | -3.3895 | 0.00096 | 0.023285 | -0.98738 |
| ZNF44 | -0.25627 | 4.484998 | -3.88326 | 0.000172 | 0.006675 | 0.580359 |
| SENP7 | -0.25638 | 5.099565 | -2.36731 | 0.019586 | 0.189667 | -3.65859 |
| ICOS | -0.25639 | 5.152659 | -2.17919 | 0.031355 | 0.252492 | -4.05802 |
| TRA2A | -0.25641 | 6.111411 | -3.21174 | 0.001711 | 0.035684 | -1.50967 |
| PIGA | -0.25645 | 4.851372 | -2.23019 | 0.027674 | 0.235195 | -3.95271 |
| ZNF431 | -0.25697 | 3.945532 | -2.39448 | 0.018258 | 0.181787 | -3.59843 |
| SGMS2 | -0.25728 | 5.997191 | -2.08407 | 0.039366 | 0.285395 | -4.24842 |
| NAT10 | -0.25739 | 6.420742 | -3.5999 | 0.000471 | 0.013903 | -0.33986 |
| SLC27A1 | -0.25782 | 6.092634 | -2.96342 | 0.003698 | 0.062479 | -2.19982 |
| SOCS3 | -0.25802 | 6.639776 | -2.01639 | 0.046087 | 0.314345 | -4.3791 |
| CTSL | -0.25812 | 7.200756 | -2.17005 | 0.032057 | 0.255711 | -4.07664 |
| ANXA1 | -0.25813 | 9.101887 | -1.98827 | 0.049155 | 0.327304 | -4.43222 |
| ZNF347 | -0.25823 | 3.218118 | -4.28855 | 3.76E-05 | 0.002067 | 1.987126 |
| ANKRD50 | -0.25862 | 4.961371 | -3.26154 | 0.001458 | 0.031877 | -1.36568 |
| EXT1 | -0.25863 | 5.102138 | -3.00144 | 0.003295 | 0.057097 | -2.09721 |
| LINC0026( | -0.25864 | 5.116216 | -2.58755 | 0.01091 | 0.128819 | -3.15322 |
| PIK3C2B | -0.25902 | 6.558408 | -2.48065 | 0.01456 | 0.158471 | -3.40354 |
| CBX7 | -0.25913 | 7.36169 | -2.1141 | 0.036665 | 0.273811 | -4.18915 |
| UBP1 | -0.25951 | 7.809013 | -2.05519 | 0.042123 | 0.297936 | -4.30467 |
| LOC10013 | -0.26017 | 4.227 | -3.64049 | 0.000409 | 0.012548 | -0.21138 |
| NSUN2 | -0.26054 | 7.878438 | -3.34361 | 0.001116 | 0.02593 | -1.12443 |
| RBM6 | -0.26093 | 6.351924 | -2.37917 | 0.018996 | 0.18625 | -3.63242 |
| ECHDC1 | -0.26128 | 7.588157 | -2.83047 | 0.005486 | 0.081883 | -2.54987 |
| PAN2 | -0.26134 | 5.991219 | -2.40029 | 0.017985 | 0.180219 | -3.58549 |
| FAU | -0.26144 | 11.37077 | -3.48153 | 0.000705 | 0.018843 | -0.708 |
| MYBL1 | -0.26174 | 5.376995 | -2.46604 | 0.015135 | 0.16168 | -3.43701 |
| LOC15756 | -0.26184 | 5.188261 | -4.24055 | 4.52E-05 | 0.002374 | 1.815206 |
| P2RX7 | -0.26199 | 5.504003 | -2.28657 | 0.024049 | 0.216769 | -3.83371 |
| TCF7 | -0.26255 | 7.809949 | -2.71983 | 0.007544 | 0.100358 | -2.83053 |
| PTCD3 | -0.26273 | 5.83388 | -2.26765 | 0.025216 | 0.221635 | -3.87394 |
| RPS6KA3 | -0.26296 | 9.085077 | -3.7218 | 0.000308 | 0.010284 | 0.049371 |
| HMGN3 | -0.26359 | 8.807905 | -1.98174 | 0.049891 | 0.32976 | -4.44445 |
| RPL34 | -0.26397 | 12.24046 | -3.67717 | 0.00036 | 0.011381 | -0.09431 |
| CHKB | -0.26401 | 8.051305 | -2.55183 | 0.012026 | 0.138555 | -3.2379 |
| PPFIBP2 | -0.26414 | 5.114353 | -2.57746 | 0.011216 | 0.131742 | -3.17725 |
| MTA1 | -0.26421 | 5.238417 | -2.27417 | 0.024808 | 0.219942 | -3.86011 |
| DFFB | -0.26427 | 5.153268 | -2.41919 | 0.017121 | 0.175776 | -3.54317 |
| PRDX1 | -0.26436 | 8.816191 | -2.46632 | 0.015124 | 0.16168 | -3.43638 |
| TTC9 | -0.26438 | 4.845624 | -2.98498 | 0.003464 | 0.059222 | -2.14177 |
| ERCC1 | -0.26448 | 5.766657 | -3.74412 | 0.000284 | 0.00979 | 0.121739 |
| BBS4 | -0.2645 | 5.163033 | -3.47827 | 0.000713 | 0.018952 | -0.718 |
| ZNF83 | -0.26464 | 4.726903 | -2.15022 | 0.033629 | 0.260752 | -4.11683 |
| VENTX | -0.26489 | 6.258976 | -3.77562 | 0.000254 | 0.008964 | 0.224454 |
| TRIM44 | -0.26521 | 6.232333 | -2.77013 | 0.006534 | 0.091251 | -2.70416 |
| SLC39A1 | -0.26523 | 5.699678 | -3.52193 | 0.000615 | 0.016966 | -0.58345 |
| RNF146 | -0.26529 | 8.544233 | -2.07735 | 0.039993 | 0.288468 | -4.26156 |
| RPLP0 | -0.2653 | 11.26669 | -2.62903 | 0.009732 | 0.119913 | -3.05357 |
| KCNN4 | -0.26547 | 4.813095 | -3.14079 | 0.002142 | 0.042098 | -1.71162 |
| RPL32 | -0.26562 | 12.69195 | -3.35827 | 0.001064 | 0.025013 | -1.0808 |
| BANP | -0.26576 | 8.105169 | -3.41007 | 0.000896 | 0.022122 | -0.92547 |
| MAT2A | -0.26617 | 6.710242 | -2.79406 | 0.006099 | 0.087204 | -2.6433 |
| NDUFAF2 | -0.26632 | 5.908261 | -3.1455 | 0.00211 | 0.041801 | -1.69832 |

| | | | | | | |
|---|---|---|---|---|---|---|
| CREM | -0.26641 | 5.229286 | -2.38506 | 0.018709 | 0.184686 | -3.61936 |
| TBC1D9 | -0.26642 | 5.977132 | -2.16394 | 0.032535 | 0.256639 | -4.08906 |
| LINC0009⁄ | -0.26677 | 4.544123 | -2.08645 | 0.039145 | 0.284646 | -4.24374 |
| PTGER4 | -0.2668 | 6.716753 | -3.04885 | 0.00285 | 0.051571 | -1.96769 |
| HS3ST3B1 | -0.26703 | 5.723731 | -2.82087 | 0.005642 | 0.083442 | -2.5746 |
| FAM118A | -0.26733 | 4.978393 | -2.45724 | 0.015492 | 0.163993 | -3.45711 |
| LYRM9 | -0.26738 | 4.563913 | -6.22782 | 7.96E-09 | 3.39E-06 | 9.923295 |
| PFKFB2 | -0.26738 | 7.034916 | -2.07733 | 0.039995 | 0.288468 | -4.26161 |
| XAB2 | -0.26796 | 5.132676 | -2.41439 | 0.017337 | 0.177106 | -3.55396 |
| CD33 | -0.26833 | 7.245009 | -2.25256 | 0.026182 | 0.226223 | -3.90582 |
| SLAMF6 | -0.26871 | 6.353515 | -2.8603 | 0.005027 | 0.077111 | -2.47255 |
| COX20 | -0.2691 | 6.431321 | -2.36544 | 0.01968 | 0.190313 | -3.66272 |
| KIAA0907 | -0.26953 | 6.292931 | -2.76476 | 0.006636 | 0.092058 | -2.71774 |
| P2RY10 | -0.26958 | 4.647328 | -2.84779 | 0.005215 | 0.079219 | -2.50506 |
| LOC10028 | -0.26987 | 4.791575 | -2.85119 | 0.005163 | 0.078846 | -2.49621 |
| LY9 | -0.26998 | 5.862224 | -2.46236 | 0.015284 | 0.162422 | -3.44543 |
| RPS10 | -0.27002 | 10.66595 | -3.62528 | 0.000431 | 0.013008 | -0.25968 |
| MBOAT7 | -0.27007 | 8.802932 | -2.16957 | 0.032094 | 0.255711 | -4.07761 |
| MEN1 | -0.27023 | 6.334139 | -2.35648 | 0.020139 | 0.193218 | -3.68241 |
| CYSTM1 | -0.27036 | 12.20289 | -2.66705 | 0.008754 | 0.111397 | -2.96099 |
| TLR6 | -0.27081 | 6.926483 | -2.21791 | 0.028524 | 0.23922 | -3.97826 |
| MAN1C1 | -0.27087 | 5.301786 | -2.38534 | 0.018696 | 0.184686 | -3.61875 |
| SPP1 | -0.27102 | 3.325151 | -2.59379 | 0.010725 | 0.127368 | -3.13831 |
| PRKCH | -0.27108 | 5.994699 | -3.68895 | 0.000345 | 0.011074 | -0.05652 |
| LOC93622 | -0.27109 | 5.676039 | -2.4123 | 0.017432 | 0.177632 | -3.55865 |
| NDNL2 | -0.27125 | 5.149157 | -2.25805 | 0.025827 | 0.224583 | -3.89425 |
| RBM5 | -0.27134 | 7.7065 | -2.40573 | 0.017732 | 0.179355 | -3.57333 |
| GOLGA8A | -0.27149 | 5.893372 | -2.28563 | 0.024106 | 0.217103 | -3.83572 |
| PEPD | -0.27193 | 6.473937 | -3.07046 | 0.002667 | 0.049291 | -1.90806 |
| IL27RA | -0.27195 | 5.95487 | -2.89115 | 0.004589 | 0.072629 | -2.39181 |
| MICAL2 | -0.27205 | 6.305354 | -2.99266 | 0.003385 | 0.058296 | -2.12101 |
| CNNM3 | -0.27232 | 6.092542 | -2.65281 | 0.009109 | 0.114777 | -2.9958 |
| PRELID1 | -0.27233 | 8.174742 | -2.52531 | 0.01292 | 0.145735 | -3.30011 |
| ZFP30 | -0.27289 | 4.454067 | -3.77499 | 0.000255 | 0.008968 | 0.222392 |
| CD48 | -0.27294 | 9.140235 | -2.95431 | 0.003801 | 0.063744 | -2.22426 |
| TCN2 | -0.27305 | 5.543449 | -3.27017 | 0.001418 | 0.031067 | -1.34054 |
| VAMP2 | -0.27329 | 6.81401 | -2.01939 | 0.045769 | 0.3128 | -4.37338 |
| TIGD1 | -0.27356 | 4.164483 | -2.07915 | 0.039824 | 0.287743 | -4.25805 |
| ZNF320 | -0.27392 | 4.879319 | -2.20927 | 0.029135 | 0.242444 | -3.99618 |
| MAP3K4 | -0.27403 | 6.992989 | -2.40537 | 0.017749 | 0.179438 | -3.57416 |
| PPRC1 | -0.27466 | 5.077245 | -3.1088 | 0.002367 | 0.045144 | -1.80143 |
| SLC12A7 | -0.27477 | 7.810025 | -2.76566 | 0.006619 | 0.091946 | -2.71547 |
| C10orf2 | -0.2748 | 5.014349 | -3.75864 | 0.00027 | 0.009345 | 0.169006 |
| KCTD12 | -0.27523 | 10.06535 | -2.30461 | 0.022981 | 0.210398 | -3.79507 |
| ACSL3 | -0.27588 | 7.934327 | -2.02239 | 0.045454 | 0.311271 | -4.36768 |
| ARHGAP1 | -0.27597 | 8.335362 | -3.9663 | 0.000127 | 0.005332 | 0.860141 |
| SH3BP5 | -0.27601 | 10.01882 | -3.43362 | 0.000829 | 0.021113 | -0.8542 |
| DPH5 | -0.27609 | 6.418095 | -4.00508 | 0.00011 | 0.004819 | 0.992316 |
| PEA15 | -0.27618 | 6.100366 | -2.79365 | 0.006106 | 0.087248 | -2.64436 |
| ATG4D | -0.27651 | 6.416354 | -3.14595 | 0.002107 | 0.041782 | -1.69706 |
| RUFY3 | -0.27673 | 4.674997 | -2.63893 | 0.009468 | 0.117663 | -3.02957 |
| ZNF641 | -0.27704 | 6.738575 | -2.37255 | 0.019324 | 0.188465 | -3.64705 |
| RPS18 | -0.27735 | 12.53477 | -3.11279 | 0.002338 | 0.044753 | -1.79028 |
| CAMK2D | -0.27763 | 5.097433 | -2.81592 | 0.005724 | 0.084199 | -2.58732 |
| LAMTOR5 | -0.27854 | 10.06131 | -3.34422 | 0.001114 | 0.025907 | -1.12261 |
| LOC49415 | -0.27865 | 4.34309 | -2.95199 | 0.003827 | 0.063856 | -2.23045 |
| ZNF419 | -0.27877 | 6.009576 | -4.96497 | 2.4E-06 | 0.000265 | 4.549655 |
| ZBTB16 | -0.27881 | 5.616505 | -2.0392 | 0.043719 | 0.304695 | -4.33549 |

| | | | | | | |
|---|---|---|---|---|---|---|
| LOC28595 | -0.27907 | 4.887283 | -2.56639 | 0.011559 | 0.134851 | -3.20352 |
| VPS51 | -0.27952 | 8.02839 | -2.0665 | 0.041024 | 0.292803 | -4.28271 |
| GLO1 | -0.27975 | 7.908941 | -2.3964 | 0.018167 | 0.181223 | -3.59416 |
| NPIPA1 | -0.2802 | 8.513031 | -2.41364 | 0.017371 | 0.177363 | -3.55563 |
| IMPDH2 | -0.28083 | 7.597124 | -2.35336 | 0.020301 | 0.19394 | -3.68925 |
| CCR9 | -0.28086 | 4.597336 | -2.60729 | 0.010334 | 0.124096 | -3.10597 |
| DENND4B | -0.2813 | 8.95291 | -2.53277 | 0.012663 | 0.143945 | -3.28267 |
| TNFRSF8 | -0.28181 | 5.305291 | -3.64451 | 0.000403 | 0.01243 | -0.1986 |
| ADAMTSL4 | -0.28183 | 5.645175 | -2.22549 | 0.027996 | 0.236424 | -3.9625 |
| MINA | -0.2827 | 5.152035 | -2.98398 | 0.003475 | 0.059279 | -2.14447 |
| CLMN | -0.28337 | 5.096514 | -3.35648 | 0.00107 | 0.025095 | -1.08613 |
| TBC1D14 | -0.28377 | 9.359569 | -2.7114 | 0.007727 | 0.101935 | -2.85153 |
| E4F1 | -0.28422 | 5.810407 | -3.36798 | 0.00103 | 0.024374 | -1.05185 |
| BBS2 | -0.28429 | 5.626437 | -2.36969 | 0.019466 | 0.189136 | -3.65335 |
| BIN1 | -0.28442 | 6.615795 | -3.14833 | 0.002092 | 0.041632 | -1.69034 |
| LOC10192 | -0.2845 | 4.177351 | -2.52931 | 0.012781 | 0.144651 | -3.29076 |
| TMEM206 | -0.28471 | 5.04123 | -3.38554 | 0.000972 | 0.023422 | -0.99928 |
| BCL11B | -0.28471 | 6.145234 | -2.22019 | 0.028364 | 0.238545 | -3.97352 |
| SCML1 | -0.28512 | 4.771198 | -2.10724 | 0.037267 | 0.276584 | -4.20275 |
| CAMK4 | -0.28562 | 4.862892 | -2.20763 | 0.029253 | 0.242829 | -3.99957 |
| DUSP2 | -0.28584 | 5.744732 | -2.43752 | 0.016318 | 0.169925 | -3.50187 |
| COL18A1 | -0.28597 | 6.275535 | -2.2128 | 0.028884 | 0.241136 | -3.98887 |
| LINC00954 | -0.2862 | 4.381122 | -3.08867 | 0.00252 | 0.047226 | -1.85755 |
| CD36 | -0.2867 | 7.630428 | -2.34425 | 0.020779 | 0.197485 | -3.70917 |
| CLN8 | -0.28676 | 4.770184 | -5.0213 | 1.89E-06 | 0.000217 | 4.774115 |
| ZNF805 | -0.28702 | 4.819445 | -2.94378 | 0.003923 | 0.064889 | -2.2524 |
| LSM6 | -0.28727 | 8.59785 | -3.06622 | 0.002702 | 0.049672 | -1.91979 |
| ST3GAL5 | -0.28729 | 6.325346 | -2.77489 | 0.006446 | 0.09052 | -2.69209 |
| JUP | -0.28752 | 5.659712 | -2.1637 | 0.032554 | 0.256688 | -4.08956 |
| KRI1 | -0.28804 | 5.97912 | -3.98146 | 0.00012 | 0.00515 | 0.911698 |
| NABP1 | -0.2883 | 8.877668 | -2.81109 | 0.005805 | 0.0846 | -2.59973 |
| LOC10272 | -0.28846 | 3.205455 | -3.5137 | 0.000633 | 0.017342 | -0.60894 |
| BCL11A | -0.2891 | 6.437003 | -2.0613 | 0.041526 | 0.294832 | -4.29282 |
| KLHL5 | -0.2893 | 5.832572 | -2.31798 | 0.022217 | 0.205789 | -3.76625 |
| RABEP2 | -0.29017 | 5.832739 | -4.6235 | 9.93E-06 | 0.000748 | 3.22442 |
| HLA-DOB | -0.29035 | 5.63425 | -4.01323 | 0.000107 | 0.004726 | 1.02023 |
| PITPNM1 | -0.29051 | 6.579705 | -2.13924 | 0.034528 | 0.264626 | -4.13893 |
| MSL3 | -0.29055 | 7.688083 | -3.2528 | 0.0015 | 0.032551 | -1.39108 |
| GPR35 | -0.29161 | 5.826362 | -3.60331 | 0.000465 | 0.01378 | -0.32912 |
| YAE1D1 | -0.29163 | 5.023917 | -2.87544 | 0.004808 | 0.074808 | -2.43302 |
| NRROS | -0.2917 | 7.054114 | -2.62421 | 0.009863 | 0.120482 | -3.06521 |
| TRAK1 | -0.29171 | 6.20069 | -5.82132 | 5.37E-08 | 1.41E-05 | 8.121514 |
| CCDC50 | -0.29178 | 4.750241 | -2.72928 | 0.007344 | 0.098476 | -2.80696 |
| TAF1A | -0.29192 | 4.105353 | -2.29437 | 0.023582 | 0.214043 | -3.81704 |
| NAGPA | -0.29232 | 6.456467 | -3.49765 | 0.000668 | 0.018032 | -0.65845 |
| PDE3B | -0.29264 | 6.494161 | -2.54986 | 0.01209 | 0.13922 | -3.24254 |
| MRPS35 | -0.29281 | 7.078768 | -2.14841 | 0.033776 | 0.261231 | -4.12047 |
| ZFAS1 | -0.29332 | 9.107437 | -3.54383 | 0.000571 | 0.016061 | -0.51547 |
| MDM4 | -0.29369 | 6.492225 | -3.6744 | 0.000363 | 0.011421 | -0.10319 |
| DUSP6 | -0.294 | 10.22204 | -2.14749 | 0.033851 | 0.26158 | -4.12234 |
| NDUFAF7 | -0.29472 | 5.706988 | -3.01547 | 0.003158 | 0.055601 | -2.05905 |
| RBM10 | -0.29486 | 7.117669 | -2.8686 | 0.004906 | 0.075708 | -2.45089 |
| CLEC4D | -0.29494 | 10.41698 | -2.65032 | 0.009173 | 0.115315 | -3.00187 |
| SLC9A7 | -0.295 | 3.925337 | -4.92413 | 2.86E-06 | 0.000301 | 4.387965 |
| WWC3 | -0.29513 | 8.173315 | -3.10907 | 0.002365 | 0.045144 | -1.80068 |
| CXCR1 | -0.29522 | 10.41838 | -2.2539 | 0.026095 | 0.225889 | -3.90301 |
| LOXHD1 | -0.29589 | 4.092059 | -2.95365 | 0.003808 | 0.063818 | -2.22602 |
| C17orf62 | -0.29612 | 9.112992 | -2.71028 | 0.007751 | 0.102127 | -2.85432 |

| | | | | | | |
|---|---|---|---|---|---|---|
| POGLUT1 | -0.2963 | 6.082052 | -2.16256 | 0.032644 | 0.256841 | -4.09187 |
| MANBAL | -0.29636 | 6.279751 | -2.4738 | 0.014828 | 0.160191 | -3.41927 |
| SAT2 | -0.29644 | 7.595737 | -2.82855 | 0.005517 | 0.082186 | -2.55482 |
| DNAJB1 | -0.29661 | 7.393406 | -1.99946 | 0.047914 | 0.322155 | -4.41117 |
| MLH3 | -0.29673 | 5.669239 | -2.84598 | 0.005243 | 0.079291 | -2.50973 |
| TAF7 | -0.29713 | 10.03163 | -3.42499 | 0.000853 | 0.021463 | -0.88036 |
| UTRN | -0.29733 | 6.345716 | -3.71217 | 0.000318 | 0.010587 | 0.01825 |
| TRIT1 | -0.29776 | 5.834915 | -2.78417 | 0.006275 | 0.088863 | -2.66851 |
| SDC2 | -0.29781 | 4.109195 | -3.77165 | 0.000258 | 0.009045 | 0.211462 |
| SYTL3 | -0.29797 | 5.012467 | -3.2939 | 0.001313 | 0.02943 | -1.27113 |
| CRIPAK | -0.29851 | 6.796367 | -3.42447 | 0.000854 | 0.021463 | -0.88193 |
| PCK2 | -0.29851 | 5.816239 | -3.69824 | 0.000334 | 0.010924 | -0.02663 |
| B3GNT5 | -0.29855 | 5.911354 | -3.38533 | 0.000973 | 0.023422 | -0.99989 |
| RPS12 | -0.29878 | 12.23673 | -3.27855 | 0.00138 | 0.030465 | -1.31609 |
| HCLS1 | -0.29882 | 11.46312 | -2.12549 | 0.035683 | 0.270026 | -4.16646 |
| FNIP2 | -0.29915 | 5.650861 | -2.33242 | 0.021416 | 0.201386 | -3.73495 |
| ZNF232 | -0.29988 | 5.144044 | -3.0312 | 0.003009 | 0.053824 | -2.01611 |
| CERK | -0.29991 | 7.729026 | -2.4575 | 0.015481 | 0.163993 | -3.45651 |
| CCNJL | -0.3003 | 7.031249 | -3.15439 | 0.002052 | 0.041095 | -1.67319 |
| NUP85 | -0.3003 | 6.546868 | -2.50317 | 0.013711 | 0.152636 | -3.3516 |
| ST20 | -0.30047 | 6.615784 | -2.48394 | 0.014433 | 0.157848 | -3.39599 |
| SMAP2 | -0.30051 | 10.84072 | -2.12388 | 0.035821 | 0.270366 | -4.16968 |
| ZNF580 | -0.30059 | 7.014449 | -2.98884 | 0.003424 | 0.058778 | -2.13134 |
| PRKDC | -0.30106 | 5.600178 | -2.41163 | 0.017462 | 0.177854 | -3.56015 |
| POLM | -0.30126 | 5.393972 | -2.2044 | 0.029485 | 0.244103 | -4.00625 |
| PRKX | -0.30253 | 5.090594 | -2.47626 | 0.014731 | 0.159651 | -3.41362 |
| SETBP1 | -0.30281 | 4.898975 | -2.20318 | 0.029573 | 0.244103 | -4.00876 |
| EPB41L4A· | -0.30291 | 5.228575 | -3.76479 | 0.000264 | 0.009182 | 0.189052 |
| LOC10028 | -0.30331 | 5.422273 | -2.0327 | 0.044383 | 0.307645 | -4.34796 |
| PFDN5 | -0.30334 | 10.32224 | -2.20165 | 0.029684 | 0.244621 | -4.01191 |
| FCER2 | -0.30402 | 4.917803 | -3.44491 | 0.000798 | 0.020612 | -0.81988 |
| HSPA5 | -0.30433 | 8.29488 | -3.32617 | 0.001182 | 0.027146 | -1.17608 |
| ARSG | -0.30444 | 5.61692 | -4.36499 | 2.79E-05 | 0.001625 | 2.263731 |
| CIB1 | -0.30469 | 7.50678 | -2.33161 | 0.02146 | 0.201639 | -3.7367 |
| PBX4 | -0.3048 | 5.225849 | -4.48083 | 1.76E-05 | 0.001135 | 2.689484 |
| C19orf70 | -0.30483 | 5.876477 | -2.3582 | 0.02005 | 0.192704 | -3.67864 |
| ZNF227 | -0.3055 | 4.78946 | -3.18824 | 0.001844 | 0.03799 | -1.57696 |
| SPNS1 | -0.30591 | 6.242528 | -2.88049 | 0.004737 | 0.074195 | -2.4198 |
| RPS3 | -0.30592 | 11.37779 | -2.18693 | 0.03077 | 0.250329 | -4.04217 |
| HSCB | -0.30641 | 6.346148 | -3.40766 | 0.000903 | 0.022246 | -0.93272 |
| NLRP1 | -0.30653 | 5.53642 | -2.7925 | 0.006126 | 0.087354 | -2.64728 |
| LOC10012 | -0.30668 | 4.894695 | -2.39864 | 0.018062 | 0.180553 | -3.58917 |
| TP53 | -0.30689 | 5.065475 | -2.72719 | 0.007388 | 0.098871 | -2.81218 |
| CDK5R1 | -0.30732 | 5.231539 | -3.08613 | 0.00254 | 0.047513 | -1.86462 |
| OPN3 | -0.3076 | 6.331598 | -2.08162 | 0.039594 | 0.28628 | -4.25322 |
| SOWAHC | -0.30822 | 4.15116 | -2.53283 | 0.012661 | 0.143945 | -3.28254 |
| GLIPR1 | -0.30845 | 8.928625 | -3.03423 | 0.002981 | 0.053556 | -2.00781 |
| ABCB4 | -0.30859 | 3.954484 | -3.57826 | 0.000507 | 0.014704 | -0.40791 |
| HNRNPA1 | -0.30974 | 6.674969 | -2.8203 | 0.005651 | 0.083494 | -2.57607 |
| C3orf14 | -0.31035 | 3.694556 | -4.09386 | 7.91E-05 | 0.003712 | 1.298516 |
| ADAM28 | -0.31038 | 4.578959 | -4.60676 | 1.06E-05 | 0.000783 | 3.161062 |
| DDX21 | -0.31048 | 8.877407 | -2.49843 | 0.013886 | 0.153587 | -3.36257 |
| RSL1D1 | -0.31057 | 6.069569 | -2.42017 | 0.017077 | 0.175506 | -3.54099 |
| HVCN1 | -0.31059 | 8.305863 | -3.07567 | 0.002624 | 0.048611 | -1.89363 |
| ASPH | -0.31092 | 5.68902 | -2.83609 | 0.005397 | 0.080727 | -2.53536 |
| LOC10272 | -0.31112 | 4.840648 | -2.17115 | 0.031972 | 0.255711 | -4.07441 |
| PWAR6 | -0.3112 | 3.663484 | -2.35416 | 0.020259 | 0.19394 | -3.68751 |
| ZNF274 | -0.31122 | 6.971645 | -2.87337 | 0.004837 | 0.075042 | -2.43843 |

| | | | | | | |
|---|---|---|---|---|---|---|
| RPL36 | -0.31139 | 9.823171 | -2.34406 | 0.020789 | 0.197489 | -3.70959 |
| PTER | -0.3115 | 5.067585 | -2.01487 | 0.046248 | 0.314904 | -4.38199 |
| ZNF331 | -0.31168 | 5.222166 | -3.97356 | 0.000124 | 0.005269 | 0.884819 |
| C1orf162 | -0.31179 | 10.28633 | -3.21903 | 0.001671 | 0.035041 | -1.4887 |
| PVRIG | -0.31183 | 7.413643 | -2.56764 | 0.01152 | 0.134468 | -3.20055 |
| RFX3 | -0.31218 | 4.496618 | -4.54125 | 1.39E-05 | 0.000948 | 2.914637 |
| GK5 | -0.31223 | 4.084983 | -3.23024 | 0.001613 | 0.034192 | -1.4564 |
| ZNF189 | -0.31251 | 7.657455 | -3.06541 | 0.002709 | 0.049708 | -1.92204 |
| ARL14EP | -0.31274 | 6.337348 | -2.27615 | 0.024686 | 0.219715 | -3.85592 |
| METRNL | -0.31361 | 7.67002 | -2.38005 | 0.018953 | 0.186072 | -3.63046 |
| LHX4-AS1 | -0.31391 | 4.630245 | -3.00227 | 0.003287 | 0.057001 | -2.09495 |
| TIAM1 | -0.31407 | 6.175363 | -2.71591 | 0.007628 | 0.101163 | -2.84032 |
| FAM49A | -0.31425 | 7.644268 | -3.00645 | 0.003246 | 0.056584 | -2.08359 |
| SIGLEC7 | -0.3147 | 7.128512 | -3.62945 | 0.000425 | 0.012866 | -0.24645 |
| GPR155 | -0.31487 | 6.899647 | -2.62264 | 0.009905 | 0.120862 | -3.069 |
| PUS1 | -0.31555 | 5.274917 | -3.74215 | 0.000286 | 0.009826 | 0.115328 |
| ZNF571 | -0.31559 | 3.539292 | -2.48748 | 0.014298 | 0.156951 | -3.38783 |
| ID3 | -0.31593 | 4.463188 | -3.67442 | 0.000363 | 0.011421 | -0.10313 |
| ARID5A | -0.31613 | 7.338822 | -2.22974 | 0.027704 | 0.235195 | -3.95364 |
| PTPRCAP | -0.31616 | 7.579674 | -2.26922 | 0.025118 | 0.221389 | -3.87063 |
| C14orf28 | -0.31655 | 4.513961 | -3.4902 | 0.000685 | 0.018395 | -0.68139 |
| ZNF506 | -0.31656 | 4.464192 | -3.45904 | 0.000761 | 0.019884 | -0.77684 |
| BEST1 | -0.31666 | 7.157891 | -3.35812 | 0.001064 | 0.025013 | -1.08125 |
| MAF | -0.3168 | 4.317736 | -4.79971 | 4.81E-06 | 0.000448 | 3.900561 |
| ZNF439 | -0.31704 | 4.324669 | -2.92931 | 0.004097 | 0.066793 | -2.29096 |
| RRN3P2 | -0.31707 | 5.071284 | -3.87527 | 0.000178 | 0.006776 | 0.553693 |
| KPNA5 | -0.31736 | 4.599895 | -2.75576 | 0.006809 | 0.09389 | -2.74045 |
| PYHIN1 | -0.31739 | 5.722856 | -1.98477 | 0.049549 | 0.328963 | -4.43879 |
| AKR1B1 | -0.3176 | 7.487554 | -3.16877 | 0.001961 | 0.039807 | -1.63242 |
| HAVCR2 | -0.31778 | 5.604355 | -5.01925 | 1.91E-06 | 0.000217 | 4.765924 |
| IDI1 | -0.31785 | 8.975286 | -3.59228 | 0.000483 | 0.014151 | -0.36388 |
| MCOLN1 | -0.3188 | 6.812118 | -2.16292 | 0.032615 | 0.256841 | -4.09114 |
| IL18RAP | -0.31895 | 11.62501 | -3.05486 | 0.002798 | 0.050805 | -1.95114 |
| C9orf91 | -0.31945 | 5.099229 | -2.84992 | 0.005183 | 0.078934 | -2.49953 |
| S100Z | -0.31947 | 5.200884 | -3.12985 | 0.002217 | 0.043121 | -1.74241 |
| C12orf5 | -0.31968 | 7.365401 | -2.46849 | 0.015038 | 0.161115 | -3.43142 |
| CECR5 | -0.31995 | 6.08678 | -3.38339 | 0.000979 | 0.023545 | -1.00571 |
| CRISP2 | -0.32 | 4.041208 | -2.3406 | 0.020974 | 0.198595 | -3.71713 |
| C6orf48 | -0.32072 | 8.039622 | -3.16248 | 0.002 | 0.040325 | -1.65027 |
| MERTK | -0.32087 | 5.049135 | -2.83625 | 0.005394 | 0.080727 | -2.53493 |
| ZNF514 | -0.32093 | 4.824799 | -3.09809 | 0.002448 | 0.046374 | -1.83134 |
| LOC10050 | -0.3216 | 6.490341 | -2.94043 | 0.003962 | 0.065333 | -2.26134 |
| TRIB2 | -0.32182 | 6.243201 | -2.10463 | 0.037499 | 0.27749 | -4.20792 |
| KLF11 | -0.32229 | 4.403939 | -3.72519 | 0.000304 | 0.010228 | 0.060352 |
| MTSS1 | -0.3226 | 5.314974 | -6.2678 | 6.57E-09 | 2.99E-06 | 10.10371 |
| MYLIP | -0.32268 | 7.404361 | -2.7629 | 0.006671 | 0.092362 | -2.72244 |
| CCDC6 | -0.3233 | 5.45081 | -2.69559 | 0.008079 | 0.105167 | -2.89072 |
| ZNF432 | -0.32335 | 5.827963 | -2.06784 | 0.040896 | 0.292387 | -4.28012 |
| PITPNA | -0.32338 | 7.250476 | -3.13501 | 0.002181 | 0.042664 | -1.72789 |
| C12orf10 | -0.32377 | 5.94427 | -2.76084 | 0.006711 | 0.092721 | -2.72764 |
| RPL3 | -0.32397 | 11.04511 | -3.52758 | 0.000603 | 0.016708 | -0.56594 |
| CCDC109E | -0.3248 | 7.665934 | -2.3013 | 0.023174 | 0.211243 | -3.80217 |
| ARFIP1 | -0.32481 | 6.536639 | -2.39114 | 0.018417 | 0.182793 | -3.60586 |
| C19orf60 | -0.32483 | 6.292537 | -3.54727 | 0.000564 | 0.01599 | -0.50475 |
| ZMYND15 | -0.3251 | 4.23792 | -3.74278 | 0.000286 | 0.00982 | 0.117382 |
| ZNF14 | -0.3254 | 6.094754 | -3.43802 | 0.000816 | 0.020954 | -0.84084 |
| COL8A2 | -0.32552 | 4.587165 | -4.96023 | 2.45E-06 | 0.000268 | 4.53086 |
| ZNF600 | -0.32568 | 5.55308 | -2.102 | 0.037733 | 0.278736 | -4.21312 |

| | | | | | | |
|---|---|---|---|---|---|---|
| ZNF137P | -0.32569 | 5.249752 | -2.35394 | 0.02027 | 0.19394 | -3.68798 |
| CHCHD7 | -0.32618 | 8.228328 | -2.61126 | 0.010222 | 0.123178 | -3.09642 |
| SLC16A6 | -0.32646 | 7.613059 | -2.85943 | 0.00504 | 0.077192 | -2.4748 |
| TRG-AS1 | -0.3265 | 6.709676 | -2.2546 | 0.026049 | 0.225703 | -3.90152 |
| DNMBP | -0.32651 | 6.012129 | -2.47114 | 0.014932 | 0.160614 | -3.42536 |
| FBXW4 | -0.32704 | 6.564812 | -4.60284 | 1.08E-05 | 0.000784 | 3.146238 |
| EPPK1 | -0.32713 | 4.721594 | -4.19257 | 5.44E-05 | 0.002735 | 1.64474 |
| IRF2BPL | -0.32746 | 10.90432 | -3.04023 | 0.002927 | 0.052722 | -1.99137 |
| P2RX4 | -0.32819 | 6.551072 | -2.71462 | 0.007656 | 0.101336 | -2.84353 |
| FBL | -0.32927 | 8.157113 | -2.51907 | 0.013139 | 0.147715 | -3.31466 |
| PID1 | -0.32934 | 3.841232 | -4.73604 | 6.26E-06 | 0.000536 | 3.654297 |
| POU2F2 | -0.33032 | 5.892482 | -4.71299 | 6.89E-06 | 0.000571 | 3.565698 |
| PCSK5 | -0.33229 | 4.821176 | -2.54759 | 0.012165 | 0.139983 | -3.24788 |
| TNFRSF10I | -0.33251 | 4.980764 | -3.95411 | 0.000133 | 0.005479 | 0.818784 |
| FBXL15 | -0.33299 | 5.502658 | -3.4251 | 0.000852 | 0.021463 | -0.88003 |
| SH3PXD2E | -0.33361 | 5.032946 | -4.36697 | 2.77E-05 | 0.00162 | 2.270971 |
| PLXNB2 | -0.33362 | 6.030457 | -4.26693 | 4.09E-05 | 0.002195 | 1.909497 |
| LOC28483 | -0.3338 | 5.796614 | -2.46586 | 0.015143 | 0.16168 | -3.43743 |
| QRSL1 | -0.3338 | 4.808655 | -3.18055 | 0.001889 | 0.03854 | -1.59889 |
| TAF1D | -0.33613 | 5.590543 | -2.79141 | 0.006146 | 0.087509 | -2.65007 |
| CIRBP | -0.33622 | 6.931954 | -3.14773 | 0.002095 | 0.041669 | -1.69201 |
| ZSCAN18 | -0.33627 | 5.518223 | -5.12569 | 1.21E-06 | 0.000151 | 5.194211 |
| CDC42EP2 | -0.33636 | 6.694858 | -3.31106 | 0.001242 | 0.028265 | -1.2207 |
| RPS5 | -0.3368 | 11.04088 | -2.02887 | 0.044778 | 0.308682 | -4.3553 |
| CHD3 | -0.33729 | 6.317446 | -3.22081 | 0.001662 | 0.034915 | -1.48359 |
| ARHGEF3 | -0.33845 | 8.418044 | -3.05254 | 0.002818 | 0.051124 | -1.95753 |
| HNMT | -0.33852 | 4.64786 | -4.66754 | 8.3E-06 | 0.000661 | 3.39182 |
| LINC01272 | -0.33881 | 5.557197 | -2.84458 | 0.005264 | 0.079441 | -2.51337 |
| PRPF3 | -0.33884 | 6.54562 | -3.51202 | 0.000636 | 0.01738 | -0.61411 |
| CECR1 | -0.33891 | 9.322539 | -2.06436 | 0.041231 | 0.293857 | -4.28689 |
| CD300C | -0.33965 | 6.350116 | -3.43073 | 0.000837 | 0.021214 | -0.86296 |
| CD93 | -0.33974 | 9.911591 | -3.38243 | 0.000982 | 0.023579 | -1.00861 |
| TBXAS1 | -0.33989 | 7.953522 | -3.13903 | 0.002154 | 0.042291 | -1.71658 |
| TLE3 | -0.34058 | 8.160773 | -2.1788 | 0.031384 | 0.252628 | -4.0588 |
| APBA2 | -0.34071 | 6.20304 | -3.85736 | 0.000189 | 0.007094 | 0.49404 |
| LDOC1L | -0.34089 | 6.067999 | -2.60434 | 0.010419 | 0.124669 | -3.11305 |
| CCDC104 | -0.34127 | 5.009103 | -3.20264 | 0.001761 | 0.036623 | -1.53578 |
| EFNA1 | -0.3428 | 4.627728 | -4.03295 | 9.94E-05 | 0.004489 | 1.087896 |
| CLK1 | -0.34355 | 9.299319 | -2.94945 | 0.003857 | 0.064209 | -2.23726 |
| SATB1 | -0.34457 | 7.1219 | -2.52492 | 0.012934 | 0.14581 | -3.30103 |
| SESN1 | -0.34469 | 6.552724 | -2.64719 | 0.009253 | 0.115661 | -3.00949 |
| BEX4 | -0.34507 | 5.603451 | -2.02789 | 0.044881 | 0.308764 | -4.35718 |
| SLC25A6 | -0.34531 | 9.259364 | -2.06512 | 0.041156 | 0.29343 | -4.2854 |
| USP11 | -0.34578 | 6.868365 | -2.52209 | 0.013032 | 0.146679 | -3.30761 |
| EPHB6 | -0.34583 | 5.196131 | -3.2294 | 0.001617 | 0.034249 | -1.45882 |
| FUCA1 | -0.34616 | 5.314898 | -4.91441 | 2.98E-06 | 0.000309 | 4.349573 |
| RUNX3 | -0.34617 | 6.196184 | -3.68421 | 0.000351 | 0.01119 | -0.07174 |
| MYC | -0.34688 | 8.134325 | -2.38289 | 0.018814 | 0.185533 | -3.62417 |
| CIPC | -0.34763 | 5.161819 | -2.93667 | 0.004007 | 0.065809 | -2.27136 |
| SFMBT2 | -0.3485 | 4.891777 | -4.26551 | 4.11E-05 | 0.002201 | 1.904405 |
| TMCO6 | -0.34865 | 6.568885 | -3.23122 | 0.001607 | 0.034139 | -1.45355 |
| ZNF121 | -0.34887 | 7.276182 | -2.12252 | 0.035937 | 0.270768 | -4.17238 |
| PPP1R16B | -0.34956 | 5.992598 | -3.21843 | 0.001675 | 0.035073 | -1.49042 |
| LINC00936 | -0.34982 | 7.218003 | -2.48938 | 0.014226 | 0.156329 | -3.38346 |
| SFXN3 | -0.35003 | 6.050418 | -4.59951 | 1.09E-05 | 0.000786 | 3.133661 |
| GPER1 | -0.35042 | 4.861884 | -4.25027 | 4.36E-05 | 0.002302 | 1.849888 |
| MAML2 | -0.35065 | 5.407768 | -2.57523 | 0.011284 | 0.132318 | -3.18255 |
| CARM1 | -0.35092 | 5.599163 | -2.80985 | 0.005826 | 0.084668 | -2.60291 |

| | | | | | | |
|---|---|---|---|---|---|---|
| BTNL8 | -0.35114 | 7.43368 | -2.18478 | 0.030931 | 0.25071 | -4.04658 |
| MAP3K8 | -0.35223 | 7.432579 | -2.87818 | 0.004769 | 0.074473 | -2.42585 |
| NCEH1 | -0.35359 | 4.065073 | -2.14647 | 0.033934 | 0.261924 | -4.12439 |
| TBCB | -0.35497 | 8.133827 | -2.40783 | 0.017636 | 0.178557 | -3.56866 |
| DPYSL2 | -0.35611 | 9.250872 | -2.38742 | 0.018595 | 0.183817 | -3.61413 |
| MIR3682 | -0.35747 | 4.332863 | -2.59651 | 0.010645 | 0.126789 | -3.13181 |
| S100A10 | -0.35798 | 8.321429 | -3.24378 | 0.001544 | 0.033151 | -1.41725 |
| LCK | -0.35808 | 7.400069 | -2.14975 | 0.033667 | 0.260793 | -4.11777 |
| RPS24 | -0.35815 | 8.301032 | -3.79535 | 0.000237 | 0.008485 | 0.289101 |
| RBM4 | -0.3582 | 6.221373 | -2.27142 | 0.02498 | 0.220983 | -3.86596 |
| RNASE4 | -0.36115 | 4.936383 | -2.32428 | 0.021864 | 0.204192 | -3.75262 |
| LOC10050 | -0.36229 | 5.104513 | -2.41987 | 0.01709 | 0.175552 | -3.54165 |
| MRVI1 | -0.36397 | 7.822387 | -2.31258 | 0.022523 | 0.207443 | -3.77791 |
| GNG7 | -0.3641 | 5.551717 | -6.30877 | 5.4E-09 | 2.67E-06 | 10.28913 |
| CTSB | -0.36417 | 9.728481 | -3.2267 | 0.001631 | 0.034403 | -1.46659 |
| RPS21 | -0.36451 | 9.231672 | -4.31332 | 3.41E-05 | 0.001924 | 2.076359 |
| MKNK1 | -0.36573 | 9.056458 | -3.15079 | 0.002075 | 0.041432 | -1.68339 |
| CD22 | -0.36621 | 5.308964 | -4.64138 | 9.24E-06 | 0.000714 | 3.292252 |
| CHD8 | -0.36626 | 6.963223 | -2.89269 | 0.004569 | 0.072467 | -2.38778 |
| TAPT1-AS | -0.36654 | 5.259688 | -2.88498 | 0.004674 | 0.07374 | -2.40801 |
| SCAI | -0.3675 | 3.857448 | -3.30537 | 0.001265 | 0.028698 | -1.23746 |
| FAM198B | -0.36752 | 7.176303 | -2.76615 | 0.00661 | 0.091941 | -2.71424 |
| ACOX2 | -0.36755 | 4.247631 | -5.25917 | 6.76E-07 | 0.0001 | 5.73907 |
| SWAP70 | -0.36789 | 6.75179 | -3.81033 | 0.000224 | 0.008165 | 0.338374 |
| NR1D2 | -0.36835 | 5.058882 | -2.0726 | 0.040441 | 0.290457 | -4.27083 |
| ZNF738 | -0.36844 | 4.350009 | -2.87679 | 0.004789 | 0.074628 | -2.42949 |
| ZNF107 | -0.36854 | 5.286832 | -3.40143 | 0.000922 | 0.022603 | -0.9515 |
| IGHD | -0.36922 | 5.993891 | -2.95182 | 0.003829 | 0.063856 | -2.23091 |
| UCP2 | -0.37045 | 9.632659 | -2.77294 | 0.006482 | 0.090905 | -2.69703 |
| TAGLN | -0.37051 | 5.220302 | -5.20655 | 8.51E-07 | 0.000116 | 5.523288 |
| HEATR3 | -0.37099 | 5.092955 | -3.68591 | 0.000349 | 0.011158 | -0.06627 |
| LMBRD1 | -0.37113 | 6.954552 | -3.65462 | 0.000389 | 0.01209 | -0.16638 |
| CCR5 | -0.37129 | 7.404349 | -3.26057 | 0.001463 | 0.031942 | -1.36849 |
| ZAP70 | -0.37192 | 6.475573 | -2.50036 | 0.013814 | 0.153211 | -3.35809 |
| KRT10 | -0.37202 | 7.982957 | -3.72235 | 0.000307 | 0.01028 | 0.051155 |
| CAPN3 | -0.37203 | 6.838856 | -2.12621 | 0.035622 | 0.269758 | -4.16502 |
| PPARG | -0.37371 | 4.276015 | -3.91075 | 0.000156 | 0.006243 | 0.672482 |
| CD2 | -0.37382 | 7.716631 | -2.33669 | 0.021184 | 0.199813 | -3.72567 |
| ZNF264 | -0.37424 | 5.921933 | -3.83132 | 0.000208 | 0.007672 | 0.407674 |
| PPCDC | -0.37488 | 6.297995 | -2.75944 | 0.006738 | 0.093033 | -2.73119 |
| AMPD2 | -0.37531 | 7.957338 | -3.52675 | 0.000605 | 0.016733 | -0.56854 |
| TNFSF8 | -0.37536 | 6.104319 | -3.43692 | 0.000819 | 0.020986 | -0.84419 |
| GDPD3 | -0.37663 | 6.655983 | -3.30963 | 0.001248 | 0.028365 | -1.22491 |
| ANXA2P2 | -0.37682 | 9.124721 | -4.63618 | 9.43E-06 | 0.00072 | 3.272511 |
| TSEN34 | -0.37689 | 9.577871 | -3.45941 | 0.00076 | 0.019884 | -0.77571 |
| ZBTB4 | -0.37774 | 6.963992 | -4.62338 | 9.94E-06 | 0.000748 | 3.223954 |
| DDIT4 | -0.378 | 7.718657 | -2.18528 | 0.030893 | 0.250651 | -4.04555 |
| TRAPPC12 | -0.37828 | 6.998476 | -2.88387 | 0.004689 | 0.073741 | -2.41092 |
| ZNF302 | -0.37831 | 5.638517 | -2.26288 | 0.025518 | 0.222853 | -3.88404 |
| LOC10013 | -0.37846 | 5.577747 | -2.60608 | 0.010369 | 0.124311 | -3.10888 |
| FZD2 | -0.37873 | 5.029994 | -4.32879 | 3.21E-05 | 0.001837 | 2.132326 |
| ZFP36 | -0.37891 | 11.23041 | -2.66103 | 0.008903 | 0.112798 | -2.97573 |
| LOC10193 | -0.37942 | 5.518784 | -4.25069 | 4.35E-05 | 0.002302 | 1.851399 |
| AGPAT4 | -0.37983 | 4.902585 | -5.67102 | 1.07E-07 | 2.28E-05 | 7.471372 |
| CD74 | -0.37985 | 7.238552 | -3.41309 | 0.000887 | 0.022061 | -0.91635 |
| LOC10193 | -0.37993 | 4.935086 | -2.50607 | 0.013605 | 0.151913 | -3.34488 |
| TRAF5 | -0.38 | 4.398617 | -3.70491 | 0.000326 | 0.010774 | -0.00517 |
| GPR65 | -0.38056 | 9.696325 | -3.67461 | 0.000363 | 0.011421 | -0.10251 |

| | | | | | | |
|---|---|---|---|---|---|---|
| SMCO4 | -0.38104 | 8.064464 | -2.81871 | 0.005678 | 0.08364 | -2.58017 |
| SLC1A3 | -0.38124 | 4.464037 | -4.13334 | 6.81E-05 | 0.00332 | 1.436251 |
| CD300LF | -0.38181 | 9.525723 | -2.91937 | 0.00422 | 0.068425 | -2.31734 |
| C15orf37 | -0.38203 | 7.882369 | -3.0422 | 0.002909 | 0.052542 | -1.98594 |
| MYO1G | -0.38315 | 8.139248 | -2.37593 | 0.019156 | 0.187275 | -3.63958 |
| ZNF32 | -0.38366 | 5.53998 | -3.00971 | 0.003214 | 0.056297 | -2.07475 |
| ZNF585A | -0.38398 | 4.649634 | -4.65911 | 8.59E-06 | 0.000676 | 3.359707 |
| FAM20A | -0.38414 | 5.413961 | -2.04511 | 0.043123 | 0.302602 | -4.32412 |
| ANXA2 | -0.3859 | 8.795196 | -5.02037 | 1.9E-06 | 0.000217 | 4.770391 |
| ZNF818P | -0.386 | 5.803851 | -2.76579 | 0.006616 | 0.091946 | -2.71513 |
| GLCE | -0.38616 | 4.253883 | -3.09477 | 0.002473 | 0.046725 | -1.84058 |
| PNPLA6 | -0.38654 | 8.284286 | -2.00781 | 0.047005 | 0.317957 | -4.39538 |
| ENPP2 | -0.38691 | 4.031015 | -3.46976 | 0.000734 | 0.01937 | -0.74408 |
| STIM2 | -0.38707 | 5.252974 | -3.97144 | 0.000125 | 0.005278 | 0.877608 |
| BIRC3 | -0.38797 | 6.652574 | -2.90551 | 0.004398 | 0.07058 | -2.35399 |
| RBM3 | -0.388 | 6.645398 | -3.72344 | 0.000306 | 0.010257 | 0.054694 |
| IER5 | -0.39003 | 8.36755 | -3.12511 | 0.00225 | 0.043551 | -1.75574 |
| PLEKHO1 | -0.39013 | 6.923401 | -2.14125 | 0.034362 | 0.264099 | -4.13489 |
| EGR1 | -0.39014 | 9.274634 | -2.03228 | 0.044426 | 0.307736 | -4.34877 |
| TRMT13 | -0.39033 | 5.218429 | -2.12019 | 0.036137 | 0.27145 | -4.17702 |
| TMEM128 | -0.39064 | 5.584264 | -2.84246 | 0.005297 | 0.0797 | -2.51886 |
| MAP4K1 | -0.39074 | 6.082639 | -3.68473 | 0.00035 | 0.011187 | -0.07007 |
| RPS6KA2 | -0.39108 | 5.429776 | -5.49845 | 2.34E-07 | 4.16E-05 | 6.736474 |
| COX16 | -0.39364 | 6.911407 | -3.4596 | 0.000759 | 0.019884 | -0.77511 |
| SPIB | -0.39369 | 5.382148 | -4.52278 | 1.49E-05 | 0.000994 | 2.845587 |
| ZNF117 | -0.39518 | 5.205041 | -3.11306 | 0.002336 | 0.044753 | -1.78951 |
| KIAA0355 | -0.39641 | 6.232329 | -3.04595 | 0.002876 | 0.051986 | -1.97564 |
| GIMAP8 | -0.398 | 6.357312 | -4.05598 | 9.12E-05 | 0.004202 | 1.167264 |
| C1orf54 | -0.39845 | 5.386235 | -5.12088 | 1.23E-06 | 0.000153 | 5.174753 |
| BLK | -0.39913 | 4.999317 | -4.50424 | 1.61E-05 | 0.00105 | 2.77648 |
| THBD | -0.39938 | 6.673836 | -2.67773 | 0.008496 | 0.109299 | -2.93476 |
| CLIP4 | -0.39973 | 5.643839 | -3.90459 | 0.00016 | 0.006327 | 0.65179 |
| GIMAP4 | -0.39992 | 9.315351 | -2.46894 | 0.01502 | 0.161113 | -3.43039 |
| MALT1 | -0.40072 | 6.14598 | -2.73906 | 0.007142 | 0.09685 | -2.78246 |
| SIGLEC10 | -0.40116 | 7.388973 | -3.60565 | 0.000462 | 0.013748 | -0.32173 |
| CSTF2T | -0.40169 | 6.484938 | -2.58122 | 0.011101 | 0.130697 | -3.16831 |
| CYTIP | -0.40175 | 9.98237 | -4.52618 | 1.47E-05 | 0.000991 | 2.858291 |
| C5orf28 | -0.402 | 4.358667 | -2.67085 | 0.008662 | 0.110678 | -2.95168 |
| KLRC3 | -0.40235 | 4.198634 | -2.2663 | 0.025301 | 0.221906 | -3.8768 |
| KBTBD8 | -0.40267 | 5.830727 | -2.60602 | 0.01037 | 0.124311 | -3.10901 |
| BAG3 | -0.40288 | 5.914987 | -2.30384 | 0.023026 | 0.21062 | -3.79673 |
| DRAM2 | -0.4031 | 7.1191 | -3.46287 | 0.000751 | 0.019731 | -0.76514 |
| PLCL1 | -0.40348 | 4.178055 | -3.63138 | 0.000422 | 0.012837 | -0.24033 |
| CAMK1 | -0.40448 | 5.214914 | -2.8253 | 0.00557 | 0.082645 | -2.5632 |
| STAP1 | -0.4046 | 5.060601 | -2.56942 | 0.011464 | 0.133972 | -3.19634 |
| ZBTB20 | -0.40549 | 5.948578 | -3.33601 | 0.001145 | 0.026463 | -1.14696 |
| ZNF304 | -0.40638 | 3.823883 | -3.86641 | 0.000183 | 0.006959 | 0.524154 |
| EDNRB | -0.40788 | 3.197099 | -5.24254 | 7.27E-07 | 0.000105 | 5.670745 |
| CD180 | -0.4081 | 6.279646 | -3.63969 | 0.00041 | 0.012565 | -0.21394 |
| ACADVL | -0.40824 | 8.510979 | -2.43407 | 0.016466 | 0.171033 | -3.50966 |
| MEF2C | -0.4086 | 6.189189 | -3.53399 | 0.00059 | 0.016472 | -0.54607 |
| SH3TC1 | -0.40882 | 5.674097 | -4.52324 | 1.49E-05 | 0.000994 | 2.847304 |
| DYRK2 | -0.40888 | 6.129272 | -2.81265 | 0.005779 | 0.084459 | -2.59574 |
| NAGA | -0.40984 | 7.341092 | -3.59829 | 0.000473 | 0.01394 | -0.34496 |
| SLC46A2 | -0.4108 | 6.028953 | -2.71081 | 0.007739 | 0.102038 | -2.85299 |
| A2M-AS1 | -0.4126 | 4.496196 | -2.26783 | 0.025205 | 0.221631 | -3.87356 |
| ZNF420 | -0.41695 | 5.177964 | -2.90955 | 0.004345 | 0.070067 | -2.34332 |
| ZNF211 | -0.41707 | 6.467365 | -3.18727 | 0.001849 | 0.037997 | -1.57973 |

| | | | | | | |
|---|---|---|---|---|---|---|
| PAX5 | -0.41863 | 5.951674 | -4.2388 | 4.56E-05 | 0.002377 | 1.808941 |
| STAB1 | -0.41901 | 5.846624 | -4.12651 | 6.99E-05 | 0.003375 | 1.412355 |
| ADRBK2 | -0.41942 | 6.645248 | -3.62455 | 0.000432 | 0.013009 | -0.26197 |
| LOC73142 | -0.41969 | 5.934326 | -2.70416 | 0.007887 | 0.103181 | -2.86952 |
| ZNF383 | -0.42029 | 4.647671 | -3.62865 | 0.000426 | 0.012883 | -0.24898 |
| LOC10013 | -0.42051 | 6.059091 | -3.9884 | 0.000117 | 0.005062 | 0.935334 |
| ZNF260 | -0.42189 | 5.034951 | -2.52432 | 0.012954 | 0.145882 | -3.30241 |
| ATP1A1 | -0.42231 | 7.962797 | -3.16377 | 0.001992 | 0.040202 | -1.64663 |
| HAUS7 | -0.4235 | 5.119502 | -3.84126 | 0.000201 | 0.007429 | 0.440564 |
| ZNF559 | -0.4237 | 6.222412 | -2.37311 | 0.019296 | 0.188283 | -3.64581 |
| ADAP2 | -0.42459 | 6.64835 | -3.9621 | 0.000129 | 0.005374 | 0.845876 |
| LPL | -0.42488 | 3.743553 | -4.50558 | 1.6E-05 | 0.001048 | 2.781454 |
| SLC2A6 | -0.4259 | 6.498009 | -4.28124 | 3.87E-05 | 0.002105 | 1.960857 |
| PCDH9 | -0.42622 | 3.56502 | -4.23836 | 4.56E-05 | 0.002377 | 1.807401 |
| SERPINB9 | -0.42668 | 6.882694 | -4.04506 | 9.5E-05 | 0.004338 | 1.129584 |
| SLC25A43 | -0.42743 | 4.354234 | -2.21151 | 0.028976 | 0.241507 | -3.99154 |
| BLCAP | -0.42773 | 7.516827 | -3.80428 | 0.000229 | 0.00829 | 0.318463 |
| ZNF266 | -0.42832 | 7.595602 | -3.68169 | 0.000354 | 0.011237 | -0.07982 |
| KLHL15 | -0.42844 | 6.519052 | -2.49433 | 0.014039 | 0.154639 | -3.37203 |
| CRTAM | -0.42945 | 5.315929 | -2.35392 | 0.020271 | 0.19394 | -3.68801 |
| KCTD6 | -0.42945 | 5.550371 | -4.21687 | 4.96E-05 | 0.002535 | 1.730892 |
| FGD2 | -0.42958 | 6.302008 | -6.20502 | 8.87E-09 | 3.63E-06 | 9.820636 |
| WDR11 | -0.43021 | 6.328063 | -3.47552 | 0.00072 | 0.019104 | -0.72643 |
| KLF4 | -0.43033 | 7.20272 | -3.59505 | 0.000479 | 0.014047 | -0.35516 |
| FCRL2 | -0.43137 | 5.055277 | -4.98117 | 2.24E-06 | 0.00025 | 4.614035 |
| PLEKHA1 | -0.43207 | 6.297682 | -2.79927 | 0.006007 | 0.086358 | -2.63 |
| HAL | -0.43341 | 5.874486 | -3.55055 | 0.000558 | 0.015877 | -0.49453 |
| LRIF1 | -0.4335 | 7.062494 | -2.07317 | 0.040388 | 0.290375 | -4.26973 |
| EVL | -0.43548 | 7.379834 | -2.94242 | 0.003939 | 0.06506 | -2.25605 |
| TRIM27 | -0.43732 | 7.838951 | -3.85752 | 0.000189 | 0.007094 | 0.494555 |
| ZMAT1 | -0.43946 | 3.96107 | -4.60176 | 1.08E-05 | 0.000784 | 3.142155 |
| HNRNPU- | -0.43986 | 6.327872 | -2.85009 | 0.00518 | 0.078934 | -2.49908 |
| GIMAP7 | -0.44036 | 9.549411 | -2.83927 | 0.005347 | 0.080154 | -2.52713 |
| MTMR11 | -0.44084 | 6.28637 | -3.80238 | 0.000231 | 0.008318 | 0.312216 |
| DDHD2 | -0.44085 | 5.411266 | -2.44772 | 0.015886 | 0.16687 | -3.47876 |
| GVINP1 | -0.44179 | 5.370369 | -3.32128 | 0.001201 | 0.027491 | -1.19054 |
| SCN3A | -0.44256 | 3.674169 | -2.7475 | 0.006972 | 0.095303 | -2.76127 |
| CASS4 | -0.44259 | 4.027515 | -4.90161 | 3.14E-06 | 0.000325 | 4.299136 |
| SIGIRR | -0.44292 | 6.745258 | -3.24644 | 0.001531 | 0.032903 | -1.40953 |
| SLA | -0.4438 | 10.81966 | -3.70089 | 0.000331 | 0.010845 | -0.0181 |
| LDLRAD3 | -0.44392 | 6.174913 | -3.90078 | 0.000162 | 0.00639 | 0.639018 |
| ZNF558 | -0.44687 | 6.09877 | -4.60178 | 1.08E-05 | 0.000784 | 3.142227 |
| AKR1C3 | -0.44703 | 5.504169 | -2.53836 | 0.012473 | 0.142281 | -3.26957 |
| ZNF329 | -0.44964 | 4.40027 | -3.43012 | 0.000838 | 0.021229 | -0.8648 |
| FCGR2B | -0.45097 | 5.904899 | -3.39832 | 0.000932 | 0.022783 | -0.96086 |
| KIAA0226L | -0.45302 | 8.662187 | -2.93535 | 0.004023 | 0.065963 | -2.27487 |
| FCGRT | -0.45348 | 8.423325 | -2.62187 | 0.009927 | 0.120975 | -3.07085 |
| SLC38A6 | -0.45433 | 4.325226 | -3.01666 | 0.003146 | 0.055495 | -2.05581 |
| FAM129C | -0.45476 | 4.986043 | -5.19063 | 9.12E-07 | 0.000122 | 5.458257 |
| ABCA1 | -0.45536 | 5.624127 | -4.13081 | 6.88E-05 | 0.003336 | 1.427408 |
| GBGT1 | -0.45542 | 6.910895 | -4.73407 | 6.32E-06 | 0.000536 | 3.646711 |
| CRYBG3 | -0.45581 | 5.24842 | -2.8035 | 0.005934 | 0.085631 | -2.61918 |
| BTLA | -0.45686 | 5.852337 | -2.70326 | 0.007907 | 0.103377 | -2.87175 |
| ZFP3 | -0.45774 | 5.207743 | -3.44247 | 0.000804 | 0.020704 | -0.82733 |
| ATHL1 | -0.4616 | 7.630268 | -3.24654 | 0.00153 | 0.032903 | -1.40923 |
| POR | -0.46399 | 7.339193 | -2.07083 | 0.04061 | 0.29126 | -4.27429 |
| SLC46A3 | -0.46554 | 7.602839 | -3.43071 | 0.000837 | 0.021214 | -0.86301 |
| KMO | -0.46847 | 5.210366 | -4.15181 | 6.35E-05 | 0.003125 | 1.501043 |

| | | | | | | |
|---|---|---|---|---|---|---|
| CD1C | -0.46924 | 6.681672 | -3.81867 | 0.000218 | 0.007972 | 0.365874 |
| JADE1 | -0.47016 | 7.109532 | -3.95507 | 0.000133 | 0.00547 | 0.822032 |
| FCRL3 | -0.47268 | 5.564996 | -3.81017 | 0.000225 | 0.008165 | 0.337865 |
| RIN2 | -0.47297 | 5.856315 | -4.07094 | 8.62E-05 | 0.003991 | 1.218975 |
| LINC0087 | -0.474 | 4.771173 | -3.38903 | 0.000961 | 0.023285 | -0.98878 |
| SNX30 | -0.47525 | 6.64727 | -3.25062 | 0.00151 | 0.032635 | -1.3974 |
| ZNF542P | -0.47741 | 4.654175 | -3.46912 | 0.000736 | 0.01937 | -0.74603 |
| PHACTR1 | -0.47829 | 5.696814 | -4.82476 | 4.34E-06 | 0.000422 | 3.99804 |
| ASGR1 | -0.47915 | 6.135126 | -3.93916 | 0.000141 | 0.005742 | 0.768194 |
| IL7R | -0.47918 | 9.144758 | -2.81433 | 0.00575 | 0.084347 | -2.59141 |
| CD200 | -0.47953 | 4.177424 | -4.19905 | 5.31E-05 | 0.002674 | 1.667671 |
| GRAMD1C | -0.48014 | 3.740984 | -3.88351 | 0.000172 | 0.006675 | 0.581194 |
| ASGR2 | -0.48105 | 7.905078 | -3.39437 | 0.000944 | 0.023027 | -0.97275 |
| EBF1 | -0.48196 | 3.932749 | -3.90913 | 0.000157 | 0.006243 | 0.667045 |
| HOTAIRM1 | -0.48368 | 5.534463 | -3.41155 | 0.000892 | 0.02212 | -0.92099 |
| FYN | -0.48382 | 7.532431 | -3.05837 | 0.002768 | 0.050302 | -1.94146 |
| SGPP1 | -0.48444 | 6.41498 | -3.6098 | 0.000455 | 0.013572 | -0.30865 |
| CAMK1D | -0.48471 | 6.194393 | -3.01348 | 0.003177 | 0.055892 | -2.06447 |
| CCR2 | -0.48848 | 7.556864 | -2.818 | 0.005689 | 0.083752 | -2.58198 |
| VPREB3 | -0.48906 | 5.299745 | -4.10053 | 7.71E-05 | 0.003662 | 1.321711 |
| SIGLEC16 | -0.48959 | 5.129417 | -4.79716 | 4.86E-06 | 0.00045 | 3.890668 |
| FOS | -0.4912 | 10.64116 | -2.10459 | 0.037503 | 0.27749 | -4.20801 |
| ECHDC3 | -0.49147 | 6.754973 | -3.14634 | 0.002105 | 0.041771 | -1.69594 |
| LOC20077 | -0.4915 | 4.295923 | -4.83999 | 4.07E-06 | 0.000399 | 4.057481 |
| CPNE5 | -0.49187 | 6.841791 | -3.27205 | 0.001409 | 0.030995 | -1.33507 |
| CYP1B1 | -0.49308 | 8.404705 | -3.44805 | 0.000789 | 0.020422 | -0.81033 |
| CRTC3 | -0.49368 | 6.295235 | -4.70907 | 7E-06 | 0.000578 | 3.550669 |
| CXXC5 | -0.49424 | 6.943692 | -3.85784 | 0.000189 | 0.007094 | 0.495615 |
| ITK | -0.49472 | 7.971819 | -2.32061 | 0.022069 | 0.205379 | -3.76055 |
| TMEM243 | -0.4948 | 7.550573 | -3.30353 | 0.001273 | 0.028838 | -1.24287 |
| SLC15A3 | -0.49813 | 7.707322 | -3.01824 | 0.003131 | 0.055324 | -2.05151 |
| PLXDC2 | -0.49937 | 6.736216 | -3.15822 | 0.002027 | 0.040711 | -1.66235 |
| CCR6 | -0.50138 | 4.439772 | -3.43548 | 0.000823 | 0.021061 | -0.84857 |
| CD52 | -0.50143 | 9.395664 | -2.39432 | 0.018266 | 0.181787 | -3.59879 |
| RALGPS2 | -0.50248 | 5.379392 | -5.53724 | 1.96E-07 | 3.56E-05 | 6.900559 |
| LBH | -0.50294 | 7.111836 | -2.90321 | 0.004428 | 0.070954 | -2.36006 |
| CALHM2 | -0.50534 | 6.85446 | -4.4324 | 2.14E-05 | 0.001334 | 2.510557 |
| SLC39A10 | -0.50625 | 5.789892 | -2.6143 | 0.010136 | 0.122699 | -3.08911 |
| BCAT1 | -0.50659 | 5.528669 | -3.61065 | 0.000454 | 0.013552 | -0.30594 |
| AHR | -0.51016 | 7.504439 | -3.23796 | 0.001573 | 0.0336 | -1.43408 |
| 1-Mar | -0.51017 | 6.826182 | -3.88302 | 0.000173 | 0.006675 | 0.579573 |
| CD19 | -0.51043 | 6.330744 | -3.98996 | 0.000117 | 0.005043 | 0.940666 |
| RTN1 | -0.51383 | 5.65359 | -3.10008 | 0.002433 | 0.046173 | -1.8258 |
| HLA-DPB1 | -0.51522 | 6.497623 | -4.74247 | 6.1E-06 | 0.000527 | 3.679093 |
| SYNE2 | -0.51697 | 5.116543 | -7.19805 | 6.71E-11 | 9.81E-08 | 14.43878 |
| HLA-DMB | -0.51746 | 7.012342 | -3.70418 | 0.000327 | 0.010784 | -0.00753 |
| KLRF1 | -0.52161 | 6.203245 | -1.99169 | 0.048773 | 0.326255 | -4.4258 |
| THBS1 | -0.52281 | 5.464122 | -3.22685 | 0.00163 | 0.034403 | -1.46616 |
| MAFB | -0.52532 | 9.571196 | -3.9666 | 0.000127 | 0.005332 | 0.861164 |
| MAK | -0.52848 | 5.405934 | -3.98566 | 0.000118 | 0.005092 | 0.926 |
| NAAA | -0.52953 | 6.592235 | -4.58706 | 1.15E-05 | 0.000812 | 3.086693 |
| SGK223 | -0.53205 | 7.196689 | -3.95741 | 0.000131 | 0.005434 | 0.829975 |
| LINC01127 | -0.53347 | 6.734512 | -2.51489 | 0.013287 | 0.148974 | -3.32439 |
| BLNK | -0.53392 | 5.796565 | -2.55195 | 0.012022 | 0.138555 | -3.23763 |
| LIPA | -0.53472 | 9.406565 | -2.75304 | 0.006863 | 0.094363 | -2.74732 |
| GAPT | -0.5355 | 8.213335 | -2.29102 | 0.023781 | 0.215221 | -3.82419 |
| KYNU | -0.54074 | 6.15345 | -4.36389 | 2.8E-05 | 0.001625 | 2.259742 |
| PCTP | -0.54292 | 8.211831 | -4.4306 | 2.15E-05 | 0.001336 | 2.503944 |

| | | | | | | |
|---|---|---|---|---|---|---|
| ENTPD7 | -0.54374 | 6.649297 | -3.54482 | 0.000569 | 0.016061 | -0.51238 |
| UBE2E2 | -0.54438 | 6.247323 | -3.53357 | 0.000591 | 0.016472 | -0.54736 |
| FCRLA | -0.54535 | 4.651156 | -4.30991 | 3.46E-05 | 0.001945 | 2.064057 |
| GIMAP6 | -0.54976 | 8.343934 | -3.5086 | 0.000644 | 0.017513 | -0.62469 |
| FCRL1 | -0.55351 | 7.166238 | -3.43478 | 0.000825 | 0.021084 | -0.85068 |
| MS4A14 | -0.55649 | 5.800494 | -3.58303 | 0.000499 | 0.014534 | -0.39293 |
| CLEC10A | -0.55906 | 5.294908 | -3.95808 | 0.000131 | 0.005434 | 0.832228 |
| RHOU | -0.56217 | 6.615313 | -4.39938 | 2.44E-05 | 0.001475 | 2.389328 |
| GIMAP1 | -0.56502 | 7.186187 | -4.6019 | 1.08E-05 | 0.000784 | 3.142683 |
| ZNF708 | -0.56964 | 4.09019 | -4.38472 | 2.58E-05 | 0.001537 | 2.335694 |
| GPRASP1 | -0.57364 | 5.218197 | -3.21922 | 0.00167 | 0.035041 | -1.48816 |
| KRT23 | -0.57569 | 8.222108 | -2.63421 | 0.009593 | 0.118846 | -3.04102 |
| IL10RA | -0.57763 | 9.412988 | -5.08542 | 1.44E-06 | 0.000176 | 5.031519 |
| HLA-DQA | -0.58254 | 5.388531 | -2.04843 | 0.042792 | 0.301104 | -4.31773 |
| SASH1 | -0.58423 | 5.526219 | -5.37645 | 4.03E-07 | 6.39E-05 | 6.224687 |
| LINC0092€ | -0.59261 | 6.320618 | -5.04078 | 1.74E-06 | 0.000203 | 4.852108 |
| ZC3H12D | -0.59281 | 5.734804 | -3.00249 | 0.003285 | 0.057001 | -2.09437 |
| TPK1 | -0.59338 | 6.694887 | -3.31178 | 0.001239 | 0.02823 | -1.21858 |
| P2RX5 | -0.59929 | 7.852213 | -3.5911 | 0.000485 | 0.014168 | -0.36756 |
| CCR7 | -0.60203 | 7.109787 | -3.45456 | 0.000772 | 0.020109 | -0.7905 |
| ST6GALNA | -0.6041 | 6.045115 | -3.0684 | 0.002684 | 0.049471 | -1.91376 |
| LOC72839 | -0.60713 | 7.953577 | -4.41848 | 2.26E-05 | 0.00138 | 2.45939 |
| SIDT2 | -0.60855 | 7.848247 | -5.24332 | 7.24E-07 | 0.000105 | 5.673956 |
| KLRB1 | -0.60991 | 7.37801 | -3.35061 | 0.001091 | 0.025485 | -1.10361 |
| SLC16A7 | -0.61205 | 5.438062 | -4.8394 | 4.08E-06 | 0.000399 | 4.055178 |
| L3MBTL3 | -0.6154 | 6.185243 | -3.94607 | 0.000137 | 0.00562 | 0.791569 |
| CPVL | -0.62045 | 7.778828 | -3.03376 | 0.002986 | 0.053556 | -2.00909 |
| LOC28307 | -0.62099 | 7.074649 | -3.52965 | 0.000599 | 0.016636 | -0.55955 |
| C1QC | -0.62132 | 5.177266 | -3.18235 | 0.001878 | 0.038437 | -1.59378 |
| BANK1 | -0.62963 | 6.416274 | -3.49097 | 0.000683 | 0.018371 | -0.67902 |
| EGR3 | -0.63852 | 5.607159 | -2.29684 | 0.023436 | 0.213082 | -3.81174 |
| ZNF658 | -0.64148 | 3.893962 | -4.44708 | 2.02E-05 | 0.001274 | 2.564665 |
| CNTNAP3 | -0.64574 | 2.951102 | -5.01692 | 1.93E-06 | 0.000218 | 4.756593 |
| FAIM3 | -0.64595 | 7.769827 | -4.17328 | 5.86E-05 | 0.002894 | 1.5766 |
| BACH2 | -0.65355 | 5.814297 | -4.04449 | 9.52E-05 | 0.004338 | 1.127619 |
| CD79A | -0.65628 | 6.823026 | -5.05797 | 1.62E-06 | 0.000195 | 4.921079 |
| LILRA1 | -0.65674 | 6.405104 | -6.48984 | 2.25E-09 | 1.59E-06 | 11.11533 |
| C1QA | -0.67442 | 5.44863 | -3.54355 | 0.000571 | 0.016061 | -0.51634 |
| SLC7A7 | -0.6757 | 9.454433 | -4.34437 | 3.02E-05 | 0.001733 | 2.188774 |
| EPB41L3 | -0.67729 | 5.916628 | -3.21378 | 0.0017 | 0.035489 | -1.50381 |
| GPR18 | -0.68065 | 6.039617 | -3.0864 | 0.002538 | 0.047513 | -1.86386 |
| RNASE6 | -0.68202 | 8.478632 | -4.1894 | 5.51E-05 | 0.002755 | 1.633534 |
| ATP2B1 | -0.68461 | 7.162119 | -3.53771 | 0.000583 | 0.016339 | -0.5345 |
| RGL1 | -0.68629 | 6.560795 | -5.04249 | 1.73E-06 | 0.000203 | 4.858947 |
| CD1D | -0.6955 | 7.865613 | -4.12351 | 7.07E-05 | 0.003405 | 1.401886 |
| CDKN1C | -0.69588 | 5.549921 | -4.51472 | 1.54E-05 | 0.001015 | 2.815517 |
| VCAN | -0.70445 | 10.50405 | -4.02366 | 0.000103 | 0.004606 | 1.055968 |
| LY86 | -0.70467 | 7.849362 | -5.28739 | 5.97E-07 | 9.12E-05 | 5.855345 |
| LINC0109: | -0.7063 | 4.021834 | -3.69677 | 0.000336 | 0.01095 | -0.03137 |
| GPR183 | -0.70772 | 7.411772 | -3.9263 | 0.000147 | 0.005971 | 0.724806 |
| ARHGEF4( | -0.71058 | 6.241534 | -5.42176 | 3.29E-07 | 5.48E-05 | 6.414012 |
| MSANTD2 | -0.71548 | 5.181888 | -3.64559 | 0.000402 | 0.012421 | -0.19518 |
| MS4A7 | -0.71959 | 7.302803 | -4.22941 | 4.72E-05 | 0.002453 | 1.775509 |
| TENM1 | -0.72649 | 4.413469 | -6.21826 | 8.33E-09 | 3.48E-06 | 9.880215 |
| HLA-DMA | -0.72755 | 7.3732 | -3.9097 | 0.000157 | 0.006243 | 0.66897 |
| ABCG1 | -0.72772 | 4.819768 | -5.28148 | 6.13E-07 | 9.29E-05 | 5.830995 |
| MRC1 | -0.72881 | 4.523743 | -4.42657 | 2.19E-05 | 0.001345 | 2.489094 |
| LOC10260 | -0.74213 | 5.87768 | -5.40032 | 3.62E-07 | 5.98E-05 | 6.324317 |

| | | | | | | |
|---|---|---|---|---|---|---|
| CD72 | -0.75396 | 5.037504 | -4.7697 | 5.45E-06 | 0.000482 | 3.784244 |
| MARCO | -0.76759 | 6.304527 | -5.97926 | 2.58E-08 | 7.75E-06 | 8.81429 |
| PDK4 | -0.77189 | 5.142556 | -5.18971 | 9.15E-07 | 0.000122 | 5.454487 |
| IRF8 | -0.78106 | 8.271364 | -6.56747 | 1.54E-09 | 1.17E-06 | 11.47271 |
| MPEG1 | -0.79052 | 10.03147 | -4.93545 | 2.72E-06 | 0.000292 | 4.432691 |
| IL1B | -0.79089 | 9.022501 | -4.37697 | 2.66E-05 | 0.001574 | 2.307424 |
| KIAA1598 | -0.79564 | 6.75247 | -3.7814 | 0.000249 | 0.008857 | 0.243347 |
| HLA-DPA1 | -0.81123 | 8.126387 | -5.1643 | 1.02E-06 | 0.000131 | 5.350939 |
| FBP1 | -0.82309 | 7.18363 | -5.50975 | 2.22E-07 | 3.99E-05 | 6.78421 |
| IL1RAP | -0.8304 | 7.626801 | -5.27124 | 6.41E-07 | 9.64E-05 | 5.788787 |
| HLA-DRA | -0.83441 | 9.390913 | -3.7194 | 0.00031 | 0.010354 | 0.041619 |
| C1QB | -0.83459 | 5.042051 | -4.39224 | 2.51E-05 | 0.001508 | 2.363209 |
| CASP5 | -0.83521 | 6.20488 | -4.63161 | 9.61E-06 | 0.000731 | 3.25514 |
| S1PR1 | -0.84946 | 8.104904 | -5.98744 | 2.48E-08 | 7.69E-06 | 8.850428 |
| CSF1R | -0.85746 | 7.526296 | -4.81458 | 4.52E-06 | 0.000431 | 3.95838 |
| GPR137B | -0.93018 | 6.496392 | -4.53554 | 1.42E-05 | 0.000963 | 2.89325 |
| VSIG4 | -0.98259 | 6.023503 | -4.80656 | 4.68E-06 | 0.000439 | 3.927202 |
| MS4A1 | -1.00672 | 7.047121 | -4.62403 | 9.91E-06 | 0.000748 | 3.226412 |
| PLA2G7 | -1.01108 | 4.403841 | -6.25426 | 7.01E-09 | 3.12E-06 | 10.04254 |
| NAPSB | -1.06233 | 5.977982 | -5.85268 | 4.65E-08 | 1.28E-05 | 8.258328 |
| HMOX1 | -1.06959 | 8.095556 | -6.79319 | 5.09E-10 | 4.53E-07 | 12.52203 |
| TCL1A | -1.07948 | 6.503327 | -5.72262 | 8.46E-08 | 1.94E-05 | 7.693564 |
| FGF13 | -1.09956 | 6.38809 | -3.30195 | 0.001279 | 0.028891 | -1.24751 |
| ADAMTS3 | -1.12624 | 3.925413 | -5.6388 | 1.24E-07 | 2.56E-05 | 7.333217 |
| TGFBI | -1.17635 | 8.624055 | -5.34989 | 4.53E-07 | 7.14E-05 | 6.114184 |
| CACNA2D | -1.18622 | 4.579053 | -7.81338 | 2.87E-12 | 8.78E-09 | 17.4232 |
| HPGD | -1.20551 | 7.059436 | -4.88913 | 3.31E-06 | 0.000339 | 4.25004 |
| OLAH | -1.3635 | 6.227015 | -4.7414 | 6.13E-06 | 0.000527 | 3.674954 |
| CD163 | -1.37285 | 7.167102 | -6.6537 | 1.01E-09 | 8.63E-07 | 11.87179 |
