# Supplementary material for: Altered Genes and Biological Functions in Response to Severe Burns
Source: Biomed Res Int. 2021 May 24;2021:8836243. doi: 10.1155/2021/8836243 (PMC8168476; doi:10.1155/2021/8836243)
Supplement: Supplementary 4 — Table S2: 12171 DEGs in GSE37069, DEG_sort_0.05. [file 8836243.f4.pdf]

Table S2 12171 DEGs in GSE37069, DEG_sort_0.05

DEG	logFC	AveExpr	t	P.Value	adj.P.Val	B
MMP8	5.857141	10.22379	13.58835	8.59E-37	1.06E-34	72.76888
CD177	5.816698	11.62053	15.0068	2.30E-43	7.05E-41	87.75724
OLFM4	4.391749	9.102089	9.909747	1.61E-21	2.51E-20	38.00619
GPR84	4.224358	9.385966	13.38405	7.15E-36	7.71E-34	70.67198
HP	4.034793	10.6258	15.66065	1.71E-46	7.95E-44	94.89221
MMP9	3.738589	13.19933	17.84995	2.53E-57	1.15E-53	119.6044
ALPL	3.659173	10.38256	14.79378	2.33E-42	5.95E-40	85.46159
LCN2	3.652286	10.68905	10.42462	1.79E-23	3.49E-22	42.44319
MCEMP1	3.556992	12.10045	16.45125	2.40E-50	2.07E-47	103.6826
BMX	3.449387	9.082218	17.41034	4.13E-55	8.31E-52	114.5546
ANXA3	3.436569	11.46834	16.92048	1.15E-52	1.36E-49	108.9754
LTF	3.376496	11.15328	9.914511	1.55E-21	2.41E-20	38.04652
RETN	3.261178	9.458068	10.58243	4.39E-24	9.04E-23	43.83424
ANKRD22	3.197594	9.707876	15.01845	2.02E-43	6.32E-41	87.88318
PGLYRP1	3.121475	10.42195	12.04421	4.97E-30	2.19E-28	57.36221
LRRN1	3.084677	6.652829	12.3869	1.72E-31	9.03E-30	60.68955
CRISP3	3.05976	8.202418	9.485298	5.83E-20	7.56E-19	34.4695
GADD45A	2.971185	10.6369	15.08375	9.90E-44	3.26E-41	88.59014
TCN1	2.928205	9.953451	10.76221	8.66E-25	1.92E-23	45.43623
TDRD9	2.927319	8.617241	11.60938	3.27E-28	1.15E-26	53.22212
CA4	2.914606	9.340817	13.69005	2.97E-37	3.99E-35	73.81886
ZDHHC19	2.755434	7.077191	9.70114	9.53E-21	1.34E-19	36.25398
GYG1	2.739173	12.11093	16.68697	1.65E-51	1.57E-48	106.3348
VNN1	2.725236	10.79375	12.23506	7.68E-31	3.75E-29	59.20837
CEACAM8	2.718766	8.903998	7.139318	2.76E-12	1.59E-11	17.10759
SLC26A8	2.68886	8.296565	15.372	4.18E-45	1.58E-42	91.72636
DEFA4	2.674477	9.614439	6.734308	3.91E-11	2.01E-10	14.51918
CYSTM1	2.601443	11.65964	14.96434	3.65E-43	1.02E-40	87.29847
RGL4	2.591451	10.20717	13.44899	3.65E-36	4.06E-34	71.33672
HK3	2.577763	10.85101	11.48733	1.04E-27	3.45E-26	52.07707
ELANE	2.574402	7.876265	6.710045	4.56E-11	2.33E-10	14.36821
IL18R1	2.568405	9.486932	13.57341	1.00E-36	1.22E-34	72.61498
FCAR	2.563901	8.381467	15.23783	1.83E-44	6.63E-42	90.26336
CLEC5A	2.549877	8.276769	11.05364	6.01E-26	1.56E-24	48.07121
PGD	2.520773	11.75758	15.03338	1.72E-43	5.56E-41	88.0447
PFKFB3	2.50936	11.08453	15.52722	7.51E-46	3.12E-43	93.42572
GJB6	2.499183	6.719233	9.906008	1.66E-21	2.59E-20	37.97454
CEACAM1	2.474171	9.045431	13.23496	3.32E-35	3.10E-33	69.15241
MAPK14	2.462884	10.15358	16.79431	4.86E-52	5.18E-49	107.5471
ST3GAL4-,	2.449516	8.698999	13.45447	3.45E-36	3.91E-34	71.39282
CEACAM6	2.434192	8.164487	6.816311	2.31E-11	1.22E-10	15.03288
IL1R2	2.428355	11.74415	11.88956	2.23E-29	9.05E-28	55.87901
FFAR2	2.387733	10.23447	9.957554	1.07E-21	1.69E-20	38.4115
SLC2A3	2.338206	11.02253	17.66844	2.08E-56	5.39E-53	117.5148
SLC51A	2.325353	7.12523	9.839229	2.95E-21	4.43E-20	37.41087
GALNT14	2.300592	8.49348	13.33848	1.14E-35	1.17E-33	70.20648
DACH1	2.289878	7.742221	12.67324	9.92E-33	6.11E-31	63.51145
FGF13	2.287512	7.487501	7.19301	1.93E-12	1.13E-11	17.46031
FLOT2	2.280204	9.873225	10.55809	5.45E-24	1.11E-22	43.61882
METTL7B	2.266834	7.824669	10.1047	2.99E-22	5.03E-21	39.66771
UPP1	2.258545	9.953773	14.58932	2.13E-41	4.70E-39	83.27227
PCOLCE2	2.234139	6.64636	7.969253	8.26E-15	6.13E-14	22.80265
CST7	2.21727	12.54278	13.38055	7.41E-36	7.94E-34	70.63616
OPLAH	2.216982	7.301871	11.20689	1.45E-26	4.09E-25	49.4754
CTSD	2.201046	9.162612	10.41968	1.88E-23	3.63E-22	42.39995
HIP1	2.20067	8.79616	14.97897	3.11E-43	8.94E-41	87.45653

SERPINB2	2.17961	7.765188	10.41247	2.00E-23	3.85E-22	42.33671
NFE2	2.157055	10.83463	11.02553	7.79E-26	1.98E-24	47.81502
GRINA	2.147827	9.079202	10.04237	5.14E-22	8.44E-21	39.13394
HBD	2.13855	9.180196	5.991441	3.62E-09	1.48E-08	10.11205
DHRS9	2.127606	10.54783	10.87837	3.01E-25	7.16E-24	46.4809
MS4A4A	2.126703	7.783956	11.27346	7.79E-27	2.28E-25	50.08919
DYSF	2.106062	11.73127	13.6718	3.60E-37	4.73E-35	73.6302
OLAH	2.104727	7.002144	7.996982	6.74E-15	5.05E-14	23.00172
UGCG	2.104554	10.48635	13.82624	7.14E-38	1.02E-35	75.23123
S100A12	2.087304	13.4454	17.3874	5.38E-55	9.74E-52	114.2923
ST6GALNA	2.086552	6.958829	10.77166	7.95E-25	1.78E-23	45.52094
ORM1	2.046765	8.392433	9.452404	7.66E-20	9.84E-19	34.20013
LILRA6	2.039957	9.70611	9.153474	8.90E-19	1.01E-17	31.78401
APOBEC3E	2.021364	7.789212	4.974739	8.58E-07	2.68E-06	4.827695
TSPO	2.019406	11.79978	10.6673	2.04E-24	4.38E-23	44.58825
SLC37A3	1.997683	9.171667	14.58699	2.18E-41	4.76E-39	83.24744
FAM20A	1.994267	6.525215	10.7832	7.15E-25	1.61E-23	45.62447
FCGR1B	1.976457	10.63739	14.49626	5.78E-41	1.22E-38	82.28056
TLR5	1.96304	10.21774	13.57924	9.45E-37	1.16E-34	72.67502
POR	1.953591	8.03874	9.909745	1.61E-21	2.51E-20	38.00617
TP53I3	1.930786	8.266513	11.6411	2.42E-28	8.68E-27	53.52092
PTX3	1.929454	6.806672	7.477757	2.75E-13	1.75E-12	19.36767
MPO	1.92834	7.250741	6.314316	5.33E-10	2.42E-09	11.97241
SIPA1L2	1.924898	9.08718	13.52937	1.59E-36	1.85E-34	72.16178
IRAK3	1.902165	9.39866	13.67856	3.35E-37	4.44E-35	73.70001
DDAH2	1.899266	8.750392	10.07249	3.96E-22	6.58E-21	39.39157
LOC10013	1.89348	9.632768	12.38765	1.71E-31	8.99E-30	60.69685
PADI4	1.889999	8.083973	11.79843	5.36E-29	2.10E-27	55.0105
FOLR3	1.889538	9.244234	8.111425	2.90E-15	2.25E-14	23.82918
SMPDL3A	1.869957	9.239566	8.246685	1.06E-15	8.56E-15	24.81924
FPR2	1.866639	10.50776	10.9859	1.12E-25	2.83E-24	47.45462
NAIP	1.864497	9.634505	12.86181	1.49E-33	1.06E-31	65.38991
APMAP	1.86269	11.76327	15.1232	6.43E-44	2.20E-41	89.01785
SAMSN1	1.86053	11.30341	14.33769	3.16E-40	5.97E-38	80.59764
MGAM	1.853107	12.26469	14.16634	1.97E-39	3.49E-37	78.789
NBEAL2	1.852661	9.519886	6.552425	1.23E-10	6.02E-10	13.39887
PYGL	1.846768	11.62982	17.20493	4.40E-54	7.25E-51	112.2088
IL4R	1.844323	10.73265	13.1991	4.79E-35	4.39E-33	68.78825
CR1	1.836516	9.471418	17.12521	1.10E-53	1.58E-50	111.3007
EXOSC4	1.823199	8.291398	9.224832	4.98E-19	5.84E-18	32.35551
PLBD1	1.821542	12.64035	14.87902	9.23E-43	2.46E-40	86.37846
SPI1	1.817235	10.52439	7.946806	9.73E-15	7.16E-14	22.64191
NR2E1	1.808691	6.555174	11.4593	1.36E-27	4.38E-26	51.81521
GPR160	1.798004	10.81294	12.70437	7.26E-33	4.53E-31	63.82045
WIPI1	1.792447	8.87053	13.9698	1.58E-38	2.46E-36	76.72778
LILRA5	1.791778	8.200983	15.09436	8.82E-44	2.96E-41	88.70504
LMNB1	1.790354	10.36565	15.13189	5.85E-44	2.04E-41	89.11213
1-Mar	1.789451	10.02634	14.33857	3.13E-40	5.97E-38	80.60691
CAMP	1.784641	9.663165	8.096636	3.24E-15	2.49E-14	23.72173
LOC10192	1.774387	7.739222	12.94594	6.34E-34	4.82E-32	66.23302
GPR97	1.774322	10.2516	12.4711	7.46E-32	4.09E-30	61.5154
NCF4	1.772455	11.59493	12.3823	1.80E-31	9.39E-30	60.64451
IL10RB-AS	1.771012	7.066824	12.98026	4.47E-34	3.59E-32	66.57782
S100P	1.752714	12.80548	11.41817	2.00E-27	6.30E-26	51.43163
PLSCR1	1.744969	9.065911	15.94732	6.99E-48	3.73E-45	98.06003
GRB10	1.739568	7.393012	12.04393	4.98E-30	2.19E-28	57.35951
BCL6	1.73578	11.48306	15.48645	1.18E-45	4.75E-43	92.97874
SULT1B1	1.732798	9.143861	12.74062	5.04E-33	3.24E-31	64.18085

LIMK2	1.719017	9.309242	12.27164	5.36E-31	2.66E-29	59.5642
LRG1	1.717096	10.50612	11.09495	4.10E-26	1.08E-24	48.44846
NEDD4	1.716924	6.892344	10.62467	3.00E-24	6.31E-23	44.20905
PGS1	1.714193	9.016988	11.55241	5.63E-28	1.92E-26	52.68663
ATP8B4	1.707656	7.251793	8.868277	8.76E-18	8.77E-17	29.53339
SLC22A4	1.706647	9.851919	12.73384	5.40E-33	3.44E-31	64.11344
ZNF438	1.704564	8.017477	14.40899	1.48E-40	3.04E-38	81.35324
PADI2	1.704469	10.78632	12.42375	1.19E-31	6.36E-30	61.05055
SLC4A1	1.70375	8.187567	6.943162	1.01E-11	5.52E-11	15.83795
ARRB2	1.698078	10.66238	8.514372	1.39E-16	1.22E-15	26.81666
CDA	1.698036	10.87657	11.32488	4.81E-27	1.46E-25	50.565
CD24	1.697235	9.081354	7.595659	1.20E-13	7.98E-13	20.17536
CEBPA	1.691181	9.642586	9.275961	3.28E-19	3.93E-18	32.76704
FKBP5	1.687152	10.29907	11.16208	2.20E-26	6.01E-25	49.06349
CSF3R	1.678803	11.56262	7.096202	3.69E-12	2.09E-11	16.82596
DDIAS	1.66895	8.286238	10.53764	6.55E-24	1.32E-22	43.438
RAC2	1.668734	12.13384	10.17016	1.69E-22	2.92E-21	40.2307
PFKFB2	1.668174	7.388204	10.75621	9.14E-25	2.02E-23	45.38248
BST1	1.664017	9.871357	11.97251	9.98E-30	4.26E-28	56.67309
STXBP2	1.658747	10.24636	8.039821	4.92E-15	3.74E-14	23.31036
SIGLEC5	1.653685	10.88114	12.35505	2.36E-31	1.21E-29	60.37794
F5	1.65356	9.260816	12.5244	4.39E-32	2.48E-30	62.04
CAPG	1.652482	9.834777	9.787187	4.59E-21	6.71E-20	36.97349
SLC1A3	1.65233	7.154746	10.85579	3.69E-25	8.73E-24	46.2772
AP5B1	1.650184	8.600887	10.79254	6.57E-25	1.49E-23	45.70825
GCLM	1.648441	8.006466	11.09764	4.00E-26	1.06E-24	48.47311
AHSP	1.642123	7.363805	6.135043	1.56E-09	6.69E-09	10.92887
IL18RAP	1.64024	11.67681	10.41768	1.91E-23	3.68E-22	42.38238
RNASE2	1.62547	11.29242	9.628979	1.75E-20	2.40E-19	35.65414
GBAP1	1.623302	9.018042	11.68609	1.57E-28	5.75E-27	53.94558
SERPINB1(	1.621564	6.429617	7.447072	3.40E-13	2.15E-12	19.15918
ATP6V1C1	1.621542	8.427805	13.63026	5.55E-37	7.13E-35	73.20111
HPGD	1.618994	7.261706	5.986893	3.71E-09	1.52E-08	10.08646
STX11	1.618627	9.067818	12.38335	1.78E-31	9.32E-30	60.65472
ZYX	1.608388	11.00487	7.459983	3.11E-13	1.97E-12	19.24682
NATD1	1.597936	9.718764	12.7446	4.84E-33	3.15E-31	64.22045
WBP2	1.596663	9.504013	8.70779	3.10E-17	2.92E-16	28.29084
RNASE3	1.593769	8.620981	6.94033	1.03E-11	5.62E-11	15.81984
ATP9A	1.593747	7.404734	11.94093	1.35E-29	5.68E-28	56.37036
ACSL1	1.592105	12.53722	15.61396	2.87E-46	1.24E-43	94.37847
G0S2	1.591095	8.81714	7.165246	2.32E-12	1.35E-11	17.27764
RNF10	1.584771	10.11298	14.31284	4.13E-40	7.71E-38	80.33471
PRKCD	1.582921	10.20639	9.729628	7.49E-21	1.07E-19	36.49169
HGF	1.565324	6.549665	9.415059	1.04E-19	1.32E-18	33.89514
FCER1G	1.55764	12.59056	13.78225	1.13E-37	1.58E-35	74.77428
LIN7A	1.556087	7.855622	11.51623	7.93E-28	2.66E-26	52.3475
CLU	1.551235	8.142314	9.296549	2.77E-19	3.34E-18	32.93322
KL	1.544293	5.154984	8.423824	2.77E-16	2.38E-15	26.1354
TNFAIP6	1.544137	10.71908	9.7093	8.89E-21	1.26E-19	36.32202
CD59	1.540118	8.362453	14.38571	1.89E-40	3.85E-38	81.1063
B4GALT5	1.536226	11.09141	14.76461	3.20E-42	7.74E-40	85.14844
PLIN3	1.525284	9.975181	13.15299	7.69E-35	6.94E-33	68.32081
GGH	1.517281	6.635383	9.178209	7.28E-19	8.37E-18	31.98174
RABAC1	1.506497	9.363077	7.75197	3.97E-14	2.77E-13	21.26213
ROPN1L	1.505416	7.848372	14.80203	2.13E-42	5.52E-40	85.55025
PHTF1	1.496232	7.048615	13.85371	5.35E-38	7.70E-36	75.517
ANKRD55	1.495386	9.002225	7.533335	1.86E-13	1.22E-12	19.74712
WDFY3	1.492656	8.736804	13.85873	5.08E-38	7.36E-36	75.56928

FUT7	1.488862	7.330663	8.414411	2.98E-16	2.54E-15	26.06491
ENTPD7	1.481423	7.009736	9.030845	2.39E-18	2.57E-17	30.80969
ALOX5AP	1.475688	12.81714	12.89947	1.01E-33	7.47E-32	65.767
INSC	1.470184	6.281285	9.366002	1.57E-19	1.94E-18	33.49586
CD55	1.466559	11.71282	14.73724	4.30E-42	1.03E-39	84.85478
GNB2	1.466348	10.03419	6.403752	3.09E-10	1.44E-09	12.50282
SIGLEC9	1.46446	7.922144	8.856066	9.65E-18	9.62E-17	29.43824
ADAM9	1.461742	9.29535	9.972738	9.37E-22	1.49E-20	38.54052
GK3P	1.454525	7.837343	11.075	4.93E-26	1.29E-24	48.26614
RRM2	1.453527	7.508653	5.678133	2.14E-08	8.04E-08	8.38965
CKAP4	1.452158	9.395484	14.98388	2.95E-43	8.62E-41	87.50952
KIF1B	1.451349	8.318678	13.07138	1.77E-34	1.52E-32	67.49573
PLAC8	1.441018	12.71553	10.11636	2.70E-22	4.57E-21	39.76777
KLF5	1.441006	6.576944	10.83858	4.32E-25	1.00E-23	46.12219
PPP1R3B	1.43949	10.50234	12.76439	3.97E-33	2.64E-31	64.41752
PRDM5	1.437134	6.833847	8.625391	5.88E-17	5.39E-16	27.65968
AZU1	1.436847	6.738213	4.352738	1.58E-05	4.19E-05	2.039742
SRPK1	1.432931	9.395623	14.11767	3.30E-39	5.74E-37	78.27726
MAP2K6	1.430958	7.243565	12.89025	1.11E-33	8.14E-32	65.67458
NQO2	1.430386	8.770626	12.06527	4.05E-30	1.81E-28	57.56501
DRAM1	1.429544	8.815	12.9805	4.46E-34	3.59E-32	66.58021
EMB	1.426957	9.221369	12.21838	9.05E-31	4.38E-29	59.04638
PPP1R3D	1.424002	9.121391	12.86936	1.38E-33	9.93E-32	65.46549
PSTPIP2	1.422846	10.00411	11.88538	2.32E-29	9.40E-28	55.8391
DHRS13	1.422646	9.127469	11.31731	5.17E-27	1.56E-25	50.49481
B3GNT5	1.414666	9.725524	9.064933	1.82E-18	1.99E-17	31.07953
TTN-AS1	1.41107	6.899983	10.851	3.86E-25	9.07E-24	46.23404
CAB39	1.409801	10.49015	14.36161	2.45E-40	4.77E-38	80.85087
ECRP	1.408365	7.410962	9.995029	7.73E-22	1.24E-20	38.73018
SLPI	1.40353	9.21844	6.956266	9.30E-12	5.10E-11	15.92183
NUCB1	1.398722	8.275257	6.792263	2.70E-11	1.41E-10	14.88168
HIST2H2BI	1.396634	9.81684	11.27868	7.42E-27	2.18E-25	50.13741
HCK	1.393482	12.11183	9.042308	2.18E-18	2.36E-17	30.90034
LTB4R	1.390835	8.855925	8.974825	3.75E-18	3.94E-17	30.3679
ADM	1.390153	11.49814	11.91306	1.77E-29	7.34E-28	56.10366
STOM	1.388751	8.885518	11.96406	1.08E-29	4.59E-28	56.59201
STAT5B	1.384308	9.58728	13.05349	2.12E-34	1.80E-32	67.31528
RALB	1.382763	10.65991	14.62266	1.48E-41	3.40E-39	83.62833
CTSG	1.379993	7.428894	5.187275	2.94E-07	9.68E-07	5.858823
FLOT1	1.378818	9.181402	11.26904	8.12E-27	2.37E-25	50.04835
MCTP2	1.37792	9.513325	12.4743	7.22E-32	4.00E-30	61.54685
BCL2A1	1.374999	12.0176	12.33819	2.78E-31	1.41E-29	60.21317
SBNO2	1.37425	8.463175	5.809	1.03E-08	4.00E-08	9.099065
ERLIN1	1.369241	8.118455	10.52275	7.48E-24	1.51E-22	43.3065
CHIT1	1.3688	6.44701	6.889877	1.43E-11	7.69E-11	15.49823
FES	1.368386	9.00865	8.639709	5.26E-17	4.85E-16	27.76902
MPP1	1.367741	10.68573	13.43774	4.10E-36	4.53E-34	71.22138
OSM	1.366784	7.634676	10.44277	1.53E-23	2.99E-22	42.60249
GAS7	1.365833	9.109728	12.60594	1.95E-32	1.14E-30	62.84487
CARD6	1.361881	9.904499	11.82753	4.05E-29	1.61E-27	55.28742
CORO2A	1.354465	7.363257	10.22385	1.06E-22	1.88E-21	40.69439
TMEM120A	1.351879	9.066222	9.747998	6.41E-21	9.22E-20	36.64523
PRTN3	1.344153	5.548521	4.210067	2.95E-05	7.55E-05	1.449289
FEM1C	1.342562	8.009626	9.896064	1.81E-21	2.79E-20	37.89044
C15orf65	1.339767	5.796875	8.954182	4.42E-18	4.59E-17	30.20563
MANSC1	1.335662	10.27102	11.9373	1.40E-29	5.87E-28	56.33565
OLR1	1.334458	5.132963	4.937513	1.03E-06	3.18E-06	4.651167
DGAT2	1.333204	9.803122	9.860722	2.45E-21	3.71E-20	37.59199

ACN9	1.321063	7.340186	9.153819	8.88E-19	1.01E-17	31.78676
LILRA3	1.321023	9.546352	5.245876	2.17E-07	7.27E-07	6.150061
FBXO6	1.317788	8.657613	9.032925	2.35E-18	2.53E-17	30.82613
SIRPA	1.314227	9.062459	9.005743	2.93E-18	3.12E-17	30.61146
ATP11B	1.314201	8.900592	11.8926	2.16E-29	8.80E-28	55.9081
ARG1	1.313741	6.149954	13.33146	1.23E-35	1.24E-33	70.13485
EHBP1L1	1.31219	8.663571	5.72132	1.68E-08	6.39E-08	8.622162
SLC25A40	1.301787	8.538541	10.81549	5.33E-25	1.22E-23	45.91445
JAK2	1.299308	8.663543	12.91975	8.26E-34	6.16E-32	65.97019
PDGFC	1.29833	7.405616	6.623587	7.89E-11	3.92E-10	13.83405
DPY19L3	1.292741	8.94249	9.112634	1.24E-18	1.38E-17	31.45842
KLHL2	1.292365	11.33245	11.98749	8.63E-30	3.71E-28	56.81682
BEX1	1.290796	6.762082	6.017712	3.10E-09	1.29E-08	10.26021
ITGAM	1.289374	12.65626	14.60148	1.86E-41	4.17E-39	83.40218
APBB1IP	1.288301	11.03402	11.34116	4.13E-27	1.26E-25	50.71587
CD82	1.28593	8.870817	10.15427	1.94E-22	3.33E-21	40.09376
PRUNE2	1.285602	5.984217	5.954772	4.47E-09	1.81E-08	9.906222
TIMP1	1.279275	11.09881	8.970474	3.88E-18	4.07E-17	30.33367
TMCO3	1.27874	6.811251	13.89973	3.30E-38	4.98E-36	75.99641
PROK2	1.276809	12.14173	10.06577	4.19E-22	6.96E-21	39.33408
CTSA	1.27633	10.51143	8.80212	1.48E-17	1.44E-16	29.01908
AIM2	1.275465	9.63535	7.577941	1.36E-13	8.98E-13	20.05332
BSG	1.27485	8.627353	6.546087	1.28E-10	6.25E-10	13.3603
LINC0026(	1.272834	6.532873	8.914772	6.05E-18	6.20E-17	29.89661
ECHDC3	1.271044	7.655652	8.491846	1.65E-16	1.44E-15	26.64665
MBOAT2	1.270292	8.822887	13.94617	2.02E-38	3.13E-36	76.48097
ALOX5	1.269952	9.691515	11.64128	2.41E-28	8.68E-27	53.52259
SELENBP1	1.269266	6.150663	5.329037	1.41E-07	4.81E-07	6.568458
NTNG2	1.26809	7.285687	9.190355	6.60E-19	7.63E-18	32.07897
CD63	1.26643	11.83502	12.43676	1.05E-31	5.66E-30	61.17815
TRIM25	1.265801	9.765821	12.95015	6.07E-34	4.66E-32	66.2753
DIRC2	1.262325	7.6523	11.8092	4.83E-29	1.90E-27	55.113
KREMEN1	1.259804	6.297745	10.23432	9.63E-23	1.72E-21	40.78495
BIK	1.257303	7.447317	11.54329	6.13E-28	2.08E-26	52.60112
H2BFS	1.257148	9.676717	9.158063	8.57E-19	9.80E-18	31.82066
CEP55	1.253208	5.173751	6.515392	1.55E-10	7.51E-10	13.174
EIF4E3	1.251925	9.054507	11.89621	2.09E-29	8.54E-28	55.94259
TKT	1.250388	10.07409	10.7578	9.01E-25	2.00E-23	45.39673
TSPAN2	1.249867	7.647581	8.647262	4.96E-17	4.58E-16	27.82676
VPS9D1	1.249522	7.773076	8.286116	7.87E-16	6.43E-15	25.1103
RAB13	1.241777	8.49503	7.249582	1.31E-12	7.84E-12	17.83434
CYP4F2	1.240984	8.395365	9.305517	2.57E-19	3.12E-18	33.0057
HBM	1.240389	7.767352	4.525998	7.27E-06	2.01E-05	2.781562
LGALS1	1.235348	11.05999	7.671705	7.03E-14	4.77E-13	20.70181
FAR2	1.232105	8.946215	7.986735	7.27E-15	5.43E-14	22.92809
NME8	1.23033	7.765032	9.319086	2.30E-19	2.80E-18	33.11546
DNAJC5	1.229223	8.355088	11.37157	3.11E-27	9.59E-26	50.99816
IL10RB	1.228956	10.09226	13.14372	8.45E-35	7.54E-33	68.22694
ITGA7	1.227407	6.694389	7.45046	3.32E-13	2.10E-12	19.18216
SORT1	1.227117	7.71316	10.66133	2.16E-24	4.61E-23	44.53511
MTF1	1.226047	8.940768	13.12455	1.03E-34	9.04E-33	68.03295
AGFG1	1.225765	8.357285	11.54638	5.96E-28	2.02E-26	52.63013
DHCR7	1.22447	6.733392	9.068698	1.77E-18	1.94E-17	31.10938
KIF3C	1.224273	7.788801	12.21276	9.56E-31	4.60E-29	58.99178
JMJD6	1.222984	8.290035	12.46294	8.09E-32	4.40E-30	61.43527
ETS2	1.22291	7.829847	13.32301	1.34E-35	1.35E-33	70.04869
LDHA	1.221268	12.45843	10.87458	3.11E-25	7.40E-24	46.44674
PPP1R15A	1.221093	8.575576	6.925713	1.14E-11	6.15E-11	15.72646

CCNA1	1.220369	5.658573	6.070941	2.27E-09	9.57E-09	10.56214
TPD52L2	1.219855	9.868261	8.174889	1.81E-15	1.44E-14	24.2921
G6PD	1.218244	9.49918	6.9194	1.18E-11	6.40E-11	15.68618
CXCR1	1.21761	11.0718	9.513255	4.62E-20	6.05E-19	34.69898
RARA-AS1	1.216043	8.784967	10.01565	6.47E-22	1.05E-20	38.90593
WASF1	1.212946	5.980371	9.892149	1.87E-21	2.88E-20	37.85734
LRRC4	1.211918	8.919322	11.85947	2.98E-29	1.20E-27	55.59178
LILRB1	1.210338	9.362236	8.821842	1.26E-17	1.24E-16	29.17209
NLRP12	1.20755	9.516775	11.77132	6.96E-29	2.68E-27	54.75302
P2RX1	1.207214	9.570819	8.970345	3.89E-18	4.07E-17	30.33265
CYBA	1.206788	9.597497	7.268357	1.16E-12	6.95E-12	17.95901
MTMR3	1.202058	8.564937	10.78693	6.92E-25	1.56E-23	45.65793
CYP1B1	1.199224	8.808184	8.659137	4.53E-17	4.20E-16	27.91761
GBGT1	1.197087	7.919875	11.8293	3.98E-29	1.59E-27	55.3043
ANO10	1.195061	7.986959	10.18531	1.48E-22	2.58E-21	40.36132
LILRB4	1.194226	6.459512	9.552414	3.33E-20	4.44E-19	35.02124
HIST1H2BI	1.194215	9.604276	10.78151	7.26E-25	1.64E-23	45.60931
EMILIN2	1.193766	7.901939	9.899397	1.76E-21	2.72E-20	37.91862
FAM126B	1.193668	9.682828	10.25942	7.73E-23	1.40E-21	41.00245
C1QC	1.193352	5.995291	7.078181	4.16E-12	2.35E-11	16.70868
GALNT2	1.193308	7.196178	8.295441	7.34E-16	6.03E-15	25.1793
C3AR1	1.190732	10.2803	7.758633	3.79E-14	2.65E-13	21.30885
PECR	1.185342	8.234805	12.21292	9.55E-31	4.60E-29	58.99337
SPTLC2	1.18498	8.167419	11.55735	5.37E-28	1.83E-26	52.73299
SLC25A39	1.184695	7.749948	5.842568	8.50E-09	3.34E-08	9.283358
KCNJ15	1.179762	9.402499	9.779653	4.90E-21	7.12E-20	36.91031
EXOC6	1.17905	7.803342	10.42902	1.73E-23	3.37E-22	42.48181
TM6SF1	1.177776	9.644067	11.59314	3.82E-28	1.33E-26	53.0693
LILRA2	1.177236	9.758976	7.159566	2.41E-12	1.39E-11	17.24035
LSP1	1.177087	11.07436	6.080122	2.16E-09	9.09E-09	10.61446
USB1	1.177075	8.77405	9.721772	8.00E-21	1.14E-19	36.42609
LCP1	1.175323	12.50517	9.183625	6.97E-19	8.04E-18	32.02508
TLR4	1.175309	9.591934	10.7713	7.97E-25	1.78E-23	45.51774
PXN	1.170681	8.632557	6.411451	2.95E-10	1.38E-09	12.54878
CDK5RAP2	1.167633	7.961662	11.28375	7.08E-27	2.08E-25	50.18427
CDC42EP3	1.165834	10.34262	13.63517	5.27E-37	6.83E-35	73.25177
RAB24	1.16431	10.60063	7.692479	6.07E-14	4.14E-13	20.84638
XRN2	1.164209	8.977951	8.940099	4.95E-18	5.11E-17	30.09508
PKM	1.164175	8.997666	7.573781	1.40E-13	9.23E-13	20.0247
CCNJL	1.16294	7.97454	10.20526	1.24E-22	2.19E-21	40.53365
PRDX5	1.161268	9.693245	8.995625	3.18E-18	3.37E-17	30.53169
ADAMTS3	1.157164	4.887137	5.578539	3.70E-08	1.35E-07	7.859471
CORO1A	1.156476	12.21211	5.672275	2.21E-08	8.29E-08	8.358231
EMR1	1.154962	9.926394	8.062051	4.18E-15	3.19E-14	23.47104
RAB31	1.154669	11.73561	12.83138	2.02E-33	1.41E-31	65.08575
MSRB1	1.154355	12.72444	11.89728	2.07E-29	8.47E-28	55.95277
UBAP1	1.153372	9.411782	13.5489	1.29E-36	1.55E-34	72.36266
HIST1H2BI	1.152924	7.527415	9.741263	6.78E-21	9.74E-20	36.58892
GK	1.149755	8.639844	9.594974	2.33E-20	3.16E-19	35.37259
GRAMD1A	1.149657	8.537546	8.543803	1.11E-16	9.85E-16	27.03932
FOSL2	1.148955	8.397532	9.678789	1.15E-20	1.60E-19	36.06784
BLOC1S1	1.147149	10.16861	9.017537	2.66E-18	2.85E-17	30.70455
CLEC4E	1.146407	9.880595	9.313071	2.42E-19	2.94E-18	33.06679
TSHZ3	1.142979	8.59383	8.860197	9.34E-18	9.32E-17	29.47042
POTEKP	1.140788	7.605081	8.382897	3.79E-16	3.21E-15	25.82935
PLP2	1.138961	11.42709	8.5726	8.86E-17	7.99E-16	27.25775
SH3GLB1	1.138146	11.11818	13.37275	8.03E-36	8.46E-34	70.55642
MAFG	1.137023	8.015315	11.21329	1.37E-26	3.87E-25	49.53428

CHPT1	1.136649	8.16052	9.942898	1.21E-21	1.90E-20	38.28709
MKNK1	1.135193	10.14226	10.31122	4.90E-23	9.06E-22	41.45252
DOK3	1.134329	9.702241	9.266699	3.54E-19	4.22E-18	32.69236
SERPINB1	1.134323	10.60355	10.63649	2.70E-24	5.72E-23	44.31402
ALDOA	1.128294	11.11565	8.323379	5.94E-16	4.94E-15	25.38638
GRK6	1.127599	9.32735	8.038607	4.97E-15	3.77E-14	23.30159
MRVI1	1.126365	8.526112	8.013142	5.99E-15	4.51E-14	23.11799
RGS14	1.124674	8.17969	6.835311	2.04E-11	1.08E-10	15.15266
CSGALNA(	1.124373	9.620466	12.85819	1.54E-33	1.09E-31	65.35373
GRN	1.122262	11.04464	7.483173	2.64E-13	1.69E-12	19.40455
PFN1	1.121864	12.46563	5.723907	1.66E-08	6.31E-08	8.636143
NSUN7	1.121795	6.02578	10.08108	3.67E-22	6.13E-21	39.46514
CEACAM2	1.121745	7.232674	7.740051	4.32E-14	3.01E-13	21.17862
QPCT	1.120977	11.45917	10.42384	1.81E-23	3.50E-22	42.43635
BEND7	1.120898	5.996319	6.915181	1.22E-11	6.57E-11	15.65928
LILRB3	1.117752	10.92555	9.887391	1.95E-21	2.99E-20	37.81713
MMRN1	1.116423	5.060669	5.422264	8.59E-08	3.02E-07	7.044599
AKIRIN2	1.113159	9.002658	9.754726	6.05E-21	8.73E-20	36.70152
SHKBP1	1.112325	9.924926	7.321462	8.06E-13	4.93E-12	18.31311
ACSL4	1.111636	8.462642	8.775677	1.82E-17	1.76E-16	28.81432
METTL9	1.111151	9.487035	10.41427	1.97E-23	3.79E-22	42.35251
VSTM1	1.110562	7.34734	6.723879	4.18E-11	2.14E-10	14.45423
HPSE	1.109573	8.561043	8.30005	7.09E-16	5.84E-15	25.21342
CDKN3	1.108895	5.957863	7.44297	3.49E-13	2.20E-12	19.13136
ADORA2B	1.108869	6.432797	10.49791	9.34E-24	1.87E-22	43.08744
SSH1	1.108036	9.059193	10.83444	4.49E-25	1.04E-23	46.0849
EHD1	1.107849	9.170167	8.73253	2.55E-17	2.43E-16	28.48125
IMPA2	1.107144	10.35426	8.711541	3.01E-17	2.84E-16	28.31969
TRIQK	1.104999	7.261692	10.77904	7.43E-25	1.67E-23	45.58708
KCNE1	1.104753	5.854792	9.616669	1.94E-20	2.65E-19	35.55213
GBE1	1.104162	9.614966	9.543945	3.57E-20	4.75E-19	34.95146
ATP6V0D1	1.103883	11.42272	7.957469	9.00E-15	6.65E-14	22.71822
MAN1A1	1.103508	8.851997	10.73331	1.13E-24	2.46E-23	45.17744
LINC0100(	1.097267	10.02371	9.442352	8.32E-20	1.07E-18	34.11795
MILR1	1.096978	7.030619	8.343451	5.11E-16	4.27E-15	25.53549
ATP6AP1	1.093459	10.04982	7.693661	6.02E-14	4.11E-13	20.85462
BASP1	1.093122	10.04943	12.78241	3.31E-33	2.25E-31	64.59704
CEBPD	1.092117	10.53738	11.47849	1.13E-27	3.73E-26	51.99442
DLGAP5	1.091068	5.230077	5.865825	7.44E-09	2.94E-08	9.411599
CNIH4	1.085793	8.336413	14.76436	3.21E-42	7.74E-40	85.14569
DSC2	1.083296	7.504038	6.971156	8.43E-12	4.64E-11	16.01731
ADCY3	1.082483	6.610648	7.172386	2.21E-12	1.29E-11	17.32456
RAB27A	1.082295	11.34679	13.02519	2.83E-34	2.34E-32	67.02998
ALAS2	1.080107	6.098657	6.810353	2.40E-11	1.26E-10	14.99538
TAGLN2	1.078545	11.13505	5.722119	1.67E-08	6.37E-08	8.626482
AP3B2	1.072688	5.879098	5.642145	2.61E-08	9.70E-08	8.197101
CPNE5	1.072634	7.504722	8.020485	5.68E-15	4.28E-14	23.17089
UBE2S	1.071031	7.205621	5.844777	8.39E-09	3.30E-08	9.295518
TIFA	1.070644	7.712904	9.088974	1.50E-18	1.66E-17	31.27031
TTC8	1.068566	5.664392	7.100121	3.59E-12	2.04E-11	16.8515
YOD1	1.067333	7.171009	8.656268	4.63E-17	4.29E-16	27.89565
MBOAT7	1.066688	9.697532	8.9901	3.32E-18	3.51E-17	30.48815
HIST1H2BI	1.064512	11.47838	11.78011	6.39E-29	2.48E-27	54.83645
CA1	1.063978	6.342474	6.076936	2.20E-09	9.25E-09	10.5963
DENND3	1.062637	9.165379	6.647821	6.77E-11	3.39E-10	13.98317
TLR8	1.062558	10.91721	10.82485	4.90E-25	1.12E-23	45.99863
STX10	1.056495	9.020318	6.909504	1.26E-11	6.80E-11	15.62311
PLEC	1.053933	7.737013	5.340266	1.33E-07	4.54E-07	6.625413

RPS6KA1	1.051288	9.856395	6.843156	1.94E-11	1.03E-10	15.2022
LAMTOR5	1.05095	10.39918	11.94765	1.27E-29	5.35E-28	56.43474
UBE2J1	1.049669	9.019438	12.5325	4.05E-32	2.31E-30	62.1198
GYS1	1.047585	7.947749	7.193557	1.92E-12	1.12E-11	17.46391
KLF6	1.046814	9.543861	8.977617	3.67E-18	3.86E-17	30.38987
KLF7	1.046114	8.48218	11.3055	5.77E-27	1.71E-25	50.38552
AGPAT9	1.04559	10.57312	8.568159	9.16E-17	8.24E-16	27.22403
TMEM185I	1.045452	7.197988	10.99526	1.03E-25	2.61E-24	47.53967
FPR1	1.043248	11.45266	9.727811	7.60E-21	1.08E-19	36.47651
OGDH	1.040736	8.086525	6.54125	1.32E-10	6.44E-10	13.3309
RNF24	1.039878	10.08707	10.19705	1.34E-22	2.34E-21	40.4627
FAM214B	1.038409	8.384364	8.936978	5.07E-18	5.22E-17	30.0706
OS9	1.037461	10.18358	8.438583	2.48E-16	2.14E-15	26.24605
RHOG	1.03598	11.92759	8.39815	3.37E-16	2.87E-15	25.94328
UHRF1	1.034339	6.387497	6.25212	7.76E-10	3.46E-09	11.60738
B3GNT8	1.031652	7.682484	8.717509	2.87E-17	2.72E-16	28.3656
FCGR2C	1.03112	9.438863	11.46485	1.29E-27	4.18E-26	51.86697
ANLN	1.030347	4.184711	5.343663	1.30E-07	4.47E-07	6.642663
WSB1	1.029073	8.944164	11.63355	2.60E-28	9.29E-27	53.44973
SEMA4A	1.028845	8.972505	11.1993	1.56E-26	4.35E-25	49.40556
ELMO2	1.027424	8.167148	8.316121	6.28E-16	5.21E-15	25.33252
SUCNR1	1.027322	5.904823	5.583727	3.59E-08	1.31E-07	7.886881
IFI35	1.026932	8.841029	5.734174	1.56E-08	5.97E-08	8.69167
HLX	1.025748	9.650837	9.919813	1.48E-21	2.31E-20	38.09141
SLCO4C1	1.023255	6.908679	11.72426	1.09E-28	4.07E-27	54.30677
LAIR1	1.023081	8.548793	7.874677	1.64E-14	1.19E-13	22.12787
FGR	1.022236	12.23459	10.14282	2.14E-22	3.66E-21	39.99527
HPCAL1	1.021699	9.291316	7.757473	3.82E-14	2.67E-13	21.30072
BATF	1.020875	8.64686	10.34685	3.58E-23	6.71E-22	41.76296
ENO1	1.020758	8.359691	11.53583	6.58E-28	2.23E-26	52.53115
ABHD17C	1.020191	6.374999	8.042672	4.82E-15	3.67E-14	23.33095
CCPG1	1.019619	9.464955	10.73841	1.07E-24	2.36E-23	45.22314
CCND3	1.019088	11.8604	9.220434	5.16E-19	6.03E-18	32.32019
NEU1	1.018614	7.887443	8.104364	3.06E-15	2.36E-14	23.77785
CHMP4B	1.017323	9.570519	10.98824	1.10E-25	2.77E-24	47.47589
ULK1	1.016781	8.781157	7.943936	9.93E-15	7.31E-14	22.62139
NDUFAF1	1.016758	8.4382	10.0898	3.40E-22	5.70E-21	39.53987
MEF2A	1.014631	7.974104	10.16904	1.71E-22	2.94E-21	40.22106
PTK2B	1.012715	8.26241	6.006153	3.32E-09	1.37E-08	10.19495
ROMO1	1.012039	8.019756	6.326278	4.96E-10	2.26E-09	12.04297
FKBP9	1.010434	7.51879	8.956173	4.35E-18	4.53E-17	30.22126
STAB1	1.009198	8.740141	6.01923	3.08E-09	1.28E-08	10.26879
TRPM2	1.008631	7.095188	7.604254	1.13E-13	7.54E-13	20.23465
FGD4	1.008346	6.810884	9.728006	7.59E-21	1.08E-19	36.47814
MYADM	1.007195	10.72756	10.15713	1.89E-22	3.25E-21	40.11839
RHBDD2	1.006202	7.868172	7.031023	5.68E-12	3.17E-11	16.40295
IL1RN	1.005081	7.507049	12.09917	2.91E-30	1.32E-28	57.89207
MPDU1	1.003977	7.163767	6.353232	4.21E-10	1.93E-09	12.2024
CYYR1	1.002684	5.70758	6.944049	1.01E-11	5.49E-11	15.84363
METRNL	1.002625	8.237229	7.954949	9.17E-15	6.77E-14	22.70018
UBE2F	1.000643	9.143721	10.82794	4.76E-25	1.10E-23	46.0264
ORMDL2	1.000506	8.724968	8.720913	2.79E-17	2.65E-16	28.3918
PCMT1	1.000068	10.29159	11.14799	2.51E-26	6.77E-25	48.93422
DUSP13	0.999911	7.221757	8.119704	2.73E-15	2.12E-14	23.88941
CEBPZOS	0.999462	7.16853	8.987967	3.38E-18	3.57E-17	30.47135
SOCS3	0.999457	7.066484	8.413506	3.00E-16	2.56E-15	26.05814
SQRDL	0.999103	11.94675	12.17138	1.44E-30	6.74E-29	58.59048
SNX3	0.999045	11.883	10.7019	1.49E-24	3.25E-23	44.89683

PNPLA6	0.998763	8.903032	5.990253	3.64E-09	1.49E-08	10.10537
HIST1H2BI	0.997695	8.189056	9.710502	8.80E-21	1.24E-19	36.33205
UTS2	0.996576	5.35593	2.896949	0.003908	0.007251	-3.10092
HMMR	0.994115	5.939984	5.830255	9.11E-09	3.56E-08	9.215649
LOC10050	0.992589	6.111797	9.246348	4.18E-19	4.94E-18	32.52848
CMTM4	0.992382	6.908496	8.333017	5.53E-16	4.60E-15	25.45794
NDUFA1	0.99224	10.62601	8.532937	1.20E-16	1.07E-15	26.95704
QSOX1	0.991082	6.898224	11.73674	9.69E-29	3.64E-27	54.42501
NUSAP1	0.991009	6.875675	6.34843	4.34E-10	1.99E-09	12.17395
GAPDH	0.9895	12.91977	9.433353	8.97E-20	1.14E-18	34.04443
SIAH2	0.98707	8.943414	9.586105	2.51E-20	3.38E-19	35.29928
ATP6V0C	0.986146	10.85916	7.613184	1.06E-13	7.09E-13	20.29631
IGLL5	0.985572	7.874858	5.146149	3.62E-07	1.18E-06	5.656219
CDKN2D	0.983284	7.206449	7.219257	1.61E-12	9.53E-12	17.63354
MEGF9	0.981641	10.52213	10.6232	3.04E-24	6.38E-23	44.19599
S1PR4	0.981326	9.759098	6.96677	8.68E-12	4.77E-11	15.98917
STX5	0.981005	7.301446	9.25625	3.85E-19	4.57E-18	32.60819
ZNF282	0.980592	7.299306	7.773782	3.40E-14	2.39E-13	21.41522
TOR1AIP1	0.98004	8.560222	12.35401	2.38E-31	1.21E-29	60.36776
E2F8	0.979746	4.790737	4.895393	1.27E-06	3.88E-06	4.452903
ZDHHC20	0.977646	8.687585	9.187661	6.74E-19	7.79E-18	32.0574
EPB42	0.977186	6.72834	4.501213	8.14E-06	2.24E-05	2.673785
FAM53C	0.975918	10.61891	11.10984	3.57E-26	9.48E-25	48.58467
B3GNT2	0.975547	7.366751	12.88167	1.22E-33	8.84E-32	65.58866
C1QA	0.975105	6.541063	5.480262	6.29E-08	2.25E-07	7.344584
TFDP1	0.972906	7.645557	11.32436	4.84E-27	1.46E-25	50.56017
MARCKS	0.972826	9.744542	10.95038	1.55E-25	3.86E-24	47.13226
ARHGDIA	0.971789	7.910382	5.379503	1.08E-07	3.74E-07	6.825274
PLEKHG1	0.970713	5.39299	5.112907	4.29E-07	1.39E-06	5.493534
IL17RA	0.970025	10.58822	11.68099	1.65E-28	6.03E-27	53.89739
KBTBD7	0.968681	7.672045	6.099818	1.92E-09	8.15E-09	10.72692
GNS	0.968657	9.917915	12.40505	1.44E-31	7.61E-30	60.86731
FAM160A2	0.96529	8.462625	7.634815	9.13E-14	6.13E-13	20.4459
DTL	0.964968	5.341126	6.047086	2.62E-09	1.09E-08	10.42653
RRAGD	0.96448	9.352786	9.365807	1.57E-19	1.94E-18	33.49427
VAMP3	0.964189	10.12307	10.65996	2.18E-24	4.66E-23	44.52287
FERMT3	0.964183	10.01797	6.393995	3.28E-10	1.53E-09	12.44464
TMEM88	0.964015	7.674045	10.9086	2.28E-25	5.51E-24	46.75402
ASPH	0.963941	6.484333	8.391947	3.54E-16	3.00E-15	25.89693
SLC25A11	0.961042	7.651355	6.779196	2.93E-11	1.53E-10	14.79972
C1QB	0.96083	5.990294	5.236726	2.28E-07	7.60E-07	6.10439
NRBP1	0.959972	7.822106	6.17873	1.20E-09	5.24E-09	11.18073
PPM1M	0.956956	9.605783	7.392053	4.97E-13	3.09E-12	18.78712
SHCBP1	0.955998	5.105169	5.160874	3.36E-07	1.10E-06	5.728594
KIF11	0.955326	5.117772	4.614769	4.83E-06	1.37E-05	3.172098
MAPKAPK	0.952767	7.88665	8.544168	1.10E-16	9.82E-16	27.04208
CAMKK2	0.952662	8.040738	10.79963	6.16E-25	1.41E-23	45.77194
IL2RG	0.950886	10.90741	5.691002	1.99E-08	7.51E-08	8.458772
PCYT1A	0.950408	7.962591	9.601694	2.20E-20	2.99E-19	35.42817
IL10	0.948894	5.477961	7.940228	1.02E-14	7.51E-14	22.59487
NRN1	0.948173	5.754631	6.256314	7.57E-10	3.38E-09	11.6319
COLGALT1	0.947313	8.213342	8.489193	1.68E-16	1.47E-15	26.62665
RAB3D	0.946866	9.046609	10.0628	4.30E-22	7.12E-21	39.30869
AP1M1	0.945966	8.455184	6.707361	4.64E-11	2.37E-10	14.35154
IGK	0.944611	6.87243	6.625499	7.80E-11	3.87E-10	13.8458
TOP2A	0.942999	5.14765	6.781617	2.89E-11	1.50E-10	14.81489
SOS2	0.94243	7.376815	12.74709	4.72E-33	3.09E-31	64.24521
TGFB1I1	0.942041	5.606568	5.003878	7.42E-07	2.34E-06	4.966726

CAPN3	0.939653	7.301096	5.8937	6.35E-09	2.52E-08	9.565893
RBM38	0.938854	8.070648	5.194888	2.82E-07	9.32E-07	5.896492
ZNF106	0.937834	9.725105	11.15029	2.45E-26	6.65E-25	48.95533
NFIL3	0.937169	10.89285	9.495191	5.37E-20	6.99E-19	34.55065
ASGR2	0.933436	8.557645	7.030259	5.71E-12	3.19E-11	16.39801
SLC36A1	0.932172	9.075338	9.139067	1.00E-18	1.13E-17	31.66903
LINC0109[illegible]	0.931371	6.267709	5.483803	6.18E-08	2.21E-07	7.36299
THEMIS2	0.92923	10.55615	6.382612	3.52E-10	1.63E-09	12.37686
CCDC125	0.928862	6.313441	7.04292	5.25E-12	2.94E-11	16.47991
KIAA0040	0.928611	8.572979	9.757319	5.92E-21	8.55E-20	36.72322
SYNE1	0.927488	6.77869	10.81104	5.55E-25	1.27E-23	45.87442
SLC11A1	0.926555	8.564273	8.667364	4.25E-17	3.95E-16	27.98061
ROGDI	0.926103	7.793818	5.023283	6.74E-07	2.13E-06	5.059724
IFITM3	0.925603	13.40764	9.435774	8.79E-20	1.12E-18	34.0642
NDUFB3	0.92546	9.955362	14.37671	2.08E-40	4.15E-38	81.01089
RTN3	0.924739	11.20515	12.1783	1.34E-30	6.33E-29	58.65752
CKLF	0.924358	10.34846	11.24138	1.05E-26	3.03E-25	49.79315
CLPTM1	0.924248	7.936914	6.385656	3.46E-10	1.60E-09	12.39498
CEBPE	0.923792	7.22065	4.931605	1.06E-06	3.27E-06	4.623263
PLXNC1	0.922543	9.48715	10.61823	3.18E-24	6.65E-23	44.15183
KIF14	0.919842	4.839081	6.598195	9.26E-11	4.57E-10	13.67831
HN1	0.919229	9.845235	11.30737	5.67E-27	1.69E-25	50.40278
OAT	0.918225	10.46574	8.562631	9.56E-17	8.58E-16	27.18207
ITGA2B	0.916268	6.728166	5.623537	2.89E-08	1.07E-07	8.097969
KDM6B	0.914843	9.72416	6.998556	7.04E-12	3.89E-11	16.19347
TGFA	0.914817	7.269146	12.9908	4.02E-34	3.25E-32	66.68382
ARF5	0.913268	9.222349	6.395331	3.26E-10	1.52E-09	12.4526
PPP4C	0.912694	9.938358	8.291296	7.57E-16	6.21E-15	25.14862
MMP25	0.911953	8.886154	7.063698	4.58E-12	2.58E-11	16.6146
HHEX	0.911498	9.961588	10.16617	1.75E-22	3.02E-21	40.19626
MICU1	0.911495	9.234052	9.745634	6.54E-21	9.40E-20	36.62546
MXD3	0.910616	8.076968	8.204949	1.45E-15	1.16E-14	24.51236
COL17A1	0.910275	5.683082	5.965202	4.21E-09	1.71E-08	9.964652
SLC25A37	0.90545	9.632623	9.239835	4.41E-19	5.19E-18	32.47609
ACTR1A	0.905431	8.43012	8.254266	1.00E-15	8.11E-15	24.87512
TPX2	0.905247	5.915169	6.193949	1.10E-09	4.81E-09	11.26884
APOBR	0.904848	9.230787	5.061273	5.57E-07	1.78E-06	5.242752
SLC22A15	0.904579	8.922102	8.403757	3.23E-16	2.75E-15	25.9852
TTC7B	0.904344	6.575939	6.524898	1.46E-10	7.10E-10	13.23162
RIN3	0.903862	8.286506	6.668858	5.93E-11	2.99E-10	14.11301
PGK1	0.90328	10.08483	10.46798	1.22E-23	2.42E-22	42.82398
JUNB	0.902963	10.48797	6.819501	2.26E-11	1.19E-10	15.05297
MKI67	0.901758	6.209132	7.17174	2.22E-12	1.29E-11	17.32032
NECAB1	0.901204	4.195269	5.589421	3.48E-08	1.28E-07	7.916991
MSRB3	0.899757	6.037571	9.48355	5.91E-20	7.67E-19	34.45517
CSF2RA	0.899693	8.246733	8.112646	2.88E-15	2.23E-14	23.83806
ENTPD1	0.898567	8.71202	10.60975	3.43E-24	7.16E-23	44.07656
TIMP2	0.897978	10.54381	12.78925	3.09E-33	2.11E-31	64.66529
MS4A3	0.893736	8.148007	2.713871	0.006844	0.012193	-3.6061
SMAD1	0.892084	6.14043	6.444161	2.41E-10	1.14E-09	12.7446
CDK5	0.890814	7.107188	8.005986	6.31E-15	4.74E-14	23.06648
MAPK13	0.889903	7.535341	9.976586	9.07E-22	1.44E-20	38.57324
ZDHHC3	0.88967	7.29777	11.41959	1.98E-27	6.23E-26	51.44485
PLA2G4A	0.889441	6.428996	6.405689	3.06E-10	1.43E-09	12.51438
YKT6	0.889033	7.165374	7.56946	1.45E-13	9.51E-13	19.99499
PDZD8	0.88764	7.630828	7.971111	8.15E-15	6.05E-14	22.81597
GSR	0.887623	8.48007	9.115333	1.21E-18	1.36E-17	31.47991
ADAM8	0.885541	8.954445	4.946168	9.88E-07	3.06E-06	4.692101

CD14	0.885455	11.65857	6.657615	6.37E-11	3.20E-10	14.04357
R3HDM4	0.882536	11.65975	6.115388	1.75E-09	7.46E-09	10.81606
CHP1	0.881525	9.352757	9.224422	5.00E-19	5.86E-18	32.35222
DEDD	0.881171	7.797063	9.350656	1.78E-19	2.19E-18	33.37128
TREML2	0.880931	8.968654	7.503843	2.29E-13	1.48E-12	19.54548
UBE2M	0.88077	8.259438	5.432626	8.13E-08	2.87E-07	7.097982
CPD	0.880591	8.62522	10.35018	3.47E-23	6.53E-22	41.79204
SELPLG	0.880136	10.25894	6.620874	8.03E-11	3.98E-10	13.81739
MFN2	0.879619	8.044439	7.912551	1.25E-14	9.12E-14	22.39732
GNAI2	0.878764	11.23445	6.518445	1.52E-10	7.38E-10	13.1925
RTN2	0.878098	6.922533	10.58399	4.32E-24	8.92E-23	43.84809
STEAP4	0.877897	9.69471	7.833354	2.21E-14	1.58E-13	21.8351
RPH3A	0.877664	7.152618	3.945124	8.93E-05	0.000214	0.402075
PPARG	0.877573	5.400693	8.125564	2.61E-15	2.04E-14	23.93207
CD44	0.876826	8.490938	10.60854	3.47E-24	7.22E-23	44.06582
YIPF1	0.876749	9.041082	10.90402	2.38E-25	5.74E-24	46.71262
TBC1D8	0.876698	7.233295	9.350152	1.78E-19	2.20E-18	33.36719
REPS2	0.87668	7.983087	7.55095	1.65E-13	1.08E-12	19.86786
TLR1	0.876422	10.83039	9.233895	4.63E-19	5.44E-18	32.42833
PGM2	0.875804	9.019667	8.277516	8.40E-16	6.84E-15	25.04673
CFLAR	0.874469	9.769568	10.68541	1.74E-24	3.76E-23	44.74962
GPSM3	0.874325	10.73431	5.993523	3.57E-09	1.46E-08	10.12377
NDST2	0.872742	8.897864	8.249512	1.04E-15	8.39E-15	24.84007
CEACAM4	0.87259	8.518597	9.525148	4.18E-20	5.51E-19	34.79676
SESN2	0.871824	7.549067	9.260597	3.72E-19	4.43E-18	32.6432
NAPA	0.871681	8.331556	4.895122	1.27E-06	3.88E-06	4.451632
PDLIM7	0.871152	7.057667	7.013595	6.38E-12	3.54E-11	16.2904
UBA1	0.86822	10.39885	5.447931	7.49E-08	2.65E-07	7.177
CDC20	0.865595	5.979639	6.447192	2.37E-10	1.12E-09	12.76279
FAM105A	0.865382	7.675627	8.376711	3.97E-16	3.35E-15	25.7832
CENPW	0.864198	6.510443	7.71312	5.24E-14	3.60E-13	20.99033
LAMTOR1	0.863185	10.77014	9.157528	8.61E-19	9.83E-18	31.81639
LOC44112	0.862685	8.248696	6.809357	2.42E-11	1.27E-10	14.98911
GPR27	0.861773	8.993789	11.63706	2.51E-28	9.00E-27	53.4828
PGM1	0.860192	9.663189	10.37201	2.86E-23	5.41E-22	41.98271
P4HB	0.860075	9.391147	5.374956	1.10E-07	3.82E-07	6.802049
RANBP9	0.85994	7.57574	9.992391	7.91E-22	1.27E-20	38.70773
MAP1S	0.858601	7.099161	5.389081	1.02E-07	3.57E-07	6.874265
ORAI2	0.857886	7.493556	8.911083	6.24E-18	6.37E-17	29.86774
SH3PXD2E	0.857146	6.971861	6.509464	1.61E-10	7.78E-10	13.13811
ARHGAP2!	0.857072	5.535513	5.438755	7.86E-08	2.78E-07	7.1296
IDI1	0.855835	9.447528	9.063569	1.84E-18	2.01E-17	31.06872
HOMER3	0.85437	6.475854	7.609127	1.09E-13	7.29E-13	20.26828
FMNL1	0.853655	10.27275	5.133558	3.86E-07	1.26E-06	5.594489
CPEB4	0.852074	8.62272	8.808697	1.40E-17	1.37E-16	29.07007
TOM1	0.851676	7.241905	5.167325	3.25E-07	1.07E-06	5.76036
SCPEP1	0.851254	10.11696	8.305435	6.81E-16	5.62E-15	25.25331
SLA	0.850452	11.6052	9.248651	4.10E-19	4.86E-18	32.54701
CNN2	0.850219	9.956169	6.671465	5.83E-11	2.94E-10	14.12911
PET100	0.850083	9.002425	5.651295	2.48E-08	9.25E-08	8.245951
ARPC4	0.849832	9.916458	6.76827	3.15E-11	1.63E-10	14.73129
TPM4	0.849711	8.803995	7.486091	2.59E-13	1.66E-12	19.42443
CMTM5	0.849262	7.461377	5.394445	9.96E-08	3.47E-07	6.901737
GM2A	0.848839	7.288513	8.204774	1.45E-15	1.16E-14	24.51107
MOB3A	0.847538	9.464716	7.605036	1.13E-13	7.50E-13	20.24005
RAB5C	0.846938	9.274242	6.038826	2.75E-09	1.14E-08	10.37969
ME2	0.846786	9.651168	9.094563	1.43E-18	1.59E-17	31.31471
GLT1D1	0.84634	11.08983	8.674612	4.01E-17	3.74E-16	28.03615

ACAP1	0.845939	7.766986	7.026352	5.86E-12	3.26E-11	16.37276
PLB1	0.845046	7.79471	8.988697	3.36E-18	3.55E-17	30.4771
CYTH4	0.844706	9.056694	5.214233	2.56E-07	8.49E-07	5.99243
FLVCR2	0.843882	7.288366	9.311803	2.44E-19	2.97E-18	33.05653
TRPS1	0.84338	7.540882	8.101126	3.13E-15	2.42E-14	23.75433
MVP	0.840856	9.671103	5.322694	1.45E-07	4.97E-07	6.536333
SERPINB8	0.839701	7.621878	9.312754	2.43E-19	2.95E-18	33.06422
RAB8B	0.839458	9.812843	10.42706	1.76E-23	3.42E-22	42.46464
SLC9A8	0.838773	8.189738	8.240362	1.11E-15	8.96E-15	24.77267
MCU	0.837499	8.057013	8.859816	9.37E-18	9.34E-17	29.46745
EMC2	0.83676	8.678364	7.740491	4.31E-14	3.00E-13	21.18171
ATG9A	0.836346	8.351268	8.529438	1.24E-16	1.09E-15	26.93057
FCN1	0.835975	12.63746	5.937584	4.94E-09	1.98E-08	9.810126
INSL3	0.835382	6.081035	6.779954	2.92E-11	1.52E-10	14.80447
MCOLN1	0.834551	7.701768	7.28858	1.01E-12	6.10E-12	18.0936
PRKACA	0.834121	7.16376	7.352075	6.54E-13	4.02E-12	18.51821
ITPRIP	0.832993	8.587641	11.80383	5.09E-29	2.00E-27	55.06185
PPP1CA	0.83191	10.14308	5.404987	9.41E-08	3.30E-07	6.955795
CXCL16	0.831708	9.217753	6.412907	2.92E-10	1.37E-09	12.55748
TBC1D14	0.83061	9.772622	8.429172	2.66E-16	2.28E-15	26.17548
CDADC1	0.82913	6.508945	7.081795	4.06E-12	2.30E-11	16.73218
TBCB	0.827916	9.062713	5.481303	6.26E-08	2.23E-07	7.349994
CFP	0.826791	9.899081	5.04081	6.17E-07	1.96E-06	5.144008
PLOD1	0.826293	7.924736	7.042671	5.26E-12	2.94E-11	16.4783
ENSA	0.82574	7.477091	10.69669	1.57E-24	3.40E-23	44.85029
LOC10013	0.825624	6.875124	8.334381	5.47E-16	4.56E-15	25.46807
TMEM52B	0.825564	4.114604	4.543688	6.71E-06	1.87E-05	2.858822
SSFA2	0.825391	7.155142	8.983536	3.50E-18	3.69E-17	30.43646
LOC10192	0.824742	6.540375	7.347323	6.75E-13	4.15E-12	18.48632
MSRB2	0.824173	7.92718	9.021878	2.57E-18	2.76E-17	30.73883
SIRPD	0.821474	7.308731	10.76349	8.56E-25	1.90E-23	45.44773
LPCAT2	0.820818	7.905264	11.30584	5.75E-27	1.71E-25	50.38864
DAAM2	0.820685	6.591498	5.376617	1.09E-07	3.79E-07	6.810528
MAD2L2	0.820451	7.615979	6.489933	1.82E-10	8.72E-10	13.02006
CCDC126	0.818666	8.078802	8.181319	1.73E-15	1.37E-14	24.33915
LRP10	0.81846	9.875937	9.10527	1.31E-18	1.47E-17	31.39984
KIF20A	0.81832	5.306818	6.385861	3.45E-10	1.60E-09	12.39619
GLIPR2	0.817909	10.72855	11.16149	2.21E-26	6.04E-25	49.05809
NFKB1	0.817223	9.306697	9.192705	6.47E-19	7.50E-18	32.09779
TMEM260	0.816778	8.281518	8.769916	1.90E-17	1.83E-16	28.76978
GNAQ	0.815658	9.528177	10.21634	1.13E-22	2.00E-21	40.62939
AQP9	0.815297	12.89112	8.594428	7.48E-17	6.80E-16	27.42371
GFOD2	0.814892	7.384914	10.42429	1.80E-23	3.49E-22	42.44034
TRAPPC1	0.814202	9.846471	5.53658	4.64E-08	1.68E-07	7.638631
DHCR24	0.813588	6.234917	6.820356	2.25E-11	1.19E-10	15.05836
STX3	0.811557	9.236805	7.822372	2.40E-14	1.71E-13	21.75749
LPPR2	0.811295	8.724967	6.86603	1.67E-11	8.94E-11	15.34692
PRC1	0.810924	5.637223	4.522535	7.39E-06	2.04E-05	2.766468
TUBA4A	0.810408	11.00219	9.921947	1.45E-21	2.27E-20	38.10949
GGTLC1	0.810049	6.856963	8.312413	6.46E-16	5.35E-15	25.30503
UBTD1	0.808806	6.376038	8.349341	4.89E-16	4.08E-15	25.5793
C10orf54	0.807179	10.96802	7.44968	3.34E-13	2.11E-12	19.17687
MICAL1	0.806695	9.67812	6.218741	9.48E-10	4.18E-09	11.41279
HIATL1	0.805494	7.087136	9.865582	2.35E-21	3.56E-20	37.63299
CPNE2	0.805169	8.88923	6.648801	6.73E-11	3.37E-10	13.98921
AGTRAP	0.804121	8.695916	8.863653	9.08E-18	9.07E-17	29.49735
PRMT5	0.803599	7.906117	7.112187	3.31E-12	1.89E-11	16.93021
GCNT1	0.802481	7.356656	8.300275	7.08E-16	5.83E-15	25.21509

SDHC	0.802407	7.464037	10.67462	1.91E-24	4.12E-23	44.65346
HIST1H4H	0.802293	6.684911	6.045508	2.64E-09	1.10E-08	10.41758
PFKFB4	0.801838	8.185227	8.464238	2.04E-16	1.77E-15	26.43876
MERTK	0.801275	5.709967	7.486045	2.59E-13	1.66E-12	19.42411
TK1	0.801128	6.394679	7.235378	1.45E-12	8.58E-12	17.7402
FAM217B	0.801122	10.18907	7.070579	4.37E-12	2.47E-11	16.65928
PICALM	0.79949	9.238668	9.080928	1.60E-18	1.77E-17	31.20641
S100A6	0.798758	9.406101	9.350683	1.78E-19	2.19E-18	33.37149
CDC37	0.798728	8.80053	4.633446	4.43E-06	1.26E-05	3.255166
ARL8A	0.798403	8.210549	10.68269	1.78E-24	3.84E-23	44.72541
PROS1	0.797977	5.980628	4.073512	5.26E-05	0.00013	0.901513
LPCAT3	0.797734	6.570136	8.812885	1.36E-17	1.33E-16	29.10256
ARHGAP2(	0.796965	7.993588	8.873934	8.37E-18	8.42E-17	29.57751
KAZN	0.796062	6.557642	7.694921	5.97E-14	4.07E-13	20.8634
AP2B1	0.79597	8.62172	9.895008	1.83E-21	2.81E-20	37.88151
NR1H2	0.795706	7.509527	5.176253	3.11E-07	1.02E-06	5.804379
MRVI1-AS	0.795683	7.387245	9.725309	7.77E-21	1.11E-19	36.45561
SNX20	0.795456	8.325743	6.192457	1.11E-09	4.85E-09	11.2602
SRXN1	0.792798	9.567579	7.737495	4.40E-14	3.06E-13	21.16073
TPST2	0.791913	10.50719	8.44405	2.38E-16	2.05E-15	26.28708
ANKRD33I	0.791787	7.425216	7.677339	6.76E-14	4.59E-13	20.74099
JAK3	0.791736	7.166312	7.014744	6.33E-12	3.51E-11	16.29781
VAT1	0.790019	8.490383	7.119904	3.15E-12	1.80E-11	16.9806
CDK14	0.789884	7.145402	11.99982	7.65E-30	3.30E-28	56.93523
BUB1B	0.78791	5.789673	4.851224	1.57E-06	4.75E-06	4.246677
ACER3	0.787343	7.161367	6.64644	6.83E-11	3.41E-10	13.97466
BAZ1A	0.786866	10.81144	9.972901	9.36E-22	1.49E-20	38.5419
TENM1	0.786445	6.105069	3.195069	0.001472	0.002945	-2.20986
EGR1	0.786427	9.755941	4.181515	3.33E-05	8.47E-05	1.333346
TUBA1C	0.786045	12.76561	10.84898	3.93E-25	9.22E-24	46.21591
GMPR2	0.78601	8.796378	6.469128	2.07E-10	9.85E-10	12.89464
INHBA	0.785178	4.801466	5.724122	1.65E-08	6.30E-08	8.637306
IL1R1	0.785066	6.714352	8.55139	1.04E-16	9.32E-16	27.09681
NCAPG	0.78501	5.021609	5.358386	1.20E-07	4.15E-07	6.717549
EDEM2	0.784834	8.247011	9.104903	1.32E-18	1.47E-17	31.39692
KDELR1	0.784636	6.816886	5.657201	2.40E-08	8.97E-08	8.277523
UBE2C	0.780402	7.405888	7.810655	2.61E-14	1.85E-13	21.6748
PSEN1	0.779797	8.401752	10.163	1.80E-22	3.09E-21	40.169
TBC1D10B	0.779485	8.509818	7.076785	4.19E-12	2.37E-11	16.6996
MAP1LC3/	0.776616	7.607198	7.45834	3.14E-13	2.00E-12	19.23566
PYCARD	0.776534	10.74263	6.65214	6.59E-11	3.30E-10	14.0098
AGPAT1	0.776256	8.658363	6.454158	2.27E-10	1.08E-09	12.80462
DNAH10	0.775892	5.891998	8.115819	2.81E-15	2.18E-14	23.86114
PIM3	0.775837	9.69065	7.736358	4.44E-14	3.08E-13	21.15277
DSE	0.775277	9.547389	6.610672	8.56E-11	4.24E-10	13.75477
TMEM45B	0.773963	6.88681	5.111159	4.33E-07	1.40E-06	5.485005
CCR1	0.773466	10.80361	7.743632	4.22E-14	2.94E-13	21.2037
COPA	0.773108	6.957362	13.36528	8.67E-36	9.08E-34	70.48015
ZNF608	0.773084	5.856088	5.550493	4.31E-08	1.57E-07	7.711695
SH3BP5L	0.771868	8.241254	7.748287	4.08E-14	2.85E-13	21.23631
TTK	0.769226	4.728422	4.930833	1.07E-06	3.28E-06	4.619617
ABTB1	0.766114	7.173832	5.555028	4.20E-08	1.53E-07	7.735546
LSMEM1	0.764017	6.783491	6.188303	1.14E-09	4.96E-09	11.23613
AKTIP	0.763816	8.645621	9.381471	1.38E-19	1.73E-18	33.6216
HBQ1	0.762707	6.421692	5.01156	7.14E-07	2.25E-06	5.0035
STK17B	0.761869	9.501249	9.263785	3.62E-19	4.32E-18	32.66888
C1RL	0.761536	7.987815	10.72488	1.21E-24	2.65E-23	45.10205
TBC1D7	0.760554	7.25681	7.895402	1.41E-14	1.03E-13	22.27519

VASP	0.760428	11.13228	8.297455	7.23E-16	5.94E-15	25.19421
UQCRC1	0.760394	9.380277	6.014764	3.16E-09	1.31E-08	10.24355
HDAC4	0.759209	8.694428	8.125543	2.61E-15	2.04E-14	23.93191
PLD1	0.758598	5.734357	9.33623	2.00E-19	2.45E-18	33.2543
CD164	0.757892	10.63672	9.590237	2.42E-20	3.28E-19	35.33343
TMOD3	0.75773	7.409008	10.47385	1.16E-23	2.30E-22	42.87567
LOC10050	0.756506	7.799209	8.749422	2.24E-17	2.14E-16	28.6115
SERPINA1	0.754071	9.20163	10.04138	5.18E-22	8.51E-21	39.1255
RHBDF2	0.753316	8.572724	5.597269	3.34E-08	1.23E-07	7.958536
GNG5	0.752215	12.32705	11.68986	1.52E-28	5.56E-27	53.98121
SEC61A1	0.752201	7.83055	6.491556	1.80E-10	8.64E-10	13.02986
PLEKHO2	0.751647	9.59379	6.054128	2.51E-09	1.05E-08	10.46652
ECE1	0.750714	8.025277	7.912183	1.25E-14	9.13E-14	22.3947
CCDC71L	0.750326	6.902051	10.01285	6.63E-22	1.08E-20	38.88204
PEF1	0.74997	8.785467	7.081193	4.07E-12	2.31E-11	16.72826
MCTP1	0.749442	8.546796	5.789103	1.15E-08	4.45E-08	8.990277
FLII	0.749112	9.026823	6.675096	5.70E-11	2.88E-10	14.15157
MZB1	0.748731	6.899369	3.636111	0.000301	0.000669	-0.73761
FBXW2	0.748571	7.001205	8.982717	3.52E-18	3.71E-17	30.43001
XPO6	0.74821	11.36935	7.578622	1.36E-13	8.95E-13	20.05801
CTDP1	0.747929	7.990373	7.539724	1.78E-13	1.16E-12	19.79088
CLEC1B	0.747513	7.722874	4.216021	2.88E-05	7.37E-05	1.47356
HIATL2	0.747189	7.238933	6.90365	1.31E-11	7.05E-11	15.58583
CUX1	0.746921	8.530584	10.7036	1.47E-24	3.21E-23	44.91199
FURIN	0.746025	8.486758	5.776811	1.23E-08	4.75E-08	8.923233
MAP2K2	0.745009	7.214018	5.63396	2.73E-08	1.01E-07	8.153458
AMPH	0.743725	5.617415	6.431842	2.60E-10	1.23E-09	12.67075
CDK1	0.743656	5.409709	5.836683	8.78E-09	3.44E-08	9.250983
TBC1D20	0.743335	7.120454	10.38997	2.44E-23	4.66E-22	42.13973
ZDHHC12	0.743218	7.923897	7.178567	2.12E-12	1.24E-11	17.36521
AMPD3	0.742304	9.290538	8.394177	3.48E-16	2.95E-15	25.91359
SYTL1	0.741764	9.554561	3.746045	0.000197	0.000451	-0.3423
TBC1D2	0.740494	7.028176	7.905107	1.32E-14	9.60E-14	22.34428
RALGAPA2	0.740468	7.004684	10.07936	3.73E-22	6.22E-21	39.45047
ADIPOR1	0.739501	10.57338	8.651134	4.82E-17	4.45E-16	27.85637
VIM	0.739435	13.42153	10.15235	1.97E-22	3.38E-21	40.07726
MARK2	0.739352	6.821939	6.629828	7.59E-11	3.77E-10	13.87241
TP53I11	0.738218	6.610929	7.237341	1.43E-12	8.47E-12	17.7532
TUBA1B	0.738127	12.585	9.85371	2.60E-21	3.93E-20	37.53287
MAPK3	0.738087	9.047538	7.561896	1.53E-13	1.00E-12	19.94301
TMEM2	0.737871	9.176405	10.39094	2.42E-23	4.62E-22	42.1482
FLNA	0.737281	9.605189	3.781479	0.000172	0.000396	-0.21249
TFF3	0.736806	7.202829	7.315617	8.39E-13	5.12E-12	18.27403
VSIG4	0.735639	6.931088	3.742678	0.0002	0.000456	-0.35458
KIAA1715	0.735258	6.916819	5.587864	3.51E-08	1.29E-07	7.908754
PRG2	0.734978	5.748245	4.30721	1.94E-05	5.07E-05	1.849315
HSD3B7	0.734292	6.747742	7.114176	3.27E-12	1.87E-11	16.94319
XK	0.733591	6.293578	3.013527	0.002693	0.005147	-2.76254
ZBTB48	0.731081	6.655893	5.107394	4.41E-07	1.43E-06	5.466647
GMIP	0.730478	8.303754	4.950712	9.66E-07	3.00E-06	4.713617
RAB32	0.729911	8.212552	7.928309	1.11E-14	8.16E-14	22.50973
IGF2BP3	0.729443	5.638134	4.723288	2.90E-06	8.47E-06	3.65909
EBPL	0.729145	8.886058	8.122144	2.68E-15	2.09E-14	23.90716
LPAR2	0.729035	8.660294	4.547268	6.60E-06	1.84E-05	2.874491
RALY	0.728096	8.282588	4.922847	1.11E-06	3.41E-06	4.581956
SLC24A3	0.727598	6.236861	6.251027	7.81E-10	3.48E-09	11.601
LAT2	0.727562	8.744695	10.96317	1.38E-25	3.45E-24	47.24822
PXK	0.727239	7.465391	13.32003	1.38E-35	1.38E-33	70.0183

STK16	0.725718	6.984961	10.34951	3.49E-23	6.56E-22	41.78618
TRIB1	0.725667	6.909863	9.368382	1.53E-19	1.91E-18	33.5152
ILK	0.725117	10.09441	7.289237	1.00E-12	6.07E-12	18.09798
NME6	0.724789	6.919671	7.959952	8.84E-15	6.54E-14	22.736
SRA1	0.724204	8.682867	7.447006	3.40E-13	2.15E-12	19.15873
TSEN34	0.722585	10.32761	7.703298	5.62E-14	3.85E-13	20.92179
TUBB3	0.722542	7.902934	6.727977	4.07E-11	2.09E-10	14.47974
NSUN3	0.72243	6.291387	9.66917	1.25E-20	1.73E-19	35.98783
BAX	0.722192	6.951601	4.416713	1.19E-05	3.20E-05	2.310502
PDXK	0.721621	8.148098	9.831979	3.14E-21	4.69E-20	37.34984
SLC12A9	0.72117	7.066287	6.662524	6.17E-11	3.11E-10	14.07388
DMTN	0.720629	7.638767	4.319912	1.83E-05	4.81E-05	1.902254
LDLR	0.71934	6.297024	8.880887	7.92E-18	7.98E-17	29.63176
FOSB	0.719219	7.554067	3.734088	0.000207	0.000471	-0.38584
TYMP	0.719059	9.376149	5.019647	6.86E-07	2.17E-06	5.042274
RNF144B	0.717783	8.237597	9.536535	3.80E-20	5.03E-19	34.89045
ADAMTSL4	0.716504	6.876111	6.527242	1.44E-10	7.01E-10	13.24584
SDF2	0.716041	8.908831	10.93441	1.80E-25	4.41E-24	46.98757
RBMS1	0.714956	10.91664	9.819486	3.49E-21	5.20E-20	37.24475
RNF141	0.714795	9.651021	8.652078	4.78E-17	4.42E-16	27.86359
CENPE	0.714171	5.277812	6.041866	2.70E-09	1.13E-08	10.39692
TBXAS1	0.713781	8.730974	7.545197	1.72E-13	1.12E-12	19.8284
S100A11	0.713498	12.99727	9.059311	1.90E-18	2.08E-17	31.03497
TOLLIP	0.713358	6.93875	8.532864	1.20E-16	1.07E-15	26.95649
SETD8	0.713338	7.420864	12.52667	4.29E-32	2.44E-30	62.06232
ARHGEF11	0.712964	6.696064	8.408458	3.12E-16	2.66E-15	26.02036
ATP6V0A1	0.712578	7.805565	9.086833	1.53E-18	1.69E-17	31.2533
RASGRP4	0.71248	7.569414	9.477735	6.20E-20	8.03E-19	34.40751
LOC10193	0.711448	6.7138	9.984505	8.47E-22	1.35E-20	38.6406
RFXANK	0.711355	8.265448	6.296307	5.95E-10	2.69E-09	11.86639
ERI1	0.711347	6.296813	7.008907	6.58E-12	3.65E-11	16.26016
LY96	0.708912	11.36468	8.058709	4.28E-15	3.26E-14	23.44686
ZNF787	0.70851	6.43274	3.900262	0.000107	0.000254	0.231136
AATK	0.708126	9.12642	9.505863	4.91E-20	6.42E-19	34.63826
MLF2	0.707938	9.098785	5.402088	9.56E-08	3.34E-07	6.940918
SNCA	0.707105	7.495828	4.304331	1.96E-05	5.13E-05	1.837337
ARPC1A	0.706793	9.430588	8.354523	4.70E-16	3.93E-15	25.61787
NADK	0.705913	9.055634	5.311948	1.54E-07	5.24E-07	6.481992
C17orf62	0.70492	9.813991	7.701853	5.68E-14	3.89E-13	20.91172
TBKBP1	0.704854	6.767869	6.289877	6.18E-10	2.79E-09	11.8286
ZNF516	0.704598	8.026735	7.251777	1.30E-12	7.73E-12	17.8489
CRISP2	0.704183	4.844326	5.497335	5.74E-08	2.06E-07	7.433438
KIF18B	0.704068	6.084542	5.998106	3.48E-09	1.43E-08	10.14959
HSDL2	0.703901	7.836746	7.423787	3.99E-13	2.51E-12	19.00144
UBE2H	0.703398	7.454391	9.45542	7.47E-20	9.61E-19	34.2248
PLGRKT	0.703167	8.025352	7.9324	1.08E-14	7.93E-14	22.53895
PJA2	0.701813	10.36421	9.73622	7.08E-21	1.01E-19	36.54676
NARF	0.701182	8.265191	9.796834	4.23E-21	6.23E-20	37.05444
ACTN1	0.701064	9.610009	7.679554	6.65E-14	4.52E-13	20.7564
HIST1H2A	0.700913	7.526694	7.869529	1.71E-14	1.23E-13	22.09133
TM9SF2	0.699306	11.39053	10.29954	5.43E-23	1.00E-21	41.35091
RNF146	0.699126	9.239958	7.306797	8.91E-13	5.42E-12	18.21511
KIAA0319L	0.698589	8.806019	6.979585	7.98E-12	4.40E-11	16.07144
DCAF11	0.698585	7.646448	7.766828	3.57E-14	2.51E-13	21.36637
ZMAT5	0.697694	6.746437	7.277859	1.08E-12	6.54E-12	18.02221
SIRPB1	0.697517	7.257941	9.735474	7.12E-21	1.02E-19	36.54053
MYH9	0.697226	10.43074	6.865194	1.68E-11	8.98E-11	15.34162
PPP4R2	0.697156	8.393842	8.699444	3.31E-17	3.11E-16	28.2267

C8orf88	0.696143	5.472609	7.335691	7.31E-13	4.48E-12	18.40835
MSRA	0.695552	6.734515	10.26042	7.66E-23	1.39E-21	41.01117
HSPB1	0.694999	7.140494	3.609478	0.000333	0.000735	-0.83169
PIK3AP1	0.694796	11.89564	9.26146	3.69E-19	4.40E-18	32.65015
C1orf162	0.694624	10.74827	6.702674	4.78E-11	2.44E-10	14.32244
GLUL	0.694473	9.848116	6.775965	2.99E-11	1.55E-10	14.77947
CLN3	0.69414	7.966021	5.034901	6.36E-07	2.02E-06	5.11556
FZD5	0.694083	5.113999	10.54126	6.34E-24	1.29E-22	43.46997
OIP5	0.693661	5.20988	5.292393	1.70E-07	5.77E-07	6.38336
GPR132	0.693378	6.867384	6.096308	1.96E-09	8.30E-09	10.70686
FLI1	0.692459	8.134755	8.272374	8.73E-16	7.10E-15	25.00874
LAMP1	0.691647	9.553595	7.399812	4.71E-13	2.94E-12	18.83945
ZAK	0.690624	6.842967	6.718783	4.32E-11	2.21E-10	14.42253
FBXO38	0.690106	8.759683	9.285744	3.03E-19	3.64E-18	32.84597
PINK1	0.689976	8.938804	8.481077	1.79E-16	1.56E-15	26.5655
TMED2	0.689758	8.639945	7.216234	1.65E-12	9.71E-12	17.61356
MOSPD3	0.689284	7.572246	6.025948	2.96E-09	1.23E-08	10.30677
CD58	0.688568	8.260603	9.648386	1.49E-20	2.05E-19	35.81514
SHISA5	0.688114	10.47983	5.420532	8.67E-08	3.05E-07	7.035686
ERO1L	0.687598	9.485101	6.97862	8.03E-12	4.43E-11	16.06524
SELP	0.687351	8.268382	4.630282	4.49E-06	1.28E-05	3.241069
FAM63B	0.686897	6.249456	7.560143	1.54E-13	1.01E-12	19.93097
KIAA0930	0.686529	8.699736	7.66473	7.39E-14	4.99E-13	20.65335
MTX1	0.686481	9.660546	8.397334	3.39E-16	2.89E-15	25.93718
FAR1	0.686272	9.548948	9.200357	6.08E-19	7.07E-18	32.15912
RENBP	0.686244	7.178398	5.403569	9.49E-08	3.32E-07	6.948518
KCNE3	0.685829	9.040633	6.64483	6.90E-11	3.45E-10	13.96474
MMADHC	0.685327	11.76827	10.21342	1.16E-22	2.05E-21	40.60418
DNAJC13	0.685065	9.153288	7.815361	2.52E-14	1.80E-13	21.708
VCAN	0.684967	11.16651	5.434532	8.04E-08	2.84E-07	7.107813
TESC	0.684912	6.712417	9.421547	9.89E-20	1.25E-18	33.94806
FAM110B	0.684776	5.907893	9.804175	3.97E-21	5.88E-20	37.11608
TMEM180	0.684739	6.904496	9.324431	2.20E-19	2.69E-18	33.15872
KIAA0101	0.684703	6.790095	4.838782	1.67E-06	5.03E-06	4.188895
LRRC6	0.684443	6.686266	5.681853	2.09E-08	7.88E-08	8.409618
CMTM2	0.684074	11.75774	5.359837	1.20E-07	4.12E-07	6.724937
RSPH9	0.683834	5.74256	7.79986	2.82E-14	2.00E-13	21.5987
OSBPL1A	0.68338	7.232146	4.125411	4.23E-05	0.000106	1.107691
ZNF784	0.68314	6.601727	10.2021	1.28E-22	2.25E-21	40.50633
JHDM1D-/	0.681976	6.813616	9.59219	2.39E-20	3.22E-19	35.34958
CYB5D1	0.681842	6.992157	5.698677	1.91E-08	7.21E-08	8.500059
HK2	0.681747	10.39816	9.261765	3.68E-19	4.39E-18	32.65261
HIPK3	0.681578	7.716309	9.463182	7.00E-20	9.02E-19	34.28832
MAP1LC3I	0.681416	10.68035	9.89575	1.82E-21	2.80E-20	37.88778
PLOD2	0.680926	4.611047	4.867582	1.45E-06	4.41E-06	4.322851
MYL9	0.679623	6.278656	5.051902	5.84E-07	1.86E-06	5.197484
PSMC3	0.679266	7.558541	4.531231	7.10E-06	1.97E-05	2.804387
FRAT2	0.678972	11.49574	6.541339	1.32E-10	6.44E-10	13.33144
ASAP1	0.678439	9.713235	8.723504	2.74E-17	2.60E-16	28.41174
TMED8	0.677215	8.063444	10.5778	4.57E-24	9.38E-23	43.79322
PNPLA2	0.67702	9.080748	4.972823	8.66E-07	2.70E-06	4.81858
TMEM158	0.67677	6.405032	4.168813	3.52E-05	8.92E-05	1.282008
COPE	0.676119	7.669758	6.072316	2.26E-09	9.49E-09	10.56997
NMNAT1	0.676098	5.618396	8.117359	2.78E-15	2.16E-14	23.87234
DHRS7B	0.675539	7.237441	7.576621	1.38E-13	9.06E-13	20.04424
F8	0.675097	7.518567	5.338799	1.34E-07	4.58E-07	6.617963
PRO2852	0.674908	8.737753	4.912885	1.16E-06	3.57E-06	4.535052
SGSH	0.674563	8.174143	6.087828	2.06E-09	8.72E-09	10.65842

FAM89B	0.674352	8.82744	5.206121	2.67E-07	8.83E-07	5.95216
KIF15	0.67406	4.967385	4.529438	7.16E-06	1.98E-05	2.796564
CREB5	0.673677	9.802239	4.825481	1.78E-06	5.35E-06	4.127278
PSTPIP1	0.673362	9.431268	5.99644	3.51E-09	1.44E-08	10.1402
ZNF222	0.672768	7.306859	6.793895	2.67E-11	1.39E-10	14.89193
FADD	0.672054	8.548353	8.528811	1.24E-16	1.10E-15	26.92582
TOR1A	0.671428	8.900487	9.654107	1.42E-20	1.96E-19	35.86265
ARID5A	0.671281	7.996908	5.447406	7.51E-08	2.66E-07	7.174289
MYO10	0.671259	5.001241	8.288379	7.74E-16	6.34E-15	25.12704
EIF4G3	0.671198	8.199065	10.49791	9.34E-24	1.87E-22	43.08745
MIAT	0.671041	7.742778	4.350057	1.60E-05	4.24E-05	2.028478
E2F2	0.670989	6.428786	9.128997	1.09E-18	1.22E-17	31.58874
LOC10192	0.670853	6.75789	7.564119	1.50E-13	9.85E-13	19.95828
MGST1	0.669939	6.84079	9.629935	1.74E-20	2.39E-19	35.66206
ANAPC15	0.669208	8.385622	7.841467	2.09E-14	1.50E-13	21.89248
CBL	0.668994	8.704463	10.28284	6.29E-23	1.15E-21	41.20577
ARFGEF1	0.668472	8.861374	8.41871	2.88E-16	2.47E-15	26.0971
RAB1B	0.667537	9.731175	6.398206	3.20E-10	1.49E-09	12.46974
TCIRG1	0.667315	10.58557	5.468273	6.71E-08	2.39E-07	7.282338
GADD45G	0.666131	6.113115	8.513088	1.40E-16	1.23E-15	26.80696
RBM47	0.666003	8.11584	9.268503	3.49E-19	4.16E-18	32.7069
HSPA6	0.665588	10.44683	4.780297	2.21E-06	6.55E-06	3.919127
APPL2	0.665335	7.197926	5.60632	3.17E-08	1.17E-07	8.006511
DEGS1	0.664995	9.944003	9.617422	1.93E-20	2.63E-19	35.55837
ARPC1B	0.664218	12.33996	8.429798	2.65E-16	2.27E-15	26.18017
ZEB1-AS1	0.663739	6.177394	5.890958	6.45E-09	2.56E-08	9.550688
FCGRT	0.663691	9.399909	3.920255	9.88E-05	0.000236	0.307085
CANT1	0.663103	9.718135	8.060864	4.22E-15	3.21E-14	23.46245
ASPM	0.6631	4.690878	5.613736	3.05E-08	1.13E-07	8.045875
FOXM1	0.662403	5.895976	6.298327	5.88E-10	2.65E-09	11.87827
PLEK	0.661764	10.87952	7.676812	6.78E-14	4.61E-13	20.73732
SYT2	0.661631	7.078543	9.56044	3.11E-20	4.16E-19	35.08741
IFITM2	0.661583	13.9854	7.961881	8.72E-15	6.46E-14	22.74982
COL4A3BF	0.661266	9.437493	8.695068	3.42E-17	3.21E-16	28.19309
MAP4K4	0.661115	8.774326	9.036483	2.29E-18	2.46E-17	30.85426
CLTCL1	0.661025	6.911065	6.187838	1.14E-09	4.97E-09	11.23345
CDCA5	0.660764	6.381869	6.642204	7.02E-11	3.50E-10	13.94857
BCL7B	0.658492	7.812456	6.228446	8.95E-10	3.96E-09	11.46927
SEC23A	0.65815	7.930639	9.300217	2.69E-19	3.25E-18	32.96286
PHF23	0.657563	9.039352	8.352306	4.78E-16	4.00E-15	25.60136
IFNAR1	0.65745	7.956222	8.815179	1.33E-17	1.30E-16	29.12036
UBL5	0.657424	10.0292	8.492744	1.64E-16	1.43E-15	26.65342
ITGB2	0.65728	10.09066	4.424134	1.15E-05	3.10E-05	2.342146
AREL1	0.656631	6.772932	8.006479	6.29E-15	4.73E-14	23.07003
TMEM176	0.656166	8.271335	3.457462	0.000584	0.001243	-1.35596
CETP	0.656095	5.722575	6.384492	3.48E-10	1.61E-09	12.38805
PAG1	0.655754	9.796442	6.33312	4.76E-10	2.17E-09	12.08338
STIP1	0.655686	7.259897	4.915306	1.15E-06	3.53E-06	4.546439
IQSEC1	0.655145	9.785589	7.769018	3.52E-14	2.47E-13	21.38175
SEMA4B	0.654998	8.999196	6.300812	5.79E-10	2.62E-09	11.89288
AGTPBP1	0.654301	9.610523	6.508049	1.63E-10	7.84E-10	13.12955
ATXN7L3	0.65392	8.174063	6.063818	2.37E-09	9.95E-09	10.5216
UNC13D	0.653802	6.993221	5.491418	5.93E-08	2.12E-07	7.402614
TMUB2	0.652473	8.162617	8.250758	1.03E-15	8.32E-15	24.84925
TNFRSF10	0.651728	9.20984	5.186505	2.95E-07	9.71E-07	5.85502
CPNE1	0.6514	8.687128	6.010147	3.24E-09	1.34E-08	10.21748
INSIG2	0.650974	7.86419	6.763906	3.24E-11	1.67E-10	14.70398
TRAPPC5	0.650312	8.647824	4.903508	1.22E-06	3.73E-06	4.490978

TMEM45A	0.650205	4.834825	3.861729	0.000125	0.000294	0.085793
KDM7A	0.649125	8.873644	9.800647	4.10E-21	6.05E-20	37.08646
PPP1R12A	0.648955	10.13506	9.209541	5.64E-19	6.57E-18	32.23276
PPP4R1	0.648324	11.11894	8.492902	1.64E-16	1.43E-15	26.65461
ARF3	0.647535	9.344494	7.767244	3.56E-14	2.50E-13	21.36929
MYD88	0.647421	12.44608	11.0329	7.27E-26	1.87E-24	47.88218
ZNHIT1	0.647413	8.290748	5.374886	1.10E-07	3.82E-07	6.801689
CAPNS1	0.647302	10.67971	4.491859	8.49E-06	2.33E-05	2.633256
CSRNP1	0.64721	8.255543	5.363059	1.18E-07	4.06E-07	6.741355
RAB43	0.646012	7.285004	7.603662	1.14E-13	7.56E-13	20.23057
RFT1	0.645947	6.921128	9.49578	5.34E-20	6.96E-19	34.55548
SLC25A44	0.645171	9.442881	9.400994	1.17E-19	1.48E-18	33.78051
RNASEL	0.644928	8.739151	8.062973	4.15E-15	3.17E-14	23.47771
CRK	0.642898	8.736899	8.288897	7.71E-16	6.32E-15	25.13087
ACVR1B	0.642734	6.654485	11.36925	3.17E-27	9.76E-26	50.97658
DUSP1	0.64222	9.396861	5.802489	1.07E-08	4.14E-08	9.063427
TRIM21	0.641124	9.387054	6.245106	8.10E-10	3.60E-09	11.56642
SZRD1	0.640166	7.902438	7.188598	1.98E-12	1.16E-11	17.43125
RNF166	0.639512	9.046654	4.560311	6.21E-06	1.74E-05	2.931682
UCHL1	0.639264	6.228343	7.655818	7.87E-14	5.30E-13	20.59148
CEACAM3	0.639	7.979053	7.415639	4.22E-13	2.64E-12	18.94634
NAGK	0.63898	10.62785	4.478744	9.01E-06	2.46E-05	2.576559
RAB20	0.63859	7.378637	8.158926	2.04E-15	1.61E-14	24.17539
MAPRE1	0.63798	10.14176	8.783747	1.71E-17	1.65E-16	28.87676
CYP4F3	0.637884	11.24996	4.245617	2.53E-05	6.52E-05	1.594685
PEAK1	0.637711	7.74102	9.736471	7.06E-21	1.01E-19	36.54886
C4orf3	0.637413	10.37232	9.883319	2.02E-21	3.09E-20	37.78273
H1FX	0.636797	8.420652	3.426514	0.000654	0.001382	-1.46005
IL6R	0.636609	9.562046	6.131246	1.60E-09	6.83E-09	10.90705
LINC0096٤	0.636466	5.132259	5.726093	1.64E-08	6.23E-08	8.647959
LAMC1	0.636162	6.202425	7.200614	1.83E-12	1.07E-11	17.51044
MEIS3P1	0.636132	5.622712	6.778941	2.94E-11	1.53E-10	14.79812
TPM1	0.635628	6.60317	6.481338	1.92E-10	9.17E-10	12.9682
SLC28A3	0.635516	4.934006	5.888242	6.55E-09	2.60E-08	9.535631
TMEM92	0.635407	6.375298	7.232754	1.47E-12	8.73E-12	17.72282
CYB5R1	0.635257	8.60189	6.74999	3.54E-11	1.82E-10	14.61701
MMP27	0.635095	4.974905	6.686317	5.31E-11	2.69E-10	14.22102
GAA	0.634988	8.331585	3.958315	8.47E-05	0.000203	0.452689
BLVRB	0.634708	8.964109	5.843865	8.43E-09	3.31E-08	9.290498
LRRFIP2	0.634072	6.373837	10.59624	3.87E-24	8.02E-23	43.95664
ATP2A3	0.634003	7.633266	4.048331	5.84E-05	0.000144	0.802363
UBE2R2	0.632474	10.1207	8.967663	3.97E-18	4.16E-17	30.31156
CD274	0.631399	7.41645	3.997187	7.22E-05	0.000176	0.602779
C1orf226	0.631213	5.265509	7.632626	9.28E-14	6.22E-13	20.43074
RAB4B	0.630566	6.80744	7.421844	4.04E-13	2.54E-12	18.98829
LOC10272	0.630382	4.65548	4.731314	2.79E-06	8.17E-06	3.695523
NFYC	0.62991	7.171566	6.677269	5.62E-11	2.84E-10	14.16501
TRPM6	0.629881	6.939016	7.308122	8.83E-13	5.37E-12	18.22396
ACOX2	0.629631	5.189836	7.565584	1.49E-13	9.76E-13	19.96834
H1F0	0.62954	7.449951	4.878913	1.37E-06	4.19E-06	4.375755
TPP1	0.629056	9.272835	7.467734	2.94E-13	1.88E-12	19.29949
RAB10	0.629012	11.05765	9.373587	1.47E-19	1.83E-18	33.5575
ARID3A	0.628725	8.140005	7.710746	5.33E-14	3.66E-13	20.97376
XRCC4	0.628442	6.3926	7.207365	1.75E-12	1.03E-11	17.55499
ZBTB7B	0.62818	7.739068	5.406788	9.32E-08	3.27E-07	6.965041
PCNX	0.628112	7.249255	7.316141	8.36E-13	5.10E-12	18.27753
GPI	0.627827	9.77927	3.909039	0.000103	0.000246	0.264434
VPS18	0.627523	7.101337	6.124642	1.66E-09	7.08E-09	10.86913

EGR3	0.626588	6.343807	2.198302	0.028315	0.044561	-4.85536
MED15	0.625181	8.112168	4.044345	5.94E-05	0.000146	0.78672
ZNF281	0.624696	9.8263	6.948894	9.76E-12	5.33E-11	15.87462
PTBP3	0.624099	8.877994	9.207359	5.74E-19	6.69E-18	32.21526
EAF2	0.622996	7.896305	5.716047	1.73E-08	6.58E-08	8.593688
OXSR1	0.622949	9.449259	9.665386	1.29E-20	1.79E-19	35.95637
KIAA0922	0.621916	9.415969	8.198854	1.51E-15	1.21E-14	24.46764
RGS19	0.621439	11.42476	8.369461	4.19E-16	3.53E-15	25.72914
GABARAPI	0.62095	11.74951	8.980234	3.59E-18	3.78E-17	30.41046
SGMS2	0.620733	6.726745	5.195388	2.82E-07	9.30E-07	5.898965
C16orf72	0.620677	10.10916	7.469767	2.90E-13	1.85E-12	19.31332
CCNDBP1	0.620453	11.14752	8.628659	5.74E-17	5.26E-16	27.68463
STAT3	0.620082	9.652336	8.6069	6.79E-17	6.19E-16	27.51868
VAV3	0.619334	7.365892	8.445738	2.35E-16	2.03E-15	26.29975
PPAP2B	0.619039	5.529419	7.368338	5.85E-13	3.62E-12	18.62746
GCA	0.619002	12.73908	8.29833	7.18E-16	5.91E-15	25.20069
TNPO3	0.61878	8.460775	8.432277	2.60E-16	2.24E-15	26.19876
SIPA1	0.618591	9.230261	4.51467	7.66E-06	2.11E-05	2.732235
SCYL2	0.618254	8.615362	6.97498	8.22E-12	4.52E-11	16.04186
RBM23	0.61817	9.789144	6.588214	9.86E-11	4.85E-10	13.61723
DNM2	0.618128	6.713195	5.824487	9.41E-09	3.67E-08	9.183974
TUBB4B	0.617797	9.093081	5.385414	1.04E-07	3.63E-07	6.855499
MLKL	0.617408	10.28214	7.889883	1.47E-14	1.07E-13	22.23593
CRADD	0.617249	7.5059	9.793271	4.36E-21	6.41E-20	37.02454
VPS8	0.616369	7.463619	6.913054	1.23E-11	6.65E-11	15.64572
PTGR1	0.616115	5.194919	7.623137	9.92E-14	6.64E-13	20.3651
MXD1	0.616063	10.9415	6.429628	2.64E-10	1.24E-09	12.65749
FPGT	0.615663	7.635171	5.435817	7.99E-08	2.82E-07	7.114441
RNF181	0.615635	9.44939	5.803435	1.06E-08	4.12E-08	9.068602
PRAM1	0.615579	9.16914	4.979055	8.40E-07	2.63E-06	4.848241
PRKAG1	0.615041	8.462163	6.60933	8.63E-11	4.27E-10	13.74654
MFF	0.614767	8.275389	7.714099	5.20E-14	3.58E-13	20.99717
TCAIM	0.614636	5.586696	9.563465	3.03E-20	4.06E-19	35.11237
TLN1	0.614335	7.668782	7.592949	1.23E-13	8.12E-13	20.15668
MAF1	0.613722	8.206003	4.966523	8.93E-07	2.78E-06	4.788628
LOC10272	0.613327	5.604456	8.017895	5.78E-15	4.36E-14	23.15222
LONRF3	0.612196	6.248738	7.369039	5.82E-13	3.60E-12	18.63217
ALDH3B1	0.612071	7.86559	5.859342	7.72E-09	3.05E-08	9.375804
TLE3	0.611937	8.819128	5.130879	3.92E-07	1.27E-06	5.58137
EXT1	0.611863	5.754605	7.087029	3.92E-12	2.22E-11	16.76623
PAFAH1B1	0.611664	8.353164	9.983335	8.55E-22	1.37E-20	38.63065
PLEKHF2	0.61124	9.363608	8.392194	3.53E-16	3.00E-15	25.89877
BCL2L1	0.611198	6.523221	5.579888	3.67E-08	1.34E-07	7.866596
SYN2	0.611188	5.726971	13.37773	7.63E-36	8.13E-34	70.6073
NTSR1	0.610912	6.482945	7.147247	2.62E-12	1.51E-11	17.15954
HGS	0.610856	7.775911	6.149731	1.43E-09	6.16E-09	11.01337
FKBP8	0.610752	7.015158	5.231856	2.33E-07	7.79E-07	6.08011
CAPZB	0.610668	9.401469	8.77908	1.77E-17	1.72E-16	28.84065
FIBP	0.610159	9.040748	6.890155	1.43E-11	7.68E-11	15.5
THBS1	0.61007	6.05191	4.349185	1.61E-05	4.25E-05	2.024816
ELL2	0.609888	6.2137	7.450569	3.31E-13	2.10E-12	19.1829
LIMD2	0.609295	8.728291	2.908681	0.003766	0.007007	-3.06745
TMEM8A	0.609171	8.378808	5.59575	3.36E-08	1.24E-07	7.950489
TMEM30A	0.608706	9.246402	8.843596	1.06E-17	1.06E-16	29.34118
DAGLB	0.608446	6.281073	5.201102	2.73E-07	9.04E-07	5.927275
ABO	0.607706	6.223344	9.419413	1.01E-19	1.27E-18	33.93065
TRIM6	0.606903	5.265245	3.708319	0.000228	0.000517	-0.47923
TMEM55A	0.606128	9.214465	5.158762	3.40E-07	1.11E-06	5.7182

APLP2	0.606031	9.62281	7.746789	4.12E-14	2.87E-13	21.22582
CLIC1	0.605999	12.27985	7.795326	2.91E-14	2.06E-13	21.56676
LITAF	0.605987	12.82018	8.216704	1.33E-15	1.06E-14	24.59867
STAT6	0.605807	9.169299	4.218708	2.84E-05	7.28E-05	1.484524
DNAJB6	0.605797	8.059847	6.544082	1.30E-10	6.33E-10	13.34811
TNFAIP8L2	0.605607	9.256333	6.441447	2.45E-10	1.16E-09	12.72832
PSENEN	0.605273	8.349581	5.477055	6.40E-08	2.28E-07	7.32792
BCKDK	0.604791	7.645532	6.34522	4.42E-10	2.03E-09	12.15495
ACIN1	0.604469	7.311786	3.913003	0.000102	0.000242	0.279496
MELK	0.603162	5.963332	3.988845	7.47E-05	0.000181	0.570453
PSAT1	0.60312	6.275844	5.204617	2.69E-07	8.89E-07	5.944701
NABP1	0.602975	9.082192	5.829263	9.16E-09	3.58E-08	9.210201
SYAP1	0.60256	8.268057	8.204919	1.45E-15	1.16E-14	24.51214
GDE1	0.601545	9.00333	7.154131	2.50E-12	1.44E-11	17.20468
VPS25	0.601545	7.984935	6.186511	1.15E-09	5.01E-09	11.22576
VPS37B	0.601386	8.575217	5.491027	5.94E-08	2.13E-07	7.400578
ATOX1	0.601363	8.545316	6.408002	3.01E-10	1.41E-09	12.52818
ZNF354A	0.599914	8.473242	4.638525	4.32E-06	1.23E-05	3.277808
ZFP36	0.599715	12.03895	5.804931	1.05E-08	4.09E-08	9.076791
VNN2	0.598409	12.78473	5.997171	3.50E-09	1.44E-08	10.14432
IRAK4	0.598301	8.301166	6.522609	1.49E-10	7.20E-10	13.21774
POLD4	0.597842	8.802387	4.567393	6.01E-06	1.68E-05	2.962795
GADD45B	0.597091	8.199146	7.624262	9.84E-14	6.59E-13	20.37288
ARL4A	0.596989	8.058964	5.984998	3.75E-09	1.53E-08	10.0758
LMO2	0.596047	8.124641	6.649252	6.71E-11	3.36E-10	13.99199
HPD	0.595959	5.054378	6.213213	9.80E-10	4.31E-09	11.38065
NOP10	0.595923	11.5095	7.835202	2.19E-14	1.56E-13	21.84816
NUF2	0.595822	4.457116	3.818756	0.000148	0.000345	-0.07468
TNFSF14	0.595758	8.071817	5.921395	5.42E-09	2.17E-08	9.719841
YWHAE	0.595622	8.625318	4.636983	4.35E-06	1.24E-05	3.270932
MRPL28	0.594873	8.330799	6.066645	2.33E-09	9.79E-09	10.53769
PSME3	0.594546	7.942565	7.787304	3.08E-14	2.18E-13	21.51029
DHFR	0.594486	6.566406	4.992224	7.87E-07	2.47E-06	4.911032
WDR1	0.594447	8.925332	8.355174	4.67E-16	3.92E-15	25.62271
DNAJC3	0.593184	8.996584	6.580781	1.03E-10	5.07E-10	13.57179
EDEM3	0.593172	8.0546	6.907741	1.28E-11	6.87E-11	15.61188
KIF4A	0.593079	5.511426	5.537113	4.63E-08	1.68E-07	7.641428
CHIC2	0.592942	10.16093	7.492534	2.48E-13	1.59E-12	19.46833
IQGAP1	0.592538	11.36658	7.365271	5.97E-13	3.69E-12	18.60683
IGHM	0.592179	7.934594	3.366186	0.000812	0.001691	-1.66035
NFAM1	0.592135	9.155915	6.168955	1.28E-09	5.53E-09	11.12424
NRGN	0.592085	9.983905	2.849789	0.004527	0.008326	-3.23412
AZIN1	0.591419	9.676242	7.337213	7.24E-13	4.44E-12	18.41854
LGALS8	0.591395	8.068172	7.423565	4.00E-13	2.51E-12	18.99993
SLC6A6	0.591302	7.327732	7.308219	8.82E-13	5.37E-12	18.2246
POLR2E	0.590644	8.333327	4.144679	3.90E-05	9.82E-05	1.184865
GOLGA1	0.590637	7.531694	6.059555	2.43E-09	1.02E-08	10.49736
CCNB1	0.590259	4.78316	4.278835	2.19E-05	5.69E-05	1.731584
SPTLC1	0.590177	8.684761	8.142148	2.31E-15	1.81E-14	24.05292
UPB1	0.590111	5.858865	8.072362	3.87E-15	2.97E-14	23.54569
TMTC1	0.590066	5.824188	3.020377	0.002634	0.005041	-2.74225
MAOB	0.590017	6.106713	5.322472	1.46E-07	4.97E-07	6.53521
SNAP23	0.587547	9.109669	6.803345	2.51E-11	1.32E-10	14.9513
CTDSP1	0.586212	9.796311	5.156898	3.43E-07	1.12E-06	5.709033
PIK3CG	0.586031	8.639975	7.521972	2.02E-13	1.31E-12	19.66935
UBALD2	0.585893	9.223737	5.331005	1.39E-07	4.76E-07	6.57843
SKAP2	0.585546	8.811869	8.340918	5.21E-16	4.34E-15	25.51665
MOSPD2	0.585082	9.679682	6.352721	4.22E-10	1.94E-09	12.19937

NFKBIA	0.584761	8.891331	6.86221	1.72E-11	9.15E-11	15.32272
HIST1H2A	0.584668	5.205415	5.127517	3.98E-07	1.29E-06	5.564917
SLC27A2	0.584093	5.105023	4.59683	5.25E-06	1.48E-05	3.092608
C19orf33	0.584082	5.776055	4.103817	4.64E-05	0.000115	1.021604
NDRG1	0.584073	9.55473	6.753725	3.45E-11	1.78E-10	14.64034
RCHY1	0.583722	7.396226	6.181547	1.18E-09	5.15E-09	11.19703
RP2	0.583143	9.258676	5.268483	1.93E-07	6.50E-07	6.263205
DUSP3	0.582644	6.882023	8.3164	6.27E-16	5.20E-15	25.33459
TTPAL	0.582614	7.594699	9.311209	2.46E-19	2.98E-18	33.05172
CCDC88B	0.582478	8.86186	4.490491	8.55E-06	2.34E-05	2.627333
AOC1	0.58184	5.313709	3.474489	0.000549	0.001172	-1.29832
RNPEPL1	0.581595	7.831302	5.096481	4.66E-07	1.50E-06	5.413502
GNA15	0.581392	7.907574	6.913126	1.23E-11	6.65E-11	15.64618
OSBPL9	0.581246	9.986285	7.190692	1.96E-12	1.14E-11	17.44504
CHMP4C	0.581061	4.788721	4.622229	4.66E-06	1.33E-05	3.205241
CDCA3	0.580913	6.337545	7.771214	3.46E-14	2.43E-13	21.39717
C8orf60	0.580702	8.556779	5.010617	7.18E-07	2.26E-06	4.998985
TECPR2	0.579998	8.81581	6.479858	1.94E-10	9.25E-10	12.95928
CCR2	0.579207	8.773283	4.441815	1.06E-05	2.88E-05	2.417748
TWF2	0.578976	8.356366	5.315807	1.51E-07	5.14E-07	6.501496
MYL6B	0.578386	6.568985	6.510003	1.61E-10	7.75E-10	13.14137
PTPRJ	0.578136	7.370864	9.770057	5.31E-21	7.69E-20	36.82989
TNFRSF1A	0.57804	10.87938	5.094999	4.70E-07	1.51E-06	5.406292
WBP5	0.577977	5.288032	4.343038	1.65E-05	4.36E-05	1.999013
ETHE1	0.577944	8.09357	5.4357	7.99E-08	2.82E-07	7.113838
EPAS1	0.577719	6.0658	6.983367	7.78E-12	4.29E-11	16.09575
SELT	0.577699	9.577338	7.283416	1.04E-12	6.30E-12	18.0592
PHF21A	0.576754	8.752845	9.758657	5.85E-21	8.46E-20	36.73442
PSAP	0.576638	12.29726	4.93606	1.04E-06	3.21E-06	4.644302
SLC43A3	0.576248	7.232963	6.966596	8.69E-12	4.77E-11	15.98806
PPP2R1A	0.575921	8.319329	4.382393	1.39E-05	3.70E-05	2.164792
KLF1	0.575572	5.183493	5.346095	1.29E-07	4.41E-07	6.655018
SEC24A	0.575509	6.884919	5.052869	5.81E-07	1.85E-06	5.202154
ZNF467	0.575332	7.965475	7.53031	1.90E-13	1.24E-12	19.72641
CDYL2	0.57427	7.767861	5.344844	1.29E-07	4.44E-07	6.648664
SLC25A24	0.574013	9.858088	5.305808	1.59E-07	5.40E-07	6.450985
CHSY1	0.574013	10.75886	6.302966	5.71E-10	2.58E-09	11.90555
CDK2AP2	0.573705	7.970685	3.795967	0.000162	0.000375	-0.15908
SPR	0.5737	7.008716	10.80947	5.63E-25	1.29E-23	45.86031
AZI2	0.573573	6.772686	6.388781	3.39E-10	1.58E-09	12.41358
C1GALT1C	0.572821	7.407115	7.468121	2.94E-13	1.87E-12	19.30213
EFCAB2	0.572375	5.273326	6.081358	2.14E-09	9.03E-09	10.6215
ZFAND3	0.572094	8.143876	5.746009	1.46E-08	5.60E-08	8.755797
RAB5A	0.57173	7.892827	7.595234	1.21E-13	7.99E-13	20.17243
TCN2	0.570681	6.742045	6.847218	1.89E-11	1.00E-10	15.22787
DEPDC1B	0.570249	3.75473	4.903912	1.22E-06	3.73E-06	4.492877
ARHGAP4	0.569436	8.385692	3.893842	0.00011	0.000261	0.206826
TDRD7	0.569211	8.484836	6.851381	1.84E-11	9.78E-11	15.25419
RXRA	0.568803	9.149239	6.952699	9.52E-12	5.20E-11	15.89898
TOMM40L	0.568634	7.271101	6.824632	2.19E-11	1.16E-10	15.0853
DNTTIP1	0.568474	8.727317	5.061354	5.57E-07	1.78E-06	5.243141
NDUFB9	0.567811	10.06123	5.216766	2.52E-07	8.39E-07	6.005016
C9orf16	0.567559	7.30361	4.994478	7.78E-07	2.44E-06	4.921794
ST3GAL4	0.567452	5.717174	7.276429	1.10E-12	6.60E-12	18.0127
SLC44A2	0.567232	8.719348	3.708503	0.000228	0.000516	-0.47856
BCAP31	0.56635	9.845584	4.929297	1.07E-06	3.31E-06	4.612368
CDC123	0.565881	9.505123	6.804662	2.49E-11	1.31E-10	14.95958
SNX17	0.565715	9.319108	4.959526	9.25E-07	2.87E-06	4.755406

MOB2	0.565337	7.377613	6.968556	8.58E-12	4.71E-11	16.00063
GYPB	0.565055	6.160928	5.705017	1.84E-08	6.97E-08	8.534204
S100A9	0.564671	14.61889	9.24188	4.33E-19	5.11E-18	32.49254
ARHGAP2	0.564315	6.353676	7.319223	8.18E-13	5.00E-12	18.29814
NT5C3A	0.563496	10.61604	5.972426	4.04E-09	1.64E-08	10.00518
GNAI3	0.563241	9.871377	7.686112	6.35E-14	4.32E-13	20.80204
RAD23B	0.562692	8.513729	9.078812	1.63E-18	1.79E-17	31.18962
BIRC5	0.562631	5.952301	6.114586	1.76E-09	7.50E-09	10.81146
RHOU	0.56229	7.229363	4.843394	1.63E-06	4.92E-06	4.210295
ERGIC1	0.562032	7.583345	6.430544	2.62E-10	1.24E-09	12.66298
DCUN1D3	0.561356	7.124314	8.238004	1.13E-15	9.11E-15	24.75531
SERINC3	0.561178	10.09644	10.07662	3.82E-22	6.36E-21	39.42694
NIN	0.560807	7.686824	9.993859	7.81E-22	1.25E-20	38.72022
STK3	0.560335	5.859809	7.220951	1.60E-12	9.42E-12	17.64474
BPGM	0.560123	7.14072	3.799601	0.00016	0.00037	-0.14566
FAM129A	0.56006	12.07105	6.721092	4.25E-11	2.18E-10	14.43689
DESI1	0.560004	7.063429	6.306273	5.60E-10	2.54E-09	11.92502
IRF2	0.559746	10.34522	6.051391	2.55E-09	1.07E-08	10.45098
PTPRN2	0.559642	7.287698	5.491786	5.92E-08	2.12E-07	7.40453
CHCHD1	0.559462	8.455044	7.680889	6.59E-14	4.48E-13	20.76569
DUSP18	0.558571	8.554894	6.964379	8.81E-12	4.84E-11	15.97383
MARCO	0.558492	7.168313	4.420959	1.17E-05	3.14E-05	2.328603
FOS	0.557594	11.41169	2.994557	0.002863	0.005445	-2.81848
PGAM1	0.55755	12.05993	8.969992	3.90E-18	4.08E-17	30.32988
DGAT1	0.557396	7.762947	4.67699	3.61E-06	1.04E-05	3.450036
TSPAN14	0.556657	9.537385	7.187662	2.00E-12	1.16E-11	17.42508
STARD3	0.5564	6.803045	4.539387	6.84E-06	1.90E-05	2.840014
SCCPDH	0.556299	6.924164	6.089393	2.04E-09	8.64E-09	10.66736
LIG4	0.556207	7.280176	5.90513	5.95E-09	2.37E-08	9.629352
ATP13A3	0.555803	7.292682	6.440332	2.47E-10	1.17E-09	12.72163
MS4A6A	0.555798	9.624236	7.927279	1.12E-14	8.21E-14	22.50238
NLRP3	0.555118	6.429112	4.536906	6.92E-06	1.92E-05	2.829168
TAZ	0.554646	7.889443	6.281243	6.51E-10	2.93E-09	11.77791
MAPK1	0.55437	8.603417	11.60844	3.30E-28	1.16E-26	53.21321
GDPD3	0.55426	7.69929	5.394322	9.96E-08	3.47E-07	6.901104
SRPR	0.554012	9.179866	8.152693	2.14E-15	1.68E-14	24.12987
LYN	0.553759	11.94662	6.764137	3.23E-11	1.67E-10	14.70543
C5AR1	0.553316	12.15647	6.493858	1.78E-10	8.52E-10	13.04376
GLTP	0.553195	9.32953	7.690244	6.17E-14	4.21E-13	20.83081
PANX2	0.552906	6.594873	5.847851	8.24E-09	3.24E-08	9.312451
NEDD8	0.552743	9.764607	5.042176	6.13E-07	1.95E-06	5.150588
LEMD2	0.552565	6.698416	5.053241	5.80E-07	1.85E-06	5.20395
ACOX1	0.552219	7.261455	10.18582	1.47E-22	2.57E-21	40.36576
TMBIM6	0.552019	11.23902	9.040345	2.22E-18	2.39E-17	30.88482
PPP3R1	0.551937	8.523619	5.560013	4.09E-08	1.49E-07	7.761781
HJURP	0.5517	5.5781	5.953964	4.49E-09	1.81E-08	9.901695
HMGB3	0.551682	6.233631	6.984392	7.73E-12	4.27E-11	16.10234
CD37	0.551132	7.950075	5.004752	7.39E-07	2.33E-06	4.970906
KLC1	0.550731	7.174907	7.64526	8.48E-14	5.71E-13	20.51826
NAMPT	0.549243	12.73912	6.331095	4.82E-10	2.19E-09	12.07142
ESAM	0.548863	6.504021	4.526841	7.24E-06	2.00E-05	2.785236
COL9A3	0.548622	6.747623	4.265252	2.32E-05	6.02E-05	1.675485
COPS7A	0.548438	7.602504	4.607357	5.00E-06	1.41E-05	3.139222
TRIM58	0.548212	6.044434	3.563314	0.000396	0.000865	-0.99318
MYL4	0.548181	7.008897	6.72892	4.05E-11	2.08E-10	14.48561
MYO1F	0.548091	11.7837	4.003435	7.04E-05	0.000171	0.627031
MOB1A	0.54748	10.03368	8.283469	8.03E-16	6.56E-15	25.09073
NETO2	0.547271	5.854019	5.402212	9.55E-08	3.34E-07	6.941555

TINF2	0.547121	9.354967	8.52863	1.24E-16	1.10E-15	26.92445
TANK	0.546836	8.169622	7.711548	5.30E-14	3.64E-13	20.97936
STYXL1	0.546824	7.389619	9.220754	5.15E-19	6.02E-18	32.32276
ATXN1	0.546768	7.768529	7.675255	6.86E-14	4.65E-13	20.7265
E2F4	0.546564	8.125518	8.993266	3.24E-18	3.43E-17	30.5131
NRBF2	0.546403	10.33134	5.499662	5.67E-08	2.04E-07	7.445569
SASH3	0.546367	10.29691	3.522981	0.00046	0.000992	-1.13265
ITGB3	0.54596	6.055301	4.989805	7.96E-07	2.50E-06	4.899487
TADA3	0.545936	7.072214	7.44194	3.52E-13	2.22E-12	19.12438
ARPC3	0.54536	12.07652	7.689325	6.21E-14	4.23E-13	20.82441
AGPAT2	0.545048	7.502226	4.572421	5.88E-06	1.65E-05	2.984914
TYW5	0.545018	6.512044	5.945636	4.71E-09	1.90E-08	9.855109
INPP5D	0.544896	8.561012	4.045055	5.93E-05	0.000145	0.789506
WDR45	0.544244	8.554891	5.620608	2.94E-08	1.09E-07	8.082393
AGO4	0.544191	8.630495	7.350323	6.62E-13	4.07E-12	18.50645
STRN3	0.543904	7.016627	5.852328	8.04E-09	3.17E-08	9.337117
SH2B2	0.543814	8.997398	5.550615	4.30E-08	1.56E-07	7.712334
CCNE2	0.543731	4.780885	4.706735	3.14E-06	9.12E-06	3.584129
FAM160B1	0.543704	10.00387	6.904072	1.31E-11	7.03E-11	15.58851
ATG7	0.543618	7.656015	8.034938	5.10E-15	3.87E-14	23.27511
RDH5	0.543045	7.544619	6.342684	4.49E-10	2.06E-09	12.13994
NRM	0.542996	8.427858	7.572519	1.42E-13	9.31E-13	20.01602
TMEM169	0.542599	6.275123	5.97885	3.89E-09	1.58E-08	10.04125
ANP32A-I	0.542083	5.109088	4.525614	7.28E-06	2.02E-05	2.77989
CD38	0.542073	7.406029	3.01584	0.002673	0.005111	-2.75569
LAMTOR2	0.541671	8.223378	5.244374	2.19E-07	7.32E-07	6.142558
CITED4	0.541496	6.695645	6.408171	3.01E-10	1.41E-09	12.52919
HELZ2	0.541285	7.171929	5.71521	1.74E-08	6.61E-08	8.589174
PKHD1L1	0.54117	4.642945	4.710429	3.08E-06	8.97E-06	3.600835
TNNI2	0.541149	6.452503	6.428747	2.65E-10	1.25E-09	12.65221
ATP2C2	0.54073	5.92596	6.149639	1.43E-09	6.16E-09	11.01284
SCAND1	0.540672	7.856969	4.284986	2.13E-05	5.55E-05	1.757043
ITPKC	0.540437	6.625776	6.069204	2.30E-09	9.65E-09	10.55225
PTPN6	0.539693	11.42322	4.385646	1.37E-05	3.65E-05	2.178557
PSMB3	0.539398	10.98198	8.831068	1.18E-17	1.16E-16	29.24375
KPNA4	0.538862	8.719122	6.846503	1.90E-11	1.01E-10	15.22335
SLC7A5	0.538835	7.291902	4.754216	2.51E-06	7.37E-06	3.799805
PLEKHA2	0.53738	8.442349	7.108164	3.40E-12	1.94E-11	16.90396
MFSD5	0.537284	7.858344	5.802966	1.06E-08	4.13E-08	9.066037
MGAT1	0.537264	9.331537	5.351446	1.25E-07	4.30E-07	6.682227
POLE2	0.53708	5.58294	4.685354	3.47E-06	1.00E-05	3.487664
TMEM167	0.537052	10.05729	7.902595	1.34E-14	9.78E-14	22.32639
BAMBI	0.537019	5.798921	5.087396	4.88E-07	1.57E-06	5.369337
AIG1	0.536568	5.315839	7.522881	2.00E-13	1.30E-12	19.67557
RBM42	0.536179	8.083855	4.787031	2.14E-06	6.36E-06	3.950032
FHOD1	0.535862	7.842784	5.039959	6.20E-07	1.97E-06	5.139909
AP2A1	0.535624	6.746117	4.823898	1.79E-06	5.38E-06	4.119952
IFI27L1	0.53544	6.740971	8.524549	1.28E-16	1.13E-15	26.89359
MIIP	0.535279	8.304679	7.084303	3.99E-12	2.26E-11	16.74849
SLC25A28	0.535147	8.348689	5.019091	6.88E-07	2.18E-06	5.039605
NFKBID	0.534646	6.948185	6.662391	6.18E-11	3.11E-10	14.07306
FBN2	0.534483	5.776775	6.072605	2.25E-09	9.48E-09	10.57162
CTSB	0.534253	10.19494	5.81584	9.88E-09	3.85E-08	9.136541
WFDC1	0.534143	5.670115	5.23059	2.35E-07	7.84E-07	6.073803
GMPR	0.534112	6.595546	3.05837	0.002326	0.004491	-2.62893
ADRBK1	0.534072	8.289104	5.374988	1.10E-07	3.82E-07	6.80221
MKL1	0.534057	6.87302	6.622882	7.93E-11	3.93E-10	13.82972
LOC10050	0.53309	6.58093	9.061929	1.86E-18	2.03E-17	31.05572

IGLJ3	0.532659	7.046429	3.933364	9.37E-05	0.000224	0.357088
UBL7	0.53234	7.911167	4.941521	1.01E-06	3.13E-06	4.670113
EPHA8	0.532169	6.567069	9.092858	1.45E-18	1.61E-17	31.30116
GNB4	0.531614	7.878944	7.743001	4.23E-14	2.95E-13	21.19928
YWHAZ	0.531607	10.78289	8.753082	2.17E-17	2.08E-16	28.63974
RNASE4	0.531393	5.77684	3.771081	0.000179	0.000411	-0.25071
IVNS1ABP	0.531253	8.450028	6.984306	7.73E-12	4.27E-11	16.10178
LOH12CR1	0.53101	7.215845	7.245432	1.35E-12	8.05E-12	17.80682
IFITM1	0.530983	12.9459	7.150918	2.56E-12	1.47E-11	17.18361
CALCOCO	0.5305	8.851611	4.677956	3.59E-06	1.04E-05	3.454378
ABHD2	0.530305	8.759565	6.096576	1.96E-09	8.29E-09	10.70839
GDI1	0.530287	9.978582	5.800468	1.08E-08	4.18E-08	9.052374
TYMS	0.52946	6.364725	4.390192	1.34E-05	3.58E-05	2.197809
FBXW5	0.529438	7.748163	5.481773	6.24E-08	2.23E-07	7.352435
ANXA5	0.528914	11.34113	7.602233	1.15E-13	7.64E-13	20.2207
ST3GAL2	0.528528	7.340907	7.451347	3.30E-13	2.09E-12	19.18818
LTBP1	0.528247	7.026168	4.297181	2.02E-05	5.28E-05	1.80762
STAT5A	0.52784	8.32069	5.896895	6.23E-09	2.48E-08	9.583624
SPECC1L	0.526623	7.728009	6.202967	1.04E-09	4.57E-09	11.32115
P2RX4	0.526565	7.683379	4.936302	1.04E-06	3.20E-06	4.645446
LMTK2	0.525497	5.807751	10.51727	7.86E-24	1.58E-22	43.25815
GRPEL1	0.523459	8.312302	5.848271	8.22E-09	3.24E-08	9.314761
RARA	0.52342	8.00352	4.768844	2.34E-06	6.90E-06	3.866654
FOXN2	0.523319	8.263547	7.280998	1.06E-12	6.40E-12	18.04311
TMEM176I	0.523229	6.363483	3.459563	0.00058	0.001234	-1.34887
UBE2L3	0.523003	8.503888	6.879385	1.54E-11	8.22E-11	15.4316
5-Mar	0.522981	7.78276	6.303253	5.70E-10	2.58E-09	11.90725
HCLS1	0.522806	12.24751	5.445158	7.60E-08	2.69E-07	7.162672
UEVLD	0.522713	7.106977	4.820419	1.82E-06	5.47E-06	4.103867
BCAT1	0.522638	6.154823	3.771757	0.000178	0.00041	-0.24823
DCAF10	0.522506	6.624449	5.93618	4.98E-09	2.00E-08	9.802283
AKIRIN1	0.521933	8.607386	7.215418	1.66E-12	9.76E-12	17.60817
BNIP2	0.521697	9.869466	6.802332	2.53E-11	1.32E-10	14.94493
TAPBP	0.521381	9.189555	5.040631	6.18E-07	1.96E-06	5.143145
ACTN4	0.521325	9.264543	3.632418	0.000305	0.000677	-0.75069
ZNF319	0.521185	7.858095	6.838803	2.00E-11	1.06E-10	15.1747
PHKA2	0.521122	7.942079	7.683372	6.48E-14	4.41E-13	20.78296
TMX1	0.520906	9.491144	7.445862	3.42E-13	2.16E-12	19.15097
JDP2	0.520793	7.497824	7.243322	1.37E-12	8.16E-12	17.79283
TRPC4AP	0.520132	7.798313	5.427142	8.37E-08	2.95E-07	7.069717
AKT1S1	0.51967	6.094451	4.954042	9.50E-07	2.95E-06	4.729396
SRF	0.519119	6.691442	8.037523	5.01E-15	3.80E-14	23.29377
WDFY3-AS	0.518352	4.00363	6.834341	2.06E-11	1.09E-10	15.14653
ANKS1A	0.518349	8.731327	5.70517	1.84E-08	6.97E-08	8.535028
ZWINT	0.518216	7.015119	2.451785	0.014503	0.02428	-4.27326
MAX	0.517753	8.691634	6.079796	2.16E-09	9.11E-09	10.6126
MED12	0.517192	7.629012	6.365199	3.92E-10	1.81E-09	12.27338
TAF8	0.517054	6.43125	6.265637	7.16E-10	3.20E-09	11.68645
FAM198B	0.516755	7.69731	4.286348	2.12E-05	5.52E-05	1.762686
WAS	0.516639	9.61442	4.641122	4.27E-06	1.22E-05	3.289394
PPP1R18	0.51663	11.47545	4.859741	1.51E-06	4.57E-06	4.286307
KIF2C	0.516293	5.780586	6.086879	2.07E-09	8.77E-09	10.65301
DEF6	0.516187	8.569357	3.906496	0.000104	0.000248	0.254777
SNX1	0.515841	7.772044	7.318326	8.23E-13	5.03E-12	18.29214
GAS6	0.515745	7.071302	8.121695	2.69E-15	2.10E-14	23.9039
PLAUR	0.515078	8.956171	5.415218	8.92E-08	3.13E-07	7.008349
ADAM15	0.514972	6.109629	4.953589	9.52E-07	2.95E-06	4.72725
LILRB2	0.51455	10.87001	6.774058	3.03E-11	1.57E-10	14.76752

PTAFR	0.514238	10.13974	5.376067	1.10E-07	3.80E-07	6.807722
TDRG1	0.514141	6.955111	8.092408	3.34E-15	2.57E-14	23.69103
ALPK1	0.514134	7.839302	5.770349	1.28E-08	4.92E-08	8.888039
UBXN2B	0.513575	9.350624	6.144883	1.47E-09	6.33E-09	10.98546
DRAP1	0.513479	7.910822	3.697565	0.000238	0.000537	-0.51802
NCAPH	0.513248	6.049134	6.014302	3.17E-09	1.31E-08	10.24094
AURKA	0.513112	5.338764	6.269011	7.01E-10	3.14E-09	11.7062
CD163	0.512794	7.995252	2.760674	0.005947	0.010705	-3.48001
COX8A	0.512743	10.3673	4.356535	1.56E-05	4.13E-05	2.05571
SH3BGRL3	0.512474	12.23575	4.084567	5.02E-05	0.000124	0.945222
AP2M1	0.512318	9.679547	4.059843	5.57E-05	0.000137	0.847618
ATP6V1B2	0.512158	11.58283	5.623207	2.89E-08	1.07E-07	8.096213
BCL2L15	0.511705	5.523738	4.516305	7.60E-06	2.10E-05	2.739346
SMIM3	0.511073	8.052622	5.544566	4.45E-08	1.61E-07	7.680551
2-Mar	0.509869	8.434479	4.486501	8.70E-06	2.38E-05	2.610074
DNAJB11	0.50985	8.471026	5.903988	5.99E-09	2.38E-08	9.623005
PSMD3	0.50925	6.562103	3.636948	0.0003	0.000667	-0.73464
CASP5	0.508276	7.42554	3.040259	0.002468	0.004742	-2.68312
HTRA1	0.508113	6.593203	4.989246	7.98E-07	2.50E-06	4.896821
SNX12	0.508048	6.79415	9.026997	2.47E-18	2.65E-17	30.77927
SNRPB	0.507891	9.071121	4.40307	1.27E-05	3.39E-05	2.25245
ADAMTS2	0.507725	5.842169	6.503642	1.67E-10	8.05E-10	13.10289
PHF12	0.507525	6.552093	5.836631	8.79E-09	3.44E-08	9.250696
HMOX1	0.507264	8.848957	3.517677	0.000469	0.00101	-1.15088
SELL	0.507215	13.35716	7.389355	5.06E-13	3.15E-12	18.76893
GPD2	0.506969	6.057353	7.853207	1.92E-14	1.38E-13	21.9756
PLK3	0.506726	6.645274	6.628566	7.65E-11	3.80E-10	13.86465
THBS3	0.505291	7.159013	5.806509	1.04E-08	4.05E-08	9.085426
SEL1L3	0.504738	6.890828	5.207129	2.65E-07	8.78E-07	5.95716
NEIL3	0.504371	4.727182	4.851683	1.57E-06	4.74E-06	4.248811
GLDC	0.503855	5.609253	4.446668	1.04E-05	2.82E-05	2.438549
FBRS	0.503749	6.773254	3.861955	0.000125	0.000294	0.086644
LHX4	0.503742	5.146036	5.66208	2.34E-08	8.75E-08	8.303623
RAPGEF1	0.50353	7.249436	4.406358	1.25E-05	3.34E-05	2.266423
BTG2	0.503528	9.270611	3.770146	0.00018	0.000413	-0.25414
IRS2	0.503226	10.77452	5.648364	2.52E-08	9.40E-08	8.230292
CHMP1A	0.502924	8.45797	3.809539	0.000154	0.000357	-0.10887
CLIP1	0.502839	6.809741	7.608359	1.10E-13	7.33E-13	20.26298
GYPA	0.502813	4.500593	3.992408	7.36E-05	0.000179	0.584251
SAMD8	0.502759	7.068626	6.526434	1.45E-10	7.04E-10	13.24094
JAZF1	0.502235	8.57806	6.788873	2.76E-11	1.44E-10	14.86041
NMI	0.502085	11.36813	7.417206	4.18E-13	2.62E-12	18.95693
NDUFA13	0.501724	9.067671	3.148762	0.001722	0.003403	-2.3538
TACC3	0.501262	9.265857	4.260174	2.38E-05	6.14E-05	1.654556
TMEM38A	0.501173	6.250984	6.510699	1.60E-10	7.72E-10	13.14559
SLC8A1	0.501171	6.086026	7.479527	2.71E-13	1.73E-12	19.37973
DDX60L	0.501139	10.44052	5.017034	6.95E-07	2.20E-06	5.029742
EBLN2	0.501104	8.017791	5.360393	1.19E-07	4.11E-07	6.727768
CDC34	0.501006	7.325188	5.530772	4.79E-08	1.73E-07	7.608181
ZFAND2B	0.500871	7.886665	5.027148	6.61E-07	2.09E-06	5.078285
TMEM11	0.500384	8.427385	6.759511	3.33E-11	1.72E-10	14.67649
CARHSP1	0.500012	7.519227	6.125348	1.65E-09	7.06E-09	10.87318
ZNF780A	0.499817	5.52334	4.699272	3.25E-06	9.44E-06	3.550408
EDNRB	0.499265	4.471371	5.029442	6.53E-07	2.07E-06	5.089311
ALAS1	0.499226	8.330719	5.432402	8.14E-08	2.87E-07	7.096828
IGLV1-44	0.499191	6.866593	3.90701	0.000104	0.000248	0.256731
CDC45	0.498899	5.685422	5.800638	1.08E-08	4.18E-08	9.053304
TTYH3	0.498687	7.626928	4.220176	2.83E-05	7.24E-05	1.490516

TPST1	0.498438	7.241902	2.846041	0.00458	0.008419	-3.24461
SVIL	0.498435	7.911137	8.683218	3.75E-17	3.51E-16	28.10214
PLIN5	0.498225	6.958077	8.131259	2.51E-15	1.96E-14	23.97354
TM9SF1	0.4982	6.360416	7.812641	2.57E-14	1.83E-13	21.68881
SEPHS2	0.498015	10.07527	7.304586	9.04E-13	5.50E-12	18.20035
IER3	0.497383	10.23391	4.618091	4.76E-06	1.35E-05	3.186848
PATL1	0.497219	7.08295	7.271184	1.14E-12	6.82E-12	17.97781
GLRX	0.496943	11.14327	8.01052	6.11E-15	4.60E-14	23.09911
BTBD10	0.49659	8.912959	6.66754	5.98E-11	3.01E-10	14.10486
ZNF687	0.496287	7.639872	5.730014	1.60E-08	6.10E-08	8.66916
DEPDC1	0.495857	4.46213	5.355015	1.23E-07	4.22E-07	6.700385
HMGB2	0.495779	8.404781	9.858958	2.49E-21	3.76E-20	37.57712
SLC22A16	0.495442	5.77564	4.936747	1.03E-06	3.20E-06	4.647549
NEK2	0.495184	4.411578	5.632554	2.75E-08	1.02E-07	8.145969
TMEM165	0.495081	6.662902	11.6066	3.36E-28	1.17E-26	53.19589
RNF123	0.494684	7.293012	4.880597	1.36E-06	4.16E-06	4.383626
TFE3	0.494241	9.098473	6.510084	1.61E-10	7.75E-10	13.14187
H6PD	0.493925	6.95952	7.451028	3.30E-13	2.10E-12	19.18602
TFEB	0.493056	8.721031	5.07331	5.24E-07	1.68E-06	5.301003
CSGALNA(	0.492855	9.561693	3.477067	0.000544	0.001162	-1.28957
SLC25A1	0.492767	6.942726	4.636072	4.37E-06	1.25E-05	3.266869
MVB12A	0.492629	6.547041	3.696098	0.000239	0.00054	-0.5233
RFX2	0.492185	6.633275	7.161758	2.38E-12	1.38E-11	17.25474
TRMT6	0.491544	7.060341	4.429203	1.13E-05	3.04E-05	2.363791
BIN3	0.491175	8.555539	5.754672	1.39E-08	5.36E-08	8.802808
CD300C	0.491149	7.480914	5.158912	3.40E-07	1.11E-06	5.718941
ORAI1	0.491032	7.928479	3.408949	0.000697	0.001467	-1.51872
UHRF1BP1	0.490591	7.046508	6.062487	2.39E-09	1.00E-08	10.51403
PCGF3	0.490372	7.040035	7.44422	3.46E-13	2.19E-12	19.13984
KIAA1958	0.490097	6.578826	6.725901	4.13E-11	2.11E-10	14.46682
MYBPC3	0.490045	7.648864	6.789116	2.75E-11	1.44E-10	14.86193
OSBP2	0.489848	6.695575	5.380063	1.07E-07	3.73E-07	6.828136
MAP7D1	0.489547	8.752407	3.054774	0.002354	0.00454	-2.63971
SLC12A6	0.489409	9.320295	6.268529	7.03E-10	3.15E-09	11.70338
BAK1	0.489232	7.121267	5.1571	3.43E-07	1.12E-06	5.710026
GTPBP1	0.488693	6.800983	6.489101	1.83E-10	8.76E-10	13.01504
PPP2R5A	0.488043	7.745429	7.483841	2.63E-13	1.69E-12	19.4091
CD93	0.488018	10.51405	5.657877	2.39E-08	8.94E-08	8.281138
BAG4	0.487832	7.217728	6.523275	1.48E-10	7.17E-10	13.22178
EXTL3	0.487821	6.852466	6.845329	1.92E-11	1.02E-10	15.21593
CLASP1	0.487438	7.590672	5.716852	1.72E-08	6.55E-08	8.598035
FOSL1	0.487275	5.604396	7.497855	2.39E-13	1.54E-12	19.50462
EGF	0.48704	5.321002	3.090126	0.002095	0.004083	-2.53316
GTPBP2	0.486987	6.489449	6.54827	1.27E-10	6.17E-10	13.37358
KIF5B	0.486692	9.568757	8.294292	7.40E-16	6.08E-15	25.1708
PLD3	0.485536	7.495513	2.906476	0.003792	0.007054	-3.07375
TMEM110	0.485297	6.80117	6.945893	9.95E-12	5.43E-11	15.85542
ATP2B4	0.485188	8.013333	7.193316	1.92E-12	1.12E-11	17.46232
SNX27	0.485162	9.18806	6.501808	1.69E-10	8.13E-10	13.0918
NUS1P3	0.485051	4.437667	7.688497	6.24E-14	4.25E-13	20.81865
USF2	0.485047	9.202195	6.183221	1.17E-09	5.10E-09	11.20672
BIN3-IT1	0.484915	6.303429	6.42741	2.68E-10	1.26E-09	12.64421
RELT	0.484479	8.146919	5.59554	3.37E-08	1.24E-07	7.949379
TMLHE	0.484178	6.482885	6.630315	7.56E-11	3.76E-10	13.8754
KIAA1107	0.483876	5.206115	3.974181	7.94E-05	0.000192	0.513781
BICD2	0.482972	9.280837	6.876729	1.56E-11	8.35E-11	15.41475
TM9SF4	0.482476	7.497598	5.602502	3.24E-08	1.19E-07	7.986267
PRRC2A	0.482475	8.042709	4.432351	1.11E-05	3.00E-05	2.377245

LOC10050	0.48243	6.598147	7.978278	7.73E-15	5.76E-14	22.86738
CEBPB	0.482413	13.33484	7.734368	4.50E-14	3.12E-13	21.13885
CDT1	0.482274	5.726794	5.205764	2.67E-07	8.84E-07	5.95039
PHACTR2	0.482135	7.933267	4.261285	2.37E-05	6.12E-05	1.659132
NAPRT	0.481994	8.033725	3.849404	0.000131	0.000308	0.039596
MCM2	0.481671	7.204531	3.804816	0.000157	0.000363	-0.12636
SPC25	0.481517	4.127626	4.299092	2.01E-05	5.24E-05	1.815558
ARHGAP1!	0.481369	7.735672	5.547147	4.38E-08	1.59E-07	7.694106
FAM46C	0.480596	7.812937	2.845223	0.004592	0.00844	-3.2469
ACAD8	0.480313	6.668429	3.613971	0.000327	0.000723	-0.81586
PIK3CD	0.480212	9.357371	4.165014	3.58E-05	9.06E-05	1.266682
LOC64551	0.479992	5.289509	5.118767	4.17E-07	1.35E-06	5.522141
CALML4	0.479935	7.198871	5.790275	1.14E-08	4.42E-08	8.996677
NCKAP5L	0.479239	6.627928	6.973984	8.28E-12	4.55E-11	16.03547
CD151	0.47905	7.33091	4.735154	2.74E-06	8.03E-06	3.712977
LOC10004	0.478669	6.597403	5.896401	6.25E-09	2.48E-08	9.580878
PTPN12	0.478654	7.860591	4.800853	2.00E-06	5.98E-06	4.0136
EGLN1	0.478426	8.208728	5.81373	1.00E-08	3.89E-08	9.124978
CSNK1D	0.477845	8.074126	7.479233	2.72E-13	1.74E-12	19.37772
AGER	0.477774	6.362854	4.475925	9.13E-06	2.49E-05	2.564392
ARRDC1	0.477686	6.4396	5.166985	3.26E-07	1.07E-06	5.758686
HMG20B	0.477615	6.01755	4.480019	8.96E-06	2.45E-05	2.582062
ASNA1	0.477435	7.798095	3.98234	7.67E-05	0.000186	0.545288
CLIC4	0.477328	6.815406	5.607243	3.16E-08	1.16E-07	8.01141
KCND1	0.477161	6.228732	7.760665	3.73E-14	2.62E-13	21.32311
MPZ	0.476575	6.568523	7.185703	2.02E-12	1.18E-11	17.41218
B9D2	0.47596	7.379654	4.797053	2.04E-06	6.08E-06	3.996103
SIAE	0.475914	5.99872	7.05946	4.71E-12	2.65E-11	16.5871
FBXO30	0.475737	6.677107	4.373528	1.44E-05	3.84E-05	2.127327
WASF2	0.475433	9.112585	5.537847	4.61E-08	1.67E-07	7.645278
LOXL1	0.475338	5.46891	4.936091	1.04E-06	3.21E-06	4.64445
HBB	0.475052	13.94974	3.81672	0.000149	0.000348	-0.08224
ABCD1	0.474648	7.074133	4.096932	4.77E-05	0.000119	0.994245
PTPN22	0.474523	6.650327	5.230979	2.34E-07	7.82E-07	6.075743
SLC40A1	0.47403	8.885421	6.48197	1.91E-10	9.14E-10	12.97201
ATP11A	0.473599	6.808605	8.950991	4.54E-18	4.70E-17	30.18056
SLC8B1	0.473511	6.79301	4.938871	1.02E-06	3.17E-06	4.657584
RNF7	0.47344	7.398804	8.319773	6.11E-16	5.08E-15	25.35962
PIWIL4	0.473267	6.372907	3.594414	0.000352	0.000775	-0.8846
MPP7	0.473218	6.577911	5.522384	5.02E-08	1.81E-07	7.564259
FAM101B	0.472946	11.62515	4.835718	1.69E-06	5.10E-06	4.174688
GPAA1	0.472744	8.521156	4.873488	1.41E-06	4.29E-06	4.350414
RELB	0.472425	6.737705	3.731021	0.000209	0.000476	-0.39699
RRBP1	0.472358	6.347203	8.166928	1.92E-15	1.52E-14	24.23387
TSSC4	0.472164	6.886256	5.944776	4.74E-09	1.91E-08	9.850304
HIST1H4D	0.471806	5.429	6.111795	1.79E-09	7.61E-09	10.79547
OAZ1	0.471681	13.01671	5.120426	4.13E-07	1.34E-06	5.530247
ACTA2	0.471006	6.183472	9.044446	2.15E-18	2.32E-17	30.91726
UNC79	0.470812	5.983333	8.941298	4.90E-18	5.06E-17	30.10448
DPP3	0.470515	7.541708	4.979016	8.40E-07	2.63E-06	4.848053
C1orf85	0.470172	6.786748	4.110702	4.50E-05	0.000112	1.049006
HCFC1R1	0.46993	7.269148	5.306418	1.58E-07	5.38E-07	6.454068
ACAA1	0.469795	9.646361	6.778064	2.95E-11	1.54E-10	14.79262
GPSM2	0.469487	6.186405	6.654816	6.48E-11	3.25E-10	14.0263
LINC0026(	0.469088	5.607715	5.021209	6.81E-07	2.15E-06	5.04977
GLYR1	0.468984	7.24703	5.930018	5.16E-09	2.06E-08	9.767903
HEBP2	0.468522	8.019307	9.485265	5.83E-20	7.56E-19	34.46923
RBPJ	0.468501	9.307086	7.288788	1.01E-12	6.09E-12	18.09499

TRIP13	0.468369	5.902012	5.114132	4.26E-07	1.38E-06	5.499512
SYK	0.468129	9.082935	6.454482	2.26E-10	1.07E-09	12.80656
SRGN	0.468066	13.64162	8.371615	4.13E-16	3.48E-15	25.7452
CDCA2	0.467906	5.343321	6.007791	3.29E-09	1.36E-08	10.20419
RIPK3	0.467881	7.554825	5.727764	1.62E-08	6.18E-08	8.656992
ARL8B	0.467865	9.421335	7.020301	6.10E-12	3.39E-11	16.33368
KDM1B	0.467388	8.40529	5.145314	3.64E-07	1.19E-06	5.652124
SLC35B1	0.467241	7.987211	5.916783	5.56E-09	2.22E-08	9.694161
GALNS	0.466972	6.752592	9.349729	1.79E-19	2.20E-18	33.36375
NCF2	0.466868	12.78195	6.957594	9.22E-12	5.06E-11	15.93034
TUBG1	0.466296	6.014384	4.297551	2.02E-05	5.27E-05	1.809157
CARS	0.46621	7.465417	10.51517	8.01E-24	1.61E-22	43.23968
TMEM63B	0.465935	6.886277	6.369727	3.81E-10	1.76E-09	12.30026
LRP1	0.465666	7.364254	3.999044	7.17E-05	0.000174	0.609983
KCNH7	0.464371	5.51761	4.510693	7.80E-06	2.15E-05	2.714942
IPMK	0.464325	5.85553	5.786044	1.17E-08	4.52E-08	8.97358
FUT4	0.464082	7.079609	4.480154	8.96E-06	2.45E-05	2.582646
SPAG11A	0.46339	5.995827	8.735742	2.49E-17	2.38E-16	28.50601
C2orf76	0.462888	6.962401	4.138664	4.00E-05	0.000101	1.160738
C1orf106	0.462753	5.443113	6.376601	3.65E-10	1.69E-09	12.34111
GPBP1L1	0.462225	8.965159	5.821283	9.58E-09	3.74E-08	9.16639
DCTN1	0.462028	8.29288	6.386549	3.44E-10	1.60E-09	12.40029
NUP98	0.461575	7.941807	6.243937	8.15E-10	3.62E-09	11.55959
POMP	0.461517	8.494656	6.51362	1.57E-10	7.59E-10	13.16327
ZDHHC17	0.461368	8.087655	6.647013	6.81E-11	3.40E-10	13.97819
SELO	0.461005	7.190748	3.321896	0.000949	0.001957	-1.80523
C5orf30	0.460918	6.914718	3.319491	0.000957	0.001972	-1.81305
CPQ	0.46083	8.793704	5.163609	3.31E-07	1.09E-06	5.742055
MYPOP	0.460681	6.867196	6.444141	2.41E-10	1.14E-09	12.74447
DNASE1L1	0.460555	8.697204	5.741615	1.50E-08	5.74E-08	8.731976
SH3BP5	0.460531	10.26258	6.325305	4.99E-10	2.27E-09	12.03723
TNFSF13B	0.460316	11.96322	5.120707	4.12E-07	1.34E-06	5.531622
ALOX12	0.460308	7.142171	2.714586	0.00683	0.01217	-3.60419
CRISPLD2	0.460245	10.80477	4.015058	6.71E-05	0.000164	0.672246
APAF1	0.460083	7.841965	8.49165	1.65E-16	1.45E-15	26.64517
GINS1	0.460053	4.950638	3.119212	0.001902	0.003727	-2.44459
OPRL1	0.459378	6.123103	6.076284	2.20E-09	9.28E-09	10.59258
PRB1	0.459353	7.353685	8.521197	1.32E-16	1.16E-15	26.86824
RHD	0.459288	5.829504	6.005584	3.33E-09	1.37E-08	10.19174
SCN9A	0.459248	3.904526	6.458599	2.21E-10	1.05E-09	12.83131
VWA5A	0.459228	6.714438	4.537104	6.91E-06	1.92E-05	2.830034
IFNGR1	0.458536	10.70273	5.851331	8.08E-09	3.18E-08	9.331625
PTOV1	0.458499	7.541862	3.066326	0.002266	0.004386	-2.60502
GP9	0.458317	7.478368	4.998313	7.63E-07	2.40E-06	4.940114
PELO	0.458159	5.83407	9.702705	9.40E-21	1.32E-19	36.26703
CLEC12B	0.457961	5.416944	2.600539	0.009541	0.016541	-3.90272
TLR6	0.457686	7.819364	4.505393	7.99E-06	2.20E-05	2.691926
FLJ36848	0.457659	5.166294	5.834164	8.91E-09	3.49E-08	9.237128
WDFY4	0.457538	8.390314	5.895833	6.27E-09	2.49E-08	9.577729
AGAP2	0.456776	5.661653	6.20561	1.03E-09	4.50E-09	11.33649
HIST1H1E	0.456607	5.320346	3.175656	0.001573	0.003133	-2.27045
RAF1	0.456499	10.20986	4.249588	2.49E-05	6.42E-05	1.610999
PRL	0.456049	5.46114	5.171812	3.18E-07	1.04E-06	5.782473
PTH2R	0.455778	4.891714	4.423824	1.15E-05	3.11E-05	2.340823
MGC12916	0.455178	5.22517	6.861281	1.73E-11	9.20E-11	15.31684
IGLL3P	0.455176	8.744706	2.852347	0.004492	0.008266	-3.22695
MYL6	0.454648	9.871278	7.435527	3.68E-13	2.32E-12	19.08092
SLCO3A1	0.45464	8.924346	7.134575	2.85E-12	1.64E-11	17.07654

PRKAR1A	0.454592	9.827664	6.937247	1.05E-11	5.73E-11	15.80013
ANG	0.454493	5.803735	4.775074	2.27E-06	6.71E-06	3.895184
DYNLT1	0.454386	11.82828	6.924118	1.15E-11	6.21E-11	15.71628
DHRSX	0.454297	7.281748	6.12777	1.63E-09	6.96E-09	10.88708
PRKCSH	0.454289	8.880739	3.280429	0.001097	0.002238	-1.93919
CNR2	0.454174	6.485318	9.155838	8.73E-19	9.94E-18	31.80289
KIDINS220	0.454136	6.962709	11.47507	1.17E-27	3.83E-26	51.96252
SLMAP	0.454036	7.16426	5.586252	3.54E-08	1.30E-07	7.900228
ATF7	0.453895	6.276656	10.75724	9.06E-25	2.01E-23	45.3917
EDC3	0.453716	6.798214	7.335849	7.30E-13	4.48E-12	18.40941
WDR26	0.45356	8.480931	8.351384	4.81E-16	4.02E-15	25.5945
PPP2R5D	0.453501	7.154952	6.460091	2.19E-10	1.04E-09	12.84028
CYBB	0.453217	7.478927	9.04037	2.22E-18	2.39E-17	30.88501
TMEM33	0.452927	8.533666	5.243609	2.20E-07	7.35E-07	6.138739
ZDHHC2	0.452863	7.886067	4.961725	9.15E-07	2.84E-06	4.765845
HOMER2	0.452837	6.085793	4.939257	1.02E-06	3.16E-06	4.659409
ACADVL	0.452346	9.234909	3.094141	0.002067	0.004033	-2.52098
ANXA1	0.452205	9.37211	3.987895	7.50E-05	0.000182	0.566776
NT5DC2	0.451745	4.999459	4.844886	1.62E-06	4.89E-06	4.217225
DENND5A	0.45171	11.30498	6.01904	3.08E-09	1.28E-08	10.26771
B4GALT4	0.451709	6.662243	6.0385	2.75E-09	1.15E-08	10.37784
DHX38	0.451678	7.11216	3.665808	0.000269	0.000602	-0.63193
CCDC47	0.451489	8.712026	6.289518	6.20E-10	2.79E-09	11.82649
GPR137	0.45132	6.350007	7.642195	8.67E-14	5.83E-13	20.49702
TANC2	0.451267	5.687296	6.637039	7.25E-11	3.61E-10	13.91677
GPR37L1	0.451137	5.539456	7.292479	9.82E-13	5.95E-12	18.11959
LOC10050	0.451129	6.440468	5.444365	7.63E-08	2.70E-07	7.158573
TUBBP5	0.450831	5.252659	3.696855	0.000239	0.000538	-0.52057
CDKN2C	0.450792	6.505707	5.043103	6.10E-07	1.94E-06	5.155053
TOR1B	0.450717	9.140303	4.688392	3.42E-06	9.90E-06	3.501346
ZMAT2	0.450119	10.02595	7.507882	2.23E-13	1.44E-12	19.57305
ADAM17	0.450079	8.115279	7.157886	2.44E-12	1.41E-11	17.22932
OASL	0.450076	7.971573	2.355311	0.018833	0.030797	-4.50214
PREX1	0.449495	10.51189	4.116307	4.40E-05	0.00011	1.071347
IDNK	0.449438	7.516753	3.714579	0.000223	0.000505	-0.4566
UBE4B	0.44909	6.911876	7.408652	4.43E-13	2.77E-12	18.89912
TCEANC2	0.448416	6.781849	7.411496	4.34E-13	2.72E-12	18.91834
PIP5K1A	0.448386	6.644189	7.520581	2.04E-13	1.32E-12	19.65984
ZNF230	0.447974	7.398058	4.538891	6.85E-06	1.90E-05	2.837846
DOK1	0.447888	7.744852	4.591481	5.38E-06	1.52E-05	3.068965
PSMB6	0.447266	9.316962	4.88263	1.35E-06	4.12E-06	4.393135
ZBED6	0.44664	6.906298	2.40405	0.016521	0.027355	-4.38764
TGOLN2	0.446353	9.6455	8.838203	1.11E-17	1.10E-16	29.29923
TIPARP	0.446303	9.619772	3.787509	0.000168	0.000387	-0.19029
CD300A	0.446098	8.18337	4.143639	3.92E-05	9.86E-05	1.180691
INHBB	0.445986	5.79374	4.865738	1.46E-06	4.45E-06	4.314254
DDIT4	0.445708	8.510356	3.088772	0.002104	0.004098	-2.53726
STK40	0.445656	8.193586	5.380654	1.07E-07	3.72E-07	6.83116
SPINT2	0.44556	8.537611	5.390567	1.02E-07	3.54E-07	6.881875
SYNGR2	0.445381	7.155181	5.942764	4.79E-09	1.93E-08	9.839062
NABP2	0.444549	5.456046	5.660401	2.36E-08	8.82E-08	8.294638
MBOAT1	0.444239	8.635706	6.343361	4.47E-10	2.05E-09	12.14395
PDK3	0.443909	8.123075	6.513908	1.57E-10	7.57E-10	13.16502
DCTN4	0.44384	6.983692	8.100834	3.14E-15	2.42E-14	23.75221
ST14	0.443594	5.878765	5.851268	8.09E-09	3.18E-08	9.331274
RXRB	0.44312	6.541258	5.243554	2.20E-07	7.35E-07	6.138464
EREG	0.443099	4.26123	2.58861	0.009874	0.017074	-3.93322
CDC6	0.443042	4.553139	4.451196	1.02E-05	2.77E-05	2.457976

INO80B	0.442736	7.453132	6.445854	2.39E-10	1.13E-09	12.75476
CTNNA1	0.442394	8.304556	6.947677	9.83E-12	5.37E-11	15.86684
UFD1L	0.441986	9.220215	6.345853	4.41E-10	2.02E-09	12.1587
HIPK2	0.441971	7.340561	7.282363	1.05E-12	6.35E-12	18.0522
ATP6V1A	0.441967	9.974695	5.901453	6.07E-09	2.42E-08	9.608925
RAB1A	0.441852	8.683304	8.067728	4.01E-15	3.06E-14	23.51213
TLE4	0.441816	7.392507	5.530324	4.80E-08	1.74E-07	7.605836
SLC15A3	0.441651	8.669936	3.127815	0.001848	0.003627	-2.41825
SFT2D1	0.441062	7.969674	8.946009	4.72E-18	4.89E-17	30.14145
CHCHD7	0.440867	8.602193	4.396959	1.30E-05	3.48E-05	2.226503
MYO9B	0.440684	8.07855	3.603179	0.000341	0.000751	-0.85384
TPRA1	0.440629	6.271221	4.806503	1.95E-06	5.82E-06	4.039631
ABCC13	0.440358	4.960924	3.381977	0.000767	0.001606	-1.60825
SFXN5	0.440243	6.039906	10.08519	3.54E-22	5.93E-21	39.50041
NBN	0.440063	8.650972	5.856642	7.84E-09	3.09E-08	9.360908
RBPMS2	0.439942	6.174712	3.920218	9.88E-05	0.000236	0.306947
C4BPA	0.439865	6.687898	2.468512	0.013849	0.023278	-4.23266
GLTSCR1	0.439727	8.121248	7.298797	9.41E-13	5.71E-12	18.16172
RNF145	0.43962	7.739625	6.728824	4.05E-11	2.08E-10	14.48501
RAB11B	0.439278	6.239796	5.693624	1.96E-08	7.41E-08	8.472871
TPM3	0.439267	7.241458	8.363723	4.38E-16	3.68E-15	25.68638
MSL1	0.439174	9.924893	7.811343	2.59E-14	1.85E-13	21.67965
KCTD21	0.438954	7.272461	6.143111	1.49E-09	6.39E-09	10.97526
C11orf71	0.438537	6.839791	6.977271	8.10E-12	4.46E-11	16.05658
GSN	0.438367	6.347505	6.57741	1.05E-10	5.17E-10	13.5512
UBE2T	0.438265	5.537333	4.858395	1.52E-06	4.60E-06	4.280041
SIL1	0.438038	7.253977	5.30019	1.64E-07	5.55E-07	6.422645
LOC10272	0.437871	5.694299	6.385318	3.46E-10	1.61E-09	12.39296
DNAJC3-A	0.437622	5.028591	6.58112	1.03E-10	5.06E-10	13.57386
SLC4A1AP	0.437501	8.03941	5.592765	3.42E-08	1.25E-07	7.934686
GTDC1	0.437461	6.265096	8.153485	2.12E-15	1.67E-14	24.13565
GRB2	0.437451	9.602805	8.006178	6.30E-15	4.74E-14	23.06786
SP100	0.437428	8.14667	5.263373	1.98E-07	6.67E-07	6.237593
SLC6A8	0.437381	6.241368	4.979825	8.36E-07	2.62E-06	4.851907
SMARCD3	0.436803	6.468456	5.284277	1.78E-07	6.01E-07	6.342517
ARPC5	0.436782	12.70987	7.451549	3.29E-13	2.09E-12	19.18955
TRPV2	0.436667	6.92015	4.977693	8.45E-07	2.64E-06	4.841755
SUOX	0.436178	7.109969	5.121683	4.10E-07	1.33E-06	5.536391
SF3B4	0.436163	8.86119	4.237426	2.62E-05	6.74E-05	1.561082
TAF13	0.436049	6.307371	4.289078	2.10E-05	5.46E-05	1.774
ACSL3	0.43591	8.281857	3.884667	0.000114	0.00027	0.17215
ABL2	0.435707	6.725629	7.862325	1.80E-14	1.29E-13	22.04023
ACSS2	0.435517	8.150158	5.514552	5.23E-08	1.88E-07	7.523294
UBAC1	0.435403	7.716678	3.67653	0.000258	0.000579	-0.59358
LRSAM1	0.435271	5.661031	4.530449	7.12E-06	1.97E-05	2.800975
PDSS1	0.435224	6.85494	4.314072	1.88E-05	4.93E-05	1.877896
EMID1	0.435155	6.856181	6.66318	6.15E-11	3.10E-10	14.07792
SQLE	0.434768	6.078082	6.084669	2.10E-09	8.88E-09	10.64039
TRAFD1	0.43459	7.224194	5.752832	1.41E-08	5.41E-08	8.792816
N4BP1	0.434491	9.896746	7.587909	1.27E-13	8.40E-13	20.12195
DDA1	0.434444	6.410449	7.046901	5.11E-12	2.87E-11	16.50569
MAP3K3	0.434393	8.594962	5.908215	5.84E-09	2.33E-08	9.646496
RAB35	0.434174	7.497588	5.633701	2.73E-08	1.01E-07	8.152079
HRH2	0.433667	6.266023	5.032019	6.45E-07	2.05E-06	5.101701
RRAGA	0.433419	9.345036	6.689926	5.19E-11	2.63E-10	14.24338
PSMD9	0.433328	7.329976	7.475913	2.78E-13	1.78E-12	19.35513
APH1B	0.433296	7.903149	6.144966	1.47E-09	6.33E-09	10.98594
LINC00652	0.433271	6.134728	8.420978	2.83E-16	2.43E-15	26.11408

ISY1	0.433057	7.425918	10.53961	6.44E-24	1.30E-22	43.4554
PIM1	0.432981	9.884909	6.066162	2.34E-09	9.82E-09	10.53494
PTPLA	0.432716	5.061754	5.979271	3.88E-09	1.58E-08	10.04361
CNOT8	0.43267	8.869127	7.714572	5.19E-14	3.57E-13	21.00047
KLF16	0.432303	7.003974	4.301378	1.99E-05	5.19E-05	1.825058
PITPNM1	0.431447	7.483023	3.473576	0.000551	0.001176	-1.30141
FAXDC2	0.430877	7.746521	4.6093	4.95E-06	1.40E-05	3.147835
GNG2	0.430823	8.270841	8.241056	1.10E-15	8.92E-15	24.77777
CHCHD5	0.430701	6.000818	5.480354	6.29E-08	2.24E-07	7.34506
ABHD5	0.430664	7.576008	3.988878	7.47E-05	0.000181	0.57058
TGFB1	0.430372	8.209099	3.926145	9.65E-05	0.00023	0.329532
BAD	0.430221	6.160273	6.115859	1.75E-09	7.45E-09	10.81876
HLA-J	0.430095	11.25902	3.052448	0.002372	0.004572	-2.64668
PARVB	0.430038	6.247029	4.684546	3.48E-06	1.01E-05	3.484024
OAZ2	0.429998	9.410208	3.2723	0.001129	0.002299	-1.96527
TBC1D8B	0.428736	4.271908	4.798348	2.03E-06	6.04E-06	4.002066
ZFP92	0.428336	7.391898	5.967158	4.16E-09	1.69E-08	9.97562
VAV1	0.427908	9.241804	4.458685	9.87E-06	2.68E-05	2.490145
PPP1R10	0.427603	8.569776	6.496133	1.75E-10	8.41E-10	13.0575
CPSF2	0.427143	7.763118	6.035276	2.80E-09	1.17E-08	10.35957
TMEM234	0.42708	6.530395	7.501992	2.32E-13	1.50E-12	19.53285
CHRNB1	0.426872	5.227014	6.245431	8.08E-10	3.59E-09	11.56831
SUSD1	0.426847	8.055066	5.052333	5.83E-07	1.86E-06	5.199566
KLLN	0.42637	5.902319	8.062454	4.17E-15	3.18E-14	23.47395
BRF2	0.426268	6.865468	6.523312	1.48E-10	7.17E-10	13.222
KIF21B	0.42604	9.551169	5.083185	4.99E-07	1.60E-06	5.348889
BTNL3	0.425453	7.315923	3.314647	0.000974	0.002004	-1.82877
SPTY2D1-/	0.424942	5.169769	5.88237	6.77E-09	2.68E-08	9.503098
HK1	0.424369	10.01644	6.572879	1.09E-10	5.32E-10	13.52354
ADAM19	0.424256	7.744124	8.101388	3.13E-15	2.41E-14	23.75623
DENND2C	0.423348	5.584433	5.464652	6.85E-08	2.43E-07	7.263557
PTTG1	0.423208	8.129863	3.694473	0.000241	0.000543	-0.52915
DRC1	0.423195	6.616033	7.974142	7.97E-15	5.93E-14	22.83771
CAP1	0.423012	13.27505	7.858683	1.84E-14	1.33E-13	22.01441
COTL1	0.42297	11.90515	4.570606	5.92E-06	1.66E-05	2.976924
LILRP2	0.422909	5.02882	6.671761	5.82E-11	2.94E-10	14.13095
TLR2	0.422858	11.6727	4.561676	6.17E-06	1.73E-05	2.937671
PTGES2	0.422853	7.248302	5.473438	6.53E-08	2.32E-07	7.309139
LOC10012	0.422535	5.496434	9.065869	1.81E-18	1.98E-17	31.08695
CHST2	0.422187	6.083558	6.677928	5.60E-11	2.83E-10	14.16909
SHC1	0.42201	8.013004	5.022508	6.76E-07	2.14E-06	5.056003
ASGR1	0.421952	7.122605	3.972903	7.98E-05	0.000193	0.508852
GOLGA2	0.421763	7.274909	6.101599	1.90E-09	8.07E-09	10.73711
DIAPH1	0.421312	8.978843	7.379127	5.43E-13	3.36E-12	18.70004
AGO2	0.421283	8.301887	4.588223	5.46E-06	1.54E-05	3.054572
XIAP	0.421178	7.66737	7.878144	1.60E-14	1.16E-13	22.1525
HLA-G	0.420297	11.81146	3.316203	0.000968	0.001994	-1.82372
ACOT9	0.419974	9.303989	5.354757	1.23E-07	4.23E-07	6.69907
SENP2	0.419494	7.087098	8.619861	6.14E-17	5.61E-16	27.61749
DHX34	0.419046	6.678075	4.641043	4.27E-06	1.22E-05	3.289042
TMEM208	0.418933	7.903899	4.893769	1.28E-06	3.91E-06	4.445289
MTHFD2	0.418834	7.410713	6.034321	2.82E-09	1.17E-08	10.35416
AP3B1	0.418564	7.545668	7.031962	5.65E-12	3.15E-11	16.40902
LINC01272	0.418089	6.300517	4.109371	4.53E-05	0.000113	1.043708
GSTO1	0.41771	10.73732	5.361671	1.18E-07	4.08E-07	6.734281
LAPTM4B	0.41751	6.692613	3.098474	0.002038	0.003979	-2.50782
HIST1H3G	0.41739	7.319567	6.449494	2.34E-10	1.11E-09	12.77661
GALNT3	0.41716	7.364889	6.41684	2.85E-10	1.34E-09	12.58098

ZNF581	0.416621	7.974918	4.173871	3.45E-05	8.74E-05	1.302433
SCD	0.416569	6.362186	5.28617	1.76E-07	5.95E-07	6.352038
LHFPL4	0.416499	6.149007	7.197612	1.87E-12	1.09E-11	17.49064
SCN5A	0.416475	6.16051	5.965467	4.20E-09	1.70E-08	9.966136
PVRL2	0.416298	6.321038	4.808053	1.94E-06	5.79E-06	4.046774
ETV6	0.416186	8.057605	6.746927	3.61E-11	1.86E-10	14.59788
RLIM	0.415956	8.141666	4.661577	3.88E-06	1.11E-05	3.380865
ABHD16A	0.415762	6.772362	6.176847	1.22E-09	5.29E-09	11.16984
TXNDC11	0.415295	7.794691	4.930102	1.07E-06	3.30E-06	4.616169
ID1	0.414894	4.498032	4.874888	1.40E-06	4.27E-06	4.356949
CDCA8	0.413567	6.98709	6.280685	6.54E-10	2.94E-09	11.77464
RAB3IL1	0.41346	7.851408	5.08789	4.87E-07	1.57E-06	5.371735
TMEM258	0.413048	10.33412	4.124124	4.25E-05	0.000107	1.102551
CNPPD1	0.412907	7.622087	5.047842	5.96E-07	1.90E-06	5.177897
TAAR5	0.412609	5.942076	9.818018	3.53E-21	5.25E-20	37.2324
CAMTA2	0.412514	7.58102	4.675217	3.64E-06	1.05E-05	3.44207
ESPL1	0.412131	6.119445	6.185275	1.16E-09	5.05E-09	11.2186
SLC2A5	0.411792	5.726152	5.145376	3.64E-07	1.19E-06	5.652427
ZC3H3	0.411624	5.93014	5.404918	9.42E-08	3.30E-07	6.95544
ATCAY	0.411543	6.045943	6.76498	3.21E-11	1.66E-10	14.7107
NXPE3	0.411279	6.764206	5.254427	2.08E-07	6.97E-07	6.192805
LOC44043	0.410871	8.788056	5.958306	4.38E-09	1.77E-08	9.926007
VLDLR	0.410692	4.690081	3.169697	0.001605	0.003189	-2.28898
KAT5	0.410616	7.910358	4.922557	1.11E-06	3.41E-06	4.580589
LOC10192	0.410429	6.51496	6.213706	9.77E-10	4.30E-09	11.38351
FBXO34	0.410374	9.272053	5.549517	4.33E-08	1.57E-07	7.706562
PEX6	0.410277	6.074888	3.72216	0.000216	0.000492	-0.42914
SCAF1	0.409791	5.912127	3.908643	0.000104	0.000246	0.262928
PGLYRP4	0.409728	6.393033	6.690476	5.17E-11	2.62E-10	14.24679
FLJ30064	0.409678	6.261081	6.86091	1.73E-11	9.22E-11	15.31449
CCNA2	0.409448	5.73956	3.063331	0.002289	0.004426	-2.61403
ZDHHC5	0.40939	7.526616	4.428298	1.13E-05	3.05E-05	2.359927
LBR	0.40934	12.1115	6.954715	9.39E-12	5.14E-11	15.9119
THTPA	0.409079	7.151598	4.589199	5.44E-06	1.53E-05	3.058884
PRR13	0.408938	11.55008	6.607969	8.71E-11	4.30E-10	13.73819
ETFDH	0.40878	5.974922	4.102312	4.66E-05	0.000116	1.015622
LOC10272	0.408063	3.768168	3.656434	0.000279	0.000622	-0.66538
FCRL1	0.406899	7.316378	2.266986	0.023752	0.037967	-4.70379
LAPTM5	0.406851	12.83922	4.277531	2.20E-05	5.72E-05	1.726192
ANK1	0.4065	5.776679	5.409321	9.20E-08	3.22E-07	6.978046
DRP2	0.40643	6.805788	8.762835	2.01E-17	1.94E-16	28.71506
KBTBD2	0.406245	8.518251	5.085561	4.93E-07	1.59E-06	5.360427
ASB7	0.405034	7.153191	7.169898	2.25E-12	1.31E-11	17.30821
TMEM205	0.404968	8.041669	3.652868	0.000282	0.000629	-0.67808
NUP214	0.404717	7.807984	8.124402	2.64E-15	2.06E-14	23.9236
CNOT3	0.404604	5.742065	4.159604	3.66E-05	9.26E-05	1.244879
PBK	0.4046	4.24593	3.137258	0.00179	0.003523	-2.38925
TRIP6	0.403992	6.545976	5.472433	6.57E-08	2.34E-07	7.303921
SWT1	0.403595	6.706734	7.22615	1.54E-12	9.11E-12	17.67912
LMBR1	0.40342	5.258664	6.516099	1.55E-10	7.48E-10	13.17829
TYK2	0.403118	9.801441	2.838628	0.004686	0.008595	-3.26533
AIP	0.402795	8.166117	4.396347	1.31E-05	3.49E-05	2.223904
EP300	0.402708	9.29676	5.494656	5.83E-08	2.09E-07	7.41948
GNG10	0.402471	12.5723	6.155957	1.38E-09	5.94E-09	11.04924
CR1L	0.402403	4.398984	6.340416	4.55E-10	2.08E-09	12.12652
LYPLA2	0.402283	8.274302	6.693352	5.08E-11	2.58E-10	14.26461
LOC10050	0.402283	7.079143	3.61687	0.000324	0.000716	-0.80564
RAB6B	0.401754	6.289149	6.372611	3.74E-10	1.73E-09	12.31739

GP1BA	0.401619	7.228403	3.578541	0.000374	0.000819	-0.94013
SH2D4A	0.401467	5.834243	8.696586	3.38E-17	3.18E-16	28.20475
MIDN	0.401383	6.061067	5.691628	1.98E-08	7.49E-08	8.462137
PAPSS1	0.401007	9.876492	3.938979	9.16E-05	0.000219	0.378552
RIOK3	0.400721	8.735087	7.849855	1.97E-14	1.41E-13	21.95186
DBN1	0.400132	6.796686	5.411826	9.08E-08	3.18E-07	6.990919
E2F1	0.399876	6.263632	7.183064	2.06E-12	1.20E-11	17.3948
KIAA2013	0.399513	8.619407	8.00779	6.23E-15	4.69E-14	23.07946
B3GALNT1	0.3995	4.083196	5.892115	6.41E-09	2.54E-08	9.557101
BRE	0.398869	8.358921	7.122402	3.09E-12	1.77E-11	16.99693
CYB5R3	0.398775	10.44415	6.175733	1.23E-09	5.32E-09	11.16341
FCGR2A	0.398712	11.85453	5.30877	1.56E-07	5.32E-07	6.46594
FBXL19	0.398694	6.296958	5.268276	1.93E-07	6.51E-07	6.262169
PYGM	0.398563	6.480224	4.490366	8.55E-06	2.34E-05	2.62679
FAM107B	0.398501	10.70394	5.360031	1.19E-07	4.12E-07	6.725925
TMEM91	0.39798	8.12458	4.475289	9.16E-06	2.50E-05	2.56165
CIDEB	0.397853	8.771185	3.397913	0.000725	0.001523	-1.55544
RTN4	0.39758	12.0661	7.301147	9.26E-13	5.62E-12	18.1774
TNNT1	0.397011	6.404374	3.592475	0.000355	0.00078	-0.8914
TYROBP	0.396849	12.71386	3.946116	8.90E-05	0.000213	0.405878
RAB3A	0.396747	5.897325	6.955023	9.37E-12	5.13E-11	15.91387
GGA3	0.396521	7.530909	6.171326	1.26E-09	5.46E-09	11.13793
CYB5D2	0.396513	6.807527	5.054319	5.77E-07	1.84E-06	5.209152
SIRT5	0.396512	6.4003	5.875444	7.05E-09	2.79E-08	9.464767
NFKBIZ	0.396461	10.90384	4.964549	9.02E-07	2.81E-06	4.779254
SH3GLB2	0.395927	6.263043	5.86672	7.41E-09	2.93E-08	9.416543
CLEC2L	0.395651	5.81464	7.097812	3.65E-12	2.07E-11	16.83645
TUBA4B	0.39564	7.350192	6.732765	3.95E-11	2.03E-10	14.50956
OR7A5	0.395528	5.80756	7.06151	4.64E-12	2.61E-11	16.6004
SLC44A1	0.395176	6.685532	5.987104	3.71E-09	1.52E-08	10.08765
MTPN	0.394968	6.338178	4.148234	3.84E-05	9.68E-05	1.19914
CD80	0.39496	5.724517	8.372918	4.09E-16	3.45E-15	25.75491
FNDC3A	0.394812	6.19512	9.062624	1.85E-18	2.02E-17	31.06123
SUPT6H	0.394642	7.275782	3.86251	0.000125	0.000293	0.088727
CD46	0.394614	9.889149	4.038691	6.08E-05	0.000149	0.764561
ELK2AP	0.39422	6.427578	6.223062	9.24E-10	4.08E-09	11.43793
TIMM8B	0.394097	7.928176	5.940304	4.86E-09	1.95E-08	9.825316
RILPL1	0.393938	7.025329	4.868191	1.45E-06	4.40E-06	4.32569
ACPT	0.393656	6.952037	7.160635	2.40E-12	1.39E-11	17.24737
ATL3	0.393315	7.015945	6.438876	2.49E-10	1.18E-09	12.7129
MAFB	0.39309	10.25924	3.480448	0.000537	0.001149	-1.27808
ERGIC2	0.393089	7.880423	5.303084	1.61E-07	5.47E-07	6.437243
LEPREL1	0.393017	6.032801	4.616815	4.78E-06	1.36E-05	3.181182
OSTF1	0.392931	10.35415	5.840921	8.58E-09	3.36E-08	9.274293
GYPC	0.392771	8.672037	2.619789	0.009024	0.015735	-3.85321
DRD4	0.392723	5.058515	6.896871	1.37E-11	7.36E-11	15.5427
GLYCTK	0.392707	6.524715	6.166832	1.29E-09	5.60E-09	11.11198
PPP1R14B	0.392601	8.128977	3.483088	0.000532	0.001139	-1.2691
EPDR1	0.39238	5.478722	4.343727	1.65E-05	4.35E-05	2.001904
GSK3A	0.391867	8.056804	5.695803	1.94E-08	7.32E-08	8.484594
ZSWIM6	0.390933	9.940358	5.796159	1.10E-08	4.28E-08	9.028819
MESDC1	0.390161	8.748278	4.491855	8.49E-06	2.33E-05	2.633235
RNF167	0.390092	9.070634	6.221288	9.34E-10	4.12E-09	11.4276
SLC4A2	0.389782	7.101215	3.581479	0.00037	0.000811	-0.92987
AURKB	0.389663	5.141874	5.731121	1.59E-08	6.07E-08	8.675149
TSACC	0.389375	6.292688	6.840269	1.98E-11	1.05E-10	15.18396
LINC00937	0.389252	5.857668	6.490111	1.82E-10	8.71E-10	13.02113
PARP9	0.388845	9.105476	4.20071	3.07E-05	7.85E-05	1.411211

TGFBR1	0.38868	8.057871	6.178374	1.21E-09	5.25E-09	11.17868
CSMD2	0.388553	5.57787	7.301503	9.24E-13	5.61E-12	18.17978
ACPP	0.388194	6.364324	4.836978	1.68E-06	5.07E-06	4.18053
UBE2D1	0.387984	7.858685	5.904057	5.98E-09	2.38E-08	9.623392
MYLK2	0.387475	7.457631	7.496455	2.41E-13	1.55E-12	19.49507
TIMP4	0.387336	5.028484	4.849928	1.58E-06	4.78E-06	4.240651
ANKRD6	0.387178	5.591675	3.962315	8.33E-05	0.0002	0.468073
VPS39	0.386882	7.763414	3.45476	0.00059	0.001255	-1.36509
LOC10029	0.386877	4.893135	3.846819	0.000133	0.000311	0.029922
RNF208	0.386752	5.866783	5.713303	1.76E-08	6.67E-08	8.57888
TGM3	0.386727	6.224047	3.386068	0.000756	0.001584	-1.59472
AP1M2	0.386644	6.315771	8.084386	3.54E-15	2.72E-14	23.63283
MMP19	0.386476	6.395048	10.16959	1.70E-22	2.93E-21	40.22576
PREB	0.386285	7.921886	4.078893	5.15E-05	0.000127	0.922774
POLK	0.386212	7.1559	4.167359	3.54E-05	8.97E-05	1.276139
DNAJC4	0.385991	6.400655	4.769695	2.33E-06	6.87E-06	3.870549
LOC10050	0.385911	10.24821	5.20348	2.70E-07	8.94E-07	5.93906
LINC0017[illegible]	0.385772	6.18674	8.033762	5.15E-15	3.90E-14	23.26663
MGAT4B	0.385592	7.838021	3.822064	0.000146	0.000341	-0.06239
LOC10192	0.385489	5.734177	7.614969	1.05E-13	7.01E-13	20.30864
TMEM115	0.385488	6.398843	4.13404	4.08E-05	0.000102	1.142211
MYBL2	0.385186	6.815207	4.461873	9.73E-06	2.65E-05	2.503854
B3GAT3	0.384319	6.645645	4.408751	1.24E-05	3.31E-05	2.276603
BTN2A3P	0.384116	5.946836	8.188469	1.64E-15	1.30E-14	24.39152
NXT2	0.383807	7.198076	4.182725	3.32E-05	8.43E-05	1.338246
ATP7B	0.383753	5.524503	6.920601	1.17E-11	6.35E-11	15.69384
BTNL8	0.383374	8.119645	2.208395	0.0276	0.043561	-4.83337
COMMD4	0.383305	6.776344	6.371507	3.77E-10	1.74E-09	12.31083
TUBB	0.38294	9.452152	2.800847	0.005264	0.00957	-3.3701
PCSK9	0.382472	5.993977	5.41346	9.00E-08	3.16E-07	6.999315
WDTC1	0.382446	8.304692	6.082582	2.12E-09	8.98E-09	10.62849
PPBP	0.382439	12.16889	2.215153	0.02713	0.042872	-4.81859
BUB1	0.382182	4.647356	7.603987	1.14E-13	7.55E-13	20.23281
BGLT3	0.382056	5.905818	8.076442	3.76E-15	2.88E-14	23.57525
BCL2L2	0.381386	6.573159	5.238282	2.26E-07	7.55E-07	6.112149
USF1	0.381362	6.480064	3.371862	0.000795	0.001661	-1.64165
DCAF15	0.381278	6.629847	3.650214	0.000285	0.000635	-0.68753
ICAM1	0.381259	7.656155	4.315583	1.87E-05	4.90E-05	1.884195
NCKAP1L	0.381137	9.285455	5.612436	3.07E-08	1.13E-07	8.038973
LOC10192	0.380771	5.178612	8.012392	6.02E-15	4.54E-14	23.11259
CALU	0.380729	6.570097	7.427035	3.90E-13	2.46E-12	19.02342
PDLIM5	0.380608	5.919947	9.778814	4.93E-21	7.15E-20	36.90328
PSMD13	0.380425	7.756297	3.545521	0.000423	0.000919	-1.0549
GP6	0.380319	7.169379	2.359089	0.018644	0.030511	-4.49335
NKIRAS2	0.380276	7.748787	6.314316	5.33E-10	2.42E-09	11.9724
ZKSCAN7	0.380229	5.845731	4.691487	3.37E-06	9.76E-06	3.515291
GMFB	0.379954	8.802334	4.141822	3.95E-05	9.93E-05	1.1734
MCMBP	0.379829	7.421352	6.539858	1.33E-10	6.49E-10	13.32244
EIF4H	0.379451	10.26078	4.778166	2.23E-06	6.62E-06	3.909353
MOB3C	0.379401	7.342269	6.698158	4.92E-11	2.50E-10	14.29441
ZNF276	0.379136	7.012312	3.633143	0.000304	0.000676	-0.74813
GANAB	0.378953	8.673241	3.166559	0.001622	0.003219	-2.29872
MND1	0.378862	3.755401	4.698933	3.26E-06	9.44E-06	3.54888
VRK3	0.378798	7.581522	8.071436	3.90E-15	2.98E-14	23.53898
ABLIM3	0.378709	6.071123	3.502419	0.000496	0.001064	-1.20317
PLIN4	0.37856	6.430745	3.548907	0.000418	0.000908	-1.04318
ASB12	0.378533	6.961452	3.427022	0.000653	0.00138	-1.45835
STIM1	0.378527	7.430057	5.407815	9.27E-08	3.25E-07	6.970311

ADCY10P1	0.378512	6.2473	4.714599	3.02E-06	8.80E-06	3.619709
TET2	0.378411	8.677194	2.742326	0.006285	0.01127	-3.52969
TACSTD2	0.37837	5.449928	4.459803	9.82E-06	2.67E-05	2.49495
GPR182	0.378312	5.858146	7.799811	2.82E-14	2.00E-13	21.59835
STX16	0.378203	8.435999	5.226235	2.40E-07	8.01E-07	6.052115
CREG1	0.37819	10.32355	4.892491	1.29E-06	3.93E-06	4.4393
CALM1	0.378162	7.442845	3.065512	0.002272	0.004397	-2.60747
ATP5H	0.378118	10.41532	3.796939	0.000162	0.000374	-0.15549
RBX1	0.378097	9.523327	4.071207	5.31E-05	0.000131	0.892411
ARNTL	0.378093	8.805478	4.442672	1.06E-05	2.87E-05	2.421419
KLHL12	0.377843	7.711195	5.660099	2.36E-08	8.84E-08	8.293025
SPNS2	0.377807	6.838066	5.741651	1.50E-08	5.74E-08	8.732171
DLC1	0.37775	4.644562	5.77474	1.25E-08	4.80E-08	8.91195
EFHD2	0.37706	10.59984	4.052114	5.75E-05	0.000141	0.81722
CORT	0.377031	5.778249	6.609004	8.65E-11	4.28E-10	13.74454
CD99L2	0.376975	6.244041	6.230396	8.84E-10	3.91E-09	11.48063
PPFIA1	0.376881	7.267795	6.529129	1.43E-10	6.93E-10	13.25729
PRICKLE3	0.376865	5.685901	5.029582	6.53E-07	2.07E-06	5.089984
ACOT13	0.376843	7.116227	4.93976	1.02E-06	3.15E-06	4.661787
NHLH1	0.376747	6.89316	6.031859	2.86E-09	1.19E-08	10.34022
PLAU	0.376385	5.665077	5.339447	1.33E-07	4.56E-07	6.621253
CCDC186	0.376207	7.194547	3.069785	0.00224	0.004342	-2.59461
VEGFA	0.376197	6.83588	6.353849	4.20E-10	1.93E-09	12.20606
CYP19A1	0.376168	4.433519	7.350002	6.63E-13	4.08E-12	18.5043
CYP2B7P	0.376078	6.07777	9.588997	2.45E-20	3.31E-19	35.32318
TSG101	0.375528	10.02024	6.000847	3.42E-09	1.41E-08	10.16503
ZBP1	0.375443	6.653434	2.661149	0.007999	0.014065	-3.74563
C11orf68	0.375344	8.52839	5.506006	5.48E-08	1.97E-07	7.47866
ABCA4	0.375223	5.567869	7.847492	2.00E-14	1.43E-13	21.93513
NICN1	0.375186	7.285301	7.00577	6.71E-12	3.72E-11	16.23994
ZC3H18	0.374687	6.319308	5.532189	4.76E-08	1.72E-07	7.615607
R3HCC1L	0.374643	6.192224	7.647898	8.33E-14	5.60E-13	20.53654
DSCR3	0.374601	7.188276	6.766249	3.19E-11	1.65E-10	14.71864
UBXN4	0.374562	9.072326	6.581716	1.03E-10	5.04E-10	13.5775
TALDO1	0.373944	12.88397	5.878331	6.93E-09	2.74E-08	9.480739
PCBP1	0.373827	11.39188	4.582421	5.61E-06	1.58E-05	3.028969
TNS1	0.373401	5.614926	3.696809	0.000239	0.000538	-0.52074
PROSC	0.373323	7.022865	6.18977	1.13E-09	4.92E-09	11.24463
CHRNA2	0.373308	6.851998	6.415539	2.88E-10	1.35E-09	12.57321
PPP1R14A	0.373154	4.518819	3.746963	0.000197	0.000449	-0.33896
KBTBD6	0.373043	6.12256	2.008337	0.045062	0.067847	-5.25065
SPATA1	0.373012	4.306282	5.017938	6.92E-07	2.19E-06	5.034074
KHDC1	0.372995	5.885129	7.951623	9.39E-15	6.93E-14	22.67637
ATOH1	0.372511	5.304878	6.260622	7.38E-10	3.30E-09	11.65709
RCOR1	0.372289	8.019974	8.55876	9.85E-17	8.83E-16	27.1527
KIAA1522	0.372226	6.699268	7.422599	4.02E-13	2.53E-12	18.9934
BRMS1	0.372223	7.652405	3.707442	0.000229	0.000518	-0.48239
UNC93B1	0.372179	6.550202	3.74736	0.000196	0.000448	-0.33751
TSPO2	0.372156	6.080716	6.244144	8.14E-10	3.62E-09	11.5608
DR1	0.372085	7.763461	6.236348	8.53E-10	3.78E-09	11.51532
C20orf27	0.372059	6.78848	3.579775	0.000372	0.000816	-0.93582
PSMD6	0.371805	10.22789	5.933453	5.05E-09	2.03E-08	9.787064
RAB5B	0.371805	9.057186	3.623419	0.000316	0.000699	-0.78253
SPRED2	0.371225	4.770964	8.836279	1.13E-17	1.12E-16	29.28427
ERG	0.370717	4.519718	4.292127	2.07E-05	5.39E-05	1.786644
FCGR1A	0.369925	4.486118	3.962633	8.32E-05	0.0002	0.469295
CPNE3	0.369885	9.633995	3.146419	0.001736	0.003427	-2.36103
YBX2	0.369882	6.267107	8.119199	2.74E-15	2.13E-14	23.88573

ERV3-2	0.369499	8.179414	2.325954	0.020358	0.033029	-4.57
F7	0.36939	6.340627	8.005465	6.34E-15	4.76E-14	23.06273
CERS2	0.369294	9.599132	5.081212	5.04E-07	1.62E-06	5.339317
TWIST2	0.368949	4.869725	3.109076	0.001967	0.003848	-2.47555
DPH3	0.368842	8.325537	4.40959	1.23E-05	3.30E-05	2.280172
GRIPAP1	0.368621	7.648448	3.824602	0.000145	0.000338	-0.05295
IDH1	0.368591	7.533061	6.653826	6.52E-11	3.27E-10	14.02019
PDAP1	0.368254	5.968145	6.609044	8.65E-11	4.28E-10	13.74478
CCRL2	0.368203	6.585703	4.046627	5.89E-05	0.000145	0.795675
PCBD1	0.367928	5.749799	4.805192	1.96E-06	5.86E-06	4.033587
FBXO9	0.367889	8.926258	4.300191	2.00E-05	5.22E-05	1.820125
ZFP36L1	0.367726	7.487276	5.517724	5.14E-08	1.85E-07	7.539878
TAF12	0.367674	8.002213	3.875216	0.000118	0.00028	0.136509
RCVRN	0.367567	5.39909	6.342917	4.48E-10	2.05E-09	12.14132
SGTB	0.367524	7.099646	4.295642	2.04E-05	5.31E-05	1.80123
ADAM10	0.367469	9.11316	4.655849	3.99E-06	1.14E-05	3.355214
ITGA9	0.367375	5.307944	4.39165	1.33E-05	3.56E-05	2.203988
HNRNPF	0.367296	8.981902	4.479411	8.99E-06	2.46E-05	2.57944
LRPAP1	0.366869	7.304204	4.72401	2.89E-06	8.45E-06	3.662365
POLE	0.366863	6.697029	4.479226	8.99E-06	2.46E-05	2.578641
ACP6	0.366697	6.575533	5.255103	2.07E-07	6.94E-07	6.196184
FBXL5	0.366671	11.09188	5.035388	6.34E-07	2.01E-06	5.117908
CASP9	0.366638	6.723232	8.686474	3.66E-17	3.43E-16	28.12712
LY6G5C	0.366529	5.947915	4.246122	2.53E-05	6.51E-05	1.596759
PHC2	0.366189	8.433035	7.569122	1.45E-13	9.52E-13	19.99266
APP	0.365849	8.123438	5.503007	5.57E-08	2.00E-07	7.463012
CAPZA2	0.365781	11.08901	5.386706	1.04E-07	3.61E-07	6.862112
ZNF254	0.365522	4.983764	5.403148	9.51E-08	3.32E-07	6.94636
FLT3	0.365419	6.499023	2.851711	0.0045	0.008279	-3.22873
SSH2	0.36517	10.93477	4.868737	1.44E-06	4.39E-06	4.328237
ZFYVE21	0.365161	6.468033	5.40368	9.48E-08	3.32E-07	6.949088
GPKOW	0.365131	7.816868	4.695152	3.31E-06	9.60E-06	3.531818
ASCC2	0.365065	6.887598	3.107224	0.001979	0.003871	-2.48119
CASP4	0.364816	9.150712	3.558446	0.000403	0.000879	-1.0101
TTC9C	0.364671	7.526684	5.05201	5.83E-07	1.86E-06	5.198006
ZNF155	0.364261	4.418285	5.690641	1.99E-08	7.52E-08	8.45683
CDK16	0.363966	6.884779	5.575926	3.75E-08	1.37E-07	7.845676
CHMP1B	0.363853	9.25627	4.877584	1.38E-06	4.21E-06	4.369544
PTGES	0.3637	6.534553	5.909611	5.80E-09	2.31E-08	9.654257
LOC10050	0.363696	6.122095	6.665791	6.05E-11	3.05E-10	14.09405
UTY	0.363342	6.132057	2.979217	0.003008	0.005702	-2.86347
ZNF341	0.363212	5.205605	5.683513	2.07E-08	7.81E-08	8.418527
VPS35	0.363007	9.61324	4.923207	1.11E-06	3.41E-06	4.583653
VKORC1	0.362947	7.842108	3.315298	0.000972	0.001999	-1.82665
IMPDH1	0.36283	9.578858	6.217221	9.57E-10	4.21E-09	11.40395
HLA-B	0.362819	13.38332	2.667376	0.007854	0.013839	-3.72928
NLK	0.362599	6.938232	4.137961	4.01E-05	0.000101	1.157919
FKBP15	0.362584	7.027697	6.525516	1.46E-10	7.08E-10	13.23537
PRUNE	0.362492	6.778763	5.683831	2.07E-08	7.80E-08	8.420236
MROH6	0.362355	7.873684	3.655362	0.00028	0.000624	-0.6692
C9orf69	0.362311	7.963952	3.931246	9.45E-05	0.000226	0.348996
PYCR1	0.362296	6.751309	6.832683	2.08E-11	1.10E-10	15.13607
NOTCH1	0.362044	7.765723	3.960267	8.40E-05	0.000202	0.460194
RASA2	0.36183	9.407624	4.689311	3.41E-06	9.85E-06	3.505482
NR6A1	0.361724	6.10074	9.786296	4.63E-21	6.76E-20	36.96602
HIST1H1C	0.361704	8.515565	2.911325	0.003735	0.006957	-3.05989
ZKSCAN2	0.36156	6.398323	7.104645	3.48E-12	1.98E-11	16.881
CUTC	0.361237	8.79994	3.974215	7.93E-05	0.000192	0.513915

RIT1	0.361062	9.10437	3.661268	0.000273	0.000611	-0.64814
HAT1	0.361039	8.796725	4.349371	1.61E-05	4.25E-05	2.025596
ASAP1-IT1	0.361018	7.473264	2.764859	0.005872	0.010578	-3.46863
TIMM17B	0.360951	7.757543	3.90722	0.000104	0.000247	0.257527
FANCD2O	0.36068	5.814244	7.129509	2.95E-12	1.69E-11	17.04339
SHISA7	0.360679	6.258074	6.357343	4.11E-10	1.89E-09	12.22677
CMTM1	0.360657	6.135173	4.343496	1.65E-05	4.35E-05	2.000936
SH3GL1	0.360559	8.275344	6.138249	1.53E-09	6.57E-09	10.9473
DOK4	0.360402	5.613319	5.305964	1.59E-07	5.40E-07	6.451772
HTRA2	0.360164	7.95238	4.230996	2.70E-05	6.92E-05	1.534747
RGL2	0.359719	9.694807	4.316223	1.86E-05	4.88E-05	1.886865
CEP97	0.359531	5.662777	3.950046	8.76E-05	0.00021	0.420942
CLK3	0.359287	6.929702	5.647701	2.53E-08	9.42E-08	8.226755
PP13	0.359094	6.685323	6.281159	6.52E-10	2.93E-09	11.77742
ADCK4	0.358987	6.430394	6.725161	4.14E-11	2.12E-10	14.46221
PTPRE	0.358969	8.759256	8.709163	3.06E-17	2.89E-16	28.3014
S100A2	0.358931	5.542938	7.70538	5.54E-14	3.80E-13	20.93631
CACUL1	0.358661	6.841164	7.998011	6.69E-15	5.02E-14	23.00912
WBP4	0.358571	6.733641	4.416163	1.19E-05	3.21E-05	2.308156
LOC10272	0.358372	5.734051	7.401165	4.66E-13	2.91E-12	18.84858
SNF8	0.358369	8.59007	4.787149	2.14E-06	6.36E-06	3.950576
PLEKHG6	0.358303	5.640957	6.36843	3.84E-10	1.77E-09	12.29256
POLQ	0.358204	5.759521	5.602548	3.24E-08	1.19E-07	7.986509
DCAF12	0.35786	9.221329	4.608164	4.98E-06	1.41E-05	3.142798
NDUFAF7	0.357793	6.025972	4.093819	4.83E-05	0.00012	0.981891
CTSH	0.357616	10.5154	3.343858	0.000879	0.001819	-1.73362
3-Mar	0.356992	6.202624	4.976021	8.52E-07	2.66E-06	4.833798
CMIP	0.356941	7.996675	4.65449	4.01E-06	1.15E-05	3.349129
PRKAR2A	0.356218	8.825696	5.656047	2.41E-08	9.02E-08	8.271352
C9orf89	0.355953	8.162845	3.007707	0.002744	0.005238	-2.77974
HINT3	0.3555	6.572476	3.369104	0.000803	0.001675	-1.65074
RRP12	0.355315	6.723741	6.242628	8.22E-10	3.65E-09	11.55195
ACTR2	0.355229	9.187023	5.628606	2.81E-08	1.04E-07	8.124944
HMGB3P1	0.355146	6.490351	6.237914	8.45E-10	3.75E-09	11.52445
IKBKG	0.355125	8.814388	5.542559	4.50E-08	1.63E-07	7.670008
LOC15840	0.355106	6.03789	2.800069	0.005276	0.00959	-3.37225
GOLPH3	0.354824	10.36547	5.496216	5.78E-08	2.07E-07	7.427608
LRRC32	0.354554	6.096383	6.099186	1.93E-09	8.17E-09	10.72331
CHMP2B	0.354428	8.960772	4.62829	4.54E-06	1.29E-05	3.2322
SLC38A2	0.354335	11.30085	5.535208	4.68E-08	1.69E-07	7.631439
GLRX2	0.354277	7.693862	5.642208	2.61E-08	9.70E-08	8.197433
RNF19B	0.354121	9.720368	2.874994	0.004186	0.007734	-3.16319
HCRT	0.354086	5.133625	7.556889	1.58E-13	1.03E-12	19.90862
ELL	0.353273	7.178097	5.503144	5.56E-08	2.00E-07	7.463726
KEAP1	0.353206	7.211456	5.306752	1.58E-07	5.38E-07	6.45575
C7orf60	0.353108	8.442066	3.518328	0.000468	0.001008	-1.14864
DVL3	0.352726	7.221745	5.129684	3.94E-07	1.28E-06	5.575518
IL1B	0.352674	9.763768	2.139396	0.032813	0.050915	-4.98169
MPG	0.35251	7.107703	4.398332	1.29E-05	3.46E-05	2.23233
GNA13	0.35246	9.103843	5.993033	3.58E-09	1.47E-08	10.12101
BTBD3	0.352381	6.022338	2.725782	0.006605	0.011791	-3.57421
KAT6A	0.35228	9.183477	4.30157	1.98E-05	5.19E-05	1.825854
CAPNS2	0.352231	6.114117	5.971905	4.05E-09	1.65E-08	10.00225
LOC10192	0.351836	6.247427	4.551681	6.46E-06	1.80E-05	2.893823
CLDN17	0.351816	6.827493	7.876101	1.63E-14	1.18E-13	22.13799
OR7E156P	0.351761	6.124649	6.284326	6.39E-10	2.88E-09	11.796
TMC4	0.351594	6.659065	4.705606	3.16E-06	9.17E-06	3.579024
FYB	0.351542	10.06533	4.478543	9.02E-06	2.46E-05	2.575691

ZNRF4	0.351216	6.271682	7.779883	3.25E-14	2.29E-13	21.4581
LRRC10B	0.351165	5.574564	5.318323	1.49E-07	5.07E-07	6.514219
OGFR	0.351148	7.469306	3.680994	0.000254	0.00057	-0.57758
SKIL	0.351016	5.944382	5.535645	4.67E-08	1.69E-07	7.633728
CXorf23	0.350623	6.041676	2.53511	0.011498	0.019621	-4.06833
E2F7	0.350597	4.936479	6.383724	3.50E-10	1.62E-09	12.38348
C6orf25	0.350385	5.133265	3.380581	0.000771	0.001613	-1.61287
TGM5	0.350228	6.71688	6.467048	2.10E-10	9.97E-10	12.88212
SLC17A5	0.350104	7.702579	5.046122	6.01E-07	1.91E-06	5.169606
LASP1	0.349959	11.31043	5.715787	1.73E-08	6.59E-08	8.592286
PILRA	0.349798	9.519823	2.311844	0.021129	0.034146	-4.60232
CNEP1R1	0.34971	9.536183	4.448249	1.03E-05	2.80E-05	2.44533
JAG1	0.349186	5.679146	6.160728	1.34E-09	5.78E-09	11.07676
PPP1R11	0.349166	10.4518	5.669193	2.25E-08	8.43E-08	8.341711
PAM	0.348972	8.174009	2.798981	0.005294	0.009619	-3.37524
AMFR	0.34883	7.219439	3.066477	0.002265	0.004384	-2.60457
BFAR	0.348363	8.140303	4.738416	2.70E-06	7.92E-06	3.727813
MLLT1	0.348215	6.915862	4.600315	5.16E-06	1.46E-05	3.108028
CACNA1F	0.348024	6.136793	5.547654	4.37E-08	1.59E-07	7.696773
IGFBP2	0.347933	5.0723	3.490595	0.000518	0.00111	-1.24354
ARAF	0.347812	6.576182	5.034915	6.36E-07	2.02E-06	5.115628
GPCPD1	0.347716	9.61545	4.058859	5.59E-05	0.000138	0.843745
ZER1	0.347421	6.455206	4.486505	8.70E-06	2.38E-05	2.61009
KRT1	0.347287	5.503905	3.001054	0.002804	0.005342	-2.79936
C1orf105	0.347199	5.682866	5.134917	3.84E-07	1.25E-06	5.601143
KIFC1	0.347076	7.263507	4.380227	1.40E-05	3.73E-05	2.155632
TSC22D3	0.346728	8.533306	3.37307	0.000792	0.001654	-1.63767
MFAP3	0.346647	6.759338	7.190457	1.96E-12	1.14E-11	17.44349
SUPT4H1	0.346563	9.264036	5.237639	2.26E-07	7.57E-07	6.108942
UBE2A	0.346558	9.310231	6.116161	1.74E-09	7.44E-09	10.82049
FBN1	0.346529	4.555704	5.211552	2.59E-07	8.60E-07	5.979112
RNF39	0.346318	5.912137	7.524553	1.98E-13	1.29E-12	19.687
TK2	0.346163	6.768794	6.434321	2.56E-10	1.21E-09	12.6856
STRADA	0.345928	8.881991	6.201403	1.05E-09	4.61E-09	11.31207
PYY2	0.345386	6.517373	3.807431	0.000155	0.00036	-0.11668
MSL3	0.34506	8.111538	4.24916	2.49E-05	6.43E-05	1.609239
SYCP2	0.344658	4.181448	4.932788	1.06E-06	3.26E-06	4.62885
IER2	0.344559	11.92073	2.915805	0.003682	0.006867	-3.04706
NDRG3	0.344511	7.494418	5.283994	1.78E-07	6.02E-07	6.341095
AXIN1	0.344463	7.782666	5.27005	1.91E-07	6.46E-07	6.271065
ADRM1	0.344323	8.242907	3.666258	0.000268	0.000601	-0.63033
HSPB6	0.344168	5.541345	5.657639	2.39E-08	8.95E-08	8.279865
PDE4D	0.344152	5.449778	3.804703	0.000157	0.000363	-0.12678
PEX11G	0.344	6.606607	6.021473	3.04E-09	1.26E-08	10.28146
GIT1	0.343628	8.08692	4.974421	8.59E-07	2.68E-06	4.826181
TRAPPC6B	0.343275	7.721078	4.007773	6.91E-05	0.000168	0.643891
RALY-AS1	0.343075	5.48642	6.248508	7.93E-10	3.53E-09	11.58628
GDAP2	0.343056	6.518775	4.353118	1.58E-05	4.18E-05	2.04134
ETF1	0.342995	8.857254	4.941496	1.01E-06	3.13E-06	4.669995
SPAG5	0.34297	5.801284	3.617555	0.000323	0.000714	-0.80323
LOC10192	0.3426	7.101417	2.930095	0.003519	0.006589	-3.00603
CBX4	0.342584	8.021331	4.119578	4.34E-05	0.000109	1.084398
SMURF1	0.342435	5.636451	7.378156	5.46E-13	3.38E-12	18.6935
RTFDC1	0.342244	8.109911	6.332831	4.77E-10	2.17E-09	12.08168
PAMR1	0.342121	6.167709	5.316598	1.50E-07	5.12E-07	6.505498
CORO7	0.342049	7.220835	2.787954	0.005475	0.009923	-3.40554
HOTS	0.342045	4.628348	8.193491	1.58E-15	1.26E-14	24.42833
EIF2AK1	0.341704	8.289743	4.112648	4.47E-05	0.000112	1.056761

NRD1	0.341615	9.112402	5.344963	1.29E-07	4.44E-07	6.649267
DLK2	0.34148	6.689168	6.653285	6.54E-11	3.28E-10	14.01686
LOC10272	0.341114	5.087963	5.956646	4.42E-09	1.79E-08	9.916714
RSBN1	0.34109	8.719871	4.415286	1.20E-05	3.22E-05	2.304421
CD99P1	0.340901	6.287524	4.988191	8.02E-07	2.52E-06	4.891786
IFNGR2	0.340731	11.89292	5.77605	1.24E-08	4.77E-08	8.919085
NOL3	0.340714	5.660039	7.113088	3.29E-12	1.88E-11	16.93609
SMOX	0.340539	6.746279	4.664945	3.82E-06	1.10E-05	3.395961
P2RY1	0.340496	4.381652	4.329628	1.75E-05	4.62E-05	1.942849
TEX2	0.340356	7.087733	3.95944	8.43E-05	0.000203	0.457014
LSM6	0.340181	9.078395	4.195708	3.14E-05	8.01E-05	1.39089
CEP135	0.340033	6.136245	3.579288	0.000373	0.000817	-0.93753
DHTKD1	0.339985	8.017031	4.961144	9.17E-07	2.85E-06	4.763083
KCNIP2-A:	0.339627	7.027628	4.32917	1.76E-05	4.62E-05	1.940932
S100A16	0.339617	7.027908	6.651079	6.63E-11	3.32E-10	14.00325
DGKD	0.339463	9.471308	4.548717	6.55E-06	1.83E-05	2.880838
AKR1C1	0.339292	6.377438	4.484297	8.79E-06	2.40E-05	2.600545
POLDIP2	0.339241	7.720543	5.474564	6.49E-08	2.31E-07	7.314984
GBF1	0.339087	7.041399	3.283612	0.001085	0.002216	-1.92897
IL18	0.338832	5.71092	2.88393	0.00407	0.007533	-3.1379
AP2A2	0.33882	7.235895	4.499488	8.20E-06	2.25E-05	2.666306
TNF	0.338083	7.376071	3.199218	0.001452	0.002906	-2.19686
ZDHHC18	0.337724	7.133057	4.196549	3.13E-05	7.98E-05	1.394302
CCNL1	0.337615	8.136497	3.751116	0.000193	0.000442	-0.3238
MAP3K19	0.337196	7.066291	5.944016	4.76E-09	1.91E-08	9.846054
SOX21	0.337143	5.382955	4.690476	3.39E-06	9.80E-06	3.510734
PKP2	0.337134	4.705157	5.587864	3.51E-08	1.29E-07	7.908752
ZBTB20-A	0.337122	5.502574	6.42546	2.71E-10	1.27E-09	12.63254
SERINC1	0.337029	11.30464	5.26536	1.96E-07	6.61E-07	6.247548
JOSD2	0.337	7.232188	3.381684	0.000768	0.001607	-1.60922
ARFGAP3	0.336811	9.503753	4.30537	1.95E-05	5.11E-05	1.841659
POLR2C	0.336691	7.878132	6.460772	2.18E-10	1.04E-09	12.84437
KIAA1109	0.336671	6.482144	7.224485	1.56E-12	9.21E-12	17.66811
HBE1	0.33657	5.603906	4.314223	1.88E-05	4.92E-05	1.878525
KCNMA1	0.336421	4.812243	4.661796	3.88E-06	1.11E-05	3.381845
TBC1D10C	0.336023	9.537845	2.099811	0.036169	0.055613	-5.06469
LOC40094	0.335929	5.60886	7.451276	3.30E-13	2.09E-12	19.1877
THOC5	0.335884	6.4331	6.016832	3.12E-09	1.29E-08	10.25523
OR2H1	0.335815	5.426382	8.065712	4.07E-15	3.11E-14	23.49753
ABCC2	0.335801	5.602795	5.411052	9.12E-08	3.20E-07	6.986941
PKMYT1	0.335345	6.93014	5.07345	5.24E-07	1.68E-06	5.301685
EIF2AK2	0.335134	8.148823	3.715626	0.000222	0.000503	-0.45281
MYO15B	0.335041	7.482554	3.081146	0.002158	0.004195	-2.56034
KCNE1L	0.334871	5.078914	6.143169	1.49E-09	6.39E-09	10.97559
OPN4	0.334865	5.698901	8.247227	1.05E-15	8.53E-15	24.82323
ELFN1-AS	0.334412	6.108662	6.124255	1.66E-09	7.10E-09	10.86691
ARHGEF2	0.334184	9.110622	4.483549	8.82E-06	2.41E-05	2.597311
SIGLEC7	0.334175	7.895436	4.059664	5.58E-05	0.000137	0.846914
CREM	0.334112	5.84858	3.960065	8.41E-05	0.000202	0.459418
SLC8A2	0.334006	5.67547	5.749078	1.44E-08	5.51E-08	8.772441
RHAG	0.33398	5.061219	3.608643	0.000334	0.000737	-0.83462
SF3B6	0.333832	10.50582	6.720786	4.26E-11	2.18E-10	14.43498
SSNA1	0.333514	7.123132	3.534792	0.00044	0.000954	-1.09197
SUMF1	0.333311	8.96911	4.109317	4.53E-05	0.000113	1.043492
LOC28307	0.333262	3.81939	5.944154	4.75E-09	1.91E-08	9.846828
ATG13	0.333107	8.018533	4.783365	2.18E-06	6.46E-06	3.933201
NPLOC4	0.332943	8.721441	4.009799	6.86E-05	0.000167	0.651773
SLC39A1	0.332561	6.821588	4.837914	1.68E-06	5.05E-06	4.18487

MAP3K2	0.332553	8.328425	4.197547	3.11E-05	7.95E-05	1.398356
ARMC12	0.332402	5.483252	6.15877	1.36E-09	5.85E-09	11.06546
NCSTN	0.332078	7.28116	5.706882	1.82E-08	6.91E-08	8.544254
TRABD	0.331924	7.533045	3.917291	0.0001	0.000238	0.295802
LOC10193	0.331837	4.680166	3.180618	0.001547	0.003086	-2.25499
GRAMD4	0.331757	7.800841	4.712175	3.06E-06	8.90E-06	3.608737
NDUFB7	0.331719	5.651493	3.909603	0.000103	0.000245	0.266577
TMCC2	0.33138	6.553096	2.797906	0.005311	0.009645	-3.3782
RPUSD1	0.331194	6.747496	6.243095	8.19E-10	3.64E-09	11.55468
ABHD8	0.330846	5.697681	3.656124	0.000279	0.000622	-0.66648
HM13	0.330702	7.145202	4.553174	6.42E-06	1.79E-05	2.900368
MXRA7	0.330353	6.515317	3.298279	0.001031	0.002112	-1.88173
MALL	0.330334	5.440566	5.484708	6.15E-08	2.20E-07	7.367695
HTR1E	0.330176	5.213408	7.013149	6.39E-12	3.55E-11	16.28752
ANKRD35	0.329704	5.69884	4.64621	4.17E-06	1.19E-05	3.312111
CMAHP	0.329387	6.176119	3.417921	0.000674	0.001423	-1.48879
LOC10159	0.32938	5.542788	5.485688	6.11E-08	2.19E-07	7.372794
ANXA6	0.329353	7.457824	4.190547	3.21E-05	8.17E-05	1.369944
CNN1	0.329129	5.438175	6.156966	1.37E-09	5.91E-09	11.05506
GNB1	0.329051	10.64124	5.551197	4.29E-08	1.56E-07	7.715394
MON1B	0.328994	8.423168	5.692756	1.97E-08	7.44E-08	8.468202
TULP2	0.328837	5.660984	4.891604	1.29E-06	3.94E-06	4.435145
HOXC10	0.328604	4.73621	8.185375	1.68E-15	1.33E-14	24.36886
ANPEP	0.328548	6.484027	6.340972	4.54E-10	2.08E-09	12.12981
SNX13	0.328437	6.994487	4.947468	9.82E-07	3.04E-06	4.698255
CYP8B1	0.328433	4.889682	7.80441	2.73E-14	1.94E-13	21.63077
SURF4	0.328197	8.446596	4.105551	4.60E-05	0.000115	1.028501
LINC0066ℇ	0.328136	6.678278	6.223366	9.22E-10	4.07E-09	11.4397
RNF149	0.328089	11.99587	5.129869	3.94E-07	1.28E-06	5.576426
TNFSF10	0.327883	11.16852	4.540505	6.80E-06	1.89E-05	2.8449
DNAJB13	0.327645	5.952421	6.597616	9.29E-11	4.58E-10	13.67476
MAZ	0.327151	7.040377	2.606803	0.00937	0.016274	-3.88665
DHRS12	0.327034	6.428997	4.501526	8.13E-06	2.23E-05	2.675144
TEX28	0.326954	5.012273	6.976437	8.14E-12	4.48E-11	16.05122
FOXC1	0.326946	6.138266	4.658006	3.95E-06	1.13E-05	3.364871
BAP1	0.326876	8.145576	3.368671	0.000804	0.001678	-1.65217
PRB4	0.326645	6.21065	5.572432	3.82E-08	1.40E-07	7.827235
RRAGC	0.326574	8.191324	8.260472	9.55E-16	7.74E-15	24.92088
ZEB2	0.32657	7.422682	4.906724	1.20E-06	3.68E-06	4.506086
STX1B	0.32651	6.511118	6.219955	9.41E-10	4.15E-09	11.41985
PGRMC1	0.326245	8.374732	3.128753	0.001842	0.003617	-2.41537
STK11IP	0.325982	7.017119	3.660925	0.000274	0.000612	-0.64936
LYPD8	0.325932	6.066705	7.0491	5.04E-12	2.83E-11	16.51994
MS4A5	0.325802	5.531846	6.260318	7.39E-10	3.30E-09	11.65532
AQP5	0.325794	5.841954	4.805967	1.96E-06	5.84E-06	4.03716
IGFLR1	0.325577	8.882713	3.367602	0.000808	0.001683	-1.65569
PRPF18	0.325467	6.87028	6.500933	1.70E-10	8.17E-10	13.08651
KBTBD4	0.325302	6.196327	6.278447	6.63E-10	2.98E-09	11.76151
DISC1	0.325248	5.583576	4.466234	9.54E-06	2.60E-05	2.522622
CHMP2A	0.325226	10.58894	4.203529	3.03E-05	7.76E-05	1.422676
CC2D2B	0.325185	6.87791	3.449671	0.000601	0.001277	-1.38225
STX1A	0.325159	6.97083	5.979224	3.88E-09	1.58E-08	10.04335
INCENP	0.324958	5.817912	6.282021	6.48E-10	2.92E-09	11.78247
ARHGAP2	0.324897	6.684922	4.130987	4.13E-05	0.000104	1.129991
SULT1A2	0.324891	9.023708	3.048442	0.002403	0.004628	-2.65867
SEC23B	0.324841	8.6126	4.206699	2.99E-05	7.65E-05	1.435574
LOC10013	0.324613	5.378834	2.864521	0.004325	0.007978	-3.19274
TRMT1L	0.324602	7.244946	3.724544	0.000214	0.000487	-0.4205

RPS6KA3	0.324581	9.422388	4.586728	5.50E-06	1.55E-05	3.047972
PTCHD2	0.3243	6.52111	7.169567	2.26E-12	1.31E-11	17.30603
HIST1H2B.	0.32429	4.812657	3.800298	0.00016	0.000369	-0.14308
LSM2	0.324036	7.019398	2.535509	0.011485	0.019602	-4.06733
GDPD2	0.323804	6.187545	6.083818	2.11E-09	8.92E-09	10.63554
FBLIM1	0.32349	5.682113	6.253646	7.69E-10	3.43E-09	11.6163
FAM200B	0.32347	8.412655	4.200025	3.08E-05	7.87E-05	1.408427
CDX1	0.3234	7.14536	5.822561	9.52E-09	3.71E-08	9.173405
ARHGAP9	0.323397	10.95549	3.058112	0.002328	0.004494	-2.6297
DDX59	0.323376	8.056893	5.35375	1.23E-07	4.25E-07	6.693947
ABR	0.323372	7.735066	3.546603	0.000421	0.000915	-1.05115
TAB2	0.323043	9.461533	4.772212	2.30E-06	6.80E-06	3.882074
VCP	0.323012	8.893264	4.509295	7.85E-06	2.16E-05	2.708871
GPER1	0.322887	5.474859	4.549296	6.53E-06	1.82E-05	2.883375
SERAC1	0.322741	6.357896	2.956615	0.003235	0.006093	-2.92935
PTEN	0.322647	9.755518	4.194767	3.15E-05	8.04E-05	1.387065
KCTD5	0.322483	6.553016	5.258556	2.03E-07	6.83E-07	6.213466
KDM4B	0.321955	7.675387	5.085302	4.93E-07	1.59E-06	5.359168
CREB3	0.321526	7.119009	4.474798	9.18E-06	2.50E-05	2.559531
ERVH-4	0.321506	5.212428	5.388524	1.03E-07	3.57E-07	6.871416
ITGAX	0.32149	9.96517	2.470787	0.013763	0.023146	-4.22712
FMN1	0.321316	5.597421	5.274172	1.87E-07	6.33E-07	6.291748
ZNF770	0.321244	6.579391	3.52209	0.000461	0.000996	-1.13571
PNKP	0.321188	6.761163	2.7805	0.0056	0.01013	-3.42596
LINC0020?	0.321094	6.412904	5.41239	9.05E-08	3.18E-07	6.993815
ROCK1	0.320864	8.464149	5.834365	8.90E-09	3.49E-08	9.238234
STRA6	0.320828	6.342892	6.853713	1.81E-11	9.64E-11	15.26894
NAV2-AS	0.320669	6.890181	6.651993	6.60E-11	3.31E-10	14.00889
FNDC4	0.32062	7.205109	5.300883	1.63E-07	5.53E-07	6.426142
ACTC1	0.320604	6.943567	5.515915	5.19E-08	1.87E-07	7.530419
LTBP2	0.320573	6.428112	6.424405	2.73E-10	1.28E-09	12.62623
FLYWCH2	0.320546	6.601268	4.927637	1.08E-06	3.33E-06	4.604537
ZFPL1	0.320431	6.009101	4.05966	5.58E-05	0.000137	0.846896
SDCBP	0.320403	13.39762	5.977812	3.91E-09	1.59E-08	10.03542
MFSD11	0.320357	7.356234	7.099815	3.60E-12	2.05E-11	16.84951
DYNLRB1	0.320271	9.667457	3.800776	0.000159	0.000368	-0.14131
SLC25A20	0.320253	8.521548	4.093469	4.84E-05	0.00012	0.980505
ARHGDIB	0.320061	13.15782	5.041909	6.14E-07	1.95E-06	5.149302
SPSB4	0.319968	5.959887	5.182413	3.01E-07	9.91E-07	5.834797
HPSE2	0.319921	5.061505	6.349883	4.30E-10	1.97E-09	12.18256
PLEK2	0.319769	7.074081	4.78528	2.16E-06	6.41E-06	3.941993
DPY30	0.319745	8.720714	4.841606	1.65E-06	4.96E-06	4.201996
CD2BP2	0.31948	8.32531	5.513849	5.25E-08	1.89E-07	7.519621
CSTA	0.319389	11.90637	4.830113	1.74E-06	5.23E-06	4.148718
REM1	0.319328	6.101004	4.905679	1.21E-06	3.70E-06	4.501175
CHAD	0.319301	5.875233	6.799637	2.57E-11	1.34E-10	14.928
RAI2	0.319099	4.683379	3.56738	0.00039	0.000852	-0.97904
FAS	0.319051	9.147878	2.840873	0.004654	0.008544	-3.25906
DEFB118	0.318965	6.453263	6.094312	1.98E-09	8.40E-09	10.69545
ABT1	0.318933	7.707508	3.828608	0.000143	0.000333	-0.03804
GBA2	0.318871	7.595381	3.804795	0.000157	0.000363	-0.12644
DAPK2	0.31881	7.447872	4.104757	4.62E-05	0.000115	1.025345
C19orf83	0.318786	6.363354	4.656078	3.98E-06	1.14E-05	3.35624
HIST1H1B	0.318761	4.119452	4.655654	3.99E-06	1.14E-05	3.354342
NDEL1	0.318736	7.577544	7.06042	4.68E-12	2.63E-11	16.59333
PRB3	0.318497	6.205793	6.595072	9.44E-11	4.66E-10	13.65918
STEAP3	0.318344	6.759405	3.878303	0.000117	0.000276	0.148141
LINC0059?	0.318332	4.84058	2.513912	0.012205	0.020731	-4.1211

ANGPT4	0.318089	6.614407	7.44297	3.49E-13	2.20E-12	19.13136
USP38	0.318028	7.594744	3.7953	0.000163	0.000376	-0.16155
DIP2B	0.317946	7.987312	4.961961	9.14E-07	2.84E-06	4.766963
ALDH4A1	0.317703	6.251734	5.98885	3.67E-09	1.50E-08	10.09747
EIF4G1	0.317528	7.702234	3.282056	0.001091	0.002227	-1.93397
SLC38A10	0.317477	6.577076	5.47409	6.51E-08	2.32E-07	7.312522
OCRL	0.317447	5.941998	5.987312	3.70E-09	1.51E-08	10.08882
MLX	0.317423	8.589104	6.188703	1.13E-09	4.95E-09	11.23845
CCM2	0.317318	8.490156	2.797671	0.005315	0.009651	-3.37885
LOC10192	0.317288	7.259519	5.825041	9.38E-09	3.66E-08	9.187013
ZSWIM8	0.317182	7.154541	4.084025	5.04E-05	0.000125	0.943077
ABCB6	0.316985	5.699064	5.296087	1.67E-07	5.67E-07	6.401964
SNAP29	0.316915	7.261095	6.68251	5.44E-11	2.75E-10	14.19744
CYTH1	0.316896	8.394248	3.193268	0.001482	0.002963	-2.21549
KCTD11	0.316733	6.179469	5.594862	3.38E-08	1.24E-07	7.945787
ARG2	0.316642	5.353347	3.591626	0.000356	0.000783	-0.89438
NFKB2	0.316576	5.911797	2.958288	0.003217	0.006066	-2.92449
MTMR1	0.316516	7.519535	3.571399	0.000384	0.00084	-0.96504
GIT2	0.316424	9.342696	6.052358	2.54E-09	1.06E-08	10.45647
HIF1AN	0.31638	7.527226	5.259443	2.02E-07	6.80E-07	6.21791
CCKBR	0.316136	5.241129	7.69643	5.90E-14	4.04E-13	20.87391
ATF6B	0.315899	6.294237	5.680422	2.11E-08	7.94E-08	8.401935
RASSF2	0.315781	11.85311	4.536292	6.94E-06	1.93E-05	2.826489
CTDSPL	0.315519	5.71822	4.346362	1.63E-05	4.30E-05	2.012961
LINC01016	0.315464	6.313084	5.803925	1.06E-08	4.11E-08	9.071284
FAH	0.315423	5.28401	5.916091	5.58E-09	2.23E-08	9.690306
PSMD4	0.315158	8.781926	5.00375	7.43E-07	2.34E-06	4.966111
GALR3	0.315053	7.157596	5.992964	3.58E-09	1.47E-08	10.12063
LRRC45	0.31471	5.83706	3.862886	0.000124	0.000293	0.090138
CYB5R2	0.31452	4.874565	4.683495	3.50E-06	1.01E-05	3.479296
MAGIX	0.314485	4.910371	9.996338	7.65E-22	1.23E-20	38.74133
HBEGF	0.314369	5.789369	5.747507	1.45E-08	5.56E-08	8.763919
P4HA3	0.314009	6.444203	5.454767	7.22E-08	2.56E-07	7.212357
LOC34017	0.31388	4.964061	5.538768	4.59E-08	1.66E-07	7.650109
HSD11B1L	0.313761	6.496693	5.871999	7.19E-09	2.84E-08	9.445717
HIF1A	0.313672	12.00288	5.489093	6.00E-08	2.15E-07	7.39051
GINS2	0.31343	5.178171	4.990937	7.92E-07	2.48E-06	4.904888
CCL26	0.313023	4.963381	5.677668	2.14E-08	8.05E-08	8.387155
CRKL	0.312919	7.88106	5.38536	1.04E-07	3.63E-07	6.855226
CA14	0.312909	6.059524	5.434066	8.06E-08	2.85E-07	7.105411
TTTY5	0.312841	5.050478	6.784985	2.83E-11	1.47E-10	14.83601
EXOC5	0.312821	6.14316	4.869209	1.44E-06	4.38E-06	4.330439
TXN	0.312784	8.737658	3.365417	0.000814	0.001695	-1.66289
AP1B1	0.312639	7.362336	3.365903	0.000812	0.001692	-1.66128
PTP4A1	0.312632	7.962765	4.930821	1.07E-06	3.28E-06	4.619562
CCDC159	0.312547	7.246417	3.399647	0.00072	0.001514	-1.54968
ATP5J	0.312493	10.2284	4.417975	1.19E-05	3.19E-05	2.315878
PSMD1	0.312479	8.58435	4.809912	1.92E-06	5.74E-06	4.055348
MAPK8IP1	0.312478	4.852205	5.516885	5.17E-08	1.86E-07	7.535491
MPZL3	0.312358	9.491557	2.993645	0.002872	0.00546	-2.82116
UBQLN2	0.312312	8.209973	3.15988	0.001659	0.003288	-2.31942
ADD1	0.312219	8.344551	6.165646	1.30E-09	5.63E-09	11.10514
OSBPL2	0.312188	8.662486	4.527395	7.22E-06	2.00E-05	2.787651
PGLYRP2	0.311884	4.974537	5.253647	2.08E-07	6.99E-07	6.188901
LSM12	0.311862	7.185045	4.653004	4.04E-06	1.16E-05	3.342483
IL36RN	0.311818	5.059499	7.036653	5.47E-12	3.06E-11	16.43936
CTNNAL1	0.311794	6.412845	2.107071	0.035532	0.054746	-5.04958
CCNB2	0.311738	5.579752	5.662804	2.33E-08	8.72E-08	8.307497

FRAT1	0.311518	9.681765	3.523577	0.000459	0.00099	-1.1306
SORCS3-A	0.311471	5.870147	6.728076	4.07E-11	2.09E-10	14.48036
NDST1	0.311318	6.284555	4.467259	9.50E-06	2.59E-05	2.527038
INPPL1	0.311219	7.828795	3.243786	0.001246	0.00252	-2.05623
LHFP	0.311197	4.703652	3.781566	0.000172	0.000396	-0.21218
LAMA5-AS	0.311042	5.48664	3.026983	0.002578	0.00494	-2.72265
HIST1H2A	0.311039	5.574333	2.427749	0.01549	0.02578	-4.33113
KCNH3	0.310792	6.559686	4.116381	4.40E-05	0.00011	1.07164
PKN1	0.31077	7.564085	1.985852	0.047511	0.071122	-5.2951
TLX3	0.310492	4.526601	5.995343	3.54E-09	1.45E-08	10.13402
MKNK1-A	0.310361	5.582939	7.028043	5.79E-12	3.23E-11	16.38369
CCDC22	0.310185	6.647889	3.1778	0.001561	0.003112	-2.26377
ZNF746	0.309573	9.179868	2.919505	0.003639	0.006795	-3.03646
MPST	0.309451	7.679727	2.955592	0.003245	0.006111	-2.93232
SEMA5B	0.309385	8.481679	5.874662	7.08E-09	2.80E-08	9.46044
TMEM259	0.30931	6.077582	2.345529	0.01933	0.031524	-4.52485
KLHDC3	0.309109	7.70462	2.976332	0.003037	0.005752	-2.87191
NCOA6	0.308971	9.4487	6.077961	2.18E-09	9.20E-09	10.60214
KLK4	0.308893	6.224619	5.865623	7.45E-09	2.94E-08	9.410481
RNF13	0.308799	11.2638	4.459194	9.85E-06	2.68E-05	2.492333
LINC0049	0.308797	9.488599	3.345648	0.000873	0.001808	-1.72777
KCTD2	0.308702	7.838789	4.560675	6.20E-06	1.74E-05	2.933277
ARHGEF5	0.308692	5.8505	6.484554	1.88E-10	9.00E-10	12.9876
CLEC1A	0.30863	5.893022	3.689993	0.000245	0.000552	-0.54526
OXT	0.308501	5.81695	4.803537	1.98E-06	5.90E-06	4.025961
PHKG2	0.308459	7.110835	2.603204	0.009468	0.016428	-3.89588
TCTA	0.308458	7.709436	4.358932	1.54E-05	4.09E-05	2.065795
HAPLN4	0.308298	5.753654	7.017525	6.21E-12	3.45E-11	16.31575
PRCP	0.308154	6.46925	7.631678	9.34E-14	6.26E-13	20.42418
WDR34	0.307877	5.7211	4.480496	8.94E-06	2.44E-05	2.584124
CD163L1	0.30782	5.27642	4.939313	1.02E-06	3.16E-06	4.659673
ANTXR2	0.307751	8.121631	4.67477	3.65E-06	1.05E-05	3.440059
OR2S2	0.307632	5.72286	7.012548	6.42E-12	3.56E-11	16.28364
FIZ1	0.307455	7.171006	5.72542	1.64E-08	6.26E-08	8.644321
ABCA2	0.307421	6.0898	3.481591	0.000535	0.001145	-1.27419
MAP3K1	0.307399	9.358461	3.39228	0.000739	0.001551	-1.57413
AAAS	0.30721	7.144502	5.452602	7.30E-08	2.59E-07	7.201158
TMEM56	0.307176	4.13039	3.946864	8.87E-05	0.000213	0.408742
ZFAT	0.307169	6.843057	5.509651	5.37E-08	1.93E-07	7.497692
C22orf24	0.30712	5.53454	7.494808	2.44E-13	1.57E-12	19.48384
CLTB	0.306969	7.170844	4.699775	3.24E-06	9.42E-06	3.55268
NAT16	0.306952	5.747128	5.967963	4.14E-09	1.68E-08	9.980133
NUTM2B	0.306872	6.493707	4.967249	8.90E-07	2.77E-06	4.79208
LMX1B	0.306847	5.704237	5.999241	3.46E-09	1.42E-08	10.15598
TMEM222	0.306741	7.81085	3.20354	0.001431	0.002865	-2.1833
GMFG	0.306734	11.87689	3.521679	0.000462	0.000997	-1.13713
IGKC	0.306718	6.640636	3.148849	0.001722	0.003402	-2.35353
SEL1L	0.306595	7.502316	5.386357	1.04E-07	3.61E-07	6.860327
TSPAN7	0.306545	5.057997	5.825205	9.37E-09	3.66E-08	9.187915
ADAP1	0.306268	8.539425	3.261516	0.001172	0.002381	-1.99976
MCM10	0.306168	4.535665	3.73793	0.000204	0.000464	-0.37187
LOC10013	0.30609	5.820959	6.114148	1.76E-09	7.51E-09	10.80896
FHL2	0.306073	5.535217	3.155184	0.001686	0.003337	-2.33396
NADSYN1	0.305903	7.815488	2.605696	0.0094	0.016321	-3.88949
MUC3	0.305875	5.582447	6.700455	4.85E-11	2.47E-10	14.30867
MOV10	0.305614	5.940398	2.577584	0.01019	0.017573	-3.96129
LOC10050	0.305599	4.512922	8.904616	6.56E-18	6.69E-17	29.81715
VIPAS39	0.305515	7.337331	5.962653	4.27E-09	1.73E-08	9.950366

TFPT	0.305498	6.462454	4.121628	4.30E-05	0.000108	1.092581
CCL25	0.305305	5.186057	5.672015	2.21E-08	8.30E-08	8.356836
OLFML2A	0.305267	5.638919	5.34277	1.31E-07	4.48E-07	6.638128
KIR2DS4	0.30513	6.0825	5.192069	2.86E-07	9.45E-07	5.882536
CCL24	0.305063	5.510971	4.681094	3.54E-06	1.02E-05	3.46849
CLCNKB	0.304993	5.832374	4.944055	9.98E-07	3.09E-06	4.682103
FAM69C	0.304973	6.267279	6.177054	1.22E-09	5.28E-09	11.17104
LOC10050	0.304732	6.224483	5.703798	1.85E-08	7.02E-08	8.527638
NMUR2	0.304694	6.238903	5.940237	4.86E-09	1.95E-08	9.824941
STRN4	0.304685	6.894079	2.418061	0.015905	0.026412	-4.35429
PDE6G	0.304517	6.883144	4.530485	7.12E-06	1.97E-05	2.801131
GCGR	0.304432	6.190198	4.999975	7.57E-07	2.38E-06	4.948059
MLPH	0.304345	6.77225	5.391177	1.01E-07	3.53E-07	6.884995
RPS6KA2	0.304314	6.398846	2.940322	0.003407	0.006393	-2.97654
HOXA10	0.303917	5.63445	5.140854	3.72E-07	1.21E-06	5.630244
IFRD1	0.303765	8.730176	3.02106	0.002628	0.005031	-2.74022
VCL	0.303715	7.907405	6.123172	1.67E-09	7.14E-09	10.86069
CINP	0.303636	5.85832	6.048631	2.59E-09	1.08E-08	10.43531
CITED2	0.303588	7.676959	4.748426	2.58E-06	7.57E-06	3.773395
LOC10050	0.303567	5.773808	6.582433	1.02E-10	5.02E-10	13.58189
PGAP2	0.303484	7.719511	5.136166	3.81E-07	1.24E-06	5.607264
TTR	0.303444	6.753975	6.356653	4.12E-10	1.90E-09	12.22268
LYRM1	0.303415	9.775289	3.766333	0.000182	0.000418	-0.26812
WWP2	0.30338	8.283608	7.512817	2.15E-13	1.39E-12	19.60677
CTSZ	0.30335	7.249839	3.582263	0.000369	0.000809	-0.92713
CCDC151	0.303342	6.132254	4.767805	2.35E-06	6.93E-06	3.8619
VBP1	0.303208	10.22404	4.879921	1.37E-06	4.17E-06	4.380469
LGALS12	0.302972	7.146193	2.500186	0.012683	0.021472	-4.15504
RAD51	0.302553	5.103165	4.978041	8.44E-07	2.64E-06	4.843411
FNDC7	0.302532	5.73558	5.354254	1.23E-07	4.24E-07	6.696511
CACNA1E	0.302482	4.887083	7.833373	2.21E-14	1.58E-13	21.83523
HBBP1	0.302471	5.365541	4.635797	4.38E-06	1.25E-05	3.265641
NLRX1	0.302405	8.593188	4.729806	2.81E-06	8.23E-06	3.688674
SHOC2	0.302111	10.48675	4.60676	5.01E-06	1.42E-05	3.136574
OVOL3	0.302004	5.732142	6.398424	3.20E-10	1.49E-09	12.47104
C2orf57	0.301852	5.818287	6.012818	3.19E-09	1.32E-08	10.23256
KRT83	0.301628	5.791986	6.536162	1.36E-10	6.64E-10	13.29999
ACOT8	0.301521	6.584011	6.063776	2.37E-09	9.95E-09	10.52136
DTNBP1	0.301439	7.092531	4.265443	2.32E-05	6.01E-05	1.676271
LOC10192	0.301095	6.384898	5.566217	3.95E-08	1.44E-07	7.794462
CD276	0.300902	6.547936	5.544194	4.46E-08	1.62E-07	7.678593
MIR4755	0.300777	6.204995	5.652276	2.47E-08	9.20E-08	8.251191
GTF3C5	0.300751	7.092182	4.462283	9.71E-06	2.64E-05	2.50562
VAPA	0.300749	7.671078	5.381731	1.07E-07	3.70E-07	6.836662
PRDX3	0.300712	7.836849	6.057506	2.46E-09	1.03E-08	10.48571
ELK1	0.300371	6.651455	5.198793	2.77E-07	9.15E-07	5.91583
LOC10050	0.300284	4.971229	6.209311	1.00E-09	4.40E-09	11.35798
PIP5K1B	0.300275	6.03322	5.029481	6.53E-07	2.07E-06	5.089498
CECR6	0.300228	7.122722	2.24229	0.025313	0.040228	-4.75881
CWF19L1	0.300108	7.015537	4.982458	8.26E-07	2.58E-06	4.864453
CORO2B	0.300069	5.077477	4.182674	3.32E-05	8.43E-05	1.33804
KRTAP4-1	0.299928	4.87284	4.81592	1.86E-06	5.58E-06	4.083078
F12	0.29987	6.098301	6.082431	2.13E-09	8.98E-09	10.62763
DOPEY2	0.299667	5.306224	5.185763	2.96E-07	9.74E-07	5.851352
SPRY4	0.29958	6.413619	6.465917	2.11E-10	1.00E-09	12.87532
LINC00967	0.299567	5.033667	4.515843	7.62E-06	2.10E-05	2.737335
ELOVL3	0.29945	4.79024	4.499543	8.20E-06	2.25E-05	2.666546
RNF182	0.299386	4.552694	2.110124	0.035267	0.054365	-5.04321

PALM3	0.299253	6.097963	5.888923	6.52E-09	2.59E-08	9.539407
SMPD1	0.299195	6.239657	6.320696	5.13E-10	2.33E-09	12.01003
PRKCDBP	0.299136	4.783709	4.283423	2.15E-05	5.58E-05	1.750571
TLR10	0.298729	6.66655	3.309864	0.00099	0.002034	-1.84427
FAM127A	0.298623	8.816503	4.486126	8.72E-06	2.39E-05	2.608451
AFF2	0.298543	5.236807	8.609933	6.63E-17	6.05E-16	27.54179
SUCLG1	0.298237	6.937061	6.569829	1.11E-10	5.41E-10	13.50492
BABAM1	0.298062	8.045995	2.808555	0.005141	0.009363	-3.34884
YIF1B	0.298056	6.395316	4.990796	7.92E-07	2.48E-06	4.904217
HIST1H3A	0.298034	4.521515	6.643632	6.95E-11	3.47E-10	13.95736
SMCR8	0.29774	8.211337	5.592423	3.43E-08	1.26E-07	7.932876
OTUD5	0.297645	7.942595	3.518014	0.000468	0.001009	-1.14972
LATS2	0.297561	7.299802	6.456201	2.24E-10	1.06E-09	12.81689
PSPH	0.297393	4.420079	2.52031	0.011987	0.020392	-4.10522
ZNF672	0.297375	6.267804	4.366548	1.49E-05	3.96E-05	2.097879
MVD	0.29727	5.675533	3.833807	0.00014	0.000326	-0.01867
KCNK5	0.297245	4.92511	5.255615	2.06E-07	6.93E-07	6.19875
WSB2	0.297181	10.00525	3.329907	0.000923	0.001905	-1.77916
PRELID1	0.297063	8.947963	3.209323	0.001403	0.002813	-2.16514
CD34	0.296874	6.039674	6.007609	3.29E-09	1.36E-08	10.20316
FKBP1B	0.296805	6.125752	1.978489	0.048337	0.072233	-5.30956
MAP3K5	0.296636	9.901817	4.595556	5.28E-06	1.49E-05	3.086974
MYL12A	0.296548	11.55797	5.620263	2.94E-08	1.09E-07	8.08056
S100A1	0.296509	5.835452	5.787347	1.16E-08	4.49E-08	8.980689
NEK6	0.296476	6.426005	8.113203	2.86E-15	2.22E-14	23.84211
GLRA2	0.296418	5.776173	4.812967	1.89E-06	5.66E-06	4.069449
LOC93444	0.296294	6.627436	5.04077	6.17E-07	1.96E-06	5.143814
LRRC29	0.296233	5.759637	4.855424	1.54E-06	4.66E-06	4.266213
RTF1	0.296201	7.629539	5.932776	5.07E-09	2.03E-08	9.78329
C17orf53	0.296001	6.050574	5.079447	5.08E-07	1.63E-06	5.330753
LRRC42	0.295876	5.908583	4.099704	4.72E-05	0.000117	1.005258
NCAPG2	0.295806	6.048655	2.238342	0.02557	0.040595	-4.76755
SCAMP4	0.295689	5.934561	3.94095	9.09E-05	0.000218	0.386092
MAOA	0.295676	4.638864	3.709834	0.000227	0.000514	-0.47376
PAX7	0.295625	5.479832	6.003539	3.37E-09	1.39E-08	10.18021
USP32	0.295567	9.185324	4.184119	3.30E-05	8.39E-05	1.343891
ATP6V1D	0.29522	7.364806	5.793603	1.12E-08	4.34E-08	9.014848
OPTC	0.295213	5.628918	5.062545	5.53E-07	1.77E-06	5.248899
NPL	0.295108	8.222143	3.456734	0.000586	0.001246	-1.35842
TMED1	0.295102	7.12462	4.695126	3.32E-06	9.60E-06	3.5317
ZFYVE1	0.294934	7.287585	5.390355	1.02E-07	3.54E-07	6.880786
ATP6V1H	0.294887	8.514193	4.514073	7.68E-06	2.12E-05	2.729639
CENPBD1	0.29476	7.65053	4.876856	1.39E-06	4.23E-06	4.366141
HSF1	0.294642	6.058224	3.845356	0.000133	0.000313	0.024452
PIP	0.294625	5.665683	7.138223	2.78E-12	1.60E-11	17.10042
TRAPPC3	0.294615	8.35192	5.526553	4.90E-08	1.77E-07	7.58608
WDR13	0.294529	6.434659	6.522159	1.49E-10	7.21E-10	13.21501
TPBGL	0.294448	5.676578	5.515646	5.20E-08	1.87E-07	7.529016
COL8A2	0.294385	5.596184	4.609119	4.96E-06	1.40E-05	3.147032
SYNDIG1	0.29438	4.891458	6.245104	8.10E-10	3.60E-09	11.5664
PPP2R5B	0.2943	7.09671	4.473932	9.21E-06	2.51E-05	2.555797
HIST1H2BI	0.294223	4.722881	6.27219	6.88E-10	3.09E-09	11.72483
TACR2	0.294002	5.774003	5.216365	2.53E-07	8.40E-07	6.003024
VEPH1	0.29386	5.007678	4.464147	9.63E-06	2.62E-05	2.513637
PSG7	0.293585	6.000401	6.806804	2.46E-11	1.29E-10	14.97305
BCORL1	0.293253	6.173132	5.634257	2.72E-08	1.01E-07	8.155042
C6orf1	0.293225	6.862265	2.401855	0.01662	0.027508	-4.39284
RMI2	0.29317	6.35235	2.990507	0.002901	0.00551	-2.83038

MYADML2	0.293067	5.40614	5.144336	3.66E-07	1.19E-06	5.647324
UNC13B	0.292961	5.305051	5.293848	1.69E-07	5.73E-07	6.390683
BRAF	0.292615	7.050438	3.311423	0.000985	0.002024	-1.83922
ARF4	0.292269	9.999038	4.474598	9.19E-06	2.50E-05	2.558667
NMT1	0.292222	7.277433	4.50036	8.17E-06	2.25E-05	2.670085
SPTSSA	0.292171	7.886007	2.312399	0.021098	0.034102	-4.60105
CYP26A1	0.292109	5.63786	6.586616	9.96E-11	4.90E-10	13.60745
SECTM1	0.292069	9.911008	2.479409	0.013438	0.022651	-4.20606
TRIM71	0.291998	4.570371	4.473182	9.24E-06	2.52E-05	2.552562
RELA	0.291835	8.260228	3.132015	0.001822	0.003579	-2.40536
LUZP2	0.291819	5.026657	7.421748	4.05E-13	2.54E-12	18.98765
Igk	0.291744	5.845932	5.865494	7.46E-09	2.94E-08	9.409766
ANKRD32	0.291733	7.170207	2.617769	0.009077	0.015816	-3.85842
SLC24A4	0.2917	7.331303	2.214639	0.027165	0.042924	-4.81972
IGF1R	0.291677	7.447715	4.910092	1.18E-06	3.62E-06	4.521915
CISH	0.29155	8.271844	2.72594	0.006602	0.011786	-3.57379
TOX4	0.291465	7.562987	5.349971	1.26E-07	4.33E-07	6.674724
CNTLN	0.291409	4.552453	4.610477	4.93E-06	1.40E-05	3.153056
FAM195B	0.291288	7.692683	4.029701	6.31E-05	0.000154	0.729382
SLC17A7	0.291248	6.481189	7.20604	1.76E-12	1.04E-11	17.54624
PTGS1	0.291026	7.876255	2.310516	0.021203	0.034253	-4.60535
TFF1	0.290754	5.155448	6.070765	2.28E-09	9.57E-09	10.56114
FSTL3	0.290744	6.555191	3.177021	0.001566	0.003119	-2.2662
CDK9	0.29058	5.66336	4.11343	4.45E-05	0.000111	1.059877
PML	0.290501	6.34929	5.193714	2.84E-07	9.38E-07	5.890678
TMEM173	0.290356	8.114639	2.467044	0.013906	0.023368	-4.23623
ARID1A	0.290333	8.804131	4.031992	6.25E-05	0.000153	0.738339
RNASE1	0.290201	5.284661	5.280486	1.81E-07	6.13E-07	6.32346
IGLC1	0.289973	7.701228	2.338856	0.019675	0.032027	-4.54028
ARSD	0.289916	6.34572	5.321418	1.46E-07	5.00E-07	6.529877
PCNXL3	0.289825	6.999581	2.663738	0.007939	0.013969	-3.73884
LDHC	0.289691	5.133351	5.207018	2.65E-07	8.79E-07	5.956611
C7orf49	0.289524	7.37614	3.774984	0.000176	0.000405	-0.23638
KRT8P12	0.289457	6.166959	6.604505	8.90E-11	4.40E-10	13.71696
MAPKAP1	0.289292	6.427509	5.59157	3.44E-08	1.26E-07	7.92836
GFI1B	0.289164	6.293851	2.807737	0.005154	0.009384	-3.3511
BMP15	0.289056	4.702371	6.0739	2.24E-09	9.41E-09	10.579
PTGFR	0.28903	4.027528	4.143397	3.92E-05	9.87E-05	1.17972
P2RY13	0.288859	11.51914	2.923257	0.003596	0.006724	-3.02569
ATP12A	0.288822	6.045651	5.978791	3.89E-09	1.58E-08	10.04092
YWHAB	0.288777	12.03896	5.682224	2.09E-08	7.86E-08	8.411609
PRKAB1	0.28863	6.934429	3.689448	0.000245	0.000553	-0.54722
C7orf69	0.288453	4.769203	6.393109	3.30E-10	1.54E-09	12.43936
OPA3	0.288438	5.935456	5.942879	4.79E-09	1.92E-08	9.8397
DAXX	0.288165	7.604077	4.248291	2.50E-05	6.45E-05	1.60567
EPHA5-AS	0.288145	5.564249	4.351771	1.59E-05	4.21E-05	2.03568
C5AR2	0.28813	6.684236	3.637764	0.000299	0.000665	-0.73175
AREG	0.28791	4.079975	2.98688	0.002935	0.005572	-2.84103
TOR1AIP2	0.287811	6.397188	5.665012	2.30E-08	8.62E-08	8.319321
ZNF837	0.287625	5.269064	5.291824	1.71E-07	5.79E-07	6.380494
TUBB6	0.287501	6.733839	2.525253	0.011822	0.020134	-4.09292
LRCH4	0.287364	8.571472	3.866724	0.000123	0.000289	0.104557
DGCR11	0.287305	6.770828	5.038009	6.26E-07	1.99E-06	5.130521
NEUROD4	0.287275	4.731279	6.472826	2.02E-10	9.64E-10	12.91691
POU5F1B	0.287216	7.656546	5.003374	7.44E-07	2.34E-06	4.964314
CASQ2	0.287091	5.381332	5.486669	6.08E-08	2.17E-07	7.377899
MMP24-A	0.286806	7.812444	4.90185	1.23E-06	3.76E-06	4.483195
MAN2A2	0.286797	9.017712	3.121664	0.001886	0.003698	-2.43709

ARFGAP1	0.286716	7.065547	3.332897	0.000913	0.001887	-1.76942
LOC10028	0.286583	5.824467	6.035317	2.80E-09	1.17E-08	10.35981
KCTD3	0.286497	4.098391	2.962917	0.00317	0.005984	-2.91103
TMEM144	0.286363	4.753277	3.934688	9.32E-05	0.000223	0.362147
MEDAG	0.28627	5.879439	3.908801	0.000103	0.000246	0.26353
SNX11	0.285934	9.263894	5.130694	3.92E-07	1.27E-06	5.580465
FBXW11	0.285656	7.044193	5.967777	4.15E-09	1.68E-08	9.979089
RRAS	0.285436	6.282035	2.906442	0.003793	0.007054	-3.07385
LOC39990	0.285404	5.372859	5.294327	1.69E-07	5.72E-07	6.3931
PSPC1	0.285394	6.627	5.327757	1.42E-07	4.84E-07	6.561973
ANKRD13I	0.285381	7.903149	2.692228	0.007299	0.012938	-3.6637
HAGLR	0.285197	4.638615	8.476291	1.86E-16	1.62E-15	26.52946
AAMP	0.285006	8.568836	3.071989	0.002224	0.004312	-2.58797
PIK3CB	0.284724	7.502483	3.155895	0.001681	0.003329	-2.33176
FRMD4B	0.284555	5.331732	4.731785	2.79E-06	8.16E-06	3.697664
TWF1	0.284535	5.575031	6.203498	1.04E-09	4.55E-09	11.32423
TBC1D22B	0.284355	5.666781	7.804953	2.72E-14	1.93E-13	21.63459
DOT1L	0.284292	5.92367	4.494067	8.41E-06	2.31E-05	2.642815
SETDB1	0.284284	7.083904	4.78175	2.20E-06	6.51E-06	3.925793
BAI1	0.284247	5.735901	4.220263	2.82E-05	7.24E-05	1.490871
DNAH17	0.284179	5.1643	4.40495	1.26E-05	3.36E-05	2.260438
SERTAD1	0.284166	7.656523	3.94578	8.91E-05	0.000214	0.404588
GPR52	0.283978	5.662298	4.135022	4.06E-05	0.000102	1.146143
TSPAN33	0.283911	7.871417	2.693839	0.007264	0.012882	-3.65943
TMEM170I	0.283631	8.942402	2.910633	0.003743	0.006971	-3.06187
GRIP2	0.283512	6.820742	4.817423	1.85E-06	5.54E-06	4.090025
USP35	0.283273	6.384786	4.620702	4.70E-06	1.34E-05	3.198453
TNIP2	0.283069	8.325646	4.56846	5.98E-06	1.68E-05	2.967488
FAM134A	0.283038	8.785385	4.96741	8.89E-07	2.77E-06	4.792845
HTATIP2	0.283033	7.768841	5.387876	1.03E-07	3.59E-07	6.8681
LOC44014	0.282866	6.05385	4.799223	2.02E-06	6.02E-06	4.006092
C16orf59	0.282812	6.321838	4.975847	8.53E-07	2.66E-06	4.832969
ASL	0.282764	6.847991	2.801077	0.00526	0.009566	-3.36947
ATP6V0B	0.282681	11.05303	3.214563	0.001378	0.002765	-2.14865
C1GALT1	0.282532	7.794171	3.334796	0.000907	0.001876	-1.76322
REG1P	0.282501	5.216386	5.537169	4.63E-08	1.68E-07	7.641723
LOC34488	0.282352	4.901131	5.029139	6.54E-07	2.07E-06	5.087853
KCNS1	0.282143	6.601574	3.747981	0.000196	0.000447	-0.33524
CCS	0.281896	7.071887	2.483448	0.013288	0.022415	-4.19618
HIST1H3E	0.281841	5.285498	3.156435	0.001678	0.003324	-2.33009
RCN3	0.281816	6.891435	3.116627	0.001918	0.003758	-2.4525
SSH3	0.281712	7.686283	4.719508	2.95E-06	8.61E-06	3.641951
SMPD2	0.281688	6.377409	3.696615	0.000239	0.000539	-0.52144
RB1	0.281246	6.818136	4.58475	5.55E-06	1.56E-05	3.039243
AOAH	0.281164	9.148368	2.721823	0.006684	0.011926	-3.58483
HSPA4L	0.281124	3.980521	4.336666	1.70E-05	4.48E-05	1.972306
HAGH	0.280971	7.408189	3.022154	0.002618	0.005014	-2.73698
KLHDC8A	0.280796	5.545188	6.501878	1.69E-10	8.13E-10	13.09222
CC2D1A	0.280685	5.61752	4.084962	5.02E-05	0.000124	0.946787
KRTAP3-1	0.28065	7.019622	5.857825	7.79E-09	3.07E-08	9.367433
LOC10050	0.280631	5.741074	6.122635	1.68E-09	7.16E-09	10.85761
GALNT1	0.280423	9.199948	3.47968	0.000539	0.001152	-1.28069
ELF4	0.280067	9.54658	5.269899	1.92E-07	6.46E-07	6.27031
CBS	0.280032	5.53044	3.808147	0.000155	0.000359	-0.11403
ICA1	0.279989	5.407807	7.796568	2.89E-14	2.04E-13	21.57551
GSX1	0.279833	5.322265	3.969321	8.09E-05	0.000195	0.495043
TUSC2	0.279497	7.358615	3.334095	0.000909	0.00188	-1.76551
POLR2J	0.279421	9.894745	2.570897	0.010387	0.017885	-3.97826

PLEKHM2	0.279276	7.044224	2.840364	0.004661	0.008554	-3.26048
LOC10272	0.279006	5.570402	6.000192	3.44E-09	1.41E-08	10.16134
PLEKHM1	0.278866	6.879776	5.661123	2.35E-08	8.79E-08	8.298503
DCP2	0.278817	8.358647	4.628536	4.53E-06	1.29E-05	3.233297
NMNAT2	0.278674	5.356191	5.809352	1.03E-08	3.99E-08	9.100994
PCBP3	0.278499	6.176666	3.902467	0.000106	0.000252	0.239495
MRPL23	0.278441	7.438859	2.311804	0.021131	0.034146	-4.60242
DSCC1	0.278407	5.407895	5.140724	3.73E-07	1.21E-06	5.629607
SLITRK4	0.278279	4.427374	4.157982	3.69E-05	9.31E-05	1.238346
GLDN	0.278194	5.843714	4.616908	4.78E-06	1.36E-05	3.181598
C19orf10	0.278159	7.99227	2.717574	0.006769	0.012071	-3.5962
CSAD	0.278073	7.428831	4.349242	1.61E-05	4.25E-05	2.025055
ZNF598	0.278048	6.830822	6.221181	9.34E-10	4.12E-09	11.42698
FNBP1L	0.277715	4.427849	2.111324	0.035164	0.054219	-5.04071
KRT19P2	0.277673	5.060825	5.66804	2.26E-08	8.48E-08	8.335536
SKA3	0.27763	5.778455	4.541612	6.77E-06	1.88E-05	2.84974
RNPEP	0.277431	8.918197	2.68845	0.007381	0.01307	-3.67371
TMEM86B	0.277408	6.136731	3.374801	0.000787	0.001644	-1.63196
HNRNPLL	0.277276	6.815488	3.111813	0.001949	0.003816	-2.4672
AP1G1	0.277254	8.207521	4.489751	8.58E-06	2.35E-05	2.624133
COX6B1	0.277135	9.918586	2.552522	0.010945	0.01876	-4.02466
SAP30	0.276852	6.057358	4.859225	1.51E-06	4.58E-06	4.283905
ARAP1	0.276834	8.315927	2.800905	0.005263	0.009569	-3.36994
CASKIN1	0.276688	5.145352	5.996164	3.52E-09	1.44E-08	10.13864
TNFRSF8	0.276611	6.417802	3.650405	0.000285	0.000635	-0.68685
LOC10272	0.276416	5.893866	5.654253	2.44E-08	9.10E-08	8.261761
GNAT2	0.276233	5.14852	3.913931	0.000101	0.000241	0.283021
EXOC7	0.275818	7.080135	5.877945	6.95E-09	2.75E-08	9.478607
C14orf2	0.275801	10.09437	4.533103	7.04E-06	1.95E-05	2.812561
CTSE	0.275677	5.888003	6.025347	2.97E-09	1.23E-08	10.30337
CLPB	0.275543	6.050912	5.833323	8.95E-09	3.50E-08	9.232505
PRKAR1B	0.275541	6.249883	3.911547	0.000102	0.000243	0.273961
PLK1	0.275527	5.347881	3.750428	0.000194	0.000444	-0.32631
PCIF1	0.275423	6.796241	3.965631	8.22E-05	0.000198	0.480832
ERF	0.275354	6.639837	2.76834	0.005811	0.01048	-3.45916
MMGT1	0.275272	9.266327	4.075288	5.22E-05	0.000129	0.908526
KRTAP17-	0.275188	6.067554	5.808142	1.03E-08	4.02E-08	9.094367
KLF13	0.274943	7.874662	3.09772	0.002043	0.003988	-2.51011
CSTB	0.274729	8.068192	4.170705	3.49E-05	8.85E-05	1.289646
GRIK5	0.274729	6.470291	5.187244	2.94E-07	9.68E-07	5.858673
DMWD	0.27453	6.921987	5.06671	5.42E-07	1.73E-06	5.269048
INPP1	0.274342	7.804739	3.267732	0.001147	0.002334	-1.97989
CHRNA10	0.274301	5.753946	6.472422	2.03E-10	9.66E-10	12.91448
HOOK3	0.2743	7.042643	3.157917	0.00167	0.003309	-2.3255
PSG3	0.274259	4.303319	7.514156	2.13E-13	1.38E-12	19.61591
LOC14688	0.274185	5.377588	2.834022	0.004754	0.008709	-3.27818
SNX16	0.274125	6.010113	2.016362	0.044214	0.066698	-5.23466
TMPRSS5	0.274098	6.440784	5.307028	1.58E-07	5.37E-07	6.457146
SPIDR	0.274091	6.829794	5.874878	7.07E-09	2.80E-08	9.461636
APOA2	0.274038	4.559358	3.834283	0.000139	0.000326	-0.01689
COL18A1	0.274021	6.993191	2.243597	0.025228	0.040121	-4.75591
NCAPD2	0.274019	7.247432	3.132395	0.00182	0.003576	-2.40419
CEACAM1	0.273905	6.516399	5.355825	1.22E-07	4.20E-07	6.704509
HDGF	0.273825	9.889185	3.218603	0.001359	0.002728	-2.13592
SS18	0.273217	6.049928	5.648131	2.52E-08	9.41E-08	8.229053
HSPA1L	0.27316	7.118255	5.961788	4.29E-09	1.74E-08	9.945517
PTPRN	0.272961	5.385924	5.932782	5.07E-09	2.03E-08	9.783322
NCOA3	0.272756	8.491118	3.892931	0.00011	0.000261	0.20338

ZBTB45	0.272442	5.526889	4.54037	6.81E-06	1.89E-05	2.844311
CEP19	0.272333	8.88416	2.278001	0.023083	0.036996	-4.67905
OAF	0.27231	6.636877	4.657953	3.95E-06	1.13E-05	3.364633
TUBGCP2	0.272231	6.818534	4.601019	5.15E-06	1.45E-05	3.111146
PLCB2	0.272187	7.346065	5.369292	1.14E-07	3.93E-07	6.773136
SYNC	0.272109	4.155326	2.750874	0.006126	0.011	-3.50659
LTBR	0.272083	6.732513	4.857396	1.53E-06	4.62E-06	4.275391
GLCCI1	0.271984	8.15293	3.249988	0.00122	0.002471	-2.03651
TRIM67	0.271811	6.691161	5.368311	1.14E-07	3.95E-07	6.768134
MED16	0.271807	7.245487	3.109273	0.001966	0.003846	-2.47495
GATSL2	0.271733	7.073182	4.96315	9.08E-07	2.82E-06	4.772608
CNR1	0.271716	4.990792	5.963651	4.25E-09	1.72E-08	9.955958
GPR107	0.271644	6.09191	8.391212	3.56E-16	3.01E-15	25.89144
LARP4B	0.271463	6.649003	5.969415	4.11E-09	1.67E-08	9.988279
FAM209B	0.271306	6.371035	4.125803	4.22E-05	0.000106	1.109258
HARBI1	0.271285	5.688432	5.550454	4.31E-08	1.57E-07	7.711486
GPATCH3	0.271256	7.023828	4.574732	5.81E-06	1.63E-05	2.995088
TMEM40	0.271134	6.702585	2.917814	0.003659	0.006827	-3.04131
CRAT	0.271118	7.329514	5.565427	3.97E-08	1.45E-07	7.7903
BARX2	0.271057	5.921314	4.407535	1.24E-05	3.33E-05	2.271429
GPX1	0.270669	11.36592	2.193232	0.02868	0.045098	-4.86636
MGRN1	0.270533	9.6146	3.88391	0.000114	0.000271	0.16929
CCM2L	0.270503	5.076856	5.38706	1.04E-07	3.60E-07	6.86392
ARL6IP6	0.270405	7.778348	4.554566	6.38E-06	1.78E-05	2.906474
MKRN1	0.270386	10.18192	4.33002	1.75E-05	4.61E-05	1.944486
RERE	0.270372	6.500534	4.222178	2.80E-05	7.18E-05	1.498692
SYTL4	0.270256	5.243266	3.646505	0.000289	0.000644	-0.70072
ARFRP1	0.270033	6.593332	2.775068	0.005694	0.010287	-3.44081
CDC42	0.270024	8.85545	5.496307	5.77E-08	2.07E-07	7.428079
LINC0119:	0.269824	4.715171	4.48714	8.68E-06	2.38E-05	2.612837
BHLHE23	0.269502	5.47078	6.230847	8.82E-10	3.90E-09	11.48326
CDH24	0.269437	6.4154	6.337298	4.64E-10	2.12E-09	12.10809
MTMR6	0.269377	7.424666	2.830901	0.0048	0.008783	-3.28687
MSN	0.269236	9.291899	3.844708	0.000134	0.000313	0.022027
FAM124B	0.269109	5.020516	4.840339	1.66E-06	4.99E-06	4.19612
ARR3	0.269099	6.70663	5.471837	6.59E-08	2.34E-07	7.300827
PACSIN2	0.269024	7.976254	5.587967	3.51E-08	1.29E-07	7.909299
CLEC14A	0.269006	6.083047	3.909498	0.000103	0.000245	0.266178
PPP4R1L	0.26898	5.208841	5.464657	6.85E-08	2.43E-07	7.263585
IQGAP3	0.268921	5.685978	5.186683	2.95E-07	9.70E-07	5.855897
TSC2	0.268859	6.08847	3.336061	0.000903	0.001868	-1.7591
SENCR	0.268841	7.179138	5.393279	1.00E-07	3.49E-07	6.895765
PGM2L1	0.268778	6.228886	3.178075	0.00156	0.00311	-2.26292
TPI1	0.268768	8.507707	3.543075	0.000427	0.000927	-1.06336
CTRB2	0.268732	5.996857	5.18962	2.90E-07	9.57E-07	5.87042
FLRT1	0.2687	5.591208	5.2642	1.97E-07	6.64E-07	6.241737
NXPH3	0.268673	6.798985	4.144458	3.90E-05	9.83E-05	1.183978
SIRT6	0.268642	7.365437	4.866327	1.46E-06	4.43E-06	4.316999
LOC10050	0.268591	6.459837	3.972175	8.00E-05	0.000193	0.506046
RAP1GAP	0.268589	6.578989	4.849333	1.59E-06	4.79E-06	4.237884
CNTNAP3	0.268546	3.732949	2.167589	0.030588	0.047799	-4.92165
LYVE1	0.268456	4.943463	4.035074	6.18E-05	0.000151	0.750398
ENTPD3	0.268335	5.270476	6.445171	2.40E-10	1.13E-09	12.75065
PHF20L1	0.26831	7.620094	4.298574	2.01E-05	5.25E-05	1.813404
LRRC61	0.268183	6.820003	3.560224	0.0004	0.000874	-1.00392
GPRC5D	0.268118	6.328207	4.893788	1.28E-06	3.91E-06	4.445378
ISOC2	0.267944	6.526192	3.812366	0.000152	0.000353	-0.09839
MMP1	0.267928	3.919347	2.539166	0.011367	0.019415	-4.05818

PBX2	0.267892	7.833852	3.255767	0.001196	0.002425	-2.0181
VAMP2	0.267865	7.738629	2.170505	0.030366	0.047493	-4.91539
RFC2	0.26783	6.660988	3.918098	9.97E-05	0.000237	0.298877
NUDT22	0.267827	6.625793	4.525441	7.29E-06	2.02E-05	2.779135
MTRR	0.267768	7.402993	2.488917	0.013087	0.022098	-4.18277
RAP2C	0.267762	9.546678	3.849807	0.000131	0.000307	0.041102
WNT11	0.267655	5.903984	4.397891	1.30E-05	3.46E-05	2.230457
GATA1	0.267635	6.775977	4.012372	6.78E-05	0.000165	0.661787
LOC44093	0.267602	3.843313	3.365895	0.000812	0.001692	-1.66131
VPS54	0.267299	7.985844	2.758836	0.00598	0.010763	-3.485
F11R	0.267083	8.408446	4.070742	5.32E-05	0.000131	0.890576
SYCP3	0.267043	3.521769	4.483459	8.82E-06	2.41E-05	2.596924
LOC10012	0.266946	6.908036	5.417808	8.79E-08	3.09E-07	7.021673
CCDC70	0.266876	4.838808	4.677111	3.61E-06	1.04E-05	3.450581
TMEM127	0.266796	8.635637	5.421691	8.61E-08	3.03E-07	7.041648
TMOD1	0.266778	6.224331	3.156122	0.00168	0.003327	-2.33106
GPR156	0.266778	6.925093	5.707804	1.81E-08	6.87E-08	8.549226
STAC2	0.266751	7.148236	5.257959	2.04E-07	6.85E-07	6.210477
FAM154A	0.266649	5.526032	5.069418	5.35E-07	1.71E-06	5.282155
GATSL3	0.266527	5.521	4.818957	1.84E-06	5.51E-06	4.097112
MS4A8	0.266468	6.033443	6.927032	1.13E-11	6.10E-11	15.73488
KIF13A	0.266365	6.628974	3.805077	0.000157	0.000363	-0.1254
POU5F1P4	0.266307	5.910654	4.657801	3.95E-06	1.13E-05	3.363951
RNF185-A	0.266284	5.970018	5.186387	2.95E-07	9.71E-07	5.854437
PTGIR	0.266042	6.924851	3.85822	0.000127	0.000298	0.072625
SERTAD3	0.265916	7.795981	3.190198	0.001497	0.002992	-2.22509
PRELP	0.265849	5.465132	7.371904	5.70E-13	3.53E-12	18.65143
CAV1	0.265698	4.788651	1.991251	0.046913	0.070354	-5.28447
LOC10050	0.265612	6.484487	5.183714	2.99E-07	9.84E-07	5.841225
DCST2	0.265538	4.834606	4.66699	3.78E-06	1.09E-05	3.405134
COASY	0.265457	8.426048	3.818802	0.000148	0.000345	-0.07451
ARID3B	0.265423	8.293314	4.638899	4.32E-06	1.23E-05	3.279476
C11orf85	0.26534	5.937831	5.953041	4.52E-09	1.82E-08	9.896529
NOD2	0.265241	8.735829	2.145907	0.032287	0.050177	-4.96789
LHFPL2	0.265179	7.51666	2.93559	0.003459	0.006485	-2.99019
DHRS1	0.265156	8.137777	3.228634	0.001313	0.002644	-2.10425
LOC10192	0.265056	5.134259	4.727508	2.84E-06	8.31E-06	3.678241
RDH16	0.2649	6.158546	4.552168	6.45E-06	1.80E-05	2.895956
KCNC4	0.26483	5.337299	7.41912	4.12E-13	2.59E-12	18.96987
TPSG1	0.264764	6.82355	4.842613	1.64E-06	4.94E-06	4.206671
APCDD1	0.264703	5.204196	2.018935	0.043945	0.066353	-5.22952
SLCO4A1	0.264614	5.606717	4.349117	1.61E-05	4.25E-05	2.02453
DCTN5	0.264406	6.445077	5.740506	1.51E-08	5.77E-08	8.725966
DDX3X	0.264298	8.942614	4.338705	1.69E-05	4.44E-05	1.980849
KCNE2	0.264078	5.973116	6.736876	3.85E-11	1.98E-10	14.53519
MRPL38	0.264061	5.972697	4.504174	8.03E-06	2.21E-05	2.686634
SNX18	0.263931	11.19209	3.469901	0.000559	0.001192	-1.31388
KPNA6	0.263759	7.164296	4.426382	1.14E-05	3.07E-05	2.351743
DCAF6	0.263655	6.240498	7.164613	2.33E-12	1.35E-11	17.27349
RAB33B	0.263581	8.218898	2.505328	0.012502	0.021188	-4.14234
CENPM	0.263547	5.931122	3.319019	0.000959	0.001975	-1.81458
CHMP6	0.263537	7.150856	2.74613	0.006214	0.01115	-3.51942
TMEM141	0.263494	6.92189	3.899138	0.000108	0.000255	0.226878
SDF2L1	0.263337	7.130438	1.976046	0.048614	0.072593	-5.31434
GFRA3	0.263214	5.976143	7.005091	6.74E-12	3.73E-11	16.23556
KRT33A	0.263208	5.219181	5.305803	1.59E-07	5.40E-07	6.450963
KDM6A	0.263187	6.76392	2.767447	0.005827	0.010506	-3.46159
ANP32A	0.263117	9.830564	3.979169	7.78E-05	0.000188	0.533035

ARMC7	0.262962	6.981103	5.679712	2.12E-08	7.97E-08	8.39812
IDI2-AS1	0.262832	4.898968	4.671396	3.71E-06	1.07E-05	3.424909
TFIP11	0.262133	6.850433	4.010838	6.83E-05	0.000166	0.655814
ZNF259P1	0.262079	4.40705	7.008385	6.60E-12	3.66E-11	16.2568
SLC1A5	0.261789	6.184152	2.94807	0.003324	0.00625	-2.95413
SLC22A18/	0.2617	7.139322	6.362243	3.99E-10	1.83E-09	12.25583
ANKRD9	0.261451	5.619928	3.247488	0.00123	0.002491	-2.04446
KIF12	0.261443	6.114054	4.200812	3.07E-05	7.84E-05	1.411624
HDAC5	0.261347	7.074476	3.740832	0.000201	0.000459	-0.3613
APOL1	0.261271	7.239247	2.881397	0.004103	0.007591	-3.14508
TRH	0.26124	5.660412	5.393838	9.99E-08	3.48E-07	6.898624
TRIM69	0.26124	5.354308	7.022068	6.03E-12	3.36E-11	16.34509
UBQLN1	0.261008	8.495217	4.375152	1.43E-05	3.81E-05	2.134183
NECAP1	0.261001	8.479531	3.211943	0.00139	0.002789	-2.1569
LINC01208	0.260936	6.808386	4.387972	1.36E-05	3.61E-05	2.188403
RANGAP1	0.260915	6.743224	2.768773	0.005803	0.010469	-3.45798
HADHA	0.26084	8.980948	3.951759	8.70E-05	0.000209	0.427515
WSCD2	0.260733	5.992415	4.709285	3.10E-06	9.02E-06	3.595661
NPM2	0.260591	7.249529	4.964092	9.04E-07	2.81E-06	4.777083
GDF5	0.260493	6.512472	4.383492	1.38E-05	3.68E-05	2.16944
TSSK2	0.260226	5.671083	7.016444	6.26E-12	3.48E-11	16.30878
PLA2G2D	0.260109	6.257784	4.870383	1.43E-06	4.35E-06	4.335919
CA5BP1	0.260067	6.389331	3.965366	8.23E-05	0.000198	0.479812
LINC00277	0.260003	5.981423	5.763255	1.33E-08	5.11E-08	8.849448
NAA38	0.259973	8.231606	2.598126	0.009607	0.016648	-3.9089
TOR4A	0.259957	6.154885	4.559305	6.24E-06	1.74E-05	2.927264
PLIN1	0.259926	6.333781	3.701027	0.000235	0.00053	-0.50554
CCDC17	0.259902	6.463468	3.365979	0.000812	0.001692	-1.66104
DRG2	0.259795	6.956375	4.201863	3.06E-05	7.81E-05	1.4159
HTRA3	0.25978	5.858556	4.073777	5.26E-05	0.00013	0.902557
COL10A1	0.259698	4.866903	5.01792	6.92E-07	2.19E-06	5.033987
IL1RAPL2	0.259382	5.877191	5.493814	5.85E-08	2.10E-07	7.415095
CAST	0.259367	8.962662	4.273957	2.24E-05	5.80E-05	1.711418
KIF18A	0.259365	3.904987	3.377335	0.00078	0.00163	-1.62359
TEAD3	0.259335	4.748413	5.115524	4.23E-07	1.37E-06	5.506305
UNC5B-AS	0.259305	5.20386	4.89599	1.26E-06	3.87E-06	4.455703
RAPGEFL1	0.259237	5.856111	4.276025	2.22E-05	5.75E-05	1.719962
SLC3A2	0.259139	7.717324	3.558665	0.000403	0.000879	-1.00934
LINC00454	0.259124	5.681683	6.363859	3.95E-10	1.82E-09	12.26542
HIST1H2BI	0.259017	4.918266	6.154939	1.39E-09	5.98E-09	11.04337
NEURL1B	0.258963	5.712355	3.961447	8.36E-05	0.000201	0.464732
RDH8	0.258506	5.429722	5.80406	1.06E-08	4.11E-08	9.072024
CKAP2L	0.258393	5.645065	3.161418	0.00165	0.003273	-2.31466
ZNF630	0.258328	6.218013	2.942635	0.003382	0.006349	-2.96985
WFDC21P	0.258116	6.689992	4.970398	8.76E-07	2.73E-06	4.807045
GATS	0.258115	4.850185	4.772812	2.29E-06	6.78E-06	3.884821
CDKN1A	0.257995	8.340254	2.990598	0.0029	0.005509	-2.83011
TYR	0.257863	5.95759	5.2699	1.92E-07	6.46E-07	6.270312
C16orf3	0.257757	5.049773	5.177383	3.09E-07	1.02E-06	5.809958
OR51M1	0.25768	5.315137	6.391782	3.33E-10	1.55E-09	12.43145
FAM49B	0.257662	9.162777	4.437379	1.09E-05	2.94E-05	2.398756
COLEC10	0.257598	4.678764	6.595576	9.41E-11	4.64E-10	13.66227
STAM2	0.257591	6.913741	4.299704	2.00E-05	5.23E-05	1.8181
DCPS	0.257575	7.115982	2.790856	0.005427	0.009841	-3.39758
UNC45A	0.257538	7.209733	6.241274	8.28E-10	3.67E-09	11.54405
BIN2	0.257425	11.11395	2.902592	0.003839	0.007131	-3.08483
SPHK2	0.257026	5.997724	4.672625	3.69E-06	1.06E-05	3.430426
EHD2	0.257017	6.788124	4.540519	6.80E-06	1.89E-05	2.844961

HS1BP3	0.256633	6.00489	6.586545	9.96E-11	4.90E-10	13.60702
RRAGB	0.256598	5.105052	4.940684	1.01E-06	3.14E-06	4.666156
FAM219A	0.256366	7.289974	3.768073	0.000181	0.000416	-0.26174
GML	0.25635	5.314661	3.921457	9.83E-05	0.000234	0.311666
UCK1	0.2561	6.609957	4.355581	1.56E-05	4.14E-05	2.051698
IGLV6-57	0.25603	4.830214	4.281706	2.16E-05	5.62E-05	1.743463
ZNF576	0.256026	6.774268	6.356951	4.12E-10	1.89E-09	12.22444
C1orf192	0.255794	4.783476	4.935447	1.04E-06	3.21E-06	4.641405
MCRS1	0.255748	7.370816	2.567507	0.010488	0.018045	-3.98684
Ndufaf4	0.255699	5.095097	5.44934	7.43E-08	2.63E-07	7.184284
EXO1	0.255698	5.285598	4.051843	5.76E-05	0.000142	0.816156
KDM4A	0.255579	7.584624	5.998517	3.47E-09	1.43E-08	10.1519
ATXN2L	0.255568	5.537329	3.424244	0.000659	0.001392	-1.46765
CYP2A7	0.255469	5.346054	4.768197	2.34E-06	6.92E-06	3.863692
FGF17	0.255376	5.893574	4.13528	4.06E-05	0.000102	1.147177
H2AFY	0.255318	9.227083	4.001802	7.09E-05	0.000172	0.620689
MFSD9	0.254947	5.496595	2.686944	0.007414	0.013123	-3.6777
INTS6	0.254827	7.561551	3.078904	0.002174	0.004224	-2.56711
FLCN	0.254821	5.724272	5.670748	2.23E-08	8.36E-08	8.350046
STX7	0.254818	7.791141	5.456127	7.17E-08	2.54E-07	7.219399
PLAA	0.254789	5.722199	3.623845	0.000315	0.000698	-0.78102
LOC44160	0.254726	5.751077	6.502878	1.68E-10	8.08E-10	13.09827
HNF1B	0.254722	6.607427	5.253241	2.09E-07	7.01E-07	6.186874
SNCG	0.254709	5.5134	4.325241	1.79E-05	4.70E-05	1.924506
LOC10192	0.254702	5.518707	5.563737	4.01E-08	1.46E-07	7.781395
TRIM27	0.254585	8.608406	2.904745	0.003813	0.007089	-3.07869
RAD54L	0.25455	5.808527	3.980765	7.72E-05	0.000187	0.539203
RBKS	0.254445	6.017808	4.168279	3.53E-05	8.94E-05	1.279853
CDKN2B	0.254334	4.630368	3.59648	0.00035	0.000769	-0.87736
RHOT1	0.254129	8.18063	3.42172	0.000665	0.001405	-1.47609
DRD5	0.254048	5.674487	5.077815	5.12E-07	1.64E-06	5.322838
LAMTOR4	0.254028	9.894682	2.148577	0.032074	0.049875	-4.96222
FAM127B	0.253939	6.602449	4.327622	1.77E-05	4.66E-05	1.934458
C19orf45	0.253938	6.089881	5.594546	3.39E-08	1.24E-07	7.944113
LINC01118	0.253833	5.46702	5.015256	7.01E-07	2.21E-06	5.021214
SMIM24	0.253772	6.056938	3.484421	0.00053	0.001133	-1.26456
DIAPH3	0.253599	4.461556	6.111485	1.79E-09	7.62E-09	10.7937
C10orf99	0.253535	4.681994	6.020303	3.06E-09	1.27E-08	10.27485
SMARCD2	0.253492	8.161709	2.648445	0.008302	0.014567	-3.77884
MIP	0.253456	5.003601	6.265904	7.15E-10	3.20E-09	11.68801
SLC46A1	0.253425	5.675229	5.936119	4.98E-09	2.00E-08	9.801945
CCNG2	0.253367	8.979569	3.450195	0.0006	0.001275	-1.38048
CPT1A	0.25321	6.180651	4.113612	4.45E-05	0.000111	1.0606
VN1R1	0.253196	4.194004	6.747692	3.59E-11	1.85E-10	14.60266
ADORA2A	0.253034	5.422277	7.349182	6.67E-13	4.10E-12	18.4988
CRYBB2	0.25292	6.083451	3.643557	0.000293	0.000651	-0.71119
LOC10192	0.252806	4.392564	3.043695	0.002441	0.004694	-2.67286
GABPB1	0.25278	7.388272	3.528566	0.00045	0.000974	-1.11343
PTCRA	0.252692	7.257193	3.865782	0.000123	0.00029	0.101016
4-Sep	0.25269	5.183746	4.324937	1.79E-05	4.71E-05	1.923238
ADPGK	0.252681	8.516939	3.559002	0.000402	0.000878	-1.00817
ART1	0.252675	6.222282	4.952801	9.56E-07	2.96E-06	4.723515
CASC5	0.252645	4.435897	3.112134	0.001947	0.003812	-2.46622
MMP17	0.252635	5.987983	3.533012	0.000443	0.000959	-1.09811
STARD13-	0.252588	5.113676	8.928789	5.42E-18	5.56E-17	30.0064
ABHD13	0.252488	6.407262	2.515165	0.012162	0.020668	-4.11799
KLK3	0.25239	6.36441	5.62875	2.81E-08	1.04E-07	8.12571
DGCR2	0.252355	7.896873	3.875593	0.000118	0.000279	0.137931

F3	0.252293	4.598857	4.066349	5.42E-05	0.000134	0.873248
GAS8	0.252234	7.519619	4.112552	4.47E-05	0.000112	1.056375
C6orf165	0.252075	6.072708	5.562169	4.04E-08	1.47E-07	7.773135
UPF1	0.252062	7.741401	2.967587	0.003123	0.005904	-2.89743
TTTY11	0.252024	6.459974	6.221899	9.30E-10	4.10E-09	11.43116
PLXNB1	0.251915	6.196989	5.449968	7.41E-08	2.62E-07	7.187533
DBI	0.251906	10.26379	3.434694	0.000635	0.001343	-1.43263
DUSP16	0.251905	6.895637	2.828538	0.004835	0.008842	-3.29345
SNRK-AS1	0.251884	5.553587	2.940472	0.003405	0.006391	-2.9761
SOX10	0.251837	6.120542	4.999343	7.59E-07	2.39E-06	4.945039
ACTG2	0.251674	5.896716	5.729659	1.60E-08	6.12E-08	8.667242
SAC3D1	0.25165	6.035734	2.216832	0.027014	0.042708	-4.81492
NEUROD2	0.25145	5.423181	4.729122	2.82E-06	8.25E-06	3.685567
BRAT1	0.251426	6.965918	2.512363	0.012258	0.020813	-4.12494
CACFD1	0.251381	7.843696	5.469142	6.68E-08	2.38E-07	7.286843
DPPA2	0.251368	4.467022	6.649563	6.70E-11	3.35E-10	13.99391
HDGFL1	0.25133	7.141134	5.101426	4.55E-07	1.47E-06	5.437571
DMRTB1	0.251271	5.254872	6.103109	1.88E-09	8.00E-09	10.74575
STIL	0.251206	5.217965	2.104138	0.035788	0.055093	-5.05569
PTPN1	0.251119	5.931766	7.361628	6.12E-13	3.78E-12	18.58236
EYA3	0.251096	7.223596	5.517151	5.16E-08	1.86E-07	7.536884
EFEMP2	0.250882	6.349025	3.392727	0.000738	0.001549	-1.57265
FSTL4	0.250867	5.543005	6.639749	7.13E-11	3.55E-10	13.93345
CUL4B	0.250816	7.84056	3.346573	0.00087	0.001803	-1.72474
PPP5D1	0.250685	4.45413	4.824845	1.79E-06	5.36E-06	4.124336
FAM160A1	0.250553	6.539768	6.914796	1.22E-11	6.58E-11	15.65682
EFCAB4A	0.250551	6.086304	2.578648	0.01016	0.017526	-3.95859
LRRC15	0.250308	5.225045	6.404206	3.09E-10	1.44E-09	12.50553
CCDC135	0.250289	5.521213	4.909211	1.18E-06	3.64E-06	4.517772
VPS28	0.250205	9.739082	2.209615	0.027515	0.043438	-4.83071
NAV2	0.250117	5.336853	6.670043	5.88E-11	2.97E-10	14.12033
AMHR2	0.250054	6.546682	4.176274	3.41E-05	8.66E-05	1.312145
KIF22	0.250035	5.819424	3.759625	0.000187	0.000429	-0.29269
SPATC1L	0.249833	5.364593	2.403657	0.016539	0.027382	-4.38857
PIF1	0.249752	5.14692	4.97828	8.43E-07	2.63E-06	4.84455
RNF40	0.249633	6.639346	4.629632	4.51E-06	1.28E-05	3.238178
POLR2L	0.249495	7.445201	3.143528	0.001753	0.003456	-2.36994
KRT75	0.249435	5.266051	5.517849	5.14E-08	1.85E-07	7.540534
CMTM6	0.249384	10.0851	4.40748	1.24E-05	3.33E-05	2.271198
SCN1B	0.249362	6.700996	3.259051	0.001182	0.002399	-2.00763
PKP1	0.249232	5.464235	5.985458	3.74E-09	1.53E-08	10.07839
CPSF1	0.249207	5.564104	4.516387	7.60E-06	2.10E-05	2.739704
RAB7A	0.249123	6.674472	7.727026	4.75E-14	3.28E-13	21.08749
CECR7	0.249091	4.933123	6.420492	2.79E-10	1.31E-09	12.60282
C16orf70	0.248899	6.567403	5.968941	4.12E-09	1.67E-08	9.98562
MED8	0.248887	7.190671	4.430008	1.12E-05	3.03E-05	2.367232
SLC39A11	0.248851	7.326217	2.780934	0.005593	0.010118	-3.42477
HOXD-AS	0.248838	5.738006	4.750873	2.55E-06	7.48E-06	3.784554
CETN1	0.248788	5.867947	5.734786	1.56E-08	5.95E-08	8.694985
STK19	0.24875	7.226576	4.87327	1.41E-06	4.30E-06	4.349396
C10orf10	0.248732	5.217937	5.366043	1.16E-07	4.00E-07	6.756565
C6orf89	0.248683	7.607663	4.23644	2.63E-05	6.77E-05	1.557043
CHDH	0.248654	5.723391	6.493138	1.78E-10	8.56E-10	13.03941
POLDIP3	0.248476	6.698667	4.620034	4.71E-06	1.34E-05	3.195485
MAP3K10	0.248247	7.320758	4.359153	1.54E-05	4.08E-05	2.066727
LOC10192	0.24814	5.769423	4.767156	2.36E-06	6.95E-06	3.858931
GPX3	0.248127	5.385831	2.431104	0.015349	0.025564	-4.32308
KCNJ5	0.248118	6.148188	6.233214	8.70E-10	3.85E-09	11.49705

HIST1H3F	0.247969	4.212489	6.655969	6.43E-11	3.23E-10	14.03342
BTBD2	0.247966	6.698555	2.273316	0.023365	0.037402	-4.68959
GPR35	0.247924	6.64661	3.142945	0.001756	0.003462	-2.37174
CCNO	0.247686	5.099767	6.250562	7.84E-10	3.49E-09	11.59828
CSNK1G1	0.247663	6.317036	6.585944	1.00E-10	4.92E-10	13.60335
TET3	0.247501	8.350662	4.118807	4.35E-05	0.000109	1.08132
SEC14L5	0.247303	5.734504	2.171376	0.0303	0.047418	-4.91352
C9orf3	0.247048	5.460637	5.746946	1.46E-08	5.58E-08	8.760878
AP3S1	0.246691	10.89412	4.290616	2.08E-05	5.42E-05	1.780376
SAT1	0.246433	12.42038	4.30268	1.97E-05	5.16E-05	1.830469
LURAP1L	0.246425	5.085363	4.745651	2.61E-06	7.66E-06	3.760748
MAD2L1BI	0.246337	8.45142	3.961288	8.36E-05	0.000201	0.464123
NCR1	0.246255	6.346034	4.664456	3.83E-06	1.10E-05	3.39377
RAX	0.246249	6.196822	4.96813	8.86E-07	2.76E-06	4.796264
UBQLN3	0.246162	4.353135	7.03546	5.52E-12	3.08E-11	16.43164
SMG7	0.246136	6.847432	5.404493	9.44E-08	3.30E-07	6.953258
TGM6	0.24607	6.219123	4.295115	2.04E-05	5.32E-05	1.799044
LINC00521	0.245979	6.025521	5.043013	6.10E-07	1.94E-06	5.15462
AP2S1	0.24589	9.086869	2.102893	0.035897	0.055228	-5.05828
ZBTB22	0.245731	6.992386	3.174755	0.001578	0.003141	-2.27325
VGLL2	0.245713	5.251861	5.814881	9.94E-09	3.87E-08	9.131283
OR2B6	0.245692	4.524325	7.132444	2.89E-12	1.66E-11	17.0626
SMTN	0.245604	5.499005	4.005467	6.98E-05	0.00017	0.634926
KLHL18	0.245481	6.849633	6.109359	1.82E-09	7.71E-09	10.78152
PGPEP1	0.245437	6.608307	4.175535	3.42E-05	8.68E-05	1.309157
MINK1	0.245098	7.682005	3.446765	0.000608	0.001289	-1.39204
NDUFC1	0.245026	7.342153	5.108982	4.38E-07	1.41E-06	5.474389
SAMD1	0.244884	6.776685	5.027276	6.60E-07	2.09E-06	5.078902
VEGFC	0.244719	4.645119	3.736186	0.000205	0.000467	-0.37821
PPP6R1	0.244666	8.61757	2.840751	0.004656	0.008546	-3.2594
PRAF2	0.24462	7.5579	2.956804	0.003233	0.00609	-2.9288
DCTN2	0.244599	6.673883	7.000869	6.93E-12	3.83E-11	16.20836
FCHSD1	0.24458	6.175949	3.390321	0.000745	0.001561	-1.58063
INE1	0.244557	6.758508	5.151558	3.53E-07	1.15E-06	5.682782
E2F3	0.244553	9.476142	3.077123	0.002187	0.004246	-2.57249
KLF15	0.244494	6.35043	5.849496	8.17E-09	3.22E-08	9.321509
WIBG	0.24446	7.058462	4.004237	7.01E-05	0.000171	0.630146
DAB2	0.24425	5.111583	3.358453	0.000834	0.001734	-1.68578
PADI1	0.2442	6.522998	6.87992	1.53E-11	8.19E-11	15.435
ARPC2	0.244181	12.40826	5.106315	4.44E-07	1.43E-06	5.461388
NT5C2	0.244122	10.49337	4.266664	2.31E-05	5.98E-05	1.681309
CHD7	0.24411	7.018578	4.813922	1.88E-06	5.63E-06	4.073856
LOC10272	0.244073	6.644096	4.189532	3.22E-05	8.21E-05	1.365827
SCAMP2	0.24398	8.455085	3.235107	0.001284	0.002591	-2.08376
STAC	0.243821	6.616268	3.670894	0.000264	0.000591	-0.61375
RNFT1	0.243812	6.563155	2.852787	0.004485	0.008255	-3.22571
CENPBD1F	0.24377	7.434296	4.200294	3.08E-05	7.86E-05	1.409519
CSF3	0.243737	6.797438	4.315386	1.87E-05	4.90E-05	1.883374
FKBPL	0.243699	6.276458	4.559739	6.23E-06	1.74E-05	2.929168
OR1D2	0.243651	5.883327	5.370349	1.13E-07	3.91E-07	6.778527
MAVS	0.243524	7.235334	3.936325	9.26E-05	0.000222	0.368401
ITGA1	0.243448	4.678656	2.713694	0.006848	0.012198	-3.60658
PNCK	0.243438	6.116185	5.439331	7.84E-08	2.77E-07	7.132577
APLNR	0.243346	5.728119	4.383068	1.38E-05	3.69E-05	2.167647
GTSF1L	0.243323	5.655057	5.046152	6.01E-07	1.91E-06	5.169752
LOC10050	0.243298	6.395953	2.565876	0.010537	0.018121	-3.99097
LINC00202	0.243228	6.674251	4.309419	1.92E-05	5.02E-05	1.858512
ATP13A2	0.243126	6.257018	2.155981	0.031488	0.049069	-4.94646

ZNF628	0.243013	5.476959	3.089652	0.002098	0.004088	-2.53459
CPNE7	0.242961	6.591913	3.830191	0.000142	0.000331	-0.03214
KCNQ3	0.242933	5.89728	6.289049	6.21E-10	2.80E-09	11.82373
C20orf78	0.24289	5.47005	5.608041	3.14E-08	1.16E-07	8.015644
SGPP2	0.242817	4.511482	4.567284	6.02E-06	1.68E-05	2.962314
OR7E47P	0.242781	6.049845	4.647301	4.15E-06	1.19E-05	3.316988
CDC40	0.242633	8.192611	2.913571	0.003708	0.006913	-3.05346
ACTR3	0.242613	10.59424	4.350548	1.60E-05	4.23E-05	2.030539
NINJ2	0.242469	8.78567	2.580138	0.010116	0.01746	-3.9548
ANXA2P1	0.242255	5.146977	4.090369	4.90E-05	0.000122	0.968207
HRAS	0.242193	6.141077	2.649417	0.008279	0.014531	-3.77631
MIR205HG	0.242119	4.433922	4.965596	8.98E-07	2.79E-06	4.784223
TXNRD1	0.241627	9.923873	3.630606	0.000307	0.000681	-0.75711
AIFM1	0.241542	6.88363	3.100556	0.002024	0.003954	-2.50149
CCR8	0.241468	5.349639	5.913891	5.66E-09	2.26E-08	9.678065
KCTD20	0.241459	8.126785	4.731909	2.79E-06	8.15E-06	3.698229
SLC5A9	0.241416	5.749847	2.819457	0.004972	0.009076	-3.31867
OR12D2	0.241363	5.424339	6.053731	2.52E-09	1.05E-08	10.46426
CDC25A	0.24134	5.623788	3.43758	0.000628	0.00133	-1.42294
AP1S1	0.241179	5.890998	4.071191	5.31E-05	0.000131	0.892348
ABI1	0.241101	9.794495	4.346087	1.63E-05	4.31E-05	2.011808
TEX40	0.241073	6.207148	4.322968	1.81E-05	4.75E-05	1.915014
TFDP3	0.241007	5.359799	5.094855	4.70E-07	1.52E-06	5.405591
MIXL1	0.24097	5.213191	3.957078	8.51E-05	0.000204	0.447936
ACTR10	0.240923	10.43337	4.015366	6.70E-05	0.000163	0.673444
SMIM1	0.24087	6.031697	2.953687	0.003265	0.006144	-2.93785
MYOZ1	0.240835	5.879818	4.500447	8.17E-06	2.24E-05	2.670465
ANXA4	0.24071	8.956158	2.596898	0.009641	0.016694	-3.91204
REC114	0.240586	4.959492	5.072853	5.25E-07	1.68E-06	5.298792
KIR2DS3	0.240523	5.901144	4.127445	4.20E-05	0.000105	1.115822
DAP	0.240498	8.532722	3.113087	0.001941	0.003801	-2.46331
SUN5	0.240296	5.605335	6.900505	1.34E-11	7.19E-11	15.56581
TP53BP2	0.240214	8.468668	3.508529	0.000485	0.001043	-1.18226
POLL	0.240118	6.85896	3.04677	0.002416	0.004652	-2.66367
MAPK7	0.240029	6.823083	4.04586	5.91E-05	0.000145	0.792663
WDR93	0.239897	5.665393	3.934585	9.32E-05	0.000223	0.361751
FAM132B	0.239843	5.424182	2.967783	0.003121	0.005901	-2.89686
ARHGAP33	0.239826	6.256393	5.077272	5.14E-07	1.65E-06	5.32021
PMS2L2	0.239754	5.066746	2.79501	0.005358	0.009723	-3.38617
MVB12B	0.239618	6.516641	5.434608	8.04E-08	2.84E-07	7.108206
FGD5	0.239493	5.734805	6.232833	8.72E-10	3.86E-09	11.49483
THEG	0.239451	6.097329	3.87134	0.00012	0.000284	0.121919
FTL	0.239425	13.59953	3.132719	0.001818	0.003572	-2.4032
C10orf95	0.23921	5.688836	4.382307	1.39E-05	3.70E-05	2.164428
FOXN3-AS	0.238986	5.992528	4.817817	1.85E-06	5.53E-06	4.091843
PRPH	0.238824	6.790654	4.412254	1.22E-05	3.26E-05	2.291508
DAD1	0.238821	9.530442	3.298289	0.001031	0.002112	-1.88169
FOXF1	0.238801	4.841537	5.784569	1.18E-08	4.56E-08	8.96553
C1orf194	0.238796	5.365618	4.421934	1.16E-05	3.13E-05	2.332761
SYN1	0.238768	5.887547	4.232318	2.68E-05	6.89E-05	1.540159
ZSCAN12P	0.238579	5.522993	4.71926	2.96E-06	8.62E-06	3.640827
PLCL2	0.238577	7.582171	5.209227	2.62E-07	8.70E-07	5.967571
SLMO1	0.238256	5.284723	4.513076	7.71E-06	2.13E-05	2.725304
GPR64	0.238139	4.776759	4.073318	5.27E-05	0.00013	0.900743
PAPLN	0.23789	5.170838	3.621567	0.000318	0.000704	-0.78907
VWA5B2	0.237869	5.728684	3.316411	0.000968	0.001993	-1.82304
TXNDC17	0.237824	7.708092	5.23836	2.26E-07	7.55E-07	6.112541
TTTY15	0.237714	5.460278	2.295452	0.022057	0.035476	-4.63963

MGST2	0.2376	7.925218	3.234017	0.001289	0.0026	-2.08721
RARRES2	0.237597	4.843167	3.029838	0.002554	0.004897	-2.71416
HAND1	0.237532	5.340146	5.115839	4.23E-07	1.37E-06	5.507845
HIPK1	0.237455	9.65636	3.169837	0.001604	0.003188	-2.28854
MUC1	0.237409	5.733042	4.618658	4.74E-06	1.35E-05	3.189371
TAOK2	0.237324	6.347947	3.577045	0.000376	0.000824	-0.94536
EIF6	0.237304	8.31097	2.674769	0.007685	0.013563	-3.70984
RAB21	0.237301	6.940819	3.950474	8.74E-05	0.00021	0.422583
BANF2	0.2373	4.834912	6.284989	6.37E-10	2.87E-09	11.79989
TEX14	0.237198	4.564652	5.375955	1.10E-07	3.81E-07	6.807147
PLEKHG2	0.237096	5.642448	4.089484	4.92E-05	0.000122	0.9647
ZNF296	0.237008	5.740079	3.165743	0.001626	0.003228	-2.30125
PIK3CA	0.236955	6.947054	3.497821	0.000504	0.001081	-1.21888
SEC22C	0.23695	6.146599	5.037564	6.27E-07	1.99E-06	5.128377
LINC0083(	0.236785	5.057353	5.617368	2.99E-08	1.10E-07	8.06517
BMP2	0.236746	4.520009	3.200632	0.001445	0.002893	-2.19242
CCK	0.236741	4.915877	5.258449	2.03E-07	6.83E-07	6.212931
POLD1	0.236732	6.620552	2.576633	0.010218	0.017618	-3.9637
LOH12CR:	0.236667	5.028446	3.514457	0.000474	0.001021	-1.16193
AVPR1B	0.236642	6.850738	4.757798	2.46E-06	7.25E-06	3.816157
LUZP4	0.236468	6.289302	5.224812	2.42E-07	8.06E-07	6.045035
MED12L	0.236445	4.97383	4.598449	5.21E-06	1.47E-05	3.099773
ASF1B	0.236224	7.350541	2.406045	0.016432	0.02722	-4.3829
TMCO2	0.236091	5.312304	6.279428	6.59E-10	2.96E-09	11.76726
SLC39A3	0.236035	6.556902	5.228608	2.37E-07	7.91E-07	6.063933
KCNH1	0.236024	4.647754	4.428577	1.13E-05	3.05E-05	2.361118
PHYKPL	0.235951	7.686881	4.088568	4.94E-05	0.000123	0.96107
MYBPC1	0.235911	4.313238	5.023846	6.72E-07	2.13E-06	5.06243
ADCYAP1I	0.235901	5.462851	6.58579	1.00E-10	4.92E-10	13.6024
IGFBP4	0.235799	5.973915	3.678205	0.000256	0.000575	-0.58757
ASH2L	0.235709	9.545986	4.663017	3.86E-06	1.11E-05	3.387318
PXMP2	0.235525	6.126199	3.060451	0.00231	0.004465	-2.62268
SEC61B	0.23548	7.810615	4.763206	2.40E-06	7.07E-06	3.840867
NFKBIL1	0.23542	6.399863	2.691012	0.007325	0.01298	-3.66692
GPR50	0.235331	5.170113	6.418244	2.83E-10	1.33E-09	12.58938
STARD10	0.235295	6.550969	2.17228	0.030231	0.047331	-4.91158
DYNC2LI1	0.235268	4.533802	2.690684	0.007332	0.01299	-3.66779
LOC10050	0.235222	4.652057	5.984419	3.77E-09	1.54E-08	10.07255
MED22	0.235219	6.573672	2.533656	0.011545	0.019697	-4.07196
POC1A	0.235129	5.676671	4.843647	1.63E-06	4.92E-06	4.21147
MTG2	0.234989	6.497449	5.826658	9.30E-09	3.63E-08	9.19589
SPEM1	0.234859	5.943269	4.060272	5.56E-05	0.000137	0.849306
ADPRH	0.234648	5.803884	4.676091	3.63E-06	1.05E-05	3.445996
STGC3	0.234616	5.29867	4.808198	1.93E-06	5.78E-06	4.047443
RUNDC1	0.234485	6.910923	4.343166	1.65E-05	4.36E-05	1.999553
NFKBIB	0.234437	6.623492	3.218836	0.001358	0.002727	-2.13518
LRRC41	0.234323	5.673793	4.974571	8.59E-07	2.68E-06	4.826895
HHLA1	0.234293	6.168036	6.139299	1.52E-09	6.53E-09	10.95333
PTPN7	0.234241	7.563224	2.298373	0.021889	0.035238	-4.633
FGF6	0.234238	6.277587	4.581116	5.64E-06	1.59E-05	3.023217
SPINT1	0.234159	6.508259	3.998794	7.17E-05	0.000174	0.609011
STARD8	0.234098	6.445669	4.115031	4.42E-05	0.000111	1.066257
HIST1H4J	0.234092	6.768887	2.507702	0.012419	0.021061	-4.13648
AKAP13	0.234016	7.960153	4.006638	6.95E-05	0.000169	0.639478
MRPL37	0.233819	7.298528	2.102055	0.035971	0.055337	-5.06003
NUDT16P:	0.23377	4.644218	2.407352	0.016374	0.027127	-4.3798
CCDC53	0.233759	8.591497	3.242366	0.001252	0.002531	-2.06073
PROKR2	0.233745	5.436766	5.815411	9.91E-09	3.86E-08	9.134192

GORASP1	0.233641	6.282882	5.079653	5.08E-07	1.63E-06	5.331755
CACNB1	0.233555	5.667726	5.128819	3.96E-07	1.29E-06	5.571289
BCR	0.233392	6.715358	2.754211	0.006064	0.010899	-3.49755
RNF38	0.233371	7.983127	3.964156	8.27E-05	0.000199	0.475154
SAMD4B	0.233336	6.949223	3.777022	0.000175	0.000402	-0.22889
FAM212B-	0.233244	6.557629	5.354081	1.23E-07	4.24E-07	6.695633
MTSS1L	0.233215	5.320756	3.692655	0.000242	0.000546	-0.53569
RAB3GAP1	0.233183	6.439679	5.400663	9.63E-08	3.37E-07	6.93361
SNX21	0.233156	6.37009	4.575339	5.80E-06	1.63E-05	2.997759
LINC0026:	0.233084	4.092143	5.48309	6.20E-08	2.21E-07	7.359285
FAM129B	0.233068	6.559288	3.539991	0.000432	0.000937	-1.07402
ERICH1	0.232823	6.733796	3.544855	0.000424	0.000921	-1.0572
GAB2	0.232801	9.353337	2.840988	0.004652	0.008542	-3.25874
GLIS2	0.232666	5.725089	3.346924	0.000869	0.001801	-1.72359
EPHX3	0.232648	5.248156	4.545032	6.66E-06	1.86E-05	2.864703
ZFR	0.232628	7.479452	2.635494	0.008622	0.015092	-3.81255
SHOX2	0.232604	4.66424	6.731732	3.97E-11	2.04E-10	14.50313
KIAA0556	0.232592	7.55053	3.039118	0.002478	0.004759	-2.68652
2-Mar	0.232566	4.950853	5.154664	3.47E-07	1.14E-06	5.698051
SEC61G	0.232364	8.994489	2.714624	0.006829	0.01217	-3.60409
KLHL6	0.232295	8.138553	2.303836	0.021578	0.034787	-4.62058
ITGB1BP2	0.232256	4.806385	5.370311	1.13E-07	3.91E-07	6.778337
FOXN4	0.232216	5.150914	5.709767	1.79E-08	6.80E-08	8.559807
IFT20	0.232115	8.983635	3.26276	0.001167	0.002371	-1.99578
TP53INP2	0.232112	7.477009	2.368088	0.018201	0.029837	-4.47235
TGFBR2	0.232112	8.602476	3.558013	0.000404	0.00088	-1.0116
LOC64662	0.232078	5.243012	4.460857	9.77E-06	2.66E-05	2.499482
PCTP	0.231995	8.834051	2.409702	0.01627	0.026979	-4.37421
CORO1B	0.231886	7.20908	2.535755	0.011477	0.01959	-4.06672
TRIM41	0.23183	7.541349	2.885012	0.004057	0.007511	-3.13483
AIM1L	0.231811	5.907562	5.320883	1.47E-07	5.01E-07	6.527169
TMEM102	0.231766	5.572333	3.362629	0.000822	0.00171	-1.67206
MUC5B	0.231696	5.03575	4.787924	2.13E-06	6.33E-06	3.954135
SH3TC1	0.231681	6.703623	2.852261	0.004493	0.008266	-3.22719
IGF2R	0.231459	10.61134	2.058615	0.039969	0.060908	-5.14945
RPH3AL	0.231389	5.210218	5.616308	3.01E-08	1.11E-07	8.059538
NKAP	0.231374	7.720439	3.359097	0.000832	0.001731	-1.68367
MIR670HG	0.231358	5.774254	5.830229	9.11E-09	3.56E-08	9.215504
SYNGR4	0.23135	5.310532	5.273407	1.88E-07	6.35E-07	6.28791
C1orf228	0.231301	5.927594	2.818118	0.004992	0.009111	-3.32238
HS3ST5	0.231293	5.348462	4.535157	6.97E-06	1.94E-05	2.821528
SDC1	0.231268	5.214052	3.653609	0.000282	0.000628	-0.67544
MYLPF	0.231256	6.291809	3.807514	0.000155	0.00036	-0.11638
GPC1	0.231255	5.309437	3.95397	8.62E-05	0.000207	0.436003
PEX16	0.231251	7.367187	3.826551	0.000144	0.000335	-0.04569
ZCCHC11	0.231232	6.290425	3.146095	0.001738	0.003429	-2.36203
SLC25A48	0.231179	5.1972	5.59989	3.29E-08	1.21E-07	7.972423
PMVK	0.231105	6.570332	2.651205	0.008236	0.014462	-3.77164
TRMT2B	0.230938	6.392392	3.46816	0.000562	0.001198	-1.31978
NEURL3	0.230902	7.04008	3.508086	0.000486	0.001044	-1.18377
C7orf61	0.230883	6.070791	3.91266	0.000102	0.000242	0.278192
LGALS8-A	0.230826	6.433054	4.58788	5.47E-06	1.54E-05	3.053059
TUBB7P	0.230791	4.95831	4.135762	4.05E-05	0.000102	1.14911
GNA14	0.230664	5.102757	6.911568	1.25E-11	6.71E-11	15.63625
RASD2	0.230446	4.957628	5.610583	3.10E-08	1.14E-07	8.029133
SPRR2G	0.230403	6.1917	4.805106	1.96E-06	5.86E-06	4.033188
CSPG5	0.230299	4.939218	7.732388	4.57E-14	3.16E-13	21.12499
RGS3	0.230266	6.657836	4.331049	1.74E-05	4.59E-05	1.948792

CTDSP2	0.230156	9.510284	4.364047	1.51E-05	4.00E-05	2.087335
DHRS7C	0.229881	5.184844	4.791547	2.10E-06	6.23E-06	3.970782
TFPI	0.229773	4.113876	3.10304	0.002007	0.003923	-2.49393
OCA2	0.229721	6.713704	5.968488	4.13E-09	1.68E-08	9.98308
SNX19	0.229716	7.530496	4.042031	6.00E-05	0.000147	0.777649
ENPP7	0.22969	6.161362	2.497615	0.012774	0.021609	-4.16137
FCN2	0.22956	5.422582	4.622532	4.66E-06	1.32E-05	3.206585
SLC17A9	0.229529	6.350413	4.697952	3.27E-06	9.48E-06	3.544453
ASB8	0.229508	8.580877	4.485481	8.74E-06	2.39E-05	2.605661
SPAST	0.229462	7.103331	2.363452	0.018428	0.030182	-4.48318
LINC00654	0.229416	4.837601	4.915429	1.15E-06	3.53E-06	4.547017
OR7E12P	0.22934	6.559896	3.804581	0.000157	0.000363	-0.12723
REEP5	0.229191	9.804964	4.262169	2.36E-05	6.09E-05	1.662772
KAL1	0.229147	5.119313	3.932358	9.41E-05	0.000225	0.353243
SMUG1	0.229114	5.873962	5.250867	2.11E-07	7.09E-07	6.175002
MIR4500H	0.229008	4.419378	6.179868	1.20E-09	5.20E-09	11.18732
DKK1	0.228984	4.554921	4.454875	1.00E-05	2.73E-05	2.473773
SREBF2	0.228958	7.250031	2.81504	0.00504	0.009191	-3.3309
KIF9	0.228933	5.689876	6.419429	2.81E-10	1.32E-09	12.59646
GTF2F1	0.228894	7.533944	3.383819	0.000762	0.001596	-1.60216
ZDHHC24	0.228841	6.508349	4.020237	6.57E-05	0.00016	0.692432
LOC10050	0.228683	5.673989	3.516613	0.000471	0.001014	-1.15453
KCNAB2	0.228675	7.529285	2.320447	0.020656	0.03345	-4.58264
MYH14	0.228492	6.085963	5.232032	2.33E-07	7.78E-07	6.080986
PRODH2	0.22843	5.820434	3.070902	0.002232	0.004327	-2.59125
PRAMEF1(	0.228337	5.873465	4.151923	3.78E-05	9.54E-05	1.213969
TMEM255I	0.228307	5.582743	3.363789	0.000819	0.001704	-1.66824
PLCD1	0.228303	8.264745	3.371523	0.000796	0.001662	-1.64277
UBALD1	0.228259	7.235567	2.337344	0.019754	0.03213	-4.54377
EMILIN3	0.22817	5.171474	4.940707	1.01E-06	3.14E-06	4.666265
SYNE4	0.228165	5.357677	5.00075	7.54E-07	2.37E-06	4.951767
SEC62	0.228092	9.914167	3.51505	0.000473	0.001019	-1.1599
DAPL1	0.227986	5.066059	4.227021	2.74E-05	7.04E-05	1.518486
FAM189A2	0.22793	6.03799	4.750006	2.56E-06	7.51E-06	3.780601
SLC35A5	0.22778	8.465817	2.714483	0.006832	0.012172	-3.60447
GS1-279B	0.227769	5.58131	5.095817	4.68E-07	1.51E-06	5.410271
PRKCB	0.227692	8.370997	4.53102	7.11E-06	1.97E-05	2.803467
MLXIPL	0.227629	6.668148	3.004997	0.002768	0.005281	-2.78773
USH1C	0.227567	5.651336	5.841607	8.54E-09	3.35E-08	9.278067
MCAT	0.227514	5.86074	4.28172	2.16E-05	5.62E-05	1.743521
AGT	0.227421	5.719583	4.721682	2.92E-06	8.53E-06	3.651808
MUC6	0.227412	5.771441	4.430292	1.12E-05	3.03E-05	2.368447
DDOST	0.227329	9.255816	2.131865	0.03343	0.051766	-4.9976
NOL12	0.227212	6.549883	3.719615	0.000219	0.000496	-0.43837
CACNG5	0.227197	5.423389	4.507498	7.91E-06	2.18E-05	2.701063
GNAO1	0.227182	6.037831	5.092659	4.75E-07	1.53E-06	5.394911
PIGO	0.227068	6.004288	5.289924	1.73E-07	5.84E-07	6.370926
SMO	0.226971	6.468043	5.262319	1.99E-07	6.71E-07	6.232312
NMRK1	0.226941	8.72644	2.639222	0.008529	0.014944	-3.80287
ECEL1	0.226784	6.258091	5.078976	5.09E-07	1.64E-06	5.328469
SPTB	0.226742	6.581857	4.532461	7.06E-06	1.96E-05	2.809757
SKIV2L	0.226677	8.03787	2.844851	0.004597	0.008448	-3.24794
C7orf62	0.226569	4.883186	5.417663	8.80E-08	3.09E-07	7.020924
IFT172	0.226466	5.673576	4.965811	8.97E-07	2.79E-06	4.785245
KIF1C	0.226323	6.505949	4.614334	4.84E-06	1.37E-05	3.17017
UNC119	0.226184	8.52149	2.171715	0.030274	0.047394	-4.91279
AVL9	0.226153	6.811973	5.467432	6.74E-08	2.40E-07	7.277974
MARK3	0.226134	6.878892	3.848902	0.000132	0.000308	0.037715

GKN2	0.226086	5.637437	4.519323	7.50E-06	2.07E-05	2.752483
TBC1D13	0.225955	7.475526	3.841583	0.000135	0.000317	0.010351
LURAP1	0.225524	6.324569	4.776574	2.25E-06	6.67E-06	3.902056
NAA60	0.225403	9.120396	3.367237	0.000809	0.001685	-1.65689
NFE2L2	0.225331	10.73262	2.727552	0.00657	0.011733	-3.56946
NT5DC3	0.225315	5.974514	5.018752	6.89E-07	2.18E-06	5.037981
FSCN3	0.225163	5.867133	4.523704	7.35E-06	2.03E-05	2.771563
GAMT	0.225151	5.429587	3.160956	0.001653	0.003277	-2.31609
SPAG4	0.225058	4.740605	4.276356	2.22E-05	5.75E-05	1.721333
EMR2	0.225015	7.810959	2.631994	0.00871	0.015232	-3.82163
DKKL1	0.224671	5.949665	3.750503	0.000194	0.000443	-0.32603
SLC17A3	0.224634	5.080144	4.424957	1.15E-05	3.09E-05	2.345661
AMBN	0.224574	5.592659	4.554943	6.37E-06	1.78E-05	2.908124
CDC42BPE	0.224538	4.928291	3.003983	0.002777	0.005296	-2.79073
MCAM	0.224363	5.814438	3.159945	0.001659	0.003287	-2.31922
TM4SF4	0.224353	5.429077	5.256229	2.06E-07	6.91E-07	6.201822
LYL1	0.224349	8.829008	2.870747	0.004242	0.007835	-3.17518
RAC3	0.224303	4.877826	3.506743	0.000488	0.001049	-1.18837
STK32B	0.224173	5.570346	5.295205	1.68E-07	5.69E-07	6.397522
PLCD3	0.224062	6.731163	4.097094	4.77E-05	0.000119	0.994891
MYF6	0.224015	4.930303	4.40858	1.24E-05	3.31E-05	2.275874
CYP1A1	0.223956	5.525028	5.352491	1.24E-07	4.27E-07	6.687543
KRTAP5-8	0.223917	7.482064	4.839167	1.67E-06	5.02E-06	4.190683
PPEF1	0.223815	4.711163	4.875446	1.40E-06	4.25E-06	4.359553
HIST1H3B	0.223769	4.679537	4.260056	2.38E-05	6.15E-05	1.654066
MTA2	0.22375	5.443894	2.1435	0.03248	0.050455	-4.973
FSD1L	0.223523	4.218149	2.60335	0.009464	0.016423	-3.89551
EPHX1	0.223515	5.707684	2.180772	0.029594	0.046449	-4.8933
RFFL	0.223495	6.664591	3.038101	0.002486	0.004774	-2.68956
GSG2	0.223489	5.502572	3.906958	0.000104	0.000248	0.25653
FAM20C	0.223421	5.897625	4.382494	1.39E-05	3.70E-05	2.165218
C4orf17	0.223366	5.355828	4.307364	1.93E-05	5.07E-05	1.849957
ESRRA	0.223345	7.259925	3.445038	0.000611	0.001296	-1.39786
BACE1	0.223322	5.93303	6.579673	1.04E-10	5.10E-10	13.56502
SLC5A2	0.223267	6.358834	4.856598	1.53E-06	4.63E-06	4.271675
FGD3	0.223206	11.29691	2.32513	0.020403	0.033087	-4.5719
CHEK1	0.223156	4.674861	3.394449	0.000734	0.00154	-1.56694
DSTYK	0.223121	5.865744	5.226591	2.40E-07	7.99E-07	6.053886
ATXN7L3B	0.223117	7.916063	3.336967	0.0009	0.001863	-1.75614
ZNF213	0.223095	7.604224	3.519891	0.000465	0.001003	-1.14327
WBP1L	0.223091	9.775102	4.037011	6.13E-05	0.00015	0.75798
CACNA2D	0.223076	4.579945	6.480465	1.93E-10	9.22E-10	12.96294
ADCY4	0.223069	7.414892	3.401454	0.000716	0.001505	-1.54367
SLC9A1	0.222991	6.77836	5.141263	3.72E-07	1.21E-06	5.632251
HBZ	0.222983	5.07987	3.170754	0.001599	0.003179	-2.28569
UBA6	0.222955	6.569289	3.331569	0.000917	0.001895	-1.77375
ARRDC3-A	0.222729	5.841946	5.178505	3.07E-07	1.01E-06	5.815498
ARCN1	0.222683	9.597117	3.641533	0.000295	0.000656	-0.71838
IL17RC	0.222597	6.281712	5.210816	2.60E-07	8.63E-07	5.975459
ETNK1	0.222593	6.129992	2.697747	0.00718	0.01275	-3.64906
LINC01339	0.222583	5.780172	4.270359	2.27E-05	5.89E-05	1.696557
ALPP	0.222556	4.94371	4.245322	2.53E-05	6.53E-05	1.593474
SLC16A4	0.222522	3.853535	3.555339	0.000408	0.000888	-1.02088
SPCS3	0.222386	8.97381	3.256782	0.001191	0.002418	-2.01486
ETV4	0.22208	6.114955	3.445312	0.000611	0.001295	-1.39694
CAMK1G	0.221973	5.73924	5.14837	3.58E-07	1.17E-06	5.667124
NR2C2	0.22194	7.376062	2.615704	0.009132	0.015899	-3.86374
MFAP4	0.221868	5.675941	4.599551	5.18E-06	1.46E-05	3.104649

SLC7A8	0.221855	6.031777	6.775209	3.01E-11	1.56E-10	14.77473
SEMA6C	0.221853	6.964802	3.760299	0.000187	0.000428	-0.29022
TNK2	0.221825	6.60005	2.861785	0.004362	0.00804	-3.20044
TUT1	0.22178	6.085938	2.655591	0.00813	0.014285	-3.76018
NEK9	0.22173	6.829566	6.005814	3.33E-09	1.37E-08	10.19304
ELMO1	0.22171	8.358887	2.498428	0.012745	0.02157	-4.15937
TNNI3	0.221598	4.96477	5.45809	7.09E-08	2.52E-07	7.22956
NIPSNAP1	0.221568	7.703923	4.114678	4.43E-05	0.000111	1.064852
GAPDHS	0.221458	4.596093	5.252062	2.10E-07	7.05E-07	6.180975
MTL5	0.221412	5.26563	5.457691	7.11E-08	2.52E-07	7.227495
TEC	0.221406	5.944018	5.072033	5.28E-07	1.69E-06	5.294821
KHNYN	0.221338	8.565826	3.749195	0.000195	0.000446	-0.33081
PTPN11	0.221328	6.638781	4.656294	3.98E-06	1.14E-05	3.357206
FZR1	0.221217	6.079542	3.574189	0.00038	0.000832	-0.95532
OR51B6	0.22112	5.281695	5.042771	6.11E-07	1.94E-06	5.153456
MPC2	0.221008	7.311366	3.062762	0.002293	0.004434	-2.61574
NCLN	0.220992	6.140743	3.958388	8.46E-05	0.000203	0.452973
CDV3	0.220978	8.999914	3.254123	0.001202	0.002439	-2.02334
KRT82	0.22097	5.840253	3.965256	8.23E-05	0.000198	0.479389
BEST1	0.220938	8.181514	2.473786	0.013649	0.022981	-4.2198
TMEM230	0.220885	9.545044	2.927563	0.003548	0.006639	-3.01331
RILP	0.220874	7.972748	3.062092	0.002298	0.004443	-2.61775
MCM4	0.220756	5.3557	3.328428	0.000928	0.001914	-1.78398
NME4	0.220644	7.15451	2.121159	0.034324	0.053056	-5.02012
PYY	0.220402	4.960357	4.3042	1.96E-05	5.13E-05	1.836791
OR7E37P	0.220231	7.510325	2.936276	0.003451	0.006472	-2.98821
BACH1	0.220186	7.689052	4.727142	2.85E-06	8.33E-06	3.676576
ZSWIM3	0.219948	6.501438	3.51829	0.000468	0.001008	-1.14877
KRT8	0.219877	5.603033	4.602955	5.10E-06	1.44E-05	3.119719
OGDHL	0.219757	5.259086	3.760282	0.000187	0.000428	-0.29029
IL17C	0.219728	6.593297	3.799683	0.00016	0.00037	-0.14535
CENPN	0.219704	4.976739	4.583258	5.59E-06	1.57E-05	3.032663
GSTM5	0.219645	6.436693	3.977374	7.83E-05	0.00019	0.526105
GALC	0.219179	7.519888	3.659806	0.000275	0.000614	-0.65336
FAM83D	0.219065	5.20081	2.327385	0.020282	0.032914	-4.56672
MAT1A	0.21893	6.172761	3.781768	0.000172	0.000395	-0.21143
CNRIP1	0.21887	5.218355	3.017795	0.002656	0.00508	-2.7499
HS3ST4	0.218843	5.61794	4.545118	6.66E-06	1.85E-05	2.865081
ZNF488	0.218813	5.606972	4.756098	2.48E-06	7.31E-06	3.808396
PRR30	0.218765	6.303468	5.48226	6.23E-08	2.22E-07	7.354968
ABCG4	0.218681	6.22483	4.509969	7.82E-06	2.15E-05	2.711796
MR1	0.218681	7.045921	4.153934	3.75E-05	9.46E-05	1.222055
CHKA	0.218665	5.758313	3.722199	0.000216	0.000492	-0.429
DLD	0.21849	8.230108	3.360786	0.000827	0.001721	-1.67812
TGIF2LY	0.218326	5.271418	3.817026	0.000149	0.000347	-0.0811
FOXP4	0.218308	6.32598	5.26903	1.92E-07	6.49E-07	6.26595
IL22RA2	0.218294	4.337649	2.415482	0.016017	0.026593	-4.36045
GSX2	0.218286	5.836944	4.841529	1.65E-06	4.96E-06	4.201642
COX10	0.218265	7.23801	3.655524	0.00028	0.000624	-0.66862
LGI2	0.218038	6.80519	4.064521	5.46E-05	0.000135	0.866041
GPR17	0.217981	4.406961	5.21389	2.56E-07	8.50E-07	5.990726
ERVH48-1	0.217948	4.95745	5.218085	2.51E-07	8.34E-07	6.011571
GTF2E2	0.217741	8.021822	3.325911	0.000936	0.00193	-1.79217
HABP2	0.217637	5.601383	5.42932	8.27E-08	2.91E-07	7.080939
HAS1	0.217595	5.139845	4.283859	2.14E-05	5.57E-05	1.752375
SLC30A3	0.217589	7.687274	4.79866	2.03E-06	6.03E-06	4.003501
LINC00687	0.217535	5.324657	4.520839	7.44E-06	2.06E-05	2.759082
PRG3	0.21744	5.206765	3.106754	0.001982	0.003877	-2.48263

ME1	0.217371	4.775299	2.949432	0.00331	0.006224	-2.95018
EGFLAM	0.217364	5.452024	5.019177	6.88E-07	2.17E-06	5.04002
PLSCR2	0.217349	4.19933	6.860263	1.74E-11	9.25E-11	15.31039
AURKC	0.217339	5.738215	4.06843	5.38E-05	0.000133	0.881454
JSRP1	0.216861	5.587252	2.714646	0.006828	0.01217	-3.60403
CTIF	0.216789	6.415066	5.302257	1.62E-07	5.49E-07	6.43307
WWC2-AS	0.216633	5.485618	3.371818	0.000796	0.001661	-1.6418
SLC35A4	0.216601	8.116377	2.156295	0.031463	0.04904	-4.94579
PSG2	0.216548	5.13836	5.371937	1.12E-07	3.88E-07	6.786636
A2MP1	0.216533	5.540764	4.471171	9.33E-06	2.54E-05	2.543892
BRCA2	0.216529	3.963748	3.536522	0.000437	0.000948	-1.086
STOX2	0.216227	4.446289	5.300584	1.63E-07	5.54E-07	6.424635
TSNAXIP1	0.216173	6.333956	3.631783	0.000306	0.000679	-0.75295
CYHR1	0.216091	6.331047	3.245342	0.001239	0.002507	-2.05128
LOC10050	0.216034	5.548395	3.559484	0.000401	0.000876	-1.00649
TESK2	0.215771	7.609773	2.85057	0.004516	0.008307	-3.23193
MED7	0.215679	7.162321	3.253151	0.001206	0.002446	-2.02644
ORMDL3	0.215604	6.653286	2.238389	0.025567	0.040594	-4.76745
PRR15L	0.215568	5.635834	4.772777	2.29E-06	6.78E-06	3.88466
AUNIP	0.215458	5.179113	3.639531	0.000297	0.000661	-0.72548
FLJ90680	0.215449	5.282465	5.205223	2.68E-07	8.86E-07	5.947706
CSNK2A1	0.215358	7.539646	3.664933	0.00027	0.000604	-0.63506
CLSTN3	0.215255	6.413176	2.736056	0.006405	0.011467	-3.5466
CHD8	0.215183	7.909276	2.150131	0.03195	0.0497	-4.95892
MMP2	0.215126	5.741974	3.46781	0.000563	0.0012	-1.32096
WDR5	0.215123	6.093804	3.00716	0.002749	0.005246	-2.78135
LOC64532	0.215016	5.009077	4.461298	9.75E-06	2.65E-05	2.501381
INPP5K	0.214929	7.458363	3.557589	0.000404	0.000882	-1.01307
CHST15	0.214916	8.473889	2.842167	0.004635	0.008512	-3.25545
SLC44A4	0.214893	4.76658	4.018968	6.60E-05	0.000161	0.687484
CDSN	0.214852	5.457102	6.110545	1.80E-09	7.66E-09	10.78831
ADCY6	0.214834	6.166096	5.207395	2.65E-07	8.77E-07	5.95848
TMEM174	0.21481	5.824775	5.900077	6.12E-09	2.43E-08	9.601283
MT3	0.2147	5.807141	4.394858	1.31E-05	3.51E-05	2.21759
ADRA2C	0.2146	5.805159	3.252016	0.001211	0.002455	-2.03005
HTR3A	0.214552	5.562845	5.914622	5.63E-09	2.25E-08	9.68213
MBD6	0.214389	8.181288	2.788838	0.00546	0.0099	-3.40312
KCNN1	0.214292	7.137526	3.806391	0.000156	0.000361	-0.12054
BMP1	0.214227	6.090367	5.012772	7.10E-07	2.24E-06	5.009307
RFX1	0.214187	6.403246	4.576583	5.76E-06	1.62E-05	3.003239
MYL1	0.21418	4.959521	3.352079	0.000853	0.00177	-1.7067
TEAD4	0.214134	5.494357	6.246219	8.04E-10	3.58E-09	11.57291
ANO7	0.214094	7.041692	4.215925	2.88E-05	7.37E-05	1.473168
CAPN13	0.214094	5.301652	5.990961	3.63E-09	1.49E-08	10.10935
PVRL4	0.214015	6.477011	3.96642	8.19E-05	0.000198	0.483871
PTPN18	0.213999	8.836072	2.65018	0.00826	0.014501	-3.77432
RABIF	0.213987	7.604561	4.037666	6.11E-05	0.00015	0.760545
SPOPL	0.213912	9.829208	2.448545	0.014633	0.024488	-4.28109
NRAS	0.213872	8.049238	2.580194	0.010115	0.017459	-3.95466
FBN3	0.213834	5.677001	4.614645	4.83E-06	1.37E-05	3.171548
PLVAP	0.21375	5.884804	3.827264	0.000143	0.000334	-0.04304
EAF1	0.213666	7.745053	2.421043	0.015776	0.02621	-4.34717
KCNIP4	0.213502	5.045823	6.533099	1.39E-10	6.76E-10	13.28139
RGL3	0.213433	5.795854	4.237788	2.62E-05	6.74E-05	1.562566
ARPP19	0.213424	8.312855	3.604948	0.000339	0.000746	-0.84762
NXN	0.213353	5.533196	2.542814	0.01125	0.01923	-4.04904
LYPD3	0.213307	6.40377	3.952391	8.67E-05	0.000208	0.42994
WT1-AS	0.213191	5.581951	4.057093	5.64E-05	0.000139	0.836795

HCRTR1	0.213182	4.066303	5.161355	3.35E-07	1.10E-06	5.730961
DNAJB12	0.213157	7.107778	4.722868	2.91E-06	8.49E-06	3.657185
HMGCL	0.212895	8.332886	4.324691	1.79E-05	4.71E-05	1.922212
USP10	0.21285	8.658689	2.543865	0.011217	0.019177	-4.04641
ATP13A5	0.21274	4.684245	6.776136	2.99E-11	1.55E-10	14.78054
NRSN1	0.21267	5.024681	4.910341	1.18E-06	3.62E-06	4.523087
FAM172A	0.212646	7.145365	2.338207	0.019709	0.032064	-4.54178
OR1G1	0.212608	5.598894	4.814658	1.88E-06	5.61E-06	4.077251
B3GNTL1	0.212303	6.584298	3.602191	0.000342	0.000754	-0.85731
PHACTR1	0.212269	6.642074	2.070096	0.038877	0.059392	-5.12599
DNM1	0.212204	5.192587	3.745955	0.000197	0.000451	-0.34263
LINC0115ε	0.212137	5.528919	4.768245	2.34E-06	6.92E-06	3.863911
EYA1	0.21206	5.764688	5.977005	3.93E-09	1.60E-08	10.03089
SMR3B	0.212039	4.3371	5.342984	1.31E-07	4.48E-07	6.639214
UBE2J2	0.212029	6.475032	3.768353	0.000181	0.000415	-0.26071
CIRBP-AS:	0.211894	4.78754	3.564453	0.000394	0.000861	-0.98922
ZNF613	0.211848	4.425109	3.442472	0.000617	0.001308	-1.40649
NUDT16	0.211844	6.247764	3.699454	0.000236	0.000533	-0.51121
CLPS	0.211809	5.353952	5.546788	4.39E-08	1.59E-07	7.692219
NBR1	0.211803	8.9685	3.142901	0.001757	0.003462	-2.37187
WDR47	0.211717	8.014997	2.139841	0.032776	0.050867	-4.98075
SALL4	0.211626	5.99933	4.369303	1.47E-05	3.91E-05	2.109497
PAQR6	0.211504	6.687241	2.768586	0.005807	0.010474	-3.45849
EFNB3	0.211427	6.161416	5.23958	2.24E-07	7.50E-07	6.118628
KRTAP4-8	0.211338	4.922791	4.288831	2.10E-05	5.46E-05	1.772974
IRF6	0.211313	6.584959	4.572251	5.88E-06	1.65E-05	2.984165
CACNA1S	0.211244	5.27088	3.671511	0.000263	0.00059	-0.61154
LY6G6C	0.211114	5.663479	3.971691	8.02E-05	0.000194	0.504181
FADS3	0.211048	6.423657	2.979943	0.003001	0.00569	-2.86134
PKNOX2	0.211012	5.860268	6.641759	7.04E-11	3.51E-10	13.94582
MLXIP	0.210953	6.983192	2.92142	0.003617	0.006758	-3.03096
CSDC2	0.210745	6.436387	4.17272	3.46E-05	8.78E-05	1.297782
LOC73010	0.210714	4.776898	4.226521	2.75E-05	7.05E-05	1.516442
DIRAS1	0.210699	4.963639	3.297869	0.001033	0.002115	-1.88305
PARVG	0.210678	8.39588	2.678819	0.007594	0.01342	-3.69916
MNX1	0.210565	5.475576	3.615321	0.000326	0.00072	-0.8111
AK9	0.210489	5.465862	2.956101	0.00324	0.006103	-2.93084
NCOA2	0.210469	7.360958	3.30826	0.000996	0.002045	-1.84946
ZBTB47	0.210419	6.431162	4.670271	3.73E-06	1.07E-05	3.419856
LILRB5	0.210362	5.269733	4.099258	4.72E-05	0.000118	1.003486
SORL1	0.210334	11.78035	2.574369	0.010285	0.01772	-3.96945
PRSS58	0.210331	5.316562	4.340909	1.67E-05	4.40E-05	1.990085
SNTB2	0.210299	5.924071	4.803044	1.98E-06	5.91E-06	4.023688
CXCL3	0.210284	4.553415	2.335264	0.019863	0.032284	-4.54857
SPC24	0.210159	5.622231	4.699138	3.25E-06	9.44E-06	3.549804
PQBP1	0.209937	6.794589	2.799435	0.005286	0.009606	-3.37399
TRIP10	0.20953	5.721969	3.821459	0.000147	0.000342	-0.06463
ZNF280A	0.209517	5.683402	5.692366	1.97E-08	7.46E-08	8.466105
GUCD1	0.209453	8.59648	3.311811	0.000983	0.002022	-1.83796
FOXO4	0.209418	6.261598	2.521306	0.011954	0.020339	-4.10274
LOC28464	0.209341	4.851357	5.120543	4.13E-07	1.34E-06	5.530821
RUSC2	0.209275	6.410284	4.244687	2.54E-05	6.55E-05	1.590867
HIST1H2BI	0.209193	4.328842	4.551748	6.46E-06	1.80E-05	2.894119
GJB1	0.209138	6.404816	3.245866	0.001237	0.002503	-2.04962
CYP2A13	0.209094	5.641002	3.242796	0.00125	0.002528	-2.05937
ARNT	0.209085	6.472645	5.302162	1.62E-07	5.50E-07	6.432591
OR1I1	0.209075	6.570577	4.405535	1.25E-05	3.36E-05	2.262926
AANAT	0.208887	4.494966	4.821393	1.82E-06	5.45E-06	4.108368

RPS6KA2-.	0.208874	5.429608	3.814856	0.000151	0.00035	-0.08916
GFPT2	0.208661	5.482501	4.426198	1.14E-05	3.08E-05	2.350959
TSR3	0.208572	6.070916	3.173322	0.001585	0.003155	-2.27771
EPHB3	0.208499	6.265684	4.429729	1.12E-05	3.03E-05	2.366038
TPTEP1	0.208459	5.430133	3.706526	0.00023	0.00052	-0.4857
ZNF394	0.208314	7.738071	3.902831	0.000106	0.000252	0.240873
APBB2	0.208292	4.814636	7.213789	1.67E-12	9.86E-12	17.59741
LIPH	0.20818	4.855925	4.279256	2.19E-05	5.68E-05	1.733325
TEX35	0.208106	5.049314	4.677744	3.60E-06	1.04E-05	3.453426
FLJ32154	0.208048	4.807127	4.651701	4.07E-06	1.16E-05	3.336656
STARD3NL	0.208041	9.687031	2.658618	0.008059	0.014164	-3.75225
AQP2	0.208005	5.88441	5.691177	1.99E-08	7.50E-08	8.459712
CENPF	0.207994	4.542975	3.558453	0.000403	0.000879	-1.01007
SLC6A17	0.207783	7.026858	4.709517	3.10E-06	9.01E-06	3.596707
LOC64690	0.207732	3.760084	4.554522	6.38E-06	1.78E-05	2.906277
MYCBPAP	0.207508	5.417312	3.745422	0.000198	0.000451	-0.34457
RHOA	0.207456	8.262843	5.943478	4.77E-09	1.92E-08	9.843049
TMEM54	0.20738	5.14104	4.148302	3.84E-05	9.68E-05	1.199413
VPS26B	0.207324	7.357846	3.141297	0.001766	0.003478	-2.37682
FAM83H	0.207154	4.913542	4.300249	2.00E-05	5.21E-05	1.820365
RAD51D	0.207018	5.501855	4.453057	1.01E-05	2.75E-05	2.465963
CCL22	0.206698	6.878196	3.478338	0.000542	0.001157	-1.28525
MAPK6	0.206687	6.693207	2.955835	0.003243	0.006107	-2.93161
CRMP1	0.206661	6.676668	3.426217	0.000655	0.001383	-1.46104
IFNA17	0.206594	6.036383	3.582683	0.000368	0.000808	-0.92567
STC2	0.206564	5.371056	5.713895	1.75E-08	6.65E-08	8.582074
C1orf56	0.206541	6.207917	3.20906	0.001404	0.002815	-2.16596
AFTPH	0.20645	9.727108	3.880404	0.000116	0.000274	0.156061
CALB2	0.206407	5.761305	3.396001	0.00073	0.001532	-1.56179
MX2	0.206263	10.25399	2.012953	0.044573	0.067183	-5.24146
ARMC2	0.206225	4.473756	3.377747	0.000779	0.001628	-1.62223
CDC42EP4	0.206154	6.282712	3.85111	0.00013	0.000306	0.04598
TOP3A	0.206061	6.29753	4.993438	7.82E-07	2.45E-06	4.916827
KLHL8	0.206026	9.32909	2.222904	0.0266	0.042122	-4.80159
TRIM8	0.205989	7.21058	3.208795	0.001405	0.002818	-2.1668
KCNA4	0.205786	5.286062	6.244346	8.13E-10	3.61E-09	11.56198
PSORS1C2	0.205691	5.666149	3.66039	0.000274	0.000613	-0.65127
DSCR9	0.205661	6.318339	4.500085	8.18E-06	2.25E-05	2.668895
LOC10192	0.205624	6.079498	3.795172	0.000163	0.000376	-0.16202
MED11	0.205522	8.136629	3.223318	0.001337	0.002687	-2.12105
GALK1	0.205484	5.844028	3.290377	0.00106	0.002166	-1.9072
ATP4A	0.20545	5.381608	3.543641	0.000426	0.000925	-1.0614
LOC10050	0.205427	6.613521	4.780379	2.21E-06	6.55E-06	3.919501
ERCC2	0.205293	6.292283	4.693077	3.35E-06	9.69E-06	3.522457
ATG3	0.205279	8.606213	3.103951	0.002001	0.003912	-2.49116
MLIP	0.205278	4.915081	4.239948	2.59E-05	6.68E-05	1.571422
MIER1	0.205231	7.611202	4.537326	6.90E-06	1.92E-05	2.831003
HR	0.205218	6.147049	4.873155	1.41E-06	4.30E-06	4.348857
DNAH2	0.205209	5.332842	5.712607	1.76E-08	6.70E-08	8.575128
U2AF1	0.205062	7.806011	4.014457	6.73E-05	0.000164	0.669903
CKM	0.204953	5.105393	3.4251	0.000657	0.001388	-1.46478
MFN1	0.204925	6.922572	2.552565	0.010944	0.018759	-4.02455
KANK2	0.204883	6.018878	3.077886	0.002181	0.004236	-2.57018
HHIP-AS1	0.204813	5.668263	4.598412	5.21E-06	1.47E-05	3.099606
LOC10013	0.204628	4.516753	5.301273	1.63E-07	5.52E-07	6.428105
IRF9	0.204568	10.23381	1.993819	0.046631	0.06996	-5.27941
SLC5A5	0.204445	7.010215	4.495292	8.36E-06	2.29E-05	2.648121
FAM65C	0.204407	5.370291	3.058133	0.002328	0.004494	-2.62964

PRR14	0.204348	9.282849	2.914598	0.003696	0.006891	-3.05052
FAM64A	0.204344	5.199621	3.74784	0.000196	0.000448	-0.33575
CHAC1	0.204201	5.67287	4.002432	7.07E-05	0.000172	0.623135
EMX1	0.204199	4.986064	3.966393	8.19E-05	0.000198	0.483767
DLL4	0.20419	6.756047	3.058416	0.002326	0.004491	-2.62879
ZBTB8OS	0.203979	8.451444	3.279321	0.001102	0.002246	-1.94275
SLC5A10	0.203873	5.456698	5.102796	4.52E-07	1.46E-06	5.444242
ATP1B2	0.203831	5.974642	3.803865	0.000157	0.000364	-0.12989
DEFA5	0.203813	5.322564	4.302801	1.97E-05	5.16E-05	1.830974
NNAT	0.203757	6.250661	3.503823	0.000493	0.001059	-1.19836
MUC5AC	0.203705	6.112557	4.727203	2.85E-06	8.33E-06	3.676855
PTTG3P	0.203547	4.555419	3.418013	0.000674	0.001423	-1.48848
NACC2	0.203452	7.739538	2.683541	0.007489	0.013247	-3.68669
PRPF6	0.203431	7.724981	2.482066	0.013339	0.022499	-4.19956
FAM114A1	0.203384	5.72841	3.321267	0.000951	0.001961	-1.80727
SLC7A11	0.20327	4.777974	3.164291	0.001634	0.003243	-2.30575
PPP2R4	0.203248	7.104761	2.152703	0.031746	0.049408	-4.95345
LOC10050	0.203236	3.965911	5.215024	2.55E-07	8.46E-07	5.996359
GLIS1	0.203236	5.053639	4.166031	3.56E-05	9.02E-05	1.27078
HS3ST2	0.203201	5.802742	4.107388	4.57E-05	0.000114	1.035811
LRGUK	0.203151	4.595062	4.426065	1.14E-05	3.08E-05	2.350391
CLPTM1L	0.203139	7.2973	3.30997	0.00099	0.002034	-1.84392
SLC35A2	0.203112	5.992856	4.455564	1.00E-05	2.72E-05	2.47673
PDZD3	0.203062	6.31841	4.581409	5.64E-06	1.58E-05	3.024506
MYL2	0.203032	4.723772	3.66595	0.000269	0.000601	-0.63142
PARP4	0.202848	10.46613	2.963857	0.00316	0.005968	-2.90829
APOD	0.202675	5.837237	2.338458	0.019696	0.032053	-4.5412
GPR15	0.202619	5.16734	4.483891	8.81E-06	2.41E-05	2.598791
UGT2B28	0.20259	5.462312	4.082818	5.06E-05	0.000125	0.938299
MFAP2	0.202511	5.957493	3.740459	0.000202	0.00046	-0.36266
RPS6KB2	0.20249	7.303454	2.564623	0.010575	0.018182	-3.99414
ANKRD33	0.202438	4.741139	5.316907	1.50E-07	5.11E-07	6.507057
ALG1	0.202296	5.366798	4.275629	2.22E-05	5.76E-05	1.718325
COMT	0.202218	7.274721	4.139398	3.99E-05	0.0001	1.163679
TPRXL	0.2021	6.40624	4.066333	5.42E-05	0.000134	0.873186
C11orf94	0.202096	6.035845	4.516105	7.61E-06	2.10E-05	2.738476
PHKA1	0.202058	4.747222	6.25959	7.42E-10	3.32E-09	11.65106
WIZ	0.201926	6.427499	4.191922	3.19E-05	8.13E-05	1.375521
KRTAP5-9	0.201881	5.432327	4.241679	2.57E-05	6.63E-05	1.57852
RPS6KA4	0.201715	6.281948	4.795893	2.05E-06	6.11E-06	3.99077
CLINT1	0.201675	8.22206	2.084059	0.037584	0.057603	-5.09729
FUT6	0.201628	5.664702	4.78076	2.21E-06	6.54E-06	3.92125
LY6G6E	0.201408	4.701505	4.280493	2.18E-05	5.65E-05	1.738441
ANP32D	0.201383	4.170005	4.533032	7.04E-06	1.95E-05	2.812251
NXPE4	0.201372	5.386984	4.323451	1.80E-05	4.74E-05	1.917031
LIMD1-AS	0.201352	6.317878	4.719495	2.95E-06	8.61E-06	3.641889
SLC32A1	0.201258	4.562086	5.632305	2.75E-08	1.02E-07	8.144643
PHLPP1	0.201251	4.652956	3.292688	0.001052	0.00215	-1.89976
IFT122	0.201179	7.083742	4.31718	1.85E-05	4.87E-05	1.890855
SCGB2B2	0.201123	5.285625	3.932007	9.42E-05	0.000225	0.351903
DSCR8	0.201103	4.854738	3.890711	0.000111	0.000264	0.194982
TAC3	0.20108	6.138966	4.046842	5.88E-05	0.000144	0.796519
CCDC30	0.201024	4.661105	5.641185	2.62E-08	9.75E-08	8.191979
LOC39988	0.200987	5.42639	3.301515	0.00102	0.002089	-1.87128
LOC10192	0.200982	6.156645	4.461534	9.74E-06	2.65E-05	2.502394
PRSS8	0.200826	5.284971	4.189223	3.23E-05	8.22E-05	1.364575
SLC22A13	0.200795	5.314895	2.988252	0.002922	0.005548	-2.837
SGCA	0.200739	4.985664	2.961411	0.003185	0.006009	-2.91541

ACTL7B	0.200673	5.196566	3.29757	0.001034	0.002116	-1.88402
ADORA3	0.20057	6.086876	2.000963	0.045853	0.068856	-5.26528
IL1RAPL1	0.200543	4.752436	6.260705	7.37E-10	3.30E-09	11.65758
DPEP1	0.200482	6.187191	4.022471	6.51E-05	0.000159	0.701146
TRIM23	0.200426	6.26971	2.281576	0.022869	0.036686	-4.671
PHACTR3	0.20039	3.666619	5.298952	1.65E-07	5.58E-07	6.416403
PSG5	0.200386	5.31683	4.56956	5.95E-06	1.67E-05	2.972325
UBE2O	0.200353	6.979951	2.829327	0.004823	0.008823	-3.29125
LAMB3	0.200311	5.180128	3.072577	0.00222	0.004305	-2.5862
BCAS3	0.200276	6.901437	3.232986	0.001293	0.002608	-2.09048
FOXO6	0.200021	5.652051	2.240974	0.025398	0.04035	-4.76173
NXF5	0.199782	5.190754	3.626077	0.000313	0.000693	-0.77313
FBXO17	0.199656	5.358236	2.738887	0.006351	0.011379	-3.53897
RAB3IP	0.199531	5.188162	3.531362	0.000446	0.000965	-1.1038
RECQL4	0.199528	5.925296	3.021553	0.002624	0.005023	-2.73876
LOC10192	0.199468	4.68241	4.799652	2.02E-06	6.01E-06	4.008067
LOC15354	0.199401	6.666262	2.755385	0.006043	0.010866	-3.49436
FBXO40	0.199374	5.118308	5.492228	5.90E-08	2.12E-07	7.406832
LHX3	0.199374	5.856161	3.292174	0.001053	0.002153	-1.90142
HORMAD2	0.199187	5.033337	6.050136	2.57E-09	1.07E-08	10.44385
SDF4	0.199121	8.559358	2.255177	0.024487	0.03905	-4.73017
TMEM65	0.199014	6.454543	2.502115	0.012614	0.021361	-4.15028
TNFAIP1	0.198986	6.911229	4.766938	2.36E-06	6.96E-06	3.857931
LIPE	0.198862	5.695157	3.180728	0.001546	0.003085	-2.25465
SLFNL1-AS	0.198852	4.789235	4.735496	2.74E-06	8.02E-06	3.714529
POC1B	0.198624	7.736751	2.255846	0.024445	0.038989	-4.72868
TMEM86A	0.198573	5.731343	5.963501	4.25E-09	1.72E-08	9.955116
SCN3B	0.198467	4.654825	5.735412	1.55E-08	5.93E-08	8.698374
PLK4	0.198454	4.436914	3.311845	0.000983	0.002022	-1.83785
ADAR	0.198368	12.13756	3.083721	0.00214	0.004162	-2.55255
SLC30A1	0.198306	6.877308	2.929329	0.003528	0.006603	-3.00823
TMEM53	0.198101	5.762256	4.430094	1.12E-05	3.03E-05	2.367598
RTN4R	0.198091	5.452194	3.993975	7.32E-05	0.000178	0.590324
UBC	0.198074	13.47836	2.807962	0.00515	0.009379	-3.35048
TEX13B	0.198067	5.764234	4.768052	2.35E-06	6.92E-06	3.863031
GDI2	0.197923	10.59957	2.36852	0.01818	0.029805	-4.47134
SLC39A9	0.19763	6.065296	5.42189	8.60E-08	3.03E-07	7.042673
HECTD3	0.197527	7.250697	2.038193	0.041975	0.063655	-5.19085
COG7	0.197512	6.382292	2.549999	0.011024	0.018879	-4.03101
RBFOX1	0.197489	5.80705	4.377273	1.42E-05	3.78E-05	2.143145
SBF2	0.197396	6.935512	2.995483	0.002855	0.00543	-2.81576
PRADC1	0.197381	6.590436	3.642149	0.000294	0.000655	-0.71619
SEC13	0.197304	9.437273	2.734476	0.006435	0.011517	-3.55085
DECR1	0.197247	9.975541	3.771716	0.000178	0.00041	-0.24837
NIT1	0.197236	7.378737	3.747647	0.000196	0.000448	-0.33646
XRRA1	0.197186	5.463129	4.38453	1.38E-05	3.67E-05	2.173831
HPS1	0.196985	7.083086	4.947408	9.82E-07	3.04E-06	4.697971
LOC10192	0.196956	5.035913	3.487557	0.000524	0.001121	-1.25389
HAGLROS	0.196918	4.59171	3.715973	0.000222	0.000503	-0.45156
AFF1	0.196827	7.551913	3.097181	0.002046	0.003994	-2.51175
LSM14B	0.196804	5.674865	3.889026	0.000112	0.000265	0.188614
CYP2F1	0.196693	4.668908	5.60021	3.28E-08	1.21E-07	7.974115
SPRYD4	0.196649	5.918884	4.286565	2.12E-05	5.51E-05	1.763584
KMT2D	0.196634	7.211932	3.001837	0.002797	0.00533	-2.79705
SLC22A14	0.196532	6.521754	3.787648	0.000168	0.000387	-0.18978
HSPA2	0.196518	4.607374	3.478664	0.000541	0.001156	-1.28414
GLB1L	0.196492	6.040294	3.137191	0.001791	0.003523	-2.38945
TPCN2	0.196437	6.560864	4.520343	7.46E-06	2.06E-05	2.75692

HOXC11	0.196415	6.533147	4.133036	4.10E-05	0.000103	1.138193
UQCR11	0.196355	8.72411	3.222916	0.001339	0.00269	-2.12231
LINC0111(	0.196291	5.364339	4.504888	8.01E-06	2.20E-05	2.689732
MT4	0.19628	6.076514	4.176004	3.41E-05	8.67E-05	1.311054
PCDHB11	0.196259	5.086182	2.834593	0.004745	0.008697	-3.27659
PHF2	0.19623	6.870494	5.097797	4.63E-07	1.49E-06	5.419907
GUCA2B	0.196188	5.063991	4.694589	3.32E-06	9.62E-06	3.529276
IGSF9	0.196159	5.570883	3.767708	0.000181	0.000416	-0.26308
FAM109B	0.19601	6.48136	4.241536	2.58E-05	6.63E-05	1.577937
P2RY6	0.196007	5.045654	4.615668	4.81E-06	1.36E-05	3.176091
DOC2B	0.195937	5.91203	3.15462	0.001689	0.003343	-2.3357
BDKRB1	0.195935	5.283229	3.812157	0.000152	0.000354	-0.09917
SYT13	0.195928	4.922616	5.483434	6.19E-08	2.21E-07	7.361071
HYAL1	0.195904	5.344475	3.856238	0.000128	0.0003	0.065192
MGC3479(	0.195853	6.408411	3.684814	0.00025	0.000562	-0.56387
DYRK3	0.195844	5.925649	3.23302	0.001293	0.002608	-2.09037
LHFPL3-A!	0.195827	6.437676	3.764469	0.000184	0.000421	-0.27495
RNF185	0.195807	8.110545	3.479679	0.000539	0.001152	-1.28069
MAP2K1	0.195791	9.849083	2.892543	0.003962	0.007348	-3.11345
ACTL7A	0.195784	5.943114	4.148544	3.84E-05	9.67E-05	1.200385
SNN	0.195766	8.714617	2.155667	0.031513	0.049104	-4.94713
CYP17A1	0.195752	5.209248	2.964446	0.003155	0.005959	-2.90658
LRRC48	0.195677	4.328604	4.111775	4.48E-05	0.000112	1.053281
ALG12	0.1954	6.441802	2.970099	0.003098	0.005861	-2.8901
RHBDL3	0.195353	5.627833	5.549833	4.32E-08	1.57E-07	7.708223
B4GALNT1	0.195335	5.529874	4.743191	2.64E-06	7.75E-06	3.749545
KIAA1654	0.195178	5.015659	6.115657	1.75E-09	7.45E-09	10.8176
SLC38A3	0.195164	5.749048	3.842177	0.000135	0.000316	0.01257
RPP25L	0.195101	8.783328	3.966149	8.20E-05	0.000198	0.482827
RGS8	0.195095	4.701108	7.27077	1.14E-12	6.84E-12	17.97506
SLC12A5	0.195086	4.545886	3.281023	0.001095	0.002234	-1.93729
CCDC7	0.194918	5.67637	4.224414	2.77E-05	7.12E-05	1.507828
PPM1N	0.194902	5.717826	3.232541	0.001295	0.002612	-2.09189
TFF2	0.194715	5.080223	3.239841	0.001263	0.002551	-2.06875
EVI2B	0.194708	12.59529	2.950511	0.003298	0.006204	-2.94706
MOV10L1	0.194107	5.229218	6.0642	2.37E-09	9.93E-09	10.52377
MEP1B	0.193939	4.881546	5.258114	2.04E-07	6.84E-07	6.211257
FAM124A	0.193881	4.635755	7.523808	1.99E-13	1.29E-12	19.68191
SLC35F1	0.193825	6.55148	5.984464	3.77E-09	1.54E-08	10.0728
SLC39A4	0.193742	6.49696	2.428765	0.015448	0.025716	-4.32869
SOD3	0.193718	6.648654	2.154312	0.031619	0.04924	-4.95002
MUL1	0.193644	7.599233	3.551415	0.000414	0.0009	-1.03449
C2orf73	0.193505	4.674086	5.205161	2.68E-07	8.86E-07	5.947398
NOS1AP	0.193433	6.961578	3.714499	0.000223	0.000505	-0.45689
SRGAP1	0.193365	5.190797	3.892448	0.000111	0.000262	0.20155
PTGFRN	0.193309	4.855645	3.815035	0.00015	0.00035	-0.08849
PCDHB9	0.193299	5.481103	3.383564	0.000763	0.001597	-1.60301
CPE	0.193265	4.192441	3.059018	0.002321	0.004484	-2.62698
TMUB1	0.193183	6.872159	2.498225	0.012752	0.021576	-4.15987
PNKD	0.193111	7.586829	2.892383	0.003964	0.007351	-3.11391
BPIFB2	0.193069	5.86734	3.541675	0.000429	0.000931	-1.0682
C10orf53	0.193031	5.522736	3.134437	0.001807	0.003554	-2.39792
12-Sep	0.192998	6.068032	3.605993	0.000337	0.000744	-0.84395
FSD1	0.192955	6.27877	3.715339	0.000222	0.000504	-0.45385
GABARAPI	0.192908	7.424904	2.089825	0.03706	0.056868	-5.08539
HDAC11	0.192876	6.151274	4.679269	3.57E-06	1.03E-05	3.460282
C8orf17	0.192756	5.013566	4.183755	3.30E-05	8.40E-05	1.342418
LOC10192	0.192647	5.130875	4.587057	5.49E-06	1.55E-05	3.049426

WDR74	0.192549	6.007365	3.852204	0.00013	0.000305	0.050079
ITFG3	0.19245	7.482586	3.053039	0.002367	0.004564	-2.64491
GOLGA7	0.192413	10.67111	3.808601	0.000154	0.000358	-0.11235
MYO15A	0.192393	5.107369	4.482759	8.85E-06	2.42E-05	2.5939
UBR4	0.192197	6.366939	6.010667	3.23E-09	1.34E-08	10.22042
FXR2	0.192196	7.246021	2.815655	0.00503	0.009175	-3.3292
FAM135B	0.192157	5.238503	5.619841	2.95E-08	1.09E-07	8.078317
HS3ST6	0.192139	4.541472	3.663343	0.000271	0.000607	-0.64074
TNPO1	0.192129	8.088329	3.617724	0.000323	0.000714	-0.80263
TEX37	0.192048	5.921033	4.4459	1.05E-05	2.83E-05	2.435256
C9orf40	0.191924	6.273222	2.028053	0.043003	0.065034	-5.21126
FAM219B	0.191891	6.618049	2.989102	0.002914	0.005534	-2.83451
MFI2	0.191882	5.224411	5.840763	8.58E-09	3.37E-08	9.273426
SPSB3	0.191863	8.409418	2.254422	0.024535	0.039109	-4.73185
NPHS2	0.191832	5.455273	4.122063	4.29E-05	0.000108	1.094319
PCDHA5	0.191778	5.331017	4.245601	2.53E-05	6.52E-05	1.594621
ADAM6	0.191774	4.941653	4.527811	7.21E-06	2.00E-05	2.789467
PIGR	0.191768	5.701189	5.505938	5.48E-08	1.97E-07	7.478308
CRLF2	0.191758	5.916901	3.988896	7.47E-05	0.000181	0.57065
ZNF330	0.191645	7.629765	4.340525	1.67E-05	4.41E-05	1.988475
LACTB	0.191628	9.411015	2.931722	0.003501	0.006557	-3.00134
NAV2-AS2	0.191433	5.309288	4.634819	4.40E-06	1.25E-05	3.261284
PTPLAD2	0.191409	7.40854	5.065624	5.45E-07	1.74E-06	5.263792
NKAIN4	0.191349	5.17117	6.309718	5.49E-10	2.48E-09	11.94531
KHDRBS2	0.191348	5.459031	3.807342	0.000155	0.00036	-0.11701
ADD3-AS1	0.191304	4.830225	6.083799	2.11E-09	8.92E-09	10.63543
CACNG2	0.191296	5.337224	4.74283	2.64E-06	7.76E-06	3.7479
ADPRHL1	0.191243	6.34285	5.252925	2.09E-07	7.02E-07	6.185292
LDB1	0.191206	7.820273	2.802895	0.005231	0.009515	-3.36446
CSNK2B	0.191123	10.10869	2.576549	0.010221	0.01762	-3.96392
CA12	0.191103	5.297079	5.744986	1.47E-08	5.63E-08	8.75025
ELMOD3	0.191028	6.61914	2.954077	0.003261	0.006137	-2.93671
DCAF12L1	0.191027	3.893589	6.036926	2.78E-09	1.16E-08	10.36892
DHRS7	0.191022	7.286477	3.81727	0.000149	0.000347	-0.08019
TRAPPC9	0.190807	7.053618	3.445796	0.00061	0.001293	-1.3953
SP1	0.19076	8.607746	3.238828	0.001268	0.00256	-2.07196
EVA1B	0.190513	6.550972	2.002437	0.045694	0.068657	-5.26236
VTCN1	0.19051	5.125926	4.377042	1.42E-05	3.78E-05	2.142169
FKBP4	0.190444	6.561974	2.186269	0.029187	0.045843	-4.88144
GPR1-AS	0.190442	4.299781	4.354208	1.57E-05	4.16E-05	2.045923
OVOL1-AS	0.190363	6.525997	3.752676	0.000192	0.00044	-0.3181
HPX	0.190231	6.547986	4.331616	1.74E-05	4.58E-05	1.951163
SORCS2	0.190145	5.238074	2.825777	0.004876	0.008913	-3.30112
SLC43A1	0.190137	6.048044	3.50611	0.000489	0.001051	-1.19054
RAB18	0.190064	8.251585	3.030603	0.002547	0.004886	-2.71189
DKFZP434	0.189999	4.852362	4.405265	1.25E-05	3.36E-05	2.261779
LPO	0.189975	5.416781	3.255215	0.001198	0.00243	-2.01986
OVCH1-AS	0.189945	5.703597	2.805505	0.005189	0.009443	-3.35726
FOXQ1	0.189939	5.464759	2.254932	0.024503	0.039068	-4.73072
DLGAP4	0.189938	7.299927	5.041007	6.17E-07	1.96E-06	5.144958
TEX29	0.189926	4.74378	4.142365	3.94E-05	9.91E-05	1.17558
MAPK15	0.189829	5.586438	3.920308	9.88E-05	0.000236	0.307287
ITSN1	0.189826	5.089722	4.865868	1.46E-06	4.44E-06	4.314859
CCDC124	0.189622	5.293741	3.355512	0.000843	0.001751	-1.69544
GJB4	0.189575	6.253568	3.537296	0.000436	0.000945	-1.08332
MFGE8	0.18954	6.33396	2.982332	0.002979	0.005648	-2.85435
ZBTB12	0.189504	5.02102	3.862636	0.000125	0.000293	0.0892
CLVS1	0.189391	4.865807	3.859817	0.000126	0.000296	0.078617

LOC10192	0.189386	5.638726	3.763561	0.000184	0.000423	-0.27828
LOC10272	0.18936	5.38498	4.158055	3.69E-05	9.31E-05	1.238641
MIEN1	0.189325	8.55505	2.476944	0.01353	0.022796	-4.21209
GAL	0.189243	4.878372	5.078306	5.11E-07	1.64E-06	5.325222
FGFR1OP2	0.189183	7.606462	2.557805	0.010782	0.018498	-4.01135
ASIC2	0.189159	5.096381	4.003146	7.05E-05	0.000171	0.625907
SLC10A2	0.189121	5.024323	3.887002	0.000113	0.000267	0.180966
YPEL4	0.189006	4.437829	2.457162	0.01429	0.023954	-4.26024
LOC10192	0.188909	5.388809	3.980148	7.74E-05	0.000188	0.536817
C2orf82	0.188897	5.30082	4.718518	2.97E-06	8.65E-06	3.637465
POLN	0.188889	5.719873	3.83677	0.000138	0.000323	-0.00762
ABHD4	0.188741	7.194504	3.584341	0.000366	0.000804	-0.91987
CXCL17	0.188704	5.479698	5.080014	5.07E-07	1.63E-06	5.333507
C19orf25	0.18854	6.641213	2.572963	0.010326	0.017785	-3.97302
UQCRFS1	0.188473	10.40939	2.336486	0.019799	0.032195	-4.54575
ARHGAP1	0.188401	7.430608	3.674095	0.00026	0.000584	-0.6023
SLC9A9	0.188253	6.546222	4.023831	6.47E-05	0.000158	0.706452
MMP14	0.188204	6.289474	4.650001	4.10E-06	1.17E-05	3.329054
LINC00845	0.188161	4.875558	4.626862	4.57E-06	1.30E-05	3.225846
PPP1R13L	0.188034	5.640706	3.594793	0.000352	0.000774	-0.88328
FZD4	0.187817	5.531937	4.151067	3.80E-05	9.57E-05	1.210526
CLIC6	0.187774	5.076826	4.778188	2.23E-06	6.62E-06	3.909455
MAP3K15	0.187753	4.27354	5.050969	5.87E-07	1.87E-06	5.192983
HIST1H3C	0.187704	5.40898	4.742954	2.64E-06	7.75E-06	3.748466
C3orf20	0.187676	5.586918	2.730962	0.006503	0.011623	-3.5603
DERL3	0.1876	6.21024	3.354058	0.000847	0.001758	-1.70021
CDC42EP2	0.187567	7.48631	2.105079	0.035706	0.054976	-5.05373
ADAMTS1	0.186945	5.744368	4.72561	2.87E-06	8.39E-06	3.669623
APOA5	0.186885	6.302198	5.413252	9.01E-08	3.16E-07	6.998247
SPATS2L	0.18669	4.979703	3.004455	0.002773	0.005289	-2.78933
LYPLA1	0.186649	10.11554	2.424103	0.015645	0.026005	-4.33986
ZBTB17	0.186614	6.664055	2.753608	0.006076	0.010917	-3.49918
ATOH8	0.186602	5.812702	3.194529	0.001475	0.00295	-2.21155
IFI27L2	0.186593	7.283673	2.608111	0.009334	0.016224	-3.88328
CLDN2	0.18657	5.422807	4.48933	8.59E-06	2.35E-05	2.622309
STAU2	0.186526	6.750608	2.302601	0.021648	0.034887	-4.62339
ALX3	0.186464	5.789258	3.32906	0.000926	0.00191	-1.78192
LOC10026	0.186458	4.358582	4.891626	1.29E-06	3.94E-06	4.435245
BRCA1	0.18642	6.323899	3.542125	0.000428	0.00093	-1.06664
GABARAP	0.186356	12.48156	2.909737	0.003753	0.006986	-3.06443
MAGEL2	0.186346	4.768405	4.795371	2.06E-06	6.12E-06	3.988368
SEPN1	0.186305	6.706505	2.240398	0.025436	0.040399	-4.763
LOC28424	0.186222	5.148679	4.879116	1.37E-06	4.18E-06	4.376706
FOXB1	0.186163	4.725801	3.315655	0.00097	0.001997	-1.8255
SDHAF2	0.185965	8.29941	3.138757	0.001781	0.003507	-2.38463
ARHGEF17	0.185933	7.183164	3.218412	0.00136	0.00273	-2.13652
ALDOAP2	0.185847	4.597944	3.833172	0.00014	0.000327	-0.02104
WNT8B	0.185842	5.053328	4.572788	5.87E-06	1.65E-05	2.986528
WBSCR17	0.18579	5.457395	4.670323	3.73E-06	1.07E-05	3.420089
SLC39A8	0.185765	5.436358	3.183575	0.001531	0.003057	-2.24577
RMST	0.185623	4.676641	3.968	8.14E-05	0.000196	0.489954
LMNB2	0.185578	6.154972	2.323371	0.020498	0.03322	-4.57594
SPDEF	0.185494	7.359611	3.691296	0.000244	0.000549	-0.54058
MAML3	0.185425	6.339077	4.457812	9.91E-06	2.69E-05	2.48639
CENPI	0.185336	4.291149	4.439497	1.08E-05	2.91E-05	2.407822
LOXHD1	0.185241	5.021707	2.824648	0.004893	0.008943	-3.30426
SLC22A11	0.185205	4.726376	3.134898	0.001804	0.00355	-2.3965
COX6A1	0.185182	7.817935	3.469039	0.00056	0.001195	-1.3168

ARSI	0.185149	4.899199	3.62072	0.000319	0.000706	-0.79206
LOC28493	0.185047	5.911327	2.765495	0.005861	0.010562	-3.4669
LOC10272	0.18503	5.165084	3.878801	0.000117	0.000276	0.150018
SLC17A1	0.184948	5.357919	3.265127	0.001157	0.002353	-1.98822
LOC38878	0.184933	5.608285	2.579574	0.010133	0.017485	-3.95623
ERICH6	0.184854	5.323067	3.942168	9.04E-05	0.000217	0.390756
KRTAP2-1	0.184718	4.429545	5.590504	3.46E-08	1.27E-07	7.922719
DDX25	0.184696	4.276686	4.365255	1.50E-05	3.98E-05	2.092427
TRIM7	0.184675	5.281222	3.525247	0.000456	0.000985	-1.12485
DHPS	0.184649	7.508535	2.263657	0.023957	0.038275	-4.71124
BRSK2	0.184636	5.275577	4.973205	8.64E-07	2.70E-06	4.820398
HTR3B	0.184413	4.971969	3.035226	0.002509	0.004816	-2.69812
INO80D	0.184149	7.33158	2.946029	0.003346	0.006287	-2.96004
LOC73096	0.184093	5.127611	4.055586	5.67E-05	0.000139	0.830869
GFM2	0.183977	5.623372	3.084064	0.002137	0.004157	-2.55151
WTIP	0.183922	5.902334	2.788646	0.005463	0.009905	-3.40365
HAGHL	0.183897	6.728277	3.342436	0.000883	0.001828	-1.73827
LOC10192	0.183745	6.154623	3.218882	0.001357	0.002726	-2.13504
LOC10272	0.18363	5.303027	4.453712	1.01E-05	2.74E-05	2.468774
LRRC71	0.183527	5.498059	2.767494	0.005826	0.010505	-3.46146
HOXA6	0.183513	4.975686	3.259093	0.001182	0.002399	-2.00749
HGFAC	0.183473	5.007807	3.763385	0.000184	0.000423	-0.27892
KIAA0247	0.183376	10.46489	2.33941	0.019646	0.031986	-4.539
CCL21	0.183347	5.84206	2.701437	0.007102	0.01262	-3.63925
FDPS	0.183339	8.364276	2.112122	0.035095	0.054122	-5.03904
PIR	0.183177	3.917125	3.608742	0.000334	0.000737	-0.83428
CHRNA5	0.183064	4.379441	4.084617	5.02E-05	0.000124	0.945422
SLC39A7	0.183052	6.599533	2.378802	0.017686	0.029065	-4.44724
NFATC4	0.183051	5.653806	4.149317	3.82E-05	9.64E-05	1.203493
HAVCR1P1	0.183042	6.361026	3.710282	0.000227	0.000513	-0.47213
GHITM	0.183042	10.6699	3.852433	0.00013	0.000304	0.050936
PPP1R17	0.183016	5.679906	3.240849	0.001259	0.002543	-2.06555
CAMKK1	0.182978	7.846993	2.97169	0.003082	0.005833	-2.88546
PTCHD4	0.182885	4.96729	4.602589	5.11E-06	1.44E-05	3.118094
FGGY	0.182865	4.772226	4.873764	1.41E-06	4.29E-06	4.3517
CASC4	0.182809	10.20593	2.795115	0.005357	0.009721	-3.38588
SERINC2	0.182731	5.564752	3.525301	0.000456	0.000985	-1.12467
PRDM7	0.182661	4.584432	5.395227	9.92E-08	3.46E-07	6.905744
LOC10192	0.182645	5.947608	3.0835	0.002141	0.004164	-2.55322
PRSS22	0.182572	6.169481	3.52207	0.000461	0.000996	-1.13578
MAP1LC3B	0.182551	4.7848	3.882594	0.000115	0.000272	0.164323
LOC10192	0.182501	5.060823	3.975729	7.89E-05	0.000191	0.519756
KPNA1	0.182497	7.754847	2.610675	0.009266	0.016113	-3.87669
TP53INP1	0.182284	6.930157	5.572303	3.82E-08	1.40E-07	7.826555
PPP1R14C	0.182258	5.775521	3.305653	0.001005	0.002062	-1.8579
ITIH5	0.182051	5.423951	3.058866	0.002322	0.004486	-2.62744
TMEM74B	0.182003	5.138184	4.175822	3.42E-05	8.68E-05	1.310318
VANGL2	0.181945	4.6242	4.504987	8.00E-06	2.20E-05	2.690159
ADARB2	0.181892	5.160604	3.712712	0.000224	0.000508	-0.46335
CACNG8	0.181887	5.450042	3.611567	0.00033	0.00073	-0.82433
CHRM5	0.181884	5.026423	3.52647	0.000454	0.000981	-1.12065
IGFBP7	0.181868	6.691583	2.49577	0.01284	0.021708	-4.16592
CHST4	0.181853	5.683177	4.297611	2.02E-05	5.27E-05	1.809407
HDAC8	0.18177	5.334662	4.692521	3.36E-06	9.71E-06	3.519952
FOXD3-AS	0.181758	4.980837	2.499783	0.012697	0.021494	-4.15603
SMIM14	0.181751	6.9961	2.843014	0.004623	0.008493	-3.25308
ADIRF-AS1	0.181678	5.774553	2.869071	0.004264	0.007873	-3.17991
KRTAP4-2	0.181584	5.80857	4.69491	3.32E-06	9.61E-06	3.530728

PRPH2	0.181582	5.911648	3.862758	0.000125	0.000293	0.089657
LOC10192	0.181343	5.499351	2.699581	0.007141	0.012683	-3.64418
BREA2	0.18132	5.419206	2.472552	0.013696	0.023054	-4.22281
KPTN	0.181165	6.796356	3.060934	0.002307	0.004458	-2.62123
KIAA1644	0.181104	4.885693	4.194657	3.15E-05	8.04E-05	1.38662
SHB	0.181076	5.371406	4.873433	1.41E-06	4.29E-06	4.350157
CDC73	0.181019	7.599882	2.276414	0.023178	0.037138	-4.68262
CLDN9	0.181011	6.209385	2.700992	0.007112	0.012635	-3.64043
LOC22007	0.180977	5.513842	3.986665	7.54E-05	0.000183	0.562015
MST4	0.180959	8.023973	2.385722	0.01736	0.0286	-4.43097
ACKR1	0.180938	5.371892	2.350467	0.019078	0.031158	-4.5134
ECT2	0.18092	4.5881	3.330441	0.000921	0.001902	-1.77742
MREG	0.180919	5.489873	2.272698	0.023403	0.037452	-4.69098
HSPB2	0.180855	5.479948	2.960357	0.003196	0.006029	-2.91847
MEIS3	0.180779	6.911371	3.44712	0.000607	0.001288	-1.39085
GIGYF2	0.18073	7.300822	4.363343	1.51E-05	4.01E-05	2.084371
TBC1D2B	0.18066	6.824123	3.694539	0.000241	0.000543	-0.52891
C22orf23	0.18059	5.873194	4.865587	1.47E-06	4.45E-06	4.313546
WDR87	0.180534	5.310273	3.118907	0.001904	0.00373	-2.44553
WDR37	0.180509	6.883604	2.136401	0.033057	0.051261	-4.98802
CRHBP	0.180457	5.028047	4.306299	1.94E-05	5.09E-05	1.845525
GIF	0.180392	4.570019	5.608599	3.14E-08	1.16E-07	8.018605
SNAI1	0.180346	5.693672	3.121965	0.001884	0.003695	-2.43617
SLC27A4	0.180326	5.873767	2.094525	0.036638	0.056263	-5.07566
DPYSL5	0.180207	5.553401	5.889083	6.52E-09	2.59E-08	9.540292
SPHK1	0.18017	6.273747	2.323337	0.020499	0.03322	-4.57601
TOMM34	0.180167	7.240726	4.06996	5.34E-05	0.000132	0.887492
GLE1	0.18009	6.687331	4.139717	3.98E-05	0.0001	1.164958
ITIH3	0.180041	4.612896	3.592743	0.000354	0.00078	-0.89046
MRPS36	0.179969	8.356101	2.433964	0.01523	0.025388	-4.31622
PSG9	0.17991	5.536634	4.96175	9.15E-07	2.84E-06	4.765963
LRRC16B	0.1799	6.699582	2.125261	0.033979	0.052536	-5.0115
ELMO3	0.179879	5.653945	2.279126	0.023015	0.036897	-4.67652
MYL3	0.179842	6.379022	3.161038	0.001653	0.003276	-2.31584
ZNF444	0.179772	6.79131	3.42724	0.000652	0.001379	-1.45762
SNHG10	0.179705	4.097575	3.51673	0.00047	0.001014	-1.15413
LOC10272	0.179695	5.376257	5.793843	1.12E-08	4.34E-08	9.016164
LYZL6	0.179617	5.429919	3.823014	0.000146	0.00034	-0.05885
LOC40146	0.179594	5.916797	2.558658	0.010756	0.018464	-4.0092
NOTCH3	0.1795	6.2841	4.408725	1.24E-05	3.31E-05	2.276491
SH2D5	0.179499	5.350981	4.126591	4.21E-05	0.000106	1.112408
ITIH4	0.179483	5.952194	3.543516	0.000426	0.000925	-1.06184
CA9	0.179387	6.379034	2.645873	0.008365	0.014672	-3.78555
GSN-AS1	0.179374	5.230985	4.560218	6.21E-06	1.74E-05	2.93127
ASPDH	0.179342	6.384673	2.230721	0.026074	0.041348	-4.78439
DMPK	0.179175	5.971022	2.876847	0.004162	0.007694	-3.15795
NPFF	0.179174	6.140483	3.701631	0.000234	0.000529	-0.50336
IP6K1	0.179127	8.043591	2.960209	0.003198	0.006031	-2.91891
CDK15	0.179106	6.382708	2.710689	0.006909	0.012299	-3.6146
TMEM216	0.179051	8.265944	2.690182	0.007343	0.013007	-3.66912
PAK2	0.17899	8.109568	3.513761	0.000476	0.001024	-1.16432
TRIM15	0.178906	5.633435	5.207512	2.65E-07	8.77E-07	5.959059
COL23A1	0.178722	5.701329	3.367608	0.000808	0.001683	-1.65567
RNF25	0.178667	6.223596	2.670915	0.007773	0.013704	-3.71998
SOX9-AS1	0.178626	4.883806	4.804946	1.97E-06	5.86E-06	4.032452
AKR1E2	0.178521	6.819694	3.609249	0.000333	0.000735	-0.83249
LRRC4C	0.178508	4.53151	5.772315	1.26E-08	4.87E-08	8.898746
CLDN16	0.178492	5.692972	4.260861	2.37E-05	6.13E-05	1.657386

DLGAP1-A	0.178462	5.476621	3.854825	0.000129	0.000302	0.059897
TLCD1	0.17845	5.052193	3.472791	0.000553	0.001179	-1.30408
SLC1A4	0.178391	6.257708	2.562842	0.010628	0.018264	-3.99864
MAP1A	0.178246	5.311685	3.640612	0.000296	0.000658	-0.72165
FA2H	0.178243	5.145514	5.01149	7.15E-07	2.25E-06	5.003165
SERPINH1	0.178217	5.955832	2.382229	0.017523	0.028851	-4.43919
NLGN2	0.178209	6.099523	3.364417	0.000817	0.001701	-1.66618
DQX1	0.178158	5.298023	3.98325	7.65E-05	0.000185	0.548808
PAGE5	0.178139	5.342688	3.25295	0.001207	0.002448	-2.02708
SLC22A2	0.177911	5.105847	4.121662	4.30E-05	0.000108	1.092714
VAX2	0.177882	5.1649	2.770065	0.005781	0.01043	-3.45446
LINC00589	0.177828	5.259701	3.928858	9.54E-05	0.000228	0.33988
YWHAH	0.177799	5.685568	2.980261	0.002998	0.005685	-2.86041
HSF4	0.177765	6.535705	2.910595	0.003743	0.006971	-3.06198
ACACA	0.177644	6.370479	3.93039	9.48E-05	0.000227	0.345728
HOXC9	0.177557	4.951657	3.762851	0.000185	0.000424	-0.28088
MAS1	0.177512	5.347636	3.198365	0.001456	0.002914	-2.19953
ZNF865	0.177418	6.134186	3.567792	0.000389	0.000851	-0.9776
CADM3	0.177248	5.845812	4.128764	4.17E-05	0.000105	1.121096
CACNG4	0.177165	6.308093	5.053788	5.78E-07	1.85E-06	5.20659
PSMC4	0.177005	7.979967	2.492228	0.012967	0.021903	-4.17463
OLFML2B	0.17699	5.237525	3.044105	0.002438	0.004688	-2.67164
C20orf141	0.176913	6.365979	2.919877	0.003635	0.006788	-3.03539
PIRT	0.176878	5.635068	4.00113	7.10E-05	0.000173	0.618079
DBIL5P2	0.176853	5.151793	3.352937	0.000851	0.001765	-1.70389
GLP1R	0.176822	5.832988	3.979707	7.76E-05	0.000188	0.535115
CDRT15	0.176785	5.52929	2.734059	0.006443	0.011531	-3.55197
KIF25	0.176715	5.632382	4.118052	4.37E-05	0.000109	1.078305
KDM5B	0.176678	6.613793	4.144523	3.90E-05	9.83E-05	1.184239
MYCNOS	0.176643	5.015329	4.716408	3.00E-06	8.73E-06	3.627904
CASP2	0.176536	6.688768	2.957512	0.003225	0.006078	-2.92674
TMPRSS11	0.176501	4.509647	4.765683	2.37E-06	6.99E-06	3.852194
GSTTP1	0.176498	6.359974	2.661362	0.007994	0.014057	-3.74507
TMEM125	0.176451	5.44471	3.539843	0.000432	0.000937	-1.07453
LRP2	0.176237	4.280879	5.820715	9.62E-09	3.75E-08	9.163275
PLA2G2A	0.176202	6.134376	2.696123	0.007215	0.012807	-3.65337
ABCB8	0.1762	5.816535	4.156502	3.71E-05	9.37E-05	1.232388
SEMA6B	0.176165	5.874038	2.974583	0.003054	0.005783	-2.87702
ST7L	0.176129	5.733192	5.098229	4.62E-07	1.49E-06	5.422006
LOC10192	0.17612	3.862599	4.444274	1.05E-05	2.85E-05	2.428286
SYNJ1	0.175946	5.752681	4.355717	1.56E-05	4.14E-05	2.05227
PKP3	0.175856	5.74922	4.534467	6.99E-06	1.94E-05	2.818513
LOC10192	0.175856	5.397977	4.208784	2.97E-05	7.59E-05	1.444064
UBLCP1	0.175854	9.330871	2.190923	0.028847	0.045348	-4.87136
FAM90A1	0.175809	5.240728	3.919165	9.93E-05	0.000237	0.302935
IDH3G	0.175755	8.000248	1.981256	0.048026	0.071838	-5.30413
PRELID2	0.175727	5.137311	5.479089	6.33E-08	2.26E-07	7.338486
IL17RB	0.175697	5.702744	3.044936	0.002431	0.004676	-2.66916
TEX36	0.175615	4.819699	5.791537	1.13E-08	4.39E-08	9.003568
TMEM107	0.175608	6.098784	2.059502	0.039884	0.060804	-5.14764
PDZD7	0.175547	5.272294	3.719973	0.000218	0.000496	-0.43707
ZBTB18	0.175512	8.495499	2.061854	0.039658	0.060476	-5.14284
LINC00563	0.175421	5.677149	4.119156	4.35E-05	0.000109	1.082711
HDGFRP2	0.175312	7.706963	3.278483	0.001105	0.002252	-1.94544
CCDC28A	0.175222	9.581069	2.74199	0.006292	0.011281	-3.5306
F2RL2	0.175188	4.204378	3.231763	0.001299	0.002618	-2.09435
KIAA0513	0.175146	9.095981	2.133867	0.033265	0.051532	-4.99338
GLUD2	0.175059	5.556266	3.621345	0.000318	0.000705	-0.78985

FZD10-AS	0.175021	5.4124	4.906168	1.20E-06	3.69E-06	4.503474
DIAPH2	0.174979	6.141392	2.935289	0.003462	0.006491	-2.99106
KCNS2	0.174893	4.230347	4.952799	9.56E-07	2.96E-06	4.723507
TOP3B	0.17486	5.425192	2.401808	0.016622	0.027509	-4.39296
SLC35G2	0.174741	4.502075	3.781416	0.000172	0.000396	-0.21273
THPO	0.174722	4.283986	8.294379	7.40E-16	6.07E-15	25.17144
DGCR9	0.174707	6.433815	3.440126	0.000622	0.001319	-1.41438
RAB3GAP2	0.174584	4.280214	3.235582	0.001282	0.002587	-2.08226
SEMA4G	0.174518	5.764756	3.357693	0.000836	0.001738	-1.68828
RUNDC3A	0.174471	6.068072	3.403457	0.00071	0.001495	-1.53701
FGF22	0.174462	5.152824	3.945001	8.94E-05	0.000214	0.401605
WFDC2	0.174294	6.264443	3.61657	0.000324	0.000717	-0.8067
TTLL2	0.174083	4.885208	5.142252	3.70E-07	1.21E-06	5.637099
PHYHD1	0.173798	5.375223	3.206305	0.001417	0.00284	-2.17462
PENK	0.173768	4.975826	5.217162	2.52E-07	8.37E-07	6.006985
LOC10192	0.173754	6.523743	3.778605	0.000174	0.0004	-0.22307
CLRN3	0.173733	4.251091	4.85738	1.53E-06	4.62E-06	4.275316
CDHR2	0.173492	6.623598	4.11107	4.50E-05	0.000112	1.050474
STON1	0.173473	4.959712	2.620521	0.009005	0.015703	-3.85132
TIMP3	0.173443	5.228609	3.784369	0.00017	0.000392	-0.20185
ABCF3	0.173397	7.320896	2.018593	0.043981	0.066397	-5.2302
SAPCD2	0.173338	7.017029	3.167834	0.001615	0.003206	-2.29476
LRFN1	0.173259	6.950297	2.666747	0.007869	0.013857	-3.73094
KIR2DL3	0.173123	6.25733	2.757449	0.006006	0.010804	-3.48877
PON3	0.173054	5.077965	3.839406	0.000137	0.00032	0.00222
GLYAT	0.172863	4.972403	5.727387	1.62E-08	6.19E-08	8.654952
KLHL34	0.17275	5.388039	4.448124	1.04E-05	2.81E-05	2.444791
CD209	0.172737	5.982572	3.742069	0.0002	0.000457	-0.35679
UBE2NL	0.172682	8.847019	4.482614	8.86E-06	2.42E-05	2.593273
DPCD	0.172666	6.981175	3.539458	0.000432	0.000938	-1.07586
HOXA11	0.172612	5.46503	4.442138	1.06E-05	2.88E-05	2.419131
LOC10050	0.172572	5.938275	3.610966	0.000331	0.000731	-0.82645
NOXO1	0.172567	5.560599	3.093054	0.002075	0.004046	-2.52428
CCDC69	0.172432	9.405808	2.495863	0.012837	0.021704	-4.16569
RGS18	0.172302	11.49739	2.058005	0.040028	0.060987	-5.15069
PARD6A	0.172265	6.794622	2.754788	0.006054	0.010883	-3.49598
FLJ22184	0.172238	5.950411	2.842603	0.004629	0.008502	-3.25423
SMG6	0.172232	5.746364	4.229179	2.72E-05	6.98E-05	1.527313
USP32P2	0.172204	9.774838	2.652905	0.008195	0.014393	-3.7672
LOC10028	0.172182	4.180239	5.619753	2.95E-08	1.09E-07	8.077847
UCA1	0.172175	5.440408	3.151776	0.001705	0.003372	-2.34449
ZCWPW1	0.172122	6.85315	3.944648	8.95E-05	0.000214	0.400252
VSIG10L	0.172121	4.48132	3.093975	0.002068	0.004035	-2.52148
SERPINA4	0.172027	5.529948	2.754321	0.006062	0.010897	-3.49725
CASP10	0.172018	6.67882	4.427655	1.13E-05	3.06E-05	2.357178
DNAH7	0.171996	5.084955	5.26774	1.94E-07	6.53E-07	6.259482
TUBB4A	0.171985	5.608215	3.295721	0.00104	0.002129	-1.88998
RNGTT	0.171949	6.633334	2.400574	0.016678	0.027584	-4.39588
ADRA2A	0.17189	4.194911	2.473003	0.013678	0.023027	-4.22171
GPX2	0.171841	5.41596	4.296155	2.03E-05	5.30E-05	1.80336
CHRNE	0.171677	5.650351	3.773836	0.000177	0.000407	-0.24059
ONECUT1	0.171643	4.951113	4.731295	2.79E-06	8.17E-06	3.695438
ALKBH7	0.171617	5.822819	2.037999	0.041995	0.063674	-5.19124
QRICH2	0.171597	5.417612	3.393842	0.000735	0.001543	-1.56895
EPOR	0.171583	6.226976	3.554538	0.000409	0.000891	-1.02366
XXYLT1	0.17154	6.413575	3.357164	0.000838	0.001741	-1.69002
B9D1	0.171509	5.126875	4.408561	1.24E-05	3.31E-05	2.275793
CC2D2A	0.171498	5.069484	5.088013	4.87E-07	1.57E-06	5.372334

POSTN	0.171438	3.868437	4.412314	1.22E-05	3.26E-05	2.291765
SFRP2	0.171417	4.772917	3.933635	9.36E-05	0.000224	0.358122
IRG1	0.171358	4.939167	3.306258	0.001003	0.002058	-1.85594
KIAA1161	0.171219	5.127592	4.987594	8.05E-07	2.52E-06	4.888936
LOC10013	0.171171	5.562735	3.860024	0.000126	0.000296	0.079395
NCOR1	0.171102	7.236959	3.414062	0.000684	0.001442	-1.50167
PI16	0.171087	6.354298	2.319931	0.020684	0.033489	-4.58382
YIPF5	0.170986	7.832725	1.972087	0.049066	0.073194	-5.32208
LOC10192	0.170885	4.947503	2.617715	0.009079	0.015817	-3.85856
LOC40004	0.17072	4.457172	3.970744	8.05E-05	0.000194	0.500528
C20orf173	0.170422	5.383623	3.138436	0.001783	0.00351	-2.38562
KRT27	0.170382	4.532263	5.014362	7.04E-07	2.22E-06	5.016928
TFAP2D	0.170351	5.158001	3.847741	0.000132	0.00031	0.033372
LOC10050	0.170309	4.904467	4.872025	1.42E-06	4.32E-06	4.343582
LMAN1L	0.170263	6.17723	3.903493	0.000106	0.000251	0.243385
KRT9	0.170147	5.893442	3.045256	0.002428	0.004672	-2.6682
LOC10272	0.1701	4.346683	2.411003	0.016213	0.026891	-4.37111
EVC	0.169961	5.096711	4.511727	7.76E-06	2.14E-05	2.719439
ADAMTS9	0.169944	5.2344	5.371262	1.13E-07	3.89E-07	6.78319
ITIH2	0.169931	5.224912	3.32861	0.000927	0.001913	-1.78339
ACTN3	0.169818	5.240823	3.732011	0.000208	0.000474	-0.39339
IGSF21	0.169804	4.942289	3.250023	0.00122	0.002471	-2.0364
DNM1P46	0.169776	5.418627	3.941513	9.07E-05	0.000217	0.388248
DISP2	0.169706	5.180342	4.240625	2.59E-05	6.66E-05	1.574198
SPSB1	0.169681	4.72945	4.355956	1.56E-05	4.14E-05	2.053274
GAPVD1	0.169373	7.965994	3.081724	0.002154	0.004187	-2.55859
SVOP	0.169285	4.88088	3.559328	0.000402	0.000877	-1.00704
VIL1	0.169258	5.542818	2.462207	0.014093	0.023659	-4.24799
TLK1	0.16924	5.573728	3.519655	0.000465	0.001004	-1.14408
HMGA1	0.169232	6.779339	2.033217	0.042477	0.06433	-5.20088
LINC0031Z	0.169192	5.54927	4.979374	8.38E-07	2.62E-06	4.849761
POM121L9	0.169158	4.402524	4.330308	1.75E-05	4.60E-05	1.945692
PTCH2	0.169153	5.706227	3.761959	0.000185	0.000425	-0.28415
SFTPC	0.169135	6.424935	4.449961	1.03E-05	2.78E-05	2.452672
DPM2	0.169099	6.914564	2.796162	0.00534	0.009691	-3.383
TUBA1A	0.168976	12.86343	2.625808	0.008868	0.015485	-3.83765
GJC2	0.168945	6.378591	3.695392	0.00024	0.000541	-0.52584
ANXA11	0.16888	9.527401	4.067251	5.40E-05	0.000133	0.876805
LINC00595	0.16886	5.033788	4.081763	5.08E-05	0.000126	0.934126
MSX1	0.168833	5.01366	4.299352	2.00E-05	5.23E-05	1.816638
CEP85	0.168817	6.723261	3.717108	0.000221	0.000501	-0.44745
KRT33B	0.168806	5.611298	3.555049	0.000408	0.000889	-1.02189
MVK	0.168779	6.722998	3.729362	0.00021	0.000479	-0.40302
MYOD1	0.168751	6.350449	3.488761	0.000521	0.001117	-1.24979
LOC64648	0.168681	4.81283	3.925334	9.68E-05	0.000231	0.326441
EMP2	0.168431	4.839778	3.935544	9.29E-05	0.000222	0.365417
NDUFA11	0.168425	7.673384	2.166535	0.030669	0.047917	-4.9239
ZNF821	0.168425	6.088002	4.891715	1.29E-06	3.94E-06	4.435665
GUCY2C	0.168343	4.336339	4.971009	8.74E-07	2.72E-06	4.809954
LINC0046Z	0.168335	4.096582	4.666571	3.79E-06	1.09E-05	3.403256
HMGCS2	0.168329	4.993592	3.836903	0.000138	0.000323	-0.00712
ITCH	0.168326	7.027179	2.306137	0.021448	0.034605	-4.61534
PSG6	0.16819	5.023103	4.913713	1.16E-06	3.56E-06	4.538943
THAP3	0.168154	5.901474	3.77684	0.000175	0.000403	-0.22956
DLG5-AS1	0.16813	6.118009	3.456765	0.000586	0.001246	-1.35832
CACNG1	0.168078	5.612231	3.640299	0.000296	0.000659	-0.72276
ECSCR	0.168053	5.185372	3.894469	0.00011	0.00026	0.209196
STAG3	0.168008	5.821291	3.072938	0.002217	0.0043	-2.58511

LOC10192	0.167985	4.66607	4.017226	6.65E-05	0.000162	0.680693
C20orf62	0.167949	4.383038	3.887684	0.000113	0.000267	0.183543
C8B	0.167915	5.871836	3.201687	0.00144	0.002883	-2.18912
HSD11B1	0.167902	5.385289	3.179412	0.001553	0.003097	-2.25875
TGM4	0.167873	4.726844	5.142558	3.69E-07	1.20E-06	5.638602
SPRR1A	0.167808	5.293274	4.359937	1.53E-05	4.07E-05	2.070029
TMEM60	0.16761	8.955403	2.694981	0.00724	0.012841	-3.6564
LINC0094	0.167515	5.561156	3.415401	0.000681	0.001436	-1.4972
GTF2IRD1	0.167473	5.613509	3.23154	0.0013	0.00262	-2.09506
KLC3	0.167432	5.165589	3.100501	0.002024	0.003954	-2.50166
PCDHGA3	0.167428	4.926967	4.16377	3.60E-05	9.10E-05	1.261665
SPINK5	0.167417	5.233022	3.768085	0.000181	0.000416	-0.2617
SCN8A	0.16736	4.437683	3.385984	0.000756	0.001584	-1.595
PRR16	0.167273	3.702622	3.084673	0.002133	0.00415	-2.54967
DEAF1	0.167257	5.238051	3.450656	0.000599	0.001273	-1.37893
A1CF	0.167234	4.602988	4.81276	1.89E-06	5.66E-06	4.068493
HSD17B3	0.167219	5.162499	3.702488	0.000233	0.000528	-0.50027
ACRV1	0.167141	5.379023	5.221647	2.46E-07	8.19E-07	6.029287
NXF3	0.16714	4.844132	3.228216	0.001315	0.002647	-2.10557
NAPG	0.167095	6.68978	2.343357	0.019442	0.031693	-4.52988
NKX6-1	0.167077	5.043957	2.835641	0.00473	0.008672	-3.27366
SLCO2A1	0.167036	5.370822	3.776191	0.000175	0.000404	-0.23194
ALDH1L2	0.166997	3.292479	2.505682	0.012489	0.021169	-4.14147
TTI2	0.166953	6.955406	2.428291	0.015468	0.025746	-4.32983
ARHGEF25	0.166938	6.180746	3.892012	0.000111	0.000262	0.199901
THY1	0.166906	5.991263	3.2948	0.001044	0.002135	-1.89295
FEV	0.166758	5.527362	3.134552	0.001807	0.003553	-2.39757
COQ7	0.166713	6.416211	3.801292	0.000159	0.000368	-0.1394
JPH2	0.166651	5.510577	4.472787	9.26E-06	2.52E-05	2.550859
RAD51B	0.166642	4.445314	3.059051	0.002321	0.004484	-2.62688
WDR24	0.166587	5.652692	2.162421	0.030986	0.048362	-4.93271
PRCD	0.166542	5.654831	3.292842	0.001051	0.002149	-1.89926
SDHD	0.166522	8.031771	2.690721	0.007331	0.01299	-3.6677
KIF23	0.166505	3.823615	2.819829	0.004966	0.009067	-3.31763
DLX2	0.166496	4.963816	3.610763	0.000331	0.000732	-0.82716
LOXL3	0.166479	6.771109	2.551569	0.010975	0.018804	-4.02706
CCR4	0.166425	5.536296	3.640617	0.000296	0.000658	-0.72163
BRWD1-IT	0.166383	5.086491	4.23507	2.65E-05	6.81E-05	1.551431
NEDD4L	0.166365	4.890638	4.856726	1.53E-06	4.63E-06	4.272269
LOC10050	0.166356	6.638497	3.354585	0.000846	0.001756	-1.69848
TRIM9	0.166306	4.325957	2.774271	0.005707	0.010309	-3.44298
PROK1	0.16628	5.577194	3.359864	0.00083	0.001727	-1.68115
PGK2	0.166269	4.516247	4.161639	3.63E-05	9.18E-05	1.253077
APC	0.166144	6.58841	2.624431	0.008904	0.015539	-3.84121
FKBP10	0.165974	5.590285	3.714406	0.000223	0.000505	-0.45722
LINC0057	0.16585	5.739061	4.019048	6.60E-05	0.000161	0.687796
CYTH2	0.165655	6.624642	2.407778	0.016355	0.027103	-4.37878
TTC33	0.165629	5.92925	2.607449	0.009352	0.01625	-3.88499
SH3BGRL	0.165477	11.01569	2.121527	0.034293	0.053012	-5.01935
LOC10272	0.165446	6.163733	3.597883	0.000348	0.000766	-0.87244
GPR4	0.165435	5.549456	3.788816	0.000167	0.000385	-0.18547
C19orf73	0.165342	5.440193	3.196458	0.001466	0.002932	-2.20551
MYPN	0.165296	5.07023	4.245722	2.53E-05	6.52E-05	1.595118
ITLN1	0.165219	4.721842	4.157577	3.69E-05	9.33E-05	1.236714
TBC1D29	0.165158	5.306138	2.992016	0.002887	0.005486	-2.82595
KIF7	0.165082	5.938348	3.134158	0.001809	0.003557	-2.39878
TREM2	0.164927	6.404681	3.807799	0.000155	0.000359	-0.11532
SYDE1	0.164559	6.329061	3.929726	9.51E-05	0.000227	0.343195

PVALB	0.164432	5.422714	2.250445	0.024788	0.039484	-4.74071
PLXNA2	0.164389	5.474304	4.833346	1.71E-06	5.15E-06	4.163695
OR6B1	0.164355	4.482878	4.638659	4.32E-06	1.23E-05	3.278404
PDE2A	0.164333	6.050532	2.176265	0.029931	0.046925	-4.90301
THRSP	0.164277	4.629734	2.695492	0.007229	0.012825	-3.65504
SLC12A3	0.164213	5.372514	5.362536	1.18E-07	4.07E-07	6.738689
LOC10012	0.164174	5.185683	3.499745	0.000501	0.001074	-1.21231
FHOD3	0.164171	4.676045	3.636191	0.000301	0.000669	-0.73733
PRR7	0.164147	5.272703	2.331431	0.020066	0.032593	-4.55741
LOC10050	0.164143	6.24518	4.110032	4.52E-05	0.000113	1.046337
KIAA1217	0.164082	5.388619	5.444522	7.62E-08	2.70E-07	7.159383
CHRND	0.164079	5.143415	3.685198	0.00025	0.000561	-0.56249
STX6	0.163961	6.37332	3.647544	0.000288	0.000642	-0.69702
DCTN3	0.163898	9.288819	2.60032	0.009547	0.016549	-3.90328
DDX51	0.163811	5.461663	3.367985	0.000806	0.001681	-1.65443
CXCL12	0.163758	4.910273	2.713308	0.006856	0.012211	-3.60761
OR52D1	0.163684	5.324634	3.152244	0.001702	0.003367	-2.34305
F2	0.163616	5.722888	2.695472	0.007229	0.012825	-3.6551
PHLDB1	0.16361	5.86276	3.438167	0.000627	0.001327	-1.42096
CDK2	0.163608	5.786625	3.740035	0.000202	0.00046	-0.36421
SERTAD4-	0.163506	4.26904	4.148917	3.83E-05	9.66E-05	1.201887
TMEM37	0.163421	5.709745	2.781507	0.005583	0.010102	-3.42321
APOL5	0.163313	5.343475	2.867923	0.004279	0.0079	-3.18315
WFDC3	0.163147	5.360684	3.143567	0.001753	0.003456	-2.36982
LRRC28	0.163069	4.954996	6.730998	3.99E-11	2.05E-10	14.49856
CEP170B	0.16295	6.360102	3.47521	0.000548	0.00117	-1.29587
C17orf59	0.162921	6.08508	2.065034	0.039355	0.060054	-5.13635
CD53	0.162716	8.878437	2.692772	0.007287	0.012918	-3.66226
TMEM225	0.1627	4.876774	4.699229	3.25E-06	9.44E-06	3.550217
MAU2	0.162684	6.607945	4.438162	1.08E-05	2.93E-05	2.402104
DENND1A	0.162679	6.872697	2.445865	0.014741	0.024658	-4.28756
ZRANB1	0.162619	7.728291	2.397613	0.016812	0.027788	-4.40289
ZFHX2	0.162265	6.140368	3.912729	0.000102	0.000242	0.278452
C11orf86	0.162195	6.258308	3.057898	0.00233	0.004497	-2.63034
C3orf27	0.162149	6.091201	3.185317	0.001522	0.00304	-2.24034
COQ2	0.162134	6.191225	2.229375	0.026164	0.041479	-4.78735
NACC1	0.162	6.659988	3.267379	0.001148	0.002336	-1.98102
FXYD1	0.161795	5.518066	2.954714	0.003254	0.006127	-2.93487
SPACA6P	0.161696	4.928026	3.476352	0.000546	0.001165	-1.29199
BPIFB1	0.161601	5.206884	4.245223	2.54E-05	6.53E-05	1.593069
GTSE1	0.161586	5.810275	3.225138	0.001329	0.002672	-2.1153
VASN	0.161568	5.630024	3.231753	0.001299	0.002618	-2.09438
C19orf47	0.161444	6.468046	3.604146	0.00034	0.000748	-0.85044
STOML1	0.161438	6.3521	3.604935	0.000339	0.000746	-0.84767
CREB3L1	0.161433	6.003812	3.754365	0.000191	0.000437	-0.31193
CSF2RB	0.16138	12.60501	2.304217	0.021556	0.034755	-4.61971
BMP7	0.16132	5.826706	4.736841	2.72E-06	7.97E-06	3.720649
DDX49	0.161179	6.327206	3.199034	0.001453	0.002908	-2.19743
AMBP	0.161161	5.014101	3.901412	0.000107	0.000253	0.235494
NSD1	0.161157	6.301501	3.697444	0.000238	0.000537	-0.51845
HRH1	0.161106	4.952711	5.140628	3.73E-07	1.21E-06	5.629133
FAM46B	0.161065	4.964128	3.799946	0.00016	0.00037	-0.14438
HES7	0.160973	5.744803	2.743594	0.006262	0.01123	-3.52627
RBM11	0.160954	3.961455	2.078704	0.038075	0.058258	-5.10832
SLC51B	0.160761	5.147054	3.086581	0.00212	0.004125	-2.54389
LOC44033	0.160718	6.736923	2.986391	0.00294	0.005579	-2.84246
ARL15	0.1607	6.390302	2.284567	0.022692	0.036417	-4.66425
SPATA8	0.160529	6.003686	4.114796	4.43E-05	0.000111	1.065319

LY6D	0.160441	5.142299	2.726538	0.00659	0.011767	-3.57218
KIF27	0.160384	4.224182	2.854493	0.004462	0.008217	-3.22093
GABBR2	0.160379	5.148805	4.930932	1.06E-06	3.28E-06	4.620084
ABCG2	0.160347	4.718804	2.7148	0.006825	0.012166	-3.60362
GTF2IRD2	0.160295	7.075094	2.202693	0.028002	0.044122	-4.8458
CES3	0.160242	6.536323	4.015104	6.71E-05	0.000164	0.672423
KIAA1524	0.160153	4.168598	2.172886	0.030185	0.047275	-4.91028
VPS13A-A	0.160132	5.795441	2.957883	0.003221	0.006071	-2.92566
MRPL33	0.160087	9.638422	3.557362	0.000405	0.000882	-1.01386
CERCAM	0.160049	5.944736	4.133413	4.09E-05	0.000103	1.1397
DHX8	0.160045	6.954707	4.11026	4.51E-05	0.000113	1.047245
ZNF408	0.159999	7.976455	3.553907	0.00041	0.000892	-1.02585
LBX1	0.159984	4.502635	3.655195	0.00028	0.000624	-0.66979
GRB7	0.159958	5.837308	3.145513	0.001741	0.003435	-2.36382
CAPZA1	0.159918	9.298547	2.116178	0.034747	0.053668	-5.03056
MROH2B	0.159909	4.839591	4.586642	5.50E-06	1.55E-05	3.047591
ITPKB-IT1	0.159902	6.280421	2.622798	0.008946	0.015607	-3.84544
HPR	0.159876	5.450421	3.85855	0.000127	0.000297	0.073862
MAGEA10	0.159826	4.889409	3.440608	0.000621	0.001317	-1.41276
BCL2L13	0.1598	6.729046	4.23909	2.60E-05	6.70E-05	1.567905
IGSF10	0.159682	6.390901	3.33964	0.000892	0.001845	-1.74741
MKRN7P	0.159674	5.149252	2.585741	0.009955	0.017205	-3.94053
HOXB13	0.159668	5.112971	3.533665	0.000442	0.000958	-1.09586
PRNP	0.159664	8.79262	2.709689	0.00693	0.01233	-3.61727
PSMC1	0.159525	9.795565	3.205657	0.00142	0.002845	-2.17666
TCP11	0.159462	5.099309	4.080504	5.11E-05	0.000126	0.929145
MC2R	0.159401	5.00917	4.858103	1.52E-06	4.60E-06	4.27868
CILP	0.159388	6.419087	3.569965	0.000386	0.000845	-0.97004
EPHB2	0.159352	5.872921	4.673164	3.68E-06	1.06E-05	3.432845
CRNN	0.159283	5.446517	3.396662	0.000728	0.001529	-1.55959
SRCAP	0.159062	6.299338	3.688899	0.000246	0.000554	-0.5492
KCNK9	0.159042	5.447262	4.28457	2.14E-05	5.56E-05	1.755318
ADAM20	0.159026	5.576104	4.645558	4.18E-06	1.20E-05	3.309199
SVEP1	0.159003	5.191771	4.158456	3.68E-05	9.30E-05	1.240252
CNTF	0.158982	4.749234	4.232338	2.68E-05	6.89E-05	1.540241
C16orf78	0.158911	4.738346	3.362632	0.000822	0.00171	-1.67205
GRM6	0.158701	5.206049	3.958397	8.46E-05	0.000203	0.453008
EML2	0.158692	6.307852	3.267444	0.001148	0.002335	-1.98081
NAT2	0.15869	5.159751	4.296779	2.03E-05	5.28E-05	1.80595
PCK1	0.158673	3.725462	2.07926	0.038024	0.058194	-5.10718
SLC9A3R2	0.158654	4.08645	3.383264	0.000764	0.001599	-1.604
MAGEB4	0.158564	4.117822	5.943727	4.76E-09	1.92E-08	9.844442
ATP6AP2	0.158452	8.638519	3.706014	0.00023	0.000521	-0.48755
CNTFR	0.158394	6.766165	2.378006	0.017724	0.029117	-4.44911
LINC00881	0.158334	4.968492	3.313734	0.000977	0.002009	-1.83173
FAM217A	0.157983	5.00413	4.344945	1.64E-05	4.33E-05	2.007017
CRYGD	0.157948	6.073922	3.178952	0.001555	0.003101	-2.26019
SLC9A2	0.157908	4.570628	3.793709	0.000164	0.000378	-0.16742
SLCO2B1	0.157828	5.749493	3.760834	0.000186	0.000427	-0.28827
PGC	0.157823	6.245148	3.719682	0.000218	0.000496	-0.43812
SERPINA2	0.157722	4.390212	3.493894	0.000512	0.001097	-1.23229
DRD2	0.157617	6.153986	4.449057	1.03E-05	2.80E-05	2.448796
IQCC	0.157611	5.586988	2.25571	0.024454	0.039	-4.72898
TLR9	0.157597	6.184476	2.142407	0.032569	0.050579	-4.97532
FPGS	0.157549	6.809599	2.639369	0.008525	0.014939	-3.80248
KIR3DX1	0.157518	4.992186	3.964339	8.26E-05	0.000199	0.47586
CABLES1	0.157517	6.054456	4.045125	5.92E-05	0.000145	0.789782
MBD3	0.157475	7.154056	2.895346	0.003927	0.007285	-3.10548

PRAP1	0.157449	5.761329	2.93331	0.003484	0.006528	-2.99677
IL1A	0.157447	5.594475	2.254612	0.024523	0.039093	-4.73143
RAB11A	0.157398	8.969537	2.352058	0.018997	0.031038	-4.50971
SHANK2	0.157253	4.086563	6.8198	2.26E-11	1.19E-10	15.05485
S100G	0.157158	4.446706	4.434481	1.10E-05	2.97E-05	2.386356
C1orf86	0.156999	5.520749	3.124433	0.001869	0.003666	-2.42861
OLFM2	0.156984	5.895121	4.059018	5.59E-05	0.000138	0.844372
TMEM217	0.156903	4.769807	3.505563	0.00049	0.001053	-1.19241
CXCL2	0.156785	4.774212	2.218462	0.026903	0.04255	-4.81134
CENPA	0.156724	5.606161	2.642903	0.008438	0.014794	-3.79329
CIB4	0.156704	4.609778	3.250818	0.001216	0.002465	-2.03387
BRINP1	0.156623	5.326232	3.131518	0.001825	0.003585	-2.40689
SLC29A1	0.156511	6.198647	1.99176	0.046857	0.070276	-5.28347
MLST8	0.15651	6.07361	2.173898	0.030109	0.047171	-4.9081
C15orf48	0.156485	4.393691	2.217073	0.026998	0.042689	-4.81439
THRB	0.156459	4.616781	3.407043	0.000701	0.001477	-1.52507
LYG1	0.156436	6.110358	3.077742	0.002182	0.004237	-2.57062
YIPF3	0.156382	8.403204	1.993548	0.046661	0.069999	-5.27994
LRRC57	0.156379	5.903534	2.045624	0.041236	0.062611	-5.17583
CNIH2	0.156368	6.146453	2.982272	0.002979	0.005649	-2.85453
SLC31A1	0.156345	6.348221	2.479554	0.013432	0.022644	-4.20571
SPA17	0.156327	4.558665	2.248655	0.024902	0.039655	-4.74469
ROCK2	0.156307	7.207029	2.986457	0.002939	0.005578	-2.84226
H2AFJ	0.156257	6.634997	2.885612	0.004049	0.0075	-3.13313
CCDC155	0.156239	4.809888	3.037709	0.002489	0.00478	-2.69072
BRINP2	0.156177	5.070862	3.721646	0.000217	0.000493	-0.43101
PPP1R14D	0.156108	5.382286	3.439603	0.000624	0.001321	-1.41614
ELSPBP1	0.156106	5.740232	3.643021	0.000293	0.000653	-0.71309
CLP1	0.156055	7.188105	4.425163	1.15E-05	3.09E-05	2.34654
BMP8A	0.15603	5.2243	4.120108	4.33E-05	0.000108	1.086512
RCAN1	0.155984	5.969605	4.731513	2.79E-06	8.16E-06	3.696427
RGS22	0.155954	5.058168	3.858552	0.000127	0.000297	0.073871
ZBTB37	0.155937	5.954369	3.231416	0.0013	0.00262	-2.09545
LINC0024	0.155813	4.963252	3.467113	0.000564	0.001202	-1.32333
GPR143	0.155795	4.790754	2.576098	0.010234	0.017636	-3.96506
USP2	0.155677	5.314384	4.640083	4.29E-06	1.23E-05	3.284757
SLC38A5	0.15565	6.312721	1.990003	0.047051	0.070526	-5.28693
ZYG11A	0.155625	5.271663	3.667015	0.000268	0.000599	-0.62762
TTLL12	0.155593	6.918623	2.355141	0.018842	0.030806	-4.50254
CCL19	0.155585	4.772457	3.022642	0.002614	0.005007	-2.73553
RHO	0.155496	6.177267	4.248144	2.50E-05	6.46E-05	1.605063
LOC10272	0.155457	5.545088	3.293811	0.001047	0.002143	-1.89614
LOC10272	0.15544	4.92107	3.701277	0.000235	0.00053	-0.50464
SMOC1	0.155316	5.601029	3.639038	0.000298	0.000662	-0.72723
C2CD4C	0.155278	6.701918	3.087167	0.002115	0.004118	-2.54212
CSF1	0.15526	6.223084	4.171614	3.48E-05	8.82E-05	1.293317
CRTC1	0.155231	7.173594	2.564486	0.010579	0.018187	-3.99448
CPXM1	0.155152	5.124683	2.339114	0.019662	0.032008	-4.53969
PNLIPRP1	0.154977	4.888361	4.213691	2.91E-05	7.44E-05	1.464059
LINC0131	0.15496	5.400871	4.671883	3.70E-06	1.06E-05	3.427094
LOC10192	0.15481	4.715077	3.233662	0.00129	0.002603	-2.08834
BPIFA2	0.15465	5.126303	2.712924	0.006864	0.012223	-3.60863
GAST	0.154614	6.398817	2.765539	0.00586	0.010561	-3.46678
FAM83C-/	0.154501	4.957171	2.481528	0.013359	0.022529	-4.20088
TLN2	0.15445	5.367793	3.983724	7.63E-05	0.000185	0.55064
PSMB5	0.154428	8.076218	2.438475	0.015043	0.025114	-4.30537
RIBC2	0.154378	4.598675	3.474241	0.00055	0.001173	-1.29916
FRMPD3	0.153998	5.481614	3.238669	0.001268	0.002561	-2.07247

TIMM23	0.153928	4.665931	3.868953	0.000121	0.000286	0.112937
KBTBD12	0.153879	4.286424	4.502373	8.10E-06	2.23E-05	2.678816
NETO1	0.153842	4.82647	3.934114	9.34E-05	0.000223	0.359952
OR51B2	0.153747	5.798195	3.525555	0.000455	0.000984	-1.1238
FAM228A	0.153697	4.242676	3.175394	0.001574	0.003135	-2.27126
FAM205A	0.15365	4.545378	4.297896	2.02E-05	5.26E-05	1.81059
TRPC2	0.153603	5.497786	4.033437	6.22E-05	0.000152	0.743993
LOC64320	0.153516	5.329506	3.817118	0.000149	0.000347	-0.08076
BPIFA1	0.153453	5.923685	2.975438	0.003045	0.005767	-2.87452
CNTNAP1	0.153337	5.953558	3.085409	0.002128	0.004141	-2.54744
HRG	0.153293	5.406767	3.828361	0.000143	0.000333	-0.03896
LOC10272	0.153265	7.552644	3.882833	0.000115	0.000272	0.165227
NECAB3	0.153241	6.382627	3.208263	0.001408	0.002822	-2.16847
LOC10192	0.153157	4.216948	2.976426	0.003036	0.005751	-2.87163
SLC25A51	0.153154	6.165579	2.3035	0.021597	0.034814	-4.62134
OGFOD2	0.153074	6.608439	2.673617	0.007711	0.013604	-3.71287
NPY6R	0.153052	4.920451	4.787865	2.13E-06	6.33E-06	3.953865
SRC	0.152999	6.104844	2.585356	0.009966	0.017222	-3.94152
AZGP1P1	0.15299	4.405397	4.446327	1.04E-05	2.83E-05	2.437087
KRT25	0.15283	4.343863	3.907555	0.000104	0.000247	0.258799
SLC16A11	0.152708	4.714652	2.345572	0.019328	0.031524	-4.52475
LOC10012	0.152686	5.648838	2.389554	0.017181	0.028324	-4.42193
WNT5B	0.152677	4.656139	3.722315	0.000216	0.000491	-0.42858
RETNLB	0.152649	5.444272	4.595048	5.29E-06	1.49E-05	3.084726
DMBT1	0.152553	6.398909	3.084655	0.002133	0.00415	-2.54972
CDH3	0.152544	5.266866	2.911595	0.003732	0.006952	-3.05912
FAM71F2	0.15249	5.636724	3.192428	0.001486	0.00297	-2.21812
YBX3	0.15228	7.282036	2.200313	0.028171	0.044354	-4.85098
NOX5	0.152277	6.514307	2.456324	0.014323	0.023999	-4.26227
SYCE3	0.152265	4.538711	3.358517	0.000834	0.001733	-1.68557
GATAD2A	0.15226	7.233351	4.159054	3.67E-05	9.28E-05	1.242664
VSNL1	0.152169	5.191431	5.293555	1.69E-07	5.74E-07	6.389211
SNX32	0.152126	5.450362	3.067734	0.002256	0.004368	-2.60079
PRSS3	0.152063	5.677056	3.608586	0.000334	0.000737	-0.83483
SLC23A3	0.152039	6.627905	3.197857	0.001459	0.002919	-2.20112
TPSB2	0.15203	5.607692	3.024902	0.002595	0.004972	-2.72883
DNASE2B	0.151987	4.08611	4.597398	5.23E-06	1.48E-05	3.09512
CCNI	0.151924	10.07047	2.169026	0.030479	0.047652	-4.91857
H1FNT	0.151895	5.236469	3.519961	0.000465	0.001003	-1.14303
LOC10272	0.151882	4.576883	3.273095	0.001126	0.002294	-1.96272
PHLDA1	0.151873	5.265432	3.710847	0.000226	0.000512	-0.47009
MED20	0.151838	6.233623	3.727966	0.000212	0.000481	-0.40809
HRSP12	0.151805	5.673685	3.39168	0.000741	0.001554	-1.57612
NLGN3	0.151737	5.828719	3.046179	0.002421	0.00466	-2.66544
BAIAP2	0.151689	5.879623	3.695599	0.00024	0.000541	-0.52509
LLGL1	0.151676	6.92971	3.524967	0.000456	0.000986	-1.12582
MBP	0.151662	7.239122	3.243374	0.001248	0.002523	-2.05754
ST6GALNA	0.151645	5.597305	2.487136	0.013152	0.022201	-4.18714
LINC01015	0.151603	4.761003	4.265199	2.33E-05	6.02E-05	1.675266
C6orf106	0.151554	6.751405	2.921538	0.003616	0.006756	-3.03062
FLJ21408	0.151551	5.284516	4.43115	1.12E-05	3.02E-05	2.372112
SMIM10	0.151537	3.831427	4.121612	4.30E-05	0.000108	1.092515
LACRT	0.151501	5.337759	3.712957	0.000224	0.000508	-0.46246
SDC4	0.151411	7.063181	2.846736	0.00457	0.008403	-3.24267
ALX4	0.151344	5.441565	3.316534	0.000967	0.001992	-1.82264
C9orf84	0.151199	4.430563	3.773037	0.000178	0.000408	-0.24353
FXYD3	0.151149	6.867853	3.541876	0.000429	0.000931	-1.0675
PLXNB3	0.1511	5.720908	2.85384	0.004471	0.008232	-3.22276

ZNF295-A	0.151058	5.324454	2.280827	0.022914	0.036748	-4.67269
MDK	0.151049	5.32566	3.064944	0.002276	0.004405	-2.60918
MEX3A	0.151025	5.876192	3.725923	0.000213	0.000485	-0.4155
LOC10028	0.15095	5.048239	3.132742	0.001818	0.003572	-2.40313
IER5L	0.150837	5.797282	3.119575	0.001899	0.003723	-2.44348
OLFML3	0.150822	5.16774	2.70427	0.007043	0.012521	-3.63171
LOC10192	0.150761	3.996966	3.079351	0.002171	0.004218	-2.56576
TSKU	0.150743	7.099988	3.169413	0.001606	0.003192	-2.28986
TMEM50A	0.150678	10.37277	3.110733	0.001956	0.003828	-2.47049
MYBPH	0.150644	5.412195	3.685022	0.00025	0.000562	-0.56312
IRGC	0.150562	4.65994	3.073549	0.002213	0.004292	-2.58327
LINC0114(	0.150557	5.077696	3.59525	0.000351	0.000773	-0.88167
ZG16	0.150461	5.403731	2.909964	0.003751	0.006982	-3.06378
FBXL18	0.150424	6.045991	4.685271	3.47E-06	1.00E-05	3.48729
PROP1	0.150255	5.660722	2.600646	0.009538	0.016538	-3.90244
CENPO	0.15022	5.588246	3.686801	0.000248	0.000558	-0.55673
SLC30A8	0.150217	4.432825	4.157728	3.69E-05	9.32E-05	1.237321
CDR2L	0.150105	4.903007	3.200797	0.001444	0.002891	-2.19191
PPAPDC3	0.149939	5.814755	3.148894	0.001722	0.003402	-2.35339
TMEM106/	0.149918	5.044191	5.350391	1.26E-07	4.32E-07	6.676858
C3P1	0.149818	5.887261	2.520314	0.011987	0.020392	-4.10521
ACVRL1	0.149774	5.428046	2.33441	0.019908	0.032351	-4.55054
TMEM79	0.149588	6.239507	2.649328	0.008281	0.014533	-3.77654
3-Sep	0.149561	5.958595	3.613852	0.000327	0.000724	-0.81628
KLHL25	0.149558	5.953936	3.060007	0.002314	0.00447	-2.62401
PDCD1	0.149393	6.080742	2.995532	0.002854	0.005429	-2.81561
FAM168B	0.149369	7.090199	2.700249	0.007127	0.012661	-3.64241
STARD13	0.14931	4.751465	3.008285	0.002739	0.005229	-2.77803
PNMT	0.149287	6.388931	3.07051	0.002235	0.004332	-2.59243
LCN10	0.149239	6.029306	2.339845	0.019624	0.031958	-4.538
KRT85	0.149163	5.91354	3.023001	0.002611	0.005001	-2.73447
LOC10050	0.149129	4.887191	3.575293	0.000378	0.000829	-0.95147
SSX3	0.14896	5.298457	3.68507	0.00025	0.000562	-0.56295
TNNC2	0.148936	5.393528	2.295375	0.022061	0.03548	-4.6398
STAB2	0.148924	5.478739	3.870584	0.000121	0.000285	0.11907
CARS2	0.148902	6.359458	3.836808	0.000138	0.000323	-0.00748
CRTAC1	0.148822	5.519328	3.402701	0.000712	0.001499	-1.53952
TBC1D30	0.148803	5.273441	2.569902	0.010417	0.017932	-3.98078
C10orf71-	0.148722	4.678455	3.034075	0.002519	0.004834	-2.70155
VDR	0.148721	6.979586	3.330697	0.00092	0.001901	-1.77659
FGA	0.148678	4.997428	4.315114	1.87E-05	4.91E-05	1.882241
PDE1B	0.148671	6.292439	2.456926	0.014299	0.023966	-4.26081
PTPN21	0.148582	4.604799	6.003454	3.37E-09	1.39E-08	10.17972
SRP54	0.148463	8.964261	2.171679	0.030277	0.047394	-4.91287
NCOA5	0.14844	6.832665	2.379726	0.017642	0.029006	-4.44507
LOC10013	0.148417	4.509975	4.393236	1.32E-05	3.53E-05	2.210712
CDX2	0.148416	5.662979	2.242628	0.025291	0.0402	-4.75806
CPEB3	0.14836	5.352418	2.688237	0.007386	0.013076	-3.67427
TRAF6	0.14835	7.45864	2.847843	0.004555	0.008375	-3.23957
PDE6A	0.148342	5.269686	2.661763	0.007985	0.014043	-3.74402
CDC42BP/	0.148327	5.099195	3.79908	0.00016	0.000371	-0.14758
COLCA2	0.148326	4.735625	3.720542	0.000218	0.000495	-0.43501
KIR2DS1	0.148303	5.841685	4.227014	2.74E-05	7.04E-05	1.518458
LMAN2	0.148252	6.506862	2.496897	0.0128	0.021646	-4.16314
IHH	0.148183	6.190678	2.45531	0.014363	0.024061	-4.26473
CHIA	0.148143	5.151985	3.028853	0.002562	0.004912	-2.71709
CAPN5	0.148104	5.653208	4.37295	1.45E-05	3.85E-05	2.124884
TMEM70	0.148081	7.135612	2.617783	0.009077	0.015816	-3.85838

ACO1	0.148011	5.941467	3.437184	0.000629	0.001332	-1.42427
RFWD2	0.14801	9.852314	2.126269	0.033895	0.05241	-5.00939
LINC0091(	0.147946	4.946214	3.875539	0.000118	0.000279	0.137725
UBE2Q1	0.147854	6.710106	3.701546	0.000234	0.000529	-0.50367
MB	0.147828	5.402842	2.402435	0.016594	0.027468	-4.39147
SLC39A2	0.147687	6.00805	2.924844	0.003578	0.006691	-3.02113
CCDC3	0.147642	6.670864	2.601604	0.009511	0.016495	-3.89999
HIST1H2A	0.147639	3.857599	2.875597	0.004178	0.007721	-3.16149
DLX6-AS1	0.147588	4.59246	4.085185	5.01E-05	0.000124	0.947672
4-Mar	0.147549	5.195082	2.749297	0.006155	0.011047	-3.51085
DNASE2	0.147522	7.564357	2.115216	0.034829	0.053782	-5.03257
SPAG6	0.147474	4.555172	5.042476	6.12E-07	1.95E-06	5.152035
CXorf36	0.147389	5.619316	3.242045	0.001254	0.002533	-2.06176
RNF126P1	0.147304	6.430674	2.661733	0.007985	0.014043	-3.74409
BCL2L12	0.147236	6.430058	2.273729	0.02334	0.037365	-4.68866
PART1	0.147204	5.257753	2.909783	0.003753	0.006986	-3.0643
C1QL1	0.147168	5.104307	3.802094	0.000158	0.000367	-0.13644
RIMS2	0.147025	4.693406	5.368628	1.14E-07	3.94E-07	6.76975
MAATS1	0.146993	4.820757	2.354468	0.018875	0.03085	-4.5041
ACR	0.146969	5.508128	3.273744	0.001123	0.002289	-1.96064
BTG4	0.146944	4.411673	4.099072	4.73E-05	0.000118	1.002748
GABRG3	0.146824	4.019947	4.217041	2.86E-05	7.33E-05	1.477721
SLURP1	0.146806	5.265004	2.197853	0.028347	0.044608	-4.85633
CASP14	0.146778	5.164191	3.088539	0.002106	0.004101	-2.53797
SLC25A10	0.146629	5.673586	2.780268	0.005604	0.010136	-3.4266
RETSAT	0.146622	6.83141	2.045561	0.041242	0.062611	-5.17596
TCF7L1	0.146536	5.317477	2.813172	0.005069	0.009238	-3.33607
KCNH4	0.146531	5.98328	3.032298	0.002533	0.00486	-2.70684
IQCJ-SCHI	0.146333	5.665154	3.622841	0.000316	0.000701	-0.78457
GKN1	0.146329	4.520912	4.078299	5.16E-05	0.000127	0.920427
FAM63A	0.146285	9.501667	2.372674	0.017979	0.029496	-4.46162
BMP4	0.146233	5.78772	3.053998	0.00236	0.004551	-2.64204
RANBP10	0.146154	5.884666	2.765194	0.005867	0.01057	-3.46772
PKD2L1	0.146144	6.869084	3.067495	0.002257	0.00437	-2.60151
RILPL2	0.146138	10.5703	2.617165	0.009093	0.015838	-3.85998
SLC6A3	0.146132	6.251358	3.688068	0.000247	0.000555	-0.55218
MAP3K6	0.146109	4.900178	3.099998	0.002027	0.00396	-2.50319
MRPS18A	0.145996	6.995516	2.541194	0.011302	0.019312	-4.0531
ELF5	0.145991	4.64189	4.100777	4.69E-05	0.000117	1.00952
C10orf82	0.145969	5.896023	2.425827	0.015572	0.025899	-4.33573
CELA3A	0.145907	5.251959	3.555037	0.000408	0.000889	-1.02193
DIXDC1	0.145892	4.818136	2.180009	0.029651	0.046522	-4.89495
CCDC174	0.145829	6.965651	2.891241	0.003978	0.007374	-3.11715
DPF1	0.145795	6.352967	2.626524	0.00885	0.015457	-3.8358
PCP4	0.145783	4.517854	2.942609	0.003382	0.006349	-2.96993
GNMT	0.145777	5.657842	2.357488	0.018724	0.030633	-4.49708
DLGAP1-A	0.145775	6.12441	2.309666	0.02125	0.03432	-4.6073
SLC22A8	0.14575	5.534274	2.562911	0.010626	0.018262	-3.99846
SLC6A12	0.145749	5.824848	2.505867	0.012483	0.02116	-4.14101
SOCS1	0.145658	5.16461	3.127101	0.001852	0.003635	-2.42044
GRM4	0.145535	6.739853	2.451042	0.014533	0.024328	-4.27506
OVOL1	0.145474	6.155602	3.82817	0.000143	0.000333	-0.03967
LOC10254	0.145473	4.286458	4.663846	3.84E-06	1.10E-05	3.391037
EGFR	0.145395	5.471649	4.593839	5.32E-06	1.50E-05	3.079382
PQLC1	0.145392	6.937962	2.922352	0.003607	0.006741	-3.02828
ATE1	0.145371	5.692947	3.769596	0.00018	0.000413	-0.25616
CCDC117	0.145364	7.133264	2.36413	0.018395	0.030133	-4.4816
DNAI1	0.145246	5.185056	4.04486	5.93E-05	0.000146	0.788741

LOC10050	0.145234	6.216847	2.840708	0.004656	0.008546	-3.25952
CYSLTR2	0.145216	5.627254	3.236518	0.001278	0.002579	-2.07929
REEP2	0.14517	7.083075	2.603141	0.009469	0.016428	-3.89605
LOC10029	0.145045	4.122875	2.023747	0.043446	0.065665	-5.21989
LOC10099	0.144978	5.170253	4.106758	4.58E-05	0.000114	1.033304
BMP10	0.144911	5.107018	2.597916	0.009613	0.016653	-3.90944
LAMA3	0.144901	4.876094	4.978381	8.42E-07	2.63E-06	4.84503
IZUMO2	0.144891	5.565379	2.710131	0.006921	0.012317	-3.61609
LBX2-AS1	0.144609	5.885835	2.267894	0.023696	0.037884	-4.70175
ARHGEF16	0.144601	6.282944	2.609442	0.009299	0.016166	-3.87986
PCYT1B	0.144595	4.881828	2.966216	0.003137	0.005928	-2.90142
COL1A2	0.144583	5.323829	2.275211	0.023251	0.037242	-4.68533
IST1	0.144567	10.6526	3.133305	0.001814	0.003567	-2.4014
RET	0.144516	5.677333	3.984343	7.61E-05	0.000185	0.553033
TMC7	0.144501	4.266721	3.344534	0.000876	0.001815	-1.73141
SRRM4	0.144324	5.017289	5.209266	2.62E-07	8.70E-07	5.967767
PRSS21	0.144141	5.649791	2.320911	0.020631	0.033418	-4.58158
LINC0115	0.144087	5.425931	3.172003	0.001592	0.003167	-2.28181
SUMO1	0.144032	8.546192	2.756286	0.006027	0.010837	-3.49192
LOC10024	0.1438	4.805907	3.103917	0.002001	0.003912	-2.49127
FAM149B1	0.143784	5.039618	3.530284	0.000447	0.000969	-1.10751
UCP1	0.143781	4.618152	3.71614	0.000221	0.000502	-0.45095
ADAMTS1	0.14378	4.884456	2.855372	0.00445	0.008196	-3.21846
OCLM	0.143761	4.86362	3.782088	0.000171	0.000395	-0.21025
RAB36	0.14366	6.791896	2.327951	0.020251	0.032867	-4.56541
PRDM12	0.143623	6.115229	2.015735	0.04428	0.066786	-5.23591
FAM181B	0.143529	4.982453	3.758511	0.000188	0.00043	-0.29677
ALPK3	0.143504	4.615878	3.858822	0.000126	0.000297	0.074886
GFAP	0.143432	4.753867	4.239723	2.60E-05	6.68E-05	1.570502
NDNF	0.143376	5.492962	1.988561	0.04721	0.070736	-5.28977
CSPG4P1Y	0.143321	5.715275	3.179203	0.001554	0.003099	-2.2594
VTA1	0.143236	7.650836	2.470191	0.013785	0.023177	-4.22857
ADRB3	0.143217	5.77631	3.980729	7.73E-05	0.000187	0.539061
TMEM108	0.143187	5.227297	5.057998	5.66E-07	1.81E-06	5.226921
RBM33	0.14318	6.335154	3.418244	0.000674	0.001422	-1.48771
LSM10	0.143108	8.30155	2.058178	0.040011	0.060967	-5.15034
IL1RL1	0.143066	5.765707	3.582639	0.000368	0.000808	-0.92582
RAD21	0.143061	10.17866	2.244265	0.025185	0.040063	-4.75443
DSCAM	0.143029	4.60876	4.812646	1.89E-06	5.66E-06	4.067963
MYO7A	0.14299	5.484099	3.446058	0.000609	0.001292	-1.39442
MCHR2	0.142965	5.930578	3.659515	0.000275	0.000615	-0.65439
LINC0045	0.142853	4.491981	3.756499	0.000189	0.000434	-0.30413
MIR10A	0.1427	4.726852	3.475513	0.000547	0.001168	-1.29484
CFHR4	0.142684	4.044902	3.649715	0.000286	0.000637	-0.6893
AGPAT6	0.142564	6.277249	3.253941	0.001203	0.00244	-2.02392
IL9R	0.142527	5.511759	3.137313	0.00179	0.003523	-2.38908
C1orf233	0.142483	6.488501	2.222367	0.026636	0.042171	-4.80277
GCSAML-/	0.142393	3.750324	2.474496	0.013622	0.022941	-4.21807
LIMK1	0.142391	6.071082	2.259772	0.024199	0.038622	-4.71992
LINC00911	0.142352	5.024367	3.93025	9.49E-05	0.000227	0.345194
COL4A1	0.142262	4.96964	2.111716	0.03513	0.054172	-5.03989
LIM2	0.1422	5.483243	2.355166	0.01884	0.030806	-4.50248
AP4M1	0.142151	5.841201	2.614951	0.009152	0.015929	-3.86568
C1orf159	0.142114	5.871453	2.557898	0.010779	0.018495	-4.01112
RAB38	0.142078	4.730053	2.145555	0.032315	0.050216	-4.96864
LOC10192	0.141865	4.095891	3.463986	0.000571	0.001215	-1.33391
SLC10A1	0.141853	5.616579	3.153444	0.001695	0.003355	-2.33934
RECQL5	0.141828	5.719519	3.309662	0.000991	0.002035	-1.84492

FAT2	0.141749	4.963299	2.843501	0.004616	0.008481	-3.25172
SLC6A19	0.141724	5.941428	2.71693	0.006782	0.012093	-3.59793
ADORA1	0.141721	5.481429	2.945502	0.003351	0.006296	-2.96156
GIPR	0.141674	5.657456	3.515528	0.000473	0.001018	-1.15826
PIP5KL1	0.141648	5.302271	2.41983	0.015828	0.026292	-4.35007
DKFZP434	0.141571	6.412736	3.333784	0.00091	0.001882	-1.76652
RER1	0.141483	8.793097	2.445417	0.014759	0.024686	-4.28865
RASGEF1C	0.141449	5.206408	3.419329	0.000671	0.001416	-1.48409
GBX2	0.141441	4.641813	2.378891	0.017681	0.029061	-4.44703
C1orf111	0.141405	5.456333	2.485517	0.013212	0.022295	-4.19111
BCAM	0.141336	6.17148	2.519027	0.012031	0.02046	-4.10841
LRRC8E	0.141317	4.221763	4.322463	1.81E-05	4.76E-05	1.912906
DBH	0.141293	5.69977	2.902571	0.003839	0.007131	-3.08489
LOC40132	0.141231	7.027818	3.132282	0.00182	0.003577	-2.40454
SULF1	0.141207	4.59147	4.533408	7.03E-06	1.95E-05	2.81389
CDH16	0.141125	5.308097	2.512213	0.012263	0.02082	-4.12531
PPFIA2	0.141046	4.508147	5.019503	6.87E-07	2.17E-06	5.041583
SAMD10	0.141014	6.012086	2.917243	0.003666	0.006838	-3.04294
ANO5	0.140928	4.688572	2.675776	0.007662	0.013529	-3.70718
C2orf16	0.140855	6.064213	2.909648	0.003755	0.006987	-3.06469
C17orf96	0.14081	6.285655	2.701644	0.007098	0.012615	-3.6387
STX4	0.140767	7.778105	3.068351	0.002251	0.00436	-2.59893
TSPY1	0.140669	5.91715	3.312168	0.000982	0.00202	-1.8368
BROX	0.140665	7.506417	2.583662	0.010015	0.017301	-3.94583
OR5I1	0.140401	5.032611	3.304817	0.001008	0.002067	-1.8606
PGAM2	0.140358	4.838335	3.057603	0.002332	0.0045	-2.63123
GCNT4	0.140324	4.963463	3.205715	0.00142	0.002845	-2.17647
LOC10192	0.140267	4.346809	5.202657	2.71E-07	8.97E-07	5.93498
FDX1L	0.140218	6.383184	2.217014	0.027002	0.042692	-4.81452
CCDC154	0.140143	5.732399	2.923068	0.003599	0.006727	-3.02623
BCMO1	0.140096	4.359824	3.994505	7.30E-05	0.000177	0.59238
INPP5A	0.140076	9.307501	2.108073	0.035445	0.054616	-5.04749
PVRL1	0.14007	5.611315	2.941248	0.003397	0.006375	-2.97386
ATP13A4	0.140035	4.989309	4.036427	6.14E-05	0.00015	0.755694
RASL11A	0.140009	5.569203	2.495521	0.012849	0.021721	-4.16653
COA3	0.139913	6.793726	3.449881	0.000601	0.001276	-1.38154
CCDC42	0.139894	5.46156	3.409271	0.000696	0.001466	-1.51765
RGR	0.139892	5.467018	2.597818	0.009616	0.016655	-3.90969
POLA2	0.139869	5.794125	3.503394	0.000494	0.001061	-1.19983
CDO1	0.13977	4.968403	2.607112	0.009361	0.016261	-3.88585
LINC0111€	0.139758	4.206106	3.37563	0.000785	0.001639	-1.62922
ACSBG1	0.139702	4.886154	3.108422	0.001971	0.003856	-2.47754
ZNF839	0.139678	6.489112	2.933278	0.003484	0.006528	-2.99686
AGXT2	0.139645	4.996708	3.064479	0.00228	0.004411	-2.61058
CYP1B1-A	0.139636	5.245211	3.613198	0.000328	0.000725	-0.81859
CHRNA3	0.139586	5.525099	4.49746	8.28E-06	2.27E-05	2.657513
PHYHIPL	0.139556	5.826434	2.995693	0.002853	0.005428	-2.81514
PDCD1LG2	0.139517	4.994778	4.276432	2.21E-05	5.74E-05	1.721646
SAP30L	0.13949	6.811589	3.283015	0.001088	0.00222	-1.93089
LOC10192	0.139478	4.443836	4.922881	1.11E-06	3.41E-06	4.582116
LINC0109!	0.139411	4.326482	3.924929	9.70E-05	0.000231	0.324895
SZT2	0.139405	5.608123	2.172528	0.030213	0.04731	-4.91105
KRTAP4-1	0.139374	4.635153	3.544733	0.000424	0.000921	-1.05763
MCM8	0.139368	5.180056	2.546134	0.011145	0.019067	-4.04072
ROM1	0.139263	5.758901	2.015017	0.044355	0.066889	-5.23734
WISP2	0.139229	5.458508	2.339739	0.019629	0.031962	-4.53824
QPRT	0.139223	5.214799	2.8229	0.004919	0.008988	-3.30911
P2RY4	0.139043	5.282517	2.989712	0.002909	0.005524	-2.83271

C9orf117	0.139011	4.988001	3.497022	0.000506	0.001084	-1.22161
FOXN1	0.138971	5.19217	3.087622	0.002112	0.004113	-2.54074
ABCB9	0.138784	5.538217	2.185418	0.02925	0.045938	-4.88328
TTLL3	0.13878	6.178557	2.900845	0.00386	0.007167	-3.08982
LINC0044!	0.138747	5.017839	3.642967	0.000293	0.000653	-0.71329
ALKBH4	0.138716	5.480307	2.511335	0.012293	0.020864	-4.12749
MED23	0.138664	6.833622	2.733331	0.006457	0.011551	-3.55393
SCN4A	0.138636	5.224266	2.325022	0.020408	0.033093	-4.57214
RFTN2	0.138598	4.42097	5.603458	3.22E-08	1.19E-07	7.991335
LZTR1	0.138526	7.168848	2.13679	0.033025	0.051218	-4.9872
LOC10050	0.138514	4.864027	3.172087	0.001592	0.003167	-2.28155
DGKB	0.138493	3.720214	5.186102	2.95E-07	9.73E-07	5.853027
PTPN9	0.138465	5.573947	3.820283	0.000147	0.000343	-0.06901
DAZAP2	0.138382	9.806692	3.731898	0.000208	0.000474	-0.3938
CYP4A11	0.138343	5.610383	3.717138	0.000221	0.000501	-0.44734
HAO1	0.138238	4.327516	4.095097	4.81E-05	0.000119	0.986962
ASAP3	0.138231	5.848028	3.981992	7.69E-05	0.000186	0.543946
FAIM2	0.138226	5.413171	3.970052	8.07E-05	0.000195	0.497861
GJD3	0.138204	5.307044	3.707416	0.000229	0.000518	-0.48249
TTC26	0.138189	4.462435	2.016147	0.044237	0.066727	-5.23509
ATP10B	0.138121	5.215117	3.749621	0.000195	0.000445	-0.32926
MAPT-AS1	0.138117	6.161313	3.739919	0.000202	0.00046	-0.36463
SSUH2	0.138048	5.586595	3.392978	0.000738	0.001548	-1.57182
ACOX3	0.138026	6.226919	2.902608	0.003839	0.007131	-3.08479
LYNX1	0.138026	5.31618	4.537186	6.91E-06	1.92E-05	2.830391
TBC1D1	0.138007	6.838695	3.693296	0.000242	0.000545	-0.53338
KRT37	0.137901	4.895035	3.069668	0.002241	0.004343	-2.59496
SLC45A1	0.137872	5.290482	3.429627	0.000647	0.001367	-1.44962
LOC10192	0.137772	4.723769	3.027282	0.002575	0.004936	-2.72176
CHAF1B	0.137671	5.660273	2.262795	0.02401	0.03835	-4.71317
FRS3	0.137604	6.248556	2.345551	0.019329	0.031524	-4.5248
APLP1	0.137585	5.495675	2.626216	0.008858	0.015469	-3.8366
CYP2S1	0.137464	5.224942	2.419151	0.015858	0.026336	-4.35169
LEMD1	0.13744	4.606697	3.183918	0.001529	0.003054	-2.2447
IGF2BP1	0.137416	5.234988	4.583094	5.59E-06	1.57E-05	3.03194
SIN3B	0.137362	6.74767	2.828965	0.004828	0.008832	-3.29226
HIST1H1A	0.1373	4.952385	2.332975	0.019984	0.032472	-4.55385
PAX4	0.13727	4.702829	4.660459	3.90E-06	1.12E-05	3.375856
POMC	0.137236	5.452201	2.213263	0.027261	0.043063	-4.82273
DEF8	0.13719	8.851058	2.00931	0.044958	0.067709	-5.24871
TMEM132I	0.137184	4.30472	3.715943	0.000222	0.000503	-0.45166
PEG10	0.137151	4.733671	2.821734	0.004937	0.009016	-3.31235
ZNF589	0.137129	5.709047	2.381797	0.017544	0.028878	-4.4402
IL25	0.137106	5.582458	3.26454	0.00116	0.002357	-1.9901
LINC00997	0.137033	4.640859	3.989631	7.45E-05	0.000181	0.573493
ZFP57	0.137014	5.775726	2.338214	0.019709	0.032064	-4.54177
OR7C1	0.136964	4.732492	3.780673	0.000172	0.000397	-0.21546
NCAN	0.136909	4.894481	3.469878	0.000559	0.001192	-1.31396
SOWAHA	0.136831	4.146446	3.972075	8.00E-05	0.000193	0.505658
PPP5C	0.13683	6.195787	2.535446	0.011487	0.019604	-4.06749
LOC10013	0.136554	5.446706	2.374635	0.017885	0.029355	-4.45702
POLR2F	0.136501	6.426348	2.737728	0.006373	0.011413	-3.54209
KDELR3	0.136401	4.914064	4.29967	2.00E-05	5.23E-05	1.817959
RGS16	0.136334	5.381827	3.438481	0.000626	0.001326	-1.41991
TMEM61	0.13625	5.268506	2.52877	0.011705	0.019956	-4.08416
CACNA1G	0.136246	5.151871	3.970856	8.04E-05	0.000194	0.500961
LINC00574	0.136223	5.740798	3.096586	0.00205	0.004002	-2.51356
INTS3	0.136117	7.070138	3.178872	0.001556	0.003102	-2.26043

C9orf156	0.136057	6.581119	4.257837	2.40E-05	6.20E-05	1.644929
PCOLCE	0.135782	5.701704	2.4348	0.015195	0.025337	-4.31421
LINC0028(	0.135742	4.517828	2.625248	0.008883	0.015505	-3.8391
HMP19	0.135695	5.312575	2.006106	0.0453	0.068155	-5.25508
SATB2	0.135658	4.135854	3.463855	0.000571	0.001216	-1.33435
TM4SF5	0.135647	5.144379	2.388264	0.017241	0.028415	-4.42498
DSCR4	0.135601	5.645786	3.150911	0.00171	0.00338	-2.34716
CALD1	0.135543	4.612473	2.571307	0.010375	0.017866	-3.97722
LOC10028	0.135527	6.018768	2.166295	0.030688	0.047937	-4.92442
MYO16	0.135501	4.892333	3.351481	0.000855	0.001774	-1.70866
WDR55	0.135415	7.025167	3.051117	0.002382	0.004589	-2.65067
LOC10012	0.135399	3.754588	2.324051	0.020461	0.033169	-4.57438
LOC10192	0.135334	4.359443	3.939698	9.13E-05	0.000219	0.3813
C1orf100	0.135262	4.730578	2.270943	0.023509	0.037613	-4.69491
AMOTL1	0.135242	5.312342	3.505051	0.000491	0.001055	-1.19416
MKRN2	0.135223	6.436252	3.949113	8.79E-05	0.000211	0.417364
FSD2	0.135143	5.386526	3.661561	0.000273	0.000611	-0.6471
FAM131A	0.135048	6.189142	3.138643	0.001782	0.003508	-2.38499
COX15	0.135006	7.144387	2.386985	0.017301	0.028505	-4.42799
TMPRSS6	0.134989	5.983125	3.227526	0.001318	0.002653	-2.10775
MSR1	0.134956	4.562726	2.974063	0.003059	0.005792	-2.87854
SNCAIP	0.134916	4.675874	4.145048	3.89E-05	9.81E-05	1.186345
SYNGAP1	0.134768	5.673218	3.032387	0.002533	0.004859	-2.70658
MFSD7	0.134737	5.697834	2.94715	0.003334	0.006266	-2.95679
KLK10	0.134691	4.751683	4.052379	5.75E-05	0.000141	0.818261
ACOT7	0.134644	5.953509	2.001693	0.045774	0.068749	-5.26383
PPP1R1C	0.134617	5.516338	3.127396	0.00185	0.003632	-2.41953
CSN1S1	0.134478	3.954157	2.797949	0.00531	0.009645	-3.37808
MOS	0.134455	4.701253	3.010794	0.002717	0.005189	-2.77062
C2orf54	0.134419	5.538974	2.903469	0.003828	0.007116	-3.08233
CHGA	0.134284	5.199946	2.963862	0.00316	0.005968	-2.90828
POU3F4	0.134162	4.328473	2.644438	0.0084	0.014731	-3.78929
ARHGAP1:	0.134118	3.953861	2.705026	0.007027	0.012495	-3.6297
SMARCD1	0.134103	6.874325	2.77631	0.005672	0.010252	-3.43742
NKX2-5	0.134088	4.906288	2.831022	0.004798	0.00878	-3.28653
LINC0042	0.134074	4.991097	3.51612	0.000471	0.001016	-1.15623
FSHB	0.133845	5.112009	2.922838	0.003601	0.006731	-3.02689
TUB	0.13383	5.763954	4.527341	7.23E-06	2.00E-05	2.787415
GPR123	0.13382	4.945817	3.092127	0.002081	0.004058	-2.52709
KCNK12	0.133627	4.540311	3.484654	0.000529	0.001133	-1.26377
PRDX6	0.133534	10.15545	2.448242	0.014645	0.024506	-4.28183
UCMA	0.133493	4.960135	2.839424	0.004675	0.008574	-3.26311
ATP4B	0.133454	4.58723	3.225305	0.001328	0.002671	-2.11477
HIST1H3I	0.13339	4.217391	3.46058	0.000578	0.00123	-1.34543
LOC72804	0.13336	4.967306	3.128923	0.001841	0.003615	-2.41485
HIF3A	0.133308	5.22372	3.329925	0.000923	0.001905	-1.7791
TMPRSS11	0.133283	5.998054	2.524522	0.011846	0.020171	-4.09474
GPR173	0.133278	5.415124	3.662185	0.000273	0.000609	-0.64487
GABRR2	0.133242	6.344877	2.529315	0.011687	0.019931	-4.0828
TMEM201	0.133185	5.762646	3.402416	0.000713	0.0015	-1.54047
CAP2	0.133184	4.036069	4.088176	4.95E-05	0.000123	0.959519
STXBP6	0.133116	4.302831	4.915054	1.15E-06	3.54E-06	4.545254
FAM32A	0.133091	9.469851	2.136236	0.03307	0.051262	-4.98837
GRHL3	0.13309	4.757728	3.268834	0.001143	0.002326	-1.97637
POU5F1P3	0.133072	5.105905	2.707605	0.006973	0.012405	-3.62283
FGF11	0.133017	5.955278	2.95534	0.003248	0.006115	-2.93305
PTGER4P2	0.133	5.794192	2.727238	0.006576	0.011743	-3.5703
AK7	0.132988	5.853508	3.073575	0.002213	0.004292	-2.58319

KCNK17	0.132854	5.799499	2.703114	0.007067	0.012563	-3.63479
LOC10192	0.132843	5.289599	2.756568	0.006022	0.010829	-3.49116
CPS1-IT1	0.132834	5.286063	4.448307	1.03E-05	2.80E-05	2.445576
CNTNAP4	0.132785	6.013182	2.754095	0.006067	0.010902	-3.49786
TTTY13	0.132703	4.817832	3.653094	0.000282	0.000629	-0.67728
PHRF1	0.13252	6.067319	3.630798	0.000307	0.000681	-0.75643
SSTR3	0.132501	6.402762	2.625354	0.00888	0.015502	-3.83883
HDLBP	0.132296	6.654208	3.118977	0.001903	0.00373	-2.44532
ARHGEF4	0.132282	5.043487	3.043735	0.00244	0.004694	-2.67274
NXPH4	0.13223	5.496283	2.956796	0.003233	0.00609	-2.92882
TSPAN11	0.132221	5.896719	2.862281	0.004355	0.008028	-3.19904
LOC10050	0.132188	5.442829	2.625878	0.008866	0.015483	-3.83747
FLJ44087	0.132097	4.761249	3.052483	0.002372	0.004572	-2.64658
GINS4	0.132067	4.892048	4.766032	2.37E-06	6.98E-06	3.853787
NEUROD6	0.132052	5.932112	3.08943	0.0021	0.00409	-2.53526
CHRNG	0.132011	6.306779	2.290481	0.022345	0.035905	-4.65089
SERHL2	0.131973	5.436882	4.219466	2.83E-05	7.26E-05	1.487619
PRM3	0.131876	4.948901	2.461914	0.014104	0.023673	-4.24871
LOC72848	0.131849	5.539871	3.009165	0.002731	0.005214	-2.77543
SYNPO2L	0.131801	4.920381	3.874586	0.000119	0.00028	0.134138
CCNF	0.131778	5.449178	2.840327	0.004662	0.008554	-3.26059
SFN	0.13173	7.139292	2.357454	0.018726	0.030633	-4.49716
OTOF	0.131647	4.720805	2.312078	0.021116	0.034128	-4.60179
CYP2A7P1	0.131612	4.91537	3.173338	0.001585	0.003155	-2.27766
SH3GL3	0.131599	5.09022	3.504187	0.000493	0.001058	-1.19712
GADD45G	0.13158	5.336603	2.823562	0.004909	0.008972	-3.30727
FAM230B	0.131515	4.918622	2.737751	0.006372	0.011413	-3.54203
ARHGAP2:	0.131479	5.783725	2.992708	0.002881	0.005476	-2.82392
HOXA-AS:	0.13132	5.025392	2.667128	0.00786	0.013847	-3.72994
GRPR	0.131308	6.472792	2.866495	0.004298	0.007933	-3.18717
SMTNL2	0.131296	5.699495	2.809977	0.005119	0.009323	-3.34491
ADAMTS1	0.131261	4.460334	3.126385	0.001857	0.003643	-2.42263
HCG4B	0.131223	4.583697	3.074735	0.002204	0.004278	-2.57969
SHROOM:	0.131209	4.796492	3.303605	0.001012	0.002075	-1.86452
CPA4	0.131198	6.175454	3.074563	0.002205	0.00428	-2.58021
MBD2	0.13118	6.040481	5.654519	2.43E-08	9.09E-08	8.263182
MMP15	0.131169	5.75707	3.324547	0.00094	0.001939	-1.79661
RABL6	0.131164	6.954655	1.967075	0.049642	0.073969	-5.33185
PYGB	0.131151	6.58133	2.63749	0.008572	0.015009	-3.80737
LOC10028	0.131006	4.586831	2.939565	0.003415	0.006408	-2.97872
LSAMP	0.131	4.752081	5.87373	7.12E-09	2.81E-08	9.455288
BEST3	0.130866	4.429092	4.476289	9.12E-06	2.49E-05	2.565964
TCTE1	0.13082	5.026905	2.548886	0.011058	0.018931	-4.0338
NXPH2	0.130801	4.385751	4.814009	1.88E-06	5.63E-06	4.074255
SUGP1	0.130749	7.447322	2.387337	0.017284	0.028481	-4.42716
TEX38	0.130749	4.912623	2.934062	0.003475	0.006513	-2.9946
CER1	0.130736	5.475357	2.755175	0.006047	0.010872	-3.49494
SEZ6L2	0.130634	5.791604	3.154608	0.001689	0.003343	-2.33574
PCDHB4	0.130579	3.970906	5.016254	6.98E-07	2.20E-06	5.025999
MPI	0.130518	5.646314	3.626027	0.000313	0.000693	-0.77331
LOC10050	0.130498	4.726794	2.604561	0.009431	0.016372	-3.8924
TRHR	0.130434	4.783595	2.778048	0.005642	0.010202	-3.43267
TICRR	0.130381	5.370911	2.91737	0.003664	0.006836	-3.04258
SP5	0.130344	4.719915	2.435701	0.015157	0.025291	-4.31204
APH1A	0.130286	6.689019	2.573012	0.010325	0.017784	-3.9729
GDF3	0.130236	5.672849	3.10174	0.002016	0.003939	-2.49789
SHBG	0.130065	6.367247	2.622387	0.008957	0.015624	-3.8465
TTBK1	0.12997	6.423908	3.073831	0.002211	0.004289	-2.58242

RLBP1	0.129969	5.745809	2.752024	0.006105	0.010963	-3.50347
PVR	0.129922	5.579436	3.651714	0.000284	0.000632	-0.68219
CNTD2	0.129919	7.413484	2.432329	0.015298	0.025486	-4.32014
MRPL12	0.129889	6.309572	2.047719	0.041029	0.06234	-5.17159
SCAMP5	0.129875	6.571615	3.461243	0.000576	0.001227	-1.34319
SLC47A2	0.129851	4.73679	2.810047	0.005117	0.009322	-3.34472
RPL3L	0.129798	5.265772	2.518058	0.012063	0.02051	-4.11081
KIAA0509	0.129751	5.005988	2.777855	0.005646	0.010207	-3.4332
MSANTD1	0.129733	4.954495	3.302628	0.001016	0.002082	-1.86768
CPNE9	0.129691	5.384682	2.156577	0.031441	0.049009	-4.94519
SYT5	0.129645	4.654424	2.551305	0.010983	0.018815	-4.02772
KLRG2	0.12953	5.446411	3.67225	0.000262	0.000588	-0.6089
CST4	0.129526	6.38338	2.976109	0.003039	0.005756	-2.87256
CHST5	0.129518	6.020499	3.981591	7.70E-05	0.000187	0.542393
CCDC93	0.129439	6.716769	2.14112	0.032673	0.050724	-4.97804
ABCC3	0.12941	6.672283	2.769229	0.005795	0.010456	-3.45674
IKBKE	0.129309	7.085529	2.324379	0.020443	0.033143	-4.57362
EIF4ENIF1	0.129179	5.851215	3.567476	0.00039	0.000852	-0.97871
KLK11	0.129165	5.6432	2.61854	0.009057	0.015788	-3.85643
EFNA2	0.129097	4.629416	2.891427	0.003976	0.007371	-3.11662
LINC00673	0.129024	4.488363	3.418152	0.000674	0.001422	-1.48802
HTR4	0.128913	5.508641	3.244568	0.001243	0.002513	-2.05374
MET	0.128905	4.894262	5.127788	3.98E-07	1.29E-06	5.566245
STAP2	0.128837	5.799612	2.404374	0.016507	0.027336	-4.38687
NR1I2	0.128787	5.401514	3.452752	0.000595	0.001264	-1.37186
CPN1	0.128779	5.415782	3.011114	0.002714	0.005184	-2.76967
AICDA	0.128753	4.204128	3.29239	0.001053	0.002152	-1.90072
RAMP2	0.12874	5.570842	2.412418	0.016151	0.026803	-4.36774
LOC10192	0.128658	5.946969	3.461873	0.000575	0.001224	-1.34106
FCRL2	0.12864	5.608493	1.984924	0.047615	0.071253	-5.29693
UQCC2	0.128628	5.061128	3.041788	0.002456	0.004721	-2.67856
LOC10192	0.128615	4.28392	4.02231	6.51E-05	0.000159	0.700518
NUP50	0.128587	7.374102	2.565882	0.010537	0.018121	-3.99095
LINC01102	0.128563	3.908716	4.277409	2.20E-05	5.72E-05	1.725684
GGT5	0.128552	6.121072	2.178853	0.029737	0.04665	-4.89744
PRRG1	0.128465	4.22042	2.47823	0.013482	0.022719	-4.20895
KLHL11	0.128457	5.430697	2.275203	0.023251	0.037242	-4.68535
CYTL1	0.128262	4.978598	2.189922	0.02892	0.045443	-4.87353
DDAH1	0.128261	4.355301	4.980494	8.34E-07	2.61E-06	4.855093
SFTPD	0.128166	4.844491	2.313242	0.021052	0.034032	-4.59913
XAGE3	0.128152	5.310434	2.034282	0.042369	0.064182	-5.19873
ZNF571-A	0.128152	5.155588	2.640299	0.008502	0.014903	-3.80006
PNMAL2	0.128095	5.998329	2.382846	0.017494	0.028809	-4.43774
SLC48A1	0.128012	6.341297	2.65897	0.00805	0.014151	-3.75133
MAP3K13	0.127988	5.70901	3.366179	0.000812	0.001691	-1.66038
LPGAT1	0.127957	9.623859	1.97111	0.049177	0.073355	-5.32399
REN	0.12795	5.604144	3.010174	0.002722	0.005199	-2.77245
USP39	0.127932	7.415025	2.439058	0.015019	0.025078	-4.30397
LOC10192	0.127872	5.005105	2.786488	0.005499	0.009961	-3.40956
NHLH2	0.127836	4.424002	4.58529	5.54E-06	1.56E-05	3.041626
GNG4	0.127821	5.131912	2.471629	0.013731	0.023102	-4.22506
GNG3	0.127783	6.228092	2.409042	0.016299	0.027015	-4.37578
NOVA2	0.127764	5.623182	3.175596	0.001573	0.003133	-2.27064
LOC10192	0.127752	4.451401	3.428428	0.000649	0.001373	-1.45364
MDFI	0.127736	5.576152	2.16867	0.030506	0.047687	-4.91933
LOC10049	0.127731	5.3868	3.290113	0.001061	0.002168	-1.90806
ZNRF1	0.12769	5.602507	2.689485	0.007358	0.013032	-3.67097
ZNF414	0.127638	4.9585	3.055497	0.002348	0.00453	-2.63755

JAM3	0.127625	5.570084	2.047425	0.041058	0.062379	-5.17218
TBXA2R	0.127578	6.607996	1.966246	0.049738	0.074094	-5.33347
S100A5	0.127465	5.185027	2.129587	0.033618	0.052014	-5.0024
SLC5A1	0.127464	5.128312	4.108453	4.55E-05	0.000113	1.040053
LAMC2	0.127425	4.77077	3.237247	0.001274	0.002573	-2.07698
G6PC2	0.127409	5.495915	2.929963	0.003521	0.00659	-3.0064
DOC2A	0.127395	5.453525	2.38502	0.017392	0.028651	-4.43262
CA7	0.127351	6.629873	2.522767	0.011905	0.02026	-4.09911
MKS1	0.12732	5.781644	2.596939	0.00964	0.016694	-3.91194
LGALS4	0.127261	5.015746	2.732457	0.006474	0.011578	-3.55628
SHE	0.127187	5.481896	2.439942	0.014982	0.025022	-4.30184
AGBL5	0.127027	5.319043	3.486052	0.000527	0.001127	-1.25902
MEX3B	0.126942	4.793522	4.02715	6.38E-05	0.000156	0.719416
SDCCAG8	0.126913	5.800116	2.381756	0.017546	0.028878	-4.4403
USP43	0.126882	5.112896	2.476591	0.013543	0.022816	-4.21295
ADAM5	0.126833	4.821904	3.822051	0.000146	0.000341	-0.06243
LOC10042	0.126794	4.546268	4.508406	7.88E-06	2.17E-05	2.705005
ACSM5	0.126744	5.11568	2.833061	0.004768	0.008731	-3.28085
SPG11	0.126704	7.904699	3.678668	0.000256	0.000574	-0.58591
CDC5L	0.126693	7.257509	3.143078	0.001756	0.003461	-2.37133
SMG9	0.126569	6.233604	2.552011	0.010961	0.018785	-4.02594
GBX1	0.126555	6.167366	2.73571	0.006412	0.011478	-3.54753
TBK1	0.126533	9.168674	2.349534	0.019125	0.031227	-4.51556
DES	0.126429	4.964425	3.141744	0.001763	0.003473	-2.37544
LINC0068(	0.126407	5.02799	3.964578	8.25E-05	0.000199	0.476777
PARD3B	0.126398	4.130083	4.14202	3.94E-05	9.92E-05	1.174195
CACNA1D	0.126322	4.597177	3.924937	9.70E-05	0.000231	0.324925
LOC10012	0.126315	4.82164	2.960103	0.003199	0.006033	-2.91921
RSPH6A	0.126313	6.023235	2.0047	0.045451	0.068353	-5.25787
SLC35F6	0.126207	7.371731	2.887361	0.004027	0.007462	-3.12817
LOC10013	0.126198	4.003347	2.463926	0.014026	0.023553	-4.24382
AMELY	0.126175	5.341357	3.457259	0.000585	0.001244	-1.35665
STEAP1B	0.126096	5.103804	2.15746	0.031372	0.048915	-4.94331
LOC10192	0.126073	4.175623	4.559297	6.24E-06	1.74E-05	2.927227
NPEPL1	0.126064	7.102251	2.630903	0.008738	0.01527	-3.82446
LOC55310	0.126042	6.042136	3.836488	0.000138	0.000323	-0.00867
CDC25C	0.126035	4.648235	3.773941	0.000177	0.000407	-0.24021
FBXL2	0.126009	5.134881	3.091583	0.002085	0.004064	-2.52874
SYT3	0.126	5.134414	2.978818	0.003012	0.005709	-2.86464
LDLRAD4	0.125982	6.119949	2.266478	0.023783	0.03801	-4.70493
FGFRL1	0.125958	5.626141	3.325421	0.000938	0.001933	-1.79377
SEL1L2	0.125948	4.368365	3.496447	0.000507	0.001086	-1.22357
SLC44A3	0.125869	4.257565	2.298312	0.021892	0.035238	-4.63314
C14orf119	0.125795	9.364126	2.164717	0.030809	0.048098	-4.9278
RDM1	0.125731	4.569799	3.300724	0.001022	0.002094	-1.87383
DUOX1	0.125724	4.734366	3.194865	0.001473	0.002947	-2.21049
LOC10050	0.125724	5.996292	2.04215	0.04158	0.063093	-5.18286
OMP	0.125454	4.328348	2.633766	0.008665	0.015161	-3.81703
SYCN	0.125383	5.798887	3.010083	0.002723	0.0052	-2.77272
PRMT8	0.125303	5.166364	2.918105	0.003656	0.006821	-3.04047
HCN4	0.125266	5.415824	2.986345	0.00294	0.005579	-2.84259
LOC10192	0.125256	4.153532	2.990985	0.002897	0.005503	-2.82898
ERV9-1	0.125219	5.472458	2.070078	0.038879	0.059392	-5.12603
LOC14584	0.125209	4.744744	3.257322	0.001189	0.002413	-2.01314
TLDC2	0.125202	4.759676	2.413579	0.0161	0.026723	-4.36498
ZNF219	0.125201	5.952838	2.746722	0.006203	0.011131	-3.51782
OPCML	0.125199	4.710648	3.661785	0.000273	0.00061	-0.6463
GSDMC	0.125129	5.997902	3.019889	0.002638	0.005046	-2.7437

NOS1	0.124968	5.415382	4.608867	4.96E-06	1.40E-05	3.145912
CYP2C19	0.124943	4.996377	4.048166	5.85E-05	0.000144	0.801714
TES	0.124849	8.112587	2.933533	0.003481	0.006524	-2.99612
POLD3	0.124801	6.766948	3.094134	0.002067	0.004033	-2.521
CREBBP	0.124786	7.358122	2.78811	0.005472	0.00992	-3.40512
CDH5	0.124729	4.127953	2.440657	0.014953	0.024978	-4.30012
LOC10013	0.124713	4.546372	2.523069	0.011895	0.020244	-4.09836
NCR2	0.124668	6.372259	2.844808	0.004598	0.008448	-3.24806
HHIPL2	0.124668	5.595052	2.409444	0.016282	0.026994	-4.37482
DUS2	0.124656	7.742452	2.132725	0.033359	0.051669	-4.99578
SKA1	0.124649	3.743707	2.289082	0.022426	0.036027	-4.65405
OR52A1	0.12454	4.969272	3.377143	0.000781	0.001631	-1.62423
TMEM167I	0.124468	10.32043	2.177471	0.02984	0.0468	-4.90042
RXRG	0.124432	5.087716	3.237644	0.001273	0.00257	-2.07572
C14orf1	0.124428	5.746343	2.136385	0.033058	0.051261	-4.98806
POTEM	0.124358	5.660587	2.131605	0.033451	0.051795	-4.99815
NTRK3	0.124356	5.472211	3.5817	0.000369	0.000811	-0.9291
GRID2	0.124285	4.648566	3.180337	0.001548	0.003089	-2.25587
KRTAP9-4	0.124235	5.066053	3.146148	0.001738	0.003429	-2.36187
CSH1	0.124144	5.146906	2.86038	0.004381	0.008073	-3.20439
RASL10B	0.124119	5.931892	2.344997	0.019357	0.031563	-4.52608
PCA3	0.124105	4.49231	4.762651	2.41E-06	7.09E-06	3.838329
CASZ1	0.124094	5.525082	4.27468	2.23E-05	5.78E-05	1.714404
GINS3	0.124045	4.854065	2.029844	0.04282	0.064795	-5.20766
EPB41L4B	0.123963	4.509773	2.168853	0.030492	0.047669	-4.91894
C8orf22	0.123811	4.167173	3.627687	0.000311	0.000689	-0.76744
FLJ36840	0.123805	4.937939	2.435333	0.015173	0.02531	-4.31293
GREB1L	0.123782	5.18911	4.193338	3.17E-05	8.08E-05	1.381267
FJX1	0.123689	5.256913	2.350785	0.019061	0.031134	-4.51266
PI15	0.123634	4.68461	4.83782	1.68E-06	5.05E-06	4.184434
VCX2	0.123627	5.336256	2.033682	0.04243	0.064264	-5.19994
FBXL22	0.123619	3.605182	3.111546	0.001951	0.003819	-2.46801
PLCD4	0.123587	4.894998	2.666961	0.007864	0.013851	-3.73037
DUOX2	0.123531	4.571593	3.314327	0.000975	0.002005	-1.8298
KRT71	0.123482	4.879688	2.645413	0.008376	0.014691	-3.78675
C1QTNF4	0.123439	3.737436	3.269604	0.00114	0.00232	-1.9739
HTR5A	0.123434	5.18944	2.153966	0.031646	0.049274	-4.95076
SLC35C2	0.123427	6.604652	2.373191	0.017954	0.029464	-4.4604
SRGAP3	0.123403	5.412485	3.360484	0.000828	0.001723	-1.67911
MYO1B	0.123393	4.703748	3.109901	0.001962	0.003839	-2.47303
SLC17A4	0.123387	5.167584	2.766761	0.005839	0.010526	-3.46345
SLC2A12	0.123325	4.550002	4.516323	7.60E-06	2.10E-05	2.739426
RARG	0.123251	5.683789	2.712014	0.006882	0.012254	-3.61106
IGSF11	0.123245	3.619032	3.606667	0.000336	0.000742	-0.84158
APTR	0.123219	5.720828	2.164823	0.030801	0.04809	-4.92757
STPG1	0.12319	5.528149	3.662997	0.000272	0.000608	-0.64197
NDUFA2	0.123145	6.696228	3.326806	0.000933	0.001925	-1.78926
ZZEF1	0.122945	6.896378	2.523568	0.011878	0.02022	-4.09712
LRRC36	0.122825	5.845493	2.752745	0.006091	0.010943	-3.50152
MAGEC2	0.122777	5.083566	3.608574	0.000334	0.000737	-0.83487
AMOTL2	0.122737	4.639055	2.070554	0.038834	0.059333	-5.12505
LINC00675	0.12272	5.714701	3.090892	0.002089	0.004073	-2.53083
TNP2	0.122666	4.356501	4.906513	1.20E-06	3.68E-06	4.505094
FERMT1	0.122656	5.062497	4.623929	4.63E-06	1.32E-05	3.212797
ITGB1BP1	0.1224	6.803389	2.567849	0.010478	0.018029	-3.98598
TMEM121	0.12229	4.640778	3.349016	0.000863	0.001788	-1.71674
HOXD10	0.122283	4.665318	3.370722	0.000799	0.001666	-1.64541
MYO7B	0.122264	5.160751	3.439463	0.000624	0.001322	-1.41661

GJB3	0.122163	5.900294	2.976562	0.003034	0.005749	-2.87123
PNLIP	0.122152	4.097058	3.385962	0.000756	0.001584	-1.59507
C12orf54	0.122118	4.357386	3.879924	0.000116	0.000275	0.154252
VAT1L	0.122104	5.376961	2.228931	0.026194	0.041519	-4.78833
CDS2	0.122007	7.557198	2.098418	0.036292	0.055779	-5.06758
ST8SIA6-A	0.121997	5.081863	2.618181	0.009067	0.015802	-3.85736
MS4A6E	0.121834	3.951947	2.525612	0.01181	0.020115	-4.09203
PRDM8	0.121612	5.192483	2.218169	0.026923	0.042578	-4.81199
LOC25405	0.121514	5.409936	2.756669	0.00602	0.010827	-3.49088
RGMA	0.121504	5.329316	2.942943	0.003379	0.006344	-2.96896
CCDC8	0.121468	5.204457	2.49482	0.012874	0.021756	-4.16826
FAM120A(	0.121405	6.830456	3.871713	0.00012	0.000284	0.123322
SLN	0.121379	5.674942	2.688368	0.007383	0.013072	-3.67393
GDA	0.12136	4.543509	3.557805	0.000404	0.000881	-1.01232
ICOSLG	0.121344	5.837029	3.861678	0.000125	0.000294	0.085603
ARID5B	0.121343	5.625637	2.794885	0.00536	0.009726	-3.38651
SIRT7	0.121235	9.055771	2.008429	0.045052	0.067838	-5.25046
BTN1A1	0.121234	4.917248	2.924577	0.003581	0.006696	-3.0219
ALG14	0.121234	4.829191	2.774707	0.0057	0.010298	-3.44179
HOXB1	0.121198	5.580529	3.083498	0.002141	0.004164	-2.55323
TFAP2A	0.121024	4.482508	4.553791	6.40E-06	1.79E-05	2.903073
ALOX12P2	0.121013	5.941703	2.395505	0.016908	0.027926	-4.40788
SIX3	0.120929	4.08456	4.859011	1.51E-06	4.59E-06	4.282908
RCC1	0.120923	6.662446	2.228079	0.026251	0.041602	-4.79021
ALPK2	0.120878	4.692063	3.307221	0.000999	0.002052	-1.85282
SDC3	0.120873	5.950553	2.078719	0.038074	0.058258	-5.10829
TMEM179	0.120853	5.666883	2.144038	0.032437	0.050397	-4.97186
LOR	0.120812	5.151982	2.797201	0.005323	0.009664	-3.38014
MASP2	0.120769	5.381465	3.281026	0.001095	0.002234	-1.93728
PPL	0.120748	4.853853	2.401531	0.016634	0.027527	-4.39361
CPSF3L	0.120738	5.981463	2.613309	0.009195	0.016002	-3.86991
KLK13	0.120673	5.550965	2.798198	0.005306	0.009639	-3.3774
LOC10272	0.120643	4.567051	3.07424	0.002208	0.004284	-2.58119
B4GALT1	0.120627	6.106571	2.234868	0.025799	0.040929	-4.77523
LOC10013	0.120555	5.707618	2.300258	0.021781	0.035083	-4.62872
SCN10A	0.120498	5.33029	2.481725	0.013352	0.022519	-4.2004
TDRD1	0.120418	4.146858	3.14875	0.001722	0.003403	-2.35384
C16orf82	0.120412	5.640662	2.68618	0.007431	0.013152	-3.67972
RHOJ	0.120406	4.449811	4.414669	1.20E-05	3.23E-05	2.301792
GFRA1	0.12039	4.730041	4.413889	1.21E-05	3.24E-05	2.298473
TNS4	0.120321	6.457677	2.62346	0.008929	0.01558	-3.84373
C9orf116	0.120103	5.043574	3.040691	0.002465	0.004737	-2.68183
MGC4294	0.12005	5.664302	2.523284	0.011888	0.020234	-4.09782
FAM205B	0.12001	4.423601	4.411584	1.22E-05	3.27E-05	2.288656
TRIM29	0.119946	5.512416	3.305427	0.001006	0.002063	-1.85863
SCGB1D1	0.119939	3.912344	3.936721	9.24E-05	0.000221	0.369914
CDH13	0.119917	3.991556	3.055702	0.002347	0.004528	-2.63693
MOGAT2	0.119909	5.268253	3.963592	8.29E-05	0.0002	0.472986
CPXM2	0.119881	5.96666	2.832141	0.004781	0.008754	-3.28342
TANGO2	0.119824	8.44054	2.401077	0.016655	0.027554	-4.39469
OSBPL6	0.11982	4.662161	2.629577	0.008772	0.015325	-3.8279
SCNN1D	0.119774	5.568848	2.155603	0.031518	0.049107	-4.94727
DLX3	0.119771	4.906673	2.134109	0.033245	0.051506	-4.99286
BARHL1	0.119666	6.591442	2.672238	0.007743	0.013654	-3.7165
VPS53	0.119652	5.783508	3.246023	0.001236	0.002502	-2.04912
PFKFB1	0.119622	5.167495	4.163336	3.60E-05	9.12E-05	1.259915
HOXB9	0.119534	4.967128	3.355188	0.000844	0.001752	-1.6965
OR14J1	0.119462	4.821783	3.120119	0.001896	0.003717	-2.44182

PAQR5	0.119458	4.180247	2.716498	0.006791	0.012106	-3.59908
ANXA9	0.119438	5.048723	3.067938	0.002254	0.004365	-2.60017
LOC10050	0.119273	4.675174	3.356422	0.00084	0.001745	-1.69245
CNTN2	0.119253	5.539102	3.549016	0.000417	0.000908	-1.0428
XKRX	0.119244	4.97784	3.086941	0.002117	0.004121	-2.54281
LOC10192	0.119227	5.287099	2.401324	0.016644	0.027538	-4.3941
TRIM54	0.119226	4.795595	2.342316	0.019495	0.031775	-4.53228
GYG2	0.119178	4.678124	2.896416	0.003914	0.007262	-3.10243
TLX2	0.119153	4.684572	3.146585	0.001735	0.003425	-2.36052
ZNF765	0.119131	4.628165	2.998346	0.002829	0.005384	-2.80733
IWS1	0.119107	7.769352	2.061937	0.03965	0.060473	-5.14267
BMP6	0.119039	5.215807	2.823681	0.004908	0.008969	-3.30694
FRMPD4	0.118911	4.965005	3.670846	0.000264	0.000591	-0.61392
UCKL1	0.118904	6.428823	2.016368	0.044213	0.066698	-5.23465
FGFBP1	0.118841	6.039292	2.64894	0.00829	0.014548	-3.77755
TDO2	0.118805	4.216311	4.107985	4.55E-05	0.000114	1.038187
GLI2	0.118804	4.886145	3.459272	0.000581	0.001235	-1.34985
HOXD8	0.1188	4.674855	2.529126	0.011694	0.019939	-4.08327
SLC7A13	0.118784	4.125464	4.154211	3.75E-05	9.45E-05	1.223168
SERPINE1	0.118732	5.217598	2.44288	0.014862	0.02484	-4.29476
KCNJ14	0.118727	5.411093	2.948819	0.003316	0.006236	-2.95196
PANX3	0.11863	3.573591	3.915763	0.000101	0.00024	0.289991
HMGXB3	0.118593	7.313332	2.58532	0.009967	0.017223	-3.94161
PAPPA2	0.11857	5.329441	3.73503	0.000206	0.000469	-0.38242
CPB2	0.118563	4.100893	2.876004	0.004173	0.007713	-3.16034
USP26	0.118558	4.280777	2.264525	0.023903	0.038196	-4.7093
TRIM10	0.118476	4.970103	3.477351	0.000544	0.001161	-1.2886
TBX4	0.118454	5.269408	2.557737	0.010784	0.018499	-4.01152
TTLL4	0.118368	6.293028	2.139774	0.032782	0.050871	-4.98089
LOC10192	0.118283	5.901726	2.337057	0.019769	0.032151	-4.54444
NOX1	0.118258	5.205254	4.61545	4.81E-06	1.36E-05	3.175122
NR1H3	0.118246	5.393875	2.069958	0.03889	0.059404	-5.12627
C20orf196	0.11824	5.385474	3.40129	0.000716	0.001506	-1.54421
DKFZP434	0.118232	4.180194	3.181852	0.00154	0.003074	-2.25115
GMDS	0.118175	5.347011	2.683564	0.007488	0.013247	-3.68663
ATP2B2	0.118168	4.908654	4.091759	4.88E-05	0.000121	0.97372
NOBOX	0.118147	5.756941	2.368597	0.018176	0.029802	-4.47116
MORF4L1	0.118075	10.71168	2.208431	0.027597	0.043561	-4.83329
PLK2	0.117988	3.991386	2.007776	0.045122	0.067915	-5.25176
FGF1	0.117853	5.007546	3.719008	0.000219	0.000497	-0.44057
SLC13A5	0.117812	5.425602	2.60424	0.009439	0.016384	-3.89323
HHATL	0.117753	5.115544	2.685267	0.007451	0.013182	-3.68213
IGFL1	0.117728	5.275917	2.394617	0.016948	0.02798	-4.40998
CSRP3	0.117701	4.514373	2.365558	0.018325	0.030023	-4.47826
SSR4P1	0.117623	5.76442	2.631427	0.008725	0.015253	-3.8231
OTC	0.117599	4.606543	2.675019	0.007679	0.013554	-3.70918
ZNF221	0.117577	5.188023	3.787725	0.000168	0.000387	-0.18949
ERCC6L	0.117563	4.533194	2.269827	0.023577	0.037712	-4.69742
OR6A2	0.117533	5.328775	2.966938	0.003129	0.005915	-2.89932
SLC45A2	0.117437	5.126552	3.115441	0.001926	0.003772	-2.45612
ATXN8OS	0.117413	5.785993	3.302481	0.001016	0.002083	-1.86815
NKAIN1	0.117316	5.518154	2.440799	0.014947	0.024971	-4.29978
AMPD1	0.117211	4.408245	2.916057	0.003679	0.006863	-3.04634
VN1R10P	0.117066	4.212204	2.892028	0.003968	0.007357	-3.11491
RIIAD1	0.117017	6.216573	3.669111	0.000265	0.000595	-0.62013
GPR32	0.116996	5.236005	2.196945	0.028412	0.044706	-4.8583
LAD1	0.116978	5.876189	2.392167	0.017061	0.02814	-4.41577
ARHGEF28	0.116936	5.367066	3.389971	0.000746	0.001563	-1.58179

LOC10013	0.116922	5.215156	2.029101	0.042896	0.064899	-5.20915
CYP3A7-C	0.116832	4.334661	3.506804	0.000488	0.001049	-1.18816
HID1	0.116801	5.636287	2.186692	0.029156	0.045798	-4.88052
CACNA1H	0.116791	6.171243	2.426221	0.015555	0.025876	-4.33479
SRY	0.116762	4.718104	2.562479	0.010639	0.018281	-3.99956
TIMM50	0.116685	5.919246	1.972132	0.04906	0.073193	-5.32199
GPRC5A	0.116667	5.017738	3.699044	0.000237	0.000534	-0.51269
CYGB	0.116598	4.91244	2.28756	0.022516	0.036157	-4.65749
SLC26A1	0.116573	5.307924	2.059024	0.03993	0.060863	-5.14861
SOAT2	0.116572	5.699056	2.204976	0.02784	0.043912	-4.84083
P2RX2	0.116528	5.764024	3.22697	0.00132	0.002657	-2.10951
CNNM1	0.116462	5.057367	3.196041	0.001468	0.002936	-2.20681
TTLL10	0.116454	4.914367	2.054647	0.040352	0.061406	-5.15752
CTB-174D	0.116378	5.275077	2.476127	0.013561	0.022843	-4.21409
GREB1	0.116235	4.322326	4.771164	2.31E-06	6.83E-06	3.877274
TGM1	0.116119	5.676633	2.237974	0.025594	0.040629	-4.76837
LOC10192	0.116019	5.419939	2.635592	0.00862	0.01509	-3.81229
CD1B	0.115981	5.158483	2.739634	0.006336	0.011356	-3.53695
PZP	0.115934	5.37355	2.304632	0.021533	0.034727	-4.61877
KCNN3	0.115848	4.865097	3.147059	0.001732	0.003421	-2.35906
RALYL	0.115711	5.12968	3.345772	0.000873	0.001808	-1.72736
PAK7	0.115678	4.598184	4.281371	2.17E-05	5.63E-05	1.742074
ARHGEF15	0.115639	5.942777	2.903141	0.003832	0.007122	-3.08327
RSPO1	0.115564	5.202255	2.380953	0.017584	0.028921	-4.44219
CCNE1	0.115542	5.680153	2.776259	0.005673	0.010252	-3.43756
FOLR1	0.115524	5.094724	2.103303	0.035861	0.055192	-5.05743
GSTO2	0.115478	5.045629	3.282489	0.00109	0.002224	-1.93258
MRPL44	0.115416	6.836651	2.023246	0.043497	0.065732	-5.2209
RTBDN	0.11541	6.215062	2.66625	0.00788	0.013874	-3.73224
LOC10192	0.115214	4.597301	2.992013	0.002887	0.005486	-2.82596
DNAJC27-	0.115173	4.314096	2.834203	0.004751	0.008705	-3.27767
FAM171A2	0.115059	5.916475	1.967819	0.049556	0.073865	-5.3304
TPD52L1	0.115048	4.936546	2.598072	0.009609	0.016649	-3.90904
CDH26	0.115044	4.6551	3.699768	0.000236	0.000533	-0.51008
VGLL1	0.11496	4.533126	3.399718	0.00072	0.001514	-1.54944
CPN2	0.114955	4.317824	2.75209	0.006103	0.010962	-3.50329
CCDC33	0.114922	5.477285	2.400828	0.016666	0.02757	-4.39528
GNRH2	0.11489	6.119632	2.382187	0.017525	0.028852	-4.43929
LOC10050	0.114862	4.066247	3.458623	0.000582	0.001238	-1.35204
ORC1	0.114823	4.452207	1.976798	0.048529	0.072477	-5.31287
CRHR2	0.114766	5.011775	3.345274	0.000874	0.001811	-1.72899
SLC6A9	0.114722	5.048947	2.299814	0.021806	0.035121	-4.62973
KIF1A	0.114692	4.790913	4.541822	6.76E-06	1.88E-05	2.850659
SP3P	0.114656	4.0867	2.752118	0.006103	0.010962	-3.50322
CSN3	0.114636	4.545867	2.773923	0.005713	0.010318	-3.44393
OPALIN	0.114482	5.077257	2.972194	0.003077	0.005825	-2.88399
RNF112	0.114375	5.29156	2.42827	0.015468	0.025746	-4.32988
CBX8	0.114349	5.787802	2.152951	0.031726	0.049382	-4.95292
APOM	0.114347	5.690574	2.670173	0.00779	0.013733	-3.72193
OR51E1	0.114324	5.046861	2.478956	0.013455	0.022678	-4.20717
RDX	0.114305	5.181427	2.773039	0.005729	0.010342	-3.44635
OR51I1	0.114253	5.65609	2.478468	0.013473	0.022706	-4.20836
FAM166B	0.114242	5.350103	2.677189	0.00763	0.013477	-3.70346
METTL22	0.114214	6.743853	2.852886	0.004484	0.008253	-3.22544
BHLHE41	0.114177	4.495266	2.67329	0.007719	0.013615	-3.71373
SLC9A3	0.114018	5.136403	2.396155	0.016878	0.027885	-4.40634
THAP8	0.113993	6.301229	2.39526	0.016919	0.027942	-4.40846
RPA4	0.113926	4.498025	1.995809	0.046413	0.069645	-5.27548

NR0B1	0.113903	4.462153	3.857088	0.000127	0.000299	0.06838
SHISA9	0.11386	4.793358	3.143973	0.00175	0.003452	-2.36857
PITX1	0.113844	4.504293	3.507327	0.000487	0.001047	-1.18637
STAR	0.113743	5.112716	3.150745	0.001711	0.003382	-2.34768
LOC64479	0.113674	5.699919	2.3404	0.019595	0.031916	-4.53672
SCOC-AS1	0.113621	3.802595	2.986715	0.002937	0.005575	-2.84151
KCNU1	0.113585	4.595855	3.680836	0.000254	0.00057	-0.57814
WWC2	0.113522	4.453091	2.393215	0.017012	0.028076	-4.41329
MPDZ	0.113486	4.698715	2.79419	0.005372	0.009746	-3.38842
LOC10192	0.113482	4.144212	2.205222	0.027823	0.04389	-4.84029
MED24	0.113475	5.113298	2.970009	0.003099	0.005862	-2.89037
C7orf13	0.113424	6.465015	2.958181	0.003218	0.006067	-2.9248
RGN	0.113386	5.040241	2.673602	0.007712	0.013604	-3.71291
TBC1D25	0.113345	7.316963	2.456273	0.014325	0.024	-4.26239
ARX	0.113247	5.03798	2.10895	0.035369	0.054508	-5.04566
ODF3	0.113239	6.582132	2.31304	0.021063	0.034047	-4.59959
TBX18	0.113147	4.228973	2.901957	0.003847	0.007144	-3.08665
CYP3A5	0.113131	4.262725	2.470748	0.013764	0.023146	-4.22721
PCDH18	0.113072	5.596646	2.509034	0.012373	0.020987	-4.13318
FGD1	0.113018	5.524544	2.328173	0.020239	0.032854	-4.56491
S100A7	0.112987	4.481836	3.291499	0.001056	0.002158	-1.90359
TAAR2	0.11296	4.835147	3.190402	0.001496	0.00299	-2.22445
LOC15774	0.11288	4.238424	3.850422	0.000131	0.000307	0.043404
IQSEC2	0.112836	5.753549	2.229839	0.026133	0.041437	-4.78633
LINC00347	0.112833	3.969341	3.474665	0.000549	0.001172	-1.29772
LOC10050	0.112792	6.580858	2.278341	0.023062	0.036966	-4.67829
LOC10192	0.112772	5.940076	2.302679	0.021643	0.034883	-4.62321
AVPR2	0.112763	5.170364	2.316388	0.020878	0.033773	-4.59194
C2orf81	0.112655	7.47023	1.998875	0.046079	0.069178	-5.26942
MGC45800	0.112633	5.188177	2.793649	0.005381	0.009761	-3.38991
LOC51145	0.112603	3.924155	3.346455	0.00087	0.001804	-1.72512
UPP2	0.112591	4.397933	4.24581	2.53E-05	6.52E-05	1.595476
C7orf26	0.112577	6.658496	2.377293	0.017757	0.029165	-4.45079
LCE3D	0.112116	6.06138	2.840733	0.004656	0.008546	-3.25945
LOC72929	0.112061	4.356318	2.914941	0.003692	0.006884	-3.04954
ANXA10	0.112035	4.699594	3.133924	0.00181	0.00356	-2.3995
AFMID	0.112033	5.484627	1.980704	0.048088	0.071919	-5.30521
IFNA4	0.111991	4.994751	2.634514	0.008647	0.015131	-3.81509
HSBP1	0.111934	9.342081	2.239945	0.025465	0.040442	-4.76401
TRAF7	0.111847	6.54761	2.710417	0.006915	0.012308	-3.61533
LOC72794	0.111838	4.817231	2.944594	0.003361	0.006314	-2.96419
FGF18	0.111689	5.37106	3.04748	0.002411	0.004641	-2.66155
FOXS1	0.111615	4.906853	2.66663	0.007871	0.01386	-3.73124
CLGN	0.111571	3.691566	2.100638	0.036096	0.05551	-5.06297
CPNE6	0.111514	5.836044	2.210251	0.02747	0.043379	-4.82932
SLC30A2	0.111459	5.808505	2.696345	0.00721	0.012799	-3.65278
RGS4	0.111435	4.884566	3.206769	0.001415	0.002836	-2.17316
NANOG	0.111386	5.166308	2.357039	0.018746	0.030661	-4.49812
FAM95A	0.111383	5.020569	2.435402	0.01517	0.025309	-4.31276
ERC2-IT1	0.111378	4.615722	2.317713	0.020805	0.03367	-4.5889
TAGLN3	0.11131	5.594395	2.147657	0.032147	0.049976	-4.96418
STMN4	0.1113	5.461506	3.215207	0.001375	0.002759	-2.14662
DLX5	0.11128	4.757189	2.531395	0.011619	0.019822	-4.07761
PCDHGA9	0.111109	5.710761	2.623291	0.008933	0.015586	-3.84416
SLC5A4	0.111103	5.066173	2.966154	0.003137	0.005928	-2.9016
KGFLP2	0.111066	3.913356	2.731016	0.006502	0.011622	-3.56016
IL3	0.110971	4.265314	2.886597	0.004037	0.007478	-3.13034
YRDC	0.110967	7.482939	2.130593	0.033535	0.051911	-5.00028

BSN	0.110856	4.832443	2.119898	0.034431	0.053202	-5.02277
ABHD17B	0.110838	6.454053	3.431307	0.000643	0.001359	-1.44399
SLC23A1	0.110837	4.619333	2.918884	0.003647	0.006807	-3.03824
CCL18	0.110662	4.565808	2.701448	0.007102	0.01262	-3.63922
TGM2	0.110662	5.414464	3.048316	0.002404	0.004629	-2.65905
SERPINB7	0.11062	4.689302	3.302912	0.001015	0.00208	-1.86676
BECN1	0.110483	9.237539	2.166488	0.030673	0.047919	-4.92401
KCNK7	0.110324	5.619068	2.477748	0.0135	0.022747	-4.21013
CREB3L3	0.110302	3.98787	2.394802	0.01694	0.027969	-4.40954
HOXB7	0.110257	4.938041	3.516356	0.000471	0.001015	-1.15541
CNDP1	0.11025	5.062248	2.511676	0.012281	0.020848	-4.12664
CYP2C9	0.110229	5.083807	3.658135	0.000277	0.000618	-0.65932
EVA1A	0.110166	5.359551	2.905418	0.003805	0.007076	-3.07677
HSD17B1	0.110097	5.662383	3.100247	0.002026	0.003957	-2.50243
MT1G	0.110042	6.495235	2.310892	0.021182	0.034225	-4.6045
CCDC61	0.109964	5.1956	2.272972	0.023386	0.037432	-4.69036
ERVH-6	0.109955	4.925253	2.287402	0.022525	0.036169	-4.65785
CHST3	0.109927	5.755761	3.050312	0.002388	0.0046	-2.65308
EDA2R	0.109854	6.306799	2.139348	0.032816	0.050916	-4.98179
B3GNT7	0.109799	5.113608	2.00801	0.045097	0.067889	-5.2513
NR4A1	0.109785	6.62244	2.447827	0.014662	0.024532	-4.28283
GUCY1B2	0.109783	4.976103	2.875332	0.004181	0.007727	-3.16224
FAM26E	0.109778	5.632291	2.758268	0.005991	0.010779	-3.48654
MUC17	0.109748	5.334482	3.00695	0.002751	0.005249	-2.78197
SCIMP	0.109718	4.815792	2.864221	0.004329	0.007984	-3.19358
HOXB5	0.109716	5.33317	2.911782	0.003729	0.006948	-3.05858
TNN	0.109671	4.279887	2.904877	0.003811	0.007087	-3.07832
RIMKLA	0.109585	4.79515	3.580597	0.000371	0.000814	-0.93296
OR12D3	0.109575	5.026472	2.724282	0.006635	0.011842	-3.57824
GGN	0.109561	4.993973	2.729783	0.006526	0.011662	-3.56347
HOXA2	0.109448	4.231178	3.228462	0.001314	0.002645	-2.10479
LOC78052	0.109443	4.415659	2.486653	0.01317	0.022229	-4.18832
LOC10192	0.109428	4.955576	2.346144	0.019298	0.031487	-4.52342
KDM3B	0.109423	9.858518	1.972397	0.04903	0.073171	-5.32147
SLC6A13	0.109296	5.399517	2.349003	0.019152	0.031266	-4.5168
LINC0069(	0.109286	3.899105	3.438735	0.000626	0.001325	-1.41906
RASL10A	0.109265	5.768969	2.062173	0.039628	0.060444	-5.14219
OR2F2	0.109262	3.755963	3.868745	0.000122	0.000287	0.112155
DDN	0.109252	6.606807	2.163125	0.030932	0.048282	-4.93121
CHRNB2	0.109192	6.756034	2.132511	0.033376	0.051692	-4.99624
MYH8	0.109182	3.996446	3.615218	0.000326	0.00072	-0.81147
CHRD	0.109138	5.603992	2.551565	0.010975	0.018804	-4.02707
FAM57A	0.109074	5.357592	2.260441	0.024157	0.038564	-4.71843
SSX1	0.10906	4.312986	4.559412	6.24E-06	1.74E-05	2.927735
LOC10012	0.109016	4.765812	3.626715	0.000312	0.000691	-0.77087
LOC65434	0.108965	4.616332	3.459855	0.000579	0.001233	-1.34788
TEX13A	0.108861	4.841971	2.884849	0.004059	0.007513	-3.13529
TM6SF2	0.108837	5.580052	2.439349	0.015007	0.025061	-4.30327
MAP1B	0.108819	4.240498	2.418003	0.015907	0.026414	-4.35443
SV2C	0.108734	4.075041	2.744605	0.006243	0.011199	-3.52354
CALCRL	0.108672	4.315821	3.232523	0.001295	0.002612	-2.09194
CCDC97	0.108477	7.037463	2.435734	0.015156	0.025291	-4.31197
EDN1	0.108476	5.559179	2.39856	0.016769	0.027722	-4.40065
PDX1	0.108451	5.029845	2.551811	0.010967	0.018794	-4.02645
ANKRD37	0.108447	5.371934	2.524078	0.011861	0.020195	-4.09585
GGA1	0.108393	8.255857	2.07553	0.038369	0.058678	-5.11485
MDGA1	0.10836	5.82395	2.381753	0.017546	0.028878	-4.44031
NAIF1	0.108348	6.709528	2.085368	0.037464	0.057434	-5.09459

TSSK1B	0.108337	4.510603	2.693153	0.007279	0.012905	-3.66125
CALML3	0.108329	5.480875	2.549333	0.011044	0.018911	-4.03268
UPK1A	0.108323	4.543187	2.764912	0.005872	0.010578	-3.46849
GPR75	0.108147	4.890751	2.731152	0.0065	0.01162	-3.55979
COLEC11	0.108058	4.860789	2.542775	0.011251	0.019231	-4.04914
NRAP	0.107961	4.746294	3.533109	0.000443	0.000959	-1.09777
PCP4L1	0.107956	4.660303	3.267469	0.001148	0.002335	-1.98073
TMEM132	0.107782	5.931176	2.325533	0.020381	0.033054	-4.57097
LOC10192	0.107593	4.50834	2.507035	0.012442	0.021097	-4.13813
PCAT6	0.107543	5.014449	2.898337	0.003891	0.007221	-3.09696
ARMC4	0.107486	4.675389	3.012067	0.002706	0.005169	-2.76686
NFIX	0.107444	5.406488	2.434125	0.015223	0.025379	-4.31583
ALDH1A3	0.107399	4.066337	2.370444	0.018087	0.029663	-4.46684
CRH	0.107385	4.832204	3.53793	0.000435	0.000943	-1.08114
RAB2A	0.107242	8.100092	2.335473	0.019852	0.03227	-4.54809
SEC14L3	0.107233	4.920983	2.884089	0.004068	0.00753	-3.13745
FIBIN	0.107203	4.421646	3.161936	0.001648	0.003268	-2.31306
AKAP3	0.10719	4.977404	2.27775	0.023098	0.037013	-4.67962
PPY	0.107159	4.491359	2.655972	0.008121	0.01427	-3.75918
SEMA6A	0.107149	4.791135	3.076902	0.002188	0.004248	-2.57315
MEP1A	0.107149	4.347787	2.778617	0.005633	0.010185	-3.43111
NPR1	0.107127	6.146617	2.052571	0.040554	0.061706	-5.16174
LRRC46	0.107063	5.209896	2.920092	0.003633	0.006784	-3.03477
ARL9	0.107032	5.482242	2.699914	0.007134	0.012672	-3.6433
NRSN2	0.107027	5.813739	2.297331	0.021948	0.035322	-4.63536
MYLK3	0.107025	4.419898	3.146279	0.001737	0.003428	-2.36146
CYR61	0.106972	4.668875	2.010103	0.044874	0.067599	-5.24713
NCS1	0.106969	5.781256	2.764853	0.005873	0.010578	-3.46865
LOC10192	0.106843	5.905964	3.305627	0.001005	0.002062	-1.85798
NGEF	0.106731	4.526131	3.26978	0.001139	0.002319	-1.97334
LOC10050	0.106721	4.382687	3.178593	0.001557	0.003104	-2.2613
SCUBE3	0.106701	5.66386	3.145181	0.001743	0.003439	-2.36485
TMEM254	0.106525	5.909622	2.569535	0.010428	0.017948	-3.98171
KIAA1751	0.10651	5.573145	2.992454	0.002883	0.005479	-2.82466
SLC20A2	0.106499	5.262057	2.112638	0.03505	0.054068	-5.03796
GAD2	0.106385	4.619531	3.837525	0.000138	0.000322	-0.0048
SPP2	0.106266	5.788114	2.146907	0.032207	0.050061	-4.96577
ZSCAN30	0.106263	6.443179	2.030209	0.042783	0.064744	-5.20693
ACTN2	0.106222	4.917211	3.283236	0.001087	0.002219	-1.93018
NTSR2	0.106169	5.140985	2.240628	0.025421	0.040379	-4.76249
PALD1	0.106078	5.283358	2.27181	0.023457	0.037532	-4.69297
DNASE1	0.106051	5.704191	2.378066	0.017721	0.029115	-4.44897
LINC0113	0.105954	5.24867	2.222735	0.026611	0.042137	-4.80196
PSG1	0.105829	4.946127	3.788015	0.000167	0.000386	-0.18842
CLDN3	0.105823	5.101084	3.060185	0.002312	0.004468	-2.62348
FTCD	0.105731	6.028019	1.966147	0.049749	0.074104	-5.33366
LOC44145	0.105661	3.656163	3.464422	0.00057	0.001214	-1.33243
OBSL1	0.10564	4.893231	3.358364	0.000834	0.001734	-1.68608
UNC5D	0.105429	3.802783	2.259272	0.02423	0.038667	-4.72104
ZNF775	0.105288	5.184237	2.485237	0.013222	0.02231	-4.19179
TNFSF18	0.10525	4.631301	2.430806	0.015362	0.02558	-4.3238
USP49	0.105226	5.168854	4.278185	2.20E-05	5.70E-05	1.728894
EMC7	0.105133	6.884599	2.358521	0.018673	0.030554	-4.49467
LOC10192	0.105112	6.572921	2.29518	0.022072	0.035489	-4.64024
FOXE3	0.10503	5.062431	3.014903	0.002681	0.005126	-2.75846
MYOM3	0.104975	5.477746	2.243127	0.025258	0.040159	-4.75696
PPP1R12C	0.104935	6.314286	2.031994	0.042601	0.064507	-5.20334
ABCG8	0.104912	5.211378	2.328643	0.020214	0.032822	-4.56382

TMEM136	0.104892	5.245655	3.544108	0.000425	0.000924	-1.05979
ALOXE3	0.104852	5.072465	2.996377	0.002847	0.005417	-2.81313
RTDR1	0.104695	5.495664	2.575687	0.010246	0.017655	-3.96611
KRT76	0.104606	5.612074	2.432565	0.015288	0.025472	-4.31958
ZPBP2	0.104597	4.536346	2.916037	0.00368	0.006863	-3.0464
TEK	0.104576	5.570631	2.318968	0.020737	0.033565	-4.58603
CLDN15	0.104516	7.650669	2.337522	0.019745	0.032118	-4.54336
IGSF1	0.104442	5.332071	3.231202	0.001301	0.002621	-2.09612
CHRNA4	0.104417	4.880208	2.801257	0.005257	0.009562	-3.36897
KRT4	0.104383	5.206785	3.001873	0.002797	0.00533	-2.79695
LOC10272	0.104263	4.851479	2.677702	0.007619	0.013461	-3.70211
BCAR3	0.104234	4.521713	3.040667	0.002465	0.004737	-2.6819
RYR2	0.104216	4.423956	3.46187	0.000575	0.001224	-1.34107
PSD2	0.104205	6.179651	2.615391	0.00914	0.01591	-3.86455
TMPRSS15	0.104166	5.121396	3.583108	0.000368	0.000807	-0.92418
SMLR1	0.104127	4.057805	4.598052	5.22E-06	1.47E-05	3.098015
ING1	0.104077	7.462129	2.20373	0.027928	0.04403	-4.84354
FAM107A	0.10406	5.554186	2.259758	0.0242	0.038622	-4.71995
SUFU	0.103841	5.328853	3.425689	0.000656	0.001385	-1.46281
IL20RA	0.103748	4.512742	3.246353	0.001235	0.0025	-2.04807
PLD5	0.103615	3.70719	3.482989	0.000533	0.001139	-1.26944
C6orf15	0.103614	6.879796	2.016473	0.044202	0.066692	-5.23444
FBXO18	0.103571	7.349776	2.000067	0.04595	0.06899	-5.26705
DRICH1	0.103393	5.708478	3.068866	0.002247	0.004353	-2.59738
LOC10192	0.103381	4.227288	3.206059	0.001418	0.002842	-2.17539
PIK3C2G	0.103262	4.319548	2.781033	0.005591	0.010116	-3.4245
FGF3	0.103194	4.826077	2.352541	0.018973	0.031	-4.50858
WNT4	0.103184	5.256282	2.431019	0.015353	0.025568	-4.32329
SPANXB1	0.103116	4.138781	3.020183	0.002635	0.005044	-2.74283
DNMT3L	0.103102	4.368502	2.728782	0.006546	0.011695	-3.56616
DYRK1A	0.103067	6.431625	4.082064	5.08E-05	0.000126	0.935316
CLDN14	0.10304	4.712368	2.579448	0.010136	0.01749	-3.95655
ECE2	0.103031	5.894909	2.411629	0.016185	0.02685	-4.36962
SGIP1	0.10291	3.804905	4.22249	2.80E-05	7.17E-05	1.499966
KRTAP4-1	0.102906	4.492964	3.564913	0.000393	0.00086	-0.98762
MRO	0.102806	4.576028	3.369968	0.000801	0.001671	-1.6479
PLA2G5	0.102683	4.943979	3.595701	0.000351	0.000772	-0.88009
UNC5B	0.102648	6.086114	2.434315	0.015215	0.025369	-4.31537
YIPF2	0.102601	6.401031	2.126583	0.033868	0.052374	-5.00872
BCL6B	0.102598	6.419181	2.359393	0.018629	0.030497	-4.49264
IMPG2	0.102467	3.991909	4.081073	5.10E-05	0.000126	0.931397
NAA11	0.102428	4.989039	2.866271	0.004301	0.007938	-3.18781
PRKG2	0.102419	5.19487	2.396897	0.016844	0.027839	-4.40459
PCDHGB8I	0.102403	4.203745	3.001106	0.002803	0.005342	-2.79921
TMEM198	0.102371	6.317564	2.465867	0.013951	0.023435	-4.2391
SLC17A2	0.102252	4.41968	2.885437	0.004051	0.007503	-3.13363
RCL1	0.10223	5.837807	2.89842	0.00389	0.00722	-3.09673
MYOCD	0.102223	4.483857	3.501311	0.000498	0.001068	-1.20696
IFNA1	0.102191	3.96489	3.188072	0.001508	0.003013	-2.23174
LOC10050	0.102181	4.974288	2.587437	0.009907	0.017128	-3.93621
TTC23L	0.102177	4.023455	2.032803	0.042519	0.064388	-5.20171
DSG1	0.102131	3.84874	2.804281	0.005209	0.009476	-3.36064
LOC10012	0.10204	5.019033	2.588715	0.009871	0.017073	-3.93295
CARD10	0.101998	5.17565	2.381523	0.017557	0.028893	-4.44085
CHAT	0.10195	5.302042	2.7361	0.006404	0.011467	-3.54648
TUBAL3	0.10194	4.166041	3.013633	0.002692	0.005146	-2.76222
WNT7B	0.101896	5.970061	2.443408	0.014841	0.024809	-4.29349
ITGB4	0.101804	5.339288	2.17151	0.03029	0.04741	-4.91323

NFYA	0.101782	6.285674	2.161456	0.031061	0.048466	-4.93477
LENEP	0.101698	6.152598	2.374892	0.017872	0.029337	-4.45642
PDE6H	0.101669	4.080772	2.031464	0.042655	0.064572	-5.2044
LOC10105	0.101532	4.697881	2.224074	0.026521	0.042008	-4.79902
EFCC1	0.101482	4.976078	2.566734	0.010511	0.01808	-3.9888
PCDH19	0.101406	4.180321	2.848331	0.004548	0.008363	-3.2382
RBPJL	0.101388	5.53189	2.191851	0.02878	0.04525	-4.86935
BOD1L2	0.101281	4.911488	2.81428	0.005051	0.00921	-3.33301
CCL1	0.101271	5.222355	2.392256	0.017057	0.028139	-4.41556
IL11	0.101236	5.273186	2.721406	0.006692	0.011939	-3.58594
GPR61	0.101142	4.734549	2.422714	0.015705	0.026101	-4.34318
MAGI2-AS	0.101092	3.939979	2.141033	0.03268	0.05073	-4.97823
SGOL1	0.101068	3.586078	2.400424	0.016684	0.027592	-4.39624
LOC10192	0.100988	5.666658	2.358256	0.018686	0.030573	-4.49529
LOC10192	0.100907	4.248791	2.961567	0.003184	0.006007	-2.91496
LOC10050	0.100766	5.010717	2.607124	0.009361	0.016261	-3.88582
HOXD12	0.100751	3.962191	2.173875	0.030111	0.047171	-4.90815
C10orf85	0.100733	4.958421	2.676666	0.007642	0.013496	-3.70484
LOC10050	0.100721	5.257078	2.04833	0.040969	0.062264	-5.17035
NES	0.100707	5.138544	2.112947	0.035024	0.054036	-5.03732
MBD1	0.100669	7.234512	3.19194	0.001488	0.002975	-2.21965
GAS2L3	0.100665	4.664073	2.037401	0.042055	0.063744	-5.19245
AXL	0.100663	5.372983	2.275908	0.023208	0.037184	-4.68376
MACC1	0.1006	4.309339	2.739028	0.006348	0.011376	-3.53859
NR4A3	0.100556	5.282248	2.674659	0.007688	0.013566	-3.71013
LOC72916	0.10055	5.61235	2.392961	0.017024	0.02809	-4.41389
SCGB2A1	0.100424	3.903621	3.160801	0.001654	0.003278	-2.31657
LOC10192	0.100402	3.498233	3.00348	0.002782	0.005304	-2.79221
TAS2R13	0.100399	4.639894	2.631998	0.00871	0.015232	-3.82162
THSD4	0.100335	5.133284	3.513666	0.000476	0.001024	-1.16465
MYH7	0.100322	5.010863	2.248516	0.024911	0.039666	-4.745
S100A3	0.100311	5.388707	2.663893	0.007935	0.013964	-3.73843
SOX1	0.100301	5.003635	2.551295	0.010983	0.018815	-4.02775
KCNB2	0.100211	4.868676	2.943229	0.003376	0.006339	-2.96814
SEMA5A	0.10004	5.491533	3.379619	0.000774	0.001618	-1.61605
IGFL2	0.099988	5.477296	2.083265	0.037656	0.057699	-5.09893
S100A13	0.099926	5.400167	2.403418	0.01655	0.027397	-4.38914
MMP13	0.099896	4.389236	2.514201	0.012195	0.020716	-4.12038
KCNJ9	0.099845	5.203223	2.631643	0.008719	0.015245	-3.82254
LOC10192	0.099792	5.274674	2.080739	0.037888	0.058025	-5.10413
UPK2	0.099784	4.921652	2.504568	0.012528	0.021231	-4.14422
KRTAP9-9	0.099718	3.643914	3.709078	0.000228	0.000515	-0.47649
GH2	0.099714	4.689024	4.256783	2.41E-05	6.23E-05	1.640592
ZNF174	0.099696	5.330091	2.536709	0.011446	0.019543	-4.06433
BPIFB4	0.099686	5.302486	1.980298	0.048133	0.07197	-5.30601
DIRAS3	0.099671	4.023716	3.313448	0.000978	0.002011	-1.83265
PPME1	0.099654	6.094447	2.895818	0.003922	0.007275	-3.10413
CALN1	0.099592	4.024491	3.530574	0.000447	0.000968	-1.10651
PLCXD3	0.099503	4.046576	3.147988	0.001727	0.003411	-2.35619
KRT17P5	0.099469	5.610129	2.295996	0.022025	0.035432	-4.63839
MLLT10	0.099416	6.169007	2.631676	0.008718	0.015245	-3.82246
GHRHR	0.099359	5.035286	2.6402	0.008505	0.014906	-3.80032
NFASC	0.09935	4.77465	2.786771	0.005495	0.009955	-3.40879
LINC01431	0.099329	4.919881	2.767216	0.005831	0.010512	-3.46222
RASGRF1	0.099261	4.918872	3.231334	0.001301	0.002621	-2.09571
CASC15	0.099252	4.637619	2.288108	0.022483	0.036112	-4.65625
SLC30A10	0.099231	3.930882	2.773449	0.005722	0.01033	-3.44523
LOC10050	0.099217	5.823752	3.484574	0.000529	0.001133	-1.26405

LRP3	0.099159	6.136785	2.239669	0.025483	0.040468	-4.76462
KNDC1	0.099124	5.379582	2.425634	0.01558	0.025909	-4.33619
HAAO	0.099066	6.242365	2.080478	0.037912	0.058051	-5.10467
MYL12B	0.099033	12.10913	2.278579	0.023048	0.036947	-4.67775
MYRF	0.099022	4.916905	2.138265	0.032905	0.051044	-4.98408
LOC10272	0.099005	4.894175	2.559687	0.010724	0.018417	-4.00661
GCKR	0.098966	5.345567	2.129305	0.033642	0.052036	-5.00299
NUDT10	0.09895	4.936578	3.405008	0.000706	0.001487	-1.53185
LARGE	0.098949	5.101623	3.318227	0.000962	0.001981	-1.81715
KRT38	0.098948	5.384624	2.785507	0.005516	0.009988	-3.41225
FLJ45825	0.098915	5.263243	2.367503	0.01823	0.029878	-4.47372
HRC	0.098892	4.913804	2.602312	0.009492	0.016464	-3.89817
DSG3	0.098888	4.400028	3.635601	0.000302	0.00067	-0.73942
FOXL1	0.098829	4.971552	2.949514	0.003309	0.006223	-2.94994
ODAM	0.098819	4.26652	2.588643	0.009873	0.017074	-3.93313
ADCY10	0.098699	4.275775	3.353793	0.000848	0.00176	-1.70108
SIM2	0.098695	5.53619	2.388653	0.017223	0.028388	-4.42406
PKNOX1	0.098684	5.753824	2.777533	0.005651	0.010216	-3.43408
LYPD6	0.098653	4.807104	3.876482	0.000118	0.000278	0.141278
PROM2	0.098627	5.906204	2.027453	0.043064	0.065121	-5.21246
DDR1-AS1	0.0986	4.603186	2.244815	0.025149	0.040014	-4.75321
PMFBP1	0.098544	5.177245	2.33161	0.020056	0.03258	-4.55699
LHX6	0.098519	5.893551	2.78386	0.005544	0.010037	-3.41677
LOC14869	0.098462	4.733591	2.153087	0.031716	0.049369	-4.95263
FANCC	0.098455	5.007273	2.919653	0.003638	0.006792	-3.03603
ZKSCAN5	0.09837	5.99661	2.170682	0.030353	0.047483	-4.91501
COL1A1	0.098221	5.424152	2.219714	0.026817	0.042422	-4.8086
KIAA1467	0.098213	5.842181	3.33237	0.000915	0.00189	-1.77114
EGFLAM-A	0.098185	4.578676	2.926439	0.00356	0.006659	-3.01654
TNP1	0.098183	5.576285	2.051398	0.040668	0.06187	-5.16413
B3GALT4	0.098038	6.344188	2.180021	0.02965	0.046522	-4.89492
C2orf70	0.098033	5.576293	2.546358	0.011138	0.019056	-4.04015
PIH1D3	0.097938	4.974499	2.205244	0.027821	0.04389	-4.84024
IGF1	0.09789	4.995978	1.995248	0.046474	0.069731	-5.27659
RHBDD3	0.097853	6.128466	2.153664	0.03167	0.049307	-4.9514
UPK1B	0.097826	5.019612	2.786556	0.005498	0.00996	-3.40938
JAM2	0.097669	4.633387	2.323407	0.020496	0.033219	-4.57585
IZUMO4	0.097629	5.378699	2.424738	0.015618	0.025964	-4.33834
LMCD1	0.097549	5.171583	2.706985	0.006986	0.012427	-3.62448
OR51B5	0.097545	5.174852	1.997366	0.046243	0.069396	-5.2724
AIFM2	0.097466	5.742677	2.013929	0.04447	0.067045	-5.23951
DNMT3B	0.097401	5.500159	2.394239	0.016965	0.028001	-4.41087
IRS4	0.097226	5.089238	2.174629	0.030054	0.04709	-4.90653
ANKS4B	0.097106	5.062668	2.029684	0.042836	0.064814	-5.20798
CYP1A2	0.097093	5.845114	2.425165	0.0156	0.025937	-4.33732
MN1	0.097091	4.274299	2.719962	0.006721	0.011988	-3.58981
ZMYND10	0.097076	5.380051	1.998383	0.046133	0.069253	-5.27039
GPT	0.097023	6.207082	2.074705	0.038446	0.05878	-5.11654
LIPI	0.096999	3.562114	3.035591	0.002506	0.004812	-2.69704
CDHR5	0.096923	4.931302	2.61203	0.009229	0.016053	-3.8732
FDCSP	0.09686	4.36539	3.307672	0.000998	0.002049	-1.85136
LINC00302	0.096845	4.59292	2.806578	0.005172	0.009415	-3.3543
MAGI1	0.09669	5.262601	3.259705	0.001179	0.002395	-2.00554
SLIT3	0.096673	5.148282	2.52402	0.011863	0.020196	-4.09599
SBF2-AS1	0.096654	4.830202	2.298308	0.021892	0.035238	-4.63315
LINC01304	0.096609	5.398953	2.457339	0.014283	0.023947	-4.25981
CHRM2	0.096609	4.984352	2.209473	0.027524	0.04345	-4.83102
ZNF442	0.096581	3.863645	2.057449	0.040081	0.061059	-5.15182

BCAN	0.096529	5.669729	3.043436	0.002443	0.004697	-2.67364
NANS	0.096466	6.485209	2.363218	0.01844	0.030198	-4.48372
FBLN1	0.096321	5.306537	2.605788	0.009397	0.016319	-3.88925
CDRT1	0.096173	4.677702	4.147712	3.85E-05	9.70E-05	1.197045
LOC28509	0.096169	4.414358	2.066021	0.039262	0.059916	-5.13433
C6orf201	0.096066	3.841158	2.835	0.004739	0.008688	-3.27545
SHOX	0.096001	5.021094	2.319768	0.020693	0.033501	-4.5842
ANKRD7	0.09599	3.786359	3.592858	0.000354	0.000779	-0.89006
TBR1	0.095977	4.142411	2.732992	0.006464	0.011562	-3.55484
FOXF2	0.095895	4.529301	2.002314	0.045707	0.068671	-5.2626
GSTT2	0.095767	5.243019	2.413746	0.016092	0.026714	-4.36458
RGS11	0.095678	5.21003	2.288036	0.022488	0.036115	-4.65642
SPG20OS	0.095675	4.128112	2.252047	0.024686	0.039335	-4.73714
ZP2	0.095639	4.742711	2.316907	0.02085	0.033736	-4.59075
SLITRK3	0.095625	4.013417	2.724679	0.006627	0.011829	-3.57717
TSPAN9	0.095597	6.001713	2.031856	0.042615	0.064522	-5.20361
SLC9A5	0.095572	5.270789	2.77891	0.005628	0.010177	-3.43031
CLCN2	0.09557	5.678509	2.091423	0.036916	0.056671	-5.08208
LINC0031(	0.095564	5.287903	1.985589	0.047541	0.071158	-5.29562
ADCY5	0.095444	5.39344	2.877259	0.004156	0.007685	-3.15679
BRDT	0.095412	3.872652	2.931987	0.003498	0.006553	-3.00058
DGCR5	0.095241	5.850053	2.073407	0.038567	0.05895	-5.11921
FLJ40288	0.094818	5.368084	1.984775	0.047631	0.071272	-5.29722
ADH6	0.094744	4.564117	2.934145	0.003474	0.006512	-2.99436
HRASLS2	0.094644	5.640408	2.249914	0.024822	0.039534	-4.74189
FSHR	0.094632	4.34086	2.86905	0.004264	0.007873	-3.17997
CCNB3	0.09454	5.593973	2.675457	0.00767	0.013539	-3.70802
SP8	0.094536	4.554035	2.738248	0.006363	0.011399	-3.54069
CHRM3	0.094535	4.311574	3.794772	0.000163	0.000377	-0.16349
RS1	0.094515	4.355309	2.357117	0.018743	0.030658	-4.49794
ADAM21	0.094446	4.925975	2.67772	0.007619	0.013461	-3.70206
EFR3B	0.09434	5.774081	2.375755	0.017831	0.029278	-4.45439
GAP43	0.094311	4.3555	3.439535	0.000624	0.001321	-1.41637
RHOD	0.094301	7.086162	2.112515	0.035061	0.054079	-5.03822
COPB2	0.09425	6.650876	2.73391	0.006446	0.011535	-3.55237
LOC10272	0.094226	4.429132	2.953765	0.003264	0.006143	-2.93762
PAK4	0.094211	6.349057	2.680278	0.007561	0.013371	-3.69531
EPB41L5	0.09405	4.75545	2.040381	0.041756	0.063349	-5.18643
C1orf94	0.093927	5.504998	1.98553	0.047547	0.071158	-5.29574
MYO3A	0.093722	4.380747	3.212268	0.001389	0.002786	-2.15587
F2RL3	0.093642	4.48989	2.679797	0.007572	0.013389	-3.69658
FBXO43	0.093612	5.228512	2.024465	0.043371	0.065569	-5.21845
CUL7	0.093601	5.94535	2.786118	0.005506	0.009972	-3.41058
ADH1C	0.09359	4.006579	2.189412	0.028957	0.045494	-4.87464
MAST2	0.093502	6.01609	2.338185	0.01971	0.032064	-4.54183
EVPLL	0.093473	5.731308	2.268889	0.023635	0.037797	-4.69952
FGF5	0.093348	4.781711	3.628296	0.00031	0.000687	-0.76528
LOC10192	0.093139	4.879448	3.15719	0.001674	0.003316	-2.32775
CDH10	0.093139	4.254889	2.754258	0.006064	0.010898	-3.49742
NR2F1-AS	0.093111	5.229277	2.040131	0.041781	0.063382	-5.18694
ANO2	0.093047	4.774501	3.220716	0.001349	0.00271	-2.12926
CCL11	0.093006	4.514768	2.105232	0.035693	0.05496	-5.05342
PGBD5	0.092972	4.703684	2.405013	0.016478	0.027292	-4.38535
IL22	0.092969	4.395386	3.597643	0.000348	0.000766	-0.87328
MAGEA6	0.092925	3.994288	2.732002	0.006483	0.011592	-3.55751
ENDOU	0.092877	4.344828	2.806301	0.005177	0.009422	-3.35506
GDNF	0.092862	5.56401	2.962458	0.003175	0.005991	-2.91237
GPR161	0.092856	5.010665	3.524439	0.000457	0.000988	-1.12764

PCLO	0.092821	4.228388	3.934246	9.34E-05	0.000223	0.360457
KRT2	0.092767	5.277638	2.103786	0.035819	0.055136	-5.05643
FOXA2	0.092727	4.844661	2.618525	0.009058	0.015788	-3.85647
RDH12	0.09271	5.647262	2.068344	0.039042	0.059616	-5.12958
SHC3	0.092659	4.859276	2.47048	0.013774	0.02316	-4.22786
KCNK15	0.092596	3.865747	2.559049	0.010744	0.018448	-4.00821
KLK6	0.092589	5.237921	1.989095	0.047151	0.070659	-5.28872
TMEM207	0.092564	4.767027	2.0866	0.037352	0.057277	-5.09205
DBF4B	0.092532	5.062066	3.233426	0.001291	0.002605	-2.08908
LCT	0.092369	5.314258	2.38971	0.017174	0.028315	-4.42157
LINC00545	0.092363	5.161805	2.170652	0.030355	0.047483	-4.91508
CYLC1	0.09226	4.500676	3.861512	0.000125	0.000294	0.084977
KCNC3	0.09221	5.551197	2.302909	0.02163	0.034865	-4.62269
NNMT	0.092101	4.51987	2.132461	0.033381	0.051694	-4.99634
LYPD4	0.092099	5.588157	2.274432	0.023298	0.037307	-4.68708
SLC25A18	0.092059	4.570427	2.157679	0.031355	0.048896	-4.94284
ZNF197	0.092015	4.433745	1.964141	0.049982	0.074409	-5.33756
ANKRD30A	0.091812	3.626243	3.349731	0.00086	0.001784	-1.7144
LOC14594	0.091771	6.249924	2.170396	0.030374	0.047501	-4.91563
LOC10192	0.091664	3.991362	3.020124	0.002636	0.005044	-2.743
PHOX2B	0.091627	5.094527	2.33101	0.020088	0.032626	-4.55838
SCGB1A1	0.091607	4.715856	2.678848	0.007593	0.01342	-3.69908
TMEM130	0.091588	5.162724	2.093632	0.036718	0.056381	-5.07751
LOC10050	0.091543	4.194627	2.732206	0.006479	0.011586	-3.55696
LOC14779	0.091492	5.380191	2.010616	0.04482	0.067522	-5.24611
WNT10A	0.091442	5.59124	2.277961	0.023085	0.036996	-4.67914
PCDHB18	0.09142	5.449734	2.009831	0.044903	0.067636	-5.24767
DEFB119	0.091378	4.366946	1.993386	0.046678	0.07002	-5.28026
FGF2	0.091327	4.281549	2.581671	0.010072	0.01739	-3.9509
ESRP1	0.091253	4.633654	3.686043	0.000249	0.00056	-0.55946
ARL3	0.091132	6.499004	2.215394	0.027113	0.042849	-4.81807
LINC01096	0.090971	4.351202	2.609836	0.009288	0.016149	-3.87885
PMP2	0.090965	3.507824	4.297014	2.02E-05	5.28E-05	1.806926
TUNAR	0.090911	4.700078	2.168513	0.030518	0.047701	-4.91967
MESP1	0.090878	5.898444	2.190841	0.028853	0.045353	-4.87154
COPG2	0.090805	4.908767	2.017933	0.04405	0.066478	-5.23152
PCSK6	0.090753	5.009861	2.852273	0.004493	0.008266	-3.22716
DPPA4	0.090732	3.98181	3.412876	0.000687	0.001448	-1.50563
CCDC85A	0.090563	4.79849	2.547029	0.011117	0.019022	-4.03847
IGSF5	0.090483	4.42511	2.517234	0.012091	0.020556	-4.11286
PAX9	0.090473	4.200867	3.153138	0.001697	0.003358	-2.34028
LOC10050	0.090393	4.486284	2.59354	0.009735	0.01685	-3.92063
RIPK4	0.090379	5.616076	2.203371	0.027954	0.044059	-4.84433
MMP16	0.090369	4.134409	3.739647	0.000202	0.000461	-0.36562
ANTXR1	0.090335	5.26904	3.44827	0.000604	0.001283	-1.38697
EGFL6	0.090274	4.355853	2.212973	0.027281	0.043091	-4.82336
C5orf66-A	0.090271	4.272731	2.513037	0.012235	0.020776	-4.12327
HOXA11-A	0.090111	4.424756	2.354877	0.018855	0.030822	-4.50315
FOXH1	0.090022	5.171432	2.327971	0.02025	0.032867	-4.56537
SOX2	0.089952	4.784819	3.024482	0.002599	0.004978	-2.73007
KIF5A	0.089906	5.524735	3.011499	0.002711	0.005178	-2.76853
HSD3B1	0.089883	4.15448	3.301802	0.001019	0.002087	-1.87035
LOC10012	0.089823	5.098752	2.246225	0.025058	0.039879	-4.75008
DKFZp434	0.089601	4.447365	2.443885	0.014821	0.024778	-4.29234
SHROOM3	0.089589	4.48761	3.137919	0.001786	0.003516	-2.38721
C5orf49	0.089588	3.795012	3.588362	0.00036	0.000792	-0.9058
MTRF1L	0.089411	6.304831	2.49492	0.01287	0.021751	-4.16801
KRT80	0.08934	4.983651	2.310694	0.021193	0.03424	-4.60495

TRAK1	0.089265	6.8867	2.037311	0.042064	0.063752	-5.19263
TSHB	0.089228	5.138088	2.634422	0.008649	0.015134	-3.81533
EMX2OS	0.08914	5.439984	2.169386	0.030451	0.047618	-4.91779
THRA	0.089134	5.663637	2.616305	0.009116	0.015876	-3.86219
NDST3	0.089074	4.28534	3.012782	0.002699	0.005159	-2.76474
C1orf116	0.088827	4.731251	2.831177	0.004796	0.008777	-3.2861
OR51I2	0.088765	5.862505	1.980022	0.048164	0.071998	-5.30655
ANO3	0.088676	4.009901	3.389462	0.000747	0.001566	-1.58348
CDH6	0.08855	4.791554	3.18857	0.001505	0.003008	-2.23018
DLGAP2	0.08829	4.812645	2.843169	0.004621	0.008489	-3.25264
ADD2	0.08817	5.609007	3.238019	0.001271	0.002567	-2.07453
POLR3E	0.088139	5.115359	2.025722	0.043242	0.065385	-5.21593
DIO2	0.088004	5.197941	2.685708	0.007441	0.013169	-3.68096
IGHG1	0.087978	4.977051	2.604081	0.009444	0.01639	-3.89363
CBLN1	0.087971	4.588178	2.380628	0.017599	0.028942	-4.44295
ARNTL2	0.087967	5.018961	2.593739	0.009729	0.016842	-3.92012
LOC10099	0.087913	4.261965	2.307335	0.021381	0.034509	-4.61261
SSTR2	0.087847	4.844762	2.425624	0.01558	0.025909	-4.33622
LINC0093	0.087721	4.338031	2.017576	0.044087	0.066529	-5.23223
GRK4	0.087655	4.362005	3.891365	0.000111	0.000263	0.197454
SLX4	0.087503	6.39072	2.366211	0.018293	0.029976	-4.47673
ASAH1	0.087494	9.510441	2.067247	0.039146	0.059754	-5.13182
PLEKHA6	0.087408	4.52286	2.671828	0.007752	0.013668	-3.71758
FSCB	0.087406	3.950991	2.289856	0.022381	0.035957	-4.6523
COL22A1	0.087356	4.939869	3.674565	0.00026	0.000583	-0.60061
ATP2A2	0.087352	7.38632	2.241239	0.025381	0.040326	-4.76114
LOC10192	0.087307	4.55103	2.047163	0.041084	0.062413	-5.17271
SMIM12	0.087236	7.390539	2.118839	0.034521	0.053332	-5.02499
ARHGEF38	0.087163	5.33745	2.045568	0.041241	0.062611	-5.17595
NKD1	0.087016	5.759772	2.342007	0.019511	0.031798	-4.533
TMC5	0.086984	4.181362	3.63993	0.000297	0.00066	-0.72407
KRTDAP	0.086913	4.397201	2.138494	0.032886	0.05102	-4.9836
FBP2	0.086854	5.52459	2.063092	0.03954	0.060326	-5.14032
CNOT4	0.086782	5.427716	2.215687	0.027093	0.042821	-4.81742
ZNF71	0.086749	5.596821	2.667111	0.00786	0.013847	-3.72998
ZNF142	0.086747	6.507236	2.013962	0.044466	0.067045	-5.23945
CDH19	0.086546	4.188358	3.128285	0.001845	0.003622	-2.4168
FOXR1	0.086425	4.161216	2.272705	0.023402	0.037452	-4.69096
FOXD1	0.086378	4.577066	2.475745	0.013575	0.022866	-4.21502
PCAT19	0.086209	5.43384	2.648703	0.008296	0.014557	-3.77817
ZNF497	0.086169	4.425645	2.497512	0.012778	0.021613	-4.16163
HNF4A	0.086142	5.284204	2.497864	0.012765	0.021596	-4.16076
HOXA13	0.08613	4.152161	3.610456	0.000332	0.000732	-0.82824
TUBB2B	0.085949	4.131356	2.514356	0.012189	0.020709	-4.12
SMYD1	0.085925	5.185126	2.435376	0.015171	0.025309	-4.31283
LECT2	0.085911	4.214027	2.529024	0.011697	0.019943	-4.08352
IL19	0.085868	5.684536	1.974201	0.048824	0.072894	-5.31795
FARP1	0.085833	4.950877	3.06946	0.002243	0.004345	-2.59559
NBPF8	0.085579	4.13094	2.382925	0.017491	0.028805	-4.43755
DKFZp564	0.085428	4.809032	2.376292	0.017805	0.029241	-4.45313
LRTM1	0.085412	4.480645	3.129473	0.001838	0.003609	-2.41316
MALRD1	0.085403	4.136903	2.965885	0.00314	0.005933	-2.90239
EMCN	0.085389	4.324276	1.977372	0.048464	0.072392	-5.31174
FOXRED2	0.085339	5.725489	2.248286	0.024926	0.039683	-4.74551
CXXC4	0.085295	4.856445	2.560545	0.010698	0.018375	-4.00444
PLAC1	0.085273	4.961497	1.968956	0.049425	0.073694	-5.32819
ADCY2	0.085211	5.220499	2.89225	0.003966	0.007353	-3.11428
RASAL1	0.085197	5.987217	2.518754	0.01204	0.020474	-4.10908

CTXN3	0.085181	4.213826	3.855023	0.000128	0.000301	0.060639
LOC10272	0.08497	4.080225	2.264546	0.023902	0.038196	-4.70925
SPRY3	0.084917	4.9248	2.031531	0.042648	0.064567	-5.20427
CATSPERG	0.084757	5.021012	2.434927	0.01519	0.025331	-4.31391
TRIM50	0.08475	4.894993	2.089529	0.037087	0.056904	-5.086
KRTAP1-1	0.084691	4.873975	2.442024	0.014897	0.024894	-4.29683
EFCAB12	0.084622	4.951645	1.972225	0.04905	0.073189	-5.32181
CLDN8	0.084562	3.907208	2.675358	0.007672	0.013542	-3.70829
LINC00923	0.08454	4.076434	2.379551	0.01765	0.029015	-4.44548
LRP4	0.084482	4.230316	2.503463	0.012567	0.021289	-4.14695
LOC10050	0.084422	4.439622	2.097429	0.03638	0.055899	-5.06964
AGTR1	0.084397	4.223924	2.148921	0.032046	0.049836	-4.96149
ROS1	0.084286	4.209302	2.632606	0.008695	0.01521	-3.82004
MPV17L2	0.084269	6.58102	2.048845	0.040918	0.062203	-5.16931
CHRNA9	0.0842	4.310222	2.576294	0.010228	0.017631	-3.96457
ST6GALNA	0.084143	4.026761	3.54	0.000432	0.000937	-1.07399
PCDHB1	0.084129	5.035487	2.13634	0.033062	0.051262	-4.98815
LOC10050	0.084111	4.7217	2.774105	0.00571	0.010313	-3.44344
CT55	0.08408	4.544817	2.168064	0.030552	0.047747	-4.92063
PKD1L2	0.084067	3.960601	2.530032	0.011664	0.019894	-4.08101
HES5	0.083894	4.61781	2.022496	0.043575	0.065816	-5.2224
FOXK2	0.083786	5.893325	3.167871	0.001615	0.003206	-2.29465
AADAT	0.083695	4.794927	2.135195	0.033156	0.051377	-4.99057
SLC6A4	0.083402	4.180459	1.970084	0.049295	0.073519	-5.32599
DPYSL3	0.083298	4.5432	2.722231	0.006676	0.011913	-3.58373
CYP2A6	0.083295	6.079653	2.514968	0.012169	0.020675	-4.11848
LOC10192	0.083261	4.370983	2.298381	0.021888	0.035238	-4.63298
LOC10192	0.082987	5.099291	1.989533	0.047103	0.070598	-5.28786
PCSK2	0.082953	4.08948	2.867719	0.004282	0.007904	-3.18372
CLDN11	0.082934	4.863418	2.246656	0.02503	0.039842	-4.74913
TMEM246	0.082853	4.908722	2.338421	0.019698	0.032053	-4.54129
PDGFRA	0.082683	4.543594	2.321172	0.020617	0.033398	-4.58098
PIEZO2	0.082632	4.48873	2.999334	0.00282	0.005369	-2.80443
SGCD	0.082616	4.820004	3.246666	0.001234	0.002497	-2.04707
RNF207	0.082567	5.993388	2.164443	0.03083	0.048127	-4.92839
CACNG6	0.082429	5.729024	1.970103	0.049293	0.073519	-5.32595
UGGT1	0.082424	7.268868	2.729715	0.006528	0.011663	-3.56365
PPP2R3A	0.08223	4.905964	3.285162	0.00108	0.002204	-1.92399
HES2	0.081992	5.228617	2.503403	0.012569	0.02129	-4.1471
ADAM7	0.081912	4.362214	3.358022	0.000836	0.001736	-1.6872
LOC10272	0.0819	4.544877	2.378498	0.0177	0.029087	-4.44796
STMN2	0.081857	4.286817	2.597961	0.009612	0.016653	-3.90932
ADAD1	0.081855	4.473317	2.61268	0.009212	0.016028	-3.87153
KRTAP9-8	0.081824	4.836963	2.308047	0.021341	0.034448	-4.61099
NR2F1	0.081812	3.871651	2.625396	0.008879	0.015502	-3.83872
NPBWR2	0.081793	4.268519	2.257554	0.024338	0.038835	-4.72487
MGC3958	0.081772	4.703688	2.06245	0.039601	0.060409	-5.14163
BOLL	0.081649	4.777766	2.267267	0.023734	0.037943	-4.70316
IBSP	0.081535	4.636491	2.112667	0.035048	0.054068	-5.0379
CDH20	0.081479	4.021815	2.467087	0.013904	0.023368	-4.23613
CCNK	0.081435	7.49786	2.106482	0.035583	0.054818	-5.05081
GEMIN7	0.081161	6.656461	2.171197	0.030313	0.047435	-4.91391
FAM174B	0.081088	4.770071	2.32882	0.020205	0.03281	-4.56342
PDE1C	0.081059	4.978782	3.437006	0.00063	0.001332	-1.42486
TSSK4	0.080936	4.829468	2.421083	0.015775	0.02621	-4.34708
OR2B2	0.080923	3.909765	2.407739	0.016357	0.027103	-4.37888
RIMBP2	0.080916	4.597679	3.319953	0.000956	0.00197	-1.81154
TARP	0.080838	5.186464	2.222308	0.02664	0.042171	-4.8029

ESCO2	0.08066	3.994034	3.035234	0.002509	0.004816	-2.6981
AKAP6	0.080612	4.510435	2.159323	0.031227	0.048717	-4.93933
RAB26	0.080517	5.493132	2.308832	0.021297	0.034386	-4.6092
FNDC5	0.080497	5.509734	2.10468	0.035741	0.055025	-5.05457
C4orf26	0.080473	4.167112	2.261761	0.024075	0.038443	-4.71548
TAS2R7	0.080443	4.146185	2.427558	0.015498	0.025789	-4.33158
IL21	0.080421	5.234358	2.332636	0.020002	0.032498	-4.55463
OR51B4	0.080387	4.87438	1.967347	0.049611	0.073928	-5.33132
PAX8	0.080368	5.677887	2.515813	0.01214	0.020634	-4.11638
KRT12	0.080262	4.152062	2.364498	0.018377	0.030106	-4.48074
TCL6	0.080169	5.010394	2.320067	0.020677	0.03348	-4.58351
C21orf37	0.08009	3.302987	2.64823	0.008308	0.014573	-3.77941
POU4F2	0.079946	3.964091	2.590043	0.009833	0.017015	-3.92956
TBC1D16	0.079879	5.20054	2.090753	0.036977	0.056749	-5.08347
LOC10013	0.079846	4.420292	2.273945	0.023327	0.037351	-4.68817
ADRA1D	0.079842	4.385665	2.193214	0.028681	0.045098	-4.8664
LAMA4	0.079733	4.703312	2.118874	0.034518	0.053332	-5.02491
TAF6L	0.079727	5.269361	2.579233	0.010143	0.017499	-3.9571
CCHCR1	0.079633	5.404461	2.650793	0.008246	0.014477	-3.77272
FABP7	0.079604	4.205012	3.278262	0.001106	0.002254	-1.94615
EN2	0.079578	4.539231	2.379686	0.017644	0.029007	-4.44516
TM4SF1	0.079421	4.208851	2.982914	0.002973	0.005639	-2.85265
LMO3	0.079388	4.631523	2.057621	0.040065	0.061039	-5.15147
CNTN1	0.079382	4.013944	3.728402	0.000211	0.000481	-0.4065
CBX2	0.079341	5.570953	2.267915	0.023694	0.037884	-4.70171
MGST3	0.079331	7.264185	2.057057	0.040119	0.061106	-5.15262
HFE	0.079295	5.828224	2.494536	0.012884	0.021771	-4.16896
LOC10050	0.079226	4.189901	2.018377	0.044003	0.066425	-5.23064
ART4	0.079195	3.991737	2.035016	0.042295	0.064081	-5.19725
PACRG	0.079194	4.608592	2.242462	0.025301	0.040214	-4.75843
PKDREJ	0.079155	5.234845	2.097793	0.036348	0.055859	-5.06888
KCNMB2	0.079038	4.558413	2.776396	0.005671	0.01025	-3.43718
LOC10050	0.078756	4.726861	2.012516	0.044619	0.067236	-5.24233
TBC1D5	0.078734	8.108227	2.088156	0.037211	0.05708	-5.08884
SLC5A7	0.078671	4.517385	2.810701	0.005107	0.009304	-3.34291
TAF7L	0.07863	4.216813	3.380096	0.000772	0.001616	-1.61447
RARB	0.078591	5.006431	2.806168	0.005179	0.009425	-3.35543
MAP6	0.078563	5.227831	2.522263	0.011922	0.020286	-4.10036
TRPA1	0.078547	4.585907	2.814152	0.005053	0.009212	-3.33336
SHC4	0.078546	3.654283	3.414678	0.000682	0.001439	-1.49962
DNAH6	0.078545	4.951864	2.526664	0.011775	0.020059	-4.08941
SLC13A3	0.078473	5.188649	2.709674	0.00693	0.01233	-3.61731
HOXA7	0.078289	4.202534	2.612592	0.009214	0.01603	-3.87176
LOC10272	0.078037	4.041687	2.148155	0.032107	0.049923	-4.96312
HTR1D	0.078035	4.858971	2.310298	0.021215	0.034269	-4.60585
IL22RA1	0.077994	5.153083	2.083829	0.037605	0.05763	-5.09777
HAO2	0.077986	5.009943	2.576214	0.01023	0.017632	-3.96477
LOC10106	0.077887	3.57982	2.921779	0.003613	0.006752	-3.02993
ENAH	0.07786	4.279072	2.677559	0.007622	0.013466	-3.70248
CGN	0.077801	4.919355	2.588843	0.009867	0.017069	-3.93263
DCC	0.077638	4.085507	3.553902	0.00041	0.000892	-1.02586
ASXL3	0.077452	5.018267	2.545537	0.011164	0.019094	-4.04221
PCDHB6	0.077392	4.574862	2.141696	0.032626	0.050656	-4.97682
CDH4	0.077387	5.115291	2.157965	0.031333	0.048869	-4.94223
HECW1	0.077095	4.897359	2.880724	0.004112	0.007604	-3.14698
LOC10192	0.077047	4.276633	2.696347	0.00721	0.012799	-3.65278
FIGN	0.07703	3.789028	2.346531	0.019278	0.031458	-4.52253
PAX2	0.076977	5.129699	2.145453	0.032323	0.050224	-4.96886

FLJ26850	0.076943	3.945528	2.160475	0.031137	0.048581	-4.93687
DIAPH2-A	0.076845	4.347455	2.947287	0.003332	0.006264	-2.95639
CSTL1	0.076836	4.619757	2.154527	0.031602	0.049222	-4.94956
LOC10013	0.076831	4.77652	2.011276	0.04475	0.067428	-5.2448
TAS2R3	0.076804	4.95409	1.988426	0.047225	0.07075	-5.29004
WISP3	0.076716	4.550237	2.466598	0.013923	0.023393	-4.23732
PURG	0.076677	4.623852	2.210986	0.027419	0.043302	-4.82771
NPSR1-AS	0.076429	4.771514	2.209656	0.027512	0.043437	-4.83062
ERC1	0.076366	5.699002	2.129681	0.03361	0.052006	-5.0022
APOH	0.076245	5.399481	2.085434	0.037458	0.05743	-5.09446
RD3	0.076032	4.834222	2.11732	0.03465	0.053522	-5.02817
AIF1L	0.076023	5.785289	2.011234	0.044754	0.067429	-5.24488
LOC28456	0.07594	4.169433	3.364092	0.000818	0.001702	-1.66724
SRSF12	0.07581	4.589132	2.224244	0.026509	0.041993	-4.79865
NPTX2	0.075779	4.275062	2.253873	0.02457	0.039161	-4.73308
SHISA3	0.075757	3.190014	2.985411	0.002949	0.005595	-2.84533
NR2F2-AS	0.075711	4.887915	2.003674	0.045561	0.068479	-5.25991
ZNF407	0.075692	5.028944	2.792444	0.0054	0.009796	-3.39322
C1QTNF6	0.075626	5.290106	2.242748	0.025283	0.040191	-4.7578
CYP27B1	0.075586	5.476861	2.007256	0.045177	0.067993	-5.25279
LIPF	0.075369	4.236844	2.348761	0.019164	0.031281	-4.51736
MNX1-AS	0.075353	4.390867	2.061051	0.039735	0.060587	-5.14448
ESYT3	0.075244	4.189201	2.266518	0.02378	0.038009	-4.70484
PTOV1-AS	0.075118	5.093408	2.527919	0.011733	0.02	-4.08628
LINC0090(	0.075062	4.716338	2.547369	0.011106	0.019007	-4.03761
IQCF4	0.074906	3.486025	2.796222	0.005339	0.00969	-3.38284
RNF133	0.074685	4.813008	1.966014	0.049765	0.074121	-5.33392
RNLS	0.074639	4.964714	2.170693	0.030352	0.047483	-4.91499
MYT1	0.074638	5.431986	1.985543	0.047546	0.071158	-5.29571
CCDC181	0.074565	3.793622	2.559002	0.010745	0.018449	-4.00833
PTPRZ1	0.07451	3.493038	2.99904	0.002822	0.005374	-2.80529
TMEM139	0.074475	5.388288	2.070715	0.038819	0.05932	-5.12472
IRGQ	0.074465	5.467213	2.963926	0.00316	0.005967	-2.90809
TAT	0.073937	4.777921	2.558812	0.010751	0.018457	-4.00881
LOC10192	0.07389	4.812663	2.006512	0.045257	0.068101	-5.25427
ADAM18	0.073838	4.383539	2.115039	0.034844	0.0538	-5.03294
BAIAP2L1	0.073511	4.681124	2.678999	0.00759	0.013417	-3.69868
CMYA5	0.073421	5.171601	2.09601	0.036506	0.056079	-5.07258
SI	0.073295	3.427436	2.958057	0.00322	0.006069	-2.92516
MOCOS	0.07328	4.428132	2.557999	0.010776	0.018491	-4.01086
PDE3A	0.07327	4.923674	2.511258	0.012296	0.020866	-4.12768
EDIL3	0.073134	4.093652	2.348745	0.019165	0.031281	-4.51739
SLC4A9	0.073104	4.236617	2.049963	0.040809	0.062052	-5.16704
KRTAP1-3	0.073029	5.214111	2.47189	0.013721	0.023089	-4.22443
CALCB	0.072972	3.948804	2.204807	0.027852	0.043925	-4.8412
GPX5	0.072969	4.395491	2.812757	0.005075	0.009248	-3.33722
ZNF214	0.072891	4.37302	2.687616	0.007399	0.013099	-3.67592
DIO1	0.072849	3.912554	2.380992	0.017582	0.028921	-4.4421
CSF2	0.072827	5.32751	1.976945	0.048512	0.072458	-5.31258
CCDC150	0.07282	4.145364	2.38247	0.017512	0.028835	-4.43862
CAMK2B	0.072817	5.423342	2.207532	0.02766	0.043653	-4.83525
SV2B	0.072671	5.156568	2.328299	0.020233	0.032846	-4.56461
ST8SIA5	0.072448	5.603633	2.284728	0.022682	0.036405	-4.66389
ARHGEF39	0.07243	4.814794	2.685484	0.007446	0.013176	-3.68156
EPHA1	0.072392	5.259426	2.350083	0.019097	0.031187	-4.51429
ACSM2B	0.072384	4.147478	2.161962	0.031022	0.048409	-4.93369
FAM120A	0.072368	7.951555	2.110489	0.035236	0.054321	-5.04245
ATP2B3	0.072075	4.327862	2.678828	0.007594	0.01342	-3.69914

ONECUT2	0.071973	4.409864	3.042276	0.002452	0.004714	-2.6771
NALCN	0.071952	3.847718	2.243232	0.025251	0.040155	-4.75672
PHLDA2	0.071915	4.354352	2.022818	0.043542	0.065777	-5.22175
MURC	0.071899	3.432699	2.578509	0.010164	0.017532	-3.95894
DPP6	0.07177	4.904321	2.444685	0.014789	0.024729	-4.29041
GJC1	0.071695	4.453671	2.38998	0.017162	0.028299	-4.42093
PRSS12	0.071456	3.921451	3.093422	0.002072	0.004042	-2.52316
FILIP1	0.071355	3.851586	2.629831	0.008765	0.015315	-3.82724
FKSG29	0.071214	4.996956	2.077852	0.038154	0.058373	-5.11007
KIAA0408	0.0712	3.505643	2.677346	0.007627	0.013472	-3.70304
MAGEB1	0.071101	3.8965	2.142975	0.032523	0.050513	-4.97411
LZTS1	0.071067	4.812055	2.188574	0.029018	0.045586	-4.87645
SCG2	0.070993	4.372791	2.055631	0.040257	0.061295	-5.15552
ITSN2	0.070912	8.189031	2.165572	0.030743	0.048008	-4.92597
CLSPN	0.070903	4.524147	2.453785	0.014424	0.024156	-4.26842
COL4A6	0.070808	4.476232	3.327175	0.000932	0.001922	-1.78806
CCDC68	0.070777	3.974798	2.127657	0.033779	0.052239	-5.00646
CEP152	0.070773	4.875666	2.20473	0.027858	0.04393	-4.84136
KRT6A	0.070578	3.887471	2.558028	0.010775	0.018491	-4.01079
ELAVL4	0.070552	4.286025	3.396614	0.000728	0.001529	-1.55975
ENDOV	0.070484	5.842264	2.129913	0.033591	0.051985	-5.00171
ACOXL	0.070474	4.489265	2.462095	0.014097	0.023664	-4.24827
SLC2A11	0.070448	5.069615	2.08319	0.037663	0.057705	-5.09909
APOL4	0.070406	5.26756	2.020328	0.0438	0.06614	-5.22674
SMOC2	0.070372	4.558048	2.017312	0.044114	0.066565	-5.23276
LOC10272	0.070309	4.440246	2.084966	0.037501	0.057486	-5.09542
CNGB3	0.070286	4.332231	2.506819	0.01245	0.021107	-4.13866
ADAMTS2	0.070258	3.630459	2.491028	0.013011	0.021974	-4.17758
HSD3B2	0.070244	4.773858	2.038072	0.041987	0.063668	-5.19109
UGT2B15	0.07021	5.176462	2.001132	0.045835	0.068834	-5.26495
MAGI2	0.070118	4.21268	2.665537	0.007897	0.013901	-3.73411
LOC22181	0.070104	4.140391	2.317877	0.020797	0.033659	-4.58853
LOC10272	0.070099	4.262147	2.039105	0.041884	0.063527	-5.18901
SCARB1	0.07004	5.028403	2.865374	0.004313	0.007958	-3.19033
LOC10050	0.07003	4.202285	2.088612	0.03717	0.057022	-5.0879
MAB21L2	0.069891	4.150865	3.006261	0.002757	0.00526	-2.784
ACSM1	0.069655	5.188986	2.028502	0.042957	0.064975	-5.21036
SERPINA6	0.069641	5.507507	1.967754	0.049563	0.07387	-5.33053
PDLIM3	0.069591	4.638015	2.737677	0.006374	0.011414	-3.54223
CRISP1	0.069543	4.100536	2.643143	0.008432	0.014785	-3.79266
CWH43	0.069383	3.870933	2.515125	0.012163	0.020668	-4.11809
SGK2	0.069225	4.808881	2.242964	0.025269	0.040173	-4.75732
CHKB-AS1	0.069119	5.416543	2.307016	0.021399	0.034535	-4.61334
BNC1	0.068887	4.226007	2.05614	0.040208	0.061231	-5.15449
TSPAN12	0.068802	4.603769	2.029013	0.042905	0.064901	-5.20933
LOC10065	0.068659	3.577869	2.158584	0.031284	0.048802	-4.94091
MLANA	0.068484	4.302232	3.012283	0.002704	0.005166	-2.76621
TMEM62	0.068484	5.440677	2.114929	0.034854	0.05381	-5.03317
BVES	0.068374	4.615509	2.095074	0.036589	0.056198	-5.07452
STK24-AS1	0.068307	3.619708	2.308496	0.021316	0.03441	-4.60996
SORBS2	0.068226	4.57768	2.731056	0.006502	0.011622	-3.56005
CHST9	0.068205	3.610907	2.602607	0.009484	0.016452	-3.89742
CACNA1C	0.0681	4.500075	2.471746	0.013726	0.023097	-4.22478
APBA1	0.067702	5.240536	2.106464	0.035585	0.054818	-5.05085
SALL3	0.067449	4.142421	2.457118	0.014292	0.023955	-4.26034
GABRA1	0.067269	4.060626	2.444704	0.014788	0.024729	-4.29037
GRIA4	0.067093	3.829519	2.728546	0.006551	0.011702	-3.56679
KIRREL	0.067072	5.090522	2.022839	0.043539	0.065777	-5.22171

HOXB-AS3	0.067066	4.478077	2.012524	0.044618	0.067236	-5.24231
EPHA6	0.066983	4.282634	2.054804	0.040337	0.061391	-5.1572
DEFB132	0.066876	3.386694	2.030869	0.042715	0.064653	-5.2056
FEZF2	0.066857	3.956521	3.029088	0.00256	0.004908	-2.71639
LINC00113	0.066785	4.327275	2.094806	0.036613	0.05623	-5.07508
KCNV2	0.066648	3.776196	2.076442	0.038285	0.058563	-5.11297
CTB-12O2	0.066646	4.061331	2.527471	0.011748	0.020021	-4.0874
GNAS	0.066589	8.920536	2.068057	0.039069	0.059647	-5.13017
CEP112	0.0665	4.433061	2.30597	0.021458	0.034617	-4.61572
OR2F1	0.066468	5.109057	2.075198	0.0384	0.05872	-5.11553
RASAL2	0.066456	4.657713	3.205185	0.001423	0.00285	-2.17814
PROX1	0.066309	4.866169	2.343935	0.019412	0.03165	-4.52854
C12orf50	0.066206	3.551229	2.218541	0.026897	0.042545	-4.81117
APOB	0.065905	4.678177	2.237054	0.025655	0.040715	-4.7704
LOC73010	0.065761	4.251968	2.176394	0.029921	0.046916	-4.90274
LOC10192	0.065625	3.700085	1.990119	0.047038	0.070518	-5.2867
C9orf152	0.065383	3.843734	2.007859	0.045113	0.067907	-5.2516
KLHL4	0.06538	3.773914	2.274437	0.023297	0.037307	-4.68707
TP63	0.065238	4.959843	2.691848	0.007307	0.012951	-3.66471
ITGA2	0.065229	4.246262	2.083126	0.037669	0.057709	-5.09922
LONRF2	0.065143	4.03815	2.375362	0.01785	0.029306	-4.45531
ISL1	0.065121	4.115649	1.980232	0.048141	0.071975	-5.30614
FGF14	0.065093	4.613931	2.667706	0.007847	0.013827	-3.72842
ERBB4	0.065067	4.661924	2.454019	0.014414	0.024143	-4.26785
WWC1	0.064734	5.050135	2.335762	0.019837	0.03225	-4.54743
COL20A1	0.064551	4.584566	2.154124	0.031634	0.049259	-4.95042
PTGER3	0.064496	4.344709	2.14802	0.032118	0.049935	-4.96341
ACMSD	0.064474	4.480756	2.11423	0.034914	0.053898	-5.03463
CYP3A4	0.064429	5.351126	2.558023	0.010775	0.018491	-4.0108
MIR124-2I	0.064398	4.193736	2.568982	0.010444	0.017974	-3.98311
CDCP1	0.063956	5.066461	2.316875	0.020851	0.033736	-4.59082
DAW1	0.063725	4.115051	2.222327	0.026639	0.042171	-4.80286
CHRNA1	0.063653	4.855138	2.321424	0.020603	0.033379	-4.5804
LINC00933	0.063483	3.764192	2.200343	0.028169	0.044354	-4.85092
DNAH5	0.063423	4.624111	2.456483	0.014317	0.02399	-4.26188
COL2A1	0.063325	4.158108	2.10538	0.03568	0.054945	-5.05311
CRP	0.063323	4.505677	2.140131	0.032753	0.050839	-4.98013
CFL1	0.063317	7.861305	2.037253	0.04207	0.063755	-5.19275
ASCL1	0.063255	4.660219	2.625125	0.008886	0.015509	-3.83942
LOC10050	0.062887	4.446158	2.083277	0.037655	0.057699	-5.09891
SCGB2A2	0.062799	3.548295	2.396826	0.016847	0.027839	-4.40475
CYP4F11	0.062692	3.971846	2.022853	0.043538	0.065777	-5.22168
RASIP1	0.062679	4.523081	2.000711	0.04588	0.068891	-5.26578
SOX11	0.06261	4.238249	2.563421	0.010611	0.018237	-3.99718
HOTTIP	0.062432	3.669092	2.130056	0.033579	0.051976	-5.00141
ASB9	0.062382	4.281642	2.129353	0.033638	0.052035	-5.00289
UBXN10	0.062247	5.516591	2.028232	0.042984	0.065011	-5.2109
ERBB3	0.062031	4.717892	2.060299	0.039807	0.060692	-5.14602
MCF2	0.062008	4.388411	2.300334	0.021777	0.035079	-4.62854
CPEB1	0.061795	5.340957	2.001804	0.045762	0.068737	-5.26362
TTLL7	0.061767	4.189283	2.237378	0.025634	0.040684	-4.76969
DSCAML1	0.061726	4.616623	2.099657	0.036182	0.055629	-5.06501
C1QTNF7	0.061666	4.148675	2.281145	0.022895	0.036724	-4.67197
BHMT2	0.061616	4.088411	2.11367	0.034962	0.053959	-5.03581
CECR2	0.06155	4.724947	1.977591	0.048439	0.072361	-5.31131
DMGDH	0.061411	3.852061	2.884329	0.004065	0.007525	-3.13677
AMELX	0.061405	3.795482	2.109712	0.035303	0.054415	-5.04407
CLDN18	0.061255	4.85612	2.315767	0.020912	0.033822	-4.59336

DEFB126	0.061194	4.004387	2.334678	0.019894	0.032331	-4.54992
CTD-2118	0.060909	4.016369	2.149824	0.031974	0.049729	-4.95957
SCRG1	0.060735	4.083357	2.037658	0.042029	0.063715	-5.19193
TTC23	0.059722	4.464475	2.454445	0.014397	0.024116	-4.26682
PAPPA	0.05969	4.268306	2.490439	0.013032	0.022006	-4.17903
LAMB4	0.059688	4.230763	2.190067	0.028909	0.04543	-4.87322
SOX9	0.059539	4.083536	2.055218	0.040297	0.061346	-5.15636
B3GALT5	0.059315	4.823991	2.260126	0.024177	0.038592	-4.71913
GALNT5	0.059314	4.564023	2.497408	0.012781	0.021617	-4.16188
IL12B	0.059278	3.590748	2.465843	0.013952	0.023435	-4.23916
DMRT2	0.058812	4.204358	2.045899	0.041208	0.062581	-5.17528
LOC10272	0.058744	3.693522	1.976122	0.048606	0.072586	-5.31419
TRPM3	0.05848	4.595155	2.920294	0.00363	0.006781	-3.03419
TEX15	0.058332	3.931231	2.431426	0.015336	0.025546	-4.32231
RAB9BP1	0.058167	3.493442	2.193519	0.028659	0.045075	-4.86574
F13B	0.058147	3.626919	2.023598	0.043461	0.065683	-5.22019
PDPN	0.058044	4.403917	2.261378	0.024098	0.038477	-4.71633
IL36B	0.057937	4.303585	2.244991	0.025138	0.039999	-4.75282
SPAM1	0.057919	4.404889	2.50798	0.012409	0.021047	-4.13579
LOC14583	0.05784	3.918013	2.299695	0.021813	0.035129	-4.63
NKAIN2	0.057287	3.165855	2.069532	0.03893	0.05946	-5.12715
NPY2R	0.057008	4.362226	2.134859	0.033183	0.051415	-4.99128
SLC17A6	0.056769	3.759578	2.023756	0.043445	0.065665	-5.21987
PLCH1	0.056585	3.983377	2.592985	0.00975	0.016875	-3.92205
OGN	0.056356	3.61433	2.320473	0.020655	0.03345	-4.58258
SLC2A2	0.056113	3.776526	2.157455	0.031373	0.048915	-4.94332
GUCA1A	0.056029	5.200921	2.046744	0.041125	0.062465	-5.17356
OPRK1	0.056028	4.254121	2.304404	0.021546	0.034744	-4.61929
GPR110	0.055546	4.164652	1.980784	0.048079	0.071912	-5.30506
RAG2	0.055531	3.398224	2.193339	0.028672	0.045092	-4.86613
PAX3	0.055243	4.587691	2.037489	0.042046	0.063736	-5.19227
KIF25-AS1	0.055034	4.620978	2.079917	0.037964	0.058116	-5.10583
DNAH11	0.055013	3.248455	2.301763	0.021695	0.034955	-4.6253
GHSR	0.054915	4.722226	2.081472	0.037821	0.057931	-5.10263
LOC10272	0.054784	3.700831	1.997501	0.046229	0.069379	-5.27213
KCNIP2	0.054739	5.182294	1.978745	0.048308	0.072196	-5.30905
CYP2C8	0.054736	4.340117	2.08035	0.037924	0.05806	-5.10494
COL4A5	0.054378	3.939248	2.080447	0.037915	0.058051	-5.10474
ZNF704	0.054046	4.44526	2.203363	0.027954	0.044059	-4.84434
LINC0047(	0.053199	4.320427	2.381263	0.017569	0.028905	-4.44146
RASSF9	0.053135	3.552173	2.528272	0.011722	0.019982	-4.0854
ANK2	0.05304	4.168947	2.181192	0.029563	0.046408	-4.8924
BCL2L14	0.05233	4.575574	1.975506	0.048675	0.072678	-5.31539
WDR16	0.052324	4.07604	2.16622	0.030693	0.047938	-4.92458
DLGAP1	0.052	4.230159	2.35926	0.018636	0.030505	-4.49295
CEACAM7	0.051818	4.46494	2.342578	0.019482	0.031755	-4.53168
TCF21	0.051734	4.72743	1.990418	0.047005	0.070475	-5.28612
ENPP1	0.051671	4.449439	2.245597	0.025099	0.03994	-4.75148
FREM1	0.051449	4.04949	2.070573	0.038832	0.059333	-5.12502
GREM2	0.051347	4.122714	2.347088	0.01925	0.031414	-4.52124
ATRNL1	0.050755	4.078032	2.087696	0.037253	0.057134	-5.08979
TRPC5	0.050145	3.598302	2.018269	0.044014	0.066432	-5.23085
KCNJ16	0.048218	4.101788	2.121089	0.03433	0.05306	-5.02027
RAB3B	0.047561	4.558849	2.388205	0.017244	0.028417	-4.42512
LOC44120	0.047106	4.687911	1.977783	0.048417	0.072334	-5.31094
ITGBL1	0.045972	3.886873	2.081184	0.037847	0.057967	-5.10322
SULT1C2	0.04595	4.550533	1.973715	0.048879	0.072965	-5.3189
PPP1R3A	0.04582	3.732047	2.213675	0.027232	0.043022	-4.82183

ALDOB	0.045156	4.470351	2.290496	0.022344	0.035905	-4.65085
PTHLH	0.044425	3.662153	2.473974	0.013642	0.022972	-4.21934
OTX2	0.04393	3.524035	2.0507	0.040737	0.061963	-5.16554
EDDM3A	0.038447	3.867596	2.01299	0.044569	0.067183	-5.24138
GRIA2	0.037961	3.508709	2.381093	0.017577	0.028916	-4.44186
ZNF750	-0.04131	3.281003	-1.97215	0.049059	0.073193	-5.32196
GABRA2	-0.04314	3.617581	-2.12937	0.033636	0.052035	-5.00286
PTPRD	-0.0434	3.944162	-2.03554	0.042242	0.064006	-5.1962
SAMD5	-0.04432	3.961017	-2.07695	0.038238	0.058496	-5.11193
POU6F2-A	-0.04532	3.128856	-2.03847	0.041948	0.063619	-5.1903
ABCB5	-0.0458	3.623243	-2.10095	0.036068	0.055476	-5.06231
SCEL	-0.04627	3.348118	-2.25499	0.024499	0.039065	-4.73059
SOX2-OT	-0.04766	3.022154	-2.25265	0.024647	0.039278	-4.73581
CYP4F30P	-0.04828	3.275798	-2.05027	0.040779	0.062013	-5.16641
LINC00518	-0.0483	3.618843	-2.31527	0.02094	0.033857	-4.59449
RBM46	-0.04864	3.145135	-2.54925	0.011047	0.018913	-4.03289
SLC10A4	-0.04904	3.064484	-2.00931	0.044959	0.067709	-5.24872
PTH	-0.04916	3.435088	-2.02255	0.04357	0.065814	-5.22229
PDE10A	-0.04944	4.007496	-2.20306	0.027976	0.044089	-4.84501
COL8A1	-0.04945	3.630826	-2.721	0.0067	0.011953	-3.58704
POU2F3	-0.04965	3.589191	-2.20389	0.027917	0.044016	-4.8432
VSTM2A	-0.04994	3.689272	-2.18298	0.02943	0.046208	-4.88853
COL4A3	-0.04995	4.272122	-2.91041	0.003745	0.006975	-3.0625
DIRAS2	-0.05092	3.557144	-2.60528	0.009411	0.01634	-3.89056
DUXAP10	-0.05108	3.014016	-2.38416	0.017433	0.028715	-4.43464
PDE1A	-0.05132	3.644323	-2.65092	0.008242	0.014473	-3.77239
WDR78	-0.05164	3.908862	-2.35258	0.01897	0.031	-4.50848
CNTN5	-0.05202	3.560334	-2.54316	0.011239	0.019214	-4.04818
BTC	-0.05255	3.196853	-2.82263	0.004924	0.008994	-3.30987
SLC3A1	-0.05274	3.437306	-2.4353	0.015174	0.02531	-4.31301
KIF26B	-0.05297	5.001168	-2.10129	0.036038	0.055436	-5.06161
PIH2	-0.05423	3.242356	-2.82771	0.004847	0.008863	-3.29576
LOC10192	-0.0543	3.026687	-2.29619	0.022014	0.035421	-4.63796
LOC10037	-0.05469	3.463634	-1.98046	0.048115	0.071953	-5.30568
NEUROD1	-0.05473	4.099105	-2.04399	0.041397	0.062833	-5.17913
TGFB2	-0.0549	4.168839	-2.29071	0.022332	0.03589	-4.65038
RXFP1	-0.05539	3.59504	-2.50973	0.012349	0.020953	-4.13147
LOC40076	-0.05539	3.56351	-2.00259	0.045677	0.068643	-5.26205
LINC00551	-0.05552	2.987052	-2.42014	0.015815	0.026272	-4.34933
ZNF19	-0.05553	4.705027	-2.41149	0.016191	0.026858	-4.36995
AP4E1	-0.05566	4.826486	-2.46456	0.014002	0.023514	-4.24229
NAALAD2	-0.05569	3.773456	-2.00387	0.04554	0.06846	-5.25953
CFHR5	-0.05577	3.576308	-2.09851	0.036284	0.055771	-5.06739
MTAP	-0.05596	4.920339	-2.48497	0.013232	0.022325	-4.19246
HS6ST2	-0.05599	3.117516	-2.44021	0.014971	0.025006	-4.30119
TTC29	-0.0561	3.409953	-2.20346	0.027947	0.044055	-4.84412
IYD	-0.05611	3.038675	-2.36275	0.018463	0.030233	-4.48482
DCLK3	-0.05624	3.376739	-2.07591	0.038334	0.058633	-5.11406
MYT1L	-0.05679	3.823422	-3.22527	0.001328	0.002671	-2.11489
FAXC	-0.05684	3.410009	-2.40061	0.016676	0.027583	-4.39578
PRKD1	-0.05691	3.682088	-2.20446	0.027877	0.043956	-4.84196
PTPRB	-0.05699	3.939996	-2.19608	0.028474	0.044797	-4.86018
LINC00323	-0.05704	3.256728	-2.10922	0.035345	0.054476	-5.04509
SFTA3	-0.05714	3.194493	-2.39571	0.016898	0.027913	-4.40739
LGR5	-0.05725	4.486092	-2.13558	0.033124	0.051336	-4.98976
HFM1	-0.05742	3.169605	-2.66848	0.007829	0.013799	-3.72639
CEP57L1	-0.05783	3.709335	-2.36582	0.018312	0.030005	-4.47765
NEU3	-0.05792	6.039348	-2.15828	0.031308	0.048836	-4.94157

TLL1	-0.0588	3.416006	-2.18069	0.0296	0.046455	-4.89349
PLA2R1	-0.05905	3.828013	-2.16825	0.030538	0.047729	-4.92024
MTMR7	-0.05912	4.084054	-3.00516	0.002767	0.005278	-2.78726
ARSJ	-0.05917	3.79057	-2.36115	0.018542	0.030357	-4.48855
FAM133A	-0.05918	4.032974	-2.23319	0.02591	0.041094	-4.77893
TP53AIP1	-0.05918	4.687562	-1.9884	0.047228	0.07075	-5.29009
KCNC1	-0.05928	4.675453	-2.18941	0.028957	0.045494	-4.87464
RNF180	-0.05943	3.196032	-3.25014	0.001219	0.00247	-2.03601
NUP62CL	-0.05971	3.513665	-2.83443	0.004748	0.0087	-3.27704
DPY19L2	-0.05975	3.621075	-2.28699	0.022549	0.036205	-4.65879
ST18	-0.0598	3.690457	-2.45972	0.01419	0.023802	-4.25404
SBSPON	-0.05997	4.212347	-2.17494	0.03003	0.047061	-4.90586
NRXN1	-0.0604	4.201866	-2.44303	0.014856	0.024832	-4.29441
VHL	-0.06057	3.663459	-2.07926	0.038024	0.058194	-5.10718
CCDC81	-0.06065	4.49043	-2.0059	0.045322	0.068182	-5.25548
CCDC102E	-0.06098	3.344275	-2.10311	0.035878	0.055208	-5.05782
BAI3	-0.06129	3.549732	-2.56198	0.010655	0.018306	-4.00082
KITLG	-0.0614	3.919792	-2.54562	0.011161	0.019091	-4.042
TMEM196	-0.06225	3.023856	-2.51316	0.01223	0.020771	-4.12296
CNTNAP2	-0.06231	4.410179	-2.20088	0.028131	0.044306	-4.84976
ST6GAL2	-0.06265	3.148353	-2.45233	0.014482	0.024246	-4.27195
IL2	-0.06352	3.583135	-2.18084	0.029589	0.046445	-4.89315
MAP7D3	-0.06366	5.41252	-2.34596	0.019307	0.031499	-4.52384
MAGEA4	-0.06367	3.596261	-2.34819	0.019194	0.031325	-4.51869
FLT1	-0.0638	4.962257	-2.41935	0.015849	0.026324	-4.35121
SLC6A14	-0.06384	3.424139	-2.56837	0.010462	0.018004	-3.98465
HMGCLL1	-0.06408	3.079833	-3.35919	0.000832	0.00173	-1.68336
SLITRK5	-0.0642	3.895809	-2.56724	0.010496	0.018056	-3.98753
DNAL1	-0.0645	4.515777	-2.65381	0.008173	0.014357	-3.76484
C1orf101	-0.06482	3.961636	-2.51548	0.012151	0.020652	-4.11722
TFPI2	-0.06532	3.749968	-2.15665	0.031436	0.049005	-4.94504
IFNA2	-0.06535	3.845344	-2.29604	0.022023	0.035432	-4.6383
LINC00844	-0.0655	3.46617	-2.66303	0.007955	0.013995	-3.74068
SRPX2	-0.06552	3.911436	-2.0664	0.039226	0.059871	-5.13356
MMP20	-0.06555	4.016842	-2.06945	0.038938	0.059467	-5.12732
GIPC2	-0.06558	3.538179	-2.2644	0.023911	0.038205	-4.70958
FAM86B3F	-0.06564	5.373546	-1.97883	0.048299	0.072188	-5.30889
TSLP	-0.06579	3.21002	-2.32565	0.020374	0.033047	-4.57069
CHST1	-0.06592	5.166665	-2.29933	0.021834	0.035159	-4.63083
LINC00702	-0.06604	3.619193	-2.34336	0.019442	0.031693	-4.52988
SMIM2	-0.06626	4.074308	-2.89802	0.003895	0.007227	-3.09787
MTHFR	-0.06627	6.34951	-2.02507	0.043309	0.065481	-5.21724
CYP7B1	-0.06636	3.870297	-2.20107	0.028117	0.044289	-4.84934
LOC10192	-0.06646	3.400478	-2.25603	0.024434	0.038975	-4.72828
CHL1	-0.06654	3.482195	-2.46033	0.014166	0.023768	-4.25255
UGT2B4	-0.06712	3.281662	-2.63137	0.008726	0.015254	-3.82325
IFNA8	-0.06748	3.498872	-2.83968	0.004671	0.00857	-3.2624
EXOG	-0.06756	5.363059	-2.6087	0.009319	0.016199	-3.88177
RQCD1	-0.06756	6.068119	-2.29512	0.022076	0.035491	-4.64037
FRK	-0.06757	3.766715	-2.78698	0.005491	0.00995	-3.40821
LOC10192	-0.06796	3.399887	-2.42965	0.01541	0.025659	-4.32658
SCN1A	-0.0681	3.48497	-2.68218	0.007519	0.013299	-3.69028
ZFR2	-0.06827	5.124816	-2.10034	0.036122	0.055545	-5.06359
BNIP1	-0.06832	5.323867	-2.04468	0.041329	0.062737	-5.17774
TPTE	-0.06834	3.620701	-2.69029	0.007341	0.013004	-3.66883
DNAH3	-0.06859	5.899988	-2.07878	0.038068	0.058256	-5.10816
ASPN	-0.0686	4.122952	-2.13616	0.033077	0.051267	-4.98854
ULBP2	-0.06898	4.862394	-2.05574	0.040246	0.061284	-5.1553

PTPRG-AS	-0.06902	3.365663	-2.61561	0.009134	0.015901	-3.86397
LOC10050	-0.0691	4.07669	-2.17536	0.029999	0.047016	-4.90497
C12orf60	-0.06935	4.17908	-2.28381	0.022737	0.036486	-4.66597
C15orf41	-0.06956	4.07699	-3.23144	0.0013	0.00262	-2.09537
SCN11A	-0.06965	3.487369	-3.14677	0.001734	0.003423	-2.35994
CCDC110	-0.0697	3.487956	-2.45273	0.014466	0.024222	-4.27098
CYP24A1	-0.06986	3.666276	-2.43122	0.015344	0.025558	-4.3228
PROX2	-0.07017	3.860587	-2.03119	0.042682	0.064608	-5.20495
RNF128	-0.07025	3.460192	-2.99458	0.002863	0.005445	-2.8184
MAGOH2	-0.07025	5.115865	-2.08483	0.037513	0.057499	-5.0957
KCNK2	-0.07034	4.234127	-2.24163	0.025356	0.040293	-4.76028
AJUBA	-0.07063	4.037109	-3.0976	0.002044	0.003989	-2.51048
KCNAB1	-0.07089	4.778737	-3.21926	0.001356	0.002723	-2.13384
PBRM1	-0.07096	5.959391	-2.1516	0.031833	0.049527	-4.95579
TCF24	-0.07098	3.027932	-3.46175	0.000575	0.001225	-1.34147
LAMA2	-0.07118	4.275073	-2.16912	0.030472	0.047646	-4.91837
HEY2	-0.07148	4.381802	-2.23401	0.025855	0.041012	-4.77712
LOC10192	-0.07154	4.650125	-2.05125	0.040683	0.061886	-5.16442
ZNF343	-0.07162	5.312258	-2.40767	0.01636	0.027105	-4.37903
PPEF2	-0.07184	3.582137	-2.63468	0.008642	0.015125	-3.81466
IBA57	-0.07189	5.634147	-2.23412	0.025849	0.041004	-4.77689
ARHGAP3!	-0.07191	5.663143	-2.5612	0.010678	0.018344	-4.00278
C20orf26	-0.07194	4.253097	-2.20496	0.027842	0.043912	-4.84087
RGS7	-0.07194	3.693986	-2.17241	0.030221	0.047319	-4.91129
GNA11	-0.07196	5.656117	-2.00374	0.045554	0.068475	-5.25978
LOC10192	-0.07198	4.214994	-2.3157	0.020916	0.033826	-4.59352
PDC	-0.07205	3.596375	-2.51652	0.012116	0.020595	-4.11463
ERC2	-0.07219	3.87904	-2.32443	0.020441	0.033142	-4.57351
GPR115	-0.07245	2.998	-2.93126	0.003506	0.006565	-3.00268
KRT7	-0.07257	4.277641	-2.48094	0.013381	0.022562	-4.20233
POU4F1	-0.07262	4.075677	-2.3951	0.016926	0.027951	-4.40883
ADAMTS6	-0.07265	4.121979	-2.55088	0.010996	0.018833	-4.02879
FAM47A	-0.07297	3.984491	-2.25702	0.024371	0.038882	-4.72607
MIR663AH	-0.0734	3.227081	-3.13292	0.001816	0.003571	-2.40257
ZCCHC4	-0.07353	4.394141	-2.83333	0.004764	0.008726	-3.2801
GLRA3	-0.07433	3.62655	-3.76775	0.000181	0.000416	-0.26293
LINC01351	-0.07443	4.070635	-2.32565	0.020375	0.033047	-4.5707
LETM1	-0.07474	5.761201	-2.19128	0.028821	0.045312	-4.8706
GRIK1	-0.07518	4.260617	-2.24081	0.025409	0.040364	-4.7621
KCNJ10	-0.07533	4.64079	-2.39221	0.017059	0.02814	-4.41566
LOC28555	-0.07575	3.592754	-2.00398	0.045528	0.068447	-5.2593
BRINP3	-0.0758	3.438307	-3.07139	0.002229	0.00432	-2.58977
CPNE4	-0.0758	3.66394	-3.24355	0.001247	0.002522	-2.05697
NCKAP5	-0.07609	3.077056	-3.51278	0.000477	0.001027	-1.16768
MGAT4C	-0.07609	3.340042	-3.49967	0.000501	0.001074	-1.21258
GPR39	-0.0761	4.462257	-1.96857	0.04947	0.073755	-5.32895
SPATA6L	-0.07622	3.719313	-2.94708	0.003334	0.006267	-2.95699
SEZ6L	-0.07641	4.93982	-2.58332	0.010025	0.017315	-3.94671
EIF4EBP2	-0.07645	7.868791	-2.1009	0.036072	0.055479	-5.06242
WDR86	-0.07648	4.967224	-2.39931	0.016734	0.027668	-4.39887
DUSP27	-0.07708	4.168266	-2.04824	0.040978	0.06227	-5.17053
IKZF4	-0.07723	5.584126	-2.05545	0.040274	0.061317	-5.15589
PRG4	-0.07731	3.798181	-2.52001	0.011998	0.020408	-4.10598
DNAH1	-0.07739	5.430127	-2.26251	0.024028	0.038375	-4.71381
AKAP5	-0.07741	4.075091	-2.15321	0.031706	0.049358	-4.95237
CCDC144A	-0.07743	3.699785	-2.48358	0.013283	0.022409	-4.19586
CBLN2	-0.07749	5.151641	-2.09027	0.03702	0.056811	-5.08446
LMO4	-0.0778	5.575687	-2.69553	0.007228	0.012825	-3.65496

SLC26A4	-0.0779	3.565179	-2.67954	0.007578	0.013398	-3.69727
MC4R	-0.07823	4.698419	-1.97237	0.049034	0.073171	-5.32153
IL4	-0.07834	3.997976	-2.17577	0.029968	0.046976	-4.90409
CA8	-0.07864	4.092464	-2.97371	0.003062	0.005797	-2.87957
CACNB3	-0.0788	5.447182	-2.05049	0.040757	0.06199	-5.16598
SLC4A4	-0.0789	4.586593	-2.75066	0.00613	0.011005	-3.50717
ATXN7L1	-0.07896	5.581593	-2.17638	0.029922	0.046916	-4.90277
LOC10192	-0.07966	4.141782	-2.20253	0.028013	0.044136	-4.84615
CCDC54	-0.0797	4.335215	-2.28614	0.022599	0.036278	-4.66071
OVOL2	-0.07971	4.501116	-2.73276	0.006469	0.011569	-3.55547
SLC26A7	-0.07975	3.059405	-3.53304	0.000443	0.000959	-1.09802
LRP1B	-0.07981	3.409457	-2.80091	0.005263	0.009569	-3.36993
MAGEC3	-0.07995	4.000336	-2.20279	0.027995	0.044115	-4.84559
RPS14	-0.08012	6.63902	-2.17583	0.029963	0.046972	-4.90395
MIPEPP3	-0.08019	5.266149	-2.00495	0.045424	0.068324	-5.25737
CSMD1	-0.08027	3.785859	-2.70608	0.007005	0.012458	-3.6269
LOC10050	-0.08032	4.348668	-2.31329	0.021049	0.034031	-4.59903
SPOCK3	-0.08035	3.446384	-3.84875	0.000132	0.000309	0.037142
SLC25A21	-0.08038	3.327204	-3.61773	0.000323	0.000714	-0.80262
MNS1	-0.08084	3.609405	-2.8449	0.004597	0.008448	-3.24782
MKX	-0.08111	3.824709	-4.24892	2.50E-05	6.44E-05	1.608257
OSTM1-A!	-0.08137	3.718225	-2.04566	0.041232	0.062611	-5.17575
NRF1	-0.08164	5.94224	-2.36756	0.018227	0.029876	-4.47358
ACSS1	-0.08177	5.663043	-2.66889	0.007819	0.013783	-3.7253
LOC10192	-0.08178	3.94783	-2.52787	0.011735	0.020001	-4.0864
SCIN	-0.08203	4.101871	-2.77162	0.005753	0.010384	-3.45021
SPTLC3	-0.0826	4.145046	-3.52931	0.000449	0.000972	-1.11087
RBMS2	-0.0826	5.111506	-2.54019	0.011334	0.019361	-4.05562
DCHS2	-0.08279	4.747594	-2.51382	0.012208	0.020734	-4.12132
HLA-DOA	-0.08284	6.071704	-2.29768	0.021929	0.035293	-4.63458
ANKRD34(	-0.08286	4.06161	-3.12198	0.001884	0.003695	-2.43611
C8orf34	-0.08322	3.865493	-2.73386	0.006447	0.011535	-3.5525
SIX4	-0.0835	3.7218	-2.93443	0.003471	0.006507	-2.99353
SLC22A5	-0.08352	5.388208	-2.07942	0.038009	0.058181	-5.10684
NEO1	-0.08372	5.14725	-2.29519	0.022071	0.035489	-4.64021
LY6K	-0.08386	4.468292	-2.27579	0.023215	0.037192	-4.68402
TCP10	-0.08396	5.448688	-2.20078	0.028138	0.044314	-4.84998
CLCA3P	-0.08399	3.196368	-3.52843	0.000451	0.000975	-1.11389
SPATA18	-0.08424	4.253326	-2.85988	0.004388	0.008085	-3.2058
PDCL2	-0.08431	3.040018	-3.53966	0.000432	0.000938	-1.07517
RAB15	-0.08441	6.414682	-1.96436	0.049957	0.074383	-5.33714
L3MBTL1	-0.08468	5.315657	-3.68144	0.000253	0.000569	-0.57596
LINC0111(	-0.08468	5.137839	-2.29379	0.022153	0.035609	-4.6434
CLDN1	-0.08475	4.386086	-3.26318	0.001165	0.002368	-1.99443
CADM2	-0.08481	3.21109	-4.53639	6.93E-06	1.92E-05	2.826901
KIAA2022	-0.08486	3.86516	-3.54691	0.000421	0.000915	-1.05011
XIRP2	-0.08494	3.749871	-2.116	0.034762	0.053687	-5.03092
LOC10050	-0.08526	5.032258	-2.20726	0.027679	0.043675	-4.83584
IGDCC4	-0.08529	4.073566	-2.10363	0.035833	0.055152	-5.05675
ZRANB3	-0.08535	4.543177	-3.51726	0.00047	0.001012	-1.15233
TRADD	-0.08556	7.359472	-2.13077	0.03352	0.051892	-4.9999
EFCAB14	-0.08557	7.62891	-2.22089	0.026736	0.042305	-4.80601
RDH13	-0.08562	6.255425	-2.01859	0.043981	0.066397	-5.23022
KIAA2026	-0.08573	5.975172	-2.04284	0.041512	0.062999	-5.18147
TMED3	-0.08582	5.027905	-2.9026	0.003839	0.007131	-3.08482
GRIN2C	-0.08593	5.380438	-2.25383	0.024573	0.039162	-4.73318
MTIF3	-0.08596	5.807221	-2.66704	0.007862	0.013849	-3.73018
MIR17HG	-0.08607	3.069502	-3.50928	0.000484	0.00104	-1.17967

TSGA10	-0.08609	3.931967	-3.00971	0.002726	0.005205	-2.77381
NF1	-0.08637	3.590921	-3.07846	0.002177	0.004229	-2.56844
XYLB	-0.0864	4.030767	-3.6584	0.000276	0.000617	-0.65837
MTFR1	-0.08657	4.65414	-1.98012	0.048153	0.071987	-5.30635
C3orf58	-0.08666	6.114231	-2.90317	0.003832	0.007122	-3.08319
NDUFA10	-0.08669	5.592612	-2.83132	0.004793	0.008774	-3.28571
DHX35	-0.0869	5.073528	-2.007	0.045205	0.068029	-5.25331
IL5	-0.08699	3.620247	-2.37513	0.017861	0.029321	-4.45585
DPPA5	-0.08699	4.334939	-2.11767	0.03462	0.053481	-5.02743
TMEM231	-0.08707	4.412509	-3.16321	0.00164	0.003255	-2.3091
CDK13	-0.0872	6.818265	-2.71687	0.006783	0.012094	-3.59809
NREP	-0.08734	5.500656	-2.28166	0.022864	0.036681	-4.67081
ZNF471	-0.08737	3.651798	-4.76435	2.39E-06	7.03E-06	3.846113
MCF2L2	-0.08744	3.233523	-3.9062	0.000105	0.000248	0.253669
TMEM39A	-0.08755	5.872139	-2.10871	0.03539	0.054535	-5.04616
TRPC5OS	-0.08756	3.558009	-2.6123	0.009222	0.016042	-3.8725
SLC2A4	-0.08798	4.314486	-2.42896	0.015439	0.025705	-4.32824
RPAIN	-0.08803	5.278104	-4.1291	4.17E-05	0.000105	1.122437
PLD6	-0.08819	5.067191	-2.28087	0.022911	0.036747	-4.6726
PABPC5	-0.08823	3.008075	-4.08831	4.95E-05	0.000123	0.960066
COBLL1	-0.08832	4.254756	-2.46572	0.013957	0.023441	-4.23945
SLC4A10	-0.0885	3.409262	-3.70439	0.000232	0.000524	-0.4934
DDX54	-0.08858	5.942373	-2.22609	0.026384	0.041803	-4.79458
OTUD7B	-0.0886	5.152025	-3.74454	0.000198	0.000453	-0.3478
PCDHB5	-0.08876	3.687569	-2.90297	0.003834	0.007125	-3.08376
LOC10012	-0.08882	3.213468	-2.76614	0.00585	0.010545	-3.46516
NXPH1	-0.08888	3.262607	-3.57939	0.000373	0.000817	-0.93718
PCDHB15	-0.0889	3.468156	-3.22748	0.001318	0.002653	-2.10789
ZNF653	-0.089	4.634567	-2.30433	0.02155	0.034748	-4.61946
MTTP	-0.08902	3.878478	-3.48801	0.000523	0.00112	-1.25236
LOC10013	-0.08906	3.501911	-3.28686	0.001073	0.002192	-1.91854
MATN3	-0.08909	3.295629	-4.01409	6.74E-05	0.000164	0.668494
CDK20	-0.08916	5.943981	-2.25159	0.024715	0.039374	-4.73816
ZNF527	-0.08921	6.301902	-2.2281	0.02625	0.041602	-4.79017
ZNF416	-0.08928	4.859279	-2.61009	0.009281	0.016139	-3.87821
RAB9B	-0.08932	5.45715	-2.44697	0.014696	0.024585	-4.28489
ATP1B3	-0.08953	6.340483	-2.22128	0.02671	0.042272	-4.80517
PIN4	-0.08955	6.442155	-2.28988	0.022379	0.035957	-4.65224
LOC10192	-0.08959	4.071431	-2.91234	0.003723	0.006937	-3.05697
WDR31	-0.08984	4.99827	-3.03732	0.002492	0.004786	-2.6919
TAPT1	-0.08999	5.576551	-2.88353	0.004076	0.007541	-3.13903
CNIH3	-0.09017	4.353452	-2.24408	0.025197	0.040079	-4.75484
CEP164	-0.0902	5.773967	-2.06604	0.03926	0.059916	-5.1343
SCD5	-0.09022	4.931212	-3.00474	0.002771	0.005285	-2.78851
ICMT	-0.09042	5.951517	-2.4372	0.015095	0.025197	-4.30843
RBM20	-0.09052	3.475883	-3.25008	0.001219	0.00247	-2.03622
CELF1	-0.09063	7.636081	-3.06374	0.002285	0.004421	-2.61281
AADACL2	-0.09068	3.409573	-3.12708	0.001852	0.003635	-2.42051
TMEM171	-0.09071	3.313555	-3.22421	0.001333	0.00268	-2.11824
MSH4	-0.09088	3.776724	-3.3015	0.00102	0.002089	-1.87133
PATZ1	-0.09094	5.686979	-2.21268	0.027301	0.043119	-4.824
AXIN2	-0.09123	5.580831	-3.53388	0.000442	0.000957	-1.09513
SLC22A23	-0.09132	5.761274	-2.48601	0.013194	0.022267	-4.18991
UTP11L	-0.09139	5.881753	-2.58033	0.010111	0.017454	-3.9543
PLEKHH2	-0.09159	3.646599	-3.65068	0.000285	0.000634	-0.68588
SP2	-0.09204	5.705273	-2.8598	0.004389	0.008086	-3.20602
TPH1	-0.09206	3.701129	-4.36025	1.53E-05	4.07E-05	2.071324
CXCL11	-0.09219	3.476418	-2.27988	0.02297	0.036828	-4.67482

TTC21A	-0.0922	4.900956	-2.50932	0.012363	0.020974	-4.13247
FAM115A	-0.09229	4.472569	-2.37946	0.017654	0.029019	-4.44568
ZNF582-A	-0.09265	5.217113	-2.69558	0.007227	0.012825	-3.65481
LOC10050	-0.09269	3.187019	-4.17078	3.49E-05	8.85E-05	1.289947
CTTNBP2	-0.09296	3.140508	-3.0072	0.002749	0.005246	-2.78125
TPRN	-0.09314	6.027201	-1.98419	0.047697	0.071364	-5.29837
ERI2	-0.09316	4.26927	-2.09289	0.036784	0.056478	-5.07904
PDE4A	-0.09319	5.160773	-2.00475	0.045445	0.068351	-5.25777
LINC0108ξ	-0.09331	3.997742	-2.81626	0.005021	0.009159	-3.32753
FGF14-IT1	-0.09337	4.448642	-2.34572	0.01932	0.031517	-4.5244
VN1R3	-0.09345	4.113031	-2.94337	0.003374	0.006337	-2.96774
GPR126	-0.09361	3.969972	-3.57456	0.000379	0.000831	-0.95402
NUP188	-0.09373	5.841448	-3.07798	0.002181	0.004235	-2.56989
SOX21-AS	-0.09381	3.501795	-3.63109	0.000307	0.00068	-0.75538
SLC6A11	-0.09383	4.737852	-2.03069	0.042733	0.064675	-5.20596
N6AMT1	-0.09387	4.700375	-3.97476	7.92E-05	0.000192	0.516035
GPR1	-0.09398	3.541859	-2.63754	0.008571	0.015009	-3.80724
MAP2K5	-0.09401	5.258219	-3.53365	0.000442	0.000958	-1.09592
SLC41A2	-0.09402	4.18671	-3.17644	0.001569	0.003125	-2.26799
MUC20	-0.0941	4.850777	-2.24395	0.025205	0.040089	-4.75514
TBL2	-0.09437	6.384853	-2.38313	0.017481	0.028792	-4.43707
CYP2E1	-0.09443	4.41376	-2.46118	0.014133	0.023717	-4.25049
CYMP	-0.09444	4.771768	-2.23631	0.025704	0.040781	-4.77204
ERCC8	-0.09448	5.278114	-2.50971	0.012349	0.020953	-4.13151
NMU	-0.09455	4.092857	-2.5389	0.011375	0.019428	-4.05884
LOC43993	-0.09468	4.386735	-2.86572	0.004309	0.00795	-3.18937
LINC0135	-0.09468	3.153419	-3.67135	0.000263	0.00059	-0.61213
GCM2	-0.09477	3.398933	-3.63256	0.000305	0.000677	-0.75021
IRX1	-0.0948	3.799474	-2.63119	0.008731	0.015261	-3.82372
RBM19	-0.0948	5.859886	-2.04976	0.040828	0.062077	-5.16744
ZBED3-AS	-0.09488	4.868235	-2.01266	0.044603	0.067224	-5.24203
LOC10192	-0.09492	5.108995	-2.62352	0.008927	0.015579	-3.84358
C1QTNF2	-0.09494	4.422605	-2.24895	0.024883	0.039628	-4.74402
KRT222	-0.09509	3.360682	-4.31055	1.91E-05	5.00E-05	1.863233
ISYNA1	-0.09512	5.385652	-2.00857	0.045038	0.067822	-5.25019
ZNF594	-0.09526	4.512396	-2.28879	0.022444	0.036051	-4.65472
ZFP41	-0.09526	5.516583	-3.15114	0.001709	0.003379	-2.34646
EMC3	-0.09531	7.048764	-2.09522	0.036576	0.056182	-5.07422
CPLX1	-0.09545	3.907974	-2.44211	0.014894	0.02489	-4.29661
LOC10050	-0.09567	4.057778	-2.18211	0.029494	0.046305	-4.89041
TMEM163	-0.0957	4.224959	-2.0491	0.040894	0.062171	-5.1688
SNTA1	-0.09573	6.364631	-2.03411	0.042386	0.064203	-5.19907
UGT2A3	-0.09576	3.374135	-3.38768	0.000752	0.001575	-1.58937
PCDH11X	-0.0958	3.870542	-3.02057	0.002632	0.005039	-2.74169
C15orf40	-0.09587	4.157997	-2.2447	0.025156	0.040022	-4.75346
MMP28	-0.09588	4.979285	-2.83953	0.004673	0.008573	-3.26282
RSPH1	-0.09626	4.624935	-2.13627	0.033068	0.051262	-4.9883
NPTX1	-0.09635	5.668918	-2.11396	0.034937	0.053925	-5.03521
ORF1	-0.09636	3.968845	-2.2517	0.024708	0.039367	-4.73792
CWC15	-0.09637	9.593965	-1.96535	0.049841	0.074223	-5.3352
ALKBH1	-0.09653	5.924888	-2.04674	0.041125	0.062465	-5.17357
DCAKD	-0.09671	5.452131	-2.53882	0.011378	0.01943	-4.05904
VWC2	-0.09685	2.995469	-3.65533	0.00028	0.000624	-0.66931
KRT14	-0.09693	4.208055	-2.02053	0.043779	0.066114	-5.22634
ADAM33	-0.09695	5.90958	-2.15447	0.031607	0.049225	-4.94969
CDH2	-0.09701	4.604314	-2.53024	0.011657	0.019884	-4.08048
MYO1E	-0.09706	5.528197	-2.13626	0.033068	0.051262	-4.98832
SLC25A30	-0.09737	5.111385	-3.78785	0.000168	0.000387	-0.18902

LINC00973	-0.09751	3.388399	-3.53419	0.000441	0.000956	-1.09405
ZNF41	-0.09774	3.921385	-2.80672	0.00517	0.009412	-3.35391
LOC10050	-0.09779	4.632059	-2.76386	0.00589	0.010609	-3.47135
MTHFSD	-0.09822	4.900806	-3.68051	0.000254	0.000571	-0.57931
TIMELESS	-0.0983	5.625882	-2.10636	0.035594	0.054822	-5.05106
SEMA3D	-0.09836	3.605723	-3.0555	0.002348	0.00453	-2.63754
FBXL12	-0.09841	6.592976	-3.24778	0.001229	0.002489	-2.04353
ELOVL2	-0.09842	4.026165	-3.25587	0.001195	0.002425	-2.01778
ROPN1	-0.09843	4.641379	-3.14898	0.001721	0.003401	-2.35312
PCDHGA1	-0.09854	4.893045	-2.28179	0.022857	0.036672	-4.67052
LPAR4	-0.09895	3.661026	-2.91506	0.003691	0.006882	-3.04921
SSTR1	-0.09903	3.938373	-3.27682	0.001111	0.002265	-1.95079
LOC72873	-0.09919	3.662214	-4.25306	2.45E-05	6.33E-05	1.625252
PDP2	-0.09924	4.712777	-2.40419	0.016515	0.027347	-4.3873
LOC10272	-0.09971	4.690637	-2.54044	0.011326	0.019351	-4.055
NR2F6	-0.09993	4.939616	-2.54991	0.011026	0.018881	-4.03122
FRMD7	-0.10002	3.725946	-2.55833	0.010766	0.018479	-4.01004
RNF186	-0.10007	4.982788	-2.45842	0.014241	0.023881	-4.2572
SFSWAP	-0.10013	6.786162	-2.14382	0.032455	0.05042	-4.97233
SOBP	-0.10021	4.322304	-4.39919	1.29E-05	3.44E-05	2.235951
PROSER3	-0.10024	5.168624	-2.4215	0.015757	0.026183	-4.34608
NPPC	-0.10032	4.293172	-2.46021	0.01417	0.023774	-4.25284
ZNF205-A	-0.10032	4.658758	-3.04616	0.002421	0.00466	-2.66551
ZNF229	-0.10042	3.57447	-3.29526	0.001042	0.002132	-1.89148
VASH1	-0.10043	5.61951	-2.40943	0.016282	0.026994	-4.37486
ZNF48	-0.10047	7.104769	-2.66186	0.007982	0.014041	-3.74376
CLCN4	-0.10048	4.901513	-2.0648	0.039378	0.060083	-5.13683
EFHB	-0.10071	3.180785	-2.8259	0.004874	0.008911	-3.30078
GATM	-0.10081	4.445093	-3.15762	0.001672	0.003312	-2.32644
NTPCR	-0.10089	6.439475	-2.00192	0.04575	0.068724	-5.26339
TSPAN6	-0.10098	4.734818	-2.19659	0.028437	0.044742	-4.85907
ATP5SL	-0.10098	8.066193	-2.31561	0.020921	0.03383	-4.59372
KLK5	-0.10107	4.351917	-2.32852	0.020221	0.03283	-4.56411
TRIM14	-0.10107	6.494591	-2.0229	0.043533	0.065777	-5.22159
FAF1	-0.10141	5.604362	-2.92753	0.003548	0.006639	-3.01341
CYP4B1	-0.10145	3.686562	-2.57306	0.010323	0.017783	-3.97276
C5orf42	-0.10147	4.226652	-2.1777	0.029823	0.046777	-4.89992
MPPED2	-0.10161	3.761027	-3.29731	0.001035	0.002118	-1.88485
FLJ16734	-0.10181	4.407273	-2.16493	0.030793	0.048081	-4.92735
DDX4	-0.10198	3.905463	-2.40142	0.01664	0.027534	-4.39388
CT62	-0.10199	3.95708	-2.43616	0.015138	0.025264	-4.31094
TMEM248	-0.10202	8.586853	-2.1297	0.033609	0.052006	-5.00217
SAP18	-0.10212	8.377895	-2.22015	0.026787	0.042378	-4.80764
MRPL55	-0.10216	6.32346	-2.01826	0.044015	0.066432	-5.23087
EIF2S2	-0.10218	8.252799	-2.37398	0.017916	0.029404	-4.45855
TMEM67	-0.10223	3.265141	-5.07619	5.17E-07	1.66E-06	5.314969
LOC10192	-0.1023	4.248631	-2.71004	0.006923	0.012319	-3.61634
SALL1	-0.10233	3.219573	-5.43649	7.96E-08	2.81E-07	7.117914
BRS3	-0.10233	4.876269	-2.39425	0.016965	0.028001	-4.41083
MRGPRF	-0.10233	4.723081	-2.05483	0.040335	0.061391	-5.15716
ACSM3	-0.10234	4.698352	-2.65206	0.008215	0.014427	-3.7694
PHEX	-0.1024	5.234825	-2.75871	0.005983	0.010766	-3.48534
TRAF3IP2	-0.1025	5.485887	-3.01255	0.002701	0.005162	-2.76542
DEPTOR	-0.10256	4.177682	-3.51325	0.000476	0.001025	-1.16608
PARK2	-0.10269	4.24658	-2.54729	0.011109	0.01901	-4.03781
HS3ST1	-0.10277	4.710113	-2.74147	0.006302	0.011296	-3.53199
COMMD6	-0.10287	8.527566	-2.24655	0.025037	0.03985	-4.74936
SERPINI2	-0.10301	4.628325	-3.14301	0.001756	0.003461	-2.37155

USP27X-A	-0.10306	4.761418	-2.04398	0.041398	0.062833	-5.17916
LOC10192	-0.10355	4.717719	-3.25164	0.001213	0.002458	-2.03124
CCDC113	-0.10374	3.214666	-3.55162	0.000413	0.0009	-1.03377
CDH1	-0.10374	4.033819	-2.64209	0.008458	0.014828	-3.79539
SFTA2	-0.10393	3.805569	-3.10201	0.002014	0.003936	-2.49706
LOC10192	-0.10404	6.185364	-2.45277	0.014464	0.024221	-4.27087
TRIO	-0.10416	5.223873	-3.96492	8.24E-05	0.000199	0.478103
SERGEF	-0.10435	5.325	-3.50239	0.000496	0.001064	-1.20326
ZFP28	-0.10465	4.912891	-4.24923	2.49E-05	6.43E-05	1.609524
INTS9	-0.10475	6.335399	-2.71928	0.006735	0.012011	-3.59165
NUDCD3	-0.10482	6.096345	-4.2667	2.31E-05	5.98E-05	1.68146
SNX24	-0.10485	4.593082	-4.08242	5.07E-05	0.000126	0.936715
ACSF3	-0.10511	5.680142	-2.86242	0.004353	0.008026	-3.19866
DCAF12L2	-0.10519	3.852104	-3.78247	0.000171	0.000394	-0.20886
GRID1	-0.10526	4.22014	-3.26676	0.001151	0.00234	-1.98301
UROS	-0.10531	5.834263	-2.42686	0.015528	0.025834	-4.33326
SOX30	-0.10542	3.648735	-3.63044	0.000307	0.000682	-0.7577
LINC0112{	-0.10547	4.972835	-4.79432	2.07E-06	6.15E-06	3.983528
AGPAT3	-0.10562	6.659177	-2.00538	0.045377	0.06826	-5.25651
IQCG	-0.10571	4.870724	-1.98728	0.047352	0.070925	-5.29229
ZNF213-A	-0.1058	4.086551	-2.59279	0.009756	0.016883	-3.92256
HSD17B6	-0.10585	3.783473	-3.2927	0.001052	0.00215	-1.89973
ZNF287	-0.10587	4.587769	-4.69614	3.30E-06	9.56E-06	3.536259
C10orf76	-0.10591	6.66868	-1.96939	0.049375	0.073625	-5.32734
FHL5	-0.10592	3.882221	-2.17473	0.030046	0.047082	-4.90631
PPIA	-0.10616	9.173067	-2.81468	0.005045	0.0092	-3.33191
VASH2	-0.10635	3.66553	-3.71649	0.000221	0.000502	-0.44969
MED26	-0.10648	6.810034	-3.19293	0.001483	0.002966	-2.21655
PIP4K2B	-0.10654	6.776402	-2.46865	0.013844	0.023272	-4.23233
LOC10192	-0.10674	5.065418	-1.98724	0.047357	0.070925	-5.29237
RBM45	-0.1068	5.78656	-2.88492	0.004058	0.007512	-3.13509
GNL1	-0.10687	5.316537	-3.96693	8.17E-05	0.000197	0.485847
CCDC138	-0.10704	4.083897	-3.44555	0.00061	0.001294	-1.39615
CDKN2A	-0.10722	5.289912	-2.60444	0.009434	0.016376	-3.89272
SOX13	-0.10737	5.769635	-2.34013	0.019609	0.031937	-4.53734
EFCAB4B	-0.10738	6.578984	-2.7736	0.005719	0.010326	-3.44483
ARHGAP2:	-0.10756	4.158786	-2.71105	0.006902	0.012287	-3.61364
KIAA1661	-0.10765	4.312172	-2.67649	0.007646	0.013502	-3.7053
JTB	-0.10766	11.464	-2.46051	0.014159	0.023759	-4.25212
FGF14-AS:	-0.10787	3.826571	-3.48157	0.000535	0.001145	-1.27425
RGS20	-0.10811	4.102949	-2.99112	0.002895	0.005501	-2.82859
LOC10013	-0.10814	5.192829	-2.20111	0.028114	0.044288	-4.84925
CDHR3	-0.10816	4.187942	-3.84406	0.000134	0.000314	0.019602
TIMM44	-0.10825	6.332155	-1.99809	0.046164	0.069294	-5.27096
LMBRD2	-0.10825	5.197367	-2.2636	0.023961	0.038277	-4.71137
RTN4RL2	-0.10831	4.277037	-3.25267	0.001208	0.00245	-2.02796
COPS8	-0.10834	5.989843	-2.5896	0.009845	0.017035	-3.93069
KIF17	-0.10852	5.432673	-2.42431	0.015637	0.025993	-4.33937
SYNGR3	-0.10855	4.465415	-2.70236	0.007083	0.01259	-3.63679
HSD17B2	-0.10871	4.259997	-2.77427	0.005707	0.010309	-3.44298
KLHL13	-0.10884	3.423558	-3.0642	0.002282	0.004415	-2.61143
KANK1	-0.10908	5.797019	-2.4713	0.013743	0.023119	-4.22587
FAM160B2	-0.1091	6.140977	-3.02731	0.002575	0.004936	-2.72167
CCDC157	-0.10914	5.020621	-2.3691	0.018152	0.029764	-4.46998
DAPK3	-0.10918	3.774437	-2.61731	0.00909	0.015834	-3.85961
UACA	-0.10936	5.5948	-3.58146	0.00037	0.000811	-0.92994
C9orf135	-0.10946	4.246674	-3.38356	0.000763	0.001597	-1.60303
LOC10012	-0.10963	3.321168	-4.10574	4.60E-05	0.000115	1.029268

PRSS16	-0.10968	4.350462	-2.96792	0.00312	0.005899	-2.89646
SLC4A8	-0.10979	5.151383	-3.45181	0.000597	0.001268	-1.37502
SPCS2	-0.10983	8.301866	-2.03792	0.042002	0.06368	-5.19139
PPP2R2D	-0.1099	6.485088	-2.04063	0.041732	0.063318	-5.18594
SRMS	-0.10992	4.106407	-2.74168	0.006298	0.01129	-3.53144
AEN	-0.11026	6.666564	-1.96626	0.049736	0.074094	-5.33344
SEC61A2	-0.11059	5.658301	-2.58249	0.010048	0.017353	-3.9488
SLC22A6	-0.11067	4.965132	-2.17772	0.029821	0.046777	-4.89987
TNFSF9	-0.11069	4.406196	-2.40938	0.016284	0.026995	-4.37498
PGF	-0.11071	7.515664	-2.35354	0.018922	0.030924	-4.50627
GNGT1	-0.1109	3.478092	-3.39979	0.00072	0.001514	-1.54921
SOX7	-0.11092	3.78819	-3.27872	0.001104	0.002251	-1.94467
LINC01405	-0.11097	3.629471	-2.09891	0.036248	0.055721	-5.06655
PREP	-0.11103	6.273374	-2.15546	0.031529	0.049121	-4.94758
TMEM132	-0.1111	5.191431	-2.03687	0.042108	0.063809	-5.19352
COLGALT2	-0.11116	4.932365	-3.03244	0.002532	0.004859	-2.70643
CELA3B	-0.11116	5.653537	-2.19889	0.028273	0.044503	-4.85408
GPR25	-0.1113	4.051257	-2.00207	0.045733	0.068704	-5.26308
NHLRC2	-0.11131	4.553642	-3.05102	0.002383	0.00459	-2.65095
ZNF551	-0.11151	4.937888	-3.74547	0.000198	0.000451	-0.3444
SCNN1G	-0.11151	4.345367	-4.5409	6.79E-06	1.89E-05	2.84662
CELSR3-A	-0.1117	4.890174	-2.88092	0.004109	0.007602	-3.14642
FSCN2	-0.11176	4.164433	-2.52464	0.011842	0.020167	-4.09446
FOXA3	-0.11177	4.711181	-2.55672	0.010815	0.01855	-4.0141
AHCYL1	-0.11177	7.648414	-2.5274	0.011751	0.020024	-4.08757
NINL	-0.11177	4.871104	-2.41234	0.016154	0.026806	-4.36793
TEF	-0.11178	4.569907	-3.6622	0.000272	0.000609	-0.64481
SAMM50	-0.11193	6.436961	-2.79686	0.005328	0.009672	-3.38107
EHD4	-0.11203	5.505591	-2.80006	0.005276	0.00959	-3.37227
UBE2E2-A	-0.11236	4.686342	-2.81291	0.005073	0.009245	-3.3368
MROH9	-0.11238	3.466137	-3.15278	0.001699	0.003362	-2.34139
NKAPL	-0.11256	4.207969	-3.14562	0.001741	0.003435	-2.3635
ZNF616	-0.1126	3.673128	-2.38081	0.01759	0.02893	-4.44251
PPP2R2A	-0.11267	7.099549	-2.88078	0.004111	0.007604	-3.14683
PTPN20B	-0.11268	3.347994	-4.29893	2.01E-05	5.24E-05	1.814874
GNG7	-0.11268	6.304708	-2.10313	0.035877	0.055208	-5.05779
ZNF510	-0.11269	5.94656	-2.11416	0.03492	0.053903	-5.03479
SLC24A1	-0.11291	4.911906	-4.76614	2.37E-06	6.98E-06	3.854293
ZNF257	-0.11293	3.733204	-2.6076	0.009348	0.016245	-3.88461
AQP11	-0.11296	4.863364	-2.39933	0.016734	0.027668	-4.39882
EPB41L2	-0.11305	5.038163	-2.57846	0.010165	0.017533	-3.95907
LHFPL3-A	-0.11307	3.397698	-3.66236	0.000272	0.000609	-0.64424
LSM3	-0.1132	6.21039	-3.1613	0.001651	0.003274	-2.31502
FBXL3	-0.11322	8.351695	-2.05878	0.039953	0.060889	-5.14911
NECAP2	-0.11347	7.381525	-2.23177	0.026004	0.04124	-4.78206
LCAT	-0.11347	5.13916	-2.59441	0.00971	0.016811	-3.9184
ADGB	-0.11359	3.453516	-4.8427	1.64E-06	4.94E-06	4.207078
MNAT1	-0.11361	5.357278	-2.15625	0.031467	0.049041	-4.94589
SH3PXD2A	-0.11376	5.326756	-3.82999	0.000142	0.000331	-0.03287
C2orf44	-0.11392	5.268002	-2.27214	0.023437	0.037503	-4.69223
TIFAB	-0.11393	5.407411	-3.09028	0.002094	0.004081	-2.53269
ADAMTS1	-0.11398	5.781904	-1.99088	0.046954	0.070404	-5.2852
FAM92A1	-0.11403	3.702614	-3.48934	0.00052	0.001114	-1.24783
METTL21B	-0.11403	5.052307	-2.19948	0.02823	0.044444	-4.85279
EFTUD2	-0.11406	7.329989	-2.6636	0.007942	0.013973	-3.73921
C3orf67	-0.11412	3.21842	-4.42442	1.15E-05	3.10E-05	2.343382
MCF2L	-0.11421	5.526303	-4.04989	5.81E-05	0.000143	0.808466
SPIRE1	-0.1143	5.226751	-2.23636	0.025701	0.040781	-4.77194

FAM47B	-0.11438	3.735792	-3.56209	0.000398	0.000868	-0.99743
KIRREL3	-0.11456	4.921366	-2.92715	0.003552	0.006646	-3.0145
VWDE	-0.11461	3.230864	-4.0169	6.66E-05	0.000163	0.679405
TMEM155	-0.11469	3.713852	-3.49961	0.000501	0.001075	-1.21276
IFNB1	-0.1147	4.611933	-2.29531	0.022065	0.035484	-4.63996
HPCAL4	-0.11483	5.242876	-3.97772	7.82E-05	0.000189	0.527443
THEM6	-0.11497	5.826398	-2.22631	0.026369	0.041783	-4.79409
TAF1	-0.11512	6.6591	-4.34732	1.62E-05	4.28E-05	2.016968
RBBP9	-0.11525	4.779616	-4.4102	1.23E-05	3.29E-05	2.282777
NTHL1	-0.11544	4.903037	-2.08793	0.037232	0.057107	-5.0893
LINC0108:	-0.11545	4.538847	-2.14208	0.032595	0.050612	-4.97601
LOC10050	-0.11546	3.616839	-2.72852	0.006551	0.011702	-3.56687
FOXJ3	-0.11577	7.200328	-3.48256	0.000533	0.001141	-1.2709
SPINK1	-0.11582	4.190457	-3.17231	0.001591	0.003165	-2.28084
SBSN	-0.11585	5.072795	-2.93188	0.0035	0.006554	-3.00088
TMEM177	-0.11588	5.332424	-3.26337	0.001164	0.002367	-1.99383
NACAD	-0.11591	4.776538	-2.09697	0.036421	0.055957	-5.07059
ZNF397	-0.11612	5.217089	-2.25725	0.024357	0.038862	-4.72555
ENTPD4	-0.11618	8.182923	-2.67452	0.007691	0.01357	-3.71049
CADM1	-0.1162	5.021728	-3.06759	0.002257	0.004369	-2.60122
OCM2	-0.11638	4.750084	-2.81462	0.005046	0.009201	-3.33206
WRNIP1	-0.11642	5.547939	-4.87038	1.43E-06	4.35E-06	4.335885
BRD8	-0.11645	6.31175	-3.18014	0.001549	0.003091	-2.25648
LRRC7	-0.11645	3.217848	-6.37684	3.65E-10	1.69E-09	12.34251
MMP7	-0.11646	4.152227	-2.94422	0.003365	0.006321	-2.96527
LRRC34	-0.11647	3.481918	-4.06483	5.46E-05	0.000134	0.867263
MROH1	-0.11683	5.856292	-2.90684	0.003788	0.007047	-3.07272
FDFT1	-0.11709	8.743638	-2.2738	0.023336	0.037361	-4.68849
DHX30	-0.11722	6.016505	-2.62054	0.009005	0.015703	-3.85127
PCDH1	-0.11729	5.778335	-2.13696	0.033011	0.0512	-4.98684
UROD	-0.1173	6.393511	-2.24846	0.024914	0.039668	-4.74511
DDX19B	-0.11734	5.791345	-2.09956	0.036191	0.055637	-5.06521
FLJ42627	-0.11735	6.491298	-2.97032	0.003096	0.005857	-2.88946
TM4SF20	-0.1174	3.874346	-3.17038	0.001601	0.003183	-2.28685
CCDC85C	-0.11741	5.519349	-3.181	0.001545	0.003083	-2.25379
BATF3	-0.11745	6.707598	-2.26998	0.023568	0.037701	-4.69708
NPM3	-0.11756	6.192321	-1.98635	0.047456	0.071045	-5.29413
RREB1	-0.11762	6.039832	-4.35898	1.54E-05	4.09E-05	2.065989
MYL7	-0.11763	5.59849	-2.3554	0.018829	0.030793	-4.50194
CHGB	-0.11777	4.393528	-2.84248	0.004631	0.008505	-3.25457
LOC28605	-0.11778	5.118388	-3.5594	0.000402	0.000877	-1.00679
PLXNA3	-0.11784	5.519899	-2.35121	0.01904	0.031102	-4.51168
PGM5-AS:	-0.11796	5.397722	-2.11335	0.034989	0.053991	-5.03647
RNF41	-0.11823	6.698233	-2.2618	0.024072	0.038442	-4.71539
LOC10192	-0.11828	4.566764	-3.30727	0.000999	0.002051	-1.85268
ETV3	-0.11847	6.697197	-2.75739	0.006007	0.010804	-3.48893
RBM48	-0.11851	5.584093	-2.35918	0.01864	0.030509	-4.49314
REG3A	-0.11855	4.647385	-3.38402	0.000762	0.001595	-1.60149
APLF	-0.11863	3.3933	-3.03529	0.002509	0.004816	-2.69795
ZSCAN22	-0.11865	5.380028	-2.74363	0.006261	0.011229	-3.52617
TMEM27	-0.1187	3.229313	-4.02049	6.56E-05	0.00016	0.693417
OLFM1	-0.11875	5.521411	-2.44778	0.014664	0.024533	-4.28294
SYCP2L	-0.11881	4.57868	-2.25152	0.024719	0.039378	-4.73831
USP9X	-0.11898	8.850571	-2.14698	0.032201	0.050056	-4.96561
IKZF1	-0.119	8.342985	-1.99097	0.046944	0.070395	-5.28503
SGSM1	-0.1191	5.023375	-3.52654	0.000454	0.000981	-1.1204
ATF2	-0.11911	7.021915	-2.31582	0.020909	0.033821	-4.59324
FAT4	-0.11932	3.756634	-3.33403	0.00091	0.00188	-1.76572

PANX1	-0.11938	5.93331	-2.05727	0.040098	0.061079	-5.15218
RNF151	-0.11942	5.394157	-2.47519	0.013596	0.022898	-4.21637
MSX2	-0.11943	3.925267	-5.7711	1.27E-08	4.90E-08	8.892126
COX6A2	-0.11949	5.453872	-2.58858	0.009874	0.017074	-3.9333
CLCN5	-0.11964	4.925164	-2.36269	0.018466	0.030235	-4.48496
DNAJC18	-0.11977	5.22297	-3.67249	0.000262	0.000588	-0.60803
RPL39L	-0.11977	4.3939	-2.13351	0.033294	0.051574	-4.99413
SLC35E1	-0.11977	7.630169	-2.64198	0.008461	0.014831	-3.7957
CROCCP3	-0.11977	5.024247	-2.86934	0.00426	0.007867	-3.17915
ADSSL1	-0.11978	5.510301	-3.1371	0.001791	0.003524	-2.38975
KRT81	-0.11979	6.114953	-2.05891	0.039941	0.060875	-5.14885
LAMC3	-0.11988	5.349254	-2.92684	0.003556	0.006652	-3.01539
PLA2G6	-0.11991	6.926951	-2.82242	0.004927	0.008999	-3.31045
LINC01125	-0.12009	5.267354	-2.43315	0.015263	0.025438	-4.31816
TBC1D9B	-0.12012	7.887335	-2.47203	0.013715	0.023085	-4.22409
MUC16	-0.12025	4.182841	-3.17215	0.001592	0.003166	-2.28134
DDX11	-0.12043	6.096699	-2.15492	0.031571	0.049178	-4.94872
RCBTB1	-0.12045	5.899558	-1.97802	0.04839	0.0723	-5.31047
TPTE2P6	-0.12062	3.238829	-4.11578	4.41E-05	0.00011	1.069264
STAM-AS1	-0.12068	3.371391	-4.09249	4.86E-05	0.000121	0.976637
LOC10013	-0.12076	3.753869	-2.32574	0.02037	0.033045	-4.5705
CDON	-0.12084	4.915934	-4.16755	3.54E-05	8.97E-05	1.27693
XPO5	-0.12085	6.334405	-2.94732	0.003332	0.006264	-2.95631
GRWD1	-0.12087	6.529689	-2.39836	0.016778	0.027734	-4.40112
GNAL	-0.1209	4.155236	-3.82463	0.000145	0.000338	-0.05286
FBXO15	-0.12107	5.058784	-2.6159	0.009127	0.015891	-3.86323
ELOVL5	-0.12109	8.280549	-2.38143	0.017561	0.028897	-4.44105
NAG18	-0.12123	3.806889	-3.22393	0.001334	0.002682	-2.11912
CTAGE5	-0.12126	5.996843	-2.09645	0.036467	0.056024	-5.07167
ANKMY1	-0.1213	5.274208	-3.43304	0.000639	0.001351	-1.43818
CTNNA2	-0.1218	4.428598	-3.07858	0.002176	0.004227	-2.56809
DYNC1H1	-0.12184	5.753466	-3.16992	0.001604	0.003188	-2.28829
LINC00629	-0.1219	3.793664	-4.08235	5.07E-05	0.000126	0.936437
HUS1	-0.12191	5.902621	-2.18459	0.029311	0.04603	-4.88507
IPO8	-0.122	7.556563	-2.82746	0.004851	0.008869	-3.29646
MRPS31	-0.12205	6.770881	-2.22021	0.026783	0.042375	-4.8075
SYS1	-0.12208	6.613389	-2.83591	0.004726	0.008665	-3.27291
EXPH5	-0.12212	4.548176	-5.50085	5.63E-08	2.02E-07	7.451773
MAN1A2	-0.12218	5.705209	-3.00315	0.002785	0.005309	-2.79317
CCDC136	-0.12242	4.59542	-2.56433	0.010583	0.018193	-3.99487
GEM	-0.12252	4.312586	-2.58183	0.010067	0.017384	-3.95049
LINC00487	-0.12252	3.901111	-2.93246	0.003493	0.006544	-2.99922
HIC1	-0.12254	4.56086	-2.39653	0.016861	0.027859	-4.40545
PEX11A	-0.1226	4.94383	-2.78697	0.005491	0.00995	-3.40826
DEFB1	-0.12261	5.049353	-2.81809	0.004993	0.009111	-3.32247
LOC10013	-0.12284	3.697734	-2.01329	0.044537	0.067141	-5.24079
POLR3A	-0.12285	6.464257	-2.61858	0.009056	0.015788	-3.85633
LOC10012	-0.12287	5.83995	-2.41176	0.01618	0.026843	-4.36931
SPN	-0.12292	5.622881	-2.14237	0.032571	0.050579	-4.97538
RHBDD1	-0.12308	6.046767	-4.07448	5.24E-05	0.000129	0.905342
PSMF1	-0.12311	6.901204	-2.62921	0.008781	0.01534	-3.82885
LOC10192	-0.12327	5.24372	-3.33253	0.000914	0.00189	-1.77061
MGARP	-0.12335	4.526245	-2.16627	0.03069	0.047937	-4.92448
LOC10192	-0.12397	5.429076	-3.02553	0.00259	0.004963	-2.72697
WASL	-0.12408	5.745367	-2.34105	0.019561	0.031867	-4.53521
LRRC20	-0.12429	5.709957	-2.11223	0.035086	0.054113	-5.03882
SNX25	-0.1243	4.86126	-3.69215	0.000243	0.000547	-0.53751
TNFSF11	-0.12434	4.037911	-4.61903	4.73E-06	1.34E-05	3.191039

HIST1H4E	-0.12441	5.39675	-2.13735	0.032979	0.051155	-4.98601
LPA	-0.12465	3.522752	-4.41676	1.19E-05	3.20E-05	2.310698
LRRIQ1	-0.125	4.131081	-3.17124	0.001597	0.003174	-2.28419
FARP2	-0.12509	6.141382	-3.49913	0.000502	0.001076	-1.21442
TIMM13	-0.12518	5.277146	-2.65686	0.0081	0.014236	-3.75687
LOC72861	-0.12527	4.982915	-2.06726	0.039144	0.059754	-5.13179
ADAM23	-0.12532	4.497656	-4.53391	7.01E-06	1.95E-05	2.816087
TMEM182	-0.12556	3.754437	-4.6168	4.78E-06	1.36E-05	3.181127
DNAJC27	-0.12557	5.048972	-2.15215	0.03179	0.049472	-4.95463
LINC0126(	-0.12564	5.879067	-3.1879	0.001509	0.003015	-2.23229
STAG2	-0.12574	10.20069	-2.30877	0.0213	0.034388	-4.60933
ZNF541	-0.12575	5.119363	-2.58593	0.00995	0.017197	-3.94005
FOXK1	-0.12582	5.913184	-3.80635	0.000156	0.000361	-0.12067
PKDCC	-0.12586	5.637442	-2.04615	0.041184	0.062549	-5.17477
TSPYL6	-0.12587	3.347128	-3.69135	0.000244	0.000549	-0.54037
TENM3	-0.12589	4.597134	-2.61284	0.009208	0.016022	-3.87112
DAB1	-0.12638	4.783084	-3.74558	0.000198	0.000451	-0.34401
FAF2	-0.12641	7.54325	-2.27064	0.023528	0.037639	-4.6956
LOC54944	-0.12665	4.321304	-2.96963	0.003103	0.005869	-2.89148
UBN2	-0.12676	5.953816	-3.66162	0.000273	0.000611	-0.6469
EPM2A	-0.12697	5.228789	-4.70404	3.18E-06	9.23E-06	3.571954
LIMD1	-0.12697	5.943363	-4.37619	1.43E-05	3.79E-05	2.138577
DNER	-0.12704	3.222686	-3.69904	0.000237	0.000534	-0.51272
HPS3	-0.12705	6.315352	-1.96851	0.049476	0.073759	-5.32906
6-Mar	-0.12705	7.038687	-4.2281	2.73E-05	7.01E-05	1.52289
PTPMT1	-0.12715	5.798642	-2.65625	0.008115	0.01426	-3.75845
SERPINF2	-0.12723	5.321533	-2.61595	0.009125	0.015891	-3.86311
PTGR2	-0.12736	3.034317	-3.37469	0.000787	0.001644	-1.63231
WDR12	-0.12756	5.061996	-3.00105	0.002804	0.005342	-2.79938
NAB2	-0.12763	5.889772	-2.9426	0.003382	0.006349	-2.96995
ZNF473	-0.12774	5.849257	-3.39069	0.000744	0.001559	-1.57939
EMC9	-0.12778	6.375237	-2.35004	0.019099	0.031188	-4.51438
SNHG22	-0.12782	4.174435	-3.15241	0.001701	0.003366	-2.34252
SIRT4	-0.12814	4.26951	-4.40309	1.27E-05	3.39E-05	2.252514
RIBC1	-0.12833	4.935715	-3.59506	0.000351	0.000773	-0.88233
GDAP1L1	-0.12833	5.437729	-2.05684	0.04014	0.061133	-5.15306
TIMM8A	-0.12834	4.142193	-2.7631	0.005904	0.01063	-3.47342
LOC14935	-0.12845	3.138448	-5.64882	2.51E-08	9.37E-08	8.232728
PUS7L	-0.1285	5.829835	-2.02097	0.043733	0.06605	-5.22545
IMPAD1	-0.12851	5.753255	-2.91025	0.003747	0.006978	-3.06297
FAM91A1	-0.12852	7.65363	-2.90426	0.003819	0.0071	-3.08008
LOC10013	-0.1289	5.442384	-3.09207	0.002081	0.004059	-2.52726
LMOD2	-0.12915	3.96099	-3.06045	0.00231	0.004465	-2.62268
POF1B	-0.12919	4.098828	-2.88672	0.004035	0.007476	-3.12998
LIPC	-0.12919	4.931813	-2.32227	0.020557	0.033308	-4.57847
PER2	-0.12921	6.611127	-1.98949	0.047108	0.070599	-5.28794
RMND5A	-0.12921	7.783597	-2.31674	0.020859	0.033745	-4.59113
POM121L1	-0.12925	4.685304	-2.92089	0.003623	0.006768	-3.03247
NRTN	-0.12973	3.475729	-3.24745	0.00123	0.002491	-2.04459
ATXN1L	-0.12977	8.147054	-2.46371	0.014034	0.023565	-4.24433
LOC10013	-0.12981	4.617892	-2.7658	0.005856	0.010554	-3.46607
GRK1	-0.12984	5.554224	-1.9868	0.047406	0.070994	-5.29325
XRCC5	-0.12986	8.658817	-2.2992	0.021841	0.035168	-4.63112
ZNF446	-0.12997	7.074335	-3.05772	0.002331	0.004499	-2.63088
PSMC3IP	-0.13016	5.882931	-3.37171	0.000796	0.001661	-1.64214
ZSWIM5	-0.13026	5.042739	-2.88517	0.004055	0.007508	-3.13437
TMEM25	-0.13027	5.099005	-3.52798	0.000451	0.000976	-1.11543
WDR44	-0.1303	6.449282	-2.99876	0.002825	0.005378	-2.80612

ADIPOR2	-0.13051	8.683868	-2.34141	0.019542	0.03184	-4.53438
RFESD	-0.13059	4.913017	-2.16703	0.030632	0.047862	-4.92285
LINC0054	-0.13069	4.842549	-2.36721	0.018244	0.029899	-4.47439
RASSF5	-0.13078	11.10233	-2.08918	0.037119	0.056948	-5.08672
BCKDHA	-0.13083	7.952996	-2.54507	0.011178	0.019113	-4.04337
SDHA	-0.13087	9.024889	-2.31963	0.020701	0.03351	-4.58451
KIAA1432	-0.13098	5.929768	-2.52954	0.01168	0.01992	-4.08223
USP51	-0.13101	4.628941	-3.99542	7.27E-05	0.000177	0.595923
LINC0108	-0.13106	5.56476	-2.98374	0.002965	0.005624	-2.85022
FAM47C	-0.13121	4.417205	-3.28628	0.001075	0.002196	-1.9204
DICER1	-0.13125	7.580583	-2.57622	0.01023	0.017632	-3.96475
SCRN2	-0.13135	6.197989	-2.66401	0.007932	0.01396	-3.73811
SDK2	-0.13149	5.656284	-4.45008	1.03E-05	2.78E-05	2.453181
SNIP1	-0.13151	6.439417	-2.28571	0.022624	0.036316	-4.66167
ATPAF2	-0.13156	5.709077	-3.11472	0.00193	0.00378	-2.45832
CTBP1-AS	-0.1317	5.932664	-3.1798	0.001551	0.003093	-2.25753
REST	-0.1317	7.719359	-2.75049	0.006133	0.01101	-3.50762
MAT2B	-0.1318	8.288461	-3.55463	0.000409	0.00089	-1.02333
FGF4	-0.13198	5.887853	-2.87577	0.004176	0.007718	-3.16098
LINC0085	-0.13202	5.255248	-2.00402	0.045524	0.068447	-5.25923
COA7	-0.13211	6.01992	-2.96867	0.003112	0.005886	-2.89428
CMTM3	-0.13213	8.955311	-2.2561	0.024429	0.03897	-4.7281
TMEM237	-0.13224	4.68877	-2.93715	0.003441	0.006455	-2.98568
BLOC1S3	-0.13228	6.21325	-3.11114	0.001954	0.003824	-2.46926
LOC10050	-0.13239	6.067598	-2.99942	0.002819	0.005368	-2.80416
SRP19	-0.13239	9.698841	-2.37843	0.017703	0.029089	-4.44811
AMT	-0.13252	7.159223	-2.12348	0.034128	0.052762	-5.01524
ZBTB43	-0.13253	6.623492	-2.32309	0.020513	0.033239	-4.57658
C18orf21	-0.13254	7.785761	-2.30672	0.021415	0.034559	-4.61401
HDAC2	-0.13259	6.849161	-2.56505	0.010562	0.018162	-3.99306
HEXA-AS1	-0.13265	3.810711	-4.78925	2.12E-06	6.30E-06	3.960243
ZNF883	-0.13271	3.383686	-3.86213	0.000125	0.000294	0.087298
TMEM262	-0.13291	5.497644	-2.0682	0.039055	0.059631	-5.12987
FAM192A	-0.13293	8.417331	-3.56014	0.0004	0.000874	-1.00422
ERRFI1	-0.13338	4.781778	-2.07494	0.038424	0.058751	-5.11605
TRIM46	-0.1335	5.350421	-3.74543	0.000198	0.000451	-0.34456
TDRD6	-0.13375	3.734663	-3.66737	0.000267	0.000598	-0.62635
SLC7A10	-0.13376	4.943214	-2.42737	0.015506	0.0258	-4.33203
GRAP	-0.13386	5.808211	-2.3319	0.020041	0.032559	-4.55634
PM20D1	-0.13393	4.893481	-3.11556	0.001925	0.003771	-2.45575
LOC15786	-0.13397	4.46992	-1.97286	0.048977	0.073098	-5.32056
LINC0096	-0.13398	4.966885	-2.77162	0.005753	0.010384	-3.45021
DEFB123	-0.13426	5.338665	-2.85319	0.00448	0.008246	-3.22457
EGLN2	-0.13444	4.52789	-2.3547	0.018864	0.030834	-4.50357
C1orf213	-0.1345	5.010341	-3.41656	0.000678	0.00143	-1.49333
DCTPP1	-0.13462	7.914378	-2.22785	0.026266	0.041623	-4.79072
ERCC3	-0.13464	6.925554	-1.96628	0.049734	0.074094	-5.3334
ABCA3	-0.13471	6.093847	-2.74271	0.006278	0.011258	-3.52865
PRO0471	-0.1348	3.506786	-2.58354	0.010018	0.017306	-3.94614
LOC10013	-0.13481	4.265657	-3.81285	0.000152	0.000353	-0.09661
CCDC149	-0.13486	5.967887	-2.27481	0.023274	0.037277	-4.68622
ELFN1	-0.1349	5.444634	-3.00002	0.002813	0.005359	-2.80239
TBL3	-0.13491	6.580902	-1.982	0.047942	0.071725	-5.30267
RPP25	-0.13524	5.526621	-2.91344	0.00371	0.006915	-3.05385
GUCY2D	-0.13536	5.082538	-3.2401	0.001262	0.002549	-2.06792
RAP1GAP	-0.13537	9.679064	-2.20049	0.028158	0.044342	-4.85059
ZNF3	-0.13539	5.098258	-5.10071	4.56E-07	1.47E-06	5.434094
PGBD2	-0.13541	5.802389	-3.71521	0.000222	0.000504	-0.45432

TBCEL	-0.13547	5.069389	-2.60062	0.009538	0.016538	-3.90252
RAMP1	-0.13569	6.104517	-2.41397	0.016083	0.0267	-4.36405
FASTK	-0.13576	7.887247	-2.21766	0.026957	0.042629	-4.8131
COX5B	-0.13594	9.404332	-3.08763	0.002112	0.004113	-2.54073
TSNARE1	-0.13608	6.323181	-3.22322	0.001337	0.002688	-2.12134
VPS16	-0.13629	6.477346	-3.97422	7.93E-05	0.000192	0.513943
KCNH2	-0.13638	5.685233	-2.69499	0.007239	0.012841	-3.65638
DHX37	-0.13647	5.631396	-2.4259	0.015569	0.025897	-4.33556
LOC10012	-0.13662	4.979476	-2.3101	0.021226	0.034284	-4.60631
GAB3	-0.13693	6.490275	-2.44119	0.014931	0.024946	-4.29883
NTRK1	-0.13698	3.994995	-2.64943	0.008278	0.014531	-3.77627
RAP2B	-0.13714	6.862042	-3.04328	0.002444	0.004699	-2.6741
CYB561D2	-0.13716	6.775689	-2.43758	0.01508	0.025173	-4.30753
C11orf95	-0.13722	5.531473	-2.8142	0.005053	0.009211	-3.33323
ZNF165	-0.13729	3.572288	-4.14271	3.93E-05	9.90E-05	1.176981
SF3B2	-0.13729	9.31693	-2.18045	0.029618	0.046479	-4.89399
PDRG1	-0.13733	7.308862	-3.20646	0.001416	0.002839	-2.17414
SPATA42	-0.13735	3.464839	-3.86111	0.000125	0.000295	0.083464
CTLA4	-0.13735	5.331556	-3.9822	7.68E-05	0.000186	0.544747
EIF1	-0.13737	10.30944	-2.1519	0.031809	0.049498	-4.95515
PTPRH	-0.13737	5.135081	-2.26919	0.023616	0.037771	-4.69885
GPR125	-0.13743	4.034801	-2.38138	0.017563	0.028898	-4.44117
SNTN	-0.13745	3.185706	-5.0358	6.33E-07	2.01E-06	5.119873
USP44	-0.13756	4.179669	-3.05152	0.002379	0.004583	-2.64947
AP1G2	-0.13774	6.930573	-2.11354	0.034973	0.053971	-5.03607
C3orf35	-0.13775	3.905793	-3.13234	0.00182	0.003576	-2.40435
FABP6	-0.13814	4.428166	-2.37603	0.017818	0.029259	-4.45375
SEC1P	-0.1383	5.015615	-2.74996	0.006143	0.011026	-3.50906
TMBIM4	-0.13832	11.348	-3.08967	0.002098	0.004088	-2.53455
GCNT2	-0.13839	4.940427	-2.32077	0.020639	0.033428	-4.58191
CYB561D1	-0.13852	8.478936	-2.46611	0.013942	0.023423	-4.23851
LOC10050	-0.13854	4.33797	-3.13649	0.001795	0.003531	-2.39162
LOC10050	-0.13873	4.676507	-3.51037	0.000482	0.001036	-1.17595
DMRTC2	-0.13877	4.090803	-3.74491	0.000198	0.000452	-0.34645
ZNF674	-0.13879	3.325283	-5.20533	2.68E-07	8.86E-07	5.948241
CDPF1	-0.13891	5.717734	-2.56737	0.010492	0.01805	-3.98719
HOXA3	-0.13891	4.884379	-3.68076	0.000254	0.00057	-0.57842
CLDN12	-0.13901	4.194635	-2.15715	0.031396	0.048947	-4.94396
COPS4	-0.13915	5.888084	-1.99289	0.046733	0.070095	-5.28124
NME5	-0.13921	4.484761	-3.68006	0.000254	0.000572	-0.58092
B4GALT6	-0.13924	4.472684	-3.60112	0.000343	0.000757	-0.86107
UQCRB	-0.13944	8.234769	-2.34549	0.019332	0.031525	-4.52493
WNT7A	-0.1395	5.599481	-2.51145	0.012289	0.020859	-4.12721
MTMR14	-0.13964	7.628299	-2.70535	0.00702	0.012484	-3.62883
LOC10192	-0.13965	4.483774	-3.17515	0.001576	0.003137	-2.27201
SETD7	-0.13977	6.002782	-2.37757	0.017744	0.029146	-4.45014
SUV39H1	-0.14022	6.399512	-2.52731	0.011754	0.020027	-4.08781
DKFZP434	-0.14031	5.153133	-3.21747	0.001364	0.002738	-2.1395
RSU1	-0.14043	7.246394	-2.51195	0.012272	0.020834	-4.12596
GOLIM4	-0.14053	5.889684	-2.15783	0.031343	0.048882	-4.94252
PPFIBP1	-0.14059	4.366704	-5.23061	2.35E-07	7.84E-07	6.073886
ARVCF	-0.14059	7.312136	-2.39167	0.017084	0.028173	-4.41695
RHEB	-0.1407	6.068814	-5.13277	3.88E-07	1.26E-06	5.590606
IFT57	-0.14097	5.580781	-2.10295	0.035892	0.055225	-5.05816
STK38	-0.14108	7.605879	-4.63505	4.39E-06	1.25E-05	3.26232
SFT2D3	-0.14111	6.469574	-3.8564	0.000128	0.0003	0.065788
XRCC1	-0.14115	6.881415	-2.14324	0.032501	0.050483	-4.97354
LTA	-0.14128	5.400296	-2.82304	0.004917	0.008985	-3.30873

LOC80154	-0.14134	6.078037	-3.87825	0.000117	0.000276	0.147945
TRMT44	-0.14136	4.370199	-5.66064	2.35E-08	8.81E-08	8.295928
ZNF638	-0.14147	9.543943	-2.49503	0.012866	0.021747	-4.16773
TADA2A	-0.14151	5.148425	-3.32088	0.000953	0.001963	-1.80852
ZNF80	-0.1416	3.339091	-5.37129	1.13E-07	3.89E-07	6.783332
BRK1	-0.1416	7.978713	-3.44705	0.000607	0.001288	-1.39107
LOC10050	-0.14163	5.940361	-2.76226	0.005919	0.010655	-3.47571
RPGRIP1L	-0.14171	3.789209	-5.17	3.21E-07	1.05E-06	5.773549
ADAM12	-0.14176	4.65253	-4.7546	2.50E-06	7.36E-06	3.801558
TMEM8B	-0.14194	5.817295	-3.79765	0.000161	0.000373	-0.15287
ZNF800	-0.14203	6.190684	-2.16228	0.030997	0.048375	-4.93301
C21orf33	-0.14212	7.378525	-2.03999	0.041795	0.063397	-5.18721
TNKS1BP1	-0.14218	4.552077	-2.50214	0.012614	0.021361	-4.15021
SLC45A3	-0.14235	5.701371	-3.36899	0.000804	0.001676	-1.65111
IP6K3	-0.14243	4.310212	-3.55445	0.000409	0.000891	-1.02397
SMAD9	-0.14244	4.667388	-2.61146	0.009245	0.016078	-3.87467
COL24A1	-0.14259	3.673629	-4.03889	6.08E-05	0.000149	0.765332
SGCE	-0.14262	4.264542	-2.40842	0.016327	0.027058	-4.37725
KIAA0087	-0.14267	3.561966	-5.65546	2.42E-08	9.05E-08	8.268203
OGFOD3	-0.1428	5.963514	-2.64837	0.008304	0.014568	-3.77904
MTNR1B	-0.14288	5.656667	-3.91368	0.000101	0.000242	0.282069
MIRLET7D	-0.14321	6.418394	-2.19027	0.028895	0.045411	-4.87279
FGD6	-0.14336	4.174428	-4.15803	3.69E-05	9.31E-05	1.238558
KDM8	-0.14345	5.769474	-3.12597	0.001859	0.003648	-2.4239
TXLNB	-0.14353	3.37058	-4.51305	7.71E-06	2.13E-05	2.725188
DYNC1LI2	-0.1437	7.411002	-3.04122	0.002461	0.004729	-2.68025
C12orf49	-0.14385	7.273837	-2.63793	0.008561	0.014995	-3.80623
ARAP3	-0.14389	7.52999	-2.17357	0.030133	0.047198	-4.9088
LRRC19	-0.14393	3.359707	-5.4225	8.58E-08	3.02E-07	7.045797
GET4	-0.14407	7.055367	-2.40529	0.016466	0.027273	-4.38468
C16orf62	-0.14417	7.390821	-2.67752	0.007623	0.013466	-3.70259
MRTO4	-0.14419	5.707931	-2.4582	0.014249	0.023893	-4.25773
FBXO7	-0.14456	7.170835	-2.1207	0.034363	0.053106	-5.02108
ZFP91	-0.14457	7.884281	-2.73476	0.00643	0.01151	-3.55009
C12orf42	-0.14459	4.720881	-3.31047	0.000988	0.002031	-1.84231
TPBG	-0.14471	4.221354	-3.24562	0.001238	0.002505	-2.05039
GS1-259H	-0.14473	5.01335	-3.61332	0.000328	0.000725	-0.81815
LINC0066[illegible]	-0.14477	4.45434	-4.67415	3.66E-06	1.05E-05	3.437275
GSTA4	-0.14487	5.019326	-3.45006	0.0006	0.001275	-1.38093
RBM17	-0.14491	7.509005	-3.88808	0.000112	0.000266	0.185021
LAMP5	-0.14497	5.905012	-1.9677	0.04957	0.073873	-5.33063
CXCL9	-0.14498	5.503498	-2.19841	0.028307	0.044553	-4.85513
WWOX	-0.14501	5.691042	-4.82375	1.79E-06	5.39E-06	4.119269
RASA3	-0.14507	6.228124	-2.21646	0.02704	0.042744	-4.81572
TNPO2	-0.14507	6.292646	-2.77111	0.005762	0.010399	-3.4516
TMEM220	-0.14516	4.741833	-3.14252	0.001759	0.003465	-2.37305
RPN1	-0.14518	9.185323	-2.488	0.013121	0.022152	-4.185
ISCA2	-0.14525	8.233613	-2.08649	0.037363	0.057288	-5.09229
RPN2	-0.14527	10.24136	-1.97307	0.048953	0.073068	-5.32015
GSS	-0.14543	6.828905	-2.5365	0.011453	0.019553	-4.06487
WDR6	-0.14564	6.727836	-2.35911	0.018643	0.030511	-4.4933
AGFG2	-0.14577	5.720543	-5.15517	3.46E-07	1.13E-06	5.70054
PRR34-AS	-0.14581	7.613792	-2.03454	0.042343	0.064149	-5.19822
ASS1	-0.14581	4.998234	-2.07584	0.038341	0.058639	-5.11421
INO80	-0.14587	7.418589	-2.39212	0.017063	0.028141	-4.41588
DESI2	-0.14593	5.769287	-3.80896	0.000154	0.000358	-0.11102
TNFSF15	-0.14604	4.606124	-2.93714	0.003442	0.006455	-2.98571
CCDC78	-0.14605	4.1991	-2.84156	0.004644	0.008527	-3.25714

ASPHD1	-0.14614	5.27897	-2.54222	0.011269	0.019259	-4.05052
SAMD4A	-0.14615	4.824438	-3.71049	0.000226	0.000513	-0.47138
NDUFS6	-0.14617	9.021438	-2.07335	0.038573	0.058954	-5.11933
KCNK13	-0.14625	4.260582	-3.8385	0.000137	0.000321	-0.00117
ZNF492	-0.14639	3.481036	-4.10447	4.62E-05	0.000115	1.024183
TTC30B	-0.14657	3.728199	-2.96357	0.003163	0.005972	-2.90912
PPP4R4	-0.14659	3.683972	-5.49693	5.75E-08	2.07E-07	7.431305
RTKN2	-0.1467	3.393946	-4.29781	2.02E-05	5.26E-05	1.810216
POMGNT2	-0.1468	6.137194	-3.05238	0.002372	0.004572	-2.64689
LRRC17	-0.14684	4.100931	-4.55266	6.43E-06	1.80E-05	2.898111
PHOX2A	-0.14699	5.725496	-2.65429	0.008162	0.014338	-3.76358
NUMBL	-0.14702	8.079483	-3.84386	0.000134	0.000314	0.018853
NOMO3	-0.14723	7.023806	-2.30953	0.021258	0.034326	-4.60761
MAST1	-0.14743	5.105597	-2.54512	0.011177	0.019113	-4.04326
XRCC6	-0.14769	7.224058	-2.80531	0.005192	0.009447	-3.35779
BCL9	-0.14779	6.799938	-4.06568	5.44E-05	0.000134	0.870617
SAAL1	-0.14789	6.57034	-2.17357	0.030134	0.047198	-4.90881
ISPD-AS1	-0.14813	4.105737	-4.14674	3.87E-05	9.74E-05	1.193159
SYF2	-0.14815	9.844168	-3.03032	0.00255	0.00489	-2.71272
CCDC177	-0.14815	5.084496	-3.02006	0.002636	0.005045	-2.7432
C8orf46	-0.14835	3.972398	-5.8782	6.94E-09	2.75E-08	9.480021
LARP4	-0.1484	5.705428	-2.28006	0.02296	0.036815	-4.67442
TIMM22	-0.14842	6.321433	-2.92203	0.00361	0.006747	-3.0292
FBXO44	-0.14865	6.057457	-2.13125	0.03348	0.051835	-4.99889
ZNF428	-0.14869	6.546813	-2.22403	0.026523	0.042008	-4.79911
NLRP2	-0.14881	4.469143	-2.95554	0.003246	0.006112	-2.93248
RC3H2	-0.14882	6.526344	-3.80518	0.000156	0.000363	-0.12501
EPS15	-0.14886	9.382103	-2.2432	0.025253	0.040155	-4.75678
RNF20	-0.14901	9.334275	-2.30569	0.021473	0.034639	-4.61635
MAP3K12	-0.14904	6.608392	-2.33042	0.020119	0.032674	-4.55972
WDR4	-0.14914	5.799678	-3.52741	0.000452	0.000978	-1.11742
LIX1L	-0.14944	6.922412	-3.05238	0.002372	0.004572	-2.64689
PPIP5K1	-0.14957	5.189718	-3.02516	0.002593	0.004968	-2.72805
KCNQ5	-0.14971	4.20807	-3.80872	0.000154	0.000358	-0.11191
CACNA1I	-0.14985	5.849032	-2.96394	0.00316	0.005967	-2.90805
CISD3	-0.15018	5.822872	-2.8539	0.00447	0.008231	-3.2226
LOC10027	-0.15025	3.089398	-6.20799	1.01E-09	4.44E-09	11.35032
PIP4K2C	-0.15036	8.180465	-2.79866	0.005299	0.009626	-3.37613
TPM2	-0.15037	4.985031	-3.46977	0.000559	0.001192	-1.31433
RALGPS1	-0.15072	5.259883	-4.47496	9.17E-06	2.50E-05	2.560209
LANCL2	-0.15087	5.8278	-5.1172	4.20E-07	1.36E-06	5.514502
ZBTB46	-0.1511	4.819226	-4.55607	6.33E-06	1.77E-05	2.91306
ADH4	-0.1512	4.317634	-7.06739	4.46E-12	2.52E-11	16.63856
PSMA3	-0.15127	6.412476	-2.78718	0.005488	0.009945	-3.40767
TUBGCP4	-0.15133	6.344659	-2.67563	0.007666	0.013533	-3.70756
COMMD7	-0.1514	6.931348	-4.07576	5.21E-05	0.000129	0.910391
PLCB3	-0.15141	6.424197	-2.46925	0.013821	0.023235	-4.23086
TNRC6C	-0.15148	5.917124	-4.03076	6.29E-05	0.000154	0.733518
AARS2	-0.15152	6.531031	-3.79196	0.000165	0.000381	-0.17386
GOLGA3	-0.15156	7.272672	-2.83949	0.004674	0.008573	-3.26292
RANGRF	-0.15174	6.480767	-2.4506	0.01455	0.024355	-4.27612
SPRYD7	-0.15181	4.213512	-5.12202	4.10E-07	1.33E-06	5.538021
SUB1	-0.15191	9.028508	-2.51988	0.012002	0.020413	-4.10628
C3orf18	-0.15193	6.336523	-2.56084	0.010689	0.018361	-4.00369
RPS23	-0.15198	9.765584	-1.97383	0.048866	0.072951	-5.31867
MED14	-0.15229	5.601406	-3.42698	0.000653	0.00138	-1.45849
ZADH2	-0.1523	5.445877	-2.80746	0.005158	0.009391	-3.35187
ZNF346	-0.15237	5.761037	-4.40819	1.24E-05	3.32E-05	2.274201

PRMT2	-0.15238	7.604714	-2.47307	0.013676	0.023025	-4.22155
ETFA	-0.15257	8.646687	-1.97915	0.048262	0.072139	-5.30825
SF3A1	-0.15263	8.717791	-3.09766	0.002043	0.003989	-2.51029
SBNO1	-0.15271	6.607363	-3.29034	0.00106	0.002166	-1.90731
GPR20	-0.15303	5.270683	-3.08488	0.002131	0.004147	-2.54903
DSEL	-0.15304	3.62181	-4.863	1.48E-06	4.50E-06	4.301505
LINC01364	-0.15307	4.228466	-4.09441	4.82E-05	0.00012	0.98425
LOXL4	-0.15308	6.431112	-2.93004	0.00352	0.006589	-3.00617
LOC25739	-0.15311	3.654238	-3.80705	0.000155	0.00036	-0.11811
KIAA1328	-0.15327	4.886694	-5.31915	1.48E-07	5.05E-07	6.518427
TMEM150A	-0.15331	5.263991	-2.82175	0.004937	0.009016	-3.31232
TXNL4A	-0.15333	7.674363	-3.55016	0.000416	0.000904	-1.03882
NRIP2	-0.15342	4.761612	-3.09826	0.002039	0.003982	-2.50847
DGKQ	-0.15344	6.652404	-2.47085	0.01376	0.023145	-4.22697
FMO5	-0.15346	4.964275	-2.99614	0.002849	0.00542	-2.81383
BIRC2	-0.15346	10.50525	-2.44133	0.014926	0.024939	-4.2985
STAG1	-0.15355	6.830378	-2.52726	0.011755	0.020028	-4.08793
HEXIM1	-0.15363	7.792854	-3.18347	0.001532	0.003058	-2.24609
CHST8	-0.15363	5.733154	-2.46006	0.014177	0.023782	-4.25322
GRAPL	-0.15366	5.739159	-2.77592	0.005679	0.010262	-3.43849
OSBP	-0.15379	7.739015	-4.19705	3.12E-05	7.96E-05	1.396345
GUSBP5	-0.15381	4.085337	-4.88379	1.34E-06	4.09E-06	4.398544
COPS7B	-0.15382	6.046226	-4.85362	1.55E-06	4.70E-06	4.257797
SPTAN1	-0.15396	6.575257	-2.78172	0.00558	0.010097	-3.42262
LOC10192	-0.15397	5.49151	-2.81075	0.005107	0.009304	-3.34278
TUSC1	-0.1541	5.835813	-1.98637	0.047454	0.071045	-5.29409
ITGAE	-0.15415	8.774789	-2.17105	0.030325	0.047449	-4.91423
BYSL	-0.15428	5.74936	-3.4748	0.000549	0.001171	-1.29728
HEXB	-0.15526	10.21107	-2.50678	0.012451	0.021108	-4.13875
HILPDA	-0.15568	6.21789	-2.33845	0.019696	0.032053	-4.54122
CORO1C	-0.15574	10.06098	-2.86269	0.00435	0.00802	-3.1979
RAD9A	-0.15578	7.201179	-1.98665	0.047422	0.071012	-5.29353
PSMD5-AS	-0.15593	5.793362	-2.7401	0.006328	0.011341	-3.5357
KDF1	-0.15613	4.879455	-3.3945	0.000734	0.00154	-1.56677
ENPP3	-0.15623	4.502047	-5.11073	4.34E-07	1.40E-06	5.48293
TMEM161A	-0.15629	7.907491	-4.39735	1.30E-05	3.47E-05	2.228176
GYLTL1B	-0.1563	6.205942	-3.46755	0.000563	0.001201	-1.32185
ING5	-0.15636	5.89232	-4.42251	1.16E-05	3.12E-05	2.335234
DBP	-0.15644	5.834303	-2.42764	0.015495	0.025786	-4.33139
BNIP3L	-0.15647	10.49699	-2.08257	0.03772	0.057782	-5.10036
VPS33A	-0.15652	6.286299	-4.72134	2.93E-06	8.54E-06	3.65027
CCDC130	-0.15667	7.425265	-2.32603	0.020354	0.033025	-4.56982
USP34	-0.1569	7.882006	-2.42265	0.015708	0.026103	-4.34334
TCEAL2	-0.15694	3.743023	-5.08641	4.91E-07	1.58E-06	5.364524
MTUS1	-0.15718	4.86005	-4.17573	3.42E-05	8.68E-05	1.309945
C16orf45	-0.1572	5.748595	-3.27258	0.001128	0.002297	-1.96438
ODF2	-0.15721	5.41858	-2.22218	0.026649	0.042182	-4.80319
PGBD4	-0.15721	5.179099	-2.43884	0.015028	0.025091	-4.30448
C19orf68	-0.15725	5.558632	-1.98792	0.047281	0.070824	-5.29103
ANKRD19P	-0.1573	5.830926	-3.14401	0.00175	0.003452	-2.36847
IGHV5-78	-0.15731	4.014216	-2.4716	0.013732	0.023102	-4.22515
RARS2	-0.15731	5.38477	-4.3571	1.55E-05	4.12E-05	2.05809
DNMT3A	-0.15733	6.792228	-3.33032	0.000922	0.001903	-1.77783
LINC00086	-0.15734	3.883085	-4.75803	2.46E-06	7.24E-06	3.817221
ABCD4	-0.15759	6.085248	-4.33364	1.72E-05	4.54E-05	1.95964
ATP8A1	-0.15762	6.617708	-2.2248	0.026471	0.041937	-4.79742
EIF2B4	-0.15763	7.714317	-2.83736	0.004705	0.008627	-3.26887
TLDC1	-0.15773	5.141111	-5.75245	1.41E-08	5.42E-08	8.790755

ZFP64	-0.15784	5.475103	-6.08232	2.13E-09	8.99E-09	10.62699
POLRMT	-0.15797	5.207543	-3.01443	0.002685	0.005133	-2.75987
GSTCD	-0.15804	4.986353	-3.41081	0.000692	0.001458	-1.5125
PRPF40A	-0.15806	8.130506	-3.43226	0.00064	0.001355	-1.4408
RWDD2A	-0.15807	5.217669	-4.35301	1.58E-05	4.18E-05	2.040895
LINC00641	-0.15807	4.829503	-4.17714	3.40E-05	8.63E-05	1.315646
IL15RA	-0.15835	7.07279	-2.23859	0.025554	0.040576	-4.76701
C1orf74	-0.15842	4.865293	-3.66156	0.000273	0.000611	-0.6471
PCAT4	-0.15848	4.267476	-4.26483	2.33E-05	6.03E-05	1.673749
ELF1	-0.1585	10.52084	-1.96571	0.0498	0.074168	-5.33451
RPRD1B	-0.15852	6.345775	-5.04645	6.00E-07	1.91E-06	5.171175
DTX3	-0.15854	5.638246	-3.53934	0.000433	0.000939	-1.07627
TMEM97	-0.15862	5.8239	-2.50428	0.012538	0.021246	-4.14493
HIRIP3	-0.15867	6.264568	-3.26113	0.001174	0.002384	-2.00099
CHERP	-0.15871	7.518983	-4.50764	7.91E-06	2.18E-05	2.701699
ARHGEF10	-0.15881	4.807297	-3.37008	0.0008	0.00167	-1.64754
GMEB1	-0.15894	6.68085	-3.68432	0.00025	0.000563	-0.56565
AGRN	-0.15895	6.06579	-3.03821	0.002485	0.004773	-2.68923
FAM185A	-0.15905	4.613235	-2.41229	0.016156	0.026807	-4.36805
MIB2	-0.15905	5.378519	-4.55625	6.33E-06	1.77E-05	2.913841
WDR18	-0.15908	6.131441	-2.33972	0.01963	0.031962	-4.53828
B3GALT6	-0.1591	5.303537	-3.34736	0.000868	0.001799	-1.72217
IBA57-AS1	-0.15914	4.199497	-3.33443	0.000908	0.001878	-1.7644
TELO2	-0.15916	6.218396	-2.45721	0.014288	0.023953	-4.26011
TCOF1	-0.1592	6.200808	-3.30103	0.001021	0.002092	-1.87285
PPIEL	-0.15922	5.959419	-4.84831	1.59E-06	4.82E-06	4.233112
ZNF283	-0.15923	3.616407	-3.12869	0.001842	0.003617	-2.41557
BCO2	-0.15925	5.036951	-3.53922	0.000433	0.000939	-1.07669
UBE3C	-0.15934	6.561824	-3.83322	0.00014	0.000327	-0.02085
SARM1	-0.15943	5.821937	-6.26116	7.35E-10	3.29E-09	11.66024
GOPC	-0.15945	6.097345	-3.56193	0.000398	0.000869	-0.99801
FAM173B	-0.15975	5.925785	-2.63332	0.008677	0.015179	-3.81818
LOC15756	-0.15978	5.852083	-2.58859	0.009874	0.017074	-3.93328
ZNF621	-0.15983	4.81852	-3.47754	0.000543	0.00116	-1.28796
MRPL10	-0.15997	7.577358	-2.06185	0.039659	0.060476	-5.14285
LOC10013	-0.16026	3.93547	-5.19919	2.76E-07	9.13E-07	5.917791
CENPH	-0.16042	5.005316	-2.06903	0.038977	0.059522	-5.12817
MANBA	-0.16056	8.479872	-2.55325	0.010922	0.018725	-4.02283
RFPL2	-0.16064	6.035146	-3.59734	0.000348	0.000767	-0.87434
ZFP2	-0.1608	4.086361	-4.36712	1.49E-05	3.95E-05	2.100281
GGT7	-0.16092	5.295763	-3.30463	0.001009	0.002068	-1.86122
SLC7A1	-0.16095	5.684894	-4.35815	1.55E-05	4.10E-05	2.062517
C21orf2	-0.16128	6.157499	-2.82801	0.004843	0.008856	-3.29491
NOP16	-0.16137	5.664323	-4.34924	1.61E-05	4.25E-05	2.025042
BEND6	-0.16174	3.120731	-6.50439	1.66E-10	8.01E-10	13.10741
UBE3D	-0.16179	3.491839	-6.38052	3.57E-10	1.65E-09	12.36442
GSTM3	-0.16179	5.782993	-2.57053	0.010398	0.017902	-3.97919
SNRPC	-0.16196	8.211892	-2.9252	0.003574	0.006684	-3.02011
PARD6B	-0.16198	4.069255	-5.57892	3.69E-08	1.35E-07	7.861475
LINC00475	-0.16207	4.984082	-3.37965	0.000774	0.001618	-1.61595
ABHD6	-0.16217	5.023466	-4.24934	2.49E-05	6.43E-05	1.609973
ZMYND8	-0.16251	6.994453	-3.50445	0.000492	0.001057	-1.19621
TSPAN31	-0.16265	7.697869	-2.32638	0.020336	0.032999	-4.56904
EPC1	-0.1627	7.824502	-2.98595	0.002944	0.005586	-2.84376
RASSF6	-0.1627	3.759564	-4.68289	3.51E-06	1.01E-05	3.476585
BRAP	-0.16274	6.782596	-3.55552	0.000407	0.000888	-1.02025
METTL7A	-0.16282	6.975129	-3.09632	0.002052	0.004005	-2.51436
ZNF461	-0.1631	4.644242	-5.27205	1.89E-07	6.39E-07	6.281096

SURF6	-0.16314	6.292474	-2.49446	0.012887	0.021773	-4.16915
DGKG	-0.16314	5.127168	-3.16903	0.001608	0.003195	-2.29103
NPR2	-0.16319	5.080766	-4.71998	2.95E-06	8.60E-06	3.644085
ZNF483	-0.16342	4.310707	-2.87341	0.004207	0.007771	-3.16768
DST	-0.16344	4.634445	-6.77558	3.00E-11	1.56E-10	14.77703
POLR3G	-0.16358	3.716451	-5.82341	9.47E-09	3.70E-08	9.17805
SHPK	-0.1637	5.688563	-3.83015	0.000142	0.000331	-0.03229
MYEOV2	-0.16373	9.263867	-2.44482	0.014783	0.024724	-4.29008
UBE2D4	-0.16378	7.193166	-5.41497	8.93E-08	3.13E-07	7.007073
SREK1IP1	-0.16382	6.573894	-2.00245	0.045693	0.068657	-5.26233
ZNF143	-0.1641	8.260867	-2.39485	0.016938	0.027968	-4.40943
GIGYF1	-0.16412	6.594937	-2.87878	0.004137	0.007649	-3.15249
FAM229A	-0.16428	5.674702	-3.78705	0.000168	0.000388	-0.19198
FITM2	-0.16428	5.628676	-5.48729	6.06E-08	2.17E-07	7.38115
FBXO42	-0.16433	7.683124	-4.01554	6.70E-05	0.000163	0.674127
AUH	-0.16433	7.431487	-2.07359	0.03855	0.058929	-5.11883
SDS	-0.16435	6.149431	-2.91242	0.003722	0.006936	-3.05674
CTNNBIP1	-0.16457	7.360983	-3.39694	0.000727	0.001528	-1.55868
KCNG1	-0.16463	5.485804	-4.04072	6.03E-05	0.000148	0.772504
HOXC4	-0.16464	6.146645	-3.37693	0.000781	0.001632	-1.62493
EPHX4	-0.16465	3.618481	-4.75203	2.53E-06	7.44E-06	3.789821
HOXA4	-0.16487	5.944327	-4.05489	5.69E-05	0.00014	0.828149
SP110	-0.16491	9.854408	-2.09205	0.03686	0.05659	-5.08079
H2AFV	-0.16504	8.318684	-2.66685	0.007866	0.013854	-3.73067
ZNF79	-0.16508	5.268453	-5.83987	8.63E-09	3.38E-08	9.268496
MAP3K9	-0.16517	5.190633	-4.3546	1.57E-05	4.16E-05	2.047554
LOC64621	-0.16518	8.080235	-2.48037	0.013402	0.022595	-4.2037
CASP8	-0.16521	9.30015	-2.08054	0.037906	0.058048	-5.10454
NMNAT3	-0.16521	5.528845	-3.98125	7.71E-05	0.000187	0.541079
MOB4	-0.16532	8.654208	-2.37298	0.017964	0.029477	-4.46089
ZDHHC21	-0.16533	4.02801	-4.72352	2.90E-06	8.46E-06	3.660136
LOC72988	-0.16548	4.507377	-3.40235	0.000713	0.001501	-1.54069
MRPS15	-0.16552	6.356777	-4.11588	4.41E-05	0.00011	1.069646
RALBP1	-0.16574	8.721884	-2.95278	0.003274	0.006161	-2.94048
UBAP1L	-0.16583	5.786192	-3.06254	0.002294	0.004437	-2.61641
RBFA	-0.16587	5.509573	-4.12941	4.16E-05	0.000104	1.123677
ITGB8	-0.16587	3.797768	-6.69923	4.89E-11	2.49E-10	14.30109
MRPL52	-0.16594	6.116106	-3.14862	0.001723	0.003404	-2.35425
IRS1	-0.16597	4.958533	-2.46237	0.014086	0.02365	-4.2476
TMEM101	-0.16598	5.899743	-1.98657	0.047431	0.071019	-5.29368
ECHDC1	-0.16607	8.233245	-1.99777	0.0462	0.069342	-5.2716
NUDC	-0.16616	6.831978	-3.42064	0.000668	0.00141	-1.47969
HCG4	-0.16623	3.96612	-5.84133	8.56E-09	3.36E-08	9.276549
LINC0032[illegible]	-0.16651	8.170795	-2.60314	0.009469	0.016428	-3.89604
APEH	-0.16652	6.509787	-3.39618	0.000729	0.001531	-1.5612
SYBU	-0.16657	4.080316	-3.77193	0.000178	0.00041	-0.24757
SNX29	-0.16668	5.009262	-2.36407	0.018398	0.030135	-4.48173
EXOSC1	-0.16668	5.730734	-4.31407	1.88E-05	4.93E-05	1.877884
KDM5C	-0.16671	6.918567	-3.42661	0.000654	0.001381	-1.45972
LLGL2	-0.16672	5.359034	-2.34097	0.019565	0.031871	-4.53541
ZNF135	-0.16673	3.727217	-4.45147	1.02E-05	2.77E-05	2.459159
MCM6	-0.16677	5.80565	-2.09114	0.036942	0.056706	-5.08268
FBXW8	-0.16732	4.163316	-4.8452	1.62E-06	4.89E-06	4.218692
QKI	-0.16747	7.96436	-2.6311	0.008733	0.015263	-3.82395
IGBP1	-0.16763	9.070986	-3.4022	0.000714	0.001501	-1.54118
PLXNA4	-0.16773	4.575058	-3.95409	8.61E-05	0.000207	0.436468
TUBG2	-0.16776	4.825744	-3.35532	0.000844	0.001752	-1.69608
KLRAP1	-0.16788	3.616415	-4.66337	3.85E-06	1.10E-05	3.388892

EXD3	-0.16789	5.831719	-2.89856	0.003888	0.007218	-3.09633
C12orf76	-0.16807	7.378991	-2.09075	0.036977	0.056749	-5.08347
TNS3	-0.16814	6.170639	-2.49314	0.012934	0.021851	-4.17239
USP54	-0.1682	5.83492	-4.0467	5.88E-05	0.000145	0.795971
SMARCC2	-0.16847	7.829039	-2.33648	0.019799	0.032195	-4.54576
DCUN1D2	-0.16848	6.547173	-2.97384	0.003061	0.005795	-2.87918
HLF	-0.16851	4.422828	-6.35465	4.18E-10	1.92E-09	12.21083
PMEPA1	-0.16851	5.110385	-2.99887	0.002824	0.005376	-2.80579
SCAND2P	-0.16851	5.198917	-6.9205	1.17E-11	6.35E-11	15.6932
SYT17	-0.16853	5.073861	-4.41921	1.18E-05	3.17E-05	2.321142
THRAP3	-0.16885	6.936219	-3.13967	0.001776	0.003497	-2.38183
LOC10192	-0.169	5.989637	-2.76375	0.005892	0.010612	-3.47166
NIPSNAP3	-0.16908	3.895501	-3.62632	0.000312	0.000692	-0.77227
ARSF	-0.1693	5.852337	-3.63286	0.000305	0.000677	-0.74912
AAGAB	-0.16932	6.10214	-4.86457	1.47E-06	4.47E-06	4.308805
SIGMAR1	-0.16934	5.929393	-2.69759	0.007184	0.012755	-3.64946
SETD1A	-0.16942	6.008614	-2.73357	0.006453	0.011544	-3.55327
TMEM38B	-0.16962	5.400303	-1.98898	0.047164	0.070671	-5.28894
ZNF702P	-0.1698	3.864492	-3.80343	0.000158	0.000365	-0.13151
ALG3	-0.16989	7.219721	-3.17348	0.001584	0.003154	-2.27722
PFKP	-0.16994	6.306295	-3.86414	0.000124	0.000292	0.09485
ZNF717	-0.16995	5.365316	-4.13745	4.02E-05	0.000101	1.155883
CHCHD3	-0.17003	5.457724	-3.91676	0.0001	0.000239	0.293786
POFUT2	-0.17016	5.724884	-4.47288	9.26E-06	2.52E-05	2.551273
LSS	-0.17048	5.530869	-3.51878	0.000467	0.001007	-1.1471
LINC0047(	-0.17052	4.196345	-2.50725	0.012434	0.021086	-4.13759
ELMSAN1	-0.17056	6.042658	-2.29637	0.022004	0.035407	-4.63754
PPARGC1/	-0.1706	3.998942	-3.34263	0.000882	0.001827	-1.73764
HIST3H2A	-0.17063	5.904084	-4.81064	1.91E-06	5.72E-06	4.058713
LOC10099	-0.17088	6.191695	-3.39279	0.000738	0.001549	-1.57244
HDGFRP3	-0.17091	4.899632	-2.37226	0.017999	0.029527	-4.46259
DFFA	-0.17091	6.706235	-3.37695	0.000781	0.001632	-1.62488
PECAM1	-0.17105	10.53161	-2.22909	0.026183	0.041506	-4.78799
RAD18	-0.17115	4.433597	-6.02272	3.02E-09	1.25E-08	10.28852
IL3RA	-0.17127	6.832933	-2.28634	0.022587	0.036263	-4.66026
9-Mar	-0.17127	5.634587	-3.35856	0.000834	0.001733	-1.68542
LOC10192	-0.17133	5.804188	-3.48498	0.000529	0.001132	-1.26266
BAG2	-0.17141	4.55454	-3.80571	0.000156	0.000362	-0.12307
FITM1	-0.17151	5.991398	-3.76766	0.000181	0.000416	-0.26326
ARL2	-0.17157	7.482149	-2.58741	0.009908	0.017128	-3.93628
C1orf216	-0.17168	6.439129	-2.44924	0.014605	0.024444	-4.27941
ALDH1B1	-0.1717	5.12456	-4.57723	5.75E-06	1.61E-05	3.0061
RGMB	-0.17172	4.609692	-6.16378	1.32E-09	5.69E-09	11.09438
TCF20	-0.17183	6.761492	-4.4277	1.13E-05	3.06E-05	2.357357
LOC10050	-0.17204	4.019659	-3.69287	0.000242	0.000546	-0.53491
GGCX	-0.17223	5.964044	-3.37902	0.000775	0.001621	-1.61803
ECSIT	-0.17236	6.259063	-2.00303	0.04563	0.068578	-5.26119
DDX19A	-0.17275	6.515174	-2.60788	0.009341	0.016233	-3.88388
WBSCR22	-0.17276	6.162476	-3.78705	0.000168	0.000388	-0.19198
PLLP	-0.173	5.217216	-3.41438	0.000683	0.00144	-1.5006
CATSPER3	-0.17301	4.595385	-4.6914	3.37E-06	9.76E-06	3.514882
UBL7-AS1	-0.17302	5.155183	-3.17583	0.001572	0.003131	-2.26992
RITA1	-0.17303	6.336395	-3.33073	0.00092	0.001901	-1.77649
FEM1A	-0.17321	6.854388	-5.13778	3.78E-07	1.23E-06	5.615152
HMHA1	-0.17341	9.023721	-2.55447	0.010884	0.018663	-4.01975
KNTC1	-0.17346	5.069104	-2.16586	0.030721	0.047978	-4.92536
MLEC	-0.17351	8.121805	-2.37046	0.018086	0.029663	-4.4668
MPP6	-0.17358	4.087552	-2.75076	0.006128	0.011003	-3.50689

C11orf24	-0.17377	6.320366	-2.88524	0.004054	0.007507	-3.1342
ZMYM3	-0.17381	7.133916	-2.58442	0.009993	0.017266	-3.94391
PRR3	-0.17429	6.099488	-3.08074	0.002161	0.0042	-2.56156
CELSR3	-0.17452	5.504231	-3.97314	7.97E-05	0.000193	0.50977
TRUB2	-0.17462	5.926093	-2.54742	0.011104	0.019006	-4.03749
TMED6	-0.17477	4.537846	-3.4138	0.000685	0.001443	-1.50255
PPP1R16A	-0.17503	4.638032	-2.20197	0.028053	0.044195	-4.84738
DMXL2	-0.17514	8.213429	-2.05486	0.040332	0.061391	-5.1571
ATPIF1	-0.17517	7.785921	-3.06898	0.002246	0.004352	-2.59702
MPLKIP	-0.17553	7.979369	-2.94789	0.003326	0.006253	-2.95464
ENOX2	-0.17556	5.505082	-5.52788	4.87E-08	1.76E-07	7.593051
ZNF543	-0.17556	3.490698	-4.17496	3.43E-05	8.70E-05	1.306851
SCAMP1	-0.17558	6.226637	-2.38949	0.017184	0.028326	-4.42209
HAND2	-0.17563	4.698119	-3.0719	0.002225	0.004313	-2.58825
NDUFB10	-0.17566	8.542975	-2.56044	0.010701	0.018379	-4.0047
SPOP	-0.17568	7.864892	-2.59895	0.009584	0.016613	-3.90678
ZNF24	-0.17568	7.496985	-3.30565	0.001005	0.002062	-1.85792
GLRX3	-0.1757	6.716212	-5.94165	4.82E-09	1.94E-08	9.832834
ZNF398	-0.17584	7.45046	-2.43503	0.015185	0.025326	-4.31365
SLC26A11	-0.17589	8.789817	-2.04844	0.040958	0.062253	-5.17012
LCP2	-0.17591	8.222946	-2.81823	0.00499	0.009109	-3.32205
C19orf43	-0.17594	8.461146	-2.56958	0.010426	0.017947	-3.9816
DISP1	-0.17599	4.311247	-3.27848	0.001105	0.002252	-1.94544
IPO4	-0.17606	6.567574	-2.1993	0.028243	0.04446	-4.85318
POLR2K	-0.17606	7.366759	-2.13552	0.033129	0.051339	-4.98988
UNC45B	-0.17615	4.113423	-5.07424	5.22E-07	1.67E-06	5.305498
SLC22A3	-0.1762	5.882803	-3.96957	8.09E-05	0.000195	0.495988
UNKL	-0.17625	6.87167	-4.78697	2.14E-06	6.36E-06	3.949767
CAMTA1	-0.17629	6.300783	-4.72546	2.87E-06	8.39E-06	3.66895
LIN7B	-0.17639	5.126007	-4.60855	4.97E-06	1.41E-05	3.144531
CRLS1	-0.17644	5.861654	-2.63247	0.008698	0.015214	-3.82041
CA5B	-0.1765	5.618005	-3.88299	0.000115	0.000271	0.165802
RBM41	-0.17653	5.568327	-2.82027	0.004959	0.009056	-3.31641
C1D	-0.17666	8.830044	-2.24819	0.024932	0.039689	-4.74573
C5orf51	-0.17666	6.080135	-4.87897	1.37E-06	4.19E-06	4.376004
ZDHHC8	-0.17676	5.802816	-4.42822	1.13E-05	3.05E-05	2.359607
CCDC28B	-0.17679	5.218407	-3.58306	0.000368	0.000807	-0.92434
POLR2J4	-0.17696	5.92898	-2.50172	0.012628	0.021382	-4.15125
CLNK	-0.17713	3.486633	-6.18333	1.17E-09	5.10E-09	11.20732
REEP4	-0.17726	7.35087	-3.56017	0.0004	0.000874	-1.00412
PRO1804	-0.17743	3.190059	-5.09567	4.68E-07	1.51E-06	5.409552
XKR6	-0.17749	4.05052	-4.55439	6.38E-06	1.78E-05	2.905712
TLE2	-0.1775	5.954774	-3.80824	0.000155	0.000359	-0.1137
MMP10	-0.17752	3.817435	-5.14905	3.57E-07	1.17E-06	5.670467
FANK1	-0.17766	4.514664	-4.02351	6.48E-05	0.000158	0.705184
PPP1R7	-0.17766	7.643153	-3.68357	0.000251	0.000565	-0.56834
NMB	-0.17774	6.63318	-3.56358	0.000395	0.000864	-0.99226
RPLP1	-0.17779	9.403895	-4.05895	5.59E-05	0.000138	0.844093
NKAPP1	-0.17794	5.166028	-3.52933	0.000449	0.000972	-1.11078
SSU72	-0.17799	8.383405	-3.79651	0.000162	0.000374	-0.15709
KANK3	-0.17804	4.929336	-2.60868	0.009319	0.016199	-3.88183
CYTH3	-0.17809	5.478363	-5.75127	1.42E-08	5.45E-08	8.784363
NUP37	-0.17816	6.962416	-2.48748	0.01314	0.022182	-4.18629
FRS2	-0.17821	5.267088	-4.45828	9.89E-06	2.69E-05	2.488384
TSC22D2	-0.17828	4.876918	-6.24441	8.13E-10	3.61E-09	11.56234
TRIM2	-0.17831	4.257349	-7.35318	6.49E-13	4.00E-12	18.52559
ADI1	-0.17831	7.604995	-3.15986	0.001659	0.003288	-2.31949
VSTM2L	-0.17838	5.443759	-3.22591	0.001325	0.002666	-2.11287

TTN	-0.17848	4.750577	-5.11326	4.28E-07	1.39E-06	5.495247
B4GALT7	-0.17856	5.903123	-3.27266	0.001128	0.002297	-1.9641
GOLGA7B	-0.17877	6.371556	-3.216	0.001371	0.002752	-2.14413
SLC25A4	-0.17878	5.203778	-4.8964	1.26E-06	3.86E-06	4.45764
CD1E	-0.1788	4.502552	-5.44639	7.55E-08	2.67E-07	7.16902
SRRT	-0.17894	6.738748	-4.03349	6.22E-05	0.000152	0.744212
COG8	-0.179	6.637655	-5.01452	7.04E-07	2.22E-06	5.017674
ADCK3	-0.17914	6.484592	-2.71169	0.006889	0.012265	-3.61193
FBXO33	-0.17921	8.566266	-2.41057	0.016232	0.026921	-4.37215
ZC3H15	-0.17921	7.387186	-4.49552	8.35E-06	2.29E-05	2.649115
CALCOCO	-0.17922	7.729653	-3.84549	0.000133	0.000312	0.024956
PHKB	-0.17928	6.67258	-2.43645	0.015126	0.025246	-4.31023
HSCB	-0.17934	6.192731	-2.11562	0.034795	0.053733	-5.03174
PGBD1	-0.17939	3.72621	-5.59637	3.35E-08	1.23E-07	7.95376
MTCH2	-0.17944	7.328237	-2.34178	0.019523	0.031814	-4.53352
PABPC1L	-0.17946	6.323556	-3.36477	0.000816	0.001699	-1.665
TMEM241	-0.17954	7.494841	-3.9066	0.000104	0.000248	0.255164
ACAN	-0.17961	6.024116	-3.60659	0.000336	0.000742	-0.84184
FBXO46	-0.17962	7.641442	-2.75292	0.006088	0.010938	-3.50105
KLF2	-0.17967	9.682757	-2.295	0.022082	0.035499	-4.64065
ALDH3A2	-0.17967	7.331262	-3.1477	0.001728	0.003413	-2.35707
AASS	-0.17982	3.781712	-5.14109	3.72E-07	1.21E-06	5.631423
ARHGEF19	-0.17986	5.892323	-3.30783	0.000997	0.002048	-1.85085
PEX12	-0.17986	5.927887	-2.14596	0.032283	0.050174	-4.96778
ANKRD26	-0.17987	4.802287	-4.6828	3.51E-06	1.01E-05	3.476188
IDH3B	-0.17999	8.239237	-2.63949	0.008522	0.014935	-3.80217
GLIS3	-0.17999	3.918406	-4.90298	1.22E-06	3.74E-06	4.488507
MNT	-0.18001	7.358767	-3.6822	0.000252	0.000567	-0.57327
MMAB	-0.18035	5.43919	-4.57531	5.80E-06	1.63E-05	2.997629
NFIA	-0.18042	4.778546	-3.12345	0.001875	0.003677	-2.43163
PYROXD2	-0.18051	6.11688	-3.55235	0.000412	0.000897	-1.03126
PSMA8	-0.18052	3.727673	-5.01001	7.20E-07	2.27E-06	4.996097
RNF165	-0.18053	5.034188	-5.16771	3.25E-07	1.07E-06	5.762236
IL12RB2	-0.18059	4.273886	-3.90879	0.000104	0.000246	0.263482
FRMD8	-0.1808	7.507734	-4.18412	3.30E-05	8.39E-05	1.343893
NISCH	-0.18088	7.71009	-3.66282	0.000272	0.000608	-0.64259
LOC10099	-0.18103	4.203677	-3.73474	0.000206	0.00047	-0.38348
RASA1	-0.18104	8.913089	-2.17143	0.030296	0.047415	-4.9134
TOB2	-0.18111	6.171235	-3.61571	0.000325	0.000719	-0.80972
SIKE1	-0.18113	5.531357	-3.15102	0.001709	0.00338	-2.34682
GRASP	-0.18121	6.796468	-2.95993	0.0032	0.006036	-2.91973
EXOC6B	-0.18121	4.710399	-5.74112	1.50E-08	5.75E-08	8.729311
NARFL	-0.18132	6.848633	-3.07416	0.002208	0.004285	-2.58143
MDM2	-0.18138	5.542494	-4.54102	6.79E-06	1.89E-05	2.847163
USP1	-0.18149	8.941732	-2.17549	0.029989	0.047004	-4.90468
CRIPAK	-0.18159	7.452665	-2.39318	0.017014	0.028076	-4.41337
HELZ	-0.18164	7.504024	-2.61719	0.009093	0.015838	-3.8599
FKSG49	-0.18167	8.73998	-2.17059	0.030359	0.047486	-4.91521
POP7	-0.18185	7.399336	-2.73825	0.006363	0.011399	-3.54068
TFR2	-0.18195	5.339382	-3.91788	9.98E-05	0.000238	0.298055
RFPL3S	-0.18197	3.822072	-3.17713	0.001565	0.003119	-2.26587
ZSCAN2	-0.18203	5.739749	-4.81017	1.92E-06	5.73E-06	4.056551
ZNF654	-0.18217	6.354721	-2.15005	0.031957	0.049706	-4.9591
SSR2	-0.18221	10.17452	-2.5402	0.011334	0.019361	-4.05559
CLUHP3	-0.18244	6.201813	-4.51544	7.63E-06	2.10E-05	2.735568
BTG3	-0.1827	5.598514	-2.3777	0.017738	0.029138	-4.44982
RPE	-0.18273	6.169922	-2.12859	0.033701	0.052124	-5.0045
AHCYL2	-0.18279	6.184618	-4.78978	2.11E-06	6.28E-06	3.962641

CHN2	-0.18307	5.613873	-3.5267	0.000453	0.00098	-1.11985
WAPAL	-0.18312	7.375813	-4.11077	4.50E-05	0.000112	1.049265
RIMS3	-0.18314	5.426799	-6.06727	2.32E-09	9.76E-09	10.54127
CHST14	-0.18324	7.198155	-3.59917	0.000346	0.000762	-0.86791
HIGD2A	-0.18325	7.721916	-3.18562	0.001521	0.003038	-2.2394
TRUB1	-0.18334	4.49438	-7.36069	6.16E-13	3.80E-12	18.57607
ATP5G1	-0.18342	7.777787	-2.91019	0.003748	0.006978	-3.06313
NOL4L	-0.18355	5.622705	-6.42403	2.73E-10	1.28E-09	12.624
PIAS1	-0.18358	9.507903	-2.94282	0.00338	0.006346	-2.96931
LRRC56	-0.1837	5.658503	-3.6823	0.000252	0.000567	-0.57288
PMS2P3	-0.18374	6.733735	-3.7448	0.000198	0.000452	-0.34684
PMPCA	-0.18378	7.208018	-2.17729	0.029854	0.046817	-4.90081
SUPT16H	-0.18381	7.564758	-2.1206	0.034372	0.053116	-5.0213
ENTPD6	-0.1839	6.065568	-4.04377	5.96E-05	0.000146	0.784471
MRPL41	-0.18392	5.80331	-3.90848	0.000104	0.000246	0.262321
URB1	-0.18408	4.904097	-5.32986	1.40E-07	4.79E-07	6.57262
IGFBP3	-0.18413	5.254492	-3.72972	0.00021	0.000478	-0.40172
COL13A1	-0.1842	5.267861	-5.25374	2.08E-07	6.99E-07	6.189379
SLC27A1	-0.18425	7.242856	-2.50399	0.012549	0.021261	-4.14565
CACHD1	-0.18429	5.109446	-4.35326	1.58E-05	4.18E-05	2.041931
RAP2C-AS	-0.1844	5.082448	-3.15207	0.001703	0.003369	-2.34357
NDUFB11	-0.1845	8.711715	-2.22963	0.026147	0.041455	-4.78678
GATAD2B	-0.18462	7.510637	-2.79207	0.005407	0.009806	-3.39425
PRDM15	-0.18488	5.391949	-5.59744	3.33E-08	1.22E-07	7.959451
DDX6	-0.185	7.969452	-2.69377	0.007266	0.012884	-3.65962
LOC10272	-0.18515	4.930149	-4.18311	3.31E-05	8.42E-05	1.339822
TRAPPC12	-0.18519	7.796549	-2.20984	0.027499	0.043421	-4.83022
UBFD1	-0.18521	6.394351	-2.65935	0.008041	0.014136	-3.75033
C16orf46	-0.18525	3.95459	-3.76054	0.000186	0.000427	-0.28935
DNAH12	-0.18536	4.665951	-3.57905	0.000373	0.000818	-0.93836
CNPY4	-0.18553	6.107704	-4.71727	2.99E-06	8.69E-06	3.63181
HOGA1	-0.18556	4.72482	-4.15465	3.74E-05	9.44E-05	1.224917
TNK1	-0.1857	6.157424	-3.71255	0.000225	0.000509	-0.46392
LOC10192	-0.18575	4.407139	-4.22346	2.79E-05	7.14E-05	1.503927
GNAI1	-0.18583	3.602331	-2.79698	0.005326	0.00967	-3.38076
MRPS5	-0.18596	6.301763	-4.37848	1.41E-05	3.76E-05	2.148224
CES4A	-0.18598	6.402196	-3.53173	0.000445	0.000964	-1.10251
ANKRD11	-0.18609	7.850952	-4.23962	2.60E-05	6.68E-05	1.570088
ARL2BP	-0.18615	8.109735	-2.92668	0.003558	0.006655	-3.01585
TJAP1	-0.1862	7.385274	-3.99028	7.43E-05	0.00018	0.575994
ZNF490	-0.18624	6.115205	-3.62392	0.000315	0.000698	-0.78075
OXA1L	-0.18625	9.014894	-1.96965	0.049345	0.073587	-5.32683
HNRNPC	-0.18632	8.923911	-4.37635	1.43E-05	3.79E-05	2.139255
R3HCC1	-0.18639	7.043822	-3.52576	0.000455	0.000984	-1.12309
COX4I1	-0.18652	8.220711	-4.16542	3.57E-05	9.04E-05	1.268305
HKDC1	-0.18652	4.875684	-4.81933	1.83E-06	5.50E-06	4.098847
TUBD1	-0.18681	5.894051	-2.63067	0.008744	0.015279	-3.82508
LINC00865	-0.18692	5.156214	-4.23367	2.67E-05	6.85E-05	1.545697
TRIM62	-0.18698	6.12445	-4.66403	3.84E-06	1.10E-05	3.391878
TOMM40	-0.18732	6.634913	-2.36929	0.018143	0.029752	-4.46955
ATP8B1	-0.18732	6.006966	-2.95787	0.003222	0.006071	-2.92569
ALDH1A2	-0.18741	5.358401	-3.35904	0.000832	0.001731	-1.68385
TATDN2	-0.1875	8.444646	-3.33007	0.000922	0.001904	-1.77864
HSDL1	-0.18766	6.282248	-3.04571	0.002425	0.004666	-2.66684
CLK2	-0.18769	8.819486	-2.54118	0.011302	0.019312	-4.05314
DUSP22	-0.18777	9.868736	-2.97954	0.003005	0.005697	-2.86251
TRIM4	-0.18778	7.136501	-4.53186	7.08E-06	1.96E-05	2.807137
VENTX	-0.18783	6.68802	-3.05151	0.002379	0.004583	-2.64949

CBX3	-0.18785	8.457356	-3.16136	0.001651	0.003274	-2.31485
SLC35F2	-0.18786	5.965234	-2.78795	0.005475	0.009923	-3.40555
CPSF4	-0.18791	7.955047	-4.17605	3.41E-05	8.67E-05	1.311232
FUCA1	-0.18797	6.097997	-3.15373	0.001694	0.003352	-2.33845
EDC4	-0.18798	6.929409	-2.59839	0.0096	0.016637	-3.90822
LOC10192	-0.18834	6.075682	-3.3644	0.000817	0.001701	-1.66624
RGS17	-0.18844	4.465891	-4.69793	3.27E-06	9.48E-06	3.544372
LINC00294	-0.18845	7.042717	-2.28072	0.02292	0.036754	-4.67292
PHB	-0.18849	6.551604	-2.69009	0.007345	0.013009	-3.66937
PCDHGA8	-0.18885	4.309493	-4.7652	2.38E-06	7.01E-06	3.849994
PCBP1-AS	-0.18888	5.694532	-4.99353	7.81E-07	2.45E-06	4.917283
LOC15117	-0.18899	5.384514	-3.3957	0.00073	0.001533	-1.56279
AFF3	-0.1891	4.804314	-3.65542	0.00028	0.000624	-0.66898
LARP1B	-0.18915	4.429206	-4.31066	1.91E-05	5.00E-05	1.863688
SETX	-0.18931	7.955392	-2.9968	0.002843	0.00541	-2.81188
BLVRA	-0.18936	6.83534	-3.63108	0.000307	0.00068	-0.75544
NSL1	-0.18961	6.513952	-4.30176	1.98E-05	5.18E-05	1.82666
SLAMF8	-0.18983	5.510007	-2.49604	0.01283	0.021696	-4.16526
EPB41L4A	-0.18984	4.431327	-5.60521	3.19E-08	1.18E-07	8.000603
FAM104B	-0.19017	4.577033	-5.03028	6.51E-07	2.06E-06	5.093332
TTLL1	-0.19018	5.549695	-3.67525	0.000259	0.000582	-0.59817
LINC00969	-0.19036	5.438639	-3.61275	0.000329	0.000726	-0.82018
KRT15	-0.19045	4.238083	-3.12434	0.001869	0.003667	-2.42889
EXO5	-0.19046	4.897096	-4.28457	2.14E-05	5.56E-05	1.755302
LRRFIP1	-0.19069	9.139681	-3.26303	0.001166	0.002369	-1.99491
LOC10192	-0.19085	4.86246	-5.3732	1.11E-07	3.86E-07	6.793094
ESD	-0.19091	7.253647	-4.55357	6.41E-06	1.79E-05	2.902095
PTGER2	-0.19093	8.743343	-2.12992	0.033591	0.051985	-5.0017
PRDX1	-0.19096	9.599913	-2.31757	0.020814	0.03368	-4.58924
NTN4	-0.19104	3.208269	-5.11075	4.34E-07	1.40E-06	5.483028
RAVER2	-0.19129	3.716507	-4.11627	4.40E-05	0.00011	1.071204
PRICKLE1	-0.19155	4.980984	-2.76322	0.005902	0.010627	-3.4731
DTX4	-0.19161	6.848256	-2.93812	0.003431	0.006437	-2.9829
UQCC1	-0.19164	5.706446	-6.37875	3.60E-10	1.67E-09	12.35386
MYBBP1A	-0.19167	5.620857	-3.3358	0.000904	0.00187	-1.75995
MIATNB	-0.19196	6.494606	-3.31751	0.000964	0.001985	-1.81948
MKKS	-0.19198	7.654742	-2.75924	0.005973	0.010751	-3.48391
SMIM15	-0.19231	8.244816	-2.00803	0.045094	0.067889	-5.25125
APEX2	-0.19264	6.615617	-4.09229	4.86E-05	0.000121	0.975821
ANKRD28	-0.19282	7.028945	-1.96799	0.049536	0.073841	-5.33007
RBL2	-0.19302	9.783378	-2.45672	0.014308	0.023977	-4.26132
FER	-0.19316	5.279133	-4.80726	1.94E-06	5.81E-06	4.043119
LAGE3	-0.19354	6.734388	-2.17971	0.029673	0.046553	-4.89559
TTC31	-0.19371	7.77921	-2.61441	0.009166	0.015953	-3.86709
ALS2CL	-0.19373	6.090913	-4.12665	4.21E-05	0.000106	1.112642
PCMTD1	-0.19376	7.341458	-2.59517	0.009689	0.016776	-3.91647
RNF19A	-0.19389	7.488012	-3.58195	0.000369	0.00081	-0.92823
LSR	-0.19408	4.794913	-2.75776	0.006	0.010794	-3.48792
LOC10272	-0.19411	3.547833	-7.52639	1.96E-13	1.27E-12	19.69955
TMEM256	-0.19412	7.106574	-1.98182	0.047963	0.07175	-5.30303
NLRP7	-0.19414	4.390256	-3.53186	0.000445	0.000963	-1.10207
KCTD12	-0.19429	10.58978	-2.04822	0.040979	0.06227	-5.17057
BCDIN3D	-0.19437	6.474063	-2.98264	0.002976	0.005643	-2.85344
ERP29	-0.19439	9.946602	-1.97806	0.048386	0.0723	-5.3104
NEIL2	-0.19465	5.293839	-3.74858	0.000195	0.000447	-0.33307
FAM109A	-0.19471	6.305161	-3.26112	0.001174	0.002384	-2.00101
SIGLEC15	-0.19472	6.996042	-2.89481	0.003934	0.007297	-3.10701
BOLA2	-0.19483	5.07819	-4.21265	2.92E-05	7.47E-05	1.459823

NSUN4	-0.19487	5.918486	-4.61338	4.86E-06	1.38E-05	3.165916
RPUSD2	-0.19524	5.586282	-3.63684	0.0003	0.000667	-0.73504
ATG5	-0.19524	7.015713	-2.45939	0.014203	0.023821	-4.25484
GPR180	-0.19525	4.487603	-2.57236	0.010344	0.017814	-3.97455
10-Sep	-0.19539	4.359048	-2.26341	0.023972	0.038293	-4.71179
SEMA4C	-0.19541	7.175919	-3.28291	0.001088	0.002221	-1.93124
PIDD1	-0.19543	6.283154	-3.42584	0.000655	0.001385	-1.4623
WDR7	-0.19549	5.898846	-4.33141	1.74E-05	4.58E-05	1.950303
SENP7	-0.19561	5.688058	-2.17277	0.030194	0.047285	-4.91053
CDC14B	-0.19561	5.116402	-2.88875	0.004009	0.007431	-3.12423
DDX56	-0.19564	6.545058	-4.09773	4.76E-05	0.000118	0.997427
FAHD2A	-0.19564	6.032509	-4.61716	4.78E-06	1.36E-05	3.18272
LRR1	-0.19572	5.99114	-3.61006	0.000332	0.000733	-0.82965
RNF8	-0.19576	5.611927	-5.23497	2.30E-07	7.67E-07	6.095654
OCIAD1	-0.19576	8.700463	-4.67359	3.67E-06	1.06E-05	3.434758
ABAT	-0.19606	7.439773	-2.4093	0.016288	0.026999	-4.37517
PEX14	-0.19617	6.169322	-3.49219	0.000515	0.001103	-1.23811
NOL7	-0.19636	8.363219	-3.08246	0.002149	0.004178	-2.55638
LRCH1	-0.19662	5.56556	-3.18552	0.001521	0.003038	-2.2397
G3BP2	-0.19664	8.083543	-3.54904	0.000417	0.000908	-1.04271
HECTD2	-0.1967	3.305742	-5.75166	1.42E-08	5.44E-08	8.786465
THAP4	-0.19675	6.706207	-3.58602	0.000364	0.000799	-0.914
SYNJ2	-0.19698	5.964121	-3.83932	0.000137	0.00032	0.001889
NDUFS4	-0.19703	7.298149	-2.47192	0.01372	0.023089	-4.22436
HSPD1	-0.19716	6.239906	-3.56214	0.000397	0.000868	-0.99726
NPC1	-0.19721	5.121263	-5.28304	1.79E-07	6.05E-07	6.336313
PPT1	-0.19726	11.70506	-2.67267	0.007733	0.013639	-3.71537
DTX3L	-0.19732	10.15293	-2.53767	0.011415	0.019492	-4.06192
DNHD1	-0.19745	5.323631	-6.80427	2.50E-11	1.31E-10	14.9571
TPRG1	-0.19758	4.377818	-3.37472	0.000787	0.001644	-1.63221
LPCAT4	-0.19762	5.304586	-4.48788	8.65E-06	2.37E-05	2.61604
GAN	-0.19774	4.578741	-5.00793	7.27E-07	2.29E-06	4.986111
EXOC2	-0.19783	6.40363	-2.70608	0.007005	0.012458	-3.6269
DHODH	-0.19786	4.801027	-6.40071	3.15E-10	1.47E-09	12.48469
DCAF8	-0.19788	6.146345	-7.21423	1.67E-12	9.84E-12	17.60033
CRYBA4	-0.19799	4.586467	-3.9151	0.000101	0.00024	0.28748
FBXW4P1	-0.19835	5.625197	-4.30807	1.93E-05	5.05E-05	1.852894
HINFP	-0.19838	6.461031	-3.07871	0.002175	0.004226	-2.5677
ANKRD40	-0.19863	5.780743	-5.29179	1.71E-07	5.79E-07	6.380299
ZSCAN25	-0.19891	6.251345	-5.14286	3.69E-07	1.20E-06	5.640091
CLPX	-0.19908	7.439992	-2.68547	0.007446	0.013176	-3.68159
ABHD3	-0.1992	10.2395	-2.69864	0.007161	0.012718	-3.64669
BBS9	-0.19929	4.79352	-6.01267	3.20E-09	1.32E-08	10.23171
USP8	-0.19961	8.055026	-3.05208	0.002375	0.004576	-2.6478
NHLRC3	-0.19964	5.607846	-2.81879	0.004982	0.009094	-3.3205
SETD2	-0.19964	7.334362	-3.1192	0.001902	0.003727	-2.44463
ARF6	-0.20029	9.669089	-4.84411	1.63E-06	4.91E-06	4.213628
GNPTAB	-0.20054	6.698825	-4.39118	1.34E-05	3.56E-05	2.201985
SIT1	-0.20062	7.508465	-2.57968	0.01013	0.017481	-3.95597
TARBP2	-0.20066	6.994809	-3.6579	0.000277	0.000618	-0.66016
CAPN11	-0.20073	4.547978	-5.84101	8.57E-09	3.36E-08	9.274763
CTPS1	-0.20076	5.588998	-2.35136	0.019032	0.031092	-4.51131
LNPEP	-0.2012	6.885049	-3.86881	0.000122	0.000287	0.112412
ZSCAN12	-0.20125	4.325653	-8.99332	3.24E-18	3.43E-17	30.51352
DIABLO	-0.20132	8.173566	-3.36941	0.000802	0.001674	-1.64973
ST6GALNA	-0.20139	4.275411	-2.78191	0.005577	0.010092	-3.42212
COLQ	-0.20139	5.565737	-3.24895	0.001224	0.002479	-2.03981
MRPS7	-0.20143	6.988518	-2.39441	0.016958	0.027994	-4.41047

DPH2	-0.20153	6.790611	-4.23314	2.67E-05	6.87E-05	1.543506
FBXO28	-0.20165	7.827446	-2.32393	0.020467	0.033176	-4.57464
IL26	-0.20169	3.329298	-7.10523	3.47E-12	1.98E-11	16.88481
AP4B1	-0.20174	5.606397	-4.89508	1.27E-06	3.88E-06	4.451457
MED30	-0.20189	6.563347	-4.02984	6.31E-05	0.000154	0.729931
ZCCHC6	-0.20193	8.615865	-2.02905	0.042901	0.064901	-5.20925
CELSR2	-0.20195	5.264635	-6.14351	1.48E-09	6.38E-09	10.97757
QSOX2	-0.20198	6.368097	-4.61262	4.88E-06	1.38E-05	3.162551
KANSL1L	-0.20198	5.239278	-2.24157	0.02536	0.040296	-4.76041
ZFAS1	-0.20201	9.678204	-2.54564	0.011161	0.019091	-4.04195
PRLH	-0.20208	5.535683	-3.21479	0.001377	0.002763	-2.14792
PUSL1	-0.20216	5.987884	-2.83375	0.004758	0.008715	-3.27894
COQ9	-0.20217	6.667557	-4.29473	2.04E-05	5.33E-05	1.797438
CHST13	-0.20238	6.277873	-2.37135	0.018043	0.029596	-4.46471
SETD1B	-0.20244	10.24273	-3.98002	7.75E-05	0.000188	0.536321
SIPA1L1	-0.20246	6.129475	-2.49933	0.012713	0.021518	-4.15715
CDK2AP1	-0.20253	9.633848	-2.02413	0.043406	0.065616	-5.21912
HGD	-0.20258	4.830614	-3.9137	0.000101	0.000242	0.282138
LINC0011[illegible]	-0.20263	4.110253	-4.2067	2.99E-05	7.65E-05	1.435583
GLTSCR1L	-0.20269	8.949633	-3.83353	0.00014	0.000327	-0.01968
REXO2	-0.20275	5.892379	-3.22522	0.001328	0.002671	-2.11505
UBR5	-0.20282	8.099086	-3.44775	0.000605	0.001285	-1.38873
XPR1	-0.20289	6.054847	-3.36114	0.000826	0.001719	-1.67694
C8orf44	-0.20293	5.40942	-3.56595	0.000392	0.000857	-0.984
CYB561A3	-0.20293	8.058709	-2.21603	0.02707	0.042788	-4.81668
DNMBP	-0.20296	6.87896	-2.50232	0.012607	0.021353	-4.14977
CLIC5	-0.20298	4.963666	-6.92795	1.12E-11	6.07E-11	15.74074
AHNAK	-0.20312	6.941703	-3.35081	0.000857	0.001778	-1.71085
ZNF112	-0.2032	3.677099	-6.26466	7.20E-10	3.22E-09	11.68071
CAMK2N2	-0.20326	5.257282	-4.21165	2.93E-05	7.50E-05	1.455752
BBS7	-0.20327	4.532299	-2.10579	0.035644	0.054894	-5.05225
POT1	-0.20342	6.655386	-2.41049	0.016235	0.026923	-4.37233
UBE2V2	-0.20344	5.927333	-2.63825	0.008553	0.014982	-3.80538
CMTM8	-0.20366	6.184119	-2.11111	0.035183	0.054244	-5.04116
ERBB2IP	-0.20376	8.141137	-3.95878	8.45E-05	0.000203	0.454489
LINC0088[illegible]	-0.20379	3.703897	-7.46147	3.07E-13	1.96E-12	19.25693
LRIG1	-0.20382	4.929229	-6.25982	7.41E-10	3.31E-09	11.6524
PODXL	-0.20385	4.85862	-4.38495	1.37E-05	3.66E-05	2.175593
GNPAT	-0.20397	8.720331	-3.51514	0.000473	0.001019	-1.15957
CXorf24	-0.2042	4.790714	-2.54523	0.011173	0.019108	-4.04297
PDE9A	-0.20424	4.46966	-5.75101	1.42E-08	5.46E-08	8.78293
MED4	-0.20431	6.663442	-2.22322	0.026578	0.042092	-4.8009
FOXJ1	-0.2044	5.856595	-3.28845	0.001067	0.00218	-1.91342
NBPF1	-0.20453	7.833358	-5.08477	4.95E-07	1.59E-06	5.35658
ADAP2	-0.20479	7.55603	-2.22139	0.026703	0.042263	-4.80491
LRP8	-0.2048	5.233626	-3.26967	0.001139	0.002319	-1.97369
TMX2	-0.20488	7.899176	-2.43388	0.015233	0.025391	-4.31641
KIAA0319	-0.20495	4.899586	-2.07409	0.038503	0.058863	-5.1178
PRSS35	-0.20495	3.083775	-5.92054	5.44E-09	2.18E-08	9.715094
CLCN6	-0.20501	6.319865	-3.65249	0.000283	0.00063	-0.67944
GNA12	-0.2052	7.013478	-4.35896	1.54E-05	4.09E-05	2.065921
CEP89	-0.20524	5.947143	-3.31958	0.000957	0.001972	-1.81275
STK4	-0.20527	8.761595	-2.59787	0.009614	0.016654	-3.90955
EMC6	-0.20529	8.166966	-3.10541	0.001991	0.003893	-2.48672
TRAPPC8	-0.20567	10.28199	-3.52424	0.000458	0.000988	-1.12833
PIK3C3	-0.20573	5.889087	-3.8496	0.000131	0.000308	0.040341
NBR2	-0.20608	5.920101	-4.62054	4.70E-06	1.34E-05	3.197722
HTATSF1	-0.20618	6.711219	-2.55469	0.010878	0.018654	-4.0192

DAPK1	-0.20624	6.57038	-3.4648	0.000569	0.001212	-1.33115
TRAV8-3	-0.20624	4.34946	-4.47526	9.16E-06	2.50E-05	2.561516
MRPS17	-0.20641	6.471845	-2.21377	0.027225	0.043015	-4.82162
GPATCH2L	-0.20669	6.156483	-3.76602	0.000182	0.000419	-0.26929
ARPC5L	-0.20669	7.738506	-2.96629	0.003136	0.005927	-2.9012
TBC1D19	-0.20674	4.905621	-3.72567	0.000213	0.000485	-0.41643
MBNL1	-0.2069	8.162908	-4.0199	6.58E-05	0.000161	0.691124
HN1L	-0.20714	6.296151	-6.32619	4.96E-10	2.26E-09	12.04247
TPMT	-0.20718	5.615689	-4.71109	3.07E-06	8.94E-06	3.603815
TDP1	-0.20721	5.388961	-5.69712	1.92E-08	7.27E-08	8.491687
ZNF830	-0.20724	7.588987	-4.06304	5.50E-05	0.000135	0.860199
STARD9	-0.20742	4.756703	-4.7954	2.06E-06	6.12E-06	3.988508
NR3C1	-0.20742	9.432513	-3.17384	0.001583	0.00315	-2.2761
RNPS1	-0.20745	8.685409	-3.7544	0.000191	0.000437	-0.31178
MAGT1	-0.20746	8.111797	-2.39687	0.016846	0.027839	-4.40466
NEBL-AS1	-0.20749	4.580419	-3.81609	0.00015	0.000348	-0.08458
CCDC12	-0.20766	8.318573	-2.88658	0.004037	0.007478	-3.1304
CDKAL1	-0.20772	4.611467	-4.44677	1.04E-05	2.82E-05	2.438973
REXO4	-0.20802	6.623765	-3.56747	0.00039	0.000852	-0.97874
GLUD1	-0.2081	8.907561	-3.0916	0.002085	0.004064	-2.52868
LOC38869	-0.20814	5.150419	-4.55896	6.25E-06	1.75E-05	2.925748
ZNF780B	-0.20835	4.274135	-5.1155	4.24E-07	1.37E-06	5.506178
C16orf93	-0.2084	5.74684	-3.57493	0.000379	0.00083	-0.95273
NR5A1	-0.20846	4.707998	-3.48533	0.000528	0.00113	-1.26147
GEMIN6	-0.2088	6.517086	-4.41138	1.22E-05	3.28E-05	2.287796
SPATA13	-0.20899	9.778452	-2.72181	0.006684	0.011926	-3.58485
TRAPPC6A	-0.209	6.782165	-2.20747	0.027665	0.043656	-4.83539
RPF1	-0.20914	5.504923	-7.31002	8.71E-13	5.31E-12	18.23664
PKIB	-0.20922	3.289737	-6.20976	1.00E-09	4.39E-09	11.36059
RRN3P3	-0.2094	4.607211	-4.98637	8.10E-07	2.54E-06	4.883108
DLAT	-0.20954	5.804157	-2.34146	0.01954	0.031838	-4.53426
KAT2A	-0.20955	7.158889	-3.6778	0.000257	0.000576	-0.58902
EPT1	-0.20967	6.345856	-3.10801	0.001974	0.003861	-2.47879
RBM18	-0.2097	6.412513	-1.96414	0.049982	0.074409	-5.33757
DDB1	-0.20978	8.545785	-3.08983	0.002097	0.004086	-2.53406
DSTNP2	-0.20981	6.958478	-3.76235	0.000185	0.000424	-0.28272
WWC3	-0.20984	8.798559	-2.44425	0.014807	0.024756	-4.29147
BLK	-0.20987	5.49511	-2.43298	0.015271	0.025445	-4.31858
DHX32	-0.21007	7.901506	-4.37923	1.41E-05	3.75E-05	2.151394
KANSL3	-0.21011	6.188827	-7.55159	1.64E-13	1.07E-12	19.87227
TM2D2	-0.21015	7.773209	-2.5358	0.011476	0.01959	-4.0666
CAPS2	-0.21026	3.066282	-6.20492	1.03E-09	4.52E-09	11.33246
CRBN	-0.21036	9.564277	-3.35438	0.000846	0.001756	-1.69915
STT3A	-0.21051	7.68636	-2.55098	0.010993	0.01883	-4.02854
CIITA	-0.21058	5.88753	-5.74813	1.45E-08	5.54E-08	8.767318
FMNL2	-0.21064	4.90549	-2.57691	0.01021	0.017606	-3.96301
TMA7	-0.21065	12.92231	-4.91432	1.16E-06	3.55E-06	4.541791
NUMA1	-0.21067	6.007107	-3.38788	0.000751	0.001574	-1.58873
SPON1	-0.21068	4.730672	-6.01266	3.20E-09	1.32E-08	10.23167
PLEKHN1	-0.21079	6.536972	-2.467	0.013908	0.023369	-4.23634
ITGB1	-0.21117	5.119763	-8.13899	2.37E-15	1.85E-14	24.02989
COPB1	-0.21117	9.033131	-3.85027	0.000131	0.000307	0.042846
KLF9	-0.21124	5.460608	-3.63454	0.000303	0.000672	-0.74317
WDR20	-0.21185	6.910075	-2.73019	0.006518	0.011649	-3.56237
TTC1	-0.21189	8.659874	-4.28993	2.09E-05	5.44E-05	1.777548
STRAP	-0.21198	9.889734	-3.26859	0.001144	0.002327	-1.97714
UFSP1	-0.21208	5.492851	-3.897	0.000109	0.000257	0.218766
ANXA2	-0.2122	11.46662	-2.30959	0.021255	0.034324	-4.60748

COG1	-0.21251	5.232376	-6.55691	1.20E-10	5.85E-10	13.42618
RING1	-0.21269	7.973987	-3.81858	0.000148	0.000345	-0.07533
PLXNB2	-0.2127	6.885437	-2.9866	0.002938	0.005576	-2.84186
YIPF4	-0.21297	5.413131	-3.31388	0.000976	0.002008	-1.83124
ZBED5	-0.21298	7.23428	-5.98608	3.73E-09	1.52E-08	10.0819
LSM4	-0.21334	6.277924	-3.21117	0.001394	0.002796	-2.15932
BAIAP3	-0.21338	6.340587	-4.10768	4.56E-05	0.000114	1.036962
AAED1	-0.21377	6.560045	-2.66613	0.007883	0.013878	-3.73256
TMEM147	-0.21412	8.195693	-2.71286	0.006865	0.012225	-3.60881
GPN2	-0.21413	6.135087	-4.27946	2.19E-05	5.68E-05	1.734174
N6AMT2	-0.21436	6.662729	-4.53949	6.84E-06	1.90E-05	2.840466
ABI3	-0.21438	7.521861	-2.69591	0.00722	0.012813	-3.65393
PDGFRB	-0.21449	5.399983	-2.83311	0.004767	0.008731	-3.28072
PPOX	-0.21457	5.057448	-4.91349	1.16E-06	3.56E-06	4.537912
HDHD1	-0.21466	8.04836	-2.74411	0.006252	0.011214	-3.52487
NUDT21	-0.21475	6.936232	-3.1181	0.001909	0.00374	-2.44799
ILVBL	-0.21475	5.61096	-4.89512	1.27E-06	3.88E-06	4.451599
C7orf73	-0.21483	6.673264	-2.15036	0.031932	0.049675	-4.95843
CXorf40A	-0.21488	7.8951	-2.71446	0.006832	0.012172	-3.60453
CPNE8	-0.21495	5.116573	-2.83475	0.004743	0.008694	-3.27613
RYBP	-0.21496	8.722582	-3.70256	0.000233	0.000528	-0.50003
FAM122A	-0.215	6.256835	-3.35856	0.000834	0.001733	-1.68542
CHD1	-0.21508	9.451902	-2.90363	0.003826	0.007113	-3.08187
ATP5D	-0.21509	7.632227	-2.04861	0.040942	0.062233	-5.16978
NEK3	-0.21529	5.124751	-4.18827	3.24E-05	8.25E-05	1.36069
RALGDS	-0.21542	7.465695	-2.63862	0.008544	0.014969	-3.80444
PSMB2	-0.21555	6.737061	-6.10788	1.83E-09	7.78E-09	10.77303
XBP1	-0.21565	7.107796	-3.6793	0.000255	0.000573	-0.58365
C2orf27A	-0.21568	4.79611	-7.25289	1.29E-12	7.68E-12	17.85631
GON4L	-0.21568	6.651622	-5.0043	7.41E-07	2.33E-06	4.96873
TBC1D24	-0.21575	5.571467	-2.66092	0.008005	0.014073	-3.74623
SLC10A7	-0.21575	4.496896	-4.16274	3.61E-05	9.14E-05	1.257496
KDELC1	-0.21579	4.295314	-4.21852	2.85E-05	7.29E-05	1.483765
CCL8	-0.21584	4.180583	-2.39245	0.017048	0.028127	-4.41511
ZKSCAN3	-0.21607	5.848372	-4.77034	2.32E-06	6.86E-06	3.873489
HOMEZ	-0.21614	4.561534	-4.61902	4.73E-06	1.34E-05	3.190973
MEX3C	-0.21616	6.475045	-5.5158	5.20E-08	1.87E-07	7.529802
DRG1	-0.21624	8.655696	-3.86014	0.000126	0.000296	0.079836
HECTD1	-0.21645	6.743426	-2.70058	0.00712	0.01265	-3.64154
VGLL4	-0.21647	7.166048	-3.86077	0.000125	0.000295	0.082203
PCNT	-0.21652	6.209573	-3.97437	7.93E-05	0.000192	0.514494
TRMU	-0.21659	5.993933	-3.20169	0.00144	0.002883	-2.1891
DKK3	-0.21677	3.727534	-11.1013	3.87E-26	1.02E-24	48.50689
NASP	-0.21683	7.140251	-3.39643	0.000729	0.00153	-1.56038
SELK	-0.21695	9.452377	-3.82988	0.000142	0.000331	-0.03331
SP3	-0.21708	7.634052	-5.76464	1.32E-08	5.07E-08	8.856979
PTDSS1	-0.21712	9.710801	-2.73451	0.006435	0.011517	-3.55076
HDDC3	-0.21729	7.024999	-3.57514	0.000379	0.000829	-0.95199
ACTR5	-0.21729	5.785978	-4.55484	6.37E-06	1.78E-05	2.907668
TRIM26	-0.21731	7.72762	-3.44382	0.000614	0.001302	-1.40195
SURF2	-0.21752	5.626382	-3.68988	0.000245	0.000552	-0.54567
FXYD2	-0.21765	5.576885	-3.86249	0.000125	0.000293	0.088661
SPOCK1	-0.21786	5.135082	-2.78374	0.005545	0.01004	-3.41708
COX14	-0.2179	7.511121	-2.38976	0.017172	0.028313	-4.42144
CNNM4	-0.21807	5.674919	-4.72536	2.87E-06	8.39E-06	3.668499
ASH1L	-0.21811	7.761039	-4.08466	5.02E-05	0.000124	0.945599
LOC10193	-0.21822	3.114419	-8.3819	3.82E-16	3.23E-15	25.8219
RPA3	-0.21826	7.47949	-2.79979	0.005281	0.009597	-3.37303

MTA3	-0.21852	5.513479	-5.68651	2.04E-08	7.69E-08	8.434602
ACTR8	-0.21869	6.468048	-5.15425	3.48E-07	1.14E-06	5.696033
PARP14	-0.21911	6.860341	-2.23634	0.025702	0.040781	-4.77198
PLCH2	-0.21912	5.69102	-4.03289	6.23E-05	0.000153	0.741844
LPAR1	-0.21918	5.919968	-3.01672	0.002665	0.005097	-2.75308
C12orf5	-0.21923	7.655036	-2.09764	0.036361	0.055876	-5.0692
GDPD5	-0.21932	7.247673	-3.24858	0.001226	0.002482	-2.04098
SLC2A13	-0.21933	4.531117	-5.45602	7.17E-08	2.54E-07	7.218836
TAMM41	-0.21963	5.505605	-6.86383	1.70E-11	9.05E-11	15.333
GIPC1	-0.21964	6.453151	-2.91842	0.003652	0.006816	-3.03956
LOC91548	-0.21969	7.979043	-4.85533	1.54E-06	4.66E-06	4.265792
NLN	-0.21971	5.94849	-4.67706	3.61E-06	1.04E-05	3.45037
DACT1	-0.21974	4.733867	-2.05191	0.040619	0.0618	-5.16309
TSFM	-0.21984	5.606738	-6.1511	1.42E-09	6.11E-09	11.02125
PWAR5	-0.22005	3.481783	-5.98987	3.65E-09	1.49E-08	10.10322
C4orf27	-0.22021	7.947644	-2.59715	0.009634	0.016685	-3.91139
BNC2	-0.22028	4.413984	-6.45862	2.21E-10	1.05E-09	12.83143
LOC10050	-0.22038	4.974609	-4.96393	9.05E-07	2.81E-06	4.776308
PROSER2	-0.22049	5.414721	-2.77374	0.005717	0.010323	-3.44443
PPP2R5C	-0.22068	9.113181	-4.1691	3.52E-05	8.91E-05	1.283152
PPP3CA	-0.22084	10.01443	-3.66937	0.000265	0.000594	-0.61922
FBLN5	-0.22088	5.734049	-3.05865	0.002324	0.004488	-2.62808
RNASET2	-0.22103	9.789762	-2.61783	0.009076	0.015816	-3.85825
ZNF148	-0.22107	7.511399	-3.22594	0.001325	0.002666	-2.11276
CTU2	-0.22123	5.077168	-2.87012	0.00425	0.00785	-3.17694
OSBPL7	-0.2213	5.82178	-6.73538	3.88E-11	2.00E-10	14.52585
CTPS2	-0.22134	4.820195	-4.94017	1.02E-06	3.15E-06	4.663707
TRPV6	-0.22142	6.192547	-3.87585	0.000118	0.000279	0.138883
RELN	-0.22143	5.004045	-4.75016	2.55E-06	7.51E-06	3.781284
LINC0061(	-0.22161	4.603996	-4.71721	2.99E-06	8.70E-06	3.631543
CTC-338M	-0.22167	5.734005	-3.52096	0.000463	0.000999	-1.13961
HSD17B10	-0.2217	8.195598	-3.31455	0.000974	0.002004	-1.82908
ADAMTS1	-0.22176	5.765112	-3.38862	0.000749	0.00157	-1.58626
HPS4	-0.22189	5.228443	-4.962	9.14E-07	2.84E-06	4.767153
KIAA0430	-0.22198	10.16744	-3.01871	0.002648	0.005065	-2.74718
SUN2	-0.22199	9.084576	-2.71939	0.006733	0.012008	-3.59135
TOMM22	-0.22207	6.554463	-3.55546	0.000408	0.000888	-1.02045
RMDN2	-0.22213	5.342566	-2.30535	0.021492	0.034667	-4.61713
UCHL5	-0.22214	5.514441	-3.42125	0.000666	0.001407	-1.47765
ALG2	-0.22216	6.96582	-5.00946	7.22E-07	2.27E-06	4.993455
PDZD4	-0.22218	5.707296	-3.83457	0.000139	0.000325	-0.01581
LIG3	-0.22224	5.859655	-5.60474	3.20E-08	1.18E-07	7.998132
GID8	-0.22238	8.386639	-5.00972	7.21E-07	2.27E-06	4.994681
PVRL3	-0.22261	3.807144	-5.95486	4.47E-09	1.81E-08	9.906733
PHC3	-0.22261	6.190815	-3.76415	0.000184	0.000422	-0.27612
LINC0095{	-0.22266	5.189582	-4.43201	1.11E-05	3.01E-05	2.375807
CATSPERB	-0.22284	3.985319	-5.88208	6.78E-09	2.69E-08	9.501496
FOPNL	-0.22289	6.576231	-3.22846	0.001314	0.002645	-2.10479
TCEA2	-0.2229	5.243476	-5.36165	1.18E-07	4.08E-07	6.734195
FAM81B	-0.22299	4.111365	-3.92279	9.78E-05	0.000233	0.316751
HNRNPU	-0.22299	7.644047	-4.69844	3.26E-06	9.46E-06	3.546659
PRDM11	-0.22302	4.651081	-8.54304	1.11E-16	9.90E-16	27.03352
TCAP	-0.22316	6.30726	-3.25965	0.00118	0.002395	-2.00571
MINOS1	-0.22331	7.719637	-4.79393	2.07E-06	6.16E-06	3.981758
MOB3B	-0.22332	5.728744	-2.63569	0.008617	0.015087	-3.81204
SLX4IP	-0.22341	6.413849	-2.78207	0.005574	0.010088	-3.42166
HLA-DRB6	-0.22353	6.736735	-3.51391	0.000475	0.001023	-1.16382
LIN9	-0.22353	3.305625	-8.66797	4.23E-17	3.93E-16	27.98522

STYK1	-0.22354	3.679745	-6.20643	1.02E-09	4.48E-09	11.34122
FDXACB1	-0.22358	5.866101	-4.14868	3.83E-05	9.67E-05	1.200947
CTSS	-0.22363	11.9611	-3.22608	0.001324	0.002665	-2.11231
NDUFV3	-0.22374	6.26972	-3.56416	0.000395	0.000862	-0.99026
SEC23IP	-0.22381	6.897172	-3.29315	0.00105	0.002147	-1.89828
REEP6	-0.22396	4.930333	-4.96796	8.87E-07	2.76E-06	4.795462
SEMA4F	-0.22398	6.056467	-7.72555	4.80E-14	3.31E-13	21.07717
LOC10028	-0.22406	6.284452	-3.40658	0.000702	0.00148	-1.52661
PIGG	-0.2241	5.96452	-7.27043	1.14E-12	6.85E-12	17.97283
FZD2	-0.22423	5.730384	-2.97745	0.003026	0.005733	-2.86864
RAB22A	-0.22441	8.099295	-3.75185	0.000193	0.000441	-0.32111
MCF2L-AS	-0.22468	5.257036	-5.19288	2.85E-07	9.42E-07	5.886547
CD248	-0.22472	6.526406	-3.72349	0.000215	0.000489	-0.42433
RBM26-AS	-0.22474	5.485941	-4.77986	2.22E-06	6.57E-06	3.917144
RPTOR	-0.22491	6.359253	-3.59029	0.000358	0.000787	-0.89904
FOXN3	-0.22505	7.416562	-6.43772	2.51E-10	1.18E-09	12.70594
SLC38A11	-0.22505	4.622207	-4.70411	3.18E-06	9.23E-06	3.572279
PCBP4	-0.22511	5.614387	-3.97133	8.03E-05	0.000194	0.502783
LRRK2	-0.22517	10.9405	-1.96484	0.0499	0.074305	-5.3362
ATP11C	-0.22518	5.719067	-3.58083	0.000371	0.000813	-0.93215
MAP3K8	-0.2253	7.724605	-2.4586	0.014234	0.023871	-4.25676
TNFRSF9	-0.22563	5.056839	-4.34872	1.61E-05	4.26E-05	2.02285
EFNA4	-0.22563	5.589319	-4.32984	1.75E-05	4.61E-05	1.943725
PHF1	-0.22594	7.881767	-3.05332	0.002365	0.004561	-2.64407
SLC35F3	-0.22609	3.765684	-5.15334	3.49E-07	1.14E-06	5.69154
CRAMP1L	-0.22635	5.310993	-6.93078	1.10E-11	5.97E-11	15.75879
BCL9L	-0.22644	7.123262	-2.96548	0.003144	0.00594	-2.90355
CREB1	-0.22664	8.122963	-3.78834	0.000167	0.000386	-0.18721
LOC10192	-0.22704	3.904628	-6.05492	2.50E-09	1.05E-08	10.47102
TAB3	-0.2273	8.598957	-3.31864	0.00096	0.001978	-1.81581
SLC35D3	-0.22736	3.967643	-1.99	0.047051	0.070526	-5.28694
CR2	-0.22739	4.354504	-3.1682	0.001613	0.003203	-2.29362
LOC10013	-0.22744	3.85778	-5.84528	8.37E-09	3.29E-08	9.298283
RRP8	-0.22761	5.374581	-6.37188	3.76E-10	1.74E-09	12.31308
WDFY2	-0.2277	7.662838	-3.82912	0.000142	0.000332	-0.03613
WDSUB1	-0.22773	6.935479	-2.06282	0.039566	0.06036	-5.14087
FAM178A	-0.22774	6.079006	-3.81968	0.000148	0.000344	-0.07126
DNLZ	-0.22791	5.682149	-2.01527	0.044329	0.066855	-5.23685
MEPCE	-0.22796	8.313604	-2.88013	0.004119	0.007618	-3.14868
CSTF2	-0.22828	5.559017	-5.7045	1.85E-08	6.99E-08	8.531416
HCG9	-0.22831	5.648701	-5.49829	5.71E-08	2.05E-07	7.438396
PEA15	-0.22844	7.102947	-2.30184	0.021691	0.03495	-4.62512
VDAC3	-0.22844	8.111481	-5.70136	1.88E-08	7.11E-08	8.51449
ANKZF1	-0.22846	7.481512	-2.88846	0.004013	0.007437	-3.12506
IL18BP	-0.22847	6.484156	-5.93008	5.15E-09	2.06E-08	9.768263
NCAM1	-0.2285	5.116817	-7.12461	3.05E-12	1.74E-11	17.01135
C10orf35	-0.22882	5.69439	-5.39579	9.89E-08	3.45E-07	6.908636
ZNF326	-0.22893	4.921999	-4.29348	2.06E-05	5.36E-05	1.792255
SLC9A3R1	-0.22895	9.377496	-3.77638	0.000175	0.000403	-0.23125
TSPYL4	-0.22896	7.64344	-2.19358	0.028654	0.045072	-4.8656
DNAJC30	-0.22896	5.843839	-7.40242	4.62E-13	2.89E-12	18.85706
FBXW7	-0.22897	6.862785	-3.78393	0.00017	0.000392	-0.20348
ARSK	-0.22913	5.189115	-3.62668	0.000312	0.000691	-0.77101
GDAP1	-0.2296	4.592504	-3.72624	0.000213	0.000484	-0.41434
LOC10192	-0.2297	7.520024	-2.79868	0.005299	0.009626	-3.37608
AMDHD1	-0.22974	4.197184	-4.14581	3.88E-05	9.78E-05	1.189399
TRAF3IP2-	-0.22992	4.818566	-8.75865	2.08E-17	2.00E-16	28.68272
FXR1	-0.22999	6.947037	-3.89893	0.000108	0.000255	0.226098

VPS45	-0.23031	6.383557	-4.78458	2.17E-06	6.43E-06	3.938761
ATAT1	-0.23039	5.454071	-5.97979	3.87E-09	1.58E-08	10.04654
OR7A10	-0.23051	3.785595	-6.63496	7.35E-11	3.66E-10	13.90396
LUZP1	-0.23073	7.080198	-6.63094	7.53E-11	3.75E-10	13.87927
CCDC62	-0.23086	5.431208	-6.10085	1.91E-09	8.10E-09	10.73284
FBXO11	-0.231	7.218721	-3.41232	0.000688	0.00145	-1.50749
PIK3R3	-0.23109	4.822319	-4.68461	3.48E-06	1.01E-05	3.484323
LAMB2P1	-0.23133	5.855344	-5.68503	2.06E-08	7.75E-08	8.42667
SEPW1	-0.23148	8.503853	-2.33876	0.01968	0.032032	-4.5405
CAMKMT	-0.23149	5.377402	-4.4538	1.01E-05	2.74E-05	2.46915
HNRNPAB	-0.23179	9.169415	-2.85125	0.004507	0.00829	-3.23001
CDK5RAP3	-0.23181	9.236304	-2.29571	0.022042	0.035456	-4.63904
RUVBL2	-0.2319	7.390879	-3.34119	0.000887	0.001836	-1.74233
LOC10012	-0.23205	3.90073	-3.93097	9.46E-05	0.000226	0.347932
RAD52	-0.23208	4.981347	-7.1871	2.01E-12	1.17E-11	17.42135
ITGB7	-0.23237	7.869701	-2.00403	0.045523	0.068447	-5.25921
MOCS2	-0.2324	4.674382	-3.16912	0.001608	0.003195	-2.29077
RCOR3	-0.23247	7.182301	-4.12672	4.21E-05	0.000106	1.112916
GAS1	-0.2326	4.295344	-5.73956	1.52E-08	5.80E-08	8.72084
GAS6-AS1	-0.23267	4.844669	-4.30929	1.92E-05	5.03E-05	1.857961
STK32C	-0.23278	5.89824	-3.41389	0.000684	0.001443	-1.50223
USP27X	-0.23285	5.715083	-4.83154	1.73E-06	5.20E-06	4.155312
KIAA1407	-0.23299	6.013564	-3.31495	0.000973	0.002002	-1.82777
APOBEC2	-0.233	5.778917	-4.09617	4.79E-05	0.000119	0.991229
8-Sep	-0.23306	5.361044	-5.74266	1.49E-08	5.71E-08	8.737662
ARHGAP25	-0.23309	10.7224	-2.77049	0.005773	0.010418	-3.4533
ITPR1	-0.23309	6.184416	-3.19799	0.001458	0.002918	-2.2007
CMTM7	-0.23311	9.27879	-3.93841	9.18E-05	0.00022	0.376372
KLF8	-0.23318	4.070467	-5.3605	1.19E-07	4.11E-07	6.728288
SPATA6	-0.23346	4.17452	-3.77614	0.000175	0.000404	-0.23213
WDR45B	-0.23363	9.092063	-5.15677	3.43E-07	1.12E-06	5.708417
COL16A1	-0.23371	4.899453	-4.05604	5.66E-05	0.000139	0.832641
LSM11	-0.2339	4.964595	-7.2919	9.86E-13	5.97E-12	18.1157
ST6GAL1	-0.23397	5.937135	-5.24267	2.21E-07	7.38E-07	6.134049
TTC18	-0.2343	4.637239	-6.89939	1.35E-11	7.24E-11	15.55871
RNF138	-0.23446	8.238914	-2.84659	0.004572	0.008406	-3.24308
ZNF440	-0.23462	4.163879	-6.10063	1.91E-09	8.11E-09	10.73159
AAK1	-0.23476	6.224813	-6.81143	2.38E-11	1.25E-10	15.00214
LOC10050	-0.23483	4.784228	-2.5735	0.01031	0.017763	-3.97165
ZNF518B	-0.23488	6.05222	-4.30541	1.95E-05	5.11E-05	1.841845
USP4	-0.23502	10.00879	-3.47884	0.000541	0.001155	-1.28354
C9orf64	-0.23509	8.382449	-3.08662	0.002119	0.004125	-2.54379
SLC22A18	-0.23515	7.913303	-2.60717	0.00936	0.016261	-3.8857
EFHC2	-0.2352	4.729412	-2.63538	0.008625	0.015096	-3.81284
ZNF418	-0.23549	5.616943	-4.44596	1.05E-05	2.83E-05	2.435516
MIOS	-0.23551	6.207061	-3.86549	0.000123	0.00029	0.099907
ANKS6	-0.23555	4.967295	-7.95439	9.21E-15	6.79E-14	22.69616
RFC3	-0.23574	5.205768	-3.22944	0.001309	0.002637	-2.10169
ZNF235	-0.23575	5.148179	-6.6838	5.39E-11	2.73E-10	14.20541
OTUD4	-0.23579	5.724138	-4.51862	7.52E-06	2.08E-05	2.74944
MEN1	-0.23587	7.179827	-2.55772	0.010784	0.018499	-4.01156
BIN1	-0.23603	7.559688	-2.50916	0.012368	0.020981	-4.13286
TMEM116	-0.23608	5.055494	-7.79521	2.91E-14	2.06E-13	21.56594
CDK6	-0.23616	5.511469	-4.75115	2.54E-06	7.47E-06	3.785832
ORAOV1	-0.23628	4.822075	-6.16451	1.31E-09	5.67E-09	11.09861
LOC10028	-0.23635	4.399907	-5.013	7.09E-07	2.24E-06	5.010383
YAF2	-0.23636	5.107603	-3.24206	0.001254	0.002533	-2.06172
COG6	-0.2364	4.784448	-3.67594	0.000259	0.00058	-0.59569

LINC00494	-0.23654	5.355632	-4.45497	1.00E-05	2.73E-05	2.474189
ZNF667	-0.23657	3.897692	-6.55848	1.19E-10	5.80E-10	13.43574
LOC28595	-0.23662	4.869079	-2.4338	0.015236	0.025395	-4.3166
CAPN10-A	-0.23673	5.010971	-5.42483	8.47E-08	2.98E-07	7.057797
ZFHX3	-0.23675	5.195485	-5.3486	1.27E-07	4.36E-07	6.66776
PANK4	-0.23676	6.968179	-2.62872	0.008794	0.01536	-3.83011
GFER	-0.23679	6.01282	-4.38005	1.40E-05	3.73E-05	2.154864
RAP1B	-0.23684	12.17103	-4.57492	5.81E-06	1.63E-05	2.995919
TULP3	-0.23705	6.035439	-6.58003	1.04E-10	5.09E-10	13.5672
CLN8	-0.23706	5.566721	-4.38193	1.39E-05	3.70E-05	2.162843
SHISA2	-0.23729	3.877658	-6.0319	2.86E-09	1.19E-08	10.34047
C3orf14	-0.2374	4.578018	-3.14273	0.001758	0.003463	-2.37241
SECISBP2L	-0.23762	7.752611	-2.33546	0.019853	0.03227	-4.54812
DCLRE1A	-0.23795	6.253272	-3.66873	0.000266	0.000595	-0.6215
BCAT2	-0.23795	6.310045	-4.77275	2.29E-06	6.78E-06	3.884542
ATP8A2	-0.2382	4.688461	-6.63516	7.34E-11	3.65E-10	13.90519
RBM28	-0.23826	6.159841	-3.46682	0.000565	0.001204	-1.32431
PHF3	-0.2387	7.276751	-3.83739	0.000138	0.000322	-0.0053
TMEM143	-0.23905	6.714383	-6.68765	5.26E-11	2.67E-10	14.22927
ERCC6L2	-0.23929	5.533038	-3.43569	0.000633	0.001339	-1.42928
KIAA0368	-0.23938	6.746228	-4.57739	5.74E-06	1.61E-05	3.006779
CYP11B2	-0.23947	6.013456	-3.16757	0.001616	0.003209	-2.29558
LTN1	-0.23949	6.131066	-3.02003	0.002637	0.005045	-2.74329
AP4S1	-0.23949	5.848012	-6.09664	1.96E-09	8.29E-09	10.70874
OTUD3	-0.23966	5.548975	-3.73283	0.000208	0.000473	-0.39042
TRIB3	-0.23967	6.934235	-2.56393	0.010596	0.018213	-3.99589
SRR	-0.23968	4.851351	-5.08255	5.00E-07	1.61E-06	5.345797
OLIG2	-0.23976	4.590764	-2.86415	0.00433	0.007984	-3.19377
ZFP69B	-0.23994	3.870855	-4.38339	1.38E-05	3.68E-05	2.168994
PCDH8	-0.2401	3.347636	-3.28129	0.001094	0.002233	-1.93642
MID1IP1	-0.24021	8.581397	-2.10638	0.035592	0.054822	-5.05102
PCGF1	-0.24021	7.557401	-3.72973	0.00021	0.000478	-0.40167
ARL1	-0.24023	6.248029	-3.06492	0.002277	0.004405	-2.60925
ANKRD39	-0.24038	5.309262	-3.40467	0.000707	0.001489	-1.53297
PTPDC1	-0.2404	5.043145	-5.98913	3.67E-09	1.50E-08	10.09903
MAGED2	-0.24045	7.461644	-4.48827	8.63E-06	2.37E-05	2.617707
MTM1	-0.24046	6.456342	-2.80056	0.005268	0.009577	-3.37089
GCDH	-0.24052	6.369748	-3.25527	0.001198	0.002429	-2.01967
LINC00920	-0.24091	4.914775	-5.9937	3.57E-09	1.46E-08	10.12478
POLG	-0.24096	6.408039	-4.97579	8.53E-07	2.66E-06	4.832702
TMEM80	-0.24096	7.023505	-4.6622	3.87E-06	1.11E-05	3.383651
TBC1D22A	-0.24111	7.636146	-5.61952	2.95E-08	1.09E-07	8.076621
RBM4B	-0.24112	6.887437	-3.63923	0.000297	0.000661	-0.72656
TP53TG1	-0.24138	5.92286	-3.74433	0.000199	0.000453	-0.34855
BORA	-0.24138	5.836871	-3.18007	0.001549	0.003091	-2.25669
TCTN2	-0.2414	6.06266	-5.86039	7.68E-09	3.03E-08	9.381581
ATP6V1G1	-0.24155	8.730911	-4.09608	4.79E-05	0.000119	0.990862
DNAH8	-0.24159	4.755334	-5.0243	6.70E-07	2.12E-06	5.064592
SH2D3C	-0.2416	7.714579	-2.85564	0.004446	0.00819	-3.2177
NBAS	-0.24183	5.097125	-5.5465	4.40E-08	1.60E-07	7.69072
DGKH	-0.24193	4.886992	-4.41476	1.20E-05	3.23E-05	2.302174
MEFV	-0.24226	5.273723	-3.63443	0.000303	0.000673	-0.74358
ZFYVE27	-0.24234	7.054288	-3.93029	9.49E-05	0.000227	0.345365
RSPH3	-0.24243	5.643404	-3.37686	0.000781	0.001633	-1.62517
BID	-0.24244	9.728884	-3.45918	0.000581	0.001235	-1.35018
FBXL17	-0.24272	4.792914	-5.3102	1.55E-07	5.29E-07	6.473161
RAB11FIP2	-0.24279	7.65523	-3.16855	0.001611	0.0032	-2.29254
MRPL17	-0.24281	6.478399	-2.99434	0.002865	0.005448	-2.81912

HINT2	-0.24297	7.420699	-3.23211	0.001297	0.002615	-2.09326
APRT	-0.243	7.880747	-2.1952	0.028537	0.044892	-4.86209
TIMMDC1	-0.24319	8.615526	-3.84891	0.000132	0.000308	0.037742
PACRGL	-0.24322	4.098285	-6.19471	1.09E-09	4.79E-09	11.27324
ARL6IP1	-0.24334	10.02225	-3.39791	0.000725	0.001523	-1.55546
ZNF829	-0.24335	4.102201	-8.31097	6.53E-16	5.40E-15	25.29435
ZNF697	-0.24349	6.234337	-2.00647	0.045262	0.068103	-5.25436
MCPH1	-0.24366	5.145785	-6.2254	9.11E-10	4.02E-09	11.45154
AHI1	-0.24367	4.050225	-9.62949	1.74E-20	2.40E-19	35.65834
VAPB	-0.24384	6.588592	-5.24855	2.14E-07	7.17E-07	6.163403
GP5	-0.2439	4.514846	-3.23343	0.001291	0.002605	-2.08906
MEA1	-0.24393	8.755967	-3.9085	0.000104	0.000246	0.262399
SMU1	-0.24396	6.61766	-5.07176	5.28E-07	1.69E-06	5.293474
ALDH16A1	-0.24403	6.721959	-2.49259	0.012954	0.021883	-4.17375
C6orf163	-0.24405	4.268854	-6.24768	7.97E-10	3.55E-09	11.58146
COA1	-0.24423	6.276322	-4.67238	3.69E-06	1.06E-05	3.429331
PLD4	-0.24426	5.228977	-2.86617	0.004303	0.00794	-3.18809
PSMC5	-0.24456	8.983588	-3.42077	0.000668	0.001409	-1.47928
ZC3H13	-0.24457	7.351031	-4.82154	1.81E-06	5.44E-06	4.109042
DYX1C1	-0.2449	3.467605	-4.83661	1.69E-06	5.08E-06	4.178801
NFAT5	-0.24497	8.314543	-2.37281	0.017973	0.029489	-4.46131
NUP153	-0.24511	6.982363	-3.37625	0.000783	0.001636	-1.62717
CHTF18	-0.24528	6.09398	-3.07291	0.002217	0.0043	-2.58519
INTS8	-0.24536	9.367501	-3.63212	0.000306	0.000678	-0.75175
DNA2	-0.24548	5.444761	-2.96853	0.003113	0.005888	-2.89467
CRABP2	-0.24551	5.956456	-4.1945	3.15E-05	8.04E-05	1.385979
CFC1B	-0.24559	5.453874	-5.47706	6.40E-08	2.28E-07	7.327946
PWWP2B	-0.24565	6.102298	-4.8361	1.69E-06	5.09E-06	4.176461
GAS2	-0.24583	3.645672	-5.93332	5.06E-09	2.03E-08	9.786323
ZNF362	-0.24596	5.987564	-7.05532	4.84E-12	2.72E-11	16.56023
KIAA1324L	-0.24597	5.053042	-6.96124	9.00E-12	4.94E-11	15.95368
POPDC2	-0.24621	4.772294	-6.02741	2.93E-09	1.22E-08	10.31502
HELLS	-0.24638	4.307959	-3.0986	0.002037	0.003978	-2.50745
TRAP1	-0.24645	5.390199	-6.77968	2.92E-11	1.52E-10	14.80278
MCM3	-0.24646	7.135098	-2.50346	0.012567	0.021289	-4.14697
ADCK1	-0.2465	6.434389	-5.06362	5.50E-07	1.76E-06	5.254118
YBEY	-0.24652	6.479906	-3.30313	0.001014	0.002079	-1.86606
DDX43	-0.24655	4.172223	-3.30945	0.000992	0.002037	-1.8456
ATP13A1	-0.24658	7.557788	-2.9456	0.00335	0.006295	-2.96128
C17orf67	-0.24664	6.319617	-3.82178	0.000147	0.000341	-0.06345
PIGT	-0.24669	7.807305	-4.27684	2.21E-05	5.73E-05	1.723317
PELI3	-0.2468	5.965339	-3.35495	0.000845	0.001753	-1.69728
LOC10029	-0.24682	4.534603	-5.70972	1.79E-08	6.80E-08	8.559566
CCDC15	-0.24697	4.493905	-3.9584	8.46E-05	0.000203	0.452999
CLUH	-0.24701	7.112539	-4.39272	1.33E-05	3.54E-05	2.208541
CERS6	-0.24708	5.793335	-2.81746	0.005002	0.009127	-3.3242
NAPB	-0.24722	6.609062	-3.52195	0.000461	0.000996	-1.13618
CLECL1	-0.2474	5.106027	-2.05026	0.040779	0.062013	-5.16643
FBRSL1	-0.24755	6.461417	-6.65547	6.45E-11	3.24E-10	14.03033
RPL26L1	-0.24762	8.322201	-4.32201	1.81E-05	4.77E-05	1.911026
ZNF100	-0.24766	4.341022	-3.51897	0.000467	0.001006	-1.14644
FSCN1	-0.24769	4.757483	-4.58193	5.62E-06	1.58E-05	3.026803
ZNF74	-0.24774	5.385769	-5.95115	4.56E-09	1.84E-08	9.885953
B3GALTL	-0.24785	4.579108	-5.45108	7.36E-08	2.61E-07	7.193292
COQ6	-0.24793	6.052503	-4.65732	3.96E-06	1.13E-05	3.361818
PPCS	-0.24805	8.574153	-4.43159	1.12E-05	3.01E-05	2.374001
CAHM	-0.24815	4.881258	-4.63204	4.46E-06	1.27E-05	3.248891
DYM	-0.24821	6.285797	-5.08113	5.04E-07	1.62E-06	5.338916

DCAF4	-0.24831	5.745577	-5.13193	3.90E-07	1.27E-06	5.586526
STXBP1	-0.24857	5.048986	-3.57079	0.000385	0.000842	-0.96716
NDUFB2	-0.24861	7.534247	-4.25379	2.44E-05	6.31E-05	1.628289
CASC1	-0.2489	3.55628	-8.23605	1.15E-15	9.23E-15	24.74089
ZIK1	-0.24905	5.042728	-6.30253	5.73E-10	2.59E-09	11.90296
METTL2B	-0.24905	4.472339	-3.65554	0.00028	0.000624	-0.66857
DAPP1	-0.24922	8.953889	-2.6059	0.009394	0.016315	-3.88897
TMEM194I	-0.24926	4.853536	-5.14573	3.63E-07	1.19E-06	5.654153
LINC0094	-0.2493	3.842678	-6.6096	8.62E-11	4.27E-10	13.74822
TACC1	-0.24936	7.564944	-5.76591	1.31E-08	5.04E-08	8.863895
NUDT1	-0.24937	6.347224	-2.99565	0.002853	0.005428	-2.81526
PDE12	-0.24939	5.6158	-8.52884	1.24E-16	1.10E-15	26.92605
SLC35D2	-0.24943	6.477198	-6.11268	1.78E-09	7.57E-09	10.80056
ZNF252P	-0.24961	6.191055	-5.41101	9.12E-08	3.20E-07	6.986711
LOC10272	-0.24964	3.72741	-4.78452	2.17E-06	6.43E-06	3.938521
MED10	-0.24981	7.324802	-4.59279	5.35E-06	1.51E-05	3.074736
PPARA	-0.24988	5.117172	-7.2092	1.73E-12	1.02E-11	17.5671
COIL	-0.24994	6.621958	-4.40159	1.28E-05	3.41E-05	2.246181
CXorf56	-0.24996	4.394133	-6.12272	1.68E-09	7.16E-09	10.85813
YIF1A	-0.25012	6.924429	-2.96246	0.003175	0.005991	-2.91236
RNF168	-0.25015	8.17644	-3.5466	0.000421	0.000915	-1.05115
STK36	-0.25026	6.128629	-6.73633	3.86E-11	1.98E-10	14.53177
XPC	-0.25044	8.65722	-3.81715	0.000149	0.000347	-0.08065
LINC00621	-0.25051	7.739574	-2.82274	0.004922	0.008992	-3.30956
HNRNPA2	-0.25052	9.008158	-6.31466	5.32E-10	2.41E-09	11.97444
ZNF175	-0.25055	4.998006	-5.79736	1.10E-08	4.25E-08	9.035398
ENPP2	-0.25056	4.891354	-2.26829	0.023672	0.037852	-4.70087
SIK1	-0.25057	5.892228	-4.39554	1.31E-05	3.50E-05	2.220492
FAM49A	-0.25058	8.418617	-2.96707	0.003128	0.005913	-2.89894
CSDE1	-0.25063	9.871515	-4.35582	1.56E-05	4.14E-05	2.052704
UBIAD1	-0.25066	6.054696	-8.0694	3.96E-15	3.03E-14	23.52422
NFS1	-0.2507	6.058855	-3.96039	8.40E-05	0.000202	0.460663
RFPL1S	-0.25078	3.753835	-5.66846	2.25E-08	8.46E-08	8.337789
SLC5A6	-0.2508	6.885332	-5.12082	4.12E-07	1.34E-06	5.532192
KMO	-0.2508	5.94343	-2.30044	0.021771	0.035073	-4.6283
PDE4DIP	-0.25082	4.097039	-9.70784	9.00E-21	1.27E-19	36.30983
LOC10192	-0.25082	3.454441	-7.59164	1.24E-13	8.19E-13	20.14767
LOC38983	-0.25101	4.314716	-6.29957	5.83E-10	2.63E-09	11.88557
TMEM106	-0.25107	6.186011	-4.3173	1.85E-05	4.86E-05	1.891343
ZDHHC16	-0.25119	7.211877	-3.5077	0.000486	0.001046	-1.18509
PIK3R4	-0.25134	5.087977	-10.0972	3.19E-22	5.36E-21	39.60323
INPP5B	-0.25134	6.307827	-4.97223	8.69E-07	2.71E-06	4.815769
CAND2	-0.25138	4.84151	-7.81329	2.56E-14	1.82E-13	21.69339
TNFRSF10	-0.25163	7.097906	-3.28114	0.001095	0.002234	-1.93691
TBC1D15	-0.25198	6.861442	-3.40355	0.00071	0.001495	-1.5367
SNW1	-0.25206	8.887877	-4.30993	1.91E-05	5.01E-05	1.860645
STAG3L3	-0.2523	6.209365	-2.83268	0.004773	0.008741	-3.28192
KLHL28	-0.25232	7.178322	-3.18233	0.001538	0.00307	-2.24965
SLC30A9	-0.25236	5.817319	-2.85506	0.004454	0.008203	-3.21934
EBF4	-0.25247	5.411351	-3.01369	0.002691	0.005145	-2.76204
GMEB2	-0.25295	7.82277	-5.4879	6.04E-08	2.16E-07	7.384279
FGFBP3	-0.2532	3.767177	-6.50939	1.61E-10	7.78E-10	13.13764
LOC33980	-0.25325	5.014055	-4.15686	3.70E-05	9.35E-05	1.23383
RSF1	-0.25356	7.080901	-3.40618	0.000703	0.001481	-1.52793
LOC10192	-0.25361	3.822055	-6.8659	1.68E-11	8.95E-11	15.34608
CCT8	-0.25364	7.019156	-5.69705	1.92E-08	7.27E-08	8.491322
VAMP1	-0.2539	6.734075	-4.38081	1.40E-05	3.72E-05	2.158092
C8orf76	-0.25396	6.691103	-2.90102	0.003858	0.007164	-3.08933

EP400	-0.25402	6.327657	-4.4971	8.29E-06	2.28E-05	2.65597
METTL13	-0.25406	7.009451	-5.08241	5.01E-07	1.61E-06	5.345141
TMEM64	-0.25407	5.582657	-3.39962	0.00072	0.001514	-1.54976
SLC15A4	-0.25424	7.971334	-4.56129	6.18E-06	1.73E-05	2.935957
ZNF792	-0.25428	4.803815	-3.6581	0.000277	0.000618	-0.65943
ARHGAP3:	-0.25453	4.636209	-5.6319	2.76E-08	1.02E-07	8.142459
MGAT5	-0.25468	6.818821	-6.38369	3.50E-10	1.62E-09	12.38326
FAM199X	-0.25477	7.001554	-4.00656	6.95E-05	0.000169	0.639188
SH3BP4	-0.25496	4.508953	-6.15616	1.38E-09	5.94E-09	11.05044
ZGRF1	-0.25509	3.532373	-7.16956	2.26E-12	1.31E-11	17.30599
LOC38990	-0.25539	3.619092	-6.33545	4.69E-10	2.14E-09	12.09717
SKP2	-0.25544	5.416689	-5.3483	1.27E-07	4.36E-07	6.666214
CTRL	-0.25565	5.683546	-2.8741	0.004197	0.007755	-3.16571
POLA1	-0.25573	5.599564	-3.44768	0.000606	0.001285	-1.38895
SORD	-0.25587	6.185164	-3.55596	0.000407	0.000887	-1.01874
MYLIP	-0.25593	8.164933	-2.81781	0.004997	0.009118	-3.32324
TMEM41A	-0.25604	6.740176	-5.428	8.33E-08	2.93E-07	7.074146
HAS3	-0.25625	4.518536	-6.35405	4.19E-10	1.93E-09	12.20727
CACNA2D	-0.25636	6.468287	-3.68189	0.000253	0.000568	-0.57437
TCEB3-AS	-0.25638	6.195497	-4.73914	2.69E-06	7.89E-06	3.731118
FAM19A2	-0.25642	5.03965	-2.91829	0.003653	0.006818	-3.03993
CCDC51	-0.25655	6.461096	-4.58005	5.67E-06	1.59E-05	3.018496
TBC1D23	-0.25668	8.284047	-3.72503	0.000214	0.000486	-0.41872
IMPACT	-0.25678	5.092056	-2.67221	0.007743	0.013654	-3.71656
CISD2	-0.25683	5.222339	-3.56471	0.000394	0.000861	-0.98832
TSPAN3	-0.25689	6.529529	-3.5237	0.000458	0.00099	-1.13017
CSNK2A2	-0.25691	7.412457	-4.65209	4.06E-06	1.16E-05	3.338385
NT5M	-0.257	6.104121	-3.33552	0.000905	0.001871	-1.76088
GCM1	-0.25712	4.506161	-3.84652	0.000133	0.000311	0.028821
STAMBP	-0.25752	6.209065	-6.39743	3.22E-10	1.50E-09	12.46511
TRAM1	-0.25753	8.457511	-4.00132	7.10E-05	0.000173	0.618798
RPL39	-0.25754	13.69008	-4.69912	3.25E-06	9.44E-06	3.549703
RNASEH2(	-0.25768	6.926564	-3.99175	7.38E-05	0.000179	0.581696
DTYMK	-0.25774	5.622271	-4.19841	3.10E-05	7.92E-05	1.401868
POLR3C	-0.25789	6.03878	-4.15275	3.77E-05	9.51E-05	1.217292
EFCAB7	-0.25791	4.785043	-2.14008	0.032757	0.050841	-4.98023
NUDT19	-0.25802	8.399416	-2.39577	0.016895	0.027911	-4.40724
POMZP3	-0.25822	5.054869	-2.33591	0.019829	0.03224	-4.54708
UBXN2A	-0.25825	8.142679	-3.81013	0.000153	0.000356	-0.10669
SENP3	-0.25827	6.379637	-4.59315	5.34E-06	1.51E-05	3.076331
FAHD2CP	-0.25849	4.86706	-4.71702	2.99E-06	8.70E-06	3.630684
USP33	-0.25856	7.69535	-5.70436	1.85E-08	7.00E-08	8.53069
ZNF436	-0.25863	6.204339	-4.56567	6.06E-06	1.70E-05	2.955236
COQ4	-0.25883	5.623851	-4.18733	3.25E-05	8.28E-05	1.356914
C19orf60	-0.25883	7.007554	-2.49834	0.012748	0.021571	-4.1596
TGIF1	-0.25903	5.851407	-4.28513	2.13E-05	5.55E-05	1.757656
FARS2	-0.25908	6.053922	-7.14945	2.58E-12	1.49E-11	17.174
MDM4	-0.25937	7.243787	-4.41297	1.21E-05	3.25E-05	2.294554
NCAPD3	-0.25941	6.797397	-3.52398	0.000458	0.000989	-1.12922
VMA21	-0.25965	5.7602	-7.76304	3.67E-14	2.57E-13	21.33974
USP42	-0.25976	5.622045	-7.08135	4.07E-12	2.31E-11	16.72926
RNFT2	-0.25986	4.923831	-5.7824	1.19E-08	4.61E-08	8.953713
HSPBAP1	-0.26021	8.777775	-2.69113	0.007323	0.012977	-3.66661
TTF2	-0.26027	5.249344	-2.20588	0.027776	0.043825	-4.83886
CENPJ	-0.26031	4.373543	-5.25033	2.12E-07	7.11E-07	6.172337
LOC28636	-0.26058	5.174434	-6.84254	1.95E-11	1.03E-10	15.1983
HIP1R	-0.26058	6.673763	-4.62705	4.56E-06	1.30E-05	3.226669
GPM6B	-0.26084	4.275395	-5.35739	1.21E-07	4.17E-07	6.712492

ATP5I	-0.2609	9.031213	-3.46965	0.000559	0.001192	-1.31471
LRRC75A	-0.26091	5.366143	-3.58482	0.000365	0.000802	-0.9182
MTSS1	-0.26095	6.315396	-5.48094	6.27E-08	2.24E-07	7.348097
NUP93	-0.26117	5.921447	-6.61736	8.21E-11	4.07E-10	13.79584
ACAP2	-0.26131	8.023563	-2.63835	0.00855	0.014979	-3.80512
TRIP11	-0.26159	5.290062	-4.15881	3.67E-05	9.29E-05	1.241664
ZNF674-A	-0.26169	3.842906	-7.51352	2.14E-13	1.39E-12	19.61155
CENPV	-0.26172	4.771002	-5.57747	3.72E-08	1.36E-07	7.853821
DHX58	-0.26172	6.51999	-2.91865	0.003649	0.006812	-3.0389
CWC27	-0.26178	6.366638	-3.05204	0.002375	0.004576	-2.64791
STRA13	-0.26186	7.8316	-5.35795	1.21E-07	4.16E-07	6.715328
ANKRD10	-0.26195	5.982067	-6.69383	5.06E-11	2.57E-10	14.26757
POU2F2	-0.26198	6.785678	-3.51709	0.00047	0.001012	-1.1529
CRLF3	-0.26199	11.34012	-4.71753	2.98E-06	8.69E-06	3.632967
MYO19	-0.26204	6.037106	-7.31505	8.42E-13	5.13E-12	18.27022
MEAF6	-0.26218	6.308229	-6.28346	6.43E-10	2.89E-09	11.79091
THG1L	-0.26229	5.971754	-4.79491	2.06E-06	6.14E-06	3.986253
EIF5	-0.26235	8.872793	-4.79698	2.04E-06	6.08E-06	3.99578
TUG1	-0.26237	7.520376	-6.28917	6.21E-10	2.80E-09	11.82447
TFAM	-0.26273	5.965398	-4.77527	2.27E-06	6.70E-06	3.896073
SMAD3	-0.26278	6.420788	-5.79027	1.14E-08	4.42E-08	8.996657
LHPP	-0.26295	6.626997	-4.27834	2.20E-05	5.70E-05	1.729548
DUSP28	-0.26299	7.747548	-4.02856	6.34E-05	0.000155	0.724939
EIF4A3	-0.26303	9.19633	-4.56962	5.95E-06	1.67E-05	2.972608
FLJ20021	-0.2632	6.614588	-4.21398	2.90E-05	7.43E-05	1.465255
TRA2B	-0.26328	7.329522	-4.67711	3.61E-06	1.04E-05	3.450585
LRP5L	-0.26342	5.63041	-5.6232	2.89E-08	1.07E-07	8.096198
TMEM14B	-0.26354	8.242265	-3.63246	0.000305	0.000677	-0.75056
USP48	-0.26367	6.971671	-5.30387	1.60E-07	5.45E-07	6.441215
HIBADH	-0.26386	5.198826	-5.55556	4.19E-08	1.53E-07	7.738341
EML6	-0.26396	5.127286	-4.0895	4.92E-05	0.000122	0.964777
DPY19L1	-0.26396	6.253295	-3.38055	0.000771	0.001613	-1.61298
TRIM51	-0.264	4.625942	-5.82582	9.34E-09	3.65E-08	9.191298
ITPRIPL1	-0.26417	5.601317	-5.26522	1.96E-07	6.61E-07	6.246867
MAPRE2	-0.26417	6.776858	-5.56006	4.09E-08	1.49E-07	7.76202
TET1	-0.26433	4.421752	-4.49601	8.34E-06	2.29E-05	2.651213
PUM2	-0.26455	8.114391	-6.85174	1.84E-11	9.76E-11	15.25643
FH	-0.26477	6.539532	-3.83393	0.00014	0.000326	-0.01822
SOCS2	-0.2648	4.991706	-3.88356	0.000115	0.000271	0.167954
AACS	-0.26487	6.529465	-3.37812	0.000778	0.001626	-1.621
C19orf24	-0.26499	6.555785	-3.14225	0.00176	0.003468	-2.37387
MRPL21	-0.2652	7.401903	-3.58753	0.000361	0.000795	-0.90872
LRRC37A2	-0.26547	6.150763	-4.08552	5.01E-05	0.000124	0.948977
ZNF670	-0.26568	3.447968	-4.2863	2.12E-05	5.52E-05	1.762496
TMEM57	-0.26569	6.232672	-3.66028	0.000274	0.000613	-0.65167
COG4	-0.26572	7.485111	-4.71797	2.98E-06	8.67E-06	3.634998
ZNF415	-0.2658	3.992676	-3.80059	0.000159	0.000369	-0.14199
VAMP8	-0.26583	9.238027	-2.15512	0.031556	0.049159	-4.94831
TMEM204	-0.26592	6.53194	-4.00891	6.88E-05	0.000168	0.648304
GSTA1	-0.26593	5.002414	-7.76015	3.75E-14	2.62E-13	21.31948
SLC25A15	-0.26604	5.614019	-5.27272	1.89E-07	6.37E-07	6.284466
MATR3	-0.26659	5.248859	-3.24709	0.001232	0.002494	-2.04573
TUFM	-0.26669	6.938438	-4.6871	3.44E-06	9.95E-06	3.495523
CCDC34	-0.26672	5.303748	-2.0143	0.04443	0.066996	-5.23876
BRCC3	-0.26675	6.360187	-3.06151	0.002302	0.004451	-2.61949
NME7	-0.26678	5.330274	-4.16488	3.58E-05	9.06E-05	1.266155
CEP350	-0.26683	8.476161	-3.46572	0.000567	0.001208	-1.32804
LOC10012	-0.26693	4.67149	-4.35611	1.56E-05	4.13E-05	2.053903

RGS1	-0.26706	4.482575	-2.48139	0.013364	0.022535	-4.20121
C2CD2L	-0.26725	6.394482	-6.13122	1.60E-09	6.83E-09	10.90689
SRSF1	-0.26742	7.285132	-6.93991	1.03E-11	5.64E-11	15.81718
IFRD2	-0.26757	6.726557	-3.72964	0.00021	0.000478	-0.40199
XRN1	-0.2676	9.426984	-3.25999	0.001178	0.002393	-2.00465
ZNF641	-0.26762	8.060452	-2.9288	0.003534	0.006613	-3.00975
NFATC1	-0.26766	6.673957	-7.12574	3.03E-12	1.73E-11	17.01872
BRMS1L	-0.26781	4.57173	-4.50303	8.07E-06	2.22E-05	2.681681
FKBP3	-0.26787	7.273475	-6.17266	1.25E-09	5.42E-09	11.14562
HARS2	-0.26806	8.012809	-4.26079	2.37E-05	6.13E-05	1.657085
SMC1A	-0.26814	6.81724	-6.55741	1.20E-10	5.84E-10	13.4292
PRKY	-0.26815	5.198867	-2.58892	0.009865	0.017067	-3.93244
C19orf54	-0.26833	7.152615	-4.73233	2.78E-06	8.14E-06	3.700127
KIAA0226	-0.26842	7.867977	-4.31774	1.85E-05	4.86E-05	1.893189
APIP	-0.26846	5.254775	-5.82859	9.20E-09	3.59E-08	9.206498
PLEKHA8	-0.2685	5.118605	-10.4159	1.94E-23	3.74E-22	42.36695
HSD17B4	-0.26854	8.889748	-3.89207	0.000111	0.000262	0.200138
WHSC1L1	-0.26894	7.122516	-5.30645	1.58E-07	5.38E-07	6.454225
GPATCH2	-0.26903	4.179632	-4.89812	1.25E-06	3.83E-06	4.465684
SCOC	-0.26917	6.500018	-2.55541	0.010856	0.018617	-4.0174
TTI1	-0.26922	6.676393	-3.52904	0.000449	0.000973	-1.1118
PDE7A	-0.26932	6.529774	-6.47027	2.05E-10	9.78E-10	12.90151
FKBP14	-0.26933	4.350522	-6.34581	4.41E-10	2.02E-09	12.15843
NDUFA9	-0.26933	8.700231	-3.66361	0.000271	0.000607	-0.63979
LINC-PINT	-0.26937	6.324334	-5.69297	1.97E-08	7.44E-08	8.469335
SEC63	-0.2695	6.398239	-4.79218	2.09E-06	6.21E-06	3.973713
KMT2C	-0.26956	8.008817	-3.65958	0.000275	0.000615	-0.65416
PSMD7	-0.26972	9.175665	-3.50655	0.000488	0.00105	-1.18902
ELOVL6	-0.26988	4.012499	-4.52474	7.31E-06	2.02E-05	2.776069
IVD	-0.27003	5.920512	-7.19685	1.88E-12	1.10E-11	17.4856
ADARB1	-0.2701	5.808025	-10.1223	2.56E-22	4.34E-21	39.819
CAMSAP1	-0.27021	4.914446	-9.11352	1.23E-18	1.37E-17	31.46547
ANKRD13/	-0.27042	9.294071	-3.14287	0.001757	0.003462	-2.37197
GTF2H5	-0.27051	5.013134	-7.10725	3.42E-12	1.95E-11	16.89799
EED	-0.27066	6.002655	-4.12478	4.24E-05	0.000106	1.105178
NUBP1	-0.27076	7.444685	-3.6681	0.000266	0.000597	-0.62375
EARS2	-0.27095	5.486088	-6.86535	1.68E-11	8.98E-11	15.34262
KCTD18	-0.27095	7.332174	-2.74719	0.006194	0.011117	-3.51656
CCDC101	-0.27095	7.249334	-4.54917	6.54E-06	1.82E-05	2.882838
MYSM1	-0.27137	6.444457	-4.42395	1.15E-05	3.11E-05	2.341379
LAS1L	-0.27144	6.264706	-6.50828	1.62E-10	7.83E-10	13.13094
PRKCZ	-0.27203	7.394788	-2.93133	0.003506	0.006565	-3.00248
C2orf68	-0.27206	7.113632	-3.97007	8.07E-05	0.000195	0.497922
SEMG1	-0.27212	3.940202	-5.32606	1.43E-07	4.88E-07	6.553376
DEXI	-0.27242	6.634591	-4.50924	7.85E-06	2.16E-05	2.708652
ALG5	-0.27261	6.290816	-4.63289	4.44E-06	1.27E-05	3.252697
KIAA1429	-0.27271	5.83725	-4.95885	9.28E-07	2.88E-06	4.752198
CES2	-0.27275	7.638048	-6.78345	2.85E-11	1.49E-10	14.82636
ARL6	-0.27286	3.454769	-9.0587	1.91E-18	2.08E-17	31.03014
C14orf132	-0.27294	5.284037	-6.384	3.49E-10	1.62E-09	12.38514
TOPORS-/	-0.27318	8.460488	-2.86096	0.004373	0.00806	-3.20276
ITGAL	-0.27336	9.9169	-3.07902	0.002173	0.004223	-2.56677
TMEM134	-0.27353	5.537554	-4.95502	9.46E-07	2.94E-06	4.734036
STK38L	-0.27355	8.37445	-3.38269	0.000765	0.001602	-1.60591
HIC2	-0.27359	5.672835	-6.44567	2.39E-10	1.13E-09	12.75367
RBM6	-0.27363	8.041559	-2.5986	0.009594	0.016628	-3.90769
ZNF611	-0.27366	8.325467	-3.17745	0.001563	0.003115	-2.26488
ITPR3	-0.27423	5.730481	-6.39543	3.26E-10	1.52E-09	12.4532

DARS	-0.27454	8.977926	-2.7822	0.005572	0.010086	-3.42131
NEFH	-0.27455	4.479367	-5.46946	6.67E-08	2.37E-07	7.288488
LONP1	-0.27457	7.009319	-3.3161	0.000969	0.001994	-1.82405
ZFP82	-0.27469	4.846679	-6.65312	6.55E-11	3.28E-10	14.01581
C17orf89	-0.27471	6.588316	-5.83632	8.80E-09	3.45E-08	9.248992
SCP2	-0.27473	9.454637	-3.43337	0.000638	0.00135	-1.43708
LATS1	-0.275	5.911078	-5.15411	3.48E-07	1.14E-06	5.69531
LTBP3	-0.27531	6.000707	-4.02761	6.37E-05	0.000156	0.721229
NAB1	-0.27552	5.790451	-4.13988	3.98E-05	0.0001	1.165605
ABCF2	-0.27561	6.046263	-7.42312	4.01E-13	2.52E-12	18.99689
CCDC82	-0.27563	6.515218	-4.81604	1.86E-06	5.58E-06	4.083632
INIP	-0.2757	6.780864	-5.68556	2.05E-08	7.73E-08	8.429541
CD101	-0.27583	5.830353	-3.45039	0.0006	0.001274	-1.37982
SURF1	-0.27592	8.173577	-5.75643	1.38E-08	5.31E-08	8.812351
SLC6A16	-0.27602	4.921336	-4.65063	4.09E-06	1.17E-05	3.331869
NFX1	-0.27608	5.618289	-5.3042	1.60E-07	5.44E-07	6.442847
CRY2	-0.27612	6.335069	-5.76582	1.31E-08	5.04E-08	8.86337
SNAPC4	-0.27623	6.604729	-7.23342	1.47E-12	8.69E-12	17.72726
GNB5	-0.27656	5.681152	-4.29147	2.07E-05	5.40E-05	1.783928
DLG1	-0.27678	5.462134	-6.2375	8.47E-10	3.76E-09	11.52206
LOC72968	-0.27692	6.781336	-5.90357	6.00E-09	2.39E-08	9.620697
TMEM238	-0.27697	4.267619	-4.63391	4.42E-06	1.26E-05	3.257253
PAQR7	-0.27709	5.26003	-4.79436	2.07E-06	6.15E-06	3.983706
RANBP2	-0.2773	7.898303	-3.26836	0.001145	0.002329	-1.97789
USP31	-0.27731	4.950012	-4.67337	3.67E-06	1.06E-05	3.433753
TSPY26P	-0.27753	5.33563	-7.5058	2.26E-13	1.46E-12	19.55882
EEF1E1	-0.27755	5.630479	-5.04867	5.93E-07	1.89E-06	5.181893
FLJ32255	-0.27755	6.910596	-2.66491	0.007911	0.013925	-3.73576
MGME1	-0.27756	8.347522	-3.21259	0.001387	0.002783	-2.15487
SFXN4	-0.27762	5.74317	-7.11495	3.25E-12	1.86E-11	16.94824
ANO9	-0.27796	6.050196	-3.06104	0.002306	0.004457	-2.62092
THAP9	-0.27813	3.860254	-4.97078	8.75E-07	2.73E-06	4.80888
SSPN	-0.27817	4.708771	-3.48987	0.000519	0.001112	-1.246
LYSMD4	-0.27838	5.753951	-5.93499	5.01E-09	2.01E-08	9.795639
SNRNP48	-0.2784	6.315951	-3.83462	0.000139	0.000325	-0.01565
EIF1AD	-0.27854	6.926702	-6.49753	1.74E-10	8.34E-10	13.06595
ZNF862	-0.27866	7.186371	-4.81517	1.87E-06	5.60E-06	4.07961
RSL24D1	-0.27888	9.368752	-3.82385	0.000145	0.000339	-0.05573
PCCB	-0.27911	6.426329	-3.70799	0.000229	0.000517	-0.48043
SOWAHC	-0.27943	5.078422	-2.70192	0.007092	0.012605	-3.63796
ZFP37	-0.27951	4.540983	-7.28687	1.02E-12	6.17E-12	18.08218
CALHM2	-0.27956	7.685024	-2.81743	0.005003	0.009127	-3.32428
CCDC132	-0.27961	6.061691	-4.51461	7.66E-06	2.11E-05	2.731954
EXOSC2	-0.27974	5.833534	-6.2681	7.05E-10	3.16E-09	11.70086
PTGDR2	-0.27986	6.145904	-3.3472	0.000868	0.0018	-1.72267
ZNF835	-0.28037	5.390292	-5.99086	3.63E-09	1.49E-08	10.1088
SLC47A1	-0.2804	4.593977	-5.07013	5.33E-07	1.71E-06	5.285585
PPP1R8	-0.28045	8.814033	-4.06852	5.37E-05	0.000133	0.881798
FOXP1-IT1	-0.28065	6.185421	-3.82039	0.000147	0.000343	-0.06859
PKI55	-0.28071	5.065775	-5.48943	5.99E-08	2.14E-07	7.39229
UTP18	-0.28075	6.484125	-4.30757	1.93E-05	5.06E-05	1.850831
COG3	-0.28079	6.910428	-3.64416	0.000292	0.00065	-0.70904
USP13	-0.28093	5.906756	-6.68051	5.51E-11	2.79E-10	14.18504
SUMO4	-0.28097	8.870317	-5.46542	6.82E-08	2.42E-07	7.267527
DNAJC16	-0.28098	6.57019	-5.05673	5.70E-07	1.82E-06	5.220789
ZNF618	-0.28098	5.279186	-8.38997	3.59E-16	3.04E-15	25.88216
PFDN4	-0.28106	6.186312	-6.49269	1.79E-10	8.58E-10	13.03671
MFSD2A	-0.28114	5.994555	-4.22709	2.74E-05	7.04E-05	1.518756

LOC10192	-0.28123	4.306544	-4.78889	2.12E-06	6.31E-06	3.958569
CBY1	-0.28123	6.693729	-5.66447	2.30E-08	8.64E-08	8.316416
PEX7	-0.28146	4.982653	-4.52484	7.31E-06	2.02E-05	2.776496
GPC2	-0.28163	4.978379	-4.11332	4.45E-05	0.000111	1.059451
ZNF7	-0.28166	5.002714	-9.18267	7.02E-19	8.09E-18	32.01747
RNF139-A	-0.28182	4.458402	-6.96476	8.79E-12	4.83E-11	15.97626
FAM208A	-0.2821	7.198169	-6.0046	3.35E-09	1.38E-08	10.18618
RNF114	-0.28232	8.591076	-4.71979	2.95E-06	8.60E-06	3.643232
NPC2	-0.28252	10.7591	-3.70825	0.000228	0.000517	-0.47948
UNC119B	-0.28268	6.019514	-8.26493	9.23E-16	7.50E-15	24.95376
EIF2AK4	-0.28279	4.756393	-3.96349	8.29E-05	0.0002	0.472597
DLEU1	-0.28285	6.715912	-3.48153	0.000535	0.001145	-1.2744
MED6	-0.28286	6.458791	-3.22182	0.001344	0.0027	-2.12577
LARP7	-0.28299	6.357067	-5.54829	4.36E-08	1.58E-07	7.700121
WDR91	-0.28334	5.800834	-6.27423	6.80E-10	3.05E-09	11.73678
AGL	-0.28346	7.968305	-2.30185	0.02169	0.03495	-4.6251
RUFY2	-0.28357	4.798686	-8.03929	4.94E-15	3.76E-14	23.30649
SEMA3C	-0.28364	4.994039	-2.99112	0.002895	0.005501	-2.82858
ATG12	-0.28378	7.429894	-5.05677	5.70E-07	1.82E-06	5.220969
CASP7	-0.28405	6.723797	-2.73803	0.006367	0.011405	-3.54129
C1orf64	-0.2841	5.013824	-4.58405	5.57E-06	1.57E-05	3.036173
ZNF292	-0.28426	8.338099	-3.57597	0.000377	0.000827	-0.94911
NOC4L	-0.28457	6.639225	-5.13103	3.91E-07	1.27E-06	5.582131
DAAM1	-0.2846	5.760654	-4.44053	1.07E-05	2.90E-05	2.412263
MYH3	-0.2846	4.223185	-5.83473	8.88E-09	3.48E-08	9.240262
TAOK3	-0.28482	7.740573	-5.74468	1.48E-08	5.64E-08	8.748572
TMIGD2	-0.28494	7.662434	-4.99916	7.60E-07	2.39E-06	4.944173
RIN2	-0.28496	6.399376	-2.66283	0.00796	0.014002	-3.74121
SUDS3	-0.28498	7.115752	-4.57177	5.89E-06	1.65E-05	2.982027
THYN1	-0.28502	7.886721	-4.31476	1.87E-05	4.91E-05	1.880783
SMC6	-0.28506	6.053521	-3.37876	0.000776	0.001623	-1.61889
ZNF607	-0.28559	4.053807	-6.47578	1.99E-10	9.48E-10	12.93471
TNFRSF13I	-0.28566	6.054434	-4.15443	3.74E-05	9.45E-05	1.224037
CXorf40B	-0.28579	7.786108	-3.54125	0.00043	0.000933	-1.06967
CNNM3	-0.28584	7.087625	-3.03085	0.002545	0.004882	-2.71114
RNF219	-0.28586	5.998947	-6.65437	6.50E-11	3.26E-10	14.02354
LOC10050	-0.28597	6.225749	-4.03758	6.11E-05	0.00015	0.7602
ZNF662	-0.28603	4.31999	-8.29773	7.21E-16	5.93E-15	25.19624
RNF103	-0.28622	8.703908	-4.40974	1.23E-05	3.30E-05	2.280816
MPC1	-0.28633	6.334845	-8.1309	2.51E-15	1.96E-14	23.97094
BRD3	-0.28643	8.744514	-4.56772	6.00E-06	1.68E-05	2.964246
THOC7	-0.2866	9.144115	-3.96376	8.28E-05	0.000199	0.473646
MUT	-0.2866	7.086766	-3.17284	0.001588	0.00316	-2.27922
IFFO1	-0.28665	8.680238	-3.76322	0.000184	0.000423	-0.27952
DUSP11	-0.28682	8.412762	-4.96383	9.05E-07	2.82E-06	4.775835
ARFGEF2	-0.28684	6.329648	-5.10427	4.48E-07	1.45E-06	5.451403
MTERF4	-0.28686	6.337933	-5.84157	8.54E-09	3.35E-08	9.277873
BDH1	-0.28687	6.078698	-4.43877	1.08E-05	2.92E-05	2.40472
CEBPG	-0.28711	6.945802	-3.49916	0.000502	0.001076	-1.21431
SGPP1	-0.28715	6.96623	-2.52591	0.0118	0.0201	-4.09129
PRRC1	-0.28734	7.793319	-3.63132	0.000306	0.00068	-0.75459
SMNDC1	-0.28742	9.772229	-3.50356	0.000494	0.00106	-1.19926
SPICE1	-0.28745	3.999022	-6.82894	2.13E-11	1.12E-10	15.11248
ZNF365	-0.28786	4.535698	-7.08689	3.92E-12	2.22E-11	16.76534
WDPCP	-0.28828	6.391388	-6.09928	1.93E-09	8.17E-09	10.72385
ID2B	-0.28847	4.023628	-7.02994	5.72E-12	3.19E-11	16.39593
RNF2	-0.28857	5.533483	-4.49675	8.31E-06	2.28E-05	2.654438
ATP2C1	-0.28881	6.478414	-2.78598	0.005508	0.009975	-3.41095

SLC25A12	-0.28898	6.491238	-3.60469	0.000339	0.000747	-0.84852
PGAP1	-0.28905	3.813633	-5.80143	1.07E-08	4.16E-08	9.057634
FAM204A	-0.28914	6.190527	-4.54636	6.62E-06	1.84E-05	2.870523
MIPEP	-0.28945	4.940387	-6.27947	6.58E-10	2.96E-09	11.7675
CD84	-0.28946	6.288881	-6.88364	1.49E-11	8.00E-11	15.45862
GATAD1	-0.28951	6.095954	-4.81993	1.83E-06	5.48E-06	4.101593
ABCF1	-0.28954	7.906762	-3.58661	0.000363	0.000797	-0.91194
HUWE1	-0.2897	7.558534	-11.2574	9.05E-27	2.63E-25	49.94086
SLC25A23	-0.28995	5.240824	-6.8118	2.38E-11	1.25E-10	15.0045
RASAL3	-0.29026	8.325001	-3.63264	0.000305	0.000677	-0.7499
SPAG9	-0.29027	7.390577	-3.14954	0.001718	0.003395	-2.35141
DPP8	-0.29033	8.703546	-5.60667	3.17E-08	1.17E-07	8.008363
SMC4	-0.29034	6.642909	-3.24619	0.001236	0.002501	-2.04859
ERI3	-0.29037	5.794356	-3.85269	0.00013	0.000304	0.051883
EIF2S3	-0.29057	9.564766	-4.17112	3.49E-05	8.84E-05	1.291324
KIAA0753	-0.29064	7.290496	-5.75317	1.41E-08	5.40E-08	8.794672
USP47	-0.29091	7.349772	-4.19945	3.09E-05	7.89E-05	1.406072
POGZ	-0.29095	7.430136	-4.44211	1.06E-05	2.88E-05	2.418991
KRTCAP2	-0.29096	7.242886	-6.28792	6.26E-10	2.82E-09	11.81711
MDM1	-0.29101	5.950253	-2.96183	0.003181	0.006003	-2.9142
LOC10272	-0.29124	6.245201	-4.74526	2.61E-06	7.68E-06	3.758991
PAFAH2	-0.29128	5.162608	-5.16355	3.32E-07	1.09E-06	5.741783
LFNG	-0.29134	7.10982	-3.39888	0.000722	0.001518	-1.55224
CTNNBL1	-0.29134	7.137945	-4.40219	1.27E-05	3.40E-05	2.248696
SMEK2	-0.29143	6.701046	-5.87563	7.04E-09	2.79E-08	9.465779
SLC41A3	-0.29148	6.914486	-6.16278	1.32E-09	5.72E-09	11.08857
SASH1	-0.29148	6.353577	-2.74474	0.00624	0.011195	-3.52318
ZCCHC24	-0.29161	6.375077	-3.33867	0.000895	0.001852	-1.75056
FLJ37453	-0.29188	5.95465	-8.33928	5.27E-16	4.40E-15	25.50448
DAG1	-0.29194	6.105731	-7.52003	2.04E-13	1.33E-12	19.65609
CBX1	-0.29197	8.828813	-4.53969	6.83E-06	1.90E-05	2.841355
LRRC58	-0.29205	5.717889	-4.84366	1.63E-06	4.92E-06	4.211529
ACP1	-0.29222	7.748805	-4.08536	5.01E-05	0.000124	0.948376
PHPT1	-0.29224	7.312301	-4.70151	3.22E-06	9.34E-06	3.560514
PSMB4	-0.29239	9.718854	-4.67007	3.73E-06	1.07E-05	3.418939
ZNF584	-0.29247	5.988838	-5.67948	2.12E-08	7.98E-08	8.396887
MICALL1	-0.29272	6.942836	-5.9162	5.58E-09	2.23E-08	9.690888
TTC28	-0.29279	4.555146	-8.76018	2.05E-17	1.98E-16	28.69454
SPIB	-0.29302	5.875389	-2.95415	0.00326	0.006136	-2.9365
ACP5	-0.29307	6.921145	-3.50309	0.000495	0.001062	-1.20086
ZNF354C	-0.29315	4.840263	-8.77561	1.82E-17	1.76E-16	28.81383
PMS2P1	-0.29318	7.652356	-4.94822	9.78E-07	3.03E-06	4.701828
PELP1	-0.29329	6.177917	-3.62123	0.000318	0.000705	-0.79026
ZNF577	-0.29346	5.447337	-7.18692	2.01E-12	1.17E-11	17.42019
SGCB	-0.29358	4.25245	-7.24266	1.38E-12	8.19E-12	17.78844
VPS52	-0.29363	8.983352	-5.59106	3.45E-08	1.26E-07	7.925636
OGT	-0.29384	8.016113	-5.39651	9.85E-08	3.44E-07	6.91231
PPA2	-0.29385	8.537695	-3.69619	0.000239	0.00054	-0.52295
LMLN	-0.29385	4.964497	-9.2986	2.72E-19	3.29E-18	32.94982
DNAJC19	-0.29438	6.785642	-5.38248	1.06E-07	3.68E-07	6.840493
FBXO31	-0.29456	6.041587	-6.2127	9.83E-10	4.32E-09	11.37766
USP7	-0.29461	7.82615	-6.45636	2.24E-10	1.06E-09	12.81782
ABHD15	-0.29488	6.029169	-3.81435	0.000151	0.000351	-0.09103
NUBPL	-0.29499	3.863008	-4.87696	1.39E-06	4.22E-06	4.366625
CASD1	-0.29527	4.557491	-7.48646	2.58E-13	1.66E-12	19.42691
TSR1	-0.2953	6.026858	-5.21995	2.48E-07	8.26E-07	6.020846
RFXAP	-0.29541	5.021209	-3.94824	8.82E-05	0.000212	0.41402
PSMB1	-0.29546	8.608787	-5.8429	8.48E-09	3.33E-08	9.285179

TMEM186	-0.29553	6.170322	-5.21376	2.56E-07	8.51E-07	5.990101
COLCA1	-0.29562	7.521015	-5.41653	8.85E-08	3.11E-07	7.015123
DUSP10	-0.29564	5.842993	-5.18671	2.94E-07	9.70E-07	5.856045
GART	-0.29574	5.111981	-6.83637	2.03E-11	1.07E-10	15.15933
SKI	-0.29576	7.088136	-3.15741	0.001673	0.003314	-2.32707
ETV7	-0.29577	5.081882	-2.86956	0.004257	0.007863	-3.17853
MCC	-0.29591	4.563332	-6.27453	6.78E-10	3.05E-09	11.73855
BCAS4	-0.29593	5.369229	-6.77113	3.09E-11	1.60E-10	14.74918
SEC24B	-0.29594	9.045494	-3.73246	0.000208	0.000474	-0.39174
PLCB1	-0.29608	5.347801	-5.2927	1.70E-07	5.76E-07	6.384886
DYNC2H1	-0.29618	4.934899	-6.42684	2.68E-10	1.26E-09	12.6408
ANXA7	-0.29631	9.998674	-5.13988	3.74E-07	1.22E-06	5.625449
MDH2	-0.2965	7.851633	-4.58339	5.59E-06	1.57E-05	3.03324
TUFT1	-0.29654	6.233993	-2.28221	0.022831	0.036635	-4.66957
PCDH9	-0.29664	4.519157	-2.41303	0.016124	0.026761	-4.36629
ECI1	-0.29669	6.407686	-3.97723	7.84E-05	0.00019	0.525537
ZMAT3	-0.29673	7.150202	-4.03337	6.22E-05	0.000152	0.743734
MRPL20	-0.29706	6.07682	-9.81834	3.52E-21	5.24E-20	37.2351
LGMN	-0.29713	4.660278	-4.18925	3.23E-05	8.22E-05	1.36469
LOC10012	-0.29719	5.863601	-6.54731	1.27E-10	6.20E-10	13.36777
IQCH-AS1	-0.29722	6.014317	-5.43048	8.22E-08	2.90E-07	7.086939
DHFRL1	-0.29726	6.027712	-3.16825	0.001613	0.003203	-2.29347
LLPH	-0.2974	6.319637	-4.98121	8.31E-07	2.60E-06	4.858491
CLEC10A	-0.29753	5.914514	-2.57783	0.010183	0.017562	-3.96066
TRIM66	-0.29753	5.803407	-8.83602	1.13E-17	1.12E-16	29.28221
LOC28402	-0.2976	6.488072	-5.71998	1.69E-08	6.44E-08	8.614917
TGFBRAP1	-0.29763	6.920005	-6.83249	2.08E-11	1.10E-10	15.13486
PRKCI	-0.29784	6.046079	-4.84129	1.65E-06	4.97E-06	4.200536
RABGAP1	-0.29796	7.146537	-5.9427	4.79E-09	1.93E-08	9.838718
CDK12	-0.29802	7.131256	-5.50419	5.53E-08	1.99E-07	7.469165
SMARCAL:	-0.29815	6.157239	-4.31728	1.85E-05	4.86E-05	1.89128
ZMIZ1	-0.29829	7.042	-6.88233	1.51E-11	8.07E-11	15.45028
NAGPA	-0.29839	7.287906	-3.58632	0.000363	0.000798	-0.91295
ZC3H4	-0.2984	6.598348	-10.3893	2.46E-23	4.68E-22	42.13359
ZC3HAV1L	-0.29841	5.039219	-2.05919	0.039913	0.060844	-5.14827
LOC10050	-0.2986	7.137093	-2.8534	0.004477	0.008242	-3.224
SNX22	-0.2987	6.326541	-4.74321	2.64E-06	7.75E-06	3.749652
LINC0011(	-0.2987	5.476954	-4.18583	3.27E-05	8.33E-05	1.350819
TTC22	-0.29877	5.105908	-9.80985	3.79E-21	5.61E-20	37.16373
ACAD9	-0.29886	7.011771	-4.57983	5.68E-06	1.59E-05	3.017549
ARMCX1	-0.29897	4.442843	-3.80518	0.000156	0.000363	-0.12501
PRMT1	-0.29897	7.832862	-2.29075	0.022329	0.035889	-4.65027
HEATR3	-0.29931	5.94135	-2.6939	0.007263	0.012881	-3.65927
DPH7	-0.29952	6.587052	-6.31873	5.19E-10	2.36E-09	11.99844
TCF25	-0.29957	7.826334	-5.53002	4.81E-08	1.74E-07	7.604263
HSPA13	-0.30011	6.842536	-2.23795	0.025596	0.040629	-4.76843
MICB	-0.3002	9.043121	-3.29146	0.001056	0.002158	-1.90371
NUFIP2	-0.30022	8.736559	-4.61886	4.74E-06	1.35E-05	3.19026
LOC10050	-0.3003	6.4812	-5.46602	6.80E-08	2.41E-07	7.270634
MTF2	-0.30038	7.368383	-3.85892	0.000126	0.000297	0.075247
RAB30-AS	-0.30039	5.442379	-3.57914	0.000373	0.000818	-0.93806
HSPH1	-0.3005	6.607126	-2.11318	0.035004	0.05401	-5.03684
SLC25A29	-0.30058	5.882782	-7.09184	3.79E-12	2.15E-11	16.79755
RAB8A	-0.30064	10.49108	-5.22272	2.45E-07	8.15E-07	6.034646
N4BP2L1	-0.30066	5.607853	-8.66772	4.23E-17	3.94E-16	27.98335
DYNC1LI1	-0.3007	8.63366	-4.92272	1.11E-06	3.41E-06	4.581348
C2CD3	-0.30074	6.332101	-7.57172	1.42E-13	9.36E-13	20.0105
ARPIN	-0.30075	5.748508	-6.33981	4.57E-10	2.09E-09	12.12294

ZNF34	-0.3008	5.183524	-5.02088	6.82E-07	2.16E-06	5.04818
SLC14A1	-0.30084	4.066725	-2.1836	0.029384	0.04614	-4.88721
IFT46	-0.30091	5.7986	-6.36997	3.80E-10	1.75E-09	12.30169
BLCAP	-0.30091	8.308325	-3.60883	0.000334	0.000737	-0.83398
PTRHD1	-0.30093	8.169372	-4.7434	2.64E-06	7.74E-06	3.750501
COX19	-0.30098	6.577228	-7.02801	5.80E-12	3.23E-11	16.3835
GID4	-0.30101	5.744447	-5.25172	2.11E-07	7.06E-07	6.179258
RFWD3	-0.30102	5.565183	-3.39121	0.000742	0.001557	-1.57768
GPN3	-0.30114	7.442785	-2.93565	0.003458	0.006485	-2.99002
GLIDR	-0.30119	6.6473	-3.96657	8.19E-05	0.000197	0.484448
GTSF1	-0.30139	6.107736	-3.94287	9.02E-05	0.000216	0.393433
ULK4	-0.30159	4.765257	-7.49774	2.39E-13	1.54E-12	19.50385
TTBK2	-0.30169	4.900761	-3.89289	0.00011	0.000261	0.20323
POLH	-0.30178	5.00182	-8.36985	4.18E-16	3.52E-15	25.73202
TM9SF3	-0.30205	7.16917	-8.2809	8.19E-16	6.68E-15	25.07175
RPP14	-0.30215	5.912482	-6.13468	1.56E-09	6.70E-09	10.92678
USO1	-0.30218	8.663683	-3.95534	8.57E-05	0.000206	0.441257
FCRLB	-0.30232	5.434932	-4.67111	3.71E-06	1.07E-05	3.423623
CD200	-0.30252	5.046366	-2.72772	0.006567	0.011729	-3.56901
NACAP1	-0.30262	11.58439	-7.68369	6.46E-14	4.40E-13	20.78517
AASDHPPT	-0.30266	6.785132	-4.46458	9.61E-06	2.62E-05	2.515517
INPP5F	-0.30267	4.924801	-5.21027	2.61E-07	8.65E-07	5.972746
MGAT4A	-0.30283	7.571961	-3.97569	7.89E-05	0.000191	0.519613
CNOT1	-0.30284	8.997561	-7.94724	9.70E-15	7.14E-14	22.64502
SH3KBP1	-0.30289	9.714568	-5.65066	2.49E-08	9.28E-08	8.242534
HAUS2	-0.30316	6.280113	-5.14132	3.71E-07	1.21E-06	5.632506
FUT8-AS1	-0.30321	4.589994	-7.11577	3.23E-12	1.85E-11	16.9536
PNMA6A	-0.30374	5.375508	-4.03927	6.07E-05	0.000149	0.766844
UMPS	-0.30386	5.731083	-8.09806	3.20E-15	2.47E-14	23.73209
BANF1	-0.30386	8.851036	-2.66827	0.007833	0.013806	-3.72693
CSTF1	-0.3039	6.135636	-3.9129	0.000102	0.000242	0.2791
TCF3	-0.30434	6.342397	-8.13608	2.42E-15	1.89E-14	24.0087
DPP7	-0.30449	6.215406	-5.82067	9.62E-09	3.75E-08	9.163034
ARHGAP3	-0.30461	4.638526	-6.40138	3.14E-10	1.46E-09	12.48865
C21orf59	-0.30469	5.638678	-7.72665	4.76E-14	3.29E-13	21.08483
DSTN	-0.30474	7.259228	-5.0329	6.42E-07	2.04E-06	5.10593
PTP4A2	-0.30479	11.21448	-5.61622	3.01E-08	1.11E-07	8.059057
PLA2G16	-0.30494	6.445618	-4.63645	4.37E-06	1.25E-05	3.268541
PRDX4	-0.30496	7.634402	-2.00426	0.045498	0.068419	-5.25874
ABCC5	-0.3051	7.362745	-3.67169	0.000263	0.000589	-0.61091
LYSMD1	-0.30547	5.646915	-4.56706	6.02E-06	1.69E-05	2.961321
NDUFB8	-0.30567	8.165412	-4.76991	2.32E-06	6.87E-06	3.871544
TRAF1	-0.3057	5.588037	-6.04086	2.71E-09	1.13E-08	10.39119
SMAD4	-0.30584	6.448548	-4.44803	1.04E-05	2.81E-05	2.444375
WDR52	-0.3059	4.379162	-7.85482	1.90E-14	1.36E-13	21.98705
ZNF597	-0.30592	5.111982	-4.77257	2.30E-06	6.79E-06	3.883718
CDAN1	-0.30595	5.351093	-7.48654	2.58E-13	1.66E-12	19.42747
LINC0090	-0.30602	7.889739	-3.30416	0.00101	0.002072	-1.86274
VPS13D	-0.30607	6.711908	-6.79722	2.61E-11	1.37E-10	14.91282
PNPLA4	-0.30612	5.123642	-6.48951	1.82E-10	8.74E-10	13.01752
HMGXB4	-0.30615	7.34752	-5.15263	3.51E-07	1.15E-06	5.688032
METTL21A	-0.3065	6.903165	-2.58251	0.010048	0.017353	-3.94878
MPZL1	-0.30689	6.422842	-3.83689	0.000138	0.000323	-0.00719
CTSF	-0.30693	6.129316	-6.53514	1.37E-10	6.68E-10	13.2938
RBM5	-0.30714	8.24161	-4.01651	6.67E-05	0.000163	0.677912
UBB	-0.30732	13.07017	-4.93345	1.05E-06	3.25E-06	4.631984
CCDC19	-0.30739	6.106716	-3.88642	0.000113	0.000268	0.178751
PUF60	-0.30741	8.85882	-3.98961	7.45E-05	0.000181	0.573413

LIN7C	-0.30752	7.411272	-3.87024	0.000121	0.000285	0.117761
LEO1	-0.30759	6.550979	-3.16147	0.00165	0.003273	-2.31449
KLHDC4	-0.30841	6.212198	-6.71074	4.54E-11	2.32E-10	14.37255
INTS10	-0.30846	6.04178	-6.94632	9.92E-12	5.42E-11	15.85813
PRPF8	-0.30849	8.480489	-2.69371	0.007267	0.012885	-3.65977
HSPB11	-0.30864	6.741893	-4.10154	4.68E-05	0.000116	1.012543
SPATA20	-0.30875	6.769513	-2.97117	0.003087	0.005842	-2.88698
TRPM7	-0.30879	5.309274	-7.38469	5.22E-13	3.24E-12	18.73751
RNF169	-0.30889	8.400788	-4.401	1.28E-05	3.42E-05	2.243655
TMEM30B	-0.30894	4.424511	-6.71246	4.49E-11	2.29E-10	14.38319
ACSF2	-0.30894	5.873383	-3.86339	0.000124	0.000293	0.092027
EZH2	-0.30894	5.545307	-2.35491	0.018853	0.030822	-4.50307
SCAF11	-0.30895	9.095881	-6.318	5.22E-10	2.37E-09	11.99414
GPR68	-0.30899	5.392941	-7.18443	2.04E-12	1.19E-11	17.40381
SLC35B3	-0.30905	6.897383	-4.62437	4.62E-06	1.31E-05	3.214781
ZNF850	-0.3091	3.737435	-5.34448	1.30E-07	4.45E-07	6.646819
DAP3	-0.30954	7.781341	-7.89218	1.45E-14	1.05E-13	22.25227
CBFA2T2	-0.30968	6.228816	-8.65327	4.74E-17	4.38E-16	27.8727
C11orf49	-0.30984	5.999493	-7.61634	1.04E-13	6.94E-13	20.31808
NUDT16L1	-0.30985	6.841816	-4.63022	4.49E-06	1.28E-05	3.240815
IDH3A	-0.31	6.022556	-4.28521	2.13E-05	5.54E-05	1.757977
NSMAF	-0.31008	7.290245	-3.55134	0.000414	0.0009	-1.03476
CNOT2	-0.31018	6.496552	-8.15745	2.06E-15	1.62E-14	24.16459
KCTD15	-0.31023	5.42727	-7.59858	1.18E-13	7.82E-13	20.19547
TTLL5	-0.31026	4.878754	-9.78003	4.88E-21	7.10E-20	36.91348
CARF	-0.31051	3.955973	-4.22979	2.71E-05	6.96E-05	1.52983
PDK1	-0.31056	7.15821	-4.9116	1.17E-06	3.60E-06	4.529016
KCTD10	-0.3107	7.621815	-7.16352	2.35E-12	1.36E-11	17.26632
PPP6R2	-0.31076	6.360355	-6.79495	2.65E-11	1.39E-10	14.89858
TM2D3	-0.31081	9.875965	-5.08539	4.93E-07	1.59E-06	5.359609
CEP63	-0.31125	7.174389	-3.57968	0.000372	0.000816	-0.93615
EIF2B3	-0.31131	6.537271	-5.3833	1.06E-07	3.67E-07	6.844696
ANAPC7	-0.31133	7.15659	-5.15289	3.50E-07	1.14E-06	5.689328
JAGN1	-0.31136	8.229744	-4.71748	2.98E-06	8.69E-06	3.63274
ASNSD1	-0.31138	8.587016	-2.97167	0.003082	0.005833	-2.88552
DNAJC21	-0.31147	4.643375	-6.78863	2.76E-11	1.44E-10	14.85886
MRPL22	-0.31157	7.307242	-4.69917	3.25E-06	9.44E-06	3.549954
FLJ45513	-0.31166	5.683144	-5.40009	9.66E-08	3.38E-07	6.930654
MAPKAPK	-0.31178	5.827904	-6.51949	1.51E-10	7.33E-10	13.19881
PACSIN1	-0.31188	5.157232	-6.12677	1.64E-09	7.00E-09	10.88136
SNRPB2	-0.31218	9.496237	-5.07052	5.32E-07	1.70E-06	5.287489
UCN	-0.31227	4.727044	-6.35379	4.20E-10	1.93E-09	12.2057
RAB9A	-0.31229	8.546466	-4.92289	1.11E-06	3.41E-06	4.582171
TNFSF4	-0.31234	6.600965	-2.26049	0.024154	0.038562	-4.71831
VWA9	-0.31246	6.423269	-5.17113	3.19E-07	1.05E-06	5.779095
PIGK	-0.31284	7.192102	-2.76854	0.005807	0.010474	-3.45862
GATA2	-0.31284	6.604334	-6.43785	2.51E-10	1.18E-09	12.70674
POLR2G	-0.31289	9.623756	-4.40805	1.24E-05	3.32E-05	2.273612
RFC5	-0.31296	6.157832	-4.24616	2.53E-05	6.51E-05	1.596904
QDPR	-0.31321	6.092853	-5.94758	4.66E-09	1.88E-08	9.865995
PDSS2	-0.31337	6.416432	-3.99852	7.18E-05	0.000175	0.607963
MED13	-0.31343	7.445203	-6.25422	7.67E-10	3.42E-09	11.61966
FLJ46875	-0.31371	5.725269	-5.22324	2.44E-07	8.13E-07	6.037217
AP5M1	-0.31395	7.410931	-4.39284	1.33E-05	3.54E-05	2.209014
ORC5	-0.31399	4.622306	-5.40731	9.30E-08	3.26E-07	6.967716
KLHL24	-0.31407	6.94911	-4.06544	5.44E-05	0.000134	0.869666
CEP70	-0.31442	4.286397	-9.30442	2.60E-19	3.14E-18	32.99683
ZG16B	-0.31446	5.312861	-5.3902	1.02E-07	3.55E-07	6.880001

ERP44	-0.31471	7.38556	-6.76036	3.31E-11	1.71E-10	14.68181
MRPL24	-0.31475	6.861773	-4.419	1.18E-05	3.17E-05	2.320251
EFTUD1	-0.31479	6.784688	-4.91536	1.15E-06	3.53E-06	4.546689
YTHDF2	-0.31483	9.206191	-4.51443	7.66E-06	2.11E-05	2.731212
RBM39	-0.31512	8.824273	-5.59723	3.34E-08	1.23E-07	7.958347
LAP3	-0.31544	9.225962	-2.07264	0.038639	0.05905	-5.12078
UQCRC2	-0.31563	7.659781	-4.20892	2.97E-05	7.58E-05	1.444617
WDR76	-0.31566	4.148072	-4.96285	9.10E-07	2.83E-06	4.771182
ZNF33A	-0.31569	6.260031	-5.44926	7.43E-08	2.63E-07	7.183851
FBLN7	-0.31575	5.212951	-5.44819	7.48E-08	2.65E-07	7.178329
PDPR	-0.31578	6.83389	-5.24537	2.18E-07	7.29E-07	6.147511
IRAK1BP1	-0.31592	3.761398	-5.29763	1.66E-07	5.62E-07	6.409759
ACSL6	-0.31596	4.440873	-6.10965	1.81E-09	7.70E-09	10.7832
ZNF684	-0.31641	4.049981	-2.96363	0.003163	0.005971	-2.90895
CHD9	-0.31646	5.731008	-4.59996	5.17E-06	1.46E-05	3.106445
WDR92	-0.31658	5.923214	-4.58173	5.63E-06	1.58E-05	3.025914
TMEM41B	-0.31687	8.425252	-3.41185	0.000689	0.001453	-1.50904
CCT4	-0.31691	8.08608	-6.75384	3.45E-11	1.78E-10	14.64106
SKA2	-0.31702	6.628251	-3.04527	0.002428	0.004672	-2.66815
NDUFAB1	-0.31715	9.626453	-4.13966	3.98E-05	0.0001	1.164714
LOC10192	-0.3172	3.762987	-4.92885	1.08E-06	3.31E-06	4.610258
ARL6IP5	-0.31722	11.34969	-5.17584	3.11E-07	1.02E-06	5.802356
SPRYD3	-0.31726	7.054973	-4.48376	8.81E-06	2.41E-05	2.598205
C6orf136	-0.31729	6.439332	-4.03417	6.20E-05	0.000152	0.746875
ZNF107	-0.31752	6.410236	-2.25482	0.02451	0.039076	-4.73098
LYRM2	-0.31766	6.026954	-6.52315	1.48E-10	7.17E-10	13.22104
LOC14841	-0.31769	7.631063	-5.83604	8.82E-09	3.45E-08	9.247438
PLEKHB1	-0.31781	5.246726	-7.63777	8.94E-14	6.01E-13	20.46634
RHPN2	-0.31796	3.348434	-8.96059	4.20E-18	4.38E-17	30.25593
SH2B3	-0.31798	9.047421	-4.05633	5.65E-05	0.000139	0.833788
HEXIM2	-0.31808	5.517522	-4.90161	1.23E-06	3.77E-06	4.482055
NDFIP1	-0.31835	8.570587	-5.05073	5.87E-07	1.87E-06	5.191815
PARN	-0.31849	7.751104	-4.63587	4.38E-06	1.25E-05	3.265964
MMD	-0.31856	7.413198	-2.34907	0.019149	0.031263	-4.51664
USP46	-0.31903	4.597582	-6.19944	1.06E-09	4.66E-09	11.30066
ZFYVE20	-0.3193	6.534733	-8.07731	3.74E-15	2.86E-14	23.58153
PUM1	-0.31954	9.708004	-5.35841	1.20E-07	4.15E-07	6.717688
ZDHHC14	-0.31962	5.47319	-9.37792	1.42E-19	1.77E-18	33.59276
HP1BP3	-0.31968	9.396601	-7.72848	4.70E-14	3.25E-13	21.09763
SLC25A5	-0.31985	10.62515	-3.96563	8.22E-05	0.000198	0.48082
TAF4	-0.3201	6.392775	-6.47927	1.94E-10	9.28E-10	12.95573
MED19	-0.32021	7.299447	-6.20309	1.04E-09	4.56E-09	11.32187
PIAS3	-0.32041	6.230187	-5.56855	3.90E-08	1.42E-07	7.806738
KIR2DS2	-0.32043	5.531188	-2.90834	0.00377	0.007014	-3.06844
MYNN	-0.32047	6.077246	-3.9253	9.68E-05	0.000231	0.326296
ZNF134	-0.32052	6.267052	-6.37585	3.67E-10	1.70E-09	12.33667
CBR1	-0.32063	6.370944	-3.55782	0.000404	0.000881	-1.01225
ATRN	-0.32078	6.339527	-5.23115	2.34E-07	7.82E-07	6.076574
GEMIN4	-0.32096	6.155336	-5.41539	8.91E-08	3.13E-07	7.009248
RUNX2	-0.32111	5.502683	-11.8372	3.69E-29	1.48E-27	55.37958
PTGES3	-0.32124	11.20394	-4.49935	8.21E-06	2.25E-05	2.665725
SAMHD1	-0.32127	10.85101	-3.75802	0.000188	0.000431	-0.29855
TIGD5	-0.32145	6.549525	-5.38887	1.03E-07	3.57E-07	6.87318
NUP210	-0.32149	6.770615	-4.10094	4.69E-05	0.000117	1.010169
TRAPPC13	-0.32151	4.887957	-2.55423	0.010892	0.018674	-4.02035
HNRNPH1	-0.32159	7.757516	-3.60404	0.00034	0.000749	-0.85081
NEDD1	-0.32209	7.493076	-2.78962	0.005447	0.009878	-3.40097
DENND1B	-0.32213	6.782791	-3.32162	0.00095	0.001959	-1.80612

MT2A	-0.32218	9.692739	-2.49836	0.012747	0.021571	-4.15953
ADAT1	-0.32225	6.987706	-3.73114	0.000209	0.000476	-0.39657
MON2	-0.32252	5.741904	-5.65964	2.37E-08	8.86E-08	8.290554
CDK4	-0.32253	7.196168	-3.86388	0.000124	0.000292	0.093861
SRRD	-0.32257	6.542729	-4.75054	2.55E-06	7.49E-06	3.783044
JRKL	-0.32271	6.933659	-3.17134	0.001596	0.003174	-2.28388
MCFD2	-0.32293	6.674073	-4.42906	1.13E-05	3.04E-05	2.363191
GALK2	-0.32304	5.343788	-5.08246	5.01E-07	1.61E-06	5.345393
GPR19	-0.32311	5.157529	-6.13571	1.55E-09	6.66E-09	10.93268
POLR2I	-0.32316	7.507799	-3.60551	0.000338	0.000745	-0.84565
DCP1A	-0.32329	7.805709	-5.27721	1.84E-07	6.23E-07	6.307001
ITGA6	-0.32343	7.154523	-3.4051	0.000706	0.001487	-1.53154
DOCK5	-0.32345	7.930738	-3.2952	0.001042	0.002133	-1.89167
LOC10192	-0.32346	6.388	-3.57262	0.000382	0.000837	-0.96077
MSANTD4	-0.32359	4.726032	-5.95391	4.49E-09	1.81E-08	9.901373
TAP2	-0.3236	6.870562	-4.35582	1.56E-05	4.14E-05	2.052723
EIF2AK3	-0.3237	5.723358	-5.14134	3.71E-07	1.21E-06	5.632624
VEZF1	-0.32372	8.831337	-5.95299	4.52E-09	1.82E-08	9.896263
INTS4	-0.32372	6.740136	-8.00796	6.22E-15	4.68E-14	23.08067
WTAP	-0.32375	8.277123	-6.80046	2.56E-11	1.34E-10	14.93319
SLC35G1	-0.32378	4.650612	-9.55529	3.25E-20	4.34E-19	35.04498
SRPK2	-0.32383	7.620636	-4.97936	8.38E-07	2.62E-06	4.849694
FAM161B	-0.32388	6.646729	-6.95526	9.36E-12	5.13E-11	15.9154
FAM46A	-0.32398	6.809769	-4.9767	8.50E-07	2.65E-06	4.837022
ATMIN	-0.32398	6.870067	-6.35929	4.06E-10	1.87E-09	12.2383
POU6F1	-0.324	5.341923	-10.0154	6.49E-22	1.06E-20	38.90374
ARV1	-0.3243	5.861794	-6.22305	9.24E-10	4.08E-09	11.43783
MED29	-0.32447	6.66563	-3.55473	0.000409	0.00089	-1.02301
PIGM	-0.32451	5.607251	-4.63976	4.30E-06	1.23E-05	3.283311
VPS41	-0.32451	6.356376	-3.60759	0.000335	0.00074	-0.83834
SIGIRR	-0.3246	7.641692	-2.08689	0.037326	0.057242	-5.09145
S100B	-0.32475	4.292823	-2.90936	0.003758	0.006993	-3.06551
FUK	-0.32476	6.965641	-5.6594	2.37E-08	8.87E-08	8.289259
SDHAF1	-0.32511	6.573521	-4.87824	1.38E-06	4.20E-06	4.372628
C10orf32	-0.32511	8.170601	-3.05332	0.002365	0.004561	-2.64406
TMEM87B	-0.32516	7.106574	-3.7872	0.000168	0.000387	-0.19144
TIAL1	-0.32526	7.188117	-9.33929	1.95E-19	2.39E-18	33.27907
FAIM	-0.32533	4.538868	-3.07973	0.002168	0.004214	-2.56461
ZNF665	-0.32536	6.557659	-5.33331	1.38E-07	4.71E-07	6.590095
HNMT	-0.3254	5.478009	-3.74485	0.000198	0.000452	-0.34665
AFG3L1P	-0.32575	5.455512	-4.80683	1.95E-06	5.82E-06	4.041136
MIA3	-0.32588	6.922973	-4.86156	1.49E-06	4.53E-06	4.294775
ABCD3	-0.32591	7.959685	-2.83441	0.004748	0.0087	-3.27709
SUCLA2	-0.32617	7.145084	-2.49964	0.012702	0.021501	-4.15639
GGACT	-0.32622	4.705367	-8.71873	2.84E-17	2.70E-16	28.37497
ADM5	-0.3263	5.442342	-6.45012	2.33E-10	1.10E-09	12.78036
IL27RA	-0.3264	6.923447	-4.4293	1.13E-05	3.04E-05	2.364204
EIF3B	-0.32641	6.589618	-5.00193	7.49E-07	2.36E-06	4.957394
GRIP1	-0.32642	4.030062	-9.13652	1.02E-18	1.15E-17	31.64868
PTPRS	-0.32647	5.380298	-5.60327	3.23E-08	1.19E-07	7.990345
CD79B	-0.32648	7.082744	-2.40014	0.016697	0.027611	-4.39691
LINC0127ℇ	-0.32654	5.188114	-7.74773	4.09E-14	2.86E-13	21.23238
ABRACL	-0.3267	9.430832	-4.09058	4.90E-05	0.000122	0.969041
SPTSSB	-0.32698	4.072745	-8.85433	9.78E-18	9.75E-17	29.4247
TBRG1	-0.32701	6.590727	-5.75591	1.39E-08	5.32E-08	8.809517
SLC19A2	-0.32706	5.056698	-2.76284	0.005908	0.010637	-3.47412
NOSIP	-0.32734	8.780966	-2.81095	0.005103	0.009299	-3.34221
TRNT1	-0.32744	5.672905	-4.07336	5.27E-05	0.00013	0.900897

PPIB	-0.32765	10.26674	-3.25894	0.001182	0.0024	-2.00797
NOL10	-0.32767	5.666466	-8.20434	1.45E-15	1.16E-14	24.50792
BMPR1A	-0.32803	4.395995	-9.78254	4.78E-21	6.96E-20	36.93453
ZNF681	-0.32806	4.063374	-9.15363	8.89E-19	1.01E-17	31.78529
ADAMTS1	-0.3282	4.517646	-6.77544	3.00E-11	1.56E-10	14.77616
DNM1L	-0.32828	6.199333	-5.45077	7.37E-08	2.61E-07	7.191702
RPARP-AS	-0.32839	4.638621	-7.24753	1.33E-12	7.95E-12	17.82073
TOB1	-0.32843	10.12676	-4.17548	3.42E-05	8.68E-05	1.308933
UBR2	-0.32845	8.659286	-3.94881	8.80E-05	0.000211	0.416202
TATDN3	-0.32847	6.466563	-4.58528	5.54E-06	1.56E-05	3.041594
ALS2	-0.32852	5.050901	-4.62185	4.67E-06	1.33E-05	3.203551
PRDX2	-0.32858	7.018811	-6.30777	5.55E-10	2.51E-09	11.93382
APOL2	-0.32874	7.397103	-5.21415	2.56E-07	8.49E-07	5.992036
FRMD6-AS	-0.32879	4.08759	-7.75948	3.76E-14	2.64E-13	21.31482
PPIE	-0.32882	6.459398	-6.97365	8.29E-12	4.56E-11	16.0333
INO80C	-0.32883	7.243005	-4.75779	2.46E-06	7.25E-06	3.816129
RARS	-0.32925	7.844132	-5.12143	4.11E-07	1.33E-06	5.53513
EDRF1	-0.32938	5.565775	-7.32372	7.94E-13	4.85E-12	18.32822
PPCDC	-0.32939	7.360349	-2.68116	0.007542	0.013338	-3.69298
HCG26	-0.32954	7.136451	-4.80157	2.00E-06	5.96E-06	4.016908
RAD51C	-0.32972	6.206019	-4.53264	7.05E-06	1.96E-05	2.810523
C1orf35	-0.32972	6.696182	-6.95484	9.38E-12	5.14E-11	15.91269
XPNPEP3	-0.32987	5.101813	-9.717	8.33E-21	1.18E-19	36.38626
DLL1	-0.32987	4.692127	-8.8191	1.29E-17	1.27E-16	29.15078
ZNF451	-0.33004	5.595521	-8.2314	1.19E-15	9.55E-15	24.70669
ZNF449	-0.33007	5.812922	-8.26189	9.45E-16	7.67E-15	24.93134
ENTPD1-A	-0.33021	6.292202	-6.28659	6.31E-10	2.84E-09	11.80927
CCDC106	-0.33042	4.601933	-6.91334	1.23E-11	6.64E-11	15.64754
TERF2	-0.33057	6.080016	-9.57088	2.85E-20	3.83E-19	35.17352
SCAMP3	-0.33059	8.10023	-5.06846	5.37E-07	1.72E-06	5.277495
TGS1	-0.33069	6.003991	-7.38637	5.16E-13	3.21E-12	18.74881
MRPL35	-0.33076	6.141081	-6.13486	1.56E-09	6.69E-09	10.92778
THAP7	-0.33076	6.401706	-3.95391	8.62E-05	0.000207	0.435754
LOC10028	-0.33091	4.970937	-5.21732	2.52E-07	8.37E-07	6.007781
UBXN8	-0.33112	5.763136	-3.78241	0.000171	0.000394	-0.20906
ELAVL1	-0.33115	6.349524	-7.16694	2.30E-12	1.33E-11	17.28879
PIEZO1	-0.33131	7.905527	-2.51807	0.012063	0.02051	-4.11077
NUBP2	-0.33137	7.186332	-5.11103	4.33E-07	1.40E-06	5.484362
GPR157	-0.33165	5.723373	-7.56567	1.49E-13	9.75E-13	19.96892
FUS	-0.33178	7.058962	-4.84585	1.61E-06	4.87E-06	4.22171
ENO3	-0.33182	4.366573	-10.4119	2.01E-23	3.86E-22	42.33206
MAN1B1-A	-0.33193	7.622266	-4.90104	1.23E-06	3.78E-06	4.479391
IL4I1	-0.33225	5.462925	-5.85246	8.03E-09	3.16E-08	9.337845
PEX11B	-0.33231	8.044235	-3.95278	8.66E-05	0.000208	0.431421
ZNF518A	-0.3324	6.679763	-5.26205	2.00E-07	6.71E-07	6.230987
ATP5G2	-0.33259	7.77694	-6.57248	1.09E-10	5.33E-10	13.52107
HOMER1	-0.33261	4.431787	-6.06626	2.34E-09	9.81E-09	10.53547
ISG20	-0.33286	9.749101	-2.47074	0.013764	0.023146	-4.22723
CPAMD8	-0.33308	5.323819	-5.91568	5.60E-09	2.23E-08	9.688005
CTSC	-0.3331	8.216482	-4.34346	1.65E-05	4.35E-05	2.000766
PDS5A	-0.33344	6.679241	-4.04131	6.02E-05	0.000148	0.774818
AKAP1	-0.33366	5.952843	-6.11298	1.78E-09	7.56E-09	10.80224
ZNF502	-0.33379	5.348672	-7.62297	9.93E-14	6.64E-13	20.36397
RABEP1	-0.33388	6.233593	-6.65449	6.49E-11	3.26E-10	14.02429
PSMA1	-0.33403	10.18489	-5.54459	4.45E-08	1.61E-07	7.680667
SFXN2	-0.33405	5.293413	-10.0592	4.44E-22	7.34E-21	39.27743
MRPL14	-0.3345	7.461374	-4.76814	2.34E-06	6.92E-06	3.863452
BTF3L4	-0.33475	6.564128	-5.31534	1.51E-07	5.15E-07	6.499143

ABI2	-0.33482	6.19122	-8.44044	2.44E-16	2.11E-15	26.25999
ZNF470	-0.33515	4.286951	-5.84242	8.50E-09	3.34E-08	9.282555
TP53	-0.33516	6.018127	-3.35443	0.000846	0.001756	-1.69898
TCTN1	-0.3352	6.449006	-5.75737	1.37E-08	5.28E-08	8.817479
TSPAN5	-0.33523	6.294715	-2.78207	0.005574	0.010088	-3.42168
RABGGTB	-0.33553	7.011368	-4.60983	4.94E-06	1.40E-05	3.150204
C1orf21	-0.33613	5.234004	-8.36117	4.47E-16	3.74E-15	25.66739
UCHL3	-0.33644	7.88803	-3.71779	0.00022	0.0005	-0.44497
VRK2	-0.33649	7.526199	-3.85585	0.000128	0.0003	0.063746
GALNT11	-0.33661	7.342541	-4.46602	9.55E-06	2.60E-05	2.521717
ZSCAN26	-0.33666	6.313074	-3.56631	0.000391	0.000856	-0.98278
RNF170	-0.33675	6.182419	-3.96368	8.28E-05	0.000199	0.473306
GNGT2	-0.33684	6.213402	-5.60987	3.11E-08	1.15E-07	8.025372
SH2D3A	-0.33692	5.01301	-7.26381	1.19E-12	7.16E-12	17.92878
GTF3C4	-0.33695	5.904847	-8.2865	7.85E-16	6.42E-15	25.11313
FAM161A	-0.33701	3.566024	-8.11083	2.92E-15	2.26E-14	23.82485
HHAT	-0.33704	4.352105	-7.31081	8.67E-13	5.28E-12	18.24188
CKAP5	-0.3372	7.307258	-5.07243	5.27E-07	1.69E-06	5.296753
LINS	-0.33725	6.647777	-4.84459	1.62E-06	4.90E-06	4.215868
COPRS	-0.33741	6.913371	-4.50708	7.93E-06	2.18E-05	2.699261
NCL	-0.33766	7.508685	-7.67247	7.00E-14	4.74E-13	20.70716
ANKRD44	-0.33766	7.459654	-4.27241	2.25E-05	5.84E-05	1.705015
WDR70	-0.33779	7.137577	-4.43518	1.10E-05	2.96E-05	2.389345
TSC22D1	-0.33791	5.442353	-6.70402	4.74E-11	2.42E-10	14.33076
RAP1A	-0.33801	9.216342	-6.25506	7.63E-10	3.40E-09	11.62457
FICD	-0.33806	6.582683	-6.41835	2.83E-10	1.33E-09	12.58998
RPL18	-0.3381	9.165851	-5.13701	3.80E-07	1.24E-06	5.611376
TLR3	-0.33813	3.542808	-9.82952	3.20E-21	4.79E-20	37.32911
PCK2	-0.33883	6.651213	-4.1006	4.70E-05	0.000117	1.008811
ACD	-0.33892	7.281211	-4.54745	6.59E-06	1.84E-05	2.875288
PABPC1	-0.3391	13.04241	-5.21245	2.58E-07	8.56E-07	5.983564
METTL1	-0.33922	5.889501	-6.33505	4.70E-10	2.15E-09	12.09479
KLRC4	-0.33928	3.739991	-3.30506	0.001007	0.002066	-1.85982
SOD1	-0.33948	9.851126	-3.44121	0.00062	0.001314	-1.41073
EP400NL	-0.33977	5.407463	-9.60691	2.11E-20	2.87E-19	35.47131
HEXA	-0.33981	6.506298	-5.69013	2.00E-08	7.54E-08	8.454072
S100PBP	-0.33991	7.547316	-4.16833	3.53E-05	8.94E-05	1.280065
RAB39B	-0.33996	5.276504	-2.30533	0.021494	0.034667	-4.61719
C14orf182	-0.34005	4.53272	-9.24405	4.26E-19	5.03E-18	32.50996
PDCD2L	-0.34036	5.594924	-5.23721	2.27E-07	7.59E-07	6.106814
HMCES	-0.34053	6.616423	-6.54788	1.27E-10	6.18E-10	13.37121
PERP	-0.34057	4.653843	-7.24291	1.38E-12	8.18E-12	17.79012
EAPP	-0.3406	9.168012	-4.82516	1.78E-06	5.35E-06	4.125771
MED31	-0.34062	6.498309	-6.51435	1.56E-10	7.56E-10	13.16768
S1PR5	-0.34079	5.772265	-5.00732	7.30E-07	2.30E-06	4.983184
BEND5	-0.34086	4.873107	-4.43849	1.08E-05	2.92E-05	2.403507
ACAA2	-0.34096	6.702175	-7.85079	1.95E-14	1.40E-13	21.9585
PRKDC	-0.34117	6.382494	-3.29782	0.001033	0.002115	-1.88322
ZBTB9	-0.34125	6.270485	-6.04895	2.59E-09	1.08E-08	10.43712
ZBTB42	-0.34162	5.00779	-4.67609	3.63E-06	1.05E-05	3.446008
SIK2	-0.34191	5.235159	-8.23189	1.18E-15	9.51E-15	24.71034
TRIM35	-0.342	6.237847	-6.10132	1.90E-09	8.08E-09	10.7355
CPSF3	-0.34201	7.484207	-3.95728	8.50E-05	0.000204	0.448715
CDYL	-0.34203	7.024044	-5.55787	4.14E-08	1.51E-07	7.750474
LAIR2	-0.34213	6.86995	-2.85928	0.004396	0.008099	-3.20749
PAOX	-0.34229	5.526199	-8.82004	1.28E-17	1.26E-16	29.15812
ZNRD1-AS	-0.34235	4.075311	-11.3338	4.43E-27	1.35E-25	50.64745
METTL4	-0.34237	6.405718	-3.63621	0.000301	0.000669	-0.73726

LPXN	-0.34266	7.661792	-4.67007	3.73E-06	1.07E-05	3.418957
MAPK9	-0.34269	6.852792	-6.4888	1.83E-10	8.78E-10	13.01322
EXOC1	-0.3427	9.415223	-5.39736	9.80E-08	3.42E-07	6.916695
FGF9	-0.34271	3.819292	-10.5016	9.04E-24	1.81E-22	43.12035
DOCK7	-0.34284	4.483177	-6.22775	8.98E-10	3.97E-09	11.46522
SARNP	-0.343	7.231527	-6.99638	7.14E-12	3.95E-11	16.17943
NSMCE1	-0.34302	8.321461	-4.36384	1.51E-05	4.00E-05	2.086456
SND1	-0.34319	7.763822	-4.8575	1.52E-06	4.62E-06	4.275888
ZFAND6	-0.34335	9.368979	-4.89596	1.26E-06	3.87E-06	4.455572
UHRF1BP1	-0.34342	6.276808	-5.62481	2.87E-08	1.06E-07	8.104758
LOC64285	-0.34352	5.529216	-5.37366	1.11E-07	3.85E-07	6.795417
MSL2	-0.34355	9.442137	-4.26625	2.31E-05	5.99E-05	1.679612
MID2	-0.34366	5.577455	-8.10875	2.96E-15	2.29E-14	23.8097
TECR	-0.34398	7.273667	-2.8321	0.004782	0.008754	-3.28353
CCNY	-0.34401	8.546532	-4.65422	4.02E-06	1.15E-05	3.347914
ZNF345	-0.34403	4.69657	-10.0106	6.76E-22	1.10E-20	38.86322
WDR35	-0.34404	4.960476	-6.28049	6.54E-10	2.94E-09	11.77351
LRMP	-0.34409	10.06045	-3.79154	0.000165	0.000381	-0.17543
LOC15368	-0.34417	7.129413	-5.32845	1.41E-07	4.82E-07	6.565507
ING3	-0.34438	6.407051	-5.55251	4.26E-08	1.55E-07	7.72231
CEP41	-0.34444	5.496827	-7.63676	9.01E-14	6.05E-13	20.45934
GPSM1	-0.34446	6.31148	-6.00913	3.26E-09	1.35E-08	10.21174
ZMYM4	-0.34452	7.036674	-4.61166	4.90E-06	1.39E-05	3.15832
PIM2	-0.34462	8.440018	-3.22586	0.001325	0.002666	-2.113
ISCU	-0.34465	10.79998	-7.00468	6.76E-12	3.74E-11	16.23292
RLN1	-0.34488	4.561326	-7.69583	5.93E-14	4.05E-13	20.86976
DVL2	-0.34507	6.1805	-9.11443	1.22E-18	1.36E-17	31.47273
ZNF10	-0.34516	4.870633	-10.3153	4.73E-23	8.78E-22	41.4879
PLXNA1	-0.34528	6.148174	-7.06416	4.56E-12	2.57E-11	16.61758
ERN1	-0.34529	7.013002	-6.07617	2.21E-09	9.29E-09	10.59193
FRG1B	-0.34555	5.125681	-6.48577	1.87E-10	8.93E-10	12.99493
SLC29A3	-0.34595	6.916884	-7.33323	7.44E-13	4.55E-12	18.39186
TMEM18	-0.34631	6.525804	-6.61838	8.15E-11	4.04E-10	13.80209
CD19	-0.34633	6.698021	-2.58648	0.009934	0.017172	-3.93865
ADPRHL2	-0.34636	8.220387	-5.21951	2.49E-07	8.28E-07	6.018643
SNRNP40	-0.34659	7.348813	-4.51867	7.52E-06	2.08E-05	2.749654
MICAL2	-0.34664	7.04095	-4.45586	1.00E-05	2.72E-05	2.478013
PER3	-0.34702	5.222342	-4.98075	8.33E-07	2.61E-06	4.856302
FAAH2	-0.34703	5.662479	-7.24041	1.40E-12	8.30E-12	17.77352
PPM1L	-0.34708	4.80702	-6.24251	8.22E-10	3.65E-09	11.55127
GPR174	-0.34755	4.190791	-6.21004	9.99E-10	4.39E-09	11.3622
OXTR	-0.34766	4.781019	-4.03407	6.20E-05	0.000152	0.746452
GLG1	-0.34807	8.526534	-5.62092	2.93E-08	1.09E-07	8.084065
KLHL20	-0.34809	5.561359	-6.0399	2.73E-09	1.14E-08	10.38579
CRYGS	-0.3482	5.13601	-8.83352	1.15E-17	1.14E-16	29.26279
HCG18	-0.34822	5.719972	-7.12902	2.96E-12	1.70E-11	17.04021
SIK3	-0.34823	7.673883	-5.38908	1.02E-07	3.57E-07	6.874258
ZNF322	-0.34867	6.37132	-4.07874	5.15E-05	0.000127	0.922178
MTMR12	-0.34881	7.11627	-7.10366	3.51E-12	1.99E-11	16.87458
TOPORS	-0.34882	8.231986	-2.95448	0.003257	0.006131	-2.93554
TYW1	-0.349	7.088671	-5.60382	3.22E-08	1.18E-07	7.993274
CFH	-0.34905	4.883784	-4.0604	5.56E-05	0.000137	0.849793
ASAH2B	-0.34906	4.330413	-11.4681	1.25E-27	4.07E-26	51.89712
BPHL	-0.34931	5.402722	-7.75511	3.88E-14	2.71E-13	21.28416
ERCC6	-0.34938	5.759476	-8.78456	1.70E-17	1.65E-16	28.88305
ATG2B	-0.3494	6.312404	-5.95271	4.52E-09	1.82E-08	9.894692
CHKB	-0.34941	8.851077	-4.2319	2.69E-05	6.90E-05	1.538441
TAF1B	-0.34943	4.651735	-4.16088	3.64E-05	9.21E-05	1.250019

PSMD11	-0.34948	7.330425	-5.77102	1.27E-08	4.90E-08	8.891669
ZNF507	-0.34958	5.61719	-7.80651	2.69E-14	1.91E-13	21.6456
GFOD1	-0.34963	7.413506	-4.30326	1.97E-05	5.15E-05	1.832869
BTBD1	-0.34991	9.284432	-5.05577	5.73E-07	1.83E-06	5.216178
C9orf85	-0.34992	5.959908	-8.65771	4.58E-17	4.24E-16	27.90671
KHDRBS1	-0.35003	8.676276	-9.15508	8.79E-19	1.00E-17	31.79683
RPL23AP3	-0.35004	5.127456	-5.13786	3.78E-07	1.23E-06	5.615561
SSX2IP	-0.35006	5.389617	-4.54846	6.56E-06	1.83E-05	2.879726
SNRNP200	-0.35006	7.652282	-3.71678	0.000221	0.000501	-0.44863
ZNF277	-0.3501	6.980962	-5.51154	5.32E-08	1.91E-07	7.507564
PLXND1	-0.3503	7.179572	-3.50556	0.00049	0.001053	-1.19242
HCCS	-0.35047	7.401395	-4.38144	1.40E-05	3.71E-05	2.160739
PPDPF	-0.35057	8.104442	-2.80362	0.005219	0.009494	-3.36245
ADCY9	-0.35072	5.536292	-8.00309	6.45E-15	4.84E-14	23.04566
METTL23	-0.35078	8.694802	-4.23505	2.65E-05	6.81E-05	1.551344
HOXB2	-0.35083	7.512908	-3.93249	9.40E-05	0.000225	0.353766
CCT7	-0.35087	8.520496	-4.30445	1.96E-05	5.13E-05	1.837835
PPM1F	-0.35106	9.072582	-3.88398	0.000114	0.000271	0.169557
IL5RA	-0.35117	5.248989	-4.40157	1.28E-05	3.41E-05	2.246079
CAMSAP2	-0.35141	4.641949	-5.61124	3.09E-08	1.14E-07	8.032628
TNKS	-0.35149	5.136776	-11.0972	4.02E-26	1.06E-24	48.46909
ZNF667-A	-0.3516	5.066591	-3.92929	9.53E-05	0.000227	0.341526
FCF1	-0.3517	4.86409	-5.21269	2.58E-07	8.55E-07	5.984778
HDHD2	-0.35178	8.456805	-4.03867	6.08E-05	0.000149	0.76448
ZFP1	-0.35181	4.295918	-9.97684	9.05E-22	1.44E-20	38.57537
ZNF212	-0.35181	7.212636	-6.26745	7.08E-10	3.17E-09	11.69708
MORC3	-0.35185	8.739875	-4.25976	2.38E-05	6.15E-05	1.652848
PRKD3	-0.35225	6.312651	-4.35823	1.55E-05	4.10E-05	2.062861
LOC10106	-0.3523	3.671289	-5.02444	6.70E-07	2.12E-06	5.065272
TAS2R14	-0.35236	4.059836	-7.15399	2.50E-12	1.45E-11	17.20377
CTC1	-0.35246	6.393881	-8.21856	1.31E-15	1.05E-14	24.61227
PPARD	-0.35259	5.60119	-9.8126	3.70E-21	5.49E-20	37.18691
GPATCH4	-0.35268	5.672199	-10.6671	2.05E-24	4.38E-23	44.58677
CYFIP1	-0.35273	9.074483	-3.04053	0.002466	0.004739	-2.68231
MAPKAPK	-0.35279	6.197899	-4.39925	1.29E-05	3.44E-05	2.236242
AKR1A1	-0.35283	8.778291	-4.19417	3.16E-05	8.05E-05	1.384633
TRIM61	-0.35284	5.066707	-8.10642	3.01E-15	2.33E-14	23.79282
1-Mar	-0.35302	8.485639	-2.46181	0.014108	0.023678	-4.24896
CD33	-0.35304	8.188724	-3.69994	0.000236	0.000533	-0.50946
SNRPD3	-0.35306	9.504064	-5.44094	7.77E-08	2.75E-07	7.140859
PRIM2	-0.35307	4.129882	-5.15767	3.42E-07	1.12E-06	5.712827
FAM13A	-0.35329	7.242464	-3.43665	0.00063	0.001334	-1.42605
ATXN7	-0.35332	8.204212	-4.95907	9.27E-07	2.88E-06	4.753251
TVP23B	-0.35334	7.878661	-3.69578	0.00024	0.00054	-0.52443
KIAA1324	-0.35338	6.782076	-2.22103	0.026727	0.042294	-4.8057
SSR4	-0.35339	9.979908	-3.28029	0.001098	0.002239	-1.93963
PWP2	-0.3534	6.7778	-5.07971	5.08E-07	1.63E-06	5.332049
CIR1	-0.35351	8.099152	-3.79204	0.000165	0.000381	-0.17359
SRSF5	-0.35352	9.026082	-6.14327	1.49E-09	6.39E-09	10.97618
SPATA7	-0.35359	4.427631	-6.97672	8.13E-12	4.48E-11	16.05306
MFSD12	-0.35376	6.882537	-6.00236	3.39E-09	1.40E-08	10.17355
RMND1	-0.35379	4.943754	-3.69206	0.000243	0.000548	-0.53784
CUL4A	-0.35396	6.748886	-6.9369	1.06E-11	5.74E-11	15.79793
ZNF44	-0.35399	4.907757	-6.31722	5.24E-10	2.38E-09	11.98951
C5orf24	-0.35428	6.570303	-4.65813	3.95E-06	1.13E-05	3.365407
SOX8	-0.35442	4.28746	-8.37094	4.15E-16	3.50E-15	25.74014
ASAP2	-0.35446	5.448456	-2.19051	0.028877	0.045387	-4.87226
HEBP1	-0.35462	8.000071	-3.60704	0.000336	0.000741	-0.84027

BOLA1	-0.35463	5.051765	-5.34913	1.27E-07	4.35E-07	6.670454
ZFP42	-0.35465	3.355727	-9.25136	4.01E-19	4.76E-18	32.5688
CXCR6	-0.35477	5.965243	-5.72776	1.62E-08	6.18E-08	8.656969
SAE1	-0.35477	5.991665	-7.20612	1.76E-12	1.04E-11	17.54677
SPRTN	-0.355	4.77901	-3.57839	0.000374	0.00082	-0.94068
TAP1	-0.35502	9.88261	-3.31018	0.000989	0.002032	-1.84325
SRP68	-0.35505	8.19455	-5.30093	1.63E-07	5.53E-07	6.426377
ITGB2-AS1	-0.35516	8.749627	-3.29651	0.001038	0.002124	-1.88744
COPS2	-0.3552	7.617055	-4.08506	5.01E-05	0.000124	0.947184
KRT73	-0.35523	5.897777	-7.66577	7.34E-14	4.96E-13	20.66055
ELK3	-0.3555	7.081135	-5.19134	2.88E-07	9.49E-07	5.878941
RNF111	-0.35556	9.077778	-5.15331	3.49E-07	1.14E-06	5.691407
MFSD6	-0.35579	5.837019	-4.02119	6.54E-05	0.00016	0.696146
TNFAIP2	-0.35587	8.906364	-2.83205	0.004783	0.008755	-3.28367
PIGA	-0.35596	5.195243	-4.27409	2.24E-05	5.80E-05	1.711967
MIS18A	-0.35606	5.003615	-4.81865	1.84E-06	5.51E-06	4.095678
GTPBP4	-0.35627	6.319125	-4.98571	8.12E-07	2.54E-06	4.879929
CHD1L	-0.35654	6.299294	-6.5514	1.24E-10	6.05E-10	13.39261
RPL27	-0.35661	13.03285	-6.48829	1.84E-10	8.80E-10	13.01016
LOC10027	-0.35672	5.581522	-7.43964	3.58E-13	2.25E-12	19.10878
ZHX2	-0.35696	8.50399	-5.1969	2.79E-07	9.24E-07	5.906462
GEMIN8	-0.357	4.677245	-8.97567	3.73E-18	3.92E-17	30.37457
UVRAG	-0.35702	7.185266	-5.51251	5.29E-08	1.90E-07	7.512601
UST	-0.35705	4.452625	-9.57532	2.75E-20	3.69E-19	35.21016
TMEM68	-0.35714	5.392622	-3.77635	0.000175	0.000403	-0.23134
TMEM203	-0.35719	7.752305	-4.68654	3.45E-06	9.98E-06	3.493016
GCN1L1	-0.3572	6.791238	-4.92014	1.12E-06	3.45E-06	4.569187
PCF11	-0.35721	8.361127	-6.69815	4.92E-11	2.50E-10	14.29437
CROT	-0.35744	4.502215	-10.1134	2.77E-22	4.68E-21	39.74224
AQR	-0.35746	6.066071	-5.93575	4.99E-09	2.00E-08	9.799907
NUAK1	-0.35766	4.740205	-5.30702	1.58E-07	5.37E-07	6.45709
AAR2	-0.35776	7.288877	-6.91329	1.23E-11	6.64E-11	15.64725
USP40	-0.35784	5.606556	-8.47779	1.84E-16	1.60E-15	26.54074
NSRP1	-0.35797	7.924203	-4.40437	1.26E-05	3.37E-05	2.257975
BTG1	-0.35853	11.79706	-5.01038	7.19E-07	2.27E-06	4.997839
GNE	-0.35881	6.959852	-3.26841	0.001144	0.002329	-1.97771
FTSJ1	-0.35892	6.967764	-5.88366	6.72E-09	2.67E-08	9.510221
GOSR1	-0.35904	7.286235	-4.79146	2.10E-06	6.23E-06	3.970396
ZBTB16	-0.35926	6.056538	-2.8642	0.004329	0.007984	-3.19363
ZBTB11	-0.35931	6.750514	-5.51272	5.28E-08	1.90E-07	7.513708
PSMG4	-0.35933	4.59918	-11.1918	1.67E-26	4.63E-25	49.33646
KIAA1598	-0.35936	7.318486	-2.05464	0.040353	0.061406	-5.15755
EXOSC7	-0.35939	6.664164	-7.20374	1.79E-12	1.05E-11	17.53104
RALA	-0.35946	7.806783	-4.76614	2.37E-06	6.98E-06	3.854268
SNX4	-0.35961	6.713842	-3.49325	0.000513	0.001099	-1.23448
ETS1	-0.35986	8.632558	-4.5153	7.63E-06	2.11E-05	2.734994
MRPS23	-0.36002	6.027089	-6.16168	1.33E-09	5.75E-09	11.08225
PIKFYVE	-0.36021	9.535334	-4.5319	7.08E-06	1.96E-05	2.807323
RSG1	-0.36023	5.294449	-8.89328	7.18E-18	7.27E-17	29.72853
TSPYL2	-0.36035	7.277804	-6.49435	1.77E-10	8.50E-10	13.04674
IREB2	-0.36041	6.462098	-4.69575	3.31E-06	9.58E-06	3.534521
KIAA1586	-0.36048	4.114832	-4.25705	2.41E-05	6.22E-05	1.641677
PROCR	-0.36049	3.542654	-6.66209	6.19E-11	3.11E-10	14.07121
CD244	-0.3605	5.894363	-6.19973	1.06E-09	4.65E-09	11.30235
COX18	-0.36053	5.858945	-7.97726	7.79E-15	5.80E-14	22.86005
MT1X	-0.36066	8.450771	-3.20571	0.00142	0.002845	-2.17648
CCND2	-0.36071	6.585514	-4.93957	1.02E-06	3.16E-06	4.660877
USP11	-0.36081	7.801093	-3.27299	0.001126	0.002294	-1.96306

GXYLT1	-0.36086	6.237522	-3.82109	0.000147	0.000342	-0.066
COX5A	-0.36092	7.877064	-6.036	2.79E-09	1.16E-08	10.36366
AMZ2P1	-0.36095	6.332363	-5.32114	1.47E-07	5.00E-07	6.528448
CCDC6	-0.36122	6.183123	-3.87069	0.000121	0.000285	0.119486
PTPRM	-0.3616	4.732144	-5.46549	6.81E-08	2.42E-07	7.267885
KRI1	-0.36169	6.545712	-4.78161	2.20E-06	6.52E-06	3.925151
CDIPT	-0.36182	10.81198	-7.11219	3.31E-12	1.89E-11	16.93026
ZDHHC6	-0.36199	8.945396	-6.7855	2.82E-11	1.47E-10	14.83923
ZNF528	-0.36224	5.046533	-6.69965	4.88E-11	2.48E-10	14.30367
IPP	-0.36239	5.147194	-4.71961	2.95E-06	8.61E-06	3.642422
DENR	-0.36239	6.89733	-7.19872	1.85E-12	1.09E-11	17.49796
MBNL2	-0.36257	5.650855	-5.45238	7.31E-08	2.59E-07	7.199984
SETD4	-0.36288	5.850764	-11.4257	1.87E-27	5.90E-26	51.50168
RNF34	-0.36298	7.123516	-6.92833	1.12E-11	6.06E-11	15.74315
CUL1	-0.36298	6.705419	-7.16014	2.40E-12	1.39E-11	17.24414
PCCA	-0.36317	3.959091	-11.4387	1.65E-27	5.25E-26	51.6232
ANKEF1	-0.3634	4.391583	-8.56927	9.09E-17	8.19E-16	27.2325
GBP2	-0.36342	10.32207	-3.2672	0.001149	0.002337	-1.98161
CCNL2	-0.36351	7.390268	-5.05557	5.73E-07	1.83E-06	5.215181
GPA33	-0.36352	6.371962	-4.96016	9.22E-07	2.86E-06	4.758426
PHF6	-0.36353	6.08266	-7.5211	2.03E-13	1.32E-12	19.66338
ALDH9A1	-0.3637	9.895933	-4.7616	2.42E-06	7.12E-06	3.833524
MRPL16	-0.36375	8.011492	-4.86969	1.44E-06	4.37E-06	4.332705
SREK1	-0.36383	6.104408	-5.47413	6.51E-08	2.32E-07	7.31272
DNAJC1	-0.36391	7.938042	-5.00735	7.29E-07	2.30E-06	4.983359
CAMLG	-0.36398	8.80719	-4.86396	1.48E-06	4.48E-06	4.305983
ZNRF3	-0.36405	3.734956	-7.19404	1.91E-12	1.12E-11	17.4671
RRP36	-0.36418	6.70939	-6.81519	2.33E-11	1.22E-10	15.02583
PRKAG2	-0.36431	6.147279	-6.01859	3.09E-09	1.28E-08	10.26516
BTN2A1	-0.3644	7.928896	-5.94507	4.73E-09	1.90E-08	9.851969
DHX15	-0.36453	9.755853	-5.93229	5.09E-09	2.04E-08	9.780551
MMAA	-0.36463	4.108624	-8.9969	3.14E-18	3.33E-17	30.54174
GPR137B	-0.36478	7.239955	-2.04279	0.041516	0.063001	-5.18157
OPN3	-0.36484	6.821987	-3.17134	0.001596	0.003174	-2.28386
UVSSA	-0.36489	7.154036	-7.9857	7.32E-15	5.47E-14	22.92066
SNRPD1	-0.36543	6.951817	-4.73758	2.71E-06	7.95E-06	3.723995
SAFB2	-0.36568	7.701478	-5.49118	5.94E-08	2.13E-07	7.401352
CCBL2	-0.36579	8.430438	-4.65526	4.00E-06	1.14E-05	3.352571
TNRC6C-/	-0.36584	7.343731	-4.90538	1.21E-06	3.70E-06	4.499789
ATG9B	-0.36594	5.762281	-7.25777	1.24E-12	7.44E-12	17.88868
TMEM126I	-0.36597	8.990011	-4.40503	1.26E-05	3.36E-05	2.260777
FLJ38717	-0.36602	6.405492	-6.78439	2.84E-11	1.48E-10	14.83228
MRFAP1	-0.36611	11.63138	-6.53949	1.34E-10	6.50E-10	13.32019
BET1	-0.36614	7.108409	-3.83148	0.000141	0.000329	-0.02733
DNAJC15	-0.36623	6.74259	-4.1976	3.11E-05	7.95E-05	1.398592
BPTF	-0.36628	7.198528	-6.07863	2.17E-09	9.17E-09	10.60596
ALDH6A1	-0.36636	5.584639	-4.89345	1.28E-06	3.91E-06	4.44379
CHCHD6	-0.36655	5.304769	-6.50464	1.66E-10	8.00E-10	13.10891
LCMT1	-0.36656	7.627529	-5.4793	6.33E-08	2.26E-07	7.339573
TIPRL	-0.36662	7.516696	-4.66398	3.84E-06	1.10E-05	3.391652
TMEM109	-0.36665	7.593947	-4.30748	1.93E-05	5.06E-05	1.850422
FTSJ3	-0.36671	7.708981	-6.0383	2.75E-09	1.15E-08	10.3767
CCDC134	-0.36685	5.77294	-8.88726	7.53E-18	7.61E-17	29.68154
APOA1BP	-0.36691	7.383916	-4.62664	4.57E-06	1.30E-05	3.224877
MZT1	-0.36696	5.290163	-5.84709	8.28E-09	3.26E-08	9.308245
EMC1	-0.36716	5.455481	-8.66304	4.39E-17	4.08E-16	27.94752
ISL2	-0.36745	5.954533	-4.1054	4.60E-05	0.000115	1.027903
ZNF546	-0.36748	3.74024	-6.52822	1.43E-10	6.96E-10	13.25176

RHOBTB3	-0.36755	4.729355	-6.20093	1.05E-09	4.62E-09	11.30931
HEY1	-0.36797	6.679428	-2.38055	0.017603	0.028945	-4.44313
PRRT2	-0.36811	4.421676	-5.60477	3.20E-08	1.18E-07	7.99827
GTF3C6	-0.36817	9.325943	-4.73246	2.78E-06	8.13E-06	3.700714
NDFIP2	-0.3683	4.670209	-7.03544	5.52E-12	3.08E-11	16.43153
HLA-DPB2	-0.36833	4.300404	-5.37686	1.09E-07	3.79E-07	6.811769
VDAC2	-0.36838	9.426945	-5.73182	1.59E-08	6.05E-08	8.678934
FLJ31306	-0.36838	7.230526	-3.9089	0.000103	0.000246	0.263911
COQ3	-0.36855	6.002645	-6.64993	6.68E-11	3.35E-10	13.99618
OTUD6B-A	-0.36884	5.928282	-5.11864	4.17E-07	1.35E-06	5.521528
ZNF683	-0.36904	6.760271	-5.0879	4.87E-07	1.57E-06	5.371764
PDS5B	-0.36917	6.001931	-5.19013	2.89E-07	9.54E-07	5.872937
CTBP1	-0.36934	9.451316	-4.24276	2.56E-05	6.60E-05	1.582978
MSTO1	-0.36938	5.775767	-7.78535	3.13E-14	2.21E-13	21.49655
DENND1C	-0.36951	8.437082	-6.05424	2.51E-09	1.05E-08	10.46714
SOX4	-0.36962	5.628766	-6.00747	3.29E-09	1.36E-08	10.20236
PROC	-0.36967	5.419694	-6.12656	1.64E-09	7.01E-09	10.88016
NID1	-0.36969	5.171999	-3.75546	0.00019	0.000435	-0.30794
DNPEP	-0.36977	6.212228	-6.65365	6.53E-11	3.27E-10	14.01911
TMEM223	-0.36981	5.972571	-11.7123	1.23E-28	4.54E-27	54.19327
PIGX	-0.36996	4.687243	-6.78119	2.90E-11	1.51E-10	14.81221
CSTF3	-0.37015	5.619282	-5.411	9.12E-08	3.20E-07	6.98669
ABCD2	-0.37018	3.530422	-7.14767	2.61E-12	1.51E-11	17.16234
MAGOHB	-0.37031	4.481468	-6.80461	2.49E-11	1.31E-10	14.95926
MTMR2	-0.37032	5.344181	-5.70349	1.86E-08	7.03E-08	8.525979
ATP5S	-0.37038	5.212555	-6.75755	3.37E-11	1.74E-10	14.66425
ARMCX3	-0.37043	7.274234	-3.52693	0.000453	0.00098	-1.11908
TAX1BP1	-0.37056	9.373983	-5.10919	4.37E-07	1.41E-06	5.475415
RBM8A	-0.37065	7.917072	-6.98511	7.69E-12	4.25E-11	16.10697
RPL41	-0.37084	14.04297	-7.16069	2.39E-12	1.39E-11	17.24775
SEMA4D	-0.37086	9.180993	-3.8009	0.000159	0.000368	-0.14083
SLC7A7	-0.37087	10.36297	-3.03402	0.002519	0.004834	-2.70171
WDR19	-0.37092	4.784766	-7.15436	2.50E-12	1.44E-11	17.20615
SLC35D1	-0.37112	6.214451	-5.65879	2.38E-08	8.90E-08	8.286033
MSI2	-0.37141	5.603678	-7.44395	3.47E-13	2.19E-12	19.13802
MCUR1	-0.37158	6.309195	-2.93473	0.003468	0.006501	-2.99267
UTP20	-0.37162	5.942491	-4.80811	1.94E-06	5.78E-06	4.047036
EME1	-0.3719	6.204526	-5.64143	2.62E-08	9.74E-08	8.193275
FAM213A	-0.37199	5.771519	-5.05062	5.88E-07	1.87E-06	5.191321
ZNF141	-0.37228	4.149808	-7.26264	1.20E-12	7.21E-12	17.921
PLA2G12A	-0.37233	6.273199	-3.33186	0.000917	0.001894	-1.7728
VKORC1L1	-0.37237	6.312261	-3.33342	0.000911	0.001884	-1.7677
TRIM33	-0.37255	7.860135	-5.36171	1.18E-07	4.08E-07	6.734492
VPS33B	-0.37282	7.00457	-5.89005	6.48E-09	2.57E-08	9.545641
WDR53	-0.37286	5.648101	-5.98273	3.80E-09	1.55E-08	10.06304
NDUFAF2	-0.37295	6.424291	-4.94231	1.01E-06	3.12E-06	4.673829
CRTC3	-0.37315	6.82621	-3.94934	8.78E-05	0.000211	0.418228
FBXW12	-0.37334	9.579385	-4.19506	3.15E-05	8.03E-05	1.388274
CHML	-0.37349	5.351287	-5.13224	3.89E-07	1.26E-06	5.588052
DHX57	-0.37359	4.728677	-8.31909	6.14E-16	5.10E-15	25.35451
SLC25A13	-0.37394	4.871928	-7.89652	1.40E-14	1.02E-13	22.28311
LMO7	-0.37395	4.443655	-11.7589	7.83E-29	3.01E-27	54.63553
CCDC152	-0.37397	6.519795	-7.86596	1.75E-14	1.26E-13	22.06604
RPA2	-0.37397	8.709038	-4.70693	3.14E-06	9.12E-06	3.58499
REPIN1	-0.37402	6.184187	-10.7292	1.17E-24	2.55E-23	45.14076
ZNF814	-0.37414	4.396552	-4.75545	2.49E-06	7.33E-06	3.805449
TCF15	-0.37423	5.60414	-3.32763	0.00093	0.001919	-1.78657
TSC22D1-.	-0.37441	4.40896	-11.6296	2.70E-28	9.61E-27	53.41248

MPRIP	-0.37453	6.717847	-9.40156	1.17E-19	1.47E-18	33.78514
NUP50-AS	-0.37476	6.180266	-5.17174	3.18E-07	1.04E-06	5.782118
NDUFB2-A	-0.37479	5.160227	-7.92622	1.13E-14	8.27E-14	22.4948
SF1	-0.37481	9.082002	-4.18567	3.28E-05	8.34E-05	1.350163
NBPF3	-0.37511	5.123989	-4.85731	1.53E-06	4.62E-06	4.274981
WDYHV1	-0.37512	5.519502	-3.55089	0.000415	0.000902	-1.03632
TAF9B	-0.37515	6.005358	-5.73873	1.52E-08	5.82E-08	8.716343
CCDC107	-0.37523	6.54884	-4.69935	3.25E-06	9.43E-06	3.55075
RFK	-0.37526	7.175652	-3.75648	0.000189	0.000434	-0.30419
9-Sep	-0.37536	8.484077	-4.93361	1.05E-06	3.24E-06	4.632706
LRRCC1	-0.37537	4.28633	-6.55572	1.21E-10	5.90E-10	13.41893
ZSWIM7	-0.37545	6.604977	-5.62456	2.87E-08	1.06E-07	8.10341
ZWILCH	-0.37557	5.863465	-3.16849	0.001611	0.003201	-2.29273
PTPN13	-0.37562	3.638239	-9.81989	3.48E-21	5.19E-20	37.24811
TAF4B	-0.37566	5.42408	-4.21993	2.83E-05	7.25E-05	1.489502
ZSCAN9	-0.37601	5.747505	-7.29348	9.76E-13	5.91E-12	18.12623
GPX4	-0.37604	9.573494	-3.36395	0.000818	0.001703	-1.66773
BMS1P5	-0.37609	4.833069	-9.37801	1.42E-19	1.77E-18	33.59342
HMGB1	-0.37624	10.77939	-7.62123	1.01E-13	6.72E-13	20.35188
LNX1	-0.37633	4.574614	-7.40396	4.57E-13	2.86E-12	18.86746
TRANK1	-0.37643	8.60333	-2.95222	0.00328	0.006171	-2.9421
C1QTNF3	-0.37651	4.939629	-9.33574	2.01E-19	2.46E-18	33.25036
GFPT1	-0.37671	5.654507	-3.70394	0.000232	0.000525	-0.49502
RASGRP3	-0.37673	4.760683	-6.95441	9.41E-12	5.15E-11	15.90997
CD48	-0.37675	9.562282	-5.00716	7.30E-07	2.30E-06	4.982425
ASPRV1	-0.37712	7.353893	-2.60161	0.009511	0.016495	-3.89998
MBNL3	-0.37723	6.061714	-4.19681	3.12E-05	7.97E-05	1.395346
RIC8B	-0.37725	5.70138	-9.15773	8.60E-19	9.82E-18	31.81799
CRYL1	-0.37733	6.775471	-4.88544	1.33E-06	4.06E-06	4.406267
ASNS	-0.37749	5.209062	-6.92959	1.11E-11	6.01E-11	15.75123
HCST	-0.37771	9.343198	-3.92203	9.81E-05	0.000234	0.313855
CREB3L4	-0.37785	5.377849	-7.1697	2.25E-12	1.31E-11	17.30688
KIF20B	-0.37787	5.377238	-6.30307	5.71E-10	2.58E-09	11.9062
C9orf114	-0.37788	6.234288	-7.26352	1.20E-12	7.17E-12	17.92689
NHP2L1	-0.37796	6.471717	-7.60907	1.10E-13	7.29E-13	20.26788
TTC5	-0.37802	5.385735	-8.43138	2.62E-16	2.25E-15	26.19206
IMMP1L	-0.37815	4.882551	-4.98335	8.22E-07	2.57E-06	4.868707
HDDC2	-0.3786	6.240752	-5.34712	1.28E-07	4.39E-07	6.66021
ZMYM2	-0.37886	5.816615	-6.56638	1.13E-10	5.53E-10	13.48389
NIPAL3	-0.37906	5.601495	-7.69545	5.94E-14	4.06E-13	20.86705
CACNB4	-0.37924	4.518148	-8.98215	3.54E-18	3.73E-17	30.42554
TBRG4	-0.3795	5.031213	-5.69935	1.90E-08	7.19E-08	8.50368
SF3B1	-0.37991	9.758899	-5.22697	2.39E-07	7.98E-07	6.055792
C11orf63	-0.37997	4.029641	-8.15444	2.11E-15	1.66E-14	24.14262
AKAP9	-0.37998	5.654821	-6.62958	7.60E-11	3.78E-10	13.87091
ANKRD10-	-0.38039	8.115017	-4.6594	3.92E-06	1.12E-05	3.371122
TMEM218	-0.38093	5.476794	-8.40384	3.23E-16	2.75E-15	25.98583
DNAJB9	-0.38096	6.63608	-3.26583	0.001155	0.002347	-1.98598
TSN	-0.38129	7.109187	-5.65615	2.41E-08	9.02E-08	8.2719
SLC25A17	-0.38137	5.780262	-6.93423	1.07E-11	5.84E-11	15.78084
FAM58A	-0.38146	7.246364	-6.16155	1.33E-09	5.76E-09	11.08152
TIAM2	-0.38165	5.543027	-4.9115	1.17E-06	3.60E-06	4.528543
C14orf166	-0.3818	10.05084	-5.91747	5.54E-09	2.21E-08	9.697962
IL7	-0.382	3.926593	-3.58589	0.000364	0.000799	-0.91444
ELAC1	-0.38209	4.498944	-8.49893	1.56E-16	1.37E-15	26.70006
ZNF239	-0.38241	4.792155	-6.14187	1.50E-09	6.43E-09	10.96815
TTC4	-0.38286	6.332589	-6.50051	1.70E-10	8.19E-10	13.08395
LOC10050	-0.38293	4.807121	-11.8945	2.12E-29	8.66E-28	55.92621

SPECC1	-0.38305	5.74931	-6.45096	2.31E-10	1.10E-09	12.78543
ZNF658	-0.38342	4.193962	-2.49044	0.013032	0.022006	-4.17903
AK1	-0.38343	6.181663	-5.90854	5.83E-09	2.33E-08	9.648305
GPBP1	-0.38353	9.003215	-6.26911	7.01E-10	3.14E-09	11.7068
CD3EAP	-0.38353	5.711885	-5.90866	5.83E-09	2.32E-08	9.648956
SAP130	-0.38356	7.746407	-6.22842	8.95E-10	3.96E-09	11.46914
PDCD6	-0.38364	7.604781	-6.2935	6.05E-10	2.73E-09	11.84987
NUDT12	-0.38365	3.272607	-8.68339	3.75E-17	3.51E-16	28.10347
ZBTB33	-0.38366	6.001207	-8.03078	5.26E-15	3.98E-14	23.24509
TDRKH	-0.38375	4.266247	-9.69632	9.92E-21	1.39E-19	36.21385
SNRPF	-0.38404	8.087874	-4.68102	3.54E-06	1.02E-05	3.468174
RDH11	-0.38409	6.539561	-9.10434	1.32E-18	1.47E-17	31.39244
LOC10192	-0.38411	5.095947	-8.42014	2.85E-16	2.44E-15	26.10782
HNRNPUL	-0.38426	7.511513	-7.35256	6.51E-13	4.01E-12	18.52143
MTFP1	-0.38436	6.491765	-5.98531	3.75E-09	1.53E-08	10.07753
CHN1	-0.38462	5.413223	-6.323	5.06E-10	2.30E-09	12.02362
COX7B	-0.38484	7.660123	-5.96806	4.14E-09	1.68E-08	9.980687
MRPS26	-0.38485	5.92532	-8.96491	4.06E-18	4.24E-17	30.28995
FANCD2	-0.385	6.14981	-5.75878	1.36E-08	5.24E-08	8.825146
FZD3	-0.3851	4.606288	-8.40417	3.22E-16	2.75E-15	25.98828
TMEM59	-0.38573	7.820996	-5.53113	4.78E-08	1.73E-07	7.610084
RNF115	-0.38574	6.581848	-7.078	4.16E-12	2.35E-11	16.70748
SERPINF1	-0.38589	5.618138	-4.01858	6.61E-05	0.000161	0.685963
ZNF615	-0.38608	6.957795	-4.45451	1.01E-05	2.73E-05	2.472212
FUNDC2	-0.38609	6.803322	-7.1352	2.84E-12	1.63E-11	17.08063
BICD1	-0.38612	4.51406	-9.18746	6.75E-19	7.79E-18	32.05579
TBC1D12	-0.38619	5.281573	-3.7691	0.00018	0.000414	-0.25797
MIF4GD	-0.38632	7.682339	-6.04303	2.68E-09	1.12E-08	10.40354
VPS11	-0.38644	7.689163	-8.01341	5.98E-15	4.51E-14	23.11992
CCDC92	-0.38655	7.409557	-3.39886	0.000722	0.001518	-1.5523
MRPL49	-0.38657	8.737	-6.42156	2.77E-10	1.30E-09	12.60922
PSMB9	-0.38664	10.37611	-2.95967	0.003203	0.00604	-2.92047
MAP4K5	-0.38674	5.245897	-3.71205	0.000225	0.00051	-0.46573
ATF7IP	-0.38683	8.246258	-5.985	3.75E-09	1.53E-08	10.07582
EEF1A1	-0.38684	12.46071	-6.17083	1.26E-09	5.47E-09	11.13508
NCK1	-0.38691	6.597881	-4.21353	2.91E-05	7.44E-05	1.463404
LPHN1	-0.38698	5.879119	-10.0233	6.06E-22	9.91E-21	38.97109
CLEC7A	-0.38715	8.225362	-2.90063	0.003863	0.007172	-3.09044
ASCL2	-0.38718	4.750361	-7.47767	2.75E-13	1.75E-12	19.36708
ORC2	-0.38726	6.767759	-5.37977	1.08E-07	3.73E-07	6.826658
TRAF3IP1	-0.38728	5.005099	-9.37242	1.48E-19	1.85E-18	33.54801
AGAP9	-0.38741	4.834364	-5.79254	1.13E-08	4.37E-08	9.009026
LINC0135!	-0.38745	4.640392	-9.00568	2.93E-18	3.12E-17	30.61101
IMMP2L	-0.38762	5.89612	-6.39671	3.23E-10	1.50E-09	12.4608
NUB1	-0.38772	7.394673	-6.76441	3.22E-11	1.67E-10	14.70712
WDR81	-0.38809	7.735317	-7.47076	2.88E-13	1.84E-12	19.3201
SKP1	-0.38809	9.69435	-7.59633	1.20E-13	7.94E-13	20.18001
ZNF225	-0.38835	4.877286	-8.5683	9.15E-17	8.24E-16	27.22512
TMEM200/	-0.38841	3.880967	-5.08578	4.92E-07	1.58E-06	5.361493
PHTF2	-0.38843	5.746505	-7.00794	6.62E-12	3.67E-11	16.25394
C12orf45	-0.38868	6.404402	-5.85319	8.00E-09	3.15E-08	9.341861
ZNF624	-0.3887	4.215665	-4.622	4.67E-06	1.33E-05	3.204222
NEK1	-0.38871	5.097004	-6.47163	2.04E-10	9.71E-10	12.90972
DBT	-0.38899	5.66647	-7.22017	1.60E-12	9.47E-12	17.63955
AASDH	-0.3893	6.90931	-5.40129	9.60E-08	3.36E-07	6.936851
RYK	-0.38953	6.694405	-7.83989	2.11E-14	1.51E-13	21.88129
SF3B3	-0.38964	7.050176	-8.71066	3.03E-17	2.86E-16	28.31294
C4orf29	-0.3897	7.024203	-6.45405	2.27E-10	1.08E-09	12.80394

RAB37	-0.38994	8.507556	-2.89808	0.003894	0.007226	-3.09769
RPS28	-0.38997	8.331948	-9.32401	2.21E-19	2.69E-18	33.1553
KLKB1	-0.39001	5.750352	-7.71979	5.00E-14	3.44E-13	21.03692
LCLAT1	-0.39025	8.006689	-4.53352	7.02E-06	1.95E-05	2.814385
POLD2	-0.39029	5.98029	-4.76665	2.36E-06	6.96E-06	3.856608
ACVR2B	-0.39059	5.384692	-9.21692	5.31E-19	6.20E-18	32.29198
SLC35E3	-0.39059	7.03677	-7.64525	8.48E-14	5.71E-13	20.51817
IK	-0.39061	9.355324	-7.9241	1.15E-14	8.39E-14	22.47966
C1orf52	-0.39079	6.832105	-6.47564	1.99E-10	9.48E-10	12.93384
EVI2A	-0.39123	11.54504	-4.86142	1.50E-06	4.53E-06	4.294112
RAD17	-0.39131	6.846315	-5.89429	6.33E-09	2.51E-08	9.569141
SLC11A2	-0.39132	5.544425	-8.13882	2.37E-15	1.86E-14	24.02868
HSD17B8	-0.3914	6.768588	-5.4747	6.49E-08	2.31E-07	7.315698
NUPL2	-0.39141	6.959471	-5.51564	5.20E-08	1.87E-07	7.528978
GLS	-0.39197	6.030301	-7.7335	4.53E-14	3.14E-13	21.13275
RRN3P2	-0.39231	5.776288	-5.04062	6.18E-07	1.96E-06	5.14309
CD4	-0.39245	6.223305	-5.22741	2.39E-07	7.96E-07	6.057947
PALLD	-0.3925	5.766188	-3.39707	0.000727	0.001527	-1.55824
GRHL1	-0.39258	4.699417	-3.22513	0.001329	0.002672	-2.11533
TXNDC9	-0.39276	8.759272	-4.70096	3.23E-06	9.37E-06	3.55801
PNO1	-0.39279	5.985472	-8.08939	3.42E-15	2.62E-14	23.66914
SERPINB9I	-0.39307	5.813992	-5.40412	9.46E-08	3.31E-07	6.951353
CCAR2	-0.39307	5.185635	-11.0683	5.25E-26	1.36E-24	48.20492
ATPAF1	-0.39308	6.705893	-4.43761	1.09E-05	2.93E-05	2.399744
ALG13	-0.39312	6.48597	-5.71723	1.72E-08	6.54E-08	8.600085
LOC15506	-0.39337	6.492488	-8.95509	4.39E-18	4.56E-17	30.21275
LIN52	-0.39338	6.266872	-5.31758	1.49E-07	5.09E-07	6.510463
ACOT4	-0.39345	4.606279	-7.14021	2.75E-12	1.58E-11	17.11343
GOLGA5	-0.39355	8.370964	-5.63086	2.77E-08	1.03E-07	8.13694
TIGD7	-0.39361	5.620421	-4.10713	4.57E-05	0.000114	1.034801
SMC3	-0.39384	7.564647	-3.10617	0.001986	0.003884	-2.48442
C5orf45	-0.39424	6.085778	-6.57075	1.10E-10	5.38E-10	13.51056
MAPKBP1	-0.39428	6.377534	-6.41215	2.94E-10	1.37E-09	12.55295
SOS1	-0.39429	7.568181	-5.37163	1.12E-07	3.89E-07	6.785059
MBTPS2	-0.39456	5.688053	-5.75873	1.36E-08	5.24E-08	8.824865
GATC	-0.39462	6.328824	-7.53621	1.83E-13	1.19E-12	19.76679
MAGEF1	-0.39467	6.211332	-5.40349	9.49E-08	3.32E-07	6.948127
MLH1	-0.39469	8.655989	-4.82394	1.79E-06	5.38E-06	4.120167
SPRED1	-0.39473	3.518907	-6.18242	1.18E-09	5.13E-09	11.20209
KIAA0141	-0.39497	6.336915	-8.38125	3.84E-16	3.24E-15	25.81704
TMED4	-0.39516	8.20493	-6.72104	4.26E-11	2.18E-10	14.43654
RABEP2	-0.39516	6.509456	-7.77918	3.27E-14	2.30E-13	21.45317
ZBED5-AS	-0.39529	6.975922	-4.61733	4.77E-06	1.35E-05	3.183463
CCNT2	-0.39544	6.112807	-6.13278	1.58E-09	6.77E-09	10.91586
UBQLN4	-0.39553	6.915793	-8.37838	3.92E-16	3.31E-15	25.79566
CARNS1	-0.39558	5.678186	-6.6763	5.66E-11	2.86E-10	14.159
NELFCD	-0.3957	7.861195	-4.81804	1.84E-06	5.53E-06	4.092893
G2E3	-0.39582	6.276583	-4.07349	5.26E-05	0.00013	0.901435
BRD7	-0.39583	6.31821	-6.64721	6.80E-11	3.40E-10	13.97939
SLC33A1	-0.39591	5.992073	-6.48145	1.92E-10	9.17E-10	12.96885
CASK	-0.39592	4.841197	-10.3186	4.59E-23	8.55E-22	41.51637
SLC25A16	-0.39608	5.78809	-10.4636	1.27E-23	2.51E-22	42.78578
PRDM4	-0.39614	7.377137	-8.19878	1.52E-15	1.21E-14	24.46711
LRRC37A4	-0.39616	6.798788	-6.67995	5.53E-11	2.80E-10	14.18157
SLC46A3	-0.39619	8.207426	-4.26545	2.32E-05	6.01E-05	1.676287
EDEM1	-0.39628	8.308638	-6.07966	2.16E-09	9.11E-09	10.6118
PCED1B-A	-0.39638	5.676723	-5.2727	1.89E-07	6.37E-07	6.284364
ARHGAP1:	-0.39642	6.017875	-3.72506	0.000214	0.000486	-0.41864

FAM20B	-0.39654	6.373808	-6.22325	9.23E-10	4.07E-09	11.43899
IRF2BP2	-0.39668	8.431849	-7.24646	1.34E-12	8.00E-12	17.81362
FAM86A	-0.39678	5.912244	-7.0047	6.76E-12	3.74E-11	16.23305
MORC4	-0.39681	4.773767	-6.1937	1.10E-09	4.81E-09	11.26738
ZNF614	-0.39683	4.836537	-7.87075	1.69E-14	1.22E-13	22.10003
CCDC115	-0.39685	7.842782	-4.92644	1.09E-06	3.35E-06	4.598874
SARS	-0.39699	6.659688	-6.03743	2.77E-09	1.15E-08	10.37179
NKX3-1	-0.39702	5.450881	-6.25853	7.47E-10	3.34E-09	11.64486
CDK17	-0.39702	7.152339	-6.74179	3.73E-11	1.92E-10	14.56583
NEK4	-0.39711	6.898977	-2.8295	0.00482	0.008819	-3.29077
ARHGAP1	-0.39717	9.042523	-6.39013	3.36E-10	1.56E-09	12.42163
RPL36AL	-0.39765	10.69839	-4.00545	6.98E-05	0.00017	0.634857
HSF5	-0.39772	4.206125	-6.24951	7.89E-10	3.51E-09	11.59216
MRPS22	-0.39774	7.783142	-6.95357	9.46E-12	5.18E-11	15.90455
CYTIP	-0.39785	10.55924	-4.39821	1.29E-05	3.46E-05	2.231812
NARS2	-0.39812	6.29981	-4.77528	2.27E-06	6.70E-06	3.896148
CNKSR2	-0.39827	4.061119	-5.78204	1.20E-08	4.62E-08	8.951717
DTWD1	-0.39834	4.429684	-7.34442	6.89E-13	4.23E-12	18.46684
KMT2E	-0.39837	9.12099	-5.42983	8.25E-08	2.91E-07	7.083569
UTRN	-0.39845	7.17912	-6.3499	4.30E-10	1.97E-09	12.18264
D2HGDH	-0.39853	6.983787	-5.46212	6.94E-08	2.46E-07	7.250414
ZRSR2	-0.39865	7.593198	-6.32298	5.06E-10	2.30E-09	12.0235
HEMK1	-0.39876	6.201559	-8.27542	8.53E-16	6.95E-15	25.03124
PRKCH	-0.39877	7.020273	-5.96055	4.32E-09	1.75E-08	9.938566
ERMARD	-0.39888	6.63069	-6.90383	1.31E-11	7.04E-11	15.58695
PSMD6-AS	-0.3989	7.568616	-4.07832	5.16E-05	0.000127	0.920508
PARP1	-0.39909	7.625162	-4.62863	4.53E-06	1.29E-05	3.233706
IKBIP	-0.39909	6.802077	-3.47884	0.000541	0.001155	-1.28356
TXNL1	-0.3992	6.391281	-5.99429	3.56E-09	1.46E-08	10.12808
ZNF544	-0.39935	5.945294	-10.3678	2.97E-23	5.61E-22	41.94551
YDJC	-0.39943	6.280551	-6.13576	1.55E-09	6.66E-09	10.93301
OCEL1	-0.39945	7.307811	-7.15964	2.41E-12	1.39E-11	17.24086
RCBTB2	-0.39989	10.20723	-4.31113	1.90E-05	4.99E-05	1.865623
TNFRSF10	-0.40009	6.935468	-7.12587	3.02E-12	1.73E-11	17.01958
SIAH1	-0.40032	6.284947	-4.89118	1.29E-06	3.95E-06	4.433144
FOXO1	-0.40033	6.881996	-8.2802	8.23E-16	6.71E-15	25.06656
SNX5	-0.40045	6.775462	-7.06032	4.68E-12	2.63E-11	16.59268
TMEM126	-0.40045	8.054942	-4.45155	1.02E-05	2.77E-05	2.459512
PI4KA	-0.40048	8.006358	-3.99415	7.31E-05	0.000178	0.590996
FMNL3	-0.40086	5.193219	-8.42875	2.67E-16	2.29E-15	26.17235
NPTN-IT1	-0.40112	7.476101	-4.00978	6.86E-05	0.000167	0.651688
MATK	-0.40118	6.43583	-3.46816	0.000562	0.001198	-1.31978
DCUN1D5	-0.40122	5.428054	-8.70904	3.07E-17	2.89E-16	28.30045
MORF4L2-	-0.40123	4.066507	-8.82385	1.24E-17	1.22E-16	29.18769
IPO9	-0.4013	5.297899	-9.67231	1.22E-20	1.69E-19	36.01391
B3GNT1	-0.40138	5.778022	-9.74796	6.41E-21	9.22E-20	36.6449
CDC42SE1	-0.40149	9.627509	-4.08318	5.05E-05	0.000125	0.939726
RASSF3	-0.40157	9.79693	-5.18608	2.95E-07	9.73E-07	5.852907
E2F6	-0.40167	6.313914	-4.43861	1.08E-05	2.92E-05	2.404008
RBBP5	-0.40169	6.798865	-5.00926	7.23E-07	2.28E-06	4.992471
HOXA1	-0.40195	4.946462	-5.21499	2.55E-07	8.46E-07	5.996182
CD72	-0.402	5.776879	-2.53384	0.011539	0.019689	-4.0715
GPR144	-0.40231	5.478376	-4.39128	1.34E-05	3.56E-05	2.202407
CHCHD4	-0.40237	6.509042	-6.01964	3.07E-09	1.27E-08	10.2711
COPS6	-0.40274	7.702225	-6.40573	3.06E-10	1.43E-09	12.51463
CBX5	-0.40294	5.62149	-7.12756	2.99E-12	1.71E-11	17.03062
ZNF844	-0.40295	6.956016	-4.43342	1.11E-05	2.99E-05	2.381837
TPRKB	-0.40315	7.621759	-4.64866	4.12E-06	1.18E-05	3.323076

NDUFA6	-0.40322	8.297935	-6.03057	2.88E-09	1.20E-08	10.33293
RAB2B	-0.40328	7.934763	-4.11101	4.50E-05	0.000112	1.050238
TMEM42	-0.4033	7.017283	-5.76223	1.34E-08	5.14E-08	8.843868
PSMB7	-0.40335	7.440159	-5.67475	2.18E-08	8.18E-08	8.371515
HPS5	-0.40374	7.080033	-4.45291	1.01E-05	2.75E-05	2.465335
WDR60	-0.40398	5.422836	-9.83729	3.00E-21	4.49E-20	37.39454
CPPED1	-0.40424	9.792763	-3.74817	0.000196	0.000447	-0.33456
FAN1	-0.40428	5.59048	-11.7478	8.71E-29	3.31E-27	54.53029
C11orf58	-0.40431	9.412306	-6.21466	9.72E-10	4.27E-09	11.38909
MESDC2	-0.4044	7.173796	-9.05033	2.05E-18	2.22E-17	30.96381
PSME1	-0.40441	11.43945	-5.04343	6.09E-07	1.94E-06	5.15661
LOC10192	-0.40454	5.11068	-9.85077	2.67E-21	4.02E-20	37.50807
PRPF38A	-0.40462	7.098716	-5.34849	1.27E-07	4.36E-07	6.6672
HAL	-0.40469	6.788674	-3.51013	0.000482	0.001037	-1.17676
HAX1	-0.40475	7.263692	-8.72484	2.71E-17	2.58E-16	28.42201
UBE3A	-0.40483	6.24929	-8.275	8.56E-16	6.97E-15	25.02816
RPS16	-0.40509	11.00081	-5.148	3.59E-07	1.17E-06	5.665287
ZNF500	-0.40528	5.750041	-7.39082	5.01E-13	3.12E-12	18.77879
AS3MT	-0.40532	4.258752	-7.78724	3.09E-14	2.18E-13	21.50987
UPK3A	-0.40596	5.051254	-6.66937	5.91E-11	2.98E-10	14.11614
CNDP2	-0.40616	7.059091	-7.24083	1.39E-12	8.28E-12	17.77634
C1orf27	-0.40633	5.61549	-6.44541	2.39E-10	1.13E-09	12.75212
DIS3L	-0.40638	6.739023	-4.07459	5.24E-05	0.000129	0.905776
AVEN	-0.40644	5.636834	-5.36264	1.18E-07	4.07E-07	6.739211
MDH1	-0.40654	9.434626	-4.95462	9.48E-07	2.94E-06	4.73213
ARMCX4	-0.40654	4.25064	-11.4766	1.15E-27	3.79E-26	51.97638
RIMKLB	-0.40658	4.585199	-9.97949	8.84E-22	1.41E-20	38.59792
ZNF227	-0.40666	5.572558	-4.80789	1.94E-06	5.79E-06	4.04601
LOC10028	-0.40682	7.38581	-4.74441	2.63E-06	7.71E-06	3.755099
RTN4IP1	-0.40685	5.282863	-4.76552	2.37E-06	7.00E-06	3.851468
DHX36	-0.40686	8.144483	-5.98532	3.75E-09	1.53E-08	10.07763
ATP6V0E2	-0.40709	4.67139	-8.28986	7.66E-16	6.27E-15	25.13797
KDM3A	-0.40709	6.323883	-8.52374	1.29E-16	1.14E-15	26.88747
GGNBP2	-0.40714	7.728841	-4.73827	2.70E-06	7.92E-06	3.72713
MRPS14	-0.40764	6.736547	-5.97232	4.04E-09	1.64E-08	10.00458
ANAPC13	-0.40767	6.836896	-8.74623	2.29E-17	2.19E-16	28.58686
HSBP1L1	-0.40777	5.533472	-6.14367	1.48E-09	6.38E-09	10.97848
CNIH1	-0.40822	8.392328	-4.10176	4.68E-05	0.000116	1.013436
HDAC1	-0.40823	9.293095	-6.03753	2.77E-09	1.15E-08	10.37235
LRIG2	-0.40855	5.15561	-10.8987	2.50E-25	5.99E-24	46.66467
ISM1	-0.40898	4.171642	-10.3881	2.48E-23	4.72E-22	42.12348
STT3B	-0.40905	9.840471	-5.62597	2.85E-08	1.06E-07	8.110914
NOXA1	-0.40957	6.384764	-6.16428	1.31E-09	5.67E-09	11.09725
NBEA	-0.40959	4.239268	-7.01657	6.25E-12	3.47E-11	16.30962
AKAP12	-0.40974	4.167449	-6.18422	1.17E-09	5.08E-09	11.21248
RPS6KB1	-0.40987	7.056393	-6.58282	1.02E-10	5.01E-10	13.58424
CLASP2	-0.41002	5.622352	-6.00271	3.39E-09	1.39E-08	10.17556
RHOF	-0.41016	7.102453	-6.64651	6.83E-11	3.41E-10	13.97509
NUDT7	-0.41023	5.460729	-5.5703	3.87E-08	1.41E-07	7.815978
CLN5	-0.41032	6.285621	-4.64125	4.27E-06	1.22E-05	3.289969
C2orf43	-0.41039	5.519969	-5.87226	7.18E-09	2.84E-08	9.447139
RAP1GDS1	-0.41054	6.783461	-5.9455	4.72E-09	1.90E-08	9.854355
RTCB	-0.41055	8.16926	-5.7913	1.14E-08	4.40E-08	9.002248
ZFAND2A	-0.41095	7.829502	-5.26706	1.94E-07	6.55E-07	6.25609
CCDC88C	-0.41097	6.715828	-5.59349	3.41E-08	1.25E-07	7.938502
MTCH1	-0.4112	10.4667	-6.17572	1.23E-09	5.32E-09	11.16333
ZNF45	-0.41124	5.754552	-5.61813	2.98E-08	1.10E-07	8.069201
NIPA1	-0.41143	5.553096	-5.47875	6.35E-08	2.26E-07	7.336743

POP1	-0.41158	5.197572	-8.29831	7.18E-16	5.91E-15	25.20057
NAA30	-0.41169	4.540498	-10.0818	3.65E-22	6.10E-21	39.47169
CCSER2	-0.41171	7.45147	-6.37685	3.65E-10	1.69E-09	12.34256
INTS7	-0.41194	5.174808	-5.21454	2.55E-07	8.48E-07	5.99397
TRIM13	-0.412	6.710078	-4.3532	1.58E-05	4.18E-05	2.041677
AOC2	-0.412	5.873071	-4.08866	4.94E-05	0.000123	0.961434
COMMD3	-0.41218	8.625656	-6.61836	8.16E-11	4.04E-10	13.80195
NFKBIE	-0.41219	6.689418	-5.54645	4.40E-08	1.60E-07	7.690434
KIAA1919	-0.41235	5.055941	-8.92103	5.76E-18	5.91E-17	29.94559
GEMIN2	-0.41261	5.33386	-5.55254	4.26E-08	1.55E-07	7.722431
PPFIBP2	-0.41295	6.220312	-4.59959	5.18E-06	1.46E-05	3.10481
CYLD	-0.4131	7.32339	-8.03773	5.00E-15	3.80E-14	23.29527
POLR1D	-0.41313	8.397699	-7.08438	3.99E-12	2.26E-11	16.74899
MYCL	-0.41329	6.044601	-6.71857	4.32E-11	2.21E-10	14.42117
B3GAT1	-0.41353	6.103399	-6.94512	1.00E-11	5.46E-11	15.85047
GGTA1P	-0.41426	7.310005	-2.63749	0.008572	0.015009	-3.80736
CNPY2	-0.41431	7.120533	-4.11523	4.42E-05	0.00011	1.067053
UBR7	-0.41433	7.653343	-4.84622	1.61E-06	4.86E-06	4.223405
ZKSCAN4	-0.41441	6.766444	-5.64522	2.56E-08	9.55E-08	8.213523
ZNF574	-0.41456	6.569398	-6.97787	8.07E-12	4.45E-11	16.06045
RPS15	-0.41471	12.59739	-3.31148	0.000985	0.002024	-1.83902
PARP11	-0.4149	5.604788	-5.46193	6.95E-08	2.46E-07	7.249432
ATP5A1	-0.41499	11.5601	-5.56803	3.91E-08	1.43E-07	7.804011
LMF1	-0.41502	5.221229	-10.9151	2.15E-25	5.21E-24	46.81249
CA6	-0.41546	5.180198	-8.08967	3.41E-15	2.62E-14	23.67117
YLPM1	-0.41568	7.187116	-6.44682	2.37E-10	1.12E-09	12.76053
ALOX15	-0.41572	5.343241	-3.27078	0.001135	0.002311	-1.97012
ZNF180	-0.4158	5.099736	-6.4386	2.50E-10	1.18E-09	12.71123
CCDC86	-0.41587	6.733722	-5.52075	5.06E-08	1.83E-07	7.555724
TSPAN32	-0.41588	7.156551	-5.06126	5.57E-07	1.78E-06	5.242697
NUP133	-0.41604	6.434082	-9.54194	3.63E-20	4.83E-19	34.93497
CHST11	-0.41629	8.262306	-4.05748	5.63E-05	0.000138	0.838308
SLC30A5	-0.41651	6.001592	-6.1484	1.44E-09	6.20E-09	11.00573
UBA5	-0.41652	6.349994	-4.19234	3.18E-05	8.11E-05	1.3772
LOC10192	-0.41661	5.653192	-4.33868	1.69E-05	4.44E-05	1.980723
UTP6	-0.41688	6.231065	-11.5607	5.20E-28	1.78E-26	52.7645
FBXO4	-0.4169	3.967949	-10.3189	4.58E-23	8.53E-22	41.51938
QRSL1	-0.41698	5.383851	-4.23227	2.68E-05	6.89E-05	1.539969
MRPL30	-0.4171	5.848078	-8.80793	1.41E-17	1.37E-16	29.06413
PREPL	-0.41719	5.36868	-6.93064	1.10E-11	5.98E-11	15.75789
DET1	-0.41723	5.917483	-5.30865	1.57E-07	5.33E-07	6.465329
LRRC8B	-0.41724	4.752075	-8.23663	1.14E-15	9.20E-15	24.74522
MZT2B	-0.41725	7.902984	-4.51646	7.59E-06	2.10E-05	2.74002
DDX11L2	-0.41754	7.349078	-2.99759	0.002836	0.005397	-2.80957
LINC00667	-0.41808	5.97642	-7.25952	1.23E-12	7.35E-12	17.90027
C12orf66	-0.4182	4.904949	-6.02172	3.03E-09	1.26E-08	10.28285
ELK4	-0.41841	5.631868	-9.39695	1.21E-19	1.53E-18	33.74756
ZNF207	-0.41859	7.66489	-7.492	2.49E-13	1.60E-12	19.46469
TGDS	-0.41865	5.601318	-5.32253	1.46E-07	4.97E-07	6.535502
RPL30	-0.41869	13.25311	-5.90763	5.86E-09	2.34E-08	9.643271
11-Sep	-0.4187	4.960781	-6.70275	4.78E-11	2.43E-10	14.32293
VILL	-0.41919	6.38868	-5.08741	4.88E-07	1.57E-06	5.369399
MTRF1	-0.41941	4.716266	-5.91998	5.46E-09	2.18E-08	9.711963
CUZD1	-0.41945	4.908209	-7.53209	1.88E-13	1.23E-12	19.73861
PMS2P8	-0.41956	5.697282	-6.05506	2.50E-09	1.05E-08	10.4718
FANCE	-0.41974	6.221473	-7.13231	2.90E-12	1.66E-11	17.0617
CD40LG	-0.41994	6.973628	-7.07004	4.39E-12	2.48E-11	16.65577
ZBTB14	-0.42002	5.710359	-7.74614	4.14E-14	2.89E-13	21.22124

QSER1	-0.4202	5.757598	-5.64394	2.58E-08	9.61E-08	8.206663
GCC2	-0.4202	6.151778	-9.19952	6.12E-19	7.11E-18	32.1524
PON2	-0.42057	5.347823	-6.45544	2.25E-10	1.07E-09	12.8123
BCKDHB	-0.42078	5.498249	-7.92956	1.10E-14	8.09E-14	22.51866
NRCAM	-0.42078	4.659269	-9.74431	6.61E-21	9.50E-20	36.61439
MUM1	-0.42105	5.894992	-8.17601	1.80E-15	1.43E-14	24.3003
NDUFA12	-0.42108	9.57872	-4.52257	7.39E-06	2.04E-05	2.766614
PARK7	-0.4211	10.56517	-6.50904	1.62E-10	7.79E-10	13.13554
ANKIB1	-0.42115	6.618414	-6.04341	2.67E-09	1.12E-08	10.40569
ZNF626	-0.42118	5.341264	-9.81937	3.49E-21	5.20E-20	37.24381
C12orf73	-0.42124	5.889545	-8.36327	4.40E-16	3.69E-15	25.68301
FBXO25	-0.42153	7.208335	-6.08162	2.14E-09	9.02E-09	10.62302
ROBO3	-0.42191	5.920745	-7.8616	1.81E-14	1.30E-13	22.0351
YPEL2	-0.42195	7.524808	-6.27135	6.92E-10	3.10E-09	11.71988
PMS2P5	-0.42224	4.5518	-4.16159	3.63E-05	9.18E-05	1.252865
PARP2	-0.42232	5.953575	-5.84575	8.34E-09	3.28E-08	9.30088
MCCC2	-0.42235	5.930791	-6.87202	1.61E-11	8.61E-11	15.3849
DIS3	-0.42238	6.077618	-5.38949	1.02E-07	3.56E-07	6.876342
BRPF1	-0.42239	7.320576	-9.06882	1.76E-18	1.94E-17	31.11036
FAM110C	-0.4227	3.737786	-6.81991	2.26E-11	1.19E-10	15.05551
PCNXL4	-0.42321	4.744882	-6.77372	3.04E-11	1.57E-10	14.76538
NME3	-0.42322	6.967549	-4.37918	1.41E-05	3.75E-05	2.15122
CACYBP	-0.42325	7.465658	-5.35205	1.25E-07	4.28E-07	6.685283
DIDO1	-0.42326	6.62237	-7.56549	1.49E-13	9.76E-13	19.9677
TNRC6A	-0.42348	7.001713	-7.86171	1.80E-14	1.30E-13	22.03587
GTF3C3	-0.42366	5.969942	-4.39561	1.31E-05	3.50E-05	2.220774
AEBP2	-0.42367	5.293385	-7.51613	2.10E-13	1.36E-12	19.62944
PAXBP1-A	-0.42368	5.225676	-6.9357	1.06E-11	5.79E-11	15.79026
POLR1E	-0.42374	5.546139	-10.6024	3.67E-24	7.60E-23	44.01094
ZNF558	-0.42387	6.646399	-5.25935	2.02E-07	6.80E-07	6.217463
NUP205	-0.42389	5.739615	-6.27442	6.79E-10	3.05E-09	11.73788
C11orf57	-0.42392	6.346431	-5.92109	5.43E-09	2.17E-08	9.718126
HIVEP2	-0.42392	6.254519	-7.38726	5.13E-13	3.19E-12	18.75483
NKG7	-0.42408	10.06642	-2.30633	0.021437	0.034591	-4.61491
ALDH18A1	-0.42421	5.8857	-4.9929	7.84E-07	2.46E-06	4.914253
MAPK8	-0.42436	6.003974	-7.62286	9.94E-14	6.64E-13	20.36319
LOC10192	-0.42438	4.503011	-4.47388	9.22E-06	2.51E-05	2.555592
TTC17	-0.42453	6.794435	-9.17594	7.42E-19	8.51E-18	31.96359
SPATA5	-0.42455	3.870285	-10.5814	4.42E-24	9.11E-23	43.8254
DCHS1	-0.42467	5.32838	-7.66042	7.62E-14	5.14E-13	20.62339
HABP4	-0.42498	5.635332	-11.3837	2.77E-27	8.60E-26	51.11066
CEP131	-0.42538	5.414742	-9.13455	1.04E-18	1.17E-17	31.63298
RNF175	-0.42552	5.947477	-6.72319	4.20E-11	2.15E-10	14.44994
RWDD1	-0.42567	6.539606	-5.31332	1.53E-07	5.20E-07	6.488926
ENTPD5	-0.42569	5.531105	-7.73259	4.56E-14	3.16E-13	21.12641
THAP6	-0.42571	5.596174	-7.22271	1.58E-12	9.32E-12	17.65636
CNOT10	-0.42583	6.631231	-6.18035	1.19E-09	5.19E-09	11.19013
CEP104	-0.42587	5.235817	-7.52042	2.04E-13	1.32E-12	19.65872
STX17	-0.42614	5.869632	-6.7159	4.40E-11	2.25E-10	14.40458
SH3BGR	-0.42617	4.847026	-8.58808	7.86E-17	7.13E-16	27.37543
TRMT10B	-0.42647	5.590651	-8.1184	2.76E-15	2.14E-14	23.87995
PA2G4	-0.42649	6.971872	-5.85397	7.96E-09	3.14E-08	9.346175
CAMK2N1	-0.42675	4.159534	-12.3522	2.42E-31	1.23E-29	60.35011
RAB40B	-0.4271	4.679159	-8.78404	1.70E-17	1.65E-16	28.87902
FAM159A	-0.4275	6.039383	-5.95358	4.50E-09	1.82E-08	9.899527
KIFAP3	-0.4276	6.900122	-4.2086	2.97E-05	7.59E-05	1.443298
UNK	-0.42788	6.307469	-7.92579	1.13E-14	8.29E-14	22.49176
LOC10192	-0.42803	5.492841	-10.4601	1.31E-23	2.58E-22	42.75492

IFT43	-0.42833	5.516006	-8.04026	4.91E-15	3.73E-14	23.31355
FBXO32	-0.42839	4.848456	-9.22143	5.12E-19	5.99E-18	32.32822
FGFR1OP	-0.42839	6.051177	-8.93859	5.01E-18	5.16E-17	30.08322
SEC31B	-0.42843	6.610145	-5.8813	6.81E-09	2.70E-08	9.497186
FAM102A	-0.42866	6.231769	-6.99971	6.99E-12	3.86E-11	16.20089
GALNT6	-0.42873	5.845379	-5.81797	9.77E-09	3.81E-08	9.148229
SEPHS1	-0.42876	6.535782	-5.59137	3.45E-08	1.26E-07	7.927325
RAPGEF6	-0.42884	7.320954	-7.89473	1.42E-14	1.03E-13	22.27041
KIAA1652	-0.42912	6.325556	-6.33846	4.61E-10	2.10E-09	12.11493
RAPGEF2	-0.42916	6.926448	-5.05899	5.63E-07	1.80E-06	5.231723
ZNF562	-0.42916	5.915065	-8.50542	1.49E-16	1.30E-15	26.74905
RPL7	-0.42967	10.08168	-9.27563	3.29E-19	3.93E-18	32.76433
SMEK1	-0.43018	7.630963	-5.14163	3.71E-07	1.21E-06	5.634055
PDDC1	-0.43049	6.598903	-5.82286	9.50E-09	3.71E-08	9.175064
ZNF33B	-0.4305	5.813529	-5.67536	2.17E-08	8.16E-08	8.374765
LOC10192	-0.43054	6.625883	-5.66171	2.34E-08	8.77E-08	8.301649
GTF3C2	-0.43103	7.58081	-9.25962	3.75E-19	4.46E-18	32.63537
CDC14A	-0.43107	5.059435	-8.83683	1.12E-17	1.11E-16	29.28855
VPS36	-0.43107	5.983686	-8.28768	7.78E-16	6.36E-15	25.12187
P2RX7	-0.4312	6.402618	-4.33271	1.73E-05	4.56E-05	1.955744
LRFN3	-0.4315	4.201638	-7.59905	1.18E-13	7.80E-13	20.19878
LOC10192	-0.43168	5.082838	-4.86758	1.45E-06	4.41E-06	4.322861
RHOQ	-0.43189	8.905621	-5.96869	4.13E-09	1.67E-08	9.984219
ESCO1	-0.43191	6.679628	-2.88074	0.004111	0.007604	-3.14694
ERMP1	-0.43198	6.719157	-5.96956	4.10E-09	1.67E-08	9.989085
DUSP6	-0.43201	10.97501	-4.16002	3.65E-05	9.24E-05	1.246567
FUT8	-0.43216	6.018791	-3.55151	0.000414	0.0009	-1.03414
VPS51	-0.43264	9.017742	-4.0803	5.12E-05	0.000127	0.928328
MBIP	-0.43271	5.065705	-4.45613	9.98E-06	2.71E-05	2.479162
RORC	-0.43332	5.948746	-9.22629	4.92E-19	5.78E-18	32.36726
MBTD1	-0.43332	5.972182	-5.49706	5.75E-08	2.06E-07	7.432025
URGCP	-0.43337	6.362964	-6.31622	5.27E-10	2.39E-09	11.9836
ARHGEF9	-0.43345	5.137039	-7.31532	8.40E-13	5.12E-12	18.27208
SMARCA5	-0.43347	7.402489	-5.63716	2.68E-08	9.96E-08	8.17053
EPRS	-0.43352	7.421052	-5.24684	2.16E-07	7.23E-07	6.154897
NEMF	-0.43355	8.194067	-5.12253	4.09E-07	1.33E-06	5.540507
ATL2	-0.43386	5.579672	-7.7672	3.56E-14	2.50E-13	21.36899
TNFRSF21	-0.4339	4.726702	-8.63605	5.42E-17	4.98E-16	27.74108
PLEKHA7	-0.4344	4.422693	-6.14063	1.51E-09	6.48E-09	10.96098
MOAP1	-0.43452	8.900814	-5.48145	6.25E-08	2.23E-07	7.35074
DDX1	-0.43462	8.189298	-5.17961	3.05E-07	1.00E-06	5.820957
STAMBPL1	-0.43471	5.991188	-5.75845	1.37E-08	5.25E-08	8.823321
FBXO45	-0.43478	5.935454	-6.4274	2.68E-10	1.26E-09	12.64416
CLDND1	-0.43485	6.384066	-5.20553	2.67E-07	8.85E-07	5.949253
ZBTB39	-0.43492	5.447662	-8.82185	1.26E-17	1.24E-16	29.17212
CDC23	-0.43507	5.472929	-4.93838	1.03E-06	3.17E-06	4.655254
SPEN	-0.43584	9.008288	-4.60756	4.99E-06	1.41E-05	3.140128
DUSP14	-0.43586	5.674063	-7.28634	1.02E-12	6.19E-12	18.07866
TRIM32	-0.43591	5.120232	-8.93405	5.19E-18	5.34E-17	30.04765
MIEF1	-0.43595	6.957475	-8.57902	8.43E-17	7.63E-16	27.30656
CXCL6	-0.43603	4.504168	-3.0454	0.002427	0.004671	-2.66778
FAM60A	-0.43624	7.54087	-5.8054	1.05E-08	4.08E-08	9.079357
CCDC112	-0.43633	4.517102	-5.22973	2.36E-07	7.87E-07	6.069538
TSC1	-0.43637	7.742958	-7.88029	1.58E-14	1.14E-13	22.16777
LTK	-0.43644	5.511049	-5.94718	4.67E-09	1.88E-08	9.863743
CYB5A	-0.4365	5.753496	-5.98081	3.85E-09	1.57E-08	10.05226
NUDT3	-0.43661	7.889385	-5.64589	2.55E-08	9.51E-08	8.217097
GNRH1	-0.43664	4.46939	-8.91696	5.95E-18	6.10E-17	29.91378

DFNB59	-0.43701	4.227014	-10.0533	4.67E-22	7.70E-21	39.22736
E4F1	-0.43706	6.513008	-5.13925	3.75E-07	1.22E-06	5.622401
ARRB1	-0.4372	7.123921	-5.6854	2.05E-08	7.73E-08	8.428641
EPPK1	-0.43728	5.428684	-5.99387	3.57E-09	1.46E-08	10.12575
CRIP1	-0.43735	8.89759	-2.67921	0.007585	0.01341	-3.69814
WDR73	-0.43736	7.236307	-8.28792	7.77E-16	6.35E-15	25.12365
CYB5B	-0.43749	5.642609	-6.88072	1.52E-11	8.15E-11	15.44007
OXLD1	-0.4375	6.845969	-7.58362	1.31E-13	8.64E-13	20.09244
ZSCAN18	-0.43756	6.364791	-5.75115	1.42E-08	5.45E-08	8.783674
PAPD4	-0.43777	7.687908	-4.83793	1.68E-06	5.05E-06	4.184946
METTL14	-0.43779	5.971552	-7.75419	3.91E-14	2.73E-13	21.2777
PCBP2	-0.4378	7.791296	-8.4438	2.38E-16	2.05E-15	26.28524
SEC22A	-0.438	4.940255	-5.51574	5.20E-08	1.87E-07	7.529519
ZNF605	-0.43812	5.385244	-4.21587	2.88E-05	7.37E-05	1.472956
SNRK	-0.4382	8.52827	-4.88866	1.31E-06	4.00E-06	4.421367
NLRC5	-0.43822	9.981453	-4.87461	1.40E-06	4.27E-06	4.355644
GFM1	-0.43826	5.642344	-6.23873	8.41E-10	3.73E-09	11.52922
CCT2	-0.4384	8.285362	-4.20432	3.02E-05	7.73E-05	1.425892
ALKBH2	-0.43849	5.108201	-7.50445	2.28E-13	1.47E-12	19.54965
ILF3	-0.43906	7.284981	-5.60927	3.12E-08	1.15E-07	8.02214
BIVM	-0.43914	3.890787	-9.62751	1.77E-20	2.43E-19	35.64194
SSBP4	-0.43959	6.807063	-6.19004	1.13E-09	4.92E-09	11.24622
ANKS3	-0.4397	5.874379	-7.11824	3.18E-12	1.82E-11	16.96976
ARHGAP1(	-0.43977	6.54716	-7.7213	4.94E-14	3.41E-13	21.04749
HSPA14	-0.43982	6.803154	-6.4725	2.03E-10	9.66E-10	12.91494
PRKX	-0.44007	6.164512	-3.78002	0.000173	0.000398	-0.21787
UXT	-0.4403	9.846421	-5.31907	1.48E-07	5.06E-07	6.518005
PLEKHG4	-0.44048	6.02241	-8.23655	1.14E-15	9.20E-15	24.74461
CEP83	-0.44062	3.996718	-6.56877	1.11E-10	5.45E-10	13.49845
MED1	-0.44115	7.277634	-6.31947	5.17E-10	2.35E-09	12.00279
TSHZ2	-0.44117	4.81155	-12.4379	1.04E-31	5.61E-30	61.18908
ADRBK2	-0.44121	7.362973	-4.62381	4.63E-06	1.32E-05	3.212256
NKRF	-0.44123	5.861876	-6.39475	3.27E-10	1.52E-09	12.44912
ZCCHC14	-0.44131	5.264511	-13.3328	1.21E-35	1.23E-33	70.14884
MRE11A	-0.44163	4.51337	-10.3868	2.51E-23	4.77E-22	42.1119
MRPL43	-0.4419	6.664317	-5.42416	8.50E-08	2.99E-07	7.054378
FRYL	-0.44201	8.906846	-6.37829	3.62E-10	1.67E-09	12.35114
GTF2H2B	-0.44217	7.013481	-2.86446	0.004326	0.007979	-3.19292
C17orf58	-0.44224	5.781385	-4.1736	3.45E-05	8.75E-05	1.301333
FKBP11	-0.44231	7.458024	-4.08801	4.95E-05	0.000123	0.958864
HCFC2	-0.44234	5.293495	-5.64787	2.53E-08	9.42E-08	8.22768
LRRC16A	-0.4424	5.023913	-4.69824	3.27E-06	9.47E-06	3.545746
C5orf63	-0.44254	4.320754	-14.0356	7.86E-39	1.28E-36	77.41614
LOC10192	-0.44259	4.189238	-7.41903	4.12E-13	2.59E-12	18.96929
ZBED3	-0.44261	5.22323	-7.72812	4.71E-14	3.26E-13	21.09512
NPCDR1	-0.44263	4.935362	-6.56045	1.17E-10	5.73E-10	13.44773
SIMC1	-0.4429	6.65528	-5.18725	2.94E-07	9.68E-07	5.858701
C6orf57	-0.44309	5.136317	-5.37979	1.08E-07	3.73E-07	6.826722
PFDN5	-0.4432	11.02269	-4.26676	2.31E-05	5.98E-05	1.681708
KIAA1279	-0.44334	6.048599	-4.0544	5.70E-05	0.00014	0.8262
CEBPZ	-0.44356	8.125495	-6.2277	8.99E-10	3.97E-09	11.46493
UPF2	-0.4439	8.919364	-7.24806	1.33E-12	7.92E-12	17.82426
TMEM192	-0.44401	6.363929	-5.95634	4.43E-09	1.79E-08	9.914996
SLC39A14	-0.44405	5.645659	-3.83976	0.000136	0.000319	0.003552
LRRC8D	-0.44413	8.981829	-6.60593	8.82E-11	4.36E-10	13.72568
CCDC64	-0.44501	5.527236	-7.93932	1.03E-14	7.56E-14	22.58839
RLF	-0.44502	8.207436	-5.95315	4.51E-09	1.82E-08	9.897142
NANP	-0.44541	5.893942	-5.6191	2.96E-08	1.09E-07	8.074381

SOX12	-0.44576	4.893429	-10.2466	8.65E-23	1.56E-21	40.89112
SNX30	-0.44603	7.341633	-4.0427	5.98E-05	0.000147	0.780258
ABCA17P	-0.44612	3.890968	-12.118	2.42E-30	1.11E-28	58.07438
ZNF251	-0.44616	4.77337	-10.1144	2.75E-22	4.64E-21	39.75126
POLR2D	-0.44635	5.470877	-7.96325	8.63E-15	6.40E-14	22.75963
SPDL1	-0.44653	5.456119	-4.72264	2.91E-06	8.49E-06	3.656173
TTL	-0.44693	6.210851	-6.13817	1.53E-09	6.57E-09	10.94687
PSMD14	-0.44694	8.340228	-4.54653	6.62E-06	1.84E-05	2.871259
YTHDC1	-0.44708	5.82442	-8.08716	3.47E-15	2.67E-14	23.65298
RRM2B	-0.44727	7.899345	-4.88824	1.31E-06	4.01E-06	4.419386
RPL37	-0.44727	12.80516	-7.34346	6.93E-13	4.25E-12	18.4604
RBM12	-0.44732	7.518943	-5.87228	7.17E-09	2.84E-08	9.447283
FAM104A	-0.44733	8.981808	-6.40795	3.01E-10	1.41E-09	12.52788
LRIF1	-0.44734	7.855074	-3.22117	0.001347	0.002706	-2.12782
CST3	-0.44758	7.400812	-5.39681	9.83E-08	3.43E-07	6.913859
SGSM2	-0.4477	7.254863	-8.63679	5.39E-17	4.96E-16	27.7467
DSERG1	-0.44789	6.450216	-4.24704	2.52E-05	6.49E-05	1.600536
PIGL	-0.44797	5.332716	-10.2165	1.13E-22	2.00E-21	40.63105
CD2AP	-0.44827	5.956457	-6.7772	2.97E-11	1.54E-10	14.7872
MTCP1	-0.44866	4.099603	-9.72067	8.08E-21	1.15E-19	36.41687
TCF4	-0.44897	6.491696	-3.83734	0.000138	0.000322	-0.00551
NMD3	-0.44913	7.390099	-5.17216	3.17E-07	1.04E-06	5.784182
COMMD8	-0.44927	9.563791	-5.25896	2.03E-07	6.82E-07	6.215486
DVL1	-0.44947	6.845426	-6.32184	5.10E-10	2.32E-09	12.01678
LYSMD3	-0.44958	8.085066	-4.26229	2.35E-05	6.09E-05	1.663292
SOCS7	-0.44961	5.956546	-8.16571	1.94E-15	1.53E-14	24.22497
ANKRD49	-0.44978	8.631614	-8.45809	2.14E-16	1.85E-15	26.39254
LINC0093(	-0.44988	5.555239	-8.56838	9.15E-17	8.24E-16	27.22574
SUPT7L	-0.4502	6.179431	-6.59971	9.17E-11	4.53E-10	13.68759
NOLC1	-0.45047	7.021727	-6.865	1.69E-11	8.99E-11	15.34042
PDHB	-0.45051	8.344956	-5.89107	6.44E-09	2.56E-08	9.551314
RNMT	-0.45058	5.604643	-7.25261	1.29E-12	7.69E-12	17.85445
BZRAP1	-0.45061	5.842511	-6.38385	3.49E-10	1.62E-09	12.38422
TOPBP1	-0.45068	8.760584	-5.37366	1.11E-07	3.85E-07	6.795443
SSBP1	-0.45083	7.140965	-6.57312	1.08E-10	5.31E-10	13.525
PGAP3	-0.45085	6.433178	-10.4396	1.57E-23	3.08E-22	42.57507
ANKRD13(	-0.45091	4.854671	-4.31037	1.91E-05	5.00E-05	1.862486
FXN	-0.45116	5.054543	-9.51468	4.56E-20	5.99E-19	34.71069
C19orf66	-0.45127	8.295716	-5.62264	2.90E-08	1.08E-07	8.093203
R3HDM1	-0.45136	6.172568	-10.8477	3.98E-25	9.31E-24	46.20401
NPAT	-0.45206	6.320685	-7.47819	2.74E-13	1.75E-12	19.37065
ZCCHC10	-0.45225	7.078159	-4.7397	2.68E-06	7.87E-06	3.733639
CCT6B	-0.45246	4.786564	-9.13523	1.03E-18	1.16E-17	31.6384
MITD1	-0.45252	7.546031	-4.5943	5.31E-06	1.50E-05	3.081426
KPNA3	-0.45255	8.123787	-5.49606	5.78E-08	2.07E-07	7.426774
ARMCX6	-0.45261	7.077754	-9.90083	1.74E-21	2.70E-20	37.93071
CXorf21	-0.45313	7.700097	-4.23514	2.65E-05	6.81E-05	1.551736
DPF2	-0.45352	8.499063	-7.35957	6.21E-13	3.83E-12	18.56853
IBTK	-0.45352	5.352388	-6.89603	1.38E-11	7.40E-11	15.53735
FAM200A	-0.45377	3.898893	-4.66716	3.78E-06	1.09E-05	3.405909
SLC9B2	-0.45379	5.463999	-5.59535	3.37E-08	1.24E-07	7.948373
IMP3	-0.45391	7.605488	-4.0127	6.77E-05	0.000165	0.663066
PRPS1	-0.45429	7.128847	-5.90609	5.91E-09	2.36E-08	9.6347
PLEKHA5	-0.4544	3.655769	-6.65934	6.30E-11	3.17E-10	14.05422
EXOC8	-0.45487	8.519749	-4.80481	1.97E-06	5.87E-06	4.031818
ERP27	-0.4549	7.253132	-5.34249	1.31E-07	4.49E-07	6.636724
SULF2	-0.45498	8.03161	-2.49521	0.01286	0.021738	-4.1673
LOC10013	-0.45501	5.449768	-6.12964	1.61E-09	6.89E-09	10.89784

TEX10	-0.45515	6.762654	-5.25714	2.05E-07	6.87E-07	6.206385
GSPT1	-0.45536	6.318773	-6.49814	1.73E-10	8.31E-10	13.06966
DUT	-0.45561	8.185656	-4.70205	3.21E-06	9.32E-06	3.562945
ARHGEF6	-0.45563	10.02781	-6.81352	2.35E-11	1.24E-10	15.01531
PDCD7	-0.45565	6.186602	-7.70664	5.49E-14	3.77E-13	20.94512
S100A10	-0.45601	9.083618	-4.74316	2.64E-06	7.75E-06	3.749383
SON	-0.45629	9.753659	-9.89803	1.78E-21	2.75E-20	37.90704
PQLC3	-0.45642	7.265055	-6.71991	4.29E-11	2.19E-10	14.42952
TAF1C	-0.45646	6.639436	-8.47703	1.85E-16	1.61E-15	26.53504
PLEKHF1	-0.45647	7.175474	-5.98065	3.85E-09	1.57E-08	10.05136
MPPE1	-0.45662	9.483533	-7.71947	5.01E-14	3.45E-13	21.03468
RBMX2	-0.45747	6.632678	-8.53924	1.15E-16	1.02E-15	27.00479
MRS2	-0.45779	4.80439	-7.41844	4.14E-13	2.60E-12	18.9653
C1orf54	-0.45809	6.240362	-5.40906	9.21E-08	3.23E-07	6.976689
NUP155	-0.4584	6.28444	-4.82175	1.81E-06	5.44E-06	4.110029
ANKRD20/	-0.4584	6.841727	-6.71675	4.37E-11	2.23E-10	14.4099
SYNRG	-0.45849	7.71333	-9.79261	4.39E-21	6.44E-20	37.01898
SEC14L1	-0.45876	8.982267	-5.00051	7.55E-07	2.37E-06	4.950605
USP14	-0.45877	7.899493	-5.82072	9.62E-09	3.75E-08	9.16329
OSBPL5	-0.45887	6.243175	-5.47108	6.61E-08	2.35E-07	7.29692
NPRL2	-0.45892	6.953565	-9.57351	2.79E-20	3.75E-19	35.19525
FANCM	-0.45893	4.55909	-12.1395	1.96E-30	9.11E-29	58.28188
NIP7	-0.45912	6.118642	-6.07194	2.26E-09	9.51E-09	10.56784
LOC28627	-0.45914	4.204556	-4.16494	3.58E-05	9.06E-05	1.266391
ZNF555	-0.45926	3.674409	-8.88213	7.85E-18	7.90E-17	29.64149
EIF3K	-0.45932	9.163989	-8.13277	2.48E-15	1.94E-14	23.98456
C11orf80	-0.45933	4.538981	-4.97646	8.51E-07	2.66E-06	4.835878
MDN1	-0.45934	5.518972	-10.1357	2.28E-22	3.88E-21	39.93414
ST8SIA1	-0.45938	3.66046	-6.8474	1.89E-11	1.00E-10	15.229
C11orf1	-0.45967	5.033489	-9.92377	1.43E-21	2.24E-20	38.12493
GTF2H1	-0.46025	6.136775	-7.37967	5.41E-13	3.35E-12	18.70371
DGKE	-0.46028	4.317403	-9.87464	2.18E-21	3.32E-20	37.70944
DECR2	-0.46028	7.360956	-10.4802	1.09E-23	2.18E-22	42.93162
LTB	-0.46053	10.67458	-3.66416	0.00027	0.000605	-0.63781
PLEKHH1	-0.46058	3.655469	-14.1048	3.78E-39	6.52E-37	78.1421
MEOX1	-0.46066	4.075232	-6.00005	3.44E-09	1.41E-08	10.16051
FLJ10038	-0.46072	5.518606	-4.52327	7.36E-06	2.04E-05	2.769658
NAPSB	-0.46074	7.009613	-2.4253	0.015594	0.02593	-4.337
TNRC6B	-0.46087	7.744125	-6.74985	3.54E-11	1.82E-10	14.61614
BRI3BP	-0.46102	8.464958	-4.13384	4.08E-05	0.000103	1.141425
PPAP2A	-0.46115	5.531542	-5.66954	2.24E-08	8.41E-08	8.343593
VIPR1	-0.46143	7.220843	-6.19021	1.12E-09	4.91E-09	11.24719
XYLT1	-0.4618	5.967538	-6.7409	3.75E-11	1.93E-10	14.5603
DPH5	-0.46181	6.82319	-7.25742	1.25E-12	7.45E-12	17.88633
KNOP1	-0.46209	5.775859	-11.2788	7.41E-27	2.18E-25	50.13846
DENND6A	-0.46215	9.300119	-6.17831	1.21E-09	5.25E-09	11.17832
CD5	-0.46232	6.98439	-6.08452	2.10E-09	8.88E-09	10.63955
RBM26	-0.46235	5.965681	-8.94931	4.60E-18	4.76E-17	30.16736
TSPAN18	-0.46268	6.377323	-7.24454	1.36E-12	8.09E-12	17.80087
KBTBD11	-0.46274	6.843587	-4.94497	9.94E-07	3.08E-06	4.686407
MRPL15	-0.46296	7.434994	-4.18449	3.29E-05	8.38E-05	1.345379
PAICS	-0.4631	5.452618	-6.12796	1.63E-09	6.96E-09	10.88817
ZNF395	-0.46327	5.831946	-11.7063	1.30E-28	4.80E-27	54.13657
IKZF5	-0.4633	6.496078	-5.43393	8.07E-08	2.85E-07	7.104706
USP25	-0.46342	8.324121	-4.98169	8.29E-07	2.59E-06	4.860775
SDR42E1	-0.46361	4.888181	-12.7525	4.47E-33	2.96E-31	64.29928
SLC16A7	-0.46362	5.765028	-3.92574	9.66E-05	0.000231	0.327985
SMDT1	-0.46364	7.286196	-7.52761	1.94E-13	1.26E-12	19.7079

RPUSD3	-0.46375	6.63051	-7.80542	2.71E-14	1.92E-13	21.63787
GPAT2	-0.46388	5.147981	-5.36763	1.15E-07	3.96E-07	6.764647
UTP23	-0.46404	5.831819	-8.21233	1.37E-15	1.10E-14	24.56656
DDX50	-0.46418	8.543103	-6.18953	1.13E-09	4.93E-09	11.24327
ATG16L1	-0.46428	5.688592	-8.38946	3.60E-16	3.05E-15	25.87837
MCTS1	-0.46447	7.55034	-6.77385	3.04E-11	1.57E-10	14.76622
LOC10192	-0.46457	5.326973	-8.65162	4.80E-17	4.44E-16	27.86009
LCMT2	-0.46473	5.300316	-10.1766	1.60E-22	2.77E-21	40.28625
ZBTB10	-0.46489	4.208991	-9.78715	4.59E-21	6.71E-20	36.97317
HLA-DOB	-0.46516	7.06997	-3.3428	0.000882	0.001826	-1.7371
ABCB7	-0.46559	7.201539	-5.82367	9.46E-09	3.69E-08	9.179465
SNPH	-0.4656	5.850824	-7.55245	1.63E-13	1.07E-12	19.87817
OMA1	-0.46561	7.975561	-5.44189	7.73E-08	2.73E-07	7.14577
PM20D2	-0.46561	5.772263	-5.77545	1.24E-08	4.79E-08	8.915798
DDX39A	-0.46586	7.881978	-4.40339	1.27E-05	3.38E-05	2.253791
CYP2R1	-0.46588	5.062421	-10.3151	4.73E-23	8.79E-22	41.4863
CDIP1	-0.46606	6.332542	-7.69896	5.80E-14	3.97E-13	20.89154
TRAPPC2	-0.46625	7.66972	-5.7062	1.83E-08	6.93E-08	8.540581
DDX31	-0.46644	5.546852	-10.1315	2.37E-22	4.02E-21	39.898
SLC25A3	-0.46667	8.36084	-7.97222	8.08E-15	6.01E-14	22.82392
NR2C2AP	-0.46676	6.184669	-9.33572	2.01E-19	2.46E-18	33.25013
TMEM261	-0.46708	6.568413	-7.36234	6.09E-13	3.76E-12	18.58714
HELQ	-0.46711	5.307057	-11.1783	1.89E-26	5.20E-25	49.21245
ZNF280B	-0.46712	5.063279	-8.33292	5.53E-16	4.60E-15	25.4572
CIAPIN1	-0.46754	6.051641	-8.30503	6.83E-16	5.63E-15	25.25032
SRPRB	-0.46812	6.635225	-6.41305	2.92E-10	1.37E-09	12.55831
GPR155	-0.46818	7.220302	-4.74763	2.59E-06	7.59E-06	3.769755
PDCD11	-0.4684	6.164254	-9.3563	1.70E-19	2.10E-18	33.41707
FAM111A	-0.46893	6.61202	-9.0791	1.62E-18	1.79E-17	31.1919
B2M	-0.46936	12.07082	-6.47561	1.99E-10	9.48E-10	12.93367
LRCH3	-0.46945	5.817791	-7.63632	9.04E-14	6.06E-13	20.4563
NGLY1	-0.46952	7.838704	-6.92932	1.11E-11	6.02E-11	15.74947
MIF	-0.46952	8.76774	-2.99983	0.002815	0.005362	-2.80297
KCTD6	-0.46961	6.379909	-5.47799	6.37E-08	2.27E-07	7.332763
RMDN3	-0.47002	7.523687	-8.13776	2.39E-15	1.87E-14	24.02089
PLEKHG3	-0.47003	6.790551	-6.00927	3.26E-09	1.35E-08	10.21253
C8orf33	-0.47038	5.242846	-9.02892	2.43E-18	2.61E-17	30.79445
EDAR	-0.47071	6.136381	-8.8651	8.98E-18	8.99E-17	29.5086
COPS3	-0.47075	8.198278	-6.08186	2.13E-09	9.01E-09	10.62434
DCTD	-0.47175	6.959535	-5.33643	1.35E-07	4.63E-07	6.605945
FAM98A	-0.47177	5.722889	-7.4936	2.46E-13	1.58E-12	19.47558
NCOA4	-0.47196	12.60497	-6.87745	1.55E-11	8.32E-11	15.41931
ZNF599	-0.47219	4.59011	-11.1849	1.78E-26	4.92E-25	49.27292
SRSF3	-0.47235	7.505007	-7.59559	1.20E-13	7.98E-13	20.17487
MEST	-0.47248	5.357596	-3.35944	0.000831	0.001729	-1.68253
BCS1L	-0.47248	6.359696	-6.57069	1.10E-10	5.38E-10	13.51017
WDR82	-0.47264	10.67229	-8.53223	1.21E-16	1.07E-15	26.95167
RPS6	-0.47264	10.68543	-7.58297	1.32E-13	8.68E-13	20.0879
SFMBT1	-0.47272	5.3939	-11.3934	2.53E-27	7.88E-26	51.20072
ZC3H12C	-0.4728	3.910938	-4.35814	1.55E-05	4.10E-05	2.062483
F2R	-0.47289	5.713524	-3.44701	0.000607	0.001288	-1.39121
HECA	-0.47294	7.981013	-9.34847	1.81E-19	2.22E-18	33.35355
RPAP2	-0.47305	6.167489	-8.21352	1.36E-15	1.09E-14	24.57525
LOC10028	-0.47377	5.26182	-5.34308	1.31E-07	4.48E-07	6.639695
LYRM5	-0.47429	8.371176	-5.47895	6.34E-08	2.26E-07	7.337782
LOC10050	-0.47433	5.387441	-4.18328	3.31E-05	8.42E-05	1.340474
PPIG	-0.47448	7.846041	-6.82487	2.19E-11	1.15E-10	15.08681
BLOC1S4	-0.47459	7.331726	-5.79101	1.14E-08	4.40E-08	9.000707

SPINK2	-0.47468	5.439018	-8.02479	5.50E-15	4.15E-14	23.20194
POLR2B	-0.47468	9.976364	-9.37083	1.50E-19	1.87E-18	33.53512
ZFP30	-0.47481	4.906305	-6.53086	1.41E-10	6.85E-10	13.2678
ZDHHC23	-0.47483	4.846477	-5.87403	7.10E-09	2.81E-08	9.456933
COX7C	-0.47512	9.889276	-6.51505	1.56E-10	7.52E-10	13.17192
ENGASE	-0.47526	7.656878	-7.17731	2.14E-12	1.25E-11	17.35692
SERTAD2	-0.4753	8.9487	-6.62765	7.69E-11	3.82E-10	13.85903
LOC10192	-0.47539	4.189815	-5.4181	8.78E-08	3.09E-07	7.023151
SIN3A	-0.47548	6.001079	-9.86257	2.41E-21	3.65E-20	37.60759
KLHL36	-0.47578	7.55715	-8.93829	5.02E-18	5.17E-17	30.08091
MACROD2	-0.47588	5.417766	-2.91333	0.003711	0.006917	-3.05416
PIAS2	-0.47603	4.898851	-7.60073	1.16E-13	7.71E-13	20.21036
ASUN	-0.47607	7.323626	-4.83653	1.69E-06	5.08E-06	4.17843
FDX1	-0.47649	6.477237	-6.22556	9.10E-10	4.02E-09	11.45248
CSF1R	-0.47651	8.710921	-3.08907	0.002102	0.004095	-2.53634
VPS13A	-0.47652	5.166992	-6.08259	2.12E-09	8.98E-09	10.62854
DENND4A	-0.47685	5.786022	-3.79521	0.000163	0.000376	-0.16188
AGAP1	-0.47685	4.642084	-12.236	7.61E-31	3.73E-29	59.21723
DCAF16	-0.47686	5.425333	-6.63564	7.31E-11	3.64E-10	13.90814
CLUAP1	-0.4771	5.427803	-10.7995	6.17E-25	1.41E-23	45.77083
DCAF17	-0.47713	4.718381	-6.14971	1.43E-09	6.16E-09	11.01325
EBF1	-0.47728	4.527136	-3.50648	0.000489	0.00105	-1.18929
EMG1	-0.47729	7.785954	-5.73117	1.59E-08	6.07E-08	8.675439
ERMN	-0.47737	4.533056	-5.4282	8.32E-08	2.93E-07	7.075163
RBM27	-0.47762	8.184604	-7.152	2.54E-12	1.46E-11	17.19067
UPF3A	-0.47782	6.806806	-8.03721	5.02E-15	3.81E-14	23.29151
LOC10013	-0.47838	4.91575	-6.7458	3.63E-11	1.87E-10	14.59088
CENPL	-0.47838	4.699727	-4.56446	6.09E-06	1.71E-05	2.949914
ZPR1	-0.47858	6.947808	-7.3174	8.29E-13	5.06E-12	18.28595
RBBP4	-0.4787	6.840697	-7.50015	2.35E-13	1.52E-12	19.5203
PRR5L	-0.47897	4.70945	-8.9637	4.10E-18	4.28E-17	30.28037
TOP1MT	-0.47897	5.774265	-5.66355	2.32E-08	8.68E-08	8.311496
LOC10192	-0.47902	4.53598	-8.55839	9.88E-17	8.86E-16	27.14987
PFKM	-0.47914	6.601773	-7.15714	2.45E-12	1.42E-11	17.22441
LINC00526	-0.47986	5.465524	-7.46206	3.06E-13	1.95E-12	19.2609
PCM1	-0.48066	6.925582	-7.71253	5.26E-14	3.62E-13	20.98624
CKAP2	-0.48073	5.779283	-5.41058	9.14E-08	3.20E-07	6.984523
NET1	-0.48116	5.47823	-4.58292	5.60E-06	1.57E-05	3.031175
VARS2	-0.48125	7.638066	-6.39721	3.22E-10	1.50E-09	12.46382
GOLM1	-0.48154	5.739093	-3.96791	8.14E-05	0.000196	0.489603
UTP14A	-0.48169	5.159859	-7.87441	1.65E-14	1.19E-13	22.12598
RUNX1-IT1	-0.4818	6.965502	-5.17511	3.13E-07	1.03E-06	5.798746
OTUD7A	-0.48188	4.265713	-10.0446	5.04E-22	8.29E-21	39.15346
KIAA1468	-0.48189	7.688764	-7.80446	2.73E-14	1.94E-13	21.6311
SGK494	-0.48204	5.689733	-4.33182	1.74E-05	4.58E-05	1.952007
CASP6	-0.48205	6.359479	-5.28358	1.78E-07	6.03E-07	6.339021
DAZAP1	-0.48225	8.143529	-6.78598	2.81E-11	1.46E-10	14.84228
ZNF566	-0.48238	3.476403	-7.01751	6.21E-12	3.45E-11	16.31566
RRS1	-0.48243	7.423007	-6.27045	6.95E-10	3.12E-09	11.71465
ZNF133	-0.48254	5.711637	-9.00557	2.93E-18	3.12E-17	30.61007
SRGAP2C	-0.48254	7.075372	-4.08911	4.93E-05	0.000122	0.963208
DND1	-0.48264	7.159613	-7.27398	1.11E-12	6.70E-12	17.99643
DNASE1L3	-0.4827	5.227692	-8.56184	9.62E-17	8.63E-16	27.17606
NFE2L3	-0.48309	3.881865	-8.23005	1.20E-15	9.64E-15	24.69674
EDF1	-0.48321	8.729887	-5.33098	1.39E-07	4.76E-07	6.578298
MPHOSPH	-0.48413	8.056167	-6.5852	1.00E-10	4.94E-10	13.59879
LMAN2L	-0.48417	7.082042	-9.06811	1.77E-18	1.94E-17	31.10468
GLMN	-0.48425	5.689682	-6.33026	4.84E-10	2.21E-09	12.06651

CYP4V2	-0.48432	4.95352	-10.5412	6.35E-24	1.29E-22	43.46951
RABEPK	-0.48436	6.997145	-9.44192	8.35E-20	1.07E-18	34.11445
ZC3H6	-0.48442	5.634394	-7.49649	2.41E-13	1.55E-12	19.49532
SDC2	-0.48449	4.99324	-6.40051	3.16E-10	1.47E-09	12.48346
TCTN3	-0.48459	6.836889	-7.17513	2.17E-12	1.26E-11	17.34259
CLYBL	-0.485	4.556378	-14.7817	2.66E-42	6.59E-40	85.33186
POLG2	-0.48509	6.419155	-7.27121	1.14E-12	6.82E-12	17.97795
CDKN2AIP	-0.48511	7.134044	-5.53594	4.66E-08	1.69E-07	7.635274
EIF5A2	-0.48539	4.024697	-7.82413	2.37E-14	1.69E-13	21.76992
MGC2410(	-0.48541	3.824882	-7.57615	1.38E-13	9.09E-13	20.04103
DNAJC8	-0.48605	7.319955	-8.31476	6.34E-16	5.26E-15	25.32242
SLC26A6	-0.48611	6.711978	-4.83057	1.74E-06	5.22E-06	4.15083
YARS	-0.48642	7.43026	-8.17074	1.87E-15	1.48E-14	24.26175
C20orf194	-0.48666	6.581166	-6.34234	4.50E-10	2.06E-09	12.13789
DPM1	-0.48687	9.978529	-7.39472	4.88E-13	3.04E-12	18.80511
TAPBPL	-0.48717	7.040903	-5.58701	3.53E-08	1.29E-07	7.904239
NDUFV2-/	-0.48721	4.879548	-9.90546	1.67E-21	2.60E-20	37.96993
ZNF790-A	-0.48736	4.764875	-9.035	2.32E-18	2.49E-17	30.84255
SSRP1	-0.48736	6.988043	-6.23404	8.65E-10	3.83E-09	11.50189
HOXB3	-0.48739	5.586062	-9.38854	1.30E-19	1.63E-18	33.67914
HAUS1	-0.48755	6.572195	-4.40817	1.24E-05	3.32E-05	2.274119
PHB2	-0.48767	10.28909	-6.25074	7.83E-10	3.49E-09	11.59931
APOBEC3(	-0.48774	7.904397	-7.66692	7.28E-14	4.93E-13	20.66856
SMYD3	-0.48787	6.362285	-7.04374	5.22E-12	2.92E-11	16.48525
DOCK9	-0.48829	4.908118	-12.7798	3.40E-33	2.29E-31	64.57062
MRRF	-0.48831	6.085681	-9.50567	4.92E-20	6.43E-19	34.63667
PABPC3	-0.48838	12.32754	-7.23144	1.49E-12	8.80E-12	17.71415
FAM135A	-0.4884	3.935335	-10.5015	9.05E-24	1.81E-22	43.11938
NXT1	-0.48863	7.122868	-7.57753	1.37E-13	9.00E-13	20.05053
PPP2R2B	-0.48897	4.765783	-9.05082	2.04E-18	2.22E-17	30.96772
POGK	-0.4892	5.617586	-6.63141	7.51E-11	3.74E-10	13.88214
IFNLR1	-0.48931	5.58883	-4.48624	8.71E-06	2.39E-05	2.608941
RPS15A	-0.48932	8.509266	-11.114	3.44E-26	9.16E-25	48.62302
LOC10192	-0.48937	4.158894	-10.386	2.53E-23	4.80E-22	42.10483
CDC7	-0.48943	5.734477	-4.19199	3.19E-05	8.12E-05	1.375786
PRKAG2-/	-0.48952	4.918019	-11.454	1.43E-27	4.58E-26	51.76595
TOE1	-0.48955	7.12402	-9.63242	1.70E-20	2.34E-19	35.68264
YY1	-0.48989	8.538239	-11.8086	4.86E-29	1.91E-27	55.10683
GUF1	-0.49	4.545427	-6.37961	3.59E-10	1.66E-09	12.35902
FCHO2	-0.49048	8.842	-3.83739	0.000138	0.000322	-0.00531
ZNF682	-0.49095	3.655399	-9.38647	1.32E-19	1.66E-18	33.66226
AGBL3	-0.49113	3.101866	-13.5502	1.28E-36	1.54E-34	72.37651
CWC22	-0.4912	7.854482	-6.86923	1.64E-11	8.76E-11	15.36722
TDG	-0.49124	8.486224	-5.32668	1.42E-07	4.87E-07	6.556505
MTMR9	-0.49129	7.330991	-6.57115	1.10E-10	5.37E-10	13.51296
PITPNB	-0.49133	9.48064	-9.77953	4.90E-21	7.12E-20	36.90926
TMEM50B	-0.4914	6.552791	-7.91061	1.27E-14	9.24E-14	22.38349
PDE4B	-0.49181	6.716323	-3.86836	0.000122	0.000287	0.110725
HYMAI	-0.49209	5.458708	-4.84398	1.63E-06	4.91E-06	4.213014
ZNF16	-0.49227	5.604352	-9.77914	4.92E-21	7.14E-20	36.90605
ATP5O	-0.49239	8.944708	-8.70165	3.25E-17	3.06E-16	28.24363
SENP6	-0.49269	7.288107	-5.67613	2.16E-08	8.12E-08	8.378916
SLU7	-0.4927	8.766809	-6.1159	1.75E-09	7.45E-09	10.81897
MRPL40	-0.49271	6.900157	-5.76858	1.29E-08	4.97E-08	8.87841
ELMOD2	-0.49276	7.170624	-7.35498	6.41E-13	3.95E-12	18.5377
RPS29	-0.49279	9.397694	-8.75041	2.22E-17	2.12E-16	28.61909
NCBP2-AS	-0.49284	7.355926	-7.02033	6.10E-12	3.39E-11	16.33385
CMC2	-0.49295	8.103613	-6.2206	9.38E-10	4.13E-09	11.4236

ICK	-0.49307	4.509482	-6.67232	5.80E-11	2.93E-10	14.13443
TP53BP1	-0.49315	6.880035	-9.23701	4.51E-19	5.31E-18	32.4534
EFR3A	-0.49323	9.022341	-6.48292	1.90E-10	9.09E-10	12.97775
LEPREL4	-0.49323	5.033496	-7.84499	2.04E-14	1.46E-13	21.91738
ABCA11P	-0.49332	4.424686	-8.62895	5.72E-17	5.25E-16	27.68681
WARS2	-0.49342	5.068377	-6.67276	5.78E-11	2.92E-10	14.13713
EIF4B	-0.49358	8.872824	-7.14243	2.71E-12	1.56E-11	17.12799
MS4A2	-0.49368	4.260416	-10.1882	1.44E-22	2.52E-21	40.38636
ZNF317	-0.49372	7.600154	-6.41904	2.82E-10	1.32E-09	12.59413
AHCTF1	-0.49377	7.94158	-4.80044	2.01E-06	5.99E-06	4.011711
ARL10	-0.49392	5.018393	-10.9518	1.53E-25	3.81E-24	47.14529
CDKN1B	-0.49403	10.85792	-7.0452	5.17E-12	2.90E-11	16.49465
GBAS	-0.49415	8.474149	-5.21777	2.51E-07	8.35E-07	6.010018
ATP10A	-0.49451	6.11815	-10.2505	8.36E-23	1.51E-21	40.92483
NEK7	-0.4949	10.65223	-6.29747	5.91E-10	2.67E-09	11.87325
G3BP1	-0.49507	6.591923	-8.96549	4.04E-18	4.23E-17	30.29452
NAA40	-0.49515	5.953677	-6.21313	9.81E-10	4.31E-09	11.38016
ZNF117	-0.49519	6.172409	-3.88496	0.000114	0.00027	0.17325
DHX29	-0.49536	7.224343	-5.59322	3.41E-08	1.25E-07	7.937113
NUP54	-0.49545	4.7124	-10.5782	4.55E-24	9.36E-23	43.79713
GOLGB1	-0.49576	7.742144	-10.2025	1.27E-22	2.24E-21	40.50973
THUMPD3	-0.49577	6.222026	-7.20967	1.72E-12	1.01E-11	17.57021
VPS26A	-0.49614	6.820367	-7.28542	1.03E-12	6.22E-12	18.07258
TMEM184	-0.49624	7.996897	-7.00595	6.70E-12	3.71E-11	16.2411
ACVR1	-0.49646	7.201719	-4.14519	3.89E-05	9.80E-05	1.186911
PCGF5	-0.49666	6.45736	-5.71525	1.74E-08	6.61E-08	8.589385
IRAK1	-0.49689	9.057668	-7.78946	3.04E-14	2.14E-13	21.52548
MID1	-0.49713	4.516288	-13.2096	4.30E-35	4.00E-33	68.89496
PPRC1	-0.49739	5.582467	-6.31238	5.40E-10	2.44E-09	11.96097
LOC10050	-0.49753	6.097907	-8.12273	2.67E-15	2.08E-14	23.9114
RBBP6	-0.49781	7.060077	-13.7116	2.37E-37	3.23E-35	74.04235
MED17	-0.49792	6.646105	-6.2	1.06E-09	4.64E-09	11.30394
BTF3	-0.49794	9.275416	-9.99707	7.60E-22	1.23E-20	38.74757
LXN	-0.49795	7.623507	-5.57266	3.82E-08	1.39E-07	7.828443
MRPL50	-0.49807	4.759237	-5.95363	4.50E-09	1.82E-08	9.899842
LARS2	-0.49811	6.060904	-6.45548	2.25E-10	1.07E-09	12.81256
CAPN7	-0.49815	6.632631	-5.29144	1.71E-07	5.79E-07	6.378582
SUN1	-0.49871	4.859227	-8.58545	8.02E-17	7.27E-16	27.35543
TNFSF8	-0.49874	6.961611	-5.43345	8.09E-08	2.85E-07	7.102212
LPAR5	-0.49892	5.694648	-6.80348	2.51E-11	1.31E-10	14.95215
AIDA	-0.49901	8.528323	-5.16224	3.34E-07	1.09E-06	5.735332
TOMM5	-0.49903	7.86175	-5.17298	3.16E-07	1.04E-06	5.788237
DUSP7	-0.49905	6.356718	-8.56711	9.24E-17	8.31E-16	27.21608
RPL36A	-0.49915	6.472712	-5.60999	3.11E-08	1.15E-07	8.025989
TMEM206	-0.49915	5.854131	-7.21871	1.62E-12	9.56E-12	17.62995
PDCD2	-0.49953	5.479126	-9.42885	9.31E-20	1.18E-18	34.00764
FAM73B	-0.49976	7.068646	-8.54834	1.07E-16	9.53E-16	27.07369
PMS1	-0.49977	5.74044	-9.16061	8.40E-19	9.62E-18	31.84098
TMSB10	-0.49978	12.80809	-5.86077	7.66E-09	3.02E-08	9.383668
RFX5	-0.49979	7.53959	-7.04893	5.05E-12	2.83E-11	16.51885
SIRPG	-0.49991	5.499403	-9.38695	1.32E-19	1.65E-18	33.66614
HEATR6	-0.49995	6.472283	-9.2106	5.59E-19	6.52E-18	32.24124
PDIA3	-0.50017	7.120219	-6.56611	1.13E-10	5.53E-10	13.48226
ZNF182	-0.50043	6.46909	-7.38328	5.28E-13	3.27E-12	18.72798
MRPL57	-0.50048	7.839132	-8.54026	1.14E-16	1.01E-15	27.01249
RNASEH1	-0.5006	6.309854	-7.08825	3.89E-12	2.21E-11	16.77421
SRSF10	-0.50062	7.180402	-5.52911	4.84E-08	1.75E-07	7.599497
UTP15	-0.50082	4.397082	-9.81375	3.66E-21	5.44E-20	37.1965

CRYZL1	-0.50086	7.004115	-5.23229	2.33E-07	7.78E-07	6.082299
RNASEH1-	-0.50102	4.382317	-8.10218	3.11E-15	2.40E-14	23.762
METTL5	-0.50102	6.432484	-7.11652	3.22E-12	1.84E-11	16.95853
ZCRB1	-0.50117	8.144402	-8.33643	5.39E-16	4.49E-15	25.48328
SMARCE1	-0.50151	6.876757	-7.03757	5.44E-12	3.04E-11	16.4453
PDHX	-0.50163	6.934941	-4.55237	6.44E-06	1.80E-05	2.896836
LZTFL1	-0.50199	4.939204	-9.07819	1.64E-18	1.80E-17	31.18467
ZFYVE26	-0.50214	7.109011	-6.34786	4.35E-10	1.99E-09	12.1706
AKT3	-0.50221	5.013377	-10.2828	6.29E-23	1.15E-21	41.20582
SNRPG	-0.50229	9.742379	-7.65952	7.67E-14	5.17E-13	20.61714
TMEM19	-0.50276	5.703683	-7.5309	1.90E-13	1.23E-12	19.73046
HMGN1	-0.50282	10.34491	-6.36498	3.92E-10	1.81E-09	12.2721
P4HTM	-0.50299	7.150582	-6.8578	1.77E-11	9.39E-11	15.29482
PPARGC1E	-0.503	5.361939	-4.93831	1.03E-06	3.17E-06	4.654935
ZCCHC2	-0.50309	6.977582	-3.99398	7.32E-05	0.000178	0.590352
RNF113A	-0.50313	7.963744	-7.31594	8.37E-13	5.11E-12	18.2762
LMBRD1	-0.50316	7.437508	-5.49045	5.96E-08	2.13E-07	7.397598
TCEAL8	-0.50324	6.315316	-4.4847	8.77E-06	2.40E-05	2.602274
N4BP2	-0.50328	5.245549	-5.39957	9.69E-08	3.38E-07	6.928009
TMEM117	-0.50332	4.589773	-8.77835	1.78E-17	1.72E-16	28.83501
C1orf131	-0.50334	8.205021	-6.00911	3.26E-09	1.35E-08	10.21163
DEK	-0.50347	10.5651	-6.33926	4.59E-10	2.09E-09	12.11969
OPA1	-0.50348	5.950481	-6.62251	7.95E-11	3.94E-10	13.82744
DDB2	-0.50356	6.404794	-7.07581	4.22E-12	2.39E-11	16.69325
C1orf50	-0.50363	6.471187	-6.73762	3.83E-11	1.97E-10	14.53982
NPIPB15	-0.50374	6.85301	-5.82831	9.21E-09	3.60E-08	9.204968
PRPF4B	-0.50396	7.921768	-6.12526	1.65E-09	7.06E-09	10.8727
FIS1	-0.50433	8.147976	-4.99806	7.64E-07	2.40E-06	4.938903
MRPL19	-0.50459	4.506985	-5.80164	1.07E-08	4.16E-08	9.058799
YY1AP1	-0.5048	9.435916	-7.40948	4.40E-13	2.76E-12	18.90475
ADNP2	-0.50499	7.293653	-7.55075	1.65E-13	1.08E-12	19.86649
LSM14A	-0.50505	9.220594	-11.1904	1.69E-26	4.69E-25	49.32333
CHD2	-0.50541	6.513024	-7.67475	6.88E-14	4.67E-13	20.72298
USP37	-0.50552	5.480562	-6.59017	9.74E-11	4.80E-10	13.62916
APOBEC3C	-0.50558	7.402508	-7.34607	6.81E-13	4.18E-12	18.47791
TIA1	-0.50599	8.011486	-5.84313	8.47E-09	3.33E-08	9.286444
ZNF689	-0.50609	6.249922	-6.91626	1.21E-11	6.52E-11	15.66617
GCFC2	-0.50622	4.693715	-7.16623	2.31E-12	1.34E-11	17.28411
ACTR6	-0.50687	5.908548	-6.93003	1.10E-11	6.00E-11	15.75403
PDE8A	-0.50707	5.85357	-11.2709	7.98E-27	2.33E-25	50.06559
KIAA1731	-0.50737	5.174975	-9.55512	3.25E-20	4.34E-19	35.04357
UBE2I	-0.50796	7.218618	-11.5127	8.19E-28	2.74E-26	52.31483
ZMYM6	-0.50816	6.154247	-6.03959	2.73E-09	1.14E-08	10.38402
PSTK	-0.50828	4.304988	-12.842	1.81E-33	1.28E-31	65.19188
C17orf75	-0.5084	4.636361	-4.71837	2.97E-06	8.65E-06	3.636785
MAP7	-0.50852	5.463437	-7.90053	1.36E-14	9.91E-14	22.31169
HSPE1	-0.50857	7.263316	-3.97165	8.02E-05	0.000194	0.504025
DBF4	-0.50872	5.992213	-3.76313	0.000185	0.000423	-0.27985
ATP5C1	-0.50873	9.350384	-8.04214	4.84E-15	3.68E-14	23.32711
MAGEE1	-0.50948	6.235523	-8.76094	2.04E-17	1.97E-16	28.70045
MYO6	-0.5095	4.103977	-9.23824	4.46E-19	5.26E-18	32.46327
WDR5B	-0.50989	5.698763	-6.94841	9.79E-12	5.35E-11	15.87153
MLLT6	-0.51016	7.642896	-5.19593	2.81E-07	9.28E-07	5.901658
UBP1	-0.51028	8.547666	-5.97153	4.06E-09	1.65E-08	10.00015
BCCIP	-0.51031	6.037832	-8.20221	1.48E-15	1.18E-14	24.49224
PTPRO	-0.5105	8.481419	-6.17079	1.26E-09	5.47E-09	11.13485
R3HDM2	-0.5112	8.741862	-9.15864	8.53E-19	9.76E-18	31.82529
NGDN	-0.51121	5.892928	-8.87382	8.38E-18	8.42E-17	29.57658

PTCD2	-0.51165	5.59765	-8.9031	6.64E-18	6.75E-17	29.8053
ZNF818P	-0.51165	6.497517	-4.58342	5.58E-06	1.57E-05	3.033375
MRPL48	-0.51189	7.170627	-5.58821	3.51E-08	1.28E-07	7.910592
TRAK2	-0.51203	7.584558	-5.38978	1.02E-07	3.55E-07	6.877868
SFPQ	-0.51209	7.681805	-9.48952	5.62E-20	7.32E-19	34.50411
ANAPC5	-0.51225	7.343444	-7.63668	9.01E-14	6.05E-13	20.45882
PDE3B	-0.51226	7.009501	-6.16189	1.33E-09	5.75E-09	11.08348
FKTN	-0.51226	5.368815	-6.17279	1.25E-09	5.41E-09	11.14638
PWWP2A	-0.51256	6.233902	-10.4069	2.10E-23	4.03E-22	42.28801
P2RX5	-0.51263	8.428299	-3.14456	0.001747	0.003446	-2.36677
ID2	-0.51267	9.905396	-6.1913	1.12E-09	4.88E-09	11.25348
CD27	-0.51293	7.756134	-3.34368	0.000879	0.00182	-1.73422
LOC10028	-0.5132	5.470034	-7.79097	3.00E-14	2.12E-13	21.53612
TP73-AS1	-0.51331	5.869886	-9.62234	1.85E-20	2.53E-19	35.59909
TSEN2	-0.51343	4.957925	-7.99161	7.01E-15	5.25E-14	22.96311
TMEM245	-0.51371	6.87921	-8.46434	2.04E-16	1.77E-15	26.4395
CYB561	-0.51413	6.143898	-10.8351	4.46E-25	1.03E-23	46.09109
ITFG2	-0.51438	6.00496	-9.35212	1.75E-19	2.17E-18	33.38313
OBFC1	-0.51448	6.339195	-7.91415	1.23E-14	9.02E-14	22.40873
PHF5A	-0.51448	8.041799	-8.36885	4.21E-16	3.54E-15	25.7246
ZNF587	-0.5146	7.064372	-7.021	6.07E-12	3.38E-11	16.33822
LINC0067[illegible]	-0.51484	5.986931	-7.74275	4.24E-14	2.95E-13	21.1975
RAI1	-0.51493	6.543197	-10.1652	1.76E-22	3.04E-21	40.18817
KTN1	-0.51498	9.01243	-6.3632	3.96E-10	1.83E-09	12.26152
SLC30A4	-0.51514	4.346144	-10.9315	1.85E-25	4.52E-24	46.96097
EID1	-0.51538	8.240543	-6.243	8.20E-10	3.64E-09	11.55415
FAM76B	-0.51555	6.872224	-6.33989	4.57E-10	2.09E-09	12.1234
DNAJC9	-0.51595	5.797588	-7.35742	6.30E-13	3.89E-12	18.55411
LCORL	-0.51656	6.064465	-4.83495	1.70E-06	5.11E-06	4.171129
RECQL	-0.51683	7.669708	-5.49213	5.91E-08	2.12E-07	7.406336
SDCCAG3	-0.51698	5.959536	-10.2368	9.43E-23	1.69E-21	40.80607
IL15	-0.51711	6.001522	-3.94457	8.95E-05	0.000215	0.399937
MRPL39	-0.5173	5.107675	-8.68503	3.70E-17	3.46E-16	28.11604
INADL	-0.51753	4.305194	-13.0346	2.57E-34	2.15E-32	67.12454
LINGO2	-0.51777	4.100495	-10.2385	9.28E-23	1.67E-21	40.82143
RPP30	-0.51808	7.517838	-8.24432	1.08E-15	8.71E-15	24.80181
MRPL46	-0.51821	6.847274	-6.78532	2.82E-11	1.47E-10	14.83811
PAIP1	-0.51821	6.959824	-5.39505	9.92E-08	3.46E-07	6.904861
VPS37C	-0.51842	8.396079	-7.38765	5.12E-13	3.18E-12	18.75742
KRIT1	-0.51845	6.200421	-7.24283	1.38E-12	8.18E-12	17.78957
RPL22L1	-0.51847	8.374353	-5.37457	1.11E-07	3.83E-07	6.800087
HADH	-0.51854	6.215019	-6.1631	1.32E-09	5.71E-09	11.09045
GOLT1B	-0.51878	7.593945	-5.71108	1.78E-08	6.75E-08	8.566887
GOLGA2P[illegible]	-0.51954	5.254211	-14.27	6.52E-40	1.20E-37	79.88233
FAM76A	-0.51957	4.597523	-10.1981	1.32E-22	2.33E-21	40.47163
FAM134B	-0.51969	5.373995	-7.67932	6.66E-14	4.53E-13	20.75479
LOC10013	-0.51988	5.246844	-9.77308	5.18E-21	7.50E-20	36.85525
APPBP2	-0.52018	6.208052	-8.77353	1.85E-17	1.79E-16	28.79773
FNBP1	-0.52033	8.600946	-7.69828	5.82E-14	3.98E-13	20.8868
RPS19	-0.52065	9.960394	-6.70996	4.57E-11	2.33E-10	14.36766
C2orf42	-0.52087	6.89608	-6.15429	1.39E-09	6.00E-09	11.03965
RASSF1	-0.52105	8.055724	-4.83443	1.70E-06	5.13E-06	4.168709
VPRBP	-0.52117	5.655408	-12.9543	5.82E-34	4.51E-32	66.31726
LPIN2	-0.52179	8.73047	-6.12827	1.62E-09	6.95E-09	10.88995
HSPA9	-0.52222	6.608732	-8.30978	6.59E-16	5.45E-15	25.28552
ZBTB44	-0.52228	8.299715	-7.19064	1.96E-12	1.14E-11	17.44466
MICA	-0.52231	6.560421	-6.3157	5.29E-10	2.40E-09	11.98058
DTD1	-0.52233	7.439331	-5.98136	3.83E-09	1.56E-08	10.05533

RSBN1L	-0.5224	7.252405	-7.13476	2.85E-12	1.64E-11	17.07778
ICA1L	-0.52245	4.445527	-13.3755	7.80E-36	8.27E-34	70.58466
PPP3CB-A	-0.52272	5.636103	-11.29	6.67E-27	1.97E-25	50.24222
PPP1R2	-0.5231	7.051752	-9.5705	2.86E-20	3.84E-19	35.17044
TTC13	-0.52364	7.863478	-6.63955	7.14E-11	3.56E-10	13.93223
TMED10	-0.52372	7.900259	-7.84818	1.99E-14	1.43E-13	21.94001
ALDH8A1	-0.52431	3.837223	-7.19961	1.84E-12	1.08E-11	17.50382
IPO5	-0.52457	6.12116	-7.22224	1.58E-12	9.35E-12	17.65326
WDR89	-0.52475	5.298194	-11.4375	1.67E-27	5.30E-26	51.61157
APPL1	-0.52476	6.815124	-5.22171	2.46E-07	8.19E-07	6.029622
TCL1A	-0.52477	7.366205	-2.54845	0.011072	0.018953	-4.0349
PLCG1	-0.52519	7.009565	-8.18194	1.72E-15	1.37E-14	24.34372
FUBP3	-0.52531	6.386505	-6.37405	3.71E-10	1.71E-09	12.32592
AIMP2	-0.52545	7.2055	-10.5534	5.69E-24	1.16E-22	43.57714
UFL1	-0.52566	6.012449	-7.3561	6.36E-13	3.92E-12	18.54522
PIK3IP1	-0.52595	8.297086	-5.78009	1.21E-08	4.67E-08	8.941097
PAXBP1	-0.5263	6.800347	-5.8844	6.70E-09	2.65E-08	9.514321
ARID1B	-0.52668	6.538855	-10.9757	1.23E-25	3.09E-24	47.3624
CIRBP	-0.52694	7.861966	-5.33731	1.35E-07	4.61E-07	6.610406
RAB12	-0.5274	6.650317	-6.17735	1.21E-09	5.28E-09	11.17276
ZFAND4	-0.52761	4.059415	-11.1999	1.55E-26	4.33E-25	49.41068
GPATCH8	-0.5279	7.981555	-8.83875	1.11E-17	1.10E-16	29.30349
MGMT	-0.52812	5.541831	-6.31878	5.19E-10	2.36E-09	11.99871
C3orf17	-0.5282	6.611336	-7.69923	5.79E-14	3.96E-13	20.89345
GMPS	-0.52905	6.303873	-8.19608	1.55E-15	1.23E-14	24.44733
MYO5C	-0.52927	5.705296	-8.2396	1.12E-15	9.01E-15	24.76705
UXS1	-0.52959	7.609779	-6.18683	1.15E-09	5.00E-09	11.22763
FAM210A	-0.52963	4.932347	-7.93316	1.07E-14	7.89E-14	22.54438
ATF1	-0.52995	8.779527	-5.42005	8.69E-08	3.06E-07	7.033214
AK3	-0.53019	7.026962	-4.7337	2.76E-06	8.09E-06	3.706374
SF3A2	-0.53025	6.801189	-4.53894	6.85E-06	1.90E-05	2.838057
CYP2U1	-0.53025	4.874794	-9.69207	1.03E-20	1.44E-19	36.17844
LOC10050	-0.53028	5.869971	-8.43259	2.59E-16	2.23E-15	26.20114
RBM12B	-0.53038	6.04165	-4.87317	1.41E-06	4.30E-06	4.348914
SLC44A5	-0.53061	2.919718	-9.5104	4.73E-20	6.19E-19	34.67556
TXNDC15	-0.53074	7.604752	-8.17263	1.84E-15	1.46E-14	24.2756
ADPRM	-0.53087	6.770276	-6.95236	9.54E-12	5.21E-11	15.89683
RGCC	-0.53087	6.004815	-4.90776	1.19E-06	3.66E-06	4.510937
ZNF234	-0.53092	4.163795	-8.65842	4.55E-17	4.22E-16	27.9121
SUGP2	-0.531	6.847475	-9.35067	1.78E-19	2.19E-18	33.37136
ACTR3B	-0.53114	5.204311	-7.45748	3.16E-13	2.01E-12	19.22981
PRPF3	-0.53117	7.107109	-6.80899	2.42E-11	1.27E-10	14.98682
PLAGL1	-0.53117	8.108523	-4.90679	1.20E-06	3.68E-06	4.506378
MEIS1	-0.53127	5.210463	-3.99923	7.16E-05	0.000174	0.610705
SCRIB	-0.5315	6.648479	-6.92925	1.11E-11	6.02E-11	15.74906
WDR61	-0.53156	6.537746	-8.90678	6.45E-18	6.58E-17	29.83405
ANAPC16	-0.53179	9.373647	-10.0186	6.31E-22	1.03E-20	38.93097
PINX1	-0.53186	6.272469	-10.2219	1.07E-22	1.91E-21	40.67713
KBTBD3	-0.53189	4.970631	-8.56448	9.43E-17	8.47E-16	27.19608
SIDT2	-0.53198	8.732014	-5.26106	2.01E-07	6.75E-07	6.226007
NDNL2	-0.53256	6.31991	-5.2074	2.65E-07	8.77E-07	5.958501
ICT1	-0.53294	6.542585	-6.62584	7.78E-11	3.86E-10	13.84792
ASCC3	-0.53297	5.812372	-6.63615	7.29E-11	3.63E-10	13.91132
DHRS4-AS	-0.53303	5.386508	-9.42368	9.72E-20	1.23E-18	33.96548
SLC26A2	-0.53348	6.701873	-7.01361	6.37E-12	3.54E-11	16.2905
FAM24B	-0.53375	5.373362	-8.57729	8.54E-17	7.72E-16	27.29336
1-Sep	-0.53376	6.04216	-3.16316	0.001641	0.003255	-2.30925
CHMP7	-0.53389	7.756233	-5.53342	4.72E-08	1.71E-07	7.622083

SLC12A7	-0.53401	8.540217	-5.27338	1.88E-07	6.35E-07	6.287767
ATHL1	-0.53434	8.514556	-3.75799	0.000188	0.000431	-0.29868
CD47	-0.53443	8.04527	-8.90324	6.64E-18	6.75E-17	29.80639
EIF2B1	-0.53469	8.299247	-8.03826	4.98E-15	3.78E-14	23.29912
NUP88	-0.53475	6.25342	-8.16809	1.91E-15	1.51E-14	24.24235
SRFBP1	-0.53478	3.992305	-5.52848	4.85E-08	1.75E-07	7.596195
OGFOD1	-0.53481	6.429267	-8.98562	3.44E-18	3.63E-17	30.45286
MAP9	-0.53533	3.957704	-11.1853	1.77E-26	4.90E-25	49.27705
SNX9	-0.53535	6.111688	-5.96258	4.27E-09	1.73E-08	9.94995
ZNF680	-0.5355	5.497002	-7.2645	1.19E-12	7.13E-12	17.93336
MRPL36	-0.53552	7.866025	-6.93948	1.04E-11	5.65E-11	15.81441
CD86	-0.53557	7.150888	-4.76711	2.36E-06	6.95E-06	3.85874
ZNF226	-0.53557	4.740776	-11.3804	2.86E-27	8.85E-26	51.08036
PHAX	-0.53563	6.430175	-5.75224	1.41E-08	5.42E-08	8.789606
FAM221A	-0.53597	4.216122	-6.92381	1.15E-11	6.23E-11	15.71431
DIP2C	-0.53618	5.274887	-9.71447	8.51E-21	1.20E-19	36.36513
ICAM2	-0.53623	8.313081	-4.2894	2.09E-05	5.45E-05	1.775348
PHF11	-0.5371	7.966123	-6.5866	9.96E-11	4.90E-10	13.60734
SP4	-0.53724	6.849628	-7.24124	1.39E-12	8.26E-12	17.77906
SCML2	-0.53735	6.640368	-9.46749	6.76E-20	8.71E-19	34.32359
ARGLU1	-0.53741	7.780844	-9.86795	2.31E-21	3.50E-20	37.65294
SUZ12	-0.5376	9.138533	-7.08034	4.10E-12	2.32E-11	16.72273
SLC12A2	-0.53769	5.060277	-5.70532	1.84E-08	6.96E-08	8.535826
LRRC40	-0.53793	7.145046	-7.2883	1.01E-12	6.11E-12	18.09174
ITGAV	-0.53804	6.922408	-4.4269	1.14E-05	3.07E-05	2.353965
UBA52	-0.53813	12.37332	-7.26123	1.21E-12	7.27E-12	17.91168
MORF4L2	-0.53831	6.241768	-4.99956	7.58E-07	2.38E-06	4.946065
ZNF140	-0.53833	6.48438	-5.7671	1.30E-08	5.01E-08	8.870361
RALGAPA1	-0.53847	4.953268	-12.2098	9.85E-31	4.71E-29	58.96287
PTPLB	-0.53952	6.970043	-5.307	1.58E-07	5.37E-07	6.457025
SFR1	-0.53972	5.941534	-5.98306	3.80E-09	1.55E-08	10.06491
TBCA	-0.5398	10.79094	-7.93737	1.04E-14	7.66E-14	22.57448
ZNF347	-0.54025	3.806759	-10.1246	2.51E-22	4.26E-21	39.83841
DRAM2	-0.5403	7.653569	-6.12213	1.68E-09	7.18E-09	10.8547
RPIA	-0.54033	8.023672	-5.98504	3.75E-09	1.53E-08	10.07604
CUX2	-0.54059	3.947683	-10.1394	2.21E-22	3.77E-21	39.96613
FAU	-0.54111	11.94692	-6.80527	2.48E-11	1.30E-10	14.9634
XYLT2	-0.54123	5.669996	-9.08572	1.54E-18	1.70E-17	31.24445
SLTM	-0.54131	9.297054	-7.34393	6.91E-13	4.24E-12	18.46359
PSMC2	-0.54152	7.374724	-6.22631	9.06E-10	4.00E-09	11.45686
LOC20218	-0.54211	5.794062	-7.21662	1.64E-12	9.69E-12	17.61611
TEX30	-0.54218	6.12426	-6.85128	1.84E-11	9.79E-11	15.25353
CEP44	-0.54226	5.133526	-8.72513	2.70E-17	2.57E-16	28.42422
KDM4C	-0.54228	6.075954	-8.30734	6.71E-16	5.54E-15	25.26743
ZNF202	-0.54238	5.169826	-9.59928	2.25E-20	3.05E-19	35.40819
NGRN	-0.5425	7.179346	-8.91174	6.20E-18	6.34E-17	29.8729
PKD2	-0.54258	5.403422	-5.37883	1.08E-07	3.75E-07	6.821846
TTC9	-0.54262	5.480972	-6.47534	1.99E-10	9.50E-10	12.93206
HSD17B7	-0.54267	8.527806	-7.65819	7.74E-14	5.22E-13	20.60791
TRPC1	-0.54276	3.649198	-15.7533	6.10E-47	2.99E-44	95.91325
C7orf31	-0.54285	5.516942	-6.08676	2.07E-09	8.77E-09	10.65232
ASB13	-0.54292	7.08418	-7.71858	5.04E-14	3.47E-13	21.02844
DLG5	-0.54297	4.561116	-10.6216	3.08E-24	6.47E-23	44.18196
FTSJ2	-0.54308	7.004587	-7.35238	6.52E-13	4.02E-12	18.52026
PCID2	-0.54338	6.984021	-7.05812	4.75E-12	2.67E-11	16.57838
MRPS33	-0.54358	7.382553	-6.65185	6.60E-11	3.31E-10	14.00801
PNMA1	-0.54381	7.351807	-5.29109	1.72E-07	5.80E-07	6.37681
KBTBD8	-0.54448	6.044681	-4.14242	3.94E-05	9.91E-05	1.175814

BDP1	-0.54476	5.653737	-6.17124	1.26E-09	5.46E-09	11.13744
FAM118A	-0.54548	6.00295	-4.59922	5.19E-06	1.47E-05	3.103165
MTO1	-0.54629	6.7376	-7.04763	5.09E-12	2.85E-11	16.51044
ZNF711	-0.54631	3.439679	-9.06003	1.89E-18	2.06E-17	31.04071
PAAF1	-0.54644	6.461788	-7.25932	1.23E-12	7.36E-12	17.89899
HYLS1	-0.54648	6.365809	-6.16269	1.32E-09	5.72E-09	11.08808
MAGED1	-0.54655	7.997771	-5.73608	1.55E-08	5.91E-08	8.702011
MIER3	-0.54699	5.54407	-5.57709	3.73E-08	1.36E-07	7.851818
FRA10AC1	-0.547	5.427915	-10.1736	1.64E-22	2.84E-21	40.26055
CHI3L1	-0.54713	7.231773	-2.62163	0.008976	0.015655	-3.84846
KRR1	-0.54717	5.070755	-8.4681	1.98E-16	1.72E-15	26.46778
DUS1L	-0.54734	7.835131	-6.60922	8.64E-11	4.27E-10	13.74584
EXOSC9	-0.54735	7.46018	-7.00881	6.58E-12	3.65E-11	16.25954
AQP3	-0.54766	7.165842	-7.12814	2.98E-12	1.71E-11	17.03442
RBM15	-0.54784	5.594458	-11.5599	5.24E-28	1.79E-26	52.75689
NDUFA5	-0.54799	5.808254	-7.4887	2.54E-13	1.63E-12	19.44223
TMEM99	-0.54813	6.281118	-7.39115	5.00E-13	3.11E-12	18.78105
TMOD2	-0.54865	5.083714	-5.3168	1.50E-07	5.11E-07	6.506538
GPR34	-0.5487	6.32148	-2.83034	0.004808	0.008797	-3.28843
L3HYPDH	-0.54935	4.887275	-8.62778	5.78E-17	5.29E-16	27.67795
FBXW4	-0.54954	6.973931	-7.86769	1.73E-14	1.25E-13	22.07829
SCN3A	-0.54997	4.117641	-3.5767	0.000376	0.000825	-0.94654
TERF1	-0.55029	7.057948	-6.80958	2.41E-11	1.27E-10	14.99054
ATRAID	-0.55095	9.161817	-7.88893	1.48E-14	1.07E-13	22.22918
NT5C3B	-0.55114	6.66699	-7.05462	4.86E-12	2.73E-11	16.55571
NDUFB5	-0.5515	9.243549	-7.58995	1.25E-13	8.29E-13	20.13601
ZNF738	-0.55241	4.68263	-5.19427	2.83E-07	9.35E-07	5.89341
YWHAQ	-0.55244	10.28775	-7.18977	1.97E-12	1.15E-11	17.43894
CERK	-0.55248	8.551925	-7.92818	1.11E-14	8.16E-14	22.50883
MAP3K14	-0.55263	5.947821	-6.87289	1.60E-11	8.56E-11	15.39039
ZNF284	-0.55268	6.16399	-9.24643	4.18E-19	4.94E-18	32.52914
BCAS2	-0.55275	8.536396	-8.30736	6.71E-16	5.54E-15	25.26759
METTL10	-0.55298	5.099217	-11.0045	9.45E-26	2.40E-24	47.62335
MTERF2	-0.55313	5.026292	-7.96648	8.43E-15	6.26E-14	22.78278
CUL5	-0.55343	6.131279	-7.66704	7.27E-14	4.92E-13	20.66943
TMEM161I	-0.55352	4.497852	-16.1754	5.40E-49	3.38E-46	100.5968
LACC1	-0.55366	4.813089	-3.93321	9.38E-05	0.000224	0.356512
ZNF280C	-0.55367	3.902774	-7.13886	2.77E-12	1.59E-11	17.10462
PUS1	-0.55415	6.061405	-7.05868	4.73E-12	2.66E-11	16.58205
MIR142	-0.55423	6.533526	-5.61939	2.95E-08	1.09E-07	8.075936
SLC41A1	-0.55425	5.746555	-7.46149	3.07E-13	1.96E-12	19.25704
CELF2	-0.55433	9.895843	-8.57633	8.60E-17	7.77E-16	27.28611
RBM14	-0.55434	6.975316	-7.83427	2.20E-14	1.57E-13	21.84156
ATXN10	-0.55435	7.957661	-7.76777	3.55E-14	2.49E-13	21.37296
GSDMB	-0.5545	5.674396	-7.70129	5.70E-14	3.91E-13	20.90782
DANCR	-0.55469	7.574737	-5.19556	2.81E-07	9.30E-07	5.899815
LOC10013	-0.55484	7.058542	-10.227	1.03E-22	1.84E-21	40.72138
DCAF13	-0.55502	5.621677	-6.81299	2.36E-11	1.24E-10	15.012
AMD1	-0.55519	9.05862	-6.26642	7.12E-10	3.19E-09	11.69105
7-Sep	-0.55524	7.287558	-10.9251	1.96E-25	4.77E-24	46.90326
LOC10050	-0.55533	3.958616	-7.67614	6.82E-14	4.63E-13	20.73263
C2CD2	-0.55544	5.771058	-6.88892	1.44E-11	7.74E-11	15.49215
LOC10192	-0.55558	5.858592	-8.4319	2.61E-16	2.24E-15	26.19594
MCEE	-0.55591	5.796528	-6.38658	3.44E-10	1.60E-09	12.40047
SEC31A	-0.55607	9.304333	-6.68158	5.47E-11	2.77E-10	14.19166
TMF1	-0.55661	6.718976	-6.1944	1.10E-09	4.80E-09	11.27143
ACSL5	-0.55662	7.491642	-7.49725	2.40E-13	1.54E-12	19.50049
ABHD14B	-0.55699	7.240418	-4.35982	1.54E-05	4.07E-05	2.069523

PNPO	-0.55779	6.394353	-9.95055	1.13E-21	1.79E-20	38.35201
SNRPA	-0.55779	7.843988	-6.29012	6.18E-10	2.79E-09	11.83
NMRAL1	-0.55787	6.845442	-6.06185	2.40E-09	1.01E-08	10.51043
CEP76	-0.55788	4.991136	-5.2726	1.89E-07	6.37E-07	6.283884
LOC10050	-0.55816	3.312917	-12.1306	2.14E-30	9.89E-29	58.19561
BIRC6	-0.55873	8.897086	-9.7829	4.76E-21	6.94E-20	36.93752
TMEM135	-0.55899	5.853883	-3.85884	0.000126	0.000297	0.074945
SRP72	-0.55905	7.218384	-6.70482	4.72E-11	2.40E-10	14.33574
USP24	-0.55922	6.706521	-9.32887	2.12E-19	2.59E-18	33.19465
METTL17	-0.55946	7.826651	-6.77664	2.98E-11	1.55E-10	14.78371
CEP72	-0.55965	5.188458	-9.06933	1.76E-18	1.93E-17	31.11437
AFG3L2	-0.56001	5.669711	-11.3026	5.93E-27	1.76E-25	50.35827
RNASEH2E	-0.56015	5.632727	-10.9582	1.45E-25	3.61E-24	47.20273
ZNF431	-0.56062	4.352376	-5.55095	4.30E-08	1.56E-07	7.714072
RAD51-AS	-0.5612	4.378854	-6.39084	3.35E-10	1.56E-09	12.42585
KRT10	-0.56121	8.587	-6.90934	1.26E-11	6.80E-11	15.62208
RAPH1	-0.56126	4.142453	-6.91865	1.19E-11	6.42E-11	15.68137
FAM150B	-0.56164	3.827751	-9.58016	2.64E-20	3.55E-19	35.25014
ZNF703	-0.56195	5.321786	-6.58268	1.02E-10	5.02E-10	13.58337
RRP1B	-0.56216	7.350096	-8.21912	1.30E-15	1.05E-14	24.61639
NAAA	-0.56222	7.393584	-5.54825	4.36E-08	1.58E-07	7.699911
LYRM4	-0.56241	5.994729	-8.87291	8.44E-18	8.47E-17	29.56952
SENP1	-0.56281	6.227305	-6.80724	2.45E-11	1.29E-10	14.97579
ULK2	-0.56322	5.679378	-8.15702	2.07E-15	1.63E-14	24.16148
ODF2L	-0.5637	4.130269	-11.1537	2.38E-26	6.46E-25	48.98658
INPP5E	-0.56389	7.166429	-8.64538	5.04E-17	4.65E-16	27.81238
ADTRP	-0.56396	5.651714	-5.96975	4.10E-09	1.66E-08	9.99017
SMIM20	-0.5645	7.099211	-6.45647	2.24E-10	1.06E-09	12.81852
RPS27A	-0.56451	7.623163	-11.0866	4.43E-26	1.16E-24	48.37203
STIM2	-0.56453	6.247397	-6.47079	2.05E-10	9.75E-10	12.90463
PITPNC1	-0.56482	6.236529	-8.51631	1.37E-16	1.20E-15	26.83134
TPCN1	-0.56507	7.414428	-6.70284	4.78E-11	2.43E-10	14.3235
LIMA1	-0.56543	4.804012	-9.38096	1.38E-19	1.73E-18	33.61748
LOC10099	-0.56564	4.809282	-9.44725	7.99E-20	1.03E-18	34.15799
DDX42	-0.56569	8.527159	-9.87199	2.23E-21	3.39E-20	37.68705
VRK1	-0.56581	7.001338	-4.88261	1.35E-06	4.12E-06	4.393055
TXNIP	-0.56583	12.57796	-5.36941	1.14E-07	3.93E-07	6.77374
COA4	-0.56612	7.010909	-8.71491	2.93E-17	2.77E-16	28.34563
CHST12	-0.56613	6.815207	-6.97676	8.13E-12	4.48E-11	16.05327
LOC10028	-0.56641	5.501456	-4.47462	9.18E-06	2.50E-05	2.558783
XPO4	-0.56686	5.814133	-8.59843	7.25E-17	6.60E-16	27.45414
ITPKB	-0.56752	6.542029	-8.86652	8.88E-18	8.89E-17	29.51971
TRAF3	-0.56773	6.992965	-9.17951	7.20E-19	8.28E-18	31.99214
HSF2	-0.56826	4.586343	-8.08162	3.62E-15	2.78E-14	23.61281
VEZT	-0.56833	4.966763	-6.40695	3.03E-10	1.42E-09	12.52191
PRPSAP2	-0.56867	7.595879	-6.33518	4.70E-10	2.14E-09	12.09557
OLA1	-0.56925	6.583163	-9.43568	8.80E-20	1.12E-18	34.06348
NUP160	-0.56927	4.890738	-8.88236	7.83E-18	7.89E-17	29.64323
TMEM255	-0.56982	3.354306	-6.57248	1.09E-10	5.33E-10	13.52107
SLC9A7	-0.57044	4.883718	-7.24688	1.34E-12	7.98E-12	17.81644
RPS27L	-0.5706	6.41411	-7.40478	4.55E-13	2.84E-12	18.87301
LCOR	-0.57074	7.847652	-5.9629	4.27E-09	1.73E-08	9.951721
ANAPC10	-0.57092	4.520148	-7.61639	1.04E-13	6.94E-13	20.31843
IL16	-0.57093	8.470881	-9.56928	2.89E-20	3.87E-19	35.16037
TMEM209	-0.57144	5.38238	-7.66632	7.31E-14	4.94E-13	20.66441
KLHDC7B	-0.57203	5.998101	-6.13624	1.55E-09	6.64E-09	10.93575
PRIM1	-0.57226	6.694981	-4.73484	2.75E-06	8.04E-06	3.711547
MIR1244-:	-0.5723	10.70187	-8.00352	6.43E-15	4.82E-14	23.04872

COA5	-0.57242	7.354267	-8.02599	5.45E-15	4.12E-14	23.21054
IFIH1	-0.57247	6.032746	-4.25373	2.44E-05	6.31E-05	1.628016
RBAK	-0.57254	5.492672	-7.46143	3.07E-13	1.96E-12	19.25665
PSMG2	-0.57258	9.788855	-8.14948	2.19E-15	1.72E-14	24.1064
NUFIP1	-0.57298	4.784256	-10.4447	1.50E-23	2.95E-22	42.61942
POLI	-0.57332	5.017939	-5.49501	5.81E-08	2.09E-07	7.421327
SLBP	-0.574	9.486142	-7.49915	2.37E-13	1.52E-12	19.51346
HNRNPD	-0.57403	7.241292	-10.6976	1.55E-24	3.37E-23	44.85847
ABCA5	-0.57463	5.192906	-10.3763	2.75E-23	5.21E-22	42.0204
ATP6V0E2	-0.57466	6.779402	-6.50521	1.65E-10	7.97E-10	13.11238
ZNF18	-0.57468	7.046585	-8.63505	5.46E-17	5.02E-16	27.73345
CARD11	-0.57505	7.494205	-7.04492	5.18E-12	2.90E-11	16.49289
LRRC14	-0.57542	5.595662	-12.755	4.36E-33	2.89E-31	64.32418
PDPK1	-0.57545	4.610396	-8.16076	2.01E-15	1.59E-14	24.18879
RPLP2	-0.57573	5.02005	-8.52751	1.25E-16	1.11E-15	26.91599
RCC2	-0.57581	8.195696	-7.40764	4.46E-13	2.79E-12	18.89226
ZMYND19	-0.57584	5.98753	-9.3681	1.54E-19	1.91E-18	33.51289
METTL8	-0.57598	4.253064	-10.4047	2.14E-23	4.10E-22	42.26886
BTBD18	-0.57615	4.958738	-6.48856	1.83E-10	8.79E-10	13.01179
GEMIN5	-0.5767	6.518675	-6.14273	1.49E-09	6.40E-09	10.97308
IMP4	-0.57672	7.304163	-6.51519	1.56E-10	7.52E-10	13.17281
GNL3	-0.577	4.176923	-11.2589	8.93E-27	2.60E-25	49.95492
TTC28-AS	-0.57713	5.446766	-9.99601	7.67E-22	1.24E-20	38.73854
PDGFD	-0.5773	5.47336	-7.58597	1.29E-13	8.50E-13	20.10863
MME	-0.57775	8.650407	-1.98038	0.048124	0.071962	-5.30586
FGD5-AS1	-0.57794	9.401208	-8.28591	7.89E-16	6.44E-15	25.10876
COMMD2	-0.57796	7.060657	-6.1545	1.39E-09	5.99E-09	11.04085
RWDD2B	-0.57813	6.664084	-8.67641	3.96E-17	3.69E-16	28.04992
PCED1A	-0.57821	5.912029	-11.0378	6.95E-26	1.79E-24	47.92669
CEP85L	-0.57824	6.388904	-8.81872	1.30E-17	1.27E-16	29.14783
KLHL5	-0.57831	6.264756	-5.68242	2.09E-08	7.86E-08	8.412665
EXT2	-0.57852	6.800333	-7.30828	8.82E-13	5.37E-12	18.22499
POLR1C	-0.5786	6.140671	-9.1073	1.29E-18	1.44E-17	31.41595
TCEA3	-0.57865	5.420539	-11.612	3.19E-28	1.13E-26	53.24667
RUVBL1	-0.57874	6.560775	-6.37029	3.80E-10	1.75E-09	12.30362
TRMT12	-0.57948	7.119383	-7.72732	4.74E-14	3.28E-13	21.08954
FCHSD2	-0.5795	8.597375	-7.99	7.10E-15	5.31E-14	22.95152
TASP1	-0.57954	4.315781	-10.257	7.89E-23	1.43E-21	40.9818
TMEM154	-0.57975	10.80418	-5.83309	8.96E-09	3.51E-08	9.231212
ACACB	-0.5808	5.592039	-9.92483	1.42E-21	2.22E-20	38.13387
LOC10192	-0.58095	4.718261	-6.33015	4.85E-10	2.21E-09	12.06582
ZNF253	-0.58105	5.223568	-7.48222	2.66E-13	1.70E-12	19.39809
ALG8	-0.58157	7.033397	-5.34594	1.29E-07	4.42E-07	6.654237
GGCT	-0.58192	7.891455	-7.58784	1.27E-13	8.40E-13	20.12147
LINC0009	-0.58193	5.313825	-5.90393	5.99E-09	2.38E-08	9.622665
CCNT1	-0.5824	7.729646	-8.53102	1.22E-16	1.08E-15	26.94252
THOC2	-0.58244	7.420581	-7.57522	1.39E-13	9.14E-13	20.0346
RFC1	-0.58268	6.898951	-8.57587	8.63E-17	7.79E-16	27.28261
MYEF2	-0.5828	4.074402	-12.6307	1.52E-32	9.02E-31	63.09012
ZNF138	-0.58286	5.269652	-5.5264	4.91E-08	1.77E-07	7.585305
SNORA72	-0.5835	4.082559	-6.65905	6.31E-11	3.17E-10	14.05241
SLC25A46	-0.58351	8.066832	-6.00712	3.30E-09	1.36E-08	10.20041
PCNP	-0.58357	6.898424	-8.41231	3.03E-16	2.58E-15	26.04916
PET117	-0.58386	5.120842	-8.47507	1.88E-16	1.63E-15	26.5203
POLE3	-0.5845	7.728025	-4.99595	7.72E-07	2.42E-06	4.928836
N4BP2L2	-0.58451	8.153025	-7.0616	4.64E-12	2.61E-11	16.60101
REPS1	-0.58505	5.456902	-10.5781	4.56E-24	9.37E-23	43.79568
MB21D2	-0.58513	6.247875	-9.88192	2.05E-21	3.12E-20	37.77091

NADK2	-0.58545	4.832471	-7.27514	1.11E-12	6.65E-12	18.00413
FAM120C	-0.5855	5.320431	-13.467	3.03E-36	3.45E-34	71.52083
SP140L	-0.58583	6.563692	-8.77197	1.87E-17	1.81E-16	28.78566
HNRNPH3	-0.58628	8.947377	-7.9805	7.61E-15	5.67E-14	22.88332
HAUS5	-0.58678	5.937265	-12.8233	2.19E-33	1.52E-31	65.00482
RAP2A	-0.58754	7.146928	-9.07925	1.62E-18	1.79E-17	31.19307
LOC10013	-0.58772	5.963462	-9.09521	1.43E-18	1.58E-17	31.31986
TSEN54	-0.58782	6.998769	-9.77057	5.29E-21	7.66E-20	36.83422
THNSL1	-0.58866	4.592312	-11.2223	1.26E-26	3.58E-25	49.61756
PLEKHA8P	-0.58867	6.287352	-8.82399	1.24E-17	1.22E-16	29.1888
COX16	-0.58884	7.683036	-5.99693	3.50E-09	1.44E-08	10.14296
FCGR2B	-0.5889	7.206937	-4.25118	2.47E-05	6.38E-05	1.617534
PDCD5	-0.58917	5.488446	-8.47867	1.82E-16	1.59E-15	26.54738
DZANK1	-0.58935	4.781628	-9.44438	8.18E-20	1.05E-18	34.13454
DENND4C	-0.58936	6.230726	-7.66627	7.31E-14	4.94E-13	20.66402
FAM129C	-0.5894	6.745076	-3.45027	0.0006	0.001274	-1.38022
FIP1L1	-0.58944	7.02646	-7.13402	2.86E-12	1.64E-11	17.0729
MRFAP1L1	-0.58954	8.936442	-7.19839	1.86E-12	1.09E-11	17.49576
ZNF367	-0.5896	5.00757	-3.68585	0.000249	0.00056	-0.56016
PTGS2	-0.58964	9.198467	-2.62193	0.008969	0.015643	-3.84768
SETMAR	-0.59024	4.159533	-7.23656	1.44E-12	8.51E-12	17.74803
TAF1A	-0.59026	4.558087	-4.78593	2.15E-06	6.39E-06	3.944968
TRIM68	-0.59056	6.310553	-7.13079	2.93E-12	1.68E-11	17.05176
SMAD7	-0.59062	6.152781	-6.00135	3.41E-09	1.40E-08	10.16785
ZNF2	-0.59078	5.744669	-7.99356	6.91E-15	5.18E-14	22.97715
RPLP0	-0.59101	12.09206	-6.26677	7.11E-10	3.18E-09	11.69309
IPCEF1	-0.59116	9.105963	-6.53241	1.40E-10	6.79E-10	13.2772
MEGF6	-0.59121	6.728054	-7.71937	5.01E-14	3.45E-13	21.03398
RPS3	-0.59162	12.25762	-6.44371	2.42E-10	1.14E-09	12.74192
BMS1	-0.59177	6.878812	-8.92927	5.39E-18	5.54E-17	30.01019
WDFY1	-0.592	7.972772	-6.62889	7.63E-11	3.79E-10	13.86664
NFYB	-0.59222	5.371406	-6.73088	4.00E-11	2.05E-10	14.49783
FAM117A	-0.59239	8.997594	-7.87419	1.65E-14	1.19E-13	22.12443
MTFR1L	-0.59284	7.193116	-8.02752	5.39E-15	4.07E-14	23.22159
CEP192	-0.59289	6.973456	-5.78913	1.15E-08	4.45E-08	8.990443
RCN1	-0.59319	5.779928	-6.88423	1.49E-11	7.97E-11	15.46233
ZNF137P	-0.59325	5.844049	-5.97578	3.96E-09	1.61E-08	10.02403
SPP1	-0.59335	4.825785	-3.0027	0.002789	0.005317	-2.79452
CRIM1	-0.59339	6.135377	-4.88732	1.32E-06	4.02E-06	4.415064
MLH3	-0.59345	6.246729	-6.38471	3.48E-10	1.61E-09	12.38935
ZNF419	-0.59355	6.75623	-10.9762	1.23E-25	3.08E-24	47.36654
HEG1	-0.59382	6.540084	-7.77203	3.44E-14	2.42E-13	21.40291
NAF1	-0.59391	4.470149	-5.28382	1.78E-07	6.02E-07	6.340199
ZNF224	-0.59426	5.657931	-9.80206	4.05E-21	5.98E-20	37.09834
AGMAT	-0.59432	5.291857	-8.69649	3.38E-17	3.18E-16	28.20398
SERBP1	-0.59448	8.536826	-8.18123	1.73E-15	1.37E-14	24.33854
SLC38A9	-0.59458	5.689911	-7.25956	1.23E-12	7.35E-12	17.90057
PTGDS	-0.59492	7.180486	-7.90243	1.34E-14	9.78E-14	22.32518
NAA20	-0.59543	7.882596	-7.83209	2.23E-14	1.59E-13	21.82617
HERPUD2	-0.59573	9.53937	-8.66208	4.42E-17	4.10E-16	27.94015
LOC28581	-0.59591	6.988958	-5.01701	6.95E-07	2.20E-06	5.029605
RAB30	-0.59594	5.229902	-6.17222	1.25E-09	5.43E-09	11.14309
DUS4L	-0.5962	4.54942	-9.73706	7.03E-21	1.01E-19	36.55374
GABPB1-A	-0.59629	5.25351	-6.34084	4.54E-10	2.08E-09	12.12905
PPP2R5E	-0.59641	8.042898	-7.39495	4.87E-13	3.03E-12	18.80666
C11orf83	-0.59643	5.383136	-8.80992	1.39E-17	1.35E-16	29.07957
ANGEL2	-0.59666	6.141716	-9.13105	1.07E-18	1.20E-17	31.60513
CRIPT	-0.59676	7.025897	-8.64803	4.94E-17	4.56E-16	27.83264

RIPK2	-0.59681	6.932537	-7.73334	4.54E-14	3.14E-13	21.13162
LUC7L2	-0.59707	6.900772	-7.89062	1.46E-14	1.06E-13	22.24121
LOC28616	-0.59709	5.153547	-10.1168	2.69E-22	4.55E-21	39.77124
SMAD5	-0.59754	5.936757	-5.46624	6.79E-08	2.41E-07	7.271813
GRSF1	-0.5979	6.503684	-8.26079	9.53E-16	7.73E-15	24.92325
ANKRD36	-0.59815	4.084421	-5.41456	8.95E-08	3.14E-07	7.004957
LARS	-0.5983	7.36442	-7.06204	4.63E-12	2.61E-11	16.60381
NSG1	-0.59846	5.730123	-10.0665	4.17E-22	6.92E-21	39.34016
FEZ1	-0.59851	4.946013	-12.4713	7.44E-32	4.09E-30	61.51729
FAM73A	-0.59852	4.636127	-7.36414	6.02E-13	3.72E-12	18.59922
PHF20	-0.59852	7.46719	-12.8237	2.18E-33	1.52E-31	65.00863
BTN2A2	-0.59894	5.794672	-8.45727	2.15E-16	1.86E-15	26.38638
CNOT11	-0.59941	8.004755	-10.0598	4.41E-22	7.30E-21	39.28324
NAP1L5	-0.59942	4.508204	-6.29966	5.83E-10	2.63E-09	11.8861
KIAA1551	-0.59946	10.93809	-4.57822	5.72E-06	1.61E-05	3.010447
ZNF585A	-0.59952	5.525117	-7.03538	5.52E-12	3.08E-11	16.43111
JAK1	-0.59964	9.070865	-9.2646	3.60E-19	4.29E-18	32.67541
ASXL1	-0.59986	6.812602	-9.2727	3.37E-19	4.03E-18	32.74075
S1PR1	-0.60004	8.577286	-4.18307	3.31E-05	8.42E-05	1.339646
CCL28	-0.60009	4.924441	-15.0247	1.89E-43	6.00E-41	87.95038
DCLRE1C	-0.60055	6.623536	-6.92305	1.16E-11	6.25E-11	15.70944
ADO	-0.60063	6.940518	-5.9275	5.23E-09	2.09E-08	9.753868
ADNP	-0.60077	8.381496	-9.03729	2.27E-18	2.45E-17	30.86064
ULK3	-0.6013	6.843822	-9.7058	9.16E-21	1.29E-19	36.29285
SART3	-0.60174	7.682567	-7.79764	2.86E-14	2.03E-13	21.58302
ZNF554	-0.60208	5.446593	-16.7334	9.73E-52	9.79E-49	106.859
ODC1	-0.60229	9.089937	-4.98499	8.15E-07	2.55E-06	4.876535
XPOT	-0.60262	7.312476	-6.84423	1.93E-11	1.02E-10	15.20899
RHNO1	-0.60265	5.507546	-6.98486	7.70E-12	4.25E-11	16.10536
QTRTD1	-0.60271	5.603542	-6.22216	9.29E-10	4.10E-09	11.43265
NACA	-0.6028	10.37178	-8.80713	1.42E-17	1.38E-16	29.05795
NBPF20	-0.60283	5.022167	-9.18106	7.11E-19	8.18E-18	32.00459
HEATR5B	-0.60332	7.843819	-9.34379	1.88E-19	2.31E-18	33.31562
PIK3C2A	-0.60364	6.501181	-7.18889	1.98E-12	1.16E-11	17.43314
UBE4A	-0.60416	9.918367	-9.54765	3.46E-20	4.61E-19	34.982
HERC2	-0.60453	6.863945	-5.11611	4.22E-07	1.37E-06	5.509154
RWDD4	-0.60459	7.279905	-5.90123	6.08E-09	2.42E-08	9.607703
ATAD1	-0.60479	6.923086	-7.64961	8.23E-14	5.54E-13	20.5484
GRAMD3	-0.60479	4.258932	-9.23639	4.53E-19	5.33E-18	32.44839
FAM214A	-0.60486	6.685338	-6.33507	4.70E-10	2.15E-09	12.09492
EXTL2	-0.60504	5.805361	-7.84029	2.11E-14	1.51E-13	21.88418
NIFK-AS1	-0.6051	7.005637	-6.76087	3.30E-11	1.70E-10	14.68502
YME1L1	-0.60516	7.457468	-12.9005	1.00E-33	7.43E-32	65.77743
ZNF783	-0.60538	7.10374	-11.2083	1.43E-26	4.05E-25	49.48826
BNIP3	-0.60571	5.972994	-5.77095	1.27E-08	4.90E-08	8.891319
RPS24	-0.6061	13.05298	-7.73494	4.49E-14	3.11E-13	21.14288
ZNF540	-0.6064	4.712626	-14.9964	2.57E-43	7.64E-41	87.64431
CPED1	-0.60643	5.262925	-7.8629	1.79E-14	1.29E-13	22.04434
GRPEL2	-0.60679	5.552032	-11.6606	2.01E-28	7.26E-27	53.70493
WRN	-0.60693	5.582959	-8.45451	2.19E-16	1.90E-15	26.36561
CAMK1D	-0.60729	7.291695	-4.1516	3.79E-05	9.55E-05	1.212665
STAG3L4	-0.60738	4.822779	-8.31949	6.12E-16	5.09E-15	25.35754
PITPNA	-0.60754	8.126259	-7.60167	1.15E-13	7.66E-13	20.21679
C9orf41	-0.60771	4.305319	-8.47081	1.94E-16	1.68E-15	26.4882
RPL34	-0.60797	12.52214	-8.068	4.00E-15	3.06E-14	23.51412
MTPAP	-0.60812	6.680323	-6.05451	2.50E-09	1.05E-08	10.46869
SLA2	-0.6085	7.459727	-5.94513	4.73E-09	1.90E-08	9.852277
MPHOSPH	-0.60906	7.4296	-10.2827	6.30E-23	1.15E-21	41.20429

LINC00998	-0.60921	5.465709	-7.20812	1.74E-12	1.02E-11	17.55998
DDX52	-0.60943	5.918402	-6.46743	2.09E-10	9.95E-10	12.88442
NUDCD1	-0.60944	4.806572	-8.11223	2.88E-15	2.24E-14	23.83506
OSGEP	-0.60945	6.61689	-9.60789	2.09E-20	2.84E-19	35.47947
FAM3C	-0.60971	4.467015	-6.1376	1.54E-09	6.59E-09	10.94355
ECHDC2	-0.60983	6.586443	-8.8932	7.19E-18	7.27E-17	29.72789
AUTS2	-0.60986	5.043028	-9.27623	3.27E-19	3.92E-18	32.7692
SMCHD1	-0.61001	8.579621	-4.93024	1.07E-06	3.29E-06	4.616814
CAND1	-0.61023	6.153187	-7.30421	9.07E-13	5.51E-12	18.19782
GPN1	-0.61033	7.873864	-7.60201	1.15E-13	7.64E-13	20.21919
PLRG1	-0.61042	6.838557	-8.41788	2.90E-16	2.48E-15	26.09092
GOT2	-0.61052	7.882786	-8.17339	1.83E-15	1.45E-14	24.2811
ST13	-0.61064	8.223267	-6.18565	1.16E-09	5.04E-09	11.22079
RAD54B	-0.61092	4.299613	-8.59306	7.56E-17	6.87E-16	27.41327
RIOK2	-0.61102	5.748767	-6.32943	4.87E-10	2.22E-09	12.06156
NCBP2	-0.61135	6.646714	-7.6105	1.08E-13	7.23E-13	20.27774
DPH6	-0.6115	4.064288	-11.1302	2.96E-26	7.93E-25	48.77076
ERAP1	-0.61158	6.191888	-7.29698	9.52E-13	5.78E-12	18.14963
TPD52	-0.6117	5.365222	-4.92074	1.12E-06	3.44E-06	4.572033
ALG9	-0.61233	5.136571	-7.20932	1.73E-12	1.02E-11	17.56787
HAUS6	-0.61239	5.229605	-5.5179	5.14E-08	1.85E-07	7.54079
EHBP1	-0.61266	5.697906	-6.67658	5.65E-11	2.86E-10	14.16072
MAFF	-0.61269	6.623777	-4.11513	4.42E-05	0.00011	1.066661
SS18L2	-0.61271	8.448401	-10.6344	2.75E-24	5.82E-23	44.29571
PRPF38B	-0.61317	7.999738	-7.90105	1.36E-14	9.88E-14	22.31536
CSE1L	-0.61329	6.796678	-7.81823	2.47E-14	1.76E-13	21.72825
SH2D2A	-0.61335	6.703545	-6.68998	5.19E-11	2.63E-10	14.24371
NAT10	-0.61336	7.335109	-8.55815	9.90E-17	8.87E-16	27.14809
CSPP1	-0.61346	5.187027	-11.5778	4.42E-28	1.52E-26	52.92514
CCDC43	-0.61375	6.336356	-6.36294	3.97E-10	1.83E-09	12.25994
CLCC1	-0.61379	4.911733	-7.39088	5.01E-13	3.12E-12	18.77919
MRPS18B	-0.61383	7.291043	-7.65007	8.20E-14	5.52E-13	20.5516
POLR3GL	-0.61412	7.927453	-8.88646	7.58E-18	7.65E-17	29.6753
RWDD3	-0.61419	7.159339	-6.09805	1.94E-09	8.22E-09	10.71681
CIPC	-0.61443	5.380447	-5.38278	1.06E-07	3.68E-07	6.842004
TSPYL5	-0.61473	4.410974	-7.54399	1.73E-13	1.13E-12	19.82012
RPL10L	-0.61497	5.038131	-9.58305	2.57E-20	3.47E-19	35.27407
MALAT1	-0.615	9.925928	-6.08772	2.06E-09	8.73E-09	10.65783
ERO1LB	-0.61537	5.645956	-7.55741	1.57E-13	1.03E-12	19.91217
TRIM37	-0.61551	7.668915	-6.34228	4.50E-10	2.06E-09	12.13753
AGO3	-0.61605	5.870687	-9.67733	1.17E-20	1.62E-19	36.05572
C12orf65	-0.61612	5.903137	-12.7476	4.70E-33	3.09E-31	64.2499
ZBTB38	-0.61628	6.752467	-8.27066	8.84E-16	7.19E-15	24.99607
AP3M1	-0.6168	7.483236	-7.83655	2.16E-14	1.55E-13	21.85769
JARID2	-0.6169	7.837095	-10.9067	2.32E-25	5.60E-24	46.73664
ATP1B1	-0.61691	5.234509	-4.69723	3.28E-06	9.51E-06	3.541207
ZNF256	-0.6177	3.981354	-10.8408	4.24E-25	9.88E-24	46.14208
CREBRF	-0.61785	8.771626	-5.90734	5.87E-09	2.34E-08	9.641646
SMC5	-0.61785	5.525964	-5.7837	1.18E-08	4.58E-08	8.96078
POMT1	-0.61846	5.897029	-10.1781	1.58E-22	2.74E-21	40.29901
WBP11	-0.61847	6.795072	-8.56404	9.46E-17	8.49E-16	27.19277
POLB	-0.61854	6.0843	-7.41022	4.38E-13	2.74E-12	18.90969
PPTC7	-0.61854	8.177008	-7.16029	2.40E-12	1.39E-11	17.2451
RAB11FIP3	-0.61873	6.113773	-11.0817	4.64E-26	1.21E-24	48.32721
CCSAP	-0.61931	8.355761	-6.69371	5.06E-11	2.57E-10	14.26681
ILKAP	-0.61932	6.931516	-8.5262	1.27E-16	1.12E-15	26.90605
ZNF644	-0.61938	7.712984	-6.21798	9.53E-10	4.19E-09	11.40837
RPL35	-0.61939	10.95717	-4.80433	1.97E-06	5.88E-06	4.029601

AKAP8	-0.61964	7.210233	-15.4133	2.65E-45	1.04E-42	92.17767
IL21R	-0.62005	5.641364	-7.90647	1.30E-14	9.51E-14	22.35398
C5orf34	-0.62041	3.684162	-11.254	9.34E-27	2.70E-25	49.90994
ANP32B	-0.62077	10.59001	-7.76438	3.63E-14	2.55E-13	21.34918
HACE1	-0.62092	5.836293	-4.79283	2.08E-06	6.19E-06	3.976666
FBXL14	-0.62097	7.843436	-7.52411	1.99E-13	1.29E-12	19.68398
TBCCD1	-0.62105	5.146474	-5.09386	4.73E-07	1.52E-06	5.400733
PAN3	-0.62113	10.10116	-9.98015	8.79E-22	1.40E-20	38.60358
TMA16	-0.62116	3.836184	-5.68678	2.04E-08	7.68E-08	8.436064
RPS5	-0.6212	11.60326	-5.23378	2.31E-07	7.72E-07	6.089688
SEPSECS	-0.62143	4.848955	-9.48982	5.61E-20	7.31E-19	34.50657
ZNF789	-0.62178	4.958779	-11.199	1.56E-26	4.35E-25	49.40313
ELOVL4	-0.62208	3.643838	-12.8369	1.91E-33	1.34E-31	65.14108
ZBTB1	-0.62224	6.339102	-6.86037	1.74E-11	9.25E-11	15.31108
ADCY7	-0.62249	9.600403	-8.5789	8.43E-17	7.63E-16	27.30563
TPR	-0.62256	7.53264	-7.27944	1.07E-12	6.47E-12	18.03275
METTL3	-0.62294	6.922809	-9.24695	4.16E-19	4.92E-18	32.5333
C10orf88	-0.62305	5.740313	-7.98333	7.45E-15	5.56E-14	22.90367
RAD1	-0.62313	6.098543	-8.81121	1.38E-17	1.34E-16	29.08958
AGBL2	-0.62316	3.934035	-12.3786	1.87E-31	9.71E-30	60.60824
MTMR11	-0.62327	7.108236	-4.61169	4.90E-06	1.39E-05	3.158443
USP16	-0.62378	7.891583	-8.73768	2.45E-17	2.34E-16	28.52094
ATG16L2	-0.62408	10.10049	-5.0035	7.44E-07	2.34E-06	4.964921
DHX33	-0.6243	6.634438	-10.5625	5.24E-24	1.07E-22	43.65772
TULP4	-0.62497	5.131848	-9.94827	1.16E-21	1.82E-20	38.33264
NDC1	-0.62501	6.000396	-6.75767	3.37E-11	1.74E-10	14.665
ATM	-0.62522	8.509263	-6.00498	3.34E-09	1.38E-08	10.18831
SNAPC5	-0.62565	6.262989	-13.5332	1.52E-36	1.82E-34	72.2016
CECR5	-0.62586	7.348723	-7.20913	1.73E-12	1.02E-11	17.56664
CHI3L2	-0.62592	5.818846	-6.02232	3.02E-09	1.25E-08	10.28628
UPF3B	-0.62611	5.748199	-12.0229	6.11E-30	2.64E-28	57.15757
PPP3CC	-0.62701	5.219092	-11.4137	2.09E-27	6.55E-26	51.39016
CLOCK	-0.62704	5.947977	-8.64152	5.19E-17	4.78E-16	27.78285
LINC0062:	-0.6272	9.820752	-9.15068	9.10E-19	1.03E-17	31.76174
MIB1	-0.62724	6.638226	-6.20832	1.01E-09	4.43E-09	11.35222
CUL2	-0.62769	6.63154	-6.00654	3.31E-09	1.37E-08	10.19712
TMEM161	-0.62797	5.079367	-10.5169	7.89E-24	1.59E-22	43.25489
ATP6V0A2	-0.62806	5.696659	-9.15687	8.66E-19	9.87E-18	31.81115
RBM43	-0.62812	5.76183	-6.65599	6.43E-11	3.23E-10	14.03356
FLVCR1-A	-0.6285	6.161081	-6.24552	8.08E-10	3.59E-09	11.56882
RBM3	-0.62857	7.188724	-5.97363	4.01E-09	1.63E-08	10.01195
RPA1	-0.62858	7.313247	-9.51805	4.43E-20	5.83E-19	34.73838
TBC1D9	-0.6297	6.903768	-6.51881	1.52E-10	7.36E-10	13.19468
ARID2	-0.62972	7.28084	-8.68185	3.79E-17	3.54E-16	28.09166
MDC1	-0.62979	5.797457	-9.69138	1.03E-20	1.45E-19	36.17267
GORASP2	-0.63002	8.204111	-7.95907	8.90E-15	6.58E-14	22.72967
ZNF160	-0.6302	6.722648	-8.57759	8.52E-17	7.71E-16	27.29565
YES1	-0.63022	4.154157	-8.68011	3.84E-17	3.59E-16	28.07827
ECHS1	-0.63091	8.586313	-7.69574	5.93E-14	4.05E-13	20.86909
EPHB6	-0.63117	5.900385	-6.38299	3.51E-10	1.63E-09	12.37908
RRN3P1	-0.63119	5.811757	-12.1801	1.32E-30	6.23E-29	58.67527
IFFO2	-0.63191	5.776924	-8.92098	5.76E-18	5.91E-17	29.94525
PAXIP1	-0.63214	6.565263	-6.67592	5.67E-11	2.87E-10	14.15668
CFL2	-0.63245	4.543184	-7.45248	3.27E-13	2.08E-12	19.19591
ZNF706	-0.63284	7.100469	-8.53051	1.23E-16	1.08E-15	26.9387
ISOC1	-0.63286	7.18496	-4.48724	8.67E-06	2.38E-05	2.613283
PNOC	-0.63363	6.013683	-5.79467	1.11E-08	4.32E-08	9.0207
SMKR1	-0.63404	4.050332	-9.91042	1.60E-21	2.50E-20	38.01189

THUMPD2	-0.63422	5.735124	-8.0804	3.65E-15	2.80E-14	23.60396
NAPEPLD	-0.63465	4.841542	-8.29276	7.49E-16	6.14E-15	25.15947
CPVL	-0.63472	8.902223	-3.98971	7.45E-05	0.000181	0.573796
TMEM69	-0.63478	7.456084	-5.91841	5.51E-09	2.20E-08	9.703229
LOC10193	-0.63537	5.288887	-4.1791	3.37E-05	8.56E-05	1.323566
PIGH	-0.63539	5.821401	-8.02752	5.39E-15	4.07E-14	23.22157
C11orf31	-0.63545	7.897135	-9.09949	1.38E-18	1.53E-17	31.35391
USP45	-0.63557	3.552691	-9.88709	1.96E-21	2.99E-20	37.81455
ANKRD23	-0.63614	4.918443	-11.2053	1.47E-26	4.15E-25	49.46071
NR2C1	-0.63641	4.592834	-11.0697	5.18E-26	1.35E-24	48.21737
IL23A	-0.63667	5.881498	-11.1727	1.99E-26	5.46E-25	49.1612
SFMBT2	-0.6373	5.724505	-8.62519	5.89E-17	5.39E-16	27.65818
FNTA	-0.63731	7.871488	-11.307	5.69E-27	1.69E-25	50.39903
TWISTNB	-0.63732	4.652833	-8.36556	4.32E-16	3.63E-15	25.70006
ID3	-0.63756	5.304086	-7.13312	2.88E-12	1.65E-11	17.067
CAPN2	-0.63758	8.458465	-9.89919	1.76E-21	2.73E-20	37.91684
CD83	-0.63759	7.025145	-6.86515	1.68E-11	8.98E-11	15.34135
ATP7A	-0.63839	7.151879	-6.43474	2.56E-10	1.21E-09	12.68813
RECK	-0.6387	5.656778	-12.739	5.12E-33	3.28E-31	64.16465
DUSP12	-0.63937	7.382438	-9.38125	1.38E-19	1.73E-18	33.61984
ADHFE1	-0.64025	6.873963	-11.6117	3.20E-28	1.13E-26	53.24374
FOXP1	-0.64025	7.836644	-11.4534	1.44E-27	4.60E-26	51.76017
CD1D	-0.64046	8.600059	-4.76093	2.43E-06	7.14E-06	3.830479
TMTC3	-0.64051	4.637981	-5.7132	1.76E-08	6.68E-08	8.578351
LUC7L	-0.64054	7.030486	-10.1905	1.41E-22	2.48E-21	40.40621
TTC12	-0.64068	4.857144	-16.5975	4.57E-51	4.14E-48	105.3268
FARSB	-0.64079	5.440229	-10.6133	3.32E-24	6.94E-23	44.10843
PBLD	-0.64097	5.069016	-5.77755	1.23E-08	4.73E-08	8.927256
MAN2A1	-0.64098	7.965987	-5.92033	5.45E-09	2.18E-08	9.713899
H2BFXP	-0.64105	4.109972	-15.7921	3.96E-47	1.99E-44	96.34139
ZNF791	-0.64119	7.155025	-7.35121	6.58E-13	4.05E-12	18.51238
IER3IP1	-0.64119	6.842188	-8.87994	7.98E-18	8.03E-17	29.62438
EIF3D	-0.6412	9.71975	-7.86114	1.81E-14	1.30E-13	22.0318
GGPS1	-0.64123	7.365462	-7.38207	5.32E-13	3.30E-12	18.71985
UBE2E3	-0.64125	9.189358	-7.59171	1.24E-13	8.19E-13	20.14816
DNAJC10	-0.64131	5.840592	-6.82862	2.13E-11	1.13E-10	15.11047
DYNLT3	-0.64143	8.651347	-6.92114	1.17E-11	6.33E-11	15.69729
ESYT2	-0.6416	7.95166	-7.27043	1.14E-12	6.85E-12	17.97277
BACE2	-0.64191	5.070468	-7.23751	1.43E-12	8.46E-12	17.75434
PRMT6	-0.64213	5.066888	-5.11653	4.21E-07	1.36E-06	5.51121
LOC10272	-0.64213	7.122588	-6.51632	1.54E-10	7.47E-10	13.17963
CNOT6L	-0.64275	7.619628	-8.97563	3.73E-18	3.92E-17	30.37426
APBA2	-0.64277	6.881658	-7.34645	6.79E-13	4.17E-12	18.48045
ZKSCAN8	-0.64285	5.40689	-10.215	1.14E-22	2.02E-21	40.6178
FNBP4	-0.64288	5.318434	-9.54099	3.66E-20	4.86E-19	34.92709
CXCR3	-0.64308	5.78689	-8.85028	1.01E-17	1.01E-16	29.39322
OGFRL1	-0.64317	8.955883	-6.01672	3.12E-09	1.29E-08	10.25459
GPAM	-0.64334	4.679714	-5.51813	5.13E-08	1.85E-07	7.542017
HKR1	-0.6435	4.768818	-7.97942	7.67E-15	5.71E-14	22.87557
SOCS4	-0.64356	7.275798	-6.03712	2.77E-09	1.15E-08	10.37004
FBXL16	-0.64383	5.642963	-12.1598	1.61E-30	7.51E-29	58.4779
ARSG	-0.64443	6.646471	-8.87228	8.48E-18	8.51E-17	29.56464
ZBTB20	-0.64452	6.398518	-6.91229	1.24E-11	6.68E-11	15.64084
TMEFF2	-0.64453	4.192599	-8.83928	1.10E-17	1.09E-16	29.30761
PRDM1	-0.64499	7.617567	-7.13946	2.76E-12	1.59E-11	17.10849
ZC3H14	-0.64504	5.692998	-9.04221	2.19E-18	2.36E-17	30.89958
TXLNG	-0.6452	5.668422	-8.87152	8.54E-18	8.56E-17	29.55869
CLIC3	-0.64547	5.416682	-3.74958	0.000195	0.000445	-0.32939

SCYL3	-0.64566	7.074582	-7.72108	4.95E-14	3.42E-13	21.0459
ZNF671	-0.64585	6.567233	-11.0312	7.39E-26	1.90E-24	47.86641
CFDP1	-0.64591	6.724731	-6.46027	2.19E-10	1.04E-09	12.84135
EEF2	-0.64605	11.49676	-6.04685	2.62E-09	1.09E-08	10.42518
XAF1	-0.64614	7.991454	-3.24229	0.001252	0.002531	-2.06098
SPIN1	-0.64656	6.742998	-7.24113	1.39E-12	8.27E-12	17.77827
ICE2	-0.64752	4.973671	-10.2967	5.57E-23	1.03E-21	41.3262
RNF4	-0.64778	9.596173	-12.1101	2.61E-30	1.19E-28	57.99723
EMR3	-0.6478	8.567088	-3.57526	0.000378	0.000829	-0.95157
KDM1A	-0.64785	7.255392	-11.2022	1.52E-26	4.25E-25	49.43195
TMEM5	-0.64809	5.754578	-6.06754	2.32E-09	9.75E-09	10.54279
PSMG3	-0.64893	6.720896	-9.34917	1.80E-19	2.21E-18	33.35922
KAT6B	-0.64906	6.520673	-10.7389	1.07E-24	2.35E-23	45.22759
ENO2	-0.64926	5.864405	-6.91205	1.24E-11	6.69E-11	15.63935
IPO5P1	-0.64982	4.894281	-9.52246	4.27E-20	5.63E-19	34.77467
SUGT1	-0.64986	6.466812	-7.2273	1.53E-12	9.05E-12	17.68676
LOC10099	-0.65061	8.548652	-5.33153	1.39E-07	4.75E-07	6.581093
SLC30A7	-0.65063	6.849181	-9.33888	1.96E-19	2.40E-18	33.2758
RPP40	-0.65064	5.740107	-8.02423	5.52E-15	4.17E-14	23.19785
DBR1	-0.65074	6.195476	-6.84914	1.87E-11	9.92E-11	15.24
ACBD6	-0.65087	6.327513	-9.18898	6.67E-19	7.71E-18	32.06797
DPEP3	-0.65088	5.868455	-6.47634	1.98E-10	9.45E-10	12.93809
ZNF300	-0.65097	4.387939	-10.7534	9.38E-25	2.07E-23	45.35715
NUDT15	-0.6511	4.997738	-6.26728	7.09E-10	3.17E-09	11.69607
TMEM138	-0.65149	7.131687	-7.83319	2.22E-14	1.58E-13	21.83392
PID1	-0.65173	4.685117	-9.72274	7.94E-21	1.13E-19	36.4342
PPM1K	-0.65284	5.74421	-7.40382	4.58E-13	2.86E-12	18.86652
GCSAM	-0.65289	4.899514	-8.80905	1.40E-17	1.36E-16	29.07281
NRIP1	-0.6529	7.581461	-7.03932	5.38E-12	3.01E-11	16.45662
TFB1M	-0.65302	5.554306	-15.6162	2.80E-46	1.24E-43	94.40344
ADK	-0.65333	5.817332	-6.13381	1.57E-09	6.73E-09	10.9218
AP1S2	-0.65337	9.572151	-8.70249	3.23E-17	3.04E-16	28.25014
PURA	-0.65344	5.853126	-11.6329	2.62E-28	9.33E-27	53.44329
RPL32	-0.65384	13.40371	-8.59248	7.59E-17	6.90E-16	27.40891
CXXC5	-0.65405	7.509332	-5.52206	5.02E-08	1.81E-07	7.562567
TIAM1	-0.65409	6.920288	-6.92758	1.12E-11	6.08E-11	15.73837
HCRP1	-0.65462	6.822716	-7.47367	2.82E-13	1.80E-12	19.33986
LOC28378	-0.65484	5.066539	-7.73331	4.54E-14	3.14E-13	21.13143
TNIK	-0.65557	5.717536	-8.50583	1.48E-16	1.30E-15	26.75212
CLK1	-0.65598	9.551462	-7.06789	4.45E-12	2.51E-11	16.64182
EPHA4	-0.65604	5.019325	-10.197	1.34E-22	2.34E-21	40.46201
CCDC25	-0.65605	6.410753	-10.0234	6.05E-22	9.91E-21	38.97209
GOLGA4	-0.65625	7.27846	-7.50384	2.29E-13	1.48E-12	19.54549
TTC27	-0.65636	5.69828	-9.64024	1.59E-20	2.20E-19	35.74756
THADA	-0.65697	6.681015	-10.2891	5.95E-23	1.09E-21	41.26004
AKR7A2	-0.65745	7.550867	-7.22149	1.59E-12	9.39E-12	17.6483
ZNF700	-0.65748	6.953646	-6.45477	2.26E-10	1.07E-09	12.80826
ADH5	-0.65765	7.948156	-6.86127	1.73E-11	9.20E-11	15.31678
KIAA1191	-0.65798	7.974814	-7.8782	1.60E-14	1.16E-13	22.15286
CBFA2T3	-0.65803	7.534466	-5.90147	6.07E-09	2.42E-08	9.609019
NOP2	-0.65815	7.349481	-9.65649	1.39E-20	1.92E-19	35.88242
FUBP1	-0.65816	6.543255	-9.99912	7.47E-22	1.21E-20	38.76501
SCARF1	-0.6582	6.13627	-3.46622	0.000566	0.001206	-1.32636
NSMCE4A	-0.65826	6.035249	-11.0353	7.12E-26	1.83E-24	47.9039
LAMP3	-0.65881	5.070511	-3.87032	0.000121	0.000285	0.118066
CIB1	-0.65894	8.324908	-6.16678	1.29E-09	5.60E-09	11.11166
SCARNA1?	-0.65991	6.693377	-5.63846	2.66E-08	9.89E-08	8.177425
ZNF493	-0.66052	5.679376	-7.85296	1.92E-14	1.38E-13	21.97384

MRPL9	-0.66056	7.290116	-7.7066	5.49E-14	3.77E-13	20.9448
CACNA2D	-0.66068	6.128199	-8.63206	5.59E-17	5.13E-16	27.71063
CYCS	-0.66075	6.219348	-7.66539	7.36E-14	4.97E-13	20.65797
AMIGO1	-0.66139	5.666538	-8.84117	1.09E-17	1.08E-16	29.32233
CD226	-0.66144	5.645961	-4.44994	1.03E-05	2.78E-05	2.452604
CCDC58	-0.66154	5.310638	-7.61874	1.02E-13	6.83E-13	20.33469
ZNF124	-0.66196	5.008986	-8.48734	1.71E-16	1.49E-15	26.61268
RPL23A	-0.66227	13.34171	-8.73154	2.57E-17	2.45E-16	28.47366
DMTF1	-0.66275	8.622698	-9.37106	1.50E-19	1.87E-18	33.53697
BOLA3	-0.66277	7.444481	-5.70056	1.89E-08	7.14E-08	8.510191
METTL25	-0.66297	7.18404	-7.32653	7.79E-13	4.76E-12	18.347
ZFC3H1	-0.66327	8.482925	-10.4478	1.46E-23	2.87E-22	42.64688
SCAF4	-0.66351	5.895461	-9.55899	3.15E-20	4.21E-19	35.07545
MINPP1	-0.66428	5.403688	-4.26933	2.28E-05	5.92E-05	1.692292
CBR4	-0.66437	6.803685	-8.5096	1.44E-16	1.26E-15	26.78062
NIFK	-0.66446	6.581776	-10.0722	3.96E-22	6.59E-21	39.38927
AMPD2	-0.66459	9.061454	-5.78586	1.17E-08	4.52E-08	8.972601
SUPT3H	-0.66531	5.150098	-11.4267	1.85E-27	5.85E-26	51.51115
RPP38	-0.66551	7.060799	-8.66607	4.29E-17	3.99E-16	27.97068
NOA1	-0.66576	7.817756	-8.43319	2.58E-16	2.22E-15	26.20563
PIGP	-0.66606	7.765476	-7.32259	8.00E-13	4.89E-12	18.32067
FN3KRP	-0.66628	7.172881	-8.16263	1.98E-15	1.57E-14	24.20247
CCNJ	-0.66629	4.590638	-10.3962	2.31E-23	4.42E-22	42.19403
CHAMP1	-0.6665	7.913973	-7.2034	1.80E-12	1.05E-11	17.52881
MS4A7	-0.66655	8.03104	-4.43957	1.08E-05	2.91E-05	2.408115
PYROXD1	-0.66672	6.320768	-7.37775	5.48E-13	3.39E-12	18.6908
FAIM3	-0.66677	8.811471	-4.01526	6.70E-05	0.000164	0.673019
ALKBH6	-0.66682	6.849949	-8.94228	4.86E-18	5.03E-17	30.11222
DSP	-0.66689	4.241284	-4.77909	2.22E-06	6.59E-06	3.913593
TCF12	-0.66694	5.847388	-8.06425	4.11E-15	3.14E-14	23.48693
RPGR	-0.66727	6.810498	-5.8027	1.06E-08	4.13E-08	9.064604
CNST	-0.66738	7.404135	-6.76589	3.19E-11	1.65E-10	14.71638
RPL35A	-0.66747	7.974021	-12.503	5.43E-32	3.06E-30	61.82949
MPEG1	-0.66756	10.88317	-4.96417	9.04E-07	2.81E-06	4.777475
DPYSL2	-0.66783	9.766199	-5.3817	1.07E-07	3.70E-07	6.836518
ZNF223	-0.66783	4.01991	-10.3823	2.61E-23	4.95E-22	42.07305
SCRN1	-0.66789	7.013251	-6.4314	2.61E-10	1.23E-09	12.66809
TSIX	-0.6679	5.100535	-5.55415	4.22E-08	1.54E-07	7.730903
SSBP2	-0.66839	5.036854	-10.4887	1.01E-23	2.02E-22	43.00628
IKBKAP	-0.66886	5.817808	-9.96605	9.93E-22	1.57E-20	38.48367
FAM175A	-0.66886	5.355129	-5.7776	1.23E-08	4.73E-08	8.927541
HEATR2	-0.66902	7.25105	-8.24679	1.06E-15	8.56E-15	24.81999
RPL9	-0.66921	12.84789	-9.18307	7.00E-19	8.07E-18	32.02064
LSM5	-0.66943	5.896616	-5.97223	4.04E-09	1.64E-08	10.0041
ZNF512	-0.66964	7.946741	-8.25227	1.02E-15	8.23E-15	24.86042
PHF14	-0.66968	5.166799	-8.88709	7.54E-18	7.62E-17	29.68017
ATG2A	-0.66972	8.78558	-6.1054	1.86E-09	7.89E-09	10.75888
CWF19L2	-0.66973	6.026405	-8.30995	6.58E-16	5.44E-15	25.28678
BEX4	-0.6698	6.740582	-5.01215	7.12E-07	2.25E-06	5.006336
NFATC3	-0.66981	7.33809	-10.2433	8.90E-23	1.60E-21	40.86274
SSBP3-AS	-0.67026	5.785958	-9.22167	5.11E-19	5.98E-18	32.33011
RAD50	-0.67038	5.414941	-9.05106	2.04E-18	2.21E-17	30.96965
HMG20A	-0.67044	6.188267	-6.07808	2.18E-09	9.19E-09	10.60279
TLR7	-0.67049	5.8044	-6.17465	1.23E-09	5.36E-09	11.15713
TKTL1	-0.67097	5.574935	-7.3029	9.15E-13	5.56E-12	18.18913
FLT3LG	-0.67099	5.747446	-5.55106	4.29E-08	1.56E-07	7.714668
NHP2	-0.67118	8.717476	-7.95688	9.04E-15	6.68E-14	22.71403
ZBTB5	-0.67154	6.403204	-9.51426	4.58E-20	6.00E-19	34.70727

GLIPR1	-0.67159	9.488812	-7.5871	1.28E-13	8.44E-13	20.11641
ZNF879	-0.67218	5.504509	-7.94843	9.61E-15	7.08E-14	22.65351
ABHD10	-0.67235	6.910074	-8.44687	2.33E-16	2.01E-15	26.30825
NOTCH2N	-0.67239	10.80835	-7.52744	1.94E-13	1.26E-12	19.70673
EEF1D	-0.67257	8.221489	-13.0346	2.57E-34	2.15E-32	67.12514
ZNF430	-0.67289	6.833779	-6.94214	1.02E-11	5.56E-11	15.83142
PPP1R13B	-0.67304	5.579629	-11.4895	1.02E-27	3.39E-26	52.09772
IL24	-0.67314	4.873309	-13.0927	1.42E-34	1.25E-32	67.71142
TRA2A	-0.67399	6.844844	-9.56209	3.07E-20	4.11E-19	35.101
TBP	-0.67454	6.920676	-11.5239	7.37E-28	2.48E-26	52.41974
ZXDB	-0.67569	4.248351	-10.8489	3.93E-25	9.22E-24	46.21496
ZHX1	-0.67608	6.347634	-6.66227	6.18E-11	3.11E-10	14.07233
ERCC1	-0.67618	6.515774	-9.92198	1.45E-21	2.27E-20	38.10976
LYRM9	-0.6762	5.688146	-12.0644	4.08E-30	1.82E-28	57.55652
MUTYH	-0.67633	7.232953	-12.3635	2.17E-31	1.11E-29	60.4609
NSUN2	-0.67638	8.146864	-9.05035	2.05E-18	2.22E-17	30.964
DCUN1D4	-0.67686	5.420388	-9.39624	1.22E-19	1.53E-18	33.74181
ZCCHC18	-0.67705	5.477493	-9.4794	6.12E-20	7.93E-19	34.42113
RPS18	-0.67755	13.33775	-7.94938	9.55E-15	7.04E-14	22.66032
YEATS2	-0.67756	6.489447	-10.344	3.67E-23	6.88E-22	41.73806
HDAC9	-0.67776	5.515861	-9.0676	1.78E-18	1.95E-17	31.10066
TMC8	-0.67782	7.562475	-8.43775	2.49E-16	2.15E-15	26.23983
TRABD2A	-0.67813	5.500794	-11.9856	8.79E-30	3.77E-28	56.79825
DDX18	-0.67867	7.319727	-8.29852	7.17E-16	5.90E-15	25.20209
UPRT	-0.67874	6.256057	-6.21689	9.59E-10	4.22E-09	11.402
DNAJA3	-0.67896	7.369572	-9.78712	4.60E-21	6.71E-20	36.97291
ZNF786	-0.67917	5.467836	-9.6383	1.62E-20	2.23E-19	35.73146
METAP1D	-0.67917	5.02838	-10.6589	2.20E-24	4.71E-23	44.51309
PCSK7	-0.67927	6.709547	-8.91186	6.20E-18	6.34E-17	29.87384
NCR3	-0.67993	6.284428	-8.9568	4.33E-18	4.51E-17	30.22615
PPIH	-0.68001	7.273982	-9.61699	1.94E-20	2.64E-19	35.55481
ZNF12	-0.68025	7.891523	-7.62519	9.77E-14	6.55E-13	20.37931
HMGN4	-0.6811	9.696694	-8.39347	3.49E-16	2.97E-15	25.90834
SPAG16	-0.68119	3.996561	-14.0393	7.56E-39	1.25E-36	77.45489
IGIP	-0.68121	6.076625	-10.9237	1.99E-25	4.83E-24	46.89028
ZNF77	-0.6814	5.377211	-9.20503	5.85E-19	6.81E-18	32.19655
AHR	-0.68156	7.684452	-4.83177	1.73E-06	5.19E-06	4.156406
SACM1L	-0.68181	9.802155	-9.54129	3.65E-20	4.85E-19	34.92961
PDP1	-0.68181	7.434694	-7.22634	1.54E-12	9.10E-12	17.68036
PPHLN1	-0.68186	6.519193	-10.178	1.58E-22	2.74E-21	40.29791
RPL11	-0.68218	12.25334	-7.93811	1.04E-14	7.62E-14	22.57974
PCBD2	-0.68223	5.614465	-10.2799	6.46E-23	1.18E-21	41.18
ZNF514	-0.68243	5.273395	-6.69551	5.01E-11	2.54E-10	14.27799
PATL2	-0.68253	6.738022	-7.36908	5.82E-13	3.60E-12	18.63246
EIF3A	-0.68265	8.530845	-8.95317	4.46E-18	4.62E-17	30.19764
PLXDC1	-0.68292	5.375161	-13.515	1.84E-36	2.14E-34	72.0137
C1orf174	-0.68292	6.96882	-8.36331	4.39E-16	3.69E-15	25.68331
JADE2	-0.68302	6.311194	-10.4262	1.77E-23	3.45E-22	42.45683
TEFM	-0.68357	6.380993	-8.36468	4.35E-16	3.65E-15	25.69353
LYPLAL1	-0.68359	7.289405	-7.06614	4.50E-12	2.54E-11	16.63044
REM2	-0.68378	7.449265	-4.78128	2.20E-06	6.53E-06	3.923642
TGIF2	-0.684	6.197015	-8.43018	2.64E-16	2.27E-15	26.18305
NUDT13	-0.68427	4.19556	-10.4316	1.69E-23	3.29E-22	42.50431
U2SURP	-0.68443	6.645595	-7.92707	1.12E-14	8.22E-14	22.50092
C11orf21	-0.68508	8.967629	-7.99637	6.77E-15	5.07E-14	22.99735
ZNF793	-0.68558	4.120747	-10.3793	2.68E-23	5.08E-22	42.04645
ARRDC2	-0.68624	7.304699	-10.2765	6.65E-23	1.21E-21	41.1506
RBL1	-0.68653	4.589942	-6.7299	4.02E-11	2.06E-10	14.49173

RPL32P3	-0.68678	6.750123	-11.1823	1.82E-26	5.01E-25	49.24955
TUBE1	-0.68707	4.215913	-8.41454	2.98E-16	2.54E-15	26.0659
PNPT1	-0.6873	7.191275	-5.13135	3.91E-07	1.27E-06	5.583677
CCDC59	-0.68735	8.011597	-9.18201	7.06E-19	8.13E-18	32.01217
FAM179A	-0.68749	5.380251	-9.41225	1.07E-19	1.35E-18	33.87224
UNG	-0.68757	5.134753	-6.722	4.23E-11	2.16E-10	14.44257
NDC80	-0.68775	5.033812	-4.84531	1.62E-06	4.88E-06	4.219194
OXSM	-0.6885	6.506043	-8.81271	1.36E-17	1.33E-16	29.10118
ACYP1	-0.68856	5.551707	-6.81905	2.27E-11	1.20E-10	15.05012
JADE1	-0.68873	7.637542	-7.53202	1.88E-13	1.23E-12	19.73811
GPR56	-0.68875	7.232323	-5.91285	5.69E-09	2.27E-08	9.672246
PAPD5	-0.68898	7.627138	-8.55684	1.00E-16	8.95E-16	27.13817
TESK1	-0.68931	6.586468	-11.7685	7.15E-29	2.75E-27	54.72592
DDX46	-0.68934	8.208568	-8.99928	3.08E-18	3.28E-17	30.56049
KIAA1377	-0.68947	3.787175	-18.7973	3.82E-62	5.60E-58	130.6064
RPL19	-0.68963	12.62177	-7.39633	4.82E-13	3.01E-12	18.81597
LOC39949	-0.69004	8.744757	-7.16951	2.26E-12	1.31E-11	17.30568
SF3A3	-0.69012	7.202869	-6.57461	1.07E-10	5.26E-10	13.53411
TCEA1	-0.69047	9.563164	-8.186	1.67E-15	1.33E-14	24.37346
USP36	-0.69094	5.917361	-11.7713	6.95E-29	2.68E-27	54.75317
LINC0033[illegible]	-0.69106	7.093408	-6.36414	3.94E-10	1.82E-09	12.26711
DPP4	-0.69162	5.074487	-10.4201	1.87E-23	3.62E-22	42.40388
MICU3	-0.69165	3.034188	-9.60288	2.18E-20	2.96E-19	35.43795
BRWD1	-0.69174	5.52878	-9.88776	1.95E-21	2.98E-20	37.82026
BCLAF1	-0.69204	7.769266	-7.86101	1.81E-14	1.30E-13	22.03092
SMURF2	-0.69253	6.48078	-8.09145	3.36E-15	2.59E-14	23.68407
LOC28643	-0.69256	6.912373	-8.40113	3.30E-16	2.81E-15	25.96553
BDH2	-0.69264	4.991434	-11.4379	1.66E-27	5.29E-26	51.61531
JAKMIP1	-0.693	4.894773	-6.80235	2.53E-11	1.32E-10	14.94505
UFM1	-0.69301	6.734574	-8.55136	1.04E-16	9.32E-16	27.0966
RNMTL1	-0.69337	6.586617	-9.73117	7.39E-21	1.06E-19	36.50457
SPOCK2	-0.69411	7.671626	-7.3858	5.18E-13	3.22E-12	18.74495
CRTAP	-0.69486	6.314663	-11.4484	1.51E-27	4.81E-26	51.71372
YTHDF1	-0.69541	9.045075	-11.2549	9.26E-27	2.69E-25	49.91829
ALKBH3	-0.69542	6.045817	-8.81724	1.31E-17	1.28E-16	29.13638
LGR6	-0.69563	5.154285	-8.53462	1.19E-16	1.05E-15	26.96978
MRPL42	-0.69591	6.047585	-7.06956	4.40E-12	2.49E-11	16.65264
PEX1	-0.69605	5.093773	-9.30958	2.49E-19	3.02E-18	33.03855
AK6	-0.69608	7.245022	-7.48725	2.57E-13	1.65E-12	19.43231
LOC10012	-0.69683	5.922611	-6.71366	4.46E-11	2.28E-10	14.39065
ADAT2	-0.69703	4.474995	-12.398	1.54E-31	8.14E-30	60.79774
TIMM10B	-0.69704	6.575207	-10.2928	5.76E-23	1.06E-21	41.29223
SKIV2L2	-0.69771	7.823382	-9.00799	2.88E-18	3.07E-17	30.62921
UHRF2	-0.69796	8.968394	-6.92046	1.18E-11	6.35E-11	15.69293
MRPS21	-0.69809	7.967382	-8.00658	6.28E-15	4.73E-14	23.07075
FAHD1	-0.69809	5.628336	-7.21203	1.69E-12	9.98E-12	17.58577
SLAMF7	-0.69855	7.08836	-4.68754	3.44E-06	9.93E-06	3.497505
GTPBP6	-0.69894	6.757955	-8.84736	1.03E-17	1.03E-16	29.37047
ELP2	-0.69898	5.794236	-11.9065	1.89E-29	7.79E-28	56.04068
SLC25A26	-0.69946	6.41992	-10.2978	5.51E-23	1.02E-21	41.33592
SARAF	-0.69976	11.91315	-8.82311	1.25E-17	1.23E-16	29.18193
RNF157-A	-0.70032	3.909105	-12.6251	1.61E-32	9.51E-31	63.03463
ORMDL1	-0.70053	7.591998	-9.13351	1.05E-18	1.18E-17	31.6247
PAN3-AS1	-0.70062	4.710905	-8.70971	3.05E-17	2.88E-16	28.30564
DOPEY1	-0.70072	5.046527	-10.8649	3.40E-25	8.04E-24	46.35907
MARCKSL[illegible]	-0.70095	8.649361	-5.64516	2.56E-08	9.55E-08	8.213187
SPAG1	-0.70107	4.926844	-4.61968	4.72E-06	1.34E-05	3.19393
PRRC2C	-0.70164	8.501103	-10.8466	4.02E-25	9.39E-24	46.19422

ZBTB40	-0.70166	6.546555	-12.7017	7.46E-33	4.64E-31	63.7935
C14orf159	-0.70189	8.211605	-6.1667	1.29E-09	5.60E-09	11.1112
EID3	-0.70224	3.994948	-7.04827	5.07E-12	2.84E-11	16.51457
LACTB2	-0.70245	5.667254	-5.05547	5.73E-07	1.83E-06	5.214694
SCARNA15	-0.70247	5.972361	-8.75939	2.07E-17	1.99E-16	28.68842
SLC4A7	-0.70287	4.672904	-11.1831	1.81E-26	4.99E-25	49.25649
ZXDA	-0.70314	4.905631	-9.04943	2.06E-18	2.23E-17	30.9567
UNC5CL	-0.70356	5.45198	-14.5489	3.28E-41	7.08E-39	82.84136
C12orf75	-0.70367	7.358121	-3.9503	8.75E-05	0.00021	0.421931
SLC7A6	-0.7038	6.393425	-10.8163	5.29E-25	1.21E-23	45.92181
SMIM11	-0.70401	5.455045	-8.54484	1.10E-16	9.78E-16	27.04716
BBIP1	-0.70424	7.070605	-8.71761	2.87E-17	2.72E-16	28.36635
EVA1C	-0.70437	4.681351	-10.7323	1.14E-24	2.48E-23	45.16868
YPEL1	-0.70442	5.409129	-11.1102	3.56E-26	9.46E-25	48.58833
BCL2	-0.70478	5.929944	-13.1371	9.04E-35	7.99E-33	68.15965
LEPROTL1	-0.70489	9.124875	-10.7786	7.46E-25	1.67E-23	45.58331
C9orf78	-0.70506	7.931536	-8.54967	1.06E-16	9.44E-16	27.08381
RPS25	-0.7054	11.45096	-8.11062	2.92E-15	2.26E-14	23.82333
ZNF512B	-0.70546	4.84412	-10.0166	6.42E-22	1.05E-20	38.914
JAKMIP2	-0.70557	4.201041	-13.8987	3.33E-38	4.99E-36	75.9861
LOC37444	-0.70606	5.803183	-8.36897	4.21E-16	3.54E-15	25.72546
RSAD1	-0.7063	7.041045	-8.88441	7.71E-18	7.77E-17	29.65925
ZFP90	-0.70631	6.184823	-9.47158	6.53E-20	8.44E-19	34.35709
KIAA0020	-0.70631	6.932064	-9.98734	8.26E-22	1.32E-20	38.66472
ACADM	-0.70636	7.648048	-5.99117	3.62E-09	1.48E-08	10.11053
AMMECR1	-0.70638	5.629485	-7.61477	1.05E-13	7.02E-13	20.30728
LINC0142(	-0.70662	5.605965	-8.81328	1.35E-17	1.32E-16	29.10565
TNFRSF25	-0.7076	6.336435	-7.98818	7.19E-15	5.38E-14	22.93847
SAMD12	-0.70778	4.183401	-8.84213	1.08E-17	1.07E-16	29.32976
ARMCX2	-0.70781	4.967404	-6.02302	3.01E-09	1.25E-08	10.29018
LOC10019	-0.70802	7.49512	-4.74742	2.59E-06	7.60E-06	3.768826
TMEM123	-0.70813	10.87574	-7.66961	7.14E-14	4.84E-13	20.68724
SYNCRIP	-0.70819	7.421584	-9.0455	2.13E-18	2.30E-17	30.92563
C6orf203	-0.7083	5.292181	-7.32008	8.14E-13	4.97E-12	18.30385
AK5	-0.70857	5.130586	-11.1928	1.65E-26	4.59E-25	49.34602
SLC39A6	-0.70883	7.658754	-7.29382	9.73E-13	5.90E-12	18.12854
C2CD5	-0.70938	9.272395	-9.28525	3.04E-19	3.65E-18	32.84202
CLSTN1	-0.70974	7.679285	-8.20518	1.44E-15	1.16E-14	24.51404
OR52K3P	-0.70994	7.110163	-4.28111	2.17E-05	5.64E-05	1.741004
ZNF606	-0.7103	6.136608	-13.8114	8.35E-38	1.18E-35	75.07695
RPL7L1	-0.71043	8.633425	-8.93439	5.18E-18	5.33E-17	30.05032
ZNF764	-0.71048	6.069399	-9.44504	8.14E-20	1.04E-18	34.13992
CBR3	-0.71056	5.836589	-10.1268	2.47E-22	4.18E-21	39.85779
ZNF880	-0.71067	4.406596	-9.03637	2.29E-18	2.46E-17	30.85336
LOC64373	-0.71094	4.292704	-16.2175	3.36E-49	2.23E-46	101.0667
PAN2	-0.71104	6.759477	-8.18308	1.70E-15	1.36E-14	24.35203
PTMA	-0.71119	10.82827	-9.85693	2.53E-21	3.83E-20	37.56004
FASLG	-0.7115	5.370074	-9.34045	1.93E-19	2.37E-18	33.28846
MSH2	-0.71164	5.925493	-5.15733	3.42E-07	1.12E-06	5.71116
POLR3B	-0.71183	6.685286	-6.81014	2.40E-11	1.26E-10	14.99401
QRICH1	-0.71195	7.279394	-15.8726	1.61E-47	8.34E-45	97.23256
FUT11	-0.71222	6.2507	-10.4184	1.90E-23	3.66E-22	42.38897
TCEAL1	-0.7124	5.872635	-8.55477	1.02E-16	9.09E-16	27.12244
CD74	-0.71254	11.54174	-3.88656	0.000113	0.000268	0.179288
FMR1	-0.71276	9.090758	-8.80225	1.48E-17	1.43E-16	29.02012
GAL3ST4	-0.71298	6.249129	-12.9596	5.51E-34	4.29E-32	66.37067
ORC3	-0.71306	6.440301	-11.3086	5.61E-27	1.67E-25	50.41395
TOP2B	-0.71322	9.318904	-8.5724	8.87E-17	7.99E-16	27.25625

RNPC3	-0.71328	7.135508	-7.91219	1.25E-14	9.13E-14	22.39477
IFT80	-0.71329	4.773943	-10.1706	1.68E-22	2.91E-21	40.23413
TTF1	-0.7134	6.954805	-9.34233	1.90E-19	2.34E-18	33.30376
TATDN1	-0.71381	6.821666	-7.43506	3.69E-13	2.32E-12	19.07779
PSME4	-0.71393	6.925916	-7.92693	1.12E-14	8.23E-14	22.49987
HIVEP3	-0.7141	5.721366	-10.852	3.83E-25	9.00E-24	46.24263
GTPBP8	-0.7144	5.661294	-8.64411	5.09E-17	4.69E-16	27.80264
CENPC	-0.71449	6.190729	-6.59229	9.61E-11	4.74E-10	13.64215
NT5DC1	-0.71457	5.284671	-9.28888	2.95E-19	3.55E-18	32.87131
CXorf65	-0.71481	6.600079	-7.71789	5.07E-14	3.49E-13	21.02366
KDSR	-0.71499	5.025331	-7.68629	6.34E-14	4.32E-13	20.80328
RPL6	-0.71502	12.24769	-8.63301	5.55E-17	5.10E-16	27.71784
C16orf80	-0.71537	7.608094	-7.75058	4.01E-14	2.80E-13	21.25236
ZSCAN21	-0.71557	6.495125	-8.52433	1.28E-16	1.13E-15	26.89191
SEH1L	-0.71567	5.829608	-6.46798	2.08E-10	9.92E-10	12.88772
TMEM194/	-0.71572	5.220668	-11.3663	3.26E-27	1.00E-25	50.94945
PCYOX1L	-0.71591	7.277936	-9.79796	4.19E-21	6.17E-20	37.06385
ZNF331	-0.71624	5.913931	-9.68453	1.10E-20	1.53E-19	36.11563
CHD6	-0.71636	5.214506	-10.5495	5.89E-24	1.20E-22	43.54275
CMC4	-0.71647	7.173116	-11.1227	3.17E-26	8.49E-25	48.70196
ZNF337	-0.71662	6.78879	-11.4635	1.31E-27	4.22E-26	51.85406
DDX55	-0.71698	5.911281	-8.70998	3.04E-17	2.88E-16	28.30771
ZNF91	-0.7171	5.77501	-9.82497	3.33E-21	4.98E-20	37.29083
POLR2H	-0.71721	7.293078	-9.52588	4.15E-20	5.48E-19	34.80279
COG5	-0.71723	6.830028	-9.29603	2.78E-19	3.35E-18	32.92904
BBS4	-0.71761	5.526636	-9.86809	2.30E-21	3.50E-20	37.65415
RNF157	-0.71772	6.272205	-7.61817	1.03E-13	6.86E-13	20.33076
LILRA4	-0.71782	6.129678	-6.9564	9.29E-12	5.09E-11	15.92272
TUBGCP5	-0.7182	4.325482	-14.5055	5.24E-41	1.12E-38	82.37874
RP9	-0.71838	5.575838	-10.7142	1.34E-24	2.92E-23	45.00638
BAG3	-0.71871	6.363398	-5.64731	2.53E-08	9.44E-08	8.224679
RPS4X	-0.71872	12.70671	-8.74752	2.27E-17	2.17E-16	28.59683
PIK3R1	-0.7193	8.106886	-7.75306	3.94E-14	2.75E-13	21.26976
ADAM28	-0.71979	5.232547	-9.59292	2.37E-20	3.21E-19	35.35558
ADSL	-0.72064	8.482769	-9.08167	1.59E-18	1.76E-17	31.21233
CD28	-0.72091	5.054978	-10.4538	1.38E-23	2.73E-22	42.69931
SCAMP1-/	-0.7219	6.77669	-8.31326	6.42E-16	5.32E-15	25.31134
UBA2	-0.7225	7.283137	-9.3328	2.06E-19	2.51E-18	33.22647
BAG5	-0.72309	6.64518	-7.62394	9.86E-14	6.60E-13	20.37066
RTP4	-0.72321	6.987334	-4.07841	5.16E-05	0.000127	0.920845
RICTOR	-0.72339	9.065536	-7.05826	4.74E-12	2.67E-11	16.5793
ZNF264	-0.72363	6.915943	-7.29425	9.70E-13	5.88E-12	18.13137
GOLPH3L	-0.72367	7.334914	-7.2668	1.17E-12	7.02E-12	17.94864
ANKH	-0.72416	5.568518	-12.9478	6.22E-34	4.75E-32	66.25152
EPM2AIP1	-0.72435	7.047475	-8.38528	3.72E-16	3.15E-15	25.84712
TTC16	-0.72468	5.207785	-11.31	5.53E-27	1.65E-25	50.42749
ZCCHC8	-0.72508	7.019305	-8.19997	1.50E-15	1.20E-14	24.47584
VPS13C	-0.72509	6.707694	-9.12486	1.12E-18	1.26E-17	31.5558
SS18L1	-0.72517	6.562337	-7.98604	7.31E-15	5.46E-14	22.9231
SLC25A38	-0.7252	7.240196	-7.58943	1.26E-13	8.31E-13	20.13245
FCGBP	-0.72532	5.883486	-10.4727	1.17E-23	2.33E-22	42.86521
CD3E	-0.72546	8.160904	-4.77706	2.25E-06	6.65E-06	3.904277
LIPA	-0.7256	10.14422	-4.79461	2.07E-06	6.15E-06	3.98485
FAM84B	-0.72571	6.224971	-11.2214	1.27E-26	3.60E-25	49.60912
SNHG19	-0.7265	5.636248	-7.05016	5.01E-12	2.81E-11	16.52678
ZNF268	-0.7266	5.106209	-9.60965	2.06E-20	2.80E-19	35.49397
ZNF75A	-0.72679	6.970779	-10.7956	6.39E-25	1.45E-23	45.73596
NVL	-0.72692	6.467975	-10.1986	1.32E-22	2.32E-21	40.47623

THUMPD3	-0.72702	6.483803	-9.811	3.75E-21	5.56E-20	37.1734
METAP1	-0.7272	7.729745	-9.05559	1.96E-18	2.14E-17	31.00549
IFIT5	-0.72728	8.35155	-3.77264	0.000178	0.000409	-0.24498
C6orf62	-0.7297	8.08328	-8.24301	1.09E-15	8.79E-15	24.7922
ALDH5A1	-0.73097	5.178399	-8.13275	2.48E-15	1.94E-14	23.98444
GNL2	-0.73111	6.506053	-7.47957	2.71E-13	1.73E-12	19.38002
LNX2	-0.73136	5.819976	-10.0042	7.14E-22	1.16E-20	38.80835
ALKBH8	-0.73153	4.819158	-11.2339	1.13E-26	3.22E-25	49.72379
RUSC1-AS	-0.73206	6.292196	-7.20854	1.74E-12	1.02E-11	17.56276
SFI1	-0.73236	6.469014	-11.0478	6.34E-26	1.64E-24	48.01804
EIF3M	-0.73237	6.596983	-9.46425	6.94E-20	8.94E-19	34.29704
MTHFD1	-0.73254	7.196895	-6.24935	7.89E-10	3.51E-09	11.59119
COG2	-0.73269	5.554216	-11.9313	1.49E-29	6.21E-28	56.278
RBM25	-0.73383	8.293171	-9.99518	7.72E-22	1.24E-20	38.73145
PARP8	-0.73451	9.225203	-7.92927	1.11E-14	8.11E-14	22.51659
SUCO	-0.73506	7.608951	-8.22131	1.28E-15	1.03E-14	24.6325
CYSLTR1	-0.73519	6.609804	-7.2566	1.25E-12	7.49E-12	17.88089
GPD1L	-0.73671	7.24667	-9.27719	3.25E-19	3.89E-18	32.77692
CTCF	-0.7369	9.054344	-12.3754	1.93E-31	9.97E-30	60.57652
NUDCD2	-0.7377	5.877039	-8.11997	2.72E-15	2.12E-14	23.89132
SLAIN1	-0.73797	4.655751	-5.48728	6.06E-08	2.17E-07	7.38106
XPO1	-0.7387	8.071152	-8.99552	3.18E-18	3.37E-17	30.53089
ATR	-0.73888	5.691725	-8.88565	7.63E-18	7.70E-17	29.66898
ZFP14	-0.73894	4.097406	-11.5332	6.75E-28	2.28E-26	52.50697
DDX20	-0.73919	5.930681	-8.57756	8.52E-17	7.71E-16	27.29546
LEF1	-0.73945	6.531359	-6.8452	1.92E-11	1.02E-10	15.21511
ARHGEF10	-0.73955	6.276309	-9.24306	4.29E-19	5.06E-18	32.50203
KANSL1	-0.7397	8.281793	-9.10395	1.33E-18	1.48E-17	31.3893
CEP95	-0.74012	4.789358	-10.7644	8.48E-25	1.89E-23	45.45612
C10orf2	-0.74022	5.690628	-11.2395	1.07E-26	3.08E-25	49.77564
ANKHD1	-0.74052	6.292658	-6.49545	1.76E-10	8.44E-10	13.05338
DFFB	-0.74053	5.806472	-7.98461	7.38E-15	5.51E-14	22.91282
BBX	-0.74111	8.0397	-8.61494	6.38E-17	5.83E-16	27.57998
LDLRAP1	-0.74205	8.003944	-7.45039	3.32E-13	2.10E-12	19.18168
PKN2	-0.74221	7.026135	-9.12904	1.08E-18	1.22E-17	31.58912
GCLC	-0.74225	6.62631	-6.85846	1.76E-11	9.36E-11	15.299
ABCB10	-0.74246	7.267334	-5.75306	1.41E-08	5.40E-08	8.794069
NAA35	-0.74276	6.008152	-7.41748	4.17E-13	2.61E-12	18.95875
ZNF677	-0.7428	3.738107	-11.4444	1.56E-27	4.99E-26	51.6761
FAM43A	-0.74362	7.445554	-6.33402	4.73E-10	2.16E-09	12.08871
IPO7	-0.74407	7.513992	-7.24637	1.34E-12	8.00E-12	17.81306
RPF2	-0.74412	7.124052	-8.11108	2.91E-15	2.25E-14	23.82665
B3GALNT2	-0.74422	6.603343	-6.0698	2.29E-09	9.62E-09	10.55565
ZNF37BP	-0.74428	5.148279	-7.75883	3.78E-14	2.65E-13	21.31025
ZNF404	-0.74436	3.511532	-7.44642	3.41E-13	2.16E-12	19.15475
WDR54	-0.74442	6.941466	-9.12005	1.17E-18	1.31E-17	31.51744
SMIM19	-0.74448	8.093659	-9.06947	1.76E-18	1.93E-17	31.11551
DDX5	-0.745	7.049677	-7.38722	5.13E-13	3.19E-12	18.75455
FAM220A	-0.7451	7.901641	-8.59378	7.52E-17	6.83E-16	27.41878
TMX3	-0.74804	8.293748	-8.90268	6.67E-18	6.77E-17	29.80202
SDE2	-0.74833	8.489586	-6.43639	2.53E-10	1.19E-09	12.69801
HNRNPR	-0.7485	8.1894	-9.7259	7.73E-21	1.10E-19	36.46052
YARS2	-0.74958	6.101512	-9.5218	4.30E-20	5.66E-19	34.76921
CHRAC1	-0.75099	7.162794	-9.49846	5.22E-20	6.82E-19	34.57751
ATP8B2	-0.75102	7.316719	-8.06136	4.20E-15	3.20E-14	23.46601
PEBP1	-0.75209	7.967535	-6.59865	9.23E-11	4.56E-10	13.68108
LINC01003	-0.75223	7.292023	-5.93543	5.00E-09	2.00E-08	9.798107
RPS10	-0.75254	11.31064	-10.2378	9.34E-23	1.67E-21	40.81535

LY86	-0.75256	8.911504	-6.25402	7.67E-10	3.42E-09	11.61848
UGDH	-0.75285	6.286806	-5.74018	1.51E-08	5.78E-08	8.724215
TDRD3	-0.75286	4.671166	-10.6354	2.72E-24	5.77E-23	44.30427
FGL2	-0.75307	10.98242	-5.86666	7.41E-09	2.93E-08	9.41622
NDUFA8	-0.75345	7.677852	-8.75793	2.09E-17	2.01E-16	28.67714
HLTF	-0.75516	6.407971	-6.23398	8.66E-10	3.83E-09	11.50153
SLC7A6OS	-0.75523	6.914045	-11.6241	2.84E-28	1.01E-26	53.36096
KLF12	-0.75529	5.265742	-11.4723	1.20E-27	3.93E-26	51.93626
SCAF8	-0.75622	8.887988	-9.15641	8.69E-19	9.90E-18	31.80744
NEFL	-0.7565	3.946993	-13.5983	7.74E-37	9.61E-35	72.87177
IMMT	-0.75693	8.484135	-9.90037	1.75E-21	2.70E-20	37.92683
KMT2A	-0.75709	6.184866	-11.7418	9.24E-29	3.49E-27	54.47256
PFN2	-0.75779	5.723211	-8.90753	6.41E-18	6.55E-17	29.83998
LINC00888	-0.75781	5.103788	-8.28827	7.75E-16	6.34E-15	25.12623
NOM1	-0.75806	5.282383	-8.95632	4.35E-18	4.53E-17	30.22243
ZNF639	-0.75828	5.880627	-9.22941	4.80E-19	5.64E-18	32.39226
PMPCB	-0.75909	9.135108	-9.4865	5.77E-20	7.50E-19	34.47937
THAP11	-0.75932	7.959908	-11.5584	5.31E-28	1.82E-26	52.74286
BTBD6	-0.7594	7.758784	-9.90018	1.75E-21	2.71E-20	37.92528
NUP43	-0.75969	6.047026	-10.319	4.57E-23	8.53E-22	41.52041
NAA15	-0.75984	5.455924	-8.49084	1.66E-16	1.45E-15	26.63907
NIF3L1	-0.75992	7.485807	-9.00719	2.89E-18	3.09E-17	30.6229
ZSCAN16	-0.76016	5.916275	-7.89536	1.41E-14	1.03E-13	22.27491
PTPLAD1	-0.76026	6.768706	-6.80179	2.54E-11	1.33E-10	14.94152
SLC5A3	-0.76036	6.6015	-8.6639	4.36E-17	4.05E-16	27.95411
GSAP	-0.76136	9.099697	-8.71515	2.92E-17	2.77E-16	28.34747
ZNF468	-0.76149	6.59844	-5.07311	5.25E-07	1.68E-06	5.300025
PIGB	-0.76182	7.250884	-7.96147	8.74E-15	6.47E-14	22.74688
RPS21	-0.76223	9.623795	-8.60654	6.81E-17	6.20E-16	27.51592
CREBZF	-0.76247	5.635484	-12.9668	5.13E-34	4.04E-32	66.44239
ZNF721	-0.76278	8.785482	-9.03686	2.28E-18	2.46E-17	30.85725
SNRPE	-0.76292	6.749149	-11.3142	5.32E-27	1.59E-25	50.4663
MRPS27	-0.76325	7.043722	-7.4462	3.42E-13	2.16E-12	19.15328
ZRANB2	-0.76411	8.390373	-9.79141	4.43E-21	6.49E-20	37.00895
LOC44152	-0.76426	4.40868	-9.72167	8.01E-21	1.14E-19	36.42524
ZNF22	-0.76576	7.276976	-7.4674	2.95E-13	1.88E-12	19.2972
C19orf12	-0.76631	6.522091	-9.15967	8.46E-19	9.69E-18	31.83352
LOC28335	-0.76656	7.607773	-5.62685	2.84E-08	1.05E-07	8.11562
SRRM1	-0.76678	8.976993	-11.4584	1.37E-27	4.41E-26	51.8066
LOC10272	-0.76702	5.364882	-9.5865	2.50E-20	3.38E-19	35.30253
DDX27	-0.7677	7.908613	-12.9787	4.54E-34	3.63E-32	66.56226
HNRNPA0	-0.76789	7.6583	-12.0635	4.12E-30	1.83E-28	57.54764
EI24	-0.76851	7.099564	-9.25879	3.77E-19	4.48E-18	32.62864
LOC10099	-0.76879	5.488427	-12.0552	4.46E-30	1.97E-28	57.46819
LANCL1	-0.7688	6.611639	-10.7773	7.55E-25	1.69E-23	45.57187
GSE1	-0.76893	6.506693	-11.2356	1.11E-26	3.18E-25	49.74001
CNTRL	-0.76951	7.282347	-9.98932	8.12E-22	1.30E-20	38.68154
LOC64465	-0.76968	5.312238	-9.69446	1.01E-20	1.41E-19	36.1983
MINA	-0.77019	6.130398	-7.86775	1.73E-14	1.25E-13	22.07872
NFATC2IP	-0.77121	7.145266	-9.5185	4.42E-20	5.81E-19	34.74209
DEPDC7	-0.77161	3.203546	-13.0203	2.98E-34	2.45E-32	66.98113
KANSL2	-0.77175	7.611891	-8.9887	3.36E-18	3.55E-17	30.47712
C2orf40	-0.77201	4.253983	-13.9951	1.21E-38	1.92E-36	76.99284
FAM26F	-0.77208	6.460823	-8.9514	4.52E-18	4.69E-17	30.1838
TMEM14C	-0.77239	7.595479	-6.21746	9.55E-10	4.21E-09	11.40534
ELP3	-0.77269	6.849997	-10.8387	4.32E-25	1.00E-23	46.12318
IQCB1	-0.77278	6.962885	-9.1392	9.99E-19	1.13E-17	31.67012
SLAMF1	-0.77287	5.885736	-10.7906	6.69E-25	1.51E-23	45.69087

ZNF678	-0.77305	4.388135	-10.269	7.10E-23	1.29E-21	41.0855
LTV1	-0.77317	5.28284	-9.51894	4.40E-20	5.79E-19	34.7457
CCDC14	-0.77336	4.778302	-14.031	8.26E-39	1.34E-36	77.36762
PRKAB2	-0.77345	6.067067	-9.47078	6.57E-20	8.49E-19	34.35051
TARSL2	-0.77353	5.439677	-12.9653	5.20E-34	4.08E-32	66.42772
RPS27	-0.77355	9.867273	-10.3146	4.75E-23	8.82E-22	41.482
DDX21	-0.77361	9.352268	-7.77233	3.43E-14	2.42E-13	21.40498
C1QBP	-0.77389	7.931534	-6.59777	9.28E-11	4.58E-10	13.67568
EIF3F	-0.77398	6.479502	-12.0852	3.33E-30	1.51E-28	57.75685
DDHD1	-0.77425	5.183732	-10.149	2.03E-22	3.48E-21	40.04806
ZNF708	-0.77429	5.146627	-6.76264	3.26E-11	1.69E-10	14.69604
FBXO21	-0.77437	6.416536	-10.8895	2.72E-25	6.48E-24	46.58131
ANK3	-0.77473	5.038527	-12.7407	5.04E-33	3.24E-31	64.18144
SNRPD2	-0.7748	9.522379	-6.6654	6.06E-11	3.05E-10	14.09165
MLLT11	-0.77498	6.687388	-8.73184	2.57E-17	2.45E-16	28.47597
ZNF232	-0.77539	5.769839	-8.86451	9.02E-18	9.02E-17	29.50401
ACTL6A	-0.77572	6.469664	-5.99631	3.52E-09	1.44E-08	10.13949
MRPS6	-0.7758	7.922216	-8.62172	6.05E-17	5.54E-16	27.63163
MRPL32	-0.77605	7.798985	-8.67779	3.91E-17	3.65E-16	28.06052
TMCC1	-0.77626	6.804909	-8.70253	3.23E-17	3.04E-16	28.25038
MYO9A	-0.77642	5.610875	-12.6583	1.15E-32	6.98E-31	63.36319
KCNA3	-0.77647	6.383252	-5.04264	6.12E-07	1.95E-06	5.152846
ACBD5	-0.77681	8.474557	-8.3767	3.97E-16	3.35E-15	25.7831
SLC25A32	-0.77714	6.792596	-5.66272	2.33E-08	8.72E-08	8.307038
PPP1R3E	-0.77834	6.19131	-10.1965	1.34E-22	2.35E-21	40.45792
FAM162A	-0.77865	6.648423	-11.8131	4.66E-29	1.84E-27	55.14998
SMYD4	-0.77965	6.727037	-11.1449	2.58E-26	6.95E-25	48.90626
FNIP2	-0.77982	6.876871	-6.42547	2.71E-10	1.27E-09	12.63257
OARD1	-0.78045	6.907592	-10.7839	7.11E-25	1.60E-23	45.63033
RAN	-0.78046	8.264365	-8.90433	6.58E-18	6.70E-17	29.81489
COX11	-0.78061	5.487513	-10.0911	3.37E-22	5.65E-21	39.55084
BUB3	-0.78075	7.128572	-12.9674	5.10E-34	4.03E-32	66.44832
EIF3J	-0.78203	6.801534	-7.49341	2.46E-13	1.58E-12	19.47429
LSM8	-0.7823	5.936288	-11.2353	1.11E-26	3.19E-25	49.73701
TRAF3IP3	-0.78243	8.239515	-8.21522	1.34E-15	1.07E-14	24.58775
PAX5	-0.78264	6.55327	-7.42265	4.02E-13	2.53E-12	18.99374
DZIP3	-0.78264	4.966667	-10.2245	1.05E-22	1.88E-21	40.69959
B3GALT2	-0.78285	3.267213	-18.7563	6.18E-62	5.60E-58	130.128
RNASE6	-0.78311	9.233038	-6.07014	2.29E-09	9.61E-09	10.55756
ADAMTS5	-0.78314	3.963573	-11.3171	5.18E-27	1.56E-25	50.4928
RPS12	-0.78319	12.8439	-8.87962	8.00E-18	8.05E-17	29.62188
STARD7	-0.78323	9.60074	-8.61397	6.43E-17	5.87E-16	27.57255
MRPL45	-0.7853	7.350784	-11.2201	1.28E-26	3.64E-25	49.59693
CDC37L1	-0.78606	5.106222	-11.4156	2.05E-27	6.44E-26	51.40809
FANCL	-0.78626	5.031837	-5.74645	1.46E-08	5.59E-08	8.758212
SETDB2	-0.7866	5.352271	-11.1111	3.53E-26	9.40E-25	48.5961
PTPRK	-0.78684	3.811806	-9.21248	5.51E-19	6.43E-18	32.25635
ESYT1	-0.78741	8.730323	-6.34961	4.31E-10	1.97E-09	12.18097
LINC0017[illegible]	-0.78742	7.619153	-5.1928	2.85E-07	9.42E-07	5.88616
TRIM59	-0.78836	5.018737	-6.00304	3.38E-09	1.39E-08	10.17739
CLIP4	-0.78844	5.979345	-8.58683	7.93E-17	7.20E-16	27.36589
SCAI	-0.78875	4.539186	-7.94984	9.52E-15	7.02E-14	22.66361
PTGER4	-0.78894	7.711055	-10.2418	9.02E-23	1.62E-21	40.84982
HCP5	-0.79067	8.003841	-7.11502	3.25E-12	1.86E-11	16.94868
TIMM21	-0.791	5.851589	-9.06838	1.77E-18	1.94E-17	31.10686
CXorf57	-0.7911	3.808957	-16.3235	1.02E-49	8.02E-47	102.2506
LOC64223	-0.79122	4.277358	-7.79434	2.93E-14	2.07E-13	21.5598
TCERG1	-0.79158	5.087327	-9.62888	1.75E-20	2.40E-19	35.65334

ATP10D	-0.79165	7.304831	-5.97851	3.90E-09	1.59E-08	10.03935
ZNF623	-0.79192	4.702423	-7.02578	5.88E-12	3.28E-11	16.36906
CBX7	-0.79239	8.437604	-8.90194	6.71E-18	6.80E-17	29.79624
DDX26B	-0.79274	6.90546	-9.42099	9.94E-20	1.26E-18	33.94348
EPHX2	-0.79301	5.150239	-9.61039	2.05E-20	2.79E-19	35.50013
RPS7	-0.79303	12.31383	-9.99436	7.78E-22	1.25E-20	38.72445
PIK3C2B	-0.79326	7.235793	-8.14269	2.30E-15	1.81E-14	24.05689
AIMP1	-0.79343	6.831275	-8.14357	2.29E-15	1.80E-14	24.06326
IKZF3	-0.79345	6.38423	-7.73601	4.45E-14	3.09E-13	21.15032
BRD1	-0.79365	7.507231	-11.7136	1.21E-28	4.49E-27	54.20614
TARDBP	-0.79401	7.842091	-10.3567	3.28E-23	6.18E-22	41.84857
PRPS2	-0.79426	7.166949	-6.44418	2.41E-10	1.14E-09	12.74471
ZNF550	-0.79462	5.729377	-12.4931	6.00E-32	3.35E-30	61.73137
KARS	-0.79487	10.00427	-9.10615	1.31E-18	1.46E-17	31.40681
KLHL42	-0.79506	6.058889	-10.3235	4.40E-23	8.21E-22	41.55921
CETN3	-0.79516	6.855088	-6.65063	6.65E-11	3.33E-10	14.00047
DCBLD1	-0.79519	4.531197	-9.9605	1.04E-21	1.65E-20	38.4365
HVCN1	-0.79521	9.195891	-8.3189	6.15E-16	5.10E-15	25.35314
PCYOX1	-0.79673	5.817087	-7.70004	5.75E-14	3.94E-13	20.89911
FHIT	-0.79684	5.738719	-9.06871	1.77E-18	1.94E-17	31.10944
DSC1	-0.79702	4.068985	-13.2892	1.90E-35	1.87E-33	69.70372
IFT74	-0.79779	3.5744	-12.0355	5.41E-30	2.36E-28	57.27844
BRIX1	-0.79784	5.048504	-10.9566	1.47E-25	3.66E-24	47.18883
TTYH2	-0.79825	6.062503	-11.966	1.06E-29	4.53E-28	56.61069
ZNF136	-0.80025	6.790217	-8.56587	9.33E-17	8.38E-16	27.20665
DMXL1	-0.80055	6.149498	-7.8486	1.98E-14	1.42E-13	21.943
C8orf59	-0.80064	8.63773	-9.472	6.51E-20	8.42E-19	34.3605
HINT1	-0.80123	10.05464	-10.1351	2.29E-22	3.90E-21	39.92864
DENND2D	-0.80148	8.129298	-9.6192	1.90E-20	2.60E-19	35.57306
CDK8	-0.80176	6.005567	-6.87747	1.55E-11	8.32E-11	15.41943
EIF2B5	-0.8025	7.06526	-11.7031	1.34E-28	4.92E-27	54.10682
EID2	-0.80256	6.803133	-8.15813	2.05E-15	1.62E-14	24.1696
SELM	-0.80263	5.078721	-7.79307	2.96E-14	2.09E-13	21.55091
LDOC1	-0.80267	6.127082	-12.8684	1.39E-33	9.99E-32	65.45571
PSMG1	-0.80324	6.036436	-6.91916	1.19E-11	6.40E-11	15.68464
RANBP6	-0.80335	7.535925	-9.69642	9.92E-21	1.39E-19	36.21463
KIAA1430	-0.80354	4.901808	-12.9444	6.44E-34	4.88E-32	66.21715
EBLN3	-0.8038	8.131898	-9.96465	1.00E-21	1.59E-20	38.47181
FANCF	-0.80431	6.159875	-10.4353	1.63E-23	3.19E-22	42.53669
ZNF248	-0.80485	5.407156	-12.5021	5.48E-32	3.08E-30	61.82048
ANAPC4	-0.80492	8.216998	-10.8543	3.75E-25	8.84E-24	46.26369
CEPT1	-0.80498	6.996515	-9.0147	2.73E-18	2.91E-17	30.68213
PI3	-0.80526	8.116124	-3.00149	0.0028	0.005336	-2.79807
MORC2	-0.80634	6.053491	-11.9762	9.63E-30	4.12E-28	56.70824
TMEM106I	-0.80713	6.754169	-10.6997	1.52E-24	3.31E-23	44.87718
URB2	-0.80758	5.909008	-10.7545	9.29E-25	2.05E-23	45.3669
HEATR1	-0.80775	6.027167	-12.2164	9.23E-31	4.46E-29	59.02689
GPATCH1	-0.80804	6.111087	-9.20378	5.91E-19	6.88E-18	32.18654
SMS	-0.80806	7.724113	-8.17924	1.75E-15	1.39E-14	24.32391
XIST	-0.80822	5.290897	-2.15176	0.03182	0.049511	-4.95544
PPA1	-0.80951	9.781783	-7.50501	2.27E-13	1.47E-12	19.55343
ZBTB3	-0.80964	4.426683	-9.70023	9.60E-21	1.35E-19	36.2464
CCNC	-0.81128	9.014645	-9.94194	1.22E-21	1.92E-20	38.27898
TRERF1	-0.81133	6.250036	-11.1558	2.33E-26	6.34E-25	49.00605
PPAPDC2	-0.81137	6.009841	-7.27383	1.12E-12	6.71E-12	17.99538
ZNF627	-0.81141	6.893125	-7.4439	3.47E-13	2.19E-12	19.13768
DIP2A	-0.81149	8.761039	-10.2707	7.00E-23	1.27E-21	41.10075
PTCH1	-0.81223	4.722204	-13.4523	3.53E-36	3.94E-34	71.37105

MGA	-0.81243	6.123335	-9.13148	1.06E-18	1.20E-17	31.60855
TMTC4	-0.81254	5.825397	-6.76241	3.27E-11	1.69E-10	14.69463
GIMAP2	-0.81394	9.839943	-6.89292	1.41E-11	7.54E-11	15.51757
RFC4	-0.81405	6.206875	-6.1129	1.78E-09	7.57E-09	10.80182
HNRNPDL	-0.81502	8.017946	-12.6454	1.31E-32	7.84E-31	63.23522
MRPS25	-0.81511	5.585734	-15.5264	7.59E-46	3.12E-43	93.41633
ZNF827	-0.81562	4.847637	-10.4948	9.60E-24	1.92E-22	43.06034
REV1	-0.81614	6.09108	-10.8396	4.28E-25	9.97E-24	46.13173
ZMYND11	-0.81681	7.07872	-8.25323	1.01E-15	8.17E-15	24.86748
EIF2S1	-0.81719	7.452119	-9.15886	8.52E-19	9.75E-18	31.827
ZMAT1	-0.8174	5.030762	-9.89791	1.78E-21	2.75E-20	37.90604
EOGT	-0.81771	4.878703	-6.25053	7.84E-10	3.49E-09	11.59808
SIRT1	-0.81788	7.832657	-7.72092	4.96E-14	3.42E-13	21.04479
APEX1	-0.81788	9.19982	-7.5333	1.86E-13	1.22E-12	19.74685
MYOM2	-0.81949	5.621478	-3.95675	8.52E-05	0.000205	0.446684
KLRG1	-0.81963	6.941317	-8.11354	2.86E-15	2.22E-14	23.84455
CDK5RAP1	-0.81969	7.010098	-14.0001	1.14E-38	1.83E-36	77.04486
LINC00954	-0.82005	4.606177	-8.51225	1.41E-16	1.24E-15	26.8006
RPL3	-0.8206	11.79134	-8.76366	2.00E-17	1.93E-16	28.72143
PWP1	-0.82082	7.473727	-9.78324	4.75E-21	6.93E-20	36.94041
ZNF570	-0.82083	3.821263	-11.1332	2.88E-26	7.72E-25	48.79845
C5orf56	-0.82095	7.550325	-8.53164	1.21E-16	1.08E-15	26.94722
KANSL1-A	-0.82116	5.261298	-6.81717	2.30E-11	1.21E-10	15.03829
CBLL1	-0.82116	6.841144	-8.39338	3.50E-16	2.97E-15	25.90762
PARP12	-0.82149	8.124041	-5.69203	1.98E-08	7.47E-08	8.464275
RPS17	-0.82167	12.77279	-10.1746	1.62E-22	2.82E-21	40.26934
ZNF652	-0.82191	8.607408	-7.24538	1.35E-12	8.05E-12	17.80646
SECISBP2	-0.82266	7.924642	-11.5826	4.22E-28	1.46E-26	52.96984
RPL12	-0.82305	11.99867	-9.45119	7.73E-20	9.93E-19	34.19022
PCNXL2	-0.82334	5.288284	-17.7866	5.28E-57	1.91E-53	118.8742
NPHP3	-0.82347	6.500829	-6.23734	8.48E-10	3.76E-09	11.52109
GLO1	-0.82366	8.511334	-8.4654	2.02E-16	1.75E-15	26.44753
RPUSD4	-0.82451	7.25502	-10.5959	3.89E-24	8.04E-23	43.95363
PDIK1L	-0.82547	5.646562	-7.07607	4.21E-12	2.38E-11	16.69494
ZBTB2	-0.82653	6.475408	-10.6049	3.58E-24	7.44E-23	44.03373
HDC	-0.82697	6.430721	-7.96204	8.71E-15	6.45E-14	22.75093
CCDC109E	-0.82738	8.70603	-7.30209	9.20E-13	5.59E-12	18.18372
FAM216A	-0.82741	5.740464	-9.19271	6.47E-19	7.50E-18	32.09786
ZNF101	-0.82754	8.437059	-8.97274	3.81E-18	4.00E-17	30.3515
IER5	-0.82781	9.15063	-7.28345	1.04E-12	6.30E-12	18.05945
METAP2	-0.82801	6.163051	-8.55311	1.03E-16	9.21E-16	27.10989
LOC10192	-0.82838	5.841562	-8.58208	8.23E-17	7.46E-16	27.32981
DIS3L2	-0.82846	5.278414	-11.4658	1.28E-27	4.15E-26	51.87605
ARMC1	-0.82894	7.22155	-7.11773	3.19E-12	1.82E-11	16.96642
PAK1IP1	-0.8305	6.10478	-7.96609	8.45E-15	6.27E-14	22.78
LPIN1	-0.83085	6.727317	-9.89566	1.82E-21	2.80E-20	37.88706
LOC38976	-0.83106	5.178207	-6.56611	1.13E-10	5.53E-10	13.48224
CEP57	-0.83185	6.723	-9.37865	1.41E-19	1.76E-18	33.59865
UTP3	-0.8319	8.159933	-8.96573	4.03E-18	4.22E-17	30.29635
MEF2C	-0.83199	7.024229	-8.84508	1.05E-17	1.05E-16	29.35272
ITGA4	-0.83249	7.651621	-5.92644	5.26E-09	2.11E-08	9.747928
ZNF587B	-0.83284	7.157649	-9.53478	3.86E-20	5.10E-19	34.87597
MTERF1	-0.83289	5.649515	-11.7404	9.36E-29	3.52E-27	54.45973
CDC25B	-0.83349	8.28016	-5.96418	4.23E-09	1.71E-08	9.95894
SESN1	-0.83395	7.574822	-8.63022	5.67E-17	5.20E-16	27.69657
TNFAIP8L1	-0.83409	6.077995	-9.001	3.04E-18	3.23E-17	30.57406
CASP8AP2	-0.83418	7.473608	-6.84577	1.91E-11	1.01E-10	15.21871
MRPS30	-0.83495	5.778603	-9.07155	1.73E-18	1.90E-17	31.13197

ZNF25	-0.83556	5.544744	-8.11187	2.89E-15	2.24E-14	23.83244
RGS10	-0.83573	7.08195	-10.6838	1.76E-24	3.80E-23	44.73487
TTC19	-0.83578	7.710995	-8.09513	3.27E-15	2.52E-14	23.71076
CEP120	-0.83607	8.009339	-8.45717	2.15E-16	1.86E-15	26.3856
ZNF204P	-0.83634	3.694957	-17.1226	1.13E-53	1.58E-50	111.2713
DPEP2	-0.83641	9.413983	-5.61937	2.96E-08	1.09E-07	8.075813
CCDC146	-0.83719	6.206264	-8.14077	2.33E-15	1.83E-14	24.04285
FASTKD2	-0.83723	5.030112	-13.2849	1.99E-35	1.94E-33	69.66041
NT5E	-0.83798	4.759485	-8.83257	1.16E-17	1.15E-16	29.25544
AKAP7	-0.83826	6.198358	-11.7341	9.94E-29	3.73E-27	54.40017
STRBP	-0.83831	5.021731	-6.32094	5.12E-10	2.33E-09	12.01146
LAX1	-0.83872	6.635415	-7.03188	5.65E-12	3.15E-11	16.4085
MRI1	-0.83878	7.592163	-16.995	4.90E-53	6.34E-50	109.8211
MIS12	-0.83879	7.172321	-7.95681	9.04E-15	6.68E-14	22.7135
AOC3	-0.8388	5.750725	-6.60238	9.02E-11	4.45E-10	13.70394
ARHGEF18	-0.83953	9.930527	-11.2637	8.54E-27	2.49E-25	49.99889
EFHC1	-0.8398	5.131739	-13.7275	2.01E-37	2.76E-35	74.20608
IDO1	-0.84012	6.480212	-6.04659	2.62E-09	1.10E-08	10.42372
CCNG1	-0.84023	9.563163	-9.03246	2.36E-18	2.54E-17	30.82244
CD69	-0.84039	7.42999	-4.42382	1.15E-05	3.11E-05	2.340798
TIMM23B	-0.84162	5.913743	-7.46689	2.96E-13	1.89E-12	19.29375
ATG14	-0.84195	6.951625	-9.54015	3.69E-20	4.89E-19	34.92024
RPAP3	-0.84198	7.11902	-6.17021	1.27E-09	5.49E-09	11.13152
CMPK2	-0.84213	8.60517	-3.024	0.002603	0.004986	-2.73149
RRAS2	-0.8422	5.491707	-6.42431	2.73E-10	1.28E-09	12.62565
RUFY3	-0.84271	5.34114	-8.57715	8.55E-17	7.72E-16	27.29229
NAA16	-0.84279	8.194409	-9.0659	1.81E-18	1.98E-17	31.0872
PRKRIR	-0.84402	8.962681	-10.2912	5.84E-23	1.07E-21	41.27849
ACCS	-0.84444	6.4019	-8.85429	9.78E-18	9.75E-17	29.42444
ZDBF2	-0.84449	4.047891	-12.3634	2.17E-31	1.11E-29	60.45951
MIR3682	-0.84498	4.700616	-7.34347	6.93E-13	4.25E-12	18.4605
IFNG	-0.84626	4.830941	-8.628	5.77E-17	5.28E-16	27.67961
CREBL2	-0.84764	7.836157	-11.0306	7.43E-26	1.90E-24	47.86145
FAM13B	-0.84839	8.84708	-9.12914	1.08E-18	1.22E-17	31.58989
PPWD1	-0.84863	5.710082	-9.89065	1.90E-21	2.91E-20	37.84469
NUDT9	-0.84864	6.683144	-8.91171	6.20E-18	6.34E-17	29.87265
REV3L	-0.84903	6.851193	-7.55125	1.64E-13	1.07E-12	19.86992
FAM208B	-0.85097	6.070798	-10.5254	7.31E-24	1.48E-22	43.32962
BTN3A2	-0.8516	8.469956	-5.63279	2.74E-08	1.02E-07	8.147232
ZNF767P	-0.85174	6.147812	-10.9122	2.20E-25	5.34E-24	46.78687
BOD1	-0.85214	6.687625	-9.79022	4.48E-21	6.56E-20	36.9989
SLC35A3	-0.85303	5.948842	-7.72389	4.85E-14	3.35E-13	21.0656
UBE2E2	-0.85306	6.514525	-6.40654	3.04E-10	1.42E-09	12.51944
BTLA	-0.8531	6.406748	-5.61922	2.96E-08	1.09E-07	8.075016
GPATCH1	-0.85333	6.366631	-9.28645	3.01E-19	3.62E-18	32.85164
WDR36	-0.85373	5.683929	-8.90243	6.68E-18	6.78E-17	29.80005
RPL24	-0.85379	11.83989	-9.79987	4.12E-21	6.08E-20	37.07989
HNRNPA3	-0.8538	8.548642	-10.83	4.67E-25	1.08E-23	46.04491
ZNF736	-0.85406	4.1857	-8.59736	7.31E-17	6.65E-16	27.44601
LOC10272	-0.85494	3.991682	-8.83633	1.13E-17	1.12E-16	29.28469
MRPL1	-0.85549	6.992118	-7.05952	4.70E-12	2.65E-11	16.58751
TAF2	-0.8557	8.011772	-9.60139	2.21E-20	2.99E-19	35.42567
RUNX3	-0.8563	7.113696	-9.12558	1.12E-18	1.25E-17	31.56153
RPL29	-0.85677	11.70286	-7.27848	1.08E-12	6.51E-12	18.02632
SSB	-0.85721	7.893493	-8.68535	3.69E-17	3.46E-16	28.11853
TAF11	-0.85753	7.584623	-9.81243	3.70E-21	5.50E-20	37.18544
CENPK	-0.85783	5.039294	-4.27982	2.18E-05	5.67E-05	1.735653
IMPDH2	-0.85811	8.245138	-7.76754	3.55E-14	2.50E-13	21.37137

NUDT5	-0.85844	7.540819	-9.70013	9.61E-21	1.35E-19	36.24555
CCDC50	-0.85852	5.465193	-8.30503	6.83E-16	5.63E-15	25.25031
GLT8D1	-0.8586	7.260817	-10.6714	1.97E-24	4.23E-23	44.62516
EPHB1	-0.85877	5.118395	-9.08994	1.49E-18	1.65E-17	31.27794
ZNF532	-0.85964	4.910678	-10.9854	1.13E-25	2.83E-24	47.4504
GTF2H3	-0.85979	6.679424	-8.77538	1.82E-17	1.76E-16	28.81203
VSIG1	-0.85992	4.683484	-15.6629	1.67E-46	7.95E-44	94.917
RPL31	-0.8605	9.250145	-10.0331	5.57E-22	9.13E-21	39.05459
GPRIN3	-0.86081	5.854855	-7.38643	5.16E-13	3.21E-12	18.74923
TYW3	-0.86103	5.975412	-8.12004	2.72E-15	2.12E-14	23.89185
MFSD8	-0.86206	5.59544	-11.0308	7.42E-26	1.90E-24	47.86276
NIT2	-0.8621	6.413765	-8.90909	6.33E-18	6.47E-17	29.85216
MLLT3	-0.86217	5.443349	-7.51312	2.15E-13	1.39E-12	19.60883
DUSP2	-0.86333	6.747005	-6.9491	9.74E-12	5.33E-11	15.87597
BCOR	-0.8638	5.381009	-9.15432	8.84E-19	1.01E-17	31.79074
ZNF275	-0.86394	6.674531	-9.36074	1.63E-19	2.02E-18	33.45314
SERPINB9	-0.86428	7.616845	-9.65304	1.43E-20	1.98E-19	35.85376
DHRS3	-0.86435	6.774911	-9.66414	1.30E-20	1.81E-19	35.94604
PITPNA-A	-0.8657	5.612679	-10.9531	1.52E-25	3.77E-24	47.15665
KIF5C	-0.8658	5.336271	-15.6545	1.83E-46	8.29E-44	94.82458
DPY19L2P	-0.866	3.646227	-13.4286	4.51E-36	4.92E-34	71.12734
RPL15	-0.86653	9.068097	-11.6657	1.91E-28	6.95E-27	53.75271
RSAD2	-0.86705	7.294851	-2.43306	0.015267	0.025442	-4.31839
TRIT1	-0.86847	6.781007	-9.5462	3.51E-20	4.67E-19	34.97001
MDFIC	-0.86895	7.088295	-6.33215	4.79E-10	2.18E-09	12.07766
FAM174A	-0.86939	7.995331	-6.4166	2.86E-10	1.34E-09	12.57954
MCCC1	-0.86953	6.571481	-11.5857	4.10E-28	1.42E-26	52.99896
CNOT7	-0.86977	7.946904	-10.9008	2.45E-25	5.90E-24	46.68321
AKAP11	-0.86978	6.54571	-8.80519	1.44E-17	1.40E-16	29.04289
CIRH1A	-0.87001	6.767481	-8.03215	5.21E-15	3.94E-14	23.25499
DIEXF	-0.87016	5.598221	-9.47401	6.40E-20	8.28E-19	34.37698
PPIL3	-0.87038	8.077762	-8.36457	4.35E-16	3.65E-15	25.69265
OPHN1	-0.87065	9.294111	-8.99862	3.10E-18	3.29E-17	30.55532
CXCL10	-0.87084	5.389819	-5.64482	2.57E-08	9.57E-08	8.211385
LEF1-AS1	-0.87167	4.336291	-12.4878	6.31E-32	3.52E-30	61.68006
TSPYL1	-0.87183	8.943387	-9.53592	3.82E-20	5.06E-19	34.88535
SLC25A36	-0.87229	6.290662	-9.79899	4.15E-21	6.12E-20	37.07251
FAM122B	-0.87262	7.474784	-9.07401	1.69E-18	1.86E-17	31.1515
ZNHIT3	-0.87273	7.547273	-9.28782	2.98E-19	3.58E-18	32.86276
PIBF1	-0.87277	5.505811	-7.35747	6.30E-13	3.89E-12	18.55442
ZNF274	-0.87366	7.793095	-11.2883	6.78E-27	2.00E-25	50.22611
EIF3G	-0.87369	9.963063	-9.99378	7.82E-22	1.25E-20	38.71955
ZNF567	-0.87451	4.709064	-10.2663	7.28E-23	1.32E-21	41.06202
MPHOSPH	-0.87499	5.014845	-9.14713	9.37E-19	1.06E-17	31.73333
EID2B	-0.87569	4.338229	-9.84862	2.72E-21	4.09E-20	37.48996
L3MBTL3	-0.8768	6.957417	-7.97244	8.07E-15	6.00E-14	22.82547
GAR1	-0.87707	7.172158	-10.9714	1.28E-25	3.21E-24	47.32257
CMPK1	-0.87983	8.975375	-9.42918	9.28E-20	1.18E-18	34.01039
TCF7	-0.88015	8.504115	-8.43196	2.61E-16	2.24E-15	26.19635
EIF3L	-0.88036	11.62889	-10.2089	1.20E-22	2.13E-21	40.56473
SASS6	-0.88071	4.519639	-11.4644	1.29E-27	4.19E-26	51.86327
CPSF6	-0.88076	6.659955	-11.2015	1.52E-26	4.27E-25	49.42598
ADA	-0.88082	6.71759	-8.07483	3.80E-15	2.91E-14	23.56358
LOC28605	-0.88088	7.793879	-7.93018	1.10E-14	8.06E-14	22.52308
NOB1	-0.88133	7.19414	-10.0896	3.41E-22	5.71E-21	39.53854
GTF3A	-0.88172	9.511734	-11.2031	1.50E-26	4.22E-25	49.44086
ANKRA2	-0.88198	7.902459	-9.76261	5.66E-21	8.18E-20	36.76754
GALNT12	-0.88252	4.790763	-12.6505	1.25E-32	7.50E-31	63.28566

NSUN5P1	-0.88316	7.484067	-11.3286	4.65E-27	1.41E-25	50.59938
AMMECR1	-0.88316	7.680906	-8.89478	7.10E-18	7.19E-17	29.74027
LOC10192	-0.88326	6.574839	-7.80202	2.77E-14	1.97E-13	21.61392
ABCB1	-0.88327	4.463143	-12.2627	5.86E-31	2.89E-29	59.47705
LIAS	-0.88342	5.41505	-8.1674	1.92E-15	1.52E-14	24.23733
RSL1D1	-0.88524	6.835384	-9.00542	2.94E-18	3.12E-17	30.60893
SHQ1	-0.8854	6.331422	-10.9306	1.86E-25	4.55E-24	46.95287
PHLDB2	-0.88562	4.038634	-11.623	2.88E-28	1.02E-26	53.35005
AKR1B1	-0.88594	8.151826	-9.02023	2.61E-18	2.79E-17	30.72583
ARRDC3	-0.88649	9.047347	-6.48827	1.84E-10	8.80E-10	13.01005
SMYD2	-0.88663	6.074576	-16.9164	1.20E-52	1.36E-49	108.9294
CCT6A	-0.88708	7.882502	-8.13647	2.41E-15	1.89E-14	24.01154
SPIN4	-0.88711	5.112721	-6.25476	7.64E-10	3.41E-09	11.62282
MGC12488	-0.88758	4.834969	-9.19557	6.32E-19	7.33E-18	32.12076
PPAT	-0.88793	5.461664	-9.01416	2.74E-18	2.92E-17	30.67792
RBBP7	-0.88799	8.749747	-9.90105	1.74E-21	2.69E-20	37.93263
ZNF805	-0.8894	5.210083	-10.4482	1.45E-23	2.86E-22	42.65047
OTUD6B	-0.89096	5.087298	-6.63238	7.47E-11	3.72E-10	13.88809
ZNF565	-0.89117	5.731826	-8.81709	1.31E-17	1.29E-16	29.13518
EIF3E	-0.89154	8.076868	-9.70502	9.22E-21	1.30E-19	36.28636
ASF1A	-0.89232	6.910818	-8.03305	5.17E-15	3.92E-14	23.26151
NSUN6	-0.89317	6.005864	-10.4643	1.26E-23	2.50E-22	42.79129
GLOD4	-0.89338	7.471036	-8.64532	5.04E-17	4.65E-16	27.81189
TOX	-0.8935	4.706965	-9.39981	1.18E-19	1.49E-18	33.77086
KIAA1143	-0.89363	7.88133	-11.603	3.48E-28	1.21E-26	53.16201
UFSP2	-0.89398	6.554036	-9.22424	5.01E-19	5.86E-18	32.35076
GKAP1	-0.89521	5.207044	-11.2098	1.41E-26	3.99E-25	49.50254
THOC1	-0.89609	7.530099	-12.6647	1.08E-32	6.57E-31	63.42697
SMARCAD	-0.89613	6.923813	-10.056	4.56E-22	7.53E-21	39.25071
SDAD1	-0.89672	6.103665	-10.8372	4.38E-25	1.01E-23	46.10941
PAQR3	-0.89685	4.99084	-6.589	9.81E-11	4.83E-10	13.62204
IFI44L	-0.89743	6.848806	-2.58062	0.010102	0.017441	-3.95356
C12orf29	-0.89827	5.409549	-8.45562	2.18E-16	1.88E-15	26.374
IL10RA	-0.89868	9.95093	-8.68946	3.57E-17	3.35E-16	28.15006
SH2D1A	-0.90004	6.181672	-9.44608	8.07E-20	1.03E-18	34.14844
ZC2HC1A	-0.90028	4.734939	-9.25755	3.81E-19	4.53E-18	32.61865
NAA25	-0.90153	5.859756	-9.37007	1.51E-19	1.88E-18	33.52892
C15orf61	-0.90211	7.432293	-7.97486	7.93E-15	5.90E-14	22.84283
STAT1	-0.90218	10.27088	-6.02001	3.06E-09	1.27E-08	10.27322
ZNF506	-0.9034	5.178732	-9.9637	1.01E-21	1.60E-20	38.46371
CCR5	-0.90391	8.412141	-7.2625	1.20E-12	7.21E-12	17.9201
LOC10013	-0.90415	4.791245	-11.9435	1.32E-29	5.55E-28	56.39529
LOC10050	-0.90565	5.946841	-12.2599	6.02E-31	2.96E-29	59.44957
TRIM24	-0.90579	6.850752	-8.13929	2.36E-15	1.85E-14	24.03205
COQ10A	-0.90581	6.119347	-14.0603	6.05E-39	1.02E-36	77.67556
COX20	-0.90732	6.982737	-10.9204	2.05E-25	4.97E-24	46.86099
PTRH2	-0.9079	7.144599	-10.3345	3.99E-23	7.47E-22	41.65545
MAP4K1	-0.90808	7.142791	-8.11565	2.81E-15	2.18E-14	23.85995
TRAPPC10	-0.90835	6.886905	-8.46344	2.05E-16	1.78E-15	26.43277
ZSCAN29	-0.90836	6.151154	-10.799	6.20E-25	1.41E-23	45.76661
TAF15	-0.90952	6.88559	-10.1088	2.88E-22	4.86E-21	39.70328
PALB2	-0.91035	6.401325	-10.3255	4.32E-23	8.07E-22	41.57692
SRSF6	-0.9111	8.339893	-9.3604	1.64E-19	2.03E-18	33.45038
CDC16	-0.91125	7.693143	-10.284	6.22E-23	1.14E-21	41.21594
RFX7	-0.91125	6.140562	-6.80521	2.48E-11	1.30E-10	14.96302
FAM19A1	-0.91144	4.024016	-11.3728	3.07E-27	9.49E-26	51.00992
SNRPA1	-0.91167	6.895937	-10.3121	4.86E-23	9.00E-22	41.46019
IRF8	-0.91175	8.868316	-8.1739	1.82E-15	1.45E-14	24.28488

TCEAL4	-0.91276	5.924694	-7.6245	9.82E-14	6.58E-13	20.37452
PRKXP1	-0.91286	4.885114	-6.08065	2.15E-09	9.07E-09	10.61744
ZZZ3	-0.91339	6.767817	-10.0564	4.55E-22	7.51E-21	39.25403
ATF7IP2	-0.91345	6.282731	-11.6513	2.19E-28	7.92E-27	53.61732
LOC10012	-0.91396	6.687735	-10.1441	2.12E-22	3.62E-21	40.00654
BTBD11	-0.9142	4.926694	-12.1246	2.27E-30	1.05E-28	58.13767
TAGAP	-0.91482	9.273322	-7.86948	1.71E-14	1.23E-13	22.09098
USP53	-0.91572	4.211616	-11.501	9.16E-28	3.06E-26	52.20504
SCFD2	-0.91579	6.000458	-10.6338	2.76E-24	5.84E-23	44.28994
POP5	-0.91629	7.453198	-8.47914	1.82E-16	1.59E-15	26.55093
PCSK5	-0.91645	5.188219	-7.96003	8.83E-15	6.54E-14	22.73658
RPGRIP1	-0.9166	6.119546	-11.0638	5.47E-26	1.42E-24	48.16367
C14orf169	-0.91753	7.287239	-11.5958	3.73E-28	1.30E-26	53.09385
PAPD7	-0.91805	7.737926	-11.4846	1.07E-27	3.53E-26	52.0518
LOC15368	-0.91956	4.291481	-9.87077	2.25E-21	3.42E-20	37.67674
PSMA5	-0.92061	6.811393	-11.3483	3.86E-27	1.18E-25	50.78203
LOC10192	-0.9214	3.391101	-9.41749	1.02E-19	1.29E-18	33.915
CMTR2	-0.92144	6.683959	-6.66359	6.13E-11	3.09E-10	14.08048
ACVR2A	-0.92168	5.557817	-10.6314	2.82E-24	5.95E-23	44.26922
CDC42SE2	-0.92171	11.4306	-12.7084	6.97E-33	4.38E-31	63.86083
CD1C	-0.92206	7.222881	-7.77713	3.32E-14	2.34E-13	21.43871
NSA2	-0.92207	10.2204	-10.9374	1.75E-25	4.31E-24	47.01423
NPIPA1	-0.92372	9.35226	-8.52619	1.27E-16	1.12E-15	26.90602
PBX4	-0.9242	5.783917	-13.0122	3.23E-34	2.65E-32	66.89909
EPS8	-0.92527	4.487381	-5.62276	2.90E-08	1.07E-07	8.093811
MAP4K3	-0.92694	4.431716	-6.36407	3.94E-10	1.82E-09	12.2667
PHYH	-0.92724	5.496566	-7.0628	4.60E-12	2.59E-11	16.60876
ZNF320	-0.92729	5.171522	-8.90314	6.64E-18	6.75E-17	29.8056
MRPS9	-0.92819	6.064389	-8.62123	6.08E-17	5.56E-16	27.62793
EIF3J-AS1	-0.92837	5.856499	-11.3239	4.86E-27	1.47E-25	50.55555
GPR133	-0.92933	4.406609	-10.4604	1.31E-23	2.58E-22	42.7577
ACADSB	-0.9294	4.867853	-12.4694	7.58E-32	4.15E-30	61.49916
DTD2	-0.92995	5.904575	-7.79606	2.90E-14	2.05E-13	21.57192
RABGAP1L	-0.93014	7.48517	-9.82239	3.40E-21	5.08E-20	37.26921
LOC10013	-0.93168	7.487987	-8.45577	2.17E-16	1.88E-15	26.37509
SH3BP5-A	-0.9327	6.466294	-15.361	4.71E-45	1.74E-42	91.60678
KIT	-0.93314	5.2747	-10.1731	1.65E-22	2.85E-21	40.25624
KLF3-AS1	-0.93372	4.112145	-12.3314	2.98E-31	1.50E-29	60.1464
DDX10	-0.93372	6.657672	-9.84674	2.76E-21	4.16E-20	37.47411
PAQR8	-0.93407	7.175896	-11.7479	8.71E-29	3.31E-27	54.53104
SLC25A45	-0.93451	6.942765	-13.068	1.83E-34	1.56E-32	67.46179
YAE1D1	-0.93624	5.719276	-9.95383	1.10E-21	1.74E-20	38.37985
C9orf91	-0.93909	5.856063	-8.03257	5.19E-15	3.93E-14	23.25802
CTSW	-0.93915	8.234675	-5.25864	2.03E-07	6.83E-07	6.21387
UAP1	-0.93922	6.588452	-5.54633	4.40E-08	1.60E-07	7.689798
NAP1L1	-0.9402	10.09637	-8.66468	4.34E-17	4.03E-16	27.96002
GBP1	-0.94187	8.101213	-4.50786	7.90E-06	2.17E-05	2.702632
LOC10027	-0.94204	7.09184	-7.8683	1.72E-14	1.24E-13	22.08259
OXCT1	-0.94207	6.25615	-9.32487	2.20E-19	2.68E-18	33.16226
MYC	-0.9421	8.939841	-7.39522	4.86E-13	3.03E-12	18.8085
POLR1B	-0.9427	6.590543	-10.6437	2.53E-24	5.37E-23	44.37849
APOL3	-0.94357	7.125251	-8.16297	1.98E-15	1.56E-14	24.20495
DCK	-0.94371	8.226156	-8.72773	2.65E-17	2.52E-16	28.44427
LETMD1	-0.94467	7.446848	-12.0648	4.07E-30	1.82E-28	57.56013
XPA	-0.94499	7.001677	-10.4377	1.60E-23	3.13E-22	42.55798
PHOSPHO	-0.94517	4.009933	-8.27489	8.57E-16	6.97E-15	25.0273
ANGPT1	-0.9454	4.577125	-7.6667	7.29E-14	4.93E-13	20.66703
KIZ	-0.94629	6.100978	-10.6839	1.76E-24	3.80E-23	44.73605

LRRC69	-0.94703	4.884627	-11.1827	1.82E-26	5.00E-25	49.25304
TMEM128	-0.94773	6.467217	-9.1762	7.40E-19	8.50E-18	31.96569
CEP68	-0.94781	6.266831	-12.6958	7.91E-33	4.91E-31	63.73492
DIMT1	-0.94795	6.214419	-11.7057	1.30E-28	4.81E-27	54.13138
DNAAF2	-0.94799	5.957188	-8.17318	1.83E-15	1.45E-14	24.27959
PLAG1	-0.94899	4.764419	-9.17022	7.77E-19	8.90E-18	31.91784
C6orf48	-0.94927	8.834082	-9.37869	1.41E-19	1.76E-18	33.59894
MCM3AP-	-0.94953	4.276246	-13.6081	7.00E-37	8.74E-35	72.97216
SLC16A10	-0.95055	4.23715	-12.7069	7.07E-33	4.43E-31	63.84576
MRPS35	-0.9508	7.651641	-8.75123	2.20E-17	2.11E-16	28.62542
TESPA1	-0.95171	5.795927	-11.3452	3.98E-27	1.22E-25	50.75315
HLA-F-AS	-0.95193	6.348496	-9.67907	1.15E-20	1.60E-19	36.07016
SEPT7P2	-0.95223	3.934983	-16.2155	3.44E-49	2.23E-46	101.0438
MIR302B	-0.95223	5.046579	-8.30126	7.02E-16	5.79E-15	25.2224
DPY19L4	-0.95308	4.30003	-10.6265	2.95E-24	6.21E-23	44.22565
TRMT10C	-0.95382	7.347895	-9.4414	8.39E-20	1.07E-18	34.11017
HLA-DMA	-0.9539	8.636284	-6.30212	5.74E-10	2.60E-09	11.90057
NOP14	-0.95435	6.690395	-10.0198	6.24E-22	1.02E-20	38.9415
EIF1AX	-0.95436	7.138575	-7.87851	1.60E-14	1.16E-13	22.15511
SRSF7	-0.95437	8.091291	-11.7726	6.87E-29	2.66E-27	54.76474
TAF3	-0.95458	5.69971	-11.9043	1.93E-29	7.94E-28	56.01958
ZNF14	-0.95735	6.563263	-9.17382	7.55E-19	8.65E-18	31.9466
SBK1	-0.95766	6.117729	-11.2918	6.56E-27	1.94E-25	50.25872
RPL36	-0.95873	10.35162	-6.92455	1.14E-11	6.20E-11	15.71905
ETAA1	-0.95906	4.911357	-10.8716	3.20E-25	7.58E-24	46.41986
KIAA0907	-0.96011	6.906136	-13.3525	9.90E-36	1.02E-33	70.3495
NFATC2	-0.96026	6.097492	-12.866	1.42E-33	1.02E-31	65.43228
LOC10192	-0.96033	4.622923	-12.9259	7.77E-34	5.81E-32	66.03184
LOC10050	-0.96101	5.468583	-14.6891	7.24E-42	1.68E-39	84.33862
GDPD1	-0.96118	4.113901	-9.87723	2.13E-21	3.25E-20	37.73134
DDX24	-0.9625	7.889793	-10.9318	1.84E-25	4.51E-24	46.96429
EIF2D	-0.96295	7.979032	-11.146	2.56E-26	6.89E-25	48.91554
TOMM70A	-0.96349	6.944241	-9.04697	2.10E-18	2.28E-17	30.9372
TAF5	-0.96381	6.747773	-10.2156	1.13E-22	2.01E-21	40.62331
CPOX	-0.96408	6.50654	-8.34648	4.99E-16	4.17E-15	25.55804
PRMT3	-0.96408	5.617089	-7.17528	2.17E-12	1.26E-11	17.34359
ZNF823	-0.96432	3.948791	-10.2446	8.80E-23	1.59E-21	40.87391
ARHGAP5	-0.96513	4.456204	-10.9355	1.78E-25	4.37E-24	46.99723
CCDC91	-0.96531	6.775869	-7.72452	4.83E-14	3.34E-13	21.06996
STMN3	-0.96587	5.783536	-14.1906	1.52E-39	2.72E-37	79.04472
EXOSC6	-0.96684	6.802046	-8.90679	6.45E-18	6.58E-17	29.83415
TMEM200	-0.96687	5.671896	-8.43714	2.51E-16	2.16E-15	26.23521
TTC37	-0.96959	6.631019	-7.85178	1.94E-14	1.39E-13	21.96553
CKMT2-AS	-0.97155	4.604599	-12.037	5.33E-30	2.33E-28	57.29258
PHC1	-0.97215	6.681584	-11.1558	2.33E-26	6.34E-25	49.00631
ABCG1	-0.97315	5.72679	-7.12789	2.98E-12	1.71E-11	17.03282
PASK	-0.97339	5.859786	-10.2197	1.10E-22	1.95E-21	40.65823
EIF2A	-0.97349	8.878566	-9.79408	4.33E-21	6.37E-20	37.03137
C1orf109	-0.97365	4.432776	-8.69576	3.40E-17	3.19E-16	28.19837
ECI2	-0.97366	6.212048	-9.19201	6.51E-19	7.54E-18	32.09219
RNF144A	-0.97397	6.917162	-8.84235	1.08E-17	1.07E-16	29.33146
NFXL1	-0.97409	6.162234	-5.8126	1.01E-08	3.92E-08	9.118762
ANKRD12	-0.97429	7.728958	-11.0915	4.23E-26	1.11E-24	48.41684
RCN2	-0.97526	7.344933	-9.05043	2.05E-18	2.22E-17	30.96459
PLA2G7	-0.97567	5.033832	-5.69054	1.99E-08	7.52E-08	8.456293
LINC00926	-0.97575	6.711862	-8.09184	3.35E-15	2.58E-14	23.68691
LSG1	-0.97678	6.45532	-12.7805	3.37E-33	2.28E-31	64.5779
NPM1	-0.97858	10.12607	-10.9	2.47E-25	5.94E-24	46.67605

LOC14547	-0.97916	5.269962	-5.77881	1.22E-08	4.70E-08	8.934124
ASTE1	-0.98016	6.848897	-10.8945	2.59E-25	6.21E-24	46.62682
LOC10013	-0.98046	6.360878	-11.9185	1.68E-29	6.98E-28	56.15609
GABPB2	-0.98187	7.661811	-10.9301	1.87E-25	4.57E-24	46.94821
LRRC47	-0.98203	8.986603	-12.5571	3.17E-32	1.83E-30	62.36254
BANP	-0.98328	8.627851	-10.9412	1.69E-25	4.18E-24	47.04892
NOL9	-0.98574	7.669897	-12.0753	3.67E-30	1.65E-28	57.66207
ANXA2R	-0.9862	6.85606	-9.99771	7.56E-22	1.22E-20	38.75298
LPAR6	-0.98775	8.606647	-7.19699	1.88E-12	1.10E-11	17.48653
PGRMC2	-0.98813	5.989079	-9.47008	6.61E-20	8.53E-19	34.34477
KLF10	-0.98814	8.197211	-7.05813	4.75E-12	2.67E-11	16.57845
PNISR	-0.9884	8.293309	-11.4722	1.20E-27	3.93E-26	51.93559
TCEAL3	-0.98855	6.90203	-11.3249	4.81E-27	1.46E-25	50.56487
LCK	-0.98866	8.578131	-6.12133	1.69E-09	7.21E-09	10.85011
TTC14	-0.98932	6.64978	-10.0408	5.20E-22	8.54E-21	39.12079
ADRB2	-0.99194	8.083127	-8.96362	4.10E-18	4.28E-17	30.27977
KIF3A	-0.99429	4.622357	-12.27	5.45E-31	2.70E-29	59.54827
MTX3	-0.99522	5.448546	-13.0321	2.64E-34	2.19E-32	67.10006
GNPNAT1	-0.99568	5.881787	-9.70167	9.49E-21	1.33E-19	36.2584
PRKRA	-0.99576	5.301317	-10.6469	2.45E-24	5.23E-23	44.40702
BZW2	-0.99596	8.213864	-9.13865	1.00E-18	1.13E-17	31.66571
NRROS	-0.99667	7.648495	-8.72793	2.65E-17	2.52E-16	28.44583
RPL10A	-0.99761	12.05296	-10.4248	1.79E-23	3.48E-22	42.44523
NCALD	-1.00118	5.627699	-11.7864	6.02E-29	2.34E-27	54.89575
PEX3	-1.00126	5.177178	-11.8705	2.68E-29	1.08E-27	55.69706
NUCKS1	-1.00142	7.101879	-12.5008	5.55E-32	3.11E-30	61.80784
CLEC2D	-1.00237	5.728811	-8.6075	6.76E-17	6.16E-16	27.52327
CUTA	-1.00334	9.088339	-10.9406	1.70E-25	4.20E-24	47.04346
IKZF2	-1.00481	5.073292	-9.63281	1.70E-20	2.33E-19	35.68591
LAT	-1.00489	7.552263	-8.77197	1.87E-17	1.81E-16	28.78564
PVRIG	-1.00524	8.244974	-8.08168	3.62E-15	2.78E-14	23.6132
SUMF2	-1.00555	7.233347	-12.4321	1.10E-31	5.90E-30	61.13281
MALT1	-1.00561	6.771892	-9.29921	2.71E-19	3.27E-18	32.95476
ZMYM1	-1.00634	5.29238	-9.04919	2.07E-18	2.24E-17	30.95485
EPB41L4A-	-1.007	5.80037	-12.1134	2.53E-30	1.16E-28	58.02947
ERAP2	-1.00732	6.660759	-4.62221	4.67E-06	1.33E-05	3.205148
MS4A14	-1.00835	6.396376	-7.29637	9.57E-13	5.80E-12	18.14551
CBLB	-1.00841	5.215617	-10.3669	2.99E-23	5.65E-22	41.93812
BBS10	-1.00848	5.226424	-8.12212	2.68E-15	2.09E-14	23.90697
PHF10	-1.01095	6.974494	-10.1384	2.23E-22	3.80E-21	39.95724
IL32	-1.01134	8.534864	-4.84167	1.65E-06	4.96E-06	4.202309
SLC38A1	-1.01451	7.086438	-11.4989	9.34E-28	3.11E-26	52.18516
LINC0095[illegible]	-1.01466	5.052253	-12.5882	2.32E-32	1.35E-30	62.66986
HOXB-AS[illegible]	-1.01566	5.087527	-13.2626	2.50E-35	2.39E-33	69.43334
CCR6	-1.01612	4.678116	-7.27468	1.11E-12	6.67E-12	18.00103
FAM98B	-1.01706	6.796121	-9.01855	2.64E-18	2.83E-17	30.71251
MRPL3	-1.01717	8.782248	-8.54732	1.08E-16	9.60E-16	27.06595
CECR1	-1.0173	10.43362	-7.66127	7.57E-14	5.11E-13	20.62933
TMEM243	-1.01771	8.235625	-8.15962	2.03E-15	1.60E-14	24.18046
RPL22	-1.01775	11.12405	-11.3696	3.16E-27	9.75E-26	50.97958
GTPBP3	-1.02061	6.570872	-16.2228	3.17E-49	2.21E-46	101.1256
ZNF766	-1.02109	6.886845	-11.5177	7.82E-28	2.63E-26	52.36137
EBAG9	-1.02162	7.359191	-8.82259	1.26E-17	1.24E-16	29.17793
AGPAT5	-1.02226	5.653081	-8.44604	2.34E-16	2.02E-15	26.30201
CD247	-1.02228	8.571942	-6.42432	2.73E-10	1.28E-09	12.62572
ENPP5	-1.02274	3.401186	-13.237	3.25E-35	3.05E-33	69.17346
NOL8	-1.02286	7.505909	-10.3318	4.09E-23	7.65E-22	41.63151
URI1	-1.02314	6.317459	-11.1707	2.03E-26	5.56E-25	49.14246

TMEM181	-1.02434	7.336236	-9.68937	1.05E-20	1.47E-19	36.15589
ANKMY2	-1.02496	7.585666	-10.2413	9.06E-23	1.63E-21	40.84529
ZNF85	-1.02528	6.398928	-12.7184	6.30E-33	4.01E-31	63.95959
CD52	-1.02704	10.27402	-5.65836	2.38E-08	8.91E-08	8.283727
PPP1CC	-1.02708	10.54512	-10.9921	1.06E-25	2.68E-24	47.51123
SATB1	-1.02755	7.461054	-8.95471	4.40E-18	4.57E-17	30.20978
ALDH1A1	-1.02785	5.1123	-6.90436	1.31E-11	7.02E-11	15.59034
NDUFAF4	-1.02822	5.620317	-11.1215	3.21E-26	8.57E-25	48.69131
TADA1	-1.02839	6.443397	-9.33699	1.99E-19	2.43E-18	33.26048
LYAR	-1.02928	7.017136	-11.4692	1.24E-27	4.03E-26	51.90804
ST3GAL5	-1.03065	7.186908	-9.86923	2.28E-21	3.47E-20	37.66378
CCDC66	-1.03135	5.668694	-9.48711	5.74E-20	7.46E-19	34.48434
EIF3H	-1.03242	7.801472	-10.4868	1.03E-23	2.06E-22	42.98954
WHAMM	-1.03316	7.113591	-12.3534	2.39E-31	1.22E-29	60.36172
ICOS	-1.03385	5.401923	-8.81347	1.35E-17	1.32E-16	29.10712
LOC72839	-1.03513	8.911385	-7.63906	8.86E-14	5.96E-13	20.47527
ZNF304	-1.03575	4.361209	-10.0132	6.61E-22	1.08E-20	38.88488
PPIL1	-1.03597	5.682965	-8.34253	5.14E-16	4.29E-15	25.52862
ATIC	-1.03603	7.858265	-8.33725	5.35E-16	4.46E-15	25.48943
ZBTB41	-1.03731	5.817133	-6.95581	9.32E-12	5.11E-11	15.91893
RPL37A	-1.03745	8.864357	-12.1625	1.57E-30	7.33E-29	58.50396
SETBP1	-1.03772	5.357226	-7.66364	7.45E-14	5.03E-13	20.6458
GIMAP4	-1.04008	10.3522	-7.96429	8.56E-15	6.35E-14	22.76709
SLC20A1	-1.0401	8.325917	-12.1159	2.47E-30	1.13E-28	58.05378
AHSA2	-1.04029	6.628231	-13.6858	3.11E-37	4.14E-35	73.77517
TRIM52	-1.04059	7.271834	-8.67999	3.85E-17	3.59E-16	28.07736
HLA-DQB:	-1.04074	5.999853	-6.35447	4.18E-10	1.92E-09	12.20974
ABCE1	-1.04304	6.758046	-7.98104	7.58E-15	5.65E-14	22.8872
GPALPP1	-1.04526	5.050939	-10.8524	3.81E-25	8.98E-24	46.24673
NAE1	-1.04582	7.819778	-10.3161	4.69E-23	8.72E-22	41.49537
POC5	-1.04676	6.651774	-13.5313	1.56E-36	1.83E-34	72.18152
MAF	-1.04731	5.855402	-10.6096	3.44E-24	7.16E-23	44.07537
ZNF32	-1.04745	6.000577	-9.99559	7.70E-22	1.24E-20	38.73493
METTL18	-1.04934	6.692864	-8.72783	2.65E-17	2.52E-16	28.44505
EXOSC10	-1.04949	6.848199	-11.0825	4.60E-26	1.21E-24	48.33471
XCL1	-1.05034	5.00162	-12.7954	2.90E-33	1.99E-31	64.72638
PUS7	-1.0517	5.355119	-8.6753	3.99E-17	3.72E-16	28.04146
FAM171A:	-1.05197	5.803846	-11.8774	2.50E-29	1.01E-27	55.76293
MAML2	-1.05241	6.374972	-8.8699	8.65E-18	8.66E-17	29.54605
CAAP1	-1.05405	5.549511	-9.30845	2.51E-19	3.04E-18	33.02938
FTO	-1.05426	7.374501	-11.6104	3.24E-28	1.14E-26	53.23123
ZNF542P	-1.05624	4.713847	-8.7925	1.59E-17	1.55E-16	28.94457
ZNF813	-1.05628	4.45856	-11.5034	8.95E-28	2.99E-26	52.22752
TRIAP1	-1.05742	7.545335	-11.0374	6.98E-26	1.80E-24	47.92357
HLA-DPB1	-1.05786	7.114311	-9.24851	4.11E-19	4.86E-18	32.54592
OPTN	-1.05796	6.967946	-8.54518	1.09E-16	9.76E-16	27.04976
TIGIT	-1.05975	4.96331	-11.8293	3.98E-29	1.59E-27	55.30431
MAP3K4	-1.06156	7.52996	-11.0266	7.71E-26	1.97E-24	47.82521
RPL14	-1.06214	9.715657	-14.0797	4.93E-39	8.35E-37	77.87844
CAMK2D	-1.06308	5.51171	-10.6295	2.87E-24	6.05E-23	44.25179
SUPV3L1	-1.06377	6.217741	-12.7423	4.96E-33	3.21E-31	64.19734
GPR114	-1.06786	5.884524	-8.77333	1.85E-17	1.79E-16	28.79616
CLNS1A	-1.06856	7.286936	-12.3645	2.14E-31	1.11E-29	60.47066
DDX60	-1.06862	8.017935	-5.17655	3.10E-07	1.02E-06	5.805844
NUP107	-1.0687	7.628556	-10.6206	3.11E-24	6.52E-23	44.17321
HOOK1	-1.06893	4.49154	-12.7454	4.80E-33	3.13E-31	64.22853
TGFBI	-1.06966	9.708638	-5.66439	2.31E-08	8.65E-08	8.316016
ZNF383	-1.07025	5.153515	-10.574	4.73E-24	9.70E-23	43.7592

CRYBG3	-1.07055	6.275885	-7.55944	1.55E-13	1.02E-12	19.92616
KIAA0355	-1.07064	6.928157	-9.53011	4.01E-20	5.30E-19	34.83758
CLC	-1.07088	11.13193	-4.39077	1.34E-05	3.57E-05	2.20026
LOC28307	-1.07498	7.889299	-6.76678	3.18E-11	1.64E-10	14.72198
TFB2M	-1.07505	6.543599	-9.50515	4.94E-20	6.45E-19	34.63242
ICE1	-1.0755	7.914773	-10.6089	3.46E-24	7.20E-23	44.069
AGK	-1.07569	6.252459	-11.0818	4.63E-26	1.21E-24	48.32815
EIF5B	-1.07666	5.653524	-12.7164	6.43E-33	4.07E-31	63.94029
INTS2	-1.07704	5.519151	-8.1635	1.97E-15	1.56E-14	24.2088
P2RY8	-1.0779	9.376747	-10.2909	5.86E-23	1.08E-21	41.27591
LOC10028	-1.07907	4.192712	-12.7501	4.58E-33	3.02E-31	64.275
LOC10050	-1.08126	5.446529	-8.57981	8.38E-17	7.59E-16	27.31251
PCMTD2	-1.08157	6.883913	-13.0861	1.52E-34	1.33E-32	67.6441
BCL11A	-1.08232	7.000702	-8.82404	1.24E-17	1.22E-16	29.18918
LYRM7	-1.08234	5.768607	-9.9051	1.68E-21	2.60E-20	37.96687
BLNK	-1.08278	5.900432	-5.261	2.01E-07	6.75E-07	6.225706
ZNHIT6	-1.08327	4.795259	-11.3861	2.71E-27	8.42E-26	51.13313
TOMM20	-1.08417	8.741372	-11.6493	2.24E-28	8.06E-27	53.5984
KATNBL1	-1.0842	7.339982	-6.78338	2.86E-11	1.49E-10	14.82593
ALMS1	-1.08505	6.509339	-12.0402	5.17E-30	2.26E-28	57.32373
FYN	-1.08629	8.346122	-9.19193	6.51E-19	7.54E-18	32.09155
LINC0034‌2	-1.08826	4.748631	-11.2049	1.48E-26	4.15E-25	49.45751
SIGLEC17F	-1.08989	5.840386	-14.3634	2.40E-40	4.74E-38	80.86945
BTN3A1	-1.09031	8.10423	-9.03689	2.28E-18	2.46E-17	30.8575
OLIG1	-1.09176	7.762753	-5.83315	8.96E-09	3.51E-08	9.231571
DNMT1	-1.09274	8.080224	-10.6754	1.90E-24	4.09E-23	44.66051
TPP2	-1.09417	7.864668	-11.2253	1.22E-26	3.48E-25	49.6453
ZBTB21	-1.0973	6.476725	-8.82649	1.22E-17	1.20E-16	29.20818
MOB1B	-1.09743	7.49344	-8.34116	5.20E-16	4.34E-15	25.51845
PSIP1	-1.09767	7.855855	-12.0761	3.64E-30	1.64E-28	57.66918
LDOC1L	-1.0981	7.09736	-10.3845	2.56E-23	4.86E-22	42.09169
TIGD2	-1.09879	5.563284	-8.36588	4.31E-16	3.62E-15	25.70243
RNF125	-1.10098	6.690966	-9.01041	2.82E-18	3.01E-17	30.64832
ZC3H8	-1.10124	5.270895	-11.92	1.66E-29	6.89E-28	56.17036
TSHZ1	-1.10201	6.939833	-9.53111	3.98E-20	5.26E-19	34.84579
BANK1	-1.10207	6.773729	-6.4155	2.88E-10	1.35E-09	12.57299
RTTN	-1.10217	5.911035	-11.8739	2.59E-29	1.05E-27	55.72968
ZNF559	-1.10306	6.889685	-8.27952	8.27E-16	6.74E-15	25.06157
ZNF529	-1.10504	5.270598	-12.236	7.61E-31	3.73E-29	59.21785
RINT1	-1.10515	5.178062	-9.43876	8.57E-20	1.09E-18	34.08862
DROSHA	-1.10533	6.809902	-11.3375	4.28E-27	1.30E-25	50.68166
ZNF211	-1.10698	7.156615	-11.7247	1.09E-28	4.06E-27	54.31088
ZNF84	-1.10773	5.706599	-12.9514	6.00E-34	4.62E-32	66.28735
CDKN1C	-1.10841	6.290852	-7.1047	3.48E-12	1.98E-11	16.88135
MYCBP2	-1.10863	9.632846	-12.845	1.76E-33	1.25E-31	65.22199
CPA3	-1.11046	6.026951	-6.55809	1.19E-10	5.81E-10	13.43337
ZNF195	-1.11085	5.757209	-9.85166	2.65E-21	3.99E-20	37.51556
POGLUT1	-1.11101	6.745889	-10.6463	2.47E-24	5.26E-23	44.40088
KISS1R	-1.1111	5.04681	-9.12628	1.11E-18	1.24E-17	31.56711
C14orf28	-1.11191	5.14211	-12.2326	7.87E-31	3.84E-29	59.18402
HACL1	-1.11241	6.572017	-12.5262	4.31E-32	2.45E-30	62.0574
HERC6	-1.11292	6.304644	-7.41285	4.30E-13	2.69E-12	18.92746
CHST7	-1.11574	6.450967	-9.13243	1.06E-18	1.19E-17	31.61609
KIAA1671	-1.11599	4.866267	-9.197	6.25E-19	7.25E-18	32.13222
FIGNL1	-1.11758	5.924227	-7.57845	1.36E-13	8.95E-13	20.0568
ESF1	-1.11851	5.226773	-8.91654	5.97E-18	6.12E-17	29.91049
SUCLG2	-1.11855	7.261068	-9.42086	9.95E-20	1.26E-18	33.94249
PTCD3	-1.11871	6.1801	-11.7953	5.53E-29	2.16E-27	54.98034

WWP1	-1.1193	7.067079	-9.14073	9.87E-19	1.12E-17	31.6823
GBP4	-1.12101	5.905106	-6.9672	8.65E-12	4.75E-11	15.99193
TTC39C	-1.12241	6.573353	-11.7017	1.36E-28	4.98E-27	54.09279
SERINC5	-1.12274	8.987289	-10.1615	1.82E-22	3.13E-21	40.1563
ZNF184	-1.12297	5.999683	-8.53491	1.18E-16	1.05E-15	26.97198
CFD	-1.12381	9.294168	-5.61209	3.08E-08	1.14E-07	8.037131
ZFAND1	-1.12391	6.800239	-7.97148	8.13E-15	6.04E-14	22.81864
C10orf128	-1.12545	7.574489	-6.83429	2.06E-11	1.09E-10	15.14622
FAM213B	-1.12622	6.006004	-13.362	8.97E-36	9.34E-34	70.44675
ERCC5	-1.1271	7.778717	-13.6081	7.00E-37	8.74E-35	72.97216
ARMCX5	-1.12819	5.354141	-10.2095	1.20E-22	2.12E-21	40.57048
MAK16	-1.12831	6.592817	-10.5593	5.39E-24	1.10E-22	43.62976
CTDSPL2	-1.12987	6.40098	-12.6192	1.70E-32	1.01E-30	62.97599
EVL	-1.13137	8.076242	-7.21797	1.63E-12	9.60E-12	17.62504
MFHAS1	-1.1314	6.908186	-9.3627	1.61E-19	1.99E-18	33.46901
TRIM44	-1.13191	7.953031	-11.6637	1.95E-28	7.06E-27	53.73458
RHOH	-1.13224	7.544498	-9.08382	1.56E-18	1.73E-17	31.22936
LOC20202	-1.13538	7.664348	-12.4053	1.43E-31	7.61E-30	60.86956
IFIT3	-1.13571	9.318545	-4.13624	4.04E-05	0.000102	1.151015
LINC0087	-1.13714	5.826712	-7.5163	2.10E-13	1.36E-12	19.63056
IARS	-1.13743	8.211023	-9.40824	1.10E-19	1.39E-18	33.83958
BZRAP1-A	-1.13885	7.120611	-10.4121	2.01E-23	3.85E-22	42.33358
ZNF664	-1.13919	7.441715	-10.6072	3.51E-24	7.29E-23	44.05386
CMSS1	-1.1409	5.61247	-9.83397	3.08E-21	4.62E-20	37.36663
EPSTI1	-1.14143	7.271835	-4.6755	3.64E-06	1.05E-05	3.443333
SET	-1.14178	10.0768	-13.4792	2.67E-36	3.06E-34	71.64675
LRPPRC	-1.14254	7.076369	-11.3977	2.43E-27	7.57E-26	51.24145
DKFZP586	-1.14292	7.819203	-13.5314	1.55E-36	1.83E-34	72.18282
SRSF11	-1.14301	6.672062	-13.1429	8.52E-35	7.57E-33	68.21847
TIGD3	-1.14319	6.120962	-9.1462	9.44E-19	1.07E-17	31.72595
ZBTB4	-1.14336	7.447743	-13.6577	4.17E-37	5.44E-35	73.4842
CCR7	-1.14573	8.171165	-6.89863	1.35E-11	7.28E-11	15.55391
SDR39U1	-1.14584	8.085008	-11.4827	1.09E-27	3.59E-26	52.03377
SLFN5	-1.14767	7.634501	-9.33416	2.03E-19	2.49E-18	33.23753
MAGEH1	-1.14777	6.680307	-9.62642	1.79E-20	2.45E-19	35.63292
OXNAD1	-1.15007	7.276541	-9.89083	1.90E-21	2.91E-20	37.84619
PJA1	-1.15283	7.47172	-11.6919	1.49E-28	5.46E-27	54.0009
ARHGEF3	-1.15291	9.271272	-10.7666	8.32E-25	1.86E-23	45.47538
6-Sep	-1.15329	6.957959	-12.4683	7.67E-32	4.18E-30	61.48804
DCP1B	-1.15482	6.565777	-9.67518	1.19E-20	1.65E-19	36.03783
ANKRD46	-1.15511	5.85727	-7.74162	4.28E-14	2.98E-13	21.18959
DOCK10	-1.15552	6.131842	-10.7933	6.52E-25	1.48E-23	45.71543
FASTKD1	-1.15561	7.163054	-10.8331	4.54E-25	1.05E-23	46.07323
NHS	-1.15596	5.031695	-9.98589	8.37E-22	1.34E-20	38.65237
TBX21	-1.15863	7.150706	-7.80254	2.76E-14	1.96E-13	21.61756
KLF11	-1.16187	6.626922	-7.33578	7.31E-13	4.48E-12	18.40892
PRORSD1I	-1.16221	4.367755	-14.3429	2.99E-40	5.77E-38	80.65301
LOC33862	-1.16223	3.953235	-11.152	2.42E-26	6.56E-25	48.97071
KIAA1147	-1.16323	6.548424	-13.0564	2.06E-34	1.75E-32	67.3443
CCAR1	-1.16428	6.58322	-13.0797	1.62E-34	1.41E-32	67.58017
ENOSF1	-1.16722	5.676016	-14.8295	1.58E-42	4.15E-40	85.84589
TSPAN13	-1.1709	7.043909	-6.68262	5.43E-11	2.75E-10	14.1981
ACKR3	-1.17191	4.773351	-8.90223	6.69E-18	6.79E-17	29.79848
HLA-DRA	-1.17324	10.68307	-6.63671	7.27E-11	3.62E-10	13.91475
LDHB	-1.17324	10.95305	-10.311	4.91E-23	9.07E-22	41.45047
AP3M2	-1.17433	6.404673	-13.8062	8.82E-38	1.24E-35	75.02262
GOLGA8N	-1.1754	10.23234	-13.7286	1.99E-37	2.75E-35	74.21812
PCED1B	-1.17645	7.68779	-8.75063	2.21E-17	2.12E-16	28.62085

SRSF8	-1.17678	6.748472	-12.4519	9.02E-32	4.89E-30	61.32692
GVINP1	-1.17919	8.059164	-8.90488	6.55E-18	6.68E-17	29.81922
ENOPH1	-1.17954	7.508862	-10.0746	3.88E-22	6.47E-21	39.40925
TAF1D	-1.18245	6.44666	-10.1395	2.21E-22	3.77E-21	39.96693
RIOK1	-1.1843	6.297755	-9.75462	6.06E-21	8.73E-20	36.70061
ITGB3BP	-1.18806	6.621141	-10.5424	6.28E-24	1.28E-22	43.47967
OSBPL3	-1.18867	5.42036	-10.7603	8.81E-25	1.96E-23	45.41914
CMC1	-1.19138	6.902934	-10.5629	5.23E-24	1.07E-22	43.66114
LINC00936	-1.19154	8.297556	-8.83206	1.17E-17	1.15E-16	29.25144
ZCCHC7	-1.19305	7.026987	-9.31756	2.33E-19	2.84E-18	33.10311
BACH2	-1.19426	6.409149	-7.49987	2.35E-13	1.52E-12	19.51836
MPHOSPH	-1.19714	7.269637	-12.0582	4.34E-30	1.92E-28	57.49667
TTC39B	-1.1996	4.226661	-14.7282	4.74E-42	1.12E-39	84.75763
MS4A1	-1.20097	7.581297	-5.23385	2.31E-07	7.72E-07	6.090068
WDR75	-1.20106	6.128282	-12.2891	4.52E-31	2.25E-29	59.73401
ZNF146	-1.20411	8.579451	-11.315	5.28E-27	1.58E-25	50.47366
CRYZ	-1.2048	6.401726	-8.02156	5.63E-15	4.25E-14	23.17863
ARL4C	-1.20537	7.649375	-10.8934	2.62E-25	6.26E-24	46.61676
NUP35	-1.20683	5.418621	-8.86428	9.04E-18	9.03E-17	29.5022
PTGDR	-1.2079	4.467077	-12.8165	2.35E-33	1.62E-31	64.93761
LRBA	-1.20825	6.985574	-9.2441	4.26E-19	5.03E-18	32.51042
PKIA	-1.21029	5.039952	-10.0474	4.92E-22	8.10E-21	39.17688
DNAJC2	-1.21171	7.419596	-13.1528	7.70E-35	6.94E-33	68.31879
ZNF26	-1.21254	6.371986	-13.9319	2.35E-38	3.58E-36	76.33215
PRIMPOL	-1.21608	6.66512	-11.4041	2.29E-27	7.16E-26	51.30052
JMY	-1.21704	5.336432	-9.81157	3.73E-21	5.53E-20	37.17821
FAM179B	-1.2178	5.651451	-8.68366	3.74E-17	3.50E-16	28.1055
PFAS	-1.22086	5.349715	-9.97927	8.86E-22	1.41E-20	38.59604
TXNDC16	-1.22165	5.394332	-10.2148	1.14E-22	2.02E-21	40.61636
NAP1L2	-1.22241	3.839377	-13.9778	1.45E-38	2.28E-36	76.811
LOC72782	-1.22295	6.731281	-13.2459	2.97E-35	2.81E-33	69.26319
RLN2	-1.22482	4.056355	-17.6932	1.56E-56	4.72E-53	117.7995
TBC1D32	-1.22809	3.216642	-13.4792	2.67E-36	3.06E-34	71.64618
BTAF1	-1.22936	7.953747	-14.9274	5.46E-43	1.48E-40	86.89983
OCIAD2	-1.2296	7.158053	-11.2473	9.95E-27	2.87E-25	49.84804
NKTR	-1.2315	7.023462	-12.6189	1.71E-32	1.01E-30	62.97286
HLA-DPA1	-1.23273	9.192673	-7.43968	3.57E-13	2.25E-12	19.10908
CCR3	-1.23594	6.811273	-5.79874	1.09E-08	4.22E-08	9.042944
MTIF2	-1.23616	7.187133	-10.9836	1.15E-25	2.88E-24	47.43341
TIMM9	-1.23692	6.797926	-12.1761	1.37E-30	6.45E-29	58.63648
ZFP3	-1.23921	5.837427	-11.0634	5.49E-26	1.42E-24	48.16063
KRBOX4	-1.24149	6.186088	-10.754	9.32E-25	2.06E-23	45.36288
GIMAP7	-1.24175	9.796876	-9.11755	1.19E-18	1.33E-17	31.49759
CCL5	-1.24212	9.842053	-6.84251	1.95E-11	1.03E-10	15.19812
RFTN1	-1.24511	8.162013	-9.50596	4.90E-20	6.42E-19	34.63902
WDR11	-1.24618	6.890423	-11.0262	7.74E-26	1.97E-24	47.82144
RRN3	-1.24635	6.301231	-9.62456	1.82E-20	2.49E-19	35.61749
SNX29P2	-1.24744	4.697966	-11.0963	4.05E-26	1.07E-24	48.46122
ZNF30	-1.24968	4.353051	-12.0978	2.95E-30	1.33E-28	57.87892
ZNF260	-1.25004	6.48747	-9.86598	2.34E-21	3.56E-20	37.63632
KLHDC2	-1.25054	8.224901	-13.8635	4.83E-38	7.06E-36	75.61842
NARS	-1.25187	9.668133	-13.1751	6.13E-35	5.58E-33	68.54477
SEC14L1P1	-1.25417	4.710354	-16.3166	1.10E-49	8.31E-47	102.1736
ATP2B1	-1.25969	7.947427	-7.44919	3.35E-13	2.12E-12	19.17357
STAP1	-1.26035	5.807402	-6.92583	1.13E-11	6.15E-11	15.72718
CCNB1IP1	-1.26075	6.079929	-11.6293	2.71E-28	9.62E-27	53.40964
SIDT1	-1.26194	6.042932	-8.51236	1.41E-16	1.24E-15	26.80145
SKAP1	-1.26262	6.938398	-10.4698	1.20E-23	2.38E-22	42.8397

TC2N	-1.26444	4.587609	-14.9502	4.26E-43	1.17E-40	87.14589
SLC39A10	-1.2657	6.536388	-8.47236	1.91E-16	1.67E-15	26.49989
ZBTB26	-1.26672	5.531703	-10.2819	6.34E-23	1.16E-21	41.19779
LYSMD2	-1.26839	9.807185	-8.89343	7.17E-18	7.27E-17	29.72972
PPP1R16B	-1.26864	6.332437	-10.9939	1.04E-25	2.64E-24	47.52707
C12orf57	-1.27008	8.246851	-8.48783	1.70E-16	1.49E-15	26.61635
LUC7L3	-1.27054	7.866688	-14.6035	1.82E-41	4.13E-39	83.42356
ZNF439	-1.27771	4.473253	-12.602	2.02E-32	1.18E-30	62.80628
TRIB2	-1.27901	7.156703	-8.73471	2.51E-17	2.39E-16	28.49805
THUMPD1	-1.28088	7.8479	-10.95	1.56E-25	3.86E-24	47.12907
FBL	-1.28184	9.038605	-11.5911	3.90E-28	1.35E-26	53.04978
FYCO1	-1.28549	6.314185	-11.4575	1.38E-27	4.44E-26	51.79818
TARBP1	-1.28549	6.769372	-16.0477	2.27E-48	1.28E-45	99.1749
LOC10099	-1.29025	9.404508	-11.7477	8.73E-29	3.31E-27	54.52882
GNPDA2	-1.294	5.334492	-10.9382	1.74E-25	4.28E-24	47.02233
TRMT11	-1.29436	5.580211	-10.2465	8.65E-23	1.56E-21	40.89071
THEM4	-1.29656	5.29106	-12.6426	1.35E-32	8.03E-31	63.2079
PRPF39	-1.30226	6.380711	-10.1884	1.44E-22	2.52E-21	40.38761
ZNF709	-1.30663	5.065089	-12.4748	7.19E-32	3.99E-30	61.55206
ABLIM1	-1.30761	7.664218	-10.2462	8.68E-23	1.56E-21	40.88813
ZBTB25	-1.30834	5.764159	-13.2915	1.86E-35	1.84E-33	69.72711
RRP15	-1.31097	4.561541	-10.992	1.06E-25	2.68E-24	47.51026
HOTAIRM1	-1.31124	7.763575	-9.27867	3.21E-19	3.85E-18	32.78891
KPNA5	-1.31136	4.993312	-11.149	2.49E-26	6.72E-25	48.9432
ZNF92	-1.31561	6.328715	-9.36992	1.52E-19	1.88E-18	33.52768
ZNF266	-1.31649	8.321101	-12.1984	1.10E-30	5.24E-29	58.85232
RPL38	-1.31714	9.511513	-15.9605	6.03E-48	3.31E-45	98.20608
ZFP62	-1.31741	6.701415	-12.6667	1.06E-32	6.46E-31	63.44686
HERC5	-1.32179	8.103024	-4.58641	5.51E-06	1.55E-05	3.046551
INPP4B	-1.32479	4.949096	-12.5639	2.96E-32	1.72E-30	62.429
NOL11	-1.32612	8.116955	-10.7363	1.10E-24	2.40E-23	45.20397
CAPRIN2	-1.32762	6.9048	-13.9319	2.35E-38	3.58E-36	76.3319
PRKACB	-1.32918	6.987997	-10.1046	2.99E-22	5.03E-21	39.66687
PRSS23	-1.32947	4.588789	-12.0979	2.94E-30	1.33E-28	57.87958
KIF21A	-1.33166	3.645028	-14.2641	6.94E-40	1.27E-37	79.81984
HAUS3	-1.33191	7.310871	-9.71835	8.24E-21	1.17E-19	36.39748
THAP9-AS	-1.33401	6.732643	-10.8375	4.36E-25	1.01E-23	46.11244
TRDV3	-1.33414	5.460278	-10.7993	6.18E-25	1.41E-23	45.7692
SYTL2	-1.33515	5.456815	-12.1499	1.77E-30	8.25E-29	58.38205
BIRC3	-1.33719	7.191286	-10.55	5.86E-24	1.19E-22	43.54732
CX3CR1	-1.33749	11.05299	-7.78001	3.25E-14	2.29E-13	21.45902
STK39	-1.33784	6.684591	-9.95127	1.13E-21	1.78E-20	38.35815
GPR171	-1.3422	7.612507	-7.47978	2.71E-13	1.73E-12	19.38142
OSGEPL1	-1.34234	5.751586	-12.8932	1.08E-33	7.93E-32	65.70441
ZBTB24	-1.34406	6.673839	-12.67	1.02E-32	6.29E-31	63.47978
ZAP70	-1.34549	7.334714	-9.83833	2.97E-21	4.46E-20	37.40333
HNRNPU-	-1.34678	6.553845	-11.1208	3.23E-26	8.61E-25	48.68539
GIMAP6	-1.34851	9.093461	-10.0181	6.34E-22	1.03E-20	38.92671
USPL1	-1.35231	7.508205	-13.2581	2.62E-35	2.49E-33	69.38757
GBP3	-1.35256	7.821583	-6.16526	1.30E-09	5.64E-09	11.1029
RARRES3	-1.36621	7.939074	-8.94476	4.77E-18	4.93E-17	30.13162
KDM2B	-1.36811	7.525043	-11.7907	5.77E-29	2.25E-27	54.9372
IFIT2	-1.37144	9.367958	-4.95049	9.67E-07	3.00E-06	4.712551
H3F3A	-1.37162	6.823099	-8.30506	6.83E-16	5.63E-15	25.25056
CTSO	-1.37242	6.951651	-10.3967	2.30E-23	4.40E-22	42.19897
ZNF329	-1.37469	4.775565	-10.8334	4.53E-25	1.05E-23	46.07546
UBAP2	-1.37605	7.338349	-12.5328	4.04E-32	2.31E-30	62.12225
PRKCQ	-1.3763	7.307768	-11.4864	1.05E-27	3.48E-26	52.06799

MAN1C1	-1.37689	6.150191	-12.7864	3.18E-33	2.17E-31	64.63681
GORAB	-1.37808	4.961829	-9.13032	1.07E-18	1.21E-17	31.59926
CCL4	-1.37876	8.326787	-7.66229	7.52E-14	5.08E-13	20.63644
SLC18B1	-1.3807	7.008412	-9.70788	9.00E-21	1.27E-19	36.31021
PDCD4-AS	-1.38163	6.636959	-11.589	3.97E-28	1.37E-26	53.03082
EXOSC8	-1.3827	6.620767	-11.7309	1.02E-28	3.84E-27	54.36991
PTER	-1.38437	5.756707	-10.8652	3.39E-25	8.03E-24	46.36233
HNRNPA1	-1.38601	7.300492	-13.2691	2.34E-35	2.25E-33	69.49908
GOLGA8A	-1.39019	6.442879	-11.7417	9.24E-29	3.49E-27	54.47219
NOC3L	-1.39067	6.167055	-9.65391	1.42E-20	1.96E-19	35.86101
SCML1	-1.39284	5.181994	-12.0788	3.55E-30	1.60E-28	57.69541
CROCCP2	-1.39594	7.901848	-11.6743	1.76E-28	6.41E-27	53.83381
SGK1	-1.39756	10.18509	-8.37424	4.04E-16	3.41E-15	25.76475
RCAN3	-1.39813	6.250456	-11.7539	8.22E-29	3.15E-27	54.58801
IL11RA	-1.39861	6.280175	-15.1318	5.85E-44	2.04E-41	89.11137
ITM2A	-1.40024	8.169439	-8.77174	1.88E-17	1.81E-16	28.78391
RELL1	-1.40191	9.344561	-11.1408	2.68E-26	7.21E-25	48.86836
FUNDC1	-1.40328	5.609739	-10.5869	4.21E-24	8.70E-23	43.87425
FAM35A	-1.40415	8.44088	-11.8555	3.09E-29	1.24E-27	55.55441
RORA	-1.40527	5.130837	-13.3578	9.36E-36	9.70E-34	70.40425
SHPRH	-1.41083	5.536126	-8.4358	2.53E-16	2.18E-15	26.22516
KLHL3	-1.41142	5.870341	-16.412	3.74E-50	3.08E-47	103.2426
AKR1C3	-1.41177	5.902581	-8.84368	1.06E-17	1.06E-16	29.34182
EIF3C	-1.42052	3.733989	-14.7852	2.56E-42	6.43E-40	85.36986
TMEM14A	-1.42097	5.926469	-11.9306	1.50E-29	6.24E-28	56.27173
RPS11	-1.42201	7.582554	-7.25144	1.30E-12	7.75E-12	17.84667
USP28	-1.42261	6.279706	-13.426	4.63E-36	5.02E-34	71.10127
GIMAP1	-1.42532	7.439616	-11.7476	8.74E-29	3.31E-27	54.5275
UBE2Q2	-1.42716	7.740231	-9.9424	1.22E-21	1.91E-20	38.28287
ZNF83	-1.43123	4.974171	-12.5452	3.57E-32	2.06E-30	62.2448
BBS2	-1.43262	6.388557	-14.1257	3.03E-39	5.32E-37	78.36148
BCL11B	-1.43694	6.741354	-11.5466	5.94E-28	2.02E-26	52.63233
CAMK4	-1.44295	5.296754	-11.0751	4.93E-26	1.29E-24	48.26718
RBM4	-1.44388	6.490979	-11.193	1.65E-26	4.59E-25	49.34719
BEX5	-1.44567	4.970103	-12.9712	4.90E-34	3.90E-32	66.48705
NAP1L3	-1.44641	3.967152	-12.997	3.77E-34	3.07E-32	66.74577
TRBC1	-1.44697	10.06937	-8.23656	1.14E-15	9.20E-15	24.74469
CXCL8	-1.45069	8.542254	-5.04116	6.16E-07	1.96E-06	5.145678
WDR3	-1.45327	6.807157	-11.4017	2.34E-27	7.30E-26	51.27873
MKL2	-1.45618	5.503155	-12.2944	4.28E-31	2.14E-29	59.78633
LOC93622	-1.4564	6.433089	-12.9053	9.56E-34	7.10E-32	65.82568
OFD1	-1.4609	5.975281	-16.1627	6.23E-49	3.77E-46	100.455
GSPT2	-1.46454	6.289216	-9.10507	1.32E-18	1.47E-17	31.39821
LY9	-1.47162	6.323174	-12.8743	1.31E-33	9.48E-32	65.51534
CCDC104	-1.47492	5.023291	-13.149	8.01E-35	7.18E-33	68.28017
CEP290	-1.47609	4.681106	-13.8636	4.83E-38	7.06E-36	75.61954
BEX2	-1.48125	6.340223	-10.5083	8.51E-24	1.71E-22	43.17923
ITPK1-AS1	-1.48332	6.790073	-9.4526	7.64E-20	9.83E-19	34.20174
ZNF571	-1.50041	4.11632	-13.0486	2.23E-34	1.88E-32	67.26562
MAL	-1.50733	7.635139	-10.4506	1.42E-23	2.81E-22	42.67143
ZNF23	-1.50824	4.88208	-12.3769	1.90E-31	9.85E-30	60.59166
PYHIN1	-1.50878	6.084646	-9.63985	1.60E-20	2.20E-19	35.74431
FAM69A	-1.5111	5.847212	-8.93897	4.99E-18	5.15E-17	30.08624
HMGN3	-1.51328	9.314512	-12.0348	5.45E-30	2.37E-28	57.27129
TRMT61B	-1.51451	5.976601	-10.637	2.68E-24	5.70E-23	44.3189
ZNF302	-1.52287	5.769029	-11.621	2.93E-28	1.03E-26	53.33146
LY75	-1.5239	8.78751	-10.1072	2.93E-22	4.93E-21	39.68916
TMEM263	-1.52397	6.160183	-8.93984	4.96E-18	5.12E-17	30.09301

FLVCR1	-1.52438	5.951524	-9.5416	3.64E-20	4.84E-19	34.93214
ARL14EP	-1.52892	6.282876	-12.544	3.61E-32	2.08E-30	62.23322
PRKCQ-AS	-1.52941	6.070103	-13.2045	4.53E-35	4.19E-33	68.8433
LOC20077	-1.53155	5.23202	-13.273	2.24E-35	2.17E-33	69.53921
SGK223	-1.54101	7.549781	-10.8956	2.57E-25	6.15E-24	46.63644
MTERF3	-1.54278	6.259253	-11.2394	1.07E-26	3.08E-25	49.77465
HLA-DMB	-1.5443	8.402359	-10.9499	1.56E-25	3.86E-24	47.12813
LBH	-1.55106	8.392211	-9.42799	9.38E-20	1.19E-18	34.00061
BTN3A3	-1.55787	7.138469	-9.30191	2.65E-19	3.21E-18	32.97651
ITK	-1.56802	8.860066	-8.95653	4.34E-18	4.52E-17	30.2241
NOP58	-1.57221	8.637475	-11.9083	1.86E-29	7.67E-28	56.05854
MBLAC2	-1.57517	4.900444	-14.9969	2.56E-43	7.64E-41	87.65026
ANKRD36E	-1.57558	6.692169	-9.7251	7.78E-21	1.11E-19	36.45384
WDR43	-1.57804	5.967101	-11.8195	4.38E-29	1.73E-27	55.21116
MTR	-1.58787	6.37545	-12.6671	1.05E-32	6.46E-31	63.45032
MSANTD2	-1.59037	5.740294	-9.57549	2.74E-20	3.69E-19	35.21156
CD3D	-1.59146	9.433748	-9.79163	4.42E-21	6.49E-20	37.01079
RPS6KA5	-1.59512	7.68035	-9.95169	1.12E-21	1.77E-20	38.36173
LIPT1	-1.59932	6.110206	-12.9596	5.52E-34	4.29E-32	66.37033
PLCXD2	-1.60221	5.108232	-10.007	6.97E-22	1.13E-20	38.8323
RASGRF2	-1.60244	5.902641	-14.0472	6.96E-39	1.16E-36	77.53733
SETD6	-1.60573	4.906361	-15.4075	2.82E-45	1.09E-42	92.11433
MYBL1	-1.60886	6.119177	-14.0951	4.19E-39	7.16E-37	78.04026
UBASH3A	-1.61149	5.850895	-12.0435	5.00E-30	2.19E-28	57.35572
TBC1D31	-1.61646	5.387258	-13.2449	2.99E-35	2.83E-33	69.25366
CCDC84	-1.61712	7.127744	-18.6879	1.38E-61	8.36E-58	129.3291
PAXIP1-AS	-1.6204	4.974588	-14.9684	3.49E-43	9.88E-41	87.34236
GBP5	-1.62704	7.924092	-7.41706	4.18E-13	2.62E-12	18.95596
MGC40069	-1.63037	4.427243	-14.3793	2.03E-40	4.08E-38	81.03831
KLRC3	-1.63282	4.641766	-8.27639	8.47E-16	6.90E-15	25.0384
PWAR6	-1.63972	3.964721	-12.6541	1.20E-32	7.26E-31	63.32126
RTN1	-1.64939	6.298536	-9.71279	8.63E-21	1.22E-19	36.35114
MCOLN2	-1.65486	5.99158	-10.7458	1.00E-24	2.21E-23	45.28932
DYRK2	-1.6589	6.624364	-12.137	2.01E-30	9.31E-29	58.25747
C5orf28	-1.67052	4.251795	-11.9547	1.19E-29	5.02E-28	56.50192
CD8A	-1.67175	8.339522	-10.0655	4.20E-22	6.97E-21	39.33137
GPR82	-1.68194	4.076457	-8.75102	2.21E-17	2.12E-16	28.62386
AMIGO2	-1.68523	6.441504	-11.4244	1.89E-27	5.96E-26	51.48985
S100A8	-1.68658	9.524851	-11.2388	1.08E-26	3.09E-25	49.76923
MAP3K7CI	-1.68979	6.898503	-7.29987	9.34E-13	5.67E-12	18.1689
PTPN4	-1.69007	4.942867	-14.2362	9.35E-40	1.69E-37	79.52497
ZNF420	-1.69591	5.333666	-12.7741	3.60E-33	2.41E-31	64.51462
DNAJC24	-1.70389	6.293062	-12.7139	6.60E-33	4.16E-31	63.91486
CEP78	-1.72089	5.959127	-13.2848	1.99E-35	1.94E-33	69.65977
PMAIP1	-1.72286	7.191222	-9.29232	2.87E-19	3.45E-18	32.8991
CHIC1	-1.72916	4.366687	-13.4523	3.53E-36	3.94E-34	71.37089
NMT2	-1.72966	4.848771	-12.1837	1.27E-30	6.03E-29	58.70965
STAT4	-1.7307	7.402986	-13.8978	3.37E-38	5.00E-36	75.97621
FCRL3	-1.73103	7.598793	-8.71623	2.90E-17	2.75E-16	28.35572
ZNF432	-1.7413	5.989314	-12.6493	1.26E-32	7.56E-31	63.27448
LINC01215	-1.742	5.928498	-9.05155	2.03E-18	2.20E-17	30.97352
FAM117B	-1.74482	7.230596	-8.69784	3.35E-17	3.15E-16	28.21437
KLRD1	-1.74562	6.718193	-10.6701	1.99E-24	4.28E-23	44.61282
CRY1	-1.74814	5.616139	-12.3354	2.86E-31	1.44E-29	60.18554
CRTAM	-1.75317	5.594675	-10.1852	1.48E-22	2.58E-21	40.35998
TRG-AS1	-1.76442	7.137975	-13.3225	1.35E-35	1.35E-33	70.04332
TRAT1	-1.78184	6.922991	-8.73779	2.45E-17	2.34E-16	28.52182
P2RY10	-1.78631	4.829421	-13.0009	3.63E-34	2.96E-32	66.78495

NOV	-1.78932	5.6744	-10.6341	2.76E-24	5.83E-23	44.29275
SACS	-1.78991	5.340453	-10.8994	2.48E-25	5.96E-24	46.67092
NR1D2	-1.79501	5.611811	-10.8719	3.19E-25	7.57E-24	46.4224
TRMT13	-1.80088	5.794483	-11.8184	4.42E-29	1.75E-27	55.20088
DDHD2	-1.80512	5.964435	-12.0319	5.60E-30	2.43E-28	57.24397
FAM169A	-1.80923	4.399459	-16.1151	1.06E-48	6.22E-46	99.92504
TRAC	-1.81057	9.454259	-12.4725	7.35E-32	4.06E-30	61.52954
LOC10272	-1.81404	5.407171	-12.929	7.53E-34	5.66E-32	66.0626
TXK	-1.82602	6.317089	-10.1644	1.78E-22	3.06E-21	40.18097
PRF1	-1.83888	9.960986	-9.43166	9.09E-20	1.16E-18	34.03062
PLEKHA1	-1.8427	6.76206	-12.935	7.08E-34	5.35E-32	66.12302
GATA3	-1.84475	5.987199	-13.203	4.60E-35	4.23E-33	68.82817
GPR183	-1.85861	7.524651	-10.8833	2.87E-25	6.85E-24	46.52548
HOPX	-1.85889	7.559996	-10.7699	8.07E-25	1.80E-23	45.5052
GZMB	-1.86766	8.259085	-8.97273	3.81E-18	4.00E-17	30.35137
GZMH	-1.88176	7.518168	-7.74	4.33E-14	3.01E-13	21.1783
GPR18	-1.8984	6.728461	-9.40688	1.12E-19	1.41E-18	33.82849
TBC1D4	-1.90842	6.83323	-12.7962	2.88E-33	1.98E-31	64.73448
IL7R	-1.91085	9.978259	-11.7159	1.18E-28	4.40E-27	54.22774
GZMA	-1.94528	8.31018	-8.95516	4.39E-18	4.56E-17	30.2133
P2RY14	-1.95039	7.593011	-7.10805	3.41E-12	1.94E-11	16.90323
CD2	-1.95167	8.661401	-12.2752	5.18E-31	2.58E-29	59.59898
IL2RB	-1.95246	8.3871	-12.4283	1.14E-31	6.12E-30	61.09481
CACNA2D	-1.95576	5.420485	-10.2478	8.56E-23	1.55E-21	40.90181
IFIT1	-1.98367	8.470707	-4.84966	1.58E-06	4.79E-06	4.239411
CD3G	-1.98921	7.214015	-10.8442	4.10E-25	9.58E-24	46.17316
NLRC3	-1.99194	7.596841	-12.6774	9.51E-33	5.88E-31	63.55304
NR3C2	-1.99209	4.731678	-17.5496	8.25E-56	1.87E-52	116.1502
GNLY	-2.0348	8.63782	-8.48449	1.74E-16	1.52E-15	26.59119
SH3YL1	-2.06103	7.162407	-12.4239	1.19E-31	6.36E-30	61.0522
FGFBP2	-2.08662	7.183698	-8.95691	4.33E-18	4.51E-17	30.22707
GPRASP1	-2.09404	5.891952	-12.7766	3.51E-33	2.36E-31	64.53907
TRAF5	-2.09447	7.028594	-11.4882	1.03E-27	3.43E-26	52.08547
RPL27A	-2.13229	7.441136	-13.0756	1.69E-34	1.46E-32	67.5387
ZNF573	-2.13902	5.017476	-12.2119	9.64E-31	4.62E-29	58.98358
GZMK	-2.1418	7.846808	-11.012	8.82E-26	2.25E-24	47.69158
RASGRP1	-2.142	8.501879	-11.7478	8.72E-29	3.31E-27	54.52998
SAMD3	-2.14257	7.100875	-10.9372	1.75E-25	4.31E-24	47.01277
LGALS2	-2.21526	6.851527	-8.89076	7.33E-18	7.41E-17	29.70883
NELL2	-2.24579	7.583513	-13.6275	5.71E-37	7.29E-35	73.17254
EOMES	-2.27641	6.884027	-11.9513	1.23E-29	5.17E-28	56.4698
KLRB1	-2.32081	8.341197	-12.2016	1.07E-30	5.09E-29	58.88298
ZNF600	-2.3425	6.108355	-16.2355	2.75E-49	1.99E-46	101.2674
CD96	-2.35852	6.250403	-13.6128	6.66E-37	8.44E-35	73.02071
LRRN3	-2.45047	6.553483	-11.3231	4.90E-27	1.48E-25	50.5483
CD160	-2.45218	5.782009	-13.708	2.47E-37	3.33E-35	74.00486
GRAMD1C	-2.45963	4.151354	-13.4301	4.44E-36	4.87E-34	71.14363
TGFBR3	-2.59011	6.596022	-12.223	8.65E-31	4.20E-29	59.09086
NOG	-2.7709	4.421923	-14.4178	1.34E-40	2.80E-38	81.44681
KLRF1	-2.80291	6.826676	-12.3093	3.70E-31	1.86E-29	59.93114
FCER1A	-2.99442	6.570954	-11.9651	1.07E-29	4.56E-28	56.60189
