# Supplementary material for: Altered Genes and Biological Functions in Response to Severe Burns
Source: Biomed Res Int. 2021 May 24;2021:8836243. doi: 10.1155/2021/8836243 (PMC8168476; doi:10.1155/2021/8836243)
Supplement: Supplementary 5 — Table S3: 9991 DEGs in GSE77791, DEG_sort_0.05. [file 8836243.f5.pdf]

Table S3 9991 DEGs in GSE77791, DEG_sort_0.05

DEG	logFC	AveExpr	t	P.Value	adj.P.Val	B
MMP8	7.405815	9.954424	25.18831	1.74E-49	3.24E-46	101.9743
CD177	6.667866	10.60484	29.0545	7.81E-56	4.00E-52	116.2455
RETN	5.41513	8.596075	22.14521	5.34E-44	7.28E-41	89.56556
LCN2	5.156569	10.95776	17.07743	8.84E-34	4.02E-31	66.32933
HP	5.152575	9.131692	25.99648	7.22E-51	1.85E-47	105.0894
GPR84	5.127676	8.656219	25.8931	1.08E-50	2.46E-47	104.695
OLFM4	4.904571	7.84915	6.492377	2.05E-09	2.00E-08	10.78415
MCEMP1	4.87249	10.69388	39.72001	1.84E-70	3.76E-66	148.6417
TDRD9	4.521989	7.177049	20.57897	5.45E-41	6.20E-38	82.73738
ANXA3	4.491936	10.35818	26.90883	2.16E-52	7.37E-49	108.5201
CRISP3	4.263769	7.266649	12.2125	1.01E-22	9.99E-21	41.08692
S100A12	4.031256	12.72488	38.42296	7.17E-69	7.33E-65	145.1604
CEACAM8	4.015357	8.030226	7.19142	6.18E-11	7.84E-10	14.22188
LTF	3.973238	11.0998	11.24343	2.02E-20	1.33E-18	35.82502
CLEC4D	3.878387	8.479419	18.10062	5.89E-36	3.77E-33	71.28843
TCN1	3.859155	9.105888	13.06016	1.01E-24	1.42E-22	45.64882
RNASE2	3.79827	9.879669	17.52852	9.54E-35	5.01E-32	68.53224
DEFA4	3.774987	9.041336	5.378769	3.81E-07	2.52E-06	5.678746
ELANE	3.705907	7.807267	6.190511	8.84E-09	7.71E-08	9.350289
MMP9	3.601746	12.08834	24.54824	2.27E-48	3.88E-45	99.45481
GADD45A	3.514062	9.050256	23.1142	8.51E-46	1.24E-42	93.63683
RGL4	3.493748	8.819403	20.53749	6.58E-41	7.09E-38	82.5523
ANKRD22	3.475031	7.290084	19.61829	4.44E-39	4.13E-36	78.39408
CLEC5A	3.45031	6.342694	15.38717	4.61E-30	1.39E-27	57.8487
GYG1	3.421533	11.05836	27.60244	1.59E-53	6.49E-50	111.069
CYSTM1	3.408072	11.10124	26.70369	4.72E-52	1.38E-48	107.7565
VNN1	3.385254	8.433849	14.97977	3.81E-29	9.76E-27	55.7536
BMX	3.274731	6.943132	20.07669	5.37E-40	5.49E-37	80.48143
MS4A4A	3.270393	7.372517	14.26428	1.63E-27	3.40E-25	52.02986
METTL7B	3.259487	6.539311	8.659587	2.75E-14	6.14E-13	21.83561
IL1R2	3.24993	10.87535	10.99896	7.75E-20	4.61E-18	34.49332
PGLYRP1	3.241069	9.480829	14.00073	6.59E-27	1.31E-24	50.64475
RNASE3	3.172946	7.623337	8.974004	5.04E-15	1.31E-13	23.51351
S100P	3.155603	10.92539	14.78367	1.06E-28	2.56E-26	54.73848
ZDHHC19	3.132582	7.391246	15.59413	1.59E-30	5.35E-28	58.90567
AZU1	3.111343	6.176301	5.212282	7.96E-07	5.00E-06	4.963293
DACH1	3.036797	5.773547	17.20638	4.66E-34	2.27E-31	66.9617
PRTN3	3.028912	5.693872	4.822541	4.24E-06	2.31E-05	3.344899
PCOLCE2	2.976063	4.469383	9.659803	1.21E-16	4.19E-15	27.20844
PFKFB3	2.965114	9.393551	19.27059	2.25E-38	1.92E-35	76.79267
SLC51A	2.891394	6.719495	12.70868	6.78E-24	8.06E-22	43.76314
MPO	2.869276	6.309311	5.622885	1.26E-07	9.07E-07	6.752141
CEACAM6	2.849513	6.977021	4.649614	8.69E-06	4.46E-05	2.653745
UGCG	2.832212	9.385607	17.15725	5.95E-34	2.77E-31	66.72103
MS4A3	2.830969	7.054443	4.666213	8.11E-06	4.19E-05	2.719339
SERPINB1(	2.827695	5.344333	9.061862	3.13E-15	8.40E-14	23.98444
OPLAH	2.825223	6.561567	17.86861	1.81E-35	1.06E-32	70.17579
CD24	2.820793	8.202918	8.299148	1.90E-13	3.66E-12	19.92877
HPGD	2.795617	6.589816	8.52753	5.59E-14	1.17E-12	21.13473
CA4	2.766328	8.298983	15.5933	1.59E-30	5.35E-28	58.90147
SAMSN1	2.744731	7.978307	13.97256	7.65E-27	1.51E-24	50.49631
DHRS9	2.73385	8.653036	13.73204	2.75E-26	4.94E-24	49.22573
HK3	2.729802	10.29885	21.00113	8.18E-42	1.05E-38	84.60842
SERPINB2	2.634882	6.436398	9.877098	3.67E-17	1.41E-15	28.38645
PLAC8	2.627224	12.10923	18.43004	1.21E-36	8.51E-34	72.85624
ATP8B4	2.546976	6.198949	10.96837	9.17E-20	5.37E-18	34.32662

IRAK3	2.528524	7.103595	17.0711	9.12E-34	4.06E-31	66.29823
LRRN1	2.52395	4.734758	12.48013	2.34E-23	2.58E-21	42.53235
CST7	2.522625	11.03135	15.55698	1.92E-30	6.34E-28	58.7163
IL18R1	2.516906	7.59025	10.61298	6.47E-19	3.30E-17	32.38998
GJB6	2.51625	5.04265	7.020261	1.48E-10	1.75E-09	13.36643
RRM2	2.504694	6.569215	5.802176	5.53E-08	4.23E-07	7.55797
FGF13	2.477967	6.005768	4.796737	4.72E-06	2.56E-05	3.240681
ST6GALNA	2.458088	5.718636	12.8509	3.14E-24	3.94E-22	44.52724
CEACAM1	2.443445	7.777689	17.98763	1.02E-35	6.12E-33	70.74742
ECRP	2.434406	5.615246	11.55006	3.76E-21	2.92E-19	37.4936
ST3GAL4-,	2.413471	6.85318	13.55549	7.07E-26	1.16E-23	48.28964
UPP1	2.402914	9.056515	24.00882	2.05E-47	3.23E-44	97.29483
TP53I3	2.394282	6.854769	10.9272	1.15E-19	6.52E-18	34.10226
SLC26A8	2.391952	5.973859	17.60574	6.54E-35	3.62E-32	68.9067
ORM1	2.341775	6.828381	6.677048	8.23E-10	8.61E-09	11.67728
OLAH	2.325786	5.399863	5.349511	4.34E-07	2.85E-06	5.552012
DPY19L3	2.30369	7.655562	15.89523	3.39E-31	1.29E-28	60.43443
FOLR3	2.294297	8.509358	6.156611	1.04E-08	8.92E-08	9.191401
FCGR1B	2.271903	9.011864	14.79306	1.01E-28	2.46E-26	54.78717
GRB10	2.250472	6.412856	12.80132	4.10E-24	5.03E-22	44.26105
PFKFB2	2.249949	6.139638	14.73839	1.35E-28	3.20E-26	54.5035
CEP55	2.231082	3.843949	8.511025	6.11E-14	1.27E-12	21.04731
IL10RB-AS	2.223308	5.883039	13.79333	1.99E-26	3.63E-24	49.55002
GPR160	2.217576	9.12359	13.82626	1.67E-26	3.16E-24	49.72411
NLRC4	2.200354	7.020212	13.5777	6.28E-26	1.05E-23	48.40752
TSPO	2.193616	11.21314	18.00323	9.43E-36	5.85E-33	70.82223
NEDD4	2.129951	5.738849	14.00563	6.42E-27	1.29E-24	50.6706
BCL2A1	2.11063	8.895759	8.169316	3.78E-13	6.94E-12	19.24698
EMR1	2.095166	8.357973	11.22687	2.22E-20	1.44E-18	35.73485
GGH	2.079343	6.2295	10.72078	3.58E-19	1.89E-17	32.97741
VSTM1	2.075805	6.417902	10.79136	2.43E-19	1.31E-17	33.36202
PTX3	2.075649	4.570842	4.979963	2.18E-06	1.25E-05	3.988744
CTSG	2.075221	6.211088	4.039652	9.53E-05	0.000399	0.360502
EXOSC4	2.074008	6.932292	17.71549	3.82E-35	2.17E-32	69.43765
LDHA	2.07327	11.48637	17.59398	6.93E-35	3.73E-32	68.84974
ATP9A	2.053999	5.707649	17.1619	5.81E-34	2.77E-31	66.74381
BST1	2.046103	8.587334	15.13642	1.69E-29	4.67E-27	56.56144
NAIP	2.045422	7.50473	11.95865	4.02E-22	3.54E-20	39.71225
ENTPD7	2.045047	5.97211	15.23964	9.89E-30	2.81E-27	57.0922
SUCNR1	2.036735	5.501806	6.109829	1.30E-08	1.10E-07	8.972878
TLR5	2.036553	7.990011	15.84396	4.41E-31	1.61E-28	60.17486
YOD1	2.028502	6.840949	9.131797	2.14E-15	5.92E-14	24.35987
FCER1G	2.027588	11.06365	19.96889	8.80E-40	8.58E-37	79.993
FAM20A	2.027582	5.029358	7.496621	1.29E-11	1.83E-10	15.76671
GCLM	2.023182	6.237076	13.21691	4.35E-25	6.60E-23	46.48671
XK	2.01477	6.704315	4.922609	2.78E-06	1.57E-05	3.752587
RAB13	2.011018	6.645264	9.015444	4.03E-15	1.06E-13	23.73553
CLEC1B	2.001122	6.133317	7.713463	4.17E-12	6.43E-11	16.8781
G0S2	1.998189	6.723872	10.33751	2.94E-18	1.35E-16	30.88954
PSTPIP2	1.98957	7.883558	13.07017	9.60E-25	1.36E-22	45.70241
CENPW	1.984244	5.65523	8.544256	5.11E-14	1.08E-12	21.22337
PLSCR1	1.982985	7.799989	12.69636	7.25E-24	8.52E-22	43.69691
SMPDL3A	1.970718	5.101062	12.87306	2.78E-24	3.56E-22	44.64618
DDAH2	1.96937	7.51063	15.51836	2.34E-30	7.49E-28	58.51929
MAP2K6	1.963594	5.82571	13.51854	8.62E-26	1.39E-23	48.09336
MMRN1	1.960044	3.661955	6.801399	4.43E-10	4.88E-09	12.28504
SERPINB1	1.959306	9.444079	16.01358	1.86E-31	7.17E-29	61.03234
STOM	1.95896	7.620458	18.81513	1.92E-37	1.46E-34	74.67107

LOC10013	1.943285	7.296013	9.10887	2.43E-15	6.65E-14	24.23674
GALNT14	1.935252	7.332338	9.193751	1.53E-15	4.36E-14	24.69287
CLIC2	1.934932	5.308665	7.08728	1.05E-10	1.28E-09	13.7004
IL18RAP	1.90179	10.55924	12.39927	3.64E-23	3.92E-21	42.09605
CYP1B1	1.898363	7.535818	7.025023	1.44E-10	1.71E-09	13.39012
CARD6	1.893558	8.365243	19.48709	8.18E-39	7.28E-36	77.79168
LGALS1	1.881179	10.97068	13.20068	4.75E-25	7.15E-23	46.40009
ATP11B	1.872986	6.481517	15.01417	3.19E-29	8.37E-27	55.93127
PRC1	1.871454	4.761294	7.163652	7.12E-11	8.93E-10	14.08254
SULT1B1	1.867187	6.439472	12.84423	3.25E-24	4.03E-22	44.49147
LILRA5	1.861539	6.372279	11.97637	3.65E-22	3.29E-20	39.80834
EIF1AY	1.853893	5.246834	2.748255	0.006926	0.018941	-3.63827
TXN	1.843828	6.802267	11.50948	4.70E-21	3.51E-19	37.27294
RNF182	1.824679	5.570771	3.022595	0.00307	0.009296	-2.89585
FCAR	1.811578	6.676115	13.33148	2.35E-25	3.67E-23	47.09792
BLOC1S1	1.808723	8.425877	15.45488	3.25E-30	9.93E-28	58.19508
CKAP4	1.797785	7.382117	16.50271	1.56E-32	6.52E-30	63.48525
BEX1	1.787169	5.263278	4.316419	3.30E-05	0.000153	1.372066
PLA2G4A	1.785414	4.543272	10.90564	1.29E-19	7.30E-18	33.9848
CD63	1.781991	11.05015	16.17654	8.11E-32	3.25E-29	61.8528
ZWINT	1.780223	6.122933	5.745753	7.19E-08	5.39E-07	7.302835
FAM132B	1.772682	4.827699	4.314964	3.32E-05	0.000153	1.366617
KIF11	1.772068	4.056935	5.814891	5.22E-08	4.01E-07	7.615658
DLGAP5	1.768736	4.122243	6.080702	1.49E-08	1.25E-07	8.837256
CAPG	1.76551	8.761768	14.65437	2.09E-28	4.81E-26	54.06688
C1QA	1.756532	5.181325	5.453191	2.73E-07	1.85E-06	6.002984
ALPL	1.755108	7.800478	9.436487	4.09E-16	1.28E-14	26.00093
METTL9	1.752867	8.387567	17.30485	2.87E-34	1.43E-31	67.4432
C3AR1	1.749186	8.894209	8.771095	1.51E-14	3.58E-13	22.42927
CAMP	1.743213	7.554317	5.181181	9.13E-07	5.67E-06	4.83119
ACER3	1.738887	5.97349	13.62703	4.83E-26	8.30E-24	48.6693
PLBD1	1.738052	11.79704	15.47376	2.95E-30	9.14E-28	58.29155
BPGM	1.736922	7.998751	4.803199	4.60E-06	2.49E-05	3.266746
LIN7A	1.726775	6.304689	12.44093	2.90E-23	3.14E-21	42.32091
S100A9	1.723303	13.44659	30.36751	7.58E-58	5.17E-54	120.7485
KCNE1	1.713136	4.618616	10.34755	2.78E-18	1.28E-16	30.9442
PDGFC	1.702935	5.354798	8.93096	6.37E-15	1.62E-13	23.2831
SNX3	1.700274	10.94869	20.84168	1.67E-41	2.01E-38	83.90442
TNFAIP6	1.698815	8.658079	7.319381	3.21E-11	4.29E-10	14.86666
SLC22A4	1.694634	8.929155	13.08868	8.69E-25	1.25E-22	45.80142
GSTO1	1.691184	9.579802	15.17239	1.40E-29	3.93E-27	56.74655
LAMTOR5	1.686057	9.662469	18.21993	3.31E-36	2.19E-33	71.85787
LOC44108	1.684504	7.177596	6.660635	8.93E-10	9.28E-09	11.59744
KIAA0101	1.681071	5.408573	6.385982	3.44E-09	3.24E-08	10.27496
AHSP	1.679985	9.112849	4.270166	3.95E-05	0.000179	1.19957
LOC10192	1.679242	7.295834	12.31119	5.88E-23	6.04E-21	41.62038
NDUFA4	1.675129	8.853312	8.947428	5.82E-15	1.49E-13	23.37123
GBAP1	1.674618	7.904329	10.79117	2.43E-19	1.31E-17	33.36096
SLC37A3	1.665881	7.335008	9.854669	4.15E-17	1.57E-15	28.26474
CDKN3	1.646405	4.474864	6.263376	6.23E-09	5.58E-08	9.693297
BIK	1.645095	6.570915	8.534891	5.38E-14	1.13E-12	21.17373
PROS1	1.642618	5.352174	7.013125	1.53E-10	1.81E-09	13.33094
LMO2	1.639843	7.364566	12.07649	2.11E-22	2.00E-20	40.3508
ARG1	1.638801	4.455027	13.67699	3.69E-26	6.46E-24	48.93415
C1QB	1.630105	4.603948	5.049056	1.62E-06	9.56E-06	4.275601
SLC25A40	1.628312	7.375028	10.38954	2.21E-18	1.04E-16	31.17283
AIM2	1.623803	7.78413	7.538652	1.04E-11	1.50E-10	15.98128
CKS2	1.61317	5.49866	4.91494	2.87E-06	1.61E-05	3.721146

MAPK14	1.600512	8.397437	10.99577	7.89E-20	4.68E-18	34.47593
SLC2A3	1.599299	9.508096	14.16533	2.75E-27	5.63E-25	51.51065
WIPI1	1.597076	7.463465	12.14898	1.42E-22	1.38E-20	40.74326
MKNK1	1.591531	7.964308	10.48746	1.29E-18	6.28E-17	31.70617
OSCAR	1.590478	7.216328	11.84939	7.31E-22	6.23E-20	39.11962
PADI4	1.588901	6.921066	11.22813	2.20E-20	1.43E-18	35.7417
KLHL2	1.582597	9.184019	8.80363	1.27E-14	3.06E-13	22.60279
PCMT1	1.581705	8.768673	12.69348	7.36E-24	8.61E-22	43.68143
NUSAP1	1.580908	5.721039	6.249632	6.65E-09	5.93E-08	9.628444
ADAMTS3	1.580427	3.870153	5.446717	2.81E-07	1.89E-06	5.97467
HMMR	1.578258	4.705702	6.384572	3.46E-09	3.26E-08	10.26824
AGFG1	1.573168	6.933132	12.23527	8.89E-23	8.96E-21	41.21002
TMTC1	1.569158	5.090355	5.557832	1.70E-07	1.19E-06	6.46336
ANXA1	1.56093	8.839855	10.21961	5.61E-18	2.45E-16	30.24789
ATP6V1C1	1.560258	6.946716	9.461153	3.57E-16	1.13E-14	26.13413
OAT	1.559752	9.327052	10.24379	4.92E-18	2.17E-16	30.37943
PGS1	1.546715	8.589152	12.94697	1.87E-24	2.50E-22	45.0426
ROPN1L	1.545137	6.95058	11.77049	1.13E-21	9.36E-20	38.69137
F5	1.543083	6.984767	10.74942	3.06E-19	1.63E-17	33.13348
CSTA	1.541087	9.505653	6.82418	3.96E-10	4.40E-09	12.39692
COL17A1	1.540851	5.201637	7.314581	3.29E-11	4.38E-10	14.84239
SORT1	1.540634	6.445087	10.83712	1.89E-19	1.04E-17	33.6114
CCNA1	1.540526	5.560325	5.589069	1.48E-07	1.04E-06	6.601781
PGM2	1.53945	7.430731	12.17437	1.24E-22	1.22E-20	40.88064
ROMO1	1.536489	6.353548	11.58605	3.09E-21	2.41E-19	37.68927
HGF	1.535245	4.57786	10.68603	4.33E-19	2.27E-17	32.78801
HEPACAM	1.530777	4.354488	4.316567	3.30E-05	0.000153	1.372617
CDA	1.528282	9.26255	11.58123	3.17E-21	2.47E-19	37.66307
LRG1	1.525265	8.65627	11.44529	6.69E-21	4.73E-19	36.92375
MILR1	1.520147	5.340282	10.18879	6.65E-18	2.87E-16	30.08023
CNIH4	1.517265	7.20416	17.35173	2.28E-34	1.16E-31	67.67196
CHIT1	1.516138	5.254406	4.898602	3.08E-06	1.72E-05	3.654273
FKBP5	1.515526	8.804451	7.52008	1.14E-11	1.64E-10	15.88642
CAPN3	1.510659	5.970381	9.58049	1.86E-16	6.22E-15	26.77918
ZDHHC20	1.505185	6.742177	9.269708	1.01E-15	2.99E-14	25.10163
ANKRD55	1.503819	6.932434	5.349546	4.34E-07	2.85E-06	5.552165
CRISP2	1.503024	4.508615	4.904867	3.00E-06	1.68E-05	3.679898
PYGL	1.498945	10.20417	13.63122	4.72E-26	8.18E-24	48.69149
CEBPE	1.491954	5.740129	5.023238	1.81E-06	1.06E-05	4.168108
MIAT	1.487403	7.390925	7.91296	1.47E-12	2.46E-11	17.90972
DYSF	1.485315	9.840065	12.99454	1.44E-24	1.96E-22	45.29746
TSPAN2	1.47637	6.841333	7.971915	1.07E-12	1.84E-11	18.21614
GYPB	1.474732	6.466012	4.969302	2.28E-06	1.30E-05	3.944711
ACN9	1.471438	5.916807	8.240964	2.58E-13	4.87E-12	19.62287
CORO2A	1.465687	5.655008	8.577326	4.28E-14	9.17E-13	21.39873
DDIAS	1.465491	6.002505	8.173612	3.69E-13	6.79E-12	19.26949
MGST1	1.455515	6.13728	12.20281	1.06E-22	1.05E-20	41.03446
DAAM2	1.454497	5.553518	3.702175	0.000325	0.001231	-0.80342
LOC10272	1.453862	4.439764	5.055877	1.57E-06	9.29E-06	4.304059
FAR2	1.450805	5.882947	10.01953	1.68E-17	6.81E-16	29.15992
IDI1	1.44351	7.837966	10.08622	1.17E-17	4.83E-16	29.52241
GALNT2	1.442464	6.730584	9.670686	1.14E-16	3.98E-15	27.26738
GLRX	1.436468	9.064152	9.29022	9.06E-16	2.70E-14	25.21211
NDUFAF1	1.436316	6.298436	13.24118	3.82E-25	5.84E-23	46.61629
C1QC	1.425317	5.303677	5.248522	6.79E-07	4.31E-06	5.117842
NRN1	1.422986	4.258649	5.973022	2.49E-08	2.00E-07	8.338851
NECAB1	1.41794	3.106559	5.193594	8.64E-07	5.40E-06	4.883855
HIST1H1C	1.415108	7.432693	5.570105	1.61E-07	1.13E-06	6.517689

GPR97	1.414907	8.48069	11.51868	4.47E-21	3.39E-19	37.32298
WSB1	1.412643	7.526791	9.143542	2.01E-15	5.57E-14	24.42297
PLP2	1.40739	10.80441	15.73742	7.61E-31	2.73E-28	59.63452
RBX1	1.403081	8.435298	7.757916	3.30E-12	5.20E-11	17.10724
ANO10	1.401691	5.73172	12.86646	2.88E-24	3.67E-22	44.61077
TFF3	1.398123	5.663814	8.769891	1.52E-14	3.59E-13	22.42285
RAB32	1.396568	6.925312	13.77094	2.24E-26	4.05E-24	49.43159
CD55	1.39572	10.59917	10.96765	9.21E-20	5.37E-18	34.32272
ECHDC3	1.39013	7.360663	5.928266	3.07E-08	2.44E-07	8.133094
ITGA7	1.386608	5.521612	5.550842	1.76E-07	1.23E-06	6.432443
DRAM1	1.381149	7.457283	11.42234	7.58E-21	5.35E-19	36.79892
ADAM9	1.376556	5.135127	12.38169	4.00E-23	4.27E-21	42.00117
RAB31	1.376519	10.57742	14.52186	4.19E-28	9.22E-26	53.37671
SLC22A15	1.37549	7.031293	11.07549	5.09E-20	3.15E-18	34.91027
UBE2F	1.374911	7.702207	14.68595	1.77E-28	4.12E-26	54.2311
IDNK	1.371464	6.42221	8.374366	1.27E-13	2.53E-12	20.32505
NAA38	1.361217	7.407291	11.13803	3.61E-20	2.27E-18	35.251
AP5B1	1.361011	6.14677	6.44598	2.57E-09	2.47E-08	10.5616
SHCBP1	1.35678	4.21951	4.818805	4.31E-06	2.35E-05	3.329788
POR	1.352667	6.812086	10.9661	9.29E-20	5.40E-18	34.31427
PTTG1	1.348221	7.573297	7.758607	3.29E-12	5.18E-11	17.11081
RAB27A	1.341669	9.072135	11.75239	1.24E-21	1.03E-19	38.59307
CDC20	1.34098	5.313461	6.885983	2.90E-10	3.30E-09	12.70125
GAS7	1.33827	7.794127	10.4968	1.22E-18	6.01E-17	31.75703
EMB	1.335741	8.158257	11.76129	1.18E-21	9.80E-20	38.6414
PPP1R3D	1.333437	7.66904	12.79849	4.17E-24	5.08E-22	44.24584
NQO2	1.3331	7.571599	8.010574	8.76E-13	1.52E-11	18.41745
SLPI	1.332888	7.501489	4.320872	3.24E-05	0.00015	1.388741
COX7A2	1.331409	9.553414	8.645551	2.97E-14	6.56E-13	21.761
KIAA1715	1.324708	5.887892	8.149793	4.19E-13	7.62E-12	19.14471
CHPT1	1.323856	7.335588	8.584031	4.13E-14	8.87E-13	21.4343
UBL5	1.322908	7.823613	10.66193	4.94E-19	2.55E-17	32.6567
TPX2	1.322134	4.71534	6.078847	1.51E-08	1.26E-07	8.82863
TREML1	1.317543	7.170554	5.056748	1.57E-06	9.26E-06	4.307692
CR1	1.317049	7.352903	11.50307	4.87E-21	3.61E-19	37.2381
PPM1M	1.315649	8.566593	10.84403	1.82E-19	1.01E-17	33.64905
GBE1	1.313576	7.954684	8.801523	1.28E-14	3.09E-13	22.59154
CD59	1.313052	6.684677	10.5028	1.19E-18	5.83E-17	31.78969
KIF1B	1.30946	6.08944	16.77314	4.02E-33	1.71E-30	64.82867
UQCRQ	1.309456	6.540394	6.241034	6.94E-09	6.16E-08	9.58791
TMEM260	1.300548	7.292682	10.40031	2.08E-18	9.90E-17	31.23151
MMADHC	1.296578	9.629682	12.61902	1.10E-23	1.26E-21	43.28071
FABP5	1.294474	4.815292	6.519807	1.79E-09	1.77E-08	10.91607
SIGLEC5	1.293847	9.101135	8.968537	5.20E-15	1.34E-13	23.48424
ASGR2	1.291057	7.150624	6.956561	2.03E-10	2.37E-09	13.05021
NME8	1.290912	5.993028	7.647309	5.89E-12	8.86E-11	16.5379
EXOC6	1.290882	6.075832	12.23284	9.00E-23	9.03E-21	41.19692
C1orf162	1.290777	9.948363	10.57086	8.15E-19	4.08E-17	32.16049
CTNNAL1	1.289808	5.430585	3.947468	0.000134	0.000547	0.0348
TYMS	1.287709	4.756672	5.198428	8.46E-07	5.29E-06	4.904386
PECR	1.287638	6.368443	10.45686	1.53E-18	7.38E-17	31.53946
CKLF	1.286081	8.428864	11.48792	5.29E-21	3.90E-19	37.15566
ARL4A	1.28382	7.582615	7.098349	9.93E-11	1.22E-09	13.75568
PF4	1.281426	11.06259	6.054984	1.69E-08	1.40E-07	8.717791
FEM1C	1.279028	6.382129	9.443417	3.93E-16	1.23E-14	26.03835
ANAPC15	1.277759	6.396212	12.54476	1.65E-23	1.84E-21	42.88076
FAM105A	1.277164	5.78167	10.57099	8.15E-19	4.08E-17	32.16119
ASPH	1.275803	4.650565	7.551045	9.71E-12	1.41E-10	16.04463

NDUFB3	1.275456	7.205747	7.144917	7.84E-11	9.75E-10	13.98865
MCTP2	1.26556	7.350266	10.87913	1.50E-19	8.35E-18	33.84033
OLR1	1.265442	3.424494	2.87364	0.004808	0.013804	-3.30657
TIMP1	1.262247	10.60039	9.459991	3.59E-16	1.13E-14	26.12785
ADM	1.261644	9.160617	8.879502	8.41E-15	2.09E-13	23.00793
TPST2	1.257232	8.870675	13.46374	1.16E-25	1.85E-23	47.80203
PET100	1.255841	7.274635	7.225404	5.20E-11	6.71E-10	14.39269
HTATSF1P	1.2552	3.952714	3.43479	0.000817	0.002833	-1.66821
TOP2A	1.254048	3.902531	5.519088	2.03E-07	1.40E-06	6.2923
ZNF438	1.253557	6.267111	9.641305	1.34E-16	4.60E-15	27.10829
CCPG1	1.253142	8.382943	9.205849	1.43E-15	4.11E-14	24.75794
FKBP9	1.252102	5.831115	10.03252	1.57E-17	6.36E-16	29.23055
C14orf2	1.251119	7.797559	7.602837	7.42E-12	1.09E-10	16.30975
TMEM45A	1.25056	3.075403	5.90285	3.46E-08	2.73E-07	8.016622
VPS9D1	1.249672	6.187269	11.48081	5.50E-21	4.02E-19	37.11698
FGD4	1.24929	6.105834	8.433257	9.26E-14	1.88E-12	20.63594
DTL	1.246251	3.794508	4.199494	5.19E-05	0.00023	0.938649
CDKN2C	1.244842	5.444614	10.99012	8.14E-20	4.81E-18	34.44515
MYL6B	1.242364	5.516702	9.512818	2.69E-16	8.75E-15	26.41326
C19orf33	1.241962	5.167607	6.472595	2.25E-09	2.18E-08	10.68917
SQRDL	1.238518	9.888897	12.33306	5.22E-23	5.45E-21	41.73853
FIG4	1.235625	7.409788	7.612649	7.05E-12	1.04E-10	16.36005
TTK	1.233394	3.420208	4.197851	5.23E-05	0.000231	0.932623
NSUN7	1.230729	4.182989	11.05479	5.71E-20	3.47E-18	34.79749
SIAH2	1.228334	9.101279	4.110688	7.29E-05	0.000313	0.615365
JAK2	1.227498	6.309628	10.98972	8.16E-20	4.81E-18	34.44297
CTSD	1.225226	6.961174	8.985763	4.73E-15	1.23E-13	23.57649
HBD	1.224823	11.447	3.227743	0.001613	0.005236	-2.30128
ERLIN1	1.223501	6.927933	7.820083	2.39E-12	3.85E-11	17.42841
LILRA3	1.221935	7.486763	5.127214	1.15E-06	7.01E-06	4.603153
E2F8	1.220948	3.691755	4.195957	5.27E-05	0.000232	0.925675
PIWIL4	1.21781	4.865276	5.275344	6.03E-07	3.86E-06	5.232656
ITGA2B	1.216529	5.557836	5.678465	9.80E-08	7.18E-07	7.00041
GMNN	1.215633	5.539414	4.792439	4.81E-06	2.60E-05	3.223359
BCAT1	1.215619	4.844475	6.702909	7.24E-10	7.66E-09	11.80326
LTB4R	1.215346	7.359509	11.46457	6.02E-21	4.31E-19	37.02864
CCNB1	1.210534	3.946657	6.19642	8.59E-09	7.51E-08	9.378028
RNF11	1.210103	9.530985	6.714169	6.85E-10	7.28E-09	11.85819
PNPLA1	1.210035	4.645596	8.921865	6.69E-15	1.70E-13	23.23445
AGPAT9	1.209555	8.477219	9.685308	1.05E-16	3.70E-15	27.34657
B4GALT5	1.201584	8.755194	9.215734	1.36E-15	3.92E-14	24.81112
ANLN	1.19789	3.191636	4.971296	2.26E-06	1.30E-05	3.952943
CEBPA	1.197852	7.990266	11.94044	4.44E-22	3.88E-20	39.61352
GYPA	1.195572	4.532242	4.282727	3.76E-05	0.000172	1.246283
MEF2A	1.194901	6.055755	8.589936	4.00E-14	8.62E-13	21.46564
UBE2C	1.19228	6.292641	6.870632	3.13E-10	3.54E-09	12.62555
TMEM167/	1.192116	8.418849	8.901481	7.47E-15	1.87E-13	23.12542
LAIR1	1.191615	7.358509	7.877256	1.77E-12	2.92E-11	17.72449
PRDM5	1.185942	3.908562	8.676736	2.51E-14	5.63E-13	21.9268
APMAP	1.185857	10.49219	9.886119	3.50E-17	1.35E-15	28.43541
HAT1	1.184529	6.95592	5.950189	2.77E-08	2.21E-07	8.23378
CYYR1	1.182567	3.453404	6.798237	4.50E-10	4.95E-09	12.26953
AP3B2	1.180698	4.649173	6.071776	1.56E-08	1.30E-07	8.795764
CLU	1.178653	5.882455	9.203235	1.45E-15	4.16E-14	24.74388
GAPDH	1.178595	12.24801	17.01131	1.23E-33	5.34E-31	66.00422
ALOX5AP	1.177068	12.19571	13.55147	7.23E-26	1.17E-23	48.26827
CMTM5	1.17475	6.594755	5.00363	1.97E-06	1.14E-05	4.086714
LOC10050	1.173361	6.761039	6.855904	3.37E-10	3.79E-09	12.55299

GCA	1.168854	10.83533	9.730738	8.19E-17	2.95E-15	27.5927
C8orf88	1.167132	5.079281	5.946192	2.82E-08	2.25E-07	8.215408
TTC7B	1.166334	5.249212	5.842614	4.58E-08	3.55E-07	7.741677
MSMO1	1.165347	6.54456	6.851798	3.44E-10	3.87E-09	12.53277
CHCHD1	1.162144	7.751109	9.402389	4.92E-16	1.51E-14	25.81688
MARCO	1.161947	6.368577	5.645619	1.14E-07	8.23E-07	6.853525
NCAPG	1.161583	3.13311	4.382969	2.54E-05	0.00012	1.622618
MCTP1	1.1612	6.466885	8.354745	1.41E-13	2.79E-12	20.22159
KIAA0930	1.159728	7.826659	11.68053	1.84E-21	1.50E-19	38.20277
UHRF1	1.158215	4.597458	4.48893	1.67E-05	8.16E-05	2.027217
1-Mar	1.157297	8.460404	7.119085	8.94E-11	1.10E-09	13.85934
CREG1	1.155231	9.383334	8.265579	2.27E-13	4.31E-12	19.75221
CSGALNA(	1.154614	7.957224	6.328809	4.54E-09	4.18E-08	10.00303
RRAGD	1.152681	7.530236	8.879676	8.40E-15	2.09E-13	23.00886
VSIG4	1.152557	5.453358	3.146527	0.002088	0.006604	-2.54062
TSHZ3	1.151738	6.688272	7.224524	5.22E-11	6.74E-10	14.38826
P2RX1	1.150937	7.657422	7.184484	6.40E-11	8.09E-10	14.18705
PDCD10	1.147157	8.876198	6.791958	4.65E-10	5.09E-09	12.23873
ISCA1	1.14612	7.851578	3.834284	0.000203	0.000798	-0.35718
FLOT1	1.145269	7.861465	15.01153	3.23E-29	8.37E-27	55.9176
SYNE1	1.144055	5.156368	12.22486	9.41E-23	9.39E-21	41.15377
HIST1H2BI	1.143973	7.847581	7.595931	7.69E-12	1.13E-10	16.27436
CA1	1.14337	6.188898	4.300982	3.51E-05	0.000161	1.314343
DHFR	1.143124	5.247995	6.954324	2.06E-10	2.40E-09	13.03912
HIST1H4J	1.142348	5.602437	9.600438	1.67E-16	5.65E-15	26.8871
UBE2J1	1.141115	7.211487	10.45227	1.56E-18	7.53E-17	31.51446
C2orf76	1.138624	4.693352	5.90637	3.40E-08	2.69E-07	8.03274
NOP10	1.138141	9.495802	9.361253	6.16E-16	1.87E-14	25.59497
SRPK1	1.136038	8.053104	12.30074	6.22E-23	6.36E-21	41.56392
GSR	1.135493	7.216076	9.347312	6.64E-16	2.00E-14	25.5198
BCL6	1.134763	10.12799	7.719651	4.04E-12	6.26E-11	16.90997
SLC40A1	1.133037	7.691017	9.34618	6.68E-16	2.01E-14	25.51369
CTSH	1.13088	8.938937	8.192678	3.34E-13	6.15E-12	19.36944
TGFB1I1	1.12918	4.46021	4.058118	8.89E-05	0.000375	0.426432
GNG5	1.128385	10.83635	12.06317	2.27E-22	2.13E-20	40.27862
CMTM4	1.126944	5.121027	9.017556	3.98E-15	1.05E-13	23.74685
CR1L	1.122983	5.432744	4.258703	4.13E-05	0.000187	1.157033
TMEM120,	1.122973	7.872916	10.59292	7.22E-19	3.64E-17	32.28066
MERTK	1.120595	4.09755	7.065157	1.18E-10	1.42E-09	13.59002
GMFG	1.118665	10.04687	7.066606	1.17E-10	1.41E-09	13.59724
MAFG	1.117469	6.101115	16.2593	5.33E-32	2.18E-29	62.26823
VNN2	1.117038	10.61364	6.52096	1.78E-09	1.76E-08	10.92162
C5orf30	1.117022	5.552169	4.699255	7.08E-06	3.71E-05	2.850384
SIGLEC9	1.116955	7.049039	11.26407	1.81E-20	1.20E-18	35.9374
IL10	1.116265	4.55316	5.424385	3.11E-07	2.08E-06	5.877166
EMC2	1.115291	6.045923	5.825876	4.96E-08	3.82E-07	7.665554
PLGRKT	1.110417	6.456151	8.694113	2.29E-14	5.19E-13	22.01925
PHTF1	1.107955	5.32814	11.19075	2.70E-20	1.73E-18	35.53811
DUSP13	1.107324	5.04719	6.289592	5.49E-09	4.95E-08	9.817204
DHCR7	1.106128	5.40559	8.771414	1.51E-14	3.58E-13	22.43097
OIP5	1.104868	3.881998	5.297928	5.46E-07	3.52E-06	5.329612
CDK5RAP2	1.101556	6.215161	10.8853	1.45E-19	8.12E-18	33.87397
AGTPBP1	1.100011	7.778526	7.297841	3.59E-11	4.75E-10	14.75782
PRDX4	1.098493	6.782427	4.966333	2.31E-06	1.32E-05	3.93246
PNP	1.094845	8.248518	5.365625	4.04E-07	2.66E-06	5.621761
ABCA13	1.094633	2.509298	3.412677	0.00088	0.003031	-1.73737
TBC1D8	1.094078	5.825046	12.89637	2.45E-24	3.22E-22	44.77125
MOSPD3	1.092797	7.115916	9.931531	2.73E-17	1.07E-15	28.68194

MBOAT2	1.091898	7.118673	15.35857	5.34E-30	1.58E-27	57.70224
ITGAM	1.088694	9.919835	9.258437	1.08E-15	3.15E-14	25.04095
DHRS13	1.087582	7.57994	11.14252	3.52E-20	2.23E-18	35.27543
NXT2	1.085343	6.183926	5.947499	2.81E-08	2.24E-07	8.221413
FOXM1	1.08528	4.169101	5.675257	9.95E-08	7.28E-07	6.986044
RHAG	1.082679	4.484721	4.377028	2.60E-05	0.000122	1.600137
NABP1	1.080958	7.385708	5.571461	1.60E-07	1.12E-06	6.523697
FAM101B	1.080733	10.50014	8.275146	2.15E-13	4.13E-12	19.80251
AZI2	1.076873	5.540666	9.653376	1.25E-16	4.32E-15	27.17364
WDFY3	1.076508	6.78301	10.39208	2.18E-18	1.03E-16	31.1867
CCNA2	1.075256	4.103937	4.829109	4.13E-06	2.26E-05	3.371488
FES	1.07264	7.158408	10.47202	1.40E-18	6.80E-17	31.62204
FLOT2	1.070848	8.576594	9.318005	7.79E-16	2.34E-14	25.36182
IGFBP2	1.068182	5.172815	6.066263	1.60E-08	1.33E-07	8.770153
ORMDL2	1.067949	7.841158	11.12218	3.94E-20	2.47E-18	35.16463
CENPE	1.067397	3.055326	5.788602	5.89E-08	4.49E-07	7.496463
ACSL4	1.067369	5.455068	6.844713	3.57E-10	4.00E-09	12.4979
NDUFA1	1.066716	7.76426	7.184745	6.40E-11	8.08E-10	14.18836
TFDP1	1.066306	6.616099	9.547521	2.23E-16	7.36E-15	26.60087
LOC64598	1.066022	3.550057	6.48086	2.16E-09	2.10E-08	10.72883
MTF1	1.06567	7.6119	13.02222	1.24E-24	1.71E-22	45.44571
LAMTOR1	1.065377	8.79997	13.80611	1.86E-26	3.45E-24	49.61757
CDADC1	1.064756	4.565643	8.062273	6.66E-13	1.18E-11	18.6871
KLF5	1.064609	4.593625	6.122022	1.23E-08	1.04E-07	9.029748
IL4R	1.063799	8.987514	7.624762	6.62E-12	9.83E-11	16.42217
DNASE1L1	1.062415	8.091132	11.97521	3.67E-22	3.30E-20	39.80204
CPEB4	1.061567	7.950717	10.44926	1.59E-18	7.64E-17	31.4981
FKBP1B	1.061477	7.109345	4.239874	4.45E-05	0.0002	1.087341
PSAT1	1.060672	5.058162	5.655366	1.09E-07	7.89E-07	6.897061
KIF4A	1.060618	4.166203	5.999517	2.19E-08	1.78E-07	8.461046
SMIM3	1.059979	7.599934	11.23486	2.12E-20	1.38E-18	35.77835
LINC0026€	1.059743	5.114259	5.459094	2.66E-07	1.80E-06	6.028815
SPI1	1.059575	8.36059	7.755658	3.34E-12	5.25E-11	17.09559
TMEM256	1.05808	5.189847	6.956854	2.03E-10	2.37E-09	13.05166
ATP5J	1.05687	7.639041	6.741149	5.99E-10	6.45E-09	11.98995
HHEX	1.053946	8.389137	7.19514	6.07E-11	7.70E-10	14.24056
SH3GLB1	1.053534	9.105011	9.873118	3.76E-17	1.43E-15	28.36485
POMP	1.05274	6.198217	11.25601	1.89E-20	1.25E-18	35.89351
SELP	1.051879	6.512264	5.143968	1.07E-06	6.56E-06	4.673786
BUB1B	1.049385	4.691493	4.197467	5.24E-05	0.000231	0.931214
SEC24A	1.047242	5.152958	8.95305	5.65E-15	1.45E-13	23.40132
PLIN3	1.044351	8.602038	9.089056	2.70E-15	7.34E-14	24.13037
BAZ1A	1.044116	9.180592	8.101978	5.40E-13	9.67E-12	18.89453
PKM	1.040069	7.806387	9.282341	9.46E-16	2.80E-14	25.16967
ROGDI	1.031238	7.396933	9.020455	3.92E-15	1.04E-13	23.76239
CPNE3	1.031071	8.704844	5.13377	1.12E-06	6.83E-06	4.630777
KIF20A	1.029714	3.828457	5.693292	9.16E-08	6.75E-07	7.066879
VWA5A	1.027981	5.797817	5.26276	6.38E-07	4.06E-06	5.178745
PRDX5	1.027614	9.643333	9.090436	2.68E-15	7.29E-14	24.13777
CDC42EP3	1.026452	8.783897	8.495891	6.63E-14	1.37E-12	20.96719
CLIC1	1.026385	10.4839	11.37385	9.90E-21	6.84E-19	36.53501
RHOU	1.026151	5.785897	4.54991	1.30E-05	6.50E-05	2.263169
KIF14	1.024294	3.652209	5.665835	1.04E-07	7.57E-07	6.943871
F8	1.023918	5.821429	5.678976	9.78E-08	7.17E-07	7.002702
NAPRT	1.022728	7.578459	9.528634	2.47E-16	8.04E-15	26.49876
RARA-AS1	1.021554	7.505126	10.76828	2.75E-19	1.48E-17	33.23626
TRBV27	1.021154	7.071267	3.753714	0.000271	0.001041	-0.6308
MMP27	1.020001	3.203908	5.758559	6.78E-08	5.10E-07	7.360618

ACOX2	1.019249	4.273971	6.588155	1.28E-09	1.30E-08	11.24592
DPH3	1.018893	6.622529	9.417481	4.53E-16	1.40E-14	25.89833
CA2	1.018524	7.560025	3.250737	0.001498	0.004894	-2.23259
MSRB2	1.016163	6.302841	8.779337	1.44E-14	3.45E-13	22.47321
NIPSNAP3	1.01481	6.016789	5.290687	5.64E-07	3.63E-06	5.298498
EBPL	1.014507	7.185132	9.613996	1.55E-16	5.30E-15	26.96047
ARHGAP18	1.011884	5.489931	7.420312	1.91E-11	2.64E-10	15.37824
GMFB	1.008248	7.031298	6.231257	7.27E-09	6.42E-08	9.54185
SOCS3	1.00779	5.094537	9.920778	2.89E-17	1.14E-15	28.62356
CEBPZOS	1.005907	4.593257	9.405964	4.83E-16	1.48E-14	25.83617
SMAD1	1.005716	4.631172	5.294406	5.55E-07	3.58E-06	5.314473
PPP4R2	1.004798	6.217469	8.628944	3.24E-14	7.10E-13	21.67276
RAB10	1.003977	9.750817	12.34428	4.91E-23	5.15E-21	41.79916
TIPARP	1.002485	7.778915	7.855978	1.98E-12	3.25E-11	17.61422
TRPM2	1.000883	5.385709	12.37012	4.26E-23	4.52E-21	41.93869
ALCAM	0.997908	5.337748	6.388995	3.39E-09	3.20E-08	10.28933
LYPLA1	0.997847	8.617328	8.521606	5.77E-14	1.21E-12	21.10335
PDZD8	0.996169	5.597471	9.471101	3.38E-16	1.08E-14	26.18786
ELOVL7	0.99526	5.579351	3.76044	0.000265	0.001019	-0.60813
PRUNE2	0.993879	3.47921	3.970186	0.000123	0.000506	0.114533
TRPS1	0.991071	5.650415	8.869268	8.89E-15	2.20E-13	22.95324
PAG1	0.99072	8.663066	6.873994	3.08E-10	3.49E-09	12.64212
PPBP	0.990302	11.18437	3.883357	0.00017	0.000678	-0.18831
ANKRD9	0.986909	5.642701	5.507586	2.14E-07	1.47E-06	6.241653
USMG5	0.98688	9.050965	7.148289	7.70E-11	9.59E-10	14.00554
RAD51AP1	0.986236	3.452903	4.084252	8.06E-05	0.000343	0.520125
SCCPDH	0.986229	5.710821	9.720263	8.67E-17	3.10E-15	27.53594
IGFBP7	0.985615	5.569774	9.616552	1.53E-16	5.24E-15	26.97431
CPNE2	0.985579	7.468834	6.694132	7.56E-10	7.96E-09	11.76048
CTSL	0.985168	6.345394	5.177589	9.27E-07	5.75E-06	4.815966
VAMP7	0.984757	8.528614	9.590615	1.76E-16	5.92E-15	26.83396
SPC25	0.984583	2.474383	3.818829	0.000215	0.00084	-0.41002
VPS54	0.982742	6.555088	7.347316	2.78E-11	3.76E-10	15.00799
LOC10272	0.981768	3.022138	6.483678	2.13E-09	2.07E-08	10.74237
MGAM	0.981476	9.88588	5.824045	5.00E-08	3.85E-07	7.657232
CEBPD	0.980318	7.810487	6.454554	2.46E-09	2.37E-08	10.60267
GLRX2	0.979975	6.409092	8.300929	1.88E-13	3.62E-12	19.93814
EIF4E3	0.978833	7.138084	7.376232	2.40E-11	3.28E-10	15.1545
HIP1	0.978246	5.400565	8.741127	1.77E-14	4.14E-13	22.26956
MSRA	0.977946	5.5158	13.57744	6.29E-26	1.05E-23	48.40614
ALOX12	0.977693	6.154876	4.06017	8.82E-05	0.000372	0.433771
NDUFB9	0.977465	9.528475	9.077305	2.88E-15	7.78E-14	24.06729
TRMT6	0.977353	6.078535	6.723693	6.53E-10	6.97E-09	11.90467
FAM160A2	0.977068	7.089401	10.7368	3.27E-19	1.74E-17	33.06468
SIPA1L2	0.976448	6.201236	8.28528	2.04E-13	3.92E-12	19.85581
AMPD3	0.976094	7.747713	8.691871	2.31E-14	5.24E-13	22.00732
AQP10	0.975697	5.4992	4.654061	8.53E-06	4.38E-05	2.671302
CEACAM2	0.974706	6.244383	3.926226	0.000145	0.000587	-0.03944
MRPL13	0.974676	4.900567	4.722255	6.44E-06	3.41E-05	2.94198
PCYT1A	0.974491	6.611936	7.917534	1.43E-12	2.40E-11	17.93347
IL1R1	0.974325	4.914718	8.954743	5.60E-15	1.44E-13	23.41038
IFT20	0.97429	6.995407	6.775418	5.05E-10	5.49E-09	12.15765
NDST2	0.973175	5.910473	10.11382	1.00E-17	4.20E-16	29.67245
PROK2	0.972034	10.86534	4.971635	2.26E-06	1.29E-05	3.954343
UBE2S	0.969864	6.498071	5.448134	2.79E-07	1.88E-06	5.980865
FAM217B	0.969759	7.508536	5.911231	3.33E-08	2.63E-07	8.055001
TRIP13	0.969649	4.700021	6.0649	1.61E-08	1.34E-07	8.763821
MTX1	0.969358	7.940509	11.78982	1.01E-21	8.55E-20	38.79631

LY96	0.968663	8.197083	4.647171	8.77E-06	4.50E-05	2.644102
NCF4	0.96734	10.28107	10.49296	1.25E-18	6.11E-17	31.7361
QPCT	0.966631	10.19614	6.528954	1.71E-09	1.70E-08	10.96012
SPTSSA	0.966488	5.949268	6.187609	8.97E-09	7.80E-08	9.33667
GNA15	0.964482	6.898361	8.89168	7.87E-15	1.97E-13	23.07302
GNG10	0.963997	10.62292	6.504049	1.93E-09	1.89E-08	10.84025
TBCA	0.961499	7.750203	7.645537	5.94E-12	8.93E-11	16.5288
DIRC2	0.96136	6.001612	9.233498	1.23E-15	3.59E-14	24.90671
ADORA2B	0.960055	4.919235	7.71942	4.04E-12	6.27E-11	16.90878
SELT	0.959815	7.447917	6.796294	4.55E-10	4.99E-09	12.26
FBXO30	0.959814	5.090549	8.167039	3.83E-13	7.01E-12	19.23505
NARF	0.95959	6.441053	8.155488	4.07E-13	7.41E-12	19.17453
BATF	0.959375	7.461309	11.8699	6.53E-22	5.59E-20	39.2309
MSRB1	0.959311	11.38163	11.73558	1.36E-21	1.12E-19	38.50179
ACSL1	0.959305	11.10952	6.715243	6.81E-10	7.25E-09	11.86343
CLEC4E	0.958084	7.85824	5.806253	5.43E-08	4.16E-07	7.576459
TMCC2	0.957846	6.565267	3.554882	0.000544	0.001959	-1.28627
PRKAR2B	0.955644	8.511331	3.681579	0.00035	0.001314	-0.87188
MRPS36	0.955095	5.933426	6.683366	7.98E-10	8.37E-09	11.70804
GCNT1	0.954232	5.23373	10.35912	2.61E-18	1.21E-16	31.00722
CISD2	0.954225	4.7842	5.113033	1.23E-06	7.42E-06	4.543484
ZAK	0.953569	5.294025	10.11669	9.88E-18	4.14E-16	29.68809
MS4A6A	0.953131	8.004474	9.678409	1.09E-16	3.82E-15	27.3092
SGMS2	0.953103	4.946442	6.45967	2.40E-09	2.31E-08	10.62719
NLRP12	0.952312	8.129297	8.683514	2.42E-14	5.45E-13	21.96285
HIST1H2BI	0.95191	10.47568	7.300068	3.55E-11	4.70E-10	14.76906
S100A6	0.951774	8.07571	13.32865	2.39E-25	3.70E-23	47.08283
ETHE1	0.951351	7.007127	7.246747	4.66E-11	6.07E-10	14.50013
SLC1A3	0.949369	4.126383	6.529682	1.70E-09	1.69E-08	10.96363
DBI	0.949327	7.359784	4.668723	8.03E-06	4.15E-05	2.729271
GLT1D1	0.946989	9.527338	9.542012	2.30E-16	7.56E-15	26.57108
PLOD2	0.946035	3.194611	5.717216	8.20E-08	6.10E-07	7.17433
PDSS1	0.945735	5.505658	7.102949	9.70E-11	1.19E-09	13.77867
CETP	0.944853	4.200641	6.155804	1.04E-08	8.95E-08	9.187627
SCPEP1	0.941757	6.672479	8.726038	1.92E-14	4.46E-13	22.18919
LILRA6	0.941672	8.893605	7.839333	2.16E-12	3.51E-11	17.52802
VIM	0.941655	8.867057	7.84456	2.10E-12	3.42E-11	17.55508
HMGB2	0.940715	6.467856	9.920183	2.90E-17	1.14E-15	28.62033
GLTP	0.939713	8.004607	9.291304	9.01E-16	2.68E-14	25.21795
MOSPD1	0.938337	6.191067	5.527754	1.95E-07	1.35E-06	6.330501
GSKIP	0.93734	7.741072	4.857306	3.66E-06	2.02E-05	3.485905
TK1	0.937007	5.529782	5.090117	1.36E-06	8.12E-06	4.447279
MELK	0.936403	4.973523	4.463301	1.85E-05	8.95E-05	1.928723
CKS1B	0.935935	5.604562	4.76416	5.41E-06	2.90E-05	3.109645
BNIP2	0.935592	7.543982	6.726545	6.44E-10	6.88E-09	11.9186
LSM6	0.935411	7.035868	10.73521	3.30E-19	1.75E-17	33.056
ATOX1	0.932216	7.255789	11.46414	6.03E-21	4.31E-19	37.02631
PIK3CB	0.931538	6.793434	9.541753	2.30E-16	7.56E-15	26.56968
APOBEC3E	0.931478	5.712248	2.1443	0.034045	0.074299	-5.04928
BIRC5	0.930111	4.575425	6.461843	2.38E-09	2.29E-08	10.63761
TNFAIP8L3	0.926566	3.895305	6.546415	1.57E-09	1.57E-08	11.04429
CHCHD7	0.926532	6.835804	6.736688	6.12E-10	6.58E-09	11.96815
MCU	0.926507	6.25918	6.228829	7.36E-09	6.49E-08	9.530416
SPCS3	0.926286	6.861848	7.350592	2.74E-11	3.70E-10	15.02458
MTHFD2	0.923389	5.44012	7.957905	1.16E-12	1.98E-11	18.14327
CDK5	0.922975	6.298592	7.636922	6.21E-12	9.30E-11	16.48457
PBX1	0.920346	5.083011	3.966253	0.000125	0.000513	0.100703
CCNE2	0.919904	3.525507	4.134521	6.66E-05	0.000288	0.701619

H2BFS	0.918935	7.888216	6.840394	3.65E-10	4.08E-09	12.47665
BAMBI	0.917356	3.850155	5.046768	1.64E-06	9.64E-06	4.266058
ZDHHC3	0.916647	6.044857	14.26174	1.65E-27	3.41E-25	52.0165
RABAC1	0.916461	8.793301	9.538542	2.34E-16	7.65E-15	26.55232
OR7E37P	0.915828	6.820226	10.60719	6.68E-19	3.39E-17	32.35843
FAM228B	0.91381	5.578839	9.286239	9.26E-16	2.75E-14	25.19066
TMEM52B	0.913525	2.560072	3.026148	0.003037	0.009212	-2.88584
OSM	0.912544	5.610021	8.72247	1.96E-14	4.55E-13	22.17019
IDH1	0.912438	6.982433	8.959445	5.46E-15	1.41E-13	23.43555
TMEM170I	0.910463	6.942321	6.066211	1.60E-08	1.33E-07	8.769913
ALOX5	0.91024	8.026119	9.119717	2.29E-15	6.30E-14	24.29499
MICAL1	0.909123	8.008914	8.150438	4.18E-13	7.60E-12	19.14808
B3GNT5	0.909088	4.785841	7.199794	5.92E-11	7.54E-10	14.26394
IMPA2	0.906601	8.781346	7.632728	6.35E-12	9.47E-11	16.46305
NFE2	0.906511	10.22127	10.92273	1.18E-19	6.66E-18	34.07793
CDK1	0.906132	3.561133	4.766434	5.36E-06	2.88E-05	3.118772
SFXN1	0.904828	6.185414	4.760069	5.50E-06	2.95E-05	3.093234
TTC8	0.904407	4.086729	6.343798	4.22E-09	3.91E-08	10.07421
WASF3	0.904167	4.000794	4.353439	2.85E-05	0.000134	1.511097
PGM1	0.90414	7.819171	8.688762	2.35E-14	5.32E-13	21.99078
SNRPG	0.903919	7.228492	3.528757	0.000595	0.002127	-1.37026
H1F0	0.903637	6.382579	6.060704	1.64E-08	1.36E-07	8.744341
HIST1H2A	0.903189	4.490235	4.921612	2.79E-06	1.57E-05	3.7485
ERO1L	0.901065	7.389859	7.968395	1.09E-12	1.87E-11	18.19783
PGK1	0.9008	8.343589	7.56165	9.19E-12	1.34E-10	16.09886
NTNG2	0.899888	6.24923	8.024564	8.13E-13	1.42E-11	18.49037
CD58	0.899841	6.230592	6.428674	2.79E-09	2.67E-08	10.47879
MANSC1	0.897545	8.762646	6.001209	2.18E-08	1.77E-07	8.468862
COX8A	0.897355	9.602562	9.113571	2.37E-15	6.50E-14	24.26198
TCTEX1D1	0.89731	2.721885	4.202103	5.14E-05	0.000228	0.948225
CCNDBP1	0.895921	10.16903	7.804418	2.59E-12	4.15E-11	17.3474
TMED8	0.895016	7.088889	15.53927	2.10E-30	6.84E-28	58.62599
LOC15354	0.893965	5.104487	8.128426	4.70E-13	8.49E-12	19.03286
LPCAT2	0.893623	5.858587	9.254013	1.10E-15	3.23E-14	25.01713
LOC10013	0.89276	3.823581	5.008336	1.93E-06	1.12E-05	4.10623
WFDC1	0.888151	4.920528	3.671885	0.000362	0.001355	-0.90399
MRPL33	0.887766	8.056362	9.215509	1.36E-15	3.92E-14	24.80991
SIL1	0.887691	5.908677	10.80126	2.30E-19	1.25E-17	33.41598
MKI67	0.886856	4.524907	5.391135	3.61E-07	2.39E-06	5.732436
PADI2	0.886499	6.200792	8.442364	8.82E-14	1.80E-12	20.68407
GPI	0.885996	8.374483	4.835245	4.02E-06	2.20E-05	3.396346
BAG4	0.885966	5.590375	8.536424	5.33E-14	1.12E-12	21.18186
TIFA	0.885564	5.121111	7.648973	5.84E-12	8.79E-11	16.54644
MSRB3	0.884455	4.57802	7.272744	4.08E-11	5.35E-10	14.63116
TMEM56	0.882991	3.02611	3.816907	0.000216	0.000845	-0.41658
SLC14A1	0.882817	6.296203	2.377672	0.019018	0.045467	-4.5415
MGST2	0.882053	6.372536	7.976036	1.05E-12	1.80E-11	18.23759
MAD2L2	0.881447	6.830584	8.370393	1.30E-13	2.57E-12	20.3041
TRAPPC5	0.879959	8.100318	8.122883	4.84E-13	8.72E-12	19.00386
TMCO3	0.879052	4.832538	8.629392	3.24E-14	7.09E-13	21.67513
CD82	0.878767	7.123182	8.195647	3.29E-13	6.06E-12	19.38501
PSMB6	0.878393	7.697606	7.215633	5.46E-11	7.00E-10	14.34355
MYL6	0.877066	8.514209	12.91397	2.23E-24	2.95E-22	44.86562
ZNHIT1	0.87526	7.461258	9.807955	5.37E-17	2.00E-15	28.01132
CLEC12A	0.874772	5.488481	3.671184	0.000363	0.001358	-0.90631
C17orf62	0.8742	8.577807	9.18208	1.63E-15	4.63E-14	24.63011
CETN2	0.873013	7.02089	8.770668	1.51E-14	3.58E-13	22.42699
MFF	0.873	6.630871	8.874207	8.65E-15	2.15E-13	22.97963

SLC36A4	0.872603	4.159098	7.785257	2.86E-12	4.56E-11	17.24839
TRIQK	0.872386	4.682968	9.604707	1.63E-16	5.53E-15	26.9102
CCDC53	0.872006	6.997599	7.933804	1.31E-12	2.22E-11	18.01798
S100A11	0.870343	10.89803	10.39202	2.18E-18	1.03E-16	31.18637
AGL	0.870057	6.917502	4.117825	7.10E-05	0.000305	0.641152
LINC00597	0.869733	4.505558	5.504275	2.17E-07	1.49E-06	6.227083
TRAPPC1	0.869112	9.489506	9.355091	6.37E-16	1.92E-14	25.56174
WSB2	0.868787	8.862314	7.092968	1.02E-10	1.24E-09	13.72881
HIGD1A	0.868721	6.924606	7.236839	4.90E-11	6.36E-10	14.45024
HK2	0.867347	7.613544	8.155768	4.06E-13	7.41E-12	19.176
C15orf65	0.866934	3.77756	4.875642	3.39E-06	1.88E-05	3.560547
POLE2	0.866021	4.170132	4.044331	9.36E-05	0.000393	0.377184
SAR1B	0.865776	5.590181	8.659372	2.76E-14	6.14E-13	21.83446
AIG1	0.864836	4.149947	7.639164	6.14E-12	9.21E-11	16.49608
SPTLC2	0.864521	6.452346	7.200126	5.91E-11	7.53E-10	14.26561
CCDC126	0.864203	6.009824	5.002029	1.98E-06	1.15E-05	4.080077
SLIRP	0.863498	5.815064	4.188603	5.42E-05	0.000239	0.898726
ZNF281	0.863231	7.419118	7.308136	3.40E-11	4.52E-10	14.80982
G6PD	0.862819	7.767907	5.461317	2.63E-07	1.79E-06	6.038549
SRA1	0.860984	7.778704	10.24127	4.98E-18	2.19E-16	30.36573
SEPHS2	0.860005	8.520371	9.795899	5.73E-17	2.12E-15	27.94594
KIF2C	0.859803	4.858379	6.462741	2.37E-09	2.28E-08	10.64191
TREML3P	0.857319	4.817545	6.45287	2.48E-09	2.39E-08	10.5946
MXD3	0.857251	6.540978	10.57003	8.19E-19	4.09E-17	32.15597
COL4A3BP	0.857108	8.138258	8.729234	1.89E-14	4.39E-13	22.20621
YIPF1	0.855937	6.857151	9.491386	3.03E-16	9.73E-15	26.29744
SLCO4C1	0.855721	4.651734	7.950273	1.20E-12	2.05E-11	18.10358
FHL2	0.85484	5.979566	3.302211	0.001267	0.004219	-2.07735
LAMTOR2	0.853758	6.419754	7.807413	2.55E-12	4.09E-11	17.36289
GINS1	0.85337	3.527587	3.470119	0.000726	0.002545	-1.55696
SEC61G	0.851621	6.456113	6.504168	1.93E-09	1.89E-08	10.84083
FAR1	0.850869	7.307509	5.634843	1.20E-07	8.62E-07	6.80544
PYCARD	0.850743	9.277225	8.641246	3.04E-14	6.71E-13	21.73812
MRPL22	0.84989	4.972456	4.281869	3.78E-05	0.000172	1.243089
SVIP	0.849751	5.854228	5.238826	7.09E-07	4.48E-06	5.076425
GP9	0.847888	7.049317	7.173431	6.78E-11	8.53E-10	14.13158
C7orf73	0.847114	5.535686	6.939663	2.22E-10	2.56E-09	12.96652
APCDD1	0.846947	3.846193	2.708175	0.007764	0.020937	-3.74156
GABARAPL	0.846663	10.69822	7.791232	2.78E-12	4.43E-11	17.27925
LOC10050	0.84658	5.072816	12.73079	6.01E-24	7.24E-22	43.88204
CD163	0.845172	5.778606	2.310275	0.022598	0.052563	-4.69307
PTPN22	0.844645	5.253545	6.507994	1.89E-09	1.86E-08	10.85922
AMPH	0.843327	3.946499	4.037575	9.60E-05	0.000402	0.353101
NFKBIZ	0.840465	9.389352	7.430486	1.81E-11	2.51E-10	15.42995
NME1	0.838188	6.184502	4.146266	6.37E-05	0.000277	0.744263
IFNGR1	0.83701	9.086576	6.818169	4.08E-10	4.52E-09	12.36739
DOK3	0.835353	8.41578	9.283913	9.38E-16	2.78E-14	25.17814
PLD1	0.835284	4.028618	8.225541	2.80E-13	5.25E-12	19.54188
ANKS1A	0.834755	6.956127	5.573456	1.58E-07	1.11E-06	6.532537
SUMF1	0.833753	7.819758	6.77718	5.00E-10	5.45E-09	12.16629
STXBP2	0.832314	7.423425	11.31618	1.36E-20	9.21E-19	36.2211
SLC36A1	0.8323	6.101049	10.32905	3.08E-18	1.41E-16	30.8435
C4orf3	0.832145	8.743606	7.544797	1.00E-11	1.45E-10	16.01269
PPP1R12A	0.831045	8.43031	7.045259	1.30E-10	1.56E-09	13.49085
FCRL1	0.829981	5.893342	3.439787	0.000804	0.002793	-1.65253
LOC10050	0.829495	5.538152	5.573682	1.58E-07	1.11E-06	6.533542
NEK2	0.828961	3.156713	5.502576	2.19E-07	1.50E-06	6.219612
ARL8A	0.828438	6.833457	10.45446	1.55E-18	7.46E-17	31.52639

ZUFSP	0.825986	5.234283	4.917672	2.84E-06	1.60E-05	3.732344
ZNF354A	0.825962	6.071563	3.992471	0.000114	0.000469	0.193086
FBXO9	0.825606	6.707306	11.96621	3.86E-22	3.44E-20	39.75323
TMEM40	0.825234	6.045568	5.410311	3.31E-07	2.21E-06	5.815839
GLA	0.824681	7.564206	9.985806	2.02E-17	8.06E-16	28.97671
AURKA	0.823433	3.78461	6.203439	8.31E-09	7.28E-08	9.410999
FGR	0.823286	9.892312	7.838826	2.16E-12	3.51E-11	17.52539
TCEB1	0.822127	5.649737	6.316816	4.81E-09	4.42E-08	9.946141
GALNT1	0.821252	7.419816	7.545218	1.00E-11	1.45E-10	16.01484
VMP1	0.820226	7.083096	4.302851	3.48E-05	0.00016	1.321324
SLC22A16	0.81943	4.272813	4.682361	7.59E-06	3.95E-05	2.783303
ADCY3	0.818449	5.437118	4.184236	5.51E-05	0.000242	0.882738
VRK1	0.818336	5.634874	3.178094	0.00189	0.006042	-2.4482
RSPH9	0.817509	3.816974	7.220685	5.32E-11	6.84E-10	14.36895
IL18	0.817022	4.455856	5.899684	3.51E-08	2.77E-07	8.002135
GGTLC1	0.816915	4.526555	7.197474	5.99E-11	7.62E-10	14.25228
ATP13A3	0.816325	5.813534	6.667486	8.63E-10	8.99E-09	11.63075
AGTRAP	0.812822	7.925813	7.222081	5.29E-11	6.82E-10	14.37598
FTX	0.812046	6.604885	4.238358	4.47E-05	0.000201	1.081738
ERH	0.811595	9.019973	5.923247	3.14E-08	2.49E-07	8.110073
EMILIN2	0.810933	5.622385	6.855371	3.38E-10	3.80E-09	12.55036
FAM110B	0.810883	3.788844	9.013135	4.08E-15	1.07E-13	23.72315
IL10RB	0.810447	8.653095	6.925051	2.38E-10	2.74E-09	12.89423
C9orf72	0.809843	5.499596	5.211328	8.00E-07	5.02E-06	4.959231
LIMK2	0.809481	7.627367	6.505766	1.92E-09	1.88E-08	10.84851
ZNF282	0.809072	5.82512	7.661665	5.46E-12	8.27E-11	16.61164
UBTD1	0.8089	5.242475	12.06017	2.31E-22	2.16E-20	40.2624
FAM63B	0.808536	5.688519	6.7998	4.47E-10	4.92E-09	12.2772
CTSA	0.806707	9.373075	6.418454	2.94E-09	2.80E-08	10.42994
CLEC12B	0.806582	3.378507	3.601342	0.000463	0.001694	-1.13566
FBXL5	0.805811	9.322926	8.696471	2.26E-14	5.14E-13	22.0318
C10orf11	0.80578	5.330523	7.275472	4.02E-11	5.29E-10	14.64491
MGLL	0.805572	6.026724	4.398683	2.39E-05	0.000113	1.682182
HJURP	0.805148	3.846141	4.701507	7.02E-06	3.68E-05	2.859342
ANXA4	0.803899	7.579766	6.149012	1.08E-08	9.23E-08	9.155847
SDF2	0.803576	7.041765	10.06266	1.33E-17	5.45E-16	29.39433
THBS1	0.802897	3.961314	7.51344	1.18E-11	1.69E-10	15.85252
ACOT13	0.802888	5.88267	5.878435	3.88E-08	3.03E-07	7.904994
EBLN2	0.802331	7.01319	7.327608	3.08E-11	4.12E-10	14.90826
VAT1	0.801965	7.310379	6.147176	1.09E-08	9.30E-08	9.147262
TPM1	0.801859	5.071259	6.82573	3.93E-10	4.37E-09	12.40454
PIK3AP1	0.801542	8.255333	4.840938	3.93E-06	2.16E-05	3.419431
STARD3NL	0.800364	7.135151	5.979625	2.41E-08	1.94E-07	8.369277
FUT4	0.799897	5.473029	5.169762	9.59E-07	5.92E-06	4.782813
VBP1	0.799253	8.08196	5.90333	3.45E-08	2.72E-07	8.018823
IER3	0.799222	7.787478	6.555394	1.50E-09	1.50E-08	11.08761
SEMA4A	0.798188	7.116139	7.473112	1.45E-11	2.06E-10	15.64688
BTBD3	0.797882	4.103498	5.982346	2.38E-08	1.92E-07	8.38182
NDUFA13	0.79712	8.490396	7.097285	9.98E-11	1.22E-09	13.75037
HSD3B7	0.797042	5.834066	7.063776	1.18E-10	1.42E-09	13.58313
RHOT1	0.796661	7.311215	7.195909	6.04E-11	7.68E-10	14.24442
UBAP1	0.796187	7.703225	9.587347	1.79E-16	6.02E-15	26.81628
C11orf71	0.795751	5.703135	11.08127	4.93E-20	3.06E-18	34.94178
TUBG1	0.795521	5.096215	4.260265	4.11E-05	0.000186	1.162822
KBTBD7	0.793481	5.824818	3.588154	0.000485	0.001768	-1.17857
JHDM1D-/	0.793172	6.8546	4.121037	7.01E-05	0.000302	0.652771
EXT1	0.793097	4.670902	5.703576	8.73E-08	6.46E-07	7.113036
ASPM	0.793072	3.105359	4.717419	6.57E-06	3.47E-05	2.922695

VPS25	0.792075	6.641632	9.464279	3.51E-16	1.12E-14	26.15101
PTRHD1	0.791423	7.097205	7.742911	3.57E-12	5.61E-11	17.02985
LINC0100(	0.786259	8.220647	5.800314	5.58E-08	4.27E-07	7.549529
NUF2	0.785669	2.734009	3.621515	0.000432	0.001588	-1.06978
COL9A3	0.785609	5.037669	4.098572	7.63E-05	0.000326	0.571658
KIF3C	0.783373	6.017721	7.833578	2.22E-12	3.61E-11	17.49823
JMJD6	0.783321	5.212255	11.54	3.98E-21	3.05E-19	37.43891
LYRM1	0.782888	7.686033	6.584602	1.30E-09	1.32E-08	11.22873
COQ2	0.782832	4.717176	7.215881	5.46E-11	7.00E-10	14.34479
HES6	0.781907	5.343227	4.210494	4.98E-05	0.000221	0.979052
GM2A	0.781708	5.54364	7.38678	2.27E-11	3.12E-10	15.20799
ENTPD1	0.781027	7.115256	6.826162	3.92E-10	4.36E-09	12.40666
GNAI3	0.779839	8.102673	6.393833	3.31E-09	3.13E-08	10.3124
COLGALT1	0.779409	6.502339	7.928018	1.35E-12	2.28E-11	17.98792
LOC10192	0.778711	3.441543	4.68774	7.43E-06	3.87E-05	2.804643
ACSL3	0.777496	6.367865	5.41971	3.17E-07	2.12E-06	5.856781
FECH	0.776507	6.824794	3.104653	0.002382	0.00742	-2.662
HSBP1	0.775961	7.615791	7.410312	2.01E-11	2.77E-10	15.32744
SETD8	0.775737	5.791999	12.87643	2.73E-24	3.52E-22	44.66428
HMGB3	0.775624	4.806267	7.588118	8.01E-12	1.18E-10	16.23434
AP3S1	0.775043	8.78213	7.580494	8.33E-12	1.22E-10	16.1953
FOXN2	0.772754	6.385463	7.208555	5.66E-11	7.24E-10	14.30796
SGOL2	0.772688	2.976941	4.16822	5.86E-05	0.000256	0.824216
ALPK1	0.771805	6.707838	6.616193	1.11E-09	1.14E-08	11.38169
HEBP2	0.770601	6.672647	13.85519	1.43E-26	2.73E-24	49.87696
RDH5	0.769624	5.487685	5.88059	3.84E-08	3.01E-07	7.914838
NR2E1	0.769084	4.274195	4.178273	5.64E-05	0.000247	0.860931
SIRPD	0.768983	5.839029	8.053172	6.99E-13	1.23E-11	18.63959
PGAM1	0.767611	10.51422	7.769351	3.11E-12	4.93E-11	17.16626
PSMA7	0.767504	7.313434	7.275682	4.02E-11	5.28E-10	14.64597
DYNLT1	0.767133	9.627925	7.820816	2.38E-12	3.84E-11	17.4322
DKFZp667	0.766511	4.035129	3.480183	0.000701	0.002469	-1.5251
DERA	0.76434	6.069711	5.781256	6.10E-08	4.62E-07	7.463213
OSBPL9	0.763114	8.088341	6.264244	6.20E-09	5.56E-08	9.697399
MIR646HG	0.763058	5.891474	5.27615	6.01E-07	3.85E-06	5.236114
AGPAT2	0.762387	6.195683	13.18053	5.30E-25	7.85E-23	46.29242
THOC7	0.762127	6.952286	6.196015	8.61E-09	7.52E-08	9.376127
PRADC1	0.761866	5.411543	8.329418	1.61E-13	3.14E-12	20.08813
FLJ30064	0.761715	5.258999	6.935091	2.27E-10	2.62E-09	12.94389
LSM1	0.760639	7.057341	6.780666	4.92E-10	5.36E-09	12.18337
NDUFAF7	0.759665	4.767744	6.333618	4.43E-09	4.09E-08	10.02586
FPGT	0.759102	5.844327	4.26425	4.05E-05	0.000183	1.177606
PHACTR2	0.755196	6.089652	6.58603	1.29E-09	1.31E-08	11.23564
GOLGA1	0.754887	6.029328	5.920171	3.19E-08	2.53E-07	8.095969
POC1B	0.753524	6.575678	5.181061	9.13E-07	5.67E-06	4.830682
FERMT3	0.753129	8.933493	7.315877	3.27E-11	4.36E-10	14.84895
LILRB3	0.752105	9.7318	7.053049	1.25E-10	1.50E-09	13.52966
MAN1A1	0.751806	6.849958	5.75955	6.74E-08	5.08E-07	7.365094
ABCG2	0.751379	4.17927	2.85534	0.005075	0.014458	-3.35578
FNBP1L	0.75104	2.989669	4.411053	2.27E-05	0.000108	1.729181
TNNI2	0.750374	5.150778	6.736719	6.12E-10	6.58E-09	11.9683
CSGALNA(	0.750195	8.13468	3.360963	0.001045	0.003539	-1.89768
HN1	0.750026	8.083597	8.344877	1.49E-13	2.92E-12	20.16958
MRPL51	0.749977	8.181115	4.930307	2.69E-06	1.52E-05	3.784178
CAPZA2	0.749653	6.881995	6.353591	4.03E-09	3.75E-08	10.12076
HIST1H2BI	0.748981	6.070964	6.162216	1.01E-08	8.70E-08	9.217641
COPS5	0.747467	7.417874	6.335881	4.39E-09	4.05E-08	10.03661
OSTF1	0.746385	8.608917	8.825497	1.13E-14	2.74E-13	22.71948

TOMM40L	0.745703	5.537099	6.168556	9.82E-09	8.46E-08	9.247337
FIBP	0.744698	7.679013	7.027256	1.42E-10	1.69E-09	13.40123
RACGAP1	0.744668	5.047785	3.317062	0.001207	0.004041	-2.03218
LOC10012	0.744603	7.244736	6.105751	1.33E-08	1.12E-07	8.953869
LRRFIP2	0.744062	4.332355	9.892297	3.38E-17	1.31E-15	28.46894
PRL	0.742752	3.803475	4.042786	9.42E-05	0.000395	0.371672
HPSE	0.742302	6.917755	4.564749	1.23E-05	6.14E-05	2.320924
KIF15	0.742271	2.507196	3.702531	0.000325	0.00123	-0.80224
AMFR	0.742085	5.874693	2.648624	0.00918	0.024172	-3.89255
B3GNT8	0.742085	6.545195	7.095855	1.01E-10	1.23E-09	13.74323
PNPLA6	0.741975	8.153646	7.345944	2.80E-11	3.78E-10	15.00105
C9orf66	0.740179	3.382308	6.73612	6.14E-10	6.60E-09	11.96537
FUT7	0.739665	5.661061	6.666459	8.68E-10	9.03E-09	11.62575
MLKL	0.739378	8.707929	7.449414	1.64E-11	2.30E-10	15.52622
TNFSF13B	0.739113	10.08497	4.659237	8.35E-06	4.30E-05	2.69175
BLM	0.738778	5.006836	4.023583	0.000101	0.000421	0.303315
SPAG5	0.737584	4.641337	5.785499	5.98E-08	4.54E-07	7.482414
C11orf73	0.736961	6.110032	6.033687	1.87E-08	1.54E-07	8.619064
TYW5	0.736589	4.829333	8.039549	7.52E-13	1.32E-11	18.56851
ABCB10	0.73607	6.35223	3.968259	0.000124	0.00051	0.107754
TSTA3	0.736017	7.73849	3.897953	0.000161	0.000645	-0.13777
GADD45G	0.735918	4.859089	6.675212	8.31E-10	8.69E-09	11.66834
ARPC1B	0.735848	10.2702	8.922134	6.68E-15	1.70E-13	23.23588
LOXL1	0.735383	3.621712	5.220551	7.68E-07	4.84E-06	4.998497
SEC62	0.735201	6.978096	6.962141	1.98E-10	2.32E-09	13.07786
CCNB2	0.735102	4.061358	9.691801	1.01E-16	3.60E-15	27.38174
ARPC2	0.735079	11.0117	10.79113	2.43E-19	1.31E-17	33.36076
HPD	0.733917	3.56656	3.734354	0.00029	0.001111	-0.69586
PAPSS1	0.733828	9.148876	5.981997	2.38E-08	1.92E-07	8.380214
HIST1H2BI	0.73368	5.762739	6.078119	1.51E-08	1.26E-07	8.825247
PTGR1	0.733608	3.613428	6.491047	2.06E-09	2.01E-08	10.77776
ELL2	0.733436	4.676791	5.155571	1.02E-06	6.26E-06	4.722787
ITGB3	0.732382	4.70612	4.71708	6.58E-06	3.47E-05	2.921346
KIF18B	0.731618	3.892715	4.813408	4.41E-06	2.40E-05	3.307971
UPB1	0.729938	4.501871	7.817237	2.42E-12	3.90E-11	17.41368
ARPC3	0.727891	10.60375	8.045175	7.30E-13	1.28E-11	18.59786
FAM200B	0.727888	6.449494	6.142862	1.11E-08	9.48E-08	9.127089
GRAMD1A	0.727449	6.476954	6.689729	7.73E-10	8.13E-09	11.73903
PLB1	0.727053	4.931352	8.346307	1.47E-13	2.90E-12	20.17712
BARD1	0.726847	4.765993	4.38949	2.48E-05	0.000117	1.647319
LINC0127	0.726834	4.753956	6.888273	2.87E-10	3.26E-09	12.71254
ETS2	0.726458	5.574415	6.189373	8.89E-09	7.74E-08	9.344949
CUTC	0.724726	7.069002	6.045007	1.77E-08	1.46E-07	8.67152
FAM96A	0.722843	8.443153	6.076614	1.52E-08	1.27E-07	8.81825
UFD1L	0.722748	8.26216	8.291069	1.98E-13	3.81E-12	19.88626
GSTZ1	0.722645	4.871684	7.90191	1.55E-12	2.58E-11	17.85237
LOC10050	0.722564	6.212781	6.531909	1.69E-09	1.67E-08	10.97436
FBXO6	0.721596	6.500561	4.830897	4.09E-06	2.24E-05	3.378731
ATP5C1	0.720879	8.164517	5.702302	8.78E-08	6.50E-07	7.107316
INHBA	0.720726	2.980978	4.912759	2.90E-06	1.63E-05	3.712213
PSMA4	0.7207	7.784211	3.7238	0.000301	0.00115	-0.73122
SNRNP25	0.720457	6.835804	4.209663	5.00E-05	0.000222	0.975995
PSMD14	0.719476	7.276093	4.276073	3.86E-05	0.000176	1.221526
MICU1	0.719265	7.775045	6.50839	1.89E-09	1.86E-08	10.86113
PXK	0.718128	6.371754	8.864929	9.10E-15	2.25E-13	22.93006
GRINA	0.717909	7.891535	5.407228	3.36E-07	2.23E-06	5.802416
RHOG	0.717626	10.69657	8.212575	3.00E-13	5.58E-12	19.47382
RHD	0.715937	4.986906	3.615732	0.000441	0.001618	-1.08869

CHCHD2	0.715575	9.388176	6.49524	2.02E-09	1.97E-08	10.79791
ERI1	0.714983	4.322566	6.739191	6.05E-10	6.51E-09	11.98038
CDCA5	0.714223	5.126485	4.685163	7.51E-06	3.90E-05	2.794417
RPL22L1	0.71418	5.137224	2.372637	0.019266	0.04598	-4.55296
ETFA	0.713763	7.973696	6.440147	2.64E-09	2.53E-08	10.53368
TMEM33	0.713689	6.717314	6.366761	3.78E-09	3.53E-08	10.18341
RAP1GAP	0.713061	5.328456	3.01163	0.003175	0.009571	-2.92669
ATP2C2	0.712473	4.304936	5.410903	3.30E-07	2.20E-06	5.818414
FAM107B	0.71207	9.256128	6.78706	4.76E-10	5.20E-09	12.21471
C6orf211	0.710185	6.864236	4.166556	5.89E-05	0.000258	0.818144
SPTA1	0.709581	3.66731	3.436048	0.000814	0.002822	-1.66427
COX7A2L	0.708836	8.112606	6.007864	2.11E-08	1.72E-07	8.499606
TPM4	0.708369	5.977948	6.290988	5.45E-09	4.92E-08	9.823809
MEF2BNB	0.706204	5.873975	9.997565	1.90E-17	7.62E-16	29.0406
FBXW2	0.706187	4.952143	7.02353	1.45E-10	1.72E-09	13.38269
HBQ1	0.705435	8.799362	3.508293	0.000638	0.002264	-1.43571
TOR4A	0.705338	4.832329	10.09829	1.09E-17	4.53E-16	29.58802
WDR41	0.705204	4.892821	7.900865	1.56E-12	2.59E-11	17.84694
CDC45	0.703153	4.272629	4.462303	1.85E-05	8.98E-05	1.924897
C1orf122	0.702326	6.849364	9.8279	4.81E-17	1.80E-15	28.11951
RCHY1	0.70181	5.420716	5.69224	9.20E-08	6.78E-07	7.062158
CYBB	0.701665	5.575432	8.869688	8.87E-15	2.20E-13	22.95549
CEACAM4	0.701221	7.796931	5.373222	3.91E-07	2.58E-06	5.654684
ARL6IP6	0.700829	6.022719	7.230905	5.05E-11	6.54E-10	14.42037
RAB20	0.700681	5.265927	8.886694	8.09E-15	2.02E-13	23.04637
USB1	0.700599	6.129813	10.2013	6.21E-18	2.69E-16	30.14827
PSMA2	0.698774	6.953026	4.535828	1.38E-05	6.86E-05	2.208484
FAM65C	0.69846	4.073819	5.259591	6.47E-07	4.12E-06	5.165182
LPGAT1	0.698107	7.801145	6.909402	2.58E-10	2.95E-09	12.81687
DHCR24	0.698088	4.673462	4.432599	2.09E-05	0.0001	1.811265
HBM	0.6976	11.55829	2.78971	0.006147	0.017074	-3.53004
ACTR10	0.696815	8.716742	7.253054	4.51E-11	5.88E-10	14.5319
QSOX1	0.696521	5.26475	11.50965	4.70E-21	3.51E-19	37.27389
PGM2L1	0.695964	5.415985	7.467591	1.50E-11	2.11E-10	15.61876
CYB5R3	0.695346	8.441554	6.145662	1.10E-08	9.36E-08	9.140182
MXI1	0.694456	10.80922	3.506843	0.000641	0.002275	-1.44033
CCDC176	0.694293	5.450324	3.205401	0.001732	0.005587	-2.36763
TMEM165	0.694236	5.032183	10.78382	2.53E-19	1.36E-17	33.32093
CSF2RA	0.694124	6.488353	6.567248	1.42E-09	1.43E-08	11.14485
NAT1	0.692388	5.900207	4.507016	1.55E-05	7.63E-05	2.096965
PAM	0.692143	6.275509	4.358882	2.79E-05	0.000131	1.531612
CD36	0.69198	6.563675	4.061961	8.76E-05	0.00037	0.440179
CYP19A1	0.691217	3.308627	6.827322	3.89E-10	4.34E-09	12.41236
LOC10050	0.691215	4.518948	9.231342	1.25E-15	3.62E-14	24.8951
COX5B	0.69104	7.829075	7.912604	1.47E-12	2.46E-11	17.90787
PSMB3	0.690905	10.11408	7.349749	2.75E-11	3.72E-10	15.02031
UROD	0.690302	5.229016	6.263406	6.23E-09	5.58E-08	9.69344
FSD1L	0.689288	2.802267	5.590777	1.46E-07	1.04E-06	6.609363
PRTFDC1	0.68876	2.829274	3.363763	0.001035	0.00351	-1.88905
C9orf84	0.68855	3.607842	5.336382	4.60E-07	3.00E-06	5.495282
RNF181	0.687467	8.51778	8.492919	6.73E-14	1.40E-12	20.95146
H2AFZ	0.686864	9.154013	5.783768	6.03E-08	4.57E-07	7.474581
RNF141	0.686381	5.721539	5.342608	4.48E-07	2.93E-06	5.522173
MYL9	0.686113	5.539607	3.86627	0.000181	0.000717	-0.2473
STK3	0.685983	3.912054	8.20997	3.05E-13	5.64E-12	19.46015
DNAJC5	0.685874	7.361528	8.022249	8.23E-13	1.44E-11	18.4783
CLTC-IT1	0.685775	3.758457	4.272383	3.92E-05	0.000178	1.20781
SGTB	0.684676	6.078146	4.791846	4.82E-06	2.61E-05	3.220968

C1GALT1C	0.684592	5.708935	6.608244	1.16E-09	1.18E-08	11.34317
TXNDC17	0.684252	6.346875	7.852745	2.01E-12	3.30E-11	17.59747
TP53I11	0.683952	5.483679	10.08267	1.19E-17	4.91E-16	29.50308
GLRX5	0.683093	10.18581	2.307545	0.022754	0.052861	-4.69913
BIRC2	0.683043	8.910692	5.015955	1.87E-06	1.09E-05	4.137852
MYL12B	0.682919	10.42964	8.28698	2.02E-13	3.89E-12	19.86475
GRPEL1	0.682664	6.991192	5.787126	5.93E-08	4.51E-07	7.48978
KL	0.682473	2.335142	4.688689	7.40E-06	3.86E-05	2.808412
SRP19	0.682382	7.273205	4.704536	6.93E-06	3.64E-05	2.871389
CHP1	0.682175	7.663292	8.38452	1.20E-13	2.40E-12	20.37862
SLC15A2	0.68205	4.439438	6.946729	2.14E-10	2.48E-09	13.0015
MYL12A	0.681526	6.351789	5.398868	3.48E-07	2.31E-06	5.766046
JOSD2	0.680641	6.081788	8.689273	2.35E-14	5.31E-13	21.99349
NUCB2	0.680385	5.673626	3.083166	0.002547	0.007878	-2.72375
ACAA1	0.679011	7.253701	8.917602	6.84E-15	1.73E-13	23.21164
TMEM180	0.678959	5.267432	6.056441	1.68E-08	1.39E-07	8.724551
HIST1H4D	0.678918	4.231001	3.655924	0.000383	0.001426	-0.95672
CENPU	0.678281	3.63614	4.107362	7.38E-05	0.000316	0.603355
ETF1	0.678248	7.168654	8.404279	1.08E-13	2.17E-12	20.4829
SLC28A3	0.677933	3.806209	5.727906	7.81E-08	5.81E-07	7.222426
RBMS1	0.67765	9.112728	8.572399	4.40E-14	9.39E-13	21.3726
SLC16A14	0.674428	2.929824	3.407159	0.000896	0.003079	-1.75457
HCK	0.674266	10.74469	6.189133	8.90E-09	7.75E-08	9.343822
WBP5	0.674245	2.877839	3.645686	0.000397	0.001473	-0.99045
PPIP5K2	0.673934	7.304247	4.098119	7.65E-05	0.000327	0.570024
MAB21L3	0.673824	2.570338	3.498245	0.00066	0.002335	-1.46772
RPL26L1	0.672649	5.343457	3.910567	0.000154	0.000619	-0.09396
HIST1H2A	0.672407	3.445326	5.404224	3.40E-07	2.26E-06	5.789342
ANP32E	0.672104	6.961176	3.97457	0.000121	0.000499	0.129958
FLVCR2	0.672035	5.339562	6.512336	1.86E-09	1.83E-08	10.88011
LAPTM4B	0.670485	4.93168	4.595114	1.09E-05	5.47E-05	2.439518
SLC35B1	0.669634	7.028771	6.003957	2.15E-08	1.75E-07	8.481552
LOC10106	0.668922	4.588404	4.713772	6.67E-06	3.51E-05	2.908163
HDAC4	0.668809	6.036839	10.53261	1.01E-18	4.97E-17	31.9521
C4orf48	0.667573	8.13559	7.394527	2.18E-11	3.00E-10	15.2473
TFPI	0.66756	2.750869	6.301434	5.18E-09	4.71E-08	9.873253
MROH6	0.66714	6.32243	6.976052	1.84E-10	2.17E-09	13.14684
KREMEN1	0.664728	4.353946	5.755303	6.88E-08	5.17E-07	7.34592
GK3P	0.664545	5.98428	3.83669	0.000201	0.000791	-0.34894
ATL3	0.664133	5.196874	10.2628	4.43E-18	1.98E-16	30.48289
LACTB	0.664094	6.240765	5.131557	1.13E-06	6.89E-06	4.621451
UQCRFS1	0.663196	9.330941	6.110312	1.30E-08	1.10E-07	8.975129
ALG14	0.662974	3.654983	6.47347	2.24E-09	2.18E-08	10.69337
UQCR11	0.662827	7.512294	9.462005	3.55E-16	1.13E-14	26.13873
ACSS2	0.66252	6.249423	6.992306	1.70E-10	2.01E-09	13.22751
CLEC11A	0.662422	4.396498	4.358609	2.80E-05	0.000131	1.530581
HMBS	0.661771	6.65991	3.135533	0.002162	0.006808	-2.57262
COX6B1	0.661574	9.541896	9.896814	3.30E-17	1.29E-15	28.49347
GLIPR2	0.661473	8.623363	8.073709	6.27E-13	1.11E-11	18.74681
BRI3	0.661387	8.06216	9.871859	3.78E-17	1.44E-15	28.35802
DPCD	0.661238	5.865939	3.234454	0.001578	0.005137	-2.28128
EIF2AK1	0.660407	7.978102	3.738159	0.000286	0.001097	-0.68309
PGD	0.659078	6.801235	13.13653	6.71E-25	9.81E-23	46.05731
MFSD10	0.657777	6.173003	7.536934	1.04E-11	1.51E-10	15.9725
DCAF10	0.657299	5.756018	6.199016	8.49E-09	7.43E-08	9.39022
C15orf37	0.657184	6.303642	4.85722	3.67E-06	2.02E-05	3.485556
COMMD8	0.656923	7.540427	3.301972	0.001268	0.004222	-2.07807
IGF2BP3	0.656865	3.011529	6.259027	6.36E-09	5.69E-08	9.67277

PDE4D	0.656612	3.617773	7.586547	8.08E-12	1.18E-10	16.2263
ZDHHC2	0.65644	5.97736	6.906796	2.61E-10	2.98E-09	12.80399
RTN3	0.656282	9.645985	7.511859	1.19E-11	1.70E-10	15.84445
FADD	0.656038	7.46568	6.959088	2.01E-10	2.35E-09	13.06273
UBA3	0.655221	8.47071	4.94232	2.56E-06	1.45E-05	3.833544
TLR8	0.654773	8.942191	4.432203	2.09E-05	0.0001	1.809753
LOC10272	0.654561	3.645746	5.599131	1.41E-07	1.00E-06	6.646469
TIMM8B	0.654364	5.955507	5.614849	1.31E-07	9.37E-07	6.716364
ACPP	0.654225	4.928218	5.656309	1.09E-07	7.86E-07	6.901277
FASTKD3	0.653884	6.418312	3.70449	0.000323	0.001223	-0.79571
DYNLL1	0.652842	9.482056	5.423474	3.12E-07	2.09E-06	5.873193
C7orf55	0.652736	5.114163	4.973987	2.23E-06	1.28E-05	3.964055
LINC0109	0.65237	4.205237	3.711427	0.000315	0.001195	-0.77257
LTBP1	0.652223	5.451489	4.736595	6.07E-06	3.23E-05	2.999243
TFRC	0.652148	5.214036	6.409284	3.07E-09	2.92E-08	10.38613
KLHL8	0.650729	6.347406	4.071229	8.46E-05	0.000359	0.473377
SMIM1	0.649024	5.754099	2.792315	0.0061	0.016962	-3.52319
HIST1H4H	0.648447	4.634551	3.561186	0.000532	0.00192	-1.26592
C9orf16	0.647322	6.567742	7.911005	1.48E-12	2.48E-11	17.89957
TMEM11	0.64675	7.507142	9.891341	3.40E-17	1.31E-15	28.46375
TMEM185	0.645718	5.520772	6.642037	9.79E-10	1.01E-08	11.50707
PEF1	0.645223	7.937614	6.366939	3.77E-09	3.53E-08	10.18426
LOC10004	0.644777	5.124538	5.135269	1.11E-06	6.79E-06	4.637095
EDEM2	0.644649	6.762909	6.832117	3.80E-10	4.25E-09	12.43594
C2orf47	0.644418	6.695399	6.722877	6.56E-10	6.99E-09	11.90069
PRG2	0.644395	3.809215	2.694784	0.008064	0.021626	-3.77577
MAP1LC3	0.644256	9.424363	6.068614	1.58E-08	1.32E-07	8.781073
CDKN2D	0.644181	5.801768	10.26388	4.40E-18	1.97E-16	30.48879
CAP1	0.643972	11.78537	8.333007	1.58E-13	3.09E-12	20.10704
ATP5I	0.643901	7.241083	4.665194	8.15E-06	4.20E-05	2.715304
INSIG2	0.64365	5.974381	3.531087	0.00059	0.002112	-1.36279
UQCRC1	0.642774	8.116414	7.533069	1.07E-11	1.54E-10	15.95276
TSNAX	0.642658	7.216387	3.379297	0.000983	0.003351	-1.84108
SDHC	0.642451	6.160957	6.405391	3.13E-09	2.97E-08	10.36755
LOC10050	0.641203	3.322381	6.392941	3.32E-09	3.14E-08	10.30814
FIS1	0.640918	8.851955	3.688205	0.000342	0.001287	-0.84989
GYS1	0.640632	6.743815	4.98202	2.16E-06	1.24E-05	3.997247
KCTD9	0.640294	5.561026	4.051648	9.11E-05	0.000383	0.403305
RBBP8	0.640168	5.459418	3.424145	0.000847	0.002924	-1.70155
CATSPER1	0.636738	5.430813	6.744454	5.89E-10	6.35E-09	12.00611
LMNB1	0.635236	6.476037	4.054702	9.00E-05	0.000379	0.414218
NXPE3	0.635001	4.948812	5.382211	3.75E-07	2.48E-06	5.693681
ZDHHC12	0.63496	6.094498	7.962761	1.13E-12	1.93E-11	18.16852
SLC25A28	0.634783	5.561276	8.795638	1.32E-14	3.17E-13	22.56015
GUCY1B3	0.634545	4.568348	3.229154	0.001605	0.005215	-2.29708
SCAND1	0.634432	7.31194	8.050033	7.11E-13	1.25E-11	18.62321
PRKCD	0.634377	8.374054	5.496619	2.25E-07	1.54E-06	6.193418
APH1B	0.63422	6.247807	7.303778	3.48E-11	4.61E-10	14.78781
GNG11	0.633915	4.947527	3.89974	0.00016	0.000642	-0.13157
XRCC6BP1	0.632751	4.239387	4.921572	2.79E-06	1.57E-05	3.748336
IFI35	0.632398	6.512429	6.00035	2.19E-08	1.78E-07	8.464897
MAOA	0.632152	3.192779	3.626087	0.000425	0.001566	-1.0548
GP6	0.631757	5.366843	4.014099	0.000105	0.000435	0.269645
SEL1L3	0.631579	5.310615	5.355475	4.23E-07	2.78E-06	5.577813
HIST2H2B	0.631185	7.447071	3.766836	0.000259	0.000997	-0.58654
ZKSCAN7	0.630325	4.018513	5.893566	3.61E-08	2.84E-07	7.974145
REEP5	0.629778	8.280929	6.24893	6.68E-09	5.95E-08	9.625133
TAF13	0.628825	4.362858	6.968817	1.91E-10	2.25E-09	13.11096

MPC2	0.628801	5.918228	4.971871	2.25E-06	1.29E-05	3.955316
USP12	0.628571	5.392566	3.535225	0.000582	0.002083	-1.34951
XRCC4	0.628526	3.687378	4.795035	4.76E-06	2.57E-05	3.233821
CPNE5	0.62845	6.546571	3.190974	0.001814	0.00583	-2.41027
AURKB	0.628324	3.594856	4.689691	7.37E-06	3.84E-05	2.812388
TMEM208	0.628229	6.620999	6.148521	1.08E-08	9.24E-08	9.153549
INTS6	0.628	5.657108	6.215494	7.84E-09	6.88E-08	9.467667
ARG2	0.627964	4.579708	3.721742	0.000304	0.001157	-0.7381
PRO2852	0.627537	8.147753	4.582626	1.14E-05	5.73E-05	2.390679
NATD1	0.627461	7.076466	5.44253	2.86E-07	1.93E-06	5.956374
CHMP4C	0.626907	2.603694	3.645515	0.000397	0.001473	-0.99101
EAF2	0.626305	2.758652	6.313513	4.89E-09	4.47E-08	9.930485
UBE2H	0.626142	6.326565	7.638344	6.17E-12	9.24E-11	16.49187
SH3BGRL2	0.626057	6.148384	2.770939	0.006489	0.017902	-3.57922
ZEB1-AS1	0.624945	4.403201	4.23637	4.51E-05	0.000202	1.074395
DHRS7B	0.623437	5.973242	8.099432	5.47E-13	9.78E-12	18.88121
TUBA4A	0.623253	9.731593	6.789424	4.71E-10	5.15E-09	12.2263
GNAQ	0.623217	7.609389	6.642964	9.75E-10	1.01E-08	11.51157
CD300LF	0.62312	9.204102	3.607143	0.000454	0.001663	-1.11674
WRB	0.623117	6.331908	4.896284	3.11E-06	1.73E-05	3.644799
BNIP3L	0.623085	10.89041	3.433443	0.000821	0.002845	-1.67244
CISD1	0.622364	5.077287	4.439779	2.03E-05	9.75E-05	1.838684
YWHAE	0.621956	7.332986	4.8793	3.34E-06	1.86E-05	3.575459
C8orf76	0.621689	4.930155	4.856449	3.68E-06	2.03E-05	3.482419
LOC10192	0.621605	7.257421	4.712189	6.71E-06	3.53E-05	2.901854
GALNT3	0.620547	4.969895	7.462384	1.54E-11	2.17E-10	15.59224
GRK6	0.619501	8.300744	9.182244	1.63E-15	4.63E-14	24.631
CARD16	0.618851	5.329049	5.964676	2.59E-08	2.08E-07	8.300421
HCFC1R1	0.617931	6.633436	5.805345	5.45E-08	4.17E-07	7.572343
CDCA3	0.616873	4.354445	3.97728	0.00012	0.000494	0.139502
LPP-AS2	0.616791	2.513555	4.637336	9.13E-06	4.67E-05	2.60533
LILRB2	0.616001	8.665717	4.596484	1.08E-05	5.45E-05	2.444881
SGSH	0.614356	6.357293	4.728492	6.28E-06	3.33E-05	2.96687
TTN-AS1	0.613514	5.456603	3.604151	0.000459	0.00168	-1.1265
CMTM2	0.612879	10.15439	2.783153	0.006264	0.017361	-3.54725
NKAP	0.612451	6.006118	5.850844	4.41E-08	3.42E-07	7.779152
ENO1	0.612366	6.95377	7.862781	1.91E-12	3.14E-11	17.64946
BTBD10	0.610346	6.32783	5.850588	4.42E-08	3.42E-07	7.777985
LOC64551	0.610289	3.445009	5.388343	3.65E-07	2.42E-06	5.720305
RNF7	0.610031	5.791873	9.467056	3.46E-16	1.10E-14	26.16601
TBC1D2	0.608448	6.124933	7.995694	9.47E-13	1.64E-11	18.33993
CD151	0.608343	6.822631	4.667198	8.08E-06	4.17E-05	2.723233
SLC7A5	0.608298	6.088611	2.729627	0.007305	0.019847	-3.68644
ABCC13	0.607932	4.368417	3.028515	0.003015	0.009159	-2.87916
ZBTB8OS	0.607332	5.422361	3.458227	0.000755	0.002642	-1.59451
SAT1	0.607122	10.77326	6.876125	3.05E-10	3.45E-09	12.65263
DEPDC1	0.606554	2.737694	4.518046	1.48E-05	7.34E-05	2.139598
ARL8B	0.605122	8.04762	5.930076	3.04E-08	2.42E-07	8.1414
DSC2	0.604076	5.284917	2.904737	0.004384	0.012742	-3.22231
AGPS	0.604052	5.42211	4.067251	8.59E-05	0.000363	0.459121
RGS19	0.603708	9.167456	6.731783	6.27E-10	6.72E-09	11.94418
STBD1	0.603662	3.810049	4.290351	3.65E-05	0.000167	1.274681
TCTEX1D2	0.602818	4.499992	4.725144	6.36E-06	3.37E-05	2.953509
ATP6V0E1	0.602715	7.418312	7.669629	5.24E-12	7.96E-11	16.65257
ETFDH	0.602373	4.443634	4.638875	9.08E-06	4.64E-05	2.611394
BASP1	0.601828	7.82228	9.355975	6.34E-16	1.92E-14	25.56651
ENPP4	0.601255	4.534053	2.849354	0.005165	0.014676	-3.37182
SRP54	0.601169	7.480835	5.356189	4.22E-07	2.77E-06	5.580905

C19orf10	0.600669	6.71647	4.266872	4.00E-05	0.000181	1.187338
SPATA1	0.60008	2.407971	5.152796	1.03E-06	6.33E-06	4.711064
PSENEN	0.599796	6.603548	6.482881	2.14E-09	2.08E-08	10.73854
GNG2	0.5997	6.384281	4.808641	4.49E-06	2.44E-05	3.288712
KBTBD6	0.599631	4.939884	2.675165	0.008522	0.022691	-3.82562
LOC10192	0.59941	5.207373	4.871108	3.46E-06	1.92E-05	3.54207
NDUFV2	0.599311	6.891522	5.622338	1.27E-07	9.09E-07	6.749708
INSC	0.598952	3.616868	3.496857	0.000663	0.002345	-1.47214
CD164	0.596266	9.269786	4.421233	2.18E-05	0.000104	1.767928
RBPJ	0.595992	8.030652	6.248235	6.70E-09	5.97E-08	9.621857
SYCP3	0.595641	2.920513	5.710372	8.46E-08	6.28E-07	7.143563
TMEM258	0.594596	9.038822	5.072156	1.47E-06	8.73E-06	4.372073
PLEKHG1	0.59426	4.245167	2.779923	0.006323	0.017493	-3.55572
FAM214B	0.594252	7.500822	6.187319	8.98E-09	7.80E-08	9.335311
IRAK4	0.594235	6.932787	5.688187	9.37E-08	6.90E-07	7.043979
PTPLA	0.594205	3.834774	4.922667	2.78E-06	1.57E-05	3.752824
FAM102B	0.593566	5.96113	4.595153	1.08E-05	5.47E-05	2.439671
CDK14	0.593552	5.389524	7.094228	1.01E-10	1.24E-09	13.7351
ATG9A	0.593197	7.091645	5.70308	8.75E-08	6.48E-07	7.110808
SLC11A1	0.592822	6.247688	6.077983	1.51E-08	1.26E-07	8.824615
HSDL2	0.592601	5.902496	5.665636	1.04E-07	7.57E-07	6.942982
ESAM	0.591779	5.127804	3.348911	0.001087	0.00367	-1.93475
ANKRD33I	0.591572	4.950063	4.755611	5.61E-06	3.00E-05	3.075358
POLQ	0.590764	3.888734	3.817064	0.000216	0.000845	-0.41604
NDUFB1	0.590678	7.214319	6.205701	8.22E-09	7.20E-08	9.421626
GK	0.589594	6.714824	3.635284	0.000412	0.001521	-1.02464
RFXANK	0.587487	6.885879	8.053412	6.98E-13	1.23E-11	18.64084
RIOK3	0.587417	7.778947	5.217727	7.78E-07	4.89E-06	4.98647
S100A4	0.587401	11.94907	4.614618	1.00E-05	5.09E-05	2.515983
MSL3	0.586445	6.024437	5.035111	1.72E-06	1.01E-05	4.217497
PSMC6	0.586337	7.898466	2.896188	0.004497	0.013032	-3.24555
ZNF467	0.586175	5.815445	6.900141	2.70E-10	3.08E-09	12.77113
TCAIM	0.585123	4.300516	5.45923	2.66E-07	1.80E-06	6.029413
JDP2	0.58465	5.490302	6.466673	2.32E-09	2.24E-08	10.66076
TENM1	0.584394	3.570981	3.80995	0.000222	0.000865	-0.4403
BCL2L15	0.583704	3.650792	3.180385	0.001876	0.006007	-2.44146
NFU1	0.583274	6.023826	3.430913	0.000828	0.002866	-1.68036
ALDOA	0.58292	10.01405	6.688356	7.78E-10	8.18E-09	11.73234
PRAM1	0.582316	7.012698	4.453364	1.92E-05	9.28E-05	1.890644
STK17B	0.582136	6.964841	4.608673	1.03E-05	5.21E-05	2.492652
EZH2	0.581813	3.718643	2.656815	0.008972	0.02371	-3.87196
KLF14	0.581393	3.741241	5.658513	1.07E-07	7.79E-07	6.911126
FBXL4	0.581335	4.605632	5.021982	1.82E-06	1.06E-05	4.162889
YWHAH	0.580945	4.920979	6.185673	9.05E-09	7.85E-08	9.327587
PTGS1	0.580879	6.771991	2.719555	0.007517	0.020352	-3.71237
TMEM55A	0.580285	7.234472	3.296793	0.00129	0.004287	-2.09378
SNCA	0.579961	8.86897	2.362365	0.019783	0.046983	-4.57627
NAPG	0.579938	5.072129	5.486246	2.35E-07	1.61E-06	6.147852
GFOD2	0.579272	6.119068	7.209496	5.64E-11	7.21E-10	14.31269
CRLS1	0.579206	5.016893	6.583829	1.31E-09	1.32E-08	11.22499
YPEL4	0.579134	4.366177	2.484027	0.014384	0.035723	-4.29428
SLC44A1	0.579118	5.047211	6.844497	3.57E-10	4.00E-09	12.49684
PPAP2B	0.578716	3.780434	5.143942	1.07E-06	6.56E-06	4.673675
DONSON	0.578554	4.473957	3.34085	0.001117	0.003761	-1.95948
TROAP	0.578447	4.445403	6.73143	6.28E-10	6.73E-09	11.94246
3-Mar	0.578089	4.624925	6.241809	6.91E-09	6.14E-08	9.591562
RIPK3	0.57781	6.291628	7.071964	1.14E-10	1.37E-09	13.62397
JKAMP	0.577719	6.290154	3.892642	0.000164	0.000657	-0.15618

LRPAP1	0.577007	5.761571	6.43022	2.77E-09	2.65E-08	10.48619
DPY30	0.57674	6.597007	3.695366	0.000333	0.001258	-0.82609
MBNL3	0.576467	6.797734	2.541352	0.01233	0.031298	-4.15698
PPP1CB	0.576354	8.080144	4.338104	3.03E-05	0.000141	1.453401
GRN	0.576214	9.021188	4.67724	7.75E-06	4.02E-05	2.763002
APOO	0.576183	5.430951	4.196758	5.25E-05	0.000232	0.928612
BSG	0.575658	9.086661	2.487788	0.01424	0.035414	-4.28536
NEDD8	0.575564	8.401329	8.178899	3.59E-13	6.61E-12	19.2972
TSPO2	0.574928	5.624274	3.04416	0.002873	0.008786	-2.83491
CHSY1	0.574077	7.970791	3.420866	0.000856	0.002954	-1.7118
PPARG	0.574048	3.38847	6.08805	1.44E-08	1.21E-07	8.871439
SRP14	0.573747	10.07803	5.098616	1.31E-06	7.86E-06	4.482929
FAM64A	0.573484	4.083052	5.285502	5.77E-07	3.71E-06	5.276235
TACSTD2	0.572973	3.815176	4.252844	4.23E-05	0.000191	1.135321
NBEAL2	0.572412	6.368302	8.117805	4.97E-13	8.93E-12	18.97729
ENY2	0.572114	5.536943	4.625248	9.60E-06	4.89E-05	2.557751
ORAI2	0.571734	6.364462	6.746128	5.84E-10	6.30E-09	12.0143
RPS27L	0.571481	4.067202	3.57659	0.000505	0.001829	-1.21609
LGALS12	0.571142	5.832809	4.209963	4.99E-05	0.000222	0.977098
SLC2A1	0.571019	5.368945	2.915678	0.004243	0.012384	-3.19247
CSRP1	0.570752	7.48749	5.532244	1.91E-07	1.32E-06	6.350304
PNPLA8	0.570527	7.361639	4.416476	2.23E-05	0.000106	1.749813
RUNDC3A	0.570152	6.94033	2.788366	0.006171	0.017136	-3.53357
WBP4	0.569873	4.30773	4.694529	7.22E-06	3.77E-05	2.831603
ZYX	0.56985	9.394727	4.352348	2.87E-05	0.000134	1.506988
MRPL36	0.56958	6.105631	4.391266	2.46E-05	0.000116	1.654049
RAP2C	0.569371	7.352623	3.975433	0.000121	0.000497	0.132996
RALGAPA2	0.569091	5.751173	7.384584	2.30E-11	3.15E-10	15.19685
TBCB	0.567094	7.621954	5.13889	1.10E-06	6.69E-06	4.652362
PRR11	0.566076	7.736047	6.494954	2.02E-09	1.97E-08	10.79653
ANXA5	0.565657	9.103955	4.713719	6.67E-06	3.51E-05	2.90795
C1RL	0.565097	6.464906	6.770256	5.18E-10	5.62E-09	12.13237
GBGT1	0.56478	7.351678	4.733231	6.15E-06	3.27E-05	2.985798
LOC72987	0.564761	5.67974	2.973849	0.003562	0.010592	-3.03223
DNTTIP1	0.564458	7.27344	5.098114	1.31E-06	7.88E-06	4.480823
PCGF3	0.564348	5.049056	6.441074	2.63E-09	2.52E-08	10.53812
SESN2	0.563019	5.998353	5.722095	8.02E-08	5.96E-07	7.196276
C2orf69	0.562984	6.691671	4.666395	8.11E-06	4.19E-05	2.720056
HENMT1	0.562837	7.005424	4.972636	2.25E-06	1.29E-05	3.958475
NRAS	0.562391	6.497594	4.532983	1.40E-05	6.93E-05	2.197448
FZD5	0.561899	3.489729	6.706624	7.11E-10	7.54E-09	11.82138
GP1BA	0.561767	5.524826	4.067933	8.57E-05	0.000363	0.461567
ENSA	0.561555	5.867315	7.932911	1.32E-12	2.23E-11	18.01334
CCDC23	0.561053	7.288219	4.315849	3.31E-05	0.000153	1.36993
TSPAN33	0.56092	7.014931	3.018584	0.003108	0.009398	-2.90715
SLA	0.559015	10.54433	5.002444	1.98E-06	1.15E-05	4.081798
PCNA	0.558989	7.400749	2.401454	0.01788	0.043113	-4.48707
TOR1A	0.558808	5.701194	8.916139	6.90E-15	1.74E-13	23.20382
UBAC1	0.558325	7.600103	5.620724	1.28E-07	9.14E-07	6.742517
C14orf119	0.558074	8.009231	6.221172	7.63E-09	6.71E-08	9.494376
LDLR	0.558013	4.297039	6.489814	2.07E-09	2.02E-08	10.77184
RAB24	0.557669	8.294286	5.064723	1.51E-06	8.97E-06	4.340998
FDPS	0.557649	7.244575	4.675652	7.80E-06	4.05E-05	2.756707
TMEM45B	0.556895	5.371748	2.592751	0.010716	0.027644	-4.0315
RAB1B	0.555603	8.670813	7.377756	2.38E-11	3.26E-10	15.16222
MAPK6	0.555399	4.769083	7.251844	4.54E-11	5.92E-10	14.5258
ARPC4	0.55521	8.474558	6.872268	3.11E-10	3.51E-09	12.63361
CBS	0.555089	4.343379	5.332098	4.69E-07	3.05E-06	5.476791

LAMTOR3	0.555069	6.492197	3.319418	0.001198	0.004014	-2.025
LINC0141(	0.554834	6.452511	3.631564	0.000417	0.001538	-1.03685
SLC35A5	0.554535	6.598277	4.095692	7.72E-05	0.000329	0.561281
RMI1	0.554285	6.665296	5.055275	1.58E-06	9.31E-06	4.301543
SLC2A5	0.55409	4.30061	3.291042	0.001314	0.004357	-2.11121
MPLKIP	0.553815	6.492693	5.712418	8.38E-08	6.23E-07	7.152758
PLOD1	0.553092	6.365262	5.338266	4.57E-07	2.98E-06	5.503418
S1PR4	0.552768	8.630986	6.706149	7.12E-10	7.55E-09	11.81906
C16orf72	0.552634	7.803691	5.188123	8.85E-07	5.51E-06	4.860632
GPN3	0.55211	6.200903	3.725115	0.0003	0.001145	-0.72682
RAB2B	0.551834	8.576583	2.280986	0.02433	0.055956	-4.7577
DCTN3	0.5511	8.031709	7.689364	4.73E-12	7.24E-11	16.75406
CDC6	0.550646	2.527846	2.898634	0.004464	0.012954	-3.23891
ERG	0.5506	2.911533	3.75014	0.000275	0.001054	-0.64283
ASAP1-IT2	0.55058	5.093053	4.334113	3.08E-05	0.000143	1.43841
RSBN1	0.55054	7.665906	6.318571	4.77E-09	4.38E-08	9.954466
RTN4	0.550317	9.645919	6.257022	6.42E-09	5.74E-08	9.663305
C1D	0.550076	7.466628	4.373813	2.63E-05	0.000124	1.587984
POLE4	0.550048	4.460962	6.305839	5.07E-09	4.62E-08	9.894119
TYROBP	0.549962	10.84483	5.68787	9.39E-08	6.91E-07	7.042558
CENPM	0.549942	4.949514	4.123296	6.95E-05	0.0003	0.660946
ASL	0.549329	6.057402	4.820407	4.28E-06	2.33E-05	3.336268
ACVR1B	0.548815	4.299808	9.168911	1.75E-15	4.93E-14	24.55932
MIIP	0.54869	7.25634	8.295905	1.93E-13	3.72E-12	19.9117
GPAA1	0.548192	7.181456	9.637316	1.36E-16	4.69E-15	27.08669
COL6A3	0.548087	3.39739	2.69056	0.008161	0.021856	-3.78653
PGLYRP2	0.547798	3.726422	6.484513	2.13E-09	2.07E-08	10.74637
MKKS	0.547396	5.553281	4.635815	9.19E-06	4.69E-05	2.599339
RAB8B	0.547253	7.957338	4.629119	9.45E-06	4.82E-05	2.572978
LILRB1	0.547045	7.797702	5.230313	7.36E-07	4.64E-06	5.040105
PIN1	0.546799	5.505805	5.183201	9.05E-07	5.62E-06	4.839756
SSH1	0.5466	5.449362	7.436096	1.76E-11	2.45E-10	15.45847
NFIL3	0.545982	8.495069	3.80092	0.000229	0.000891	-0.47104
MFSD1	0.544461	9.459594	4.516823	1.49E-05	7.37E-05	2.134866
NUPL1	0.543873	5.94123	5.146745	1.06E-06	6.48E-06	4.685509
PLTP	0.543588	4.678348	5.839005	4.66E-08	3.61E-07	7.725254
FBN2	0.543184	3.426236	5.883453	3.79E-08	2.97E-07	7.92792
SIRPA	0.543002	6.836374	4.249286	4.29E-05	0.000193	1.122148
ENDOD1	0.542858	5.483535	2.756858	0.006757	0.018544	-3.61593
CDT1	0.542836	4.398022	4.631438	9.36E-06	4.77E-05	2.582105
PUS3	0.542539	5.687677	4.900695	3.05E-06	1.70E-05	3.662834
SLC35A1	0.542273	7.676005	3.579999	0.000499	0.001812	-1.20504
CASC5	0.541708	2.671961	5.347917	4.37E-07	2.87E-06	5.54512
CDC40	0.541571	6.325222	3.889654	0.000166	0.000664	-0.16653
MPP1	0.541298	10.05181	3.346807	0.001095	0.003694	-1.94121
STXBP5	0.541074	5.862459	4.074008	8.37E-05	0.000355	0.483346
MRVI1-AS	0.540203	5.755699	6.556348	1.49E-09	1.50E-08	11.09221
SERPINB8	0.540011	5.479512	9.666641	1.16E-16	4.05E-15	27.24547
CC2D2B	0.539897	4.761943	4.834456	4.03E-06	2.21E-05	3.393149
HIATL1	0.53944	5.13128	7.297585	3.59E-11	4.75E-10	14.75653
GPD2	0.538379	4.205255	6.640305	9.87E-10	1.02E-08	11.49866
PEAK1	0.538066	5.654066	8.371818	1.29E-13	2.56E-12	20.31161
LOC10050	0.537921	4.163947	4.928221	2.71E-06	1.53E-05	3.775617
COX6C	0.537783	6.854601	2.715865	0.007596	0.020546	-3.72185
HLX	0.537415	7.376116	5.272575	6.11E-07	3.90E-06	5.220786
TEX2	0.537365	5.777395	3.986227	0.000116	0.000479	0.171044
SLC16A6	0.535798	6.606004	3.877092	0.000174	0.000692	-0.20996
DPP3	0.535737	6.162645	4.693916	7.24E-06	3.78E-05	2.829168

ME2	0.53573	7.679913	4.316516	3.30E-05	0.000153	1.372429
DIAPH2	0.535511	4.642987	5.906409	3.40E-08	2.69E-07	8.032919
ARPC1A	0.535484	7.195126	5.143255	1.08E-06	6.57E-06	4.670777
PSMD12	0.534664	6.796697	4.69412	7.23E-06	3.78E-05	2.829976
DEPDC1B	0.53458	2.21515	3.853355	0.000189	0.000748	-0.29175
HTATIP2	0.534062	6.337895	7.736577	3.70E-12	5.77E-11	16.99719
FPR1	0.533922	9.864476	4.22045	4.79E-05	0.000214	1.015686
RENBP	0.533444	5.633315	5.449708	2.77E-07	1.87E-06	5.987747
SPINT2	0.533229	6.91952	4.827772	4.15E-06	2.27E-05	3.366075
RAB6B	0.533162	4.747333	4.979245	2.18E-06	1.26E-05	3.985777
TADA3	0.532792	6.027464	9.108649	2.43E-15	6.65E-14	24.23555
PIGF	0.532576	6.417192	4.80306	4.60E-06	2.49E-05	3.266184
CYC1	0.532457	6.648197	6.123048	1.22E-08	1.04E-07	9.034538
PFN1	0.532457	11.71194	5.894758	3.59E-08	2.83E-07	7.9796
SNF8	0.532368	6.93283	7.910407	1.48E-12	2.48E-11	17.89647
ETFB	0.532319	6.821683	6.011626	2.07E-08	1.69E-07	8.516993
LOC10192	0.532179	6.637061	3.506793	0.000641	0.002275	-1.44049
TAX1BP1	0.532133	7.23263	3.947684	0.000134	0.000547	0.035554
RAD23B	0.531061	7.122267	5.988177	2.32E-08	1.87E-07	8.408711
KDM7A	0.530911	7.266544	6.655816	9.14E-10	9.48E-09	11.57401
PCSK9	0.53084	4.470523	3.939598	0.000138	0.000562	0.007259
RANBP9	0.530517	5.731676	5.822962	5.02E-08	3.87E-07	7.652313
TCN2	0.530372	5.591824	4.67344	7.88E-06	4.08E-05	2.747944
NRD1	0.530106	8.117821	6.845167	3.56E-10	3.99E-09	12.50013
HAUS1	0.52941	4.906244	2.348849	0.020482	0.048367	-4.60681
JUNB	0.529271	6.992358	4.649063	8.71E-06	4.47E-05	2.651569
HNRNPLL	0.529005	4.903537	6.025118	1.94E-08	1.59E-07	8.579392
RALB	0.528984	9.045515	4.580617	1.15E-05	5.78E-05	2.382829
AGA	0.528758	5.684309	3.718269	0.000307	0.00117	-0.74972
POLK	0.528681	4.666845	5.98364	2.37E-08	1.91E-07	8.387786
RLIM	0.528157	5.88629	4.072035	8.44E-05	0.000358	0.47627
GPER1	0.527763	4.131881	4.860101	3.62E-06	2.00E-05	3.497269
MRPL23	0.527051	5.191259	9.765151	6.78E-17	2.47E-15	27.77922
NDUFB7	0.526863	4.887932	6.869413	3.15E-10	3.56E-09	12.61954
SUB1	0.526628	6.897315	2.840029	0.005308	0.015021	-3.39675
SUCLG1	0.525625	5.668328	6.880344	2.99E-10	3.39E-09	12.67343
POP7	0.525448	5.708068	5.69288	9.17E-08	6.76E-07	7.065028
COQ5	0.525359	5.896859	3.815797	0.000217	0.000848	-0.42036
LOC10192	0.525135	5.032191	5.090494	1.35E-06	8.11E-06	4.448863
CAPZA1	0.524963	7.035191	9.603382	1.64E-16	5.57E-15	26.90304
TDG	0.524449	6.28889	3.770426	0.000255	0.000986	-0.57441
TIMP4	0.524309	3.419299	3.278554	0.001369	0.004516	-2.14895
CCNH	0.524295	6.517298	4.22921	4.63E-05	0.000207	1.047972
PRKCDBP	0.524109	3.046548	4.083633	8.08E-05	0.000344	0.517901
IFITM1	0.522664	11.98798	5.852702	4.37E-08	3.40E-07	7.787616
C1orf226	0.522324	3.560411	4.283026	3.76E-05	0.000171	1.247394
ABLIM3	0.521985	5.128773	3.094707	0.002457	0.007628	-2.69063
ZBTB7B	0.521406	6.207931	7.818431	2.41E-12	3.88E-11	17.41986
AZIN1	0.520821	7.890992	3.356419	0.001061	0.003588	-1.91167
TCEB2	0.5206	8.036084	8.704571	2.16E-14	4.95E-13	22.0749
ATG3	0.519072	6.575664	5.273986	6.07E-07	3.88E-06	5.226836
ZNF230	0.518928	4.702773	4.036049	9.66E-05	0.000404	0.347663
DYRK4	0.518869	6.394117	4.58388	1.14E-05	5.71E-05	2.395578
FAM173B	0.51858	4.280746	6.378622	3.56E-09	3.35E-08	10.23989
MRPL28	0.518371	7.204733	8.212019	3.01E-13	5.59E-12	19.4709
CASP3	0.517745	6.153126	3.650114	0.000391	0.001452	-0.97587
RTN2	0.517476	5.307845	4.806463	4.54E-06	2.46E-05	3.27992
KPNA2	0.517451	7.473804	4.46362	1.84E-05	8.94E-05	1.92995

CLTCL1	0.517303	5.898606	3.829031	0.000207	0.000812	-0.37516
LOC10192	0.516441	4.853714	6.734866	6.18E-10	6.63E-09	11.95925
LOC14293	0.516017	3.809376	5.81137	5.30E-08	4.07E-07	7.599677
SF3B6	0.515812	5.664815	5.480644	2.41E-07	1.65E-06	6.123265
ANG	0.515467	4.320924	3.877452	0.000174	0.000692	-0.20872
SWSAP1	0.514817	5.109858	5.150783	1.04E-06	6.38E-06	4.702558
FGF13-AS	0.514168	3.291788	2.930089	0.004064	0.011921	-3.15303
RBM38	0.513965	9.343915	2.381779	0.018817	0.045074	-4.53213
ACP6	0.513597	4.955746	5.240351	7.04E-07	4.45E-06	5.082939
JAK3	0.51339	5.550686	9.152643	1.91E-15	5.35E-14	24.47188
MAD2L1	0.513184	3.470533	3.142461	0.002115	0.006681	-2.55246
IL1RN	0.512779	5.755831	7.037008	1.36E-10	1.62E-09	13.44977
LGALS8	0.512646	6.518627	3.878687	0.000173	0.000689	-0.20445
KLF7	0.512547	6.340744	5.202292	8.32E-07	5.21E-06	4.920804
SPX	0.512279	3.980989	2.976215	0.003536	0.010535	-3.02565
TUSC1	0.511809	4.177137	5.404935	3.39E-07	2.25E-06	5.792438
CCDC125	0.511504	5.146961	2.982664	0.003468	0.010349	-3.00771
SKA1	0.51146	2.156471	3.001363	0.003276	0.009842	-2.95549
NDUFS6	0.51145	7.609725	4.283054	3.76E-05	0.000171	1.247499
SLC9A6	0.511377	7.053839	4.387941	2.49E-05	0.000118	1.641447
ZEB2	0.510653	5.61614	4.104306	7.47E-05	0.00032	0.59233
NLK	0.51062	5.567565	5.479716	2.42E-07	1.65E-06	6.119191
PDXK	0.510454	6.071878	6.5376	1.64E-09	1.63E-08	11.00178
SLC25A24	0.510136	5.618196	5.151803	1.04E-06	6.35E-06	4.706866
MGAT4B	0.510022	6.935939	5.043383	1.66E-06	9.77E-06	4.251949
ZNF787	0.509704	5.111016	8.540244	5.22E-14	1.10E-12	21.2021
MMP1	0.509623	2.157838	2.669399	0.008661	0.023023	-3.84021
CYB5R2	0.509244	3.387925	4.143726	6.43E-05	0.000279	0.735032
PSMD9	0.509236	6.252072	8.631489	3.20E-14	7.03E-13	21.68628
COX6A1	0.509199	6.241473	8.199024	3.23E-13	5.97E-12	19.40272
ERLIN2	0.509184	5.048957	4.296959	3.56E-05	0.000163	1.299326
FAM198B	0.50902	5.652344	2.198188	0.029873	0.066653	-4.93631
ATP6V1D	0.508633	5.73336	7.586522	8.08E-12	1.18E-10	16.22617
CHMP2B	0.508286	7.184958	3.670317	0.000364	0.001362	-0.90918
NTSR1	0.507696	4.868125	4.535001	1.38E-05	6.88E-05	2.205276
MYBL2	0.507687	5.428087	3.359033	0.001052	0.003561	-1.90362
FUCA2	0.507245	6.073637	4.074561	8.36E-05	0.000355	0.485327
EIF4G3	0.506875	4.462783	7.017477	1.50E-10	1.77E-09	13.35259
MYB	0.506709	3.669954	3.440016	0.000803	0.002791	-1.65181
CMTM1	0.50615	4.34485	6.215549	7.84E-09	6.88E-08	9.467926
ZMPSTE24	0.505709	8.098374	5.562758	1.66E-07	1.17E-06	6.485159
PHKA2	0.505581	5.8246	7.513231	1.18E-11	1.69E-10	15.85145
TUBA1A	0.505422	11.47599	4.836571	4.00E-06	2.19E-05	3.401723
VAMP5	0.505318	4.758079	7.98664	9.94E-13	1.71E-11	18.29279
RPA3	0.505039	6.007998	3.006193	0.003228	0.00971	-2.94195
SSFA2	0.504965	4.759732	5.549832	1.76E-07	1.23E-06	6.427981
SUMO1	0.504677	6.778359	5.745642	7.19E-08	5.39E-07	7.302333
CTDP1	0.504577	6.382847	5.577829	1.55E-07	1.10E-06	6.551923
ATP6V1E1	0.504226	8.422766	5.521608	2.01E-07	1.39E-06	6.303402
LILRB4	0.504184	3.518747	5.928865	3.06E-08	2.43E-07	8.135844
TAL1	0.503723	5.661408	4.179897	5.60E-05	0.000246	0.866867
SFXN5	0.503369	4.400926	9.572407	1.95E-16	6.48E-15	26.73546
SLC38A2	0.503266	9.306437	3.894258	0.000163	0.000653	-0.15058
ARHGAP2	0.502999	4.303549	5.459465	2.65E-07	1.80E-06	6.030441
STX10	0.502922	7.37536	5.025371	1.79E-06	1.05E-05	4.176975
RNF146	0.502535	7.181775	3.005003	0.00324	0.009741	-2.94529
TMEM60	0.501699	7.53344	5.868451	4.06E-08	3.17E-07	7.859421
TMEM169	0.501165	4.287301	4.337164	3.04E-05	0.000142	1.44987

LINC00493	0.501147	7.816943	4.848969	3.80E-06	2.09E-05	3.45203
DCTN6	0.501092	6.572535	4.080616	8.17E-05	0.000347	0.507064
COA6	0.501055	5.981006	4.144478	6.41E-05	0.000278	0.737763
PPP2R3C	0.500672	5.69058	4.948354	2.49E-06	1.41E-05	3.858372
LHFPL2	0.500609	5.23691	5.090399	1.35E-06	8.12E-06	4.448465
RMDN1	0.500344	4.922372	5.161161	9.96E-07	6.13E-06	4.74642
C14orf142	0.500159	5.10021	3.183899	0.001855	0.005947	-2.43112
PIM3	0.500133	8.463277	4.173795	5.73E-05	0.000251	0.844567
EDNRB	0.499342	2.408312	3.951261	0.000132	0.00054	0.048088
NEXN	0.498951	4.214524	2.835727	0.005376	0.015178	-3.40823
ACTR1A	0.498451	7.449518	7.097411	9.98E-11	1.22E-09	13.751
DLD	0.498423	6.580253	4.94976	2.48E-06	1.41E-05	3.864157
ATP6V0D1	0.498229	10.52748	5.735802	7.53E-08	5.62E-07	7.257983
OST4	0.498204	9.183151	4.239579	4.45E-05	0.0002	1.086249
GTDC1	0.498058	4.943307	7.686987	4.79E-12	7.32E-11	16.74183
PIGK	0.497753	4.49903	5.014932	1.88E-06	1.09E-05	4.133603
FSTL3	0.497687	5.327692	3.45824	0.000755	0.002642	-1.59447
YKT6	0.497159	4.868007	5.599587	1.41E-07	1.00E-06	6.648494
POLR2K	0.497157	5.378774	2.074849	0.040159	0.085206	-5.19105
ATP5L	0.496987	8.669828	4.732634	6.17E-06	3.27E-05	2.983412
ELOVL3	0.496888	3.316937	3.594894	0.000474	0.00173	-1.15665
OSBPL1A	0.496675	4.823308	3.773166	0.000253	0.000977	-0.56515
SULT1A2	0.496019	7.912773	3.383588	0.00097	0.003309	-1.82779
DNAJC4	0.495569	5.530305	8.491824	6.77E-14	1.40E-12	20.94566
TMLHE	0.495155	4.905342	6.466057	2.33E-09	2.25E-08	10.65781
ACOT9	0.495126	7.218174	4.325758	3.18E-05	0.000148	1.407057
SUOX	0.494729	4.478287	6.492894	2.04E-09	1.99E-08	10.78663
CYP4F2	0.494586	5.999554	3.283288	0.001348	0.004456	-2.13465
SIRT5	0.494375	4.669134	6.064367	1.61E-08	1.34E-07	8.761346
NDUFB10	0.4939	7.466606	5.795781	5.70E-08	4.35E-07	7.528983
ARID5A	0.493746	6.651097	4.002786	0.000109	0.000452	0.22956
CEBPB	0.493584	11.46151	4.003927	0.000109	0.000451	0.2336
PPP1R14A	0.49327	3.298638	3.38597	0.000962	0.003284	-1.82041
MPST	0.492775	6.576253	6.926418	2.37E-10	2.72E-09	12.90098
FAM53C	0.492396	8.884781	4.772284	5.23E-06	2.81E-05	3.142267
CTSK	0.492382	5.484591	5.267025	6.26E-07	3.99E-06	5.19701
B3GNT2	0.492027	5.033499	3.894797	0.000163	0.000652	-0.14871
ANKDD1A	0.491975	4.151875	3.355691	0.001063	0.003596	-1.91391
MTRR	0.491906	6.110587	3.639535	0.000406	0.001501	-1.01068
SHKBP1	0.491811	8.508192	4.333717	3.08E-05	0.000144	1.436921
FURIN	0.491776	8.076672	4.699562	7.07E-06	3.71E-05	2.851606
SLK	0.491622	7.537171	3.840944	0.000198	0.00078	-0.33436
C2orf88	0.491555	6.17362	2.243264	0.026732	0.060663	-4.83982
LOC72817	0.491008	3.276818	7.143873	7.88E-11	9.79E-10	13.98341
CAMKK2	0.490948	6.507364	6.072912	1.55E-08	1.29E-07	8.801041
LIG4	0.490777	4.765915	2.780042	0.006321	0.01749	-3.55541
TIMP2	0.490709	8.346568	4.056015	8.96E-05	0.000378	0.418909
ZDHHC16	0.489849	6.072623	5.46289	2.61E-07	1.78E-06	6.045435
THBS3	0.489693	6.102765	5.874462	3.95E-08	3.09E-07	7.886853
ST3GAL4	0.489625	3.838717	7.652307	5.74E-12	8.64E-11	16.56357
HYAL3	0.488762	4.295849	3.321595	0.001189	0.003987	-2.01836
NUDT1	0.488541	4.758141	5.317811	5.00E-07	3.24E-06	5.41518
FAM160B1	0.488465	5.306659	6.669914	8.53E-10	8.89E-09	11.64256
CWF19L1	0.488331	5.661846	6.433095	2.73E-09	2.62E-08	10.49994
CTDSPL	0.488121	4.239774	4.929446	2.70E-06	1.53E-05	3.780643
SLC12A9	0.487275	5.757431	6.314775	4.86E-09	4.46E-08	9.936467
GPSM2	0.486669	4.286097	5.869624	4.04E-08	3.15E-07	7.864773
TMEM126A	0.486559	6.201666	2.997787	0.003312	0.009932	-2.96549

BAD	0.486353	5.458067	9.506686	2.79E-16	9.00E-15	26.38012
TRAPPC6B	0.486119	6.175624	3.120381	0.002267	0.0071	-2.61657
ARL11	0.486108	5.593785	5.682681	9.61E-08	7.06E-07	7.019301
DLC1	0.486068	3.118971	6.307875	5.02E-09	4.58E-08	9.903765
FBN1	0.485706	2.925027	4.166568	5.89E-05	0.000258	0.818187
SH3PXD2B	0.485517	4.561133	6.136449	1.15E-08	9.76E-08	9.097119
ALAS1	0.485204	6.978537	4.217735	4.84E-05	0.000216	1.005689
C9orf89	0.485138	5.137123	8.434988	9.18E-14	1.86E-12	20.64509
CLINT1	0.485059	7.256722	3.636713	0.00041	0.001514	-1.01995
TXNDC9	0.48464	5.207941	4.030034	9.88E-05	0.000412	0.326253
DNAJB11	0.484595	6.947179	3.364929	0.001031	0.003498	-1.88546
UCHL3	0.484505	6.452466	2.82328	0.005575	0.015682	-3.44135
NMI	0.484462	9.060107	4.652306	8.59E-06	4.42E-05	2.66437
TK2	0.484258	4.967224	7.060165	1.21E-10	1.45E-09	13.56513
COX7C	0.482862	6.564975	2.772039	0.006468	0.017855	-3.57635
CHIC2	0.482317	8.403622	4.290828	3.65E-05	0.000167	1.276461
STX11	0.482291	7.3888	3.438166	0.000808	0.002805	-1.65762
NMNAT1	0.482007	4.219104	5.643678	1.15E-07	8.29E-07	6.84486
ERMAP	0.481722	4.171319	4.136988	6.60E-05	0.000286	0.710568
SFT2D1	0.48168	6.14807	7.51553	1.17E-11	1.67E-10	15.86319
ARRB2	0.481608	9.749148	4.906965	2.97E-06	1.66E-05	3.688484
ASGR1	0.481401	4.703156	3.095471	0.002451	0.007614	-2.68843
PTTG3P	0.481328	3.381248	4.790522	4.85E-06	2.62E-05	3.215634
TBKBP1	0.480819	5.577336	6.109016	1.31E-08	1.10E-07	8.969087
ZFYVE16	0.480512	4.632071	5.589628	1.47E-07	1.04E-06	6.604263
UBXN2B	0.48033	7.450571	5.498303	2.23E-07	1.53E-06	6.200823
ARF4	0.480012	8.71401	5.77927	6.16E-08	4.66E-07	7.454226
WDR26	0.479968	6.886287	4.567449	1.21E-05	6.08E-05	2.331447
E2F2	0.479617	5.747296	4.292336	3.63E-05	0.000166	1.282083
ABCC4	0.479596	4.4342	2.995165	0.003339	0.010004	-2.97283
KCTD21	0.479481	5.743391	5.224612	7.54E-07	4.76E-06	5.015798
SNRNP27	0.479352	6.820623	4.35465	2.84E-05	0.000133	1.515662
LINC00884	0.478981	3.963209	6.735231	6.17E-10	6.62E-09	11.96103
HOMER3	0.478913	4.934277	7.146213	7.78E-11	9.69E-10	13.99514
SLC27A2	0.478851	3.096871	2.780131	0.006319	0.017488	-3.55517
CDC123	0.478848	7.526419	3.77834	0.000248	0.000962	-0.54765
ESPL1	0.478462	4.723021	4.970269	2.27E-06	1.30E-05	3.948702
RPS19BP1	0.478085	6.30879	5.752327	6.97E-08	5.24E-07	7.332492
MB21D1	0.477574	3.419558	7.845729	2.09E-12	3.41E-11	17.56113
FLJ36848	0.477309	3.993513	4.660042	8.32E-06	4.28E-05	2.694932
POLR2L	0.476983	6.084306	7.496624	1.29E-11	1.83E-10	15.76672
LOC10192	0.476966	4.936902	4.324891	3.19E-05	0.000148	1.403807
AQP9	0.476091	11.38326	4.324005	3.20E-05	0.000149	1.400483
LINC00968	0.476084	3.080099	5.67192	1.01E-07	7.37E-07	6.971104
PFKFB4	0.476041	6.326707	6.442142	2.62E-09	2.51E-08	10.54323
TBPL1	0.475303	7.460563	4.466724	1.82E-05	8.84E-05	1.941857
TMEM70	0.474939	5.756911	6.259681	6.34E-09	5.67E-08	9.675856
CEL	0.474836	3.138182	4.540792	1.35E-05	6.74E-05	2.227747
SMIM5	0.474729	7.03412	2.171709	0.031864	0.070289	-4.99214
LOC10029	0.474607	2.844776	3.86711	0.00018	0.000715	-0.24441
LBR	0.474503	9.986964	3.803418	0.000227	0.000884	-0.46254
DNAJC6	0.474319	2.576025	3.115629	0.002301	0.007196	-2.63032
YIPF6	0.474136	6.173126	5.343867	4.45E-07	2.91E-06	5.527615
CDCA2	0.473946	3.394149	3.809642	0.000222	0.000866	-0.44135
TSG101	0.473845	8.723963	7.454087	1.60E-11	2.25E-10	15.55001
KRT8P12	0.473778	5.059248	7.168551	6.95E-11	8.73E-10	14.1071
HPRT1	0.473742	4.571341	5.610468	1.34E-07	9.55E-07	6.69687
DEGS1	0.473621	8.091256	3.609098	0.000451	0.001652	-1.11037

2-Mar	0.473546	9.082262	3.178877	0.001885	0.00603	-2.4459
PSMB5	0.473393	6.887646	6.962867	1.97E-10	2.31E-09	13.08146
CDC25A	0.473378	3.608236	3.676511	0.000356	0.001335	-0.88867
TBC1D7	0.473088	4.129118	7.521803	1.13E-11	1.62E-10	15.89521
PP13439	0.472902	3.226109	3.61114	0.000448	0.001642	-1.1037
HOOK3	0.472844	4.360725	5.210634	8.02E-07	5.03E-06	4.95628
RNF135	0.472687	7.702788	4.055011	8.99E-05	0.000379	0.415323
MRPS28	0.471904	4.840981	2.625317	0.009795	0.025586	-3.95083
MYOF	0.471368	4.849273	3.57093	0.000514	0.001862	-1.23442
TNFSF10	0.471232	8.702685	4.146783	6.36E-05	0.000276	0.746141
CD99P1	0.47103	4.87179	5.153279	1.03E-06	6.32E-06	4.713103
CDYL2	0.470755	5.133243	7.512542	1.19E-11	1.69E-10	15.84794
LOC73142	0.470048	5.010718	4.149876	6.28E-05	0.000273	0.757387
PCTP	0.469926	7.885303	3.757568	0.000267	0.001029	-0.61781
CENPBD1	0.469439	5.262242	4.761521	5.47E-06	2.93E-05	3.099056
TMX1	0.469059	7.152899	2.828646	0.005488	0.015468	-3.42709
SEC61B	0.468932	6.300525	5.742673	7.29E-08	5.46E-07	7.288948
HMGN2	0.468566	11.4383	3.980242	0.000119	0.000489	0.149938
UBE2T	0.468051	4.637565	2.880857	0.004706	0.01355	-3.28708
SELK	0.468051	8.514604	2.768711	0.006531	0.018003	-3.58504
SRGN	0.467848	10.38504	5.04948	1.62E-06	9.54E-06	4.277369
RAB27B	0.467657	4.096074	3.389296	0.000951	0.003251	-1.8101
COMMD1	0.467606	6.463754	4.159396	6.06E-05	0.000264	0.79204
ADAMTS2	0.467442	4.049117	3.581679	0.000496	0.001803	-1.19959
ASB7	0.466913	5.443729	5.424695	3.10E-07	2.08E-06	5.878517
ZMAT5	0.466865	5.614631	6.488649	2.08E-09	2.03E-08	10.76624
TM6SF1	0.46649	7.655669	3.400796	0.000916	0.003142	-1.77438
ALDH3B1	0.466119	6.651937	7.154631	7.46E-11	9.30E-10	14.03731
VAMP8	0.466087	8.17085	2.766052	0.006581	0.018121	-3.59198
LAPTM4A	0.464564	9.346007	3.646579	0.000396	0.001469	-0.98751
VAMP3	0.462859	8.731305	3.600811	0.000464	0.001697	-1.13739
NME4	0.462822	6.386317	3.569166	0.000518	0.001872	-1.24013
JAZF1	0.462344	8.582783	2.680222	0.008402	0.022414	-3.81281
LIMS1	0.462242	4.396117	4.995028	2.04E-06	1.18E-05	4.051071
NCAPH	0.462052	4.539967	4.051081	9.13E-05	0.000384	0.401278
NSMCE2	0.461963	6.548051	3.767657	0.000258	0.000995	-0.58377
NUDT22	0.461944	4.86043	7.354693	2.68E-11	3.63E-10	15.04535
VAMP4	0.461935	5.527477	3.656789	0.000382	0.001423	-0.95387
H2AFJ	0.461791	5.472447	7.893275	1.62E-12	2.69E-11	17.80756
PPP2R5B	0.461542	6.528099	3.554108	0.000545	0.001964	-1.28876
LGALSL	0.461356	5.752071	2.713653	0.007644	0.020663	-3.72752
TMOD3	0.461133	5.475554	4.477747	1.74E-05	8.51E-05	1.984192
ZCCHC17	0.460607	6.353016	4.969492	2.28E-06	1.30E-05	3.945494
NT5C3A	0.460228	8.991732	3.698761	0.000329	0.001245	-0.81479
ATXN1	0.460103	6.085523	6.164413	1.00E-08	8.62E-08	9.227932
MCM10	0.459467	2.890709	3.116999	0.002291	0.007169	-2.62636
HINT3	0.459367	2.947522	6.366474	3.78E-09	3.54E-08	10.18204
IMPA1	0.459004	7.603286	2.740729	0.007077	0.01931	-3.65777
TMCO1	0.45884	6.365674	3.521135	0.00061	0.002176	-1.39467
COPS4	0.458405	4.477214	5.706562	8.61E-08	6.38E-07	7.126449
WASF1	0.458251	3.402403	4.840495	3.93E-06	2.16E-05	3.417636
CYB5D2	0.458164	5.719784	4.663796	8.19E-06	4.23E-05	2.709776
HBE1	0.458095	6.350266	3.742853	0.000282	0.00108	-0.66733
SLC39A8	0.457396	4.254148	5.153442	1.03E-06	6.31E-06	4.71379
PRDX1	0.457317	8.067284	3.770505	0.000255	0.000986	-0.57415
RASGRP4	0.457128	5.332429	7.854956	1.99E-12	3.26E-11	17.60892
SERTAD3	0.457086	5.809554	4.666752	8.10E-06	4.18E-05	2.721468
ALDH4A1	0.45699	5.129725	7.945942	1.23E-12	2.09E-11	18.08107

UEVLD	0.456699	5.489527	4.835647	4.01E-06	2.20E-05	3.397979
TESC	0.456068	6.378143	4.904996	3.00E-06	1.68E-05	3.680426
NDUFA8	0.455443	6.409316	4.36882	2.69E-05	0.000126	1.569115
MYEOV2	0.455221	4.74923	7.436061	1.76E-11	2.45E-10	15.4583
KCNMA1	0.454907	3.245866	3.551207	0.000551	0.001982	-1.29811
CHMP4B	0.454588	7.669221	5.606263	1.36E-07	9.71E-07	6.67817
MRPL27	0.454311	6.885479	3.497306	0.000662	0.002342	-1.47071
STEAP3	0.45408	3.95832	4.985943	2.12E-06	1.22E-05	4.013468
C6orf25	0.453885	3.562737	3.543352	0.000566	0.00203	-1.3234
ATP5E	0.453742	7.52611	6.184133	9.12E-09	7.89E-08	9.320361
MYO10	0.453163	2.830212	7.512066	1.19E-11	1.70E-10	15.84551
FAH	0.452558	4.072561	5.349758	4.34E-07	2.84E-06	5.553083
PSMD10	0.452275	7.023077	4.621999	9.72E-06	4.95E-05	2.544979
MAPRE1	0.452254	8.730942	3.888557	0.000167	0.000667	-0.17032
ADORA3	0.451168	3.84286	2.862643	0.004967	0.014193	-3.33618
LOC44112	0.450988	7.31428	3.729932	0.000295	0.001127	-0.71069
NSUN3	0.450774	4.326669	5.647885	1.13E-07	8.15E-07	6.863642
SHOC2	0.450587	8.678587	4.424196	2.16E-05	0.000103	1.779218
NDUFAB1	0.450285	8.416773	3.427364	0.000838	0.002898	-1.69148
PPP3CB	0.450122	6.458126	5.267851	6.24E-07	3.98E-06	5.200545
LEPROT	0.44981	6.302373	6.729683	6.34E-10	6.78E-09	11.93393
RASSF1-A	0.449746	4.060631	4.830594	4.10E-06	2.24E-05	3.377502
DESI1	0.449633	6.130599	7.453172	1.61E-11	2.26E-10	15.54535
OIP5-AS1	0.449538	7.120092	4.290805	3.65E-05	0.000167	1.276372
PIK3CG	0.449358	7.14624	5.393109	3.58E-07	2.37E-06	5.741013
UBE2A	0.449347	8.074388	6.104389	1.33E-08	1.13E-07	8.947523
ATP2C1	0.449165	4.739377	5.274116	6.07E-07	3.88E-06	5.227395
MLLT1	0.449152	5.764792	6.194516	8.67E-09	7.57E-08	9.369091
GTSE1	0.449099	4.765792	4.550341	1.30E-05	6.49E-05	2.264845
PKHD1L1	0.449005	2.590714	2.930111	0.004064	0.011921	-3.15297
CRADD	0.44873	5.265833	5.420085	3.17E-07	2.12E-06	5.858417
CACNA1E	0.448698	3.21273	7.22104	5.31E-11	6.84E-10	14.37074
KAT2B	0.447979	8.566425	3.020974	0.003086	0.00934	-2.90042
ZNF222	0.447396	4.79552	3.362544	0.00104	0.003523	-1.89281
GAS6	0.447396	5.00075	5.568893	1.62E-07	1.14E-06	6.512323
SH3RF1	0.446743	3.379649	4.109105	7.33E-05	0.000314	0.609647
ITGAE	0.446654	6.946	4.308058	3.41E-05	0.000157	1.340782
CITED4	0.44664	5.307104	4.71597	6.61E-06	3.49E-05	2.91692
USP35	0.44659	5.350692	6.172524	9.64E-09	8.32E-08	9.265928
MRPL52	0.446314	4.779928	5.755287	6.88E-08	5.17E-07	7.34585
MARCKS	0.446245	7.766092	3.059711	0.002739	0.008419	-2.79074
TMEM110	0.446099	5.596252	5.935244	2.97E-08	2.36E-07	8.165122
IRX3	0.445529	3.318214	4.025468	0.0001	0.000418	0.310015
PPP3R1	0.445257	6.084632	3.562662	0.000529	0.001912	-1.26116
NT5M	0.445239	5.172333	2.365859	0.019606	0.04666	-4.56835
NPL	0.445097	7.729208	4.562508	1.24E-05	6.19E-05	2.312192
FAM126B	0.444962	6.955292	2.710824	0.007706	0.020807	-3.73478
SP100	0.444385	6.110919	5.321429	4.92E-07	3.19E-06	5.430771
PRKAR2A	0.444266	7.270236	7.411157	2.00E-11	2.76E-10	15.33173
F12	0.444133	4.044832	6.236065	7.10E-09	6.29E-08	9.564497
CHUK	0.444078	6.593139	2.969234	0.003612	0.01073	-3.04504
NHLRC3	0.443637	4.303578	4.33383	3.08E-05	0.000144	1.437346
PIP5K1B	0.443576	4.30235	4.134935	6.65E-05	0.000288	0.703119
C11orf83	0.443259	3.980699	3.69796	0.00033	0.001248	-0.81745
SNX16	0.443181	4.083743	3.147497	0.002082	0.006585	-2.53779
FANCI	0.442988	3.591424	2.628921	0.009697	0.025384	-3.94185
RAB33A	0.442984	5.026804	3.379355	0.000983	0.003351	-1.8409
TMEM2	0.442963	6.700142	3.690103	0.00034	0.001279	-0.84358

BLOC1S2	0.442835	7.928136	3.255878	0.001473	0.00482	-2.21718
SMARCD3	0.442811	5.130074	3.625366	0.000426	0.001569	-1.05717
SPOCD1	0.442645	4.491334	4.694083	7.23E-06	3.78E-05	2.829829
KIF5B	0.442557	7.507051	5.290229	5.65E-07	3.64E-06	5.29653
SDF2L1	0.442521	5.588944	2.62409	0.009828	0.025667	-3.95389
CENPF	0.442002	3.012969	5.532975	1.90E-07	1.32E-06	6.353532
PARK7	0.441851	9.952092	4.860439	3.62E-06	2.00E-05	3.498646
GPX7	0.441768	5.023014	2.503113	0.013668	0.034224	-4.24888
TMEM216	0.441563	7.2026	5.170674	9.55E-07	5.89E-06	4.786674
IGSF6	0.441468	7.873443	3.664418	0.000372	0.001389	-0.92868
CATIP-AS1	0.441326	5.256309	4.139559	6.53E-05	0.000283	0.719897
LDLRAD3	0.441081	4.691322	2.828061	0.005498	0.015486	-3.42864
TRPM6	0.440947	4.975715	4.875474	3.39E-06	1.88E-05	3.559862
LOC10013	0.440526	5.759574	2.699	0.007968	0.0214	-3.76502
AP2S1	0.440413	8.845586	6.69807	7.42E-10	7.82E-09	11.77967
C7orf49	0.440358	6.562451	5.493883	2.27E-07	1.56E-06	6.181395
MUT	0.440269	5.63902	4.136456	6.61E-05	0.000286	0.708638
MRPL15	0.440267	6.17212	2.945165	0.003884	0.011444	-3.11159
LRRC29	0.440108	4.052729	5.47932	2.43E-07	1.65E-06	6.117455
COX7B	0.439923	4.920905	2.880407	0.004713	0.013565	-3.2883
RIN3	0.439718	6.351441	4.670019	7.99E-06	4.13E-05	2.7344
TMEM92	0.439238	4.400353	4.035621	9.67E-05	0.000404	0.34614
SLCO4A1	0.439158	4.089046	4.967736	2.29E-06	1.31E-05	3.938248
RAB18	0.439094	6.646279	4.89678	3.10E-06	1.73E-05	3.646825
RP2	0.438835	5.530272	3.211939	0.001697	0.005475	-2.34825
STYXL1	0.43859	6.072817	4.910626	2.93E-06	1.64E-05	3.703474
DPM3	0.43853	5.423488	4.075583	8.32E-05	0.000353	0.488996
FHOD1	0.438275	5.75289	4.383999	2.53E-05	0.000119	1.626516
CCDC22	0.43712	5.58645	3.857948	0.000186	0.000737	-0.27596
ZNRF1	0.436994	4.302939	5.932462	3.01E-08	2.39E-07	8.15235
NDUFA7	0.436893	5.105854	7.471905	1.46E-11	2.07E-10	15.64073
RMI2	0.436705	4.644899	2.434662	0.016392	0.040044	-4.41024
EPDR1	0.436627	3.274125	3.943861	0.000136	0.000554	0.02217
PRKAA1	0.436592	4.944749	4.622494	9.71E-06	4.94E-05	2.546922
C1orf106	0.436492	3.70652	3.568343	0.000519	0.001876	-1.24279
SYCP2	0.436224	2.411081	4.260892	4.10E-05	0.000185	1.165149
ILK	0.436214	8.66703	5.810995	5.31E-08	4.08E-07	7.597973
IMPDH1	0.436075	7.865357	7.236993	4.90E-11	6.36E-10	14.45101
TNPO3	0.435917	6.814575	5.359793	4.15E-07	2.73E-06	5.596503
CYB5R4	0.435697	6.182284	3.95326	0.000131	0.000537	0.055095
C9orf40	0.435069	5.480996	2.607084	0.010301	0.026726	-3.99611
DSE	0.434908	3.92466	7.440776	1.72E-11	2.40E-10	15.48228
MTMR6	0.434665	5.502651	3.496089	0.000665	0.002351	-1.47459
PSMA1	0.43466	8.571983	3.930476	0.000143	0.000579	-0.02461
CHKA	0.434571	4.85881	5.012714	1.89E-06	1.10E-05	4.124398
PSMD8	0.433422	8.133082	4.991545	2.07E-06	1.20E-05	4.036649
RAD51	0.433075	3.445847	4.020496	0.000102	0.000426	0.292348
PDE6H	0.433064	2.338638	5.039694	1.69E-06	9.91E-06	4.236579
LRRC4	0.431729	7.18465	4.048498	9.22E-05	0.000387	0.392056
FOXQ1	0.431502	3.622279	2.558582	0.011766	0.030004	-4.11517
DGAT1	0.43137	6.523099	4.124279	6.92E-05	0.000299	0.664506
LSM10	0.431361	7.307812	4.599423	1.07E-05	5.39E-05	2.456393
CHST13	0.430221	4.713307	3.757286	0.000268	0.00103	-0.61876
METTL22	0.430018	5.712396	7.610438	7.13E-12	1.05E-10	16.34872
MRPL40	0.429795	4.977622	2.824851	0.005549	0.015619	-3.43718
KAZN	0.429579	4.783114	3.123273	0.002247	0.007046	-2.6082
SLC38A6	0.429502	3.674069	2.438509	0.016227	0.039679	-4.40128
DR1	0.429452	5.567276	4.30237	3.49E-05	0.00016	1.319529

RNF14	0.429106	4.910822	3.527044	0.000598	0.002138	-1.37575
ITPKC	0.42891	5.013915	3.635227	0.000412	0.001521	-1.02483
CGRRF1	0.428868	5.293464	3.685547	0.000345	0.001298	-0.85871
RPL23AP3.	0.428825	3.818534	3.01486	0.003144	0.009488	-2.91762
PPA2	0.428576	5.00442	5.069594	1.48E-06	8.80E-06	4.361359
NT5DC2	0.428157	3.632732	7.298705	3.57E-11	4.73E-10	14.76218
BEND2	0.427938	4.460244	2.26402	0.025386	0.058026	-4.79479
LOC10193	0.427616	4.376495	4.645388	8.84E-06	4.53E-05	2.637068
TWF2	0.426953	7.37522	6.685912	7.88E-10	8.28E-09	11.72044
ATP11A	0.426873	4.731333	4.884909	3.26E-06	1.81E-05	3.59834
RGS18	0.426754	9.173908	2.485391	0.014332	0.03561	-4.29104
FGFR1OP2	0.426692	5.891261	4.36901	2.69E-05	0.000126	1.569835
RAI2	0.426602	2.647777	3.209187	0.001712	0.005522	-2.35642
RFESD	0.426358	3.683967	2.532537	0.012627	0.031966	-4.17828
PREB	0.425826	6.62888	4.275743	3.87E-05	0.000176	1.220299
CDCA7	0.425769	3.857742	3.17169	0.001929	0.006153	-2.46701
CENPBD1F	0.425749	6.409083	4.829914	4.11E-06	2.25E-05	3.374748
GINS2	0.425647	4.657354	4.247635	4.31E-05	0.000194	1.116037
UGP2	0.425582	7.245226	4.427419	2.13E-05	0.000102	1.791503
MRPL47	0.425333	4.952622	3.84895	0.000192	0.000759	-0.30689
STX7	0.425307	5.874105	4.419268	2.20E-05	0.000105	1.760444
SLC44A3	0.425258	2.303742	3.993073	0.000113	0.000468	0.195214
CRBN	0.425067	7.996463	3.202346	0.001749	0.005639	-2.37667
CD44	0.424836	7.058341	4.535396	1.38E-05	6.87E-05	2.206806
SLC1A5	0.424766	6.973217	2.199416	0.029783	0.066495	-4.9337
VPS29	0.424475	6.54155	5.101946	1.29E-06	7.76E-06	4.496906
RBKS	0.424239	4.219038	4.915296	2.87E-06	1.61E-05	3.722605
PAM16	0.423981	5.7639	5.576578	1.56E-07	1.10E-06	6.546376
TGFA	0.423864	5.418897	4.849557	3.79E-06	2.09E-05	3.454415
TRABD	0.423754	6.353958	7.484319	1.37E-11	1.95E-10	15.70399
CAB39	0.42284	8.173963	2.768009	0.006544	0.018029	-3.58688
H2AFY	0.422158	8.485105	4.440924	2.02E-05	9.71E-05	1.843059
CMBL	0.421671	5.626416	3.547467	0.000558	0.002006	-1.31016
EFHD2	0.421557	8.552436	4.024507	0.000101	0.00042	0.306599
AHCY	0.421006	6.23121	3.426093	0.000841	0.002907	-1.69546
TRIM25	0.420758	8.085139	4.9719	2.25E-06	1.29E-05	3.955436
RNF144B	0.420612	6.357768	3.31404	0.001219	0.004077	-2.04138
PPCS	0.420289	6.966153	3.884157	0.000169	0.000677	-0.18555
PHF19	0.42008	5.891552	4.453927	1.92E-05	9.27E-05	1.892801
RNF10	0.418693	9.648221	2.862076	0.004975	0.014208	-3.3377
CLEC4A	0.418575	7.705242	2.271199	0.024934	0.057102	-4.77913
CINP	0.41851	4.000683	6.613059	1.13E-09	1.16E-08	11.3665
CHMP5	0.418421	6.989181	2.006784	0.047042	0.097397	-5.32577
SLC9A8	0.418227	6.332852	3.871493	0.000177	0.000705	-0.22929
MRPL11	0.4182	5.849762	3.540887	0.00057	0.002047	-1.33133
SLC31A1	0.417407	4.767752	5.952101	2.74E-08	2.20E-07	8.242569
DGAT2	0.417228	8.606973	3.165836	0.001965	0.006256	-2.48418
C17orf99	0.41707	2.857163	2.963938	0.00367	0.010888	-3.05972
ABHD13	0.417058	4.586298	2.153182	0.033324	0.073015	-5.03084
ANAPC11	0.416629	4.974357	6.974988	1.85E-10	2.18E-09	13.14156
NME6	0.416073	5.68146	4.996462	2.03E-06	1.17E-05	4.057009
RABGEF1	0.415998	4.780694	4.961092	2.36E-06	1.35E-05	3.910842
CAPNS2	0.415649	4.412007	4.385099	2.52E-05	0.000119	1.630683
LOC10192	0.415547	6.637674	2.23028	0.027606	0.062321	-4.8678
CHMP2A	0.415544	9.205245	4.706847	6.86E-06	3.61E-05	2.880585
LRRC6	0.415428	5.36914	2.59969	0.010513	0.027197	-4.01439
VPS8	0.41514	5.171813	4.29404	3.60E-05	0.000165	1.288437
BUB1	0.414628	3.094801	4.101037	7.56E-05	0.000323	0.58054

LOH12CR2	0.414474	2.926246	5.137666	1.10E-06	6.72E-06	4.647201
ATG4D	0.413858	6.235832	5.190493	8.76E-07	5.46E-06	4.870692
NDUFA6	0.413682	6.517564	3.78141	0.000246	0.000952	-0.53725
CXorf23	0.413618	4.397678	3.08585	0.002525	0.007824	-2.71606
NDST1	0.413584	4.686145	5.010468	1.91E-06	1.11E-05	4.115076
COMMD3	0.413445	6.713642	3.079313	0.002577	0.00796	-2.73478
SNX4	0.413362	5.108279	2.747942	0.006932	0.018953	-3.63909
EFEMP2	0.413322	4.979397	3.826314	0.000209	0.000819	-0.38445
PTPLAD2	0.412467	5.457466	5.629848	1.23E-07	8.81E-07	6.783171
PARVB	0.41234	5.206537	3.085575	0.002528	0.007828	-2.71685
NDUFC1	0.412013	5.89597	5.329499	4.75E-07	3.08E-06	5.465575
VLDLR	0.411866	2.89628	2.446437	0.015892	0.038994	-4.38277
TRIP4	0.411073	6.78167	4.045291	9.33E-05	0.000392	0.380611
TIMM17B	0.410862	5.583819	7.449954	1.64E-11	2.29E-10	15.52897
YIF1B	0.410787	5.726413	7.220779	5.32E-11	6.84E-10	14.36942
RPA4	0.410392	3.92909	4.306321	3.43E-05	0.000158	1.33429
CIDEB	0.410279	7.637674	4.99016	2.08E-06	1.20E-05	4.030917
TOM1	0.409904	6.146571	4.503638	1.57E-05	7.72E-05	2.083923
MEA1	0.409475	7.166662	5.164311	9.82E-07	6.05E-06	4.759743
CALML5	0.409377	3.633709	5.042601	1.66E-06	9.80E-06	4.248693
ZDHHC13	0.409093	5.232332	3.818765	0.000215	0.00084	-0.41024
YTHDF3-A	0.408552	4.454184	4.785181	4.96E-06	2.67E-05	3.194128
LOC10050	0.408436	4.967321	4.18767	5.44E-05	0.00024	0.89531
TMEM205	0.408427	7.051139	3.920955	0.000148	0.000598	-0.05781
GHITM	0.408359	8.392324	5.181443	9.12E-07	5.66E-06	4.832304
PRR13	0.408251	9.830943	5.447347	2.80E-07	1.89E-06	5.977425
TOLLIP	0.40824	5.70268	7.181112	6.52E-11	8.22E-10	14.17013
GLYCTK	0.408116	5.407569	8.016554	8.49E-13	1.48E-11	18.44861
METRNL	0.407877	6.596925	2.4631	0.015207	0.03752	-4.34369
TGFBR1	0.407667	7.080914	3.354864	0.001066	0.003604	-1.91645
GAS2L1	0.407339	5.299922	2.737222	0.007148	0.019471	-3.66684
SRP9	0.406755	9.778579	2.513253	0.013301	0.033456	-4.22463
GNS	0.405765	7.665868	3.581214	0.000497	0.001805	-1.2011
FSTL1	0.405693	3.866694	3.255323	0.001476	0.004828	-2.21884
LZIC	0.405668	5.395773	4.808896	4.49E-06	2.44E-05	3.289743
HMGB3P1	0.405443	4.706013	4.681694	7.61E-06	3.95E-05	2.780657
CCDC71L	0.405203	5.087018	5.121219	1.19E-06	7.18E-06	4.577916
LOC10013	0.405026	3.320215	3.180164	0.001877	0.006009	-2.44212
MAP3K8	0.405013	6.446128	2.975353	0.003546	0.010553	-3.02805
ARHGEF37	0.404908	4.06817	3.636625	0.00041	0.001515	-1.02024
SKAP2	0.404853	7.084775	4.426423	2.14E-05	0.000102	1.787707
PXMP2	0.404685	5.037903	3.317677	0.001205	0.004034	-2.03031
RTCA	0.404593	5.23169	4.483747	1.70E-05	8.32E-05	2.007266
HPS1	0.404297	6.590777	3.579297	0.0005	0.001814	-1.20732
PQLC1	0.404281	5.896387	4.350026	2.89E-05	0.000135	1.498244
LHFP	0.404169	2.786018	4.27428	3.89E-05	0.000177	1.214858
FBXW5	0.404141	7.109244	5.57992	1.54E-07	1.09E-06	6.561191
MND1	0.403874	2.303794	3.183761	0.001856	0.005948	-2.43153
ATP5O	0.403857	5.516759	5.111811	1.23E-06	7.45E-06	4.538348
SERPINA1	0.403807	7.361896	5.772983	6.34E-08	4.79E-07	7.425793
EPAS1	0.403309	4.210876	3.579369	0.0005	0.001814	-1.20708
TXNL4B	0.403307	5.239471	4.992239	2.07E-06	1.19E-05	4.039523
E2F3	0.403274	6.987323	5.123731	1.17E-06	7.11E-06	4.588487
KRT8	0.403118	4.255512	5.890703	3.66E-08	2.88E-07	7.961056
WDR34	0.402714	4.40664	2.861389	0.004985	0.014233	-3.33955
VOPP1	0.402592	8.679177	3.922239	0.000147	0.000595	-0.05334
UQCR10	0.402172	6.788583	5.674785	9.97E-08	7.29E-07	6.983928
FRMD4B	0.402142	3.527607	5.455261	2.71E-07	1.83E-06	6.012041

CDKL1	0.402057	3.79746	4.147522	6.34E-05	0.000275	0.748826
CSTB	0.401787	6.431693	4.739657	5.99E-06	3.19E-05	3.011485
B3GNTL1	0.401487	5.352144	5.645409	1.14E-07	8.23E-07	6.852587
LRRC42	0.401448	4.072547	4.101552	7.55E-05	0.000323	0.582397
CUX1	0.401046	6.539031	5.062936	1.53E-06	9.04E-06	4.333535
SCFD1	0.401017	5.696516	5.003378	1.97E-06	1.14E-05	4.085668
ZNF254	0.401007	3.244837	6.557298	1.49E-09	1.49E-08	11.0968
PHF21A	0.400798	6.482907	4.279815	3.81E-05	0.000173	1.235445
SULT1A1	0.400701	7.677115	3.239717	0.001552	0.005055	-2.26556
MYD88	0.399204	11.02495	5.44963	2.77E-07	1.87E-06	5.987409
PRKACA	0.399063	5.792545	4.294971	3.59E-05	0.000164	1.291908
B9D2	0.399059	6.016377	5.473419	2.49E-07	1.70E-06	6.091573
ALG6	0.398976	6.984728	4.657929	8.39E-06	4.32E-05	2.68658
IFI27L2	0.398798	5.660227	4.889523	3.20E-06	1.78E-05	3.617175
TNFSF13	0.398648	7.464968	3.1342	0.002171	0.006835	-2.57649
ISCA2	0.398327	6.978604	4.838769	3.96E-06	2.18E-05	3.410635
LINC0026(	0.398323	5.911951	2.92159	0.004169	0.012197	-3.17631
UBE2K	0.39831	6.836705	6.029418	1.91E-08	1.56E-07	8.5993
BCKDK	0.398267	6.69399	4.401815	2.36E-05	0.000112	1.694072
AIM1	0.398065	8.325015	4.028012	9.95E-05	0.000415	0.31906
NTAN1	0.398041	7.348103	3.671879	0.000362	0.001355	-0.90401
CCBL2	0.397789	6.916453	2.581756	0.011044	0.028415	-4.05853
POLR2J	0.397501	9.133017	5.948022	2.80E-08	2.24E-07	8.223819
TMEM91	0.397473	5.592744	3.848544	0.000193	0.00076	-0.30828
SKIL	0.3973	3.841274	6.842575	3.61E-10	4.04E-09	12.48738
ERP27	0.397177	6.493283	3.178214	0.001889	0.006041	-2.44785
TKT	0.396637	8.262414	3.657503	0.000381	0.00142	-0.95151
CCDC167	0.396517	5.54565	3.217992	0.001664	0.005381	-2.33029
ANKRD35	0.396389	4.288897	4.313392	3.34E-05	0.000154	1.360733
IL17RA	0.39629	8.837512	4.153337	6.20E-05	0.00027	0.769979
JAG1	0.395983	4.149042	6.267989	6.09E-09	5.47E-08	9.715084
LMBR1	0.395588	3.610751	4.572475	1.19E-05	5.96E-05	2.351045
PTMS	0.39545	4.651949	4.090304	7.87E-05	0.000336	0.541889
XXYLT1	0.395197	4.187546	4.963549	2.34E-06	1.33E-05	3.920977
TMEM38A	0.395172	4.493213	3.850174	0.000192	0.000756	-0.30269
SSR3	0.39517	4.685011	3.924465	0.000146	0.000591	-0.04558
KIF18A	0.395079	2.180537	3.26501	0.00143	0.004695	-2.18975
UBQLN1	0.39498	7.094779	5.363967	4.07E-07	2.68E-06	5.614578
CLEC4G	0.394694	4.198756	5.235514	7.19E-07	4.54E-06	5.062292
PLSCR4	0.394602	2.114027	3.240642	0.001547	0.005042	-2.2628
RALY-AS1	0.394545	3.533167	4.614765	1.00E-05	5.09E-05	2.51656
NCSTN	0.394526	5.819182	4.874762	3.40E-06	1.89E-05	3.556959
DCP2	0.394515	6.591749	4.310154	3.38E-05	0.000156	1.348622
LDHC	0.393976	3.962548	4.563904	1.23E-05	6.16E-05	2.317633
LOC44014	0.393615	4.367916	5.507315	2.14E-07	1.47E-06	6.240459
PLK1	0.393505	3.635779	3.957977	0.000129	0.000528	0.071639
HBBP1	0.393128	4.67901	4.25716	4.16E-05	0.000188	1.151312
ORC6	0.392945	5.126284	3.170758	0.001934	0.006169	-2.46975
PSMC1	0.392775	8.543185	5.766282	6.54E-08	4.93E-07	7.395501
TPD52L2	0.39268	8.358147	4.826322	4.17E-06	2.28E-05	3.360203
DAD1	0.392325	8.804189	4.912023	2.91E-06	1.63E-05	3.709197
NOL3	0.392243	4.104579	7.562426	9.15E-12	1.33E-10	16.10283
PJA2	0.392184	9.025202	3.875606	0.000175	0.000696	-0.2151
TUBBP5	0.392164	3.768527	2.028377	0.044757	0.093384	-5.28349
B4GALT4	0.392056	5.31954	4.209659	5.00E-05	0.000222	0.97598
FAM206A	0.392006	6.803251	4.517276	1.49E-05	7.36E-05	2.136619
SUMO3	0.391722	7.523321	4.286292	3.71E-05	0.000169	1.259556
NETO2	0.391662	3.653866	3.408455	0.000893	0.003069	-1.75053

PKMYT1	0.391524	5.971545	4.449666	1.95E-05	9.41E-05	1.876488
FAM172A	0.391273	5.861758	3.474164	0.000716	0.002514	-1.54416
TPI1	0.390931	7.192319	5.649256	1.12E-07	8.11E-07	6.869763
NAT8B	0.390866	4.66843	3.059614	0.00274	0.008421	-2.79101
GUSB	0.390537	5.725776	5.515479	2.06E-07	1.42E-06	6.276401
TAF12	0.390227	6.057441	3.519777	0.000613	0.002184	-1.39902
MVP	0.390106	8.747074	3.874342	0.000175	0.000698	-0.21946
ABCG5	0.390096	2.725108	4.692911	7.27E-06	3.79E-05	2.825177
VRK2	0.390055	6.369614	3.499499	0.000657	0.002327	-1.46373
SERINC2	0.389757	3.907057	6.550727	1.54E-09	1.54E-08	11.06509
TMBIM4	0.38975	8.681581	6.905538	2.63E-10	3.00E-09	12.79778
CEACAM3	0.389654	6.654508	4.250264	4.27E-05	0.000192	1.125768
FATE1	0.389606	5.233487	4.08802	7.94E-05	0.000339	0.53367
EIF1B	0.389376	5.99132	5.040305	1.68E-06	9.89E-06	4.239125
CAT	0.389336	8.268215	3.086473	0.002521	0.007811	-2.71427
C2orf57	0.388898	4.852759	4.7544	5.64E-06	3.01E-05	3.070505
ITGA9	0.388792	2.727361	5.022257	1.82E-06	1.06E-05	4.164033
ZMAT2	0.388599	8.276469	2.706982	0.00779	0.020994	-3.74462
TWF1	0.388599	4.226081	4.515201	1.50E-05	7.40E-05	2.128592
ZCCHC10	0.388423	4.790294	2.404131	0.017755	0.042849	-4.48091
RECQL4	0.388412	5.120083	5.074452	1.45E-06	8.65E-06	4.381677
LOC10050	0.388365	4.632401	4.506879	1.55E-05	7.63E-05	2.096435
GPCPD1	0.387696	7.595374	2.606588	0.010315	0.026755	-3.99733
C21orf119	0.387673	4.822002	3.979569	0.000119	0.00049	0.147567
MANF	0.387649	7.583059	2.334709	0.021236	0.04989	-4.63858
RAD23A	0.38736	8.003611	2.308471	0.022701	0.052761	-4.69708
IL22RA2	0.387068	2.298817	2.616243	0.010044	0.026154	-3.9734
AUNIP	0.386905	3.725009	3.844386	0.000196	0.00077	-0.32256
TPM3	0.386903	5.665929	7.442784	1.70E-11	2.37E-10	15.49249
NT5C2	0.386802	9.16473	4.103604	7.49E-05	0.00032	0.589798
TMPO-AS	0.386741	2.811061	4.79584	4.74E-06	2.57E-05	3.237065
EMC8	0.386741	6.536422	5.244382	6.92E-07	4.38E-06	5.100153
MTFR2	0.386673	2.772815	3.715033	0.000311	0.001182	-0.76053
SOS2	0.386513	5.481559	5.324189	4.86E-07	3.15E-06	5.442672
STRN3	0.386305	4.733586	3.859799	0.000185	0.000732	-0.26959
RAC2	0.386183	11.20897	4.213385	4.92E-05	0.000219	0.98968
DPY19L1P	0.386098	2.867042	3.113663	0.002316	0.007234	-2.636
ACTR3	0.385999	9.123609	5.114158	1.22E-06	7.38E-06	4.548212
LOC10192	0.385955	2.83352	4.246483	4.33E-05	0.000195	1.111776
SUSD1	0.385857	6.183564	3.199537	0.001765	0.005682	-2.38498
E2F1	0.385258	5.12708	4.304458	3.46E-05	0.000159	1.327326
SPTLC1	0.385208	5.657346	3.856023	0.000188	0.000742	-0.28258
H2AFX	0.385098	4.909003	4.553124	1.29E-05	6.42E-05	2.275668
TBC1D20	0.38503	6.100633	5.954186	2.72E-08	2.18E-07	8.252158
GAS2L3	0.384438	2.8273	5.035582	1.72E-06	1.01E-05	4.219459
TTC33	0.384124	4.373414	4.224033	4.73E-05	0.000211	1.028883
PBK	0.383453	2.25043	2.087222	0.039005	0.08324	-5.16611
C1orf85	0.383353	5.135137	3.045888	0.002858	0.008744	-2.83001
ETNK1	0.383217	4.670497	3.428585	0.000834	0.002886	-1.68765
HIST1H2A.	0.383079	6.561801	5.036656	1.71E-06	1.00E-05	4.22393
LOC10028	0.382966	3.628181	4.050978	9.13E-05	0.000384	0.400911
FLJ20021	0.38291	4.856771	4.094292	7.76E-05	0.000331	0.556241
CDK2AP1	0.382873	8.084172	2.848091	0.005184	0.014726	-3.37521
NUMB	0.382815	6.000991	2.314215	0.022373	0.052114	-4.68432
SMIM12	0.382708	5.933197	7.620793	6.76E-12	1.00E-10	16.40182
CLCN3	0.382059	5.925985	3.927974	0.000144	0.000584	-0.03334
ARMC8	0.381481	4.861757	6.264269	6.20E-09	5.56E-08	9.697515
CALML4	0.381067	4.075087	5.047105	1.63E-06	9.63E-06	4.267465

C4orf27	0.381004	4.281546	4.919205	2.82E-06	1.59E-05	3.738626
ATF7	0.380969	4.403396	8.631772	3.20E-14	7.03E-13	21.68778
SCARNA1!	0.380842	4.254647	4.056856	8.93E-05	0.000377	0.421917
SEC23B	0.380773	6.75847	3.829524	0.000206	0.000811	-0.37347
CLIC4	0.380491	4.746834	3.801162	0.000229	0.000891	-0.47021
RRAGA	0.380454	8.020144	5.21742	7.79E-07	4.89E-06	4.985161
RNASE1	0.380188	3.38141	5.330246	4.73E-07	3.08E-06	5.468798
MRPL21	0.3794	5.959688	3.69782	0.000331	0.001248	-0.81792
EXOSC3	0.379286	4.306727	5.288394	5.70E-07	3.66E-06	5.288653
LRSAM1	0.379225	4.646749	4.697794	7.12E-06	3.73E-05	2.844579
SPNS2	0.379037	5.622671	4.171811	5.78E-05	0.000253	0.837321
VEGFA	0.378794	4.825451	4.737935	6.03E-06	3.21E-05	3.004601
ACOT8	0.37878	4.885524	5.768422	6.47E-08	4.89E-07	7.405174
DUSP22	0.378485	6.991841	4.951556	2.46E-06	1.40E-05	3.871551
SRD5A3	0.378383	3.1687	2.422848	0.016908	0.041119	-4.43768
PCDHA5	0.378367	3.439276	5.336318	4.61E-07	3.00E-06	5.495005
SRD5A1	0.378137	5.028492	2.928428	0.004084	0.011975	-3.15758
GPR27	0.377825	7.023145	4.185147	5.49E-05	0.000242	0.886073
DCUN1D1	0.377562	5.256886	4.633857	9.26E-06	4.73E-05	2.591625
P4HB	0.37736	8.011872	3.108757	0.002351	0.007337	-2.65017
SYN2	0.377117	3.41321	7.107357	9.49E-11	1.16E-09	13.8007
NAA60	0.376961	7.225875	5.371985	3.93E-07	2.59E-06	5.649322
KIF23	0.376829	2.557515	2.609932	0.010221	0.026563	-3.98905
ICT1	0.376734	4.598494	2.823486	0.005572	0.015675	-3.4408
DNAJC13	0.37637	4.160488	6.264062	6.21E-09	5.56E-08	9.696539
ISY1	0.376233	5.792507	8.116382	5.00E-13	8.99E-12	18.96985
PTEN	0.375942	7.946565	3.59842	0.000468	0.00171	-1.14518
SLC16A5	0.375834	6.00413	3.579604	0.000499	0.001813	-1.20632
TRIB1	0.375394	4.456999	4.82284	4.24E-06	2.31E-05	3.346112
EMC6	0.37493	6.258458	5.056161	1.57E-06	9.28E-06	4.305243
SLC30A1	0.374661	5.185724	4.603468	1.05E-05	5.31E-05	2.472243
RNF217	0.374589	2.967507	5.143879	1.07E-06	6.56E-06	4.67341
DRAP1	0.374058	6.545452	4.432208	2.09E-05	0.0001	1.809772
TMEM17	0.373822	2.432018	4.518263	1.48E-05	7.33E-05	2.140435
ABHD16A	0.373587	5.309212	6.231945	7.25E-09	6.40E-08	9.545091
SEC11A	0.372988	8.891464	3.594776	0.000474	0.00173	-1.15704
UNC79	0.372773	4.794819	4.712954	6.69E-06	3.52E-05	2.904905
STAB1	0.372701	4.673407	3.436572	0.000812	0.002819	-1.66262
ATP5G3	0.372242	7.110924	4.810002	4.47E-06	2.43E-05	3.294209
MREG	0.372184	4.126787	3.775228	0.000251	0.000971	-0.55818
GNPAT	0.372062	7.08746	3.440849	0.000801	0.002784	-1.6492
DNAJA1	0.3719	7.946324	2.838777	0.005328	0.015062	-3.4001
GADD45B	0.37176	6.439024	4.696409	7.17E-06	3.75E-05	2.839073
NDUFS3	0.371608	6.769527	3.576144	0.000505	0.001832	-1.21754
PLA2G15	0.371419	4.632678	3.192046	0.001808	0.005813	-2.40711
CHMP6	0.371416	5.775684	5.809876	5.34E-08	4.09E-07	7.592897
CYBA	0.37113	7.907869	5.25505	6.60E-07	4.19E-06	5.145752
MCMBP	0.371051	5.603287	5.168929	9.63E-07	5.94E-06	4.779286
CUEDC2	0.370987	7.470423	4.386108	2.51E-05	0.000118	1.634504
PDLIM7	0.370801	5.463808	5.592445	1.45E-07	1.03E-06	6.616772
TUBA1C	0.370768	10.83026	4.872898	3.43E-06	1.90E-05	3.549364
NANS	0.370766	5.639669	6.308012	5.02E-09	4.58E-08	9.904415
LINC0065i	0.370759	7.198084	3.378469	0.000986	0.003359	-1.84364
SLC50A1	0.370527	6.234418	4.483457	1.70E-05	8.33E-05	2.006152
RHBDF2	0.370035	7.133491	3.682654	0.000349	0.001309	-0.86831
NSF	0.369943	4.563311	4.651378	8.62E-06	4.43E-05	2.660708
PTPRN2	0.369938	5.33862	3.650661	0.00039	0.00145	-0.97407
RELT	0.369833	6.869725	6.732025	6.26E-10	6.72E-09	11.94537

TM2D1	0.369301	5.756989	3.325837	0.001173	0.003937	-2.00541
ATP6V0B	0.368995	9.983324	4.431613	2.10E-05	0.0001	1.807501
BMP2K	0.368949	4.631106	5.35052	4.32E-07	2.84E-06	5.556376
STAU2	0.368824	3.777224	5.961874	2.62E-08	2.11E-07	8.287523
LRRC57	0.368558	4.437434	3.579942	0.000499	0.001812	-1.20523
GTF2A2	0.36848	6.001967	4.802662	4.61E-06	2.50E-05	3.264577
SH3BGRL3	0.367909	10.71768	4.096011	7.71E-05	0.000329	0.562431
PRKAG1	0.367793	7.275725	5.454585	2.71E-07	1.84E-06	6.009082
ART4	0.367313	2.283744	3.100956	0.002409	0.007498	-2.67265
NEDD4L	0.367239	4.101561	2.675835	0.008506	0.022654	-3.82393
PHGDH	0.367178	5.165927	3.158505	0.002011	0.006385	-2.50564
SAP30	0.366893	4.415303	4.835137	4.02E-06	2.21E-05	3.39591
TSPAN14	0.366824	7.880212	4.480479	1.72E-05	8.42E-05	1.994696
CDC42	0.365902	6.91981	3.227299	0.001615	0.005243	-2.3026
NUDT19	0.365475	7.086637	3.028115	0.003019	0.009166	-2.88029
TPO	0.364866	3.195377	3.711839	0.000315	0.001194	-0.7712
LOXL3	0.364731	5.287964	4.751013	5.72E-06	3.05E-05	3.056934
SLC24A3	0.364415	4.25106	2.422916	0.016905	0.041116	-4.43753
ANKRD50	0.364338	3.650631	4.349734	2.90E-05	0.000135	1.497147
SLC39A4	0.36373	5.124398	4.34516	2.95E-05	0.000138	1.479931
LOC10260	0.363355	5.086189	2.128732	0.035341	0.076628	-5.08144
ZWILCH	0.363353	4.186617	1.986824	0.049242	0.101084	-5.36449
TOLLIP-AS	0.363131	3.315255	3.302544	0.001266	0.004216	-2.07634
FAM228A	0.363121	2.816686	4.712793	6.70E-06	3.53E-05	2.904264
LSM2	0.363106	4.957	3.091548	0.002481	0.007695	-2.69971
C6orf164	0.363016	2.602577	4.608225	1.03E-05	5.21E-05	2.490892
TMEM234	0.362707	5.179327	7.964826	1.11E-12	1.91E-11	18.17926
ST3GAL2	0.362321	5.55235	6.695894	7.50E-10	7.90E-09	11.76907
IFNGR2	0.362235	9.852851	3.696852	0.000332	0.001252	-0.82114
SAPCD2	0.362229	4.950263	4.144603	6.41E-05	0.000278	0.738219
RABIF	0.362134	6.624296	5.480689	2.41E-07	1.65E-06	6.123459
INSL3	0.362048	4.475231	4.603509	1.05E-05	5.31E-05	2.472401
ARL6IP4	0.362009	6.350114	4.686816	7.45E-06	3.88E-05	2.800975
TFPT	0.361982	5.518457	4.697768	7.13E-06	3.73E-05	2.844474
FKBP8	0.361674	7.054341	2.741337	0.007064	0.019279	-3.6562
ENPP7	0.361402	5.591713	3.528419	0.000595	0.002129	-1.37135
LOC15896	0.361149	6.884781	4.624721	9.62E-06	4.90E-05	2.555676
ABHD17C	0.360395	4.530994	2.140849	0.034329	0.074743	-5.05643
FAM96B	0.360254	7.118278	3.925741	0.000145	0.000588	-0.04113
KDM1B	0.360206	4.932056	2.477976	0.014618	0.036246	-4.30861
MALSU1	0.360061	5.728205	5.202628	8.31E-07	5.21E-06	4.922232
LAT2	0.359873	7.43901	5.30047	5.40E-07	3.49E-06	5.340539
RAB11A	0.359799	7.119989	3.297374	0.001287	0.00428	-2.09202
LOC10050	0.359498	4.861086	2.559731	0.011729	0.029933	-4.11237
TMUB2	0.359369	6.178685	4.56727	1.22E-05	6.09E-05	2.330747
H6PD	0.359323	4.587965	5.786563	5.95E-08	4.52E-07	7.487232
NUDT18	0.35923	5.118173	4.593593	1.09E-05	5.50E-05	2.433566
SH3BGRL	0.359075	9.890299	2.699166	0.007965	0.021393	-3.76459
RAB1A	0.358948	6.544291	4.103963	7.48E-05	0.00032	0.591092
MBOAT1	0.358948	7.18315	3.896561	0.000162	0.000648	-0.14259
ABCC2	0.358935	3.723582	3.610672	0.000448	0.001644	-1.10523
DKFZp564	0.358704	3.55692	4.097816	7.65E-05	0.000327	0.568934
KANK2	0.358671	4.446968	3.136168	0.002158	0.006796	-2.57077
FAM83D	0.35858	3.310891	2.449099	0.01578	0.038763	-4.37655
GATA1	0.358564	6.653666	2.666382	0.008735	0.023176	-3.84784
SCP2	0.35833	8.398791	3.062648	0.002714	0.008347	-2.78238
NDUFA3	0.358085	8.614534	4.434808	2.07E-05	9.92E-05	1.819696
RAB33B	0.357965	6.216489	2.157225	0.033001	0.072414	-5.02242

DENND2C	0.3577	3.994499	4.503853	1.57E-05	7.72E-05	2.084754
LOC10192	0.357659	4.2074	3.174772	0.00191	0.006099	-2.45797
RNF145	0.357645	5.420476	4.764705	5.40E-06	2.90E-05	3.111831
DNAJC3-A	0.357468	3.041153	4.906589	2.98E-06	1.67E-05	3.686946
HIST1H3B	0.357306	2.712525	4.339661	3.01E-05	0.00014	1.459253
TREML4	0.356933	3.731247	3.574778	0.000508	0.001839	-1.22196
LINC00853	0.35685	3.50936	2.796611	0.006025	0.016776	-3.51188
OTUD1	0.356844	5.622733	3.362114	0.001041	0.003527	-1.89413
DNAH10	0.356751	3.768947	6.021943	1.97E-08	1.62E-07	8.564701
ZNF106	0.356737	8.118303	4.431092	2.10E-05	0.000101	1.805513
SPA17	0.356644	2.432067	4.314512	3.33E-05	0.000154	1.364925
ARID3A	0.356508	6.068389	3.87671	0.000174	0.000693	-0.21129
ATOH1	0.356198	3.982671	4.241073	4.43E-05	0.000199	1.091771
ASAP1	0.356095	8.186542	4.18773	5.43E-05	0.00024	0.895528
LOC10192	0.355851	3.780062	3.474528	0.000715	0.002511	-1.54301
LOC10031	0.355817	4.994102	3.523955	0.000604	0.002158	-1.38565
SESTD1	0.355779	5.213011	3.161091	0.001994	0.006339	-2.49808
LINC00672	0.355304	4.522986	3.163274	0.001981	0.006299	-2.49169
TRIP6	0.355187	4.526019	3.902375	0.000158	0.000636	-0.12242
PTGES	0.354892	5.048844	4.836663	4.00E-06	2.19E-05	3.402096
WNT11	0.354812	4.64246	4.317809	3.28E-05	0.000152	1.377269
HK1	0.35481	8.835276	2.74777	0.006936	0.01896	-3.63953
WDR83OS	0.354762	8.099645	5.297173	5.48E-07	3.53E-06	5.326366
SERPINI1	0.354641	4.644758	2.259217	0.025692	0.058621	-4.80525
FAM20C	0.354388	4.142885	5.892208	3.64E-08	2.86E-07	7.967936
TUBB6	0.354314	4.927919	2.345772	0.020644	0.048672	-4.61374
GCKR	0.353651	3.395706	3.940452	0.000138	0.00056	0.010245
C1orf112	0.353378	2.950989	3.221722	0.001644	0.005327	-2.3192
KPNA4	0.353317	6.253221	3.926511	0.000145	0.000587	-0.03844
FGGY	0.353296	3.440658	6.184427	9.10E-09	7.88E-08	9.321739
MIEN1	0.353295	8.044182	5.996007	2.23E-08	1.81E-07	8.444843
SETD3	0.353244	5.327749	5.05675	1.57E-06	9.26E-06	4.3077
TAZ	0.352848	6.510485	6.80891	4.27E-10	4.71E-09	12.32191
WDR37	0.35275	5.266353	5.356324	4.21E-07	2.77E-06	5.581489
RPS6KB2	0.352697	5.777124	5.218458	7.75E-07	4.88E-06	4.989583
ZCRB1	0.352537	5.982812	3.624295	0.000428	0.001574	-1.06067
NDUFAF3	0.352383	6.960302	3.864627	0.000182	0.000721	-0.25296
MTDH	0.352322	7.601614	3.529301	0.000593	0.002123	-1.36852
HSPB1	0.35224	6.234139	2.495022	0.013968	0.03485	-4.26816
RFT1	0.351999	4.668189	6.801222	4.44E-10	4.89E-09	12.28418
OR7E47P	0.351992	3.710508	4.939457	2.59E-06	1.47E-05	3.821773
ABO	0.35186	4.858396	3.39956	0.000919	0.003154	-1.77823
EGFL7	0.351477	5.0977	3.300803	0.001273	0.004237	-2.08162
CNGA4	0.351127	3.129107	3.919511	0.000149	0.0006	-0.06284
LINC00671	0.350486	2.882305	6.106432	1.32E-08	1.12E-07	8.95704
EIF2B2	0.350392	6.40285	4.741276	5.95E-06	3.17E-05	3.01796
MFSD11	0.350375	6.074948	5.407606	3.35E-07	2.23E-06	5.80406
VPS35	0.349963	7.176164	3.959933	0.000128	0.000524	0.078503
COPE	0.34976	6.391209	6.593324	1.25E-09	1.27E-08	11.27093
GNB4	0.34973	6.233064	4.053412	9.05E-05	0.000381	0.409606
TCIRG1	0.349467	8.155578	3.310588	0.001233	0.004118	-2.05189
SPPL2A	0.349415	6.76829	2.757008	0.006754	0.018541	-3.61554
RASGEF1A	0.349278	4.363744	2.594888	0.010653	0.027506	-4.02623
GAL	0.34907	3.200264	4.588418	1.12E-05	5.61E-05	2.413318
TMEM141	0.348902	5.68013	5.12798	1.15E-06	6.99E-06	4.606382
TMEM144	0.348707	2.630193	4.792531	4.81E-06	2.60E-05	3.223729
ABRACL	0.34853	8.349237	2.800025	0.005965	0.01663	-3.50288
CMAS	0.348455	5.133826	3.980433	0.000119	0.000489	0.150611

DCAF11	0.348449	6.821216	3.924705	0.000146	0.00059	-0.04474
PMM1	0.348257	4.141444	3.633762	0.000414	0.001528	-1.02964
BCAP29	0.347631	4.421857	5.66518	1.04E-07	7.58E-07	6.940942
IFNAR1	0.347557	5.887393	4.52069	1.47E-05	7.27E-05	2.149826
SWT1	0.347266	5.106202	3.900274	0.00016	0.00064	-0.12971
CHEK1	0.347213	3.034757	3.051619	0.002808	0.0086	-2.81375
KRTAP13-	0.346987	5.206711	4.128486	6.81E-05	0.000294	0.679742
SH3BP5	0.346963	8.492801	2.679415	0.008421	0.022462	-3.81485
CTSLP8	0.346678	2.659751	2.72665	0.007367	0.020003	-3.69412
PAQR4	0.346492	4.722824	4.037484	9.61E-05	0.000402	0.352775
CDK7	0.346154	6.042433	2.874945	0.00479	0.013763	-3.30305
SUPT4H1	0.346132	7.398781	3.965697	0.000125	0.000514	0.098748
SCYL2	0.345885	7.012342	3.058523	0.002749	0.008439	-2.79412
PHF20L1	0.345768	6.171639	4.213946	4.91E-05	0.000219	0.991746
CYB5R1	0.34528	5.824329	5.785424	5.98E-08	4.54E-07	7.482076
ADAM15	0.344864	6.374393	4.809484	4.48E-06	2.43E-05	3.292116
CAMTA2	0.344771	6.446937	4.361976	2.76E-05	0.000129	1.543282
COCH	0.344423	4.629025	2.44577	0.01592	0.039049	-4.38433
OR7E12P	0.344194	4.554276	4.522749	1.45E-05	7.21E-05	2.157796
SPTB	0.343849	5.395335	2.750892	0.006874	0.018814	-3.63143
RFNG	0.343598	6.330136	4.326277	3.18E-05	0.000147	1.409002
NBN	0.343487	6.765676	3.44169	0.000799	0.002778	-1.64656
STX18	0.343334	5.95155	5.097143	1.32E-06	7.91E-06	4.476747
INPP1	0.343316	6.189535	2.821552	0.005603	0.01574	-3.44594
TMEM88	0.343309	5.402542	4.017209	0.000104	0.00043	0.280679
ANKLE1	0.342878	3.070797	5.094612	1.33E-06	7.99E-06	4.466128
APP	0.342744	5.821707	3.082421	0.002553	0.00789	-2.72588
SELL	0.342692	12.21477	4.378127	2.59E-05	0.000122	1.604294
CDK2AP2	0.342109	6.617237	3.417022	0.000867	0.00299	-1.72381
MYF6	0.341706	3.359668	3.876058	0.000174	0.000695	-0.21353
ASH2L	0.341684	7.948949	6.310334	4.96E-09	4.53E-08	9.915417
SLC4A1AP	0.341364	5.850429	3.259683	0.001455	0.004768	-2.20576
STAT5B	0.341296	7.944283	3.803341	0.000227	0.000884	-0.4628
DYNC2LI1	0.341259	2.319727	3.638431	0.000407	0.001506	-1.0143
PCSK1N	0.341216	4.452408	3.002761	0.003262	0.009805	-2.95157
STX5	0.340891	5.789893	4.901334	3.04E-06	1.70E-05	3.665447
PCBD1	0.340832	5.149731	3.408809	0.000892	0.003066	-1.74943
VTI1B	0.340698	7.48931	3.140629	0.002128	0.006716	-2.5578
PSTPIP1	0.340448	7.911115	3.314918	0.001216	0.004066	-2.03871
MED7	0.340121	5.093997	3.909922	0.000154	0.00062	-0.09621
EIF5A	0.339887	6.817884	2.01412	0.046255	0.095981	-5.31146
PELO	0.339856	3.271674	6.172739	9.63E-09	8.32E-08	9.266935
UBE2D1	0.3398	5.553537	2.613604	0.010117	0.026322	-3.97995
COPS2	0.339767	5.684409	2.391875	0.01833	0.04407	-4.50905
APOA1	0.33975	3.296269	4.31538	3.31E-05	0.000153	1.368174
GPT2	0.339452	3.028705	3.508191	0.000638	0.002265	-1.43603
GIN1	0.339177	3.79469	2.108381	0.037098	0.079805	-5.12314
HAMP	0.338891	3.692131	3.281055	0.001358	0.004484	-2.1414
PCYT1B	0.338675	3.183366	3.172622	0.001923	0.006137	-2.46428
CCDC159	0.338547	5.891543	4.824207	4.21E-06	2.30E-05	3.35164
MGME1	0.338457	6.817215	2.673072	0.008572	0.02281	-3.83092
ALYREF	0.33844	4.66685	2.542618	0.012287	0.031198	-4.15392
ACAD8	0.33834	6.328894	2.37673	0.019064	0.04556	-4.54364
SNTB2	0.33812	4.220328	6.352814	4.04E-09	3.76E-08	10.11706
CHAF1B	0.337883	3.812594	3.972398	0.000122	0.000503	0.122316
RAD54L	0.337749	4.39398	3.586489	0.000488	0.001777	-1.18398
TMEM8A	0.337482	7.278596	5.158718	1.01E-06	6.18E-06	4.73609
CKAP2L	0.337394	3.894525	4.044854	9.34E-05	0.000392	0.379052

RIT1	0.33739	6.425199	2.39518	0.018174	0.04375	-4.50148
SNX12	0.337378	5.496543	6.679821	8.12E-10	8.51E-09	11.69078
SYTL4	0.337287	3.397776	4.663167	8.22E-06	4.24E-05	2.707286
FAM83A	0.337227	4.31924	2.643606	0.009309	0.024474	-3.90514
ZNF780A	0.337169	4.470665	2.38153	0.018829	0.045096	-4.5327
RPL39L	0.337016	3.061758	3.480741	0.0007	0.002466	-1.52333
RHCG	0.336933	4.219069	4.036059	9.66E-05	0.000404	0.347701
POLD4	0.336828	7.077023	6.079943	1.50E-08	1.25E-07	8.833725
CREB5	0.336576	9.080224	2.187774	0.030643	0.068043	-4.95834
UBE2W	0.336389	5.026156	3.519678	0.000613	0.002185	-1.39933
MOGAT1	0.336311	3.738984	4.083101	8.09E-05	0.000344	0.515988
LOC10050	0.335887	2.754223	3.666729	0.000369	0.001379	-0.92105
ZDHHC17	0.335707	6.068689	2.89835	0.004468	0.012963	-3.23967
LINC0126	0.335699	2.443425	2.781031	0.006303	0.017447	-3.55282
CD300C	0.335316	6.671024	3.274097	0.001389	0.004572	-2.16239
GALC	0.335286	5.818429	2.907378	0.00435	0.012653	-3.21511
MPDU1	0.335201	5.684592	6.950124	2.10E-10	2.44E-09	13.01831
CLTB	0.335012	5.726918	4.203709	5.11E-05	0.000226	0.954123
AQP1	0.334781	4.73132	2.243991	0.026684	0.060581	-4.83825
TMED1	0.334637	6.168905	5.001612	1.99E-06	1.15E-05	4.078348
GALK1	0.334598	4.329223	6.341704	4.26E-09	3.94E-08	10.06426
LINC0048	0.334289	4.704882	3.49811	0.00066	0.002336	-1.46815
TNNT1	0.334273	5.02623	3.158841	0.002009	0.00638	-2.50466
LOC10192	0.334251	3.082107	3.755617	0.000269	0.001035	-0.62439
SRI	0.334225	6.163102	2.438543	0.016225	0.039679	-4.4012
NINJ2	0.334011	8.239512	2.184581	0.030882	0.068487	-4.96508
SNAPIN	0.333765	7.473092	3.88691	0.000168	0.00067	-0.17602
BEND7	0.33342	3.4375	4.897907	3.09E-06	1.72E-05	3.651431
PSMD1	0.333212	7.554256	4.018477	0.000103	0.000428	0.285179
LINC0083	0.333178	2.473089	4.625464	9.59E-06	4.88E-05	2.5586
PARPBP	0.333177	2.294048	3.993491	0.000113	0.000467	0.196689
CENPN	0.332894	3.733823	3.895394	0.000163	0.000651	-0.14664
CYP2W1	0.332627	4.45157	3.649646	0.000391	0.001454	-0.97741
GINS3	0.332617	3.548437	2.592056	0.010736	0.027692	-4.03321
MAD2L1B	0.332542	6.848613	5.366202	4.03E-07	2.66E-06	5.624261
SF3B5	0.332314	8.45524	3.588917	0.000484	0.001764	-1.17609
LPCAT3	0.332208	5.165672	3.907818	0.000155	0.000624	-0.10352
DUSP3	0.332155	5.712496	3.250287	0.0015	0.004899	-2.23394
ZRANB1	0.331691	6.458613	2.671026	0.008622	0.022933	-3.8361
ANKRD6	0.331647	3.521781	2.972838	0.003573	0.01062	-3.03504
CFL1	0.330823	7.574193	7.905309	1.53E-12	2.54E-11	17.87
USP32	0.33082	7.657808	3.892181	0.000164	0.000658	-0.15777
RAPGEFL1	0.330716	4.189422	3.446601	0.000786	0.002735	-1.63112
SPIDR	0.330239	4.702952	4.923336	2.77E-06	1.56E-05	3.755569
HTRA1	0.330131	4.483901	3.437937	0.000809	0.002807	-1.65834
IFI27L1	0.329257	4.863289	3.229222	0.001605	0.005215	-2.29688
TLR1	0.329206	9.308527	2.070357	0.040585	0.086012	-5.20007
BLOC1S6	0.328914	7.069003	3.125008	0.002235	0.007014	-2.60317
RAX2	0.32867	5.405569	4.642012	8.96E-06	4.59E-05	2.623758
CDC34	0.328634	7.11908	2.91925	0.004198	0.01227	-3.18271
SDC1	0.328625	3.421097	2.558622	0.011765	0.030004	-4.11507
CEPT1	0.328415	6.034627	2.984506	0.003449	0.010296	-3.00258
SPATA21	0.328124	4.581079	4.321829	3.23E-05	0.00015	1.39233
HDGF	0.328062	9.732551	2.126258	0.03555	0.076969	-5.08653
CSRP3	0.328013	3.528501	3.830021	0.000206	0.000809	-0.37177
ITGB5	0.327947	3.939053	3.585618	0.000489	0.001781	-1.18681
ZC3H3	0.327937	4.140146	5.675843	9.92E-08	7.26E-07	6.988668
NRIP3	0.327902	4.129629	3.430066	0.00083	0.002873	-1.68302

PLIN5	0.327889	5.262032	4.662347	8.24E-06	4.25E-05	2.704047
STRA13	0.327629	7.001566	3.54253	0.000567	0.002036	-1.32604
LOC10192	0.327592	3.268239	4.695142	7.20E-06	3.77E-05	2.834037
ARPC5	0.327368	8.583165	4.591431	1.10E-05	5.55E-05	2.425102
DSCC1	0.327362	4.254881	4.018962	0.000103	0.000428	0.2869
TMEM71	0.327187	9.221961	2.08905	0.038837	0.082961	-5.16241
SPAG4	0.327151	2.96507	3.042124	0.002891	0.008832	-2.84068
OXER1	0.326539	6.612662	5.119823	1.19E-06	7.22E-06	4.57204
DDX59	0.326303	5.668296	3.839373	0.000199	0.000784	-0.33975
IGLON5	0.32623	4.317576	3.343213	0.001108	0.003734	-1.95224
SPR	0.325936	5.760354	3.682103	0.000349	0.001312	-0.87014
PPP1R14B	0.325815	7.022127	3.436054	0.000814	0.002822	-1.66425
GLTPD2	0.325742	2.795994	2.998672	0.003303	0.009909	-2.96302
SNX14	0.325684	5.912065	2.44125	0.01611	0.039451	-4.39489
GUCY1A3	0.325665	3.980803	2.161944	0.032627	0.071747	-5.01258
OSTC	0.325631	7.813151	2.195224	0.03009	0.067012	-4.94259
STX8	0.325513	4.584583	6.035847	1.85E-08	1.52E-07	8.629071
FAM114A2	0.325377	5.852977	3.709059	0.000318	0.001205	-0.78047
SLC7A11	0.32519	3.138617	4.265495	4.03E-05	0.000182	1.182226
NDUFA11	0.325047	6.56351	5.491096	2.30E-07	1.58E-06	6.16915
KIAA1033	0.324936	8.062267	2.887977	0.004608	0.013301	-3.26782
MRPS22	0.324894	6.347019	3.323186	0.001183	0.003971	-2.01351
FBXL19	0.324791	5.723117	4.110307	7.30E-05	0.000313	0.613988
NOS1AP	0.324501	4.323865	5.15875	1.01E-06	6.18E-06	4.736226
ATG4A	0.323589	5.92133	3.658591	0.000379	0.001415	-0.94792
SYK	0.323555	8.213661	3.836843	0.000201	0.000791	-0.34841
C10orf54	0.323443	9.946078	2.974722	0.003553	0.010568	-3.0298
UBA6	0.323391	4.520416	3.109554	0.002345	0.007323	-2.64786
NTN3	0.323298	3.744917	4.673367	7.88E-06	4.08E-05	2.747657
WDR13	0.32317	4.448689	6.108159	1.31E-08	1.11E-07	8.96509
NEK6	0.323021	4.748315	6.314494	4.87E-09	4.46E-08	9.935135
CSRNP1	0.322858	6.088097	2.891065	0.004566	0.013204	-3.25945
ZNF524	0.322641	5.239163	5.924984	3.12E-08	2.47E-07	8.11804
PSMD4	0.322426	7.464303	6.560139	1.47E-09	1.47E-08	11.11052
LAMA5-AS	0.322021	4.896982	3.25288	0.001487	0.004864	-2.22617
RWDD3	0.321983	3.251848	2.368361	0.01948	0.046408	-4.56267
KIN	0.321845	3.467012	3.139631	0.002134	0.006734	-2.5607
ACVRL1	0.321578	4.054975	5.344223	4.45E-07	2.91E-06	5.529155
CDCA8	0.321166	5.320178	2.950687	0.00382	0.011268	-3.09636
PRELID1	0.321047	7.227248	2.89665	0.004491	0.013018	-3.24429
CEBPG	0.321015	5.286398	2.839955	0.005309	0.015022	-3.39695
CNRIP1	0.321013	3.498421	3.704964	0.000322	0.001221	-0.79413
CCZ1B	0.320823	5.847075	2.547851	0.012114	0.030793	-4.14124
NUDT2	0.320669	5.10006	3.695023	0.000334	0.001259	-0.82723
TUBA4B	0.320009	5.510163	4.720893	6.48E-06	3.42E-05	2.936548
DNAJB6	0.319961	7.219623	3.283295	0.001348	0.004456	-2.13463
LOC10272	0.3197	5.60759	2.740628	0.007079	0.01931	-3.65803
NDUFV3	0.319128	5.273834	3.454987	0.000764	0.002667	-1.60473
NOS2	0.318998	4.168394	3.693467	0.000336	0.001265	-0.8324
ECT2	0.318931	2.717912	4.180105	5.60E-05	0.000246	0.867629
GLDN	0.318798	4.190756	3.288572	0.001325	0.004389	-2.11868
THYN1	0.318648	6.652606	2.889943	0.004581	0.013235	-3.26249
EMC7	0.318232	5.438249	4.668556	8.04E-06	4.15E-05	2.728607
PAGE5	0.318188	3.940996	3.773141	0.000253	0.000977	-0.56524
MRPS15	0.318132	4.873346	4.176038	5.68E-05	0.000249	0.852761
GSN	0.317765	4.775773	5.02213	1.82E-06	1.06E-05	4.163506
MYLK	0.31775	3.818322	3.022897	0.003067	0.009289	-2.895
FAM188A	0.317668	4.896639	3.506127	0.000642	0.002279	-1.44261

RPL27	0.317518	10.99529	2.191322	0.030379	0.067566	-4.95085
FAM132A	0.317503	3.677262	2.960039	0.003714	0.010994	-3.07052
STK16	0.317419	4.658837	3.675235	0.000358	0.00134	-0.8929
CDC37	0.31741	8.083028	3.543819	0.000565	0.002028	-1.3219
CBX3	0.31731	6.36313	2.801171	0.005946	0.016582	-3.49986
ANKRD37	0.317262	3.966869	4.816379	4.35E-06	2.37E-05	3.319977
EGF	0.317156	3.604476	2.024281	0.045183	0.094167	-5.29154
CCNJL	0.317132	6.461205	3.013871	0.003153	0.009514	-2.9204
DAPK3	0.31664	2.181102	4.05075	9.14E-05	0.000384	0.400095
EMC9	0.316533	5.082769	3.213958	0.001686	0.005442	-2.34226
PINK1	0.316317	8.186889	2.200086	0.029735	0.066401	-4.93228
C8orf44	0.316243	3.94255	3.155524	0.00203	0.006442	-2.51436
SRF	0.316005	5.009805	5.206329	8.17E-07	5.13E-06	4.937966
EFCAB4A	0.315461	4.655791	4.252566	4.23E-05	0.000191	1.134292
ACTR2	0.315425	7.654175	3.218059	0.001664	0.005381	-2.33009
B3GALNT1	0.315089	2.228081	2.375959	0.019102	0.045635	-4.5454
LOC28569	0.315039	2.33325	3.39509	0.000933	0.003198	-1.79212
DEGS2	0.315037	3.892082	3.020454	0.00309	0.009353	-2.90188
BRAF	0.314858	5.374424	3.91134	0.000153	0.000617	-0.09128
ALG10	0.314785	2.472581	4.76049	5.49E-06	2.94E-05	3.094922
MCM4	0.314614	4.18013	2.975059	0.003549	0.010561	-3.02887
C21orf91	0.314483	6.653061	2.773755	0.006437	0.017779	-3.57186
MED20	0.314475	4.080434	5.034696	1.72E-06	1.01E-05	4.21577
ST20	0.314274	6.808219	2.122377	0.035881	0.077546	-5.0945
CTSZ	0.314264	5.352876	3.169074	0.001945	0.006198	-2.47469
SBNO2	0.314162	6.490235	2.790689	0.006129	0.017031	-3.52747
LOC10192	0.314041	3.819271	4.221598	4.77E-05	0.000213	1.019914
GGCX	0.313734	4.542088	5.959297	2.65E-08	2.13E-07	8.275667
RAP2C-AS	0.313674	3.226313	4.052025	9.10E-05	0.000383	0.40465
STAM2	0.31357	5.460858	3.45226	0.000771	0.002689	-1.61331
MFAP3	0.313326	5.424543	3.39517	0.000933	0.003198	-1.79187
ZNF185	0.313271	7.516264	2.094785	0.038314	0.082006	-5.1508
NAPB	0.312963	4.142704	3.772705	0.000253	0.000979	-0.56671
HTRA3	0.312699	4.515964	3.218354	0.001662	0.005378	-2.32921
IFITM2	0.312679	12.77485	4.519463	1.47E-05	7.30E-05	2.145079
EFCAB2	0.312584	2.961327	3.709976	0.000317	0.001201	-0.77741
ZNF784	0.312537	5.180363	4.627016	9.53E-06	4.85E-05	2.564703
SUSD3	0.312237	5.285567	3.027728	0.003022	0.009174	-2.88138
SSR4	0.312237	9.508002	2.625746	0.009783	0.025562	-3.94976
ASB8	0.312084	7.307548	4.07928	8.21E-05	0.000349	0.502265
GDF15	0.312075	3.097072	5.425168	3.10E-07	2.08E-06	5.880582
E2F7	0.312027	2.975136	3.14473	0.0021	0.006638	-2.54585
TBCE	0.311993	5.030589	3.00816	0.003209	0.009663	-2.93644
CYTL1	0.311548	3.033236	2.877403	0.004755	0.013674	-3.29641
NDUFA2	0.311272	4.953022	6.679743	8.12E-10	8.51E-09	11.69039
LOC21969	0.311125	4.827742	3.740931	0.000284	0.001087	-0.67379
MVB12B	0.31099	5.167139	6.730177	6.32E-10	6.77E-09	11.93634
LOC10192	0.310971	2.860348	4.180969	5.58E-05	0.000245	0.870787
LRRN4	0.310941	3.277474	3.638657	0.000407	0.001505	-1.01356
OSTM1	0.310401	5.85306	2.729873	0.0073	0.019836	-3.68581
DAP	0.310396	7.666051	2.963374	0.003677	0.010903	-3.06129
TCF7L2	0.310386	6.083561	2.469253	0.01496	0.037002	-4.3292
COMMD4	0.310272	5.725521	4.69917	7.08E-06	3.71E-05	2.85005
KRCC1	0.309772	6.82665	2.165734	0.032329	0.071166	-5.00466
FLJ45482	0.309727	3.466708	3.176708	0.001898	0.006067	-2.45228
BRCA2	0.30971	2.455707	2.768744	0.00653	0.018003	-3.58496
UBLCP1	0.309281	7.363884	2.627804	0.009727	0.025443	-3.94463
ID1	0.309049	2.653548	2.882736	0.00468	0.013483	-3.282

AGO4	0.309005	5.866759	3.376057	0.000994	0.003383	-1.8511
MOSPD2	0.308891	7.63667	3.218231	0.001663	0.005379	-2.32958
AK9	0.308792	3.566624	3.490539	0.000677	0.002391	-1.49223
RSRC1	0.308692	3.511115	4.086083	8.00E-05	0.000341	0.526708
LOC10192	0.308663	2.390624	2.989764	0.003394	0.010147	-2.98791
DNAJC3	0.30836	6.907856	3.052408	0.002801	0.008583	-2.81151
TWIST2	0.308291	2.578657	3.175025	0.001908	0.006096	-2.45722
MEGF9	0.308269	8.754324	3.409686	0.000889	0.003059	-1.7467
C1orf116	0.308238	3.079883	3.184759	0.00185	0.005933	-2.42859
VPS37B	0.307837	6.961999	3.347968	0.001091	0.003681	-1.93765
CTTN	0.307757	3.795187	2.573729	0.01129	0.028952	-4.0782
PSMD7	0.307726	8.08515	3.022914	0.003067	0.009289	-2.89495
MATR3	0.307693	2.930489	2.686262	0.00826	0.022097	-3.79747
UBE2M	0.307652	7.505497	2.491997	0.014081	0.03509	-4.27536
PSPH	0.307481	2.26244	2.515296	0.013228	0.033294	-4.21974
ECM1	0.307257	4.958199	3.45699	0.000759	0.00265	-1.59841
NMRK1	0.307143	6.147073	2.578023	0.011158	0.028671	-4.06769
KNSTRN	0.307112	4.946721	2.765805	0.006586	0.018129	-3.59262
FLJ46026	0.307064	4.361681	4.313362	3.34E-05	0.000154	1.36062
EME1	0.306718	4.510441	2.854827	0.005082	0.01447	-3.35716
B4GALT2	0.306364	4.524064	3.770413	0.000255	0.000986	-0.57446
ELMOD2	0.306289	3.936798	4.166048	5.91E-05	0.000258	0.816289
PSMC3IP	0.306281	4.811279	4.715597	6.62E-06	3.49E-05	2.915435
C3P1	0.306184	4.545736	3.749648	0.000275	0.001055	-0.64448
ACRBP	0.305995	6.273707	2.046111	0.042952	0.090113	-5.24844
SWI5	0.305844	4.792326	3.456868	0.000759	0.002651	-1.5988
CTC-338M	0.305785	4.542782	3.270471	0.001405	0.004622	-2.17331
DOK1	0.305698	6.200236	5.750527	7.03E-08	5.28E-07	7.324369
LDLRAD4	0.305563	4.467573	4.259243	4.12E-05	0.000186	1.159033
CCS	0.305554	6.668008	2.34392	0.020742	0.048869	-4.6179
SAMD14	0.305507	4.196972	3.809794	0.000222	0.000865	-0.44083
VASP	0.305451	9.306411	3.561816	0.000531	0.001917	-1.26389
NLRX1	0.305213	6.600463	3.993834	0.000113	0.000467	0.1979
NEDD1	0.304994	5.561875	2.133641	0.034927	0.075869	-5.07132
ATG10	0.304989	3.667216	4.655899	8.46E-06	4.35E-05	2.67856
PPM1A	0.304973	6.750066	3.410165	0.000887	0.003055	-1.74521
C17orf58	0.304364	3.798069	2.407476	0.017601	0.042532	-4.47321
PHYKPL	0.30419	6.379749	4.977036	2.21E-06	1.27E-05	3.976648
DNAJA2	0.304148	6.939335	4.120341	7.03E-05	0.000303	0.650253
15-Sep	0.304104	9.464338	2.380499	0.018879	0.045203	-4.53505
ATP5F1	0.303904	6.691714	3.569338	0.000517	0.001871	-1.23957
TOR1AIP1	0.303706	6.132088	3.837105	0.000201	0.00079	-0.34752
GADD45G	0.303626	4.292262	5.952946	2.73E-08	2.19E-07	8.246455
FAM207A	0.303608	4.107239	4.477463	1.74E-05	8.51E-05	1.983102
GPR35	0.303393	5.437569	3.529482	0.000593	0.002122	-1.36794
TUBB4B	0.303146	7.684452	3.025549	0.003043	0.009223	-2.88753
POLDIP2	0.303008	6.490218	5.330149	4.73E-07	3.08E-06	5.46838
RB1	0.30286	5.617918	2.821919	0.005597	0.01573	-3.44496
TDRP	0.302721	3.359443	3.322064	0.001188	0.003983	-2.01693
C1orf192	0.302401	2.595924	3.64157	0.000403	0.001492	-1.00399
C20orf27	0.302119	6.403352	3.645674	0.000397	0.001473	-0.99049
BCRP3	0.302059	2.737944	5.563393	1.66E-07	1.16E-06	6.487969
TTC32	0.301992	6.502481	2.254753	0.02598	0.059191	-4.81495
ACAP1	0.30192	6.544194	5.983158	2.37E-08	1.91E-07	8.385565
FKBP15	0.301751	5.471865	3.30846	0.001242	0.004145	-2.05836
TRMT1L	0.301577	5.451513	2.608363	0.010265	0.026662	-3.99294
DCTN2	0.301518	5.244429	6.605159	1.17E-09	1.20E-08	11.32823
TINF2	0.301322	7.912233	3.639661	0.000405	0.001501	-1.01026

LOC10192	0.301162	4.476995	4.148359	6.32E-05	0.000275	0.75187
KCNJ15	0.300806	6.992651	2.078084	0.039854	0.0847	-5.18454
TBCEL	0.300755	4.329174	2.326237	0.0217	0.050856	-4.65753
GTF2IRD2	0.300639	4.769813	4.156182	6.13E-05	0.000267	0.780336
HIF1A	0.300623	10.36071	2.281139	0.024321	0.055947	-4.75737
BUD31	0.300538	5.285416	5.51245	2.09E-07	1.44E-06	6.26306
DHRS7	0.300404	5.519734	3.810278	0.000221	0.000864	-0.43918
GPR132	0.30035	5.465235	3.568523	0.000519	0.001875	-1.24221
KCNK5	0.300266	3.176779	2.99002	0.003391	0.010142	-2.9872
LOC10192	0.300211	3.445645	4.492978	1.64E-05	8.03E-05	2.042812
CKM	0.300011	4.099309	3.959119	0.000129	0.000526	0.075645
LOC10192	0.299874	3.858388	4.676024	7.79E-06	4.04E-05	2.758181
LOC81691	0.299832	2.986525	4.431306	2.10E-05	0.0001	1.80633
LOC10192	0.299783	2.717408	4.821359	4.26E-06	2.32E-05	3.340119
MTFMT	0.299779	4.439961	2.835575	0.005378	0.015183	-3.40864
SPRED2	0.299747	2.829438	5.511651	2.10E-07	1.45E-06	6.259545
NUP37	0.299691	5.710312	3.760153	0.000265	0.00102	-0.6091
ABCB6	0.299678	4.026812	3.570826	0.000515	0.001863	-1.23476
FAM32A	0.299677	7.418401	3.917274	0.00015	0.000605	-0.07063
LOC10012	0.29967	4.396436	2.890973	0.004567	0.013204	-3.2597
SREK1IP1	0.299626	5.558517	2.504519	0.013617	0.034109	-4.24552
IL17C	0.299617	5.42502	4.355828	2.83E-05	0.000132	1.5201
POLR2G	0.299506	8.45689	3.316784	0.001208	0.004044	-2.03303
ARL6IP1	0.299369	8.271608	2.81272	0.005749	0.016095	-3.46935
LAMB3	0.299261	3.321167	3.874514	0.000175	0.000698	-0.21887
FLJ25758	0.299149	4.567639	3.939433	0.000138	0.000562	0.006682
APOBEC3F	0.299095	3.844341	4.323902	3.21E-05	0.000149	1.400098
TEKT5	0.299008	3.976297	3.443599	0.000793	0.002761	-1.64056
VTA1	0.298952	5.862934	3.682941	0.000348	0.001308	-0.86736
SH3RF3	0.298814	4.655976	2.112142	0.036768	0.079219	-5.11546
JTB	0.298495	10.07937	4.972037	2.25E-06	1.29E-05	3.956002
PPP2R5A	0.298456	6.132602	3.515311	0.000623	0.002216	-1.4133
P2RY13	0.298423	10.02152	2.847572	0.005192	0.014743	-3.37659
MCEE	0.298083	4.409437	2.194087	0.030174	0.067168	-4.945
VAPA	0.298034	6.171033	3.943312	0.000136	0.000555	0.020251
GZF1	0.298032	4.246399	3.641474	0.000403	0.001492	-1.0043
C4orf45	0.297827	2.906019	3.732845	0.000292	0.001116	-0.70092
CCNYL1	0.297664	4.750874	3.027864	0.003021	0.009171	-2.881
LPL	0.297489	3.017169	2.798076	0.005999	0.016711	-3.50802
SKP1	0.297459	8.772929	4.706672	6.87E-06	3.61E-05	2.879892
OMP	0.297425	3.68589	3.143067	0.002111	0.00667	-2.5507
TUBA1B	0.297172	9.607782	4.360545	2.78E-05	0.00013	1.537883
ATP5H	0.297058	6.862512	4.912704	2.90E-06	1.63E-05	3.711984
ZNF114	0.296506	2.316769	4.532099	1.40E-05	6.96E-05	2.194022
CTDSP1	0.29629	8.548243	3.813664	0.000219	0.000854	-0.42764
ASAH1	0.296249	7.971065	3.12162	0.002259	0.007075	-2.61298
PRG3	0.296144	4.349239	3.27474	0.001386	0.004565	-2.16045
GTPBP1	0.295928	4.865697	5.550031	1.76E-07	1.23E-06	6.42886
DEDD	0.295639	6.480952	3.806318	0.000225	0.000876	-0.45267
CD8B	0.295274	3.133177	3.689387	0.00034	0.001282	-0.84596
STK11	0.295207	6.541809	2.557725	0.011793	0.030071	-4.11725
SOX6	0.295	3.605602	3.275838	0.001381	0.004553	-2.15714
NICN1	0.294846	5.740536	3.954686	0.000131	0.000534	0.060093
TMEM255I	0.29454	4.936336	2.579967	0.011099	0.028548	-4.06292
CCDC17	0.294472	4.438149	2.611102	0.010188	0.026488	-3.98615
HIST1H3G	0.294407	5.322801	4.364755	2.73E-05	0.000128	1.553768
PSMD6	0.29438	5.447481	4.325278	3.19E-05	0.000148	1.405256
CCM2L	0.294139	4.733221	4.699234	7.08E-06	3.71E-05	2.850301

PRCP	0.294136	4.183948	5.120432	1.19E-06	7.20E-06	4.574603
CNTLN	0.294072	2.762374	5.470934	2.52E-07	1.71E-06	6.08068
GYPE	0.294047	2.368104	3.426941	0.000839	0.002901	-1.6928
BRCA1	0.294039	4.330432	2.940189	0.003943	0.011592	-3.12529
NUP214	0.293988	6.598067	4.946112	2.52E-06	1.43E-05	3.849144
SLC12A8	0.293846	3.221552	4.083497	8.08E-05	0.000344	0.517414
ALPP	0.293422	3.035331	3.570145	0.000516	0.001866	-1.23696
LILRP2	0.293393	2.740618	3.492855	0.000672	0.002375	-1.48487
SHOX2	0.293373	3.317917	4.952515	2.45E-06	1.39E-05	3.875501
FOSL2	0.293285	6.264167	2.943489	0.003904	0.011495	-3.1162
DNASE1	0.293241	4.414732	5.217449	7.79E-07	4.89E-06	4.985285
SNX8	0.293199	4.338407	2.688623	0.008205	0.021968	-3.79146
SFT2D2	0.293089	6.799415	2.761307	0.006671	0.018338	-3.60435
KLHL7	0.293037	4.79393	2.517678	0.013144	0.033118	-4.21402
LOC10192	0.292889	4.495968	4.939578	2.59E-06	1.47E-05	3.822271
LGALS3	0.292793	6.657074	3.389741	0.00095	0.003248	-1.80872
NEURL1B	0.292646	4.158606	3.439266	0.000805	0.002796	-1.65417
NCOA7	0.292523	4.6105	4.301568	3.50E-05	0.000161	1.316532
KIAA0895L	0.292513	3.286628	3.597535	0.000469	0.001715	-1.14806
LOC10028	0.292468	3.057794	3.509854	0.000634	0.002253	-1.43072
MICU2	0.292361	7.77309	2.002394	0.047519	0.098185	-5.33432
CELP	0.291671	3.936605	3.150013	0.002066	0.006538	-2.53045
RFWD2	0.291519	7.762915	3.447856	0.000782	0.002725	-1.62717
AFMID	0.291261	4.118661	3.670952	0.000363	0.001359	-0.90708
MMP17	0.291109	5.093015	2.657076	0.008966	0.023699	-3.8713
C7orf61	0.291023	4.962863	3.091505	0.002481	0.007695	-2.69983
WDFY3-AS	0.290629	2.74615	4.391977	2.45E-05	0.000116	1.656746
PCBP3	0.290548	4.610667	3.733604	0.000291	0.001114	-0.69838
CLIP1	0.29015	4.547433	4.425988	2.14E-05	0.000102	1.786046
TFR2	0.289946	4.48666	2.508695	0.013465	0.033802	-4.23554
DYRK3	0.289911	4.413739	3.053183	0.002795	0.008566	-2.80931
GMPPA	0.289817	5.341052	3.031355	0.002989	0.009092	-2.87114
GCGR	0.289386	5.008483	2.880498	0.004711	0.013563	-3.28805
TOP1	0.289315	7.552466	2.427208	0.016716	0.040734	-4.42757
LOC10192	0.289185	3.464336	4.113559	7.21E-05	0.00031	0.625733
MARK3	0.28885	5.90724	3.041068	0.002901	0.008854	-2.84367
YIPF5	0.288824	6.516739	2.426149	0.016762	0.040823	-4.43003
LOC10192	0.288249	2.361204	2.736793	0.007157	0.019489	-3.66795
LOC10192	0.288222	3.795286	3.55098	0.000551	0.001983	-1.29884
NFKBIA	0.288206	6.59855	3.902306	0.000158	0.000636	-0.12266
ARF5	0.288145	7.906358	3.954996	0.000131	0.000534	0.06118
SLC25A51	0.287953	4.707038	3.813583	0.000219	0.000855	-0.42792
S100A2	0.28791	4.331571	3.65993	0.000378	0.001409	-0.94351
FOSL1	0.287781	3.518931	3.578315	0.000502	0.00182	-1.2105
CD9	0.287651	4.517781	3.525738	0.000601	0.002146	-1.37994
TCTA	0.287622	6.227066	4.196545	5.25E-05	0.000232	0.927833
PLEKHN1	0.287465	5.52512	2.657358	0.008958	0.023689	-3.87059
NACC2	0.287364	5.964875	3.1512	0.002058	0.00652	-2.52698
HBS1L	0.287272	4.642832	2.987438	0.003418	0.01021	-2.9944
PRODH2	0.28704	4.174662	4.27786	3.84E-05	0.000175	1.228172
FGF3	0.286776	3.913364	3.480374	0.000701	0.002468	-1.52449
ABHD8	0.286608	4.727266	4.15475	6.17E-05	0.000269	0.775121
C10orf10	0.286548	4.379532	3.94024	0.000138	0.00056	0.009506
MRPS16	0.286384	5.670409	3.280516	0.00136	0.004491	-2.14303
POLR2I	0.286169	6.812897	3.28766	0.001329	0.004399	-2.12144
NKX1-1	0.286067	5.544228	3.07301	0.002628	0.008107	-2.75281
SH3BP5L	0.285852	5.547304	2.559742	0.011729	0.029933	-4.11235
CCRL2	0.285563	4.961288	2.946704	0.003866	0.011394	-3.10735

ORC1	0.285212	2.734741	2.639553	0.009415	0.024714	-3.91529
SDC4	0.285106	3.781833	3.367652	0.001022	0.003471	-1.87706
PSRC1	0.285096	5.046196	2.828107	0.005497	0.015486	-3.42852
B3GALT4	0.284991	4.820518	5.065337	1.51E-06	8.96E-06	4.343564
IL17RB	0.2849	3.743432	2.838134	0.005338	0.015082	-3.40181
LATS2	0.284889	5.553406	5.571889	1.60E-07	1.12E-06	6.525594
LOC10272	0.28484	2.731727	3.333329	0.001145	0.003847	-1.98251
NKIRAS2	0.284837	6.029409	4.661884	8.26E-06	4.26E-05	2.702216
NUDT16P1	0.284783	4.182983	3.198584	0.00177	0.005697	-2.3878
SLITRK4	0.28473	2.590942	3.189837	0.001821	0.00585	-2.41363
HPDL	0.284661	2.797147	3.124904	0.002235	0.007015	-2.60347
NADSYN1	0.284535	5.124099	5.864583	4.14E-08	3.22E-07	7.841777
SLC22A31	0.284518	6.134561	2.80498	0.00588	0.016415	-3.48981
COX5A	0.284417	6.064929	3.683658	0.000347	0.001305	-0.86498
TRAF7	0.284348	5.316137	6.793291	4.62E-10	5.06E-09	12.24527
FAM118B	0.284276	3.752458	3.049613	0.002825	0.00865	-2.81944
FNDC3B	0.284249	3.811448	2.835398	0.005381	0.015189	-3.40911
KIAA1107	0.28401	2.315964	3.473514	0.000717	0.002519	-1.54622
TBC1D8B	0.283998	2.591588	3.621545	0.000432	0.001588	-1.06968
RCVRN	0.283904	4.118476	2.816237	0.005691	0.015958	-3.46003
IFITM3	0.283478	11.92073	3.96563	0.000126	0.000514	0.098514
DCLRE1A	0.283245	5.31701	3.249209	0.001505	0.004912	-2.23717
PNPLA2	0.282831	8.110699	2.874174	0.0048	0.013788	-3.30513
IGSF21	0.282383	3.063875	3.868642	0.000179	0.000712	-0.23912
COTL1	0.282288	8.781185	2.862453	0.00497	0.014198	-3.33669
STX1A	0.282239	5.217085	3.674811	0.000358	0.001342	-0.89431
MSANTD1	0.28203	3.768557	4.540318	1.36E-05	6.75E-05	2.225906
SLC17A5	0.281827	5.886792	4.001674	0.00011	0.000454	0.225626
SORCS2	0.281827	3.663845	3.51727	0.000618	0.002201	-1.40703
SCD	0.281632	4.667266	3.230086	0.001601	0.005203	-2.2943
CACNA1S	0.28154	4.4267	2.790164	0.006138	0.017054	-3.52885
SPAG11A	0.281535	4.526317	2.811434	0.005771	0.016151	-3.47275
SNRK-AS1	0.281336	3.883177	2.843298	0.005258	0.014907	-3.38802
OR10C1	0.281209	3.660956	3.566167	0.000523	0.00189	-1.24983
PRO1483	0.281142	3.186586	3.722808	0.000303	0.001153	-0.73454
CDK16	0.280868	5.111992	5.042142	1.67E-06	9.81E-06	4.246779
FOXB1	0.280597	2.790834	3.664547	0.000372	0.001389	-0.92826
C18orf21	0.280371	4.188946	4.633171	9.29E-06	4.74E-05	2.588926
PLK4	0.280318	2.451006	3.037978	0.002929	0.008925	-2.85242
ICAM5	0.280304	3.306912	3.792777	0.000236	0.000916	-0.49871
CHCHD5	0.28018	4.757283	6.410855	3.05E-09	2.90E-08	10.39364
CORO1C	0.280168	8.372791	3.270744	0.001404	0.004618	-2.17249
LRP3	0.280159	5.218862	4.041473	9.46E-05	0.000396	0.366993
KIF9	0.280109	4.188543	5.067988	1.49E-06	8.86E-06	4.354645
IGLL1	0.280084	3.927627	2.909627	0.004321	0.012581	-3.20898
HIST1H1E	0.279962	2.973191	3.643724	0.0004	0.001482	-0.9969
LOC64898	0.27996	4.765593	3.675634	0.000357	0.001339	-0.89158
CMAHP	0.279896	3.387327	2.177191	0.031443	0.06949	-4.98063
TBX19	0.279342	4.445107	3.459592	0.000752	0.002631	-1.59021
NDUFA9	0.27932	7.06551	2.297123	0.023362	0.054046	-4.72219
C1orf228	0.279241	4.57005	3.982927	0.000118	0.000485	0.159404
TEPP	0.279237	3.093536	2.982398	0.003471	0.010356	-3.00845
KCNE1L	0.279149	3.318486	3.274378	0.001387	0.00457	-2.16154
NCR1	0.279129	4.668871	4.400009	2.38E-05	0.000113	1.687215
EMID1	0.279126	4.637425	3.960544	0.000128	0.000523	0.08065
LAMC1	0.279006	3.986395	3.220353	0.001651	0.005348	-2.32327
LOC10050	0.278613	2.7611	3.441288	0.0008	0.002781	-1.64782
CDH26	0.278601	3.268166	6.24435	6.83E-09	6.07E-08	9.60354

LINC0140[illegible]	0.27842	2.203485	3.225865	0.001622	0.005265	-2.30687
MOB1A	0.278124	8.163202	2.793797	0.006074	0.016901	-3.51929
MTMR3	0.277942	6.822297	3.236088	0.00157	0.005112	-2.2764
GPR37L1	0.277803	4.057667	3.699967	0.000328	0.00124	-0.81077
ZBTB47	0.277785	5.512163	5.179788	9.18E-07	5.69E-06	4.825284
TRIB3	0.277745	5.182818	2.652407	0.009083	0.023954	-3.88305
SPEF1	0.277565	4.400206	3.443745	0.000793	0.00276	-1.6401
KLHL12	0.277458	6.110395	3.043832	0.002876	0.008791	-2.83584
CYP4F62P	0.2774	2.834822	3.588535	0.000484	0.001766	-1.17733
NPB	0.277366	6.047786	3.171893	0.001927	0.00615	-2.46642
PSPC1	0.277305	4.769843	3.72134	0.000304	0.001159	-0.73945
RGS16	0.276871	3.644962	3.436242	0.000813	0.002821	-1.66366
CAPNS1	0.276827	9.768277	3.096265	0.002445	0.007596	-2.68615
GTF3C6	0.276825	8.033629	3.270096	0.001407	0.004626	-2.17444
FAM127A	0.276792	7.895674	3.353858	0.00107	0.003615	-1.91954
NRM	0.276384	7.543086	4.509909	1.53E-05	7.55E-05	2.108139
ITGB1BP1	0.276356	5.295969	3.772409	0.000254	0.00098	-0.56771
ARPC5L	0.276115	6.380827	2.656907	0.00897	0.023707	-3.87173
NOL12	0.276063	5.368916	3.752032	0.000273	0.001047	-0.63646
B4GALNT[illegible]	0.275997	3.186091	3.066932	0.002678	0.008248	-2.77016
PRSS36	0.275382	4.865129	3.855411	0.000188	0.000743	-0.28468
KCNJ2-AS	0.275268	2.365266	2.190027	0.030475	0.06775	-4.95358
LMO1	0.275154	2.854078	4.49302	1.64E-05	8.03E-05	2.042972
SYNGR2	0.275152	5.43843	3.830586	0.000206	0.000808	-0.36984
C16orf70	0.275097	4.987368	5.456372	2.69E-07	1.82E-06	6.016901
CDX4	0.275054	4.503674	3.229959	0.001601	0.005204	-2.29468
TLR8-AS1	0.274921	3.024857	3.88279	0.00017	0.00068	-0.19027
ARL5C	0.274786	4.579942	4.159499	6.05E-05	0.000264	0.792418
DCTN4	0.274495	5.481749	4.463916	1.84E-05	8.93E-05	1.931085
OR52D1	0.274313	3.642299	3.621844	0.000431	0.001587	-1.0687
CNTFR	0.274238	5.38919	3.201562	0.001754	0.005651	-2.37899
LST1	0.274034	9.142022	3.116411	0.002296	0.007182	-2.62806
MAPK8IP3	0.273891	4.912271	5.328798	4.76E-07	3.09E-06	5.462552
TYMP	0.273263	6.556888	2.350339	0.020404	0.048217	-4.60345
FCGR1A	0.273097	2.060607	3.3193	0.001198	0.004015	-2.02536
NACC1	0.273009	5.499757	4.890005	3.19E-06	1.78E-05	3.619143
HIST1H4G	0.272887	2.657837	3.624317	0.000428	0.001574	-1.0606
ATP12A	0.272748	5.205944	3.775927	0.00025	0.000969	-0.55581
SEMA7A	0.272735	4.993257	4.000539	0.00011	0.000456	0.221607
NUCB1	0.272371	7.352043	2.737637	0.00714	0.019453	-3.66577
CDC26	0.272128	7.338554	3.052788	0.002798	0.008574	-2.81043
SSNA1	0.272024	5.455982	3.647563	0.000394	0.001464	-0.98427
STRADA	0.271879	6.316794	5.265276	6.31E-07	4.02E-06	5.189519
CCKBR	0.271878	3.28151	4.426819	2.14E-05	0.000102	1.789214
GALNS	0.271824	5.216739	4.743654	5.89E-06	3.14E-05	3.027473
FBXO38	0.271534	6.503951	2.242825	0.026762	0.060716	-4.84077
LOC10272	0.271521	4.958846	2.365157	0.019642	0.046739	-4.56994
SMUG1	0.271515	4.562903	6.050964	1.72E-08	1.42E-07	8.699143
LOC10192	0.271468	4.547976	2.183534	0.030961	0.06863	-4.96728
LRTM2	0.271447	3.987586	2.650626	0.009129	0.024052	-3.88752
MTRF1L	0.27133	4.479954	6.278206	5.80E-09	5.23E-08	9.763358
LOC10192	0.271308	2.525206	3.340873	0.001117	0.003761	-1.95941
KIAA0754	0.271248	4.576496	3.122093	0.002255	0.007068	-2.61161
SARNP	0.271173	4.814705	1.997326	0.048074	0.099122	-5.34416
TMED9	0.271035	7.423257	2.703761	0.007862	0.021156	-3.75285
TEC	0.270828	4.201603	3.279262	0.001366	0.004506	-2.14681
ARHGDIB	0.270797	10.52561	4.22744	4.66E-05	0.000209	1.041444
FAM89B	0.270539	7.91929	4.070414	8.49E-05	0.000359	0.470456

LOC10013	0.270514	2.326345	3.624217	0.000428	0.001575	-1.06093
DUSP9	0.270342	3.327965	3.02816	0.003018	0.009166	-2.88016
MTHFD1L	0.270311	2.582074	3.553924	0.000545	0.001965	-1.28936
CALD1	0.270136	2.825545	3.900871	0.000159	0.00064	-0.12764
UHRF1BP1	0.269805	3.743024	3.914141	0.000152	0.000612	-0.08154
TRAPPC3	0.269761	6.861661	4.234982	4.53E-05	0.000203	1.069273
HIST1H2A	0.26971	1.93824	3.766042	0.000259	0.001	-0.58922
IQCF6	0.269602	3.501644	2.924596	0.004132	0.012098	-3.16808
CNR1	0.269432	3.148542	3.762684	0.000263	0.001012	-0.60056
PRPF18	0.269346	4.35726	3.690987	0.000339	0.001276	-0.84064
PDHA1	0.269275	6.320959	2.151631	0.033449	0.073257	-5.03406
CA3	0.268813	1.689539	2.995005	0.00334	0.010007	-2.97327
PYY	0.268805	3.506377	3.797562	0.000232	0.000901	-0.48245
PSMB4	0.26873	8.63305	3.261639	0.001446	0.004741	-2.19988
ARHGAP6	0.268636	3.39714	2.231707	0.027509	0.062129	-4.86473
SLC10A3	0.268592	5.95425	3.482301	0.000696	0.002454	-1.51838
LOC44105	0.268585	3.521765	3.121609	0.002259	0.007075	-2.61301
STXBP3	0.268518	6.612321	2.123097	0.03582	0.077454	-5.09302
MAPKAPK	0.268474	6.354276	2.941273	0.00393	0.011557	-3.1223
MIER1	0.268415	5.634116	4.02759	9.97E-05	0.000416	0.317559
ERICH1	0.268025	4.76539	3.649919	0.000391	0.001453	-0.97652
PRDX2	0.267981	6.437957	2.033389	0.04424	0.092504	-5.27361
SDHAF2	0.267945	7.001885	3.270123	0.001407	0.004626	-2.17436
CRYBB2	0.267934	5.081328	3.030441	0.002997	0.009111	-2.87372
RPE	0.267821	4.154381	2.616466	0.010038	0.026144	-3.97285
COX7A1	0.267813	3.479238	3.073219	0.002627	0.008102	-2.75221
C6orf57	0.267749	3.916025	2.485049	0.014345	0.035639	-4.29185
LOC28421	0.267502	2.842674	4.197054	5.24E-05	0.000232	0.929698
FAM104B	0.267278	3.569236	2.976265	0.003536	0.010535	-3.02551
SPTY2D1-.	0.267217	2.891246	4.45845	1.88E-05	9.11E-05	1.910128
EHD1	0.267156	7.520833	3.009861	0.003192	0.009619	-2.93166
RPN1	0.267145	7.900448	2.821591	0.005602	0.01574	-3.44583
PNMAL2	0.267104	4.339068	3.380352	0.00098	0.003342	-1.83781
APOA2	0.26697	2.765293	3.756244	0.000269	0.001033	-0.62227
FOXRED1	0.266793	5.871548	3.470556	0.000725	0.002542	-1.55558
MSS51	0.266754	4.256079	2.670302	0.008639	0.022976	-3.83793
COX4I2	0.266747	4.083121	3.084083	0.002539	0.00786	-2.72112
LRGUK	0.266632	2.414455	3.152789	0.002047	0.00649	-2.52234
TBL1XR1	0.266609	6.149942	3.628006	0.000422	0.001557	-1.04851
ECE1	0.266575	6.031023	3.93974	0.000138	0.000561	0.007756
MS4A5	0.266565	3.249465	3.15857	0.00201	0.006385	-2.50545
ZFAND2A	0.266467	5.716612	2.354227	0.020202	0.04781	-4.59468
PLD2	0.266428	5.491498	3.817086	0.000216	0.000845	-0.41597
KAL1	0.266359	2.702816	3.222921	0.001638	0.005308	-2.31563
RHOV	0.266346	4.600337	3.01776	0.003116	0.009419	-2.90946
LOC28401	0.266115	3.041087	3.545229	0.000562	0.002019	-1.31736
PEAR1	0.266023	3.913011	2.157585	0.032972	0.072367	-5.02167
TADA2B	0.265985	7.229394	3.546333	0.00056	0.002012	-1.31381
GSG1L	0.26591	5.229754	3.068142	0.002668	0.008224	-2.76671
LOC10050	0.265732	2.999123	3.528139	0.000596	0.00213	-1.37224
SMPD2	0.265254	4.707773	3.265987	0.001426	0.004682	-2.18681
COQ7	0.265073	4.452425	4.212672	4.94E-05	0.00022	0.987057
KPTN	0.264998	4.870863	3.599994	0.000465	0.001701	-1.14005
KHDC1	0.264842	4.222981	3.665158	0.000371	0.001386	-0.92624
PPP1R3B	0.264745	6.278338	2.849991	0.005155	0.014651	-3.37012
MFSD9	0.26465	3.69804	2.204479	0.029416	0.065805	-4.92295
FNDC7	0.264335	3.930313	3.31325	0.001222	0.004085	-2.04379
DEF8	0.264002	7.772616	3.911566	0.000153	0.000617	-0.09049

MAPK1	0.263958	7.251894	3.784023	0.000243	0.000944	-0.5284
IGLV6-57	0.263562	3.512517	2.319388	0.022081	0.051572	-4.67281
POC1A	0.263342	3.966097	3.517431	0.000618	0.0022	-1.40652
TRPV4	0.262987	4.12304	3.168383	0.001949	0.006211	-2.47671
CENPA	0.262871	5.004466	3.619782	0.000434	0.001597	-1.07545
C18orf12	0.262739	3.288161	3.24723	0.001515	0.004942	-2.24309
MXRA7	0.262691	4.870432	2.533815	0.012584	0.031875	-4.1752
CEBPZ	0.262504	6.275187	2.060967	0.041488	0.08759	-5.21887
SLC22A18,	0.262445	5.65506	3.633743	0.000414	0.001528	-1.0297
CRYM	0.262027	5.449917	2.324704	0.021785	0.05102	-4.66096
MR1	0.261985	5.840406	3.390194	0.000949	0.003243	-1.80731
PGP	0.261935	4.792718	4.071488	8.45E-05	0.000358	0.474306
AKR1C1	0.261896	4.387874	3.929183	0.000144	0.000582	-0.02912
UNC50	0.261887	6.981765	2.532916	0.012614	0.031937	-4.17737
CREM	0.261793	4.091805	3.513099	0.000627	0.00223	-1.42036
SMOX	0.261711	5.490827	2.034443	0.044132	0.092297	-5.27153
CCDC3	0.261576	5.537542	2.548439	0.012095	0.030748	-4.13982
ZSWIM6	0.261555	8.130253	2.443103	0.016032	0.039277	-4.39056
COX6B2	0.261532	4.198061	3.512009	0.00063	0.002238	-1.42384
FOXC1	0.261057	3.271313	2.922998	0.004151	0.012151	-3.17246
RPS6KA1	0.261015	8.515626	2.52921	0.012741	0.032211	-4.1863
LINC01351	0.260855	2.176855	3.78062	0.000246	0.000955	-0.53993
CDC73	0.26076	5.613506	2.502512	0.01369	0.034275	-4.25031
A4GALT	0.260484	4.971819	2.709297	0.007739	0.020881	-3.73869
STAC3	0.26047	4.346649	3.309345	0.001238	0.004134	-2.05567
CPT2	0.260402	5.068282	2.915383	0.004247	0.01239	-3.19328
SPATA41	0.260399	3.098419	3.452032	0.000771	0.00269	-1.61403
ZNF654	0.260336	4.437334	2.215796	0.02861	0.064296	-4.89883
TMEM59	0.260328	7.557153	3.480944	0.0007	0.002464	-1.52269
HIST1H1B	0.260322	2.824396	2.915872	0.004241	0.012384	-3.19194
FAM187B	0.260303	2.883982	3.108854	0.002351	0.007337	-2.64989
THOC5	0.260241	4.736853	4.344577	2.96E-05	0.000138	1.477735
SNX18	0.260118	7.082172	2.991965	0.003371	0.01009	-2.98177
WDR47	0.259877	6.600606	2.170668	0.031944	0.070429	-4.99433
ERGIC2	0.259724	5.900579	2.559796	0.011727	0.029933	-4.11221
MMP24-A	0.259634	6.923732	4.116418	7.13E-05	0.000307	0.636068
CPSF3	0.259614	6.319196	2.232447	0.027459	0.062042	-4.86314
TMED5	0.2595	5.250414	3.339446	0.001122	0.003777	-1.96378
DCTPP1	0.259488	6.686637	3.407395	0.000896	0.003078	-1.75384
R3HDML	0.259388	4.041776	3.409098	0.000891	0.003063	-1.74853
NCKAP5L	0.259257	4.555027	4.130668	6.76E-05	0.000292	0.687648
ZNF213	0.259208	5.767864	3.780701	0.000246	0.000955	-0.53965
BPNT1	0.259174	3.175585	4.34923	2.90E-05	0.000136	1.495248
Ndufaf4	0.259164	3.324404	2.441338	0.016107	0.039446	-4.39468
RHBDD2	0.25894	6.7264	3.139374	0.002136	0.006735	-2.56145
LOC10050	0.258671	5.030662	3.449127	0.000779	0.002714	-1.62318
KRT79	0.258606	3.049618	3.329388	0.001159	0.003893	-1.99456
IVNS1ABP	0.258593	6.624532	2.079291	0.039741	0.084539	-5.18211
FN3K	0.25853	2.968541	3.773232	0.000253	0.000977	-0.56493
RPS6KA2	0.258526	4.973954	2.30563	0.022865	0.05307	-4.70337
CST6	0.258457	2.750613	3.000116	0.003289	0.009871	-2.95898
GNG8	0.258306	3.694649	2.453683	0.01559	0.038333	-4.36581
ADAMTS4	0.25824	4.496344	4.169212	5.83E-05	0.000255	0.827836
NEU1	0.258136	6.637012	2.854031	0.005094	0.014494	-3.35929
C1orf105	0.258086	3.559238	3.005939	0.003231	0.009716	-2.94266
NXF5	0.257938	3.28732	3.621709	0.000432	0.001587	-1.06914
PDZRN3-/	0.257931	2.406057	3.460718	0.000749	0.002622	-1.58666
MPL	0.25786	4.007527	2.864853	0.004934	0.01412	-3.33023

TIMMDC1	0.257737	7.448056	3.109391	0.002347	0.007325	-2.64834
KHDC3L	0.257736	3.323012	3.026169	0.003037	0.009212	-2.88578
IL27	0.25765	4.820006	3.844428	0.000196	0.00077	-0.32241
BROX	0.257454	5.440546	4.272212	3.92E-05	0.000178	1.207172
MYADML2	0.257392	4.020399	3.229297	0.001605	0.005214	-2.29665
RYR2	0.257024	2.482742	4.61303	1.01E-05	5.12E-05	2.509746
SLC39A3	0.256992	5.150874	4.037308	9.61E-05	0.000402	0.352148
COL8A2	0.256923	3.712961	2.607224	0.010297	0.026726	-3.99576
TRIM17	0.256458	4.182408	2.587245	0.010879	0.028044	-4.04505
C16orf91	0.256285	5.488659	4.397216	2.40E-05	0.000114	1.676616
ANKRD32	0.256188	3.772073	2.831924	0.005436	0.015329	-3.41836
ZNF319	0.256143	6.29364	2.890029	0.00458	0.013235	-3.26226
CTSB	0.256112	9.029134	2.81443	0.005721	0.016023	-3.46482
HPR	0.256095	4.079673	3.653852	0.000386	0.001435	-0.96355
ARL3	0.255998	4.745817	4.870831	3.46E-06	1.92E-05	3.540941
SMIM20	0.255968	5.123058	2.100701	0.037781	0.081009	-5.13878
SERPINA4	0.255874	4.58078	3.026688	0.003032	0.009201	-2.88431
NFIX	0.255846	4.625583	2.020635	0.045565	0.094838	-5.2987
MAP2K2	0.255778	5.83457	4.181577	5.56E-05	0.000245	0.873012
PSG7	0.255749	4.047633	3.520692	0.000611	0.002179	-1.39609
POU5F2	0.255736	2.734272	3.492037	0.000674	0.002381	-1.48747
RTFDC1	0.255718	6.345005	6.113628	1.28E-08	1.08E-07	8.990592
TPTEP1	0.2556	3.238092	3.480065	0.000702	0.00247	-1.52547
SIGLEC16	0.25524	4.535364	2.111039	0.036864	0.079377	-5.11772
LOC10028	0.255211	2.500743	3.099475	0.002421	0.007529	-2.67691
STAG3	0.255094	4.975402	2.725643	0.007388	0.020055	-3.69671
DUS2	0.254901	6.489838	3.409126	0.000891	0.003063	-1.74844
LOC10192	0.254647	3.754239	3.042254	0.00289	0.00883	-2.84031
GDE1	0.254619	7.744095	2.132436	0.035028	0.076048	-5.07381
PTCSC1	0.254542	4.788432	2.739197	0.007108	0.019382	-3.66173
KRT5	0.254529	3.578803	3.365221	0.00103	0.003496	-1.88456
TMC4	0.254518	4.993366	2.896966	0.004487	0.013007	-3.24344
NKX2-1-A	0.254478	3.835324	2.630597	0.009652	0.025282	-3.93767
PSMD13	0.254179	6.59054	3.03599	0.002947	0.008977	-2.85804
EIF6	0.254163	6.89565	3.257418	0.001466	0.004799	-2.21256
LSMEM1	0.253792	4.510343	2.190894	0.03041	0.067629	-4.95175
ORC4	0.253666	5.880327	2.366352	0.019581	0.046611	-4.56723
TLX3	0.253613	2.453113	3.292581	0.001308	0.004338	-2.10654
LOC28519	0.25359	4.105432	3.500906	0.000654	0.002318	-1.45925
PLAU	0.253484	3.526164	2.685757	0.008272	0.02212	-3.79875
APOM	0.253444	4.501952	3.536203	0.00058	0.002077	-1.34637
STMN1	0.25333	3.586571	2.3686	0.019468	0.046385	-4.56213
LOC10028	0.253253	4.819569	2.81917	0.005642	0.015839	-3.45226
LHX1	0.25318	3.651352	2.920535	0.004182	0.012227	-3.1792
ALX4	0.253163	3.906054	3.954507	0.000131	0.000534	0.059467
FRMD8P1	0.252869	3.145209	3.412238	0.000881	0.003035	-1.73874
SPATC1	0.252767	3.93283	3.848882	0.000193	0.000759	-0.30712
COPB2	0.252729	5.058741	3.737936	0.000287	0.001098	-0.68384
ATPIF1	0.252513	6.643437	2.604117	0.010386	0.026918	-4.00345
CCND3	0.25244	8.083819	4.158982	6.07E-05	0.000265	0.790532
KIFC3	0.252148	3.87364	3.824991	0.00021	0.000823	-0.38897
DYNC1LI2	0.25214	6.025795	3.696327	0.000332	0.001254	-0.82289
ECSCR	0.252096	3.09338	4.733704	6.14E-06	3.26E-05	2.98769
FOXD3-AS	0.252014	3.526706	2.766986	0.006563	0.018078	-3.58955
LOC10192	0.251926	3.13119	2.997672	0.003313	0.009933	-2.96582
CYP1B1-A	0.251806	2.772436	2.858337	0.00503	0.014341	-3.34774
LGR4	0.251727	2.741551	3.522278	0.000608	0.002169	-1.39102
MAPK13	0.25154	5.924029	2.627819	0.009727	0.025443	-3.9446

RNF31	0.251532	5.268066	3.374469	0.000999	0.0034	-1.85601
COL23A1	0.251428	3.700912	2.980349	0.003492	0.010416	-3.01415
PTH2R	0.25115	2.17572	2.418866	0.017085	0.041481	-4.44691
PLK3	0.251044	4.502795	4.409366	2.29E-05	0.000109	1.722765
S100A5	0.251029	4.572973	2.837968	0.00534	0.015087	-3.40225
LOC10192	0.250853	2.191927	3.56453	0.000526	0.0019	-1.25512
TRIM71	0.25068	2.861707	3.058356	0.00275	0.008442	-2.7946
PUS10	0.250483	3.548665	3.306474	0.00125	0.004169	-2.0644
FAM114A1	0.250458	3.703369	4.344203	2.96E-05	0.000138	1.476329
HYAL2	0.2504	6.047273	3.508365	0.000637	0.002264	-1.43548
PTK2B	0.249913	7.076915	3.5405	0.000571	0.002048	-1.33257
PPP2R4	0.249783	6.528748	4.074079	8.37E-05	0.000355	0.4836
LOC10013	0.249637	2.517797	3.145788	0.002093	0.006618	-2.54277
MDH1	0.249361	7.956397	2.174909	0.031617	0.069835	-4.98543
ATXN7L3	0.249238	6.822948	4.186679	5.46E-05	0.00024	0.891681
DCAF6	0.249162	4.863898	2.868204	0.004886	0.014	-3.32121
HIST1H2B(	0.249007	2.196013	2.989946	0.003392	0.010143	-2.9874
TXNDC11	0.248912	5.604149	2.092697	0.038504	0.082352	-5.15503
DDRGK1	0.248896	6.108365	4.020892	0.000102	0.000425	0.293755
TANK	0.248748	6.712159	3.003007	0.00326	0.009799	-2.95088
RNASEH2(	0.248702	5.845875	2.750444	0.006883	0.018836	-3.63259
ETV4	0.248457	4.007547	3.251749	0.001493	0.00488	-2.22956
ADCK4	0.248434	4.590584	3.774725	0.000251	0.000973	-0.55988
MRPL54	0.248251	6.566413	2.085749	0.039141	0.083462	-5.16909
LOC10192	0.248182	3.641016	4.410371	2.28E-05	0.000108	1.726586
C16orf3	0.248141	3.671298	2.855031	0.005079	0.014465	-3.35661
ATP6V1G1	0.248083	6.715607	2.659262	0.008911	0.023582	-3.8658
PDGFA	0.248057	3.976658	2.507806	0.013497	0.033871	-4.23767
TRMT5	0.248056	6.775522	3.052277	0.002802	0.008585	-2.81188
RAB3GAP2	0.247965	2.177071	4.117914	7.09E-05	0.000305	0.641476
APAF1	0.247902	5.382041	3.697107	0.000331	0.001251	-0.82029
SSR4P1	0.247893	4.696604	2.841583	0.005284	0.014961	-3.3926
LOC10192	0.247836	3.1864	3.340535	0.001118	0.003764	-1.96045
SLC5A10	0.247829	3.835585	3.814469	0.000218	0.000852	-0.42489
COMMD5	0.247821	4.870871	5.696468	9.02E-08	6.66E-07	7.081127
MAGT1	0.247808	6.685259	2.245794	0.026565	0.06033	-4.83436
HIATL2	0.247808	6.492164	3.189785	0.001821	0.00585	-2.41378
RBP2	0.247743	3.80431	2.781482	0.006295	0.017434	-3.55163
FAM90A1	0.247714	4.063523	2.730848	0.007279	0.019789	-3.6833
REXO2	0.247647	4.386248	2.05832	0.041745	0.088034	-5.22415
PLEKHO2	0.247511	8.028643	3.165616	0.001966	0.006258	-2.48483
LCN1	0.247494	3.880879	2.707248	0.007784	0.020983	-3.74393
P2RY2	0.247325	4.102657	2.017293	0.045918	0.095417	-5.30525
SDSL	0.247287	4.529417	2.48732	0.014258	0.035453	-4.28647
MOB3C	0.247234	5.85883	4.161401	6.01E-05	0.000263	0.799348
NDUFB2	0.247115	5.795318	2.495539	0.013948	0.034815	-4.26693
BRK1	0.247078	7.166109	3.768311	0.000257	0.000992	-0.58156
EBI3	0.246977	3.539928	2.720107	0.007505	0.020325	-3.71095
RPS6KA2-,	0.246769	3.518861	3.167516	0.001954	0.006226	-2.47926
ARHGAP2!	0.246765	2.295575	4.850976	3.76E-06	2.07E-05	3.460181
HAVCR1P1	0.246717	4.982503	3.805825	0.000225	0.000877	-0.45435
COMMD1(	0.246698	4.84829	2.819839	0.005631	0.015812	-3.45048
LOC10099	0.246653	3.178532	3.642212	0.000402	0.001489	-1.00188
SPC24	0.246642	3.883606	2.938055	0.003968	0.011661	-3.13115
5-Mar	0.246598	4.978285	2.380132	0.018897	0.04524	-4.53589
OCRL	0.246594	4.48311	3.913726	0.000152	0.000612	-0.08298
FLJ33544	0.246263	3.370603	3.300208	0.001276	0.004243	-2.08343
MUS81	0.246247	6.088695	3.034499	0.00296	0.009016	-2.86226

TNFRSF12.	0.245988	5.833593	3.47471	0.000715	0.002511	-1.54243
LOC10192	0.245872	2.894494	3.775678	0.000251	0.00097	-0.55666
LOC10192	0.245816	2.165315	2.499379	0.013806	0.034517	-4.25779
OLFML2B	0.24572	3.230768	2.862959	0.004962	0.014185	-3.33533
EFNB2	0.245199	2.404214	4.953728	2.44E-06	1.39E-05	3.880499
LOC72794	0.244962	3.824545	2.989099	0.003401	0.010165	-2.98977
LOC38878	0.244908	4.713883	2.708525	0.007756	0.020924	-3.74067
MPG	0.244899	6.23636	3.583542	0.000493	0.001792	-1.19355
MIR4435-:	0.244819	3.623512	3.052922	0.002797	0.008572	-2.81005
MMEL1	0.244757	5.137751	3.392518	0.000941	0.003223	-1.8001
DCUN1D3	0.244755	5.43955	4.133343	6.69E-05	0.000289	0.697345
SEMA6B	0.244711	4.350104	4.005138	0.000108	0.000449	0.237886
ANXA2P1	0.244676	3.397468	2.972499	0.003577	0.010629	-3.03598
CHRNA2	0.244431	5.25676	2.866194	0.004915	0.014072	-3.32663
PSMD2	0.244135	7.531816	3.358286	0.001054	0.003569	-1.90592
PTGFR	0.244079	2.108008	3.167707	0.001953	0.006223	-2.4787
OPRL1	0.243988	4.470518	3.783531	0.000244	0.000945	-0.53007
SIAE	0.243977	2.999183	5.022326	1.82E-06	1.06E-05	4.164318
KIAA1958	0.243918	4.225313	3.949572	0.000133	0.000543	0.042167
KRT3	0.243884	3.540346	2.230749	0.027574	0.062255	-4.86679
ZNF394	0.243815	6.417316	4.165821	5.91E-05	0.000258	0.815461
LOC10050	0.243651	3.826923	3.106613	0.002367	0.007384	-2.65635
VASN	0.243571	4.414102	2.84006	0.005308	0.015021	-3.39667
BARX1	0.243559	3.365816	2.753599	0.006821	0.018691	-3.6244
FTL	0.243372	12.65469	6.47704	2.21E-09	2.14E-08	10.7105
TTLL3	0.243331	4.772324	3.724061	0.000301	0.001149	-0.73035
LINC00261	0.243201	3.045397	3.629509	0.00042	0.001549	-1.04359
ADAM19	0.242913	5.854513	4.477807	1.74E-05	8.51E-05	1.984423
LPO	0.242689	3.623554	3.031343	0.002989	0.009092	-2.87118
NDNF	0.242622	3.808374	3.065769	0.002688	0.008274	-2.77348
LINC01352	0.242581	2.885992	3.041573	0.002896	0.008843	-2.84224
TALDO1	0.242464	12.02027	3.312134	0.001227	0.004099	-2.04719
LOC64601	0.242337	5.049257	2.327105	0.021652	0.05075	-4.6556
TTC1	0.242071	6.357921	3.448602	0.00078	0.002718	-1.62483
CAMSAP3	0.241765	3.103404	3.47925	0.000704	0.002475	-1.52805
RHBDD3	0.241742	5.122602	5.426625	3.08E-07	2.06E-06	5.886936
DNAH9	0.241714	3.168687	4.233653	4.55E-05	0.000204	1.064366
CCDC34	0.241534	2.610211	2.527227	0.01281	0.032352	-4.19107
ACTR3BP2	0.241511	3.168814	3.064229	0.002701	0.008311	-2.77787
TNPO1	0.241337	6.692022	3.922601	0.000147	0.000594	-0.05207
KIAA0513	0.241253	5.447275	2.866102	0.004916	0.014074	-3.32687
PTGER1	0.241153	5.179072	3.632029	0.000416	0.001536	-1.03532
TTC39A	0.241109	3.468053	3.187198	0.001836	0.005894	-2.4214
TPRXL	0.241018	4.531871	3.05895	0.002745	0.008432	-2.7929
MRPS18A	0.240876	5.90706	3.981678	0.000118	0.000487	0.154999
NDUFAF5	0.240871	3.641399	3.344342	0.001104	0.003723	-1.94877
CRHR2	0.24087	2.934772	3.973317	0.000122	0.000501	0.125548
GBX1	0.240807	4.933821	3.597075	0.00047	0.001718	-1.14956
LOH12CR1	0.240806	5.340808	2.426448	0.016749	0.040805	-4.42933
TUSC2	0.240631	6.086606	4.286871	3.70E-05	0.000169	1.261714
MCTS1	0.2406	4.839518	4.118417	7.08E-05	0.000305	0.643294
TSNAXIP1	0.240478	4.956529	3.092109	0.002477	0.007683	-2.69809
ROCK1	0.240342	5.350021	3.572963	0.000511	0.00185	-1.22784
SLC48A1	0.240302	5.567107	2.011125	0.046575	0.096508	-5.31731
LOC10012	0.24025	2.520047	3.924128	0.000146	0.000591	-0.04675
C20orf96	0.240208	2.801403	3.006445	0.003226	0.009704	-2.94124
CDC27	0.240197	5.741563	3.072624	0.002631	0.008115	-2.75391
SNORA68	0.24007	3.830801	2.863087	0.00496	0.014181	-3.33498

HS3ST6	0.239815	2.763371	3.050736	0.002816	0.008621	-2.81626
SLC30A9	0.239613	4.067854	2.042757	0.043288	0.090764	-5.25509
MT1M	0.239571	2.10458	2.363224	0.01974	0.046912	-4.57433
LINC0135€	0.239498	3.664416	3.419129	0.000861	0.00297	-1.71723
TLN1	0.239467	6.170835	3.871361	0.000177	0.000705	-0.22975
BMP2	0.239402	2.617227	2.804102	0.005895	0.016452	-3.49213
DEFA5	0.239382	2.996722	2.678394	0.008445	0.022515	-3.81744
AURKAIP1	0.239037	6.107804	6.504255	1.93E-09	1.89E-08	10.84124
SLURP1	0.238771	4.918483	3.105035	0.002379	0.007413	-2.6609
PAFAH1B2	0.238636	5.25185	5.490342	2.31E-07	1.58E-06	6.165839
LRR1	0.238587	4.320821	2.27091	0.024953	0.057137	-4.77976
FBXO45	0.238582	4.46062	2.510859	0.013387	0.033635	-4.23036
TNF	0.238578	5.157835	2.046491	0.042914	0.090071	-5.24769
PNKD	0.238397	5.769463	4.431628	2.10E-05	0.0001	1.80756
FAM63A	0.238334	7.500984	2.800265	0.005961	0.016621	-3.50225
PSMC3	0.238248	6.657289	2.160196	0.032765	0.071988	-5.01623
CES1P1	0.238235	3.387903	2.261881	0.025522	0.058278	-4.79945
LRFN4	0.238194	5.429514	4.639989	9.04E-06	4.63E-05	2.615784
F2	0.238013	3.869496	2.332799	0.02134	0.05011	-4.64286
CHIA	0.237739	3.915633	2.742326	0.007045	0.019227	-3.65364
SLC25A44	0.237438	7.402039	2.412005	0.017394	0.042116	-4.46276
LINC0016!	0.237383	4.297436	2.539076	0.012406	0.031479	-4.16249
LOC64485	0.237356	2.111798	3.227807	0.001612	0.005236	-2.30109
LOC10024	0.237308	3.550636	2.645172	0.009269	0.024383	-3.90121
BCAP31	0.237252	8.862533	2.372709	0.019263	0.045977	-4.5528
C21orf90	0.237001	3.684212	3.615239	0.000441	0.00162	-1.0903
LYZL6	0.236779	4.178182	3.609694	0.00045	0.00165	-1.10842
GSTT2	0.236763	3.28292	3.439305	0.000805	0.002796	-1.65405
GTF2B	0.236701	7.326803	2.195963	0.030036	0.066942	-4.94102
LOC44033	0.236248	5.363787	3.430958	0.000828	0.002866	-1.68022
FAM134A	0.23608	7.619083	2.854354	0.00509	0.014484	-3.35843
ITIH4	0.23595	4.619106	2.889392	0.004589	0.013255	-3.26398
SH2D4B	0.235932	2.620795	3.115485	0.002302	0.007198	-2.63073
PLIN4	0.235841	5.330253	2.66625	0.008738	0.023182	-3.84817
TBC1D23	0.235704	6.97332	2.275479	0.024669	0.056569	-4.76977
CRYBA4	0.235625	3.142872	3.040184	0.002909	0.008875	-2.84618
TNIP2	0.235382	7.016122	4.097443	7.67E-05	0.000327	0.567589
LIN37	0.235344	5.36171	4.275292	3.88E-05	0.000176	1.218622
BTK	0.235336	7.460884	2.560214	0.011714	0.029912	-4.11119
PIF1	0.235073	2.6973	4.552807	1.29E-05	6.43E-05	2.274434
ERI2	0.235049	2.392292	4.016053	0.000104	0.000432	0.276577
C17orf53	0.235022	5.536629	2.411576	0.017414	0.042159	-4.46375
LOC10012	0.234936	2.696071	3.125077	0.002234	0.007014	-2.60297
ZNF189	0.234907	6.698812	2.497809	0.013864	0.034642	-4.26153
PPM1N	0.234821	3.923164	2.884094	0.004661	0.013438	-3.27833
KCND1	0.234799	3.995918	3.090254	0.002491	0.007724	-2.70342
FBXO34	0.234796	7.012528	2.408876	0.017537	0.042402	-4.46998
GTF2E2	0.234651	6.627916	2.699292	0.007962	0.021388	-3.76427
IQCF3	0.2343	3.082805	2.867322	0.004899	0.014029	-3.32359
DBH	0.234297	4.194158	3.291788	0.001311	0.004347	-2.10895
C12orf74	0.234204	1.849809	3.795158	0.000234	0.000909	-0.49062
CLN5	0.234143	5.0627	2.136236	0.034711	0.075511	-5.06596
LOC10192	0.233897	3.903608	2.921236	0.004173	0.012206	-3.17728
ANKRD39	0.233632	4.242993	2.257618	0.025795	0.058836	-4.80872
ACOT7	0.233598	4.81493	2.431535	0.016527	0.040331	-4.41752
DNM3	0.233583	3.185694	2.962323	0.003688	0.01093	-3.0642
SLC25A37	0.233581	9.860603	2.454592	0.015553	0.038255	-4.36368
ANKRD19I	0.233554	4.68214	2.991224	0.003379	0.010108	-2.98384

LOC78052	0.233467	2.692526	2.660664	0.008876	0.023505	-3.86226
CSRP2	0.233374	3.41313	3.286544	0.001334	0.004414	-2.12481
AKR1B10	0.232812	2.220588	3.707617	0.000319	0.00121	-0.78528
GMIP	0.232806	6.545989	2.562312	0.011647	0.02977	-4.10608
LGALS8-A	0.232802	4.389036	3.165163	0.001969	0.006266	-2.48615
C7orf69	0.23258	3.31297	3.356801	0.001059	0.003585	-1.91049
NXF3	0.232569	2.842791	2.528531	0.012765	0.03225	-4.18794
LRRC2	0.232548	1.922012	3.377489	0.000989	0.003368	-1.84667
VCL	0.232528	6.012844	3.014167	0.00315	0.009507	-2.91957
LOC10028	0.232488	4.977118	2.885451	0.004643	0.013392	-3.27466
KIAA1524	0.232445	2.093503	2.833878	0.005405	0.015252	-3.41316
ARRDC3-A	0.232372	4.190877	2.462526	0.01523	0.037564	-4.34504
SNX13	0.232287	4.962547	2.129261	0.035296	0.076539	-5.08035
TMEM9B	0.23207	7.503038	2.251513	0.02619	0.059631	-4.82197
RNF13	0.232028	9.344753	2.123994	0.035743	0.077313	-5.09118
PDE2A	0.232014	4.161458	2.024089	0.045203	0.094199	-5.29192
ARSD	0.231973	4.934769	3.702241	0.000325	0.001231	-0.8032
SLCO2B1	0.23191	3.892854	4.042057	9.44E-05	0.000396	0.369076
FAM204A	0.231876	4.588811	3.18372	0.001856	0.005948	-2.43165
STX4	0.23182	6.332555	5.575684	1.57E-07	1.10E-06	6.542411
RAB43	0.231333	5.470038	2.444605	0.015968	0.039141	-4.38706
C16orf95	0.231325	3.947331	4.498261	1.61E-05	7.88E-05	2.063177
KRT14	0.231293	2.48598	2.893972	0.004527	0.01311	-3.25156
MAST4	0.23129	4.912205	2.827916	0.0055	0.015491	-3.42903
NR6A1	0.231229	4.369465	4.716807	6.59E-06	3.47E-05	2.920259
GSC	0.23113	3.057142	2.806766	0.00585	0.016345	-3.48509
LIPH	0.231027	3.004086	3.358967	0.001052	0.003561	-1.90383
CWC15	0.23098	7.841621	2.487204	0.014262	0.03546	-4.28674
HIPK2	0.230926	5.591628	3.635405	0.000411	0.001521	-1.02424
TEX101	0.230922	4.408306	2.793056	0.006087	0.01693	-3.52124
PRSS50	0.230723	3.876055	2.051163	0.042449	0.089278	-5.23841
TEKT2	0.230657	3.519995	2.235205	0.027272	0.061682	-4.85721
NOXO1	0.23026	4.253387	2.565372	0.01155	0.029543	-4.09862
ING2	0.230232	3.883518	3.315383	0.001214	0.004061	-2.0373
PDE5A	0.230084	3.624247	3.852322	0.00019	0.000751	-0.2953
SLC52A1	0.2299	3.714955	2.891866	0.004555	0.013176	-3.25728
GSG2	0.229869	3.229091	2.792972	0.006089	0.016932	-3.52146
LOC10192	0.22981	4.417827	2.497718	0.013867	0.034646	-4.26174
SEC24D	0.229728	3.958542	2.452916	0.015622	0.038397	-4.36761
TSGA13	0.22946	2.896471	3.245677	0.001522	0.004965	-2.24774
LCE1B	0.229411	3.420361	3.364144	0.001034	0.003507	-1.88788
FGF21	0.229327	4.025248	3.082774	0.00255	0.007883	-2.72487
NFKBID	0.229222	5.073781	5.218287	7.76E-07	4.88E-06	4.988856
PPAP2C	0.229174	3.53415	2.448408	0.015809	0.038829	-4.37816
SLC47A1	0.228942	3.275168	2.307934	0.022732	0.052821	-4.69827
FKBP2	0.228931	6.755673	2.339883	0.020958	0.049308	-4.62697
PRIMA1	0.228866	3.081114	2.806727	0.00585	0.016345	-3.4852
SCN8A	0.228835	2.547289	4.033729	9.74E-05	0.000407	0.339402
OPA1	0.228771	4.536443	2.431557	0.016526	0.040331	-4.41747
C22orf23	0.228723	4.147516	2.963566	0.003675	0.010898	-3.06075
SSH3	0.228618	5.994822	3.949089	0.000133	0.000544	0.040476
MMGT1	0.228567	7.758404	2.369847	0.019406	0.046263	-4.5593
VMO1	0.228403	3.55583	2.067254	0.040881	0.086533	-5.20629
ACPT	0.228365	6.226033	2.454124	0.015572	0.038298	-4.36477
ARC	0.228305	2.977784	2.417138	0.017163	0.041647	-4.4509
TM9SF2	0.228296	9.80615	2.786142	0.00621	0.017235	-3.53941
KIF25	0.228042	4.775818	3.466259	0.000735	0.002576	-1.56916
LINC0047(	0.228009	2.858545	2.203249	0.029505	0.065968	-4.92557

SKA3	0.227996	4.382998	2.204711	0.0294	0.065782	-4.92246
LINC00922	0.227901	4.457687	2.953	0.003794	0.011205	-3.08997
LNP1	0.227848	5.112824	3.039216	0.002917	0.008896	-2.84892
SLC39A1	0.227812	5.352565	2.830895	0.005452	0.015371	-3.4211
ADAMTSL4	0.227723	4.845104	2.293761	0.023561	0.054431	-4.72961
RAD51B	0.227579	2.991288	4.401007	2.37E-05	0.000112	1.691006
DLGAP1-A	0.227536	3.412053	3.243836	0.001531	0.004992	-2.25325
MYL5	0.227477	4.621639	2.443027	0.016035	0.03928	-4.39074
CRNDE	0.227444	2.152747	2.690884	0.008153	0.021842	-3.78571
PICK1	0.227413	5.432252	3.085785	0.002526	0.007824	-2.71624
RFC2	0.227062	5.284013	2.800544	0.005956	0.01661	-3.50152
ZG16	0.227054	3.855032	2.352675	0.020282	0.047962	-4.59818
VTI1A	0.226968	4.823457	3.802862	0.000227	0.000885	-0.46443
RAC3	0.226945	3.555123	2.869532	0.004867	0.013951	-3.31764
ZNF630	0.22688	3.260286	2.255761	0.025915	0.059063	-4.81276
DDA1	0.226875	5.399563	5.609507	1.34E-07	9.57E-07	6.692596
SOX18	0.226839	2.665909	3.074744	0.002614	0.008067	-2.74785
TSSC1	0.226641	5.672161	2.959698	0.003718	0.011003	-3.07146
ELMO3	0.226477	4.207024	2.96687	0.003638	0.010796	-3.0516
PLA1A	0.226382	3.289525	2.915559	0.004245	0.012385	-3.1928
LPPR3	0.226381	5.018198	2.307091	0.022781	0.052898	-4.70013
IQCF2	0.226263	3.954944	2.607845	0.01028	0.026693	-3.99422
KRT15	0.225962	2.968064	2.673141	0.008571	0.022809	-3.83075
AQP5	0.225919	5.505804	2.36191	0.019807	0.047027	-4.5773
IQCD	0.225841	3.541164	2.757684	0.006741	0.018508	-3.61378
IL22RA1	0.225791	3.731248	3.611337	0.000447	0.001641	-1.10305
CORO6	0.225731	3.74266	2.710647	0.00771	0.02081	-3.73523
RBM12B-A	0.22571	2.416356	3.078192	0.002586	0.007986	-2.73799
LRRC16B	0.22571	4.326325	2.385656	0.018629	0.044681	-4.52328
SLC35B3	0.225615	5.383015	2.048298	0.042733	0.089784	-5.2441
TTLL13	0.225507	3.313897	3.293654	0.001303	0.004327	-2.10329
BAAT	0.225463	3.272073	2.66996	0.008648	0.022995	-3.8388
HTR1E	0.225454	4.335355	3.032542	0.002978	0.009064	-2.86779
MYNN	0.225319	3.596083	3.617036	0.000439	0.001611	-1.08443
ICA1	0.225213	3.783041	4.439298	2.03E-05	9.76E-05	1.836846
NANOS3	0.225182	4.636129	2.565866	0.011535	0.029507	-4.09742
ABCC3	0.224995	5.270539	3.056243	0.002768	0.008495	-2.80061
ITIH3	0.224917	2.702537	2.420325	0.01702	0.041342	-4.44353
HIST1H3F	0.22469	2.023825	4.052081	9.09E-05	0.000383	0.404851
CASP16	0.224539	3.066154	2.756945	0.006756	0.018542	-3.6157
CLGN	0.224531	1.95041	2.685629	0.008275	0.022125	-3.79907
DEFA6	0.224519	4.808384	3.307351	0.001246	0.004159	-2.06173
FANCD2O	0.224518	5.462124	3.343804	0.001106	0.003728	-1.95042
ARF3	0.224428	7.595462	2.827594	0.005505	0.015501	-3.42988
NUPR1	0.224399	3.761157	2.322156	0.021926	0.051281	-4.66664
SAP30L	0.224369	5.366014	4.123303	6.95E-05	0.0003	0.66097
TAB3	0.224241	4.126239	2.619637	0.00995	0.025933	-3.96497
FAM170A	0.224215	3.537333	3.659426	0.000378	0.001411	-0.94517
DLGAP1-A	0.224031	4.948034	2.372363	0.01928	0.046007	-4.55358
ZMYND10	0.223895	3.768482	3.589354	0.000483	0.001762	-1.17467
ADRA1B	0.22388	2.623688	2.906655	0.004359	0.012677	-3.21708
TYRO3	0.223854	3.425987	3.691919	0.000337	0.001272	-0.83755
COPB1	0.223819	7.606966	2.91355	0.00427	0.012453	-3.19828
BSN-AS2	0.223804	3.855515	2.816991	0.005678	0.015927	-3.45803
LOC10192	0.223772	1.968049	3.305979	0.001252	0.004175	-2.0659
ACADVL	0.223651	7.60912	2.264795	0.025337	0.057933	-4.79311
LINC00927	0.223377	3.518292	2.94198	0.003922	0.011539	-3.12036
CXCL3	0.223377	2.560857	2.407026	0.017622	0.042577	-4.47424

SLC7A4	0.223355	3.798076	3.537803	0.000576	0.002066	-1.34123
CPNE6	0.223292	4.790705	3.495493	0.000666	0.002354	-1.47648
SNRPD3	0.22327	7.88761	2.192731	0.030274	0.067356	-4.94787
AIFM2	0.223237	3.872248	3.409901	0.000888	0.003057	-1.74603
CLPB	0.223167	5.007197	3.27957	0.001364	0.004502	-2.14588
NUDT4	0.222938	3.209323	2.970255	0.003601	0.0107	-3.04221
SPRY3	0.222835	3.15268	2.661864	0.008846	0.023441	-3.85924
ATP13A5	0.222647	3.155807	4.163217	5.97E-05	0.000261	0.805966
LOC10192	0.222646	4.599411	2.678598	0.00844	0.022508	-3.81692
YIPF2	0.222638	5.237449	2.706795	0.007794	0.020999	-3.74509
ASNA1	0.222637	7.422994	3.097235	0.002437	0.007576	-2.68336
ABHD16B	0.222614	4.406897	2.39457	0.018203	0.043804	-4.50288
CDC25C	0.222595	3.037054	3.108778	0.002351	0.007337	-2.65011
PROSER2	0.222491	3.973308	2.236882	0.027159	0.061494	-4.85359
LRRC40	0.222356	5.977968	1.989472	0.048945	0.100594	-5.35937
LOC10193	0.222235	3.979672	2.939623	0.003949	0.01161	-3.12684
SLC25A11	0.222118	6.323549	3.051447	0.00281	0.008603	-2.81424
DHRS7C	0.222112	3.053963	3.530169	0.000592	0.002118	-1.36573
FDXR	0.222103	3.692445	2.731235	0.007271	0.019777	-3.6823
KCP	0.222091	3.970861	4.087913	7.95E-05	0.000339	0.533286
PTS	0.221892	4.47905	2.004372	0.047303	0.097829	-5.33047
LMAN2	0.221867	5.142889	4.516777	1.49E-05	7.37E-05	2.134688
CCP110	0.221852	5.037594	2.198556	0.029846	0.066613	-4.93553
LINC01181	0.221841	2.713247	3.439391	0.000805	0.002795	-1.65378
OR12D2	0.221824	5.323461	3.134022	0.002172	0.006837	-2.57701
FAM83F	0.221688	3.717425	2.087186	0.039008	0.08324	-5.16618
TBC1D14	0.221655	7.522638	2.174834	0.031623	0.06984	-4.98559
ATP13A2	0.221445	5.282828	3.202304	0.00175	0.005639	-2.3768
OXSR1	0.221321	7.549333	3.585918	0.000489	0.00178	-1.18583
LINC00961	0.221181	4.107606	3.392204	0.000942	0.003225	-1.80108
LTB4R2	0.221008	4.285056	2.04998	0.042566	0.08947	-5.24076
LOC10050	0.220929	2.109467	4.029288	9.90E-05	0.000413	0.323597
KIF13A	0.220909	4.551141	3.21931	0.001657	0.005363	-2.32637
FTCD	0.22075	4.487821	3.292949	0.001306	0.004334	-2.10543
FGF8	0.220664	2.728288	2.58435	0.010966	0.028246	-4.05216
INTS6-AS1	0.220573	3.949366	2.378475	0.018978	0.045385	-4.53967
KRTAP8-1	0.220546	3.488181	2.815486	0.005703	0.015984	-3.46202
C7orf13	0.22047	4.404085	3.393427	0.000938	0.003214	-1.79728
COLEC10	0.220381	3.311676	2.981073	0.003485	0.010394	-3.01214
VIPAS39	0.220361	6.044558	3.429267	0.000833	0.00288	-1.68552
FAM195B	0.220269	5.879085	3.186253	0.001841	0.005909	-2.42419
LOC28464	0.220229	3.48486	2.360307	0.019888	0.047161	-4.58093
TNNC2	0.219974	4.509615	2.609328	0.010238	0.026604	-3.99055
LOC10272	0.219863	2.737908	3.336236	0.001134	0.003812	-1.97361
VEPH1	0.219765	3.135589	2.623038	0.009857	0.025732	-3.95651
LOC10028	0.21974	3.857153	3.029054	0.00301	0.009145	-2.87764
GOLPH3	0.219649	8.370071	2.415732	0.017226	0.041758	-4.45416
HIGD1B	0.219647	4.544121	3.10218	0.0024	0.007472	-2.66913
SLCO2A1	0.219464	2.698165	3.356787	0.001059	0.003585	-1.91054
KRT35	0.219462	2.119942	3.136374	0.002156	0.006794	-2.57017
TMEM114	0.219435	3.375235	2.888293	0.004604	0.01329	-3.26696
LOC10050	0.219401	3.176483	2.742391	0.007043	0.019226	-3.65347
ST14	0.219167	4.177881	3.378346	0.000987	0.003359	-1.84402
TSR3	0.219132	4.620772	5.085059	1.39E-06	8.29E-06	4.426086
LINC00092	0.219028	4.008553	4.02013	0.000102	0.000426	0.291047
INTS12	0.21891	6.04122	2.378913	0.018957	0.045345	-4.53867
DNAH17	0.218893	2.976136	4.028906	9.92E-05	0.000414	0.322238
PMFBP1	0.218879	3.546745	2.596092	0.010618	0.027439	-4.02326

PDGFRL	0.218654	2.819464	2.47805	0.014615	0.036243	-4.30843
MUC1	0.218595	4.192124	4.231165	4.60E-05	0.000206	1.055182
RAB2A	0.218295	6.28163	3.005484	0.003235	0.009728	-2.94394
DERL3	0.218238	4.519166	2.129685	0.03526	0.07647	-5.07947
DGKI	0.218182	2.065351	2.705569	0.007822	0.021067	-3.74823
LOC10272	0.218084	2.659314	3.141145	0.002124	0.006706	-2.55629
EPO	0.218027	3.26469	3.123972	0.002242	0.007034	-2.60617
MUC2	0.21801	3.960129	2.994088	0.00335	0.010034	-2.97584
SLC9A2	0.21797	2.930221	2.523865	0.012926	0.03263	-4.19916
PRSS58	0.217918	3.319366	2.407986	0.017578	0.042495	-4.47203
C1GALT1	0.217781	5.985073	2.089653	0.038782	0.082851	-5.16119
LOC10013	0.217759	4.737983	2.507178	0.01352	0.033923	-4.23917
MAPKAPK	0.217735	7.784784	2.18454	0.030885	0.068487	-4.96517
DICER1-AS	0.217724	3.064207	3.678142	0.000354	0.001328	-0.88327
LOC34488	0.217698	2.632367	2.20738	0.029208	0.065417	-4.91678
ATP6V0A1	0.217448	6.504693	3.726422	0.000299	0.00114	-0.72244
GALE	0.21737	4.765407	4.508355	1.54E-05	7.59E-05	2.102135
ATXN3	0.217352	4.018774	3.710425	0.000316	0.001199	-0.77591
TMEM200	0.217305	2.898171	3.884192	0.000169	0.000677	-0.18543
FAM109B	0.217233	5.062254	2.897081	0.004485	0.013007	-3.24312
WNT9B	0.217058	4.05971	2.8144	0.005721	0.016023	-3.4649
KRT36	0.217049	3.303188	2.500195	0.013776	0.034451	-4.25584
COL4A1	0.217031	2.567352	4.243055	4.39E-05	0.000197	1.099098
CTSV	0.217016	3.80759	2.782715	0.006272	0.017376	-3.5484
RNF215	0.216884	3.520241	2.839767	0.005312	0.015029	-3.39745
HSD11B2	0.216856	2.430855	3.037923	0.002929	0.008925	-2.85258
ROM1	0.216832	4.427484	2.357603	0.020027	0.047446	-4.58705
NFIB	0.216732	2.345836	3.58066	0.000498	0.001808	-1.2029
FSD1	0.21661	4.516973	2.360847	0.019861	0.047118	-4.57971
LOC10028	0.216461	2.748073	3.454324	0.000765	0.002671	-1.60681
MOS	0.216447	3.949954	2.343831	0.020747	0.048875	-4.6181
SH3GL1	0.216113	6.517793	2.650637	0.009128	0.024052	-3.88749
DCAF4L1	0.216086	2.275243	2.207884	0.029172	0.065357	-4.91571
NNMT	0.21601	2.767091	2.25203	0.026156	0.059561	-4.82085
MLX	0.215896	6.848226	4.374357	2.63E-05	0.000124	1.590039
ATF4	0.215882	8.733302	2.059811	0.0416	0.087773	-5.22118
SREBF2	0.215611	5.673748	2.621019	0.009912	0.02585	-3.96153
OR7E156P	0.215587	4.813505	3.31322	0.001223	0.004085	-2.04388
HAPLN2	0.215573	4.458442	2.62094	0.009914	0.025852	-3.96173
TVP23A	0.215568	2.405274	3.653737	0.000386	0.001436	-0.96393
WFDC3	0.215355	3.831959	2.685833	0.00827	0.022118	-3.79856
SMTN	0.215318	4.267674	3.741191	0.000283	0.001087	-0.67291
PIM1	0.215271	8.665703	2.122568	0.035865	0.077519	-5.09411
DENND1B	0.215258	4.679475	2.89004	0.00458	0.013235	-3.26223
REG1A	0.215235	4.366981	2.508554	0.01347	0.033811	-4.23588
EFCAB11	0.215156	2.376371	3.11885	0.002278	0.00713	-2.621
MAGEA10	0.21508	3.81862	2.219712	0.028335	0.063763	-4.89046
NKX2-8	0.215041	3.5242	2.569767	0.011412	0.029227	-4.08789
FBXL15	0.214953	5.196497	2.064613	0.041135	0.086953	-5.21158
DDC-AS1	0.214946	3.065712	2.77156	0.006477	0.017877	-3.5776
CIDEC	0.214941	4.642976	2.850507	0.005147	0.014633	-3.36874
APOE	0.214727	4.696371	3.388829	0.000953	0.003255	-1.81155
EPHA5-AS	0.214673	3.288241	3.260832	0.00145	0.004753	-2.20231
MFN2	0.214672	5.711918	2.85807	0.005034	0.014351	-3.34846
POMC	0.214511	3.863583	2.44313	0.016031	0.039277	-4.3905
PLEKHH3	0.214416	3.381809	3.615203	0.000441	0.00162	-1.09042
RAB36	0.214409	4.998769	2.385823	0.01862	0.044672	-4.5229
C1orf64	0.214175	4.058658	2.640879	0.00938	0.024632	-3.91197

DACT2	0.214098	4.278543	3.461133	0.000748	0.00262	-1.58535
BCL7B	0.213954	6.416738	3.333436	0.001144	0.003846	-1.98219
GGT5	0.21394	4.754948	2.681121	0.008381	0.022369	-3.81052
KCNC4	0.213938	3.802676	4.152822	6.21E-05	0.00027	0.768103
IGSF10	0.213857	3.474278	2.755064	0.006792	0.01862	-3.62059
KCNMB3	0.21384	3.481326	3.217835	0.001665	0.005383	-2.33075
ZDHHC24	0.213738	5.202706	4.873531	3.42E-06	1.90E-05	3.551944
MAP1LC3A	0.213704	5.836841	2.555074	0.011879	0.030266	-4.1237
INE1	0.213645	5.370884	3.16636	0.001961	0.006248	-2.48264
TDRD7	0.213542	6.699959	2.236518	0.027183	0.061529	-4.85438
PANK3	0.2134	5.393995	2.407583	0.017596	0.04253	-4.47296
MST1R	0.213354	4.75526	3.063591	0.002706	0.008325	-2.77969
EXTL1	0.213325	3.75193	2.409884	0.017491	0.042325	-4.46766
SNTB1	0.213282	3.973428	3.578114	0.000502	0.001821	-1.21115
DYNLRB1	0.21328	5.789523	5.944908	2.84E-08	2.26E-07	8.209509
CLTC	0.213266	4.541807	4.267808	3.99E-05	0.000181	1.190815
LINC0024	0.213244	2.950985	2.772091	0.006467	0.017855	-3.57621
LOC39070	0.213112	5.620139	2.667578	0.008706	0.023128	-3.84482
C3orf58	0.213001	4.273889	4.442585	2.01E-05	9.66E-05	1.849404
SCNN1B	0.212925	4.187693	2.256871	0.025843	0.058906	-4.81035
KRT37	0.212766	3.518927	2.749777	0.006896	0.018867	-3.63432
SURF4	0.212699	7.251251	2.156781	0.033036	0.072476	-5.02335
SLC22A13	0.212633	3.514697	2.296579	0.023394	0.054095	-4.72339
GPR4	0.21263	3.954832	3.368596	0.001019	0.003461	-1.87415
LRRK1	0.212588	4.002183	3.264825	0.001431	0.004697	-2.1903
MT4	0.212522	4.844674	3.067358	0.002675	0.00824	-2.76895
MTSS1L	0.212473	2.754913	5.310021	5.18E-07	3.34E-06	5.381632
RAB3IL1	0.2124	5.960906	2.307303	0.022768	0.052881	-4.69966
POU5F1P4	0.21213	4.158324	2.680992	0.008384	0.022375	-3.81085
C4orf32	0.212064	4.958353	2.49674	0.013904	0.034724	-4.26407
ADARB2	0.212004	3.528227	2.511507	0.013364	0.033597	-4.22882
SYCE2	0.211999	3.013732	2.707838	0.007771	0.020954	-3.74243
DPEP1	0.211884	3.981221	2.477263	0.014645	0.036302	-4.31029
COPA	0.211853	4.857652	3.255194	0.001476	0.004829	-2.21923
AAMDC	0.211784	3.749796	4.308461	3.41E-05	0.000157	1.342289
MCAT	0.211733	4.865723	2.474685	0.014746	0.036538	-4.31638
NDUFS7	0.211707	5.343485	3.891954	0.000165	0.000659	-0.15856
CHADL	0.21164	4.199568	2.120765	0.03602	0.077804	-5.09781
CCL24	0.211633	3.637001	2.788333	0.006171	0.017136	-3.53366
UNC5B-AS	0.211632	3.240677	2.457125	0.015449	0.038023	-4.35773
GAGE3	0.211628	2.606394	3.225214	0.001626	0.005275	-2.30881
LOC25565	0.211626	5.378789	2.353921	0.020217	0.047842	-4.59537
BLVRA	0.211622	5.05066	2.266088	0.025255	0.057766	-4.79029
MRPL55	0.211571	4.802736	4.468481	1.81E-05	8.80E-05	1.948599
DCDC2B	0.211553	4.69952	2.708401	0.007759	0.020929	-3.74098
FOXL1	0.211536	3.766557	3.269402	0.00141	0.004635	-2.17653
SLC7A1	0.211535	4.593771	2.498511	0.013838	0.034594	-4.25985
FAM71A	0.2115	4.15423	3.253608	0.001484	0.004853	-2.22398
PGAM2	0.21149	3.373902	2.569024	0.011436	0.029279	-4.08971
COX15	0.211397	5.53944	3.176191	0.001901	0.006075	-2.45379
IGFBP6	0.211188	4.492287	2.862233	0.004973	0.014203	-3.33728
LOC15005	0.211098	3.281601	2.405382	0.017698	0.042729	-4.47803
MKL1	0.210877	4.900531	4.755152	5.62E-06	3.00E-05	3.07352
HIST1H3C	0.21075	3.332921	3.674812	0.000358	0.001342	-0.8943
LINC0017	0.210726	3.457362	2.505435	0.013583	0.034037	-4.24333
UNC13D	0.210648	5.137352	3.918327	0.000149	0.000603	-0.06696
PRM2	0.210572	3.858788	2.077084	0.039948	0.084882	-5.18656
LOC10192	0.210383	3.479822	2.872257	0.004828	0.013851	-3.3103

NFAM1	0.210253	7.128813	2.699874	0.007949	0.021356	-3.76279
HBEGF	0.210004	3.464058	5.177063	9.29E-07	5.76E-06	4.813738
SEMA4G	0.209883	3.789105	2.885277	0.004645	0.013396	-3.27513
METRN	0.209788	4.988866	3.27656	0.001378	0.004544	-2.15496
MAGIX	0.209785	3.301156	4.732729	6.17E-06	3.27E-05	2.983793
LOC10192	0.209774	5.463728	2.875145	0.004787	0.013758	-3.30251
EML2	0.209752	4.522575	3.816294	0.000217	0.000847	-0.41867
APLP2	0.209745	7.567605	2.643028	0.009324	0.02451	-3.90658
LOC10099	0.209713	3.374914	3.05944	0.002741	0.008423	-2.79151
LOC10028	0.209649	3.714541	2.521226	0.013019	0.032826	-4.20551
ZNF488	0.209563	4.042693	2.701791	0.007906	0.02126	-3.75789
ARL16	0.209483	4.304459	2.715206	0.007611	0.02058	-3.72354
COASY	0.209467	6.423115	3.112976	0.002321	0.007247	-2.63798
ADAP1	0.209369	7.453101	3.454713	0.000764	0.002669	-1.60559
ARMC12	0.209327	4.857293	2.529598	0.012728	0.032189	-4.18536
RASL11A	0.209152	3.878303	2.517578	0.013147	0.033122	-4.21426
DENND1A	0.209077	5.955396	2.83818	0.005337	0.015082	-3.40169
LINC00851	0.209071	4.887439	2.89419	0.004524	0.013104	-3.25097
SOX21	0.208799	3.273255	2.288047	0.023902	0.055127	-4.74219
CECR5-AS	0.208772	3.196229	2.75178	0.006856	0.018779	-3.62913
HOTS	0.20861	4.754849	2.070157	0.040604	0.086034	-5.20047
LOC72988	0.208539	2.942096	2.612092	0.01016	0.026419	-3.9837
SSR2	0.208499	8.700227	2.144535	0.034026	0.074265	-5.04879
PRDM12	0.208468	4.759216	2.234944	0.027289	0.061715	-4.85777
LSM12	0.208445	5.404148	2.913171	0.004275	0.012465	-3.19932
NDUFA4L2	0.208391	4.688105	2.391973	0.018326	0.044064	-4.50883
NPPC	0.208369	3.450173	2.369748	0.019411	0.04627	-4.55953
CHID1	0.208301	5.327451	2.137292	0.034623	0.075336	-5.06378
LOC10192	0.208261	4.76031	3.219629	0.001655	0.005359	-2.32542
CACNA1G	0.208242	3.697997	2.594002	0.010679	0.027559	-4.02842
AMER3	0.208058	3.289375	3.489147	0.00068	0.002402	-1.49666
STARD13	0.207992	2.693583	2.595027	0.010649	0.027506	-4.02589
ADAM17	0.207971	6.160502	2.260363	0.025619	0.058487	-4.80276
CCDC135	0.207962	4.254694	2.854387	0.005089	0.014484	-3.35834
KRT19P2	0.207923	3.794995	2.268148	0.025126	0.057495	-4.78579
NPPB	0.207664	3.755205	2.654026	0.009042	0.023868	-3.87898
NAA10	0.207646	6.234187	4.247182	4.32E-05	0.000194	1.114362
KCNH7	0.20763	2.766524	3.525196	0.000602	0.002149	-1.38167
PHYHD1	0.207628	4.116965	3.151316	0.002057	0.006519	-2.52665
PHACTR3	0.207622	1.848921	3.269092	0.001411	0.004639	-2.17746
BMP3	0.207456	2.864462	2.623035	0.009857	0.025732	-3.95651
HOXA10	0.207328	4.208987	3.039849	0.002912	0.008882	-2.84712
CAST	0.207279	6.44759	2.384267	0.018696	0.044816	-4.52645
PKP2	0.207137	2.844637	3.268047	0.001416	0.004654	-2.18061
LOC10192	0.207137	3.302805	2.891052	0.004566	0.013204	-3.25948
FAM209B	0.207076	4.827208	2.656301	0.008985	0.023741	-3.87325
HAUS8	0.207053	4.250378	2.202992	0.029524	0.065987	-4.92611
LOC10050	0.20693	2.440005	3.301201	0.001271	0.004232	-2.08041
TBC1D10B	0.20692	7.112213	2.863255	0.004958	0.014178	-3.33453
COMP	0.206827	2.816302	2.314915	0.022333	0.052043	-4.68277
ALAD	0.206778	5.644676	2.283395	0.024183	0.055671	-4.75241
VENTXP1	0.20669	2.46315	4.555825	1.27E-05	6.36E-05	2.286176
LINC01220	0.206647	2.224864	2.92719	0.0041	0.012013	-3.16098
ERLEC1	0.206637	5.478709	2.321196	0.02198	0.051377	-4.66878
PARL	0.206548	5.099465	2.227138	0.027821	0.062765	-4.87455
MYOM1	0.206438	3.258478	2.55099	0.012011	0.030554	-4.13363
ZBED8	0.206005	2.234157	2.184608	0.03088	0.068487	-4.96502
SENCR	0.20594	5.71448	3.087757	0.002511	0.007783	-2.71059

TRIM67	0.205924	5.254396	2.198036	0.029884	0.066669	-4.93663
NPW	0.205869	2.934964	2.037769	0.043793	0.091709	-5.26496
RSPH1	0.205806	2.823633	2.505836	0.013569	0.034018	-4.24238
PARD6A	0.205733	5.630726	2.418017	0.017123	0.041563	-4.44887
LINC00917	0.20573	2.740099	2.465281	0.015119	0.037336	-4.33856
HIST1H1D	0.205716	4.567043	2.702061	0.0079	0.021246	-3.7572
AZGP1P1	0.205709	2.708208	3.659518	0.000378	0.001411	-0.94487
LINC00654	0.20569	2.492815	3.181425	0.00187	0.005989	-2.4384
TECTB	0.205643	5.172199	2.909151	0.004327	0.012594	-3.21028
IL1RL2	0.205486	3.222632	2.942171	0.003919	0.011534	-3.11983
PSKH1	0.205431	5.578787	3.401193	0.000914	0.003139	-1.77315
SNAP29	0.205401	5.628426	2.147724	0.033766	0.073818	-5.04218
KCNRG	0.205359	3.01307	3.280051	0.001362	0.004497	-2.14443
NBL1	0.205354	4.757845	2.726928	0.007361	0.019992	-3.6934
NOL7	0.205146	7.29822	2.165705	0.032331	0.071166	-5.00472
YWHAG	0.205042	8.98201	2.106453	0.037269	0.080087	-5.12707
LOC10012	0.20499	3.152306	2.667447	0.008709	0.023134	-3.84515
KAAG1	0.204974	2.431221	2.844799	0.005234	0.014853	-3.38401
CARS-AS1	0.204972	3.265682	2.810126	0.005793	0.016202	-3.47621
SNRNP35	0.204965	5.482598	2.782967	0.006268	0.017366	-3.54774
SP5	0.204905	2.714334	2.636591	0.009493	0.024906	-3.9227
CLCN1	0.204831	3.958601	2.290183	0.023774	0.054868	-4.73749
DNAH2	0.204826	3.875628	2.510682	0.013393	0.033643	-4.23079
FBXL22	0.204815	3.703372	2.479711	0.01455	0.036101	-4.3045
LINC00911	0.204699	3.019248	2.676584	0.008488	0.02262	-3.82203
OR2L1P	0.204605	3.59979	2.975385	0.003545	0.010553	-3.02796
TFE3	0.204507	5.603588	2.858662	0.005025	0.01433	-3.34687
STEAP1	0.204076	2.697574	2.710844	0.007705	0.020807	-3.73473
PNCK	0.204031	4.713426	2.899781	0.004449	0.012914	-3.23579
RRBP1	0.204018	4.329739	4.450462	1.94E-05	9.38E-05	1.879537
SYT13	0.203935	3.520822	2.90239	0.004415	0.012826	-3.22869
CDO1	0.20391	2.912541	3.807613	0.000223	0.000872	-0.44826
NIN	0.203791	5.758029	2.660196	0.008888	0.023526	-3.86344
DLX4	0.203744	2.265244	3.771659	0.000254	0.000982	-0.57025
LOC10028	0.203688	2.397396	3.321625	0.001189	0.003987	-2.01827
CXorf36	0.203639	3.933856	3.82667	0.000209	0.000818	-0.38323
HPS6	0.203553	5.060276	3.847693	0.000193	0.000762	-0.3112
LRP2BP	0.203479	2.490653	2.812767	0.005748	0.016095	-3.46922
CD163L1	0.20343	4.912527	2.631273	0.009634	0.025238	-3.93598
GPR182	0.203395	3.752718	3.900641	0.000159	0.00064	-0.12844
CRYBA2	0.203201	2.317244	2.651228	0.009113	0.024027	-3.88601
MBNL1-AS	0.203185	3.796865	2.99928	0.003297	0.009893	-2.96132
PTTG1IP	0.203124	9.255258	2.121677	0.035942	0.077651	-5.09594
P4HA1	0.202999	4.001457	3.019976	0.003095	0.009364	-2.90323
MUC3	0.202822	3.850555	3.027667	0.003023	0.009174	-2.88155
IFT57	0.20279	3.964961	2.178339	0.031355	0.069338	-4.97822
PPP1R1B	0.202772	4.032387	2.480141	0.014533	0.036064	-4.30348
CYP1A1	0.202515	4.531946	2.240045	0.026947	0.061068	-4.84677
LOC10012	0.202502	5.049122	3.100646	0.002412	0.007504	-2.67354
TREM2	0.202491	5.202867	2.78345	0.006259	0.017349	-3.54647
LOC28373	0.202426	2.886448	2.015674	0.046089	0.095705	-5.30842
LOC10192	0.202203	2.420518	2.514038	0.013273	0.03339	-4.22275
DUSP23	0.202061	6.528539	2.900988	0.004433	0.012869	-3.2325
LINC00592	0.202034	3.301719	2.725576	0.007389	0.020056	-3.69688
RGS3	0.201999	4.829626	2.599631	0.010515	0.027198	-4.01453
ACTR3C	0.201661	2.664118	2.720834	0.00749	0.020288	-3.70908
CENPQ	0.20156	2.154543	2.067631	0.040845	0.086465	-5.20554
RSU1	0.20156	4.286859	4.430072	2.11E-05	0.000101	1.801623

LOC10192	0.201381	2.492671	2.738745	0.007117	0.019399	-3.6629
CALU	0.201266	5.226243	2.883805	0.004665	0.013444	-3.27911
MBD6	0.201231	6.736457	2.197808	0.029901	0.066692	-4.93711
CLDN3	0.201181	2.911001	3.771593	0.000254	0.000982	-0.57047
ANPEP	0.201123	4.808645	3.021779	0.003078	0.009318	-2.89815
FLJ33534	0.201097	2.989559	3.00685	0.003222	0.009696	-2.94011
B9D1	0.20108	3.304328	2.83094	0.005451	0.015371	-3.42098
RSPO4	0.201049	3.497954	2.622328	0.009876	0.025774	-3.95827
11-Mar	0.20102	2.563864	2.52007	0.013059	0.032917	-4.20828
LOXHD1	0.201009	2.703424	2.329451	0.021523	0.050494	-4.65035
GPR64	0.200956	2.856553	2.720521	0.007497	0.020304	-3.70989
LINC00202	0.2009	5.093945	3.54388	0.000565	0.002028	-1.3217
ZNF513	0.200861	4.684754	2.448125	0.015821	0.038849	-4.37883
LOC10192	0.200818	3.536504	3.041593	0.002896	0.008843	-2.84219
HCN4	0.200727	4.225596	2.842273	0.005273	0.014937	-3.39076
EXOC3L1	0.200536	4.870733	3.02929	0.003008	0.00914	-2.87697
CTIF	0.200494	4.882835	3.124519	0.002238	0.007023	-2.60458
MRPS18C	0.200408	3.431092	3.039192	0.002918	0.008896	-2.84898
EIF2S2	0.200278	6.742586	3.129189	0.002205	0.006931	-2.59104
MORF4L1	0.200268	9.442732	2.871037	0.004845	0.013897	-3.31358
GINS4	0.200219	3.02498	3.991835	0.000114	0.00047	0.190838
DNM2	0.200181	4.976452	3.712656	0.000314	0.001191	-0.76847
IGH	0.199807	4.533857	2.689402	0.008187	0.021922	-3.78948
STIM1	0.199775	6.198849	2.257569	0.025798	0.058836	-4.80883
LOC72950	0.199708	4.216508	2.726687	0.007366	0.020003	-3.69402
CEND1	0.199659	5.544511	3.023318	0.003063	0.00928	-2.89381
TYMSOS	0.199595	3.561102	2.250628	0.026248	0.059736	-4.82389
GNB2	0.199587	9.246712	2.060539	0.041529	0.08765	-5.21972
LINC00929	0.199465	3.151528	2.498428	0.013841	0.034597	-4.26005
CYSRT1	0.199452	4.287852	2.667088	0.008718	0.023139	-3.84605
PINLYP	0.199432	4.749127	2.27662	0.024598	0.056458	-4.76727
LOC10106	0.199312	3.304183	3.017447	0.003119	0.009426	-2.91034
FAM69B	0.199275	3.797539	3.330567	0.001155	0.003881	-1.99096
CT55	0.199247	3.54182	2.080938	0.039587	0.084265	-5.17879
BRSK1	0.19896	4.918786	2.618661	0.009977	0.025997	-3.96739
FLJ34521	0.198923	2.208213	2.628136	0.009718	0.025426	-3.94381
DRC1	0.19888	4.132036	2.595769	0.010627	0.027457	-4.02406
TMEM107	0.19887	3.81835	3.058732	0.002747	0.008436	-2.79353
ACAT1	0.198795	3.935239	3.230503	0.001598	0.005197	-2.29306
TBXA2R	0.198784	5.288354	2.282127	0.024261	0.055821	-4.7552
AUP1	0.198746	6.701892	2.099307	0.037906	0.081252	-5.14162
KLHL9	0.198678	5.049371	3.522366	0.000608	0.002169	-1.39073
SNX21	0.198536	4.072895	2.438685	0.016219	0.03967	-4.40087
PGK2	0.198395	3.394703	2.819508	0.005637	0.015826	-3.45136
PLCD3	0.198315	5.450587	2.862631	0.004967	0.014193	-3.33621
ADRA2A	0.198309	2.530831	2.376636	0.019068	0.045566	-4.54386
PCK1	0.198266	1.859326	3.150026	0.002065	0.006538	-2.53041
LINC01141	0.198242	3.186848	3.187008	0.001837	0.005896	-2.42196
PHKA1	0.198098	2.813841	3.511162	0.000631	0.002243	-1.42655
ZNRF2P1	0.198089	1.822488	3.521698	0.000609	0.002173	-1.39287
REXO1	0.198085	4.728135	2.238964	0.027019	0.061219	-4.84911
ZNF628	0.198054	2.705448	2.950053	0.003827	0.011288	-3.09811
HCCS	0.197952	6.044256	2.242227	0.026801	0.060786	-4.84206
RNASEL	0.197923	7.041135	2.064755	0.041121	0.086933	-5.21129
CLDN17	0.197862	5.066166	2.632358	0.009605	0.025178	-3.93327
LOC10192	0.19782	2.963931	3.19739	0.001777	0.005717	-2.39133
LOC10192	0.197784	3.28	2.71825	0.007545	0.020424	-3.71572
IZUMO1	0.197706	1.8117	2.867754	0.004892	0.014015	-3.32243

CHRNA4	0.197655	3.434279	2.888296	0.004604	0.01329	-3.26695
COA3	0.197654	5.424158	3.98173	0.000118	0.000487	0.155181
LOC10192	0.19762	2.421981	2.468684	0.014983	0.037039	-4.33055
LINC0101(	0.197604	3.803046	2.67206	0.008597	0.022869	-3.83349
OPRD1	0.197596	3.860131	2.249002	0.026354	0.059918	-4.82741
CDC42EP4	0.197587	4.682526	3.856842	0.000187	0.000739	-0.27976
KLHL18	0.197568	5.075784	3.864413	0.000182	0.000721	-0.2537
UBXN11	0.19754	6.358715	2.131625	0.035097	0.076156	-5.07548
C19orf80	0.197525	2.846424	2.3398	0.020962	0.049313	-4.62716
DEFB123	0.197496	3.769075	2.17407	0.031682	0.069932	-4.98719
EVA1B	0.197355	5.324844	2.901055	0.004432	0.012868	-3.23232
LOC10192	0.197335	4.110072	2.39179	0.018335	0.044074	-4.50925
BRE	0.197217	6.736713	2.925173	0.004124	0.012081	-3.1665
PIGZ	0.196983	4.217976	2.715841	0.007597	0.020546	-3.72191
EDA2R	0.196962	4.519152	2.832479	0.005427	0.015306	-3.41688
ETV6	0.196845	6.320993	2.107656	0.037162	0.079909	-5.12462
CYP4F8	0.196575	2.398483	2.653435	0.009057	0.023901	-3.88046
CBR4	0.196521	6.389707	2.303721	0.022976	0.053272	-4.7076
OGDHL	0.196498	4.15073	2.253506	0.02606	0.059362	-4.81765
CAPN8	0.196483	2.585001	2.908709	0.004332	0.012607	-3.21148
SLC8A2	0.196477	3.979272	2.166263	0.032287	0.071108	-5.00355
SLCO3A1	0.196412	6.863008	2.422754	0.016912	0.041124	-4.4379
HHATL	0.196391	3.602505	2.363126	0.019745	0.046917	-4.57455
RAMP2-A:	0.196388	2.429094	2.956519	0.003754	0.011096	-3.08025
CADM3	0.196215	4.602132	2.765	0.006601	0.018164	-3.59472
DLX5	0.196207	3.371836	2.570182	0.0114	0.029198	-4.08687
SLITRK2	0.196086	2.667565	3.006977	0.00322	0.009694	-2.93975
LOC10192	0.195974	3.620449	2.511289	0.013372	0.033606	-4.22934
RS1	0.195895	2.652533	3.689387	0.00034	0.001282	-0.84596
SPHK1	0.195881	4.468323	2.070177	0.040602	0.086034	-5.20043
LOC28488	0.195745	3.808073	2.193955	0.030184	0.067169	-4.94528
SOD3	0.195697	4.793043	2.440379	0.016147	0.039532	-4.39692
GSTA3	0.195658	3.059965	2.606774	0.01031	0.026745	-3.99687
LILRB5	0.195637	3.743928	2.626671	0.009758	0.0255	-3.94746
LY6G6C	0.195615	4.500931	2.793367	0.006082	0.016918	-3.52042
PCP2	0.195595	4.194914	2.039508	0.043616	0.091367	-5.26152
LOC10192	0.195567	2.678879	3.025429	0.003044	0.009225	-2.88786
SAA3P	0.195563	2.4426	2.623846	0.009835	0.025681	-3.9545
SNX11	0.195559	7.302327	2.979945	0.003497	0.010427	-3.01528
TONSL	0.195431	3.911787	2.954154	0.00378	0.011172	-3.08679
LOC34010	0.195374	3.635898	3.113353	0.002318	0.00724	-2.6369
APC2	0.195369	4.559373	2.641576	0.009362	0.024597	-3.91022
SYT6	0.195311	2.299884	3.172973	0.001921	0.006132	-2.46325
LOC10013	0.195262	5.809026	2.279227	0.024438	0.056166	-4.76156
LOC25405	0.195261	4.224874	3.189609	0.001822	0.005852	-2.4143
DGCR5	0.195252	4.012345	3.129986	0.0022	0.006916	-2.58873
CASC11	0.195147	2.892946	2.822288	0.005591	0.015719	-3.44398
AKAP10	0.195135	6.351279	2.053399	0.042228	0.088896	-5.23396
HYI	0.195106	5.326104	2.198179	0.029874	0.066653	-4.93633
PMVK	0.195084	5.7696	2.367055	0.019546	0.046549	-4.56564
ZNF271	0.194831	4.113706	2.402924	0.017811	0.042973	-4.48369
EHMT1-IT	0.194799	4.048015	2.923629	0.004144	0.01213	-3.17073
DGCR9	0.194748	4.722458	2.754789	0.006797	0.018632	-3.62131
LOC10106	0.194557	2.438722	2.50136	0.013733	0.034364	-4.25306
FAM196A	0.194494	1.486579	3.249244	0.001505	0.004912	-2.23706
NCKAP1L	0.194461	7.541751	2.222512	0.028141	0.063381	-4.88447
VKORC1	0.194438	6.24935	2.179217	0.031288	0.069242	-4.97637
PIR	0.194432	1.895836	2.731334	0.007269	0.019774	-3.68204

NPBWR1	0.194417	5.438968	2.323826	0.021833	0.051116	-4.66292
CASC4	0.194386	5.923107	2.944448	0.003893	0.011465	-3.11356
IZUMO2	0.194348	3.464401	2.447413	0.015851	0.038913	-4.38049
PGPEP1	0.194331	5.268146	2.710698	0.007709	0.02081	-3.7351
GJB1	0.194295	5.282902	2.174212	0.031671	0.069916	-4.98689
RAB40C	0.194165	5.070258	4.392575	2.45E-05	0.000116	1.659013
GSDMD	0.194001	7.341693	2.370448	0.019376	0.046197	-4.55794
GORASP1	0.193903	5.213448	4.180489	5.59E-05	0.000245	0.869032
MALL	0.193674	4.047383	2.2067	0.029257	0.065505	-4.91823
PHPT1	0.193634	6.0509	2.135997	0.034731	0.075524	-5.06646
DNAJB8	0.193632	3.468527	2.577495	0.011174	0.028706	-4.06898
GNRH2	0.193612	5.504711	2.468909	0.014974	0.03703	-4.33002
WDR53	0.193478	4.394614	2.2655	0.025292	0.057838	-4.79157
SMR3B	0.193447	2.230175	2.614505	0.010092	0.02627	-3.97771
LYPLA2	0.193437	6.714876	4.515496	1.50E-05	7.40E-05	2.129736
CHDH	0.193415	3.692355	3.491777	0.000674	0.002382	-1.4883
MTIF3	0.193372	3.591339	3.120773	0.002265	0.007093	-2.61544
SLIT2-IT1	0.193359	1.860955	3.401655	0.000913	0.003135	-1.77171
C9orf62	0.193277	4.265163	2.666446	0.008733	0.023175	-3.84767
LOC10050	0.193205	2.92133	3.309514	0.001237	0.004132	-2.05516
PTCRA	0.193197	6.199579	2.118835	0.036186	0.07812	-5.10177
BMP10	0.193183	2.917987	2.896608	0.004491	0.013018	-3.24441
FCN2	0.193153	3.009827	3.734608	0.00029	0.00111	-0.69501
STAB2	0.193001	3.305045	3.45796	0.000756	0.002644	-1.59535
TULP2	0.192988	3.051481	2.141188	0.034301	0.074723	-5.05573
MAP3K6	0.192852	3.378502	3.311069	0.001231	0.004112	-2.05043
HHIPL1	0.192827	4.128221	2.337874	0.021065	0.049534	-4.63148
ZAN	0.192761	4.831548	3.184397	0.001852	0.005939	-2.42966
RXRA	0.192635	8.318876	2.559518	0.011736	0.029947	-4.11289
ARRDC1	0.192511	5.349596	2.865853	0.00492	0.014082	-3.32754
TBX1	0.192468	3.337054	3.34594	0.001098	0.003704	-1.94387
LOC14679	0.192457	2.604952	2.659188	0.008913	0.023582	-3.86598
FAM69C	0.192411	5.198038	2.58922	0.01082	0.027899	-4.04019
LINC0089(	0.192343	2.854713	3.220726	0.001649	0.005342	-2.32216
NPHS1	0.192317	2.654984	2.660201	0.008887	0.023526	-3.86343
FGFR4	0.192265	3.810064	3.685579	0.000345	0.001298	-0.8586
PRR22	0.192137	4.101044	3.148572	0.002075	0.006565	-2.53465
TMEM105	0.192107	5.629011	3.017478	0.003119	0.009426	-2.91026
LINC00881	0.192098	3.035896	2.761103	0.006675	0.018347	-3.60488
TBATA	0.192034	4.81085	3.015044	0.003142	0.009484	-2.9171
CA5BP1	0.19174	5.610126	2.65787	0.008946	0.023658	-3.8693
HSD17B12	0.191728	3.640885	4.300158	3.52E-05	0.000161	1.311267
CHAC1	0.191687	4.529595	2.603738	0.010397	0.026943	-4.00438
LINC0046	0.191584	2.597415	2.141234	0.034297	0.074723	-5.05563
SLC16A4	0.191486	1.902901	2.686036	0.008265	0.022108	-3.79804
LOC10192	0.191255	3.876806	2.075013	0.040143	0.085182	-5.19072
GPR12	0.191252	3.434001	3.000131	0.003288	0.009871	-2.95894
TULP1	0.191136	3.687413	2.292006	0.023665	0.054648	-4.73347
SYT8	0.191126	3.439325	2.783494	0.006258	0.017349	-3.54636
LINC0110	0.190858	2.653038	2.422703	0.016915	0.041125	-4.43802
FAM71E1	0.190838	3.19725	2.36239	0.019782	0.046983	-4.57621
PIN1P1	0.190771	4.521744	3.251644	0.001493	0.004881	-2.22987
NR2E3	0.190612	3.118999	2.822976	0.00558	0.015692	-3.44216
LOC10013	0.190544	4.116615	2.29665	0.02339	0.054091	-4.72323
LOC10192	0.190485	2.293667	3.442387	0.000797	0.002772	-1.64437
LINC01121	0.19047	4.90416	3.370201	0.001014	0.003443	-1.86919
LOC10192	0.190428	2.41895	2.69431	0.008075	0.021649	-3.77698
DCST1	0.190267	4.338694	2.385349	0.018643	0.044712	-4.52398

CCDC78	0.190256	2.924709	2.703893	0.007859	0.021153	-3.75252
TRAM2-AS	0.190254	3.95476	2.952146	0.003803	0.01123	-3.09233
CEACAM5	0.190139	4.010702	3.90317	0.000158	0.000635	-0.11966
SLC9A3	0.190098	3.007204	2.733637	0.007222	0.019658	-3.6761
AVP	0.190034	3.876084	2.531702	0.012656	0.032026	-4.18029
LINC01341	0.189962	4.508416	2.49581	0.013938	0.034794	-4.26629
MIR7-3HG	0.189893	2.721845	3.828618	0.000207	0.000813	-0.37657
LAD1	0.189828	4.388742	2.88012	0.004717	0.013573	-3.28907
PGM5-AS1	0.189821	4.371542	2.177602	0.031411	0.06944	-4.97977
FAM71E2	0.189815	3.384557	3.575491	0.000506	0.001835	-1.21965
VN1R4	0.189814	2.40179	2.749627	0.006899	0.018872	-3.63472
ATPAF2	0.18981	4.156496	2.738879	0.007114	0.019394	-3.66256
NLRP10	0.189723	2.932636	2.977654	0.003521	0.010495	-3.02165
LOC10192	0.189686	2.184251	3.648107	0.000394	0.001462	-0.98248
C17orf89	0.189636	5.266614	2.245366	0.026593	0.060381	-4.83528
LOC10012	0.189628	4.238263	3.164957	0.00197	0.006269	-2.48676
ZGLP1	0.189582	4.881775	2.635617	0.009518	0.024967	-3.92513
ABCB11	0.189557	2.859814	3.652613	0.000387	0.001441	-0.96764
LINC00520	0.189542	6.436885	2.368742	0.019461	0.046379	-4.56181
LOC10192	0.189534	2.612972	2.804913	0.005881	0.016416	-3.48999
NAPA-AS1	0.189445	3.895164	2.680806	0.008388	0.022384	-3.81132
LOC10192	0.189225	3.847784	3.02997	0.003002	0.009123	-2.87505
PLAC1	0.189181	3.251987	2.885241	0.004646	0.013396	-3.27522
CCL21	0.189034	4.082298	2.366589	0.019569	0.046594	-4.5667
REEP4	0.188985	7.02938	2.843836	0.005249	0.014885	-3.38658
LOC10050	0.188926	3.557156	2.207709	0.029184	0.065378	-4.91608
KLLN	0.188732	3.537871	2.547097	0.012139	0.030848	-4.14307
ABCA3	0.188646	4.925779	2.133491	0.03494	0.07588	-5.07163
HOOK2	0.188633	4.915602	2.807814	0.005832	0.0163	-3.48232
DDX4	0.188596	2.020549	3.288117	0.001327	0.004394	-2.12006
OR10A4	0.188496	3.645959	2.651159	0.009115	0.024029	-3.88618
IFITM10	0.188495	3.901773	2.880928	0.004705	0.013549	-3.28689
STOML1	0.188479	5.000123	3.933386	0.000141	0.000574	-0.01445
FAM3A	0.188471	5.802633	3.002069	0.003269	0.009824	-2.95351
KRT19	0.188443	3.228411	2.712169	0.007677	0.020739	-3.73133
KRTAP4-1	0.188416	2.867491	2.148219	0.033725	0.073751	-5.04115
TMPRSS11	0.188397	4.679379	2.366136	0.019592	0.046632	-4.56772
KLK15	0.188395	3.663824	3.106065	0.002371	0.007394	-2.65793
ARHGAP20	0.188359	3.532469	2.381501	0.01883	0.045096	-4.53277
ABI1	0.188342	6.329345	1.998292	0.047968	0.098957	-5.34229
DNAAF1	0.188163	4.256918	2.505579	0.013578	0.034028	-4.24299
XKRX	0.188131	3.539228	2.578593	0.01114	0.028641	-4.06629
VPS9D1-A	0.187999	5.352001	2.773671	0.006438	0.017781	-3.57208
KCNK2	0.187948	2.601678	2.808272	0.005824	0.016283	-3.48111
LOC10192	0.187946	5.808171	2.725514	0.007391	0.020057	-3.69704
CHRNG	0.18792	4.404387	2.387775	0.018527	0.044473	-4.51844
COX14	0.187881	6.492887	1.992244	0.048636	0.10007	-5.35401
SMTNL1	0.187837	5.038944	2.345649	0.020651	0.048681	-4.61401
LRRC59	0.187696	3.873595	4.087753	7.95E-05	0.000339	0.532712
IL12A-AS1	0.187673	2.94558	2.585452	0.010933	0.028167	-4.04946
LCN12	0.187665	3.765658	2.655497	0.009005	0.023782	-3.87528
YJEFN3	0.187548	4.247767	2.076713	0.039983	0.084911	-5.1873
ELF3	0.18746	3.613355	3.223709	0.001634	0.005296	-2.31329
C17orf64	0.187394	2.94425	2.213736	0.028755	0.064545	-4.90323
ATP5S	0.187364	4.084045	2.910491	0.00431	0.012551	-3.20663
KCNK13	0.187363	2.736225	2.36243	0.01978	0.046983	-4.57613
PCDH12	0.187225	5.011337	2.728161	0.007335	0.019925	-3.69023
KRTAP7-1	0.187195	2.969983	2.319398	0.022081	0.051572	-4.67279

ZNF613	0.187164	2.189972	2.584082	0.010974	0.028263	-4.05282
MYCT1	0.186964	2.818953	3.154048	0.002039	0.006469	-2.51867
LOC10192	0.186911	2.764785	2.49788	0.013861	0.034639	-4.26136
C10orf113	0.186903	3.349962	2.297114	0.023362	0.054046	-4.72221
NEURL3	0.186901	5.314949	2.730508	0.007286	0.019803	-3.68417
MCM8	0.186831	3.466488	2.24805	0.026417	0.06004	-4.82948
RAB11B-A	0.186752	3.074352	3.013003	0.003162	0.009535	-2.92284
NME5	0.186743	2.202222	2.697251	0.008008	0.021489	-3.76948
LOC72804	0.186712	2.416277	2.322529	0.021906	0.05125	-4.66581
RPH3AL	0.186562	3.699279	3.251426	0.001494	0.004884	-2.23053
OR10H1	0.18648	4.503889	2.154929	0.033184	0.072762	-5.0272
MGC1081	0.186443	3.499763	2.318183	0.022149	0.051683	-4.67549
TEX22	0.186397	4.236395	2.941011	0.003933	0.011565	-3.12302
ADCY4	0.186341	5.268877	2.49388	0.01401	0.034944	-4.27088
DTNBP1	0.186298	6.127768	2.469641	0.014945	0.036969	-4.32829
KCNJ9	0.186269	3.645253	2.842217	0.005274	0.014937	-3.39091
PLEKHA4	0.186172	4.049132	2.353286	0.02025	0.047898	-4.5968
MLST8	0.186126	5.284636	2.283377	0.024184	0.055671	-4.75245
SLC5A4	0.186034	2.962686	2.694147	0.008078	0.021656	-3.7774
ARHGAP4	0.185797	8.03518	2.637645	0.009465	0.02484	-3.92006
LINC0020	0.185794	4.269204	2.757811	0.006739	0.018504	-3.61345
SYAP1	0.185733	6.4995	2.490467	0.014139	0.035217	-4.27899
SLC39A2	0.185619	4.024711	2.735404	0.007185	0.019562	-3.67154
POMK	0.185575	4.519081	2.476363	0.01468	0.03638	-4.31242
CCDC93	0.185484	5.37001	2.536301	0.012499	0.031685	-4.16919
STOX2	0.185287	2.724174	3.233033	0.001586	0.005159	-2.28551
VPS26B	0.185275	6.381761	2.171873	0.031851	0.070268	-4.9918
C1orf100	0.185227	3.683849	2.01891	0.045747	0.095119	-5.30208
LRRTM1	0.185224	3.622353	2.752982	0.006833	0.018722	-3.626
HYPK	0.185101	4.837472	2.157283	0.032996	0.072412	-5.0223
LOC10272	0.184892	3.797635	2.543403	0.012261	0.031136	-4.15202
IAH1	0.184877	5.803427	3.086944	0.002517	0.007801	-2.71292
LINC0120	0.184796	4.442935	2.910673	0.004307	0.012547	-3.20613
TRNAU1A	0.184692	4.083875	4.466148	1.83E-05	8.86E-05	1.939647
PDX1	0.184619	3.620128	2.777688	0.006364	0.017592	-3.56157
TEX14	0.184512	2.56224	2.686695	0.00825	0.022073	-3.79637
AGAP2-A	0.18449	4.232264	2.324911	0.021773	0.051	-4.66049
PRSS3	0.184472	4.389689	2.607435	0.010291	0.02672	-3.99524
BPIFA1	0.184442	4.036635	2.517316	0.013157	0.033142	-4.21489
ACOT6	0.184388	1.870769	3.153852	0.002041	0.006472	-2.51924
ADORA1	0.184385	4.140696	2.959913	0.003715	0.010997	-3.07087
LOC44079	0.184291	4.044445	2.526226	0.012844	0.032435	-4.19348
CHST9	0.184133	2.069284	4.08865	7.92E-05	0.000338	0.535937
LINC0118	0.184128	2.857097	2.30734	0.022766	0.052881	-4.69958
OBP2B	0.184091	3.976131	2.180945	0.031157	0.068997	-4.97274
LINC0002	0.184086	3.403434	2.367558	0.019521	0.046494	-4.5645
DNALI1	0.184033	2.307881	3.122586	0.002252	0.007058	-2.61018
AMN	0.184033	4.237448	3.000846	0.003281	0.009852	-2.95694
MLN	0.183985	4.447273	2.485995	0.014308	0.035557	-4.28961
C19orf26	0.1839	3.744065	3.170825	0.001934	0.006169	-2.46955
TICRR	0.183845	4.042718	2.999353	0.003296	0.009893	-2.96111
CTXN1	0.183815	4.046406	2.1188	0.036189	0.07812	-5.10184
CPA4	0.18374	4.578707	2.134164	0.034884	0.075782	-5.07024
LAMA5	0.183684	4.239436	2.11222	0.036761	0.079213	-5.1153
AIF1L	0.183647	4.170781	3.216631	0.001671	0.005402	-2.33433
NCKAP1	0.183586	2.450571	2.842436	0.005271	0.014933	-3.39032
LINC0042	0.183583	3.730087	2.2633	0.025432	0.058098	-4.79636
MAP1LC3	0.183552	3.036924	2.619694	0.009948	0.025932	-3.96482

ADD3-AS1	0.183518	2.891866	3.513967	0.000625	0.002224	-1.41759
LOC10012	0.18348	2.525827	3.082842	0.002549	0.007883	-2.72468
DYNLRB2	0.183434	2.423171	2.0622	0.041368	0.08741	-5.2164
LOC10050	0.183422	4.035944	3.312903	0.001224	0.004089	-2.04484
STRIP2	0.183389	2.218737	3.275583	0.001382	0.004554	-2.15791
PEX11G	0.183364	4.746723	2.336275	0.021152	0.049708	-4.63507
SOX9-AS1	0.183342	2.500408	3.656734	0.000382	0.001423	-0.95405
PYCARDO	0.183272	4.064603	2.723149	0.007441	0.020171	-3.70313
BTBD19	0.183204	4.268892	2.657133	0.008964	0.023698	-3.87116
AIM1L	0.183098	4.281122	2.789151	0.006157	0.0171	-3.53151
ALDH3A1	0.183073	3.811998	2.362012	0.019801	0.04702	-4.57707
C5orf27	0.18292	2.371232	2.698963	0.007969	0.0214	-3.76511
SH3GL1P2	0.182917	3.757371	2.572232	0.011336	0.029053	-4.08186
PSMB7	0.182811	6.791119	2.030453	0.044542	0.093012	-5.2794
HAS1	0.182781	3.8216	2.108198	0.037114	0.079823	-5.12352
MYO7A	0.182764	3.51289	3.469922	0.000726	0.002546	-1.55758
MUC5AC	0.182743	4.135694	2.409296	0.017518	0.042365	-4.46901
CTB-174D	0.182701	4.052956	2.118515	0.036214	0.078165	-5.10242
DIO1	0.182524	1.939048	3.128314	0.002212	0.006949	-2.59358
TLN2	0.182434	3.428715	3.000986	0.00328	0.009849	-2.95654
OR1D5	0.182413	3.008086	2.893115	0.004538	0.013134	-3.25389
MRGBP	0.182354	4.972442	2.002526	0.047504	0.098175	-5.33406
ZIC5	0.182292	3.183238	2.80245	0.005924	0.016527	-3.49649
UTP11L	0.182246	4.03084	3.200095	0.001762	0.005674	-2.38333
CLSTN2-A	0.182069	2.171278	2.561214	0.011682	0.029846	-4.10876
CCDC85B	0.182036	3.914001	3.471879	0.000721	0.002531	-1.55139
IQCF5-AS1	0.182017	3.086486	2.216339	0.028572	0.064217	-4.89767
OR51B2	0.181944	3.938264	2.851347	0.005135	0.014603	-3.36649
PACSIN3	0.181916	3.566864	2.451987	0.01566	0.038487	-4.36978
LOC15148	0.181864	2.733968	2.667182	0.008715	0.023139	-3.84582
C8orf74	0.181832	3.540172	3.297597	0.001287	0.004278	-2.09134
GDF5	0.181828	5.280902	2.232036	0.027486	0.062092	-4.86403
MPC1	0.181783	4.494629	2.875014	0.004789	0.013762	-3.30286
ALDH1L2	0.181762	2.007586	3.114803	0.002307	0.007211	-2.63271
SPINK4	0.181672	4.498404	2.049312	0.042632	0.089591	-5.24209
LOC10012	0.181656	2.315281	2.601058	0.010474	0.027111	-4.01101
LOC10028	0.181559	3.756607	2.127903	0.035411	0.076748	-5.08314
FAM26E	0.181547	4.68013	2.862233	0.004973	0.014203	-3.33728
DKFZp547	0.181526	3.531414	2.874819	0.004791	0.013766	-3.30339
NTSR2	0.181517	3.600651	2.271758	0.0249	0.057035	-4.77791
HOXA6	0.181444	3.715471	2.068378	0.040773	0.086331	-5.20404
CHRNB2	0.181438	4.672138	2.457135	0.015449	0.038023	-4.35771
CALHM3	0.181403	2.3993	2.573285	0.011303	0.028976	-4.07929
LCT	0.181359	3.182267	2.114096	0.036597	0.078901	-5.11147
GRB2	0.181349	8.147313	3.125381	0.002232	0.00701	-2.60209
GDNF	0.181229	3.709038	2.963097	0.00368	0.010911	-3.06205
RNF5	0.181222	3.983742	2.127084	0.03548	0.076857	-5.08483
GCK	0.180911	4.135238	2.562286	0.011648	0.02977	-4.10615
FZD9	0.180852	3.883894	2.215445	0.028635	0.064331	-4.89958
WDR90	0.180796	4.155023	3.094999	0.002455	0.007624	-2.68979
NSMCE1	0.180587	7.294244	2.074512	0.04019	0.085264	-5.19173
FBXW9	0.180553	4.769186	2.717106	0.00757	0.020486	-3.71866
LURAP1	0.180527	4.526935	2.312432	0.022474	0.052324	-4.68828
TIRAP	0.180343	4.440884	4.71718	6.58E-06	3.47E-05	2.921746
LRRC32	0.180329	4.692967	2.143085	0.034145	0.074453	-5.0518
LOC28584	0.180203	3.815291	3.603949	0.000459	0.001681	-1.12716
ABCA2	0.180157	4.432399	2.957584	0.003742	0.011063	-3.07731
MTL5	0.180078	3.389245	2.998826	0.003302	0.009906	-2.96259

NAV2-IT1	0.18006	2.725229	2.701714	0.007907	0.021262	-3.75809
TRMT10A	0.180058	2.531443	2.791039	0.006123	0.017016	-3.52654
C1orf86	0.18002	4.02201	4.956184	2.41E-06	1.37E-05	3.890615
LDLRAD4-	0.179934	3.226294	2.528835	0.012754	0.032235	-4.1872
PCDHGA3	0.179823	3.08908	2.576379	0.011208	0.028773	-4.07171
ZNF366	0.17973	2.620405	2.43375	0.016431	0.040126	-4.41237
LINGO3	0.179723	7.012875	2.812288	0.005757	0.016113	-3.47049
LOC10192	0.179693	4.415418	2.893626	0.004531	0.013119	-3.2525
PDHA2	0.179683	3.427135	2.072815	0.040351	0.085564	-5.19514
SPATA3	0.179648	4.627148	2.304704	0.022919	0.053176	-4.70542
ARSI	0.179577	3.916392	2.087122	0.039014	0.083244	-5.16631
GMPR2	0.179514	7.14975	1.983204	0.04965	0.101776	-5.37147
LOC10192	0.179451	3.8348	2.904977	0.004381	0.012735	-3.22165
HECTD3	0.179405	6.339151	2.069476	0.040669	0.086145	-5.20184
HOXD10	0.179334	2.616056	3.361962	0.001042	0.003528	-1.8946
RFX2	0.179252	4.734393	2.717285	0.007566	0.020478	-3.7182
KRT78	0.179177	3.890021	2.846659	0.005206	0.014779	-3.37904
PRICKLE3	0.179167	3.912705	2.691142	0.008147	0.021829	-3.78505
SLC27A5	0.1791	4.364507	2.773096	0.006449	0.017808	-3.57359
ZBTB12	0.178939	3.709117	2.505624	0.013576	0.034028	-4.24288
FADS2	0.178912	4.487109	2.571836	0.011348	0.02908	-4.08283
MAML3	0.178895	4.701823	2.614308	0.010098	0.026274	-3.9782
ATP6V1B1	0.178882	3.212448	3.067303	0.002675	0.00824	-2.7691
ARHGDIG	0.178857	4.145658	2.325426	0.021745	0.050944	-4.65935
PRKG2	0.178821	3.645455	2.667095	0.008717	0.023139	-3.84604
GALR2	0.178795	3.030352	2.14378	0.034088	0.074345	-5.05036
ASB12	0.178749	4.014034	1.992058	0.048657	0.100083	-5.35437
IMPAD1	0.178683	4.170703	2.691895	0.00813	0.021786	-3.78313
BSPRY	0.178565	3.525	2.70349	0.007868	0.021166	-3.75355
A1BG	0.178472	4.050062	2.739701	0.007098	0.019359	-3.66043
EIF4E2	0.178386	5.939188	2.746809	0.006955	0.019007	-3.64202
LOC10192	0.178379	2.003038	2.8616	0.004982	0.014226	-3.33898
LOC10192	0.178325	3.416669	3.389414	0.000951	0.003251	-1.80974
FCGR2C	0.178114	8.058561	2.552764	0.011954	0.030426	-4.12932
ZFPL1	0.178103	4.587705	3.041683	0.002895	0.008843	-2.84193
ST7L	0.178077	3.395075	3.778056	0.000248	0.000962	-0.54861
TMEM72-/	0.178066	4.487856	2.648357	0.009187	0.024183	-3.89322
SQLE	0.178007	3.769573	2.316039	0.02227	0.051924	-4.68026
TMEM213	0.178001	3.15221	3.329363	0.00116	0.003893	-1.99464
FOXI1	0.177987	2.749776	1.994901	0.048341	0.099513	-5.34886
IGFN1	0.177848	3.4593	3.055371	0.002776	0.008516	-2.80309
PRM3	0.177809	2.944288	2.304478	0.022932	0.0532	-4.70592
C9orf131	0.177756	4.109745	2.107062	0.037215	0.079981	-5.12583
ATP5B	0.177718	9.444289	2.18904	0.030548	0.067877	-4.95567
MIR10A	0.177691	3.150659	1.998958	0.047894	0.098832	-5.341
MYBPC3	0.177671	5.989465	2.322784	0.021891	0.051234	-4.66524
LINC00937	0.17765	4.512687	2.74662	0.006958	0.019013	-3.64251
FGF17	0.177641	4.487217	2.161611	0.032653	0.071797	-5.01327
POP4	0.177625	5.956951	3.506125	0.000642	0.002279	-1.44262
IFNA1	0.177602	1.769209	3.365275	0.00103	0.003496	-1.88439
GPRC5B	0.177532	3.121824	3.65684	0.000382	0.001423	-0.9537
FAM183CF	0.177529	2.668566	3.180074	0.001878	0.00601	-2.44238
ARHGEF17	0.177478	5.542306	2.321728	0.02195	0.051313	-4.66759
ZP2	0.17741	4.109074	2.503473	0.013655	0.034195	-4.24802
SOCS2-AS	0.177398	2.075037	3.266478	0.001423	0.004675	-2.18533
NAT16	0.177326	4.201898	3.030499	0.002997	0.009111	-2.87356
LOC33896	0.1773	4.581845	2.53324	0.012603	0.031917	-4.17658
LOC10272	0.177197	4.083723	2.479125	0.014573	0.036144	-4.30589

COL19A1	0.177121	3.697628	2.522205	0.012984	0.032748	-4.20315
KRT33B	0.177119	3.600056	2.083467	0.039352	0.083835	-5.17369
TMEM79	0.176967	4.905169	2.498209	0.013849	0.034613	-4.26057
STRA6	0.176939	3.331483	3.341018	0.001116	0.00376	-1.95897
NSL1	0.176879	4.868731	4.002362	0.00011	0.000453	0.228057
LOC10192	0.176783	2.807396	2.676385	0.008493	0.022628	-3.82253
ITGB4	0.176726	3.69578	3.178189	0.001889	0.006041	-2.44792
HRASLS2	0.176576	3.764328	2.864528	0.004939	0.014129	-3.33111
LOC10013	0.176467	2.659798	2.79991	0.005967	0.016633	-3.50319
WNT5B	0.176437	2.894935	2.85932	0.005016	0.014312	-3.3451
CYP2A7P1	0.176403	2.963652	3.123056	0.002248	0.00705	-2.60882
LOC40054	0.176382	3.743814	2.231987	0.02749	0.062093	-4.86413
HIST1H2BI	0.176277	2.064953	2.880046	0.004718	0.013573	-3.28927
MIA	0.176259	5.335332	2.215172	0.028654	0.064367	-4.90017
CCL7	0.176242	2.569331	2.627532	0.009735	0.025455	-3.94531
DBN1	0.176228	5.01033	2.030516	0.044536	0.093008	-5.27927
LINC01342	0.176218	4.485035	2.76522	0.006597	0.018155	-3.59415
ASPG	0.176065	3.955613	2.582555	0.01102	0.028364	-4.05657
MYRF	0.176032	3.375385	2.993769	0.003353	0.010039	-2.97673
TMEM44-/	0.176023	4.089631	2.236171	0.027207	0.061555	-4.85513
LOC10192	0.17601	2.00135	3.067803	0.002671	0.00823	-2.76768
LOC10012	0.175883	5.595557	2.388104	0.018511	0.044446	-4.51768
C17orf78	0.175832	2.291926	2.424384	0.01684	0.040993	-4.43412
C14orf180	0.175773	4.403603	2.5995	0.010519	0.027204	-4.01486
LINC00857	0.175771	3.034825	2.298507	0.02328	0.053881	-4.71913
LYG1	0.175735	4.919327	2.918611	0.004206	0.01229	-3.18446
NTS	0.175734	2.054588	2.62696	0.00975	0.025489	-3.94674
LINC0115(	0.175619	4.028439	2.205617	0.029334	0.065672	-4.92053
MNDA	0.175558	12.23487	2.492346	0.014068	0.035066	-4.27453
KPNA1	0.17552	6.129988	2.517091	0.013165	0.033154	-4.21543
WISP2	0.175494	3.465451	2.391351	0.018355	0.044119	-4.51025
ELN	0.175435	4.002742	2.844033	0.005246	0.014879	-3.38606
MAGEA8	0.175389	2.926557	2.332385	0.021363	0.050146	-4.64379
GRIN2D	0.175285	3.129885	2.506538	0.013543	0.033965	-4.2407
CPE	0.175255	2.161347	2.916337	0.004235	0.012372	-3.19067
CCDC168	0.175242	2.313265	4.305561	3.44E-05	0.000159	1.331449
ADM2	0.174992	5.545823	2.01114	0.046573	0.096508	-5.31728
RNPEPL1	0.174963	6.740639	2.385169	0.018652	0.044727	-4.52439
SNHG18	0.174947	3.630026	2.102177	0.037649	0.080777	-5.13578
PRDX3	0.174934	6.470851	2.233012	0.02742	0.061976	-4.86193
GTPBP2	0.174876	5.213619	2.826026	0.00553	0.01557	-3.43405
OPTC	0.174685	3.139614	1.989286	0.048966	0.100617	-5.35973
SGSM3	0.174676	5.210412	3.028173	0.003018	0.009166	-2.88013
RAE1	0.174646	5.573925	2.921696	0.004168	0.012195	-3.17602
MFAP4	0.174625	3.058353	2.592026	0.010737	0.027692	-4.03329
TEX19	0.17452	2.575715	2.77105	0.006487	0.017899	-3.57893
LOC64626	0.174492	2.382793	4.00023	0.00011	0.000456	0.220516
DOT1L	0.174461	4.265217	3.303489	0.001262	0.004207	-2.07347
AVPR2	0.174418	4.514844	2.282292	0.02425	0.055809	-4.75484
GPSM3	0.174399	9.569002	2.014041	0.046263	0.095987	-5.31161
GJC2	0.174135	5.129314	2.115474	0.036477	0.078676	-5.10865
CRYGA	0.174037	2.904863	2.465191	0.015123	0.03734	-4.33877
PDLIM4	0.174023	3.715538	3.39176	0.000944	0.003229	-1.80245
LOC10012	0.173939	2.688657	3.223966	0.001632	0.005293	-2.31253
FGFR3	0.173924	2.442143	3.570149	0.000516	0.001866	-1.23695
BHLHE23	0.173869	4.305975	2.822304	0.005591	0.015719	-3.44394
TRIM7	0.17382	3.720452	2.978607	0.003511	0.010468	-3.019
LOC10050	0.173819	2.756938	2.543768	0.012249	0.031109	-4.15114

TAAR3	0.173811	2.342093	2.118446	0.03622	0.07817	-5.10256
HPYR1	0.173741	2.854848	2.892194	0.004551	0.013165	-3.25639
FDX1L	0.173547	4.596296	2.464025	0.015169	0.037438	-4.34152
HIST1H2A	0.173497	3.426225	2.411505	0.017417	0.042162	-4.46392
COL4A2	0.173436	2.485474	3.260196	0.001453	0.004761	-2.20421
CDH7	0.173334	2.547053	3.788794	0.000239	0.000929	-0.51222
SLMO1	0.173322	3.292458	2.37164	0.019316	0.046071	-4.55523
C20orf144	0.173292	3.086925	2.169819	0.03201	0.070551	-4.9961
PDE3A	0.17326	3.100126	3.755412	0.000269	0.001036	-0.62507
GRIN3B	0.173076	4.134877	2.169189	0.032059	0.070651	-4.99742
TIMM17A	0.173072	4.16017	2.425271	0.016801	0.040907	-4.43207
AWAT1	0.17299	2.867073	2.319101	0.022097	0.051597	-4.67345
UROC1	0.172977	2.498291	2.366476	0.019575	0.046602	-4.56695
MECOM	0.172937	1.915759	4.045473	9.32E-05	0.000391	0.381259
FAM131C	0.172927	4.350173	2.258698	0.025726	0.058684	-4.80638
NEURL1	0.172848	4.420191	2.951691	0.003809	0.011242	-3.09359
PENK	0.172816	3.40864	2.60917	0.010242	0.026612	-3.99094
LOC10105	0.172745	2.398786	2.22255	0.028138	0.063381	-4.88439
GPR137	0.172725	4.685691	2.567144	0.011495	0.029422	-4.0943
SIGLEC11	0.172724	3.325704	2.54872	0.012086	0.030728	-4.13913
PROKR2	0.172723	3.995884	2.341643	0.020863	0.049126	-4.62302
EMILIN1	0.1727	3.137397	2.004513	0.047288	0.097817	-5.3302
LOC40332	0.172685	3.505647	2.050571	0.042507	0.089356	-5.23958
LOC64676	0.172669	3.87568	2.645225	0.009267	0.024383	-3.90108
AGBL5	0.172644	3.755962	2.488753	0.014204	0.03534	-4.28306
GSC2	0.172622	2.034613	2.554683	0.011891	0.030294	-4.12465
NT5C1A	0.172577	4.562209	2.470248	0.014921	0.036914	-4.32686
CLRN1-AS	0.172565	2.404484	2.424268	0.016845	0.041	-4.43439
GCNT2	0.17252	3.67363	2.013342	0.046338	0.096104	-5.31298
TTTY10	0.172458	2.344796	2.030204	0.044568	0.093056	-5.27989
COMTD1	0.172421	5.278611	2.36309	0.019746	0.046917	-4.57463
ZCCHC13	0.172419	2.237145	2.73072	0.007282	0.019793	-3.68363
NIPA2	0.172335	6.889342	2.576366	0.011208	0.028773	-4.07174
OR2S2	0.172266	5.12685	2.153184	0.033324	0.073015	-5.03083
SPG21	0.17224	7.609919	2.94369	0.003901	0.01149	-3.11565
MRGPRX3	0.172198	2.784379	2.201085	0.029662	0.066246	-4.93016
AMOTL1	0.17216	3.728015	2.654561	0.009029	0.023841	-3.87763
C5orf66-A	0.172084	2.281597	2.673392	0.008565	0.022796	-3.83011
NPAS1	0.172001	4.114942	2.10607	0.037302	0.080143	-5.12786
KAZALD1	0.171954	4.433297	2.76833	0.006538	0.018017	-3.58604
HSD11B1	0.171952	3.845846	2.273698	0.024779	0.056797	-4.77367
OR51M1	0.171934	3.406705	2.479615	0.014554	0.036106	-4.30473
LRP12	0.171931	2.139162	3.336954	0.001131	0.003804	-1.97142
PLXNB1	0.171896	4.814572	2.092914	0.038484	0.082318	-5.15459
PRKAR1B	0.171794	5.207514	2.000577	0.047717	0.098546	-5.33785
LOC10050	0.17167	3.057335	2.352581	0.020287	0.047968	-4.59839
SLC22A24	0.171557	3.170799	2.464076	0.015167	0.037437	-4.3414
PRR34-AS	0.171497	7.004397	2.010738	0.046616	0.096584	-5.31806
SLC7A2	0.171463	2.915452	3.080546	0.002567	0.007933	-2.73125
GXYLT2	0.171459	2.437115	2.629542	0.00968	0.025346	-3.9403
LOC10012	0.171436	2.738919	2.504501	0.013617	0.034109	-4.24557
CXCL14	0.171321	2.823275	3.214463	0.001683	0.005437	-2.34077
CAPN1	0.171297	5.92621	2.500733	0.013756	0.034414	-4.25456
SNRPB2	0.171185	5.205381	2.162704	0.032567	0.071638	-5.01099
SMIM10	0.171121	2.081095	2.423405	0.016883	0.041073	-4.43639
NDST3	0.171104	2.017373	2.93589	0.003994	0.011734	-3.1371
LOC10050	0.171062	3.505896	3.016837	0.003125	0.009439	-2.91206
NELL1	0.171018	2.681684	3.106593	0.002367	0.007384	-2.65641

NFKBIL1	0.170975	4.879671	2.183926	0.030932	0.068572	-4.96646
TAS1R1	0.170946	3.267605	3.10492	0.00238	0.007415	-2.66123
POM121L9	0.170912	2.513489	2.818743	0.005649	0.015854	-3.45339
ZBED5-AS	0.170905	5.87548	2.184526	0.030886	0.068487	-4.96519
ALMS1P	0.170813	3.001665	2.820038	0.005628	0.015805	-3.44996
AA06	0.170781	3.522375	2.135037	0.034811	0.075656	-5.06844
SDS	0.170643	4.284309	2.667927	0.008697	0.023109	-3.84393
LOC10013	0.17064	3.610892	2.785938	0.006214	0.017241	-3.53995
TG	0.17062	4.09404	2.044403	0.043123	0.090444	-5.25183
LOC15391	0.170509	4.177684	2.393069	0.018274	0.043952	-4.50632
COL26A1	0.170497	4.926347	2.653029	0.009068	0.023921	-3.88148
HIST1H2BI	0.170356	3.287203	2.379197	0.018943	0.045323	-4.53802
WDR62	0.170328	3.616994	2.342199	0.020834	0.049068	-4.62177
LMTK2	0.170072	4.111564	2.92796	0.00409	0.01199	-3.15887
GUSBP11	0.170065	5.9839	2.214101	0.028729	0.064508	-4.90245
MSX1	0.170062	3.116731	2.976738	0.003531	0.010521	-3.0242
LOC15774	0.170032	2.212368	2.696937	0.008015	0.021504	-3.77028
PLA2G5	0.169987	3.363812	3.025612	0.003042	0.009223	-2.88735
PROM2	0.169915	4.081263	3.366093	0.001027	0.003488	-1.88187
SLC23A3	0.169885	3.241959	2.75648	0.006765	0.01856	-3.61691
CASC10	0.169873	3.714386	3.684953	0.000346	0.0013	-0.86068
TFAP2A-A	0.169811	4.040158	3.519986	0.000613	0.002183	-1.39835
LOC10028	0.169795	3.031324	2.065457	0.041054	0.086833	-5.20989
ERVH-6	0.169657	4.318843	3.184968	0.001849	0.00593	-2.42797
ZSCAN10	0.169655	4.46555	2.927319	0.004098	0.01201	-3.16062
LMCD1	0.169461	3.812007	2.914808	0.004254	0.012409	-3.19485
LOC10192	0.169458	4.352105	2.593383	0.010697	0.027599	-4.02994
PCAT18	0.16938	2.300622	2.320041	0.022045	0.051499	-4.67136
LINC00673	0.169377	2.746446	2.535107	0.01254	0.031771	-4.17208
PLSCR2	0.169369	2.167465	3.68184	0.00035	0.001313	-0.87101
CASC6	0.169357	2.014918	2.228105	0.027755	0.062622	-4.87247
MAPK3	0.169306	7.814123	2.275776	0.02465	0.05654	-4.76912
LOC10192	0.169167	2.589568	2.439081	0.016203	0.039638	-4.39995
TEKT1	0.169125	3.693347	2.087299	0.038998	0.083235	-5.16596
THAP2	0.168962	3.494868	2.406581	0.017642	0.04262	-4.47527
PPP1R35	0.168941	6.866413	2.168746	0.032094	0.070719	-4.99835
ARHGAP1:	0.168924	1.950589	2.454681	0.015549	0.038251	-4.36347
IPPK	0.168917	4.815097	3.826618	0.000209	0.000818	-0.38341
GS1-259H	0.168873	2.64109	2.436449	0.016315	0.039881	-4.40608
HSPA12B	0.168809	3.39656	2.747544	0.00694	0.01897	-3.64012
RNF187	0.168808	4.401117	2.438346	0.016234	0.039692	-4.40166
LOC28619	0.168764	1.575894	3.184206	0.001853	0.005942	-2.43022
KRTCAP3	0.168744	3.843165	2.076706	0.039984	0.084911	-5.18732
C1QTNF4	0.168733	2.505712	2.128476	0.035362	0.076659	-5.08196
SDHB	0.168731	5.175544	2.81578	0.005698	0.015974	-3.46124
HSPB6	0.168635	3.385229	2.372015	0.019297	0.046032	-4.55437
SHB	0.168562	4.475273	2.828448	0.005491	0.015475	-3.42761
PNPLA5	0.168543	4.581478	2.296732	0.023385	0.054089	-4.72305
RUFY1	0.168542	5.515466	2.322701	0.021896	0.051234	-4.66543
HFE2	0.168435	3.400089	2.009263	0.046775	0.096873	-5.32094
TARS	0.168329	4.750788	2.284384	0.024123	0.055568	-4.75024
ISM1-AS1	0.168269	2.478216	2.527919	0.012786	0.032299	-4.18941
CHRM1	0.168266	2.243658	2.585421	0.010934	0.028167	-4.04953
MITF	0.168252	3.405275	2.576404	0.011207	0.028773	-4.07165
DDR1-AS1	0.168238	2.814667	2.777958	0.006359	0.017583	-3.56086
LRRC18	0.168047	2.975578	2.581938	0.011039	0.028405	-4.05808
NOTCH4	0.168037	3.955353	2.839398	0.005318	0.015041	-3.39844
C5orf47	0.167997	2.408011	3.007596	0.003214	0.009678	-2.93802

ROPN1B	0.167899	2.756836	2.111879	0.036791	0.079252	-5.116
C8B	0.167755	3.549488	2.481699	0.014473	0.035928	-4.29979
PKLR	0.167699	3.212732	2.311903	0.022505	0.052382	-4.68946
TAS2R38	0.167653	3.069422	2.304067	0.022956	0.053238	-4.70683
PRICKLE2	0.167441	2.529211	2.3818	0.018816	0.045074	-4.53209
IRGC	0.167293	3.391533	2.153771	0.033277	0.072942	-5.02961
SERAC1	0.167269	2.888112	2.413096	0.017345	0.042017	-4.46024
TBC1D2B	0.167208	5.341591	3.864791	0.000182	0.000721	-0.2524
ZNF821	0.167204	4.949114	3.149833	0.002067	0.006541	-2.53097
ZYG11A	0.167025	3.965935	2.086699	0.039053	0.08331	-5.16717
FER1L4	0.167025	3.294056	2.868324	0.004884	0.013997	-3.32089
KCNS3	0.166944	2.508294	2.111488	0.036825	0.079309	-5.1168
KIDINS220	0.166917	5.220443	3.398586	0.000922	0.003164	-1.78125
RNF152	0.166881	1.925419	3.200335	0.001761	0.005672	-2.38262
LY6G6E	0.166877	2.90974	2.502314	0.013698	0.034281	-4.25079
HOXB5	0.166865	4.820033	2.429024	0.016637	0.040555	-4.42335
MAFG-AS1	0.16674	5.192813	2.571621	0.011355	0.02909	-4.08336
FAM124B	0.16661	3.058862	2.221781	0.028191	0.063467	-4.88603
MARVELD3	0.16656	3.459088	2.551824	0.011984	0.030496	-4.1316
NR5A1	0.166531	3.602934	2.050913	0.042474	0.089307	-5.2389
KIAA1211	0.166478	2.265507	2.751235	0.006867	0.0188	-3.63054
RAMP1	0.166463	4.968034	2.220724	0.028265	0.063619	-4.8883
PPP1R1A	0.166396	2.605117	2.926565	0.004107	0.012034	-3.16269
IL1RAPL2	0.16638	4.093082	2.696579	0.008023	0.021522	-3.7712
CACNG3	0.166322	2.772877	2.036048	0.043968	0.092029	-5.26836
AGRP	0.166223	4.945267	2.82308	0.005578	0.015689	-3.44188
CDH16	0.166198	4.18376	2.090791	0.038677	0.082646	-5.15889
DPPA2	0.166156	2.99803	2.865166	0.00493	0.014109	-3.32939
HOMER2	0.166152	2.596623	2.489346	0.014181	0.035292	-4.28166
LOC10192	0.166054	2.404359	3.11896	0.002277	0.007129	-2.62068
KRBA2	0.165917	3.872097	2.42401	0.016857	0.041018	-4.43499
RNF122	0.165813	5.447174	2.198908	0.02982	0.066563	-4.93478
RRAGC	0.165799	6.811191	2.577157	0.011184	0.028725	-4.06981
HOXB-AS3	0.165654	2.7031	3.042718	0.002886	0.008818	-2.839
LOC10013	0.165541	3.747717	2.1905	0.03044	0.067687	-4.95258
RNF207	0.165526	4.121897	3.009763	0.003193	0.00962	-2.93194
LOC10192	0.1655	3.507537	2.068485	0.040763	0.086322	-5.20382
CPB1	0.165405	2.296307	2.09486	0.038307	0.082	-5.15065
ANTXR1	0.165368	3.440089	3.937783	0.000139	0.000565	0.000914
LOC10192	0.165316	4.017886	1.998274	0.04797	0.098957	-5.34232
ESR2	0.165265	2.925767	4.116854	7.12E-05	0.000306	0.637642
INMT	0.165258	3.155301	2.2941	0.023541	0.054391	-4.72886
OR10A3	0.165207	2.298415	3.250416	0.001499	0.004897	-2.23355
CFC1	0.165205	3.637412	2.891016	0.004567	0.013204	-3.25958
LOC10192	0.165157	2.184142	3.01244	0.003167	0.009549	-2.92442
BCAR1	0.165099	3.212757	2.614383	0.010096	0.026272	-3.97802
C11orf94	0.165053	4.115315	2.363313	0.019735	0.046907	-4.57413
TFF2	0.165049	3.816404	2.024874	0.045121	0.094057	-5.29038
MAST2	0.164999	4.560858	3.139382	0.002136	0.006735	-2.56143
KCNK16	0.164945	5.183096	2.287454	0.023938	0.055184	-4.7435
SLC5A1	0.164923	3.524163	2.77499	0.006414	0.01772	-3.56863
C9orf3	0.164852	3.671961	2.953273	0.00379	0.011199	-3.08922
SPRY1	0.164748	3.691874	2.395042	0.01818	0.04376	-4.50179
LOC10065	0.164748	3.981735	2.318599	0.022126	0.051635	-4.67457
EXOC3L2	0.164709	6.92242	2.259358	0.025683	0.058607	-4.80494
DRG1	0.164586	7.234625	1.99667	0.048146	0.099221	-5.34543
DLL3	0.164564	4.027369	3.257621	0.001465	0.004797	-2.21195
ACY1	0.164518	3.634307	2.004918	0.047244	0.097746	-5.32941

VN1R10P	0.164499	1.783896	2.723506	0.007433	0.020153	-3.70221
ZNF445	0.164419	5.545121	3.05765	0.002756	0.008459	-2.79661
MGAT5B	0.164345	3.529568	2.217314	0.028503	0.064078	-4.89559
MED8	0.16427	5.770249	2.360743	0.019866	0.047125	-4.57994
PDK3	0.164168	6.103122	2.020392	0.045591	0.094873	-5.29917
LOC10050	0.164129	4.404763	1.981809	0.049808	0.102051	-5.37416
CMTM6	0.1641	7.290082	2.130573	0.035185	0.07634	-5.07764
STAC	0.164043	5.400185	2.030554	0.044532	0.093008	-5.2792
SETD9	0.163933	3.215424	2.177535	0.031416	0.069444	-4.97991
SAMD1	0.163904	5.527236	2.573513	0.011296	0.028962	-4.07873
P2RX6	0.163775	4.305761	2.795592	0.006043	0.016818	-3.51457
BFSP2	0.163737	3.184836	2.202053	0.029592	0.06611	-4.92811
LOC10192	0.1636	3.249983	2.630057	0.009667	0.025317	-3.93902
ITIH5	0.163546	3.074969	3.775848	0.00025	0.000969	-0.55608
LCN15	0.163519	5.127794	2.300446	0.023167	0.053672	-4.71485
VPS4A	0.163497	6.985169	2.602491	0.010433	0.027022	-4.00747
LOC10193	0.163298	2.620055	2.061537	0.041432	0.0875	-5.21773
MYO18B	0.163297	2.931482	2.308811	0.022682	0.052722	-4.69632
HTR2A	0.163287	2.144093	4.070667	8.48E-05	0.000359	0.471363
SLC5A2	0.163219	5.237693	1.99043	0.048838	0.100395	-5.35752
PHF7	0.163151	3.323733	2.822243	0.005592	0.015719	-3.4441
PHKG1	0.163095	3.776339	2.363979	0.019701	0.046837	-4.57261
KIAA1644	0.163076	3.507822	3.249776	0.001502	0.004904	-2.23547
OS9	0.163059	7.960924	2.477723	0.014627	0.036266	-4.3092
SLC28A1	0.163018	3.251399	2.66275	0.008824	0.023389	-3.85701
SV2A	0.163018	4.131461	2.198499	0.02985	0.066616	-4.93565
MAPK8IP1	0.162977	3.941647	2.028466	0.044748	0.093374	-5.28331
GABBR2	0.162956	3.186757	3.807537	0.000224	0.000872	-0.44852
CH25H	0.162903	2.29076	2.704574	0.007844	0.021115	-3.75078
PROP1	0.162891	4.059943	2.501029	0.013745	0.034391	-4.25385
ZP1	0.162839	3.150067	2.044521	0.043111	0.090429	-5.2516
LOC10192	0.162761	2.781545	2.210234	0.029004	0.065017	-4.9107
LOC10050	0.162747	2.991308	2.231479	0.027524	0.06215	-4.86522
MLIP	0.162739	3.295962	1.983732	0.049591	0.101696	-5.37045
CCDC150	0.162674	2.776272	3.338594	0.001125	0.003786	-1.9664
KIRREL2	0.162659	2.864355	2.015819	0.046074	0.095683	-5.30813
CENPI	0.162645	2.214011	3.82881	0.000207	0.000812	-0.37592
SEC13	0.162579	8.265505	2.179392	0.031275	0.069221	-4.97601
JARID2-AS	0.162568	3.15752	2.348161	0.020518	0.048431	-4.60836
LOC10099	0.162462	1.784832	2.815055	0.00571	0.016002	-3.46316
PCDHGB5	0.162443	4.80239	2.31867	0.022122	0.051632	-4.67441
LINC01432	0.16232	2.67763	2.283732	0.024163	0.055634	-4.75167
DIAPH3	0.162295	2.598886	2.631622	0.009625	0.02522	-3.93511
LOC44093	0.162292	1.962305	2.567427	0.011486	0.029403	-4.09361
LOC10050	0.162287	2.851884	2.409489	0.017509	0.042354	-4.46857
SEPP1	0.162184	2.19983	2.159894	0.032789	0.072003	-5.01686
LOC72811	0.162177	2.284399	2.60064	0.010486	0.027136	-4.01204
TBL2	0.162146	4.879139	2.752661	0.006839	0.018736	-3.62684
RMND5B	0.162127	5.276198	3.105886	0.002373	0.007396	-2.65845
PCDHGA1	0.16205	2.887955	2.050993	0.042466	0.089304	-5.23874
AGAP2	0.162001	3.868458	2.648822	0.009175	0.024167	-3.89205
LINC00839	0.161961	3.628576	2.080614	0.039617	0.084301	-5.17945
HAND1	0.161869	3.845436	1.98671	0.049255	0.101089	-5.36471
APOBEC1	0.161809	3.688599	2.287176	0.023955	0.055217	-4.74411
NFATC4	0.16176	4.51846	2.155799	0.033115	0.072633	-5.02539
SFTPC	0.161759	4.676457	2.821653	0.005601	0.01574	-3.44567
AFAP1-AS	0.161642	2.852762	2.559077	0.01175	0.029975	-4.11396
MUM1L1	0.161578	1.645089	2.906469	0.004362	0.012682	-3.21759

SLC10A2	0.16154	2.859363	2.48465	0.01436	0.035672	-4.2928
GHRHR	0.161434	3.347681	2.91574	0.004242	0.012384	-3.1923
AQP6	0.161399	4.612494	2.751398	0.006864	0.018797	-3.63012
PAQR9	0.161277	2.100835	2.429729	0.016606	0.040494	-4.42172
NDOR1	0.161276	5.08147	2.938466	0.003963	0.011648	-3.13002
IGFL2	0.161258	2.61046	2.174628	0.031639	0.069868	-4.98602
BTBD7	0.161217	5.292529	2.937709	0.003972	0.011671	-3.1321
FKBP3	0.161163	5.605874	2.09462	0.038329	0.08203	-5.15113
SCN9A	0.161162	1.773183	4.203414	5.12E-05	0.000227	0.953038
RAB3A	0.161095	4.536136	2.095613	0.038239	0.081871	-5.14912
ABCD1	0.160988	5.540809	2.307691	0.022746	0.052848	-4.6988
ZNF100	0.160867	3.144385	2.084981	0.039212	0.083578	-5.17064
ITFG3	0.160761	5.562374	2.756467	0.006765	0.01856	-3.61695
FAM149B1	0.160713	3.593395	2.674992	0.008526	0.022696	-3.82606
C15orf26	0.160697	1.96203	2.232515	0.027454	0.062039	-4.863
ABCA4	0.160679	4.319769	2.421908	0.01695	0.041196	-4.43986
ZNF775	0.160631	3.990869	3.183568	0.001857	0.00595	-2.4321
C2orf61	0.160628	3.358282	2.186604	0.03073	0.068222	-4.96081
LOC10012	0.160625	1.834798	2.821957	0.005596	0.01573	-3.44486
PLP1	0.160605	1.778344	2.951899	0.003806	0.011237	-3.09302
ASMT	0.160592	2.922172	2.841335	0.005288	0.01497	-3.39326
ZNF576	0.160347	5.222044	3.80471	0.000226	0.00088	-0.45814
SLC27A4	0.160333	4.677719	2.057447	0.041831	0.088196	-5.22589
PTCHD2	0.160318	5.699294	2.204607	0.029407	0.065791	-4.92268
IL13	0.16026	3.701374	2.175317	0.031586	0.069774	-4.98457
ADCY5	0.160109	3.908128	2.489997	0.014156	0.035248	-4.28011
LOC10192	0.160099	3.084492	2.394802	0.018192	0.043782	-4.50234
LINC00427	0.160072	3.031377	2.504182	0.013629	0.034135	-4.24633
LOC10099	0.160011	3.454141	2.087814	0.03895	0.083175	-5.16491
LOC10192	0.159968	3.551978	2.309097	0.022665	0.052696	-4.69569
DLX2	0.15991	3.657292	2.566096	0.011527	0.029492	-4.09686
PCDH18	0.159818	3.702424	2.654165	0.009039	0.023865	-3.87863
CTAGE5	0.159754	4.621292	2.247045	0.026483	0.06017	-4.83165
CNTD2	0.15959	5.886947	2.046257	0.042937	0.090108	-5.24815
ACOT12	0.159572	1.684309	2.467872	0.015015	0.037107	-4.33246
MST1	0.159571	5.368636	2.337602	0.02108	0.049557	-4.63209
GOLT1A	0.159494	3.342811	2.334073	0.021271	0.049965	-4.64001
LOC10192	0.159465	2.336418	2.607872	0.010279	0.026693	-3.99416
ARHGEF12	0.159442	2.768596	2.438936	0.016209	0.039649	-4.40029
AMPD1	0.159385	2.384733	2.135068	0.034808	0.075656	-5.06838
LOC10192	0.159364	2.160717	2.531782	0.012653	0.032023	-4.1801
TRIP10	0.159279	4.47691	1.999311	0.047856	0.098772	-5.34031
POLD3	0.159269	5.05056	2.859506	0.005013	0.014308	-3.34461
EGFLAM	0.159256	3.93322	1.997195	0.048088	0.099132	-5.34442
SEMA6C	0.159159	5.342954	2.116283	0.036407	0.078532	-5.10699
LOC10192	0.15911	2.644103	2.281656	0.024289	0.055881	-4.75623
ZNRF4	0.159086	4.865664	2.280638	0.024351	0.055986	-4.75847
ACTL9	0.159073	2.423684	2.066547	0.040949	0.086658	-5.20771
GRTP1	0.159006	2.649837	2.658056	0.008941	0.023649	-3.86883
KRTAP4-8	0.15898	2.512017	2.416442	0.017194	0.041685	-4.45251
CNPY1	0.158963	1.627722	3.180708	0.001874	0.006001	-2.44051
PPME1	0.158923	4.795729	2.566562	0.011513	0.029462	-4.09572
NR1H2	0.158901	6.142581	2.123315	0.035801	0.077422	-5.09257
RTKN	0.158787	3.555136	2.424047	0.016855	0.041018	-4.43491
TBX22	0.158762	1.59609	3.012743	0.003164	0.009541	-2.92357
DKFZP434	0.158736	2.396642	2.360403	0.019883	0.047161	-4.58072
CHRNE	0.158622	4.068989	2.205562	0.029338	0.065673	-4.92065
PRRX2-AS	0.158607	5.18643	2.225658	0.027923	0.062974	-4.87773

TPSB2	0.158589	4.071121	2.092612	0.038511	0.08236	-5.1552
GPHB5	0.158505	3.943464	2.288522	0.023874	0.055073	-4.74114
NTPCR	0.158419	4.869007	2.126563	0.035524	0.076937	-5.0859
OPN1SW	0.158315	2.311615	3.082712	0.00255	0.007884	-2.72505
LOC28493	0.158308	4.42241	2.157082	0.033012	0.072432	-5.02272
MSL1	0.158222	7.84314	2.354845	0.020169	0.047739	-4.59328
SPINK6	0.158122	3.391977	2.412573	0.017369	0.042064	-4.46145
HSPB7	0.15811	3.030772	2.473594	0.014789	0.036618	-4.31896
FLJ37453	0.158106	4.359094	3.094491	0.002458	0.007632	-2.69125
LOC10192	0.158094	2.917653	3.265883	0.001426	0.004683	-2.18712
LINC00527	0.158091	3.834878	2.560535	0.011704	0.029897	-4.11041
CD109	0.158061	2.40737	3.432182	0.000824	0.002855	-1.67639
SPATA19	0.157948	3.297089	2.961722	0.003695	0.010947	-3.06586
GLI4	0.157892	3.188773	3.044403	0.002871	0.008782	-2.83422
LOC10193	0.157848	4.853497	2.298554	0.023278	0.053881	-4.71903
LOC10192	0.157838	3.123705	2.624188	0.009825	0.025663	-3.95364
MED11	0.15766	6.07043	2.21378	0.028752	0.064545	-4.90314
LIMD1-AS	0.157653	5.409862	2.5857	0.010925	0.028152	-4.04885
KLHDC10	0.15759	5.590196	3.013625	0.003156	0.00952	-2.92109
CENPO	0.15755	3.335581	2.021642	0.045459	0.094647	-5.29672
SNORA65	0.157397	3.061428	2.381176	0.018846	0.045129	-4.53351
CHAD	0.157347	4.154114	2.196643	0.029986	0.066846	-4.93958
LOC10192	0.157312	3.555809	2.841994	0.005278	0.014945	-3.39151
LOC10192	0.157311	2.393509	2.263148	0.025441	0.058114	-4.79669
PLEKHG2	0.157239	4.603545	3.00875	0.003203	0.009647	-2.93478
PNLIP	0.15716	2.171659	2.45856	0.015391	0.03791	-4.35436
CCL13	0.157149	3.753615	2.439436	0.016187	0.039611	-4.39912
TMEM198	0.15711	4.786168	2.495345	0.013955	0.034828	-4.26739
LOC10192	0.157103	4.570518	2.326161	0.021704	0.05086	-4.6577
ZACN	0.157058	4.818538	2.368887	0.019454	0.046367	-4.56148
BCORL1	0.157005	4.348627	3.511216	0.000631	0.002243	-1.42638
GAPDHS	0.156926	3.389447	2.372302	0.019283	0.046009	-4.55372
TMEM87A	0.156911	5.054545	2.707217	0.007785	0.020983	-3.74402
CECR9	0.156714	2.921501	2.816006	0.005694	0.015966	-3.46064
LOC64690	0.156668	1.78998	2.781259	0.006299	0.017438	-3.55222
HYAL4	0.156618	3.622516	2.212739	0.028826	0.064668	-4.90536
CCNF	0.156605	3.872415	2.08295	0.0394	0.083927	-5.17474
TMEM95	0.156542	5.634303	2.349622	0.020442	0.048289	-4.60506
MMP15	0.156458	4.207715	2.339916	0.020956	0.049308	-4.6269
SBSN	0.156377	3.668217	2.235558	0.027248	0.061635	-4.85645
LOC10192	0.156358	2.524276	2.292945	0.023609	0.054531	-4.7314
SIRT2	0.156335	6.013836	2.742605	0.007039	0.019217	-3.65291
XG	0.156312	2.474609	2.645443	0.009262	0.024371	-3.90053
LOC49725	0.156269	1.777375	2.968667	0.003618	0.010744	-3.04661
PLA2G2F	0.156258	3.004087	2.287739	0.023921	0.055157	-4.74287
TFCP2L1	0.156232	3.975316	2.395765	0.018146	0.043688	-4.50013
HOXA11	0.156107	3.426416	2.332394	0.021362	0.050146	-4.64377
COL10A1	0.156105	2.608592	2.517212	0.01316	0.033147	-4.21514
LINC00882	0.156056	2.370701	2.250971	0.026225	0.059705	-4.82315
SAG	0.15591	4.942483	2.109393	0.037009	0.079646	-5.12108
CCDC37	0.155842	3.269574	2.505989	0.013563	0.034011	-4.24201
MYLPF	0.155794	4.658309	2.028639	0.04473	0.093346	-5.28297
LOC10192	0.155793	1.970779	3.001786	0.003272	0.009831	-2.9543
LOC10192	0.155742	3.969011	2.514508	0.013256	0.03336	-4.22163
TSSC4	0.155703	5.914909	2.606199	0.010326	0.026781	-3.9983
H1FOO	0.15563	3.141567	2.057058	0.041869	0.088249	-5.22667
LOC28605	0.15555	3.873067	2.280838	0.024339	0.055971	-4.75803
FBP2	0.155515	4.391632	1.997275	0.048079	0.099124	-5.34426

IL17RC	0.155451	4.4794	2.55768	0.011795	0.030071	-4.11737
LOC10192	0.155445	2.302852	1.982273	0.049756	0.101953	-5.37326
CPEB3	0.155416	3.386891	3.13908	0.002138	0.006741	-2.56231
OSCP1	0.155416	2.198484	2.522706	0.012967	0.032712	-4.20195
CSPG4P5	0.155357	4.056882	1.994843	0.048348	0.099517	-5.34898
AKAP3	0.155277	3.068541	2.105812	0.037325	0.080175	-5.12838
CYMP	0.155239	3.703918	2.392238	0.018313	0.044039	-4.50822
MIP	0.155149	3.38041	2.212152	0.028867	0.064754	-4.90661
P2RY1	0.155146	1.94846	2.679358	0.008422	0.022463	-3.815
BCL2L2	0.15505	4.102588	2.941836	0.003923	0.011543	-3.12075
IL5	0.155024	1.784406	2.556212	0.011842	0.03018	-4.12094
LINC00535	0.154998	3.167868	2.992709	0.003364	0.010068	-2.97969
CPNE7	0.154968	4.780448	2.076632	0.039991	0.084911	-5.18746
FGF20	0.154952	1.875681	2.414579	0.017278	0.041874	-4.45682
EIF4E	0.154925	4.562927	2.300893	0.02314	0.053624	-4.71386
SOHLH2	0.154905	2.514191	2.268547	0.0251	0.057444	-4.78492
SARDH	0.154861	3.829193	2.799033	0.005983	0.016671	-3.5055
KCNC3	0.154662	4.308684	2.959056	0.003725	0.011021	-3.07324
CRIPT	0.154558	6.485385	2.040113	0.043555	0.091258	-5.26033
KIAA1161	0.154543	3.813646	2.442558	0.016055	0.039324	-4.39183
TAS2R3	0.154505	2.941959	2.242582	0.026778	0.060739	-4.8413
LOC10192	0.154501	1.889072	2.712521	0.007669	0.020724	-3.73043
APOC3	0.154495	3.714309	2.641168	0.009373	0.024616	-3.91124
RPRML	0.154463	3.290655	2.144956	0.033991	0.074222	-5.04792
PON3	0.154419	3.191591	2.108468	0.037091	0.079797	-5.12297
FITM1	0.154397	4.541884	2.019425	0.045693	0.095026	-5.30107
AVL9	0.154367	4.216217	2.872798	0.00482	0.013831	-3.30884
TMEM132	0.154218	2.292469	2.467267	0.015039	0.037157	-4.33388
LINC01207	0.154174	1.866964	2.812755	0.005749	0.016095	-3.46925
LOC10106	0.154156	2.739641	3.335063	0.001138	0.003826	-1.97721
CABP1	0.154066	2.649357	2.456772	0.015464	0.038049	-4.35856
MAFK	0.15404	5.217669	2.220376	0.028289	0.063666	-4.88904
CARS2	0.154029	4.210023	4.026633	0.0001	0.000417	0.314157
DIRAS1	0.153933	3.279205	2.183011	0.031	0.068688	-4.96839
IQGAP3	0.153923	3.117401	3.153802	0.002041	0.006472	-2.51939
RER1	0.153868	7.329231	2.859064	0.005019	0.014319	-3.34579
LINC01339	0.153829	4.234365	2.388519	0.018491	0.044414	-4.51673
CASZ1	0.153796	3.488982	3.577114	0.000504	0.001826	-1.21439
C21orf58	0.15373	3.887389	2.652529	0.00908	0.023949	-3.88274
ABCC11	0.153628	3.339163	3.523947	0.000604	0.002158	-1.38567
SNORA78	0.153596	5.745577	2.328223	0.021591	0.050634	-4.6531
TAAR9	0.153412	2.947812	2.364807	0.019659	0.046759	-4.57074
APBB2	0.153351	2.864643	3.558863	0.000536	0.001935	-1.27342
RCOR1	0.153339	6.317364	2.556298	0.011839	0.030176	-4.12073
BSND	0.153319	4.238342	2.639961	0.009404	0.024689	-3.91427
NLRP5	0.153165	3.782064	2.271961	0.024887	0.057019	-4.77746
SEC14L2	0.153098	2.941725	2.477622	0.014631	0.036272	-4.30944
PPFIA4	0.152966	2.325795	2.323264	0.021865	0.051178	-4.66417
NPTX1	0.152827	3.723603	2.798394	0.005994	0.0167	-3.50718
PRSS2	0.152773	3.510818	2.347709	0.020542	0.048476	-4.60938
ADCY10	0.152768	2.108499	3.156664	0.002023	0.00642	-2.51102
PCP4L1	0.152737	3.181005	2.359654	0.019922	0.047235	-4.58241
KRAS	0.152694	5.598284	3.222706	0.001639	0.005311	-2.31627
LINC00452	0.152528	3.06029	2.215551	0.028627	0.064328	-4.89936
TPGS1	0.152464	4.124771	2.553832	0.011919	0.030353	-4.12672
NLRP9	0.152459	2.921908	2.663925	0.008795	0.023315	-3.85404
OR4D1	0.152362	4.321102	2.046182	0.042945	0.090108	-5.2483
VCAM1	0.15234	2.173269	2.08869	0.03887	0.083023	-5.16314

FLJ90680	0.152222	2.912332	2.18323	0.030984	0.068673	-4.96793
JMJD1C-A	0.152071	2.856074	2.259799	0.025655	0.058556	-4.80398
TXN2	0.15207	6.338298	2.79167	0.006112	0.016989	-3.52489
FOXN3-AS	0.152067	4.23974	2.548735	0.012085	0.030728	-4.1391
PDIA2	0.152005	3.640703	2.1599	0.032788	0.072003	-5.01684
C17orf50	0.151969	4.172506	1.999423	0.047843	0.098768	-5.34009
GPR3	0.151967	2.535589	2.949456	0.003834	0.011307	-3.09976
P2RX3	0.151893	2.452477	2.016724	0.045978	0.095507	-5.30636
ADORA2A	0.151821	2.417905	3.353699	0.00107	0.003615	-1.92004
PEX13	0.151742	2.776069	2.739523	0.007101	0.019366	-3.66089
LOC10192	0.151684	2.419103	2.466284	0.015079	0.03725	-4.3362
MIR194-2	0.15163	4.581553	2.889997	0.004581	0.013235	-3.26234
LAMTOR5	0.151578	3.016479	2.18134	0.031127	0.068949	-4.97191
LOC10050	0.151499	1.957084	2.195527	0.030068	0.066985	-4.94195
EVPLL	0.151352	4.260034	2.085925	0.039125	0.083436	-5.16873
SLC27A6	0.151337	1.947511	2.462263	0.01524	0.037581	-4.34566
LOC10192	0.151265	4.728087	2.046201	0.042943	0.090108	-5.24827
LSM14B	0.151204	3.917151	3.009508	0.003196	0.009626	-2.93265
LOC64409	0.151175	3.262574	2.00298	0.047455	0.098083	-5.33318
SSPO	0.151133	4.708706	2.12582	0.035588	0.077033	-5.08743
2-Mar	0.15102	3.079807	3.500601	0.000654	0.00232	-1.46023
FAM83H	0.150848	3.052437	2.177128	0.031447	0.06949	-4.98077
KRBOX1-A	0.150836	2.235079	2.179173	0.031291	0.069242	-4.97647
LOC10050	0.150781	4.59919	2.225178	0.027956	0.063021	-4.87875
AGT	0.15072	4.601133	2.116489	0.036389	0.07851	-5.10657
LMTK3	0.150718	2.762032	2.107055	0.037215	0.079981	-5.12585
UGT1A1	0.150717	2.312107	2.086541	0.039068	0.083332	-5.16749
FLJ32790	0.150711	2.384531	2.165815	0.032322	0.071162	-5.00449
ELF5	0.150655	2.944316	2.661595	0.008853	0.023456	-3.85992
SLC22A6	0.150506	3.362177	2.277804	0.024525	0.056316	-4.76468
OBSL1	0.150452	3.220248	2.842409	0.005271	0.014933	-3.3904
KLHL30-A	0.150375	4.225758	2.195062	0.030102	0.067032	-4.94293
CLDN16	0.150371	3.85482	2.124904	0.035666	0.077161	-5.08931
SSTR5	0.150286	3.683404	2.022766	0.045341	0.09444	-5.29452
GPRIN1	0.150221	3.355141	2.427205	0.016716	0.040734	-4.42758
ARMC7	0.150158	5.560514	2.843127	0.00526	0.01491	-3.38848
PVR	0.150131	4.304171	3.390875	0.000946	0.003237	-1.8052
SPDYA	0.150129	1.938115	2.407509	0.0176	0.042532	-4.47313
SEC14L3	0.149951	3.113058	3.21936	0.001657	0.005363	-2.32622
FAM163A	0.149943	2.511598	2.059419	0.041638	0.087835	-5.22196
SCAMP4	0.149879	4.481371	2.500597	0.013761	0.034422	-4.25488
BBOX1	0.149793	2.293488	2.257089	0.025829	0.058887	-4.80987
LOC10192	0.149767	1.855407	2.225329	0.027946	0.063004	-4.87843
WBSCR28	0.149719	2.24723	2.20245	0.029563	0.066061	-4.92726
DMRTA2	0.149588	2.621819	2.206853	0.029246	0.065487	-4.9179
C18orf54	0.149503	2.573843	2.805345	0.005874	0.0164	-3.48885
OR10D3	0.149482	3.836873	2.924674	0.004131	0.012097	-3.16787
PES1	0.149443	4.452057	2.398077	0.018037	0.043447	-4.49483
PSG3	0.149361	2.267613	2.191628	0.030356	0.067523	-4.9502
LMNA	0.149277	4.98216	2.049695	0.042594	0.08952	-5.24133
MFSD7	0.149265	4.613117	2.328268	0.021588	0.050634	-4.653
LINC00445	0.149241	3.062012	2.03734	0.043836	0.091782	-5.26581
CUEDC1	0.149215	5.068072	2.346888	0.020585	0.048565	-4.61122
SCG5	0.149149	2.251294	2.00583	0.047145	0.097581	-5.32763
C22orf34	0.149069	4.499233	2.886937	0.004622	0.013338	-3.27063
UBE2J2	0.149013	5.444587	3.687622	0.000343	0.001289	-0.85182
MAP10	0.14899	2.036022	2.598536	0.010547	0.027273	-4.01724
NUTM1	0.148982	4.842779	2.302407	0.023052	0.053431	-4.71051

LOC28550	0.14895	1.608503	2.400055	0.017945	0.043249	-4.49028
GPR135	0.148931	2.826907	2.540228	0.012367	0.031389	-4.1597
PCSK6	0.14892	3.377054	2.814509	0.005719	0.016023	-3.46461
RASIP1	0.148879	3.208346	2.270598	0.024972	0.057169	-4.78044
SGOL1	0.148867	2.781279	2.810352	0.005789	0.016196	-3.47561
NOTCH3	0.148863	4.70364	2.412897	0.017354	0.042033	-4.4607
LOC40004	0.148777	2.28123	2.185584	0.030807	0.068347	-4.96296
COX7B2	0.148719	2.787038	2.236416	0.02719	0.061538	-4.8546
ARNTL2	0.148557	2.742953	2.665844	0.008748	0.023202	-3.8492
OAZ3	0.148456	2.962184	2.401591	0.017873	0.043102	-4.48675
DERL2	0.148235	6.865138	2.530929	0.012682	0.032086	-4.18216
ARHGAP28	0.148218	2.181698	3.226555	0.001619	0.005254	-2.30482
ANKRD18B	0.148202	3.334814	2.028749	0.044718	0.093343	-5.28275
MNT	0.148171	5.242737	2.758812	0.006719	0.018458	-3.61085
ODF3	0.148111	3.646866	2.232914	0.027427	0.061985	-4.86214
AGPAT6	0.148046	4.691115	3.093843	0.002463	0.007645	-2.69311
PTPN5	0.148036	2.938466	2.46844	0.014993	0.037055	-4.33112
FOXF2	0.147996	2.694214	2.085422	0.039171	0.083517	-5.16975
C10orf53	0.147973	3.759102	2.366591	0.019569	0.046594	-4.56669
CCDC18	0.147838	2.755572	2.594915	0.010652	0.027506	-4.02617
C10orf62	0.147809	4.623054	2.146891	0.033833	0.07394	-5.04391
RBP4	0.147731	3.262863	2.341981	0.020845	0.04909	-4.62226
RDX	0.147685	3.394377	2.272356	0.024862	0.056975	-4.7766
AFAP1L1	0.14762	3.073675	2.522249	0.012983	0.032748	-4.20305
CCDC30	0.147569	2.488077	2.961807	0.003694	0.010945	-3.06562
SNAI2	0.147507	1.626844	3.052176	0.002803	0.008587	-2.81217
ADIG	0.147469	4.661752	2.040383	0.043528	0.091219	-5.25979
CAND1.11	0.14742	3.338588	2.458045	0.015412	0.037958	-4.35557
LOC10192	0.147415	3.28586	2.011224	0.046564	0.096505	-5.31711
KLK13	0.147414	3.844458	2.492241	0.014072	0.035072	-4.27478
GPANK1	0.147408	6.765196	2.593555	0.010692	0.02759	-4.02952
PLAC9	0.147322	4.252971	2.255142	0.025955	0.059147	-4.8141
CACNA2D	0.147099	3.039591	2.798352	0.005994	0.0167	-3.5073
HDDC3	0.147047	6.114402	2.018409	0.0458	0.09521	-5.30306
LHX4	0.147044	3.044629	2.388923	0.018471	0.044377	-4.51581
C2orf54	0.147038	4.363403	2.14118	0.034301	0.074723	-5.05574
IFT27	0.14696	4.121929	3.356354	0.001061	0.003588	-1.91187
AATK	0.14694	7.811192	2.03305	0.044275	0.092567	-5.27428
AREL1	0.146906	5.012102	2.934587	0.00401	0.011778	-3.14068
OR2H2	0.14686	2.81843	2.382384	0.018787	0.045025	-4.53075
RRN3P3	0.146837	2.831678	2.088015	0.038932	0.083147	-5.16451
TMEM254	0.146823	4.972485	2.041571	0.043408	0.090977	-5.25744
SPIRE2	0.146809	2.99252	3.853489	0.000189	0.000748	-0.29129
TAGLN3	0.146798	3.959665	1.982758	0.049701	0.101854	-5.37233
TTLL9	0.146755	3.462241	2.417048	0.017167	0.041647	-4.45111
C21orf62	0.146712	3.935048	2.344672	0.020702	0.048786	-4.61621
DIAPH2-A	0.146708	2.193682	2.622106	0.009882	0.025782	-3.95882
10-Mar	0.146697	2.984766	2.866218	0.004915	0.014072	-3.32656
APCDD1L	0.146691	3.89855	2.040116	0.043555	0.091258	-5.26032
DGCR12	0.14647	3.397456	2.457461	0.015435	0.038003	-4.35695
NMNAT2	0.146412	3.328807	3.313461	0.001222	0.004084	-2.04315
IDI2	0.146383	3.820969	2.134789	0.034832	0.075693	-5.06895
GPR156	0.146375	4.086112	2.379052	0.01895	0.045335	-4.53835
LOC10192	0.146328	2.350222	2.427105	0.01672	0.04074	-4.42781
LOC10012	0.146307	4.009934	2.222958	0.02811	0.063339	-4.88351
NPAS4	0.146304	3.549417	2.594967	0.010651	0.027506	-4.02604
SYN1	0.146223	4.668117	2.275575	0.024663	0.056562	-4.76956
MGC16025	0.146217	1.809862	2.49652	0.013912	0.03474	-4.2646

COL1A1	0.146116	3.43778	2.845357	0.005226	0.014831	-3.38252
LOC10028	0.146079	1.988356	2.211873	0.028887	0.064777	-4.90721
ZDHHC9	0.145853	4.385591	2.603431	0.010406	0.026962	-4.00514
RBMXL2	0.145747	2.572566	2.483853	0.01439	0.035735	-4.29469
RSPO3	0.145711	2.319957	2.229169	0.027682	0.062478	-4.87019
MIR670HG	0.145693	4.130917	2.047179	0.042845	0.089963	-5.24632
GRIK5	0.145594	4.149962	2.528684	0.01276	0.032241	-4.18757
ST6GALNA	0.145558	3.106393	3.13942	0.002136	0.006735	-2.56131
LOC10192	0.145535	5.38566	2.298453	0.023283	0.053882	-4.71925
NRAP	0.145473	3.123114	2.783743	0.006254	0.01734	-3.54571
HIF3A	0.14547	3.044725	2.858681	0.005025	0.01433	-3.34682
DRP2	0.14542	3.39041	2.88518	0.004647	0.013397	-3.27539
SIRT6	0.145378	5.908128	3.123457	0.002246	0.007044	-2.60766
C9	0.145378	1.671433	2.66052	0.00888	0.023511	-3.86263
NPFFR1	0.145342	4.284346	2.163339	0.032517	0.071552	-5.00966
HMGN5	0.145339	2.10841	2.310517	0.022584	0.052536	-4.69254
RGS22	0.145301	3.576285	2.041873	0.043377	0.090923	-5.25684
PGLS	0.145296	6.352167	3.055243	0.002777	0.008519	-2.80345
USP2	0.145219	3.233913	2.745539	0.00698	0.019066	-3.64531
NKPD1	0.145185	4.294096	2.074463	0.040195	0.085265	-5.19183
FNDC1	0.145154	1.911605	2.320191	0.022036	0.051497	-4.67102
LOC10272	0.145148	3.178129	2.081355	0.039548	0.084199	-5.17795
ATG7	0.145148	5.146156	2.202793	0.029538	0.066012	-4.92653
WRAP73	0.145015	5.095443	2.178471	0.031345	0.069323	-4.97794
BDNF	0.144927	2.788432	3.03129	0.002989	0.009092	-2.87133
SAMD11	0.144813	6.484678	1.988308	0.049076	0.100791	-5.36162
IL21	0.144761	3.626855	2.323686	0.021841	0.051129	-4.66323
KRT12	0.144745	1.939569	2.481781	0.01447	0.035924	-4.2996
B4GALNT3	0.144743	3.886294	2.497269	0.013884	0.034679	-4.26281
CHRD	0.144657	4.206311	2.15127	0.033478	0.073305	-5.03481
LINC00588	0.14465	1.683748	2.655497	0.009005	0.023782	-3.87528
TXNL1	0.14464	5.41399	2.322422	0.021912	0.051258	-4.66605
PABPC4L	0.1446	1.78045	2.468881	0.014975	0.03703	-4.33008
4-Sep	0.144467	3.622825	2.08209	0.03948	0.084071	-5.17647
LOC28493	0.144452	3.946402	2.014726	0.04619	0.095876	-5.31027
MAPK15	0.14444	4.275765	2.461645	0.015265	0.037625	-4.34712
GPR75	0.1444	2.239775	2.032511	0.04433	0.092663	-5.27534
ZBTB8B	0.144395	1.971527	2.376451	0.019078	0.045582	-4.54428
OR2F1	0.144351	3.335125	2.346852	0.020587	0.048565	-4.61131
LINC01116	0.144301	2.480195	2.27345	0.024794	0.056826	-4.77421
WFIKKN2	0.144277	3.78599	2.106141	0.037296	0.080138	-5.12771
TTC36	0.144185	4.965526	2.141098	0.034308	0.07473	-5.05591
C1orf127	0.144063	5.151768	2.076367	0.040016	0.084946	-5.188
PARVG	0.144053	6.770309	2.294245	0.023532	0.054377	-4.72854
LINC00900	0.144022	2.46637	2.614438	0.010094	0.026271	-3.97788
CLRN3	0.143786	1.946163	2.155285	0.033156	0.072708	-5.02646
USH1C	0.143785	3.718225	2.164982	0.032388	0.071275	-5.00623
MYO7B	0.143721	3.320638	2.703672	0.007864	0.021158	-3.75308
LINC00669	0.143709	1.731286	2.677201	0.008474	0.022585	-3.82047
LIPI	0.143648	1.777562	2.98887	0.003403	0.01017	-2.99041
C1orf53	0.143491	2.16688	2.683968	0.008314	0.022211	-3.8033
KIAA0825	0.143483	2.208019	2.681315	0.008376	0.02236	-3.81003
PSG6	0.143468	3.183459	2.690335	0.008166	0.021867	-3.78711
EIF1B-AS1	0.143467	4.141145	2.73557	0.007182	0.019555	-3.67111
OR7C1	0.143446	2.46291	2.072743	0.040358	0.085564	-5.19528
HOXA2	0.143342	2.213782	2.399155	0.017987	0.043346	-4.49235
KLK11	0.143072	3.119304	2.053075	0.04226	0.088945	-5.2346
CDSN	0.142836	3.799275	2.259159	0.025696	0.058623	-4.80537

CLDN14	0.142809	3.187075	2.294755	0.023502	0.054319	-4.72741
INF2	0.142783	3.990809	2.544846	0.012213	0.031022	-4.14852
C1orf115	0.142733	3.932981	2.199609	0.029769	0.066471	-4.93329
SERPINA2	0.142697	2.072565	2.353771	0.020225	0.047853	-4.59571
TEX38	0.142497	3.526628	2.018082	0.045834	0.095263	-5.3037
RSPO1	0.142442	3.10837	1.983169	0.049654	0.101776	-5.37154
FAM229A	0.142441	4.88387	2.722406	0.007456	0.020206	-3.70504
RAB7A	0.142414	4.970034	2.910572	0.004308	0.012549	-3.20641
CACNA1D	0.142378	3.142982	2.535891	0.012513	0.031717	-4.17018
LOC10192	0.142351	1.976455	2.433503	0.016442	0.040147	-4.41294
LOC10050	0.142351	2.934781	2.094505	0.038339	0.082043	-5.15137
CDKL2	0.142289	2.994369	2.761509	0.006668	0.018333	-3.60382
LINC0034:	0.14219	1.844614	2.920827	0.004178	0.012218	-3.1784
LOC10192	0.14218	1.879475	2.312168	0.02249	0.052353	-4.68887
SIGLECL1	0.142161	1.969605	2.393936	0.018233	0.04386	-4.50433
MAGOH2	0.142145	2.665274	2.783728	0.006254	0.01734	-3.54574
PRAC1	0.142129	2.002698	2.48946	0.014177	0.035286	-4.28139
TMSB4Y	0.142057	4.934324	2.396409	0.018116	0.043621	-4.49866
PRAMEF11	0.14199	3.779949	2.003187	0.047432	0.098056	-5.33278
LINC0098:	0.141953	3.631845	2.978227	0.003515	0.010478	-3.02006
TTC9B	0.141649	4.630279	2.082635	0.039429	0.083981	-5.17537
NR1H4	0.14159	2.273108	2.854864	0.005082	0.01447	-3.35706
GTF2A1	0.141513	5.698593	2.975652	0.003543	0.01055	-3.02722
STK4-AS1	0.141465	4.719094	2.15062	0.033531	0.073394	-5.03617
MORN1	0.141456	4.082009	2.555307	0.011871	0.03025	-4.12314
SHROOM:	0.141344	2.557362	2.13826	0.034543	0.075169	-5.06178
LOC15727	0.141293	1.878191	2.285984	0.024026	0.055357	-4.74673
FRS3	0.141256	4.181649	2.025805	0.045024	0.093893	-5.28855
NT5DC4	0.141225	2.31387	3.78039	0.000246	0.000955	-0.54071
ADAMTS1	0.141033	3.787508	2.578088	0.011156	0.02867	-4.06753
IL9	0.140873	2.128855	2.185643	0.030802	0.068345	-4.96284
SYT5	0.140821	3.981645	2.215053	0.028662	0.064379	-4.90042
CFC1B	0.140782	3.793205	2.00667	0.047054	0.097413	-5.326
ITGAX	0.140739	5.891239	2.154715	0.033202	0.072792	-5.02765
KCNJ1	0.140731	2.642598	2.465792	0.015098	0.03729	-4.33736
GVQW1	0.140666	4.866353	2.025564	0.045049	0.093926	-5.28902
HIST3H3	0.140618	2.532586	2.32014	0.022039	0.051498	-4.67114
PPP4R1L	0.140425	2.827233	3.552979	0.000547	0.001971	-1.2924
HSPC081	0.140418	3.457344	2.261056	0.025575	0.058392	-4.80125
RTDR1	0.140354	2.991802	2.449321	0.015771	0.038745	-4.37603
LOC10192	0.140346	2.037226	2.380076	0.0189	0.045242	-4.53602
TMEM174	0.140205	4.543227	2.14771	0.033767	0.073818	-5.04221
LOC10050	0.140192	2.616287	2.131777	0.035084	0.076136	-5.07516
MAPK11	0.140102	3.835843	2.340024	0.02095	0.049302	-4.62666
JPH3	0.140046	3.088228	2.414374	0.017287	0.041886	-4.45729
PRSS16	0.140004	2.489733	1.987535	0.049162	0.100959	-5.36311
T	0.139984	6.010511	2.13342	0.034946	0.075885	-5.07178
OR6B1	0.139886	2.011783	2.24647	0.026521	0.060242	-4.83289
CYP26A1	0.139854	3.269392	2.492664	0.014056	0.035045	-4.27377
RHBDL3	0.139792	3.685597	2.27187	0.024893	0.057026	-4.77766
SLC39A5	0.13969	3.839076	2.119924	0.036092	0.077944	-5.09953
DUSP5P1	0.139655	1.692288	2.888579	0.0046	0.013283	-3.26618
LOC10192	0.139598	3.361289	2.237646	0.027107	0.061385	-4.85195
POLR2F	0.139585	5.050135	2.595365	0.010639	0.027484	-4.02506
HAGLR	0.139392	2.684064	3.18639	0.001841	0.005907	-2.42379
FAM171B	0.139341	2.19495	3.648913	0.000392	0.001458	-0.97983
D21S2090I	0.13933	3.517879	2.391269	0.018359	0.044124	-4.51044
NBR2	0.139314	4.522908	2.934486	0.004011	0.01178	-3.14096

VN1R3	0.139229	1.959118	2.031161	0.044469	0.092916	-5.27801
HMGCL	0.139107	6.902719	2.341405	0.020876	0.049151	-4.62356
MEGF10	0.138949	2.727707	2.893468	0.004534	0.013123	-3.25293
STON1	0.138898	2.53198	2.409776	0.017496	0.042327	-4.4679
MESP1	0.138861	4.92167	1.989155	0.048981	0.100627	-5.35998
GPRC5C	0.13882	4.173648	2.033617	0.044217	0.092464	-5.27316
GGT1	0.138786	4.697441	2.090124	0.038738	0.082768	-5.16024
KNDC1	0.138734	3.509612	2.303978	0.022961	0.053244	-4.70703
CLIP1-AS1	0.13858	2.806912	1.99926	0.047861	0.098773	-5.34041
RGS11	0.138495	3.209425	3.025896	0.003039	0.009216	-2.88655
LOC10192	0.13839	1.598015	4.095238	7.73E-05	0.00033	0.559646
SLC13A2	0.138361	3.458057	2.203063	0.029519	0.065987	-4.92596
PDE6D	0.138207	4.951387	2.493821	0.014013	0.034945	-4.27102
FFAR1	0.138126	3.025988	2.760337	0.00669	0.018382	-3.60688
CHGB	0.138079	2.397401	2.036002	0.043973	0.092029	-5.26845
HCN3	0.138047	4.248949	2.140941	0.034321	0.074743	-5.05624
LINC0029(	0.137821	3.287992	2.003902	0.047354	0.097925	-5.33139
H1FNT	0.137722	3.857959	2.263592	0.025413	0.058069	-4.79573
RRP1	0.137698	5.054108	2.425723	0.016781	0.040864	-4.43102
FAM189A1	0.13762	2.739442	2.247705	0.026439	0.060078	-4.83022
LDHAL6A	0.137486	3.200771	2.447944	0.015828	0.038863	-4.37925
KCNIP4	0.1374	3.069364	2.684307	0.008306	0.022196	-3.80244
IHH	0.1374	4.961508	2.029225	0.044669	0.093258	-5.28182
CDC42BPA	0.137382	3.730624	2.213224	0.028791	0.0646	-4.90432
C9orf116	0.137369	3.336197	2.372834	0.019257	0.045972	-4.55251
DDX49	0.137175	4.460398	2.920089	0.004188	0.012241	-3.18042
ASIC2	0.137058	2.736512	2.035262	0.044048	0.092159	-5.26992
MPHOSPH	0.136967	3.797557	2.064076	0.041187	0.087044	-5.21265
PROSC	0.136947	5.369908	2.316724	0.022231	0.051845	-4.67874
LOC10050	0.136942	3.630056	2.496169	0.013925	0.034764	-4.26543
LOC10192	0.136917	2.014669	2.201582	0.029626	0.06618	-4.92911
CHAT	0.136872	3.841199	2.010287	0.046665	0.096656	-5.31894
MOGAT3	0.136867	2.161398	2.247938	0.026424	0.06005	-4.82972
TMEM108	0.136843	3.459151	2.462075	0.015248	0.037591	-4.3461
LOC28479	0.136788	4.428267	2.17874	0.031324	0.069293	-4.97738
R3HCC1	0.13677	5.308496	2.144057	0.034065	0.074327	-5.04978
HES2	0.136739	3.642437	2.561591	0.01167	0.029819	-4.10784
HSF1	0.136598	4.655297	2.35635	0.020092	0.047588	-4.58988
HSPA12A	0.136379	3.364169	2.032473	0.044334	0.092663	-5.27542
TMEM37	0.136298	3.979268	2.197227	0.029943	0.066772	-4.93835
EFCAB12	0.136278	2.535817	2.062044	0.041383	0.087432	-5.21671
TMEM53	0.136261	4.278958	2.506792	0.013534	0.03395	-4.24009
MAG	0.136205	2.669716	2.153708	0.033282	0.072946	-5.02975
SCTR	0.136087	3.375019	2.532274	0.012636	0.031985	-4.17891
TERT	0.136022	4.059947	2.288689	0.023864	0.055063	-4.74078
CELA3A	0.136004	3.497468	2.030737	0.044513	0.092989	-5.27884
MTMR9LP	0.135985	4.250341	2.170422	0.031964	0.070463	-4.99484
LOC10050	0.13588	2.343965	2.192369	0.030301	0.067408	-4.94863
B3GNT6	0.135814	3.963687	2.521193	0.01302	0.032826	-4.20559
SPDEF	0.135793	5.649636	2.242723	0.026768	0.060724	-4.84099
OR2K2	0.135775	2.321448	2.701654	0.007909	0.021263	-3.75824
MPV17L2	0.135749	5.116173	2.136146	0.034718	0.075511	-5.06615
IQUB	0.135708	1.781419	2.509426	0.013439	0.033744	-4.23379
FA2H	0.135669	3.387647	2.491449	0.014102	0.035137	-4.27666
SNX7	0.13554	2.434483	2.494886	0.013973	0.034858	-4.26849
C11orf58	0.135535	5.220843	2.14089	0.034325	0.074743	-5.05634
SPATS1	0.135433	3.818286	2.655503	0.009005	0.023782	-3.87526
CHMP1A	0.135321	7.717289	2.407813	0.017586	0.042509	-4.47243

VWA1	0.135138	3.563515	2.641398	0.009367	0.024606	-3.91067
TMEM86A	0.13505	4.324012	2.154509	0.033218	0.072821	-5.02808
DBIL5P	0.135038	3.338436	2.031317	0.044453	0.092892	-5.2777
RXRG	0.135019	2.770506	2.179989	0.031229	0.069128	-4.97475
MTHFD2L	0.134935	2.38713	3.643023	0.000401	0.001485	-0.99921
EVI5L	0.134865	3.911846	2.3979	0.018046	0.043462	-4.49523
MMP2	0.134864	3.178472	2.816581	0.005685	0.015944	-3.45912
RBP1	0.134857	3.748956	2.313448	0.022417	0.052201	-4.68603
C6orf223	0.134829	4.402048	2.669778	0.008652	0.023004	-3.83926
ZCWPW2	0.134816	1.724025	2.622669	0.009867	0.025755	-3.95743
LOC22194	0.13479	2.320693	2.173109	0.031756	0.070088	-4.98921
RGS6	0.134774	3.571825	2.05064	0.042501	0.089351	-5.23945
LOC10192	0.134725	2.093465	2.07861	0.039805	0.08464	-5.18348
FGD5	0.134642	3.932866	2.061659	0.041421	0.087484	-5.21748
C9orf57	0.134566	1.794104	2.218727	0.028404	0.063897	-4.89257
SDCCAG8	0.13449	3.743933	2.448348	0.015812	0.038831	-4.3783
RAB39A	0.134484	3.063122	2.016706	0.04598	0.095507	-5.3064
SLC4A11	0.134477	3.433572	2.250072	0.026284	0.059812	-4.8251
FSHB	0.134013	4.01171	2.071956	0.040432	0.085707	-5.19686
MYRFL	0.133992	1.975811	2.194581	0.030138	0.067103	-4.94395
ZNF324B	0.133888	3.952467	2.423632	0.016873	0.041054	-4.43587
DNAJC8	0.133848	5.590209	2.144698	0.034012	0.074252	-5.04846
LOC10192	0.133841	4.062695	3.098476	0.002428	0.007551	-2.67979
GPD1	0.133839	3.351086	2.41696	0.017171	0.041647	-4.45132
NEUROG1	0.133804	3.393008	2.06847	0.040765	0.086322	-5.20385
DNAJB8-A	0.133798	2.015096	2.047979	0.042765	0.089815	-5.24474
HS1BP3	0.133777	4.000341	3.525317	0.000602	0.002149	-1.38128
C1QTNF9E	0.133762	2.279687	2.034518	0.044124	0.09229	-5.27138
B3GAT3	0.133754	5.311877	2.838428	0.005333	0.015075	-3.40103
SERPINB12	0.133748	3.635336	2.245484	0.026586	0.06037	-4.83503
PGBD4	0.133718	2.988305	2.046982	0.042865	0.089995	-5.24671
FBLIM1	0.133704	4.323405	2.422026	0.016945	0.041188	-4.43959
MFI2	0.133679	3.386722	2.602627	0.010429	0.027015	-4.00713
SDC3	0.133604	3.31166	2.213208	0.028793	0.0646	-4.90436
PAX7	0.133539	3.155809	2.304152	0.022951	0.053232	-4.70665
CCDC61	0.133534	3.79978	2.444948	0.015954	0.039127	-4.38625
ADAT3	0.133499	5.248405	2.743841	0.007014	0.019152	-3.64971
LOC22112	0.133393	3.640789	2.09364	0.038418	0.082194	-5.15312
KHNYN	0.133298	7.72616	2.563028	0.011624	0.029717	-4.10434
OR51I2	0.133262	4.286068	2.013237	0.046349	0.096118	-5.31318
HERC2P7	0.133215	3.086668	2.181321	0.031128	0.068949	-4.97195
SLC22A7	0.133202	3.964116	2.243291	0.026731	0.060663	-4.83977
TAT	0.133101	2.943483	2.319066	0.022099	0.051597	-4.67353
PRDM10	0.133088	4.466439	2.096448	0.038163	0.081727	-5.14742
C11orf97	0.132941	2.192238	2.354965	0.020163	0.04773	-4.59301
THY1	0.132845	4.443713	2.058739	0.041704	0.087966	-5.22332
STMN4	0.132804	3.630101	2.075284	0.040118	0.085136	-5.19018
GSX1	0.132798	3.207563	2.064244	0.041171	0.087019	-5.21232
OR8G2	0.132706	2.437676	2.108288	0.037106	0.079814	-5.12333
LOC10050	0.13263	2.510273	2.170783	0.031936	0.070417	-4.99408
GUCA1A	0.132541	3.377201	2.77704	0.006376	0.017621	-3.56327
C1QTNF1	0.132535	3.474773	2.03079	0.044507	0.092987	-5.27874
TCP11	0.132362	2.766498	2.093312	0.038448	0.082249	-5.15378
UBR4	0.132283	3.936708	3.621044	0.000433	0.00159	-1.07132
PHLDA2	0.132278	2.750018	2.449709	0.015755	0.03871	-4.37512
FLJ34503	0.132251	2.191918	2.076648	0.039989	0.084911	-5.18743
LOC10192	0.132044	3.816317	2.035183	0.044056	0.092167	-5.27007
PAX9	0.132014	2.377894	2.527334	0.012806	0.032346	-4.19082

LOC33980	0.132008	4.623116	2.307125	0.022779	0.052898	-4.70006
PODNL1	0.131864	5.258062	2.23354	0.027384	0.061916	-4.86079
PMCH	0.13182	2.847056	2.059547	0.041626	0.087818	-5.2217
CLYBL-AS:	0.131803	2.94148	2.186377	0.030747	0.068241	-4.96129
PGAM5	0.131667	3.554791	2.505034	0.013598	0.034069	-4.24429
LOC10050	0.131664	2.953889	2.604362	0.010379	0.026903	-4.00284
GABRP	0.131634	2.138615	2.036935	0.043878	0.091859	-5.26661
ENTPD3	0.131613	2.62367	2.814782	0.005715	0.016012	-3.46389
KCNK4	0.131521	4.064998	2.143939	0.034075	0.074339	-5.05003
XCR1	0.131486	3.074807	2.158691	0.032884	0.072197	-5.01937
NKAIN4	0.131479	3.548031	2.105025	0.037395	0.080283	-5.12998
TRIM29	0.131449	3.914912	2.361294	0.019838	0.047075	-4.5787
SOX9	0.131402	2.247175	2.852919	0.005111	0.014539	-3.36228
PDLIM5	0.131371	4.389666	2.602292	0.010438	0.027033	-4.00796
TMEM184,	0.131345	4.375851	2.148389	0.033712	0.073737	-5.0408
PLCE1	0.131262	2.269427	3.412427	0.000881	0.003033	-1.73815
LOC72791	0.131225	1.734315	3.047521	0.002844	0.008703	-2.82538
DNM1P35	0.131213	2.096602	2.257042	0.025832	0.058888	-4.80998
RIMS2	0.131105	2.630555	2.914771	0.004255	0.012409	-3.19495
CHST6	0.130966	3.192349	2.142375	0.034203	0.074549	-5.05327
SIM2	0.130832	3.085113	2.632722	0.009595	0.025156	-3.93237
TRIP12	0.130801	7.206305	1.995939	0.048227	0.099337	-5.34685
C6orf118	0.130755	2.260169	2.607207	0.010298	0.026726	-3.9958
COL22A1	0.130738	2.715179	2.416606	0.017187	0.041674	-4.45213
CACNA1G	0.130726	3.44829	2.912065	0.004289	0.012499	-3.20234
DAW1	0.130725	2.023885	2.274782	0.024712	0.056656	-4.7713
CTD-2118	0.130629	2.022685	2.665014	0.008768	0.023247	-3.85129
LRRN2	0.130542	2.712934	2.737926	0.007134	0.019439	-3.66502
SLC6A11	0.130509	2.996321	2.626676	0.009758	0.0255	-3.94745
CLDN20	0.130366	1.839292	2.290907	0.023731	0.054787	-4.73589
WNT6	0.130227	3.552591	2.522711	0.012967	0.032712	-4.20194
LINC0064	0.129991	1.757224	2.279879	0.024398	0.056087	-4.76013
EFS	0.129991	3.362041	2.243453	0.02672	0.060648	-4.83942
LINC0099	0.129982	2.576457	2.468784	0.014979	0.037035	-4.33031
VPREB1	0.129819	1.976869	2.333272	0.021314	0.050061	-4.6418
LINC0068	0.12977	3.084369	2.825841	0.005533	0.015576	-3.43455
ROR1	0.129723	2.426698	2.975592	0.003543	0.01055	-3.02738
BAIAP2L2	0.129646	3.14009	2.283251	0.024192	0.055683	-4.75273
TBC1D16	0.129605	4.157086	2.327812	0.021613	0.050682	-4.65401
LOC10099	0.129604	2.507687	2.309225	0.022658	0.052685	-4.6954
LRRC61	0.129574	5.338861	1.984569	0.049496	0.101512	-5.36884
SPATA16	0.129543	3.147535	2.076286	0.040023	0.084954	-5.18816
TNXB	0.129447	3.583897	2.141212	0.034299	0.074723	-5.05568
GGT6	0.129316	3.641913	2.114497	0.036562	0.078842	-5.11065
RBFADN	0.129306	3.334143	2.784826	0.006234	0.017292	-3.54286
CNTNAP5	0.129117	3.188529	2.23973	0.026968	0.061109	-4.84745
LOC10050	0.129103	2.909359	1.99674	0.048138	0.099215	-5.3453
MFI2-AS1	0.129078	3.919031	2.433463	0.016444	0.040147	-4.41303
LOC10050	0.128951	2.519658	1.992017	0.048661	0.100083	-5.35445
LRTM1	0.128855	3.071991	2.456826	0.015461	0.038048	-4.35844
SIX3	0.128665	1.932542	3.096367	0.002444	0.007595	-2.68586
POLR3K	0.128619	3.854502	2.129953	0.035237	0.076437	-5.07892
SLC4A9	0.128612	2.76897	2.510558	0.013398	0.03365	-4.23109
TRIM36	0.128275	2.790868	2.702159	0.007897	0.021243	-3.75695
GRIK3	0.128207	1.94334	2.873201	0.004814	0.013818	-3.30775
AMZ1	0.12817	4.644446	2.052293	0.042337	0.089089	-5.23616
PPP1R12C	0.128028	4.908945	2.295188	0.023476	0.054268	-4.72646
PRR35	0.127897	5.758087	1.981127	0.049886	0.102189	-5.37547

LOC40094	0.127838	2.506805	3.281314	0.001357	0.004482	-2.14062
FAM71F1	0.127766	2.417267	2.805412	0.005873	0.016399	-3.48867
LOC10192	0.127701	1.758896	2.159252	0.03284	0.072107	-5.0182
NAV2	0.127691	3.292242	2.349977	0.020423	0.048256	-4.60426
ABHD11	0.127599	4.3034	2.710948	0.007703	0.020806	-3.73446
OPRK1	0.127503	2.426004	2.538667	0.01242	0.031507	-4.16348
BCL2L10	0.127425	2.847086	2.551542	0.011993	0.030516	-4.13228
CNBD2	0.127391	2.960751	2.632873	0.009591	0.025148	-3.93199
DAB2IP	0.127361	3.758501	2.102066	0.037659	0.08079	-5.13601
CBLN2	0.127285	3.522235	2.012265	0.046453	0.096294	-5.31508
TRABD2B	0.127236	2.864519	2.684118	0.00831	0.022205	-3.80292
SMG7-AS1	0.127161	2.362663	2.324383	0.021803	0.051056	-4.66167
LOC10050	0.127125	1.872304	2.197723	0.029907	0.066698	-4.93729
OR2C1	0.127108	3.831127	1.983303	0.049639	0.101772	-5.37128
FNDC5	0.127014	3.991722	2.197179	0.029947	0.066773	-4.93845
CYP2F1	0.126922	2.589335	1.982743	0.049703	0.101854	-5.37236
GJB3	0.126915	4.428551	2.696912	0.008016	0.021504	-3.77035
ANGPTL3	0.126837	1.717269	3.374082	0.001001	0.003403	-1.8572
S100A13	0.126748	3.918693	2.101227	0.037734	0.080925	-5.13771
PPM1E	0.126691	2.224087	2.595935	0.010623	0.027448	-4.02365
SLC6A5	0.126655	3.378586	2.143847	0.034082	0.074341	-5.05022
GABRB1	0.126622	1.700095	2.465894	0.015094	0.037284	-4.33712
CASP9	0.126574	5.298349	2.767475	0.006554	0.018055	-3.58827
GNB1L	0.126572	5.254759	2.379582	0.018924	0.04529	-4.53715
TACO1	0.126532	4.280027	2.364011	0.0197	0.046837	-4.57254
RAPSN	0.126418	2.489569	1.999411	0.047845	0.098768	-5.34012
MIR34A	0.126394	2.747922	2.082489	0.039443	0.084001	-5.17567
CAMK2N2	0.126199	3.80337	2.160337	0.032754	0.071972	-5.01593
ATP8B3	0.126061	3.238611	2.701334	0.007916	0.021279	-3.75906
OR1Q1	0.126019	3.063475	2.660832	0.008872	0.023497	-3.86184
CXCL12	0.12597	2.910158	2.266357	0.025238	0.05774	-4.7897
TMEM221	0.125957	4.071774	2.025263	0.04508	0.093982	-5.28961
NOX3	0.125873	1.876364	2.27131	0.024928	0.057093	-4.77889
PTPN7	0.125665	5.291838	2.018503	0.04579	0.095199	-5.30288
ANKRD45	0.125587	2.096979	2.314618	0.02235	0.05207	-4.68343
IBSP	0.125547	3.026092	2.104116	0.037476	0.08044	-5.13184
LOC10192	0.125477	1.885018	2.384612	0.018679	0.044781	-4.52567
SLC38A10	0.125444	4.663107	3.129387	0.002204	0.006928	-2.59047
CASKIN1	0.125429	4.266948	2.277863	0.024522	0.056314	-4.76455
FAM181B	0.125349	3.256473	2.042658	0.043298	0.090775	-5.25529
FLRT3	0.125305	2.19521	2.277428	0.024548	0.056357	-4.7655
KATNB1	0.125238	4.85128	2.631804	0.00962	0.02521	-3.93466
CACFD1	0.125197	6.413667	2.322709	0.021896	0.051234	-4.66541
ATN1	0.125087	5.24748	2.277503	0.024544	0.056352	-4.76534
USP49	0.124929	3.136703	3.900279	0.00016	0.00064	-0.1297
CDKL5	0.124869	3.355313	2.014004	0.046267	0.095987	-5.31168
MAP6D1	0.12483	4.088522	2.138406	0.034531	0.07515	-5.06148
SYT12	0.124813	4.032273	2.111978	0.036782	0.079242	-5.1158
KLK3	0.124728	4.640186	1.985839	0.049353	0.101239	-5.36639
CX3CL1	0.124653	3.924116	2.222407	0.028148	0.06339	-4.88469
FER1L5	0.124358	2.31969	2.072711	0.040361	0.085564	-5.19535
PCGEM1	0.124261	2.267205	2.318667	0.022122	0.051632	-4.67442
SLC9A3R2	0.124256	2.215007	2.145774	0.033925	0.074113	-5.04623
PRKG1	0.124189	2.669268	2.288422	0.02388	0.055081	-4.74136
RACGAP1I	0.123966	1.812189	2.000573	0.047718	0.098546	-5.33786
LOC10192	0.123885	2.29034	2.420088	0.017031	0.041363	-4.44408
HTR7P1	0.12388	2.372152	2.026106	0.044993	0.093838	-5.28796
B3GALT1	0.123874	2.347272	2.056373	0.041936	0.088353	-5.22803

GJA4	0.123842	3.359389	2.186444	0.030742	0.068241	-4.96115
PGBD5	0.123798	2.801919	2.022146	0.045406	0.094546	-5.29573
GPR111	0.123741	2.212599	2.22185	0.028187	0.063463	-4.88589
ISM2	0.123689	3.473919	2.124328	0.035715	0.077259	-5.09049
IKBKG	0.123683	7.195928	2.601758	0.010454	0.027068	-4.00928
SMIM17	0.123659	1.912834	2.240146	0.02694	0.06106	-4.84656
FOXA1	0.123652	1.981839	2.653002	0.009068	0.023921	-3.88155
NETO1	0.123636	2.304817	3.256107	0.001472	0.004817	-2.21649
AKAP6	0.123518	2.35677	2.214281	0.028717	0.064487	-4.90207
KIAA1755	0.123461	3.767612	2.163285	0.032521	0.071553	-5.00978
ST8SIA2	0.123382	2.629876	2.394448	0.018208	0.043812	-4.50315
NPY1R	0.123373	1.770948	2.299254	0.023236	0.053804	-4.71748
PITPNM3	0.123368	2.815402	2.224063	0.028033	0.063173	-4.88114
COMT	0.12326	5.762854	2.683706	0.00832	0.022225	-3.80396
SERTAD1	0.123223	7.193398	2.125124	0.035647	0.077129	-5.08886
CASR	0.12315	3.535134	2.601349	0.010465	0.027093	-4.01029
LOC10192	0.123041	1.749523	2.109221	0.037024	0.079671	-5.12143
POU3F3	0.123008	2.463818	2.750953	0.006873	0.018813	-3.63127
MAP3K13	0.122878	3.821257	3.397153	0.000927	0.003178	-1.78571
LOC10050	0.12276	2.105876	2.012933	0.046381	0.096175	-5.31377
CCDC13	0.122743	4.300317	2.172392	0.031811	0.070203	-4.99071
SPG7	0.122469	2.521408	2.065377	0.041061	0.086833	-5.21005
ANKRD34(	0.122438	2.365617	2.651731	0.0091	0.023997	-3.88475
MRPL41	0.122344	4.329022	2.890375	0.004575	0.013225	-3.26132
RRAD	0.122338	2.716996	1.99557	0.048268	0.099391	-5.34757
TCEANC2	0.122329	3.407837	2.533319	0.012601	0.031914	-4.17639
TMEM121	0.122273	3.109747	2.264032	0.025385	0.058026	-4.79477
CLTA	0.122107	6.658413	2.324306	0.021807	0.05106	-4.66185
PROSER2-	0.122047	2.380116	2.018239	0.045818	0.095238	-5.30339
TOR1AIP2	0.121996	3.980666	3.073372	0.002625	0.0081	-2.75178
RCBTB1	0.12197	3.468063	2.109032	0.037041	0.079698	-5.12181
ATP5SL	0.121922	5.726012	2.313383	0.02242	0.052204	-4.68617
BOC	0.121919	4.188796	2.24944	0.026325	0.059879	-4.82647
MMP19	0.121896	4.492982	2.099998	0.037844	0.081136	-5.14021
TACC2	0.121894	2.989906	2.661217	0.008862	0.023477	-3.86087
ADAMTS7	0.121887	3.693363	1.996572	0.048157	0.099233	-5.34562
CCKAR	0.121798	3.514798	2.002232	0.047536	0.098212	-5.33463
LDHAL6B	0.121767	1.773438	2.05999	0.041583	0.087745	-5.22082
DNAH5	0.121732	2.895132	2.724391	0.007414	0.020108	-3.69993
C12orf50	0.121678	1.809238	2.343436	0.020768	0.048919	-4.61899
TOR2A	0.12164	4.357885	2.643628	0.009309	0.024474	-3.90508
ITCH	0.121546	4.640927	2.019705	0.045663	0.094984	-5.30052
BTC	0.121536	1.482607	3.177173	0.001895	0.006059	-2.45091
MGST3	0.121379	5.916384	2.471419	0.014875	0.036812	-4.32409
COL9A1	0.121323	2.582926	2.576296	0.011211	0.028775	-4.07192
BRMS1	0.121285	6.918036	2.240519	0.026915	0.06101	-4.84575
CEP152	0.121234	3.434758	2.27622	0.024623	0.0565	-4.76815
TTC40	0.121215	2.817127	2.892585	0.004545	0.013152	-3.25533
IZUMO4	0.121171	4.133241	2.158001	0.032939	0.07231	-5.0208
PCDHB13	0.121083	3.092819	2.080651	0.039614	0.084301	-5.17937
AREG	0.121025	1.790896	2.248259	0.026403	0.060015	-4.82902
STPG1	0.120778	4.380783	2.189616	0.030505	0.067804	-4.95445
SMC1B	0.120708	2.143449	2.077439	0.039915	0.08482	-5.18584
DTNB	0.120708	4.147522	2.022161	0.045405	0.094546	-5.29571
ACKR4	0.120472	1.820449	2.04544	0.043019	0.090245	-5.24977
MYT1	0.120417	4.209109	2.647381	0.009212	0.024243	-3.89567
MYH14	0.120336	4.617257	2.107171	0.037205	0.079976	-5.12561
TRAPPC2L	0.120228	5.630125	2.413517	0.017326	0.041975	-4.45927

RPS9	0.120169	7.500858	2.072788	0.040354	0.085564	-5.19519
SLC45A2	0.120042	3.110253	2.222107	0.028169	0.06343	-4.88533
CMYA5	0.119885	3.458451	2.211942	0.028882	0.064773	-4.90706
RSU1P2	0.119816	1.838585	2.371164	0.01934	0.046123	-4.55631
NSD1	0.119812	5.206539	2.688021	0.008219	0.022002	-3.793
LRRC15	0.119704	3.076434	2.21359	0.028766	0.064561	-4.90354
EGFR	0.119465	3.49146	2.121421	0.035964	0.077691	-5.09646
OR6W1P	0.119387	2.638848	2.123013	0.035827	0.077461	-5.09319
APOA5	0.119272	4.59891	2.99789	0.003311	0.009931	-2.96521
SNORA71/	0.119237	2.941367	2.145549	0.033943	0.074132	-5.04669
LRRC31	0.119215	1.858955	2.024807	0.045128	0.094062	-5.29051
GYG2	0.119211	3.445709	2.290478	0.023756	0.054834	-4.73684
LOC10192	0.119188	1.980864	2.832821	0.005422	0.015293	-3.41597
LOC40079	0.119183	3.245869	1.9933	0.048519	0.099839	-5.35197
TAS1R2	0.119037	2.581234	1.997744	0.048028	0.099057	-5.34335
DMC1	0.11894	2.040871	2.453973	0.015578	0.038309	-4.36513
EYA3	0.118926	3.879173	2.609135	0.010243	0.026612	-3.99103
LOC10050	0.118887	2.960858	2.186641	0.030728	0.068222	-4.96073
PANK2	0.118866	3.654224	2.34671	0.020595	0.048572	-4.61163
PLK2	0.118696	2.017361	1.986747	0.049251	0.101089	-5.36463
EMP1	0.118642	2.317995	2.117294	0.036319	0.078368	-5.10492
RAB3C	0.118641	2.379547	2.714335	0.00763	0.020628	-3.72577
DIO2	0.118605	3.415458	2.424683	0.016827	0.040965	-4.43343
GPRC5A	0.118393	2.989229	2.26453	0.025354	0.057965	-4.79368
PLCB4	0.118312	2.071183	2.572461	0.011329	0.029038	-4.0813
RANBP3L	0.118208	1.762305	2.122621	0.035861	0.077517	-5.094
C17orf105	0.118064	1.8534	2.085076	0.039203	0.083568	-5.17045
LOC55420	0.117871	1.951451	2.056496	0.041924	0.088337	-5.22779
PGR	0.117851	1.821358	2.877803	0.004749	0.01366	-3.29533
TTLL2	0.117824	2.859571	1.986374	0.049293	0.101136	-5.36536
NR2F6	0.117695	3.337663	2.148943	0.033667	0.073654	-5.03965
FBXL6	0.117691	4.551941	2.196894	0.029968	0.066812	-4.93905
WFDC13	0.117456	2.309464	2.24938	0.026329	0.059881	-4.8266
TBX2	0.117284	4.049196	2.155384	0.033148	0.072698	-5.02626
TP53AIP1	0.117245	2.864469	2.749418	0.006903	0.018881	-3.63526
AGR3	0.117226	1.737642	2.340931	0.020902	0.049199	-4.62462
MIB2	0.117109	4.124823	2.462612	0.015226	0.03756	-4.34484
ANO3	0.116984	2.054544	2.148304	0.033719	0.073744	-5.04098
LINC00347	0.11696	1.658089	2.091403	0.038622	0.082544	-5.15765
TRO	0.116906	3.181493	2.350957	0.020372	0.048157	-4.60205
LOC10192	0.116794	1.995421	2.13576	0.034751	0.075557	-5.06695
GABARAP	0.1167	10.9493	2.516255	0.013194	0.033224	-4.21744
CSPG4	0.116372	2.405169	2.357691	0.020023	0.047441	-4.58685
ZNF70	0.116127	5.200621	2.103454	0.037535	0.080549	-5.13318
SNCAIP	0.115879	2.701265	2.130233	0.035214	0.076394	-5.07834
ITSN1	0.115846	3.158407	2.886449	0.004629	0.013354	-3.27195
FFAR4	0.115832	1.772714	2.219027	0.028383	0.063857	-4.89193
LOC10192	0.115827	2.403226	2.416588	0.017187	0.041674	-4.45218
TYR	0.115616	2.742961	2.620295	0.009932	0.025892	-3.96333
TSPAN12	0.11553	2.778468	2.011464	0.046538	0.096462	-5.31664
RRP12	0.115466	4.414519	2.421674	0.01696	0.041211	-4.4404
LINC00284	0.115383	1.803697	2.194006	0.03018	0.067168	-4.94517
LINC00354	0.11534	2.447109	2.188074	0.03062	0.068	-4.95771
UNC93A	0.115276	2.078106	2.621524	0.009898	0.025817	-3.96027
UBE2NL	0.115274	7.473825	2.174222	0.03167	0.069916	-4.98687
GAS2L2	0.114976	5.039758	2.038211	0.043748	0.091624	-5.26409
CD99L2	0.114955	4.212227	2.047982	0.042765	0.089815	-5.24473
CBR3-AS1	0.114848	3.775357	2.551204	0.012004	0.03054	-4.13311

C19orf47	0.114624	4.812183	2.000507	0.047725	0.098551	-5.33799
PTPN21	0.114595	2.736798	2.632227	0.009609	0.025184	-3.9336
RIPK4	0.11454	3.67026	2.204969	0.029381	0.065754	-4.92191
LOC10192	0.11441	1.501689	2.278856	0.024461	0.056199	-4.76237
DRICH1	0.114398	3.802225	2.553729	0.011922	0.030358	-4.12697
DPYSL5	0.114278	3.932104	2.076738	0.039981	0.084911	-5.18725
MUC7	0.114205	2.227512	2.355706	0.020125	0.047653	-4.59134
CAMKK1	0.114067	5.03658	1.980268	0.049984	0.102369	-5.37712
NTN1	0.114	2.77902	2.050895	0.042475	0.089307	-5.23894
TEAD4	0.113985	4.01551	1.997624	0.048041	0.099064	-5.34358
RAD21L1	0.113957	1.612286	2.968281	0.003622	0.010755	-3.04768
LOC10192	0.113919	1.877455	2.157746	0.032959	0.072347	-5.02133
LOC10272	0.113911	1.811325	2.076953	0.03996	0.0849	-5.18682
ARR3	0.113661	4.955887	2.065918	0.041009	0.086768	-5.20897
NRG2	0.113647	3.813652	2.08534	0.039179	0.083525	-5.16991
ITGA1	0.113549	2.338922	2.071181	0.040506	0.085854	-5.19842
FAM219B	0.113489	5.245913	2.34002	0.02095	0.049302	-4.62667
UGGT2	0.113459	2.014027	3.318921	0.0012	0.004019	-2.02651
FARP1	0.113405	3.144828	2.577664	0.011169	0.028696	-4.06857
KDM4D	0.113284	2.727399	2.283766	0.024161	0.055634	-4.7516
GREB1	0.113133	2.47137	2.666521	0.008732	0.023173	-3.84749
PQLC2	0.112801	4.397934	2.355647	0.020128	0.047653	-4.59147
TENM3	0.112668	2.657858	2.310668	0.022575	0.052522	-4.6922
PAPPA2	0.112652	3.580899	2.249977	0.02629	0.059819	-4.8253
KLF15	0.112567	4.436522	2.132207	0.035048	0.076074	-5.07428
WNT4	0.112512	3.626725	2.180976	0.031154	0.068997	-4.97267
EXOC7	0.11246	5.699275	2.706862	0.007793	0.020998	-3.74492
DDC	0.112446	3.397969	2.140902	0.034324	0.074743	-5.05632
MAOB	0.112341	2.652568	2.165961	0.032311	0.071145	-5.00418
LINC0011З	0.112211	1.771706	2.032618	0.044319	0.09265	-5.27513
LINC0048Ɛ	0.11217	1.778143	2.006474	0.047076	0.097447	-5.32638
KCNIP3	0.11209	3.757151	2.317432	0.022191	0.051758	-4.67716
FERMT2	0.112031	2.59133	2.360321	0.019888	0.047161	-4.5809
PAX4	0.111996	2.799252	2.317471	0.022189	0.051758	-4.67708
HMCN2	0.111972	4.531333	2.444781	0.015961	0.039137	-4.38664
CAPN13	0.111894	3.287491	2.080717	0.039608	0.084299	-5.17924
LOC10272	0.111837	3.585072	2.12034	0.036056	0.077875	-5.09868
TMEM136	0.111577	3.267027	2.260095	0.025636	0.058519	-4.80334
FLJ40288	0.111507	4.288694	2.208813	0.029105	0.06523	-4.91373
MED12L	0.111316	2.931608	2.051659	0.0424	0.089203	-5.23742
AVPR1A	0.111279	2.80564	2.51127	0.013372	0.033606	-4.22938
ARHGAP4:	0.111224	1.993242	2.123313	0.035801	0.077422	-5.09258
CCDC124	0.111174	3.919725	2.095469	0.038252	0.081891	-5.14941
MASP2	0.111152	4.052243	1.991306	0.048741	0.100224	-5.35583
DIXDC1	0.111084	3.368437	2.186292	0.030754	0.068244	-4.96147
SLC44A4	0.111048	2.777596	2.163039	0.03254	0.071588	-5.01029
TNFRSF19	0.110874	2.623649	2.783047	0.006266	0.017364	-3.54753
OIT3	0.110865	2.055533	2.034596	0.044117	0.092283	-5.27123
OR2H1	0.110695	2.968577	2.758684	0.006722	0.018462	-3.61118
SCRT1	0.110677	3.175926	1.998891	0.047902	0.098837	-5.34113
LOC33987	0.110525	2.376556	2.225614	0.027926	0.062974	-4.87782
FGF18	0.110442	3.573217	2.004941	0.047242	0.097746	-5.32936
EPG5	0.110365	4.629927	2.061209	0.041464	0.087549	-5.21838
LOC22007	0.110271	4.077274	1.994125	0.048427	0.09966	-5.35037
CLDN11	0.110227	2.90396	2.838772	0.005328	0.015062	-3.40011
SHC3	0.109736	3.182134	2.048216	0.042741	0.089792	-5.24426
LOC10192	0.109712	2.256864	2.168554	0.032109	0.07073	-4.99875
HOTTIP	0.109707	2.989748	2.148737	0.033683	0.073683	-5.04008

FAM71B	0.109496	3.371698	2.304211	0.022947	0.05323	-4.70652
ISPD-AS1	0.109326	1.798567	2.666075	0.008742	0.02319	-3.84861
LZTS1	0.108982	3.082922	2.278508	0.024482	0.056242	-4.76314
DLGAP2	0.108834	2.83793	2.086955	0.03903	0.083268	-5.16665
PNPLA3	0.108725	2.987872	2.026864	0.044914	0.093692	-5.28646
FBLN1	0.10858	3.214789	2.128588	0.035353	0.076646	-5.08173
WDR12	0.108478	3.648354	2.20208	0.02959	0.06611	-4.92805
HAPLN1	0.108396	2.780069	2.685137	0.008286	0.02215	-3.80033
TNNT2	0.108214	3.762236	2.061834	0.041404	0.087466	-5.21714
SRC	0.108068	3.828287	2.152299	0.033396	0.073163	-5.03267
LOC10050	0.108018	1.810431	2.020806	0.045547	0.09481	-5.29836
MRO	0.108007	2.702006	2.022325	0.045388	0.094526	-5.29538
ST7-AS2	0.107892	1.863613	2.053953	0.042173	0.088799	-5.23286
PLEKHM1	0.107661	5.222653	2.105414	0.037361	0.080234	-5.12919
CEP89	0.107644	3.210767	2.960498	0.003709	0.010982	-3.06925
COL4A6	0.107408	2.644012	2.629878	0.009671	0.025326	-3.93946
PTPN1	0.107243	4.244117	2.412147	0.017388	0.042106	-4.46244
CYP39A1	0.106832	2.212485	2.226826	0.027843	0.062806	-4.87522
ANKRD65	0.106793	4.440809	2.135541	0.034769	0.075589	-5.0674
FETUB	0.106508	1.777831	2.28478	0.024099	0.055519	-4.74937
OTUB2	0.106368	3.215291	2.500318	0.013771	0.034443	-4.25555
CLDN18	0.106265	3.372308	2.348341	0.020509	0.048414	-4.60795
SERPINB5	0.105951	2.269114	2.126414	0.035537	0.076956	-5.08621
CLSPN	0.105932	2.553784	2.462645	0.015225	0.03756	-4.34476
PPP2R2C	0.10586	2.976665	2.574066	0.011279	0.028929	-4.07738
GPR115	0.10575	2.371197	2.444921	0.015955	0.039127	-4.38632
C17orf104	0.105575	2.34855	3.185403	0.001846	0.005923	-2.42669
OR51E2	0.105252	2.652562	2.301692	0.023094	0.053522	-4.71209
SHROOM3	0.105187	3.036249	2.17092	0.031925	0.070401	-4.9938
DPH6-AS1	0.105077	1.618981	2.306763	0.0228	0.05293	-4.70086
TIE1	0.104857	2.169469	2.440174	0.016156	0.039545	-4.3974
C11orf70	0.104197	1.903726	2.506992	0.013527	0.033936	-4.23961
BCAN	0.104119	4.225544	2.056037	0.041969	0.088414	-5.2287
GABRG1	0.103952	1.811714	2.224538	0.028	0.063113	-4.88013
C6orf58	0.103569	2.640383	2.143117	0.034142	0.074453	-5.05173
LOC28559	0.103511	2.348997	2.091892	0.038577	0.082457	-5.15666
WFDC2	0.103424	3.853192	2.039853	0.043581	0.091304	-5.26084
WDR88	0.103359	2.381211	2.015327	0.046126	0.095763	-5.3091
DBF4B	0.103303	3.921028	2.327327	0.02164	0.050733	-4.6551
DLGAP1	0.10314	2.511977	2.721222	0.007482	0.020269	-3.70809
L2HGDH	0.103041	2.962242	2.243495	0.026717	0.060648	-4.83933
LOC28307	0.103012	1.838887	2.022902	0.045327	0.09442	-5.29425
APOPT1	0.10287	6.346379	2.160524	0.032739	0.071947	-5.01554
CNTNAP4	0.102722	2.839366	2.41725	0.017158	0.041642	-4.45064
C21orf88	0.102646	3.046155	2.101879	0.037675	0.080817	-5.13639
MUSK	0.102554	2.313707	2.344987	0.020686	0.048753	-4.6155
AR	0.102338	3.149124	2.108141	0.037119	0.079825	-5.12363
ADCY2	0.102325	3.443096	2.098691	0.037961	0.081353	-5.14287
ESCO2	0.102278	2.246037	2.402313	0.01784	0.043032	-4.48509
RCAN1	0.102075	4.118641	2.016971	0.045952	0.095472	-5.30588
ABCC12	0.101847	2.261525	2.28796	0.023907	0.055133	-4.74238
NXPH2	0.101732	2.828422	2.263516	0.025418	0.058073	-4.79589
LOC15176	0.101499	2.537642	2.081657	0.03952	0.084148	-5.17734
CCDC129	0.101406	2.14134	2.084204	0.039284	0.083714	-5.17221
EBF2	0.101125	2.2207	2.675111	0.008523	0.022692	-3.82576
KCNT1	0.100798	3.500275	2.150592	0.033533	0.073394	-5.03622
CTXN3	0.100794	2.137047	2.066171	0.040985	0.086726	-5.20846
GPLD1	0.100045	2.673292	2.281093	0.024324	0.055948	-4.75747

ZNF81	0.100032	2.911304	2.579316	0.011118	0.028592	-4.06452
TTLL7	0.099608	2.028473	2.126329	0.035544	0.076964	-5.08638
LINC00606	0.099513	1.838006	2.257188	0.025823	0.058879	-4.80966
ATE1	0.099299	4.060343	2.030578	0.044529	0.093008	-5.27915
DIAPH3-A	0.099267	2.918965	2.09956	0.037883	0.081212	-5.1411
ZNF688	0.099149	3.852378	2.70076	0.007929	0.021308	-3.76053
ANO4	0.099128	2.820395	1.990502	0.04883	0.100388	-5.35738
HM13	0.099061	4.676384	2.008676	0.046838	0.096994	-5.32209
ACTN2	0.099054	2.948948	1.989799	0.048909	0.10053	-5.35874
PTN	0.098653	3.312171	2.147056	0.03382	0.073919	-5.04357
BICC1	0.098615	2.043996	2.058471	0.041731	0.088012	-5.22385
DLX6	0.098111	2.304998	2.149498	0.033622	0.073564	-5.0385
TSHR	0.098	1.994901	2.109635	0.036988	0.079609	-5.12058
GAPVD1	0.097911	6.43877	1.986463	0.049283	0.101136	-5.36518
WWC1	0.097907	3.228458	2.47429	0.014762	0.036572	-4.31732
ATP9B	0.097209	4.255602	2.346792	0.02059	0.048567	-4.61144
EFCAB6	0.09718	2.549396	2.696504	0.008025	0.021524	-3.77139
GPC4	0.096666	2.378748	2.078257	0.039838	0.084684	-5.18419
TACR1	0.096633	2.463644	2.248645	0.026378	0.059964	-4.82819
NPAS2	0.096418	2.889022	2.496217	0.013923	0.034764	-4.26532
RNF17	0.09633	2.129001	2.582978	0.011007	0.028335	-4.05553
ZNF672	0.096246	4.736753	1.996261	0.048191	0.099274	-5.34623
KIAA1217	0.095735	3.442336	2.471742	0.014862	0.036785	-4.32333
ADH1B	0.095639	2.1557	2.628218	0.009716	0.025424	-3.9436
UBE3B	0.095564	4.69603	2.511285	0.013372	0.033606	-4.22935
ETV1	0.095495	3.213298	2.086116	0.039107	0.083407	-5.16835
DPPA4	0.095441	2.064376	2.249552	0.026318	0.059869	-4.82622
ACOXL	0.095329	2.951922	2.008473	0.04686	0.09703	-5.32248
CCDC144A	0.095318	1.704265	2.2628	0.025464	0.058158	-4.79745
PCDP1	0.095197	2.925887	2.054191	0.04215	0.088759	-5.23238
KCNK3	0.094883	3.882403	2.051782	0.042388	0.089186	-5.23718
RUNX1T1	0.094783	2.576135	2.364554	0.019672	0.046784	-4.57131
CCDC148	0.094695	2.152661	2.111621	0.036813	0.079292	-5.11653
OR1J4	0.094511	2.160377	2.013383	0.046333	0.096104	-5.3129
KRT7	0.09419	3.144029	2.529379	0.012736	0.032204	-4.18589
LINC00521	0.094103	3.321409	2.065385	0.041061	0.086833	-5.21003
ACRV1	0.093949	3.156525	1.996512	0.048164	0.099237	-5.34574
PLCH1	0.093662	2.141196	2.358123	0.02	0.047394	-4.58587
LINC00643	0.09366	1.927794	2.101647	0.037696	0.080853	-5.13686
DKK2	0.093637	1.671241	2.526038	0.012851	0.032448	-4.19394
ARNT	0.093457	4.938717	2.087779	0.038954	0.083175	-5.16498
ATP1A4	0.093346	2.417831	1.989207	0.048975	0.100625	-5.35988
CSMD1	0.092976	1.982428	2.989376	0.003398	0.010158	-2.989
FBXL21	0.092732	1.81961	2.491361	0.014105	0.035141	-4.27687
RAB3B	0.09257	2.909727	2.217552	0.028487	0.064047	-4.89508
ABCB5	0.092569	2.341892	2.386942	0.018567	0.044564	-4.52034
CDH8	0.092535	2.069649	2.377658	0.019018	0.045467	-4.54153
TAF10	0.092175	5.407642	2.322362	0.021915	0.05126	-4.66618
LOC28466	0.092097	1.955559	1.991551	0.048713	0.100178	-5.35535
NOL4	0.092074	2.16256	2.01028	0.046665	0.096656	-5.31896
PMCHL1	0.091211	2.397045	2.060343	0.041548	0.087682	-5.22011
ADAMTS9	0.090946	3.317831	1.987005	0.049222	0.101061	-5.36414
GH2	0.090792	3.101766	2.132837	0.034995	0.075983	-5.07298
UGT3A1	0.090397	2.441549	1.997949	0.048005	0.099021	-5.34295
CYP2C8	0.090262	2.469964	2.119663	0.036115	0.077984	-5.10007
SEMA6D	0.090208	2.930681	2.514174	0.013268	0.033382	-4.22243
PCDH15	0.090003	1.50644	2.36177	0.019814	0.047033	-4.57762
EPHA6	0.089901	1.691052	2.054465	0.042123	0.088712	-5.23184

CNKSR3	0.089302	2.135044	2.398259	0.018029	0.043432	-4.49441
LOC10192	0.089285	1.891024	2.019944	0.045638	0.094948	-5.30005
LYPD6	0.088735	2.538771	2.020157	0.045615	0.094914	-5.29964
CDH6	0.08852	2.643846	2.121812	0.03593	0.077643	-5.09566
PYGO1	0.088111	2.297375	2.047813	0.042782	0.08984	-5.24506
PARD3B	0.087945	2.593165	2.177651	0.031407	0.069439	-4.97967
SCIN	0.087805	1.975256	2.290485	0.023756	0.054834	-4.73682
CCDC181	0.085979	1.939191	2.249767	0.026304	0.059844	-4.82576
ADAMTSL:	0.085854	2.77943	2.159912	0.032787	0.072003	-5.01682
RHOJ	0.085419	2.910631	2.406281	0.017656	0.042639	-4.47596
RFX4	0.085128	1.867288	2.18134	0.031127	0.068949	-4.97191
PDZRN3	0.085096	2.62308	2.175632	0.031562	0.069728	-4.98391
BNC1	0.08476	1.689861	2.060821	0.041502	0.087602	-5.21916
CPNE4	0.084038	1.756834	2.194458	0.030147	0.067116	-4.94421
SRGAP1	0.083972	2.45121	2.069528	0.040664	0.086143	-5.20173
STK32A	0.083765	2.651575	2.012281	0.046451	0.096294	-5.31505
THSD4	0.083725	2.771464	2.184846	0.030862	0.068463	-4.96452
PDPN	0.083271	2.540017	2.014554	0.046209	0.095904	-5.31061
EBF3	0.082885	2.677709	2.078186	0.039845	0.084689	-5.18434
ARID5B	0.08199	3.404829	2.083832	0.039318	0.083779	-5.17296
NPY2R	0.081846	2.488082	2.023369	0.045278	0.094327	-5.29333
CELF4	0.081776	2.585022	2.046813	0.042881	0.090012	-5.24705
MPDZ	0.081674	2.664066	2.014272	0.046239	0.095957	-5.31116
C3orf65	0.080683	2.372689	2.150692	0.033525	0.073392	-5.03602
DEPDC4	0.079818	1.754603	2.315759	0.022286	0.051955	-4.68089
LINC00301	0.077834	1.869009	2.188946	0.030555	0.067885	-4.95587
FKBP7	0.077583	1.832736	2.001308	0.047637	0.09841	-5.33643
ASB4	0.077371	2.203646	2.195257	0.030088	0.067012	-4.94252
TNC	0.077186	1.962884	2.035544	0.04402	0.092109	-5.26936
SLC6A2	0.077109	2.926181	2.116372	0.036399	0.078524	-5.10681
TUSC3	0.0765	2.164629	2.216634	0.028551	0.064178	-4.89704
OFCC1	0.074735	2.391341	2.363509	0.019725	0.046889	-4.57368
TM4SF1	0.07367	2.341058	1.98853	0.049051	0.10075	-5.36119
LAMB1	0.072855	1.737963	2.102221	0.037645	0.080777	-5.13569
FOXP2	0.071839	2.367239	2.703766	0.007862	0.021156	-3.75284
FILIP1	0.071496	1.735933	2.167699	0.032175	0.070869	-5.00054
LINC0136(	0.070162	1.644555	2.092301	0.03854	0.082395	-5.15583
CUL7	0.069172	4.263708	1.992073	0.048655	0.100083	-5.35434
LINC0047ε	0.068792	1.755642	2.122883	0.035838	0.077477	-5.09346
RASAL2	0.066768	2.504292	2.112634	0.036725	0.079151	-5.11446
CCDC67	0.060772	1.624354	2.044333	0.04313	0.09045	-5.25197
MTBP	-0.06504	2.006711	-1.98628	0.049303	0.101147	-5.36553
C7orf26	-0.06562	5.155831	-2.30333	0.022998	0.053319	-4.70846
DKK3	-0.06598	1.967248	-2.3155	0.0223	0.051983	-4.68147
AJUBA	-0.07213	2.154626	-2.05315	0.042252	0.088938	-5.23445
SCAND2P	-0.07608	3.628132	-2.02747	0.044851	0.09357	-5.28527
PPFIBP1	-0.07835	3.084898	-2.32893	0.021552	0.050555	-4.65152
TRIO	-0.0793	3.561236	-2.19526	0.030088	0.067012	-4.94252
PDE4DIP	-0.07984	2.379621	-2.07686	0.039969	0.084909	-5.187
C8orf46	-0.08106	2.220141	-2.10476	0.037418	0.080325	-5.13052
ZNF781	-0.08106	1.870216	-2.10582	0.037325	0.080175	-5.12837
C15orf41	-0.08355	2.490571	-2.13521	0.034796	0.075641	-5.06808
C5orf42	-0.08361	2.034636	-2.16858	0.032107	0.07073	-4.9987
PIH2	-0.08387	1.556026	-2.19555	0.030067	0.066985	-4.9419
SUGT1P3	-0.0845	1.671777	-2.23835	0.02706	0.061287	-4.85044
TRIM2	-0.08597	2.408227	-2.58199	0.011037	0.028405	-4.05797
ICA1L	-0.08762	2.772555	-2.49003	0.014155	0.035248	-4.28003
RP9P	-0.08885	4.311866	-2.3175	0.022188	0.051758	-4.67702

CNNM2	-0.08887	3.474598	-2.0788	0.039787	0.08461	-5.18309
LINC00491	-0.08929	3.222116	-2.01379	0.04629	0.096024	-5.31209
ADAMTS6	-0.08963	2.186189	-2.33306	0.021326	0.050083	-4.64228
MDM2	-0.0899	3.742143	-2.01268	0.046409	0.096222	-5.31428
TLK2	-0.09011	4.675149	-2.14512	0.033978	0.0742	-5.04758
VPS52	-0.09042	6.977126	-2.04888	0.042675	0.089671	-5.24294
DYNC2H1	-0.09072	2.243989	-1.99062	0.048817	0.100371	-5.35715
NUP188	-0.09145	4.256029	-2.0165	0.046002	0.095543	-5.3068
CDHR3	-0.09148	2.285821	-2.26804	0.025133	0.057504	-4.78603
AASS	-0.09154	1.902164	-2.21765	0.028479	0.064045	-4.89486
FARP2	-0.09271	4.026677	-2.43385	0.016427	0.04012	-4.41213
PTGR2	-0.0934	1.475172	-2.08455	0.039252	0.083655	-5.17152
C9orf156	-0.09364	4.570392	-2.74458	0.006999	0.019116	-3.6478
GATM	-0.09456	2.342442	-2.7141	0.007635	0.020639	-3.72638
MID1	-0.09463	2.914217	-2.1051	0.037388	0.080277	-5.12983
PITX2	-0.09476	1.438335	-2.00025	0.047752	0.098598	-5.33848
JRK	-0.09535	5.021415	-3.04309	0.002883	0.00881	-2.83795
DFNB31	-0.0954	3.323253	-2.208	0.029163	0.065346	-4.91546
MTAP	-0.09544	3.220419	-2.38578	0.018622	0.044672	-4.52299
RREB1	-0.09548	4.453446	-2.5099	0.013421	0.033705	-4.23265
SCLY	-0.09628	3.733891	-2.45794	0.015416	0.037964	-4.35582
FUT8-AS1	-0.09651	2.952056	-1.99768	0.048035	0.099062	-5.34348
SLC3A1	-0.09682	1.974141	-2.2626	0.025476	0.05818	-4.79788
IL26	-0.09702	1.582219	-2.12657	0.035524	0.076937	-5.08588
HDAC7	-0.0971	4.085571	-2.06334	0.041258	0.087185	-5.21412
CCHCR1	-0.09717	3.873051	-2.52348	0.01294	0.03266	-4.20008
INTS4	-0.09795	2.95203	-3.30309	0.001264	0.004211	-2.07468
MPP6	-0.09811	2.073942	-2.0566	0.041914	0.088326	-5.22759
PDE12	-0.09825	3.680271	-2.32186	0.021943	0.051303	-4.66731
ZNF17	-0.09832	2.443502	-2.67656	0.008489	0.02262	-3.82208
PGBD2	-0.09863	4.167099	-2.23625	0.027202	0.06155	-4.85496
RRP8	-0.09882	3.130037	-2.94474	0.003889	0.011457	-3.11276
TTBK2	-0.09885	2.990001	-2.53656	0.012491	0.031668	-4.16858
TRUB1	-0.09928	2.791921	-2.69385	0.008085	0.021671	-3.77815
LRRC41	-0.09963	3.897389	-2.10995	0.03696	0.079565	-5.11994
DXO	-0.09992	4.886648	-2.06088	0.041496	0.087599	-5.21905
ZNF365	-0.09995	2.485432	-2.1566	0.033051	0.072501	-5.02373
ZDBF2	-0.10031	1.920565	-2.16087	0.032712	0.071903	-5.01482
CEP250	-0.10054	4.747192	-2.43587	0.01634	0.039936	-4.40742
GATAD2A	-0.10068	5.472486	-2.21785	0.028466	0.064022	-4.89445
PLEKHA8	-0.10106	2.896398	-2.95118	0.003814	0.011258	-3.09499
OTUD7B	-0.10112	3.245785	-2.44979	0.015751	0.038706	-4.37493
DCAF8	-0.10112	4.852948	-2.79917	0.00598	0.016667	-3.50513
NUMBL	-0.10121	6.199808	-2.37219	0.019289	0.046017	-4.55398
ABHD17B	-0.10126	4.689344	-2.10551	0.037352	0.080224	-5.12899
ZNF250	-0.10127	3.257457	-2.34415	0.02073	0.048846	-4.61738
TTC28	-0.10139	2.74853	-2.4282	0.016673	0.040638	-4.42527
SLC25A30	-0.10174	3.291854	-2.85408	0.005094	0.014494	-3.35917
FAM168B	-0.10235	5.117125	-2.00384	0.047361	0.097928	-5.3315
SPATS2L	-0.10255	2.963957	-2.20303	0.029521	0.065987	-4.92603
FAM161A	-0.10278	2.032305	-3.39091	0.000946	0.003237	-1.8051
LOC39990	-0.10298	3.201022	-1.98327	0.049642	0.101772	-5.37133
LOC10192	-0.10305	1.670327	-2.71607	0.007592	0.02054	-3.72132
TCEAL2	-0.10315	1.998928	-2.05746	0.041829	0.088196	-5.22586
TTLL5	-0.10339	3.081041	-2.83727	0.005351	0.015114	-3.40412
UBE3A	-0.1041	4.437968	-1.9836	0.049605	0.101716	-5.3707
B4GALT7	-0.10444	4.064057	-2.36806	0.019495	0.046439	-4.56335
TNPO2	-0.10448	4.739063	-2.13456	0.034851	0.075727	-5.06943

SRCAP	-0.10453	4.493437	-2.17775	0.0314	0.06943	-4.97946
KSR1	-0.10468	4.549981	-2.06149	0.041437	0.0875	-5.21782
NKAPL	-0.10479	2.3948	-2.00303	0.04745	0.098082	-5.33309
C2CD2L	-0.10501	4.004858	-2.31168	0.022517	0.052405	-4.68995
ZNF19	-0.10508	3.50239	-2.40542	0.017696	0.042729	-4.47795
FITM2	-0.10532	3.717903	-2.36184	0.01981	0.04703	-4.57746
ZNF93	-0.10534	3.435371	-2.17713	0.031447	0.06949	-4.98076
C10orf12	-0.10706	3.604364	-2.36151	0.019827	0.04706	-4.57821
FBF1	-0.10711	4.112762	-2.55299	0.011946	0.030414	-4.12876
ZDHHC14	-0.10732	3.613089	-2.4264	0.016752	0.040806	-4.42946
JUN	-0.10739	3.466735	-2.09587	0.038216	0.081831	-5.14861
MZF1	-0.1075	5.374096	-2.64166	0.00936	0.024594	-3.91
SLC35E1	-0.10772	5.940623	-2.34601	0.020632	0.048648	-4.6132
MLLT10	-0.10811	4.787879	-2.38629	0.018598	0.044624	-4.52184
KDM4A	-0.10856	5.272782	-2.09392	0.038392	0.082148	-5.15255
ANXA11	-0.10877	7.941846	-2.00444	0.047295	0.097823	-5.33033
LMLN	-0.10917	3.418656	-2.6504	0.009134	0.024064	-3.88808
ZNF549	-0.11052	1.869794	-2.0813	0.039554	0.084202	-5.17807
SMG9	-0.11077	4.318261	-2.54154	0.012323	0.031285	-4.15653
RPGRIP1L	-0.1108	1.809614	-3.12506	0.002234	0.007014	-2.60303
LOC10192	-0.11093	1.646286	-2.29309	0.023601	0.054518	-4.73109
KIAA0226	-0.11112	4.416323	-3.22425	0.001631	0.005289	-2.31167
ATG13	-0.11134	6.77771	-2.20127	0.029649	0.066224	-4.92978
EXOSC2	-0.11135	4.004823	-2.15139	0.033469	0.073292	-5.03457
TMEM222	-0.11138	6.642726	-2.56035	0.011709	0.029907	-4.11085
RHPN2	-0.11181	1.593813	-2.09114	0.038646	0.082588	-5.15819
DYRK1A	-0.11188	4.488369	-2.50582	0.013569	0.034018	-4.24242
APEX2	-0.11203	5.270891	-2.27539	0.024674	0.056575	-4.76996
ICMT	-0.11206	4.370552	-2.38946	0.018446	0.044326	-4.51458
NUP50	-0.11236	5.189144	-2.10759	0.037168	0.079914	-5.12476
ARMCX4	-0.11251	2.855329	-2.92986	0.004067	0.011927	-3.15366
ZNF547	-0.11274	1.884698	-2.64412	0.009296	0.024446	-3.90386
RAD18	-0.11279	2.830233	-2.73099	0.007276	0.019783	-3.68293
SMPD1	-0.1129	4.942942	-2.23866	0.027039	0.061258	-4.84976
ALS2CL	-0.11297	4.024501	-2.05714	0.04186	0.08824	-5.2265
PRO2214	-0.11343	2.407254	-2.29872	0.023268	0.05387	-4.71866
GOLGA3	-0.11363	4.687918	-2.18952	0.030513	0.067813	-4.95466
PPP2R2D	-0.11378	4.825491	-2.4356	0.016351	0.03996	-4.40805
SIRT3	-0.11379	5.680917	-2.13225	0.035044	0.076074	-5.07418
MACROD1	-0.114	2.544803	-2.23217	0.027478	0.062079	-4.86375
KIF1C	-0.11407	4.062034	-2.27071	0.024965	0.057159	-4.78019
N6AMT1	-0.11417	2.928386	-2.95904	0.003725	0.011021	-3.07329
ANKRD26	-0.11454	2.50313	-2.18981	0.030491	0.06778	-4.95405
KIAA0895	-0.11456	1.565364	-2.22432	0.028016	0.063141	-4.8806
TAS2R19	-0.11462	1.687511	-2.24212	0.026808	0.060795	-4.84229
ZNF451	-0.11479	5.035452	-2.19332	0.03023	0.067266	-4.94661
GAN	-0.115	2.844308	-2.51577	0.013212	0.03326	-4.21861
TPTE2P6	-0.11512	1.704804	-1.99715	0.048094	0.099133	-5.34451
LINC0024(	-0.11531	1.705412	-2.37958	0.018924	0.04529	-4.53716
RNF103	-0.11552	4.93151	-2.18815	0.030615	0.067995	-4.95755
ULK4	-0.11569	3.30136	-2.59992	0.010507	0.027186	-4.01382
CCDC120	-0.11613	2.940467	-2.41088	0.017446	0.04222	-4.46536
HDAC6	-0.11637	4.724244	-2.03587	0.043987	0.092049	-5.26872
POFUT2	-0.11637	4.3148	-2.51257	0.013326	0.033514	-4.22627
OCIAD1	-0.1165	7.100593	-2.1615	0.032662	0.071809	-5.0135
LOC10192	-0.11651	1.627185	-2.34609	0.020627	0.048643	-4.61301
SPTBN1	-0.11653	3.392336	-2.29219	0.023654	0.054628	-4.73306
TRAP1	-0.11654	4.151933	-2.77301	0.00645	0.01781	-3.57382

ZFHX3	-0.1166	3.254475	-2.53508	0.012541	0.031771	-4.17215
GPR55	-0.11676	2.427749	-2.3478	0.020537	0.04847	-4.60918
LOC14841	-0.11681	6.047284	-2.33764	0.021078	0.049557	-4.632
GGA3	-0.11683	6.313568	-2.30025	0.023178	0.05368	-4.71527
SMURF1	-0.1169	3.836749	-2.10392	0.037493	0.080468	-5.13223
TBL3	-0.11695	4.300262	-2.53092	0.012683	0.032086	-4.18218
FOXK1	-0.11706	4.613102	-2.56032	0.01171	0.029907	-4.11094
DZANK1	-0.11707	3.004992	-2.19798	0.029888	0.066671	-4.93675
ZNF696	-0.11738	3.946576	-2.08757	0.038973	0.083199	-5.1654
PIGO	-0.11765	4.277212	-2.44046	0.016144	0.039527	-4.39672
ZNF287	-0.11794	2.402421	-3.25051	0.001499	0.004897	-2.23327
RBMS3-AS	-0.11805	1.636416	-2.16016	0.032768	0.071988	-5.0163
TSC22D1-	-0.11812	2.524967	-2.3775	0.019026	0.04548	-4.5419
POLR2C	-0.11831	6.009984	-2.4802	0.014531	0.036063	-4.30335
WASL	-0.11834	4.260244	-2.54623	0.012168	0.030913	-4.14517
CCDC132	-0.11837	3.312896	-2.16066	0.032728	0.071932	-5.01526
AHI1	-0.1184	2.334975	-3.19756	0.001776	0.005715	-2.39084
ANKRD10	-0.11841	4.778657	-1.98125	0.049872	0.10217	-5.37523
DLG3	-0.11853	4.126781	-2.65383	0.009047	0.023878	-3.87947
PLCXD1	-0.11855	5.195445	-2.05729	0.041846	0.088218	-5.2262
SKIV2L	-0.11862	5.901588	-2.40239	0.017836	0.043028	-4.48491
FUT10	-0.11863	3.405754	-2.12819	0.035387	0.076704	-5.08256
SEMA4F	-0.11873	4.617771	-2.21388	0.028745	0.064537	-4.90293
GOLGA2	-0.11889	5.303801	-1.99467	0.048367	0.099546	-5.34931
TRIM69	-0.1189	4.713645	-2.14026	0.034377	0.074841	-5.05765
LOC10272	-0.11895	3.46825	-2.6124	0.010151	0.0264	-3.98294
GPM6B	-0.11916	2.237198	-2.6695	0.008659	0.023019	-3.83996
TEF	-0.11922	2.526956	-2.69224	0.008122	0.021767	-3.78225
KMT2B	-0.11933	5.033295	-2.26924	0.025057	0.057352	-4.78342
LRRC16A	-0.11941	3.062543	-2.72148	0.007476	0.020257	-3.70743
ZNF740	-0.1195	3.961867	-2.99777	0.003312	0.009932	-2.96554
TMEM8B	-0.11954	4.100318	-2.42324	0.016891	0.041087	-4.43678
ENOX2	-0.11967	3.745598	-3.1991	0.001768	0.005689	-2.38627
ZNF883	-0.11991	1.870888	-1.99937	0.047849	0.098768	-5.34019
SIPA1L3	-0.11999	4.178719	-3.0297	0.003004	0.009129	-2.87582
LOC10192	-0.12016	2.27679	-2.785	0.006231	0.017285	-3.54241
HHLA3	-0.12018	3.169502	-2.34743	0.020557	0.048505	-4.61001
NRF1	-0.12021	4.873011	-2.01548	0.04611	0.095739	-5.3088
CLASRP	-0.12047	6.578233	-2.19568	0.030057	0.066974	-4.94163
PDCD7	-0.12088	5.400731	-2.02571	0.045034	0.093905	-5.28874
MIER2	-0.12131	4.366108	-2.25078	0.026238	0.059726	-4.82357
TYSND1	-0.12166	4.311328	-3.02022	0.003093	0.009358	-2.90254
TCP10	-0.12175	2.487902	-2.03183	0.0444	0.092791	-5.27669
ARID4B	-0.12177	4.575719	-2.06478	0.041118	0.086933	-5.21124
ZNF616	-0.12179	1.780683	-2.55668	0.011827	0.030149	-4.11981
KANSL3	-0.12182	4.249685	-3.46932	0.000728	0.002551	-1.5595
SDK2	-0.12191	4.031135	-2.64861	0.00918	0.024172	-3.89259
COPS7B	-0.12215	4.335046	-2.87457	0.004795	0.013774	-3.30406
FBXL17	-0.1222	3.144432	-2.31475	0.022343	0.052058	-4.68313
C11orf45	-0.12238	1.938322	-2.02676	0.044925	0.093706	-5.28667
ZNF473	-0.12257	3.729866	-2.06802	0.040807	0.086394	-5.20475
ANGEL1	-0.12258	4.753243	-2.44346	0.016017	0.039249	-4.38973
ARHGAP32	-0.12266	2.580963	-3.23585	0.001571	0.005115	-2.2771
ARHGEF7	-0.12294	4.142396	-2.81774	0.005666	0.015894	-3.45605
BCAT2	-0.12295	4.866506	-2.06557	0.041043	0.08683	-5.20967
ZKSCAN3	-0.12316	2.992413	-3.12183	0.002257	0.007073	-2.61238
PROSER3	-0.1232	4.151755	-2.23326	0.027404	0.061953	-4.8614
SLC39A14	-0.12387	3.012325	-2.1336	0.034931	0.075869	-5.07141

DNASE1L3	-0.12393	3.135806	-2.14245	0.034197	0.074543	-5.05312
TCEB3	-0.12421	4.722454	-2.9756	0.003543	0.01055	-3.02735
SLC35G3	-0.12428	1.864807	-2.33142	0.021415	0.050258	-4.64594
C1orf210	-0.1243	2.033618	-2.23156	0.027519	0.062144	-4.86505
C18orf8	-0.12438	4.601343	-3.03459	0.002959	0.009015	-2.862
ZC3HAV1L	-0.1244	2.042007	-2.01945	0.04569	0.095026	-5.30103
PIK3C3	-0.12442	4.124488	-2.35072	0.020384	0.048175	-4.60258
HDLBP	-0.12452	4.651316	-2.34955	0.020446	0.048292	-4.60524
COL4A3	-0.12468	2.509015	-2.80645	0.005855	0.016356	-3.48592
SZT2	-0.12513	3.844998	-3.04144	0.002898	0.008845	-2.84262
TAF2	-0.12531	5.78322	-1.99523	0.048305	0.099458	-5.34822
IRS1	-0.12535	2.859684	-2.9773	0.003525	0.010505	-3.02262
CCAR2	-0.12549	3.522482	-2.31129	0.02254	0.052452	-4.69083
LOC10029	-0.12564	2.612057	-2.29866	0.023271	0.053872	-4.71879
LOC10050	-0.12574	3.331674	-2.30028	0.023176	0.05368	-4.71522
KLHL13	-0.12578	1.705094	-2.12755	0.03544	0.07678	-5.08386
UBN2	-0.12581	4.401458	-2.74398	0.007011	0.019147	-3.64936
ALG2	-0.12592	5.009267	-2.17013	0.031986	0.070505	-4.99545
GNPTAB	-0.12708	5.442161	-2.38511	0.018655	0.044729	-4.52453
FAM160B2	-0.12713	4.5404	-2.70472	0.00784	0.021112	-3.7504
DNAJC21	-0.12749	2.786005	-2.9915	0.003376	0.010103	-2.98308
NCOR2	-0.12752	4.599432	-2.60173	0.010454	0.027068	-4.00934
LARP4	-0.1277	3.79548	-2.16553	0.032345	0.071189	-5.00509
OSBPL10	-0.12777	4.106472	-2.45741	0.015438	0.038003	-4.35707
CTLA4	-0.12786	3.5034	-2.73273	0.00724	0.019704	-3.67845
TSC2	-0.12792	4.679202	-2.45983	0.015339	0.037793	-4.35139
TRAF4	-0.12801	3.938049	-3.17316	0.00192	0.006129	-2.4627
CNP	-0.12812	4.552578	-2.43257	0.016482	0.040237	-4.41512
INO80D	-0.12817	5.298776	-3.04904	0.00283	0.008664	-2.82107
SLC35F3	-0.12824	2.241278	-2.18314	0.030991	0.068681	-4.96812
MRPS26	-0.12831	4.504051	-2.07382	0.040255	0.085376	-5.19311
VANGL1	-0.12839	3.312802	-2.68254	0.008347	0.022289	-3.80693
PACRGL	-0.12855	2.573337	-2.74795	0.006932	0.018953	-3.63905
SCD5	-0.12895	2.663439	-3.2961	0.001293	0.004296	-2.09589
POLR1E	-0.12903	3.541498	-2.35114	0.020362	0.04814	-4.60164
C9orf85	-0.12911	4.222882	-2.72529	0.007396	0.020065	-3.69763
KLHL32	-0.12912	1.507389	-2.68265	0.008345	0.022285	-3.80663
RHOT2	-0.12915	6.727505	-3.06077	0.00273	0.008393	-2.78773
LOC28555	-0.12925	1.80207	-2.00474	0.047263	0.097776	-5.32975
IPO5P1	-0.1293	2.579759	-3.06806	0.002669	0.008224	-2.76694
LOC25739	-0.12943	1.966396	-2.28693	0.023969	0.055244	-4.74464
WARS2	-0.12947	3.013582	-2.47262	0.014827	0.036704	-4.32127
ZNF496	-0.12969	3.595011	-2.77014	0.006504	0.017938	-3.58132
CTNNBIP1	-0.12979	6.396303	-2.4446	0.015969	0.039141	-4.38707
TRAFD1	-0.1299	5.572252	-2.51221	0.013339	0.033538	-4.22714
ANKS6	-0.12993	3.299667	-2.46345	0.015193	0.03749	-4.34287
POMGNT1	-0.13007	3.03421	-2.45669	0.015467	0.038053	-4.35876
ZNF836	-0.13013	3.748784	-2.2289	0.027701	0.062513	-4.87077
CASP10	-0.13023	4.874669	-2.51847	0.013116	0.033051	-4.21213
RPS14	-0.13036	5.164986	-3.31639	0.00121	0.004049	-2.03423
ZNF717	-0.13038	3.749507	-2.46135	0.015277	0.037645	-4.3478
LATS1	-0.13046	3.319584	-2.52044	0.013047	0.032889	-4.20741
MAP2K5	-0.13058	3.236738	-3.64698	0.000395	0.001467	-0.98618
AKAP5	-0.13074	2.172345	-2.74565	0.006978	0.019063	-3.64503
ZDHHC21	-0.13077	2.490015	-3.68749	0.000343	0.00129	-0.85225
FBXW8	-0.13082	3.172793	-2.41696	0.017171	0.041647	-4.45131
ARFGEF2	-0.13082	4.735302	-2.28384	0.024156	0.055634	-4.75143
RIMS3	-0.13094	3.497563	-2.80593	0.005864	0.016376	-3.4873

NDUFV1	-0.13143	5.946347	-2.14273	0.034173	0.074508	-5.05253
ZNF778	-0.13162	2.578561	-2.09812	0.038013	0.081438	-5.14403
TCEA1	-0.13166	5.293264	-3.15731	0.002018	0.006408	-2.50912
PPP6R2	-0.13193	4.734081	-2.7525	0.006842	0.018742	-3.62726
KMT2D	-0.13202	5.36928	-2.96558	0.003652	0.010837	-3.05518
BNIP1	-0.13215	3.822373	-2.80903	0.005811	0.016251	-3.4791
TPTE2P5	-0.13255	3.098511	-2.82938	0.005476	0.015437	-3.42512
LOC10050	-0.13263	3.727078	-2.11436	0.036574	0.07886	-5.11093
UGGT1	-0.13306	4.899821	-2.95868	0.003729	0.01103	-3.07429
KDM4B	-0.13308	5.946967	-2.83373	0.005407	0.015256	-3.41354
ZNF77	-0.13324	3.368654	-1.98682	0.049243	0.101084	-5.3645
PTPN11	-0.13341	4.296199	-2.80182	0.005934	0.016555	-3.49816
WBSCR16	-0.13346	3.739996	-2.94323	0.003907	0.011502	-3.11691
LOC10013	-0.13379	1.656356	-2.52341	0.012942	0.032662	-4.20025
CDK12	-0.13398	5.014671	-3.41797	0.000865	0.002981	-1.72085
DPP7	-0.13407	4.677898	-2.36486	0.019657	0.046758	-4.57061
COLCA1	-0.13427	6.504965	-2.33266	0.021347	0.050122	-4.64316
LOC10027	-0.13431	1.705023	-3.37226	0.001007	0.003422	-1.86284
ZNF626	-0.13431	2.863244	-3.20571	0.001731	0.005582	-2.36672
LOC40096	-0.1345	2.460432	-2.07567	0.040081	0.085067	-5.18939
SH3BP4	-0.13457	2.644572	-2.17861	0.031334	0.069307	-4.97765
TMEM177	-0.13463	3.682915	-2.59431	0.01067	0.027539	-4.02765
NOC2L	-0.13465	4.032589	-2.78126	0.006299	0.017438	-3.55221
AMBRA1	-0.13477	3.77823	-3.62669	0.000424	0.001563	-1.05284
PIK3R3	-0.13481	2.78657	-2.22549	0.027935	0.062987	-4.87809
TRIM66	-0.13485	4.31464	-2.32186	0.021943	0.051303	-4.66729
TATDN2	-0.1351	4.839767	-3.30233	0.001267	0.004218	-2.07698
TMEM248	-0.13511	6.857122	-2.25749	0.025803	0.058841	-4.809
ZNF692	-0.13514	5.571859	-2.11323	0.036673	0.079056	-5.11325
ALS2	-0.13514	2.967872	-2.66719	0.008715	0.023139	-3.84579
SPATA7	-0.13519	2.501657	-3.23161	0.001593	0.00518	-2.28975
DHX8	-0.13546	5.488367	-2.80723	0.005842	0.016326	-3.48387
LINC0066	-0.13561	2.356344	-3.46555	0.000737	0.002582	-1.5714
SLC4A5	-0.13563	3.125311	-2.70731	0.007783	0.020982	-3.74377
RHOB	-0.1357	3.845159	-2.42113	0.016984	0.04126	-4.44167
SYCE1L	-0.13591	2.791945	-2.509	0.013454	0.033779	-4.23481
RABEPK	-0.13608	4.000209	-2.87399	0.004803	0.013792	-3.30561
PLEKHM2	-0.13611	5.400957	-2.06664	0.04094	0.086649	-5.20753
IPO8	-0.13618	6.020527	-2.14775	0.033764	0.073818	-5.04213
LOC10106	-0.13634	2.010206	-2.07425	0.040215	0.085299	-5.19225
SOX7	-0.13659	1.765873	-3.27994	0.001363	0.004498	-2.14476
ZNF276	-0.1366	5.748616	-2.44592	0.015913	0.039038	-4.38398
KCTD15	-0.13673	3.285558	-2.69064	0.008159	0.021854	-3.78632
MOB3B	-0.13679	4.038703	-2.32735	0.021639	0.050733	-4.65506
ZMYND8	-0.13683	5.028407	-2.96244	0.003687	0.010928	-3.06388
GLUD2	-0.13698	3.551517	-2.16221	0.032605	0.071708	-5.01201
TMEM231	-0.137	2.358153	-3.11922	0.002276	0.007124	-2.61993
GNAL	-0.13702	2.512108	-3.06908	0.00266	0.008201	-2.76403
RNFT2	-0.13718	3.332474	-2.56533	0.011552	0.029543	-4.09873
PEX14	-0.13727	4.380137	-2.266	0.025261	0.057771	-4.79047
TNFSF15	-0.13738	2.466106	-2.78687	0.006197	0.017202	-3.53751
CTTNBP2N	-0.13751	2.199137	-3.49992	0.000656	0.002324	-1.4624
EIF1AD	-0.13759	5.390495	-2.19402	0.030179	0.067168	-4.94515
GDPD1	-0.13761	2.644617	-2.91564	0.004244	0.012384	-3.19257
NEUROD6	-0.1378	3.04629	-2.08761	0.038969	0.083199	-5.16533
TEX264	-0.13783	5.35361	-2.18876	0.030569	0.067909	-4.95627
PAOX	-0.13792	4.025191	-2.79593	0.006037	0.016804	-3.51367
SREK1	-0.13804	3.94775	-2.04797	0.042766	0.089815	-5.24475

SPTSSB	-0.13809	2.486345	-2.11907	0.036166	0.078087	-5.10129
BRAP	-0.13819	5.631183	-2.56222	0.01165	0.029771	-4.10631
SAMD10	-0.13822	4.41636	-2.55992	0.011723	0.029929	-4.11192
GSTA1	-0.13838	3.311695	-2.33897	0.021007	0.049407	-4.62903
ILVBL	-0.13843	4.527867	-2.89424	0.004523	0.013104	-3.25082
EXOSC7	-0.13862	5.555196	-2.24052	0.026915	0.06101	-4.84574
ASAH2B	-0.13864	2.230018	-2.60711	0.010301	0.026726	-3.99605
SNX5	-0.13873	5.182785	-2.37273	0.019262	0.045977	-4.55274
USP51	-0.13876	2.573566	-2.35835	0.019989	0.047372	-4.58537
TSNARE1	-0.1392	4.433593	-2.92081	0.004179	0.012218	-3.17843
MGC5734(	-0.1392	2.999001	-2.22118	0.028233	0.063554	-4.88731
UBE3D	-0.13959	1.777655	-4.16371	5.96E-05	0.00026	0.80778
SETDB2	-0.13963	2.987204	-2.95318	0.003792	0.0112	-3.08948
LOC28378	-0.13998	3.191362	-2.11006	0.03695	0.079553	-5.11971
ZNF711	-0.14005	1.7388	-2.52994	0.012716	0.032163	-4.18454
PER2	-0.1402	4.160835	-2.41446	0.017283	0.041882	-4.45709
SFT2D3	-0.14022	4.49764	-2.61607	0.010049	0.026163	-3.97382
LINC00307	-0.14031	1.68689	-2.90685	0.004357	0.012671	-3.21655
LOC72965	-0.14035	1.610761	-2.41705	0.017166	0.041647	-4.4511
GAS2	-0.14051	1.980361	-2.1051	0.037389	0.080277	-5.12983
TIMD4	-0.141	2.606399	-2.00521	0.047212	0.0977	-5.32884
CEP70	-0.14104	2.086075	-4.06666	8.61E-05	0.000364	0.457008
CLN8	-0.14107	4.827384	-1.98642	0.049288	0.101136	-5.36527
RORC	-0.14119	4.158876	-2.57717	0.011184	0.028725	-4.06977
KCNQ1	-0.14135	6.444885	-1.99201	0.048662	0.100083	-5.35446
TRAV12-2	-0.14135	2.92202	-2.01695	0.045954	0.095472	-5.30591
OLFM2	-0.14151	3.822103	-2.43075	0.016561	0.0404	-4.41934
ZNF554	-0.14163	3.759672	-2.56654	0.011513	0.029462	-4.09576
LINC00638	-0.14163	5.237273	-2.36504	0.019648	0.046748	-4.57021
SYBU	-0.14169	2.185264	-2.41808	0.017121	0.041562	-4.44873
ZBTB49	-0.14185	5.00061	-2.58645	0.010903	0.028101	-4.04699
LIN7B	-0.14215	3.416414	-2.52926	0.01274	0.032211	-4.18619
ZC4H2	-0.14219	4.276393	-2.70832	0.007761	0.020931	-3.74119
CACNB3	-0.14223	3.784395	-2.43023	0.016584	0.040451	-4.42055
TBC1D5	-0.14227	6.697404	-2.58132	0.011057	0.028446	-4.0596
SFXN2	-0.14258	3.933668	-2.58598	0.010917	0.028134	-4.04815
DPH5	-0.14273	5.25784	-2.23317	0.02741	0.061959	-4.86159
TIFAB	-0.14274	3.997382	-2.48866	0.014207	0.035344	-4.28329
RBL1	-0.14275	2.738697	-3.13851	0.002142	0.006751	-2.56397
HGS	-0.14277	5.070032	-2.89394	0.004527	0.01311	-3.25166
LUZP1	-0.14281	4.632289	-2.88913	0.004592	0.013263	-3.2647
CRAMP1L	-0.14302	3.574637	-2.91897	0.004202	0.012279	-3.18349
ZNF257	-0.14312	1.563268	-3.14747	0.002082	0.006585	-2.53786
MICAL3	-0.14313	3.800298	-2.89371	0.00453	0.013117	-3.25229
POU2F1	-0.14326	4.76699	-3.46091	0.000749	0.002621	-1.58606
HEMK1	-0.14335	4.359765	-3.42509	0.000844	0.002916	-1.69861
ZMIZ2	-0.14335	5.239476	-3.22724	0.001615	0.005243	-2.30279
GRIP1	-0.14362	2.324102	-2.90223	0.004417	0.012827	-3.22914
LOC10050	-0.14378	3.222453	-2.26387	0.025395	0.058041	-4.79511
LOC10192	-0.14393	3.561714	-2.41499	0.017259	0.041833	-4.45586
CCDC14	-0.144	2.822697	-2.3352	0.02121	0.049839	-4.63748
COX10	-0.14401	5.76187	-2.05687	0.041887	0.088278	-5.22704
CA6	-0.14419	2.451767	-2.87136	0.004841	0.013886	-3.31273
ZNF709	-0.14487	2.524354	-4.03347	9.75E-05	0.000407	0.338488
GCC1	-0.14493	4.110306	-3.30366	0.001261	0.004206	-2.07296
PXMP4	-0.14505	4.635097	-3.42988	0.000831	0.002875	-1.68361
TMEM104	-0.14555	5.166994	-2.52908	0.012746	0.032219	-4.18662
KIAA0100	-0.14562	5.539425	-2.17896	0.031308	0.069263	-4.97692

GPATCH4	-0.1457	3.883003	-3.00649	0.003225	0.009704	-2.94111
LINC00293	-0.14571	3.477404	-2.02039	0.045591	0.094873	-5.29918
MAPK1IP1	-0.14597	6.806207	-2.2414	0.026857	0.060898	-4.84386
ACSS1	-0.14603	4.28137	-3.38252	0.000973	0.00332	-1.83111
SGPL1	-0.14634	4.925378	-2.16011	0.032772	0.071988	-5.0164
C1orf204	-0.14667	4.485253	-2.65917	0.008913	0.023582	-3.86603
MRPL38	-0.14704	4.743052	-2.40523	0.017705	0.042741	-4.47839
TSPAN6	-0.14731	2.200557	-2.79703	0.006017	0.016759	-3.51077
ZFP37	-0.14734	3.247193	-2.00357	0.047391	0.097981	-5.33204
MLF1	-0.14737	1.932907	-3.04684	0.00285	0.00872	-2.82732
PDP2	-0.14753	2.988006	-3.42026	0.000858	0.00296	-1.71371
DCAF5	-0.14764	4.810783	-3.47662	0.00071	0.002495	-1.53637
AAAS	-0.14767	5.957678	-2.36304	0.019749	0.046918	-4.57474
BTF3P11	-0.14772	1.827052	-2.77818	0.006355	0.017575	-3.5603
ZFYVE19	-0.14773	5.131518	-2.49519	0.013961	0.034839	-4.26777
NUP54	-0.14789	3.122798	-2.56501	0.011562	0.029564	-4.0995
ZNF649	-0.14806	2.781281	-2.40635	0.017653	0.042637	-4.47581
SPG20	-0.14808	3.904163	-3.27242	0.001396	0.004595	-2.16744
SLC35G1	-0.14834	2.647766	-3.61998	0.000434	0.001596	-1.07481
ERCC4	-0.14847	4.029875	-2.53005	0.012712	0.032157	-4.18427
CEP83	-0.14861	2.278253	-1.98937	0.048957	0.100608	-5.35957
TBC1D12	-0.14876	2.409392	-2.25506	0.02596	0.059152	-4.81428
ZNF490	-0.14878	4.156108	-2.14734	0.033797	0.073875	-5.04297
TRIP11	-0.14879	3.334839	-2.52421	0.012914	0.032604	-4.19832
ADAM12	-0.14907	2.562659	-3.35006	0.001083	0.003658	-1.93123
STX18-AS1	-0.14928	2.385724	-2.14579	0.033923	0.074113	-5.04619
ZNF615	-0.14946	4.825006	-2.0442	0.043143	0.090468	-5.25222
USP7	-0.14952	6.140082	-2.86833	0.004884	0.013997	-3.32088
HERPUD1	-0.14954	6.394174	-2.48041	0.014523	0.036047	-4.30286
ZNF213-A	-0.14962	2.5241	-2.57171	0.011352	0.029087	-4.08315
TSPY26P	-0.14972	4.045128	-2.7551	0.006791	0.01862	-3.62051
CCDC65	-0.14984	2.133916	-4.21312	4.93E-05	0.000219	0.988716
SMEK2	-0.14986	4.306384	-2.65097	0.00912	0.024038	-3.88665
METAP1D	-0.14987	2.564256	-3.75104	0.000274	0.001051	-0.63978
FLJ42627	-0.14996	4.784802	-2.69444	0.008072	0.021644	-3.77664
GOLGA6A	-0.15029	2.408873	-2.42238	0.016929	0.041155	-4.43877
LOC10272	-0.15034	2.02206	-2.25955	0.025671	0.058585	-4.80452
LINC00910	-0.15041	2.543392	-2.99381	0.003352	0.010039	-2.97661
C1orf56	-0.15047	4.107737	-2.43156	0.016526	0.040331	-4.41746
CDYL	-0.15048	5.400161	-2.52876	0.012757	0.032238	-4.18738
LINC00886	-0.15052	4.460481	-2.02389	0.045224	0.094233	-5.29231
GRWD1	-0.15083	5.674298	-2.00241	0.047517	0.098185	-5.3343
KIFC2	-0.15089	4.76258	-2.279	0.024452	0.056186	-4.76206
WDR5	-0.15104	4.321307	-2.4019	0.017859	0.043073	-4.48605
CISD3	-0.15107	4.672011	-2.89699	0.004486	0.013007	-3.24338
C19orf60	-0.1511	5.579461	-2.03652	0.04392	0.091937	-5.26742
CUX2	-0.1511	3.213317	-3.21719	0.001668	0.005393	-2.33267
FRS2	-0.15122	3.266015	-2.91566	0.004243	0.012384	-3.19251
ZNF582-A	-0.15123	3.4683	-2.47386	0.014779	0.036601	-4.31834
ZNF568	-0.15141	2.153893	-4.14436	6.41E-05	0.000278	0.737325
DHPS	-0.15147	6.386784	-2.54594	0.012177	0.030933	-4.14587
PGAP1	-0.15147	2.124677	-3.26651	0.001423	0.004675	-2.18525
PDCD2	-0.15162	4.097836	-2.27001	0.025008	0.057246	-4.78172
FBRS	-0.15168	5.638158	-2.20486	0.029389	0.065765	-4.92214
ZNF440	-0.15185	2.360888	-3.23541	0.001573	0.005122	-2.27842
LOC28606	-0.15192	3.127076	-2.35842	0.019985	0.047369	-4.5852
SERGEF	-0.15198	3.342747	-3.6337	0.000414	0.001528	-1.02983
NTMT1	-0.15204	5.016355	-2.75534	0.006787	0.018613	-3.61989

TMEM63A	-0.15219	5.663347	-2.06969	0.040649	0.08612	-5.20142
MED26	-0.15237	5.53166	-2.9746	0.003554	0.010569	-3.03013
SLC25A17	-0.15248	4.525801	-2.87328	0.004813	0.013817	-3.30753
FGFR1OP	-0.15267	4.078557	-2.70459	0.007843	0.021115	-3.75073
ANKRD52	-0.15283	6.66353	-1.99627	0.04819	0.099274	-5.34621
RNF38	-0.15288	6.193964	-2.13902	0.03448	0.075048	-5.06021
LOC10013	-0.1529	3.834199	-2.03749	0.043821	0.091759	-5.26552
AGBL2	-0.15329	2.271395	-2.01938	0.045698	0.095027	-5.30117
PHLDB3	-0.15345	2.975059	-2.69738	0.008005	0.021484	-3.76915
VGLL4	-0.15369	6.261119	-2.28857	0.023871	0.055073	-4.74103
IFT43	-0.15409	3.939655	-3.61325	0.000444	0.001631	-1.0968
COL4A4	-0.15433	2.454477	-3.32199	0.001188	0.003984	-2.01716
PLA2G6	-0.15441	5.211707	-3.14421	0.002104	0.006648	-2.54736
WWP2	-0.15449	5.477608	-2.38802	0.018515	0.04445	-4.51788
LOC10012	-0.15453	3.770906	-2.13599	0.034732	0.075524	-5.06648
PAEP	-0.15454	2.675098	-2.00579	0.04715	0.097581	-5.32772
GPR180	-0.15468	2.438816	-3.30304	0.001264	0.004211	-2.07482
ZNF581	-0.15471	6.856852	-2.50237	0.013695	0.034279	-4.25064
NCAM1	-0.15487	3.330687	-3.5314	0.000589	0.00211	-1.36179
TUBGCP6	-0.1549	4.363685	-2.81025	0.005791	0.016198	-3.47588
WDR55	-0.15491	5.492254	-4.4577	1.89E-05	9.14E-05	1.907269
LZTS3	-0.15492	4.445148	-2.09845	0.037983	0.081391	-5.14336
CCDC130	-0.15496	5.903583	-2.63131	0.009633	0.025238	-3.93588
LOC28636	-0.15497	2.649002	-2.05143	0.042423	0.089232	-5.23788
GDI1	-0.15531	7.984907	-2.18051	0.03119	0.069055	-4.97366
PLXNA3	-0.1555	3.721455	-3.08763	0.002511	0.007785	-2.71094
HECW2	-0.15557	2.620749	-2.55413	0.011909	0.030332	-4.126
RELL2	-0.15575	5.667903	-2.76602	0.006582	0.018121	-3.59208
ZNF765	-0.15577	4.490604	-2.39304	0.018275	0.043952	-4.50639
RBMS2	-0.15592	3.645429	-3.14888	0.002073	0.00656	-2.53377
SDR42E1	-0.15604	3.140703	-3.13984	0.002133	0.006731	-2.56008
ZSCAN12	-0.1561	2.573367	-4.21092	4.97E-05	0.000221	0.980601
UBE2D4	-0.15612	5.349375	-3.43479	0.000817	0.002833	-1.6682
COL9A2	-0.15616	4.537877	-2.80637	0.005856	0.016357	-3.48613
LOC10192	-0.15622	2.560842	-3.38699	0.000959	0.003274	-1.81726
FAM120A	-0.15631	4.807447	-3.60152	0.000463	0.001694	-1.13509
KLF8	-0.15634	2.298865	-3.16184	0.00199	0.006325	-2.49589
PSMB2	-0.15635	5.609461	-2.94129	0.00393	0.011557	-3.12225
LOC10192	-0.15635	3.720544	-2.29518	0.023477	0.054268	-4.72648
ZNF461	-0.15636	2.896435	-2.43939	0.016189	0.039611	-4.39922
MCM9	-0.15638	3.681053	-2.84746	0.005194	0.014746	-3.37689
C8orf37	-0.15659	3.454938	-2.53579	0.012517	0.031721	-4.17042
ZNF599	-0.15668	2.714368	-4.47628	1.75E-05	8.55E-05	1.978549
ACO1	-0.15685	3.473332	-2.99046	0.003387	0.01013	-2.98597
ZNF148	-0.1571	6.052918	-2.30073	0.02315	0.05364	-4.71421
UBIAD1	-0.15736	4.645052	-3.17928	0.001883	0.006023	-2.44472
DGCR2	-0.1576	6.62467	-2.71593	0.007595	0.020546	-3.72168
TMEM184	-0.15786	6.473235	-2.15339	0.033308	0.072993	-5.0304
SMARCD1	-0.15803	5.36535	-2.96034	0.00371	0.010986	-3.06967
CLCF1	-0.15804	4.888657	-2.55937	0.011741	0.029955	-4.11326
CSDE1	-0.15807	8.387604	-2.57471	0.011259	0.028892	-4.07579
SYMPK	-0.15809	4.326592	-4.13162	6.73E-05	0.000291	0.691107
PBRM1	-0.15817	4.670948	-2.71954	0.007518	0.020352	-3.71241
PTK2	-0.15849	2.971898	-2.62231	0.009877	0.025774	-3.95833
SAV1	-0.1587	3.614638	-2.24916	0.026344	0.0599	-4.82707
DNHD1	-0.15879	3.342405	-4.42468	2.15E-05	0.000103	1.781059
EPB41L4A	-0.15922	2.322256	-3.49051	0.000677	0.002391	-1.49231
VPS41	-0.15937	4.395212	-2.23658	0.027179	0.061527	-4.85425

CYP2R1	-0.15948	3.596478	-2.35946	0.019932	0.047253	-4.58285
GAB1	-0.15965	3.827381	-2.85264	0.005115	0.014549	-3.36303
RPL35A	-0.15968	5.742176	-2.44694	0.01587	0.038951	-4.38159
LOC10192	-0.15969	2.761246	-2.22285	0.028117	0.063349	-4.88374
CCT4	-0.15987	6.945004	-2.18828	0.030605	0.067981	-4.95728
ZNF555	-0.15988	2.784012	-4.06743	8.58E-05	0.000363	0.459766
DHX30	-0.15992	4.355498	-3.95118	0.000132	0.00054	0.04779
KRT18	-0.16029	3.950442	-2.04693	0.04287	0.089997	-5.24682
ELF4	-0.16033	7.48834	-2.20409	0.029445	0.065854	-4.92379
PCNXL4	-0.16046	2.755829	-3.56074	0.000533	0.001923	-1.26737
SPINK1	-0.16047	2.14161	-2.66577	0.00875	0.023204	-3.84938
CALCOCO	-0.1605	7.020292	-3.06684	0.002679	0.008248	-2.77042
DTD1	-0.16054	4.676814	-3.09414	0.002461	0.007639	-2.69226
EIF3K	-0.16059	7.856064	-2.645	0.009273	0.024389	-3.90164
LINC0086[illegible]	-0.16067	3.192515	-2.88345	0.00467	0.013456	-3.28007
TLR3	-0.16081	1.848231	-4.13365	6.68E-05	0.000289	0.698455
AFG3L1P	-0.16096	4.695615	-2.58301	0.011006	0.028335	-4.05546
PPM1L	-0.16097	2.977279	-3.49211	0.000674	0.002381	-1.48725
CLPTM1	-0.16101	6.392554	-2.60717	0.010299	0.026726	-3.9959
RBFA	-0.16101	4.081556	-3.15219	0.002051	0.006502	-2.5241
DDX11	-0.16105	4.75224	-2.12171	0.035939	0.077651	-5.09587
ZNF48	-0.16108	5.847166	-2.65721	0.008962	0.023696	-3.87097
GSS	-0.1611	5.9204	-2.61628	0.010043	0.026154	-3.97331
CCNI	-0.16124	8.365139	-2.29618	0.023417	0.054143	-4.72426
SPRR2G	-0.16141	4.623516	-2.31351	0.022413	0.052199	-4.68589
KLHL25	-0.16156	4.310533	-2.2361	0.027212	0.061559	-4.85529
MRPL4	-0.16164	4.607801	-2.11454	0.036559	0.078842	-5.11057
USP9X	-0.16167	6.920766	-2.65987	0.008896	0.023545	-3.86427
DPH2	-0.16167	5.776418	-2.20443	0.02942	0.065806	-4.92306
TMEM170[illegible]	-0.16168	4.561869	-2.68054	0.008394	0.022398	-3.81201
GOLGA7B	-0.16177	4.310142	-2.21	0.02902	0.065047	-4.9112
TSFM	-0.16195	3.853353	-3.4143	0.000875	0.003015	-1.7323
SUV39H1	-0.1621	4.941302	-2.0535	0.042218	0.088884	-5.23375
RNF34	-0.1621	5.591297	-2.27838	0.02449	0.056255	-4.76343
NDUFV2-[illegible]	-0.16221	3.712584	-2.2637	0.025406	0.058059	-4.79548
DEFB127	-0.16222	2.348493	-2.21916	0.028374	0.063843	-4.89164
ZFP64	-0.16244	3.733388	-4.23662	4.50E-05	0.000202	1.075303
GLI1	-0.16265	5.072641	-2.2155	0.028631	0.064329	-4.89947
ZNF142	-0.16281	5.310373	-3.96258	0.000127	0.00052	0.087808
SPATA2	-0.16291	5.140133	-2.57854	0.011142	0.028642	-4.06643
ADAM23	-0.16307	2.519653	-4.40772	2.30E-05	0.00011	1.716498
FAM193B	-0.16311	5.507665	-3.04541	0.002862	0.008756	-2.83138
SYNGAP1	-0.16323	4.138656	-2.27975	0.024406	0.056099	-4.76042
EFCAB14	-0.16336	5.726065	-3.23669	0.001567	0.005104	-2.27461
TCTN2	-0.1634	4.76056	-2.20336	0.029497	0.065957	-4.92533
TSC22D2	-0.16342	3.132782	-3.81861	0.000215	0.000841	-0.41076
RABL3	-0.16342	4.514402	-2.05453	0.042117	0.088708	-5.23171
PRICKLE1	-0.16343	3.531234	-3.24762	0.001513	0.004936	-2.24194
ZSCAN22	-0.16344	3.550158	-2.61017	0.010214	0.026549	-3.98846
ZNRF3	-0.1635	1.896847	-2.37524	0.019137	0.045704	-4.54703
DGKH	-0.16373	3.421527	-3.37857	0.000986	0.003358	-1.84331
GNB1	-0.16388	8.941052	-2.20833	0.02914	0.0653	-4.91475
LOC20083	-0.16391	1.960117	-3.05895	0.002745	0.008432	-2.79289
GUSBP2	-0.16396	2.354579	-3.12758	0.002217	0.006964	-2.59571
TTLL4	-0.16408	4.578495	-3.35194	0.001077	0.003636	-1.92544
TBC1D19	-0.16436	3.244416	-2.17441	0.031656	0.069898	-4.98648
TMEM198[illegible]	-0.16459	5.112662	-2.66508	0.008767	0.023246	-3.85112
PEX5	-0.1646	4.002888	-2.85447	0.005088	0.014483	-3.35811

CNOT2	-0.16464	4.704922	-3.56114	0.000532	0.00192	-1.26606
POM121	-0.16474	4.702208	-2.42926	0.016626	0.040534	-4.4228
CTSF	-0.16491	4.657525	-2.56906	0.011435	0.029279	-4.08963
ZNF415	-0.16506	1.793278	-2.73197	0.007256	0.019744	-3.6804
FRA10AC1	-0.16514	3.406013	-2.59451	0.010664	0.027528	-4.02716
PPARGC1E	-0.1652	4.124789	-3.48479	0.000691	0.002435	-1.51049
HNRNPM	-0.16534	4.987084	-3.88609	0.000168	0.000672	-0.17886
ZNF667	-0.16539	2.188145	-3.46456	0.000739	0.00259	-1.57453
SDC2	-0.1654	3.133926	-1.98725	0.049195	0.101016	-5.36367
PPM1D	-0.16562	4.973709	-2.40134	0.017885	0.043121	-4.48733
NPR2	-0.16565	3.053366	-3.28308	0.001349	0.004458	-2.1353
PVRL3	-0.16566	2.015181	-3.16599	0.001964	0.006254	-2.48374
LOC64285	-0.16579	3.84447	-2.44016	0.016157	0.039545	-4.39743
CYTH2	-0.16603	4.566543	-3.69177	0.000338	0.001272	-0.83806
C11orf30	-0.16627	3.969759	-3.71212	0.000314	0.001193	-0.77026
CCDC62	-0.16638	3.697083	-2.61725	0.010016	0.026094	-3.97089
PCNXL3	-0.16638	4.792702	-2.83624	0.005368	0.015158	-3.40687
ZNF519	-0.16658	2.048087	-4.68359	7.55E-06	3.93E-05	2.788181
CBX6	-0.1667	6.850234	-2.11283	0.036708	0.079123	-5.11406
SARM1	-0.16676	3.40776	-4.29246	3.62E-05	0.000166	1.282561
LOC10012	-0.16687	3.359508	-2.1457	0.033931	0.074113	-5.04638
NGRN	-0.16702	6.131369	-2.23479	0.0273	0.061732	-4.85811
MFSD12	-0.16709	5.721535	-2.77016	0.006504	0.017938	-3.58126
UBE2Q1	-0.16771	5.259516	-3.07539	0.002609	0.008052	-2.746
LOC10099	-0.16773	3.152299	-2.33749	0.021086	0.049565	-4.63234
SIRT4	-0.16786	2.194156	-3.14843	0.002076	0.006567	-2.53506
XYLT2	-0.16787	3.05193	-3.40915	0.00089	0.003063	-1.74836
GINM1	-0.16808	6.61782	-2.1511	0.033492	0.073327	-5.03516
PRKAB2	-0.16811	4.460048	-2.21342	0.028777	0.06458	-4.9039
B3GNT1	-0.16815	4.646211	-2.86962	0.004866	0.01395	-3.3174
TRMT12	-0.16817	4.884794	-1.98566	0.049373	0.101269	-5.36672
HELQ	-0.16834	4.248898	-2.8492	0.005167	0.014681	-3.37223
COIL	-0.16844	5.499359	-2.12769	0.035429	0.076771	-5.08358
BTBD2	-0.16852	6.290188	-2.17131	0.031895	0.07035	-4.99299
MRPL44	-0.16856	5.128014	-2.0686	0.040752	0.086313	-5.20359
USP40	-0.16866	4.39555	-2.59459	0.010662	0.027526	-4.02698
LOC10013	-0.16895	1.63013	-2.97009	0.003603	0.010704	-3.04267
SAMD12	-0.16941	1.948781	-3.71727	0.000309	0.001173	-0.75305
ZMYM6	-0.16947	3.650575	-2.31615	0.022264	0.051915	-4.68002
ITFG2	-0.16957	2.932358	-2.36087	0.01986	0.047118	-4.57966
UGT8	-0.16959	2.436673	-3.11401	0.002313	0.007227	-2.635
SENP2	-0.16967	5.252626	-2.93299	0.004029	0.011831	-3.14508
LARP7	-0.16968	5.004069	-2.61514	0.010074	0.026227	-3.97613
ZNF814	-0.1697	3.15231	-2.14982	0.033596	0.073515	-5.03784
ZNF8	-0.16978	4.637232	-2.67609	0.0085	0.022644	-3.82328
THUMPD3	-0.16995	4.862526	-2.37385	0.019206	0.045858	-4.55021
CELSR3	-0.17011	3.441223	-2.56329	0.011616	0.0297	-4.1037
PCBD2	-0.17023	4.070167	-3.48688	0.000686	0.002419	-1.50385
WNT7A	-0.17039	3.971575	-2.13201	0.035065	0.076102	-5.07469
TMEM161I	-0.17043	3.003726	-3.63272	0.000415	0.001533	-1.03306
CEP85	-0.17053	4.949593	-3.51365	0.000626	0.002226	-1.41859
TRAF2	-0.1706	5.048065	-2.62682	0.009754	0.025496	-3.94708
MLYCD	-0.1706	5.029466	-3.03809	0.002928	0.008924	-2.8521
RNF26	-0.17064	5.25686	-2.40466	0.017731	0.0428	-4.4797
DOCK4-AS	-0.17064	1.959397	-2.53686	0.012481	0.03165	-4.16786
DNASE2	-0.17065	6.183237	-2.55295	0.011948	0.030414	-4.12886
FBXO28	-0.17094	5.008959	-2.12614	0.03556	0.076981	-5.08676
AGAP9	-0.1711	2.941826	-2.12521	0.035639	0.07712	-5.08867

HUWE1	-0.17113	5.06534	-5.67271	1.01E-07	7.35E-07	6.974636
LOC10192	-0.17115	1.641603	-3.44383	0.000793	0.00276	-1.63985
NEK8	-0.17133	3.567549	-2.96883	0.003617	0.010742	-3.04617
C16orf58	-0.17134	4.185487	-3.05328	0.002794	0.008565	-2.80902
SGSM1	-0.17147	3.345235	-3.4557	0.000762	0.002661	-1.60247
FLYWCH1	-0.17164	5.388758	-3.2318	0.001592	0.005178	-2.2892
DSTYK	-0.17166	4.502414	-2.81854	0.005653	0.015862	-3.45393
ARHGAP3!	-0.17197	3.723618	-4.42795	2.13E-05	0.000102	1.793516
TTC17	-0.17197	5.192004	-3.15112	0.002058	0.006521	-2.52721
SETDB1	-0.17223	5.976183	-3.85731	0.000187	0.000738	-0.27814
AP1AR	-0.17229	2.365911	-2.40654	0.017644	0.04262	-4.47536
CDH2	-0.17235	2.373393	-3.53747	0.000577	0.002068	-1.3423
HKDC1	-0.17241	3.683353	-2.98694	0.003423	0.010224	-2.9958
MAP3K9	-0.17241	3.583768	-2.92126	0.004173	0.012206	-3.1772
XRRA1	-0.17243	3.777448	-2.23834	0.027061	0.061287	-4.85046
MRPL30	-0.17243	5.020813	-2.33898	0.021006	0.049407	-4.62901
SLC38A7	-0.17247	4.401639	-3.65199	0.000388	0.001444	-0.9697
WDR7	-0.17249	3.601686	-3.09791	0.002432	0.007563	-2.68142
LOC54147	-0.17269	3.480638	-2.58348	0.010992	0.028306	-4.0543
ZNF638	-0.17282	6.163218	-3.06459	0.002698	0.008303	-2.77685
TMEM161/	-0.17288	6.420489	-3.50044	0.000655	0.002321	-1.46075
AGBL3	-0.17302	1.550892	-4.99064	2.08E-06	1.20E-05	4.032891
SLC35E3	-0.17328	5.300282	-2.5374	0.012462	0.031607	-4.16654
BBS9	-0.17347	2.74327	-4.74189	5.94E-06	3.16E-05	3.020404
ZNF132	-0.17357	2.128687	-2.29671	0.023386	0.054089	-4.72309
DDX54	-0.17358	4.678471	-3.33871	0.001125	0.003785	-1.96604
THAP4	-0.17359	5.550221	-2.83389	0.005405	0.015252	-3.41313
RAB12	-0.17363	4.557594	-2.07837	0.039828	0.084671	-5.18398
STX2	-0.17363	5.034436	-3.55135	0.00055	0.001981	-1.29765
OLIG2	-0.17376	2.831532	-2.49071	0.014129	0.035198	-4.27841
TOP3A	-0.17382	4.171213	-3.65196	0.000388	0.001444	-0.9698
PABPC1L	-0.17423	4.85449	-2.20502	0.029377	0.065753	-4.9218
SMG7	-0.17424	5.405957	-2.6877	0.008227	0.022019	-3.79382
HLF	-0.17425	2.208773	-4.69205	7.30E-06	3.81E-05	2.821768
RAD54L2	-0.17429	5.318736	-3.73257	0.000292	0.001117	-0.70184
ABT1	-0.1743	6.463169	-2.86311	0.00496	0.014181	-3.33493
ROCK2	-0.17446	4.80685	-2.87282	0.00482	0.013831	-3.30877
SREBF1	-0.1746	4.456023	-3.29517	0.001297	0.004307	-2.09871
ANKRD20/	-0.1746	5.38167	-2.59762	0.010573	0.027334	-4.01949
ZNF592	-0.1747	6.024273	-3.23387	0.001581	0.005146	-2.28303
RASSF7	-0.17471	6.955235	-4.01289	0.000105	0.000437	0.265358
CORO1B	-0.17481	5.974294	-2.48663	0.014284	0.035506	-4.28812
ZNF182	-0.17488	5.247664	-2.31097	0.022558	0.052488	-4.69152
FER	-0.17496	3.65094	-4.32425	3.20E-05	0.000148	1.401412
ZNF436	-0.17511	4.414552	-2.72477	0.007407	0.020092	-3.69896
IRF3	-0.17523	7.144257	-2.19891	0.02982	0.066563	-4.93478
DOCK7	-0.17528	2.174847	-3.22464	0.001629	0.005284	-2.31051
ZNF343	-0.17535	3.340167	-3.32288	0.001184	0.003974	-2.01445
IQSEC1	-0.17535	7.91515	-2.43099	0.016551	0.04038	-4.41878
ZNF470	-0.17539	2.084177	-2.9507	0.00382	0.011268	-3.09632
LOC10013	-0.17578	2.724711	-2.40935	0.017516	0.042364	-4.46889
PREP	-0.1759	4.913954	-3.10589	0.002373	0.007396	-2.65844
GOLGA5	-0.17599	6.937049	-1.98087	0.049915	0.102238	-5.37596
RAB40B	-0.17609	2.788096	-3.27564	0.001382	0.004554	-2.15773
METTL10	-0.17616	3.201517	-3.01301	0.003162	0.009535	-2.92282
GET4	-0.17621	5.892326	-2.79335	0.006082	0.016918	-3.52046
SPEF2	-0.17631	2.72877	-3.5914	0.000479	0.00175	-1.16802
KIF27	-0.17634	2.254388	-2.61865	0.009977	0.025997	-3.96741

POLG	-0.17646	4.619394	-3.58415	0.000492	0.001789	-1.19157
ANAPC16	-0.17664	7.793793	-2.57818	0.011153	0.028666	-4.06729
CCSAP	-0.17691	3.501782	-3.92464	0.000146	0.00059	-0.04498
METTL14	-0.17696	4.249552	-2.48859	0.01421	0.035346	-4.28345
MXRA8	-0.17701	4.327507	-2.04224	0.04334	0.090854	-5.25612
NAT9	-0.17701	5.735913	-3.05479	0.002781	0.008528	-2.80475
NFYC	-0.17709	5.660685	-2.49259	0.014059	0.035048	-4.27395
ANKRD16	-0.17725	4.551237	-2.32576	0.021726	0.050906	-4.65859
FGFBP3	-0.17727	2.318632	-2.54938	0.012064	0.030684	-4.13754
IWS1	-0.17734	5.641314	-3.01653	0.003128	0.009445	-2.91291
SYT17	-0.17758	3.44347	-3.27428	0.001388	0.004571	-2.16185
UBAP2L	-0.17762	5.485055	-3.20799	0.001718	0.005542	-2.35995
DRG2	-0.17807	5.828463	-3.62463	0.000427	0.001573	-1.05959
SCRN2	-0.17819	4.972697	-3.2882	0.001327	0.004393	-2.11982
RHEBL1	-0.17829	3.197894	-3.81243	0.00022	0.000858	-0.43185
TTI1	-0.17842	6.127303	-2.27658	0.024601	0.056458	-4.76736
NOP9	-0.17851	4.260081	-3.57322	0.00051	0.001849	-1.22701
PIAS2	-0.17863	3.210071	-2.9467	0.003866	0.011394	-3.10734
MUC20	-0.17888	3.223144	-3.48186	0.000697	0.002458	-1.5198
C5AR2	-0.1789	5.077629	-2.22595	0.027903	0.062936	-4.8771
PRDM15	-0.17894	3.760676	-3.37185	0.001008	0.003426	-1.8641
SUSD4	-0.17915	3.273605	-2.84267	0.005267	0.014926	-3.3897
USE1	-0.17919	4.855139	-2.41942	0.017061	0.041426	-4.44563
SPAG16	-0.17924	2.327124	-4.30654	3.43E-05	0.000158	1.335126
FBXW4P1	-0.17928	5.498584	-2.57487	0.011254	0.028883	-4.0754
KAT8	-0.17955	6.013758	-2.61342	0.010123	0.026332	-3.98041
LRRFIP1	-0.17991	7.556171	-2.10071	0.03778	0.081009	-5.13877
IL5RA	-0.18005	2.935473	-3.29569	0.001295	0.004301	-2.09714
MKL2	-0.18019	2.908078	-4.69797	7.12E-06	3.73E-05	2.845274
IL24	-0.18021	4.351198	-2.14611	0.033897	0.074072	-5.04553
NUDCD3	-0.18024	4.07443	-4.65838	8.38E-06	4.31E-05	2.68837
RUSC1	-0.18042	5.799733	-2.30922	0.022658	0.052685	-4.69541
IREB2	-0.18053	4.450708	-2.13616	0.034717	0.075511	-5.06612
H2BFXP	-0.18058	2.739709	-2.78064	0.00631	0.017464	-3.55384
TRADD	-0.18076	6.709714	-5.51043	2.11E-07	1.46E-06	6.254147
PMEPA1	-0.18077	3.060577	-2.91565	0.004244	0.012384	-3.19256
ZNF789	-0.18085	3.621826	-3.4571	0.000758	0.00265	-1.59806
RABGGTB	-0.18091	4.579577	-2.09245	0.038526	0.082383	-5.15553
LOC10192	-0.18091	3.715825	-2.42183	0.016953	0.0412	-4.44005
YPEL2	-0.18109	5.525211	-2.97471	0.003553	0.010568	-3.02984
GDPGP1	-0.18109	1.849248	-3.61824	0.000437	0.001605	-1.08049
HIRIP3	-0.18111	4.901011	-2.57421	0.011275	0.028921	-4.07701
MIATNB	-0.18112	5.414116	-2.39899	0.017995	0.043359	-4.49273
GGACT	-0.18113	2.947874	-3.34253	0.00111	0.003742	-1.95434
NACAP1	-0.18119	9.421527	-3.06235	0.002716	0.008353	-2.78322
TAS2R4	-0.18146	2.334455	-2.27212	0.024877	0.057002	-4.77711
FDXACB1	-0.18182	4.003623	-2.25268	0.026114	0.059472	-4.81945
VPS39	-0.18186	6.364286	-3.47063	0.000724	0.002542	-1.55533
NOL6	-0.18195	4.310208	-3.87009	0.000178	0.000709	-0.23412
LRCH3	-0.18219	4.247345	-2.51612	0.013199	0.033232	-4.21776
BAZ1B	-0.18219	4.20146	-3.92986	0.000143	0.00058	-0.02677
ZC3H6	-0.18221	4.113137	-2.14256	0.034188	0.074531	-5.05289
CYTH3	-0.18226	3.704836	-4.18465	5.50E-05	0.000242	0.884249
SEPT7P2	-0.18227	2.711328	-2.9541	0.003781	0.011172	-3.08695
PPIE	-0.1823	5.069266	-3.07949	0.002576	0.007957	-2.73427
HSD17B7	-0.18231	6.228694	-2.08058	0.039621	0.084301	-5.17952
MPND	-0.18254	5.86054	-2.24668	0.026507	0.060218	-4.83244
EDARADD	-0.18263	3.323428	-2.91273	0.004281	0.012478	-3.20051

NRCAM	-0.18305	2.952804	-3.10222	0.0024	0.007472	-2.66902
PYGB	-0.18308	5.661722	-3.47334	0.000718	0.00252	-1.54677
PER3	-0.18375	2.356266	-5.64587	1.14E-07	8.23E-07	6.854639
SLC35D2	-0.18376	4.976152	-3.14285	0.002113	0.006673	-2.55134
ATXN2L	-0.18377	4.165455	-2.97484	0.003551	0.010566	-3.02946
NSUN4	-0.18386	3.992054	-3.51799	0.000617	0.002197	-1.40474
B4GALT1	-0.18388	4.407535	-3.37309	0.001004	0.003413	-1.86027
CDK6	-0.18388	4.124677	-2.5217	0.013002	0.032789	-4.20436
MAMDC4	-0.18394	3.68714	-2.25312	0.026085	0.059412	-4.81848
KIAA1804	-0.18399	2.531054	-2.96875	0.003617	0.010743	-3.04639
COA1	-0.184	4.493046	-3.77817	0.000248	0.000962	-0.54822
LOC10272	-0.18408	2.91623	-2.64126	0.00937	0.024612	-3.91101
FANCM	-0.18416	3.076937	-3.62756	0.000423	0.001559	-1.04999
EGR2	-0.1844	2.052763	-2.85875	0.005024	0.01433	-3.34663
TCEA2	-0.1845	4.023676	-4.09911	7.62E-05	0.000326	0.573602
TMEM229I	-0.18452	4.248229	-3.52068	0.000611	0.002179	-1.39613
GAB3	-0.18463	4.802245	-2.53816	0.012437	0.031546	-4.1647
CANT1	-0.18481	7.085888	-2.68721	0.008238	0.022046	-3.79507
APTR	-0.18506	3.301018	-3.42224	0.000852	0.002941	-1.70752
HLA-DPB2	-0.18532	1.831235	-2.38197	0.018807	0.045063	-4.5317
PITRM1	-0.18533	4.989902	-3.09926	0.002422	0.007533	-2.67754
TCF12	-0.18558	4.155683	-2.02104	0.045522	0.094768	-5.2979
MANBAL	-0.18577	5.862335	-2.59672	0.0106	0.027399	-4.02172
SPON1	-0.1858	2.776889	-5.3366	4.60E-07	3.00E-06	5.496212
SBF1	-0.18585	4.68618	-4.78339	4.99E-06	2.69E-05	3.18693
PNO1	-0.18602	4.870439	-3.27359	0.001391	0.004578	-2.16392
RPAIN	-0.18634	3.30011	-5.34066	4.52E-07	2.95E-06	5.513773
AP3D1	-0.18651	6.475152	-2.6845	0.008301	0.022187	-3.80195
RBMX2	-0.18657	4.878444	-2.5057	0.013574	0.034025	-4.2427
LEAP2	-0.18658	4.533628	-2.7265	0.00737	0.020009	-3.6945
DEDD2	-0.18661	8.507283	-2.82015	0.005626	0.015802	-3.44966
HEXDC	-0.18674	4.662111	-4.21928	4.81E-05	0.000215	1.011389
BOLA1	-0.1868	3.609854	-2.40978	0.017496	0.042327	-4.4679
SDHAF1	-0.18686	5.370246	-3.42653	0.00084	0.002904	-1.69409
PRKAG2-A	-0.18687	3.399406	-2.56651	0.011514	0.029462	-4.09584
ZNF76	-0.18713	5.643189	-3.13954	0.002135	0.006735	-2.56097
C14orf132	-0.18736	3.40907	-3.13402	0.002172	0.006837	-2.57702
BLOC1S3	-0.18756	4.344938	-3.86913	0.000179	0.000711	-0.23746
B3GNT7	-0.18757	2.84008	-4.45937	1.88E-05	9.08E-05	1.913645
ZC2HC1A	-0.18762	2.750444	-3.11589	0.002299	0.007192	-2.62955
KIAA1430	-0.18782	2.977382	-3.75712	0.000268	0.00103	-0.61932
FGD6	-0.18796	2.086364	-4.36526	2.73E-05	0.000128	1.555661
CRELD1	-0.18806	4.802717	-2.30894	0.022674	0.052711	-4.69604
YDJC	-0.18814	5.106451	-2.31778	0.022172	0.051731	-4.67639
GPM6A	-0.18828	2.505	-4.11345	7.21E-05	0.00031	0.62534
ATG4B	-0.18836	6.428195	-4.42538	2.15E-05	0.000103	1.783725
CACTIN	-0.18843	4.085315	-4.46773	1.81E-05	8.81E-05	1.945721
PRRT3-AS	-0.18867	2.807756	-2.37083	0.019356	0.046156	-4.55706
LOC10027	-0.18867	3.392583	-2.57989	0.011101	0.02855	-4.0631
MESDC1	-0.18872	6.597892	-2.51421	0.013267	0.033382	-4.22233
OSBPL7	-0.18872	4.329264	-3.95974	0.000128	0.000525	0.07782
SMC1A	-0.18881	4.051944	-4.15849	6.08E-05	0.000265	0.788738
ATF2	-0.1889	4.010464	-2.38653	0.018586	0.044601	-4.52127
EWSR1	-0.18893	5.781738	-3.13263	0.002182	0.006866	-2.58106
CCDC170	-0.18915	3.101217	-2.44726	0.015857	0.038923	-4.38084
CARF	-0.18919	2.654908	-3.12032	0.002268	0.007101	-2.61675
UPF3B	-0.1892	3.972431	-2.29752	0.023338	0.054002	-4.7213
SNX25	-0.1893	2.792185	-4.74916	5.76E-06	3.07E-05	3.049516

NCOA6	-0.18932	4.375478	-3.94577	0.000135	0.00055	0.028858
ZFP91	-0.18935	6.129208	-2.48646	0.014291	0.035517	-4.2885
GHRL	-0.18944	3.952336	-2.7247	0.007408	0.020093	-3.69914
CNDP2	-0.18955	5.426026	-3.9354	0.00014	0.00057	-0.00742
LYRM9	-0.18998	4.459427	-2.17221	0.031826	0.070227	-4.9911
LOC10192	-0.19008	3.06336	-2.46432	0.015157	0.037417	-4.34082
DDX56	-0.1901	5.09259	-3.44036	0.000802	0.002788	-1.65074
PHLPP1	-0.19012	2.814361	-2.84642	0.00521	0.014787	-3.37968
PIP5K1A	-0.19054	4.871819	-3.24197	0.001541	0.005021	-2.25884
SUGP2	-0.19071	5.377509	-2.90797	0.004342	0.012633	-3.21351
TNS3	-0.19091	3.721513	-2.24607	0.026547	0.060295	-4.83375
WDR35	-0.19097	2.838672	-3.29792	0.001285	0.004275	-2.09037
LRRCC1	-0.19104	1.845495	-3.10233	0.002399	0.007471	-2.6687
RNF139-A	-0.19109	2.174458	-3.01872	0.003107	0.009396	-2.90677
HIGD2A	-0.19121	6.056264	-3.11022	0.002341	0.007309	-2.64593
SNW1	-0.19124	7.107009	-2.02345	0.04527	0.09432	-5.29318
BDH2	-0.19134	3.474615	-2.79416	0.006068	0.016886	-3.51833
FAM122C	-0.19135	2.611071	-5.13184	1.13E-06	6.88E-06	4.622642
UNC5CL	-0.19138	3.633891	-2.85537	0.005074	0.014458	-3.35569
C14orf93	-0.19194	3.855507	-2.37928	0.018939	0.045319	-4.53784
COX18	-0.19218	4.854148	-3.02609	0.003037	0.009212	-2.886
MORF4L2-	-0.1922	2.968294	-4.18276	5.54E-05	0.000244	0.877334
UTP20	-0.19222	4.177272	-2.35374	0.020227	0.047853	-4.59578
LOC25402	-0.19232	3.852099	-2.73107	0.007275	0.019782	-3.68273
LINC00957	-0.19252	5.15167	-3.77345	0.000253	0.000977	-0.56418
SLC52A2	-0.19258	4.743006	-2.96163	0.003696	0.010948	-3.06613
LINC01138	-0.19324	3.412174	-4.54571	1.33E-05	6.61E-05	2.246864
SEC63	-0.19333	4.640628	-2.33116	0.021429	0.050285	-4.64652
WRN	-0.19339	3.035094	-2.22946	0.027662	0.06244	-4.86956
BTBD6	-0.19345	7.201909	-2.38842	0.018496	0.04442	-4.51697
PRIM2	-0.19374	2.746729	-2.24114	0.026874	0.06093	-4.84442
TMTC4	-0.19386	3.764052	-2.84787	0.005187	0.014732	-3.37579
C12orf45	-0.19401	4.252914	-2.60087	0.010479	0.027122	-4.01148
FMO4	-0.19405	4.523832	-2.51069	0.013393	0.033643	-4.23078
ZNF606	-0.19406	4.567662	-2.9093	0.004325	0.01259	-3.20986
PIK3R6	-0.19407	3.899372	-2.22279	0.028121	0.06335	-4.88386
LINC00845	-0.19422	2.501663	-2.54771	0.012119	0.030801	-4.14158
CPSF4	-0.19443	6.627154	-3.79664	0.000232	0.000904	-0.48559
VAPB	-0.19467	5.123501	-3.09333	0.002467	0.007655	-2.69458
SGCB	-0.19473	2.818001	-4.30182	3.49E-05	0.000161	1.317473
MTERF1	-0.19505	3.935165	-2.30691	0.022791	0.052916	-4.70054
SSBP1	-0.19506	5.581445	-2.23857	0.027045	0.061265	-4.84996
NSUN5P1	-0.19519	6.266601	-2.09205	0.038563	0.082436	-5.15635
B3GAT1	-0.19538	4.481542	-2.35588	0.020116	0.04764	-4.59094
KCNH8	-0.19541	2.047275	-2.47952	0.014558	0.036111	-4.30495
TMEM219	-0.19553	5.865019	-3.29624	0.001292	0.004294	-2.09545
RAPH1	-0.19576	2.355299	-3.47868	0.000705	0.002479	-1.52986
FTH1	-0.19576	9.930993	-2.09916	0.037919	0.081271	-5.14192
NAALADL1	-0.1959	4.388129	-3.37984	0.000982	0.003347	-1.8394
ZBTB24	-0.19593	3.111647	-4.02683	1.00E-04	0.000417	0.314851
PHTF2	-0.19598	3.759342	-2.73112	0.007274	0.019781	-3.68261
ZZEF1	-0.19603	5.022441	-3.99799	0.000111	0.00046	0.212607
ZFP28	-0.19613	3.487729	-4.44047	2.02E-05	9.73E-05	1.841334
CERS6	-0.19617	3.951064	-2.07898	0.03977	0.084592	-5.18274
ATL2	-0.19622	3.373877	-2.57458	0.011263	0.028899	-4.07611
ADARB1	-0.19623	4.002359	-3.94126	0.000137	0.000559	0.013061
LINC00629	-0.1963	1.671904	-3.47411	0.000716	0.002514	-1.54432
XPNPEP3	-0.19631	3.479782	-5.34354	4.46E-07	2.92E-06	5.526216

AGO1	-0.19643	4.989957	-4.05423	9.02E-05	0.00038	0.412543
FBXW4	-0.19643	5.989902	-2.3484	0.020506	0.048412	-4.60781
TBC1D10A	-0.19653	5.523234	-3.0724	0.002633	0.008119	-2.75456
TTLL1	-0.1966	3.906401	-2.28625	0.02401	0.055326	-4.74613
HEXA	-0.19663	4.916407	-3.79423	0.000234	0.000912	-0.49379
ASS1	-0.19673	3.966489	-2.08027	0.039649	0.084353	-5.18014
DENND4B	-0.19682	7.795989	-2.30017	0.023182	0.053685	-4.71545
ANKIB1	-0.19683	5.442913	-3.2812	0.001357	0.004483	-2.14097
SMKR1	-0.19688	2.200945	-3.27847	0.001369	0.004516	-2.14919
RGS9	-0.19716	2.821608	-2.85084	0.005142	0.014622	-3.36784
SUV420H1	-0.19722	6.684922	-2.89697	0.004487	0.013007	-3.24342
GDAP1	-0.19723	3.106158	-3.08508	0.002531	0.007838	-2.71825
PAK1	-0.19723	6.081221	-2.81076	0.005782	0.016179	-3.47454
MPV17L	-0.19726	5.314844	-2.77472	0.006419	0.017732	-3.56934
RPS29	-0.19726	7.496121	-2.41693	0.017172	0.041647	-4.45139
HDHD3	-0.19727	5.052496	-2.30551	0.022872	0.05308	-4.70364
TMED10	-0.19728	6.78156	-2.46248	0.015232	0.037564	-4.34516
CDK18	-0.19743	3.803913	-2.37555	0.019122	0.045678	-4.54634
ACAA2	-0.19747	5.523266	-2.76845	0.006536	0.018014	-3.58573
VPS36	-0.19751	4.4198	-2.66717	0.008716	0.023139	-3.84585
LOC15506	-0.19756	5.499908	-3.45771	0.000757	0.002646	-1.59614
FAM150B	-0.19771	1.853057	-2.99348	0.003356	0.010046	-2.97752
MYO6	-0.19771	2.682588	-3.7568	0.000268	0.001031	-0.62038
SAP18	-0.19778	6.90657	-2.63429	0.009554	0.025056	-3.92845
C2orf68	-0.19784	6.152186	-2.20693	0.02924	0.065482	-4.91774
CIC	-0.19784	6.792264	-2.97539	0.003545	0.010553	-3.02796
B3GALNT2	-0.19787	3.564853	-3.37162	0.001009	0.003428	-1.86482
TERF2	-0.19788	3.594093	-5.85622	4.30E-08	3.34E-07	7.80365
MRS2	-0.19802	3.761581	-2.73714	0.00715	0.019472	-3.66704
POLH	-0.19803	3.328306	-5.01472	1.88E-06	1.09E-05	4.132718
PRMT1	-0.19817	5.267688	-2.75515	0.00679	0.01862	-3.62036
CCDC86	-0.19823	4.550129	-2.03908	0.043659	0.091448	-5.26237
GIGYF2	-0.19831	4.569754	-4.40944	2.29E-05	0.000109	1.723044
ANGPT1	-0.19835	2.368683	-2.76962	0.006514	0.01796	-3.58266
SPRYD3	-0.19857	6.424127	-3.23092	0.001596	0.005191	-2.29181
DUS4L	-0.1986	2.975849	-2.59972	0.010512	0.027197	-4.01431
AP3M1	-0.19871	5.678649	-2.36443	0.019679	0.046794	-4.5716
ARFIP2	-0.19873	5.908752	-2.58782	0.010862	0.028003	-4.04363
SRR	-0.19878	3.043087	-3.1598	0.002003	0.006363	-2.50187
CA11	-0.19895	4.830583	-2.85928	0.005016	0.014312	-3.34522
ERI3	-0.19895	4.652134	-2.30509	0.022896	0.053131	-4.70458
TSR2	-0.19896	6.159553	-3.20013	0.001762	0.005674	-2.38323
C14orf182	-0.19896	2.770439	-3.4316	0.000826	0.00286	-1.6782
UCKL1	-0.19909	4.92748	-3.94806	0.000134	0.000546	0.036861
TTC7A	-0.19927	4.196137	-3.69593	0.000333	0.001255	-0.82422
SPTY2D1	-0.19928	5.636773	-2.42634	0.016754	0.040807	-4.42958
MAP3K7	-0.19929	6.132998	-2.1614	0.03267	0.071819	-5.01372
ZGPAT	-0.19946	5.230788	-2.68717	0.008239	0.022046	-3.79515
FAM76B	-0.1995	3.689346	-2.02864	0.044729	0.093346	-5.28296
TRMT112	-0.19957	8.672541	-2.06486	0.041112	0.08693	-5.21109
PCBP1-AS	-0.19963	3.780557	-4.90867	2.95E-06	1.65E-05	3.695468
TAF1A-AS	-0.19984	2.835225	-3.29242	0.001308	0.00434	-2.10703
CCDC106	-0.19994	3.013088	-2.89894	0.00446	0.012944	-3.23807
EFTUD2	-0.19997	5.754096	-3.55185	0.000549	0.001978	-1.29605
AGPAT3	-0.20013	5.055812	-3.09733	0.002437	0.007575	-2.68307
CAMSAP2	-0.20043	3.050001	-4.13892	6.55E-05	0.000284	0.71758
ZSCAN32	-0.2005	5.133341	-3.71793	0.000308	0.001171	-0.75086
COL6A2	-0.20055	4.205559	-3.45088	0.000774	0.0027	-1.61766

TMEM18	-0.20055	4.360808	-2.70038	0.007937	0.021328	-3.7615
SSR1	-0.20063	7.319671	-2.56006	0.011719	0.029921	-4.11158
HSPD1	-0.20063	4.57542	-2.7681	0.006542	0.018027	-3.58664
RPL37A	-0.20068	7.176596	-2.14422	0.034051	0.074305	-5.04944
ALKBH2	-0.20071	3.207512	-2.2943	0.023529	0.054376	-4.72842
GLMN	-0.20101	4.346943	-2.51091	0.013385	0.033635	-4.23024
GLIDR	-0.20108	4.457396	-3.22422	0.001631	0.005289	-2.31176
CCM2	-0.20115	6.821502	-3.36503	0.001031	0.003497	-1.88513
ELAC2	-0.20119	3.951286	-4.21486	4.90E-05	0.000218	0.995119
ZNF236	-0.20124	4.387245	-4.59682	1.08E-05	5.44E-05	2.44621
PTPRA	-0.20125	4.887122	-3.85718	0.000187	0.000739	-0.27859
C11orf24	-0.20129	5.027084	-2.37708	0.019046	0.045524	-4.54284
ENTPD6	-0.20132	4.528622	-3.55087	0.000551	0.001983	-1.29921
RPARP-AS	-0.20146	2.824216	-3.7242	0.000301	0.001149	-0.72988
TGFBRAP1	-0.20169	5.51393	-4.05146	9.11E-05	0.000383	0.402639
SH3PXD2A	-0.20172	3.275197	-4.1829	5.54E-05	0.000243	0.877836
USP34	-0.2018	6.162754	-2.74068	0.007078	0.01931	-3.65791
NDFIP2	-0.20181	2.67651	-3.58009	0.000498	0.001811	-1.20473
FAHD2CP	-0.2019	3.17253	-3.42683	0.000839	0.002901	-1.69316
ZBTB42	-0.20191	3.677145	-2.42963	0.01661	0.0405	-4.42195
PRKAB1	-0.20205	5.464057	-2.77872	0.006345	0.01755	-3.55888
PKDCC	-0.20208	3.828683	-2.33632	0.021149	0.049708	-4.63498
TRAF3IP1	-0.20223	3.498309	-3.79368	0.000235	0.000913	-0.49564
HGH1	-0.20223	5.754661	-3.81899	0.000214	0.00084	-0.40947
TP53I13	-0.20234	4.929932	-3.36295	0.001038	0.003519	-1.89154
TJAP1	-0.20235	5.889067	-4.0819	8.13E-05	0.000346	0.511677
LOC93444	-0.20246	4.603138	-2.4935	0.014024	0.034971	-4.27178
PHF8	-0.20249	3.639719	-4.67858	7.71E-06	4.00E-05	2.768313
SLC37A1	-0.20252	3.946242	-3.40644	0.000899	0.003086	-1.75682
LYSMD1	-0.20253	4.138688	-2.45942	0.015355	0.037828	-4.35234
PIAS4	-0.20259	4.898144	-3.70155	0.000326	0.001234	-0.8055
NFIC	-0.20277	4.856255	-3.29298	0.001306	0.004334	-2.10532
DOCK11	-0.2028	6.888797	-1.99494	0.048337	0.099513	-5.34879
EHMT1	-0.20285	4.392192	-2.90337	0.004402	0.012791	-3.22602
MTOR	-0.20287	3.933854	-3.87562	0.000175	0.000696	-0.21503
IFT46	-0.203	4.972177	-2.65852	0.008929	0.023622	-3.86768
DYNC1H1	-0.20312	3.964128	-5.67848	9.80E-08	7.18E-07	7.000496
ZNF300	-0.20332	2.885167	-2.64505	0.009272	0.024388	-3.90152
SS18	-0.20342	4.036608	-2.43551	0.016356	0.039965	-4.40828
HLA-DOA	-0.20352	4.398783	-4.40679	2.31E-05	0.00011	1.712958
LRRC3B	-0.20372	2.091138	-2.9483	0.003848	0.011345	-3.10294
UNKL	-0.20377	5.279815	-4.31822	3.28E-05	0.000152	1.378814
MPZL2	-0.20407	2.954597	-2.79631	0.00603	0.016787	-3.51267
TRPT1	-0.20424	5.385098	-3.2499	0.001502	0.004904	-2.23508
TNK1	-0.20464	4.812498	-3.70032	0.000328	0.001239	-0.80961
SIK2	-0.20465	3.824702	-4.49622	1.62E-05	7.94E-05	2.055311
WBSCR22	-0.20469	4.305222	-3.58368	0.000492	0.001792	-1.19309
2-Sep	-0.20479	6.612018	-2.76457	0.006609	0.018182	-3.59585
ZNF546	-0.20494	1.96427	-4.3792	2.58E-05	0.000121	1.608365
TAF1L	-0.20509	3.945627	-2.7601	0.006695	0.018392	-3.60749
SSPN	-0.20523	2.256781	-2.86043	0.004999	0.014271	-3.34213
GNRH1	-0.20538	3.646011	-2.51234	0.013334	0.03353	-4.22681
IGF1R	-0.2056	5.731104	-2.65406	0.009041	0.023868	-3.87889
GPN1	-0.20572	6.59816	-2.09237	0.038533	0.082389	-5.15569
USP3-AS1	-0.20578	2.202159	-2.44419	0.015986	0.039179	-4.38803
DEAF1	-0.20587	3.588988	-4.21262	4.94E-05	0.00022	0.986885
UMPS	-0.20591	4.309755	-3.72626	0.000299	0.001141	-0.72299
UTP15	-0.20591	3.015343	-3.21639	0.001673	0.005405	-2.33505

RNASEH1-	-0.20591	2.477727	-3.03144	0.002988	0.009092	-2.8709
DOCK2	-0.20592	5.418988	-2.99564	0.003334	0.009991	-2.97151
ANKLE2	-0.20605	4.07727	-3.21746	0.001667	0.005389	-2.33186
TNKS	-0.20621	3.37859	-4.82719	4.16E-06	2.27E-05	3.363735
KIAA1919	-0.20623	4.055745	-3.51447	0.000624	0.002222	-1.41597
SPTBN5	-0.20624	3.703291	-3.20249	0.001749	0.005637	-2.37625
LINC0086§	-0.2063	3.166304	-3.66445	0.000372	0.001389	-0.92857
DVL1	-0.20632	5.439963	-2.27909	0.024446	0.056179	-4.76185
SYNC	-0.20656	2.417219	-2.57444	0.011268	0.028906	-4.07646
NOMO3	-0.20661	5.830457	-2.20758	0.029193	0.065392	-4.91635
CMKLR1	-0.20662	4.023318	-2.73905	0.007111	0.019387	-3.66211
FAM161B	-0.20674	4.759611	-3.99111	0.000114	0.000471	0.188267
MCPH1	-0.2068	3.826973	-3.96154	0.000127	0.000522	0.084152
PRR24	-0.20711	5.011114	-2.07892	0.039776	0.084596	-5.18287
NFRKB	-0.20712	4.389908	-5.17321	9.45E-07	5.84E-06	4.797394
CXorf40A	-0.20714	6.78722	-2.24929	0.026335	0.059888	-4.82679
CPAMD8	-0.20714	3.530899	-2.88384	0.004665	0.013444	-3.27901
BIVM	-0.20715	2.429841	-4.89941	3.07E-06	1.71E-05	3.657576
TOP1MT	-0.20733	3.425448	-3.53941	0.000573	0.002055	-1.33607
NKAPP1	-0.20743	3.593023	-2.78744	0.006187	0.017175	-3.536
ANKRD40	-0.20748	4.330122	-5.0427	1.66E-06	9.80E-06	4.249122
MED25	-0.20748	4.176346	-3.15509	0.002033	0.006448	-2.51562
LINC0112ℇ	-0.20749	3.660819	-4.45027	1.95E-05	9.39E-05	1.878802
ELAC1	-0.2075	2.611217	-3.28642	0.001334	0.004415	-2.12518
SCGB2B2	-0.2076	3.004265	-2.37844	0.01898	0.045385	-4.53975
FARS2	-0.20762	4.481019	-4.27692	3.85E-05	0.000175	1.224662
NAA15	-0.20767	3.417097	-3.01715	0.003122	0.009433	-2.91118
EXOSC5	-0.2078	6.368821	-3.18768	0.001833	0.005886	-2.41998
FKRP	-0.20786	4.52509	-2.63736	0.009473	0.024856	-3.92078
SAMD4B	-0.20792	5.653491	-4.32226	3.23E-05	0.000149	1.393929
NUDC	-0.20793	5.17703	-3.8251	0.00021	0.000823	-0.38862
SMARCE1	-0.208	5.132162	-2.78388	0.006251	0.017337	-3.54534
ANKRD36(	-0.20804	2.090903	-3.22863	0.001608	0.005223	-2.29863
BFSP1	-0.20804	2.685753	-3.03309	0.002973	0.009051	-2.86625
OVGP1	-0.20818	4.845207	-2.09794	0.038028	0.081463	-5.14439
ALKBH4	-0.2082	4.070233	-2.46512	0.015125	0.037343	-4.33894
PLAUR	-0.20832	6.905731	-2.3412	0.020887	0.049171	-4.62401
NAA50	-0.20839	5.185481	-2.15164	0.033449	0.073257	-5.03405
ZC3H18	-0.20845	4.149729	-3.85508	0.000188	0.000744	-0.28582
EMC3-AS1	-0.20855	4.623897	-2.4039	0.017766	0.042869	-4.48144
DNAJC16	-0.20862	4.734221	-3.72497	0.0003	0.001146	-0.72731
PALLD	-0.20876	3.518736	-2.66832	0.008688	0.023086	-3.84294
ARMC10	-0.20883	5.883285	-2.2758	0.024649	0.05654	-4.76907
WDR19	-0.20895	3.204053	-5.06165	1.53E-06	9.09E-06	4.328167
C17orf100	-0.20897	3.017346	-2.64206	0.009349	0.024573	-3.909
TMEM106/	-0.20903	4.273842	-2.98125	0.003483	0.01039	-3.01166
WWOX	-0.20905	4.462948	-4.96227	2.35E-06	1.34E-05	3.915685
TBC1D17	-0.2091	5.582989	-2.27824	0.024498	0.056267	-4.76372
SYPL1	-0.2091	7.122305	-2.01991	0.045641	0.094948	-5.30011
NUDT3	-0.20912	5.813573	-2.96499	0.003659	0.010855	-3.05681
CAPN15	-0.20924	4.472834	-3.17626	0.001901	0.006074	-2.45358
PPP2R5E	-0.20932	6.513423	-2.62749	0.009736	0.025455	-3.94541
PDDC1	-0.20933	5.396615	-2.93282	0.004031	0.011835	-3.14552
NISCH	-0.20939	5.648334	-3.13824	0.002144	0.006755	-2.56475
ACBD4	-0.2095	5.552842	-3.1357	0.002161	0.006805	-2.57214
RAI1	-0.20954	5.272265	-3.96455	0.000126	0.000516	0.094702
YAF2	-0.21005	2.946386	-2.8039	0.005899	0.01646	-3.49265
HDAC3	-0.21064	6.74642	-3.45538	0.000763	0.002664	-1.60348

ABL1	-0.21066	6.24475	-2.53894	0.01241	0.031487	-4.16281
SPICE1	-0.21066	1.985115	-4.41474	2.24E-05	0.000107	1.743189
MIDN	-0.21069	3.735769	-2.18056	0.031186	0.069054	-4.97355
TRPM7	-0.21073	3.803827	-5.17283	9.47E-07	5.85E-06	4.795785
TRIM37	-0.21075	4.328746	-3.15645	0.002024	0.006424	-2.51165
KIR2DS3	-0.21113	3.857825	-2.47285	0.014818	0.036686	-4.32072
DHX38	-0.21145	6.008667	-3.40246	0.000911	0.003127	-1.76922
PFKL	-0.21172	6.526104	-3.32993	0.001157	0.003888	-1.99292
EIF1	-0.21197	7.944434	-2.51561	0.013217	0.033269	-4.21898
IVD	-0.21204	4.34383	-5.26724	6.25E-07	3.99E-06	5.197925
IPCEF1	-0.21219	7.024558	-2.35077	0.020382	0.048175	-4.60248
TRIM78P	-0.21232	3.242555	-2.21824	0.028438	0.063966	-4.8936
SP140L	-0.21236	3.969655	-3.90882	0.000155	0.000622	-0.10005
FBXL3	-0.21252	6.69419	-2.39841	0.018022	0.04342	-4.49407
PLOD3	-0.2126	5.707169	-2.88725	0.004618	0.013328	-3.2698
TANGO6	-0.21264	4.764077	-2.78151	0.006294	0.017434	-3.55156
DIAPH1	-0.21269	5.808114	-4.13701	6.60E-05	0.000286	0.710661
GRHPR	-0.21296	6.346151	-2.38203	0.018804	0.045061	-4.53156
SH3KBP1	-0.21299	7.966607	-2.89758	0.004478	0.012991	-3.24176
FBXL12	-0.21307	4.832762	-4.09736	7.67E-05	0.000327	0.567301
CDC23	-0.21311	3.757301	-2.35641	0.020088	0.047586	-4.58974
ZNF345	-0.21323	3.094834	-3.10511	0.002378	0.007413	-2.66069
SERPINB7	-0.21331	2.800204	-3.12567	0.00223	0.007005	-2.60126
STK40	-0.2135	6.317304	-3.0111	0.00318	0.009585	-2.92819
ANXA6	-0.21351	6.306802	-3.16184	0.00199	0.006325	-2.49588
EIF4EBP2	-0.21356	5.372561	-4.741	5.96E-06	3.17E-05	3.016866
GDF11	-0.21384	3.731094	-4.87479	3.40E-06	1.89E-05	3.557092
XRCC6	-0.214	5.082069	-3.7085	0.000318	0.001206	-0.78233
ENKD1	-0.21411	4.576875	-2.7648	0.006605	0.018172	-3.59525
PRR14	-0.21421	6.686423	-2.28754	0.023933	0.055178	-4.7433
PAAF1	-0.21459	4.939208	-3.9445	0.000136	0.000553	0.024391
ZNF85	-0.2146	3.436348	-4.34299	2.97E-05	0.000139	1.471751
ZSWIM5	-0.21464	2.94137	-3.13883	0.00214	0.006745	-2.56303
PACSIN1	-0.21487	3.571808	-3.17564	0.001905	0.006085	-2.4554
ZNF518B	-0.21491	3.743827	-4.00413	0.000109	0.00045	0.234326
TM9SF3	-0.21503	5.494119	-3.36534	0.00103	0.003496	-1.88418
ZMYM3	-0.21507	4.05072	-3.81331	0.000219	0.000855	-0.42886
TLE4	-0.2154	6.213067	-2.69881	0.007973	0.021406	-3.76549
FDPSP2	-0.21556	3.42128	-2.29122	0.023712	0.054749	-4.73519
AXIN2	-0.21561	3.918531	-3.86819	0.000179	0.000713	-0.24067
TMEM50B	-0.21567	5.047771	-2.53656	0.012491	0.031668	-4.16858
RMND1	-0.2157	4.284825	-2.47407	0.01477	0.03659	-4.31785
PLCG1-AS	-0.21609	1.929731	-3.82257	0.000212	0.00083	-0.39724
TRAM2	-0.21612	4.921787	-2.1904	0.030447	0.067696	-4.95279
LOC10192	-0.21614	2.669131	-2.66709	0.008718	0.023139	-3.84604
U2AF2	-0.21618	4.670844	-4.79768	4.71E-06	2.55E-05	3.244468
LMBR1L	-0.21618	4.956793	-3.46208	0.000746	0.002612	-1.58236
RBM19	-0.21627	4.464039	-3.48748	0.000684	0.002414	-1.50194
ZNF850	-0.21634	1.903665	-3.78729	0.00024	0.000934	-0.51733
SLC35D1	-0.21643	4.806734	-3.31828	0.001202	0.004027	-2.02846
THOC2	-0.21649	5.327554	-2.48328	0.014412	0.035785	-4.29604
NBPF3	-0.2166	3.854365	-3.55039	0.000552	0.001986	-1.30073
ZNF480	-0.21671	1.804155	-4.27628	3.86E-05	0.000175	1.222301
TCTN1	-0.2168	4.6391	-3.57918	0.0005	0.001815	-1.20771
SIGLEC1	-0.21686	3.846058	-3.2929	0.001306	0.004334	-2.10557
PLLP	-0.2169	3.438953	-2.32009	0.022042	0.051498	-4.67125
LSS	-0.217	4.215442	-3.3671	0.001024	0.003477	-1.87876
PRPF31	-0.2171	5.201708	-4.16065	6.03E-05	0.000263	0.79662

CCDC71	-0.21713	5.100953	-3.0334	0.00297	0.009044	-2.86538
AEBP2	-0.21714	3.522805	-3.10156	0.002405	0.007485	-2.6709
USO1	-0.21724	6.747397	-2.46865	0.014984	0.037039	-4.33061
ANKFY1	-0.21732	4.904887	-5.56598	1.64E-07	1.15E-06	6.499426
ARHGAP2(	-0.21735	5.652457	-2.33159	0.021406	0.050242	-4.64556
TRAK1	-0.21762	5.186043	-3.92951	0.000143	0.000581	-0.02798
AMMECR1	-0.21786	4.055452	-3.48343	0.000694	0.002446	-1.51481
MOCS3	-0.21802	4.86729	-3.54652	0.00056	0.002011	-1.3132
BRD9	-0.21814	5.961438	-3.91204	0.000153	0.000616	-0.08883
GLCCI1	-0.21815	5.376046	-2.21196	0.028881	0.064773	-4.90701
MFSD5	-0.21829	6.627454	-2.32715	0.02165	0.05075	-4.65549
CROT	-0.21836	2.546533	-5.90328	3.45E-08	2.72E-07	8.018588
YARS	-0.21838	6.416856	-2.49757	0.013873	0.034656	-4.2621
ZW10	-0.21891	5.441012	-2.91157	0.004296	0.012516	-3.20369
PTDSS1	-0.21895	8.181877	-2.12755	0.03544	0.07678	-5.08386
ZNF880	-0.21915	2.54436	-2.91563	0.004244	0.012384	-3.19261
CACNA2D	-0.21917	4.804439	-2.84445	0.00524	0.014862	-3.38494
ZNF844	-0.21922	3.652656	-3.69639	0.000332	0.001254	-0.82267
DEPDC7	-0.21948	1.784984	-3.32165	0.001189	0.003987	-2.0182
CBFB	-0.21957	6.747959	-2.243	0.02675	0.060696	-4.84039
NUP88	-0.21967	5.512079	-2.41097	0.017442	0.042216	-4.46516
ARL1	-0.21969	4.751231	-2.09667	0.038143	0.081691	-5.14696
INTS7	-0.21983	3.115877	-3.07789	0.002589	0.007992	-2.73886
ICK	-0.2199	2.066439	-4.51616	1.49E-05	7.38E-05	2.132318
ZNF571	-0.21994	2.072835	-2.44621	0.015901	0.039013	-4.3833
CD1E	-0.22008	3.037694	-4.27098	3.94E-05	0.000179	1.20258
SORD	-0.22019	4.326375	-2.96284	0.003683	0.010918	-3.06276
CLCN6	-0.22035	5.226168	-3.02528	0.003045	0.009228	-2.88829
FAAH2	-0.22039	4.200396	-2.71236	0.007672	0.02073	-3.73083
PRKCB	-0.22048	7.056889	-2.87548	0.004782	0.013746	-3.30159
C21orf49	-0.22049	4.944808	-2.76966	0.006513	0.01796	-3.58257
DDX28	-0.22052	4.429626	-5.69491	9.09E-08	6.71E-07	7.074144
TFAP2E	-0.22058	4.888215	-3.65938	0.000378	0.001411	-0.94533
PACS2	-0.22071	5.737246	-4.31602	3.31E-05	0.000153	1.370564
WDR59	-0.22075	3.993173	-5.01716	1.86E-06	1.08E-05	4.142841
FBRSL1	-0.22085	4.947894	-5.61742	1.30E-07	9.27E-07	6.727801
PRO1082	-0.22098	3.762162	-2.01055	0.046636	0.096616	-5.31843
THAP6	-0.221	3.91536	-4.3834	2.54E-05	0.00012	1.62426
DTX2P1-U	-0.22118	6.738111	-4.9815	2.16E-06	1.25E-05	3.995084
WHAMMP	-0.22122	5.75389	-2.28989	0.023791	0.054902	-4.73813
CLDND1	-0.2214	4.399582	-3.07049	0.002649	0.008166	-2.76
TRMT10B	-0.22149	3.980005	-3.71461	0.000311	0.001184	-0.76194
POLR2J2	-0.22163	5.324104	-3.0101	0.00319	0.009613	-2.93098
INIP	-0.22178	4.736482	-4.41909	2.20E-05	0.000105	1.75978
ADCK1	-0.22187	5.012248	-3.26471	0.001432	0.004697	-2.19064
PDCD5	-0.22191	3.860833	-2.94595	0.003875	0.011419	-3.10942
SNRNP40	-0.22193	5.44492	-2.25069	0.026244	0.059733	-4.82375
PLA2G16	-0.22207	4.688671	-2.75134	0.006865	0.018797	-3.63027
FBXO4	-0.22235	2.60144	-3.60095	0.000464	0.001696	-1.13695
TIMM22	-0.22241	4.818334	-3.1416	0.002121	0.006698	-2.55497
TRIM33	-0.2226	6.078423	-3.326	0.001172	0.003935	-2.00493
MYH3	-0.2226	1.981805	-3.19636	0.001783	0.005735	-2.39436
SH2B1	-0.22263	5.421413	-4.6391	9.07E-06	4.64E-05	2.61227
ACAD9	-0.22264	5.356791	-2.3193	0.022086	0.051578	-4.673
SLC23A2	-0.223	4.0007	-4.48493	1.69E-05	8.28E-05	2.011813
TRIM21	-0.22301	7.157356	-3.54737	0.000558	0.002006	-1.31048
IDO1	-0.22311	5.320645	-2.46186	0.015257	0.037608	-4.34661
C10orf88	-0.22311	4.268375	-2.78138	0.006296	0.017436	-3.5519

TSEN15	-0.22314	5.542196	-2.31509	0.022324	0.052026	-4.68239
CCDC122	-0.2232	1.906633	-3.05955	0.00274	0.008421	-2.7912
PCGF6	-0.2232	4.280365	-2.48897	0.014195	0.035324	-4.28256
ELP4	-0.22337	5.720463	-2.27612	0.024629	0.056504	-4.76837
GNGT2	-0.22362	4.718908	-2.50806	0.013488	0.033852	-4.23706
DHX35	-0.22369	3.198026	-4.93644	2.62E-06	1.48E-05	3.809361
CRIPAK	-0.22373	6.159573	-2.02874	0.044719	0.093343	-5.28277
MFAP1	-0.22383	7.01531	-2.65551	0.009005	0.023782	-3.87524
MYBBP1A	-0.22388	4.0335	-4.67324	7.88E-06	4.08E-05	2.74714
PPIP5K1	-0.2239	3.448615	-3.87792	0.000173	0.000691	-0.20709
LOC10272	-0.22412	2.487936	-3.54234	0.000568	0.002037	-1.32667
LINC0054(	-0.22413	2.852729	-2.95852	0.003731	0.011034	-3.07471
YBEY	-0.22417	5.635408	-2.14223	0.034215	0.074566	-5.05356
AKAP17A	-0.22436	6.144447	-2.98588	0.003434	0.010256	-2.99875
PABPN1	-0.2244	5.874947	-2.77914	0.006337	0.01753	-3.55776
BBS4	-0.22443	3.402878	-3.67856	0.000354	0.001327	-0.8819
DCAF15	-0.22466	4.705786	-4.88889	3.21E-06	1.78E-05	3.614608
UBE3C	-0.22495	3.491798	-5.86022	4.22E-08	3.28E-07	7.821857
PLEKHA8P	-0.22503	4.309717	-2.9309	0.004054	0.011897	-3.15081
CXorf40B	-0.22506	6.708152	-2.70109	0.007921	0.021291	-3.75969
ARIH2	-0.22521	5.495235	-4.08915	7.91E-05	0.000337	0.537719
ALDH3A2	-0.22524	5.906141	-3.4834	0.000694	0.002446	-1.51489
LMO7	-0.22533	2.654245	-5.20229	8.32E-07	5.21E-06	4.920785
CNKSR2	-0.2254	2.282364	-4.97967	2.18E-06	1.26E-05	3.987544
SERPINH1	-0.2255	4.31909	-3.15904	0.002007	0.006377	-2.50407
LIMD1	-0.22563	4.060863	-5.18187	9.10E-07	5.65E-06	4.834123
OTUD5	-0.22573	6.143653	-3.91706	0.00015	0.000605	-0.07137
ELOVL5	-0.22584	5.907529	-3.86407	0.000182	0.000722	-0.25488
XPR1	-0.2259	4.483827	-3.90028	0.00016	0.00064	-0.12968
RNF126	-0.22601	5.431515	-3.94695	0.000134	0.000548	0.03297
RTTN	-0.22604	3.386304	-4.3627	2.75E-05	0.000129	1.546025
TTC5	-0.22611	3.216864	-5.14659	1.06E-06	6.48E-06	4.684861
WDR86-A:	-0.22644	2.178684	-3.33893	0.001124	0.003783	-1.96535
HOXB4	-0.22652	4.600681	-3.33847	0.001125	0.003786	-1.96677
LINC00852	-0.22666	3.022563	-2.14461	0.034019	0.074259	-5.04863
MON1A	-0.22686	4.36956	-3.69175	0.000338	0.001272	-0.8381
ZFP1	-0.22688	2.259898	-5.44166	2.88E-07	1.93E-06	5.952556
PPP2R5D	-0.22693	6.123357	-4.79751	4.71E-06	2.55E-05	3.243811
CXXC1	-0.22702	7.163353	-4.06799	8.57E-05	0.000363	0.461783
MKRN2	-0.22704	4.752649	-3.78286	0.000244	0.000948	-0.53235
UNC119	-0.22708	6.932269	-3.92046	0.000148	0.000599	-0.05953
MSTO1	-0.22714	4.499318	-4.27045	3.95E-05	0.000179	1.200643
LEO1	-0.22715	4.962895	-2.05595	0.041978	0.088423	-5.22888
METTL2B	-0.22726	3.037981	-2.48438	0.01437	0.035693	-4.29343
NAB2	-0.22735	3.989212	-4.18039	5.59E-05	0.000245	0.868668
RBM8A	-0.22759	5.902897	-3.99915	0.000111	0.000458	0.216701
IQCH-AS1	-0.22787	3.865275	-2.72303	0.007443	0.020173	-3.70343
PPP1R16A	-0.22809	3.312323	-2.49432	0.013994	0.034907	-4.26982
KRBA1	-0.2282	5.701241	-3.21437	0.001683	0.005437	-2.34105
MCM3AP	-0.22838	4.097337	-7.03773	1.35E-10	1.61E-09	13.45337
STAG3L3	-0.22845	4.881284	-2.62821	0.009716	0.025424	-3.94361
CHCHD4	-0.22865	5.298466	-3.47466	0.000715	0.002511	-1.5426
TNFRSF21	-0.22877	2.827081	-3.97238	0.000122	0.000503	0.12224
GGPS1	-0.22884	5.722634	-2.6531	0.009066	0.02392	-3.8813
ZNF609	-0.22886	5.04077	-3.54732	0.000558	0.002006	-1.31064
SUPT5H	-0.22887	6.454965	-4.04514	9.33E-05	0.000392	0.380086
UAP1L1	-0.22893	4.065061	-2.17685	0.031468	0.069529	-4.98134
PHLDB2	-0.22896	2.041921	-5.95451	2.71E-08	2.17E-07	8.253636

ELP5	-0.22901	5.478412	-3.45701	0.000758	0.00265	-1.59836
BTF3	-0.22904	7.866749	-3.88109	0.000171	0.000684	-0.19616
RNF169	-0.22916	6.550438	-2.9939	0.003351	0.010038	-2.97636
LOC10050	-0.22936	4.352934	-2.69829	0.007984	0.021432	-3.76682
ETV7	-0.22964	3.089203	-4.1995	5.19E-05	0.00023	0.938683
PRR3	-0.22972	4.377927	-2.93065	0.004057	0.011904	-3.15149
CIAPIN1	-0.22982	4.818222	-3.13617	0.002158	0.006796	-2.57077
KRR1	-0.22995	3.59455	-3.33857	0.001125	0.003786	-1.96646
MYSM1	-0.23002	3.666588	-2.86436	0.004942	0.014134	-3.33156
TBCC	-0.23033	7.35617	-2.96094	0.003704	0.010969	-3.06802
VPS37C	-0.23035	6.142729	-4.33422	3.08E-05	0.000143	1.438801
TMEM99	-0.23043	4.084077	-3.5208	0.000611	0.002178	-1.39573
ZNF680	-0.23052	2.864473	-2.27664	0.024597	0.056458	-4.76722
LOC10042	-0.23059	4.744045	-3.15537	0.002031	0.006444	-2.5148
LOC10192	-0.23064	2.531376	-2.53309	0.012609	0.031927	-4.17696
NME3	-0.23077	6.061092	-3.37684	0.000992	0.003375	-1.84868
GAS6-AS1	-0.23086	3.419925	-3.02452	0.003052	0.009248	-2.89042
OLFM1	-0.23087	3.403451	-3.798	0.000231	0.0009	-0.48095
SLC15A4	-0.23089	6.428264	-2.71067	0.007709	0.02081	-3.73518
DTX4	-0.23122	5.035752	-2.73306	0.007234	0.019688	-3.67759
TUBD1	-0.23126	4.477177	-2.85509	0.005078	0.014465	-3.35645
HEATR9	-0.23136	2.291792	-3.72322	0.000302	0.001152	-0.73316
CD3EAP	-0.23148	4.481538	-3.5846	0.000491	0.001787	-1.19011
SRSF4	-0.23151	5.323291	-2.70533	0.007827	0.021078	-3.74884
CEP350	-0.23153	6.843948	-3.29739	0.001287	0.00428	-2.09196
RPP30	-0.23186	3.437684	-4.47265	1.78E-05	8.66E-05	1.96462
DOLK	-0.23188	4.602249	-3.37947	0.000983	0.00335	-1.84055
PMS1	-0.23199	2.894629	-4.92749	2.72E-06	1.54E-05	3.772633
CHTOP	-0.23204	6.844566	-3.34397	0.001105	0.003727	-1.94992
SOX12	-0.23208	3.720559	-3.59545	0.000473	0.001727	-1.15484
HDAC9	-0.23218	2.984906	-5.31382	5.09E-07	3.29E-06	5.397973
PAK2	-0.23243	6.088529	-3.13207	0.002186	0.006876	-2.58268
SEMA4C	-0.23298	5.824897	-3.17062	0.001935	0.006171	-2.47016
ZBTB10	-0.23306	2.327318	-6.02151	1.98E-08	1.62E-07	8.562693
KIAA1671	-0.23317	3.472946	-4.93142	2.68E-06	1.51E-05	3.788747
DIS3	-0.2332	4.499473	-2.71633	0.007586	0.020528	-3.72065
MRE11A	-0.2334	2.593431	-4.30159	3.50E-05	0.000161	1.316633
SOS1	-0.23351	5.193607	-4.27681	3.85E-05	0.000175	1.22425
R3HDM1	-0.23374	4.5914	-4.68368	7.55E-06	3.93E-05	2.788546
RBM26-AS	-0.23389	3.147221	-3.29231	0.001309	0.004341	-2.10736
CCDC174	-0.23392	4.880732	-3.43672	0.000812	0.002818	-1.66214
PABPC1	-0.23393	6.843378	-5.70483	8.68E-08	6.43E-07	7.118662
WAC	-0.23395	6.936233	-4.21362	4.92E-05	0.000219	0.990548
PCGF5	-0.23404	6.060987	-2.00109	0.047661	0.09845	-5.33686
RLTPR	-0.23419	4.324249	-3.65682	0.000382	0.001423	-0.95377
SCML2	-0.2342	5.128455	-3.19162	0.00181	0.00582	-2.40838
SOX4	-0.23434	3.785569	-3.66141	0.000376	0.001403	-0.93861
TOE1	-0.23435	5.521505	-3.38153	0.000976	0.00333	-1.83416
MRPL19	-0.23437	2.790933	-2.67748	0.008467	0.02257	-3.81976
MOCS2	-0.23443	3.268098	-3.66073	0.000377	0.001406	-0.94088
ISM1	-0.23446	2.646475	-4.10752	7.38E-05	0.000316	0.603923
ZSCAN21	-0.23465	4.337435	-2.99612	0.003329	0.009979	-2.97016
GUF1	-0.23476	3.752865	-2.94151	0.003927	0.011552	-3.12165
ANP32B	-0.23482	9.381161	-2.09711	0.038103	0.081615	-5.14607
CYB5D1	-0.2349	4.668475	-4.40603	2.32E-05	0.00011	1.710076
GTF2H1	-0.23508	4.686956	-4.34648	2.93E-05	0.000137	1.484898
DAPP1	-0.23509	6.753737	-2.60286	0.010422	0.027001	-4.00655
DTWD1	-0.2351	3.181843	-5.15992	1.00E-06	6.16E-06	4.741159

CYCS	-0.23524	4.701883	-2.25365	0.026051	0.059348	-4.81734
B3GALT2	-0.23525	1.616613	-5.09348	1.34E-06	8.02E-06	4.461382
HOXA1	-0.23539	2.800128	-2.32197	0.021937	0.051299	-4.66706
C2orf44	-0.23541	2.838554	-3.27603	0.00138	0.004551	-2.15656
C11orf84	-0.23549	4.429158	-2.96274	0.003684	0.010919	-3.06305
WDR60	-0.23573	3.242567	-4.60968	1.02E-05	5.19E-05	2.496585
SLC2A4RG	-0.23589	4.038757	-4.1169	7.12E-05	0.000306	0.637816
CALCOCO	-0.2359	5.405157	-6.19276	8.75E-09	7.63E-08	9.360849
CLCN4	-0.23606	2.865475	-4.76163	5.47E-06	2.93E-05	3.099512
GPR162	-0.23611	4.085668	-3.16093	0.001995	0.006341	-2.49854
EML4	-0.2362	6.071445	-2.06168	0.041418	0.087484	-5.21744
DDX31	-0.23622	3.716949	-5.59263	1.45E-07	1.03E-06	6.617588
DNAJC2	-0.23625	3.421802	-3.28932	0.001322	0.004379	-2.11642
EIF2B1	-0.2363	6.968961	-2.46211	0.015247	0.037591	-4.34602
GMEB2	-0.23654	6.057989	-4.78665	4.93E-06	2.66E-05	3.200047
METTL21B	-0.23658	3.201225	-3.10427	0.002385	0.007428	-2.66312
ZNF234	-0.23667	2.117267	-4.46986	1.80E-05	8.75E-05	1.953897
MAP2K1	-0.23674	7.718757	-2.11781	0.036275	0.07828	-5.10387
TMF1	-0.23679	5.50264	-2.23675	0.027168	0.061508	-4.85389
ARL6	-0.2368	1.948672	-4.59851	1.07E-05	5.41E-05	2.452835
C15orf57	-0.2369	4.225899	-3.39402	0.000937	0.003208	-1.79543
FPR3	-0.2369	2.974629	-2.76617	0.006579	0.018118	-3.59167
KIF3B	-0.23691	5.743469	-3.55837	0.000537	0.001938	-1.27502
GLT8D1	-0.23696	5.741049	-2.26629	0.025243	0.057743	-4.78985
ZMAT3	-0.23733	5.42236	-3.04428	0.002872	0.008784	-2.83456
ZNF829	-0.23751	2.405874	-5.55056	1.76E-07	1.23E-06	6.4312
ZCCHC8	-0.23764	5.147649	-2.32085	0.021999	0.051417	-4.66956
UBXN7	-0.23764	6.087127	-2.73756	0.007141	0.019454	-3.66595
SRRT	-0.23774	5.312244	-5.19281	8.67E-07	5.41E-06	4.880541
TSPAN32	-0.238	5.932033	-4.18436	5.51E-05	0.000242	0.8832
CASD1	-0.23805	2.773611	-4.52479	1.44E-05	7.16E-05	2.165695
FCHSD2	-0.2381	5.536511	-2.89554	0.004506	0.013055	-3.2473
STK36	-0.23824	4.038765	-4.50044	1.59E-05	7.81E-05	2.071573
PITPNM2	-0.23833	4.113566	-3.72082	0.000305	0.00116	-0.74118
ARSK	-0.23845	3.611119	-4.68768	7.43E-06	3.87E-05	2.80441
GART	-0.23884	3.805251	-4.11305	7.23E-05	0.00031	0.623899
USP5	-0.23885	5.167519	-3.72045	0.000305	0.001161	-0.74242
LOC10192	-0.23887	3.717384	-2.35565	0.020128	0.047653	-4.59147
SPRTN	-0.23906	3.524672	-2.01513	0.046147	0.095796	-5.30948
PTPN13	-0.23914	2.050426	-5.03417	1.73E-06	1.01E-05	4.213582
ZNF853	-0.23914	3.201374	-4.21805	4.84E-05	0.000216	1.006836
SLC35F2	-0.23922	3.246616	-2.78611	0.006211	0.017235	-3.53949
LOC10192	-0.23935	2.861815	-3.28807	0.001327	0.004394	-2.12019
PRORSD1F	-0.23952	2.958202	-2.75003	0.006891	0.018855	-3.63367
LOC10272	-0.23973	1.8567	-4.17514	5.70E-05	0.00025	0.849475
RTKN2	-0.2399	1.759981	-4.59544	1.08E-05	5.47E-05	2.440804
KIR2DL4	-0.23992	3.907985	-3.18158	0.001869	0.005987	-2.43794
SURF6	-0.23994	4.82549	-3.97993	0.000119	0.00049	0.148839
ZNF687	-0.24001	5.608356	-2.83784	0.005342	0.015091	-3.4026
APOBEC3C	-0.24027	6.781994	-3.62559	0.000426	0.001568	-1.05642
PEPD	-0.2404	5.484299	-2.16258	0.032576	0.071652	-5.01125
PRKXP1	-0.24048	3.32836	-2.18947	0.030516	0.067813	-4.95476
GPR19	-0.24051	3.634769	-2.83309	0.005417	0.015283	-3.41526
CPSF6	-0.24052	5.311913	-2.35322	0.020254	0.0479	-4.59695
NOL4L	-0.24055	3.940921	-6.38994	3.37E-09	3.18E-08	10.29385
MARK2	-0.24064	5.285062	-3.12277	0.00225	0.007055	-2.60965
FLJ27354	-0.24075	3.806694	-3.07979	0.002573	0.007951	-2.73341
SDHA	-0.24087	7.881224	-4.22512	4.71E-05	0.00021	1.032892

LMF1	-0.24114	4.149088	-5.37291	3.91E-07	2.58E-06	5.653351
LOC10334	-0.2415	4.76904	-3.086	0.002524	0.007821	-2.71564
CCDC15	-0.24162	3.480329	-3.00724	0.003218	0.009687	-2.93901
HPS3	-0.24168	4.771873	-3.45974	0.000752	0.00263	-1.58974
USP46	-0.24174	2.881521	-5.3216	4.92E-07	3.18E-06	5.4315
LOC72968	-0.24179	5.386197	-4.77939	5.08E-06	2.73E-05	3.170819
KCTD10	-0.24181	6.360089	-4.3119	3.36E-05	0.000155	1.355168
DDX55	-0.24185	3.775917	-2.38902	0.018467	0.044372	-4.51559
SRSF1	-0.24192	5.598954	-4.07064	8.48E-05	0.000359	0.471273
VAV2	-0.242	4.23397	-4.18119	5.57E-05	0.000245	0.871595
NMRAL1	-0.24208	5.871262	-3.22352	0.001635	0.005299	-2.31386
CCDC101	-0.24232	6.147583	-4.8766	3.38E-06	1.88E-05	3.564452
SP2	-0.24242	3.758997	-5.05818	1.56E-06	9.22E-06	4.313663
LOC10028	-0.24253	4.894856	-2.79225	0.006102	0.016963	-3.52336
THUMPD3	-0.24256	4.967775	-3.35437	0.001068	0.003609	-1.91796
ATG9B	-0.24277	3.355543	-3.71557	0.00031	0.00118	-0.75875
NAGK	-0.24288	8.652626	-2.36131	0.019837	0.047075	-4.57866
RANBP2	-0.24303	5.953698	-2.52298	0.012957	0.032697	-4.2013
MGAT4A	-0.24308	5.02437	-2.12525	0.035636	0.07712	-5.0886
AP4S1	-0.24309	4.268531	-5.80716	5.41E-08	4.14E-07	7.580575
TOM1L2	-0.24338	5.142002	-5.41025	3.31E-07	2.21E-06	5.815557
ZNF681	-0.24345	2.347161	-4.41897	2.20E-05	0.000105	1.759326
CSE1L	-0.24351	5.110587	-2.32491	0.021774	0.051	-4.66051
WDR5B	-0.24356	3.951455	-2.50666	0.013539	0.033958	-4.24041
ZNF141	-0.24358	2.095107	-3.84834	0.000193	0.00076	-0.309
CCR9	-0.24359	3.243461	-2.46777	0.015019	0.037112	-4.3327
RNF165	-0.24396	2.576735	-4.81137	4.44E-06	2.42E-05	3.299728
GPR68	-0.24397	3.84974	-3.73148	0.000293	0.001121	-0.70551
NANP	-0.244	4.067256	-2.30665	0.022806	0.052939	-4.70111
CCT6B	-0.24426	3.376067	-2.88398	0.004663	0.013441	-3.27863
AKT2	-0.24432	4.77134	-5.4883	2.33E-07	1.60E-06	6.156849
PRSS30P	-0.2445	3.945544	-3.41792	0.000865	0.002981	-1.72101
WDPCP	-0.24468	4.947317	-4.25628	4.17E-05	0.000188	1.148054
ORAI3	-0.24474	7.109492	-3.25667	0.001469	0.004809	-2.21479
RFX1	-0.24479	4.668881	-4.06713	8.59E-05	0.000363	0.458686
RANBP3	-0.24481	5.457513	-5.62103	1.28E-07	9.13E-07	6.743895
NADK2	-0.24483	3.263764	-2.8701	0.004859	0.013934	-3.31612
CC2D1B	-0.24487	4.089396	-3.87239	0.000177	0.000703	-0.22619
NME7	-0.24497	3.396934	-4.05835	8.88E-05	0.000375	0.427271
TCOF1	-0.24523	4.27722	-4.60273	1.05E-05	5.32E-05	2.469346
RAB14	-0.24526	5.690342	-2.59775	0.01057	0.027328	-4.01917
THRAP3	-0.24528	4.793996	-3.8805	0.000172	0.000685	-0.19818
TMEM116	-0.2453	3.169034	-5.50252	2.19E-07	1.50E-06	6.21935
DCHS1	-0.24542	3.912225	-3.58649	0.000488	0.001777	-1.18397
RALGAPB	-0.24566	5.127685	-3.04002	0.00291	0.008879	-2.84664
COX2	-0.24585	12.3191	-2.53533	0.012532	0.031757	-4.17154
KDF1	-0.24595	3.031632	-2.84807	0.005184	0.014726	-3.37527
DEPDC5	-0.24604	5.164892	-4.26978	3.96E-05	0.00018	1.198129
ACTN4	-0.24632	7.652522	-2.68324	0.008331	0.022251	-3.80514
DDX1	-0.24639	6.961436	-2.1719	0.031849	0.070268	-4.99175
RBM27	-0.24647	6.72443	-3.58596	0.000488	0.00178	-1.18569
LYRM4	-0.24651	4.59686	-3.1316	0.002189	0.006883	-2.58405
GHDC	-0.24656	5.285545	-2.83951	0.005316	0.015038	-3.39814
TIMM21	-0.24667	4.537499	-2.48811	0.014228	0.035387	-4.28458
ZNF610	-0.24676	2.101147	-3.743	0.000282	0.00108	-0.66684
ABCF2	-0.24679	4.955292	-5.05328	1.59E-06	9.39E-06	4.293239
PDCD6	-0.24679	6.451973	-3.54023	0.000572	0.00205	-1.33345
ALG9	-0.2469	3.955395	-2.75795	0.006736	0.018499	-3.6131

RASSF4	-0.24691	4.519083	-2.12566	0.035601	0.077054	-5.08776
DHX33	-0.24718	5.438925	-3.21261	0.001693	0.005465	-2.34627
B2M	-0.24733	10.63174	-3.83006	0.000206	0.000809	-0.37163
MGAT5	-0.24733	5.585696	-4.73895	6.01E-06	3.20E-05	3.008678
SOX13	-0.24748	3.790216	-4.41118	2.27E-05	0.000108	1.729672
BMPR1A	-0.24762	2.685444	-7.07088	1.14E-10	1.38E-09	13.61854
IFT88	-0.24766	5.274869	-3.51437	0.000625	0.002222	-1.41632
SUPT7L	-0.24766	4.80019	-2.74663	0.006958	0.019013	-3.64249
ZDHHC18	-0.24767	5.659557	-3.67544	0.000358	0.00134	-0.89221
ZNF418	-0.2477	4.445259	-3.28492	0.001341	0.004434	-2.12971
YTHDF2	-0.2477	7.661044	-3.00106	0.003279	0.009849	-2.95633
GPATCH11	-0.24776	3.152146	-3.14325	0.00211	0.006667	-2.55016
TSR1	-0.2479	4.496817	-3.38872	0.000953	0.003256	-1.81189
RAB8A	-0.24796	8.297089	-2.82366	0.005569	0.01567	-3.44035
INPP5F	-0.24806	3.258918	-4.45403	1.92E-05	9.27E-05	1.89319
SMYD3	-0.24812	4.450468	-2.48981	0.014163	0.035257	-4.28054
DAZAP2	-0.24813	8.07724	-3.86761	0.00018	0.000714	-0.24268
ZNF354C	-0.24814	3.243956	-4.68771	7.43E-06	3.87E-05	2.804522
PTGDR2	-0.24816	4.816778	-2.90224	0.004417	0.012827	-3.2291
DYNC1LI1	-0.24818	7.039669	-2.98326	0.003462	0.010332	-3.00604
LRRC75A	-0.24824	4.985548	-2.18305	0.030998	0.068688	-4.96831
ENDOG	-0.24841	4.196481	-2.1439	0.034078	0.074339	-5.0501
TSHZ2	-0.24851	3.093446	-4.74446	5.87E-06	3.13E-05	3.030697
QRICH1	-0.24869	5.742073	-5.68901	9.34E-08	6.87E-07	7.047683
ZBTB33	-0.2487	4.131366	-3.7086	0.000318	0.001206	-0.78199
ZNF493	-0.2491	4.983318	-3.1063	0.002369	0.00739	-2.65725
MBTPS1	-0.24919	6.269945	-3.19607	0.001785	0.005739	-2.39523
EXOSC9	-0.24935	5.673778	-3.11486	0.002307	0.007211	-2.63253
QDPR	-0.24935	4.826035	-3.14546	0.002095	0.006624	-2.54372
TRIM38	-0.24958	7.648277	-3.4677	0.000732	0.002564	-1.56462
CD37	-0.24962	6.228203	-4.46275	1.85E-05	8.97E-05	1.926627
USP20	-0.24965	6.098812	-3.28267	0.001351	0.004463	-2.13652
IRAK1BP1	-0.24972	1.896769	-4.21067	4.98E-05	0.000221	0.979711
LCMT2	-0.2498	3.68268	-4.14483	6.40E-05	0.000278	0.739027
DPY19L2P	-0.24982	2.111043	-3.55502	0.000543	0.001958	-1.28582
LOC10028	-0.24986	2.587816	-3.56873	0.000518	0.001874	-1.24155
LOC10192	-0.24988	1.751246	-3.70895	0.000318	0.001205	-0.78085
DAXX	-0.24991	6.34975	-4.21445	4.90E-05	0.000218	0.993609
ZNF791	-0.24992	7.117179	-2.75414	0.00681	0.018665	-3.623
AARS	-0.25001	6.806881	-2.41971	0.017048	0.041399	-4.44495
CELF1	-0.25034	6.248245	-5.41002	3.31E-07	2.21E-06	5.814553
AKAP9	-0.25042	4.129024	-3.18578	0.001844	0.005917	-2.42558
MTCP1	-0.25043	2.311957	-2.99142	0.003377	0.010103	-2.98328
PARP2	-0.25054	4.44061	-2.38886	0.018474	0.044379	-4.51595
NBAS	-0.25066	4.029745	-5.23181	7.31E-07	4.61E-06	5.046501
LINC0067	-0.25068	4.400013	-4.44497	1.99E-05	9.58E-05	1.858529
ZNF133	-0.2507	4.653043	-3.98542	0.000117	0.00048	0.168199
TP73-AS1	-0.25079	4.371514	-4.32571	3.18E-05	0.000148	1.406884
CAD	-0.25104	4.113357	-4.61529	1.00E-05	5.08E-05	2.518605
BCKDHB	-0.25115	3.575382	-4.5028	1.58E-05	7.75E-05	2.080678
C1QTNF3	-0.25119	3.340272	-4.37875	2.58E-05	0.000122	1.606635
TFB1M	-0.25136	3.63771	-6.23915	7.00E-09	6.21E-08	9.579018
IL18BP	-0.25152	5.006166	-4.19812	5.22E-05	0.000231	0.933614
SLC46A3	-0.25165	6.347388	-2.10991	0.036963	0.079565	-5.12002
FAM135A	-0.25177	2.273726	-5.94532	2.83E-08	2.26E-07	8.211404
TRAPPC6A	-0.25182	6.170056	-2.87612	0.004773	0.013722	-3.29987
KANK1	-0.2519	3.702419	-3.63311	0.000415	0.001531	-1.03178
RAB9B	-0.25193	3.537355	-4.46599	1.83E-05	8.86E-05	1.939028

CCDC107	-0.25198	4.988009	-3.04399	0.002875	0.008788	-2.8354
MANBA	-0.25207	6.430684	-3.13097	0.002193	0.006896	-2.58587
USP14	-0.25253	6.826313	-2.76141	0.006669	0.018335	-3.60407
MEF2D	-0.25254	5.322103	-5.24612	6.86E-07	4.35E-06	5.107585
FAM184B	-0.25289	3.550916	-2.67564	0.008511	0.022664	-3.82442
NR2C2	-0.25298	5.212505	-2.80798	0.005829	0.016295	-3.4819
KDM6B	-0.25336	6.96451	-2.71045	0.007714	0.020819	-3.73574
EZH1	-0.25341	5.532686	-5.96304	2.61E-08	2.10E-07	8.292909
ELL	-0.25346	4.669809	-3.47263	0.00072	0.002525	-1.54901
SQSTM1	-0.25357	6.214772	-4.72154	6.46E-06	3.42E-05	2.939122
CLYBL	-0.25359	3.377475	-5.44515	2.83E-07	1.91E-06	5.967804
ABHD6	-0.25377	3.450653	-4.70207	7.00E-06	3.67E-05	2.861588
HSPA5	-0.25379	6.468238	-2.72534	0.007394	0.020064	-3.69749
SLC38A11	-0.2538	2.672963	-3.11399	0.002313	0.007227	-2.63507
PDHB	-0.25404	6.296568	-2.84287	0.005264	0.014919	-3.38918
ATP6	-0.25406	8.167823	-3.13187	0.002187	0.006878	-2.58327
SCARB2	-0.25421	4.569013	-2.48712	0.014266	0.035464	-4.28694
LSM14A	-0.25424	6.685271	-3.6396	0.000405	0.001501	-1.01046
RBM5	-0.25432	7.089666	-2.44664	0.015883	0.038977	-4.38229
FLJ38379	-0.25438	1.854988	-3.88245	0.00017	0.00068	-0.19146
CLUH	-0.25454	5.595836	-3.6792	0.000353	0.001324	-0.87976
KCTD6	-0.25471	4.102783	-2.36866	0.019465	0.046384	-4.562
IGFBP4	-0.25497	4.370565	-3.90996	0.000154	0.00062	-0.09609
ZNF587B	-0.25518	4.902239	-2.05148	0.042417	0.089231	-5.23778
LMF2	-0.25526	7.160937	-5.54477	1.81E-07	1.26E-06	6.405589
SPDYE2	-0.2553	4.473604	-2.38822	0.018505	0.044438	-4.51743
NSMCE4A	-0.2555	4.529778	-2.76157	0.006666	0.018332	-3.60366
TRIM51	-0.25559	3.141394	-3.00667	0.003224	0.0097	-2.94062
ALG12	-0.25572	5.007285	-2.95899	0.003726	0.011021	-3.07341
LINC0018[illegible]	-0.25589	2.136549	-2.59665	0.010602	0.027401	-4.02188
CDK13	-0.256	5.082396	-5.1007	1.30E-06	7.80E-06	4.491693
FARSA	-0.25608	5.261651	-3.44716	0.000784	0.002731	-1.62937
TFIP11	-0.25611	5.335505	-3.9365	0.00014	0.000567	-0.00357
RAD54B	-0.2562	2.868119	-2.33086	0.021446	0.050319	-4.6472
CCNY	-0.2562	5.979522	-3.07598	0.002604	0.008038	-2.74431
SEC31B	-0.25625	5.10195	-2.90342	0.004401	0.012791	-3.22588
TRAF6	-0.25627	5.984421	-4.47265	1.78E-05	8.66E-05	1.964599
ZCCHC4	-0.25629	2.860206	-4.75844	5.54E-06	2.97E-05	3.086698
SMARCAL[illegible]	-0.2563	5.002139	-2.79109	0.006122	0.017016	-3.52641
NBEA	-0.25652	2.348866	-4.75569	5.61E-06	3.00E-05	3.075656
CCNT2	-0.25656	4.638333	-1.99563	0.048261	0.099388	-5.34745
TAF1B	-0.25679	2.903818	-3.40732	0.000896	0.003079	-1.75406
CEP41	-0.25697	3.46113	-6.29443	5.36E-09	4.85E-08	9.840092
PSTK	-0.25717	2.847331	-4.71593	6.61E-06	3.49E-05	2.916757
RGMB	-0.2572	3.122623	-4.5637	1.23E-05	6.16E-05	2.316837
PIP4K2B	-0.25721	4.832039	-7.26234	4.30E-11	5.62E-10	14.5787
IFNLR1	-0.25729	4.944369	-4.53981	1.36E-05	6.76E-05	2.223942
SALL2	-0.2575	4.667275	-3.04073	0.002904	0.008862	-2.84464
ZNF614	-0.25793	2.98652	-5.13435	1.12E-06	6.82E-06	4.633211
NEIL2	-0.25793	4.387204	-2.08346	0.039353	0.083835	-5.17371
ARFRP1	-0.25799	5.427233	-4.10555	7.43E-05	0.000318	0.596824
SFMBT1	-0.25803	3.557709	-4.50133	1.59E-05	7.79E-05	2.075016
CAPRIN1	-0.25803	5.084833	-4.89614	3.11E-06	1.73E-05	3.64422
SLC25A15	-0.25819	3.977612	-3.85043	0.000191	0.000755	-0.30181
CCNK	-0.25845	5.324997	-4.75476	5.63E-06	3.01E-05	3.071929
SLC25A29	-0.25855	4.617944	-3.84452	0.000196	0.00077	-0.32209
METTL17	-0.25856	5.341524	-3.25763	0.001465	0.004797	-2.21191
HOXB3	-0.25865	3.784353	-4.21165	4.96E-05	0.00022	0.983283

VCP	-0.2588	7.385109	-3.47665	0.00071	0.002495	-1.53629
ZNF746	-0.25895	5.405827	-3.30322	0.001263	0.00421	-2.07427
ABHD12	-0.25917	4.656722	-4.66773	8.06E-06	4.17E-05	2.725344
ZNRD1-AS	-0.25918	2.702477	-5.71193	8.40E-08	6.24E-07	7.150582
OCLN	-0.2593	3.304134	-2.7559	0.006776	0.018585	-3.61842
LINS	-0.2593	5.94946	-2.86275	0.004965	0.014192	-3.33588
SHISA2	-0.25941	1.664529	-4.52564	1.44E-05	7.13E-05	2.168988
FMO5	-0.25962	3.314426	-4.22889	4.64E-05	0.000207	1.046782
BCL7A	-0.25968	4.38945	-3.73888	0.000286	0.001095	-0.68068
ATP10A	-0.26005	4.823391	-5.47266	2.50E-07	1.70E-06	6.08824
LRIG1	-0.26011	3.483393	-5.46119	2.63E-07	1.79E-06	6.037985
KLRAP1	-0.2602	1.784202	-3.30257	0.001266	0.004216	-2.07626
SPATA5	-0.26033	2.124602	-5.6765	9.89E-08	7.24E-07	6.991611
EEFSEC	-0.26041	5.680109	-3.65048	0.00039	0.001451	-0.97467
FRG1B	-0.26046	3.78589	-3.43393	0.00082	0.002841	-1.67092
DFNB59	-0.26073	2.32944	-3.64077	0.000404	0.001495	-1.00662
UNK	-0.26087	4.637911	-5.10175	1.29E-06	7.77E-06	4.496077
ZNF585A	-0.26091	3.647403	-2.80121	0.005945	0.016582	-3.49975
CCDC94	-0.2611	5.625599	-3.95271	0.000132	0.000538	0.053181
SLC35A4	-0.26123	6.979563	-3.12519	0.002233	0.007013	-2.60263
LOC10099	-0.26138	1.874579	-4.90043	3.05E-06	1.71E-05	3.661761
GTPBP3	-0.26153	3.974323	-5.09022	1.36E-06	8.12E-06	4.447709
TP53RK	-0.26155	5.140994	-3.37863	0.000986	0.003358	-1.84315
TRIM4	-0.26157	5.269207	-5.01673	1.86E-06	1.09E-05	4.141062
FAM26F	-0.26207	4.457139	-2.40898	0.017532	0.042395	-4.46973
LSM11	-0.26215	3.167174	-5.03793	1.70E-06	9.98E-06	4.229233
LTK	-0.26231	3.644875	-4.21632	4.87E-05	0.000217	1.000471
DLL1	-0.26238	2.491257	-5.12469	1.17E-06	7.09E-06	4.592515
ZNF398	-0.26252	5.946516	-3.83407	0.000203	0.000798	-0.35791
ZC3H13	-0.26256	5.433638	-5.2529	6.66E-07	4.23E-06	5.136577
FAM76A	-0.26277	2.993908	-4.60034	1.06E-05	5.37E-05	2.459997
PPM1G	-0.26285	6.342793	-3.60945	0.00045	0.001651	-1.10923
PPP3CA	-0.2629	7.978593	-2.94242	0.003916	0.011527	-3.11915
FAM210A	-0.2629	3.294507	-4.85414	3.71E-06	2.05E-05	3.473046
LPCAT4	-0.26295	3.739437	-5.85227	4.38E-08	3.40E-07	7.785657
EDEM1	-0.263	7.885279	-2.77782	0.006362	0.017588	-3.56124
PM20D2	-0.26318	4.121795	-2.91288	0.004279	0.012474	-3.20011
PDE9A	-0.26321	2.815329	-4.62433	9.63E-06	4.90E-05	2.554149
CLIC5	-0.26322	3.276563	-7.25978	4.36E-11	5.70E-10	14.56581
ZFYVE28	-0.26328	3.440277	-5.07406	1.45E-06	8.66E-06	4.380044
PCED1A	-0.26331	4.107971	-4.56962	1.20E-05	6.03E-05	2.339918
NEURL4	-0.26342	4.059021	-3.27559	0.001382	0.004554	-2.1579
LINC00965	-0.26343	2.714299	-3.63895	0.000406	0.001504	-1.01259
DACT1	-0.26345	2.902217	-2.4382	0.01624	0.039702	-4.40201
TRIM13	-0.26346	5.752392	-2.55241	0.011965	0.030452	-4.13018
KIAA1407	-0.26352	4.028744	-3.39241	0.000942	0.003223	-1.80044
RPL30	-0.26392	11.45062	-2.92733	0.004098	0.01201	-3.16059
ZCCHC14	-0.26394	3.483639	-5.21744	7.79E-07	4.89E-06	4.98524
OPHN1	-0.26396	8.818943	-3.77822	0.000248	0.000962	-0.54804
NUBP1	-0.26397	6.163555	-2.53983	0.012381	0.031419	-4.16068
NFE2L1	-0.26401	6.316516	-5.34489	4.43E-07	2.90E-06	5.532018
NEFH	-0.26415	2.575568	-4.47852	1.74E-05	8.48E-05	1.987181
LOC10272	-0.26428	1.982946	-3.82133	0.000213	0.000833	-0.40149
HNRNPC	-0.26433	6.487005	-4.13519	6.64E-05	0.000287	0.704045
GAK	-0.26462	7.204752	-4.43541	2.06E-05	9.90E-05	1.821986
SDF4	-0.26473	7.361165	-4.58354	1.14E-05	5.71E-05	2.394241
PRCC	-0.26479	5.706068	-2.89309	0.004539	0.013134	-3.25396
FAM122B	-0.26482	5.711655	-2.43138	0.016534	0.040343	-4.41787

ZNF621	-0.26497	3.882726	-3.54445	0.000563	0.002024	-1.31986
NTN4	-0.26511	1.790194	-4.32899	3.14E-05	0.000146	1.419161
SLC45A3	-0.26519	3.507524	-4.62322	9.68E-06	4.92E-05	2.549767
GRAPL	-0.26571	2.985972	-6.11554	1.27E-08	1.07E-07	8.999529
ATXN2	-0.26584	6.042014	-3.7219	0.000304	0.001157	-0.73758
DUSP12	-0.26634	6.108768	-2.60449	0.010375	0.026897	-4.00253
DNMT3A	-0.26643	4.993627	-5.28804	5.70E-07	3.67E-06	5.287133
AAK1	-0.26646	4.684689	-5.67464	9.98E-08	7.29E-07	6.98327
TSPYL2	-0.26649	6.164546	-3.2567	0.001469	0.004809	-2.2147
AAMP	-0.26656	7.077556	-3.88698	0.000168	0.00067	-0.17577
ARL10	-0.26657	3.563392	-5.83176	4.82E-08	3.72E-07	7.692305
PRRC1	-0.26666	6.323642	-3.33749	0.001129	0.003798	-1.96978
LINC0049	-0.26677	3.836239	-3.24655	0.001518	0.004952	-2.24511
ADRBK2	-0.26684	5.657717	-2.40048	0.017925	0.043207	-4.48931
CPSF7	-0.26692	4.436936	-3.90641	0.000156	0.000627	-0.1084
EFHC2	-0.26709	2.613789	-2.43423	0.016411	0.040085	-4.41125
SERPINB9	-0.2673	5.012265	-2.38393	0.018712	0.04485	-4.52722
AKAP8	-0.2674	5.488258	-3.96789	0.000124	0.00051	0.106451
ANKZF1	-0.26765	6.266163	-5.12111	1.19E-06	7.18E-06	4.577454
FBXW7	-0.26772	4.759196	-4.72379	6.40E-06	3.39E-05	2.948086
CELSR2	-0.26773	2.993699	-5.65904	1.07E-07	7.78E-07	6.913476
ZNF510	-0.26777	4.0111	-3.43268	0.000823	0.002852	-1.67483
TRAPPC2	-0.26819	4.285705	-4.11354	7.21E-05	0.00031	0.625675
THG1L	-0.26838	3.920232	-3.26323	0.001438	0.004719	-2.19511
ZNF721	-0.2684	5.87232	-3.26982	0.001408	0.004629	-2.17527
ABAT	-0.26873	5.408432	-1.98896	0.049002	0.100661	-5.36035
VPS11	-0.26893	6.781684	-5.43306	2.99E-07	2.01E-06	5.915022
COG8	-0.26919	4.865786	-4.96948	2.28E-06	1.30E-05	3.945432
PIP5K1C	-0.26921	5.920981	-4.704	6.94E-06	3.65E-05	2.869256
BRF2	-0.26924	4.966293	-5.06642	1.50E-06	8.92E-06	4.348091
TLR10	-0.26965	4.93506	-2.57647	0.011205	0.028773	-4.07148
LOC28402	-0.26973	4.990963	-3.97514	0.000121	0.000498	0.131969
ACACB	-0.26982	4.345748	-5.42824	3.05E-07	2.05E-06	5.893993
MEOX1	-0.26992	2.634871	-2.81132	0.005773	0.016155	-3.47306
ERBB2IP	-0.26995	5.748863	-3.03251	0.002978	0.009064	-2.86788
ZNF347	-0.26996	2.027842	-3.91197	0.000153	0.000616	-0.08909
ARHGEF1	-0.27003	4.112349	-4.25446	4.20E-05	0.00019	1.141292
EI24	-0.2701	5.77653	-3.49109	0.000676	0.002387	-1.49049
PPARA	-0.27023	3.405485	-7.80336	2.61E-12	4.17E-11	17.34192
CD200R1	-0.27026	3.731811	-3.58008	0.000499	0.001811	-1.20479
ZNF639	-0.27064	4.476222	-2.87409	0.004802	0.01379	-3.30535
GATA2	-0.27066	4.955146	-3.92453	0.000146	0.00059	-0.04534
NFYA	-0.27071	4.495456	-4.73845	6.02E-06	3.20E-05	3.006659
RBM15	-0.2708	4.897009	-3.7796	0.000247	0.000958	-0.54337
KLC2	-0.2708	3.932814	-3.62193	0.000431	0.001587	-1.06843
SAMD9L	-0.2708	6.279298	-2.20388	0.029459	0.06588	-4.92422
RSPH3	-0.27082	4.890879	-4.17929	5.61E-05	0.000246	0.864643
CD300A	-0.27084	7.023178	-4.7186	6.54E-06	3.45E-05	2.927408
RBM28	-0.27086	5.082281	-2.87875	0.004736	0.013624	-3.29278
GMCL1	-0.2709	6.344804	-2.18443	0.030893	0.068495	-4.9654
HMBOX1	-0.27091	5.449756	-3.42371	0.000848	0.002928	-1.7029
RHOBTB3	-0.27109	3.079074	-5.08323	1.40E-06	8.35E-06	4.41844
SGSM2	-0.27147	5.531815	-4.13236	6.71E-05	0.00029	0.693791
FAM222B	-0.27147	5.617687	-5.12126	1.18E-06	7.18E-06	4.578083
AATF	-0.27151	7.144492	-3.60396	0.000459	0.001681	-1.12712
SLC25A25	-0.27152	4.544507	-3.87457	0.000175	0.000698	-0.21867
ZNF585B	-0.27152	2.951699	-4.56452	1.23E-05	6.15E-05	2.320036
SMARCD2	-0.27169	6.171825	-3.86218	0.000183	0.000727	-0.26139

PAN3-AS1	-0.27172	4.37232	-2.14484	0.034001	0.074235	-5.04817
SH2B2	-0.27182	6.488501	-2.62878	0.009701	0.02539	-3.9422
COG3	-0.27189	3.290694	-2.92564	0.004119	0.012066	-3.16523
KLHL20	-0.27197	4.082416	-4.27269	3.91E-05	0.000178	1.208948
NIFK	-0.27235	4.671558	-2.9427	0.003913	0.011519	-3.11837
LOC64465	-0.27265	4.732044	-2.37531	0.019134	0.045701	-4.54688
EPB41L2	-0.27273	3.063974	-5.58764	1.49E-07	1.05E-06	6.595437
DENR	-0.27285	4.955997	-3.71954	0.000306	0.001165	-0.74546
USP42	-0.2731	3.833165	-7.67943	4.98E-12	7.59E-11	16.70298
EDRF1	-0.27317	3.68525	-5.08366	1.39E-06	8.34E-06	4.420227
GTF2H3	-0.27341	4.808379	-2.40412	0.017756	0.042849	-4.48093
PHLPP2	-0.27358	3.805983	-3.75957	0.000265	0.001022	-0.61106
IDH3A	-0.27362	4.602786	-3.25937	0.001456	0.004772	-2.2067
SRP72	-0.27366	5.576011	-2.4214	0.016972	0.041236	-4.44104
FGF9	-0.27374	2.112775	-6.08784	1.44E-08	1.21E-07	8.870473
ATG14	-0.27379	5.794161	-2.27415	0.024751	0.056739	-4.77267
SLC5A6	-0.27383	5.328325	-4.05315	9.06E-05	0.000381	0.408678
G2E3	-0.27391	4.661486	-2.27619	0.024625	0.0565	-4.76821
MTO1	-0.27393	5.255929	-2.85519	0.005077	0.014463	-3.35619
ATG2B	-0.27412	4.292409	-4.99282	2.06E-06	1.19E-05	4.041908
STAT5A	-0.27413	6.888614	-3.58759	0.000486	0.001771	-1.18042
SLC25A53	-0.27451	2.137235	-5.16066	9.98E-07	6.14E-06	4.744305
IL23A	-0.27474	4.705586	-3.44128	0.0008	0.002781	-1.64785
CYB561D1	-0.27487	7.037648	-4.33365	3.09E-05	0.000144	1.436669
EIF3J-AS1	-0.27496	4.397546	-2.90892	0.00433	0.012601	-3.21091
ZNF738	-0.27499	2.782774	-3.05514	0.002778	0.00852	-2.80374
CNOT3	-0.27499	3.691085	-4.11833	7.08E-05	0.000305	0.642987
KNOP1	-0.27508	4.606862	-5.13175	1.13E-06	6.88E-06	4.622243
SF1	-0.27511	5.622371	-3.60225	0.000462	0.00169	-1.13269
SLAIN2	-0.27514	4.85267	-5.25628	6.56E-07	4.17E-06	5.151014
ZNF235	-0.27519	2.661183	-5.64155	1.16E-07	8.37E-07	6.835339
PNPLA4	-0.27531	3.551254	-4.03134	9.83E-05	0.00041	0.330909
TMEM64	-0.2754	4.235909	-2.64803	0.009195	0.024202	-3.89403
FAM168A	-0.27541	4.965755	-4.22578	4.69E-05	0.00021	1.035311
ARAP1	-0.27545	6.167924	-4.81476	4.38E-06	2.38E-05	3.313438
BAZ2A	-0.27545	5.021675	-6.60501	1.18E-09	1.20E-08	11.32752
BCAS4	-0.27564	3.358457	-5.66613	1.04E-07	7.56E-07	6.945212
AS3MT	-0.27573	2.003174	-4.49821	1.61E-05	7.88E-05	2.062968
AQR	-0.27575	5.210514	-3.31474	0.001216	0.004068	-2.03926
NUP50-AS	-0.27608	3.687834	-3.71391	0.000312	0.001186	-0.76427
HBP1	-0.27626	5.638342	-3.39432	0.000936	0.003205	-1.79452
ASB2	-0.27636	3.231148	-4.20262	5.13E-05	0.000227	0.950128
RYK	-0.2764	5.136146	-3.43669	0.000812	0.002818	-1.66224
RDH11	-0.27645	5.074597	-4.29266	3.62E-05	0.000166	1.28328
AMZ2P1	-0.27651	4.072492	-3.06683	0.002679	0.008248	-2.77046
FAM111A	-0.27656	5.242585	-3.9679	0.000124	0.00051	0.106483
PIK3R4	-0.27661	3.096105	-6.78333	4.85E-10	5.29E-09	12.19641
ARIH1	-0.27682	5.385295	-5.2941	5.55E-07	3.58E-06	5.313178
FXYD7	-0.27685	3.587994	-3.24979	0.001502	0.004904	-2.23542
AHCYL2	-0.2769	4.714093	-6.01621	2.03E-08	1.66E-07	8.53818
WDR36	-0.27694	3.909179	-2.62775	0.009729	0.025443	-3.94476
LOC10192	-0.27697	3.361976	-5.29086	5.63E-07	3.63E-06	5.299242
TBC1D32	-0.27708	1.863996	-4.13961	6.53E-05	0.000283	0.72008
ZNF2	-0.27715	4.085894	-3.53535	0.000581	0.002082	-1.34912
TLE2	-0.27729	4.756071	-4.45085	1.94E-05	9.37E-05	1.881015
PSMD5-AS	-0.27734	4.42815	-2.1501	0.033573	0.073473	-5.03725
NR2C2AP	-0.2775	5.190136	-4.22136	4.78E-05	0.000213	1.019019
HOXC4	-0.27751	4.677118	-3.43943	0.000805	0.002795	-1.65365

PAPOLG	-0.27754	4.717691	-4.30764	3.42E-05	0.000157	1.339222
CHKB	-0.27761	6.826818	-3.71694	0.000309	0.001175	-0.75414
MIPEP	-0.27772	3.611503	-4.63903	9.07E-06	4.64E-05	2.61202
TFAM	-0.27775	4.659144	-3.90347	0.000158	0.000634	-0.11862
TRMT10C	-0.27792	5.38274	-2.30818	0.022718	0.052795	-4.69773
EPPK1	-0.27815	3.430799	-4.74216	5.93E-06	3.16E-05	3.021505
BRD4	-0.27835	6.069499	-4.06408	8.69E-05	0.000367	0.447769
GMEB1	-0.27836	5.304288	-5.69997	8.88E-08	6.56E-07	7.096855
DTHD1	-0.27836	1.76799	-4.27749	3.84E-05	0.000175	1.226791
CLUHP3	-0.27838	4.904822	-4.37504	2.62E-05	0.000123	1.592613
TTC12	-0.27862	3.479953	-5.82695	4.93E-08	3.80E-07	7.670418
ETV3	-0.27871	4.985156	-4.11747	7.11E-05	0.000306	0.639853
ILKAP	-0.27886	5.303313	-2.76216	0.006655	0.018303	-3.60212
ASF1B	-0.27887	5.398204	-2.01777	0.045868	0.095322	-5.30432
PRKD3	-0.27899	4.188572	-3.51954	0.000614	0.002185	-1.39978
RLN2	-0.27905	2.41124	-3.76838	0.000257	0.000992	-0.58134
PRDM2	-0.27928	5.440272	-6.5122	1.86E-09	1.83E-08	10.87946
TP53BP1	-0.27928	3.725481	-5.73649	7.50E-08	5.61E-07	7.261062
MAPKAPK	-0.27929	4.713462	-4.61839	9.87E-06	5.02E-05	2.530807
DISP1	-0.27947	2.84756	-3.67962	0.000352	0.001323	-0.87836
CBX5	-0.27958	4.473629	-3.69491	0.000334	0.001259	-0.82761
USP16	-0.2796	6.053566	-2.14571	0.03393	0.074113	-5.04637
OCEL1	-0.27976	5.298983	-3.74053	0.000284	0.001089	-0.67514
EARS2	-0.27981	3.844567	-5.03184	1.74E-06	1.02E-05	4.203891
SARS2	-0.28	4.097503	-4.00492	0.000108	0.000449	0.237102
SMAD9	-0.28013	2.838559	-4.49858	1.60E-05	7.87E-05	2.064403
C1orf21	-0.28021	3.379743	-5.21699	7.80E-07	4.90E-06	4.983347
ARHGAP3:	-0.2805	2.744073	-4.68228	7.59E-06	3.95E-05	2.782975
RAD9A	-0.2806	5.722018	-5.02383	1.80E-06	1.06E-05	4.17056
MAT2B	-0.28068	6.673265	-4.20496	5.09E-05	0.000225	0.958703
VPS13D	-0.28069	4.531813	-6.02832	1.92E-08	1.57E-07	8.594214
YES1	-0.28079	2.455049	-4.11141	7.27E-05	0.000312	0.61798
CRYL1	-0.28083	5.237942	-2.77108	0.006486	0.017899	-3.57887
KLHL28	-0.28095	6.053682	-2.78818	0.006174	0.017141	-3.53407
KRIT1	-0.28105	4.691473	-3.30363	0.001261	0.004206	-2.07303
HIVEP1	-0.28113	4.113481	-3.38736	0.000957	0.00327	-1.81609
ITPK1	-0.28121	5.800315	-5.63446	1.20E-07	8.63E-07	6.803713
ITPA	-0.28126	6.947366	-3.51119	0.000631	0.002243	-1.42645
ARHGEF2	-0.28142	6.560263	-5.5773	1.56E-07	1.10E-06	6.549592
HLA-DRB6	-0.28145	5.399572	-3.17939	0.001882	0.006022	-2.44439
ODF2L	-0.28164	2.481352	-4.92129	2.80E-06	1.57E-05	3.747185
PON2	-0.28182	3.733612	-3.61378	0.000444	0.001628	-1.09507
BBS12	-0.28187	3.400383	-2.67851	0.008443	0.022511	-3.81716
SPPL3	-0.28188	5.90279	-7.03298	1.38E-10	1.65E-09	13.42971
ICAM3	-0.28208	10.23852	-2.64174	0.009358	0.024593	-3.90982
CIAO1	-0.28224	5.21101	-4.17138	5.79E-05	0.000253	0.835754
ENG	-0.28229	4.731698	-5.14788	1.06E-06	6.45E-06	4.690309
MADCAM:	-0.2824	4.729282	-3.99137	0.000114	0.000471	0.189203
INTS5	-0.2825	5.468634	-4.99895	2.01E-06	1.16E-05	4.067314
UBQLN4	-0.2827	4.962448	-5.04507	1.65E-06	9.70E-06	4.258979
RPS15	-0.2828	7.138408	-5.04744	1.63E-06	9.62E-06	4.26884
USP10	-0.28284	6.891752	-4.01322	0.000105	0.000436	0.266518
STARD4	-0.283	4.162863	-2.18636	0.030749	0.068241	-4.96134
COX11	-0.28305	3.872242	-3.50403	0.000647	0.002294	-1.44931
SLC7A6OS	-0.28312	5.807521	-4.25802	4.14E-05	0.000187	1.154514
DCP1A	-0.28331	6.443275	-4.28339	3.76E-05	0.000171	1.248762
POLG2	-0.28339	5.533583	-4.08667	7.98E-05	0.00034	0.528803
TDRKH	-0.28354	2.752861	-5.54496	1.80E-07	1.26E-06	6.406446

PIEZO1	-0.28371	4.908996	-5.17232	9.49E-07	5.86E-06	4.793632
TMEM206	-0.284	3.869754	-3.35536	0.001065	0.003599	-1.91494
ST8SIA1	-0.28402	1.775967	-5.31135	5.14E-07	3.33E-06	5.38734
UQCC1	-0.28411	4.439813	-5.98013	2.41E-08	1.94E-07	8.371622
ZNF671	-0.28437	5.068811	-3.9249	0.000146	0.00059	-0.04405
NEK1	-0.28438	3.526569	-5.63489	1.20E-07	8.62E-07	6.805648
TMEM194I	-0.2846	3.22233	-3.93912	0.000138	0.000562	0.005581
POLRMT	-0.28462	4.302795	-4.82665	4.17E-06	2.28E-05	3.361527
UTP6	-0.28465	4.293927	-5.19826	8.47E-07	5.29E-06	4.903659
DAG1	-0.28481	4.671509	-5.66912	1.02E-07	7.46E-07	6.958587
ITGB1	-0.2851	4.232349	-4.52353	1.45E-05	7.19E-05	2.160805
C5orf45	-0.2852	4.94725	-4.15104	6.25E-05	0.000272	0.761603
NUFIP1	-0.28524	3.759235	-3.55721	0.000539	0.001945	-1.27875
MTMR2	-0.28554	3.874901	-3.86197	0.000184	0.000727	-0.26212
DPH6	-0.28557	2.321544	-4.21893	4.82E-05	0.000215	1.010096
LAS1L	-0.28557	4.328001	-6.1543	1.05E-08	9.00E-08	9.180582
FBXO46	-0.28576	5.862532	-3.88413	0.000169	0.000677	-0.18565
SMC6	-0.2858	3.976433	-2.73157	0.007264	0.019764	-3.68144
DPAGT1	-0.28592	4.770596	-2.43508	0.016374	0.040005	-4.40927
IMMP2L	-0.28598	2.86994	-5.70932	8.51E-08	6.31E-07	7.138842
EXD2	-0.28599	4.035248	-5.00151	1.99E-06	1.15E-05	4.077928
DMXL1	-0.28608	5.559716	-2.45296	0.01562	0.038397	-4.36749
ABCA5	-0.28612	3.183559	-2.2363	0.027198	0.061549	-4.85485
CHD1L	-0.28621	5.113493	-3.81067	0.000221	0.000863	-0.43785
FAM221A	-0.28625	2.401368	-3.59214	0.000478	0.001746	-1.16561
FDX1	-0.28633	4.432188	-3.26281	0.00144	0.004725	-2.19636
CDIP1	-0.28637	5.07461	-3.69705	0.000331	0.001251	-0.82048
GAA	-0.28643	6.220338	-2.75641	0.006766	0.018561	-3.6171
LOC10050	-0.28677	2.59615	-5.83294	4.80E-08	3.70E-07	7.697666
SMAD5	-0.28683	4.696235	-2.60728	0.010296	0.026726	-3.99562
AGAP6	-0.28684	3.697397	-4.8445	3.87E-06	2.13E-05	3.433886
LINC0029	-0.28685	5.770016	-3.50079	0.000654	0.002319	-1.45962
WDR48	-0.28694	6.105956	-6.16928	9.79E-09	8.44E-08	9.250718
GGT7	-0.287	3.562876	-5.52959	1.93E-07	1.34E-06	6.338577
PHF11	-0.28724	6.964491	-3.25894	0.001459	0.004778	-2.20799
LOC38869	-0.28728	3.489543	-5.17532	9.36E-07	5.80E-06	4.806336
SLC29A3	-0.2876	5.251162	-4.5535	1.28E-05	6.41E-05	2.277124
POLR3GL	-0.28762	6.484544	-2.66116	0.008864	0.023478	-3.86102
TARP	-0.28781	3.46376	-4.84185	3.91E-06	2.15E-05	3.423144
KHSRP	-0.28789	4.97649	-5.57482	1.57E-07	1.11E-06	6.538584
ZNF597	-0.28801	3.216802	-3.9436	0.000136	0.000554	0.021273
GNA13	-0.28804	6.764836	-2.13933	0.034454	0.075	-5.05957
CYB5B	-0.28815	4.157101	-3.72185	0.000304	0.001157	-0.73775
VEGFB	-0.28825	4.143413	-3.64339	0.0004	0.001483	-0.99801
GABARAPI	-0.28838	5.488337	-2.88652	0.004628	0.013353	-3.27175
LRRC8D	-0.28838	6.699868	-3.48759	0.000684	0.002414	-1.50161
ZNF223	-0.28853	2.188978	-3.14022	0.00213	0.006724	-2.55898
SF3B4	-0.28854	6.962865	-4.00749	0.000107	0.000445	0.246208
SUPT6H	-0.28855	4.447984	-4.4116	2.27E-05	0.000108	1.731243
ZNF485	-0.28875	1.697748	-4.72999	6.24E-06	3.31E-05	2.972852
RAP1GDS1	-0.28877	5.654629	-4.39107	2.46E-05	0.000116	1.653316
PERP	-0.28881	2.884081	-5.26331	6.36E-07	4.05E-06	5.181083
EIF4A3	-0.28884	7.736123	-3.13246	0.002183	0.006869	-2.58154
NHP2	-0.2889	7.811106	-2.47389	0.014777	0.036601	-4.31827
PLXNB2	-0.28924	4.473006	-3.16575	0.001965	0.006256	-2.48442
SCMH1	-0.28924	4.931074	-4.14891	6.30E-05	0.000274	0.753859
ZNF37BP	-0.28927	3.371612	-3.31576	0.001212	0.004056	-2.03616
LPHN1	-0.28939	3.812534	-5.99728	2.22E-08	1.80E-07	8.450706

TLE3	-0.28941	6.896869	-2.90609	0.004366	0.012695	-3.21863
TIGD7	-0.28951	3.091217	-2.61024	0.010212	0.026548	-3.9883
MPP5	-0.28982	3.702282	-3.27536	0.001383	0.004557	-2.15859
NIPA1	-0.29003	3.360014	-4.44506	1.99E-05	9.58E-05	1.858884
KIAA0141	-0.29006	4.989147	-5.0896	1.36E-06	8.14E-06	4.445103
SKIV2L2	-0.29009	4.997318	-3.45022	0.000776	0.002705	-1.61974
LOC10013	-0.29012	6.165635	-4.03973	9.52E-05	0.000399	0.360766
SYS1	-0.29023	5.099332	-5.41675	3.22E-07	2.15E-06	5.843888
ISG20L2	-0.29044	4.98105	-6.03534	1.85E-08	1.52E-07	8.626708
DTX1	-0.29051	4.235564	-3.38242	0.000973	0.00332	-1.8314
CECR5	-0.29056	5.354091	-2.11111	0.036858	0.079371	-5.11756
LOC28451	-0.29068	3.576269	-3.95428	0.000131	0.000535	0.058666
KIAA0753	-0.29085	5.943122	-5.07242	1.46E-06	8.72E-06	4.373198
BOK	-0.29088	3.629096	-3.49865	0.000659	0.002332	-1.46642
METTL3	-0.29094	5.662405	-3.40743	0.000896	0.003078	-1.75373
APPBP2	-0.29095	4.413936	-4.08527	8.03E-05	0.000342	0.523791
PORCN	-0.29099	4.097527	-4.20824	5.02E-05	0.000223	0.97078
LZTFL1	-0.29122	2.866824	-4.77146	5.25E-06	2.82E-05	3.138942
RPL37	-0.29162	11.27189	-2.99601	0.00333	0.009981	-2.97047
TMEM252	-0.292	2.920238	-4.15409	6.18E-05	0.000269	0.772701
NID1	-0.29202	3.303163	-4.21034	4.98E-05	0.000221	0.978492
KDM5A	-0.29207	6.322175	-4.02066	0.000102	0.000425	0.292941
ATMIN	-0.29211	5.130042	-4.79141	4.83E-06	2.61E-05	3.219223
ZIK1	-0.29213	3.14669	-5.85187	4.39E-08	3.41E-07	7.783812
RC3H2	-0.29216	5.391356	-5.76778	6.49E-08	4.90E-07	7.402286
PRPS2	-0.2923	5.114659	-2.15175	0.03344	0.073252	-5.03382
MED10	-0.29236	5.848995	-4.14239	6.46E-05	0.00028	0.730187
CCNG1	-0.2927	8.490681	-2.63764	0.009465	0.02484	-3.92009
C2CD3	-0.29279	4.121424	-7.55123	9.70E-12	1.41E-10	16.04556
ILF3-AS1	-0.293	4.339716	-3.09689	0.00244	0.007584	-2.68436
SENP3	-0.29329	4.733349	-5.56684	1.63E-07	1.15E-06	6.503222
DGKE	-0.29335	2.378847	-7.50818	1.21E-11	1.73E-10	15.82568
CENPB	-0.29346	4.617529	-2.30988	0.02262	0.052609	-4.69395
TET3	-0.2935	5.56025	-5.89522	3.59E-08	2.82E-07	7.981699
LLGL2	-0.2938	3.588045	-5.41825	3.19E-07	2.13E-06	5.850402
DAP3	-0.29439	5.777982	-5.69099	9.25E-08	6.82E-07	7.056533
EIF2A	-0.29445	7.802342	-2.40069	0.017915	0.043188	-4.48883
DGKK	-0.29456	3.550548	-4.2619	4.08E-05	0.000185	1.168892
NPC1	-0.29458	3.949488	-4.74915	5.76E-06	3.07E-05	3.04948
XPA	-0.29464	4.885967	-2.77764	0.006365	0.017592	-3.5617
ABCB4	-0.29478	2.943027	-3.20368	0.001742	0.005617	-2.37273
ZNF397	-0.29492	3.890003	-4.43029	2.11E-05	0.000101	1.802463
RIMKLB	-0.29497	2.697398	-7.37765	2.38E-11	3.26E-10	15.16166
POMT1	-0.29503	4.53796	-3.27378	0.00139	0.004576	-2.16336
ZNF691	-0.29504	4.841525	-3.89831	0.000161	0.000645	-0.13654
CUL1	-0.29504	5.431811	-5.08365	1.39E-06	8.34E-06	4.42018
ZNF232	-0.29555	4.420076	-2.96758	0.00363	0.010775	-3.04964
DDX42	-0.29569	4.707164	-3.72057	0.000305	0.001161	-0.74202
ZNF264	-0.2957	4.656537	-3.58908	0.000483	0.001763	-1.17556
BRD7	-0.29574	4.494386	-4.28977	3.66E-05	0.000167	1.272499
ATXN7L3B	-0.29578	6.080666	-4.79008	4.86E-06	2.62E-05	3.213852
ZNF268	-0.29581	3.489013	-3.5644	0.000526	0.001901	-1.25554
SPINT1	-0.29588	4.800894	-4.48626	1.68E-05	8.24E-05	2.016937
SH3BP1	-0.29601	5.791543	-4.10315	7.50E-05	0.000321	0.588173
LINC0052€	-0.29605	3.580242	-4.19853	5.21E-05	0.000231	0.935108
PELP1	-0.29609	4.861088	-4.34821	2.91E-05	0.000136	1.491391
C17orf67	-0.29614	4.500383	-3.98681	0.000116	0.000478	0.173102
PPHLN1	-0.29619	4.594337	-5.42291	3.13E-07	2.09E-06	5.870734

RPP14	-0.29628	4.282899	-4.60768	1.03E-05	5.22E-05	2.488748
DMTF1	-0.29635	7.126412	-1.99571	0.048252	0.099378	-5.34729
GALNT6	-0.29641	5.10492	-2.13439	0.034865	0.07575	-5.06978
NASP	-0.29646	5.55954	-3.90897	0.000155	0.000622	-0.09951
ERBB2	-0.29655	3.171934	-5.70661	8.61E-08	6.38E-07	7.126683
RNF216	-0.29667	4.945602	-5.25586	6.58E-07	4.18E-06	5.149199
YTHDF1	-0.29696	7.504002	-4.64571	8.83E-06	4.53E-05	2.638354
CEP95	-0.29714	3.026388	-4.86536	3.54E-06	1.96E-05	3.518648
RABGAP1	-0.29714	5.515765	-6.65706	9.09E-10	9.43E-09	11.58006
FCHSD1	-0.29725	4.256275	-4.87	3.47E-06	1.92E-05	3.537568
FAN1	-0.29726	3.942946	-5.23208	7.30E-07	4.61E-06	5.047657
YEATS2	-0.29729	5.314408	-6.67879	8.16E-10	8.55E-09	11.68574
C1orf216	-0.29739	4.917492	-3.42438	0.000846	0.002922	-1.70082
TGM3	-0.2975	4.488871	-2.94818	0.003849	0.011347	-3.10329
DSTN	-0.29781	5.427836	-4.19177	5.35E-05	0.000236	0.91033
GEMIN8	-0.29783	3.072622	-5.23838	7.10E-07	4.49E-06	5.074512
LOC10106	-0.29794	4.637753	-3.76967	0.000256	0.000988	-0.57698
ZNF785	-0.29807	2.757591	-4.72678	6.32E-06	3.35E-05	2.960055
PTP4A3	-0.2981	4.92788	-3.2836	0.001346	0.004452	-2.13371
ZNF813	-0.2981	2.493263	-2.83838	0.005334	0.015075	-3.40115
U2AF1L4	-0.29814	5.48152	-3.9494	0.000133	0.000544	0.041577
ZNF322	-0.29844	4.342698	-2.11235	0.03675	0.079197	-5.11504
LRCH1	-0.29846	3.882546	-4.71855	6.54E-06	3.45E-05	2.92721
SCAMP3	-0.29846	6.840755	-4.61423	1.00E-05	5.10E-05	2.514461
ZNF500	-0.29847	4.517162	-4.67011	7.98E-06	4.13E-05	2.734748
FBXW11	-0.29851	5.065025	-3.97807	0.00012	0.000493	0.142286
LPP	-0.29858	4.796397	-5.42957	3.04E-07	2.04E-06	5.899793
HOMER1	-0.29874	3.024626	-5.27978	5.92E-07	3.80E-06	5.251662
EGLN1	-0.29877	6.594517	-2.9121	0.004289	0.012499	-3.20223
NUDT7	-0.29884	3.788007	-3.42233	0.000852	0.002941	-1.70722
XKR8	-0.29923	7.933104	-5.74948	7.07E-08	5.30E-07	7.319637
MEIS1	-0.29932	3.310813	-2.31521	0.022317	0.052016	-4.68211
ORC5	-0.29939	3.35034	-4.24496	4.36E-05	0.000196	1.106149
BAIAP2-AS	-0.29956	4.198155	-4.36658	2.71E-05	0.000127	1.560672
SLC30A4	-0.29956	2.623974	-6.34816	4.13E-09	3.83E-08	10.09495
ZBTB40	-0.29985	5.025268	-4.92123	2.80E-06	1.57E-05	3.746935
DOPEY1	-0.30002	3.168653	-6.38131	3.52E-09	3.31E-08	10.25268
RHNO1	-0.30007	4.019153	-2.67602	0.008502	0.022646	-3.82346
OSBP	-0.30011	5.269981	-5.27601	6.02E-07	3.85E-06	5.235499
SLC11A2	-0.30014	3.951297	-4.97196	2.25E-06	1.29E-05	3.95568
CHCHD10	-0.30015	7.324001	-2.9508	0.003819	0.011268	-3.09605
TPCN1	-0.30025	5.121668	-5.89548	3.58E-08	2.82E-07	7.98292
KIAA2026	-0.30026	4.386277	-5.94653	2.82E-08	2.25E-07	8.216939
C6orf203	-0.30026	4.349852	-2.57017	0.0114	0.029198	-4.08691
ZNF528	-0.30026	3.8385	-3.86023	0.000185	0.000731	-0.2681
SDCCAG3	-0.30027	4.797251	-4.67876	7.71E-06	4.00E-05	2.76901
MAP3K12	-0.30054	4.889877	-5.11737	1.21E-06	7.29E-06	4.561725
HN1L	-0.30091	4.563835	-5.9701	2.52E-08	2.03E-07	8.325408
ZNF441	-0.301	1.811766	-3.58276	0.000494	0.001796	-1.19607
THAP11	-0.30112	5.542167	-3.33042	0.001156	0.003882	-1.9914
CIITA	-0.30125	4.30527	-5.74379	7.25E-08	5.44E-07	7.293984
KIAA1468	-0.30144	6.234742	-2.12773	0.035426	0.076771	-5.0835
WHSC1L1	-0.30166	5.473241	-6.93316	2.29E-10	2.64E-09	12.93433
PAQR3	-0.30171	2.924163	-3.71296	0.000313	0.00119	-0.76747
CYP2S1	-0.30208	4.335147	-3.35538	0.001064	0.003599	-1.91486
TNFRSF9	-0.3021	3.549207	-4.95593	2.41E-06	1.37E-05	3.889579
TAF5	-0.30214	4.766889	-3.0167	0.003126	0.009442	-2.91244
TIAM2	-0.30215	3.534709	-5.32276	4.89E-07	3.17E-06	5.436494

PSMD11	-0.30226	5.806541	-3.38886	0.000953	0.003255	-1.81144
RBM6	-0.30246	5.756462	-3.11578	0.0023	0.007193	-2.62987
MKLN1	-0.30251	4.661303	-5.02653	1.78E-06	1.04E-05	4.181798
ADCY7	-0.30274	6.145742	-3.83881	0.0002	0.000785	-0.34167
LILRA4	-0.30296	4.809187	-3.53053	0.000591	0.002115	-1.36459
CSNK2A2	-0.30304	6.042787	-4.29226	3.63E-05	0.000166	1.281809
ANKRD13(	-0.30314	3.416528	-2.4399	0.016168	0.039567	-4.39803
ZNF706	-0.30319	5.305937	-3.3619	0.001042	0.003529	-1.89478
PCCA	-0.30321	2.389966	-6.26764	6.10E-09	5.48E-08	9.713451
TMEM135	-0.30344	3.033386	-2.97305	0.003571	0.010615	-3.03444
PRPF19	-0.30355	5.896965	-4.01587	0.000104	0.000432	0.275919
OBFC1	-0.30366	4.582967	-3.1633	0.001981	0.006299	-2.49161
AKAP12	-0.30368	2.129834	-9.17459	1.70E-15	4.79E-14	24.58987
HAPLN3	-0.30374	6.104857	-4.28429	3.74E-05	0.000171	1.252094
PARS2	-0.30381	4.653577	-4.37085	2.67E-05	0.000125	1.57677
ATF7IP	-0.30389	6.14801	-4.77318	5.21E-06	2.80E-05	3.145849
C21orf33	-0.30407	5.807436	-4.20725	5.04E-05	0.000224	0.967119
GTF3C4	-0.30414	4.205972	-6.0861	1.46E-08	1.22E-07	8.862366
RPP38	-0.3042	5.001734	-3.32643	0.001171	0.00393	-2.0036
FAM24B	-0.30421	3.664855	-3.97011	0.000123	0.000506	0.114276
ZNF570	-0.30422	2.669134	-5.35687	4.20E-07	2.76E-06	5.58383
ZNF74	-0.30425	3.874256	-5.63606	1.19E-07	8.57E-07	6.810856
MIB1	-0.30436	5.147227	-2.67258	0.008584	0.022839	-3.83217
SEC14L1P1	-0.30444	2.868096	-3.79736	0.000232	0.000902	-0.48315
SLMO2	-0.30457	5.562265	-3.12326	0.002247	0.007046	-2.60822
MRPS35	-0.30468	6.467455	-2.19588	0.030042	0.066949	-4.94121
ZNF618	-0.30479	3.298645	-6.44057	2.64E-09	2.53E-08	10.53573
UBE4B	-0.30483	5.189549	-4.38935	2.48E-05	0.000117	1.646785
PIAS3	-0.30501	4.628281	-3.48432	0.000692	0.002439	-1.51199
HAVCR1	-0.30506	2.30324	-4.4684	1.81E-05	8.80E-05	1.948282
DPY19L4	-0.30513	2.82466	-3.21393	0.001686	0.005442	-2.34236
ZNF432	-0.30517	4.186983	-2.07846	0.039819	0.084661	-5.18379
ESCO1	-0.3053	5.172823	-3.05382	0.002789	0.008552	-2.80749
RNASET2	-0.30534	6.755086	-3.48018	0.000701	0.002469	-1.5251
LMBRD1	-0.30541	6.894733	-2.39406	0.018227	0.043852	-4.50405
PI4KB	-0.30587	6.36902	-5.49572	2.25E-07	1.55E-06	6.189472
MTA3	-0.30606	3.674396	-5.19231	8.69E-07	5.42E-06	4.878421
MDH2	-0.30621	6.825275	-3.85176	0.00019	0.000752	-0.29722
EAF1	-0.30634	5.582792	-2.42997	0.016595	0.040473	-4.42116
FUT8	-0.30638	3.953546	-2.84113	0.005291	0.014977	-3.3938
TACC1	-0.30678	5.432243	-4.63743	9.13E-06	4.67E-05	2.605685
SMARCA4	-0.30686	4.969305	-6.1279	1.19E-08	1.02E-07	9.057174
GRK5	-0.30697	5.176673	-6.5211	1.78E-09	1.76E-08	10.9223
CASP6	-0.30704	5.511889	-3.42685	0.000839	0.002901	-1.6931
PHF2	-0.30719	4.222389	-5.73175	7.67E-08	5.72E-07	7.239752
MSH6	-0.30735	4.12686	-3.39674	0.000928	0.003182	-1.78699
SF3B2	-0.30749	5.998519	-6.08987	1.43E-08	1.20E-07	8.87992
PLCD1	-0.30752	6.025208	-5.0768	1.44E-06	8.57E-06	4.391498
YTHDC1	-0.30753	4.449116	-5.20225	8.32E-07	5.21E-06	4.920636
SMNDC1	-0.30767	7.980484	-2.36489	0.019655	0.046758	-4.57055
MYO1G	-0.30777	7.473718	-2.55453	0.011896	0.030303	-4.12502
FLJ35934	-0.30792	2.612351	-3.71362	0.000313	0.001187	-0.76525
ABCB7	-0.30811	5.049998	-3.37393	0.001001	0.003405	-1.85768
POLE3	-0.30824	6.752858	-2.70967	0.007731	0.020862	-3.73774
IFT74	-0.30845	1.805217	-4.76473	5.40E-06	2.90E-05	3.111932
NUP93	-0.30847	4.339419	-5.55057	1.76E-07	1.23E-06	6.431244
ACVR2B	-0.3085	3.281904	-5.48123	2.41E-07	1.64E-06	6.125822
ZNF273	-0.30873	2.346754	-3.11835	0.002282	0.00714	-2.62246

EEPD1	-0.30892	4.263872	-7.16657	7.02E-11	8.81E-10	14.09715
GALNT11	-0.30909	5.994209	-3.24527	0.001524	0.004971	-2.24896
ZDHHC5	-0.30935	6.059593	-5.51339	2.08E-07	1.44E-06	6.267204
CTDSP2	-0.30945	7.932826	-4.02968	9.89E-05	0.000413	0.324993
FKSG49	-0.30949	7.808593	-4.95463	2.43E-06	1.38E-05	3.88421
ZNF502	-0.30965	3.891372	-4.50902	1.54E-05	7.58E-05	2.104709
PHF12	-0.30974	4.624719	-3.57605	0.000505	0.001832	-1.21785
DEXI	-0.30978	5.748627	-3.68493	0.000346	0.0013	-0.86076
COX19	-0.3099	5.655314	-4.38344	2.54E-05	0.00012	1.624389
PMS2L2	-0.31009	4.056964	-3.09479	0.002456	0.007628	-2.6904
PAIP2B	-0.31011	3.773558	-4.24239	4.40E-05	0.000198	1.096634
USP18	-0.3102	3.662848	-3.32248	0.001186	0.003979	-2.01567
APBA3	-0.3104	5.332263	-5.33626	4.61E-07	3.00E-06	5.494776
LOC10013	-0.31048	4.858187	-3.69806	0.00033	0.001248	-0.81713
PWWP2A	-0.31068	4.92095	-5.28478	5.79E-07	3.72E-06	5.273153
STRIP1	-0.31073	5.87968	-4.57201	1.19E-05	5.97E-05	2.349242
KRT73	-0.31079	3.2062	-5.33642	4.60E-07	3.00E-06	5.495461
VARS	-0.31082	4.333274	-4.26632	4.01E-05	0.000182	1.185303
COX20	-0.31083	5.817743	-3.46038	0.00075	0.002625	-1.58771
RNF157-A	-0.31119	2.147859	-4.67979	7.67E-06	3.98E-05	2.773103
NDRG2	-0.31138	3.861653	-5.49225	2.29E-07	1.57E-06	6.174212
TSPAN4	-0.31162	4.290573	-5.25885	6.49E-07	4.13E-06	5.162027
LOC10192	-0.31162	3.66156	-3.08316	0.002547	0.007878	-2.72377
ADAT1	-0.31166	5.34631	-4.14535	6.39E-05	0.000277	0.740922
ZNF678	-0.31186	2.842506	-4.66067	8.30E-06	4.27E-05	2.69743
PIGL	-0.31188	3.012564	-6.52257	1.76E-09	1.75E-08	10.92939
DEF6	-0.31201	6.859766	-3.87735	0.000174	0.000692	-0.20906
ORMDL3	-0.31222	5.612173	-2.68183	0.008364	0.022331	-3.80872
ADAT2	-0.31223	3.368493	-4.59981	1.06E-05	5.38E-05	2.457901
KIAA1429	-0.31223	3.865803	-6.51448	1.84E-09	1.81E-08	10.89043
S1PR3	-0.31232	4.470009	-3.0586	0.002748	0.008438	-2.79391
BTAF1	-0.3124	6.224913	-2.28076	0.024344	0.055975	-4.75819
CASP8AP2	-0.31268	3.926518	-3.34354	0.001107	0.003731	-1.95124
FNIP2	-0.3127	4.690559	-2.85049	0.005148	0.014633	-3.36878
B3GALTL	-0.31281	3.098884	-5.50317	2.18E-07	1.50E-06	6.222204
NHSL2	-0.31293	4.366322	-3.57434	0.000508	0.001842	-1.2234
XPO1	-0.313	7.384126	-3.01702	0.003123	0.009435	-2.91154
ZNF532	-0.31307	3.096412	-4.73299	6.16E-06	3.27E-05	2.984821
LOC15357	-0.31335	4.01675	-2.21136	0.028924	0.064852	-4.9083
ADPRM	-0.31343	5.002658	-3.19864	0.00177	0.005697	-2.38763
RBM26	-0.31349	4.213978	-6.36107	3.88E-09	3.63E-08	10.15631
ALG8	-0.31354	6.063539	-2.62948	0.009682	0.025347	-3.94045
APOL2	-0.31363	5.694008	-6.76019	5.44E-10	5.90E-09	12.08308
PTPDC1	-0.31369	3.339545	-4.62096	9.77E-06	4.96E-05	2.540886
KIF16B	-0.31385	3.606815	-4.42376	2.16E-05	0.000103	1.77756
HHAT	-0.31387	2.428356	-4.50432	1.57E-05	7.71E-05	2.08656
ULK2	-0.31389	3.696086	-4.04865	9.21E-05	0.000387	0.392583
TSC1	-0.31393	6.28956	-4.50783	1.54E-05	7.61E-05	2.100117
SLFN11	-0.31431	5.290876	-2.31879	0.022115	0.051627	-4.67413
TMCO6	-0.31439	5.420457	-2.88191	0.004692	0.013512	-3.28423
MCC	-0.3144	2.500776	-5.86105	4.21E-08	3.27E-07	7.825682
NUP85	-0.3144	5.77816	-2.84321	0.005259	0.014908	-3.38825
HEATR6	-0.31453	4.868213	-4.75441	5.64E-06	3.01E-05	3.070526
RNPEP	-0.31454	7.927447	-2.44471	0.015964	0.03914	-4.38682
HNRNPD	-0.31455	5.49077	-4.0354	9.68E-05	0.000405	0.345345
CD84	-0.31464	4.880771	-3.90909	0.000155	0.000622	-0.09911
ZNF569	-0.31465	2.685037	-4.14686	6.35E-05	0.000276	0.746422
POLM	-0.31484	4.423432	-3.93361	0.000141	0.000573	-0.01366

RANGRF	-0.31486	5.345028	-4.35461	2.84E-05	0.000133	1.515519
CLEC4F	-0.31486	3.083827	-3.86904	0.000179	0.000711	-0.23774
CLCC1	-0.31526	4.137942	-3.38586	0.000962	0.003285	-1.82076
RHOF	-0.31534	5.106639	-6.15942	1.03E-08	8.81E-08	9.204563
ZNF286A	-0.31542	3.026914	-6.13784	1.14E-08	9.70E-08	9.103604
THADA	-0.31552	3.230013	-9.20108	1.47E-15	4.21E-14	24.7323
GPRIN3	-0.31557	3.612897	-2.95282	0.003796	0.011209	-3.09047
ZNF57	-0.31571	2.652062	-3.17366	0.001917	0.00612	-2.46124
CERS2	-0.31573	8.114909	-4.9184	2.83E-06	1.59E-05	3.735314
UNC119B	-0.31583	4.485011	-7.22343	5.25E-11	6.77E-10	14.38275
CD40	-0.31585	4.911223	-3.6551	0.000384	0.00143	-0.95944
FARSB	-0.31593	4.0518	-3.87963	0.000172	0.000687	-0.20119
ZSCAN16	-0.31659	3.970648	-2.33834	0.02104	0.049481	-4.63044
NXT1	-0.31677	5.804073	-3.1502	0.002064	0.006537	-2.52992
DTD2	-0.31678	3.303841	-3.86653	0.000181	0.000717	-0.24639
CAND1	-0.31707	4.449984	-4.47594	1.76E-05	8.56E-05	1.977251
TMIGD2	-0.31728	4.29177	-4.06438	8.68E-05	0.000367	0.448833
USP45	-0.3174	2.007921	-4.99425	2.05E-06	1.18E-05	4.047837
CLASP2	-0.3175	4.293576	-5.26571	6.30E-07	4.01E-06	5.191371
BRAT1	-0.31752	6.03534	-3.39528	0.000933	0.003197	-1.79153
HDAC5	-0.31757	5.767993	-4.97943	2.18E-06	1.26E-05	3.986559
RAB11FIP5	-0.31758	4.143805	-4.35006	2.89E-05	0.000135	1.498387
YARS2	-0.31762	5.041098	-3.1035	0.00239	0.007445	-2.66533
B3GALT6	-0.31771	4.287333	-4.85783	3.66E-06	2.02E-05	3.488028
KDM5C	-0.31788	4.855687	-4.23562	4.52E-05	0.000203	1.071636
HEY1	-0.31805	4.700472	-3.19099	0.001814	0.00583	-2.41023
PHACTR4	-0.31806	5.281235	-3.37032	0.001013	0.003443	-1.86881
TAMM41	-0.31845	3.458146	-6.72924	6.35E-10	6.79E-09	11.93174
ANKRD23	-0.31846	3.17781	-3.40112	0.000915	0.003139	-1.77338
AAR2	-0.31847	6.208053	-4.28759	3.69E-05	0.000169	1.2644
PPP1R15A	-0.31861	6.262114	-4.01409	0.000105	0.000435	0.269613
DNAJC11	-0.31866	5.659307	-5.00863	1.93E-06	1.12E-05	4.107458
INPP5B	-0.31867	3.806211	-5.40833	3.34E-07	2.22E-06	5.807195
COX10-AS	-0.31881	2.695292	-4.17698	5.66E-05	0.000248	0.856195
SLC26A2	-0.31903	5.206329	-2.92978	0.004068	0.011928	-3.15387
TMEM259	-0.31906	4.427489	-5.12302	1.18E-06	7.13E-06	4.585508
DHX58	-0.31932	4.364223	-4.15472	6.17E-05	0.000269	0.775017
SERBP1	-0.31954	7.330029	-4.12345	6.95E-05	0.0003	0.661493
DNAJC30	-0.31961	4.343326	-6.18667	9.01E-09	7.82E-08	9.332261
DDB1	-0.31963	7.418437	-3.16369	0.001978	0.006293	-2.49047
TRPC1	-0.31964	2.025365	-5.43808	2.92E-07	1.96E-06	5.936939
IL2RA	-0.31973	4.033423	-4.12196	6.98E-05	0.000301	0.656127
EPC1	-0.31973	5.765308	-5.05839	1.56E-06	9.21E-06	4.314528
GTF3A	-0.31983	8.741247	-3.95217	0.000132	0.000538	0.051281
HARS2	-0.3199	6.469342	-4.18752	5.44E-05	0.00024	0.894749
STARD7	-0.31997	7.278953	-2.86735	0.004898	0.014029	-3.32351
MRPS25	-0.32025	4.626233	-4.57888	1.16E-05	5.82E-05	2.376035
RBAK	-0.32027	3.26374	-3.7537	0.000271	0.001041	-0.63083
SAE1	-0.32027	4.914676	-4.26934	3.97E-05	0.00018	1.196512
USP13	-0.32043	4.154948	-6.67806	8.19E-10	8.57E-09	11.68222
LOC91548	-0.32052	6.673889	-5.20234	8.32E-07	5.21E-06	4.920992
HAUS5	-0.32058	4.518189	-6.86924	3.16E-10	3.56E-09	12.61867
METTL13	-0.32062	5.549436	-5.45575	2.70E-07	1.83E-06	6.014171
FAM134C	-0.32064	7.492811	-3.88005	0.000172	0.000686	-0.19976
ACYP1	-0.32082	3.909065	-2.21436	0.028711	0.064481	-4.90189
EXTL2	-0.32085	3.812506	-4.15514	6.16E-05	0.000268	0.776552
KLHL24	-0.32085	4.818259	-3.52619	0.0006	0.002143	-1.37849
ABCA11P	-0.32093	2.847409	-3.82057	0.000213	0.000836	-0.40409

ZNF225	-0.32098	2.843604	-5.54676	1.79E-07	1.25E-06	6.414423
LSM8	-0.32109	4.254041	-2.79658	0.006025	0.016776	-3.51195
UBE2G2	-0.32114	5.492772	-4.91126	2.92E-06	1.64E-05	3.706081
IFNG	-0.32137	2.36966	-4.52761	1.43E-05	7.08E-05	2.176633
RAVER1	-0.32138	5.287405	-4.32442	3.20E-05	0.000148	1.402051
ZNF385A	-0.32144	4.718287	-4.04192	9.45E-05	0.000396	0.368574
SH2D3A	-0.32174	3.561619	-6.10599	1.32E-08	1.12E-07	8.954996
TMA16	-0.32179	1.910611	-3.15376	0.002041	0.006472	-2.5195
POLR2B	-0.32257	5.92997	-3.45127	0.000773	0.002697	-1.61644
CAMSAP1	-0.32274	3.331183	-7.61101	7.11E-12	1.05E-10	16.35162
RASGRP3	-0.32291	3.236073	-4.11679	7.12E-05	0.000306	0.637426
PLEKHG4	-0.32296	4.583425	-4.69049	7.34E-06	3.83E-05	2.815572
DLG1	-0.32312	4.162292	-5.6282	1.23E-07	8.87E-07	6.775818
RALGAPA1	-0.32321	3.501054	-6.23561	7.12E-09	6.30E-08	9.562353
LOC38990	-0.32337	3.215345	-7.22545	5.20E-11	6.71E-10	14.39291
PLXND1	-0.32371	5.596436	-4.48905	1.67E-05	8.16E-05	2.027697
GSPT1	-0.32387	6.325208	-2.62211	0.009882	0.025782	-3.95882
MED15	-0.32432	6.768745	-5.23972	7.06E-07	4.47E-06	5.080254
ZNF224	-0.32445	4.214733	-3.293	0.001306	0.004334	-2.10528
DGKQ	-0.32478	5.251208	-4.78747	4.91E-06	2.65E-05	3.203331
GTPBP8	-0.32488	4.452967	-4.76393	5.42E-06	2.90E-05	3.108733
CCT8	-0.3249	5.343322	-4.95376	2.44E-06	1.39E-05	3.880639
RNF115	-0.32495	5.418984	-6.18536	9.06E-09	7.86E-08	9.326131
BAG2	-0.32505	3.269397	-4.87279	3.43E-06	1.90E-05	3.548927
C5orf63	-0.32509	3.338486	-7.04014	1.33E-10	1.60E-09	13.46535
IDH3B	-0.32515	6.881212	-4.58763	1.12E-05	5.63E-05	2.410241
TM2D3	-0.32516	7.639665	-2.61668	0.010032	0.026132	-3.97231
KLHDC4	-0.32522	4.050447	-7.20662	5.72E-11	7.31E-10	14.29826
ARCN1	-0.32539	8.162918	-4.45336	1.92E-05	9.28E-05	1.890638
LINC00173	-0.32561	4.555701	-2.9508	0.003819	0.011268	-3.09606
RNA45S5	-0.32561	4.758178	-3.58274	0.000494	0.001796	-1.19614
DPH7	-0.32573	5.077148	-4.69764	7.13E-06	3.73E-05	2.843951
LOC10050	-0.32573	1.871558	-4.83031	4.10E-06	2.25E-05	3.376368
MTA1	-0.32588	4.546621	-4.36929	2.68E-05	0.000126	1.570885
LOC10013	-0.32601	3.487075	-4.26608	4.02E-05	0.000182	1.184412
C9orf142	-0.32625	5.252815	-4.96773	2.29E-06	1.31E-05	3.938239
FMNL1	-0.32655	6.887219	-3.21857	0.001661	0.005375	-2.32857
LOC20077	-0.32667	3.832942	-2.65087	0.009122	0.024042	-3.88691
GOLGA8N	-0.32672	8.306129	-2.08751	0.038978	0.083202	-5.16553
USP19	-0.32673	5.8937	-7.49171	1.32E-11	1.88E-10	15.74168
MRPL45	-0.32676	5.059531	-3.44964	0.000778	0.00271	-1.62157
RPL18	-0.32683	7.992537	-5.41846	3.19E-07	2.13E-06	5.851346
RCN1	-0.32731	3.254341	-3.01589	0.003134	0.009462	-2.91472
RNF220	-0.32732	6.779444	-5.28162	5.87E-07	3.77E-06	5.259575
MECP2	-0.32738	6.04752	-7.72531	3.92E-12	6.08E-11	16.93911
ABCF3	-0.32761	6.031722	-5.57366	1.58E-07	1.11E-06	6.533435
KLHDC8B	-0.32769	5.804951	-2.4771	0.014652	0.036313	-4.31068
ANKRD44-	-0.32783	2.576307	-3.60017	0.000465	0.0017	-1.13948
G3BP2	-0.32795	6.221445	-4.15165	6.24E-05	0.000272	0.763829
AIMP1	-0.32799	5.068709	-2.1804	0.031198	0.069066	-4.97389
MED6	-0.32824	4.921342	-3.16197	0.001989	0.006324	-2.49549
SCFD2	-0.32824	4.819529	-3.99809	0.000111	0.00046	0.212953
DDX19B	-0.32835	4.092656	-3.76244	0.000263	0.001012	-0.60139
C9orf38	-0.3284	2.790352	-3.56222	0.00053	0.001915	-1.2626
PHF14	-0.32841	3.643568	-4.51849	1.48E-05	7.33E-05	2.141303
CERK	-0.32845	7.65504	-3.02446	0.003053	0.009249	-2.8906
CNNM4	-0.32862	3.615289	-5.11806	1.20E-06	7.27E-06	4.564614
C8orf33	-0.32866	4.338594	-3.48164	0.000698	0.002459	-1.52047

CWC25	-0.32881	5.48969	-5.91295	3.30E-08	2.61E-07	8.06287
GMPS	-0.32883	4.549326	-6.04924	1.73E-08	1.43E-07	8.691156
INPP4A	-0.32889	6.020044	-3.63855	0.000407	0.001505	-1.01392
DDX23	-0.32899	6.60825	-4.71226	6.71E-06	3.53E-05	2.902142
COQ6	-0.32901	4.401242	-4.46782	1.81E-05	8.81E-05	1.946063
ZNF589	-0.32911	4.333005	-5.9512	2.76E-08	2.20E-07	8.238425
AARS2	-0.32925	5.139583	-4.95417	2.43E-06	1.38E-05	3.88233
CXCR1	-0.3293	9.375077	-2.85648	0.005058	0.014416	-3.35272
ZFYVE27	-0.32936	5.84932	-6.57246	1.38E-09	1.39E-08	11.17002
TBL1X	-0.3294	6.00178	-4.06535	8.65E-05	0.000366	0.452304
WDR6	-0.32959	4.840912	-5.39384	3.56E-07	2.36E-06	5.744178
LRRC37A4	-0.32969	5.296698	-3.76914	0.000257	0.00099	-0.57875
ANAPC13	-0.32976	4.879685	-6.30255	5.15E-09	4.69E-08	9.878519
S100B	-0.32988	2.181609	-3.73747	0.000287	0.001099	-0.68541
UBA52	-0.33008	12.08799	-5.0477	1.63E-06	9.61E-06	4.269956
FCHO1	-0.33018	5.195596	-4.5399	1.36E-05	6.76E-05	2.224288
EPS8	-0.33021	2.452605	-3.03913	0.002918	0.008896	-2.84917
ZNF138	-0.33027	2.367645	-4.50879	1.54E-05	7.58E-05	2.103827
USP21	-0.33027	5.83459	-5.12975	1.14E-06	6.94E-06	4.613826
RPS19	-0.33027	8.182605	-3.75678	0.000268	0.001031	-0.62046
STYX	-0.33031	3.615742	-3.52694	0.000598	0.002138	-1.37607
RPA3OS	-0.33046	4.653956	-2.17115	0.031908	0.07037	-4.99333
ADCK2	-0.33049	4.522421	-6.26927	6.05E-09	5.44E-08	9.721143
TESK1	-0.33054	5.369135	-4.42205	2.18E-05	0.000104	1.771051
RAPGEF2	-0.33056	5.574912	-3.49122	0.000676	0.002387	-1.49006
N4BP2L1	-0.33059	3.915142	-6.95199	2.08E-10	2.42E-09	13.02757
SEPSECS	-0.33066	3.313737	-5.62309	1.26E-07	9.07E-07	6.753035
NACA	-0.33074	9.509657	-4.97103	2.26E-06	1.30E-05	3.951847
MEFV	-0.33079	5.572699	-3.30708	0.001247	0.004162	-2.06257
GTF2H4	-0.33089	4.171286	-3.49873	0.000659	0.002332	-1.46619
SRPR	-0.33096	7.194429	-3.862	0.000184	0.000727	-0.26202
PPM1F	-0.33105	6.031145	-4.23161	4.59E-05	0.000206	1.056814
PLEKHA5	-0.33108	1.977801	-5.97894	2.42E-08	1.95E-07	8.366119
NNT	-0.33109	4.565367	-5.1746	9.39E-07	5.81E-06	4.803294
CYFIP2	-0.33124	7.841104	-3.08296	0.002548	0.007881	-2.72433
DDX50	-0.33134	5.043109	-4.51611	1.49E-05	7.38E-05	2.132101
MPPE1	-0.33149	7.538931	-4.15801	6.09E-05	0.000265	0.78699
C15orf40	-0.33163	3.890251	-3.67526	0.000358	0.00134	-0.89283
RSF1	-0.33165	5.076985	-4.75849	5.54E-06	2.97E-05	3.086898
EXPH5	-0.3317	2.698765	-7.33039	3.03E-11	4.07E-10	14.92232
GOLM1	-0.33183	4.150005	-4.28662	3.71E-05	0.000169	1.260762
SCO2	-0.33189	6.848171	-2.28629	0.024008	0.055326	-4.74605
PAX5	-0.33191	4.589755	-4.4979	1.61E-05	7.88E-05	2.061792
ZC3HAV1	-0.33203	7.298204	-4.36142	2.77E-05	0.00013	1.5412
GATAD1	-0.33227	4.992383	-4.26238	4.07E-05	0.000184	1.170662
FZD3	-0.3325	2.923767	-6.3044	5.11E-09	4.65E-08	9.887304
RFXAP	-0.33276	3.706131	-3.5579	0.000538	0.00194	-1.27653
ZBED1	-0.33278	5.578685	-3.67061	0.000364	0.00136	-0.90821
NOD2	-0.33281	6.572238	-2.06545	0.041055	0.086833	-5.20991
NOTCH1	-0.33294	5.61937	-5.6648	1.04E-07	7.60E-07	6.939232
ZNF416	-0.33294	3.132244	-4.96826	2.29E-06	1.31E-05	3.940425
SEC23IP	-0.33328	4.550283	-5.39685	3.52E-07	2.33E-06	5.757253
JAKMIP1	-0.33367	3.843024	-3.65571	0.000383	0.001427	-0.95742
HPS4	-0.33387	3.974725	-8.69601	2.26E-14	5.14E-13	22.02932
UBR7	-0.33391	5.748329	-3.7193	0.000306	0.001166	-0.74625
EIF3M	-0.33391	5.121886	-4.63781	9.12E-06	4.66E-05	2.607211
IPO4	-0.33405	3.603997	-3.66023	0.000377	0.001408	-0.94251
UBP1	-0.33408	6.776741	-2.62534	0.009794	0.025586	-3.95078

RXRB	-0.3342	5.427811	-5.78727	5.93E-08	4.51E-07	7.490455
PRAF2	-0.33425	6.234014	-4.98838	2.10E-06	1.21E-05	4.023552
RAPGEF6	-0.33433	5.483755	-4.04584	9.31E-05	0.000391	0.382581
MMAA	-0.33439	3.034622	-5.82876	4.89E-08	3.77E-07	7.678675
CMPK1	-0.3347	5.514626	-4.21966	4.81E-05	0.000214	1.01279
ANGEL2	-0.33497	4.280914	-3.93291	0.000142	0.000574	-0.0161
SIK1	-0.33515	4.193334	-5.11239	1.23E-06	7.44E-06	4.540779
ADPRHL2	-0.33524	6.402436	-5.07192	1.47E-06	8.73E-06	4.371102
KCTD13	-0.33529	4.707537	-6.03098	1.89E-08	1.55E-07	8.606516
GLYR1	-0.3353	5.506223	-5.25585	6.58E-07	4.18E-06	5.149171
ATP13A1	-0.33567	6.542439	-4.19915	5.20E-05	0.00023	0.9374
MAP4K2	-0.33584	4.392488	-3.27237	0.001396	0.004595	-2.16759
L3HYPDH	-0.33634	3.200238	-3.74528	0.000279	0.001072	-0.65918
DCBLD1	-0.3364	2.889982	-4.98312	2.15E-06	1.24E-05	4.001786
ACAP3	-0.33658	4.272095	-5.65915	1.07E-07	7.78E-07	6.913985
ZNF92	-0.33664	2.689748	-4.96753	2.30E-06	1.31E-05	3.937418
ATP6V0A2	-0.3367	3.964319	-5.64872	1.12E-07	8.12E-07	6.867386
ZFX	-0.33672	3.976963	-4.18871	5.41E-05	0.000239	0.899135
SMAD4	-0.33696	4.047749	-5.7935	5.76E-08	4.39E-07	7.51863
FAM213A	-0.33696	3.845307	-4.2385	4.47E-05	0.000201	1.082275
APRT	-0.33696	6.740315	-3.54048	0.000571	0.002048	-1.33265
DCAF4	-0.3372	4.583848	-5.49452	2.27E-07	1.55E-06	6.184186
MS4A2	-0.33744	2.087407	-6.45099	2.51E-09	2.41E-08	10.58558
FAM134B	-0.33777	3.398867	-3.75077	0.000274	0.001052	-0.6407
NIP7	-0.33796	5.29163	-3.29433	0.0013	0.004319	-2.10126
TSPAN3	-0.33799	3.846334	-6.29578	5.33E-09	4.82E-08	9.846487
DENND4A	-0.33828	5.316645	-3.00103	0.003279	0.009849	-2.95641
LOC10050	-0.33839	4.66568	-3.37747	0.000989	0.003368	-1.84672
ZNF180	-0.33857	3.154478	-5.21595	7.84E-07	4.92E-06	4.978889
CA5B	-0.3386	4.198271	-5.77466	6.29E-08	4.76E-07	7.433384
RPS18	-0.3387	11.21236	-2.16856	0.032108	0.07073	-4.99873
ZNF160	-0.33884	4.62967	-4.06245	8.75E-05	0.000369	0.441914
OPN3	-0.33887	4.793693	-2.71274	0.007664	0.020714	-3.72986
CDV3	-0.33921	6.953039	-3.9276	0.000144	0.000585	-0.03464
AVIL	-0.33949	4.448635	-4.34631	2.94E-05	0.000137	1.484265
GXYLT1	-0.33984	4.191198	-3.52153	0.000609	0.002174	-1.39341
LOC10050	-0.33989	5.613922	-4.46868	1.81E-05	8.79E-05	1.949381
LYAR	-0.33994	5.600359	-3.47888	0.000704	0.002478	-1.52921
MAT2A	-0.33998	6.07879	-2.93141	0.004048	0.01188	-3.14939
ZNF551	-0.34002	3.469145	-8.40411	1.08E-13	2.17E-12	20.48201
ICAM1	-0.34012	5.233315	-3.38539	0.000964	0.00329	-1.8222
EIF5A2	-0.34029	2.641935	-5.25622	6.56E-07	4.17E-06	5.150771
NEK7	-0.34059	8.114556	-2.38672	0.018577	0.044585	-4.52085
SERTAD2	-0.34061	7.185476	-3.60588	0.000456	0.00167	-1.12086
RRM2B	-0.34065	6.363221	-2.21757	0.028486	0.064047	-4.89505
ST6GALNA	-0.34093	5.41421	-3.9166	0.00015	0.000606	-0.07299
LGMN	-0.34097	3.312338	-3.60201	0.000462	0.001691	-1.13347
TSPAN18	-0.34102	4.999832	-4.53761	1.37E-05	6.81E-05	2.215394
FTSJ2	-0.34151	5.833846	-3.43249	0.000824	0.002853	-1.67542
RBM23	-0.34163	8.168262	-4.26539	4.03E-05	0.000182	1.181826
LPAR6	-0.34166	4.313133	-2.88249	0.004684	0.013491	-3.28267
CNOT10	-0.34166	5.544692	-4.81725	4.34E-06	2.36E-05	3.323498
WDR52	-0.34167	2.297112	-5.57666	1.56E-07	1.10E-06	6.546743
HKR1	-0.34199	3.002474	-6.49658	2.00E-09	1.96E-08	10.80433
MTIF2	-0.342	5.135983	-2.10721	0.037202	0.079976	-5.12553
CAMK1	-0.34205	5.266157	-2.19601	0.030033	0.066942	-4.94093
VDAC1	-0.34236	5.1659	-4.42315	2.17E-05	0.000103	1.775222
MTHFD1	-0.34244	5.232052	-2.66212	0.00884	0.023427	-3.85859

MLH3	-0.34275	5.274644	-3.42607	0.000842	0.002907	-1.69554
C22orf39	-0.34294	5.457185	-5.11047	1.24E-06	7.49E-06	4.532721
NR2C1	-0.34296	2.865572	-5.16556	9.77E-07	6.02E-06	4.765031
TYK2	-0.3432	7.149549	-4.27637	3.86E-05	0.000175	1.222638
RELB	-0.3436	5.383071	-4.18322	5.53E-05	0.000243	0.879016
CDK19	-0.34367	5.722385	-4.8252	4.19E-06	2.29E-05	3.355681
KIF13B	-0.34372	5.300304	-4.02567	0.0001	0.000418	0.310719
SLC25A23	-0.34375	3.796944	-5.18376	9.02E-07	5.61E-06	4.842141
MEX3C	-0.34398	3.532685	-7.98371	1.01E-12	1.74E-11	18.27754
CDKAL1	-0.34407	3.067841	-5.18374	9.02E-07	5.61E-06	4.842024
PPARD	-0.34408	3.834919	-6.91563	2.50E-10	2.86E-09	12.84763
MRFAP1	-0.34414	9.567833	-4.08251	8.11E-05	0.000345	0.513855
SRM	-0.34429	5.555513	-2.22836	0.027737	0.062589	-4.87192
KCNN4	-0.34442	5.293711	-3.51431	0.000625	0.002222	-1.41649
ATRN	-0.34448	3.89118	-4.92507	2.75E-06	1.55E-05	3.76269
TWISTNB	-0.34456	3.071469	-4.04413	9.37E-05	0.000393	0.376468
FUBP3	-0.34456	5.193366	-3.53551	0.000581	0.002081	-1.34861
PAXIP1OS	-0.34494	3.890264	-4.69364	7.25E-06	3.78E-05	2.828079
REV1	-0.34514	4.238297	-4.95278	2.45E-06	1.39E-05	3.876606
BCL9	-0.34519	3.016033	-6.07487	1.54E-08	1.28E-07	8.810124
UBAC2	-0.3452	6.640299	-5.27764	5.97E-07	3.83E-06	5.242482
RNF214	-0.34534	5.146162	-4.69699	7.15E-06	3.74E-05	2.841396
FLJ16734	-0.34561	3.313482	-4.23095	4.60E-05	0.000206	1.054373
MYADM	-0.34579	9.19079	-3.01809	0.003113	0.009411	-2.90855
NIPSNAP1	-0.3458	6.020741	-5.45899	2.66E-07	1.80E-06	6.028341
FAM98B	-0.34593	3.862237	-3.79205	0.000236	0.000918	-0.50116
TMEM150/	-0.34653	3.257028	-4.49072	1.65E-05	8.11E-05	2.034125
MRPL9	-0.34659	6.362234	-3.17025	0.001937	0.006177	-2.47124
CD101	-0.34665	3.691707	-4.07119	8.46E-05	0.000359	0.473252
RIOK1	-0.34669	4.563705	-2.83915	0.005322	0.01505	-3.39911
CD320	-0.3468	5.369953	-4.24801	4.31E-05	0.000194	1.117445
ZNF607	-0.34682	2.402025	-5.14368	1.07E-06	6.56E-06	4.672581
CYB561D2	-0.34696	5.483808	-6.72251	6.57E-10	7.00E-09	11.89889
GALK2	-0.34711	4.129179	-5.87206	4.00E-08	3.12E-07	7.875878
MYO1F	-0.34717	10.07672	-3.55071	0.000552	0.001984	-1.29972
ZNF226	-0.34723	2.646745	-6.06629	1.60E-08	1.33E-07	8.770274
FRYL	-0.34731	4.512642	-9.29241	8.96E-16	2.67E-14	25.22392
IGFBP3	-0.34756	3.634917	-4.59987	1.06E-05	5.38E-05	2.458145
ADAM8	-0.34771	6.386347	-2.57367	0.011291	0.028953	-4.07835
CYP20A1	-0.34811	4.455283	-3.78013	0.000247	0.000956	-0.5416
GOSR1	-0.34817	4.733552	-5.20074	8.38E-07	5.24E-06	4.914219
VPS45	-0.34833	5.045827	-5.76598	6.55E-08	4.94E-07	7.394155
SLC27A1	-0.34834	5.621586	-4.23862	4.47E-05	0.0002	1.082723
PPIH	-0.34841	6.536517	-3.2602	0.001453	0.004761	-2.2042
GUCD1	-0.34841	9.089252	-2.60513	0.010357	0.026856	-4.00094
CHD8	-0.34859	6.565371	-4.22943	4.63E-05	0.000207	1.048776
IFT172	-0.34882	3.899488	-5.09653	1.32E-06	7.92E-06	4.474163
SNRPA1	-0.34883	6.024184	-3.24392	0.001531	0.004992	-2.253
LOC28066	-0.34895	3.083846	-5.11452	1.22E-06	7.38E-06	4.549719
FAM13A	-0.34898	4.099021	-4.22317	4.74E-05	0.000212	1.025693
FAM86A	-0.34905	4.327401	-5.37482	3.88E-07	2.56E-06	5.661633
KIAA1324L	-0.34907	3.401626	-6.8171	4.10E-10	4.54E-09	12.36214
RPL7L1	-0.34911	7.905151	-3.47975	0.000702	0.002472	-1.52647
MAPK9	-0.34913	5.23208	-4.78383	4.99E-06	2.69E-05	3.188686
TRIM62	-0.34916	4.146946	-7.09201	1.03E-10	1.25E-09	13.72403
CLUAP1	-0.3493	3.674155	-6.64175	9.80E-10	1.01E-08	11.50566
LINC0099(	-0.34931	1.93965	-5.96605	2.57E-08	2.07E-07	8.306745
ZNF544	-0.34973	3.849506	-7.1106	9.33E-11	1.15E-09	13.81692

RUNX2	-0.35044	3.642391	-8.49065	6.81E-14	1.41E-12	20.93946
FBXO25	-0.35048	5.970626	-5.459	2.66E-07	1.80E-06	6.02841
RYR1	-0.35096	2.728027	-4.19197	5.35E-05	0.000236	0.911078
CRKL	-0.35098	5.82142	-4.90259	3.03E-06	1.69E-05	3.670575
AFF3	-0.35098	3.173014	-5.41779	3.20E-07	2.14E-06	5.848414
ZNF22	-0.3511	5.582893	-2.349	0.020474	0.048354	-4.60646
CR2	-0.35117	2.418046	-4.19934	5.20E-05	0.00023	0.938082
TBC1D1	-0.35117	4.853333	-4.50765	1.55E-05	7.61E-05	2.099402
CRY2	-0.35137	5.076742	-6.37348	3.65E-09	3.43E-08	10.2154
EPS15L1	-0.35146	4.69667	-6.61292	1.13E-09	1.16E-08	11.36585
CROCCP3	-0.3515	4.39905	-6.56162	1.46E-09	1.46E-08	11.11765
FCGBP	-0.35154	3.535383	-4.45395	1.92E-05	9.27E-05	1.8929
SNRPA	-0.35164	6.80633	-2.93213	0.004039	0.011859	-3.14744
PUS1	-0.35177	4.827944	-3.04404	0.002874	0.008788	-2.83526
TBP	-0.35185	6.270955	-4.59512	1.09E-05	5.47E-05	2.439539
CDK10	-0.35188	4.589294	-5.68629	9.46E-08	6.95E-07	7.035466
ANAPC5	-0.35194	6.225261	-4.32903	3.14E-05	0.000146	1.419313
VPS13A	-0.35197	3.719108	-4.56181	1.24E-05	6.21E-05	2.309456
KAT2A	-0.352	5.86424	-4.57791	1.16E-05	5.84E-05	2.372241
PHF3	-0.35201	5.141648	-6.35702	3.96E-09	3.69E-08	10.13706
NINL	-0.35205	3.995191	-4.15474	6.17E-05	0.000269	0.775092
TMCO4	-0.35206	4.482362	-6.62311	1.08E-09	1.10E-08	11.41525
RPTOR	-0.35212	4.552082	-4.37635	2.61E-05	0.000123	1.597588
LOC64253	-0.35214	4.556218	-4.31315	3.34E-05	0.000154	1.359818
PTPRM	-0.35235	2.297365	-6.43006	2.77E-09	2.65E-08	10.48544
NUMA1	-0.35243	4.04765	-4.4902	1.66E-05	8.12E-05	2.032123
RAD17	-0.35248	5.644319	-4.57401	1.18E-05	5.93E-05	2.35704
ZNF790-A	-0.35258	3.001936	-7.29007	3.73E-11	4.92E-10	14.7186
GPX3	-0.35263	4.076351	-4.19676	5.25E-05	0.000232	0.928629
UBR2	-0.35273	5.455732	-4.78615	4.94E-06	2.66E-05	3.198045
DND1	-0.35347	6.206553	-4.71089	6.75E-06	3.55E-05	2.896682
GPHN	-0.35359	3.323709	-5.99435	2.25E-08	1.82E-07	8.437206
NHP2L1	-0.35363	4.967258	-6.77554	5.04E-10	5.49E-09	12.15825
DENND1C	-0.35366	6.678383	-4.4036	2.34E-05	0.000111	1.700845
PLCB1	-0.3537	3.594035	-5.81344	5.25E-08	4.04E-07	7.609071
ZNF43	-0.35374	4.010653	-2.90227	0.004416	0.012827	-3.22902
MKNK2	-0.35377	8.741206	-4.44432	1.99E-05	9.60E-05	1.856049
RAB22A	-0.35377	5.843875	-4.03335	9.76E-05	0.000407	0.338051
SFPQ	-0.35385	6.298386	-4.02245	0.000102	0.000423	0.299291
HNRNPF	-0.3539	6.535569	-3.50565	0.000643	0.002282	-1.44413
GNE	-0.35401	4.43605	-2.61256	0.010147	0.026392	-3.98255
WDR92	-0.35415	4.532485	-4.22095	4.78E-05	0.000213	1.017525
PRKCSH	-0.35415	7.0562	-5.33749	4.58E-07	2.99E-06	5.500055
LUC7L	-0.35418	4.718159	-6.49079	2.06E-09	2.01E-08	10.77654
NUP98	-0.35418	6.049696	-5.63159	1.22E-07	8.74E-07	6.790942
PRUNE	-0.35418	5.103124	-3.89663	0.000162	0.000648	-0.14236
NUBPL	-0.35433	2.357434	-4.15883	6.07E-05	0.000265	0.78998
FAM53B	-0.35438	3.867614	-7.35732	2.64E-11	3.59E-10	15.05864
ZNF296	-0.35453	4.668214	-5.09844	1.31E-06	7.87E-06	4.482208
PFKP	-0.35476	4.813377	-6.24645	6.76E-09	6.01E-08	9.613461
CORO7	-0.35496	5.376282	-5.09239	1.34E-06	8.06E-06	4.456821
ARID1B	-0.35501	5.194118	-9.61026	1.58E-16	5.41E-15	26.94024
TUBB	-0.35506	8.586591	-2.9855	0.003438	0.010266	-2.9998
CXCR5	-0.35508	4.645092	-5.03102	1.75E-06	1.03E-05	4.20046
DDX46	-0.35516	6.22311	-4.51105	1.52E-05	7.52E-05	2.112557
BRWD1	-0.35521	3.475948	-6.09497	1.40E-08	1.17E-07	8.903656
CBX1	-0.35561	7.043408	-3.18032	0.001877	0.006007	-2.44167
USP25	-0.35571	4.392658	-5.32937	4.75E-07	3.09E-06	5.465033

SLC25A26	-0.35589	5.188759	-4.67343	7.88E-06	4.08E-05	2.747902
ZC3H4	-0.35592	4.235016	-7.36403	2.55E-11	3.47E-10	15.09263
THAP7	-0.35604	5.030858	-4.27971	3.81E-05	0.000173	1.235064
BTBD18	-0.35609	3.654415	-3.75428	0.000271	0.00104	-0.62888
RABEP2	-0.3565	5.430208	-5.66239	1.06E-07	7.67E-07	6.928476
PSMF1	-0.35663	6.72983	-2.45318	0.015611	0.03838	-4.367
NUP43	-0.35672	4.728532	-4.52894	1.42E-05	7.04E-05	2.181757
DDX21	-0.35699	7.323389	-2.48989	0.014161	0.035254	-4.28037
UBE2Q2L	-0.35705	3.572657	-4.85422	3.71E-06	2.05E-05	3.473379
ZNF565	-0.35711	4.361127	-3.7276	0.000297	0.001136	-0.7185
MTCH1	-0.35716	8.633303	-4.78931	4.87E-06	2.63E-05	3.210736
ADAMTS1	-0.35718	2.555967	-5.50068	2.20E-07	1.51E-06	6.211252
AKAP13	-0.35749	6.174733	-4.87705	3.37E-06	1.87E-05	3.566287
ATAD1	-0.3577	5.491118	-2.85945	0.005014	0.014309	-3.34477
LOC10192	-0.35793	1.988936	-4.83538	4.02E-06	2.20E-05	3.39689
SIDT2	-0.35848	6.847867	-2.59154	0.010752	0.027725	-4.03448
TMEM138	-0.35855	6.19204	-3.25265	0.001488	0.004867	-2.22685
ANKRD36	-0.35861	2.700926	-2.90938	0.004324	0.012589	-3.20966
ZNF239	-0.35878	2.866898	-4.23823	4.47E-05	0.000201	1.081274
TNRC6A	-0.35903	5.307899	-6.64977	9.42E-10	9.75E-09	11.54464
PNPO	-0.35904	5.261071	-5.50458	2.17E-07	1.49E-06	6.228439
HACL1	-0.3593	5.348438	-3.58423	0.000491	0.001789	-1.1913
ZCCHC18	-0.35942	4.194044	-4.07845	8.23E-05	0.00035	0.499296
MAPK8	-0.35948	4.057059	-6.70106	7.31E-10	7.72E-09	11.79422
CHN1	-0.35971	4.45367	-5.76445	6.59E-08	4.97E-07	7.387207
PDE8A	-0.35973	4.35347	-4.58607	1.13E-05	5.66E-05	2.404138
CSPP1	-0.35989	3.343341	-7.10677	9.51E-11	1.17E-09	13.79778
TUG1	-0.35997	6.149804	-4.74817	5.78E-06	3.09E-05	3.045551
NCOA2	-0.36044	5.924368	-4.93258	2.67E-06	1.51E-05	3.793504
LOC64223	-0.36052	3.299346	-2.9222	0.004161	0.012178	-3.17465
QSER1	-0.36063	4.110082	-4.98631	2.12E-06	1.22E-05	4.014974
PPP1R15B	-0.36101	7.371972	-3.01522	0.00314	0.00948	-2.91661
MTUS1	-0.36104	2.692376	-7.63103	6.41E-12	9.54E-11	16.45435
PMS2P3	-0.36109	5.248211	-6.07607	1.53E-08	1.27E-07	8.815719
PPP3CB-A	-0.36127	4.121539	-5.72285	7.99E-08	5.95E-07	7.19969
GRAP	-0.36129	4.656867	-5.80186	5.54E-08	4.24E-07	7.556546
FCHO2	-0.36147	4.490562	-3.01946	0.0031	0.009378	-2.90469
SFI1	-0.36148	4.838189	-7.15496	7.44E-11	9.30E-10	14.03897
PCBP4	-0.36151	4.717906	-4.19636	5.26E-05	0.000232	0.927135
RBM12	-0.36182	5.7347	-3.3027	0.001265	0.004215	-2.07588
ATXN1L	-0.36194	6.199574	-3.678	0.000354	0.001329	-0.88374
GTF2F1	-0.36194	5.150011	-6.0553	1.69E-08	1.39E-07	8.719259
CEP57	-0.36202	5.069593	-2.88011	0.004717	0.013573	-3.2891
SLC30A5	-0.3621	4.460158	-4.14834	6.32E-05	0.000275	0.751803
ZNF10	-0.36218	2.658798	-7.07092	1.14E-10	1.38E-09	13.61874
F11R	-0.36226	6.51652	-5.54245	1.82E-07	1.27E-06	6.395382
ZNF468	-0.36231	4.563985	-2.34881	0.020484	0.048367	-4.6069
TULP3	-0.3625	4.761245	-5.82457	4.99E-08	3.84E-07	7.659633
CXCL10	-0.36253	3.780624	-4.29404	3.60E-05	0.000165	1.288445
RUNDC1	-0.3626	4.949239	-4.18736	5.44E-05	0.00024	0.894161
SMPD3	-0.36283	4.339755	-4.43923	2.03E-05	9.76E-05	1.836574
AVEN	-0.36289	4.081798	-2.69863	0.007977	0.021414	-3.76597
GTPBP6	-0.36305	5.840189	-4.2087	5.01E-05	0.000222	0.972459
PSMB8-AS	-0.36333	6.156738	-4.47907	1.73E-05	8.47E-05	1.98926
STIP1	-0.36355	5.965481	-3.35719	0.001058	0.003581	-1.90928
MSANTD4	-0.36359	3.015036	-4.32882	3.14E-05	0.000146	1.418534
RBM48	-0.36382	3.664119	-5.17286	9.46E-07	5.85E-06	4.795939
DDB2	-0.3639	5.138975	-3.87888	0.000173	0.000689	-0.20379

BAHD1	-0.36395	4.762729	-6.27289	5.95E-09	5.36E-08	9.738216
IRF2BP2	-0.36395	7.051391	-4.04114	9.47E-05	0.000397	0.365787
ZNF792	-0.36427	1.963994	-6.39664	3.27E-09	3.09E-08	10.32581
TCEAL3	-0.3645	5.040379	-3.81597	0.000217	0.000848	-0.41977
FAM120C	-0.3647	3.410156	-6.23966	6.98E-09	6.20E-08	9.581419
LIG1	-0.36481	5.084399	-3.85146	0.000191	0.000753	-0.29826
SORBS3	-0.36487	4.85371	-5.46962	2.54E-07	1.72E-06	6.0749
OGT	-0.36515	6.596945	-3.83275	0.000204	0.000802	-0.36242
ZNF563	-0.36516	2.824475	-5.73962	7.40E-08	5.53E-07	7.275209
ZNF280C	-0.3653	2.518929	-4.56705	1.22E-05	6.09E-05	2.32989
ERCC6	-0.36547	4.012799	-6.2763	5.85E-09	5.27E-08	9.754337
NFYB	-0.36548	3.351048	-4.00956	0.000107	0.000442	0.253535
P2RX5	-0.36551	7.323388	-2.30307	0.023014	0.053348	-4.70905
ZNF512B	-0.36561	2.794876	-5.51292	2.09E-07	1.44E-06	6.265149
DNAJC7	-0.36609	6.35701	-3.59502	0.000473	0.001729	-1.15623
BLK	-0.36627	4.470173	-3.02663	0.003032	0.009201	-2.88448
PAXBP1	-0.36627	5.268403	-3.66401	0.000372	0.00139	-0.93002
ZSCAN25	-0.36652	4.295486	-6.77503	5.06E-10	5.50E-09	12.15575
VWA9	-0.36655	3.872732	-7.15187	7.56E-11	9.43E-10	14.02349
ZNF542P	-0.36663	3.081506	-3.63034	0.000419	0.001545	-1.04085
SEC24C	-0.3668	6.568228	-5.67278	1.01E-07	7.35E-07	6.974951
ARMCX5	-0.36685	3.622005	-4.33275	3.10E-05	0.000144	1.433276
ZNF736	-0.36702	2.213752	-5.18036	9.16E-07	5.68E-06	4.827707
TUBGCP4	-0.36709	5.105744	-5.41719	3.21E-07	2.14E-06	5.845788
RBBP6	-0.36713	5.051597	-6.6387	9.95E-10	1.03E-08	11.49086
11-Sep	-0.36728	3.198037	-5.61486	1.31E-07	9.37E-07	6.716423
STK25	-0.36737	5.745952	-4.2449	4.36E-05	0.000196	1.105922
TSEN2	-0.36754	3.430554	-5.01162	1.90E-06	1.11E-05	4.119844
DBT	-0.36804	3.888816	-8.05705	6.85E-13	1.21E-11	18.65982
PCF11	-0.36807	6.292846	-4.57907	1.16E-05	5.81E-05	2.376796
RNASEH1	-0.3681	5.175063	-6.34104	4.28E-09	3.95E-08	10.06109
ALDH6A1	-0.3683	4.018306	-5.86068	4.21E-08	3.28E-07	7.823976
ASCL2	-0.36842	3.223192	-6.86293	3.26E-10	3.67E-09	12.5876
NLRP6	-0.36869	3.000772	-3.18797	0.001831	0.005882	-2.41912
TMEM218	-0.36873	4.409621	-4.2288	4.64E-05	0.000207	1.046474
SPOP	-0.36879	6.408239	-6.72221	6.58E-10	7.01E-09	11.89742
ZBTB20	-0.36881	5.445086	-3.30758	0.001245	0.004156	-2.06103
NCAPD3	-0.36884	5.16696	-3.52753	0.000597	0.002134	-1.37418
GLIPR1	-0.36886	8.107673	-2.55878	0.01176	0.029996	-4.11469
HEATR1	-0.36886	3.9234	-6.5809	1.32E-09	1.34E-08	11.21083
GPR157	-0.369	4.042838	-5.1989	8.45E-07	5.28E-06	4.906377
CCDC134	-0.36902	4.286009	-6.17035	9.74E-09	8.40E-08	9.255745
ALOX15	-0.36926	2.575292	-3.9858	0.000116	0.00048	0.169553
MICALL1	-0.36941	5.483932	-5.34756	4.38E-07	2.87E-06	5.54359
ANKEF1	-0.36944	2.702271	-6.82737	3.89E-10	4.34E-09	12.41258
TSPYL1	-0.36947	3.815962	-8.33346	1.58E-13	3.09E-12	20.10941
SNHG17	-0.36949	5.061198	-2.73866	0.007119	0.019401	-3.66313
TNFRSF10	-0.3696	7.794149	-3.45767	0.000757	0.002646	-1.59628
TMEM43	-0.36962	5.83328	-4.62385	9.65E-06	4.91E-05	2.552272
RPL8	-0.36967	10.66687	-3.40813	0.000894	0.003072	-1.75154
NT5E	-0.36968	2.861508	-5.46628	2.57E-07	1.75E-06	6.060302
ZFP36L1	-0.3697	5.657766	-4.55315	1.29E-05	6.42E-05	2.27575
CEP104	-0.36974	3.69945	-5.33671	4.60E-07	3.00E-06	5.496686
INTS9	-0.3699	5.490539	-7.08707	1.05E-10	1.28E-09	13.69935
UBA2	-0.36997	5.67965	-4.26399	4.05E-05	0.000183	1.176656
SETD1A	-0.37056	4.389517	-4.64162	8.97E-06	4.60E-05	2.622224
C10orf2	-0.37081	4.403881	-4.40933	2.29E-05	0.000109	1.722613
RAP1GAP2	-0.3712	7.494758	-4.03998	9.52E-05	0.000399	0.361668

PRNP	-0.37129	6.538141	-2.47054	0.014909	0.036892	-4.32616
ERO1LB	-0.37132	4.64337	-3.47559	0.000712	0.002504	-1.53966
FAM162A	-0.37161	5.073216	-4.16124	6.01E-05	0.000263	0.798751
LRIG2	-0.3718	3.434506	-7.57658	8.51E-12	1.24E-10	16.17527
SMCR8	-0.37201	5.174257	-6.03313	1.87E-08	1.54E-07	8.616478
CD244	-0.37208	4.152929	-4.68138	7.62E-06	3.96E-05	2.779411
AKR7A2	-0.3721	6.143704	-3.43218	0.000824	0.002855	-1.67639
RNASEH2E	-0.37212	4.218964	-5.84292	4.58E-08	3.55E-07	7.743087
C9orf114	-0.37222	4.830727	-5.22458	7.55E-07	4.76E-06	5.015684
LOC15756	-0.37226	4.679369	-4.65209	8.60E-06	4.42E-05	2.663502
POLD2	-0.37232	5.402522	-3.57797	0.000502	0.001821	-1.21163
MTSS1	-0.37249	4.327175	-5.29199	5.61E-07	3.61E-06	5.304095
EMC1	-0.37249	3.503667	-8.22895	2.75E-13	5.16E-12	19.55975
BET1L	-0.37255	5.154699	-5.9129	3.30E-08	2.61E-07	8.062656
GFM1	-0.37268	4.096004	-4.67441	7.84E-06	4.06E-05	2.751801
GNL3	-0.3727	3.145197	-4.73716	6.05E-06	3.22E-05	3.001512
HNRNPUL	-0.37272	6.570916	-4.63086	9.38E-06	4.78E-05	2.579813
WDR77	-0.37282	5.904457	-6.27176	5.98E-09	5.38E-08	9.732916
TCF25	-0.37298	6.019945	-7.32292	3.15E-11	4.22E-10	14.88457
RNPS1	-0.37339	7.130046	-5.61593	1.31E-07	9.33E-07	6.721194
CCDC64	-0.37353	4.046152	-6.32438	4.64E-09	4.27E-08	9.982023
SMYD5	-0.37364	4.740466	-4.74503	5.86E-06	3.13E-05	3.032966
ZNF507	-0.37366	3.483067	-7.70414	4.38E-12	6.73E-11	16.83011
BNC2	-0.37371	2.410186	-8.1336	4.57E-13	8.27E-12	19.05993
CYSLTR1	-0.37377	5.068388	-3.49559	0.000666	0.002354	-1.47618
FAM86C1	-0.37407	2.943421	-4.94468	2.53E-06	1.44E-05	3.843255
SIGMAR1	-0.37407	4.763432	-4.89724	3.10E-06	1.73E-05	3.648688
ZNF41	-0.37409	2.472919	-6.55729	1.49E-09	1.49E-08	11.09677
GLO1	-0.37417	7.239703	-3.03446	0.00296	0.009016	-2.86238
LILRA1	-0.37425	6.090994	-2.16615	0.032296	0.071119	-5.00378
TTC31	-0.37454	6.175479	-4.69664	7.16E-06	3.75E-05	2.840007
MEAF6	-0.3746	4.774394	-7.68649	4.80E-12	7.33E-11	16.73929
ZNF862	-0.37492	6.05934	-4.18409	5.51E-05	0.000242	0.8822
LOC10192	-0.37493	2.227794	-3.90881	0.000155	0.000622	-0.10007
CBR3	-0.375	4.577591	-4.25998	4.11E-05	0.000186	1.161768
SPATA20	-0.37523	5.496174	-3.99016	0.000115	0.000472	0.184941
TRIM5	-0.37526	3.633168	-5.44862	2.79E-07	1.88E-06	5.98297
SCAMP1	-0.37529	4.481784	-5.32698	4.80E-07	3.12E-06	5.45471
CTRL	-0.3753	3.98268	-5.44976	2.77E-07	1.87E-06	5.987964
GOPC	-0.37536	4.541849	-5.08333	1.40E-06	8.35E-06	4.418855
TGS1	-0.37584	4.233415	-6.06414	1.62E-08	1.34E-07	8.760308
TMEM161I	-0.37596	4.310889	-4.23941	4.45E-05	0.0002	1.085616
E2F6	-0.37619	4.387693	-3.96724	0.000125	0.000511	0.104178
ST6GALNA	-0.37626	3.062339	-4.11801	7.09E-05	0.000305	0.641836
GAR1	-0.37645	5.574538	-3.57264	0.000511	0.001852	-1.22889
ADK	-0.37707	4.1622	-3.76257	0.000263	0.001012	-0.60093
MAP7	-0.37712	3.294216	-5.19237	8.69E-07	5.42E-06	4.87866
PAPD5	-0.37721	6.2016	-4.3704	2.67E-05	0.000125	1.575086
AASDHPPT	-0.37728	4.553569	-4.44369	2.00E-05	9.62E-05	1.853639
ALKBH3	-0.37729	4.846933	-3.96385	0.000126	0.000517	0.092252
ZNF682	-0.37733	1.884851	-6.38516	3.45E-09	3.25E-08	10.27104
PRPSAP2	-0.37764	6.506261	-3.95814	0.000129	0.000528	0.072209
KLF3	-0.37775	6.564112	-4.20377	5.11E-05	0.000226	0.954357
ZDHHC8	-0.3778	4.666306	-6.50888	1.89E-09	1.86E-08	10.86351
CTPS2	-0.37798	3.137624	-6.1221	1.23E-08	1.04E-07	9.030116
TCF20	-0.37798	4.982443	-9.12213	2.26E-15	6.22E-14	24.30796
GABPB1-A	-0.378	4.412038	-2.47368	0.014785	0.036613	-4.31875
PRMT7	-0.3784	4.556374	-4.44581	1.98E-05	9.55E-05	1.861741

FAM49A	-0.37849	6.643996	-3.57957	0.000499	0.001813	-1.20644
LOC10013	-0.37874	1.783413	-5.6454	1.14E-07	8.23E-07	6.852532
MYEF2	-0.37913	2.329139	-8.63443	3.15E-14	6.94E-13	21.70188
MADD	-0.37954	6.074358	-9.2289	1.27E-15	3.66E-14	24.88194
MGMT	-0.37985	4.582816	-3.98458	0.000117	0.000482	0.165225
PLEKHB1	-0.37997	3.826664	-7.36632	2.52E-11	3.43E-10	15.10427
OARD1	-0.38001	5.500142	-4.37664	2.61E-05	0.000123	1.598675
RPL15	-0.38008	7.534733	-4.38196	2.55E-05	0.00012	1.6188
PCM1	-0.38019	5.287541	-7.94922	1.21E-12	2.06E-11	18.09811
TMEM19	-0.38036	4.212041	-4.51961	1.47E-05	7.30E-05	2.145653
FXN	-0.38092	3.643734	-5.32486	4.85E-07	3.14E-06	5.445549
BMS1P6	-0.38098	5.166249	-4.59357	1.09E-05	5.50E-05	2.433478
TMEM39B	-0.3817	4.953123	-3.75316	0.000272	0.001043	-0.63266
RPAP1	-0.38194	5.304765	-6.06628	1.60E-08	1.33E-07	8.77025
ZNF184	-0.38198	4.092013	-2.85644	0.005058	0.014416	-3.35284
LINC00667	-0.38203	4.481839	-4.38788	2.49E-05	0.000118	1.64122
ZNF75A	-0.38219	5.027124	-4.05813	8.89E-05	0.000375	0.426476
TBRG1	-0.38269	5.072947	-4.5032	1.57E-05	7.74E-05	2.082247
ZNF12	-0.38277	6.347828	-2.50245	0.013692	0.034276	-4.25045
IL6R	-0.38283	7.594087	-3.64558	0.000397	0.001473	-0.99081
TMEM192	-0.38288	5.699607	-5.08722	1.37E-06	8.22E-06	4.435156
LOC10012	-0.38305	4.822948	-4.17987	5.60E-05	0.000246	0.866766
ARL2BP	-0.38309	6.661004	-5.73	7.73E-08	5.76E-07	7.231848
ZNF575	-0.38314	5.015497	-5.77691	6.22E-08	4.71E-07	7.443553
TNRC6C	-0.38362	3.798259	-10.3724	2.43E-18	1.13E-16	31.07925
LOC10099	-0.38367	4.181706	-5.10512	1.27E-06	7.66E-06	4.510249
WIPF2	-0.38369	6.139023	-6.25156	6.59E-09	5.88E-08	9.637526
CHST14	-0.38379	5.793789	-5.60998	1.34E-07	9.56E-07	6.69471
C3orf18	-0.38411	5.397235	-4.73895	6.01E-06	3.20E-05	3.008675
FAM159A	-0.3842	4.187099	-6.78565	4.80E-10	5.23E-09	12.20782
GOLGB1	-0.38431	5.790526	-5.17228	9.49E-07	5.86E-06	4.793481
TMEM55B	-0.38437	6.536459	-5.98678	2.33E-08	1.89E-07	8.402285
MID2	-0.38443	4.270332	-6.87725	3.03E-10	3.44E-09	12.65816
CEP164	-0.38457	4.906085	-7.37606	2.40E-11	3.28E-10	15.15362
FAM120B	-0.38467	5.581378	-6.86441	3.23E-10	3.64E-09	12.5949
TMEM156	-0.38471	2.86479	-5.60993	1.34E-07	9.56E-07	6.6945
SCRIB	-0.38471	6.026311	-5.36109	4.12E-07	2.72E-06	5.6021
C12orf79	-0.38494	2.29477	-4.64301	8.92E-06	4.57E-05	2.627688
FAM60A	-0.38517	6.487556	-3.77842	0.000248	0.000962	-0.54738
EP400	-0.38524	3.935952	-7.64097	6.09E-12	9.13E-11	16.50536
IPO9	-0.38528	3.84002	-8.13327	4.58E-13	8.28E-12	19.05821
LOC72908	-0.38549	3.014659	-4.64622	8.81E-06	4.52E-05	2.640335
ZNF764	-0.38556	4.148736	-5.95729	2.68E-08	2.15E-07	8.266448
ODF2	-0.38583	3.980142	-4.6901	7.35E-06	3.83E-05	2.814004
LOC10050	-0.38592	5.286062	-4.44366	2.00E-05	9.62E-05	1.853504
NADK	-0.3861	7.447552	-4.72221	6.44E-06	3.41E-05	2.941788
RALGPS2	-0.38621	4.14172	-3.22152	0.001645	0.005329	-2.31981
MIR3682	-0.38638	2.964743	-3.06379	0.002704	0.008321	-2.77913
NKTR	-0.38686	5.012014	-5.20498	8.22E-07	5.15E-06	4.932243
ZNF830	-0.38687	5.993518	-5.53603	1.88E-07	1.30E-06	6.367004
UPF2	-0.38699	6.722924	-4.46468	1.84E-05	8.91E-05	1.93401
YBX1	-0.38719	7.608418	-4.32687	3.17E-05	0.000147	1.41121
RBM10	-0.38731	5.919182	-4.64602	8.81E-06	4.52E-05	2.639548
TBC1D22A	-0.38744	5.896767	-8.08203	6.00E-13	1.07E-11	18.79026
COX1	-0.38769	12.5874	-3.73734	0.000287	0.0011	-0.68584
NLRP2	-0.38793	2.715517	-5.39344	3.57E-07	2.37E-06	5.742461
MRPL49	-0.38857	7.120978	-5.67886	9.79E-08	7.17E-07	7.002165
HECA	-0.38871	6.711311	-7.94545	1.23E-12	2.10E-11	18.07849

UST	-0.38872	2.583193	-7.2659	4.22E-11	5.54E-10	14.59666
PDLIM2	-0.38885	5.132138	-5.18965	8.79E-07	5.48E-06	4.867095
CYP4V2	-0.38899	3.3582	-6.6677	8.62E-10	8.98E-09	11.63178
TCEA3	-0.389	3.679543	-5.89238	3.63E-08	2.86E-07	7.968703
RPS5	-0.38905	9.998195	-3.08312	0.002547	0.007878	-2.72387
ACTR1B	-0.38941	6.738771	-6.39104	3.36E-09	3.17E-08	10.29906
POLR2D	-0.38946	4.436517	-4.62839	9.47E-06	4.83E-05	2.570111
HMOX2	-0.38971	5.899786	-6.95221	2.08E-10	2.42E-09	13.02863
CS	-0.38972	7.513268	-4.21566	4.88E-05	0.000217	0.998042
ZNF664	-0.38977	4.566779	-8.24146	2.58E-13	4.86E-12	19.62548
FAM50B	-0.3899	5.214077	-5.03601	1.71E-06	1.01E-05	4.22126
MRI1	-0.3901	5.964344	-6.86548	3.22E-10	3.63E-09	12.60017
BZW2	-0.39021	6.94673	-3.4791	0.000704	0.002476	-1.52854
LOC10192	-0.39038	1.895262	-5.86789	4.07E-08	3.17E-07	7.85687
HPCAL4	-0.39051	3.332547	-8.45901	8.07E-14	1.66E-12	20.77205
GAL3ST4	-0.39053	3.950216	-4.63053	9.39E-06	4.79E-05	2.578544
CDC42EP2	-0.39073	5.953101	-4.46684	1.82E-05	8.84E-05	1.942313
HLTF	-0.39073	5.382364	-2.62058	0.009924	0.025875	-3.96262
ROBO3	-0.39095	4.534972	-5.03564	1.72E-06	1.01E-05	4.219688
ZBED4	-0.39115	3.797508	-4.95876	2.38E-06	1.36E-05	3.901216
ITPR3	-0.39119	4.288874	-7.93055	1.34E-12	2.26E-11	18.00105
EIF3E	-0.39141	5.953061	-3.58526	0.00049	0.001783	-1.18796
METAP2	-0.39145	4.417177	-3.28599	0.001336	0.00442	-2.12649
ORC2	-0.39149	5.416354	-4.44306	2.00E-05	9.64E-05	1.851205
PRPF3	-0.39158	6.338436	-3.9708	0.000123	0.000505	0.116691
SF3A2	-0.3918	5.988745	-4.81131	4.44E-06	2.42E-05	3.299488
RPL31	-0.39181	6.799471	-2.51935	0.013085	0.032977	-4.21001
LRRK2	-0.39201	8.188982	-2.37887	0.018959	0.045345	-4.53876
HOXB-AS1	-0.39207	3.528052	-5.99161	2.28E-08	1.85E-07	8.424572
RMDN3	-0.39209	6.125252	-4.29887	3.54E-05	0.000162	1.30646
NPIPB15	-0.39214	5.032931	-3.65459	0.000385	0.001432	-0.96113
EXOC2	-0.39226	5.003021	-3.89801	0.000161	0.000645	-0.13758
RNF20	-0.39235	7.685631	-4.68647	7.46E-06	3.88E-05	2.799607
SAFB	-0.39269	4.660294	-7.23737	4.89E-11	6.35E-10	14.45292
TMEM42	-0.39275	5.636039	-4.43861	2.04E-05	9.79E-05	1.834232
CD47	-0.39306	6.543119	-4.27661	3.86E-05	0.000175	1.223513
POU6F1	-0.39326	3.965353	-8.40722	1.06E-13	2.15E-12	20.49841
RDH13	-0.39336	4.143925	-6.01814	2.01E-08	1.64E-07	8.547106
LNPEP	-0.39356	5.390463	-5.98766	2.32E-08	1.88E-07	8.406319
MBNL2	-0.39362	3.712462	-5.48126	2.41E-07	1.64E-06	6.125966
TAPBPL	-0.39366	6.116777	-4.00604	0.000108	0.000447	0.241088
CSTF1	-0.39367	4.553928	-4.86356	3.57E-06	1.97E-05	3.51135
GSDMB	-0.39371	4.131837	-7.27304	4.07E-11	5.35E-10	14.63264
NBR1	-0.39375	6.333586	-5.32904	4.76E-07	3.09E-06	5.46358
ATP6V0E2	-0.39379	3.038245	-4.99432	2.05E-06	1.18E-05	4.048154
SNRNP70	-0.39447	5.171088	-5.03633	1.71E-06	1.00E-05	4.222554
HOPX	-0.39478	3.708503	-4.84111	3.92E-06	2.16E-05	3.420149
ABHD10	-0.39481	5.75368	-3.45448	0.000765	0.00267	-1.60631
LPAR2	-0.39499	6.579591	-3.44529	0.000789	0.002747	-1.63526
GPAM	-0.39509	3.558674	-3.6192	0.000435	0.001599	-1.07735
MAGEF1	-0.39512	5.267314	-4.07568	8.32E-05	0.000353	0.489327
RABEP1	-0.39513	4.472844	-6.09298	1.41E-08	1.18E-07	8.894377
CABLES2	-0.3952	4.532636	-4.77549	5.16E-06	2.78E-05	3.155131
TECR	-0.39537	4.538384	-5.15721	1.01E-06	6.22E-06	4.729696
ADH5	-0.39554	6.005053	-3.33116	0.001153	0.003874	-1.98915
PCBP2	-0.39575	6.945513	-6.46734	2.31E-09	2.24E-08	10.66398
HCG18	-0.3958	4.52385	-7.20602	5.74E-11	7.32E-10	14.29524
ASPSCR1	-0.396	4.830617	-4.42931	2.11E-05	0.000101	1.798732

RIPK2	-0.39615	4.629399	-4.07716	8.28E-05	0.000352	0.494648
RNMTL1	-0.39623	5.133098	-4.47977	1.73E-05	8.45E-05	1.991977
PTPRO	-0.39634	4.695322	-5.06914	1.48E-06	8.82E-06	4.359458
XPO4	-0.39637	4.490105	-6.03124	1.89E-08	1.55E-07	8.607739
CD19	-0.39667	5.536743	-3.23986	0.001551	0.005054	-2.26513
COX17	-0.3967	4.114519	-3.68618	0.000344	0.001295	-0.85661
FNTA	-0.39675	6.948241	-4.02857	9.93E-05	0.000414	0.321049
TM9SF4	-0.39699	6.080539	-4.0201	0.000103	0.000426	0.290927
SRRM2	-0.39729	5.366888	-6.34199	4.26E-09	3.94E-08	10.06561
THEM6	-0.39741	3.774819	-4.90776	2.96E-06	1.66E-05	3.691736
PMPCA	-0.39773	6.1096	-5.18654	8.91E-07	5.55E-06	4.853916
IER2	-0.39803	9.825056	-6.27491	5.89E-09	5.31E-08	9.747799
PLAGL1	-0.39837	6.234524	-2.64064	0.009386	0.024645	-3.91256
PLGLB2	-0.39842	2.594102	-4.47406	1.77E-05	8.62E-05	1.970038
PRMT3	-0.39851	4.353403	-2.13696	0.034651	0.075388	-5.06447
CCNG2	-0.39857	6.104216	-3.09371	0.002464	0.007647	-2.6935
PPP1R8	-0.39902	7.298774	-3.70267	0.000325	0.00123	-0.80177
CD248	-0.39903	4.417301	-5.01203	1.90E-06	1.10E-05	4.121562
TMEM106I	-0.39924	5.765637	-4.54217	1.35E-05	6.70E-05	2.233101
OTUD4	-0.39947	3.832547	-7.09081	1.03E-10	1.26E-09	13.71805
GFPT1	-0.39969	3.964706	-3.57933	0.0005	0.001814	-1.20721
CWF19L2	-0.39993	3.605056	-7.04018	1.33E-10	1.60E-09	13.46555
VPRBP	-0.40007	4.311525	-7.63499	6.28E-12	9.39E-11	16.47466
ND4	-0.40029	12.75379	-4.59369	1.09E-05	5.50E-05	2.433952
RANGAP1	-0.40043	5.79127	-6.25627	6.45E-09	5.76E-08	9.659737
EDAR	-0.40046	4.088732	-4.93366	2.65E-06	1.50E-05	3.797969
TTC9	-0.40047	3.758091	-4.17212	5.77E-05	0.000253	0.838446
PNMA6A	-0.40049	3.803044	-4.80292	4.60E-06	2.49E-05	3.265637
RAPGEF1	-0.40079	5.78499	-7.84996	2.04E-12	3.34E-11	17.58306
ZNF514	-0.40079	4.26428	-2.96816	0.003624	0.010757	-3.04801
RAB5C	-0.4008	7.528307	-2.6855	0.008278	0.02213	-3.7994
CD180	-0.40096	6.023492	-3.41999	0.000859	0.002962	-1.71455
TMEM245	-0.401	6.20333	-3.84531	0.000195	0.000768	-0.31938
SNRK	-0.40118	4.562656	-5.54901	1.77E-07	1.24E-06	6.424362
C12orf65	-0.40135	4.527897	-7.31495	3.28E-11	4.38E-10	14.84425
CXorf57	-0.40151	2.156638	-6.77087	5.16E-10	5.61E-09	12.13539
DOCK4	-0.40152	3.202609	-3.64443	0.000399	0.001478	-0.99459
YAE1D1	-0.40165	3.349184	-3.54061	0.000571	0.002048	-1.33222
TMOD2	-0.40172	3.458275	-4.23968	4.45E-05	0.0002	1.086611
GRPEL2	-0.40177	3.75961	-5.08322	1.40E-06	8.35E-06	4.418401
KIR2DL5A	-0.40192	6.073158	-2.12984	0.035247	0.07645	-5.07916
GPR133	-0.40196	2.523305	-4.87083	3.46E-06	1.92E-05	3.540926
PP12719	-0.40218	4.765629	-2.7056	0.007821	0.021067	-3.74815
GTPBP4	-0.40232	4.787197	-4.31188	3.36E-05	0.000155	1.355074
RING1	-0.40254	6.756596	-7.75944	3.28E-12	5.16E-11	17.11511
STXBP1	-0.40258	2.854596	-3.73907	0.000286	0.001094	-0.68002
RASAL3	-0.40282	7.050067	-5.22448	7.55E-07	4.76E-06	5.015223
CHI3L2	-0.403	4.588945	-2.76039	0.006689	0.018382	-3.60673
HEATR2	-0.40303	3.828029	-5.87889	3.87E-08	3.03E-07	7.907075
OXA1L	-0.40311	7.271219	-4.39403	2.43E-05	0.000115	1.664534
MDM4	-0.40315	5.748453	-4.72908	6.26E-06	3.32E-05	2.96923
IP6K2	-0.40319	5.545456	-7.39981	2.12E-11	2.92E-10	15.27412
AMMECR1	-0.40345	5.766915	-3.6565	0.000382	0.001424	-0.95484
IDS	-0.40347	5.370408	-5.99954	2.19E-08	1.78E-07	8.461175
TTC4	-0.4035	4.987729	-4.76101	5.48E-06	2.94E-05	3.097013
CNBP	-0.40392	8.007315	-4.54024	1.36E-05	6.75E-05	2.225602
TOB2	-0.40401	4.898541	-6.64486	9.65E-10	9.99E-09	11.52079
DIP2C	-0.40403	3.481463	-8.26405	2.28E-13	4.34E-12	19.74417

IKZF1	-0.40407	6.097847	-6.21064	8.03E-09	7.04E-08	9.44483
CYP2U1	-0.40414	2.728998	-9.73016	8.21E-17	2.95E-15	27.58955
PSMA5	-0.40433	5.287687	-4.07211	8.43E-05	0.000358	0.476537
SMARCA2	-0.4049	4.592995	-7.43123	1.81E-11	2.50E-10	15.43375
OSGIN2	-0.40494	3.619499	-7.29233	3.69E-11	4.87E-10	14.72998
NAB1	-0.40496	3.771164	-5.57146	1.60E-07	1.12E-06	6.523692
CHERP	-0.40496	5.921321	-9.74362	7.63E-17	2.76E-15	27.66249
ZNF782	-0.40498	2.61776	-3.29084	0.001315	0.004359	-2.11181
CTBP1	-0.40524	6.90576	-5.5214	2.01E-07	1.39E-06	6.302467
KPNB1	-0.40526	7.948655	-5.07828	1.43E-06	8.52E-06	4.397677
WDR43	-0.40531	4.798251	-3.01885	0.003106	0.009394	-2.90639
UBE2Z	-0.40536	6.730466	-6.57591	1.36E-09	1.37E-08	11.1867
WDR81	-0.4057	6.210044	-6.41399	3.00E-09	2.86E-08	10.40859
C20orf194	-0.40596	4.89536	-4.33693	3.05E-05	0.000142	1.448987
AHNAK	-0.406	4.722935	-7.07584	1.11E-10	1.35E-09	13.64333
RNMT	-0.40681	4.114417	-5.91651	3.24E-08	2.57E-07	8.07918
FCER2	-0.40727	4.184192	-3.28087	0.001358	0.004486	-2.14197
CNTRL	-0.40749	5.573869	-4.83116	4.09E-06	2.24E-05	3.379803
CHMP1B	-0.40753	6.986572	-4.21498	4.89E-05	0.000218	0.995559
ZNF383	-0.40753	2.551299	-3.92012	0.000148	0.000599	-0.06073
PDS5A	-0.40764	4.987719	-4.6946	7.22E-06	3.77E-05	2.831884
CTR9	-0.40777	7.306618	-3.62187	0.000431	0.001587	-1.06863
SND1	-0.40779	6.455498	-4.46831	1.81E-05	8.80E-05	1.947928
OSBPL5	-0.4078	4.751319	-5.61481	1.31E-07	9.37E-07	6.716178
DDX27	-0.4079	6.342979	-6.5016	1.96E-09	1.91E-08	10.82849
RNF114	-0.40802	6.532631	-4.71934	6.52E-06	3.44E-05	2.930348
VEZF1	-0.4081	7.287262	-5.47086	2.52E-07	1.71E-06	6.080339
CCDC25	-0.40813	4.621609	-4.43964	2.03E-05	9.76E-05	1.838152
TP53INP2	-0.40816	5.263867	-2.81557	0.005702	0.015982	-3.46179
NBPF1	-0.40816	5.824681	-6.23816	7.03E-09	6.23E-08	9.574362
FAM35A	-0.40822	6.415926	-2.47052	0.01491	0.036892	-4.32623
ST6GALNA	-0.40832	5.688226	-2.39742	0.018068	0.043511	-4.49634
POP5	-0.40835	5.635474	-2.86816	0.004887	0.014	-3.32134
HYLS1	-0.40836	4.996372	-4.36723	2.70E-05	0.000127	1.563099
U2SURP	-0.40839	5.106383	-3.52366	0.000605	0.002159	-1.3866
OCM2	-0.40843	2.845621	-5.33922	4.55E-07	2.97E-06	5.507538
PRPF4	-0.40854	5.950194	-4.99583	2.03E-06	1.18E-05	4.054404
SRFBP1	-0.40865	3.048928	-3.4993	0.000657	0.002328	-1.46435
MAP9	-0.40881	2.312096	-7.77761	2.98E-12	4.74E-11	17.20887
CCSER2	-0.40896	4.071316	-7.45337	1.61E-11	2.26E-10	15.54636
TDP1	-0.40897	3.911823	-7.00883	1.56E-10	1.85E-09	13.30962
FIGNL1	-0.40909	3.475156	-2.72835	0.007331	0.019917	-3.68973
CCDC66	-0.40923	3.220415	-6.12287	1.22E-08	1.04E-07	9.033685
TRAF1	-0.40936	4.171857	-5.61891	1.29E-07	9.22E-07	6.734424
PPP2R1A	-0.40997	6.781192	-5.09741	1.31E-06	7.90E-06	4.477862
KRT77	-0.41009	2.965114	-2.37444	0.019177	0.045793	-4.54885
HNRNPK	-0.41014	9.42493	-5.21441	7.89E-07	4.95E-06	4.972346
EBAG9	-0.41014	5.416569	-3.21183	0.001697	0.005477	-2.34858
TADA1	-0.41024	3.417587	-5.58685	1.49E-07	1.05E-06	6.591937
ATRX	-0.4103	5.851913	-4.91382	2.89E-06	1.62E-05	3.716552
PTPRK	-0.41032	2.144648	-4.74924	5.76E-06	3.07E-05	3.049843
FAM129C	-0.41033	4.615244	-5.01467	1.88E-06	1.09E-05	4.132514
RPS28	-0.4105	6.94938	-6.62591	1.06E-09	1.09E-08	11.4288
SLC2A6	-0.41054	5.019488	-3.23637	0.001569	0.005109	-2.27556
WDR11	-0.41062	6.334661	-2.95677	0.003751	0.011089	-3.07957
RPS6	-0.41063	8.538652	-4.25742	4.15E-05	0.000188	1.152267
ZNF18	-0.41077	5.695145	-5.10906	1.25E-06	7.53E-06	4.526799
ZMYM4	-0.41082	5.483827	-4.26177	4.08E-05	0.000185	1.168416

PMS2P8	-0.41092	5.361994	-4.6071	1.03E-05	5.23E-05	2.486462
RCN2	-0.41101	5.718073	-2.60504	0.010359	0.026859	-4.00116
CAAP1	-0.41104	4.161575	-3.50526	0.000644	0.002285	-1.44539
ACKR3	-0.41112	3.163617	-4.39548	2.42E-05	0.000115	1.670035
ZNF667-A	-0.41135	3.238757	-3.1004	0.002414	0.007509	-2.67425
ACLY	-0.41153	6.306335	-4.81368	4.40E-06	2.40E-05	3.309056
WDFY1	-0.41175	6.061702	-4.94223	2.56E-06	1.45E-05	3.833185
GALT	-0.41177	4.269521	-6.27021	6.03E-09	5.42E-08	9.72556
ZBTB14	-0.41193	4.641161	-5.2492	6.77E-07	4.30E-06	5.120748
FUBP1	-0.41197	4.848218	-3.85098	0.000191	0.000754	-0.29993
EVA1C	-0.41211	2.989977	-5.38592	3.69E-07	2.44E-06	5.709787
UBTF	-0.41215	4.800267	-7.57492	8.58E-12	1.25E-10	16.16678
TRIM32	-0.41218	2.997364	-6.59233	1.25E-09	1.27E-08	11.2661
CDK5RAP1	-0.41232	5.55863	-4.81641	4.35E-06	2.37E-05	3.320119
EIF3B	-0.41264	5.271833	-5.69232	9.20E-08	6.78E-07	7.062496
P4HTM	-0.41272	6.033884	-5.48691	2.35E-07	1.60E-06	6.150756
MAML1	-0.41278	7.760146	-5.14984	1.05E-06	6.40E-06	4.698564
SFXN3	-0.41301	5.090398	-4.08877	7.92E-05	0.000338	0.536372
EXT2	-0.41325	5.145171	-5.89618	3.57E-08	2.81E-07	7.986081
ERCC3	-0.41327	5.389648	-4.60033	1.06E-05	5.37E-05	2.459942
HLA-F-AS	-0.4135	2.8483	-6.71235	6.91E-10	7.34E-09	11.84933
POU2F2	-0.41425	5.35229	-6.39966	3.22E-09	3.05E-08	10.34021
AGO3	-0.41428	4.398451	-5.15665	1.02E-06	6.24E-06	4.72734
ZNF354B	-0.41431	3.433617	-4.4425	2.01E-05	9.66E-05	1.849067
CBFA2T2	-0.41443	4.941384	-8.54685	5.04E-14	1.07E-12	21.23712
PDCD11	-0.41455	4.916762	-7.72773	3.87E-12	6.03E-11	16.95157
ZNF24	-0.41475	5.675022	-5.64942	1.12E-07	8.11E-07	6.8705
SOAT1	-0.41487	5.734647	-3.49996	0.000656	0.002324	-1.46228
C10orf128	-0.41493	5.202726	-4.01535	0.000104	0.000433	0.27408
POP1	-0.41515	3.22301	-6.07149	1.56E-08	1.30E-07	8.794451
ENGASE	-0.41534	6.261792	-6.97755	1.83E-10	2.16E-09	13.15427
FTSJ1	-0.41541	5.470146	-5.24243	6.98E-07	4.42E-06	5.091836
MICU3	-0.41546	1.833477	-3.93766	0.000139	0.000565	0.000492
UBE2I	-0.41547	5.484879	-6.7041	7.20E-10	7.62E-09	11.80906
TNIP1	-0.41548	7.668096	-5.92771	3.08E-08	2.44E-07	8.130562
CREBBP	-0.41552	5.195548	-7.90477	1.53E-12	2.54E-11	17.86721
POGK	-0.41558	4.943233	-3.86053	0.000185	0.000731	-0.26709
LINC01278	-0.41575	3.191829	-6.56474	1.43E-09	1.44E-08	11.13272
DNAJC14	-0.41579	5.364702	-5.78001	6.13E-08	4.65E-07	7.457559
MORF4L2	-0.4158	5.283664	-3.46941	0.000727	0.00255	-1.5592
HADH	-0.41616	4.480096	-4.56626	1.22E-05	6.11E-05	2.3268
BCL2L11	-0.41646	5.303553	-7.48163	1.39E-11	1.97E-10	15.69028
H1FX	-0.41647	7.15395	-2.72309	0.007442	0.020172	-3.70329
CNOT11	-0.4166	6.573443	-5.69539	9.07E-08	6.69E-07	7.076291
SENP6	-0.41683	4.868825	-3.32106	0.001192	0.003993	-2.02
TAF1D	-0.41684	5.667364	-2.97365	0.003564	0.010597	-3.03278
AP3M2	-0.41709	5.089742	-5.78376	6.03E-08	4.57E-07	7.474563
CD40LG	-0.41709	5.48949	-5.59766	1.42E-07	1.01E-06	6.639912
ISL2	-0.41714	3.868367	-2.64839	0.009186	0.024183	-3.89314
LOC10192	-0.41718	2.927875	-5.74295	7.28E-08	5.45E-07	7.2902
CEP290	-0.41726	2.306079	-5.67418	1.00E-07	7.30E-07	6.981235
ZNF767P	-0.41782	5.452712	-4.87	3.47E-06	1.92E-05	3.537554
SLC25A36	-0.41794	5.234438	-3.72964	0.000295	0.001128	-0.71165
ZNF320	-0.41808	3.161526	-4.29918	3.53E-05	0.000162	1.307627
ZNF195	-0.41816	4.099995	-2.8278	0.005502	0.015494	-3.42935
TTC22	-0.41835	2.958417	-9.45939	3.61E-16	1.14E-14	26.1246
MRPS27	-0.41875	4.23894	-7.09012	1.04E-10	1.26E-09	13.71458
PUF60	-0.41877	6.960356	-5.33239	4.69E-07	3.05E-06	5.478053

METTL4	-0.41887	4.586279	-4.8746	3.41E-06	1.89E-05	3.5563
CEP85L	-0.41899	4.324703	-4.42171	2.18E-05	0.000104	1.769743
ZC3H14	-0.41911	4.437945	-6.43607	2.69E-09	2.58E-08	10.51415
LINC0056	-0.41938	4.125457	-5.94358	2.86E-08	2.28E-07	8.203406
STARD9	-0.41951	2.85309	-6.40111	3.19E-09	3.03E-08	10.34713
RWDD2B	-0.41962	5.180561	-5.17716	9.29E-07	5.76E-06	4.814159
ZBTB5	-0.4198	5.109617	-6.62787	1.05E-09	1.08E-08	11.43833
PHF13	-0.41989	5.958315	-5.18948	8.80E-07	5.48E-06	4.86639
FBXL14	-0.42005	3.546275	-9.98886	1.99E-17	7.96E-16	28.99332
QKI	-0.42005	6.039298	-4.75273	5.68E-06	3.03E-05	3.063797
CST3	-0.42026	5.32812	-4.50988	1.53E-05	7.55E-05	2.108034
PIGM	-0.4203	3.793957	-5.62168	1.27E-07	9.11E-07	6.74679
KRT72	-0.42032	4.255069	-4.6413	8.99E-06	4.60E-05	2.620956
LOC10028	-0.42063	5.036786	-5.58005	1.54E-07	1.09E-06	6.561791
PDIK1L	-0.42064	4.038518	-2.63368	0.00957	0.025095	-3.92997
PREPL	-0.42068	3.99193	-5.5693	1.61E-07	1.13E-06	6.514129
LOC10013	-0.42098	3.782309	-4.21519	4.89E-05	0.000218	0.996326
RPS12	-0.4211	11.86082	-5.32431	4.86E-07	3.15E-06	5.443185
LPCAT1	-0.42118	7.835265	-5.171	9.54E-07	5.89E-06	4.788039
KIAA1279	-0.42122	4.915825	-3.17022	0.001938	0.006177	-2.47133
PSMD6-A	-0.4215	6.524659	-4.59951	1.07E-05	5.39E-05	2.456731
NAA35	-0.42156	4.966785	-3.99594	0.000112	0.000463	0.205358
TYW3	-0.42184	3.942394	-3.21412	0.001685	0.005441	-2.3418
KDM2A	-0.42201	6.899863	-6.00715	2.12E-08	1.73E-07	8.496323
UBR5	-0.42205	5.69907	-4.02051	0.000102	0.000426	0.292386
MUM1	-0.4222	4.442515	-6.03676	1.84E-08	1.51E-07	8.633289
ZMAT1	-0.42223	2.525536	-3.92982	0.000143	0.00058	-0.02688
KAT6A	-0.42228	6.452766	-5.07268	1.46E-06	8.71E-06	4.374285
THOC1	-0.42253	5.951148	-4.58602	1.13E-05	5.66E-05	2.403945
POLA1	-0.42267	4.164924	-3.39034	0.000948	0.003242	-1.80687
ELOVL4	-0.42291	2.125583	-6.46774	2.31E-09	2.23E-08	10.6659
RAD1	-0.42305	5.039262	-5.54697	1.79E-07	1.25E-06	6.415324
WDR91	-0.42305	4.267884	-6.42096	2.90E-09	2.76E-08	10.44191
C2CD5	-0.42318	7.208659	-3.05904	0.002744	0.008432	-2.79264
BICD1	-0.42339	3.064953	-8.22273	2.85E-13	5.31E-12	19.52712
RPS27A	-0.42344	6.209797	-6.22678	7.43E-09	6.55E-08	9.520768
ZBTB39	-0.42366	3.853349	-5.02947	1.76E-06	1.03E-05	4.194035
RNF113A	-0.42367	7.029398	-6.49121	2.06E-09	2.01E-08	10.77853
NKRF	-0.42372	5.003413	-5.40063	3.46E-07	2.30E-06	5.773713
ZNF644	-0.42391	4.901166	-3.13196	0.002186	0.006877	-2.58301
HYOU1	-0.42393	6.749664	-3.03206	0.002982	0.009076	-2.86914
BRD3	-0.42407	5.49855	-9.49843	2.91E-16	9.39E-15	26.33552
LAG3	-0.42421	3.825733	-5.1502	1.04E-06	6.39E-06	4.700106
ITM2B	-0.42424	11.74307	-4.88964	3.20E-06	1.78E-05	3.617635
HABP4	-0.42428	4.09021	-8.66313	2.70E-14	6.03E-13	21.85446
EHMT2	-0.42431	5.090685	-7.92431	1.38E-12	2.33E-11	17.96867
ZNF280B	-0.42438	3.35938	-8.42481	9.69E-14	1.96E-12	20.59134
SORL1	-0.42461	10.2372	-3.68408	0.000347	0.001304	-0.86358
RBBP4	-0.4247	5.817852	-6.78912	4.71E-10	5.16E-09	12.2248
SLC35A3	-0.425	4.665256	-3.08469	0.002535	0.007846	-2.71938
ECHS1	-0.42503	7.354851	-4.81662	4.35E-06	2.37E-05	3.320951
NUP107	-0.42534	6.013135	-3.27419	0.001388	0.004571	-2.16211
NOTCH2	-0.42543	7.322951	-4.32393	3.21E-05	0.000149	1.400186
TRAF3IP2	-0.42551	3.753993	-7.43446	1.78E-11	2.47E-10	15.45014
BPTF	-0.42557	5.216941	-6.31949	4.75E-09	4.37E-08	9.958826
SNIP1	-0.42565	4.937986	-6.08878	1.44E-08	1.21E-07	8.874843
CACNA2D	-0.42625	4.984816	-5.42312	3.13E-07	2.09E-06	5.871647
IL1B	-0.42655	7.310223	-2.35891	0.01996	0.047315	-4.5841

LIG3	-0.42678	4.253271	-6.96073	1.99E-10	2.33E-09	13.07087
URB2	-0.42682	3.662265	-4.50173	1.58E-05	7.78E-05	2.076576
PIK3C2A	-0.42692	4.220263	-5.66046	1.06E-07	7.73E-07	6.919839
DDX17	-0.42709	8.250213	-2.92431	0.004135	0.012107	-3.16887
DDX10	-0.42711	3.332379	-7.99849	9.33E-13	1.62E-11	18.35449
FAM65B	-0.42727	9.346629	-3.55541	0.000543	0.001956	-1.28457
GCC2	-0.4274	4.357763	-6.81141	4.22E-10	4.66E-09	12.33418
PKI55	-0.42779	3.381219	-4.93935	2.59E-06	1.47E-05	3.821347
PSMB10	-0.42787	7.881545	-4.01377	0.000105	0.000435	0.26848
LOC10013	-0.42817	5.375023	-4.06918	8.53E-05	0.000361	0.466046
XKR6	-0.42846	2.965801	-8.33688	1.55E-13	3.04E-12	20.12743
TMEM176I	-0.42865	4.595108	-2.21024	0.029003	0.065017	-4.91069
ZC3H7A	-0.42867	6.119988	-3.54493	0.000563	0.002021	-1.31831
CRTC2	-0.42869	5.757774	-6.69932	7.37E-10	7.78E-09	11.78577
PTAFR	-0.42872	8.165195	-4.67812	7.73E-06	4.01E-05	2.766494
IGHM	-0.42905	6.539415	-2.35364	0.020232	0.047859	-4.596
TBC1D10C	-0.4291	7.846235	-4.481	1.72E-05	8.41E-05	1.996687
ASCC3	-0.42915	4.007888	-5.28048	5.90E-07	3.79E-06	5.254672
AHSA2	-0.42916	5.196037	-4.06858	8.55E-05	0.000362	0.46387
FCRLB	-0.42916	3.789014	-3.85873	0.000186	0.000735	-0.27327
ICE2	-0.42956	3.262077	-4.83807	3.97E-06	2.18E-05	3.407793
MPI	-0.42965	3.826613	-6.92237	2.42E-10	2.77E-09	12.88098
MIEF1	-0.42973	5.631587	-6.13333	1.16E-08	9.90E-08	9.082533
MED17	-0.42974	4.96774	-4.51089	1.53E-05	7.53E-05	2.111926
EIF2AK3	-0.42977	3.924324	-5.33401	4.65E-07	3.03E-06	5.485023
THBD	-0.42992	4.921869	-2.81825	0.005657	0.015873	-3.4547
ZNF134	-0.42997	4.538915	-5.38613	3.69E-07	2.44E-06	5.7107
IKBKAP	-0.43011	3.972198	-6.36064	3.89E-09	3.63E-08	10.1543
EEF1A1	-0.43016	9.957409	-5.60514	1.37E-07	9.75E-07	6.673178
SLC9B2	-0.43078	3.454391	-6.32121	4.71E-09	4.34E-08	9.966961
ELK4	-0.43084	4.000383	-9.33369	7.15E-16	2.15E-14	25.44637
ZNF248	-0.43114	4.511106	-9.6904	1.02E-16	3.61E-15	27.37416
LONP1	-0.43127	5.662469	-4.84435	3.87E-06	2.13E-05	3.433283
YPEL3	-0.43163	8.417551	-4.693	7.27E-06	3.79E-05	2.825525
CRYGS	-0.43187	3.552105	-6.50417	1.93E-09	1.89E-08	10.84085
DUSP5	-0.43206	3.644927	-3.08342	0.002545	0.007875	-2.72302
CCL28	-0.43213	3.761324	-8.54631	5.06E-14	1.07E-12	21.23425
SF3B3	-0.43215	5.401486	-5.7895	5.87E-08	4.47E-07	7.500519
SSB	-0.43216	5.928472	-2.84464	0.005237	0.014856	-3.38445
SRSF10	-0.43269	5.5562	-3.03078	0.002994	0.009104	-2.87276
FAM73A	-0.43271	3.274678	-4.36417	2.74E-05	0.000128	1.551563
LPXN	-0.43288	6.327954	-4.63444	9.24E-06	4.72E-05	2.593916
CDK5RAP3	-0.433	7.687767	-6.30982	4.98E-09	4.54E-08	9.912983
PFKM	-0.43319	5.425885	-4.3008	3.51E-05	0.000161	1.313656
TLR7	-0.43337	3.284995	-4.99361	2.05E-06	1.19E-05	4.045188
IGHD	-0.43353	5.320262	-2.30031	0.023174	0.05368	-4.71515
FAM110A	-0.43356	5.35369	-4.96264	2.35E-06	1.34E-05	3.917216
SYNCRIP	-0.43364	5.419162	-4.39807	2.39E-05	0.000113	1.679868
RBM14	-0.43369	4.744318	-5.89001	3.67E-08	2.88E-07	7.957889
ZNF641	-0.43373	6.488135	-4.38148	2.56E-05	0.00012	1.617
MBP	-0.43404	6.007989	-9.98722	2.01E-17	8.01E-16	28.9844
GRSF1	-0.43415	4.660077	-6.00412	2.15E-08	1.75E-07	8.482328
MATN1-A	-0.43421	2.855088	-6.7638	5.35E-10	5.80E-09	12.10074
ELK3	-0.43423	5.520738	-4.51454	1.50E-05	7.42E-05	2.126023
PURA	-0.43434	4.490397	-6.70166	7.29E-10	7.70E-09	11.79718
LINC0092C	-0.43435	3.054938	-6.15849	1.03E-08	8.84E-08	9.200211
NFKB2	-0.43438	3.875891	-8.32502	1.65E-13	3.21E-12	20.06499
TTC37	-0.4344	4.480489	-3.68908	0.000341	0.001283	-0.84699

PIP4K2C	-0.43453	6.061806	-7.32944	3.05E-11	4.09E-10	14.91752
LOC28335	-0.43479	5.147557	-3.37552	0.000996	0.003388	-1.85276
AGMAT	-0.43482	3.721492	-6.18648	9.01E-09	7.82E-08	9.331393
PUM2	-0.4356	6.38078	-7.93078	1.33E-12	2.26E-11	18.00227
VAMP2	-0.43568	5.917133	-5.09097	1.35E-06	8.10E-06	4.450849
CNOT1	-0.43587	6.12337	-8.88926	7.98E-15	1.99E-13	23.0601
REPIN1	-0.43588	4.431763	-5.8864	3.74E-08	2.93E-07	7.941371
PPAT	-0.43631	4.28875	-3.85291	0.00019	0.000749	-0.29328
ZNF431	-0.43638	3.382844	-4.0572	8.92E-05	0.000376	0.423155
GON4L	-0.43658	4.998348	-7.84186	2.13E-12	3.47E-11	17.54111
GCFC2	-0.43668	3.082335	-5.26657	6.27E-07	4.00E-06	5.195068
ALDH8A1	-0.43688	2.284367	-3.29007	0.001318	0.004369	-2.11415
ZNF677	-0.43739	2.366948	-6.53899	1.63E-09	1.62E-08	11.0085
DOCK5	-0.43746	5.032641	-5.54158	1.83E-07	1.27E-06	6.391518
POC5	-0.43772	5.539774	-4.91354	2.89E-06	1.62E-05	3.715405
CD83	-0.43773	5.969228	-6.36947	3.73E-09	3.50E-08	10.19629
VWCE	-0.43802	6.019873	-2.34518	0.020675	0.048734	-4.61506
SFMBT2	-0.43809	3.684682	-3.68585	0.000345	0.001297	-0.85772
NBPF20	-0.4383	3.236445	-7.01426	1.52E-10	1.80E-09	13.33659
CD52	-0.43861	8.458334	-2.07646	0.040006	0.084936	-5.1878
USPL1	-0.43866	3.50721	-6.3692	3.73E-09	3.50E-08	10.19502
HSF5	-0.4387	2.370494	-4.58807	1.12E-05	5.62E-05	2.411952
SP1	-0.43872	6.56814	-4.93254	2.67E-06	1.51E-05	3.793332
GGA2	-0.43876	4.577671	-9.06037	3.16E-15	8.46E-14	23.97645
FAM175A	-0.43911	3.720593	-3.26674	0.001422	0.004672	-2.18453
PMS2P1	-0.43915	6.108896	-5.93362	2.99E-08	2.38E-07	8.157643
ATXN10	-0.43973	6.655996	-4.92129	2.80E-06	1.57E-05	3.747161
DLG5	-0.43991	3.029115	-7.22127	5.31E-11	6.84E-10	14.3719
ZFP90	-0.43991	4.961157	-6.18873	8.92E-09	7.76E-08	9.341952
CLOCK	-0.43993	4.422495	-6.62334	1.07E-09	1.10E-08	11.41632
NUP153	-0.43996	5.618758	-4.39411	2.43E-05	0.000115	1.664843
DBP	-0.44001	4.42077	-7.19386	6.11E-11	7.75E-10	14.23412
USP4	-0.44004	8.45896	-5.22818	7.43E-07	4.69E-06	5.031007
YY1	-0.44013	6.993949	-8.59286	3.94E-14	8.50E-13	21.48113
NSUN6	-0.44042	5.390684	-4.93308	2.66E-06	1.50E-05	3.79556
BMF	-0.44043	5.13613	-5.74745	7.13E-08	5.35E-07	7.310493
AIP	-0.44049	7.014025	-6.31416	4.87E-09	4.46E-08	9.933541
NKX3-1	-0.44057	3.562187	-6.82076	4.02E-10	4.47E-09	12.38011
RPS16	-0.44062	9.71495	-5.16386	9.84E-07	6.06E-06	4.75785
LIAS	-0.44064	3.769019	-3.59005	0.000482	0.001758	-1.17241
RALGDS	-0.44066	6.789587	-4.43693	2.05E-05	9.85E-05	1.827815
FBXO31	-0.44068	4.346158	-6.31651	4.82E-09	4.42E-08	9.944709
ZNF91	-0.4407	3.261445	-5.75607	6.85E-08	5.15E-07	7.349391
ZFP82	-0.44073	3.786886	-7.72637	3.90E-12	6.06E-11	16.94456
JADE1	-0.44074	5.493495	-4.83833	3.97E-06	2.18E-05	3.408842
SUN1	-0.44075	3.189179	-6.51046	1.87E-09	1.85E-08	10.87107
KIR3DL1	-0.44089	4.828367	-5.0986	1.31E-06	7.86E-06	4.482856
HDAC1	-0.4413	7.527502	-4.02603	0.0001	0.000418	0.311996
KRI1	-0.44179	5.300445	-6.81015	4.24E-10	4.68E-09	12.32798
SHISA5	-0.4418	8.930296	-4.28411	3.74E-05	0.000171	1.251433
GKAP1	-0.44198	3.193266	-6.14058	1.12E-08	9.58E-08	9.11641
ETAA1	-0.44208	2.45743	-3.65568	0.000383	0.001427	-0.95753
RPL7	-0.44209	8.123954	-3.29401	0.001302	0.004322	-2.10222
ZMYM1	-0.44215	3.419025	-2.81892	0.005646	0.015848	-3.45291
LTA	-0.44219	4.126006	-4.97584	2.22E-06	1.27E-05	3.971722
PPP2R5C	-0.44222	6.27405	-5.86789	4.07E-08	3.17E-07	7.856844
PPP2R2B	-0.44231	2.343054	-7.71372	4.16E-12	6.43E-11	16.87943
MCM3AP-	-0.44231	2.340383	-6.07429	1.54E-08	1.28E-07	8.807425

SOCS2	-0.44239	3.395164	-5.58111	1.53E-07	1.08E-06	6.566491
ARRDC2	-0.44244	5.879746	-5.12182	1.18E-06	7.17E-06	4.580435
HIP1R	-0.4428	5.216267	-6.69209	7.64E-10	8.04E-09	11.75055
FAM65A	-0.44307	5.349852	-7.66433	5.39E-12	8.17E-11	16.62533
SMC5	-0.44326	3.811249	-4.84244	3.90E-06	2.15E-05	3.42553
SEH1L	-0.44333	4.431582	-3.26229	0.001443	0.004732	-2.19792
SLC27A3	-0.44333	5.373587	-3.62672	0.000424	0.001563	-1.05274
GBF1	-0.44354	4.834923	-5.26032	6.45E-07	4.10E-06	5.168282
LOC28581	-0.44361	5.64522	-4.17744	5.65E-05	0.000248	0.857887
RNF157	-0.44381	4.123021	-5.3144	5.08E-07	3.28E-06	5.400472
PDIA3	-0.44389	5.426497	-4.9991	2.01E-06	1.16E-05	4.067921
BCR	-0.44389	5.604461	-6.54107	1.61E-09	1.61E-08	11.01851
KLHDC1	-0.44419	1.795709	-8.27079	2.20E-13	4.21E-12	19.77958
MIRLET7D	-0.44423	4.933196	-5.57965	1.54E-07	1.09E-06	6.56
LOC10050	-0.44429	2.229367	-5.55544	1.72E-07	1.20E-06	6.452775
ORMDL1	-0.44433	5.872425	-4.30513	3.45E-05	0.000159	1.32982
SF3B1	-0.44444	7.887827	-5.0936	1.34E-06	8.02E-06	4.461869
STAP1	-0.44458	3.731858	-2.09812	0.038013	0.081438	-5.14404
ZNF136	-0.44472	4.922813	-4.76762	5.33E-06	2.86E-05	3.123552
ZNF526	-0.44486	3.83239	-7.08167	1.08E-10	1.31E-09	13.67238
KMT2C	-0.44515	5.566817	-8.05163	7.05E-13	1.24E-11	18.63154
HERPUD2	-0.44525	6.86984	-4.90553	2.99E-06	1.67E-05	3.682615
NFE2L3	-0.44535	1.918156	-6.6309	1.03E-09	1.06E-08	11.45303
TARSL2	-0.44558	4.191118	-5.06979	1.48E-06	8.80E-06	4.362177
REXO4	-0.44576	5.19107	-7.57218	8.70E-12	1.27E-10	16.15273
CEP120	-0.44617	4.311198	-3.78673	0.000241	0.000935	-0.51924
LCP2	-0.44648	6.700634	-5.35272	4.28E-07	2.81E-06	5.56589
ZNF793	-0.44656	2.730346	-5.24538	6.89E-07	4.36E-06	5.104397
NSA2	-0.44669	7.697285	-3.40717	0.000896	0.003079	-1.75453
GIPC1	-0.44679	4.580843	-5.53117	1.92E-07	1.33E-06	6.345577
POLR3C	-0.44692	4.887997	-6.47049	2.28E-09	2.20E-08	10.67907
IKZF5	-0.44694	4.584044	-4.12095	7.01E-05	0.000302	0.652462
MAPRE2	-0.44703	5.16332	-8.28571	2.04E-13	3.91E-12	19.85806
ADAMTS5	-0.44741	2.043876	-10.5955	7.12E-19	3.60E-17	32.29487
SETD2	-0.44763	5.014265	-6.25619	6.45E-09	5.76E-08	9.659403
PPIL3	-0.44768	6.150859	-2.46139	0.015276	0.037645	-4.34773
SUPT3H	-0.44773	3.131497	-7.43544	1.77E-11	2.46E-10	15.45515
RNF144A	-0.44794	3.727036	-6.95797	2.02E-10	2.36E-09	13.0572
SFSWAP	-0.44795	5.031082	-7.57259	8.68E-12	1.27E-10	16.15484
UFM1	-0.44803	5.112955	-3.06331	0.002708	0.008331	-2.78048
RASSF1	-0.44811	6.034323	-5.79557	5.71E-08	4.35E-07	7.528024
RSBN1L	-0.44836	4.957495	-6.75881	5.48E-10	5.94E-09	12.07633
PLCG1	-0.44884	4.931572	-6.78856	4.73E-10	5.17E-09	12.22209
DHX15	-0.44893	7.826314	-3.79912	0.00023	0.000897	-0.47716
TRIM35	-0.44897	4.563648	-5.70844	8.54E-08	6.33E-07	7.134866
GOLGA2P!	-0.44971	3.863702	-8.49729	6.58E-14	1.37E-12	20.97457
CHD4	-0.4498	6.080378	-6.68564	7.89E-10	8.29E-09	11.71912
PPP1R9B	-0.44991	4.904526	-6.13487	1.15E-08	9.83E-08	9.089718
GPBAR1	-0.45	6.153356	-4.47652	1.75E-05	8.54E-05	1.979462
LMAN1	-0.45001	4.469025	-5.1647	9.81E-07	6.04E-06	4.761388
PACS1	-0.45052	4.891361	-7.79028	2.79E-12	4.45E-11	17.27432
TMEM41A	-0.45076	5.122523	-8.95847	5.49E-15	1.41E-13	23.43035
FBXO32	-0.45082	3.327473	-6.25349	6.53E-09	5.83E-08	9.646622
GPBP1	-0.45096	7.230597	-4.15041	6.27E-05	0.000273	0.75933
FLJ45513	-0.45146	4.160016	-5.55637	1.71E-07	1.20E-06	6.456911
CCNB1IP1	-0.45156	5.222174	-4.12209	6.98E-05	0.000301	0.656594
MORC2-A	-0.45158	5.632149	-6.23765	7.05E-09	6.25E-08	9.571959
HERC6	-0.45167	3.243251	-7.42011	1.91E-11	2.64E-10	15.37721

CARKD	-0.45188	6.151983	-4.24887	4.29E-05	0.000193	1.120617
NOD1	-0.45214	4.869957	-8.26909	2.22E-13	4.23E-12	19.77064
GPR114	-0.45232	4.437725	-6.69742	7.44E-10	7.84E-09	11.77653
ABI3	-0.45236	6.398414	-4.32716	3.17E-05	0.000147	1.412297
MAN2B2	-0.45261	5.869506	-3.86301	0.000183	0.000725	-0.25852
GPD1L	-0.45272	5.969362	-5.27784	5.97E-07	3.83E-06	5.243352
MYO9B	-0.45284	6.098576	-8.07307	6.29E-13	1.12E-11	18.74347
L3MBTL2	-0.4529	5.795699	-7.99827	9.35E-13	1.62E-11	18.35335
PARP16	-0.45302	6.196777	-6.40152	3.19E-09	3.03E-08	10.34907
CKAP5	-0.45363	5.200013	-4.89688	3.10E-06	1.73E-05	3.647242
HERC2	-0.45371	3.951707	-2.85033	0.00515	0.014639	-3.36922
FCGRT	-0.4539	7.90044	-4.2406	4.43E-05	0.000199	1.090022
MTMR12	-0.45404	5.012181	-5.98485	2.35E-08	1.90E-07	8.393381
ANKH	-0.45434	4.188565	-5.29559	5.52E-07	3.56E-06	5.319556
HTATSF1	-0.45479	5.129433	-3.93096	0.000143	0.000579	-0.0229
EID2B	-0.45541	2.392761	-4.46674	1.82E-05	8.84E-05	1.941928
SLC25A38	-0.45577	6.405463	-2.93157	0.004046	0.011876	-3.14896
FOXN3	-0.45579	5.749005	-11.0214	6.85E-20	4.14E-18	34.61558
ERAP1	-0.45603	4.512139	-5.98979	2.30E-08	1.86E-07	8.416173
NPRL2	-0.45609	5.219478	-9.03826	3.56E-15	9.49E-14	23.85786
PNRC2	-0.45624	6.845938	-4.25447	4.20E-05	0.00019	1.141351
MRPL1	-0.45635	4.719206	-2.54902	0.012076	0.03071	-4.1384
SRGAP2C	-0.45641	5.7798	-3.17192	0.001927	0.00615	-2.46632
STX17	-0.45665	4.313972	-8.98091	4.86E-15	1.26E-13	23.55051
CREBZF	-0.45705	3.903294	-4.3048	3.45E-05	0.000159	1.328617
ALDH16A1	-0.45742	5.119034	-5.54128	1.83E-07	1.27E-06	6.390202
SSBP4	-0.45762	5.841216	-5.54614	1.79E-07	1.25E-06	6.411681
SCAF11	-0.45771	7.083435	-5.68047	9.71E-08	7.13E-07	7.009394
EIF3F	-0.45775	4.78239	-6.29425	5.37E-09	4.85E-08	9.839241
HERC1	-0.458	3.909536	-7.03461	1.37E-10	1.64E-09	13.4378
NUP62	-0.45852	4.429674	-5.93224	3.01E-08	2.39E-07	8.151336
PARN	-0.45892	6.67492	-6.0936	1.40E-08	1.18E-07	8.89727
C12orf43	-0.45893	4.779836	-5.62098	1.28E-07	9.13E-07	6.743669
RECK	-0.45899	3.095699	-10.0435	1.48E-17	6.00E-16	29.29013
TYW1	-0.459	5.22621	-5.56281	1.66E-07	1.17E-06	6.485374
KLHDC7B	-0.45908	4.447789	-5.81869	5.13E-08	3.94E-07	7.632886
WDR45B	-0.45916	7.5552	-4.29122	3.64E-05	0.000167	1.277922
LY86	-0.45929	7.384273	-2.31419	0.022374	0.052114	-4.68437
FAM214A	-0.45952	5.050981	-5.00017	2.00E-06	1.16E-05	4.072369
RAB11FIP4	-0.4597	4.874899	-5.9432	2.86E-08	2.28E-07	8.201681
DZIP3	-0.4598	3.664034	-5.19979	8.41E-07	5.26E-06	4.910186
CPED1	-0.45991	3.08253	-6.41309	3.01E-09	2.87E-08	10.40431
CCNT1	-0.45997	6.346126	-6.77339	5.10E-10	5.54E-09	12.14771
PWAR5	-0.46001	1.758504	-6.79431	4.59E-10	5.04E-09	12.25024
RPL32	-0.46013	11.48527	-4.60951	1.02E-05	5.19E-05	2.495915
SCAF4	-0.46071	4.620285	-5.94144	2.89E-08	2.30E-07	8.193571
PPP3CC	-0.46108	3.510803	-8.62424	3.33E-14	7.26E-13	21.64778
POLR1B	-0.46142	4.234581	-7.57349	8.64E-12	1.26E-10	16.15945
GOLPH3L	-0.46144	6.064991	-3.36357	0.001036	0.003512	-1.88965
EIF2D	-0.4621	6.340875	-4.90621	2.98E-06	1.67E-05	3.685384
LETM2	-0.46277	3.476922	-5.59709	1.42E-07	1.01E-06	6.637391
MFNG	-0.46292	6.005875	-7.67424	5.12E-12	7.78E-11	16.67628
URGCP	-0.4631	5.24316	-5.50071	2.20E-07	1.51E-06	6.211413
CCDC115	-0.46315	6.82984	-4.53418	1.39E-05	6.90E-05	2.202094
PCBP1	-0.46315	9.561684	-4.83185	4.08E-06	2.23E-05	3.382602
APOL6	-0.46344	4.363741	-5.29775	5.46E-07	3.53E-06	5.32884
CSTF2T	-0.46353	5.344772	-3.36637	0.001026	0.003485	-1.881
RPAP2	-0.46359	4.962124	-6.95689	2.03E-10	2.37E-09	13.05183

INPP5D	-0.46369	6.638167	-6.03036	1.90E-08	1.56E-07	8.603667
ABR	-0.46374	6.002556	-6.94326	2.18E-10	2.52E-09	12.98434
ZNF567	-0.46409	3.601755	-4.18805	5.43E-05	0.000239	0.896702
STK4	-0.46429	6.676549	-6.00736	2.12E-08	1.72E-07	8.497263
EXOSC10	-0.46431	4.364413	-7.18494	6.39E-11	8.08E-10	14.18932
CD72	-0.46446	5.013669	-2.60855	0.01026	0.026652	-3.99248
GEMIN4	-0.46467	4.611798	-5.69805	8.96E-08	6.62E-07	7.088231
DHX57	-0.46478	3.435652	-6.75494	5.59E-10	6.04E-09	12.05738
MALAT1	-0.46499	8.910926	-3.35374	0.00107	0.003615	-1.91992
DNAJB1	-0.46506	6.821105	-5.58346	1.51E-07	1.07E-06	6.576887
MPHOSPH	-0.46523	3.723473	-4.85292	3.73E-06	2.06E-05	3.468096
N4BP2L2-	-0.46531	3.910907	-7.00082	1.63E-10	1.92E-09	13.2698
ELAVL1	-0.46554	4.948078	-7.03933	1.34E-10	1.60E-09	13.46132
PRKY	-0.46559	3.577882	-2.45742	0.015437	0.038003	-4.35704
NVL	-0.46576	4.317657	-4.54064	1.35E-05	6.74E-05	2.227162
YWHAQ	-0.46579	8.446617	-4.16155	6.01E-05	0.000262	0.799897
CRYBG3	-0.46592	4.041702	-3.66408	0.000372	0.00139	-0.92981
TCEAL1	-0.46604	3.570278	-4.07185	8.44E-05	0.000358	0.475617
KIAA0226L	-0.46615	7.144725	-2.54668	0.012153	0.030879	-4.14407
HLA-A	-0.46626	12.61791	-8.02462	8.13E-13	1.42E-11	18.49065
MRPS2	-0.46647	5.069368	-4.4401	2.03E-05	9.74E-05	1.83992
CHN2	-0.46669	3.878079	-5.11866	1.20E-06	7.25E-06	4.567138
C9orf41	-0.46676	2.419663	-6.60724	1.16E-09	1.19E-08	11.33829
EBF1	-0.46682	3.140399	-5.04718	1.63E-06	9.63E-06	4.267792
KIR2DL3	-0.46698	4.412566	-4.66503	8.15E-06	4.21E-05	2.714676
9-Mar	-0.46716	4.315809	-7.16006	7.25E-11	9.09E-10	14.06452
CEP19	-0.46733	6.278959	-2.77662	0.006384	0.017639	-3.56436
ZRSR2	-0.46742	6.208407	-5.45264	2.74E-07	1.85E-06	6.000579
FIP1L1	-0.46764	4.061606	-5.38791	3.66E-07	2.42E-06	5.71843
NCK1	-0.46789	4.466473	-4.96551	2.32E-06	1.32E-05	3.929063
MYO5C	-0.46834	3.939284	-6.16533	9.98E-09	8.59E-08	9.232239
KIR3DL3	-0.46866	4.585097	-4.01822	0.000103	0.000429	0.284284
SPTAN1	-0.46931	4.94364	-8.56648	4.54E-14	9.67E-13	21.34118
CEP192	-0.46933	4.553785	-2.66923	0.008665	0.023031	-3.84064
COG1	-0.46949	3.644947	-8.97594	4.99E-15	1.30E-13	23.52389
MAST3	-0.4697	6.973739	-4.70425	6.94E-06	3.64E-05	2.870257
AKAP7	-0.47016	4.782718	-4.99561	2.04E-06	1.18E-05	4.05348
HAUS3	-0.47022	4.86057	-3.26477	0.001431	0.004697	-2.19046
VNN3	-0.47022	4.320775	-3.0392	0.002918	0.008896	-2.84896
HSPA9	-0.47061	4.609353	-6.6699	8.53E-10	8.89E-09	11.64247
TRAPPC10	-0.47066	6.798117	-4.52922	1.42E-05	7.04E-05	2.182873
ZNF140	-0.47081	4.632021	-2.90205	0.004419	0.012832	-3.22961
PPCDC	-0.47096	5.446697	-3.28613	0.001335	0.004419	-2.12607
PKD2	-0.471	3.601481	-3.56211	0.00053	0.001915	-1.26294
BOD1	-0.47101	5.258185	-3.87225	0.000177	0.000703	-0.22669
TAS2R10	-0.4712	2.208645	-5.80871	5.37E-08	4.11E-07	7.587591
CHIC1	-0.47139	2.901324	-8.58076	4.20E-14	9.02E-13	21.41696
DFFA	-0.47151	5.174608	-7.62238	6.70E-12	9.94E-11	16.40998
ANO9	-0.47157	5.030526	-6.30043	5.21E-09	4.73E-08	9.868512
LINC0009	-0.47169	4.162953	-3.61254	0.000446	0.001635	-1.09911
MPRIP	-0.47185	5.223462	-8.48852	6.89E-14	1.42E-12	20.92818
RBM43	-0.4721	3.842002	-5.1545	1.03E-06	6.29E-06	4.718261
MGC1627	-0.4722	3.82326	-3.72081	0.000305	0.00116	-0.74121
ATF7IP2	-0.47231	4.455205	-5.67487	9.97E-08	7.29E-07	6.984333
TAF1A	-0.47243	2.543044	-4.12346	6.95E-05	0.0003	0.661544
MAK16	-0.47246	5.568248	-3.96755	0.000125	0.000511	0.10528
DCAF7	-0.47265	5.957535	-9.69064	1.02E-16	3.61E-15	27.37546
CDC16	-0.47268	6.723151	-6.20591	8.21E-09	7.20E-08	9.422619

LTV1	-0.47271	3.929692	-4.45219	1.93E-05	9.32E-05	1.886159
MAK	-0.47283	3.816003	-3.4522	0.000771	0.002689	-1.61351
S100A10	-0.47334	7.820506	-2.4996	0.013798	0.034502	-4.25727
POLR2J4	-0.47368	4.777179	-6.54687	1.57E-09	1.56E-08	11.04647
ZBP1	-0.4743	4.35147	-4.85839	3.65E-06	2.02E-05	3.49032
CXCL6	-0.47492	1.81258	-5.72849	7.79E-08	5.80E-07	7.225039
CIR1	-0.47492	5.161269	-4.73302	6.16E-06	3.27E-05	2.984946
RERE	-0.47502	3.919995	-8.93764	6.14E-15	1.57E-13	23.31885
SLC25A43	-0.47529	3.050025	-2.28374	0.024163	0.055634	-4.75167
RRP15	-0.47532	2.813856	-4.53928	1.36E-05	6.77E-05	2.221884
DVL2	-0.47534	4.433175	-9.20754	1.42E-15	4.08E-14	24.76702
PRPF39	-0.47536	4.196228	-3.08123	0.002562	0.007918	-2.7293
SIAH1	-0.47547	3.825575	-7.03045	1.40E-10	1.67E-09	13.41714
DBR1	-0.47565	4.82059	-4.36478	2.73E-05	0.000128	1.553866
CASP8	-0.47572	6.164896	-4.95914	2.38E-06	1.36E-05	3.902814
ZNF574	-0.47575	5.415704	-6.68399	7.95E-10	8.35E-09	11.71108
DNMBP	-0.47616	4.699945	-5.27328	6.09E-07	3.89E-06	5.223803
GLTSCR1L	-0.47619	7.096212	-5.81282	5.27E-08	4.05E-07	7.606255
PIGP	-0.47619	5.751427	-3.78716	0.000241	0.000934	-0.51777
ARSG	-0.47628	4.796152	-5.0652	1.51E-06	8.96E-06	4.342996
NUP133	-0.47669	5.027811	-7.73277	3.77E-12	5.87E-11	16.97759
DLAT	-0.47685	4.719738	-3.92679	0.000145	0.000586	-0.03747
LRRC37A2	-0.47699	5.540957	-4.46646	1.82E-05	8.85E-05	1.94086
NSMAF	-0.47707	4.996374	-6.55599	1.50E-09	1.50E-08	11.09049
AFG3L2	-0.4772	5.019478	-7.24249	4.76E-11	6.19E-10	14.47868
VPS13C	-0.47722	5.169609	-6.10278	1.34E-08	1.13E-07	8.940046
APEX1	-0.47727	7.973405	-3.39228	0.000942	0.003224	-1.80084
FLNA	-0.47805	7.402277	-4.71267	6.70E-06	3.53E-05	2.903772
SETBP1	-0.47809	3.868494	-4.72064	6.48E-06	3.43E-05	2.935521
KLHL22	-0.47816	4.313515	-10.2333	5.21E-18	2.29E-16	30.3226
CKMT2-AS	-0.47816	3.582441	-6.35279	4.04E-09	3.76E-08	10.11694
PABPC3	-0.47819	10.5884	-5.021	1.83E-06	1.07E-05	4.158818
SYNE2	-0.47827	4.348704	-7.89466	1.61E-12	2.67E-11	17.81477
NUDT5	-0.4783	6.386821	-4.25446	4.20E-05	0.00019	1.14129
LOC28616	-0.47841	4.382854	-6.16083	1.02E-08	8.76E-08	9.211152
TIGD2	-0.47849	3.244972	-3.9929	0.000113	0.000468	0.194605
EFTUD1	-0.47866	4.791627	-5.77473	6.29E-08	4.76E-07	7.433701
ABI2	-0.47885	4.285339	-8.6815	2.45E-14	5.50E-13	21.95213
SLA2	-0.47888	5.38427	-4.11646	7.13E-05	0.000307	0.636204
MB21D2	-0.4792	4.895462	-6.09073	1.42E-08	1.20E-07	8.883931
RAD50	-0.47922	3.674539	-6.8937	2.79E-10	3.18E-09	12.73932
MCL1	-0.4794	7.327531	-4.05092	9.13E-05	0.000384	0.400718
GTF3C2	-0.47965	6.105192	-7.71729	4.09E-12	6.33E-11	16.8978
IL11RA	-0.47969	4.607721	-7.21491	5.48E-11	7.02E-10	14.33992
IL12RB1	-0.48006	4.669283	-9.45034	3.79E-16	1.19E-14	26.07571
TAF1	-0.48007	4.791195	-9.72897	8.27E-17	2.97E-15	27.58312
ACSF2	-0.48008	4.678993	-5.6276	1.24E-07	8.89E-07	6.773149
DDX3X	-0.48013	5.799863	-6.35144	4.07E-09	3.78E-08	10.11051
MTERF3	-0.4808	4.822243	-2.82404	0.005562	0.015654	-3.43932
PTMA	-0.48082	8.722005	-4.16746	5.87E-05	0.000257	0.821444
RHOQ	-0.48107	6.973747	-5.79516	5.72E-08	4.36E-07	7.526182
TARBP1	-0.48112	6.347351	-5.96101	2.63E-08	2.11E-07	8.283553
VPREB3	-0.48128	4.750846	-2.62185	0.009889	0.025797	-3.95947
ZNF26	-0.48131	3.429424	-5.0918	1.35E-06	8.07E-06	4.454357
FLJ32255	-0.4814	5.311233	-4.79869	4.69E-06	2.54E-05	3.24854
RPUSD4	-0.48146	5.735325	-5.20202	8.33E-07	5.21E-06	4.919638
IL2RG	-0.48162	9.292317	-4.47711	1.75E-05	8.52E-05	1.981735
PPP1CC	-0.48282	9.017478	-4.32504	3.19E-05	0.000148	1.404373

SPIN3	-0.48291	2.496254	-8.85563	9.57E-15	2.35E-13	22.88037
UPF3A	-0.48348	5.008938	-5.54862	1.77E-07	1.24E-06	6.42263
NUB1	-0.48354	4.175715	-9.04341	3.46E-15	9.24E-14	23.88544
ITPRIPL1	-0.48365	2.907782	-6.11529	1.27E-08	1.07E-07	8.998353
SECISBP2	-0.48403	6.904578	-8.32966	1.61E-13	3.14E-12	20.08939
ZNF101	-0.48404	5.590377	-3.7612	0.000264	0.001017	-0.60557
ZNF419	-0.48418	5.258105	-7.87141	1.82E-12	3.01E-11	17.69419
BMS1P5	-0.48421	3.606645	-7.07828	1.10E-10	1.33E-09	13.65547
CMPK2	-0.48441	6.921191	-2.05291	0.042276	0.088969	-5.23492
CFL2	-0.48443	2.937288	-5.00252	1.98E-06	1.15E-05	4.082119
MUTYH	-0.48443	5.540006	-7.18659	6.34E-11	8.02E-10	14.19765
DANCR	-0.48457	6.192648	-2.91622	0.004236	0.012375	-3.191
ULK3	-0.48488	5.829718	-6.02635	1.93E-08	1.58E-07	8.585093
HNRNPH1	-0.48505	4.758759	-6.1919	8.78E-09	7.66E-08	9.356807
BUB3	-0.48533	5.281312	-6.43281	2.74E-09	2.62E-08	10.49859
DAZAP1	-0.48544	6.76837	-5.02348	1.81E-06	1.06E-05	4.169122
MRPL10	-0.48612	6.580072	-5.43848	2.92E-07	1.96E-06	5.938692
GIMAP2	-0.48665	7.874993	-2.58338	0.010995	0.028311	-4.05455
AKNA	-0.48699	5.464877	-6.67362	8.37E-10	8.75E-09	11.66059
TMEM209	-0.48711	3.602259	-5.45405	2.72E-07	1.84E-06	6.006732
LOC10012	-0.48719	2.608255	-5.79937	5.61E-08	4.28E-07	7.545236
UVRAG	-0.48729	5.467409	-5.6664	1.04E-07	7.55E-07	6.94641
IKZF2	-0.48748	3.44149	-8.6467	2.95E-14	6.54E-13	21.76711
TSPAN13	-0.4876	5.456535	-2.72417	0.007419	0.020118	-3.7005
CHAMP1	-0.48781	5.690877	-5.44924	2.78E-07	1.87E-06	5.985707
NARS	-0.48781	8.033212	-4.55794	1.26E-05	6.30E-05	2.294403
KIAA1377	-0.48786	2.092952	-11.4734	5.73E-21	4.17E-19	37.07669
CCAR1	-0.48822	5.669135	-3.98967	0.000115	0.000473	0.183195
TMEM128	-0.48838	5.06859	-2.76338	0.006632	0.018242	-3.59895
ERP29	-0.48865	8.002215	-4.81249	4.42E-06	2.41E-05	3.304261
PIK3R5	-0.48889	6.014142	-7.80371	2.60E-12	4.16E-11	17.34374
KBTBD2	-0.48889	6.324392	-3.70661	0.00032	0.001214	-0.78864
TBCCD1	-0.48932	3.914703	-4.27914	3.82E-05	0.000174	1.232926
CHD6	-0.48937	3.201928	-9.78852	5.97E-17	2.20E-15	27.90595
MDS2	-0.48944	3.666015	-9.59937	1.68E-16	5.67E-15	26.88132
TFCP2	-0.48946	5.323753	-6.46459	2.34E-09	2.26E-08	10.65078
NAA25	-0.48947	4.660956	-5.13068	1.14E-06	6.91E-06	4.617751
ATP6V1B2	-0.48959	9.542575	-5.00729	1.94E-06	1.13E-05	4.101881
MAN2A1	-0.48959	6.481785	-4.37329	2.64E-05	0.000124	1.585989
CELF2	-0.48961	8.011214	-5.05751	1.56E-06	9.24E-06	4.310884
MCCC2	-0.4897	3.73728	-9.14438	2.00E-15	5.56E-14	24.42746
SOCS7	-0.48971	4.363216	-7.94954	1.21E-12	2.06E-11	18.09977
SEC24B	-0.49031	7.108805	-3.67502	0.000358	0.001341	-0.89361
TULP4	-0.49037	3.031815	-7.81658	2.43E-12	3.91E-11	17.41028
LOC28605	-0.49062	5.701239	-2.87645	0.004768	0.013711	-3.299
RPA2	-0.49088	6.961932	-4.26513	4.03E-05	0.000182	1.180889
ACSL5	-0.49098	6.114527	-4.08103	8.16E-05	0.000347	0.508562
ZNF665	-0.49112	4.308222	-5.1518	1.04E-06	6.35E-06	4.706846
PLD4	-0.4915	3.659922	-7.65319	5.71E-12	8.61E-11	16.5681
ELF2	-0.49221	6.399062	-6.17004	9.75E-09	8.41E-08	9.254307
IFI6	-0.49247	5.266238	-2.98441	0.00345	0.010297	-3.00285
NOA1	-0.4927	6.731329	-6.07459	1.54E-08	1.28E-07	8.808858
GPALPP1	-0.49281	4.598768	-4.45198	1.93E-05	9.33E-05	1.885329
HOOK1	-0.49298	2.70415	-6.0769	1.52E-08	1.27E-07	8.819573
ZNF805	-0.49299	3.765867	-5.8854	3.75E-08	2.94E-07	7.936824
KIR2DL1	-0.4932	5.623184	-5.17357	9.43E-07	5.84E-06	4.798946
RFC1	-0.4933	5.779214	-5.95203	2.75E-08	2.20E-07	8.24222
KLHL21	-0.49334	6.724439	-5.60981	1.34E-07	9.56E-07	6.693939

RIPK1	-0.4936	6.173276	-7.83114	2.25E-12	3.65E-11	17.48561
IK	-0.49364	7.718127	-5.90454	3.43E-08	2.71E-07	8.024376
PRO0471	-0.49415	2.08246	-4.78013	5.06E-06	2.73E-05	3.173802
NFKBIE	-0.49424	5.250019	-5.47939	2.43E-07	1.65E-06	6.117756
LARS2	-0.49441	4.572717	-6.70469	7.18E-10	7.60E-09	11.81193
HP1BP3	-0.49459	7.237579	-7.1115	9.29E-11	1.14E-09	13.82142
EPRS	-0.49474	6.031729	-4.55661	1.27E-05	6.34E-05	2.289247
AK5	-0.49505	4.05629	-7.36442	2.55E-11	3.47E-10	15.09464
SLC9A7	-0.49508	2.886732	-7.8975	1.59E-12	2.64E-11	17.82949
LOC10192	-0.4952	3.213765	-3.73687	0.000288	0.001101	-0.6874
AGAP1	-0.49522	2.844985	-9.01271	4.09E-15	1.07E-13	23.72086
NIPBL	-0.49535	6.478625	-6.15397	1.05E-08	9.01E-08	9.179046
C2orf40	-0.49594	2.41958	-6.53133	1.69E-09	1.68E-08	10.97156
OAZ2	-0.49595	8.089621	-6.31469	4.86E-09	4.46E-08	9.936044
PGAP3	-0.49636	5.055151	-9.36935	5.89E-16	1.79E-14	25.63862
NFX1	-0.49664	4.623155	-6.6331	1.02E-09	1.05E-08	11.46368
PHF20	-0.49712	4.965238	-10.9416	1.06E-19	6.11E-18	34.18067
TMEM184I	-0.49739	6.142114	-5.35457	4.25E-07	2.79E-06	5.573885
DNAJC24	-0.49741	3.890683	-6.51011	1.88E-09	1.85E-08	10.86939
CMTR2	-0.49758	5.504897	-2.64862	0.00918	0.024172	-3.89255
CDK4	-0.49758	6.196054	-4.40726	2.31E-05	0.00011	1.714742
MOB3A	-0.49795	7.399831	-6.98869	1.73E-10	2.04E-09	13.20957
WDR3	-0.49837	3.196855	-5.8706	4.02E-08	3.14E-07	7.869221
PROCR	-0.4985	1.876468	-7.63368	6.32E-12	9.44E-11	16.46793
PDE7A	-0.49859	4.928862	-6.34885	4.12E-09	3.82E-08	10.09822
TCERG1	-0.49867	3.922151	-4.37876	2.58E-05	0.000122	1.606678
OPTN	-0.49875	7.00834	-2.34962	0.020442	0.048289	-4.60508
SNPH	-0.49883	4.099843	-6.28428	5.63E-09	5.08E-08	9.792074
ASH1L	-0.49912	5.903604	-7.70281	4.41E-12	6.78E-11	16.82326
OTUD3	-0.49923	3.995853	-5.27968	5.92E-07	3.80E-06	5.251243
DNPEP	-0.49944	4.724193	-7.41729	1.94E-11	2.68E-10	15.36288
RPRD2	-0.49946	4.872802	-8.91285	7.02E-15	1.77E-13	23.18623
IST1	-0.4997	5.973217	-7.73374	3.75E-12	5.85E-11	16.98255
ZNF382	-0.5	2.378185	-6.9288	2.34E-10	2.69E-09	12.91278
SIN3B	-0.50011	4.689097	-8.48018	7.21E-14	1.48E-12	20.88404
MTERF4	-0.50016	5.019505	-6.29352	5.38E-09	4.87E-08	9.83581
LINC00847	-0.50035	5.331387	-5.22334	7.59E-07	4.78E-06	5.010363
TARDBP	-0.50062	7.03867	-4.81242	4.42E-06	2.41E-05	3.303996
VEZT	-0.50081	2.955976	-4.57636	1.17E-05	5.87E-05	2.366201
TCEAL4	-0.50105	4.240598	-3.29207	0.00131	0.004343	-2.10809
MAGEE1	-0.50126	4.145324	-8.8082	1.24E-14	3.00E-13	22.62716
EDC4	-0.50152	5.709413	-7.46111	1.55E-11	2.18E-10	15.58577
PITPNB	-0.50155	7.614832	-10.1049	1.05E-17	4.39E-16	29.62399
KIR2DS5	-0.50187	5.511798	-4.91854	2.83E-06	1.59E-05	3.735895
TRA2A	-0.502	6.749412	-5.65685	1.08E-07	7.85E-07	6.903681
MMS19	-0.50212	6.212941	-6.02073	1.99E-08	1.62E-07	8.559091
CHTF8	-0.50222	7.03903	-6.34977	4.10E-09	3.81E-08	10.10257
STAT2	-0.50226	6.020052	-9.60821	1.60E-16	5.44E-15	26.92917
PPAP2A	-0.50229	4.076961	-5.99711	2.22E-08	1.80E-07	8.449947
TEX10	-0.50256	4.389813	-5.65841	1.07E-07	7.79E-07	6.910657
HIC2	-0.50271	4.432403	-7.67543	5.08E-12	7.74E-11	16.68238
RPL38	-0.50299	8.989503	-6.04967	1.73E-08	1.43E-07	8.693157
ZKSCAN4	-0.50304	4.991443	-5.73104	7.69E-08	5.74E-07	7.236535
ZNF200	-0.50325	5.92522	-4.30317	3.48E-05	0.00016	1.322518
ASXL1	-0.50332	5.599728	-8.247	2.50E-13	4.73E-12	19.65455
FAM13A-A	-0.50333	5.721102	-5.00864	1.93E-06	1.12E-05	4.107498
ARF6	-0.5034	7.63548	-6.02476	1.95E-08	1.59E-07	8.577757
PCED1B-A	-0.50367	5.741549	-4.0111	0.000106	0.000439	0.259019

LOC10099	-0.50404	5.85909	-6.83266	3.79E-10	4.24E-09	12.43862
PHYH	-0.50443	4.147392	-3.63797	0.000408	0.001508	-1.01583
CD22	-0.50457	4.420475	-5.06967	1.48E-06	8.80E-06	4.361684
SRSF11	-0.50497	5.511761	-4.30448	3.46E-05	0.000159	1.327401
LOC44152	-0.505	3.21919	-6.67101	8.48E-10	8.85E-09	11.64791
CAPN7	-0.50509	4.945577	-3.63249	0.000416	0.001534	-1.03381
ESF1	-0.50532	3.513398	-4.40431	2.34E-05	0.000111	1.703565
HSD17B8	-0.50562	5.497769	-5.64297	1.15E-07	8.32E-07	6.84168
PDCD2L	-0.50568	3.679489	-5.45167	2.75E-07	1.86E-06	5.99634
ZBED3	-0.50595	3.698678	-6.81525	4.14E-10	4.58E-09	12.35303
EHBP1	-0.5062	3.635171	-6.16692	9.90E-09	8.53E-08	9.239652
TNFRSF14	-0.50632	6.406198	-6.81368	4.17E-10	4.61E-09	12.34533
ARHGAP2!	-0.50634	6.009588	-8.53969	5.24E-14	1.11E-12	21.19914
ZNF550	-0.50647	3.514493	-7.02614	1.43E-10	1.70E-09	13.39568
DDX26B	-0.50653	5.321789	-3.80583	0.000225	0.000877	-0.45434
DCP1B	-0.50704	4.79164	-4.05735	8.92E-05	0.000376	0.423693
BCL2	-0.50722	4.42099	-6.95719	2.03E-10	2.37E-09	13.05333
NINJ1	-0.50746	7.929561	-4.76955	5.29E-06	2.84E-05	3.13127
TASP1	-0.50769	2.687331	-6.70382	7.21E-10	7.63E-09	11.80772
TAF6	-0.50808	5.550541	-5.17081	9.55E-07	5.89E-06	4.787261
ANKRA2	-0.50858	5.803696	-3.16582	0.001965	0.006256	-2.48424
AGO2	-0.50877	7.785385	-4.59356	1.09E-05	5.50E-05	2.433452
FAM43A	-0.50878	5.625873	-8.21219	3.01E-13	5.59E-12	19.47183
SOX8	-0.50906	2.707148	-5.05692	1.57E-06	9.26E-06	4.308398
C2orf43	-0.50908	4.674887	-6.06509	1.61E-08	1.33E-07	8.764683
XPO5	-0.50916	4.584361	-9.40093	4.96E-16	1.52E-14	25.80902
SMURF2	-0.50922	4.654106	-5.08115	1.41E-06	8.42E-06	4.409713
ERMP1	-0.50928	4.740105	-7.18285	6.46E-11	8.15E-10	14.17886
RIC8B	-0.50935	3.723006	-8.3109	1.78E-13	3.44E-12	19.99063
SP110	-0.50943	8.248527	-5.35436	4.25E-07	2.79E-06	5.57297
CHRAC1	-0.50951	5.848484	-6.34301	4.24E-09	3.92E-08	10.07044
ENPP5	-0.50968	1.75644	-8.75399	1.66E-14	3.89E-13	22.33808
UCP2	-0.50981	9.480555	-4.29675	3.56E-05	0.000163	1.298532
CYLD	-0.51009	5.716668	-6.53371	1.67E-09	1.66E-08	10.98304
KARS	-0.51035	8.796264	-5.54782	1.78E-07	1.24E-06	6.41907
NOC3L	-0.51082	5.054127	-2.76572	0.006587	0.018131	-3.59284
EIF1AX	-0.51087	6.09379	-3.23279	0.001587	0.005162	-2.28623
POLR1C	-0.51101	4.934824	-5.88852	3.70E-08	2.90E-07	7.951082
NGDN	-0.51105	4.801877	-6.14303	1.11E-08	9.48E-08	9.127878
RAD51-AS	-0.51167	4.552935	-5.04501	1.65E-06	9.70E-06	4.258748
CDKN2AIP	-0.51189	5.262558	-4.12622	6.87E-05	0.000297	0.671531
EPHB6	-0.5119	4.02403	-5.03547	1.72E-06	1.01E-05	4.218999
INO80	-0.512	5.797497	-7.58392	8.19E-12	1.20E-10	16.21282
PDPR	-0.51212	5.248335	-6.50117	1.96E-09	1.92E-08	10.8264
ANKRD44	-0.51224	5.883509	-5.98545	2.35E-08	1.90E-07	8.396117
CAMK2G	-0.51224	5.944352	-5.73136	7.68E-08	5.73E-07	7.237991
CECR6	-0.51259	4.983658	-4.61216	1.01E-05	5.14E-05	2.506329
RBM15B	-0.51261	5.326263	-9.2709	1.01E-15	2.97E-14	25.10807
TIMM10B	-0.51262	4.824548	-5.72326	7.98E-08	5.94E-07	7.201521
ZNF512	-0.51291	6.895967	-6.96509	1.95E-10	2.29E-09	13.0925
CCDC6	-0.5131	4.985228	-4.39194	2.45E-05	0.000116	1.65661
RAB30	-0.51375	3.889099	-5.73664	7.50E-08	5.60E-07	7.261744
SIN3A	-0.51375	3.876958	-9.28523	9.31E-16	2.76E-14	25.18522
CMTM8	-0.5138	3.344973	-3.34722	0.001093	0.00369	-1.93995
KLF3-AS1	-0.51393	3.790922	-7.48235	1.39E-11	1.96E-10	15.69393
CRLF3	-0.514	9.151034	-5.47356	2.49E-07	1.70E-06	6.092171
ARAP3	-0.51405	5.764219	-6.72497	6.49E-10	6.93E-09	11.91093
SRSF8	-0.51435	6.103002	-3.55622	0.000541	0.001951	-1.28194

ZNF212	-0.51477	5.829415	-8.23011	2.74E-13	5.14E-12	19.56586
CAPRIN2	-0.51488	4.894698	-7.5167	1.16E-11	1.66E-10	15.86919
G3BP1	-0.51527	5.120338	-8.43533	9.16E-14	1.86E-12	20.64688
TMEM203	-0.51576	6.982338	-5.24216	6.98E-07	4.42E-06	5.090651
CDC37L1	-0.51581	3.543927	-5.39506	3.54E-07	2.35E-06	5.749495
UPF1	-0.51635	5.85859	-8.75338	1.66E-14	3.90E-13	22.33483
TRAF3	-0.51672	5.094548	-7.39059	2.23E-11	3.06E-10	15.22731
KLHL26	-0.51682	4.492435	-6.22037	7.66E-09	6.74E-08	9.490623
KANSL2	-0.51683	6.018039	-4.38816	2.49E-05	0.000118	1.642268
PPP1R13B	-0.51703	4.352422	-5.83996	4.64E-08	3.59E-07	7.729606
THNSL1	-0.51724	2.654467	-7.34023	2.88E-11	3.89E-10	14.97211
KIF5C	-0.51727	4.176335	-8.26913	2.22E-13	4.23E-12	19.77087
COLQ	-0.51737	4.157159	-5.60995	1.34E-07	9.56E-07	6.694554
DNAAF2	-0.51781	3.879445	-5.08287	1.40E-06	8.36E-06	4.416928
ADCY9	-0.51783	3.75637	-8.25115	2.45E-13	4.63E-12	19.67636
ATP10D	-0.51832	5.410022	-3.15023	0.002064	0.006537	-2.52982
PRR12	-0.51843	4.808766	-7.30503	3.46E-11	4.59E-10	14.79414
FKTN	-0.51862	4.173857	-4.95214	2.45E-06	1.39E-05	3.873955
TPP2	-0.51865	4.065975	-7.40175	2.10E-11	2.90E-10	15.28398
PTPLB	-0.51868	5.185346	-4.63905	9.07E-06	4.64E-05	2.61208
KDM1A	-0.51876	5.921548	-7.25744	4.41E-11	5.76E-10	14.55402
ABCC1	-0.51892	5.682291	-6.16519	9.98E-09	8.59E-08	9.231585
TMX2	-0.51896	7.073987	-5.11533	1.22E-06	7.35E-06	4.553136
SLC44A2	-0.51928	7.345901	-5.64125	1.16E-07	8.37E-07	6.834041
CTSS	-0.51935	9.633038	-4.47376	1.77E-05	8.62E-05	1.968856
ERGIC1	-0.51941	4.808807	-4.10251	7.52E-05	0.000322	0.585851
ADAM28	-0.51959	3.805341	-4.95693	2.40E-06	1.37E-05	3.893684
TDRD3	-0.51966	3.165639	-9.10104	2.53E-15	6.92E-14	24.19467
NUP205	-0.51999	4.57651	-6.52557	1.74E-09	1.72E-08	10.94381
KDM4C	-0.52004	4.919423	-6.298	5.27E-09	4.78E-08	9.856999
BAG5	-0.52014	5.159673	-6.33691	4.36E-09	4.03E-08	10.04148
PALB2	-0.52031	4.66289	-5.35022	4.33E-07	2.84E-06	5.555087
EID2	-0.5205	4.853687	-3.92985	0.000143	0.00058	-0.0268
SLC7A7	-0.52053	8.466288	-2.28226	0.024253	0.055809	-4.75491
RPUSD2	-0.52086	5.003127	-6.18183	9.22E-09	7.97E-08	9.309552
ZNF573	-0.521	3.014268	-5.81201	5.29E-08	4.06E-07	7.6026
USP53	-0.52119	2.535336	-6.34919	4.11E-09	3.82E-08	10.09983
RELL1	-0.52119	5.237647	-5.84094	4.62E-08	3.58E-07	7.734057
SH2D3C	-0.52155	6.379525	-5.14331	1.08E-06	6.57E-06	4.671025
PATZ1	-0.52156	4.158556	-12.3544	4.64E-23	4.90E-21	41.85404
ZSCAN26	-0.52182	4.620398	-5.68091	9.69E-08	7.11E-07	7.011369
LOC33862	-0.522	2.54129	-6.23568	7.12E-09	6.30E-08	9.562671
UTP3	-0.52222	6.089604	-3.67855	0.000354	0.001327	-0.88192
CCNL2	-0.52271	5.716494	-8.5341	5.40E-14	1.14E-12	21.16956
ATXN7	-0.52276	6.888971	-6.61291	1.13E-09	1.16E-08	11.36579
RPS3	-0.52292	11.16494	-5.53808	1.86E-07	1.29E-06	6.376043
IPO5	-0.52301	4.812344	-6.30015	5.21E-09	4.73E-08	9.86719
LTBP3	-0.52304	5.222757	-8.21399	2.98E-13	5.55E-12	19.48124
LSG1	-0.52324	5.147395	-4.99801	2.02E-06	1.17E-05	4.063411
NUDT9	-0.5237	4.865362	-4.20565	5.07E-05	0.000225	0.961252
CCT6A	-0.52377	6.113783	-4.03589	9.66E-05	0.000404	0.347095
MTERF2	-0.52379	2.992198	-5.10118	1.29E-06	7.78E-06	4.493683
FYCO1	-0.52384	4.820349	-8.60818	3.63E-14	7.86E-13	21.56246
HLA-C	-0.52392	11.86866	-7.76105	3.25E-12	5.14E-11	17.1234
KIR3DS1	-0.52401	2.973033	-4.81249	4.42E-06	2.41E-05	3.304258
TTC27	-0.52426	4.114664	-6.18917	8.90E-09	7.75E-08	9.344012
SND1-IT1	-0.52429	5.312735	-5.19188	8.71E-07	5.43E-06	4.876574
LOC10050	-0.52452	4.637667	-5.06316	1.52E-06	9.03E-06	4.334469

ZNF202	-0.52454	3.799921	-7.64121	6.08E-12	9.12E-11	16.5066
SNX9	-0.52592	5.166106	-4.47433	1.77E-05	8.61E-05	1.97105
SESN1	-0.52594	5.504747	-4.94166	2.56E-06	1.45E-05	3.83083
HSPH1	-0.52616	4.887342	-3.86536	0.000181	0.000719	-0.25043
TNFRSF1B	-0.52649	8.815688	-5.28573	5.76E-07	3.70E-06	5.277227
CIB1	-0.52718	7.520925	-4.20839	5.02E-05	0.000223	0.971319
ZBTB9	-0.52729	5.073924	-7.32198	3.17E-11	4.23E-10	14.8798
DUSP10	-0.5274	4.424111	-7.2158	5.46E-11	7.00E-10	14.34439
MIR1244-3	-0.52919	8.153857	-4.6552	8.49E-06	4.37E-05	2.675791
ERN1	-0.52932	5.317965	-7.60208	7.45E-12	1.10E-10	16.30587
SLC41A3	-0.52935	5.505463	-8.86897	8.90E-15	2.20E-13	22.95165
SAFB2	-0.52941	5.970519	-7.86628	1.87E-12	3.08E-11	17.66757
LOC10192	-0.52956	4.967914	-6.20336	8.31E-09	7.28E-08	9.41061
GORAB	-0.52976	3.43204	-2.82807	0.005497	0.015486	-3.42862
RBM22	-0.52988	7.344651	-6.55534	1.50E-09	1.50E-08	11.08737
ADCK3	-0.52989	4.701792	-7.76003	3.27E-12	5.15E-11	17.11816
MAPKAPK	-0.53031	4.487647	-4.04546	9.32E-05	0.000391	0.381227
RPL12	-0.53064	10.6066	-5.16715	9.70E-07	5.98E-06	4.771742
BBS10	-0.53066	3.037805	-3.45371	0.000767	0.002676	-1.60875
RNF213	-0.53102	5.829349	-7.26679	4.21E-11	5.52E-10	14.60112
P2RX7	-0.53123	4.483998	-3.74043	0.000284	0.001089	-0.67546
TRIM24	-0.53179	3.58571	-8.86244	9.22E-15	2.27E-13	22.91675
ANKMY2	-0.53191	5.290322	-4.97595	2.22E-06	1.27E-05	3.972153
WDR73	-0.53206	5.657157	-7.12615	8.62E-11	1.07E-09	13.89469
ADAR	-0.53218	9.103948	-7.16569	7.05E-11	8.85E-10	14.09277
DCUN1D4	-0.53238	3.369731	-5.87822	3.88E-08	3.04E-07	7.904019
NT5DC1	-0.53239	3.698255	-8.05796	6.82E-13	1.20E-11	18.66456
SLC18B1	-0.53253	5.489418	-3.15024	0.002064	0.006537	-2.52978
MIS12	-0.53273	6.012975	-4.13516	6.64E-05	0.000287	0.703942
APOL1	-0.53276	5.415306	-8.34723	1.47E-13	2.89E-12	20.182
NFATC2IP	-0.53294	6.044829	-6.34348	4.23E-09	3.91E-08	10.07268
DHRS4-AS	-0.53295	3.363143	-7.23261	5.01E-11	6.49E-10	14.42896
KIF2A	-0.53298	5.510452	-4.3468	2.93E-05	0.000137	1.486117
TSC22D3	-0.53301	6.796713	-4.58285	1.14E-05	5.73E-05	2.391571
RPL6	-0.53303	9.964347	-3.94352	0.000136	0.000554	0.020969
ZSCAN29	-0.53332	5.644931	-5.78216	6.07E-08	4.61E-07	7.467317
CHD3	-0.5334	5.860896	-8.01778	8.43E-13	1.47E-11	18.45502
NSUN2	-0.53341	6.491581	-6.05689	1.67E-08	1.39E-07	8.726646
SCML1	-0.53342	3.812	-3.49562	0.000666	0.002354	-1.47608
LOC37444	-0.5337	3.717131	-6.12234	1.23E-08	1.04E-07	9.031234
ZNF675	-0.53412	1.803813	-5.95808	2.67E-08	2.14E-07	8.270085
SMARCC2	-0.53424	5.150411	-8.38923	1.17E-13	2.34E-12	20.40345
ARHGAP5	-0.53426	2.54916	-6.81744	4.09E-10	4.53E-09	12.36383
PFN2	-0.5345	4.109148	-5.1902	8.77E-07	5.47E-06	4.869436
CNST	-0.53454	4.668399	-6.84339	3.59E-10	4.02E-09	12.49137
SUPV3L1	-0.53475	4.24504	-5.37779	3.83E-07	2.53E-06	5.674508
DDX19A	-0.53546	3.838301	-8.83759	1.05E-14	2.58E-13	22.78405
PRR5	-0.53563	5.988567	-3.67799	0.000354	0.001329	-0.88377
C11orf80	-0.53574	4.066463	-4.45625	1.90E-05	9.19E-05	1.901691
ZNF117	-0.53581	3.849619	-3.18752	0.001834	0.005889	-2.42046
MICAL2	-0.53692	6.683504	-4.16985	5.82E-05	0.000255	0.830145
AKT3	-0.53707	3.444972	-9.83441	4.64E-17	1.74E-15	28.1548
FN3KRP	-0.53708	5.310422	-5.09202	1.35E-06	8.07E-06	4.455256
BTN2A2	-0.5373	3.199844	-11.5095	4.70E-21	3.51E-19	37.27306
NDUFAF4	-0.53752	3.985604	-5.34306	4.47E-07	2.92E-06	5.524111
TTC16	-0.53797	3.499851	-4.72194	6.45E-06	3.41E-05	2.940727
FRY	-0.5381	4.032991	-8.71582	2.03E-14	4.70E-13	22.1348
UTP23	-0.53821	3.942935	-5.50375	2.17E-07	1.50E-06	6.224784

ERCC1	-0.5384	5.320478	-5.58922	1.47E-07	1.04E-06	6.60247
ZMYND11	-0.53898	4.093854	-8.89825	7.60E-15	1.90E-13	23.10813
FAM3C	-0.53914	3.17211	-4.46503	1.83E-05	8.89E-05	1.935355
USP24	-0.53952	5.146774	-8.40466	1.08E-13	2.17E-12	20.48489
BRPF1	-0.53961	4.821366	-6.94557	2.15E-10	2.49E-09	12.99574
LOC10013	-0.54088	3.872258	-5.74526	7.20E-08	5.40E-07	7.300625
DIDO1	-0.54098	5.2755	-9.11258	2.38E-15	6.53E-14	24.25665
CLK2	-0.54099	6.759367	-8.14298	4.35E-13	7.88E-12	19.10902
REPS1	-0.54102	4.309035	-8.00813	8.87E-13	1.54E-11	18.4047
CEP63	-0.54113	4.703881	-6.12788	1.19E-08	1.02E-07	9.057098
TIMM23B	-0.54136	4.156883	-4.28456	3.74E-05	0.000171	1.253092
ASCC2	-0.54138	8.185145	-2.6358	0.009513	0.024957	-3.92466
FEZ1	-0.54182	2.917647	-7.09922	9.89E-11	1.21E-09	13.76003
ZNF217	-0.54196	8.233663	-5.25232	6.68E-07	4.24E-06	5.134083
IFT80	-0.54244	3.131178	-6.16004	1.02E-08	8.79E-08	9.207464
CMTR1	-0.54264	5.808323	-6.07718	1.52E-08	1.27E-07	8.820861
CSF2RB	-0.5429	10.38098	-4.34353	2.97E-05	0.000138	1.473792
GTF3C1	-0.5431	6.13627	-9.53346	2.41E-16	7.85E-15	26.52486
ZXDB	-0.54319	2.42672	-7.71753	4.08E-12	6.32E-11	16.89902
ACOT4	-0.54341	3.059373	-6.63501	1.01E-09	1.04E-08	11.47296
BZRAP1	-0.5435	4.738371	-6.85279	3.43E-10	3.85E-09	12.53766
PRIMPOL	-0.54393	5.110818	-4.86737	3.51E-06	1.94E-05	3.526868
MEPCE	-0.54419	7.125934	-7.79133	2.77E-12	4.43E-11	17.27979
NFATC1	-0.54436	5.120121	-9.81417	5.19E-17	1.93E-15	28.04502
SARS	-0.54463	5.361786	-5.33719	4.59E-07	2.99E-06	5.49877
TAF4	-0.54475	4.637561	-7.73604	3.71E-12	5.78E-11	16.99444
PARP11	-0.54483	3.821469	-7.70844	4.28E-12	6.59E-11	16.85225
HLA-DOB	-0.54495	4.506771	-5.59504	1.44E-07	1.02E-06	6.628292
GLS	-0.54514	4.53283	-9.06197	3.13E-15	8.40E-14	23.98502
PSAP	-0.54531	10.657	-5.77133	6.39E-08	4.83E-07	7.418336
NPCDR1	-0.54601	3.834782	-5.01446	1.88E-06	1.09E-05	4.131638
DOK2	-0.54624	7.32182	-4.31198	3.36E-05	0.000155	1.355469
CDKN1C	-0.54627	4.794085	-2.8648	0.004935	0.01412	-3.33038
SLC43A2	-0.54659	5.324596	-6.59051	1.26E-09	1.28E-08	11.25733
CDKN1B	-0.54678	9.128522	-4.50421	1.57E-05	7.71E-05	2.086118
MTPAP	-0.54689	5.112513	-5.74144	7.33E-08	5.49E-07	7.283389
RBL2	-0.5469	7.856644	-6.19332	8.72E-09	7.61E-08	9.363471
ANKRD17	-0.54726	6.189607	-6.51003	1.88E-09	1.85E-08	10.86901
MARS2	-0.54741	3.63718	-6.88198	2.96E-10	3.36E-09	12.68149
ADNP2	-0.5475	5.76893	-7.69487	4.59E-12	7.05E-11	16.78236
SNX29P2	-0.54762	3.690755	-5.73287	7.63E-08	5.70E-07	7.244765
AKAP1	-0.54778	4.262583	-7.20886	5.66E-11	7.23E-10	14.30948
TBRG4	-0.54784	3.556946	-5.84427	4.55E-08	3.52E-07	7.749199
ZNF324	-0.54787	6.041294	-8.27958	2.10E-13	4.03E-12	19.82583
NUDT13	-0.54792	2.760264	-5.79951	5.60E-08	4.28E-07	7.545892
YME1L1	-0.54798	5.837676	-9.83606	4.60E-17	1.73E-15	28.16378
SRP68	-0.54872	7.058285	-5.83848	4.67E-08	3.61E-07	7.722874
NAA16	-0.54936	5.650301	-4.47386	1.77E-05	8.62E-05	1.969263
PTGDS	-0.54973	5.542614	-7.36777	2.50E-11	3.41E-10	15.11159
HNRNPU	-0.54978	5.544148	-9.59548	1.72E-16	5.78E-15	26.86025
ZNF823	-0.55053	2.168052	-5.76622	6.54E-08	4.93E-07	7.395204
TCTN3	-0.55075	5.156214	-7.93988	1.27E-12	2.16E-11	18.04957
PIGX	-0.55076	4.396449	-6.9362	2.25E-10	2.60E-09	12.94936
ZMIZ1	-0.551	5.114984	-8.69683	2.25E-14	5.13E-13	22.0337
ZCCHC6	-0.55115	7.126005	-4.63696	9.15E-06	4.67E-05	2.603838
PNISR	-0.55126	7.261348	-5.36223	4.10E-07	2.70E-06	5.607041
LOC38976	-0.5513	3.605664	-4.25357	4.22E-05	0.00019	1.138002
RCC2	-0.5516	6.708116	-5.53643	1.87E-07	1.30E-06	6.368779

C1orf174	-0.55164	4.964096	-5.23937	7.07E-07	4.47E-06	5.078763
PRPF4B	-0.55179	6.225927	-4.39903	2.38E-05	0.000113	1.683498
PAN2	-0.55218	5.047674	-6.12558	1.21E-08	1.03E-07	9.046359
HDC	-0.55286	4.29806	-6.31046	4.96E-09	4.53E-08	9.916019
KIR2DS4	-0.5529	4.245797	-6.5681	1.41E-09	1.42E-08	11.14897
RBM4B	-0.55312	5.242383	-6.73372	6.21E-10	6.67E-09	11.95366
DTX3L	-0.5537	7.961129	-7.12267	8.77E-11	1.08E-09	13.87727
NPM1	-0.55379	7.776061	-4.02633	0.0001	0.000417	0.313076
MYH9	-0.55408	7.989813	-4.70825	6.82E-06	3.59E-05	2.886189
GPATCH8	-0.55427	6.166147	-9.41358	4.63E-16	1.43E-14	25.8773
MDC1	-0.55428	4.297974	-6.56433	1.44E-09	1.44E-08	11.13077
ARMC1	-0.5544	6.642771	-4.29406	3.60E-05	0.000165	1.288497
SMARCA5	-0.55442	5.002125	-4.13183	6.73E-05	0.000291	0.691857
ATP6V1A	-0.55451	8.176457	-4.43926	2.03E-05	9.76E-05	1.836695
DIS3L	-0.55452	3.754656	-4.91447	2.88E-06	1.62E-05	3.719224
PRRC2B	-0.55455	5.89781	-10.9285	1.14E-19	6.51E-18	34.10959
CCND2	-0.55465	4.970837	-4.66178	8.26E-06	4.26E-05	2.701823
SCAMP1-/	-0.55474	4.650108	-6.15459	1.05E-08	8.99E-08	9.181945
KIF21A	-0.55497	1.86609	-8.32596	1.64E-13	3.20E-12	20.06993
CHD2	-0.55535	5.068871	-7.907	1.51E-12	2.52E-11	17.87879
PUM1	-0.55547	7.341262	-7.90648	1.52E-12	2.53E-11	17.87606
MPHOSPH	-0.55573	5.235343	-4.72333	6.41E-06	3.39E-05	2.946262
PWP1	-0.55598	6.034159	-5.89407	3.60E-08	2.84E-07	7.976456
NUCKS1	-0.55598	5.312613	-6.48762	2.09E-09	2.04E-08	10.7613
IL12RB2	-0.55604	2.430863	-7.79084	2.78E-12	4.44E-11	17.27724
FNBP1	-0.55605	7.366353	-5.95895	2.66E-08	2.13E-07	8.274088
PBX4	-0.55612	4.668441	-5.48458	2.37E-07	1.62E-06	6.140556
SMYD4	-0.55613	5.405339	-7.63391	6.31E-12	9.44E-11	16.4691
MS4A7	-0.55722	6.891748	-2.33482	0.021231	0.049882	-4.63834
QRSL1	-0.55751	4.235788	-5.68327	9.59E-08	7.04E-07	7.021944
ZNF337	-0.55758	5.574705	-7.35244	2.71E-11	3.67E-10	15.03395
OGFOD1	-0.55771	4.796393	-8.58918	4.02E-14	8.64E-13	21.46165
SNN	-0.5578	6.617483	-5.3589	4.16E-07	2.74E-06	5.592642
IGSF8	-0.55805	4.837679	-6.46735	2.31E-09	2.24E-08	10.66402
COG2	-0.5581	4.279327	-6.23095	7.28E-09	6.43E-08	9.540419
HIBADH	-0.55819	3.473735	-10.2862	3.90E-18	1.76E-16	30.61005
DOCK9	-0.55825	3.424172	-7.83871	2.16E-12	3.51E-11	17.52481
PLXDC1	-0.55828	3.503847	-11.6496	2.18E-21	1.74E-19	38.03475
RFX7	-0.55889	4.404356	-5.5429	1.82E-07	1.27E-06	6.397337
PPP1R12B	-0.55904	3.838513	-9.09579	2.61E-15	7.10E-14	24.1665
HMHA1	-0.55959	6.763985	-7.98276	1.01E-12	1.74E-11	18.27259
ICOS	-0.55965	4.05501	-4.3982	2.39E-05	0.000113	1.680336
LOC10192	-0.55969	3.627227	-5.11102	1.24E-06	7.47E-06	4.535033
SON	-0.56006	7.507286	-6.61262	1.13E-09	1.16E-08	11.36436
TUFT1	-0.56057	4.065914	-6.63936	9.92E-10	1.02E-08	11.49409
CDC14A	-0.5612	3.464538	-8.84167	1.03E-14	2.53E-13	22.80583
SERPINF1	-0.56143	4.737053	-5.88329	3.79E-08	2.97E-07	7.927177
EIF3G	-0.56155	8.103453	-5.62894	1.23E-07	8.84E-07	6.779119
LIPA	-0.56161	8.362997	-2.57922	0.011121	0.028596	-4.06476
ZNF33B	-0.56161	3.108581	-7.84898	2.05E-12	3.36E-11	17.57796
ALDOC	-0.56166	5.925772	-7.99813	9.35E-13	1.62E-11	18.35264
NUP160	-0.56185	3.186738	-6.82015	4.04E-10	4.48E-09	12.37712
NPAT	-0.56192	4.634173	-10.5903	7.33E-19	3.68E-17	32.26625
MPHOSPH	-0.56194	5.469422	-7.12939	8.48E-11	1.05E-09	13.91088
DDX20	-0.562	3.932231	-6.50668	1.91E-09	1.87E-08	10.85289
SIK3	-0.56235	4.914093	-9.80338	5.50E-17	2.04E-15	27.9865
BMS1	-0.56264	5.30724	-7.10847	9.43E-11	1.16E-09	13.80625
TUBGCP5	-0.56315	2.600626	-8.45211	8.38E-14	1.71E-12	20.73559

TPPP3	-0.56326	4.020098	-5.29888	5.44E-07	3.51E-06	5.333694
LEF1-AS1	-0.56341	2.57736	-6.89192	2.82E-10	3.20E-09	12.73053
NAPEPLD	-0.56385	3.828087	-7.52633	1.10E-11	1.59E-10	15.91832
MSI2	-0.56385	4.630982	-9.08414	2.78E-15	7.53E-14	24.10396
CCDC104	-0.56386	3.455283	-4.51514	1.50E-05	7.40E-05	2.128362
BTLA	-0.56401	5.314791	-3.42455	0.000846	0.002921	-1.7003
DOCK8	-0.56416	6.87696	-8.03601	7.66E-13	1.34E-11	18.55004
KHDRBS1	-0.56422	6.786058	-8.79881	1.30E-14	3.13E-13	22.57705
SLC26A6	-0.56427	5.560685	-4.68049	7.65E-06	3.97E-05	2.775875
PPP1R2	-0.56477	5.837579	-6.26767	6.10E-09	5.48E-08	9.713591
C15orf39	-0.56489	5.824051	-6.75861	5.49E-10	5.94E-09	12.07537
ZKSCAN8	-0.5649	3.724411	-8.62008	3.40E-14	7.39E-13	21.62566
CSNK1G2	-0.56559	7.551422	-7.51349	1.18E-11	1.69E-10	15.8528
IPO7	-0.56571	6.05722	-4.66419	8.18E-06	4.22E-05	2.711345
PREX1	-0.56576	8.411388	-5.1679	9.67E-07	5.96E-06	4.774925
MYCL	-0.56581	4.584758	-7.63223	6.37E-12	9.49E-11	16.4605
PLD3	-0.56596	6.685574	-5.44756	2.80E-07	1.89E-06	5.978362
C14orf64	-0.56672	3.787198	-8.64632	2.96E-14	6.54E-13	21.7651
SLC12A7	-0.56681	7.467563	-4.29154	3.64E-05	0.000166	1.279129
ZNF786	-0.56709	4.170129	-6.75322	5.64E-10	6.09E-09	12.04897
RAB11FIP3	-0.56713	4.6545	-8.77418	1.48E-14	3.53E-13	22.44573
RPL29	-0.56715	9.872913	-4.48511	1.69E-05	8.28E-05	2.012515
KIFAP3	-0.56729	5.423362	-6.10065	1.36E-08	1.14E-07	8.930092
NCL	-0.56797	5.375353	-8.22736	2.78E-13	5.20E-12	19.55143
RAD21	-0.56799	8.018728	-6.04717	1.75E-08	1.45E-07	8.68156
RASGRF2	-0.56805	4.819408	-7.82608	2.31E-12	3.74E-11	17.45942
LOC10192	-0.56881	2.741571	-7.07462	1.12E-10	1.35E-09	13.63722
EIF5B	-0.56889	4.030342	-6.6986	7.40E-10	7.81E-09	11.78225
C3orf17	-0.56892	5.130335	-7.68728	4.78E-12	7.31E-11	16.74332
METTL18	-0.56916	4.718149	-3.69879	0.000329	0.001245	-0.81469
FASTKD2	-0.56922	3.6296	-6.18709	8.99E-09	7.81E-08	9.334217
PTPLAD1	-0.56958	5.610836	-4.58511	1.13E-05	5.68E-05	2.400392
MBLAC2	-0.57038	2.421211	-6.95134	2.09E-10	2.43E-09	13.02434
ARID1A	-0.57057	6.683186	-8.72231	1.96E-14	4.55E-13	22.16934
KLHL36	-0.57107	5.895508	-7.21784	5.40E-11	6.94E-10	14.35465
LIMA1	-0.57109	3.209611	-8.75531	1.64E-14	3.87E-13	22.34515
MBNL1	-0.57182	6.556565	-7.55905	9.32E-12	1.35E-10	16.08557
HCG11	-0.57252	2.518686	-5.01896	1.84E-06	1.08E-05	4.15033
NOM1	-0.57258	3.697465	-6.21849	7.73E-09	6.79E-08	9.481743
MAP4K3	-0.57269	2.518646	-3.72679	0.000298	0.001139	-0.72119
RPS25	-0.57321	9.644789	-4.89646	3.11E-06	1.73E-05	3.6455
DAPK1	-0.57324	4.429815	-5.87676	3.91E-08	3.06E-07	7.897344
CHST12	-0.57386	5.730878	-6.02999	1.90E-08	1.56E-07	8.601956
TIMM9	-0.57424	4.927169	-4.71964	6.51E-06	3.44E-05	2.931533
CLEC10A	-0.57442	4.62802	-3.83999	0.000199	0.000782	-0.33764
RRN3P1	-0.57473	4.358974	-9.14459	2.00E-15	5.56E-14	24.42862
PRPS1	-0.57484	5.84988	-5.76056	6.71E-08	5.06E-07	7.369662
AGK	-0.57519	3.520993	-7.68131	4.93E-12	7.52E-11	16.71264
CNOT6L	-0.57545	4.675719	-7.0663	1.17E-10	1.41E-09	13.59572
ITPR1	-0.57563	5.198434	-6.04218	1.79E-08	1.48E-07	8.658407
ELF1	-0.57663	8.451903	-6.34732	4.15E-09	3.85E-08	10.09095
MTX3	-0.57678	4.095021	-6.52242	1.77E-09	1.75E-08	10.92865
MYO9A	-0.57723	4.136429	-9.54183	2.30E-16	7.56E-15	26.57008
FANCF	-0.57766	5.024613	-6.36377	3.83E-09	3.58E-08	10.1692
CXorf65	-0.57772	4.236151	-4.64439	8.87E-06	4.55E-05	2.633135
EXOSC6	-0.57775	4.458994	-7.82075	2.38E-12	3.84E-11	17.43184
NEFL	-0.57792	2.381087	-10.1616	7.72E-18	3.29E-16	29.93213
FOXJ3	-0.57867	4.67773	-9.03803	3.57E-15	9.49E-14	23.85664

NDRG3	-0.57908	5.793131	-5.41787	3.20E-07	2.14E-06	5.848744
SH3BP5-A	-0.57908	5.095856	-7.68906	4.74E-12	7.25E-11	16.75247
LANCL1	-0.57918	5.393769	-7.42196	1.89E-11	2.62E-10	15.38663
LUC7L3	-0.57924	6.373716	-5.68396	9.56E-08	7.02E-07	7.025019
SP4	-0.57933	4.567701	-7.69335	4.63E-12	7.10E-11	16.77458
EIF3H	-0.57951	5.82855	-5.39925	3.48E-07	2.31E-06	5.767694
LOC10012	-0.57959	6.056663	-3.4886	0.000682	0.002406	-1.49839
EFHC1	-0.57963	4.008062	-8.2695	2.22E-13	4.23E-12	19.77281
ZNF879	-0.57965	4.574657	-4.39384	2.43E-05	0.000115	1.663803
LOC10192	-0.58019	4.086727	-5.07306	1.46E-06	8.70E-06	4.375873
CTC1	-0.58032	4.431853	-11.4652	6.00E-21	4.31E-19	37.03205
TMEM123	-0.58049	8.958152	-3.80551	0.000225	0.000878	-0.45541
FAM193A	-0.58054	5.335679	-7.26405	4.26E-11	5.58E-10	14.5873
GCSAM	-0.58056	3.55952	-6.05231	1.71E-08	1.41E-07	8.705387
BLCAP	-0.58066	7.368416	-8.65012	2.90E-14	6.43E-13	21.78529
TAGLN2	-0.58118	10.03676	-4.31305	3.34E-05	0.000154	1.359466
FAM223B	-0.58179	4.796579	-6.75974	5.46E-10	5.91E-09	12.08087
FLVCR1-A	-0.58187	4.907254	-4.77175	5.24E-06	2.82E-05	3.140104
HIVEP3	-0.58247	3.965397	-8.77874	1.45E-14	3.46E-13	22.47001
SLC25A45	-0.58253	3.617531	-7.60336	7.40E-12	1.09E-10	16.31244
TRIM59	-0.58265	3.629184	-4.04195	9.45E-05	0.000396	0.368707
FAM179B	-0.58313	3.766399	-3.41575	0.000871	0.003001	-1.72779
TOP2B	-0.5838	7.884291	-6.39286	3.33E-09	3.14E-08	10.30777
PRKAG2	-0.5842	4.053817	-9.71646	8.85E-17	3.16E-15	27.51534
CD86	-0.58426	5.739977	-4.91891	2.82E-06	1.59E-05	3.737423
WARS	-0.58457	7.376869	-4.53959	1.36E-05	6.76E-05	2.223077
STMN3	-0.58464	4.644864	-8.1181	4.96E-13	8.93E-12	18.97883
TMEM176/	-0.58495	6.382707	-2.17906	0.0313	0.069254	-4.97671
MLLT11	-0.58569	5.271506	-6.35509	4.00E-09	3.73E-08	10.1279
FGD3	-0.58619	7.50924	-6.84931	3.49E-10	3.91E-09	12.52051
ZNF107	-0.58629	4.018371	-6.1278	1.19E-08	1.02E-07	9.056706
IL16	-0.58681	5.688388	-10.073	1.26E-17	5.17E-16	29.45065
FHL1	-0.58741	4.706772	-3.70199	0.000326	0.001232	-0.80402
CBX4	-0.58777	6.421853	-6.93444	2.27E-10	2.62E-09	12.94065
SHQ1	-0.58808	4.421794	-6.71041	6.98E-10	7.40E-09	11.83983
DIMT1	-0.58898	4.808753	-6.09759	1.38E-08	1.16E-07	8.915841
FAM84B	-0.589	4.54828	-7.31529	3.28E-11	4.37E-10	14.84599
TXLNG	-0.58924	4.335582	-8.7375	1.81E-14	4.22E-13	22.25026
ZNF783	-0.5893	4.775816	-10.3305	3.05E-18	1.40E-16	30.85163
RNASE6	-0.58965	7.252709	-3.31764	0.001205	0.004034	-2.0304
DPEP3	-0.58977	4.164442	-6.29493	5.35E-09	4.84E-08	9.842472
NAF1	-0.58982	3.113099	-5.14102	1.09E-06	6.63E-06	4.661362
SECTM1	-0.59011	8.831143	-3.71193	0.000314	0.001194	-0.7709
PCNXL2	-0.59022	3.650735	-9.76512	6.78E-17	2.47E-15	27.77903
KLHL5	-0.59041	4.603721	-3.8955	0.000162	0.000651	-0.14629
LAP3	-0.59073	7.285569	-2.71194	0.007682	0.02075	-3.73192
ZNF284	-0.59092	4.561994	-9.14368	2.01E-15	5.57E-14	24.42374
SELM	-0.59097	4.239621	-6.08196	1.48E-08	1.24E-07	8.843096
ZNF45	-0.59178	3.946807	-5.15317	1.03E-06	6.32E-06	4.712632
CASK	-0.59195	3.250235	-8.62876	3.25E-14	7.10E-13	21.67178
SAP130	-0.59306	5.779536	-7.278	3.97E-11	5.22E-10	14.65768
USP44	-0.5931	2.445334	-7.08007	1.09E-10	1.32E-09	13.6644
FNBP4	-0.59347	4.704329	-6.52957	1.70E-09	1.69E-08	10.96307
C2CD2	-0.59375	4.359138	-6.39272	3.33E-09	3.14E-08	10.30707
MOAP1	-0.59382	5.913891	-4.61204	1.01E-05	5.14E-05	2.505881
MST4	-0.59447	6.412228	-3.86764	0.00018	0.000714	-0.24257
CCDC50	-0.59458	3.924684	-6.23271	7.22E-09	6.38E-08	9.548711
SSBP3-AS:	-0.5951	4.327887	-7.04034	1.33E-10	1.60E-09	13.46636

LENG8	-0.59523	5.583053	-5.15572	1.02E-06	6.26E-06	4.723415
RPL14	-0.59531	7.789364	-6.54723	1.56E-09	1.56E-08	11.0482
INADL	-0.59591	2.817027	-8.62006	3.40E-14	7.39E-13	21.62554
ARHGAP1:	-0.59603	3.87686	-3.07847	0.002584	0.00798	-2.7372
NCALD	-0.59644	3.080988	-10.173	7.25E-18	3.10E-16	29.99444
HLA-G	-0.59663	10.02788	-7.37291	2.44E-11	3.33E-10	15.13765
BTN2A1	-0.59663	5.690955	-7.4836	1.38E-11	1.95E-10	15.70031
SS18L1	-0.59696	5.1606	-4.22392	4.73E-05	0.000211	1.028479
TSPYL5	-0.59708	3.316852	-6.18755	8.97E-09	7.80E-08	9.336411
PRKD2	-0.59731	5.899767	-10.318	3.27E-18	1.48E-16	30.78338
RUFY3	-0.59746	3.918707	-6.22782	7.39E-09	6.52E-08	9.525667
ZNF84	-0.59747	4.437312	-5.74368	7.26E-08	5.44E-07	7.293503
PNRC1	-0.59764	9.8417	-7.08259	1.08E-10	1.30E-09	13.67697
RBM18	-0.59778	4.607719	-6.72556	6.47E-10	6.91E-09	11.91377
ZNF689	-0.5978	5.258101	-6.31331	4.89E-09	4.48E-08	9.92954
TRIM26	-0.59787	6.030411	-7.84118	2.14E-12	3.48E-11	17.53759
RAB37	-0.59834	7.181358	-5.60685	1.36E-07	9.69E-07	6.680786
ALMS1	-0.59842	4.053576	-8.65866	2.77E-14	6.16E-13	21.8307
PITPNC1	-0.59873	4.685579	-9.06004	3.16E-15	8.47E-14	23.97467
DUSP7	-0.59896	5.162882	-7.2299	5.08E-11	6.57E-10	14.41531
PQLC3	-0.59936	6.105689	-4.82503	4.20E-06	2.29E-05	3.354991
CCDC88C	-0.60095	5.331124	-6.39524	3.29E-09	3.11E-08	10.31912
SHPRH	-0.60141	4.323148	-2.9884	0.003408	0.010183	-2.99171
MRPS18B	-0.60164	5.888686	-5.33132	4.71E-07	3.06E-06	5.47345
FAM220A	-0.60177	6.227972	-5.32453	4.85E-07	3.15E-06	5.444129
SIGLEC10	-0.60186	6.417471	-2.98835	0.003409	0.010183	-2.99185
RPL19	-0.60221	11.21217	-5.06988	1.48E-06	8.80E-06	4.362558
SRSF7	-0.60232	6.409708	-5.85445	4.34E-08	3.37E-07	7.795564
RRP1B	-0.60242	5.639199	-7.44924	1.65E-11	2.30E-10	15.52535
TRABD2A	-0.60253	3.887127	-7.83943	2.16E-12	3.51E-11	17.52853
PAN3	-0.60261	8.055457	-5.60612	1.37E-07	9.71E-07	6.67752
LINC0142(	-0.6027	3.836537	-6.82566	3.93E-10	4.37E-09	12.40421
FAM19A1	-0.6029	2.385851	-4.94853	2.49E-06	1.41E-05	3.859094
ASF1A	-0.6031	5.728699	-4.30426	3.46E-05	0.000159	1.326582
SLC25A6	-0.60341	9.578148	-4.93598	2.63E-06	1.49E-05	3.807484
LINC0135!	-0.60347	4.491413	-6.80372	4.38E-10	4.83E-09	12.29642
MAP2K4	-0.60355	5.83157	-6.31638	4.82E-09	4.43E-08	9.944092
ACTR5	-0.60377	4.701411	-8.10183	5.41E-13	9.67E-12	18.89376
FAM208B	-0.60421	4.265532	-7.84653	2.08E-12	3.40E-11	17.56531
PRKRIR	-0.60455	6.709751	-4.11494	7.17E-05	0.000308	0.630711
ZNF256	-0.60463	2.220558	-6.47162	2.26E-09	2.19E-08	10.68447
TMC6	-0.60467	6.466055	-7.45404	1.60E-11	2.25E-10	15.54976
EPB41L4A-	-0.60486	4.208191	-5.55072	1.76E-07	1.23E-06	6.43192
SNX30	-0.60502	5.009017	-3.73122	0.000294	0.001122	-0.70635
HNRNPU-	-0.60511	6.606525	-3.84303	0.000197	0.000774	-0.32721
IKBIP	-0.60684	4.568946	-5.06994	1.48E-06	8.80E-06	4.362807
SEC31A	-0.60695	7.137271	-6.20168	8.38E-09	7.33E-08	9.402716
POLB	-0.60711	4.239485	-10.6167	6.34E-19	3.24E-17	32.41009
PIK3CD	-0.60727	6.780886	-7.3379	2.92E-11	3.93E-10	14.96034
STK10	-0.60731	7.620086	-8.08066	6.05E-13	1.07E-11	18.78312
XYLT1	-0.60751	4.056497	-9.29713	8.73E-16	2.61E-14	25.24932
CNOT7	-0.60753	6.250041	-5.91089	3.33E-08	2.63E-07	8.053459
CD99	-0.60764	8.760132	-4.72898	6.26E-06	3.32E-05	2.968828
TAB2	-0.60766	7.310184	-5.80269	5.52E-08	4.22E-07	7.56031
SUMF2	-0.60767	5.836966	-6.51793	1.80E-09	1.78E-08	10.90705
CRTAP	-0.60778	5.747607	-6.74771	5.79E-10	6.26E-09	12.02202
IMMT	-0.60815	6.824559	-6.17891	9.35E-09	8.08E-08	9.295845
BTG1	-0.60829	7.697534	-7.52359	1.12E-11	1.61E-10	15.90432

GOT2	-0.60833	6.651362	-7.10715	9.49E-11	1.16E-09	13.79967
LOC10028	-0.60834	3.809662	-5.17982	9.18E-07	5.69E-06	4.825411
DUSP2	-0.60841	4.838421	-6.39361	3.31E-09	3.13E-08	10.31134
VPS51	-0.60865	8.047583	-7.76013	3.27E-12	5.15E-11	17.11868
MCOLN2	-0.6088	3.879631	-7.65569	5.64E-12	8.51E-11	16.58093
PUS7	-0.60887	3.568411	-4.68681	7.45E-06	3.88E-05	2.800971
MIA3	-0.60888	4.723304	-7.76057	3.26E-12	5.15E-11	17.12093
PECAM1	-0.60889	7.493167	-7.81174	2.49E-12	4.01E-11	17.38526
ARHGEF9	-0.60914	3.122846	-7.99446	9.54E-13	1.65E-11	18.3335
LGR6	-0.60924	4.607277	-6.92503	2.38E-10	2.74E-09	12.8941
PIK3R1	-0.60937	6.447732	-5.1723	9.49E-07	5.86E-06	4.793554
ARHGEF1(	-0.6094	4.954006	-7.12018	8.89E-11	1.10E-09	13.86484
M6PR	-0.61014	7.017998	-4.73906	6.01E-06	3.20E-05	3.009088
MRPS30	-0.61017	4.414649	-5.79319	5.77E-08	4.40E-07	7.517224
LOC33998	-0.61027	5.735367	-8.4029	1.09E-13	2.19E-12	20.47564
BIRC6	-0.61047	6.117632	-6.65401	9.23E-10	9.55E-09	11.56525
RINT1	-0.61052	4.305467	-4.09876	7.63E-05	0.000326	0.572321
RPA1	-0.61114	6.126969	-7.81757	2.42E-12	3.90E-11	17.41541
FOXO1	-0.61158	4.774764	-9.56588	2.02E-16	6.70E-15	26.70017
FLJ31306	-0.612	6.50209	-3.78725	0.00024	0.000934	-0.51745
ZNF32	-0.61275	4.893063	-5.24598	6.87E-07	4.35E-06	5.106961
FXYD5	-0.61384	8.876119	-6.37428	3.64E-09	3.42E-08	10.21921
LTB	-0.61408	7.335671	-7.18861	6.27E-11	7.94E-10	14.20779
DENND4C	-0.61484	4.766266	-8.71007	2.10E-14	4.82E-13	22.10418
MDN1	-0.61522	3.261909	-9.02104	3.91E-15	1.03E-13	23.76555
DNAJC10	-0.6156	4.011178	-5.26709	6.26E-07	3.99E-06	5.197281
RPL11	-0.61603	10.24494	-5.22564	7.51E-07	4.74E-06	5.020174
PNMA1	-0.61652	5.997794	-4.48812	1.67E-05	8.18E-05	2.024108
BLMH	-0.61661	4.832631	-7.59851	7.59E-12	1.12E-10	16.2876
PCYOX1L	-0.61667	5.807189	-6.74917	5.75E-10	6.21E-09	12.02917
NOL9	-0.61674	5.148895	-8.62689	3.28E-14	7.16E-13	21.66184
ACADSB	-0.61706	3.025464	-6.35781	3.94E-09	3.68E-08	10.1408
ENO2	-0.61731	4.202224	-6.04537	1.77E-08	1.46E-07	8.673218
ELMSAN1	-0.61744	3.763747	-10.5554	8.88E-19	4.41E-17	32.07608
UFSP2	-0.61782	5.324203	-5.41942	3.18E-07	2.12E-06	5.85553
ZNF395	-0.61789	4.865288	-9.4238	4.38E-16	1.37E-14	25.93243
FAM179A	-0.61796	3.055601	-7.2658	4.23E-11	5.54E-10	14.59613
RPL36	-0.618	8.862208	-4.0296	9.89E-05	0.000413	0.324711
ZBTB38	-0.61852	4.952749	-7.54	1.03E-11	1.49E-10	15.98819
NIPAL3	-0.6189	4.49685	-11.0029	7.59E-20	4.53E-18	34.51465
ST3GAL1	-0.61893	5.830179	-7.8297	2.27E-12	3.68E-11	17.47814
KLF2	-0.61941	7.18047	-8.0437	7.35E-13	1.29E-11	18.59015
NOL8	-0.61958	5.843943	-5.0745	1.45E-06	8.65E-06	4.381863
SLC9A3R1	-0.61992	7.594949	-6.98723	1.74E-10	2.05E-09	13.20233
PEX12	-0.62009	5.306977	-7.0572	1.22E-10	1.47E-09	13.55036
RAF1	-0.62071	7.515982	-7.11889	8.94E-11	1.10E-09	13.85839
CD200	-0.62077	3.548085	-4.10929	7.33E-05	0.000314	0.610326
MED29	-0.62129	5.223902	-4.44149	2.01E-05	9.70E-05	1.845212
C12orf66	-0.62136	2.920232	-6.21845	7.73E-09	6.79E-08	9.481587
KIAA0430	-0.62138	6.009012	-6.39838	3.24E-09	3.07E-08	10.3341
NAE1	-0.62164	6.203792	-4.56704	1.22E-05	6.09E-05	2.329858
PID1	-0.62215	2.75973	-9.05874	3.19E-15	8.51E-14	23.96768
ZNF137P	-0.62219	4.225532	-3.85209	0.00019	0.000751	-0.29609
URI1	-0.62221	4.156916	-9.00439	4.28E-15	1.12E-13	23.6763
SNHG22	-0.62225	2.634193	-8.9018	7.45E-15	1.87E-13	23.12713
SET	-0.62265	8.331385	-6.79144	4.66E-10	5.10E-09	12.23617
NRROS	-0.62274	5.72922	-5.02469	1.80E-06	1.05E-05	4.174165
LOC15165	-0.62282	5.13897	-7.78228	2.91E-12	4.63E-11	17.23304

MGC4006(	-0.62299	2.185313	-9.72575	8.41E-17	3.02E-15	27.56566
HLA-E	-0.62314	9.929526	-8.59378	3.92E-14	8.47E-13	21.48604
ADO	-0.62345	5.436862	-4.64869	8.72E-06	4.48E-05	2.650091
ZNF430	-0.62354	4.37513	-4.80546	4.56E-06	2.47E-05	3.275857
NAA40	-0.62368	4.492995	-7.3622	2.58E-11	3.50E-10	15.0834
ATG16L1	-0.62376	5.057367	-8.20985	3.05E-13	5.64E-12	19.45951
KIT	-0.62391	2.766399	-7.62702	6.54E-12	9.72E-11	16.43378
NCBP2	-0.62391	5.081987	-5.46033	2.64E-07	1.79E-06	6.034244
C14orf28	-0.62402	3.675456	-6.97205	1.88E-10	2.21E-09	13.12701
BNIP3	-0.62436	4.216886	-4.8427	3.90E-06	2.14E-05	3.426576
ZC3H8	-0.62456	4.256354	-6.76325	5.36E-10	5.81E-09	12.09807
GPR155	-0.62486	5.943371	-4.8461	3.84E-06	2.12E-05	3.440379
PSME1	-0.62559	9.430572	-9.17245	1.72E-15	4.84E-14	24.57835
ALKBH8	-0.62638	3.514242	-7.55635	9.45E-12	1.37E-10	16.07177
METTL8	-0.62646	3.518185	-6.91318	2.53E-10	2.90E-09	12.83552
KDSR	-0.62674	2.994112	-6.70879	7.03E-10	7.46E-09	11.83194
APOBEC3(	-0.62708	6.549902	-7.17258	6.81E-11	8.56E-10	14.12733
MRPS9	-0.62744	4.444349	-4.59573	1.08E-05	5.46E-05	2.441922
DHX9	-0.62764	5.383754	-6.49174	2.05E-09	2.00E-08	10.78109
MSH2	-0.62774	4.42239	-3.94108	0.000137	0.000559	0.012431
ASTE1	-0.62783	5.586831	-6.55766	1.48E-09	1.49E-08	11.09855
BEX5	-0.62849	3.868443	-4.43567	2.06E-05	9.89E-05	1.822973
ADNP	-0.62861	6.606179	-7.8087	2.53E-12	4.07E-11	17.36952
ZNF623	-0.62885	3.223569	-4.92394	2.76E-06	1.56E-05	3.758044
PIGB	-0.62895	6.045276	-5.312	5.13E-07	3.32E-06	5.390136
USP36	-0.62933	4.357933	-12.1421	1.48E-22	1.43E-20	40.70611
ZSCAN18	-0.62941	5.015624	-7.12777	8.55E-11	1.06E-09	13.90278
SMS	-0.63064	4.976459	-5.3807	3.78E-07	2.50E-06	5.687127
SART3	-0.63085	4.955278	-9.60918	1.59E-16	5.43E-15	26.93439
NARS2	-0.63093	5.097927	-5.2769	5.99E-07	3.84E-06	5.23931
PRRG4	-0.63103	5.584745	-5.45355	2.73E-07	1.84E-06	6.004565
SMARCAD	-0.63124	5.411384	-5.64314	1.15E-07	8.31E-07	6.842469
BOD1L1	-0.63148	6.151437	-5.02775	1.77E-06	1.04E-05	4.186859
LARS	-0.63312	5.987414	-7.0922	1.02E-10	1.25E-09	13.72497
SMAD3	-0.6332	4.622512	-12.0479	2.47E-22	2.28E-20	40.19577
PPRC1	-0.63346	4.311544	-7.2863	3.80E-11	5.01E-10	14.69955
PSD4	-0.63363	5.488627	-9.94193	2.58E-17	1.02E-15	28.7384
SPIN1	-0.63379	5.31369	-5.67754	9.84E-08	7.21E-07	6.996268
DKFZP586	-0.6338	7.271371	-6.49155	2.05E-09	2.00E-08	10.78017
CREBL2	-0.63408	6.477259	-6.3642	3.82E-09	3.57E-08	10.17122
EID3	-0.63414	2.428578	-5.54501	1.80E-07	1.26E-06	6.406653
ZNF253	-0.63467	3.569946	-8.2233	2.84E-13	5.30E-12	19.53011
PAXIP1-A:	-0.63476	2.63498	-5.99362	2.26E-08	1.83E-07	8.433848
SLAMF7	-0.63504	5.239189	-3.28532	0.001339	0.004429	-2.1285
MCM3	-0.63558	5.850314	-5.13176	1.13E-06	6.88E-06	4.62232
RBBP7	-0.63561	7.305165	-5.61122	1.33E-07	9.52E-07	6.700225
PDGFD	-0.63562	3.859483	-7.14025	8.02E-11	9.97E-10	13.96526
NUP210	-0.63602	5.669895	-9.89357	3.36E-17	1.31E-15	28.47587
JAKMIP2	-0.6361	2.618174	-9.07932	2.85E-15	7.71E-14	24.07809
KIZ	-0.63652	4.608921	-8.5696	4.46E-14	9.52E-13	21.35774
HOXB2	-0.63734	5.791074	-5.27436	6.06E-07	3.88E-06	5.228448
MAFF	-0.63761	4.537453	-4.68574	7.49E-06	3.90E-05	2.796718
UBN1	-0.63764	7.570213	-5.56402	1.65E-07	1.16E-06	6.490762
CYB561	-0.63776	4.406115	-11.6549	2.12E-21	1.70E-19	38.06351
SMYD2	-0.63808	4.886222	-6.80094	4.44E-10	4.89E-09	12.2828
UBAP2	-0.63812	5.35266	-6.09618	1.39E-08	1.17E-07	8.909263
RBM25	-0.63849	6.232957	-7.19331	6.12E-11	7.77E-10	14.23135
MGC2410:	-0.63871	2.065101	-7.33086	3.03E-11	4.06E-10	14.92472

FYTTD1	-0.63883	6.896214	-5.27439	6.06E-07	3.88E-06	5.228548
RUVBL1	-0.63895	5.306807	-6.6233	1.07E-09	1.10E-08	11.41615
PP7080	-0.63902	4.158151	-6.51933	1.79E-09	1.77E-08	10.91379
CXCR6	-0.63948	4.481	-8.82327	1.14E-14	2.77E-13	22.7076
MAF	-0.64052	3.510213	-5.50594	2.15E-07	1.48E-06	6.234402
IMPDH2	-0.64114	7.15809	-4.60088	1.06E-05	5.36E-05	2.462108
BRD2	-0.64158	7.51306	-7.74072	3.62E-12	5.66E-11	17.01856
DROSHA	-0.64172	5.04135	-6.54831	1.55E-09	1.55E-08	11.05344
RCSD1	-0.64207	8.123301	-6.74217	5.96E-10	6.42E-09	11.99496
SDR39U1	-0.64222	6.722555	-6.87148	3.12E-10	3.53E-09	12.62971
PIM2	-0.64278	6.45753	-4.2932	3.61E-05	0.000165	1.285307
ZDHHC23	-0.64335	3.160232	-7.06845	1.16E-10	1.39E-09	13.60644
GATAD2B	-0.64439	5.243525	-8.61888	3.43E-14	7.42E-13	21.61928
LOC10027	-0.64497	6.795026	-5.07045	1.48E-06	8.79E-06	4.36492
ZZZ3	-0.64599	5.058634	-6.22672	7.43E-09	6.55E-08	9.520496
SSRP1	-0.64634	5.247016	-6.0936	1.40E-08	1.18E-07	8.897291
ZNF540	-0.6466	3.283604	-9.74157	7.72E-17	2.79E-15	27.6514
SLC45A4	-0.64679	6.803214	-5.07885	1.42E-06	8.50E-06	4.400066
RPL23A	-0.64695	11.12954	-7.32516	3.12E-11	4.17E-10	14.89588
NOP14	-0.64728	5.126269	-7.30743	3.41E-11	4.54E-10	14.80625
FAM13B	-0.64731	6.331233	-5.21957	7.71E-07	4.86E-06	4.994333
GAB2	-0.64735	7.827487	-5.41124	3.30E-07	2.20E-06	5.819866
CAPN2	-0.64757	7.031207	-5.79019	5.85E-08	4.46E-07	7.503642
ENOPH1	-0.64761	6.137435	-4.10812	7.36E-05	0.000316	0.606081
ARHGAP1	-0.64779	6.670353	-8.11557	5.03E-13	9.02E-12	18.96562
KLHL3	-0.64795	3.255154	-12.4523	2.73E-23	2.97E-21	42.38208
GLG1	-0.64849	6.712335	-7.74123	3.61E-12	5.65E-11	17.02115
COQ10A	-0.64877	4.678781	-7.77506	3.02E-12	4.79E-11	17.19574
DIS3L2	-0.64899	3.62942	-6.3529	4.04E-09	3.76E-08	10.11749
LOC10050	-0.64955	3.611943	-6.35212	4.05E-09	3.77E-08	10.11376
ADHFE1	-0.64985	5.462596	-9.57148	1.96E-16	6.51E-15	26.73043
RPGRIP1	-0.65006	3.778118	-8.67079	2.59E-14	5.79E-13	21.89517
RASSF3	-0.65028	7.491388	-6.78208	4.88E-10	5.32E-09	12.19031
C1orf109	-0.65046	3.313993	-5.7656	6.56E-08	4.94E-07	7.39242
ST8SIA4	-0.65056	5.512661	-4.95222	2.45E-06	1.39E-05	3.874284
ARNTL	-0.65092	6.578395	-3.91922	0.000149	0.000601	-0.06386
SLC4A7	-0.65111	3.15468	-7.91048	1.48E-12	2.48E-11	17.89684
ACVR2A	-0.65124	3.387742	-5.8208	5.08E-08	3.91E-07	7.642493
INPP5E	-0.65127	5.154718	-6.88406	2.93E-10	3.33E-09	12.69174
ACVR1C	-0.65148	2.838342	-7.39968	2.12E-11	2.92E-10	15.27348
NOLC1	-0.65189	5.217836	-8.25935	2.34E-13	4.44E-12	19.71949
ZNF506	-0.65192	2.96676	-6.60591	1.17E-09	1.19E-08	11.33185
TMEM204	-0.6524	4.843797	-7.89773	1.59E-12	2.63E-11	17.83065
NCR3	-0.6534	5.347847	-8.51134	6.10E-14	1.27E-12	21.04896
ZNF30	-0.65341	3.263315	-7.15851	7.31E-11	9.14E-10	14.05675
HNRNPR	-0.65359	6.380431	-6.17133	9.69E-09	8.37E-08	9.260312
LRRC69	-0.65398	4.190265	-6.29817	5.26E-09	4.78E-08	9.857788
SCARF1	-0.65405	3.267312	-6.65643	9.12E-10	9.46E-09	11.57701
SLC5A3	-0.65514	4.650392	-7.67774	5.02E-12	7.65E-11	16.69429
CTDSPL2	-0.65549	4.479157	-5.53318	1.90E-07	1.32E-06	6.354435
LDOC1	-0.65631	4.374971	-6.92942	2.33E-10	2.68E-09	12.91585
GTF2I	-0.6565	3.355106	-6.90066	2.70E-10	3.07E-09	12.77368
C7orf31	-0.65735	4.78492	-5.57964	1.54E-07	1.09E-06	6.559939
GBP2	-0.65778	7.549293	-3.95248	0.000132	0.000538	0.052345
TGIF1	-0.6581	3.991418	-6.82764	3.89E-10	4.34E-09	12.4139
TNFAIP3	-0.65837	7.228885	-4.23026	4.61E-05	0.000207	1.051828
NCOA5	-0.65892	5.212464	-8.1213	4.88E-13	8.79E-12	18.99559
SRSF5	-0.65894	6.480655	-6.43651	2.69E-09	2.58E-08	10.51626

ELP2	-0.65981	4.231692	-10.7803	2.58E-19	1.38E-17	33.30201
ZNF548	-0.65984	3.666654	-9.01397	4.06E-15	1.07E-13	23.72762
HNRNPUL	-0.66023	5.720855	-10.8803	1.49E-19	8.32E-18	33.84678
UHRF2	-0.66023	7.285868	-6.29659	5.31E-09	4.81E-08	9.850323
SLC20A1	-0.66043	7.670094	-5.94362	2.86E-08	2.28E-07	8.20361
ECHDC2	-0.66044	5.444782	-7.58909	7.97E-12	1.17E-10	16.23934
ELMO1	-0.66068	6.547083	-7.22584	5.19E-11	6.71E-10	14.3949
MCCC1	-0.66075	5.766425	-6.90709	2.61E-10	2.98E-09	12.80544
C2orf42	-0.66075	4.790264	-7.23777	4.88E-11	6.34E-10	14.45493
NAP1L1	-0.66093	7.491007	-4.99961	2.00E-06	1.16E-05	4.070051
ST3GAL5	-0.66094	5.40753	-7.29074	3.72E-11	4.91E-10	14.72197
TNRC6B	-0.66133	6.273933	-7.75236	3.40E-12	5.34E-11	17.07856
C19orf66	-0.66152	5.9623	-9.25956	1.07E-15	3.14E-14	25.04697
SIPA1L1	-0.66161	4.363571	-9.23859	1.20E-15	3.49E-14	24.93409
SPAG9	-0.66162	5.427182	-7.70479	4.36E-12	6.72E-11	16.83346
RABGAP1L	-0.662	5.03118	-9.68358	1.06E-16	3.72E-15	27.33723
MLLT6	-0.66276	5.241717	-9.88455	3.53E-17	1.36E-15	28.42691
FMNL3	-0.66286	3.303882	-11.5417	3.94E-21	3.03E-19	37.44829
FMR1	-0.66351	6.952449	-4.43835	2.04E-05	9.79E-05	1.833236
PLEKHG3	-0.66359	5.063127	-10.0998	1.08E-17	4.51E-16	29.59637
ATM	-0.66371	5.331013	-9.80034	5.59E-17	2.07E-15	27.97002
ALDH18A1	-0.6638	4.676032	-6.7109	6.96E-10	7.39E-09	11.84222
MED1	-0.66388	5.475587	-9.07342	2.94E-15	7.92E-14	24.04645
DNAJA3	-0.66395	5.895658	-7.75631	3.33E-12	5.24E-11	17.09897
IKBKB	-0.66414	5.267109	-6.94357	2.17E-10	2.52E-09	12.98588
GPA33	-0.66455	4.847953	-7.66759	5.30E-12	8.03E-11	16.64209
SSBP2	-0.66493	3.432296	-13.052	1.06E-24	1.47E-22	45.60513
SF3A3	-0.66496	5.342582	-5.31353	5.10E-07	3.30E-06	5.39676
ZNF827	-0.66519	3.383608	-10.4243	1.82E-18	8.72E-17	31.36204
C6orf48	-0.66591	7.482933	-5.24403	6.93E-07	4.39E-06	5.098661
FAM213B	-0.66595	5.318019	-6.64171	9.81E-10	1.01E-08	11.50548
ANXA2R	-0.66599	5.587871	-7.13885	8.08E-11	1.00E-09	13.95828
STRBP	-0.66772	4.001257	-5.64449	1.15E-07	8.27E-07	6.848491
TIGD1	-0.66794	2.924145	-4.45445	1.91E-05	9.25E-05	1.894787
TOB1	-0.66831	7.107291	-3.87003	0.000178	0.000709	-0.23433
PKIA	-0.66842	3.222127	-6.96731	1.93E-10	2.26E-09	13.10351
E2F5	-0.6688	3.697195	-4.5911	1.10E-05	5.55E-05	2.423805
ZXDA	-0.6689	3.00855	-7.65363	5.70E-12	8.60E-11	16.57034
RNF19B	-0.66932	7.278036	-5.52019	2.02E-07	1.40E-06	6.297162
C19orf12	-0.66994	5.396783	-6.54262	1.60E-09	1.59E-08	11.02599
BCLAF1	-0.67008	5.895393	-5.95496	2.71E-08	2.17E-07	8.255732
PPP1R3E	-0.67011	4.726153	-7.64437	5.98E-12	8.98E-11	16.52282
CARD11	-0.6702	5.031133	-10.4388	1.68E-18	8.07E-17	31.4411
ILF3	-0.67032	5.452086	-7.43793	1.74E-11	2.43E-10	15.46782
ZNFX1	-0.67039	7.799262	-9.54011	2.32E-16	7.60E-15	26.56078
MEGF6	-0.67161	4.881531	-8.81042	1.22E-14	2.96E-13	22.63902
EIF3C	-0.67236	3.068088	-4.71512	6.63E-06	3.50E-05	2.913519
R3HDM2	-0.67239	6.450948	-9.15927	1.85E-15	5.16E-14	24.50752
SLC46A2	-0.67336	4.477786	-3.44197	0.000798	0.002776	-1.64568
CIRH1A	-0.67339	5.57002	-5.48821	2.33E-07	1.60E-06	6.156462
TTF1	-0.67383	5.472686	-8.92688	6.51E-15	1.66E-13	23.26127
ACD	-0.67388	5.251682	-7.26519	4.24E-11	5.55E-10	14.59304
ZBTB18	-0.6744	6.460865	-5.17395	9.42E-07	5.83E-06	4.800535
ABCF1	-0.67465	6.216368	-7.71409	4.16E-12	6.42E-11	16.88132
SERPINB9	-0.67465	6.019441	-4.47754	1.74E-05	8.51E-05	1.983379
LRMP	-0.67543	7.844542	-6.04432	1.78E-08	1.46E-07	8.668313
NAAA	-0.67589	6.033551	-4.41187	2.27E-05	0.000108	1.732286
LINC00926	-0.6763	5.733502	-4.49986	1.60E-05	7.83E-05	2.069335

SLBP	-0.67649	7.315758	-5.66093	1.06E-07	7.72E-07	6.921928
TGFBR2	-0.67733	7.183304	-6.27207	5.97E-09	5.37E-08	9.734364
JARID2	-0.6775	5.491663	-12.2548	7.99E-23	8.13E-21	41.31547
MS4A14	-0.67758	4.936633	-3.08529	0.00253	0.007834	-2.71767
ZBTB6	-0.67767	3.119919	-5.48748	2.34E-07	1.60E-06	6.153281
HLA-B	-0.67868	11.9276	-7.72761	3.87E-12	6.03E-11	16.95099
TAF3	-0.67879	3.718523	-8.97518	5.01E-15	1.30E-13	23.5198
PEX1	-0.67933	3.5322	-6.44971	2.52E-09	2.42E-08	10.57948
MFSD8	-0.67936	4.008419	-6.30089	5.20E-09	4.72E-08	9.870696
LOC10192	-0.67972	3.308396	-7.46589	1.51E-11	2.13E-10	15.61012
AMD1	-0.67997	6.770874	-6.59348	1.24E-09	1.27E-08	11.27169
ZNF33A	-0.67997	4.656221	-10.1873	6.70E-18	2.89E-16	30.07228
RPS27	-0.68147	8.452245	-6.2538	6.52E-09	5.83E-08	9.648095
IRF8	-0.68148	7.558864	-3.94541	0.000135	0.000551	0.027607
ERCC5	-0.68245	5.841962	-5.75646	6.84E-08	5.15E-07	7.35113
ADRB2	-0.68267	6.491012	-5.1329	1.13E-06	6.86E-06	4.627118
TOMM20	-0.68285	7.607106	-5.99173	2.28E-08	1.85E-07	8.425101
KLF4	-0.68321	5.86105	-3.38139	0.000977	0.003331	-1.83459
PDP1	-0.68341	5.88562	-4.96196	2.35E-06	1.34E-05	3.914409
ANK3	-0.68387	3.589814	-6.59189	1.25E-09	1.27E-08	11.26397
C14orf169	-0.68426	5.470392	-7.45728	1.58E-11	2.22E-10	15.56628
NELFCD	-0.68429	6.440093	-7.71667	4.10E-12	6.34E-11	16.89462
HLA-J	-0.68437	9.420541	-9.46391	3.52E-16	1.12E-14	26.14902
L3MBTL3	-0.68466	4.820591	-4.70109	7.03E-06	3.69E-05	2.857695
ABCD2	-0.68531	1.746259	-8.71296	2.06E-14	4.75E-13	22.11956
KIAA0247	-0.68564	8.513066	-5.48513	2.36E-07	1.62E-06	6.142972
ARRB1	-0.68616	5.389497	-7.92033	1.41E-12	2.37E-11	17.94798
SIRT1	-0.68625	6.077806	-4.60851	1.03E-05	5.21E-05	2.491994
CLNS1A	-0.68664	6.524384	-5.5772	1.56E-07	1.10E-06	6.549143
TMEM194/	-0.68706	3.640789	-7.30673	3.43E-11	4.55E-10	14.80271
FOS	-0.68724	7.01133	-4.11906	7.06E-05	0.000304	0.645611
TMC8	-0.68726	5.905958	-9.03141	3.70E-15	9.82E-14	23.82114
PRPF6	-0.68753	5.707307	-11.4173	7.80E-21	5.48E-19	36.77148
LPAR5	-0.68757	4.20802	-6.56611	1.42E-09	1.43E-08	11.13934
WWC3	-0.68766	7.039009	-6.45249	2.49E-09	2.39E-08	10.59279
HNRNPA3	-0.68771	6.814588	-6.33017	4.51E-09	4.16E-08	10.00949
NAGPA	-0.68783	5.808067	-5.72635	7.86E-08	5.85E-07	7.215432
CYB561A3	-0.68787	6.951146	-8.51329	6.04E-14	1.26E-12	21.0593
PCSK5	-0.68793	2.956385	-7.35019	2.74E-11	3.71E-10	15.02253
TRIM22	-0.68853	9.330132	-3.39505	0.000933	0.003198	-1.79225
AKAP11	-0.68855	5.300802	-6.24643	6.76E-09	6.01E-08	9.61334
HIVEP2	-0.68866	4.109463	-11.7893	1.02E-21	8.55E-20	38.79345
VIPR1	-0.68887	5.816392	-8.10928	5.20E-13	9.31E-12	18.93272
FUNDC1	-0.68945	3.811899	-4.30672	3.43E-05	0.000158	1.335772
TNRC6C-/	-0.68986	5.214328	-9.26146	1.06E-15	3.12E-14	25.0572
MGEA5	-0.69004	6.571494	-6.94155	2.19E-10	2.54E-09	12.97586
CUTA	-0.69056	7.687491	-6.0807	1.49E-08	1.25E-07	8.83727
PELI1	-0.69082	8.179957	-3.90719	0.000156	0.000626	-0.1057
YLPM1	-0.69167	4.835843	-9.87145	3.79E-17	1.44E-15	28.3558
TTC39B	-0.69205	2.587053	-8.99972	4.39E-15	1.15E-13	23.65128
CREBRF	-0.69298	7.223641	-6.9311	2.31E-10	2.66E-09	12.92416
LINC0062:	-0.69437	8.611751	-9.2292	1.26E-15	3.66E-14	24.88355
PRSS23	-0.69488	3.24056	-9.54115	2.31E-16	7.57E-15	26.56642
SIRPG	-0.69489	3.627151	-9.83639	4.59E-17	1.73E-15	28.16559
PAFAH2	-0.69509	4.028689	-11.5053	4.81E-21	3.58E-19	37.25013
CDK17	-0.69509	5.344361	-6.41835	2.94E-09	2.80E-08	10.42942
SMAGP	-0.69513	4.682027	-8.26206	2.31E-13	4.38E-12	19.73371
IGIP	-0.69513	4.787104	-8.53897	5.26E-14	1.11E-12	21.19533

AHCTF1	-0.69515	5.187565	-5.87798	3.89E-08	3.04E-07	7.902924
SDAD1	-0.69522	4.489859	-7.33837	2.91E-11	3.92E-10	14.96269
DPYSL2	-0.69524	6.675642	-3.19965	0.001764	0.005681	-2.38466
DGKZ	-0.69554	6.254413	-10.3912	2.19E-18	1.03E-16	31.1821
SMEK1	-0.69604	5.868551	-7.57318	8.66E-12	1.26E-10	16.15786
WDR89	-0.69614	3.896367	-10.0016	1.86E-17	7.46E-16	29.06261
LOC39949	-0.69645	7.033164	-8.44109	8.88E-14	1.81E-12	20.67733
WHAMM	-0.69671	5.046508	-7.38201	2.33E-11	3.19E-10	15.18379
LYRM7	-0.69697	4.920198	-7.25172	4.54E-11	5.92E-10	14.52519
ICE1	-0.69717	5.547472	-5.11222	1.23E-06	7.44E-06	4.540051
ZBTB21	-0.69747	4.787921	-5.17789	9.26E-07	5.74E-06	4.817252
CLIP4	-0.69802	4.261332	-5.70503	8.68E-08	6.42E-07	7.119557
KRBOX4	-0.69802	4.457702	-5.72221	8.01E-08	5.96E-07	7.196794
ZNF204P	-0.6984	2.104246	-7.9349	1.31E-12	2.21E-11	18.02368
SUPT16H	-0.69939	5.634849	-8.67614	2.52E-14	5.64E-13	21.92365
NDNL2	-0.69957	3.983791	-5.2037	8.27E-07	5.18E-06	4.926786
TTYH2	-0.69961	4.515319	-6.72025	6.64E-10	7.07E-09	11.88784
NDE1	-0.69994	5.597182	-13.0191	1.26E-24	1.73E-22	45.42885
ANKRD12	-0.701	5.137802	-4.8272	4.16E-06	2.27E-05	3.363753
CRIP1	-0.70118	7.824856	-3.30049	0.001274	0.00424	-2.08256
BLOC1S4	-0.70196	6.18916	-6.22219	7.59E-09	6.68E-08	9.499166
FLI1	-0.702	6.326136	-6.57077	1.39E-09	1.41E-08	11.16188
WDR75	-0.70206	4.541362	-4.86714	3.52E-06	1.95E-05	3.525894
DDX5	-0.70208	5.544707	-5.42247	3.13E-07	2.10E-06	5.868794
KDM3A	-0.70228	4.64086	-10.6125	6.48E-19	3.30E-17	32.38738
IL21R	-0.70244	4.356484	-6.79652	4.54E-10	4.99E-09	12.26109
ND2	-0.70275	10.57835	-5.49475	2.26E-07	1.55E-06	6.185219
TIGIT	-0.70324	4.288997	-7.55218	9.65E-12	1.40E-10	16.05041
PRRC2C	-0.70325	6.269575	-8.11481	5.05E-13	9.05E-12	18.96165
HMGN4	-0.70418	8.018666	-6.5839	1.30E-09	1.32E-08	11.22534
IL27RA	-0.70438	4.975948	-7.43222	1.80E-11	2.49E-10	15.43878
ZNF317	-0.70445	4.603447	-8.39663	1.13E-13	2.26E-12	20.44253
DCAF17	-0.70448	2.878246	-8.62281	3.35E-14	7.31E-13	21.64018
SUCLG2	-0.70704	5.504513	-5.35909	4.16E-07	2.74E-06	5.593442
PDE3B	-0.70778	5.356472	-6.29203	5.42E-09	4.90E-08	9.828719
TSEN54	-0.70797	5.666113	-11.9189	5.00E-22	4.35E-20	39.49687
DET1	-0.70821	4.734742	-7.94959	1.21E-12	2.06E-11	18.10003
KIR2DL2	-0.70825	5.764667	-6.16299	1.01E-08	8.67E-08	9.221243
PGRMC2	-0.70842	4.414725	-5.93471	2.98E-08	2.37E-07	8.162659
PHF1	-0.70854	6.429236	-10.9323	1.12E-19	6.39E-18	34.13021
GORASP2	-0.70879	6.826245	-6.91754	2.48E-10	2.84E-09	12.8571
REV3L	-0.70977	5.57165	-6.97552	1.85E-10	2.17E-09	13.1442
ZNF566	-0.71015	2.013755	-7.53291	1.07E-11	1.54E-10	15.95195
TGIF2	-0.7111	4.459349	-8.49057	6.82E-14	1.41E-12	20.93902
ENOSF1	-0.7113	4.864835	-7.28252	3.88E-11	5.11E-10	14.68046
PCID2	-0.71136	5.389314	-6.82411	3.96E-10	4.40E-09	12.39658
KATNBL1	-0.71165	5.085342	-5.61406	1.32E-07	9.40E-07	6.712853
PRDM1	-0.71216	6.344454	-9.19789	1.50E-15	4.27E-14	24.71513
ELP3	-0.7123	5.339509	-8.6218	3.37E-14	7.34E-13	21.63481
SPIB	-0.71254	4.185957	-6.93862	2.23E-10	2.57E-09	12.96136
TMEM14A	-0.71331	4.799217	-5.73961	7.40E-08	5.53E-07	7.275157
PRKCQ-AS	-0.71338	4.020463	-7.11605	9.07E-11	1.12E-09	13.84418
GNPDA2	-0.71363	3.402748	-5.17628	9.32E-07	5.77E-06	4.810407
ZCCHC7	-0.71416	4.891026	-7.17993	6.56E-11	8.26E-10	14.1642
PARP8	-0.71481	7.193821	-5.0675	1.50E-06	8.88E-06	4.3526
MATK	-0.71506	5.132758	-7.9287	1.35E-12	2.28E-11	17.99148
LOC28595	-0.7151	3.070693	-4.85694	3.67E-06	2.03E-05	3.484405
PTCD3	-0.71542	4.762271	-7.03229	1.39E-10	1.65E-09	13.42627

SYNRG	-0.71608	5.519542	-11.4617	6.11E-21	4.36E-19	37.01328
CALM1	-0.71628	5.17205	-6.56763	1.41E-09	1.42E-08	11.14669
CD1C	-0.71694	5.477325	-8.45862	8.09E-14	1.66E-12	20.76999
NOSIP	-0.71782	7.42651	-7.37547	2.41E-11	3.29E-10	15.15064
LOC10192	-0.71803	3.554012	-8.6346	3.15E-14	6.94E-13	21.70281
EBLN3	-0.71825	6.650371	-5.43171	3.01E-07	2.02E-06	5.90912
GANAB	-0.71842	6.816889	-8.67657	2.51E-14	5.63E-13	21.92591
RELA	-0.71847	6.392224	-9.74677	7.50E-17	2.72E-15	27.67959
MAP7D1	-0.71851	7.518647	-6.60362	1.18E-09	1.21E-08	11.32079
C6orf136	-0.71881	5.262283	-7.72563	3.91E-12	6.08E-11	16.94077
TBC1D31	-0.71887	3.128551	-8.91131	7.08E-15	1.78E-13	23.178
GNPNAT1	-0.71957	4.43845	-6.30358	5.13E-09	4.67E-08	9.883409
ZNF302	-0.71979	4.539251	-4.38931	2.48E-05	0.000117	1.646641
WIPF1	-0.7206	7.327668	-7.99957	9.28E-13	1.61E-11	18.36012
ZNF274	-0.72064	5.937807	-6.34498	4.20E-09	3.89E-08	10.07983
APBA2	-0.7208	5.8	-7.45023	1.64E-11	2.29E-10	15.5304
SMAP2	-0.72093	9.822834	-4.24906	4.29E-05	0.000193	1.121306
TXNDC16	-0.72114	3.333728	-5.28915	5.68E-07	3.65E-06	5.291881
LAMP3	-0.72131	2.682039	-6.00614	2.13E-08	1.73E-07	8.491658
CLK1	-0.72168	5.665857	-3.39724	0.000927	0.003178	-1.78543
CEP68	-0.72195	5.109492	-8.49236	6.75E-14	1.40E-12	20.94847
KMT2A	-0.72212	4.014494	-12.5576	1.54E-23	1.73E-21	42.94981
PTCH1	-0.72218	2.737416	-13.2528	3.59E-25	5.52E-23	46.67821
ZNF211	-0.72271	5.046657	-6.53548	1.66E-09	1.65E-08	10.99156
MFHAS1	-0.72333	6.170063	-3.70349	0.000324	0.001227	-0.79905
MTMR11	-0.72334	5.409622	-3.8781	0.000173	0.00069	-0.20647
CCDC146	-0.72383	4.085696	-7.48684	1.35E-11	1.92E-10	15.71685
CD1D	-0.72399	6.629106	-2.80831	0.005824	0.016283	-3.48101
IARS	-0.72438	6.907746	-4.42447	2.16E-05	0.000103	1.780266
LOC15368	-0.72463	3.048049	-7.09698	1.00E-10	1.22E-09	13.74886
TNFRSF10I	-0.72512	4.809002	-7.86274	1.91E-12	3.14E-11	17.64923
LINC0095(	-0.72528	2.67918	-7.34866	2.76E-11	3.73E-10	15.0148
LOC20202	-0.72533	6.633419	-4.01538	0.000104	0.000433	0.274204
TRIM56	-0.72606	6.846204	-8.68328	2.42E-14	5.45E-13	21.96162
STAT6	-0.72725	7.714034	-8.68721	2.37E-14	5.36E-13	21.98252
OLIG1	-0.72726	6.242942	-2.87002	0.00486	0.013935	-3.31633
TMEM154	-0.72735	8.204478	-6.31348	4.89E-09	4.47E-08	9.930347
PITPNA	-0.72765	6.504986	-9.51102	2.72E-16	8.82E-15	26.40355
EPHA4	-0.72876	3.036715	-11.651	2.17E-21	1.73E-19	38.0421
ERAP2	-0.72908	3.920841	-3.60249	0.000461	0.001689	-1.1319
RPS23	-0.72914	8.607973	-6.24521	6.80E-09	6.05E-08	9.607605
NSG1	-0.72961	4.615287	-8.56549	4.56E-14	9.71E-13	21.33597
AGPAT5	-0.72968	4.18088	-6.31125	4.94E-09	4.52E-08	9.919745
POM121C	-0.73005	5.826346	-8.90344	7.39E-15	1.86E-13	23.13591
SMARCC1	-0.7303	5.470697	-11.5156	4.55E-21	3.43E-19	37.30643
LOC10013	-0.73063	5.613019	-8.36381	1.34E-13	2.66E-12	20.26936
PLEKHF1	-0.73129	5.908714	-8.69722	2.25E-14	5.13E-13	22.03578
FUS	-0.73141	5.116279	-9.43403	4.14E-16	1.30E-14	25.98768
LRPPRC	-0.73146	5.124704	-7.19072	6.20E-11	7.86E-10	14.21835
HEATR5B	-0.73227	5.864759	-6.90303	2.66E-10	3.04E-09	12.78539
BBIP1	-0.7324	5.173755	-7.1701	6.89E-11	8.67E-10	14.11488
PEX3	-0.73249	4.057893	-5.64929	1.12E-07	8.11E-07	6.869918
NONO	-0.73256	8.053836	-7.71226	4.20E-12	6.47E-11	16.87191
SLC25A32	-0.73287	5.119891	-3.85838	0.000186	0.000736	-0.27448
SRGAP2	-0.73355	5.303546	-5.88681	3.73E-08	2.93E-07	7.94326
PNPT1	-0.73377	4.764239	-4.59217	1.10E-05	5.53E-05	2.427995
MGA	-0.73438	3.418709	-9.19102	1.55E-15	4.42E-14	24.67817
CD6	-0.73476	4.502749	-10.9569	9.77E-20	5.65E-18	34.26413

ATP1A1	-0.73543	7.106663	-6.32043	4.73E-09	4.35E-08	9.963262
ZNF529	-0.73544	4.085526	-7.53323	1.07E-11	1.54E-10	15.95359
CALHM2	-0.73666	6.252462	-3.74981	0.000275	0.001055	-0.64394
LETMD1	-0.73895	6.254091	-8.73687	1.82E-14	4.23E-13	22.24686
KAT6B	-0.7391	4.407475	-10.6514	5.24E-19	2.70E-17	32.59955
GRAMD3	-0.73954	2.466933	-10.6769	4.55E-19	2.37E-17	32.73836
CIRBP	-0.73992	6.139075	-6.95876	2.01E-10	2.35E-09	13.06112
OCIAD2	-0.74017	5.607108	-6.65723	9.08E-10	9.43E-09	11.58086
UPRT	-0.74088	4.129965	-4.90625	2.98E-06	1.67E-05	3.685573
WDR54	-0.74145	5.80052	-8.57241	4.40E-14	9.39E-13	21.37265
CIPC	-0.74164	4.045624	-6.61483	1.12E-09	1.15E-08	11.37508
NAP1L2	-0.74175	2.798683	-6.1974	8.55E-09	7.48E-08	9.382622
SETD1B	-0.74244	7.531837	-11.0365	6.31E-20	3.83E-18	34.69772
ITM2A	-0.74267	7.037177	-3.70476	0.000323	0.001222	-0.79481
ND6	-0.74293	6.594349	-4.77077	5.26E-06	2.83E-05	3.136191
NOP58	-0.74315	6.964742	-4.04545	9.32E-05	0.000391	0.381191
EZR	-0.74317	5.587914	-8.70145	2.20E-14	5.03E-13	22.05828
EIF2S3	-0.74326	7.395176	-6.22284	7.57E-09	6.67E-08	9.502213
PLCXD2	-0.74509	3.72883	-8.51517	5.98E-14	1.25E-12	21.06926
CLEC2B	-0.74525	5.403243	-4.17719	5.66E-05	0.000248	0.856984
KAT7	-0.74528	5.998513	-10.9781	8.70E-20	5.11E-18	34.37951
SCAP	-0.74584	7.009932	-11.178	2.90E-20	1.84E-18	35.46856
CCDC84	-0.74592	5.620272	-6.09671	1.38E-08	1.17E-07	8.911737
RPL10A	-0.74594	10.49658	-5.80613	5.43E-08	4.16E-07	7.57589
THAP9-AS	-0.74632	4.984431	-4.40611	2.32E-05	0.00011	1.710373
NCOA1	-0.74708	7.503519	-8.31994	1.70E-13	3.29E-12	20.03823
ZFP3	-0.7476	4.467191	-6.30163	5.18E-09	4.71E-08	9.874183
PARP15	-0.74763	2.767887	-8.701	2.20E-14	5.03E-13	22.05589
ZNF703	-0.7481	4.059853	-7.45774	1.57E-11	2.22E-10	15.56862
SIMC1	-0.74885	4.644929	-6.39524	3.29E-09	3.11E-08	10.3191
RPS16P5	-0.74894	6.701181	-4.79006	4.86E-06	2.62E-05	3.21379
TOMM70A	-0.7506	5.619063	-5.65139	1.11E-07	8.04E-07	6.879306
TRAF3IP3	-0.75293	6.729611	-6.43924	2.65E-09	2.54E-08	10.52932
LOC10050	-0.75297	3.562483	-6.54576	1.57E-09	1.57E-08	11.04111
ZNF766	-0.75322	5.700444	-8.45313	8.33E-14	1.71E-12	20.74096
RFTN1	-0.75368	4.177014	-8.62005	3.40E-14	7.39E-13	21.62552
GPATCH1	-0.75369	4.570301	-7.00777	1.57E-10	1.86E-09	13.30432
KB-431C1.	-0.7538	5.991156	-4.00971	0.000107	0.000442	0.254094
PTCD2	-0.754	4.181169	-7.37136	2.46E-11	3.35E-10	15.12982
PCMTD2	-0.75495	6.001989	-7.91201	1.47E-12	2.47E-11	17.90477
USP28	-0.75531	3.790617	-11.5945	2.95E-21	2.31E-19	37.73536
METAP1	-0.75538	6.19036	-7.3145	3.29E-11	4.38E-10	14.84197
SPEN	-0.75621	5.75827	-9.80021	5.60E-17	2.07E-15	27.96931
RPL3	-0.75769	8.854259	-5.86087	4.21E-08	3.28E-07	7.824841
ZFAND1	-0.7583	4.482524	-4.76493	5.39E-06	2.90E-05	3.112747
DIEXF	-0.76018	4.323567	-8.8645	9.12E-15	2.25E-13	22.92778
AES	-0.76071	6.360895	-6.00827	2.11E-08	1.72E-07	8.501476
HCG26	-0.76179	5.409565	-7.69992	4.48E-12	6.87E-11	16.80835
CYTH1	-0.76225	6.53756	-10.6475	5.35E-19	2.75E-17	32.57795
ZBTB41	-0.76308	4.228601	-4.05506	8.99E-05	0.000379	0.415484
TRMT61B	-0.76371	4.552115	-4.67422	7.85E-06	4.07E-05	2.751048
PAPD7	-0.76417	6.130421	-8.78111	1.43E-14	3.42E-13	22.48268
RPL34	-0.76437	7.987237	-2.84466	0.005237	0.014856	-3.38438
HMG20A	-0.7644	4.868759	-6.12809	1.19E-08	1.01E-07	9.058077
CARNS1	-0.76447	3.8442	-9.47242	3.36E-16	1.07E-14	26.19496
ZNF439	-0.76452	2.675224	-4.77005	5.28E-06	2.84E-05	3.133273
TNFAIP8L1	-0.76536	4.093541	-8.33686	1.55E-13	3.04E-12	20.12736
MDFIC	-0.76549	4.380536	-6.23532	7.13E-09	6.31E-08	9.560974

RGS2	-0.76556	11.34775	-4.88546	3.25E-06	1.81E-05	3.600591
PAXIP1	-0.76748	5.34156	-5.67513	9.95E-08	7.28E-07	6.985456
C16orf80	-0.76774	6.53407	-6.37754	3.58E-09	3.37E-08	10.23472
MAGEH1	-0.76803	4.971045	-5.48204	2.40E-07	1.64E-06	6.1294
C14orf159	-0.76808	6.404549	-11.7759	1.09E-21	9.12E-20	38.72095
CRTC3	-0.76809	5.611232	-4.44117	2.02E-05	9.71E-05	1.843991
PSIP1	-0.76946	5.353265	-8.47956	7.23E-14	1.49E-12	20.88077
ATR	-0.76977	3.987232	-7.15922	7.29E-11	9.12E-10	14.06032
EIF3D	-0.76998	8.88184	-7.87909	1.75E-12	2.89E-11	17.73399
ITPKB	-0.77001	5.153324	-14.4139	7.40E-28	1.59E-25	52.8132
CD300LB	-0.77143	5.899757	-9.97351	2.17E-17	8.61E-16	28.9099
RPGR	-0.77234	4.044721	-5.39659	3.52E-07	2.34E-06	5.756134
SUN2	-0.77427	7.398328	-11.2373	2.09E-20	1.37E-18	35.79143
HNRNPDL	-0.77435	5.941122	-6.31986	4.74E-09	4.36E-08	9.960576
PRSS33	-0.77497	4.666776	-4.51807	1.48E-05	7.34E-05	2.139672
OAS1	-0.77497	5.659525	-4.07577	8.32E-05	0.000353	0.489678
RICTOR	-0.77555	6.247981	-7.04261	1.32E-10	1.58E-09	13.47765
CLEC7A	-0.77582	5.488948	-5.76818	6.48E-08	4.89E-07	7.404059
TPD52	-0.7761	4.128145	-5.12084	1.19E-06	7.19E-06	4.576339
TRIT1	-0.77635	4.507125	-5.7969	5.67E-08	4.33E-07	7.534063
ARL14EP	-0.77692	5.699492	-5.48074	2.41E-07	1.65E-06	6.123697
HDDC2	-0.77693	4.54705	-5.8155	5.20E-08	4.00E-07	7.618444
C12orf75	-0.77714	5.332003	-3.21439	0.001683	0.005437	-2.34098
DUSP14	-0.77716	4.020081	-10.5092	1.14E-18	5.64E-17	31.82444
ABHD3	-0.77767	7.560651	-4.91959	2.82E-06	1.58E-05	3.740196
PPIF	-0.77786	6.553459	-7.13641	8.18E-11	1.02E-09	13.94606
PRR5L	-0.77813	3.500053	-11.0137	7.15E-20	4.29E-18	34.57383
SDE2	-0.77819	5.438248	-6.67137	8.47E-10	8.84E-09	11.64963
JMJD1C	-0.77869	5.251798	-8.89807	7.61E-15	1.90E-13	23.10716
C16orf54	-0.77942	9.416263	-5.76884	6.46E-08	4.88E-07	7.407063
KIAA0907	-0.77994	6.006904	-8.78845	1.37E-14	3.29E-13	22.52182
PRMT6	-0.77997	3.606272	-4.51629	1.49E-05	7.38E-05	2.132786
TRIM44	-0.7802	5.368589	-8.07579	6.20E-13	1.10E-11	18.75767
ZNF260	-0.78175	3.79809	-5.36694	4.02E-07	2.65E-06	5.627451
MALT1	-0.78216	4.886748	-5.25101	6.72E-07	4.26E-06	5.128496
LRRC47	-0.7825	6.962675	-6.81122	4.22E-10	4.66E-09	12.33324
SH2B3	-0.78292	7.057011	-6.81341	4.17E-10	4.62E-09	12.34403
UBE2E2	-0.78429	5.017739	-4.51605	1.49E-05	7.38E-05	2.131862
LOC10019	-0.78575	6.704938	-4.72284	6.42E-06	3.40E-05	2.944317
UXS1	-0.78649	6.246173	-6.71145	6.94E-10	7.37E-09	11.84493
CLSTN1	-0.78664	5.570311	-10.535	9.93E-19	4.92E-17	31.96514
RNF4	-0.78682	7.806603	-8.7712	1.51E-14	3.58E-13	22.42983
GPR174	-0.78713	2.114906	-9.5094	2.74E-16	8.89E-15	26.3948
KANSL1	-0.78802	7.596066	-7.24107	4.80E-11	6.24E-10	14.47153
IRF9	-0.78888	8.200283	-8.68098	2.45E-14	5.51E-13	21.94937
SLC16A10	-0.78903	2.427663	-10.0109	1.76E-17	7.12E-16	29.11289
KIF3A	-0.78918	3.42345	-8.33336	1.58E-13	3.09E-12	20.10892
MRPS6	-0.7897	6.984949	-6.65864	9.02E-10	9.37E-09	11.58773
XCL1	-0.78984	3.477549	-7.81115	2.50E-12	4.02E-11	17.3822
DDHD1	-0.7901	3.626249	-9.68376	1.06E-16	3.72E-15	27.3382
AKT1	-0.79296	6.240236	-7.95588	1.17E-12	2.00E-11	18.13272
ABCE1	-0.79327	5.047418	-4.32222	3.23E-05	0.000149	1.393794
SRRM1	-0.79383	6.911521	-10.2053	6.07E-18	2.63E-16	30.17008
PHF10	-0.79446	5.645364	-6.4593	2.41E-09	2.32E-08	10.62542
ZNF227	-0.79453	4.148527	-7.21775	5.40E-11	6.94E-10	14.35418
NOP2	-0.79455	5.517415	-7.15873	7.30E-11	9.14E-10	14.05785
ZFP62	-0.79519	5.099202	-4.4941	1.63E-05	8.00E-05	2.047147
NHS	-0.79744	2.338054	-13.9338	9.40E-27	1.83E-24	50.29215

VSIG1	-0.79853	3.218316	-10.3257	3.14E-18	1.43E-16	30.82519
SRPK2	-0.79872	5.725386	-9.02277	3.87E-15	1.03E-13	23.77478
KMT2E	-0.79926	7.158867	-7.80094	2.64E-12	4.22E-11	17.32944
PI4KA	-0.80008	5.432491	-7.51561	1.17E-11	1.67E-10	15.86362
GEMIN5	-0.80057	5.085532	-9.395	5.12E-16	1.57E-14	25.77702
ZBTB1	-0.80166	4.165048	-5.6232	1.26E-07	9.06E-07	6.753542
TNFRSF25	-0.80258	4.854534	-10.1816	6.92E-18	2.97E-16	30.04106
SLAMF1	-0.80262	4.371425	-8.23439	2.68E-13	5.04E-12	19.58835
FHIT	-0.80332	4.477648	-6.08415	1.47E-08	1.23E-07	8.853312
EIF3L	-0.80334	9.197107	-5.90483	3.43E-08	2.71E-07	8.025682
HNRNPA1	-0.80529	5.865565	-6.56721	1.42E-09	1.43E-08	11.14467
CYP27A1	-0.80712	5.807921	-7.45627	1.59E-11	2.23E-10	15.5611
POGLUT1	-0.80737	4.992303	-6.56929	1.40E-09	1.41E-08	11.15473
NPIPA1	-0.80756	7.216976	-9.17968	1.65E-15	4.67E-14	24.61721
MAGED1	-0.80788	6.075208	-7.11028	9.35E-11	1.15E-09	13.8153
RPS24	-0.80827	7.579107	-5.2753	6.03E-07	3.86E-06	5.23245
NUP35	-0.80987	4.728563	-5.78852	5.90E-08	4.49E-07	7.496093
PKN2	-0.81069	5.894786	-7.37186	2.45E-11	3.35E-10	15.13232
RPAP3	-0.811	4.628099	-6.94802	2.12E-10	2.46E-09	13.0079
BTG2	-0.81261	6.723575	-7.06038	1.20E-10	1.45E-09	13.56619
PCYOX1	-0.81425	4.249883	-8.14651	4.27E-13	7.75E-12	19.12753
PANK4	-0.81484	5.457405	-9.53088	2.44E-16	7.95E-15	26.51088
ARFIP1	-0.81622	5.271723	-6.24187	6.91E-09	6.14E-08	9.591829
OAS3	-0.81685	4.309187	-8.16328	3.90E-13	7.14E-12	19.21534
OASL	-0.8172	5.306392	-4.77837	5.10E-06	2.75E-05	3.166728
GCN1L1	-0.81749	4.883529	-10.3741	2.40E-18	1.12E-16	31.08877
ANKRD46	-0.81795	4.199316	-5.52793	1.95E-07	1.35E-06	6.331287
CTCF	-0.81815	7.140889	-10.4958	1.23E-18	6.03E-17	31.7518
CREB1	-0.81894	6.86806	-11.316	1.36E-20	9.21E-19	36.22036
TMEM109	-0.81968	6.162104	-8.3874	1.18E-13	2.37E-12	20.39381
TPR	-0.81984	4.893292	-8.79537	1.32E-14	3.17E-13	22.55873
GALNT12	-0.82171	2.935136	-7.0243	1.45E-10	1.72E-09	13.38654
HCRP1	-0.8221	5.151624	-9.08167	2.81E-15	7.62E-14	24.0907
ALDH9A1	-0.82274	8.044097	-9.48687	3.10E-16	9.95E-15	26.27307
HEG1	-0.82331	4.460076	-9.50208	2.86E-16	9.22E-15	26.35523
FBL	-0.82347	7.835473	-6.04274	1.79E-08	1.47E-07	8.660992
PRKCZ	-0.82551	5.562035	-8.73859	1.80E-14	4.20E-13	22.25605
TTC39C	-0.82687	4.609976	-7.37041	2.47E-11	3.37E-10	15.125
KLF11	-0.82701	3.64661	-7.67952	4.98E-12	7.59E-11	16.70341
LIME1	-0.82717	5.990305	-7.42512	1.86E-11	2.58E-10	15.40268
ZNF420	-0.82731	3.923964	-7.45904	1.56E-11	2.20E-10	15.57521
TTC19	-0.82758	6.577674	-5.66434	1.05E-07	7.61E-07	6.93718
DCTD	-0.82815	5.425426	-6.90769	2.60E-10	2.97E-09	12.80839
SGPP1	-0.82954	5.294805	-4.38298	2.54E-05	0.00012	1.622677
SLC39A10	-0.83114	4.818105	-4.33168	3.11E-05	0.000145	1.429256
FBXL16	-0.83118	4.520513	-10.097	1.10E-17	4.56E-16	29.58104
NAT10	-0.8314	5.471911	-9.55221	2.17E-16	7.18E-15	26.62624
CBFA2T3	-0.83161	4.721588	-7.33489	2.97E-11	3.99E-10	14.9451
SLAIN1	-0.83236	3.511345	-5.15516	1.02E-06	6.27E-06	4.721049
TNFSF8	-0.83251	5.100916	-5.04929	1.62E-06	9.55E-06	4.276573
TAF15	-0.83275	5.90782	-8.35704	1.39E-13	2.75E-12	20.23367
SEC14L1	-0.83286	7.141062	-9.58551	1.81E-16	6.07E-15	26.80633
LOC10050	-0.83288	4.704218	-8.84071	1.04E-14	2.54E-13	22.80073
APBB1	-0.83318	4.586585	-11.377	9.73E-21	6.75E-19	36.55198
LOC72782	-0.83342	6.822909	-8.16557	3.86E-13	7.06E-12	19.22734
SENP7	-0.83443	3.728647	-4.97839	2.19E-06	1.26E-05	3.982256
LINC00342	-0.8347	4.632401	-8.44462	8.72E-14	1.78E-12	20.69601
SH2D1A	-0.83494	4.688901	-9.68492	1.05E-16	3.71E-15	27.34445

CRYZ	-0.83551	4.61098	-3.81928	0.000214	0.000839	-0.40847
PIK3C2B	-0.83624	6.047027	-8.97154	5.11E-15	1.32E-13	23.50032
ITGA5	-0.83636	6.611286	-10.5977	7.03E-19	3.56E-17	32.3067
DCAF16	-0.83647	3.595245	-8.65493	2.82E-14	6.28E-13	21.81083
PFAS	-0.83651	3.810405	-6.22306	7.56E-09	6.66E-08	9.503285
RTP4	-0.83798	5.410956	-5.18574	8.95E-07	5.57E-06	4.850516
ZBTB25	-0.83811	3.982179	-8.34194	1.51E-13	2.96E-12	20.15412
ATG16L2	-0.83895	8.176	-6.36758	3.76E-09	3.52E-08	10.18729
RSL1D1	-0.83966	4.775657	-7.15477	7.45E-11	9.30E-10	14.03802
PEA15	-0.83971	5.710713	-5.66284	1.05E-07	7.66E-07	6.930477
SETX	-0.83982	6.003036	-11.8419	7.62E-22	6.47E-20	39.07876
HSF2	-0.84073	2.765673	-8.5193	5.84E-14	1.23E-12	21.09116
ARMCX2	-0.84082	3.209248	-7.60313	7.41E-12	1.09E-10	16.31123
NOL11	-0.84143	5.917081	-6.43226	2.74E-09	2.62E-08	10.49593
CCDC109E	-0.84197	6.937493	-5.49352	2.28E-07	1.56E-06	6.179811
PPFIBP2	-0.84305	4.620954	-6.72524	6.48E-10	6.92E-09	11.91223
FASLG	-0.84321	3.421412	-11.07	5.25E-20	3.22E-18	34.88027
EIF3A	-0.84417	6.776309	-8.85048	9.84E-15	2.42E-13	22.85289
TP53	-0.84513	4.243844	-7.62831	6.50E-12	9.67E-11	16.44037
BCL11A	-0.84537	5.537128	-6.6645	8.76E-10	9.11E-09	11.61622
FBXO21	-0.84704	4.919388	-11.0119	7.22E-20	4.32E-18	34.56387
FOXP1	-0.84748	6.260178	-12.0559	2.36E-22	2.20E-20	40.23933
ISG20	-0.84761	7.740543	-5.1102	1.24E-06	7.50E-06	4.531577
TAP2	-0.84901	4.694127	-12.878	2.71E-24	3.51E-22	44.67278
LOC10272	-0.85099	6.611295	-5.54045	1.84E-07	1.28E-06	6.386535
LOC10192	-0.85127	4.168955	-6.42233	2.88E-09	2.75E-08	10.44847
KLRC4	-0.85131	1.835916	-8.39702	1.12E-13	2.25E-12	20.44456
TMEM181	-0.85186	6.959758	-6.7873	4.76E-10	5.20E-09	12.21589
UNG	-0.85322	4.187	-6.39372	3.31E-09	3.13E-08	10.31185
ACVR1	-0.85686	5.307442	-5.42377	3.12E-07	2.09E-06	5.874462
OSGEPL1	-0.85715	4.361453	-8.19328	3.33E-13	6.14E-12	19.37261
MEN1	-0.85831	5.851434	-10.2137	5.80E-18	2.52E-16	30.2156
MID1IP1	-0.85866	6.893431	-9.73772	7.88E-17	2.84E-15	27.63051
IL15	-0.85881	3.197416	-6.18526	9.07E-09	7.86E-08	9.325651
ATG2A	-0.85883	6.304389	-9.87602	3.70E-17	1.42E-15	28.3806
OXCT1	-0.86009	5.322143	-6.30716	5.04E-09	4.60E-08	9.900369
ADA	-0.86014	5.735276	-6.81888	4.06E-10	4.51E-09	12.3709
PHOSPHO	-0.86126	6.542268	-3.34994	0.001084	0.003659	-1.93159
SLC39A6	-0.86191	4.882728	-8.95821	5.49E-15	1.41E-13	23.42894
PPM1K	-0.86204	3.874896	-9.24286	1.17E-15	3.42E-14	24.95708
HMCES	-0.86239	4.888019	-12.6161	1.12E-23	1.27E-21	43.26478
RPS4X	-0.86284	11.49415	-7.63066	6.42E-12	9.56E-11	16.45241
NEDD9	-0.86288	5.729532	-10.9607	9.56E-20	5.54E-18	34.28508
MAD1L1	-0.86294	5.104981	-4.67129	7.95E-06	4.11E-05	2.739448
THUMPD1	-0.86298	5.930793	-6.67901	8.15E-10	8.54E-09	11.68681
HAL	-0.86378	4.753787	-6.81973	4.04E-10	4.49E-09	12.37504
GZMM	-0.86463	4.458624	-7.34313	2.84E-11	3.83E-10	14.98679
LSR	-0.86532	4.014344	-7.55015	9.76E-12	1.41E-10	16.04003
ABCB1	-0.86606	2.886806	-12.0001	3.21E-22	2.93E-20	39.93703
SAMHD1	-0.86636	7.303959	-5.16644	9.73E-07	5.99E-06	4.768767
RRS1	-0.86817	5.618886	-6.61243	1.13E-09	1.16E-08	11.36344
DFFB	-0.86883	3.751566	-5.50423	2.17E-07	1.49E-06	6.226879
BHLHE40	-0.86978	4.943384	-8.79747	1.31E-14	3.15E-13	22.5699
BRD1	-0.87014	5.634008	-8.34885	1.45E-13	2.87E-12	20.19054
MX2	-0.87174	8.002434	-7.67185	5.18E-12	7.87E-11	16.66401
CENPC	-0.87253	4.554628	-6.29451	5.36E-09	4.85E-08	9.840453
ITGAL	-0.87402	6.960178	-8.06004	6.74E-13	1.19E-11	18.67544
MYLIP	-0.87843	6.63322	-8.44138	8.87E-14	1.81E-12	20.67887

BEX4	-0.87911	4.251816	-4.70954	6.79E-06	3.57E-05	2.891295
NOB1	-0.87945	6.28711	-8.31164	1.77E-13	3.44E-12	19.99451
CD74	-0.88038	6.422297	-8.34751	1.46E-13	2.89E-12	20.18345
BANP	-0.88056	6.956635	-10.8222	2.05E-19	1.13E-17	33.53022
HNRNPA0	-0.88059	5.456593	-10.2272	5.39E-18	2.35E-16	30.28904
RFX5	-0.88183	5.997042	-10.148	8.32E-18	3.52E-16	29.85856
DPF2	-0.88201	7.224783	-13.0686	9.68E-25	1.37E-22	45.69385
NNT-AS1	-0.88254	3.751734	-5.57576	1.57E-07	1.10E-06	6.542739
PCSK7	-0.88269	5.658626	-9.22516	1.29E-15	3.73E-14	24.86184
ICAM2	-0.88347	7.26365	-6.1846	9.10E-09	7.88E-08	9.32253
EPHB1	-0.88624	2.866812	-15.1175	1.86E-29	5.08E-27	56.46396
JUP	-0.88751	4.523328	-6.20864	8.10E-09	7.11E-08	9.435435
LINC00936	-0.88967	6.093573	-5.98124	2.39E-08	1.93E-07	8.37673
LPIN2	-0.8913	7.033087	-7.22079	5.32E-11	6.84E-10	14.36951
LIPT1	-0.89341	5.231302	-5.81007	5.33E-08	4.09E-07	7.593776
SSBP3	-0.89471	5.622248	-6.11216	1.29E-08	1.09E-07	8.983735
AOC2	-0.89659	3.955136	-8.42355	9.76E-14	1.97E-12	20.58465
RSAD1	-0.89674	5.808938	-8.11839	4.95E-13	8.92E-12	18.98034
OFD1	-0.89717	5.435208	-6.66214	8.86E-10	9.21E-09	11.60477
MORC2	-0.89759	4.870953	-11.3314	1.25E-20	8.55E-19	36.30371
ATHL1	-0.89783	5.909386	-6.53647	1.65E-09	1.64E-08	10.99633
GABPB2	-0.89842	5.187657	-8.80755	1.24E-14	3.00E-13	22.62369
SCAF8	-0.89845	6.77647	-8.41932	9.98E-14	2.02E-12	20.5623
LOC28483	-0.9006	6.34904	-4.1131	7.22E-05	0.00031	0.624063
ZNF275	-0.90148	5.473557	-8.38468	1.20E-13	2.40E-12	20.37948
ESYT2	-0.90201	4.393207	-10.2736	4.17E-18	1.88E-16	30.5417
MOV10	-0.90229	4.186271	-12.3965	3.69E-23	3.96E-21	42.08124
CXCR3	-0.90281	4.656446	-12.4634	2.57E-23	2.81E-21	42.44204
SIGLEC17F	-0.9029	4.924057	-7.29774	3.59E-11	4.75E-10	14.75729
HNRNPH3	-0.90441	6.837887	-9.25242	1.11E-15	3.25E-14	25.00853
TRAF5	-0.90546	3.543166	-8.16749	3.82E-13	7.00E-12	19.23739
SF3A1	-0.90728	6.630637	-13.818	1.74E-26	3.27E-24	49.68048
KIR2DS2	-0.90835	3.411082	-6.4258	2.83E-09	2.70E-08	10.46504
EIF4B	-0.9088	7.532503	-10.481	1.34E-18	6.49E-17	31.67119
BBX	-0.90911	6.122552	-8.65377	2.84E-14	6.31E-13	21.8047
C5orf56	-0.9096	5.58991	-10.1076	1.04E-17	4.34E-16	29.63861
UTRN	-0.91023	4.905373	-8.93243	6.32E-15	1.61E-13	23.29096
RUNX1-IT1	-0.9112	6.834418	-7.20586	5.74E-11	7.33E-10	14.29441
PRKRA	-0.91168	4.793174	-6.79492	4.58E-10	5.03E-09	12.25323
TAP1	-0.91186	7.351342	-10.2715	4.22E-18	1.90E-16	30.53049
IGF2R	-0.91195	7.870575	-6.58585	1.29E-09	1.31E-08	11.23476
AMIGO1	-0.91334	4.566266	-9.36106	6.16E-16	1.87E-14	25.59395
TRRAP	-0.91449	4.692744	-9.87248	3.77E-17	1.44E-15	28.36137
TRIM28	-0.91529	6.88939	-7.77488	3.02E-12	4.79E-11	17.19479
OR52K3P	-0.91665	4.946077	-5.71498	8.29E-08	6.16E-07	7.164291
CD97	-0.91855	8.288718	-5.45675	2.69E-07	1.82E-06	6.018569
TMEM243	-0.91925	6.369947	-5.69779	8.97E-08	6.63E-07	7.087056
MYOM2	-0.92161	3.678054	-5.83842	4.68E-08	3.61E-07	7.722597
DGKA	-0.92289	6.687901	-8.21982	2.89E-13	5.39E-12	19.51185
SH3YL1	-0.92291	5.213708	-8.01972	8.35E-13	1.45E-11	18.46513
RASA3	-0.92315	4.794894	-11.876	6.32E-22	5.43E-20	39.26421
SBK1	-0.92367	5.158101	-11.3546	1.10E-20	7.55E-19	36.42999
ITGB7	-0.92481	5.616574	-10.1365	8.86E-18	3.73E-16	29.79559
ASPRV1	-0.92512	5.290056	-8.80421	1.26E-14	3.05E-13	22.60586
SIT1	-0.92628	6.0561	-8.35877	1.38E-13	2.73E-12	20.24282
FAM117B	-0.92636	4.208933	-11.2499	1.95E-20	1.29E-18	35.86019
TRERF1	-0.92723	4.191327	-11.3625	1.05E-20	7.26E-19	36.47307
LEPROTL1	-0.92784	7.16127	-8.91758	6.85E-15	1.73E-13	23.21151

ZNF14	-0.92848	4.293494	-5.99659	2.23E-08	1.81E-07	8.447556
ZNF708	-0.92894	3.035771	-5.66272	1.05E-07	7.66E-07	6.929918
LIX1L	-0.9292	5.036993	-12.1355	1.53E-22	1.47E-20	40.67017
NFXL1	-0.93065	4.798379	-4.11997	7.04E-05	0.000303	0.648916
ZNHIT6	-0.93113	3.39822	-7.87091	1.83E-12	3.01E-11	17.69157
AGAP4	-0.93174	4.786806	-7.12962	8.47E-11	1.05E-09	13.91208
WDR82	-0.93364	8.272233	-10.5639	8.47E-19	4.22E-17	32.1226
PEBP1	-0.93368	6.151271	-7.52314	1.12E-11	1.61E-10	15.90206
S1PR5	-0.93472	4.265359	-11.5221	4.39E-21	3.34E-19	37.34148
CD79A	-0.93579	6.339875	-5.34103	4.51E-07	2.95E-06	5.515353
PILRA	-0.9358	7.306864	-6.08497	1.46E-08	1.23E-07	8.857088
MFSD6	-0.93604	4.031019	-7.77822	2.97E-12	4.72E-11	17.21204
LGALS3BP	-0.9377	4.066717	-9.65452	1.24E-16	4.30E-15	27.17985
HCG27	-0.93971	6.940324	-5.41235	3.28E-07	2.19E-06	5.824701
BCOR	-0.93998	3.666936	-11.786	1.03E-21	8.67E-20	38.77578
GIMAP8	-0.94087	5.471314	-7.18028	6.54E-11	8.25E-10	14.16594
SLC7A6	-0.94136	4.877895	-9.41434	4.61E-16	1.43E-14	25.88138
ATP6V0E2	-0.94419	5.682427	-9.49257	3.01E-16	9.68E-15	26.30382
BIN1	-0.94458	6.059029	-10.3402	2.90E-18	1.33E-16	30.90424
SCARNA1[illegible]	-0.9447	5.108677	-8.96765	5.22E-15	1.35E-13	23.47948
CSF1R	-0.94532	6.607428	-5.38459	3.71E-07	2.46E-06	5.704029
MORC4	-0.94674	2.690073	-10.7613	2.86E-19	1.53E-17	33.19827
FAM102A	-0.94678	4.592403	-10.8147	2.13E-19	1.17E-17	33.48935
CNNM3	-0.94682	6.03978	-11.1847	2.79E-20	1.78E-18	35.50527
RASSF5	-0.94721	8.122129	-7.33661	2.94E-11	3.95E-10	14.95378
HLA-F	-0.94736	8.327545	-9.76574	6.76E-17	2.47E-15	27.7824
MPZL1	-0.94813	5.164333	-8.31112	1.78E-13	3.44E-12	19.9918
LINC0095[illegible]	-0.94903	4.491413	-7.82563	2.32E-12	3.75E-11	17.45711
PARP14	-0.94989	5.060159	-13.6228	4.93E-26	8.41E-24	48.64708
MRFAP1L1	-0.94996	6.833766	-7.35527	2.67E-11	3.62E-10	15.04826
GPR171	-0.95011	6.085567	-4.24335	4.39E-05	0.000197	1.100202
FAM69A	-0.95028	3.525045	-6.95442	2.06E-10	2.40E-09	13.03961
TRMT13	-0.9513	5.204976	-5.18492	8.98E-07	5.59E-06	4.847031
SLC41A1	-0.95314	4.625874	-9.55595	2.13E-16	7.05E-15	26.64645
KISS1R	-0.95374	3.437186	-7.65947	5.53E-12	8.36E-11	16.60038
CD4	-0.95385	4.935689	-11.4061	8.29E-21	5.79E-19	36.71054
JMY	-0.95486	3.846113	-8.44424	8.74E-14	1.78E-12	20.694
YPEL1	-0.95561	4.068168	-13.9212	1.01E-26	1.94E-24	50.22533
TMCC1	-0.95569	5.003361	-15.8905	3.48E-31	1.29E-28	60.41057
TMCC3	-0.9565	6.23598	-5.70943	8.50E-08	6.31E-07	7.139344
MSL2	-0.95656	6.188076	-8.75556	1.64E-14	3.87E-13	22.34646
NFATC2	-0.95678	4.38186	-12.8916	2.52E-24	3.28E-22	44.74576
SETD6	-0.95708	4.042741	-9.01896	3.95E-15	1.04E-13	23.75436
AUTS2	-0.95817	3.303486	-13.0863	8.80E-25	1.26E-22	45.78845
MEF2C	-0.95972	5.360407	-7.2935	3.67E-11	4.84E-10	14.73592
TOX	-0.95984	2.71382	-11.9827	3.53E-22	3.21E-20	39.8428
AKR1B1	-0.96012	7.190867	-6.93183	2.30E-10	2.66E-09	12.92777
CYTIP	-0.96112	8.598348	-6.90113	2.69E-10	3.07E-09	12.776
KCNA3	-0.9623	4.770825	-5.68515	9.51E-08	6.99E-07	7.030358
CXCR4	-0.9639	9.365177	-7.73791	3.67E-12	5.74E-11	17.00405
C5orf28	-0.96448	4.42558	-4.97893	2.19E-06	1.26E-05	3.984487
C12orf57	-0.96592	6.336284	-6.19656	8.59E-09	7.51E-08	9.378671
ID3	-0.96681	3.921957	-8.82014	1.16E-14	2.82E-13	22.6909
CCR5	-0.9687	6.117207	-6.11599	1.26E-08	1.07E-07	9.00159
ATP8B2	-0.96896	4.979583	-7.59298	7.81E-12	1.15E-10	16.25922
INPP4B	-0.96946	2.800608	-9.26848	1.02E-15	3.00E-14	25.09502
IL13RA1	-0.96993	6.424688	-5.59522	1.44E-07	1.02E-06	6.629084
IFIH1	-0.97058	2.989945	-9.35539	6.36E-16	1.92E-14	25.56337

RAB11FIP1	-0.97335	7.48651	-10.7967	2.36E-19	1.28E-17	33.39118
LRFN3	-0.97403	3.397552	-9.56134	2.07E-16	6.85E-15	26.67562
RAB39B	-0.97428	3.21851	-8.25675	2.38E-13	4.50E-12	19.70581
ZBTB4	-0.97476	6.537883	-11.1735	2.97E-20	1.88E-18	35.4442
ZNF331	-0.97605	4.658043	-8.41341	1.03E-13	2.08E-12	20.53111
MAP3K4	-0.97679	5.958	-10.215	5.76E-18	2.51E-16	30.223
PTPRCAP	-0.97797	6.853501	-6.58806	1.28E-09	1.30E-08	11.24544
TRIM68	-0.9794	4.871742	-9.86417	3.94E-17	1.49E-15	28.31629
CCDC92	-0.9797	6.076253	-6.30027	5.21E-09	4.73E-08	9.867727
LRBA	-0.98041	4.960558	-9.0728	2.95E-15	7.94E-14	24.04312
ZNF304	-0.98133	3.206694	-8.82719	1.12E-14	2.72E-13	22.7285
PARP12	-0.98201	6.686614	-8.36352	1.34E-13	2.67E-12	20.26787
LOC10272	-0.98207	3.923858	-6.50743	1.90E-09	1.87E-08	10.85652
EEF2	-0.9822	10.85382	-9.16773	1.76E-15	4.95E-14	24.55298
TBC1D9	-0.98353	5.151119	-7.84533	2.09E-12	3.41E-11	17.55908
CDC42SE2	-0.98364	7.510639	-7.66024	5.50E-12	8.33E-11	16.60432
PLA2G7	-0.98405	3.20622	-4.06397	8.70E-05	0.000367	0.447364
MAP4K1	-0.98501	5.332808	-9.75436	7.20E-17	2.62E-15	27.72072
SIGIRR	-0.98583	6.1499	-9.23297	1.24E-15	3.59E-14	24.90386
ST6GAL1	-0.98898	4.490815	-11.4971	5.03E-21	3.72E-19	37.2055
TCL1A	-0.98956	6.154166	-4.10652	7.41E-05	0.000317	0.600333
TKTL1	-0.99057	4.009571	-8.92609	6.54E-15	1.66E-13	23.25703
DUSP6	-0.99125	8.014057	-4.51577	1.50E-05	7.39E-05	2.130781
CBLL1	-0.99247	4.913862	-8.46348	7.88E-14	1.62E-12	20.79568
BANK1	-0.99328	5.745109	-5.0352	1.72E-06	1.01E-05	4.217864
AQP3	-0.99372	5.521211	-8.17785	3.61E-13	6.64E-12	19.29168
STK39	-0.99409	4.855206	-6.3869	3.42E-09	3.23E-08	10.27935
CD69	-0.99441	3.728998	-3.92419	0.000146	0.000591	-0.04654
IMP3	-0.99571	6.574457	-8.80654	1.25E-14	3.02E-13	22.61833
ABHD15	-0.99637	4.481406	-7.91034	1.49E-12	2.48E-11	17.89612
FCGR3B	-0.99762	11.82701	-5.62185	1.27E-07	9.11E-07	6.747535
FUT11	-0.99797	4.470104	-13.1559	6.05E-25	8.90E-23	46.16079
RNF44	-1.00021	7.851503	-9.07532	2.91E-15	7.85E-14	24.05665
FCRLA	-1.00048	3.893253	-5.93232	3.01E-08	2.39E-07	8.151716
DDX18	-1.00162	5.862969	-8.70127	2.20E-14	5.03E-13	22.05733
CPVL	-1.00184	7.383322	-3.20122	0.001756	0.005656	-2.38001
BEX2	-1.00359	4.671193	-6.27866	5.79E-09	5.22E-08	9.765486
SLC38A1	-1.00379	5.708532	-9.66253	1.19E-16	4.14E-15	27.22319
KLF10	-1.00581	6.101998	-4.63401	9.26E-06	4.73E-05	2.592216
KPNA5	-1.00592	3.784226	-6.87782	3.02E-10	3.43E-09	12.66099
RBM4	-1.00669	5.76957	-6.34202	4.26E-09	3.94E-08	10.06576
ITGA6	-1.00803	5.3246	-6.36896	3.74E-09	3.50E-08	10.19387
CEP78	-1.00854	4.482703	-10.6657	4.84E-19	2.51E-17	32.67718
PHC1	-1.0099	4.868546	-10.7429	3.17E-19	1.68E-17	33.09812
CABIN1	-1.01036	6.376161	-12.9186	2.18E-24	2.89E-22	44.89065
SARAF	-1.01084	9.71128	-8.58679	4.07E-14	8.75E-13	21.44896
NFATC3	-1.01091	5.622216	-15.6008	1.53E-30	5.32E-28	58.93973
PPAPDC2	-1.01187	4.966198	-6.23279	7.22E-09	6.38E-08	9.549056
CHMP7	-1.01204	6.655131	-11.8812	6.14E-22	5.31E-20	39.29215
SMCHD1	-1.01279	6.762745	-7.69634	4.56E-12	7.00E-11	16.78994
DDX58	-1.0143	5.681259	-9.36534	6.02E-16	1.83E-14	25.61701
RPL22	-1.01696	8.923383	-8.08968	5.76E-13	1.03E-11	18.83027
BBS2	-1.01727	5.032657	-8.56531	4.57E-14	9.71E-13	21.33501
1-Sep	-1.0177	5.315316	-7.6336	6.32E-12	9.44E-11	16.4675
ISG15	-1.02311	6.406112	-5.6105	1.34E-07	9.55E-07	6.697027
PATL2	-1.02373	4.969579	-11.5438	3.90E-21	3.01E-19	37.45956
SKI	-1.02458	5.806746	-10.9278	1.15E-19	6.51E-18	34.10572
IRF1	-1.02474	8.207232	-10.0656	1.31E-17	5.37E-16	29.41055

STIM2	-1.03347	4.819432	-8.08879	5.79E-13	1.03E-11	18.8256
GLOD4	-1.03523	5.29192	-6.18216	9.20E-09	7.96E-08	9.311105
MYCBP2	-1.03575	7.65562	-12.8603	2.98E-24	3.77E-22	44.57759
WWP1	-1.03927	5.421226	-8.06338	6.63E-13	1.17E-11	18.69288
NAP1L3	-1.04282	2.397839	-8.22981	2.74E-13	5.15E-12	19.56428
PAQR8	-1.04435	6.062531	-10.2485	4.79E-18	2.13E-16	30.40523
ZHX2	-1.04447	5.183782	-10.715	3.69E-19	1.94E-17	32.94562
PHOSPHO	-1.04833	3.068568	-6.17164	9.68E-09	8.36E-08	9.261793
WBP11	-1.04833	5.204896	-11.204	2.51E-20	1.62E-18	35.61033
PRKDC	-1.04976	4.106576	-12.3229	5.51E-23	5.70E-21	41.68349
ZCCHC2	-1.05012	4.798728	-10.6789	4.50E-19	2.35E-17	32.74905
PRKACB	-1.05066	5.144301	-7.02317	1.45E-10	1.73E-09	13.38092
PRPF8	-1.05172	6.757688	-10.9543	9.91E-20	5.71E-18	34.25007
REM2	-1.05229	4.827471	-7.91778	1.43E-12	2.40E-11	17.93477
PARP1	-1.05249	6.273268	-10.0278	1.61E-17	6.52E-16	29.20471
S1PR1	-1.05344	5.738342	-4.39019	2.47E-05	0.000117	1.649968
LPIN1	-1.05399	4.690634	-9.42163	4.43E-16	1.38E-14	25.92072
RORA	-1.05456	3.641955	-9.6087	1.60E-16	5.43E-15	26.9318
RRAS2	-1.05488	3.821202	-6.8614	3.28E-10	3.69E-09	12.58005
PSMB9	-1.05506	8.649325	-10.6327	5.80E-19	2.98E-17	32.49759
DENND2D	-1.05603	6.476737	-8.59147	3.97E-14	8.56E-13	21.47378
GPR56	-1.05778	5.048603	-10.8025	2.28E-19	1.25E-17	33.423
TC2N	-1.05931	2.80481	-8.13968	4.42E-13	8.02E-12	19.09177
LOC10099	-1.06086	7.856446	-8.63623	3.12E-14	6.89E-13	21.71149
HSP90AB1	-1.06114	7.633553	-6.15763	1.03E-08	8.88E-08	9.196193
JPX	-1.06147	3.274472	-7.12657	8.60E-11	1.06E-09	13.89678
SH2D2A	-1.06155	5.765094	-9.18053	1.65E-15	4.66E-14	24.6218
ATIC	-1.06317	6.494978	-7.53969	1.03E-11	1.49E-10	15.9866
SERPING1	-1.0636	4.166336	-6.95129	2.09E-10	2.43E-09	13.02408
6-Sep	-1.06366	5.985773	-10.049	1.43E-17	5.84E-16	29.31991
SCRN1	-1.06388	5.181688	-7.7664	3.16E-12	5.00E-11	17.15104
LAX1	-1.06505	5.298418	-6.30372	5.13E-09	4.67E-08	9.884069
NLRP1	-1.06613	4.93924	-12.7267	6.15E-24	7.36E-22	43.85986
PTER	-1.0662	5.258854	-6.59083	1.26E-09	1.28E-08	11.25887
CBLB	-1.06639	3.922979	-11.0893	4.72E-20	2.93E-18	34.98577
SOD2	-1.07074	7.520115	-5.29297	5.58E-07	3.60E-06	5.308286
LFNG	-1.07304	6.015575	-9.0957	2.61E-15	7.10E-14	24.16605
HMGN3	-1.07957	6.970883	-6.73992	6.02E-10	6.49E-09	11.98396
ZNF329	-1.08046	3.051747	-8.09306	5.66E-13	1.01E-11	18.84791
DHRS3	-1.08066	5.350915	-8.87458	8.64E-15	2.14E-13	22.98162
FLJ12120	-1.0807	3.574144	-6.2413	6.93E-09	6.15E-08	9.589165
XPC	-1.08083	6.405482	-14.4088	7.60E-28	1.62E-25	52.78637
ZNF559	-1.08136	4.902761	-5.97078	2.51E-08	2.02E-07	8.328508
HLA-DPB1	-1.08169	5.787088	-6.99051	1.71E-10	2.02E-09	13.21861
ARRDC3	-1.08315	7.005643	-6.78845	4.73E-10	5.17E-09	12.22154
GSE1	-1.08366	4.728558	-15.2695	8.47E-30	2.48E-27	57.24541
EPHX2	-1.08413	3.253631	-10.1601	7.78E-18	3.31E-16	29.92413
DSC1	-1.09052	2.202408	-10.1446	8.47E-18	3.57E-16	29.83994
CAMK2D	-1.09084	4.003976	-9.37679	5.66E-16	1.73E-14	25.67876
CD96	-1.09192	3.480856	-8.15625	4.05E-13	7.39E-12	19.17855
ZBTB2	-1.09672	4.343864	-9.90109	3.22E-17	1.26E-15	28.51666
OAS2	-1.09682	4.690822	-10.2638	4.40E-18	1.97E-16	30.48858
TESPA1	-1.09889	5.093795	-10.2459	4.86E-18	2.15E-16	30.39111
LOC10192	-1.09905	2.438317	-6.38696	3.42E-09	3.23E-08	10.27963
BIRC3	-1.09941	5.419188	-7.20192	5.86E-11	7.47E-10	14.27463
KIAA1147	-1.10043	5.248252	-9.79445	5.78E-17	2.13E-15	27.9381
PTGDR	-1.10179	3.340178	-9.14107	2.04E-15	5.64E-14	24.40967
IFFO2	-1.10266	4.836078	-11.0569	5.64E-20	3.44E-18	34.809

SMAD7	-1.10323	4.194834	-8.71998	1.99E-14	4.60E-13	22.15693
PIK3IP1	-1.10352	6.010127	-8.595	3.89E-14	8.42E-13	21.49251
USP11	-1.10398	6.28351	-11.6584	2.08E-21	1.68E-19	38.08275
TNIK	-1.10409	4.087817	-12.1487	1.42E-22	1.38E-20	40.74192
PRKCH	-1.1093	5.231463	-11.286	1.60E-20	1.08E-18	36.05698
FCGR2B	-1.10955	5.641418	-6.42376	2.86E-09	2.73E-08	10.45528
TOPORS	-1.11238	5.992116	-8.75967	1.61E-14	3.79E-13	22.36839
LY6E	-1.11322	6.141569	-9.46945	3.41E-16	1.09E-14	26.17896
CXCR2	-1.11425	9.612161	-7.11503	9.12E-11	1.12E-09	13.83907
C9orf91	-1.11889	4.258046	-9.12935	2.17E-15	5.99E-14	24.34675
ISOC1	-1.12007	5.07181	-6.47875	2.19E-09	2.12E-08	10.71872
MLLT3	-1.12018	3.869101	-9.97137	2.19E-17	8.69E-16	28.89828
TRMT11	-1.12031	3.729893	-6.01325	2.06E-08	1.68E-07	8.524485
CTSO	-1.12065	5.447594	-6.99256	1.70E-10	2.00E-09	13.22876
ABHD14B	-1.12624	6.075681	-9.41961	4.48E-16	1.39E-14	25.90981
GVINP1	-1.12866	5.209832	-5.266	6.29E-07	4.01E-06	5.192604
WLS	-1.12941	5.052916	-4.94539	2.52E-06	1.43E-05	3.846177
ZNF652	-1.1319	6.499396	-11.4849	5.38E-21	3.95E-19	37.13904
JADE2	-1.13251	4.873458	-13.4038	1.59E-25	2.51E-23	47.48336
PJA1	-1.13401	6.111979	-11.273	1.72E-20	1.15E-18	35.9862
GOLGA8A	-1.1382	4.91309	-8.02692	8.03E-13	1.40E-11	18.50265
DDX24	-1.14003	6.210646	-11.3129	1.38E-20	9.34E-19	36.20338
CD5	-1.14087	5.650514	-12.0723	2.16E-22	2.04E-20	40.32784
FTO	-1.14118	6.064055	-10.8264	2.00E-19	1.10E-17	33.55323
JAK1	-1.14389	7.277324	-12.7023	7.02E-24	8.30E-22	43.7288
RSAD2	-1.14552	5.603803	-3.41627	0.00087	0.002997	-1.72617
9-Sep	-1.1463	5.994454	-11.7163	1.51E-21	1.23E-19	38.39714
BCL9L	-1.14645	5.258694	-13.5859	6.01E-26	1.02E-23	48.4508
AOC3	-1.14667	2.760576	-10.0529	1.40E-17	5.73E-16	29.34117
LAT	-1.14818	6.050135	-8.63056	3.22E-14	7.05E-13	21.68136
KIAA0355	-1.14881	4.841675	-10.7045	3.91E-19	2.05E-17	32.8887
KLF12	-1.15013	4.000559	-11.1032	4.37E-20	2.73E-18	35.06148
PDCD4	-1.15071	3.488979	-5.86843	4.06E-08	3.17E-07	7.859345
BLNK	-1.15201	5.522042	-4.31567	3.31E-05	0.000153	1.369254
HVCN1	-1.1525	7.609112	-10.3744	2.40E-18	1.12E-16	31.09027
TIGD3	-1.15262	4.573901	-12.1296	1.58E-22	1.51E-20	40.63823
CD46	-1.15484	7.890337	-7.20269	5.84E-11	7.44E-10	14.27849
MAL	-1.155	6.953242	-5.83032	4.85E-08	3.75E-07	7.68577
TIAM1	-1.1578	5.098049	-8.63161	3.20E-14	7.03E-13	21.68693
CRTAM	-1.15839	3.70502	-6.25102	6.61E-09	5.90E-08	9.634991
FBLN5	-1.15929	3.953392	-8.23114	2.72E-13	5.12E-12	19.5713
TMEM140	-1.1607	7.231765	-7.87103	1.83E-12	3.01E-11	17.69221
UBE2L6	-1.16074	7.110599	-11.4456	6.68E-21	4.73E-19	36.92523
XAF1	-1.16162	5.128286	-7.15661	7.38E-11	9.23E-10	14.04723
CXXC5	-1.16276	6.332274	-7.62044	6.77E-12	1.00E-10	16.4
PLEKHO1	-1.16797	6.610618	-8.97372	5.05E-15	1.31E-13	23.51201
GSPT2	-1.17102	4.561454	-8.31061	1.78E-13	3.44E-12	19.98911
FAM171A1	-1.1729	3.957491	-11.1884	2.74E-20	1.75E-18	35.52524
RHOH	-1.17356	6.938238	-8.50372	6.35E-14	1.32E-12	21.00865
CPPED1	-1.18915	7.927218	-8.51266	6.06E-14	1.27E-12	21.05596
CD28	-1.19013	3.615139	-8.74681	1.72E-14	4.03E-13	22.29983
ZNF121	-1.19105	6.079505	-6.67365	8.37E-10	8.75E-09	11.66073
ZNF831	-1.19107	2.708397	-13.7998	1.92E-26	3.54E-24	49.5843
CHST11	-1.19417	6.334183	-12.2492	8.24E-23	8.34E-21	41.28527
THEM4	-1.1952	4.311755	-9.45203	3.75E-16	1.18E-14	26.08484
LOC10193	-1.19635	4.501833	-6.04922	1.73E-08	1.43E-07	8.691041
CACNA2D	-1.19697	4.576224	-4.95833	2.39E-06	1.36E-05	3.899455
RRN3	-1.20037	4.660254	-7.04573	1.30E-10	1.56E-09	13.49322

MTR	-1.2006	4.397326	-9.87107	3.80E-17	1.44E-15	28.35373
STAT1	-1.20107	6.424492	-9.42054	4.46E-16	1.39E-14	25.91483
CD81	-1.20351	7.516901	-7.9223	1.39E-12	2.35E-11	17.9582
ARHGEF18	-1.20399	8.627247	-15.2648	8.68E-30	2.50E-27	57.22155
ZNF83	-1.20405	4.195733	-7.46919	1.48E-11	2.10E-10	15.62688
ECI2	-1.20442	5.188072	-7.98446	1.01E-12	1.73E-11	18.28144
SLAMF6	-1.2053	5.888684	-11.7394	1.33E-21	1.10E-19	38.52242
OSBPL3	-1.20817	3.785158	-9.17785	1.67E-15	4.71E-14	24.60736
F2RL1	-1.21173	5.015789	-5.84912	4.45E-08	3.45E-07	7.771278
HCP5	-1.21743	6.164586	-8.69178	2.31E-14	5.24E-13	22.00685
DNMT1	-1.21975	6.538167	-9.29828	8.68E-16	2.60E-14	25.25551
SCML4	-1.21984	4.438177	-12.1262	1.61E-22	1.53E-20	40.62001
OGFRL1	-1.22163	6.804093	-10.3472	2.79E-18	1.28E-16	30.94217
NR3C2	-1.22976	3.206227	-11.0742	5.13E-20	3.16E-18	34.90314
HLA-DQB1	-1.23243	4.305498	-5.43031	3.03E-07	2.03E-06	5.903003
IFIT5	-1.23249	5.756852	-7.41566	1.96E-11	2.70E-10	15.35461
TRAV8-3	-1.23328	2.735337	-15.081	2.25E-29	5.98E-27	56.27614
PTGS2	-1.23597	4.622995	-5.56074	1.68E-07	1.18E-06	6.476225
IKZF3	-1.23762	4.458583	-10.8435	1.82E-19	1.01E-17	33.64633
SACS	-1.23901	2.868465	-6.93031	2.32E-10	2.67E-09	12.92024
SAMD3	-1.24138	3.704313	-9.78341	6.14E-17	2.25E-15	27.87823
PBXIP1	-1.24144	6.260796	-14.6452	2.19E-28	4.99E-26	54.01918
IFI44	-1.24414	4.065314	-5.76083	6.70E-08	5.05E-07	7.370893
LOC93622	-1.24572	5.386182	-9.09961	2.55E-15	6.97E-14	24.18702
FLVCR1	-1.24612	4.306794	-7.45038	1.64E-11	2.29E-10	15.53112
FCRL3	-1.24678	4.601743	-7.65574	5.63E-12	8.51E-11	16.58121
CHI3L1	-1.25618	5.416695	-2.95542	0.003766	0.011131	-3.0833
TNFAIP2	-1.26194	6.153096	-13.4227	1.44E-25	2.29E-23	47.58351
BZRAP1-A	-1.26339	5.786513	-10.156	7.96E-18	3.38E-16	29.90195
SERINC5	-1.26384	7.028572	-8.33464	1.57E-13	3.07E-12	20.11565
CCR6	-1.26517	3.94134	-8.57425	4.35E-14	9.32E-13	21.3824
PTPN4	-1.26604	3.570002	-13.1977	4.83E-25	7.21E-23	46.38407
SATB1	-1.26622	6.688109	-8.23465	2.67E-13	5.03E-12	19.58969
DPP4	-1.26659	3.874387	-9.68727	1.04E-16	3.67E-15	27.35717
AMIGO2	-1.26709	4.770301	-6.65422	9.22E-10	9.55E-09	11.56624
MYBL1	-1.26901	4.50895	-7.33342	2.99E-11	4.01E-10	14.93766
CLEC2D	-1.26924	4.410705	-8.68704	2.37E-14	5.36E-13	21.98161
CBX7	-1.26925	6.905492	-12.748	5.48E-24	6.63E-22	43.97444
SEMA4D	-1.27261	7.198722	-12.845	3.24E-24	4.03E-22	44.49569
RNF125	-1.27407	4.766173	-10.3572	2.64E-18	1.22E-16	30.99671
IER5	-1.27992	6.715084	-6.81036	4.24E-10	4.68E-09	12.32904
PWAR6	-1.2868	3.271838	-8.40647	1.07E-13	2.15E-12	20.49444
LOC10013	-1.29116	3.926803	-9.20725	1.42E-15	4.08E-14	24.7655
LDOC1L	-1.29326	4.787851	-9.8732	3.75E-17	1.43E-15	28.36532
GIMAP1	-1.29431	6.378711	-7.33375	2.98E-11	4.01E-10	14.93936
ABLIM1	-1.29521	5.80694	-9.89324	3.36E-17	1.31E-15	28.47406
PMAIP1	-1.2991	4.360333	-8.50068	6.46E-14	1.34E-12	20.99253
MAP3K14	-1.30116	4.427887	-12.6525	9.19E-24	1.06E-21	43.46084
ARHGEF3	-1.30646	6.03467	-8.35334	1.42E-13	2.80E-12	20.21419
KIAA1551	-1.30941	8.478229	-8.26991	2.21E-13	4.23E-12	19.775
FAM169A	-1.31238	3.312118	-8.51151	6.09E-14	1.27E-12	21.0499
RRN3P2	-1.31239	5.018801	-8.74309	1.76E-14	4.11E-13	22.28002
MSANTD2	-1.3135	4.662966	-6.56779	1.41E-09	1.42E-08	11.14745
TGFBI	-1.31447	7.89118	-4.13679	6.60E-05	0.000286	0.709831
CRY1	-1.31726	4.1651	-8.32329	1.67E-13	3.24E-12	20.05586
UBE2Q2	-1.31727	5.639582	-7.03445	1.37E-10	1.64E-09	13.43704
KDM2B	-1.31739	5.907233	-9.41393	4.62E-16	1.43E-14	25.87918
ITPK1-AS1	-1.32207	4.106798	-11.9617	3.96E-22	3.50E-20	39.72856

GBP3	-1.32282	5.22262	-4.1377	6.58E-05	0.000285	0.713164
FLT3LG	-1.32754	4.742201	-7.98596	9.97E-13	1.72E-11	18.28922
GIMAP7	-1.33211	7.462168	-6.61166	1.14E-09	1.16E-08	11.35972
LY75	-1.33233	6.286981	-7.88889	1.66E-12	2.75E-11	17.78484
MARCKSL1	-1.3336	6.969531	-8.99668	4.46E-15	1.16E-13	23.63496
RCAN3	-1.33725	4.412336	-9.64152	1.33E-16	4.60E-15	27.10943
DPEP2	-1.33878	7.481698	-8.44269	8.81E-14	1.80E-12	20.68579
MS4A1	-1.33944	6.64429	-4.96823	2.29E-06	1.31E-05	3.940271
LDHB	-1.34385	9.274734	-8.75711	1.63E-14	3.84E-13	22.35474
IL10RA	-1.34693	7.896734	-7.77732	2.99E-12	4.74E-11	17.20742
P2RY8	-1.34785	7.308959	-11.2837	1.62E-20	1.09E-18	36.04418
PPP1R16B	-1.35129	5.448384	-9.16542	1.79E-15	5.01E-14	24.54053
OXNAD1	-1.35659	5.705724	-9.76107	6.94E-17	2.53E-15	27.75709
ZNF266	-1.3581	6.694222	-8.19666	3.27E-13	6.04E-12	19.39031
MYC	-1.36211	6.991952	-6.35419	4.01E-09	3.74E-08	10.1236
TREM1	-1.36812	8.044612	-5.9997	2.19E-08	1.78E-07	8.461871
ZNF146	-1.37024	5.035207	-9.78353	6.13E-17	2.25E-15	27.87886
RUNX3	-1.3748	5.685078	-11.5245	4.33E-21	3.31E-19	37.35475
EPSTI1	-1.37912	4.668715	-8.0317	7.83E-13	1.37E-11	18.5276
KRT23	-1.38207	6.30453	-4.82449	4.21E-06	2.30E-05	3.352781
MPEG1	-1.38535	8.600437	-6.31568	4.84E-09	4.44E-08	9.940757
CD7	-1.38743	5.300677	-8.22445	2.82E-13	5.27E-12	19.53613
DDX60	-1.39198	4.04684	-7.16511	7.07E-11	8.87E-10	14.08984
DOCK10	-1.39286	4.699144	-10.2278	5.37E-18	2.35E-16	30.29257
ETS1	-1.39827	5.79363	-11.9581	4.03E-22	3.54E-20	39.70933
PYHIN1	-1.39891	3.828626	-9.41167	4.68E-16	1.44E-14	25.86696
LDLRAP1	-1.40076	6.898336	-11.2486	1.97E-20	1.29E-18	35.85295
MAN1C1	-1.40076	3.763478	-11.8808	6.16E-22	5.31E-20	39.29001
PPTC7	-1.40103	6.890126	-14.2829	1.48E-27	3.12E-25	52.12722
TRAT1	-1.40111	5.266968	-4.8935	3.15E-06	1.75E-05	3.633425
HLA-DMA	-1.40132	7.16432	-6.60334	1.19E-09	1.21E-08	11.31943
CAMK4	-1.40178	4.162049	-8.71415	2.05E-14	4.73E-13	22.1259
CHRM3-A	-1.40391	4.283882	-5.87353	3.97E-08	3.10E-07	7.882589
BAG3	-1.41613	4.772915	-8.48787	6.92E-14	1.43E-12	20.92472
ZFP36L2	-1.41623	6.470757	-14.8286	8.39E-29	2.12E-26	54.97162
PASK	-1.41693	3.95925	-13.1274	7.05E-25	1.02E-22	46.00856
CD3D	-1.41964	7.446104	-6.9495	2.11E-10	2.45E-09	13.01522
CHST7	-1.42007	4.95617	-13.0309	1.19E-24	1.64E-22	45.49213
FAM174A	-1.42079	5.700141	-8.33284	1.58E-13	3.09E-12	20.10614
FGL2	-1.43315	8.821639	-7.45258	1.62E-11	2.26E-10	15.54236
KLRC3	-1.44204	2.498813	-11.0154	7.08E-20	4.26E-18	34.58295
BTBD11	-1.4453	3.300013	-12.3311	5.27E-23	5.48E-21	41.72773
LGALS2	-1.45164	4.668888	-5.81774	5.15E-08	3.96E-07	7.628584
TSHZ1	-1.45219	5.225615	-10.9375	1.09E-19	6.23E-18	34.1585
TCF7	-1.45959	6.272829	-9.81702	5.11E-17	1.91E-15	28.06048
RTN1	-1.46134	4.542011	-8.7991	1.30E-14	3.12E-13	22.57861
PDCD4-AS	-1.46479	4.2549	-12.575	1.40E-23	1.58E-21	43.04348
KLRG1	-1.46672	5.534373	-10.0611	1.34E-17	5.48E-16	29.38563
GBP4	-1.4714	3.743428	-14.6896	1.74E-28	4.09E-26	54.25013
CFD	-1.48283	6.968168	-4.7923	4.81E-06	2.60E-05	3.222784
CPA3	-1.48322	3.964778	-6.64171	9.81E-10	1.01E-08	11.50548
ANKRD36I	-1.48372	5.797437	-6.87289	3.10E-10	3.51E-09	12.63668
MAML2	-1.48715	4.991369	-12.98	1.56E-24	2.10E-22	45.21955
TRIB2	-1.48751	5.101201	-8.75737	1.63E-14	3.84E-13	22.35613
EVL	-1.48956	6.991897	-8.12389	4.81E-13	8.69E-12	19.00911
AMPD2	-1.49302	6.806789	-12.8361	3.40E-24	4.19E-22	44.44777
GIMAP6	-1.49465	7.089828	-6.50369	1.94E-09	1.90E-08	10.83854
APOL3	-1.49894	5.160516	-11.9777	3.62E-22	3.28E-20	39.8156

CAMK1D	-1.4991	6.013877	-8.54771	5.02E-14	1.07E-12	21.2417
LINC0121[illegible]	-1.50794	4.455174	-5.57739	1.56E-07	1.10E-06	6.549957
PCED1B	-1.51114	6.540821	-7.08811	1.05E-10	1.27E-09	13.70456
CCL4	-1.5133	6.075932	-9.02894	3.75E-15	9.94E-14	23.80789
CDC25B	-1.51614	7.152699	-11.4138	7.95E-21	5.57E-19	36.75261
GRAMD1C	-1.52048	1.857757	-18.8648	1.52E-37	1.20E-34	74.90377
LOC72839	-1.52404	5.966128	-9.37705	5.65E-16	1.73E-14	25.6802
ABCG1	-1.52752	3.645069	-11.2226	2.27E-20	1.47E-18	35.71154
DYRK2	-1.52775	5.447173	-8.92511	6.57E-15	1.67E-13	23.25179
LY9	-1.52903	5.631385	-11.0657	5.37E-20	3.29E-18	34.85695
HLA-DMB	-1.53429	5.963157	-8.15409	4.10E-13	7.46E-12	19.16723
DDHD2	-1.53818	4.146574	-7.73877	3.65E-12	5.71E-11	17.00849
LEF1	-1.54355	5.129905	-8.0368	7.63E-13	1.34E-11	18.55415
RPS6KA5	-1.54793	4.442795	-16.0834	1.30E-31	5.12E-29	61.38408
SPOCK2	-1.54878	5.807438	-13.5741	6.40E-26	1.06E-23	48.38833
ESYT1	-1.55113	7.021213	-14.8152	9.00E-29	2.22E-26	54.90219
BACH2	-1.5529	4.909654	-8.83861	1.05E-14	2.56E-13	22.7895
TBX21	-1.55333	5.526329	-12.5281	1.81E-23	2.01E-21	42.79113
ARL4C	-1.55805	5.863439	-11.0263	6.67E-20	4.04E-18	34.64246
CASS4	-1.55902	3.939708	-11.471	5.81E-21	4.21E-19	37.06367
TRDV3	-1.56318	3.424014	-8.8433	1.02E-14	2.51E-13	22.81452
SKAP1	-1.56609	5.521203	-10.2783	4.07E-18	1.84E-16	30.56704
TXNIP	-1.56923	10.70247	-9.1611	1.83E-15	5.12E-14	24.51734
TMEM263	-1.56998	4.773718	-7.09477	1.01E-10	1.23E-09	13.73781
SIDT1	-1.5703	4.888593	-9.42157	4.43E-16	1.38E-14	25.92039
HLA-DRA	-1.57216	9.080765	-6.36868	3.74E-09	3.50E-08	10.19254
ATP2B1	-1.5876	5.712988	-8.37229	1.28E-13	2.55E-12	20.31408
BTN3A1	-1.60261	6.166699	-15.4756	2.92E-30	9.14E-28	58.30105
SYTL2	-1.61654	3.915071	-10.3853	2.26E-18	1.06E-16	31.1497
GBP1	-1.61673	5.73665	-9.14801	1.96E-15	5.47E-14	24.44699
FCER1A	-1.61998	3.241894	-10.2527	4.68E-18	2.08E-16	30.428
SH2D1B	-1.626	3.344418	-10.2533	4.67E-18	2.08E-16	30.43141
KIAA1324	-1.62992	4.088828	-11.6421	2.27E-21	1.80E-19	37.99409
PI3	-1.63695	6.498092	-4.98405	2.14E-06	1.23E-05	4.005643
CD27	-1.63832	7.452775	-8.70907	2.11E-14	4.84E-13	22.09884
ZC3H12D	-1.64361	5.225945	-7.25435	4.48E-11	5.85E-10	14.53841
ITGA4	-1.64719	5.29204	-6.51549	1.83E-09	1.80E-08	10.89529
TRG-AS1	-1.65351	6.851222	-8.25759	2.36E-13	4.48E-12	19.71022
GIMAP4	-1.66722	8.177829	-9.19436	1.53E-15	4.35E-14	24.69615
MX1	-1.66973	6.697932	-7.15055	7.61E-11	9.49E-10	14.01685
RARRES3	-1.67425	6.608535	-9.66696	1.16E-16	4.05E-15	27.24721
CECR1	-1.67671	7.843146	-8.70649	2.14E-14	4.90E-13	22.0851
ZAP70	-1.67844	5.784626	-9.63818	1.36E-16	4.67E-15	27.09138
PTGER4	-1.68243	5.5183	-11.5975	2.90E-21	2.28E-19	37.75173
KLRD1	-1.68369	5.445968	-9.59111	1.76E-16	5.91E-15	26.83664
ZNF600	-1.6854	4.963556	-7.94706	1.22E-12	2.08E-11	18.08686
GPR183	-1.69225	5.875589	-8.90887	7.18E-15	1.81E-13	23.16496
CD160	-1.69471	4.121677	-8.71416	2.05E-14	4.73E-13	22.12594
NOV	-1.69624	3.539673	-25.2612	1.30E-49	2.66E-46	102.2583
P2RY10	-1.70155	4.17493	-10.0052	1.82E-17	7.34E-16	29.08181
LYSMD2	-1.702	7.7132	-11.2627	1.82E-20	1.21E-18	35.92969
PRKCQ	-1.71867	5.756954	-11.1312	3.75E-20	2.35E-18	35.21362
CXCL8	-1.72449	3.143889	-10.1754	7.16E-18	3.06E-16	30.00716
IL32	-1.72836	7.217065	-6.92824	2.35E-10	2.70E-09	12.91
GATA3	-1.72958	4.004269	-14.493	4.88E-28	1.06E-25	53.22639
PDE4B	-1.73323	4.324651	-13.7203	2.93E-26	5.17E-24	49.16354
SLFN5	-1.7384	5.291042	-12.0247	2.80E-22	2.57E-20	40.07029
TAGAP	-1.73938	6.919117	-15.1023	2.02E-29	5.43E-27	56.3858

BTN3A2	-1.74849	5.837969	-10.6689	4.76E-19	2.47E-17	32.69485
EMR3	-1.75181	5.554059	-8.16118	3.95E-13	7.21E-12	19.20435
CCL5	-1.75766	8.164539	-6.61638	1.11E-09	1.14E-08	11.38259
THEMIS	-1.75872	3.478135	-7.10956	9.38E-11	1.15E-09	13.81169
LOC28358	-1.76626	2.603514	-18.2932	2.33E-36	1.59E-33	72.20654
FYN	-1.77617	6.733648	-9.37748	5.64E-16	1.72E-14	25.68247
PRKX	-1.77869	4.219852	-9.93296	2.71E-17	1.07E-15	28.68969
TBC1D4	-1.78401	4.753776	-8.00861	8.85E-13	1.54E-11	18.40719
CCR7	-1.80647	6.99898	-6.10263	1.35E-08	1.13E-07	8.939349
CD3G	-1.8151	5.981944	-8.54933	4.98E-14	1.06E-12	21.25028
STAT4	-1.81975	6.245202	-12.0485	2.46E-22	2.28E-20	40.19944
GPRASP1	-1.83291	4.421135	-9.57819	1.89E-16	6.29E-15	26.76673
NMT2	-1.83407	3.084112	-14.5831	3.04E-28	6.83E-26	53.69592
CX3CR1	-1.83728	6.92155	-6.86468	3.23E-10	3.64E-09	12.59621
HLA-DPA1	-1.83759	7.600736	-7.92367	1.38E-12	2.33E-11	17.96535
HLA-DQA	-1.83896	5.219041	-4.80927	4.48E-06	2.43E-05	3.291273
NOG	-1.85786	2.920327	-14.5614	3.40E-28	7.57E-26	53.58304
LOC28307	-1.8656	6.042974	-10.4127	1.94E-18	9.27E-17	31.29908
GPR18	-1.87081	5.531713	-8.22491	2.81E-13	5.26E-12	19.53857
UBASH3A	-1.87547	4.950477	-9.7094	9.20E-17	3.28E-15	27.47706
XIST	-1.87758	4.311987	-2.49014	0.014151	0.035243	-4.27977
RASGRP1	-1.87812	6.481378	-7.76412	3.20E-12	5.06E-11	17.13927
PLEKHA1	-1.87948	5.156	-10.1827	6.88E-18	2.96E-16	30.04696
HSH2D	-1.8879	3.967368	-18.7535	2.58E-37	1.88E-34	74.38199
ALDH1A1	-1.90576	2.504196	-14.8195	8.80E-29	2.20E-26	54.92423
SGK223	-1.90728	6.423942	-9.70098	9.64E-17	3.43E-15	27.43145
CTSW	-1.90973	6.891884	-9.93738	2.64E-17	1.04E-15	28.71372
CD8A	-1.912	6.409267	-9.25862	1.08E-15	3.15E-14	25.04192
BCL11B	-1.92981	5.388066	-11.3227	1.31E-20	8.94E-19	36.25668
PVRIG	-1.92996	5.332513	-9.87762	3.66E-17	1.41E-15	28.38929
TRANK1	-1.94787	5.693139	-18.8968	1.31E-37	1.07E-34	75.05342
HERC5	-1.95067	4.938535	-10.1184	9.78E-18	4.11E-16	29.69739
LBH	-1.97206	6.891733	-10.9036	1.31E-19	7.36E-18	33.9739
GZMH	-1.99166	6.203989	-5.69045	9.28E-08	6.83E-07	7.054135
LINC00877	-1.99281	3.730015	-12.1552	1.38E-22	1.35E-20	40.77697
GZMK	-1.99668	6.339209	-7.32141	3.18E-11	4.24E-10	14.87693
CD3E	-1.99899	7.1907	-9.31017	8.13E-16	2.44E-14	25.31961
NR1D2	-2.01897	4.706586	-9.99469	1.93E-17	7.72E-16	29.02498
NLRC3	-2.01953	6.337865	-10.3258	3.13E-18	1.43E-16	30.82578
GBP5	-2.02261	5.155917	-11.6371	2.34E-21	1.85E-19	37.96661
P2RY14	-2.04227	4.447029	-7.62329	6.67E-12	9.90E-11	16.41462
BTN3A3	-2.08435	4.926797	-10.3973	2.12E-18	1.00E-16	31.21492
TXK	-2.09766	4.992104	-8.08757	5.83E-13	1.04E-11	18.8192
A2M-AS1	-2.10231	4.694963	-8.19794	3.25E-13	6.00E-12	19.39702
LCK	-2.1156	6.896328	-9.90472	3.16E-17	1.24E-15	28.53636
FAIM3	-2.12542	7.462721	-10.3754	2.39E-18	1.12E-16	31.09573
GZMA	-2.13141	6.003464	-6.97655	1.84E-10	2.17E-09	13.14932
GZMB	-2.13908	6.997454	-8.27162	2.19E-13	4.20E-12	19.78395
CD2	-2.16533	7.080605	-9.15206	1.92E-15	5.36E-14	24.46876
SULF2	-2.19649	5.573055	-11.4689	5.87E-21	4.25E-19	37.05242
KLRB1	-2.21236	6.260653	-9.84072	4.48E-17	1.69E-15	28.18903
TRBC1	-2.21863	8.372683	-8.77739	1.46E-14	3.48E-13	22.46286
EOMES	-2.23764	5.136354	-10.1548	8.01E-18	3.39E-16	29.89555
TRAC	-2.23848	7.812541	-11.9658	3.87E-22	3.44E-20	39.75083
TGFBR3	-2.25516	4.691822	-11.6575	2.09E-21	1.68E-19	38.07765
SGK1	-2.26154	6.756981	-14.117	3.55E-27	7.20E-25	51.25653
ITK	-2.27932	7.28195	-7.48966	1.33E-11	1.90E-10	15.73119
IFI44L	-2.28144	3.99218	-8.40988	1.05E-13	2.12E-12	20.51244

CCR3	-2.3842	4.853691	-8.04689	7.23E-13	1.27E-11	18.6068
IL7R	-2.40631	8.802351	-9.00324	4.31E-15	1.13E-13	23.67013
LRRN3	-2.4197	4.086082	-9.65652	1.23E-16	4.26E-15	27.19069
CLIC3	-2.43799	3.926583	-10.9677	9.21E-20	5.37E-18	34.32303
KLRF1	-2.47414	5.268602	-8.74918	1.70E-14	3.98E-13	22.31245
MAP3K7CI	-2.47941	5.001748	-9.4753	3.31E-16	1.06E-14	26.21052
IL2RB	-2.5974	6.855819	-12.5231	1.86E-23	2.05E-21	42.76415
PRF1	-2.60154	7.052027	-12.6243	1.07E-23	1.23E-21	43.3094
CD247	-2.61484	7.743765	-11.3991	8.62E-21	6.00E-19	36.6722
HCAR3	-2.65747	7.053927	-7.1127	9.23E-11	1.14E-09	13.8274
MME	-2.69169	5.91856	-8.21882	2.91E-13	5.41E-12	19.50658
FGFBP2	-2.69229	5.026726	-8.27285	2.18E-13	4.17E-12	19.79044
IFIT3	-2.73069	6.41353	-12.6636	8.66E-24	1.01E-21	43.52058
NELL2	-2.73424	5.826652	-13.7247	2.86E-26	5.10E-24	49.18668
GNLY	-2.92409	7.340767	-8.73599	1.82E-14	4.24E-13	22.24222
IFIT2	-3.07667	5.512695	-15.7317	7.84E-31	2.76E-28	59.60534
IFIT1	-3.29358	4.322892	-10.2412	4.99E-18	2.19E-16	30.36556
