# Supplementary material for: Altered Genes and Biological Functions in Response to Severe Burns
Source: Biomed Res Int. 2021 May 24;2021:8836243. doi: 10.1155/2021/8836243 (PMC8168476; doi:10.1155/2021/8836243)
Supplement: Supplementary 6 — Table S4: 2631 common upregulated genes. [file 8836243.f6.pdf]

Table S4 2631 common up-regulated genes

Symbol
MMP8
CD177
OLFM4
GPR84
HP
MMP9
ALPL
LCN2
MCEMP1
BMX
ANXA3
LTF
RETN
ANKRD22
PGLYRP1
LRRN1
CRISP3
GADD45A
TCN1
TDRD9
CA4
ZDHHC19
GYG1
VNN1
CEACAM8
SLC26A8
DEFA4
CYSTM1
RGL4
HK3
ELANE
IL18R1
FCAR
CLEC5A
PGD
PFKFB3
GJB6
CEACAM1
MAPK14
ST3GAL4-AS1
CEACAM6
IL1R2
SLC2A3
SLC51A
GALNT14
DACH1
FGF13
FLOT2
METTL7B
UPP1
PCOLCE2
CST7
OPLAH
CTSD
HIP1
SERPINB2

NFE2
GRINA
HBD
DHRS9
MS4A4A
DYSF
OLAH
UGCG
S100A12
ST6GALNAC3
ORM1
LILRA6
APOBEC3B
TSPO
SLC37A3
FAM20A
FCGR1B
TLR5
POR
TP53I3
PTX3
MPO
SIPA1L2
IRAK3
DDAH2
LOC100134822
PADI4
FOLR3
SMPDL3A
NAIP
APMAP
SAMSN1
MGAM
NBEAL2
PYGL
IL4R
CR1
EXOSC4
PLBD1
SPI1
NR2E1
GPR160
WIPI1
LILRA5
LMNB1
1-Mar
CAMP
LOC101928429
GPR97
NCF4
IL10RB-AS1
S100P
PLSCR1
GRB10
BCL6
SULT1B1
LIMK2
LRG1

NEDD4
PGS1
ATP8B4
SLC22A4
ZNF438
PADI2
ARRB2
CDA
CD24
CEBPA
FKBP5
DDIAS
RAC2
PFKFB2
BST1
STXBP2
SIGLEC5
F5
CAPG
SLC1A3
AP5B1
GCLM
AHSP
IL18RAP
RNASE2
GBAP1
SERPINB10
ATP6V1C1
HPGD
STX11
ZYX
NATD1
RNASE3
ATP9A
ACSL1
G0S2
RNF10
PRKCD
HGF
FCER1G
LIN7A
CLU
KL
TNFAIP6
CD59
B4GALT5
PLIN3
GGH
RABAC1
ROPN1L
PHTF1
ANKRD55
WDFY3
FUT7
ENTPD7
ALOX5AP
INSC
CD55

SIGLEC9
ADAM9
GK3P
RRM2
CKAP4
KIF1B
PLAC8
KLF5
PPP1R3B
PRDM5
AZU1
SRPK1
MAP2K6
NQO2
DRAM1
EMB
PPP1R3D
PSTPIP2
DHRS13
B3GNT5
TTN-AS1
CAB39
ECRP
SLPI
NUCB1
HIST2H2BE
HCK
LTB4R
ADM
STOM
STAT5B
RALB
CTSG
FLOT1
MCTP2
BCL2A1
SBNO2
ERLIN1
CHIT1
FES
MPP1
OSM
GAS7
CARD6
CORO2A
TMEM120A
PRTN3
FEM1C
C15orf65
MANSC1
OLR1
DGAT2
ACN9
LILRA3
FBXO6
SIRPA
ATP11B
ARG1

SLC25A40
JAK2
PDGFC
DPY19L3
KLHL2
BEX1
ITGAM
CD82
PRUNE2
TIMP1
TMCO3
PROK2
CTSA
AIM2
BSG
LINC00266-1
ECHDC3
MBOAT2
ALOX5
NTNG2
CD63
TRIM25
DIRC2
KREMEN1
BIK
H2BFS
CEP55
EIF4E3
TKT
TSPAN2
VPS9D1
RAB13
CYP4F2
HBM
LGALS1
FAR2
NME8
DNAJC5
IL10RB
ITGA7
SORT1
MTF1
AGFG1
DHCR7
KIF3C
JMJD6
ETS2
LDHA
CCNA1
G6PD
RARA-AS1
WASF1
LRRC4
LILRB1
NLRP12
P2RX1
CYBA
MTMR3

CYP1B1
GBGT1
ANO10
LILRB4
HIST1H2BD
EMILIN2
FAM126B
C1QC
GALNT2
C3AR1
PECR
SPTLC2
KCNJ15
EXOC6
TM6SF1
USB1
CDK5RAP2
CDC42EP3
RAB24
PKM
CCNJL
PRDX5
ADAMTS3
EMR1
RAB31
MSRB1
UBAP1
HIST1H2BH
GK
GRAMD1A
FOSL2
BLOC1S1
CLEC4E
TSHZ3
PLP2
SH3GLB1
MAFG
CHPT1
MKNK1
DOK3
SERPINB1
ALDOA
GRK6
CSGALNACT2
GRN
NSUN7
CEACAM21
QPCT
BEND7
LILRB3
MMRN1
SHKBP1
ACSL4
METTL9
VSTM1
HPSE
CDKN3
ADORA2B

SSH1
EHD1
IMPA2
TRIQK
KCNE1
GBE1
ATP6V0D1
MAN1A1
LINC01000
MILR1
BASP1
CEBPD
DLGAP5
CNIH4
DSC2
ADCY3
RAB27A
AP3B2
CPNE5
TIFA
TTC8
YOD1
HIST1H2BK
CA1
TLR8
STX10
RPS6KA1
LAMTOR5
UBE2J1
GYS1
KLF7
AGPAT9
TMEM185B
FPR1
FAM214B
OS9
RHOG
UHRF1
B3GNT8
FCGR2C
ANLN
WSB1
SEMA4A
SUCNR1
HLX
SLCO4C1
LAIR1
FGR
BATF
ENO1
ABHD17C
CCPG1
NEU1
CHMP4B
NDUFAF1
MEF2A
PTK2B
ROMO1

FKBP9
STAB1
TRPM2
FGD4
RHBDD2
IL1RN
CYYR1
METRNL
UBE2F
ORMDL2
PCMT1
DUSP13
CEBPZOS
SOCS3
SQRDL
SNX3
PNPLA6
HIST1H2BE
HMMR
LOC100506459
CMTM4
NDUFA1
QSOX1
NUSAP1
GAPDH
SIAH2
CDKN2D
MEGF9
S1PR4
STX5
ZNF282
TOR1AIP1
E2F8
ZDHHC20
FAM53C
B3GNT2
C1QA
TFDP1
MARCKS
PLEKHG1
IL17RA
KBTBD7
GNS
FAM160A2
DTL
RRAGD
VAMP3
FERMT3
TMEM88
ASPH
C1QB
PPM1M
SHCBP1
KIF11
MAPKAPK2
CAMKK2
PCYT1A
IL10

NRN1
COLGALT1
TOP2A
SOS2
TGFB1I1
CAPN3
RBM38
ZNF106
NFIL3
ASGR2
SLC36A1
LINC01094
SYNE1
SLC11A1
ROGDI
IFITM3
NDUFB3
RTN3
CKLF
CEBPE
KIF14
HN1
OAT
ITGA2B
TGFA
HHEX
MICU1
MXD3
COL17A1
SLC25A37
ACTR1A
TPX2
SLC22A15
TTC7B
RIN3
PGK1
JUNB
MKI67
NECAB1
MSRB3
CSF2RA
ENTPD1
TIMP2
MS4A3
SMAD1
CDK5
MAPK13
ZDHHC3
PLA2G4A
YKT6
PDZD8
GSR
CHP1
DEDD
MFN2
RTN2
PPARG
CD44

YIPF1
TBC1D8
TLR1
PGM2
GPSM3
NDST2
CEACAM4
SESN2
PDLIM7
CDC20
FAM105A
CENPW
LAMTOR1
LOC441124
GPR27
PGM1
RANBP9
ORAI2
SH3PXD2B
ARHGAP29
IDI1
HOMER3
CPEB4
TOM1
SCPEP1
SLA
ARPC4
TPM4
CMTM5
GM2A
ME2
GLT1D1
ACAP1
PLB1
FLVCR2
TRPS1
SERPINB8
RAB8B
SLC9A8
MCU
EMC2
ATG9A
INSL3
PRKACA
TBC1D14
CDADC1
PLOD1
ENSA
LOC100131043
TMEM52B
SSFA2
LOC101928635
MSRB2
SIRPD
LPCAT2
DAAM2
MAD2L2
CCDC126

KIF20A
GLIPR2
TMEM260
GNAQ
AQP9
GFOD2
TRAPPC1
DHCR24
PRC1
TUBA4A
GGTLC1
UBTD1
C10orf54
MICAL1
HIATL1
CPNE2
AGTRAP
GCNT1
SDHC
HIST1H4H
PFKFB4
MERTK
TK1
FAM217B
S100A6
ARL8A
PROS1
LPCAT3
KAZN
MRVI1-AS1
TPST2
ANKRD33B
JAK3
VAT1
CDK14
BUB1B
ACER3
BAZ1A
TENM1
TUBA1C
INHBA
IL1R1
NCAPG
EDEM2
UBE2C
TBC1D10B
MAP1LC3A
DNAH10
PIM3
DSE
TMEM45B
COPA
SH3BP5L
TTK
LSMEM1
HBQ1
STK17B
C1RL

TBC1D7
VASP
UQCRC1
HDAC4
PLD1
CD164
TMOD3
LOC100506119
SERPINA1
RHBDF2
GNG5
PLEKHO2
ECE1
CCDC71L
PEF1
MCTP1
FBXW2
CTDP1
CLEC1B
HIATL2
CUX1
FURIN
MAP2K2
AMPH
CDK1
TBC1D20
ZDHHC12
AMPD3
TBC1D2
RALGAPA2
VIM
TP53I11
TUBA1B
MAPK3
TMEM2
TFF3
VSIG4
KIAA1715
PRG2
HSD3B7
XK
GMIP
RAB32
IGF2BP3
EBPL
SLC24A3
LAT2
PXK
STK16
TRIB1
ILK
SRA1
NSUN3
PDXK
SLC12A9
LDLR
RNF144B
ADAMTSL4

SDF2
RBMS1
RNF141
CENPE
S100A11
TOLLIP
SETD8
ATP6V0A1
RASGRP4
LOC101930114
ERI1
LY96
ZNF787
AATK
SNCA
ARPC1A
C17orf62
TBKBP1
CRISP2
KIF18B
HSDL2
UBE2H
PLGRKT
PJA2
NARF
HIST1H2AJ
TM9SF2
RNF146
DCAF11
ZMAT5
PPP4R2
C8orf88
MSRA
HSPB1
PIK3AP1
C1orf162
FZD5
OIP5
GPR132
ZAK
FBXO38
PINK1
MOSPD3
CD58
ERO1L
SELP
FAM63B
KIAA0930
MTX1
FAR1
RENBP
MMADHC
DNAJC13
TESC
FAM110B
TMEM180
KIAA0101
LRRC6

RSPH9
OSBPL1A
ZNF784
JHDM1D-AS1
HK2
MAP1LC3B
PLOD2
MYL9
ASAP1
TMED8
PNPLA2
NMNAT1
F8
PRO2852
SGSH
KIF15
CREB5
PSTPIP1
ZNF222
FADD
TOR1A
ARID5A
MYO10
EIF4G3
MIAT
E2F2
LOC101927974
MGST1
ANAPC15
RAB1B
GADD45G
DEGS1
ARPC1B
ZEB1-AS1
ASPM
FOXM1
IFITM2
COL4A3BP
CLTCL1
CDCA5
BCL7B
IFNAR1
UBL5
AREL1
CETP
PAG1
AGTPBP1
ATXN7L3
UNC13D
TMUB2
INSIG2
TMEM45A
KDM7A
PPP1R12A
ARF3
MYD88
CAPNS1
RAB43

RFT1
SLC25A44
RNASEL
ACVR1B
CEACAM3
RAB20
MAPRE1
PEAK1
C4orf3
LINC00968
LAMC1
TPM1
SLC28A3
TMEM92
CYB5R1
MMP27
LRRFIP2
C1orf226
LOC102724587
TRPM6
ACOX2
H1F0
RAB10
ARID3A
XRCC4
ZBTB7B
GPI
ZNF281
EAF2
OXSR1
RGS19
GABARAPL2
SGMS2
C16orf72
CCNDBP1
PPAP2B
GCA
TNPO3
SCYL2
DNM2
TUBB4B
MLKL
CRADD
PTGR1
FPGT
PRAM1
MFF
TCAIM
TLN1
LOC102723845
ALDH3B1
EXT1
SYN2
NTSR1
FKBP8
FIBP
THBS1
ELL2

TMEM8A
ABO
TMEM55A
APLP2
CLIC1
DNAJB6
BCKDK
MELK
PSAT1
NABP1
GDE1
VPS25
VPS37B
ATOX1
ZNF354A
VNN2
GADD45B
ARL4A
LMO2
HPD
NOP10
NUF2
YWHAE
MRPL28
DHFR
DNAJC3
KIF4A
CHIC2
NFAM1
AZIN1
LGALS8
GOLGA1
CCNB1
SPTLC1
UPB1
TMTC1
MAOB
PIK3CG
SKAP2
MOSPD2
NFKBIA
HIST1H2AM
SLC27A2
C19orf33
RCHY1
RP2
DUSP3
GNA15
OSBPL9
CHMP4C
CDCA3
TWF2
MYL6B
WBP5
ETHE1
EPAS1
SELT
PHF21A

SEC24A
ZNF467
CDYL2
SLC25A24
CHSY1
SPR
AZI2
C1GALT1C1
EFCAB2
TCN2
DEPDC1B
TDRD7
RXRA
TOMM40L
NDUFB9
C9orf16
ST3GAL4
GYPB
S100A9
ARHGAP24
NT5C3A
GNAI3
RAD23B
BIRC5
RHOU
DCUN1D3
NIN
STK3
BPGM
DESI1
PTPRN2
MARCO
PGAM1
DGAT1
TSPAN14
SCCPDH
LIG4
ATP13A3
MS4A6A
TAZ
MAPK1
GLTP
HJURP
HMGB3
ESAM
COL9A3
MOB1A
NETO2
TINF2
TANK
STYXL1
ATXN1
ITGB3
TADA3
ARPC3
AGPAT2
TYW5
AGO4

STRN3
CCNE2
FAM160B1
RDH5
NRM
TMEM169
CITED4
PKHD1L1
TNNI2
ATP2C2
ITPKC
PSMB3
KPNA4
SLC7A5
POLE2
TMEM167A
BAMBI
AIG1
FHOD1
IFI27L1
MIIP
SLC25A28
FBN2
CTSB
WFDC1
MKL1
LOC100507507
IVNS1ABP
IFITM1
TYMS
ANXA5
ST3GAL2
LTBP1
LMTK2
GRPEL1
FOXN2
5-Mar
UEVLD
BCAT1
DCAF10
BNIP2
ZNF319
PHKA2
TMX1
JDP2
SRF
WDFY3-AS2
ANKS1A
ZWINT
FAM198B
KIF2C
GAS6
ADAM15
LILRB2
UBXN2B
NCAPH
AURKA
CD163

SH3BGRL3
BCL2L15
SMIM3
2-Mar
HTRA1
SNX12
ADAMTS2
SELL
GPD2
PLK3
THBS3
SEL1L3
LHX4
CLIP1
GYPA
JAZF1
NMI
TMEM38A
EBLN2
CDC34
TMEM11
ZNF780A
EDNRB
ALAS1
CDC45
PLIN5
SEPHS2
IER3
GLRX
BTBD10
DEPDC1
HMGB2
SLC22A16
NEK2
TMEM165
TFE3
H6PD
CSGALNACT1
RFX2
TRMT6
UHRF1BP1L
PCGF3
KIAA1958
MYBPC3
GTPBP1
PPP2R5A
BAG4
FOSL1
EGF
GTPBP2
KIF5B
TMEM110
RELT
TMLHE
KIAA1107
LOC100507540
CEBPB
CDT1

PHACTR2
NAPRT
SPC25
LOC645513
CALML4
NCKAP5L
CD151
LOC100049716
CLIC4
KCND1
B9D2
SIAE
FBXO30
LOXL1
ABCD1
PTPN22
SLC40A1
ATP11A
RNF7
PIWIL4
FAM101B
RRBP1
HIST1H4D
UNC79
DPP3
HCFC1R1
ACAA1
GPSM2
LINC00260
HEBP2
RBPJ
TRIP13
CDCA2
ARL8B
KDM1B
SLC35B1
GALNS
TUBG1
KCNH7
FUT4
SPAG11A
C2orf76
C1orf106
POMP
ZDHHC17
C5orf30
DNASE1L1
SH3BP5
TNFSF13B
ALOX12
APAF1
GINS1
OPRL1
RHD
SCN9A
VWA5A
IFNGR1
GP9

PELO
CLEC12B
FLJ36848
AGAP2
PRL
PTH2R
MYL6
SLCO3A1
ANG
DYNLT1
KIDINS220
ATF7
WDR26
CYBB
TMEM33
ZDHHC2
HOMER2
ANXA1
NT5DC2
B4GALT4
GPR137
GPR37L1
LOC100507642
TUBBP5
CDKN2C
ZMAT2
ADAM17
IDNK
TCEANC2
ZNF230
DOK1
TIPARP
SPINT2
MBOAT1
PDK3
DCTN4
ST14
CDC6
UFD1L
HIPK2
RAB1A
SFT2D1
CHCHD7
ABCC13
SFXN5
NBN
RNF145
TPM3
MSL1
KCTD21
C11orf71
GSN
UBE2T
SIL1
LOC102724782
DNAJC3-AS1
SLC4A1AP
GTDC1

GRB2
SP100
SMARCD3
ARPC5
SUOX
TAF13
ACSL3
ACSS2
LRSAM1
PDSS1
EMID1
SQLE
DDA1
RRAGA
PSMD9
APH1B
ISY1
PIM1
PTPLA
GNG2
CHCHD5
PARVB
TBC1D8B
TMEM234
KLLN
SPTY2D1-AS1
HK1
ADAM19
DENND2C
PTTG1
DRC1
CAP1
LILRP2
MTHFD2
LINC01272
GSTO1
LAPTM4B
HIST1H3G
GALNT3
SCD
ETV6
RLIM
ABHD16A
TXNDC11
ID1
CDCA8
RAB3IL1
ESPL1
SLC2A5
ZC3H3
NXPE3
VLDLR
LOC101927603
FBXO34
FLJ30064
CCNA2
LBR
PRR13

LOC102724387
FCRL1
DRP2
ASB7
NUP214
PBK
TRIP6
SWT1
LMBR1
GNG10
CR1L
LYPLA2
RAB6B
GP1BA
PAPSS1
RIOK3
DBN1
E2F1
B3GALNT1
BRE
CYB5R3
FBXL19
FAM107B
TMEM91
CIDEB
RTN4
RAB3A
SIRT5
NFKBIZ
TUBA4B
SLC44A1
TIMM8B
ACPT
ATL3
ERGIC2
OSTF1
GLYCTK
EPDR1
ZSWIM6
AURKB
LINC00937
TGFBR1
ACPP
UBE2D1
TIMP4
ANKRD6
MMP19
DNAJC4
LOC100505812
MGAT4B
MYBL2
NXT2
PCSK9
PPBP
BUB1
BCL2L2
CALU
PDLIM5

GP6
ZKSCAN7
GMFB
MCMBP
MOB3C
MND1
ABLIM3
PLIN4
ASB12
STIM1
TACSTD2
GPR182
CREG1
KLHL12
SPNS2
DLC1
EFHD2
CD99L2
PRICKLE3
PLAU
VEGFA
CYP19A1
TSG101
ABCA4
TALDO1
PROSC
CHRNA2
KBTBD6
SPATA1
KHDC1
ATOH1
RCOR1
TSPO2
DR1
C20orf27
SPRED2
ERG
FCGR1A
CPNE3
TWIST2
DPH3
IDH1
CCRL2
FBXO9
RCVRN
SGTB
ITGA9
LRPAP1
ACP6
FBXL5
CASP9
APP
CAPZA2
ZNF254
CDK16
PTGES
LOC100506299
VPS35

IMPDH1
NLK
FKBP15
MROH6
NR6A1
CUTC
RIT1
HAT1
TIMM17B
FANCD2OS
CMTM1
SH3GL1
ADCK4
S100A2
WBP4
LOC102723932
POLQ
NDUFAF7
CTSH
3-Mar
PRKAR2A
C9orf89
ACTR2
HMGB3P1
IKBKG
GOLPH3
LRRC32
CHMP2B
SLC38A2
GLRX2
BTBD3
CAPNS2
LOC101926963
CLDN17
OR7E156P
TMC4
ZNRF4
SKIL
E2F7
C6orf25
SLC17A5
JAG1
PAM
MLLT1
IGFBP2
GPCPD1
C1orf105
MFAP3
UBE2A
FBN1
TK2
STRADA
MSL3
SYCP2
HSPB6
PDE4D
PEX11G
RALY-AS1

ETF1
SPAG5
RTFDC1
HOTS
EIF2AK1
LOC102724511
RSBN1
CD99P1
IFNGR2
NOL3
SMOX
P2RY1
TEX2
LSM6
AKR1C1
POLDIP2
SOX21
PKP2
JOSD2
HBE1
KCNMA1
LOC400940
OR2H1
ABCC2
PKMYT1
KCNE1L
SLC8A2
RHAG
SF3B6
SUMF1
LOC283075
ARMC12
NCSTN
TRABD
TMCC2
ABHD8
HM13
MXRA7
MALL
HTR1E
ANKRD35
CMAHP
TULP2
ANPEP
SNX13
TNFSF10
FOXC1
ZEB2
MS4A5
AQP5
PRPF18
CC2D2B
STX1A
SEC23B
LOC100130938
TRMT1L
PTCHD2
FBLIM1

FAM200B
DDX59
GPER1
PTEN
ITGAX
LINC00202-1
ROCK1
STRA6
ZFPL1
MFSD11
DYNLRB1
ARHGDIB
CSTA
CHAD
RAI2
HIST1H1B
STEAP3
LINC00597
ALDH4A1
SLC38A10
OCRL
MLX
ABCB6
ARG2
CCKBR
CTDSPL
FAH
PSMD4
CYB5R2
MAGIX
HBEGF
HIF1A
GINS2
TXN
CCDC159
ATP5J
PSMD1
MAPK8IP1
PGLYRP2
CTNNAL1
CCNB2
NDST1
LHFP
LAMA5-AS1
TLX3
MPST
RNF13
PRCP
WDR34
CD163L1
OR2S2
ABCA2
TMEM56
NAT16
MCM10
MUC3
LOC100506860
VIPAS39

CCL24
FAM69C
LOC100505774
GCGR
RPS6KA2
HOXA10
VCL
CINP
LYRM1
VBP1
LGALS12
RAD51
FNDC7
CACNA1E
HBBP1
NLRX1
SHOC2
C2orf57
ACOT8
VAPA
PRDX3
PIP5K1B
CWF19L1
KRTAP4-12
F12
ELOVL3
RNF182
PRKCDBP
FAM127A
SUCLG1
YIF1B
LATS2
ZNF672
KCNK5
WSB2
FKBP1B
MYL12A
NEK6
LRRC29
C17orf53
SCAMP4
MAOA
PAX7
USP32
ATP6V1D
OPTC
WDR13
COL8A2
PPP2R5B
VEPH1
PSG7
BCORL1
RMI2
MYADML2
BRAF
ARF4
SPTSSA
CYP26A1

TRIM71
ANKRD32
CNTLN
PTGS1
FSTL3
RNASE1
ARSD
LDHC
C7orf49
KRT8P12
PTGFR
P2RY13
ATP12A
C7orf69
EPHA5-AS1
TOR1AIP2
MMP24-AS1
TMEM144
SNX11
HAGLR
PIK3CB
FRMD4B
TWF1
DOT1L
DNAH17
TMEM170B
USP35
HTATIP2
LOC440149
C1GALT1
LOC344887
SSH3
RB1
GALNT1
CBS
ICA1
GSX1
PLEKHM1
DCP2
NMNAT2
PCBP3
DSCC1
GLDN
KRT19P2
SKA3
HNRNPLL
SAP30
CASKIN1
EXOC7
C14orf2
CLPB
PRKAR1B
PLK1
MMGT1
GRIK5
HOOK3
PSG3
SPIDR

APOA2
HDGF
TRIM67
CNR1
FAM209B
TMEM40
CCM2L
ARL6IP6
SYTL4
CDC42
BHLHE23
MTMR6
FAM124B
ARR3
PPP4R1L
IQGAP3
SENCR
PGM2L1
SIRT6
LOC100507670
RAP1GAP
ENTPD3
PHF20L1
LRRC61
MMP1
NUDT22
MTRR
RAP2C
WNT11
GATA1
LOC440934
VPS54
SYCP3
GPR156
KIF13A
POU5F1P4
LHFPL2
LOC101929761
KCNC4
APCDD1
SLCO4A1
SNX18
DCAF6
RAB33B
CENPM
CHMP6
ARMC7
SLC1A5
SLC22A18AS
ANKRD9
LINC01208
GDF5
TOR4A
CCDC17
HTRA3
COL10A1
IL1RAPL2
CAST

KIF18A
UNC5B-AS1
RAPGEFL1
HIST1H2BB
NEURL1B
CKAP2L
ZNF630
TYR
C16orf3
OR51M1
COLEC10
HS1BP3
IGLV6-57
C1orf192
Ndufaf4
FGF17
MFSD9
INTS6
STX7
RAD54L
RBKS
RHOT1
DIAPH3
MIP
ADORA2A-AS1
CRYBB2
PTCRA
4-Sep
CASC5
MMP17
ABHD13
KLK3
PLXNB1
SNRK-AS1
CACFD1
DPPA2
PTPN1
EYA3
EFEMP2
EFCAB4A
LRRC15
CCDC135
NAV2
PIF1
RAB7A
C16orf70
C10orf10
CHDH
HIST1H3F
C9orf3
AP3S1
NCR1
LINC00521
SMTN
KLHL18
PGPEP1
SAMD1
DCTN2

INE1
E2F3
KLF15
ARPC2
NT5C2
STAC
ITGA1
PNCK
LINC00202-2
ATP13A2
ZNF628
CPNE7
OR7E47P
ACTR3
NINJ2
ANXA2P1
OR12D2
CDC25A
ABI1
ACTR10
SMIM1
ANXA4
FAM132B
MVB12B
FGD5
FOXN3-AS1
SYN1
SLMO1
GPR64
TXNDC17
MGST2
HAND1
MUC1
TEX14
PLEKHG2
LINC00836
BMP2
LOH12CR2
MED12L
SLC39A3
PHYKPL
ASH2L
PXMP2
NFKBIL1
DYNC2LI1
LOC100507277
POC1A
CCDC53
PROKR2
GORASP1
MTSS1L
SNX21
SHOX2
2-Mar
IFT20
AIM1L
RPH3AL
NKAP

MIR670HG
C1orf228
SDC1
MYLPF
NEURL3
C7orf61
LGALS8-AS1
RGS3
DHRS7C
TFPI
ENPP7
FCN2
LINC00654
OR7E12P
REEP5
KAL1
SMUG1
SREBF2
KIF9
ZDHHC24
MYH14
PRODH2
TMEM255B
SEC62
SLC35A5
USH1C
AGT
NMRK1
SPTB
AVL9
LURAP1
NAA60
SPAG4
RAC3
PLCD3
MYF6
CYP1A1
HIST1H3B
FSD1L
GSG2
FAM20C
SLC5A2
CHEK1
ZNF213
CACNA2D1
ADCY4
UBA6
ARRDC3-AS1
IL17RC
LINC01339
ALPP
SLC16A4
SPCS3
ETV4
MFAP4
SEMA6C
GAPDHS
MTL5

TEC
MCM4
NME4
PYY
OR7E37P
KRT8
OGDHL
IL17C
CENPN
FAM83D
CNRIP1
ZNF488
MR1
CHKA
DLD
IL22RA2
HAS1
PRG3
EGFLAM
PLSCR2
CTIF
BRCA2
STOX2
TSNAXIP1
AUNIP
FLJ90680
MMP2
SLC44A4
CDSN
TMEM174
TEAD4
CAPN13
RABIF
NRAS
KCNIP4
ATP13A5
B3GNTL1
SMR3B
UBE2J2
ZNF613
WDR47
KRTAP4-8
CACNA1S
LY6G6C
DIRAS1
AK9
ZBTB47
LILRB5
PRSS58
SNTB2
CXCL3
SPC24
TRIP10
LOC284648
HIST1H2BM
GJB1
ARNT
RPS6KA2-AS1

TSR3
TPTEP1
APBB2
LIPH
STARD3NL
CENPF
LOC646903
FAM83H
MAPK6
CDC42EP4
KLHL8
GALK1
ATG3
MLIP
MIER1
DNAH2
CKM
KANK2
FAM65C
FAM64A
CHAC1
ZBTB8OS
SLC5A10
DEFA5
MUC5AC
PTTG3P
FAM114A1
SLC7A11
PPP2R4
LOC100506314
LRGUK
TPRXL
C11orf94
PHKA1
CLINT1
LY6G6E
LIMD1-AS1
CCDC30
LOC101928869
SLC22A13
DPEP1
PHACTR3
LAMB3
NXF5
RECQL4
LOC153546
POC1B
TMEM86A
PLK4
SLC30A1
TMEM53
HECTD3
HPS1
LOC101929655
CYP2F1
MT4
FAM109B
SYT13

DYRK3
RHBDL3
PPM1N
TFF2
SOD3
NOS1AP
SRGAP1
CPE
PNKD
C10orf53
ITFG3
UBR4
HS3ST6
TNPO1
MFI2
PCDHA5
LACTB
PTPLAD2
NKAIN4
ADD3-AS1
DHRS7
EVA1B
SORCS2
LPO
FOXQ1
MAPK15
ITSN1
LOC102724323
GAL
FGFR1OP2
ASIC2
SLC10A2
YPEL4
HIST1H3C
DERL3
APOA5
LYPLA1
STAU2
BRCA1
FOXB1
ARHGEF17
SLC39A8
SPDEF
MAML3
CENPI
LOXHD1
COX6A1
ARSI
LOC284933
LOC388780
TRIM7
LOC102724312
HOXA6
CCL21
PIR
NFATC4
HAVCR1P1
GHITM

CAMKK1
FGGY
SERINC2
MAP1LC3B2
LOC101927552
KPNA1
ITIH5
ADARB2
IGFBP7
FOXD3-AS1
KPTN
KIAA1644
SHB
CDC73
LOC220077
ECT2
MREG
C22orf23
SLC27A4
DPYSL5
ITIH3
LRRC16B
LYZL6
NOTCH3
ITIH4
TMEM216
COL23A1
SOX9-AS1
CLDN16
DLGAP1-AS2
FA2H
PAGE5
YWHAH
CADM3
OLFML2B
KIF25
SEMA6B
ST7L
FAM90A1
IL17RB
KIAA0513
DIAPH2
DGCR9
SEMA4G
RUNDC3A
WFDC2
TTLL2
PHYHD1
PENK
CLRN3
STON1
SAPCD2
PON3
UBE2NL
DPCD
HOXA11
NOXO1
RGS18

PARD6A
LOC100288181
SERPINA4
ADRA2A
CHRNE
B9D1
KIAA1161
LOC100130502
LOC101927479
LOC400043
ADAMTS9
IGSF21
POM121L9P
SFTPC
TUBA1A
GJC2
MSX1
KRT33B
ZNF821
PSG6
ECSCR
STAG3
C8B
HSD11B1
TMEM60
PCDHGA3
SCN8A
ACRV1
NXF3
SLCO2A1
ALDH1L2
THY1
COQ7
RAD51B
KIF23
DLX2
LOXL3
NEDD4L
PGK2
TTC33
GPR4
TREM2
OR6B1
PDE2A
LOC100128988
KIAA1217
DCTN3
CXCL12
OR52D1
F2
TMEM37
WFDC3
NACC1
GTSE1
VASN
C19orf47
STOML1
LOC440330

GABBR2
ABCG2
KIAA1524
MRPL33
HPR
MAGEA10
IGSF10
TCP11
EML2
PCK1
SLC9A3R2
CNTFR
LINC00881
SLC9A2
SLCO2B1
SERPINA2
RAB11A
C1orf86
CENPA
SPA17
RCAN1
RGS22
LINC00244
USP2
ZYG11A
LOC102724842
TLN2
PSMB5
NETO1
OR51B2
FAM228A
BPIFA1
LOC101928707
SLC25A51
SRC
AZGP1P1
WNT5B
PRSS3
SLC23A3
TPSB2
H1FNT
MED20
SMIM10
SDC4
ALX4
C9orf84
LOC100288570
IRGC
ZG16
PROP1
CENPO
C3P1
ACVRL1
STARD13
TNNC2
STAB2
PTPN21
SRP54

CPEB3
CDC42BPA
IHH
CHIA
TMEM70
SLC39A2
CCDC3
HIST1H2AL
CXorf36
RIMS2
SLURP1
MAP3K6
ELF5
CELA3A
DIXDC1
EGFR
PQLC1
ATE1
LOC100291666
LOC100996671
BMP10
IZUMO2
PCYT1B
LINC01159
LOC100240734
ADAMTS15
RAB36
PRDM12
FAM181B
NDNF
VTA1
TMEM108
MYO7A
MIR10A
LINC00911
COL4A1
ADORA1
DBH
CDH16
PGAM2
RASL11A
CDO1
LINC01116
CYP1B1-AS1
SAP30L
LOC101927668
ROM1
WISP2
TTLL3
LINC00445
LOC100506022
KRT37
CHAF1B
FRS3
PAX4
POMC
TMEM132D
LINC00997

OR7C1
RGS16
CACNA1G
CALD1
LOC100288175
LOC100129112
C1orf100
AMOTL1
SNCAIP
MFSD7
ACOT7
MOS
C2orf54
ARHGAP11A
LINC00427
FSHB
LOC728040
HIF3A
TMPRSS11D
CNTNAP4
GINS4
CHRNG
PRM3
CCNF
CYP2A7P1
SHROOM2
CPA4
MMP15
LOC100286922
NXPH2
TEX38
LOC100505938
TICRR
SP5
PVR
CNTD2
MSANTD1
SYT5
CCDC93
ABCC3
KLK11
GGT5
CYTL1
PNMAL2
SLC48A1
MAP3K13
LPGAT1
ZNRF1
TBXA2R
S100A5
SLC5A1
AGBL5
GBX1
LINC00689
PARD3B
CACNA1D
LOC100129449
CDC25C

LDLRAD4
SLC44A3
C14orf119
OMP
HCN4
POLD3
SKA1
RXRG
CASZ1
GINS3
FBXL22
C1QTNF4
STPG1
TMEM121
HOXD10
MYO7B
GJB3
PNLIP
LOC254057
ARID5B
SIX3
SDC3
MASP2
KLK13
RHOJ
C9orf116
TRIM29
XKRX
GYG2
PAPPA2
DKFZP434F142
HHATL
CSRP3
SSR4P1
SLC45A2
AMPD1
VN1R10P
LAD1
GPRC5A
CTB-174D11.1
GREB1
LOC101927278
RSPO1
CDH26
GNRH2
ORC1
CRHR2
METTL22
SLC9A3
MPDZ
C7orf13
ODF3
PCDH18
LOC157740
LINC00347
LOC101928682
AVPR2
AFMID

TRAF7
LOC727944
FGF18
CLGN
CPNE6
TAGLN3
STMN4
DLX5
SLC5A4
CCDC61
ERVH-6
EDA2R
FAM26E
HOXB5
HOXA2
LOC780529
CHRNB2
CHRD
PDX1
ANKRD37
GPR75
NRAP
PCP4L1
LOC101927417
NFIX
RAB2A
SEC14L3
AKAP3
TMEM254-AS1
ACTN2
NTSR2
DNASE1
CLDN3
FTCD
OBSL1
ZNF775
USP49
EMC7
PPP1R12C
TMEM136
RTDR1
CHRNA4
RYR2
DRICH1
FGF3
WNT4
CLDN14
MRO
PLA2G5
YIPF2
PRKG2
TMEM198
IFNA1
LOC100128164
CHAT
ITGB4
PDE6H
LOC101059948

SGOL1
LOC101929459
GAS2L3
THSD4
IGFL2
KCNJ9
GH2
PPME1
GHRHR
LRP3
KNDC1
MYL12B
MYRF
GCKR
FOXL1
ADCY10
SIM2
LYPD6
PROM2
DDR1-AS1
PMFBP1
COL1A1
RHBDD3
LMCD1
AIFM2
ZMYND10
BCAN
NANS
FBLN1
FOXF2
GSTT2
RGS11
ZP2
ADCY5
DGCR5
FLJ40288
HRASLS2
RS1
COPB2
CUL7
MAST2
EVPLL
PGBD5
GDNF
SHC3
DBF4B
LCT
KCNC3
NNMT
ARL3
MESP1
PCSK6
DPPA4
PAX9
RIPK4
ANTXR1
C5orf66-AS1
LOC100128079

SHROOM3
MTRF1L
NDST3
C1orf116
OR51I2
ANO3
CDH6
DLGAP2
DIO2
ARNTL2
ASAH1
COL22A1
FBP2
FARP1
LRTM1
PLAC1
ADCY2
CTXN3
EFCAB12
ST6GALNAC5
CT55
LOC101929705
CLDN11
RNF207
HES2
IBSP
ESCO2
AKAP6
FNDC5
IL21
KRT12
TBC1D16
TM4SF1
ART4
IL22RA1
DIAPH2-AS1
TAS2R3
AIF1L
LINC00900
MYT1
CCDC181
TAT
CMYA5
PDE3A
SLC4A9
DIO1
CCDC150
PHLDA2
FILIP1
LZTS1
CLSPN
COL4A6
ACOXL
LOC100507468
BNC1
TSPAN12
CHST9
HOXB-AS3

EPHA6
LINC00113
OR2F1
RASAL2
C12orf50
WWC1
DAW1
DNAH5
CFL1
RASIP1
HOTTIP
TTLL7
CLDN18
CTD-2118P12.1
SOX9
PDPN
NPY2R
PLCH1
GUCA1A
OPRK1
CYP2C8
DLGAP1
RAB3B
