# Supplementary material for: Altered Genes and Biological Functions in Response to Severe Burns
Source: Biomed Res Int. 2021 May 24;2021:8836243. doi: 10.1155/2021/8836243 (PMC8168476; doi:10.1155/2021/8836243)
Supplement: Supplementary 7 — Table S5: 3451 common downregulated genes. [file 8836243.f7.pdf]

Table S5 3451 common down-regulated genes

Symbol
PIH2
ZNF19
MTAP
BNIP1
PBRM1
ZNF343
ZCCHC4
EIF4EBP2
RPS14
NRF1
ACSS1
TRADD
EFCAB14
RDH13
KIAA2026
DHX35
TMEM231
CDK13
RPAIN
DDX54
ZNF416
CEP164
ICMT
PATZ1
AXIN2
NUP188
MAP2K5
RBM19
C15orf40
SLC25A30
ZNF41
FBXL12
PDP2
LOC102725383
SFSWAP
PROSER3
ZNF48
CLCN4
FLJ16734
TMEM248
SAP18
TRAF3IP2
TRIO
SERGEF
ZFP28
INTS9
NUDCD3
LINC01128
AGPAT3
ZNF213-AS1
MED26
CDHR3
KLHL13
KANK1
FAM160B2
PPP2R2D

PREP
ZNF551
TEF
ZNF616
ZNF510
ZNF257
EPB41L2
C2orf44
TIFAB
EFTUD2
TAF1
FOXJ3
ZNF397
OCM2
DHX30
DDX19B
FLJ42627
RREB1
ETV3
RBM48
ZSCAN22
OLFM1
IKZF1
SLC35E1
DDX11
XPO5
GRWD1
GNAL
ELOVL5
DYNC1H1
IPO8
SYS1
EXPH5
PSMF1
WASL
SNX25
FARP2
UBN2
LIMD1
HPS3
PTGR2
ZNF473
SNHG22
SIRT4
ATXN1L
SDHA
SCRN2
SNIP1
MAT2B
LINC00852
ZNF883
GRAP
LINC00965
ERCC3
PRO0471
RAP1GAP2
CYB561D2
SF3B2

EIF1
USP44
CYB561D1
SFT2D3
ZNF638
ADAM12
TMEM8B
C21orf33
SLC45A3
SMAD9
MIRLET7D
FGD6
ARAP3
GET4
ZFP91
WWOX
RASA3
GSS
INO80
TNFSF15
RTKN2
NUMBL
NOMO3
XRCC6
LARP4
TIMM22
NLRP2
RC3H2
RNF20
LIX1L
PPIP5K1
CISD3
LOC100272217
PIP4K2C
TUBGCP4
TNRC6C
AARS2
GOLGA3
RANGRF
C3orf18
RPS23
SF3A1
LOC257396
TMEM150A
DGKQ
FMO5
OSBP
COPS7B
RAD9A
PSMD5-AS1
TMEM161A
CCDC130
USP34
MTUS1
ODF2
DNMT3A
POLRMT
ELF1

HIRIP3
CHERP
GMEB1
B3GALT6
UBE3C
SARM1
GOPC
LOC157562
ZNF621
MRPL10
MANBA
GGT7
UBE3D
ABHD6
ZMYND8
EPC1
BRAP
ZNF461
SURF6
UBE2D4
FITM2
CTNNBIP1
HOXC4
SP110
CASP8
ZDHHC21
RBFA
NUDC
LLGL2
FBXW8
QKI
KLRAP1
TNS3
SMARCC2
PMEPA1
SYT17
THRAP3
SIGMAR1
SETD1A
PFKP
LSS
ELMSAN1
DFFA
PECAM1
RAD18
9-Mar
BAG2
C1orf216
RGMB
TCF20
DDX19A
WBSCR22
HMHA1
MPP6
C11orf24
ZMYM3
PRR3
PPP1R16A

ENOX2
SCAMP1
SPOP
ZNF24
ZNF398
LCP2
DISP1
UNKL
LIN7B
CA5B
ZDHHC8
POLR2J4
XKR6
NKAPP1
FRS2
TSC22D2
CD1E
SRRT
COG8
ADCK3
CALCOCO2
PABPC1L
FBXO46
KLF2
ALDH3A2
ARHGEF19
PEX12
IDH3B
RNF165
IL12RB2
NISCH
TOB2
MDM2
CRIPAK
CLUHP3
AHCYL2
CHN2
RIMS3
CHST14
HIGD2A
TRUB1
NOL4L
PMS2P3
PMPCA
SUPT16H
ENTPD6
SLC27A1
GATAD2B
PRDM15
ARL2BP
TJAP1
ZNF490
OXA1L
HNRNPC
TUBD1
LINC00865
TRIM62
CLK2

TRIM4
SLC35F2
CPSF4
EDC4
LINC00294
PCBP1-AS1
AFF3
SETX
EPB41L4A
TTLL1
LRRFIP1
LOC101928728
NTN4
DTX4
UQCC1
MYBBP1A
MIATNB
APEX2
RBL2
FER
TTC31
LSR
ERP29
NEIL2
NSUN4
RPUSD2
GPR180
SEMA4C
WDR7
SENP7
DDX56
OCIAD1
ABAT
PEX14
LRCH1
G3BP2
THAP4
HSPD1
NPC1
DTX3L
DNHD1
GAN
EXOC2
DCAF8
FBXW4P1
ANKRD40
ZSCAN25
ABHD3
BBS9
SETD2
ARF6
GNPTAB
SIT1
LNPEP
ZSCAN12
ST6GALNAC1
COLQ
DPH2

FBXO28
ZCCHC6
CELSR2
SETD1B
SIPA1L1
GLTSCR1L
UBR5
XPR1
CYB561A3
DNMBP
AHNAK
CMTM8
ERBB2IP
PDE9A
NBPF1
TMX2
CLCN6
STK4
PIK3C3
HTATSF1
DAPK1
TRAV8-3
TBC1D19
MBNL1
HN1L
TDP1
ZNF830
STARD9
RNPS1
CDKAL1
REXO4
LOC388692
TRAPPC6A
DLAT
KAT2A
RBM18
DDB1
WWC3
BLK
KANSL3
CIITA
NUMA1
SPON1
ITGB1
COG1
RING1
PLXNB2
ABI3
ILVBL
CXorf40A
RALGDS
PSMB2
GON4L
ZKSCAN3
MEX3C
VGLL4
NASP
PTDSS1

ACTR5
TRIM26
CNNM4
ASH1L
MTA3
PARP14
TAMM41
LOC91548
DACT1
TSFM
PWAR5
BNC2
PPP2R5C
FBLN5
RNASET2
ZNF148
OSBPL7
CTPS2
HPS4
SUN2
ALG2
LIG3
PVRL3
TCEA2
HNRNPU
MOB3B
HLA-DRB6
FDXACB1
CTSS
SEC23IP
SEMA4F
RAB22A
CD248
RBM26-AS1
RPTOR
FOXN3
PCBP4
LRRK2
TNFRSF9
PHF1
CRAMP1L
BCL9L
CREB1
LOC101928767
CR2
LOC100130476
RRP8
MEPCE
PEA15
ANKZF1
IL18BP
NCAM1
SLC9A3R1
DNAJC30
FBXW7
ARSK
GDAP1
VPS45

LUZP1
CCDC62
PIK3R3
CDK5RAP3
ITGB7
MOCS2
GAS6-AS1
KIAA1407
ARHGAP25
ITPR1
KLF8
WDR45B
LSM11
ST6GAL1
ZNF440
AAK1
ZNF518B
USP4
EFHC2
ZNF418
ZNF235
OTUD4
MEN1
BIN1
TMEM116
CDK6
YAF2
ZNF667
LOC285957
ZFHX3
PANK4
TULP3
CLN8
SHISA2
RBM28
PHF3
AP4S1
OTUD3
SRR
OLIG2
MID1IP1
ARL1
PTPDC1
LINC00920
POLG
TBC1D22A
RBM4B
SH2D3C
NBAS
DGKH
ZFYVE27
RSPH3
FBXL17
APRT
PACRGL
ZNF829
MCPH1
AHI1

VAPB
ALDH16A1
COA1
PLD4
ZC3H13
NUP153
KIAA1324L
TRAP1
MCM3
ADCK1
YBEY
ATP13A1
CCDC15
CLUH
CERS6
FBRSL1
ZNF74
B3GALTL
COQ6
DCAF4
ZIK1
METTL2B
DAPP1
TACC1
SLC35D2
LOC102724275
MED10
PPARA
COIL
STK36
XPC
SIK1
FAM49A
CSDE1
UBIAD1
PDE4DIP
PIK3R4
INPP5B
SNW1
STAG3L3
KLHL28
GMEB2
FGFBP3
RSF1
CCT8
EP400
METTL13
TMEM64
SLC15A4
ZNF792
ARHGAP31
MGAT5
SH3BP4
LOC389906
CTRL
POLA1
SORD
MYLIP

TMEM41A
CACNA2D4
TSPAN3
CSNK2A2
POLR3C
SENP3
ZNF436
C19orf60
TGIF1
FARS2
MDM4
NCAPD3
USP42
RNFT2
LOC286367
HIP1R
GPM6B
LRRC75A
MTSS1
NUP93
TRIP11
DHX58
ANKRD10
POU2F2
CRLF3
MEAF6
THG1L
TUG1
TFAM
SMAD3
EIF4A3
HIBADH
ITPRIPL1
MAPRE2
PUM2
SOCS2
LRRC37A2
ZNF415
TMEM204
GSTA1
SLC25A15
NME7
CEP350
LOC100128108
C2CD2L
SRSF1
ZNF641
NFATC1
HARS2
SMC1A
PRKY
PLEKHA8
WHSC1L1
TTI1
PDE7A
SEC63
KMT2C
IVD

ADARB1
CAMSAP1
NUBP1
EARS2
CCDC101
MYSM1
LAS1L
PRKCZ
C2orf68
DEXI
KIAA1429
ARL6
ITGAL
HIC2
RBM6
ITPR3
NEFH
LONP1
ZFP82
LATS1
LTBP3
NAB1
ABCF2
INIP
CD101
NFX1
CRY2
DLG1
LOC729683
RANBP2
TSPY26P
FLJ32255
ANO9
SSPN
EIF1AD
ZNF862
ZFP37
CALHM2
CCDC132
EXOSC2
PTGDR2
PPP1R8
PKI55
COG3
USP13
DNAJC16
ZNF618
RNF139-AS1
RNF114
UNC119B
MED6
LARP7
WDR91
MYH3
TMIGD2
SMC6
ZNF607
CXorf40B

CNNM3
RNF103
BRD3
ARFGEF2
MTERF4
SGPP1
PRRC1
SMNDC1
SPICE1
WDPCP
PGAP1
MIPEP
CD84
GATAD1
ABCF1
HUWE1
SPAG9
ERI3
EIF2S3
KIAA0753
PAFAH2
LFNG
SMEK2
SLC41A3
DAG1
CBX1
MICALL1
TTC28
SPIB
ZNF354C
PMS2P1
SGCB
OGT
LMLN
FBXO31
USP7
ABHD15
NUBPL
CASD1
TSR1
RFXAP
DUSP10
GART
SKI
ETV7
MCC
BCAS4
SEC24B
PLCB1
DYNC2H1
MDH2
TUFT1
ZMAT3
LGMN
CLEC10A
TRIM66
LOC284023
TGFBRAP1

RABGAP1
CDK12
SMARCAL1
ZMIZ1
NAGPA
ZC3H4
LOC100507006
TTC22
ACAD9
PRMT1
DPH7
TCF25
LOC100507431
HSPH1
SLC25A29
RAB8A
N4BP2L1
DYNC1LI1
C2CD3
IFT46
BLCAP
COX19
GLIDR
ULK4
TTBK2
POLH
TM9SF3
RPP14
USO1
FCRLB
CD200
NACAP1
AASDHPPT
INPP5F
MGAT4A
CNOT1
SH3KBP1
FUT8-AS1
PNMA6A
UMPS
CSTF1
DPP7
ARHGAP32
DSTN
PLA2G16
TRAF1
SMAD4
WDR52
ZNF597
VPS13D
PNPLA4
MPZL1
CTSF
RBM5
PUF60
LEO1
KLHDC4
PRPF8

SPATA20
TRPM7
RNF169
ACSF2
SCAF11
GPR68
ZNF850
DAP3
CBFA2T2
IDH3A
NSMAF
CNOT2
KCTD15
TTLL5
CARF
KCTD10
PPP6R2
TM2D3
CEP63
DNAJC21
FLJ45513
MAPKAPK5-AS1
PACSIN1
VWA9
GATA2
QDPR
ORC5
KLHL24
CEP70
EFTUD1
YTHDF2
LAP3
ZNF33A
PDPR
IRAK1BP1
WDR92
CCT4
C6orf136
ZNF107
LOC148413
RHPN2
SH2B3
PARN
USP46
PUM1
ZDHHC14
HP1BP3
TAF4
PIAS3
KIR2DS2
ZNF134
ATRN
GEMIN4
RUNX2
SAMHD1
NUP210
HNRNPH1
ADAT1

CDK4
GALK2
DCP1A
ITGA6
MSANTD4
TAP2
EIF2AK3
VEZF1
INTS4
SLC35G1
SRPK2
FAM161B
ATMIN
POU6F1
MED29
PIGM
VPS41
SIGIRR
S100B
SDHAF1
ZNF665
AFG3L1P
MIA3
GGACT
IL27RA
EIF3B
GRIP1
LINC01278
SPTSSB
TBRG1
NOSIP
BMPR1A
ZNF681
ADAMTS1
RPARP-AS1
TOB1
UBR2
ALS2
APOL2
PPIE
EDRF1
PPCDC
HCG26
XPNPEP3
DLL1
ZNF451
TERF2
SCAMP3
TGS1
THAP7
ELAVL1
PIEZO1
GPR157
FUS
HOMER1
ISG20
CPAMD8
PDS5A

AKAP1
ZNF502
RABEP1
SFXN2
ABI2
ZNF470
TP53
TCTN1
RABGGTB
C1orf21
GALNT11
ZSCAN26
GNGT2
SH2D3A
GTF3C4
FAM161A
HHAT
CKAP5
LINS
NCL
ANKRD44
RPL18
TLR3
ACD
PABPC1
KLRC4
HEXA
RAB39B
C14orf182
PDCD2L
HMCES
PERP
S1PR5
ACAA2
PRKDC
ZBTB9
ZBTB42
SIK2
TRIM35
CDYL
PAOX
ZNRD1-AS1
METTL4
LPXN
MAPK9
FGF9
DOCK7
SND1
LOC642852
MSL2
MID2
TECR
CCNY
ZNF345
WDR35
LRMP
CEP41
ZMYM4

PIM2
DVL2
ZNF10
ERN1
FRG1B
SLC29A3
TMEM18
CD19
ADPRHL2
SNRNP40
MICAL2
PER3
FAAH2
PPM1L
GPR174
GLG1
KLHL20
CRYGS
HCG18
SIK3
ZNF322
MTMR12
TOPORS
TYW1
ASAH2B
ERCC6
ATG2B
CHKB
TAF1B
PSMD11
ZNF507
C9orf85
KHDRBS1
PLXND1
ADCY9
HOXB2
PPM1F
IL5RA
CAMSAP2
TNKS
ZNF667-AS1
ZFP1
ZNF212
PRKD3
LOC101060391
CTC1
PPARD
GPATCH4
MAPKAPK5
PRIM2
FAM13A
ATXN7
KIAA1324
CIR1
SRSF5
SPATA7
MFSD12
RMND1

BOLA1
CXCR6
SAE1
SPRTN
TAP1
SRP68
KRT73
ELK3
MFSD6
TNFAIP2
GTPBP4
CHD1L
LOC100270804
ZHX2
GEMIN8
UVRAG
UST
TMEM203
GCN1L1
PCF11
CROT
AQR
AAR2
USP40
BTG1
GNE
FTSJ1
GOSR1
EXOSC7
ETS1
TSPYL2
IREB2
PROCR
CD244
COX18
CCND2
USP11
GXYLT1
AMZ2P1
CCDC6
PTPRM
KRI1
DENR
MBNL2
RNF34
CUL1
PCCA
ANKEF1
GBP2
CCNL2
GPA33
ALDH9A1
SREK1
ZNRF3
PRKAG2
BTN2A1
DHX15
MMAA

OPN3
SAFB2
TNRC6C-AS1
MRFAP1
BPTF
ALDH6A1
TMEM109
CCDC134
EMC1
ISL2
ZNF546
RHOBTB3
HEY1
NDFIP2
HLA-DPB2
FLJ31306
CTBP1
MSTO1
SOX4
NID1
DNPEP
PIGX
ABCD2
MTMR2
RBM8A
SEMA4D
SLC7A7
WDR19
SLC35D1
MSI2
UTP20
FAM213A
ZNF141
TRIM33
CRTC3
DHX57
LMO7
RPA2
REPIN1
ZNF814
TSC22D1-AS1
MPRIP
NUP50-AS1
SF1
CCDC107
9-Sep
LRRCC1
PTPN13
BMS1P5
TRANK1
C1QTNF3
GFPT1
RASGRP3
ASPRV1
RIC8B
CRYL1
C9orf114
NHP2L1

TTC5
HDDC2
NIPAL3
TBRG4
SF3B1
AKAP9
TMEM218
SLC25A17
TIAM2
ELAC1
ZNF239
TTC4
GPBP1
CD3EAP
SAP130
PDCD6
ZBTB33
TDRKH
RDH11
HNRNPUL2
CHN1
MRPS26
FZD3
RNF115
SERPINF1
BICD1
TBC1D12
VPS11
CCDC92
MRPL49
PSMB9
ATF7IP
EEF1A1
NCK1
LPHN1
CLEC7A
ASCL2
ORC2
TRAF3IP1
AGAP9
LINC01355
IMMP2L
NUB1
WDR81
ZNF225
PHTF2
C12orf45
NEK1
DBT
RYK
SF3B3
RAB37
RPS28
POLD2
ACVR2B
SLC35E3
IK
RAD17

SLC11A2
HSD17B8
GLS
RRN3P2
CD4
PALLD
PNO1
SERPINB9P1
CCAR2
LOC155060
ACOT4
GOLGA5
TIGD7
C5orf45
SOS1
MAGEF1
KIAA0141
RABEP2
CCNT2
UBQLN4
CARNS1
NELFCD
G2E3
BRD7
CASK
LRRC37A4P
SLC46A3
EDEM1
PCED1B-AS1
ARHGAP12
IRF2BP2
FAM86A
MORC4
ZNF614
CCDC115
SARS
NKX3-1
CDK17
ARHGAP17
HSF5
CYTIP
NARS2
CNKSR2
DTWD1
KMT2E
UTRN
ZRSR2
HEMK1
PRKCH
PSMD6-AS2
PARP1
IKBIP
ZNF544
YDJC
OCEL1
TNFRSF10B
SIAH1
FOXO1

SNX5
PI4KA
FMNL3
MATK
MORF4L2-AS1
IPO9
B3GNT1
E2F6
HOXA1
CD72
CHCHD4
CBX5
ZNF844
TMEM42
WDR60
CPPED1
FAN1
PSME1
LOC101928403
HAL
UBE3A
RPS16
ZNF500
CNDP2
DIS3L
AVEN
RIMKLB
ZNF227
ATP6V0E2-AS1
KDM3A
ANAPC13
HDAC1
LRIG2
NBEA
AKAP12
CLASP2
RHOF
NUDT7
C2orf43
RAP1GDS1
CCDC88C
MTCH1
ZNF45
NIPA1
POP1
CCSER2
INTS7
TRIM13
AOC2
NFKBIE
KIAA1919
PPFIBP2
CYLD
MYCL
B3GAT1
UBR7
ZKSCAN4
ZNF574

RPS15
PARP11
LMF1
YLPM1
ALOX15
ZNF180
TSPAN32
NUP133
CHST11
SLC30A5
UTP6
FBXO4
QRSL1
MRPL30
PREPL
DET1
LINC00667
C12orf66
ELK4
RPL30
11-Sep
PMS2P8
CD40LG
ZBTB14
QSER1
GCC2
PON2
BCKDHB
NRCAM
MUM1
ANKIB1
ZNF626
FBXO25
ROBO3
YPEL2
PARP2
MCCC2
DIS3
BRPF1
PCNXL4
NME3
DIDO1
TNRC6A
AEBP2
POLR1E
NUP205
HIVEP2
ALDH18A1
MAPK8
LOC101928370
TTC17
SPATA5
DCHS1
HABP4
THAP6
CNOT10
CEP104
STX17

TRMT10B
RAB40B
FAM159A
KIFAP3
UNK
IFT43
FBXO32
FGFR1OP
SEC31B
FAM102A
GALNT6
RAPGEF6
RAPGEF2
RPL7
SMEK1
PDDC1
ZNF33B
LOC101929668
GTF3C2
CDC14A
VPS36
P2RX7
LRFN3
RHOQ
ESCO1
ERMP1
DUSP6
VPS51
RORC
URGCP
ARHGEF9
SMARCA5
EPRS
ATL2
TNFRSF21
MOAP1
DDX1
CLDND1
ZBTB39
CDC23
SPEN
DUSP14
TRIM32
MIEF1
CXCL6
FAM60A
TSC1
LTK
NUDT3
GNRH1
DFNB59
ARRB1
CRIP1
WDR73
CYB5B
ZSCAN18
METTL14
PCBP2

SNRK
GFM1
ALKBH2
ILF3
BIVM
SSBP4
PRKX
PLEKHG4
CEP83
MED1
TSHZ2
ADRBK2
NKRF
ZCCHC14
MRE11A
FRYL
C5orf63
LOC101927027
ZBED3
NPCDR1
SIMC1
KIAA1279
UPF2
TMEM192
LRRC8D
CCDC64
NANP
SOX12
SNX30
POLR2D
YTHDC1
RRM2B
RPL37
RBM12
CST3
SGSM2
PIGL
MTCP1
DVL1
SOCS7
SUPT7L
NOLC1
PDHB
RNMT
BZRAP1
SSBP1
PGAP3
ANKRD13C
FXN
C19orf66
R3HDM1
NPAT
CCT6B
DPF2
SLC9B2
IMP3
PRPS1
PLEKHA5

SULF2
LOC100133130
TEX10
GSPT1
PDCD7
S100A10
SON
PQLC3
PLEKHF1
MPPE1
RBMX2
MRS2
ANKRD20A11P
SYNRG
SEC14L1
USP14
OSBPL5
NPRL2
FANCM
NIP7
ZNF555
EIF3K
C11orf80
MDN1
ST8SIA1
GTF2H1
DGKE
LTB
TNRC6B
PPAP2A
VIPR1
XYLT1
DPH5
KNOP1
CD5
RBM26
TSPAN18
ZNF395
IKZF5
USP25
SDR42E1
UTP23
DDX50
ATG16L1
LOC101929112
LCMT2
ZBTB10
HLA-DOB
ABCB7
SNPH
PM20D2
CYP2R1
CDIP1
TRAPPC2
DDX31
NR2C2AP
HELQ
ZNF280B

CIAPIN1
GPR155
PDCD11
FAM111A
B2M
LRCH3
KCTD6
RMDN3
PLEKHG3
C8orf33
EDAR
DCTD
ZNF599
WDR82
RPS6
SFMBT1
HECA
RPAP2
LOC100505501
BLOC1S4
POLR2B
ZDHHC23
ENGASE
SERTAD2
SIN3A
KLHL36
PIAS2
FDX1
CSF1R
VPS13A
DENND4A
AGAP1
DCAF16
CLUAP1
DCAF17
EBF1
RBM27
UPF3A
RBBP4
PRR5L
TOP1MT
LOC101929760
PFKM
LINC00526
PCM1
GOLM1
RUNX1-IT1
KIAA1468
CASP6
DAZAP1
ZNF566
RRS1
ZNF133
SRGAP2C
DND1
NFE2L3
GLMN
CYP4V2

RABEPK
ZC3H6
SDC2
TCTN3
CLYBL
POLG2
CDKN2AIP
EIF5A2
MGC24103
SLC26A6
YARS
C20orf194
TAPBPL
NDUFV2-AS1
ZNF790-AS1
SSRP1
HOXB3
APOBEC3G
SMYD3
DOCK9
PABPC3
FAM135A
NXT1
PPP2R2B
POGK
IFNLR1
LOC101927620
PRKAG2-AS1
TOE1
YY1
GUF1
FCHO2
ZNF682
AGBL3
PITPNB
TMEM50B
PDE4B
SENP6
RPS29
ICK
TP53BP1
ABCA11P
WARS2
EIF4B
MS4A2
ZNF317
AHCTF1
ARL10
CDKN1B
ATP10A
NEK7
G3BP1
NAA40
ZNF117
NUP54
GOLGB1
THUMPD3
TMEM184C

ACVR1
PCGF5
MID1
PPRC1
LOC100505715
RBBP6
MED17
BTF3
LARS2
CAPN7
SUN1
TNFSF8
LPAR5
DUSP7
TMEM206
PDCD2
PMS1
RFX5
SIRPG
HEATR6
PDIA3
ZNF182
RNASEH1
SRSF10
UTP15
RNASEH1-AS1
SMARCE1
LZTFL1
AKT3
TMEM19
P4HTM
PPARGC1B
ZCCHC2
RNF113A
LMBRD1
DDB2
PRPF4B
MRPL19
ADNP2
LSM14A
CHD2
APOBEC3C
ZNF689
GCFC2
PDE8A
UBE2I
ZMYM6
PSTK
MAP7
MAGEE1
MYO6
WDR5B
MLLT6
UBP1
PTPRO
R3HDM2
NGDN
PTCD2

SFPQ
ANAPC5
PDE3B
FKTN
PWWP2A
P2RX5
CD27
TP73-AS1
TSEN2
TMEM245
CYB561
ITFG2
OBFC1
LINC00674
SLC30A4
FAM76B
SDCCAG3
IL15
INADL
RPP30
VPS37C
KRIT1
HADH
GOLGA2P5
FAM76A
FAM134B
APPBP2
FNBP1
RPS19
C2orf42
RASSF1
VPRBP
LPIN2
HSPA9
DTD1
RSBN1L
PPP3CB-AS1
PPP1R2
TMED10
ALDH8A1
IPO5
WDR89
TCL1A
PLCG1
FUBP3
PIK3IP1
PAXBP1
ARID1B
CIRBP
RAB12
GPATCH8
MGMT
C3orf17
GMPS
MYO5C
UXS1
FAM210A
CYP2U1

LOC100506990
ADPRM
ZNF234
SUGP2
PRPF3
PLAGL1
SCRIB
ANAPC16
SIDT2
NDNL2
ASCC3
DHRS4-AS1
SLC26A2
FAM24B
1-Sep
CHMP7
SLC12A7
ATHL1
CD47
EIF2B1
NUP88
SRFBP1
OGFOD1
MAP9
SNX9
ZNF680
CD86
ZNF226
FAM221A
DIP2C
ICAM2
PHF11
SP4
SCML2
UBA52
MORF4L2
ZNF140
RALGAPA1
PTPLB
ZNF347
CUX2
XYLT2
KDM4C
ZNF202
NGRN
PKD2
TTC9
HSD17B7
TRPC1
C7orf31
DLG5
FTSJ2
PCID2
PNMA1
MTO1
ZNF711
PAAF1
HYLS1

MAGED1
FRA10AC1
CHI3L1
KRR1
EXOSC9
AQP3
RBM15
TMEM99
TMOD2
L3HYPDH
FBXW4
ZNF738
YWHAQ
CERK
MAP3K14
ZNF284
METTL10
MTERF2
TMEM161B-AS1
ZNF280C
PUS1
SLC41A1
CELF2
RBM14
ATXN10
GSDMB
DANCR
LOC100131564
AMD1
LOC100505564
C2CD2
SEC31A
TMF1
ACSL5
ABHD14B
PNPO
SNRPA
NMRAL1
LOC100507557
BIRC6
TMEM135
SRP72
USP24
METTL17
AFG3L2
RNASEH2B
ZNF431
RAD51-AS1
RAPH1
FAM150B
ZNF703
RRP1B
NAAA
LYRM4
ULK2
ODF2L
INPP5E
RPS27A

STIM2
PITPNC1
LIMA1
LOC100996583
DDX42
TXNIP
CHST12
XPO4
ITPKB
TRAF3
HSF2
VEZT
PRPSAP2
NUP160
SLC9A7
IL16
TMEM209
KLHDC7B
MIR1244-3
IFIH1
RBAK
NUFIP1
SLBP
HNRNPD
ABCA5
ATP6V0E2
ZNF18
CARD11
RCC2
METTL8
BTBD18
GEMIN5
GNL3
PDGFD
MME
RWDD2B
PCED1A
CEP85L
KLHL5
EXT2
POLR1C
TCEA3
RUVBL1
TRMT12
FCHSD2
TASP1
TMEM154
ACACB
LOC101927365
ZNF253
ALG8
LINC00094
CCNT1
THOC2
RFC1
MYEF2
ZNF138
POLE3

REPS1
MB21D2
NADK2
FAM120C
SP140L
HNRNPH3
HAUS5
LOC100133315
TSEN54
THNSL1
PLEKHA8P1
FCGR2B
PDCD5
DENND4C
FAM129C
FIP1L1
MRFAP1L1
PTGS2
TAF1A
TRIM68
SMAD7
ZNF2
IPCEF1
MEGF6
RPS3
BMS1
WDFY1
NFYB
CEP192
RCN1
ZNF137P
MLH3
ZNF419
HEG1
NAF1
ZNF224
AGMAT
SERBP1
PTGDS
HERPUD2
LOC285812
RAB30
DUS4L
GABPB1-AS1
PPP2R5E
ANGEL2
RIPK2
LOC286161
SMAD5
GRSF1
ANKRD36
LARS
NSG1
FEZ1
FAM73A
PHF20
BTN2A2
CNOT11

KIAA1551
ZNF585A
JAK1
ASXL1
S1PR1
CCL28
ADO
ADNP
ULK3
SART3
ZNF554
RHNO1
NACA
NBPF20
HEATR5B
PIK3C2A
HERC2
ATAD1
GRAMD3
FAM214A
EXTL2
YME1L1
ZNF783
BNIP3
RPS24
ZNF540
CPED1
GRPEL2
WRN
CAMK1D
PITPNA
C9orf41
RPL34
MTPAP
MPHOSPH8
FAM3C
ECHDC2
AUTS2
SMCHD1
CAND1
GPN1
GOT2
RAD54B
NCBP2
DPH6
ERAP1
TPD52
ALG9
EHBP1
MAFF
CSE1L
SH2D2A
NAT10
CSPP1
CLCC1
MRPS18B
POLR3GL
CIPC

TSPYL5
MALAT1
ERO1LB
TRIM37
AGO3
C12orf65
ZBTB38
AP3M1
JARID2
ZNF256
CREBRF
SMC5
POMT1
WBP11
POLB
PPTC7
RAB11FIP3
CCSAP
ILKAP
ZNF644
AKAP8
IL21R
ANP32B
FBXL14
TBCCD1
PAN3
TMA16
RPS5
SEPSECS
ZNF789
ELOVL4
ZBTB1
ADCY7
TPR
METTL3
C10orf88
RAD1
AGBL2
MTMR11
USP16
ATG16L2
DHX33
TULP4
ATM
CECR5
CHI3L2
UPF3B
PPP3CC
CLOCK
LINC00623
MIB1
TMEM161B
ATP6V0A2
RBM43
FLVCR1-AS1
RPA1
TBC1D9
MDC1

GORASP2
ZNF160
YES1
ECHS1
EPHB6
RRN3P1
IFFO2
PAXIP1
CFL2
ZNF706
ISOC1
SMKR1
NAPEPLD
CPVL
LOC101930415
USP45
ANKRD23
NR2C1
IL23A
SFMBT2
FNTA
TWISTNB
ID3
CAPN2
CD83
RECK
DUSP12
ADHFE1
FOXP1
CD1D
LUC7L
TTC12
FARSB
MAN2A1
H2BFXP
ZNF791
EIF3D
GGPS1
DNAJC10
ESYT2
PRMT6
CNOT6L
APBA2
ZKSCAN8
FNBP4
CXCR3
OGFRL1
GPAM
HKR1
FBXL16
ARSG
ZBTB20
PRDM1
ZC3H14
TXLNG
CLIC3
ZNF671
EEF2

XAF1
SPIN1
ICE2
RNF4
EMR3
KDM1A
KAT6B
ENO2
IPO5P1
DBR1
DPEP3
ZNF300
TMEM138
PID1
PPM1K
GCSAM
TFB1M
ADK
PURA
RPL32
CXXC5
TIAM1
HCRP1
LOC283788
TNIK
CLK1
EPHA4
CCDC25
TTC27
THADA
AKR7A2
ADH5
CBFA2T3
NOP2
FUBP1
SCARF1
NSMCE4A
LAMP3
CIB1
SCARNA17
ZNF493
MRPL9
CACNA2D2
CYCS
AMIGO1
RPL23A
DMTF1
SCAF4
NIFK
AMPD2
SUPT3H
RPP38
NOA1
PIGP
FN3KRP
CHAMP1
MS4A7
FAIM3

TCF12
RPGR
CNST
RPL35A
MPEG1
DPYSL2
ZNF223
SCRN1
SSBP2
IKBKAP
FAM175A
HEATR2
ZNF512
PHF14
ATG2A
CWF19L2
BEX4
NFATC3
SSBP3-AS1
RAD50
HMG20A
TLR7
TKTL1
FLT3LG
NHP2
ZBTB5
GLIPR1
ZNF879
ABHD10
ZNF430
PPP1R13B
TRA2A
TBP
ZXDB
ERCC1
LYRM9
MUTYH
NSUN2
DCUN1D4
ZCCHC18
RPS18
YEATS2
HDAC9
TMC8
TRABD2A
DDX18
UPRT
DNAJA3
ZNF786
METAP1D
PCSK7
NCR3
PPIH
ZNF12
HMGN4
SPAG16
IGIP
ZNF77

PDP1
PPHLN1
RPL11
PCBD2
ZNF514
PATL2
EIF3A
PLXDC1
C1orf174
JADE2
REM2
TGIF2
NUDT13
U2SURP
ZNF793
ARRDC2
RBL1
PNPT1
FAM179A
UNG
ACYP1
JADE1
GPR56
PAPD5
TESK1
DDX46
KIAA1377
RPL19
LOC399491
SF3A3
TCEA1
USP36
DPP4
MICU3
BRWD1
BCLAF1
SMURF2
BDH2
JAKMIP1
UFM1
RNMTL1
SPOCK2
CRTAP
YTHDF1
ALKBH3
LGR6
PEX1
LOC100127972
ADAT2
TIMM10B
SKIV2L2
UHRF2
SLAMF7
GTPBP6
ELP2
SLC25A26
SARAF
RNF157-AS1

ORMDL1
PAN3-AS1
DOPEY1
MARCKSL1
PRRC2C
ZBTB40
C14orf159
EID3
SLC4A7
ZXDA
UNC5CL
C12orf75
SLC7A6
BBIP1
EVA1C
YPEL1
BCL2
LEPROTL1
RPS25
ZNF512B
JAKMIP2
LOC374443
RSAD1
ZFP90
AMMECR1
LINC01420
TNFRSF25
ARMCX2
LOC100190986
TMEM123
SYNCRIP
C6orf203
AK5
SLC39A6
C2CD5
CLSTN1
OR52K3P
ZNF606
RPL7L1
ZNF764
CBR3
ZNF880
PAN2
PTMA
FASLG
MSH2
QRICH1
FUT11
TCEAL1
CD74
FMR1
GAL3ST4
TOP2B
IFT80
TTF1
HIVEP3
GTPBP8
CENPC

NT5DC1
CXorf65
KDSR
RPL6
C16orf80
ZSCAN21
SEH1L
TMEM194A
PCYOX1L
ZNF331
CHD6
ZNF337
DDX55
ZNF91
BBS4
RNF157
LILRA4
TUBGCP5
BAG3
RPS4X
PIK3R1
ADAM28
CD28
SCAMP1-AS1
UBA2
BAG5
RTP4
RICTOR
ZNF264
GOLPH3L
ANKH
TTC16
ZCCHC8
VPS13C
SS18L1
SLC25A38
FCGBP
CD3E
LIPA
FAM84B
ZNF268
ZNF75A
NVL
THUMPD3-AS1
METAP1
IFIT5
ALKBH8
SFI1
EIF3M
MTHFD1
COG2
RBM25
PARP8
CYSLTR1
GPD1L
CTCF
SLAIN1
XPO1

ATR
DDX20
LEF1
ARHGEF10L
KANSL1
CEP95
C10orf2
DFFB
BBX
LDLRAP1
PKN2
NAA35
ZNF677
FAM43A
IPO7
B3GALNT2
ZNF37BP
WDR54
DDX5
FAM220A
SDE2
HNRNPR
YARS2
CHRAC1
ATP8B2
PEBP1
LY86
TDRD3
FGL2
HLTF
SLC7A6OS
KLF12
SCAF8
NEFL
IMMT
KMT2A
PFN2
NOM1
ZNF639
THAP11
BTBD6
NUP43
NAA15
ZSCAN16
PTPLAD1
SLC5A3
ZNF468
PIGB
CREBZF
ZNF721
MRPS27
LOC441528
ZNF22
C19orf12
LOC283357
SRRM1
DDX27
HNRNPA0

EI24
LOC100996246
LANCL1
GSE1
CNTRL
LOC644656
NFATC2IP
DEPDC7
KANSL2
C2orf40
FAM26F
ELP3
SLAMF1
ZNF678
LTV1
CCDC14
PRKAB2
TARSL2
RPS27
DDX21
EIF3F
DDHD1
ZNF708
FBXO21
ANK3
MLLT11
ZNF232
MRPS6
TMCC1
MYO9A
KCNA3
SLC25A32
PPP1R3E
FAM162A
SMYD4
FNIP2
OARD1
COX11
BUB3
LSM8
TRAF3IP3
PAX5
DZIP3
B3GALT2
RNASE6
ADAMTS5
RPS12
STARD7
MRPL45
CDC37L1
SETDB2
PTPRK
ESYT1
LINC00173
TRIM59
CLIP4
PTGER4
HCP5

TIMM21
CXorf57
LOC642236
TCERG1
ATP10D
ZNF623
CBX7
DDX26B
EPHX2
PIK3C2B
AIMP1
IKZF3
BRD1
TARDBP
PRPS2
ZNF550
KARS
DCBLD1
HVCN1
PCYOX1
FHIT
DSC1
IFT74
TTYH2
ZNF136
DMXL1
DENND2D
EID2
SELM
LDOC1
KIAA1430
EBLN3
FANCF
ZNF248
PI3
MORC2
TMEM106B
URB2
HEATR1
GPATCH1
SMS
XIST
TRERF1
PPAPDC2
PTCH1
MGA
TMTC4
GIMAP2
HNRNPDL
MRPS25
ZNF827
REV1
ZMYND11
ZMAT1
SIRT1
APEX1
MYOM2
KLRG1

CDK5RAP1
LINC00954
RPL3
PWP1
ZNF570
C5orf56
CBLL1
PARP12
ZNF652
SECISBP2
RPL12
PCNXL2
GLO1
RPUSD4
PDIK1L
ZBTB2
HDC
CCDC109B
ZNF101
IER5
METAP2
LOC101928152
DIS3L2
ARMC1
LPIN1
LOC389765
CEP57
UTP3
MEF2C
ITGA4
ZNF587B
MTERF1
CDC25B
SESN1
TNFAIP8L1
CASP8AP2
MRPS30
TTC19
CEP120
ZNF204P
DPEP2
CCDC146
FASTKD2
NT5E
AKAP7
STRBP
LAX1
MRI1
MIS12
AOC3
ARHGEF18
EFHC1
IDO1
CCNG1
CD69
TIMM23B
ATG14
RPAP3

CMPK2
RRAS2
RUFY3
NAA16
PRKRIR
ZDBF2
MIR3682
IFNG
CREBL2
FAM13B
NUDT9
REV3L
FAM208B
BTN3A2
ZNF767P
BOD1
SLC35A3
UBE2E2
BTLA
GPATCH11
WDR36
HNRNPA3
ZNF736
LOC102724611
MRPL1
RUNX3
RPL29
SSB
IMPDH2
NUDT5
CCDC50
GLT8D1
EPHB1
ZNF532
GTF2H3
VSIG1
RPL31
GPRIN3
TYW3
MFSD8
MLLT3
DUSP2
BCOR
ZNF275
SERPINB9
DHRS3
KIF5C
DPY19L2P2
RPL15
RSAD2
TRIT1
MDFIC
FAM174A
MCCC1
CNOT7
AKAP11
CIRH1A
DIEXF

PPIL3
CXCL10
LEF1-AS1
TSPYL1
SLC25A36
FAM122B
ZNF274
EIF3G
ZNF567
MPHOSPH9
EID2B
L3MBTL3
GAR1
CMPK1
TCF7
EIF3L
CPSF6
ADA
LOC286052
NOB1
GTF3A
ANKRA2
GALNT12
NSUN5P1
AMMECR1L
LOC101929787
ABCB1
LIAS
RSL1D1
SHQ1
PHLDB2
AKR1B1
ARRDC3
SMYD2
CCT6A
PPAT
RBBP7
ZNF805
ZNF565
EIF3E
ASF1A
NSUN6
GLOD4
TOX
UFSP2
GKAP1
THOC1
SMARCAD1
SDAD1
PAQR3
IFI44L
IL10RA
SH2D1A
ZC2HC1A
NAA25
STAT1
ZNF506
CCR5

LOC100130744
TRIM24
COQ10A
COX20
MAP4K1
TRAPPC10
ZSCAN29
TAF15
PALB2
CDC16
RFX7
FAM19A1
SNRPA1
IRF8
TCEAL4
PRKXP1
ZZZ3
ATF7IP2
LOC100128751
BTBD11
TAGAP
USP53
SCFD2
POP5
PCSK5
RPGRIP1
C14orf169
PAPD7
LOC153682
PSMA5
LOC101927482
CMTR2
ACVR2A
CDC42SE2
CD1C
NSA2
NPIPA1
PBX4
EPS8
MAP4K3
PHYH
ZNF320
MRPS9
EIF3J-AS1
GPR133
ACADSB
DTD2
RABGAP1L
SH3BP5-AS1
KIT
KLF3-AS1
DDX10
PAQR8
SLC25A45
YAE1D1
C9orf91
CTSW
NAP1L1

GBP1
LOC100272216
OXCT1
MYC
POLR1B
APOL3
LETMD1
XPA
PHOSPHO2
ANGPT1
KIZ
LRRC69
TMEM128
CEP68
DIMT1
DNAAF2
C6orf48
MCM3AP-AS1
SLC16A10
MRPS35
TESPA1
HLA-F-AS1
SEPT7P2
DPY19L4
TRMT10C
HLA-DMA
NOP14
EIF1AX
SRSF7
TAF3
ZNF14
SBK1
RPL36
ETAA1
KIAA0907
NFATC2
LOC101927204
LOC100506469
GDPD1
DDX24
EIF2D
TOMM70A
TAF5
PRMT3
ZNF823
ARHGAP5
STMN3
EXOSC6
TTC37
CKMT2-AS1
PHC1
ABCG1
PASK
EIF2A
C1orf109
ECI2
RNF144A
NFXL1

ANKRD12
RCN2
PLA2G7
LINC00926
LSG1
NPM1
ASTE1
LOC100132352
GABPB2
LRRC47
BANP
NOL9
ANXA2R
LPAR6
PGRMC2
KLF10
PNISR
TCEAL3
LCK
ADRB2
KIF3A
MTX3
GNPNAT1
PRKRA
BZW2
NRROS
RPL10A
NCALD
PEX3
NUCKS1
CLEC2D
CUTA
IKZF2
LAT
PVRIG
SUMF2
MALT1
ZMYM1
EPB41L4A-AS1
ERAP2
MS4A14
CBLB
BBS10
PHF10
IL32
SLC38A1
LINC00959
HOXB-AS1
CCR6
FAM98B
CECR1
TMEM243
RPL22
GTPBP3
ZNF766
EBAG9
AGPAT5
CD247

ENPP5
NOL8
URI1
TMEM181
ANKMY2
ZNF85
CD52
PPP1CC
SATB1
ALDH1A1
NDUFAF4
TADA1
LYAR
ST3GAL5
CCDC66
EIF3H
WHAMM
ICOS
LOC728392
ZNF304
ATIC
ZBTB41
RPL37A
SETBP1
GIMAP4
SLC20A1
AHSA2
HLA-DQB1
ABCE1
GPALPP1
NAE1
POC5
MAF
ZNF32
METTL18
EXOSC10
XCL1
PUS7
FAM171A1
MAML2
CAAP1
FTO
ZNF542P
ZNF813
HLA-DPB1
OPTN
TIGIT
MAP3K4
RPL14
CAMK2D
SUPV3L1
GPR114
CLNS1A
DDX60
NUP107
HOOK1
TGFBI
ZNF383

CRYBG3
KIAA0355
LOC283070
ICE1
AGK
EIF5B
P2RY8
LOC100287497
LOC100505549
PCMTD2
BCL11A
LYRM7
BLNK
ZNHIT6
TOMM20
KATNBL1
ALMS1
FYN
LINC00342
SIGLEC17P
BTN3A1
OLIG1
DNMT1
TPP2
ZBTB21
PSIP1
LDOC1L
TIGD2
RNF125
ZC3H8
TSHZ1
BANK1
RTTN
ZNF559
ZNF529
RINT1
DROSHA
ZNF211
ZNF84
CDKN1C
MYCBP2
CPA3
ZNF195
POGLUT1
KISS1R
C14orf28
HACL1
HERC6
CHST7
KIAA1671
FIGNL1
ESF1
SUCLG2
PTCD3
WWP1
GBP4
TTC39C
SERINC5

ZNF184
CFD
ZFAND1
C10orf128
FAM213B
ERCC5
ARMCX5
MAK16
CTDSPL2
EVL
MFHAS1
TRIM44
RHOH
LOC202025
IFIT3
LINC00877
IARS
BZRAP1-AS1
ZNF664
EPSTI1
SET
LRPPRC
DKFZP586I1420
SRSF11
TIGD3
ZBTB4
CCR7
SDR39U1
SLFN5
MAGEH1
OXNAD1
PJA1
ARHGEF3
6-Sep
DCP1B
ANKRD46
DOCK10
NHS
TBX21
KLF11
PRORSD1P
LOC338620
KIAA1147
CCAR1
ENOSF1
TSPAN13
ACKR3
HLA-DRA
LDHB
AP3M2
GOLGA8N
PCED1B
SRSF8
GVINP1
ENOPH1
TAF1D
RIOK1
OSBPL3

LINC00936
ZCCHC7
BACH2
MPHOSPH10
TTC39B
MS4A1
WDR75
ZNF146
CRYZ
ARL4C
NUP35
PTGDR
LRBA
PKIA
DNAJC2
ZNF26
PRIMPOL
JMY
FAM179B
PFAS
TXNDC16
NAP1L2
LOC727820
RLN2
TBC1D32
BTAF1
OCIAD2
NKTR
HLA-DPA1
CCR3
MTIF2
TIMM9
ZFP3
KRBOX4
GIMAP7
CCL5
RFTN1
WDR11
RRN3
SNX29P2
ZNF30
ZNF260
NARS
SEC14L1P1
ATP2B1
STAP1
CCNB1IP1
SIDT1
SKAP1
TC2N
SLC39A10
LYSMD2
PPP1R16B
C12orf57
LUC7L3
ZNF439
TRIB2
THUMPD1

FBL
FYCO1
TARBP1
LOC100996740
GNPDA2
TRMT11
THEM4
PRPF39
ZNF709
ABLIM1
ZBTB25
RRP15
KPNA5
ZNF92
ZNF266
RPL38
ZFP62
HERC5
INPP4B
NOL11
CAPRIN2
PRKACB
PRSS23
KIF21A
HAUS3
THAP9-AS1
TRDV3
SYTL2
BIRC3
CX3CR1
STK39
GPR171
OSGEPL1
ZBTB24
ZAP70
HNRNPU-AS1
GIMAP6
USPL1
GBP3
RARRES3
KDM2B
IFIT2
CTSO
ZNF329
UBAP2
PRKCQ
MAN1C1
GORAB
CCL4
SLC18B1
PDCD4-AS1
PTER
HNRNPA1
GOLGA8A
NOC3L
SCML1
SGK1
RCAN3

IL11RA
ITM2A
RELL1
FUNDC1
FAM35A
RORA
SHPRH
KLHL3
EIF3C
TMEM14A
USP28
GIMAP1
UBE2Q2
ZNF83
BBS2
BCL11B
CAMK4
RBM4
BEX5
NAP1L3
TRBC1
CXCL8
WDR3
MKL2
LOC93622
OFD1
GSPT2
LY9
CCDC104
CEP290
BEX2
ITPK1-AS1
ZNF571
MAL
PYHIN1
FAM69A
HMGN3
TRMT61B
ZNF302
LY75
TMEM263
FLVCR1
ARL14EP
PRKCQ-AS1
LOC200772
SGK223
MTERF3
HLA-DMB
LBH
BTN3A3
ITK
NOP58
MBLAC2
ANKRD36B
WDR43
MTR
MSANTD2
CD3D

RPS6KA5
LIPT1
PLCXD2
RASGRF2
SETD6
MYBL1
UBASH3A
TBC1D31
CCDC84
PAXIP1-AS1
GBP5
MGC40069
KLRC3
PWAR6
RTN1
MCOLN2
DYRK2
C5orf28
CD8A
AMIGO2
MAP3K7CL
PTPN4
ZNF420
DNAJC24
CEP78
PMAIP1
CHIC1
NMT2
STAT4
FCRL3
ZNF432
LINC01215
FAM117B
KLRD1
CRY1
CRTAM
TRG-AS1
TRAT1
P2RY10
NOV
SACS
NR1D2
TRMT13
DDHD2
FAM169A
TRAC
LOC102724356
TXK
PRF1
PLEKHA1
GATA3
GPR183
HOPX
GZMB
GZMH
GPR18
TBC1D4
IL7R

GZMA
P2RY14
CD2
IL2RB
CACNA2D3
IFIT1
CD3G
NLRC3
NR3C2
GNLY
SH3YL1
FGFBP2
GPRASP1
TRAF5
ZNF573
GZMK
RASGRP1
SAMD3
LGALS2
NELL2
EOMES
KLRB1
ZNF600
CD96
LRRN3
CD160
GRAMD1C
TGFBR3
NOG
KLRF1
FCER1A
