# Supplementary material for: Altered Genes and Biological Functions in Response to Severe Burns
Source: Biomed Res Int. 2021 May 24;2021:8836243. doi: 10.1155/2021/8836243 (PMC8168476; doi:10.1155/2021/8836243)
Supplement: Supplementary 8 — Table S6: module_gene. [file 8836243.f8.pdf]

Table S6 Module_Gene

Module	Symbol
m3	AAK1
m3	AMPH
m3	PIK3C2A
m3	WASL
m12	CD4
m3	ITSN1
m3	AGFG1
m3	SNX18
m12	LDLRAP1
m12	AVPR2
m1	UBA52
m3	ARPC1A
m3	LDLR
m3	TRIP10
m3	PACSIN1
m3	ARPC3
m1	RPS27A
m3	OCRL
m3	ACTR3
m3	ARPC5
m3	DNM2
m3	HIP1
m12	DVL2
m3	FNBP1
m3	CLTCL1
m3	SNX9
m3	ARRB1
m3	EGFR
m3	EGF
m12	BIN1
m3	SH3GL1
m12	CD3D
m3	ARPC2
m12	ARRB2
m3	FCHO2
m1	PRPF8
m1	EFTUD2
m8	TP53
m1	ETF1
m6	RNASEL
m1	EIF2S3
m12	ABI1
m12	ABI2
m12	SOS1
m12	WASF1
m12	NCK1
m12	MYO10
m12	ARPC4
m7	TRIO
m8	UBE2I
m12	ACKR3
m12	CXCR6
m12	ADCY9
m7	GABBR2
m12	CCR3
m7	CCL5

m7	CXCL12
m7	CNR1
m7	GAL
m7	CCL21
m12	CXCL3
m12	CXCR3
m7	CXCL10
m7	PENK
m12	S1PR1
m7	CCR5
m12	C3AR1
m12	CCR7
m12	CXCL6
m7	APP
m12	S1PR4
m12	ACTN2
m12	PROS1
m7	TMOD2
m12	HGF
m12	F8
m12	ALDOA
m7	MYH3
m7	F5
m8	AKAP9
m12	SERPINA4
m12	CLU
m7	TIMP1
m12	SERPINA1
m7	SRC
m7	TNNI2
m7	TMOD3
m7	TNNC2
m12	CFD
m7	CTSW
m12	ITIH4
m12	THBS1
m12	VEGFA
m7	MYBPC3
m7	PIK3R1
m7	TLN1
m7	VIM
m7	VCL
m7	ITIH3
m7	RASGRF2
m8	ACTR1A
m12	RAB11A
m8	HMMR
m8	PLK4
m3	KIF15
m3	TUBA1A
m8	KIF11
m8	TTBK2
m2	PLK1
m12	RAB8A
m8	KIF5B
m12	C2CD3
m3	TUBB4B
m8	PRKACA

m2	MAPRE1
m3	CEP290
m2	CDK1
m3	TUBA4A
m8	KIF3C
m12	TUBG1
m12	B9D1
m12	PCM1
m2	KIF2C
m3	DYNC2H1
m8	RAB7A
m8	DYNC1H1
m8	KIF3A
m8	KIF20A
m12	TCTN1
m10	AURKA
m3	YWHAE
m3	KIF23
m2	CKAP5
m12	TUBGCP4
m8	NEK2
m12	TUBGCP5
m8	KIF4A
m3	KIFAP3
m2	CENPE
m2	KIF18A
m8	XPC
m8	RAD23B
m12	DDB1
m12	RUVBL1
m12	YY1
m8	DDB2
m12	CCNT1
m12	CCNT2
m1	SMURF2
m5	POLR1B
m12	PKM
m12	AK9
m1	POLR2B
m12	ENTPD1
m7	ADCY2
m12	POMC
m12	FPR1
m7	ANXA1
m12	NME7
m7	P2RY14
m12	ADCY3
m7	NPY2R
m12	ENTPD3
m7	GPR18
m7	ADORA1
m12	NME4
m7	ADORA2B
m7	GHRHR
m7	PTGDR
m7	PTGER4
m12	ADCY7
m7	ADRA2A

m7	VIPR1
m12	NME3
m7	ADM
m7	GCGR
m7	CRHR2
m7	PYY
m7	P2RY13
m7	OPRL1
m7	PTH2R
m7	PTGDR2
m7	F2
m12	AEBP2
m12	DNMT1
m12	DNMT3A
m12	PHF1
m12	E2F2
m12	CDK6
m12	E2F1
m12	E2F3
m7	GNA15
m7	GRB2
m13	FGR
m7	EDNRB
m7	NTSR1
m7	LTB4R
m7	GNAQ
m7	GPER1
m7	PTGFR
m7	HCK
m7	CCKBR
m7	GNRH1
m7	P2RY1
m2	AHCTF1
m2	CENPA
m2	BUB3
m2	PPP1CC
m2	CENPI
m2	CDCA5
m2	NUF2
m2	XPO1
m2	NUP133
m2	CASC5
m2	SGOL1
m2	CCNB2
m2	CCNB1
m2	AURKB
m2	NUP107
m2	ESPL1
m2	RCC2
m2	NUP43
m1	CDC20
m2	CENPC
m2	CENPF
m2	CLASP2
m2	BUB1
m2	MIS12
m2	ZWINT
m2	CDCA8

m2	PPP2R5A
m2	SMC1A
m2	NUP160
m2	RANBP2
m2	PDS5A
m2	BIRC5
m12	B9D2
m12	SFI1
m3	OFD1
m12	CEP41
m8	CDK5RAP2
m12	ALMS1
m12	CEP164
m1	PPIH
m8	POLD2
m3	HAUS3
m8	CNTRL
m12	CEP63
m12	TPX2
m8	CEP192
m7	PIK3CB
m7	YES1
m7	CD28
m13	LCK
m7	JAK1
m13	CYP2C8
m12	ORM1
m12	QSOX1
m8	CEP78
m13	ALOX12
m13	CYP4F2
m13	PLA2G4A
m13	PTGS1
m13	PTGS2
m13	CYP2U1
m13	ALOX5
m13	ALOX15
m1	CUL1
m1	UBE3A
m8	ANAPC16
m1	FBXW7
m1	HERC5
m2	UBE2C
m2	RNF7
m2	CDC23
m1	CDC34
m1	HERC6
m2	BUB1B
m10	ANAPC15
m2	RCHY1
m1	UBE2A
m2	ANAPC5
m2	UBE2D1
m1	CDC16
m8	PSMB3
m10	CCNA2
m8	PSME1
m10	PSMD11

m8	PSMB5
m10	PTTG1
m8	PSMB2
m10	PSMA5
m8	PSMB9
m8	PSMD9
m2	UBE2D4
m2	HUWE1
m1	WWP1
m2	ASB7
m1	KLHL3
m1	UBE2H
m10	PSMD4
m1	UBE2J1
m1	UBE3C
m1	KLHL2
m10	PSMD1
m1	KLHL5
m1	NEDD4L
m1	UBA6
m2	TRIM4
m2	AREL1
m1	NEDD4
m2	TRAF7
m2	RNF4
m2	CUL7
m2	RNF144B
m2	KLHL13
m1	HERC2
m2	HECTD3
m1	CCNF
m2	KLHL20
m8	CCNA1
m1	UBE2E2
m3	ARF4
m12	GOSR1
m3	DYNC2LI1
m12	RAB1B
m3	ARF3
m8	TP53BP1
m12	DLGAP5
m8	PARP2
m8	PARP1
m8	RPA2
m8	POLD3
m8	RFC1
m12	POLE3
m7	FAM3C
m12	GAS6
m12	MMRN1
m7	CCL28
m1	AQR
m2	FIP1L1
m1	SF3A1
m1	SRSF7
m1	SNRNP40
m8	ERCC6
m1	SNRPA

m8	TCEA1
m2	CHERP
m2	HNRNPU
m1	PPIE
m2	SKIV2L2
m2	WBP11
m1	SF3A3
m1	SRSF5
m1	DDX5
m1	HNRNPH1
m1	HNRNPC
m2	GCFC2
m2	NHP2L1
m2	PCBP2
m1	DDX42
m1	PUF60
m1	SF3B3
m1	CCAR1
m1	HNRNPA1
m1	SNRPA1
m8	XPA
m1	DDX46
m2	LSM6
m1	SRRM1
m2	UPF3B
m2	PCF11
m2	CSTF1
m1	SMNDC1
m1	SRRT
m1	SNW1
m1	SRSF11
m8	USP7
m1	SRSF1
m1	DHX15
m1	ELAVL1
m8	ERCC3
m8	GTF2H3
m8	ERCC5
m2	HNRNPA3
m1	SF3B1
m1	WBP4
m2	SF1
m1	HNRNPD
m1	CPSF4
m1	HNRNPR
m1	SF3B2
m1	PRPF3
m8	GTF2H1
m2	FUS
m1	RBM5
m2	POLR2D
m8	LIG3
m7	ARAP3
m7	RHOH
m7	RHOT1
m7	RAC3
m7	RAC2
m7	RHOG

m7	RHOJ
m7	RHOF
m7	RHOQ
m7	RHOU
m2	PJA1
m2	RNF115
m2	DET1
m2	CBLB
m2	FBXO9
m2	PJA2
m2	SIAH1
m2	UBR2
m2	RBBP6
m2	ASB12
m2	RLIM
m2	UBE2Q2
m2	UBR4
m2	SIAH2
m1	LRSAM1
m2	UBE2F
m2	TRIM37
m3	KIF18B
m3	KIF21A
m12	TMED10
m3	KIF1B
m3	KIF25
m7	TIAM1
m7	ARHGAP11A
m7	ARHGAP17
m7	ARHGAP24
m7	ARHGAP25
m7	ARHGAP5
m13	ROCK1
m7	ARHGEF19
m7	ARHGEF3
m12	COG2
m12	COG3
m8	NFKBIA
m12	ASH2L
m13	WDR82
m12	CTCF
m12	KMT2C
m12	NUSAP1
m10	TOP2A
m8	RAD17
m12	PRKDC
m12	RAD51B
m12	RAD1
m8	ERCC1
m12	SUPT16H
m12	NBN
m12	WRN
m12	CHEK1
m12	CTDP1
m8	RAD9A
m12	CDK12
m12	MDC1
m12	BRCA2

m12	CLSPN
m12	RAD51
m12	PALB2
m12	SSRP1
m12	ORC1
m12	CDC25A
m12	HAUS5
m10	NUP54
m10	NUP188
m10	NUP153
m10	NUP205
m1	RPS27
m10	NUP88
m10	NUP35
m2	CENPO
m2	SPC25
m2	CENPN
m2	SPC24
m10	TPR
m2	SKA1
m10	NUP93
m7	DVL1
m13	KLRD1
m7	HLA-DRA
m6	SP100
m7	ZAP70
m7	PRKCQ
m6	GBP2
m7	CD86
m6	IRF8
m13	TREM2
m7	HLA-DPB1
m7	LCP2
m8	PSMF1
m13	PTK2B
m13	SLA
m7	PRKCD
m2	BMS1
m5	NIFK
m2	WDR43
m2	TSR1
m1	RPS15
m2	RPP38
m2	NOP14
m5	RBM28
m1	RPS3
m2	IMP3
m5	SDAD1
m2	UTP6
m5	RRS1
m2	FBL
m2	UTP15
m2	KRR1
m5	GNL3
m2	NOP58
m1	RPS29
m5	DDX27
m1	RPS19

m1	RPS18
m5	NSUN6
m2	UTP3
m1	RPS12
m2	WDR75
m1	RPS23
m1	RPS14
m2	HEATR1
m2	DIEXF
m2	PNO1
m1	RPS6
m1	RPS5
m5	PWP1
m2	RIOK1
m5	RSL1D1
m5	NOC3L
m1	RPS28
m1	RPS4X
m2	MPHOSPH10
m5	DDX24
m2	CIRH1A
m2	RIOK3
m5	NAT10
m2	NOB1
m5	KRI1
m1	RPS25
m5	GTPBP4
m2	WDR36
m2	WDR3
m1	RPS24
m2	PDCD11
m2	LTV1
m5	DDX56
m1	RPS16
m3	RINT1
m12	RBL1
m12	NELFCD
m1	RPL11
m7	MYL9
m7	MYL12B
m7	MYL6
m8	NFKBIE
m4	DAP3
m1	EIF3A
m1	RBM8A
m12	S1PR5
m12	CCR6
m12	CCNE2
m10	NUP214
m13	PI4KA
m12	VWA9
m12	INTS6
m12	INTS4
m12	NABP1
m12	CDC73
m12	LEO1
m12	INTS7
m6	IFITM1

m7	LAT
m12	TTF1
m12	GOLGB1
m7	CHN2
m7	MYO9A
m7	ECT2
m7	INPP5B
m7	FAM13B
m7	SOS2
m7	FAM13A
m7	FGD4
m3	KIF9
m3	NBAS
m9	TSG101
m8	CHD1L
m9	CHMP2B
m9	VTA1
m9	VPS37C
m9	VPS37B
m9	CHMP6
m9	CHMP4C
m9	VPS36
m9	CHMP7
m9	VPS25
m9	CHMP4B
m11	CLUAP1
m11	IFT43
m11	WDR34
m11	IFT20
m11	DYNLRB1
m11	WDR60
m11	WDR19
m11	TRAF3IP1
m11	WDR35
m11	IFT74
m11	IFT80
m1	EIF4B
m1	PABPC1
m12	SEC31A
m10	THOC1
m10	THOC2
m12	SIN3A
m13	SHOC2
m13	PPP1R12A
m4	MRPL49
m4	MRPL28
m4	MRPL9
m4	TSFM
m4	GFM1
m4	MRPS30
m4	PTCD3
m4	MRPS35
m4	MTIF2
m4	MTRF1L
m4	MRPL45
m4	MRPL19
m4	MRPL10
m4	MRPS18B

m4	MRPS6
m1	RPL19
m1	RPL23A
m1	RPL22
m1	RPL14
m1	RPL12
m1	RPL37
m1	RPL10A
m1	RPL29
m1	RPL37A
m1	RPL38
m1	RPL3
m1	RPL6
m1	RPL30
m1	RPL32
m1	RPL35A
m1	RPL15
m1	RPL18
m1	RPL36
m1	RPL34
m1	RPL7
m1	RPL31
m1	EIF3C
m1	EIF1AX
m1	EIF3G
m1	EIF3B
m1	EIF3E
m1	EIF3M
m1	EIF5B
m1	EIF3H
m1	EIF3D
m1	EIF3K
m1	EIF3L
m1	EIF3F
m1	GSPT2
m1	GSPT1
m10	NXT1
m12	TRAPPC2
m6	RSAD2
m6	IFIT3
m6	ISG20
m6	IFIT2
m6	IFIT1
m6	IFITM3
m6	SAMHD1
m6	IFITM2
m6	XAF1
m12	TRAPPC10
m12	TRAPPC6A
m12	STX17
m12	TRAPPC1
m12	SEC24A
m12	SEC24B
m13	MTMR3
m13	MTMR6
m13	INPP5E
m13	MTMR2
m13	PIP4K2C

m13 PPP4R2
