# Supplementary material for: Altered Genes and Biological Functions in Response to Severe Burns
Source: Biomed Res Int. 2021 May 24;2021:8836243. doi: 10.1155/2021/8836243 (PMC8168476; doi:10.1155/2021/8836243)
Supplement: Supplementary 9 — Table S7: Gene Ontology (GO) and Kyoto Encyclopedia of Genes and Genomes (KEGG) enrichment analysis, enrichment_all_0.05. [file 8836243.f9.pdf]

Table S7 Gene Ontology (GO) and Kyoto Encyclopedia of Genes and Genomes (KEGG) enrichmen

Module	ID	Descriptio	GeneRatio	BgRatio	pvalue	p.adjust	qvalue	geneID
GO_BP_m1	GO:00482	mRNA stal	4/122	36/17913	0.000102	0.001801	0.001466	ELAVL1/HI
GO_BP_m1	GO:00434	regulation	7/122	158/17913	0.000104	0.001819	0.00148	ELAVL1/HI
GO_BP_m1	GO:19033	negative re	5/122	69/17913	0.000107	0.001845	0.001501	ELAVL1/HI
GO_BP_m1	GO:00434	regulation	7/122	163/17913	0.000127	0.002154	0.001753	ELAVL1/HI
GO_BP_m1	GO:19032	U2-type p	2/122	3/17913	0.000137	0.002309	0.001879	SF3A1/SF3
GO_BP_m1	GO:00062	transcriptio	5/122	73/17913	0.00014	0.002324	0.001891	AQR/POLF
GO_BP_m1	GO:00003	regulation	4/122	41/17913	0.000171	0.002765	0.00225	DDX5/HNI
GO_BP_m1	GO:00434	RNA stabil	4/122	41/17913	0.000171	0.002765	0.00225	ELAVL1/HI
GO_BP_m1	GO:00063	mRNA spli	4/122	42/17913	0.000188	0.003005	0.002445	SF3A1/SF3
GO_BP_m1	GO:19023	negative re	4/122	43/17913	0.000206	0.003257	0.00265	ELAVL1/HI
GO_BP_m1	GO:00610	regulation	7/122	178/17913	0.000218	0.003411	0.002775	ELAVL1/HI
GO_BP_m1	GO:00315	protein-co	8/122	238/17913	0.000226	0.0035	0.002848	CPSF4/RBI
GO_BP_m1	GO:00439	positive re	7/122	180/17913	0.000233	0.00357	0.002905	LRSAM1/P
GO_BP_m1	GO:19023	negative re	4/122	49/17913	0.000342	0.005178	0.004213	ELAVL1/HI
GO_BP_m1	GO:00190	viral life cy	9/122	324/17913	0.000378	0.00565	0.004597	DDX5/LRS
GO_BP_m1	GO:00003	mRNA 3'-s	2/122	5/17913	0.000454	0.006643	0.005405	SF3A1/SF3
GO_BP_m1	GO:00320	ISG15-pro	2/122	5/17913	0.000454	0.006643	0.005405	HERC5/UB
GO_BP_m1	GO:00062	nucleotide	5/122	102/17913	0.000664	0.009611	0.00782	AQR/POLF
GO_BP_m1	GO:00485	positive re	5/122	103/17913	0.000694	0.009941	0.008089	PABPC1/P
GO_BP_m1	GO:00508	progestero	2/122	8/17913	0.001254	0.01778	0.014466	NEDD4/UE
GO_BP_m1	GO:19033	positive re	4/122	71/17913	0.0014	0.019637	0.015977	HNRNPD/
GO_BP_m1	GO:00450	positive re	3/122	35/17913	0.001721	0.023896	0.019443	PABPC1/P
GO_BP_m1	GO:00439	regulation	9/122	408/17913	0.001914	0.026316	0.021411	DDX5/HER
GO_BP_m1	GO:20006	negative re	2/122	10/17913	0.001998	0.027188	0.022121	NEDD4/NE
GO_BP_m1	GO:00190	virion asse	3/122	38/17913	0.002185	0.029156	0.023723	LRSAM1/R
GO_BP_m1	GO:00427	DNA dama	3/122	38/17913	0.002185	0.029156	0.023723	RPS27A/RI
GO_BP_m1	GO:00311	anaphase-	4/122	81/17913	0.002275	0.030057	0.024455	CDC16/CD
GO_BP_m1	GO:00000	ribosomal	2/122	11/17913	0.002431	0.031507	0.025635	RPL11/RPL
GO_BP_m1	GO:19023	negative re	2/122	11/17913	0.002431	0.031507	0.025635	NEDD4/NE
GO_BP_m1	GO:00507	regulation	6/122	204/17913	0.002744	0.035233	0.028667	DDX5/PAE
GO_BP_m1	GO:00610	positive re	3/122	44/17913	0.00333	0.04236	0.034466	HNRNPD/
GO_BP_m1	GO:00107	negative re	2/122	13/17913	0.003417	0.042661	0.034711	NEDD4/NE
GO_BP_m1	GO:00304	maturation	2/122	13/17913	0.003417	0.042661	0.034711	RPS14/RPS
GO_BP_m1	GO:00098	negative re	7/122	288/17913	0.003592	0.044441	0.036159	ELAVL1/HI
GO_BP_m1	GO:00063	postreplica	3/122	47/17913	0.004018	0.048741	0.039658	RPS27A/U
GO_BP_m1	GO:00514	regulation	3/122	47/17913	0.004018	0.048741	0.039658	CDC20/FB
GO_BP_m1	GO:00450	regulation	4/122	95/17913	0.004047	0.048741	0.039658	DDX5/PAE
GO_BP_m1	GO:00709	3'-UTR-me	2/122	15/17913	0.004559	0.054423	0.044281	ELAVL1/HI
GO_BP_m1	GO:00439	regulation	6/122	232/17913	0.00514	0.060831	0.049495	DDX5/PAE
GO_BP_m1	GO:00170	response t	2/122	17/17913	0.005852	0.068662	0.055866	HNRNPA1
GO_BP_m1	GO:00313	negative re	6/122	244/17913	0.006536	0.070758	0.057572	ELAVL1/HI
GO_BP_m1	GO:00422	error-pron	2/122	18/17913	0.006555	0.070758	0.057572	RPS27A/U
GO_BP_m1	GO:00190	transmissio	1/122	1/17913	0.006811	0.070758	0.057572	NEDD4
GO_BP_m1	GO:00344	90S prerib	1/122	1/17913	0.006811	0.070758	0.057572	RPL38
GO_BP_m1	GO:00350	sperm ent	1/122	1/17913	0.006811	0.070758	0.057572	UBE3A
GO_BP_m1	GO:00440	disseminat	1/122	1/17913	0.006811	0.070758	0.057572	NEDD4
GO_BP_m1	GO:00518	disseminat	1/122	1/17913	0.006811	0.070758	0.057572	NEDD4
GO_BP_m1	GO:00614	response t	1/122	1/17913	0.006811	0.070758	0.057572	RPS3
GO_BP_m1	GO:00995	regulation	1/122	1/17913	0.006811	0.070758	0.057572	RPL22
GO_BP_m1	GO:01402	regulation	1/122	1/17913	0.006811	0.070758	0.057572	RPL22
GO_BP_m1	GO:19025	positive re	1/122	1/17913	0.006811	0.070758	0.057572	RPS3
GO_BP_m1	GO:19045	positive re	1/122	1/17913	0.006811	0.070758	0.057572	RPL30
GO_BP_m1	GO:19045	response t	1/122	1/17913	0.006811	0.070758	0.057572	HNRNPD
GO_BP_m1	GO:19045	cellular res	1/122	1/17913	0.006811	0.070758	0.057572	HNRNPD
GO_BP_m1	GO:19908	hepatocyte	1/122	1/17913	0.006811	0.070758	0.057572	HNRNPD
GO_BP_m1	GO:00709	error-free	2/122	19/17913	0.007293	0.074632	0.060724	RPS27A/U

GO_BP_m	GO:19020	response t	2/122	19/17913	0.007293	0.074632	0.060724	HNRNPA1
GO_BP_m	GO:19039	positive re	3/122	59/17913	0.007593	0.077117	0.062746	PABPC1/P
GO_BP_m	GO:00170	regulation	4/122	115/17913	0.007923	0.079871	0.064987	RPS27A/SI
GO_BP_m	GO:00322	negative re	2/122	20/17913	0.008068	0.080736	0.06569	HNRNPA1
GO_BP_m	GO:19038	regulation	4/122	117/17913	0.008409	0.083533	0.067966	RPS27A/SI
GO_BP_m	GO:20012	positive re	3/122	62/17913	0.008704	0.085841	0.069844	FBXW7/RP
GO_BP_m	GO:19017	positive re	2/122	21/17913	0.008877	0.086922	0.070724	DDX5/RPL
GO_BP_m	GO:00190	viral genor	4/122	120/17913	0.009173	0.088547	0.072046	DDX5/PAE
GO_BP_m	GO:00341	erythrocyt	4/122	120/17913	0.009173	0.088547	0.072046	RPS14/RPS
GO_BP_m	GO:00007	nucleotide	2/122	22/17913	0.009722	0.091886	0.074763	RPS27A/U
GO_BP_m	GO:00007	nucleotide	2/122	22/17913	0.009722	0.091886	0.074763	RPS27A/U
GO_BP_m	GO:19046	regulation	2/122	22/17913	0.009722	0.091886	0.074763	CDC20/RP
GO_BP_m	GO:00065	protein mo	3/122	65/17913	0.009906	0.092977	0.07565	CUL1/NED
GO_BP_m	GO:00718	DNA biosy	5/122	192/17913	0.010147	0.094593	0.076965	HNRNPA1
GO_BP_m	GO:00062	nucleotide	2/122	23/17913	0.010601	0.098148	0.079857	RPS27A/U
GO_BP_m	GO:00421	regulation	7/122	354/17913	0.010716	0.098547	0.080182	FBXW7/NE
GO_BP_m	GO:00305	intracellula	6/122	272/17913	0.010839	0.099008	0.080557	DDX5/NED
GO_BP_m	GO:00313	regulation	5/122	196/17913	0.011024	0.100026	0.081385	CDC20/FB
GO_BP_m	GO:00311	SCF-deper	3/122	69/17913	0.011651	0.104325	0.084884	CCNF/CUL
GO_BP_m	GO:00518	protein au	3/122	69/17913	0.011651	0.104325	0.084884	LRSAM1/U
GO_BP_m	GO:00971	intrinsic ap	6/122	277/17913	0.011779	0.104777	0.085251	CUL1/DDX
GO_BP_m	GO:00018	liver devel	4/122	130/17913	0.012047	0.106465	0.086624	FBXW7/HN
GO_BP_m	GO:00002	spliceoson	2/122	25/17913	0.012459	0.106798	0.086896	PRPF3/PRI
GO_BP_m	GO:00610	hepaticobi	4/122	133/17913	0.013009	0.106798	0.086896	FBXW7/HN
GO_BP_m	GO:00709	global ger	2/122	26/17913	0.013438	0.106798	0.086896	RPS27A/U
GO_BP_m	GO:00107	negative re	1/122	2/17913	0.013575	0.106798	0.086896	NEDD4
GO_BP_m	GO:00441	developme	1/122	2/17913	0.013575	0.106798	0.086896	NEDD4
GO_BP_m	GO:00467	modificatio	1/122	2/17913	0.013575	0.106798	0.086896	CPSF4
GO_BP_m	GO:00602	positive re	1/122	2/17913	0.013575	0.106798	0.086896	RPS19
GO_BP_m	GO:00705	positive re	1/122	2/17913	0.013575	0.106798	0.086896	SNW1
GO_BP_m	GO:19013	response t	1/122	2/17913	0.013575	0.106798	0.086896	HNRNPD
GO_BP_m	GO:19025	regulation	1/122	2/17913	0.013575	0.106798	0.086896	RPS3
GO_BP_m	GO:19033	positive re	1/122	2/17913	0.013575	0.106798	0.086896	FBXW7
GO_BP_m	GO:19045	regulation	1/122	2/17913	0.013575	0.106798	0.086896	RPL30
GO_BP_m	GO:19050	regulation	1/122	2/17913	0.013575	0.106798	0.086896	RPS3
GO_BP_m	GO:19050	positive re	1/122	2/17913	0.013575	0.106798	0.086896	RPS3
GO_BP_m	GO:19056	positive re	1/122	2/17913	0.013575	0.106798	0.086896	HNRNPD
GO_BP_m	GO:19901	maintenan	1/122	2/17913	0.013575	0.106798	0.086896	RPS23
GO_BP_m	GO:20004	negative re	1/122	2/17913	0.013575	0.106798	0.086896	RPL11
GO_BP_m	GO:20006	negative re	1/122	2/17913	0.013575	0.106798	0.086896	FBXW7
GO_BP_m	GO:20012	positive re	1/122	2/17913	0.013575	0.106798	0.086896	RPS3
GO_BP_m	GO:00448	cell cycle (	6/122	288/17913	0.014046	0.109864	0.08939	CDC34/CU
GO_BP_m	GO:19043	negative re	2/122	27/17913	0.014449	0.112373	0.091432	HNRNPA1
GO_BP_m	GO:00723	intrinsic ap	3/122	75/17913	0.014582	0.112761	0.091747	DDX5/RPL
GO_BP_m	GO:00514	positive re	2/122	28/17913	0.015492	0.118453	0.096379	CDC20/FB
GO_BP_m	GO:00611	mRNA des	2/122	28/17913	0.015492	0.118453	0.096379	HNRNPD/
GO_BP_m	GO:00510	negative re	4/122	141/17913	0.015811	0.120216	0.097813	FBXW7/HN
GO_BP_m	GO:00305	negative re	3/122	78/17913	0.016189	0.12241	0.099598	RPS27A/SI
GO_BP_m	GO:00062	nucleotide	2/122	29/17913	0.016566	0.123883	0.100797	RPS27A/U
GO_BP_m	GO:00356	TRIF-depe	2/122	29/17913	0.016566	0.123883	0.100797	RPS27A/U
GO_BP_m	GO:00322	regulation	3/122	79/17913	0.016747	0.124547	0.101337	HNRNPA1
GO_BP_m	GO:19038	negative re	3/122	80/17913	0.017314	0.127593	0.103815	RPS27A/SI
GO_BP_m	GO:00022	myeloid ce	4/122	145/17913	0.017344	0.127593	0.103815	RPS14/RPS
GO_BP_m	GO:00507	RNA desta	2/122	30/17913	0.017671	0.129306	0.105209	HNRNPD/
GO_BP_m	GO:19039	regulation	4/122	147/17913	0.018144	0.132051	0.107442	DDX5/PAE
GO_BP_m	GO:19033	regulation	5/122	223/17913	0.018324	0.132651	0.107931	CDC20/FB
GO_BP_m	GO:00331	positive re	2/122	31/17913	0.018807	0.135431	0.110192	SNW1/SRS
GO_BP_m	GO:00466	response t	2/122	32/17913	0.019973	0.138103	0.112367	HNRNPA1

GO_BP_m	GO:00974	liver regen	2/122	32/17913	0.019973	0.138103	0.112367	RPL30/SRS
GO_BP_m	GO:00021	cap-deper	1/122	3/17913	0.020294	0.138103	0.112367	EIF3D
GO_BP_m	GO:00107	regulation	1/122	3/17913	0.020294	0.138103	0.112367	NEDD4
GO_BP_m	GO:00396	modulatio	1/122	3/17913	0.020294	0.138103	0.112367	CPSF4
GO_BP_m	GO:00721	distal tubu	1/122	3/17913	0.020294	0.138103	0.112367	KLHL3
GO_BP_m	GO:19043	response t	1/122	3/17913	0.020294	0.138103	0.112367	HNRNPD
GO_BP_m	GO:19056	regulation	1/122	3/17913	0.020294	0.138103	0.112367	HNRNPD
GO_BP_m	GO:20004	regulation	1/122	3/17913	0.020294	0.138103	0.112367	RPL11
GO_BP_m	GO:20006	regulation	1/122	3/17913	0.020294	0.138103	0.112367	FBXW7
GO_BP_m	GO:20012	positive re	1/122	3/17913	0.020294	0.138103	0.112367	NEDD4L
GO_BP_m	GO:00027	MyD88-in	2/122	33/17913	0.021168	0.14333	0.116619	RPS27A/U
GO_BP_m	GO:00902	negative r	4/122	156/1791	0.022026	0.148403	0.120747	NEDD4/RP
GO_BP_m	GO:00004	protein pe	2/122	35/17913	0.023645	0.156216	0.127104	PPIE/PPIH
GO_BP_m	GO:00063	mRNA pol	2/122	35/17913	0.023645	0.156216	0.127104	PABPC1/S
GO_BP_m	GO:00452	mRNA cis	2/122	35/17913	0.023645	0.156216	0.127104	SRSF1/WB
GO_BP_m	GO:00704	nucleotide	2/122	35/17913	0.023645	0.156216	0.127104	RPS27A/U
GO_BP_m	GO:00358	modificati	4/122	160/1791	0.023902	0.157154	0.127868	CPSF4/RPL
GO_BP_m	GO:00027	MyD88-de	2/122	36/17913	0.024926	0.161542	0.131438	RPS27A/U
GO_BP_m	GO:00322	negative r	2/122	36/17913	0.024926	0.161542	0.131438	HNRNPA1
GO_BP_m	GO:00358	nucleotide	2/122	36/17913	0.024926	0.161542	0.131438	RPS27A/U
GO_BP_m	GO:00062	nucleotide	2/122	37/17913	0.026234	0.166079	0.135129	RPS27A/U
GO_BP_m	GO:00436	RNA polya	2/122	37/17913	0.026234	0.166079	0.135129	PABPC1/S
GO_BP_m	GO:00000	ribosomal	1/122	4/17913	0.026968	0.166079	0.135129	RPS15
GO_BP_m	GO:00217	amygdala	1/122	4/17913	0.026968	0.166079	0.135129	UBA6
GO_BP_m	GO:00320	positive re	1/122	4/17913	0.026968	0.166079	0.135129	RPS3
GO_BP_m	GO:00702	renal sodi	1/122	4/17913	0.026968	0.166079	0.135129	KLHL3
GO_BP_m	GO:00902	positive re	1/122	4/17913	0.026968	0.166079	0.135129	CDC34
GO_BP_m	GO:00971	circadian r	1/122	4/17913	0.026968	0.166079	0.135129	HNRNPD
GO_BP_m	GO:20003	negative r	1/122	4/17913	0.026968	0.166079	0.135129	FBXW7
GO_BP_m	GO:20006	regulation	1/122	4/17913	0.026968	0.166079	0.135129	PABPC1
GO_BP_m	GO:20006	negative r	1/122	4/17913	0.026968	0.166079	0.135129	PABPC1
GO_BP_m	GO:20002	negative r	2/122	38/17913	0.027571	0.169025	0.137526	HNRNPA1
GO_BP_m	GO:00336	nucleotide	2/122	39/17913	0.028933	0.176585	0.143677	RPS27A/U
GO_BP_m	GO:00199	translesior	2/122	40/17913	0.030323	0.184239	0.149905	RPS27A/U
GO_BP_m	GO:00704	interleukin	3/122	101/1791	0.031711	0.187397	0.152474	CUL1/RPS
GO_BP_m	GO:00504	positive re	2/122	41/17913	0.031738	0.187397	0.152474	POLR2B/S
GO_BP_m	GO:20006	regulation	2/122	41/17913	0.031738	0.187397	0.152474	NEDD4/N
GO_BP_m	GO:00165	protein de	5/122	260/1791	0.032677	0.187397	0.152474	CDC20/EIF
GO_BP_m	GO:00003	re-entry ir	1/122	5/17913	0.033596	0.187397	0.152474	CCNF
GO_BP_m	GO:00023	T cell proli	1/122	5/17913	0.033596	0.187397	0.152474	RPS6
GO_BP_m	GO:00102	SCF compl	1/122	5/17913	0.033596	0.187397	0.152474	CUL1
GO_BP_m	GO:00108	negative r	1/122	5/17913	0.033596	0.187397	0.152474	FBXW7
GO_BP_m	GO:00305	ubiquitin-	1/122	5/17913	0.033596	0.187397	0.152474	SMURF2
GO_BP_m	GO:00320	positive re	1/122	5/17913	0.033596	0.187397	0.152474	RPS3
GO_BP_m	GO:00459	positive re	1/122	5/17913	0.033596	0.187397	0.152474	RPL30
GO_BP_m	GO:00602	negative r	1/122	5/17913	0.033596	0.187397	0.152474	RPS19
GO_BP_m	GO:00700	ubiquitin-	1/122	5/17913	0.033596	0.187397	0.152474	LRSAM1
GO_BP_m	GO:00995	regulation	1/122	5/17913	0.033596	0.187397	0.152474	RPL22
GO_BP_m	GO:01402	regulation	1/122	5/17913	0.033596	0.187397	0.152474	RPL22
GO_BP_m	GO:19022	positive re	1/122	5/17913	0.033596	0.187397	0.152474	RPL11
GO_BP_m	GO:19032	positive re	1/122	5/17913	0.033596	0.187397	0.152474	FBXW7
GO_BP_m	GO:19039	cellular res	1/122	5/17913	0.033596	0.187397	0.152474	HNRNPA1
GO_BP_m	GO:20012	negative r	1/122	5/17913	0.033596	0.187397	0.152474	FBXW7
GO_BP_m	GO:20012	regulation	1/122	5/17913	0.033596	0.187397	0.152474	RPS3
GO_BP_m	GO:00510	positive re	4/122	181/1791	0.035318	0.196195	0.159632	EIF3C/EIF3
GO_BP_m	GO:00432	response t	3/122	106/1791	0.035833	0.197442	0.160647	DHX15/EF
GO_BP_m	GO:20002	regulation	3/122	106/1791	0.035833	0.197442	0.160647	HNRNPA1
GO_BP_m	GO:00351	productior	2/122	44/17913	0.036137	0.198135	0.161211	DDX5/SRR

GO_BP_m[illegible]	GO:00436[illegible]	post-trans	6/122	360/17913	0.036701	0.198135	0.161211	CCNF/CUL
GO_BP_m[illegible]	GO:00000[illegible]	G1/S trans	5/122	269/17913	0.036993	0.198135	0.161211	CDC34/CU
GO_BP_m[illegible]	GO:00071[illegible]	transformi	4/122	184/17913	0.037167	0.198135	0.161211	RPS27A/SI
GO_BP_m[illegible]	GO:00182[illegible]	peptidyl-p	2/122	45/17913	0.037653	0.198135	0.161211	PPIE/PPIH
GO_BP_m[illegible]	GO:00085[illegible]	fibroblast	3/122	109/17913	0.038431	0.198135	0.161211	HNRNPA1
GO_BP_m[illegible]	GO:00436[illegible]	regulation	3/122	109/17913	0.038431	0.198135	0.161211	NEDD4/RF
GO_BP_m[illegible]	GO:00706[illegible]	protein mc	5/122	273/17913	0.039019	0.198135	0.161211	CDC20/EIF
GO_BP_m[illegible]	GO:00619[illegible]	selective a	2/122	46/17913	0.039193	0.198135	0.161211	KLHL3/LRS
GO_BP_m[illegible]	GO:00705[illegible]	protein K6	2/122	46/17913	0.039193	0.198135	0.161211	NEDD4/UE
GO_BP_m[illegible]	GO:00313[illegible]	positive re	3/122	110/17913	0.039319	0.198135	0.161211	CDC20/FB
GO_BP_m[illegible]	GO:00000[illegible]	ribosomal	1/122	6/17913	0.04018	0.198135	0.161211	RPS15
GO_BP_m[illegible]	GO:00015[illegible]	selenocyst	1/122	6/17913	0.04018	0.198135	0.161211	RPL30
GO_BP_m[illegible]	GO:00030[illegible]	renal sodiu	1/122	6/17913	0.04018	0.198135	0.161211	KLHL3
GO_BP_m[illegible]	GO:00064[illegible]	translation	1/122	6/17913	0.04018	0.198135	0.161211	RPL30
GO_BP_m[illegible]	GO:00321[illegible]	positive re	1/122	6/17913	0.04018	0.198135	0.161211	SNW1
GO_BP_m[illegible]	GO:00321[illegible]	positive re	1/122	6/17913	0.04018	0.198135	0.161211	SNW1
GO_BP_m[illegible]	GO:00328[illegible]	negative re	1/122	6/17913	0.04018	0.198135	0.161211	FBXW7
GO_BP_m[illegible]	GO:00337[illegible]	ribosome l	1/122	6/17913	0.04018	0.198135	0.161211	RPS15
GO_BP_m[illegible]	GO:00602[illegible]	regulation	1/122	6/17913	0.04018	0.198135	0.161211	RPS19
GO_BP_m[illegible]	GO:00602[illegible]	negative re	1/122	6/17913	0.04018	0.198135	0.161211	RPS19
GO_BP_m[illegible]	GO:00705[illegible]	regulation	1/122	6/17913	0.04018	0.198135	0.161211	SNW1
GO_BP_m[illegible]	GO:19011[illegible]	positive re	1/122	6/17913	0.04018	0.198135	0.161211	SMURF2
GO_BP_m[illegible]	GO:19021[illegible]	positive re	1/122	6/17913	0.04018	0.198135	0.161211	FBXW7
GO_BP_m[illegible]	GO:19030[illegible]	negative re	1/122	6/17913	0.04018	0.198135	0.161211	FBXW7
GO_BP_m[illegible]	GO:19039[illegible]	response t	1/122	6/17913	0.04018	0.198135	0.161211	HNRNPA1
GO_BP_m[illegible]	GO:19044[illegible]	regulation	1/122	6/17913	0.04018	0.198135	0.161211	LRSAM1
GO_BP_m[illegible]	GO:19044[illegible]	positive re	1/122	6/17913	0.04018	0.198135	0.161211	LRSAM1
GO_BP_m[illegible]	GO:20012[illegible]	regulation	1/122	6/17913	0.04018	0.198135	0.161211	NEDD4L
GO_BP_m[illegible]	GO:00902[illegible]	regulation	5/122	276/17913	0.040583	0.199397	0.162238	NEDD4/RF
GO_BP_m[illegible]	GO:00302[illegible]	erythrocyt	3/122	112/17913	0.041124	0.20133	0.163811	RPS14/RPS
GO_BP_m[illegible]	GO:00609[illegible]	regulation	3/122	113/17913	0.042042	0.204355	0.166272	DDX5/ELA
GO_BP_m[illegible]	GO:00310[illegible]	dsRNA pro	2/122	48/17913	0.042342	0.204355	0.166272	DDX5/SRR
GO_BP_m[illegible]	GO:00422[illegible]	response t	2/122	48/17913	0.042342	0.204355	0.166272	EFTUD2/U
GO_BP_m[illegible]	GO:00709[illegible]	productior	2/122	48/17913	0.042342	0.204355	0.166272	DDX5/SRR
GO_BP_m[illegible]	GO:00427[illegible]	regulation	3/122	114/17913	0.042971	0.206655	0.168143	FBXW7/HN
GO_BP_m[illegible]	GO:00436[illegible]	regulation	3/122	115/17913	0.04391	0.209852	0.170745	NEDD4/RF
GO_BP_m[illegible]	GO:00003[illegible]	spliceoson	2/122	50/17913	0.045584	0.209852	0.170745	PRPF3/PRI
GO_BP_m[illegible]	GO:00007[illegible]	DNA synth	2/122	50/17913	0.045584	0.209852	0.170745	RPS27A/U
GO_BP_m[illegible]	GO:00070[illegible]	sister chro	2/122	50/17913	0.045584	0.209852	0.170745	CDC20/FB
GO_BP_m[illegible]	GO:00362[illegible]	interstranc	2/122	50/17913	0.045584	0.209852	0.170745	RPS27A/U
GO_BP_m[illegible]	GO:00601[illegible]	regulation	3/122	117/17913	0.045818	0.209852	0.170745	DDX5/ELA
GO_BP_m[illegible]	GO:00609[illegible]	regulation	3/122	117/17913	0.045818	0.209852	0.170745	DDX5/ELA
GO_BP_m[illegible]	GO:00171[illegible]	negative re	4/122	198/17913	0.046521	0.209852	0.170745	EIF3E/HNF
GO_BP_m[illegible]	GO:19030[illegible]	regulation	4/122	198/17913	0.046521	0.209852	0.170745	FBXW7/RF
GO_BP_m[illegible]	GO:00485[illegible]	rhythmic p	5/122	287/17913	0.046638	0.209852	0.170745	DDX5/FBX
GO_BP_m[illegible]	GO:00004[illegible]	maturatior	1/122	7/17913	0.046719	0.209852	0.170745	RPS19
GO_BP_m[illegible]	GO:00025[illegible]	respiratory	1/122	7/17913	0.046719	0.209852	0.170745	RPS19
GO_BP_m[illegible]	GO:00069[illegible]	activation-	1/122	7/17913	0.046719	0.209852	0.170745	RPS6
GO_BP_m[illegible]	GO:00070[illegible]	nucleolus	1/122	7/17913	0.046719	0.209852	0.170745	RPS19
GO_BP_m[illegible]	GO:00226[illegible]	mammalia	1/122	7/17913	0.046719	0.209852	0.170745	RPS6
GO_BP_m[illegible]	GO:00483[illegible]	axial meso	1/122	7/17913	0.046719	0.209852	0.170745	RPL38
GO_BP_m[illegible]	GO:00610[illegible]	negative re	1/122	7/17913	0.046719	0.209852	0.170745	UBE3A
GO_BP_m[illegible]	GO:00714[illegible]	rRNA-con	1/122	7/17913	0.046719	0.209852	0.170745	RPS15
GO_BP_m[illegible]	GO:19020[illegible]	cellular res	1/122	7/17913	0.046719	0.209852	0.170745	HNRNPA1
GO_BP_m[illegible]	GO:19022[illegible]	positive re	1/122	7/17913	0.046719	0.209852	0.170745	RPS3
GO_BP_m[illegible]	GO:00322[illegible]	positive re	2/122	51/17913	0.047238	0.21079	0.171508	HNRNPA1
GO_BP_m[illegible]	GO:19023[illegible]	regulation	2/122	51/17913	0.047238	0.21079	0.171508	NEDD4/NI
GO_BP_m[illegible]	GO:00076[illegible]	circadian r	4/122	201/17913	0.048681	0.216142	0.175862	FBXW7/HN

GO_BP_m	GO:00075	embryo im	2/122	52/17913	0.048914	0.216142	0.175862	RPL29/SM
GO_BP_m	GO:19017	negative re	2/122	52/17913	0.048914	0.216142	0.175862	RPL11/UBI
GO_BP_m	GO:00901	negative re	3/122	121/17913	0.049756	0.219151	0.178311	RPS27A/SI
GO_BP_m	GO:00322	regulation	2月21日	13/17913	0.000101	0.000333	0.000127	NUP153/T
GO_BP_m	GO:00198	antigen pr	4月21日	220/17913	0.000112	0.000368	0.00014	PSMA5/PS
GO_BP_m	GO:00902	regulation	2月21日	14/17913	0.000118	0.00038	0.000145	ANAPC15/
GO_BP_m	GO:00902	regulation	2月21日	14/17913	0.000118	0.00038	0.000145	ANAPC15/
GO_BP_m	GO:19035	regulation	2月21日	14/17913	0.000118	0.00038	0.000145	ANAPC15/
GO_BP_m	GO:00508	antigen re	4月21日	224/17913	0.000121	0.000386	0.000147	PSMA5/PS
GO_BP_m	GO:00488	stem cell c	4月21日	237/17913	0.00015	0.000477	0.000182	PSMA5/PS
GO_BP_m	GO:00380	Fc recepto	4月21日	241/17913	0.00016	0.000504	0.000192	PSMA5/PS
GO_BP_m	GO:00421	cellular ke	4月21日	241/17913	0.00016	0.000504	0.000192	PSMA5/PS
GO_BP_m	GO:20000	regulation	4月21日	250/17913	0.000184	0.000577	0.00022	PSMA5/PS
GO_BP_m	GO:00608	regulation	4月21日	264/17913	0.000227	0.000707	0.00027	PSMA5/PS
GO_BP_m	GO:00713	cellular res	4月21日	266/17913	0.000233	0.000724	0.000276	PSMA5/PS
GO_BP_m	GO:00468	negative re	2月21日	20/17913	0.000246	0.000759	0.000289	NUP153/T
GO_BP_m	GO:00346	response t	4月21日	284/17913	0.000299	0.00092	0.000351	PSMA5/PS
GO_BP_m	GO:00002	protein po	4月21日	288/17913	0.000316	0.000965	0.000368	PSMA5/PS
GO_BP_m	GO:00027	innate imm	4月21日	298/17913	0.000359	0.001094	0.000417	PSMA5/PS
GO_BP_m	GO:00511	import into	3月21日	125/17913	0.000402	0.001213	0.000463	NUP153/N
GO_BP_m	GO:20012	negative re	3月21日	125/17913	0.000402	0.001213	0.000463	ANAPC15/
GO_BP_m	GO:00600	canonical	4月21日	311/17913	0.000422	0.001267	0.000483	PSMA5/PS
GO_BP_m	GO:00022	activation	4月21日	319/17913	0.000465	0.001388	0.000529	PSMA5/PS
GO_BP_m	GO:00070	mitotic spi	2月21日	30/17913	0.000558	0.001628	0.000621	ANAPC15/
GO_BP_m	GO:00071	female me	2月21日	30/17913	0.000558	0.001628	0.000621	AURKA/TC
GO_BP_m	GO:00315	spindle ch	2月21日	30/17913	0.000558	0.001628	0.000621	ANAPC15/
GO_BP_m	GO:00711	spindle ass	2月21日	30/17913	0.000558	0.001628	0.000621	ANAPC15/
GO_BP_m	GO:00711	mitotic spi	2月21日	30/17913	0.000558	0.001628	0.000621	ANAPC15/
GO_BP_m	GO:00301	regulation	4月21日	341/17913	0.000598	0.001734	0.000661	PSMA5/PS
GO_BP_m	GO:00458	negative re	2月21日	32/17913	0.000636	0.001826	0.000696	ANAPC15/
GO_BP_m	GO:20008	negative re	2月21日	32/17913	0.000636	0.001826	0.000696	ANAPC15/
GO_BP_m	GO:00065	cellular am	4月21日	347/17913	0.000638	0.001826	0.000696	PSMA5/PS
GO_BP_m	GO:00070	mitotic cel	3月21日	147/17913	0.000646	0.001841	0.000702	ANAPC15/
GO_BP_m	GO:19021	negative re	2月21日	33/17913	0.000676	0.001909	0.000728	ANAPC15/
GO_BP_m	GO:19058	negative re	2月21日	33/17913	0.000676	0.001909	0.000728	ANAPC15/
GO_BP_m	GO:00070	regulation	3月21日	150/17913	0.000685	0.001925	0.000734	ANAPC15/
GO_BP_m	GO:00002	nuclear div	4月21日	357/17913	0.00071	0.001986	0.000757	ANAPC15/
GO_BP_m	GO:00436	post-trans	4月21日	360/17913	0.000732	0.00204	0.000778	PSMA5/PS
GO_BP_m	GO:00330	negative re	2月21日	35/17913	0.000761	0.002109	0.000805	ANAPC15/
GO_BP_m	GO:00450	positive re	4月21日	373/17913	0.000836	0.002308	0.00088	PSMA5/PS
GO_BP_m	GO:00330	negative re	2月21日	37/17913	0.00085	0.002337	0.000891	ANAPC15/
GO_BP_m	GO:00165	histone ph	2月21日	38/17913	0.000897	0.002443	0.000932	AURKA/CC
GO_BP_m	GO:00519	negative re	2月21日	38/17913	0.000897	0.002443	0.000932	ANAPC15/
GO_BP_m	GO:00517	regulation	3月21日	172/17913	0.001019	0.002763	0.001054	ANAPC15/
GO_BP_m	GO:00482	organelle f	4月21日	395/17913	0.001035	0.002795	0.001066	ANAPC15/
GO_BP_m	GO:00024	immune re	4月21日	399/17913	0.001074	0.002889	0.001102	PSMA5/PS
GO_BP_m	GO:00323	regulation	4月21日	400/17913	0.001085	0.002903	0.001107	NUP153/N
GO_BP_m	GO:00319	mRNA exp	1月21日	1/17913	0.001172	0.003111	0.001187	TPR
GO_BP_m	GO:00482	negative re	1月21日	1/17913	0.001172	0.003111	0.001187	THOC1
GO_BP_m	GO:00300	regulation	2月21日	44/17913	0.001202	0.003177	0.001212	ANAPC15/
GO_BP_m	GO:00458	negative re	2月21日	45/17913	0.001257	0.003308	0.001262	ANAPC15/
GO_BP_m	GO:19020	regulation	2月21日	46/17913	0.001313	0.003441	0.001312	ANAPC15/
GO_BP_m	GO:00070	metaphase	2月21日	47/17913	0.001371	0.003562	0.001358	ANAPC15/
GO_BP_m	GO:00109	regulation	2月21日	47/17913	0.001371	0.003562	0.001358	ANAPC15/
GO_BP_m	GO:00027	immune re	4月21日	434/17913	0.001466	0.003794	0.001447	PSMA5/PS
GO_BP_m	GO:00447	metaphase	2月21日	49/17913	0.001489	0.003821	0.001457	ANAPC15/
GO_BP_m	GO:00513	mitotic sist	2月21日	49/17913	0.001489	0.003821	0.001457	ANAPC15/
GO_BP_m	GO:00450	regulation	4月21日	440/17913	0.001542	0.003941	0.001503	PSMA5/PS

GO_BP_m:	GO:00517	negative re	2月21日	52/17913	0.001675	0.004247	0.00162	ANAPC15/
GO_BP_m:	GO:19058	regulation	2月21日	52/17913	0.001675	0.004247	0.00162	ANAPC15/
GO_BP_m:	GO:00517	interaction	3月21日	211/17913	0.001832	0.004625	0.001764	NUP153/T
GO_BP_m:	GO:00423	regulation	2月21日	55/17913	0.001872	0.004708	0.001795	NUP54/TP
GO_BP_m:	GO:19037	regulation	4月21日	468/17913	0.001934	0.004842	0.001847	PSMA5/PS
GO_BP_m:	GO:00330	regulation	2月21日	57/17913	0.00201	0.005012	0.001911	ANAPC15/
GO_BP_m:	GO:19045	regulation	2月21日	58/17913	0.00208	0.005166	0.00197	NUP54/TP
GO_BP_m:	GO:00070	spindle ass	1月21日	2/17913	0.002343	0.005707	0.002176	AURKA
GO_BP_m:	GO:00314	regulation	1月21日	2/17913	0.002343	0.005707	0.002176	TPR
GO_BP_m:	GO:00314	positive re	1月21日	2/17913	0.002343	0.005707	0.002176	TPR
GO_BP_m:	GO:00458	positive re	1月21日	2/17913	0.002343	0.005707	0.002176	TOP2A
GO_BP_m:	GO:19001	positive re	1月21日	2/17913	0.002343	0.005707	0.002176	AURKA
GO_BP_m:	GO:00160	Wnt signal	4月21日	498/17913	0.002425	0.005882	0.002243	PSMA5/PS
GO_BP_m:	GO:01987	cell-cell sig	4月21日	500/17913	0.00246	0.005945	0.002267	PSMA5/PS
GO_BP_m:	GO:01400	mitotic nu	3月21日	237/17913	0.00255	0.006138	0.002341	ANAPC15/
GO_BP_m:	GO:00323	negative re	2月21日	65/17913	0.002604	0.006244	0.002381	NUP153/T
GO_BP_m:	GO:00513	chromoso	2月21日	68/17913	0.002846	0.006798	0.002593	ANAPC15/
GO_BP_m:	GO:00330	regulation	2月21日	70/17913	0.003013	0.007169	0.002734	ANAPC15/
GO_BP_m:	GO:00311	animal org	2月21日	75/17913	0.00345	0.00808	0.003082	AURKA/CC
GO_BP_m:	GO:00064	RNA impo	1月21日	3/17913	0.003513	0.00808	0.003082	TPR
GO_BP_m:	GO:00070	spindle ass	1月21日	3/17913	0.003513	0.00808	0.003082	AURKA
GO_BP_m:	GO:00457	positive re	1月21日	3/17913	0.003513	0.00808	0.003082	TPR
GO_BP_m:	GO:00602	positive re	1月21日	3/17913	0.003513	0.00808	0.003082	AURKA
GO_BP_m:	GO:00713	cellular res	1月21日	3/17913	0.003513	0.00808	0.003082	CCNA2
GO_BP_m:	GO:00713	cellular res	1月21日	3/17913	0.003513	0.00808	0.003082	CCNA2
GO_BP_m:	GO:19054	negative re	1月21日	3/17913	0.003513	0.00808	0.003082	TOP2A
GO_BP_m:	GO:19058	positive re	1月21日	3/17913	0.003513	0.00808	0.003082	AURKA
GO_BP_m:	GO:00070	chromoso	3月21日	275/17913	0.003879	0.008888	0.00339	ANAPC15/
GO_BP_m:	GO:00108	regulation	1月21日	4/17913	0.004681	0.010533	0.004017	TPR
GO_BP_m:	GO:00346	response t	1月21日	4/17913	0.004681	0.010533	0.004017	CCNA2
GO_BP_m:	GO:00458	negative re	1月21日	4/17913	0.004681	0.010533	0.004017	THOC1
GO_BP_m:	GO:00757	viral penet	1月21日	4/17913	0.004681	0.010533	0.004017	NUP153
GO_BP_m:	GO:19025	multi-orga	1月21日	4/17913	0.004681	0.010533	0.004017	NUP153
GO_BP_m:	GO:00311	mRNA 3'-	2月21日	89/17913	0.004821	0.010807	0.004122	THOC1/TH
GO_BP_m:	GO:00512	spindle ass	2月21日	90/17913	0.004927	0.011005	0.004197	AURKA/TP
GO_BP_m:	GO:00519	regulation	2月21日	93/17913	0.005251	0.011689	0.004458	ANAPC15/
GO_BP_m:	GO:00330	regulation	3月21日	311/17913	0.005467	0.012125	0.004624	ANAPC15/
GO_BP_m:	GO:00302	apoptotic	1月21日	5/17913	0.005849	0.012834	0.004895	TOP2A
GO_BP_m:	GO:19054	regulation	1月21日	5/17913	0.005849	0.012834	0.004895	TOP2A
GO_BP_m:	GO:20000	negative re	1月21日	5/17913	0.005849	0.012834	0.004895	THOC1
GO_BP_m:	GO:00066	protein im	2月21日	103/17913	0.006403	0.014001	0.00534	NUP54/TP
GO_BP_m:	GO:00311	RNA 3'-en	2月21日	107/17913	0.006893	0.01502	0.005728	THOC1/TH
GO_BP_m:	GO:00482	regulation	1月21日	6/17913	0.007014	0.015125	0.005768	THOC1
GO_BP_m:	GO:00903	spindle ass	1月21日	6/17913	0.007014	0.015125	0.005768	AURKA
GO_BP_m:	GO:19019	negative re	1月21日	6/17913	0.007014	0.015125	0.005768	THOC1
GO_BP_m:	GO:00106	negative re	3月21日	352/17913	0.007695	0.016534	0.006306	ANAPC15/
GO_BP_m:	GO:00062	DNA topo	1月21日	7/17913	0.008179	0.017011	0.006488	TOP2A
GO_BP_m:	GO:00071	female me	1月21日	7/17913	0.008179	0.017011	0.006488	AURKA
GO_BP_m:	GO:00400	embryonic	1月21日	7/17913	0.008179	0.017011	0.006488	TOP2A
GO_BP_m:	GO:00482	isotype sw	1月21日	7/17913	0.008179	0.017011	0.006488	THOC1
GO_BP_m:	GO:00902	positive re	1月21日	7/17913	0.008179	0.017011	0.006488	TPR
GO_BP_m:	GO:00902	positive re	1月21日	7/17913	0.008179	0.017011	0.006488	TPR
GO_BP_m:	GO:19001	regulation	1月21日	7/17913	0.008179	0.017011	0.006488	AURKA
GO_BP_m:	GO:19025	multi-orga	1月21日	7/17913	0.008179	0.017011	0.006488	NUP153
GO_BP_m:	GO:19025	multi-orga	1月21日	7/17913	0.008179	0.017011	0.006488	NUP153
GO_BP_m:	GO:19001	regulation	2月21日	117/17913	0.008191	0.017011	0.006488	NUP54/TP
GO_BP_m:	GO:00457	positive re	3月21日	373/17913	0.009017	0.018665	0.007118	AURKA/TH
GO_BP_m:	GO:00169	poly(A)+ n	1月21日	8/17913	0.009342	0.01921	0.007326	THOC2

GO_BP_m:	GO:19034:	positive re	1月21日	8/17913	0.009342	0.01921	0.007326	AURKA
GO_BP_m:	GO:00000	mitotic sist	2月21日	132/17913	0.010326	0.021042	0.008025	ANAPC15/
GO_BP_m:	GO:00072	female gar	2月21日	132/17913	0.010326	0.021042	0.008025	AURKA/TC
GO_BP_m:	GO:00000	DNA dama	2月21日	133/17913	0.010476	0.021042	0.008025	AURKA/TH
GO_BP_m:	GO:00016	regulation	1月21日	9/17913	0.010504	0.021042	0.008025	TPR
GO_BP_m:	GO:00602	regulation	1月21日	9/17913	0.010504	0.021042	0.008025	AURKA
GO_BP_m:	GO:19019	positive re	1月21日	9/17913	0.010504	0.021042	0.008025	TPR
GO_BP_m:	GO:19058	regulation	1月21日	9/17913	0.010504	0.021042	0.008025	AURKA
GO_BP_m:	GO:19903	cellular res	1月21日	9/17913	0.010504	0.021042	0.008025	CCNA2
GO_BP_m:	GO:00170	protein im	2月21日	140/17913	0.011556	0.023074	0.0088	NUP54/TP
GO_BP_m:	GO:00027	negative re	1月21日	10/17913	0.011665	0.023143	0.008826	THOC1
GO_BP_m:	GO:00028	negative re	1月21日	10/17913	0.011665	0.023143	0.008826	THOC1
GO_BP_m:	GO:00315	DNA integ	2月21日	141/17913	0.011714	0.023166	0.008835	AURKA/TH
GO_BP_m:	GO:01400	meiotic nu	2月21日	142/17913	0.011873	0.023406	0.008927	AURKA/TC
GO_BP_m:	GO:00070	spindle org	2月21日	147/17913	0.012682	0.024732	0.009432	AURKA/TP
GO_BP_m:	GO:00026	negative re	1月21日	11/17913	0.012824	0.024732	0.009432	THOC1
GO_BP_m:	GO:00071	mitotic cer	1月21日	11/17913	0.012824	0.024732	0.009432	AURKA
GO_BP_m:	GO:00315	heterochrc	1月21日	11/17913	0.012824	0.024732	0.009432	TPR
GO_BP_m:	GO:00354	histone-se	1月21日	11/17913	0.012824	0.024732	0.009432	AURKA
GO_BP_m:	GO:00354	cellular res	1月21日	11/17913	0.012824	0.024732	0.009432	TPR
GO_BP_m:	GO:00432	proteasom	1月21日	11/17913	0.012824	0.024732	0.009432	PSMD11
GO_BP_m:	GO:19030	meiotic ce	2月21日	151/17913	0.013346	0.025659	0.009786	AURKA/TC
GO_BP_m:	GO:00459	positive re	2月21日	154/17913	0.013854	0.026553	0.010127	AURKA/TP
GO_BP_m:	GO:00002	meiotic sp	1月21日	12/17913	0.013982	0.026553	0.010127	AURKA
GO_BP_m:	GO:00512	centrosom	1月21日	12/17913	0.013982	0.026553	0.010127	AURKA
GO_BP_m:	GO:00603	positive re	1月21日	12/17913	0.013982	0.026553	0.010127	NUP93
GO_BP_m:	GO:00008	sister chro	2月21日	157/17913	0.014371	0.027208	0.010377	ANAPC15/
GO_BP_m:	GO:00702	protein he	1月21日	13/17913	0.015139	0.028575	0.010898	NUP54
GO_BP_m:	GO:00705	regulation	2月21日	170/17913	0.016704	0.031435	0.011989	AURKA/TP
GO_BP_m:	GO:00450	regulation	1月21日	15/17913	0.017448	0.032735	0.012485	TOP2A
GO_BP_m:	GO:00433	neutrophil	3月21日	485/17913	0.018257	0.034149	0.013024	PSMA5/PS
GO_BP_m:	GO:00022	neutrophil	3月21日	488/17913	0.018556	0.03428	0.013074	PSMA5/PS
GO_BP_m:	GO:00396	single strai	1月21日	16/17913	0.018601	0.03428	0.013074	TOP2A
GO_BP_m:	GO:00717	cellular res	1月21日	16/17913	0.018601	0.03428	0.013074	CCNA2
GO_BP_m:	GO:20000	regulation	1月21日	16/17913	0.018601	0.03428	0.013074	THOC1
GO_BP_m:	GO:00439	positive re	2月21日	180/17913	0.018603	0.03428	0.013074	AURKA/TC
GO_BP_m:	GO:00024	neutrophil	3月21日	499/17913	0.019679	0.03605	0.013749	PSMA5/PS
GO_BP_m:	GO:00421	neutrophil	3月21日	499/17913	0.019679	0.03605	0.013749	PSMA5/PS
GO_BP_m:	GO:00062	DNA ligati	1月21日	17/17913	0.019753	0.036078	0.01376	TOP2A
GO_BP_m:	GO:00443	cellular res	1月21日	18/17913	0.020903	0.037734	0.014391	CCNA2
GO_BP_m:	GO:00459	negative re	1月21日	18/17913	0.020903	0.037734	0.014391	TPR
GO_BP_m:	GO:00708	heterochrc	1月21日	18/17913	0.020903	0.037734	0.014391	TPR
GO_BP_m:	GO:00713	cellular res	1月21日	18/17913	0.020903	0.037734	0.014391	CCNA2
GO_BP_m:	GO:00063	DNA pack	2月21日	194/17913	0.021407	0.038421	0.014653	TOP2A/TP
GO_BP_m:	GO:00310	regenerati	2月21日	194/17913	0.021407	0.038421	0.014653	AURKA/CC
GO_BP_m:	GO:00717	response t	1月21日	19/17913	0.022052	0.039351	0.015008	CCNA2
GO_BP_m:	GO:19021	cellular res	1月21日	19/17913	0.022052	0.039351	0.015008	CCNA2
GO_BP_m:	GO:00328	regulation	2月21日	199/17913	0.022448	0.039944	0.015234	AURKA/TP
GO_BP_m:	GO:19016	regulation	1月21日	20/17913	0.0232	0.041163	0.015699	TPR
GO_BP_m:	GO:00513	meiotic ce	2月21日	203/17913	0.023297	0.041218	0.01572	AURKA/TC
GO_BP_m:	GO:00354	response t	1月21日	21/17913	0.024346	0.042709	0.016289	TPR
GO_BP_m:	GO:00361	cellular res	1月21日	21/17913	0.024346	0.042709	0.016289	CCNA2
GO_BP_m:	GO:00468	positive re	1月21日	21/17913	0.024346	0.042709	0.016289	TPR
GO_BP_m:	GO:00327	positive re	1月21日	22/17913	0.025491	0.044095	0.016817	THOC1
GO_BP_m:	GO:00361	response t	1月21日	22/17913	0.025491	0.044095	0.016817	CCNA2
GO_BP_m:	GO:00443	response t	1月21日	22/17913	0.025491	0.044095	0.016817	CCNA2
GO_BP_m:	GO:00603	regulation	1月21日	22/17913	0.025491	0.044095	0.016817	NUP93
GO_BP_m:	GO:19034	regulation	1月21日	22/17913	0.025491	0.044095	0.016817	AURKA

GO_BP_m:	GO:00331!	regulation	2月21日	220/17913	0.027049	0.04653	0.017746	NUP54/TP
GO_BP_m:	GO:00988:	nuclear ch	2月21日	220/17913	0.027049	0.04653	0.017746	ANAPC15/
GO_BP_m:	GO:00345(	protein loc	2月21日	223/17913	0.027735	0.04752	0.018124	NUP54/TP
GO_BP_m:	GO:00516<	centrosom	1月21日	24/17913	0.027778	0.04752	0.018124	AURKA
GO_BP_m:	GO:00015!	oocyte ma	1月21日	25/17913	0.028919	0.049202	0.018765	AURKA
GO_BP_m:	GO:00618<	microtubu	1月21日	25/17913	0.028919	0.049202	0.018765	AURKA
GO_BP_m:	GO:00901(	regulation	1月21日	27/17913	0.031198	0.052934	0.020188	TPR
GO_BP_m:	GO:00346!	response t	1月21日	28/17913	0.032335	0.054418	0.020754	CCNA2
GO_BP_m:	GO:00396!	viral RNA (	1月21日	28/17913	0.032335	0.054418	0.020754	TOP2A
GO_BP_m:	GO:00508(	negative re	1月21日	28/17913	0.032335	0.054418	0.020754	THOC1
GO_BP_m:	GO:00312!	replication	1月21日	29/17913	0.033471	0.056177	0.021425	THOC1
GO_BP_m:	GO:00715:	protein loc	1月21日	30/17913	0.034606	0.057926	0.022092	AURKA
GO_BP_m:	GO:00302(	apoptotic	1月21日	31/17913	0.03574	0.059343	0.022633	TOP2A
GO_BP_m:	GO:00459:	negative re	1月21日	31/17913	0.03574	0.059343	0.022633	THOC1
GO_BP_m:	GO:19055(	protein loc	1月21日	31/17913	0.03574	0.059343	0.022633	AURKA
GO_BP_m:	GO:00062(	DNA replic	2月21日	260/17913	0.036763	0.060736	0.023164	CCNA2/TH
GO_BP_m:	GO:00302(	chromosor	1月21日	32/17913	0.036872	0.060736	0.023164	TOP2A
GO_BP_m:	GO:00974:	liver regen	1月21日	32/17913	0.036872	0.060736	0.023164	AURKA
GO_BP_m:	GO:00069:	cellular co	1月21日	33/17913	0.038003	0.062105	0.023686	TOP2A
GO_BP_m:	GO:00450(	DNA-depe	1月21日	33/17913	0.038003	0.062105	0.023686	THOC1
GO_BP_m:	GO:00451!	regulation	1月21日	33/17913	0.038003	0.062105	0.023686	THOC1
GO_BP_m:	GO:00713!	cellular res	1月21日	34/17913	0.039133	0.063784	0.024326	CCNA2
GO_BP_m:	GO:00027(	negative re	1月21日	35/17913	0.040262	0.065111	0.024833	THOC1
GO_BP_m:	GO:00450'	positive re	1月21日	35/17913	0.040262	0.065111	0.024833	TOP2A
GO_BP_m:	GO:00713:	cellular res	1月21日	35/17913	0.040262	0.065111	0.024833	CCNA2
GO_BP_m:	GO:00337(	response t	1月21日	36/17913	0.041389	0.066587	0.025396	CCNA2
GO_BP_m:	GO:00468:	regulation	1月21日	36/17913	0.041389	0.066587	0.025396	TPR
GO_BP_m:	GO:00711(	DNA confo	2月21日	279/17913	0.041786	0.067051	0.025573	TOP2A/TP
GO_BP_m:	GO:00423(	positive re	1月21日	37/17913	0.042515	0.067871	0.025885	TPR
GO_BP_m:	GO:00602:	regulation	1月21日	37/17913	0.042515	0.067871	0.025885	TPR
GO_BP_m:	GO:00900(	positive re	2月21日	283/17913	0.042875	0.06827	0.026037	AURKA/TP
GO_BP_m:	GO:00027(	negative re	1月21日	38/17913	0.04364	0.069311	0.026434	THOC1
GO_BP_m:	GO:00448<	cell cycle (	2月21日	288/17913	0.044251	0.070102	0.026736	AURKA/CC
GO_BP_m:	GO:00327(	regulation	1月21日	39/17913	0.044764	0.070554	0.026908	THOC1
GO_BP_m:	GO:19045!	positive re	1月21日	39/17913	0.044764	0.070554	0.026908	TPR
GO_BP_m:	GO:00028:	negative re	1月21日	40/17913	0.045886	0.07214	0.027513	THOC1
GO_BP_m:	GO:00902:	regulation	1月21日	41/17913	0.047007	0.073715	0.028114	TPR
GO_BP_m:	GO:00310'	embryonic	2月11日	36/17913	0.000214	0.003727	0.00208	TRAF3IP1/
GO_BP_m:	GO:00487(	skeletal sy:	3月11日	204/17913	0.000224	0.003727	0.00208	WDR60/IF'
GO_BP_m:	GO:00215:	neural tub	2月11日	39/17913	0.000251	0.003905	0.002179	TRAF3IP1/
GO_BP_m:	GO:00615:	protein loc	2月11日	42/17913	0.000291	0.004266	0.00238	WDR35/W
GO_BP_m:	GO:19055:	non-motil	2月11日	49/17913	0.000397	0.00549	0.003064	IFT80/IFT7
GO_BP_m:	GO:00076:	visual beha	2月11日	52/17913	0.000447	0.005859	0.003269	DYNLRB1/
GO_BP_m:	GO:00363'	opsin trans	1月11日	1/17913	0.000614	0.00695	0.003878	IFT20
GO_BP_m:	GO:19015!	response t	1月11日	1/17913	0.000614	0.00695	0.003878	WDR35
GO_BP_m:	GO:19057(	cellular res	1月11日	1/17913	0.000614	0.00695	0.003878	WDR35
GO_BP_m:	GO:00085(	regulation	2月11日	78/17913	0.001004	0.010865	0.006063	TRAF3IP1/
GO_BP_m:	GO:00487(	embryonic	2月11日	85/17913	0.00119	0.01235	0.006891	WDR60/W
GO_BP_m:	GO:00099!	dorsal/ven	2月11日	89/17913	0.001304	0.012987	0.007247	TRAF3IP1/
GO_BP_m:	GO:00073(	pattern sp	3月11日	408/17913	0.001689	0.01613	0.009001	TRAF3IP1/
GO_BP_m:	GO:00485(	embryonic	3月11日	413/17913	0.001749	0.01613	0.009001	TRAF3IP1/
GO_BP_m:	GO:00608:	ciliary rece	1月11日	3/17913	0.001841	0.016374	0.009137	WDR19
GO_BP_m:	GO:00487(	embryonic	2月11日	117/17913	0.002239	0.01922	0.010725	WDR60/W
GO_BP_m:	GO:00610!	myotome	1月11日	4/17913	0.002454	0.01922	0.010725	WDR19
GO_BP_m:	GO:00303:	embryonic	2月11日	123/17913	0.00247	0.01922	0.010725	TRAF3IP1/
GO_BP_m:	GO:00351:	embryonic	2月11日	123/17913	0.00247	0.01922	0.010725	TRAF3IP1/
GO_BP_m:	GO:00015(	skeletal sy:	3月11日	479/17913	0.002671	0.020155	0.011247	WDR60/IF'
GO_BP_m:	GO:20005(	regulation	1月11日	5/17913	0.003067	0.022343	0.012468	IFT20

GO_BP_m[illegible]	GO:00017[illegible]	morphoge	2月11日	139/17913	0.003141	0.022343	0.012468	TRAF3IP1/
GO_BP_m[illegible]	GO:00351[illegible]	appendag	2月11日	146/17913	0.003458	0.022904	0.012781	TRAF3IP1/
GO_BP_m[illegible]	GO:00351[illegible]	limb morp	2月11日	146/17913	0.003458	0.022904	0.012781	TRAF3IP1/
GO_BP_m[illegible]	GO:00357[illegible]	platelet-de	1月11日	6/17913	0.003679	0.022904	0.012781	IFT20
GO_BP_m[illegible]	GO:19016[illegible]	negative re	1月11日	6/17913	0.003679	0.022904	0.012781	TRAF3IP1
GO_BP_m[illegible]	GO:20000[illegible]	negative re	1月11日	6/17913	0.003679	0.022904	0.012781	IFT80
GO_BP_m[illegible]	GO:00219[illegible]	neural tub	2月11日	155/17913	0.003887	0.023608	0.013174	TRAF3IP1/
GO_BP_m[illegible]	GO:00506[illegible]	negative re	2月11日	163/17913	0.004289	0.02485	0.013867	IFT80/IFT7
GO_BP_m[illegible]	GO:19034[illegible]	protein loc	1月11日	7/17913	0.004291	0.02485	0.013867	WDR19
GO_BP_m[illegible]	GO:19016[illegible]	regulation	1月11日	8/17913	0.004903	0.02696	0.015044	TRAF3IP1
GO_BP_m[illegible]	GO:00487[illegible]	appendag	2月11日	176/17913	0.004981	0.02696	0.015044	TRAF3IP1/
GO_BP_m[illegible]	GO:00601[illegible]	limb devel	2月11日	176/17913	0.004981	0.02696	0.015044	TRAF3IP1/
GO_BP_m[illegible]	GO:00358[illegible]	photorece	1月11日	9/17913	0.005514	0.029215	0.016302	IFT20
GO_BP_m[illegible]	GO:00435[illegible]	ear develo	2月11日	212/17913	0.007147	0.037074	0.020688	WDR19/IF
GO_BP_m[illegible]	GO:00033[illegible]	keratinocy	1月11日	12/17913	0.007346	0.037332	0.020832	IFT74
GO_BP_m[illegible]	GO:00326[illegible]	negative re	1月11日	14/17913	0.008566	0.042659	0.023804	TRAF3IP1
GO_BP_m[illegible]	GO:00363[illegible]	post-anal	1月11日	18/17913	0.011001	0.053367	0.02978	TRAF3IP1
GO_BP_m[illegible]	GO:00018[illegible]	kidney dev	2月11日	276/17913	0.011869	0.053367	0.02978	TRAF3IP1/
GO_BP_m[illegible]	GO:00485[illegible]	embryonic	2月11日	276/17913	0.011869	0.053367	0.02978	WDR60/W
GO_BP_m[illegible]	GO:19907[illegible]	protein loc	2月11日	276/17913	0.011869	0.053367	0.02978	WDR19/IF
GO_BP_m[illegible]	GO:00336[illegible]	positive re	1月11日	20/17913	0.012217	0.053367	0.02978	IFT74
GO_BP_m[illegible]	GO:00450[illegible]	negative re	1月11日	20/17913	0.012217	0.053367	0.02978	WDR35
GO_BP_m[illegible]	GO:19044[illegible]	negative re	1月11日	20/17913	0.012217	0.053367	0.02978	WDR35
GO_BP_m[illegible]	GO:00720[illegible]	renal syste	2月11日	291/17913	0.013131	0.055012	0.030698	TRAF3IP1/
GO_BP_m[illegible]	GO:00340[illegible]	protein loc	1月11日	22/17913	0.013431	0.055012	0.030698	IFT20
GO_BP_m[illegible]	GO:20000[illegible]	regulation	1月11日	22/17913	0.013431	0.055012	0.030698	IFT80
GO_BP_m[illegible]	GO:00430[illegible]	camera-ty	2月11日	295/17913	0.013477	0.055012	0.030698	TRAF3IP1/
GO_BP_m[illegible]	GO:00094[illegible]	response t	2月11日	307/17913	0.014539	0.056973	0.031792	DYNLRB1/
GO_BP_m[illegible]	GO:00106[illegible]	regulation	1月11日	24/17913	0.014644	0.056973	0.031792	IFT20
GO_BP_m[illegible]	GO:00506[illegible]	negative re	1月11日	24/17913	0.014644	0.056973	0.031792	TRAF3IP1
GO_BP_m[illegible]	GO:00030[illegible]	regionaliza	2月11日	318/17913	0.015544	0.059545	0.033228	TRAF3IP1/
GO_BP_m[illegible]	GO:00459[illegible]	negative re	1月11日	26/17913	0.015855	0.059817	0.033379	TRAF3IP1
GO_BP_m[illegible]	GO:00016[illegible]	urogenital	2月11日	324/17913	0.016105	0.059851	0.033398	TRAF3IP1/
GO_BP_m[illegible]	GO:00601[illegible]	inner ear r	1月11日	27/17913	0.01646	0.060274	0.033634	IFT20
GO_BP_m[illegible]	GO:00902[illegible]	positive re	1月11日	28/17913	0.017065	0.061583	0.034365	WDR35
GO_BP_m[illegible]	GO:00301[illegible]	regulation	2月11日	341/17913	0.01774	0.062332	0.034782	IFT80/IFT2
GO_BP_m[illegible]	GO:00016[illegible]	eye develo	2月11日	342/17913	0.017839	0.062332	0.034782	TRAF3IP1/
GO_BP_m[illegible]	GO:01500[illegible]	visual syste	2月11日	346/17913	0.018235	0.062332	0.034782	TRAF3IP1/
GO_BP_m[illegible]	GO:00458[illegible]	negative re	1月11日	30/17913	0.018274	0.062332	0.034782	TRAF3IP1
GO_BP_m[illegible]	GO:00488[illegible]	sensory sy	2月11日	351/17913	0.018735	0.062674	0.034974	TRAF3IP1/
GO_BP_m[illegible]	GO:00458[illegible]	positive re	1月11日	31/17913	0.018878	0.062674	0.034974	IFT80
GO_BP_m[illegible]	GO:00974[illegible]	liver regen	1月11日	32/17913	0.019481	0.063827	0.035617	WDR35
GO_BP_m[illegible]	GO:20007[illegible]	regulation	1月11日	33/17913	0.020085	0.064949	0.036243	IFT20
GO_BP_m[illegible]	GO:00506[illegible]	regulation	2月11日	369/17913	0.020584	0.065711	0.036668	IFT80/IFT7
GO_BP_m[illegible]	GO:19034[illegible]	negative re	1月11日	37/17913	0.022494	0.070899	0.039563	WDR35
GO_BP_m[illegible]	GO:00440[illegible]	regulation	1月11日	38/17913	0.023095	0.071885	0.040113	IFT20
GO_BP_m[illegible]	GO:00601[illegible]	inner ear r	1月11日	39/17913	0.023697	0.072845	0.040649	IFT20
GO_BP_m[illegible]	GO:00424[illegible]	photorece	1月11日	44/17913	0.026697	0.078833	0.043991	IFT20
GO_BP_m[illegible]	GO:00487[illegible]	embryonic	1月11日	44/17913	0.026697	0.078833	0.043991	WDR19
GO_BP_m[illegible]	GO:00506[illegible]	epithelial c	2月11日	425/17913	0.026808	0.078833	0.043991	IFT80/IFT7
GO_BP_m[illegible]	GO:00336[illegible]	regulation	1月11日	45/17913	0.027297	0.078833	0.043991	IFT74
GO_BP_m[illegible]	GO:00324[illegible]	negative re	1月11日	46/17913	0.027895	0.078833	0.043991	TRAF3IP1
GO_BP_m[illegible]	GO:00901[illegible]	regulation	1月11日	46/17913	0.027895	0.078833	0.043991	WDR35
GO_BP_m[illegible]	GO:00093[illegible]	response t	2月11日	435/17913	0.027992	0.078833	0.043991	DYNLRB1/
GO_BP_m[illegible]	GO:00028[illegible]	negative re	1月11日	47/17913	0.028494	0.078833	0.043991	TRAF3IP1
GO_BP_m[illegible]	GO:00085[illegible]	visual learr	1月11日	47/17913	0.028494	0.078833	0.043991	IFT20
GO_BP_m[illegible]	GO:00326[illegible]	regulation	1月11日	48/17913	0.029092	0.079492	0.044358	TRAF3IP1
GO_BP_m[illegible]	GO:00431[illegible]	receptor c	1月11日	49/17913	0.02969	0.079492	0.044358	WDR19

GO_BP_m1	GO:00901(	cochlea de	1月11日	49/17913	0.02969	0.079492	0.044358	IFT20
GO_BP_m1	GO:00326(	interferon-	1月11日	50/17913	0.030287	0.080229	0.04477	TRAF3IP1
GO_BP_m1	GO:00601	inner ear r	1月11日	53/17913	0.032078	0.084078	0.046917	IFT20
GO_BP_m1	GO:00480(	platelet-de	1月11日	55/17913	0.03327	0.085404	0.047657	IFT20
GO_BP_m1	GO:19020	regulation	1月11日	55/17913	0.03327	0.085404	0.047657	IFT20
GO_BP_m1	GO:00018	release of	1月11日	57/17913	0.03446	0.087557	0.048859	WDR35
GO_BP_m1	GO:00424	mechanor	1月11日	58/17913	0.035055	0.088169	0.0492	IFT20
GO_BP_m1	GO:00465	photorece	1月11日	59/17913	0.03565	0.088198	0.049216	IFT20
GO_BP_m1	GO:00160	Wnt signal	2月11日	498/17913	0.035932	0.088198	0.049216	IFT80/IFT2
GO_BP_m1	GO:01987	cell-cell sig	2月11日	500/17913	0.036198	0.088198	0.049216	IFT80/IFT2
GO_BP_m1	GO:00336	cell adhesi	1月11日	61/17913	0.036838	0.088198	0.049216	IFT74
GO_BP_m1	GO:00427	embryonic	1月11日	61/17913	0.036838	0.088198	0.049216	TRAF3IP1
GO_BP_m1	GO:20003	negative re	1月11日	64/17913	0.038617	0.091245	0.050916	WDR35
GO_BP_m1	GO:00454	regulation	1月11日	65/17913	0.03921	0.091245	0.050916	WDR35
GO_BP_m1	GO:19048	cranial ske	1月11日	65/17913	0.03921	0.091245	0.050916	WDR19
GO_BP_m1	GO:00000	autophago	1月11日	70/17913	0.042167	0.097218	0.05425	IFT20
GO_BP_m1	GO:00506	regulation	1月11日	73/17913	0.043937	0.099458	0.0555	TRAF3IP1
GO_BP_m1	GO:19050	autophago	1月11日	73/17913	0.043937	0.099458	0.0555	IFT20
GO_BP_m1	GO:00311(	animal org	1月11日	75/17913	0.045116	0.100303	0.055971	WDR35
GO_BP_m1	GO:00350	embryonic	1月11日	75/17913	0.045116	0.100303	0.055971	TRAF3IP1
GO_BP_m1	GO:00068(	nitric oxide	1月11日	76/17913	0.045705	0.100713	0.0562	WDR35
GO_BP_m1	GO:00083(	associative	1月11日	77/17913	0.046293	0.101115	0.056424	IFT20
GO_BP_m1	GO:00977	negative re	1月11日	79/17913	0.047469	0.102782	0.057354	WDR35
GO_BP_m1	GO:00462(	nitric oxide	1月11日	81/17913	0.048644	0.103525	0.057769	WDR35
GO_BP_m1	GO:00610	somite dev	1月11日	81/17913	0.048644	0.103525	0.057769	WDR19
GO_BP_m1	GO:00457	positive re	11/133	373/17913	0.000108	0.004169	0.002996	BRCA2/CC
GO_BP_m1	GO:19010	guanosine	3/133	13/17913	0.000108	0.004169	0.002996	NME3/NM
GO_BP_m1	GO:00080(	regulation	7/133	147/17913	0.000114	0.004333	0.003114	ABI2/ACTN
GO_BP_m1	GO:19029(	regulation	10/133	313/17913	0.000117	0.00437	0.00314	ABI2/ACTN
GO_BP_m1	GO:00308	regulation	7/133	148/17913	0.000119	0.004409	0.003168	ABI2/ACTN
GO_BP_m1	GO:00062	CTP biosyr	3/133	14/17913	0.000137	0.0049	0.003521	NME3/NM
GO_BP_m1	GO:00092(	pyrimidine	3/133	14/17913	0.000137	0.0049	0.003521	NME3/NM
GO_BP_m1	GO:00460	CTP metab	3/133	14/17913	0.000137	0.0049	0.003521	NME3/NM
GO_BP_m1	GO:00091	nucleoside	7/133	153/17913	0.000146	0.005166	0.003712	AK9/ALDC
GO_BP_m1	GO:00508	multicellul	5/133	68/17913	0.00015	0.00523	0.003758	ADCY3/AD
GO_BP_m1	GO:00007	telomere r	7/133	154/17913	0.000152	0.005255	0.003777	BRCA2/CC
GO_BP_m1	GO:00002	microtubu	12/133	460/17913	0.000165	0.005623	0.004041	BRCA2/C2
GO_BP_m1	GO:00091	purine ribo	9/133	266/17913	0.000168	0.005623	0.004041	ADCY3/AD
GO_BP_m1	GO:00092(	pyrimidine	3/133	15/17913	0.000171	0.005623	0.004041	NME3/NM
GO_BP_m1	GO:00161	snRNA pro	3/133	15/17913	0.000171	0.005623	0.004041	INTS4/INT
GO_BP_m1	GO:00109	negative re	8/133	210/17913	0.000175	0.005698	0.004094	ARRB2/BIN
GO_BP_m1	GO:19040	epithelial c	6/133	111/17913	0.000179	0.005775	0.00415	ARRB2/DN
GO_BP_m1	GO:00070	centrosom	6/133	112/17913	0.000188	0.005992	0.004306	BRCA2/C2
GO_BP_m1	GO:00509(	leukocyte	12/133	467/17913	0.00019	0.005992	0.004306	C3AR1/CC
GO_BP_m1	GO:00075	hemostasis	10/133	336/17913	0.000207	0.006457	0.00464	ARRB2/AV
GO_BP_m1	GO:00102	response t	3/133	16/17913	0.000209	0.006457	0.00464	BRCA2/WF
GO_BP_m1	GO:00301(	water hom	5/133	74/17913	0.000224	0.006828	0.004907	ADCY3/AD
GO_BP_m1	GO:00075(	cell aging	6/133	116/17913	0.000228	0.006828	0.004907	BRCA2/CD
GO_BP_m1	GO:00322	positive re	6/133	116/17913	0.000228	0.006828	0.004907	ABI2/ARPC
GO_BP_m1	GO:00092(	ribonuclec	9/133	279/17913	0.00024	0.006975	0.005012	ADCY3/AD
GO_BP_m1	GO:00025	acute infla	8/133	220/17913	0.00024	0.006975	0.005012	C3AR1/CC
GO_BP_m1	GO:00061(	purine nuc	9/133	280/17913	0.000246	0.006975	0.005012	ADCY3/AD
GO_BP_m1	GO:00300	myeloid ce	11/133	411/17913	0.00025	0.006975	0.005012	ABI1/ASH2
GO_BP_m1	GO:00322(	telomere c	7/133	167/17913	0.000251	0.006975	0.005012	BRCA2/CC
GO_BP_m1	GO:00068	intra-Golg	3/133	17/17913	0.000252	0.006975	0.005012	COG2/CO
GO_BP_m1	GO:00091	pyrimidine	3/133	17/17913	0.000252	0.006975	0.005012	NME3/NM
GO_BP_m1	GO:00341	activation	3/133	17/17913	0.000252	0.006975	0.005012	ADCY3/AD
GO_BP_m1	GO:00325(	DNA duple	5/133	76/17913	0.000253	0.006975	0.005012	DDB1/NBN

GO_BP_m:	GO:00104(	negative re	8/133	223/17913	0.000263	0.007168	0.005151	ARRB2/BIN
GO_BP_m:	GO:00463!	ribose pho	9/133	284/17913	0.000273	0.007326	0.005265	ADCY3/AD
GO_BP_m:	GO:00030:	renal syste	6/133	120/17913	0.000274	0.007326	0.005265	ADCY3/AD
GO_BP_m:	GO:00310:	microtubu	6/133	122/17913	0.000299	0.007783	0.005593	BRCA2/C2
GO_BP_m:	GO:00106:	negative re	10/133	352/17913	0.0003	0.007783	0.005593	ARRB2/CH
GO_BP_m:	GO:00091!	cyclic nucl	3/133	18/17913	0.000301	0.007783	0.005593	ADCY3/AD
GO_BP_m:	GO:00526!	cyclic purir	3/133	18/17913	0.000301	0.007783	0.005593	ADCY3/AD
GO_BP_m:	GO:00431!	negative re	5/133	79/17913	0.000304	0.007787	0.005596	ARRB2/GA
GO_BP_m:	GO:00901:	C-5 methy	2/133	4/17913	0.000325	0.00817	0.005871	DNMT1/D
GO_BP_m:	GO:19900(	lens fiber c	2/133	4/17913	0.000325	0.00817	0.005871	E2F1/E2F2
GO_BP_m:	GO:00725:	purine-cor	9/133	291/17913	0.000327	0.00817	0.005871	ADCY3/AD
GO_BP_m:	GO:00512!	protein po	8/133	232/17913	0.000343	0.008502	0.006109	ABI2/ARP(
GO_BP_m:	GO:00018:	blastocyst	3/133	19/17913	0.000355	0.008599	0.006179	BRCA2/NE
GO_BP_m:	GO:00091/	pyrimidine	3/133	19/17913	0.000355	0.008599	0.006179	NME3/NM
GO_BP_m:	GO:00902(	negative re	3/133	19/17913	0.000355	0.008599	0.006179	ARRB2/CLU
GO_BP_m:	GO:00329!	regulation	9/133	297/17913	0.000379	0.009103	0.006541	ABI2/ACTN
GO_BP_m:	GO:19017!	regulation	7/133	179/17913	0.000382	0.009103	0.006541	CHEK1/NB
GO_BP_m:	GO:00061(	nucleoside	6/133	129/17913	0.000403	0.009335	0.006708	AK9/ALDC
GO_BP_m:	GO:00308:	regulation	6/133	129/17913	0.000403	0.009335	0.006708	ABI2/ARP(
GO_BP_m:	GO:00716:	granulocyt	5/133	84/17913	0.000404	0.009335	0.006708	C3AR1/CC
GO_BP_m:	GO:00069!	acute-pha	4/133	47/17913	0.000405	0.009335	0.006708	F8/ITIH4/C
GO_BP_m:	GO:00062(	DNA-depe	6/133	130/17913	0.00042	0.009554	0.006866	BRCA2/CC
GO_BP_m:	GO:00509:	positive re	6/133	130/17913	0.00042	0.009554	0.006866	C3AR1/CC
GO_BP_m:	GO:00427	signal tran	6/133	131/17913	0.000438	0.009804	0.007045	BRCA2/CE
GO_BP_m:	GO:00469:	nucleotide	6/133	131/17913	0.000438	0.009804	0.007045	AK9/ALDC
GO_BP_m:	GO:00323!	DNA geon	5/133	86/17913	0.00045	0.009807	0.007048	DDB1/NBN
GO_BP_m:	GO:00447	mitotic DN	5/133	86/17913	0.00045	0.009807	0.007048	CLSPN/E2I
GO_BP_m:	GO:00481/	regulation	5/133	86/17913	0.00045	0.009807	0.007048	CDC73/CD
GO_BP_m:	GO:00507:	positive re	7/133	184/17913	0.000451	0.009807	0.007048	ABI1/ARRE
GO_BP_m:	GO:00481/	fibroblast		5/133	87/17913	0.000475	0.009848	0.007077	CDC73/CD
GO_BP_m:	GO:00726	lymphocyt	5/133	87/17913	0.000475	0.009848	0.007077	CCR6/CCR
GO_BP_m:	GO:20001:	negative re	5/133	87/17913	0.000475	0.009848	0.007077	ARRB2/GA
GO_BP_m:	GO:00482(	positive re	4/133	49/17913	0.000475	0.009848	0.007077	ARRB2/CLU
GO_BP_m:	GO:00026!	regulation	7/133	186/17913	0.000481	0.009848	0.007077	C3AR1/CC
GO_BP_m:	GO:00424!	purine nuc	3/133	21/17913	0.000483	0.009848	0.007077	NME3/NM
GO_BP_m:	GO:00460!	cAMP met	3/133	21/17913	0.000483	0.009848	0.007077	ADCY3/AD
GO_BP_m:	GO:00461:	purine ribc	3/133	21/17913	0.000483	0.009848	0.007077	NME3/NM
GO_BP_m:	GO:00461:	pyrimidine	3/133	21/17913	0.000483	0.009848	0.007077	NME3/NM
GO_BP_m:	GO:00459:	negative re	9/133	308/17913	0.000494	0.010005	0.00719	CDC73/CD
GO_BP_m:	GO:00182(	peptidyl-ly	10/133	376/17913	0.000504	0.010155	0.007298	ASH2L/BR(
GO_BP_m:	GO:00102:	maintenan	2/133	5/17913	0.000539	0.010552	0.007583	CTCF/DNN
GO_BP_m:	GO:00327	DNA meth	2/133	5/17913	0.000539	0.010552	0.007583	DNMT1/D
GO_BP_m:	GO:00703/	negative re	2/133	5/17913	0.000539	0.010552	0.007583	E2F1/E2F3
GO_BP_m:	GO:00481/	positive re	4/133	51/17913	0.000554	0.010552	0.007583	CDK6/E2F:
GO_BP_m:	GO:00447	mitotic DN	5/133	90/17913	0.000555	0.010552	0.007583	CLSPN/E2I
GO_BP_m:	GO:00094:	response t	6/133	137/17913	0.000555	0.010552	0.007583	BRCA2/CD
GO_BP_m:	GO:00092:	pyrimidine	3/133	22/17913	0.000556	0.010552	0.007583	NME3/NM
GO_BP_m:	GO:00327!	positive re	3/133	22/17913	0.000556	0.010552	0.007583	CDC73/LE(
GO_BP_m:	GO:20007	negative re	3/133	22/17913	0.000556	0.010552	0.007583	CDK6/PRK
GO_BP_m:	GO:00456:	regulation	8/133	250/17913	0.000563	0.010621	0.007632	ASH2L/CD
GO_BP_m:	GO:00310!	regulation	6/133	138/17913	0.000577	0.010809	0.007767	CHEK1/CT
GO_BP_m:	GO:00325:	regulation	9/133	315/17913	0.00058	0.010809	0.007767	ABI2/ACTN
GO_BP_m:	GO:19020(	regulation	3/133	23/17913	0.000636	0.011771	0.008459	BIN1/CLU/
GO_BP_m:	GO:00327:	negative re	4/133	54/17913	0.000689	0.012682	0.009114	ARRB2/GA
GO_BP_m:	GO:00024(	dendritic c	3/133	24/17913	0.000723	0.013058	0.009383	CCR6/CCR
GO_BP_m:	GO:00092:	pyrimidine	3/133	24/17913	0.000723	0.013058	0.009383	NME3/NM
GO_BP_m:	GO:00109	positive re	3/133	24/17913	0.000723	0.013058	0.009383	RAB11A/R
GO_BP_m:	GO:00429!	amyloid pr	4/133	55/17913	0.000739	0.013273	0.009538	BIN1/CLU/

GO_BP_m	GO:00302	megakaryo	5/133	96/17913	0.000745	0.01329	0.00955	ABI1/ASH2
GO_BP_m	GO:00300	actin filam	6/133	145/17913	0.000749	0.01329	0.00955	ABI2/ARP
GO_BP_m	GO:19019	positive re	5/133	97/17913	0.000781	0.013779	0.009902	CDC73/DL
GO_BP_m	GO:00514	regulation	11/133	472/1791	0.000796	0.013967	0.010037	ABI2/ACTN
GO_BP_m	GO:00070	mitotic cel	6/133	147/1791	0.000804	0.014027	0.01008	CLSPN/E2
GO_BP_m	GO:00075	blood coa	9/133	331/1791	0.000825	0.014308	0.010282	ARRB2/EN
GO_BP_m	GO:00514	positive re	7/133	205/1791	0.000856	0.014739	0.010592	ABI2/ACTN
GO_BP_m	GO:00510	regulation	10/133	403/1791	0.00086	0.014739	0.010592	ARRB2/BR
GO_BP_m	GO:00975	granulocyt	5/133	100/1791	0.000896	0.015162	0.010895	C3AR1/CC
GO_BP_m	GO:00439	histone H3	4/133	58/17913	0.000904	0.015162	0.010895	BRCA2/CH
GO_BP_m	GO:00000	G1/S trans	8/133	269/1791	0.000907	0.015162	0.010895	CCNE2/CD
GO_BP_m	GO:00072	nitric oxide	3/133	26/17913	0.000918	0.015162	0.010895	FPR1/THB
GO_BP_m	GO:00314	regulation	3/133	26/17913	0.000918	0.015162	0.010895	CCNT1/CD
GO_BP_m	GO:00713	cellular res	3/133	26/17913	0.000918	0.015162	0.010895	ADCY3/AD
GO_BP_m	GO:00165	histone ac	6/133	151/1791	0.000925	0.015162	0.010895	BRCA2/CH
GO_BP_m	GO:00326	regulation	6/133	151/1791	0.000925	0.015162	0.010895	ARRB2/CL
GO_BP_m	GO:00508	coagulatio	9/133	337/1791	0.000936	0.0152	0.010922	ARRB2/EN
GO_BP_m	GO:00458	negative r	5/133	101/1791	0.000937	0.0152	0.010922	AEBP2/DN
GO_BP_m	GO:00432	regulation	10/133	409/1791	0.000962	0.015512	0.011147	ABI2/ARP
GO_BP_m	GO:00329	regulation	9/133	341/1791	0.001017	0.015969	0.011475	ABI2/ACTN
GO_BP_m	GO:00485	positive re	5/133	103/1791	0.001024	0.015969	0.011475	CCNT1/CD
GO_BP_m	GO:00459	positive re	6/133	154/1791	0.001024	0.015969	0.011475	BRCA2/DL
GO_BP_m	GO:00305	neutrophil	4/133	60/17913	0.001027	0.015969	0.011475	C3AR1/CC
GO_BP_m	GO:00159	nucleobas	3/133	27/17913	0.001027	0.015969	0.011475	AK9/NME
GO_BP_m	GO:00363	dendritic c	3/133	27/17913	0.001027	0.015969	0.011475	CCR6/CCR
GO_BP_m	GO:19027	positive re	3/133	27/17913	0.001027	0.015969	0.011475	RAB11A/R
GO_BP_m	GO:00602	anatomica	10/133	413/1791	0.001035	0.016007	0.011502	ALDOA/B
GO_BP_m	GO:00326	tumor nec	6/133	155/1791	0.001059	0.01621	0.011648	ARRB2/CL
GO_BP_m	GO:19035	regulation	6/133	155/1791	0.001059	0.01621	0.011648	ARRB2/CL
GO_BP_m	GO:00109	negative r	9/133	344/1791	0.001081	0.016464	0.011831	CDC73/CD
GO_BP_m	GO:00183	internal pe	6/133	156/1791	0.001095	0.01649	0.01185	BRCA2/CH
GO_BP_m	GO:00190	fusion of v	2/133	7/17913	0.001121	0.01649	0.01185	CD4/GAS6
GO_BP_m	GO:00396	membrane	2/133	7/17913	0.001121	0.01649	0.01185	CD4/GAS6
GO_BP_m	GO:00448	multi-orga	2/133	7/17913	0.001121	0.01649	0.01185	CD4/GAS6
GO_BP_m	GO:19029	mitotic DN	2/133	7/17913	0.001121	0.01649	0.01185	BRCA2/RA
GO_BP_m	GO:20005	positive re	2/133	7/17913	0.001121	0.01649	0.01185	CCR7/GAS
GO_BP_m	GO:20006	negative r	2/133	7/17913	0.001121	0.01649	0.01185	CCR7/GAS
GO_BP_m	GO:00903	negative r	3/133	28/17913	0.001144	0.016747	0.012034	CDK6/PRK
GO_BP_m	GO:00092	purine rib	6/133	159/1791	0.001208	0.017587	0.012638	AK9/ALDC
GO_BP_m	GO:00091	purine nu	6/133	160/1791	0.001247	0.017965	0.01291	AK9/ALDC
GO_BP_m	GO:00103	histone m	3/133	29/17913	0.00127	0.017965	0.01291	CDC73/DD
GO_BP_m	GO:00312	replication	3/133	29/17913	0.00127	0.017965	0.01291	BRCA2/RA
GO_BP_m	GO:00342	amyloid-b	3/133	29/17913	0.00127	0.017965	0.01291	BIN1/CLU/
GO_BP_m	GO:00460	GTP meta	3/133	29/17913	0.00127	0.017965	0.01291	NME3/NM
GO_BP_m	GO:00461	pyrimidine	3/133	29/17913	0.00127	0.017965	0.01291	NME3/NM
GO_BP_m	GO:00064	internal pr	6/133	161/1791	0.001288	0.018052	0.012972	BRCA2/CH
GO_BP_m	GO:00717	tumor nec	6/133	161/1791	0.001288	0.018052	0.012972	ARRB2/CL
GO_BP_m	GO:00510	positive re	7/133	222/1791	0.001358	0.018955	0.013621	ARRB2/DN
GO_BP_m	GO:00002	nuclear di	9/133	357/1791	0.001398	0.018955	0.013621	BRCA2/CH
GO_BP_m	GO:00448	cell cycle	8/133	288/1791	0.001403	0.018955	0.013621	CCNE2/CD
GO_BP_m	GO:00062	DNA repli	3/133	30/17913	0.001403	0.018955	0.013621	CCNE2/NE
GO_BP_m	GO:00315	G2 DNA d	3/133	30/17913	0.001403	0.018955	0.013621	CHEK1/CL
GO_BP_m	GO:00343	Arp2/3 co	3/133	30/17913	0.001403	0.018955	0.013621	ABI2/ARP
GO_BP_m	GO:00715	protein lo	3/133	30/17913	0.001403	0.018955	0.013621	C2CD3/GC
GO_BP_m	GO:19029	regulation	3/133	30/17913	0.001403	0.018955	0.013621	BIN1/CLU/
GO_BP_m	GO:00092	ribonucle	6/133	164/1791	0.001415	0.018955	0.013621	AK9/ALDC
GO_BP_m	GO:00183	peptidyl-l	6/133	164/1791	0.001415	0.018955	0.013621	BRCA2/CH
GO_BP_m	GO:19022	regulation	6/133	165/1791	0.00146	0.019239	0.013825	CHEK1/CT

GO_BP_m:	GO:00018:	negative re	8/133	290/17913	0.001465	0.019239	0.013825	ADCY7/AR
GO_BP_m:	GO:00091(	nucleotide	9/133	360/17913	0.001481	0.019239	0.013825	ADCY3/AD
GO_BP_m:	GO:00199:	second-m	9/133	360/17913	0.001481	0.019239	0.013825	ADCY3/AD
GO_BP_m:	GO:00430	DNA meth	2/133	8/17913	0.001488	0.019239	0.013825	DNMT1/D
GO_BP_m:	GO:19015:	changes tc	2/133	8/17913	0.001488	0.019239	0.013825	DNMT1/D
GO_BP_m:	GO:20005(	regulation	2/133	8/17913	0.001488	0.019239	0.013825	CCR7/GAS
GO_BP_m:	GO:20006(	positive re	2/133	8/17913	0.001488	0.019239	0.013825	ABI2/WAS
GO_BP_m:	GO:00311:	cytoplasm	3/133	31/17913	0.001545	0.019807	0.014233	PCM1/RAE
GO_BP_m:	GO:19055(	protein loc	3/133	31/17913	0.001545	0.019807	0.014233	C2CD3/GC
GO_BP_m:	GO:19012	nucleoside	9/133	365/17913	0.001628	0.020784	0.014935	ADCY3/AD
GO_BP_m:	GO:20012	positive re	6/133	169/17913	0.001649	0.020964	0.015065	DLGAP5/D
GO_BP_m:	GO:00713	cellular res	8/133	297/17913	0.001703	0.02149	0.015443	ACTN2/AD
GO_BP_m:	GO:00181(	peptidyl-s	7/133	231/17913	0.001705	0.02149	0.015443	ARRB2/CLS
GO_BP_m:	GO:00327:	negative re	4/133	69/17913	0.001729	0.021613	0.015531	ARRB2/GA
GO_BP_m:	GO:00714	cellular res	4/133	69/17913	0.001729	0.021613	0.015531	INTS7/RAD
GO_BP_m:	GO:00072(	phospholip	4/133	70/17913	0.001823	0.022698	0.01631	ACTN2/C3
GO_BP_m:	GO:00302:	T cell diffe	7/133	234/17913	0.001834	0.022741	0.016342	CCR6/CCR
GO_BP_m:	GO:00424	ribonuclec	3/133	33/17913	0.001855	0.022808	0.01639	NME3/NM
GO_BP_m:	GO:00450(	DNA-depe	3/133	33/17913	0.001855	0.022808	0.01639	BRCA2/RA
GO_BP_m:	GO:00609	coronary a	2/133	9/17913	0.001904	0.02331	0.01675	SEC24B/VE
GO_BP_m:	GO:19035	negative re	4/133	71/17913	0.001921	0.02343	0.016837	ARRB2/GA
GO_BP_m:	GO:00331:	regulation	5/133	119/17913	0.001944	0.02361	0.016966	ARRB2/GA
GO_BP_m:	GO:00091	nucleoside	6/133	175/17913	0.001967	0.023798	0.017101	AK9/ALDC
GO_BP_m:	GO:00071	adenylate	4/133	72/17913	0.002023	0.024272	0.017442	ADCY3/AD
GO_BP_m:	GO:19902(	neutrophil	4/133	72/17913	0.002023	0.024272	0.017442	C3AR1/CC
GO_BP_m:	GO:00026	positive re	5/133	121/17913	0.002091	0.024894	0.017889	C3AR1/CC
GO_BP_m:	GO:00550(	cardiac mu	5/133	121/17913	0.002091	0.024894	0.017889	ACTN2/AR
GO_BP_m:	GO:00063	mRNA pol	3/133	35/17913	0.002201	0.025797	0.018538	CCNT1/CD
GO_BP_m:	GO:00302:	monocyte	3/133	35/17913	0.002201	0.025797	0.018538	CD4/CDK6
GO_BP_m:	GO:00461:	pyrimidine	3/133	35/17913	0.002201	0.025797	0.018538	NME3/NM
GO_BP_m:	GO:19031:	mononucl	3/133	35/17913	0.002201	0.025797	0.018538	CD4/CDK6
GO_BP_m:	GO:00712:	cellular res	8/133	310/17913	0.002224	0.025855	0.018579	CDC25A/C
GO_BP_m:	GO:01040(	cellular res	8/133	310/17913	0.002224	0.025855	0.018579	CDC25A/C
GO_BP_m:	GO:00322	regulation	6/133	180/17913	0.002266	0.026172	0.018807	ABI2/ARPC
GO_BP_m:	GO:00330	regulation	8/133	311/17913	0.002268	0.026172	0.018807	CHEK1/CT
GO_BP_m:	GO:00018	neural plat	2/133	10/17913	0.002368	0.02654	0.019071	C2CD3/DV
GO_BP_m:	GO:00107	regulation	2/133	10/17913	0.002368	0.02654	0.019071	THBS1/VE
GO_BP_m:	GO:00448(	multi-orga	2/133	10/17913	0.002368	0.02654	0.019071	CD4/GAS6
GO_BP_m:	GO:00703	fat cell prc	2/133	10/17913	0.002368	0.02654	0.019071	E2F1/E2F3
GO_BP_m:	GO:00703	regulation	2/133	10/17913	0.002368	0.02654	0.019071	E2F1/E2F3
GO_BP_m:	GO:00507:	regulation	7/133	245/17913	0.002376	0.02654	0.019071	ABI1/ARRE
GO_BP_m:	GO:00091	cyclic nucl	3/133	36/17913	0.002389	0.02654	0.019071	ADCY3/AD
GO_BP_m:	GO:00337(	response t	3/133	36/17913	0.002389	0.02654	0.019071	ADCY3/AD
GO_BP_m:	GO:00343	smooth m	3/133	36/17913	0.002389	0.02654	0.019071	ARRB2/DN
GO_BP_m:	GO:00343	regulation	3/133	36/17913	0.002389	0.02654	0.019071	ARRB2/DN
GO_BP_m:	GO:00436:	RNA polya	3/133	37/17913	0.002585	0.028484	0.020469	CCNT1/CD
GO_BP_m:	GO:00346	cellular res	4/133	77/17913	0.002586	0.028484	0.020469	CDC25A/C
GO_BP_m:	GO:19037(	regulation	10/133	468/17913	0.0026	0.028484	0.020469	ASH2L/CD
GO_BP_m:	GO:00075(	aging	8/133	318/17913	0.002602	0.028484	0.020469	BRCA2/CD
GO_BP_m:	GO:00509:	regulation	6/133	186/17913	0.002669	0.029115	0.020922	C3AR1/CC
GO_BP_m:	GO:00550:	cardiac mu	4/133	78/17913	0.00271	0.02946	0.02117	ACTN2/CT
GO_BP_m:	GO:00482	organelle f	9/133	395/17913	0.002774	0.030003	0.02156	BRCA2/CH
GO_BP_m:	GO:00182(	peptidyl-s	7/133	252/17913	0.00278	0.030003	0.02156	ARRB2/CLS
GO_BP_m:	GO:00456	regulation	4/133	79/17913	0.002839	0.030106	0.021634	ASH2L/KM
GO_BP_m:	GO:00975	lamellipod	4/133	79/17913	0.002839	0.030106	0.021634	ABI1/NCK:
GO_BP_m:	GO:00165	histone me	5/133	130/17913	0.002856	0.030106	0.021634	ASH2L/CT
GO_BP_m:	GO:00023:	pro-B cell	2/133	11/17913	0.00288	0.030106	0.021634	PRKDC/SC
GO_BP_m:	GO:00026(	regulation	2/133	11/17913	0.00288	0.030106	0.021634	CCR7/THB

GO_BP_m	GO:00314	positive re	2/133	11/17913	0.00288	0.030106	0.021634	CDC73/LE
GO_BP_m	GO:00335	histone H2	2/133	11/17913	0.00288	0.030106	0.021634	CDC73/LE
GO_BP_m	GO:00970	dendritic c	2/133	11/17913	0.00288	0.030106	0.021634	CCR7/GAS
GO_BP_m	GO:20006	regulation	2/133	11/17913	0.00288	0.030106	0.021634	CCR7/GAS
GO_BP_m	GO:19019	negative re	7/133	254/17913	0.002905	0.03026	0.021745	CDC73/CD
GO_BP_m	GO:00458	negative re	8/133	324/17913	0.002917	0.030278	0.021758	ARRB2/BIN
GO_BP_m	GO:00300	lymphocyt	8/133	325/17913	0.002972	0.030746	0.022094	CCR6/CCR
GO_BP_m	GO:00327	regulation	3/133	39/17913	0.003008	0.031012	0.022285	CDC73/LE
GO_BP_m	GO:00026	positive re	4/133	81/17913	0.003108	0.03182	0.022866	C3AR1/CC
GO_BP_m	GO:00517	interaction	4/133	81/17913	0.003108	0.03182	0.022866	CCNT1/CT
GO_BP_m	GO:00429	amyloid pr	3/133	40/17913	0.003235	0.032894	0.023638	BIN1/CLU/
GO_BP_m	GO:00504	amyloid-b	3/133	40/17913	0.003235	0.032894	0.023638	BIN1/CLU/
GO_BP_m	GO:19019	positive re	4/133	82/17913	0.003249	0.032925	0.02366	DLGAP5/R
GO_BP_m	GO:00064	protein ac	6/133	195/17913	0.00337	0.034031	0.024455	BRCA2/CH
GO_BP_m	GO:00007	telomere r	2/133	12/17913	0.003439	0.034031	0.024455	BRCA2/RA
GO_BP_m	GO:00024	dendritic c	2/133	12/17913	0.003439	0.034031	0.024455	CCR7/THB
GO_BP_m	GO:00329	positive re	2/133	12/17913	0.003439	0.034031	0.024455	CDC73/LE
GO_BP_m	GO:19024	negative re	2/133	12/17913	0.003439	0.034031	0.024455	BIN1/CLU
GO_BP_m	GO:19029	regulation	2/133	12/17913	0.003439	0.034031	0.024455	CLU/HGF
GO_BP_m	GO:00091	nucleoside	3/133	41/17913	0.003471	0.034031	0.024455	NME3/NM
GO_BP_m	GO:00310	negative re	3/133	41/17913	0.003471	0.034031	0.024455	DNMT1/PI
GO_BP_m	GO:00504	positive re	3/133	41/17913	0.003471	0.034031	0.024455	CCNT1/CT
GO_BP_m	GO:00703	negative re	3/133	41/17913	0.003471	0.034031	0.024455	CHEK1/E2I
GO_BP_m	GO:00550	cardiac cel	4/133	84/17913	0.003543	0.034624	0.024881	ACTN2/CT
GO_BP_m	GO:00062	pyrimidine	3/133	42/17913	0.003718	0.036098	0.02594	NME3/NM
GO_BP_m	GO:19016	glycosyl co	3/133	42/17913	0.003718	0.036098	0.02594	NME3/NM
GO_BP_m	GO:00450	actin nucle	3/133	43/17913	0.003976	0.038473	0.027646	ABI2/ARP
GO_BP_m	GO:00458	positive re	5/133	141/17913	0.004044	0.038578	0.027722	ARRB2/CLU
GO_BP_m	GO:00017	in utero er	8/133	342/17913	0.004044	0.038578	0.027722	B9D1/BRC
GO_BP_m	GO:00018	inner cell r	2/133	13/17913	0.004045	0.038578	0.027722	BRCA2/PA
GO_BP_m	GO:00610	negative re	2/133	13/17913	0.004045	0.038578	0.027722	CTDP1/YY
GO_BP_m	GO:00024	neutrophil	10/133	499/17913	0.004097	0.038578	0.027722	ALDOA/C3
GO_BP_m	GO:00421	neutrophil	10/133	499/17913	0.004097	0.038578	0.027722	ALDOA/C3
GO_BP_m	GO:00434	positive re	10/133	499/17913	0.004097	0.038578	0.027722	ACKR3/AR
GO_BP_m	GO:00457	positive re	6/133	203/17913	0.004102	0.038578	0.027722	C3AR1/CC
GO_BP_m	GO:00513	meiotic ce	6/133	203/17913	0.004102	0.038578	0.027722	BRCA2/NE
GO_BP_m	GO:00513	negative re	9/133	420/17913	0.004152	0.038927	0.027972	ARRB2/BIN
GO_BP_m	GO:00973	cellular res	4/133	88/17913	0.004186	0.039121	0.028112	ADCY3/AD
GO_BP_m	GO:00165	histone ub	3/133	44/17913	0.004243	0.039413	0.028322	CDC73/DD
GO_BP_m	GO:00716	regulation	3/133	44/17913	0.004243	0.039413	0.028322	C3AR1/CC
GO_BP_m	GO:00181	peptidyl-ty	8/133	346/17913	0.004335	0.040139	0.028844	ABI1/ABI2/
GO_BP_m	GO:00450	T cell selec	3/133	45/17913	0.004522	0.041479	0.029806	CCR7/CD3
GO_BP_m	GO:00703	regulation	3/133	45/17913	0.004522	0.041479	0.029806	CHEK1/E2I
GO_BP_m	GO:19010	guanosine	3/133	45/17913	0.004522	0.041479	0.029806	NME3/NM
GO_BP_m	GO:00512	spindle ass	4/133	90/17913	0.004535	0.041479	0.029806	CEP63/HA
GO_BP_m	GO:00182	peptidyl-ty	8/133	349/17913	0.004564	0.041609	0.0299	ABI1/ABI2/
GO_BP_m	GO:00069	humoral ir	7/133	276/17913	0.004577	0.041609	0.0299	C3AR1/CC
GO_BP_m	GO:00971	intrinsic ap	7/133	277/17913	0.004667	0.041932	0.030132	ACKR3/BR
GO_BP_m	GO:00319	positive re	2/133	14/17913	0.004696	0.041932	0.030132	DNMT1/SI
GO_BP_m	GO:00343	regulation	2/133	14/17913	0.004696	0.041932	0.030132	ABI2/WAS
GO_BP_m	GO:00343	negative re	2/133	14/17913	0.004696	0.041932	0.030132	ARRB2/DN
GO_BP_m	GO:00511	positive re	2/133	14/17913	0.004696	0.041932	0.030132	ABI2/WAS
GO_BP_m	GO:00713	cellular res	2/133	14/17913	0.004696	0.041932	0.030132	ADCY7/DN
GO_BP_m	GO:00901	regulation	3/133	46/17913	0.004812	0.042678	0.030668	ARRB2/CLU
GO_BP_m	GO:00070	spindle org	5/133	147/17913	0.004823	0.042678	0.030668	CEP63/HA
GO_BP_m	GO:00712	cellular res	5/133	147/17913	0.004823	0.042678	0.030668	ADCY3/AD
GO_BP_m	GO:00711	DNA confo	7/133	279/17913	0.004852	0.042811	0.030764	CTCF/DDB
GO_BP_m	GO:00086	intrinsic ap	4/133	92/17913	0.004903	0.043136	0.030998	ACKR3/BR

GO_BP_m:	GO:00344(	nucleobas	6/133	211/17913	0.004945	0.043381	0.031173	ALDOA/EN
GO_BP_m:	GO:00352{	segmentat	4/133	93/17913	0.005095	0.044325	0.031852	ABI1/DVL2
GO_BP_m:	GO:00332(	nuclear DN	3/133	47/17913	0.005112	0.044325	0.031852	BRCA2/PC
GO_BP_m:	GO:00450:	G0 to G1 t	3/133	47/17913	0.005112	0.044325	0.031852	CHEK1/E2I
GO_BP_m:	GO:00726(	protein loc	3/133	47/17913	0.005112	0.044325	0.031852	C2CD3/GC
GO_BP_m:	GO:00027(	immune re	9/133	434/17913	0.00513	0.044353	0.031872	ABI1/ARPC
GO_BP_m:	GO:00900(	positive re	7/133	283/17913	0.005238	0.045151	0.032446	CDC73/DL
GO_BP_m:	GO:0070:	Golgi orga	4/133	94/17913	0.005291	0.04535	0.032589	COG2/CO
GO_BP_m:	GO:00071{	adenylate	4/133	94/17913	0.005291	0.04535	0.032589	ADCY3/AD
GO_BP_m:	GO:00013:	formation	2/133	15/17913	0.005393	0.045359	0.032595	NBN/WRN
GO_BP_m:	GO:00017:	endoderm	2/133	15/17913	0.005393	0.045359	0.032595	CDC73/LE
GO_BP_m:	GO:00107!	positive re	2/133	15/17913	0.005393	0.045359	0.032595	C3AR1/TH
GO_BP_m:	GO:00906!	t-circle for	2/133	15/17913	0.005393	0.045359	0.032595	NBN/WRN
GO_BP_m:	GO:00907:	telomere r	2/133	15/17913	0.005393	0.045359	0.032595	NBN/WRN
GO_BP_m:	GO:19029(	negative re	2/133	15/17913	0.005393	0.045359	0.032595	BIN1/CLU
GO_BP_m:	GO:00723	protein act	5/133	151/17913	0.005398	0.045359	0.032595	C3AR1/CF
GO_BP_m:	GO:19052(	negative re	3/133	48/17913	0.005424	0.04545	0.032661	DNMT1/PI
GO_BP_m:	GO:00326!	regulation	4/133	96/17913	0.005699	0.047621	0.03422	ARRB2/CC
GO_BP_m:	GO:00900(	regulation	9/133	442/17913	0.005764	0.048036	0.034519	ABI2/ACTN
GO_BP_m:	GO:19016!	cellular res	8/133	364/17913	0.005849	0.048562	0.034896	ACTN2/AD
GO_BP_m:	GO:00350!	cardiocyte	5/133	154/17913	0.00586	0.048562	0.034896	ACTN2/AF
GO_BP_m:	GO:00443{	protein loc	3/133	50/17913	0.006081	0.050114	0.036012	C2CD3/GC
GO_BP_m:	GO:20007:	regulation	3/133	50/17913	0.006081	0.050114	0.036012	ARRB2/CT
GO_BP_m:	GO:00326(	negative re	2/133	16/17913	0.006133	0.050272	0.036125	ARRB2/TH
GO_BP_m:	GO:00514{	positive re	2/133	16/17913	0.006133	0.050272	0.036125	C3AR1/S1I
GO_BP_m:	GO:00487:	cardiac mu	6/133	221/17913	0.006171	0.050443	0.036248	ACTN2/AF
GO_BP_m:	GO:00019!	positive re	3/133	51/17913	0.006426	0.051889	0.037287	CCR7/CDK
GO_BP_m:	GO:00062:	pyrimidine	3/133	51/17913	0.006426	0.051889	0.037287	NME3/NM
GO_BP_m:	GO:00350(	regulation	3/133	51/17913	0.006426	0.051889	0.037287	CHEK1/CT
GO_BP_m:	GO:20007	regulation	3/133	51/17913	0.006426	0.051889	0.037287	CDK6/PRK
GO_BP_m:	GO:00525	regulation	8/133	370/17913	0.006434	0.051889	0.037287	ARRB2/BIN
GO_BP_m:	GO:00482!	regulation	4/133	100/17913	0.006576	0.05289	0.038006	ARRB2/CLI
GO_BP_m:	GO:00550(	muscle cel	5/133	159/17913	0.006691	0.053161	0.038201	ACTN2/BIN
GO_BP_m:	GO:00507(	regulation	3/133	52/17913	0.006783	0.053161	0.038201	CCR7/GAS
GO_BP_m:	GO:00515(	histone H3	3/133	52/17913	0.006783	0.053161	0.038201	ASH2L/DN
GO_BP_m:	GO:00725:	pyrimidine	3/133	52/17913	0.006783	0.053161	0.038201	NME3/NM
GO_BP_m:	GO:00025(	antigen pr	4/133	101/17913	0.006808	0.053161	0.038201	SEC24A/SE
GO_BP_m:	GO:00486(	positive re	4/133	101/17913	0.006808	0.053161	0.038201	DNMT1/LE
GO_BP_m:	GO:19052(	positive re	4/133	101/17913	0.006808	0.053161	0.038201	DNMT1/PI
GO_BP_m:	GO:00063	regulation	2/133	17/17913	0.006917	0.053161	0.038201	CTCF/DNM
GO_BP_m:	GO:00439:	positive re	2/133	17/17913	0.006917	0.053161	0.038201	CCNT1/CT
GO_BP_m:	GO:00726	lamellipod	2/133	17/17913	0.006917	0.053161	0.038201	ABI1/WAS
GO_BP_m:	GO:00316:	receptor ir	4/133	102/17913	0.007046	0.053161	0.038201	ACKR3/AR
GO_BP_m:	GO:00326!	regulation	3/133	53/17913	0.007151	0.053161	0.038201	ARRB2/CC
GO_BP_m:	GO:00061	dADP pho	1/133	1/17913	0.007425	0.053161	0.038201	AK9
GO_BP_m:	GO:00061{	dGDP pho	1/133	1/17913	0.007425	0.053161	0.038201	AK9
GO_BP_m:	GO:00067!	AMP phos	1/133	1/17913	0.007425	0.053161	0.038201	AK9
GO_BP_m:	GO:00069	induction I	1/133	1/17913	0.007425	0.053161	0.038201	CD4
GO_BP_m:	GO:00219!	central ner	1/133	1/17913	0.007425	0.053161	0.038201	TCTN1
GO_BP_m:	GO:00353(	helper T ce	1/133	1/17913	0.007425	0.053161	0.038201	CD4
GO_BP_m:	GO:00381:	chemokine	1/133	1/17913	0.007425	0.053161	0.038201	CCR7
GO_BP_m:	GO:00605	regulation	1/133	1/17913	0.007425	0.053161	0.038201	WRN
GO_BP_m:	GO:00615(	CDP phos	1/133	1/17913	0.007425	0.053161	0.038201	AK9
GO_BP_m:	GO:00615(	dAMP pho	1/133	1/17913	0.007425	0.053161	0.038201	AK9
GO_BP_m:	GO:00615(	CMP phos	1/133	1/17913	0.007425	0.053161	0.038201	AK9
GO_BP_m:	GO:00615(	dCMP pho	1/133	1/17913	0.007425	0.053161	0.038201	AK9
GO_BP_m:	GO:00615(	GDP phos	1/133	1/17913	0.007425	0.053161	0.038201	AK9
GO_BP_m:	GO:00615(	UDP phos	1/133	1/17913	0.007425	0.053161	0.038201	AK9

GO_BP_m	GO:00615	dCDP pho	1/133	1/17913	0.007425	0.053161	0.038201	AK9
GO_BP_m	GO:00615	TDP phos	1/133	1/17913	0.007425	0.053161	0.038201	AK9
GO_BP_m	GO:00985	positive re	1/133	1/17913	0.007425	0.053161	0.038201	WRN
GO_BP_m	GO:19001	negative r	1/133	1/17913	0.007425	0.053161	0.038201	GAS6
GO_BP_m	GO:19016	negative r	1/133	1/17913	0.007425	0.053161	0.038201	SIN3A
GO_BP_m	GO:19028	regulation	1/133	1/17913	0.007425	0.053161	0.038201	CLU
GO_BP_m	GO:19029	positive re	1/133	1/17913	0.007425	0.053161	0.038201	CLU
GO_BP_m	GO:19035	regulation	1/133	1/17913	0.007425	0.053161	0.038201	VEGFA
GO_BP_m	GO:19035	positive re	1/133	1/17913	0.007425	0.053161	0.038201	VEGFA
GO_BP_m	GO:19036	regulation	1/133	1/17913	0.007425	0.053161	0.038201	NCK1
GO_BP_m	GO:19036	positive re	1/133	1/17913	0.007425	0.053161	0.038201	NCK1
GO_BP_m	GO:19041	DN3 thym	1/133	1/17913	0.007425	0.053161	0.038201	CCR6
GO_BP_m	GO:19058	regulation	1/133	1/17913	0.007425	0.053161	0.038201	CLU
GO_BP_m	GO:19058	negative r	1/133	1/17913	0.007425	0.053161	0.038201	CLU
GO_BP_m	GO:19058	regulation	1/133	1/17913	0.007425	0.053161	0.038201	CLU
GO_BP_m	GO:19058	negative r	1/133	1/17913	0.007425	0.053161	0.038201	CLU
GO_BP_m	GO:20005	positive re	1/133	1/17913	0.007425	0.053161	0.038201	CCR7
GO_BP_m	GO:20005	positive re	1/133	1/17913	0.007425	0.053161	0.038201	CCR7
GO_BP_m	GO:20005	negative r	1/133	1/17913	0.007425	0.053161	0.038201	GAS6
GO_BP_m	GO:20012	negative r	1/133	1/17913	0.007425	0.053161	0.038201	PRKDC
GO_BP_m	GO:00108	negative r	3/133	54/17913	0.007531	0.053796	0.038658	ARRB2/CL
GO_BP_m	GO:00070	cell cycle	6/133	231/1791	0.007604	0.054187	0.038939	BIN1/CDK
GO_BP_m	GO:00063	mitotic rec	2/133	18/17913	0.007744	0.054646	0.039269	BRCA2/RA
GO_BP_m	GO:00063	methylatio	2/133	18/17913	0.007744	0.054646	0.039269	DNMT1/D
GO_BP_m	GO:00609	coronary	2/133	18/17913	0.007744	0.054646	0.039269	SEC24B/V
GO_BP_m	GO:00439	regulation	6/133	232/1791	0.00776	0.054646	0.039269	CCNT1/C
GO_BP_m	GO:19040	positive re	6/133	232/1791	0.00776	0.054646	0.039269	C3AR1/CC
GO_BP_m	GO:00026	regulation	4/133	105/1791	0.007793	0.054751	0.039344	C3AR1/CC
GO_BP_m	GO:00435	protein ac	6/133	233/1791	0.007918	0.055279	0.039723	BRCA2/C
GO_BP_m	GO:00103	response t	3/133	55/17913	0.007923	0.055279	0.039723	BRCA2/PR
GO_BP_m	GO:00326	interleukin	3/133	55/17913	0.007923	0.055279	0.039723	ARRB2/CC
GO_BP_m	GO:00199	cyclic-nucl	5/133	166/1791	0.007988	0.055603	0.039956	ADCY3/A
GO_BP_m	GO:19019	negative r	6/133	235/1791	0.00824	0.057223	0.04112	CDC73/C
GO_BP_m	GO:00610	positive re	3/133	56/17913	0.008327	0.057693	0.041458	ABI1/DVL
GO_BP_m	GO:01400	mitotic nu	6/133	237/1791	0.008572	0.0582	0.041823	CHEK1/CT
GO_BP_m	GO:00326	interleukin	4/133	108/1791	0.00859	0.0582	0.041823	ARRB2/CC
GO_BP_m	GO:00064	protein m	5/133	169/1791	0.008594	0.0582	0.041823	ASH2L/CT
GO_BP_m	GO:00082	protein alk	5/133	169/1791	0.008594	0.0582	0.041823	ASH2L/CT
GO_BP_m	GO:00025	regulation	2/133	19/17913	0.008614	0.0582	0.041823	CCR7/THB
GO_BP_m	GO:00070	mitotic G2	2/133	19/17913	0.008614	0.0582	0.041823	CLSPN/N
GO_BP_m	GO:00310	negative r	2/133	19/17913	0.008614	0.0582	0.041823	DNMT1/P
GO_BP_m	GO:00450	thymic T c	2/133	19/17913	0.008614	0.0582	0.041823	CCR7/CD
GO_BP_m	GO:00516	nucleus lo	2/133	19/17913	0.008614	0.0582	0.041823	BIN1/PCM
GO_BP_m	GO:19052	vascular a	2/133	19/17913	0.008614	0.0582	0.041823	DNMT1/E
GO_BP_m	GO:19054	regulation	2/133	19/17913	0.008614	0.0582	0.041823	DNMT1/E
GO_BP_m	GO:00018	release of	3/133	57/17913	0.008743	0.058675	0.042164	ARRB2/CL
GO_BP_m	GO:00447	cell cycle	3/133	57/17913	0.008743	0.058675	0.042164	BRCA2/PC
GO_BP_m	GO:20007	regulation	3/133	57/17913	0.008743	0.058675	0.042164	CHEK1/CT
GO_BP_m	GO:00975	myeloid le	5/133	170/1791	0.008803	0.058951	0.042362	C3AR1/CC
GO_BP_m	GO:00349	histone lys	4/133	109/1791	0.008867	0.059111	0.042477	ASH2L/D
GO_BP_m	GO:00436	regulation	4/133	109/1791	0.008867	0.059111	0.042477	CHEK1/N
GO_BP_m	GO:00486	regulation	5/133	172/1791	0.009232	0.061409	0.044128	DNMT1/L
GO_BP_m	GO:00518	positive re	5/133	173/1791	0.009451	0.062524	0.04493	ARRB2/CC
GO_BP_m	GO:00719	regulation	9/133	479/1791	0.009522	0.062524	0.04493	CCNT1/C
GO_BP_m	GO:00023	lymphoid	2/133	20/17913	0.009525	0.062524	0.04493	PRKDC/SC
GO_BP_m	GO:00070	microtubu	2/133	20/17913	0.009525	0.062524	0.04493	TUBGCP4/
GO_BP_m	GO:00456	regulation	2/133	20/17913	0.009525	0.062524	0.04493	CD4/CDK
GO_BP_m	GO:19055	positive re	2/133	20/17913	0.009525	0.062524	0.04493	C3AR1/TH

GO_BP_m1	GO:000622	pyrimidine	3/133	59/17913	0.00961	0.062537	0.044939	NME3/NM
GO_BP_m1	GO:005129	centrosom	3/133	59/17913	0.00961	0.062537	0.044939	BRCA2/C2
GO_BP_m1	GO:007267	T cell migr	3/133	59/17913	0.00961	0.062537	0.044939	CCR6/CXC
GO_BP_m1	GO:009034	regulation	3/133	59/17913	0.00961	0.062537	0.044939	CDK6/PRK
GO_BP_m1	GO:006062	regulation	9/133	480/17913	0.009645	0.062627	0.045004	ACTN2/AF
GO_BP_m1	GO:004865	smooth m	5/133	174/17913	0.009674	0.062685	0.045045	DNMT1/LD
GO_BP_m1	GO:005254	regulation	8/133	398/17913	0.009772	0.063181	0.045402	ARRB2/BIN
GO_BP_m1	GO:000242	immune re	8/133	399/17913	0.009911	0.063944	0.04595	ABI1/ARPC
GO_BP_m1	GO:005181	modificatio	4/133	113/17913	0.010033	0.064223	0.04615	CCNT1/CD
GO_BP_m1	GO:007148	cellular res	4/133	113/17913	0.010033	0.064223	0.04615	CDC25A/C
GO_BP_m1	GO:000250	somatic di	3/133	60/17913	0.010062	0.064223	0.04615	CCR6/NBN
GO_BP_m1	GO:001644	somatic ce	3/133	60/17913	0.010062	0.064223	0.04615	CCR6/NBN
GO_BP_m1	GO:005070	interleukin	3/133	60/17913	0.010062	0.064223	0.04615	CCR7/GAS
GO_BP_m1	GO:000252	leukocyte	9/133	485/17913	0.010275	0.065305	0.046928	CCR6/CCR
GO_BP_m1	GO:004331	neutrophil	9/133	485/17913	0.010275	0.065305	0.046928	ALDOA/C3
GO_BP_m1	GO:007190	positive re	7/133	322/17913	0.010307	0.065372	0.046976	CCNT1/CC
GO_BP_m1	GO:004311	receptor m	5/133	177/17913	0.010365	0.065603	0.047142	ACKR3/AR
GO_BP_m1	GO:003193	regulation	2/133	21/17913	0.010476	0.066166	0.047547	DNMT1/SI
GO_BP_m1	GO:004343	response t	8/133	404/17913	0.01063	0.06695	0.04811	ACTN2/AD
GO_BP_m1	GO:004362	regulation	4/133	115/17913	0.010651	0.06695	0.04811	CHEK1/NC
GO_BP_m1	GO:000228	neutrophil	9/133	488/17913	0.010667	0.06695	0.04811	ALDOA/C3
GO_BP_m1	GO:000300	heart morp	6/133	249/17913	0.010767	0.067437	0.04846	C2CD3/DV
GO_BP_m1	GO:000175	somitogen	3/133	62/17913	0.011002	0.06876	0.049411	ABI1/PALB
GO_BP_m1	GO:004390	positive re	5/133	180/17913	0.011089	0.069165	0.049702	CCNT1/CD
GO_BP_m1	GO:000195	regulation	4/133	117/17913	0.011294	0.070296	0.050514	CCR7/CDK
GO_BP_m1	GO:190533	regulation	5/133	181/17913	0.011338	0.070424	0.050606	ARRB2/DV
GO_BP_m1	GO:003424	regulation	2/133	22/17913	0.011468	0.070938	0.050976	CDC73/LE
GO_BP_m1	GO:005092	positive re	2/133	22/17913	0.011468	0.070938	0.050976	S1PR1/VE
GO_BP_m1	GO:000863	apoptotic	4/133	119/17913	0.011961	0.073564	0.052863	ARRB2/CL
GO_BP_m1	GO:003100	regulation	3/133	64/17913	0.011991	0.073564	0.052863	CTCF/DNM
GO_BP_m1	GO:004612	purine ribo	3/133	64/17913	0.011991	0.073564	0.052863	NME3/NM
GO_BP_m1	GO:004678	regulation	3/133	64/17913	0.011991	0.073564	0.052863	CCNT1/CT
GO_BP_m1	GO:000915	purine ribo	9/133	500/17913	0.012352	0.075625	0.054344	ADCY3/AD
GO_BP_m1	GO:001075	regulation	2/133	23/17913	0.0125	0.075629	0.054347	C3AR1/TH
GO_BP_m1	GO:004481	mitotic G2	2/133	23/17913	0.0125	0.075629	0.054347	CLSPN/NB
GO_BP_m1	GO:005092	regulation	2/133	23/17913	0.0125	0.075629	0.054347	S1PR1/VE
GO_BP_m1	GO:005112	regulation	2/133	23/17913	0.0125	0.075629	0.054347	ABI2/WAS
GO_BP_m1	GO:200072	negative re	2/133	23/17913	0.0125	0.075629	0.054347	CTDP1/YY
GO_BP_m1	GO:000651	protein mo	3/133	65/17913	0.012504	0.075629	0.054347	CDC73/DD
GO_BP_m1	GO:007037	positive re	5/133	186/17913	0.01264	0.076297	0.054827	ACKR3/AR
GO_BP_m1	GO:000914	nucleoside	7/133	337/17913	0.012988	0.078022	0.056066	AK9/ALDC
GO_BP_m1	GO:001802	peptidyl-ly	4/133	122/17913	0.013009	0.078022	0.056066	ASH2L/DN
GO_BP_m1	GO:005114	negative re	3/133	66/17913	0.01303	0.078022	0.056066	CTDP1/DN
GO_BP_m1	GO:190520	regulation	3/133	66/17913	0.01303	0.078022	0.056066	ARRB2/CT
GO_BP_m1	GO:003010	regulation	6/133	262/17913	0.013567	0.078976	0.056752	ARRB2/BIN
GO_BP_m1	GO:000220	somatic di	3/133	67/17913	0.013568	0.078976	0.056752	CCR6/NBN
GO_BP_m1	GO:004227	purine nuc	3/133	67/17913	0.013568	0.078976	0.056752	NME3/NM
GO_BP_m1	GO:005091	positive ch	3/133	67/17913	0.013568	0.078976	0.056752	HGF/S1PR
GO_BP_m1	GO:000209	positive re	2/133	24/17913	0.013571	0.078976	0.056752	ARRB2/VE
GO_BP_m1	GO:003195	positive re	2/133	24/17913	0.013571	0.078976	0.056752	NBN/VEGF
GO_BP_m1	GO:009002	positive re	2/133	24/17913	0.013571	0.078976	0.056752	C3AR1/CC
GO_BP_m1	GO:190302	positive re	2/133	24/17913	0.013571	0.078976	0.056752	CCR7/RAB
GO_BP_m1	GO:200102	regulation	2/133	24/17913	0.013571	0.078976	0.056752	THBS1/VE
GO_BP_m1	GO:200013	negative re	4/133	124/17913	0.013739	0.078976	0.056752	CDC73/CD
GO_BP_m1	GO:005067	epithelial c	8/133	425/17913	0.014077	0.078976	0.056752	BRCA2/CC
GO_BP_m1	GO:190134	regulation	8/133	425/17913	0.014077	0.078976	0.056752	C3AR1/CC
GO_BP_m1	GO:200125	negative re	4/133	125/17913	0.014113	0.078976	0.056752	DNMT1/N
GO_BP_m1	GO:005160	localizatio	4/133	126/17913	0.014494	0.078976	0.056752	RAB8A/SE

GO_BP_m	GO:00427	fibrinolysis	2/133	25/17913	0.01468	0.078976	0.056752	PROS1/TH
GO_BP_m	GO:00481	regulation	2/133	25/17913	0.01468	0.078976	0.056752	RAB11A/R
GO_BP_m	GO:00610	regulation	2/133	25/17913	0.01468	0.078976	0.056752	CTDP1/YY
GO_BP_m	GO:00715	genetic im	2/133	25/17913	0.01468	0.078976	0.056752	CTCF/DN
GO_BP_m	GO:00020	desensitiza	1/133	2/17913	0.014795	0.078976	0.056752	ARRB2
GO_BP_m	GO:00031	coronary v	1/133	2/17913	0.014795	0.078976	0.056752	VEGFA
GO_BP_m	GO:00083	olfactory l	1/133	2/17913	0.014795	0.078976	0.056752	ADCY3
GO_BP_m	GO:00092	purine dec	1/133	2/17913	0.014795	0.078976	0.056752	AK9
GO_BP_m	GO:00190	early viral	1/133	2/17913	0.014795	0.078976	0.056752	CCNT2
GO_BP_m	GO:00219	neural plat	1/133	2/17913	0.014795	0.078976	0.056752	C2CD3
GO_BP_m	GO:00346	response t	1/133	2/17913	0.014795	0.078976	0.056752	YY1
GO_BP_m	GO:00354	histone H	1/133	2/17913	0.014795	0.078976	0.056752	CHEK1
GO_BP_m	GO:00381	VEGF-acti	1/133	2/17913	0.014795	0.078976	0.056752	VEGFA
GO_BP_m	GO:00400	regulation	1/133	2/17913	0.014795	0.078976	0.056752	CTCF
GO_BP_m	GO:00439	histone H	1/133	2/17913	0.014795	0.078976	0.056752	SIN3A
GO_BP_m	GO:00482	Golgi vesic	1/133	2/17913	0.014795	0.078976	0.056752	RAB8A
GO_BP_m	GO:00515	meiotic re	1/133	2/17913	0.014795	0.078976	0.056752	RAD1
GO_BP_m	GO:00516	actin filam	1/133	2/17913	0.014795	0.078976	0.056752	ACTN2
GO_BP_m	GO:00617	chaperone	1/133	2/17913	0.014795	0.078976	0.056752	CLU
GO_BP_m	GO:00709	regulation	1/133	2/17913	0.014795	0.078976	0.056752	CXCL6
GO_BP_m	GO:00709	regulation	1/133	2/17913	0.014795	0.078976	0.056752	CXCL6
GO_BP_m	GO:00713	cellular res	1/133	2/17913	0.014795	0.078976	0.056752	GAS6
GO_BP_m	GO:00860	phospholi	1/133	2/17913	0.014795	0.078976	0.056752	ACTN2
GO_BP_m	GO:00970	renal albu	1/133	2/17913	0.014795	0.078976	0.056752	GAS6
GO_BP_m	GO:00970	lymphocyt	1/133	2/17913	0.014795	0.078976	0.056752	CCR7
GO_BP_m	GO:00971	endoplasm	1/133	2/17913	0.014795	0.078976	0.056752	STX17
GO_BP_m	GO:00972	hematopo	1/133	2/17913	0.014795	0.078976	0.056752	GAS6
GO_BP_m	GO:00976	double-st	1/133	2/17913	0.014795	0.078976	0.056752	PRKDC
GO_BP_m	GO:00989	dendritic t	1/133	2/17913	0.014795	0.078976	0.056752	WASF1
GO_BP_m	GO:19001	regulation	1/133	2/17913	0.014795	0.078976	0.056752	GAS6
GO_BP_m	GO:19013	regulation	1/133	2/17913	0.014795	0.078976	0.056752	SEC24B
GO_BP_m	GO:19016	regulation	1/133	2/17913	0.014795	0.078976	0.056752	SIN3A
GO_BP_m	GO:19023	positive re	1/133	2/17913	0.014795	0.078976	0.056752	VEGFA
GO_BP_m	GO:19029	negative r	1/133	2/17913	0.014795	0.078976	0.056752	BIN1
GO_BP_m	GO:19029	regulation	1/133	2/17913	0.014795	0.078976	0.056752	CLU
GO_BP_m	GO:19039	negative r	1/133	2/17913	0.014795	0.078976	0.056752	BIN1
GO_BP_m	GO:19041	DN2 thym	1/133	2/17913	0.014795	0.078976	0.056752	CCR6
GO_BP_m	GO:19045	response t	1/133	2/17913	0.014795	0.078976	0.056752	CLU
GO_BP_m	GO:19045	cellular res	1/133	2/17913	0.014795	0.078976	0.056752	CLU
GO_BP_m	GO:19055	positive re	1/133	2/17913	0.014795	0.078976	0.056752	LDLRAP1
GO_BP_m	GO:19056	positive re	1/133	2/17913	0.014795	0.078976	0.056752	LDLRAP1
GO_BP_m	GO:19057	protein loc	1/133	2/17913	0.014795	0.078976	0.056752	GOLGB1
GO_BP_m	GO:19059	negative r	1/133	2/17913	0.014795	0.078976	0.056752	DNMT1
GO_BP_m	GO:19059	negative r	1/133	2/17913	0.014795	0.078976	0.056752	DNMT1
GO_BP_m	GO:20005	positive re	1/133	2/17913	0.014795	0.078976	0.056752	CCR7
GO_BP_m	GO:20005	regulation	1/133	2/17913	0.014795	0.078976	0.056752	GAS6
GO_BP_m	GO:20012	regulation	1/133	2/17913	0.014795	0.078976	0.056752	PRKDC
GO_BP_m	GO:00069	compleme	4/133	128/17913	0.015276	0.081398	0.058492	C3AR1/CF
GO_BP_m	GO:00432	regulation	5/133	196/17913	0.015539	0.082656	0.059397	ARRB2/GA
GO_BP_m	GO:00716	positive re	2/133	26/17913	0.015827	0.083691	0.06014	C3AR1/CC
GO_BP_m	GO:00063	DNA alkyl	3/133	71/17913	0.015846	0.083691	0.06014	CTCF/DN
GO_BP_m	GO:00063	DNA meth	3/133	71/17913	0.015846	0.083691	0.06014	CTCF/DN
GO_BP_m	GO:19019	regulation	3/133	71/17913	0.015846	0.083691	0.06014	CHEK1/CT
GO_BP_m	GO:19028	negative r	4/133	130/17913	0.016083	0.084797	0.060935	CDC73/CD
GO_BP_m	GO:20007	regulation	3/133	72/17913	0.016447	0.086561	0.062202	CHEK1/PR
GO_BP_m	GO:00067	NADH reg	2/133	27/17913	0.017011	0.088242	0.063411	ALDOA/P
GO_BP_m	GO:00331	negative r	2/133	27/17913	0.017011	0.088242	0.063411	HGF/NCK1
GO_BP_m	GO:00439	modulatio	2/133	27/17913	0.017011	0.088242	0.063411	CCNT1/CT

GO_BP_m	GO:00524	modulatio	2/133	27/17913	0.017011	0.088242	0.063411	CCNT1/CT
GO_BP_m	GO:00616	canonical	2/133	27/17913	0.017011	0.088242	0.063411	ALDOA/PK
GO_BP_m	GO:00617	glucose ca	2/133	27/17913	0.017011	0.088242	0.063411	ALDOA/PK
GO_BP_m	GO:19026	positive re	2/133	27/17913	0.017011	0.088242	0.063411	C3AR1/CC
GO_BP_m	GO:19052	negative r	2/133	27/17913	0.017011	0.088242	0.063411	CTDP1/YY
GO_BP_m	GO:00724	signal tran	3/133	73/17913	0.01706	0.088242	0.063411	CHEK1/E2
GO_BP_m	GO:00724	signal tran	3/133	73/17913	0.01706	0.088242	0.063411	CHEK1/E2
GO_BP_m	GO:00434	macromol	6/133	276/17913	0.017114	0.088367	0.0635	ASH2L/CT
GO_BP_m	GO:20010	regulation	5/133	201/17913	0.017143	0.088367	0.0635	ACKR3/CH
GO_BP_m	GO:00421	T cell activ	8/133	443/17913	0.017628	0.090712	0.065185	CCR6/CCR
GO_BP_m	GO:00723	signal tran	3/133	74/17913	0.017687	0.090856	0.065289	CHEK1/E2
GO_BP_m	GO:00426	muscle cel	7/133	359/17913	0.01778	0.091181	0.065522	ACTN2/AR
GO_BP_m	GO:00436	post-trans	7/133	360/17913	0.018024	0.092076	0.066165	DDB1/GAS
GO_BP_m	GO:00507	regulation	5/133	204/17913	0.018157	0.092076	0.066165	CCNT1/CD
GO_BP_m	GO:00029	regulation	4/133	135/17913	0.018218	0.092076	0.066165	C3AR1/CC
GO_BP_m	GO:00457	positive re	2/133	28/17913	0.018231	0.092076	0.066165	CCNT1/CC
GO_BP_m	GO:00506	positive re	2/133	28/17913	0.018231	0.092076	0.066165	CDC73/LE
GO_BP_m	GO:00523	modulatio	2/133	28/17913	0.018231	0.092076	0.066165	CCNT1/CT
GO_BP_m	GO:00616	glycolytic	2/133	28/17913	0.018231	0.092076	0.066165	ALDOA/PK
GO_BP_m	GO:00616	glycolytic	2/133	28/17913	0.018231	0.092076	0.066165	ALDOA/PK
GO_BP_m	GO:00850	extracellul	2/133	28/17913	0.018231	0.092076	0.066165	GAS6/QSC
GO_BP_m	GO:00018	positive re	8/133	446/17913	0.018278	0.092089	0.066174	C3AR1/CC
GO_BP_m	GO:00311	animal org	3/133	75/17913	0.018326	0.092089	0.066174	GAS6/HGF
GO_BP_m	GO:00723	intrinsic ap	3/133	75/17913	0.018326	0.092089	0.066174	BRCA2/E2
GO_BP_m	GO:00356	cellular res	7/133	362/17913	0.018519	0.092904	0.066761	ADCY3/AD
GO_BP_m	GO:00068	retrograde	3/133	76/17913	0.018977	0.095045	0.068299	COG3/RAB
GO_BP_m	GO:00102	endomem	7/133	365/17913	0.019279	0.096395	0.069269	BIN1/CLU/
GO_BP_m	GO:00000	regulation	2/133	29/17913	0.019487	0.097275	0.069902	E2F1/ORC
GO_BP_m	GO:00518	modificati	3/133	77/17913	0.019642	0.097721	0.070222	CCNT1/CT
GO_BP_m	GO:00903	cellular ser	3/133	77/17913	0.019642	0.097721	0.070222	CDK6/PRK
GO_BP_m	GO:00024	immune re	4/133	139/17913	0.020046	0.099003	0.071143	ABI1/ARP
GO_BP_m	GO:00326	regulation	4/133	139/17913	0.020046	0.099003	0.071143	ARRB2/GA
GO_BP_m	GO:00380	Fc-gamma	4/133	139/17913	0.020046	0.099003	0.071143	ABI1/ARP
GO_BP_m	GO:00327	positive re	3/133	78/17913	0.020319	0.099003	0.071143	CLU/ORM
GO_BP_m	GO:00506	regulation	7/133	369/17913	0.020326	0.099003	0.071143	BRCA2/CC
GO_BP_m	GO:00346	response t	2/133	30/17913	0.020779	0.099003	0.071143	CCR7/YY1
GO_BP_m	GO:00900	regulation	2/133	30/17913	0.020779	0.099003	0.071143	C3AR1/CC
GO_BP_m	GO:00380	Fc-gamma	4/133	141/17913	0.021002	0.099003	0.071143	ABI1/ARP
GO_BP_m	GO:00024	Fc recepto	4/133	142/17913	0.021489	0.099003	0.071143	ABI1/ARP
GO_BP_m	GO:00031	outflow tra	3/133	80/17913	0.021712	0.099003	0.071143	DVL2/SEC
GO_BP_m	GO:00071	cell-matrix	5/133	214/17913	0.021818	0.099003	0.071143	ACTN2/CC
GO_BP_m	GO:00199	cAMP-me	4/133	143/17913	0.021984	0.099003	0.071143	ADCY3/AD
GO_BP_m	GO:19036	regulation	4/133	143/17913	0.021984	0.099003	0.071143	E2F2/PKM
GO_BP_m	GO:00070	centriole r	2/133	31/17913	0.022106	0.099003	0.071143	C2CD3/CE
GO_BP_m	GO:00166	Rac protei	2/133	31/17913	0.022106	0.099003	0.071143	ABI2/WAS
GO_BP_m	GO:00357	endothelia	2/133	31/17913	0.022106	0.099003	0.071143	THBS1/VE
GO_BP_m	GO:19013	negative r	2/133	31/17913	0.022106	0.099003	0.071143	ACTN2/BI
GO_BP_m	GO:19022	negative r	2/133	31/17913	0.022106	0.099003	0.071143	ACKR3/CL
GO_BP_m	GO:00007	DNA recor	1/133	3/17913	0.022111	0.099003	0.071143	RAD51
GO_BP_m	GO:00021	cap-deper	1/133	3/17913	0.022111	0.099003	0.071143	NCK1
GO_BP_m	GO:00024	immune c	1/133	3/17913	0.022111	0.099003	0.071143	CLU
GO_BP_m	GO:00025	negative r	1/133	3/17913	0.022111	0.099003	0.071143	THBS1
GO_BP_m	GO:00032	cardiac mu	1/133	3/17913	0.022111	0.099003	0.071143	S1PR1
GO_BP_m	GO:00107	negative r	1/133	3/17913	0.022111	0.099003	0.071143	THBS1
GO_BP_m	GO:00107	negative r	1/133	3/17913	0.022111	0.099003	0.071143	THBS1
GO_BP_m	GO:00107	regulation	1/133	3/17913	0.022111	0.099003	0.071143	CHEK1
GO_BP_m	GO:00107	regulation	1/133	3/17913	0.022111	0.099003	0.071143	RAB11A
GO_BP_m	GO:00140	neural plat	1/133	3/17913	0.022111	0.099003	0.071143	DVL2

GO_BP_m[illegible]	GO:00217[illegible]	cochlear n	1/133	3/17913	0.022111	0.099003	0.071143	SEC24B
GO_BP_m[illegible]	GO:00220[illegible]	convergen	1/133	3/17913	0.022111	0.099003	0.071143	DVL2
GO_BP_m[illegible]	GO:00323[illegible]	positive re	1/133	3/17913	0.022111	0.099003	0.071143	LDLRAP1
GO_BP_m[illegible]	GO:00323[illegible]	positive re	1/133	3/17913	0.022111	0.099003	0.071143	LDLRAP1
GO_BP_m[illegible]	GO:00323[illegible]	positive re	1/133	3/17913	0.022111	0.099003	0.071143	LDLRAP1
GO_BP_m[illegible]	GO:00364[illegible]	positive re	1/133	3/17913	0.022111	0.099003	0.071143	NCK1
GO_BP_m[illegible]	GO:00380[illegible]	VEGF-acti	1/133	3/17913	0.022111	0.099003	0.071143	VEGFA
GO_BP_m[illegible]	GO:00380[illegible]	positive re	1/133	3/17913	0.022111	0.099003	0.071143	VEGFA
GO_BP_m[illegible]	GO:00421[illegible]	strand inva	1/133	3/17913	0.022111	0.099003	0.071143	WRN
GO_BP_m[illegible]	GO:00426[illegible]	follicle-stir	1/133	3/17913	0.022111	0.099003	0.071143	ARRB2
GO_BP_m[illegible]	GO:00450[illegible]	double-str	1/133	3/17913	0.022111	0.099003	0.071143	RAD51
GO_BP_m[illegible]	GO:00460[illegible]	dADP met	1/133	3/17913	0.022111	0.099003	0.071143	AK9
GO_BP_m[illegible]	GO:00460[illegible]	dGDP met	1/133	3/17913	0.022111	0.099003	0.071143	AK9
GO_BP_m[illegible]	GO:00515[illegible]	response t	1/133	3/17913	0.022111	0.099003	0.071143	SIN3A
GO_BP_m[illegible]	GO:00604[illegible]	positive re	1/133	3/17913	0.022111	0.099003	0.071143	CCR6
GO_BP_m[illegible]	GO:00609[illegible]	cardiac va	1/133	3/17913	0.022111	0.099003	0.071143	VEGFA
GO_BP_m[illegible]	GO:00617[illegible]	protein tar	1/133	3/17913	0.022111	0.099003	0.071143	CLU
GO_BP_m[illegible]	GO:00709[illegible]	regulation	1/133	3/17913	0.022111	0.099003	0.071143	CXCL6
GO_BP_m[illegible]	GO:00719[illegible]	negative re	1/133	3/17913	0.022111	0.099003	0.071143	E2F1
GO_BP_m[illegible]	GO:00897[illegible]	protein kir	1/133	3/17913	0.022111	0.099003	0.071143	VEGFA
GO_BP_m[illegible]	GO:00907[illegible]	DNA repai	1/133	3/17913	0.022111	0.099003	0.071143	RAD51
GO_BP_m[illegible]	GO:00970[illegible]	renal prote	1/133	3/17913	0.022111	0.099003	0.071143	GAS6
GO_BP_m[illegible]	GO:00975[illegible]	stress resp	1/133	3/17913	0.022111	0.099003	0.071143	VEGFA
GO_BP_m[illegible]	GO:00975[illegible]	cellular str	1/133	3/17913	0.022111	0.099003	0.071143	VEGFA
GO_BP_m[illegible]	GO:01061[illegible]	negative re	1/133	3/17913	0.022111	0.099003	0.071143	BIN1
GO_BP_m[illegible]	GO:19029[illegible]	neurofibril	1/133	3/17913	0.022111	0.099003	0.071143	CLU
GO_BP_m[illegible]	GO:19036[illegible]	regulation	1/133	3/17913	0.022111	0.099003	0.071143	NCK1
GO_BP_m[illegible]	GO:19036[illegible]	positive re	1/133	3/17913	0.022111	0.099003	0.071143	NCK1
GO_BP_m[illegible]	GO:19045[illegible]	response t	1/133	3/17913	0.022111	0.099003	0.071143	CLU
GO_BP_m[illegible]	GO:19045[illegible]	cellular res	1/133	3/17913	0.022111	0.099003	0.071143	CLU
GO_BP_m[illegible]	GO:19046[illegible]	midbrain r	1/133	3/17913	0.022111	0.099003	0.071143	SOS1
GO_BP_m[illegible]	GO:19048[illegible]	negative re	1/133	3/17913	0.022111	0.099003	0.071143	BIN1
GO_BP_m[illegible]	GO:19052[illegible]	negative re	1/133	3/17913	0.022111	0.099003	0.071143	BIN1
GO_BP_m[illegible]	GO:19053[illegible]	mesenchyr	1/133	3/17913	0.022111	0.099003	0.071143	ACKR3
GO_BP_m[illegible]	GO:19053[illegible]	regulation	1/133	3/17913	0.022111	0.099003	0.071143	ACKR3
GO_BP_m[illegible]	GO:19053[illegible]	positive re	1/133	3/17913	0.022111	0.099003	0.071143	ACKR3
GO_BP_m[illegible]	GO:20005[illegible]	regulation	1/133	3/17913	0.022111	0.099003	0.071143	CCR7
GO_BP_m[illegible]	GO:20005[illegible]	regulation	1/133	3/17913	0.022111	0.099003	0.071143	CCR7
GO_BP_m[illegible]	GO:20005[illegible]	regulation	1/133	3/17913	0.022111	0.099003	0.071143	CCR7
GO_BP_m[illegible]	GO:00147[illegible]	striated m	7/133	376/1791[illegible]	0.022253	0.099493	0.071496	ACTN2/AR
GO_BP_m[illegible]	GO:00063[illegible]	double-str	3/133	81/17913	0.022428	0.09953	0.071522	MDC1/NB
GO_BP_m[illegible]	GO:00070[illegible]	cell-substr	3/133	81/17913	0.022428	0.09953	0.071522	ACTN2/TH
GO_BP_m[illegible]	GO:00480[illegible]	focal adhe	3/133	81/17913	0.022428	0.09953	0.071522	ACTN2/TH
GO_BP_m[illegible]	GO:00610[illegible]	somite dev	3/133	81/17913	0.022428	0.09953	0.071522	ABI1/PALB
GO_BP_m[illegible]	GO:19035[illegible]	positive re	3/133	81/17913	0.022428	0.09953	0.071522	CLU/ORM
GO_BP_m[illegible]	GO:00351[illegible]	tube form	4/133	144/1791[illegible]	0.022486	0.099643	0.071603	DVL2/SEC
GO_BP_m[illegible]	GO:00485[illegible]	developm	5/133	216/1791[illegible]	0.022603	0.100015	0.071871	CTDP1/RA
GO_BP_m[illegible]	GO:00550[illegible]	striated m	4/133	145/1791[illegible]	0.022995	0.101598	0.073008	ACTN2/CT
GO_BP_m[illegible]	GO:00326[illegible]	regulation	3/133	82/17913	0.023156	0.102158	0.07341	ARRB2/CC
GO_BP_m[illegible]	GO:00550[illegible]	negative re	2/133	32/17913	0.023466	0.102923	0.07396	CTDP1/YY
GO_BP_m[illegible]	GO:00611[illegible]	negative re	2/133	32/17913	0.023466	0.102923	0.07396	CTDP1/YY
GO_BP_m[illegible]	GO:00613[illegible]	heart trab	2/133	32/17913	0.023466	0.102923	0.07396	S1PR1/SO
GO_BP_m[illegible]	GO:19040[illegible]	positive re	2/133	32/17913	0.023466	0.102923	0.07396	CCNT1/CC
GO_BP_m[illegible]	GO:00000[illegible]	regulation	3/133	83/17913	0.023897	0.104506	0.075097	CCNT1/CC
GO_BP_m[illegible]	GO:00063[illegible]	chromatin	3/133	83/17913	0.023897	0.104506	0.075097	DNMT1/D
GO_BP_m[illegible]	GO:00198[illegible]	antigen pr	5/133	220/1791[illegible]	0.02423	0.105806	0.076032	CCR7/SEC
GO_BP_m[illegible]	GO:00457[illegible]	regulation	7/133	383/1791[illegible]	0.024304	0.105977	0.076155	C3AR1/CC
GO_BP_m[illegible]	GO:00488[illegible]	multicellul	8/133	472/1791[illegible]	0.024645	0.107177	0.077017	ADCY3/AD

GO_BP_m:	GO:00001:	regulation	3/133	84/17913	0.024651	0.107177	0.077017	CHEK1/RA
GO_BP_m:	GO:00480:	neurotropl	2/133	33/17913	0.024861	0.107777	0.077448	SOS1/WAS
GO_BP_m:	GO:00972	amyloid-b	2/133	33/17913	0.024861	0.107777	0.077448	CLU/RAB1
GO_BP_m:	GO:00068	receptor-r	6/133	301/17913	0.02495	0.10801	0.077616	ACKR3/AR
GO_BP_m:	GO:20001:	regulation	5/133	222/17913	0.02507	0.108345	0.077857	ARRB2/GA
GO_BP_m:	GO:00326:	interleukin	4/133	149/17913	0.0251	0.108345	0.077857	ARRB2/GA
GO_BP_m:	GO:00705	response t	3/133	85/17913	0.025417	0.109558	0.078728	CCR7/E2F:
GO_BP_m:	GO:20012:	negative re	5/133	223/17913	0.025498	0.109746	0.078863	ACKR3/AR
GO_BP_m:	GO:19030	meiotic ce	4/133	151/17913	0.026195	0.112272	0.080678	BRCA2/RA
GO_BP_m:	GO:00331:	positive re	3/133	86/17913	0.026197	0.112272	0.080678	ARRB2/GA
GO_BP_m:	GO:00610	regulation	3/133	86/17913	0.026197	0.112272	0.080678	ABI1/DVL2
GO_BP_m:	GO:00607	cell fate cc	2/133	34/17913	0.026288	0.112342	0.080728	CDC73/LE
GO_BP_m:	GO:19026:	regulation	2/133	34/17913	0.026288	0.112342	0.080728	C3AR1/CC
GO_BP_m:	GO:19040:	regulation	3/133	87/17913	0.026989	0.115036	0.082664	CCNT1/CC
GO_BP_m:	GO:00605:	muscle tiss	7/133	392/17913	0.027128	0.115036	0.082664	ACTN2/AR
GO_BP_m:	GO:00094:	response t	6/133	307/17913	0.027137	0.115036	0.082664	BRCA2/CD
GO_BP_m:	GO:00069	phagocytc	6/133	308/17913	0.027513	0.115036	0.082664	ABI1/ARP(
GO_BP_m:	GO:00482	macropha	2/133	35/17913	0.027748	0.115036	0.082664	C3AR1/TH
GO_BP_m:	GO:00985:	centriole a	2/133	35/17913	0.027748	0.115036	0.082664	C2CD3/CE
GO_BP_m:	GO:00310	positive re	3/133	88/17913	0.027794	0.115036	0.082664	DNMT1/PI
GO_BP_m:	GO:19040:	regulation	3/133	88/17913	0.027794	0.115036	0.082664	ARRB2/GA
GO_BP_m:	GO:00219:	neural tub	4/133	155/17913	0.028469	0.115036	0.082664	C2CD3/DV
GO_BP_m:	GO:00007:	non-recor	3/133	89/17913	0.028611	0.115036	0.082664	MDC1/NB
GO_BP_m:	GO:00091:	ribonuclec	3/133	89/17913	0.028611	0.115036	0.082664	NME3/NM
GO_BP_m:	GO:00311:	mRNA 3'-(	3/133	89/17913	0.028611	0.115036	0.082664	CCNT1/CD
GO_BP_m:	GO:00447:	DNA meth	3/133	89/17913	0.028611	0.115036	0.082664	CTCF/DNN
GO_BP_m:	GO:00512	protein ho	3/133	89/17913	0.028611	0.115036	0.082664	ACTN2/AL
GO_BP_m:	GO:20012:	regulation	7/133	397/17913	0.028789	0.115036	0.082664	ACKR3/AR
GO_BP_m:	GO:00060	glucose ca	2/133	36/17913	0.029239	0.115036	0.082664	ALDOA/PK
GO_BP_m:	GO:00067:	NADH me	2/133	36/17913	0.029239	0.115036	0.082664	ALDOA/PK
GO_BP_m:	GO:00081	negative re	2/133	36/17913	0.029239	0.115036	0.082664	BRCA2/TT
GO_BP_m:	GO:00988	regulation	2/133	36/17913	0.029239	0.115036	0.082664	AVPR2/GA
GO_BP_m:	GO:19055:	regulation	2/133	36/17913	0.029239	0.115036	0.082664	C3AR1/TH
GO_BP_m:	GO:00024	myeloid de	1/133	4/17913	0.029372	0.115036	0.082664	CCR7
GO_BP_m:	GO:00026	negative re	1/133	4/17913	0.029372	0.115036	0.082664	THBS1
GO_BP_m:	GO:00031	positive re	1/133	4/17913	0.029372	0.115036	0.082664	GAS6
GO_BP_m:	GO:00070	mitotic chr	1/133	4/17913	0.029372	0.115036	0.082664	DLGAP5
GO_BP_m:	GO:00072	vitellogene	1/133	4/17913	0.029372	0.115036	0.082664	SOS1
GO_BP_m:	GO:00091	purine dec	1/133	4/17913	0.029372	0.115036	0.082664	AK9
GO_BP_m:	GO:00091	deoxyribo	1/133	4/17913	0.029372	0.115036	0.082664	AK9
GO_BP_m:	GO:00107	regulation	1/133	4/17913	0.029372	0.115036	0.082664	THBS1
GO_BP_m:	GO:00107	negative re	1/133	4/17913	0.029372	0.115036	0.082664	THBS1
GO_BP_m:	GO:00159:	plasma me	1/133	4/17913	0.029372	0.115036	0.082664	THBS1
GO_BP_m:	GO:00162	autophagc	1/133	4/17913	0.029372	0.115036	0.082664	STX17
GO_BP_m:	GO:00190	late viral tr	1/133	4/17913	0.029372	0.115036	0.082664	CCNT2
GO_BP_m:	GO:00300:	microspike	1/133	4/17913	0.029372	0.115036	0.082664	ACTN2
GO_BP_m:	GO:00318	telomeric :	1/133	4/17913	0.029372	0.115036	0.082664	NBN
GO_BP_m:	GO:00325	response t	1/133	4/17913	0.029372	0.115036	0.082664	GAS6
GO_BP_m:	GO:00333:	meiotic ce	1/133	4/17913	0.029372	0.115036	0.082664	RAD1
GO_BP_m:	GO:00333:	mitotic DN	1/133	4/17913	0.029372	0.115036	0.082664	CLSPN
GO_BP_m:	GO:00336	negative re	1/133	4/17913	0.029372	0.115036	0.082664	BRCA2
GO_BP_m:	GO:00358:	negative re	1/133	4/17913	0.029372	0.115036	0.082664	AVPR2
GO_BP_m:	GO:00359	COPI-coat	1/133	4/17913	0.029372	0.115036	0.082664	TMED10
GO_BP_m:	GO:00381:	chemokine	1/133	4/17913	0.029372	0.115036	0.082664	CCR7
GO_BP_m:	GO:00381	neuropilin	1/133	4/17913	0.029372	0.115036	0.082664	VEGFA
GO_BP_m:	GO:00467:	positive re	1/133	4/17913	0.029372	0.115036	0.082664	DDB1
GO_BP_m:	GO:00482	Golgi trans	1/133	4/17913	0.029372	0.115036	0.082664	TMED10
GO_BP_m:	GO:00482	COPI coati	1/133	4/17913	0.029372	0.115036	0.082664	TMED10

GO_BP_m	GO:00603	primitive e	1/133	4/17913	0.029372	0.115036	0.082664	VEGFA
GO_BP_m	GO:00606	regulation	1/133	4/17913	0.029372	0.115036	0.082664	HGF
GO_BP_m	GO:00609	lipid tube	1/133	4/17913	0.029372	0.115036	0.082664	BIN1
GO_BP_m	GO:00610	negative r	1/133	4/17913	0.029372	0.115036	0.082664	PHF1
GO_BP_m	GO:00706	regulation	1/133	4/17913	0.029372	0.115036	0.082664	CTCF
GO_BP_m	GO:00708	phosphory	1/133	4/17913	0.029372	0.115036	0.082664	CDK12
GO_BP_m	GO:00712	protein tar	1/133	4/17913	0.029372	0.115036	0.082664	CLU
GO_BP_m	GO:00727	cellular res	1/133	4/17913	0.029372	0.115036	0.082664	RAD51
GO_BP_m	GO:00902	positive re	1/133	4/17913	0.029372	0.115036	0.082664	CLU
GO_BP_m	GO:00970	lymphocyt	1/133	4/17913	0.029372	0.115036	0.082664	CCR7
GO_BP_m	GO:00970	dendritic c	1/133	4/17913	0.029372	0.115036	0.082664	CCR7
GO_BP_m	GO:00970	mature co	1/133	4/17913	0.029372	0.115036	0.082664	CCR7
GO_BP_m	GO:00972	oligodend	1/133	4/17913	0.029372	0.115036	0.082664	GAS6
GO_BP_m	GO:19000	positive re	1/133	4/17913	0.029372	0.115036	0.082664	VEGFA
GO_BP_m	GO:19002	negative r	1/133	4/17913	0.029372	0.115036	0.082664	DNMT1
GO_BP_m	GO:19039	negative r	1/133	4/17913	0.029372	0.115036	0.082664	NCK1
GO_BP_m	GO:19046	positive re	1/133	4/17913	0.029372	0.115036	0.082664	NCK1
GO_BP_m	GO:19059	positive re	1/133	4/17913	0.029372	0.115036	0.082664	CLU
GO_BP_m	GO:19904	negative r	1/133	4/17913	0.029372	0.115036	0.082664	NCK1
GO_BP_m	GO:00343	adherens j	3/133	90/17913	0.029441	0.115155	0.08275	ACTN2/TH
GO_BP_m	GO:00973	response t	5/133	232/17913	0.029555	0.115447	0.08296	ADCY3/AD
GO_BP_m	GO:00008	sister chro	4/133	157/17913	0.02965	0.115668	0.083119	CTCF/DLG
GO_BP_m	GO:00092	purine ribo	6/133	314/17913	0.029844	0.116276	0.083556	AK9/ALDC
GO_BP_m	GO:19016	response t	8/133	491/17913	0.030188	0.117401	0.084364	ACTN2/AD
GO_BP_m	GO:00321	positive re	6/133	315/17913	0.030245	0.117401	0.084364	C3AR1/CC
GO_BP_m	GO:00022	hematopo	4/133	158/17913	0.03025	0.117401	0.084364	CDK6/PRK
GO_BP_m	GO:00106	negative r	2/133	37/17913	0.030763	0.119081	0.085571	CTDP1/YY
GO_BP_m	GO:00302	hyalurona	2/133	37/17913	0.030763	0.119081	0.085571	HGF/ITIH4
GO_BP_m	GO:00456	negative r	3/133	92/17913	0.03114	0.120386	0.086509	CDC73/CD
GO_BP_m	GO:00030	regionaliz	6/133	318/17913	0.031468	0.121207	0.087099	ABI1/DVL
GO_BP_m	GO:00026	regulation	4/133	160/17913	0.031474	0.121207	0.087099	C3AR1/CC
GO_BP_m	GO:00358	modificati	4/133	160/17913	0.031474	0.121207	0.087099	CCNT1/CD
GO_BP_m	GO:00091	ribonucle	6/133	319/17913	0.031883	0.122628	0.08812	AK9/ALDC
GO_BP_m	GO:00726	protein lo	5/133	237/17913	0.031975	0.122636	0.088126	ACTN2/G
GO_BP_m	GO:00326	interleukin	3/133	93/17913	0.032008	0.122636	0.088126	ARRB2/CC
GO_BP_m	GO:00480	vascular e	3/133	93/17913	0.032008	0.122636	0.088126	ABI1/NCK
GO_BP_m	GO:00432	negative r	2/133	38/17913	0.032316	0.123345	0.088636	ACTN2/BI
GO_BP_m	GO:00432	apoptotic	2/133	38/17913	0.032316	0.123345	0.088636	GAS6/THB
GO_BP_m	GO:19040	positive re	2/133	38/17913	0.032316	0.123345	0.088636	ARRB2/TH
GO_BP_m	GO:00073	pattern sp	7/133	408/17913	0.032686	0.124274	0.089303	ABI1/C2C
GO_BP_m	GO:00439	regulation	7/133	408/17913	0.032686	0.124274	0.089303	CCNT1/CD
GO_BP_m	GO:00091	purine nuc	6/133	321/17913	0.032724	0.124274	0.089303	AK9/ALDC
GO_BP_m	GO:00513	regulation	4/133	162/17913	0.032725	0.124274	0.089303	BRCA2/RA
GO_BP_m	GO:00486	cell morph	8/133	500/17913	0.033092	0.125408	0.090118	ABI1/ABI2
GO_BP_m	GO:00315	cell-substr	6/133	322/17913	0.033149	0.125408	0.090118	ACTN2/C
GO_BP_m	GO:00434	regulation	6/133	322/17913	0.033149	0.125408	0.090118	CDK12/D
GO_BP_m	GO:00506	negative r	4/133	163/17913	0.033362	0.126054	0.090582	BRCA2/CD
GO_BP_m	GO:00718	protein-D	5/133	240/17913	0.033485	0.126076	0.090598	CTCF/DDB
GO_BP_m	GO:00024	antigen pr	3/133	95/17913	0.033782	0.126076	0.090598	SEC24A/S
GO_BP_m	GO:00085	female go	3/133	95/17913	0.033782	0.126076	0.090598	ARRB2/BR
GO_BP_m	GO:00332	response t	3/133	95/17913	0.033782	0.126076	0.090598	CD4/DNM
GO_BP_m	GO:00725	pyrimidine	3/133	95/17913	0.033782	0.126076	0.090598	NME3/NM
GO_BP_m	GO:00032	physiologi	2/133	39/17913	0.033901	0.126076	0.090598	CTDP1/YY
GO_BP_m	GO:00033	physiologi	2/133	39/17913	0.033901	0.126076	0.090598	CTDP1/YY
GO_BP_m	GO:00147	negative r	2/133	39/17913	0.033901	0.126076	0.090598	CTDP1/YY
GO_BP_m	GO:00310	positive re	2/133	39/17913	0.033901	0.126076	0.090598	DNMT1/P
GO_BP_m	GO:00381	neurotrop	2/133	39/17913	0.033901	0.126076	0.090598	SOS1/WA
GO_BP_m	GO:00610	cell growt	2/133	39/17913	0.033901	0.126076	0.090598	CTDP1/YY

GO_BP_m1	GO:19022	regulation	2/133	39/17913	0.033901	0.126076	0.090598	ACKR3/CL
GO_BP_m1	GO:00380	Fc recepto	5/133	241/17913	0.033998	0.126076	0.090598	ABI1/ARP(
GO_BP_m1	GO:00488	homeostas	5/133	241/17913	0.033998	0.126076	0.090598	CCR7/CDK
GO_BP_m1	GO:00518	regulation	5/133	241/17913	0.033998	0.126076	0.090598	ARRB2/CC
GO_BP_m1	GO:00070	cell-substr	3/133	96/17913	0.034688	0.128316	0.092207	ACTN2/TH
GO_BP_m1	GO:00106	regulation	3/133	96/17913	0.034688	0.128316	0.092207	ARRB2/DN
GO_BP_m1	GO:00330	muscle cel	5/133	243/17913	0.035039	0.128957	0.092668	DNMT1/LI
GO_BP_m1	GO:00319	regulation	2/133	40/17913	0.035515	0.128957	0.092668	NBN/VEGI
GO_BP_m1	GO:00326	negative re	2/133	40/17913	0.035515	0.128957	0.092668	ARRB2/GA
GO_BP_m1	GO:00466	negative re	2/133	40/17913	0.035515	0.128957	0.092668	CTDP1/YY
GO_BP_m1	GO:00198	antigen pr	3/133	97/17913	0.035606	0.128957	0.092668	SEC24A/SI
GO_BP_m1	GO:00347	negative re	3/133	97/17913	0.035606	0.128957	0.092668	ACTN2/BII
GO_BP_m1	GO:19027	negative re	3/133	97/17913	0.035606	0.128957	0.092668	CHEK1/CL
GO_BP_m1	GO:00023	immunogl	3/133	98/17913	0.036537	0.128957	0.092668	CCR6/NBN
GO_BP_m1	GO:00021	cap-indep	1/133	5/17913	0.036581	0.128957	0.092668	NCK1
GO_BP_m1	GO:00025	somatic di	1/133	5/17913	0.036581	0.128957	0.092668	PRKDC
GO_BP_m1	GO:00025	regulation	1/133	5/17913	0.036581	0.128957	0.092668	THBS1
GO_BP_m1	GO:00026	somatic re	1/133	5/17913	0.036581	0.128957	0.092668	PRKDC
GO_BP_m1	GO:00032	growth inv	1/133	5/17913	0.036581	0.128957	0.092668	S1PR1
GO_BP_m1	GO:00092	deoxyribo	1/133	5/17913	0.036581	0.128957	0.092668	AK9
GO_BP_m1	GO:00215	somatic m	1/133	5/17913	0.036581	0.128957	0.092668	TCTN1
GO_BP_m1	GO:00220	interkineti	1/133	5/17913	0.036581	0.128957	0.092668	PCM1
GO_BP_m1	GO:00301	regulation	1/133	5/17913	0.036581	0.128957	0.092668	NBN
GO_BP_m1	GO:00331	T cell rece	1/133	5/17913	0.036581	0.128957	0.092668	PRKDC
GO_BP_m1	GO:00344	protein loc	1/133	5/17913	0.036581	0.128957	0.092668	STX17
GO_BP_m1	GO:00358	negative re	1/133	5/17913	0.036581	0.128957	0.092668	AVPR2
GO_BP_m1	GO:00380	positive re	1/133	5/17913	0.036581	0.128957	0.092668	VEGFA
GO_BP_m1	GO:00468	receptor-r	1/133	5/17913	0.036581	0.128957	0.092668	GAS6
GO_BP_m1	GO:00510	regulation	1/133	5/17913	0.036581	0.128957	0.092668	RAB11A
GO_BP_m1	GO:00511	positive re	1/133	5/17913	0.036581	0.128957	0.092668	RAD51
GO_BP_m1	GO:00703	lens fiber	1/133	5/17913	0.036581	0.128957	0.092668	ABI2
GO_BP_m1	GO:00709	neutrophil	1/133	5/17913	0.036581	0.128957	0.092668	CXCL6
GO_BP_m1	GO:00709	regulation	1/133	5/17913	0.036581	0.128957	0.092668	CXCL6
GO_BP_m1	GO:00902	regulation	1/133	5/17913	0.036581	0.128957	0.092668	VEGFA
GO_BP_m1	GO:00906	cell differe	1/133	5/17913	0.036581	0.128957	0.092668	DNMT1
GO_BP_m1	GO:19000	regulation	1/133	5/17913	0.036581	0.128957	0.092668	VEGFA
GO_BP_m1	GO:19015	response t	1/133	5/17913	0.036581	0.128957	0.092668	RAD51
GO_BP_m1	GO:19038	negative re	1/133	5/17913	0.036581	0.128957	0.092668	NCK1
GO_BP_m1	GO:19044	protein loc	1/133	5/17913	0.036581	0.128957	0.092668	TCTN1
GO_BP_m1	GO:19046	regulation	1/133	5/17913	0.036581	0.128957	0.092668	NCK1
GO_BP_m1	GO:19054	vascular sr	1/133	5/17913	0.036581	0.128957	0.092668	DNMT1
GO_BP_m1	GO:19056	regulation	1/133	5/17913	0.036581	0.128957	0.092668	LDLRAP1
GO_BP_m1	GO:19059	negative re	1/133	5/17913	0.036581	0.128957	0.092668	CLU
GO_BP_m1	GO:19059	regulation	1/133	5/17913	0.036581	0.128957	0.092668	DNMT1
GO_BP_m1	GO:19059	regulation	1/133	5/17913	0.036581	0.128957	0.092668	DNMT1
GO_BP_m1	GO:20011	positive re	1/133	5/17913	0.036581	0.128957	0.092668	ACTN2
GO_BP_m1	GO:00011	response t	6/133	330/17913	0.036685	0.129171	0.092822	CCR7/DNI
GO_BP_m1	GO:00105	regulation	2/133	41/17913	0.037158	0.130532	0.0938	CHEK1/RA
GO_BP_m1	GO:00457	positive re	2/133	41/17913	0.037158	0.130532	0.0938	RAB11A/V
GO_BP_m1	GO:00018	blastocyst	3/133	99/17913	0.037481	0.131511	0.094504	BRCA2/NE
GO_BP_m1	GO:00018	neural tub	3/133	100/17913	0.038437	0.134237	0.096462	DVL2/SEC
GO_BP_m1	GO:00024	antigen pr	3/133	100/17913	0.038437	0.134237	0.096462	SEC24A/SI
GO_BP_m1	GO:00106	muscle cel	3/133	100/17913	0.038437	0.134237	0.096462	ARRB2/DN
GO_BP_m1	GO:00465	developme	3/133	100/17913	0.038437	0.134237	0.096462	ARRB2/BR
GO_BP_m1	GO:00358	vascular sr	2/133	42/17913	0.03883	0.134697	0.096793	DNMT1/VI
GO_BP_m1	GO:00482	lymphocyt	2/133	42/17913	0.03883	0.134697	0.096793	CXCR3/GA
GO_BP_m1	GO:00514	regulation	2/133	42/17913	0.03883	0.134697	0.096793	CCR7/MY(
GO_BP_m1	GO:00434	positive re	5/133	250/17913	0.038838	0.134697	0.096793	DVL2/FPR

GO_BP_m1	GO:00905(	sensory or	5/133	250/17913	0.038838	0.134697	0.096793	ABI2/DVL2
GO_BP_m1	GO:20000:	regulation	5/133	250/17913	0.038838	0.134697	0.096793	ARRB2/DV
GO_BP_m1	GO:00550:	regulation	3/133	101/17913	0.039405	0.136399	0.098016	ARRB2/CT
GO_BP_m1	GO:00517(	regulation	4/133	172/17913	0.039419	0.136399	0.098016	CHEK1/DL
GO_BP_m1	GO:00322!	methylatio	6/133	336/17913	0.039489	0.136484	0.098077	ASH2L/CT
GO_BP_m1	GO:00106:	positive re	4/133	173/17913	0.040129	0.138375	0.099436	CCR6/RAB
GO_BP_m1	GO:00706:	regulation	4/133	173/17913	0.040129	0.138375	0.099436	C3AR1/CL
GO_BP_m1	GO:00062	regulation	3/133	102/17913	0.040385	0.138961	0.099857	BRCA2/NE
GO_BP_m1	GO:00511!	negative re	2/133	43/17913	0.04053	0.138961	0.099857	CTDP1/YY
GO_BP_m1	GO:00550:	negative re	2/133	43/17913	0.04053	0.138961	0.099857	CTDP1/YY
GO_BP_m1	GO:00970:	dendritic c	2/133	43/17913	0.04053	0.138961	0.099857	CCR7/GAS
GO_BP_m1	GO:19900(	cellular res	2/133	43/17913	0.04053	0.138961	0.099857	E2F1/WAS
GO_BP_m1	GO:00108:	regulation	4/133	175/17913	0.041569	0.142036	0.102066	ARRB2/CL
GO_BP_m1	GO:00359(	response t	4/133	175/17913	0.041569	0.142036	0.102066	CLU/NCK1
GO_BP_m1	GO:19033:	regulation	4/133	175/17913	0.041569	0.142036	0.102066	C3AR1/CL
GO_BP_m1	GO:00072(	positive re	5/133	255/17913	0.041699	0.142318	0.10227	C3AR1/CC
GO_BP_m1	GO:00302:	macropha	2/133	44/17913	0.042258	0.143735	0.103288	CD4/VEGF
GO_BP_m1	GO:00507(	regulation	2/133	44/17913	0.042258	0.143735	0.103288	CCR7/ORM
GO_BP_m1	GO:19035	negative re	2/133	44/17913	0.042258	0.143735	0.103288	CLU/NCK1
GO_BP_m1	GO:00190(	viral transc	4/133	177/17913	0.043039	0.143868	0.103383	CCNT1/CC
GO_BP_m1	GO:20000	regulation	4/133	177/17913	0.043039	0.143868	0.103383	CDC73/CD
GO_BP_m1	GO:00326(	interferon-	3/133	105/17913	0.043401	0.143868	0.103383	AVPR2/CC
GO_BP_m1	GO:00013(	replicative	1/133	6/17913	0.043736	0.143868	0.103383	WRN
GO_BP_m1	GO:00023:	B cell linea	1/133	6/17913	0.043736	0.143868	0.103383	PRKDC
GO_BP_m1	GO:00024:	compleme	1/133	6/17913	0.043736	0.143868	0.103383	C3AR1
GO_BP_m1	GO:00028(	positive re	1/133	6/17913	0.043736	0.143868	0.103383	CCR7
GO_BP_m1	GO:00107!	negative re	1/133	6/17913	0.043736	0.143868	0.103383	THBS1
GO_BP_m1	GO:00165(	nucleosom	1/133	6/17913	0.043736	0.143868	0.103383	CTCF
GO_BP_m1	GO:00303(	fructose 1,	1/133	6/17913	0.043736	0.143868	0.103383	ALDOA
GO_BP_m1	GO:00315	intra-S DN	1/133	6/17913	0.043736	0.143868	0.103383	MDC1
GO_BP_m1	GO:00320!	positive re	1/133	6/17913	0.043736	0.143868	0.103383	NCK1
GO_BP_m1	GO:00322(	central ner	1/133	6/17913	0.043736	0.143868	0.103383	CLU
GO_BP_m1	GO:00357(	hematopo	1/133	6/17913	0.043736	0.143868	0.103383	GAS6
GO_BP_m1	GO:00364(	eiF2alpha	1/133	6/17913	0.043736	0.143868	0.103383	NCK1
GO_BP_m1	GO:00443	canonical \	1/133	6/17913	0.043736	0.143868	0.103383	DVL2
GO_BP_m1	GO:00458	positive re	1/133	6/17913	0.043736	0.143868	0.103383	DLGAP5
GO_BP_m1	GO:00484	replication	1/133	6/17913	0.043736	0.143868	0.103383	BRCA2
GO_BP_m1	GO:00511(	regulation	1/133	6/17913	0.043736	0.143868	0.103383	RAD51
GO_BP_m1	GO:00600:	convergen	1/133	6/17913	0.043736	0.143868	0.103383	DVL2
GO_BP_m1	GO:00611!	pulmonary	1/133	6/17913	0.043736	0.143868	0.103383	SEC24B
GO_BP_m1	GO:00614:	positive re	1/133	6/17913	0.043736	0.143868	0.103383	VEGFA
GO_BP_m1	GO:00615:	centriole e	1/133	6/17913	0.043736	0.143868	0.103383	C2CD3
GO_BP_m1	GO:00615:	microglial	1/133	6/17913	0.043736	0.143868	0.103383	CLU
GO_BP_m1	GO:00726	thymocyte	1/133	6/17913	0.043736	0.143868	0.103383	CCR6
GO_BP_m1	GO:00901:	receptor-r	1/133	6/17913	0.043736	0.143868	0.103383	LDLRAP1
GO_BP_m1	GO:00974	motor neu	1/133	6/17913	0.043736	0.143868	0.103383	VEGFA
GO_BP_m1	GO:00989:	dendritic t	1/133	6/17913	0.043736	0.143868	0.103383	WASF1
GO_BP_m1	GO:19017:	positive re	1/133	6/17913	0.043736	0.143868	0.103383	VEGFA
GO_BP_m1	GO:19019	positive re	1/133	6/17913	0.043736	0.143868	0.103383	DLGAP5
GO_BP_m1	GO:00512(	protein ho	6/133	345/17913	0.043947	0.144148	0.103584	ACTN2/AL
GO_BP_m1	GO:00359(	endoderm	2/133	45/17913	0.044013	0.144148	0.103584	CDC73/LE
GO_BP_m1	GO:00716	regulation	2/133	45/17913	0.044013	0.144148	0.103584	C3AR1/TH
GO_BP_m1	GO:19030:	regulation	2/133	45/17913	0.044013	0.144148	0.103584	CCR7/RAB
GO_BP_m1	GO:00020(	columnar/	3/133	106/17913	0.04443	0.144881	0.104111	B9D1/CDK
GO_BP_m1	GO:00303:	DNA dama	3/133	106/17913	0.04443	0.144881	0.104111	BRCA2/E2
GO_BP_m1	GO:00432	response t	3/133	106/17913	0.04443	0.144881	0.104111	DNMT3A/
GO_BP_m1	GO:00550:	cardiac mu	3/133	106/17913	0.04443	0.144881	0.104111	CTDP1/S1
GO_BP_m1	GO:00165	protein de	5/133	260/17913	0.044686	0.145556	0.104596	ARRB2/CD

GO_BP_m:	GO:00508!	T cell rece|	4/133	180/17913	0.045297	0.147388	0.105913	CCR7/CD3
GO_BP_m:	GO:00311:	RNA 3'-en	3/133	107/17913	0.045472	0.147796	0.106206	CCNT1/CD
GO_BP_m:	GO:00316	protein de	2/133	46/17913	0.045795	0.148365	0.106614	CDC73/PR
GO_BP_m:	GO:19900	response t	2/133	46/17913	0.045795	0.148365	0.106614	E2F1/WAS
GO_BP_m:	GO:20001(	negative re	2/133	46/17913	0.045795	0.148365	0.106614	CCR7/GAS
GO_BP_m:	GO:00020	sprouting	4/133	181/17913	0.046065	0.149077	0.107126	E2F2/PKM
GO_BP_m:	GO:00026	regulation	7/133	441/17913	0.046447	0.150121	0.107877	ARRB2/C3
GO_BP_m:	GO:00506	regulation	3/133	108/17913	0.046526	0.150121	0.107877	CCNT1/CD
GO_BP_m:	GO:00511	striated m	5/133	263/17913	0.046538	0.150121	0.107877	ACTN2/AF
GO_BP_m:	GO:00099	anterior/p	4/133	182/17913	0.046839	0.150933	0.10846	ABI1/PALE
GO_BP_m:	GO:00485	camera-ty	3/133	109/17913	0.047592	0.151764	0.109057	ABI2/VEGF
GO_BP_m:	GO:00901	regulation	3/133	109/17913	0.047592	0.151764	0.109057	ARRB2/DV
GO_BP_m:	GO:00022(	somatic re	2/133	47/17913	0.047603	0.151764	0.109057	CCR6/NBN
GO_BP_m:	GO:00022(	somatic di	2/133	47/17913	0.047603	0.151764	0.109057	CCR6/NBN
GO_BP_m:	GO:00330	cellular pi	2/133	47/17913	0.047603	0.151764	0.109057	POMC/RA
GO_BP_m:	GO:00447(	multi-orga	2/133	47/17913	0.047603	0.151764	0.109057	CD4/GAS6
GO_BP_m:	GO:00451	isotype sw	2/133	47/17913	0.047603	0.151764	0.109057	CCR6/NBN
GO_BP_m:	GO:00481(	regulation	2/133	47/17913	0.047603	0.151764	0.109057	RAB11A/R
GO_BP_m:	GO:00613	trabecula	2/133	47/17913	0.047603	0.151764	0.109057	S1PR1/SO
GO_BP_m:	GO:19007	regulation	2/133	47/17913	0.047603	0.151764	0.109057	HGF/VEGF
GO_BP_m:	GO:00109	positive re	5/133	265/17913	0.047797	0.152221	0.109386	DVL2/HGF
GO_BP_m:	GO:00108:	positive re	3/133	110/17913	0.048669	0.154749	0.111202	CCR7/CDK
GO_BP_m:	GO:00511	regulation	4/133	185/17913	0.049207	0.154749	0.111202	ARRB2/CT
GO_BP_m:	GO:00346	nucleobas	2/133	48/17913	0.049437	0.154749	0.111202	ENTPD1/E
GO_BP_m:	GO:00433	negative re	2/133	48/17913	0.049437	0.154749	0.111202	E2F1/SIN3
GO_BP_m:	GO:00458	positive re	2/133	48/17913	0.049437	0.154749	0.111202	DLGAP5/N
GO_BP_m:	GO:00616	histone H3	2/133	48/17913	0.049437	0.154749	0.111202	CHEK1/DN
GO_BP_m:	GO:00903:	regulation	2/133	48/17913	0.049437	0.154749	0.111202	BRCA2/NE
GO_BP_m:	GO:19035	regulation	2/133	48/17913	0.049437	0.154749	0.111202	THBS1/VE
GO_BP_m:	GO:00434	protein kir	5/133	268/17913	0.049725	0.154749	0.111202	ARRB2/CC
GO_BP_m:	GO:00067	ATP gener	3/133	111/17913	0.049759	0.154749	0.111202	AK9/ALDC
GO_BP_m:	GO:00109:	regulation	4月26日	171/17913	0.000102	0.002681	0.001444	PPP1R12A
GO_BP_m:	GO:00193	cyclooxyg	2月26日	11/17913	0.000111	0.002803	0.00151	PTGS1/PT
GO_BP_m:	GO:00193	epoxygena	2月26日	11/17913	0.000111	0.002803	0.00151	CYP2C8/C
GO_BP_m:	GO:00436	inositol ph	3月26日	68/17913	0.000128	0.003123	0.001683	INPP5E/M
GO_BP_m:	GO:00508(	negative re	3月26日	68/17913	0.000128	0.003123	0.001683	MTMR2/P
GO_BP_m:	GO:00341:	positive re	2月26日	16/17913	0.00024	0.005563	0.002998	ALOX15/L
GO_BP_m:	GO:00461	polyol cata	2月26日	16/17913	0.00024	0.005563	0.002998	INPP5E/M
GO_BP_m:	GO:19002:	regulation	2月26日	16/17913	0.00024	0.005563	0.002998	ROCK1/TR
GO_BP_m:	GO:00192	regulation	5月26日	402/17913	0.000248	0.005638	0.003038	FGR/MTM
GO_BP_m:	GO:00900(	regulation	5月26日	442/17913	0.000383	0.008584	0.004625	ALOX12/A
GO_BP_m:	GO:00353:	peptidyl-t	3月26日	101/17913	0.000412	0.008923	0.004808	MTMR2/M
GO_BP_m:	GO:00714	cellular res	2月26日	21/17913	0.000418	0.008923	0.004808	PTGS2/PT
GO_BP_m:	GO:20001:	regulation	2月26日	21/17913	0.000418	0.008923	0.004808	PTK2B/RO
GO_BP_m:	GO:00102:	response t	2月26日	22/17913	0.00046	0.009653	0.005202	PTGS2/PT
GO_BP_m:	GO:00328	regulation	2月26日	23/17913	0.000503	0.010243	0.005519	PTK2B/RO
GO_BP_m:	GO:00519(	positive re	2月26日	23/17913	0.000503	0.010243	0.005519	PTGS2/PT
GO_BP_m:	GO:00301(	regulation	4月26日	262/17913	0.000519	0.010409	0.005609	ALOX15/F
GO_BP_m:	GO:00108:	positive re	3月26日	110/17913	0.000529	0.010446	0.005629	ALOX15/P
GO_BP_m:	GO:00197	polyol met	3月26日	115/17913	0.000602	0.011721	0.006316	INPP5E/M
GO_BP_m:	GO:00341:	regulation	2月26日	26/17913	0.000644	0.012199	0.006573	ALOX15/L
GO_BP_m:	GO:00701(	negative re	2月26日	26/17913	0.000644	0.012199	0.006573	PTK2B/RO
GO_BP_m:	GO:00468	phosphati	2月26日	27/17913	0.000695	0.01298	0.006994	PI4KA/PIP
GO_BP_m:	GO:00015:	prostaglan	2月26日	28/17913	0.000748	0.013387	0.007214	PTGS1/PT
GO_BP_m:	GO:00464	prostanoic	2月26日	28/17913	0.000748	0.013387	0.007214	PTGS1/PT
GO_BP_m:	GO:19907	response t	2月26日	28/17913	0.000748	0.013387	0.007214	PTGS2/RO
GO_BP_m:	GO:00352	regulation	3月26日	125/17913	0.000767	0.013387	0.007214	ALOX12/P
GO_BP_m:	GO:00977	regulation	3月26日	125/17913	0.000767	0.013387	0.007214	ALOX12/P

GO_BP_m[illegible]	GO:00105[illegible]	positive re	3月26日	129/1791[illegible]	0.00084	0.014481	0.007803	ALOX12/P
GO_BP_m[illegible]	GO:00508[illegible]	regulation	3月26日	133/1791[illegible]	0.000918	0.01562	0.008417	ALOX12/P
GO_BP_m[illegible]	GO:00351[illegible]	regulation	3月26日	134/1791[illegible]	0.000938	0.015763	0.008494	ALOX12/P
GO_BP_m[illegible]	GO:00435[illegible]	positive re	2月26日	32/17913	0.000978	0.016232	0.008746	FGR/PTK2I
GO_BP_m[illegible]	GO:00105[illegible]	regulation	4月26日	314/1791[illegible]	0.001021	0.016647	0.00897	MTMR3/PI
GO_BP_m[illegible]	GO:00972[illegible]	amyloid-b	2月26日	33/17913	0.00104	0.016647	0.00897	ROCK1/TR
GO_BP_m[illegible]	GO:20007[illegible]	regulation	2月26日	33/17913	0.00104	0.016647	0.00897	MTMR3/PI
GO_BP_m[illegible]	GO:00432[illegible]	negative re	3月26日	144/1791[illegible]	0.001155	0.017693	0.009534	CYP4F2/PT
GO_BP_m[illegible]	GO:00458[illegible]	positive re	3月26日	144/1791[illegible]	0.001155	0.017693	0.009534	FGR/PTGS
GO_BP_m[illegible]	GO:00902[illegible]	positive re	2月26日	36/17913	0.001238	0.017693	0.009534	FGR/PTK2I
GO_BP_m[illegible]	GO:19055[illegible]	regulation	2月26日	36/17913	0.001238	0.017693	0.009534	PTK2B/TRE
GO_BP_m[illegible]	GO:00458[illegible]	positive re	4月26日	333/1791[illegible]	0.001269	0.017693	0.009534	ALOX12/L
GO_BP_m[illegible]	GO:00344[illegible]	response t	2月26日	37/17913	0.001308	0.017693	0.009534	PTGS2/PTI
GO_BP_m[illegible]	GO:00432[illegible]	apoptotic	2月26日	38/17913	0.001379	0.017693	0.009534	ALOX15/T
GO_BP_m[illegible]	GO:00440[illegible]	regulation	2月26日	38/17913	0.001379	0.017693	0.009534	MTMR3/PI
GO_BP_m[illegible]	GO:00313[illegible]	positive re	4月26日	341/1791[illegible]	0.001385	0.017693	0.009534	PIP4K2C/P
GO_BP_m[illegible]	GO:00103[illegible]	response t	1月26日	1/17913	0.001451	0.017693	0.009534	PTGS2
GO_BP_m[illegible]	GO:00314[illegible]	oxylipin m	1月26日	1/17913	0.001451	0.017693	0.009534	ALOX12
GO_BP_m[illegible]	GO:00355[illegible]	positive re	1月26日	1/17913	0.001451	0.017693	0.009534	PPP1R12A
GO_BP_m[illegible]	GO:00381[illegible]	C-C chem	1月26日	1/17913	0.001451	0.017693	0.009534	TREM2
GO_BP_m[illegible]	GO:00467[illegible]	viral replic	1月26日	1/17913	0.001451	0.017693	0.009534	PI4KA
GO_BP_m[illegible]	GO:00703[illegible]	detection	1月26日	1/17913	0.001451	0.017693	0.009534	TREM2
GO_BP_m[illegible]	GO:00714[illegible]	cellular res	1月26日	1/17913	0.001451	0.017693	0.009534	PTGS2
GO_BP_m[illegible]	GO:19017[illegible]	leukotrien	1月26日	1/17913	0.001451	0.017693	0.009534	ALOX12
GO_BP_m[illegible]	GO:19030[illegible]	regulation	1月26日	1/17913	0.001451	0.017693	0.009534	TREM2
GO_BP_m[illegible]	GO:19030[illegible]	positive re	1月26日	1/17913	0.001451	0.017693	0.009534	TREM2
GO_BP_m[illegible]	GO:19040[illegible]	regulation	1月26日	1/17913	0.001451	0.017693	0.009534	TREM2
GO_BP_m[illegible]	GO:19040[illegible]	negative re	1月26日	1/17913	0.001451	0.017693	0.009534	TREM2
GO_BP_m[illegible]	GO:19052[illegible]	positive re	1月26日	1/17913	0.001451	0.017693	0.009534	TREM2
GO_BP_m[illegible]	GO:20003[illegible]	positive re	1月26日	1/17913	0.001451	0.017693	0.009534	TREM2
GO_BP_m[illegible]	GO:20013[illegible]	lipoxin B4	1月26日	1/17913	0.001451	0.017693	0.009534	ALOX12
GO_BP_m[illegible]	GO:20013[illegible]	lipoxin B4	1月26日	1/17913	0.001451	0.017693	0.009534	ALOX12
GO_BP_m[illegible]	GO:00468[illegible]	lipid phos	2月26日	39/17913	0.001452	0.017693	0.009534	PI4KA/PIP
GO_BP_m[illegible]	GO:00181[illegible]	peptidyl-t	4月26日	346/1791[illegible]	0.001461	0.017693	0.009534	FGR/LCK/F
GO_BP_m[illegible]	GO:00182[illegible]	peptidyl-t	4月26日	349/1791[illegible]	0.001508	0.0181	0.009753	FGR/LCK/F
GO_BP_m[illegible]	GO:00030[illegible]	vascular p	3月26日	161/1791[illegible]	0.001592	0.018429	0.009931	ALOX12/P
GO_BP_m[illegible]	GO:00066[illegible]	prostanoic	2月26日	41/17913	0.001604	0.018429	0.009931	PTGS1/PT
GO_BP_m[illegible]	GO:00066[illegible]	prostaglan	2月26日	41/17913	0.001604	0.018429	0.009931	PTGS1/PT
GO_BP_m[illegible]	GO:00069[illegible]	phagocyto	2月26日	41/17913	0.001604	0.018429	0.009931	ALOX15/T
GO_BP_m[illegible]	GO:00988[illegible]	modulatio	2月26日	41/17913	0.001604	0.018429	0.009931	MTMR2/PT
GO_BP_m[illegible]	GO:00323[illegible]	icosanoid	2月26日	42/17913	0.001683	0.019009	0.010243	CYP4F2/PL
GO_BP_m[illegible]	GO:00454[illegible]	positive re	2月26日	42/17913	0.001683	0.019009	0.010243	PTGS2/PTI
GO_BP_m[illegible]	GO:00717[illegible]	icosanoid	2月26日	43/17913	0.001764	0.019428	0.010469	CYP4F2/PL
GO_BP_m[illegible]	GO:19015[illegible]	fatty acid	2月26日	43/17913	0.001764	0.019428	0.010469	CYP4F2/PL
GO_BP_m[illegible]	GO:19044[illegible]	positive re	2月26日	43/17913	0.001764	0.019428	0.010469	PTGS2/PTI
GO_BP_m[illegible]	GO:00082[illegible]	regulation	3月26日	169/1791[illegible]	0.001829	0.01998	0.010766	CYP4F2/PT
GO_BP_m[illegible]	GO:00461[illegible]	alcohol ca	2月26日	44/17913	0.001846	0.020007	0.010781	INPP5E/M
GO_BP_m[illegible]	GO:00423[illegible]	regulation	4月26日	374/1791[illegible]	0.001943	0.020851	0.011235	ALOX12/M
GO_BP_m[illegible]	GO:00106[illegible]	positive re	3月26日	173/1791[illegible]	0.001955	0.020851	0.011235	ALOX12/P
GO_BP_m[illegible]	GO:19037[illegible]	positive re	2月26日	48/17913	0.002193	0.023143	0.012471	FGR/PTK2I
GO_BP_m[illegible]	GO:00015[illegible]	ossificatio	4月26日	388/1791[illegible]	0.002221	0.023143	0.012471	ALOX15/F
GO_BP_m[illegible]	GO:00457[illegible]	positive re	4月26日	388/1791[illegible]	0.002221	0.023143	0.012471	ALOX15/L
GO_BP_m[illegible]	GO:00435[illegible]	blood vess	3月26日	182/1791[illegible]	0.002258	0.023348	0.012581	ALOX12/P
GO_BP_m[illegible]	GO:00098[illegible]	positive re	4月26日	392/1791[illegible]	0.002306	0.023655	0.012746	PIP4K2C/P
GO_BP_m[illegible]	GO:00519[illegible]	regulation	2月26日	50/17913	0.002378	0.023847	0.01285	PTGS2/PTI
GO_BP_m[illegible]	GO:00990[illegible]	plasma me	2月26日	50/17913	0.002378	0.023847	0.01285	ALOX15/T
GO_BP_m[illegible]	GO:19055[illegible]	macropha	2月26日	50/17913	0.002378	0.023847	0.01285	PTK2B/TRE
GO_BP_m[illegible]	GO:00703[illegible]	positive re	3月26日	186/1791[illegible]	0.002402	0.023915	0.012887	ALOX15/P

GO_BP_m[illegible]	GO:00019[illegible]	positive re	2月26日	51/17913	0.002472	0.023988	0.012926	PTK2B/RO
GO_BP_m[illegible]	GO:00700[illegible]	chemokine	2月26日	51/17913	0.002472	0.023988	0.012926	PTK2B/TRE
GO_BP_m[illegible]	GO:01500[illegible]	regulation	2月26日	51/17913	0.002472	0.023988	0.012926	PTGS2/TRI
GO_BP_m[illegible]	GO:00323[illegible]	regulation	4月26日	400/17913	0.002481	0.023988	0.012926	FGR/MTM
GO_BP_m[illegible]	GO:00435[illegible]	regulation	2月26日	53/17913	0.002667	0.024521	0.013213	FGR/PTK2I
GO_BP_m[illegible]	GO:00068[illegible]	potassium	3月26日	194/17913	0.002707	0.024521	0.013213	MTMR6/PT
GO_BP_m[illegible]	GO:00432[illegible]	regulation	3月26日	196/17913	0.002786	0.024521	0.013213	ALOX12/L
GO_BP_m[illegible]	GO:00025[illegible]	arachidoni	1月26日	2/17913	0.002901	0.024521	0.013213	ALOX5
GO_BP_m[illegible]	GO:00025[illegible]	leukotriene	1月26日	2/17913	0.002901	0.024521	0.013213	ALOX5
GO_BP_m[illegible]	GO:00025[illegible]	positive re	1月26日	2/17913	0.002901	0.024521	0.013213	TREM2
GO_BP_m[illegible]	GO:00025[illegible]	positive re	1月26日	2/17913	0.002901	0.024521	0.013213	TREM2
GO_BP_m[illegible]	GO:00025[illegible]	positive re	1月26日	2/17913	0.002901	0.024521	0.013213	TREM2
GO_BP_m[illegible]	GO:00324[illegible]	detection	1月26日	2/17913	0.002901	0.024521	0.013213	TREM2
GO_BP_m[illegible]	GO:00423[illegible]	menaquin	1月26日	2/17913	0.002901	0.024521	0.013213	CYP4F2
GO_BP_m[illegible]	GO:00423[illegible]	vitamin K	1月26日	2/17913	0.002901	0.024521	0.013213	CYP4F2
GO_BP_m[illegible]	GO:00903[illegible]	positive re	1月26日	2/17913	0.002901	0.024521	0.013213	PTGS2
GO_BP_m[illegible]	GO:01100[illegible]	regulation	1月26日	2/17913	0.002901	0.024521	0.013213	ROCK1
GO_BP_m[illegible]	GO:19010[illegible]	regulation	1月26日	2/17913	0.002901	0.024521	0.013213	ALOX15
GO_BP_m[illegible]	GO:19019[illegible]	positive re	1月26日	2/17913	0.002901	0.024521	0.013213	TREM2
GO_BP_m[illegible]	GO:19035[illegible]	negative re	1月26日	2/17913	0.002901	0.024521	0.013213	INPP5E
GO_BP_m[illegible]	GO:19045[illegible]	phosphati	1月26日	2/17913	0.002901	0.024521	0.013213	MTMR3
GO_BP_m[illegible]	GO:19059[illegible]	positive re	1月26日	2/17913	0.002901	0.024521	0.013213	ALOX12
GO_BP_m[illegible]	GO:20005[illegible]	regulation	1月26日	2/17913	0.002901	0.024521	0.013213	PTK2B
GO_BP_m[illegible]	GO:20005[illegible]	positive re	1月26日	2/17913	0.002901	0.024521	0.013213	PTK2B
GO_BP_m[illegible]	GO:00108[illegible]	regulation	3月26日	201/17913	0.002992	0.025131	0.013542	ALOX15/P
GO_BP_m[illegible]	GO:00323[illegible]	positive re	3月26日	203/17913	0.003077	0.025539	0.013762	FGR/MTM
GO_BP_m[illegible]	GO:19034[illegible]	positive re	2月26日	57/17913	0.003078	0.025539	0.013762	PTGS2/PTI
GO_BP_m[illegible]	GO:00103[illegible]	membrane	2月26日	58/17913	0.003186	0.026266	0.014153	ALOX15/T
GO_BP_m[illegible]	GO:00106[illegible]	negative re	2月26日	59/17913	0.003294	0.026998	0.014548	ALOX12/P
GO_BP_m[illegible]	GO:19908[illegible]	response t	2月26日	60/17913	0.003405	0.027568	0.014855	PTK2B/TRE
GO_BP_m[illegible]	GO:19908[illegible]	cellular res	2月26日	60/17913	0.003405	0.027568	0.014855	PTK2B/TRE
GO_BP_m[illegible]	GO:00341[illegible]	heterotypi	2月26日	61/17913	0.003517	0.028306	0.015253	ALOX15/L
GO_BP_m[illegible]	GO:00352[illegible]	synaptic tr	2月26日	62/17913	0.003631	0.028878	0.015561	PTGS2/PTI
GO_BP_m[illegible]	GO:00435[illegible]	regulation	2月26日	62/17913	0.003631	0.028878	0.015561	FGR/PTK2I
GO_BP_m[illegible]	GO:20001[illegible]	regulation	3月26日	222/17913	0.003956	0.031272	0.016851	ALOX12/L
GO_BP_m[illegible]	GO:00454[illegible]	regulation	2月26日	65/17913	0.003984	0.031272	0.016851	PTGS2/PTI
GO_BP_m[illegible]	GO:01100[illegible]	regulation	3月26日	228/17913	0.004263	0.031272	0.016851	ALOX15/P
GO_BP_m[illegible]	GO:00509[illegible]	leukocyte	4月26日	467/17913	0.004324	0.031272	0.016851	LCK/PTK2E
GO_BP_m[illegible]	GO:00030[illegible]	pressure n	1月26日	3/17913	0.004348	0.031272	0.016851	CYP4F2
GO_BP_m[illegible]	GO:00033[illegible]	apical cons	1月26日	3/17913	0.004348	0.031272	0.016851	ROCK1
GO_BP_m[illegible]	GO:00092[illegible]	menaquin	1月26日	3/17913	0.004348	0.031272	0.016851	CYP4F2
GO_BP_m[illegible]	GO:00322[illegible]	negative re	1月26日	3/17913	0.004348	0.031272	0.016851	PTGS2
GO_BP_m[illegible]	GO:00356[illegible]	maintenan	1月26日	3/17913	0.004348	0.031272	0.016851	PTGS2
GO_BP_m[illegible]	GO:00423[illegible]	phylloquin	1月26日	3/17913	0.004348	0.031272	0.016851	CYP4F2
GO_BP_m[illegible]	GO:00423[illegible]	phylloquin	1月26日	3/17913	0.004348	0.031272	0.016851	CYP4F2
GO_BP_m[illegible]	GO:00701[illegible]	positive re	1月26日	3/17913	0.004348	0.031272	0.016851	TREM2
GO_BP_m[illegible]	GO:00903[illegible]	platelet-de	1月26日	3/17913	0.004348	0.031272	0.016851	PTGS2
GO_BP_m[illegible]	GO:00903[illegible]	regulation	1月26日	3/17913	0.004348	0.031272	0.016851	PTGS2
GO_BP_m[illegible]	GO:19016[illegible]	quinone ca	1月26日	3/17913	0.004348	0.031272	0.016851	CYP4F2
GO_BP_m[illegible]	GO:19052[illegible]	regulation	1月26日	3/17913	0.004348	0.031272	0.016851	TREM2
GO_BP_m[illegible]	GO:20006[illegible]	negative re	1月26日	3/17913	0.004348	0.031272	0.016851	MTMR2
GO_BP_m[illegible]	GO:00508[illegible]	multicellul	2月26日	68/17913	0.004351	0.031272	0.016851	ALOX12/C
GO_BP_m[illegible]	GO:00105[illegible]	regulation	3月26日	231/17913	0.004421	0.031531	0.01699	ALOX12/P
GO_BP_m[illegible]	GO:00069[illegible]	autophagy	4月26日	471/17913	0.004457	0.031531	0.01699	MTMR3/PI
GO_BP_m[illegible]	GO:00619[illegible]	process ut	4月26日	471/17913	0.004457	0.031531	0.01699	MTMR3/PI
GO_BP_m[illegible]	GO:00488[illegible]	multicellul	4月26日	472/17913	0.004491	0.031602	0.017029	ALOX12/C
GO_BP_m[illegible]	GO:00000[illegible]	autophago	2月26日	70/17913	0.004605	0.032232	0.017368	MTMR3/PI
GO_BP_m[illegible]	GO:00606[illegible]	regulation	4月26日	480/17913	0.004767	0.033195	0.017887	ALOX15/F

GO_BP_m	GO:19016	organic hy	3月26日	241/1791	0.004975	0.034443	0.01856	ALOX12/A
GO_BP_m	GO:19050	autophagc	2月26日	73/17913	0.004997	0.034443	0.01856	MTMR3/P
GO_BP_m	GO:00301	water hom	2月26日	74/17913	0.005132	0.035131	0.01893	ALOX12/C
GO_BP_m	GO:00313	negative r	3月26日	244/1791	0.005149	0.035131	0.01893	MTMR2/R
GO_BP_m	GO:00068	nitric oxide	2月26日	76/17913	0.005405	0.036504	0.01967	PTGS2/PT
GO_BP_m	GO:01500	neuroinfla	2月26日	76/17913	0.005405	0.036504	0.01967	PTGS2/TR
GO_BP_m	GO:00434	positive re	4月26日	499/1791	0.005467	0.03673	0.019792	ALOX15/P
GO_BP_m	GO:00025	regulation	1月26日	4/17913	0.005794	0.03673	0.019792	TREM2
GO_BP_m	GO:00066	platelet ac	1月26日	4/17913	0.005794	0.03673	0.019792	PLA2G4A
GO_BP_m	GO:00313	positive re	1月26日	4/17913	0.005794	0.03673	0.019792	PTGS2
GO_BP_m	GO:00323	negative r	1月26日	4/17913	0.005794	0.03673	0.019792	CYP4F2
GO_BP_m	GO:00324	detection	1月26日	4/17913	0.005794	0.03673	0.019792	TREM2
GO_BP_m	GO:00359	cellular res	1月26日	4/17913	0.005794	0.03673	0.019792	ALOX15
GO_BP_m	GO:00361	leukotrien	1月26日	4/17913	0.005794	0.03673	0.019792	CYP4F2
GO_BP_m	GO:00361	leukotrien	1月26日	4/17913	0.005794	0.03673	0.019792	CYP4F2
GO_BP_m	GO:00361	leukotrien	1月26日	4/17913	0.005794	0.03673	0.019792	CYP4F2
GO_BP_m	GO:00423	vitamin E r	1月26日	4/17913	0.005794	0.03673	0.019792	CYP4F2
GO_BP_m	GO:00712	cellular res	1月26日	4/17913	0.005794	0.03673	0.019792	PTGS2
GO_BP_m	GO:00971	epoxide m	1月26日	4/17913	0.005794	0.03673	0.019792	ALOX12
GO_BP_m	GO:00442	cellular ca	3月26日	256/1791	0.005883	0.037118	0.020001	INPP5E/M
GO_BP_m	GO:00435	positive re	2月26日	80/17913	0.005972	0.037504	0.020209	ALOX12/P
GO_BP_m	GO:00070	cell-substr	2月26日	81/17913	0.006117	0.037889	0.020416	PTK2B/RO
GO_BP_m	GO:00462	nitric oxide	2月26日	81/17913	0.006117	0.037889	0.020416	PTGS2/PT
GO_BP_m	GO:00480	focal adhe	2月26日	81/17913	0.006117	0.037889	0.020416	PTK2B/RO
GO_BP_m	GO:00069	response t	2月26日	82/17913	0.006265	0.038623	0.020812	PTGS2/PT
GO_BP_m	GO:00308	positive re	2月26日	84/17913	0.006564	0.040102	0.021609	ALOX15/P
GO_BP_m	GO:20010	reactive ni	2月26日	84/17913	0.006564	0.040102	0.021609	PTGS2/PT
GO_BP_m	GO:00725	reactive ox	3月26日	269/1791	0.006744	0.041016	0.022101	ALOX12/P
GO_BP_m	GO:00094	response t	3月26日	270/1791	0.006814	0.041041	0.022115	CYP2C8/P
GO_BP_m	GO:00703	regulation	3月26日	271/1791	0.006883	0.041041	0.022115	ALOX15/P
GO_BP_m	GO:00435	endothelia	3月26日	275/1791	0.007166	0.041041	0.022115	ALOX12/P
GO_BP_m	GO:00701	regulation	2月26日	88/17913	0.007183	0.041041	0.022115	PTK2B/RO
GO_BP_m	GO:00025	regulation	1月26日	5/17913	0.007237	0.041041	0.022115	TREM2
GO_BP_m	GO:00226	membrane	1月26日	5/17913	0.007237	0.041041	0.022115	ROCK1
GO_BP_m	GO:00342	regulation	1月26日	5/17913	0.007237	0.041041	0.022115	TREM2
GO_BP_m	GO:00342	positive re	1月26日	5/17913	0.007237	0.041041	0.022115	TREM2
GO_BP_m	GO:00355	negative r	1月26日	5/17913	0.007237	0.041041	0.022115	ROCK1
GO_BP_m	GO:00359	response t	1月26日	5/17913	0.007237	0.041041	0.022115	ALOX15
GO_BP_m	GO:00712	cellular res	1月26日	5/17913	0.007237	0.041041	0.022115	TREM2
GO_BP_m	GO:00902	positive re	1月26日	5/17913	0.007237	0.041041	0.022115	PTGS2
GO_BP_m	GO:19015	icosanoid	1月26日	5/17913	0.007237	0.041041	0.022115	CYP4F2
GO_BP_m	GO:19022	negative r	1月26日	5/17913	0.007237	0.041041	0.022115	PTGS2
GO_BP_m	GO:19033	negative r	1月26日	5/17913	0.007237	0.041041	0.022115	ROCK1
GO_BP_m	GO:19041	positive re	1月26日	5/17913	0.007237	0.041041	0.022115	TREM2
GO_BP_m	GO:00159	fatty acid t	2月26日	90/17913	0.007502	0.042009	0.022636	CYP4F2/PL
GO_BP_m	GO:00343	adherens j	2月26日	90/17913	0.007502	0.042009	0.022636	PTK2B/RO
GO_BP_m	GO:00507	regulation	2月26日	90/17913	0.007502	0.042009	0.022636	ALOX15/F
GO_BP_m	GO:00480	vascular e	2月26日	93/17913	0.007992	0.044349	0.023897	PTK2B/RO
GO_BP_m	GO:00098	negative r	3月26日	288/1791	0.008134	0.044349	0.023897	MTMR2/R
GO_BP_m	GO:00072	integrin-m	2月26日	94/17913	0.008158	0.044349	0.023897	FGR/PTK2
GO_BP_m	GO:00703	ERK1 and	3月26日	289/1791	0.008211	0.044349	0.023897	ALOX15/P
GO_BP_m	GO:00600	excitatory	2月26日	95/17913	0.008326	0.044349	0.023897	MTMR2/P
GO_BP_m	GO:00070	cell-substr	2月26日	96/17913	0.008496	0.044349	0.023897	PTK2B/RO
GO_BP_m	GO:00106	regulation	2月26日	96/17913	0.008496	0.044349	0.023897	ALOX12/P
GO_BP_m	GO:00106	regulation	3月26日	294/1791	0.008605	0.044349	0.023897	ALOX12/P
GO_BP_m	GO:00300	contractile	2月26日	97/17913	0.008667	0.044349	0.023897	PTK2B/RO
GO_BP_m	GO:00431	stress fibe	2月26日	97/17913	0.008667	0.044349	0.023897	PTK2B/RO
GO_BP_m	GO:00025	regulation	1月26日	6/17913	0.008678	0.044349	0.023897	TREM2

GO_BP_m:	GO:00029:	lipid hydrc	1月26日	6/17913	0.008678	0.044349	0.023897	CYP2C8
GO_BP_m:	GO:00342:	macrophag	1月26日	6/17913	0.008678	0.044349	0.023897	TREM2
GO_BP_m:	GO:00359(	cardiolipin	1月26日	6/17913	0.008678	0.044349	0.023897	PLA2G4A
GO_BP_m:	GO:00423'	vitamin K r	1月26日	6/17913	0.008678	0.044349	0.023897	CYP4F2
GO_BP_m:	GO:00464(	platelet ac	1月26日	6/17913	0.008678	0.044349	0.023897	PLA2G4A
GO_BP_m:	GO:00510‹	negative re	1月26日	6/17913	0.008678	0.044349	0.023897	ROCK1
GO_BP_m:	GO:00618!	positive re	1月26日	6/17913	0.008678	0.044349	0.023897	TREM2
GO_BP_m:	GO:00716(	macrophag	1月26日	6/17913	0.008678	0.044349	0.023897	TREM2
GO_BP_m:	GO:00716‹	regulation	1月26日	6/17913	0.008678	0.044349	0.023897	TREM2
GO_BP_m:	GO:19022:	regulation	1月26日	6/17913	0.008678	0.044349	0.023897	PTGS2
GO_BP_m:	GO:20003‹	regulation	1月26日	6/17913	0.008678	0.044349	0.023897	TREM2
GO_BP_m:	GO:20012{	positive re	1月26日	6/17913	0.008678	0.044349	0.023897	PTGS2
GO_BP_m:	GO:00421:	neurotrans	2月26日	98/17913	0.00884	0.044531	0.023995	PTGS2/PTI
GO_BP_m:	GO:00432(	regulation	2月26日	98/17913	0.00884	0.044531	0.023995	PTK2B/TRE
GO_BP_m:	GO:00508‹	regulation	2月26日	98/17913	0.00884	0.044531	0.023995	PTK2B/TRE
GO_BP_m:	GO:00329!	regulation	3月26日	297/1791:	0.008847	0.044531	0.023995	ALOX15/P
GO_BP_m:	GO:00988(	cellular oxi	2月26日	99/17913	0.009014	0.045204	0.024358	PTGS1/PT(
GO_BP_m:	GO:00106!	muscle cel	2月26日	100/1791:	0.00919	0.045744	0.024649	ALOX12/P
GO_BP_m:	GO:00468:	regulation	2月26日	100/1791:	0.00919	0.045744	0.024649	PPP1R12A
GO_BP_m:	GO:00486(	positive re	2月26日	101/1791:	0.009367	0.046115	0.024849	ALOX12/P
GO_BP_m:	GO:19034:	regulation	2月26日	101/1791:	0.009367	0.046115	0.024849	PTGS2/PTI
GO_BP_m:	GO:20003'	positive re	2月26日	101/1791:	0.009367	0.046115	0.024849	PTGS2/PTI
GO_BP_m:	GO:00026!	positive re	3月26日	305/1791:	0.00951	0.046649	0.025137	FGR/LCK/T
GO_BP_m:	GO:00995(	chemical s	2月26日	103/1791:	0.009726	0.047535	0.025614	MTMR2/P'
GO_BP_m:	GO:00069(	phagocyto	3月26日	308/1791:	0.009767	0.047546	0.02562	ALOX15/F(
GO_BP_m:	GO:00069:	smooth m	2月26日	105/1791:	0.010092	0.047546	0.02562	PTGS2/RO
GO_BP_m:	GO:00097!	response t	1月26日	7/17913	0.010118	0.047546	0.02562	PTGS2
GO_BP_m:	GO:00316:	positive re	1月26日	7/17913	0.010118	0.047546	0.02562	PTGS2
GO_BP_m:	GO:00329(	regulation	1月26日	7/17913	0.010118	0.047546	0.02562	PTK2B
GO_BP_m:	GO:00436!	engulfmer	1月26日	7/17913	0.010118	0.047546	0.02562	ALOX15
GO_BP_m:	GO:00617(	CAMKK-A	1月26日	7/17913	0.010118	0.047546	0.02562	TREM2
GO_BP_m:	GO:19015(	regulation	1月26日	7/17913	0.010118	0.047546	0.02562	ALOX12
GO_BP_m:	GO:19019'	regulation	1月26日	7/17913	0.010118	0.047546	0.02562	TREM2
GO_BP_m:	GO:19041:	microglial	1月26日	7/17913	0.010118	0.047546	0.02562	TREM2
GO_BP_m:	GO:19041:	regulation	1月26日	7/17913	0.010118	0.047546	0.02562	TREM2
GO_BP_m:	GO:19029(	regulation	3月26日	313/1791:	0.010203	0.047778	0.025745	ALOX15/P
GO_BP_m:	GO:00321(	positive re	3月26日	315/1791:	0.01038	0.048441	0.026102	PTGS2/PTI
GO_BP_m:	GO:19907‹	cellular de	2月26日	107/1791:	0.010463	0.048659	0.02622	PTGS1/PT(
GO_BP_m:	GO:00508(	positive re	3月26日	319/1791:	0.010741	0.049708	0.026785	FGR/LCK/T
GO_BP_m:	GO:19030!	positive re	2月26日	109/1791:	0.01084	0.049708	0.026785	PTK2B/TRE
GO_BP_m:	GO:00315{	cell-substr	3月26日	322/1791:	0.011016	0.049708	0.026785	ALOX15/P
GO_BP_m:	GO:00068(	xenobiotic	2月26日	110/1791:	0.011031	0.049708	0.026785	CYP2C8/P'
GO_BP_m:	GO:00070:	actin filam	3月26日	325/1791:	0.011295	0.049708	0.026785	ALOX15/P
GO_BP_m:	GO:00512(	release of	2月26日	112/1791:	0.011418	0.049708	0.026785	LCK/PTK2E
GO_BP_m:	GO:00512{	negative re	2月26日	112/1791:	0.011418	0.049708	0.026785	LCK/PTK2E
GO_BP_m:	GO:00071'	signal com	1月26日	8/17913	0.011555	0.049708	0.026785	PTK2B
GO_BP_m:	GO:00086:	intrinsic ap	1月26日	8/17913	0.011555	0.049708	0.026785	PTGS2
GO_BP_m:	GO:00230:	CD40 sign:	1月26日	8/17913	0.011555	0.049708	0.026785	TREM2
GO_BP_m:	GO:00316:	regulation	1月26日	8/17913	0.011555	0.049708	0.026785	PTGS2
GO_BP_m:	GO:00316‹	negative re	1月26日	8/17913	0.011555	0.049708	0.026785	MTMR2
GO_BP_m:	GO:00329!	inositol tris	1月26日	8/17913	0.011555	0.049708	0.026785	PTK2B
GO_BP_m:	GO:00356!	interleukin	1月26日	8/17913	0.011555	0.049708	0.026785	ALOX5
GO_BP_m:	GO:00357!	B cell cher	1月26日	8/17913	0.011555	0.049708	0.026785	PTK2B
GO_BP_m:	GO:00481(	autophagi	1月26日	8/17913	0.011555	0.049708	0.026785	TREM2
GO_BP_m:	GO:00600'	regulation	1月26日	8/17913	0.011555	0.049708	0.026785	TREM2
GO_BP_m:	GO:00713!	cellular res	1月26日	8/17913	0.011555	0.049708	0.026785	ALOX5
GO_BP_m:	GO:00990(	calmodulir	1月26日	8/17913	0.011555	0.049708	0.026785	TREM2
GO_BP_m:	GO:19035(	regulation	1月26日	8/17913	0.011555	0.049708	0.026785	INPP5E

GO_BP_m:	GO:20001!	negative re	1月26日	8/17913	0.011555	0.049708	0.026785	CYP4F2
GO_BP_m:	GO:20006	positive re	1月26日	8/17913	0.011555	0.049708	0.026785	MTMR2
GO_BP_m:	GO:00105(	positive re	2月26日	113/17913	0.011613	0.049708	0.026785	PIP4K2C/R
GO_BP_m:	GO:00467:	acid secret	2月26日	113/17913	0.011613	0.049708	0.026785	CYP4F2/PL
GO_BP_m:	GO:00987!	detoxificat	2月26日	113/17913	0.011613	0.049708	0.026785	PTGS1/PT(
GO_BP_m:	GO:00512!	regulation	2月26日	114/17913	0.01181	0.05039	0.027153	LCK/PTK2E
GO_BP_m:	GO:00075!	blood coa	3月26日	331/17913	0.011866	0.050469	0.027195	ALOX12/C
GO_BP_m:	GO:00322	positive re	2月26日	116/17913	0.012208	0.05176	0.027891	ALOX15/P
GO_BP_m:	GO:00100:	response t	3月26日	335/17913	0.012256	0.051797	0.027911	ALOX15/P
GO_BP_m:	GO:00075!	hemostasi:	3月26日	336/17913	0.012355	0.051797	0.027911	ALOX12/C
GO_BP_m:	GO:00019!	regulation	2月26日	117/17913	0.01241	0.051797	0.027911	PTK2B/RO
GO_BP_m:	GO:00140(	regulation	2月26日	117/17913	0.01241	0.051797	0.027911	FGR/PIP4K
GO_BP_m:	GO:00512(	sequesteri	2月26日	117/17913	0.01241	0.051797	0.027911	LCK/PTK2E
GO_BP_m:	GO:00508:	coagulatio	3月26日	337/17913	0.012454	0.05182	0.027923	ALOX12/C
GO_BP_m:	GO:00432!	positive re	2月26日	118/17913	0.012612	0.052273	0.028167	ALOX12/L
GO_BP_m:	GO:00060(	alcohol me	3月26日	339/17913	0.012653	0.052273	0.028167	INPP5E/M
GO_BP_m:	GO:00329	regulation	3月26日	341/17913	0.012855	0.052273	0.028167	ALOX15/P
GO_BP_m:	GO:00023:	marginal z	1月26日	9/17913	0.01299	0.052273	0.028167	PTK2B
GO_BP_m:	GO:00025	positive re	1月26日	9/17913	0.01299	0.052273	0.028167	TREM2
GO_BP_m:	GO:00421!	ketone cat	1月26日	9/17913	0.01299	0.052273	0.028167	CYP4F2
GO_BP_m:	GO:00423(	fat-soluble	1月26日	9/17913	0.01299	0.052273	0.028167	CYP4F2
GO_BP_m:	GO:00427!	long-chair	1月26日	9/17913	0.01299	0.052273	0.028167	CYP4F2
GO_BP_m:	GO:00902(	fibroblast (	1月26日	9/17913	0.01299	0.052273	0.028167	PTGS2
GO_BP_m:	GO:00902	regulation	1月26日	9/17913	0.01299	0.052273	0.028167	PTGS2
GO_BP_m:	GO:20006	regulation	1月26日	9/17913	0.01299	0.052273	0.028167	MTMR2
GO_BP_m:	GO:00026!	positive re	2月26日	121/17913	0.01323	0.053076	0.0286	PTK2B/TRE
GO_BP_m:	GO:19034(	reactive ox	2月26日	122/17913	0.013438	0.053752	0.028964	PTGS2/PTI
GO_BP_m:	GO:00031!	endotheliu	2月26日	124/17913	0.01386	0.054917	0.029592	ALOX12/R
GO_BP_m:	GO:00083(	regulation	2月26日	124/17913	0.01386	0.054917	0.029592	FGR/PTK2I
GO_BP_m:	GO:00106:	epithelial (	3月26日	354/17913	0.014209	0.054917	0.029592	ALOX12/P
GO_BP_m:	GO:00421	regulation	3月26日	354/17913	0.014209	0.054917	0.029592	PTK2B/RO
GO_BP_m:	GO:00300:	establishm	2月26日	126/17913	0.014287	0.054917	0.029592	PTK2B/RO
GO_BP_m:	GO:00515!	response t	2月26日	126/17913	0.014287	0.054917	0.029592	ALOX15/P
GO_BP_m:	GO:00975!	calcium ioı	2月26日	126/17913	0.014287	0.054917	0.029592	LCK/PTK2E
GO_BP_m:	GO:00016(	fever gene	1月26日	10/17913	0.014424	0.054917	0.029592	PTGS2
GO_BP_m:	GO:00316!	positive re	1月26日	10/17913	0.014424	0.054917	0.029592	PTGS2
GO_BP_m:	GO:00448(	multi-orga	1月26日	10/17913	0.014424	0.054917	0.029592	PI4KA
GO_BP_m:	GO:00519(	positive re	1月26日	10/17913	0.014424	0.054917	0.029592	ALOX12
GO_BP_m:	GO:00700!	regulation	1月26日	10/17913	0.014424	0.054917	0.029592	TREM2
GO_BP_m:	GO:00703!	response t	1月26日	10/17913	0.014424	0.054917	0.029592	TREM2
GO_BP_m:	GO:00712:	cellular res	1月26日	10/17913	0.014424	0.054917	0.029592	TREM2
GO_BP_m:	GO:00903:	positive re	1月26日	10/17913	0.014424	0.054917	0.029592	PTGS2
GO_BP_m:	GO:01060	regulation	1月26日	10/17913	0.014424	0.054917	0.029592	PTGS2
GO_BP_m:	GO:20004:	regulation	1月26日	10/17913	0.014424	0.054917	0.029592	ALOX15
GO_BP_m:	GO:00600	regulation	2月26日	127/17913	0.014503	0.055018	0.029646	MTMR2/P
GO_BP_m:	GO:00901:	epithelium	3月26日	357/17913	0.014532	0.055018	0.029646	ALOX12/P
GO_BP_m:	GO:00308:	regulation	2月26日	129/17913	0.014938	0.056238	0.030304	ALOX15/P
GO_BP_m:	GO:19033(	positive re	2月26日	129/17913	0.014938	0.056238	0.030304	PTK2B/TRE
GO_BP_m:	GO:00466!	response t	2月26日	130/17913	0.015158	0.056717	0.030562	PTGS2/PTI
GO_BP_m:	GO:00509:	positive re	2月26日	130/17913	0.015158	0.056717	0.030562	PTK2B/TRE
GO_BP_m:	GO:00901:	tissue migı	3月26日	363/17913	0.015192	0.056717	0.030562	ALOX12/P
GO_BP_m:	GO:00070:	vacuole or	2月26日	131/17913	0.01538	0.056977	0.030702	MTMR3/PI
GO_BP_m:	GO:00109!	regulation	3月26日	365/17913	0.015416	0.056977	0.030702	PTGS2/PTI
GO_BP_m:	GO:00072!	female gaı	2月26日	132/17913	0.015602	0.056977	0.030702	PTGS2/PTI
GO_BP_m:	GO:00313!	regulation	1月26日	11/17913	0.015855	0.056977	0.030702	PTGS2
GO_BP_m:	GO:00319:	positive re	1月26日	11/17913	0.015855	0.056977	0.030702	PTGS2
GO_BP_m:	GO:00320(	bleb assen	1月26日	11/17913	0.015855	0.056977	0.030702	ROCK1
GO_BP_m:	GO:00324!	detection (	1月26日	11/17913	0.015855	0.056977	0.030702	TREM2

GO_BP_m	GO:00324	response t	1月26日	11/17913	0.015855	0.056977	0.030702	TREM2
GO_BP_m	GO:00329	inositol tri	1月26日	11/17913	0.015855	0.056977	0.030702	PTK2B
GO_BP_m	GO:00706	response t	1月26日	11/17913	0.015855	0.056977	0.030702	ALOX5
GO_BP_m	GO:00903	negative r	1月26日	11/17913	0.015855	0.056977	0.030702	MTMR2
GO_BP_m	GO:19015	fatty acid	1月26日	11/17913	0.015855	0.056977	0.030702	CYP4F2
GO_BP_m	GO:19039	positive re	1月26日	11/17913	0.015855	0.056977	0.030702	TREM2
GO_BP_m	GO:20007	positive re	1月26日	11/17913	0.015855	0.056977	0.030702	PIP4K2C
GO_BP_m	GO:00525	regulation	3月26日	370/1791	0.015983	0.057226	0.030836	ALOX12/L
GO_BP_m	GO:00316	regulation	2月26日	134/1791	0.016052	0.057226	0.030836	MTMR2/P
GO_BP_m	GO:00343	adherens j	2月26日	134/1791	0.016052	0.057226	0.030836	PTK2B/RO
GO_BP_m	GO:20010	positive re	2月26日	135/1791	0.016279	0.057881	0.031189	ALOX12/L
GO_BP_m	GO:00510	actin filam	2月26日	136/1791	0.016508	0.058539	0.031543	PTK2B/RO
GO_BP_m	GO:00157	monocarb	2月26日	137/1791	0.016737	0.059042	0.031814	CYP4F2/P
GO_BP_m	GO:00615	actin filam	2月26日	137/1791	0.016737	0.059042	0.031814	PTK2B/RO
GO_BP_m	GO:00140	phosphati	2月26日	138/1791	0.016968	0.05911	0.031851	FGR/PIP4K
GO_BP_m	GO:00091	vitamin ca	1月26日	12/17913	0.017284	0.05911	0.031851	CYP4F2
GO_BP_m	GO:00107	regulation	1月26日	12/17913	0.017284	0.05911	0.031851	PTK2B
GO_BP_m	GO:00316	regulation	1月26日	12/17913	0.017284	0.05911	0.031851	PTGS2
GO_BP_m	GO:00381	interleukin	1月26日	12/17913	0.017284	0.05911	0.031851	PTK2B
GO_BP_m	GO:00427	exogenou	1月26日	12/17913	0.017284	0.05911	0.031851	CYP2C8
GO_BP_m	GO:00514	myoblast r	1月26日	12/17913	0.017284	0.05911	0.031851	ROCK1
GO_BP_m	GO:00903	negative r	1月26日	12/17913	0.017284	0.05911	0.031851	ALOX12
GO_BP_m	GO:19015	regulation	1月26日	12/17913	0.017284	0.05911	0.031851	ROCK1
GO_BP_m	GO:19024	negative r	1月26日	12/17913	0.017284	0.05911	0.031851	ROCK1
GO_BP_m	GO:19031	regulation	1月26日	12/17913	0.017284	0.05911	0.031851	ROCK1
GO_BP_m	GO:19041	positive re	1月26日	12/17913	0.017284	0.05911	0.031851	ALOX12
GO_BP_m	GO:00718	cellular po	2月26日	140/1791	0.017435	0.059322	0.031966	MTMR6/T
GO_BP_m	GO:00718	potassium	2月26日	140/1791	0.017435	0.059322	0.031966	MTMR6/T
GO_BP_m	GO:00457	regulation	3月26日	383/1791	0.017514	0.059442	0.03203	PTGS2/PT
GO_BP_m	GO:00620	positive re	2月26日	143/1791	0.018144	0.061004	0.032872	PTGS2/TR
GO_BP_m	GO:00224	regulation	3月26日	389/1791	0.018248	0.061004	0.032872	ALOX12/A
GO_BP_m	GO:00140	response t	2月26日	145/1791	0.018624	0.061004	0.032872	PTGS2/PT
GO_BP_m	GO:00300	actin filam	2月26日	145/1791	0.018624	0.061004	0.032872	ALOX15/P
GO_BP_m	GO:00421	neurotran	2月26日	145/1791	0.018624	0.061004	0.032872	PTGS2/PT
GO_BP_m	GO:00508	positive re	2月26日	145/1791	0.018624	0.061004	0.032872	PTGS2/PT
GO_BP_m	GO:00604	calcium io	2月26日	145/1791	0.018624	0.061004	0.032872	LCK/PTK2
GO_BP_m	GO:00330	positive re	1月26日	13/17913	0.018712	0.061004	0.032872	FGR
GO_BP_m	GO:00433	positive re	1月26日	13/17913	0.018712	0.061004	0.032872	FGR
GO_BP_m	GO:00440	positive re	1月26日	13/17913	0.018712	0.061004	0.032872	PIP4K2C
GO_BP_m	GO:00474	regulation	1月26日	13/17913	0.018712	0.061004	0.032872	PTGS2
GO_BP_m	GO:00618	regulation	1月26日	13/17913	0.018712	0.061004	0.032872	TREM2
GO_BP_m	GO:00709	oxidative	1月26日	13/17913	0.018712	0.061004	0.032872	CYP2C8
GO_BP_m	GO:00713	cellular re	1月26日	13/17913	0.018712	0.061004	0.032872	PTK2B
GO_BP_m	GO:19036	positive re	1月26日	13/17913	0.018712	0.061004	0.032872	MTMR2
GO_BP_m	GO:20012	regulation	1月26日	13/17913	0.018712	0.061004	0.032872	PTGS2
GO_BP_m	GO:00508	modulatio	3月26日	393/1791	0.018746	0.061004	0.032872	MTMR2/P
GO_BP_m	GO:00435	negative r	2月26日	146/1791	0.018866	0.061117	0.032933	PTK2B/RO
GO_BP_m	GO:00991	regulation	3月26日	394/1791	0.018872	0.061117	0.032933	MTMR2/P
GO_BP_m	GO:00080	regulation	2月26日	147/1791	0.01911	0.061739	0.033268	ALOX15/P
GO_BP_m	GO:00308	regulation	2月26日	148/1791	0.019354	0.062312	0.033577	ALOX15/P
GO_BP_m	GO:00525	regulation	3月26日	398/1791	0.01938	0.062312	0.033577	ALOX12/L
GO_BP_m	GO:00512	positive re	3月26日	403/1791	0.020026	0.062854	0.033868	FGR/PTGS
GO_BP_m	GO:00020	negative r	1月26日	14/17913	0.020137	0.062854	0.033868	MTMR2
GO_BP_m	GO:00066	phosphati	1月26日	14/17913	0.020137	0.062854	0.033868	ALOX15
GO_BP_m	GO:00091	coenzyme	1月26日	14/17913	0.020137	0.062854	0.033868	CYP4F2
GO_BP_m	GO:00320	cardiolipin	1月26日	14/17913	0.020137	0.062854	0.033868	PLA2G4A
GO_BP_m	GO:00353	regulation	1月26日	14/17913	0.020137	0.062854	0.033868	ALOX15
GO_BP_m	GO:00381	angiotensi	1月26日	14/17913	0.020137	0.062854	0.033868	ROCK1

GO_BP_m:	GO:00429	activation	1月26日	14/17913	0.020137	0.062854	0.033868	PTK2B
GO_BP_m:	GO:00459	negative re	1月26日	14/17913	0.020137	0.062854	0.033868	PTGS2
GO_BP_m:	GO:00508	positive re	1月26日	14/17913	0.020137	0.062854	0.033868	LCK
GO_BP_m:	GO:00600	regulation	1月26日	14/17913	0.020137	0.062854	0.033868	ALOX15
GO_BP_m:	GO:00706	response t	1月26日	14/17913	0.020137	0.062854	0.033868	PTK2B
GO_BP_m:	GO:00442	small mole	3月26日	404/1791	0.020156	0.062854	0.033868	CYP4F2/IN
GO_BP_m:	GO:00301	platelet ac	2月26日	152/1791	0.020346	0.063299	0.034109	ALOX12/L
GO_BP_m:	GO:00507	positive re	2月26日	153/1791	0.020597	0.063933	0.03445	PTGS2/TRI
GO_BP_m:	GO:00510	positive re	3月26日	409/1791	0.020816	0.064463	0.034736	CYP4F2/FC
GO_BP_m:	GO:00341	negative re	1月26日	15/17913	0.02156	0.065902	0.035511	ALOX12
GO_BP_m:	GO:00343	NAD biosy	1月26日	15/17913	0.02156	0.065902	0.035511	PTGS2
GO_BP_m:	GO:19029	negative re	1月26日	15/17913	0.02156	0.065902	0.035511	ROCK1
GO_BP_m:	GO:19051	regulation	1月26日	15/17913	0.02156	0.065902	0.035511	ALOX15
GO_BP_m:	GO:19052	positive re	1月26日	15/17913	0.02156	0.065902	0.035511	ROCK1
GO_BP_m:	GO:00604	cytosolic c	2月26日	157/1791	0.021616	0.065902	0.035511	LCK/PTK2E
GO_BP_m:	GO:00068	calcium ioi	3月26日	415/1791	0.021624	0.065902	0.035511	LCK/PTGS
GO_BP_m:	GO:00435	regulation	2月26日	158/1791	0.021873	0.066511	0.035839	ALOX12/P
GO_BP_m:	GO:00480	phosphati	2月26日	160/1791	0.022393	0.067937	0.036608	FGR/PIP4K
GO_BP_m:	GO:00109	positive re	2月26日	161/1791	0.022655	0.068114	0.036703	ALOX12/L
GO_BP_m:	GO:00019	renal syste	1月26日	16/17913	0.022982	0.068114	0.036703	CYP4F2
GO_BP_m:	GO:00109	regulation	1月26日	16/17913	0.022982	0.068114	0.036703	PTK2B
GO_BP_m:	GO:00316	heat gene	1月26日	16/17913	0.022982	0.068114	0.036703	PTGS2
GO_BP_m:	GO:00322	regulation	1月26日	16/17913	0.022982	0.068114	0.036703	PTGS2
GO_BP_m:	GO:00323	positive re	1月26日	16/17913	0.022982	0.068114	0.036703	CYP4F2
GO_BP_m:	GO:00361	phosphati	1月26日	16/17913	0.022982	0.068114	0.036703	PLA2G4A
GO_BP_m:	GO:00903	regulation	1月26日	16/17913	0.022982	0.068114	0.036703	PTGS2
GO_BP_m:	GO:19002	regulation	1月26日	16/17913	0.022982	0.068114	0.036703	ROCK1
GO_BP_m:	GO:19052	regulation	1月26日	16/17913	0.022982	0.068114	0.036703	ROCK1
GO_BP_m:	GO:19013	regulation	3月26日	425/1791	0.023009	0.068114	0.036703	PTGS2/PTI
GO_BP_m:	GO:00310	actomyosi	2月26日	163/1791	0.023182	0.068175	0.036736	PTK2B/RO
GO_BP_m:	GO:00480	inositol lip	2月26日	163/1791	0.023182	0.068175	0.036736	FGR/PIP4K
GO_BP_m:	GO:20012	regulation	2月26日	163/1791	0.023182	0.068175	0.036736	LCK/PTGS
GO_BP_m:	GO:00714	cellular res	2月26日	164/1791	0.023447	0.068806	0.037076	CYP2C8/P
GO_BP_m:	GO:00162	regulation	2月26日	166/1791	0.023982	0.069926	0.037679	MTMR3/PI
GO_BP_m:	GO:20012	regulation	2月26日	166/1791	0.023982	0.069926	0.037679	PTK2B/TRE
GO_BP_m:	GO:00712	cellular res	2月26日	167/1791	0.024251	0.069926	0.037679	ALOX15/P
GO_BP_m:	GO:00189	ether meta	1月26日	17/17913	0.024401	0.069926	0.037679	ALOX12
GO_BP_m:	GO:00305	negative re	1月26日	17/17913	0.024401	0.069926	0.037679	PTK2B
GO_BP_m:	GO:00436	linoleic aci	1月26日	17/17913	0.024401	0.069926	0.037679	ALOX12
GO_BP_m:	GO:00487	positive re	1月26日	17/17913	0.024401	0.069926	0.037679	TREM2
GO_BP_m:	GO:19038	positive re	1月26日	17/17913	0.024401	0.069926	0.037679	TREM2
GO_BP_m:	GO:19039	regulation	1月26日	17/17913	0.024401	0.069926	0.037679	TREM2
GO_BP_m:	GO:19039	positive re	1月26日	17/17913	0.024401	0.069926	0.037679	TREM2
GO_BP_m:	GO:20006	regulation	1月26日	17/17913	0.024401	0.069926	0.037679	MTMR2
GO_BP_m:	GO:00481	regulation	2月26日	169/1791	0.024793	0.070899	0.038204	PTGS2/PTI
GO_BP_m:	GO:00620	regulation	3月26日	439/1791	0.025028	0.071075	0.038298	PTGS2/PTI
GO_BP_m:	GO:00081	actin polyr	2月26日	170/1791	0.025066	0.071075	0.038298	ALOX15/P
GO_BP_m:	GO:00487	tissue rem	2月26日	170/1791	0.025066	0.071075	0.038298	PTK2B/RO
GO_BP_m:	GO:00975	myeloid le	2月26日	170/1791	0.025066	0.071075	0.038298	PTK2B/TRE
GO_BP_m:	GO:19049	positive re	3月26日	440/1791	0.025175	0.071233	0.038384	FGR/PTGS
GO_BP_m:	GO:00069	response t	3月26日	442/1791	0.025473	0.071253	0.038394	PTGS1/PT(
GO_BP_m:	GO:00071	establishm	2月26日	172/1791	0.025616	0.071253	0.038394	PTK2B/RO
GO_BP_m:	GO:00486	regulation	2月26日	172/1791	0.025616	0.071253	0.038394	ALOX12/P
GO_BP_m:	GO:00068	cellular zin	1月26日	18/17913	0.025819	0.071253	0.038394	LCK
GO_BP_m:	GO:00322	myelin ass	1月26日	18/17913	0.025819	0.071253	0.038394	MTMR2
GO_BP_m:	GO:00330	positive re	1月26日	18/17913	0.025819	0.071253	0.038394	FGR
GO_BP_m:	GO:00361	phosphati	1月26日	18/17913	0.025819	0.071253	0.038394	PLA2G4A
GO_BP_m:	GO:00380	peptidyl-t	1月26日	18/17913	0.025819	0.071253	0.038394	PTK2B

GO_BP_m:	GO:00713:	cellular res	1月26日	18/17913	0.025819	0.071253	0.038394	PTGS2
GO_BP_m:	GO:00903:	regulation	1月26日	18/17913	0.025819	0.071253	0.038394	ALOX12
GO_BP_m:	GO:00985:	detection (	1月26日	18/17913	0.025819	0.071253	0.038394	TREM2
GO_BP_m:	GO:20001:	positive re	1月26日	18/17913	0.025819	0.071253	0.038394	CYP4F2
GO_BP_m:	GO:00016(	ameboidal	3月26日	446/1791:	0.026072	0.071659	0.038613	ALOX12/P
GO_BP_m:	GO:00018:	positive re	3月26日	446/1791:	0.026072	0.071659	0.038613	FGR/PTGS
GO_BP_m:	GO:00486:	smooth m	2月26日	174/1791:	0.026171	0.071783	0.03868	ALOX12/P
GO_BP_m:	GO:00023:	mature B c	1月26日	19/17913	0.027234	0.073352	0.039525	PTK2B
GO_BP_m:	GO:00025:	regulation	1月26日	19/17913	0.027234	0.073352	0.039525	TREM2
GO_BP_m:	GO:00031:	morphoge	1月26日	19/17913	0.027234	0.073352	0.039525	ALOX12
GO_BP_m:	GO:00105:	negative re	1月26日	19/17913	0.027234	0.073352	0.039525	ALOX12
GO_BP_m:	GO:00193:	leukotriene	1月26日	19/17913	0.027234	0.073352	0.039525	ALOX5
GO_BP_m:	GO:00323(	regulation	1月26日	19/17913	0.027234	0.073352	0.039525	CYP4F2
GO_BP_m:	GO:00328:	negative re	1月26日	19/17913	0.027234	0.073352	0.039525	CYP4F2
GO_BP_m:	GO:00611:	endothelia	1月26日	19/17913	0.027234	0.073352	0.039525	ALOX12
GO_BP_m:	GO:01401:	positive re	1月26日	19/17913	0.027234	0.073352	0.039525	PTK2B
GO_BP_m:	GO:00708:	divalent m	3月26日	454/1791:	0.027295	0.073369	0.039535	LCK/PTGS:
GO_BP_m:	GO:00109:	positive re	2月26日	180/1791:	0.027864	0.074596	0.040196	ALOX12/L(
GO_BP_m:	GO:00322:	regulation	2月26日	180/1791:	0.027864	0.074596	0.040196	ALOX15/P
GO_BP_m:	GO:00725:	divalent in	3月26日	458/1791:	0.027918	0.074596	0.040196	LCK/PTGS:
GO_BP_m:	GO:00020:	sprouting	2月26日	181/1791:	0.028151	0.075053	0.040442	PTGS2/PTI
GO_BP_m:	GO:00100:	response t	1月26日	20/17913	0.028647	0.075053	0.040442	PTGS2
GO_BP_m:	GO:00325:	positive re	1月26日	20/17913	0.028647	0.075053	0.040442	PPP1R12A
GO_BP_m:	GO:00353:	peroxisom	1月26日	20/17913	0.028647	0.075053	0.040442	ALOX15
GO_BP_m:	GO:00457:	positive re	1月26日	20/17913	0.028647	0.075053	0.040442	PTGS2
GO_BP_m:	GO:00550(	zinc ion hc	1月26日	20/17913	0.028647	0.075053	0.040442	LCK
GO_BP_m:	GO:00716:	positive re	1月26日	20/17913	0.028647	0.075053	0.040442	PTGS2
GO_BP_m:	GO:00970(	dendritic s	1月26日	20/17913	0.028647	0.075053	0.040442	MTMR2
GO_BP_m:	GO:01400:	neuron prc	1月26日	20/17913	0.028647	0.075053	0.040442	ROCK1
GO_BP_m:	GO:19055:	positive re	1月26日	20/17913	0.028647	0.075053	0.040442	TREM2
GO_BP_m:	GO:00507:	positive re	2月26日	184/1791:	0.029018	0.075875	0.040885	PTK2B/TRI
GO_BP_m:	GO:19021:	regulation	2月26日	185/1791:	0.029309	0.076339	0.041135	MTMR3/PI
GO_BP_m:	GO:19029(	positive re	2月26日	185/1791:	0.029309	0.076339	0.041135	ALOX15/P
GO_BP_m:	GO:00026:	regulation	2月26日	186/1791:	0.029601	0.076804	0.041385	PTK2B/TRI
GO_BP_m:	GO:00509:	regulation	2月26日	186/1791:	0.029601	0.076804	0.041385	PTK2B/TRI
GO_BP_m:	GO:00307:	ovulation	1月26日	21/17913	0.030059	0.077245	0.041623	PTGS2
GO_BP_m:	GO:00361:	phosphatic	1月26日	21/17913	0.030059	0.077245	0.041623	PLA2G4A
GO_BP_m:	GO:00510:	regulation	1月26日	21/17913	0.030059	0.077245	0.041623	ROCK1
GO_BP_m:	GO:00519(	regulation	1月26日	21/17913	0.030059	0.077245	0.041623	ALOX12
GO_BP_m:	GO:00977(	connective	1月26日	21/17913	0.030059	0.077245	0.041623	ROCK1
GO_BP_m:	GO:00514:	regulation	3月26日	472/1791:	0.030158	0.077286	0.041645	ALOX15/P
GO_BP_m:	GO:00197:	calcium-m	2月26日	188/1791:	0.03019	0.077286	0.041645	PTK2B/TRI
GO_BP_m:	GO:00156:	monovaler	3月26日	477/1791:	0.030981	0.079161	0.042656	MTMR6/P
GO_BP_m:	GO:00457:	positive re	2月26日	192/1791:	0.031381	0.079499	0.042838	PTK2B/TRI
GO_BP_m:	GO:00095:	detection (	1月26日	22/17913	0.031468	0.079499	0.042838	TREM2
GO_BP_m:	GO:00433(	positive re	1月26日	22/17913	0.031468	0.079499	0.042838	FGR
GO_BP_m:	GO:00463:	phosphatic	1月26日	22/17913	0.031468	0.079499	0.042838	ALOX15
GO_BP_m:	GO:00510(	positive re	1月26日	22/17913	0.031468	0.079499	0.042838	PTK2B
GO_BP_m:	GO:20008:	regulation	1月26日	22/17913	0.031468	0.079499	0.042838	ROCK1
GO_BP_m:	GO:00712:	cellular res	2月26日	194/1791:	0.031983	0.080497	0.043376	ALOX15/P
GO_BP_m:	GO:20003:	regulation	2月26日	194/1791:	0.031983	0.080497	0.043376	PTGS2/PTI
GO_BP_m:	GO:00097:	response t	2月26日	195/1791:	0.032286	0.080897	0.043591	PTGS2/PTI
GO_BP_m:	GO:00025:	leukocyte (	3月26日	485/1791:	0.032323	0.080897	0.043591	LCK/PTK2I
GO_BP_m:	GO:00433:	neutrophil	3月26日	485/1791:	0.032323	0.080897	0.043591	ALOX5/FG
GO_BP_m:	GO:00022:	neutrophil	3月26日	488/1791:	0.032834	0.081074	0.043686	ALOX5/FG
GO_BP_m:	GO:00102:	response t	1月26日	23/17913	0.032876	0.081074	0.043686	PTGS2
GO_BP_m:	GO:00107:	regulation	1月26日	23/17913	0.032876	0.081074	0.043686	PTK2B
GO_BP_m:	GO:00508:	positive re	1月26日	23/17913	0.032876	0.081074	0.043686	LCK

GO_BP_m1	GO:00518	mitochonc	1月26日	23/17913	0.032876	0.081074	0.043686	ALOX12
GO_BP_m1	GO:00601	positive re	1月26日	23/17913	0.032876	0.081074	0.043686	TREM2
GO_BP_m1	GO:00614	establishm	1月26日	23/17913	0.032876	0.081074	0.043686	ALOX12
GO_BP_m1	GO:19020	regulation	1月26日	23/17913	0.032876	0.081074	0.043686	ROCK1
GO_BP_m1	GO:19030	regulation	2月26日	198/17913	0.033202	0.081728	0.044039	PTK2B/TRE
GO_BP_m1	GO:19016	response t	3月26日	491/17913	0.033349	0.081939	0.044153	PTGS2/RO
GO_BP_m1	GO:00026	regulation	3月26日	492/17913	0.033521	0.082213	0.0443	FGR/LCK/T
GO_BP_m1	GO:00096	response t	2月26日	200/17913	0.033818	0.08264	0.04453	PTGS2/PTI
GO_BP_m1	GO:00342	response t	2月26日	200/17913	0.033818	0.08264	0.04453	PTGS2/PTI
GO_BP_m1	GO:00329	inositol ph	1月26日	24/17913	0.034281	0.082718	0.044573	PTK2B
GO_BP_m1	GO:00466	decidualiz	1月26日	24/17913	0.034281	0.082718	0.044573	PTGS2
GO_BP_m1	GO:00481	astrocyte a	1月26日	24/17913	0.034281	0.082718	0.044573	TREM2
GO_BP_m1	GO:01500	positive re	1月26日	24/17913	0.034281	0.082718	0.044573	TREM2
GO_BP_m1	GO:19018	negative re	1月26日	24/17913	0.034281	0.082718	0.044573	ROCK1
GO_BP_m1	GO:19034	regulation	1月26日	24/17913	0.034281	0.082718	0.044573	ROCK1
GO_BP_m1	GO:19036	regulation	1月26日	24/17913	0.034281	0.082718	0.044573	MTMR2
GO_BP_m1	GO:00024	neutrophil	3月26日	499/17913	0.034744	0.083106	0.044781	ALOX5/FG
GO_BP_m1	GO:00096	response t	3月26日	499/17913	0.034744	0.083106	0.044781	PTGS1/PTG
GO_BP_m1	GO:00421	neutrophil	3月26日	499/17913	0.034744	0.083106	0.044781	ALOX5/FG
GO_BP_m1	GO:00508	regulation	3月26日	499/17913	0.034744	0.083106	0.044781	ALOX12/C
GO_BP_m1	GO:00457	positive re	2月26日	203/17913	0.034751	0.083106	0.044781	PTGS2/PTI
GO_BP_m1	GO:00514	positive re	2月26日	205/17913	0.035379	0.083265	0.044867	ALOX15/P
GO_BP_m1	GO:00000	very long-	1月26日	25/17913	0.035685	0.083265	0.044867	CYP4F2
GO_BP_m1	GO:00015	oocyte ma	1月26日	25/17913	0.035685	0.083265	0.044867	PTK2B
GO_BP_m1	GO:00019	synaptic tr	1月26日	25/17913	0.035685	0.083265	0.044867	PTGS2
GO_BP_m1	GO:00023	mature B c	1月26日	25/17913	0.035685	0.083265	0.044867	PTK2B
GO_BP_m1	GO:00030	renal syste	1月26日	25/17913	0.035685	0.083265	0.044867	CYP4F2
GO_BP_m1	GO:00341	regulation	1月26日	25/17913	0.035685	0.083265	0.044867	ALOX12
GO_BP_m1	GO:00352	ionotropic	1月26日	25/17913	0.035685	0.083265	0.044867	PTK2B
GO_BP_m1	GO:00456	positive re	1月26日	25/17913	0.035685	0.083265	0.044867	TREM2
GO_BP_m1	GO:00459	negative re	1月26日	25/17913	0.035685	0.083265	0.044867	PTGS2
GO_BP_m1	GO:00509	leukocyte	1月26日	25/17913	0.035685	0.083265	0.044867	ROCK1
GO_BP_m1	GO:00518	positive re	1月26日	25/17913	0.035685	0.083265	0.044867	ROCK1
GO_BP_m1	GO:19016	regulation	1月26日	25/17913	0.035685	0.083265	0.044867	PTK2B
GO_BP_m1	GO:19043	cellular res	1月26日	25/17913	0.035685	0.083265	0.044867	ROCK1
GO_BP_m1	GO:19012	negative re	2月26日	207/17913	0.036011	0.083879	0.045198	PTK2B/RO
GO_BP_m1	GO:00435	regulation	2月26日	210/17913	0.036967	0.085059	0.045834	PTK2B/RO
GO_BP_m1	GO:00467	protein au	2月26日	210/17913	0.036967	0.085059	0.045834	FGR/PTK2I
GO_BP_m1	GO:00199	cGMP-me	1月26日	26/17913	0.037087	0.085059	0.045834	PTK2B
GO_BP_m1	GO:00316	negative re	1月26日	26/17913	0.037087	0.085059	0.045834	MTMR2
GO_BP_m1	GO:00335	regulation	1月26日	26/17913	0.037087	0.085059	0.045834	ALOX12
GO_BP_m1	GO:00361	phosphatio	1月26日	26/17913	0.037087	0.085059	0.045834	PLA2G4A
GO_BP_m1	GO:00601	regulation	1月26日	26/17913	0.037087	0.085059	0.045834	TREM2
GO_BP_m1	GO:19010	positive re	1月26日	26/17913	0.037087	0.085059	0.045834	TREM2
GO_BP_m1	GO:19037	negative re	1月26日	26/17913	0.037087	0.085059	0.045834	CYP4F2
GO_BP_m1	GO:00071	cell-matrix	2月26日	214/17913	0.038257	0.086934	0.046844	PTK2B/RO
GO_BP_m1	GO:00343	cell junctio	2月26日	214/17913	0.038257	0.086934	0.046844	PTK2B/RO
GO_BP_m1	GO:00359	response t	1月26日	27/17913	0.038486	0.086934	0.046844	PTK2B
GO_BP_m1	GO:00361	phosphatio	1月26日	27/17913	0.038486	0.086934	0.046844	PLA2G4A
GO_BP_m1	GO:00433	regulation	1月26日	27/17913	0.038486	0.086934	0.046844	FGR
GO_BP_m1	GO:00459	positive re	1月26日	27/17913	0.038486	0.086934	0.046844	PTGS2
GO_BP_m1	GO:19016	quinone m	1月26日	27/17913	0.038486	0.086934	0.046844	CYP4F2
GO_BP_m1	GO:20001	regulation	1月26日	27/17913	0.038486	0.086934	0.046844	CYP4F2
GO_BP_m1	GO:20004	positive re	1月26日	27/17913	0.038486	0.086934	0.046844	PTK2B
GO_BP_m1	GO:00031	aortic valv	1月26日	28/17913	0.039884	0.088896	0.047901	ROCK1
GO_BP_m1	GO:00327	positive re	1月26日	28/17913	0.039884	0.088896	0.047901	PTK2B
GO_BP_m1	GO:00396	viral RNA g	1月26日	28/17913	0.039884	0.088896	0.047901	PI4KA
GO_BP_m1	GO:00482	negative re	1月26日	28/17913	0.039884	0.088896	0.047901	MTMR2

GO_BP_m:	GO:00504{	arachidoni	1月26日	28/17913	0.039884	0.088896	0.047901	PLA2G4A
GO_BP_m:	GO:00602	long-term	1月26日	28/17913	0.039884	0.088896	0.047901	PTK2B
GO_BP_m:	GO:0061	mRNA des	1月26日	28/17913	0.039884	0.088896	0.047901	ROCK1
GO_BP_m:	GO:19039	arachidona	1月26日	28/17913	0.039884	0.088896	0.047901	PLA2G4A
GO_BP_m:	GO:00316	cellular res	2月26日	220/1791	0.040225	0.089507	0.04823	MTMR3/P
GO_BP_m:	GO:00028	positive re	1月26日	29/17913	0.04128	0.09125	0.04917	FGR
GO_BP_m:	GO:00105	positive re	1月26日	29/17913	0.04128	0.09125	0.04917	PTGS2
GO_BP_m:	GO:00330	regulation	1月26日	29/17913	0.04128	0.09125	0.04917	FGR
GO_BP_m:	GO:00342	amyloid-b	1月26日	29/17913	0.04128	0.09125	0.04917	ROCK1
GO_BP_m:	GO:00097	response t	2月26日	224/1791	0.041558	0.091563	0.049339	PTGS2/PT
GO_BP_m:	GO:00464	organopho	2月26日	224/1791	0.041558	0.091563	0.049339	INPP5E/M
GO_BP_m:	GO:00105	regulation	1月26日	30/17913	0.042673	0.093409	0.050333	ALOX12
GO_BP_m:	GO:00506	regulation	1月26日	30/17913	0.042673	0.093409	0.050333	LCK
GO_BP_m:	GO:00507	RNA desta	1月26日	30/17913	0.042673	0.093409	0.050333	ROCK1
GO_BP_m:	GO:19029	regulation	1月26日	30/17913	0.042673	0.093409	0.050333	ROCK1
GO_BP_m:	GO:19033	regulation	2月26日	230/1791	0.043589	0.094758	0.05106	PTK2B/TR
GO_BP_m:	GO:00181	peptidyl-s	2月26日	231/1791	0.043931	0.094758	0.05106	PTGS2/RO
GO_BP_m:	GO:00026	positive re	1月26日	31/17913	0.044065	0.094758	0.05106	PTGS2
GO_BP_m:	GO:00033	epithelial c	1月26日	31/17913	0.044065	0.094758	0.05106	ROCK1
GO_BP_m:	GO:00066	phosphatic	1月26日	31/17913	0.044065	0.094758	0.05106	PLA2G4A
GO_BP_m:	GO:00308	cortical act	1月26日	31/17913	0.044065	0.094758	0.05106	ROCK1
GO_BP_m:	GO:00331	response t	1月26日	31/17913	0.044065	0.094758	0.05106	PTGS2
GO_BP_m:	GO:00459	positive re	1月26日	31/17913	0.044065	0.094758	0.05106	PTGS2
GO_BP_m:	GO:00464	phosphatic	1月26日	31/17913	0.044065	0.094758	0.05106	PLA2G4A
GO_BP_m:	GO:19033	positive re	1月26日	31/17913	0.044065	0.094758	0.05106	ROCK1
GO_BP_m:	GO:19039	regulation	1月26日	31/17913	0.044065	0.094758	0.05106	TREM2
GO_BP_m:	GO:00512	protein po	2月26日	232/1791	0.044274	0.094902	0.051138	ALOX15/P
GO_BP_m:	GO:19040	positive re	2月26日	232/1791	0.044274	0.094902	0.051138	PTGS2/PT
GO_BP_m:	GO:00324	regulation	2月26日	234/1791	0.044963	0.096226	0.051851	PTK2B/TR
GO_BP_m:	GO:00031	aortic valv	1月26日	32/17913	0.045455	0.096818	0.05217	ROCK1
GO_BP_m:	GO:00327	positive re	1月26日	32/17913	0.045455	0.096818	0.05217	TREM2
GO_BP_m:	GO:20011	positive re	1月26日	32/17913	0.045455	0.096818	0.05217	TREM2
GO_BP_m:	GO:00726	protein loc	2月26日	237/1791	0.046004	0.097832	0.052717	ROCK1/TR
GO_BP_m:	GO:00514	neuron ap	2月26日	238/1791	0.046354	0.098371	0.053007	PTK2B/RO
GO_BP_m:	GO:00313	positive re	2月26日	239/1791	0.046704	0.098371	0.053007	ALOX15/P
GO_BP_m:	GO:00109	positive re	1月26日	33/17913	0.046843	0.098371	0.053007	PPP1R12A
GO_BP_m:	GO:00328	receptor ca	1月26日	33/17913	0.046843	0.098371	0.053007	MTMR2
GO_BP_m:	GO:00328	positive re	1月26日	33/17913	0.046843	0.098371	0.053007	CYP4F2
GO_BP_m:	GO:00425	superoxide	1月26日	33/17913	0.046843	0.098371	0.053007	ALOX12
GO_BP_m:	GO:00430	positive re	1月26日	33/17913	0.046843	0.098371	0.053007	TREM2
GO_BP_m:	GO:19018	positive re	1月26日	33/17913	0.046843	0.098371	0.053007	ROCK1
GO_BP_m:	GO:00421	cellular ket	2月26日	241/1791	0.047407	0.099244	0.053478	CYP4F2/P
GO_BP_m:	GO:00972	cellular res	2月26日	241/1791	0.047407	0.099244	0.053478	PTGS1/PT
GO_BP_m:	GO:00228	regulation	2月26日	242/1791	0.04776	0.099828	0.053792	PTK2B/TR
GO_BP_m:	GO:00330	muscle cel	2月26日	243/1791	0.048114	0.100031	0.053901	ALOX12/P
GO_BP_m:	GO:00105	regulation	1月26日	34/17913	0.048229	0.100031	0.053901	PTGS2
GO_BP_m:	GO:00326	negative re	1月26日	34/17913	0.048229	0.100031	0.053901	TREM2
GO_BP_m:	GO:20002	regulation	1月26日	34/17913	0.048229	0.100031	0.053901	PTK2B
GO_BP_m:	GO:20004	positive re	1月26日	34/17913	0.048229	0.100031	0.053901	PTK2B
GO_BP_m:	GO:00519	regulation	2月26日	244/1791	0.048469	0.100374	0.054086	PTGS2/PT
GO_BP_m:	GO:00507	regulation	2月26日	245/1791	0.048825	0.100956	0.0544	PTK2B/TR
GO_BP_m:	GO:00018	maternal p	1月26日	35/17913	0.049613	0.101647	0.054772	PTGS2
GO_BP_m:	GO:00308	cortical cyt	1月26日	35/17913	0.049613	0.101647	0.054772	ROCK1
GO_BP_m:	GO:00459	positive re	1月26日	35/17913	0.049613	0.101647	0.054772	PTGS2
GO_BP_m:	GO:00482	macropha	1月26日	35/17913	0.049613	0.101647	0.054772	PTK2B
GO_BP_m:	GO:00487	regulation	1月26日	35/17913	0.049613	0.101647	0.054772	TREM2
GO_BP_m:	GO:20003	regulation	1月26日	35/17913	0.049613	0.101647	0.054772	PTK2B
GO_BP_m	GO:00601	regulation	6/116	117/1791	0.000113	0.001081	0.000761	NUP107/N

GO_BP_m…	GO:00609(	regulation	6/116	117/17913	0.000113	0.001081	0.000761	NUP107/N
GO_BP_m…	GO:00061:	regulation	5/116	75/17913	0.000126	0.001194	0.000841	NUP107/N
GO_BP_m…	GO:00070‹	regulation	3/116	16/17913	0.000139	0.001316	0.000927	CDC23/CD
GO_BP_m…	GO:00902…	regulation	4/116	41/17913	0.000141	0.001318	0.000928	HNRNPU/I
GO_BP_m…	GO:00308:	regulation	5/116	77/17913	0.000142	0.001326	0.000934	NUP107/N
GO_BP_m…	GO:00169…	protein su	5/116	78/17913	0.000151	0.0014	0.000986	NUP107/N
GO_BP_m…	GO:19000…	regulation	5/116	79/17913	0.000161	0.001478	0.00104	NUP107/N
GO_BP_m…	GO:20012!	negative re	6/116	125/1791…	0.000162	0.001484	0.001044	AURKB/CC
GO_BP_m…	GO:00616‹	cytoskelet	5/116	82/17913	0.000192	0.001739	0.001224	AURKB/CE
GO_BP_m…	GO:00069‹	nuclear en	4/116	45/17913	0.000203	0.001816	0.001279	CCNB1/CC
GO_BP_m…	GO:00458…	negative re	4/116	45/17913	0.000203	0.001816	0.001279	AURKB/CC
GO_BP_m…	GO:00434‾	regulation	5/116	84/17913	0.000214	0.00191	0.001345	NUP107/N
GO_BP_m…	GO:00190‹	viral gene	7/116	191/1791…	0.000246	0.002121	0.001493	NUP107/N
GO_BP_m…	GO:00063…	nucleosom	6/116	135/1791…	0.000247	0.002121	0.001493	KNL1/CEN
GO_BP_m…	GO:00309!	establishm	2/116	4/17913	0.000247	0.002121	0.001493	CKAP5/KIF
GO_BP_m…	GO:00482!	snoRNA lo	2/116	4/17913	0.000247	0.002121	0.001493	FBL/NOP5
GO_BP_m…	GO:19028‹	regulation	2/116	4/17913	0.000247	0.002121	0.001493	SIAH1/SIA
GO_BP_m…	GO:19028‹	negative re	2/116	4/17913	0.000247	0.002121	0.001493	SIAH1/SIA
GO_BP_m…	GO:00458‹	positive re	4/116	48/17913	0.000261	0.002223	0.001565	CDCA5/ES
GO_BP_m…	GO:19016‾	regulation	3/116	20/17913	0.000278	0.002358	0.00166	HNRNPU/I
GO_BP_m…	GO:00311…	mRNA 3'-(	5/116	89/17913	0.000281	0.002367	0.001666	CCNB1/CS
GO_BP_m…	GO:00609(	regulation	6/116	139/1791…	0.000289	0.002418	0.001703	NUP107/N
GO_BP_m…	GO:00511‹	regulation	5/116	90/17913	0.000296	0.002435	0.001714	NUP107/N
GO_BP_m…	GO:00512…	spindle ass	5/116	90/17913	0.000296	0.002435	0.001714	AURKB/HN
GO_BP_m…	GO:20011(	regulation	5/116	90/17913	0.000296	0.002435	0.001714	NUP107/N
GO_BP_m…	GO:00070(	regulation	3/116	21/17913	0.000323	0.002642	0.00186	BUB1/CDC
GO_BP_m…	GO:00517‹	negative re	4/116	52/17913	0.000356	0.002859	0.002013	AURKB/CC
GO_BP_m…	GO:00903(	mitotic spi	4/116	52/17913	0.000356	0.002859	0.002013	AURKB/HN
GO_BP_m…	GO:00513…	meiotic ce	7/116	203/1791…	0.000356	0.002859	0.002013	BUB3/ESPI
GO_BP_m…	GO:00315…	positive re	2/116	5/17913	0.00041	0.003261	0.002296	CDCA5/UE
GO_BP_m…	GO:19054‹	positive re	2/116	5/17913	0.00041	0.003261	0.002296	CCNB1/CD
GO_BP_m…	GO:00106…	negative re	9/116	352/1791…	0.000475	0.003752	0.002641	AURKB/CC
GO_BP_m…	GO:00314‹	chromatin	6/116	153/1791…	0.000482	0.003788	0.002666	KNL1/CEN
GO_BP_m…	GO:00109‾	positive re	3/116	24/17913	0.000485	0.003788	0.002666	CCNB1/CD
GO_BP_m…	GO:00900(	positive re	8/116	283/1791…	0.000512	0.003977	0.0028	AURKB/CC
GO_BP_m…	GO:00063…	chromatin	6/116	156/1791…	0.000535	0.004129	0.002907	KNL1/CEN
GO_BP_m…	GO:00066(	NLS-beari	2/116	6/17913	0.000613	0.004677	0.003293	CBLB/RAN
GO_BP_m…	GO:00309!	establishm	2/116	6/17913	0.000613	0.004677	0.003293	CKAP5/KIF
GO_BP_m…	GO:00517‹	positive re	4/116	60/17913	0.000616	0.004677	0.003293	CDCA5/ES
GO_BP_m…	GO:00311…	RNA 3'-en	5/116	107/1791…	0.000656	0.00496	0.003492	CCNB1/CS
GO_BP_m…	GO:19027!	positive re	3/116	27/17913	0.000691	0.005194	0.003656	CCNB1/CD
GO_BP_m…	GO:00063(	transcripti	4/116	62/17913	0.000697	0.005213	0.00367	HEATR1/U
GO_BP_m…	GO:19003‾	regulation	5/116	109/1791…	0.000714	0.005309	0.003738	NUP107/N
GO_BP_m…	GO:00347…	nucleosom	6/116	165/1791…	0.000718	0.005311	0.003739	KNL1/CEN
GO_BP_m…	GO:00060‹	glycolytic	5/116	110/1791…	0.000744	0.005419	0.003815	NUP107/N
GO_BP_m…	GO:00308(	regulation	5/116	110/1791…	0.000744	0.005419	0.003815	NUP107/N
GO_BP_m…	GO:00313‹	positive re	5/116	110/1791…	0.000744	0.005419	0.003815	HUWE1/PI
GO_BP_m…	GO:00400(	establishm	3/116	28/17913	0.00077	0.005579	0.003928	CENPA/ES
GO_BP_m…	GO:00067!	ATP gener	5/116	111/1791…	0.000775	0.005587	0.003933	NUP107/N
GO_BP_m…	GO:00004(	cleavage i	2/116	7/17913	0.000855	0.006097	0.004292	NOB1/NO
GO_BP_m…	GO:19018!	positive re	2/116	7/17913	0.000855	0.006097	0.004292	CCNB1/CD
GO_BP_m…	GO:00428(	pyruvate b	5/116	114/1791…	0.000875	0.006175	0.004347	NUP107/N
GO_BP_m…	GO:00511‹	regulation	5/116	114/1791…	0.000875	0.006175	0.004347	NUP107/N
GO_BP_m…	GO:00459…	negative re	8/116	308/1791…	0.000889	0.006248	0.004399	AURKB/BU
GO_BP_m…	GO:00346(	cellular res	5/116	117/1791…	0.000983	0.006871	0.004837	NUP107/N
GO_BP_m…	GO:00514‹	regulation	10/116	472/1791…	0.000991	0.006893	0.004853	CLASP2/H
GO_BP_m…	GO:00190‹	viral trans	6/116	177/1791…	0.001034	0.007152	0.005035	NUP107/N
GO_BP_m…	GO:00063(	terminatio	3/116	31/17913	0.001042	0.007152	0.005035	CSTF1/FIP:

GO_BP_m2	GO:00028	mitotic cyt	4/116	69/17913	0.001044	0.007152	0.005035	CENPA/CL
GO_BP_m2	GO:00063	chromatin	6/116	178/17913	0.001065	0.007261	0.005112	KNL1/CEN
GO_BP_m2	GO:00850	protein K6	2/116	8/17913	0.001135	0.007619	0.005364	RNF4/UBE
GO_BP_m2	GO:19038	positive re	2/116	8/17913	0.001135	0.007619	0.005364	CCNB1/CD
GO_BP_m2	GO:19054	regulation	2/116	8/17913	0.001135	0.007619	0.005364	CCNB1/CD
GO_BP_m2	GO:00460	ADP metal	5/116	121/17913	0.001143	0.007619	0.005364	NUP107/N
GO_BP_m2	GO:00302	chromoso	3/116	32/17913	0.001144	0.007619	0.005364	CCNB1/CD
GO_BP_m2	GO:00190	viral life cy	8/116	324/17913	0.00123	0.008151	0.005739	CDK1/NUF
GO_BP_m2	GO:00512	nuclear pc	2/116	9/17913	0.001453	0.009581	0.006745	AHCTF1/N
GO_BP_m2	GO:19033	positive re	5/116	128/17913	0.001467	0.00963	0.00678	HUWE1/PL
GO_BP_m2	GO:00061	nucleoside	5/116	129/17913	0.001519	0.00992	0.006984	NUP107/N
GO_BP_m2	GO:00512	establishm	3/116	36/17913	0.001617	0.010513	0.007401	CENPA/ES
GO_BP_m2	GO:00469	nucleotide	5/116	131/17913	0.001625	0.010519	0.007406	NUP107/N
GO_BP_m2	GO:00091	purine nuc	5/116	133/17913	0.001737	0.011118	0.007827	NUP107/N
GO_BP_m2	GO:00091	purine ribc	5/116	133/17913	0.001737	0.011118	0.007827	NUP107/N
GO_BP_m2	GO:00313	regulation	6/116	196/17913	0.001742	0.011118	0.007827	HUWE1/PL
GO_BP_m2	GO:00602	regulation	3/116	37/17913	0.001751	0.011128	0.007834	HNRNPU/
GO_BP_m2	GO:00109	negative re	8/116	344/17913	0.001795	0.011356	0.007995	AURKB/CC
GO_BP_m2	GO:00091	ribonuclec	5/116	135/17913	0.001855	0.011683	0.008225	NUP107/N
GO_BP_m2	GO:00165	histone ph	3/116	38/17913	0.001892	0.011863	0.008352	AURKB/CC
GO_BP_m2	GO:00324	regulation	4/116	83/17913	0.00207	0.012918	0.009094	AHCTF1/A
GO_BP_m2	GO:00354	histone-se	2/116	11/17913	0.002201	0.013555	0.009543	AURKB/CC
GO_BP_m2	GO:00451	protein ne	2/116	11/17913	0.002201	0.013555	0.009543	RNF7/UBE
GO_BP_m2	GO:00550	ventricular	2/116	11/17913	0.002201	0.013555	0.009543	CCNB1/CD
GO_BP_m2	GO:00193	nicotinami	5/116	142/17913	0.002314	0.014062	0.0099	NUP107/N
GO_BP_m2	GO:00193	pyridine nu	5/116	142/17913	0.002314	0.014062	0.0099	NUP107/N
GO_BP_m2	GO:01400	meiotic nu	5/116	142/17913	0.002314	0.014062	0.0099	ESPL1/KIF
GO_BP_m2	GO:00516	spindle loc	3/116	41/17913	0.002358	0.014269	0.010046	CENPA/ES
GO_BP_m2	GO:00436	post-trans	8/116	360/17913	0.002381	0.014345	0.010099	ASB12/ASI
GO_BP_m2	GO:00725	pyridine-c	5/116	145/17913	0.002533	0.0152	0.010701	NUP107/N
GO_BP_m2	GO:00004	maturatior	2/116	12/17913	0.00263	0.015646	0.011015	NOP14/M
GO_BP_m2	GO:19046	positive re	2/116	12/17913	0.00263	0.015646	0.011015	PLK1/UBE2
GO_BP_m2	GO:00460	ATP metak	7/116	287/17913	0.002659	0.015754	0.011091	CCNB1/CD
GO_BP_m2	GO:00060	pyruvate n	5/116	147/17913	0.002688	0.015855	0.011162	NUP107/N
GO_BP_m2	GO:00165	histone ub	3/116	44/17913	0.002888	0.016966	0.011945	HUWE1/TI
GO_BP_m2	GO:00067	ATP biosyr	5/116	150/17913	0.002933	0.017154	0.012077	NUP107/N
GO_BP_m2	GO:00457	positive re	8/116	373/17913	0.002959	0.017236	0.012134	AURKB/CC
GO_BP_m2	GO:19030	meiotic ce	5/116	151/17913	0.003018	0.017505	0.012324	ESPL1/KIF
GO_BP_m2	GO:00380	netrin-acti	2/116	13/17913	0.003095	0.017806	0.012536	SIAH1/SIA
GO_BP_m2	GO:00620	positive re	2/116	13/17913	0.003095	0.017806	0.012536	ESPL1/HN
GO_BP_m2	GO:00512	protein de	4/116	93/17913	0.00313	0.017934	0.012626	CKAP5/CL
GO_BP_m2	GO:00091	nucleoside	5/116	153/17913	0.003193	0.01822	0.012827	NUP107/N
GO_BP_m2	GO:00316	protein de	3/116	46/17913	0.00328	0.018563	0.013069	UTP25/PLI
GO_BP_m2	GO:00705	protein K6	3/116	46/17913	0.00328	0.018563	0.013069	RNF115/RI
GO_BP_m2	GO:00345	protein loc	6/116	223/17913	0.003314	0.018609	0.013101	CBLB/CDK
GO_BP_m2	GO:19033	regulation	6/116	223/17913	0.003314	0.018609	0.013101	HUWE1/PL
GO_BP_m2	GO:00977	ciliary basa	4/116	95/17913	0.003379	0.018897	0.013304	CDK1/CKA
GO_BP_m2	GO:00353	protein loc	2/116	14/17913	0.003596	0.020027	0.014099	HNRNPU/
GO_BP_m2	GO:00094	response t	5/116	158/17913	0.003663	0.020324	0.014308	NUP107/N
GO_BP_m2	GO:00092	purine ribc	5/116	159/17913	0.003763	0.020795	0.01464	NUP107/N
GO_BP_m2	GO:00091	purine nuc	5/116	160/17913	0.003865	0.021272	0.014976	NUP107/N
GO_BP_m2	GO:00469	pore comp	2/116	15/17913	0.004131	0.02265	0.015946	AHCTF1/N
GO_BP_m2	GO:00092	ribonuclec	5/116	164/17913	0.004291	0.023435	0.016499	NUP107/N
GO_BP_m2	GO:00092	purine ribc	7/116	314/17913	0.004357	0.023613	0.016624	CCNB1/CD
GO_BP_m2	GO:00062	regulation	4/116	102/17913	0.004358	0.023613	0.016624	CDK1/PDS
GO_BP_m2	GO:00091	purine ribc	7/116	317/17913	0.004587	0.02476	0.017432	CCNB1/CD
GO_BP_m2	GO:00091	purine nuc	7/116	318/17913	0.004666	0.025088	0.017662	CCNB1/CD
GO_BP_m2	GO:00090	dosage co	2/116	16/17913	0.004702	0.025088	0.017662	HNRNPU/

GO_BP_m	GO:00550	ventricular	2/116	16/17913	0.004702	0.025088	0.017662	CCNB1/CD
GO_BP_m	GO:00091	ribonuclec	7/116	319/17913	0.004746	0.025225	0.017759	CCNB1/CD
GO_BP_m	GO:00091	nucleotide	5/116	169/17913	0.004871	0.025792	0.018158	NUP107/N
GO_BP_m	GO:00091	purine nuc	7/116	321/17913	0.004908	0.025892	0.018228	CCNB1/CD
GO_BP_m	GO:00071	establishm	5/116	172/17913	0.005244	0.027558	0.019402	CENPA/CK
GO_BP_m	GO:00091	purine nuc	5/116	174/17913	0.005503	0.028707	0.02021	NUP107/N
GO_BP_m	GO:00091	purine ribo	5/116	174/17913	0.005503	0.028707	0.02021	NUP107/N
GO_BP_m	GO:00091	nucleoside	5/116	175/17913	0.005636	0.029184	0.020546	NUP107/N
GO_BP_m	GO:19012	nucleoside	5/116	175/17913	0.005636	0.029184	0.020546	NUP107/N
GO_BP_m	GO:00091	ribonuclec	7/116	331/17913	0.005784	0.029838	0.021006	CCNB1/CD
GO_BP_m	GO:00075	dosage co	2/116	18/17913	0.005944	0.030329	0.021352	HNRNPU/
GO_BP_m	GO:00344	ncRNA 5'-	2/116	18/17913	0.005944	0.030329	0.021352	NOP14/RP
GO_BP_m	GO:00518	negative re	2/116	18/17913	0.005944	0.030329	0.021352	CLASP2/R
GO_BP_m	GO:00193	pyridine nu	5/116	179/17913	0.006192	0.031364	0.02208	NUP107/N
GO_BP_m	GO:00464	nicotinami	5/116	179/17913	0.006192	0.031364	0.02208	NUP107/N
GO_BP_m	GO:00091	nucleoside	7/116	337/17913	0.006363	0.031544	0.022207	CCNB1/CD
GO_BP_m	GO:00007	karyogamy	1/116	1/17913	0.006476	0.031544	0.022207	CDK1
GO_BP_m	GO:00073	pronuclear	1/116	1/17913	0.006476	0.031544	0.022207	CDK1
GO_BP_m	GO:00466	response t	1/116	1/17913	0.006476	0.031544	0.022207	CCNB1
GO_BP_m	GO:00996	microtubu	1/116	1/17913	0.006476	0.031544	0.022207	CENPE
GO_BP_m	GO:00996	lateral atta	1/116	1/17913	0.006476	0.031544	0.022207	CENPE
GO_BP_m	GO:19028	protein loc	1/116	1/17913	0.006476	0.031544	0.022207	HNRNPU
GO_BP_m	GO:19902	histone glu	1/116	1/17913	0.006476	0.031544	0.022207	FBL
GO_BP_m	GO:19902	RNA locali	1/116	1/17913	0.006476	0.031544	0.022207	HNRNPU
GO_BP_m	GO:19903	protein K3	1/116	1/17913	0.006476	0.031544	0.022207	KLHL20
GO_BP_m	GO:20007	histone H3	1/116	1/17913	0.006476	0.031544	0.022207	CCNB1
GO_BP_m	GO:19033	negative re	2/116	19/17913	0.006616	0.032113	0.022608	CLASP2/R
GO_BP_m	GO:00725	pyridine-c	5/116	184/17913	0.006939	0.033568	0.023632	NUP107/N
GO_BP_m	GO:00160	carbohydr	5/116	185/17913	0.007096	0.03409	0.024	NUP107/N
GO_BP_m	GO:19021	regulation	5/116	185/17913	0.007096	0.03409	0.024	HNRNPU/
GO_BP_m	GO:00329	circadian r	3/116	61/17913	0.007249	0.034707	0.024434	HNRNPU/
GO_BP_m	GO:00009	RNA 5'-en	2/116	20/17913	0.00732	0.034925	0.024588	NOP14/RP
GO_BP_m	GO:00091	ribonuclec	5/116	188/17913	0.007581	0.036048	0.025378	NUP107/N
GO_BP_m	GO:00091	nucleoside	7/116	349/17913	0.00765	0.036252	0.025522	CCNB1/CD
GO_BP_m	GO:00067	oxidoredu	5/116	192/17913	0.008263	0.039025	0.027474	NUP107/N
GO_BP_m	GO:19046	regulation	2/116	22/17913	0.008825	0.041538	0.029243	PLK1/UBE2
GO_BP_m	GO:00091	nucleoside	5/116	196/17913	0.008986	0.042102	0.02964	NUP107/N
GO_BP_m	GO:00467	microtubu	3/116	66/17913	0.009005	0.042102	0.02964	CKAP5/CLA
GO_BP_m	GO:00061	regulation	5/116	198/17913	0.009364	0.043579	0.03068	NUP107/N
GO_BP_m	GO:00451	meiotic ch	3/116	67/17913	0.009383	0.043579	0.03068	ESPL1/PLK
GO_BP_m	GO:00102	endomem	7/116	365/17913	0.009653	0.044687	0.03146	KNL1/CCN
GO_BP_m	GO:00324	positive re	3/116	68/17913	0.00977	0.045079	0.031737	DET1/PLK1
GO_BP_m	GO:00076	circadian r	5/116	201/17913	0.009951	0.04576	0.032215	CDK1/HN
GO_BP_m	GO:00063	DNA-temp	3/116	69/17913	0.010167	0.046298	0.032595	CSTF1/FIP1
GO_BP_m	GO:00311	regulation	3/116	69/17913	0.010167	0.046298	0.032595	CLASP2/M
GO_BP_m	GO:00518	protein au	3/116	69/17913	0.010167	0.046298	0.032595	RNF115/R
GO_BP_m	GO:00001	establishm	2/116	24/17913	0.010455	0.046998	0.033087	CENPA/PL
GO_BP_m	GO:00020	regulation	2/116	24/17913	0.010455	0.046998	0.033087	CCNB1/CD
GO_BP_m	GO:00506	negative re	2/116	24/17913	0.010455	0.046998	0.033087	PCBP2/RIC
GO_BP_m	GO:19018	negative re	2/116	24/17913	0.010455	0.046998	0.033087	CLASP2/R
GO_BP_m	GO:00905	RNA phos	3/116	70/17913	0.010573	0.047375	0.033353	NOB1/NO
GO_BP_m	GO:00905	RNA phos	4/116	132/17913	0.010692	0.047756	0.033621	NOB1/NO
GO_BP_m	GO:00335	histone H2	2/116	25/17913	0.011317	0.050385	0.035472	TRIM37/U
GO_BP_m	GO:00724	signal tran	3/116	73/17913	0.011847	0.05241	0.036898	CCNB1/CD
GO_BP_m	GO:00724	signal tran	3/116	73/17913	0.011847	0.05241	0.036898	CCNB1/CD
GO_BP_m	GO:00344	nucleobas	5/116	211/17913	0.012089	0.053312	0.037532	NUP107/N
GO_BP_m	GO:00723	signal tran	3/116	74/17913	0.01229	0.05403	0.038038	CCNB1/CD
GO_BP_m	GO:00004	endonucle	1/116	2/17913	0.01291	0.054691	0.038503	NOP14

GO_BP_m2	GO:00090	rRNA 5'-e	1/116	2/17913	0.01291	0.054691	0.038503	NOP14
GO_BP_m2	GO:00608	random in	1/116	2/17913	0.01291	0.054691	0.038503	RLIM
GO_BP_m2	GO:00701	synaptone	1/116	2/17913	0.01291	0.054691	0.038503	PLK1
GO_BP_m2	GO:00712	cellular res	1/116	2/17913	0.01291	0.054691	0.038503	CCNB1
GO_BP_m2	GO:00723	chromoso	1/116	2/17913	0.01291	0.054691	0.038503	RCC2
GO_BP_m2	GO:19024	regulation	1/116	2/17913	0.01291	0.054691	0.038503	HNRNPU
GO_BP_m2	GO:19024	positive re	1/116	2/17913	0.01291	0.054691	0.038503	HNRNPU
GO_BP_m2	GO:19030	regulation	1/116	2/17913	0.01291	0.054691	0.038503	MAPRE1
GO_BP_m2	GO:19030	positive re	1/116	2/17913	0.01291	0.054691	0.038503	MAPRE1
GO_BP_m2	GO:19907	response t	1/116	2/17913	0.01291	0.054691	0.038503	RIOK3
GO_BP_m2	GO:19907	cellular res	1/116	2/17913	0.01291	0.054691	0.038503	RIOK3
GO_BP_m2	GO:00068	retrograde	3/116	76/17913	0.013205	0.055772	0.039264	CENPE/KIF
GO_BP_m2	GO:01400	organelle l	4/116	141/17913	0.01336	0.056255	0.039604	CDK1/CKA
GO_BP_m2	GO:00092	response t	5/116	219/17913	0.014009	0.058585	0.041245	NUP107/N
GO_BP_m2	GO:00512	establishm	2/116	28/17913	0.014081	0.058585	0.041245	CENPA/PL
GO_BP_m2	GO:00514	positive re	2/116	28/17913	0.014081	0.058585	0.041245	PLK1/UBE2
GO_BP_m2	GO:00711	protein loc	2/116	28/17913	0.014081	0.058585	0.041245	CDCA5/PL
GO_BP_m2	GO:00550	cardiac mu	3/116	78/17913	0.014158	0.058732	0.041348	CCNB1/CD
GO_BP_m2	GO:00224	cellular co	8/116	492/17913	0.014706	0.060825	0.042821	CCNB1/CC
GO_BP_m2	GO:00434	regulation	2/116	29/17913	0.01506	0.062107	0.043724	CCNB1/CD
GO_BP_m2	GO:00464	organopho	5/116	224/17913	0.015309	0.062945	0.044314	NUP107/N
GO_BP_m2	GO:00510	regulation	7/116	403/17913	0.015918	0.065259	0.045943	AURKB/CD
GO_BP_m2	GO:00315	G2 DNA d	2/116	30/17913	0.016068	0.065683	0.046242	CDK1/PLK
GO_BP_m2	GO:20000	positive re	3/116	82/17913	0.016178	0.065939	0.046422	DET1/PLK1
GO_BP_m2	GO:00224	membrane	4/116	150/17913	0.016418	0.06672	0.046972	CDK1/CKA
GO_BP_m2	GO:00550	cardiac cel	3/116	84/17913	0.017246	0.069883	0.049199	CCNB1/CD
GO_BP_m2	GO:00311	negative re	2/116	32/17913	0.018169	0.07341	0.051681	CLASP2/M
GO_BP_m2	GO:19019	negative re	5/116	235/17913	0.018446	0.074314	0.052318	AURKB/CC
GO_BP_m2	GO:00004	endonucle	1/116	3/17913	0.019303	0.07623	0.053667	NOP14
GO_BP_m2	GO:00140	regulation	1/116	3/17913	0.019303	0.07623	0.053667	CDK1
GO_BP_m2	GO:00183	peptidyl-g	1/116	3/17913	0.019303	0.07623	0.053667	FBL
GO_BP_m2	GO:00439	histone H3	1/116	3/17913	0.019303	0.07623	0.053667	AURKB
GO_BP_m2	GO:00902	regulation	1/116	3/17913	0.019303	0.07623	0.053667	RNF4
GO_BP_m2	GO:00989	dendritic t	1/116	3/17913	0.019303	0.07623	0.053667	HNRNPU
GO_BP_m2	GO:00989	dendritic t	1/116	3/17913	0.019303	0.07623	0.053667	HNRNPU
GO_BP_m2	GO:00165	histone m	7/116	420/17913	0.019495	0.076565	0.053903	AURKB/CC
GO_BP_m2	GO:00071	meiosis I	3/116	88/17913	0.019497	0.076565	0.053903	ESPL1/PLK
GO_BP_m2	GO:00513	regulation	4/116	162/17913	0.02113	0.082745	0.058254	AHCTF1/A
GO_BP_m2	GO:00619	meiosis I c	3/116	91/17913	0.021287	0.083127	0.058523	ESPL1/PLK
GO_BP_m2	GO:00091	coenzyme	5/116	245/17913	0.021643	0.084281	0.059335	NUP107/N
GO_BP_m2	GO:19018	positive re	3/116	93/17913	0.022529	0.087468	0.061579	DET1/PLK1
GO_BP_m2	GO:00165	covalent c	7/116	433/17913	0.022587	0.087468	0.061579	AURKB/CC
GO_BP_m2	GO:00081	negative re	2/116	36/17913	0.022694	0.087641	0.061701	PDS5A/SM
GO_BP_m2	GO:00070	Golgi orga	3/116	94/17913	0.023165	0.089212	0.062807	CDK1/CLA
GO_BP_m2	GO:00620	regulation	7/116	439/17913	0.024122	0.092646	0.065224	CCNB1/CD
GO_BP_m2	GO:20012	positive re	4/116	169/17913	0.024226	0.092788	0.065324	AURKB/CC
GO_BP_m2	GO:19019	negative re	5/116	254/17913	0.024812	0.094631	0.066621	AURKB/CC
GO_BP_m2	GO:00198	antigen pr	3/116	97/17913	0.025129	0.094631	0.066621	CENPE/KIF
GO_BP_m2	GO:19027	negative re	3/116	97/17913	0.025129	0.094631	0.066621	AURKB/CD
GO_BP_m2	GO:00000	ribosomal	1/116	4/17913	0.025655	0.094631	0.066621	XPO1
GO_BP_m2	GO:00000	ribosomal	1/116	4/17913	0.025655	0.094631	0.066621	XPO1
GO_BP_m2	GO:00004	endonucle	1/116	4/17913	0.025655	0.094631	0.066621	NOP14
GO_BP_m2	GO:00070	mitotic chr	1/116	4/17913	0.025655	0.094631	0.066621	CENPE
GO_BP_m2	GO:00331	positive re	1/116	4/17913	0.025655	0.094631	0.066621	RANBP2
GO_BP_m2	GO:00395	negative re	1/116	4/17913	0.025655	0.094631	0.066621	RIOK3
GO_BP_m2	GO:00448	mitotic cyt	1/116	4/17913	0.025655	0.094631	0.066621	AURKB
GO_BP_m2	GO:00606	regulation	1/116	4/17913	0.025655	0.094631	0.066621	CCNB1
GO_BP_m2	GO:00719	regulation	1/116	4/17913	0.025655	0.094631	0.066621	CDCA5

GO_BP_m	GO:00719	mitotic sis	1/116	4/17913	0.025655	0.094631	0.066621	SGO1
GO_BP_m	GO:19036	negative r	1/116	4/17913	0.025655	0.094631	0.066621	CLASP2
GO_BP_m	GO:00600	positive re	2/116	39/17913	0.02636	0.096977	0.068273	CCNB1/CD
GO_BP_m	GO:00024	antigen pr	3/116	100/17913	0.027181	0.099735	0.070215	CENPE/KIF
GO_BP_m	GO:00108	regulation	2/116	40/17913	0.027632	0.10086	0.071007	TRIM37/X
GO_BP_m	GO:00400	positive re	2/116	40/17913	0.027632	0.10086	0.071007	CLASP2/U
GO_BP_m	GO:00025	antigen pr	3/116	101/17913	0.027884	0.101516	0.071469	CENPE/KIF
GO_BP_m	GO:19035	positive re	2/116	41/17913	0.028928	0.105042	0.073951	CCNB1/CD
GO_BP_m	GO:00091	purine rib	5/116	266/17913	0.029483	0.10678	0.075175	NUP107/N
GO_BP_m	GO:00019	negative r	2/116	42/17913	0.030248	0.109268	0.076926	CLASP2/R
GO_BP_m	GO:00003	mRNA 3'-	1/116	5/17913	0.031966	0.110888	0.078067	SF1
GO_BP_m	GO:00004	endonucle	1/116	5/17913	0.031966	0.110888	0.078067	NOP14
GO_BP_m	GO:00004	endonucle	1/116	5/17913	0.031966	0.110888	0.078067	NOP14
GO_BP_m	GO:00004	box C/D s	1/116	5/17913	0.031966	0.110888	0.078067	SNU13
GO_BP_m	GO:00100	response t	1/116	5/17913	0.031966	0.110888	0.078067	CCNB1
GO_BP_m	GO:00439	histone H	1/116	5/17913	0.031966	0.110888	0.078067	CCNB1
GO_BP_m	GO:00443	protein K2	1/116	5/17913	0.031966	0.110888	0.078067	UBE2D4
GO_BP_m	GO:00458	negative r	1/116	5/17913	0.031966	0.110888	0.078067	ESPL1
GO_BP_m	GO:00709	CRD-medi	1/116	5/17913	0.031966	0.110888	0.078067	HNRNPU
GO_BP_m	GO:01100	regulation	1/116	5/17913	0.031966	0.110888	0.078067	CLASP2
GO_BP_m	GO:19033	positive re	1/116	5/17913	0.031966	0.110888	0.078067	RANBP2
GO_BP_m	GO:19042	regulation	1/116	5/17913	0.031966	0.110888	0.078067	CLASP2
GO_BP_m	GO:19042	positive re	1/116	5/17913	0.031966	0.110888	0.078067	CLASP2
GO_BP_m	GO:19056	regulation	1/116	5/17913	0.031966	0.110888	0.078067	CDCA5
GO_BP_m	GO:20005	regulation	1/116	5/17913	0.031966	0.110888	0.078067	CBLB
GO_BP_m	GO:20011	basement	1/116	5/17913	0.031966	0.110888	0.078067	CLASP2
GO_BP_m	GO:00071	male meio	2/116	44/17913	0.032958	0.114049	0.080292	KIF18A/UB
GO_BP_m	GO:19030	positive re	3/116	109/17913	0.033857	0.116869	0.082277	DET1/PLK
GO_BP_m	GO:00324	regulation	3/116	110/17913	0.034647	0.1193	0.083989	DET1/PLK
GO_BP_m	GO:00092	ribonucle	5/116	279/17913	0.035133	0.120678	0.084959	NUP107/N
GO_BP_m	GO:00061	purine nu	5/116	280/17913	0.035594	0.12196	0.085862	NUP107/N
GO_BP_m	GO:00140	response t	2/116	46/17913	0.03576	0.122041	0.085919	CDK1/RAN
GO_BP_m	GO:00718	DNA biosy	4/116	192/17913	0.036255	0.122041	0.085919	AURKB/CE
GO_BP_m	GO:00028	negative r	2/116	47/17913	0.037195	0.122041	0.085919	PCBP2/RI
GO_BP_m	GO:00451	homologo	2/116	47/17913	0.037195	0.122041	0.085919	ESPL1/PLK
GO_BP_m	GO:00514	regulation	2/116	47/17913	0.037195	0.122041	0.085919	PLK1/UBE
GO_BP_m	GO:00726	protein lo	2/116	47/17913	0.037195	0.122041	0.085919	HNRNPU/
GO_BP_m	GO:00463	ribose ph	5/116	284/17913	0.037473	0.122041	0.085919	NUP107/N
GO_BP_m	GO:00427	regulation	3/116	114/17913	0.0379	0.122041	0.085919	CDK1/PPP
GO_BP_m	GO:00436	cellular pr	4/116	195/17913	0.038037	0.122041	0.085919	CKAP5/CL
GO_BP_m	GO:00000	ribosomal	1/116	6/17913	0.038236	0.122041	0.085919	XPO1
GO_BP_m	GO:00004	maturatio	1/116	6/17913	0.038236	0.122041	0.085919	NOP14
GO_BP_m	GO:00004	small nucl	1/116	6/17913	0.038236	0.122041	0.085919	SNU13
GO_BP_m	GO:00098	abscission	1/116	6/17913	0.038236	0.122041	0.085919	AURKB
GO_BP_m	GO:00109	free ubiqu	1/116	6/17913	0.038236	0.122041	0.085919	UBE2C
GO_BP_m	GO:00163	female me	1/116	6/17913	0.038236	0.122041	0.085919	PLK1
GO_BP_m	GO:00328	negative r	1/116	6/17913	0.038236	0.122041	0.085919	SMC1A
GO_BP_m	GO:00337	ribosome	1/116	6/17913	0.038236	0.122041	0.085919	XPO1
GO_BP_m	GO:00341	positive re	1/116	6/17913	0.038236	0.122041	0.085919	PJA2
GO_BP_m	GO:00355	protein K2	1/116	6/17913	0.038236	0.122041	0.085919	UBE2D4
GO_BP_m	GO:00357	platelet-d	1/116	6/17913	0.038236	0.122041	0.085919	CBLB
GO_BP_m	GO:00458	positive re	1/116	6/17913	0.038236	0.122041	0.085919	ESPL1
GO_BP_m	GO:00708	aggresom	1/116	6/17913	0.038236	0.122041	0.085919	TRIM37
GO_BP_m	GO:00719	cohesin lo	1/116	6/17913	0.038236	0.122041	0.085919	CDCA5
GO_BP_m	GO:00901	Golgi disa	1/116	6/17913	0.038236	0.122041	0.085919	CDK1
GO_BP_m	GO:00902	regulation	1/116	6/17913	0.038236	0.122041	0.085919	RNF4
GO_BP_m	GO:00989	dendritic t	1/116	6/17913	0.038236	0.122041	0.085919	HNRNPU
GO_BP_m	GO:19019	positive re	1/116	6/17913	0.038236	0.122041	0.085919	ESPL1

GO_BP_m2	GO:190452	positive re	1/116	6/17913	0.038236	0.122041	0.085919	MAPRE1
GO_BP_m2	GO:200037	regulation	1/116	6/17913	0.038236	0.122041	0.085919	HNRNPU
GO_BP_m2	GO:200037	positive re	1/116	6/17913	0.038236	0.122041	0.085919	HNRNPU
GO_BP_m2	GO:004851	rhythmic p	5/116	287/17913	0.038922	0.123948	0.087261	CDK1/HNI
GO_BP_m2	GO:004598	positive re	2/116	49/17913	0.040129	0.126922	0.089355	CCNB1/CD
GO_BP_m2	GO:005502	positive re	2/116	49/17913	0.040129	0.126922	0.089355	CCNB1/CD
GO_BP_m2	GO:190054	positive re	2/116	49/17913	0.040129	0.126922	0.089355	CCNB1/CD
GO_BP_m2	GO:007252	purine-co	5/116	291/17913	0.040907	0.129092	0.090883	NUP107/N
GO_BP_m2	GO:004438	protein lo	2/116	50/17913	0.041627	0.13107	0.092275	HNRNPU/I
GO_BP_m2	GO:005500	cardiac mu	3/116	121/17913	0.043956	0.135139	0.09514	CCNB1/CD
GO_BP_m2	GO:000915	purine ribo	7/116	500/17913	0.043965	0.135139	0.09514	CCNB1/CD
GO_BP_m2	GO:000046	maturation	1/116	7/17913	0.044466	0.135139	0.09514	NOP14
GO_BP_m2	GO:001099	ubiquitin r	1/116	7/17913	0.044466	0.135139	0.09514	UBE2C
GO_BP_m2	GO:003246	negative re	1/116	7/17913	0.044466	0.135139	0.09514	AURKB
GO_BP_m2	GO:003408	establishm	1/116	7/17913	0.044466	0.135139	0.09514	CDCA5
GO_BP_m2	GO:003608	cleavage fu	1/116	7/17913	0.044466	0.135139	0.09514	AURKB
GO_BP_m2	GO:003953	regulation	1/116	7/17913	0.044466	0.135139	0.09514	RIOK3
GO_BP_m2	GO:005125	mitotic spi	1/116	7/17913	0.044466	0.135139	0.09514	AURKB
GO_BP_m2	GO:005130	chromoso	1/116	7/17913	0.044466	0.135139	0.09514	CENPE
GO_BP_m2	GO:007142	rRNA-con	1/116	7/17913	0.044466	0.135139	0.09514	XPO1
GO_BP_m2	GO:009021	negative re	1/116	7/17913	0.044466	0.135139	0.09514	PPP2R5A
GO_BP_m2	GO:009023	regulation	1/116	7/17913	0.044466	0.135139	0.09514	HNRNPU
GO_BP_m2	GO:009043	protein loc	1/116	7/17913	0.044466	0.135139	0.09514	PLK1
GO_BP_m2	GO:190210	positive re	1/116	7/17913	0.044466	0.135139	0.09514	ESPL1
GO_BP_m2	GO:190368	regulation	1/116	7/17913	0.044466	0.135139	0.09514	CLASP2
GO_BP_m2	GO:003221	regulation	2/116	53/17913	0.046248	0.139944	0.098522	AURKB/HN
GO_BP_m2	GO:006042	positive re	2/116	53/17913	0.046248	0.139944	0.098522	CCNB1/CD
GO_BP_m2	GO:004660	regulation	2/116	54/17913	0.047828	0.144413	0.101669	TRIM37/XI
GO_BP_m3	GO:003295	regulation	6/46	297/17913	0.000106	0.002283	0.001636	ACTR3/AR
GO_BP_m3	GO:001988	antigen pr	4/46	97/17913	0.000111	0.002351	0.001685	DNM2/KIF
GO_BP_m3	GO:003250	cytokinetic	3/46	37/17913	0.000116	0.002352	0.001686	KIF23/SNX
GO_BP_m3	GO:004848	synaptic ve	3/46	37/17913	0.000116	0.002352	0.001686	ITSN1/PAC
GO_BP_m3	GO:014023	presynapti	3/46	37/17913	0.000116	0.002352	0.001686	ITSN1/PAC
GO_BP_m3	GO:000859	regulation	4/46	99/17913	0.00012	0.002399	0.00172	AAK1/ARR
GO_BP_m3	GO:000249	antigen pr	4/46	100/17913	0.000125	0.002443	0.001751	DNM2/KIF
GO_BP_m3	GO:004749	vesicle tra	3/46	38/17913	0.000126	0.002443	0.001751	KIF1B/KIF2
GO_BP_m3	GO:000250	antigen pr	4/46	101/17913	0.00013	0.00247	0.00177	DNM2/KIF
GO_BP_m3	GO:003608	cleavage fu	2/46	7/17913	0.000134	0.00247	0.00177	SNX18/SN
GO_BP_m3	GO:007238	plus-end-	2/46	7/17913	0.000134	0.00247	0.00177	KIF23/KIFA
GO_BP_m3	GO:005165	establishm	7/46	448/17913	0.000134	0.00247	0.00177	ACTR3/DN
GO_BP_m3	GO:004574	negative re	3/46	39/17913	0.000136	0.00247	0.00177	ARRB1/EG
GO_BP_m3	GO:003573	intraciliary	3/46	40/17913	0.000146	0.002631	0.001886	DYNC2H1/
GO_BP_m3	GO:005165	spindle loc	3/46	41/17913	0.000158	0.002798	0.002006	ACTR3/KIF
GO_BP_m3	GO:007238	plus-end-	2/46	8/17913	0.000179	0.003133	0.002245	KIF23/KIFA
GO_BP_m3	GO:005149	regulation	7/46	472/17913	0.000185	0.003208	0.0023	ACTR3/AR
GO_BP_m3	GO:003297	regulation	6/46	341/17913	0.000224	0.003835	0.002749	ACTR3/AR
GO_BP_m3	GO:004207	intraciliary	3/46	48/17913	0.000253	0.004267	0.003059	DYNC2H1/
GO_BP_m3	GO:003640	synaptic ve	3/46	50/17913	0.000285	0.004761	0.003412	ITSN1/PAC
GO_BP_m3	GO:005099	regulation	3/46	51/17913	0.000303	0.00499	0.003577	DNM2/EG
GO_BP_m3	GO:001025	endomem	6/46	365/17913	0.000323	0.005267	0.003775	AGFG1/DN
GO_BP_m3	GO:001032	membrane	3/46	58/17913	0.000443	0.007133	0.005113	FCHO2/SN
GO_BP_m3	GO:005165	establishm	5/46	253/17913	0.000462	0.007349	0.005267	DNM2/KIF
GO_BP_m3	GO:000242	immune re	6/46	399/17913	0.000519	0.008165	0.005852	ACTR3/AR
GO_BP_m3	GO:000091	cytokinesis	4/46	147/17913	0.000544	0.008461	0.006064	ACTR3/KIF
GO_BP_m3	GO:190372	regulation	2/46	14/17913	0.000576	0.008863	0.006353	DNM2/WA
GO_BP_m3	GO:003276	regulation	3/46	64/17913	0.000592	0.009011	0.006459	DNM2/EG
GO_BP_m3	GO:005164	vesicle loc	5/46	270/17913	0.00062	0.009342	0.006696	DNM2/KIF
GO_BP_m3	GO:000203	G protein-	2/46	15/17913	0.000663	0.009884	0.007084	ARRB1/DN

GO_BP_m:	GO:00028	mitotic cyt	3/46	69/17913	0.000737	0.010876	0.007795	KIF23/SNX
GO_BP_m:	GO:00027	immune re	6/46	434/17913	0.000806	0.011757	0.008427	ACTR3/AR
GO_BP_m:	GO:01200	regulation	4/46	166/17913	0.000857	0.012379	0.008873	ARPC2/DN
GO_BP_m:	GO:00723	organelle t	3/46	73/17913	0.000869	0.012426	0.008907	KIF1B/KIF2
GO_BP_m:	GO:00604	regulation	4/46	168/17913	0.000896	0.012682	0.00909	ARPC2/DN
GO_BP_m:	GO:00071	establishm	4/46	172/17913	0.000978	0.013703	0.009822	ACTR3/AR
GO_BP_m:	GO:00518	positive re	4/46	173/17913	0.000999	0.013863	0.009937	EGF/EGFR/
GO_BP_m:	GO:00024	antigen pr	4/46	174/17913	0.001021	0.013888	0.009954	DNM2/KIF
GO_BP_m:	GO:00990	vesicle-me	4/46	174/17913	0.001021	0.013888	0.009954	DNM2/ITS
GO_BP_m:	GO:00198	antigen pr	4/46	176/17913	0.001065	0.014181	0.010165	DNM2/KIF
GO_BP_m:	GO:00020	desensitiza	2/46	19/17913	0.001073	0.014181	0.010165	ARRB1/DN
GO_BP_m:	GO:00224	negative a	2/46	19/17913	0.001073	0.014181	0.010165	ARRB1/DN
GO_BP_m:	GO:00975	lamellipod	3/46	79/17913	0.001093	0.014315	0.010261	ARPC2/AR
GO_BP_m:	GO:00072	Notch sign	4/46	179/17913	0.001134	0.014711	0.010544	AAK1/ARR
GO_BP_m:	GO:00105	positive re	2/46	20/17913	0.00119	0.015298	0.010965	ARPC2/DN
GO_BP_m:	GO:00510	regulation	5/46	316/17913	0.001254	0.015977	0.011452	ARRB1/DN
GO_BP_m:	GO:00420	regulation	3/46	84/17913	0.001306	0.016428	0.011775	EGF/EGFR/
GO_BP_m:	GO:00230	adaptation	2/46	21/17913	0.001313	0.016428	0.011775	ARRB1/DN
GO_BP_m:	GO:00480	antigen pr	4/46	188/17913	0.001359	0.01685	0.012078	DNM2/KIF
GO_BP_m:	GO:00902	positive re	3/46	91/17913	0.001644	0.020027	0.014355	ARRB1/EG
GO_BP_m:	GO:19011	regulation	3/46	91/17913	0.001644	0.020027	0.014355	EGF/EGFR/
GO_BP_m:	GO:00435	positive re	5/46	339/17913	0.00171	0.020393	0.014618	AGFG1/AR
GO_BP_m:	GO:00020	positive re	2/46	24/17913	0.001718	0.020393	0.014618	ARRB1/EG
GO_BP_m:	GO:00481	astrocyte a	2/46	24/17913	0.001718	0.020393	0.014618	EGFR/LDLI
GO_BP_m:	GO:00609	dendritic s	3/46	94/17913	0.001804	0.02124	0.015224	ARF4/ITSN
GO_BP_m:	GO:01200	positive re	3/46	95/17913	0.00186	0.021711	0.015562	ARPC2/DN
GO_BP_m:	GO:00163	dendrite d	4/46	216/17913	0.002257	0.025948	0.018599	ARF4/ITSN
GO_BP_m:	GO:00313	positive re	5/46	362/17913	0.002277	0.025948	0.018599	ARPC2/DN
GO_BP_m:	GO:00316	receptor ir	3/46	102/17913	0.002278	0.025948	0.018599	ARRB1/DN
GO_BP_m:	GO:19027	positive re	2/46	28/17913	0.002337	0.026406	0.018927	ARPC2/DN
GO_BP_m:	GO:00198	antigen pr	4/46	220/17913	0.002412	0.027038	0.01938	DNM2/KIF
GO_BP_m:	GO:00513	regulation	3/46	105/17913	0.002474	0.027255	0.019536	DNM2/EG
GO_BP_m:	GO:00465	positive re	1/46	1/17913	0.002568	0.027255	0.019536	KIFAP3
GO_BP_m:	GO:00466	negative re	1/46	1/17913	0.002568	0.027255	0.019536	KIF25
GO_BP_m:	GO:00712	cellular res	1/46	1/17913	0.002568	0.027255	0.019536	DNM2
GO_BP_m:	GO:19034	regulation	1/46	1/17913	0.002568	0.027255	0.019536	DNM2
GO_BP_m:	GO:19034	positive re	1/46	1/17913	0.002568	0.027255	0.019536	DNM2
GO_BP_m:	GO:19046	response t	1/46	1/17913	0.002568	0.027255	0.019536	KIF1B
GO_BP_m:	GO:00105	regulation	2/46	31/17913	0.002861	0.029912	0.021441	ARPC2/DN
GO_BP_m:	GO:00457	positive re	2/46	31/17913	0.002861	0.029912	0.021441	EGF/HIP1
GO_BP_m:	GO:00381	ERBB2 sign	2/46	32/17913	0.003047	0.031618	0.022663	EGF/EGFR
GO_BP_m:	GO:00066	phosphatid	3/46	114/17913	0.003123	0.031934	0.02289	ARF3/OCR
GO_BP_m:	GO:00073	determina	3/46	114/17913	0.003123	0.031934	0.02289	DYNC2H1/
GO_BP_m:	GO:19011	positive re	2/46	33/17913	0.003238	0.032873	0.023563	EGF/HIP1
GO_BP_m:	GO:00518	regulation	4/46	241/17913	0.003349	0.033752	0.024193	EGF/EGFR/
GO_BP_m:	GO:00450	glycerolipi	4/46	246/17913	0.003604	0.036061	0.025848	ARF3/LDLI
GO_BP_m:	GO:19000	positive re	2/46	36/17913	0.003845	0.038072	0.027289	ARPC2/DN
GO_BP_m:	GO:00098	determina	3/46	123/17913	0.003867	0.038072	0.027289	DYNC2H1/
GO_BP_m:	GO:00430	regulation	5/46	410/17913	0.003886	0.038072	0.027289	AGFG1/AR
GO_BP_m:	GO:00097	specificatio	3/46	124/17913	0.003956	0.038489	0.027588	DYNC2H1/
GO_BP_m:	GO:00468	positive re	3/46	125/17913	0.004046	0.039094	0.028022	ARRB1/EG
GO_BP_m:	GO:00434	protein kir	4/46	268/17913	0.004882	0.046851	0.033582	EGF/EGFR/
GO_BP_m:	GO:00140	astrocyte c	2/46	41/17913	0.004964	0.047306	0.033908	EGFR/LDLI
GO_BP_m:	GO:00030	regulation	1/46	2/17913	0.005129	0.047592	0.034113	YWHAE
GO_BP_m:	GO:00430	activation	1/46	2/17913	0.005129	0.047592	0.034113	EGFR
GO_BP_m:	GO:00465	regulation	1/46	2/17913	0.005129	0.047592	0.034113	KIFAP3
GO_BP_m:	GO:19025	regulation	1/46	2/17913	0.005129	0.047592	0.034113	RINT1
GO_BP_m:	GO:00615	protein loc	2/46	42/17913	0.005203	0.047959	0.034376	ARF4/DYN

GO_BP_m:	GO:19907	protein loc	4/46	276/17913	0.005415	0.049245	0.035298	EGFR/FCH
GO_BP_m:	GO:19027	regulation	2/46	43/17913	0.005448	0.049245	0.035298	ARPC2/DN
GO_BP_m:	GO:19900	cellular res	2/46	43/17913	0.005448	0.049245	0.035298	ARPC3/KIF
GO_BP_m:	GO:00512	protein ins	2/46	44/17913	0.005698	0.051176	0.036682	EGFR/YWH
GO_BP_m:	GO:19900	response t	2/46	46/17913	0.006214	0.055453	0.039748	ARPC3/KIF
GO_BP_m:	GO:00420	negative re	2/46	47/17913	0.00648	0.057458	0.041185	EGF/EGFR
GO_BP_m:	GO:00072	small GTPa	5/46	465/17913	0.006588	0.05805	0.041609	ARRB1/DN
GO_BP_m:	GO:00001	activation	2/46	49/17913	0.007026	0.060146	0.043111	EGF/EGFR
GO_BP_m:	GO:19055	non-motil	2/46	49/17913	0.007026	0.060146	0.043111	DNM2/DY
GO_BP_m:	GO:00066	glyceroph	4/46	300/17913	0.007241	0.060146	0.043111	ARF3/LDLF
GO_BP_m:	GO:00609	coronary v	2/46	50/17913	0.007307	0.060146	0.043111	DNM2/DY
GO_BP_m:	GO:00990	plasma me	2/46	50/17913	0.007307	0.060146	0.043111	SNX18/SN
GO_BP_m:	GO:00457	negative re	2/46	51/17913	0.007593	0.060146	0.043111	ARRB1/DN
GO_BP_m:	GO:19000	regulation	2/46	51/17913	0.007593	0.060146	0.043111	ARPC2/DN
GO_BP_m:	GO:00108	regulation	1/46	3/17913	0.007685	0.060146	0.043111	LDLR
GO_BP_m:	GO:00163	meiotic ch	1/46	3/17913	0.007685	0.060146	0.043111	ACTR3
GO_BP_m:	GO:00426	follicle-stir	1/46	3/17913	0.007685	0.060146	0.043111	ARRB1
GO_BP_m:	GO:00617	response t	1/46	3/17913	0.007685	0.060146	0.043111	LDLR
GO_BP_m:	GO:00724	signal tran	1/46	3/17913	0.007685	0.060146	0.043111	RINT1
GO_BP_m:	GO:19023	negative re	1/46	3/17913	0.007685	0.060146	0.043111	YWHAE
GO_BP_m:	GO:19027	positive re	1/46	3/17913	0.007685	0.060146	0.043111	EGFR
GO_BP_m:	GO:19028	negative re	1/46	3/17913	0.007685	0.060146	0.043111	DNM2
GO_BP_m:	GO:19042	regulation	1/46	3/17913	0.007685	0.060146	0.043111	RINT1
GO_BP_m:	GO:19051	positive re	1/46	3/17913	0.007685	0.060146	0.043111	LDLR
GO_BP_m:	GO:19059	negative re	1/46	3/17913	0.007685	0.060146	0.043111	YWHAE
GO_BP_m:	GO:20005	positive re	1/46	3/17913	0.007685	0.060146	0.043111	HIP1
GO_BP_m:	GO:20012	positive re	1/46	3/17913	0.007685	0.060146	0.043111	ITSN1
GO_BP_m:	GO:00464	phosphatid	3/46	159/17913	0.007878	0.061026	0.043742	ARF3/OCR
GO_BP_m:	GO:19011	negative re	2/46	52/17913	0.007884	0.061026	0.043742	EGF/EGFR
GO_BP_m:	GO:00995	synaptic ve	3/46	162/17913	0.008291	0.063826	0.045749	ITSN1/PAC
GO_BP_m:	GO:00020	regulation	2/46	55/17913	0.008787	0.06654	0.047695	ARRB1/EG
GO_BP_m:	GO:00480	platelet-de	2/46	55/17913	0.008787	0.06654	0.047695	HIP1/PIK3
GO_BP_m:	GO:19020	regulation	2/46	55/17913	0.008787	0.06654	0.047695	DNM2/DY
GO_BP_m:	GO:19038	positive re	4/46	318/17913	0.008852	0.066673	0.04779	CEP290/E
GO_BP_m:	GO:00105	positive re	2/46	57/17913	0.009413	0.07052	0.050547	ARF4/EGFI
GO_BP_m:	GO:00619	glial cell ac	2/46	59/17913	0.010058	0.071684	0.051382	EGFR/LDLI
GO_BP_m:	GO:00009	assembly c	1/46	4/17913	0.010233	0.071684	0.051382	KIF23
GO_BP_m:	GO:00009	actomyosi	1/46	4/17913	0.010233	0.071684	0.051382	KIF23
GO_BP_m:	GO:00217	orbitofron	1/46	4/17913	0.010233	0.071684	0.051382	ARPC5
GO_BP_m:	GO:00324	activation	1/46	4/17913	0.010233	0.071684	0.051382	EGFR
GO_BP_m:	GO:00335	response t	1/46	4/17913	0.010233	0.071684	0.051382	EGFR
GO_BP_m:	GO:00335	response t	1/46	4/17913	0.010233	0.071684	0.051382	EGFR
GO_BP_m:	GO:00344	response t	1/46	4/17913	0.010233	0.071684	0.051382	DNM2
GO_BP_m:	GO:00609	lipid tube a	1/46	4/17913	0.010233	0.071684	0.051382	SNX9
GO_BP_m:	GO:19001	positive re	1/46	4/17913	0.010233	0.071684	0.051382	EGF
GO_BP_m:	GO:19059	regulation	1/46	4/17913	0.010233	0.071684	0.051382	YWHAE
GO_BP_m:	GO:20006	regulation	1/46	4/17913	0.010233	0.071684	0.051382	NBAS
GO_BP_m:	GO:20006	negative re	1/46	4/17913	0.010233	0.071684	0.051382	NBAS
GO_BP_m:	GO:00458	positive re	4/46	333/17913	0.010361	0.072215	0.051762	ARRB1/EG
GO_BP_m:	GO:00431	receptor m	3/46	177/17913	0.010547	0.073147	0.05243	ARRB1/DN
GO_BP_m:	GO:00300	lamellipod	2/46	61/17913	0.010723	0.074004	0.053045	ARPC2/DN
GO_BP_m:	GO:19010	regulation	2/46	62/17913	0.011062	0.075972	0.054456	DNM2/YW
GO_BP_m:	GO:19021	regulation	3/46	185/17913	0.011881	0.080624	0.05779	DNM2/DY
GO_BP_m:	GO:00161	endosoma	3/46	188/17913	0.012406	0.080624	0.05779	CLTCL1/SN
GO_BP_m:	GO:00105	regulation	2/46	66/17913	0.012467	0.080624	0.05779	ARF4/EGFI
GO_BP_m:	GO:00421	regulation	4/46	354/17913	0.012741	0.080624	0.05779	EGF/EGFR/
GO_BP_m:	GO:00107	positive re	1/46	5/17913	0.012775	0.080624	0.05779	EGFR
GO_BP_m:	GO:00161	synaptic ve	1/46	5/17913	0.012775	0.080624	0.05779	SH3GL1

GO_BP_m	GO:00300	vesicle trai	1/46	5/17913	0.012775	0.080624	0.05779	WASL
GO_BP_m	GO:00315	activation	1/46	5/17913	0.012775	0.080624	0.05779	ARF4
GO_BP_m	GO:00448	actomyosi	1/46	5/17913	0.012775	0.080624	0.05779	KIF23
GO_BP_m	GO:00516	actin filam	1/46	5/17913	0.012775	0.080624	0.05779	ARPC5
GO_BP_m	GO:00618	negative re	1/46	5/17913	0.012775	0.080624	0.05779	LDLR
GO_BP_m	GO:00801	response t	1/46	5/17913	0.012775	0.080624	0.05779	EGFR
GO_BP_m	GO:19000	regulation	1/46	5/17913	0.012775	0.080624	0.05779	EGFR
GO_BP_m	GO:19000	positive re	1/46	5/17913	0.012775	0.080624	0.05779	EGFR
GO_BP_m	GO:19023	regulation	1/46	5/17913	0.012775	0.080624	0.05779	YWHAE
GO_BP_m	GO:19043	positive re	1/46	5/17913	0.012775	0.080624	0.05779	LDLR
GO_BP_m	GO:19059	negative re	1/46	5/17913	0.012775	0.080624	0.05779	LDLR
GO_BP_m	GO:19900	retrograde	1/46	5/17913	0.012775	0.080624	0.05779	KIF1B
GO_BP_m	GO:00002	nuclear div	4/46	357/17913	0.013107	0.082091	0.058842	ACTR3/EG
GO_BP_m	GO:00457	positive re	3/46	192/17913	0.013125	0.082091	0.058842	EGF/LDLR/
GO_BP_m	GO:00426	ovulation	2/46	68/17913	0.013197	0.082175	0.058901	ARRB1/EG
GO_BP_m	GO:20003	regulation	3/46	194/17913	0.013494	0.08365	0.059959	ARF4/DNM
GO_BP_m	GO:19000	positive re	2/46	69/17913	0.013569	0.083746	0.060028	ITSN1/PAC
GO_BP_m	GO:00432	regulation	3/46	196/17913	0.013869	0.085218	0.061083	ARRB1/HIF
GO_BP_m	GO:00601	positive re	2/46	70/17913	0.013946	0.085318	0.061154	ARF4/EGFI
GO_BP_m	GO:00423	vasoconstr	2/46	71/17913	0.014327	0.087268	0.062552	EGFR/PIK3
GO_BP_m	GO:00902	regulation	3/46	199/17913	0.014441	0.087587	0.06278	ARRB1/EG
GO_BP_m	GO:00309	forebrain	4/46	371/17913	0.014906	0.088641	0.063536	ARPC5/DY
GO_BP_m	GO:00106	positive re	1/46	6/17913	0.015311	0.088641	0.063536	HIP1
GO_BP_m	GO:00324	positive re	1/46	6/17913	0.015311	0.088641	0.063536	EGFR
GO_BP_m	GO:00332	meiotic cy	1/46	6/17913	0.015311	0.088641	0.063536	ACTR3
GO_BP_m	GO:00606	regulation	1/46	6/17913	0.015311	0.088641	0.063536	LDLR
GO_BP_m	GO:00701	response t	1/46	6/17913	0.015311	0.088641	0.063536	EGFR
GO_BP_m	GO:00704	prolactin s	1/46	6/17913	0.015311	0.088641	0.063536	EGFR
GO_BP_m	GO:00723	clathrin co	1/46	6/17913	0.015311	0.088641	0.063536	SH3GL1
GO_BP_m	GO:00901	receptor-r	1/46	6/17913	0.015311	0.088641	0.063536	LDLR
GO_BP_m	GO:19052	regulation	1/46	6/17913	0.015311	0.088641	0.063536	ITSN1
GO_BP_m	GO:20012	regulation	1/46	6/17913	0.015311	0.088641	0.063536	ITSN1
GO_BP_m	GO:01500	neuroinfla	2/46	76/17913	0.016298	0.093967	0.067354	EGFR/LDLI
GO_BP_m	GO:00487	astrocyte	2/46	77/17913	0.016706	0.095922	0.068755	EGFR/LDLI
GO_BP_m	GO:00464	glycerolipi	4/46	385/17913	0.016854	0.09638	0.069083	ARF3/LDLI
GO_BP_m	GO:00464	glyceroph	3/46	213/17913	0.017292	0.097637	0.069984	ARF3/OCR
GO_BP_m	GO:00431	negative re	2/46	79/17913	0.017534	0.097637	0.069984	ARRB1/YW
GO_BP_m	GO:00977	negative re	2/46	79/17913	0.017534	0.097637	0.069984	EGFR/PIK3
GO_BP_m	GO:00465	regulation	3/46	215/17913	0.017723	0.097637	0.069984	ARRB1/DN
GO_BP_m	GO:00512	mitotic spi	1/46	7/17913	0.017841	0.097637	0.069984	KIF23
GO_BP_m	GO:00513	chromoso	1/46	7/17913	0.017841	0.097637	0.069984	ACTR3
GO_BP_m	GO:00723	vesicle unc	1/46	7/17913	0.017841	0.097637	0.069984	SH3GL1
GO_BP_m	GO:00902	positive re	1/46	7/17913	0.017841	0.097637	0.069984	ARRB1
GO_BP_m	GO:01500	amyloid-b	1/46	7/17913	0.017841	0.097637	0.069984	LDLR
GO_BP_m	GO:19002	positive re	1/46	7/17913	0.017841	0.097637	0.069984	SH3GL1
GO_BP_m	GO:19900	anterograc	1/46	7/17913	0.017841	0.097637	0.069984	KIF1B
GO_BP_m	GO:00508	synapse or	4/46	394/17913	0.018188	0.09915	0.071069	ARF4/ITSN
GO_BP_m	GO:00482	organelle f	4/46	395/17913	0.01834	0.09943	0.071269	ACTR3/EG
GO_BP_m	GO:00508	regulation	3/46	218/17913	0.018381	0.09943	0.071269	ARF4/ITSN
GO_BP_m	GO:00988	nuclear ch	3/46	220/17913	0.018828	0.100105	0.071753	ACTR3/KIF
GO_BP_m	GO:00508	regulation	3/46	222/17913	0.01928	0.100105	0.071753	ARF4/ITSN
GO_BP_m	GO:20001	regulation	3/46	222/17913	0.01928	0.100105	0.071753	ARRB1/HIF
GO_BP_m	GO:00066	phospholip	4/46	402/17913	0.019428	0.100105	0.071753	ARF3/LDLI
GO_BP_m	GO:00512	positive re	4/46	403/17913	0.019586	0.100105	0.071753	ARRB1/CE
GO_BP_m	GO:19013	regulation	2/46	84/17913	0.019679	0.100105	0.071753	DNM2/YW
GO_BP_m	GO:00030	regulation	1/46	8/17913	0.020364	0.100105	0.071753	YWHAE
GO_BP_m	GO:00072	neuron-ne	1/46	8/17913	0.020364	0.100105	0.071753	KIF1B
GO_BP_m	GO:00219	positive re	1/46	8/17913	0.020364	0.100105	0.071753	EGF

GO_BP_m3	GO:003091	otic vesicle	1/46	8/17913	0.020364	0.100105	0.071753	CEP290
GO_BP_m3	GO:003225	dense core	1/46	8/17913	0.020364	0.100105	0.071753	KIF1B
GO_BP_m3	GO:003463	phosphatid	1/46	8/17913	0.020364	0.100105	0.071753	LDLR
GO_BP_m3	GO:004660	regulation	1/46	8/17913	0.020364	0.100105	0.071753	KIF25
GO_BP_m3	GO:004879	pronephro	1/46	8/17913	0.020364	0.100105	0.071753	CEP290
GO_BP_m3	GO:009915	regulation	1/46	8/17913	0.020364	0.100105	0.071753	ITSN1
GO_BP_m3	GO:009951	actin filam	1/46	8/17913	0.020364	0.100105	0.071753	WASL
GO_BP_m3	GO:009951	dense core	1/46	8/17913	0.020364	0.100105	0.071753	KIF1B
GO_BP_m3	GO:190012	regulation	1/46	8/17913	0.020364	0.100105	0.071753	EGF
GO_BP_m3	GO:190195	dense core	1/46	8/17913	0.020364	0.100105	0.071753	KIF1B
GO_BP_m3	GO:190290	regulation	1/46	8/17913	0.020364	0.100105	0.071753	EGF
GO_BP_m3	GO:190290	positive re	1/46	8/17913	0.020364	0.100105	0.071753	EGF
GO_BP_m3	GO:190510	regulation	1/46	8/17913	0.020364	0.100105	0.071753	LDLR
GO_BP_m3	GO:190590	regulation	1/46	8/17913	0.020364	0.100105	0.071753	LDLR
GO_BP_m3	GO:199003	calcium ion	1/46	8/17913	0.020364	0.100105	0.071753	YWHAE
GO_BP_m3	GO:200058	regulation	1/46	8/17913	0.020364	0.100105	0.071753	HIP1
GO_BP_m3	GO:200060	positive re	1/46	8/17913	0.020364	0.100105	0.071753	WASL
GO_BP_m3	GO:003313	positive re	2/46	86/17913	0.020567	0.100397	0.071962	ARRB1/EG
GO_BP_m3	GO:003444	substrate a	2/46	86/17913	0.020567	0.100397	0.071962	ARPC2/DN
GO_BP_m3	GO:200011	negative re	2/46	87/17913	0.021017	0.102237	0.073281	ARRB1/YW
GO_BP_m3	GO:005122	spindle ass	2/46	90/17913	0.022391	0.107933	0.077364	HAUS3/KIF
GO_BP_m3	GO:007265	protein loc	3/46	237/17913	0.022864	0.107933	0.077364	EGFR/FCH
GO_BP_m3	GO:014001	mitotic nuc	3/46	237/17913	0.022864	0.107933	0.077364	EGF/KIF23/
GO_BP_m3	GO:000002	mitotic spi	1/46	9/17913	0.022881	0.107933	0.077364	KIF23
GO_BP_m3	GO:003438	chylomicro	1/46	9/17913	0.022881	0.107933	0.077364	LDLR
GO_BP_m3	GO:006012	positive re	1/46	9/17913	0.022881	0.107933	0.077364	ITSN1
GO_BP_m3	GO:007183	triglyceride	1/46	9/17913	0.022881	0.107933	0.077364	LDLR
GO_BP_m3	GO:190294	protein loc	1/46	9/17913	0.022881	0.107933	0.077364	EGF
GO_BP_m3	GO:190342	positive re	1/46	9/17913	0.022881	0.107933	0.077364	SH3GL1
GO_BP_m3	GO:005120	protein de	2/46	93/17913	0.023802	0.111736	0.08009	KIF18B/SH
GO_BP_m3	GO:003007	peptide ho	3/46	241/17913	0.023877	0.111736	0.08009	ARRB1/EG
GO_BP_m3	GO:000703	Golgi orga	2/46	94/17913	0.02428	0.111736	0.08009	DNM2/DY
GO_BP_m3	GO:006019	regulation	2/46	94/17913	0.02428	0.111736	0.08009	ARF4/EGF
GO_BP_m3	GO:009732	response t	2/46	96/17913	0.025248	0.111736	0.08009	EGFR/KIF1
GO_BP_m3	GO:001074	regulation	1/46	10/17913	0.025391	0.111736	0.08009	EGFR
GO_BP_m3	GO:002192	cell prolife	1/46	10/17913	0.025391	0.111736	0.08009	EGF
GO_BP_m3	GO:002193	cerebellar	1/46	10/17913	0.025391	0.111736	0.08009	EGF
GO_BP_m3	GO:002193	regulation	1/46	10/17913	0.025391	0.111736	0.08009	EGF
GO_BP_m3	GO:003242	regulation	1/46	10/17913	0.025391	0.111736	0.08009	EGFR
GO_BP_m3	GO:003537	sterol impo	1/46	10/17913	0.025391	0.111736	0.08009	LDLR
GO_BP_m3	GO:004435	macropino	1/46	10/17913	0.025391	0.111736	0.08009	DNM2
GO_BP_m3	GO:004574	positive re	1/46	10/17913	0.025391	0.111736	0.08009	EGF
GO_BP_m3	GO:005123	spindle elo	1/46	10/17913	0.025391	0.111736	0.08009	KIF23
GO_BP_m3	GO:005125	spindle mi	1/46	10/17913	0.025391	0.111736	0.08009	KIF23
GO_BP_m3	GO:006028	epithelial c	1/46	10/17913	0.025391	0.111736	0.08009	OFD1
GO_BP_m3	GO:007050	cholestero	1/46	10/17913	0.025391	0.111736	0.08009	LDLR
GO_BP_m3	GO:007148	cellular res	1/46	10/17913	0.025391	0.111736	0.08009	DNM2
GO_BP_m3	GO:007160	otic vesicle	1/46	10/17913	0.025391	0.111736	0.08009	CEP290
GO_BP_m3	GO:190380	positive re	1/46	10/17913	0.025391	0.111736	0.08009	EGFR
GO_BP_m3	GO:190435	regulation	1/46	10/17913	0.025391	0.111736	0.08009	LDLR
GO_BP_m3	GO:000761	learning or	3/46	247/17913	0.025442	0.111736	0.08009	ARF4/EGF
GO_BP_m3	GO:190495	positive re	4/46	440/17913	0.026028	0.113834	0.081594	ARRB1/CE
GO_BP_m3	GO:003030	cholestero	2/46	98/17913	0.026232	0.113834	0.081594	EGF/LDLR
GO_BP_m3	GO:004320	regulation	2/46	98/17913	0.026232	0.113834	0.081594	DNM2/YW
GO_BP_m3	GO:004340	positive re	3/46	250/17913	0.026244	0.113834	0.081594	ARRB1/EG
GO_BP_m3	GO:000865	phospholip	3/46	251/17913	0.026515	0.114653	0.082181	ARF3/OCR
GO_BP_m3	GO:006084	artery dev	2/46	100/17913	0.027231	0.116314	0.083372	DNM2/LD
GO_BP_m3	GO:004688	regulation	3/46	254/17913	0.027336	0.116314	0.083372	ARRB1/EG

GO_BP_m	GO:20003	positive re	2/46	101/17913	0.027737	0.116314	0.083372	DNM2/EG
GO_BP_m	GO:00071	mitotic ce	1/46	11/17913	0.027895	0.116314	0.083372	KIF25
GO_BP_m	GO:00215	cell prolife	1/46	11/17913	0.027895	0.116314	0.083372	EGF
GO_BP_m	GO:00451	apical prot	1/46	11/17913	0.027895	0.116314	0.083372	ARF4
GO_BP_m	GO:00725	caveolin-n	1/46	11/17913	0.027895	0.116314	0.083372	ITSN1
GO_BP_m	GO:19016	calcium io	1/46	11/17913	0.027895	0.116314	0.083372	YWHAE
GO_BP_m	GO:19039	negative r	1/46	11/17913	0.027895	0.116314	0.083372	LDLR
GO_BP_m	GO:19051	lysosomal	1/46	11/17913	0.027895	0.116314	0.083372	LDLR
GO_BP_m	GO:19052	regulation	1/46	11/17913	0.027895	0.116314	0.083372	ITSN1
GO_BP_m	GO:19056	positive re	1/46	11/17913	0.027895	0.116314	0.083372	EGF
GO_BP_m	GO:00002	microtubu	4/46	460/17913	0.029986	0.122712	0.087957	HAUS3/KI
GO_BP_m	GO:00217	limbic syst	2/46	106/17913	0.03032	0.122712	0.087957	ARPC5/YW
GO_BP_m	GO:00071	activation	1/46	12/17913	0.030393	0.122712	0.087957	EGF
GO_BP_m	GO:00109	magnesiur	1/46	12/17913	0.030393	0.122712	0.087957	EGFR
GO_BP_m	GO:00322	secretory	1/46	12/17913	0.030393	0.122712	0.087957	KIF1B
GO_BP_m	GO:00421	lipoprotei	1/46	12/17913	0.030393	0.122712	0.087957	LDLR
GO_BP_m	GO:00512	centrosom	1/46	12/17913	0.030393	0.122712	0.087957	KIF25
GO_BP_m	GO:00988	modificati	1/46	12/17913	0.030393	0.122712	0.087957	ITSN1
GO_BP_m	GO:19020	negative r	1/46	12/17913	0.030393	0.122712	0.087957	DNM2
GO_BP_m	GO:19028	regulation	1/46	12/17913	0.030393	0.122712	0.087957	DNM2
GO_BP_m	GO:19056	regulation	1/46	12/17913	0.030393	0.122712	0.087957	EGF
GO_BP_m	GO:00159	sterol tran	2/46	107/17913	0.030848	0.124189	0.089016	EGF/LDLR
GO_BP_m	GO:00725	reactive o	3/46	269/17913	0.031644	0.127031	0.091053	ARF4/DN
GO_BP_m	GO:00108	positive re	2/46	110/17913	0.032453	0.128333	0.091987	ARPC2/D
GO_BP_m	GO:00074	midgut de	1/46	13/17913	0.032885	0.128333	0.091987	EGFR
GO_BP_m	GO:00300	maintenan	1/46	13/17913	0.032885	0.128333	0.091987	ARPC5
GO_BP_m	GO:00302	intestinal	1/46	13/17913	0.032885	0.128333	0.091987	LDLR
GO_BP_m	GO:00350	regulation	1/46	13/17913	0.032885	0.128333	0.091987	DNM2
GO_BP_m	GO:00601	regulation	1/46	13/17913	0.032885	0.128333	0.091987	ITSN1
GO_BP_m	GO:00606	regulation	1/46	13/17913	0.032885	0.128333	0.091987	RINT1
GO_BP_m	GO:00610	eyelid dev	1/46	13/17913	0.032885	0.128333	0.091987	EGFR
GO_BP_m	GO:00618	regulation	1/46	13/17913	0.032885	0.128333	0.091987	LDLR
GO_BP_m	GO:00902	regulation	1/46	13/17913	0.032885	0.128333	0.091987	ARRB1
GO_BP_m	GO:00217	glial cell d	2/46	111/17913	0.032995	0.128407	0.09204	EGFR/LDL
GO_BP_m	GO:00027	positive re	3/46	275/17913	0.033462	0.129097	0.092534	ARRB1/EG
GO_BP_m	GO:00070	chromoso	3/46	275/17913	0.033462	0.129097	0.092534	ACTR3/KI
GO_BP_m	GO:00070	centrosom	2/46	112/17913	0.033541	0.129097	0.092534	HAUS3/KI
GO_BP_m	GO:00219	cerebral c	2/46	112/17913	0.033541	0.129097	0.092534	EGFR/YW
GO_BP_m	GO:00108	positive re	1/46	14/17913	0.03537	0.131442	0.094215	LDLR
GO_BP_m	GO:00302	hyalurona	1/46	14/17913	0.03537	0.131442	0.094215	EGF
GO_BP_m	GO:00343	regulation	1/46	14/17913	0.03537	0.131442	0.094215	WASL
GO_BP_m	GO:00357	platelet-d	1/46	14/17913	0.03537	0.131442	0.094215	HIP1
GO_BP_m	GO:00466	negative r	1/46	14/17913	0.03537	0.131442	0.094215	KIF25
GO_BP_m	GO:00487	negative r	1/46	14/17913	0.03537	0.131442	0.094215	LDLR
GO_BP_m	GO:00511	positive re	1/46	14/17913	0.03537	0.131442	0.094215	WASL
GO_BP_m	GO:00718	regulation	1/46	14/17913	0.03537	0.131442	0.094215	KIF9
GO_BP_m	GO:00724	signal tran	1/46	14/17913	0.03537	0.131442	0.094215	RINT1
GO_BP_m	GO:00996	protein tra	1/46	14/17913	0.03537	0.131442	0.094215	HIP1
GO_BP_m	GO:00996	neurotrans	1/46	14/17913	0.03537	0.131442	0.094215	HIP1
GO_BP_m	GO:19033	cellular res	1/46	14/17913	0.03537	0.131442	0.094215	DNM2
GO_BP_m	GO:20006	positive re	1/46	14/17913	0.03537	0.131442	0.094215	DNM2
GO_BP_m	GO:00900	positive re	3/46	283/17913	0.03597	0.133317	0.095559	EGF/EGFR
GO_BP_m	GO:00432	positive re	2/46	118/17913	0.036891	0.1355	0.097124	ARRB1/HI
GO_BP_m	GO:00508	cognition	3/46	286/17913	0.036935	0.1355	0.097124	ARF4/EGF
GO_BP_m	GO:00331	regulation	2/46	119/17913	0.037461	0.1355	0.097124	ARRB1/EG
GO_BP_m	GO:00098	negative r	3/46	288/17913	0.037585	0.1355	0.097124	EGFR/KIF2
GO_BP_m	GO:00070	protein ca	1/46	15/17913	0.037849	0.1355	0.097124	LDLR
GO_BP_m	GO:00510	positive re	1/46	15/17913	0.037849	0.1355	0.097124	SNX9

GO_BP_m3	GO:00702(	peptidyl-s	1/46	15/17913	0.037849	0.1355	0.097124	YWHAE
GO_BP_m3	GO:00715!	otic vesicle	1/46	15/17913	0.037849	0.1355	0.097124	CEP290
GO_BP_m3	GO:00988!	intestinal li	1/46	15/17913	0.037849	0.1355	0.097124	LDLR
GO_BP_m3	GO:19033!	response t	1/46	15/17913	0.037849	0.1355	0.097124	DNM2
GO_BP_m3	GO:19033!	regulation	1/46	15/17913	0.037849	0.1355	0.097124	DNM2
GO_BP_m3	GO:19900(	amyloid fil	1/46	15/17913	0.037849	0.1355	0.097124	LDLR
GO_BP_m3	GO:20004(	negative re	1/46	15/17913	0.037849	0.1355	0.097124	WASL
GO_BP_m3	GO:00703	ERK1 and		3/46	289/17913	0.037913	0.1355	0.097124	ARRB1/EG
GO_BP_m3	GO:19054	positive re	2/46	120/17913	0.038035	0.13559	0.097188	EGFR/YWH
GO_BP_m3	GO:00467	viral entry	2/46	121/17913	0.038612	0.137298	0.098413	EGFR/LDLI
GO_BP_m3	GO:00310	microtubu	2/46	122/17913	0.039193	0.13901	0.099639	HAUS3/KII
GO_BP_m3	GO:00016	acrosome	1/46	16/17913	0.040322	0.139284	0.099836	AGFG1
GO_BP_m3	GO:00080!	retrograde	1/46	16/17913	0.040322	0.139284	0.099836	KIF1B
GO_BP_m3	GO:00302!	growth ho	1/46	16/17913	0.040322	0.139284	0.099836	ITSN1
GO_BP_m3	GO:00350	positive re	1/46	16/17913	0.040322	0.139284	0.099836	ARRB1
GO_BP_m3	GO:00457	positive re	1/46	16/17913	0.040322	0.139284	0.099836	EGFR
GO_BP_m3	GO:00468!	positive re	1/46	16/17913	0.040322	0.139284	0.099836	EGFR
GO_BP_m3	GO:00717	cellular res	1/46	16/17913	0.040322	0.139284	0.099836	DNM2
GO_BP_m3	GO:00990	modificati	1/46	16/17913	0.040322	0.139284	0.099836	ITSN1
GO_BP_m3	GO:19002	regulation	1/46	16/17913	0.040322	0.139284	0.099836	SH3GL1
GO_BP_m3	GO:20000(	regulation	1/46	16/17913	0.040322	0.139284	0.099836	RINT1
GO_BP_m3	GO:00350	regulation	2/46	124/17913	0.040364	0.139284	0.099836	ARRB1/ITS
GO_BP_m3	GO:00352!	regulation	2/46	125/17913	0.040954	0.140629	0.100801	EGFR/PIK3
GO_BP_m3	GO:00977	regulation	2/46	125/17913	0.040954	0.140629	0.100801	EGFR/PIK3
GO_BP_m3	GO:00468	hormone s	3/46	301/17913	0.041958	0.143724	0.103019	ARRB1/EG
GO_BP_m3	GO:00321	activation	3/46	303/17913	0.042653	0.144103	0.103291	ARRB1/EG
GO_BP_m3	GO:00025	platelet de	2/46	128/17913	0.042745	0.144103	0.103291	EGF/TUBA
GO_BP_m3	GO:00170	response t	1/46	17/17913	0.042789	0.144103	0.103291	KIF1B
GO_BP_m3	GO:00329	positive re	1/46	17/17913	0.042789	0.144103	0.103291	EGFR
GO_BP_m3	GO:00343	high-dens	1/46	17/17913	0.042789	0.144103	0.103291	LDLR
GO_BP_m3	GO:00346	cellular pr	1/46	17/17913	0.042789	0.144103	0.103291	WASL
GO_BP_m3	GO:00442	lipid diges	1/46	17/17913	0.042789	0.144103	0.103291	LDLR
GO_BP_m3	GO:19033(	positive re	2/46	129/17913	0.043349	0.14564	0.104392	EGF/LDLR
GO_BP_m3	GO:00094	response t	3/46	307/17913	0.04406	0.147674	0.10585	ARRB1/DN
GO_BP_m3	GO:00459	negative re	3/46	308/17913	0.044415	0.14851	0.106449	EGFR/KIF2
GO_BP_m3	GO:00000	mitotic sist	2/46	132/17913	0.045179	0.149163	0.106917	KIF23/KIF2
GO_BP_m3	GO:00072	neuromusc	1/46	18/17913	0.045249	0.149163	0.106917	KIF1B
GO_BP_m3	GO:00380	peptidyl-t	1/46	18/17913	0.045249	0.149163	0.106917	EGFR
GO_BP_m3	GO:00607	mammary	1/46	18/17913	0.045249	0.149163	0.106917	EGF
GO_BP_m3	GO:00613	mammary	1/46	18/17913	0.045249	0.149163	0.106917	EGF
GO_BP_m3	GO:00718(	podosome	1/46	18/17913	0.045249	0.149163	0.106917	KIF9
GO_BP_m3	GO:00099	hormone t	3/46	311/17913	0.04549	0.149604	0.107234	ARRB1/EG
GO_BP_m3	GO:00508	regulation	2/46	133/17913	0.045795	0.150255	0.1077	EGFR/PIK3
GO_BP_m3	GO:00302(	entry into	2/46	134/17913	0.046415	0.150525	0.107894	EGFR/LDLI
GO_BP_m3	GO:00351!	regulation	2/46	134/17913	0.046415	0.150525	0.107894	EGFR/PIK3
GO_BP_m3	GO:00444(	entry into	2/46	134/17913	0.046415	0.150525	0.107894	EGFR/LDLI
GO_BP_m3	GO:00518(	entry into	2/46	134/17913	0.046415	0.150525	0.107894	EGFR/LDLI
GO_BP_m3	GO:00518	entry into	2/46	134/17913	0.046415	0.150525	0.107894	EGFR/LDLI
GO_BP_m3	GO:00902(	positive re	2/46	135/17913	0.047037	0.150863	0.108136	EGF/EGFR
GO_BP_m3	GO:20010!	positive re	2/46	135/17913	0.047037	0.150863	0.108136	ARRB1/HII
GO_BP_m3	GO:00070!	mitotic G2	1/46	19/17913	0.047703	0.150863	0.108136	RINT1
GO_BP_m3	GO:00346	establishm	1/46	19/17913	0.047703	0.150863	0.108136	KIF1B
GO_BP_m3	GO:00464	glyceropho	1/46	19/17913	0.047703	0.150863	0.108136	LDLR
GO_BP_m3	GO:00474!	mitochonc	1/46	19/17913	0.047703	0.150863	0.108136	KIF1B
GO_BP_m3	GO:00516	nucleus lo	1/46	19/17913	0.047703	0.150863	0.108136	KIF25
GO_BP_m3	GO:00717	response t	1/46	19/17913	0.047703	0.150863	0.108136	DNM2
GO_BP_m3	GO:19021	cellular res	1/46	19/17913	0.047703	0.150863	0.108136	DNM2
GO_BP_m3	GO:19023(	positive re	1/46	19/17913	0.047703	0.150863	0.108136	DNM2

GO_BP_m3	GO:190379	regulation	1/46	19/17913	0.047703	0.150863	0.108136	EGFR
GO_BP_m3	GO:00107	positive re	2/46	138/17913	0.048923	0.154193	0.110522	ARPC2/DN
GO_BP_m3	GO:00434	regulation	3/46	322/17913	0.049542	0.154193	0.110522	ARRB1/EG
GO_BP_m3	GO:007190	positive re	3/46	322/17913	0.049542	0.154193	0.110522	ARRB1/EG
GO_BP_m3	GO:00507	regulation	2/46	139/17913	0.049558	0.154193	0.110522	ITSN1/PAC
GO_BP_m4	GO:00701	mitochond	1月16日	2/17913	0.001786	0.004286	0.001316	MTIF2
GO_BP_m4	GO:00064	regulation	3月16日	397/17913	0.004881	0.01065	0.00327	MTIF2/PTC
GO_BP_m4	GO:00327	ribosome	1月16日	7/17913	0.006237	0.012474	0.00383	MTIF2
GO_BP_m4	GO:00342	regulation	3月16日	452/17913	0.006997	0.012918	0.003966	MTIF2/PTC
GO_BP_m4	GO:00329	ribonucleo	1月16日	13/17913	0.011554	0.019806	0.006081	MTIF2
GO_BP_m4	GO:00701	regulation	1月16日	25/17913	0.022107	0.035371	0.01086	TSFM
GO_BP_m4	GO:00427	DNA dama	1月16日	38/17913	0.033421	0.048404	0.014861	MRPS35
GO_BP_m4	GO:00327	regulation	1月16日	39/17913	0.034286	0.048404	0.014861	TSFM
GO_BP_m5	GO:00333	negative re	1月15日	1/17913	0.000837	0.012979	0.007492	GTPBP4
GO_BP_m5	GO:00513	tRNA acety	1月15日	1/17913	0.000837	0.012979	0.007492	NAT10
GO_BP_m5	GO:19908	RNA acety	1月15日	1/17913	0.000837	0.012979	0.007492	NAT10
GO_BP_m5	GO:00064	tRNA mod	2月15日	61/17913	0.001164	0.015973	0.00922	NAT10/NS
GO_BP_m5	GO:00063	transcriptio	2月15日	62/17913	0.001202	0.015973	0.00922	POLR1B/P
GO_BP_m5	GO:00171	nucleologe	1月15日	2/17913	0.001674	0.018317	0.010573	POLR1B
GO_BP_m5	GO:00331	negative re	1月15日	2/17913	0.001674	0.018317	0.010573	PWP1
GO_BP_m5	GO:00333	regulation	1月15日	2/17913	0.001674	0.018317	0.010573	GTPBP4
GO_BP_m5	GO:00322	regulation	2月15日	79/17913	0.001943	0.020075	0.011589	GNL3/NAT
GO_BP_m5	GO:00000	ribosomal	1月15日	4/17913	0.003346	0.028745	0.016593	SDAD1
GO_BP_m5	GO:00004	maturation	1月15日	4/17913	0.003346	0.028745	0.016593	GTPBP4
GO_BP_m5	GO:00004	maturation	1月15日	4/17913	0.003346	0.028745	0.016593	GTPBP4
GO_BP_m5	GO:00080	tRNA proc	2月15日	105/17913	0.0034	0.028745	0.016593	NAT10/NS
GO_BP_m5	GO:00510	regulation	3月15日	403/17913	0.004205	0.034005	0.019629	GNL3/GTP
GO_BP_m5	GO:00094	RNA modi	2月15日	120/17913	0.004414	0.034208	0.019747	NAT10/NS
GO_BP_m5	GO:00000	ribosomal	1月15日	6/17913	0.005014	0.034544	0.019941	SDAD1
GO_BP_m5	GO:00337	ribosome l	1月15日	6/17913	0.005014	0.034544	0.019941	SDAD1
GO_BP_m5	GO:00347	histone H4	1月15日	6/17913	0.005014	0.034544	0.019941	PWP1
GO_BP_m5	GO:00070	nucleolus	1月15日	7/17913	0.005848	0.036241	0.02092	POLR1B
GO_BP_m5	GO:00331	regulation	1月15日	7/17913	0.005848	0.036241	0.02092	PWP1
GO_BP_m5	GO:00714	rRNA-con	1月15日	7/17913	0.005848	0.036241	0.02092	SDAD1
GO_BP_m5	GO:00510	negative re	2月15日	141/17913	0.00604	0.036241	0.02092	GTPBP4/N
GO_BP_m5	GO:00007	telomere r	2月15日	154/17913	0.007165	0.041644	0.024039	GNL3/NAT
GO_BP_m5	GO:00063	tRNA meta	2月15日	160/17913	0.007713	0.04328	0.024983	NAT10/NS
GO_BP_m5	GO:00347	histone H4	1月15日	10/17913	0.008344	0.04328	0.024983	PWP1
GO_BP_m5	GO:19018	positive re	1月15日	10/17913	0.008344	0.04328	0.024983	PWP1
GO_BP_m5	GO:00322	telomere c	2月15日	167/17913	0.008377	0.04328	0.024983	GNL3/NAT
GO_BP_m5	GO:00000	ribosomal	1月15日	11/17913	0.009175	0.043759	0.02526	RRS1
GO_BP_m5	GO:00333	positive re	1月15日	11/17913	0.009175	0.043759	0.02526	GNL3
GO_BP_m5	GO:00425	serine pho	1月15日	11/17913	0.009175	0.043759	0.02526	PWP1
GO_BP_m5	GO:19048	positive re	1月15日	12/17913	0.010005	0.046525	0.026857	GNL3
GO_BP_m5	GO:00427	nucleolar l	1月15日	13/17913	0.010835	0.047984	0.027699	PWP1
GO_BP_m5	GO:19018	regulation	1月15日	13/17913	0.010835	0.047984	0.027699	PWP1
GO_BP_m5	GO:19025	protein loc	1月15日	14/17913	0.011664	0.049307	0.028462	RRS1
GO_BP_m5	GO:19048	regulation	1月15日	14/17913	0.011664	0.049307	0.028462	GNL3
GO_BP_m5	GO:00016	osteoblast	2月15日	222/17913	0.014437	0.058876	0.033986	GTPBP4/R
GO_BP_m5	GO:19033	regulation	2月15日	223/17913	0.014561	0.058876	0.033986	GNL3/GTP
GO_BP_m5	GO:00322	negative re	1月15日	20/17913	0.016624	0.064417	0.037185	NAT10
GO_BP_m5	GO:20007	positive re	1月15日	20/17913	0.016624	0.064417	0.037185	PWP1
GO_BP_m5	GO:00001	rRNA mod	1月15日	22/17913	0.018272	0.066639	0.038467	NAT10
GO_BP_m5	GO:00333	regulation	1月15日	22/17913	0.018272	0.066639	0.038467	GNL3
GO_BP_m5	GO:00459	positive re	1月15日	22/17913	0.018272	0.066639	0.038467	PWP1
GO_BP_m5	GO:00304	tRNA meth	1月15日	24/17913	0.019917	0.071243	0.041125	NSUN6
GO_BP_m5	GO:00422	ribosome a	1月15日	26/17913	0.02156	0.074337	0.042911	RRS1
GO_BP_m5	GO:00434	macromol	2月15日	276/17913	0.021761	0.074337	0.042911	NSUN6/PW

GO_BP_m5	GO:00331:	negative re	1月15日	27/17913	0.022381	0.074337	0.042911	PWP1
GO_BP_m5	GO:19043!	negative re	1月15日	27/17913	0.022381	0.074337	0.042911	NAT10
GO_BP_m5	GO:00701!	protein loc	1月15日	28/17913	0.023201	0.075708	0.043702	GNL3
GO_BP_m5	GO:00063(	transcriptio	1月15日	30/17913	0.024839	0.078305	0.045201	POLR1B
GO_BP_m5	GO:19028!	positive re	1月15日	30/17913	0.024839	0.078305	0.045201	GNL3
GO_BP_m5	GO:00063(	terminatio	1月15日	31/17913	0.025657	0.079535	0.045912	POLR1B
GO_BP_m5	GO:00063!	regulation	1月15日	32/17913	0.026474	0.080724	0.046598	PWP1
GO_BP_m5	GO:00330	regulation	2月15日	311/17913	0.02718	0.08154	0.047069	GNL3/NAT
GO_BP_m5	GO:00063(	transcriptio	1月15日	35/17913	0.028922	0.085093	0.04912	POLR1B
GO_BP_m5	GO:00081!	negative re	1月15日	36/17913	0.029737	0.085093	0.04912	GTPBP4
GO_BP_m5	GO:00322(	negative re	1月15日	36/17913	0.029737	0.085093	0.04912	NAT10
GO_BP_m5	GO:00322!	methylatio	2月15日	336/17913	0.031354	0.087071	0.050262	NSUN6/PV
GO_BP_m5	GO:20002	negative re	1月15日	38/17913	0.031364	0.087071	0.050262	NAT10
GO_BP_m5	GO:00171	stem cell c	1月15日	39/17913	0.032177	0.088014	0.050806	GNL3
GO_BP_m5	GO:19028!	regulation	1月15日	40/17913	0.032989	0.088928	0.051334	GNL3
GO_BP_m5	GO:00070	mitotic me	1月15日	41/17913	0.033801	0.089814	0.051845	RRS1
GO_BP_m5	GO:00180	peptidyl-ly	1月15日	42/17913	0.034612	0.090673	0.052341	PWP1
GO_BP_m5	GO:00616	pri-miRNA	1月15日	46/17913	0.037849	0.097165	0.056088	GNL3
GO_BP_m5	GO:00182(	peptidyl-ly	2月15日	376/17913	0.038529	0.097165	0.056088	GNL3/PWI
GO_BP_m5	GO:00458	positive re	1月15日	47/17913	0.038657	0.097165	0.056088	POLR1B
GO_BP_m5	GO:00015(	ossificatior	2月15日	388/17913	0.040795	0.101169	0.0584	GTPBP4/R!
GO_BP_m5	GO:00322(	positive re	1月15日	51/17913	0.041882	0.101169	0.0584	GNL3
GO_BP_m5	GO:20007	regulation	1月15日	51/17913	0.041882	0.101169	0.0584	RSL1D1
GO_BP_m5	GO:00075(	embryo im	1月15日	52/17913	0.042686	0.101791	0.058759	POLR1B
GO_BP_m5	GO:00322	regulation	1月15日	53/17913	0.04349	0.102395	0.059107	NAT10
GO_BP_m5	GO:00513	metaphase	1月15日	55/17913	0.045096	0.104111	0.060098	RRS1
GO_BP_m5	GO:00602	anatomica	2月15日	413/17913	0.045677	0.104111	0.060098	GNL3/NAT
GO_BP_m5	GO:00015	RNA meth	1月15日	56/17913	0.045898	0.104111	0.060098	NSUN6
GO_BP_m5	GO:00903	regulation	1月15日	59/17913	0.048301	0.108241	0.062482	RSL1D1
GO_BP_m5	GO:19043!	regulation	1月15日	61/17913	0.049899	0.110492	0.063781	NAT10
GO_BP_m6	GO:00467	viral entry	3月14日	121/17913	0.000104	0.001188	0.000608	IFITM1/IFI
GO_BP_m6	GO:00905(	RNA phos	3月14日	132/17913	0.000134	0.001348	0.000691	ISG20/RN/
GO_BP_m6	GO:00302(	entry into	3月14日	134/17913	0.00014	0.001348	0.000691	IFITM1/IFI
GO_BP_m6	GO:00444(	entry into	3月14日	134/17913	0.00014	0.001348	0.000691	IFITM1/IFI
GO_BP_m6	GO:00518(	entry into	3月14日	134/17913	0.00014	0.001348	0.000691	IFITM1/IFI
GO_BP_m6	GO:00518	entry into	3月14日	134/17913	0.00014	0.001348	0.000691	IFITM1/IFI
GO_BP_m6	GO:00713	cellular res	3月14日	153/17913	0.000208	0.001933	0.00099	GBP2/IRF8
GO_BP_m6	GO:00603	regulation	2月14日	30/17913	0.000244	0.002153	0.001103	RNASEL/S/
GO_BP_m6	GO:00511(	negative re	3月14日	162/17913	0.000246	0.002153	0.001103	IFIT1/IFIT2
GO_BP_m6	GO:00903(	nucleic aci	3月14日	268/17913	0.001067	0.009083	0.004652	ISG20/RN/
GO_BP_m6	GO:00190(	intracellula	1月14日	2/17913	0.001563	0.011641	0.005962	IFIT1
GO_BP_m6	GO:00305	symbiont i	1月14日	2/17913	0.001563	0.011641	0.005962	IFIT1
GO_BP_m6	GO:00341!	positive re	1月14日	2/17913	0.001563	0.011641	0.005962	RSAD2
GO_BP_m6	GO:00517(	intracellula	1月14日	2/17913	0.001563	0.011641	0.005962	IFIT1
GO_BP_m6	GO:19020	regulation	1月14日	2/17913	0.001563	0.011641	0.005962	SP100
GO_BP_m6	GO:00007	DNA catab	1月14日	3/17913	0.002343	0.015178	0.007774	ISG20
GO_BP_m6	GO:00062(	dGTP cata	1月14日	3/17913	0.002343	0.015178	0.007774	SAMHD1
GO_BP_m6	GO:00341!	regulation	1月14日	3/17913	0.002343	0.015178	0.007774	RSAD2
GO_BP_m6	GO:00341(	positive re	1月14日	3/17913	0.002343	0.015178	0.007774	RSAD2
GO_BP_m6	GO:00460(	dATP catal	1月14日	3/17913	0.002343	0.015178	0.007774	SAMHD1
GO_BP_m6	GO:01100	DNA stran	1月14日	3/17913	0.002343	0.015178	0.007774	SAMHD1
GO_BP_m6	GO:00510!	regulation	3月14日	368/17913	0.002645	0.016771	0.00859	IFIT1/IFIT2
GO_BP_m6	GO:00320!	negative re	2月14日	103/17913	0.002848	0.017683	0.009057	IFIT1/IFIT2
GO_BP_m6	GO:00363	Fas signali	1月14日	4/17913	0.003123	0.018612	0.009533	SP100
GO_BP_m6	GO:00460	dGTP meta	1月14日	4/17913	0.003123	0.018612	0.009533	SAMHD1
GO_BP_m6	GO:00092	purine dec	1月14日	5/17913	0.003902	0.02194	0.011237	SAMHD1
GO_BP_m6	GO:00460(	dATP meta	1月14日	5/17913	0.003902	0.02194	0.011237	SAMHD1
GO_BP_m6	GO:00510!	negative re	1月14日	5/17913	0.003902	0.02194	0.011237	IFIT1

GO_BP_m(	GO:00450(	regulation	3月14日	440/17913	0.004379	0.02405	0.012318	RNASEL/R
GO_BP_m(	GO:00091	purine nuc	1月14日	6/17913	0.004681	0.02405	0.012318	SAMHD1
GO_BP_m(	GO:00091	purine dec	1月14日	6/17913	0.004681	0.02405	0.012318	SAMHD1
GO_BP_m(	GO:00341	toll-like re	1月14日	6/17913	0.004681	0.02405	0.012318	RSAD2
GO_BP_m(	GO:00468	negative re	1月14日	6/17913	0.004681	0.02405	0.012318	SP100
GO_BP_m(	GO:00092(	deoxyribo	1月14日	7/17913	0.005459	0.025822	0.013226	SAMHD1
GO_BP_m(	GO:00467	regulation	1月14日	7/17913	0.005459	0.025822	0.013226	IFIT1
GO_BP_m(	GO:19025	multi-orga	1月14日	7/17913	0.005459	0.025822	0.013226	IFIT1
GO_BP_m(	GO:19025	multi-orga	1月14日	7/17913	0.005459	0.025822	0.013226	IFIT1
GO_BP_m(	GO:20005	positive re	1月14日	7/17913	0.005459	0.025822	0.013226	RSAD2
GO_BP_m(	GO:00063(	rRNA proc	2月14日	154/17913	0.006244	0.029075	0.014892	ISG20/RN/
GO_BP_m(	GO:00092	purine dec	1月14日	9/17913	0.007014	0.031668	0.01622	SAMHD1
GO_BP_m(	GO:00341(	regulation	1月14日	9/17913	0.007014	0.031668	0.01622	RSAD2
GO_BP_m(	GO:00019	regulation	2月14日	170/17913	0.00756	0.033624	0.017222	RNASEL/S/
GO_BP_m(	GO:00091	nucleoside	1月14日	11/17913	0.008566	0.035953	0.018415	SAMHD1
GO_BP_m(	GO:00091	purine dec	1月14日	11/17913	0.008566	0.035953	0.018415	SAMHD1
GO_BP_m(	GO:00164	somatic hy	1月14日	11/17913	0.008566	0.035953	0.018415	SAMHD1
GO_BP_m(	GO:20005	regulation	1月14日	11/17913	0.008566	0.035953	0.018415	RSAD2
GO_BP_m(	GO:00607	regulation	2月14日	183/17913	0.008713	0.036063	0.018471	RNASEL/S/
GO_BP_m(	GO:00512	negative re	2月14日	189/17913	0.009271	0.036143	0.018512	RSAD2/SP
GO_BP_m(	GO:00025(	somatic di	1月14日	12/17913	0.009341	0.036143	0.018512	SAMHD1
GO_BP_m(	GO:00510	regulation	1月14日	12/17913	0.009341	0.036143	0.018512	IFIT1
GO_BP_m(	GO:00603	negative re	1月14日	12/17913	0.009341	0.036143	0.018512	SAMHD1
GO_BP_m(	GO:00024	productior	2月14日	191/17913	0.00946	0.036143	0.018512	RSAD2/SA
GO_BP_m(	GO:00160	rRNA meta	2月14日	191/17913	0.00946	0.036143	0.018512	ISG20/RN/
GO_BP_m(	GO:19049	negative re	2月14日	193/17913	0.009651	0.036406	0.018647	RSAD2/SP
GO_BP_m(	GO:00357	T-helper 2	1月14日	13/17913	0.010116	0.037217	0.019062	RSAD2
GO_BP_m(	GO:00441	negative re	1月14日	13/17913	0.010116	0.037217	0.019062	IRF8
GO_BP_m(	GO:00422	ribosome l	2月14日	202/17913	0.010532	0.038276	0.019605	ISG20/RN/
GO_BP_m(	GO:00441	regulation	1月14日	14/17913	0.01089	0.038635	0.019788	IRF8
GO_BP_m(	GO:00441	negative re	1月14日	14/17913	0.01089	0.038635	0.019788	IRF8
GO_BP_m(	GO:00092(	deoxyribo	1月14日	15/17913	0.011664	0.040417	0.020701	SAMHD1
GO_BP_m(	GO:00441	modulatio	1月14日	15/17913	0.011664	0.040417	0.020701	IRF8
GO_BP_m(	GO:00433	regulation	2月14日	217/17913	0.012078	0.041371	0.021189	IFIT1/IFIT2
GO_BP_m(	GO:00028	positive re	1月14日	16/17913	0.012437	0.041436	0.021223	RSAD2
GO_BP_m(	GO:00441	growth of	1月14日	16/17913	0.012437	0.041436	0.021223	IRF8
GO_BP_m(	GO:00345(	protein loc	2月14日	223/17913	0.012723	0.041436	0.021223	GBP2/SP1(
GO_BP_m(	GO:00069	DNA dama	1月14日	17/17913	0.01321	0.041436	0.021223	SP100
GO_BP_m(	GO:00092(	deoxyribo	1月14日	17/17913	0.01321	0.041436	0.021223	SAMHD1
GO_BP_m(	GO:00441	growth inv	1月14日	17/17913	0.01321	0.041436	0.021223	IRF8
GO_BP_m(	GO:00441	growth of	1月14日	17/17913	0.01321	0.041436	0.021223	IRF8
GO_BP_m(	GO:00713(	cellular res	1月14日	17/17913	0.01321	0.041436	0.021223	IFIT1
GO_BP_m(	GO:00427	DNA dama	1月14日	18/17913	0.013982	0.043401	0.022229	SP100
GO_BP_m(	GO:00027	positive re	1月14日	19/17913	0.014753	0.044861	0.022977	RSAD2
GO_BP_m(	GO:00463	deoxyribo	1月14日	19/17913	0.014753	0.044861	0.022977	SAMHD1
GO_BP_m(	GO:00428	defense re	1月14日	20/17913	0.015524	0.046261	0.023694	IRF8
GO_BP_m(	GO:00468	negative re	1月14日	20/17913	0.015524	0.046261	0.023694	SP100
GO_BP_m(	GO:00015(	response t	1月14日	21/17913	0.016294	0.047604	0.024382	IRF8
GO_BP_m(	GO:00357	CD4-posit	1月14日	21/17913	0.016294	0.047604	0.024382	RSAD2
GO_BP_m(	GO:00514	maintenan	1月14日	22/17913	0.017064	0.048894	0.025043	SP100
GO_BP_m(	GO:00713	cellular res	1月14日	22/17913	0.017064	0.048894	0.025043	IFIT1
GO_BP_m(	GO:00341	positive re	1月14日	23/17913	0.017833	0.050612	0.025923	RSAD2
GO_BP_m(	GO:00328	negative re	1月14日	24/17913	0.018602	0.051806	0.026534	IFITM3
GO_BP_m(	GO:00341(	toll-like re	1月14日	24/17913	0.018602	0.051806	0.026534	RSAD2
GO_BP_m(	GO:00093	2'-deoxyri	1月14日	27/17913	0.020904	0.05768	0.029543	SAMHD1
GO_BP_m(	GO:00344	ncRNA pro	2月14日	293/17913	0.021313	0.058268	0.029844	ISG20/RN/
GO_BP_m(	GO:00092(	deoxyribo	1月14日	29/17913	0.022436	0.059696	0.030576	SAMHD1
GO_BP_m(	GO:00196	deoxyribo	1月14日	29/17913	0.022436	0.059696	0.030576	SAMHD1

GO_BP_m(	GO:00312(	replication	1月14日	29/17913	0.022436	0.059696	0.030576	SAMHD1
GO_BP_m(	GO:00028:	regulation	1月14日	30/17913	0.023201	0.061186	0.031339	RSAD2
GO_BP_m(	GO:00027:	regulation	1月14日	31/17913	0.023966	0.062104	0.031809	RSAD2
GO_BP_m(	GO:00433:	response t	1月14日	31/17913	0.023966	0.062104	0.031809	IFIT1
GO_BP_m(	GO:00063(	DNA catab	1月14日	33/17913	0.025494	0.064383	0.032976	ISG20
GO_BP_m(	GO:00450(	DNA-depe	1月14日	33/17913	0.025494	0.064383	0.032976	SAMHD1
GO_BP_m(	GO:00905(	RNA phos	1月14日	33/17913	0.025494	0.064383	0.032976	ISG20
GO_BP_m(	GO:00327:	positive re	1月14日	34/17913	0.026257	0.065205	0.033397	IRF8
GO_BP_m(	GO:00725(	maintenan	1月14日	34/17913	0.026257	0.065205	0.033397	SP100
GO_BP_m(	GO:00303:	negative re	2月14日	332/17913	0.026905	0.065462	0.033529	IFITM1/SP.
GO_BP_m(	GO:00420(	type 2 imn	1月14日	35/17913	0.027019	0.065462	0.033529	RSAD2
GO_BP_m(	GO:00450'	positive re	1月14日	35/17913	0.027019	0.065462	0.033529	IFIT1
GO_BP_m(	GO:00468:	regulation	1月14日	36/17913	0.027781	0.066765	0.034196	SP100
GO_BP_m(	GO:00463:	positive re	1月14日	37/17913	0.028543	0.068046	0.034852	RNASEL
GO_BP_m(	GO:20001	negative re	2月14日	347/17913	0.029199	0.068222	0.034943	IFITM1/SP.
GO_BP_m(	GO:00027:	positive re	1月14日	38/17913	0.029304	0.068222	0.034943	RSAD2
GO_BP_m(	GO:00433:	response t	1月14日	38/17913	0.029304	0.068222	0.034943	IFIT1
GO_BP_m(	GO:00061(	purine nuc	1月14日	39/17913	0.030064	0.06945	0.035571	SAMHD1
GO_BP_m(	GO:00023(	T cell cytol	1月14日	40/17913	0.030824	0.070657	0.03619	RSAD2
GO_BP_m(	GO:00903:	negative re	1月14日	41/17913	0.031583	0.071845	0.036798	SP100
GO_BP_m(	GO:00512'	negative re	2月14日	365/17913	0.032055	0.072366	0.037065	IFITM1/SP.
GO_BP_m(	GO:00064(	RNA catab	2月14日	368/17913	0.032541	0.072912	0.037344	ISG20/RN/
GO_BP_m(	GO:00108:	positive re	1月14日	43/17913	0.033099	0.073609	0.037702	RNASEL
GO_BP_m(	GO:00725:	purine-co	1月14日	44/17913	0.033857	0.07454	0.038178	SAMHD1
GO_BP_m(	GO:00400:	negative re	2月14日	377/17913	0.034018	0.07454	0.038178	IFITM1/SP.
GO_BP_m(	GO:00226:	ribonuclec	2月14日	381/17913	0.034683	0.075442	0.038641	ISG20/RN/
GO_BP_m(	GO:00433(	negative re	1月14日	48/17913	0.036881	0.079642	0.040792	SP100
GO_BP_m(	GO:00027:	positive re	1月14日	52/17913	0.039897	0.085535	0.04381	RSAD2
GO_BP_m(	GO:00326(	regulation	1月14日	53/17913	0.04065	0.086526	0.044317	IRF8
GO_BP_m(	GO:00164	somatic di	1月14日	55/17913	0.042153	0.088462	0.045309	SAMHD1
GO_BP_m(	GO:00326:	interleukin	1月14日	55/17913	0.042153	0.088462	0.045309	IRF8
GO_BP_m(	GO:00325(	maintenan	1月14日	57/17913	0.043654	0.089717	0.045952	SP100
GO_BP_m(	GO:00458:	negative re	1月14日	57/17913	0.043654	0.089717	0.045952	SAMHD1
GO_BP_m(	GO:19020	regulation	1月14日	57/17913	0.043654	0.089717	0.045952	SP100
GO_BP_m(	GO:19039(	positive re	1月14日	59/17913	0.045153	0.091757	0.046997	IFIT1
GO_BP_m(	GO:00026(	regulation	2月14日	441/17913	0.045263	0.091757	0.046997	IFIT1/RSAI
GO_BP_m(	GO:00463:	regulation	1月14日	60/17913	0.045902	0.092387	0.047319	RNASEL
GO_BP_m(	GO:00018:	positive re	2月14日	446/17913	0.046193	0.092387	0.047319	IRF8/RSAD
GO_BP_m(	GO:00027(	regulation	1月14日	61/17913	0.04665	0.092679	0.047469	RSAD2
GO_BP_m(	GO:00327:	positive re	1月14日	62/17913	0.047398	0.093541	0.04791	IRF8
GO_BP_m(	GO:00467(	regulation	1月14日	64/17913	0.048892	0.095853	0.049095	IFITM3
GO_BP_m(	GO:00323(	negative re	1月14日	65/17913	0.049638	0.09668	0.049518	SP100
GO_BP_m:	GO:00109:	regulation	3/104	16/17913	0.000101	0.001301	0.000776	GPER1/NT
GO_BP_m:	GO:00082(	insulin rec	6/104	128/17913	0.000101	0.001303	0.000777	GRB2/PIK3
GO_BP_m:	GO:00509:	regulation	7/104	186/17913	0.000106	0.001348	0.000803	CCL21/CC
GO_BP_m:	GO:00308:	regulation	6/104	129/17913	0.000106	0.001348	0.000803	CCL21/GR
GO_BP_m:	GO:00902(	regulation	8/104	251/17913	0.000108	0.00136	0.000811	ADORA1/
GO_BP_m:	GO:00606:	regulation	11/104	480/17913	0.000108	0.00136	0.000811	ADORA2B.
GO_BP_m:	GO:00026(	positive re	5/104	81/17913	0.000108	0.00136	0.000811	CCL21/CC
GO_BP_m:	GO:00171(	regulation	7/104	187/17913	0.000109	0.001363	0.000812	ADORA2B.
GO_BP_m:	GO:00508(	negative re	7/104	187/17913	0.000109	0.001363	0.000812	ANXA1/CN
GO_BP_m:	GO:00509:	positive re	6/104	130/17913	0.00011	0.001371	0.000817	CCL21/CC
GO_BP_m:	GO:00513:	regulation	4/104	43/17913	0.000111	0.001374	0.000819	ADORA2B.
GO_BP_m:	GO:00027(	regulation	11/104	483/17913	0.000114	0.001403	0.000836	ADORA1/
GO_BP_m:	GO:00025	monocyte	4/104	44/17913	0.000122	0.001481	0.000883	ANXA1/C(
GO_BP_m:	GO:00071(	negative re	3/104	17/17913	0.000122	0.001481	0.000883	EDNRB/G/
GO_BP_m:	GO:00451(	regulation	3/104	17/17913	0.000122	0.001481	0.000883	ADORA1/(
GO_BP_m:	GO:00420(	regulation	5/104	84/17913	0.000128	0.001555	0.000927	ADORA1/

GO_BP_m	GO:00706	positive re	6/104	134/17913	0.00013	0.001572	0.000937	ANXA1/C
GO_BP_m	GO:00303	negative r	9/104	332/17913	0.000136	0.001625	0.000968	ADORA1/
GO_BP_m	GO:00705	response t	5/104	85/17913	0.000136	0.001625	0.000968	CCL21/GN
GO_BP_m	GO:19033	regulation	6/104	136/17913	0.000141	0.001684	0.001004	ADORA2B
GO_BP_m	GO:00610	regulation	5/104	86/17913	0.000144	0.001696	0.001011	ADORA1/
GO_BP_m	GO:19037	regulation	5/104	86/17913	0.000144	0.001696	0.001011	CCKBR/CC
GO_BP_m	GO:00900	positive re	3/104	18/17913	0.000146	0.001712	0.001021	CCL5/CXC
GO_BP_m	GO:00068	calcium io	10/104	415/17913	0.00015	0.001758	0.001048	ADRA2A/
GO_BP_m	GO:00328	cellular res	7/104	197/17913	0.000151	0.001762	0.00105	GHRHR/G
GO_BP_m	GO:00106	regulation	6/104	138/17913	0.000153	0.00178	0.001061	FAM3C/G
GO_BP_m	GO:00485	circadian	4/104	47/17913	0.000158	0.001819	0.001084	ADORA1/
GO_BP_m	GO:00906	activation	4/104	47/17913	0.000158	0.001819	0.001084	CCL21/EC
GO_BP_m	GO:00347	positive re	7/104	199/17913	0.000161	0.001846	0.0011	CXCL10/F
GO_BP_m	GO:00076	rhythmic b	4/104	48/17913	0.000171	0.001946	0.00116	ADORA1/
GO_BP_m	GO:00160	detection	3/104	19/17913	0.000172	0.001946	0.00116	ADORA1/
GO_BP_m	GO:00312	negative r	3/104	19/17913	0.000172	0.001946	0.00116	EDNRB/G
GO_BP_m	GO:00450	regulation	3/104	19/17913	0.000172	0.001946	0.00116	CD28/CD
GO_BP_m	GO:00466	regulation	5/104	90/17913	0.000178	0.002003	0.001194	ANXA1/C
GO_BP_m	GO:00323	positive re	7/104	203/17913	0.000182	0.002033	0.001212	ADORA2B
GO_BP_m	GO:00071	negative r	8/104	271/17913	0.000182	0.002033	0.001212	ANXA1/C
GO_BP_m	GO:00620	positive re	6/104	143/17913	0.000186	0.002062	0.001229	ADM/ADC
GO_BP_m	GO:00171	regulation	5/104	91/17913	0.000187	0.002062	0.001229	ADORA2B
GO_BP_m	GO:00603	interferon-	5/104	91/17913	0.000187	0.002062	0.001229	HCK/HLA-
GO_BP_m	GO:19011	regulation	5/104	91/17913	0.000187	0.002062	0.001229	ADORA1/
GO_BP_m	GO:20001	negative r	9/104	347/17913	0.000189	0.002071	0.001235	ADORA1/
GO_BP_m	GO:00458	positive re	6/104	144/17913	0.000193	0.002101	0.001252	ADM/ADC
GO_BP_m	GO:19031	regulation	6/104	144/17913	0.000193	0.002101	0.001252	ADRA2A/
GO_BP_m	GO:00460	positive re	2/104	4/17913	0.000199	0.002107	0.001256	GHRHR/N
GO_BP_m	GO:00604	positive re	2/104	4/17913	0.000199	0.002107	0.001256	EDNRB/P2
GO_BP_m	GO:00715	lymphocyt	2/104	4/17913	0.000199	0.002107	0.001256	RAC2/ZAP
GO_BP_m	GO:00712	cellular res	7/104	206/17913	0.000199	0.002107	0.001256	CCL5/CCR
GO_BP_m	GO:00300	actin filam	6/104	145/17913	0.000201	0.002107	0.001256	CCL21/GR
GO_BP_m	GO:00508	positive re	6/104	145/17913	0.000201	0.002107	0.001256	ADORA1/
GO_BP_m	GO:00604	calcium io	6/104	145/17913	0.000201	0.002107	0.001256	CCL21/CC
GO_BP_m	GO:00076	copulation	3/104	20/17913	0.000202	0.002107	0.001256	CNR1/EDN
GO_BP_m	GO:00140	dopamine	3/104	20/17913	0.000202	0.002107	0.001256	CNR1/CXC
GO_BP_m	GO:00140	regulation	3/104	20/17913	0.000202	0.002107	0.001256	CNR1/CXC
GO_BP_m	GO:00336	positive re	3/104	20/17913	0.000202	0.002107	0.001256	CCL21/CC
GO_BP_m	GO:00072	integrin-m	5/104	94/17913	0.000218	0.002271	0.001354	HCK/LAT/
GO_BP_m	GO:00072	neurotrans	6/104	149/17913	0.000232	0.002395	0.001428	ADORA2B
GO_BP_m	GO:00069	inflammat	3/104	21/17913	0.000234	0.002395	0.001428	CCL5/CCR
GO_BP_m	GO:00427	regulation	3/104	21/17913	0.000234	0.002395	0.001428	ADORA1/
GO_BP_m	GO:00508	circadian s	3/104	21/17913	0.000234	0.002395	0.001428	ADORA1/
GO_BP_m	GO:00509	sensory pe	3/104	21/17913	0.000234	0.002395	0.001428	ADORA1/
GO_BP_m	GO:00996	signal rele	6/104	150/17913	0.000241	0.002456	0.001464	ADORA2B
GO_BP_m	GO:00071	cell-matri	7/104	214/17913	0.000251	0.002543	0.001516	CCL21/CC
GO_BP_m	GO:00435	regulation	4/104	53/17913	0.000252	0.002543	0.001516	CCKBR/CC
GO_BP_m	GO:00509	regulation	4/104	53/17913	0.000252	0.002543	0.001516	ADORA1/
GO_BP_m	GO:00355	purinergic	3/104	22/17913	0.00027	0.00266	0.001586	P2RY1/P2
GO_BP_m	GO:00358	regulation	3/104	22/17913	0.00027	0.00266	0.001586	ADM/EDN
GO_BP_m	GO:00420	interleukin	3/104	22/17913	0.00027	0.00266	0.001586	CD28/CD
GO_BP_m	GO:00509	negative r	3/104	22/17913	0.00027	0.00266	0.001586	ADORA1/
GO_BP_m	GO:00513	negative r	3/104	22/17913	0.00027	0.00266	0.001586	EDNRB/G
GO_BP_m	GO:19034	regulation	3/104	22/17913	0.00027	0.00266	0.001586	EDNRB/G
GO_BP_m	GO:00105	positive re	4/104	54/17913	0.000271	0.00266	0.001586	CXCL10/F
GO_BP_m	GO:00326	regulation	4/104	54/17913	0.000271	0.00266	0.001586	ANXA1/C
GO_BP_m	GO:00512	negative r	9/104	365/17913	0.000275	0.002686	0.001601	ADORA1/
GO_BP_m	GO:00466	alpha-beta	5/104	99/17913	0.000278	0.002707	0.001613	ANXA1/C

GO_BP_m	GO:00319	response t	6/104	155/17913	0.000288	0.002793	0.001664	ADM/ANX
GO_BP_m	GO:00975	granulocyt	5/104	100/17913	0.000291	0.002818	0.001679	ANXA1/C
GO_BP_m	GO:00604	cytosolic c	6/104	157/17913	0.000308	0.002944	0.001755	CCL21/CC
GO_BP_m	GO:00705	calcium io	8/104	293/17913	0.000309	0.002944	0.001755	ADRA2A/
GO_BP_m	GO:00708	divalent m	10/104	454/17913	0.000309	0.002944	0.001755	ADRA2A/
GO_BP_m	GO:00327	positive re	3/104	23/17913	0.000309	0.002944	0.001755	CD28/CD
GO_BP_m	GO:00716	positive re	3/104	23/17913	0.000309	0.002944	0.001755	CCL5/CXC
GO_BP_m	GO:00610	positive re	4/104	56/17913	0.000312	0.002962	0.001765	ADORA1/
GO_BP_m	GO:00717	response t	2/104	5/17913	0.00033	0.003108	0.001852	PTGDR/PT
GO_BP_m	GO:00717	cellular res	2/104	5/17913	0.00033	0.003108	0.001852	PTGDR/PT
GO_BP_m	GO:00725	divalent in	10/104	458/17913	0.000331	0.003108	0.001852	ADRA2A/
GO_BP_m	GO:00995	chemical s	5/104	103/17913	0.000334	0.003127	0.001864	ADORA1/
GO_BP_m	GO:00400	negative r	9/104	377/17913	0.000348	0.003244	0.001934	ADORA1/
GO_BP_m	GO:00510	negative r	7/104	226/17913	0.000349	0.003244	0.001934	ADORA1/
GO_BP_m	GO:00024	dendritic c	3/104	24/17913	0.000352	0.003244	0.001934	CCL21/CC
GO_BP_m	GO:00224	circadian s	3/104	24/17913	0.000352	0.003244	0.001934	ADORA1/
GO_BP_m	GO:00329	inositol ph	3/104	24/17913	0.000352	0.003244	0.001934	GPER1/NT
GO_BP_m	GO:00466	regulation	4/104	58/17913	0.000358	0.003273	0.001951	PIK3R1/PR
GO_BP_m	GO:00468	filopodium	4/104	58/17913	0.000358	0.003273	0.001951	CCL21/FG
GO_BP_m	GO:00425	glucose hc	7/104	227/17913	0.000359	0.003274	0.001951	ADRA2A/
GO_BP_m	GO:00335	carbohydr	7/104	228/17913	0.000368	0.003351	0.001997	ADRA2A/
GO_BP_m	GO:00451	cellular ext	4/104	59/17913	0.000382	0.003463	0.002064	CCL21/CC
GO_BP_m	GO:00071	regulation	3/104	25/17913	0.000398	0.003547	0.002114	ADORA1/
GO_BP_m	GO:00108	T cell chen	3/104	25/17913	0.000398	0.003547	0.002114	CCL21/CC
GO_BP_m	GO:00341	regulation	3/104	25/17913	0.000398	0.003547	0.002114	CCL5/PRK
GO_BP_m	GO:00509	leukocyte	3/104	25/17913	0.000398	0.003547	0.002114	CCL21/CC
GO_BP_m	GO:00900	regulation	3/104	25/17913	0.000398	0.003547	0.002114	CCL5/CXC
GO_BP_m	GO:19016	regulation	3/104	25/17913	0.000398	0.003547	0.002114	CCL21/CC
GO_BP_m	GO:00106	positive re	4/104	60/17913	0.000407	0.003614	0.002154	GPER1/NT
GO_BP_m	GO:00484	cell matura	6/104	167/17913	0.000429	0.003791	0.00226	APP/CCL2
GO_BP_m	GO:00302	T cell diffe	7/104	234/17913	0.000431	0.003797	0.002263	ANXA1/CD
GO_BP_m	GO:00604	regulation	6/104	168/17913	0.000442	0.00389	0.002318	CCL21/PR
GO_BP_m	GO:00224	negative r	6/104	169/17913	0.000457	0.00399	0.002378	ANXA1/C
GO_BP_m	GO:00380	Fc-epsilon	6/104	169/17913	0.000457	0.00399	0.002378	GRB2/LAT
GO_BP_m	GO:00326	interleukin	4/104	62/17913	0.000462	0.004024	0.002398	ANXA1/CD
GO_BP_m	GO:19040	regulation	8/104	312/17913	0.000469	0.004073	0.002427	ADRA2A/
GO_BP_m	GO:00421	positive re	4/104	63/17913	0.000491	0.004209	0.002509	APP/CD28
GO_BP_m	GO:00329	positive re	2/104	6/17913	0.000493	0.004209	0.002509	GPER1/P2
GO_BP_m	GO:00336	positive re	2/104	6/17913	0.000493	0.004209	0.002509	CXCL12/N
GO_BP_m	GO:00459	positive re	2/104	6/17913	0.000493	0.004209	0.002509	GHRHR/N
GO_BP_m	GO:20001	negative r	2/104	6/17913	0.000493	0.004209	0.002509	CCL5/CCR
GO_BP_m	GO:20003	regulation	2/104	6/17913	0.000493	0.004209	0.002509	GNRH1/SI
GO_BP_m	GO:00326	regulation	3/104	27/17913	0.000502	0.004213	0.002511	CD28/CD
GO_BP_m	GO:00363	dendritic c	3/104	27/17913	0.000502	0.004213	0.002511	CCL21/CC
GO_BP_m	GO:00427	circadian s	3/104	27/17913	0.000502	0.004213	0.002511	ADORA1/
GO_BP_m	GO:00457	regulation	3/104	27/17913	0.000502	0.004213	0.002511	EDNRB/G
GO_BP_m	GO:00485	positive re	3/104	27/17913	0.000502	0.004213	0.002511	GHRHR/N
GO_BP_m	GO:20010	positive re	3/104	27/17913	0.000502	0.004213	0.002511	CXCL12/G
GO_BP_m	GO:00068	neurotrans	7/104	241/17913	0.000513	0.00428	0.002551	ADORA2B
GO_BP_m	GO:00300	peptide hc	7/104	241/17913	0.000513	0.00428	0.002551	ADRA2A/
GO_BP_m	GO:00427	regulation	5/104	114/17913	0.000533	0.004428	0.002639	ADORA1/
GO_BP_m	GO:00330	regulation	3/104	28/17913	0.00056	0.004619	0.002753	CCL5/CCR
GO_BP_m	GO:00423	vasodilatic	3/104	28/17913	0.00056	0.004619	0.002753	ADORA1/
GO_BP_m	GO:19029	positive re	3/104	28/17913	0.00056	0.004619	0.002753	GPER1/NT
GO_BP_m	GO:00507	regulation	7/104	245/17913	0.000566	0.004653	0.002773	ADORA1/
GO_BP_m	GO:19000	regulation	4/104	66/17913	0.000586	0.004804	0.002863	PIK3R1/PR
GO_BP_m	GO:00019	regulation	5/104	117/17913	0.0006	0.004886	0.002912	CCL21/CC
GO_BP_m	GO:19001	regulation	5/104	117/17913	0.0006	0.004886	0.002912	ECT2/F2/P

GO_BP_m	GO:003009	lymphocyt	8/104	325/17913	0.000613	0.004983	0.00297	ANXA1/CD
GO_BP_m	GO:190499	regulation	3/104	29/17913	0.000622	0.005044	0.003006	CCL21/CC
GO_BP_m	GO:003227	regulation	6/104	180/17913	0.000637	0.005145	0.003066	CCL21/GR
GO_BP_m	GO:004341	positive re	10/104	499/17913	0.000646	0.005206	0.003103	ADORA1/A
GO_BP_m	GO:004667	response t	8/104	328/17913	0.000651	0.005232	0.003118	ADCY2/CN
GO_BP_m	GO:004860	cell morph	10/104	500/17913	0.000656	0.005232	0.003118	APP/CXCL
GO_BP_m	GO:004364	inositol ph	4/104	68/17913	0.000656	0.005232	0.003118	GPER1/INP
GO_BP_m	GO:007167	mononucle	4/104	68/17913	0.000656	0.005232	0.003118	ANXA1/CC
GO_BP_m	GO:000301	renal syste	5/104	120/17913	0.000673	0.005347	0.003187	ADCY2/AD
GO_BP_m	GO:003290	regulation	2/104	7/17913	0.000688	0.005385	0.003209	GPER1/P2
GO_BP_m	GO:005169	pointed-e	2/104	7/17913	0.000688	0.005385	0.003209	TMOD2/TI
GO_BP_m	GO:200060	negative re	2/104	7/17913	0.000688	0.005385	0.003209	CCL21/CX
GO_BP_m	GO:001054	regulation	3/104	30/17913	0.000689	0.005385	0.003209	F2/PRKCD
GO_BP_m	GO:007005	neuron cel	3/104	30/17913	0.000689	0.005385	0.003209	ADORA1/C
GO_BP_m	GO:009007	relaxation	3/104	30/17913	0.000689	0.005385	0.003209	ADORA1/A
GO_BP_m	GO:190303	negative re	5/104	121/17913	0.000699	0.005447	0.003246	ANXA1/CC
GO_BP_m	GO:190442	positive re	4/104	70/17913	0.000733	0.005697	0.003396	CXCL10/F2
GO_BP_m	GO:001587	dopamine	3/104	31/17913	0.000759	0.005842	0.003482	CNR1/CXC
GO_BP_m	GO:001660	Rac protei	3/104	31/17913	0.000759	0.005842	0.003482	RHOG/RH
GO_BP_m	GO:003080	cortical ac	3/104	31/17913	0.000759	0.005842	0.003482	ECT2/RHC
GO_BP_m	GO:004574	positive re	3/104	31/17913	0.000759	0.005842	0.003482	ADORA1/A
GO_BP_m	GO:200030	regulation	4/104	71/17913	0.000773	0.005931	0.003535	ADORA2B
GO_BP_m	GO:001972	calcium-m	6/104	188/17913	0.000799	0.006116	0.003645	CCR5/EDN
GO_BP_m	GO:000719	activation	3/104	32/17913	0.000834	0.006318	0.003766	ADCY2/AD
GO_BP_m	GO:003263	interleukin	3/104	32/17913	0.000834	0.006318	0.003766	CD28/CD8
GO_BP_m	GO:003302	myeloid ce	3/104	32/17913	0.000834	0.006318	0.003766	CCL5/CCR
GO_BP_m	GO:003812	ERBB2 sigr	3/104	32/17913	0.000834	0.006318	0.003766	GRB2/PIK3
GO_BP_m	GO:006035	response t	5/104	126/17913	0.000839	0.006336	0.003776	CNR1/GNA
GO_BP_m	GO:190165	response t	6/104	191/17913	0.000868	0.006498	0.003873	ADCY2/CC
GO_BP_m	GO:001715	calcium ioi	5/104	127/17913	0.000869	0.006498	0.003873	ADORA2B
GO_BP_m	GO:003133	negative re	5/104	127/17913	0.000869	0.006498	0.003873	PRKCD/PT
GO_BP_m	GO:006007	regulation	5/104	127/17913	0.000869	0.006498	0.003873	ADORA1/A
GO_BP_m	GO:001070	negative re	2/104	8/17913	0.000914	0.00671	0.003999	ADRA2A/F
GO_BP_m	GO:003162	regulation	2/104	8/17913	0.000914	0.00671	0.003999	CNR1/EDN
GO_BP_m	GO:003295	inositol tris	2/104	8/17913	0.000914	0.00671	0.003999	GPER1/P2
GO_BP_m	GO:006008	relaxation	2/104	8/17913	0.000914	0.00671	0.003999	ADORA1/A
GO_BP_m	GO:190343	negative re	2/104	8/17913	0.000914	0.00671	0.003999	EDNRB/GA
GO_BP_m	GO:005195	positive re	3/104	33/17913	0.000914	0.00671	0.003999	CXCL12/N
GO_BP_m	GO:190118	positive re	3/104	33/17913	0.000914	0.00671	0.003999	ADORA1/A
GO_BP_m	GO:000762	locomotor	6/104	194/17913	0.000941	0.00689	0.004107	APP/CXCL
GO_BP_m	GO:190280	regulation	4/104	76/17913	0.000998	0.007292	0.004346	ADORA2B
GO_BP_m	GO:006201	regulation	9/104	439/17913	0.001034	0.007536	0.004491	ADM/ADC
GO_BP_m	GO:000610	regulation	6/104	198/17913	0.001045	0.007599	0.004529	FAM3C/G
GO_BP_m	GO:009027	regulation	6/104	199/17913	0.001073	0.007779	0.004636	ADRA2A/C
GO_BP_m	GO:003080	cortical cy	3/104	35/17913	0.001087	0.007862	0.004686	ECT2/RHC
GO_BP_m	GO:004591	positive re	4/104	78/17913	0.0011	0.007903	0.00471	GPER1/NT
GO_BP_m	GO:005127	regulation	4/104	78/17913	0.0011	0.007903	0.00471	CXCL10/F2
GO_BP_m	GO:003007	insulin sec	6/104	200/17913	0.001101	0.007903	0.00471	ADRA2A/C
GO_BP_m	GO:009775	negative re	4/104	79/17913	0.001154	0.008261	0.004923	ADM/ADC
GO_BP_m	GO:190353	negative re	6/104	202/17913	0.001159	0.008277	0.004933	ADORA1/A
GO_BP_m	GO:000267	regulation	2/104	9/17913	0.00117	0.008299	0.004946	ADORA2B
GO_BP_m	GO:004455	relaxation	2/104	9/17913	0.00117	0.008299	0.004946	ADORA1/A
GO_BP_m	GO:005190	negative re	2/104	9/17913	0.00117	0.008299	0.004946	ADORA1/N
GO_BP_m	GO:004311	regulation	3/104	36/17913	0.001181	0.008313	0.004954	ADM/SRC
GO_BP_m	GO:009021	positive re	3/104	36/17913	0.001181	0.008313	0.004954	CCL21/F2/
GO_BP_m	GO:200051	positive re	3/104	36/17913	0.001181	0.008313	0.004954	ANXA1/CD
GO_BP_m	GO:200102	regulation	4/104	80/17913	0.001209	0.008489	0.005059	CNR1/CXC
GO_BP_m	GO:003134	positive re	8/104	362/17913	0.00123	0.008594	0.005122	CCL21/CN

GO_BP_m	GO:00356	cellular res	8/104	362/1791	0.00123	0.008594	0.005122	ADCY2/EC
GO_BP_m	GO:00030	skeletal mu	3/104	37/17913	0.00128	0.008857	0.005279	MYH3/TNI
GO_BP_m	GO:00423	positive re	3/104	37/17913	0.00128	0.008857	0.005279	ECT2/PIK3
GO_BP_m	GO:00515	positive re	3/104	37/17913	0.00128	0.008857	0.005279	CNR1/GPE
GO_BP_m	GO:00519	synaptic tr	3/104	37/17913	0.00128	0.008857	0.005279	ADORA1/
GO_BP_m	GO:00342	positive re	5/104	140/1791	0.001343	0.009254	0.005515	APP/CCL5
GO_BP_m	GO:19040	positive re	5/104	140/1791	0.001343	0.009254	0.005515	CXCL10/F
GO_BP_m	GO:00508	cognition	7/104	286/1791	0.001396	0.009572	0.005705	ADORA1/
GO_BP_m	GO:00512	maintenan	7/104	286/1791	0.001396	0.009572	0.005705	CCL21/CC
GO_BP_m	GO:00485	rhythmic p	7/104	287/1791	0.001424	0.009742	0.005806	ADORA1/
GO_BP_m	GO:00016	cellular glu	5/104	142/1791	0.001431	0.009761	0.005818	ADRA2A/
GO_BP_m	GO:00716	granulocyt	4/104	84/17913	0.001449	0.009782	0.00583	ANXA1/C
GO_BP_m	GO:00016	fever gene	2/104	10/17913	0.001457	0.009782	0.00583	CNR1/EDN
GO_BP_m	GO:00140	regulation	2/104	10/17913	0.001457	0.009782	0.00583	EDNRB/RA
GO_BP_m	GO:00450	negative tl	2/104	10/17913	0.001457	0.009782	0.00583	CD28/ZAP
GO_BP_m	GO:00457	positive re	2/104	10/17913	0.001457	0.009782	0.00583	ADORA1/
GO_BP_m	GO:01500	regulation	2/104	10/17913	0.001457	0.009782	0.00583	DVL1/MY
GO_BP_m	GO:20001	regulation	2/104	10/17913	0.001457	0.009782	0.00583	CCL5/CCR
GO_BP_m	GO:00610	regulation	5/104	143/1791	0.001476	0.009882	0.00589	ADRA2A/
GO_BP_m	GO:00703	ERK1 and	7/104	289/1791	0.001482	0.009903	0.005902	APP/CCL2
GO_BP_m	GO:00330	regulation	3/104	39/17913	0.001493	0.009927	0.005916	ADORA2B
GO_BP_m	GO:19045	positive re	3/104	39/17913	0.001493	0.009927	0.005916	ECT2/PIK3
GO_BP_m	GO:00423	regulation	8/104	374/1791	0.001512	0.010032	0.005979	ADORA1/
GO_BP_m	GO:00432	negative re	5/104	144/1791	0.001522	0.010075	0.006004	ADORA1/
GO_BP_m	GO:00605	developme	6/104	214/1791	0.001556	0.010277	0.006125	APP/CXCL
GO_BP_m	GO:00075	response t	6/104	215/1791	0.001593	0.010499	0.006258	CCL28/CN
GO_BP_m	GO:00065	membrane	3/104	40/17913	0.001607	0.010517	0.006268	ADRA2A/
GO_BP_m	GO:19031	negative re	3/104	40/17913	0.001607	0.010517	0.006268	ADRA2A/
GO_BP_m	GO:20002	positive re	3/104	40/17913	0.001607	0.010517	0.006268	ADORA1/
GO_BP_m	GO:00485	developme	6/104	216/1791	0.001631	0.010652	0.006349	APP/CXCL
GO_BP_m	GO:00488	multicellul	9/104	472/1791	0.001706	0.011118	0.006626	ADCY2/AD
GO_BP_m	GO:00988	modulatio	3/104	41/17913	0.001726	0.011224	0.006689	APP/DVL1
GO_BP_m	GO:00329	inositol tris	2/104	11/17913	0.001775	0.011408	0.006799	GPER1/P2
GO_BP_m	GO:00433	negative T	2/104	11/17913	0.001775	0.011408	0.006799	CD28/ZAP
GO_BP_m	GO:00456	regulation	2/104	11/17913	0.001775	0.011408	0.006799	ANXA1/CD
GO_BP_m	GO:00970	dendritic c	2/104	11/17913	0.001775	0.011408	0.006799	CCL21/CX
GO_BP_m	GO:20006	regulation	2/104	11/17913	0.001775	0.011408	0.006799	CCL21/CX
GO_BP_m	GO:00903	positive re	5/104	150/1791	0.001821	0.011683	0.006963	ECT2/PIK3
GO_BP_m	GO:00482	lymphocyt	3/104	42/17913	0.001851	0.011824	0.007047	CCL21/CC
GO_BP_m	GO:00514	regulation	3/104	42/17913	0.001851	0.011824	0.007047	CCL21/PR
GO_BP_m	GO:00357	CD4-posit	4/104	90/17913	0.001869	0.011907	0.007097	ANXA1/CD
GO_BP_m	GO:00345	protein loc	6/104	223/1791	0.001916	0.012184	0.007262	DVL1/ECT
GO_BP_m	GO:00347	positive re	5/104	152/1791	0.00193	0.012245	0.007298	CXCL10/F
GO_BP_m	GO:00458	negative re	4/104	91/17913	0.001946	0.012291	0.007326	ADORA1/
GO_BP_m	GO:19016	cellular res	4/104	91/17913	0.001946	0.012291	0.007326	ADCY2/PT
GO_BP_m	GO:00433	mast cell c	3/104	43/17913	0.001982	0.012491	0.007445	ADORA2B
GO_BP_m	GO:00507	positive re	5/104	153/1791	0.001986	0.012491	0.007445	ADORA2B
GO_BP_m	GO:00025	leukocyte	9/104	485/1791	0.002052	0.01288	0.007676	ANXA1/AP
GO_BP_m	GO:19030	regulation	4/104	93/17913	0.002107	0.013058	0.007782	GPER1/PIK
GO_BP_m	GO:00432	positive re	3/104	44/17913	0.002118	0.013058	0.007782	ADORA1/
GO_BP_m	GO:00716	regulation	3/104	44/17913	0.002118	0.013058	0.007782	CCL21/CC
GO_BP_m	GO:00028	positive re	2/104	12/17913	0.002121	0.013058	0.007782	CD28/CNR
GO_BP_m	GO:00316	regulation	2/104	12/17913	0.002121	0.013058	0.007782	CNR1/EDN
GO_BP_m	GO:00336	positive re	2/104	12/17913	0.002121	0.013058	0.007782	CXCL12/N
GO_BP_m	GO:00606	branching	2/104	12/17913	0.002121	0.013058	0.007782	ADM/GRB
GO_BP_m	GO:00704	leukocyte	2/104	12/17913	0.002121	0.013058	0.007782	RAC2/ZAP
GO_BP_m	GO:00718	macropha	2/104	12/17913	0.002121	0.013058	0.007782	CCL5/CCR
GO_BP_m	GO:00158	organic hy	6/104	228/1791	0.002142	0.013157	0.007841	ADRA2A/

GO_BP_m	GO:00432	regulation	4/104	94/17913	0.002191	0.013429	0.008004	FAM3C/GI
GO_BP_m	GO:00022	mast cell a	3/104	45/17913	0.00226	0.013678	0.008152	ADORA2B
GO_BP_m	GO:00024	mast cell n	3/104	45/17913	0.00226	0.013678	0.008152	ADORA2B
GO_BP_m	GO:00336	regulation	3/104	45/17913	0.00226	0.013678	0.008152	CCL21/CC
GO_BP_m	GO:00425	neuron ma	3/104	45/17913	0.00226	0.013678	0.008152	APP/EDNF
GO_BP_m	GO:00617	leukocyte	3/104	45/17913	0.00226	0.013678	0.008152	CCL21/CC
GO_BP_m	GO:00716	regulation	3/104	45/17913	0.00226	0.013678	0.008152	CCL5/CXC
GO_BP_m	GO:00600	excitatory	4/104	95/17913	0.002277	0.013754	0.008197	ADORA1/
GO_BP_m	GO:00512	protein po	6/104	232/17913	0.002337	0.014083	0.008393	CCL21/GR
GO_BP_m	GO:00308	negative re	3/104	46/17913	0.002407	0.014448	0.008611	PRKCD/TN
GO_BP_m	GO:00512	positive re	8/104	403/17913	0.002408	0.014448	0.008611	ECT2/GPEI
GO_BP_m	GO:00325	regulation	7/104	315/17913	0.002412	0.014448	0.008611	CCL21/CX
GO_BP_m	GO:00099	response t	9/104	498/17913	0.002453	0.014659	0.008737	ADM/ADC
GO_BP_m	GO:00030	negative re	2/104	13/17913	0.002498	0.014746	0.008789	ADORA1/N
GO_BP_m	GO:00336	regulation	2/104	13/17913	0.002498	0.014746	0.008789	PTGER4/SI
GO_BP_m	GO:00357	interleukin	2/104	13/17913	0.002498	0.014746	0.008789	GRB2/JAK
GO_BP_m	GO:00713	cellular res	2/104	13/17913	0.002498	0.014746	0.008789	GRB2/JAK
GO_BP_m	GO:19002	negative re	2/104	13/17913	0.002498	0.014746	0.008789	ADORA1/
GO_BP_m	GO:20001	regulation	2/104	13/17913	0.002498	0.014746	0.008789	GNRH1/SF
GO_BP_m	GO:00105	regulation	4/104	98/17913	0.00255	0.015024	0.008954	CXCL10/F
GO_BP_m	GO:00069	acute-pha	3/104	47/17913	0.002561	0.015028	0.008956	CNR1/EDN
GO_BP_m	GO:00466	positive re	3/104	47/17913	0.002561	0.015028	0.008956	ANXA1/CD
GO_BP_m	GO:00026	negative re	5/104	163/17913	0.002615	0.015285	0.00911	ANXA1/CN
GO_BP_m	GO:00310	actomyosi	5/104	163/17913	0.002615	0.015285	0.00911	ECT2/MYH
GO_BP_m	GO:00432	regulation	8/104	409/17913	0.002637	0.015382	0.009168	CCL21/GR
GO_BP_m	GO:00015	regulation	7/104	321/17913	0.00268	0.015598	0.009297	ADORA2B
GO_BP_m	GO:19037	positive re	3/104	48/17913	0.00272	0.015802	0.009418	CCL21/PRI
GO_BP_m	GO:00434	regulation	7/104	322/17913	0.002726	0.015807	0.009421	ADORA1/
GO_BP_m	GO:00160	synaptic ve	4/104	100/17913	0.002744	0.015875	0.009461	ADORA2B
GO_BP_m	GO:01200	regulation	5/104	166/17913	0.002829	0.016338	0.009737	CCL21/PRI
GO_BP_m	GO:00508	multicellul	3/104	49/17913	0.002885	0.016434	0.009795	MYH3/TNI
GO_BP_m	GO:00508	musculosk	3/104	49/17913	0.002885	0.016434	0.009795	MYH3/TNI
GO_BP_m	GO:00108	positive re	2/104	14/17913	0.002903	0.016434	0.009795	CCL21/CC
GO_BP_m	GO:00140	regulation	2/104	14/17913	0.002903	0.016434	0.009795	ADORA1/N
GO_BP_m	GO:00358	positive re	2/104	14/17913	0.002903	0.016434	0.009795	EDNRB/OF
GO_BP_m	GO:00450	T-helper 2	2/104	14/17913	0.002903	0.016434	0.009795	ANXA1/CD
GO_BP_m	GO:00459	negative re	2/104	14/17913	0.002903	0.016434	0.009795	ADORA1/
GO_BP_m	GO:00706	response t	2/104	14/17913	0.002903	0.016434	0.009795	GRB2/JAK
GO_BP_m	GO:00718	regulation	2/104	14/17913	0.002903	0.016434	0.009795	HCK/SRC
GO_BP_m	GO:00726	eosinophil	2/104	14/17913	0.002903	0.016434	0.009795	CCL5/PTG
GO_BP_m	GO:00507	regulation	5/104	169/17913	0.003056	0.017238	0.010274	ADRA2A/C
GO_BP_m	GO:00519	regulation	3/104	50/17913	0.003057	0.017238	0.010274	ADORA1/C
GO_BP_m	GO:00069	striated mu	5/104	170/17913	0.003134	0.017606	0.010493	ADORA1/N
GO_BP_m	GO:00507	negative re	5/104	170/17913	0.003134	0.017606	0.010493	ADORA1/F
GO_BP_m	GO:00457	negative re	3/104	51/17913	0.003234	0.018133	0.010807	ADM/CCL
GO_BP_m	GO:00016	temperatu	5/104	172/17913	0.003295	0.018439	0.010989	ADORA1/C
GO_BP_m	GO:00016	gastric acic	2/104	15/17913	0.003337	0.018493	0.011022	CCKBR/OF
GO_BP_m	GO:00140	regulation	2/104	15/17913	0.003337	0.018493	0.011022	ADRA2A/F
GO_BP_m	GO:00302	male sex d	2/104	15/17913	0.003337	0.018493	0.011022	GNRH1/PT
GO_BP_m	GO:00356	entry of ba	2/104	15/17913	0.003337	0.018493	0.011022	GRB2/SRC
GO_BP_m	GO:00485	negative re	2/104	15/17913	0.003337	0.018493	0.011022	ADORA1/N
GO_BP_m	GO:19030	regulation	5/104	173/17913	0.003378	0.018654	0.011118	ADRA2A/A
GO_BP_m	GO:00434	positive re	6/104	250/17913	0.003382	0.018654	0.011118	ADORA1/
GO_BP_m	GO:00986	regulation	4/104	106/17913	0.003385	0.018654	0.011118	ADORA2B
GO_BP_m	GO:00509	neuromusc	4/104	107/17913	0.003501	0.019256	0.011477	APP/DVL1
GO_BP_m	GO:00158	drug trans	5/104	175/17913	0.003548	0.019475	0.011607	CNR1/CXC
GO_BP_m	GO:00442	cellular ca	6/104	256/17913	0.003797	0.020532	0.012237	FAM3C/GC
GO_BP_m	GO:00017	neutrophil	2/104	16/17913	0.003799	0.020532	0.012237	ANXA1/PII

GO_BP_m⁷	GO:00316⁴	heat gener	2/104	16/17913	0.003799	0.020532	0.012237	CNR1/EDN
GO_BP_m⁷	GO:00330⁽	negative re	2/104	16/17913	0.003799	0.020532	0.012237	CCL5/CCR
GO_BP_m⁷	GO:00448⁴	estrous cyc	2/104	16/17913	0.003799	0.020532	0.012237	GNRH1/OI
GO_BP_m⁷	GO:00482⁴	norepinep	2/104	16/17913	0.003799	0.020532	0.012237	ADRA2A/F
GO_BP_m⁷	GO:00514⁸	positive re	2/104	16/17913	0.003799	0.020532	0.012237	GNA15/OI
GO_BP_m⁷	GO:00461⁷	polyol bios	3/104	54/17913	0.003804	0.020532	0.012237	GPER1/NT
GO_BP_m⁷	GO:19030⁷	positive re	3/104	54/17913	0.003804	0.020532	0.012237	GPER1/PIK
GO_BP_m⁷	GO:00093:	response t	8/104	435/17913	0.003834	0.020656	0.012311	APP/CXCL
GO_BP_m⁷	GO:00321(	negative re	7/104	343/17913	0.003861	0.020696	0.012335	ADORA1/I
GO_BP_m⁷	GO:00108:	positive re	4/104	110/17913	0.003865	0.020696	0.012335	CCL21/CC
GO_BP_m⁷	GO:00420:	regulation	4/104	110/17913	0.003865	0.020696	0.012335	APP/CD28
GO_BP_m⁷	GO:00324(	regulation	6/104	257/17913	0.00387	0.020696	0.012335	ADRA2A/A
GO_BP_m⁷	GO:00423(	regulation	3/104	55/17913	0.004007	0.021387	0.012746	ECT2/PIK3
GO_BP_m⁷	GO:19049!	positive re	8/104	440/17913	0.004106	0.021876	0.013038	ECT2/GPEI
GO_BP_m⁷	GO:00336:	membrane	3/104	56/17913	0.004216	0.022379	0.013338	ADRA2A/F
GO_BP_m⁷	GO:00713⁹	cellular res	3/104	56/17913	0.004216	0.022379	0.013338	PTGDR/PT
GO_BP_m⁷	GO:00217(	developme	6/104	262/17913	0.00425	0.02246	0.013386	APP/CCL2
GO_BP_m⁷	GO:00467:	acid secret	4/104	113/17913	0.004254	0.02246	0.013386	ADORA1/(
GO_BP_m⁷	GO:19043⁷	regulation	4/104	113/17913	0.004254	0.02246	0.013386	GPER1/PIK
GO_BP_m⁷	GO:00072:	G protein-	2/104	17/17913	0.004289	0.022521	0.013422	GNA15/GN
GO_BP_m⁷	GO:00427!	positive re	2/104	17/17913	0.004289	0.022521	0.013422	GHRHR/NI
GO_BP_m⁷	GO:00702:	negative re	2/104	17/17913	0.004289	0.022521	0.013422	CCL5/PRK(
GO_BP_m⁷	GO:00507:	positive re	5/104	184/17913	0.004389	0.023001	0.013709	ADORA1/A
GO_BP_m⁷	GO:00105:	positive re	3/104	57/17913	0.004432	0.023184	0.013817	CCL5/GNA
GO_BP_m⁷	GO:00197!	polyol met	4/104	115/17913	0.004528	0.023645	0.014092	GPER1/INI
GO_BP_m⁷	GO:00516!	establishm	8/104	448/17913	0.004572	0.023832	0.014204	ADORA2B
GO_BP_m⁷	GO:00703⁷	positive re	5/104	186/17913	0.004593	0.023901	0.014245	APP/CCL2
GO_BP_m⁷	GO:00323⁷	positive re	3/104	58/17913	0.004654	0.024042	0.014329	GAL/NTSR
GO_BP_m⁷	GO:00420⁹	T-helper c	3/104	58/17913	0.004654	0.024042	0.014329	ANXA1/CI
GO_BP_m⁷	GO:00510!	positive re	3/104	58/17913	0.004654	0.024042	0.014329	GRB2/SOS
GO_BP_m⁷	GO:19045⁸	regulation	3/104	58/17913	0.004654	0.024042	0.014329	ECT2/PIK3
GO_BP_m⁷	GO:00466⁴	positive re	2/104	18/17913	0.004807	0.024703	0.014723	CD28/ZAP
GO_BP_m⁷	GO:00718(	podosome	2/104	18/17913	0.004807	0.024703	0.014723	HCK/SRC
GO_BP_m⁷	GO:00903:	regulation	2/104	18/17913	0.004807	0.024703	0.014723	PRKCD/PR
GO_BP_m⁷	GO:00322⁷	negative re	3/104	59/17913	0.004883	0.024916	0.01485	PRKCD/TN
GO_BP_m⁷	GO:00903(	positive re	3/104	59/17913	0.004883	0.024916	0.01485	ADRA2A/A
GO_BP_m⁷	GO:20005:	regulation	3/104	59/17913	0.004883	0.024916	0.01485	ANXA1/CI
GO_BP_m⁷	GO:00347(	regulation	8/104	453/17913	0.004883	0.024916	0.01485	ADRA2A/A
GO_BP_m⁷	GO:00703⁷	regulation	6/104	271/17913	0.005002	0.025479	0.015185	APP/CCL2
GO_BP_m⁷	GO:00507(	regulation	8/104	456/17913	0.005078	0.025817	0.015387	ADRA2A/A
GO_BP_m⁷	GO:00022⁹	CD4-posit	3/104	60/17913	0.005118	0.02584	0.015401	ANXA1/CI
GO_BP_m⁷	GO:00468:	positive re	3/104	60/17913	0.005118	0.02584	0.015401	ECT2/PIK3
GO_BP_m⁷	GO:00420⁸	cytokine b	4/104	120/17913	0.005263	0.02584	0.015401	APP/CD28
GO_BP_m⁷	GO:00025⁴	chronic inf	2/104	19/17913	0.005353	0.02584	0.015401	ADORA2B
GO_BP_m⁷	GO:00105⁴	negative re	2/104	19/17913	0.005353	0.02584	0.015401	F2/PRKCD
GO_BP_m⁷	GO:00450(	thymic T c	2/104	19/17913	0.005353	0.02584	0.015401	CD28/ZAP
GO_BP_m⁷	GO:01401:	positive re	2/104	19/17913	0.005353	0.02584	0.015401	CCL21/CC
GO_BP_m⁷	GO:19059:	regulation	2/104	19/17913	0.005353	0.02584	0.015401	GNRH1/SF
GO_BP_m⁷	GO:00022⁸	alpha-beta	3/104	61/17913	0.00536	0.02584	0.015401	ANXA1/CI
GO_BP_m⁷	GO:00022⁹	alpha-beta	3/104	61/17913	0.00536	0.02584	0.015401	ANXA1/CI
GO_BP_m⁷	GO:00316⁴	positive re	3/104	61/17913	0.00536	0.02584	0.015401	DVL1/NTS
GO_BP_m⁷	GO:00336:	cell adhesi	3/104	61/17913	0.00536	0.02584	0.015401	CCL21/CC
GO_BP_m⁷	GO:19043⁷	positive re	3/104	61/17913	0.00536	0.02584	0.015401	GPER1/PIK
GO_BP_m⁷	GO:20002⁴	positive re	3/104	61/17913	0.00536	0.02584	0.015401	EDNRB/P2
GO_BP_m⁷	GO:00027⁹	positive re	6/104	275/17913	0.005366	0.02584	0.015401	ADORA1/(
GO_BP_m⁷	GO:00421(	cytokine m	4/104	121/17913	0.005419	0.02584	0.015401	APP/CD28
GO_BP_m⁷	GO:00352⁴	synaptic tr	3/104	62/17913	0.00561	0.02584	0.015401	ADORA1/(
GO_BP_m⁷	GO:00160⁴	cell growth	8/104	466/17913	0.005769	0.02584	0.015401	APP/CXCL

GO_BP_m7	GO:00316(	response t	8/104	466/17913	0.005769	0.02584	0.015401	ADM/CCL2
GO_BP_m7	GO:00015	primary ov	1/104	1/17913	0.005806	0.02584	0.015401	SRC
GO_BP_m7	GO:00022	alpha-beta	1/104	1/17913	0.005806	0.02584	0.015401	GPR18
GO_BP_m7	GO:00023(	CD8-posit	1/104	1/17913	0.005806	0.02584	0.015401	GPR18
GO_BP_m7	GO:00025	chronic inf	1/104	1/17913	0.005806	0.02584	0.015401	ADORA2B
GO_BP_m7	GO:00028	regulation	1/104	1/17913	0.005806	0.02584	0.015401	ADORA2B
GO_BP_m7	GO:00028	positive re	1/104	1/17913	0.005806	0.02584	0.015401	ADORA2B
GO_BP_m7	GO:00082	biolumines	1/104	1/17913	0.005806	0.02584	0.015401	MYO9A
GO_BP_m7	GO:00100	root morp	1/104	1/17913	0.005806	0.02584	0.015401	GAL
GO_BP_m7	GO:00100	root epide	1/104	1/17913	0.005806	0.02584	0.015401	GAL
GO_BP_m7	GO:00100	trichoblast	1/104	1/17913	0.005806	0.02584	0.015401	GAL
GO_BP_m7	GO:00226	root syster	1/104	1/17913	0.005806	0.02584	0.015401	GAL
GO_BP_m7	GO:00302(	nuclear fra	1/104	1/17913	0.005806	0.02584	0.015401	GPER1
GO_BP_m7	GO:00322	regulation	1/104	1/17913	0.005806	0.02584	0.015401	ADORA1
GO_BP_m7	GO:00322	positive re	1/104	1/17913	0.005806	0.02584	0.015401	ADORA1
GO_BP_m7	GO:00326	regulation	1/104	1/17913	0.005806	0.02584	0.015401	CD86
GO_BP_m7	GO:00327(	positive re	1/104	1/17913	0.005806	0.02584	0.015401	CD86
GO_BP_m7	GO:00329(	negative re	1/104	1/17913	0.005806	0.02584	0.015401	ADORA1
GO_BP_m7	GO:00346	establishm	1/104	1/17913	0.005806	0.02584	0.015401	RHOT1
GO_BP_m7	GO:00356	enteric sm	1/104	1/17913	0.005806	0.02584	0.015401	EDNRB
GO_BP_m7	GO:00430	regulation	1/104	1/17913	0.005806	0.02584	0.015401	CD86
GO_BP_m7	GO:00430	positive re	1/104	1/17913	0.005806	0.02584	0.015401	CD86
GO_BP_m7	GO:00483(	root devel	1/104	1/17913	0.005806	0.02584	0.015401	GAL
GO_BP_m7	GO:00487(	trichoblast	1/104	1/17913	0.005806	0.02584	0.015401	GAL
GO_BP_m7	GO:00487(	root hair c	1/104	1/17913	0.005806	0.02584	0.015401	GAL
GO_BP_m7	GO:00487(	root hair e	1/104	1/17913	0.005806	0.02584	0.015401	GAL
GO_BP_m7	GO:00515(	smooth er	1/104	1/17913	0.005806	0.02584	0.015401	APP
GO_BP_m7	GO:00518	cytolysis b	1/104	1/17913	0.005806	0.02584	0.015401	F2
GO_BP_m7	GO:00518(	general ad	1/104	1/17913	0.005806	0.02584	0.015401	PENK
GO_BP_m7	GO:00718	phospholi	1/104	1/17913	0.005806	0.02584	0.015401	ADRA2A
GO_BP_m7	GO:00726	eosinophil	1/104	1/17913	0.005806	0.02584	0.015401	PTGER4
GO_BP_m7	GO:00801	root hair c	1/104	1/17913	0.005806	0.02584	0.015401	GAL
GO_BP_m7	GO:00905	plant epid	1/104	1/17913	0.005806	0.02584	0.015401	GAL
GO_BP_m7	GO:00906	plant epid	1/104	1/17913	0.005806	0.02584	0.015401	GAL
GO_BP_m7	GO:00990	microtubu	1/104	1/17913	0.005806	0.02584	0.015401	RHOT1
GO_BP_m7	GO:00994(	plant orga	1/104	1/17913	0.005806	0.02584	0.015401	GAL
GO_BP_m7	GO:01101	positive re	1/104	1/17913	0.005806	0.02584	0.015401	PRKCD
GO_BP_m7	GO:19001(	regulation	1/104	1/17913	0.005806	0.02584	0.015401	PRKCD
GO_BP_m7	GO:19001(	positive re	1/104	1/17913	0.005806	0.02584	0.015401	PRKCD
GO_BP_m7	GO:19028	regulation	1/104	1/17913	0.005806	0.02584	0.015401	GAL
GO_BP_m7	GO:19028	negative re	1/104	1/17913	0.005806	0.02584	0.015401	GAL
GO_BP_m7	GO:19038	regulation	1/104	1/17913	0.005806	0.02584	0.015401	GAL
GO_BP_m7	GO:19038	negative re	1/104	1/17913	0.005806	0.02584	0.015401	GAL
GO_BP_m7	GO:19042(	regulation	1/104	1/17913	0.005806	0.02584	0.015401	TIAM1
GO_BP_m7	GO:19042(	positive re	1/104	1/17913	0.005806	0.02584	0.015401	TIAM1
GO_BP_m7	GO:19053	plant orga	1/104	1/17913	0.005806	0.02584	0.015401	GAL
GO_BP_m7	GO:19054	regulation	1/104	1/17913	0.005806	0.02584	0.015401	GAL
GO_BP_m7	GO:19054	negative re	1/104	1/17913	0.005806	0.02584	0.015401	GAL
GO_BP_m7	GO:19907	Schwann c	1/104	1/17913	0.005806	0.02584	0.015401	TIAM1
GO_BP_m7	GO:20000(	regulation	1/104	1/17913	0.005806	0.02584	0.015401	GAL
GO_BP_m7	GO:20002	regulation	1/104	1/17913	0.005806	0.02584	0.015401	GAL
GO_BP_m7	GO:20004	regulation	1/104	1/17913	0.005806	0.02584	0.015401	PTGER4
GO_BP_m7	GO:20004	negative re	1/104	1/17913	0.005806	0.02584	0.015401	PTGER4
GO_BP_m7	GO:20004	regulation	1/104	1/17913	0.005806	0.02584	0.015401	ECT2
GO_BP_m7	GO:20005	regulation	1/104	1/17913	0.005806	0.02584	0.015401	CCL21
GO_BP_m7	GO:20005	positive re	1/104	1/17913	0.005806	0.02584	0.015401	CCL21
GO_BP_m7	GO:20005	negative re	1/104	1/17913	0.005806	0.02584	0.015401	CCL21
GO_BP_m7	GO:20005(	regulation	1/104	1/17913	0.005806	0.02584	0.015401	PRKCQ

GO_BP_m	GO:20005	positive re	1/104	1/17913	0.005806	0.02584	0.015401	PRKCQ
GO_BP_m	GO:20007	regulation	1/104	1/17913	0.005806	0.02584	0.015401	PRKCD
GO_BP_m	GO:20007	positive re	1/104	1/17913	0.005806	0.02584	0.015401	PRKCD
GO_BP_m	GO:20007	regulation	1/104	1/17913	0.005806	0.02584	0.015401	PRKCD
GO_BP_m	GO:20007	positive re	1/104	1/17913	0.005806	0.02584	0.015401	PRKCD
GO_BP_m	GO:00466	regulation	3/104	63/17913	0.005865	0.026066	0.015535	ANXA1/CD
GO_BP_m	GO:00158	norepinep	2/104	20/17913	0.005925	0.026209	0.015621	ADRA2A/F
GO_BP_m	GO:00607	labyrinthin	2/104	20/17913	0.005925	0.026209	0.015621	ADM/GRB
GO_BP_m	GO:01400	neuron pro	2/104	20/17913	0.005925	0.026209	0.015621	DVL1/MYC
GO_BP_m	GO:00026	positive re	5/104	198/17913	0.005966	0.026353	0.015706	ADORA2B
GO_BP_m	GO:00095	detection	4/104	125/17913	0.006074	0.026747	0.015941	ADORA1/C
GO_BP_m	GO:00468	positive re	4/104	125/17913	0.006074	0.026747	0.015941	GAL/GHRH
GO_BP_m	GO:00075	excretion	3/104	64/17913	0.006128	0.026944	0.016059	ADORA1/A
GO_BP_m	GO:00072	JNK cascad	5/104	200/17913	0.006221	0.027267	0.016251	ADORA2B
GO_BP_m	GO:00096	response t	5/104	200/17913	0.006221	0.027267	0.016251	CXCL10/C
GO_BP_m	GO:00484	synaptic ve	4/104	126/17913	0.006246	0.027295	0.016268	ADORA2B
GO_BP_m	GO:00974	establishm	4/104	126/17913	0.006246	0.027295	0.016268	ADORA2B
GO_BP_m	GO:00076	circadian r	5/104	201/17913	0.00635	0.02771	0.016515	ADORA1/C
GO_BP_m	GO:00420	gliogenesi	6/104	285/17913	0.006361	0.027716	0.016518	APP/F2/P2
GO_BP_m	GO:00019	positive re	2/104	21/17913	0.006524	0.028254	0.016839	CNR1/GPE
GO_BP_m	GO:00361	cellular res	2/104	21/17913	0.006524	0.028254	0.016839	SRC/YES1
GO_BP_m	GO:00510	regulation	2/104	21/17913	0.006524	0.028254	0.016839	ADRA2A/T
GO_BP_m	GO:00713	cellular res	2/104	21/17913	0.006524	0.028254	0.016839	PTGDR/PT
GO_BP_m	GO:00095	detection	4/104	128/17913	0.006599	0.028451	0.016957	ADORA1/C
GO_BP_m	GO:00323	response t	4/104	128/17913	0.006599	0.028451	0.016957	GPER1/OP
GO_BP_m	GO:00713	cellular res	4/104	128/17913	0.006599	0.028451	0.016957	ADRA2A/C
GO_BP_m	GO:00022	T cell diffe	3/104	66/17913	0.006675	0.028608	0.01705	ANXA1/CD
GO_BP_m	GO:00105	regulation	3/104	66/17913	0.006675	0.028608	0.01705	CCL5/GNA
GO_BP_m	GO:00519	negative re	3/104	66/17913	0.006675	0.028608	0.01705	ADRA2A/N
GO_BP_m	GO:19036	positive re	3/104	66/17913	0.006675	0.028608	0.01705	ANXA1/JA
GO_BP_m	GO:00324	lysosome l	3/104	67/17913	0.006959	0.029719	0.017712	ADORA2B
GO_BP_m	GO:00517	multi-orga	3/104	67/17913	0.006959	0.029719	0.017712	APP/DVL1
GO_BP_m	GO:00713	cellular res	4/104	130/17913	0.006965	0.029719	0.017712	ADRA2A/C
GO_BP_m	GO:00346	response t	2/104	22/17913	0.007149	0.030297	0.018057	CCL21/GN
GO_BP_m	GO:00358	regulation	2/104	22/17913	0.007149	0.030297	0.018057	ADORA1/E
GO_BP_m	GO:00361	response t	2/104	22/17913	0.007149	0.030297	0.018057	SRC/YES1
GO_BP_m	GO:00456	positive re	2/104	22/17913	0.007149	0.030297	0.018057	ANXA1/CD
GO_BP_m	GO:00713	cellular res	4/104	131/17913	0.007153	0.030297	0.018057	ADRA2A/C
GO_BP_m	GO:00426	ovulation	3/104	68/17913	0.00725	0.030662	0.018274	GNRH1/OI
GO_BP_m	GO:00302	entry into	4/104	134/17913	0.007736	0.032531	0.019388	CCR5/CD8
GO_BP_m	GO:00444	entry into	4/104	134/17913	0.007736	0.032531	0.019388	CCR5/CD8
GO_BP_m	GO:00518	entry into	4/104	134/17913	0.007736	0.032531	0.019388	CCR5/CD8
GO_BP_m	GO:00518	entry into	4/104	134/17913	0.007736	0.032531	0.019388	CCR5/CD8
GO_BP_m	GO:00075	sex determ	2/104	23/17913	0.007801	0.032707	0.019493	GNRH1/PT
GO_BP_m	GO:00435	regulation	2/104	23/17913	0.007801	0.032707	0.019493	ADORA1/N
GO_BP_m	GO:00601	positive re	3/104	70/17913	0.007853	0.032877	0.019594	CCL5/GNA
GO_BP_m	GO:00335	multicellul	3/104	71/17913	0.008165	0.034036	0.020286	EDNRB/NP
GO_BP_m	GO:00423	vasoconstr	3/104	71/17913	0.008165	0.034036	0.020286	ADM/ADR
GO_BP_m	GO:19030	positive re	3/104	71/17913	0.008165	0.034036	0.020286	ADRA2A/A
GO_BP_m	GO:00343	cell junctic	5/104	214/17913	0.008217	0.034202	0.020384	ECT2/MYC
GO_BP_m	GO:00974	synaptic ve	4/104	137/17913	0.008351	0.034711	0.020687	ADORA2B
GO_BP_m	GO:00028	regulation	2/104	24/17913	0.008478	0.034916	0.02081	CD28/CNR
GO_BP_m	GO:00358	renal sodiu	2/104	24/17913	0.008478	0.034916	0.02081	ADORA1/E
GO_BP_m	GO:00439	negative re	2/104	24/17913	0.008478	0.034916	0.02081	NPY2R/OP
GO_BP_m	GO:00509	positive re	2/104	24/17913	0.008478	0.034916	0.02081	ADORA1/F
GO_BP_m	GO:00602	regulation	2/104	24/17913	0.008478	0.034916	0.02081	CNR1/NPY
GO_BP_m	GO:00900	positive re	2/104	24/17913	0.008478	0.034916	0.02081	CCL21/RA
GO_BP_m	GO:00723	response t	3/104	72/17913	0.008485	0.034916	0.02081	CNR1/PEN

GO_BP_m	GO:000975	hormone-	5/104	216/17913	0.008534	0.035069	0.020901	CRHR2/GC
GO_BP_m	GO:004339	regulation	5/104	217/17913	0.008696	0.035684	0.021267	APP/DVL1
GO_BP_m	GO:000173	morphoge	4/104	139/17913	0.008778	0.035866	0.021376	ARHGEF19
GO_BP_m	GO:004558	regulation	4/104	139/17913	0.008778	0.035866	0.021376	ANXA1/CD
GO_BP_m	GO:007132	cellular res	4/104	139/17913	0.008778	0.035866	0.021376	ADRA2A/C
GO_BP_m	GO:000664	phospholi	7/104	402/17913	0.00898	0.03664	0.021837	CCKBR/CC
GO_BP_m	GO:000926	response t	5/104	219/17913	0.009026	0.036778	0.021919	ADM/ADC
GO_BP_m	GO:003019	regulation	3/104	74/17913	0.009146	0.036939	0.022016	F2/PRKCD
GO_BP_m	GO:004330	CD4-posit	3/104	74/17913	0.009146	0.036939	0.022016	ANXA1/CD
GO_BP_m	GO:003523	ionotropic	2/104	25/17913	0.009181	0.036939	0.022016	APP/TIAM
GO_BP_m	GO:004400	regulation	2/104	25/17913	0.009181	0.036939	0.022016	ADORA1/E
GO_BP_m	GO:004593	negative re	2/104	25/17913	0.009181	0.036939	0.022016	ADORA1/A
GO_BP_m	GO:006060	embryonic	2/104	25/17913	0.009181	0.036939	0.022016	ADM/GRB
GO_BP_m	GO:009550	acetylcholi	2/104	25/17913	0.009181	0.036939	0.022016	GNA15/GN
GO_BP_m	GO:190383	signal tran	2/104	25/17913	0.009181	0.036939	0.022016	GNA15/GN
GO_BP_m	GO:190438	cellular res	2/104	25/17913	0.009181	0.036939	0.022016	PRKCD/SR
GO_BP_m	GO:003315	regulation	5/104	220/17913	0.009194	0.036943	0.022018	ECT2/PIK3
GO_BP_m	GO:000834	adult locor	3/104	75/17913	0.009487	0.037961	0.022625	APP/CXCL
GO_BP_m	GO:005114	smooth m	3/104	75/17913	0.009487	0.037961	0.022625	ADM/EDN
GO_BP_m	GO:190004	regulation	3/104	75/17913	0.009487	0.037961	0.022625	F2/PRKCD
GO_BP_m	GO:004390	regulation	7/104	408/17913	0.009693	0.038733	0.023084	CCL5/CD2
GO_BP_m	GO:001993	cGMP-me	2/104	26/17913	0.009908	0.03932	0.023435	ADORA2B
GO_BP_m	GO:003290	positive re	2/104	26/17913	0.009908	0.03932	0.023435	F2/VIM
GO_BP_m	GO:004860	collateral s	2/104	26/17913	0.009908	0.03932	0.023435	APP/DVL1
GO_BP_m	GO:007137	cellular res	2/104	26/17913	0.009908	0.03932	0.023435	ADCY2/GC
GO_BP_m	GO:007162	positive re	2/104	26/17913	0.009908	0.03932	0.023435	CCL21/RA
GO_BP_m	GO:004470	multi-mult	5/104	225/17913	0.010068	0.039901	0.023781	ADM/CNR
GO_BP_m	GO:004870	astrocyte c	3/104	77/17913	0.010192	0.040336	0.02404	APP/F2/VI
GO_BP_m	GO:006024	anatomica	7/104	413/17913	0.010319	0.040783	0.024306	ADORA1/C
GO_BP_m	GO:001072	negative re	6/104	317/17913	0.01045	0.041244	0.024581	APP/EDNR
GO_BP_m	GO:001082	regulation	3/104	78/17913	0.010556	0.041509	0.024739	PIK3R1/RH
GO_BP_m	GO:190595	positive re	3/104	78/17913	0.010556	0.041509	0.024739	GAL/NTSR
GO_BP_m	GO:007123	cellular res	4/104	147/17913	0.010627	0.041509	0.024739	ADCY2/EC
GO_BP_m	GO:001071	positive re	2/104	27/17913	0.010661	0.041509	0.024739	F2/VIM
GO_BP_m	GO:004330	regulation	2/104	27/17913	0.010661	0.041509	0.024739	ADORA2B
GO_BP_m	GO:004664	regulation	2/104	27/17913	0.010661	0.041509	0.024739	CD28/ZAP
GO_BP_m	GO:012003	negative re	2/104	27/17913	0.010661	0.041509	0.024739	CCL21/PRI
GO_BP_m	GO:190262	positive re	2/104	27/17913	0.010661	0.041509	0.024739	CCL21/RA
GO_BP_m	GO:190514	response t	2/104	27/17913	0.010661	0.041509	0.024739	GNA15/GN
GO_BP_m	GO:190514	cellular res	2/104	27/17913	0.010661	0.041509	0.024739	GNA15/GN
GO_BP_m	GO:199013	neuron pro	4/104	148/17913	0.010874	0.042284	0.025201	CXCL12/D
GO_BP_m	GO:005081	regulation	3/104	79/17913	0.010927	0.042293	0.025206	F2/PRKCD
GO_BP_m	GO:009758	lamellipod	3/104	79/17913	0.010927	0.042293	0.025206	RAC2/SRC
GO_BP_m	GO:007190	positive re	6/104	322/17913	0.011226	0.042293	0.025206	ADORA1/A
GO_BP_m	GO:190401	positive re	5/104	232/17913	0.011386	0.042293	0.025206	ADM/ANX
GO_BP_m	GO:000190	startle resp	2/104	28/17913	0.011438	0.042293	0.025206	DVL1/PEN
GO_BP_m	GO:007022	negative re	2/104	28/17913	0.011438	0.042293	0.025206	CCL5/PRK
GO_BP_m	GO:190274	positive re	2/104	28/17913	0.011438	0.042293	0.025206	RAC2/SRC
GO_BP_m	GO:199077	response t	2/104	28/17913	0.011438	0.042293	0.025206	PRKCD/SR
GO_BP_m	GO:200010	positive re	2/104	28/17913	0.011438	0.042293	0.025206	CCL5/PIK3
GO_BP_m	GO:000230	gamma-de	1/104	2/17913	0.011578	0.042293	0.025206	GPR18
GO_BP_m	GO:000230	CD8-posit	1/104	2/17913	0.011578	0.042293	0.025206	GPR18
GO_BP_m	GO:000250	antigen pr	1/104	2/17913	0.011578	0.042293	0.025206	HLA-DRA
GO_BP_m	GO:000250	polysaccha	1/104	2/17913	0.011578	0.042293	0.025206	HLA-DRA
GO_BP_m	GO:000252	leukocyte	1/104	2/17913	0.011578	0.042293	0.025206	HCK
GO_BP_m	GO:000749	posterior r	1/104	2/17913	0.011578	0.042293	0.025206	EDNRB
GO_BP_m	GO:003289	regulation	1/104	2/17913	0.011578	0.042293	0.025206	ADORA1
GO_BP_m	GO:003362	negative re	1/104	2/17913	0.011578	0.042293	0.025206	PTGER4

GO_BP_m	GO:00357	T-helper 2	1/104	2/17913	0.011578	0.042293	0.025206	PRKCQ
GO_BP_m	GO:00357	mesangial	1/104	2/17913	0.011578	0.042293	0.025206	CCL21
GO_BP_m	GO:00381	cholecysto	1/104	2/17913	0.011578	0.042293	0.025206	CCKBR
GO_BP_m	GO:00423	negative r	1/104	2/17913	0.011578	0.042293	0.025206	ADORA1
GO_BP_m	GO:00454	positive re	1/104	2/17913	0.011578	0.042293	0.025206	CD86
GO_BP_m	GO:00514	negative r	1/104	2/17913	0.011578	0.042293	0.025206	PRKCD
GO_BP_m	GO:00518	cytolysis ir	1/104	2/17913	0.011578	0.042293	0.025206	F2
GO_BP_m	GO:00518	general ad	1/104	2/17913	0.011578	0.042293	0.025206	PENK
GO_BP_m	GO:00601	somatotro	1/104	2/17913	0.011578	0.042293	0.025206	GHRHR
GO_BP_m	GO:00604	positive re	1/104	2/17913	0.011578	0.042293	0.025206	OPRL1
GO_BP_m	GO:00606	positive re	1/104	2/17913	0.011578	0.042293	0.025206	PRKCD
GO_BP_m	GO:00650	protein-ca	1/104	2/17913	0.011578	0.042293	0.025206	HLA-DRA
GO_BP_m	GO:00702	negative r	1/104	2/17913	0.011578	0.042293	0.025206	ADORA1
GO_BP_m	GO:00704	T cell aggr	1/104	2/17913	0.011578	0.042293	0.025206	ZAP70
GO_BP_m	GO:00718	protein-ca	1/104	2/17913	0.011578	0.042293	0.025206	HLA-DRA
GO_BP_m	GO:00718	cellular res	1/104	2/17913	0.011578	0.042293	0.025206	APP
GO_BP_m	GO:00860	angiotensi	1/104	2/17913	0.011578	0.042293	0.025206	SRC
GO_BP_m	GO:00989	NMDA sel	1/104	2/17913	0.011578	0.042293	0.025206	TIAM1
GO_BP_m	GO:00995	trans-syna	1/104	2/17913	0.011578	0.042293	0.025206	CNR1
GO_BP_m	GO:00995	trans-syna	1/104	2/17913	0.011578	0.042293	0.025206	CNR1
GO_BP_m	GO:19017	negative r	1/104	2/17913	0.011578	0.042293	0.025206	CXCL10
GO_BP_m	GO:19026	regulation	1/104	2/17913	0.011578	0.042293	0.025206	GAL
GO_BP_m	GO:19026	positive re	1/104	2/17913	0.011578	0.042293	0.025206	GAL
GO_BP_m	GO:19053	regulation	1/104	2/17913	0.011578	0.042293	0.025206	DVL1
GO_BP_m	GO:19053	positive re	1/104	2/17913	0.011578	0.042293	0.025206	DVL1
GO_BP_m	GO:19904	response t	1/104	2/17913	0.011578	0.042293	0.025206	CXCL12
GO_BP_m	GO:19907	condition	1/104	2/17913	0.011578	0.042293	0.025206	OPRL1
GO_BP_m	GO:19908	response t	1/104	2/17913	0.011578	0.042293	0.025206	EDNRB
GO_BP_m	GO:20001	positive re	1/104	2/17913	0.011578	0.042293	0.025206	ADM
GO_BP_m	GO:20003	positive re	1/104	2/17913	0.011578	0.042293	0.025206	SRC
GO_BP_m	GO:20004	negative r	1/104	2/17913	0.011578	0.042293	0.025206	PTGER4
GO_BP_m	GO:00156	ammoniur	3/104	81/17913	0.011692	0.042546	0.025357	CNR1/CXC
GO_BP_m	GO:19029	regulation	3/104	81/17913	0.011692	0.042546	0.025357	GPER1/NT
GO_BP_m	GO:19035	positive re	3/104	81/17913	0.011692	0.042546	0.025357	APP/CD86
GO_BP_m	GO:00324	regulation	5/104	234/1791	0.011783	0.042823	0.025523	ADRA2A/
GO_BP_m	GO:00330	regulation	2/104	29/17913	0.012239	0.044369	0.026444	ADORA2B
GO_BP_m	GO:00466	negative r	2/104	29/17913	0.012239	0.044369	0.026444	PRKCD/PR
GO_BP_m	GO:00313	negative r	5/104	237/1791	0.012396	0.044826	0.026716	ADORA1/
GO_BP_m	GO:00726	protein loc	5/104	237/1791	0.012396	0.044826	0.026716	GPER1/P2
GO_BP_m	GO:00030	regulation	3/104	83/17913	0.012487	0.045042	0.026845	ADM/ADC
GO_BP_m	GO:00455	positive re	3/104	83/17913	0.012487	0.045042	0.026845	ANXA1/CD
GO_BP_m	GO:00011	response t	6/104	330/1791	0.012551	0.045219	0.02695	CCL21/GN
GO_BP_m	GO:00716	anatomica	4/104	155/1791	0.012711	0.045682	0.027226	APP/EDNF
GO_BP_m	GO:19035	regulation	4/104	155/1791	0.012711	0.045682	0.027226	APP/CD86
GO_BP_m	GO:00017	leukocyte	3/104	84/17913	0.012895	0.046229	0.027552	ANXA1/LA
GO_BP_m	GO:00308	positive re	3/104	84/17913	0.012895	0.046229	0.027552	CCL21/GR
GO_BP_m	GO:00028	regulation	2/104	30/17913	0.013064	0.046429	0.027672	ANXA1/CD
GO_BP_m	GO:00072	activation	2/104	30/17913	0.013064	0.046429	0.027672	GNA15/GN
GO_BP_m	GO:00433	positive re	2/104	30/17913	0.013064	0.046429	0.027672	ANXA1/CD
GO_BP_m	GO:00466	alpha-beta	2/104	30/17913	0.013064	0.046429	0.027672	CD28/ZAP
GO_BP_m	GO:00506	regulation	2/104	30/17913	0.013064	0.046429	0.027672	CD28/HCK
GO_BP_m	GO:00514	positive re	2/104	30/17913	0.013064	0.046429	0.027672	CCL21/RH
GO_BP_m	GO:00900	regulation	2/104	30/17913	0.013064	0.046429	0.027672	CCL21/RA
GO_BP_m	GO:00488	homeosta	5/104	241/1791	0.013246	0.047021	0.028024	ANXA1/LA
GO_BP_m	GO:01060	neuron pr	3/104	85/17913	0.013312	0.047137	0.028094	APP/DVL1
GO_BP_m	GO:19040	negative r	3/104	85/17913	0.013312	0.047137	0.028094	ADRA2A/N
GO_BP_m	GO:00228	regulation	5/104	242/1791	0.013465	0.047622	0.028382	ADRA2A/
GO_BP_m	GO:00022	lymphocyt	4/104	158/1791	0.013556	0.047884	0.028538	ANXA1/CD

GO_BP_m	GO:00480	ephrin rec	3/104	86/17913	0.013736	0.048461	0.028882	SRC/TIAM
GO_BP_m	GO:00459	negative r	2/104	31/17913	0.013912	0.048786	0.029076	ADRA2A/
GO_BP_m	GO:00459	positive re	2/104	31/17913	0.013912	0.048786	0.029076	GPER1/NP
GO_BP_m	GO:00513	response t	2/104	31/17913	0.013912	0.048786	0.029076	GPER1/SR
GO_BP_m	GO:00519	regulation	2/104	31/17913	0.013912	0.048786	0.029076	ADORA1/
GO_BP_m	GO:19000	negative r	2/104	31/17913	0.013912	0.048786	0.029076	PRKCD/PR
GO_BP_m	GO:00026	regulation	4/104	160/1791	0.014138	0.049518	0.029512	ADORA1/
GO_BP_m	GO:00717	tumor nec	4/104	161/1791	0.014435	0.050497	0.030096	APP/CD86
GO_BP_m	GO:00076	learning o	5/104	247/1791	0.014595	0.050913	0.030344	APP/CNR1
GO_BP_m	GO:00022	T cell activ	3/104	88/17913	0.014607	0.050913	0.030344	ANXA1/CD
GO_BP_m	GO:00973	cellular res	3/104	88/17913	0.014607	0.050913	0.030344	ADCY2/PT
GO_BP_m	GO:00995	synaptic ve	4/104	162/1791	0.014736	0.051161	0.030492	ADORA2B
GO_BP_m	GO:00322	regulation	2/104	32/17913	0.014784	0.051161	0.030492	ADORA1/
GO_BP_m	GO:00435	positive re	2/104	32/17913	0.014784	0.051161	0.030492	CCL21/SR
GO_BP_m	GO:00516	actin filam	2/104	32/17913	0.014784	0.051161	0.030492	TMOD2/T
GO_BP_m	GO:00702	regulation	2/104	32/17913	0.014784	0.051161	0.030492	CCL5/PRK
GO_BP_m	GO:19039	positive re	2/104	32/17913	0.014784	0.051161	0.030492	RAC2/RH
GO_BP_m	GO:00028	positive re	3/104	89/17913	0.015054	0.052032	0.031011	ANXA1/CD
GO_BP_m	GO:00456	regulation	4/104	164/1791	0.01535	0.052992	0.031583	ANXA1/CD
GO_BP_m	GO:00427	eating beh	2/104	33/17913	0.015679	0.053999	0.032183	OPRL1/P2
GO_BP_m	GO:00480	neurotrop	2/104	33/17913	0.015679	0.053999	0.032183	GRB2/SRC
GO_BP_m	GO:20012	regulation	4/104	166/1791	0.01598	0.054969	0.032761	APP/GAL/
GO_BP_m	GO:00516	establishm	5/104	253/1791	0.016033	0.055088	0.032832	ADORA2B
GO_BP_m	GO:00140	response t	2/104	34/17913	0.016597	0.056496	0.033671	CNR1/PEN
GO_BP_m	GO:00321	activation	2/104	34/17913	0.016597	0.056496	0.033671	ADRA2A/S
GO_BP_m	GO:00432	response t	2/104	34/17913	0.016597	0.056496	0.033671	CNR1/PEN
GO_BP_m	GO:00457	respiratory	2/104	34/17913	0.016597	0.056496	0.033671	HCK/RAC2
GO_BP_m	GO:00457	positive re	2/104	34/17913	0.016597	0.056496	0.033671	ADORA1/
GO_BP_m	GO:19026	regulation	2/104	34/17913	0.016597	0.056496	0.033671	CCL21/RA
GO_BP_m	GO:00507	positive re	7/104	455/1791	0.016793	0.056496	0.033671	APP/CNR1
GO_BP_m	GO:00440	regulation	3/104	93/17913	0.016919	0.056496	0.033671	ADORA1/
GO_BP_m	GO:00456	positive re	3/104	93/17913	0.016919	0.056496	0.033671	ANXA1/CD
GO_BP_m	GO:00480	vascular e	3/104	93/17913	0.016919	0.056496	0.033671	PIK3CB/PI
GO_BP_m	GO:00487	tissue rem	4/104	170/1791	0.017288	0.056496	0.033671	ANXA1/RA
GO_BP_m	GO:00025	peptide ar	1/104	3/17913	0.017318	0.056496	0.033671	HLA-DRA
GO_BP_m	GO:00072	phospholi	1/104	3/17913	0.017318	0.056496	0.033671	GNA15
GO_BP_m	GO:00140	neural plat	1/104	3/17913	0.017318	0.056496	0.033671	DVL1
GO_BP_m	GO:00140	negative r	1/104	3/17913	0.017318	0.056496	0.033671	EDNRB
GO_BP_m	GO:00140	negative r	1/104	3/17913	0.017318	0.056496	0.033671	ADORA1
GO_BP_m	GO:00140	positive re	1/104	3/17913	0.017318	0.056496	0.033671	NTSR1
GO_BP_m	GO:00220	convergen	1/104	3/17913	0.017318	0.056496	0.033671	DVL1
GO_BP_m	GO:00230	terminatio	1/104	3/17913	0.017318	0.056496	0.033671	PRKCD
GO_BP_m	GO:00326	lymphoto	1/104	3/17913	0.017318	0.056496	0.033671	CD86
GO_BP_m	GO:00328	negative r	1/104	3/17913	0.017318	0.056496	0.033671	ADRA2A
GO_BP_m	GO:00329	regulation	1/104	3/17913	0.017318	0.056496	0.033671	ECT2
GO_BP_m	GO:00330	positive re	1/104	3/17913	0.017318	0.056496	0.033671	PIK3CB
GO_BP_m	GO:00336	negative r	1/104	3/17913	0.017318	0.056496	0.033671	CNR1
GO_BP_m	GO:00342	negative r	1/104	3/17913	0.017318	0.056496	0.033671	CXCL10
GO_BP_m	GO:00420	interleukin	1/104	3/17913	0.017318	0.056496	0.033671	CD86
GO_BP_m	GO:00421	lymphoto	1/104	3/17913	0.017318	0.056496	0.033671	CD86
GO_BP_m	GO:00433	beta selec	1/104	3/17913	0.017318	0.056496	0.033671	ZAP70
GO_BP_m	GO:00454	regulation	1/104	3/17913	0.017318	0.056496	0.033671	CD86
GO_BP_m	GO:00457	respiratory	1/104	3/17913	0.017318	0.056496	0.033671	HCK
GO_BP_m	GO:00517	positive re	1/104	3/17913	0.017318	0.056496	0.033671	GAL
GO_BP_m	GO:00713	cellular res	1/104	3/17913	0.017318	0.056496	0.033671	SRC
GO_BP_m	GO:00718	response t	1/104	3/17913	0.017318	0.056496	0.033671	APP
GO_BP_m	GO:00861	endothelir	1/104	3/17913	0.017318	0.056496	0.033671	EDNRB
GO_BP_m	GO:00989	retrograde	1/104	3/17913	0.017318	0.056496	0.033671	CNR1

GO_BP_m	GO:00989	retrograde	1/104	3/17913	0.017318	0.056496	0.033671	CNR1
GO_BP_m	GO:19004	negative re	1/104	3/17913	0.017318	0.056496	0.033671	ADORA1
GO_BP_m	GO:19040	regulation	1/104	3/17913	0.017318	0.056496	0.033671	OPRL1
GO_BP_m	GO:19059	negative re	1/104	3/17913	0.017318	0.056496	0.033671	PTGDR2
GO_BP_m	GO:19906	response t	1/104	3/17913	0.017318	0.056496	0.033671	GNRH1
GO_BP_m	GO:20001	positive re	1/104	3/17913	0.017318	0.056496	0.033671	SRC
GO_BP_m	GO:20002	negative re	1/104	3/17913	0.017318	0.056496	0.033671	PTGDR2
GO_BP_m	GO:20005	regulation	1/104	3/17913	0.017318	0.056496	0.033671	CCL21
GO_BP_m	GO:20005	positive re	1/104	3/17913	0.017318	0.056496	0.033671	SRC
GO_BP_m	GO:00028	positive re	3/104	94/17913	0.017404	0.056496	0.033671	ANXA1/CD
GO_BP_m	GO:00226	digestive s	3/104	94/17913	0.017404	0.056496	0.033671	ADRA2A/C
GO_BP_m	GO:00601	regulation	3/104	94/17913	0.017404	0.056496	0.033671	CCL5/GNA
GO_BP_m	GO:01100	regulation	3/104	94/17913	0.017404	0.056496	0.033671	ECT2/PIK3
GO_BP_m	GO:00075	neuromus	2/104	35/17913	0.017537	0.056496	0.033671	APP/DVL1
GO_BP_m	GO:00308	negative re	2/104	35/17913	0.017537	0.056496	0.033671	TMOD2/TI
GO_BP_m	GO:00420	type 2 imn	2/104	35/17913	0.017537	0.056496	0.033671	ANXA1/CD
GO_BP_m	GO:00450	positive re	2/104	35/17913	0.017537	0.056496	0.033671	CCL5/CD2
GO_BP_m	GO:00456	regulation	2/104	35/17913	0.017537	0.056496	0.033671	ANXA1/CD
GO_BP_m	GO:00482	macropha	2/104	35/17913	0.017537	0.056496	0.033671	CCL5/EDN
GO_BP_m	GO:00487	regulation	2/104	35/17913	0.017537	0.056496	0.033671	APP/F2
GO_BP_m	GO:19010	negative re	2/104	35/17913	0.017537	0.056496	0.033671	ADRA2A/C
GO_BP_m	GO:00508	protein sta	4/104	171/17913	0.017625	0.056716	0.033803	DVL1/GNA
GO_BP_m	GO:01200	positive re	3/104	95/17913	0.017897	0.05753	0.034288	CCL21/RA
GO_BP_m	GO:00080	neuron rec	2/104	36/17913	0.018499	0.0592	0.035283	APP/CNR1
GO_BP_m	GO:00337	response t	2/104	36/17913	0.018499	0.0592	0.035283	ADCY2/GC
GO_BP_m	GO:00480	insulin-like	2/104	36/17913	0.018499	0.0592	0.035283	GHRHR/PI
GO_BP_m	GO:00988	regulation	2/104	36/17913	0.018499	0.0592	0.035283	ADORA1/E
GO_BP_m	GO:00990	vesicle-me	4/104	174/17913	0.01866	0.059652	0.035552	ADORA2B
GO_BP_m	GO:00300	contractile	3/104	97/17913	0.018907	0.060241	0.035903	PIK3R1/PT
GO_BP_m	GO:00347	negative re	3/104	97/17913	0.018907	0.060241	0.035903	ADRA2A/N
GO_BP_m	GO:00431	stress fiber	3/104	97/17913	0.018907	0.060241	0.035903	PIK3R1/PT
GO_BP_m	GO:19037	regulation	7/104	468/17913	0.019279	0.061358	0.036569	ANXA1/CD
GO_BP_m	GO:00070	cell-cell ju	3/104	98/17913	0.019424	0.061683	0.036763	ECT2/TLN
GO_BP_m	GO:00432	regulation	3/104	98/17913	0.019424	0.061683	0.036763	ADORA1/A
GO_BP_m	GO:00016	behavioral	2/104	37/17913	0.019483	0.061734	0.036793	NPY2R/PE
GO_BP_m	GO:00463	positive re	2/104	37/17913	0.019483	0.061734	0.036793	PIK3R1/RH
GO_BP_m	GO:19021	regulation	5/104	267/17913	0.01975	0.062513	0.037257	ANXA1/CD
GO_BP_m	GO:00703	cellular res	3/104	99/17913	0.019948	0.063071	0.03759	ECT2/PRK
GO_BP_m	GO:00510	regulation	6/104	368/17913	0.020371	0.064147	0.038231	APP/DVL1
GO_BP_m	GO:00468	regulation	3/104	100/17913	0.02048	0.064147	0.038231	ECT2/PIK3
GO_BP_m	GO:00022	behavioral	2/104	38/17913	0.020488	0.064147	0.038231	NPY2R/PE
GO_BP_m	GO:00323	regulation	2/104	38/17913	0.020488	0.064147	0.038231	ADM/GAL
GO_BP_m	GO:00332	response t	2/104	38/17913	0.020488	0.064147	0.038231	CXCL10/PI
GO_BP_m	GO:00718	response t	2/104	38/17913	0.020488	0.064147	0.038231	APP/PENK
GO_BP_m	GO:00718	response t	2/104	38/17913	0.020488	0.064147	0.038231	APP/PENK
GO_BP_m	GO:00900	positive re	2/104	38/17913	0.020488	0.064147	0.038231	ANXA1/RH
GO_BP_m	GO:19035	negative re	2/104	38/17913	0.020488	0.064147	0.038231	ADM/ADC
GO_BP_m	GO:00516	vesicle loc	5/104	270/17913	0.020614	0.064472	0.038425	ADORA2B
GO_BP_m	GO:00439	positive re	4/104	180/17913	0.020844	0.06512	0.038811	CCL5/CD2
GO_BP_m	GO:00507	negative re	5/104	271/17913	0.020907	0.065178	0.038846	APP/EDNR
GO_BP_m	GO:00514	stress-acti	5/104	271/17913	0.020907	0.065178	0.038846	ADORA2B
GO_BP_m	GO:00486	axon exter	3/104	101/17913	0.02102	0.065388	0.038971	CXCL12/D
GO_BP_m	GO:20003	positive re	3/104	101/17913	0.02102	0.065388	0.038971	F2/GRB2/F
GO_BP_m	GO:00316	regulation	5/104	272/17913	0.021204	0.065735	0.039178	DVL1/GNA
GO_BP_m	GO:00513	negative re	5/104	272/17913	0.021204	0.065735	0.039178	DVL1/GNA
GO_BP_m	GO:19053	regulation	4/104	181/17913	0.021222	0.065735	0.039178	ARHGEF1
GO_BP_m	GO:19054	regulation	4/104	181/17913	0.021222	0.065735	0.039178	GPER1/PIK
GO_BP_m	GO:00381	neurotrop	2/104	39/17913	0.021515	0.066498	0.039633	GRB2/SRC

GO_BP_m	GO:00425	fear respo	2/104	39/17913	0.021515	0.066498	0.039633	NPY2R/PE
GO_BP_m	GO:00719	regulation	7/104	479/1791	0.021576	0.066616	0.039703	ADORA1/
GO_BP_m	GO:00457	positive re	6/104	373/1791	0.021601	0.066623	0.039707	APP/CD28
GO_BP_m	GO:00485	response t	6/104	375/1791	0.022107	0.06808	0.040575	ADM/ANX
GO_BP_m	GO:00066	protein im	3/104	103/1791	0.022123	0.06808	0.040575	ECT2/PIK3
GO_BP_m	GO:19907	protein loc	5/104	276/1791	0.022416	0.06808	0.040575	GPER1/P2
GO_BP_m	GO:00343	cell junctic	5/104	277/1791	0.022726	0.06808	0.040575	ECT2/MYC
GO_BP_m	GO:19029	positive re	4/104	185/1791	0.022779	0.06808	0.040575	APP/CCL2
GO_BP_m	GO:00009	assembly	1/104	4/17913	0.023024	0.06808	0.040575	ECT2
GO_BP_m	GO:00009	actomyosi	1/104	4/17913	0.023024	0.06808	0.040575	ECT2
GO_BP_m	GO:00022	astrocyte	1/104	4/17913	0.023024	0.06808	0.040575	APP
GO_BP_m	GO:00023	MHC class	1/104	4/17913	0.023024	0.06808	0.040575	HLA-DRA
GO_BP_m	GO:00024	myeloid de	1/104	4/17913	0.023024	0.06808	0.040575	CCL21
GO_BP_m	GO:00026	positive re	1/104	4/17913	0.023024	0.06808	0.040575	ADORA2B
GO_BP_m	GO:00066	glucosylce	1/104	4/17913	0.023024	0.06808	0.040575	PRKCD
GO_BP_m	GO:00140	microglia	1/104	4/17913	0.023024	0.06808	0.040575	APP
GO_BP_m	GO:00140	regulation	1/104	4/17913	0.023024	0.06808	0.040575	ADRA2A
GO_BP_m	GO:00148	vein smoo	1/104	4/17913	0.023024	0.06808	0.040575	EDNRB
GO_BP_m	GO:00161	axon midli	1/104	4/17913	0.023024	0.06808	0.040575	APP
GO_BP_m	GO:00320	positive re	1/104	4/17913	0.023024	0.06808	0.040575	PRKCD
GO_BP_m	GO:00328	neurotropl	1/104	4/17913	0.023024	0.06808	0.040575	ADORA1
GO_BP_m	GO:00329	regulation	1/104	4/17913	0.023024	0.06808	0.040575	MYBPC3
GO_BP_m	GO:00358	negative re	1/104	4/17913	0.023024	0.06808	0.040575	ADORA1
GO_BP_m	GO:00381	chemokine	1/104	4/17913	0.023024	0.06808	0.040575	CCL21
GO_BP_m	GO:00381	chemokine	1/104	4/17913	0.023024	0.06808	0.040575	CXCL12
GO_BP_m	GO:00426	catagen	1/104	4/17913	0.023024	0.06808	0.040575	GAL
GO_BP_m	GO:00456	negative re	1/104	4/17913	0.023024	0.06808	0.040575	ANXA1
GO_BP_m	GO:00482	epinephrir	1/104	4/17913	0.023024	0.06808	0.040575	ADRA2A
GO_BP_m	GO:00514	positive re	1/104	4/17913	0.023024	0.06808	0.040575	GAL
GO_BP_m	GO:00517	regulation	1/104	4/17913	0.023024	0.06808	0.040575	GAL
GO_BP_m	GO:00607	regulation	1/104	4/17913	0.023024	0.06808	0.040575	RAC2
GO_BP_m	GO:00707	D-asparta	1/104	4/17913	0.023024	0.06808	0.040575	NTSR1
GO_BP_m	GO:00707	D-asparta	1/104	4/17913	0.023024	0.06808	0.040575	NTSR1
GO_BP_m	GO:00970	dendritic c	1/104	4/17913	0.023024	0.06808	0.040575	CCL21
GO_BP_m	GO:00995	positive re	1/104	4/17913	0.023024	0.06808	0.040575	CNR1
GO_BP_m	GO:00997	induction	1/104	4/17913	0.023024	0.06808	0.040575	CNR1
GO_BP_m	GO:19001	positive re	1/104	4/17913	0.023024	0.06808	0.040575	TIAM1
GO_BP_m	GO:19007	positive re	1/104	4/17913	0.023024	0.06808	0.040575	F2
GO_BP_m	GO:19059	positive re	1/104	4/17913	0.023024	0.06808	0.040575	APP
GO_BP_m	GO:19059	regulation	1/104	4/17913	0.023024	0.06808	0.040575	PTGDR2
GO_BP_m	GO:19904	adrenome	1/104	4/17913	0.023024	0.06808	0.040575	ADM
GO_BP_m	GO:20001	regulation	1/104	4/17913	0.023024	0.06808	0.040575	ADM
GO_BP_m	GO:20002	regulation	1/104	4/17913	0.023024	0.06808	0.040575	PTGDR2
GO_BP_m	GO:00310	stress-acti	5/104	278/1791	0.023038	0.06808	0.040575	ADORA2B
GO_BP_m	GO:00140	astrocyte	2/104	41/17913	0.023631	0.06976	0.041577	APP/VIM
GO_BP_m	GO:00432	response t	3/104	106/1791	0.023836	0.070294	0.041895	CNR1/PEN
GO_BP_m	GO:00600	Wnt signal	3/104	107/1791	0.024423	0.071877	0.042838	ARHGEF1
GO_BP_m	GO:19046	glucose tra	3/104	107/1791	0.024423	0.071877	0.042838	PIK3R1/RH
GO_BP_m	GO:00019	negative re	2/104	42/17913	0.02472	0.072238	0.043053	PIK3R1/SR
GO_BP_m	GO:00108	positive re	2/104	42/17913	0.02472	0.072238	0.043053	GNA15/GN
GO_BP_m	GO:00140	glutamate	2/104	42/17913	0.02472	0.072238	0.043053	ADORA1/N
GO_BP_m	GO:00353	positive re	2/104	42/17913	0.02472	0.072238	0.043053	ADORA1/F
GO_BP_m	GO:00358	vascular sr	2/104	42/17913	0.02472	0.072238	0.043053	ADM/GPE
GO_BP_m	GO:00990	presynaps	2/104	42/17913	0.02472	0.072238	0.043053	APP/DVL1
GO_BP_m	GO:19002	regulation	2/104	42/17913	0.02472	0.072238	0.043053	ADORA1/
GO_BP_m	GO:00097	response t	4/104	190/1791	0.02482	0.072456	0.043183	ADRA2A/C
GO_BP_m	GO:00324	positive re	3/104	108/1791	0.025017	0.072959	0.043483	GAL/NTSR
GO_BP_m	GO:00901	regulation	3/104	109/1791	0.025619	0.074639	0.044485	ARHGEF1

GO_BP_m	GO:00094	response t	2/104	43/17913	0.025829	0.074874	0.044624	ADM/CXC
GO_BP_m	GO:00108	positive re	2/104	43/17913	0.025829	0.074874	0.044624	PIK3R1/RH
GO_BP_m	GO:00350	response t	2/104	43/17913	0.025829	0.074874	0.044624	CNR1/PEN
GO_BP_m	GO:00433	regulation	2/104	43/17913	0.025829	0.074874	0.044624	ADORA2B
GO_BP_m	GO:19027	regulation	2/104	43/17913	0.025829	0.074874	0.044624	RAC2/SRC
GO_BP_m	GO:00075	female pre	4/104	193/1791	0.026096	0.075571	0.04504	ADM/CNR
GO_BP_m	GO:00096	response t	7/104	499/1791	0.026229	0.075881	0.045225	CCL5/CNR
GO_BP_m	GO:00160	carbohydr	4/104	194/1791	0.02653	0.076598	0.045652	FAM3C/G
GO_BP_m	GO:20003	regulation	4/104	194/1791	0.02653	0.076598	0.045652	F2/GRB2/
GO_BP_m	GO:00024	inflammat	2/104	44/17913	0.026958	0.077477	0.046176	CD28/CN
GO_BP_m	GO:00329	regulation	2/104	44/17913	0.026958	0.077477	0.046176	F2/VIM
GO_BP_m	GO:19002	regulation	2/104	44/17913	0.026958	0.077477	0.046176	GNA15/G
GO_BP_m	GO:19032	regulation	2/104	44/17913	0.026958	0.077477	0.046176	RAC2/RH
GO_BP_m	GO:00097	response t	4/104	195/1791	0.026968	0.077477	0.046176	ADRA2A/
GO_BP_m	GO:00519	negative r	5/104	290/1791	0.02701	0.07752	0.046202	APP/EDN
GO_BP_m	GO:00086	hexose tra	3/104	112/1791	0.027472	0.078769	0.046946	PIK3R1/RH
GO_BP_m	GO:00109	regulation	3/104	113/1791	0.028106	0.079143	0.047169	FAM3C/G
GO_BP_m	GO:00308	regulation	2/104	45/17913	0.028107	0.079143	0.047169	TMOD2/T
GO_BP_m	GO:00450	T cell sele	2/104	45/17913	0.028107	0.079143	0.047169	CD28/ZAP
GO_BP_m	GO:00991	presynaps	2/104	45/17913	0.028107	0.079143	0.047169	APP/DVL1
GO_BP_m	GO:00025	peptide ar	1/104	5/17913	0.028697	0.079143	0.047169	HLA-DRA
GO_BP_m	GO:00140	microglia	1/104	5/17913	0.028697	0.079143	0.047169	APP
GO_BP_m	GO:00140	regulation	1/104	5/17913	0.028697	0.079143	0.047169	NTSR1
GO_BP_m	GO:00161	axon choic	1/104	5/17913	0.028697	0.079143	0.047169	APP
GO_BP_m	GO:00302	apoptotic	1/104	5/17913	0.028697	0.079143	0.047169	GPER1
GO_BP_m	GO:00315	activation	1/104	5/17913	0.028697	0.079143	0.047169	CCL5
GO_BP_m	GO:00319	negative r	1/104	5/17913	0.028697	0.079143	0.047169	CNR1
GO_BP_m	GO:00320	positive re	1/104	5/17913	0.028697	0.079143	0.047169	PRKCD
GO_BP_m	GO:00330	negative r	1/104	5/17913	0.028697	0.079143	0.047169	GNRH1
GO_BP_m	GO:00356	receptor t	1/104	5/17913	0.028697	0.079143	0.047169	ADRA2A
GO_BP_m	GO:00361	Schwann c	1/104	5/17913	0.028697	0.079143	0.047169	TIAM1
GO_BP_m	GO:00381	cannabino	1/104	5/17913	0.028697	0.079143	0.047169	CNR1
GO_BP_m	GO:00423	negative r	1/104	5/17913	0.028697	0.079143	0.047169	ADORA1
GO_BP_m	GO:00434	cortisol se	1/104	5/17913	0.028697	0.079143	0.047169	GAL
GO_BP_m	GO:00434	response t	1/104	5/17913	0.028697	0.079143	0.047169	CRHR2
GO_BP_m	GO:00448	actomyosi	1/104	5/17913	0.028697	0.079143	0.047169	ECT2
GO_BP_m	GO:00453	positive re	1/104	5/17913	0.028697	0.079143	0.047169	APP
GO_BP_m	GO:00482	epinephrir	1/104	5/17913	0.028697	0.079143	0.047169	ADRA2A
GO_BP_m	GO:00486	collateral	1/104	5/17913	0.028697	0.079143	0.047169	APP
GO_BP_m	GO:00488	positive re	1/104	5/17913	0.028697	0.079143	0.047169	GAL
GO_BP_m	GO:00507	positive re	1/104	5/17913	0.028697	0.079143	0.047169	APP
GO_BP_m	GO:00514	regulation	1/104	5/17913	0.028697	0.079143	0.047169	GAL
GO_BP_m	GO:00601	somatotro	1/104	5/17913	0.028697	0.079143	0.047169	GHRHR
GO_BP_m	GO:00617	leukotrien	1/104	5/17913	0.028697	0.079143	0.047169	LTB4R
GO_BP_m	GO:00709	neutrophil	1/104	5/17913	0.028697	0.079143	0.047169	F2
GO_BP_m	GO:00713	cellular re	1/104	5/17913	0.028697	0.079143	0.047169	CRHR2
GO_BP_m	GO:00718	adenylate	1/104	5/17913	0.028697	0.079143	0.047169	ADRA2A
GO_BP_m	GO:00973	neutrophil	1/104	5/17913	0.028697	0.079143	0.047169	ANXA1
GO_BP_m	GO:00995	trans-syna	1/104	5/17913	0.028697	0.079143	0.047169	CNR1
GO_BP_m	GO:00995	trans-syna	1/104	5/17913	0.028697	0.079143	0.047169	CNR1
GO_BP_m	GO:01500	regulation	1/104	5/17913	0.028697	0.079143	0.047169	APP
GO_BP_m	GO:19001	regulation	1/104	5/17913	0.028697	0.079143	0.047169	TIAM1
GO_BP_m	GO:19007	regulation	1/104	5/17913	0.028697	0.079143	0.047169	F2
GO_BP_m	GO:20004	regulation	1/104	5/17913	0.028697	0.079143	0.047169	PTGER4
GO_BP_m	GO:20007	positive re	1/104	5/17913	0.028697	0.079143	0.047169	GPER1
GO_BP_m	GO:20008	positive re	1/104	5/17913	0.028697	0.079143	0.047169	GAL
GO_BP_m	GO:00157	monosacc	3/104	114/1791	0.028747	0.079179	0.04719	PIK3R1/RH
GO_BP_m	GO:00712	cellular re	4/104	199/1791	0.028765	0.079179	0.04719	PTGDR/PT

GO_BP_m	GO:003428	response t	4/104	200/17913	0.029225	0.080278	0.047845	ADRA2A/C
GO_BP_m	GO:005507	sodium ior	2/104	46/17913	0.029275	0.080278	0.047845	ADORA1/E
GO_BP_m	GO:006071	labyrinthin	2/104	46/17913	0.029275	0.080278	0.047845	ADM/GRB
GO_BP_m	GO:007023	T cell apop	2/104	46/17913	0.029275	0.080278	0.047845	CCL5/PRKC
GO_BP_m	GO:001082	positive re	3/104	115/17913	0.029395	0.080531	0.047996	GPER1/RA
GO_BP_m	GO:003227	positive re	3/104	116/17913	0.030052	0.082174	0.048975	CCL21/GR
GO_BP_m	GO:003421	carbohydr	3/104	116/17913	0.030052	0.082174	0.048975	PIK3R1/RH
GO_BP_m	GO:001491	positive re	2/104	47/17913	0.030463	0.082984	0.049458	CCL5/SRC
GO_BP_m	GO:004337	regulation	2/104	47/17913	0.030463	0.082984	0.049458	ANXA1/CD
GO_BP_m	GO:004470	multi-orga	2/104	47/17913	0.030463	0.082984	0.049458	CCR5/F2
GO_BP_m	GO:190311	regulation	2/104	47/17913	0.030463	0.082984	0.049458	ADORA1/N
GO_BP_m	GO:004570	positive re	4/104	203/17913	0.030632	0.083365	0.049685	ADM/ANX
GO_BP_m	GO:004572	positive re	3/104	117/17913	0.030716	0.083516	0.049775	CCL5/CD2
GO_BP_m	GO:003004	actin filam	2/104	48/17913	0.031669	0.085704	0.051079	TMOD2/TI
GO_BP_m	GO:004222	response t	2/104	48/17913	0.031669	0.085704	0.051079	CNR1/TIAM
GO_BP_m	GO:004593	positive re	2/104	48/17913	0.031669	0.085704	0.051079	GPER1/NP
GO_BP_m	GO:005197	regulation	2/104	48/17913	0.031669	0.085704	0.051079	PRKCQ/SR
GO_BP_m	GO:190330	positive re	2/104	48/17913	0.031669	0.085704	0.051079	ADORA2B/
GO_BP_m	GO:190547	positive re	3/104	120/17913	0.032755	0.08844	0.05271	GPER1/PIK
GO_BP_m	GO:000325	regulation	2/104	49/17913	0.032894	0.08844	0.05271	NTSR1/SRC
GO_BP_m	GO:001071	regulation	2/104	49/17913	0.032894	0.08844	0.05271	F2/VIM
GO_BP_m	GO:003019	negative re	2/104	49/17913	0.032894	0.08844	0.05271	F2/PRKCD
GO_BP_m	GO:003152	ruffle orga	2/104	49/17913	0.032894	0.08844	0.05271	CCL21/RH
GO_BP_m	GO:004394	regulation	2/104	49/17913	0.032894	0.08844	0.05271	NPY2R/OF
GO_BP_m	GO:190379	positive re	2/104	49/17913	0.032894	0.08844	0.05271	NTSR1/PR
GO_BP_m	GO:001095	negative re	4/104	210/17913	0.034067	0.089415	0.053291	APP/ITIH3/
GO_BP_m	GO:000834	glial cell m	2/104	50/17913	0.034137	0.089415	0.053291	P2RY1/TIA
GO_BP_m	GO:190004	negative re	2/104	50/17913	0.034137	0.089415	0.053291	F2/PRKCD
GO_BP_m	GO:190551	macrophag	2/104	50/17913	0.034137	0.089415	0.053291	CCL5/EDN
GO_BP_m	GO:003230	regulation	3/104	122/17913	0.034152	0.089415	0.053291	GAL/NTSR
GO_BP_m	GO:003470	negative re	3/104	122/17913	0.034152	0.089415	0.053291	ADRA2A/N
GO_BP_m	GO:005500	monovaler	3/104	122/17913	0.034152	0.089415	0.053291	ADORA1/C
GO_BP_m	GO:190290	negative re	3/104	122/17913	0.034152	0.089415	0.053291	PRKCD/TM
GO_BP_m	GO:000217	male germ	1/104	6/17913	0.034338	0.089415	0.053291	PTGDR2
GO_BP_m	GO:000718	transmeml	1/104	6/17913	0.034338	0.089415	0.053291	TRIO
GO_BP_m	GO:001053	positive re	1/104	6/17913	0.034338	0.089415	0.053291	CCL5
GO_BP_m	GO:001064	positive re	1/104	6/17913	0.034338	0.089415	0.053291	SRC
GO_BP_m	GO:003222	negative re	1/104	6/17913	0.034338	0.089415	0.053291	ADORA1
GO_BP_m	GO:003224	positive re	1/104	6/17913	0.034338	0.089415	0.053291	ADORA1
GO_BP_m	GO:003302	regulation	1/104	6/17913	0.034338	0.089415	0.053291	PIK3CB
GO_BP_m	GO:003363	positive re	1/104	6/17913	0.034338	0.089415	0.053291	CCL5
GO_BP_m	GO:003411	positive re	1/104	6/17913	0.034338	0.089415	0.053291	CCL5
GO_BP_m	GO:003609	germ cell p	1/104	6/17913	0.034338	0.089415	0.053291	PTGDR2
GO_BP_m	GO:004294	D-amino a	1/104	6/17913	0.034338	0.089415	0.053291	NTSR1
GO_BP_m	GO:004562	positive re	1/104	6/17913	0.034338	0.089415	0.053291	ANXA1
GO_BP_m	GO:004608	adenosine	1/104	6/17913	0.034338	0.089415	0.053291	PTGDR
GO_BP_m	GO:005104	negative re	1/104	6/17913	0.034338	0.089415	0.053291	TIMP1
GO_BP_m	GO:005190	negative re	1/104	6/17913	0.034338	0.089415	0.053291	SRC
GO_BP_m	GO:006002	convergen	1/104	6/17913	0.034338	0.089415	0.053291	DVL1
GO_BP_m	GO:006069	regulation	1/104	6/17913	0.034338	0.089415	0.053291	PRKCD
GO_BP_m	GO:006189	positive re	1/104	6/17913	0.034338	0.089415	0.053291	APP
GO_BP_m	GO:007170	nuclear me	1/104	6/17913	0.034338	0.089415	0.053291	GPER1
GO_BP_m	GO:009023	positive re	1/104	6/17913	0.034338	0.089415	0.053291	NTSR1
GO_BP_m	GO:009764	amylin rec	1/104	6/17913	0.034338	0.089415	0.053291	ADM
GO_BP_m	GO:011011	regulation	1/104	6/17913	0.034338	0.089415	0.053291	PRKCD
GO_BP_m	GO:190110	negative re	1/104	6/17913	0.034338	0.089415	0.053291	TIMP1
GO_BP_m	GO:190527	regulation	1/104	6/17913	0.034338	0.089415	0.053291	TIAM1
GO_BP_m	GO:200050	positive re	1/104	6/17913	0.034338	0.089415	0.053291	CCL5

GO_BP_m	GO:200072	regulation	1/104	6/17913	0.034338	0.089415	0.053291	GPER1
GO_BP_m	GO:200128	regulation	1/104	6/17913	0.034338	0.089415	0.053291	SRC
GO_BP_m	GO:005170	interaction	4/104	211/17913	0.034576	0.089953	0.053611	CCR5/CD8
GO_BP_m	GO:000150	action pot	3/104	123/17913	0.034863	0.090456	0.053911	CNR1/GPE
GO_BP_m	GO:000173	establishm	3/104	123/17913	0.034863	0.090456	0.053911	ARHGEF19
GO_BP_m	GO:000716	establishm	3/104	123/17913	0.034863	0.090456	0.053911	ARHGEF19
GO_BP_m	GO:000195	positive re	2/104	51/17913	0.035398	0.091435	0.054495	CCL21/CC
GO_BP_m	GO:003261	interleukin	2/104	51/17913	0.035398	0.091435	0.054495	CD28/PRK
GO_BP_m	GO:004559	negative re	2/104	51/17913	0.035398	0.091435	0.054495	GPER1/TRI
GO_BP_m	GO:005088	neuromusc	2/104	51/17913	0.035398	0.091435	0.054495	APP/RAC3
GO_BP_m	GO:007022	regulation	2/104	51/17913	0.035398	0.091435	0.054495	CCL5/PRKC
GO_BP_m	GO:004633	positive re	3/104	124/17913	0.035581	0.091824	0.054727	APP/CCL2
GO_BP_m	GO:005117	import into	3/104	125/17913	0.036306	0.093613	0.055793	ECT2/PIK3
GO_BP_m	GO:004211	B cell activ	4/104	215/17913	0.036654	0.094234	0.056163	CD28/PIK3
GO_BP_m	GO:000288	regulation	2/104	52/17913	0.036677	0.094234	0.056163	ADORA2B/
GO_BP_m	GO:007132	cellular res	2/104	52/17913	0.036677	0.094234	0.056163	APP/PENK
GO_BP_m	GO:200024	negative re	2/104	52/17913	0.036677	0.094234	0.056163	PTGDR2/T
GO_BP_m	GO:190134	regulation	6/104	425/17913	0.037463	0.096166	0.057314	ADM/ANX
GO_BP_m	GO:004547	response t	3/104	127/17913	0.03778	0.096896	0.057749	CNR1/GNI
GO_BP_m	GO:000690	cellular de	2/104	53/17913	0.037974	0.096964	0.05779	ADORA2B/
GO_BP_m	GO:001922	regulation	2/104	53/17913	0.037974	0.096964	0.05779	ADM/ADR
GO_BP_m	GO:003221	regulation	2/104	53/17913	0.037974	0.096964	0.05779	PRKCQ/SR
GO_BP_m	GO:003290	collagen b	2/104	53/17913	0.037974	0.096964	0.05779	F2/VIM
GO_BP_m	GO:005081	negative re	2/104	53/17913	0.037974	0.096964	0.05779	F2/PRKCD
GO_BP_m	GO:001401	regulation	3/104	128/17913	0.038529	0.097936	0.058369	APP/F2/TI
GO_BP_m	GO:004521	cell-cell ju	3/104	128/17913	0.038529	0.097936	0.058369	ECT2/TLN1
GO_BP_m	GO:005149	negative re	3/104	128/17913	0.038529	0.097936	0.058369	PRKCD/TM
GO_BP_m	GO:000803	cell recogn	3/104	129/17913	0.039284	0.097936	0.058369	APP/CCL2
GO_BP_m	GO:003051	intracellula	3/104	129/17913	0.039284	0.097936	0.058369	GHRHR/GI
GO_BP_m	GO:000154	ovarian fol	2/104	54/17913	0.039289	0.097936	0.058369	GNRH1/SF
GO_BP_m	GO:006117	regulation	2/104	54/17913	0.039289	0.097936	0.058369	ADRA2A/T
GO_BP_m	GO:000252	acute infla	4/104	220/17913	0.039351	0.097936	0.058369	ADORA1/C
GO_BP_m	GO:000178	neutrophil	1/104	7/17913	0.039946	0.097936	0.058369	PIK3CB
GO_BP_m	GO:000183	neural plat	1/104	7/17913	0.039946	0.097936	0.058369	DVL1
GO_BP_m	GO:000224	connective	1/104	7/17913	0.039946	0.097936	0.058369	TIMP1
GO_BP_m	GO:000239	MHC prote	1/104	7/17913	0.039946	0.097936	0.058369	HLA-DRA
GO_BP_m	GO:000260	positive re	1/104	7/17913	0.039946	0.097936	0.058369	CCL21
GO_BP_m	GO:000668	sphingomy	1/104	7/17913	0.039946	0.097936	0.058369	PRKCD
GO_BP_m	GO:000670	progestero	1/104	7/17913	0.039946	0.097936	0.058369	ADM
GO_BP_m	GO:001585	nucleoside	1/104	7/17913	0.039946	0.097936	0.058369	ADORA1
GO_BP_m	GO:001900	fusion of v	1/104	7/17913	0.039946	0.097936	0.058369	CCR5
GO_BP_m	GO:002189	cerebral co	1/104	7/17913	0.039946	0.097936	0.058369	RAC3
GO_BP_m	GO:003162	positive re	1/104	7/17913	0.039946	0.097936	0.058369	CNR1
GO_BP_m	GO:003303	positive re	1/104	7/17913	0.039946	0.097936	0.058369	PIK3CB
GO_BP_m	GO:003960	membrane	1/104	7/17913	0.039946	0.097936	0.058369	CCR5
GO_BP_m	GO:004001	embryonic	1/104	7/17913	0.039946	0.097936	0.058369	PIK3CB
GO_BP_m	GO:004480	multi-orga	1/104	7/17913	0.039946	0.097936	0.058369	CCR5
GO_BP_m	GO:004563	positive re	1/104	7/17913	0.039946	0.097936	0.058369	CD86
GO_BP_m	GO:004590	negative re	1/104	7/17913	0.039946	0.097936	0.058369	ADM
GO_BP_m	GO:004647	glycosylce	1/104	7/17913	0.039946	0.097936	0.058369	PRKCD
GO_BP_m	GO:004884	positive re	1/104	7/17913	0.039946	0.097936	0.058369	CXCL12
GO_BP_m	GO:005112	synaptic gr	1/104	7/17913	0.039946	0.097936	0.058369	APP
GO_BP_m	GO:006002	Bergmann	1/104	7/17913	0.039946	0.097936	0.058369	VIM
GO_BP_m	GO:007010	negative re	1/104	7/17913	0.039946	0.097936	0.058369	CCL5
GO_BP_m	GO:007094	neutrophil	1/104	7/17913	0.039946	0.097936	0.058369	F2
GO_BP_m	GO:007138	cellular res	1/104	7/17913	0.039946	0.097936	0.058369	GPER1
GO_BP_m	GO:009064	modulatio	1/104	7/17913	0.039946	0.097936	0.058369	APP
GO_BP_m	GO:009715	positive re	1/104	7/17913	0.039946	0.097936	0.058369	NTSR1

GO_BP_m	GO:00976	calcitonin	1/104	7/17913	0.039946	0.097936	0.058369	ADM
GO_BP_m	GO:00988	modulatio	1/104	7/17913	0.039946	0.097936	0.058369	NTSR1
GO_BP_m	GO:00989	retrograde	1/104	7/17913	0.039946	0.097936	0.058369	CNR1
GO_BP_m	GO:19015	regulation	1/104	7/17913	0.039946	0.097936	0.058369	CXCL10
GO_BP_m	GO:19024	positive re	1/104	7/17913	0.039946	0.097936	0.058369	DVL1
GO_BP_m	GO:19033	positive re	1/104	7/17913	0.039946	0.097936	0.058369	PENK
GO_BP_m	GO:19039	positive re	1/104	7/17913	0.039946	0.097936	0.058369	SRC
GO_BP_m	GO:19040	positive re	1/104	7/17913	0.039946	0.097936	0.058369	OPRL1
GO_BP_m	GO:19041	negative re	1/104	7/17913	0.039946	0.097936	0.058369	SRC
GO_BP_m	GO:20003	positive re	1/104	7/17913	0.039946	0.097936	0.058369	SRC
GO_BP_m	GO:20005	positive re	1/104	7/17913	0.039946	0.097936	0.058369	CCL21
GO_BP_m	GO:20008	positive re	1/104	7/17913	0.039946	0.097936	0.058369	GAL
GO_BP_m	GO:20009	positive re	1/104	7/17913	0.039946	0.097936	0.058369	PENK
GO_BP_m	GO:00466	response t	3/104	130/17913	0.040048	0.098102	0.058468	APP/P2RY
GO_BP_m	GO:00096	response t	5/104	323/17913	0.040096	0.098137	0.058489	CCL5/CXC
GO_BP_m	GO:00510	positive re	4/104	222/17913	0.040461	0.098834	0.058904	CD28/PRK
GO_BP_m	GO:00458	negative re	5/104	324/17913	0.040544	0.098834	0.058904	APP/F2/ITI
GO_BP_m	GO:00316	lipopolysa	2/104	55/17913	0.04062	0.098834	0.058904	CCL5/HCK
GO_BP_m	GO:00327	positive re	2/104	55/17913	0.04062	0.098834	0.058904	ADORA2B
GO_BP_m	GO:00353	positive re	2/104	55/17913	0.04062	0.098834	0.058904	ADORA1/
GO_BP_m	GO:00609	dendritic s	2/104	55/17913	0.04062	0.098834	0.058904	DVL1/TIAN
GO_BP_m	GO:00989	regulation	2/104	55/17913	0.04062	0.098834	0.058904	CNR1/NTS
GO_BP_m	GO:00104	negative re	4/104	223/17913	0.041022	0.099729	0.059438	APP/ITIH3
GO_BP_m	GO:00097	response t	4/104	224/17913	0.041588	0.100788	0.060069	ADRA2A/C
GO_BP_m	GO:00075	digestion	3/104	132/17913	0.041597	0.100788	0.060069	ADRA2A/C
GO_BP_m	GO:00485	digestive t	3/104	132/17913	0.041597	0.100788	0.060069	CCKBR/ED
GO_BP_m	GO:00512	negative re	3/104	132/17913	0.041597	0.100788	0.060069	ANXA1/GA
GO_BP_m	GO:19018	negative re	2/104	56/17913	0.041968	0.101602	0.060554	TMOD2/TI
GO_BP_m	GO:00328	regulation	2/104	57/17913	0.043333	0.104644	0.062367	ADORA1/N
GO_BP_m	GO:00454	bone resor	2/104	57/17913	0.043333	0.104644	0.062367	RAC2/SRC
GO_BP_m	GO:19037	positive re	2/104	57/17913	0.043333	0.104644	0.062367	RAC2/RHO
GO_BP_m	GO:00026	regulation	6/104	441/17913	0.043535	0.105044	0.062606	ADORA2B
GO_BP_m	GO:00028	regulation	3/104	135/17913	0.043978	0.106023	0.063189	ANXA1/CD
GO_BP_m	GO:00458	positive re	5/104	333/17913	0.044708	0.106392	0.063409	ADRA2A/A
GO_BP_m	GO:00713	cellular res	2/104	58/17913	0.044715	0.106392	0.063409	ANXA1/GP
GO_BP_m	GO:00510	actin filam	3/104	136/17913	0.044786	0.106392	0.063409	PIK3R1/PT
GO_BP_m	GO:00506	cytokine se	4/104	230/17913	0.045078	0.106392	0.063409	ANXA1/LC
GO_BP_m	GO:00022	wound hea	1/104	8/17913	0.045522	0.106392	0.063409	TIMP1
GO_BP_m	GO:00028	positive re	1/104	8/17913	0.045522	0.106392	0.063409	CNR1
GO_BP_m	GO:00071	signal com	1/104	8/17913	0.045522	0.106392	0.063409	SRC
GO_BP_m	GO:00072	neuron-ne	1/104	8/17913	0.045522	0.106392	0.063409	TMOD2
GO_BP_m	GO:00105	regulation	1/104	8/17913	0.045522	0.106392	0.063409	CCL5
GO_BP_m	GO:00107	positive re	1/104	8/17913	0.045522	0.106392	0.063409	ADORA2B
GO_BP_m	GO:00140	gamma-ar	1/104	8/17913	0.045522	0.106392	0.063409	NTSR1
GO_BP_m	GO:00312	positive re	1/104	8/17913	0.045522	0.106392	0.063409	ADORA2B
GO_BP_m	GO:00320	regulation	1/104	8/17913	0.045522	0.106392	0.063409	PRKCD
GO_BP_m	GO:00320	positive re	1/104	8/17913	0.045522	0.106392	0.063409	PRKCD
GO_BP_m	GO:00330	regulation	1/104	8/17913	0.045522	0.106392	0.063409	GNRH1
GO_BP_m	GO:00336	positive re	1/104	8/17913	0.045522	0.106392	0.063409	SRC
GO_BP_m	GO:00343	negative re	1/104	8/17913	0.045522	0.106392	0.063409	PRKCD
GO_BP_m	GO:00381	interleukin	1/104	8/17913	0.045522	0.106392	0.063409	JAK1
GO_BP_m	GO:00420	epithelial f	1/104	8/17913	0.045522	0.106392	0.063409	EDNRB
GO_BP_m	GO:00508	progestero	1/104	8/17913	0.045522	0.106392	0.063409	SRC
GO_BP_m	GO:00517	cytolysis ir	1/104	8/17913	0.045522	0.106392	0.063409	F2
GO_BP_m	GO:00600	convergen	1/104	8/17913	0.045522	0.106392	0.063409	DVL1
GO_BP_m	GO:00704	positive re	1/104	8/17913	0.045522	0.106392	0.063409	GPER1
GO_BP_m	GO:00709	neutrophil	1/104	8/17913	0.045522	0.106392	0.063409	F2
GO_BP_m	GO:00714	cellular res	1/104	8/17913	0.045522	0.106392	0.063409	PRKCD

GO_BP_m7	GO:00902	regulation	1/104	8/17913	0.045522	0.106392	0.063409	NTSR1
GO_BP_m7	GO:00987	response t	1/104	8/17913	0.045522	0.106392	0.063409	JAK1
GO_BP_m7	GO:00987	cellular res	1/104	8/17913	0.045522	0.106392	0.063409	JAK1
GO_BP_m7	GO:00991	regulation	1/104	8/17913	0.045522	0.106392	0.063409	TIAM1
GO_BP_m7	GO:01500	positive re	1/104	8/17913	0.045522	0.106392	0.063409	DVL1
GO_BP_m7	GO:19026	positive re	1/104	8/17913	0.045522	0.106392	0.063409	CXCL12
GO_BP_m7	GO:19059	regulation	1/104	8/17913	0.045522	0.106392	0.063409	APP
GO_BP_m7	GO:20002	negative re	1/104	8/17913	0.045522	0.106392	0.063409	NPY2R
GO_BP_m7	GO:20004	negative re	1/104	8/17913	0.045522	0.106392	0.063409	ANXA1
GO_BP_m7	GO:20005	regulation	1/104	8/17913	0.045522	0.106392	0.063409	CCL21
GO_BP_m7	GO:20005	regulation	1/104	8/17913	0.045522	0.106392	0.063409	SRC
GO_BP_m7	GO:20008	regulation	1/104	8/17913	0.045522	0.106392	0.063409	GAL
GO_BP_m7	GO:00305	adult beha	3/104	137/17913	0.045602	0.106392	0.063409	APP/CXCL
GO_BP_m7	GO:00507	negative re	3/104	137/17913	0.045602	0.106392	0.063409	ADRA2A/A
GO_BP_m7	GO:00615	actin filam	3/104	137/17913	0.045602	0.106392	0.063409	PIK3R1/PT
GO_BP_m7	GO:19039	positive re	2/104	59/17913	0.046112	0.107496	0.064067	CCL5/CD2
GO_BP_m7	GO:00107	positive re	3/104	138/17913	0.046425	0.108051	0.064398	CXCL12/RA
GO_BP_m7	GO:00308	mammary	3/104	138/17913	0.046425	0.108051	0.064398	ARHGAP5/
GO_BP_m7	GO:00513	response t	3/104	139/17913	0.047255	0.109895	0.065497	ADM/ANX
GO_BP_m7	GO:00305	neutrophil	2/104	60/17913	0.047526	0.110347	0.065766	CCL21/RA
GO_BP_m7	GO:00463	regulation	2/104	60/17913	0.047526	0.110347	0.065766	PIK3R1/RH
GO_BP_m7	GO:00060	alcohol me	5/104	339/17913	0.047622	0.110483	0.065847	APP/GPER
GO_BP_m7	GO:00170	protein im	3/104	140/17913	0.048093	0.111485	0.066444	ECT2/PIK3
GO_BP_m7	GO:00301	regulation	5/104	341/17913	0.048619	0.112613	0.067117	APP/DVL1
GO_BP_m7	GO:00300	lamellipod	2/104	61/17913	0.048955	0.113031	0.067366	RAC2/VCL
GO_BP_m7	GO:00357	insulin sec	2/104	61/17913	0.048955	0.113031	0.067366	ADRA2A/T
GO_BP_m7	GO:00468	negative re	2/104	61/17913	0.048955	0.113031	0.067366	ADORA1/A
GO_BP_m7	GO:19043	regulation	2/104	61/17913	0.048955	0.113031	0.067366	PRKCQ/SR
GO_BP_m7	GO:00027	negative re	3/104	142/17913	0.049791	0.114346	0.06815	ADRA2A/A
GO_BP_m7	GO:00076	learning	3/104	142/17913	0.049791	0.114346	0.06815	APP/NTSR
GO_BP_m8	GO:00063	transcripti	4/55	79/17913	0.000101	0.000992	0.00066	ERCC3/GT
GO_BP_m8	GO:00322	regulation	4/55	79/17913	0.000101	0.000992	0.00066	ERCC1/NE
GO_BP_m8	GO:00063	terminatio	3/55	31/17913	0.000116	0.001134	0.000754	ERCC3/GT
GO_BP_m8	GO:00182	peptidyl-s	6/55	252/17913	0.000119	0.001158	0.00077	AKAP9/ER
GO_BP_m8	GO:00064	RNA catab	7/55	368/17913	0.000127	0.001227	0.000816	PSMB2/PS
GO_BP_m8	GO:00702	protein po	2/55	6/17913	0.000138	0.001326	0.000882	PARP1/PA
GO_BP_m8	GO:00063	7-methylg	3/55	33/17913	0.00014	0.00134	0.000891	ERCC3/GT
GO_BP_m8	GO:00094	7-methylg	3/55	34/17913	0.000153	0.00145	0.000964	ERCC3/GT
GO_BP_m8	GO:00362	RNA cappi	3/55	34/17913	0.000153	0.00145	0.000964	ERCC3/GT
GO_BP_m8	GO:00063	transcripti	3/55	35/17913	0.000167	0.001574	0.001046	ERCC3/GT
GO_BP_m8	GO:00086	intrinsic ap	4/55	92/17913	0.000182	0.001694	0.001126	ERCC6/RA
GO_BP_m8	GO:00081	negative re	3/55	36/17913	0.000182	0.001694	0.001126	LIG3/RAD1
GO_BP_m8	GO:00723	plus-end-	2/55	7/17913	0.000192	0.001782	0.001185	KIF3A/KIF5
GO_BP_m8	GO:00311	microtubu	4/55	94/17913	0.000198	0.001819	0.00121	AKAP9/CD
GO_BP_m8	GO:00070	mitotic spi	4/55	95/17913	0.000206	0.001885	0.001253	CEP192/D
GO_BP_m8	GO:00466	regulation	2/55	8/17913	0.000256	0.00232	0.001543	KIF11/NEK
GO_BP_m8	GO:00723	plus-end-	2/55	8/17913	0.000256	0.00232	0.001543	KIF3A/KIF5
GO_BP_m8	GO:00105	regulation	3/55	41/17913	0.000269	0.002422	0.00161	PARP1/RP
GO_BP_m8	GO:00481	Golgi vesic	6/55	304/17913	0.000328	0.002937	0.001953	ACTR1A/D
GO_BP_m8	GO:00007	double-str	4/55	108/17913	0.000336	0.002999	0.001994	LIG3/PARP
GO_BP_m8	GO:00007	recombina	4/55	109/17913	0.000348	0.00309	0.002055	LIG3/PARP
GO_BP_m8	GO:00712	cellular res	6/55	310/17913	0.000364	0.003192	0.002123	DDB2/PAR
GO_BP_m8	GO:01040	cellular res	6/55	310/17913	0.000364	0.003192	0.002123	DDB2/PAR
GO_BP_m8	GO:00330	regulation	6/55	311/17913	0.00037	0.003231	0.002148	CDK5RAP2
GO_BP_m8	GO:00062	regulation	4/55	112/17913	0.000386	0.00335	0.002227	ERCC6/PA
GO_BP_m8	GO:00069	response t	7/55	442/17913	0.000388	0.00335	0.002227	ERCC1/ER
GO_BP_m8	GO:00714	cellular res	4/55	113/17913	0.0004	0.003436	0.002285	DDB2/PAR
GO_BP_m8	GO:00709	UV-damag	2/55	10/17913	0.00041	0.003508	0.002332	DDB2/XPC

GO_BP_m8	GO:000007	DNA replic	2/55	11/17913	0.0005	0.004236	0.002817	RAD17/RA
GO_BP_m8	GO:000710	mitotic cer	2/55	11/17913	0.0005	0.004236	0.002817	KIF11/NEK
GO_BP_m8	GO:009030	mitotic spi	3/55	52/17913	0.000544	0.004586	0.003049	CEP192/KI
GO_BP_m8	GO:005149	regulation	7/55	472/17913	0.000574	0.004803	0.003193	AKAP9/CD
GO_BP_m8	GO:001623	telomere c	3/55	53/17913	0.000576	0.004803	0.003193	ERCC1/NE
GO_BP_m8	GO:001045	centriole-c	2/55	12/17913	0.000599	0.00495	0.003291	KIF3A/NEK
GO_BP_m8	GO:005129	centrosom	2/55	12/17913	0.000599	0.00495	0.003291	KIF11/NEK
GO_BP_m8	GO:000701	microtubu	5/55	224/17913	0.000612	0.00503	0.003344	KIF11/KIF3
GO_BP_m8	GO:001033	response t	3/55	55/17913	0.000642	0.005252	0.003492	ERCC6/PA
GO_BP_m8	GO:000028	nuclear div	6/55	357/17913	0.000765	0.006229	0.004141	CCNA1/CD
GO_BP_m8	GO:001605	Wnt signal	7/55	498/17913	0.000787	0.006334	0.004211	PSMB2/PS
GO_BP_m8	GO:005129	centrosom	3/55	59/17913	0.000788	0.006334	0.004211	CDK5RAP2
GO_BP_m8	GO:014001	mitotic nu	5/55	237/17913	0.000789	0.006334	0.004211	CDK5RAP2
GO_BP_m8	GO:019873	cell-cell sig	7/55	500/17913	0.000806	0.006439	0.004281	PSMB2/PS
GO_BP_m8	GO:004660	negative re	2/55	14/17913	0.000823	0.006542	0.00435	CDK5RAP2
GO_BP_m8	GO:000631	DNA recor	5/55	244/17913	0.000899	0.007116	0.004731	ERCC1/LIG
GO_BP_m8	GO:004299	cytoplasm	2/55	15/17913	0.000947	0.007464	0.004963	NFKBIA/NI
GO_BP_m8	GO:001097	transport a	4/55	143/17913	0.000968	0.00756	0.005027	KIF3A/KIF3
GO_BP_m8	GO:009911	microtubu	4/55	143/17913	0.000968	0.00756	0.005027	KIF3A/KIF3
GO_BP_m8	GO:001022	response t	2/55	16/17913	0.001081	0.008396	0.005583	ERCC5/TP5
GO_BP_m8	GO:004678	microtubu	3/55	66/17913	0.001093	0.008455	0.005621	AKAP9/CD
GO_BP_m8	GO:003520	multicellul	4/55	149/17913	0.001128	0.008686	0.005775	ERCC1/ER
GO_BP_m8	GO:001022	response t	2/55	17/17913	0.001222	0.009368	0.006229	ERCC6/XP
GO_BP_m8	GO:000635	DNA-temp	3/55	69/17913	0.001243	0.009403	0.006252	ERCC3/GT
GO_BP_m8	GO:003111	regulation	3/55	69/17913	0.001243	0.009403	0.006252	AKAP9/CD
GO_BP_m8	GO:007147	cellular res	3/55	69/17913	0.001243	0.009403	0.006252	RAD9A/TP
GO_BP_m8	GO:003070	cytoskelet	4/55	154/17913	0.001274	0.009595	0.00638	KIF3A/KIF3
GO_BP_m8	GO:004828	organelle f	6/55	395/17913	0.001289	0.009661	0.006424	CCNA1/CD
GO_BP_m8	GO:004227	error-pror	2/55	18/17913	0.001372	0.010241	0.006809	RFC1/RPA
GO_BP_m8	GO:200077	regulation	3/55	72/17913	0.001406	0.010448	0.006947	PARP1/RP
GO_BP_m8	GO:007238	organelle f	3/55	73/17913	0.001463	0.010824	0.007197	KIF3A/KIF3
GO_BP_m8	GO:000705	chromoso	5/55	275/17913	0.001529	0.011177	0.007431	CDK5RAP2
GO_BP_m8	GO:004659	regulation	2/55	19/17913	0.001531	0.011177	0.007431	CDK5RAP2
GO_BP_m8	GO:007098	error-free	2/55	19/17913	0.001531	0.011177	0.007431	RFC1/RPA
GO_BP_m8	GO:009719	intrinsic ap	5/55	277/17913	0.001578	0.011474	0.007629	ERCC6/PA
GO_BP_m8	GO:000702	microtubu	2/55	20/17913	0.001697	0.012288	0.00817	AKAP9/CE
GO_BP_m8	GO:006113	regulation	4/55	167/17913	0.001717	0.012374	0.008228	PRKACA/P
GO_BP_m8	GO:001083	telomere r	3/55	79/17913	0.001836	0.013182	0.008765	NEK2/PAR
GO_BP_m8	GO:004682	positive re	2/55	21/17913	0.001872	0.013383	0.008898	PRKACA/T
GO_BP_m8	GO:005122	cytoplasm	2/55	22/17913	0.002056	0.014631	0.009728	NFKBIA/NI
GO_BP_m8	GO:000636	transcripti	4/55	178/17913	0.002166	0.015353	0.010208	ERCC3/GT
GO_BP_m8	GO:000001	regulation	3/55	84/17913	0.002189	0.015451	0.010273	PARP1/RP
GO_BP_m8	GO:190179	regulation	4/55	179/17913	0.002211	0.015538	0.010331	RAD17/RA
GO_BP_m8	GO:004447	mitotic DN	3/55	86/17913	0.002341	0.016387	0.010895	RPA2/TP53
GO_BP_m8	GO:005164	centrosom	2/55	24/17913	0.002446	0.017054	0.011339	AKAP9/KIF
GO_BP_m8	GO:006184	microtubu	2/55	25/17913	0.002654	0.018421	0.012248	AKAP9/KIF
GO_BP_m8	GO:004447	mitotic DN	3/55	90/17913	0.002664	0.018421	0.012248	RPA2/TP53
GO_BP_m8	GO:200010	positive re	3/55	92/17913	0.002836	0.019527	0.012983	ERCC6/PA
GO_BP_m8	GO:001099	response t	2/55	26/17913	0.002869	0.0196	0.013032	XPA/XPC
GO_BP_m8	GO:190357	negative re	2/55	26/17913	0.002869	0.0196	0.013032	PARP1/TP5
GO_BP_m8	GO:005198	regulation	3/55	93/17913	0.002924	0.019891	0.013225	CDK5RAP2
GO_BP_m8	GO:001842	peptidyl-g	1/55	1/17913	0.00307	0.020082	0.013352	PARP1
GO_BP_m8	GO:005141	microtubu	1/55	1/17913	0.00307	0.020082	0.013352	CEP192
GO_BP_m8	GO:009022	centrosom	1/55	1/17913	0.00307	0.020082	0.013352	CEP192
GO_BP_m8	GO:190191	regulation	1/55	1/17913	0.00307	0.020082	0.013352	TCEA1
GO_BP_m8	GO:190191	positive re	1/55	1/17913	0.00307	0.020082	0.013352	TCEA1
GO_BP_m8	GO:190312	negative re	1/55	1/17913	0.00307	0.020082	0.013352	NEK2
GO_BP_m8	GO:190318	regulation	1/55	1/17913	0.00307	0.020082	0.013352	UBE2I

GO_BP_m8	GO:190375	positive re	1/55	1/17913	0.00307	0.020082	0.013352	UBE2I
GO_BP_m8	GO:190515	positive re	1/55	1/17913	0.00307	0.020082	0.013352	KIF5B
GO_BP_m8	GO:190577	positive re	1/55	1/17913	0.00307	0.020082	0.013352	TCEA1
GO_BP_m8	GO:190305	regulation	4/55	198/17913	0.003181	0.020724	0.013779	PRKACA/P
GO_BP_m8	GO:003111	positive re	2/55	28/17913	0.003324	0.021494	0.014291	AKAP9/CD
GO_BP_m8	GO:190054	negative re	2/55	28/17913	0.003324	0.021494	0.014291	PARP1/TP5
GO_BP_m8	GO:003238	positive re	4/55	203/17913	0.003478	0.0224	0.014893	DYNC1H1/
GO_BP_m8	GO:004598	negative re	2/55	29/17913	0.003564	0.022866	0.015203	PARP1/TP5
GO_BP_m8	GO:005149	positive re	4/55	205/17913	0.003602	0.023023	0.015307	AKAP9/CD
GO_BP_m8	GO:000627	regulation	3/55	102/17913	0.003794	0.024163	0.016066	LIG3/RAD1
GO_BP_m8	GO:000647	protein AD	2/55	30/17913	0.003811	0.024176	0.016074	PARP1/PA
GO_BP_m8	GO:000709	centriole re	2/55	31/17913	0.004065	0.025601	0.017022	CDK5RAP2
GO_BP_m8	GO:190197	regulation	2/55	31/17913	0.004065	0.025601	0.017022	CDK5RAP2
GO_BP_m8	GO:003111	positive re	2/55	32/17913	0.004328	0.027153	0.018054	AKAP9/CD
GO_BP_m8	GO:004217	regulation	5/55	354/17913	0.004539	0.028371	0.018864	PRKACA/P
GO_BP_m8	GO:009881	nuclear ch	4/55	220/17913	0.004628	0.02882	0.019162	CDK5RAP2
GO_BP_m8	GO:003450	protein loc	4/55	223/17913	0.004854	0.03012	0.020027	NFKBIA/PA
GO_BP_m8	GO:001025	multicellul	2/55	34/17913	0.004876	0.030145	0.020043	ERCC1/TP5
GO_BP_m8	GO:009853	centriole a	2/55	35/17913	0.005161	0.031781	0.021131	CDK5RAP2
GO_BP_m8	GO:000630	DNA modi	3/55	114/17913	0.005178	0.031781	0.021131	PARP1/PA
GO_BP_m8	GO:005109	regulation	5/55	368/17913	0.005341	0.032661	0.021716	NEK2/NFK
GO_BP_m8	GO:190330	regulation	4/55	230/17913	0.005411	0.032764	0.021784	PRKACA/P
GO_BP_m8	GO:003220	negative re	2/55	36/17913	0.005454	0.032764	0.021784	ERCC1/PA
GO_BP_m8	GO:004591	positive re	2/55	36/17913	0.005454	0.032764	0.021784	PARP1/TP5
GO_BP_m8	GO:004682	regulation	2/55	36/17913	0.005454	0.032764	0.021784	PRKACA/T
GO_BP_m8	GO:005129	establishm	2/55	36/17913	0.005454	0.032764	0.021784	CDK5RAP2
GO_BP_m8	GO:000635	DNA-temp	4/55	231/17913	0.005494	0.032773	0.02179	ERCC3/GT
GO_BP_m8	GO:001810	peptidyl-s	4/55	231/17913	0.005494	0.032773	0.02179	AKAP9/ER
GO_BP_m8	GO:005110	regulation	3/55	120/17913	0.00597	0.034972	0.023253	NEK2/NFK
GO_BP_m8	GO:004749	vesicle trar	2/55	38/17913	0.006062	0.034972	0.023253	KIF3A/KIF5
GO_BP_m8	GO:000717	epidermal	1/55	2/17913	0.006132	0.034972	0.023253	RAB7A
GO_BP_m8	GO:002193	cerebellar	1/55	2/17913	0.006132	0.034972	0.023253	TTBK2
GO_BP_m8	GO:004294	D-serine tı	1/55	2/17913	0.006132	0.034972	0.023253	NFKBIE
GO_BP_m8	GO:007230	regulation	1/55	2/17913	0.006132	0.034972	0.023253	TP53
GO_BP_m8	GO:009061	DNA clamp	1/55	2/17913	0.006132	0.034972	0.023253	RFC1
GO_BP_m8	GO:190351	positive re	1/55	2/17913	0.006132	0.034972	0.023253	PARP1
GO_BP_m8	GO:190452	negative re	1/55	2/17913	0.006132	0.034972	0.023253	TTBK2
GO_BP_m8	GO:190470	positive re	1/55	2/17913	0.006132	0.034972	0.023253	PARP1
GO_BP_m8	GO:190497	negative re	1/55	2/17913	0.006132	0.034972	0.023253	RAB7A
GO_BP_m8	GO:190530	regulation	1/55	2/17913	0.006132	0.034972	0.023253	RAB7A
GO_BP_m8	GO:190530	negative re	1/55	2/17913	0.006132	0.034972	0.023253	RAB7A
GO_BP_m8	GO:200125	negative re	3/55	125/17913	0.006682	0.037833	0.025154	CDK5RAP2
GO_BP_m8	GO:001082	regulation	2/55	40/17913	0.0067	0.037833	0.025154	CDK5RAP2
GO_BP_m8	GO:003573	intraciliary	2/55	40/17913	0.0067	0.037833	0.025154	KIF3A/KIF3
GO_BP_m8	GO:005165	spindle loc	2/55	41/17913	0.00703	0.039563	0.026305	CDK5RAP2
GO_BP_m8	GO:005149	negative re	3/55	128/17913	0.007133	0.04001	0.026602	CDK5RAP2
GO_BP_m8	GO:009025	regulation	4/55	251/17913	0.007337	0.041021	0.027274	AKAP9/PA
GO_BP_m8	GO:000808	anterograd	2/55	43/17913	0.007711	0.042688	0.028383	KIF4A/KIF5
GO_BP_m8	GO:001061	positive re	2/55	43/17913	0.007711	0.042688	0.028383	PARP1/PA
GO_BP_m8	GO:009951	vesicle cyt	2/55	43/17913	0.007711	0.042688	0.028383	KIF3A/KIF5
GO_BP_m8	GO:000007	mitotic sist	3/55	132/17913	0.007761	0.042826	0.028474	CDK5RAP2
GO_BP_m8	GO:001474	positive re	2/55	44/17913	0.008062	0.0442	0.029388	PARP1/PA
GO_BP_m8	GO:004320	positive re	2/55	44/17913	0.008062	0.0442	0.029388	AKAP9/KIF
GO_BP_m8	GO:007233	signal tran	4/55	264/17913	0.008731	0.046577	0.030968	RAD17/RA
GO_BP_m8	GO:006191	selective a	2/55	46/17913	0.008787	0.046577	0.030968	RAB7A/TP
GO_BP_m8	GO:000220	somatic re	2/55	47/17913	0.009159	0.046577	0.030968	ERCC1/TP5
GO_BP_m8	GO:000220	somatic di	2/55	47/17913	0.009159	0.046577	0.030968	ERCC1/TP5
GO_BP_m8	GO:003111	regulation	2/55	47/17913	0.009159	0.046577	0.030968	AKAP9/CD

GO_BP_m8	GO:00451	isotype sw	2/55	47/17913	0.009159	0.046577	0.030968	ERCC1/TP5
GO_BP_m8	GO:00726	protein loc	2/55	47/17913	0.009159	0.046577	0.030968	CEP192/TT
GO_BP_m8	GO:00007	pyrimidine	1/55	3/17913	0.009183	0.046577	0.030968	ERCC1
GO_BP_m8	GO:00062	base-excis	1/55	3/17913	0.009183	0.046577	0.030968	LIG3
GO_BP_m8	GO:00219	hindbrain	1/55	3/17913	0.009183	0.046577	0.030968	TTBK2
GO_BP_m8	GO:00309	regulation	1/55	3/17913	0.009183	0.046577	0.030968	NEK2
GO_BP_m8	GO:00351	post-embr	1/55	3/17913	0.009183	0.046577	0.030968	ERCC1
GO_BP_m8	GO:00618	telomeric l	1/55	3/17913	0.009183	0.046577	0.030968	ERCC1
GO_BP_m8	GO:00706	proteasom	1/55	3/17913	0.009183	0.046577	0.030968	PSMD9
GO_BP_m8	GO:00723	regulation	1/55	3/17913	0.009183	0.046577	0.030968	TP53
GO_BP_m8	GO:00727	cellular res	1/55	3/17913	0.009183	0.046577	0.030968	TP53
GO_BP_m8	GO:00902	negative re	1/55	3/17913	0.009183	0.046577	0.030968	LIG3
GO_BP_m8	GO:00986	regulation	1/55	3/17913	0.009183	0.046577	0.030968	TP53
GO_BP_m8	GO:19026	negative re	1/55	3/17913	0.009183	0.046577	0.030968	TP53
GO_BP_m8	GO:19035	regulation	1/55	3/17913	0.009183	0.046577	0.030968	PARP1
GO_BP_m8	GO:19035	negative re	1/55	3/17913	0.009183	0.046577	0.030968	RAB7A
GO_BP_m8	GO:19040	negative re	1/55	3/17913	0.009183	0.046577	0.030968	TP53
GO_BP_m8	GO:19057	regulation	1/55	3/17913	0.009183	0.046577	0.030968	ERCC1
GO_BP_m8	GO:19057	negative re	1/55	3/17913	0.009183	0.046577	0.030968	ERCC1
GO_BP_m8	GO:19057	regulation	1/55	3/17913	0.009183	0.046577	0.030968	TCEA1
GO_BP_m8	GO:00420	intraciliary	2/55	48/17913	0.009539	0.047948	0.03188	KIF3A/KIF3
GO_BP_m8	GO:00433	negative re	2/55	48/17913	0.009539	0.047948	0.03188	NEK2/NFK
GO_BP_m8	GO:00519	regulation	2/55	48/17913	0.009539	0.047948	0.03188	NEK2/TP53
GO_BP_m8	GO:00164	somatic re	2/55	49/17913	0.009925	0.049743	0.033074	ERCC1/TP5
GO_BP_m8	GO:00362	interstranc	2/55	50/17913	0.010319	0.05141	0.034182	ERCC1/RPA
GO_BP_m8	GO:00443	protein loc	2/55	50/17913	0.010319	0.05141	0.034182	CEP192/TT
GO_BP_m8	GO:00322	positive re	2/55	51/17913	0.010719	0.053092	0.0353	ERCC1/NE
GO_BP_m8	GO:00989	axonal trar	2/55	51/17913	0.010719	0.053092	0.0353	KIF4A/KIF5
GO_BP_m8	GO:00070	regulation	3/55	150/17913	0.010987	0.054106	0.035975	CDK5RAP2
GO_BP_m8	GO:00903	positive re	3/55	150/17913	0.010987	0.054106	0.035975	KIF5B/PRK
GO_BP_m8	GO:00900	positive re	4/55	283/17913	0.011065	0.05433	0.036123	DYNC1H1/
GO_BP_m8	GO:19017	negative re	2/55	52/17913	0.011126	0.054471	0.036217	PSMF1/US
GO_BP_m8	GO:00512	maintenan	4/55	286/17913	0.011467	0.055979	0.03722	AKAP9/NF
GO_BP_m8	GO:00516	establishm	5/55	448/17913	0.011936	0.05628	0.03742	CDK5RAP2
GO_BP_m8	GO:00108	negative re	2/55	54/17913	0.01196	0.05628	0.03742	LIG3/TP53
GO_BP_m8	GO:00226	protein to	1/55	4/17913	0.012226	0.05628	0.03742	RAB7A
GO_BP_m8	GO:00356	histone H2	1/55	4/17913	0.012226	0.05628	0.03742	USP7
GO_BP_m8	GO:00704	nucleotide	1/55	4/17913	0.012226	0.05628	0.03742	NFKBIA
GO_BP_m8	GO:00706	intralumer	1/55	4/17913	0.012226	0.05628	0.03742	RAB7A
GO_BP_m8	GO:00727	response t	1/55	4/17913	0.012226	0.05628	0.03742	TP53
GO_BP_m8	GO:00902	regulation	1/55	4/17913	0.012226	0.05628	0.03742	LIG3
GO_BP_m8	GO:00904	oxidative s	1/55	4/17913	0.012226	0.05628	0.03742	TP53
GO_BP_m8	GO:00972	oligodend	1/55	4/17913	0.012226	0.05628	0.03742	TP53
GO_BP_m8	GO:00977	de novo ce	1/55	4/17913	0.012226	0.05628	0.03742	PLK4
GO_BP_m8	GO:00985	de novo ce	1/55	4/17913	0.012226	0.05628	0.03742	PLK4
GO_BP_m8	GO:19010	negative re	1/55	4/17913	0.012226	0.05628	0.03742	TP53
GO_BP_m8	GO:19015	positive re	1/55	4/17913	0.012226	0.05628	0.03742	USP7
GO_BP_m8	GO:19018	negative re	1/55	4/17913	0.012226	0.05628	0.03742	LIG3
GO_BP_m8	GO:19044	positive re	1/55	4/17913	0.012226	0.05628	0.03742	ERCC1
GO_BP_m8	GO:19045	regulation	1/55	4/17913	0.012226	0.05628	0.03742	ERCC1
GO_BP_m8	GO:19045	negative re	1/55	4/17913	0.012226	0.05628	0.03742	ERCC1
GO_BP_m8	GO:19049	regulation	1/55	4/17913	0.012226	0.05628	0.03742	RAB7A
GO_BP_m8	GO:19051	regulation	1/55	4/17913	0.012226	0.05628	0.03742	KIF5B
GO_BP_m8	GO:19909	ATP gener	1/55	4/17913	0.012226	0.05628	0.03742	PARP1
GO_BP_m8	GO:00023	immunogl	2/55	55/17913	0.012387	0.056712	0.037707	ERCC1/TP5
GO_BP_m8	GO:00164	somatic di	2/55	55/17913	0.012387	0.056712	0.037707	ERCC1/TP5
GO_BP_m8	GO:00008	sister chro	3/55	157/17913	0.012422	0.056719	0.037712	CDK5RAP2
GO_BP_m8	GO:00022	lymphocyt	3/55	158/17913	0.012635	0.057538	0.038256	ERCC1/TP5

GO_BP_m8	GO:00511(	negative re	3/55	162/17913	0.01351	0.061355	0.040794	NEK2/NFK
GO_BP_m8	GO:200124	regulation	3/55	163/17913	0.013733	0.062204	0.041359	PARP1/RA
GO_BP_m8	GO:00025(	somatic di	2/55	60/17913	0.014621	0.064787	0.043076	ERCC1/TP5
GO_BP_m8	GO:00164	somatic ce	2/55	60/17913	0.014621	0.064787	0.043076	ERCC1/TP5
GO_BP_m8	GO:00468	positive re	2/55	60/17913	0.014621	0.064787	0.043076	PRKACA/T
GO_BP_m8	GO:00080	axo-dendr	2/55	61/17913	0.015087	0.064787	0.043076	KIF4A/KIF5
GO_BP_m8	GO:19043	regulation	2/55	61/17913	0.015087	0.064787	0.043076	NEK2/PAR
GO_BP_m8	GO:20012	positive re	3/55	169/17913	0.015121	0.064787	0.043076	ERCC1/NE
GO_BP_m8	GO:00023(	T cell proli	1/55	5/17913	0.01526	0.064787	0.043076	TP53
GO_BP_m8	GO:00097	response t	1/55	5/17913	0.01526	0.064787	0.043076	ERCC1
GO_BP_m8	GO:00102	maintenan	1/55	5/17913	0.01526	0.064787	0.043076	USP7
GO_BP_m8	GO:00196	glucose ca	1/55	5/17913	0.01526	0.064787	0.043076	TP53
GO_BP_m8	GO:00196(	glycolytic f	1/55	5/17913	0.01526	0.064787	0.043076	TP53
GO_BP_m8	GO:00196(	glucose ca	1/55	5/17913	0.01526	0.064787	0.043076	TP53
GO_BP_m8	GO:00196(	nitrogenou	1/55	5/17913	0.01526	0.064787	0.043076	TP53
GO_BP_m8	GO:00331	positive re	1/55	5/17913	0.01526	0.064787	0.043076	ERCC6
GO_BP_m8	GO:00342	response t	1/55	5/17913	0.01526	0.064787	0.043076	ERCC1
GO_BP_m8	GO:00356	stress grar	1/55	5/17913	0.01526	0.064787	0.043076	KIF5B
GO_BP_m8	GO:00510	negative re	1/55	5/17913	0.01526	0.064787	0.043076	TP53
GO_BP_m8	GO:00702	positive re	1/55	5/17913	0.01526	0.064787	0.043076	TP53
GO_BP_m8	GO:19028	regulation	1/55	5/17913	0.01526	0.064787	0.043076	TTBK2
GO_BP_m8	GO:19028	negative re	1/55	5/17913	0.01526	0.064787	0.043076	TTBK2
GO_BP_m8	GO:19040	regulation	1/55	5/17913	0.01526	0.064787	0.043076	TP53
GO_BP_m8	GO:19040	response t	1/55	5/17913	0.01526	0.064787	0.043076	PARP1
GO_BP_m8	GO:19047(	regulation	1/55	5/17913	0.01526	0.064787	0.043076	PARP1
GO_BP_m8	GO:19058	positive re	1/55	5/17913	0.01526	0.064787	0.043076	DYNC1H1
GO_BP_m8	GO:19900	retrograde	1/55	5/17913	0.01526	0.064787	0.043076	KIF5B
GO_BP_m8	GO:00457	positive re	2/55	62/17913	0.01556	0.06573	0.043703	ERCC6/PA
GO_BP_m8	GO:20012	positive re	2/55	62/17913	0.01556	0.06573	0.043703	RAD9A/TP
GO_BP_m8	GO:00434	negative re	3/55	171/17913	0.015601	0.06574	0.04371	NFKBIA/NI
GO_BP_m8	GO:00517	regulation	3/55	172/17913	0.015844	0.066599	0.04428	CDK5RAP2
GO_BP_m8	GO:00315	mitotic G1	2/55	63/17913	0.016038	0.067084	0.044603	RPA2/TP53
GO_BP_m8	GO:00448	mitotic G1	2/55	63/17913	0.016038	0.067084	0.044603	RPA2/TP53
GO_BP_m8	GO:19038	positive re	4/55	318/17913	0.016348	0.068211	0.045352	KIF5B/PAR
GO_BP_m8	GO:00447	G1 DNA d	2/55	64/17913	0.016523	0.068773	0.045726	RPA2/TP53
GO_BP_m8	GO:00069(	vesicle fusi	2/55	66/17913	0.017512	0.072367	0.048116	KIF5B/RAB
GO_BP_m8	GO:00022(	somatic di	2/55	67/17913	0.018016	0.072367	0.048116	ERCC1/TP5
GO_BP_m8	GO:00070(	telomere r	2/55	67/17913	0.018016	0.072367	0.048116	NEK2/RFC
GO_BP_m8	GO:19030	negative re	2/55	67/17913	0.018016	0.072367	0.048116	PSMF1/US
GO_BP_m8	GO:00013(	replicative	1/55	6/17913	0.018284	0.072367	0.048116	ERCC1
GO_BP_m8	GO:00023	B cell linea	1/55	6/17913	0.018284	0.072367	0.048116	TP53
GO_BP_m8	GO:00061	fermentati	1/55	6/17913	0.018284	0.072367	0.048116	TP53
GO_BP_m8	GO:00109	regulation	1/55	6/17913	0.018284	0.072367	0.048116	PARP1
GO_BP_m8	GO:00315	intra-S DN	1/55	6/17913	0.018284	0.072367	0.048116	XPC
GO_BP_m8	GO:00323	serine tran	1/55	6/17913	0.018284	0.072367	0.048116	NFKBIE
GO_BP_m8	GO:00364	myofibrob	1/55	6/17913	0.018284	0.072367	0.048116	PARP1
GO_BP_m8	GO:00429	D-amino a	1/55	6/17913	0.018284	0.072367	0.048116	NFKBIE
GO_BP_m8	GO:00434(	regulation	1/55	6/17913	0.018284	0.072367	0.048116	TP53
GO_BP_m8	GO:00458	positive re	1/55	6/17913	0.018284	0.072367	0.048116	TP53
GO_BP_m8	GO:00466(	positive re	1/55	6/17913	0.018284	0.072367	0.048116	PLK4
GO_BP_m8	GO:00516(	maintenan	1/55	6/17913	0.018284	0.072367	0.048116	AKAP9
GO_BP_m8	GO:00607(	regulation	1/55	6/17913	0.018284	0.072367	0.048116	TCEA1
GO_BP_m8	GO:00614	positive re	1/55	6/17913	0.018284	0.072367	0.048116	TP53
GO_BP_m8	GO:00617	lipophagy	1/55	6/17913	0.018284	0.072367	0.048116	RAB7A
GO_BP_m8	GO:19016	negative re	1/55	6/17913	0.018284	0.072367	0.048116	PRKACA
GO_BP_m8	GO:19026	regulation	1/55	6/17913	0.018284	0.072367	0.048116	TP53
GO_BP_m8	GO:00320	positive re	2/55	68/17913	0.018526	0.073154	0.048639	KIF5B/PSM
GO_BP_m8	GO:00002	mitotic cyt	2/55	69/17913	0.019042	0.075016	0.049877	KIF20A/KIF

GO_BP_m	GO:19021	regulation	3/55	185/17913	0.019198	0.075455	0.050169	CDK5RAP
GO_BP_m	GO:00023	B cell activ	2/55	71/17913	0.020092	0.078604	0.052262	ERCC1/TP
GO_BP_m	GO:00901	organelle	2/55	71/17913	0.020092	0.078604	0.052262	KIF5B/RAB
GO_BP_m	GO:19021	positive re	2/55	72/17913	0.020626	0.080002	0.053192	DYNC1H1/
GO_BP_m	GO:00062	DNA topo	1/55	7/17913	0.021299	0.080002	0.053192	ERCC3
GO_BP_m	GO:00072	cytoplasm	1/55	7/17913	0.021299	0.080002	0.053192	NFKBIA
GO_BP_m	GO:00331	regulation	1/55	7/17913	0.021299	0.080002	0.053192	ERCC6
GO_BP_m	GO:00512	mitotic spi	1/55	7/17913	0.021299	0.080002	0.053192	KIF4A
GO_BP_m	GO:00714	cellular res	1/55	7/17913	0.021299	0.080002	0.053192	TP53
GO_BP_m	GO:00902	regulation	1/55	7/17913	0.021299	0.080002	0.053192	DYNC1H1
GO_BP_m	GO:00903	phagosom	1/55	7/17913	0.021299	0.080002	0.053192	RAB7A
GO_BP_m	GO:19015	negative r	1/55	7/17913	0.021299	0.080002	0.053192	TP53
GO_BP_m	GO:19015	regulation	1/55	7/17913	0.021299	0.080002	0.053192	USP7
GO_BP_m	GO:19018	regulation	1/55	7/17913	0.021299	0.080002	0.053192	LIG3
GO_BP_m	GO:19022	positive re	1/55	7/17913	0.021299	0.080002	0.053192	RAD9A
GO_BP_m	GO:19025	multi-orga	1/55	7/17913	0.021299	0.080002	0.053192	RAB7A
GO_BP_m	GO:19025	multi-orga	1/55	7/17913	0.021299	0.080002	0.053192	RAB7A
GO_BP_m	GO:19032	multi-ciliat	1/55	7/17913	0.021299	0.080002	0.053192	PLK4
GO_BP_m	GO:19044	regulation	1/55	7/17913	0.021299	0.080002	0.053192	ERCC1
GO_BP_m	GO:19900	anterogra	1/55	7/17913	0.021299	0.080002	0.053192	KIF5B
GO_BP_m	GO:19901	intrinsic a	1/55	7/17913	0.021299	0.080002	0.053192	TP53
GO_BP_m	GO:00062	RNA-depe	2/55	74/17913	0.021711	0.081368	0.054101	NEK2/RFC
GO_BP_m	GO:00106	regulation	2/55	76/17913	0.02282	0.085335	0.056738	PARP1/PA
GO_BP_m	GO:00096	response t	3/55	200/17913	0.023521	0.086681	0.057633	NFKBIA/X
GO_BP_m	GO:00015	microtubu	2/55	78/17913	0.023952	0.086681	0.057633	CDK5RAP
GO_BP_m	GO:19030	organelle	2/55	78/17913	0.023952	0.086681	0.057633	KIF5B/TP5
GO_BP_m	GO:00007	DNA stran	1/55	8/17913	0.024306	0.086681	0.057633	TP53
GO_BP_m	GO:00074	negative r	1/55	8/17913	0.024306	0.086681	0.057633	TP53
GO_BP_m	GO:00108	positive re	1/55	8/17913	0.024306	0.086681	0.057633	PLK4
GO_BP_m	GO:00192	lactate bio	1/55	8/17913	0.024306	0.086681	0.057633	TP53
GO_BP_m	GO:00318	protection	1/55	8/17913	0.024306	0.086681	0.057633	ERCC1
GO_BP_m	GO:00320	positive re	1/55	8/17913	0.024306	0.086681	0.057633	TCEA1
GO_BP_m	GO:00322	dense core	1/55	8/17913	0.024306	0.086681	0.057633	KIF5B
GO_BP_m	GO:00344	microtubu	1/55	8/17913	0.024306	0.086681	0.057633	KIF3A
GO_BP_m	GO:00364	neuron int	1/55	8/17913	0.024306	0.086681	0.057633	PARP1
GO_BP_m	GO:00466	negative r	1/55	8/17913	0.024306	0.086681	0.057633	CDK5RAP
GO_BP_m	GO:00485	bone marr	1/55	8/17913	0.024306	0.086681	0.057633	TP53
GO_BP_m	GO:00904	stress-ind	1/55	8/17913	0.024306	0.086681	0.057633	TP53
GO_BP_m	GO:00989	regulation	1/55	8/17913	0.024306	0.086681	0.057633	AKAP9
GO_BP_m	GO:00995	dense core	1/55	8/17913	0.024306	0.086681	0.057633	KIF5B
GO_BP_m	GO:19016	regulation	1/55	8/17913	0.024306	0.086681	0.057633	PRKACA
GO_BP_m	GO:19019	dense core	1/55	8/17913	0.024306	0.086681	0.057633	KIF5B
GO_BP_m	GO:19033	regulation	1/55	8/17913	0.024306	0.086681	0.057633	PARP1
GO_BP_m	GO:19043	negative r	1/55	8/17913	0.024306	0.086681	0.057633	ERCC1
GO_BP_m	GO:19051	positive re	1/55	8/17913	0.024306	0.086681	0.057633	PARP1
GO_BP_m	GO:00147	regulation	2/55	79/17913	0.024526	0.087101	0.057912	PARP1/PA
GO_BP_m	GO:20010	negative r	2/55	79/17913	0.024526	0.087101	0.057912	ERCC1/TP
GO_BP_m	GO:19033	negative r	2/55	80/17913	0.025106	0.088974	0.059158	PSMF1/US
GO_BP_m	GO:00063	double-str	2/55	81/17913	0.025692	0.09067	0.060285	ERCC1/TP
GO_BP_m	GO:00551	regulation	2/55	81/17913	0.025692	0.09067	0.060285	AKAP9/PR
GO_BP_m	GO:00616	cytoskelet	2/55	82/17913	0.026283	0.092372	0.061417	KIF20A/KI
GO_BP_m	GO:00711	positive re	2/55	82/17913	0.026283	0.092372	0.061417	PRKACA/T
GO_BP_m	GO:00000	mitotic spi	1/55	9/17913	0.027303	0.094197	0.06263	KIF4A
GO_BP_m	GO:00313	positive re	1/55	9/17913	0.027303	0.094197	0.06263	KIF5B
GO_BP_m	GO:00355	monoubiq	1/55	9/17913	0.027303	0.094197	0.06263	USP7
GO_BP_m	GO:00458	regulation	1/55	9/17913	0.027303	0.094197	0.06263	TP53
GO_BP_m	GO:00511	DNA ligati	1/55	9/17913	0.027303	0.094197	0.06263	LIG3
GO_BP_m	GO:00704	cellular res	1/55	9/17913	0.027303	0.094197	0.06263	NFKBIA

GO_BP_m	GO:00713	cellular res	1/55	9/17913	0.027303	0.094197	0.06263	PRKACA
GO_BP_m	GO:00996	anterograd	1/55	9/17913	0.027303	0.094197	0.06263	KIF5B
GO_BP_m	GO:19031	negative r	1/55	9/17913	0.027303	0.094197	0.06263	TP53
GO_BP_m	GO:00457	positive re	4/55	373/1791	0.027444	0.094494	0.062827	DYNC1H1/
GO_BP_m	GO:00451	maintenan	2/55	85/17913	0.028089	0.096518	0.064174	NFKBIA/N
GO_BP_m	GO:00421	B cell activ	3/55	215/1791	0.028331	0.097151	0.064594	ERCC1/TP
GO_BP_m	GO:00331	positive re	2/55	86/17913	0.028703	0.098226	0.065309	AKAP9/ER
GO_BP_m	GO:00092	response t	3/55	219/1791	0.029696	0.100613	0.066896	NFKBIA/P
GO_BP_m	GO:00331	regulation	3/55	220/1791	0.030042	0.100613	0.066896	KIF5B/PRK
GO_BP_m	GO:00000	single stra	1/55	10/17913	0.030291	0.100613	0.066896	PARP1
GO_BP_m	GO:00062	mitochonc	1/55	10/17913	0.030291	0.100613	0.066896	LIG3
GO_BP_m	GO:00070	cytoplasm	1/55	10/17913	0.030291	0.100613	0.066896	KIF5B
GO_BP_m	GO:00466	positive re	1/55	10/17913	0.030291	0.100613	0.066896	PLK4
GO_BP_m	GO:00512	spindle elc	1/55	10/17913	0.030291	0.100613	0.066896	KIF4A
GO_BP_m	GO:00512	spindle mi	1/55	10/17913	0.030291	0.100613	0.066896	KIF4A
GO_BP_m	GO:00516	maintenan	1/55	10/17913	0.030291	0.100613	0.066896	AKAP9
GO_BP_m	GO:00519	positive re	1/55	10/17913	0.030291	0.100613	0.066896	PARP1
GO_BP_m	GO:00714	cellular res	1/55	10/17913	0.030291	0.100613	0.066896	TP53BP1
GO_BP_m	GO:00723	microtubu	1/55	10/17913	0.030291	0.100613	0.066896	KIF3A
GO_BP_m	GO:19037	negative r	1/55	10/17913	0.030291	0.100613	0.066896	TP53
GO_BP_m	GO:19038	positive re	1/55	10/17913	0.030291	0.100613	0.066896	TP53
GO_BP_m	GO:19045	regulation	1/55	10/17913	0.030291	0.100613	0.066896	TTBK2
GO_BP_m	GO:00007	non-recor	2/55	89/17913	0.030574	0.100959	0.067126	ERCC1/TP
GO_BP_m	GO:00320	negative r	2/55	89/17913	0.030574	0.100959	0.067126	NFKBIA/U
GO_BP_m	GO:00447	DNA meth	2/55	89/17913	0.030574	0.100959	0.067126	PARP1/US
GO_BP_m	GO:20011	regulation	2/55	90/17913	0.031208	0.102853	0.068385	PARP1/TP
GO_BP_m	GO:00097	response t	3/55	224/1791	0.03145	0.103449	0.068782	ERCC1/KIF
GO_BP_m	GO:00902	positive re	2/55	91/17913	0.031848	0.104554	0.069516	KIF5B/PS
GO_BP_m	GO:19012	positive re	2/55	93/17913	0.033142	0.106539	0.070836	PARP1/TP
GO_BP_m	GO:00018	phagolyso	1/55	11/17913	0.03327	0.106539	0.070836	RAB7A
GO_BP_m	GO:00072	activation	1/55	11/17913	0.03327	0.106539	0.070836	ERCC6
GO_BP_m	GO:00331	positive re	1/55	11/17913	0.03327	0.106539	0.070836	PARP1
GO_BP_m	GO:00332	positive re	1/55	11/17913	0.03327	0.106539	0.070836	UBE2I
GO_BP_m	GO:00425	serine pho	1/55	11/17913	0.03327	0.106539	0.070836	ERCC6
GO_BP_m	GO:00432	telomere r	1/55	11/17913	0.03327	0.106539	0.070836	ERCC1
GO_BP_m	GO:00432	proteasom	1/55	11/17913	0.03327	0.106539	0.070836	PSMD9
GO_BP_m	GO:00519	negative r	1/55	11/17913	0.03327	0.106539	0.070836	TP53
GO_BP_m	GO:00702	protein au	1/55	11/17913	0.03327	0.106539	0.070836	PARP1
GO_BP_m	GO:00702	regulation	1/55	11/17913	0.03327	0.106539	0.070836	TP53
GO_BP_m	GO:19053	protein loc	1/55	11/17913	0.03327	0.106539	0.070836	KIF5B
GO_BP_m	GO:19904	positive re	1/55	11/17913	0.03327	0.106539	0.070836	TP53
GO_BP_m	GO:00482	organelle	2/55	94/17913	0.033797	0.108023	0.071823	KIF5B/RAB
GO_BP_m	GO:00323	regulation	4/55	400/1791	0.034215	0.109154	0.072575	DYNC1H1/
GO_BP_m	GO:00512	protein po	3/55	232/1791	0.034368	0.109436	0.072762	AKAP9/CD
GO_BP_m	GO:00512	positive re	4/55	403/1791	0.035023	0.11121	0.073942	KIF5B/PRK
GO_BP_m	GO:00324	regulation	3/55	234/1791	0.035119	0.11121	0.073942	AKAP9/KIF
GO_BP_m	GO:00069	regulation	2/55	96/17913	0.035122	0.11121	0.073942	AKAP9/PR
GO_BP_m	GO:00007	telomere r	1/55	12/17913	0.03624	0.111308	0.074007	ERCC1
GO_BP_m	GO:00069	ER overloa	1/55	12/17913	0.03624	0.111308	0.074007	TP53
GO_BP_m	GO:00108	negative r	1/55	12/17913	0.03624	0.111308	0.074007	CDK5RAP
GO_BP_m	GO:00216	cerebellar	1/55	12/17913	0.03624	0.111308	0.074007	TTBK2
GO_BP_m	GO:00322	positive re	1/55	12/17913	0.03624	0.111308	0.074007	KIF5B
GO_BP_m	GO:00322	secretory	1/55	12/17913	0.03624	0.111308	0.074007	KIF5B
GO_BP_m	GO:00343	high-dens	1/55	12/17913	0.03624	0.111308	0.074007	PRKACA
GO_BP_m	GO:00510	regulation	1/55	12/17913	0.03624	0.111308	0.074007	TP53
GO_BP_m	GO:00519	regulation	1/55	12/17913	0.03624	0.111308	0.074007	NEK2
GO_BP_m	GO:00603	positive re	1/55	12/17913	0.03624	0.111308	0.074007	PARP1
GO_BP_m	GO:00607	trophobla	1/55	12/17913	0.03624	0.111308	0.074007	PLK4

GO_BP_m8	GO:00711	response t	1/55	12/17913	0.03624	0.111308	0.074007	PRKACA
GO_BP_m8	GO:00989	regulation	1/55	12/17913	0.03624	0.111308	0.074007	AKAP9
GO_BP_m8	GO:00996	axo-dendr	1/55	12/17913	0.03624	0.111308	0.074007	KIF5B
GO_BP_m8	GO:19004	regulation	1/55	12/17913	0.03624	0.111308	0.074007	TP53
GO_BP_m8	GO:19041	positive re	1/55	12/17913	0.03624	0.111308	0.074007	PARP1
GO_BP_m8	GO:00020	regulation	2/55	98/17913	0.036467	0.111308	0.074007	AKAP9/PR
GO_BP_m8	GO:00023	immunogl	2/55	98/17913	0.036467	0.111308	0.074007	ERCC1/TP
GO_BP_m8	GO:00215	cerebellum	2/55	98/17913	0.036467	0.111308	0.074007	TP53/TTBK
GO_BP_m8	GO:00432	regulation	2/55	98/17913	0.036467	0.111308	0.074007	AKAP9/KIF
GO_BP_m8	GO:00485	embryonic	4/55	413/17913	0.037795	0.114853	0.076364	ERCC1/PLI
GO_BP_m8	GO:00324	positive re	2/55	100/17913	0.037832	0.114853	0.076364	AKAP9/KIF
GO_BP_m8	GO:00468	regulation	2/55	100/17913	0.037832	0.114853	0.076364	PRKACA/T
GO_BP_m8	GO:00228	regulation	3/55	242/17913	0.038208	0.115787	0.076985	AKAP9/KIF
GO_BP_m8	GO:00313	negative re	3/55	244/17913	0.039002	0.116707	0.077597	PSMF1/TP
GO_BP_m8	GO:00060	lactate me	1/55	13/17913	0.039201	0.116707	0.077597	TP53
GO_BP_m8	GO:00071	SMAD pro	1/55	13/17913	0.039201	0.116707	0.077597	PARP1
GO_BP_m8	GO:00107	negative re	1/55	13/17913	0.039201	0.116707	0.077597	NFKBIA
GO_BP_m8	GO:00215	cell migrat	1/55	13/17913	0.039201	0.116707	0.077597	TTBK2
GO_BP_m8	GO:00329	ribonuclec	1/55	13/17913	0.039201	0.116707	0.077597	KIF5B
GO_BP_m8	GO:00704	nucleotide	1/55	13/17913	0.039201	0.116707	0.077597	NFKBIA
GO_BP_m8	GO:00718	mitotic cel	1/55	13/17913	0.039201	0.116707	0.077597	TP53
GO_BP_m8	GO:00718	cellular res	1/55	13/17913	0.039201	0.116707	0.077597	PRKACA
GO_BP_m8	GO:19024	protein loc	1/55	13/17913	0.039201	0.116707	0.077597	KIF3A
GO_BP_m8	GO:00066	protein im	2/55	103/17913	0.039916	0.118627	0.078873	NFKBIA/TF
GO_BP_m8	GO:00435	regulation	2/55	106/17913	0.042042	0.122273	0.081297	PARP1/PA
GO_BP_m8	GO:20002	regulation	2/55	106/17913	0.042042	0.122273	0.081297	NEK2/TP5
GO_BP_m8	GO:00353	protein loc	1/55	14/17913	0.042153	0.122273	0.081297	TTBK2
GO_BP_m8	GO:00485	post-embr	1/55	14/17913	0.042153	0.122273	0.081297	ERCC1
GO_BP_m8	GO:00511	RNA polyr	1/55	14/17913	0.042153	0.122273	0.081297	TP53
GO_BP_m8	GO:00602	positive re	1/55	14/17913	0.042153	0.122273	0.081297	TP53
GO_BP_m8	GO:00608	smoothen	1/55	14/17913	0.042153	0.122273	0.081297	PRKACA
GO_BP_m8	GO:00610	positive re	1/55	14/17913	0.042153	0.122273	0.081297	PARP2
GO_BP_m8	GO:00702	positive re	1/55	14/17913	0.042153	0.122273	0.081297	TP53
GO_BP_m8	GO:00902	regulation	1/55	14/17913	0.042153	0.122273	0.081297	CDK5RAP2
GO_BP_m8	GO:00902	regulation	1/55	14/17913	0.042153	0.122273	0.081297	CDK5RAP2
GO_BP_m8	GO:00903	replicative	1/55	14/17913	0.042153	0.122273	0.081297	TP53
GO_BP_m8	GO:19035	regulation	1/55	14/17913	0.042153	0.122273	0.081297	CDK5RAP2
GO_BP_m8	GO:20006	positive re	1/55	14/17913	0.042153	0.122273	0.081297	KIF5B
GO_BP_m8	GO:00510	regulation	4/55	429/17913	0.042488	0.123035	0.081804	NFKBIA/NI
GO_BP_m8	GO:00220	metencepl	2/55	107/17913	0.04276	0.123401	0.082047	TP53/TTBK
GO_BP_m8	GO:00620	negative re	2/55	107/17913	0.04276	0.123401	0.082047	PARP1/TP
GO_BP_m8	GO:00033	cardiac mu	2/55	108/17913	0.043483	0.124848	0.08301	PARP1/PA
GO_BP_m8	GO:00324	positive re	2/55	108/17913	0.043483	0.124848	0.08301	AKAP9/KIF
GO_BP_m8	GO:00711	regulation	2/55	108/17913	0.043483	0.124848	0.08301	PRKACA/T
GO_BP_m8	GO:19003	regulation	2/55	109/17913	0.044211	0.126061	0.083816	PARP1/TP
GO_BP_m8	GO:00324	regulation	3/55	257/17913	0.044363	0.126061	0.083816	AKAP9/KIF
GO_BP_m8	GO:00308	regulation	2/55	110/17913	0.044942	0.126061	0.083816	PARP1/TP
GO_BP_m8	GO:00324	regulation	2/55	110/17913	0.044942	0.126061	0.083816	RAD23B/U
GO_BP_m8	GO:19035	regulation	2/55	110/17913	0.044942	0.126061	0.083816	PARP1/TP
GO_BP_m8	GO:00013	formation	1/55	15/17913	0.045096	0.126061	0.083816	ERCC1
GO_BP_m8	GO:00308	negative re	1/55	15/17913	0.045096	0.126061	0.083816	TP53
GO_BP_m8	GO:00343	glial cell ap	1/55	15/17913	0.045096	0.126061	0.083816	TP53
GO_BP_m8	GO:00511	negative re	1/55	15/17913	0.045096	0.126061	0.083816	TP53
GO_BP_m8	GO:00906	t-circle for	1/55	15/17913	0.045096	0.126061	0.083816	ERCC1
GO_BP_m8	GO:00907	telomere r	1/55	15/17913	0.045096	0.126061	0.083816	ERCC1
GO_BP_m8	GO:00988	protein tra	1/55	15/17913	0.045096	0.126061	0.083816	KIF5B
GO_BP_m8	GO:00991	microtubu	1/55	15/17913	0.045096	0.126061	0.083816	KIF5B
GO_BP_m8	GO:19001	positive re	1/55	15/17913	0.045096	0.126061	0.083816	TP53

GO_BP_m8	GO:19033	regulation	1/55	15/17913	0.045096	0.126061	0.083816	AKAP9
GO_BP_m8	GO:19035	positive re	1/55	15/17913	0.045096	0.126061	0.083816	RAB7A
GO_BP_m8	GO:00148	striated m	2/55	111/17913	0.045679	0.127479	0.084759	PARP1/PA
GO_BP_m8	GO:19049	positive re	4/55	440/17913	0.045899	0.127884	0.085028	KIF5B/PRK
GO_BP_m8	GO:00148	muscle hy	2/55	113/17913	0.047165	0.131021	0.087114	PARP1/PA
GO_BP_m8	GO:00427	regulation	2/55	114/17913	0.047915	0.131021	0.087114	TP53/USP
GO_BP_m8	GO:00080	retrograde	1/55	16/17913	0.048031	0.131021	0.087114	KIF5B
GO_BP_m8	GO:00083	determina	1/55	16/17913	0.048031	0.131021	0.087114	TP53
GO_BP_m8	GO:00108	positive re	1/55	16/17913	0.048031	0.131021	0.087114	NFKBIA
GO_BP_m8	GO:00165	protein au	1/55	16/17913	0.048031	0.131021	0.087114	PARP1
GO_BP_m8	GO:00302	hyalurona	1/55	16/17913	0.048031	0.131021	0.087114	HMMR
GO_BP_m8	GO:00609	negative r	1/55	16/17913	0.048031	0.131021	0.087114	TP53
GO_BP_m8	GO:00702	thymocyte	1/55	16/17913	0.048031	0.131021	0.087114	TP53
GO_BP_m8	GO:00718	response t	1/55	16/17913	0.048031	0.131021	0.087114	PRKACA
GO_BP_m8	GO:19035	regulation	1/55	16/17913	0.048031	0.131021	0.087114	RAB7A
GO_BP_m8	GO:20000	regulation	1/55	16/17913	0.048031	0.131021	0.087114	RPA2
GO_BP_m8	GO:20011	negative r	1/55	16/17913	0.048031	0.131021	0.087114	PARP1
GO_BP_m8	GO:00075	cell aging	2/55	116/17913	0.049428	0.134399	0.08936	ERCC1/TP
GO_BP_m8	GO:00322	positive re	2/55	116/17913	0.049428	0.134399	0.08936	AKAP9/CD
GO_BP_m8	GO:00000	G1/S trans	3/55	269/17913	0.049623	0.134713	0.089569	CCNA1/R
GO_BP_m9	GO:00104	exit from r	2月11日	28/17913	0.000128	0.000305	9.17E-05	CHMP4B/
GO_BP_m9	GO:00705	regulation	3月11日	170/17913	0.000131	0.000308	9.25E-05	CHMP2B/
GO_BP_m9	GO:00517	regulation	3月11日	172/17913	0.000136	0.000316	9.47E-05	CHMP2B/
GO_BP_m9	GO:00224	cellular co	4月11日	492/17913	0.000159	0.000366	0.00011	CHMP2B/
GO_BP_m9	GO:00328	regulation	3月11日	199/17913	0.000209	0.000475	0.000143	CHMP2B/
GO_BP_m9	GO:00064	negative r	3月11日	222/17913	0.000288	0.000649	0.000195	CHMP6/T
GO_BP_m9	GO:00069	nuclear en	2月11日	45/17913	0.000335	0.000746	0.000224	CHMP4B/
GO_BP_m9	GO:00336	negative r	3月11日	244/17913	0.00038	0.000838	0.000251	CHMP6/T
GO_BP_m9	GO:00507	regulation	3月11日	245/17913	0.000385	0.00084	0.000252	CHMP6/T
GO_BP_m9	GO:00083	endosome	2月11日	49/17913	0.000397	0.000858	0.000257	CHMP2B/
GO_BP_m9	GO:00513	negative r	3月11日	272/17913	0.000522	0.001117	0.000335	CHMP6/T
GO_BP_m9	GO:00453	late endos	1月11日	1/17913	0.000614	0.001276	0.000383	CHMP7
GO_BP_m9	GO:19048	ESCRT con	1月11日	1/17913	0.000614	0.001276	0.000383	CHMP6
GO_BP_m9	GO:19049	ESCRT III c	1月11日	1/17913	0.000614	0.001276	0.000383	CHMP6
GO_BP_m9	GO:00181	peptidyl-t	3月11日	346/17913	0.001051	0.002162	0.000649	CHMP6/T
GO_BP_m9	GO:00182	peptidyl-t	3月11日	349/17913	0.001077	0.002195	0.000659	CHMP6/T
GO_BP_m9	GO:00617	multivesic	1月11日	2/17913	0.001228	0.002433	0.00073	CHMP2B
GO_BP_m9	GO:20003	regulation	1月11日	2/17913	0.001228	0.002433	0.00073	TSG101
GO_BP_m9	GO:20003	positive re	1月11日	2/17913	0.001228	0.002433	0.00073	TSG101
GO_BP_m9	GO:00070	lysosomal	2月11日	94/17913	0.001453	0.002853	0.000856	CHMP2B/
GO_BP_m9	GO:00019	negative r	3月11日	416/17913	0.001786	0.003474	0.001042	CHMP6/T
GO_BP_m9	GO:00068	extracellul	1月11日	3/17913	0.001841	0.00355	0.001065	TSG101
GO_BP_m9	GO:00423	negative r	3月11日	455/17913	0.002307	0.004409	0.001323	CHMP6/T
GO_BP_m9	GO:00002	microtubu	3月11日	460/17913	0.00238	0.004508	0.001353	CHMP2B/
GO_BP_m9	GO:00364	maintenan	1月11日	4/17913	0.002454	0.004567	0.00137	CHMP4B
GO_BP_m9	GO:00448	mitotic cyt	1月11日	4/17913	0.002454	0.004567	0.00137	CHMP4C
GO_BP_m9	GO:00514	regulation	3月11日	472/17913	0.002562	0.004726	0.001418	CHMP2B/
GO_BP_m9	GO:00700	ubiquitin-	1月11日	5/17913	0.003067	0.00561	0.001683	TSG101
GO_BP_m9	GO:00066	posttransl	1月11日	6/17913	0.003679	0.006507	0.001952	CHMP4B
GO_BP_m9	GO:00098	abscission	1月11日	6/17913	0.003679	0.006507	0.001952	CHMP4C
GO_BP_m9	GO:00325	endosome	1月11日	6/17913	0.003679	0.006507	0.001952	CHMP2B
GO_BP_m9	GO:19035	regulation	1月11日	6/17913	0.003679	0.006507	0.001952	TSG101
GO_BP_m9	GO:00324	negative r	1月11日	7/17913	0.004291	0.007466	0.00224	CHMP4C
GO_BP_m9	GO:00719	extracellul	1月11日	7/17913	0.004291	0.007466	0.00224	TSG101
GO_BP_m9	GO:00901	membrane	1月11日	8/17913	0.004903	0.008394	0.002519	CHMP4B
GO_BP_m9	GO:00991	regulation	1月11日	8/17913	0.004903	0.008394	0.002519	CHMP2B
GO_BP_m9	GO:19052	regulation	1月11日	11/17913	0.006736	0.011441	0.003433	CHMP2B
GO_BP_m9	GO:19029	negative r	1月11日	13/17913	0.007956	0.013407	0.004023	CHMP4B

GO_BP_m[illegible]	GO:00431[illegible]	ubiquitin-[illegible]	1月11日	15/17913	0.009175	0.015221	0.004567	TSG101
GO_BP_m[illegible]	GO:19035[illegible]	positive re	1月11日	15/17913	0.009175	0.015221	0.004567	TSG101
GO_BP_m[illegible]	GO:00990[illegible]	modificatio	1月11日	16/17913	0.009784	0.015983	0.004796	CHMP2B
GO_BP_m[illegible]	GO:19035[illegible]	regulation	1月11日	16/17913	0.009784	0.015983	0.004796	TSG101
GO_BP_m[illegible]	GO:00517[illegible]	negative re	1月11日	18/17913	0.011001	0.017835	0.005351	CHMP4C
GO_BP_m[illegible]	GO:19901[illegible]	exosomal [illegible]	1月11日	19/17913	0.011609	0.018679	0.005605	TSG101
GO_BP_m[illegible]	GO:00977[illegible]	extracellula	1月11日	20/17913	0.012217	0.01951	0.005854	TSG101
GO_BP_m[illegible]	GO:00995[illegible]	modificatio	1月11日	21/17913	0.012824	0.020328	0.006099	CHMP2B
GO_BP_m[illegible]	GO:01401[illegible]	extracellula	1月11日	22/17913	0.013431	0.021134	0.006341	TSG101
GO_BP_m[illegible]	GO:00711[illegible]	protein loc	1月11日	28/17913	0.017065	0.026657	0.007998	CHMP7
GO_BP_m[illegible]	GO:00700[illegible]	neuron cel	1月11日	30/17913	0.018274	0.028338	0.008503	CHMP2B
GO_BP_m[illegible]	GO:00162[illegible]	negative re	1月11日	33/17913	0.020085	0.030701	0.009212	CHMP4B
GO_BP_m[illegible]	GO:20007[illegible]	regulation	1月11日	33/17913	0.020085	0.030701	0.009212	CHMP4B
GO_BP_m[illegible]	GO:00973[illegible]	autophago	1月11日	34/17913	0.020687	0.031398	0.009421	TSG101
GO_BP_m[illegible]	GO:00440[illegible]	regulation	1月11日	38/17913	0.023095	0.034806	0.010443	CHMP4B
GO_BP_m[illegible]	GO:19021[illegible]	negative re	1月11日	39/17913	0.023697	0.035462	0.01064	CHMP4B
GO_BP_m[illegible]	GO:00602[illegible]	anatomica	2月11日	413/1791[illegible]	0.025416	0.037771	0.011333	CHMP2B/[illegible]
GO_BP_m[illegible]	GO:00725[illegible]	establishm	2月11日	452/1791[illegible]	0.030054	0.044356	0.013309	CHMP4B/[illegible]
GO_BP_m[illegible]	GO:00065[illegible]	protein mo	1月11日	65/17913	0.03921	0.057472	0.017244	TSG101
GO_BP_m[illegible]	GO:00000[illegible]	autophago	1月11日	70/17913	0.042167	0.061386	0.018419	CHMP4B
GO_BP_m[illegible]	GO:00901[illegible]	organelle [illegible]	1月11日	71/17913	0.042757	0.061825	0.018551	CHMP2B
GO_BP_m[illegible]	GO:19050[illegible]	autophago	1月11日	73/17913	0.043937	0.063105	0.018935	CHMP4B
GO_BP_m[illegible]	GO:00345[illegible]	protein loc	1月11日	82/17913	0.049231	0.06968	0.020907	CHMP7
GO_BP_m[illegible]	GO:00459[illegible]	positive re	1月11日	82/17913	0.049231	0.06968	0.020907	TSG101
GO_BP_m[illegible]	GO:00105[illegible]	negative re	1月11日	83/17913	0.049818	0.06968	0.020907	CHMP4B
GO_BP_m[illegible]	GO:00324[illegible]	regulation	1月11日	83/17913	0.049818	0.06968	0.020907	CHMP4C
GO_CC_m	GO:00057[illegible]	rough end	5/121	74/18678	0.000118	0.000485	0.000299	RPL18/RPL
GO_CC_m	GO:19904[illegible]	Parkin-FB[illegible]	2/121	3/18678	0.000124	0.000493	0.000304	CUL1/FBX[illegible]
GO_CC_m	GO:00150[illegible]	Cajal body	5/121	75/18678	0.000126	0.000493	0.000304	DDX42/DD
GO_CC_m	GO:00985[illegible]	cytoplasm	2/121	5/18678	0.000411	0.001567	0.000966	RPS28/RPS
GO_CC_m	GO:00190[illegible]	SCF ubiqu	3/121	35/18678	0.001492	0.005561	0.003427	CCNF/CUL
GO_CC_m	GO:00985[illegible]	cytoplasm	2/121	15/18678	0.004136	0.015073	0.009287	RPS28/RPS
GO_CC_m	GO:00364[illegible]	cytoplasm	5/121	176/1867[illegible]	0.005794	0.020655	0.012727	ELAVL1/PA
GO_CC_m	GO:00332[illegible]	eukaryotic	1/121	1/18678	0.006478	0.021249	0.013093	RPL38
GO_CC_m	GO:00346[illegible]	U11/U12 s	1/121	1/18678	0.006478	0.021249	0.013093	SF3B1
GO_CC_m	GO:00709[illegible]	translation	1/121	1/18678	0.006478	0.021249	0.013093	RPL38
GO_CC_m	GO:00715[illegible]	eukaryotic	1/121	1/18678	0.006478	0.021249	0.013093	EIF3E
GO_CC_m	GO:00351[illegible]	exon-exor	2/121	19/18678	0.006622	0.021296	0.013122	RBM8A/SF
GO_CC_m	GO:00357[illegible]	ribonuclec	5/121	188/1867[illegible]	0.007608	0.023996	0.014786	ELAVL1/PA
GO_CC_m	GO:00056[illegible]	anaphase-	2/121	21/18678	0.008065	0.024954	0.015376	CDC16/CD
GO_CC_m	GO:00308[illegible]	rough end	2/121	23/18678	0.009634	0.02926	0.018029	RPS28/RPS
GO_CC_m	GO:00711[illegible]	NF-kappa	1/121	2/18678	0.012915	0.03851	0.023729	RPS3
GO_CC_m	GO:00710[illegible]	U4/U6 snR	1/121	3/18678	0.01931	0.052781	0.032522	PPIH
GO_CC_m	GO:00710[illegible]	U2-type p	1/121	3/18678	0.01931	0.052781	0.032522	SF3A1
GO_CC_m	GO:00710[illegible]	U2-type p	1/121	3/18678	0.01931	0.052781	0.032522	DHX15
GO_CC_m	GO:00710[illegible]	prespliceo	1/121	3/18678	0.01931	0.052781	0.032522	SF3A1
GO_CC_m	GO:00710[illegible]	post-mRN	1/121	3/18678	0.01931	0.052781	0.032522	DHX15
GO_CC_m	GO:00314[illegible]	Cul3-RING	2/121	34/18678	0.020398	0.054841	0.033792	KLHL2/KLH
GO_CC_m	GO:00001[illegible]	nuclear ub	2/121	38/18678	0.025137	0.065764	0.040522	CDC16/CD
GO_CC_m	GO:00058[illegible]	eukaryotic	1/121	4/18678	0.025664	0.065764	0.040522	EIF2S3
GO_CC_m	GO:00335[illegible]	HULC com	1/121	4/18678	0.025664	0.065764	0.040522	UBE2A
GO_CC_m	GO:00080[illegible]	cyclin/CDK	1/121	5/18678	0.031977	0.080682	0.049714	SNW1
GO_CC_m	GO:00710[illegible]	U2-type ca	1/121	7/18678	0.044483	0.108883	0.067091	RBM8A
GO_CC_m	GO:00710[illegible]	catalytic st	1/121	7/18678	0.044483	0.108883	0.067091	RBM8A
GO_CC_m	GO:00104[illegible]	cytoplasm	2/121	55/18678	0.049471	0.119313	0.073518	ELAVL1/PA
GO_CC_m	GO:00451[illegible]	pronucleus	2月21日	15/18678	0.000125	0.000547	0.000201	AURKA/CC
GO_CC_m	GO:19908[illegible]	cytoplasm	1月21日	1/18678	0.001124	0.004666	0.001716	NUP214
GO_CC_m	GO:00093[illegible]	DNA topo	1月21日	2/18678	0.002247	0.008479	0.003118	TOP2A

GO_CC_m	GO:0019O	viral integr	1月21日	2/18678	0.002247	0.008479	0.003118	TOP2A
GO_CC_m	GO:00425	germinal v	1月21日	3/18678	0.003369	0.011652	0.004286	AURKA
GO_CC_m	GO:00971	cyclin A2-	1月21日	3/18678	0.003369	0.011652	0.004286	CCNA2
GO_CC_m	GO:00162	inclusion b	2月21日	81/18678	0.003698	0.012276	0.004515	NUP153/T
GO_CC_m	GO:00016	female ger	1月21日	4/18678	0.00449	0.014334	0.005272	AURKA
GO_CC_m	GO:00726	mitotic spi	2月21日	97/18678	0.005256	0.015759	0.005796	AURKA/TP
GO_CC_m	GO:00347	secretory g	3月21日	321/18678	0.005316	0.015759	0.005796	PSMA5/PS
GO_CC_m	GO:00986	chromosor	3月21日	337/18678	0.006084	0.016557	0.00609	THOC1/TH
GO_CC_m	GO:00602	cytoplasm	3月21日	338/18678	0.006134	0.016557	0.00609	PSMA5/PS
GO_CC_m	GO:00319	vesicle lum	3月21日	339/18678	0.006184	0.016557	0.00609	PSMA5/PS
GO_CC_m	GO:00019	male pron	1月21日	6/18678	0.006728	0.01745	0.006418	CCNA2
GO_CC_m	GO:00007	nuclear ch	2月21日	121/18678	0.008065	0.019572	0.007199	THOC1/TH
GO_CC_m	GO:00058	centriole	2月21日	124/18678	0.008454	0.019572	0.007199	AURKA/TC
GO_CC_m	GO:19048	ficolin-1-r	2月21日	124/18678	0.008454	0.019572	0.007199	PSMA5/PS
GO_CC_m	GO:00166	nuclear sp	3月21日	386/18678	0.008835	0.019572	0.007199	NXT1/THC
GO_CC_m	GO:00197	proteasom	1月21日	8/18678	0.008961	0.019572	0.007199	PSMA5
GO_CC_m	GO:00432	axon hilloc	1月21日	8/18678	0.008961	0.019572	0.007199	AURKA
GO_CC_m	GO:00019	female prc	1月21日	9/18678	0.010076	0.021443	0.007886	CCNA2
GO_CC_m	GO:00058	proteasom	1月21日	10/18678	0.011189	0.022651	0.008331	PSMD1
GO_CC_m	GO:00726	meiotic sp	1月21日	10/18678	0.011189	0.022651	0.008331	AURKA
GO_CC_m	GO:00056	nuclear lar	1月21日	11/18678	0.012301	0.02431	0.008941	NUP35
GO_CC_m	GO:00007	chromosor	2月21日	156/18678	0.013117	0.025318	0.009312	THOC1/TH
GO_CC_m	GO:00316	spindle po	1月21日	13/18678	0.014523	0.027395	0.010075	AURKA
GO_CC_m	GO:00444	microtubu	2月21日	170/18678	0.015439	0.028476	0.010473	AURKA/TC
GO_CC_m	GO:01010	ficolin-1-r	2月21日	185/18678	0.018108	0.032674	0.012017	PSMA5/PS
GO_CC_m	GO:00058	cytoplasm	1月21日	17/18678	0.01895	0.033466	0.012308	TPR
GO_CC_m	GO:00430	germ cell r	1月21日	19/18678	0.021157	0.036585	0.013455	AURKA
GO_CC_m	GO:00058	proteasom	1月21日	20/18678	0.022259	0.037704	0.013867	PSMA5
GO_CC_m	GO:00056	anaphase-	1月21日	21/18678	0.023359	0.038777	0.014262	ANAPC15
GO_CC_m	GO:00974	mitotic spi	1月21日	26/18678	0.028844	0.046942	0.017265	AURKA
GO_CC_m	GO:00003	cyclin-dep	1月21日	29/18678	0.032121	0.05127	0.018856	CCNA2
GO_CC_m	GO:00001	nuclear ub	1月21日	38/18678	0.041888	0.065598	0.024126	ANAPC15
GO_CC_m	GO:00302	dynein cor	1月21日	39/18678	0.042967	0.066042	0.02429	TPR
GO_CC_m	GO:00315	motile ciliu	3月11日	162/18678	0.0001	0.00028	3.77E-05	WDR19/IF
GO_CC_m	GO:00444	microtubu	3月11日	170/18678	0.000116	0.000284	3.83E-05	WDR60/W
GO_CC_m	GO:00058	centrosom	4月11日	473/18678	0.000116	0.000284	3.83E-05	WDR60/W
GO_CC_m	GO:00328	plasma me	3月11日	181/18678	0.000139	0.00032	4.32E-05	TRAF3IP1/
GO_CC_m	GO:00323	photorece	2月11日	36/18678	0.000196	0.000426	5.75E-05	WDR19/IF
GO_CC_m	GO:00017	photorece	2月11日	75/18678	0.000855	0.001754	0.000237	WDR19/IF
GO_CC_m	GO:00310	interphase	1月11日	2/18678	0.001178	0.002187	0.000295	WDR60
GO_CC_m	GO:19026	kinociliary	1月11日	2/18678	0.001178	0.002187	0.000295	IFT20
GO_CC_m	GO:00995	cytoplasm	3月11日	402/18678	0.001436	0.002409	0.000325	TRAF3IP1/
GO_CC_m	GO:00977	photorece	2月11日	98/18678	0.001453	0.002409	0.000325	WDR19/IF
GO_CC_m	GO:00977	9+0 non-r	2月11日	99/18678	0.001483	0.002409	0.000325	WDR19/IF
GO_CC_m	GO:00058	centriole	2月11日	124/18678	0.002312	0.003468	0.000468	WDR34/IF
GO_CC_m	GO:00977	non-motil	2月11日	124/18678	0.002312	0.003468	0.000468	WDR19/IF
GO_CC_m	GO:00600	kinocilium	1月11日	6/18678	0.003529	0.004915	0.000663	IFT20
GO_CC_m	GO:00977	9+2 non-r	1月11日	6/18678	0.003529	0.004915	0.000663	IFT20
GO_CC_m	GO:00442	dendrite te	1月11日	12/18678	0.007046	0.009476	0.001279	IFT20
GO_CC_m	GO:00002	pericentric	1月11日	20/18678	0.011719	0.015234	0.002056	WDR60
GO_CC_m	GO:00058	cis-Golgi r	1月11日	39/18678	0.022736	0.028603	0.00386	IFT20
GO_CC_m	GO:00324	stereociliu	1月11日	51/18678	0.029636	0.036119	0.004874	IFT20
GO_CC_m	GO:00324	stereociliu	1月11日	54/18678	0.031355	0.037055	0.005001	IFT20
GO_CC_m	GO:00059	microvillus	1月11日	80/18678	0.04613	0.052914	0.007141	IFT20
GO_CC_m	GO:00058	spindle	10/133	326/18678	0.000116	0.001309	0.00087	ALMS1/CE
GO_CC_m	GO:00331	endoplasm	5/133	70/18678	0.000142	0.001515	0.001006	F8/RAB1B/
GO_CC_m	GO:00360	ciliary basa	6/133	111/18678	0.000143	0.001515	0.001006	B9D1/B9D
GO_CC_m	GO:00320	integrator	3/133	15/18678	0.000151	0.00155	0.00103	INTS4/INT

GO_CC_m	GO:19029	protein kir	5/133	77/18678	0.000222	0.002218	0.001474	CCNE2/CC
GO_CC_m	GO:00009	gamma-tu	3/133	18/18678	0.000266	0.002508	0.001667	TUBG1/TU
GO_CC_m	GO:00324	filopodium	3/133	18/18678	0.000266	0.002508	0.001667	ABI1/ABI2/
GO_CC_m	GO:00347	methyltrar	5/133	88/18678	0.000414	0.003796	0.002523	AEBP2/ASI
GO_CC_m	GO:00358	site of dou	4/133	51/18678	0.000474	0.004227	0.002809	ARPC4/NE
GO_CC_m	GO:00080	cyclin/CDK	2/133	5/18678	0.000496	0.004313	0.002866	CCNT1/CC
GO_CC_m	GO:00056	replication	4/133	54/18678	0.00059	0.004999	0.003322	DNMT1/N
GO_CC_m	GO:00444	ciliary part	10/133	415/18678	0.000784	0.00648	0.004306	B9D1/B9D
GO_CC_m	GO:00306	Golgi-assc	5/133	102/18678	0.000814	0.006569	0.004365	SEC24A/SI
GO_CC_m	GO:00358	ciliary tran	4/133	61/18678	0.000937	0.007384	0.004907	B9D1/B9D
GO_CC_m	GO:00550	recycling e	6/133	164/18678	0.001145	0.008822	0.005862	ACKR3/LD
GO_CC_m	GO:00907	site of DN/	4/133	65/18678	0.001189	0.008956	0.005951	ARPC4/NE
GO_CC_m	GO:00009	gamma-tu	2/133	8/18678	0.00137	0.009676	0.00643	TUBGCP4/
GO_CC_m	GO:00082	gamma-tu	2/133	8/18678	0.00137	0.009676	0.00643	TUBGCP4/
GO_CC_m	GO:00165	Cdc73/Paf	2/133	8/18678	0.00137	0.009676	0.00643	CDC73/LE
GO_CC_m	GO:00446	MLL3/4 co	2/133	9/18678	0.001753	0.01213	0.00806	ASH2L/KM
GO_CC_m	GO:00301	clathrin-cc	6/133	182/18678	0.001946	0.013194	0.008768	AVPR2/CD
GO_CC_m	GO:00007	condensec	4/133	75/18678	0.002021	0.013431	0.008925	BRCA2/CH
GO_CC_m	GO:00171	Golgi trans	2/133	11/18678	0.002654	0.016791	0.011158	COG2/CO
GO_CC_m	GO:00300	TRAPP cor	2/133	11/18678	0.002654	0.016791	0.011158	TRAPPC10
GO_CC_m	GO:00058	cis-Golgi r	3/133	39/18678	0.002675	0.016791	0.011158	COG3/GO
GO_CC_m	GO:00007	chromatin	10/133	500/18678	0.003099	0.0191	0.012692	ASH2L/CH
GO_CC_m	GO:00008	lateral eler	2/133	14/18678	0.00433	0.025751	0.017112	BRCA2/RA
GO_CC_m	GO:00310	platelet de	2/133	14/18678	0.00433	0.025751	0.017112	ITIH4/SERI
GO_CC_m	GO:00301	vesicle coa	3/133	48/18678	0.004832	0.027636	0.018364	SEC24A/SI
GO_CC_m	GO:00310	Ino80 com	2/133	15/18678	0.004973	0.027636	0.018364	RUVBL1/Y
GO_CC_m	GO:00328	carboxy-te	2/133	15/18678	0.004973	0.027636	0.018364	CCNT1/CC
GO_CC_m	GO:00332	DNA helica	2/133	15/18678	0.004973	0.027636	0.018364	RUVBL1/Y
GO_CC_m	GO:00301	filopodium	4/133	99/18678	0.005491	0.030022	0.01995	ABI1/ABI2/
GO_CC_m	GO:00350	ESC/E(Z) c	2/133	16/18678	0.005657	0.030439	0.020227	AEBP2/PH
GO_CC_m	GO:00057	endoplasm	7/133	306/18678	0.006365	0.033281	0.022115	CD4/CLU/I
GO_CC_m	GO:00421	T cell rece	2/133	17/18678	0.006381	0.033281	0.022115	CD3D/CD4
GO_CC_m	GO:00007	nuclear ch	7/133	309/18678	0.006702	0.033527	0.022278	ASH2L/DN
GO_CC_m	GO:19047	tertiary gra	3/133	55/18678	0.00707	0.033527	0.022278	ALDOA/OI
GO_CC_m	GO:00335	BRCA2-M/	1/133	1/18678	0.007121	0.033527	0.022278	BRCA2
GO_CC_m	GO:00609	lipid tube	1/133	1/18678	0.007121	0.033527	0.022278	BIN1
GO_CC_m	GO:00971	cyclin D2-	1/133	1/18678	0.007121	0.033527	0.022278	CDK6
GO_CC_m	GO:00990	perinuclea	1/133	1/18678	0.007121	0.033527	0.022278	CLU
GO_CC_m	GO:19029	pyruvate k	1/133	1/18678	0.007121	0.033527	0.022278	PKM
GO_CC_m	GO:00306	clathrin-cc	4/133	110/18678	0.007933	0.036838	0.024479	AVPR2/CD
GO_CC_m	GO:00002	pericentric	2/133	20/18678	0.008792	0.040275	0.026763	PCM1/TUE
GO_CC_m	GO:00428	platelet de	2/133	21/18678	0.009672	0.043718	0.02905	ITIH4/SERI
GO_CC_m	GO:01010	ficolin-1-r	5/133	185/18678	0.010479	0.046624	0.030981	ALDOA/CI
GO_CC_m	GO:00973	INO80-typ	2/133	22/18678	0.01059	0.046624	0.030981	RUVBL1/Y
GO_CC_m	GO:00306	transport v	5/133	188/18678	0.011177	0.048579	0.03228	SEC24A/SI
GO_CC_m	GO:19048	ficolin-1-r	4/133	124/18678	0.01195	0.051278	0.034074	ALDOA/CI
GO_CC_m	GO:00301	transport v	7/133	349/18678	0.012562	0.053232	0.035372	GOSR1/RA
GO_CC_m	GO:00007	condensec	5/133	199/18678	0.014011	0.057271	0.038056	BRCA2/CH
GO_CC_m	GO:00029	cyclin K-CI	1/133	2/18678	0.014191	0.057271	0.038056	CDK12
GO_CC_m	GO:00351	Rb-E2F co	1/133	2/18678	0.014191	0.057271	0.038056	E2F1
GO_CC_m	GO:00971	cyclin E2-(	1/133	2/18678	0.014191	0.057271	0.038056	CCNE2
GO_CC_m	GO:00312	SNARE cor	2/133	28/18678	0.016855	0.06722	0.044667	GOSR1/ST
GO_CC_m	GO:00446	MLL1/2 co	2/133	29/18678	0.01802	0.070215	0.046657	ASH2L/RU
GO_CC_m	GO:00713	MLL1 com	2/133	29/18678	0.01802	0.070215	0.046657	ASH2L/RU
GO_CC_m	GO:00310	extracellul	8/133	468/18678	0.018924	0.072899	0.048441	CLU/ITIH4/
GO_CC_m	GO:00344	centriolar s	2/133	30/18678	0.019218	0.073201	0.048641	C2CD3/PC
GO_CC_m	GO:00162	inclusion b	3/133	81/18678	0.020123	0.075796	0.050366	CLU/DVL2
GO_CC_m	GO:00725	blood micr	4/133	147/18678	0.021011	0.078272	0.052012	CLU/ITIH4/

GO_CC_m	GO:00007!	euchromat	2/133	32/18678	0.021712	0.080003	0.053161	ASH2L/DN
GO_CC_m	GO:00325!	trans-Golg	3/133	85/18678	0.022825	0.082969	0.055132	COG2/CO
GO_CC_m	GO:19025(	H4 histone	2/133	33/18678	0.023006	0.082969	0.055132	POLE3/RU
GO_CC_m	GO:00301:	membrane	3/133	86/18678	0.02353	0.083089	0.055212	SEC24A/SE
GO_CC_m	GO:00484	coated me	3/133	86/18678	0.02353	0.083089	0.055212	SEC24A/SE
GO_CC_m	GO:00620:	collagen-c	7/133	399/1867	0.024221	0.083317	0.055364	CLU/ITIH4
GO_CC_m	GO:00007!	synaptone	2/133	34/18678	0.024332	0.083317	0.055364	BRCA2/RA
GO_CC_m	GO:00990!	synaptone	2/133	34/18678	0.024332	0.083317	0.055364	BRCA2/RA
GO_CC_m	GO:00059!	DNA-depe	1/133	4/18678	0.028182	0.090988	0.060461	PRKDC
GO_CC_m	GO:00320:	NELF com	1/133	4/18678	0.028182	0.090988	0.060461	NELFCD
GO_CC_m	GO:00330(	Rad51B-R	1/133	4/18678	0.028182	0.090988	0.060461	RAD51B
GO_CC_m	GO:00708	SOSS com	1/133	4/18678	0.028182	0.090988	0.060461	NABP1
GO_CC_m	GO:00972!	R2TP com	1/133	4/18678	0.028182	0.090988	0.060461	RUVBL1
GO_CC_m	GO:00988:	postsynap	1/133	4/18678	0.028182	0.090988	0.060461	RAB11A
GO_CC_m	GO:00708:	tertiary gr	4/133	164/1867	0.029842	0.095437	0.063417	ALDOA/FF
GO_CC_m	GO:00329!	protein-lip	2/133	39/18678	0.031408	0.099508	0.066122	BIN1/CLU
GO_CC_m	GO:0015€	actin cytos	7/133	432/1867	0.035096	0.105311	0.069978	ACTN2/AL
GO_CC_m	GO:00058:	polar micr	1/133	5/18678	0.035104	0.105311	0.069978	TUBG1
GO_CC_m	GO:00086:	epsilon DN	1/133	5/18678	0.035104	0.105311	0.069978	POLE3
GO_CC_m	GO:00314(	Cul4B-RIN	1/133	5/18678	0.035104	0.105311	0.069978	DDB1
GO_CC_m	GO:00707(	gamma-se	1/133	5/18678	0.035104	0.105311	0.069978	TMED10
GO_CC_m	GO:00974:	neurofibril	1/133	5/18678	0.035104	0.105311	0.069978	CLU
GO_CC_m	GO:00315:	PcG protei	2/133	42/18678	0.035996	0.10704	0.071127	AEBP2/PH
GO_CC_m	GO:00163(	nuclear ma	3/133	106/1867	0.040075	0.118133	0.078499	DNMT3A/
GO_CC_m	GO:00308(	smooth er	1/133	6/18678	0.041976	0.120593	0.080133	STX17
GO_CC_m	GO:00974:	smooth er	1/133	6/18678	0.041976	0.120593	0.080133	STX17
GO_CC_m	GO:00975:	sperm plas	1/133	6/18678	0.041976	0.120593	0.080133	CCR6
GO_CC_m	GO:00058	microtubu	6/133	357/1867	0.042643	0.121479	0.080722	HAUS5/RA
GO_CC_m	GO:00007	chromoso	4/133	185/1867	0.043379	0.122546	0.081431	CTCF/DNN
GO_CC_m	GO:00056(	transcripti	5/133	270/1867	0.044069	0.123467	0.082043	E2F1/E2F2
GO_CC_m	GO:00300:	lamellipod	4/133	191/1867	0.047788	0.132346	0.087943	ABI1/ABI2
GO_CC_m	GO:00058:	kinetochor	1/133	7/18678	0.0488	0.132346	0.087943	RAB11A
GO_CC_m	GO:00308	Mre11 cor	1/133	7/18678	0.0488	0.132346	0.087943	NBN
GO_CC_m	GO:00330(	DNA recor	1/133	7/18678	0.0488	0.132346	0.087943	RAD51B
GO_CC_m	GO:00312!	cell leadin	5月26日	384/1867	0.000165	0.00455	0.003158	FGR/INPP!
GO_CC_m	GO:00312:	extrinsic cc	2月26日	54/18678	0.00255	0.04266	0.029611	ALOX15/P
GO_CC_m	GO:00198!	extrinsic cc	3月26日	199/1867	0.002585	0.04266	0.029611	ALOX15/N
GO_CC_m	GO:00190:	viral replic	1月26日	2/18678	0.002782	0.04266	0.029611	PI4KA
GO_CC_m	GO:01060(	amyloid-b	1月26日	2/18678	0.002782	0.04266	0.029611	ROCK1
GO_CC_m	GO:00420:	host cell n	1月26日	3/18678	0.00417	0.048824	0.03389	PI4KA
GO_CC_m	GO:00440!	host cell n	1月26日	3/18678	0.00417	0.048824	0.03389	PI4KA
GO_CC_m	GO:00058:	lipid dropl	2月26日	70/18678	0.004246	0.048824	0.03389	ALOX15/P
GO_CC_m	GO:00302!	protein ph	1月26日	4/18678	0.005557	0.056964	0.03954	PPP4R2
GO_CC_m	GO:00319	organelle (	2月26日	82/18678	0.005779	0.056964	0.03954	ALOX5/FG
GO_CC_m	GO:00336	host intrac	1月26日	5/18678	0.006941	0.057322	0.039788	PI4KA
GO_CC_m	GO:00336	host intrac	1月26日	5/18678	0.006941	0.057322	0.039788	PI4KA
GO_CC_m	GO:00325!	ruffle mem	2月26日	91/18678	0.007071	0.057322	0.039788	FGR/MTM
GO_CC_m	GO:00451:	membrane	3月26日	309/1867	0.008796	0.057322	0.039788	LCK/PTGS:
GO_CC_m	GO:00198!	extrinsic cc	2月26日	102/1867	0.008812	0.057322	0.039788	ALOX15/P
GO_CC_m	GO:00988!	membrane	3月26日	310/1867	0.008874	0.057322	0.039788	LCK/PTGS:
GO_CC_m	GO:00347	secretory (	3月26日	321/1867	0.009757	0.057322	0.039788	ALOX5/FG
GO_CC_m	GO:00985!	membrane	3月26日	321/1867	0.009757	0.057322	0.039788	LCK/PTGS:
GO_CC_m	GO:00098!	cytoplasm	2月26日	114/1867	0.010907	0.057322	0.039788	ALOX15/P
GO_CC_m	GO:00602(	cytoplasm	3月26日	338/1867	0.011219	0.057322	0.039788	ALOX5/FG
GO_CC_m	GO:00319!	vesicle lum	3月26日	339/1867	0.011309	0.057322	0.039788	ALOX5/FG
GO_CC_m	GO:00001(	protein ph	1月26日	9/18678	0.012461	0.057322	0.039788	SHOC2
GO_CC_m	GO:00056	nuclear en	1月26日	9/18678	0.012461	0.057322	0.039788	ALOX5
GO_CC_m	GO:00320!	bleb	1月26日	9/18678	0.012461	0.057322	0.039788	ROCK1

GO_CC_m	GO:00336	host intrac	1月26日	9/18678	0.012461	0.057322	0.039788	PI4KA
GO_CC_m	GO:00436	intracellula	1月26日	9/18678	0.012461	0.057322	0.039788	PI4KA
GO_CC_m	GO:00985	side of me	3月26日	359/18678	0.013194	0.058733	0.040768	ALOX15/K
GO_CC_m	GO:00171	NMDA sel	1月26日	11/18678	0.01521	0.063606	0.04415	PTK2B
GO_CC_m	GO:00481	Set1C/COI	1月26日	11/18678	0.01521	0.063606	0.04415	WDR82
GO_CC_m	GO:00985	cytoplasm	2月26日	138/18678	0.015684	0.063659	0.044187	ALOX15/P
GO_CC_m	GO:00336	host cell p	1月26日	12/18678	0.016582	0.065182	0.045245	PI4KA
GO_CC_m	GO:00059	focal adhe	3月26日	397/18678	0.017246	0.065182	0.045245	PI4KA/PPP
GO_CC_m	GO:00059	cell-substr	3月26日	399/18678	0.017476	0.065182	0.045245	PI4KA/PPP
GO_CC_m	GO:00300	cell-substr	3月26日	404/18678	0.018061	0.065588	0.045526	PI4KA/PPP
GO_CC_m	GO:00312	leading ed	2月26日	153/18678	0.019049	0.067403	0.046786	FGR/MTM
GO_CC_m	GO:00444	microtubu	2月26日	170/18678	0.023196	0.078803	0.054699	LCK/ROCK
GO_CC_m	GO:00974	apical den	1月26日	17/18678	0.023412	0.078803	0.054699	PTK2B
GO_CC_m	GO:00058	centrosom	3月26日	473/18678	0.027242	0.08822	0.061236	LCK/PPP1F
GO_CC_m	GO:00002	pericentric	1月26日	20/18678	0.027489	0.08822	0.061236	LCK
GO_CC_m	GO:00300	lamellipod	2月26日	191/18678	0.028783	0.090275	0.062662	PTK2B/RO
GO_CC_m	GO:00059	adherens j	3月26日	496/18678	0.030768	0.094356	0.065495	PI4KA/PPP
GO_CC_m	GO:00316	A band	1月26日	31/18678	0.042297	0.12689	0.088078	PPP1R12A
GO_CC_m	GO:00017	immunolo	1月26日	36/18678	0.048955	0.143742	0.099775	LCK
GO_CC_m	GO:00056	annulate la	2/117	5/18678	0.000384	0.002157	0.001287	RANBP2/X
GO_CC_m	GO:19907	microtubu	3/117	26/18678	0.000561	0.003077	0.001836	CKAP5/KIF
GO_CC_m	GO:00444	nuclear ch	10/117	469/18678	0.000736	0.003951	0.002357	AURKB/BU
GO_CC_m	GO:00306	90S prerib	2/117	8/18678	0.001063	0.005585	0.003332	IMP3/NOF
GO_CC_m	GO:00343	nuclear pe	5/117	126/18678	0.001183	0.006088	0.003632	AHCTF1/C
GO_CC_m	GO:00056	nuclear en	9/117	420/18678	0.001312	0.006616	0.003947	AHCTF1/C
GO_CC_m	GO:00316	nuclear pe	2/117	9/18678	0.001361	0.006723	0.004011	CENPA/CE
GO_CC_m	GO:00082	cohesin cc	2/117	12/18678	0.002464	0.011936	0.007121	CDCA5/SM
GO_CC_m	GO:00103	chromoce	2/117	14/18678	0.00337	0.016009	0.009551	AURKB/CD
GO_CC_m	GO:00353	microtubu	2/117	16/18678	0.004408	0.020341	0.012136	CKAP5/KIF
GO_CC_m	GO:00163	nuclear ma	4/117	106/18678	0.004447	0.020341	0.012136	AHCTF1/C
GO_CC_m	GO:00451	intercellula	3/117	59/18678	0.006035	0.026224	0.015646	CDCA8/CE
GO_CC_m	GO:00306	Noc comp	1/117	1/18678	0.006264	0.026224	0.015646	NOP14
GO_CC_m	GO:00306	Noc4p-Nc	1/117	1/18678	0.006264	0.026224	0.015646	NOP14
GO_CC_m	GO:00345	chromoso	1/117	1/18678	0.006264	0.026224	0.015646	RCC2
GO_CC_m	GO:00985	inactive se	1/117	1/18678	0.006264	0.026224	0.015646	HNRNPU
GO_CC_m	GO:00057	pericentric	2/117	20/18678	0.006866	0.028045	0.016733	CENPA/CE
GO_CC_m	GO:00058	cytoplasm	3/117	62/18678	0.006926	0.028045	0.016733	CLASP2/KI
GO_CC_m	GO:00319	nuclear me	6/117	281/18678	0.008559	0.034096	0.020343	AHCTF1/N
GO_CC_m	GO:00150	Cajal body	3/117	75/18678	0.011662	0.044069	0.026293	FBL/NOP5
GO_CC_m	GO:00056	U2-type sp	3/117	76/18678	0.012087	0.044069	0.026293	GCFC2/LSI
GO_CC_m	GO:00009	condensec	1/117	2/18678	0.012489	0.044069	0.026293	CENPA
GO_CC_m	GO:00320	small-subu	1/117	2/18678	0.012489	0.044069	0.026293	NOP14
GO_CC_m	GO:00344	t-UTP com	1/117	2/18678	0.012489	0.044069	0.026293	UTP4
GO_CC_m	GO:00344	Mpp10 co	1/117	2/18678	0.012489	0.044069	0.026293	IMP3
GO_CC_m	GO:00446	nuclear pc	1/117	2/18678	0.012489	0.044069	0.026293	RANBP2
GO_CC_m	GO:19907	cytoplasm	1/117	2/18678	0.012489	0.044069	0.026293	RANBP2
GO_CC_m	GO:00007	chromatin	8/117	500/18678	0.01341	0.046652	0.027834	AHCTF1/C
GO_CC_m	GO:00003	cyclin-dep	2/117	29/18678	0.014145	0.048525	0.028951	CCNB1/CD
GO_CC_m	GO:00710	catalytic st	3/117	82/18678	0.014822	0.050151	0.029922	HNRNPA3
GO_CC_m	GO:00057	nuclear he	2/117	33/18678	0.018099	0.056949	0.033978	CENPA/CE
GO_CC_m	GO:00056	U4atac snI	1/117	3/18678	0.018676	0.056949	0.033978	SNU13
GO_CC_m	GO:00309	cortical mi	1/117	3/18678	0.018676	0.056949	0.033978	MAPRE1
GO_CC_m	GO:00314	TRAMP co	1/117	3/18678	0.018676	0.056949	0.033978	MTREX
GO_CC_m	GO:00710	U2-type p	1/117	3/18678	0.018676	0.056949	0.033978	GCFC2
GO_CC_m	GO:00710	post-mRN	1/117	3/18678	0.018676	0.056949	0.033978	GCFC2
GO_CC_m	GO:19057	mitotic spi	1/117	3/18678	0.018676	0.056949	0.033978	MAPRE1
GO_CC_m	GO:19904	mitotic spi	1/117	3/18678	0.018676	0.056949	0.033978	HNRNPU
GO_CC_m	GO:00314	Cul3-RING	2/117	34/18678	0.019153	0.057691	0.034421	KLHL13/KI

GO_CC_m	GO:00058	microtubu	3/117	92/18678	0.020097	0.059805	0.035682	AURKB/BII
GO_CC_m	GO:00465	U4/U6 x U	2/117	37/18678	0.022463	0.065275	0.038945	LSM6/SNU
GO_CC_m	GO:00975	spliceoson	2/117	37/18678	0.022463	0.065275	0.038945	LSM6/SNU
GO_CC_m	GO:00082	protein ser	2/117	38/18678	0.023615	0.067045	0.040001	PPP1CC/P
GO_CC_m	GO:19032	phosphata	2/117	38/18678	0.023615	0.067045	0.040001	PPP1CC/P
GO_CC_m	GO:00004	MIS12/MIN	1/117	4/18678	0.024824	0.068893	0.041104	MIS12
GO_CC_m	GO:00314	Cul7-RING	1/117	4/18678	0.024824	0.068893	0.041104	CUL7
GO_CC_m	GO:00016	granular c	1/117	5/18678	0.030934	0.08305	0.04955	FBL
GO_CC_m	GO:00451	basal corte	1/117	5/18678	0.030934	0.08305	0.04955	CLASP2
GO_CC_m	GO:19903	3M compl	1/117	5/18678	0.030934	0.08305	0.04955	CUL7
GO_CC_m	GO:00710	U2-type p	2/117	48/18678	0.036394	0.090499	0.053995	LSM6/SNU
GO_CC_m	GO:00710	precatalyti	2/117	48/18678	0.036394	0.090499	0.053995	LSM6/SNU
GO_CC_m	GO:00308	meiotic co	1/117	6/18678	0.037006	0.090499	0.053995	SMC1A
GO_CC_m	GO:00314	Cul5-RING	1/117	6/18678	0.037006	0.090499	0.053995	RNF7
GO_CC_m	GO:00443	dendritic s	1/117	6/18678	0.037006	0.090499	0.053995	FUS
GO_CC_m	GO:00616	mitotic spi	1/117	6/18678	0.037006	0.090499	0.053995	MAPRE1
GO_CC_m	GO:00707	pre-snoRN	1/117	6/18678	0.037006	0.090499	0.053995	NOP58
GO_CC_m	GO:00709	CRD-medi	1/117	6/18678	0.037006	0.090499	0.053995	HNRNPU
GO_CC_m	GO:19901	messenger	1/117	6/18678	0.037006	0.090499	0.053995	HNRNPA3
GO_CC_m	GO:00723	PTW/PP1 p	1/117	7/18678	0.04304	0.103212	0.06158	PPP1CC
GO_CC_m	GO:01201	Lsm2-8 cc	1/117	7/18678	0.04304	0.103212	0.06158	LSM6
GO_CC_m	GO:00321	cleavage f	2/117	55/18678	0.046585	0.110638	0.066011	CENPC/PP
GO_CC_m	GO:00975	ciliary tran	1/117	8/18678	0.049036	0.114652	0.068405	CENPF
GO_CC_m	GO:00321	cell divisio	2/117	57/18678	0.049667	0.114652	0.068405	CENPC/PP
GO_CC_m	GO:00321	cell divisio	2/117	57/18678	0.049667	0.114652	0.068405	CENPC/PP
GO_CC_m	GO:00198	extrinsic c	4/47	102/18678	0.000125	0.000842	0.000292	AAK1/HIP1
GO_CC_m	GO:00058	spindle	6/47	326/18678	0.000159	0.001036	0.00036	CLTCL1/HA
GO_CC_m	GO:00360	ciliary basa	4/47	111/18678	0.000173	0.001096	0.000381	CEP290/DY
GO_CC_m	GO:00975	ciliary tip	3/47	45/18678	0.000197	0.001209	0.00042	DYNC2H1/
GO_CC_m	GO:00312	extrinsic c	3/47	54/18678	0.000339	0.002021	0.000702	HIP1/SNX1
GO_CC_m	GO:00358	ciliary tran	3/47	61/18678	0.000485	0.002817	0.000979	CEP290/DY
GO_CC_m	GO:00058	cytoplasm	3/47	62/18678	0.000509	0.002875	0.000999	DYNC2LI1/
GO_CC_m	GO:00444	ciliary part	6/47	415/18678	0.000577	0.003146	0.001093	CEP290/DY
GO_CC_m	GO:00312	leading ed	4/47	153/18678	0.000587	0.003146	0.001093	AMPH/AR
GO_CC_m	GO:00304	growth co	4/47	166/18678	0.000797	0.004047	0.001406	ARPC3/AR
GO_CC_m	GO:00058	cytoplasm	2/47	17/18678	0.000823	0.004047	0.001406	DYNC2H1/
GO_CC_m	GO:00907	growth co	2/47	17/18678	0.000823	0.004047	0.001406	ARPC3/YW
GO_CC_m	GO:00017	ruffle	4/47	168/18678	0.000833	0.004047	0.001406	ARF4/DNM
GO_CC_m	GO:00304	site of pol	4/47	169/18678	0.000852	0.004047	0.001406	ARPC3/AR
GO_CC_m	GO:00990	postsynap	2/47	18/18678	0.000924	0.004292	0.001492	DNM2/SH
GO_CC_m	GO:00990	postsynap	2/47	21/18678	0.001262	0.005736	0.001993	DNM2/SH
GO_CC_m	GO:00058	trans-Golg	4/47	196/18678	0.001474	0.006434	0.002236	CLTCL1/DI
GO_CC_m	GO:00301	clathrin ve	2/47	23/18678	0.001516	0.006434	0.002236	CLTCL1/HI
GO_CC_m	GO:00325	ruffle mem	3/47	91/18678	0.001553	0.006434	0.002236	ARF4/DNM
GO_CC_m	GO:00198	extrinsic c	4/47	199/18678	0.001559	0.006434	0.002236	AAK1/HIP1
GO_CC_m	GO:00319	vesicle lun	5/47	339/18678	0.00157	0.006434	0.002236	ARPC5/CE
GO_CC_m	GO:00977	photorece	3/47	98/18678	0.001921	0.007722	0.002683	CEP290/KI
GO_CC_m	GO:00977	9+0 non-r	3/47	99/18678	0.001978	0.007799	0.00271	CEP290/KI
GO_CC_m	GO:00018	phagocytic	2/47	27/18678	0.00209	0.007957	0.002765	DNM2/TRI
GO_CC_m	GO:00436	axon termi	3/47	101/18678	0.002094	0.007957	0.002765	AAK1/ITSN
GO_CC_m	GO:00021	podosome	2/47	29/18678	0.00241	0.008993	0.003125	KIF9/SH3G
GO_CC_m	GO:00618	growth co	1/47	1/18678	0.002516	0.009048	0.003145	ARPC3
GO_CC_m	GO:00974	multivesic	1/47	1/18678	0.002516	0.009048	0.003145	EGFR
GO_CC_m	GO:00344	centriolar	2/47	30/18678	0.002578	0.009048	0.003145	CEP290/O
GO_CC_m	GO:00059	cell cortex	4/47	229/18678	0.002598	0.009048	0.003145	FNBP1/SN
GO_CC_m	GO:00098	cytoplasm	3/47	114/18678	0.002953	0.010119	0.003516	HIP1/SNX1
GO_CC_m	GO:00443	neuron pro	3/47	120/18678	0.003413	0.011505	0.003998	AAK1/ITSN
GO_CC_m	GO:00323	photorece	2/47	36/18678	0.003697	0.012222	0.004247	CEP290/KI

GO_CC_m:	GO:00977:	non-motil	3/47	124/18678	0.003743	0.012222	0.004247	CEP290/KI
GO_CC_m:	GO:00302(	dynein cor	2/47	39/18678	0.004328	0.013916	0.004836	DYNC2H1/
GO_CC_m:	GO:00306(	clathrin-cc	2/47	40/18678	0.004548	0.014403	0.005005	HIP1/LDLR
GO_CC_m:	GO:00301:	clathrin co	2/47	41/18678	0.004774	0.014448	0.005021	CLTCL1/HI
GO_CC_m:	GO:00361(	muscle cel	1/47	2/18678	0.005026	0.014448	0.005021	ARPC2
GO_CC_m:	GO:00361(	muscle cel	1/47	2/18678	0.005026	0.014448	0.005021	ARPC2
GO_CC_m:	GO:00704:	Shc-EGFR	1/47	2/18678	0.005026	0.014448	0.005021	EGFR
GO_CC_m:	GO:00907:	peripheral	1/47	2/18678	0.005026	0.014448	0.005021	ARPC3
GO_CC_m:	GO:19906(	PCSK9-LD	1/47	2/18678	0.005026	0.014448	0.005021	LDLR
GO_CC_m:	GO:00985(	cytoplasm	3/47	138/18678	0.005046	0.014448	0.005021	HIP1/SNX:
GO_CC_m:	GO:00431(	dendritic s	3/47	142/18678	0.005463	0.01543	0.005362	ARF4/ARR
GO_CC_m:	GO:00057(	Golgi-assc	3/47	145/18678	0.005789	0.015919	0.005532	CLTCL1/O
GO_CC_m:	GO:00443(	neuron spi	3/47	145/18678	0.005789	0.015919	0.005532	ARF4/ARR
GO_CC_m:	GO:00100(	endosome	5/47	464/18678	0.006021	0.016343	0.005679	EGFR/LDLI
GO_CC_m:	GO:00319(	early endo	3/47	148/18678	0.006126	0.016415	0.005704	EGFR/OCR
GO_CC_m:	GO:00301:	vesicle coa	2/47	48/18678	0.006494	0.017181	0.005971	CLTCL1/HI
GO_CC_m:	GO:00058	plus-end k	1/47	3/18678	0.00753	0.019193	0.00667	KIF15
GO_CC_m:	GO:00169:	kinesin II c	1/47	3/18678	0.00753	0.019193	0.00667	KIFAP3
GO_CC_m:	GO:00971	centralspir	1/47	3/18678	0.00753	0.019193	0.00667	KIF23
GO_CC_m:	GO:00058	spindle mi	2/47	53/18678	0.007871	0.019819	0.006887	KIF18B/KII
GO_CC_m:	GO:00312!	cell projec	4/47	317/18678	0.008183	0.020361	0.007076	ARF4/ARP
GO_CC_m:	GO:00990:	tethering c	2/47	55/18678	0.008455	0.020763	0.007216	NBAS/RIN
GO_CC_m:	GO:00347	secretory (	4/47	321/18678	0.008544	0.020763	0.007216	ARPC5/CE
GO_CC_m:	GO:00308(	cortical ac	2/47	57/18678	0.009058	0.021759	0.007562	SNX9/WAS
GO_CC_m:	GO:00451	intercellula	2/47	59/18678	0.00968	0.022989	0.007989	HAUS3/KII
GO_CC_m:	GO:00304	actin cap	1/47	4/18678	0.010028	0.023288	0.008093	WASL
GO_CC_m:	GO:00974(	multivesicu	1/47	4/18678	0.010028	0.023288	0.008093	EGFR
GO_CC_m:	GO:00602(	cytoplasm	4/47	338/18678	0.010192	0.023408	0.008135	ARPC5/CE
GO_CC_m:	GO:00057(	early endo	4/47	340/18678	0.010399	0.023623	0.00821	EGFR/LDLI
GO_CC_m:	GO:00328:	plasma me	3/47	181/18678	0.010618	0.023862	0.008292	DYNC2LI1/
GO_CC_m:	GO:00985!	side of me	4/47	359/18678	0.012498	0.026976	0.009375	HIP1/LDLR
GO_CC_m:	GO:00907:	central reg	1/47	5/18678	0.01252	0.026976	0.009375	YWHAE
GO_CC_m:	GO:00988:	presynapti	1/47	5/18678	0.01252	0.026976	0.009375	FCHO2
GO_CC_m:	GO:00988(	extrinsic cc	1/47	5/18678	0.01252	0.026976	0.009375	HIP1
GO_CC_m:	GO:00451	apical part	4/47	365/18678	0.013214	0.028181	0.009793	DYNC2H1/
GO_CC_m:	GO:00163:	basolatera	3/47	201/18678	0.014066	0.029696	0.01032	ARRB1/EG
GO_CC_m:	GO:00306	phagocytic	2/47	74/18678	0.01492	0.030746	0.010685	DNM2/OC
GO_CC_m:	GO:00988	postsynap	1/47	6/18678	0.015005	0.030746	0.010685	ITSN1
GO_CC_m:	GO:19900	periciliary	1/47	6/18678	0.015005	0.030746	0.010685	KIFAP3
GO_CC_m:	GO:00970(	synaptic m	4/47	395/18678	0.017185	0.03487	0.012118	ARRB1/DN
GO_CC_m:	GO:00986(	photorece	1/47	7/18678	0.017485	0.035137	0.012211	PACSIN1
GO_CC_m:	GO:00308(	cortical cy	2/47	81/18678	0.017699	0.03523	0.012243	SNX9/WAS
GO_CC_m:	GO:00301:	membrane	2/47	86/18678	0.019809	0.038268	0.013299	CLTCL1/HI
GO_CC_m:	GO:00484	coated me	2/47	86/18678	0.019809	0.038268	0.013299	CLTCL1/HI
GO_CC_m:	GO:00324:	cuticular p	1/47	8/18678	0.019958	0.038268	0.013299	SNX9
GO_CC_m:	GO:00706!	HAUS com	1/47	8/18678	0.019958	0.038268	0.013299	HAUS3
GO_CC_m:	GO:00995	postsynap	1/47	9/18678	0.022425	0.042237	0.014678	ITSN1
GO_CC_m:	GO:00057	late endos	3/47	240/18678	0.022432	0.042237	0.014678	CLTCL1/EC
GO_CC_m:	GO:00726(	mitotic spi	2/47	97/18678	0.024798	0.045625	0.015855	HAUS3/KII
GO_CC_m:	GO:00002:	astral micr	1/47	10/18678	0.024886	0.045625	0.015855	KIF18B
GO_CC_m:	GO:00058:	aster	1/47	10/18678	0.024886	0.045625	0.015855	KIF18B
GO_CC_m:	GO:00059:	axoneme	2/47	100/18678	0.026239	0.047686	0.016572	DYNC2LI1/
GO_CC_m:	GO:00970:	ciliary plas	2/47	101/18678	0.026727	0.048019	0.016688	DYNC2LI1/
GO_CC_m:	GO:00301:	AP-2 adap	1/47	11/18678	0.027341	0.048019	0.016688	HIP1
GO_CC_m:	GO:00301:	clathrin co	1/47	11/18678	0.027341	0.048019	0.016688	CLTCL1
GO_CC_m:	GO:00974	ribbon syn	1/47	11/18678	0.027341	0.048019	0.016688	PACSIN1
GO_CC_m:	GO:00301:	clathrin co	1/47	12/18678	0.02979	0.051456	0.017882	HIP1
GO_CC_m:	GO:00992	extrinsic cc	1/47	12/18678	0.02979	0.051456	0.017882	HIP1

GO_CC_m:	GO:00125:	trans-Golg	1/47	13/18678	0.032233	0.054328	0.01888	CLTCL1
GO_CC_m:	GO:00360:	endolysos	1/47	13/18678	0.032233	0.054328	0.01888	LDLR
GO_CC_m:	GO:00360:	MKS comp	1/47	13/18678	0.032233	0.054328	0.01888	CEP290
GO_CC_m:	GO:00343	low-densit	1/47	14/18678	0.03467	0.057968	0.020145	LDLR
GO_CC_m:	GO:00140	postsynap	3/47	290/18678	0.036376	0.060338	0.020969	ARRB1/DN
GO_CC_m:	GO:00322	asymmetri	3/47	294/18678	0.037647	0.061955	0.02153	ARRB1/DN
GO_CC_m:	GO:00058:	centriole	2/47	124/18678	0.038921	0.063545	0.022083	CEP290/O
GO_CC_m:	GO:00301:	clathrin co	1/47	16/18678	0.039526	0.063545	0.022083	HIP1
GO_CC_m:	GO:00353	microtubu	1/47	16/18678	0.039526	0.063545	0.022083	KIF18B
GO_CC_m:	GO:00444	cell cortex	2/47	128/18678	0.041223	0.06527	0.022682	SNX9/WAS
GO_CC_m:	GO:00453:	phagocytic	2/47	128/18678	0.041223	0.06527	0.022682	DNM2/OC
GO_CC_m:	GO:00311	pseudopo	1/47	17/18678	0.041945	0.065421	0.022735	ARRB1
GO_CC_m:	GO:00974	apical den	1/47	17/18678	0.041945	0.065421	0.022735	ITSN1
GO_CC_m:	GO:00009:	gamma-tu	1/47	18/18678	0.044358	0.068167	0.02369	CEP290
GO_CC_m:	GO:00360:	endolysos	1/47	18/18678	0.044358	0.068167	0.02369	LDLR
GO_CC_m:	GO:00989	neuron to	3/47	315/18678	0.044689	0.068176	0.023692	ARRB1/DN
GO_CC_m:	GO:00995	postsynap	3/47	316/18678	0.04504	0.068213	0.023705	ARRB1/DN
GO_CC_m:	GO:00319	filamentou	1/47	19/18678	0.046765	0.070315	0.024436	ARPC3
GO_CC_m:	GO:00427:	presynapti	2/47	141/18678	0.049045	0.073218	0.025445	FCHO2/HI
GO_CC_m!	GO:00007	condensec	2月16日	75/18678	0.001841	0.025771	0.021314	NIFK/RRS1
GO_CC_m!	GO:00007	condensec	2月16日	199/18678	0.012285	0.075407	0.062367	NIFK/RRS1
GO_CC_m!	GO:00056	telomerase	1月16日	19/18678	0.016159	0.075407	0.062367	NAT10
GO_CC_m	GO:00166	PML body	2月14日	99/18678	0.002428	0.08012	0.071559	ISG20/SP1
GO_CC_m	GO:00308	Mre11 cor	1月14日	7/18678	0.005236	0.082192	0.073409	SP100
GO_CC_m	GO:00971	tetraspanir	1月14日	10/18678	0.007472	0.082192	0.073409	SAMHD1
GO_CC_m	GO:00358	site of dou	1月14日	51/18678	0.037569	0.150674	0.134573	SAMHD1
GO_CC_m	GO:00907:	site of DN/	1月14日	65/18678	0.04765	0.150674	0.134573	SAMHD1
GO_CC_m	GO:00602	cytoplasm	9/104	338/18678	0.000114	0.001464	0.000779	APP/CTSW
GO_CC_m	GO:00319	vesicle lur	9/104	339/18678	0.000116	0.001464	0.000779	APP/CTSW
GO_CC_m	GO:00058	muscle my	3/104	18/18678	0.000129	0.001551	0.000825	MYBPC3/N
GO_CC_m	GO:00431	dendritic s	6/104	142/18678	0.000143	0.001645	0.000875	ADORA1/
GO_CC_m	GO:00363	myofilame	3/104	19/18678	0.000152	0.001645	0.000875	MYBPC3/T
GO_CC_m	GO:00057	Golgi-asso	6/104	145/18678	0.00016	0.001645	0.000875	APP/F5/GI
GO_CC_m	GO:00443	neuron spi	6/104	145/18678	0.00016	0.001645	0.000875	ADORA1/
GO_CC_m	GO:00428:	platelet de	3/104	21/18678	0.000207	0.002051	0.001091	CTSW/FAN
GO_CC_m	GO:00164	myosin II c	3/104	22/18678	0.000239	0.002284	0.001215	MYBPC3/N
GO_CC_m	GO:00487	presynapti	4/104	60/18678	0.000348	0.003214	0.00171	ADORA1/
GO_CC_m	GO:00989	neuron to	8/104	315/18678	0.000379	0.00339	0.001804	ADORA1/I
GO_CC_m	GO:00021	podosome	3/104	29/18678	0.000552	0.004774	0.00254	RHOU/SR(
GO_CC_m	GO:00301:	COPII-coa	4/104	69/18678	0.000594	0.004985	0.002652	APP/F5/HL
GO_CC_m	GO:00058	troponin c	2/104	7/18678	0.000633	0.005159	0.002745	TNNC2/TN
GO_CC_m	GO:00444	contractile	6/104	192/18678	0.000719	0.005689	0.003026	MYBPC3/N
GO_CC_m	GO:00987	presynaps	9/104	438/18678	0.00076	0.005846	0.00311	ADORA1/
GO_CC_m	GO:00300	myofibril	6/104	197/18678	0.000822	0.006153	0.003273	MYBPC3/N
GO_CC_m	GO:00430:	neuronal c	9/104	454/18678	0.000978	0.00713	0.003793	ADORA1/
GO_CC_m	GO:00431	dendritic s	3/104	36/18678	0.001048	0.007259	0.003862	APP/GPER
GO_CC_m	GO:00985	cytoplasm	5/104	138/18678	0.001048	0.007259	0.003862	GNA15/GN
GO_CC_m	GO:00432	contractile	6/104	209/18678	0.001116	0.007538	0.00401	MYBPC3/N
GO_CC_m	GO:00164	unconvent	2/104	10/18678	0.001342	0.008852	0.004709	MYL6/MY(
GO_CC_m	GO:00322	symmetric	2/104	11/18678	0.001634	0.010429	0.005548	NTSR1/PEI
GO_CC_m	GO:00312	leading ed	5/104	153/18678	0.001657	0.010429	0.005548	ADORA1/(
GO_CC_m	GO:00995	postsynap	7/104	316/18678	0.001945	0.01197	0.006368	ADORA1/I
GO_CC_m	GO:00436	axon termi	4/104	101/18678	0.002449	0.014746	0.007845	ADORA1/(
GO_CC_m	GO:00198	extrinsic c	4/104	102/18678	0.002538	0.014958	0.007958	ANXA1/GI
GO_CC_m	GO:00300	sarcomere	5/104	174/18678	0.002899	0.016728	0.0089	MYBPC3/N
GO_CC_m	GO:00312	extrinsic c	3/104	54/18678	0.003385	0.018876	0.010043	GNA15/H(
GO_CC_m	GO:00058	striated m	2/104	16/18678	0.003502	0.018876	0.010043	TNNC2/TN
GO_CC_m	GO:00426	MHC class	2/104	16/18678	0.003502	0.018876	0.010043	HLA-DPB1

GO_CC_m	GO:00989	glutamater	7/104	352/18678	0.003544	0.018876	0.010043	ADORA2B/
GO_CC_m	GO:00098	cytoplasmi	4/104	114/18678	0.003788	0.019798	0.010533	GNA15/H(
GO_CC_m	GO:00300	lamellipod	5/104	191/18678	0.004313	0.022122	0.011769	APP/ARAP
GO_CC_m	GO:00443(	neuron pr(	4/104	120/18678	0.004547	0.022898	0.012182	ADORA1/(
GO_CC_m	GO:00098	external si	5/104	199/18678	0.005125	0.023939	0.012736	ANXA1/C(
GO_CC_m	GO:00198	extrinsic cc	5/104	199/18678	0.005125	0.023939	0.012736	ANXA1/GN
GO_CC_m	GO:00432(	perikaryon	4/104	127/18678	0.005554	0.023939	0.012736	APP/GNRH
GO_CC_m	GO:00147(	C zone	1/104	1/18678	0.005568	0.023939	0.012736	MYBPC3
GO_CC_m	GO:00345	synaptic ve	1/104	1/18678	0.005568	0.023939	0.012736	PENK
GO_CC_m	GO:00363	TCR signal	1/104	1/18678	0.005568	0.023939	0.012736	LCP2
GO_CC_m	GO:00988	dense core	1/104	1/18678	0.005568	0.023939	0.012736	PENK
GO_CC_m	GO:00990	neuronal c	1/104	1/18678	0.005568	0.023939	0.012736	PENK
GO_CC_m	GO:19900(	neurosecre	1/104	1/18678	0.005568	0.023939	0.012736	GNRH1
GO_CC_m	GO:00140(	postsynap	6/104	290/18678	0.005671	0.023939	0.012736	ADORA1/I
GO_CC_m	GO:00443(	main axon	3/104	65/18678	0.005704	0.023939	0.012736	ADORA1/A
GO_CC_m	GO:00990	postsynap	2/104	21/18678	0.006018	0.024656	0.013117	SRC/YES1
GO_CC_m	GO:00322	asymmetri	6/104	294/18678	0.006053	0.024656	0.013117	ADORA1/I
GO_CC_m	GO:00310	platelet alp	3/104	67/18678	0.006206	0.024913	0.013254	APP/F5/TII
GO_CC_m	GO:00970(	synaptic m	7/104	395/18678	0.006589	0.025377	0.013501	ADORA1/A
GO_CC_m	GO:00329	myosin fila	2/104	22/18678	0.006596	0.025377	0.013501	MYBPC3/M
GO_CC_m	GO:00426	mast cell g	2/104	22/18678	0.006596	0.025377	0.013501	ANXA1/LA
GO_CC_m	GO:00427	presynapti	4/104	141/18678	0.007993	0.030329	0.016136	ADORA1/(
GO_CC_m	GO:00990	integral cc	3/104	74/18678	0.008166	0.030566	0.016262	ADORA1/(
GO_CC_m	GO:00426	MHC prote	2/104	25/18678	0.008474	0.031297	0.016651	HLA-DPB1
GO_CC_m	GO:00059	cell-cell ac	3/104	79/18678	0.009764	0.03541	0.018839	ANXA1/TN
GO_CC_m	GO:00018	phagocyti(	2/104	27/18678	0.009843	0.03541	0.018839	ANXA1/AF
GO_CC_m	GO:00442	axonal grc	2/104	28/18678	0.010562	0.036837	0.019598	MYO9A/TI
GO_CC_m	GO:00059	phosphati(	1/104	2/18678	0.011105	0.036837	0.019598	PIK3R1
GO_CC_m	GO:00380	G protein-	1/104	2/18678	0.011105	0.036837	0.019598	GABBR2
GO_CC_m	GO:00704	Grb2-EGFI	1/104	2/18678	0.011105	0.036837	0.019598	GRB2
GO_CC_m	GO:19900(	granular v	1/104	2/18678	0.011105	0.036837	0.019598	GNRH1
GO_CC_m	GO:00988	intrinsic cc	3/104	83/18678	0.011166	0.036837	0.019598	ADORA1/(
GO_CC_m	GO:00715	integral cc	2/104	29/18678	0.011304	0.036837	0.019598	HLA-DPB1
GO_CC_m	GO:00985	lumenal si	2/104	29/18678	0.011304	0.036837	0.019598	HLA-DPB1
GO_CC_m	GO:00325	trans-Golg	3/104	85/18678	0.011908	0.038353	0.020405	APP/HLA-
GO_CC_m	GO:00306(	endocytic	4/104	161/18678	0.01256	0.03999	0.021276	HLA-DPB1
GO_CC_m	GO:00310	platelet alp	3/104	91/18678	0.0143	0.044508	0.023679	APP/F5/TII
GO_CC_m	GO:00325	ruffle mem	3/104	91/18678	0.0143	0.044508	0.023679	SRC/TIAM
GO_CC_m	GO:00017	ruffle	4/104	168/18678	0.014484	0.044578	0.023716	FGD4/SRC
GO_CC_m	GO:00301	coated ves	5/104	261/18678	0.01539	0.046847	0.024923	APP/DVL1
GO_CC_m	GO:00081	COP9 sign	2/104	35/18678	0.016212	0.048438	0.02577	GRB2/LAT
GO_CC_m	GO:00058(	striated mu	1/104	3/18678	0.016612	0.048438	0.02577	MYBPC3
GO_CC_m	GO:00971	centralspir	1/104	3/18678	0.016612	0.048438	0.02577	ECT2
GO_CC_m	GO:19907(	growth co	1/104	3/18678	0.016612	0.048438	0.02577	APP
GO_CC_m	GO:00431	terminal b	2/104	37/18678	0.018016	0.051984	0.027656	ADORA1/N
GO_CC_m	GO:00306(	Golgi-assc	3/104	102/18678	0.019347	0.055249	0.029393	HLA-DPB1
GO_CC_m	GO:00306(	clathrin-cc	2/104	40/18678	0.020874	0.059	0.031389	HLA-DPB1
GO_CC_m	GO:00163(	nuclear ma	3/104	106/18678	0.021396	0.059267	0.031531	GHRHR/PF
GO_CC_m	GO:00448	plasma me	3/104	106/18678	0.021396	0.059267	0.031531	HCK/LCP2
GO_CC_m	GO:19905	perinuclea	1/104	4/18678	0.022089	0.059986	0.031913	PIK3R1
GO_CC_m	GO:19908	growth co	1/104	4/18678	0.022089	0.059986	0.031913	APP
GO_CC_m	GO:00301	endocytic	5/104	289/18678	0.022792	0.061295	0.03261	HLA-DPB1
GO_CC_m	GO:00432	receptor c	5/104	292/18678	0.023701	0.062705	0.03336	ADRA2A/A
GO_CC_m	GO:00325	neuron pr(	2/104	43/18678	0.023907	0.062705	0.03336	ADORA1/(
GO_CC_m	GO:00058(	trans-Golg	4/104	196/18678	0.023995	0.062705	0.03336	APP/GPER
GO_CC_m	GO:00163	basolatera	4/104	201/18678	0.026012	0.067341	0.035826	ADORA1/A
GO_CC_m	GO:00976	phosphati(	1/104	5/18678	0.027535	0.069338	0.036889	PIK3R1
GO_CC_m	GO:00985	cytoplasmi	1/104	5/18678	0.027535	0.069338	0.036889	GNRH1

GO_CC_m	GO:00991	extrinsic co	1/104	5/18678	0.027535	0.069338	0.036889	TIAM1
GO_CC_m	GO:00057	endoplasm	5/104	306/18678	0.028251	0.070501	0.037508	APP/F2/F5
GO_CC_m	GO:00056	nuclear en	6/104	420/18678	0.030002	0.074201	0.039476	APP/EDNF
GO_CC_m	GO:00312	cell projec	5/104	317/18678	0.032192	0.077351	0.041152	ADORA1/(
GO_CC_m	GO:00423	sarcolemm	3/104	125/18678	0.032691	0.077351	0.041152	ANXA1/GH
GO_CC_m	GO:00380	G protein-	1/104	6/18678	0.032951	0.077351	0.041152	GABBR2
GO_CC_m	GO:00443	dendritic s	1/104	6/18678	0.032951	0.077351	0.041152	GPER1
GO_CC_m	GO:00988	extrinsic co	1/104	6/18678	0.032951	0.077351	0.041152	TIAM1
GO_CC_m	GO:00989	neuronal c	1/104	6/18678	0.032951	0.077351	0.041152	PENK
GO_CC_m	GO:00343	nuclear pe	3/104	126/18678	0.033357	0.077645	0.041308	GHRHR/PF
GO_CC_m	GO:00453	phagocytic	3/104	128/18678	0.034709	0.08012	0.042625	INPP5B/RA
GO_CC_m	GO:00125	ER to Golg	2/104	55/18678	0.037664	0.086222	0.045872	HLA-DPB1
GO_CC_m	GO:00708	cell body f	1/104	7/18678	0.038337	0.086337	0.045933	PENK
GO_CC_m	GO:00975	cardiac my	1/104	7/18678	0.038337	0.086337	0.045933	MYBPC3
GO_CC_m	GO:00987	plasma me	6/104	451/18678	0.040267	0.089952	0.047856	GABBR2/G
GO_CC_m	GO:00163	lateral plas	2/104	58/18678	0.041479	0.091002	0.048414	ANXA1/DV
GO_CC_m	GO:00453	clathrin-co	2/104	59/18678	0.042782	0.091002	0.048414	HLA-DPB1
GO_CC_m	GO:00058	heterotrim	1/104	8/18678	0.043694	0.091002	0.048414	GNA15
GO_CC_m	GO:00312	extrinsic co	1/104	8/18678	0.043694	0.091002	0.048414	ANXA1
GO_CC_m	GO:00313	extrinsic co	1/104	8/18678	0.043694	0.091002	0.048414	ANXA1
GO_CC_m	GO:00976	G protein-	1/104	8/18678	0.043694	0.091002	0.048414	GABBR2
GO_CC_m	GO:00988	extrinsic co	1/104	8/18678	0.043694	0.091002	0.048414	TIAM1
GO_CC_m	GO:19027	GABA rece	1/104	8/18678	0.043694	0.091002	0.048414	GABBR2
GO_CC_m	GO:19053	GTPase co	1/104	8/18678	0.043694	0.091002	0.048414	GNA15
GO_CC_m	GO:00301	transport v	5/104	349/18678	0.045546	0.094151	0.05009	APP/HCK/
GO_CC_m	GO:00325	integral co	2/104	63/18678	0.048143	0.098783	0.052554	CNR1/RHC
GO_CC_m	GO:00056	nuclear en	1/104	9/18678	0.049021	0.099844	0.053119	APP
GO_CC_m	GO:00985	intrinsic co	2/104	64/18678	0.04952	0.100124	0.053268	CNR1/RHC
GO_CC_m	GO:00444	ciliary part	7/55	415/18678	0.000206	0.001132	0.000582	CEP78/CN
GO_CC_m	GO:00166	PML body	4/55	99/18678	0.000206	0.001132	0.000582	RPA2/TP5
GO_CC_m	GO:00058	spindle	6/55	326/18678	0.000381	0.002042	0.001049	CDK5RAP2
GO_CC_m	GO:00007	nuclear ch	4/55	121/18678	0.000443	0.002314	0.001189	ERCC1/PA
GO_CC_m	GO:00058	spindle mi	3/55	53/18678	0.00051	0.002599	0.001335	KIF11/KIF3
GO_CC_m	GO:19041	axon cytop	3/55	54/18678	0.000539	0.002681	0.001377	KIF3A/KIF4
GO_CC_m	GO:00058	microtubu	6/55	357/18678	0.000615	0.002992	0.001537	CDK5RAP2
GO_CC_m	GO:00002	pericentric	2/55	20/18678	0.001563	0.007427	0.003815	CDK5RAP2
GO_CC_m	GO:00304	midbody	4/55	171/18678	0.001608	0.007469	0.003837	CNTRL/KIF
GO_CC_m	GO:01201	neuron pro	3/55	80/18678	0.001691	0.007683	0.003947	KIF3A/KIF4
GO_CC_m	GO:00328	plasma me	4/55	181/18678	0.001979	0.008801	0.004521	KIF3A/KIF4
GO_CC_m	GO:00226	proteasom	2/55	23/18678	0.00207	0.009014	0.004631	PSMD9/PS
GO_CC_m	GO:00058	microtubu	3/55	92/18678	0.002521	0.010753	0.005524	ACTR1A/D
GO_CC_m	GO:00007	condensed	4/55	199/18678	0.00279	0.011397	0.005855	ANAPC16/
GO_CC_m	GO:00313	Rad17 RFC	1/55	1/18678	0.002945	0.011397	0.005855	RAD17
GO_CC_m	GO:00324	extrinsic co	1/55	1/18678	0.002945	0.011397	0.005855	RAB7A
GO_CC_m	GO:00436	delta DNA	1/55	1/18678	0.002945	0.011397	0.005855	POLD3
GO_CC_m	GO:19903	sumoylate	1/55	1/18678	0.002945	0.011397	0.005855	UBE2I
GO_CC_m	GO:00007	condensed	3/55	100/18678	0.003193	0.012133	0.006233	ANAPC16/
GO_CC_m	GO:00007	condensed	3/55	110/18678	0.004174	0.015579	0.008003	ANAPC16/
GO_CC_m	GO:00001	nucleotide	1/55	2/18678	0.005881	0.021191	0.010886	ERCC1
GO_CC_m	GO:00971	cyclin A1-(	1/55	2/18678	0.005881	0.021191	0.010886	CCNA1
GO_CC_m	GO:00995	cytoplasm	5/55	402/18678	0.006485	0.022974	0.011802	ACTR1A/K
GO_CC_m	GO:00007	kinetochor	3/55	131/18678	0.006781	0.023621	0.012134	ANAPC16/
GO_CC_m	GO:00975	ciliary tip	2/55	45/18678	0.007772	0.026629	0.01368	KIF3A/KIF3
GO_CC_m	GO:00003	extrinsic co	1/55	3/18678	0.008808	0.026681	0.013706	RAB7A
GO_CC_m	GO:00004	core TFIIH	1/55	3/18678	0.008808	0.026681	0.013706	GTF2H3
GO_CC_m	GO:00085	proteasom	1/55	3/18678	0.008808	0.026681	0.013706	PSME1
GO_CC_m	GO:00085	proteasom	1/55	3/18678	0.008808	0.026681	0.013706	PSMD9
GO_CC_m	GO:00169	kinesin II c	1/55	3/18678	0.008808	0.026681	0.013706	KIF3A

GO_CC_m	GO:00313	Elg1 RFC-l	1/55	3/18678	0.008808	0.026681	0.013706	RFC1
GO_CC_m	GO:00443	dendritic b	1/55	3/18678	0.008808	0.026681	0.013706	AKAP9
GO_CC_m	GO:00971	cyclin A2-	1/55	3/18678	0.008808	0.026681	0.013706	CCNA1
GO_CC_m	GO:00009	spindle po	3/55	152/18678	0.010177	0.030385	0.015609	CDK5RAP2
GO_CC_m	GO:00321	cleavage f	2/55	55/18678	0.011441	0.033576	0.017249	KIF20A/PL
GO_CC_m	GO:00332	I-kappaB/I	1/55	4/18678	0.011728	0.033576	0.017249	NFKBIA
GO_CC_m	GO:00705	ERCC4-ER	1/55	4/18678	0.011728	0.033576	0.017249	ERCC1
GO_CC_m	GO:00321	cell divisio	2/55	57/18678	0.012251	0.034139	0.017538	KIF20A/PL
GO_CC_m	GO:00321	cell divisio	2/55	57/18678	0.012251	0.034139	0.017538	KIF20A/PL
GO_CC_m	GO:00056	DNA replic	1/55	5/18678	0.014638	0.038243	0.019646	RFC1
GO_CC_m	GO:00314	Cul4B-RIN	1/55	5/18678	0.014638	0.038243	0.019646	DDB2
GO_CC_m	GO:00985	deuteroso	1/55	5/18678	0.014638	0.038243	0.019646	PLK4
GO_CC_m	GO:00991	extrinsic c	1/55	5/18678	0.014638	0.038243	0.019646	AKAP9
GO_CC_m	GO:19901	spermatop	1/55	5/18678	0.014638	0.038243	0.019646	PSMB9
GO_CC_m	GO:00007	chromosoı	3/55	185/18678	0.017206	0.043643	0.02242	ANAPC16/
GO_CC_m	GO:00004	transcripti	1/55	6/18678	0.017541	0.043643	0.02242	GTF2H3
GO_CC_m	GO:00975	transcripti	1/55	6/18678	0.017541	0.043643	0.02242	GTF2H3
GO_CC_m	GO:00988	extrinsic c	1/55	6/18678	0.017541	0.043643	0.02242	AKAP9
GO_CC_m	GO:00058	dynactin c	1/55	7/18678	0.020435	0.050052	0.025713	ACTR1A
GO_CC_m	GO:00007	condensed	2/55	75/18678	0.020596	0.050052	0.025713	NEK2/UBE
GO_CC_m	GO:00009	gamma-tu	1/55	8/18678	0.02332	0.053559	0.027515	CDK5RAP2
GO_CC_m	GO:00021	manchette	1/55	8/18678	0.02332	0.053559	0.027515	ACTR1A
GO_CC_m	GO:00059	cAMP-dep	1/55	8/18678	0.02332	0.053559	0.027515	PRKACA
GO_CC_m	GO:00082	gamma-tu	1/55	8/18678	0.02332	0.053559	0.027515	CDK5RAP2
GO_CC_m	GO:00988	extrinsic c	1/55	8/18678	0.02332	0.053559	0.027515	AKAP9
GO_CC_m	GO:01201	centriolar	1/55	9/18678	0.026197	0.059513	0.030574	CNTRL
GO_CC_m	GO:00058	proteasom	1/55	10/18678	0.029066	0.064626	0.0332	PSMD9
GO_CC_m	GO:00352	ciliary root	1/55	10/18678	0.029066	0.064626	0.0332	KIF5B
GO_CC_m	GO:00726	mitotic spi	2/55	97/18678	0.03317	0.072973	0.037488	CDK5RAP2
GO_CC_m	GO:00058	kinesin cor	1/55	12/18678	0.034779	0.074936	0.038497	KIF3A
GO_CC_m	GO:00992	extrinsic c	1/55	12/18678	0.034779	0.074936	0.038497	AKAP9
GO_CC_m	GO:00017	XY body	1/55	13/18678	0.037623	0.079427	0.040803	PLK4
GO_CC_m	GO:00425	DNA polyr	1/55	13/18678	0.037623	0.079427	0.040803	POLD3
GO_CC_m	GO:00163	nuclear ma	2/55	106/18678	0.038989	0.081307	0.041769	KIF4A/TP5
GO_CC_m	GO:00331	melanosor	1/55	14/18678	0.040459	0.081307	0.041769	RAB7A
GO_CC_m	GO:00340	phagopho	1/55	14/18678	0.040459	0.081307	0.041769	RAB7A
GO_CC_m	GO:00450	chitosome	1/55	14/18678	0.040459	0.081307	0.041769	RAB7A
GO_CC_m	GO:00907	pigment g	1/55	14/18678	0.040459	0.081307	0.041769	RAB7A
GO_CC_m	GO:00360	ciliary basa	2/55	111/18678	0.042378	0.084352	0.043334	CEP78/TTE
GO_CC_m	GO:00353	microtubu	1/55	16/18678	0.046106	0.090907	0.046701	CDK5RAP2
GO_CC_m	GO:00058	cytoplasm	1/55	17/18678	0.048917	0.095549	0.049086	DYNC1H1
GO_CC_m	GO:00905	Flemming	2月11日	28/18678	0.000118	0.000245	9.78E-05	CHMP4C/
GO_CC_m	GO:00057	multivesicu	1月11日	41/18678	0.023889	0.043969	0.017556	TSG101
GO_CC_m	GO:00056	nuclear en	2月11日	420/18678	0.024259	0.043969	0.017556	CHMP4B/C
GO_CC_m	GO:00301	membrane	1月11日	86/18678	0.049511	0.079767	0.031849	CHMP4B
GO_CC_m	GO:00484	coated me	1月11日	86/18678	0.049511	0.079767	0.031849	CHMP4B
GO_MF_m	GO:00037	mRNA 3'-l	5/122	67/16969	0.00012	0.002253	0.001524	DDX5/ELA
GO_MF_m	GO:00037	single-stra	5/122	68/16969	0.000128	0.002253	0.001524	HNRNPA1
GO_MF_m	GO:00422	ISG15 tran	2/122	3/16969	0.000153	0.002273	0.001537	HERC5/UB
GO_MF_m	GO:00508	phosphoth	2/122	3/16969	0.000153	0.002273	0.001537	FBXW7/NE
GO_MF_m	GO:00551	ubiquitin-	3/122	16/16969	0.00019	0.002614	0.001768	CDC20/FB
GO_MF_m	GO:00452	cadherin b	9/122	297/16969	0.000296	0.003808	0.002575	EIF2S3/EIF
GO_MF_m	GO:00082	poly(U) RN	3/122	20/16969	0.000378	0.00456	0.003084	HNRNPC/I
GO_MF_m	GO:00081	poly-pyrin	3/122	22/16969	0.000505	0.005135	0.003473	HNRNPC/I
GO_MF_m	GO:00037	translation	2/122	5/16969	0.000505	0.005135	0.003473	ETF1/GSPT
GO_MF_m	GO:00080	translation	2/122	5/16969	0.000505	0.005135	0.003473	ETF1/GSPT
GO_MF_m	GO:00313	translation	3/122	23/16969	0.000578	0.005578	0.003773	EIF3B/EIF3
GO_MF_m	GO:00198	sodium ch	2/122	6/16969	0.000755	0.006936	0.004691	NEDD4/NE

GO_MF_m	GO:00430	ribonuclec	5/122	107/16969	0.001048	0.008824	0.005968	DDX5/EIF3
GO_MF_m	GO:00970	ubiquitin-|	2/122	7/16969	0.001052	0.008824	0.005968	CDC20/FB
GO_MF_m	GO:00043	helicase ac	5/122	128/16969	0.002316	0.018623	0.012595	AQR/DDX
GO_MF_m	GO:00080	5S rRNA b	2/122	11/16969	0.002703	0.020864	0.014111	RPL11/RPL
GO_MF_m	GO:00160	cyclosporin	2/122	13/16969	0.003797	0.028185	0.019062	PPIE/PPIH
GO_MF_m	GO:00508	cell adhesi	9/122	444/16969	0.004782	0.033699	0.02279	EIF2S3/EIF
GO_MF_m	GO:00451	translation	3/122	48/16969	0.004955	0.033699	0.02279	PABPC1/R
GO_MF_m	GO:00081	poly(A) bir	2/122	15/16969	0.005064	0.033699	0.02279	PABPC1/P
GO_MF_m	GO:00197	FAT10 acti	1/122	1/16969	0.00719	0.041297	0.02793	UBA6
GO_MF_m	GO:00323	oxidized p	1/122	1/16969	0.00719	0.041297	0.02793	RPS3
GO_MF_m	GO:00701	large ribos	1/122	1/16969	0.00719	0.041297	0.02793	RPL23A
GO_MF_m	GO:19048	TORC2 cor	1/122	1/16969	0.00719	0.041297	0.02793	RPL23A
GO_MF_m	GO:00700	proline-ric	2/122	18/16969	0.007275	0.041297	0.02793	NEDD4/W
GO_MF_m	GO:00707	poly-purir	2/122	21/16969	0.009846	0.054295	0.03672	PABPC1/P
GO_MF_m	GO:00355	MH2 dom	1/122	2/16969	0.014328	0.072771	0.049215	DDX5
GO_MF_m	GO:00617	telomeric r	1/122	2/16969	0.014328	0.072771	0.049215	HNRNPA1
GO_MF_m	GO:00988	mRNA cap	1/122	2/16969	0.014328	0.072771	0.049215	EIF3D
GO_MF_m	GO:00037	peptidyl-p	2/122	26/16969	0.014887	0.072798	0.049234	PPIE/PPIH
GO_MF_m	GO:00463	SMAD bin	3/122	72/16969	0.015088	0.072798	0.049234	DDX5/SMU
GO_MF_m	GO:00168	cis-trans is	2/122	28/16969	0.017154	0.078825	0.053309	PPIE/PPIH
GO_MF_m	GO:00170	sodium ch	2/122	28/16969	0.017154	0.078825	0.053309	NEDD4/NE
GO_MF_m	GO:00616	ubiquitin p	4/122	139/16969	0.018013	0.080847	0.054677	LRSAM1/N
GO_MF_m	GO:00616	ubiquitin-l	4/122	145/16969	0.020685	0.086107	0.058235	LRSAM1/N
GO_MF_m	GO:00351	miRNA bir	2/122	31/16969	0.020809	0.086107	0.058235	ELAVL1/HI
GO_MF_m	GO:00316	beta-2 adr	1/122	3/16969	0.021415	0.086107	0.058235	NEDD4
GO_MF_m	GO:00353	selenocyst	1/122	3/16969	0.021415	0.086107	0.058235	RPL30
GO_MF_m	GO:00701	small ribos	1/122	3/16969	0.021415	0.086107	0.058235	RPS3
GO_MF_m	GO:00037	RNA helica	2/122	34/16969	0.024757	0.095564	0.06463	DDX5/DH
GO_MF_m	GO:00421	telomeric l	2/122	34/16969	0.024757	0.095564	0.06463	HNRNPA1
GO_MF_m	GO:00082	ion channe	2/122	35/16969	0.026136	0.098142	0.066374	NEDD4/NE
GO_MF_m	GO:00162	channel in	2/122	36/16969	0.027546	0.098142	0.066374	NEDD4/NE
GO_MF_m	GO:00306	U1 snRNA	1/122	4/16969	0.028452	0.098142	0.066374	SNRPA
GO_MF_m	GO:00445	DNA topo	1/122	4/16969	0.028452	0.098142	0.066374	SRSF1
GO_MF_m	GO:00508	phosphose	1/122	4/16969	0.028452	0.098142	0.066374	NEDD4
GO_MF_m	GO:00325	ribonuclec	6/122	322/16969	0.028877	0.098142	0.066374	EFTUD2/EI
GO_MF_m	GO:00619	regulatory	2/122	37/16969	0.028985	0.098142	0.066374	ELAVL1/HI
GO_MF_m	GO:00018	nucleoside	6/122	329/16969	0.031603	0.105162	0.071121	EFTUD2/EI
GO_MF_m	GO:00198	potassium	1/122	5/16969	0.035439	0.112126	0.075831	NEDD4L
GO_MF_m	GO:00971	supercoile	1/122	5/16969	0.035439	0.112126	0.075831	RPS3
GO_MF_m	GO:19909	ubiquitin li	1/122	5/16969	0.035439	0.112126	0.075831	RPL11
GO_MF_m	GO:00453	protein ph	2/122	44/16969	0.039859	0.122108	0.082582	FBXW7/NE
GO_MF_m	GO:00506	androgen	2/122	44/16969	0.039859	0.122108	0.082582	DDX5/SNW
GO_MF_m	GO:00551	ubiquitin-|	1/122	6/16969	0.042376	0.125823	0.085094	RPL11
GO_MF_m	GO:19904	U1 snRNP	1/122	6/16969	0.042376	0.125823	0.085094	SNRPA
GO_MF_m	GO:00430	ribosome l	2/122	47/16969	0.044922	0.131003	0.088598	EIF3K/ETF
GO_MF_m	GO:00316	ubiquitin p	5/122	270/16969	0.045478	0.131003	0.088598	FBXW7/HE
GO_MF_m	GO:00084	translation	1/122	7/16969	0.049263	0.137794	0.09319	PABPC1
GO_MF_m	GO:19902	N6-methy	1/122	7/16969	0.049263	0.137794	0.09319	HNRNPC
GO_MF_m	GO:00039	DNA topo	1月19日	2/16969	0.002238	0.034319	0.021989	TOP2A
GO_MF_m	GO:00615	DNA topo	1月19日	2/16969	0.002238	0.034319	0.021989	TOP2A
GO_MF_m	GO:00039	DNA topo	1月19日	5/16969	0.005587	0.064246	0.041164	TOP2A
GO_MF_m	GO:00050	nuclear ex	1月19日	8/16969	0.008924	0.076931	0.049292	NUP214
GO_MF_m	GO:00434	protein me	1月19日	9/16969	0.010035	0.076931	0.049292	NUP153
GO_MF_m	GO:00974	cyclin-dep	1月19日	15/16969	0.016671	0.09007	0.05771	CCNA2
GO_MF_m	GO:00083	DNA bindi	1月19日	19/16969	0.021072	0.09007	0.05771	TOP2A
GO_MF_m	GO:00042	threonine-	1月19日	21/16969	0.023266	0.09007	0.05771	PSMA5
GO_MF_m	GO:00700	threonine-	1月19日	21/16969	0.023266	0.09007	0.05771	PSMA5
GO_MF_m	GO:00510	mitogen-a	1月19日	22/16969	0.024361	0.09007	0.05771	TPR

GO_MF_m	GO:00708	dynein cor	1月19日	22/16969	0.024361	0.09007	0.05771	TPR
GO_MF_m	GO:01401	nucleocytc	1月19日	23/16969	0.025454	0.09007	0.05771	NUP214
GO_MF_m	GO:00085	Ran GTPas	1月19日	28/16969	0.030906	0.101549	0.065066	NXT1
GO_MF_m	GO:00316	ubiquitin p	2月19日	270/16969	0.036098	0.108468	0.069499	AURKA/PS
GO_MF_m	GO:01401	molecular	1月19日	36/16969	0.039569	0.108468	0.069499	NUP214
GO_MF_m	GO:00443	ubiquitin-l	2月19日	286/16969	0.040086	0.108468	0.069499	AURKA/PS
GO_MF_m	GO:00047	protein sei	1月19日	40/16969	0.043873	0.112119	0.071838	AURKA
GO_MF_m	GO:00020	opsin bind	1月10日	3/16969	0.001767	0.010602	0.00651	IFT20
GO_MF_m	GO:00156	tubulin bir	2月10日	252/16969	0.009138	0.036552	0.022444	TRAF3IP1/
GO_MF_m	GO:00484	beta-tubu	1月10日	38/16969	0.022175	0.060144	0.03693	IFT74
GO_MF_m	GO:00037	microtubu	1月10日	43/16969	0.02506	0.060144	0.03693	DYNLRB1
GO_MF_m	GO:00451	bioactive l	3/129	13/16969	0.000116	0.003112	0.00208	S1PR1/S1F
GO_MF_m	GO:00036	chromatin	12/129	437/16969	0.000126	0.003112	0.00208	ASH2L/CC
GO_MF_m	GO:01400	catalytic ac	7/129	152/16969	0.000162	0.003112	0.00208	DNMT1/D
GO_MF_m	GO:00038	DNA (cyto	2/129	3/16969	0.000171	0.003112	0.00208	DNMT1/D
GO_MF_m	GO:01024	dATP phos	2/129	3/16969	0.000171	0.003112	0.00208	ENTPD1/E
GO_MF_m	GO:01024	dCTP phos	2/129	3/16969	0.000171	0.003112	0.00208	ENTPD1/E
GO_MF_m	GO:01024	dUTP phos	2/129	3/16969	0.000171	0.003112	0.00208	ENTPD1/E
GO_MF_m	GO:01024	dTTP phos	2/129	3/16969	0.000171	0.003112	0.00208	ENTPD1/E
GO_MF_m	GO:01024	GTP phosp	2/129	3/16969	0.000171	0.003112	0.00208	ENTPD1/E
GO_MF_m	GO:01024	8-oxo-dG	2/129	3/16969	0.000171	0.003112	0.00208	ENTPD1/E
GO_MF_m	GO:01024	dGTP phos	2/129	3/16969	0.000171	0.003112	0.00208	ENTPD1/E
GO_MF_m	GO:00306	protein bir	7/129	156/16969	0.00019	0.003302	0.002207	ABI2/ARPC
GO_MF_m	GO:00080	DNA-depe	5/129	72/16969	0.000219	0.003577	0.002391	NBN/RAD5
GO_MF_m	GO:00004	four-way j	3/129	16/16969	0.000224	0.003577	0.002391	RAD51B/W
GO_MF_m	GO:00600	molecular	8/129	216/16969	0.000247	0.003799	0.002539	ABI2/ARPC
GO_MF_m	GO:00168	phosphoru	3/129	17/16969	0.00027	0.004	0.002673	ADCY3/AD
GO_MF_m	GO:00099	cyclase act	3/129	18/16969	0.000322	0.004603	0.003076	ADCY3/AD
GO_MF_m	GO:00090	DNA-meth	2/129	5/16969	0.000565	0.007532	0.005035	DNMT1/D
GO_MF_m	GO:00164	C-C chem	2/129	5/16969	0.000565	0.007532	0.005035	CCR6/CCR
GO_MF_m	GO:00002	DNA secor	3/129	23/16969	0.00068	0.008775	0.005866	RAD51B/W
GO_MF_m	GO:00199	C-X-C che	2/129	6/16969	0.000843	0.01054	0.007045	ACKR3/CX
GO_MF_m	GO:00016	G protein-	7/129	205/16969	0.000978	0.011851	0.007922	ARRB2/CX
GO_MF_m	GO:00036	damaged	4/129	60/16969	0.001118	0.013159	0.008795	DDB1/NBN
GO_MF_m	GO:00973	7SK snRN	2/129	7/16969	0.001175	0.013424	0.008973	CCNT1/CC
GO_MF_m	GO:00700	RNA polyr	4/129	63/16969	0.001343	0.014917	0.009971	BIN1/CCN
GO_MF_m	GO:00040	ATP-depe	3/129	31/16969	0.001651	0.017852	0.011933	NBN/RUVI
GO_MF_m	GO:00054	SNAP rece	2/129	9/16969	0.001994	0.020985	0.014027	GOSR1/ST
GO_MF_m	GO:00150	corecepto	3/129	40/16969	0.003453	0.03542	0.023676	ACKR3/CD
GO_MF_m	GO:00036	DNA helica	3/129	42/16969	0.003969	0.039687	0.026528	NBN/RUVI
GO_MF_m	GO:00080	cytoskelet	2/129	13/16969	0.004234	0.040326	0.026955	ABI2/NCK
GO_MF_m	GO:00428	histone me	2/129	13/16969	0.004234	0.040326	0.026955	ASH2L/KM
GO_MF_m	GO:00045	nucleoside	2/129	14/16969	0.004915	0.044739	0.029905	AK9/NME
GO_MF_m	GO:00036	single-stra	4/129	90/16969	0.004921	0.044739	0.029905	BRCA2/NA
GO_MF_m	GO:00426	ATPase ac	6/129	218/16969	0.006451	0.046073	0.030796	MYO10/N
GO_MF_m	GO:00483	Rac GTPas	3/129	51/16969	0.006851	0.046073	0.030796	ABI2/DVL
GO_MF_m	GO:00080	ATP-depe	3/129	52/16969	0.007231	0.046073	0.030796	NBN/RUVI
GO_MF_m	GO:00700	purine NTI	3/129	52/16969	0.007231	0.046073	0.030796	NBN/RUVI
GO_MF_m	GO:00314	myosin V	2/129	17/16969	0.007238	0.046073	0.030796	RAB11A/R
GO_MF_m	GO:00010	TFIIF-class	1/129	1/16969	0.007602	0.046073	0.030796	CTDP1
GO_MF_m	GO:00048	compleme	1/129	1/16969	0.007602	0.046073	0.030796	C3AR1
GO_MF_m	GO:00049	N-formyl	1/129	1/16969	0.007602	0.046073	0.030796	FPR1
GO_MF_m	GO:00169	flavin-link	1/129	1/16969	0.007602	0.046073	0.030796	QSOX1
GO_MF_m	GO:00318	type 2A se	1/129	1/16969	0.007602	0.046073	0.030796	ARRB2
GO_MF_m	GO:00318	platelet ac	1/129	1/16969	0.007602	0.046073	0.030796	ARRB2
GO_MF_m	GO:00357	chemokine	1/129	1/16969	0.007602	0.046073	0.030796	CCR7
GO_MF_m	GO:00357	chemokine	1/129	1/16969	0.007602	0.046073	0.030796	CCR7
GO_MF_m	GO:00381	C-C motif	1/129	1/16969	0.007602	0.046073	0.030796	CCR7

GO_MF_m	GO:00381	C-C motif	1/129	1/16969	0.007602	0.046073	0.030796	CCR7
GO_MF_m	GO:00420	interleukin	1/129	1/16969	0.007602	0.046073	0.030796	CD4
GO_MF_m	GO:00420	interleukin	1/129	1/16969	0.007602	0.046073	0.030796	CD4
GO_MF_m	GO:00430	chromatin	1/129	1/16969	0.007602	0.046073	0.030796	CTCF
GO_MF_m	GO:00700	collagen V	1/129	1/16969	0.007602	0.046073	0.030796	THBS1
GO_MF_m	GO:00703	3'-flap-str	1/129	1/16969	0.007602	0.046073	0.030796	WRN
GO_MF_m	GO:00709	FHA doma	1/129	1/16969	0.007602	0.046073	0.030796	MDC1
GO_MF_m	GO:19901	euchroma	1/129	1/16969	0.007602	0.046073	0.030796	ASH2L
GO_MF_m	GO:00010	RNA polyr	2/129	18/16969	0.008102	0.048371	0.032332	CTDP1/RU
GO_MF_m	GO:00302	protein tyr	2/129	19/16969	0.00901	0.053003	0.035428	ABI1/GAS
GO_MF_m	GO:00010	basal trans	3/129	60/16969	0.010716	0.061234	0.04093	CDC73/CT
GO_MF_m	GO:00010	basal RNA	3/129	60/16969	0.010716	0.061234	0.04093	CDC73/CT
GO_MF_m	GO:00507	low-densit	2/129	21/16969	0.010956	0.061727	0.041259	CLU/LDLR
GO_MF_m	GO:00301	receptor si	2/129	22/16969	0.011993	0.065713	0.043924	LDLRAP1/
GO_MF_m	GO:00430	gamma-tu	2/129	22/16969	0.011993	0.065713	0.043924	B9D2/BRC
GO_MF_m	GO:00001	SNARE bir	3/129	66/16969	0.013867	0.068076	0.045503	RAB11A/S
GO_MF_m	GO:00019	fibronectir	2/129	24/16969	0.014188	0.068076	0.045503	THBS1/VE
GO_MF_m	GO:00047	pyruvate k	1/129	2/16969	0.015147	0.068076	0.045503	PKM
GO_MF_m	GO:00081	hedgehog	1/129	2/16969	0.015147	0.068076	0.045503	B9D1
GO_MF_m	GO:00093	four-way j	1/129	2/16969	0.015147	0.068076	0.045503	WRN
GO_MF_m	GO:00169	thiol oxida	1/129	2/16969	0.015147	0.068076	0.045503	QSOX1
GO_MF_m	GO:00316	alpha-1A	1/129	2/16969	0.015147	0.068076	0.045503	ARRB2
GO_MF_m	GO:00317	follicle-stir	1/129	2/16969	0.015147	0.068076	0.045503	ARRB2
GO_MF_m	GO:00354	histone kir	1/129	2/16969	0.015147	0.068076	0.045503	CHEK1
GO_MF_m	GO:00431	vascular er	1/129	2/16969	0.015147	0.068076	0.045503	VEGFA
GO_MF_m	GO:00617	forked DN	1/129	2/16969	0.015147	0.068076	0.045503	WRN
GO_MF_m	GO:00618	telomeric	1/129	2/16969	0.015147	0.068076	0.045503	WRN
GO_MF_m	GO:00700	titin Z dom	1/129	2/16969	0.015147	0.068076	0.045503	ACTN2
GO_MF_m	GO:00703	flap-struct	1/129	2/16969	0.015147	0.068076	0.045503	WRN
GO_MF_m	GO:00710	eukaryotic	1/129	2/16969	0.015147	0.068076	0.045503	NCK1
GO_MF_m	GO:00987	FBXO fami	1/129	2/16969	0.015147	0.068076	0.045503	CDK6
GO_MF_m	GO:00703	lipoproteir	2/129	26/16969	0.016543	0.073523	0.049145	CLU/LDLR
GO_MF_m	GO:00303	cyclin binc	2/129	28/16969	0.019052	0.083744	0.055976	CDK12/CD
GO_MF_m	GO:00087	S-adenosy	4/129	136/1696	0.020137	0.085736	0.057308	ASH2L/DN
GO_MF_m	GO:00168	ATPase ac	6/129	280/1696	0.020178	0.085736	0.057308	MYO10/N
GO_MF_m	GO:00329	protein-cc	2/129	29/16969	0.020362	0.085736	0.057308	LDLRAP1/
GO_MF_m	GO:00420	chemoattr	2/129	29/16969	0.020362	0.085736	0.057308	HGF/VEGF
GO_MF_m	GO:00080	chemokine	2/129	30/16969	0.02171	0.086227	0.057636	CXCL3/CX
GO_MF_m	GO:00043	fructose-b	1/129	3/16969	0.022635	0.086227	0.057636	ALDOA
GO_MF_m	GO:00046	DNA-depe	1/129	3/16969	0.022635	0.086227	0.057636	PRKDC
GO_MF_m	GO:00316	alpha-1B	1/129	3/16969	0.022635	0.086227	0.057636	ARRB2
GO_MF_m	GO:00318	G protein-	1/129	3/16969	0.022635	0.086227	0.057636	ARRB2
GO_MF_m	GO:00513	FATZ bind	1/129	3/16969	0.022635	0.086227	0.057636	ACTN2
GO_MF_m	GO:00600	plus-end	1/129	3/16969	0.022635	0.086227	0.057636	MYO10
GO_MF_m	GO:00700	fibrinogen	1/129	3/16969	0.022635	0.086227	0.057636	THBS1
GO_MF_m	GO:00709	type 1 mel	1/129	3/16969	0.022635	0.086227	0.057636	POMC
GO_MF_m	GO:19057	8-hydroxy	1/129	3/16969	0.022635	0.086227	0.057636	WRN
GO_MF_m	GO:19907	protein tyr	3/129	80/16969	0.023068	0.087049	0.058185	CD4/GAS
GO_MF_m	GO:00510	actin filam	4/129	143/1696	0.023697	0.088588	0.059214	ACTN2/A
GO_MF_m	GO:00170	Rho GTPa	4/129	146/1696	0.025333	0.093826	0.062716	ABI2/DVL
GO_MF_m	GO:00167	phosphotr	2/129	33/16969	0.025965	0.095286	0.063691	AK9/NME
GO_MF_m	GO:00048	endopepti	4/129	149/1696	0.027036	0.095448	0.063799	GAS6/ITIH
GO_MF_m	GO:00017	phosphati	2/129	35/16969	0.028974	0.095448	0.063799	GAS6/THB
GO_MF_m	GO:00302	clathrin bir	2/129	35/16969	0.028974	0.095448	0.063799	BIN1/LDL
GO_MF_m	GO:00435	ADP bindi	2/129	35/16969	0.028974	0.095448	0.063799	PKM/RUV
GO_MF_m	GO:00611	endopepti	4/129	154/1696	0.030022	0.095448	0.063799	GAS6/ITIH
GO_MF_m	GO:00004	Y-form DN	1/129	4/16969	0.030066	0.095448	0.063799	WRN
GO_MF_m	GO:00048	compleme	1/129	4/16969	0.030066	0.095448	0.063799	C3AR1

GO_MF_m	GO:00050(	vasopressi	1/129	4/16969	0.030066	0.095448	0.063799	AVPR2
GO_MF_m	GO:00051:	scavenger	1/129	4/16969	0.030066	0.095448	0.063799	FPR1
GO_MF_m	GO:00088!	exodeoxyr	1/129	4/16969	0.030066	0.095448	0.063799	RAD1
GO_MF_m	GO:00104!	H3 histone	1/129	4/16969	0.030066	0.095448	0.063799	BRCA2
GO_MF_m	GO:00303	thyroid ho	1/129	4/16969	0.030066	0.095448	0.063799	ACTN2
GO_MF_m	GO:00317	melanocor	1/129	4/16969	0.030066	0.095448	0.063799	POMC
GO_MF_m	GO:00317!	type 3 mel	1/129	4/16969	0.030066	0.095448	0.063799	POMC
GO_MF_m	GO:00317!	type 4 mel	1/129	4/16969	0.030066	0.095448	0.063799	POMC
GO_MF_m	GO:00618:	telomeric I	1/129	4/16969	0.030066	0.095448	0.063799	WRN
GO_MF_m	GO:00620:	D-loop DN	1/129	4/16969	0.030066	0.095448	0.063799	WRN
GO_MF_m	GO:00192(	nucleobas	2/129	36/16969	0.030529	0.096153	0.064271	AK9/NME7
GO_MF_m	GO:00304:	peptidase	4/129	157/16969	0.031904	0.098871	0.066087	GAS6/ITIH
GO_MF_m	GO:00170(	snRNA bin	2/129	37/16969	0.032115	0.098871	0.066087	CCNT1/CC
GO_MF_m	GO:00510!	chaperone	3/129	91/16969	0.032133	0.098871	0.066087	BIN1/CDC:
GO_MF_m	GO:00084:	RNA polyr	1/129	5/16969	0.037441	0.110121	0.073607	CTDP1
GO_MF_m	GO:00166	oxidoredu	1/129	5/16969	0.037441	0.110121	0.073607	QSOX1
GO_MF_m	GO:00356!	AP-1 adap	1/129	5/16969	0.037441	0.110121	0.073607	LDLRAP1
GO_MF_m	GO:00431	ATP-depe	1/129	5/16969	0.037441	0.110121	0.073607	RUVBL1
GO_MF_m	GO:00700(	fructose bi	1/129	5/16969	0.037441	0.110121	0.073607	ALDOA
GO_MF_m	GO:00719!	WD40-rep	1/129	5/16969	0.037441	0.110121	0.073607	DDB1
GO_MF_m	GO:00168:	lyase activi	4/129	168/16969	0.039388	0.115002	0.07687	ADCY3/AD
GO_MF_m	GO:00180:	histone-ly:	2/129	43/16969	0.042285	0.121682	0.081335	ASH2L/KM
GO_MF_m	GO:00508	extracellul:	2/129	43/16969	0.042285	0.121682	0.081335	THBS1/VE(
GO_MF_m	GO:00156:	tubulin bir	5/129	252/16969	0.043433	0.124095	0.082948	ALDOA/B9
GO_MF_m	GO:00084(	3'-5' exon	2/129	44/16969	0.044083	0.125057	0.083591	RAD1/WRI
GO_MF_m	GO:00324(	MutLalpha	1/129	6/16969	0.044761	0.125205	0.08369	WRN
GO_MF_m	GO:00703:	thyroid ho	1/129	6/16969	0.044761	0.125205	0.08369	PKM
GO_MF_m	GO:00481!	tau proteir	2/129	45/16969	0.045908	0.127524	0.085239	BIN1/CLU
GO_MF_m	GO:19908	promoter-	2/129	46/16969	0.047762	0.131757	0.088069	DNMT1/RI
GO_MF_m	GO:00423	chemokine	2/129	47/16969	0.049642	0.136006	0.090909	CXCL3/CX
GO_MF_m	GO:00168(	hydrolase	2月26日	10/16969	0.000101	0.000892	0.000356	ALOX12/A
GO_MF_m	GO:00192(	phosphata	3月26日	77/16969	0.000217	0.001826	0.000728	PPP1R12A
GO_MF_m	GO:00044!	monooxyg	3月26日	86/16969	0.0003	0.002417	0.000963	CYP2C8/C
GO_MF_m	GO:00047:	protein tyr	3月26日	92/16969	0.000366	0.002819	0.001123	MTMR2/M
GO_MF_m	GO:00703:	aromatase	2月26日	21/16969	0.000466	0.003434	0.001368	CYP2C8/C
GO_MF_m	GO:00200:	heme binc	3月26日	106/16969	0.000555	0.003812	0.001519	CYP4F2/PT
GO_MF_m	GO:00167:	oxidoredu	2月26日	23/16969	0.00056	0.003812	0.001519	CYP2C8/C
GO_MF_m	GO:00469(	tetrapyrrol	3月26日	116/16969	0.000722	0.00473	0.001885	CYP4F2/PT
GO_MF_m	GO:00047:	protein tyr	3月26日	120/16969	0.000796	0.005034	0.002006	FGR/LCK/F
GO_MF_m	GO:00199(	protein ph	3月26日	132/16969	0.001049	0.006405	0.002552	LCK/MTMI
GO_MF_m	GO:00017!	phosphoty	2月26日	37/16969	0.001455	0.007137	0.002844	FGR/LCK
GO_MF_m	GO:00046(	peroxidase	2月26日	37/16969	0.001455	0.007137	0.002844	PTGS1/PT(
GO_MF_m	GO:00047:	non-mem	2月26日	37/16969	0.001455	0.007137	0.002844	FGR/PTK2I
GO_MF_m	GO:00040!	arachidon:	1月26日	1/16969	0.001532	0.007137	0.002844	ALOX5
GO_MF_m	GO:00434:	3-phosph(	1月26日	1/16969	0.001532	0.007137	0.002844	PTK2B
GO_MF_m	GO:00528(	arachidoni	1月26日	1/16969	0.001532	0.007137	0.002844	CYP4F2
GO_MF_m	GO:00528	tocophero	1月26日	1/16969	0.001532	0.007137	0.002844	CYP4F2
GO_MF_m	GO:00528	alpha-toc(	1月26日	1/16969	0.001532	0.007137	0.002844	CYP4F2
GO_MF_m	GO:00528	tocotrieno	1月26日	1/16969	0.001532	0.007137	0.002844	CYP4F2
GO_MF_m	GO:00166!	oxidoredu	2月26日	40/16969	0.001699	0.00771	0.003072	PTGS1/PT(
GO_MF_m	GO:00453(	protein ph	2月26日	44/16969	0.002053	0.009083	0.003619	FGR/LCK
GO_MF_m	GO:00199(	phosphata	3月26日	177/16969	0.002432	0.010497	0.004183	LCK/MTMI
GO_MF_m	GO:00161(	linoleate 1	1月26日	2/16969	0.003062	0.011532	0.004595	ALOX12
GO_MF_m	GO:00230:	MHC class	1月26日	2/16969	0.003062	0.011532	0.004595	KLRD1
GO_MF_m	GO:00349!	Fc-gamma	1月26日	2/16969	0.003062	0.011532	0.004595	FGR
GO_MF_m	GO:00504	arachidon:	1月26日	2/16969	0.003062	0.011532	0.004595	ALOX15
GO_MF_m	GO:00511:	hepoxilin /	1月26日	2/16969	0.003062	0.011532	0.004595	ALOX15
GO_MF_m	GO:00725:	Rho-depe	1月26日	2/16969	0.003062	0.011532	0.004595	ROCK1

GO_MF_m	GO:00162(	antioxidan	2月26日	65/16969	0.004425	0.015328	0.006108	PTGS1/PT(
GO_MF_m	GO:00198(	protein ph	2月26日	66/16969	0.004559	0.015328	0.006108	PPP4R2/SI
GO_MF_m	GO:00044:	1-phospha	1月26日	3/16969	0.00459	0.015328	0.006108	PI4KA
GO_MF_m	GO:00163(	1-phospha	1月26日	3/16969	0.00459	0.015328	0.006108	PIP4K2C
GO_MF_m	GO:00341(	very-low-(	1月26日	3/16969	0.00459	0.015328	0.006108	TREM2
GO_MF_m	GO:00426:	CD8 recep	1月26日	3/16969	0.00459	0.015328	0.006108	LCK
GO_MF_m	GO:00512:	phosphop	2月26日	76/16969	0.006001	0.017458	0.006956	FGR/LCK
GO_MF_m	GO:00083(	arachidoni	1月26日	4/16969	0.006115	0.017458	0.006956	CYP4F2
GO_MF_m	GO:00083(	arachidoni	1月26日	4/16969	0.006115	0.017458	0.006956	CYP4F2
GO_MF_m	GO:00186(	alkane 1-r	1月26日	4/16969	0.006115	0.017458	0.006956	CYP4F2
GO_MF_m	GO:00230:	MHC class	1月26日	4/16969	0.006115	0.017458	0.006956	KLRD1
GO_MF_m	GO:00336(	oxidoredu	1月26日	4/16969	0.006115	0.017458	0.006956	CYP2C8
GO_MF_m	GO:00348	caffeine ox	1月26日	4/16969	0.006115	0.017458	0.006956	CYP2C8
GO_MF_m	GO:00500!	leukotriene	1月26日	4/16969	0.006115	0.017458	0.006956	CYP4F2
GO_MF_m	GO:00708(	lipoteichoi	1月26日	4/16969	0.006115	0.017458	0.006956	TREM2
GO_MF_m	GO:00044	inositol-pc	1月26日	5/16969	0.007638	0.0208	0.008288	INPP5E
GO_MF_m	GO:00341(	apolipopro	1月26日	5/16969	0.007638	0.0208	0.008288	TREM2
GO_MF_m	GO:01010:	estrogen 1	1月26日	5/16969	0.007638	0.0208	0.008288	CYP2C8
GO_MF_m	GO:00044:	phosphati(	1月26日	6/16969	0.009159	0.023841	0.0095	INPP5E
GO_MF_m	GO:00230:	MHC class	1月26日	6/16969	0.009159	0.023841	0.0095	KLRD1
GO_MF_m	GO:00460:	inositol tris	1月26日	6/16969	0.009159	0.023841	0.0095	INPP5E
GO_MF_m	GO:00049	NMDA glu	1月26日	7/16969	0.010678	0.02625	0.01046	PTK2B
GO_MF_m	GO:00167:	oxidoredu	1月26日	7/16969	0.010678	0.02625	0.01046	CYP4F2
GO_MF_m	GO:00426(	T cell rece	1月26日	7/16969	0.010678	0.02625	0.01046	LCK
GO_MF_m	GO:19904(	protein an	1月26日	7/16969	0.010678	0.02625	0.01046	KLRD1
GO_MF_m	GO:00167:	oxidoredu	1月26日	8/16969	0.012195	0.028779	0.011467	CYP2C8
GO_MF_m	GO:00230:	MHC class	1月26日	8/16969	0.012195	0.028779	0.011467	KLRD1
GO_MF_m	GO:00426(	CD4 recep	1月26日	8/16969	0.012195	0.028779	0.011467	LCK
GO_MF_m	GO:00345(	phosphati(	1月26日	9/16969	0.013709	0.031109	0.012395	INPP5E
GO_MF_m	GO:00349(	immunogl	1月26日	9/16969	0.013709	0.031109	0.012395	FGR
GO_MF_m	GO:00527	phosphati(	1月26日	9/16969	0.013709	0.031109	0.012395	PI4KA
GO_MF_m	GO:01060:	phosphati(	1月26日	10/16969	0.015221	0.034102	0.013588	INPP5E
GO_MF_m	GO:00055	phospholi	3月26日	363/1696(	0.017533	0.038791	0.015456	ALOX15/P
GO_MF_m	GO:00152(	calcium-ac	1月26日	12/16969	0.018238	0.038893	0.015497	MTMR6
GO_MF_m	GO:00163(	phosphati(	1月26日	12/16969	0.018238	0.038893	0.015497	PIP4K2C
GO_MF_m	GO:00170	GTP-Rho k	1月26日	12/16969	0.018238	0.038893	0.015497	ROCK1
GO_MF_m	GO:00046(	calmodulir	1月26日	13/16969	0.019744	0.040635	0.016191	PTK2B
GO_MF_m	GO:00428(	histone me	1月26日	13/16969	0.019744	0.040635	0.016191	WDR82
GO_MF_m	GO:00527	inositol ph	1月26日	13/16969	0.019744	0.040635	0.016191	INPP5E
GO_MF_m	GO:00428:	peptidogly	1月26日	14/16969	0.021247	0.043226	0.017224	TREM2
GO_MF_m	GO:00301(	low-densit	1月26日	15/16969	0.022747	0.045753	0.018231	TREM2
GO_MF_m	GO:00306	protein bir	2月26日	156/1696(	0.023622	0.046648	0.018587	PPP4R2/SI
GO_MF_m	GO:00046:	lysophospl	1月26日	16/16969	0.024246	0.046648	0.018587	PLA2G4A
GO_MF_m	GO:00081!	protein ph	1月26日	16/16969	0.024246	0.046648	0.018587	SHOC2
GO_MF_m	GO:00341(	apolipopro	1月26日	16/16969	0.024246	0.046648	0.018587	TREM2
GO_MF_m	GO:00080:	high-dens	1月26日	17/16969	0.025743	0.048994	0.019522	TREM2
GO_MF_m	GO:00049	ionotropic	1月26日	18/16969	0.027237	0.051287	0.020435	PTK2B
GO_MF_m	GO:00480:	cofactor b	3月26日	438/1696(	0.028601	0.052969	0.021106	CYP4F2/PT
GO_MF_m	GO:00083(	steroid hyc	1月26日	19/16969	0.028729	0.052969	0.021106	CYP2C8
GO_MF_m	GO:00503:	tau-protei	1月26日	21/16969	0.031706	0.056687	0.022587	ROCK1
GO_MF_m	GO:01025(	phospholi	1月26日	21/16969	0.031706	0.056687	0.022587	PLA2G4A
GO_MF_m	GO:01025(	phospholi	1月26日	21/16969	0.031706	0.056687	0.022587	PLA2G4A
GO_MF_m	GO:00080:	protein C-	2月26日	186/1696(	0.03269	0.057862	0.023055	LCK/PTK2E
GO_MF_m	GO:00052:	calcium ac	1月26日	23/16969	0.034675	0.060767	0.024213	MTMR6
GO_MF_m	GO:00230:	MHC prote	1月26日	25/16969	0.037635	0.065308	0.026022	KLRD1
GO_MF_m	GO:00046:	phospholi	1月26日	26/16969	0.039112	0.065931	0.026271	PLA2G4A
GO_MF_m	GO:00055	calcium-de	1月26日	26/16969	0.039112	0.065931	0.026271	PLA2G4A
GO_MF_m	GO:00080(	glutamate	1月26日	26/16969	0.039112	0.065931	0.026271	PTK2B

GO_MF_m	GO:00435	phosphati	1月26日	28/16969	0.042059	0.07023	0.027984	LCK
GO_MF_m	GO:00600	molecular	2月26日	216/1696	0.042907	0.070976	0.028281	PPP4R2/SI
GO_MF_m	GO:00015	lipopolysa	1月26日	30/16969	0.044997	0.073069	0.029114	TREM2
GO_MF_m	GO:00718	14-3-3 pr	1月26日	30/16969	0.044997	0.073069	0.029114	PPP1R12A
GO_MF_m	GO:00167	oxidoredu	1月26日	33/16969	0.049388	0.07947	0.031665	CYP4F2
GO_MF_m	GO:00010	RNA polyr	3/117	18/16969	0.000242	0.004329	0.003284	FBL/HNRN
GO_MF_m	GO:00616	ubiquitin c	3/117	21/16969	0.000388	0.006371	0.004834	UBE2C/UB
GO_MF_m	GO:00197	NEDD8 tra	2/117	6/16969	0.000694	0.010522	0.007984	RNF7/UBE
GO_MF_m	GO:00085	Ran GTPas	3/117	28/16969	0.000923	0.012986	0.009853	BIRC5/RAN
GO_MF_m	GO:00010	TFIID-class	2/117	11/16969	0.002489	0.032691	0.024804	FBL/NOP5
GO_MF_m	GO:00036	chromatin	9/117	437/1696	0.003268	0.040235	0.030529	CDCA5/CD
GO_MF_m	GO:00351	histone kir	2/117	14/16969	0.004063	0.047085	0.035726	AURKB/CD
GO_MF_m	GO:00171	poly(C) RN	1/117	1/16969	0.006895	0.061741	0.046846	HNRNPU
GO_MF_m	GO:00199	interferon-	1/117	1/16969	0.006895	0.061741	0.046846	KLHL20
GO_MF_m	GO:00616	NEDD8 co	1/117	1/16969	0.006895	0.061741	0.046846	UBE2F
GO_MF_m	GO:00704	tubulin-de	1/117	1/16969	0.006895	0.061741	0.046846	KIF18A
GO_MF_m	GO:19902	histone-gl	1/117	1/16969	0.006895	0.061741	0.046846	FBL
GO_MF_m	GO:00010	basal trans	3/117	60/16969	0.008217	0.067448	0.051176	FBL/HNRN
GO_MF_m	GO:00010	basal RNA	3/117	60/16969	0.008217	0.067448	0.051176	FBL/HNRN
GO_MF_m	GO:00010	TFIIH-class	1/117	2/16969	0.013743	0.091509	0.069432	HNRNPU
GO_MF_m	GO:00199	interferon	1/117	2/16969	0.013743	0.091509	0.069432	KLHL20
GO_MF_m	GO:00345	box C/D sr	1/117	2/16969	0.013743	0.091509	0.069432	SNU13
GO_MF_m	GO:00360	protein-gl	1/117	2/16969	0.013743	0.091509	0.069432	FBL
GO_MF_m	GO:19908	C-rich sing	1/117	2/16969	0.013743	0.091509	0.069432	PCBP2
GO_MF_m	GO:00443	ubiquitin-l	6/117	286/1696	0.014346	0.091509	0.069432	CCNB1/CU
GO_MF_m	GO:00046	protein ser	7/117	371/1696	0.0144	0.091509	0.069432	AURKB/BU
GO_MF_m	GO:00511	ATPase bir	3/117	77/16969	0.016147	0.099404	0.075423	FBL/SNU1
GO_MF_m	GO:00345	U3 snoRN	1/117	3/16969	0.020544	0.115632	0.087735	SNU13
GO_MF_m	GO:00510	nucleic aci	1/117	3/16969	0.020544	0.115632	0.087735	HNRNPA3
GO_MF_m	GO:00510	RNA trans	1/117	3/16969	0.020544	0.115632	0.087735	HNRNPA3
GO_MF_m	GO:00036	single-stra	3/117	90/16969	0.024323	0.131164	0.099521	HNRNPU/
GO_MF_m	GO:00170	snRNA bin	2/117	37/16969	0.02683	0.131164	0.099521	HNRNPU/
GO_MF_m	GO:00306	U4atac snR	1/117	4/16969	0.027298	0.131164	0.099521	SNU13
GO_MF_m	GO:00351	histone ser	1/117	4/16969	0.027298	0.131164	0.099521	AURKB
GO_MF_m	GO:00615	cyclin-dep	1/117	4/16969	0.027298	0.131164	0.099521	CCNB1
GO_MF_m	GO:00991	RNA polyr	1/117	4/16969	0.027298	0.131164	0.099521	HNRNPU
GO_MF_m	GO:00192	centromer	1/117	5/16969	0.034006	0.152256	0.115524	KIF2C
GO_MF_m	GO:00321	SUMO pol	1/117	5/16969	0.034006	0.152256	0.115524	RNF4
GO_MF_m	GO:00360	mediator c	1/117	5/16969	0.034006	0.152256	0.115524	SMC1A
GO_MF_m	GO:00037	microtubu	2/117	43/16969	0.035421	0.155066	0.117656	CENPE/KIF
GO_MF_m	GO:00080	protein C-	4/117	186/1696	0.039867	0.160235	0.121579	CENPF/MA
GO_MF_m	GO:00435	kinetochor	1/117	6/16969	0.040669	0.160235	0.121579	CENPE
GO_MF_m	GO:00451	pre-mRNA	1/117	6/16969	0.040669	0.160235	0.121579	SF1
GO_MF_m	GO:00707	leucine bir	1/117	6/16969	0.040669	0.160235	0.121579	UBR2
GO_MF_m	GO:19907	protein bir	1/117	6/16969	0.040669	0.160235	0.121579	DET1
GO_MF_m	GO:00010	RNA polyr	3/117	113/1696	0.043312	0.167304	0.126942	FBL/HNRN
GO_MF_m	GO:00051	patched bi	1/117	7/16969	0.047286	0.172505	0.130888	CCNB1
GO_MF_m	GO:00085	ATP-depe	1/117	7/16969	0.047286	0.172505	0.130888	KIF18A
GO_MF_m	GO:00340	poly(G) bir	1/117	7/16969	0.047286	0.172505	0.130888	HNRNPU
GO_MF_m	GO:00045	endoribon	2/117	52/16969	0.049986	0.17904	0.135846	NOB1/RPP
GO_MF_m	GO:00302	clathrin bir	3/47	35/16969	0.000122	0.001643	0.000806	ARRB1/HIF
GO_MF_m	GO:00198	kinesin bin	3/47	40/16969	0.000183	0.002268	0.001112	KIF18B/KIF
GO_MF_m	GO:00356	clathrin ad	2/47	9/16969	0.000267	0.002866	0.001405	ARRB1/HIF
GO_MF_m	GO:00987	endocytic	2/47	9/16969	0.000267	0.002866	0.001405	ARRB1/HIF
GO_MF_m	GO:00350	phosphati	5/47	217/1696	0.000324	0.003256	0.001597	FCHO2/HI
GO_MF_m	GO:00316	adrenergic	2/47	17/16969	0.000994	0.009418	0.004618	ARRB1/SH
GO_MF_m	GO:00380	cargo rece	3/47	85/16969	0.001678	0.015006	0.007358	ARRB1/HIF
GO_MF_m	GO:19029	phosphati	3/47	96/16969	0.002376	0.020132	0.009872	FCHO2/HI

GO_MF_m	GO:00318(	V2 vasopre	1/47	1/16969	0.00277	0.022297	0.010933	ARRB1
GO_MF_m	GO:00306(	GTPase re	4/47	235/16969	0.004011	0.029676	0.014552	AGFG1/AR
GO_MF_m	GO:00443	ion channe	3/47	116/16969	0.004055	0.029676	0.014552	ARRB1/SH
GO_MF_m	GO:00708	growth fac	3/47	121/16969	0.004563	0.031809	0.015598	ARF4/EGF/
GO_MF_m	GO:00050(	epidermal	1/47	2/16969	0.005532	0.031809	0.015598	EGFR
GO_MF_m	GO:00316(	alpha-1A a	1/47	2/16969	0.005532	0.031809	0.015598	ARRB1
GO_MF_m	GO:00317(	follicle-stir	1/47	2/16969	0.005532	0.031809	0.015598	ARRB1
GO_MF_m	GO:00350(	1-phospha	1/47	2/16969	0.005532	0.031809	0.015598	PIK3C2A
GO_MF_m	GO:01201	intraciliary	1/47	2/16969	0.005532	0.031809	0.015598	KIFAP3
GO_MF_m	GO:00080	enzyme ac	5/47	419/16969	0.005864	0.032182	0.015781	AGFG1/AR
GO_MF_m	GO:00510	GTPase bir	5/47	426/16969	0.006282	0.032182	0.015781	ARF4/EGF/
GO_MF_m	GO:00605	nucleoside	4/47	267/16969	0.006287	0.032182	0.015781	AGFG1/AR
GO_MF_m	GO:00316	ubiquitin p	4/47	270/16969	0.006537	0.032182	0.015781	ARRB1/EG
GO_MF_m	GO:00453(	protein ph	2/47	44/16969	0.006596	0.032182	0.015781	ARRB1/YW
GO_MF_m	GO:19019	phosphati	3/47	143/16969	0.007248	0.032541	0.015957	FCHO2/HI
GO_MF_m	GO:00168	ATPase ac	4/47	280/16969	0.007416	0.032541	0.015957	DYNC2H1/
GO_MF_m	GO:00443	ubiquitin-l	4/47	286/16969	0.00798	0.032541	0.015957	ARRB1/EG
GO_MF_m	GO:00302	very-low-(	1/47	3/16969	0.008287	0.032541	0.015957	LDLR
GO_MF_m	GO:00316(	alpha-1B a	1/47	3/16969	0.008287	0.032541	0.015957	ARRB1
GO_MF_m	GO:00317	D2 dopam	1/47	3/16969	0.008287	0.032541	0.015957	DNM2
GO_MF_m	GO:00318(	vasopressi	1/47	3/16969	0.008287	0.032541	0.015957	ARRB1
GO_MF_m	GO:00320	clathrin lig	1/47	3/16969	0.008287	0.032541	0.015957	HIP1
GO_MF_m	GO:00452	cadherin b	4/47	297/16969	0.009085	0.034825	0.017077	EGFR/SH3
GO_MF_m	GO:00316(	beta-1 adı	1/47	4/16969	0.011034	0.040237	0.019731	SH3GL1
GO_MF_m	GO:00508	phosphose	1/47	4/16969	0.011034	0.040237	0.019731	YWHAE
GO_MF_m	GO:00971	scaffold pr	2/47	58/16969	0.011246	0.040237	0.019731	KIF1B/YWH
GO_MF_m	GO:00199(	phosphata	3/47	177/16969	0.012928	0.045249	0.022188	EGFR/KIFA
GO_MF_m	GO:00719	Arp2/3 cor	1/47	5/16969	0.013774	0.046889	0.022992	SNX9
GO_MF_m	GO:00055	phosphati	2/47	65/16969	0.013979	0.046889	0.022992	FCHO2/SN
GO_MF_m	GO:00044	phosphati	1/47	6/16969	0.016506	0.052479	0.025733	OCRL
GO_MF_m	GO:00320	clathrin he	1/47	6/16969	0.016506	0.052479	0.025733	LDLR
GO_MF_m	GO:00050	guanyl-nu	3/47	196/16969	0.016955	0.052479	0.025733	ARF4/EGF/
GO_MF_m	GO:00016	virus recep	2/47	74/16969	0.017869	0.052479	0.025733	EGFR/LDLI
GO_MF_m	GO:00192(	kinase acti	2/47	74/16969	0.017869	0.052479	0.025733	EGF/ITSN1
GO_MF_m	GO:01040(	hijacked m	2/47	74/16969	0.017869	0.052479	0.025733	EGFR/LDLI
GO_MF_m	GO:00050(	GTPase ac	3/47	203/16969	0.018597	0.052479	0.025733	AGFG1/AR
GO_MF_m	GO:00512	phosphop	2/47	76/16969	0.018789	0.052479	0.025733	ARRB1/YW
GO_MF_m	GO:00016(	G protein-	3/47	205/16969	0.019082	0.052479	0.025733	ARRB1/DN
GO_MF_m	GO:00302	nitric-oxid	1/47	7/16969	0.019231	0.052479	0.025733	EGFR
GO_MF_m	GO:00302(	transmeml	1/47	7/16969	0.019231	0.052479	0.025733	EGF
GO_MF_m	GO:00600(	molecular	3/47	216/16969	0.021874	0.057931	0.028407	ARRB1/HII
GO_MF_m	GO:00317(	angiotensi	1/47	8/16969	0.021949	0.057931	0.028407	ARRB1
GO_MF_m	GO:00426	ATPase ac	3/47	218/16969	0.022404	0.058179	0.028528	DYNC2H1/
GO_MF_m	GO:00345(	phosphati	1/47	9/16969	0.024659	0.062033	0.030418	OCRL
GO_MF_m	GO:19907(	arrestin far	1/47	9/16969	0.024659	0.062033	0.030418	ARRB1
GO_MF_m	GO:00363	phosphati	1/47	10/16969	0.027362	0.066747	0.03273	DNM2
GO_MF_m	GO:01060	phosphati	1/47	10/16969	0.027362	0.066747	0.03273	OCRL
GO_MF_m	GO:00305	receptor a	1/47	11/16969	0.030058	0.070134	0.034391	EGF
GO_MF_m	GO:00314	mitogen-a	1/47	11/16969	0.030058	0.070134	0.034391	ARRB1
GO_MF_m	GO:00350(	phosphati	1/47	11/16969	0.030058	0.070134	0.034391	PIK3C2A
GO_MF_m	GO:00156	tubulin bir	3/47	252/16969	0.032497	0.07222	0.035414	DNM2/KIF
GO_MF_m	GO:00050	low-densit	1/47	12/16969	0.032746	0.07222	0.035414	LDLR
GO_MF_m	GO:00163(	phosphati	1/47	12/16969	0.032746	0.07222	0.035414	PIK3C2A
GO_MF_m	GO:00507	dopamine	1/47	12/16969	0.032746	0.07222	0.035414	DNM2
GO_MF_m	GO:00508	cell adhesi	4/47	444/16969	0.034069	0.074122	0.036346	EGFR/SH3
GO_MF_m	GO:00527	inositol ph	1/47	13/16969	0.035427	0.07605	0.037291	OCRL
GO_MF_m	GO:00055	1-phospha	1/47	14/16969	0.0381	0.078643	0.038563	SNX9
GO_MF_m	GO:00302	lipoprotei	1/47	14/16969	0.0381	0.078643	0.038563	LDLR

GO_MF_m	GO:00509	nitric-oxid	1/47	14/16969	0.0381	0.078643	0.038563	DNM2
GO_MF_m	GO:00051	insulin-like	1/47	15/16969	0.040767	0.082043	0.040231	ARRB1
GO_MF_m	GO:00301	low-densit	1/47	15/16969	0.040767	0.082043	0.040231	LDLR
GO_MF_m	GO:00050	ARF guany	1/47	16/16969	0.043426	0.084236	0.041306	ARF4
GO_MF_m	GO:00230	MHC class	1/47	16/16969	0.043426	0.084236	0.041306	YWHAE
GO_MF_m	GO:00422	MHC class	1/47	16/16969	0.043426	0.084236	0.041306	TUBB4B
GO_MF_m	GO:00171	SH3 doma	2/47	122/1696	0.044922	0.0861	0.04222	DNM2/SH
GO_MF_m	GO:00050	Ras guany	2/47	125/1696	0.046923	0.088878	0.043582	EGF/ITSN1
GO_MF_m	GO:00700	proline-ric	1/47	18/16969	0.048723	0.091214	0.044727	ITSN1
GO_MF_m	GO:00081	translation	2月14日	67/16969	0.001355	0.003423	NA	GFM1/MTI
GO_MF_m	GO:00055	GTP bindir	3月14日	313/1696	0.001946	0.003423	NA	DAP3/GFN
GO_MF_m	GO:00325	purine ribo	3月14日	318/1696	0.002036	0.003423	NA	DAP3/GFN
GO_MF_m	GO:00018	purine nuc	3月14日	321/1696	0.002091	0.003423	NA	DAP3/GFN
GO_MF_m	GO:00325	ribonuclec	3月14日	322/1696	0.00211	0.003423	NA	DAP3/GFN
GO_MF_m	GO:00018	nucleoside	3月14日	329/1696	0.002243	0.003423	NA	DAP3/GFN
GO_MF_m	GO:00190	guanyl nuc	3月14日	331/1696	0.002282	0.003423	NA	DAP3/GFN
GO_MF_m	GO:00325	guanyl ribo	3月14日	331/1696	0.002282	0.003423	NA	DAP3/GFN
GO_MF_m	GO:00430	ribonuclec	2月14日	107/1696	0.003412	0.004652	NA	MTIF2/PTC
GO_MF_m	GO:00037	translation	1月14日	12/16969	0.009859	0.012323	NA	GFM1
GO_MF_m	GO:00039	GTPase ac	2月14日	224/1696	0.014221	0.016408	NA	GFM1/MTI
GO_MF_m	GO:00037	translation	1月14日	41/16969	0.033313	0.035692	NA	MTIF2
GO_MF_m	GO:00198	rRNA bind	1月14日	44/16969	0.035709	0.035709	NA	PTCD3
GO_MF_m	GO:00480	mRNA 5'-(	2月16日	23/16969	0.000208	0.004807	0.0023	GNL3/RSL
GO_MF_m	GO:00043	helicase ac	3月16日	128/1696	0.000219	0.004807	0.0023	DDX24/DD
GO_MF_m	GO:00037	RNA helica	2月16日	34/16969	0.000459	0.006739	0.003224	DDX24/DD
GO_MF_m	GO:19908	H4K20me3	1月16日	1/16969	0.000943	0.010372	0.004963	PWP1
GO_MF_m	GO:01400	catalytic ac	3月16日	268/1696	0.001873	0.016485	0.007887	DDX24/DD
GO_MF_m	GO:00325	ribonuclec	3月16日	322/1696	0.003156	0.021076	0.010084	GNL3/GTP
GO_MF_m	GO:00018	nucleoside	3月16日	329/1696	0.003353	0.021076	0.010084	GNL3/GTP
GO_MF_m	GO:00164	tRNA (cyto	1月16日	5/16969	0.004706	0.025884	0.012385	NSUN6
GO_MF_m	GO:00164	tRNA (cyto	1月16日	8/16969	0.00752	0.036764	0.01759	NSUN6
GO_MF_m	GO:00080	5S rRNA b	1月16日	11/16969	0.010326	0.045435	0.021739	RRS1
GO_MF_m	GO:00701	DNA polyr	1月16日	17/16969	0.015916	0.063665	0.030462	NAT10
GO_MF_m	GO:00081	tRNA meth	1月16日	24/16969	0.022401	0.078942	0.037772	NSUN6
GO_MF_m	GO:00040	ATP-depe	1月16日	25/16969	0.023324	0.078942	0.037772	DDX56
GO_MF_m	GO:00081	RNA-depe	1月16日	28/16969	0.026088	0.081992	0.03923	DDX56
GO_MF_m	GO:00055	GTP bindir	2月16日	313/1696	0.034323	0.087891	0.042053	GNL3/GTP
GO_MF_m	GO:00325	purine ribo	2月16日	318/1696	0.035333	0.087891	0.042053	GNL3/GTP
GO_MF_m	GO:00018	purine nuc	2月16日	321/1696	0.035945	0.087891	0.042053	GNL3/GTP
GO_MF_m	GO:00190	guanyl nuc	2月16日	331/1696	0.038015	0.087891	0.042053	GNL3/GTP
GO_MF_m	GO:00325	guanyl ribo	2月16日	331/1696	0.038015	0.087891	0.042053	GNL3/GTP
GO_MF_m	GO:00198	rRNA bind	1月16日	44/16969	0.040708	0.087891	0.042053	RRS1
GO_MF_m	GO:00000	tRNA bind	1月16日	47/16969	0.043426	0.087891	0.042053	NSUN6
GO_MF_m	GO:00081	RNA meth	1月16日	48/16969	0.044331	0.087891	0.042053	NSUN6
GO_MF_m	GO:00080	ATP-depe	1月16日	52/16969	0.04794	0.087891	0.042053	DDX56
GO_MF_m	GO:00700	purine NTI	1月16日	52/16969	0.04794	0.087891	0.042053	DDX56
GO_MF_m	GO:00045	nuclease a	3月13日	171/1696	0.000267	0.005582	0.001267	ISG20/RNA
GO_MF_m	GO:00088	dGTPase a	1月13日	1/16969	0.000766	0.005582	0.001267	SAMHD1
GO_MF_m	GO:00088	exoribonu	1月13日	1/16969	0.000766	0.005582	0.001267	ISG20
GO_MF_m	GO:00167	triphospho	1月13日	1/16969	0.000766	0.005582	0.001267	SAMHD1
GO_MF_m	GO:00325	guanyl dec	1月13日	1/16969	0.000766	0.005582	0.001267	SAMHD1
GO_MF_m	GO:00325	dGTP bind	1月13日	1/16969	0.000766	0.005582	0.001267	SAMHD1
GO_MF_m	GO:01400	catalytic ac	3月13日	268/1696	0.000991	0.006317	0.001434	ISG20/RNA
GO_MF_m	GO:00306	U2 snRNA	1月13日	2/16969	0.001532	0.008679	0.001971	ISG20
GO_MF_m	GO:00345	U3 snoRN	1月13日	3/16969	0.002297	0.011713	0.002659	ISG20
GO_MF_m	GO:00083	single-stra	1月13日	4/16969	0.003061	0.01301	0.002954	ISG20
GO_MF_m	GO:00306	U1 snRNA	1月13日	4/16969	0.003061	0.01301	0.002954	ISG20
GO_MF_m	GO:00325	purine dec	1月13日	5/16969	0.003825	0.013765	0.003125	SAMHD1

GO_MF_m	GO:00082!	3'-5'-exoc	1月13日	6/16969	0.004588	0.013765	0.003125	ISG20
GO_MF_m	GO:00082!	single-stra	1月13日	6/16969	0.004588	0.013765	0.003125	ISG20
GO_MF_m	GO:00325!	deoxyribo	1月13日	6/16969	0.004588	0.013765	0.003125	SAMHD1
GO_MF_m	GO:00700(	chromo sh	1月13日	6/16969	0.004588	0.013765	0.003125	SP100
GO_MF_m	GO:00045	exodeoxyr	1月13日	14/16969	0.010676	0.028657	0.006506	ISG20
GO_MF_m	GO:00168!	exodeoxyr	1月13日	14/16969	0.010676	0.028657	0.006506	ISG20
GO_MF_m	GO:00305	snoRNA bi	1月13日	20/16969	0.01522	0.03881	0.008811	ISG20
GO_MF_m	GO:00001	3'-5'-exori	1月13日	29/16969	0.021998	0.043586	0.009896	ISG20
GO_MF_m	GO:00055	GTP bindir	2月13日	313/1696	0.023132	0.043586	0.009896	GBP2/SAM
GO_MF_m	GO:00168!	exoribonu	1月13日	31/16969	0.023499	0.043586	0.009896	ISG20
GO_MF_m	GO:00325!	purine ribo	2月13日	318/1696	0.023827	0.043586	0.009896	GBP2/SAM
GO_MF_m	GO:00018	purine nuc	2月13日	321/1696	0.024248	0.043586	0.009896	GBP2/SAM
GO_MF_m	GO:00325	ribonuclec	2月13日	322/1696	0.024389	0.043586	0.009896	GBP2/SAM
GO_MF_m	GO:00045	exoribonu	1月13日	33/16969	0.024997	0.043586	0.009896	ISG20
GO_MF_m	GO:00018	nucleoside	2月13日	329/1696	0.025386	0.043586	0.009896	GBP2/SAM
GO_MF_m	GO:00190(	guanyl nuc	2月13日	331/1696	0.025674	0.043586	0.009896	GBP2/SAM
GO_MF_m	GO:00325(	guanyl ribo	2月13日	331/1696	0.025674	0.043586	0.009896	GBP2/SAM
GO_MF_m	GO:00515	4 iron, 4 su	1月13日	35/16969	0.026494	0.043586	0.009896	RSAD2
GO_MF_m	GO:00170(	snRNA bin	1月13日	37/16969	0.027988	0.044605	0.010127	ISG20
GO_MF_m	GO:00045	deoxyribo	1月13日	41/16969	0.03097	0.047034	0.010679	ISG20
GO_MF_m	GO:00084(	3'-5' exonu	1月13日	44/16969	0.033201	0.047034	0.010679	ISG20
GO_MF_m	GO:00167!	exonucleas	1月13日	44/16969	0.033201	0.047034	0.010679	ISG20
GO_MF_m	GO:00198	rRNA bind	1月13日	44/16969	0.033201	0.047034	0.010679	RNASEL
GO_MF_m	GO:00045	endoribon	1月13日	52/16969	0.039127	0.053931	0.012244	RNASEL
GO_MF_m	GO:00436	protein sel	1月13日	56/16969	0.042077	0.054587	0.012393	RSAD2
GO_MF_m	GO:00515	iron-sulfur	1月13日	57/16969	0.042813	0.054587	0.012393	RSAD2
GO_MF_m	GO:00515	metal clust	1月13日	57/16969	0.042813	0.054587	0.012393	RSAD2
GO_MF_m	GO:00355	purinergic	3/103	19/16969	0.000196	0.001614	0.000815	ADORA1/
GO_MF_m	GO:00327!	heterotrim	2/103	4/16969	0.000217	0.001743	0.00088	ADORA1/
GO_MF_m	GO:00422	peptide bi	7/103	215/1696	0.000337	0.002636	0.001331	CCKBR/ED
GO_MF_m	GO:00192(	kinase reg	6/103	154/1696	0.000351	0.002684	0.001356	CCKBR/CC
GO_MF_m	GO:00306	protein bir	6/103	156/1696	0.000376	0.002809	0.001419	CD28/CHN
GO_MF_m	GO:00051	hormone a	5/103	103/1696	0.000408	0.00298	0.001505	ADM/GAL
GO_MF_m	GO:00480	CCR chem	3/103	26/16969	0.00051	0.003602	0.001819	CCL21/CC
GO_MF_m	GO:00051(	neurotropl	2/103	6/16969	0.000539	0.003602	0.001819	GRB2/PIK3
GO_MF_m	GO:00051(	neurotropl	2/103	6/16969	0.000539	0.003602	0.001819	GRB2/PIK3
GO_MF_m	GO:00350	phosphati	2/103	6/16969	0.000539	0.003602	0.001819	CCKBR/PIK
GO_MF_m	GO:00317	CCR5 cher	2/103	7/16969	0.000751	0.004921	0.002485	CCL5/JAK1
GO_MF_m	GO:00332	amide bin	7/103	266/1696	0.001185	0.007605	0.003841	CCKBR/ED
GO_MF_m	GO:00016(	G protein-	2/103	9/16969	0.001278	0.008041	0.004061	P2RY1/P2F
GO_MF_m	GO:00083(	structural	3/103	36/16969	0.001341	0.008276	0.004179	MYBPC3/N
GO_MF_m	GO:00081	neuropept	3/103	38/16969	0.00157	0.009284	0.004688	GAL/NPY2
GO_MF_m	GO:00051(	neurotropl	2/103	10/16969	0.001591	0.009284	0.004688	GRB2/PIK3
GO_MF_m	GO:00452	CXCR cher	2/103	10/16969	0.001591	0.009284	0.004688	CXCL10/C
GO_MF_m	GO:00150	corecepto	3/103	40/16969	0.001823	0.010449	0.005277	CCR5/CD2
GO_MF_m	GO:00600!	molecular	6/103	216/1696	0.002036	0.011464	0.00579	CD28/CHN
GO_MF_m	GO:00320	myosin he	2/103	12/16969	0.002315	0.01281	0.006469	MYBPC3/N
GO_MF_m	GO:00039	GTPase ac	6/103	224/1696	0.002442	0.013286	0.00671	ARHGAP5/
GO_MF_m	GO:00018	purine nuc	7/103	321/1696	0.003417	0.018282	0.009233	ADORA1/
GO_MF_m	GO:00018	nucleoside	7/103	329/1696	0.00391	0.020576	0.010391	ADORA1/
GO_MF_m	GO:00046!	protein kir	2/103	16/16969	0.004142	0.021105	0.010658	PRKCD/PR
GO_MF_m	GO:00165(	nucleotide	2/103	16/16969	0.004142	0.021105	0.010658	P2RY1/P2F
GO_MF_m	GO:00986	cadherin b	2/103	17/16969	0.004676	0.023453	0.011844	ANXA1/TN
GO_MF_m	GO:00971	scaffold pr	3/103	58/16969	0.005262	0.024664	0.012455	P2RY1/SRC
GO_MF_m	GO:00044	phosphati	2/103	19/16969	0.005833	0.024664	0.012455	CCL5/CCR
GO_MF_m	GO:00016	nociceptin	1/103	1/16969	0.00607	0.024664	0.012455	OPRL1
GO_MF_m	GO:00150!	gastrin rec	1/103	1/16969	0.00607	0.024664	0.012455	CCKBR
GO_MF_m	GO:00316	A1 adenos	1/103	1/16969	0.00607	0.024664	0.012455	P2RY1

GO_MF_m	GO:00317(	adrenome	1/103	1/16969	0.00607	0.024664	0.012455	ADM
GO_MF_m	GO:00317:	cholecysto	1/103	1/16969	0.00607	0.024664	0.012455	CCKBR
GO_MF_m	GO:00317‹	type B gas	1/103	1/16969	0.00607	0.024664	0.012455	CCKBR
GO_MF_m	GO:00317(	galanin re(	1/103	1/16969	0.00607	0.024664	0.012455	GAL
GO_MF_m	GO:00317(	type 1 gala	1/103	1/16969	0.00607	0.024664	0.012455	GAL
GO_MF_m	GO:00317(	type 2 gala	1/103	1/16969	0.00607	0.024664	0.012455	GAL
GO_MF_m	GO:00317(	type 3 gala	1/103	1/16969	0.00607	0.024664	0.012455	GAL
GO_MF_m	GO:00450:	ADP-activ	1/103	1/16969	0.00607	0.024664	0.012455	P2RY1
GO_MF_m	GO:00468:	chemokine	1/103	1/16969	0.00607	0.024664	0.012455	CCL5
GO_MF_m	GO:00995(	neurotrans	1/103	1/16969	0.00607	0.024664	0.012455	ADORA1
GO_MF_m	GO:00708!	growth fac	4/103	121/16969	0.006322	0.025367	0.01281	APP/GRB2
GO_MF_m	GO:00051!	insulin rec	2/103	20/16969	0.006456	0.025584	0.01292	PIK3R1/SR
GO_MF_m	GO:00051(	neuropept	2/103	21/16969	0.007107	0.027822	0.01405	GAL/PENK
GO_MF_m	GO:00718!	neuropept	2/103	23/16969	0.008495	0.032855	0.016592	CCKBR/GA
GO_MF_m	GO:00046:	phospholi	2/103	24/16969	0.009231	0.035276	0.017814	CCL5/CCR
GO_MF_m	GO:00468'	ephrin rec	2/103	25/16969	0.009994	0.03772	0.019049	GRB2/SRC
GO_MF_m	GO:00469(	protein he	8/103	497/1696(	0.01075	0.03772	0.019049	ADCY2/AD
GO_MF_m	GO:00055:	GTP bindir	6/103	313/1696(	0.012061	0.03772	0.019049	ARHGAP5/
GO_MF_m	GO:00016(	peptide YY	1/103	2/16969	0.012103	0.03772	0.019049	NPY2R
GO_MF_m	GO:00046(	calcium-in	1/103	2/16969	0.012103	0.03772	0.019049	PRKCD
GO_MF_m	GO:00049!	cholecysto	1/103	2/16969	0.012103	0.03772	0.019049	CCKBR
GO_MF_m	GO:00049(	endothelir	1/103	2/16969	0.012103	0.03772	0.019049	EDNRB
GO_MF_m	GO:00049(	G protein-	1/103	2/16969	0.012103	0.03772	0.019049	GABBR2
GO_MF_m	GO:00049(	glucagon r	1/103	2/16969	0.012103	0.03772	0.019049	GCGR
GO_MF_m	GO:00049(	vasoactive	1/103	2/16969	0.012103	0.03772	0.019049	VIPR1
GO_MF_m	GO:00164(	G protein-	1/103	2/16969	0.012103	0.03772	0.019049	NTSR1
GO_MF_m	GO:00316(	alpha-2C a	1/103	2/16969	0.012103	0.03772	0.019049	ADRA2A
GO_MF_m	GO:00317:	CCR4 cher	1/103	2/16969	0.012103	0.03772	0.019049	CCL5
GO_MF_m	GO:00317:	CCR7 cher	1/103	2/16969	0.012103	0.03772	0.019049	CCL21
GO_MF_m	GO:00324:	GBD doma	1/103	2/16969	0.012103	0.03772	0.019049	RHOQ
GO_MF_m	GO:00450:	UDP-activ	1/103	2/16969	0.012103	0.03772	0.019049	P2RY14
GO_MF_m	GO:00450:	ATP-activa	1/103	2/16969	0.012103	0.03772	0.019049	P2RY1
GO_MF_m	GO:00469:	1-phospha	1/103	2/16969	0.012103	0.03772	0.019049	CCKBR
GO_MF_m	GO:00717(	chemokine	1/103	2/16969	0.012103	0.03772	0.019049	CCR5
GO_MF_m	GO:00325!	purine ribo	6/103	318/1696(	0.012959	0.039999	0.0202	ARHGAP5/
GO_MF_m	GO:00051!	epidermal	2/103	29/16969	0.013315	0.040321	0.020362	GRB2/YES:
GO_MF_m	GO:00420!	chemoattr	2/103	29/16969	0.013315	0.040321	0.020362	CCL5/CXC
GO_MF_m	GO:00325‹	ribonuclec	6/103	322/1696(	0.013711	0.041133	0.020772	ARHGAP5/
GO_MF_m	GO:00426(	peptide ar	2/103	30/16969	0.01421	0.042234	0.021328	HLA-DPB1
GO_MF_m	GO:00190(	guanyl nu(	6/103	331/1696(	0.015513	0.04527	0.022861	ARHGAP5/
GO_MF_m	GO:00325(	guanyl ribo	6/103	331/1696(	0.015513	0.04527	0.022861	ARHGAP5/
GO_MF_m	GO:00986:	cell-cell ac	2/103	33/16969	0.017046	0.047625	0.024051	ANXA1/TN
GO_MF_m	GO:00049:	alpha2-ad	1/103	3/16969	0.0181	0.047625	0.024051	ADRA2A
GO_MF_m	GO:00049‹	cannabino	1/103	3/16969	0.0181	0.047625	0.024051	CNR1
GO_MF_m	GO:00049!	prostaglan	1/103	3/16969	0.0181	0.047625	0.024051	PTGER4
GO_MF_m	GO:00150(	uridine nu(	1/103	3/16969	0.0181	0.047625	0.024051	P2RY14
GO_MF_m	GO:00165:	growth ho	1/103	3/16969	0.0181	0.047625	0.024051	GHRHR
GO_MF_m	GO:00198:	phospholi	1/103	3/16969	0.0181	0.047625	0.024051	ANXA1
GO_MF_m	GO:00302(	receptor si	1/103	3/16969	0.0181	0.047625	0.024051	CCL5
GO_MF_m	GO:00310:	troponin T	1/103	3/16969	0.0181	0.047625	0.024051	TNNI2
GO_MF_m	GO:00316(	alpha-1B a	1/103	3/16969	0.0181	0.047625	0.024051	ADRA2A
GO_MF_m	GO:00514:	PTB doma	1/103	3/16969	0.0181	0.047625	0.024051	APP
GO_MF_m	GO:00715!	G protein-	1/103	3/16969	0.0181	0.047625	0.024051	P2RY14
GO_MF_m	GO:00421(	SH2 doma	2/103	35/16969	0.019059	0.049739	0.025118	ARHGAP5/
GO_MF_m	GO:00015:	opioid pep	1/103	4/16969	0.024062	0.057213	0.028893	PENK
GO_MF_m	GO:00049(	galanin re(	1/103	4/16969	0.024062	0.057213	0.028893	GAL
GO_MF_m	GO:00049'	leukotrien(	1/103	4/16969	0.024062	0.057213	0.028893	LTB4R
GO_MF_m	GO:00170:	myosin ph	1/103	4/16969	0.024062	0.057213	0.028893	MYH3

GO_MF_m	GO:00316	G-protein	1/103	4/16969	0.024062	0.057213	0.028893	ADORA1
GO_MF_m	GO:00431	ErbB-3 cla	1/103	4/16969	0.024062	0.057213	0.028893	PIK3R1
GO_MF_m	GO:00469	phosphati	1/103	4/16969	0.024062	0.057213	0.028893	PIK3CB
GO_MF_m	GO:00513	norepinep	1/103	4/16969	0.024062	0.057213	0.028893	ADRA2A
GO_MF_m	GO:00526	inositol-1,	1/103	4/16969	0.024062	0.057213	0.028893	INPP5B
GO_MF_m	GO:00528	phosphati	1/103	4/16969	0.024062	0.057213	0.028893	PIK3CB
GO_MF_m	GO:00700	thrombosp	1/103	4/16969	0.024062	0.057213	0.028893	F2
GO_MF_m	GO:00996	voltage-ga	1/103	4/16969	0.024062	0.057213	0.028893	CNR1
GO_MF_m	GO:00037	motor acti	3/103	103/16969	0.024806	0.058549	0.029567	MYH3/MY
GO_MF_m	GO:00986	cell adhesi	2/103	42/16969	0.026835	0.062876	0.031752	ANXA1/TN
GO_MF_m	GO:00049	alpha-adre	1/103	5/16969	0.029987	0.065039	0.032845	ADRA2A
GO_MF_m	GO:00051	growth ho	1/103	5/16969	0.029987	0.065039	0.032845	JAK1
GO_MF_m	GO:00164	C-C chem	1/103	5/16969	0.029987	0.065039	0.032845	CCR5
GO_MF_m	GO:00302	estrogen r	1/103	5/16969	0.029987	0.065039	0.032845	GPER1
GO_MF_m	GO:00316	adenosine	1/103	5/16969	0.029987	0.065039	0.032845	P2RY1
GO_MF_m	GO:00317	CCR1 cher	1/103	5/16969	0.029987	0.065039	0.032845	CCL5
GO_MF_m	GO:00435	insulin bin	1/103	5/16969	0.029987	0.065039	0.032845	PIK3R1
GO_MF_m	GO:00482	CXCR3 che	1/103	5/16969	0.029987	0.065039	0.032845	CXCL10
GO_MF_m	GO:00513	epinephrir	1/103	5/16969	0.029987	0.065039	0.032845	ADRA2A
GO_MF_m	GO:00528	phosphati	1/103	5/16969	0.029987	0.065039	0.032845	PIK3CB
GO_MF_m	GO:19902	keratin fila	1/103	5/16969	0.029987	0.065039	0.032845	VIM
GO_MF_m	GO:00051	integrin bi	3/103	113/16969	0.031457	0.067769	0.034223	CXCL12/SI
GO_MF_m	GO:00452	cadherin b	5/103	297/16969	0.034727	0.074316	0.03753	ANXA1/SR
GO_MF_m	GO:00044	phosphati	1/103	6/16969	0.035876	0.074781	0.037765	INPP5B
GO_MF_m	GO:00460	inositol tris	1/103	6/16969	0.035876	0.074781	0.037765	INPP5B
GO_MF_m	GO:00712	connexin b	1/103	6/16969	0.035876	0.074781	0.037765	SRC
GO_MF_m	GO:19902	steroid hor	1/103	6/16969	0.035876	0.074781	0.037765	GPER1
GO_MF_m	GO:00483	Rac GTPas	2/103	51/16969	0.038369	0.079461	0.040128	DVL1/TIAM
GO_MF_m	GO:00086	cAMP-dep	1/103	7/16969	0.041731	0.083722	0.04228	CXCL10
GO_MF_m	GO:00302	LIM domai	1/103	7/16969	0.041731	0.083722	0.04228	TLN1
GO_MF_m	GO:00317	type 1 ang	1/103	7/16969	0.041731	0.083722	0.04228	EDNRB
GO_MF_m	GO:00995	voltage-ga	1/103	7/16969	0.041731	0.083722	0.04228	CNR1
GO_MF_m	GO:00996	voltage-ga	1/103	7/16969	0.041731	0.083722	0.04228	CNR1
GO_MF_m	GO:00199	protein ph	3/103	132/16969	0.046398	0.092508	0.046717	GRB2/JAK1
GO_MF_m	GO:00049	adrenergic	1/103	8/16969	0.04755	0.09307	0.047	ADRA2A
GO_MF_m	GO:00317	angiotensi	1/103	8/16969	0.04755	0.09307	0.047	EDNRB
GO_MF_m	GO:00450	G protein-	1/103	8/16969	0.04755	0.09307	0.047	P2RY1
GO_MF_m	GO:00309	receptor ty	2/103	58/16969	0.048412	0.093615	0.047276	PIK3R1/TIA
GO_MF_m	GO:00483	calcium-de	2/103	58/16969	0.048412	0.093615	0.047276	ANXA1/RA
GO_MF_m	GO:00198	protein kir	3/103	136/16969	0.049919	0.095952	0.048456	CCL5/CXC
GO_MF_m	GO:00046	protein ser	7/53	371/16969	0.000146	0.002621	0.00161	ERCC3/GT
GO_MF_m	GO:00199	phosphata	5/53	177/16969	0.000223	0.003666	0.002253	CEP192/KI
GO_MF_m	GO:00004	bubble DN	2/53	8/16969	0.000265	0.003918	0.002407	ERCC5/XP
GO_MF_m	GO:00045	deoxyribo	3/53	41/16969	0.000282	0.003918	0.002407	ERCC1/ER
GO_MF_m	GO:00037	motor acti	4/53	103/16969	0.000298	0.003918	0.002407	DYNC1H1/
GO_MF_m	GO:00037	microtubu	3/53	43/16969	0.000325	0.004002	0.002459	DYNC1H1/
GO_MF_m	GO:00010	TFIID-class	2/53	11/16969	0.000517	0.005992	0.003682	ERCC1/TP5
GO_MF_m	GO:19909	ATP-depe	2/53	12/16969	0.000619	0.006777	0.004164	DYNC1H1/
GO_MF_m	GO:00199	protein ph	4/53	132/16969	0.000763	0.007908	0.004859	KIF3A/NEK
GO_MF_m	GO:00010	basal trans	3/53	60/16969	0.000868	0.008145	0.005005	ERCC1/ER
GO_MF_m	GO:00010	basal RNA	3/53	60/16969	0.000868	0.008145	0.005005	ERCC1/ER
GO_MF_m	GO:19904	protein AD	2/53	15/16969	0.000979	0.008768	0.005388	PARP1/PA
GO_MF_m	GO:00020	p53 bindir	3/53	65/16969	0.001096	0.009388	0.005769	TP53/TP53
GO_MF_m	GO:00342	protein kir	2/53	17/16969	0.001263	0.010369	0.006372	AKAP9/PR
GO_MF_m	GO:00010	RNA polyr	2/53	18/16969	0.001418	0.011176	0.006868	ERCC1/TP5
GO_MF_m	GO:00316	ubiquitin p	5/53	270/16969	0.001512	0.011455	0.007039	NFKBIA/PF
GO_MF_m	GO:00443	ubiquitin-l	5/53	286/16969	0.001946	0.014199	0.008725	NFKBIA/PF
GO_MF_m	GO:00002	DNA secor	2/53	23/16969	0.002322	0.016337	0.010039	ERCC5/XP

GO_MF_m	GO:00080	protein C-	4/53	186/1696	0.002692	0.018288	0.011238	ERCC1/ER
GO_MF_m	GO:00004	heterodup	1/53	1/16969	0.003123	0.019848	0.012196	XPC
GO_MF_m	GO:00616	SUMO cor	1/53	1/16969	0.003123	0.019848	0.012196	UBE2I
GO_MF_m	GO:00041	endopepti	5/53	322/1696	0.003248	0.019998	0.012288	PSMB2/PS
GO_MF_m	GO:00045	endodeoxy	2/53	28/16969	0.003435	0.020504	0.0126	ERCC1/ER
GO_MF_m	GO:00428	histone de	3/53	102/1696	0.003973	0.023018	0.014144	PARP1/RA
GO_MF_m	GO:00421	telomeric	2/53	34/16969	0.005037	0.028351	0.017421	RPA2/TP5
GO_MF_m	GO:00010	RNA polyr	3/53	113/1696	0.005288	0.028939	0.017782	ERCC1/TP
GO_MF_m	GO:00618	DNA clam	1/53	2/16969	0.006237	0.032335	0.019869	RFC1
GO_MF_m	GO:00996	microtubu	1/53	2/16969	0.006237	0.032335	0.019869	KIF5B
GO_MF_m	GO:00036	DNA helic	2/53	42/16969	0.007608	0.038432	0.023616	CHD1L/ER
GO_MF_m	GO:00167	transferase	2/53	45/16969	0.008696	0.040894	0.025129	PARP1/PA
GO_MF_m	GO:00510	protein kir	2/53	45/16969	0.008696	0.040894	0.025129	AKAP9/PR
GO_MF_m	GO:00039	DNA ligas	1/53	3/16969	0.009341	0.040894	0.025129	LIG3
GO_MF_m	GO:00433	HLH doma	1/53	3/16969	0.009341	0.040894	0.025129	UBE2I
GO_MF_m	GO:00616	ubiquitin r	1/53	3/16969	0.009341	0.040894	0.025129	TP53BP1
GO_MF_m	GO:19905	3' overhan	1/53	3/16969	0.009341	0.040894	0.025129	ERCC1
GO_MF_m	GO:00088	exodeoxyr	1/53	4/16969	0.012436	0.048037	0.029518	RAD9A
GO_MF_m	GO:00168	ligase acti	1/53	4/16969	0.012436	0.048037	0.029518	LIG3
GO_MF_m	GO:00350	histone de	1/53	4/16969	0.012436	0.048037	0.029518	TP53
GO_MF_m	GO:00519	dynein ligl	1/53	4/16969	0.012436	0.048037	0.029518	DYNC1H1
GO_MF_m	GO:00715	RING-like	1/53	4/16969	0.012436	0.048037	0.029518	UBE2I
GO_MF_m	GO:19053	retromer c	1/53	4/16969	0.012436	0.048037	0.029518	RAB7A
GO_MF_m	GO:00080	microtubu	3/53	166/1696	0.015064	0.056624	0.034794	CDK5RAP
GO_MF_m	GO:00085	ATP-depe	1/53	5/16969	0.015521	0.056624	0.034794	DYNC1H1
GO_MF_m	GO:00443	small prot	1/53	5/16969	0.015521	0.056624	0.034794	UBE2I
GO_MF_m	GO:00045	nuclease a	3/53	171/1696	0.016298	0.058376	0.035871	ERCC1/ER
GO_MF_m	GO:00081	nuclear lo	1/53	6/16969	0.018597	0.063166	0.038814	NFKBIA
GO_MF_m	GO:00084	JUN kinase	1/53	6/16969	0.018597	0.063166	0.038814	KIF5B
GO_MF_m	GO:00321	DNA inser	1/53	6/16969	0.018597	0.063166	0.038814	XPC
GO_MF_m	GO:00036	DNA clam	1/53	7/16969	0.021663	0.067741	0.041626	RFC1
GO_MF_m	GO:00046	cAMP-de	1/53	7/16969	0.021663	0.067741	0.041626	PRKACA
GO_MF_m	GO:00085	ATP-depe	1/53	7/16969	0.021663	0.067741	0.041626	KIF3A
GO_MF_m	GO:00331	protein-D	1/53	7/16969	0.021663	0.067741	0.041626	RFC1
GO_MF_m	GO:00431	3'-5' DNA	1/53	7/16969	0.021663	0.067741	0.041626	ERCC3
GO_MF_m	GO:00000	single-stra	1/53	8/16969	0.02472	0.074922	0.046038	ERCC1
GO_MF_m	GO:00973	MDM2/MI	1/53	8/16969	0.02472	0.074922	0.046038	TP53
GO_MF_m	GO:00046	cyclic nucl	1/53	9/16969	0.027768	0.082883	0.05093	PRKACA
GO_MF_m	GO:00309	mismatche	1/53	10/16969	0.030806	0.086697	0.053274	XPC
GO_MF_m	GO:00985	G-rich stra	1/53	10/16969	0.030806	0.086697	0.053274	RPA2
GO_MF_m	GO:01400	ubiquitina	1/53	10/16969	0.030806	0.086697	0.053274	TP53BP1
GO_MF_m	GO:01400	ubiquitin-	1/53	10/16969	0.030806	0.086697	0.053274	TP53BP1
GO_MF_m	GO:00430	single-stra	1/53	11/16969	0.033835	0.09388	0.057688	RPA2
GO_MF_m	GO:00706	proteasor	1/53	12/16969	0.036855	0.100838	0.061963	PSMF1
GO_MF_m	GO:00162	RNA polyr	1/53	13/16969	0.039865	0.103334	0.063497	GTF2H3
GO_MF_m	GO:00510	microtubu	1/53	13/16969	0.039865	0.103334	0.063497	TTBK2
GO_MF_m	GO:00988	sequence-	1/53	13/16969	0.039865	0.103334	0.063497	RPA2
GO_MF_m	GO:19903	Lys48-spe	1/53	13/16969	0.039865	0.103334	0.063497	USP7
GO_MF_m	GO:00045	endonucle	2/53	105/1696	0.042602	0.106894	0.065684	ERCC1/ER
GO_MF_m	GO:00045	exodeoxyr	1/53	14/16969	0.042866	0.106894	0.065684	RAD9A
GO_MF_m	GO:00168	exodeoxyr	1/53	14/16969	0.042866	0.106894	0.065684	RAD9A
GO_MF_m	GO:00156	tubulin bir	3/53	252/1696	0.044033	0.108431	0.066629	CDK5RAP
GO_MF_m	GO:00039	NAD+ AD	1/53	15/16969	0.045858	0.110171	0.067698	PARP1
GO_MF_m	GO:01402	general tr	1/53	15/16969	0.045858	0.110171	0.067698	GTF2H3
GO_MF_m	GO:00431	ubiquitin b	2月11日	54/16969	0.000537	0.004831	0.00226	TSG101/V
GO_MF_m	GO:00321	ubiquitin-l	2月11日	70/16969	0.000901	0.005404	0.002528	TSG101/V
GO_MF_m	GO:00474	protein N-	2月11日	109/1696	0.002165	0.009743	0.004558	CHMP6/V
GO_MF_m	GO:00467	virion binc	1月11日	8/16969	0.005175	0.018476	0.008644	TSG101

GO_MF_m	GO:00080	protein C-	2月11日	186/1696	0.006159	0.018476	0.008644	VPS36/VT
GO_MF_m	GO:00452	cadherin b	2月11日	297/1696	0.015129	0.038904	0.018201	CHMP2B/
GO_MF_m	GO:00322	phosphati	1月11日	38/16969	0.024366	0.054824	0.025649	VPS36
GO_MF_m	GO:00303	nuclear re	1月11日	45/16969	0.028795	0.057591	0.026943	TSG101
GO_MF_m	GO:00508	cell adhesi	2月11日	444/1696	0.032136	0.057844	0.027062	CHMP2B/
KEGG_Path	hsa03015	mRNA sur	7/104	91/7946	0.000177	0.001519	0.001413	CPSF4/ETF
KEGG_Path	hsa05169	Epstein-Ba	4月20日	201/7946	0.001401	0.009337	0.006388	CCNA2/PS
KEGG_Path	hsa04914	Progester	2月20日	99/7946	0.025234	0.126171	0.086328	AURKA/CC
KEGG_Path	hsa04110	Cell cycle	2月20日	124/7946	0.038214	0.127392	0.087163	CCNA2/PT
KEGG_Path	hsa04114	Oocyte me	2月20日	128/7946	0.040487	0.127392	0.087163	AURKA/PT
KEGG_Path	hsa03040	Spliceosor	2月20日	135/7946	0.044587	0.127392	0.087163	THOC1/TH
KEGG_Path	hsa05166	Human T-	10/86	219/7946	0.000117	0.001723	0.001199	ADCY3/AD
KEGG_Path	hsa05163	Human cy	10/86	225/7946	0.000146	0.001988	0.001383	ADCY3/AD
KEGG_Path	hsa04610	Compleme	6/86	85/7946	0.000298	0.00357	0.002484	C3AR1/CF
KEGG_Path	hsa05169	Epstein-Ba	9/86	201/7946	0.000303	0.00357	0.002484	CCNE2/CD
KEGG_Path	hsa05206	MicroRNA	11/86	310/7946	0.000478	0.005286	0.003678	CCNE2/CD
KEGG_Path	hsa05223	Non-small	5/86	66/7946	0.000703	0.007319	0.005093	CDK6/E2F
KEGG_Path	hsa05224	Breast can	7/86	147/7946	0.00102	0.009746	0.006781	BRCA2/CD
KEGG_Path	hsa05218	Melanoma	5/86	72/7946	0.001046	0.009746	0.006781	CDK6/E2F
KEGG_Path	hsa05226	Gastric car	7/86	149/7946	0.001104	0.009766	0.006795	CCNE2/DV
KEGG_Path	hsa04962	Vasopressi	4/86	44/7946	0.001248	0.010123	0.007043	ADCY3/AD
KEGG_Path	hsa05214	Glioma	5/86	75/7946	0.001258	0.010123	0.007043	CDK6/E2F
KEGG_Path	hsa05220	Chronic m	5/86	76/7946	0.001335	0.010277	0.007151	CDK6/E2F
KEGG_Path	hsa05130	Pathogeni	8/86	202/7946	0.001474	0.010874	0.007566	ABI1/ARP
KEGG_Path	hsa05225	Hepatocel	7/86	168/7946	0.002204	0.015602	0.010856	CDK6/DVL
KEGG_Path	hsa04926	Relaxin sig	6/86	129/7946	0.002652	0.018056	0.012564	ADCY3/AD
KEGG_Path	hsa05165	Human pa	10/86	330/7946	0.002849	0.018678	0.012997	CCNE2/CD
KEGG_Path	hsa05222	Small cell	5/86	92/7946	0.003106	0.019636	0.013663	CCNE2/CD
KEGG_Path	hsa05167	Kaposi sar	7/86	186/7946	0.003892	0.023028	0.016023	CCR3/CDK
KEGG_Path	hsa05215	Prostate c	5/86	97/7946	0.003903	0.023028	0.016023	CCNE2/E2
KEGG_Path	hsa04060	Cytokine-	9/86	294/7946	0.004343	0.024798	0.017255	ACKR3/CC
KEGG_Path	hsa04916	Melanoge	5/86	101/7946	0.004639	0.025657	0.017852	ADCY3/AD
KEGG_Path	hsa04972	Pancreatic	5/86	102/7946	0.004837	0.025943	0.018052	ADCY3/AD
KEGG_Path	hsa04927	Cortisol sy	4/86	65/7946	0.005243	0.027296	0.018993	ADCY3/AD
KEGG_Path	hsa05203	Viral carcir	7/86	201/7946	0.005934	0.030011	0.020882	CCNE2/CC
KEGG_Path	hsa04015	Rap1 signa	7/86	210/7946	0.007494	0.036	0.025049	ADCY3/AD
KEGG_Path	hsa04115	p53 signal	4/86	72/7946	0.007525	0.036	0.025049	CCNE2/CD
KEGG_Path	hsa05161	Hepatitis B	6/86	162/7946	0.008038	0.037442	0.026052	CCNE2/DD
KEGG_Path	hsa04918	Thyroid hc	4/86	74/7946	0.008281	0.037582	0.02615	ADCY3/AD
KEGG_Path	hsa01521	EGFR tyros	4/86	79/7946	0.010382	0.045942	0.031967	GAS6/HGF
KEGG_Path	hsa04540	Gap juncti	4/86	88/7946	0.014979	0.064664	0.044994	ADCY3/AD
KEGG_Path	hsa04913	Ovarian st	3/86	49/7946	0.015765	0.066438	0.046228	ADCY3/AD
KEGG_Path	hsa04915	Estrogen s	5/86	138/7946	0.016654	0.067403	0.046899	ADCY3/AD
KEGG_Path	hsa05032	Morphine	4/86	91/7946	0.016756	0.067403	0.046899	ADCY3/AD
KEGG_Path	hsa04666	Fc gamma	4/86	93/7946	0.018011	0.069302	0.048221	ARPC4/BIN
KEGG_Path	hsa04912	GnRH sign	4/86	93/7946	0.018011	0.069302	0.048221	ADCY3/AD
KEGG_Path	hsa03460	Fanconi ar	3/86	54/7946	0.020423	0.076776	0.053421	BRCA2/PA
KEGG_Path	hsa04925	Aldosteror	4/86	98/7946	0.0214	0.076776	0.053421	ADCY3/AD
KEGG_Path	hsa04923	Regulatior	3/86	55/7946	0.021436	0.076776	0.053421	ADCY3/AD
KEGG_Path	hsa04072	Phospholi	5/86	148/7946	0.021849	0.076776	0.053421	ADCY3/AD
KEGG_Path	hsa04914	Progester	4/86	99/7946	0.022122	0.076776	0.053421	ADCY3/AD
KEGG_Path	hsa04660	T cell rece	4/86	104/7946	0.025953	0.087007	0.06054	CD3D/CD
KEGG_Path	hsa05160	Hepatitis C	5/86	155/7946	0.026053	0.087007	0.06054	CDK6/E2F
KEGG_Path	hsa04928	Parathyroi	4/86	106/7946	0.027591	0.090437	0.062927	ADCY3/AD
KEGG_Path	hsa04213	Longevity	3/86	62/7946	0.029286	0.094249	0.065579	ADCY3/AD
KEGG_Path	hsa05211	Renal cell	3/86	69/7946	0.038459	0.121557	0.08458	HGF/SOS1
KEGG_Path	hsa04935	Growth hc	4/86	119/7946	0.039728	0.123367	0.08584	ADCY3/AD
KEGG_Path	hsa04976	Bile secreti	3/86	72/7946	0.042788	0.130577	0.090856	ADCY3/AD

KEGG_Patł	hsa04971	Gastric aci	3/86	75/7946	0.047351	0.142054	0.098842	ADCY3/AD
KEGG_Patł	hsa04114	Oocyte me	4/86	128/7946	0.049663	0.146506	0.10194	ADCY3/AD
KEGG_Patł	hsa04913	Ovarian st	3月24日	49/7946	0.000407	0.004848	0.003544	ALOX5/PL
KEGG_Patł	hsa04611	Platelet ac	4月24日	124/7946	0.000471	0.004848	0.003544	PLA2G4A/
KEGG_Patł	hsa04921	Oxytocin s	4月24日	153/7946	0.00104	0.00936	0.006842	PLA2G4A/
KEGG_Patł	hsa05135	Yersinia in	3月24日	120/7946	0.005394	0.04315	0.031543	LCK/PTK2E
KEGG_Patł	hsa04650	Natural kil	3月24日	131/7946	0.006881	0.045996	0.033623	KLRD1/LCI
KEGG_Patł	hsa04270	Vascular sı	3月24日	132/7946	0.007027	0.045996	0.033623	PLA2G4A/
KEGG_Patł	hsa04923	Regulatior	2月24日	55/7946	0.011777	0.070663	0.051654	PTGS1/PT(
KEGG_Patł	hsa04370	VEGF signa	2月24日	59/7946	0.01347	0.074605	0.054536	PLA2G4A/
KEGG_Patł	hsa04664	Fc epsilon	2月24日	68/7946	0.017641	0.089098	0.06513	ALOX5/PL
KEGG_Patł	hsa04062	Chemokin	3月24日	189/7946	0.018562	0.089098	0.06513	FGR/PTK2I
KEGG_Patł	hsa05204	Chemical (	2月24日	82/7946	0.025068	0.107531	0.078604	CYP2C8/P
KEGG_Patł	hsa04810	Regulatior	3月24日	213/7946	0.025389	0.107531	0.078604	PIP4K2C/P
KEGG_Patł	hsa05163	Human cy	3月24日	225/7946	0.029251	0.117004	0.085529	PTGS2/PTI
KEGG_Patł	hsa04912	GnRH sign	2月24日	93/7946	0.03165	0.119936	0.087673	PLA2G4A/
KEGG_Patł	hsa04750	Inflammat	2月24日	100/7946	0.036157	0.128532	0.093956	ALOX12/P
KEGG_Patł	hsa04064	NF-kappa	2月24日	102/7946	0.037489	0.128532	0.093956	LCK/PTGS
KEGG_Patł	hsa04670	Leukocyte	2月24日	112/7946	0.044424	0.145387	0.106277	PTK2B/RO
KEGG_Patł	hsa03015	mRNA sur	6/67	91/7946	0.000109	0.000902	0.000769	CSTF1/FIP
KEGG_Patł	hsa04115	p53 signal	5/67	72/7946	0.000331	0.002403	0.002049	CCNB1/CC
KEGG_Patł	hsa03040	Spliceosor	6/67	135/7946	0.000918	0.005917	0.005047	CHERP/HN
KEGG_Patł	hsa05166	Human T-	7/67	219/7946	0.002344	0.013597	0.011598	ANAPC5/E
KEGG_Patł	hsa04218	Cellular se	4/67	160/7946	0.045634	0.240614	0.205243	CCNB1/CC
KEGG_Patł	hsa04145	Phagosom	4月27日	152/7946	0.001603	0.013266	0.008342	DYNC2H1/
KEGG_Patł	hsa05160	Hepatitis (	4月27日	155/7946	0.001723	0.013266	0.008342	EGF/EGFR/
KEGG_Patł	hsa05219	Bladder ca	2月27日	41/7946	0.008403	0.058823	0.036991	EGF/EGFR
KEGG_Patł	hsa04962	Vasopressi	2月27日	44/7946	0.009634	0.061819	0.038874	DYNC2H1/
KEGG_Patł	hsa04072	Phospholi	3月27日	148/7946	0.013351	0.075751	0.047636	DNM2/EG
KEGG_Patł	hsa04961	Endocrine	2月27日	53/7946	0.013773	0.075751	0.047636	CLTCL1/DI
KEGG_Patł	hsa05213	Endometri	2月27日	58/7946	0.016351	0.083933	0.052781	EGF/EGFR
KEGG_Patł	hsa04530	Tight junct	3月27日	169/7946	0.01901	0.091486	0.057531	ACTR3/TU
KEGG_Patł	hsa05223	Non-small	2月27日	66/7946	0.020867	0.093605	0.058863	EGF/EGFR
KEGG_Patł	hsa04520	Adherens j	2月27日	71/7946	0.023925	0.093605	0.058863	EGFR/WAS
KEGG_Patł	hsa05218	Melanoma	2月27日	72/7946	0.024558	0.093605	0.058863	EGF/EGFR
KEGG_Patł	hsa00562	Inositol ph	2月27日	74/7946	0.025844	0.093605	0.058863	OCRL/PIK3
KEGG_Patł	hsa05214	Glioma	2月27日	75/7946	0.026497	0.093605	0.058863	EGF/EGFR
KEGG_Patł	hsa05212	Pancreatic	2月27日	76/7946	0.027156	0.093605	0.058863	EGF/EGFR
KEGG_Patł	hsa04721	Synaptic v	2月27日	78/7946	0.028496	0.093605	0.058863	CLTCL1/DI
KEGG_Patł	hsa01521	EGFR tyros	2月27日	79/7946	0.029175	0.093605	0.058863	EGF/EGFR
KEGG_Patł	hsa04012	ErbB signa	2月27日	85/7946	0.033389	0.101027	0.06353	EGF/EGFR
KEGG_Patł	hsa05210	Colorectal	2月27日	86/7946	0.034113	0.101027	0.06353	EGF/EGFR
KEGG_Patł	hsa05235	PD-L1 exp	2月27日	89/7946	0.036323	0.103587	0.06514	EGF/EGFR
KEGG_Patł	hsa05215	Prostate ca	2月27日	97/7946	0.042479	0.113127	0.071139	EGF/EGFR
KEGG_Patł	hsa05231	Choline m	2月27日	98/7946	0.043274	0.113127	0.071139	EGF/EGFR
KEGG_Patł	hsa04070	Phosphatic	2月27日	99/7946	0.044075	0.113127	0.071139	OCRL/PIK3
KEGG_Patł	hsa04928	Parathyroi	2月27日	106/7946	0.049838	0.123791	0.077845	ARRB1/EG
KEGG_Patł	hsa03020	RNA polyr	1月5日	31/7946	0.01936	0.01936	NA	POLR1B
KEGG_Patł	hsa05160	Hepatitis (	3月8日	155/7946	0.000379	0.003035	0.001997	IFIT1/RNA
KEGG_Patł	hsa05164	Influenza /	2月8日	170/7946	0.011706	0.035203	0.02316	RNASEL/R
KEGG_Patł	hsa04621	NOD-like	2月8日	181/7946	0.013201	0.035203	0.02316	GBP2/RNA
KEGG_Patł	hsa04014	Ras signali	10/81	232/7946	0.000113	0.000867	0.000427	GRB2/LAT
KEGG_Patł	hsa05131	Shigellosis	10/81	236/7946	0.00013	0.000959	0.000472	CCL5/MYL
KEGG_Patł	hsa04020	Calcium si	9/81	193/7946	0.000141	0.000975	0.00048	ADCY2/AD
KEGG_Patł	hsa01521	EGFR tyros	6/81	79/7946	0.000143	0.000975	0.00048	GRB2/JAK
KEGG_Patł	hsa04662	B cell rece	6/81	82/7946	0.000176	0.001156	0.000569	GRB2/PIK3
KEGG_Patł	hsa04935	Growth hc	7/81	119/7946	0.000197	0.00125	0.000615	ADCY2/GH
KEGG_Patł	hsa05210	Colorectal	6/81	86/7946	0.000229	0.001402	0.00069	GRB2/PIK3

KEGG_Path	hsa04370	VEGF signa	5/81	59/7946	0.000316	0.001877	0.000923	PIK3CB/PII
KEGG_Path	hsa04658	Th1 and Th	6/81	92/7946	0.00033	0.001896	0.000933	HLA-DPB1
KEGG_Path	hsa04666	Fc gamma	6/81	93/7946	0.00035	0.001896	0.000933	HCK/LAT/I
KEGG_Path	hsa05323	Rheumato	6/81	93/7946	0.00035	0.001896	0.000933	CCL5/CD2
KEGG_Path	hsa05231	Choline m	6/81	98/7946	0.000465	0.002442	0.001202	GRB2/PIK3
KEGG_Path	hsa04360	Axon guid	8/81	181/7946	0.000483	0.002471	0.001216	CXCL12/M
KEGG_Path	hsa04061	Viral prote	6/81	100/7946	0.000518	0.002574	0.001267	CCL21/CC
KEGG_Path	hsa05330	Allograft r	4/81	38/7946	0.000568	0.002752	0.001354	CD28/CD8
KEGG_Path	hsa04917	Prolactin s	5/81	70/7946	0.0007	0.00326	0.001604	GRB2/PIK3
KEGG_Path	hsa04072	Phospholi	7/81	148/7946	0.000742	0.00326	0.001604	ADCY2/F2
KEGG_Path	hsa04659	Th17 cell c	6/81	107/7946	0.000742	0.00326	0.001604	HLA-DPB1
KEGG_Path	hsa04520	Adherens j	5/81	71/7946	0.000748	0.00326	0.001604	RAC2/RAC
KEGG_Path	hsa05332	Graft-vers	4/81	41/7946	0.000762	0.00326	0.001604	CD28/CD8
KEGG_Path	hsa05100	Bacterial ir	5/81	73/7946	0.000849	0.00355	0.001747	PIK3CB/PII
KEGG_Path	hsa04940	Type I diab	4/81	43/7946	0.000914	0.003738	0.001839	CD28/CD8
KEGG_Path	hsa05212	Pancreatic	5/81	76/7946	0.00102	0.004078	0.002006	JAK1/PIK3
KEGG_Path	hsa04071	Sphingolip	6/81	119/7946	0.001293	0.005061	0.00249	ADORA1/(
KEGG_Path	hsa04530	Tight junct	7/81	169/7946	0.001611	0.006177	0.003039	ARHGAP1
KEGG_Path	hsa05164	Influenza A	7/81	170/7946	0.001667	0.006203	0.003052	CCL5/CXC
KEGG_Path	hsa04012	ErbB signa	5/81	85/7946	0.001686	0.006203	0.003052	GRB2/PIK3
KEGG_Path	hsa04540	Gap juncti	5/81	88/7946	0.001966	0.007093	0.00349	ADCY2/GN
KEGG_Path	hsa05320	Autoimmu	4/81	53/7946	0.002008	0.007107	0.003497	CD28/CD8
KEGG_Path	hsa04923	Regulation	4/81	55/7946	0.002304	0.007997	0.003935	ADCY2/AD
KEGG_Path	hsa05167	Kaposi sar	7/81	186/7946	0.002777	0.009368	0.004609	CCR5/CD8
KEGG_Path	hsa05213	Endometri	4/81	58/7946	0.0028	0.009368	0.004609	GRB2/PIK3
KEGG_Path	hsa04064	NF-kappa	5/81	102/7946	0.003742	0.011871	0.005841	CCL21/CX
KEGG_Path	hsa05142	Chagas dis	5/81	102/7946	0.003742	0.011871	0.005841	CCL5/GNA
KEGG_Path	hsa05146	Amoebiasi	5/81	102/7946	0.003742	0.011871	0.005841	GNA15/GN
KEGG_Path	hsa04620	Toll-like re	5/81	104/7946	0.004067	0.012683	0.00624	CCL5/CD8
KEGG_Path	hsa05169	Epstein-Ba	7/81	201/7946	0.004268	0.013089	0.00644	CXCL10/H
KEGG_Path	hsa05223	Non-small	4/81	66/7946	0.004473	0.013494	0.006639	GRB2/PIK3
KEGG_Path	hsa05221	Acute mye	4/81	67/7946	0.004721	0.014011	0.006893	GRB2/PIK3
KEGG_Path	hsa05160	Hepatitis C	6/81	155/7946	0.004871	0.014227	0.006999	CXCL10/G
KEGG_Path	hsa05211	Renal cell	4/81	69/7946	0.005244	0.015076	0.007417	GRB2/PIK3
KEGG_Path	hsa05161	Hepatitis E	6/81	162/7946	0.006028	0.017065	0.008396	GRB2/JAK1
KEGG_Path	hsa05166	Human T-	7/81	219/7946	0.006782	0.018908	0.009302	ADCY2/HL
KEGG_Path	hsa04022	cGMP-PK(	6/81	167/7946	0.00697	0.019054	0.009374	ADCY2/AD
KEGG_Path	hsa05214	Glioma	4/81	75/7946	0.007042	0.019054	0.009374	GRB2/PIK3
KEGG_Path	hsa04722	Neurotrop	5/81	119/7946	0.007176	0.019137	0.009415	GRB2/PIK3
KEGG_Path	hsa05220	Chronic m	4/81	76/7946	0.007376	0.019389	0.009539	GRB2/PIK3
KEGG_Path	hsa04380	Osteoclast	5/81	128/7946	0.009685	0.0251	0.012349	GRB2/JAK1
KEGG_Path	hsa04930	Type II dia	3/81	46/7946	0.011307	0.028895	0.014216	PIK3CB/PII
KEGG_Path	hsa04910	Insulin sigr	5/81	137/7946	0.012743	0.032118	0.015802	GRB2/PIK3
KEGG_Path	hsa05418	Fluid shea	5/81	139/7946	0.013502	0.033572	0.016517	PIK3CB/PII
KEGG_Path	hsa04550	Signaling p	5/81	140/7946	0.013892	0.034083	0.016768	DVL1/GRB
KEGG_Path	hsa05203	Viral carcir	6/81	201/7946	0.016399	0.039703	0.019533	CCR5/GRB
KEGG_Path	hsa05224	Breast can	5/81	147/7946	0.016843	0.040038	0.019698	DVL1/GRB
KEGG_Path	hsa05215	Prostate ca	4/81	97/7946	0.016973	0.040038	0.019698	GRB2/PIK3
KEGG_Path	hsa05205	Proteoglyc	6/81	204/7946	0.017519	0.040804	0.020075	GRB2/PIK3
KEGG_Path	hsa05226	Gastric car	5/81	149/7946	0.017757	0.040841	0.020094	DVL1/GRB
KEGG_Path	hsa04916	Melanoger	4/81	101/7946	0.019402	0.043634	0.021468	ADCY2/DV
KEGG_Path	hsa04150	mTOR sigr	5/81	153/7946	0.019683	0.043634	0.021468	DVL1/GRB
KEGG_Path	hsa04921	Oxytocin s	5/81	153/7946	0.019683	0.043634	0.021468	ADCY2/GN
KEGG_Path	hsa05170	Human im	6/81	212/7946	0.020763	0.045481	0.022376	CCR5/GNA
KEGG_Path	hsa04625	C-type lec	4/81	104/7946	0.021358	0.046234	0.022747	PIK3CB/PII
KEGG_Path	hsa04931	Insulin resi	4/81	108/7946	0.024149	0.051668	0.02542	PIK3CB/PII
KEGG_Path	hsa04630	JAK-STAT	5/81	162/7946	0.024507	0.05183	0.0255	GRB2/JAK1
KEGG_Path	hsa04213	Longevity	3/81	62/7946	0.025091	0.052462	0.025811	ADCY2/PII

KEGG_Patl	hsa04668	TNF signal	4/81	112/7946	0.027152	0.054901	0.027011	CCL5/CXC
KEGG_Patl	hsa04725	Cholinergi	4/81	112/7946	0.027152	0.054901	0.027011	ADCY2/GN
KEGG_Patl	hsa05145	Toxoplasm	4/81	112/7946	0.027152	0.054901	0.027011	CCR5/HLA
KEGG_Patl	hsa05225	Hepatocel	5/81	168/7946	0.028113	0.056227	0.027663	DVL1/GRB
KEGG_Patl	hsa04924	Renin secr	3/81	69/7946	0.033052	0.065393	0.032173	ADORA1/C
KEGG_Patl	hsa05310	Asthma	2/81	31/7946	0.039436	0.077194	0.037979	HLA-DPB1
KEGG_Patl	hsa04971	Gastric aci	3/81	75/7946	0.040802	0.079028	0.038881	ADCY2/CC
KEGG_Patl	hsa05140	Leishmani	3/81	77/7946	0.043573	0.083514	0.041088	HLA-DPB1
KEGG_Patl	hsa04068	FoxO signa	4/81	131/7946	0.044381	0.084187	0.041419	GRB2/PIK3
KEGG_Patl	hsa05322	Systemic lu	4/81	133/7946	0.046483	0.087274	0.042938	CD28/CD8
KEGG_Patl	hsa05169	Epstein-Ba	6/38	201/7946	0.000342	0.007416	0.006265	CCNA1/DI
KEGG_Patl	hsa03440	Homologo	3/38	41/7946	0.000949	0.018031	0.015234	POLD2/PC
KEGG_Patl	hsa03022	Basal trans	3/38	45/7946	0.001247	0.021055	0.017789	ERCC3/GT
KEGG_Patl	hsa04210	Apoptosis	4/38	136/7946	0.00386	0.058677	0.049575	NFKBIA/PA
KEGG_Patl	hsa01524	Platinum c	3/38	73/7946	0.004984	0.068869	0.058186	ERCC1/TP5
KEGG_Patl	hsa05220	Chronic m	3/38	76/7946	0.005578	0.070657	0.059696	DDB2/NFK
KEGG_Patl	hsa05161	Hepatitis E	4/38	162/7946	0.007165	0.083772	0.070777	CCNA1/DI
KEGG_Patl	hsa05222	Small cell l	3/38	92/7946	0.009456	0.102663	0.086737	DDB2/NFK
KEGG_Patl	hsa04064	NF-kappa	3/38	102/7946	0.012513	0.126795	0.107126	NFKBIA/PA
KEGG_Patl	hsa05216	Thyroid ca	2/38	37/7946	0.013349	0.126816	0.107144	DDB2/TP5
KEGG_Patl	hsa04962	Vasopressi	2/38	44/7946	0.018568	0.159285	0.134576	DYNC1H1/
KEGG_Patl	hsa04722	Neurotrop	3/38	119/7946	0.018878	0.159285	0.134576	NFKBIA/NI
KEGG_Patl	hsa05166	Human T-	4/38	219/7946	0.019911	0.159285	0.134576	CCNA1/NI
KEGG_Patl	hsa04340	Hedgehog	2/38	50/7946	0.023618	0.179494	0.15165	KIF3A/PRK
KEGG_Patl	hsa03460	Fanconi ar	2/38	54/7946	0.027262	0.197326	0.166716	ERCC1/RP
KEGG_Patl	hsa05213	Endometri	2/38	58/7946	0.031119	0.215001	0.181649	DDB2/TP5
KEGG_Patl	hsa05217	Basal cell c	2/38	63/7946	0.036224	0.236348	0.199684	DDB2/TP5
KEGG_Patl	hsa04137	Mitophagy	2/38	65/7946	0.038351	0.236348	0.199684	RAB7A/TP
KEGG_Patl	hsa05223	Non-small	2/38	66/7946	0.039432	0.236348	0.199684	DDB2/TP5
KEGG_Patl	hsa04218	Cellular se	3/38	160/7946	0.040428	0.236348	0.199684	CCNA1/RA
KEGG_Patl	hsa04920	Adipocyto	2/38	69/7946	0.042745	0.240636	0.203308	NFKBIA/NI
KEGG_Patl	hsa04115	p53 signal	2/38	72/7946	0.046158	0.241934	0.204404	DDB2/TP5
KEGG_Patl	hsa05218	Melanoma	2/38	72/7946	0.046158	0.241934	0.204404	DDB2/TP5
KEGG_Patl	hsa05214	Glioma	2/38	75/7946	0.04967	0.249387	0.210701	DDB2/TP5

t analysis, Enrichment_all_0.05

Count
4
7
5
7
2
5
4
4
4
4
7
8
7
4
9
2
2
5
5
2
4
3
9
2
3
3
4
2
2
6
3
2
2
7
3
3
4
2
6
2
6
2
1
1
1
1
1
1
1
1
1
1
1
1
1
2

2
3
4
2
4
3
2
4
4
2
2
2
3
5
2
7
6
5
3
3
6
4
2
4
2
1
1
1
1
1
1
1
1
1
1
1
1
1
1
1
1
6
2
3
2
2
4
3
2
2
3
3
4
2
4
5
2
2

2
1
1
1
1
1
1
1
1
1
2
4
2
2
2
2
4
2
2
2
2
2
1
1
1
1
1
1
1
1
1
2
2
2
3
2
2
5
1
1
1
1
1
1
1
1
1
1
1
1
1
1
1
1
4
3
3
2

6
5
4
2
3
3
5
2
2
3
1
1
1
1
1
1
1
1
1
1
1
1
1
1
1
1
1
1
5
3
3
2
2
2
3
3
2
2
2
2
3
3
4
4
5
1
1
1
1
1
1
1
1
1
1
2
2
4

2
2
3
2
4
2
2
2
4
4
4
4
4
4
4
2
4
4
4
3
3
4
4
2
2
2
2
2
4
2
2
4
3
2
2
3
4
4
2
4
2
2
2
3
4
4
4
1
1
2
2
2
2
2
4
2
2
4

2
2
3
2
4
2
2
1
1
1
1
1
4
4
3
2
2
2
2
1
1
1
1
1
1
1
1
3
1
1
1
1
1
2
2
2
3
1
1
1
2
2
1
1
1
3
1
1
1
1
1
1
1
1
1
2
3
1

1
2
2
2
1
1
1
1
1
2
1
1
2
2
2
1
1
1
1
1
1
2
2
1
1
1
2
1
2
1
3
3
1
1
1
2
3
3
1
1
1
1
1
2
2
1
1
2
1
2
1
1
1
1
1
1
1
1

2
2
2
1
1
1
1
1
1
1
1
1
1
1
1
2
1
1
1
1
1
1
1
1
1
1
1
2
1
1
2
1
2
1
1
1
1
2
3
2
2
2
2
1
1
1
2
2
2
3
3
1
2
1
2
2
3
1

2
2
2
1
1
1
2
2
1
1
2
2
1
2
1
1
1
2
2
2
1
1
1
2
1
1
2
2
1
1
2
1
2
1
1
2
2
2
1
2
1
1
1
2
1
1
1
1
1
2
1
1
1
2
1
1
1
1

1
1
1
1
1
1
1
1
2
2
1
1
1
1
1
1
1
1
1
1
1
1
1
1
1
11
3
7
10
7
3
3
3
7
5
7
12
9
3
3
8
6
6
12
10
3
5
6
6
9
8
9
11
7
3
3
3
5

8
9
6
6
10
3
3
5
2
2
9
8
3
3
3
9
7
6
6
5
4
6
6
6
6
5
5
5
7
5
5
5
4
7
3
3
3
3
9
10
2
2
2
4
5
6
3
3
3
8
6
9
3
4
3
3
3
4

5
6
5
11
6
9
7
10
5
4
8
3
3
3
6
6
9
5
10
9
5
6
4
3
3
3
10
6
6
9
6
2
2
2
2
2
2
3
6
6
3
3
3
3
3
6
6
7
9
8
3
3
3
3
3
6
6
6

8
9
9
2
2
2
2
3
3
9
6
8
7
4
4
4
7
3
3
2
4
5
6
4
4
5
5
3
3
3
3
8
8
6
8
2
2
2
2
2
7
3
3
3
3
3
4
10
8
6
4
9
7
4
4
5
2
2

2
2
2
2
7
8
8
3
4
4
3
3
4
6
2
2
2
2
2
3
3
3
3
4
3
3
3
5
8
2
2
10
10
10
6
6
9
4
3
3
8
3
3
3
4
8
7
7
2
2
2
2
2
3
5
5
7
4

6
4
3
3
3
9
7
4
4
2
2
2
2
2
2
5
3
4
9
8
5
3
3
2
2
6
3
3
3
3
8
4
5
3
3
3
4
4
4
2
2
2
4
3
1
1
1
1
1
1
1
1
1
1
1
1
1
1

1
1
1
1
1
1
1
1
1
1
1
1
1
1
1
1
1
1
1
1
3
6
2
2
2
6
6
4
6
3
3
5
6
3
6
4
5
5
2
2
2
2
2
2
2
3
3
3
5
4
4
5
5
9
2
2
2
2

3
3
3
3
9
5
8
8
4
4
3
3
3
9
9
7
5
2
8
4
9
6
3
5
4
5
2
2
4
3
3
3
9
2
2
2
2
2
3
5
7
4
3
3
6
3
3
3
2
2
2
2
2
4
8
8
4
4

2
2
2
2
1
1
1
1
1
1
1
1
1
1
1
1
1
1
1
1
1
1
1
1
1
1
1
1
1
1
1
1
1
1
1
1
1
1
1
1
1
1
1
1
1
1
1
4
5
2
3
3
3
4
3
2
2
2

2
2
2
2
2
3
3
6
5
8
3
7
7
5
4
2
2
2
2
2
2
8
3
3
7
3
7
2
3
3
4
4
4
3
7
2
2
4
4
3
5
4
4
2
2
2
2
2
1
1
1
1
1
1
1
1
1
1

1
1
1
1
1
1
1
1
1
1
1
1
1
1
1
1
1
1
1
1
1
1
1
1
1
1
1
1
1
1
1
1
1
1
1
1
1
1
1
7
3
3
3
3
3
4
5
4
3
2
2
2
2
3
3
5
7
8

3
2
2
6
5
4
3
5
4
3
3
2
2
3
7
6
6
2
2
3
3
4
3
3
3
3
3
7
2
2
2
2
2
1
1
1
1
1
1
1
1
1
1
1
1
1
1
1
1
1
1
1
1
1
1
1
1
1

1
1
1
1
1
1
1
1
1
1
1
1
1
1
1
1
1
1
1
3
5
4
6
8
6
4
2
2
3
6
4
4
6
5
3
3
2
2
2
7
7
6
4
8
6
6
4
5
3
3
3
3
2
2
2
2
2
2

2
5
5
5
3
3
5
2
2
2
3
3
3
3
1
1
1
1
1
1
1
1
1
1
1
1
1
1
1
1
1
1
1
1
1
1
1
1
1
1
1
1
1
1
1
1
1
6
2
2
3
3
3
3
3
2
2
2
5

5
5
3
4
6
4
4
3
2
2
2
2
4
4
4
5
2
2
2
4
4
3
1
1
1
1
1
1
1
1
1
1
1
1
1
1
1
1
1
1
1
1
1
1
1
1
1
1
6
2
2
2
3
3
3
3
5

4
3
2
2
2
4
7
3
5
4
3
3
2
2
2
2
2
2
2
2
5
3
4
2
2
2
2
2
2
5
3
4
2
2
3
3
2
2
2
5
5
3
2
2
2
2
2
4
3
3
2
2
2
2
2
2
3
3

3
3
3
2
4
2
2
3
3
2
2
4
2
2
2
4
1
1
1
1
1
1
1
1
1
1
1
1
1
1
1
1
2
4
4
3
2
2
2
2
2
2
2
2
2
3
2
4
3
2
4
4
3
4
2
2
2
3

2
2
2
4
2
3
3
1
1
1
1
1
1
1
1
1
1
1
1
1
1
1
1
1
3
3
2
2
2
2
2
2
2
2
3
2
3
4
1
1
1
1
1
1
1
1
1
1
1
1
1
2
3
4
4
4
2
4

3
2
2
3
2
2
4
1
1
1
1
1
1
1
1
1
1
1
1
3
2
2
2
2
2
2
2
3
3
3
3
2
1
1
1
1
1
1
1
1
1
1
1
1
2
2
2
2
3
2
3
2
2
2
3
2
2
1

1
1
1
1
1
1
1
1
1
1
1
1
2
2
2
3
2
2
2
2
2
2
3
2
3
2
1
1
1
1
1
1
1
1
1
3
3
2
3
2
3
2
3
2
2
1
1
1
1
1
1
1
1
1
1
1
1
1

1
1
2
2
2
2
3
2
3
3
2
2
2
3
2
3
3
1
1
1
1
1
1
1
1
2
2
2
2
3
3
2
2
2
1
1
1
1
1
1
1
1
1
1
2
3
2
2
2
2
3
2
3
2
1
1
1
1

1
1
1
1
1
1
1
3
2
2
2
2
2
2
2
1
1
1
1
1
1
1
1
1
1
1
2
2
3
2
3
2
2
2
2
2
1
1
1
1
1
1
1
1
1
3
2
3
2
2
3
3
1
1
1
1
1
1

1
1
1
1
1
3
2
2
3
1
1
1
1
1
2
3
2
2
2
1
1
1
1
1
1
1
1
1
3
2
2
2
2
2
2
2
1
1
1
1
1
1
1
1
2
3
2
2
2
3
3
2
2
1
1
1
1
1

1
1
1
1
3
3
2
1
1
1
1
1
1
1
1
1
3
2
2
3
2
1
1
1
1
1
1
1
1
1
2
2
2
2
2
1
1
1
1
1
3
2
3
2
1
1
1
1
1
2
2
2
3
3
3
1
1
1

1
1
1
1
2
3
3
2
2
1
1
1
1
1
1
1
3
3
3
3
2
2
1
1
1
1
1
1
1
1
1
1
1
1
1
2
2
2
1
1
1
1
1
1
1
2
2
1
1
1
1
1
1
1
1
1
1
1

1
1
1
1
2
1
1
1
1
2
2
1
1
1
1
2
2
1
1
1
1
1
1
1
1
1
2
2
2
1
1
1
2
2
2
1
1
1
1
1
1
2
2
2
2
1
1
1
1
2
2
1
1
1
1
1
1
6

6
5
3
4
5
5
5
6
5
4
4
5
7
6
2
2
2
2
4
3
5
6
5
5
5
3
4
4
7
2
2
9
6
3
8
6
2
2
4
5
3
4
5
6
5
5
5
3
5
2
2
5
5
8
5
10
6
3

4
6
2
2
2
5
3
8
2
5
5
3
5
5
5
6
3
8
5
3
4
2
2
2
5
5
5
3
8
5
2
2
7
5
3
5
8
5
2
2
4
5
3
3
6
6
4
2
5
5
5
2
5
7
4
7
7
2

2
7
5
7
5
5
5
5
5
7
2
2
2
5
5
7
1
1
1
1
1
1
1
1
1
1
2
5
5
5
3
2
5
7
5
2
5
3
5
3
7
3
5
3
3
3
2
2
2
2
3
4
2
3
3
5
3
1

1
1
1
1
1
1
1
1
1
1
1
3
4
5
2
2
2
3
8
2
5
7
2
3
4
3
2
5
1
1
1
1
1
1
1
7
3
4
3
5
3
7
2
3
7
4
5
3
3
1
1
1
1
1
1
1
1
1

1
1
2
3
2
2
3
2
5
2
1
1
1
1
1
1
1
1
1
1
1
1
1
1
1
1
2
3
3
5
5
2
4
2
2
2
2
5
3
4
1
1
1
1
1
1
1
1
1
1
1
1
1
1
1
1
1
1

1
1
1
5
2
2
2
5
2
3
7
1
1
1
1
1
1
1
1
1
1
1
1
1
1
2
2
2
6
4
3
3
3
4
4
3
4
2
2
7
3
3
3
2
7
6
3
3
3
6
3
5
6
4
2
3
5
2

3
6
4
3
4
4
4
4
4
4
2
2
3
4
2
5
3
2
4
3
3
5
2
2
3
3
4
5
3
2
4
3
1
1
1
1
1
1
2
2
2
3
3
2
4
4
2
3
5
3
3
4
2
1
1
1
1
2

4
2
2
2
2
2
5
2
2
4
2
2
2
2
1
1
1
1
1
1
1
1
1
1
1
1
1
3
2
3
2
2
2
4
2
2
1
1
1
1
1
1
1
1
1
1
1
1
4
3
2
2
3
3
2
4
1
1

1
1
1
1
1
1
1
1
1
1
1
1
4
3
2
3
2
3
2
2
3
4
1
1
1
1
1
1
1
1
1
1
2
2
4
3
2
2
3
1
1
1
1
1
1
1
4
4
3
3
3
3
4
4
2
1
1
1

1
1
1
1
1
1
1
1
1
1
1
1
1
1
1
1
1
2
2
2
2
3
3
1
1
1
1
1
1
2
3
2
2
2
1
1
1
1
1
1
1
1
1
1
1
1
1
1
1
1
3
4
2
2
3
3
2
3

2
1
1
1
1
1
1
1
1
1
4
2
1
1
1
1
1
1
1
1
1
2
3
2
1
1
1
1
1
1
1
1
1
2
3
3
2
2
1
1
1
1
1
1
1
1
1
1
1
1
1
3
2
3
2
3
1
1

1
1
1
1
1
1
1
3
2
2
2
1
1
1
1
1
1
1
1
1
1
2
2
2
3
3
2
1
1
1
1
1
2
3
3
2
1
1
1
1
1
3
2
2
2
2
2
2
2
2
1
1
1
1
1
1
1
1

1
2
3
3
2
1
3
1
3
1
1
1
1
1
1
1
2
2
1
1
1
2
1
1
1
2
3
2
1
1
1
1
1
1
2
2
2
1
1
2
1
1
1
1
1
1
1
1
2
2
1
1
1
1
1
1
1
2

1
1
1
1
1
1
1
2
1
1
1
2
1
1
1
1
1
1
2
1
2
1
1
1
1
1
2
1
1
1
3
3
3
3
3
3
3
2
3
3
1
1
1
1
1
1
1
1
1
1
1
3
2
1
1
1
1
1

3
1
1
1
1
1
1
1
1
1
2
1
1
2
1
1
1
1
2
2
1
1
1
2
2
2
1
1
2
1
1
1
1
2
1
1
2
1
1
1
1
1
1
1
1
1
1
1
1
1
1
1
1
1
1
2
1
1

1
1
1
1
1
1
1
1
1
2
1
1
1
1
2
1
1
1
1
1
2
2
1
1
2
2
1
1
1
1
1
1
1
1
1
2
1
2
1
1
1
1
3
6
7
6
8
11
5
7
7
6
4
11
4
3
3
5

6
9
5
6
5
5
3
10
7
6
4
4
7
4
3
3
3
5
7
8
6
5
5
5
9
6
6
2
2
2
7
6
6
6
3
3
3
3
5
6
3
3
3
3
6
7
4
4
3
3
3
3
3
3
4
4
9
5

6
5
6
8
10
3
3
4
2
2
10
5
9
7
3
3
3
4
4
7
7
4
3
3
3
3
3
3
4
6
7
6
6
6
4
8
4
2
2
2
2
2
3
3
3
3
3
3
7
7
5
3
3
3
7
4
5
5

8
3
6
10
8
10
4
4
5
2
2
2
3
3
3
5
4
3
3
3
3
4
6
3
3
3
3
5
6
5
5
5
2
2
2
2
2
3
3
6
4
9
6
6
3
4
4
6
4
6
2
2
2
3
3
3
4
8

8
3
3
3
3
5
5
7
7
7
5
4
2
2
2
2
2
2
5
7
3
3
8
5
6
6
3
3
3
6
9
3
2
2
2
2
2
5
3
3
4
6
5
4
4
3
5
9
4
3
3
2
2
2
2
2
2
6

4
3
3
3
3
3
3
4
6
3
8
7
9
2
2
2
2
2
2
4
3
3
5
5
8
7
3
7
4
5
3
3
2
2
2
2
2
2
2
2
5
3
5
5
3
5
2
2
2
2
2
5
6
4
4
5
6
2

2
2
2
2
2
3
3
8
7
4
4
6
3
8
3
3
6
4
4
2
2
2
5
3
4
8
5
3
3
3
3
2
2
2
3
3
3
8
6
8
3
3
4
2
2
2
2
2
3
3
3
3
3
3
6
4
3
8

8
1
1
1
1
1
1
1
1
1
1
1
1
1
1
1
1
1
1
1
1
1
1
1
1
1
1
1
1
1
1
1
1
1
1
1
1
1
1
1
1
1
1
1
1
1
1
1
1
1
1
1
1
1
1
1
1
1

1
1
1
1
1
3
2
2
2
5
4
4
3
5
5
4
4
5
6
2
2
2
2
4
4
4
3
3
3
3
3
3
4
2
2
2
2
4
3
4
4
4
4
2
2
3
3
3
3
5
4
2
2
2
2
2
2
3

5
5
4
4
4
7
5
3
3
2
2
2
2
2
2
2
5
3
3
3
7
2
2
2
2
2
5
3
7
6
3
3
4
2
2
2
2
2
2
2
4
3
3
6
5
2
2
2
2
2
1
1
1
1
1
1
1
1

1
1
1
1
1
1
1
1
1
1
1
1
1
1
1
1
1
1
1
1
1
1
1
1
1
1
1
1
1
1
1
3
3
3
5
2
2
5
5
3
3
6
4
4
3
3
2
2
2
2
2
2
2
5
3
3
5
4

3
2
2
2
2
2
4
4
5
3
3
4
2
2
2
2
2
3
4
2
2
4
5
2
2
2
2
2
2
7
3
3
3
4
1
1
1
1
1
1
1
1
1
1
1
1
1
1
1
1
1
1
1
1
1
1
1
1

1
1
1
1
1
1
1
1
1
3
3
3
3
2
2
2
2
2
2
2
2
4
3
2
2
2
2
4
3
3
3
7
3
3
2
2
5
3
6
3
2
2
2
2
2
2
2
5
4
5
5
3
3
5
5
4
4
2

2
7
6
6
3
5
5
4
1
1
1
1
1
1
1
1
1
1
1
1
1
1
1
1
1
1
1
1
1
1
1
1
1
1
1
1
1
1
1
1
1
1
1
5
2
3
3
3
2
2
2
2
2
2
2
4
3
3

2
2
2
2
2
4
7
4
4
2
2
2
2
4
5
3
3
2
2
2
1
1
1
1
1
1
1
1
1
1
1
1
1
1
1
1
1
1
1
1
1
1
1
1
1
1
1
1
1
1
1
1
1
1
1
1
1
1
3
4

4
2
2
2
3
3
3
2
2
2
2
4
3
2
2
2
2
2
3
2
2
2
2
2
2
4
2
2
2
3
3
3
3
1
1
1
1
1
1
1
1
1
1
1
1
1
1
1
1
1
1
1
1
1
1
1
1
1

1
1
4
3
3
3
2
2
2
2
2
3
3
4
2
2
2
6
3
2
2
2
2
2
3
3
3
3
3
2
2
4
1
1
1
1
1
1
1
1
1
1
1
1
1
1
1
1
1
1
1
1
1
1
1
1
1
1

1
1
1
1
1
1
1
1
1
1
1
1
1
3
5
4
5
2
2
2
2
2
4
4
3
3
3
2
2
2
2
6
3
5
2
3
4
1
1
1
1
1
1
1
1
1
1
1
1
1
1
1
1
1
1
1
1
1

1
1
1
1
1
1
1
1
1
1
1
1
3
3
3
2
3
3
3
2
2
5
3
5
2
2
2
2
3
3
4
4
3
6
7
2
3
3
3
3
4
3
2
4
4
2
2
3
6
4
4
6
6
6
4
7
4
2

2
2
3
7
3
2
2
5
3
6
7
3
5
7
2
5
2
4
4
2
3
4
2
3
3
3
4
6
2
3
3
5
2
2
5
2
4
3
2
2
4
3
4
3
2
2
3
3
2
2
3
1
1
1
1
1
1
1

1
1
1
4
2
2
4
2
4
3
2
2
2
2
5
4
4
2
2
3
5
4
2
2
2
2
4
4
3
2
1
1
1
1
1
1
1
1
1
1
1
3
2
2
2
3
4
2
2
2
3
2
2
4
2
2
2
2

2
2
1
1
1
1
1
1
1
1
1
1
1
1
1
1
1
1
1
1
2
2
2
2
2
2
2
2
3
3
4
2
4
5
2
1
1
1
1
1
1
1
1
1
1
1
1
1
1
1
1
1
1
1
2
2
3
3

3
3
2
2
2
2
2
3
1
1
1
1
1
1
1
1
1
1
1
1
1
1
1
1
1
1
1
2
2
3
3
2
2
4
2
2
2
2
2
1
1
1
1
1
1
1
1
1
1
1
1
1
1
1
1
1
2
2

3

2

2

2

1

1

1

1

1

1

1

1

1

1

1

1

1

1

1

1

1

2

2

3

2

2

1

1

1

1

1

1

1

1

1

1

1

1

1

1

1

1

1

1

1

2

2

2

2

2

2

2

1

1

1

1

1

1

1
1
1
4
2
3
2
3
3
1
1
1
1
1
1
1
1
1
1
1
1
1
2
2
2
2
3
2
2
1
1
1
1
1
1
1
1
1
1
1
1
2
4
3
4
3
2
1
1
1
1
1
1
1
1
1
1
1

1
1
1
1
1
2
2
2
2
4
2
2
3
3
1
1
1
1
1
1
1
1
1
2
2
2
1
1
1
1
1
1
1
1
1
1
1
1
4
2
2
2
2
2
2
3
2
2
2
1
1
1
1
1
1
1
1
1

1
1
2
4
2
2
1
1
1
1
1
1
1
1
1
1
1
2
2
3
2
3
3
4
3
3
2
3
3
2
3
1
1
1
3
3
1
1
1
2
3
1
3
3
1
1
3
1
1
1
1
1
1
1
1
1
1
1

1
1
1
1
1
1
1
1
1
1
1
1
1
1
1
1
2
2
1
1
1
1
1
1
1
1
5
2
5
2
3
2
5
1
1
1
1
2
5
2
2
1
1
1
1
1
1
2
2
1
1
1
1
1
2
2
1
1

1
1
1
2
1
2
3
3
3
3
1
2
2
2
3
1
1
1
1
1
1
2
1
2
2
1
1
1
1
1
1
1
1
3
3
4
3
2
2
1
1
3
2
2
2
2
1
1
1
1
1
1
1
1
10
5
6
3

5
3
3
5
4
2
4
10
5
4
6
4
2
2
2
2
6
4
2
2
3
10
2
2
3
2
2
2
4
2
7
2
7
3
1
1
1
1
1
4
2
2
5
2
5
4
7
5
1
1
1
2
2
2
8
2
3
4

2
3
2
3
3
7
2
2
1
1
1
1
1
1
4
2
7
1
1
1
1
1
2
3
1
1
1
6
4
5
4
1
1
1
5
2
3
1
1
1
1
2
1
2
1
1
2
3
2
3
3
3
2
3
3
1
1
1

1
1
3
1
1
2
1
3
3
3
2
2
1
3
1
2
3
1
1
2
3
10
2
5
9
2
2
2
2
4
3
1
1
1
1
2
3
6
3
3
1
1
1
1
1
1
8
2
3
2
1
1
1
1
1
1
1
2

3
2
2
2
2
1
1
1
1
1
2
2
1
1
1
1
1
1
1
1
1
2
1
2
2
4
6
4
3
3
3
3
6
4
4
2
2
4
4
2
2
4
2
3
4
5
3
3
2
3
2
1
1
2
4
3
3
2

3
2
2
2
1
1
1
1
1
3
3
3
3
5
3
2
1
1
1
2
4
2
4
2
2
1
1
4
4
3
4
1
1
1
4
3
2
1
1
4
1
2
2
2
1
1
1
3
2
1
1
2
2
1
1
1
1
1

1
1
1
1
3
3
2
1
1
2
2
1
1
1
1
3
3
1
2
2
2
1
2
1
1
1
1
9
9
3
6
3
6
6
3
3
4
8
3
4
2
6
9
6
9
3
5
6
2
2
5
7
4
4
5
3
2
2

7
4
5
4
5
5
4
1
1
1
1
1
1
6
3
2
6
3
7
2
2
4
3
2
3
2
2
1
1
1
1
3
2
2
3
4
3
3
4
5
2
1
1
1
2
3
2
3
3
1
1
5
5
2
4
4
1
1

1
5
6
5
3
1
1
1
1
3
3
2
1
1
6
2
2
1
1
1
1
1
1
1
5
2
1
2
7
4
6
4
3
3
6
2
4
3
4
2
3
4
1
1
1
1
3
3
1
1
5
3
2
1
1
1
1
1

1
1
1
3
2
1
1
2
2
1
1
1
1
1
3
1
1
1
1
2
1
1
1
1
1
1
1
1
2
1
1
1
1
2
1
1
1
1
2
1
1
2
1
2
1
1
5
5
2
2
3
9
3
3
2
2
3
2

5
2
5
2
2
9
3
2
1
1
1
1
2
2
1
1
1
2
3
2
2
4
4
2
1
1
1
2
2
2
2
1
1
1
6
2
6
1
1
1
2
2
1
1
2
5
1
1
1
1
1
1
1
1
1
1
1
1

1
1
1
2
1
2
1
1
2
1
1
3
12
7
2
2
2
2
2
2
2
2
7
5
3
8
3
3
2
2
3
2
7
4
2
4
3
2
3
3
2
2
2
4
6
3
3
3
2
1
1
1
1
1
1
1
1
1

1
1
1
1
1
1
1
1
2
2
3
3
2
2
2
3
2
1
1
1
1
1
1
1
1
1
1
1
1
1
1
2
2
4
6
2
2
2
1
1
1
1
1
1
1
1
1
3
4
4
2
4
2
2
2
4
1
1

1
1
1
1
1
1
1
1
1
1
2
4
2
3
1
1
1
1
1
1
4
2
2
5
2
1
1
2
2
2
2
3
3
3
2
3
2
3
3
3
2
2
2
1
1
1
1
1
1
2
2
3
1
1
1
1
1
1

2
2
1
1
1
1
2
1
1
1
1
1
1
1
1
1
1
1
1
1
1
1
1
1
1
1
1
1
1
1
1
1
3
1
1
1
1
1
1
1
1
2
1
1
1
1
1
3
1
1
1
1
2
1
1
1
1
1

1
2
1
1
1
3
3
2
3
2
9
2
1
1
1
1
1
3
3
1
1
1
1
1
6
7
3
1
1
1
3
2
1
1
1
1
1
1
1
2
4
1
1
1
1
3
1
1
1
2
3
3
2
2
5
2
3
3

1
4
3
3
1
1
1
1
1
5
5
4
4
2
3
4
4
1
1
1
1
1
4
1
1
2
3
1
2
1
1
3
2
2
2
3
2
3
1
1
3
1
3
1
1
1
1
1
1
1
3
1
1
1
4
1
1
1

1
1
1
1
1
1
2
2
1
2
3
3
3
3
3
3
3
2
1
2
1
1
2
3
2
1
3
3
3
1
1
1
1
1
1
1
2
2
2
2
2
1
1
1
1
1
3
1
1
1
1
1
3
1
1
1
1
1

1
1
1
1
1
1
1
1
2
1
2
2
2
1
2
2
2
1
1
1
1
1
1
1
1
1
1
3
2
7
6
6
5
3
2
2
2
2
7
2
3
3
2
2
3
6
2
6
7
7
2
2
2
3
2
1
1
1

1
1
1
1
1
1
1
1
1
1
4
2
2
2
2
2
8
6
1
1
1
1
1
1
1
1
1
1
1
1
1
1
1
1
6
2
2
6
2
6
6
2
1
1
1
1
1
1
1
1
1
1
1
2
1
1
1
1

1
1
1
1
1
1
1
1
3
2
1
1
1
1
1
1
1
1
1
1
1
3
5
1
1
1
1
2
1
1
1
1
1
3
1
1
1
2
2
3
7
5
2
3
4
3
2
2
4
3
3
2
3
2
2
5
5
2

4
1
1
5
2
3
2
3
1
1
2
2
2
1
1
1
1
1
1
1
1
1
1
3
1
1
3
1
1
1
1
1
1
1
1
1
1
1
1
1
1
1
1
1
1
1
1
1
1
2
1
1
3
1
1
2
2
2
1

2
2
1
1
2
7
4
2
2
2
2
10
10
6
9
11
5
7
5
7
4
5
5
8
7
6
10
5
7
5
9
5
5
4
7
7
4
6
4
4
4
3
5
4
4
4
3
4
3
5
4
4
5
4
3
3
4
3

3
4
3
4
4
3
3
3
2
2
2
3
2
3
3
2
2
2
2
6
5
6
7
4
4
4
2
2
3
2
2
3
2
2
2
2
2
2
2
2
2
2
2
2
2
2
2
1
3
2
2
10
10
9
6
6
7
6

5
6
6
6
6
8
6
4
5
7
6
5
4
5
4
5
6
7
7
5
5
4
4
7
4
5
5
5
5
7
4
4
6
4
6
7
6
4
5
4
5
3
5
5
5
6
5
4
6
5
4
5
5
6
4
4
5
3

4
4
4
5
3
2
3
3
4
4
6
3
3
4
3
3
4
3
3
2
2
3
4
2
2
2
2
2
2
3
2
2
2
2
